# Reference Whole Genome Sequence Analyses and Characterization of a Novel *Carnobacterium maltaromaticum* Distinct Sequence Type Isolated from a North American Gray Wolf (*Canis lupus*) Gastrointestinal Tract

**DOI:** 10.3390/vetsci12050410

**Published:** 2025-04-27

**Authors:** C. Cristoph Klews, Jessika L. Bryant, Jennifer McCabe, Ariel N. Atchley, Thomas W. Cousins, Maya Barnard-Davidson, Mark R. Ackermann, Michael Netherland, Nur A. Hasan, Peter A. Jordan, Evan S. Forsythe, Patrick N. Ball, Bruce S. Seal

**Affiliations:** 1 Biology Program, Oregon State University-Cascades, 1500 SW Chandler Avenue, Bend, OR 97702, USA; cck011@bucknell.edu (C.C.K.); jgrindstaff1996@gmail.com (J.L.B.); mccabeyj@gmail.com (J.M.); arielatchley@outlook.com (A.N.A.); thomascuzz@gmail.com (T.W.C.); barnamay@oregonstate.edu (M.B.-D.); peter.jordan@osucascades.edu (P.A.J.); evan.forsythe@osucascades.edu (E.S.F.); 2 Oregon Veterinary Diagnostic Laboratory, OSU Carlson College of Veterinary Medicine, 134 Magruder Hall, 700 SW 30th, Corvallis, OR 97331, USA; mark.r.ackermann@gmail.com; 3 EzBiome Inc., 704 Quince Orchard Rd Suite 250, Gaithersburg, MD 20878, USA; mjnetherland19@gmail.com (M.N.J.); nurahasan@gmail.com (N.A.H.); 4 Integrative Biology Department, Oregon State University, 2403 Cordley Hall, Corvallis, OR 97331, USA

**Keywords:** Firmicutes, Bacillota, Carnobacteriaceae, Lactic acid bacteria, probiotic, bacteriocin

## Abstract

We hypothesize that bacteria isolated from free-ranging animals could potentially be useful for practical applications. To meet this objective a Gram-positive bacterium was isolated from the gastrointestinal (GI) tract of a Gray Wolf (*Canis lupus*) using Brucella broth with hemin and vitamin K (BBHK). By small ribosomal RNA (16S) gene sequencing the bacterium was initially identified as a novel *Carnobacterium maltaromaticum* strain. The bacterium could be propagated both anaerobically and aerobically and was both catalase/oxidase negative and negative by the starch hydrolysis as well as negative using lipase assays. The reference whole genome sequence (WGS) was obtained using both Illumina and Nanopore sequencing. The genome assembly was 3,512,202 bp in length, encoding core bacterial genes with a GC% content of 34.48. No lysogenic bacteriophage genes were detected, although the genome harbors genes for the expression of bacteriocin and other secondary metabolites with potential antimicrobial properties. Multilocus sequence typing (MLST), WGS phylogenetics, average nucleotide identity (ANI), and single nucleotide polymorphism (SNP) analyses of the isolate’s genome indicate this bacterium is a newly identified *Carnobacterium maltaromaticum* sequence type (ST). Members of the Carnobacteria have anti-*listeria* activities, highlighting their potential functional properties. Consequently, the isolate could be a potential probiotic for canids and this is the first report on an axenic *C. maltaromaticum* culture from the genus *Canis*.

## Simple Summary

Worldwide, the animal kingdom contains a largely untapped resource of unknown bacteria that could significantly contribute to the health and safety of human and animal well-being. Specifically, free-ranging species of animals harbor diverse, yet-to-be-discovered bacteria that may have potentially useful applications. Across species, analyzing and categorizing microbes and their genomic components will increase the discovery of potential antibacterial and anti-fungal applications. These applications include use as new probiotics, that is, microbes that contribute to an animal’s gastroenteric health. During this investigation, a member of the Carnobacteria was discovered in the gastrointestinal tract of a North American Gray Wolf. These bacteria have known anti-*listeria* properties and may help support the gut health of animals. Genotypically, the bacterial isolate was demonstrated to be a unique member of the Carnobacteria. This is the first report of these types of bacteria isolated from any member of the genus *Canis*, including dogs and their progenitor, the Gray wolf. Because only a small percentage of bacteria have been discovered, this investigation also further supports the vital need to protect the biodiversity of our planet.

## 1. Introduction

Lactic acid bacteria (LAB) are a group of Gram-positive, non-sporulating, facultative anaerobic bacteria that include at least 25 genera utilized during food production and preservation [1,2]. The bacteria produce bioactive compounds [3], and many LAB are commonly applied as probiotics to help maintain gastrointestinal equilibrium and health [4,5]. Among the LAB are Carnobacteria, which have potential probiotic and bioprotective attributes encoding antimicrobials active against various pathogens, including *Listeria monocytogenes* and other pathogenic Gram-positive bacteria [4,6]. Consequently, these bacteria have been investigated for applications such as biopreservatives of foods because they produce antimicrobial peptides such as carnocyclin and piscicolin reviewed in [7,8].

Probiotics are defined by the Food and Agriculture Organization of the United Nations and the WHO (FAO/WHO) as “live microorganisms which when administered in adequate amounts confer a health benefit on the host” [5]. There are a wide variety of microorganisms classified as probiotics that include both bacteria and yeasts. Members of the probiotic bacteria include organisms such as *Lactobacillus* spp., *Lacticaseibacillus* spp., *Bifidobacterium* spp., *Enterococcus* spp., *Streptococcus* spp., *Bacillus* spp., and types of yeast such as *Saccharomyces cerevisiae* var. *boulardii* [9,10,11]. Not only have probiotics been used for humans, but they have also been developed for use in companion [12,13,14,15] and free-ranging animals [16]. The development of new probiotic microbial strains includes concerns of safety and efficacy that can potentially be resolved by using whole genome sequencing (WGS) to determine potential virulence and antibiotic resistances along with delineating genus and species identity [17].

Members of the *Carnobacterium* spp. are found in various ecological niches, including foods, animal organs, feces, and different natural environments [4,6]. One of the first discoveries of *Carnobacterium* spp. as a potential probiotic was its use to reduce bacterial pathogenesis in rainbow trout [18,19,20]. Using a colorectal epithelial cell line, HT29, it was subsequently demonstrated that bacteriocin-producing Carnobacteria strains reduced *Listeria monocytogenes* invasion of eukaryotic cells [21]. Although there have been investigations to reduce bacterial disease in aquaculture using Carnobacteria [22,23], very little research has been conducted among monogastric animals other than chickens [24,25]. Our research hypothesis and objectives involve investigating free-ranging animals as a source of new potential probiotic bacteria [26,27,28]. Herein, we describe the isolation and characterization of a *Carnobacterium maltaromaticum* unique sequence type (ST) from a North American Gray wolf. This is the first report of a Carnobacteria isolated from any member of the genus *Canis,* and based on genomic analyses, the bacterium may have potential probiotic properties.

## 2. Materials and Methods

### 2.1. Isolation of Bacteria and Phenotypic Characterization

Bacterial isolation was made from the ileum of a deceased North American Gray Wolf (*Canis lupus*) that had been killed when hit by a car (Oregon Veterinary Diagnostic Laboratory, Corvallis, OR; OVDL case 20V15449) [https://vetmed.oregonstate.edu/ovdl] (accessed on 13 November 2024). Briefly, several bacteria were isolated from the ileum of the wolf’s digestive material as described in previous publications [26,28]. Initial bacterial isolations were made using brucella broth agar with hemin and vitamin K (BBHK) at 37 °C in an anaerobe chamber using a Thermo Scientific™ AnaeroPack™ 2.5L Rectangular Container with sachets [29]. Subsequently, a bacterial isolate designated ClWan1 was propagated anaerobically and aerobically on brain heart infusion (BHI) agar. Characterizing the isolate using basic bacteriologic assays such as Gram stains and starch hydrolysis, with catalase, lipase, and oxidase assays were completed via standard techniques [30,31].

### 2.2. Bacterial Genomic DNA Isolation and Whole Genome Sequencing

The bacterial genomic DNA was purified from ClWan1 3 mL cultures in BHI (Illustra Nucleic Acid Purification^TM^ system, Cytiva, Marlborough, MA, USA) as described [26,28]. Purified bacterial genomic DNA was quantified by a fluorescence-based Qubit dsDNA system (ThermoFisher, Waltham, MA, USA). Genome sequencing was completed at EzBiome using the NEBNext^®^ Ultra™ II FS DNA library kit for an Illumina library, while the v14 library prep chemistry without fragmentation or size selection for the Nanopore sequencing. Genome sequences were obtained by Illumina NextSeq2000 (2 × 150 bp) and an R10.4.1 flow cell of a Nanopore PromethION (Eugene, OR, USA).

Filtering of sequencing reads was completed using Filtlong v0.2.1 min_length 1000 keep_percent 95 [https://github.com/rrwick/Filtlong] (accessed on 13 November 2024) by removing the 5% worst FASTQ reads. Flye v2.9.2 was used to assemble sequences with Nanopore reads [32]. Draft Illumina reads were aligned [33] to produce polished assemblies [34] with CheckM to detect any contamination [35]. Genomes were annotated with Prokka v1.14.6 [36], and GenoVi v0.4.3 was used to generate circular genome maps [37]. BAGEL4 [38] was used to identify bacteriocin genes and other ribosomally synthesized and post-translationally modified peptides (RiPPs) encoded in the genome. The antiSMASH program [39] was used to search for genes potentially encoding bioactive compounds, such as antimicrobials synthesized by the isolate. The PHAge Search Tool with Enhanced Sequence Translation or PHASTEST [40] was employed to search the genome for putative prophage sequences.

Using a pre-built database [41] composed of NCBI’s National Database of Antibiotic-Resistant Organisms (NDARO, www.ncbi.nlm.nih.gov/pathogens/antimicrobial-resistance/) (accessed on 15 November 2024), reference genes were used to generate a putative antibiotic resistance profile. SAMtools mpileup script [42] was used to calculate the depth and coverage of genes, and virulence factor-encoding genes were identified using a pre-built bowtie2 [43] database composed of reference factors obtained from the Virulence Factors of Pathogenic Bacteria (VFDB) database [44]. Multilocus Sequence Typing (MLST) was conducted using mlst (https://github.com/tseemann/mlst, accessed on 19 December 2024), which relies on the PubMLST website (https://pubmlst.org/, accessed on 19 December 2024) that contains data for diversity among *C. maltaromaticum* [45].

### 2.3. Phylogenetic Analyses of the Wolf Bacterial Isolate

The first assay for determining the ClWan1 species identification was utilizing the 16S rRNA gene via SpeciesFinder 2.0 [46]. Subsequently, bacterial core genes were extracted from the Up-to-date Bacterial Core Genes (UBCG) system [47]. Core genes were concatenated and aligned with MAFFT v7.508 [48] using the G-INS-i strategy, and phylogenies were constructed with 1000 bootstrap replicates using RAxML-NG v. 1.1.0 [49]. OrthoANIu [50] was used to calculate average nucleotide identity (ANI) values, and a neighbor-joining tree was generated from the ANI values using the “ape” R library [51]. Reference and the ClWan1 genomes were analyzed for single nucleotide polymorphism (SNP) comparisons using parsnp [52], and a matrix was produced using a custom script [41]. The genome was also submitted to the Type (Strain) Genome Server (TYGS) in tandem with the List of Prokaryotic names coupled to the Standing in Nomenclature (LPSN) [53], followed by preparing phylogenetic trees in MEGA12 [54] to confirm phylogenetic relationships among other bacteria.

## 3. Results

### 3.1. Bacterial Isolation and Phenotypic Characterization

Bacterial isolates were plated on BBHK and then incubated anaerobically for 48 hr. One isolate designated ClWan1 was characterized as a rod-shaped, nonmotile, Gram-positive bacterium (Supplementary Figure S1) that was facultatively anaerobic. Subsequently, ClWan1 was propagated on brain heart infusion (BHI) agar and was cultured aerobically. The isolate was both catalase and oxidase negative and could be propagated on BHI agar media. However, ClWan1 did not metabolize lipids, nor did it hydrolyze starch.

### 3.2. Whole Genome Sequence Characteristics of Bacterium ClWan1

Sequencing and assembly of the ClWan1 genomic DNA resulted in a whole genome reference sequence with a coverage of 99.45%. The assembly resulted in a single contig that was 3,512,202 base pairs in length with the same N50. The whole genome sequence (WGS) had a GC content of 34.48% (Table 1). This is very similar in size and GC content to the *Carnobacterium maltaromaticum* SF2022 strain reference genome (e.g., RefSeq: GCF_949790605.1; NZ_OX460976, Ref [55] and reviews [6,7,8]).

ClWan1 was identified as a novel MLST sequence type with three novel alleles, using the schema corresponding to *Carnobacterium maltaromaticum*. The results are displayed in Table 2. The ST data is curated by Frédéric Borges [56] on the PubMLST website [45].

The MSLT of isolate ClWan1 was most similar to a *C. maltaromaticum* ST63 with three exact matches found at *glpQ*, *leuS*, and *pyrE* genes. The *dapE* gene had three sequence differences, including a G → A at position 2559336, an A → G at 2559337, and a T → C at 2559354. The *pyc* gene had a C → T at position 944654. Isolate ClWan1 had two unique full-length alleles of *ddlA* and *ilvE*. These genes encode a D-alanine-D-alanine ligase A and a branched-chain-amino-acid aminotransferase, respectively. Consequently, isolate ClWan1 is potentially a unique ST of the species.

**Figure 1:**
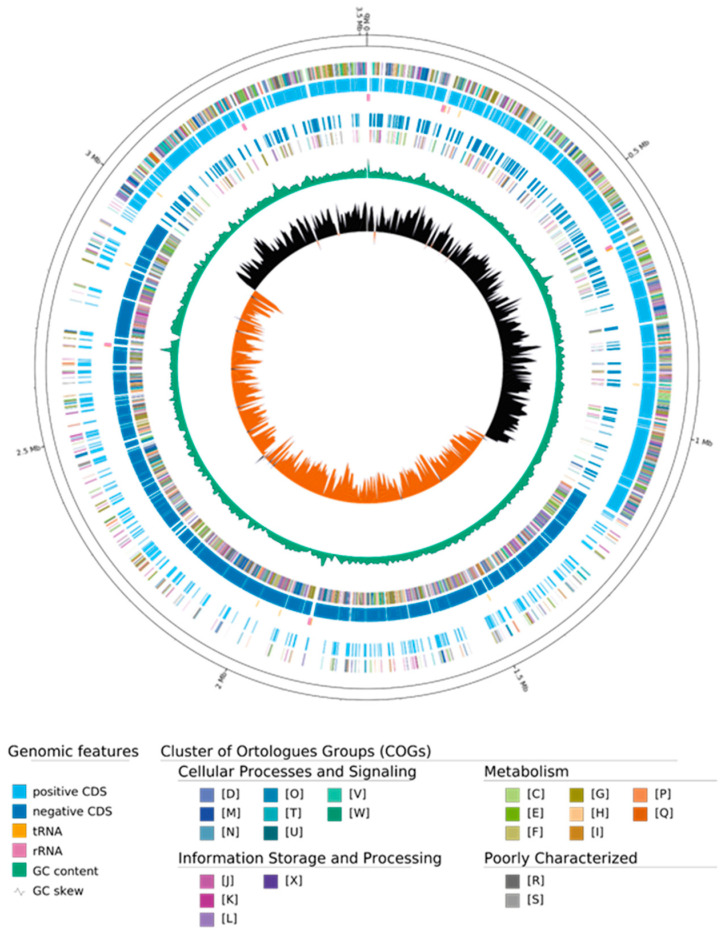
Genome Map and Genome Composition of Bacterial Isolate ClWan1. The map represents gene content as a circle plot from inner to outer circles: GC skew, GC content, negative sequence gene content, negative coding sequences, tRNA location, positive coding sequences, and positive sequence gene content with genomic coordinates.

The 3.5 Mb WGS of ClWan1 single contig mapped as a circular genome (Figure 1). The figure also illustrates the clusters of orthologous groups (COG) and gene features, such as coding sequences and GC skew. Using the PHAge Search Tool with Enhanced Sequence Translation (PHASTEST) program [40], no bacteriophage sequences were detected in the ClWan1 genome.

The core bacterial genes for ClWan1 are listed in Table 3 and as a GenBank-type file in Supplementary Table S1. The genes are delineated into COG categories, with the transcription category having the most genes. The defense mechanism had a high percentage of genes in its category, and many of these have been studied for their antimicrobial peptides and bacteriocins [57,58,59]. There are 310 carbohydrate transport and metabolism genes, including genes involved in starch digestion that are important to probiotics [3].

The secondary metabolites biosynthesis, transport, and metabolism gene category has 42 genes. The antiSMASH program, a secondary metabolite gene prediction tool [39], detected five genes associated with secondary metabolism (Supplementary Table S2). Among these were a phytoene synthase, type III polyketide synthase (T3PK), cyclic lactone autoinducer, thiopeptide genes, non-ribosomal peptide synthetase, and a ribosomally synthesized and post-translationally modified peptide-like gene (Ripp-like). This points to the clear capacity of the organism to produce bioactive metabolites that impact its environment.

The bacteriocin gene was also detected using the BAGEL software (http://bagel4.molgenrug.nl/) [38]; it encoded an antimicrobial peptide CarnobacteriocinB1 from position 2791273 to 2791458 that is 100% similar to the class II bacteriocin found among the genus *Carnobacterium* [WP_010051997]. The peptide contained the characteristic YGNGV motif of the family of class II bacteriocins [59,60]. A putative Carnobacteriocin-BM1 bacteriocin immunity protein gene encoded from positions 2791473 to 2791739 is 99% similar to other members of the genus [WP_010051996]. A lantibiotic dehydratase C-terminal domain-containing protein of *Carnobacterium maltaromaticum* was detected from positions 2715970 to 2716767 of the ClWan1 genome encoding a peptide that is 98% similar to a previously reported protein [WP_414024519].

Table 4 shows the genome location of several antimicrobial resistance genes. These include genes encoding an ABC-F type ribosomal protection protein, class A beta-lactamase, and a NAD(+)-rifampin ADP-ribosyltransferase.

### 3.3. Phylogenetic Analyses of Gray Wolf Isolate ClWan1

**Figure 2:**
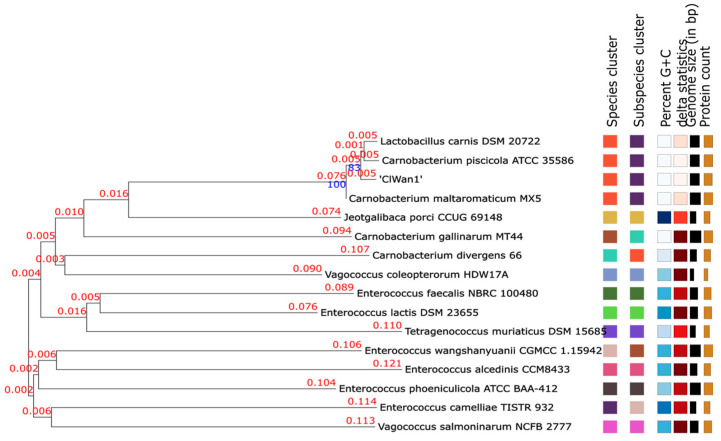
Phylogeny of Isolate ClWan1 Based on Whole Genome Sequences. Whole genome sequences were submitted to the type strain genome server (TYGS). The output was also saved as a Newick file and then analyzed using the molecular evolutionary genetics analysis (MEGA) program described in the methods (Supplementary Figure S3). The branch lengths in substitutions per site, and bootstrap confidences are above the lines of interest in blue.

Phylogeny utilizing the 16S rRNA gene was used to initially classify isolate ClWan1 as a unique *Carnobacterium maltaromaticum*. Also, phylogenetics of the 16S rRNA gene using Species Finder [46] resulted in ClWan1 being most closely related to *C. maltaromaticum* strain S_T_MRS_58 [JX860544]. Subsequently, the WGS was used to confirm this result (Figure 2 and Supplementary Figures S2 and S3). This analysis resulted in ClWan1 being closely related to *C. maltaromaticum* and *C. piscicola*. Interestingly, a bacterium originally classified as *Lactobacillus carnis* strain DSM20722 isolated from vacuum-packaged meat appears to be a *Carnobacterium* sp. by the TYGS-LPSN analysis [61]. This bacterium was reclassified as a *C. maltaromaticum* [62], while *C. maltaromaticum* MX5, originally isolated from milk as *Lactobacillus maltaromicus* [63], was also reclassified as *C. maltaromaticum* [62]. These isolates are closely related to ClWan1 isolated from a Gray wolf gastrointestinal tract. The *C. maltaromaticum* MX5 is considered a type strain (also ATCC^®^ 27865™) for the species [JQMX00000000] and is the nearest neighbor for ClWan1 (Figure 2).

**Figure 3:**
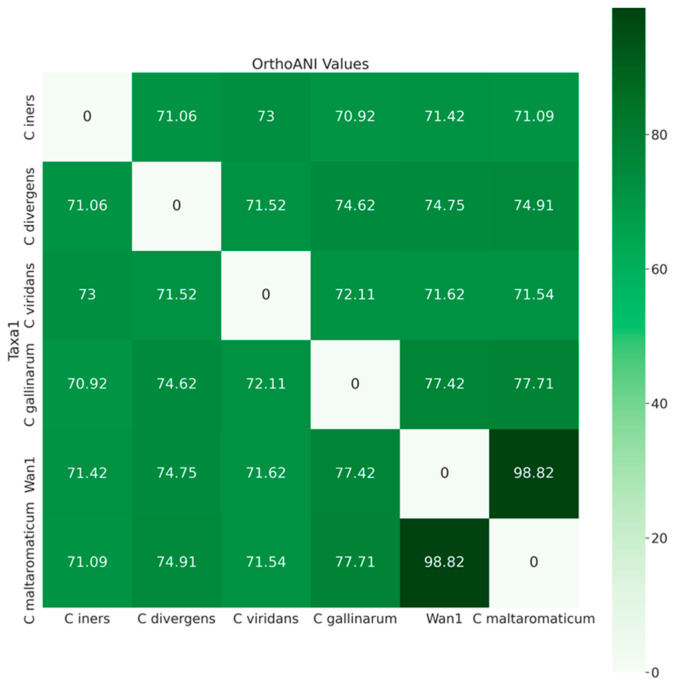
Average Nucleotide Identities Among *Carnobacterium* spp. Average nucleotide identity (ANI) values, query, and reference coverage values were calculated with OrthoANIu, and a neighbor-joining tree was constructed from the ANI values using the ‘ape’ R library functions described in the methods.

Further analysis utilizing average nucleotide identities (orthoANI) classified isolate ClWan1 as a *C. maltaromaticum* (Figure 3). The ANI of ClWan1 was 98.82% similar to *C. maltaromaticum* and only 77.71% similar to its nearest neighbor species, a *C. gallinarum* isolate.

**Figure 4:**
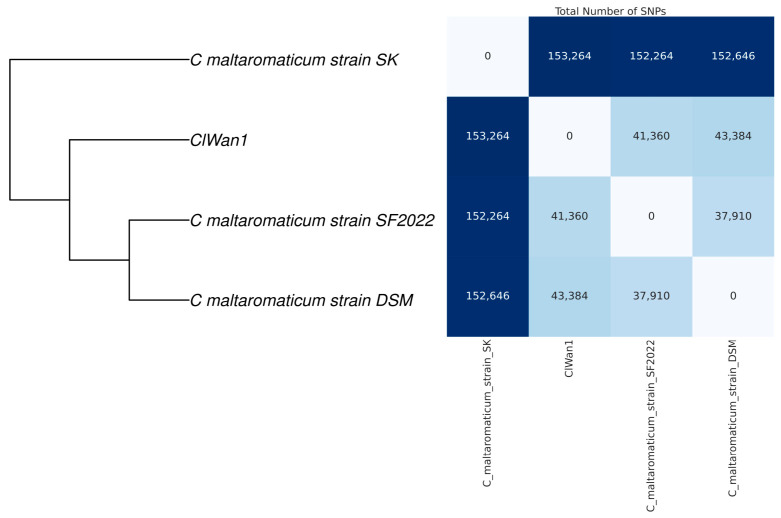
Single Nucleotide Polymorphism Matrix Among *Carnobacterium* maltaromaticum Isolates. Reference and the ClWan1 genomes were analyzed for single nucleotide polymorphisms (SNP) comparisons using parsnp with default parameters. The matrix was produced using a custom script described in the methods.

Using single nucleotide polymorphism (SNP) analysis of the core genome, it was determined that ClWan1 was a unique ST among *C. maltaromaticum* isolates (Figure 4). The core genome SNP analysis demonstrated that ClWan1 had 41,360 SNPs from its nearest neighbor *C. maltaromaticum* strain SF2022.

Digital DNA-DNA hybridization (dDDH) using the TYGS and LPSN database ([53]; Supplementary Table S3) resulted in the ClWan1 genome being 90.6% similar to *C. maltaromaticum* MX5 [Accession GCA_000744945.1] and 90.6% to the misnamed *L. carnis* DSM 20722, now *C. maltaromaticum* DSM 20722 [Accession GCA_001437035.1]. The G+C content difference was 0.05% with *C. maltaromaticum* MX5 and 0.17% with *L. carnis* DSM 20722. The results indicate that the ClWan1 from a North American Gray wolf isolate is a unique ST among the *C. maltaromaticum* isolates reported in GenBank. Also of interest is a bacterium reported as *Lactobacillus carnis* isolate that phylogenetically groups with the *Carnobacterium* spp.

## 4. Discussion

Carnobacteria are a group of Lactic acid bacteria that reportedly have anti-listerial and other probiotic properties [4,6,7,8]. *Carnobacterium* spp. whether they are or are not known bacteriocin producers, reduced eukaryotic cell invasion by *Listeria monocytogenes* [21]. *L. monocytogenes* is found in waterways and can colonize in the intestines of mammals, including ruminants [64,65,66], which wolves are known to consume alongside the rest of the carcass, except the rumen and possibly abomasa contents [67]. Thus, *C. maltaromaticum*, which has anti-listeria properties in vitro [21], is likely to confer increased resistance to listeriosis in the animal, in vivo. Furthermore, should this pattern hold, *C. maltaromaticum* might show potential benefit as a probiotic for other, similar, omnivorous monogastric animals that interface with humans and potentially human waste, such as dogs [68,69,70].

In studies with rats performed by Li, et al. [71], it was demonstrated that *C. maltaromaticum* could also stimulate Vitamin D production in mice intestines, which reduces the rate of colorectal cancer. This type of cancer is also common in domestic dogs and humans and can be fatal to the animal [72]. With further research, it is possible to determine if this mechanism is also functional in dogs and thus a potential form of preventive veterinary healthcare. It is possible to characterize, extract, isolate, purify, and concentrate the extracellular secretion of these, and related, strains for use as an anti-listeria treatment [7,8]. Given the tendency for *L. monocytogenes* to develop unique ribotypes [65], which are associated with listeria outbreaks among livestock, a strong anti-listeria treatment is valuable, especially as ampicillin and broader antibiotic resistances become more prevalent in the wild-type strains of *L. monocytogenes* [73,74,75].

Several other bioactive compounds identified in the ClWan1 genome include thiopeptides, a diverse class of secondary metabolites with broad bioactivity [76]. While the large number of hypothetical genes clustered among the thiopeptide biosynthetic genes makes predicting the exact structure difficult, the top two cluster similarity scores in the MIBiG analysis (an optional antiSMASH 4.0 output), are for cutimycin and microcin P1, which respectively have *Staphylococcus* spp. and *L. monocytogenes*, inhibiting activity [77,78]. Also encoded was a single non-ribosomal peptide synthetase gene with two synthetic modules predicted to generate a tyrosine-threonine dipeptide product. As with thiopeptides, cyclic dipeptides are widespread and possess diverse biological activities [79]. A BLAST (Version 2.13.0) search of this NRPS yields distantly related proteins with at most 41% amino acid identity to the *S. aureus* NRPS [HDP5831075.1]. Among the similar, albeit distantly related clusters in the MIBiG analysis are the genes encoding the biosynthesis of the aureusimines, tyrosine-valine cyclic dipeptides conserved among *S. aureus* strains, which are thought to play a role in virulence and host colonization [80,81]. A lantibiotic dehydratase was detected in the genome, and this enzyme participates in the biosynthesis of lantibiotics, a class of peptide antibiotics that contain one or more thioether bonds [82]. The biosynthetic gene clusters (BGCs) were previously inferred by WGS analyses in the Carnobacteria [7,8] and were detected in the ClWan1 genome. Potential antibiotic resistance genes were detected in the genome of the *C. maltaromaticum* isolate reported herein. Previous resistance to antibiotics has been reported in other isolates of the species but retained probiotic properties [83].

Carnobacteria are found in diverse environments, with *C. maltaromaticum* followed by *C. divergens* being the most common members of the genus. Genes found among these species are primarily adapted to the gastrointestinal environment of animals, specifically the resistance of *C. maltaromaticum* to bile [55]. Furthermore, as noted, these bacteria can be “mined” by genomics techniques to discover new natural products with practical applications [7,8]. The isolate from a Gray wolf described herein encodes several bioactive compounds, such as a bacteriocin that potentially has anti-listerial properties. The earliest domesticated animals were dogs (*Canis familiaris*) that are descendants of the Gray wolf (*Canis lupus*) that coevolved with humans (reviewed in [84]). Therefore, it could be argued that a dog diet might be enhanced by utilizing useful bacteria from free-ranging wolves [26,28]. Consequently, it is important to protect worldwide biodiversity for many reasons, including improving our understanding of microbial health and disease among free-ranging and domestic species [85].

## 5. Conclusions

Isolate ClWan1 was identified as a *Carnobacterium maltaromaticum* with a genomic length of 3.51 Mbp, consistent with other species of this genus with genome sizes ranging between 3.33 to 3.87 Mbp [8,55,56]. MSLT analysis, WGS phylogeny, SNP, and ANI comparisons confirmed that isolate ClWan1 is a unique ST of the species. This investigation represents the first reported isolation and axenic culture of a *C. maltaromaticum* from a free-ranging Gray wolf (*Canis lupus*). This expands the understanding of its ecological presence and potential beneficial applications for the gut health of animals. There is a knowledge gap in the wolf enteric microbiome that is not fully known and is compelling compared to domesticated dogs and other carnivores [86,87]. Additional investigations could be conducted to determine how widespread this organism is in wolves and dogs to understand if colonization is affected by age, sex, and diet.

## Figures and Tables

**Table 1:** Whole Genome Assembly Statistics for Bacterial Isolate ClWan1 and SF2022. *Carnobacterium maltaromaticum* strain SF2022 isolate SF2022 chromosome SF2022_c1 from locus GenBank NZ_OX460976.

ID#	# of Contigs	Longest Contig	Assembly Length	GC %	N50
Wan1	1	3,512,202	3,512,202	34.48	3,512,202
SF2022	2	3,410,292	3,410,292	34.50	3,400,000

**Table 2:** PubMLST Sequence Typing of Bacterial Isolate ClWan1. Allele numbers with a * indicate a partial match to the allele with at least 10% coverage of the reference allele length. Allele numbers with a ^ indicate a novel full-length allele.

Multilocus Sequence Type Genes								
Bacterial Species	*dapE*	*ddlA*	*glpQ*	*ilvE*	*leuS*	*pyc*	*pyrE*	ST
*Carnobacterium maltaromaticum*	20 *	2 ^	19	2 ^	22	10 *	16	63

NOTE: UniProt designations: *dapE*, Succinyl-diaminopimelate desuccinylase [K8ELJ0_CARML]; *ddlA*, D-alanine-D-alanine ligase A [K8E630_CARML]; *glpQ*, Glycerophosphodiester phosphodiesterase [K8EKY9_CARML]; *ilvE*, Branched-chain-amino-acid aminotransferase [K8EVZ0_CARML]; *leuS*, Leucine-tRNA ligase [A0AAW9K734_CARML]; *pyc*, Pyruvate carboxylase [K8E3L8_CARML]; *pyrE*, Orotate phosphoribosyltransferase [K8ERG2_CARML].

**Table 3:** Gene Composition of the Bacterial Isolate ClWan1 Genome. Clusters of orthologous groups (COG) and gene function are tabulated by the number of genes and percentage of each genome total in respective columns.

COG	Category Type	Number of Genes	% of Total
D	Cell cycle control, cell division, chromosome partitioning	89	2.6
M	Cell wall/membrane/envelope biogenesis	261	7.5
N	Cell motility	44	1.3
O	Post-translational modification, protein turnover, chaperones	145	4.2
T	Signal transduction mechanisms	192	5.5
U	Intracellular trafficking, secretion, and vesicular transport	49	1.4
V	Defense mechanisms	125	3.6
W	Extracellular structures	25	0.7
Y	Nuclear structure	0	0.0
Z	Cytoskeleton	3	0.1
A	RNA processing and modification	0	0.0
B	Chromatin structure and dynamics	0	0.0
J	Translation, ribosomal structure, and biogenesis	263	7.5
K	Transcription	353	10.1
L	Replication, recombination, and repair	146	4.2
X	Mobilome: prophages, transposons	64	1.8
C	Energy production and conversion	137	3.9
E	Amino acid transport and metabolism	232	6.6
F	Nucleotide transport and metabolism	105	3.0
G	Carbohydrate transport and metabolism	310	8.9
H	Coenzyme transport and metabolism	123	3.5
I	Lipid transport and metabolism	124	3.6
P	Inorganic ion transport and metabolism	149	4.3
Q	Secondary metabolites biosynthesis, transport, and catabolism	42	1.2
R	General function prediction only	290	8.3
S	Function unknown	218	6.2
Unclassified	Unclassified	0	0.0

**Table 4:** Antimicrobial Resistance Gene Profile for ClWan1.

Location	Sequence Name	Class
602492…604366	ABC-F type ribosomal protection protein	MACROLIDE
313798…314697	class A beta-lactamase	BETA-LACTAM
508539…508952	NAD(+)—rifampin ADP-ribosyltransferase	RIFAMYCIN

## Data Availability

The data are retrievable at the National Center for Biotechnology Information (NCBI) as BioProject PRJNA1125382 *Carnobacterium maltaromaticum* ClWan1 with BioSample accession SAMN38498379. The Sequence Read Archive (SRA) is SRS19889843 with Illumina reads as SRX22921895 and nanopore reads as SRX22921894. The genome submission is ID SUB14546156 with a temporary Accession CP158642.

## Supplementary Materials

The following supporting information can be downloaded at: https://www.mdpi.com/article/10.3390/vetsci12050410/s1, Figure S1: Culture of Bacterial Isolate ClWan1 and Gram-stain. Ileal contents from a North American Gray Wolf were cultured on Brucella broth with Hemin and Vitamin K agar (A). Subsequently, the isolate was Gram-stained following culture on brain-heart infusion agar (B). Figure S2: Initial Whole Genome Sequence Phylogenetics Analysis of the ClWan1 genome. The UBCG: Up-to-date bacterial core gene: 92 core bacteria genes was used to classify the isolate ClWan1 [https://www.ezbiocloud.net/tools/ubcg], accessed on 13 October 2024. Figure S3: Phylogeny of Isolate ClWan1 Based on Whole Genome Sequences. Whole genome sequences were submitted to the type strain genome server (TYGS). The output was saved as a Newick file and then analyzed using the molecular evolutionary genetics analysis (MEGA) program described in the methods [54]. The scale bar indicates branch lengths in substitutions per site, and bootstrap confidences are above each line. Tables S1 and S2: Results from the antiSMASH Bacterial Version for the ClWan1 Genome. Table S3: Type Strain Genome Server.
