# Supplementary material for: Reference Whole Genome Sequence Analyses and Characterization of a Novel Carnobacterium maltaromaticum Distinct Sequence Type Isolated from a North American Gray Wolf (Canis lupus) Gastrointestinal Tract
Source: Vet Sci. 2025 Apr 27;12(5):410. doi: 10.3390/vetsci12050410 (PMC12115997; doi:10.3390/vetsci12050410)
Supplement: Supplementary file 1 [file vetsci-12-00410-s001.zip › KlewsEtAl_Supplementary Figures S1-S3ver2.pdf]

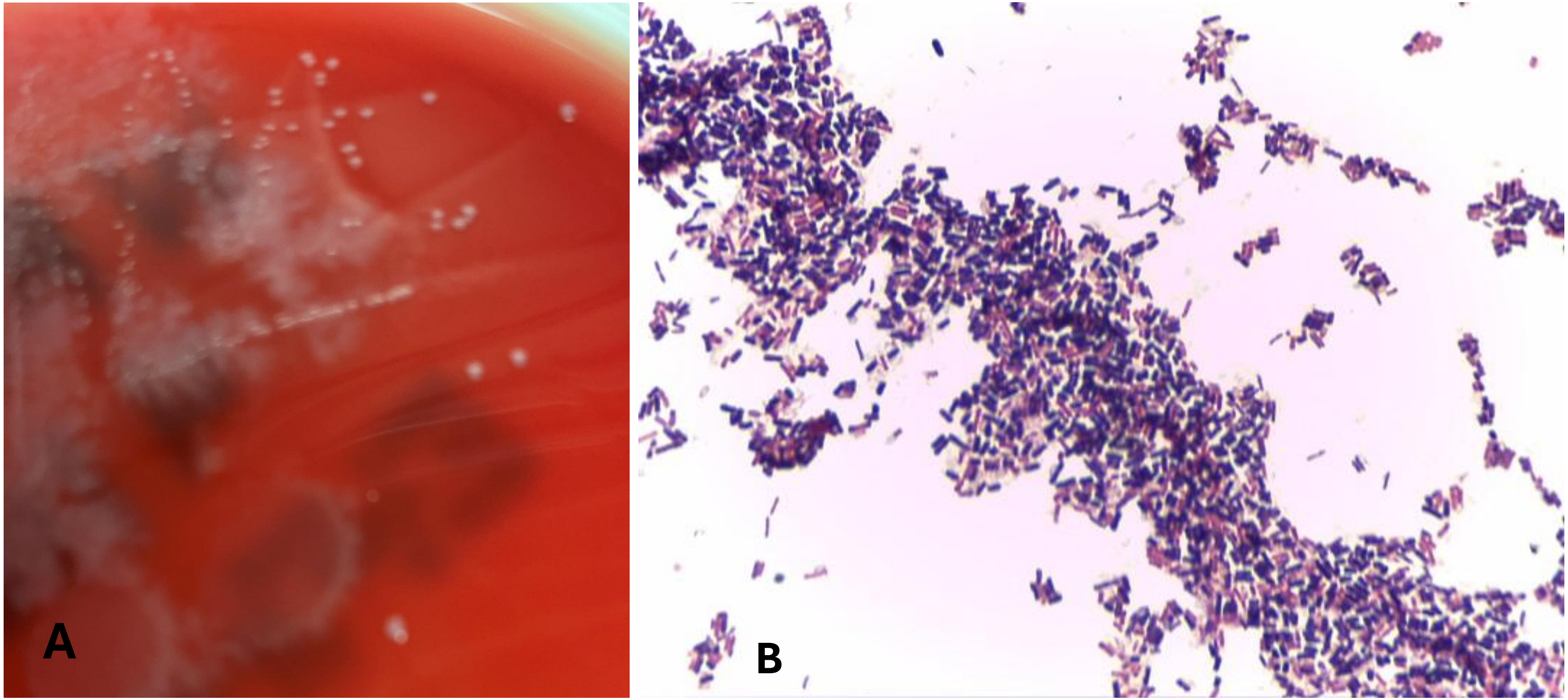

Supplementary Figure S1. Culture of Bacterial Isolate ClWan1 and Gram-stain. Ileal contents from a North American Gray Wolf were cultured on Brucella broth with Hemin and Vitamin K agar (A). Subsequently, the isolate was Gram-stained following culture on brain-heart infusion agar (B).

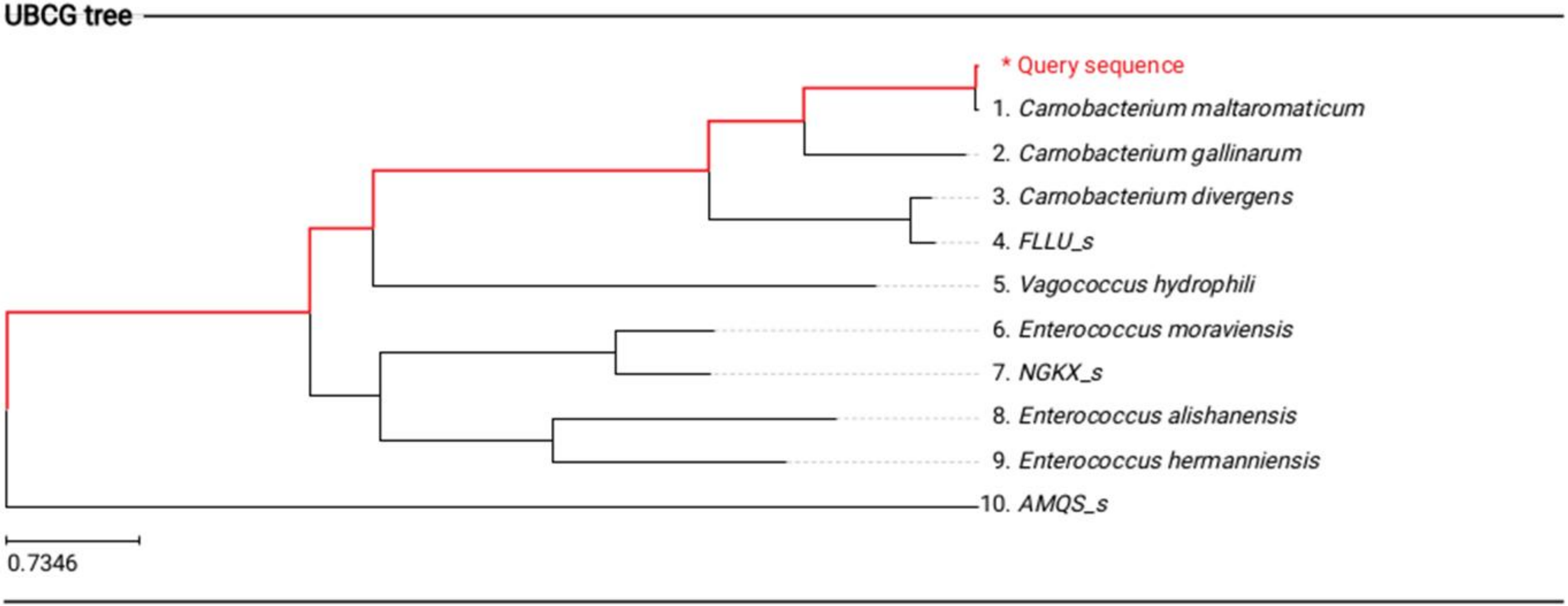

Supplemental Figure *S*2. Initial Whole Genome Sequence Phylogenetics Analysis of the ClWan1 genome. The UBCG: Up-to-date bacterial core gene: 92 core bacteria genes was used to classify the isolate ClWan1 [https://www.ezbiocloud.net/tools/ubcg]

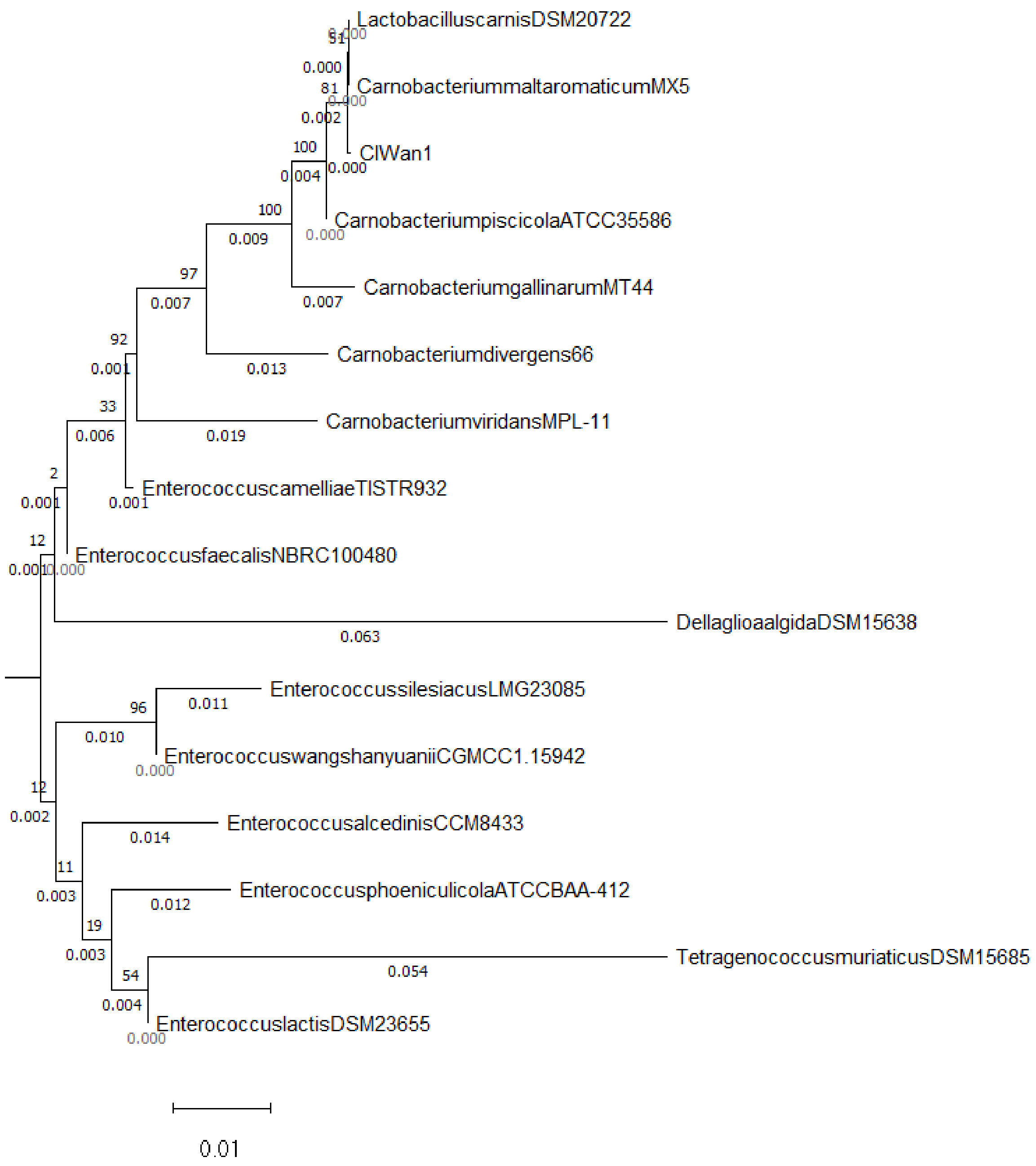

Supplementary Figure S3. Phylogeny of Isolate ClWan1 Based on Whole Genome Sequences. Whole genome sequences were submitted to the type strain genome server (TYGS). The output was saved as a Newick file and then analyzed using the molecular evolutionary genetics analysis (MEGA) program described in the methods [54]. The scale bar indicates branch lengths in substitutions per site, and bootstrap confidences are above each line.
