# Supplementary material for: Reference Whole Genome Sequence Analyses and Characterization of a Novel Carnobacterium maltaromaticum Distinct Sequence Type Isolated from a North American Gray Wolf (Canis lupus) Gastrointestinal Tract
Source: Vet Sci. 2025 Apr 27;12(5):410. doi: 10.3390/vetsci12050410 (PMC12115997; doi:10.3390/vetsci12050410)
Supplement: Supplementary file 1 [file vetsci-12-00410-s001.zip › KlewsEtAl_Supplementary Table S1_ClWan1_GenBank_File.pdf]

LOCUS: ClWan1 3512202 bp DNA linear

DEFINITION Genus species strain *Carnobacterium maltaromaticum*nClWan1.

ACCESSION to be assigned

KEYWORDS: Firmicutes; Bacillota; Carnobacteriaceae; Lactic acid bacteria; probiotic; bacteriocin

SOURCE: Gray Wolf (*Canis lupus*)

ORGANISM: *Carnobacterium maltaromaticum*

COMMENT Annotated using prokka 1.14.6 from https://github.com/tseemann/prokka

FEATURES Location/Qualifiers

source 1..3512202

/organism "*Carnobacterium maltaromaticum*"

/mol_type="genomic DNA"

/strain="strain"

rRNA 213..1764

/locus_tag="JFBMFIFI_00001"

/product="16S ribosomal RNA"

rRNA 2158..5075

/locus_tag="JFBMFIFI_00002"

/product="23S ribosomal RNA"

rRNA 5338..5447

/locus_tag="JFBMFIFI_00003"

/product="5S ribosomal RNA"

CDS 5746..5991

/locus_tag="JFBMFIFI_00004"

/inference="ab initio prediction:Prodigal:002006"

/codon_start=1

/transl_table=11

/product="hypothetical protein"

/translation="MEKNTIIEKVCYTDLVFERVNKKLSKTMTQNEIKFLVLAILNDN

RSQFEKIGKNYYISNIPKGIKLVINSSTYRLITVDKI"

CDS complement(6212..6763)

/locus_tag="JFBMFIFI_00005"

/inference="ab initio prediction:Prodigal:002006"

/codon_start=1

/transl_table=11

/product="hypothetical protein"

/translation="MHLYLVLTATSSLLSKTIKLYTRASYNHASIALMPSLNDLASFG

RKDLNNPFIGGFVREDISHDFFLSARCSIYSCEITLDEYKHIQEQINYFHQHKEDLRY

NLLGLITLAVNIDYQRNRAFFCSEFIATILEDSGLYEFSKKPHMVTPQDLQELAIFTH

IYTGTISNYLRQLQKQALILEYA"

CDS 7175..7468

/locus_tag="JFBMFIFI_00006"

/inference="ab initio prediction:Prodigal:002006"

/codon_start=1

/transl_table=11

/product="hypothetical protein"

/translation="MSQMQLKCFIEIMLNLIEENEWEDTCEILGIEDQDEFRIINEMR

QDQMNIFTETASAYMEVCMKKHDGIDYEDEHFKEMTLHEWLIHNRPEVPFLYI"

CDS complement(7509..8765)

/gene="eriC"

/locus_tag="JFBMFIFI_00007"

/inference="ab initio prediction:Prodigal:002006"

/inference="similar to AA sequence:UniProtKB:Q87WD2"

/codon_start=1

/transl_table=11

/product="Chloride/fluoride channel protein"

/db_xref="COG:COG0038"

/translation="MHSICHKIKQLEFTQILFYLIKWFIYAGFVGILTGALSAFFLKS

LEKVTALQVHYSWLLFLLPLAGAFISYLYKDFGKNAHKGNNLVIEQAIGGEEKIPLRL

IPLTLIGTLATHLFGGSAGREGTAVQMGGATADFVGRIIKINAKERRILIICGMSAGF

SSVFGTPIAGTLFAMEVLVLGYFREEALFPSLWSALVANWVTLALGATHHIYQMGLIP

TSTPLLILKLILAAILFGLTGRLFSRLTAYLKTKFSEWFPNPIFKSFFGGIIIIILVI

VVGSRAYLGLSLPMLEQSFNGDLPPLSFLIKLIFTSVTLGAGFQGGEVTPLFVIGATL

GSTLAPFLALPVSFLAGLGFIGVFSGATNTPIACFVMGLELFGTAGLPYLFLICVISY

LFSGNLGIYHSQLIQIKKGTLFEEDN"

CDS complement(8891..9727)

/gene="rhaS_1"

/locus_tag="JFBMFIFI_00008"

/inference="ab initio prediction:Prodigal:002006"

/inference="protein motif:HAMAP:MF_01534"

/codon_start=1

/transl_table=11

/product="HTH-type transcriptional activator RhaS"

/translation="MLQLNTKTFNPEILYIFDCYNNGPSEGQHHHHDFLELSIIIDGA

VDYVINDETVFLEKETILLFNPGVYHHERYQQGMESTQIHIGFRNFALQGFSRDHFPF

NTSILHLTKFKTEFFDVCQEILMEKAKGEAGYDLMLKALVMKLIIYILRDADSSQLEI

NALKLSYEEQEKQTIVNDIIHYLENHHTEEVSLSSLSQTLYISPTYISRVFKEETGES

PINYLIKLRLNRAKELMENEKDITVKEAANLVGYNDAYYFSKLFKKYYGLPPSAFLKR

TS"

CDS 10125..11879

/locus_tag="JFBMFIFI_00009"

/inference="ab initio prediction:Prodigal:002006"

/inference="similar to AA sequence:UniProtKB:Q9WYC4"

/codon_start=1

/transl_table=11

/product="putative ABC transporter ATP-binding protein"

/db_xref="COG:COG1132"

/translation="MGSIKWVWQYAKVYRWKTILATTLVLITSILSIVYPLLGGRIVD

EVIDQGKSELLLPLLGAMIGVTFIRTVLRYSYQILFERIGQNSLFQIREDLYMKLQEL

DFNFFNQTRVGDIMARMTGDTDAIRHFVSWVFYNILENLLLFICAVVVMARIDWRLML

ALVAITPLIGFLTMRMSQKAQPIFYEIRESFSRLNSMVEENISGNRVVKAFTREEFEI

EKFNHHNDDFKQRNIDSAEVSKQYLPILDSLASSLTIITLVLGGYFVIIGQMTLGDLV

AFNGFLWMLNMPMRMSGWLINDVQRFIASSFKIREMLGSKSRIPIVSEKPATALKGYV

EFDDVSFHFDDDPETEVLSHISLKAEPGQTIGILGETGSGKSTLVNLIARFYDPTSGT

VLIDGVDAKKWHVRELRDHIAIVMQDIFLFSDTIEENISFGVPGADSQEIERMARIAD

ANHFIEKMPDGYETVVGERGVGLSGGQKQRISLARALMKNPAILILDDTTSAVDMETE

TKIQGELDQITVDKTTFIIAHRVSSVKDADEILIMDHGKIVERGTHESLLAYKGYYYE

VYNKQLGNFDVTEVEPNG"

CDS 11872..13653

/locus_tag="JFBMFIFI_00010"

/inference="ab initio prediction:Prodigal:002006"

/inference="similar to AA sequence:UniProtKB:Q9WYC4"

/codon_start=1

/transl_table=11

/product="putative ABC transporter ATP-binding protein"

/db_xref="COG:COG1132"

/translation="MARNKFDVDEELEQEFNKEHYQRLFQYVKPYKKPMLTTLFVILI

ANIATMLGPYFTKLVIDDVIPNKNMTLLGWLAVAFIVSVFVTGWCMRYRIQSITLIGQ

DMLKDMRSDIFEHLQKLPFSYFDSRPHGKILIRVVNYINTLSDLLSNGLINLISDVIS

VVITLIFMFAIDFKLTLYSLALLPVLFIFVMIIKNKQRKAYQVLSNKQSNLNAYIHES

IAGIKVTQSFSREDENYKIFSEVSNEYRSSWMHAVKVQFLLWPGVQNIAVITTCLIYF

VGIRGLGVEVSTGTLIAFIGYVNNFWNPVINIGNFYNSLITATAYLERIFETLDVEPD

IKDAPDAFELPPIKGDVRFRHVTFRYEEGKDILKDISFHVLPGESIALVGPTGAGKTT

VINLLSRFYDINEGEILIDGVDIRRVTLHSLRKQMGVMLQDTFIFSGTILDNIRYGKL

DATEEEVIAAAKVVRAHDFIVDLKEGYHTAVKERGSTLSAGQRQLISFARALLADPKI

LILDEATSSIDTKTETLLQDGLDKLLEGRTSFIIAHRLSTIKNSSRIFYIDQGTVQEA

GSHDELMAEKGYYYQLYQSQFDLLKEI"

CDS 13778..14155

/locus_tag="JFBMFIFI_00011"

/inference="ab initio prediction:Prodigal:002006"

/codon_start=1

/transl_table=11

/product="hypothetical protein"

/translation="MELIEDENGLELVHRNGNWVLKKEVGFSPVQTMVASVAACSTYV

YQSVLENSNIAYQLHGVEFDYETASEGTVHPVTKINIQFKVTIDEGARERATRGLKLI

AKHCPVIQSLADSVVVEEILVFS"

CDS 14376..14576

/gene="cspLA_1"

/locus_tag="JFBMFIFI_00012"

/inference="ab initio prediction:Prodigal:002006"

/inference="similar to AA sequence:UniProtKB:P0A355"

/codon_start=1

/transl_table=11

/product="Cold shock-like protein CspLA"

/db_xref="COG:COG1278"

/translation="MTKGNVKWFNAEKGFGFIESEDGGDVFVHFSAIQGDGFKSLDEG

QAVEFDTEEGNRGPQAVNVTKA"

CDS complement(14690..15142)

/locus_tag="JFBMFIFI_00013"

/inference="ab initio prediction:Prodigal:002006"

/codon_start=1

/transl_table=11

/product="hypothetical protein"

/translation="MEQEDINFDKNLALFYFAYRAFSKTSDELVGKYGITKVHRRILF

FVHRFPGLTIKELLIVLDISKQALNRPMQELTELGWIEKKSHETDKRSKKIYLTESGL

AIDQQISQAQNDKMKLLFKESGDPLGTNWTKVMTLYAKETGIPLIDQI"

CDS 15329..16975

/gene="ipdC"

/locus_tag="JFBMFIFI_00014"

/EC_number="4.1.1.74"

/inference="ab initio prediction:Prodigal:002006"

/inference="similar to AA sequence:UniProtKB:P23234"

/codon_start=1

/transl_table=11

/product="Indole-3-pyruvate decarboxylase"

/db_xref="COG:COG3961"

/translation="MYTVGNYLLDRLTELGIRDIFGVPGDYNLKFLDHVMTHKELNWI

GNANELNAAYAADGYARTKGIAALVTTFGVGELSAANGTAGSYAEKVPVVQIVGTPTT

AVQNSHKLVHHTLGDGRFDHFEKMQTEINGAIAHLTADNALAEIDRVLRIAVTERCPV

YINLAIDVAEVVAEKPLKPLMEESKKVEEETTLVLNKIEKALQDSKNPVVLIGNEIAS

FHLESALADFVKKFNLPVTVLPFGKGGFDEEDAHFIGVYTGAPTAESIKERVEKADLI

LIIGAKLTDSATAGFSYDFEDRQVISVGSDEVSFYGEIMKPVAFAQFVNGLNSLNYLG

YTGEIKQVERVADIEAKASNLTQNNFWKFVEKYLSNGDTLVAEQGTSFFGASLVPLKS

KMKFIGQPLWGSIGYTFPAMLGSQIANPASRHLLFIGDGSLQLTIQELGMTFREKLTP

IVFVINNDGYTVEREIHGPNELYNDIPMWDYQNLPYVFGGNKGNVATYKVTTEEELVA

AMSQARQDTTRLQWIEVVMGKQDSPDLLVQLGKVFAKQNS"

CDS complement(17014..18075)

/locus_tag="JFBMFIFI_00015"

/inference="ab initio prediction:Prodigal:002006"

/codon_start=1

/transl_table=11

/product="hypothetical protein"

/translation="MLQMVKEVLANLSILISSAYLLSKISKTLLTHQSPLIHKLVMGL

GSGITGIILMNFSISYQDSFLIDIRLVPLVMASFSYGGITPLIAGVIIGISRLFYGLS

PPIIGICLTYCLIGLSQYFISSWVQDKKVYLRLTFIFTSSIVLVILNFLIHSPYQNNT

PITLVFIIYNLLGMLISYQFSKDIETEKEAFLHYESVSKYDYLTSVFNRYSFDKDLTA

LFKQEKLVTIFLLDINNFKHFNDTYGHDIGDLILKKVSSSLLNASFPQHKVYRIGGDE

FVLVIPSALSNKAIIETKAKIKQQISQLTIQLDNQFVLRISTSIGSSSSKPSQTIDEL

YRQADIELYIDKKSKSTSS"

CDS 18264..19013

/locus_tag="JFBMFIFI_00016"

/inference="ab initio prediction:Prodigal:002006"

/codon_start=1

/transl_table=11

/product="hypothetical protein"

/translation="MSGLIYEQKHRIKYYECDATKKISVPMLLNIMLYVSGKQGKLLN

VGDDVVAQHGLSWIILQTEINVERLPKANEEITVVTQPQSYNKFFTYREFKVYDEAGE

LCVTTHCTFAMMDFNKRKMVRIVDELVAPYEVEATKKLVRTPKPEIVDQEAAKTIDYR

VRYLDIDANQHVNNAKYLDWFLDSLGLEFIKNHELKSINVKYEKEVSYGNWVQCEVSQ

KVQEDGRILTAHQINNDGIPSCEASMTWLPL"

CDS 19056..20033

/gene="ccpA_1"

/locus_tag="JFBMFIFI_00017"

/inference="ab initio prediction:Prodigal:002006"

/inference="similar to AA sequence:UniProtKB:P25144"

/codon_start=1

/transl_table=11

/product="Catabolite control protein A"

/db_xref="COG:COG1609"

/translation="MVTINDIARLAGVAKSTVSRYLNGGSVSAKTKAKIDKIVAETGY
KPNTFAQSLKAKNTAMIGTIVPRLDSYATTQGLKGIDEQLRAVDYQLLITNSNQDIQR
EIEAIYAYAKQKVAGIILFATVITDEHRKAIDSSKVPVIILGQQADGYHSVVHDDYQA
GYMLGKYAVSLGHRIFTYFGVFEDDIAVGQKRKQGILDGIKPNGKIKIIETTFAIEAA
YQKALQILPKMESSYVICATDNIALGVLKAAHELKMVIPDQFSLSGFGDYDVTTAVFP
MITTIKFPYYQSGVIAAENLYQLIEGEKIPEIIKLDNQLVERESTKRLK"

CDS 20242..21678

/gene="treP"

/locus_tag="JFBMFIFI_00018"

/inference="ab initio prediction:Prodigal:002006"

/inference="similar to AA sequence:UniProtKB:P39794"

/codon_start=1

/transl_table=11

/product="PTS system trehalose-specific EIIBC component"

/db_xref="COG:COG1263"

/translation="MNYKEIAQDLLVNLGGKENIVAATHCATRLRLVLQDETKINQIE
IENNASVKGTFSTGGQYQIILGSGVVDEVYKEFVQLANIESMSTQDVKNLGTKKLNPI
QQFVKVLSDIFIPIIPAIVACGLMMGINNVLTAEDLFISGNSLIGAYPQMAGLASMIN
TFSNAAYVFLPVLIGFSAAQKFGGNAYLGAVLGMLMVHPDLLTAFSYPQAVAENSVPY
WNVFGLHIAQVGYQNTVLPILVATYILAKIEKLCHKVIPTVLDNLLTPLFTLFITAFL
TFTVVGPITRTAGDMLTNGLAWLYDSTGFIGGGIIGLFYAPLVITGMHHSFIAVETQL
LADIAKTGGTFIFPIASMSNVAQGAACLAMIFITKNSKTKSLATASSISAYLGITEPA
MFGVNLKLRYPFYAAMIGSAVGSAYVTFFKVKAVALGATGLPGIIAITPGYMTYFIIG
IIMTTILTFVLTYIFSIRGAKLTINQSE"

CDS 21697..23202

/gene="scrB"

/locus_tag="JFBMFIFI_00019"

/EC_number="3.2.1.26"

/inference="ab initio prediction:Prodigal:002006"

/inference="similar to AA sequence:UniProtKB:P13394"

/codon_start=1

/transl_table=11

/product="Sucrose-6-phosphate hydrolase"

/db_xref="COG:COG1621"

/translation="MKEWTRKERYRKIEEVSPEYLVNLANQVEKSVYRQTYHIQPPTG

LLNDPNGFSYFNGQWHLFYQWFPLGPVHGLKYWYHVVSDDLVHWENCGVGISPSTKYD

SHGAYSGSGLVDKNQLHLMYTGNTRSKNWSRTPHQLMAIMDKNGQIKKIEEPVIEGIP

EGYTDNFRDPKMWKHEGYYYAIIGAERKNHTGTALVYQSENLTDWVLLGEFNPQVQDF

GFMWECPDFFELDGSPCFLFSPQGIEPIGDSYQNIYQTGYFLGTKFNYETLRLEHNGF

HELDLGFDFYAAQTTIAPDGRRILIAWMGLPEIKYPSDQDGWAHCLTIPRELSVLEGQ

LIQRPITELTTLRGAYVKRQDSQYGRVHLGNLLGTSYEVKIKLRVEEKTKFGLCLFED

SVTNKGLYLEVDLNKGNFKVERKDCGIPFATEYGTSRQMKYNKKEISLHIFVDTTSAE

IFANDGLAVFTTRVFPENQQSGIGLFVEKGLMEYELEGWKLNKMDIKSTEK"

CDS complement(23227..24195)

/gene="ansZ"

/locus_tag="JFBMFIFI_00020"

/EC_number="3.5.1.1"

/inference="ab initio prediction:Prodigal:002006"

/inference="similar to AA sequence:UniProtKB:O34482"

/codon_start=1

/transl_table=11

/product="L-asparaginase 2"

/db_xref="COG:COG0252"

/translation="MKNILVLHTGGTIAMSEDQTTGKVAPNSENPLLTQGHLFEKEAH

LIVEDIFQHPSPHMTPTEMLQLKNRIEQAVLSEAIDGVIITHGTDTLEETAYFLDITL

EQHIPIVMTGAMRSSNEIGSDGLYNFQSAVWVATSDEAKDKGVLVVMNDEIHTARYVT

KTHTTNVATFRTPTFGPIGLIAKNKVLFFQELIEEEKFDLTSVDKNVYLLKAYAGMDS

TIFDALDNPTTDGLVVEALGAGNLPPATLSGLSKLVARNIPIVLVSRCFNGVAQDVYD

YEGGGKRLREMGVIFTNGLTGPKARIKLLVALNSTNEHDELERYFY"

CDS complement(24432..25244)

/gene="metAA"

/locus_tag="JFBMFIFI_00021"

/EC_number="2.3.1.31"

/inference="ab initio prediction:Prodigal:002006"

/inference="similar to AA sequence:UniProtKB:A5IIQ2"

/codon_start=1

/transl_table=11

/product="Homoserine O-acetyltransferase"

/db_xref="COG:COG1897"

/translation="MSSKPILSIAILNLMPTKEATATQLASLLSQNNLFKVEITLLYP

KTHYQAKAIPDDLQENYLTFDDVRDQLFDGFIITGAPVEQLPFEHVDYWLELKTIFTH

LKATHTHSLFICWGAQAALYEYYQIPKIHLPTKLVGIFQHRFCVAEHWLANTTKTDFH

APHSRNTTVDSCLVSQHPELIPVAQSDAAGLYLATNYNGLETYVFGHPEYATETLEKE

YLRDVQTLTKPILPLYYFPGNNPLNTPINTWNEHGKNLFKNWMEFIRTKKLN"

CDS complement(25386..26675)

/locus_tag="JFBMFIFI_00022"

/EC_number="2.5.1.-"

/inference="ab initio prediction:Prodigal:002006"

/inference="similar to AA sequence:UniProtKB:Q9WZY4"

/codon_start=1

/transl_table=11

/product="O-acetyl-L-homoserine sulfhydrylase"

/db_xref="COG:COG2873"

/translation="MTKARQAYGKGTLAVQGTYQPKNGEPRVLPLYQSTTFAYEQAED

VAALFDLSAEGHLYSRISNPTVAVFEEKIALLEDGVAAVATSSGMAAIFNSILNIAKA

GDNILSSKTIYGGTTNLFAVTLPKFGITTRFFDQDASPEEIIALADQNTKLVFGETIA

NPGLNVFDFEKFHSICKILDLPLIIDSSLTGPALCNPLKHGANIVVHSTTKYIDGHAT

ALGGIVIDGGNFNWDNGKFDELVLPDDSYHGVRYVADFGQAAYATKLRVQLVRDLGNI

QSPFNAYLSNLGSETLVLRSKKHSENALAVAKWLQEQKDVSFVRYPGLETDSHYPLAQ

KYLPNGASGMVTFGIKGGASAAKQFINHLNFIQLVIHCADIRTSILHPESSTHRQLNS

EQLLESGVTEDLIRLSVGIEDTHDIIQDLAQALQTVH"

CDS 27250..28701

/gene="dbpA"

/locus_tag="JFBMFIFI_00023"

/EC_number="3.6.4.13"

/inference="ab initio prediction:Prodigal:002006"

/inference="similar to AA sequence:UniProtKB:Q814I2"

/codon_start=1

/transl_table=11

/product="ATP-dependent RNA helicase DbpA"

/db_xref="COG:COG0513"

/translation="MGKKEFADYGIGEDVLKALNGLGYFQPTKVQEEVIPVALADMDV

IVTSQTGSGKTASFGIPLCEKILWEENRPQALVLVPTRELALQVNEDIANIGRFRRIK

ATSVFGKASFERQKSELKQKSHIVVGTPGRVLDHLKKGTFPVDKLEYLILDEADEMLN

MGFIDQVEEIIGFLPKERQTLLFSATMPVEVERLASHYMKDEAVSIKIETTEDSKPKI

LKSFLMVSGDKKMPTLLDLLTVENPDSCIVFCNRQETVNNVYEYLNKAGLPVDKLHGG

MIQEDRFDVMDDFRKGKFRYLVATDVAARGIDVDNITHVVNYDVPVEKESFVHRIGRT

GRAGKTGVALTLVEPYEKERWAEIKRYSASDVSEITAPSARFVLRHKPAFEKKIAERP

RLKKDKGTVLSQDITKLYFNGGKKKKIRAIDFVGTISKIPGIQADDIGIITIGDQGSY

VEILNGKGPLVLNAMKTKNVKGKTLKVNIARRK"

CDS complement(29068..29592)

/locus_tag="JFBMFIFI_00024"

/inference="ab initio prediction:Prodigal:002006"

/inference="similar to AA sequence:ISfinder:ISEfa10"

/codon_start=1

/transl_table=11

/product="IS3 family transposase ISEfa10"

/translation="MTKYSVEFKMNIVQDYLNGEGGTPYLAKKYGFKGDSQVRNWINA

YKEFGKEGLLHSRRNKTYSVQFKLDAIELYLTTEMSYREVANQFGVSTFSTIANWYIA

YQKNGVDGLSQPKGRPSKMPKKKNKLPVSDSSKTELTYLEELEKENRRLKIELAYLKE

LRRLRLKEQQNNKR"

CDS complement(29699..32359)

/locus_tag="JFBMFIFI_00025"

/inference="ab initio prediction:Prodigal:002006"

/codon_start=1

/transl_table=11

/product="hypothetical protein"

/translation="MKKRKLVPYLLLSSLVLSSISGSVIVFAESAFVENGSELAGEQE

VPTKTDEENRAVTPKTGKSGTCSWELDSDGVLTIGAGTLGDRGTTPDNEATKIIFDGP

IVLLAGNSRFMGSTAESYENIENLDTSKVESMRAMFKDNKNLKNIDLSHMDTSKLTDL

YGMFQYCSSLETINLDGFNTSKVINFAAMFSGCSSLTALDLTSFDMRSCGPYGWGSFI

GQTSNMKTLTLGEYTGIQGTSLPDIDTTSNLYTGAWIKTDGSKIFKDSKTFMNEYNQQ

TDAGTYVWETTEVSPVTVNYVDEAGAELATSDTLIGELGEDYTTSAKDITGYMLKETP
ANATGTFTTFNQTVTYVYERPKWGTCPWVLEDGVLTIESGNLVKGTNSSTATVAGIKK
ADVSKVVFKGDIVADEDSSSLFSNWSNLETIEGIENLDTSKVTNMNSMFYKCSSLTSF
DTSHFDTSKVTNMQDIFSNCSSLTELDSSGLNTSQAIQMNGMFEECTNLKNLDVSQFT
TTSVTTMKGMFNKCTSLTSLNLSGFDTSNVTNMTNMLGQTTNLTTLSLGSKFTFGENT
NLPEIEITSVYTGKWASEESEKTFSSSTDLMSTYSGATDAGTYTWEVVKAAPVTVNYV
DTVGNTLAESEVLTGELGASFESEAKTIKGYALKETPENATGIFTEEAQTVTFVYKKN
AKPFAVNYVGVQQQQVVGFVPEGVKTSQVSLLIKKDGTDEWVDTGYTATPAAETGYFA
IESRFVKDTRDFLLNNKDQIKVAFTDENGENYEGVQVVQPYTAPIVDDFTEGDEYLTG
SVPNGANQVRISVNGKTMRVVNNGPIIEEDAGINAVTGAFSIWAKTWFEGLYGSSPIQ
QTKAGDVITVDYGVQIYGDFNNPSTTIIVK"

CDS complement(32945..33115)

/locus_tag="JFBMFIFI_00026"

/inference="ab initio prediction:Prodigal:002006"

/codon_start=1

/transl_table=11

/product="hypothetical protein"

/translation="MLNKDEIHRILGHKWKELKLSITIEYFSDYETFTKITNGNFRLI
QRLFSQIEYALQ"

CDS complement(34574..34864)

/locus_tag="JFBMFIFI_00027"

/inference="ab initio prediction:Prodigal:002006"

/inference="similar to AA sequence:ISfinder:ISEfa8"

/codon_start=1

/transl_table=11

/product="IS3 family transposase ISEfa8"

/translation="MSKRKRRTYSEAFKKQMVDLYLAGKSRQELIREYELTPSAFDKW
LLQQRTSGSFKEKDNLTPEQVELKELRKQNKQLKMENDILKQAALIFGRKSK"

CDS 34978..35187

/locus_tag="JFBMFIFI_00028"

/inference="ab initio prediction:Prodigal:002006"

/codon_start=1

/transl_table=11

/product="hypothetical protein"

/translation="MKRIEDCWVDTVSYYIQFETTKNKKVSKMIILDVTYTVKEVEEI

ILLNFDQVKKVCFIEEVEDCLSLKK"

CDS 35204..36055

/locus_tag="JFBMFIFI_00029"

/inference="ab initio prediction:Prodigal:002006"

/codon_start=1

/transl_table=11

/product="hypothetical protein"

/translation="MVAKKRGNKKKPASTKREQKKALKQGNHLMTEYNQWLAKGYSLE

GEMRETVKNPILPEKYKDYWNEIASQTSKHVREGQRLKGKLRRSRFLDPSLLEDPMRQ

PLVAKKKNWIESFVFYGIIVVVIVLISTLFSGPGDSGVPRNIMGYAPLTVLTKSMHSV

YPKDSFLLIQVEDSKKLTIGDDITFLKENNTTVTHRIIGIQENFENQGQRGFETKGVD

NPLADEDIVIADNVIGKVIYSNYTIGRLLLIVRNNLLMVAVLMIVSIFFLDALITLIK

SYRKTKY"

CDS 36092..37129

/locus_tag="JFBMFIFI_00030"

/inference="ab initio prediction:Prodigal:002006"

/codon_start=1

/transl_table=11

/product="hypothetical protein"

/translation="MEKRTKVALATALMVGIMGVSGTFAWTSISQRAINEVSMTKQPG

GRVHDDYEGIRGLDTTTEGDINKNIYAENYSNKPLYVRIKLTEFFESGLGAGQYMVDS

TDHIMKPTSDNQATVLPNSGLDNAALSDRTSWPAYLPNGLLSNGSPSELRQVIQWSLG

DTNDDRKVFMPTFNQNNMDISADVTGEAIDELTWKKNLYYEDLSKVLNGTFEQWSLGE

TYTSTLIKNNGDTAAPVETPGVTHTAKETVVPTNGGYMTMTDWLSQGKPTGYFWVHDS

DGWFYWATELEPYSATSLVLDRFHVVVPQDDFYYAINVISDFSTEEDVVQWSGKTLNA

DDLLTAIQKAK"

CDS 37349..39091

/locus_tag="JFBMFIFI_00031"

/inference="ab initio prediction:Prodigal:002006"

/codon_start=1

/transl_table=11

/product="hypothetical protein"

/translation="MTKVTKRNRKKTAITVAGLGALLLTTGTFAWTSISQQALNEKSG

KIEAGGRVHDDYNKESGNKDIYAENYQTQNVIVRIKLSEYMEDNLTPMVAGTVKTDKS

TWTPFTVPGSKNSEAYRNYVTWDLGGEKVFLPTFNKVETDKSVDASGDAIDVLTNGQT

APGTGEHIDFTAGTEYNATGAVVTTPGTGHVAKSTLTQSKAPLSMADWLALPAADQKG

DFWVIDEDGWAYWANILAPGESTSLLLSQLNWDQTALGDLDDWYYGIDVLGEFATKSD

VGKFATDSAYGAPSTNANQILDMLTVEPVSLTITASGVNADSNTIDAGTSRQFTATAA

TRATTEWSVEPASAGTIDNTGKLTINRNAVKGTKVTINAKQGRNTGSKELTVTSMNGA

VVGDTIQFNGQDYIYLKDLQDGNRLIMSKATVGTAATSLTYNGSALDTLMKSHYASLS

EAAKTEVQPVQASFDVGAPKGTTAIGRDADGFLVTSPTPDRAVVTEGGEKKAFALSVD

ELNDVSGPGKVFETVASREALNASGAVTYWWLRSPVTSTHAWYVSTGSNAGSLYASTM

TTASGARPAYIINR"

CDS 39229..39603

/locus_tag="JFBMFIFI_00032"

/inference="ab initio prediction:Prodigal:002006"

/codon_start=1

/transl_table=11

/product="hypothetical protein"

/translation="MSYSQFKVSGQVTQLCEEVLRLTSKAPKKFRFSLCQRMDSLALS

CLSDIYRANSLSLSNESSQIQRQSFQSNALVTLKILGTMGRIGTENQCFTIGEYGVLS

KKITACEQLIRRWIESDNQRVH"

CDS 40294..40635

/locus_tag="JFBMFIFI_00033"

/inference="ab initio prediction:Prodigal:002006"

/codon_start=1

/transl_table=11

/product="hypothetical protein"

/translation="MTKHHDIEIQGLFEELPKETVTKRELKILRKKVANECNVSPFII

FNNLVLENIEQKNPTTRDEFLTINGLAEAKWGQFGEQEGNLLGIKSELTNTRNHRGCF

NKYRFILFKKE"

CDS 40681..43584

/locus_tag="JFBMFIFI_00034"

/inference="ab initio prediction:Prodigal:002006"

/codon_start=1

/transl_table=11

/product="hypothetical protein"

/translation="MRTIKKVMTKMNPIVKRQRVLFIGVVSLLLTILIVTNTLGWQSS

QQKALNTLTVNRDPQSVTLIKFARDKEDQETDHVLEGAVFALYQVTKDGDKQIGGYYT

TNDKGQINVDVPPGEYYFEEITPPSGYTFDLDKNGKVTKHYLFTVSSEDKEAAIEVKV

FNRLSKGQLEVVKTVKNEDDSLLSDQQKQQDFLFTITYSDKGTYSYQIDDSVNRYEVK

SGNTITLKHGQQAIFKDIPEGIHYTVTEHPLLGVVSEASHSSGTIHSKASQAAFTNTF

LTTTSELRLQKQVVSGSGKPLTEEQLQQEFTFRFTFSDKETAFPYETTTGRTGELKSD

DTLTLKHDETVTFKNMSIRTTYTVTEEPTVGYISGQTYWEGQLVKTDGVLIVVTNEVD

TDSTATNDLTFTKQVVAEKPDEDILFSFEVTFSDSKNYEYQIDDGLVLSHKSGDVIQL

KANQKVTFKDLPVGVGYQIKELETPHYRGELTEVTGTILSSEDTNYTFMNHFEGNPFL

QIKKVGEGNEFDPTKEFTFTVYVNDQALEEKVVLKAGEMSDAVPLTIGDTWRVVEDIS

EDPTYQQTGIVNSIGQITQVDQNVLVTQTNTHITQKQVTIAGQKHWIIPAEYQEKQPE

SIIVRLMNGDRLAATQEVKGPGWSYEFKVPEVDSEGNLIVYRIEEESIPGFAMSSIED

SYDITNTYVTPVQVAPLTVVKKIKGDTPLSSSTFEFTLSPGDETVSIQGSGRVDIPSP

TFDSPGTYHYTIREKNLGLVGYTYDIAVYQWTIVVEAEGDALTITSETLTKDGEAYNS

RELVFVNQFKTSLDEKQTIRGTKTWHHGTNSTKSTPTSIIVLLLADNEVIQQKEIMEP

TDWSYQFTVPVYTVSGEKIVYSIQEVPVSGYQTKVSGYDLINTYIESTPKPPGKKNPD

KGILAQTGEKISMIAFIAGLILVIGVSGFYLHQRNHKKQKNKRVKNIRSKKRR"

CDS 43726..44118

/locus_tag="JFBMFIFI_00035"

/inference="ab initio prediction:Prodigal:002006"

/codon_start=1

/transl_table=11

/product="hypothetical protein"

/translation="MKINRQEAIVQNFEYKTLTKIHKAQTSFQTVIDPLDLSKLNTSF

SGKHSVFGLRMTYNLVIGKRIVIVGNITQLVEIEEKVIDKIEDFSKTELNKLFQPLFR

MLEELVYEVTQITLDKKGIALNFTADYD"

CDS 44614..45276

/locus_tag="JFBMFIFI_00036"

/inference="ab initio prediction:Prodigal:002006"

/codon_start=1

/transl_table=11

/product="hypothetical protein"

/translation="MADLEKWYYGIDVVGEFSSKSDVSQLATSDYGSPSSNGKKIIYV

ILSSVSSLSIRSKEEDLTTISAGTSREFYAEVNYRASLDWSMTPEINSLYEKYEDLNP

FSLSKKLNIEIKFVPFKDRPYGNIKIIFGEPIIFINMSKENEHFFICAHELYHAIAHK

QLRYNYQTNIVAKAKMEIEANLFASHLIMNDYLLKYKTPPKNFLKLQTVYGLFDEMKD

FF"

CDS 45365..45949

/locus_tag="JFBMFIFI_00037"

/inference="ab initio prediction:Prodigal:002006"

/codon_start=1

/transl_table=11

/product="hypothetical protein"

/translation="MSTVVYSKKYDYVYSYQTKKGKLWGYRFNYYSSSNIRKEIGKRK

FITEKEAYRDLLTVQEQVEKGNFKAIENNKLTIKEYCYVWLESQQSKNPSINTIRGAK

TTIELHIIPRIGHLKIQAISRFEYKRLFLEDLLKTTSARSVETHHKRFIAIINSAVKN

EIILANNFKGFNFSDYEVENIKNKYFSKKKPLNF"

CDS 46167..46532

/gene="xerC_1"

/locus_tag="JFBMFIFI_00038"

/inference="ab initio prediction:Prodigal:002006"

/inference="protein motif:HAMAP:MF_01808"

/codon_start=1

/transl_table=11

/product="Tyrosine recombinase XerC"

/translation="MLNKYKINERKFLLSNGQKLNETDLFATNRYGNPISYCGITEQF

RLFCNDNDLPYITLHGLRHTHATLLLKANANIKYISRRLGHKNIEITLNTYSHVLIEK

ELETAEIFSNVVTNLAKLG"

CDS 46697..48325

/gene="ybiT_1"

/locus_tag="JFBMFIFI_00039"

/inference="ab initio prediction:Prodigal:002006"

/inference="similar to AA sequence:UniProtKB:P0A9U3"

/codon_start=1

/transl_table=11

/product="putative ABC transporter ATP-binding protein

YbiT"

/db_xref="COG:COG0488"

/translation="MITVSNVSLLFSDRNLFEDVNIKFTPGNCYGLIGANGAGKSTFL

KILSGDIQPTTGDISMSSGERLTFLKQNHFDYEEFGVLETVIMGHKRLYDIMKEKDAI

YMKEEFTDEDGIRAAELEGEFAELDGWEAEPEAAVLLQGLGIDESLQQKTMSELTGGQ

KVKVLLAQALFGKPDVLLLDEPTNGLDKQSIEWLEEFLINFSNTVIVVSHDRHFLNKV

CTHMADVDFGKIKLYVGNYDFWLESSQLASRLANDSNSKKEEQIKELQDFIARFSANA

SKSKQATSRKKMLEKITLDDIQPSSRRYPFVGFTPDREIGNDLIRVENVSKTIDGVKI

LDNISFSLNREDKTAFISQHDIAITTLFKIIMGEMEPDSGSVKWGVTTTQSYLPKDNT

AEFEKGDLTVVDWLRQFASKEESDNTFLRSFLGRMLFSGEDVMKKVSVLSGGEKVRCM

LSKMMLSKSNVLVLDDPTNHLDLESITALNDGLIAFKGALLFGSHDHQFIQTIANRII

EVSSNGVVDRADTTYDDFLEDKNVQEQVAKLYAN"

CDS complement(48461..48994)

/locus_tag="JFBMFIFI_00040"

/inference="ab initio prediction:Prodigal:002006"

/codon_start=1

/transl_table=11

/product="hypothetical protein"

/translation="MVIDFDSLETIKNSSTIGVEGIVVAEEPYVYDQEGGPSTPYIKL

TLEITKVLSGDANLVGQTIIILESGGEITKEELGFKEKFPEMSEEELKEEITVLSDGI

ENTRVGEEIVGFLTDDMGTMETDFSFSSFMGEYNSRFKKIQKQIDLNESYQKKDTKKV

QNHVEVLLMKRKLEIMR"

CDS 49778..50707

/gene="ptxB"

/locus_tag="JFBMFIFI_00041"

/inference="ab initio prediction:Prodigal:002006"

/inference="similar to AA sequence:UniProtKB:O69052"

/codon_start=1

/transl_table=11

/product="putative phosphite transport system-binding

protein PtxB"
/translation="MKFVKNGFLLVFGLVLVVSLAACGAKKESGAKEESYTPKELTVQ
FVPTIEANTIEAKAKPLEKLLSDELGIPVKVTVSTDYNTIIEAMASKQTDVGIMPPNA
YVLGHEQEAADAILQAQRFAIKQPGGETSTELADGYRAMVVVKKGSPIKNLKDLKGKK
IAAQDVVSASGYVFPVAEMQKAGINVDKEIQFATVKGIDAGLMAVLDGSVDAAFSFED
GRNLLKKDLPDVFDKLDAMYFTEAKIPNDAIAVRPDLSDKWTKVQDAFVAIGKSDKG
RELISSLYAHEGYVPADDSSFDIVREYAKEVGQ"

CDS 50780..52276

/gene="yfkN_1"
/locus_tag="JFBMFIFI_00042"
/inference="ab initio prediction:Prodigal:002006"
/inference="similar to AA sequence:UniProtKB:O34313"
/codon_start=1
/transl_table=11
/product="Trifunctional nucleotide phosphoesterase protein
YfkN"
/db_xref="COG:COG0737"
/translation="MEVTLLVTSDIHGYIRGTNELTGEATADGFARVATYIKKRKKID
PNLILIENGDFLEGSMLANTLATHQDKWSIHPFIQLMNQLNYDSAVPGNHEFNYGINY
LKKALKGLKAPYLAANIVLENGKTCWEAYQIVERKGIRIAIVGLVTSYVTRWEKVENI
KGLRFLDPILTAKKWVPFIQETEQPDLIVLAYHGGLERDLVTGEATEYMTGENVGYRL
ATEIPGINGVITGHQHRTLAGAVNGVPLLQPGTRGASIGEMIFELQQKEQKWHVKSAK
AQTISLASEKEDTAVLKEIEEWEHLSANDRKKPIIKGLDSVEPDAYQFLHKVQKVASR
RKISVVSLVKSSVGSTALTMEMILQNYTFPCTFAILRFTGQELKHLIIKGRDYYQLNQ
AGRLSPYKLGRDEQTENELIDGLTYRFYFDSKSDSYLVELVDVADDELIELSFNHYLG
ALNGERVIGMEKIIQEIMIYMPDLIREYQLEECVAIPVHGQGFYPEIS"

CDS complement(52345..53379)

/locus_tag="JFBMFIFI_00043"
/inference="ab initio prediction:Prodigal:002006"
/codon_start=1
/transl_table=11
/product="hypothetical protein"
/translation="MEKEHILTTKITINGTFYDSQLYSGKLYLWLETNQLAIYNWNNW

MLALNEIERPVYFEPRPSEAMQFDLKDLTPYLERSLAFEEAPIDTAIFNHQLYFSDST
GFYSYSLAQLKATKKKLWDLPVLQINIAKNGRMALACGANGLYEYDNTKRFTDHTADE
VAPSIYQLTNQYASRSEWSRNDLIQFGAHLEKADYICFFHLHEKQIILEKVQAANELT
YKEHVSIEELPLSLEMTKPLIDEGLLFSFNPLNRQLKKEKQYRSKSLTVKAALSELNQ
QPPFSLNTIYIKEKQHKLTAQLNDQPILQIKMDFTKWRVYDRTLYYRNQLHVLTNNAC
ILYLFTELIH"

CDS 53574..54608

/locus_tag="JFBMFIFI_00044"
/inference="ab initio prediction:Prodigal:002006"
/inference="similar to AA sequence:UniProtKB:P43951"
/codon_start=1
/transl_table=11
/product="hypothetical protein"
/db_xref="COG:COG1840"
/translation="MKKFVSLMVVLTVALVGLSACGSGEKTKGASKKEENTLIVYSPN
SEDIVNTLIPMFEKETGIKVELVSAGTGELLKRISSEKENPYADVMFGGLNKSKLTNE
DLFEKYTSKNDKDMIEGHQNIAGVLTPYITDGSNILINTDLIGDIKIEGYEDLLNAQL
KGKIAAADPASSSSGFAQLTNMLLAMGGDYESDKGWDYVGSLIKNLDGKVANGSGAVH
KGVADGEFVVGLTYEDPSASYIRDGAPVKIVYPKEGAVYLDAALAIIKDAKHKENAEK
FIDFMTSKEAQDIFGEELTNRPLRKDAALGNHMVPMKDIKILTEDEDYVQKNKDKIVN
QYTDLFTELQ"

CDS 54636..55751

/gene="msiK"
/locus_tag="JFBMFIFI_00045"
/EC_number="7.5.2.-"
/inference="ab initio prediction:Prodigal:002006"
/inference="similar to AA sequence:UniProtKB:Q9L0Q1"
/codon_start=1
/transl_table=11
/product="Diacetylchitobiose uptake system ATP-binding
protein MsiK"
/db_xref="COG:COG3839"
/translation="MSTTIKIKNVLKKYGESVVIPDLSVEIKPSEFFTLLGPSGCGKT

TLLRMVAGFNSIEGGDICFNETKINHVTPQNRNIGMVFQNYAIFPHLTVKQNIAFGLQ

NRKIKIAKDEIEKKVEKMMDVVQIQKFKDRLPENLSGGQQQRIALARAIVIEPSVLLM

DEPLSNLDAKLRVDMRNAIRNIQKEVGITTIYVTHDQEEAMAVSDRIAVMNLGSIQHV

GTPQNIYHRPANVFVANFIGRTNIIERELNMEGTVGCLHLDQDNSVKITNLADPKNRQ

SGKVKVSIRPEEFIIVEDGLGIRAKVLNNMFLGLNTHYNVELASGEQVEIIQESTLEK

VIEPGTWIGLHIKKDKINLFDSQTEVNLTKGVINDVW"

CDS 55738..57396

/locus_tag="JFBMFIFI_00046"

/inference="ab initio prediction:Prodigal:002006"

/codon_start=1

/transl_table=11

/product="hypothetical protein"

/translation="MMFGKGQKKDVWTYITIGIFLLYLILLVLPLFTLLKAGFTNPDG

TGFSIAYFTKFFGKNYYYDALFNSLKVTISVTLLAVVLATPLAYIMTTVKIKGKVFLQ

ILILISSMAPPFIGAYSWILLLGRNGAITKFISSMFGVKVPDIYGFTGIVIVLTLQLV

PLIYMYLMGALKSIDNSIIEAAESMGCTGIKKMVKVIFPLIMPTLLAGALLVFMRALA

DFGTPMLIGEGYRTVPVLIFNEFISEMGGDDGFAAAISVIVIVFAIAVFLIQKYISNR

KSFSMNALHPMEAKKAKGIRNILAHTYIYGFIVAAIAPQLYVIYTAFLKTSGRIFIKG

YSLDSFRLAFDRSGDAIKNTFSLAIASIVIVVFLAILIAYVTVRRKNTLTNGLDIVTM

IPYIVPGSVMGIALLLTFNKPPLLLSGTALILIVSYVIRRLPYTIRSSAAIVHQISPS

IEEASVSLGASNLKTFFRVTFPMMLPGVISGAILSWVTIITELSTTIILYTAKTRTMS

VAIYTEVIRGNYGTAAALSTILTLITVSSLLLFFKLTGKKEISI"

CDS 57417..59114

/locus_tag="JFBMFIFI_00047"

/inference="ab initio prediction:Prodigal:002006"

/codon_start=1

/transl_table=11

/product="hypothetical protein"

/translation="MMDKKQHKFKEKTRLLFLKYTFIPIVFLFVIFGISIFSYASWKV

RNDTVKASQETMLAFNRVLATYEKEMIRMANNEYVLDYLTQQKKSNLVFEEFYGFNLQ

QEVKSIFHLVDMKRFSLTSSSSEDYLANEIPSYRLKRWKNPKQMVIDVQTNYFSYNKV

TGLTLIQPVGKDGKIVGYLIYRLYDEDFQKLLFGKNVDIVVIADQYERIIATTSNRVK

GLMGKFSVDEREGNEILLDSKKYILAENQHSLYGVQVKTLIQKPNYQEVLVSYLLLTT

ISSLVLYFLVNYLAKKMSERNSQSVSVLMSGLDSLKTGELTNFVEIKTGDEFEELADH

YNKMLKNLNQLLLRNEELTYVLQRDEIKFLQSQFNPHFLFNMLETIRYTMVSDQDKAQ

RIILTLARLLRYSIHQGGQNWEILQKDMDYISDYLALHQYRYNNRLTYQIDLDPSLTKV

EIPKLLLQPIVENSIKYGYQNQTNLTIKISVQAQQDKIRITVEDDGPGIATKQLSEIE

ANLRQEIINLSSIGLYNTDRKIKLIYGEKYGLKIKSTLGLGTIVQIELPLIKDDGDV"

CDS 59107..59907

/locus_tag="JFBMFIFI_00048"

/inference="ab initio prediction:Prodigal:002006"

/inference="similar to AA sequence:UniProtKB:Q7A7X9"

/codon_start=1

/transl_table=11

/product="putative response regulatory protein"

/translation="MFKVLIIEDEDIIREGLKFSLDWGKENCQIVGEAANGVEALEKI

TKLKPDILLLDLNMPLKNGLMVLKESYQIALYSTIIISGYDDFEKAKEAIKYGVTEYL

LKPVDHEELIVALRKAREAIELKQQYQFLQEKLNGVEDVDILDFKRITNRHKLSKEVA

RLISFIEEEYSQKIRLQDLVFELNRSKTYLNQKFKLETGYTFNEYLNRYRVYQAIYLL

KNSDEKISSIALEVGFSNYRYFIDIFKKYTQVLPSDFASYSKGYVPEA"

CDS complement(59965..61401)

/gene="ycaM"

/locus_tag="JFBMFIFI_00049"

/inference="ab initio prediction:Prodigal:002006"

/inference="similar to AA sequence:UniProtKB:P75835"

/codon_start=1

/transl_table=11

/product="Inner membrane transporter YcaM"

/db_xref="COG:COG0531"

/translation="MKEEKKLRWYNVALIAFVSVWGLGNVVNNYATQGISVITSWILI

MLLYFVPYALIVGQLGSTFKDGNGGVSTWIKETTTKKLAYYAAWTYWVVHIPYLAQKP

QGILIALGWAFKQNGNLVNDTPALTVSLISLVIFLVFLWVSSKGLTTLKRIGSIAGMA

MFVMSLLFILLAVTAPALRGASFATPNMGDIKTYLPTFNFAYFTTISMLVFAVGGAEK

ISPYVNNTKNPSKEFPKGMLALAAMVAVCALLGSFAMGMLFDSNNIPDDLMANGAYDA

FQRLGAYYGVGNFFMILYAIANTMAQISALAFSIDAPLKILLGDADEEYIPRKLAKLN

SKGTPVNGYAMTGILVSILIIVPAIGIGSMNDLYKWLLNLNAVVMPLRYLWVFFAYMM

LNKLHKNFSSEYKFVKNHKIGYVIGAWCFLFTAFACILGMVPKIDYAADPKAWVFQFT

LNIITPIIFIALGMILPAIAKKEVAKKA"

CDS 61944..62906

/gene="ghrB"

/locus_tag="JFBMFIFI_00050"

/EC_number="1.1.1.81"

/inference="ab initio prediction:Prodigal:002006"

/inference="protein motif:HAMAP:MF_01667"

/codon_start=1

/transl_table=11

/product="Glyoxylate/hydroxypyruvate reductase B"

/translation="MKNKGIWLLQETTPEQLQRVKDLAPNYEVIDGFSDSTLNFPAAD

IEIVYGWSSAKADFLLENKDSHLKWIQAKSAGVDTMNLSLLNEKNIILTNASGIHGVP

IAESVFGMLLADTRGIKKAINQQTNKVWSQTESLVELKGKTMMIIGMGQVGKEVARLA

QAFGLNVIGVNRSGNPVTEVSEIIKQDQIPKHIKRADFVVNILPLTSETTNYYDDSFF

TSMKKGAGFINVGRGPSVDTDALIRQIKNGQIGFAGLDVFKEEPLAKDSPLWDLPEVL

ITPHISGVAEHFKKRLFAIFEENLTAYVAGEELPRNVIDYKHNY"

CDS 63505..64377

/gene="yckB"

/locus_tag="JFBMFIFI_00051"

/inference="ab initio prediction:Prodigal:002006"

/inference="similar to AA sequence:UniProtKB:P42400"

/codon_start=1

/transl_table=11

/product="putative ABC transporter extracellular-binding

protein YckB"

/db_xref="COG:COG0834"

/translation="MKKKSSWLVISFIALSLVVAGCTGGNSDKATSADKKEKNGWEQI

KEKGVLTVATSGTLFPTSYYAKDEKELTGYEVEIVKEIAKRLDVKVKFVEMGFDGMLS

AINSGQVDTAANNIDLSDKRKEKFAFSDPYKYSFASMVVRKSDQSGIHSLEDLKGKKS

AGAATTTYMKIAEKFGAESVVYDNVTNDQYLLDVANGRTDVILNDYYLQKMATEALPE

IPVEINPGLFYNPSQAGLVMKKENTELKTKVDDAITEMKKDGTLKKLSEQFFGGADVS

KEADVKITKVVEVE"

CDS 64389..65069

/gene="yecS"

/locus_tag="JFBMFIFI_00052"

/inference="ab initio prediction:Prodigal:002006"

/inference="similar to AA sequence:UniProtKB:P0AFT2"

/codon_start=1

/transl_table=11

/product="L-cystine transport system permease protein

YecS"

/db_xref="COG:COG0765"

/translation="MSGIQWQYLFNPSLALESLPFILQGLGYTLWISVVSMTFGTILG

FFLALFRLSKFKVLNLLSRIYISFMRGTPMLVFLFILYFGFPFIGIKFDAVTAAVLGF

SLHSSAYIAEILRACLNSIDKGQWEASYALGMPYFFIMRKIILPQALRTAIGPLSNVL

LDLIKGTSLAAMITVPEIFQQAKIVGGREFDYMTMYILVALVYWGICSLFTILQTILE

KKFSLYTN"

CDS complement(65146..66117)

/gene="ccpB_1"

/locus_tag="JFBMFIFI_00053"

/inference="ab initio prediction:Prodigal:002006"

/inference="similar to AA sequence:UniProtKB:P37517"

/codon_start=1

/transl_table=11

/product="Catabolite control protein B"

/translation="MANINEIAKIAGVSSATVSRVINRNGYVSDDTRAKVEAVIKKLD

YVPNRNAVFLKTGMTKMLGIIAPDFSDSLTVFLRSFTLAAQKEGYNVTLFITGGDKKK

ELDAFEMLRHKQLDGLVLVIRLNDWDQLEPFTKYGPIVTWQRVDSKHIPSVFMNHYQG

YMLGLEHLYASGCRNIANLYGSTRGLNTKSRMQAYADFCQKYKLDPKLEQQFKGLNSI

EDGEAILDWYAEQSDKPDGFATSSDAVAAGLVAESRRRGYNIPTDFSVVGFDNIEISR

LLDITTIDYPIAKQAQNAFTIIFNQLLYKNLPLLPLEFDLIKRKTTR"

CDS 66321..67676

/gene="brnQ"

/locus_tag="JFBMFIFI_00054"

/inference="ab initio prediction:Prodigal:002006"

/inference="similar to AA sequence:UniProtKB:P0AD99"

/codon_start=1

/transl_table=11

/product="Branched-chain amino acid transport system 2

carrier protein"

/db_xref="COG:COG1114"

/translation="MDKRLSLSSYIFIGSMLFGLFFGAGNLIFPVHMGQEAGTNILPA

TLGFLVTGIGLPFLGVVAIGVSKSDGLFDLASRVHPIYGIFMTVALYMTIGPFFALPR

TGTVSYEIGIAPYLPSQYQTVGLLLFTVIFFAIALAFSMKPTKILIWVGKILNPLFLV

FLAILIVTAFLKPMGVISEAAVHGNYVTEPFITGFTQGYNTMDALASLAFGIIVVQTI

KGLGVRNPSNIAIDTIKSGIVSVILMAVIYGSLAYIGATSVGQFEVSENGGIALAQIA

QHYFGSFGSVLLAIIVTVACLKTAIGLITACSETFCEMFPNSFSYRTYVILFTLLACG

IANVGLTKIISLSIPVLMFLYPLAITLIFLALLSPLFKNRQVVYVTTTIFTIFVSIAD

GLNALPAGIKSISFIDNILAFYSRYLPLFDIGMGWVFPAILGLIIGWIISLVKKQELR

F"

CDS 67816..68574

/gene="mta"

/locus_tag="JFBMFIFI_00055"

/inference="ab initio prediction:Prodigal:002006"

/inference="similar to AA sequence:UniProtKB:P71039"

/codon_start=1

/transl_table=11

/product="HTH-type transcriptional activator mta"

/db_xref="COG:COG0789"

/translation="MEYTVKKLSELAGISGRTLRFYDQIDLLKPNRINSSGYRIYGGK

EVDRLQQILFYRNLGLPLEEIKEVLDAPNFDSETALTAHYQHLLEQQQQLNKLIATVE

KTLQVKRGMSSMTDKEKFEGFKKELIEKNETQYGDEIREKYGNKAVEESNQKMASMSK

EKYEAFEKLGNELLEELEVAMNDQNPQGTAGQRVAALHKEWLSFTWGSYSKEAHRGLA

EMYVSDPRFTAYYDEKAGDGAASYLRDAIVAYTK"

CDS complement(68646..69191)

/gene="chrR"

/locus_tag="JFBMFIFI_00056"

/EC_number="1.6.5.2"

/inference="ab initio prediction:Prodigal:002006"

/inference="similar to AA sequence:UniProtKB:Q88FF8"

/codon_start=1

/transl_table=11

/product="Quinone reductase"

/db_xref="COG:COG0431"

/translation="MTKKIGILVGSLRKDSLNKIVAKQLAELFPADYTVSFIEIGDLP

VYNEDLEIDGVESYNRFRKEMKEQDAYIFITPEYNRGMPASLKNAIDVGSRPWGESVW

PGKPVLVGSVSPAGISGALANHQIRQSLVFGDSPVMAQPELYIDASKAIGEDGKVVPA

SVGFLQTIADSFVAFINKNSN"

CDS 69752..70021

/locus_tag="JFBMFIFI_00057"

/inference="ab initio prediction:Prodigal:002006"

/codon_start=1

/transl_table=11

/product="hypothetical protein"

/translation="MVKKFKYTLIFLSIGVLISLLFVFKIDEKGFLISITNILSLLIF

KTDSLYNLVSSQSIAVQNIIYGVTNVLSLMIFGLLIDFFRDVKKR"

CDS 70539..71423

/locus_tag="JFBMFIFI_00058"

/inference="ab initio prediction:Prodigal:002006"

/codon_start=1

/transl_table=11

/product="hypothetical protein"

/translation="MKSLGEAIKAGRLKKNMTQQELAEGICTQATISNIEKAGKIPAI

TLLLAIADRLDIDIDELYYLMGENTTKNGKIMKKVKVLCSQSNHKEAATLLKEINEAE

LETINEKKEYYYYKGITSLVAFHNFSDALFYFNLSNDTQGEGYISIYDVLGLSGVSIA

YSMNDEDEKALVYTERTLNILDEFVAEGYEKNDTNDIVRTYFNSAKIYSKMKNYEKAV

SLSSMGIALQQLNDSMNGLEYLMYEKAYNLQQLEQVTEAEKFYFYAAAMAMMNKNNEV

IETVKSDMKLYNVSHFMY"

CDS 71560..71811

/locus_tag="JFBMFIFI_00059"

/inference="ab initio prediction:Prodigal:002006"

/codon_start=1

/transl_table=11

/product="hypothetical protein"

/translation="MQKVKVRRIGNSLGVILPKDRGVKVGECLTYQKNGSIIQLDLSE

AQKSQDRKLIEESFRDLEEGNTLTEKEMIKEFGKYGWGN"

CDS complement(72512..74263)

/locus_tag="JFBMFIFI_00060"

/inference="ab initio prediction:Prodigal:002006"

/inference="similar to AA sequence:UniProtKB:Q9WYC4"

/codon_start=1

/transl_table=11

/product="putative ABC transporter ATP-binding protein"

/db_xref="COG:COG1132"

/translation="MQNKKINPSDLKRLLPFMTHYKMPIFAALFCGLIGGSATVAATY

YTGKGIDAIVGKGQVEFSKLFHVLWLLAAVYLLAIITQWMINYFSNQVSYNAIKDLRI

KTFDKLNELPLSFFDNTPHGTIISRFTNDLDSISDAVSLTLNNLFSGVIVVFASLGFM

LYLSPLLTLIVLVTTPLIFLIAWIVARLSQKNFTQQQRIVGEISGFVSEMVTNQKVVK

AFNQEDYVQENFEQINNRLYIWGRKAQFTSSITNPSARFVDHLAYLLIGTIGGILVVT

GNGSITVGVVSSFIIYSSQFSKPFIELSGITTQIQTAAAGLRRVFEVLDAVPEIPDAT

NATTLTTINGNVAFDHVYFSYQQDRPLIQDLNLSVAAGETIAIVGQTGAGKSTLVNLL

MRFYELDQGSIKIDGTDIKKYTRDSLRRSFGMVLQDTWLFSGSIKENIAYGHPTASEE

EIIQAAKAALAHAFIIKMPQGYDTIIGSGGVSISSGQRQLLTIARTMLSDPPMLILDE

ATSSVDTLTEQQIQTAFLRIMKGRTSFVIAHRLSTIREADLILVMDKGMIVETGTHDE

LLKIDSYYHRLYAAQFE"

CDS complement(74265..76010)

/locus_tag="JFBMFIFI_00061"

/inference="ab initio prediction:Prodigal:002006"

/inference="similar to AA sequence:UniProtKB:P9WQJ1"

/codon_start=1

/transl_table=11

/product="putative ABC transporter ATP-binding protein"

/db_xref="COG:COG1132"

/translation="MISLLRYAKKYRLQMVIGPFFKFVEAVFELFLPLLMAKLIDNGI

NKGDTAYIYKMGGLMLAMSVIGLISVFICQYSASIASQGFGTELRNALMRKINTFSHK
EIDQFGTATLITRATNDINQMQLALAMLIRLVIRAPFLSIGSLIMAIYVSPKLALVFA
VLLPLFCLILYFIMTKTIPLYKIVQTKVDRLTQVVSENLSGVRVIRAFARSKKEKQRA
EDVSDELAKSYIRVANLSALMTPATSLIMNAGILIILYVGGFQVNTGSLMQGEVLALI
NYIMQMLLALIILANLVVIFTKSAASAARINEVLDTDVSVVENESPTYLTSIENQTAI
QFENVSFKYAAASANALENIEFNLSKGQILGVTGPTGSGKSTLINLIPRFYDVTAGTL
LINGTNVNEFDLQSLRRSIGYVPQNSSLFTGTIAENLRWGKADATEAELQMALEIAQS
ADFVASLKGGLNSPVFEGGKNFSGGQRQRLTIARALVSQPDILILDDSLSALDYQTDL
NLRRALASRLTNTTVVIVSQRISSIQTADQILVLNDGKLANSGTHKQLLAESDIYRQI
YDSQSENETDVKEVH"

CDS 76206..76964

/locus_tag="JFBMFIFI_00062"

/inference="ab initio prediction:Prodigal:002006"

/codon_start=1

/transl_table=11

/product="hypothetical protein"

/translation="MEITFEKIVLLFFIYAFIGWLWETVYCSIKAKKFVYRGFLIGPY
CPIYGFGVLAVLSLISPYQQNIILLYVFSALVVTLLEYVTSYGLEKLFHTTWWDYHDV
PLNLNGRIALPISLFWGVGCVFIVKVVQPIVSEWVQFLINQFGIFLPVAIVVLMTVDL
IYSVFTLAAFQKELAKLSVEIEERKNNLVEKITETSQTLMEELKQSTELKKAFAKLNF
NQKRMLRAYSKLNMPTIKNFKELKEIQERFRKKG"

CDS complement(77158..77634)

/locus_tag="JFBMFIFI_00063"

/inference="ab initio prediction:Prodigal:002006"

/codon_start=1

/transl_table=11

/product="hypothetical protein"

/translation="MIKNSYFASPLFSEMELHYNQQLVQQIRAAYPQLELYVPQEQME
INDKNAYADSKTIAQYDTKALLNSQLMIAVLDGATIDVGVATEIGVAYQAGIPILGLY
TDSRQQGATNPHKLAALQEVAESQFSYVNLYTVGLVKLNGAIHSTSSDLITAIGKY"

CDS 77799..78893

/locus_tag="JFBMFIFI_00064"

/inference="ab initio prediction:Prodigal:002006"

/codon_start=1

/transl_table=11

/product="hypothetical protein"

/translation="MKLRKLVVSVVLTFLSVNLIANVGYAASLDEINKEQQAKQAKMA

TVDSEISQTLVSLNDKNKEIEALNQQVSEKQESLKDTAEKIKAKQANVDERVAQAKKR

LQSLQTSEASKSMVLMILESESLTDFLNRAYVINRLQSADNDNLEEAKKDQEELTNLE

AKLREDAATLAEQKEQVNKDTTELNTKMASLQKTMDENKDALNALDEQKRTEQARIDD

EAAKKKQAEELAKKAEAAKVSTTAVAQNTAQNDAVAPAAPSNPTDQSGAGGAGKTIIV

ESTAYSYAENGSSFFTANGTDLRVNPMVIAVDPRVIPIGSRVEVSGYGVAIAADTGGA

IKGNKIDVHFSSVAECLQWGRRTVTIKILD"

CDS 78924..79172

/locus_tag="JFBMFIFI_00065"

/inference="ab initio prediction:Prodigal:002006"

/codon_start=1

/transl_table=11

/product="hypothetical protein"

/translation="MAVDIDFYHEKDEQAFLDKWEAEKGSIEDIDLFYSELAEVVEEA

YQNGTVKLGKKFVYQDVVVGYVDYNTFNNLFLFSLAKQ"

CDS 79567..80538

/gene="ceo"

/locus_tag="JFBMFIFI_00066"

/EC_number="1.5.1.24"

/inference="ab initio prediction:Prodigal:002006"

/inference="similar to AA sequence:UniProtKB:A5HZ59"

/codon_start=1

/transl_table=11

/product="N(5)-(carboxyethyl)ornithine synthase"

/translation="MRSVKKKTMGFVISHKENEHRRALLPKDIKIIQNPTQLFFEKNY

GNVLNIADEEYLALGCQIVSREVALQQDIICEPKIGDAEFLQNLLPGQLIFGWVHAVQ

NRDITDLLIEQKSSAYAWEKMYYMNRHSYWRNNELAGESGIVHAYLLNGRMPYDTKVA

ILGRGSTAQGANRILTKLGADVCIYNRNQEALFKKELGDYDVIVNAILWDTTRHDHII

YEKDLKRMKKGAMIIDISCDEHGAIETTSPTTFEDPTYYVDGILHYAVDHTPSIFYQT

ATAAISEETSKYIDDLIEGNPNPILRDALIIEDGKIMDQDINKFQDR"

CDS complement(80602..82068)

/gene="bglH_1"

/locus_tag="JFBMFIFI_00067"

/EC_number="3.2.1.86"

/inference="ab initio prediction:Prodigal:002006"

/inference="similar to AA sequence:UniProtKB:P40740"

/codon_start=1

/transl_table=11

/product="Aryl-phospho-beta-D-glucosidase BglH"

/db_xref="COG:COG2723"

/translation="MNWGAFFMTTTKSIFPKNFLWGGATAANQLEGGYLEDGRGLSVA

DALPGGKERFKIVAQPDFDFEIDETKYVYPNHNGIDHYHRYQEDIALFAKMGFKVYRL

SIAWSRIFPKGDETTPNEAGLAFYDKIFDECLKYGIEPVVTISHYEMPLHLAKEYGGW

GNRKLIDFYERYAQVVLERYHAKVKYWMTFNEINSALHFPVMSQGLTMKSGAADKQTI

YQGLHHQFVASSKATKIAHELDPNLQVGCMIIYATTYAYDSNPVNHLEALEYNQRFNF

YCADVQAKGEYPHYAKRIWEENNVTLDIQPGDLEILKAHPVDYIGFSYYMSTVVTTDA

DKLAQSSGNLLSGVSNPFLEASEWGWQIDPLGLRLALNELYGRYQKPLFIVENGLGAY

DTPDENHYVADDYRIDYLRSHISAMGEAIEDGVDLMGYTPWGCIDLVSASTGEMSKRY

GFIYVDLDDNGQGTGNRYEKKSFNWYKKVIDTNGSDLA"

CDS 82197..83396

/gene="rhaR_1"

/locus_tag="JFBMFIFI_00068"

/inference="ab initio prediction:Prodigal:002006"

/inference="protein motif:HAMAP:MF_01533"

/codon_start=1

/transl_table=11

/product="HTH-type transcriptional activator RhaR"

/translation="MEVEKIIKKIRRELIAGNRLLGVATGSGMSASQVQKGGADFILA

LNSGRFRVRGRSSLAGFLPFENSNQEVLNFATKEILPLLQHTPIIFGLNATDPTIDLV

NFIGTIKANGFSGVNNYPTVGLLDGQFSEALEESGCSYQLEVEAIKLAHDQKLFTIAF

VFNQEQARQMTEVGADVICFHCGLTRGGKLGAKKVLSLANVIASANEIVKVSREIRPN

VITMIYGGPVSSLIDIRYIYNQIPGLNGYIGGSTFDRITPEELIIQKAKEFKSPRDSQ

DTSLMSQMLDGIDKHYDYVVFVEKYIEQNYHEGIYLEDLAQVAHVTSSYLSTLFKKKT

GASFTEYLIKFRLNKAIELMAKPQRLSFKEIAFLVGYPDYAQFNKIFKKYMGTSPSEY
SNARTKE"

CDS 83494..84708

/locus_tag="JFBMFIFI_00069"
/inference="ab initio prediction:Prodigal:002006"
/codon_start=1
/transl_table=11
/product="hypothetical protein"
/translation="MKKTVALVGTLDTKGEEYLYVKKLLQQKGLEVLTVHTGVFKAQF
KPDIDNAVIAEAAGITIAELVERKDRAFATEKLAQGLKEVLPELYKKDKFQGVLSFGG
SGGTSLVTPAMQKLPIGVPKIMVSTMAAGDVTPYVGTSDIIMIPSIVDVAGLNSISTK
LFTNAVYAISGMLTTNFKEEVRHKPLIAASMFGVTTPAVTVAREFLEQSGYEVLVFHT
TGTGGKIMESLVQDGFIEGVLDLTITEWADELFGGVLSAGPTRLEAAALTGTPQVVSV
GALDMVNFGPIETVPEKYKQRNLYQHNPTITLMRTTKAENQQLGEKIAEKLNLATGKT
VLILPLKGISAIDVEGQPFYGPIEDQQLFKSLKENLRNPLVKIIQRDEAINDSTFAQY
AAEQLINLIENK"

CDS 84727..85554

/locus_tag="JFBMFIFI_00070"
/inference="ab initio prediction:Prodigal:002006"
/codon_start=1
/transl_table=11
/product="hypothetical protein"
/translation="MNKQTRNEIMNSFYNKRKKGEYLVGVGAGTGITAKSSEAGGADM
LIIYNSGRYRMAGRGSLAGLLSYGDANQIVVEMGQEVLPVVEHTPVLAGVCGTDPFRV
MSVYLQQLQAQGFNGVQNFPTVGLIDGNFRQNLEETNMGYELEVEMIREAHQLNLLTT
PYVFDEKQAQDMAEAGADILVAHMGLTTKGSIGAKTALTLDDCVERIEKIIAAGKAVN
PEILVICHGGPIAEPEDAQYVLNKTKDLDGFFGASSIERFAAEKGIREQTEKFKAIK"

CDS 85811..86215

/locus_tag="JFBMFIFI_00071"
/inference="ab initio prediction:Prodigal:002006"
/codon_start=1
/transl_table=11
/product="hypothetical protein"

/translation="MKYFVKIEDVETQGFSWGTLQWLNEPQVTGSKVMVTGVVNLFPG
EGHQRHNHEGCEEVLYVLKGTGVQSLELDTGKVEKEIEAGELIRIPAGVYHSTINNGK
DPLEILAIYHYSGPEIAMKNDPDCQITAPQKN"

CDS 86328..87296

/gene="ccpB_2"

/locus_tag="JFBMFIFI_00072"

/inference="ab initio prediction:Prodigal:002006"

/inference="similar to AA sequence:UniProtKB:P37517"

/codon_start=1

/transl_table=11

/product="Catabolite control protein B"

/translation="MSNINEIAKLAGVSRATVSRVINSKGYVKEETRQKVQKVVDDLD
YTPNQNAIYLKNGETKTIGLVSPDFSDILSHFLSSFATIAKKYGYQVTLLLTDYHQES
EIEAFQKLKQKQIDGIFQIVRSNEWEVIEKYSKYGPIVTWQRVTSPKIESVFMDHYEG
FKLGLDHLYEKGHREIVPFFGIEKSLNTVARYKAWQDFCQEKGVALQAEKSRYNLHTS
TEGRELARWWEAEKEKPTALLFPTDNVAVGFLAEARERGISVPKDVAVVGFDNSSIAE
LFNLTTIDYPMHLQAENAFFKLYNQLKHKNLPLHPLSFKLITRGTT"

CDS 87354..89126

/gene="uvrC_1"

/locus_tag="JFBMFIFI_00073"

/inference="ab initio prediction:Prodigal:002006"

/inference="similar to AA sequence:UniProtKB:Q5KWH6"

/codon_start=1

/transl_table=11

/product="UvrABC system protein C"

/db_xref="COG:COG0322"

/translation="MSKEQLRNKLTLLPELPGCYIMKNKTDDIIYVGKAKNLKNRVTS
YFRGTKDGKTFLLVEEIVDFETIITSTDKEALLLEITLIQKHHPKYNIKLKEGSSYPY
IKITGDRDPRLIITSDVEKDGGHYFGPYPNVYAATETMHFIEKVYPLKRCVGRQRRAC
LYYHMGQCLGPCDHEVPVEVYEAQIKRIKAFLNGDTAKVKAELKEKMLKAAEEQVYER
AAEYRDQLRYIEATVEKQKIISTDYTTRDIFSFYVDKGWISIQVFLIRQATLIKREAA
LFPCIQSSGEEDLATFILQFYNEKNHLLPKEILVPEGVEKELISDILSVAVRVPVRGQ
KKNLLDLATQNSQISLNERFNLIEMDERKTIGAAEELSEALGLPSLKRIEAFDHSNIQ

GTSPVSAMVVFENGRPSKKNYRKYKIKTVVGSNEAATTEEVIRRRYTRLLKEKQELPG

LILMDGGIVQVRAALNVLENELGLTIPVAGMVKDDKHRTSNLLFGEELTVVPIDSKSP

AFYLVQRIQDEVHRFAITFHRQVRSKNSLSSLLDQIPGVGPKTRTKLLKHFGSLKKLK

EADLKDIQALGVSKNVALLIKIRL"

CDS 89272..89670

/locus_tag="JFBMFIFI_00074"

/inference="ab initio prediction:Prodigal:002006"

/codon_start=1

/transl_table=11

/product="hypothetical protein"

/translation="MKKLVVFCIIVPVFLLSGCSENREVEYENEIKELKTELKELKKE

NADLMATLRELALANTETAQKENEESQVNNQEDVPKQETTEEVEPNIDDKKRDLKEEI

IELKKQLGGDLTREQRMEIKKKIKELAEQF"

CDS complement(90085..90816)

/gene="glnQ_1"

/locus_tag="JFBMFIFI_00075"

/inference="ab initio prediction:Prodigal:002006"

/inference="similar to AA sequence:UniProtKB:P27675"

/codon_start=1

/transl_table=11

/product="Glutamine transport ATP-binding protein GlnQ"

/translation="MVKLNVQELKKSYGSLEVLKGLNLEVQEGEVVCLIGPSGSGKST

FLRCMNHLEEINGGTIIIDGYDLTDTSLDINKVRENIGMVFQHFNLFPHLTILENITL

APIELKKLTKEEARQKAFDLLETVGLREKADAYPSSLSGGQKQRVAIARALAMSPDIM

LFDEPTSALDPEMVGDVLAVMQQLAKEGMTMIVVTHEMGFAKEVADRVVFMDGGLIVE

EGKPEEVFNQPKNERTKNFLDKVLI"

CDS complement(90809..92245)

/gene="mltF_1"

/locus_tag="JFBMFIFI_00076"

/EC_number="4.2.2.-"

/inference="ab initio prediction:Prodigal:002006"

/inference="protein motif:HAMAP:MF_02016"

/codon_start=1

/transl_table=11

/product="Membrane-bound lytic murein transglycosylase F"

/translation="MNTRKTYLFLFSLLLTLGVLLNFTLTTDAHAETKEKYIIGTDVT

FAPFEFQDANNNFVGIDMDLLNAIAEDQGFEVEIKPLGFNAAVQALQADQVDAVIAGM

SITDERKQTFDFSDPYFESGVVMAVADTNDTIKSYEDLKGKTVAIKTGTEGATFANSI

KDKYGFKVSTFDDSANMYEAVKAGSADAAFEDYPVMAYAITQNAPLKLVGEKEKGGQY

GFAVNKGQNPELLTKFNNGLTNLKNSGKYKEITEKYLGEEKTEKQSIFALVKENYKQL

LSGLGTTLFVTLVSIAIASVLGILFGLMSVAPSKILRGISMVYVDIMRGIPMMVLAFF

VFFTIPQLLGIKMVATIAAIIVLSLNAGAYIAELVRGGINAVDKGQLEAARSLGLPYS

KAMRKIILPQAIKIMIPSFINQFVITLKDTTILSVIGLVELTQSGKIVVARTYDSNMW

FVIAFIYIVVITALTRISNRIERRVNNG"

CDS 92535..93113

/gene="yvdT"

/locus_tag="JFBMFIFI_00077"

/inference="ab initio prediction:Prodigal:002006"

/inference="similar to AA sequence:UniProtKB:O07001"

/codon_start=1

/transl_table=11

/product="putative HTH-type transcriptional regulator

YvdT"

/translation="MKLDKKNRLISAAIEAFNEKGLEQTKIADIVKIAGVAQGTYYLY

FPSKLAIMPAIAEVVVEKNIAELHQGMDSTLPYSEQLNQLIEISFSITEKYADVIALT

YAGLASSEHLNEWEKIYEPYYLYVTDFLEQGIAKEAIKAKMNPKATARLLIGLIENAA

EQLYLYAETDDTAVEDQKEEVYQFAKRALGII"

CDS 93147..93710

/locus_tag="JFBMFIFI_00078"

/inference="ab initio prediction:Prodigal:002006"

/codon_start=1

/transl_table=11

/product="hypothetical protein"

/translation="MIREARKEDAKAIAPLIMVILEDMELEVFKEYSKETIEELLVEG

IQTETYRYSYRHAHVCMRDGEIAGVVFGYRGEKEPVIDEPLIDILEAKGLNSDSRLFI

EKETFAGEWYLDSLVTAEKFRGRGVGTELLDALGEFAKEDGEEIIGLNCDQSNAGAQK

LYEKMGFKKTGEVTISGHEYNHMQKQV"

CDS complement(93774..94283)

/locus_tag="JFBMFIFI_00079"

/inference="ab initio prediction:Prodigal:002006"

/codon_start=1

/transl_table=11

/product="hypothetical protein"

/translation="MIHIYMKEQYVARKERIIIKDEAGKDIYLITGKWGNVGDGLYLY

KIDGTLVAEVKQTKLSLMPKFDIYLSGKKVCSVSKYPGIHNPYFRVSRIHWIISGDFA

NHHYKILHLTKTVMTMDKVYLASGDFYALSIKREQDLPLCVCLAVILDHWVIPRAPAK

DGPLNLPAY"

CDS 95120..95350

/locus_tag="JFBMFIFI_00080"

/inference="ab initio prediction:Prodigal:002006"

/codon_start=1

/transl_table=11

/product="hypothetical protein"

/translation="MMDHKAKNTFALREIKKIIENHEDKIDFVAEERKTMDEIKRIIE

EVYMWDNKASEYEEKQSNQKFENEMKILFKRN"

CDS 95408..96934

/locus_tag="JFBMFIFI_00081"

/inference="ab initio prediction:Prodigal:002006"

/codon_start=1

/transl_table=11

/product="hypothetical protein"

/translation="MNIEQFLGKRQCIKYSIVKELLTLQEHQISKNQLLSCLSISTTM

LNQIVQEINSDISFLNLENEIKLVEYRKYGQKFYELKLTKSVSLDHFLLHYLTTSPSF

FILVDIFFEKISSISEGASRYYFSESTFRRELNLLNKILKPYGLSINIQNKIYLMGSE

WDLRLFSTSFLSKSFGTKRWPFKFISLSDSQKYIDLIPDNFFDTNPLKNNLSVSYLIA

ISIYRMRKEKNTNFIMESDYLYSEESTCFKEFLIKGSQFFENYSPKTARDRFEIENRI

IFTSLLLNGNFTNVKKLPYFFYRDPAFQKEELFQLGFTFLTQLESCLSSPLTMEEYTL

LTYQFSLIAYRHFFLNQIDSTQMIPIKKIDSCTVDKVEVMTKYLLEREDNNFSEKKQQ

MLEKEYSTILLNTINWLRYKPILKLYLISGQDVEQLKKTILALHLEVTFNLIIVDKVD

KEVDLIVSDAAYIENYLSEYPNVPVFYWESLSSSNNIRLYEKLSILNEKNWDKLKITL

"

CDS 97181..100453

/locus_tag="JFBMFIFI_00082"

/inference="ab initio prediction:Prodigal:002006"

/codon_start=1

/transl_table=11

/product="hypothetical protein"

/translation="MKKRKIVPYILVSSLVLSSLSGSLTVFAEEALTGNEINATIDSR

DSENQEVESFVEAETFATSGTWGTCPWTIDSKGVLTIKSGTLGRGQSGSTPTAPGLNR

QDVTKVIIEENVKTGTSAAELFMNWGKLTEIENLINLDTSNTRDMSRMFSGCQKLTTI

DVSGFNTSKVTNMDSMFSSLTSVNSLDLSNFDMSNVTNTNTMLYTALESLTLGETCQF

PNDTNLASIKTTSGIYTGGWTQENGTKVFPSSSAFIADYNGATDAGTYVWEKVKSAPV

IVKYIDEQGKELAESDTLSGGLGDAYITKAKDIKGYELKETPINATGSYTSKEQTVNY

VYEKSVAKWGTCPWILEDGVLTIEAGILGKGTATNIVADIPKNEVTKVVFEGSIEAQS

DSTNLFSSFPNLQSFENMKNFDTSNVTMMQGMFENCTSLKTIDVSNFNTSKVITMNSM

FSGTGLTNLDMSQLDMGEIQYKGSMFSGSNNLSTLTLSDKNQIDGTSLPAINTDSGKY

TGAWISTDEKKIFPSSSDFMSTYNGKTDAGTYIWEVAKVAPVTVNYIDGKGGTLAEPD

TLIGEMGDVYTTKAKAIKGYELKETPENATGTFSKEAQTVTYVYEAIEGGEVTVNYED

TEGNKLAESDVLTGKFGTSFESNAVEITGYELKETPENATGTFSETAQTVTYVYQAVQ

GGDITVNYVDTAGKVLDTQTVTGKFNESYTTEAKMIEGYTLKTTPKNHTGLFTLEAQT

VTYVYQASVGAPVTVRYINQVGNEIAASTSLTGKVGQTVTATAKTPGGYTLTSASNTQ

EATIGTTEQTITFEYQGNAASKVTTEYVDTEGVRIAPVQTITGGNIGDAYATEAETIQ

GYTLQTTPENASGFRTQGAITVTYVYKKKDSKPFMVNYVGVQQQQVVGFVPEGITTTK

VSLLIKKDGSNEWVDTGYTGTPDADTGYFAIESRFVKGTKDFLLNNKDQIKVVFTDGT

GENYEGVQVVQPYTAPVVNDFTEGDDYLTGSVPNGANQVRVSINGNRLRVVNSGPIIT

DDAGINAVTGEFSIWAKTWCEGSYGSSPMHQTKAGDVITVDYGAQIYGAFNNPSTTIV

VK"

CDS 101188..101415

/locus_tag="JFBMFIFI_00083"

/inference="ab initio prediction:Prodigal:002006"

/codon_start=1

/transl_table=11

/product="hypothetical protein"

/translation="MQIDDYWQDKDLYEVIFKAKRDGGDKQFTKYFIFPDVDKVEKIK

KCIEKKLPGKIEVISINHLFMAFEERKTSNR"

CDS 101495..102175

/gene="ywqC_1"

/locus_tag="JFBMFIFI_00084"

/inference="ab initio prediction:Prodigal:002006"

/inference="similar to AA sequence:UniProtKB:P96715"

/codon_start=1

/transl_table=11

/product="putative capsular polysaccharide biosynthesis

protein YwqC"

/db_xref="COG:COG3944"

/translation="MNEIKKIFQVFKAIRKRFILFLLFVILGVSLVAGYSIFVVNPKY

SSTVQMIVQADEADTPNSVNTINANVLLINTYKDLIKGDVVLDEVQRNLGKEGFTIST

SELKNVLQITQSANSQMFMIGATSDSPQHAAQIVNATAQVFKEKAFDMLSVKKVTISS

YGKENVNPTSPNIRNNIVISLFLAVFISGGICLLLELLGDNFKGKEAIEDELGLSLLG

TVTLIEKK"

CDS 102211..102981

/gene="yveL_1"

/locus_tag="JFBMFIFI_00085"

/EC_number="2.7.10.2"

/inference="ab initio prediction:Prodigal:002006"

/inference="similar to AA sequence:UniProtKB:P71051"

/codon_start=1

/transl_table=11

/product="Putative tyrosine-protein kinase YveL"

/db_xref="COG:COG0489"

/translation="MHQGNGNETSPKVKSKEKDKSRIKRNPEKKKAGNKNYRPPSMIV

TDNRILEQYSLIRSNIKFKLEEYEMKTVSVTSSIMGEGKTTTSLNLATSFARDGYKVL

LIDADLRRPNIGRSLKISEVRGLSTLLTERLKNVGAFIVKVPKIENLWVLPSGLVPNN

PAELLGSKSMKSLLKELKLHFDLLIFDLPPVGIVTDAQILSAQTDATVLVVRDDFVKK

QEVLRAKELLENANANLIGVVLNGVKKSEDGTNYNSYY"

CDS 103006..103767

/gene="ywqE_1"

/locus_tag="JFBMFIFI_00086"

/EC_number="3.1.3.48"

/inference="ab initio prediction:Prodigal:002006"

/inference="similar to AA sequence:UniProtKB:P96717"

/codon_start=1

/transl_table=11

/product="Tyrosine-protein phosphatase YwqE"

/db_xref="COG:COG4464"

/translation="MIDLHCHLLPNVDDGPESLEESIKQGQLAKKNNISHIVCTPHHL

DGNYNNPKEQILDSFFNLKAEFERNAIDVSLILSQEVRLTPDLILALEKDELVFIDSA

SRYIQIEFPSHHIPRFSQQIITDLVLRGHIPIIVHPERNSGFQKNPNALIEFLEIGAL

AEVTVPSYLGYFGKKIQQLAVRYVHHGLVQLMASDTHGYKRQIVLGSGYSLMKKKEGI

EMVQQFKKNARCVIEGEELTELNYSKLKKRNWQFY"

CDS 103779..105587

/gene="pglF_1"

/locus_tag="JFBMFIFI_00087"

/EC_number="4.2.1.135"

/inference="ab initio prediction:Prodigal:002006"

/inference="similar to AA sequence:UniProtKB:Q0P9D4"

/codon_start=1

/transl_table=11

/product="UDP-N-acetyl-alpha-D-glucosamine C6 dehydratase"

/db_xref="COG:COG1086"

/translation="MTRKKKTVLLAIIDGLIIIFGNILTTIILKNEGIGNYFIFSTCL

MISIYFILVHYTNFYKTSYRYMKIKESSIFFIAILTTGFIEWLASLFLTIHYGFYIVI

SNLIVFLFLVGSRLAWKFIFELRHDVRYLNKNKKKNVLIIGAGNAGTLLIQHIQQDTK

TTMNIKGFIDDDSEKQQMRYRGIKVIGTTKMIQEICRQEKIQTIILAIPSLNEKRKKE

LVDSLSNEPIELLQMPSFEKMLLESVEKKQPQKINILDVLGREEVQLDCEILHKNLKG

KTILITGAGGSIGSEIVRQVCHFRPKTILLLGHGENSIYQILHEVKSIDVAPFSTIKF

IPIIVSISDKTMINKVMKMYQPEIVYHAAAHKHVPLMECNIDEAIKNNIYGTLNVAEA

AKRNNVETFVMISTDKAVRPTNVMGSTKRIAEMLVTELNDVGKTNFCAVRFGNVLGSR

GSVIPLFKRQILAGGPVTITDKKMTRYFMTIPEASRLVIQAGALSIGGEIFVLDMGEP
TKIIDLAKRMIELSGYSEMEITIKETGIRSGEKLYEELLVTKEKKPKQVFEKIFVGEI
KGFLREEIDVFLNQLMTTDDQLKKINLIQFANESLN"

CDS 105611..106741

/gene="epsN_1"

/locus_tag="JFBMFIFI_00088"

/EC_number="2.6.1.-"

/inference="ab initio prediction:Prodigal:002006"

/inference="similar to AA sequence:UniProtKB:Q795J3"

/codon_start=1

/transl_table=11

/product="Putative pyridoxal phosphate-dependent
aminotransferase EpsN"

/db_xref="COG:COG0399"

/translation="MTRNILLASPHMSKEGYEEMFVKEAFTSNWIAPVGENINQFEKK
VSERIGSKGAIALSSGTAALHLALKAAGVKAGDIVFCSSLTFAASVNPINYLGAIPVF
IGSEEKTWGMDPKVLKKAFQKYPECKAVIVVHLYGLVADLDPILEICDQHNAIVIEDA
AEALGAKYKEKAVGSFGDYGIFSFNGNKIITTSSGGMIVSEDSEKLEKVRFWSTQSRE
VERHYEHKEIGYNYRMSNVLAGIGRGQLMVLDERIQQKRAIYQFYQKALADIEGIQFM
PEINGIYATYWLSCVTFTDSKLPSKIIETLGNCGIEARPIWKPMHSQPIFNQYDSLGI
EFDDWIFKHGVCLPSDTKMEELDLLLISKLIHEICRKESEGK"

CDS 106744..107895

/locus_tag="JFBMFIFI_00089"

/inference="ab initio prediction:Prodigal:002006"

/codon_start=1

/transl_table=11

/product="hypothetical protein"

/translation="MWLFLLSLVLIVYTMVGYPLLLKFLVKHVKKKLIYRNTRFFPTV
TCIITAHNEEAVILKKLQNLESCVYPDELLDIIIASDFSEDKTNSIVQKYIDEYKGKR
RIYLHQTKEHKGKTNAQNEAVALAKGEILVFSDANALLDSRSICRLAQAFSDDSIGYV
TGKLVYTNAVESTTSNSECNYWDYDLQMRKWESDIGSITAGNGALYAIRRSIYTNIPL
TYSHDSYFPPYVVVQGKRAIFDEQAIAYEKAGETNEDEFKRKIRMSRNIFSLNYILPQ
KYNVVRNGWFTIFYLSHRTFRNNLYLFHIILLLTNIYLVNQSPLFSFILIGQCLVYGC

ALLGIVWKNPILHMVYYYVLTLCAQLVGVIKEITGQSKPTWGKAESTRK"

CDS 107921..109207

/locus_tag="JFBMFIFI_00090"

/inference="ab initio prediction:Prodigal:002006"

/codon_start=1

/transl_table=11

/product="hypothetical protein"

/translation="MIKGTIVILMIVSIGLIVNKYFQNKNWLTVENIAIVSFLILFDL

PIFLQTPNWAMDEQFLILILIGNVGFIGAFLFMQPTKEMAFIVKTEKATFSLKKDGIR

MLTLLVFCYLAYELYQLQAYNFSIVRSTLLNDRVGEYFEDAGTQNLGFIKQGFTTLNL

LLISYYWKHKNHLGTLLWICYFIITLLSAHTRFVVVVAAVIPLVYYNYQIKPIKLSTL

IGLGIIFLLFITISNYARGGVLGKGDSRFSIDEIMTGHNMKDQLFRASAGSTEHFHTL

FTSDVPNDWLRQYYYFIPLSFVPRVLWSNKPVVSYFWRVTQSITGAYPNGKMNPVLTT

TYLGEAYHQIGWIGVLIATFIYFKLLFFCLNTFKKFEYSELLIYSSILWIPMSLRGGF

SSLILTQVPTFLILYGLKVIHVYEVGQIKYKKEGQL"

CDS 109204..110289

/locus_tag="JFBMFIFI_00091"

/inference="ab initio prediction:Prodigal:002006"

/codon_start=1

/transl_table=11

/product="hypothetical protein"

/translation="MKILALSDSYPVHPRLKKVSRYFGKTETTTFFLWNRGDDEILGD

RSETVFNLPSSLGNKWKKALNLIRFRKAVKVHLSEFTYDLVICRHWQQLVLLSTIRNK

QKYKIVYDVCDMPNNKWVRHLESRLINKTSIVVLASRFFILDYTSKIDELVVLENRSW

SHFSKKKFVPTKVETPINVTIQLTFLGRIRYGEILARLIECVQKDHEISLNFYGTGPD

ESKLILLVNQLEIKNVYFHGEYIEEKIPNIYEKSDYIWAAYDNQLLNVKKAISNKFFE

TLTYQTPGIFSEDTELGELITTQEIGFIVNPYSTESIVECFEKMKDKSMSRKIKNNQK

KWNEPLYFDDYLVESPYLKIDEMKVEK"

CDS 110286..111455

/gene="ugd"

/locus_tag="JFBMFIFI_00092"

/EC_number="1.1.1.22"

/inference="ab initio prediction:Prodigal:002006"

/inference="similar to AA sequence:UniProtKB:P76373"
/codon_start=1
/transl_table=11
/product="UDP-glucose 6-dehydrogenase"
/db_xref="COG:COG1004"
/translation="MKISVVGLGYVGLSNALLLARNNEVTAYDISAEKIKQLQNGKTP
ILEKEIIDFMNNLKLTIEYTCDEKYLSDLGELVILAVPTNYDEDTRNFDTSILEEMIQ
KIMKVNFKGMVIIKSTVPIGFTKKMRLLYKTNQIIFCPEFLREGKSLFDNLHPSRIII
GDESNSGKLIGQLFLDAATEKNIPLLYMSATEAESVKLFSNTYLAMRIAYFNELDTYC
AIEELNTKKIIEGMSHDERIGSKYNNPSFGYGGYCLPKDSKQLVHTLKGVPNQLISSI
PTSNEVRKNFIVSEVLKEKPKVIGIYKLSMKKNSDNFRQASILDIITELKQKKMKVII
YEPGLDDVEYAGVEIRKNLASFKDECNLILANRMDNELKSVEENVYTRDIFGKDL"
CDS 111458..112669
/gene="wecC_1"
/locus_tag="JFBMFIFI_00093"
/EC_number="1.1.1.336"
/inference="ab initio prediction:Prodigal:002006"
/inference="similar to AA sequence:UniProtKB:P27829"
/codon_start=1
/transl_table=11
/product="UDP-N-acetyl-D-mannosamine dehydrogenase"
/db_xref="COG:COG0677"
/translation="MINVIGLGYIGLPTALILAANGQEIVGTDINKDTIAQLKNKEIP
FEEEGLSELFQDALENNIHFQVNYAQTDCYIITVPTPYLEESRKVDSSYLIAAVRSVL
ENCLPETTIIIESTISPGTIDRVVTPLIEQEGLSLDRDIYLVHAPERILPGQMISELK
NNSRTIGSDNPESAIEVKKIYQSFCKGEIICTTIKIAEMTKVVENTYRAVNIAFSNEL
SLLCKEGQMNVYDIIEIANMHPRVNILSPGPGVGGHCIPVDPWFLVGDYPEQTELICN
ALKINENRPKYVFEKATEIAREHGLSQTEIGIYGMTYKKNVEDIRESPTIQLLDYLQE
KRNDPFIVYDPLVKTKILDSQIMNFEEFLKKAKLVIVMVDHQHLLDHKVFLKEKLILD
TRNCLAQSYIL"
CDS 112700..114217
/locus_tag="JFBMFIFI_00094"
/inference="ab initio prediction:Prodigal:002006"

/codon_start=1

/transl_table=11

/product="hypothetical protein"

/translation="MARNENRDRLTEATQVGMKKQMISGMIWTIADIFGNQVAKLIVQ

IILARLLLPKDFGVISIISVFIALSQSLMDSGFQNALIREEKTCEEDLATVFYFNFFM

SIVLYGALFFAAPSLSLFFHEPKLELILRVVGIILIINSFGLIQRTILSKQLKFKTQM

IINMLSSIISGLIAIFCAFRGFGIWSLVIQTIVMQFLQASLFTMKNRWWPKTGFYWKS

FKRLYKFGWKIMISGLLNTAYQNIYFVLIGRLYSASDLGYYTNAQKLRDAADFTITGA

VRKVSYPALSKVKDQQEVLKRGYRKILRYTVFFTLPISLGLAVIAPQLFPVLFGTKWD

YSIPYFQILCLAGIFFPLHAINLNILQVKGRSDLVLKLELINKGFAVALIVLSLIFHG

GMMGLVWMSVLSSFVSYLINSYYSGFFLSYSTKNQIKDSGGSFISAILMAVMLLIFSN

FIKVSGLGGVLLLSCSGFFSYLFCHILFQTAESKEIMNYVKQFVKHEVKVNEDSV"

CDS 114201..114839

/gene="epsL"

/locus_tag="JFBMFIFI_00095"

/EC_number="2.-.-.-"

/inference="ab initio prediction:Prodigal:002006"

/inference="similar to AA sequence:UniProtKB:P71062"

/codon_start=1

/transl_table=11

/product="putative sugar transferase EpsL"

/db_xref="COG:COG2148"

/translation="MKIVYKKGMYQGRIKRTLDVILSLSALVILFPFLLVVSLFIRVK

IGSPLFFCQERPGKNGEVFTMYKFRSMTNERDAKGNFLPDSERLHFFGKCLRASSIDE

LPELVNILKGDMSIVGPRPLLVQYLPLYTDEQKIRHFVRPGLTGLAQVNGRNTLSWEE

KFLLDAQYVGNISFSQDMRIILLTMKKVFQKDTIHSDNHCTMEEFRGSKAGD"

CDS 114842..115333

/gene="epsM_1"

/locus_tag="JFBMFIFI_00096"

/EC_number="2.3.1.-"

/inference="ab initio prediction:Prodigal:002006"

/inference="similar to AA sequence:UniProtKB:P71063"

/codon_start=1

/transl_table=11

/product="Putative acetyltransferase EpsM"

/db_xref="COG:COG0110"

/translation="MKQLVILGGGGHGQVCAEIAYLMNKWQTIVFLDDLEIPNTKMYK

WDGQFHEVQKYDSEKTEFFVAVGNSSIRKKVVEDLLVKLKKFATLIHPSAIISANSSI

EHGCVLMAGVIVNPNIKIGTFCIFNTSYTVAHDCILSSYVHLSPGVHLAGSVVIIEKR

GLE"

CDS 115526..115786

/locus_tag="JFBMFIFI_00097"

/inference="ab initio prediction:Prodigal:002006"

/codon_start=1

/transl_table=11

/product="hypothetical protein"

/translation="MKEKIYNQIILKLEEHKKILLKELNEDLLVFSHQVEKHLKTNKG

YDKRKCNCYFCSKILDCQKNINLTINEVKDIEQRIANIDSEK"

CDS complement(115892..116716)

/gene="pdxK"

/locus_tag="JFBMFIFI_00098"

/EC_number="2.7.1.35"

/inference="ab initio prediction:Prodigal:002006"

/inference="similar to AA sequence:UniProtKB:P39610"

/codon_start=1

/transl_table=11

/product="Pyridoxine kinase"

/db_xref="COG:COG0351"

/translation="MTLIPKTLTIAGSDSSGGAGIQADLKTFGEYSTYGLAALTTIVT

MDPDNGWHHQVHPIDVAIVEEQLKTILAGGEIAALKTGMLGSVSIIDLVAKTIKEQQL

KNIVIDPVMVCKGEDEVLNPDSAEALRDELIPLATVATPNLFEAGVLSGLGKLTTLED

MKAAAKKIHLLGAKNVVIKGGKALAGDKAIDLFYDGSEFLVLENQKITPAYNHGAGCT

FAAAITAGLANGLTVRDSVLKAKEFVSEAIRSGFAFNEYVGPVFHGGYRLSKQEKI"

CDS complement(117062..117931)

/gene="ywpJ"

/locus_tag="JFBMFIFI_00099"

/EC_number="3.1.3.-"

/inference="ab initio prediction:Prodigal:002006"

/inference="similar to AA sequence:UniProtKB:P94592"

/codon_start=1

/transl_table=11

/product="Phosphatase YwpJ"

/db_xref="COG:COG0561"

/translation="MIELIASDMDGTLLNEKMVISKANAQAIAEAERRGIKFMVSTGR

GYTEAHPLLTEVGINCPMITLNGAQVYDGQGKIIDNIGIEKDTVRTILQLLKEHNIYA

EVVTSKGIYSDNKVKRIESVASLLVNLNPETTYKMGVVLAAARLELMNINYIDDYKEL

VNDHSIEVLKLIAFSDDGQHTLAPIKEKLETLGSLAITSSFINNIEINHKDAQKGIAL

ARTAKRLGIPMENVMAIGDNFNDVSMLQVAGVSFAMENAEEEVKSHAKYLTSSNNENG

VAEAIMRCINEDL"

CDS 118142..118825

/gene="lldR"

/locus_tag="JFBMFIFI_00100"

/inference="ab initio prediction:Prodigal:002006"

/inference="similar to AA sequence:UniProtKB:P0ACL7"

/codon_start=1

/transl_table=11

/product="Putative L-lactate dehydrogenase operon

regulatory protein"

/db_xref="COG:COG2186"

/translation="MLESSQQSGQSLAQQVANKIESLIKEQRYKTGDKLPNEFELATE

LTVGRGTIREAIKLLAARNVVVIQRGKGTFVSKTPGITEDPLGLSFINDKKRLSSDLM

DVRVMIEPEIAKLAAENATSEEVEQMEKICDEIATLIHANENHEMKDIELHSAIAKSS

KNVVVPSLIPIIQTAISLFINLTNRSLKEETIETHRQIVEAIKNKDGESAKKAMQRHL

GYNKEELNL"

CDS complement(118910..120220)

/locus_tag="JFBMFIFI_00101"

/inference="ab initio prediction:Prodigal:002006"

/codon_start=1

/transl_table=11

/product="hypothetical protein"

/translation="MDLKINHKQRTIFLWCTLIFIVFLYQLRFNFIEGSDDGWFALIK

EKYSLADYLSLRYNTWSSRIFPETMLYYIFHIPINLWRMLNTAFIFLLATSIVRIFKG

EIKIYNVVVVILLFGYSSFDVINSGFFWMTGSVNYLWPLALGAYVLIPFADKFFRKKT

TEFSLKQLVRVIITCIFAISNEQLVLCAFGIMFVYHIYLFTIKEKPSIYLIILTLIVF

AGILLMTFAPGNTVRFESEIVRWFPEFNELSFLGHMKTGIFWLFEQIITHFKLFILLI

SLLTINLLQSEKLKKIFYIMGTIILSLTIVKPTLLTDLNRINFVPFDSFENLLTRNFI

IGVFPYIFWSIVLIYIIFCSCLVTRNPIFTFFCYLAGLLSCVLMFFSPTIYASGPRVL

NALVLFLVLISFMLFQKNMEKKNSNPNFPLFLLSIFPLFSFLLN"

CDS 120588..121481

/locus_tag="JFBMFIFI_00102"

/EC_number="1.1.1.343"

/inference="ab initio prediction:Prodigal:002006"

/inference="similar to AA sequence:UniProtKB:G5EBD7"

/codon_start=1

/transl_table=11

/product="6-phosphogluconate dehydrogenase,

NAD(+)-dependent, decarboxylating"

/db_xref="COG:COG1023"

/translation="MKIGIIGLGKMGLNLAENCVDAGHKVVGFDLKPPVLNQKQKNIA

IVTDLVEMIKQLPKPRIIWSMVPSGKPTEAIFQEMNELLDTNDIFIDGGNSNYKESVV

RGKIMEAKKIHFFDVGTSGGIEGAREGGCFMIGGNQDVFKQIEPLFVDIAVENGYYYA

GAVGSGHYLKMVHNGIEYGMMQAIGEGFDLLEHAPYEFDYEKVAKVWNNGSVIRSWLM

ELTESAFRKDQKLEEIQGIMYSSGEGKWTVEESLEQQVATPIITMSLMMRYRSLYPDT

FSGKVVAALRNEFGGHDVEKK"

CDS 121493..123031

/gene="xylB"

/locus_tag="JFBMFIFI_00103"

/EC_number="2.7.1.17"

/inference="ab initio prediction:Prodigal:002006"

/inference="similar to AA sequence:UniProtKB:P35850"

/codon_start=1

/transl_table=11

/product="Xylulose kinase"
/db_xref="COG:COG1070"
/translation="MKDYLIGVDIGTTSTKAVLYDAKGNVKGYSNKEYPLYQELPDMA
EQDPDEIFETVIDVLTAVVRKSGADVTRIAGVSFSSAMHSLILLDQEKQLLTRCITWA
DNRAYHQAEELKNHKDGLAIYHRTGTPIHPMSPLSKILWLKKEQPELIAKTAHFIGIK
EYVFYKLFGQFKVDISIASATGLFNIHQLDWDQEVLALLDISVDQLSQLVETDYQVTG
LNESYAEVIGLPVETPFIVGASDGCLSNLGVNAIDGKTLALTIGTSGAVRMVTDKPVT
DPKGRTFCYALTKDKWVIGGPVNNGGIVFRWVRDQLFAPEKITAEQMQVDSYEILTQI
AEKIPAGSDGLLFHPYLGGERAPLWDANAKGAFLGLTTRHTRAHMVRASLEGIVFNLY
SVMLILEELVERPERIHATGGFARSALWRQLLADIFEQEVSIPESYESSCLGAAVIGM
ESLGLIDSIEEVSKMIGVTNQHTPNEAHFPVYRELLPIFIRTTRLLQNEFQAIAEFQR
KYSK"
CDS 123340..123915
/gene="gntT"
/locus_tag="JFBMFIFI_00104"
/inference="ab initio prediction:Prodigal:002006"
/inference="similar to AA sequence:UniProtKB:P39835"
/codon_start=1
/transl_table=11
/product="High-affinity gluconate transporter"
/db_xref="COG:COG2610"
/translation="MELLIVGIGVLILLFLIMKVKLNTFVSLIIVSILVAIGLGMPLT
QIITTVEEGVGSQLGHLALVFGFGAILGKLVSDAGGAYRISTTLINKFGRKKIQIAVL
IASFIIGIALFFEVGLVLLIPIIFTIASELGISILYLGIPMAAALSVTHGFLPPHPAP
TAIAGIYGANIGMVLSLLFQRVFWQVLCILK"
CDS 123900..124676
/gene="idnT"
/locus_tag="JFBMFIFI_00105"
/inference="ab initio prediction:Prodigal:002006"
/inference="similar to AA sequence:UniProtKB:P39344"
/codon_start=1
/transl_table=11
/product="Gnt-II system L-idonate transporter"

/db_xref="COG:COG2610"

/translation="MYTKVAQKIVPDAFNRVGNIKALGEQKKFTLEDTPGFGISTLTA

LFPVILMALATCYELLSGGKITDKSPLYEQVIALIGTPGMAMLLSLLFAIYTMGVRRQ

QTIPAIMKSAEEAIKQIAMMLLIIGGGGAFKQVLLDGGVGDYVAQLFAGSTLSPLILG

WVIAVVLRVCLGSATVAALTAAGLAAPLMAATGTDPALMVIATGAGSLFASHVNDAGF

WMFKEYFNLSIKETFATWTVLESIISVAGLLGALALNLIV"

CDS 124859..125548

/gene="ung"

/locus_tag="JFBMFIFI_00106"

/EC_number="3.2.2.27"

/inference="ab initio prediction:Prodigal:002006"

/inference="similar to AA sequence:UniProtKB:P39615"

/codon_start=1

/transl_table=11

/product="Uracil-DNA glycosylase"

/db_xref="COG:COG0692"

/translation="MIIMKTIIHNDWQDILQEEFESTYYQELREFLKTEYAEQTIYPS

MNHIWEAFEWTPYEQVKVVILGQDPYHGPNQAHGLSFSVQPNVKVPPSLVNIYKELAD

DLGITPVNHGYLKSWAEQGVLLMNTVLTVRGGSANSHRGQGWERLTDAVIKKLNEHEK

PLVFILWGNPSISKKQLIDTEKHVVITSPHPSPLSSYRGFFGSKPFSKANQALIDFGE

TPIDWRLPKQV"

CDS 125727..126707

/gene="pta"

/locus_tag="JFBMFIFI_00107"

/EC_number="2.3.1.8"

/inference="ab initio prediction:Prodigal:002006"

/inference="similar to AA sequence:UniProtKB:P39646"

/codon_start=1

/transl_table=11

/product="Phosphate acetyltransferase"

/db_xref="COG:COG0280"

/translation="MELFDSLKFKIVRKKIRIVFPEGTEPRILGAAVRLASEELVTPV

LIGSEVAVKEAAKKRGFVVDNIEIIDPNNYPAMDEMVAAFVERRKGKATEEQAREMLK

DENYFGTMLTYMGLTDGLVSGAVHSTGDTVRPALQIIKTKPGVSRTSGAFIMLRGRDN
EKYLFSDCAINVNPNAQELAEIAVESAKTAALFDIDPKVAMLSFSTKGSANAPEAKKV
EEATKIAQEMAPEIQIDGELQFDAAYVASVGQQKAPDSEVAGHATVFVFPELQSGNIG
YKIAQRFGNFEAIGPILQGLNKPVSDLSRGCNEEDVYKLSIITAAQSLMN"

CDS complement(126791..128086)
/locus_tag="JFBMFIFI_00108"
/inference="ab initio prediction:Prodigal:002006"
/inference="similar to AA sequence:ISfinder:ISSau8"
/codon_start=1
/transl_table=11
/product="ISL3 family transposase ISSau8"
/translation="MSLTHCIRTLLDLKDKNISFPQNYCEERVIKGRRTKLIYATLTY
NPVCCTRCGCLNTQFSIVKNGFLTSNIKWISTTHFPTYLVLKKQRFLCRECHCSFLAE
STEIAKHCFISNRVKQSIAFELEDNSSRKDIAKRHFISDTTVLRVLVARGKSCLNRFD
SLPAHLCFDEFKSVKRVEGKMSFIYCDAITHSLIDILPDRKLVTLRAHFLRYSLKKRS
KVQSIVVDMNAGYFTLAKELFPNANVIIDRFHLVQLLTVALNKTRIQLMNQFKRWKPE
EMKDYRKLKRYWKLLLQDSQKVNYTDYQYHRLFRKVMPHSEIVDYLLSLNSTLKATYT
CYQDLLYAMSQNNSDLLKSVLSSPHEQVSDYMSTALQTLRKHEERIRNTFQSKRSNGP
LEGSINKIKVIKRTAYGYTSFLNYKYRILISFQEKEKTI"

CDS 128349..128780
/locus_tag="JFBMFIFI_00109"
/inference="ab initio prediction:Prodigal:002006"
/codon_start=1
/transl_table=11
/product="hypothetical protein"
/translation="MKNNINYYIQAVNTAITDTEDNGELLNEHFEVVRTALDNDKISE
ISIEQLTTIKEKFAAGTEKYRGINTTLNGLKPPIKVLGVHKQLLKAYQDFVEGCQAMT
DSIDATKQSVDVEAFERSEKLQDESSDAMMKNIQRITKILF"

CDS complement(128828..129208)
/locus_tag="JFBMFIFI_00110"
/inference="ab initio prediction:Prodigal:002006"
/codon_start=1
/transl_table=11

/product="hypothetical protein"

/translation="MIESVGQVMLYVNDQKAAAEFWTQKVGFTKSTEVDNGDGTFSIE

IAPKESSDTSFVLHDRKLVEKLHPELNLGTPSILFSASSKLETLYAEFQEKGITVGDL

IEMPQMKVFNFADSEGNYFAVREV"

CDS 129537..129953

/locus_tag="JFBMFIFI_00111"

/inference="ab initio prediction:Prodigal:002006"

/codon_start=1

/transl_table=11

/product="hypothetical protein"

/translation="MQNSQAPETCEAWAFGDSKEMADELSGLVLEGLKTATSSAFEVY

QVENEPIPTEGSYSIILDGNQEAVGIIQIQKVTTCAFNQVTEELAIAEGEGDRTLEYW

RRVHEEFFKRHLATYGLSFNEEMLIVCEQFELVYAA"

CDS 129986..130657

/gene="rpiA"

/locus_tag="JFBMFIFI_00112"

/EC_number="5.3.1.6"

/inference="ab initio prediction:Prodigal:002006"

/inference="similar to AA sequence:UniProtKB:Q8DTT9"

/codon_start=1

/transl_table=11

/product="Ribose-5-phosphate isomerase A"

/db_xref="COG:COG0120"

/translation="MNLKQMVGEKAAEYVKEGMVVGLGTGSTAYYMVEAVGKMVKEGL

TITGVTTSNRTAEQAKALGIPLKSVDEVDHIDITIDGSDEISSDFQGIKGGGGALLFE

KIVATYSDKVIWIVDESKMVEELGAFPLPVEVMEYGSLQLFRLFEEKGYKPAFRLNEE

GQKYETDGGHLLIDLHLNEIKKPFELAEFLDGLTGVVEHGLFLDCVNTIIVGYTDGPK

IIEAR"

CDS 130798..131310

/locus_tag="JFBMFIFI_00113"

/inference="ab initio prediction:Prodigal:002006"

/codon_start=1

/transl_table=11

/product="hypothetical protein"

/translation="MNGKLTKQANGEINVSFELNLEASLEDVWNLLTTNAGLAQWFHE

LEVGELGEEGYFNFIMTPTERIKMPILKYVKKTKIGFEWDQDSVLFQLEPLDNDNVKL

IFSEELKNITEHTPRDIAGWNLCLRRIQLVLIKKIATTVNQDEFDELVGKYKSELVKL

EGNKDSNREE"

CDS 131399..133075

/locus_tag="JFBMFIFI_00114"

/inference="ab initio prediction:Prodigal:002006"

/codon_start=1

/transl_table=11

/product="hypothetical protein"

/translation="MNSIELTFKLFKEQKILDAKQEVTYLKNSFLHDVVNQVARELVG

DQSDNHELSDVHYPDWLNYPSDLTKQVLVEFNFDGDESDSEMIAKVADSYAQNFMENN

PSLVVYQIFTLSDKHKVQLFLNFFVQFGYLNKRFFAISLAEAIDKQKTSRNLSIIEWK

KKELSEIDSLLHTEGTTQVIQIEEDEEQTRQLPTMQKVALSQLQLKLDDLLTETVDST

VEKQYDSMEKSGTSSHYNAKSNSGRNLTQTVNANDNELFLADKELITTLNLQILRLEE

LNSKLERKLIKAESEKSKTGKIVDNKENDEIGLQDEFHQKLLTLQTENNQLIKKIAQL

ENERSDYKQEIKAKIEELMLLNKNYENQITELVQTIKIKEGLLADLKEKNKKFKQKKK

IEGPITLVKPSHLQSDEEKMEMSSSISEQNDLLNDFPEPDFEDMTKKKISSKEYKELI

KTVGYIEHIWSIYQTKLKNMPVENMKEQIELEVLFEHIDEAAVETFIFSHELSEIKDQ

VMIKNSVLLGRSTVVINNYDLQSLRYIEKQYYLMNEAIKELSKVEVKENN"

CDS complement(133187..133309)

/locus_tag="JFBMFIFI_00115"

/inference="ab initio prediction:Prodigal:002006"

/codon_start=1

/transl_table=11

/product="hypothetical protein"

/translation="MAEKIDLSSARRRLKSPNIKTKKRALKVILDDKKRKKGIK"

CDS complement(133336..134097)

/locus_tag="JFBMFIFI_00116"

/inference="ab initio prediction:Prodigal:002006"

/codon_start=1

/transl_table=11

/product="hypothetical protein"

/translation="MINVLVLLAILLFVLMKLIGGEKGTRSFIALLFNFAILFCTILL

LTLNQSPLLITFGACILISCINLFYINGWSVKTISAFIATLLTLLFSLIFVFLASYHT

HTQGFGMEEIEELGAFSLYVDINFIQIGICAIIMGMIGAITDTAMAIASAMNEVFLHN

SDISRTKLIKSGMNIGKDILGTTTNTLFFAFLGSYLALIIWFTDLSYSFGRIINSKVF

ASEAISIFCIGISAVLIIPITAIIMAYYLTKKKKP"

CDS complement(134094..135197)

/locus_tag="JFBMFIFI_00117"

/inference="ab initio prediction:Prodigal:002006"

/codon_start=1

/transl_table=11

/product="hypothetical protein"

/translation="MKASMNFKKITVLLVLICSAALLLFVHTNYELYTTPIGKITAIK

ENNNSVSDSNSGNEKQLFSQDLTVTIMNGTYKNKQLLIENTYSLSGANDYHFAVGDEV

FLQPITNDSKTGIIKEVKRDKFVALLACVFILVLVAVGGKKGLLSAVSLIVNILILLV

AMYCYTQLDSSSLVPICALLVLLFTPISLLLVSGNHPKTYTAIIATLLGTFSAFLIAA

FVLHITNEKGLRYEEMAFITRSPQRIFLASILIGSLGAVMDIAITMASSMYELYEKDP

DISSDKLIKSGLEIGKDIMGTMINILFFAYVSGAIPMILLYLKNGAAWGYTISMTLSL

EIARALAGSIGIVLTIPIGLYTSLFFIERGRKK"

CDS complement(135258..136562)

/locus_tag="JFBMFIFI_00118"

/inference="ab initio prediction:Prodigal:002006"

/codon_start=1

/transl_table=11

/product="hypothetical protein"

/translation="MKISRKLKLLLPLLVVILVVVGCTQPGKTTAPQEKTKLQVVTTF

FPMYDFTRNVTKEHADVTMLMKAGVEPHDYEPSAKDIAKIADADVFIYNSEYMETWVP

SVLKNIDSKKTTVIDASKNIPLLAGSDEHSEEDSEHHHDTDEHEEEPHFHLHGLKDHY

HTGDTIELEGHAPDNLTFDHYQWFKKEVTETEFTEIKNETKASLTLKASEELANTEIR

AKLIDASGKVVAETDSNKIILTDHHDADEGSDTDEHDHEGHSHAFDPHIWLDPVIAQQ

QVQTIKDGLVKADDVNKNSYEKNAASYIEKLKKLDKDFENELKDAKNRTFVTQHTAFA

YLANRYNLEQVAISGLSPDLEPSPAKLAELSDFVEENNISVIYFENSASPKIAKTLAS

GTGAVLEVLSPIEGVSQSDQDKGIDYIKVMEANLKALKKTIK"

CDS complement(136594..136743)

/gene="rpmG2"

/locus_tag="JFBMFIFI_00119"

/inference="ab initio prediction:Prodigal:002006"

/inference="similar to AA sequence:UniProtKB:P66231"

/codon_start=1

/transl_table=11

/product="50S ribosomal protein L33 2"

/translation="MRVQIILECVETGERNYHTTKNKRTNPERIELMKYSPKLRKKAL

YRETK"

CDS 136978..137247

/gene="rpsN2"

/locus_tag="JFBMFIFI_00120"

/inference="ab initio prediction:Prodigal:002006"

/inference="similar to AA sequence:UniProtKB:O31587"

/codon_start=1

/transl_table=11

/product="Alternate 30S ribosomal protein S14"

/db_xref="COG:COG0199"

/translation="MAKKSKIAKARRQQATVLKYQDLREELKASGDIEALRRLPRDSS

PTRLRNRDLIDGRPRAYMRKFGMSRIHFRELAHKGQIPGVKKASW"

CDS 137372..137770

/locus_tag="JFBMFIFI_00121"

/inference="ab initio prediction:Prodigal:002006"

/codon_start=1

/transl_table=11

/product="hypothetical protein"

/translation="MKKTKKGPLVIAIFFLLALYSGIFLSYRKGNLNQAIADSTWVYQ

TSEGIHGEIKFLKNQRAKIVTSEGTNYSGTYQVNNYSRGIIIKTKKGLLELSIMDEYK

ENKGFVGAIKMVNEEEYTSVDFYKENSQNK"

CDS complement(138160..138819)

/gene="dedA"

/locus_tag="JFBMFIFI_00122"

/inference="ab initio prediction:Prodigal:002006"

/inference="similar to AA sequence:UniProtKB:P0ABP6"

/codon_start=1

/transl_table=11

/product="Protein DedA"

/db_xref="COG:COG0586"

/translation="MEFLTRWIEFFLTIDEHLNTFVNHYGFWTYLVLFLIVFIETGFI

IFPFLPGNSLLFAAGAIAALPNELNLLWLILTFSAANFIGASINYSIGKKLGLDFLSN

SFFRHILKPQQVQDTIAFFYKYGGKTILFARLMPFVWTITPFIAGASKMSLKIFAFNN

FIGSLIWVSLCCLSGFFLGRVPFIAEHFSIVLLFVIFLSLIPILVSFFKKKNTYCTKN

T"

CDS 139165..139629

/locus_tag="JFBMFIFI_00123"

/inference="ab initio prediction:Prodigal:002006"

/codon_start=1

/transl_table=11

/product="hypothetical protein"

/translation="MGDGKISDNIITIMSKNVFRFSEYWYSNYIVKSLTKKAEDRTIY

IEKYRGPIVSTITNGIKAIDSKRIDADSYLKMGRDHFYLGLSLVEMEKNQVLMEQAIF

DFLQIEKNQNNAAITDKEINEYLLEFRQLNNTVTPLIIEGYLEAAKESKSER"

CDS 139864..140598

/gene="strA"

/locus_tag="JFBMFIFI_00124"

/EC_number="3.4.22.-"

/inference="ab initio prediction:Prodigal:002006"

/inference="similar to AA sequence:UniProtKB:Q8E5N2"

/codon_start=1

/transl_table=11

/product="Sortase A"

/db_xref="COG:COG3764"

/translation="MTTKKQKNKTKKSNIKNRIINTVAVLLLIIGLFLIFLNPIKDWL

VKNMSNNNNVSTVTAAQIKKNHTRDGEFDFSQVVDLDAENIIRARLYQDDMAVIGGIA

VPDVAINLPIIKGISNYNLAVGAGTMKPDQVMGEGNYALAGHNMIDKGLLFSPLDKLA

MGDEIYLTDLEYVYVYKTSFIETVNPDRVDLIDDVEGQTLVTLVTCNPDGSKRLVVQG

TFEKKVPMKKATKAMKAAFDIEKNTY"

CDS complement(140689..141459)

/locus_tag="JFBMFIFI_00125"

/inference="ab initio prediction:Prodigal:002006"

/codon_start=1

/transl_table=11

/product="hypothetical protein"

/translation="MYFIKHNLFTVPAISIPSQPFYKNYRIQDLPVSYLNLLDEQNGG

YLAFNLVPTTEPTRDGLDSVGIHYLFGLHEDFRTSILFQEHYHKTFSLPEYLIFFSVN

DDQLWAFDYSNITNGEPSIRYIDMDTDQWLTIADNFATFLDLLQVAPPDINEEKILSR

LEANHSFLLGSDDDISRLLTRFETEPDKVWYFNWLVELLTHKNQDVQQAVFSAFETQV

LYFNPVLPPTSLTIANSFKKLDPNLIDQERLAILLKEL"

CDS 141736..142209

/gene="dps"

/locus_tag="JFBMFIFI_00126"

/EC_number="1.16.-.-"

/inference="ab initio prediction:Prodigal:002006"

/inference="similar to AA sequence:UniProtKB:P80725"

/codon_start=1

/transl_table=11

/product="DNA protection during starvation protein"

/db_xref="COG:COG0783"

/translation="MQKTNIDVKGILNKLVANHGVLYTKLHQHHWYVQGASFYTLHEK

FEELFGSVDSNNDVIAERLIAIGGQPFSTLSEYLEHASIEEKPYTKKVSAEDMVASVV

HDYRILRDELVEGIELTGEAGDDCTQDMLIGYKTEIDKNIWMLQAFLGQSPLEGE"

CDS 142416..142970

/gene="lemA_1"

/locus_tag="JFBMFIFI_00127"

/inference="ab initio prediction:Prodigal:002006"

/inference="similar to AA sequence:UniProtKB:A8AVK0"

/codon_start=1

/transl_table=11

/product="Protein LemA"

/db_xref="COG:COG1704"

/translation="MGWIIGIVVIVLLVLFYASIYNGLVKARVWVQEAWSQIDVQLKR

RNDLIPNLVETVKGYAAHEKDTLTKVVEMRNQLTQVPAGNHEEAMQVSNQISDSLKTI

FALSESYPDLKANQNFQQLQEELTATENKIAYSRQLYNSSVATYNIKIQAFPSNIVAG

IHKFTEEKMLETPTEEKAVPKVSF"

CDS 143192..144094

/gene="htpX"

/locus_tag="JFBMFIFI_00128"

/EC_number="3.4.24.-"

/inference="ab initio prediction:Prodigal:002006"

/inference="similar to AA sequence:UniProtKB:O30795"

/codon_start=1

/transl_table=11

/product="Protease HtpX"

/db_xref="COG:COG0501"

/translation="MLRQQIEQNKRKTIVVMFAFFVLVALIGAAVGYANWNNAVSGVV

LAAVLALIYMAMMIFNSTKVVMGLNRGREITSKEQYPMLWNVVEELSIVARIPMPKIY

IIDDPSPNAFAAGNSPENASVACTTGLLDKLNREELEGVMAHEVSHIRNYDIRLSTIA

LALVAVIALLASIGTRMMIWGGGGRRSSSNNKDSGGAIMLVLSIVFMILAPLAATMVQ

MALSRNREYLADASAVELTRNPQGLISALRKISESEPMESADPSSAALYIENPFKKEK

DETKDSFFATHPATSNRIERLEKM"

CDS 144235..144999

/locus_tag="JFBMFIFI_00129"

/inference="ab initio prediction:Prodigal:002006"

/codon_start=1

/transl_table=11

/product="hypothetical protein"

/translation="MATEYIYVHLDSVTNHVLSKGMDINHFQQSLRKIPKNILLLDAK

KGVGEFESHTGFRIIRGRENVRRYLDDCSKFPEKAHKWIDFESIELLRQLTPVEISEL

LYIAHAHTHLHSPFYYKLQNNFIYLTLPDAMSKVYYRYLDQFYALLSRTLTSALAEKM

NEKRSFFQKPQTIEPIPTCLINELVPYLKEGALFSLKQVEVRDNLYCIPVFLVEDRLR

EVGKPFSDRERLATICYDRKDASWSVEEVESLLTSM"

CDS 145207..145533

/gene="gdx_1"

/locus_tag="JFBMFIFI_00130"

/inference="ab initio prediction:Prodigal:002006"

/inference="similar to AA sequence:UniProtKB:P69937"

/codon_start=1

/transl_table=11

/product="Guanidinium exporter"

/db_xref="COG:COG2076"

/translation="MEWIYLIAAGICEIIWAYYLDASYGFSKLIPSIIAIVFIVISFI

FLEKAMKKLGVGVAYAVFTGIGTAGTAIMGMLFLGESVSLLKITALSLLLVGILGLKL

SEGGEA"

CDS 145533..145850

/gene="gdx_2"

/locus_tag="JFBMFIFI_00131"

/inference="ab initio prediction:Prodigal:002006"

/inference="similar to AA sequence:UniProtKB:P69937"

/codon_start=1

/transl_table=11

/product="Guanidinium exporter"

/db_xref="COG:COG2076"

/translation="MGFISLVVAGFCELGFVVLLKKSEGFRLKSYGILSMIVMGMSLY

LLSIASKTIPIGTAYAIWAGIGASASVLWGMIFFGESRSFKKIIFVTLIIIGVVGLKL

TSH"

CDS 145986..147065

/gene="ddl"

/locus_tag="JFBMFIFI_00132"

/EC_number="6.3.2.4"

/inference="ab initio prediction:Prodigal:002006"

/inference="similar to AA sequence:UniProtKB:P95803"

/codon_start=1

/transl_table=11

/product="D-alanine--D-alanine ligase"

/db_xref="COG:COG1181"

/translation="MKISLIYGGKSAEHDISILTAFSIIKEVYYKYYEVQPIYITKAG

QWIKGPVLTEPVTNHEQLKFKFNDETTNDQQLSGVEIQPTSLKEEDAVIFPVLHGPNG

EDGTVQGLFEVINMPYVGTGVLASACGMDKIISKYLFQQAGIPQVPYVAVLKNEWLRD

EEQVFMRCEGSLLYPMFVKPANMGSSVGITKAENRFELISAIKTALKYDRRIVVEQGI

EAREIEVAVLGNADVHTSVPGEIVKSVDFYDYDSKYLTNDVQLQIPALVPDSVSAQLR

EYAAAAFEVLDGSGLSRCDFFVTANDEIFINEVNTMPGFTQFSMYPLLWENTGLSYGD

LVEELIQLALRRFEERQAYQMKSDE"

CDS 147395..148771

/gene="murF"

/locus_tag="JFBMFIFI_00133"

/EC_number="6.3.2.10"

/inference="ab initio prediction:Prodigal:002006"

/inference="similar to AA sequence:UniProtKB:P11880"

/codon_start=1

/transl_table=11

/product="UDP-N-acetylmuramoyl-tripeptide--D-alanyl-D-

alanine ligase"

/db_xref="COG:COG0770"

/translation="MDVLSLKEIALAVGATNDLSDWTTIEVDKVEFDSRKLEKGSLFV

PLKGDNDGHDYIQSAIEKGASAAFWSEAIEKAPVGFPVLVVEDTLKALQDLAKYYLKV

VNPKVVGITGSNGKTTTKDMTEAVIRSKFKVHKTQGNFNNHIGLPMTILEMPSSTEVI

ILEMGMNHAGEIKVLSELAEPDVAVITMIGESHIEYLGSRKGIAAAKMEIVAGLKKNG

TLIYPGEEELLSPLVQELEPKQRITFGTGKTNNMYPVTIEPGMYQTKFTTNVAPEIEC

SIPVLGTYNVTNAMAALMVGFTLGISVEVASPELAKFNLTKNRTEWLAGINDSMILND

AYNANPTAMKAVLDNFSELMNEGRKIAVLGDMLELGETSCELHASVAGHIQSTKIDEV

FLFGHEMAVLYEALKETYPVEQLHYFEDDKLTLITSLKKSIKKDDFVLVKSSLGTDLL

AVVVELTK"

CDS 148878..149573

/gene="ybhL"

/locus_tag="JFBMFIFI_00134"

/inference="ab initio prediction:Prodigal:002006"

/inference="similar to AA sequence:UniProtKB:P0AAC4"

/codon_start=1

/transl_table=11

/product="Inner membrane protein YbhL"

/db_xref="COG:COG0670"

/translation="MQTQRKEVATAGLSRFFATVYGYMTLALAISGITAFYAAQSPFI

LNLVLGNPFGFIALFLVEIFLVMKLASNGNKLRSVGSSIVGFIAFSIVNGILLSSIFI

IYPLGDIAAAFMITAGTFAGMSIFGFVTKKDMTGLGGHLRSALIGLIIATLVNGFILR

SGPGELILSYITVLIFVGLTAYDTQKLKQIYFHYSDSQSLGAIAISGALSLYLDFINI

FLAVLRIFGGGRN"

CDS 149992..151617

/gene="cshA"

/locus_tag="JFBMFIFI_00135"

/EC_number="3.6.4.13"

/inference="ab initio prediction:Prodigal:002006"

/inference="similar to AA sequence:UniProtKB:Q8Y8N0"

/codon_start=1

/transl_table=11

/product="ATP-dependent RNA helicase CshA"

/db_xref="COG:COG0513"

/translation="MKFSELGLDPELLQSVERLGFEEATPIQEQTIPLALEGKDVIGQ

AQTGTGKTAAFGLPMLQKIDLKNRAVQGLVIAPTRELAIQTQEELFRLSRDKKVRVQV

VYGGADISRQIRSLKDQPHIVVGTPGRLLDHINRRTLKLDQVETLVLDEADEMLNMGF

LEDIEKIIKEVPKTRQTLLFSATMPDAIKRIGVKFMNEPEHVRIQATAMSDSLIDQYY

VRCKDFEKFDIMTRLLDVQTPELTIVFGRTKRRVDELSKGLEMRGYRAEGIHGDLSQQ

KRMSVLKSFKKGDLDILVATDVAARGLDISGVTHVYNYDIPQDPESYVHRIGRTGRAG

KEGMSVTFITPNEMGYLRVIEDLTKKKMTTLRPPTNAEAFEGQVKAAMETAEELVKSN

DLERYEKAAAQLLEDYDTTDIAAAFLKSISKDASEIPVKITPERPLPSRKGGSGGGGG

NRGGASRGKGGYRGGSGGGERRGGAQRGGKDNRRSGGGAGSGGSWNKDAKRGSNAGGS

SSSEARKGGRGNSSDNRRSGGGDRNFTIRNKEN"

CDS 151885..152235

/gene="acpS"

/locus_tag="JFBMFIFI_00136"

/EC_number="2.7.8.7"

/inference="ab initio prediction:Prodigal:002006"

/inference="similar to AA sequence:UniProtKB:P0A2W7"

/codon_start=1

/transl_table=11

/product="Holo-[acyl-carrier-protein] synthase"

/db_xref="COG:COG0736"

/translation="MIVGIGLDIADISRIEKAHIRQNTFAKRVLTEKEWDCFMGFHGK

RQIEYLAGRFAAKEAFSKALGTGIGKVGFQDIEILNSSNGKPEITKSPFKGTCFISLT

HTDTIVAAQVILEK"

CDS 152268..153395

/gene="alr"

/locus_tag="JFBMFIFI_00137"

/EC_number="5.1.1.1"

/inference="ab initio prediction:Prodigal:002006"

/inference="similar to AA sequence:UniProtKB:Q9RLU5"

/codon_start=1

/transl_table=11

/product="Alanine racemase"

/db_xref="COG:COG0787"

/translation="MVSGKHRNTYALIDRNAIFNNIKNQMNLLEPDTEVYAVVKADGY

GHGALEVATIAREAGVQGFCVALIDEALELRQAGFKEPILIMGLVEAKYAKLLLDQRI

SVAVGYLKWLEEAEFYLKREKSFSENRKLDIHLAIDTGMGRVGFRTSAELAEVESYLT

HSTSFNCQGVFTHFATADSKDTKQFHQQVEKFQKLVEGMTVKPTYIHSANSATSLWHQ

KHQKRIVRLGIAMYGLNPSGRELDLPIKLEPAMSVETTLVQVKQMSAGETVSYGATYK

AKEGEWIGTLPIGYADGWRRSLQGQTVLVEGERCEIVGRICMDQCMIRLNKEVPIGAK

VVLIGKDQNDEISAQEIAEYLDTINYEIVCGFTQRLPRVYC"

CDS 153546..153797

/locus_tag="JFBMFIFI_00138"

/inference="ab initio prediction:Prodigal:002006"

/codon_start=1

/transl_table=11

/product="hypothetical protein"

/translation="MEPKTEKFIKITLDNLNFQKVHRYCIKYGIDENEFVNTVMKRFF

KENQKKHDTMIKGYKEMSEINLDICNEFEDSEKDSNSHV"

CDS 153825..154175

/gene="ndoA"

/locus_tag="JFBMFIFI_00139"

/EC_number="3.1.27.-"

/inference="ab initio prediction:Prodigal:002006"

/inference="similar to AA sequence:UniProtKB:P96622"

/codon_start=1

/transl_table=11

/product="Endoribonuclease EndoA"

/db_xref="COG:COG2337"

/translation="MVKRGEVYFADLSPVIGSEQGGMRPVLIIQNNVGNHYSPTVIVA

AITAKIQKAKMPTHVEVSAEKHGLERDSVVLLEQIRTIDKQRLKDKVTQLDYQLMQKV

DEALEISVGLSGSV"

CDS 154711..155199

/gene="queT_1"

/locus_tag="JFBMFIFI_00140"

/inference="ab initio prediction:Prodigal:002006"

/inference="similar to AA sequence:UniProtKB:A2RM05"

/codon_start=1

/transl_table=11

/product="Queuosine precursor transporter QueT"

/db_xref="COG:COG4708"

/translation="MKTKALLINAIIATIYVVLTLSLAFISYGAVQFRVAEMLNYLVV

FNKKYIPGIVGGVVISNLWSPMIQYDMIFGVGQSLLSLIIAALIIKNMKNIWLKMTIV

LIIFGLSSALIAWELLLAFDLPFWAGYVSVAVGEVTVLLIGMPIMVALDKKLHFSERI

EG"

CDS complement(155246..155890)

/locus_tag="JFBMFIFI_00141"

/inference="ab initio prediction:Prodigal:002006"

/codon_start=1

/transl_table=11

/product="hypothetical protein"

/translation="MLIKSLCIPKKKLTTVKETVTLQEAIEILESSGYRCVPILDETG
TIFRGNIYKMHIYRHKANGGDMTLPVTHLLKNATKFISIESSFFKVFFTIKELPYISV
LDENNRFYGILTHSSLLSMLQQSWNVDVGSYVLTIASSGLKGDLSNMTKIINKYCTIA
SCITLDVERDELVRRTMITLPAGIDQETLDQIIEHLDKRGFRVVEIEDLRHMAK"

CDS 156145..156855

/gene="trmR"

/locus_tag="JFBMFIFI_00142"

/EC_number="2.1.1.-"

/inference="ab initio prediction:Prodigal:002006"

/inference="similar to AA sequence:UniProtKB:O32036"

/codon_start=1

/transl_table=11

/product="tRNA 5-hydroxyuridine methyltransferase"

/db_xref="COG:COG4122"

/translation="MAINEMMDRPVVNKDVLEFIRKKQKPLAGKLGEIERGANERGVP
IIPHETVVFLNLLLGQLQPKNILEIGAAIGFSSSLMAQHVGKDGHVTTIDRFDVMIEK
ARANYESLGLTEKVTLLEGQAADILPTLDGPYDFIFMDSAKSKYYDFFPECMRLLRVG
GMLVVDDVLQGGTILLPKEEIPKRSRAIHKKLNAFLDVVMDHPSIESSIIPLGDGLLL
IVKQDETDFSYLIEEAKI"

rRNA 157277..158828

/locus_tag="JFBMFIFI_00143"

/product="16S ribosomal RNA"

tRNA 158918..158990

/locus_tag="JFBMFIFI_00144"

/product="tRNA-Ala"

/inference="COORDINATES:profile:Aragorn:001002"

/note="tRNA-Ala(tgc)"

rRNA 159304..162221

/locus_tag="JFBMFIFI_00145"

/product="23S ribosomal RNA"

rRNA 162482..162591

/locus_tag="JFBMFIFI_00146"

/product="5S ribosomal RNA"

tRNA 162609..162684

/locus_tag="JFBMFIFI_00147"

/product="tRNA-Asn"

/inference="COORDINATES:profile:Aragorn:001002"

/note="tRNA-Asn(gtt)"

tRNA 162727..162799

/locus_tag="JFBMFIFI_00148"

/product="tRNA-Thr"

/inference="COORDINATES:profile:Aragorn:001002"

/note="tRNA-Thr(ggt)"

CDS 162982..163254

/gene="yjdJ"

/locus_tag="JFBMFIFI_00149"

/inference="ab initio prediction:Prodigal:002006"

/inference="similar to AA sequence:UniProtKB:P39274"

/codon_start=1

/transl_table=11

/product="putative protein YjdJ"

/db_xref="COG:COG2388"

/translation="MEFEKSENRFYKNNEEGKMIAEVTYVPAGDTKVILDHTFVDPSL

RGQGIAGQLVKRVVDEMRVEGKKIVPLCPFAKAEFDRKPEYKDIEA"

CDS complement(163296..165122)

/gene="kimA"

/locus_tag="JFBMFIFI_00150"

/inference="ab initio prediction:Prodigal:002006"

/inference="similar to AA sequence:UniProtKB:P96589"

/codon_start=1

/transl_table=11

/product="Potassium transporter KimA"

/db_xref="COG:COG0531"

/translation="MWSVLKRYLIGKPLKSSEEGDQKLGRFKALALLSSDALSSIAYG

TEQIVLVLVTLSTGAIWYSLPLAGLVLILLLALVISYRQIIHAYPHGGGAYMVSSENL

GKTSGLIAGGSLLVDYMLTVAVSVSAGTEAITSAIPALYNQRVPLAILIVFLITAMNL

RGLRESASFLMFPVYLFVAVITLLIITGFLKIALGIAPFHATATVGAVVPGITLGLLL

RAFSSGSSSLTGVEAISNAVPFFKKPQAKNAAATLALMGGILGFFFAGITFLNYWYGI

IPTEKVTVLSQIGKAIFGNGAFYYLLQFSTAFILALAANTGFSAFPVLAYNLAKDKYM

PHMYLAKGDRLGYSNGIMTLSLGSILLIALFNGETSRLIPLYAVGVFIPFTLSQTGML

VKWHRERPKNWLVKSISNFIGALISFAIFAILFFYRLNEIWPFFLIMPIMLLIFYKIH

DHYKKTALQLKLEDSVPITYTGSTVIVLISNITLVTNQALNYAYTIGDNVIAMHVSFD

DNPDKETEFDNEFKMKYPEIRLVNLHSSYRSVIHPVLRFVKLIEAQSTKKGYTTTVLI

PQFIPKKAWQHILHNQTSLRLRAALATKENIVVATYSHHLKK"

tRNA 165421..165494

/locus_tag="JFBMFIFI_00151"

/product="tRNA-Glu"

/inference="COORDINATES:profile:Aragorn:001002"

/note="tRNA-Glu(ttc)"

CDS complement(165613..166236)

/locus_tag="JFBMFIFI_00152"

/inference="ab initio prediction:Prodigal:002006"

/codon_start=1

/transl_table=11

/product="hypothetical protein"

/translation="MKKQVKTLAIIGVLICSLSIYLSFSQSNQNQATSKHKSPQPAKI

LTKNEITPVNKTTIDSSSPITIIQPEAENAVTPEPEILEPTTDTQTITKMPENSDDTA

VEKIPVQLTADEASAAQTDPIVIDLSYQGNWKSLDGSRTITGTQYGFKFENSPVGWLN

RYSKNDTGLILGDTNYFTFYVRLTENDHLIIEKTDGTEFWEFTRNYQ"

CDS 166464..167699

/gene="sauU"

/locus_tag="JFBMFIFI_00153"

/inference="ab initio prediction:Prodigal:002006"

/inference="similar to AA sequence:UniProtKB:Q0K843"

/codon_start=1

/transl_table=11

/product="putative sulfoacetate transporter SauU"

/translation="MAIKQSEQLNRKNNLMIATLFLGYIMIYIDKLGVGVALVPIAKE

FSLSTTQTGWIMSSFFVGYTLMQIPMGLLNNKIGSRKILVFSILFVAIFMLLFGLGKG

LIYFMLIRFLTGAIAHSGYPASSGKEISLNLPLNKRTFAQGILLASSGIAGIVGPVMV
SPMIETIGWRKAYFVMAGLASLVMLVLLVFLPKEKHTNDKVLLDIPSKNKVPLLSIWK
DSRVWILTVCAFFINSLVYGFTNWLPTFFTSAKGMSLTDAAKINSIAGIFALIGALGG
SYIVGRYFAGKEKIVIAIFCIVGGCSMLGVYTFDSIILITLLLGIANFSMLVAFVTLM
SIPLKLFINERFSPSYATIGTGGVMGGIFAPLIIGELIRVSNGNFLSTFIFFILMGIG
AAGAIIFLKMNRQGGAINE"

CDS 167692..168864

/locus_tag="JFBMFIFI_00154"

/EC_number="3.5.1.47"

/inference="ab initio prediction:Prodigal:002006"

/inference="protein motif:HAMAP:MF_01692"

/codon_start=1

/transl_table=11

/product="N-acetyldiaminopimelate deacetylase"

/translation="MNSQTNLPIEDMVKWRRHLHENPELSFHEVETAKYIYQLLKTFP
NLELTTPTENSVVAILKGAKPGKTIALRADIDALPIVEEADVAFPSQNEGVMHACGHD
THTAMLLGACKVLTSMQEKIAGTVKFIFQPAEEVPPGGAKFLVEAGVMKDVDLVFGIH
IFPKIPVGVVAIRTGALTAAADIFELKIQGVGSHGSTPELAIDPILVGVEIITNLNNI
ISRNIAAFDNAVLSIGEFTSGNSANVIPDTAKIQGTVRTNDPAVRTLVRKRIEEIIEH
VTKMYGASYELNYIMGYSPVVNDSEATELVRKAALKVVGEKGLITAPQMMGGEDFSAY
TDVVPGSFFVVGGGTAEEGCGYMNHHPKFKINEGALVVGAEMEIQLITDLLINDEE"

CDS 169055..170497

/gene="bglC_1"

/locus_tag="JFBMFIFI_00155"

/EC_number="3.2.1.86"

/inference="ab initio prediction:Prodigal:002006"

/inference="similar to AA sequence:UniProtKB:P42403"

/codon_start=1

/transl_table=11

/product="Aryl-phospho-beta-D-glucosidase BglC"

/db_xref="COG:COG2723"

/translation="MDYSKKVSFPSTFLWGSASAAYQVEGAWNVDGKGKSVWDNYAKV
PGATFQGTNGDVAVDHYHRYKEDVKLMAEQGLKAYRFSIAWSRIFPDGTGEINEAGLA

FYDRLIDELLSYNIVPVVTLYHWDIPQALMDAYGGWENRQVIEDFNNYAVTLFKRYGD
RVKHWVSLNEQNIFVGFGYRYGSHPPAVKDEKRFYEVNHIANLANAKVIQSFKALNIP
EALIGPSFAYSPVYPATPNPNDVLASENAEELLAHFWMDVYVWGEYPKATMRYLSENG
IAPTILPGDMELLKAGKPDFMGLNYYQTTTVEYNPLIGGVGVAEMNFSGKKGTSKDSG
IPGMYKTVQNPYTETTDWDWTIDPQGLHVALRRIENRYHLPILITENGLGAFDTLEAD
DQINDDYRIDYLSQHVSAIQEAITDGVEVLGYCTWSYTDLLSWLNGYQKRYGMVYIDR
DEHDEKTLRRIPKKSYFWFKELIEKNGEQN"

CDS 170920..171882

/gene="ldh_1"
/locus_tag="JFBMFIFI_00156"
/EC_number="1.1.1.27"
/inference="ab initio prediction:Prodigal:002006"
/inference="similar to AA sequence:UniProtKB:P00343"
/codon_start=1
/transl_table=11
/product="L-lactate dehydrogenase"
/db_xref="COG:COG0039"
/translation="MKQADKNQEKIILIGAGDVGSSYAFALVAQNIGRELGIIDLDTG
KSEGDALDLSDGLAFTSPKKIYAATYADCKDADLVVITAGAAQAENETRLDLIKKNFK
IAKIIVDQVMASGFDGIFLVASNPVDILTYAVWKFSGLPTQRVIGSGTSLDSARFRGA
LAGITDVDARDVHAYIIGEHGDTQFPVWSHANIGGLQIYEWMREHPEVTEESLIKLVT
DVRDAAYKIIQRKGSTHYGIAIALARITKAIFDDENAILPLSVYLDGAYGEKDIYIGS
PAVVNREGIKSIIEIPLNDLEKQKMHESVSTIRLMQEDAFKNLL"

tRNA 172146..172229

/locus_tag="JFBMFIFI_00157"
/product="tRNA-Tyr"
/inference="COORDINATES:profile:Aragorn:001002"
/note="tRNA-Tyr(gta)"

tRNA 172233..172306

/locus_tag="JFBMFIFI_00158"
/product="tRNA-Gln"
/inference="COORDINATES:profile:Aragorn:001002"
/note="tRNA-Gln(ttg)"

rRNA 172408..172517

/locus_tag="JFBMFIFI_00159"

/product="5S ribosomal RNA"

tRNA 172538..172610

/locus_tag="JFBMFIFI_00160"

/product="tRNA-Lys"

/inference="COORDINATES:profile:Aragorn:001002"

/note="tRNA-Lys(ttt)"

CDS 172956..175553

/locus_tag="JFBMFIFI_00161"

/inference="ab initio prediction:Prodigal:002006"

/codon_start=1

/transl_table=11

/product="hypothetical protein"

/translation="MNKETRRKQILFQVLSLILPLLILVPVFLSMKVTPFGDSNLLVS

DLGTQYVPFFAYFKEMIIGDGSPLYSFSSGMGDDFLPLAAYYLMSPFNVLFLVTPNAY

LATSVTIVIMLKICFISGSLFFYLSQTYRKVEWAQLFFALAYAFCGFVGIYLYNIMWL

DALIWLPILALGIHYLVEQNKKLFYCLSLFAVIVSNYYLGYMSCLFALCYFIYWTAKK

TEFTTIKEYFKQTKVKWRNFIVYSVIGAGLSGFILVPAALGMLQTGKSAVDWHIFLPI

PRFGLDFLVQLGLENTGFNSRLDHLPTIFVGSFVLILALSYFFIKTISRKEKWLSFSL

IAIFFFSFWLQSFNTVWHMFQLAAGFPYRNTYMLSFVLIFLAYESWQKRSDLSNKTIL

KVTGSLVGLLAIGYVYSWLILPWLVKKSQFFKDLPFQSAVPLQLSLFFISVGIFLIMG

LLLVWVRNSKKRHWVLFLALFLELGFNFYNMLGETPLADEPRFRSDMTNYNYLIDEMK

AKDSDLFRIENAINGVDNGYNESFLYSYNSTPYYSSTLNENLRLSLARLGLFSRNERR

ISDVGQTPFLDYLFNTRYSLDEPNPEEAEFAIVKSDFGQIVPKNPSMATSIGYTVPLA

FADVKLKDNQPFANQNALAESIYAFDQPLFEPAEVMQSIAPNSYFVTAKADGPLYLYL

PNNRLEKIQVFVNHEKIVPRVNIKNQALLDLGDFKKGTQILLDFSSSKKIKMTGENVQ

TMNGSTYYQTIRTLEQNRLIVSDWKASKVEGKVNVQSDHELLFVSIPYDEKWKASVDG

EKVKLQKVAGNFIGIPVSKGEHQIELNYSPEGFQIGSFISYGALAGLVSLSLVDWYQK

RKRIKNRAN"

CDS 175661..176503

/gene="dacA_1"

/locus_tag="JFBMFIFI_00162"

/EC_number="2.7.7.85"

/inference="ab initio prediction:Prodigal:002006"

/inference="similar to AA sequence:UniProtKB:Q8Y5E4"

/codon_start=1

/transl_table=11

/product="Diadenylate cyclase"

/db_xref="COG:COG1624"

/translation="MSIDWTQLFTWRNFFNVIDILVVWFFVYKLITLLKGTKAIQLLK

GIAVIIVIKMVSFLLNLETVDWLMDQVISWGVIATIIVFQPEVRRGLEHLGRGAIFGR

TKRQVDPTKRLIEALDKSVQYMAKRRIGALISIEMETGLDEYIETGIPLDADISGELL

INIFIPNTPLHDGAVIIKDYKIAAAAGYLPLSESSLIPKELGTRHRAAIGLSEVTDAV

TIIVSEETGGISITRNSELLRDLTQEEFVNYLIKEIVVKEEEHKKNPIQVFIDGFSKK

EDSK"

CDS 176500..177642

/locus_tag="JFBMFIFI_00163"

/inference="ab initio prediction:Prodigal:002006"

/codon_start=1

/transl_table=11

/product="hypothetical protein"

/translation="MIEKIYNSPWFLRIVALGFSLLLFGYVNFEKNGLLNTNDAASSL

NPRGSEVISNVPVYVDVDHDKYYVSGVPQTVSVKLEGLKSIISQTVTSQNFKVVAKNL

TKLGVGEHTIQLTPEGFSDKLGVSVTPSEAKITIENKKVKTFDVNVEFNDALLARGYE

SGTPVLDKNTVEISGAESEVDKVATVKAVVSVENGVKKDIKTKVAVQVEDADGNSLDV

VVNPSEVSVTIPVIAANKDLPISLNQTGTPVAGKTYRLSLKNDISTINVAGKSTDLDE

FTNFVVNVDVTGITETTTKEIALSLPSGISSVSPQTVQVVITVSNTSNGSSSGGSGNS

PPPESGNGSSSSSSSSESSSESSSSSSAESESSSKDSSQVSSKETD"

CDS 177722..179080

/gene="glmM"

/locus_tag="JFBMFIFI_00164"

/EC_number="5.4.2.10"

/inference="ab initio prediction:Prodigal:002006"

/inference="similar to AA sequence:UniProtKB:O34824"

/codon_start=1

/transl_table=11

/product="Phosphoglucosamine mutase"

/db_xref="COG:COG1109"

/translation="MGKYFGTDGVRGVANSELTPELAFKLGRFGGYVLTQHSEGAAHP

RVLVGRDTRISGQMLESALVAGLLSVGIEVMQLGVISTPGVAYLTRVQGAAAGVMISA

SHNPAPDNGIKFFGSDGFKLSDEQELEIEALLDHEIDDLPRPSAVGLGTVDEYLEGSL

KYTQFLQQTIPSDLAGIQVCLDGANGATAPLLNRLFADLETDFDVMGASPNGININDG

VGSTHPEKLAEFVLEKGADAGLAFDGDGDRVIAVDELGQIVDGDKIMYICGKYLMEKG

RLKKDTIVATVMSNLGFHKAVESAGMTALQTQVGDRYVVEEMRKNGYNFGGEQSGHMV

FLDYNTTGDGMLSGIQLLNVMKQTGKKLSELAAEVSNYPQKLVNIRVSDKDGAMDVPA

IKAIIDEVEKEMNGDGRILVRASGTEPLLRVMGEAPTEEKVNLYVDKISDVVRQEIGL

AE"

CDS 179515..181323

/gene="glmS"

/locus_tag="JFBMFIFI_00165"

/EC_number="2.6.1.16"

/inference="ab initio prediction:Prodigal:002006"

/inference="similar to AA sequence:UniProtKB:P0CI73"

/codon_start=1

/transl_table=11

/product="Glutamine--fructose-6-phosphate aminotransferase

[isomerizing]"

/db_xref="COG:COG0449"

/translation="MCGIVGFIGEQDAKEVLLQGLEKLEYRGYDSAGIYVVDEGGAGH

LFKEKGRIAALRKIVSDDVAGTKGIGHTRWATHGIPSVNNAHPHQSTSGRFTLVHNGV

IENYREVRDAYLTDVDFVSETDTEVIVQLIAWFAENEGLATLDAFKKAIVTLKGSYAL

ALMDLENPDAIYVAKNKSPLLIGVGENFNAVCSDAMAMIQVTNQFVELMDGEVVTVTK

NDISIENLAGEKIDRASYTAEIDLNDLEKGTYAYYMLKEIDEQPAVMRKIIQHYQGMD

GKLAIDSTITQTLLDSDRVYIVACGTSYNAGWAGKQLIENLAGIPTEVHLSSEFGYNM

PLLTKKPFFIFLTQSGETADSRQVLVRINQMGYPTLTITNVPGSTLSREAQFTLLLHA

GPEIAVASTKAYTAQIAVLAILADIAAKAKGLSQTVDMAHEIGIAAAAMEAIVSDKER

LEELAADYLSISRNAFYIGRGLDYYVAMEAALKLKEISYVQTEGFAAGELKHGTIALI

EEGTPVIAIITEESIAGHTRGNVSEVNSRGAHSLVIAMENVAHEDDQFVLPQVHELLT

PLVSVVPTQLLSYYTSLHRGNDVDKPRNLAKSVTVE"

CDS 181569..182696

/locus_tag="JFBMFIFI_00166"

/inference="ab initio prediction:Prodigal:002006"

/codon_start=1

/transl_table=11

/product="hypothetical protein"

/translation="MELLKIMEPYITGTTSILGIFFIQVMWITIFRVTLKEKMSEKKY

HYLLELSSGIIMSIFSIYLLYMSQKENYYYLYSNLRLVIILLPTIFISARVSFISVFL

AGVFSFFTNGITLVSVAYIVTFSVFLLTVAITKKLVKGNQIKLFFLSCLFVIPIWFAV

YFINFDGVHVYSLDYMVPDYLNFVIISSITFFSVRYLAYLSEHFFQTMADNLTDELTK

LGNLKAFNQLYQVLFQQVEKNKYQCNLILFDIDHFKQVNDTYGHLAGNYVLVELASLL

KQMSVTHYGNRLFRIGGEEFALLIENESLEETLKIANEICDAVANYPFYYDGRRIKVS

ISLGVACSVGKENQREFYMKADKALYQAKELGRNQVSYWKE"

CDS 182946..184337

/gene="tcyP"

/locus_tag="JFBMFIFI_00167"

/inference="ab initio prediction:Prodigal:002006"

/inference="similar to AA sequence:UniProtKB:P54596"

/codon_start=1

/transl_table=11

/product="L-cystine uptake protein TcyP"

/db_xref="COG:COG1823"

/translation="MTNLYIIIILALFAAAIYGFYWMQKKHVKFSTRVFSALGAGIVL

GAIIQVILGAESEVTIGAISWINIVGNGYVSLLQMLVMPLIFVSIVGAFTKLGVSKNL

GKISFTVIGTLLATTAVAALIGILSVFIFNLDGATFVQGEAETARIEALGERQEAVEN

LSIPEQVVSFIPTNVFADLSGSRSTSTIAVVIFSAFIGIAYMGVNKKDPETGEFFAKL

INSLYKIVMRIVTLVLRLTPYGILALMIKVTATSDVRAIWNLGKFVLASYSALAVVFI

MHLLILLVVKVNPLSYIKKASTVLGFAFTSRSSAGAIPLNIETQTKALGVDEASANFA

ASFGATIGQNGCAGVYPAMLAAIVAPTVGIDVFSPLYILTIIAVVTISSFGVAGVGGG

ATFASLIVLGSLNLPIAIVGLVISVEPLIDMARTLVNVSDSMIAGIVTSKRIKEFDET

VLNDEAAVIESSI"

CDS 185204..185872

/gene="tupC"

/locus_tag="JFBMFIFI_00168"

/EC_number="7.3.2.6"

/inference="ab initio prediction:Prodigal:002006"

/inference="similar to AA sequence:UniProtKB:Q93KD4"

/codon_start=1

/transl_table=11

/product="Tungstate uptake system ATP-binding protein

TupC"

/translation="MKLNIINLTKSFRKNIFFYQQNLMIDFDNQLIAFITGENGTGKS

TLMKIISGILMVDSGEFQIESNVNYFKWSKENCYYMPATERGMTYKLTGRENIYYLCS

LKGSKKEVITNRLKYYATLFDATEILDKRVEELSTGQKRKIHLLSAFCSNCKVLLLDE

PSVGLDTYNLGLLEDILIKINNEKKQKIILVSHDQDIKKKLMGEEIYVNKSKTIIMKG

EGNL"

CDS 185869..186651

/locus_tag="JFBMFIFI_00169"

/inference="ab initio prediction:Prodigal:002006"

/codon_start=1

/transl_table=11

/product="hypothetical protein"

/translation="MKFLYSIIGTMKADVIILFRYRKSIISDIITFLALYLGIIIFND

ASNFSDFYNVQISDGPILLLIGYIFWTVSNIALSYSSSIIVGDAKSGMLEVKLQGIIP

YPVLSFFSVLVSIIESIIVLFFACLISLVIGYIEFTHIPFILLSIVLTIPSIIGMYGV

GLILAGFALKEKSIGQFVSIVSAILLFLSNTLILNLPNYIYIIPFTSGIDISRNLFST

GVFDSYLFIIYICVCLLWLVVGILIFNILLKKERTQGSFDSF"

CDS complement(186805..188031)

/gene="macB_1"

/locus_tag="JFBMFIFI_00170"

/EC_number="3.6.3.-"

/inference="ab initio prediction:Prodigal:002006"

/inference="similar to AA sequence:UniProtKB:Q2EHL8"

/codon_start=1

/transl_table=11

/product="Macrolide export ATP-binding/permease protein

MacB"

/translation="MNIKENFKMALDSIFANKMRSFLTMLGIIIGIASVIAILAVGNG

ATSEITGTFDDLGASTISLSVDNKATKGDYLTNADIQALKSGIPKIERISPDKNLTGT

LSAGDENRMAIAFGGTPDIQYVNQAMDKTIVYGRFFNQNEYQDSKNVTVISEDTAAAL

FNGRKDVVGETVNLTTQGGTNLSLKVIGVVKGTFSKMQGSFDTSSMPVYLAMPITTMA

KLDSSATNFTSLTIQVADKNDIESVSKQMVRLLETRHDAVGKDLYTATNFLQALDQVN

NVLGLFINFIAAVAGIALLVGGIGVMNIMLVSVTERTREIGTRKALGATTNTILFQFL

MESVILSLIGGIIGLILGILLANGVAGVLNIVPRITPGAILLVLLFSSAVGVFFGIYP

ARKAAKLDPIEALRYE"

CDS complement(188047..188739)

/gene="yknY_1"

/locus_tag="JFBMFIFI_00171"

/EC_number="3.6.3.-"

/inference="ab initio prediction:Prodigal:002006"

/inference="similar to AA sequence:UniProtKB:O31711"

/codon_start=1

/transl_table=11

/product="putative ABC transporter ATP-binding protein

YknY"

/db_xref="COG:COG1136"

/translation="MIDLKDIVKIYRTGGEELVALKEISLHIAEGEFTSIMGPSGSGK

STMMNILGLLDRFDSGTYILNGQNVTGLSDNESAHVRNKEIGFVFQSFNLMPRMTILE

NVELPMVYAGIPAKERKERALKALDRVGLSDRVKHRPNEISGGQKQRVAIARAIVNTP

AVLMADEPTGNLDSKTTLEIMRIFQELNAEGTTIVMVTHEPEVAAYTKRIVSFRDGEI

QDDQPQINQKLV"

CDS complement(188755..189615)

/locus_tag="JFBMFIFI_00172"

/inference="ab initio prediction:Prodigal:002006"

/codon_start=1

/transl_table=11

/product="hypothetical protein"

/translation="MNWKKIIGWLIALAALGFIGFSVFGPKEKAKAPTVETTTVKEEN

IVETLSTTGTLQPNATQNGFGTGLVSEVNFAVGDKVAKDDVIVRYLDGTTITATIDGT
ITALNVKKDQVDLNTQTGQPSVTIDDLGNLKVAILLSKSDANLVKVDQPVTLTSGNTT
YTGKVSALDPVATTTTGATGATTALGGTITFDTPPTGLFAGFEIDADITTNTAEKALT
MPIETLVYDKENKPYVYIVKNDKAKKVEIETGIQSDTKVQIIKGLEKGQKVIISPADS
LKNGTAVTVK"

CDS 189911..190645

/gene="pphA"

/locus_tag="JFBMFIFI_00173"

/EC_number="3.1.3.16"

/inference="ab initio prediction:Prodigal:002006"

/inference="similar to AA sequence:UniProtKB:Q8ZNY9"

/codon_start=1

/transl_table=11

/product="Serine/threonine-protein phosphatase 1"

/db_xref="COG:COG0639"

/translation="MKKEVFAIGDIHGQIQLFEKMLTNWNPEKQQLVLIGDLGDRGEN
PKACFLLGKKLVEEHGAIYLKGNHEDLLLRFLENPEERYENYCLNGGMRTLESLLHPG
LNGDYSPTEMSMMVKSHYPDLIAFLENLPLYFEWEDYLFVHAGVDLSKKEWRNTSDHD
FMWIREPFHIAPNKTGKIIVFGHTITPSLNGDNVTTDLWIKDHKIGIDGGAVYGGVLH
GVLFSSDKLEEDIILENTGYVWDGLV"

CDS 190750..191910

/gene="napA"

/locus_tag="JFBMFIFI_00174"

/inference="ab initio prediction:Prodigal:002006"

/inference="similar to AA sequence:UniProtKB:P26235"

/codon_start=1

/transl_table=11

/product="Na(+)/H(+) antiporter"

/translation="MAFLGSLCLILVFTKFAGHFCNRIGVPAVIGELLVGIILGPALL
GWIQPDEFIHYFSEIGVVILMFIAGLESDLNLLKKYWKPALSVALLGIIFPATFGFLT
GSLFQLGFQSSVFLGVLFSATSVSISVQVLKDLKKVDTKEGATILGAAVVDDIVVVIL
LGILVSLFHTNGGTDEPLGESLIRKVIFFIVVFALSKWIVPTLMKISSKLLMIESVVT
MALIICLSFSYFADLMGMGSIIGAFFAGIALSQTKYKHTIEEKIEPIGYAVFIPVFFV

SIGLNMDFSGIGKQWLFILVLTAIAVLTKLVGGAIGAKITGFNWKSAAVIGAGMVSRG
EMALIIGKIGLESNLLSETYYSSVIVVVIATTVIAPFLLKYTIVKQDESLRL"

CDS 192066..194249

/gene="yhgF"
/locus_tag="JFBMFIFI_00175"
/inference="ab initio prediction:Prodigal:002006"
/inference="similar to AA sequence:UniProtKB:P46837"
/codon_start=1
/transl_table=11
/product="Protein YhgF"
/db_xref="COG:COG2183"
/translation="MAENNDATILQLLKKELKIYSDKQLKTVLDLLAEGNTVPFIARY
RKEMTGTLDEVQIREIEDRYTYLQNLEKRKGEVLRIIEEQGKLTPELQKSIETADKMQ
LVEDLYRPYKQKRRTKATIAKEKGLEPLAKWLLTFPMNVDVEKEAQNYLDAEKEVTTV
EEALAGAHEILAESISDEPKYRIWIREFTMRNGIVTSAVKDAEKDEKGVYEMYYEFSE
PVAKLVSHRILALNRGEKEDILKVSVLVDEERIGKYFARELIKNEDSPATPYIVGAYE
DSYKRFIGPAIEREIRGELTEKADEQAINIFGENLRNLLLQAPLKGKMVLGLDPAYRT
GCKLAVIDPTGKVTDIDVIYPHKPAGASARSEAATKFKKIVKDFGIEMIAIGNGTASR
ESEQFVVDNLKEIDQEVYYVIVNEAGASVYSASDLARKEFPDLQVEQRSAVSIARRLQ
DPLAELVKIDPKAVGVGQYQHDVSQKRLEERLEFVVETAVNQVGVNVNTASAPLLQHV
AGLNKTTATNVVAYRDENGRFESRAALKKVPRLGPKAYEQAVGFMRIVDGKNILDNTG
IHPETYKEAKEILEMAELDLKDVGSTTAKEALAKLNFQQVADQTGLGKETVKDMIEAL
SRPGRDLRDELPAPLLRKDVLTMEDLKAGMELQGTVRNVVDFGAFVDIGVKQDGMVHI
SKLSNGYVKHPTDVVAVGDVVTVWVDSIDLKKGRIALTMLNQEQK"

CDS 194344..194832

/locus_tag="JFBMFIFI_00176"
/inference="ab initio prediction:Prodigal:002006"
/codon_start=1
/transl_table=11
/product="hypothetical protein"
/translation="MNVRIEERPTFKVVGVKEEISLKDGANLWEIPKFWERVNLDGTA
NKISDLSHDELGGLLGICTNMRDQRMDYWIGVATTEVTPLEFEEIAIEAQIWAVFEVV
GPLPTAIQAAWKFVFSKWLPHSDYLHAEGPEVEWYSIGNPTAENYVSEIWIPIMMKEA

TN"

CDS 194960..195412

/locus_tag="JFBMFIFI_00177"

/inference="ab initio prediction:Prodigal:002006"

/inference="protein motif:HAMAP:MF_00745"

/codon_start=1

/transl_table=11

/product="Protein SprT-like protein"

/translation="MLQQLVEQISLEFFDMPFKHQATFNPRLRTTGGRYHLRSHDLDF

NLQVLETFGMSEFIGVIKHELCHYHLHLANRGYQHKDADFKRLLKKVGGSRFVQSLRQ

PDKKKKLWVYCCQNCQKNVYRQRRFDTKKYVCGGCQGKLILLEKRIEV"

tRNA 195475..195560

/locus_tag="JFBMFIFI_00178"

/product="tRNA-Leu"

/inference="COORDINATES:profile:Aragorn:001002"

/note="tRNA-Leu(gag)"

CDS 196222..196719

/gene="sipS_1"

/locus_tag="JFBMFIFI_00179"

/EC_number="3.4.21.89"

/inference="ab initio prediction:Prodigal:002006"

/inference="similar to AA sequence:UniProtKB:P28628"

/codon_start=1

/transl_table=11

/product="Signal peptidase I S"

/db_xref="COG:COG0681"

/translation="MKKFLANNKYFILVLVCIVLVRIFWITGVRVSGESMEPTFHNNN

RVFINKLSKLERFDVVVLDAPDAESKEYIKRIIGMPGDDVRFEDNQLYINDKPVAEPF

LKGTNIVTEGFQLKEKVPENSYFVMGDNRGNSNDSRFFGFVSEDEMQGEVFFRYWPVT

QLKGF"

CDS 196745..196909

/locus_tag="JFBMFIFI_00180"

/inference="ab initio prediction:Prodigal:002006"

/codon_start=1

/transl_table=11

/product="hypothetical protein"

/translation="MKNETVKQTVKTGVSFGSVLAIVISYVHWQSIFWAIIHGVFGWF

YVIYYILFYG"

CDS complement(196981..199278)

/locus_tag="JFBMFIFI_00181"

/inference="ab initio prediction:Prodigal:002006"

/codon_start=1

/transl_table=11

/product="hypothetical protein"

/translation="MKKIFQLIILGFLCFILFPSPVLAENQPLYKYIKNPDGSYGYNQ

SLPVPLYSGSSELIQIPTEYQQPKNQFRSSWVATINNLNIAKPSSETDFKANYTKILS

DFSDWNMNAMIFQVRPLLDAYYPSELNPWSEFLSGTQGDNPGYDPLAWMIETTHQAGM

EYHAWFNPYRVTNTKLSTQAILTKTGLTKEEILSLSIPEQIAALHNAGILASTNYAVQ

HPENVLIFDEKLFLNPGIPAVRDYVIASIAEVVQNYDVDAIHFDDYFYPYRITVDGVN

VLFGEKGEDDATFAEFGMGYDSIDDWRRDNITSLVEGIKLVIDVHNQQNQKAVQFGIS

PFGIWEHKANDDRGSNTPTGSSQSYSTSIYADTYKWIKEETLDYVIPQIYWSFDQSAA

PYGELTKWWNDVSEGSKTQIYIGHANYKHVSNGGWDAAWMNPEEIPNQMRFNQTLPNI

LGSSLFSYNDISLSNVESLPSDQQERHKAKNASIEQLKTTYFNLPALVPAKPWLAQGT

LSAPTSMNKNMDNPDAPTLTWQDSETNPARYYVLYRGIGSAVEITSNPTSIVAKIWRT

ASETTFSYTDTRLTKYDATEEYYVSAIDAAGNESEPTLLTAPEIIVEPQQGAPVTVLY

LDEKQSEIAEKEQLTGILNTEYNSVAKEVTGYTLLKQPTNNSGIFTETEQTVIYQYKK

DTAITIIEPEIKPAVPVPEEKQDVANVIPDKTNFIPKEKQATKEQADLPRTGEAELIT

RSFLGFIVTLGTLYWFFKRKYQKTN"

CDS 199607..200506

/gene="murQ_1"

/locus_tag="JFBMFIFI_00182"

/EC_number="4.2.1.126"

/inference="ab initio prediction:Prodigal:002006"

/inference="similar to AA sequence:UniProtKB:P44862"

/codon_start=1

/transl_table=11

/product="N-acetylmuramic acid 6-phosphate etherase"
/db_xref="COG:COG2103"
/translation="MLDALTTEKRNKTTMNLDTYSVKEIINKMNQEDEQVAKAVKSEL
DSITELVEAVIQSQKKGGRLFYIGAGTSGRLGVLDAAECIPTFNTKPEMVQGIIAGGI
KAMTVAVEGAEDSLDLAEIDLKERDLNETDFVLGIAASGRTPYVIGGLDYANKIGATT
GALSCNRGAIISQHASYIVEVPVGPEILTGSTRLKSGTAQKFVLNMISTATMIRLGKV
YQNLMVDVQPTNEKLVERSKKIIMEATDCTYEEADFYFNDSNKQPKLAIVRLLSGLDK
EQAQQLLIKNAGFVGKAIEASKK"

CDS 200538..200846
/gene="celA_1"
/locus_tag="JFBMFIFI_00183"
/EC_number="2.7.1.205"
/inference="ab initio prediction:Prodigal:002006"
/inference="similar to AA sequence:UniProtKB:Q45399"
/codon_start=1
/transl_table=11
/product="PTS system cellobiose-specific EIIB component"
/translation="MKVVMVCSGGMSSAIVVKAILKEAEKQNFDLDMIAVGSGEIEDI
LKTGGYELLLVAPQVKHQYATFEGYANNAGVPIDKVEPMGYTPIGAPKTLLQIKKYSK
"

CDS 200971..202275
/gene="licC_1"
/locus_tag="JFBMFIFI_00184"
/inference="ab initio prediction:Prodigal:002006"
/inference="similar to AA sequence:UniProtKB:P46317"
/codon_start=1
/transl_table=11
/product="Lichenan permease IIC component"
/db_xref="COG:COG1455"
/translation="MTNFLNWIETKFMPPMAKLAEQKHLKAIRDGVISTLSLILVGSF
FLIIANPPIPSWAEAIAPYTANIVIPFRITMGLMSIYAAYGMGYSLAKSYKLDGVTGG
VLSLATFLMLTVPVNVDALLPEGQGIGWVLPMNNLGGAGMFSAILAMIFAVESLRFFK
SRNLMIKMPEQVPSSVARSFEALIPGAFVITVVWIIRVLLDFDLNQFILSIFSPINDI

LGNNLLGVLVPVIFITLLWAAGVHGMSVIGSIARPMWLVMLDANGQALADGTAPNALP
YIAPEQFYQWLVWVGGAGSTLALCIILLFCKSVYLKQVGRFSIVPSIFNINEPLIFGV
PLVMNPILAIPFVIAPVVTTTLSYFAVKLGVINGFVSNVPWTLPAPIGAFLSASNDWK
AAILVLINIAIAGLIYYPFVKVYDKKLLEEELNQVSEEQAIL"

CDS 202363..202668

/locus_tag="JFBMFIFI_00185"

/inference="ab initio prediction:Prodigal:002006"

/codon_start=1

/transl_table=11

/product="hypothetical protein"

/translation="MLQIIGLIVFFIVLVLSGVSFVHLLRKKGIFINRWYFGFGAFLI
ILIPSFFFQQVYSVISIVFYLISSILAIMFFETTRLKLENNEFRGVVRSEQYPSKKD"

CDS 202673..203578

/gene="murK"

/locus_tag="JFBMFIFI_00186"

/EC_number="2.7.1.-"

/inference="ab initio prediction:Prodigal:002006"

/inference="similar to AA sequence:UniProtKB:Q97ML3"

/codon_start=1

/transl_table=11

/product="N-acetylmuramic acid/N-acetylglucosamine kinase"

/db_xref="COG:COG2971"

/translation="MNYMIGIDSGGTKTEAIAYDLNGSELARCQTGFGNLLIDKKRGL
ANLEEAMKILFDKLDEKYCQIVVVGLAGLDGGNFKAELATYFSHYQPDIVFINDAWLS
YYALVKEKDGCLVISGTGSICIGQKGQETARVGGWGNLLGDEGSGYWIAKKMIQQLLK
EEDRSEGYSSLSKKLMSALEAKNIFDVVSYTYTHEKDQIAQLATLVAESAIAEDPWAI
ELLEAAGRELANQIKLLIAKLGFTQQVTIGLSGSVLEKNPIVYQSFSEVLAQLPIEIC
LITEAKSNASGGYYYYKNKVVGNEE"

CDS 203583..204671

/locus_tag="JFBMFIFI_00187"

/inference="ab initio prediction:Prodigal:002006"

/codon_start=1

/transl_table=11

/product="hypothetical protein"

/translation="MRRLGISVYPNHSELNEIKEYIVLAAKYNFKRIFTCLLSVEEGK

EKIVAEFTEIIEFAKENGMEVIADVSPKVFGELGISYKDLSFFKEIGADGIRLDMGFT

GNEESIMTFNPQDLKIELNISSGTKYLENILSYQANVDNLLGCHNFYPHRYTGLSYAH

FIETTKQYKDKGLTTAAFISSPTAKVGPWPVSEGLCTLEMHRELPIVVQAKHLWATEL

IDDVIIANSFASEAELKALSEIDAYQLTLNCELVPSIPETEKKIVLEEFHFNRGDVSD

YVIRSTQSRVKYKGHEFPPFHTPNMKRGAIIVETSLYAHYAGELQLALLEMENSGKTN

VVGYVVEEEQMLLDYIKPWQKFKFKEVN"

CDS 204700..205011

/gene="licA_1"

/locus_tag="JFBMFIFI_00188"

/inference="ab initio prediction:Prodigal:002006"

/inference="similar to AA sequence:UniProtKB:P46319"

/codon_start=1

/transl_table=11

/product="Lichenan-specific phosphotransferase enzyme IIA

component"

/db_xref="COG:COG1447"

/translation="MEERIFEIIIHGGNARGMAYEALEKARAGEYEEVERLLAECKSE

MTLAHNTQTKLVQDEIRGDEIKISLLLIHAQDQLMTAMAEQTLILQMIDMQKEINLLK

K"

CDS 205031..206161

/gene="anmK"

/locus_tag="JFBMFIFI_00189"

/EC_number="2.7.1.170"

/inference="ab initio prediction:Prodigal:002006"

/inference="similar to AA sequence:UniProtKB:Q8EHB5"

/codon_start=1

/transl_table=11

/product="Anhydro-N-acetylmuramic acid kinase"

/db_xref="COG:COG2377"

/translation="MGYAVGLMSGTSLDGVDAALVDISEENEKTKVQLVAFETYSIPA

ETLKRINQVLSIEESNVERICSLNVELGYLFSDAVKQICQKAEIETEELEFVASHGQT

IYHLPNPGHQQFSSTLQIGESAVICEETKTTVVSNFRERDMANGGQGAPIVPYSEYVL
YRDEKRTRLLQNIGGIGNVTVIPKNANLNQLIAFDTGPGNMIIDELCRYFYQKEYDHG
GAYAALGTVNQELLAELMAHPFISKSYPKTTGREDFGNEFTKQLLAKWPMTANDLIAT
VTEFTAASIAENLKPFIDSETDLIVAGGGSYNGTLIQMLKAQLPDVHVVIQEDIGYSS
EAKEAIAMTILGNQTLHHKPSNVPSATGAKKPVILGKITYYN"

CDS 206279..207130

/gene="ybbH_1"
/locus_tag="JFBMFIFI_00190"
/inference="ab initio prediction:Prodigal:002006"
/inference="similar to AA sequence:UniProtKB:Q45581"
/codon_start=1
/transl_table=11
/product="putative HTH-type transcriptional regulator
YbbH"
/db_xref="COG:COG1737"
/translation="MIGENVQGRIRSIINELSNSERKIGELILEIPSEAINMTAIELS
TRAGTSPATVIRFCKSIDIPSFTQLKVKLAAEIESPVPEGYSDITANEPVHEIKSKLL
GNAYQSMKETIEQLNDQRVEEVVEVLLEAPIIYVYGIGASHLVAEDIAQKWNRIGKPT
ICASDPHILISILVAAPKEAVFIGISNSGETKEVLKLVEVAKSQGIKSIGLTQFGTNP
VASKVDYSIQTVRSNEAELRSAATSSLHTQFIAVDVIFYTYASKQYDQNIAMIHDSKA
EIKKYMR"

CDS 207384..207740

/locus_tag="JFBMFIFI_00191"
/inference="ab initio prediction:Prodigal:002006"
/codon_start=1
/transl_table=11
/product="hypothetical protein"
/translation="MEEAAYLFSGFFFIFIFIFYMALIVGGIFLWYFVIKKAIVKGIE
QSVLNKDQSQYERELYLLKLELNQLKSLVKSEELVDQAQQEIAESGKITLEKEQVTQS
EEFNELVTSEFDEKKE"

CDS 207765..208280

/locus_tag="JFBMFIFI_00192"
/inference="ab initio prediction:Prodigal:002006"

/codon_start=1

/transl_table=11

/product="hypothetical protein"

/translation="MERKTEFILTLIGAILSGLFSLLMIGITFLIGIGISATSYTASD

DYYYDSYNYSDSLSASEASIIIGAFAVISAIFIATAIFGFIAAFKVKKDSRGWGIAVF

ICGILSISTLHGILWLIAGIMMLARKAPKQEPMTSHTLKEDMEKLSSLHDQGVLSDEE

YEAKKNEWLDF"

CDS complement(208372..209175)

/gene="yofA"

/locus_tag="JFBMFIFI_00193"

/inference="ab initio prediction:Prodigal:002006"

/inference="similar to AA sequence:UniProtKB:O34685"

/codon_start=1

/transl_table=11

/product="HTH-type transcriptional regulator YofA"

/db_xref="COG:COG0583"

/translation="METNDMKIFIEVAHSSSVSKTATKLGYVQSNISKRIEKIETELN

CTLFLRTNKGMKLLPEGELFMTYCNEILTIMTRVNASFSMNSKILKIGATQNISHHYL

SSALFSETISVYTKPIPDLISFLKQSVIDLLIINREIDDDSLKENFSFNEEICWVQSK

ESEFKIENHPIVINRDSECPYRKATLAYIKANQMESTRLIQVDTLDVLISMLEKENVS

SILPKKILKNNDKLKKVNTTTELSPIKILGYTLVTTNQVINLDQILTIE"

CDS 209290..209805

/locus_tag="JFBMFIFI_00194"

/inference="ab initio prediction:Prodigal:002006"

/codon_start=1

/transl_table=11

/product="hypothetical protein"

/translation="MIGMQYKITLPNDYDMEIIKNRVKTNGSKTDGFEGLLFKAYLVV

NSPTKKQYAPLYIWKNSEGMNRFIFDGFYKNILNSFGWQTINIGVSSYIDLSDQFDKS

QFVLEIEHEIKETQNLATPELTFPDEPSLGKVIIYNPDKWKYTEFYFYEENPVEKSER

GSIYEIVHLSL"

CDS complement(209856..211631)

/locus_tag="JFBMFIFI_00195"

/inference="ab initio prediction:Prodigal:002006"

/codon_start=1

/transl_table=11

/product="hypothetical protein"

/translation="MKPLKIIKTSLKTIWYHPTEYIKSILFLQGIRLIVALPLITFLF

YRILAASSLNGITETTIYTIFKHPWALFFLLLLALVSLFFIYYELGYYFLLAHFQQTK

ENYRFRDIIKKLNQKAKYFISFQLLFFFFYFILILPIASIGLNAALTENLRIPNFITD

ELAKSTSGMLLYLGVLVVLFYLSMRLIYTVPFFVIETNLTIWQAIKKSWNYSRLKLFK

ALFTLGLLILIHSLIVAFVLFISFLPLIVIEKILNGAAPVIAGITLTFIQWFLFIFYG

LLQALLADALTHLAYPIAESSHFEINREQNPAFLFKPKRFMYWGVFAAFLGMSIVNTF

SVSKVIYQPTTAIIAHRGYTAEGVENTISSLDAAARIGADYVEMDIQETKDHQFVVFH

DSSLSRLSNRSERISDLTLAELQKIDVSSGGFTDKIPSFEDYIAAAKKDNIKLLVEIK

LHGRESKDMEKNLVALLQKEKVTETYLVQSLDEATIKKVKKLDPVIKTGYLVALNIGN

LPQISADFLVLEEFSFNTRLLEQARQQDKMVFVWTVNDEDLMRRYLALDVDGLITNYP

SDAIGIRDSFDENKTFVERIEDLLN"

CDS 211994..213715

/locus_tag="JFBMFIFI_00196"

/inference="ab initio prediction:Prodigal:002006"

/inference="similar to AA sequence:UniProtKB:P9WQJ1"

/codon_start=1

/transl_table=11

/product="putative ABC transporter ATP-binding protein"

/db_xref="COG:COG1132"

/translation="MGILFQQMKKFKLHTLLSVFFTAIMVLSQLWQPKLLQQVLEAIM

KDDMDNINSIGIMLISVAGIGLVAGILNTITSAKVAQEVGANLREESFRKIQTFSFSN

IERFSTGNLVVRLTNDITQVQNLVMLGLQSLVRIPLLFIGSFILALYTLPKLWWIIIV

LVILVFLIVMFSFGSMGKHFGKIQGYLDKVNGIAKENLGGMRVVKSFVQEDNELKRFT

TVSDLLTKHTIIVGTIFSVMIPAFMLVSNLTIVAAIYLVGDMVKDDPAVIGAIASFMS

YLMQIMMAIIIGGMLMMMASRAFISLKRISEILDTEPDITYENTSGDEVLAGSVEFKH

VSFQYDGDDQPTLKDISFKAEPGELVGIVGATGSGKSTLAQLIPRLYDPTEGEILIGN

ESLKKVSKKSLRKTVALVSQRAILFSGTIAENLRQGKKDATLPEMEKASAIAQAKEFI

EKQAERYDSMVEERGLNYSGGQKQRLSITRGIIGDPKVLILDDSTSALDARSEKLVKE

ALERDLKDTTTFIIAQKISSVIHADKILVLDEGKLVDVGSHKELVQESAVYREIYETQ

KGKEVAE"

CDS 213715..215487

/locus_tag="JFBMFIFI_00197"

/inference="ab initio prediction:Prodigal:002006"

/inference="similar to AA sequence:UniProtKB:Q9WYC4"

/codon_start=1

/transl_table=11

/product="putative ABC transporter ATP-binding protein"

/db_xref="COG:COG1132"

/translation="MAEFKRITRFFWDYLGQFKWKLSVIILAIIGSTYLQVKAPKYIG

EAIQELANYATRYFTTGVDDKTDFIHIIWLLVMFYVLLAVGTFIQSILMTGVAGKSTN

NMRLDLFKRMENLSIRFFDSHRDGEMLSRFTSDLDNISNTLNQALTQVLSNLALMIGV

VIMMFQQNVMLSWITLAASPVAVIAATLVIRKARKYVNMQQDYIGDLNGYIDEKISGQ

KIVITNGLEEETIEGFIEHNEAVKKVSYKGQVYSGLLFPMMQGISLFNTAIVIFFGGY

MALNGQIERSVALGLIVMFVQYSQQFYMPLTQISSQYSMLQLAITGARRVSEIFEEKP

ETERPDVKLIDGVHEEVRLDHVDFSYVPDKPILKDVNITAKKGQMVALVGPTGSGKTT

IMNLLNRFYNVDSGGIYIDGVDIRDIQLASLRKQVGIVLQDSVLFSGTIRDNIVFGKP

EATDAEVEDAAKQAHIHEFIMGLEKGYQTEISDEHSVFSVGQKQLISIARTIITDPSL

LILDEATSNVDTVTESKIQKAMENIIAGRTSFVIAHRLKTILNADYIVVLKEGEVIEE

GTHESLLRQKGFYSELYHNQFVLE"

CDS complement(215562..216437)

/locus_tag="JFBMFIFI_00198"

/inference="ab initio prediction:Prodigal:002006"

/codon_start=1

/transl_table=11

/product="hypothetical protein"

/translation="MKRIGKLTIFSLVIISSFFFYTNEVKATGNRDDIVRTAKNYLGV

PYVTGGTTPSGFDCSGLVQYVFKLNGYNLPRVTTQQENAGIIIPVSEAKPGDLYFFGN

KGNTTHDAIAIGNGDFIHAPQPGQNVKITNIKWYTPSFAVRILTDDIDYPHPAIDESI

ATVAYIPGYGVFGFRSNGENIPDSNLNLKHGTQWKTFGSKMINGEEMFSIGTDYYLPR

RYTNLDASVITINYIPDYGIKSYRKDGSWTGKYLSTGTSFQTFGFEIINDKIMYLVGS

NEYVPKQYTQFGNGK"

CDS complement(216460..217521)

/locus_tag="JFBMFIFI_00199"

/inference="ab initio prediction:Prodigal:002006"

/codon_start=1

/transl_table=11

/product="hypothetical protein"

/translation="MTTQKKNSLLFLFICLFTILIFPTTSSATSDESPVAQNTEIGPG

YYSKSFSNRTIRATPDIFLNQIKQGAIDGWKNYQILPSITAAQAILESGWGNSSLSQS

ANNLFGIKGRYNGQYILLPTQEYINGQWIVVNAEFRKYPNWSASVEDHGKFFHDNSRY

SNLIGIKDYKQVANLLKQDGYATDPEYPAKLISLIESNNLASWDSEAFKQSDYPHVAI

DESIAVVNYIPGYGVFGFRSNGETILDSNLNLKHGTQWKTYGSKMINGEEMFSIGTDY

YLPRRYTNLDASIITINYSPGYGVKSYRKDGSWTGNYLTTGTSFQTFGFEIINDKIMY

LVGSNEYVPKQYTQFGNGK"

CDS 218333..218422

/locus_tag="JFBMFIFI_00200"

/inference="ab initio prediction:Prodigal:002006"

/codon_start=1

/transl_table=11

/product="hypothetical protein"

/translation="MTLIYLVVGLVGLVLLGYLFYILFWGDHK"

CDS 218419..220101

/gene="kdpA"

/locus_tag="JFBMFIFI_00201"

/inference="ab initio prediction:Prodigal:002006"

/inference="similar to AA sequence:UniProtKB:P03959"

/codon_start=1

/transl_table=11

/product="Potassium-transporting ATPase potassium-binding

subunit"

/db_xref="COG:COG2060"

/translation="MSAIVVNQILFLVILIGLGIPLGIYSYRVMSGKKVFLSRVIEPI

ENQVYKFIGKPAKLEMTAKKYALAIVSFSLFSFLAVFLLMLGQGFLPANPNGFSGTSI

GLAFNTAMSFVTNTNWQAYSGEVTLSPFTQMVALTVQNFVSAGVGIAVLFALLRGFIA

RNKETLGNFWQDLTKAVLYILVPVSFVIALLLVSQGVVQSFSADVPVFSLETGQKILI

PLGAAASQIAIKQLGTNGGGYFGANSAFPFENPTVFSNFIENLAILLIPVALIVAFGL

FVKNAKQGRTIFIVSLVLLGLALVGVTMSESYTGPSYPGVVESGSLEGKETRFGVGWT

SLWAVSTTAASNGSVNGMHDSLTPLGGMIPMFLMQLGEIVFGGAGSGLYGMIAFVILT

VFIAGLLVGRTPEYLGKKIDPFDMKMVCLLILTPPLLVLLGTMSFVMLPNGMEWLTNS

GPHGFSEVLYAFSSLANNNGSAFAGLTSDTPFLNVVGGVIMLVVRFVPMFAALFLGGN

LAKKKMVAVSDGTLSTTNGTFVGMLIGVILLIGALSFLPAMALGPIAEYFMH"

CDS 220418..222475

/gene="kdpB"

/locus_tag="JFBMFIFI_00202"

/EC_number="7.2.2.6"

/inference="ab initio prediction:Prodigal:002006"

/inference="similar to AA sequence:UniProtKB:P03960"

/codon_start=1

/transl_table=11

/product="Potassium-transporting ATPase ATP-binding

subunit"

/db_xref="COG:COG2216"

/translation="MQEEQSRKMVFQDAFKQSFVKLAPMIQLKNPVMFVVYLGAVLTT

CLYFLSFAGINDAPAGFILVIALILWVTILFANFAEAIAEGRGRAQADSLKSARKEVM

AKKLKSIKAIDQFTEVVSSDLKRGDIVYVATGEQIPMDGDVVDGAASVDESAITGESA

PVIRESGGDRSAVTGGTTVVSDFLVIQVTAESGESFLDKMIAMVEGSARKKTPNEISL

QILLVTLTIIFLVVSATLLPFTEFSSQLAGNGSALSLTTIIALLVCLAPTTIGALLSS

IGIAGMSRLNKANVLAMSGRAIEAAGDVDILMLDKTGTITLGNRKASEFLPVAGVEEF

ELADAAQLSSLADETAEGRSIVILAKERFEIRGRDFQELNATFIDFSAKTRMSGIDYQ

GNEIRKGAADTVKQFVVAAGEVYPEECDQIVAKIAGLGGTPLVVVKNKKVLGVVYLKD

IVKNGVKERFDDLRKMGIKTIMITGDNPMTAAAIAAEAGVDDFLAEATPEAKLELIRQ

YQEEGHLVAMTGDGTNDAPALAQADVAVAMNTGTQAAKEAGNMIDLDSSPTKLIDIVQ

IGKQLLMTRGALTTFSIANDVAKYFAIIPVLFYGIYPQLNALNIMHLTSPTSAILSAI

IYNALIIIALIPLSLKGVAYKELPASQILRHNLFVYGLGGLIAPFIAIKLIDMALTVV

GLV"

CDS 222498..223076

/gene="kdpC"

/locus_tag="JFBMFIFI_00203"

/inference="ab initio prediction:Prodigal:002006"

/inference="similar to AA sequence:UniProtKB:P9WKF1"

/codon_start=1

/transl_table=11

/product="Potassium-transporting ATPase KdpC subunit"

/db_xref="COG:COG2156"

/translation="MKTFKAALMPGFKMLMVMTVICGFAYTLLVTGIAQLFFPEEANG

SIVTVALAEGGEKQIGSKLIGQGFTEPKYLLGRPNTGASASQLSPVSSEQKELVLERI

ANLEKIDPMNKAEIPMELVTASGSGVDPEISLATALYQVDRIANERGISEKEVEKVIK

NNTIGTIFGKIGEPRVNVLGVNMQLDGLKIVK"

CDS 223424..226117

/gene="kdpD"

/locus_tag="JFBMFIFI_00204"

/EC_number="2.7.13.3"

/inference="ab initio prediction:Prodigal:002006"

/inference="similar to AA sequence:UniProtKB:P21865"

/codon_start=1

/transl_table=11

/product="Sensor protein KdpD"

/db_xref="COG:COG2205"

/translation="MQEEDMRPDPDQLLLQVGQDEETTMGKLRVFFGYAAGVGKTYGM

LKEAIEQQETGKVVLAGYVEPHARPETMALLDHLNQLPSKKIPYKNMMLEEFDLDEAL

RLQPEIILVDELAHTNALGSRNKKRYQDIEELLQAGIDVYTTVNVQHIESLNDIVEDI

THVRVHETVPDSFFDEALIKLIDVEPDELLGRLKEGKIYRPEHAKKAMNHFFTKENLK

LLREIAIRRAADHIGMDNRIEEAYTEKRAHVKLLTCIDSEHQTGAEKCIRWTARLANA

FRAEWVVLHVDESGDNEKSEAELALLKRNNELAERLGAEVVTLSGHNPVTTIAQYAKL

TGVTDIIIGKHRRKLDYQRLLKDDIEDRLIKHLSNVELHIVPFIEKYPRIKNRFFSKE

RLIPKNLTIADSLKTMVLLLMATLLSEIFSFLGIGDQNVIVVYLLFILIISQVTTGYF

YGVLSSIISVIFFNWFFVDPLFSLTVYKAGYPITLLIMLMVALFTSNMMIRVKSQARE

SIKKEHQMGVLYDFNKKLLATRNLNGIINLTNDYLVSTMNHSVIFYTEQGKNLLNHKA

LAFENKQDIEVLTGSDEEAVANWVFNNQKVAGYGTDTLMGAKGFYLPVLSQGRVLAVI

GVLLSKENPLTHENLNFLKLIASQLSLALERQYLVTEQQQIMLETEKEKMRSNLLRAI

SHDLRTPLTGILGASSAILENKDKFDEELKLSLIKDIKEDSEWLIRMVENLLSVTRID

EGTMKVKKMPEAVEEIVAGAVRRIRKRFKEQELIVKVPDEFLLVPMDGTLIEQVLINL

MENAIKHSGSKGAISVIVKRNRNKAIFEVTDNGIGIPEKKLASLFDQFGKESDTPIDA

TRGMGIGLSICKTIVAAHSGTIEVTNKQQGGAVFRFTLPID"

CDS 226167..226853

/gene="kdpE"

/locus_tag="JFBMFIFI_00205"

/inference="ab initio prediction:Prodigal:002006"

/inference="similar to AA sequence:UniProtKB:P21866"

/codon_start=1

/transl_table=11

/product="KDP operon transcriptional regulatory protein

KdpE"

/db_xref="COG:COG0745"

/translation="MSIKTVLVIEDDPAISNFMTAILTGNDYKVLTADTGKLGISMCA

TWSPDVVILDLGLPDIDGIEVLQTIRGWSEIPILIVSARGNEVEKVSALDEGADDYIT

KPFGTSELLARIRTALRHSTKKDSENSQLINGELTIDFEKRQVEIAGEKIHLTPNEYK

IIQLLAINIGKVLTHDFISKHVWGPYVSENQALRVNMSNIRRKIEVNPAEPHFILTEI

GIGYRMVELT"

CDS 226975..227568

/gene="plsY_1"

/locus_tag="JFBMFIFI_00206"

/EC_number="2.3.1.275"

/inference="ab initio prediction:Prodigal:002006"

/inference="similar to AA sequence:UniProtKB:Q45064"

/codon_start=1

/transl_table=11

/product="Glycerol-3-phosphate acyltransferase"

/db_xref="COG:COG0344"

/translation="MPKEIQLSLILLLGYLVGTINGGYLLVKFRKKQDIREQFSGNAG

ARNVYRVMGKYGFIGTILIDGLKTIGIYLVINYYFPDWTMAKIIVFIGVFLGHCFPVF

LGFRGGMGVVVYLASTLLILPFAIVILVAVTLLVYAIFRNKTLAGLLGMACIPVTSFY

SPLSISWTYYFAGILLAILAFYVIPRNKKKEGTDLVE"

CDS 227702..228940

/gene="pbpX"

/locus_tag="JFBMFIFI_00207"

/inference="ab initio prediction:Prodigal:002006"

/inference="similar to AA sequence:UniProtKB:O31773"

/codon_start=1

/transl_table=11

/product="Putative penicillin-binding protein PbpX"

/db_xref="COG:COG1680"

/translation="MKKKIRTILALMLLLICVGAVGFFSAKYSTSLFNQPDSKNIKKV

LATSEENSKESSNEASSESSQEPGNPVVPDTQGAEMMANLDVPNDGSQYPELTQEIEK

ALNKDTFIGSVLVVKDGKVMLNKGYGFADKSQNIGNSFQSLFYIGSVQKSVTAVGIMK

LVDEGKLKVDDPVSNFYPNLKGGAGITVRQLLTHMSGIQGRKKPTVAMTHQQVVEDIV

NQTVLKDSGQWHYSNDNYSLLAGIIEQVSGKGYSEFIRDTILAPAGLSHTGFYDTFNS

ALFHANPYQKANTDPYQLASISDASLSQEFGAGNMYMTPGDLYKFNQALITGKLVSPE

SLAEIQTPGNSKNGYGFGMYMSNDTKYSHGVLGSYNVLVLTSLDAKDAVIIMANEKPE

FDILKVAGSIYEAAKKMENS"

CDS 229144..229671

/locus_tag="JFBMFIFI_00208"

/inference="ab initio prediction:Prodigal:002006"

/codon_start=1

/transl_table=11

/product="hypothetical protein"

/translation="MKLTKGLEQAVCIITLLATQEKEIPVSSFIINQRLQGSPTYIKK

LMRKLVVNDLVNSVSGINGGFTLAKRPQNITLLDIIEALEGPIVTYPNTGLINMVFQD

MQPVATQGDLVLTDVFQKADTYYTKYLNQQTVEDLIQETLGLHEIPVLNWNDLAEKKG

LLRKVLNNVNGNDEK"

CDS 229646..232231

/locus_tag="JFBMFIFI_00209"

/inference="ab initio prediction:Prodigal:002006"

/codon_start=1

/transl_table=11

/product="hypothetical protein"

/translation="MSMEMMKNEWKKLVNNKILLISFIVILFIPILYAAFFLKSVWDP

YGKTGELPVAVVNMDKSVDYEGKTLDVGDQLVENLKKNELLDWQFVSQKQAEQGMKDK
KYYMVVTLPENFSKNASTLLDKNPKKMNITYETNGSLNYIGEVIGETAAKQLQSEVSA
NVTKAYAESIFAQIEKVGDGFTQAADGSKKLQEGSTKLNDGNKTITENLQKLATSTIT
FSDGANKLEVGLDQYTDGVTQLNDGATKLNDGIGQLASNVGPLQAGVGQLSDGSSKLS
NGLNDLNNGQNDLDNGLTQLLAGSQTLQAGLTQLDNAITATDVEQPMGKLKAGLNQMK
AGMEKLNTQVNGPELTAEIAQLEAQIETQLGVVTQNLQGLGGTISPQNPENNAAKVIG
TINASPSITADEKAKLIAEVQPVLVAEAQEQGAYIQEIKTALESLSPADGSSILKKIG
ELQAGVQELYNGFNGQLGLYNGTNQALDTIHMALNNPNATAENPSLILGATSLSQGLE
GAKNGSSKLVGGVSQLSQGATALNGGIGQISGKLPELISGVTQLNDGSSQLAAGTGKL
TENSPQLKDGIGQLASGATQIQSGSGQLADGSKTLGDGLGTLNSGTKELSTKLGDGAK
EVNGIKATDKTFDMLASPDKLTHKEFSHVDNYGAALAPYVMSLALYVGSLVFNFIFPI
RKISMEGQSSRAWWLSKFSIGTVVAIAMAVIEVGIMLVLGLNVQSVGQMFTMAIVTSL
AYMFIIMFLAMTFDNPGRFVAMVLLIVQLGGAGGTFPMPLTNGFFNAIHPFLPMSHSI
YGFREAISGGLGQGTFNQSVFILLAIFVVFSGLLMVSMNWLQKRHLDDVSQLDNNQKL
QAVEE"

CDS 232449..233612

/gene="galK_1"
/locus_tag="JFBMFIFI_00210"
/EC_number="2.7.1.6"
/inference="ab initio prediction:Prodigal:002006"
/inference="similar to AA sequence:UniProtKB:Q9R7D7"
/codon_start=1
/transl_table=11
/product="Galactokinase"
/db_xref="COG:COG0153"
/translation="MELEQLKQQFENVFKEEQGKEFFAPGRINLIGEHIDYNGGHVFP
CAITIGTYGIASKREDSLIKLYSENFPELGIISFDVGDLDYRKEDAWTNYPKGMIRYL
KENGHEIQHGMNILYYGTIPNGAGLSSSASIELLTGVILQNLFKLELEELELILIGKK
VENQFIGVNSGIMDQFAIGKGQKNQAILLDCETLEYEMVPVELGHHKIVIMNTNKRRE
LADSKYNERRSECDQALKELQTKLNINSLGQLDEATFNENKFLLSAPNLVKRARHAVT
ENQRTLKAATALKSGDLNGFGKLMNASHRSLRDDYEVSGIELDTLVESAWLQPGVIGA
RMTGAGFGGCGIAIVAEDQINSFIKIVGASYNEKIGYQADFYIASISDGAKYL"

CDS 233636..234637

/gene="galE_1"

/locus_tag="JFBMFIFI_00211"

/EC_number="5.1.3.2"

/inference="ab initio prediction:Prodigal:002006"

/inference="similar to AA sequence:UniProtKB:Q7WTB1"

/codon_start=1

/transl_table=11

/product="UDP-glucose 4-epimerase"

/db_xref="COG:COG1087"

/translation="MITILVLGGAGYIGSHAVDQLISEGFSVAVVDNLQTGHVESLHK

KARFYQGDIRDKEFLESVFLKEEIQGVLHFAANSLVGESMELPLMYFNNNVHGTQVVL

EVMEKFGVKHIVFSSSAATYGEPKMIPIRENAATNPESPYGESKLMMEKMLKWCDKAY

GMRYVALRYFNVAGAKLDGTIGEDHNPESHLLPLILQTALGQREQLTIFGEDYDTKDG

TCVRDYVHVVDLINAHILALRYLIDGKESTILNLGSSNGFSVKEMVAAARKITRREIP

IISAPRRIGDPSTLIAASDKAYQILGWNPQYTDIDTIIESAWNWHCRHPFGYETKNE"

CDS 234681..236171

/gene="galT"

/locus_tag="JFBMFIFI_00212"

/EC_number="2.7.7.12"

/inference="ab initio prediction:Prodigal:002006"

/inference="similar to AA sequence:UniProtKB:E8MF11"

/codon_start=1

/transl_table=11

/product="Galactose-1-phosphate uridylyltransferase"

/translation="MDQLIIDFIEKGIKNGQIAAIDRIYIMNQLGMISHCTELNGNLV

PSTVLLSQLELLDGIIGGMIEKGIIDETLADREISEAQIMNLLTPLPSVVNHEFHRYY

QKSPELATNYFYRLSQENNYIKTREIAKNSHFSVPTKYGELEITINLSKPEKDPKQII

LEKSALQANYPKCLLCMENEGYRGRVNHPARSNHRIVRLSLNDERWGLQYSPYAYYSE

HCIFLSEEHRPMKIQQATFRKLLAIVKEFPHYFAGSNADLPIVGGSILAHDHYQGGKH

TFAMAQAPIMEHFKLKEFPEISMGIVKWPMSVIRLSGKDVSELELAATHVLKKWENYS

DQAVGILANDEDGTPHNTITPIARKNGAYFELDLVLRNNRRTDDYPDGIFHPHQDVQH

IKKENIGLIEVMGLAILPPRLKNELQEVEDFLLGKVTKVTECHQSWAEKLKNKERMIT

PENVHEIVEQAVGKVFLRVLEDAGVYKQNDIGLAAFKKFIMYANQK"

CDS 236201..237244

/gene="mro"

/locus_tag="JFBMFIFI_00213"

/EC_number="5.1.3.3"

/inference="ab initio prediction:Prodigal:002006"

/inference="similar to AA sequence:UniProtKB:P05149"

/codon_start=1

/transl_table=11

/product="Aldose 1-epimerase"

/db_xref="COG:COG2017"

/translation="MKISEKMVEEMDGQQVIEYCITNKNGASLRAINYGATLTAIEMP

DRNGQLANVVLGFATVKEYIEHKAFFGATVGRVAGRVKDGQFELNGEKYQLILNEGKH

HLHGGGHFNQQIWESKSKITKEGGELHFNYFSPTGEKGYPGNLNVSVVFTLTEQNQWK

IKYQAETDQATLFDPTNHVYFNLSGQPHSTILDHELTLKSDKIVELQTDLIPTGELTE

VKGTAFDLNKGHKLRAGIESRHPQNQLVKGYDHGFVLANKINEEAAILVDSSSGRRLK

MYTDRPAVVVYSGNQLNGEFELNGLPVQRYAGMTLETQGLPDAMNHQGFGSIVLKPGE

KYTSETSYTFDIV"

CDS 237356..238351

/gene="melR"

/locus_tag="JFBMFIFI_00214"

/inference="ab initio prediction:Prodigal:002006"

/inference="similar to AA sequence:UniProtKB:O34829"

/codon_start=1

/transl_table=11

/product="HTH-type transcriptional repressor MelR"

/db_xref="COG:COG1609"

/translation="MATIKDIAEKAGVSSATVSRVLNYDETLSVGDETKKRVFEVAER

LDYKKYQKKKPKKNAKIAIIQWYTEKEELDDLYYLSLRMGVEKRAEEEGYDTLRVFHD

TDFELEEEVKGIIALGKFSDKQVAKLANWTKNICFVDSDQLSNKYDSVVVDFEQAVES

VLSYFLQTNHEKIGFIGGKEKFADQSEDIMDKRTYLVENYLRRTNKYQKRYMFNGTFE

VKSGYELMQKAISELGEDLPTAFFAANDAIAVGCLRALQEAQISVPDKVSIVGFNDIS

IAKYVFPSLSTVKVYTELMGETGFDLLMDRIQSEREVAKKITLSTDLILRESTFK"

CDS complement(238401..240107)

/locus_tag="JFBMFIFI_00215"

/inference="ab initio prediction:Prodigal:002006"

/codon_start=1

/transl_table=11

/product="hypothetical protein"

/translation="MINMLKKHFYNYQLFFEIMIIIMIFMTTLSVILTTVTINLSSES
YIDSYVDSNKLILERTTIEYENLHSSINTMLTSFKDSQEVKLFLTTTEKESVTDNYTI
FYQFGAFMDRQSYQFYKNIPYQLVLVGINGQTFYNNNAIQNISTTELLAKPFVTSLKA
DPSLIASIPADYELIKRSINGPTILSGTAITDEKNHTIIGYAFIELTETDFSSIYDKL
ISPDVNTILVANQYNEVLSSNKKELLQTKKKKYTALNHIRQSLTIPSFNFSITSEIQL
DSLKKEMAIWPKVITLMLLIFLLLIILLFFYLKKILSPLYKLIETTPKIVEGDFNQKI
LVSGTYEIKKLISSYNYLQDGLNIYMQQLLYTEKEKRKAEIKSLQLHIKPHFIYNTLT
SIKFLIFENRNTEALTALNAFINLLEQTIYNKNELIPLKQELLILKDFSTILNTRYGN
RIEFMSIIPDELQEFKLPKLLLQPFVENAFIHAFPNEIAGSICVTAKQTDEWFRIDIM
DTGIGFNQDTTSQTEKQNFSGIGIHNVQERISLFYNNQATLTIESVKNQGTIVTIKIP
IL"

CDS complement(240100..241530)

/gene="rhaS_2"

/locus_tag="JFBMFIFI_00216"

/inference="ab initio prediction:Prodigal:002006"

/inference="protein motif:HAMAP:MF_01534"

/codon_start=1

/transl_table=11

/product="HTH-type transcriptional activator RhaS"

/translation="MTRKTIMIVDDEFYIRNGLAKILPWEDFGYSVIALANDGKDALD
FLAKKQPDILFTDIMMPKIDGIELTKTIFEKWPTIIVIVLSSYNDFHYVKTALKNGAS
EYLLKPSILPENLLELVRKVCPPDHSFKQKFSYNTEKKSNKIDSLVENTFNFPYFFGF
LIGENTFFTSHEIQELVTPFLINTSYKIINLQVNESYFILINHTIENFKLRLTPLKIP
YIFFQNTTDLIFLKEQIELATQFYQNYTYYFTKSYFIWDSKQIISQDANFLPEQKKLA
TLIKREHFSDAIILLRELTLGDKLFLKEELQNFFNSMIISLLSKMEDSLNQSMGMKKI
DFLTINQQSNTLKQTLQNIRIFLDLIHQDFLEENEDTIKQQILDYIQKHYAEGINLAS
IAEQFHFNYNYLSRIFTTYTGSSFTDYLNNIRIKEAKIMLKQSNLSIAKISEKVGYTD
TSYFSKVFKKIALCSPSDYRRSHSND"

CDS 241858..243147

/gene="lacE"

/locus_tag="JFBMFIFI_00217"

/inference="ab initio prediction:Prodigal:002006"

/inference="similar to AA sequence:UniProtKB:P29822"

/codon_start=1

/transl_table=11

/product="Lactose-binding protein"

/translation="MKFITKLVLLLSVALVATACGNQADSSKEGNKDDKLTIWAWDES

FNIKAANEAIKAYKEKENAKDAEFEVVTMAQEDIVQKLNTSLASGNTEGLPEIVLIED

YRIQGYLTSFPDAFEPLTDIVNEKDFSSYKFGINKVGKEIYGVPFDSGVAGLFYRTDY

LEEAGYQAKDLQNITWETYINIAKDVKVKTGKNMIGLNPSDLGTLRIMMQSAGGWYLE

DGKTVNIANNNILKEALKVYVELMNSGTVTLTPDWNAGVSAINNGEVASSPTGAWYAG

TIMGSEEQSGKWAISEIPRLGAVKDSINASSIGGGGWYVLKGVGNSETAKDFLKETFA

SDADLMNTLAKEIGLVSTLNAAAETENYQSPSEFFSNEKIFQNFSTWSGEVPVVNYGL

HTYAIENIFTEYVVQKIVDGADINKTLEDAQKQIEASI"

CDS 243223..244104

/gene="lacF_1"

/locus_tag="JFBMFIFI_00218"

/inference="ab initio prediction:Prodigal:002006"

/inference="similar to AA sequence:UniProtKB:P29823"

/codon_start=1

/transl_table=11

/product="Lactose transport system permease protein LacF"

/translation="MRIKPQLTKKSTGRYFYLPSVIIISVLVFFPMLQALMMSFQTGS

PVDMQFSGLSNYIRMLSDATFKKAALNTSLYLLVQVPVMILLSMVISVLLNQKNLKFR

SFFRIAIFLPCITSLVSYSLIMKSLFMESGLVNTILLNSHLISEPIMWLTHPVWAKVL

IIISITWRWTGYNMIFFLSGLQNIDPSIYEAADIDGTTEWQKFFRITVPMLKPIILFT

TITSTIGTLQLFDEVVNITNGGPANATMTLSQYIYNLSYKFTPNFGYAAAISFVIVIA

IVLLTMIQMKLGGDRDD"

CDS 244097..244930

/gene="lacG"

/locus_tag="JFBMFIFI_00219"

/inference="ab initio prediction:Prodigal:002006"

/inference="similar to AA sequence:UniProtKB:P29824"

/codon_start=1

/transl_table=11

/product="Lactose transport system permease protein LacG"

/translation="MTNKGSSIFKYGFLSVAAFVSVFPFLFMLLGVTNKSIDISTGNL

KIGQEFLTNLSNLLSNDLNFQRAFLNSLLVSLITTICALIISSIAGYGFEIYRSKKSD

KLFNFILLSMMVPFASLMIPLYRMFGQLNKIGLSNFGLNSYFSVIIPAVSTAFLIFFF

RQNAKAFPKELVEAARIDGLSEIGIFFKIFMPAAKNTYAAGAIITFMSSWNNYLWPLV

ALQSPEKRTLPLVLSAMGASYTPDYGMMMVGIVISTLPTAIIFFVMQKQFVQGMLGSI

K"

CDS 244945..247131

/gene="agaA_1"

/locus_tag="JFBMFIFI_00220"

/EC_number="3.2.1.22"

/inference="ab initio prediction:Prodigal:002006"

/inference="similar to AA sequence:UniProtKB:Q9ALJ4"

/codon_start=1

/transl_table=11

/product="Alpha-galactosidase AgaA"

/translation="MISITFQEEQKIFHLATETMSYIFKIEEGNIVAHHYFGRRIVHY

HGSRNYPRVDRSFSPNFYDAKDRFFSKDIVLQEYSDFGTGDFRTPALVLEQENGSKMT

DFRFASYQIIDGKNVVLGLPQIHSENSQTLELLFVDSTLPKIQLYLYFSIVEGKDVVI

RSAKLVNQSSESVTIKKMDSCQLDLPESSFELISLPGTWARERQLVREEIHSGIHTIG

STRGASSHQQNPFAALVRPETTELSGEVFGFSFIYSGSFQFTFEKDPFDQLRINMGFN

SLNFNWILGSKQEFQTPESVLAYSDQGLNGLSQIYHQLYRENLMRGAYQKCERPILVN

NWEATYFDFDEAKIKTLIEESSALGIELFVLDDGWFGKRNSDKTSLGDWEINLEKLPN

GLNGMNETAKECQMKFGLWFEPEMISEESELYKEHPDWVLRVPNRPMSRSRDQFVLDF

SRADVRIYIYQKMAEILSTHSISYVKWDFNRHLSEVYSDALPAKQQGEVHHRYLLGLY

EFLEKLTTSFPTVLFESCSGGGGRFDPGMLYYMPQTWTSDNTDAVARLKIQYSTSLVY

PMITMGSHVSAIPNHQTGRETSLEMRGAVAMSGVFGYELDVNSLTKEEKKEIKEQVAF

YKEHRQLIQFGLFYRLLSPFEGNQTAWCFVSKDQKECLVFYFNVLEEASAPLTLLKLA

GLQDNELYHSDELGTFSGSELMHAGFYTSTKKIGDFRSECYYFKTE"

CDS 247143..249149

/gene="bgaB"

/locus_tag="JFBMFIFI_00221"

/EC_number="3.2.1.23"

/inference="ab initio prediction:Prodigal:002006"

/inference="similar to AA sequence:UniProtKB:Q9RFN0"

/codon_start=1

/transl_table=11

/product="Beta-galactosidase BgaB"

/translation="MLQQKRLFYGGDYNPEQWSKAIILEDMRLMKKANVNYVSLNIFG
WASIQPTEEGFDFSFLDEMLDLLWENGIGIDLANGTASPPAWLVKKHPEILPVTSQGT
PLVHGSRQHYCPSNKVYRSYVIRLTEEVAKRYATHPGIVMWHVNNEYTCHISECYCES
CEKSFRQWLQMKYKKINTLNECWSTKFWSQSYSQWDEIFLPKEMPTFKNPAHQLDYKR
FISDQNLTLFKAEKKAIRSYSKDIPVMTNLMGLHKHVDGFAFAEEMDVVGWDSYPNPF
EEKPYPQFLANDLTRSLKKKPFLVMEQAPSAVNWRRANGAKSPGQMRLWSYEALAHGA
DGILFFQWRQSQGGAEKFHSGMVSHNQDTNSRIFKEVVQLGTEMSQLDELVGTNYNAE
VAIVFDWENWWALELDAKPSGEINYIKQMRDLYTIFHELNIGVDFIHPKEDLSNYKLV
LSIAQYLVTDDFLAKVKRYIKAGGHFLTTFFSGIVDEYDRVYLGGYPGAFKEVLGIYV
EEFDPMPIGRKSQIKYGETYYTTELWKEVIHLQGAETIATFTEGYLMGQPALTKFDYG
KGKTYYMGTKLAKDGNMKFIQTILAESKIQPLNQVEIESENSKISMTCRSNSSHDYIF
LLNYGQTSEKVKLKKGGQSLLDGSMVEGEVSVKANDVKIIKLTK"

CDS 249170..250930

/gene="bga"

/locus_tag="JFBMFIFI_00222"

/EC_number="3.2.1.23"

/inference="ab initio prediction:Prodigal:002006"

/inference="similar to AA sequence:UniProtKB:P48982"

/codon_start=1

/transl_table=11

/product="Beta-galactosidase"

/translation="MSNFAIREEFYLNEQPFKIISGAIHYFRVVPEYWEHRLKLLKNM
GCNTVETYVAWNQHEPKKGQYVFSDALDLRRFIQLADSLGLKVILRPSPYICAEFEFG
GLPAWLLKDRHMRVRSTYPPFMERVRLYYRELFKEVIDLQITSGGPIILMQVENEYGG

YGSEKKYLQELVTMMKENGVTVPLVTSDGPWGDMLENGSLQESALPTVNCGSAIPEHF
DRLAAFQQKKGPLMVMEYWIGWFDAWQDKKHHTTDVKSSVESLEEILKRGSVNFYMFH
GGTNFGFMNGANYYGKLLPDTTSYDYDAPLNEYGEQTEKYKAFKEVIARYSDPILEEE
IMPIPRIHSEEIQVKEEVSLFNTLDTLAKKIDSTYPLTMEDLDQNYGYTFYSSSIGAA
REVNDFRLIDVADRAQIFINQTLIATKYDQEMEANIVFTLTEPKNQLGILVENMGRVN
YSVTMDQQRKGISGGIVVNGAFQTNWSQYSLSLEDPTLIDFSREWIPDTPTFSRFVFE
LEESGDTFIDMSKWGKGVVFVNGFNLGRYWNVGPQQKLYIPGPKLKIGVNELIIFETE
GVSQRSIQLSSQPDHLNGIK"

CDS complement(251042..252850)

/gene="zosA_1"

/locus_tag="JFBMFIFI_00223"

/EC_number="3.6.3.-"

/inference="ab initio prediction:Prodigal:002006"

/inference="similar to AA sequence:UniProtKB:O31688"

/codon_start=1

/transl_table=11

/product="Zinc-transporting ATPase"

/db_xref="COG:COG2217"

/translation="MKNEKKLFLTFGIALLALILQFILKQDLYAQILITVSGIFVSST
MFVGMVKTIRSGNYGVDILAITAIIATLAVGEYWASLIILIMLTGGDALEDYAAKKAR
SELKALLDNSPQIAHKSEGNKLTDIKVEDVKINDILVVKPGEVVPVDGSILAGQSLFD
EASLTGESKPVNKEVGDKLMSGTINGDGSITMTVEKLAKDSQFQNIIRLVKESEEQPA
HFVRLADRYAVPFTIIAYLIAGIAWYVSKDPKRFAEVLVVASPCPLILAAPIAMVSGM
SRSSRNGIIIKTGTIIEKLASAKSIAFDKTGTLTNGILAVDTIHSTATFSKEELAILA
ASVEQQSSHILAKSLVAHVGTEHLLPVTASKETIGNGIQGEVDGKIVKVGKYAFVAPN
KEIPLLNQTALFIAVDNNYVGYVTFTDTVRPEAKQTISQLKKQGLEHVMMVTGDNKDI
ASTIANQVGIVDIKSDCLPEDKINVLKNIAEDARPVIMVGDGVNDAPSLAVSDIGIAM
GAHGSTAASESADVVILTDDLSKVSTSIQISKDTMRIAKQSVLIGIIICILLMLIAST
GVIPALLGAALQEVVDVVSIFSSLRARKSKEVIHLK"

CDS complement(253033..253452)

/locus_tag="JFBMFIFI_00224"

/inference="ab initio prediction:Prodigal:002006"

/codon_start=1

/transl_table=11

/product="hypothetical protein"

/translation="MHLLVPMDLDKTSQSALKEAILLTEKFKETGKITILHVVNSELS

APVVDSAEDLETKLLTEARDYLAKIKAIVEKKNILVEVVIFEGFVATKILDYEKEHAI

DLIVIGHHNRMGFDRLVVGSVSKKVIHEAICPVVVVK"

CDS complement(253468..254691)

/gene="yfmO"

/locus_tag="JFBMFIFI_00225"

/inference="ab initio prediction:Prodigal:002006"

/inference="similar to AA sequence:UniProtKB:O06473"

/codon_start=1

/transl_table=11

/product="Multidrug efflux protein YfmO"

/translation="MEIKPDFVEELDSLPDQKQESILKGLLKQPKPAWAVAFACVIAF

MGIGLVDPILKSISEQLHATPAETSLLFTSYMLVTGIVMLFSGYISSRIGAKKTILYG

LVIIIIFAVLGGFSNSVGELVGFRAGWGLGNALFISTALSAIVGVSVGGTEQSIIMYE

AAMGLGMSVGPLLGGLLGSISWRAPFFGVATLMAVAFISVTILLEKIPKPTKKVAFWD

GLRALKHKGLLIMGITALFYNFGFFTLLAYSPFRMESYSAMQVGFVFFGWGLCLAISS

VFLAPKLQEKFGTKNMMYAALLLFALDLMIMGFGANNSTIISICIIIAGLFQGINNTL

ITTAVMEVSPVDRSIASSAYSFIRFTGGALAPWLAGKLADWYNPHVTFWFAAVAVACG

ALVLFIGKAHLEQIE"

CDS 254864..255406

/locus_tag="JFBMFIFI_00226"

/inference="ab initio prediction:Prodigal:002006"

/codon_start=1

/transl_table=11

/product="hypothetical protein"

/translation="MNSLGYTLLSMLVRKPCSGYELKGYLELFWQAHHSQIYPLLTKL

EASGYVVYISENDPVHKKIYSITDEGKAFLQEWIEGKTAKPITRDEFLGKLYAVSIID

SNRVKELFEERKSYFQKLAEIHKKTLTEIETQFGPDLDGEGWESSFGRYLICKRRYDM

DLSELEWCEWAENLYEKKFK"

CDS 255596..255982

/locus_tag="JFBMFIFI_00227"

/inference="ab initio prediction:Prodigal:002006"

/codon_start=1

/transl_table=11

/product="hypothetical protein"

/translation="MLHHIDIYVKDLEKQSNFWSWFLGELGYQEFQKWETGISWKKAD

FYYVLSIGDQGLIQAPYQKGGIGLNHIAFGTEKRATVDRLKEEIQAHGGKVLYVEDYP

YAGGPDHYALYFNDPEGMKMELVATE"

CDS complement(256110..256616)

/locus_tag="JFBMFIFI_00228"

/inference="ab initio prediction:Prodigal:002006"

/codon_start=1

/transl_table=11

/product="hypothetical protein"

/translation="MSPEEIETPGIRLENSLFFVDQIKEKVDYIHLSMGSYKRTSLND

KKNKATLISQFSTHTKGVVPLIGIGSVEHPDEAEEVLHDGADLIAIGRELIREPKWVQ

KVAIGDIASIRTKLSPLDMDELAIPSVMQVYLTESFNSVMHFTTDSANVQNYQATLAP

MEGFEKKI"

CDS complement(256697..257293)

/locus_tag="JFBMFIFI_00229"

/EC_number="1.-.-.-"

/inference="ab initio prediction:Prodigal:002006"

/inference="similar to AA sequence:UniProtKB:P0DN73"

/codon_start=1

/transl_table=11

/product="putative oxidoreductase"

/translation="MSQNFLSNYTFSNGLSVKNRVVMSPMTTMSSFYNGMVTSDEINY

YGLRAGGPGIIITGVANVSENGKGFEGELSVTTDSMIPGLTALAKAIKKDGTKAILQI

FHAGRKSTSKVLRGEQPVSASAISATYPADSEVPRELSHEEILQIITDFGEATRRAIE

AGFDGVELHGANTYLLQQFFLKTQINALMNGEGLRKNV"

CDS 257441..258130

/locus_tag="JFBMFIFI_00230"

/inference="ab initio prediction:Prodigal:002006"

/inference="similar to AA sequence:ISfinder:ISBth20"

/codon_start=1

/transl_table=11

/product="IS6 family transposase ISBth20"

/translation="MRNVFKGKQFDKRIIIEAVGLYCRFSLSYRDVSEILRHRGIQVN

PTTIMRWVHQYGNLLFVIWKKKNKPTSTSWRMDETYIKIKRKQHYLYRAIDSHGNTLD

MCLRKHRDTNAAYAFMKRLIRRYGEPRALVTDKCAATIAAVNKLKNEGFLKSVDYRLS

KYLNNVIEQDHRQIKKRFSKSLGFQSMKSAATTIQGIEVVNALYKKSRRIMSLFDFSV

WDEIRYLLEIA"

CDS complement(258585..259517)

/locus_tag="JFBMFIFI_00231"

/inference="ab initio prediction:Prodigal:002006"

/codon_start=1

/transl_table=11

/product="hypothetical protein"

/translation="MKVSRLVSIIMTLIDKKRINAKELSDIFEVSILTIYRDMEAINM

AGIPVHSTPGVGGGFEIMENYKIDKNTFSETDITTLLVGISSIPSAVKSKEFSNTLTK

IRSLIPVDKVDSIHLQTEQLHIDFSHWMGNRDLEAYLSTIKMALQECKVLSFGYINHK

GNKSKRQVEPYQLVLKSSQWYFQGYCYERNDFRLFKLTRLSNLEMEDSLFIPKDYQKP

FLNKPEIWTKLQVRIKLRIHISIMEQLLDYCEYGKFLPDGTNYYIVYFPFIENDYHYG

ILLSFGKRCECLEPLHIRSELKRKIADLSKIYEI"

CDS 259616..260254

/gene="ydeA"

/locus_tag="JFBMFIFI_00232"

/EC_number="3.2.-.-"

/inference="ab initio prediction:Prodigal:002006"

/inference="similar to AA sequence:UniProtKB:P96658"

/codon_start=1

/transl_table=11

/product="putative protease YdeA"

/db_xref="COG:COG0693"

/translation="MKTIYFYVLDTLADWEVGYITAELNSRRFFKEDAPLLTIKTVSH

SKQPIYTMGGVKISPDCLVADIELSESNVLLLPGADTWNDSKNNAILTVARVLHSVGG

TVGAICGATVAIANIGLLNEYVHTSNGSGFLEMFCHSYQGQKFYVDQPSISDNNLITA

SSTGALMWARQIIENLDVFEQDTLDAWYNYFNTGDSNYFFELMQRLPPENKS"

CDS complement(260449..260637)

/locus_tag="JFBMFIFI_00233"

/inference="ab initio prediction:Prodigal:002006"

/codon_start=1

/transl_table=11

/product="hypothetical protein"

/translation="MGIPSINGYVNDDKEKNFSADIYHQHFEGDIINSNLTETDENLK

NSAKNVFDIEKNEEIQSK"

CDS complement(260760..262220)

/locus_tag="JFBMFIFI_00234"

/inference="ab initio prediction:Prodigal:002006"

/inference="similar to AA sequence:UniProtKB:Q97P44"

/codon_start=1

/transl_table=11

/product="Putative trans-acting regulator"

/translation="MKTILAKHLTRQLNLLEYLDSKQYVEQSLIIQELGYDSKTLNKD

IFAINHYIKPLAIYTNFKKGIQLTIPQNYSFDYIYSQILTESTEISFIEKIFFNETYS

FNSLSELLLISNSTLRRIINGLNIKLKKEDIYIQTSPLKLVGNEKKICTFLSQILIEK

YFNSFLPFSEVQINVVEKILNIAANNNKQTLNYAEFERVKIWMLVSIIRIQNNNEVCN

SFDFPNSIDVSLLKNSALKRLFKATFLIPLNKQNALHIFSPFLNGSYAIDEQHLENLT

HSVASIKDFVNQIDNLIFYLSKTFKIPIYNHKNLTLQVYNLFTLRYKSNFIFTYRRQH

FVNEITNQHSFFIDTLEKEMNKNKNHSIWENDEIYLLAYFLIINWNQLASELNKASPS

MSVGLFLKTDIGHDAFIKEKLLYYFKNRLTITILNNLSLTSLNIKSVDYDLIITNISG

LEFLKTPHLCFPMYPTMKEFELIEIHYKNFVFCRNL"

CDS 262498..263997

/locus_tag="JFBMFIFI_00235"

/inference="ab initio prediction:Prodigal:002006"

/codon_start=1

/transl_table=11

/product="hypothetical protein"

/translation="MLSFIDTHAIRSMKVLLFINSSFNPVSLDEIINEIGSVKRTTYT

LLELLKTEVNQCDFNLVEDKNRNYYLVAKDKRREVDLNLYLLVCGESSLFFTMIKEIY

ERGFINIYNFCREHYISHPTFSRAKANLNKLLKLCYLNITIQKDYKIVGNEYRQRIFY
YQFFEGFYGTLRWPFEKKVEKEIIDFCTFSRAIFFEQVSEENQYKIYYMLAIMRDRNL
KGRKVKKTTYSFENHSLYEDVYKFVQIFLKKWTNQDHETIKRETTFFFCFLYTEDLLS
SRNEPIENLLSAAIDNQQMYYLNSLWIKVFTSCFERVLTEKQKINWFKEVTNIHLKLE
LIYFDRELFMKNQYQIKDVLFNDEIYKKTESFVSHLLENDLYFNYKEKNLKEISITTI
INHYYHYIYKFFIGEKKNDLVTLFVSDSIEVIEKQILEKKLKLLFGEKIKISKFLGQG
EELVVTSRNLNLLTAKSIVVYSVSDLRNFKKIVNAVERELYSKAFDTIN"

CDS 264139..264399

/locus_tag="JFBMFIFI_00236"
/inference="ab initio prediction:Prodigal:002006"
/codon_start=1
/transl_table=11
/product="hypothetical protein"
/translation="MRIEDKWIDGFLYYIEFKVIQEEVFKTIRKTIVIFEELDYIEVE
RIIRSSFGNVIEVNKIDSYEDILFLKGNEKQALKNKIGIALE"

CDS 264422..265402

/locus_tag="JFBMFIFI_00237"
/inference="ab initio prediction:Prodigal:002006"
/codon_start=1
/transl_table=11
/product="hypothetical protein"
/translation="MKIAKHVLLWGLSLIFLFNFSLISLANTTTSQAGIIFTEKEEAK
EETNTFQKNNDLANTENLRTSLKLPKTGEQKDGNLFMIGLILLVASMLIEKLRKKRGG
RKMKVKTMTILGLIGSGMLIGATSASAAIEDEIIGSSDGKGGTSHGYINLTPGKDDGV
TNPVEPTVPPGGTVNKGVLTLDNIAPLLFSTHKLDGKKQVYTSVVENSNIQVSDKRGE
EAGWHVQVSQTPFIDKVDNTKVLKGTTLVLPAGVVKAADSDNVSLAPTVSSVEVNADL
ATLMSSAKGTGAGTWLTVFDKNEVKLTVAAGNKKGEYMSIVTWALMDAPK"

CDS 265429..266085

/locus_tag="JFBMFIFI_00238"
/inference="ab initio prediction:Prodigal:002006"
/codon_start=1
/transl_table=11
/product="hypothetical protein"

/translation="MKISKYFLVGLGLSMSVVNLGNHSVEAAEASKGSSKANFELQAG
DGTTIPELLDPEIQPPTLNPGPLSLDSVSSFNFSTKKLGSESQKPLEATPVLGTKLGL
QVTDSRGQDLGWNLKVSATSFETSDKKLTLKGAIMTIPAGKLTTEEGVDPLLTPTAYK
VDLSSTAATIMSATTTQGRSRWSNAFEGKGEKVTLAVPSGNKVASYVSTITWSLEDAP
"

CDS 266121..266819

/locus_tag="JFBMFIFI_00239"

/inference="ab initio prediction:Prodigal:002006"

/codon_start=1

/transl_table=11

/product="hypothetical protein"

/translation="MKKSSLVIATVASAAFVFGGTVAQAAVSSTHTSENKINFTPGVD
IITPPVDPTNPDNPDVPNPVDATDPTNPGTGQKGPLSIDYVSNIKFGQHKITGKDIAY
KALNANPFVQVTDLRGAGDGWNLSAKMTEFKNGNKVLKGATLTVKDGVVKAGSSSNIS
IAPTKSDFVFDNQESKQVMGAKDKGGRGTWLTVWSGTDQANESIQLVVVAGTPEANTE
YTSSITWELEDAPK"

CDS 266905..268023

/locus_tag="JFBMFIFI_00240"

/inference="ab initio prediction:Prodigal:002006"

/codon_start=1

/transl_table=11

/product="hypothetical protein"

/translation="MKKTFNILVVFLIGFLHIPMTGLAVESMAYSVKANIPENQINKT
LTYFDLKMEPNQRQEISLTVSNSGDDKVTIIISPNIAITNQNGVIDYSQLDAKKDSSL
KNPLTAIISKKQEVTLEAKETRDVPFTVQMPEKEFDGLILGGFYISKKEDAPKDKDKD
KNVQIKNKYSYVIGLQMRENTNEVKPVMELNDVKPTLLNYRTAITANLQNTEATIIKD
LTVDAKVLKRGTTKIIHEISKENLSMAPNSNFDFPINWDNKSLDPGNYTLQLVAQSGE
EKWEFEKHFKITSKESNNLNKEAVELEKSEPNWLLVISIVFSGILVLIVIVSFFIYQH
QKKKAAEKRAHLMKQRKRKKKQELRKRRNSIEKMNTHS"

CDS 268200..270719

/locus_tag="JFBMFIFI_00241"

/inference="ab initio prediction:Prodigal:002006"

/codon_start=1

/transl_table=11

/product="hypothetical protein"

/translation="MKKKLILLILCSVLVLIVSIDSSVKASPLPPFSLPLDNVFTVPT

GSNSYVEGDVTVITKDINYQIGSIFSTDINKLDLTQSFHAEMYVYLGNKLGNVADGMT

FVMHTDKKRIDNFTGIAGEHLGVYAKIGSDTNKGLQEQMERSFAIEFDTYYNFDRFHN

SATMDRDLSANSNKGHIAYSFPDLKSSYNLTASGTILSLIHRGLYYPSDYLSNGKWHL

FSVDWNADTKKLDYKFDDAPTVSVPINPMTVFGSTSVYWGFTGSTGGSSQESKVAFKQ

IPGLVNLSSDMKITKDEKDITKTGVSGSEGDIKVQFDLKYDGGKQNLLNPTFNLKLDD

LLTFKPGTLTVNGTVVRDEYFVNGQLTYNFPKNLNAEEDMFTISFEGTPKVITDKERV

SSINYSVVAKNYIGNDLKTTFPIKKDTIVKSIDFENQSWLINEINRQFSPRKINQDIY

LSDLAKITIINSATGVTYPTEYIPAAINKLPNLTHLQIENLNLIGSLPIELGDLDKLT

TLSISGNKFDSEIPVSLGKLSQIEFLSLNNNQLKGLVPTTISSLPNLKKINLNDNPLS

GQIPDFSMSMEQINLSNTQLTYNLATVPLFLKNVEGRNYSNTFIEGLKLTGNSKVSSK

NTQLQPFNESDSGYFNLQALQENGVSQELHNEHTYTIKNTANGTIYYQGLKNIEATIP

YDKNISYTIILDEAEQNPNNVFVVLGKERELKFEEVPATLVLKIKLGMEKQPISLNGN

LSVFDDRENKDWKLSITLSELTQGQKKLQGEYSYTSKDGISHSIANEQKFLLETGVSD

TLNEVIPISWDNKHGLSYTAYSSNLIGNYTGDVTWTLEDTP"

CDS 271058..271972

/locus_tag="JFBMFIFI_00242"

/inference="ab initio prediction:Prodigal:002006"

/codon_start=1

/transl_table=11

/product="hypothetical protein"

/translation="MKIRHKVLIFGGILASLFVEGIVSNTYLKDNIFAEENNFKNVAE

KKEKILRVPLQTSISSFFNVYCKHDGLRKCSMRNVFKGKQFDKRIIIEAVGLYCRFSL

SYRDVSEILRHRGIQVNPTTIMRWVHQYGNLLFVIWKKKNKPTSTSWRMDETYIKIKR

KQHYLYRAIDSHGNTLDMCLRKHRDTNAAYAFMKRLIRRYGEPRALVTDKCAATIAAV

NKLKNEGFLKSVDYRLSKYLNNVIEQDHRQIKKRFSKSLGFQSMKSAATTIQGVEVVN

ALYKKSRRIMSLFDFSVWDEIRYLLEIA"

CDS 272116..272466

/gene="licA_2"

/locus_tag="JFBMFIFI_00243"

/inference="ab initio prediction:Prodigal:002006"

/inference="similar to AA sequence:UniProtKB:P46319"

/codon_start=1

/transl_table=11

/product="Lichenan-specific phosphotransferase enzyme IIA

component"

/db_xref="COG:COG1447"

/translation="MDGLELIAFTIISTVGTAKSNVMESMSLARSGKFEEAEAKVKAA

NEFLRTSEKEHFKVITQEAKEKNVELSLLFIHAEDQLMTTVCLRDVAVELIETNKKIA

CLSDTIDQLVEAQK"

CDS 272463..272723

/locus_tag="JFBMFIFI_00244"

/inference="ab initio prediction:Prodigal:002006"

/inference="similar to AA sequence:UniProtKB:B0SR19"

/codon_start=1

/transl_table=11

/product="Sugar transporter SemiSWEET"

/db_xref="COG:COG4095"

/translation="MIAFLGILAGLCTSISFVPQAIQVIKTKKTEDISLVTYCLFSVG

VFFWIIYGGIQKDPAVFLTNIVTFIPAVIILTLKFKSVRRMK"

CDS complement(272831..273481)

/gene="cat"

/locus_tag="JFBMFIFI_00245"

/EC_number="2.3.1.28"

/inference="ab initio prediction:Prodigal:002006"

/inference="similar to AA sequence:UniProtKB:P26841"

/codon_start=1

/transl_table=11

/product="Chloramphenicol acetyltransferase"

/db_xref="COG:COG0110"

/translation="MKEFTNWSDTKQLKELIKNSLIEVGEGSYYSGYYGNQNFEDGCV

RYLWGDEKTKDLFNPIEDFGWKMDKLIIGNYVCIAGGATILMGGNHNHHPDWITVYPF

AGHIKESYAPKGDTIIKSDAWIGMNAMIMPGVTIGEGAIVASGSIVVGDVPPYTIVGG

NPAKAIKKRFTDEEIKQLLTIRWFDWTADEIKNAEPILMSQSIDKLYEYYQENILN"

CDS complement(273933..274808)

/locus_tag="JFBMFIFI_00246"

/inference="ab initio prediction:Prodigal:002006"

/codon_start=1

/transl_table=11

/product="hypothetical protein"

/translation="MKEKKTLAINGYIGIIILILMALLGVWLFISGTGSLGGSVSVPL

LVSGGVLLILSGIFASTVTIVAPNQAKIIVLFGRYLGTIRNNGMFITVPFTNKLHISL

RVRNFNSSKLKVNDVDGNPIEIAAVVVFRVIDTAKAAFDVDFYEDFIEIQSETAIRHV

ATKYPYDSFQDTDVTLRGNSAEVSQELTEELQRRLEVAGVEIIEARLTHLAYSTEIAS

AMLQRQQAKAILSARQIIVEGAVGMTQLAIAQLEEEGVLKLDDERRLQLVNNLLVSII

TDKGTQPVINTGDVS"

CDS complement(274836..275231)

/locus_tag="JFBMFIFI_00247"

/inference="ab initio prediction:Prodigal:002006"

/codon_start=1

/transl_table=11

/product="hypothetical protein"

/translation="MGKKNGLKVLVGLSMLAGVTGSVLFKKQQRKRVRIGKPVFEHFQ

GQWRKKDDADTQLIFLSISSEGEFFLNQKELAGKIIHATDQKLVFEDSFGYQLVLELT

APGELTLYDDAEEQDYFLEKIVDSEEFSD"

CDS 275480..276832

/gene="yclM"

/locus_tag="JFBMFIFI_00248"

/EC_number="2.7.2.4"

/inference="ab initio prediction:Prodigal:002006"

/inference="similar to AA sequence:UniProtKB:P94417"

/codon_start=1

/transl_table=11

/product="Aspartokinase 3"

/db_xref="COG:COG0527"

/translation="MKVVKFGGSSLASGVQLKKVFQIVKEDPTRKFVVVSAPGKRSKA

DEKVTDLLIDCATKSLINEDVQPILEKIIARYASIAEELDLGSEIIAEIKQNLTELIA

GDKTQPEYYLDAFKASGENNNAKLIATYFNREGLKASYVNPLEAGLFVTDEPGNAQVL

PEAYTNLYNLREKDGIVIIPGFFGYTKSGKISTFSRGGSDITGAIVANGVKADLYENF

TDVDAIYAANPNVVKDPAEIVNLTYKEMRELSYAGFSVFHDEALYPAFRGGIPVQIKN

TNNPTAEGTRITPERKQTNQAVVGIASSNGFSSIYIEKYLMNREIGFGRKVLEILELD

GLSYEHTPSGIDDLTVILRSDQLTKEKEAKLVSQLKNELGADIVTIQHHIALMMVVGE

DMRCNIGTMSKASEALSKNNINIEMINQGSSEVSMMFGVSEADEEKALTALYEAFFTK

"

CDS complement(276879..277202)

/locus_tag="JFBMFIFI_00249"

/inference="ab initio prediction:Prodigal:002006"

/codon_start=1

/transl_table=11

/product="hypothetical protein"

/translation="MGHKKINWKNLVGIILFNLLIASLIVLFFYSIAAILWVSIFTLT

VSPFLVIIADVLHYLPFSMENLIIGTLFYALAMLLVPIVAKYTTKLIKLTKDYILYTI

KFLYY"

CDS complement(277398..277721)

/locus_tag="JFBMFIFI_00250"

/inference="ab initio prediction:Prodigal:002006"

/codon_start=1

/transl_table=11

/product="hypothetical protein"

/translation="MKNKKINKGAFIGVVGFNSSIGLGALIVLYAILACLWVITLLCI

ACPAIMIFAGVIHLQTVTAIRIFISVLVCSGGVELAPITLKLTHHLINLTKQYLNLSK

KMFYS"

CDS 278049..278930

/locus_tag="JFBMFIFI_00251"

/inference="ab initio prediction:Prodigal:002006"

/codon_start=1

/transl_table=11

/product="hypothetical protein"

/translation="MEHFGQKVKETRIVKGVTQKGLAEGICTQATISNLENGSSFPGL

GTLLAIASRLDIDFSEIYEHTELNGNGYSEVFKQIKMLCGKEKYKEAYKSLTTEIKVE

NLETNSEIKHYYYYLGITSLLAKNNFSDAFYNLNLALFTETGKHITSIDVLISTGIGL

AYQLNSEESKAKTYFERGLNQLDSLKNQHQDNVEMITIYCRVAKFYRKIEEYKKALNL

INLGIAIQKNEQVFFCLDTLIYEKGSSLAALGKKKEAEQQFFYAAAMAEFNQNTTLVK

LIKEQVGNYQLEGYRYW"

CDS 279128..280015

/locus_tag="JFBMFIFI_00252"

/inference="ab initio prediction:Prodigal:002006"

/codon_start=1

/transl_table=11

/product="hypothetical protein"

/translation="MKRIGLRVKKSRLANGLTQKELAKGICAQATISHLEKGNSQPSL

ALIVAIYKRLGLKVDNLYEGITTKARYTKIYAHVRTLISQQHYQESLAFLTEKIKIDQ

LENPSEIKQYHYYYGVNTLLGYHTYSDAFYHFSLTLLTKTTNQADHLDLLATNGMGVA

YLLNSEPRKAHTYFKKSEQLVKQSLKTVSTLQASSEIAKIYYTLAKYYSRDKEYTKAV

EFCEKGIKLQNKHQLKDYLGILYYEKGFNLKKLNRLSEAEDYLVNAFYAAKESKNQCL

MDVLKNNQKNDSLDDYPYW"

CDS complement(280155..282017)

/gene="zosA_2"

/locus_tag="JFBMFIFI_00253"

/EC_number="3.6.3.-"

/inference="ab initio prediction:Prodigal:002006"

/inference="similar to AA sequence:UniProtKB:O31688"

/codon_start=1

/transl_table=11

/product="Zinc-transporting ATPase"

/db_xref="COG:COG2217"

/translation="MKLLKNNPAMIATLISGLLILLGWILKLNAQETIATFIFLTSFI

IGGFKQAKEGFIDTYQNKNLNVDILMVLAAIGASIIGYWMEGALLIFIFSLSGSLEEY

ATNKSTEAIASLMHMQPETALKIQKDGSTKEVSIKSLAIGDSIFVPKGASIPIDGIIE

KGNGIIDEAAISGEPLPVEKSVGDPIFGGTINLYEGLTFTVSKDVQDTLFSKIIRLVE

EAQNTPSKTATLIKKIESTYVKIVLTFVPIMIAIFYFGLHWGWNESFYRGMVLLVVAS

PCALVASATPATLAAISNGAKHGILFKGGAHLENFSQIKAIAFDKTGTLTQGMPVVTD

SYFTPDCTIDETINAVIAMEKTSTHPLAKAIVNFLEPKMTESIDLQFIKDQTGHGLAC

QAYADQWKIGKKEYIGADIQGNSVLFQEAELLQKQGKTVVYITRNAQTVGYLGLLDVP
KSDAKEMIAFFKKQHIQTIMITGDNEATGQTIGKQLGLDKIYANCLPETKTTIIADLK
EEYGMIAMVGDGINDAPALASAAIGIAMGAGTDIAMDVADVVLMKNELNKLNYSYELS
KKLKKITIQNMIFSITIISVLILSNLFQLINLPLGVIGHEGSTILVILNGLRLL"

CDS 282456..283520

/locus_tag="JFBMFIFI_00254"

/inference="ab initio prediction:Prodigal:002006"

/codon_start=1

/transl_table=11

/product="hypothetical protein"

/translation="MLGISVFLGEDLSNETKAFIREMNENGFTGVFSSLHIPEDDVNQ
YLQRLEDLGSFTKKLGMDLMVDISGAALSKIGLSFKRPSEILATGITGLRMDYAIENK
IMAELSQHMKVALNASTITKKDVAELQEFNANFDNMEAWHNYYPRPETGLGKEVFVAK
NQWLKELGFRVMAFVPGNKKLRGPLFSGLPTLEKHRFSNPLSGMIELKEECHVDDVYL
GDPEIDSETMNQFNLYQSEKRILLHAIPTESKSYLELIEGDHQNRFDPARDVIRSADA
RFKKIKKVEAQQTVPRKIGSVTIDNQDYGRYMGEIQISLVDLPSDEKVNVVAVIVLAD
IDLLKQCSRAGQLFRIQIQS"

CDS 283541..284428

/gene="murQ_2"

/locus_tag="JFBMFIFI_00255"

/EC_number="4.2.1.126"

/inference="ab initio prediction:Prodigal:002006"

/inference="similar to AA sequence:UniProtKB:P44862"

/codon_start=1

/transl_table=11

/product="N-acetylmuramic acid 6-phosphate etherase"

/db_xref="COG:COG2103"

/translation="MQLEKLTTEARNKDTMGLDQLSTSEMLILMNNEDKKVANAVEKE
LKSIHAVVDQIIAAFKQNGRLIYLGAGTSGRLGVLDAAECVPTFGVEPSMVQGLIAGG
MKAMTIAVEGAEDSKELGIADLKEIQLTKNDVVVGIAASGRTPYVIGGLEYANQIGAT
TATISCNKDAEISQFATFPIEVNVGPEILTGSTRLKSGTAQKLILNMLSTVSMIGIGK
VYQNLMVDVKPTNEKLEERSKRIIMEATECSYELASQKFEEAGQQVKLAIVMILTESS
KSEAEKKLAKANGFIRDTL"

CDS 284447..285907

/locus_tag="JFBMFIFI_00256"

/inference="ab initio prediction:Prodigal:002006"

/inference="similar to AA sequence:UniProtKB:Q7A804"

/codon_start=1

/transl_table=11

/product="PTS system EIIBC component"

/translation="MVELKEDRLAREIYEKVGGTGNVSKVIHCMTRVRMDIRDESLVQ

MEDLKAIDGVMGVVEDDNLQVIVGPGTVNKVANKMVEMVGVKLGDPIPVDSNRSETAK

GNSGLEEAERVAAATKAAQKKKNNTPVKRALKSIANIFVPMIPAFVGAGIIGGIAAII

SNLLLAGNISGDNWVTIVTVLNIIKNGLFVYLQIYVGINAAKEFGATEALGGIVGGIV

YLAGMTPEMPLPNIFTGGDLVAGQGGIIGVIFAVWLIAIVEKRLHKVIPEAIDIIITP

TISLLVIGMLTIFLIMPVAGLISGGLVGAINWVLNVGGAFAGFVLGAAFLPMVMFGLH

QVLTPIHIEMITTTGMTLLLPILAMAGAGQVGAAIALWMKCQKNKKLTNMIKGALPVG

ILGIGEPLIYGVTLPLGRPFVTACIGGGIGGAVIGAFGGIGAIAIGPSGVALLPLIAN

GKWLGYLIGLLAAYLGGFLATYFFGVPKEAMEATEL"

CDS 286095..287498

/gene="gadB_1"

/locus_tag="JFBMFIFI_00257"

/EC_number="4.1.1.15"

/inference="ab initio prediction:Prodigal:002006"

/inference="similar to AA sequence:UniProtKB:O30418"

/codon_start=1

/transl_table=11

/product="Glutamate decarboxylase"

/db_xref="COG:COG0076"

/translation="MLYGKENQAKKNDYLEPIFGTGAEEQEIPKYKLGKNPIEPRVAY

RLVKDEFLDEGNARQNLATFCQTYMEDEAALLMAETLEKNAIDKSEYPRTAELENRCV

NILADLWNAPKEKSYTGTSTVGSSEACMLAGMAMKFRWRNQAKKLGLNIQAASPNLVI

SSGYQVCWEKFCVYWDIEMRRVPMDEKHMSMDMDKVMDYVDEYTIGIVGILGITYTGK

YDDIKALDALVETYNSQTDYKISIHVDGASGAMFTPFIEPELQWDFRLKNVASINTSG

HKYGLVYPGIGWVLWRDKAYLPEELVFNVSYLGGEMPTMAINFSRSASQIIGQYYNFL

RFGFEGYRAIHQRTKDVAMYLAAEVEKTGLFKMYNDGVNLPIVCYALKVETEVEWTLY

DLADRLLMKGWQVPAYPLPADLEDVLVQRYVVRADFGMNTAVDFMEDFNEALKDLNQA

HVLFHSKEEHGAQGFTH"

CDS complement(287550..288368)

/gene="uppP"

/locus_tag="JFBMFIFI_00258"

/EC_number="3.6.1.27"

/inference="ab initio prediction:Prodigal:002006"

/inference="similar to AA sequence:UniProtKB:Q5WNX2"

/codon_start=1

/transl_table=11

/product="Undecaprenyl-diphosphatase"

/translation="MIDILKAILLGIIEGITEWLPISSTGHMILVDEFLKLNVSAEFM

KMFLVVIQLGAILAVVVLYFHKLNPFSPKKTKQENQDTFSLWFKVIVAVLPAAVLGFL

FDDYLDEKFYNFQTVAATLIIYGVLFIVIETWNKDRKPSITNFNELSYKMALFIGFFQ

VLALIPGTSRSGATILGAILLGTSRFVATEFSFFLSIPVMFGASLLKLVKFGFVFTGM

EYAILFTGMITAFIVSIIAIKFLLGYIKNNDFKAFGYYRIILGIILLGYLFFIK"

CDS complement(288511..289491)

/locus_tag="JFBMFIFI_00259"

/inference="ab initio prediction:Prodigal:002006"

/inference="similar to AA sequence:UniProtKB:P62723"

/note="UPF0324 inner membrane protein YeiH"

/codon_start=1

/transl_table=11

/product="hypothetical protein"

/translation="MKIIKGLTLVLFIALVATWLGSLFPIIGSAVFAIIFGILIKNTV

DVAPEFKPGIAFSSKKILQASIVLLGFSLSIQDIGKTGLSSLKVTLVTIVVAFIIAFL

VGKWLKVPNKMQILIGVGTAICGGSAIAAVSPIIEADDDEIALSISTIFLFNIIAVFL

FPFLGHYFQMTDAGFGLWAGTAINDTSSVVAAGYSFSQPAGDFATIVKLTRATLIIPI

SLIIAGITFYQKKKTAKKVALKQIFPWFILYFLIASIISSTGILPATFIATTSWLAKF

MIAMALAAIGLSANLKDMLKTGPKPILLGLITWFFVAGSSLLIQIIEKQW"

CDS 290157..292094

/gene="thrS_1"

/locus_tag="JFBMFIFI_00260"

/EC_number="6.1.1.3"

/inference="ab initio prediction:Prodigal:002006"

/inference="similar to AA sequence:UniProtKB:Q97PI4"

/codon_start=1

/transl_table=11

/product="Threonine--tRNA ligase"

/db_xref="COG:COG0441"

/translation="MKMIKLTFQDNSSKEFEAGITTRQIVAEIYPKLVKKALAGKFNQ

QLIEMNQKISEDGSLIIVTSEAEDAPIFLKNSAILMLGMALIKLYPEIKLASYGMDEI

GFYYDFENSEAVIEERLSDVENEMKELVKANEKINGEVMTQKEAIEQFKENPYKLELI

KGSINQESYSLAKIGEHIDLVSGPQLQTTGRNQNFKLLTVSGAYWQGNSKNKMLQRIY

GVAFFSKKELKDYLQKREEAKERDHRKIGKELNLFMTSQEVGLGLPFWLPKGATIRRI

IERYIVDKEISLGYQHVYTPIMANTEIYKTSGHWEHYHEDMFPPMDIGDGELLVLRPM

NCPHHMMLYKNDVHSYRELPIRIAELGMMHRYEKSGAVSGLQRVREMTLNDGHTFVRP

DQVLAEFKRTIALMVAVYEDFNITEYRFRLSLPDLLNTEKYHGDQKVWSETADLLRKA

LKDLNLTYFEAEGEAAFYGPKLDVQVKTAMGMEETLSTIQLDRFLPEKFDLTYVGEDG

EKHRAIVIHRGIVSTMERFVAYLTEIYKGAFPTWLAPVQVMILPVSVAHHAEYAETLK

ESMLEKGIRVEVDYRNEKLGYKIRSAQMNKVPFQVVVGDEEMLNHTLTVRRYQSKENR

TMAKNDFIQMLISEINQYSRG"

CDS complement(292149..292670)

/locus_tag="JFBMFIFI_00261"

/inference="ab initio prediction:Prodigal:002006"

/codon_start=1

/transl_table=11

/product="hypothetical protein"

/translation="MKNVFKYLLLISAILMMAMGFWFIFNPTASLKTTTILIGLLLGG

NGLVEIFSFLQERKVWNISKWILFDGFASLIAGIFILFNPGIAQSTLIIAFAIWVLFS

GVMRLLTAISIKDFPGWTWLLTLGIIAILFAIFSFFTPILVGIVLGFILGFFFIIQGF

NILMIYLMVRNKS"

CDS complement(292778..293671)

/locus_tag="JFBMFIFI_00262"

/inference="ab initio prediction:Prodigal:002006"

/codon_start=1

/transl_table=11

/product="hypothetical protein"

/translation="MDYLGNKLKERRLSKGLTQKQLADGICTQATISNLENNSSIPTF

PVLMSLATKLDIEFKEIYDYVEEQSHKNTKIFNELRILCSEIKYKEAFELLNNKIDYN

QLEDSLELRKYYYYKGITSLLAYQNFSDAHYNFHLSLYSDEKRELDFFDVLSTNGIAM

TYFLDSETDKARTYFDKALLQLNQLMDYLNLNQESLEIIKIYYNTAKFHSSVKEYQKA

IELCTLGIALQKQQNMNFELDKLYYEKGFNLYQLGRTKEAEEYYYYAAALAKMDNHDL

ILDIIKIDMQEFNLTGYTYWE"

CDS 294072..294950

/gene="rhaR_2"

/locus_tag="JFBMFIFI_00263"

/inference="ab initio prediction:Prodigal:002006"

/inference="protein motif:HAMAP:MF_01533"

/codon_start=1

/transl_table=11

/product="HTH-type transcriptional activator RhaR"

/translation="MGVYLEIPELNEHFLFRSFVNEGDILVYPHWHKEIEIIYVKEGN

VNIGVNDVPIQLKRNDIYFINGGDVHYFLASPESERIVIQFDLSFFQDISSLEKTNKE

MRNLFSSIVQASSLWSQEVADQMRQLLMTVHEENNERKSGYRYVIKAKMFEMLAILSR

EVPQNENWNDQVREEISSTKQMENLERLDKIFMYIEAHYQDTITLNDISAYMGFSSFY

FTKFFKKNTGTTFIQFLTEYRLNKAKWILLNEDATVTEVAERTGFSSVKTFHHQFKDL

MGISPLKYKKTISGNN"

CDS 295123..296202

/gene="iolG_1"

/locus_tag="JFBMFIFI_00264"

/EC_number="1.1.1.369"

/inference="ab initio prediction:Prodigal:002006"

/inference="protein motif:HAMAP:MF_01671"

/codon_start=1

/transl_table=11

/product="Inositol 2-dehydrogenase/D-chiro-inositol

3-dehydrogenase"

/translation="MTLKIGIIGCGGIANGKHMPSLKAVEEGEMVAFCDIIEERALKA

KEEFGTDDAKVYTDYKEMLKDPTIDVIHVCTPNSSHSVISIAAMEADKHVMCEKPMAK

TTAEAEAMLEAAKKTGKKLTIGYQNRFRKDSSYLHTVCENNELGDIYYAKAHAIRRRA

VPTWGVFLDEEAQGGGPLIDIGTHALDLTLWMMDNYKPKYVVGNSYHKLSKKENAANA

WGPWDPAKFTVEDSAFGFITMENGATIALEASWALNSLDVREAQTTLCGTEGGADMQD

GLRINGEAHGQMYTKKIELEAGGVDFYDGAGDDPAVLEARQWLQAILNDTEPVVKPEQ

ALVVTQILEAIYQSSQTGEPVFFTK"

CDS 296224..296676

/gene="yvbK"

/locus_tag="JFBMFIFI_00265"

/EC_number="2.3.1.-"

/inference="ab initio prediction:Prodigal:002006"

/inference="similar to AA sequence:UniProtKB:O32248"

/codon_start=1

/transl_table=11

/product="putative N-acetyltransferase YvbK"

/translation="MEIKKVTPAEYPMDLLYLADPSKEMLDKYLGNSQVYGLIKNNQI

IGVCVVQIKTSKKAEIMNIAINEKNQNQGLGRYLLEQILIEMKQQGIQQVEIATGNSS

VGQLHLYQSLGFKIFKRQENYFIEHYSEPIFENGIQCIDQIWLEKIIT"

CDS 296695..297435

/gene="iolE_1"

/locus_tag="JFBMFIFI_00266"

/EC_number="4.2.1.44"

/inference="ab initio prediction:Prodigal:002006"

/inference="protein motif:HAMAP:MF_01672"

/codon_start=1

/transl_table=11

/product="Inosose dehydratase"

/translation="MTKPIALQLWSVQDETEKDFFGTLEKVAEMGYDGVEFAGYYGKT

ADEIQVKLAELGLKVAGSHIQYEQFLTNLDEVLAFEKVLGNKNLIVPWAAFDTVEEWQ

LFADNMKTIAEKVTAAGFNFSYHNHNHEFEEINGQPILTWLLQEVPTLNVELDTYWVQ

FAGVDAVEYMANYAGRMKLVHLKDQKENPIESAEIGNGVLDIASYVKQAEENGVDWFV

IEQEAFTQPTLKSVEIGLINLKRIIAEG"

CDS 297455..298177

/locus_tag="JFBMFIFI_00267"

/inference="ab initio prediction:Prodigal:002006"

/codon_start=1

/transl_table=11

/product="hypothetical protein"

/translation="MVKVTVWNEYRHEKKDEAVAKVYPKGIHSQIASFLTEAGIDTTT

ATLDEPEHGLTEAVLAETDVLIWWGHIAHQEVSDEIVNRVQKRVLEGMGLIVLHSGHM

SKIFMKLMGTSCDLKWREAEEKERLWVIDPSHPIADGIGEYIDLDVEEMYGEHFDVPN

PDELIFVGWYEGGNVFRSGMTYKRGSGKIFYFQPGHETYPTYYNKDIQKVIINGVKWA

EPTKRNYPVYGHSKALEEIKPK"

CDS 298213..299181

/locus_tag="JFBMFIFI_00268"

/inference="ab initio prediction:Prodigal:002006"

/codon_start=1

/transl_table=11

/product="hypothetical protein"

/translation="MKLGVFTPLFADLSFEEMLDRAKSAGLDAVEIGTGGNPGNHHCP

TDELLASPEKRQAYLAELEKRGLTISAFSCHSNPISPDATEARESDEILRKSIQLAAL

MGVPVVNTFSGTAGDSEDAKAPNWPVIPWPTVYSDIKTWQWENKLIPYWKEIGEFATE

HGIKIGIELHGGFLCHTPYTILKLREETCDAIGVNLDPSHLWWQGIDPVGAIKILGKA

GAIHHFHAKDTYLDQENINMYGVLDMQPYGDVQTRAWTFRSVGCGHSMSEWSDMMSAL

RTYGYDYVVSIEHEDPLMSIDEGFNRAVTNLQSILIKDAPLDMWWA"

CDS 299222..300298

/gene="iolG_2"

/locus_tag="JFBMFIFI_00269"

/EC_number="1.1.1.369"

/inference="ab initio prediction:Prodigal:002006"

/inference="protein motif:HAMAP:MF_01671"

/codon_start=1

/transl_table=11

/product="Inositol 2-dehydrogenase/D-chiro-inositol

3-dehydrogenase"

/translation="MKQDKLIVVIVGFGGMGHYHSTLIEKDQNLKVKGIFDTKQDRLD

YGTSLGYEAYPDYSAVLNDPEVDIVLIATPNDVHKELAIAALKARKHVICEKPVTLSS
QDFIEIDAVAKENAKVFMTHQNRRWDEDFLIVKEMVDQQTIGDIFQLESRVHGANGIP
GDWRHELAHGGGMLLDWGVHLLDQMLYMVDSKVTSVSAQLSFILGNEVDDGFISTLQF
ENGVTAIVEVGTTNYIPLPRWYVKGTKGTALIEDWSLKGKIVKRNEAVEKVEPKPIQA
GVGLTKTMAPPSEEATIIAELPEAQKNPQSFYENFAAVVAGTAEPIVKNSEVLRNLRL
IEAIFEAAHTNQVVTDFDQYSKTK"

CDS complement(300351..301826)

/locus_tag="JFBMFIFI_00270"

/inference="ab initio prediction:Prodigal:002006"

/inference="similar to AA sequence:UniProtKB:Q97P44"

/codon_start=1

/transl_table=11

/product="Putative trans-acting regulator"

/translation="MKHLLNSTDLRRLEFLKLLFDTDEWLTLNELATQLNCSAKSLRN
DLRAINEIFAPFSISTSVKRGLQLSYPENFTFDYIYSVILSNSLEFNFLELIFFNEHL
KKRDLEEYLYVSTPTVSRLIKKCADFFAPLAIKIQSSPYRITGNEIQIQNFYVAYFSE
KYLATDFPYSKKHESIFLELLNFVPQPFKNKSTHLNIKHLTYLSMVCLTRLSHGNLLE
SEPLPFLGKDLENKIMTNNLFMTDFQKTIGFKLTPLILSQLFYLYYKNDYLHATNFLP
EKIVACSEKKKINDLTTFLTAFQKKIGVNLNNKEELVNRLYKVQFHSLDINFILYDKR
QHFSTFIKTEYPFLIELLLNELPDYLTSNSRYFNEFIYTLIIYWPNLLEGLGNTQTPI
SIGIFSTFDYSHTLFICDLINYHFHKNVLLTIIEPNTAPESLNLEEKYDLIISNNSYS
LNIPLISINSIPTKHDWKNIKKSINRLLSSKNKITTKYLAI"

CDS 302273..302830

/locus_tag="JFBMFIFI_00271"

/inference="ab initio prediction:Prodigal:002006"

/codon_start=1

/transl_table=11

/product="hypothetical protein"

/translation="MKKRIVYSVLVSLAFVLTSCGTPEKKKTAETKTAEANESSQIRI
DTPYLPKSGSIKVSGENVAIGDIDFSIKKVSKLDGVNGTKLAFIQYKVTNNTQQKNRP
SEFWYRYVRVTQDSNIPLTVGALPFEEKENKNGKLVKKANRFLEKEQTTDGAVLYVLE
NDSDVKITYLDTDYQEISSQNYSLD"

CDS 302847..304787

/locus_tag="JFBMFIFI_00272"

/inference="ab initio prediction:Prodigal:002006"

/codon_start=1

/transl_table=11

/product="hypothetical protein"

/translation="MKKRMKFIGMSAAVLLALSPVVMPAIGATSTVVYADESYDGILK

NAIQSNYTVEDWTAERILGLKTLVNSGATNQLYTGSITNNSFYTDILSSKIFNNQTLN

QILGVAGASAKAQILVDGKSQVTEADLSKAQLTLTVKIFATNGKTAEAEVLLKTNAPE

LAVNDEMLSLTQAFGADYVDFELNPELLTVANNDSNGSWSVDTVVSVTPTKERLVRDG

ILDSEGRYIKAGEYTQTITASLISTTGTEVLRTIDVTISVLAPNAADLKFIAKTADAK

EYANGSTVLQTAIENMDATVAATSPYKFEDNLILNSPVFFNFDTKVTNYGELDEVLKN

RFIELFQVSNEISGTVATLPIQIDKLTVDSTSIDFSKAGIATVPFKYTTPNGQEMTLS

LQLKMKNPGNPVFEFSQNQDITISIGNAFNLYDFKVYPNTNDLNNGNAAGNEGIGLLG

GVTINGSVNTSKVGVYKITYTATNLNHLTTTLVRTINVIDPNEGNEKPEITNFKSVGY

VDYITGYGIRVWGTPKGNGSSQFLPHATAWKIDQKATFKNGDIWYRVGKDQWVDGSYI

KFSPVANDPGMNELKGVGTINYVPGYSINVYKGAEASSANWTGKQLEHGTKWRVYGEK

NGFYNLGGNQWVASNYVTFIKD"

CDS 304823..306697

/locus_tag="JFBMFIFI_00273"

/inference="ab initio prediction:Prodigal:002006"

/codon_start=1

/transl_table=11

/product="hypothetical protein"

/translation="MNKVKFITLNAVLVLGLLPIGTTLASVETVHAEEISQRAGVGSL

LDDKLKQTSSIGVDVLAQHADALAAIVNQQANEGILTNYSAEPERKAAMESLYKTILI

SEDGTFTLHSLTNQISSAQLIINGKTSITANEIKEAQSLTIEFKLFEFNLPDPSTSQM

VTVNVNPYSVKSTTINTKIESSYQAETGISVKTPAGIPVESDDIEVKHSGGPVNANGI

PLDLIEAGIADATGNFINVGSYNEELEVKVAGTVVASNLKRTIIVGIGEFQPSFRGEA

GQGTRYDHGSTVANSIDFLAIEGDQVTAEQLEQKIKSDVITNFHISDKNAIASTSYQE

LTNPAALSVDTSSINLFKSGTYQIPVTYSKPGSKIVSTITVPTKVTAVSPPVIRFSEG

QNLTIKVGESFDMTKNLNVFENQAAAANPTNSGKNVTWSIEGNIDTNKEGVYTLRYIA

TNIKGAKTELTRIITVEKSSETPKVENYTTVGYVNYVPGYGIRVWEAPNQTATDYFLP

HASAWKIDQKATFKDGSVWYRVGKNQWVDGKYISLSPVTSGEEVSVDGIATINYVAGY

SVNVYSSPLSSEASWTGKQLKHGTRWRTFKKTTLNGTTFYNLGGNQWVDSQYIIFSEN

"

CDS 306771..312473

/locus_tag="JFBMFIFI_00274"

/inference="ab initio prediction:Prodigal:002006"

/codon_start=1

/transl_table=11

/product="hypothetical protein"

/translation="MMKKSKWQLALLILISSIFGFSGAKLAVADTVAREGLIDVGEDL

QLSYYFGNKPGNVEITPNRVQVANVNFLTKGKRVEHLYSNGGNKGTISMGFNGANSYV

DPTNITVSSITGGGINSAVTATYTANYAGNQFEITSTLAPRGNNIIVSDTIKNISGKD

LENVWIARSYDTKLNGNDAVPVKYLGSNNGLYIAQDDFKLSYNFHMPIGPEGWAADQY

NKQIVPVVFSNPTNLGQASLNKPEGDIAFSNGDSGIFMKWSPKDFPKDSSREIGYIVG

VTPENIDAPEVTVEQEEVEYRGGDLALNGTVLSKDSSVTNLDLYYQVDGNTAKKFKTL

PNNPVNTAFPWELTLPAEELTGAMPKQISIYAIDSNGYYSDSVSVDLVLPKSQPVTIE

YKNQEGATLKASAKIEKKYGEEYDVETDNGGTLLPNFDYYTIEALPGNKSGTVTDEPQ

TVVIKYLGDLVGETTGKVTAQYINQAQDELTTEIPATGNGRYGSPYSTVQKDFSHYDF

VEVDPTGATQNGTYSNTPQTVKFKYKGHPVGDDGKVHVTYINQEGVAIADEVEAIGDE

LVGSHYTTVKHEIENYEFKSAELDGQAVTEIPVSGIYTMDPQFVTYHYQGQQVGENKG

QVKVRYVNQEGVDISPEVSATGNGLFGSPYTTEEKEIDHYQFIDVDPAGASATGFYTA

TQQIVTYRYQGEDVGEAGKITVNYKNQAGTMLATESFTGIYGQTYNLSTDNNGVLLKE

FEHYTRNELPGNAVGTITDVPQTITLTYTGDQVGNNIGRVTAKYLNQEGVVISPEVEA

NGDGLFGSPYTTEQKVIENYEFVGNDVENAPPTGFYKETPQTVIYQYQGRDVSEEGKI

TIEYRNQIGKLLDKAYITGHYGQTYNLETDQNGSLLKTFEHYTRDTLPENASGTISDT

PQTIIVTYTGNQVGNELGRVTVRYVNQEGATIAPEVDAEGDGLYGSPYTTKNKTIANY

DLESVDVNGVVTTEIPAKGHYSAEPQVVTYTYKGQEVGESRGKVIAKYINQEGVTISP

ENPASGNGLYGSPYTTEQKDIPHYEFVDIDPSGSSATGLYSDKPQTVIYRYKGEEVGE

AGKITIEYRNQAGTLLDTAYITGIYGQPYNVATDNNGELLKDFDHYTRNELPGNSVGT

VTDMPQTVVITYTGELVGNEAGAVVARYINQEGVAIAEEKAADGDKLYGSPYTTEQKT

IPNYDFVKLAENGAPATGFYTDEPQTVIYQYKGNKIGTVTVTYKNQAGDDLAAPITLK

GDFGSNYTAELKTFDHYQIDSLPGNETGTFLTTPQTVALTYTGEDVTGKGDVTVRYVD

QNGIQLTNDTITEPIKLTGRYGDHFRTTEEVFPDYNFSQATLNGETTSSPVTGRLNDQ

PQEVIYKYKGQLAGKLTIEYQNQIGKILKTEEITGSVGDEVTLTPPTEGIFQNYTLNP

NQNLTFSLEKQPQTKVIYYTGKVAKPVLEKHIGADGTIFKEEYKRGAFGDAYEFLPLL

ATETVGYTFDRLIGSKVGFLTNEEKTVTFIYKKDKIEKGLIKVTYQDEFGKQLALPVT

IKGNVGTEYTAKAKKIKGYVANAESQTATYTNEETVISFVYTREPVAGANVQVKFVKE

TGEALAGLSPLTLSGNLGVGYDATSGNNEEVQTEIEKAAALGYSVKEIPANVTGTFTN

KKQIVVYVFEKGEIPAGAVTVNYVDKDTLVEVADSDSLTGTIGAHYQSEAKIIPGYRL

VEMPSNASGSFTNEDQSVNYQYEKATGGEVTIRFVNEDGVELAEPEKRTGLVGDSIWP

ENNAKEILGYTVIGNSLQELKGQRFTENNQTLDLTYKFNSTNPDIKPVKVVCIDLYGN

ILKSNVKVVQSGQTLAVLAPVIEKYTLLGTVSKVTVNWSLGDKDPETIYFTYRGIGSK

"

CDS 312570..312815

/locus_tag="JFBMFIFI_00275"

/inference="ab initio prediction:Prodigal:002006"

/codon_start=1

/transl_table=11

/product="hypothetical protein"

/translation="MKFEEKWLDVEIINIEFLSFEKKRHNRVFIIPVGLTKKEIVTFL

QKEFYLVEEYLHIDFIEEGFYYKGNYFSTINQEKKTI"

CDS 313459..313641

/locus_tag="JFBMFIFI_00276"

/inference="ab initio prediction:Prodigal:002006"

/codon_start=1

/transl_table=11

/product="hypothetical protein"

/translation="MGIGAIGVGLAVYFLSFFYGVLVDEFNLETNPLIIGSVLYSAGI

FVSQLILFVTTFVRKR"

CDS 313798..314697

/gene="penPC"

/locus_tag="JFBMFIFI_00277"

/EC_number="3.5.2.6"

/inference="ab initio prediction:Prodigal:002006"

/inference="similar to AA sequence:UniProtKB:P10424"

/codon_start=1

/transl_table=11

/product="Beta-lactamase 1"

/translation="MNYKSYALVGVCLAVVGGYLLSQKPVGAQVKTVQKISNKAIDSD

LKKLEKEYEATLGVYAIDTETNKTINYNGEQRFAFASTYKALAGGLVLDKFNWDELNQ

TIMINQEDLVDYSPITEKYVGVGMPLKDIIGAAMEYSDNTAGNILFDQLGGPAGFQKE

LEKLGDHTTESVRYEPELNEAIPGDRRDTSTPKAIALDLKKLTAEKTLAPDKLTFYKK

QLVENKTGGNLIRAGIPTEVLVGDKSGAASYGTRNDIAVLYPPNRKPIVLVIFSNKTN

KDDAYQDELIAKSAKLVGDYFKL"

CDS complement(314780..315682)

/gene="bglK_1"

/locus_tag="JFBMFIFI_00278"

/EC_number="2.7.1.85"

/inference="ab initio prediction:Prodigal:002006"

/inference="similar to AA sequence:UniProtKB:Q93LQ8"

/codon_start=1

/transl_table=11

/product="Beta-glucoside kinase"

/db_xref="COG:COG1940"

/translation="MTNYIGIDIGGTAVKYALLDEKGTITQAGKKQSPKTLEALITCL

KNIQNEFSSLPIVGIALSMPGLIDSSTGHAVHGGALTFIRDCNIRDILTEACQTTIHI

ENDGKCAALGEYWQGRLAGHSNGVALVLGTGIGGGIIVNDQLLKGAHLSAGEFSFIQT

SQDFGELKDLFAYRNSVPMLVKNYAEEKDIPLKDMDGKIFFKAVDDEEPLAVKLLEDY

CQSLTQQIMNLQTILDPEVITIGGGISKQGRLFELIDYYLDSYLKKVPYPIVRPTVCQ

STLGNHANLLGALKNYFIQTELNS"

CDS 315759..316523

/locus_tag="JFBMFIFI_00279"

/inference="ab initio prediction:Prodigal:002006"

/codon_start=1

/transl_table=11

/product="hypothetical protein"

/translation="MFDLFDTETYTDLDQLLIKYINGNLEQVAFMRIRELAEAVHVSP

ATVMRFSQKAGFASFPEMKVAIKNYVEQQKSILKNEERRRGILPLDVFSNDLEEQIDL

ITAKLIEAPLIYCLGMGSSGVMAEYAAKQFTTIGLRSFHATSLYLPYLKESQNHSPKD

VTLLFSVSGETFEIIHIANLLKEAEAFSVSITNHIENHLAGLTSINLSYDTSYDRLQY

NVDISSQLPVVFIIETLVKKLYLLKHSGKKEELNQK"

CDS complement(316564..317904)

/gene="licC_2"

/locus_tag="JFBMFIFI_00280"

/inference="ab initio prediction:Prodigal:002006"

/inference="similar to AA sequence:UniProtKB:P46317"

/codon_start=1

/transl_table=11

/product="Lichenan permease IIC component"

/db_xref="COG:COG1455"

/translation="MMDKLEIFLNKFLGPIANWMNKSKFFSALSEAFMRTTPVTLGAA

VLMIIGNFPIPAWVAFVESSGLKVHFDSVIGATLNAISIFIVFNFAYVYAKKENYNPL

SAGLLAVGSFFILMPQQVAVPTLETAVTKFPSQGIVTGTTNVEAFQTLYTGGTGLLVA

IIVGYIVARLYVFLNKKNLVVKMPDSVPPNVAESLSPSLLAGAIFIFFFIIRLLFSFT

PFGSVFAFVFGSIQAPLQALTGSPITIIIIFTIANMLWFFGIHPNMVYGVVMPMLLAN

SVANMNAFREGQPLPFLLATVIAQVCGNGFGGQGSTYGLVVAMFTAKSERYKQLLKLA

GPPSIFNVNEPLVFGAPLMLNPIFFIPMAITPLIMGSTAWILVKLLNFTELNPLISLP

WTTPAPILMGLQGGWKYLIIFAVVFILNLIIWFPFFRIADNRAVDEEKAFVESN"

CDS complement(318048..319493)

/gene="bglC_2"

/locus_tag="JFBMFIFI_00281"

/EC_number="3.2.1.86"

/inference="ab initio prediction:Prodigal:002006"

/inference="similar to AA sequence:UniProtKB:P42403"

/codon_start=1

/transl_table=11

/product="Aryl-phospho-beta-D-glucosidase BglC"

/db_xref="COG:COG2723"

/translation="MIHTKLDPFPKDFLWGSASAAYQVEGAWNLDGKGKSVWDEFVRI

PNKTFKGSNGDVAVDHYHRFKEDIALMAEQGLKTYRFSIAWTRILPDGRGEINQKGLD

FYSDLINELLKYGIEPIVTLYHWDLPQALEDAYGGWESRQVIQDFTNYAKILFDAYSD

RVNYWVSLNEQNVFMMHGFLMASHPPAVTDPKRMYAANHIANLANASVIKAFRDGEYP

GKIGPSFAMSPAYAVDCQPENVIATENMLDLFSNFWMDVYVYGRYPKVALKNLAKNGL

APVFEAGDEDLLKAGKPDFMGVNYYQSMTIASNPLDGVTMTGEANYSGKKGTTKEAGQ
PGMYKIVSNPYLEKTNWDWTIDPAGLRISLRRISSRYDLPILITENGLGDFDTLEADG
QVHDQPRIDYLKTHCLAIQEAITDGVEVLGYCTWSFTDLLSWLNGYQKRYGFVYVDRD
ETNEKELKRYKKDSFNWYRDVIRTNGASLSE"

CDS 319936..320589

/locus_tag="JFBMFIFI_00282"

/inference="ab initio prediction:Prodigal:002006"

/codon_start=1

/transl_table=11

/product="hypothetical protein"

/translation="MNLVEKDFRLFNREVVLFVKLKQQFDYDEIEWIKQQYKELWQKW
KSLNLKAYEKSIRYIPYNKPKIESWTNGWQIRKHYWASYRMEGRESEATCIGVLLNRQ
NFRITIMWQKYKKNQSTVSASQYNRNLKQIGEWATKINQDNYFIWTTKSDEYDSYYSL
SDYLTNSEIQATIKAEIDKDETFQIGYILKRQPEIKNVEEVVTEKLIELGELYKIFQ"

CDS 320644..321624

/gene="afr"

/locus_tag="JFBMFIFI_00283"

/EC_number="1.1.1.292"

/inference="ab initio prediction:Prodigal:002006"

/inference="similar to AA sequence:UniProtKB:Q92KZ3"

/codon_start=1

/transl_table=11

/product="1,5-anhydro-D-fructose reductase"

/translation="MKKINYGILSTASIVPRFVAGVNASQHGFVKGIASRSIEKAELL
AKELAIPNVYGSYEAICQDPEIEIIYVATYNKAHYSAAMLALKNEKHVLLEKPFCLNV
KEAKELFAYAKEHNLFIMEAQKAVFLPATLKVKELIETGIIGKIRYVNASSSHLGVER
IKWFDSLESGGGALFGSGTYPLEYLQFVLGNQFSSWTGTCLGKPNQSDSQCTLSLKLG
EDTLATIFITTLVDAPSALTIYGEKGSITIPNYWKATELTVTINGEESEIFKFPMLSE
FAYEVDHVNNCLEKGFIQSPWMSESVTVSTITIIETMYQEWIGNGLLTIK"

CDS 322204..323817

/gene="malP_1"

/locus_tag="JFBMFIFI_00284"

/inference="ab initio prediction:Prodigal:002006"

/inference="similar to AA sequence:UniProtKB:P54715"

/codon_start=1

/transl_table=11

/product="PTS system maltose-specific EIICB component"

/db_xref="COG:COG1263"

/translation="MMEKIQRFGGAMFTPVLLFSFSGIMLALTIIFKNPMLVGSIANE

GTFWYNAWSVVESGAWTVFNQMELLFVIGLPIGLAKKANARAAMEAVVTYLTFNYFVG

SILEHFGKTFGVDYAGSADSGTGLKMIAGIKTLDTGIVGAILISAIVVWIHNRYFDVK

LPEFLGIFQGSSLVVIIGFFMMLPIAILTAWIWPKIQLGIGSMQGFLAASGVFGVWLY

TFLERILIPTGLHHFIYTPFIFGPAVVEGGITRYWMDHLNEFSRSTEPLKKLFPEGGF

ALHGNSKIFAAPGIAAAFYATAKPEKRKKVLAILIPVTLTAVLAGITEPLEFTFLFIA

PVLFVVHALLAATMAATMYAFGVVGDMGGGFIDLLAKNWIPLGANHWQTYAIQLVIGL

AFTAIYFLVFRFLILKFNFATPGREADTADVKLYSKKEYKEKMAGAGGPAIEGANQYT

EAAAIYLEGLGGSDNIANVNNCATRLRIGVHDETLVQPDEVFREGGAHGVVRNGKAFQ

IIVGLSVPQVREQFENLLKFNQNLEGDGQ"

CDS 323817..325148

/gene="malH"

/locus_tag="JFBMFIFI_00285"

/EC_number="3.2.1.122"

/inference="ab initio prediction:Prodigal:002006"

/inference="similar to AA sequence:UniProtKB:Q97LM4"

/codon_start=1

/transl_table=11

/product="Maltose-6'-phosphate glucosidase MalH"

/db_xref="COG:COG1486"

/translation="MKKHSIVVAGGGSTFTPGIVLMLLANLEKLPIRQIKFFDNDPAR

QKQIADACAVIIKEQAPDIKFVATSDPETAFTDVDFVMAHIRVGKYAMREKDEKIPLK

YGVVGQETCGPGGIAYGMRSIGGVLELVDFMEKYSPDAWMLNYSNPAAIVAEATRKLR

PTAKILNICDMPIGIEERMAHSIGLKSRKEMVVRYYGLNHFGWWTDIRDLKGNDLMPQ

IKAHVAEHGYALKEEVEASQHTDASWMHTFSKAKDIYALDPETLPNTYLKYYLYASDE

VAHANPEYTRANEVMAGREKHVFSECNRIAELGSTEGSTLEIDEHASYIVDLARAIAY

NTHERMLLIVENNGAIVNFEETAMVEVPCIVGTNGPEPLVQGKIPRFQKGLMEQQVAV

EKLVVEAWAEKSYQKLWQAITMSKIVPNATVAKQILDELVEVNQEFWPELS"

CDS 325457..326215

/gene="glvR_1"

/locus_tag="JFBMFIFI_00286"

/inference="ab initio prediction:Prodigal:002006"

/inference="similar to AA sequence:UniProtKB:P54717"

/codon_start=1

/transl_table=11

/product="HTH-type transcriptional regulator GlvR"

/db_xref="COG:COG1737"

/translation="MELEALVNQYHDKLNENDLAIISYVLMHKTQCETLSIVDLAKLC

HTSKSSILRLTQKLGFSGFSEFKYSLKKASKEKAPKLNLLDLQREDMEATLKLMQQTD

VATIVQKMHQADRIIGYGTGWGQQNAVKELNRNFMGCGKYMLSIPAKTEFDLNMPIIT

KNDFVVIISLSGDAKDLEQNIQTLNLRGVPILSITTFKNNYLASLAPFNLYYQVSPMH

QTERNEMVSFITLNLVCDALFREYASYLIESGEL"

CDS 326252..326731

/locus_tag="JFBMFIFI_00287"

/inference="ab initio prediction:Prodigal:002006"

/codon_start=1

/transl_table=11

/product="hypothetical protein"

/translation="MNEKNQVITKVRKMSFYSSLIAYWLILPILFSFYLAMKMLMMGI

DFQTLLTQNLTVTVLLLIALLNPFSAYFLLNTSEKDRKRNRSVGFYLKAMLVQQLLVG

NLVGAVLVFFSFREMPYSQDGVDSQMKMTSVYILVGIQYILSLVAIFALWMMVKNGS"

CDS 326755..327285

/gene="crr_1"

/locus_tag="JFBMFIFI_00288"

/inference="ab initio prediction:Prodigal:002006"

/inference="similar to AA sequence:UniProtKB:P60857"

/codon_start=1

/transl_table=11

/product="PTS system glucose-specific EIIA component"

/translation="MFGFFKKKEAKKEETKTPVTSVSIEGTLFSPANGKVIAIGEVSD

PVFGEKMMGDGYAVVPTDGKIYAPVNGKVTSVFPTKHAIGLLMDNGTEVLLHMGLDTV

ELKGAPFEIAVTEGQVVTPETLVATVDLDALATAGKDNAMVVVITNMDKIASFAVTEL

GETTASKAIGTFTTKG"

CDS 327397..328083

/gene="yfnB"

/locus_tag="JFBMFIFI_00289"

/EC_number="3.-.-.-"

/inference="ab initio prediction:Prodigal:002006"

/inference="similar to AA sequence:UniProtKB:O06480"

/codon_start=1

/transl_table=11

/product="Putative HAD-hydrolase YfnB"

/db_xref="COG:COG1011"

/translation="MKNYQTLLFDVDDTLLDFGAAEDNALRLLFEEQKVSLTVEIENY

YKKMNKGMWQEFELGKLDRDELVNTRFSKLFKAFGQEVDGVLLEQKYRGYLEEGHELI

DGAQSLIQNLKKEYDLYIVTNGVSKTQYKRLQDSGLHSAFKDVFVSEDTGYQKPMKEY

FDYVFARIPNFKRENSLIIGDTLGSDILGGKNVGMDTCWFNPTNKSNTSEIVPTYQIT

ELEKLYDILN"

CDS 328217..329632

/locus_tag="JFBMFIFI_00290"

/inference="ab initio prediction:Prodigal:002006"

/codon_start=1

/transl_table=11

/product="hypothetical protein"

/translation="MNLIQENQRTLEHIFPSYQTSEGKAQVLKETEELFTQFKQQHFL

YPIRYQLKKNTFHIKLKRDYSIAPSLEVTSINEVVPVWKYKLDQELMKLEKRTKKVFL

SDFGGIADGKTDCTKAFKRAFSVGYRHVILSSGTYLTRGLKIPSNVILAGEGSAETCL

KLHPEAPKSEQLLTNKSHFKGNHHIQIKGISLDWNVERLTKGETTATGGIASSGITLA

HVKYARIVDVVVKNPGLHGVDITSPAYSYAGDGTRSRLGSQFIWVDQIEAFGFGDDGI

TTHHSKNILISNCYLHHPSGLAHRTGFSNSNGIEIDDGSEHISLVGNRTAYCFGGVEI

KAHETSSAASDTQIFGHFSYRDNRSYNFRHIGHHKDQDLLSQSAYGIRASFLAAYYPQ

KTDLYKASEPRGIVVSAYQRVVVNQFIAREEPTTNHEKVAIAIQYRARDVRIVNSQLK

NYYGAKQPIKISKDTSDICII"

CDS complement(329695..330369)

/locus_tag="JFBMFIFI_00291"

/inference="ab initio prediction:Prodigal:002006"

/codon_start=1

/transl_table=11

/product="hypothetical protein"

/translation="MIQLKKLVIIGGSGFLGQVICQTALTENWTIVSISKHGRPPSNK

LLKSLRDAPIEWVQADIFTSQDWQAHLLHAFAIIDLVGIIKERPKEGITYQKMIADSA

KIIGTVASDNDRLQHFIFLSANAGPTKYIEAKRQAEQSLISMPLPLTIIRPGLIVGAG

RPSSIRAKWALEFLLKIPLLSVFVTQIKPISVDTIAKKILAILLSPEPLTTRVLAVDD

LDNLAD"

CDS 330594..331217

/locus_tag="JFBMFIFI_00292"

/inference="ab initio prediction:Prodigal:002006"

/codon_start=1

/transl_table=11

/product="hypothetical protein"

/translation="MKIVKPKQTITALVEDQIVKIEEVESHTHYKKCQITSNFGIKKI

NEVIFENCTFDRLDFKQMEFLDVIFDNCNLASGDFSEGLIYRSEFKNCQLSGANFIDS

KQKNVKWQGCLLSYANYSSSNMESVHFIEGRMTESYFQDCQLKNVIFEEIDANGVDLV

GSKLTGLDISKAHFDFLNLDISLAKGLKIGYDQAYKLITLSGIEIVE"

CDS 332295..333695

/gene="nhaC_1"

/locus_tag="JFBMFIFI_00293"

/inference="ab initio prediction:Prodigal:002006"

/inference="similar to AA sequence:UniProtKB:O07553"

/codon_start=1

/transl_table=11

/product="Na(+)/H(+) antiporter NhaC"

/db_xref="COG:COG1757"

/translation="MEKQQSFKEALGILILLLVVIGVSVIKFGVAPQTPLIIAIGLLI

LWGRFRKKSWDSIHKGIQEGISTGLVPMIIFILIGALIGIWIAAGIIPSMMVFGFKIL

NPGIFVPSVFIICAIVGTSIGSAFTTVSTVGIALLGMGTSMGFNPALVAGAIVSGAVF

GDKMSPLSDSTNLSAAVSGVDLFRHIKNLMWTTVPALIISLILFFILGNGETHASTSN

IDELVKILTANFSVNIFAIIPIVVMFACSWKKIPAIPTLLLSIIVALVMLVIEHSGLN

FTEISSIIQDGFIAQTGDKNIDALLSRGGVQSMMWSISLIILALALGGLLVEFKIIDS

VMAPISNSLNSTGKLILATALSCIGVNLLVGEQYLSIILPGKAFKNSFDKAGLHPLAM

SRVLEDAGAVVNSLVPWSVSGVFIAGALSIQTIDYLPFAFFCLLCPVITVFCGFTGIG

IQKKKESSIAKELREV"

CDS 333827..334780

/gene="hemH"

/locus_tag="JFBMFIFI_00294"

/EC_number="4.99.1.1"

/inference="ab initio prediction:Prodigal:002006"

/inference="similar to AA sequence:UniProtKB:P23871"

/codon_start=1

/transl_table=11

/product="Ferrochelatase"

/db_xref="COG:COG0276"

/translation="MKQKKGILLANLGTPDKPEKKEVRAFLKLFLADKRVIDTPRYIW

LPILHGIILNTRPKKSAKLYKEIWRPEGSPLLIYTKEQTRQLQDRFPDVVVRFGMSYS

HPSIVTSLTEMDDLGVTDLTIIPLYPQYSTTTTASIYDAIATYYLKKENIPSLHFIRE

FTEHPLYIDLLVDQIKTELAKHPVDHLLFSYHGIPVSYAEKGDPYPEQCAATTKAVMA

KLKQKIAYSETYQSKFGPAEWLTPATDTTLKRLPTDEGVKKIMVITPGFVSDCLETIE

EIESENRGYFMENGGEEFYYIHPFNEDPRFVDLLAQLVSDK"

CDS complement(335083..336027)

/gene="rutD_1"

/locus_tag="JFBMFIFI_00295"

/EC_number="3.5.1.-"

/inference="ab initio prediction:Prodigal:002006"

/inference="protein motif:HAMAP:MF_00832"

/codon_start=1

/transl_table=11

/product="Putative aminoacrylate hydrolase RutD"

/translation="MLIKKIGRISLKIIISFLLVVIIIVAGMFVTNKILNRLDDKKIT

DYGQKISVDDKKINVSIHGNGQETIVLLPGFGTAAPALDFTPLVNELKAYYKVVVVEP

LGYGLSDLPTSERSTKNIVSEIHETLQKLDITSYTLMGHSIAGIYGLDYSRTYPDEVT

AFVGIDTSFPTQSVDPFPASSYKLLRDLGVFRLLLKPDSLMAPDVDSKTKEQIRLISL
KNTMNTAILSEGEQMEANFNAAKNMNFPKELPVLFFLAEASIKDTEGWEPKHEEQIKG
LNYGKVEIFEGEHYLHYTKSKEMVESYRNFIKNAPSIK"

CDS 336504..337934

/locus_tag="JFBMFIFI_00296"

/inference="ab initio prediction:Prodigal:002006"

/codon_start=1

/transl_table=11

/product="hypothetical protein"

/translation="MNTNIIGIVLSFSFIFIVLALVTVSQKKANLSEEFSRKAIHIAV
GNWILIAVYFFDSILWAAFVPLCFIVLNYISYRKQLFKAMERSADDSLGTVWYAVSLF
VLTVVAFYLNMPFIAIGGILSMAYGDGFAAVIGSKWGNVTYPEKFGKKSLEGAVTVFI
FIFLITSILAYLYLPVNSLLVGLVCGSIGTILELLTPNGFDNLSVPLSVGALLYLFTI
GSTMTGLILNTTLTICILFLAWVVGALTAKSCVTSFILGMSLYLLGGWLVYAGLIVFA
ILGSGISKIGRQKKIDAAALHEREGTRGSVQVLANGLPALFFAIIYFVTQVEAFQLAA
LTTFAAANADTFSSEIGMLSQKNPISILTFKRLAKGLSGGVSFLGLISGLIGALLIGS
LALGSYPIRIILLISLIGFCGTLIDSILGSTLQAKYQIGKKITEQTTENGQKLKRISG
YSVINNDVVNFISVLLTGILACFVFN"

CDS 338061..339341

/locus_tag="JFBMFIFI_00297"

/inference="ab initio prediction:Prodigal:002006"

/codon_start=1

/transl_table=11

/product="hypothetical protein"

/translation="MPKQRLYIISLDAFGSQDLEYAKNLPNFKRLLARSALVSEVETV
YPSLTYMAHTSIVTGVYPNKHGIINNTYLQPHLDSPDWHWYAKDIKVPTLFDVAHQAG
YKIASLLWPVTGRSKSIHWNFVEIFPNRKWKTQIGVSLWSSSPKYIFDLNNKFGKLRN
GIQQPQLDEFITASMVDTIRTKNPDLFAVHLVDLDSTRHKYGVNTPETQAAIQRMDQH
LGQMIVAMEEKGIFDDTVIAILGDHYQIDVHTVIRLNQLFNEQGWITLNKDGKIQDWQ
VIAKGADGSCYIYTQPHVDLSEVKAALGKLEKEIDHIYTGEEAAAMGADSACQFLIEA
KSGYYFESGINGPLVEKTAENPTLLKGTHGFSPKKENYTTMLFISGPGINSAAKIPYA
HLIDEGPTFLHAIGLSYPQPVDGRILIELFNEVN"

CDS 339362..340642

/locus_tag="JFBMFIFI_00298"

/inference="ab initio prediction:Prodigal:002006"

/codon_start=1

/transl_table=11

/product="hypothetical protein"

/translation="MEQIKQSENDRFRYTKLERSWIMQDWANSAYSVMITTVVFPLFF

KVVSENGGLSDATSTAYWGYANSLATLVVSLLAPLLGAIADYKGYRNPLFTLATGLGV

VATLSFAFVPDENWQLLLFLYVLSAIGFSASNIFYDASLMDVTSNKRMDRVSSAGFGW

GYIGSTIPFLLFIVLQLTEILPISTGTLIKGSFIMTALWWFIFTIPYWKNVKQTTYIE

KQPKIVAKSFSRLWQTIKHIRQHRNVFLFLAAYFFYIDGVGTIITMAMTFGTDVGLSA

NNLVVIMLVVQIVAFPCSILYGVLAKRVGNKEMIFFGILTYIGICIFAIQLDSLLKFI

VLAILVGTAQGGIQSLSRSLFAQLIPVERANEFFGFYNIFGKFAAVIGPVLVAVTTQI

TGHAKDGVFSLIILFVVGGILLYFVKVDHEIENL"

CDS complement(340686..341864)

/gene="rtcB"

/locus_tag="JFBMFIFI_00299"

/EC_number="6.5.1.-"

/inference="ab initio prediction:Prodigal:002006"

/inference="similar to AA sequence:UniProtKB:P46850"

/codon_start=1

/transl_table=11

/product="RNA-splicing ligase RtcB"

/db_xref="COG:COG1690"

/translation="MLTLKGKYNEAVVYTDMLDDHTISQIISLCNQEFAKDSQIRIMP

DTHSGKGCVIGTSMTITNQVVPNLVGVDIGCGLYVVKLKPGRLKLNFDKLDQLIRNRI

PSGQSNHDTAMHQFDFTKLHAPVHEGWAMRSIATLGGGNHFIEVNQGADGIYLVIHSG

SRSLGKEVAEYHQEIAYQTLATRRKELKIELKHQMKKGLIAEANRLATLRETIKIPYE

LSYVEGSNFENYLNDMKIAQAYAALNRKLMAETILKGMKWDNFVISTFDCTHNYIDTE

NLMLRKGATSAQKGEALIVPLNMRDGSILATGLGNPAWNYSAPHGAGRILSRSKAKAQ

ISLDSYQKTMRHVWSTSVSKHTLDEAPSAYKPSKQLQKDVTGTMKIQEIIKPLYNFKG

"

CDS complement(342286..342423)

/locus_tag="JFBMFIFI_00300"

/inference="ab initio prediction:Prodigal:002006"

/codon_start=1

/transl_table=11

/product="hypothetical protein"

/translation="MKNAVTQLQIVITKQARFDLCFEFKAPLICLSGNSFPSVVNALN

K"

CDS 342683..343315

/gene="yodC_1"

/locus_tag="JFBMFIFI_00301"

/EC_number="1.-.-.-"

/inference="ab initio prediction:Prodigal:002006"

/inference="similar to AA sequence:UniProtKB:P81102"

/codon_start=1

/transl_table=11

/product="Putative NAD(P)H nitroreductase YodC"

/db_xref="COG:COG0778"

/translation="MGMVSNDFHKIMMERRSIRAYDEAVKIQQEEMAQILTEATSAPS

SVNMQPWRFVVVESSEGKAKLRPLVGSNVLQNDTSAAMILLFGDRNCFAYSADIYGVA

VERGLMPKEVEERQLKNLKHHYETISPDHLKETLLIDGGLVAMQLMLVAREHGYDTNA

IGGFKKELLAEAFNMDAARYVPIMIVSIGKAVEAGFPSVRLPIEKITEWV"

CDS complement(343402..343956)

/locus_tag="JFBMFIFI_00302"

/inference="ab initio prediction:Prodigal:002006"

/codon_start=1

/transl_table=11

/product="hypothetical protein"

/translation="MLNSNKNKTYMIVLTGLFAAITFLGVQVFRIPLPAAVGAPFIHF

GNALVVLSVLLLGFKYGAAAGAIGLGIFDLLNGYASFAVFTMIEAVIVAAVVATIFRL

FKHDDSKFYVILLIAIAAGITKIIESYVQGVIVSLIAGTTLQVASIASFVSLYATVIN

SISTVILVSLLYYPLKKIMLKNSH"

CDS complement(344095..344934)

/gene="pdxY"

/locus_tag="JFBMFIFI_00303"

/EC_number="2.7.1.35"

/inference="ab initio prediction:Prodigal:002006"

/inference="protein motif:HAMAP:MF_01639"

/codon_start=1

/transl_table=11

/product="Pyridoxal kinase PdxY"

/translation="MQKKVLVVHDLSGVGKVALTVTLPILATLGIESCVLPTALLSTH

TGGLGANTYLDLTEEMKKIIAHWKSLDLKFDAIYTGYLGNPKQIDLVIDTIDTMTASQ

CPVIIDPVMADSGKLYRGFQPDYPAKMRKLCEKATVIIPNLTEAALLLEEPFQTGPYT

KDYIEDLLKRLGQLGPKKIILTGVYFDEFEIGAASFDVETNRINYALDKKMIGHYFGT

GDIFASIVTGSVLSGKDIHAASEIAVRFIHDAISKTLQQKQDPKFGVCFETELPKLLK

IIN"

CDS 345865..347196

/locus_tag="JFBMFIFI_00304"

/inference="ab initio prediction:Prodigal:002006"

/codon_start=1

/transl_table=11

/product="hypothetical protein"

/translation="MRSGNVLFLTIIGTIIIGAGTAILVQAKEEEIVIKPIEIPYGKN

INAAVIEKQLEKQGIKDVKIIKGKIEPEKIGEYSLTLQLTKEDNEIFEKVVKIDVTDE

VPPEITVKKVEIEAGSNFNLTDYVDVIDNADGDLTGIITYKPVDTKDLGEQKLEIAST

DYSGNKTNKKIILSVIDTTKPTLNVTDEKINFGAKFNAMENSSATDLIDGNLTSEMQV

TGTVDPNTAGTYTLLYTVSDKSGNKVEKERNVIVEAAPVADNVEPEATNENANETNPE

TDTSSDVNSEAEAVTPAPISNPDPIAPPAPSYAPMTIYVGGSAIPYQNGGQGTGQSII

DNAPVASTWGGAAIQSGSDGLNTHIIGHNPGVFSALFNVGIGSQVTITDGNGTPTNYT

INRIYEVNDNAVGVRDGVDYWDQMIGTGGGERVTFQTCENDNVNWVYEAVS"

CDS 347366..347689

/gene="gdx_3"

/locus_tag="JFBMFIFI_00305"

/inference="ab initio prediction:Prodigal:002006"

/inference="similar to AA sequence:UniProtKB:P69937"

/codon_start=1

/transl_table=11

/product="Guanidinium exporter"

/db_xref="COG:COG2076"

/translation="MNRSWVYVIIGAILEVAWVTGLAHSTTIWQWIATIISVTISYGL

FTLAAKNLPVGTLYAVYTGLGTVGTILVGIWLFNESIYPLKAFLMMTLLIGIIGLKLV

EAKGK"

CDS 347706..348023

/gene="gdnD"

/locus_tag="JFBMFIFI_00306"

/inference="ab initio prediction:Prodigal:002006"

/inference="similar to AA sequence:UniProtKB:P49857"

/codon_start=1

/transl_table=11

/product="putative guanidinium efflux system subunit GdnD"

/db_xref="COG:COG2076"

/translation="MTLAWSALFVAGLIEIIGVMNIKRLADGKKSALFFAILLMGISL

LLLSFALQSIPMGTGYGIWTGIGTVGTTIVGIVVYKDSKSWPRLFFIALILLSTIGLK

LLS"

CDS complement(348075..350315)

/gene="uvrA_1"

/locus_tag="JFBMFIFI_00307"

/inference="ab initio prediction:Prodigal:002006"

/inference="similar to AA sequence:UniProtKB:P63383"

/codon_start=1

/transl_table=11

/product="UvrABC system protein A"

/translation="MTQEYIYLNGARENNLKNISLAIPKRKITIFTGVSGSGKSSIVF

GTIATESQRQLNETYSAYLRNFLPKYQQPDADSIENLSTSVIIDQKRLGGNSRSTLGT

ITDINPLLRLLFSRVGSPYVGTANYFSFNDPQGMCPECQGVGKQITVNLDKLLDRTKS

LNDGAILFPTFSVDSWYWKIYALSGFFDNDKPINDYTAEELDLLLYGKDIKITLGSDF

GALNSNYEGLVEKFNRLYLKKEGEMAESTKKRVAPFTHEAGCTLCHGARFSQATLNCR

INNYSIADYLAMQMDELLNALAEITDPIAKPLIASLLERIQHLVDIGLDYVSLDRETT

TLSGGESQRVKMVRHLSSSLTDLLYIFDEPSVGLHPKDVHRLNELLVKLRDKGNTVLV

VEHDPDVIKIADHIVDVGPLAGKKGGEIMFEGSYADLLTSDTLTGNYLQKNLALKSNP

RKATGGFSVANANLHNLKNIDVTIPKGILTVVTGVAGSGKSTLIEDIFLKEHPDSIVI
DQSAAHASSRSNPATYCGIMDTIRKEFGTANQVSPSLFSFNSKGACEDCKGLGFTTMD
LAFMDSIRTVCETCQGKRFQETVLTYHLQGKSISDVLEMTVTEALDFFTTPKITKKLQ
ALEEVGIGYLTLGQSLNTLSGGECQRLKLANELHKKGSIYIMDEPSTGLHMSDIEKII
DIMNTLVEKGNTVIVIEHNLDIIRSADWLIDLGPAGGSKGGQLIYSGPPAQIKEQANS
VTGKYV"

CDS 350745..352484

/gene="bmrA"

/locus_tag="JFBMFIFI_00308"

/EC_number="3.6.3.-"

/inference="ab initio prediction:Prodigal:002006"

/inference="similar to AA sequence:UniProtKB:O06967"

/codon_start=1

/transl_table=11

/product="Multidrug resistance ABC transporter
ATP-binding/permease protein BmrA"

/db_xref="COG:COG1132"

/translation="MEERATPKIGSWKNFFTLLNDTKPRKILIFFGLFFSLITTLIGL
VVPLFTKNLIDNFSIESLSWMVIVGIVLAFIFQAITDGVSMYMLSYMGQQMVARLREK
VWKKLVHLPVSYFDETKTGEIVSRLINDTTLVKDLITQHFPQFVSGIISIIGAVTILM
IMDWKMTLMMLIAIPITALFITPLGSKMAKISKATQNETADFTGIVSQTLAEIRLMKS
SNGEETEIKNGEAGIHRLFKFGLKEAKLFSIIGPIIFLVMMAIIVAIIVYGGIRVQEG
TLTTGTLIAFLLYLFQIIFPVTSFVTFFTQLQKAKGATERITEILSMESEDLEKGLDF
DVAGKMIQMKDVSFEYNPNEPILADINFETNPGEVIAFAGPSGSGKTTLFSLLERYYS
VKEGSIEVGSTPLESISLHSWRRQIGYVSQESAMLSGSIRDNLTYGLEQEFSEDELWK
VAKLAYAETFIRELPKGLDTEVGERGMKLSGGQRQRISIARAFLRDPKILMLDEATAS
LDSQSEGVVQQALSNLMEGRTTFVIAHRLSTIVHADKIIFIEHGRITGMGTHSELVAN
HELYRSFVEHQLT"

CDS 352841..353665

/locus_tag="JFBMFIFI_00309"

/inference="ab initio prediction:Prodigal:002006"

/codon_start=1

/transl_table=11

/product="hypothetical protein"

/translation="MKFENKKEEKKYYETVASEEEFLKWYKAQDLPKYETPSVTVDNV

MFCYNREEDQLKMLLIKRKAHPFKDSWALPGGFVNPTESTNESCLRETQEETGVKLTE

LNIEQLYTFSTPNRDPRGWVITTSYIAFLNQEVLTAGDDASDVKWFNIDIEGDLVTLT

EGDIVIQLDIQKEATLISSQERLAFDHGEIISTAFKRIRGKMYSQPRVLTILGESFTI

TEARKVFAKFLGLDYKKIDHSNFKKDLLKFVKEVDERPTGVGRPSKFYAVDSPYLT"

CDS 353894..354652

/locus_tag="JFBMFIFI_00310"

/inference="ab initio prediction:Prodigal:002006"

/inference="similar to AA sequence:UniProtKB:Q9I310"

/codon_start=1

/transl_table=11

/product="putative signaling protein"

/db_xref="COG:COG2200"

/translation="MKEADIRVAITAQNYELYYQPKFESIEKKLIGAEALMRLKVNEK

VIGPDDFMPIVNEMEQMETMQNFLIHEVVTKLNSEELVNYDFSISLNMNSSEFVHKEH

MDEVLLFFKNELINPNKLEIEITERREFPDFDLAVSYLEKLKETGIKVSLDDFGRGFN

SLAYLRMLPIDVLKIDRAFMNEVLTDKKVQMIVKAVIQLAHDLDILVLAEGMETKQQV

EFMDQMGCDTYQGFYFGSPVPYAVFQETWLPFLQ"

CDS complement(354711..355331)

/locus_tag="JFBMFIFI_00311"

/inference="ab initio prediction:Prodigal:002006"

/codon_start=1

/transl_table=11

/product="hypothetical protein"

/translation="MIHFTSDKLSKKQQYKFLSGSIIPRPIAWITTLTADGKTVNLAP

FSFFSGVSNELPLLSIAILRQNGEMKDSARNLLATKEAVIHIVDESLIHAMNQTSRSL

APDQSEVELTNLTLEPSHSVQVPGISEAKIRFETTLHQYVPIKNQQDQIVTDLFILAV

SDFHFSESVFDQKNDYILTEKLLPVARLAGNKYATLADEYTVKRPK"

CDS complement(355328..355948)

/gene="mhqD_1"

/locus_tag="JFBMFIFI_00312"

/EC_number="3.1.-.-"

/inference="ab initio prediction:Prodigal:002006"

/inference="similar to AA sequence:UniProtKB:O34842"

/codon_start=1

/transl_table=11

/product="Putative hydrolase MhqD"

/db_xref="COG:COG0400"

/translation="MEHYFKAGLTTAPPLLLLHGTGGDEHSLLKIAEELSPNSTILSL

RGTVSEQGANRFFKRFAEGQFDLENLELKTDELLATVKELSKKYQVSFDQWVLVGYSN

GANIAGHLLLERENSFKRGLLFHAMSLGKHEATFDLSTSSIWLSAGINDPIVPKSASE

TLRDSFLQRDGNVDMVWTNAGHQLTYPELEKAKEWLKKEFPKDEFV"

CDS complement(355952..356908)

/gene="mhqA_1"

/locus_tag="JFBMFIFI_00313"

/EC_number="1.13.11.-"

/inference="ab initio prediction:Prodigal:002006"

/inference="similar to AA sequence:UniProtKB:O34689"

/codon_start=1

/transl_table=11

/product="Putative ring-cleaving dioxygenase MhqA"

/db_xref="COG:COG0346"

/translation="MNNLKGIHHVTAMTSSAERNYQFFTEVLGMRLVKKTVNQDDIQT

YHTFFADDKGSAGTDMTFFDFPNIQKGIKGTNSISRTSFRVPTDAAIDFWLKRFDEFS

VEHGEIETKFGKKILTFSDFDDQHYQLISDEFNHGVPAGIPWQKGPVPYEFAIVGLGP

IFLTVADLEPMKLMIEEVMGFKQIETEAGFHLFEVGEGGNGAQVIVEERTDLLPAREG

FGNVHHVAFRVENRQVLDEWIERIHSFGLPQSGYVNRFYFESLYVRVGPILFEFATDG

PGFMQDEPYETVGESLSLPPFLEEQREYIESAVRPFNTKRSE"

CDS complement(356909..357796)

/gene="mhqA_2"

/locus_tag="JFBMFIFI_00314"

/EC_number="1.13.11.-"

/inference="ab initio prediction:Prodigal:002006"

/inference="similar to AA sequence:UniProtKB:O34689"

/codon_start=1

/transl_table=11

/product="Putative ring-cleaving dioxygenase MhqA"

/db_xref="COG:COG0346"

/translation="MVHNIHHISGFTKSAKESIYFYTEILGLRLVKNTVNQENTKMRH

LFFGDYQGTPGTLLTFFEISKLGQRRDFNNYFSTNYLAIPKGSLQFWKERLLQFNVES

HQDDEGLEFTDFDDFKLKLIETDEQIQPEKATQHSTVPAHYQIIRIAGTSLVVENPTL

TNQFLHTYLGIKSTETLPLNNRASFTIVKKSSELAKSRIGRGSIDHIAYSAATAADLE

ELYQTALANQFKIEEYVDRGYFKSLYVKEPNGLRIEIATELPGFLIDEDLATLGENLA

LPAFLETKRTTITKTLEEL"

CDS complement(358178..359209)

/gene="pgl"

/locus_tag="JFBMFIFI_00315"

/EC_number="3.1.1.31"

/inference="ab initio prediction:Prodigal:002006"

/inference="similar to AA sequence:UniProtKB:O34499"

/codon_start=1

/transl_table=11

/product="6-phosphogluconolactonase"

/db_xref="COG:COG2706"

/translation="MKETLFLGTYTKRESEGVYTITLDTEKKQLENLTLIAKVDSPTY

LGLSKNQDFLYAVAKVDGDGGMSSYKKDASGTYQLVNSVTAAGAPPCYVGVDDARGFL

YTANYHKGEIGVLKVVEDGTLTLVDTVTHTGSSVHENQTSPHAHYSDLTPDHNYIVAC

DLGTDSVYSYSVSAEGKLTEVSRYNAAPGTGPRHLVFNPNGKIAYLFGELSSVVIVLG

YDATTGAFTEIQTISTIPNDFTDFNGGAAIRVSSDGKFLYASNRGHDSIVVYSISNEG

KTLEQIQLIASEGQIPRDFNLDSTEDFVIVAHQDSDNLTLFSRDRETGLLNMIQKDVY

APECVCVLR"

CDS complement(359350..361470)

/locus_tag="JFBMFIFI_00316"

/inference="ab initio prediction:Prodigal:002006"

/codon_start=1

/transl_table=11

/product="hypothetical protein"

/translation="MSLRSSIKDSHLFLNRHKTELILKHPSFSIRTQGDQIVTTVTLN

KDHPVYFKGFFILHRETGEKYDFSTTEIDPTNFSFQFNLTDFIHQIVPQGGREHFEFY
FGISYFVDNQWVDKEEPLSLDLFEHFDSYGLTQFKDREDDIYPYFSRKTNGFCFTVNV
PVRSLRYIKGSYITDIKTKQQKINIHGKISSKAIAINRIDTIMVGKRSGLKRTIHSNI
MLDNLESGTHLYHYDYHILIDTKDFAQELLLIEFPDDDFDLYFEVYLNGLFEPTVVRV
GNPKALKSKGIYKNAFYSYATTSYTFAPNFTSQSQSLSICVTRFEKAVFSYFREMLVL
FWFIRPFYLKNKIWVIGEKPHTANNNGFAFYRYMRENHPERNVFYVIDPASPDYQKLE
KYGSDHILPFKSKKHIWHLFMSRVILTAYHPHEIYPTRTQQLLNFIRGKKIYLQNEVI
GLQNEIETLGHSSLQFETDLFLVSSKNELRLVTDVLHYVKEQVAITGLARFDELFDPA
KPMIIENQLLFFPVDHETGLHFQTEFIDILAANYLTFLASSDFTEYLKHNQLNLVIAL
PPAFNKYATEFQKLATKLVYQEDGNLIELLKTSKIMITDYASEAFDFSFLDKPVLFYQ
IEAQFQQVDSDEAVHLHHSYQNELPGEIATDADSLMHLLEIAQITHFEISKQNKQKAN
SLIEYRDTNACQRIFETVTRFTKS"

CDS 361711..362070

/locus_tag="JFBMFIFI_00317"

/inference="ab initio prediction:Prodigal:002006"

/codon_start=1

/transl_table=11

/product="hypothetical protein"

/translation="MTIGTVTSIGPQAISEQDPIIILFGEEATEDIRKVSVIQAFEDG
REMIELKKGQTIKFDNQSYTIEAVGSLAVDNLNSIGHITLSFTEVPAEDMLGNGIYLS
PQELPTISVGTKISYEA"

CDS 362241..363452

/locus_tag="JFBMFIFI_00318"

/inference="ab initio prediction:Prodigal:002006"

/inference="similar to AA sequence:UniProtKB:P9WFM5"

/codon_start=1

/transl_table=11

/product="Putative transport protein"

/db_xref="COG:COG0628"

/translation="MEDLDQKTEKRKNFSWFWRWFLNNKFVTVLLVNLLILLNIFVLS
KMGYLFQPIGSFFSVIGLPIIMAGILYYLINPLVDWLESKKIPRVFGITIVFIIILGL
IVWGVAILIPLMQKQTMSFITNWPSYWERANSELDNLLRSDLFSQLQDQLSSTNGDIV
KSVTGQLNSMLNSTVNGLGSVVGAVTNVVIAVVTMPFILFYLLKDGKNLPHHLMKFVP

TKLRDTTFRLLTDMNTQISQYIRGQLIVAFFVAIMFMAGYAIVGLEYAVTLGILAGFL
NLVPYLGSFLAMVPAVIIALVDSPEMLIKVMIIFVIEQTIEGRVISPQVLGSNLEVHP
VTIIIVLLTAGKLFGIPGVILGIPGYAVLKVLLTYIFNWYKEVSGLYHHDYNPAPLPD
SGVKNENDSAG"

CDS 363793..365325

/locus_tag="JFBMFIFI_00319"

/inference="ab initio prediction:Prodigal:002006"

/codon_start=1

/transl_table=11

/product="hypothetical protein"

/translation="MKKIINIKGETLAASILLIINIGFVIFSLKSNPFSHVLNGHDSS
MFLYFGNGMQNGLAPYSDMFDHKGIVLFWIQHLGVLIGQGNFSLGIWVLEILFYGIAV
VFLVKSVLLLTKNFLAASMSVVLFTGGIISSFAGGNLSEQYAITFISIALYLFLKICI
KEKNNKIDLLLIGIMGGLTFFIRSNMVALWLVFCLYLLIKGLVSKNYLVLSKQVLYIF
IGGVSVCLVVLLYGIYVGNLTDMIYQTFTLNLKYSSGTSSGERLLAGKAFFEFASTVG
IVSFVVLSLYYIINNTEKLKGNIYKTSLILIVYVIVNFITVILSGRFYLHYFTTMFPG
IIVLTAIGIDGVNSSIKGIGRKLLIMATLFLIPISFFSKTYNEMAKPILNTMPNERID
SYKVSSANFIKEHSSEKDTIYVHNIDANIYLLSERFSNSRFFVLPDIDYRTFNNLRQE
FKDALENNPPKFVVIRKAIYLNQENLTDARLDKTITENLNKYYEIALEFESDGILIFS
RK"

CDS complement(365629..366249)

/gene="azoR_1"

/locus_tag="JFBMFIFI_00320"

/EC_number="1.7.1.17"

/inference="ab initio prediction:Prodigal:002006"

/inference="similar to AA sequence:UniProtKB:Q831B2"

/codon_start=1

/transl_table=11

/product="FMN-dependent NADH-azoreductase"

/db_xref="COG:COG1182"

/translation="MSKVLVIKAHPLTGEVSRSMQVVDTFITAYKEKNHFDHINEINL
YTSFVPEIDLDILAAWDALRTGTEFETLSQEQQDKVSRFNELTELFLDADKVIIANPL
WNLNVPTRLKAWIDTVNVAGKTFKYTAEGPVGLVTDKKVLHIQSSGGVYKGQDPASQY

LKMIFNFIGVTDYHQLAVEGMDHDPEHAPAIMAEGFAAAKDLAQTF"

CDS 366613..367257

/locus_tag="JFBMFIFI_00321"

/inference="ab initio prediction:Prodigal:002006"

/codon_start=1

/transl_table=11

/product="hypothetical protein"

/translation="MPTQTFFNLPIDKQKRLLDAASTEFSRVPLTEASINNIIKLAEI

SRGSFYQYFKDKEDLYYYFFATLKQDSKKMLEDCLKEANGDLFLGYQNYFPKMLTMIT

EDKQASFFKHMFLHMDYRTSREVTPEAMKKRECEHKKDRHHNHFADFVNADLLKIDSD

EDFHSLFKVMMTFLFQTIGDAFAKELPRDEVIASFNKKLFWLRDGVYVKSEGEK"

CDS 367307..369028

/locus_tag="JFBMFIFI_00322"

/inference="ab initio prediction:Prodigal:002006"

/inference="similar to AA sequence:UniProtKB:P9WQJ1"

/codon_start=1

/transl_table=11

/product="putative ABC transporter ATP-binding protein"

/db_xref="COG:COG1132"

/translation="MLKLAKRMSFWAVAGAVVFMIIQVVSDLYLPTLTADIINNGVAK

GDTDYIWSVGFKMLGFSLISIAAATGNVFLAARQSQKLGKTLRSEIYTKVTNFSNDEI

DEIGTASLITRTTNDVIQIQNVVMMFLRMMIMAPIMLIGASFLAYSKDGQLTQIFLYV

LPILAIFIGVVMFFAVPLFKAIQKKTDKLNLVFREGLTGIRVIRAFNRSKSETERFAE

ANKDFMDTSIKVNTIMSLMLPVMTFIMSGTNIAIIWFGGQYIGNGTLEVGNLLAFMTY

AMQILMSFMMLSMVFIFIPRGQASAVRINEVLNMDSKIKDPENPAKLPAEIQGTLDFN

HVDFRYSGAENLALKDIDFHGKNGQTIAIIGGTGSGKSTLSNLLGRFYDVENGSIEIN

GVDIRQMKQQDLRDAVGYVPQKAMLFTGTIRENLKYGNPDATDEELWHALEIAQSKNF

VAAMPEGLDSYVEQGGGNFSGGQKQRLCIARALVKKADIYVFDDSFSALDFKTDAALR

KALKPETRESVVVLVAQRISTVIDADIILVLDEGQLVGQGTHEDLKATNETYQEIINS

QLRGEEI"

CDS 369030..370928

/locus_tag="JFBMFIFI_00323"

/EC_number="7.6.2.-"

/inference="ab initio prediction:Prodigal:002006"

/inference="similar to AA sequence:UniProtKB:P9WQJ3"

/codon_start=1

/transl_table=11

/product="Fatty acid ABC transporter ATP-binding/permease

protein"

/db_xref="COG:COG1132"

/translation="MSDKNVSQPKGGQGRGPMGGGPGARMPVQKAKNFWPTIKRLFGY

MSKRSIAIIAVFVLAITATVFQIRTPKILGEATTLIFEGVQKGFIQRQAGIEVDKLPI

DYDKIASILLVVGIMYVASALFSFLQQFIMTRVSQRTVYELRRDLKGKMNTVPMEYFD

THSNGDVMSRAVNDMDNISNTLQQSLTQLVTSIVTFFGVLYMMLTISWQLTLVAVATV

PLSLIVVGIVAPKSQRFFASQQKSLGLLNNQIEETYSGHVVVKSFNREAASIETFEKE

NEVLFKSSWKAQFISGLIMPLMNFIKNLGYIFVAVLGGIKVADGSMTLGNVQAFLQYT

NQFSQPITQLANLMNTIQSTVASAERVFEVLDEKDMDNSPSGIEPIPNSPYKISFDHV

QFGYGEGTDDLLMKDFNLDVKEGQMVAIVGPTGAGKSTLINLLERFYDVKGGSIKFNG

IDTRDFSREELRSHFAMVLQDTWLFNGSIYDNIKYGNEKKEADDFVVEAAVAAHVDDF

VRKLPHGYDTILNEDASNISQGQRQLITIARAFLVDPEVLILDEATSSVDTRTEVLIQ

KAMRKLLQGRTSFVVAHRLSTIRDADNIIVMDKGSVVETGTHDELMEKAGFYADLYNS

QFSEEAVS"

CDS 371077..372273

/gene="eis_1"

/locus_tag="JFBMFIFI_00324"

/EC_number="2.3.1.-"

/inference="ab initio prediction:Prodigal:002006"

/inference="protein motif:HAMAP:MF_01812"

/codon_start=1

/transl_table=11

/product="N-acetyltransferase Eis"

/translation="MTDYRIITPEYKDEMFELAKYAFNIPITENLKNQFNLIFDNSIS

VGAFQETGTLSGQVMITAYEVYLHKQVYKMGGIGLVANYPEYRGDGDIASLMKLALKT

MNEKEDDLSYLAPFLYSFYRKYGYEHAFDQVKLTLTPDELPKAHKQKGTMKRVDWKTG

SHELKKLYQMYQKDSIGPVRRADWWWSYNYDYNQELKIAIYYNPENQPYGYLTYETDG

QNKTFRIKELVYLDYHAYDALWGFIASHNGSFSYFEYSAGINQNQSYLLSNPRIKQEI

IPSMMARIVNMKRFIEKFNFISEIDSPLYLKVKDEVAPWNDGLWKLEIKSGQTSLTEV

ANEPEEGLENQLLTGDIGTWTQVFMNYRSLNELHFFNRIEGSEAAVQTLAAQLPDGSP

TLYDYF"

CDS complement(372331..372792)

/gene="yehR"

/locus_tag="JFBMFIFI_00325"

/inference="ab initio prediction:Prodigal:002006"

/inference="similar to AA sequence:UniProtKB:P33354"

/codon_start=1

/transl_table=11

/product="putative lipoprotein YehR"

/db_xref="COG:COG4808"

/translation="MGKMKKVLPLLAVVCSLFILVGCGGSKEETKTFETNQNGVEMSL

TYTYKGDIVSKQTATSKIPYSSLGVSTKEEAQAVTKAIEEQYQGVKGLKETMDFKEDH

AIEKVEVDYEKADLEELSAIPGMNFSDTSKDGISMKKTQEMLEKMNFKEKK"

CDS 372964..373824

/gene="catE"

/locus_tag="JFBMFIFI_00326"

/EC_number="1.13.11.2"

/inference="ab initio prediction:Prodigal:002006"

/inference="similar to AA sequence:UniProtKB:P54721"

/codon_start=1

/transl_table=11

/product="Catechol-2,3-dioxygenase"

/db_xref="COG:COG2514"

/translation="MKKMNSETSIGKVVLNVENLEAMKTFYQDKIGLEIKSETENEVS

LGAVDESQTTLLQLKRVPITAKVGRRTGLYHTAFLLPSRSALGDVLYHLVKTEYPITG

ASDHGYSEAIYLDDPEGNGIEIYHDKAREVWDINPDGTINGITIAMDAEGVLGAATGS

FTGLPKGTTVGHIHLTVADLDATEKFYIDTLKMNLQTDFPGQAKFIAAGNYHHHIGTN

VWSGRNIPPMEKDTKGLAYFTMEVPSLEALLEVKDNLDEKSYPYEYDEKHFSLRMLDP

NGINLEITVK"

CDS complement(373955..374386)

/gene="yiaC"

/locus_tag="JFBMFIFI_00327"

/EC_number="2.3.1.-"

/inference="ab initio prediction:Prodigal:002006"

/inference="similar to AA sequence:UniProtKB:P37664"

/codon_start=1

/transl_table=11

/product="Peptidyl-lysine N-acetyltransferase YiaC"

/db_xref="COG:COG0454"

/translation="MIKKMENEHLDTVMELWLSGNNSGHPFIPAQHWEDSYDLVKEML

PQADIYIYEDTISIKGFIGISENSYIAGLFVSPNFQGQGIGEKLLTYVKAHYNNLSLH

VYSENNRAVTFYQKQGFTITSESINNSTGEKEYLMNFHSDQ"

CDS complement(374479..374907)

/locus_tag="JFBMFIFI_00328"

/inference="ab initio prediction:Prodigal:002006"

/codon_start=1

/transl_table=11

/product="hypothetical protein"

/translation="MDYYKISPDGLKNMLGMELYLGKIVSLDSKLKELVKIRVSQING

CAYCITTHSKEARNNGETEQRIYGLNAWQECRFYSKKEQLALQLAENITLISEKKVPD

ALYQEIRKEFNEKEYTDLVLLITQINSWNRISIAMGNIVG"

CDS complement(375010..375810)

/gene="ybjI"

/locus_tag="JFBMFIFI_00329"

/EC_number="3.1.3.104"

/inference="ab initio prediction:Prodigal:002006"

/inference="similar to AA sequence:UniProtKB:P75809"

/codon_start=1

/transl_table=11

/product="5-amino-6-(5-phospho-D-ribitylamino)uracil

phosphatase YbjI"

/db_xref="COG:COG0561"

/translation="MTIKMIAVDMDGTFLNDQKKYDEERFRKIFTQMNEQSIKFVVAS

GNQYYQLRSFFLDYHEHISFVAENGAYILEGTTPLYNGEMSKKLIAEVLDQLYSMTPE

NLIVCGKKGAYVHEEITSDIYESAKQYYPKIKKVSSLYQIDDTIFKFALSFEENTISN

TLKLLNMKLAGRLTAVSSGHGDIDLIIPGIHKANGLKLLQERWLINENEIAAFGDSGN

DLEMIQHVSFSYAMGNAQPAIKKAAKYQIGDNNTDAVLKTIEQLLKTN"

CDS 375941..376897

/gene="ccpB_3"

/locus_tag="JFBMFIFI_00330"

/inference="ab initio prediction:Prodigal:002006"

/inference="similar to AA sequence:UniProtKB:P37517"

/codon_start=1

/transl_table=11

/product="Catabolite control protein B"

/translation="MTNIKDIARLANVSVSTVSRVMNQHPHVSDSVRKDVLKIAEKMN

YVANENAVKLSRGKSGMIGVIIPYNNNSCYDELIQAILKETKERGYQVLLLPTYFEKK

AEEYYCSLLSKKVVDGMIITSKISDDAFIEKLAKNGEIISTEKLKTNEVTAIFPDRKK

AYSDVFSYLKNQNYSSIVFTINREVEESTSSREKVLSYTEIFGEARKNQQYFSQLNSY

QGGYELAQKLFKQQLVPDAIFSNGDEVAAGIIGGAAELGYHYKNDFEIIGEGNLSYSK

LLNFSSIDFHQGEIGKAAVQQLLSESKPMHQIKLATFINRRI"

CDS 376973..377929

/gene="ldh_2"

/locus_tag="JFBMFIFI_00331"

/EC_number="1.1.1.27"

/inference="ab initio prediction:Prodigal:002006"

/inference="similar to AA sequence:UniProtKB:Q9EVR0"

/codon_start=1

/transl_table=11

/product="L-lactate dehydrogenase"

/translation="MNQNKLVLIGVGHVGSQVLTEVLHLQLFAKIALIDNQDKVAEGE

ALDHRHAAAFHSQANPEIYAGSYEDCQDATVIICAAGPSIVPNTGELMPDRSQLAMQN

AIVLREIMTRVTEYTKEAVLILITNPLDSMVYLAENEFNYAKGRIFGTGTMLDSARFR

QILAENYQVDPKSISGIMMGEHGLTAFPVLSRVSIEGFKTDELAFKGSNSLSYKNIQG

AVVKTAYDVLNAKGWTNAGIAQATVALVRAVVLNERSIYPVSTTVTNAYGYQGDVAFS

LPCIIGKNGIEEQLELPLDKIETEALKHSVAAIKETMKNCGI"

CDS 378024..379058

/gene="yfeW"

/locus_tag="JFBMFIFI_00332"

/EC_number="3.4.16.4"

/inference="ab initio prediction:Prodigal:002006"

/inference="protein motif:HAMAP:MF_01034"

/codon_start=1

/transl_table=11

/product="Putative D-alanyl-D-alanine carboxypeptidase"

/translation="MYPKTQALISEFIQEEIFPGASFAFINPIEKNSYRIGNTAVYPV

KEQIRDDQLYDLASVTKVLMTTTLILQLWEAGKLNLTDSVSLYLPAFSEPRVTIQHLL

THTSALNGFIQNRDQLTAGELKEAMLHLSIGPDFGKKVVYTDTSMILLGFIVEECTKK

NLALVFQERVSGPLKLSNTVFEPTDPLSCAPTENHPTRGIIRGVVHDPKAFTLRPNCG

SAGLFSTIEDVSRFSQMLLNDGELDGVRVLNKETVEKLKQDWTPTGNLNRSLGWDFLK

SGDKSHPKRYLFHSGFTGTFIFLDMIKKEGFVFLSNRLHLQNDTPHYLKRRNELLDVY

IRELEEGQTN"

CDS 379465..380427

/gene="gmuF"

/locus_tag="JFBMFIFI_00333"

/EC_number="5.3.1.8"

/inference="ab initio prediction:Prodigal:002006"

/inference="similar to AA sequence:UniProtKB:O05511"

/codon_start=1

/transl_table=11

/product="putative mannose-6-phosphate isomerase GmuF"

/db_xref="COG:COG1482"

/translation="MTEPLFLEAVLQEKIWGGTKLKDVFGYAIPSDKVGECWAISAHP

NGPSVVKNGPLKGLTLAEVWEQHREVFGNVTGDVFPLLTKILDAADDLSVQVHPDDAY

GLAHEGELGKTECWYVIEADEGAEIIYGHHAKTKAELETMIREGQWDHLLRRIKVNKG

DFFHVPSGTIHAIGAGIMILETQQSSDTTYRVYDYDRKDDSGQPRELHIQQSVDVTTV

PHIDPVLHVETINKGNVEITTYVETDFFDVYQWDIKGRAEFMATAPYTLVSVLEGNGF

LNLADGASYEINKGTHFILPNDIKAWEIDGHLQIIASTPGKANR"

CDS 380605..381471

/gene="dacF"

/locus_tag="JFBMFIFI_00334"

/EC_number="3.4.16.4"

/inference="ab initio prediction:Prodigal:002006"

/inference="similar to AA sequence:UniProtKB:P38422"

/codon_start=1

/transl_table=11

/product="D-alanyl-D-alanine carboxypeptidase DacF"

/db_xref="COG:COG1686"

/translation="MIKNKKRILYVLAILVAIISLSFYLLGIYGKQLNQGSKTKIGET

SELVVDLNNQKVLYQKNPNKARPLASLTKLMTIYLTYEALENNQLNSSEELELPYLND

SQAVSLRSITADEKSQWSVADLMAAAMVMSANDAAEALGNRLGGENEFIQQMNQKAQE

LKLSKKTNFTSASGLPTEKGESLATAKDLAILAEALVTDYPQVLEQTRAASWTLSTGG

IIDSTNGLLTSNQHDFEVDGLKTGYTEDAGYCYIGTASKGEQRLLVIVLGTNNSEERF

LKADKLLQKYFD"

CDS complement(381522..382181)

/gene="gatD"

/locus_tag="JFBMFIFI_00335"

/EC_number="6.3.5.13"

/inference="ab initio prediction:Prodigal:002006"

/inference="similar to AA sequence:UniProtKB:Q8DNZ8"

/codon_start=1

/transl_table=11

/product="Lipid II isoglutaminyl synthase

(glutamine-hydrolyzing) subunit GatD"

/db_xref="COG:COG3442"

/translation="MEELAVCHLYGNLLNTYGDNGNLLMLNHRAKQMGIHLNTEIISL

KDDFDPKKYDLVFFGGGQDYEQLIVSRDIQSKKAALTEYIEAGGVTLAICGGYQLLGH

YYMDASGNKIDGIGALDHYTLSQDNSRFIGDIIIKNEEFDETYVGFENHNGMTFLGKG

EKPLGVVQQGKGNNGEDKTEGAIYKQTYCSYFHGPLLVKNTILADRLLHAAMDRKKQK

N"

CDS complement(382184..383533)

/gene="murT"

/locus_tag="JFBMFIFI_00336"

/EC_number="6.3.5.13"

/inference="ab initio prediction:Prodigal:002006"

/inference="similar to AA sequence:UniProtKB:Q8DNZ9"

/codon_start=1

/transl_table=11

/product="Lipid II isoglutaminyl synthase

(glutamine-hydrolyzing) subunit MurT"

/db_xref="COG:COG0769"

/translation="MSIRSSFAIMVGKTTRWGLHTFFKGGSSLPGMITQKIDPNVLGA

LAKDYEVVIVTGTNGKTLTTALTFQVLKQKYPNIITNPTGANMQQGIISTFLMHDSAN

QKDQKKIAVLEVDEASLVHVTKYIKPKAILFTNVFRDQMDRFGEIYTIYQMMVDGAAL

APTAKIISNGDAPIFNSLDTVNERIYFGFDDQPDGELMAHYNTDGILCPHCQNILHYK

FITYSNLGKYYCPNCDFKRPELTYRLTKMLKMDHQSSKFEIDGEAFQINVGGLYNVYN

ALSAYSIGRLFDVSPAEIRAGFNLAEKVFGRQEVIKIGEKEITINLVKNPVGLNQILE

MIALDPRPFSLVSILNANYADGIDVSWIWDANYEKIMEMDIKQFTVSGERVNDIATRL

EVAGVPKDELQVLPNLSDVIKSFEKAPTEHIYVLATYTAVLQLRKELAAAGYVKEGM"

CDS 383860..385665

/locus_tag="JFBMFIFI_00337"

/inference="ab initio prediction:Prodigal:002006"

/codon_start=1

/transl_table=11

/product="hypothetical protein"

/translation="MEAINIQIKVFSQEKIKVNAEDKNVYFYKNDFLPEVILELAPEV

KNKSLFGKSKLGRNSEWISNINELVKQIVIECHPLTNKMSLMKVLGEYSQTFNEENPE

LIVYQIFLKEEADKVVLVLNFVTRFIYQDGEFLDSGIRKALMQQNVDQEGNILINWKN

SEIKKITNKLLQIQELSETTIPFVDQKSSIKLAETVVNIDQEIRQLADSLPSVSKLEE

RVNTCQSEVDQVSKQLTNAKDKKIKLDKQSKEIASFLSSLEQQVELLKVKMEQLLPNM

ELQNEKSNQVKQQEEQNELQELREKITKLEKANEEVDQLVTDSIARIAKQTQIESPLK

KELVEMNKRLIGITENETTLKQQVKKLNLIISQQNQKHEAERQRLEGTIRQLELAKLD

NTKKISNEMVDESPSKELNLLTIKVNENNESKAVQEKTELGHGNNDIVEFVNTNEGQL

VENESLETEVNLLEKQSKKTSNWLKTKKMEAAEFNEQFRKVQYLEHAWMINRDLSKKI

NQENQDFDENQEQEMIEEKLKNEINQVEIENFILNGEAANLRERVTFKNNRPFAKDRV

ILSNADYELLEKKARQYELIDYMNKQMTVKAREQA"

CDS 385888..386460

/gene="tdk"

/locus_tag="JFBMFIFI_00338"

/EC_number="2.7.1.21"

/inference="ab initio prediction:Prodigal:002006"

/inference="similar to AA sequence:UniProtKB:Q81JX0"

/codon_start=1

/transl_table=11

/product="Thymidine kinase"

/db_xref="COG:COG1435"

/translation="MAQLFFKYGAMNSGKTIEILKVAHNYEEQNKPVVIMTSGIDDRD

EVGFVSSRIGLRREAVPVFDETNIYHIIDEMAIKPACILIDESQFLTKEHVYQLAHIV

DDLDIPVMAFGLKNDFRNELFEGSHYLLIYADKIEEMKTICWYCHKKAIMNMRLVDGQ

PVYTGEQIQIGGNESYLPVCRKHYHHPPHH"

CDS 386523..387599

/gene="prfA"

/locus_tag="JFBMFIFI_00339"

/inference="ab initio prediction:Prodigal:002006"

/inference="similar to AA sequence:UniProtKB:Q8DU64"

/codon_start=1

/transl_table=11

/product="Peptide chain release factor 1"

/db_xref="COG:COG0216"

/translation="MYDKLQGVEGRYEELGELLSDPEVISDTKRLMALTKEEANLRET

VSVYRRYKVVVDDIADTEEMLGENLDSDMAEMAKEELSALKKEKEALEETIKILLLPK

DENDDKNIIMEIRGAAGGDEAALFAGDLFGMYQKYAEGQGWKTEVLEANITGIGGYKE

IIFMITGNNVFSKLKYESGAHRVQRVPSTESQGRIHTSTATVVVMPEAEEVEINIADK

DIRTDIYHASGAGGQHVNKTASAVRLTHLPTGIAVAMQDERSQIKNREKAMKILRARV

YDQIRNEAQSEYDANRKSAIGTGDRSERIRTYNFPQNRVTDHRIGLTIQKLDQILAGK

LDEIVDALIVYDQTEKLEQMKNGN"

CDS 387589..388440

/gene="prmC"

/locus_tag="JFBMFIFI_00340"

/EC_number="2.1.1.297"

/inference="ab initio prediction:Prodigal:002006"

/inference="similar to AA sequence:UniProtKB:P0ACC1"

/codon_start=1

/transl_table=11

/product="Release factor glutamine methyltransferase"

/db_xref="COG:COG2890"

/translation="MATNSSYKEVLNWASSFLESHGLEGYAAERFLLERMEWTKTDLI

LNLATKIPEKVKEQLESDVIEHSKGKPVQHILGYEWFYDRPFKVTADTLIPRPETEEI

VAKFLARNGQKQDGLTVLDVGTGTGAIAITVKKERPMDIVTAIDISSEALSVAKENAL

KLDADIRFLEGDLSAPVSHEKFDVVLSNPPYISEAERPLVDDIVFFNEPHLALFAKHD

GLFIYERLAKELPYLMKPAGQIILEIGFQQGVAVKKLFTTAFPFAKVTVEKDMSGLDR

MIYVQLP"

CDS 388501..389523

/gene="ywlC"

/locus_tag="JFBMFIFI_00341"

/EC_number="2.7.7.87"

/inference="ab initio prediction:Prodigal:002006"

/inference="similar to AA sequence:UniProtKB:P39153"

/codon_start=1

/transl_table=11

/product="Threonylcarbamoyl-AMP synthase"

/db_xref="COG:COG0009"

/translation="METSFFKENNLSEAAKLIQAGELVAFPTETVYGLGADALNEEAV

KKVYAAKGRPSDNPLIVHIASREELESLVAEIPAIAEPLIKKFWPGPLTLIFKAKPGF

FSTSVTAGLESVAIRMPDNSLTLKLIQLAERPLVGPSANTSGRPSPTSAEHVYHDLEG

KIAGIIDGGATGIGLESTVLDLTSPENPLILRPGAITQQDLKEVLGKDVTYDHHLIGK

EEAPKAPGMKYTHYAPEEPVILVKGTDYNLWRKAITHYLEQGETIGILANEEIISKVK

SEVEDVFSLGLKANVNEASHSLYAGLRFFGNTKVTLILAEAYEINDNTRAYMNRLEKS

AGGKYF"

CDS 389597..390847

/gene="glyA"

/locus_tag="JFBMFIFI_00342"

/EC_number="2.1.2.1"

/inference="ab initio prediction:Prodigal:002006"

/inference="similar to AA sequence:UniProtKB:P99091"

/codon_start=1

/transl_table=11

/product="Serine hydroxymethyltransferase"

/translation="MLNLTFKDFDPELWAAIDQEKERQEHNIELIASENFVSKAVREA

QGSILTNKYAEGYPGKRYYGGCEYIDIVENLAIDRVKELFGAGYANVQPHSGSQANMA

AFNALIEPGDTVLGMDLTHGGHLTHGSPVNFSGKTYNFIAYGVDPTTEKIDYDEVRRI

AIEGQPKLIIAGASAYSRAIDFAKFKEIADEVSAYFLVDMAHIAGLVAAGAHQNPVLY

ADIVTSTTHKTLRGPRGGLILAKEKYGKALNSAIFPGIQGGPLEHVIAGKAVAFKEAM

TPEFKTYSEQVIKNAQAMQSVFNESVGHLISEGTDNHLLLLDVTNFGLNGKEAEAILD

SVGITVNKNTIPFEKLSPFKTSGIRIGTPAITTRGFKEEDAEEVARLIVAALTAKDDT

EQLKSISKEVRALTIKHPLYDEND"

CDS 391192..391821

/gene="upp"

/locus_tag="JFBMFIFI_00343"

/EC_number="2.4.2.9"

/inference="ab initio prediction:Prodigal:002006"

/inference="similar to AA sequence:UniProtKB:P70881"

/codon_start=1

/transl_table=11

/product="Uracil phosphoribosyltransferase"

/translation="MGKFQVLDHPLIQHKLTMIRESKCGTKVFREVVNEIAMLMAYEV

SRDMPLEDVEIETPLVKTVQKQLSGKKVAIVPILRAGLGMVDGMLEMIPAAKVGHIGL

YRDHESLEPVEYFVKLPSDIQERQMFVVDPMLATGGSAIMAIDALKRRGATTIKFVCL

VAAPEGVQALQDAHPDVDIYVASLDEKLDENGYIVPGLGDAGDRLFGTK"

CDS 392357..393073

/gene="atpB"

/locus_tag="JFBMFIFI_00344"

/inference="ab initio prediction:Prodigal:002006"

/inference="similar to AA sequence:UniProtKB:P09218"

/codon_start=1

/transl_table=11

/product="ATP synthase subunit a"

/translation="MDHEAPIIHLFGLAFNVANMITVVAACLIVFLVTFFCTRKLQIK

PTGGQNFIEWVVDFVRTIISGPMSWKEGQQFHLLGFTLLLFVFVSNMMGLPLVITIGG

KGYWKSPTADPVVCLALALMVILLTHYFGVREQGFKNYFMNSYIRPVSFLLPIKLLEE

FTNALTLALRLYGNVYAGEILLGLIASLGLSHGPWTWIVGLPLEMVWQGFSIFIGSIQ

AFVFTTLTMVYMAHKVQVEH"

CDS 393129..393350

/gene="atpE"

/locus_tag="JFBMFIFI_00345"

/inference="ab initio prediction:Prodigal:002006"

/inference="similar to AA sequence:UniProtKB:P00845"

/codon_start=1

/transl_table=11

/product="ATP synthase subunit c"

/translation="MNGMGLIGAAIAVAGAAIGASLGNGKVISKTIESVARQPELQSR

LQMMMFIGVGLIEAVPIMAVVIAFILVFK"

CDS 393430..393936

/gene="atpF"

/locus_tag="JFBMFIFI_00346"

/inference="ab initio prediction:Prodigal:002006"

/inference="similar to AA sequence:UniProtKB:P09221"

/codon_start=1

/transl_table=11

/product="ATP synthase subunit b"

/translation="MYGLVLGESTSAGDTIFVLVSFLLLLLVLKKFAWKPLMNIMEDR

EKQIAKNIDSAENSRIEANRLAVESKDSLEATRIEASAIIAKARENGENIQDEMLVKA

KAEAERIIVDAKKDIELEKQKAIESVKNDVSHLSIDIATKLIGKELTSEDHAALIDKY

IEGLADDE"

CDS 393933..394475

/gene="atpH"

/locus_tag="JFBMFIFI_00347"

/inference="ab initio prediction:Prodigal:002006"

/inference="similar to AA sequence:UniProtKB:Q9RGY4"

/codon_start=1

/transl_table=11

/product="ATP synthase subunit delta"

/db_xref="COG:COG0712"

/translation="MKLDKHTVASRYAKAYYKLAEEHDTVEEAFDNMMDIRQIFADNP

GLGLVLSDARLTLNQKRPIVENLMKNFPELMQNFVQMIFDYDRMNDMELIVDEFEKLY

DYKNRTILAKVTTAVPLTDVQKERLAVSFAKKMDGKKVMFEEVVDSAILGGVIIEAEH

QIFDGSIRLNLETLKKQIMK"

CDS 394505..396022

/gene="atpA"

/locus_tag="JFBMFIFI_00348"

/EC_number="7.1.2.2"

/inference="ab initio prediction:Prodigal:002006"

/inference="similar to AA sequence:UniProtKB:Q7CRB1"

/codon_start=1

/transl_table=11

/product="ATP synthase subunit alpha"

/db_xref="COG:COG0056"

/translation="MGIKAEEISALIKQQVEGYRNELLIDEVGTVTYIGDGIARAYGL

ENAMSGELLEFSNGVFGMAQNLESNDVGIIILGPFEEIREGDKVRRTGRIMEVPVGEA

LIGRVVNSLGQPVDGLGEIVTTKTRPVETLAPGVMQRQSVGEPLQTGLKAIDALVPIG

RGQRELVIGDRKTGKTSIAIDTIINQKDQDMICIYVAIGQKESTVRNQVETLKRYGAM

DYTIVVTASASQPAPMLFIAPYAGTAMGEEFMYNGKHVLIVFDDLSKQAAAYREISLL

LRRPPGREAYPGDVFYLHSRLLERAAKLNQELGGGSMTALPFVETQAGDISAYIPTNV

ISITDGQIFLESDLFYGGTRPAVAAGLSVSRVGGAAQIKAMKKVAGTLRLDLASFREL

EAFTQFGSDLDAATQAKLNRGRRTVEILKQDLHSPLPVAKQVMILYALTHGFLDSVPV

DEILRFEGEFFDYLDSQRPEILKTIVETKDLPDSEELDAAITNFKEMFIPVKSSI"

CDS 396059..396976

/gene="atpG"

/locus_tag="JFBMFIFI_00349"

/inference="ab initio prediction:Prodigal:002006"

/inference="similar to AA sequence:UniProtKB:Q7CRB2"

/codon_start=1

/transl_table=11

/product="ATP synthase gamma chain"

/db_xref="COG:COG0224"

/translation="MGGSLIDIKKRIASTKKTSQITSAMQMVAGAKLGKAEATAKNFQ

IYASKVRGVVTHIAATQLALIEDGNLIGTNSPSNVDFHDMLIERPVKKTGYIVISSDK

GLAGNYNSSVIKSTVDMITRDHKSSDEYVFMAVGSSAANFFKSRGMNLAYELRGISDQ

PSFEEIREIARTATEMYKNEVFDELYVCYNHHVNSLSFQYRAEKMLPLSDLDASESVD

YEVEYLFEPSKEAILDILLPQYAESLIYGAILDAKAAEFAARMSAMKSATDNAHSIID

DLTIHYNRARQAAITQEITEIVGGASALE"

CDS 397262..398689

/gene="atpD"

/locus_tag="JFBMFIFI_00350"

/EC_number="7.1.2.2"

/inference="ab initio prediction:Prodigal:002006"

/inference="similar to AA sequence:UniProtKB:Q8DP44"

/codon_start=1

/transl_table=11

/product="ATP synthase subunit beta"

/db_xref="COG:COG0055"

/translation="MSIGQIVQVIGPVVDVEFPLDGSLPDINNALIVKKIKNKNKDTN

EVPETVVLEVALELGDGIIRTIAMESTDGLQRGMDVEDTGKAISVPVGSETLGRVFNV

LGETIDLKEPFPADARRDVIHRAAPTFDELSSSTEILETGIKVIDLLAPYLKGGKIGL

FGGAGVGKTVLIQELIHNIAEEHGGISVFTGVGERTREGNDLYFEMKESGVIEKTAMV

FGQMNEPPGARMRVALTGLTIAEYFRDDEGQDVLLFIDNIFRFTQAGSEVSALLGRMP

SAVGYQPTLATEMGQLQERITSTRKGSITSIQAIYVPADDYTDPAPATAFAHLDATTN

LERKLTEQGIYPAVDPLASSSSALAPEIVGKEHYKVAMEVQQTLQRYRELQDIIAILG

MDELSDSEKILVSRARRIQFFLSQNFHVAEAFTGQPGSYVPVKETIRGFKDILDGKYD

DLPEDAFRSVGRIEEVVAKAEAQGY"

CDS 398705..399127

/gene="atpC"

/locus_tag="JFBMFIFI_00351"

/inference="ab initio prediction:Prodigal:002006"

/inference="similar to AA sequence:UniProtKB:P63668"

/codon_start=1

/transl_table=11

/product="ATP synthase epsilon chain"

/db_xref="COG:COG0355"

/translation="MSVLRVNIVTPNGLVYDHNANMVVAKTLDGEIGILPQHAPIIVP

LQINEVRVKRTDNPEHEDAVAVNGGVMEVRDNICTIIADSAERERDIDLSRAERAKER

AEKRIEEAKEHEDRDEIDRATVALHKAINRISVSRHGR"

CDS 399375..399608

/locus_tag="JFBMFIFI_00352"

/inference="ab initio prediction:Prodigal:002006"

/codon_start=1

/transl_table=11

/product="hypothetical protein"

/translation="MSFLGVQSLLGIVSHIIFIMMSFWALRGIRIENWIKKGYERQAR

VLYIFLSIVIGYTVSSFFLEFISLSKSLPYLLK"

CDS 399746..401053

/gene="murA1_1"

/locus_tag="JFBMFIFI_00353"

/EC_number="2.5.1.7"

/inference="ab initio prediction:Prodigal:002006"

/inference="similar to AA sequence:UniProtKB:Q8Y4C4"

/codon_start=1

/transl_table=11

/product="UDP-N-acetylglucosamine

1-carboxyvinyltransferase 1"

/db_xref="COG:COG0766"

/translation="MEKIIVRGGKRLEGTVKVEGAKNAVLPILAATILASVGESKLTN

VPTLSDVFTINEVLHHLNLDINFSEKNKEVVVNATHELNFEAPFEYVSKMRASIVVMG

PLLARLGHAKVALPGGCAIGTRPIDLHLKGFEAMGARVHIENGFIEAFADELKGARIY

LDFPSVGATQNIMMAATLAKGTTVIENVAREPEIVDLANFLNRMGAKVIGAGTETIRI

EGVEVLTGTEHGIIPDRIEAGTFMVAAAMTKGNIFIEDAVAEHNKPLISKLKEMNVSI

TEEENGMRVIGPEKLKATDVKTMPHPGFPTDMQAQMTAVQLLAEGTSTMTETVFENRY

MHMEELRRMNANFKVEGPSLVMYGPTKLQGAEVAATDLRAAAALILSGLCAEGYTRVT

HLEYLDRGYFEFHKKLQALGADVERVNEEENQLTEAELEKMFN"

CDS 401362..402348

/gene="mbl"

/locus_tag="JFBMFIFI_00354"

/inference="ab initio prediction:Prodigal:002006"

/inference="similar to AA sequence:UniProtKB:P39751"

/codon_start=1

/transl_table=11

/product="Cell shape-determining protein Mbl"

/db_xref="COG:COG1077"

/translation="MAKDIGIDLGTANVLIHVKGKGIVLNEPAVVAIDTTNGQVLAVG

EEAYRMVGRTPGNIRAIRPLEDGVIADFDITEAMLSYFINKLNVKGFLSKPNILICCP

TNITSIEQKAIIQAAEKSGGKNVYLEEEPKVAAIGAGMDIFQPSGNMVIDIGGGTSDI

AVLSMGDIVTSRSLKLAGDKLDSEITQYVKKKYKLLIGERTAESIKIEIGTVFPKNRS

DSMDVRGRDMVSGLPRTITITSDEVQEALNESMMLIVQQAKDVLEQTPPELSADIIDR

GIILTGGGALLDGIDQLFAEELKVPVFVAERPLDAVALGTGILLENISKKRR"

CDS 402423..402623

/locus_tag="JFBMFIFI_00355"

/inference="ab initio prediction:Prodigal:002006"

/codon_start=1

/transl_table=11

/product="hypothetical protein"

/translation="MPNKKEKMGAIPTPLKVLIVIVVIILTFLIGAMIGFGLLGDGNP

FAVFSQNTWEHIFSFFTKGPKN"

CDS 402738..403085

/gene="ssbB"

/locus_tag="JFBMFIFI_00356"

/inference="ab initio prediction:Prodigal:002006"

/inference="similar to AA sequence:UniProtKB:C0SPB6"

/codon_start=1

/transl_table=11

/product="Single-stranded DNA-binding protein B"

/db_xref="COG:COG0629"

/translation="MNQVTLVGRLVREIQLQEVGESKSVVNNTIAVQRIFKTEQQQQA

DFIPLVAWGKVAKLLDLYCEKGDLIGINGKMQSRSYINSENITIFVVEMRVEEIQFLQ

NKRTEETIAIVEP"

CDS 403496..404392

/locus_tag="JFBMFIFI_00357"

/inference="ab initio prediction:Prodigal:002006"

/codon_start=1

/transl_table=11

/product="hypothetical protein"

/translation="MKKRILSIAIASTLFVGIAGVSSALAATLDDNSEQVVPKNVATL

SGQTVLANLTTQKNSNNDSKTIVQELLAAQTLKDQTPSMSKVTKVEKTPTVANYTVEA

GDTIENIAKAFEVSLADLMKWNAGLTIDSIIQVGQTIKIQTEKDPTTIETSKLSWEAP

APVVEVAPEPAVVAEAPVIQPVEAVAEVAPAPTVETTPTAAAPSGRVVTVSATAYSTS

EPGMSSFTALGIDLRQTSNVIAVDPNVIPLGSRVFVEGYGEAIAGDTGGAIVGNKIDV

HFATVDACYQWGVRSVQVTILD"

CDS 404597..405316

/gene="srrA_1"

/locus_tag="JFBMFIFI_00358"

/inference="ab initio prediction:Prodigal:002006"

/inference="similar to AA sequence:UniProtKB:Q9L524"

/codon_start=1

/transl_table=11

/product="Transcriptional regulatory protein SrrA"

/db_xref="COG:COG0745"

/translation="MKILMIEDNQSVCEMMEMFFLKENWDATFKQDGKEGLDAFLEQP

DSWDIVTLDLNLPTMDGIQICREIRKVSETMPIIMLTAKDSESDQVIGLEMGADDYVT

KPFSPLTLIARLKALHRRADLTSHEEVPKHKLDEDFEVATDHLKINTKTREAFLFEKP

IEGLTPKEFELLYTLAKSPKQVFSREQLLTLVWDYQYYGDERTVDAHIKKLRQKIEKT

GPQVIQTVWGVGYKFDDSGVE"

CDS 405319..406770

/gene="rcsC_1"

/locus_tag="JFBMFIFI_00359"

/EC_number="2.7.13.3"

/inference="ab initio prediction:Prodigal:002006"

/inference="protein motif:HAMAP:MF_00979"

/codon_start=1

/transl_table=11

/product="Sensor histidine kinase RcsC"

/translation="MKYLYQQMLAFFAVIMTVLVILGFSFLKFTTDTVQENLESQLSS

YANTIIENRLVENAEWAELLLHNQGVRIYVTDTNKQIISPKRDKGKYLNISDADLKEL

TKGNEVSSKGDSPIDLLGNKNKQLWVLVPFVYTETQEFAGIVYVDAPMSNIKGSISEL

KKNLYFALVISTICALILSFVLAQYQVKRINRLRSATHRISKGDFDVHLESKDRDEFD

DLAEDFNTMASSLKESNEEIERQENRRRQFMADAAHEMRTPLTTINGLLEGLEYDVFP

EEQKKRSITLMQNETRRLIRLVNENLDYEKIRSNQITLSRYTFNVHDALESIVQQLSG

KAEESGNKLTLDCPRELTVYADYDRFVQVIVNIVQNAIQFTKDGQISVMGLAGLKETV

IEITDTGIGMTAEEVKNIWERYYKADVSRKNTKFGESGLGLAIVQQLMNLHDGKVTVE

SVPDQGTTFRLSFPKEVIDEGTTEKLENPDLKA"

CDS complement(406885..407433)

/locus_tag="JFBMFIFI_00360"

/inference="ab initio prediction:Prodigal:002006"

/codon_start=1

/transl_table=11

/product="hypothetical protein"

/translation="MMTHLKSKNTAFPFTEQSLYTIAIGNDKQEKKVIQSLTRLFSSS

EREKIGYILKEPAFIHYFTILQNILLTVKLAKPKLRSPELLILDALEESYISPDLANK

KIDSLTDEQKKEVQLIASLCAEKKIILVDDWLKNENGTQLKNWIMIFKSLIKQKGLSI

IVLTSDDNIANLGDIQLSPADL"

CDS complement(407606..408373)

/locus_tag="JFBMFIFI_00361"

/inference="ab initio prediction:Prodigal:002006"

/codon_start=1

/transl_table=11

/product="hypothetical protein"

/translation="MTIFERAVLRIRHTPMRSILVFFLYVAFSLILVFLFSMLTQLNF

SLDTINETLNLKQSDLTINTDFFQSLAEKIKHKQDIVGQLLLFTIVFGAIGFTALHGL

LLFFRRKELLNFRLIGEKKRKIFFQLVLENLILLNVFLLFLFVTSVFFRQPVAQQLNQ

IEQNFVGKTEKHFLIQTNSTIISATQDDKLPSENTQPYKEGVSKFNTVTLTDANQTIG

TNPQRFVQFALGMNLFTLLCMSPAALWFLNRNPLKYG"

CDS complement(408542..410716)

/gene="ltaS1_1"

/locus_tag="JFBMFIFI_00362"

/inference="ab initio prediction:Prodigal:002006"

/inference="similar to AA sequence:UniProtKB:Q797B3"

/codon_start=1

/transl_table=11

/product="Lipoteichoic acid synthase 1"

/db_xref="COG:COG1368"

/translation="MKLIKKTQAILNTRLGFFTLAVLLFWAKTYYSYQTAFSLGVTGP

LQQFILFINPVATTLFIFSIALYFKKPRRAYIALFVLYLLATALLYSNVIYYREFTDF

LTVNTMLGAKSVAGGLNTSAFALTSFADIFYILDFIILLGLLLSKQVKMDPRPFKKRY

AFAMTVLSVAIFGANLSIAESNRPQLLTRTFDRNYIVKYLGLNVFTVYDGIKTAQANQ

VKANADSSDMDEVISYLNEHRAAANPATFGSAKGKNVIYIHLESFQQFLIDYKLKDEN

GVEHEVTPFLNSLYHDQSTTSYENFFHQVGQGKTSDAETLLENSLFGLPQGSAFTQAG

SSNVFQAAPQILRDNGNYTSAVFHGNVGSFWNRDNTYKSFGFNYFFDASYYDVSDGNT

LEYGLKDKLLFQESTQYLEQLPQPFYAKFLTVTNHFPYPLDDDNVEFPRATTGDSTID

GYFATAHYLDQSVKEFFDYLKASGLYDNSMIVLYGDHYGISNSRNPSLAPLLGKTSEE

WTDYDNALVQRVPFMYHIPGQTNGGIKSEFGGQIDVLPTLLHLLGVDTTNEDGFIQFG

TDLTSPEHDQTVAFRNGSFVTPKYTVMGTKIYDTKTGTLITDPSEEVLAEIEVLRTGV

EKQLNLSDSIINSDLLRYYTPANFTPVDPTVFNYKNQVDQLKEAAKKLGDENTSLINQ

KDGQSTVELYKTDAPELMTDAEKAAKAAADSAAEAAKEDATK"

CDS 411013..411663

/locus_tag="JFBMFIFI_00363"

/inference="ab initio prediction:Prodigal:002006"

/codon_start=1

/transl_table=11

/product="hypothetical protein"

/translation="MFFLQNLYEFTESNFGDTINHFPLIKLIFYSVDRTLRYFLVWLL

IRGIYLLYKHLKGTKIKVAREFVVQFFVFYVILLFQLTVFRGEYSITNVYIELRPLSE

VNWQPFTETFKLTQGTSLFDFYYNLYGNIFWFIPLGFCVAYLSQNNHPFLRTFFIGLF
CSIAIETMQFLFRTGIADVDDVMFNTTGALIGFVLYEVYFLLKKLFSKQKRRKEAV"

CDS 411766..412974

/gene="sbnD"

/locus_tag="JFBMFIFI_00364"

/inference="ab initio prediction:Prodigal:002006"

/inference="similar to AA sequence:UniProtKB:Q2G1N0"

/codon_start=1

/transl_table=11

/product="Staphyloferrin B transporter"

/translation="MKREIWQVNLKILWFGCFLAGVGFSLVMPFMALYIDTLGDFTSK
EVSMWSGITFSSTFFVTAIVAPFWGKLADRKGRKLMLLRTALGMAIVIALMGTVQNVY
QLIGLRLLQGFFSGFISNATALIATQVPREKSGATLGTLTTGSVTGTLMGPLAGGTIA
EFFGFRITFFITGFLLFLVFLLCLFFVKENFEPVLKEDELSGKAVLAELKFPRVIIGM
FVTTMMIQVAANSISPILSLYVKQLAGESSNVAFISGIVASVPGIATLLAAPRLGALG
DRIGSERVLKAGLLFSVIILIPMAFVTQVWQLAVLRFLLGVSDAALIPAVQAILTKNT
PHAVAGRIFSYNQSFQAMGNVMGPLLGSVISASLGFSGVFLITPLFIISNFFLVQRNT
KNIKKIQSNK"

CDS 413022..414188

/gene="rlmI"

/locus_tag="JFBMFIFI_00365"

/EC_number="2.1.1.191"

/inference="ab initio prediction:Prodigal:002006"

/inference="similar to AA sequence:UniProtKB:P75876"

/codon_start=1

/transl_table=11

/product="Ribosomal RNA large subunit methyltransferase I"

/db_xref="COG:COG1092"

/translation="MVKMQATNKIKSGYPLLVKGDFVKEPNVTEGTLVELIDQQKKFV
ASAYLAKQNKGDGWILSLDPQEKINQKFFEKLFHLAGSNRTFLEANGDTTAYRFFNGE
GDGLGGLTIDFYADYYVFSWYSEGIYTHQKMIIEAFKSAVPAIKGIYQKYRYDTKGQN
DESHVFGEKAPEPLLVQENGIQYATYMNDGLMTGIFLDQREVREAIREKYAVGKTILN
TFSYTGAFSVAAAMGGALETTSVDLANRSLPKTKEQFEVNGIDPETQKIIVMDVFDYF

KYASKKELSFDTVIIDPPSFARSKKRTFSVAKDYSKLLEEVIEITNKNGVIVASTNAA

NVTMEKFEGFIKKAFANKNCQFDWLEKYQLPNDFKINTHFPEGDYLKVWILRKK"

CDS 414374..414616

/locus_tag="JFBMFIFI_00366"

/inference="ab initio prediction:Prodigal:002006"

/inference="similar to AA sequence:ISfinder:ISSpn6"

/codon_start=1

/transl_table=11

/product="IS200/IS605 family transposase ISSpn6"

/translation="MKKDNQSLSHTVWKCKYHIVFAPKYRRQIIYGKYKQSIGKILRE

LCERKGVEILEANACKDHIHLLVSIPPKLSISSFIG"

CDS 414638..414844

/locus_tag="JFBMFIFI_00367"

/inference="ab initio prediction:Prodigal:002006"

/inference="similar to AA sequence:ISfinder:IS657"

/codon_start=1

/transl_table=11

/product="IS200/IS605 family transposase IS657"

/translation="MIFDRHANLKYRYGNRKFWCRGYYVDTVGRNKKQIEEYIRNQVQ

EDYVADQLTLFEEYDPFTGNKNTK"

CDS 415078..416598

/locus_tag="JFBMFIFI_00368"

/inference="ab initio prediction:Prodigal:002006"

/codon_start=1

/transl_table=11

/product="hypothetical protein"

/translation="MNFIKRALLSTKVKKGRSFLLLFIFSAILIFVLAGLTIQSAANV

ATSEARKSMGGSVTLGVNRENTLKKSTEETSSSETTETQRPTPGSYESTPVDLTVATK

IAALDHVASYNFISTTSAGAESFEPISSSDSTEATTETQQPTGGPGGEGSESERGSNF

GPGAAQGDLNVQGVLSTENLAAFTDGTSTLTTGTALTKEDVNKNVVLIEKSLAEANDL

AVGDTITISNPTDDTVTYELRIQGIYETTTSDNAMAANFNFLNPSNQIYVPYTFANTL

KGATYDNGVDSVIFNLDDPENVESFVKAAEATGLDTETYSLQTDTAVYEQMIKPIENV

SSFAKKVVLLVAIAGVVILSLIIMMTIRERKYEMGVLLSLGEKRWKLIAQFFTEILMI

FLVAMCVAGISGKYVGTIVGQQLLDQQTATTTETSENTGPGGGNSEPRGGNMGGFRNF
GAESSTAAKQIDELNVTVSTNDLVKLGGIGLGICFLSIMISSIGVIRLEPKKILTM"

CDS 416618..417277

/gene="lolD"

/locus_tag="JFBMFIFI_00369"

/EC_number="3.6.3.-"

/inference="ab initio prediction:Prodigal:002006"

/inference="similar to AA sequence:UniProtKB:P75957"

/codon_start=1

/transl_table=11

/product="Lipoprotein-releasing system ATP-binding protein
LolD"

/db_xref="COG:COG1136"

/translation="MITAKNVGYWYDKGNIPLYQHVNLTFEKGVLYSILGSSGSGKTT
FLSLLAGLEKPKEGDILYEGTSISKMGLTKFRNQKVSIVFQAYNLLPYMTALQNIVTA
MEITRSKETDKKAYALKMLKKVGISEDLANKNVMKLSGGQQQRIAIVRAMCTDTELIV
ADEPTGNLDEETSRDIIHLFQELAHKENKCIILVTHEKEVANESDVCIQLKNKEFTTI
I"

CDS complement(417331..418374)

/gene="lacD"

/locus_tag="JFBMFIFI_00370"

/EC_number="4.1.2.40"

/inference="ab initio prediction:Prodigal:002006"

/inference="similar to AA sequence:UniProtKB:P0A010"

/codon_start=1

/transl_table=11

/product="Tagatose 1,6-diphosphate aldolase"

/translation="MTKKRISRGKFEKMQQLSNSNGVIAALAIDQRGSMKKMMEQVVG
SENYTIEMVYEFKGLVSQELTKYVSAILLDEELGFKGISDKNEQAGLILSYEKTGYDV
HTPGRLPELIPEMSAQRLIAKGADAAKVLVYFNPDEEDEIIQKKLGFLERLGDEARAA
DIPVFVEPIVYDNDITDDHSPEFAKIKPKKVIDTIKELTKDKYHIDVLKVEVPVLFKY
VEGYTENGDPQVYSRDEALAYFKEASDAATRPFIYLSAGVPTKTFQAELRFAKEAGAQ
YSGILGGRATWFDGISAYAQGGKTGLIQWLDTTGKNNVETLNRILADGAVPWYDIYGG

LDNIDVFDLNVAD"

CDS 418520..419014

/gene="ybaK"

/locus_tag="JFBMFIFI_00371"

/EC_number="4.2.-.-"

/inference="ab initio prediction:Prodigal:002006"

/inference="similar to AA sequence:UniProtKB:P0AAR3"

/codon_start=1

/transl_table=11

/product="Cys-tRNA(Pro)/Cys-tRNA(Cys) deacylase YbaK"

/db_xref="COG:COG2606"

/translation="MKAKKIHKTNACRILDKAKIPYEIYEFPWSEDHVDAKTVAKELG

IPNESIYKTIVTIGDKTGVTIACLPGTEELNLKALAKASGNKKIEMLPMKELEETTGY

IRGGCSPIGMKKNYPTFLASEAQERSEIIISAGKRGMQLALNPTDLAKVVNLKIMDIT

ENKV"

CDS complement(419049..419858)

/locus_tag="JFBMFIFI_00372"

/inference="ab initio prediction:Prodigal:002006"

/codon_start=1

/transl_table=11

/product="hypothetical protein"

/translation="MMFKSFNEVSPTARTKVWNNGFSDYLVPINMTEVQLDNRLSSLS

ISEELSKVFFIDNQPAGIYLHAEGTFSDQKIAWLGGMAVDSAFRNQQVSVKMLREFER

VAVERSTSILYLEAIDGNERAISIYKKFGFAPVKKVLFLESSTNFTSETDYQLKKVVS

LKSIGVNQSSDILWQNKAIHGYDSIGIYDQTKLIGYTVVSLQGNQLVIHQLELDSPKL

QIKAALASLQKLYTPTKWIGSNLVADSPITKLLVQNGFSEKLSQHQYSKQL"

CDS complement(420220..421485)

/locus_tag="JFBMFIFI_00373"

/inference="ab initio prediction:Prodigal:002006"

/inference="similar to AA sequence:UniProtKB:P44941"

/codon_start=1

/transl_table=11

/product="hypothetical protein"

/db_xref="COG:COG2081"

/translation="MKTYTVIVIGGGTSGMMAAIAAAEEGAKVLVIEKNKKLGRKLLV

TGGGRCNVTNNRDADEIIAHIPGNGRFLYSAFHQFDNYDIINFFESNGVRLKEEDHGR

MFPVTDKSKTILEALMAKMEQLNVTILTDNPVATVLYGDGAVKGVILEDGEEIHGSAI

ILSTGGRAMPRTGSTGDGYKWAKKAGHTLKPLYPTEAPILSDEPFILDKTLQGLSLRD

VALSVLNKKEKKVITHQMDMIFTHFGVSGPAALRCSMFVHQTMGRDKSDFVTMTLDVL

PTFSSGQLNQKFQQLIKNEGDKSLKNALKGLVPERYLLFAFDRLGLLETMPLKQCTPD

QIQALAEFFKDFRFTANGTQPLEKAFVTGGGINTKEINPKTMESKLAKGLYFTGEILD

INGYTGGYNITAAFVTGRIAGMHAGKQTD"

CDS 421673..422524

/gene="glcU"

/locus_tag="JFBMFIFI_00374"

/inference="ab initio prediction:Prodigal:002006"

/inference="similar to AA sequence:UniProtKB:P40419"

/codon_start=1

/transl_table=11

/product="putative glucose uptake protein GlcU"

/db_xref="COG:COG4975"

/translation="MEIIIALIPAIAWGSIGLVSNKVGGTAYNQTLGMTIGAFIFSIG

IYLFYQPVIDMKIMITGLLSGLFWVLGQQQQFQSMKFMGVSGALPISTGCQLIANTLA

GALLFHEWKSGRDVTLGVLALVILIVGARFTAVSDGGESNGSGEKNKGYRAIAISTVG

YALYTIIVNAAGVDALAVILPQSAGMLIGGILFSVKQDVLNKYTVRNLITGLLWAVGN

IFMLISMKNVGLAVSFSLSQTGIIISTLGSIWLLGEKKTKREFRYVVMGCILVIIGGV

LLGYLKS"

CDS 422651..424039

/gene="yhdG"

/locus_tag="JFBMFIFI_00375"

/inference="ab initio prediction:Prodigal:002006"

/inference="similar to AA sequence:UniProtKB:O07576"

/codon_start=1

/transl_table=11

/product="putative amino acid permease YhdG"

/db_xref="COG:COG0531"

/translation="MDYMRKKPLDPTAHHSSKLKKELKTMDLILLGLGAMVGTGIFVI
TGTAAAKYAGPSLIISFAIAAFSCVLSALCYAEFASRVPIAGGAYSYAYTIFGELIGW
ITGWLVVCEYLLANASVASGWSGYVHGFLDGLGIPFPNALRASYNAENGTYVDVIAIC
ITFIVMFVVMQGAKKALRLNNIMVIIKFALVILFLVVGVFYVKPDNWTPFAPFGMAGI
TTGAAIVFFAFLGFDAVSMAAEEVVNPQRDIPRGIIGSLAIATILYIGVTLVLTGMVP
FSNLNVKDPVAFAMRFIDQNFVAGLISVGAILTLLTVLISMMYGLTRMIYAIGRDGLL
PKGLSKVDSKTKTPKNATLVIGITSAILSGLVPLENLAQLTNIVTLMAFAIIAAGIIK
LRKDFGDPKINEFKVPFVPVFPIVSMLVCFYLMIQLELSTWIVFGIWLVLGLAIYFLY
GYRNSELGKSKE"

CDS 424213..424551

/locus_tag="JFBMFIFI_00376"

/inference="ab initio prediction:Prodigal:002006"

/codon_start=1

/transl_table=11

/product="hypothetical protein"

/translation="MNPEVTNFINELDSPWKITLVNELRELVHSTVPDIEERIQYKKP
HFLKDGHYAAVISPSKDAITFMIMNTENIEFPKEFAGPKERRWIKISEGQKVDFDLLS
KLLAEAVNSL"

CDS 424767..425468

/locus_tag="JFBMFIFI_00377"

/inference="ab initio prediction:Prodigal:002006"

/inference="similar to AA sequence:UniProtKB:P9WQK1"

/codon_start=1

/transl_table=11

/product="putative ABC transporter ATP-binding protein"

/db_xref="COG:COG1136"

/translation="MAYVSIKDEYKRYQMGDSEIIANDGISFDIEKGEFAVIIGPSGA
GKSTVLNILGGMDNADEGQVLVDNIDIAKFTKKQLTGYRRNDVGFVFQFYNLVPNLTT
KENVELAAQISPHALDAEMVLREVGLGERLNNFPAQLSGGEQQRVAIARALAKQPKLL
LCDEPTGALDYETGKQVLKLLQDTCLNTGTTVIVITHNSAIAPMANRVIEINNAKVRK
VTLNENPLSVSEIEW"

CDS 425487..428792

/locus_tag="JFBMFIFI_00378"

/inference="ab initio prediction:Prodigal:002006"

/codon_start=1

/transl_table=11

/product="hypothetical protein"

/translation="MKKGALYKDIFREIWRTKARFLSIFAIITLGVGFFSGIKATGPN

MIDTADHYFKDKSLMDSKVLSTYGLDKEDISILKKLKGTEILPSYTQDVFLGNSGIVA

KVMSLPEQNSLNQYRVIEGRLPEKSGEIALDNVAKLKNNFKIGDEITFTSEGTTTDLK

DSFKELTYKVTGFVNSPMYIETLTRGTSSIGKGTADGFAVIPENDFSLDYYTEAYLTF

QNTKKLQAYSTEYEDTIEKNNKIVEKALADQPEKRLAEIKTKGEEKIAEGEKKIADAK

AAISDGEQQLATAEQKLADGEAAYTAGVNQLQTELAKAQASIDENQTKIDTGKSEIAR

NEATLSDGEAQLNAAKEEFQTKKVAAETGINQGRDFVAAVNQAMGVPIQQVPTTTQQS

LIQAGNTIDPAMGQVLTGYFAGVVPIEQVQGAINGLNQKLAASSAELVAAESQLQQKE

QELANGRSTLEAAKSDLVAGEEALASGKAQLETERVNGEAQLASSRSELDTGQVSFKE

ATTEFATKKADGEKEIAKGEQEIADAKKQLADLKEPEYFVLDRTTNPGYEEFSDNATR

ISAIAQVFPVFFFLIAALVSLTTMTRMVEEQRLQIGTLKALGYTNGNIAMKFIIYALL

ASVSATIVGLFIGYQLFPTIIFNAYGALYNLPDIRITYYVSYALISLVVALLCTVISA

IVVTWVELKSNAAELMRPKAPKSGKRILLERIPFIWNRFGFNQKVTARNLFRYKQRML

MTVLGVAGCTALILTGFGLKNSIADIAGLQYGKIMRYQAVVAFNPEASKADEESYAKL

IQGTPEITGTLNVRQENFKVAKKGVNTQEAAIFVPETTKDFDKFVGLQNRKTGEKYTI

PANGVLVTEKLAKLFDLEPGKTFEITNTDNQKYKLVVKGITENYAGHSIYMNPTYYEE

IFKTPVVFNSQLLTFNESSKWENSFGEKLTANPRVASTIFVNRVGDNFEDTMSSLNIV

TLVLIISAALLAFVVLYNLTNINVSERIRELSTIKVLGFYDGEVSLYIYRENIILTLM

GIVVGLGLGVVLHGFVLATAEVDIMMFSPTIKTLSYLYSAILTLLFSGIVMIAMHVKL

KKIDMIEALKSVD"

CDS 428911..429438

/locus_tag="JFBMFIFI_00379"

/inference="ab initio prediction:Prodigal:002006"

/codon_start=1

/transl_table=11

/product="hypothetical protein"

/translation="MKKIIVIGPSGSGKSTLARKIAPILNLPLYHLDQLFWQAGWKSI

SQEAFTEKLQEIIKKEEWIIDGNFNHSLESRIEKADTIIFLDVPRRLYFPRIFKRYFQ

NYGRVRVDMSPGCVEQLDKEFLKFVWTFSKKVRPSLIEKLKGLKPEQTLIVLRSKKEV

NFFIENLPSSKSDKT"

CDS 429578..430093

/gene="yuaF"

/locus_tag="JFBMFIFI_00380"

/inference="ab initio prediction:Prodigal:002006"

/inference="similar to AA sequence:UniProtKB:O32077"

/codon_start=1

/transl_table=11

/product="putative membrane protein YuaF"

/translation="MLWGTTYETVYFYILIVTAILAVITFFIGDIIDFDGPIDPVLIV

PFFAFTSLFGYLGEAHTGFGSAWILLVSLVISTIIIFLLNFYLVVPMRDAESTLSTSE

KDMEGRVGIVVTPIPEKGMGEISLKSVTGSSTRPASRYQAGEAIKAGEKVLVIEIKER

VAYVVPYEEAF"

CDS 430115..431572

/gene="yqiK"

/locus_tag="JFBMFIFI_00381"

/inference="ab initio prediction:Prodigal:002006"

/inference="similar to AA sequence:UniProtKB:P77306"

/codon_start=1

/transl_table=11

/product="Inner membrane protein YqiK"

/db_xref="COG:COG2268"

/translation="MSYLVIGLCVFVVLMCLVIFISKYQTANPDEALIISGSYLGVKN

VFKDDSGNKIKIVRGGGAFVLPVFQRSNRLSLLSSKLDVSTPEVYTEQGVPVMADGTS

IIKIGSSVEEIATAAEQFLSKTRDELENEAREVLEGHLRSILGSMTVEEIYQNRDKFS

QSVQEVASVDLAKMGLIIVSFTIKEVKDKNGYLDSLGKPRIAQVKRDADIAIAEADKE

TRIKRAEAEKNSKKSELERETEVAEALKEKELKLAAYKQEQDIAKAQADQAYGLESAK

AQQKVTEEEMTVKIIERQKQIELEEKEITRREKQYDSEVKKKADADRYAKEQEALANK

AREVAEAEADQFRVEAMATANANQVRLEGQAIADAILAKGQADAEAKAKIAEAFKQYG

EAAILSMVVEMLPNMVKEAAQPMGNIEKITVIDNGQGEGSGSGANRVANYATNLLATS

QETLKETTGLDVKDLIEKFVTKAPQTIHQDIHQDK"

CDS complement(431606..432193)

/locus_tag="JFBMFIFI_00382"

/inference="ab initio prediction:Prodigal:002006"

/codon_start=1

/transl_table=11

/product="hypothetical protein"

/translation="MELGQFRLGMRTVKTGIAVAICILIFHYSGRGTPMIASLSAVFA

LREDVETTVKFGKSRILGNSIGALIAALFIFIQGQLGSSFLIELIGIPFCIMFIIIIC

DGINYNSGIIGAIATLLIIYFSIPNNETFIYALERVGDTFIGTFIAITINHLIRSPAH

EEVKEIKADLKTLDEEKAELEELLNDTKPKNSKKK"

CDS complement(432214..432879)

/locus_tag="JFBMFIFI_00383"

/inference="ab initio prediction:Prodigal:002006"

/codon_start=1

/transl_table=11

/product="hypothetical protein"

/translation="MNFKLLNKKVYFVLLPLLFLVLASCGNTEERAGAKKIAEENNER

IENINTHLEKYEALEEDMMTALSNDLKDKNAEKIIRKKTGEVGENFKARKKELASIQK

NMTKLKENQDTLLDYQAKKAVDMPETELENAIQTLKQLSITYDLFYSYYEKTVDAESE

FYQNFSSDMKKEELAEAISLINISHGAAFQQLEVLSADLSSTAFAMTNLADAIKEKDK

KTQ"

CDS 433068..434042

/locus_tag="JFBMFIFI_00384"

/inference="ab initio prediction:Prodigal:002006"

/codon_start=1

/transl_table=11

/product="hypothetical protein"

/translation="MRIGLRTIKTAVGAAIAIFIAEKFQLNYAVSAGIITILSVQNTK

KKSITLAGQRILSTLLALGISSIFFNLVGFNPLAFGLYLVVFIPLAVRLSLTDGIVVS

SVLVTHFLIEESTSVDWWTNSFGLMLIGLSIAILANLYMPSSEGRLQELQMECENQMK

LILLDFSGTLRNGHFHSEENPLLIKLNQLLNEAVNLAKNQHNNQFFGQSYYYIKYFDM

RQMQTYVLHQIKEDLSQCHLPTMENEKLAEMLIRTAEGLHENNSADQLMADIQKLFEH

FRNSPLPETRAEFENRAMLFKMLNDFMHFIEIKQVFYKEFASEKKMSV"

CDS 434232..435377

/gene="braC"

/locus_tag="JFBMFIFI_00385"

/inference="ab initio prediction:Prodigal:002006"

/inference="similar to AA sequence:UniProtKB:P21175"

/codon_start=1

/transl_table=11

/product="Leucine-, isoleucine-, valine-, threonine-, and

alanine-binding protein"

/db_xref="COG:COG0683"

/translation="MKKIVASLAVLAIMATGCGAGGGAAKDSKTIKIGANLELSGPVS

AYGTAEYEGAKLAVEEINKNGGVLGKDLELVKLDNKSDKAESASVATKLATKENVVAI

VGPATSGAVKAATPSVTKAKVPMVTPSGTDDSVTVENGKVWDYVYRVCFQDSFQGVAL

ANFATKDLNAKKAVIIGDSSSDYAKGLTKSFKDSFKGDIVDEVNFTTGDKDFKAVLTK

IKDKNFDVLYMPSYYEEAGLIIKQARELGITQPILGADGFGNEKLVDLAQAKNLNDVY

YTAHYSDKSENPKVTDFVAAFKKENDKTPDAFSALAYDSVYMIAKAIENAGEADPEKV

KDELAKIKDFDGVTGKITMDKEHNPVKSTLVIKLEDGKEVSNTVVEP"

CDS 435462..436340

/gene="livH"

/locus_tag="JFBMFIFI_00386"

/inference="ab initio prediction:Prodigal:002006"

/inference="similar to AA sequence:UniProtKB:P0AEX7"

/codon_start=1

/transl_table=11

/product="High-affinity branched-chain amino acid

transport system permease protein LivH"

/db_xref="COG:COG0559"

/translation="MENFIQQLINGLSLGSIYALIALGYTMVYGIIKLINFAHGEVYM

VGSYVGYTVIRNFNLGLIPALLFSMVFCAILGVVVERVAYKPLRKATRVAALITAIGV

SFLLQYIVLFIMGPEVKAFPETLQNKSYSLGGITIDEQQITIFVVTIVLMLALQFIVK

KTKMGKAMRAVSVDADAAKLMGINVDNTISFTFALGSALAGAAGVLVGIYYNSISPMM

GTAPGLKAFIAAVLGGIGLIPGAMFGGFTIGIIETLISGYGNSMIKDAVVYGILIVIL

IVKPSGLLGKNTKEKV"

CDS 436350..437321

/locus_tag="JFBMFIFI_00387"

/inference="ab initio prediction:Prodigal:002006"

/codon_start=1

/transl_table=11

/product="hypothetical protein"

/translation="MKQFNKTNLGWLIFIAAIYGIIGAGVYLGIIDAFYEITLITIGI

NIILALGLNLIIGFSGQFSLGHAGFMSIGAYCCGIMTLKMPNWTGFFAGIILGIIMAS

IVALIVGIPTLRLKGDYLAIATLGVSEIIRIAILNMGDLTNGAAGLSGIPMFTTWPVL

FIFIVLVTIVIVNYIRSSSGRATVAIREDEIAAESMGINTTKYKTIAFMIGAATASVG

GALYASYFSVITPSDFGFMKSVDILIIVVFGGLGSITGTFIAAVVLGIINVLLQDFGA

VRMIIYAVALIAIMVFKPSGLLGTKEFTISGLIAKFSKKTPTKKEEK"

CDS 437322..438095

/gene="lptB_1"

/locus_tag="JFBMFIFI_00388"

/EC_number="3.6.3.-"

/inference="ab initio prediction:Prodigal:002006"

/inference="similar to AA sequence:UniProtKB:P45073"

/codon_start=1

/transl_table=11

/product="Lipopolysaccharide export system ATP-binding

protein LptB"

/db_xref="COG:COG1137"

/translation="MSLLEVKKLTKNFGGLAAVSMVSLELGENELIGLIGPNGAGKTT

LFNLLTGVYVPTEGTVELNTNGKKNVLNGQKPYKITDLGLARTFQNIRLFKELSVLEN

VLIAMHAKNKVGGLASVMRTPGFYRSEAKMRQEAIDLLAIFQLENKLDHLAKNLPYGE

QRRLEIVRALATKPKILFLDEPAAGMNPQETAELTALIRQIQKDFKITILLIEHDMSL

VMNVCERIYVLEYGRMIAHGTPEEIKNNQAVIKAYLGGD"

CDS 438099..438803

/gene="livF"

/locus_tag="JFBMFIFI_00389"

/inference="ab initio prediction:Prodigal:002006"

/inference="similar to AA sequence:UniProtKB:P22731"

/codon_start=1

/transl_table=11

/product="High-affinity branched-chain amino acid

transport ATP-binding protein LivF"

/db_xref="COG:COG0410"

/translation="MLKVKDLSVHYGVIQAINKINFEVHEGEIVSLIGANGAGKSTIL

KAISGLYRPSEGEILFKDTPIQKASTKKIVESGISLVPEGRHVFSGMTVLENLELGAY

LRKDKDGIKQDLENIYERFPVLAERKKQDTATLSGGEQQMVAMGRAMMSRPKLLLLDE

PSMGLAPIFIKEIFSIIKDINQQGTTVLLIEQNAKVALSISNRGYVLETGKVVLSGTG

EELLASDEVQKAYLGG"

CDS 438807..439454

/gene="IMPDH_1"

/locus_tag="JFBMFIFI_00390"

/EC_number="1.1.1.205"

/inference="ab initio prediction:Prodigal:002006"

/inference="protein motif:HAMAP:MF_01964"

/codon_start=1

/transl_table=11

/product="Inosine-5'-monophosphate dehydrogenase"

/translation="MYVKNYMTAEVITISEETKILEALDLMKDHQIHRLPVTKDGQIK

GLVTEGIIQENSPSTATSLSIHEMNYLLTKTAVGDIMLKNVITIAPDALLEEAADTMR

KNQVSVLPVIGTDHKLVGIITEKDIFAAFLDLLGYYNQGTRVVVDIKDDHTGILEKLT

HLFTEEQMNIDQIAVYRQAGLTQVVIQVASVEVAKIKQKLMDNGYTVSSCSLKEG"

CDS complement(439510..440475)

/locus_tag="JFBMFIFI_00391"

/inference="ab initio prediction:Prodigal:002006"

/codon_start=1

/transl_table=11

/product="hypothetical protein"

/translation="MTPIGDSMKKDIRLTKELYFINNRVKFNLRDLMDEFSISKSTAL

RDISSLEEIGVPLYAEYGKNGGYQVINQHFLPPVYFSDDEVFSIFFAMQMLAFFITTP

FQSEYDFITKKLIESLPDNQKNALNIMKKRLSFNGSPQHYPTFFLKELLLYSMNQNVV

QISYQKKDLTLTKRWIQPLGLEAMDGKWYCNAFDFEKNDYRIFRCDLILELTESKRYV

AKDFADFDIRQNHLKKNKSQAATEFTVQLTTFGVERFNQRHYPNMSLVEEKGDFYIQG

WFEPHELNFMVDYLITFGDSAKIIAPQSLIALYKDKLKIMLSHYQ"

CDS 440558..441001

/locus_tag="JFBMFIFI_00392"

/inference="ab initio prediction:Prodigal:002006"

/codon_start=1

/transl_table=11

/product="hypothetical protein"

/translation="MTVQLTTFLSMNGRAKEAISFYQEYLKAKVLFIKNYVELKEMDP

TFEFIEVEANYIAHSVLEIGGGMLMVADEIMSANKKEIIGTNFSLCITGEVEEIKKMY

AKLNTHPNVKIVIPLEPNIFGNAYGIVEDPFGITIQLVNEKQAKK"

CDS complement(441053..442021)

/gene="iolU_1"

/locus_tag="JFBMFIFI_00393"

/EC_number="1.1.1.371"

/inference="ab initio prediction:Prodigal:002006"

/inference="similar to AA sequence:UniProtKB:O05265"

/codon_start=1

/transl_table=11

/product="scyllo-inositol 2-dehydrogenase (NADP(+)) IolU"

/db_xref="COG:COG0673"

/translation="MKTINWAIIGLGAIADSFARSFNVNGGTLYAAGSRSLDKALTFT

QKHAIEKAYGDYDQLVQDPKVDVVYIATPHSHHFDLIIKCLEHGKHVLCEKAITMNAT

QLQEAMELAENKGLILAEAMTIFHMPLFHKLKALIEGGTLGKLKMLQVSFGSLKETDP

TNRFYNKNLAGGALLDIGTYALSFARFFLNSQPNEILTTMNSFETGVDEQSAIILKNE

LNEMAIVALTFRAKMPKRGIIACEDGYITVDEYPRATRATITRPDGTTEIVEAGVEAE

ALNYEAQNMTNLILEGQGENYLTLSHDVLKMMDSARKQWGLHYDFE"

CDS 442229..443278

/locus_tag="JFBMFIFI_00394"

/inference="ab initio prediction:Prodigal:002006"

/codon_start=1

/transl_table=11

/product="hypothetical protein"

/translation="MVMWENFKRNDSLRRFSVFLVICLALYFIREAISMILLTFIFTF

LILRLVKSVQRWVKIPQTLIVIVTYAVIVSLFYFTLTHYVPKLIYQTGQMIESVYRFY

QSPARSSMIVEWIGQNIDATEIKDQLKNGLSLALTYLTSIGSMGATFLLSFILSFFFS
IEEKRVREFSRLFFTSKIGWFSQDVAYLGGKFVETFGVVLEAQFLIALANTVITTIGL
YFLGLPQLVSLSIMIFILSLIPVAGVIISCIPLALIGYSIGGFTDVVYILGMIAIVHA
LESYVLNPKLMSSKTELPVFFTFVILLFSERFFGVWGLIVGIPIFVFLLDLLDVKAIR
KKIGTKKEEKTSPTI"

CDS 443425..443964

/locus_tag="JFBMFIFI_00395"

/inference="ab initio prediction:Prodigal:002006"

/codon_start=1

/transl_table=11

/product="hypothetical protein"

/translation="MNEALVKIVLMAIKEQFISEKAFYKDQLGITPQSWDRWKKGEQG
LKAENMQKIALLFTDYEWMLVQKVCRNALILPEVAENPVSEFQHMKFYVAKKWVNSGI
ADLDFKRNDSKEETENSYRKPAVTTLRVSVNYEFWSYKDSIELRLPGIIQQQIEREKQ
DLLEWFNENIGEKFPPMTK"

CDS 444215..444988

/gene="vanYB"

/locus_tag="JFBMFIFI_00396"

/EC_number="3.4.17.-"

/inference="ab initio prediction:Prodigal:002006"

/inference="similar to AA sequence:UniProtKB:Q47746"

/codon_start=1

/transl_table=11

/product="D-alanyl-D-alanine carboxypeptidase"

/db_xref="COG:COG1876"

/translation="MNKKIIISLFSLTLLSGCQFIGENTAKDSTDNTNKTTKTSTTEK
DKATEEKVAIALLPNVSTADWNLLLVNSDFPMDPTREIPLAVVDNGLQIDQRMEADYQ
AWMEAARNAGFNMELISAYRSIDLQQTVYDQSIQDNVAKGMSQEEALKETKEYVAFPG
ASEHHSALAIDIVDDQWLATGKGLIPEYDQTDSQKWLVKTMQDYGFILRFPKDKESYT
KISYESWHFRYVGKENAKYIVDNHLSLEEYIERLKAAGK"

CDS 445448..446080

/gene="lytG_1"

/locus_tag="JFBMFIFI_00397"

/EC_number="3.2.1.-"

/inference="ab initio prediction:Prodigal:002006"

/inference="similar to AA sequence:UniProtKB:O32083"

/codon_start=1

/transl_table=11

/product="Exo-glucosaminidase LytG"

/db_xref="COG:COG1705"

/translation="MVLKKGNKNLKKLKSIKIIPSYLVASIFISCLVFFGTLSFLSSR

ENAQQAQVAVEIDQEQAFIDKISAYAKVLQSEYGVLPSISIAQAILESNWGNSELSEK

YNNFYGIKGSEEANTVLMNTKEFVDEEWIEINGRFRIYESWQDSMNAHTKLFVEGTTW

NPQQYAAVLAADNYRDAAFALQASGYATDPTYPEKLIELIQQYKLDQFDK"

CDS 446270..446956

/locus_tag="JFBMFIFI_00398"

/inference="ab initio prediction:Prodigal:002006"

/codon_start=1

/transl_table=11

/product="hypothetical protein"

/translation="MSEKKNIPIITTDLRKDIIEVPKEIEAASGMRVFGKLIKSIIFT

TDISIIANTDADAVIAVYPFTPHPAIIQSITMVANIPVFSGVGGGLTHGTRSANISLF

AEAQGSIGVVVNAPTPISTIKEISDNIDIPIVGTIVSEYTNIKEKLDAGVSILNVSGG

ADTARIVRKIRQDFPDVAIMATGGPTPESISETIEAGANTITYTPPTNGVLFSRKMEK

YRDQEEAKYS"

CDS 446993..449023

/gene="pbp"

/locus_tag="JFBMFIFI_00399"

/inference="ab initio prediction:Prodigal:002006"

/inference="similar to AA sequence:UniProtKB:P07944"

/codon_start=1

/transl_table=11

/product="Beta-lactam-inducible penicillin-binding

protein"

/db_xref="COG:COG0768"

/translation="MRQVKKEKNKTVLMIVASILLVIAMVGGTFYYNWNNSQKSDRAA

LETGKNFTKALEKQDFKKLSQLVTSESLKKVDYTKESLVDKYTVIFDGINASKLKISD
LSVTPIENKSEFKLTYTLNMQTNLGKFDEEKYEAILVPNGENFLVEWTAQLIFPEMEA
TDKINITSTFGKRGSIVDRNGNPLAYLAQYPEVGMIPGELGSGADKAANLKAISEQFD
VSVESLENSLTAEWVKPNIFVPIKVLPNGETPVMTGIQYASKDMRSYPLNQAAAHLIG
YVGEVSAEDIEKDPNLNVGDTIGKAGLEATFEKRLRGKNGGRIVMNDQDGNLKKVLQE
SEKTDGETISLTIDSKLQQQAFDQLNGEKSSAVIMNPQDGSLLALVSTPSYDANLMAT
GISSKEYDAYANDPNLPFLARYASGYAPGSTFKAITAGIGLDAGVTKENKEREISGLK
WQKNSSWGNYFVTRVSDVSPVNMDQALIYSDNIYFAQEGLEMGKKTFEAGLNKFIFGE
KFDLPLAMDPAQIANKKGLNTDILLADTAYGQGQLLLNPIQQAVSYSPFADDGMIMYP
KLTADQKTAKSKQAITPESAAIVKNALIQVVDDPNGTARTLAIPGHKIAAKTGTAELK
QEQDTKGDENGFLLAIDADNASYLMVAMIEGKSSHVVIEKMKPVLESIYAGQ"

CDS 449052..449693

/locus_tag="JFBMFIFI_00400"

/inference="ab initio prediction:Prodigal:002006"

/codon_start=1

/transl_table=11

/product="hypothetical protein"

/translation="MMPLVLPENKDNRQAMEAYMKNRFIFKGIKAPERRLLEKKLLKE
SQKWPIERVLKEIESYYQEEAREYQYIAADLLLKNYKRMSYPELKSLFPLINQKAWWD
TVDVLRSSFSKWCLLNPAYFHEIFTYFTRSESFWDRRVALNLQLGFKEQTNEAYLKQS
ILQDLTTHEFFIQKAIGWALRDYSKTNPEWVTKFITENPGLSTLAVREGSKYI"

CDS 449833..450552

/gene="gmuR_1"

/locus_tag="JFBMFIFI_00401"

/inference="ab initio prediction:Prodigal:002006"

/inference="similar to AA sequence:UniProtKB:O05509"

/codon_start=1

/transl_table=11

/product="HTH-type transcriptional regulator GmuR"

/db_xref="COG:COG2188"

/translation="MAKYSQIADEIRKRILAGRYAVDHALPDQGKLAAEFETSRITIK
KAIELLVVEGLVYSRQGSGTYVRKNALEMSKLDSKMDEYVGVTHQLADRSTITSQILH
FDVRFPTDVEAEKLMLTKTQPVYAIDRLRLLSGEPFLIEHTIMPVEVIPNITKEILLN

SIYHYIKGELGLKFGGANQHIRADKPNELDQKYLKCAQDDPVLEIDQVVYLTDGVPFE

YSQTRHRYDQGNILVVTINSV"

CDS 451410..451994

/gene="xpt"

/locus_tag="JFBMFIFI_00402"

/EC_number="2.4.2.22"

/inference="ab initio prediction:Prodigal:002006"

/inference="similar to AA sequence:UniProtKB:Q831Y0"

/codon_start=1

/transl_table=11

/product="Xanthine phosphoribosyltransferase"

/db_xref="COG:COG0503"

/translation="MKLLEERILKDGVVLGDNVLKVDNFLNHQIDPVLMQAIGNEFAE

LFKGRGINKIITIESSGIAPAVFAGLAMGVPVVFARKHKSLTLVDNMYSASVYSFTKN

VTNEIAVSKSFLDSSDNVLLIDDFLANGQAAIGLLEICEKAGAKVSGMGIVIEKSFQK

GRQIVEESGIEIASLARIAAFEDGIVKFVPQEDK"

CDS 451999..453327

/locus_tag="JFBMFIFI_00403"

/inference="ab initio prediction:Prodigal:002006"

/inference="similar to AA sequence:UniProtKB:A0A2A5JY22"

/codon_start=1

/transl_table=11

/product="Nucleobase transporter PlUacP"

/translation="MLSNGKSAALGLQHVLAMYAGAVIVPLLIGGALNFTPEEMTYLV

SIDIFMCGVATLLQLTVNKFFGIGLPVVLGCAIQAVSPLILIGSNQGIGAMYGSIIVA

GIFIILISGVFSKIKRFFPPVVTGTVITVIGLTLIPVALEKMGGGSKTMSDFGSTKFL

VLAFVTIATILIVQIYGIGFMRSIAVLIGLLVGTGLAAFLGMVNLAPVAEATWFHMPQ

PFYFGRPTFEWSSILTMILISLVSMVESTGVYFALGEITDKKIQEDDLKRGYRAEGLA

VLLGGIFNTFPYTGFSQNVGLVQLSGIKNRKPIYFSAGFLILLGLLPKIGAVATIIPD

PVLGGAMLVMFGMVATQGIRMLAHVDFTNESNLLVVAMSVGLGLGVTVVPELFAGLPE

TVQLFTSNGIVVASLTSIILNVLFNKKERGKVDLETSTVGSEFAETGNSH"

CDS 453955..454197

/locus_tag="JFBMFIFI_00404"

/inference="ab initio prediction:Prodigal:002006"

/inference="similar to AA sequence:ISfinder:ISSpn6"

/codon_start=1

/transl_table=11

/product="IS200/IS605 family transposase ISSpn6"

/translation="MKKDNQSLSHTVWKCKYHIVFAPKYRRQIIYGKYKQSIGKILRE

LCERKGVEILEANACKDHIHLLVSIPPKLSISSFIG"

CDS 454219..454425

/locus_tag="JFBMFIFI_00405"

/inference="ab initio prediction:Prodigal:002006"

/inference="similar to AA sequence:ISfinder:IS657"

/codon_start=1

/transl_table=11

/product="IS200/IS605 family transposase IS657"

/translation="MIFDRHANLKYRYGNRKFWCRGYYVDTVGRNKKQIEEYIRNQVQ

EDYVADQLTLFEEYDPFTGNKNTK"

CDS 454723..454875

/locus_tag="JFBMFIFI_00406"

/inference="ab initio prediction:Prodigal:002006"

/codon_start=1

/transl_table=11

/product="hypothetical protein"

/translation="MKKMKIKTFLVMVSDVFLSLFLFTDSVSVEEKVNINDLDQLSID

LITEAE"

CDS 454974..455615

/locus_tag="JFBMFIFI_00407"

/inference="ab initio prediction:Prodigal:002006"

/codon_start=1

/transl_table=11

/product="hypothetical protein"

/translation="MKREKINLNTIIGIQFIISRCYKLRYRNTRESIRHDSTIFLFCF

FQSILIYTLNLFLTISKSMNTQAENNEFSILIQIIELSLLFFIFVFFIFGTLKLLIFF

SQKVLRYKEEINTKYLLGVTVSHISSEIVIESFLGVPFIFIFNYLLSKFIVTKFLYLL

LDIFPIGNAFENSIHTSNLFILGISPFIFILTIIISLLFVKSKILKSTKKFKY"

CDS 455612..456031

/locus_tag="JFBMFIFI_00408"

/inference="ab initio prediction:Prodigal:002006"

/codon_start=1

/transl_table=11

/product="hypothetical protein"

/translation="MKRMFEKVVGVLSENSSDDYFYDDEFIYVQELLRDFSDNDWEKL

FQSVKSKGDKYKIRLVYCIDADFGMNGLKLLFSLLDENDEVVEYVIDSLRSFKTPEYK

ELINSNNQIIEKAESLLKNAIPPVKRVLEAFIEENKR"

CDS complement(456338..456607)

/locus_tag="JFBMFIFI_00409"

/inference="ab initio prediction:Prodigal:002006"

/codon_start=1

/transl_table=11

/product="hypothetical protein"

/translation="MSLKKDIQQVFKESYLEDLYQFLNEDKEYLLMREKIRLVENEVL

EYPTEINKKSLKQNKLKIAEYLHISIYKMGFYVGINRSQSESKES"

CDS 456712..456924

/locus_tag="JFBMFIFI_00410"

/inference="ab initio prediction:Prodigal:002006"

/codon_start=1

/transl_table=11

/product="hypothetical protein"

/translation="MKYKNIRSIREDNDVTQQKMAELLNVSQNTYSQYETGKIEWTAS

TLIKIADYFDVSVDYLLDRTKTKKFK"

CDS 457041..457208

/locus_tag="JFBMFIFI_00411"

/inference="ab initio prediction:Prodigal:002006"

/codon_start=1

/transl_table=11

/product="hypothetical protein"

/translation="MEKLSIGGKFGWFLDTLSKSGIFVKNLSDREIETYIFEDFIVGV

ISFMQTKTYKN"

CDS 457871..458236

/locus_tag="JFBMFIFI_00412"

/inference="ab initio prediction:Prodigal:002006"

/codon_start=1

/transl_table=11

/product="hypothetical protein"

/translation="MKNQAQQAAIFLLAGMCLWIGTLMVRSNITLANPILLFIVGSLP

NFGTAWMLPSFLILVNITLTKRQLSLKVVRCMLVGTFILQNLSELYYVYFAGASFDLV

DCLFGLGALLILEQAMKRI"

CDS 458253..458798

/locus_tag="JFBMFIFI_00413"

/inference="ab initio prediction:Prodigal:002006"

/inference="similar to AA sequence:UniProtKB:P61544"

/note="UPF0316 protein SA1727"

/codon_start=1

/transl_table=11

/product="hypothetical protein"

/translation="MVIDAKMLLMIFIINLSYITLNTIRFMLTMKGYRLIAPIVSVFE

ITIYVLGLSLVLNRLDNPWNLAAYAIGYGVGIGIGIRIEEFLALGYIMVTVIIPDVES

KMPEELRELGYGVTMNYAYGREGERMILEILSPRKNERTLYKEIHKLEPKAFVISYEP

KYISGGFWTKKVRRRRNQLKK"

CDS 458922..459410

/gene="purE"

/locus_tag="JFBMFIFI_00414"

/EC_number="5.4.99.18"

/inference="ab initio prediction:Prodigal:002006"

/inference="similar to AA sequence:UniProtKB:Q9WYS7"

/codon_start=1

/transl_table=11

/product="N5-carboxyaminoimidazole ribonucleotide mutase"

/db_xref="COG:COG0041"

/translation="MTEKVAVIMGSTSDWETMKFTCDILDEFGIAYQKKVVSAHRTPD

YMFEFAKGARASGLQVIIAGAGGAAHLPGMVAAQTTLPVIGVPIESAALKGMDSLLSI

VQMPGGVPVATVAIGKAGATNAGLLALEMLSMTDGEIADKLDKRRIKLKEKVMESSDE

LV"

CDS 459397..460524

/gene="purK"

/locus_tag="JFBMFIFI_00415"

/EC_number="6.3.4.18"

/inference="ab initio prediction:Prodigal:002006"

/inference="similar to AA sequence:UniProtKB:Q7A695"

/codon_start=1

/transl_table=11

/product="N5-carboxyaminoimidazole ribonucleotide

synthase"

/translation="MNLSNLIPPGKTIGIIGGGQLGRMLTLAAKEMGYFVGILDPEKN

CPAAQVADWQIVAKFNDYTALQELAFRTDVVTYEFENVDVDAISKIEHLVSLPQGSEL

LSITQDRLLEKAYLEENNINIAPYATIIDLNDISASLDGIGFPCVLKTIRGGYDGKGQ

VVLYSEEDIPKCEVLLKTGICVLEAWIPFERECSVMVARNEAGEIRVFPVVENLHRNN

ILHETIAPARVSNEISEEVERVATVIAKKLNLIGVLGIEMFLTSSGSLYVNELAPRPH

NSGHYSIEACSFSQFDLHIRAICGWPLPEVKLLSKAVMVNILGEHIEDSLFQILFKPE

WHFHYYGKSLARLGRKMGHITILTENIERTLETIEDTGIWE"

CDS 460538..461836

/gene="purB"

/locus_tag="JFBMFIFI_00416"

/EC_number="4.3.2.2"

/inference="ab initio prediction:Prodigal:002006"

/inference="similar to AA sequence:UniProtKB:P12047"

/codon_start=1

/transl_table=11

/product="Adenylosuccinate lyase"

/db_xref="COG:COG0015"

/translation="MIERYTRPEMGAIWTDENRYDTWLEVEILADEAWAELGEIPKED

AQKIRENASFDVARILEIEAETRHDVVAFTRAVSESLGAERKWVHYGLTSTDVVDTAY

GYQLKQANDILRQDLQRFLEIIGTKAKEHKMTVMMGRTHGVHAEPTTFGLKLALWYSE

MKRNIERFEHASKGVEAGKISGAVGTFANIPPFVEEYVCEKLEIRPQEISTQVLPRDL

HAEYVSAMALIATSIEKFATEIRGLQKSETREVEEFFAKGQKGSSAMPHKRNPIGSEN

MSGLARVIRGYMVTAYENVTLWHERDISHSSAERIILPDTTILLNYMLNRFGNIVKNL

TVFPENMLRNMDATFGLIYSQRVLLKLIDNGMSREAAYDLVQPKTAISWDEQVQFRPL

LEADPEVTAILSQADLDDAFDYHHHLKNVDVIFERVGLGD"

CDS 462209..463510

/gene="gltP"

/locus_tag="JFBMFIFI_00417"

/inference="ab initio prediction:Prodigal:002006"

/inference="similar to AA sequence:UniProtKB:P39817"

/codon_start=1

/transl_table=11

/product="Proton/glutamate-aspartate symporter"

/db_xref="COG:COG1301"

/translation="MVKFLKNYKSSLTLLAGIAVGAIIGVFFGEAALFLEPIGKLFLN

CVFVLIIPLIFCSMSLAVAGTGKQGVSRIKKILGLTLGTFAITTLIAVTLMYVATLVY

NPFASIDRNQFSALMDQNLVAESQSFGMMIVNTISVGNFLDLFDKSNLLALILFSLFF

GITLSLIGEKASPLLAVLTVGNDVTMKMIALVMKFAPIGLGSYFAYTLGELGPKILGG

YMKALLLFILICGIYFIFILSFYAYIAGGLRGLKLYWQNIFIPALQALGTSSSAACIP

TNLAALKKIGVPEDIRETVISLGANIHKDGSAMSGVLQVVLLFTLFNRDLMTLSAGLS

IICGGFLVGIVMGSIPSGGFTAEVLLIALFGFPVEVIPVIAVITTITDMTATLVNSTS

NITCAMMITKVIEKESLTHKFYETECSSENQGIKGSAKELS"

CDS 463568..464929

/gene="norR_1"

/locus_tag="JFBMFIFI_00418"

/inference="ab initio prediction:Prodigal:002006"

/inference="protein motif:HAMAP:MF_01314"

/codon_start=1

/transl_table=11

/product="Anaerobic nitric oxide reductase transcription

regulator NorR"

/translation="MTKTVTKLMNDDVTTLFVTDGEGNILLANEFTAMTLGMPLNTLL

NSNVYDLVASNIYNASATIETLKTHKMSEVNLTTKAGYQIKSKSTPIFYPDKTLHLVV

TKSDPLTIEELERWTKNIAETSKETEEFSELEVNEKVIAESNEMKRVLRVCKQVSKYN
SRILLLGESGVGKEVVANYIHQHSQLADCQFITVNCAAIPDNLFESEFFGYKKGTFTG
AKEDKIGLIEAADGGTLFLDEISEMPLEMQTKLLRALETMKIRKVGDIAEIPISFRLI
SASNQDILSLVEKGKFRRDLYYRINAIPVVIPPLRERKKDVLALANYFIDYFNEQHQR
SISLETQHIKHLMLSEWSGNVRELKNYIEYLVVTADLDLPVHQGGYETPSNDQIEESL
QAIAEECDYQAFIQYVEKIYFQKKLESSEWNISQTAEESQVSRPFIYKKINELELKQN
VTS"

CDS 465330..466895

/gene="carA_1"

/locus_tag="JFBMFIFI_00419"

/EC_number="2.8.3.23"

/inference="ab initio prediction:Prodigal:002006"

/inference="similar to AA sequence:UniProtKB:F1CYZ5"

/codon_start=1

/transl_table=11

/product="Caffeate CoA-transferase"

/db_xref="COG:COG4670"

/translation="MKIITREAAAALIKDNDTLAFSGFELACANEEVAIALEERFLET
NSPQALTVIHSSCWGDRKEKGMSHLSHKQLLKRWIGGIVKASSPKLSEMIQNNECEAY
NFPQGVLAQLYREIAAKRPGLITKIGLETYIDPRIEGGKMNEVTTEELIQIIELLDEE
WMFYPAFPIDVGLIRGTYADENGNLTLGKEGLHMEVLPIAQAVHNSGGIVIAQVEKIV
KNATLHPKDVVVPGILIDYLVISEPENHFQTGTTQFEPAFSGDVKIPLANVPNLALNE
RKVIARRAAMELEPKMILNLGVGIPVSVSTVAAEEGVSDQFILTTEAGTIGGIPAGLK
DFGHAYNSEMIIDHHAQFDFYDGGGLDLSVLGLAEVDQLGNVNVSKLGNQVIGCGGFI
NISQTAKKLIFTGTFTAGGLKTELKDGELLILAEGRNKKFVNKINQITFNGGFATKNK
QEVLFVTERAVFKLENGELILTEIAPGIDLSRDVLDQMEFDVRVSSDLKEMPVEIFTE
KWGKLAEIMEEKK"

CDS 466892..468097

/locus_tag="JFBMFIFI_00420"

/inference="ab initio prediction:Prodigal:002006"

/inference="similar to AA sequence:UniProtKB:A0LNN5"

/codon_start=1

/transl_table=11

/product="L-lactate transporter"
/translation="MSEIKKKVGMNRWAILFASVGIIICQGGIYAFSVFATPIAKANG
WNVSDVMFAFTIAIAISPLPMVIGGIISDKGKSRELILFSSLLLAVAFILTGFATEKW
MLYLTYGVFGGFGLNLGYIACINNSIRFFPDKKGLASGIVITGIGIGTMIFAPLSAWM
IENLTIRMTFVVLGAIYTVISLICCLIIRNAPIVAKDVEIAEDTPIDHKWNEMLRKPL
FYIIVLMYAAGGFSGLMISSNAADIGQNMFNLTPILAATFVSIYALSNCLGRVFWGGL
SDKLNRTNTMILIFISIAISLLAFIFIHSVAGFAIGMIGLGLCEGGVAAVMPPITIES
FGNKNQGVNYAFVFAGYSIAAMVAPKLSAMIGEKNDGNFTQAFMIGFGLALVGVALTF
VYKAMRKAI"
CDS 468118..469050
/locus_tag="JFBMFIFI_00421"
/EC_number="1.6.5.11"
/inference="ab initio prediction:Prodigal:002006"
/inference="similar to AA sequence:UniProtKB:Q9I4V0"
/codon_start=1
/transl_table=11
/product="NADH:quinone reductase"
/db_xref="COG:COG2070"
/translation="MKQITEILGIKYPIFQGAMAQISKHQLAAAVSNAGGLGIIASGG
MTEEQLREEVIQCKKLTDKPFAVNIMLMAPNVPDMVKVVVEEGVKIVTTGAGTPKNYM
ETFKEANIKVIPVVPSAILAKKMEAIGATAVVAEGSEAGGHIGELSTMVLLPQVVAAV
KIPVIAAGGIGCGKGMAAAYTLGAQGVQMGTAFMLANECPIPENVKEFIANAKEMDTA
VTGRNGGAPVRSLKNKMIEQYIQWEKENMPREQLEELTMGSARKAAAGDIENGSVMAG
QVSGLINEVKSAQQIVDDVLAEAKVTIKNVQLNF"
CDS 469063..470622
/gene="hutH"
/locus_tag="JFBMFIFI_00422"
/EC_number="4.3.1.3"
/inference="ab initio prediction:Prodigal:002006"
/inference="similar to AA sequence:UniProtKB:P10944"
/codon_start=1
/transl_table=11
/product="Histidine ammonia-lyase"

/db_xref="COG:COG2986"

/translation="MSVAESIKKVVLTGEDLTLDELIAISRFNVPVEISEQAIEEVNR

SRKIVDEIVNEERVVYGITTGFGSLCNVSISKEDSVQLQENLIRTHASGFGAPFSTDI

VRAIMAIRANSLTKGYSGIRLIVIEKLVEMLNKQVHPIIPEKGSLGASGDLAPLSHMV

LPILGLGEAEYQGKVLDGKVAMSEANIDTMALASKEGLALINGTQALTACGGLALYDA

IRLLKVSDIAGALSLEAHNGIIDAFKEELHVIRNHEGQVLTAKNVRNLLEGSTFVTKQ

GEIRVQDAYSLRCMPQIHGASKDAVRFVQHKVEIEINSVTDNPIITRDKEAISGGNFH

GQPMALPFDFLGIAVAEIASVSERRLERLVNAEHNNLTSFLVAKSGLNSGFMITQYAA

AALVSENKILAHPASVDSIPSCENQEDLVSMGTIAARKAKSIGENTSRVVATEVLAAC

QAIDMRRAINPDYKLGKGTEAAYNVVRKHCGFLEEDKNIEMYKELDTITNLIMSDEFV

DAVEAVVEMTY"

CDS complement(470694..470984)

/locus_tag="JFBMFIFI_00423"

/inference="ab initio prediction:Prodigal:002006"

/codon_start=1

/transl_table=11

/product="hypothetical protein"

/translation="MLKLIAEDYIKLEHLETVKPLYRELVEKTQLEPDCLAYNLFVDQ

KDPGHFIFIEEWPNELALEKHCNSEHFTRLVPLINSYQSKPGTFLRMDAFQK"

CDS 471113..471721

/locus_tag="JFBMFIFI_00424"

/inference="ab initio prediction:Prodigal:002006"

/codon_start=1

/transl_table=11

/product="hypothetical protein"

/translation="MNQSKQVNLLRENYEVIKSSGVHFIDKRMRYLIARVFTGSAQRI

QPELFLHTNLKLKQQSGLFTNLTSTVRASIASLLIANEMNTNRYYHELADNYQLLLDS

GFRRSEFTYFAAYQLLHSEAQDRKKIVREGKAIFEAIQNNHRFLKGRSSCSMAILIAQ

FNLDKKATEITEIVDYCFQELSQIGFRKNEQLYYLAALGAMF"

CDS 471782..472072

/locus_tag="JFBMFIFI_00425"

/inference="ab initio prediction:Prodigal:002006"

/codon_start=1

/transl_table=11

/product="hypothetical protein"

/translation="MEIPLKPLYYTTIGILAFTQGDQTKLMNEKELSEFIQEIRLLPG

MRFQKEISFALGLSLYTEKLNSNLSSLEMESLLLALQIQIFKENQRVYSAST"

CDS 472409..473206

/locus_tag="JFBMFIFI_00426"

/inference="ab initio prediction:Prodigal:002006"

/codon_start=1

/transl_table=11

/product="hypothetical protein"

/translation="MSFIRGLLQFTYQQVLCCLFPGAIFFALIVTKYFSIPGIARYDL

LLFICILIQVLLLYFKLETVDELKVIMLFHVIGLVLEIYKVNIGSWSYPGAGFWKLGG

VPLYSGFMYSSIGSYICKAWKVFDLQLSNWPNQKLVITLSLAIYANFFTHHFIWDFRW

ILILLVFILFYRTWVYFTVGGKVLKMPMSFSFFCIAFFIWVAENIASFFGAWAYPDQL

ARWQLVHIGKITSWYLLIIISIMIVAELKLKKTSHLFNESTEIKSNN"

CDS 473378..474052

/locus_tag="JFBMFIFI_00427"

/inference="ab initio prediction:Prodigal:002006"

/inference="similar to AA sequence:UniProtKB:Q5XDZ5"

/codon_start=1

/transl_table=11

/product="hypothetical protein"

/translation="MKYSIEGDTLPVVICELEKGEVMVSENGGRSWALGEITTETTSG

GGMKKMLGRAFSGESLFLSRYEAQSNATIAFASSFPGCIIAKELQAGESVIAQKTAFL

AATEGVELSTFFQKKGTAGFFGGEGFIMQKLQGPGTVFFEIDGSTKTYDLAAGERLVC

DTGLVALMDETCKLEIVAVKGLKNKLLGGEGFFDTVVTGPGKVTVQTMTLGGFAQSIL

PFMSSK"

CDS 474365..475450

/gene="serC"

/locus_tag="JFBMFIFI_00428"

/EC_number="2.6.1.52"

/inference="ab initio prediction:Prodigal:002006"

/inference="similar to AA sequence:UniProtKB:Q9HZ66"

/codon_start=1

/transl_table=11

/product="Phosphoserine aminotransferase"

/db_xref="COG:COG1932"

/translation="MERVYNFSAGPAVLPVPVLEQVQAELLSYNGSGMSVMELSHRSS

WFQQIMDDAEALLRELMNIPSNYQVLFLQGGGSLQFSMVPMNLAKNKKIAFVDTGSWS

KKAIEEAEKMKDMQVEVIASSKEKNYASVPEIPAVETDIDYLHITTNNTIEGTCYFTA

PTTNGVPVVADMSSNILSSEYNVADFGLIYAGAQKNIGPAGLTVVIVREDLIAESLPG

LPSMLDYKLQAKNGSMYNTPPTFAIYVAKLVFEWIKELGGVSAIEAINREKAALLYDY

LEESTLFSSPVEKAARSLTNIPFVTGNPELDKKFVSEATAAGFVNLKGHRSVGGMRAS

LYNAFPIEGVTALVAFMQKFAENEGEK"

CDS 475452..476645

/gene="pdxB"

/locus_tag="JFBMFIFI_00429"

/EC_number="1.1.1.290"

/inference="ab initio prediction:Prodigal:002006"

/inference="protein motif:HAMAP:MF_01825"

/codon_start=1

/transl_table=11

/product="Erythronate-4-phosphate dehydrogenase"

/translation="MVFQIKTYNAIAKEGLQKFDGPDYQIDQTEEPDALLIRSQNLHE

TFIPNSVLAIGRAGAGVNNIPVEECTENGVVVFNTPGANANAVKELVLASLFMAARPI

LQGAAWTKTLPTNDPAVEKTVEAEKKRFAGTELQDKKLGVIGLGAIGAMVANDAYRLG

MDVVGYDPYVSVDTAWTISSRVKRALTLEEVLATCDYLTIHIPLMPQTKHLINSEKLA

LVKEGTVLLNFARGGLVDNEAVLKSLNAGGLKQYIVDFAAPELLAEEKVLVLPHLGAS

TEEAEVNCAKMAARTLKYYLETGNIKRSVNFPTVDMKFQAPIRLALIHRNVPSMVGTM

TIELANHNINIVDMINRSKGNHAYTLIDIEDTSKEQLEKIVAKLNSVEDIIHVRTIEN

QAVHF"

CDS 476709..477956

/locus_tag="JFBMFIFI_00430"

/inference="ab initio prediction:Prodigal:002006"

/codon_start=1

/transl_table=11

/product="hypothetical protein"

/translation="MVKIKAFKGIRPQPEHAQKVASLPYDVMNSAEARVIGDENPESF

LHIDKAEIDLAKDISPYADVVYQKAAENLKAFLERQWLLKDATEKFYIYELTMNGRSQ

TGLVVCTAIDDYLDEKIKKHEFTREEKELDRIRHVDACDANTSPIFLTYRENQSVNQV

IADWTKSNAPIYDFTSFYDVEHKVWMIDDSQVTAHLENLFDQEVAALYIADGHHRTES

AVKVGLKRRIEFPEAPETAEFNYFLSVLFPKEQLEILDYNRVVNVPLSADFITQLEKS

FTLEKSGLEPYKPKEPKTIGMYFSGEWYQLKAKPEVIVTDPVAGLDVSILQDQVLTPI

FGIEDIRTDKRIDFVGGIRGLKELAELVDSGAFTVAFSMYPTTMEDLLTVADSGQIMP

PKSTWFEPKLLSGLFVHDLESSD"

CDS 478116..478718

/locus_tag="JFBMFIFI_00431"

/inference="ab initio prediction:Prodigal:002006"

/codon_start=1

/transl_table=11

/product="hypothetical protein"

/translation="MKKALIIGGNGTIGSAVSNALNDSYEIITAGRTHGDVKVDITSV

ESITRLFETVGEVDAVIITAGQAHFGALKDMTPQDNLISVNSKLLGQVNTVLIGTNYV

KDHGSFTLVTGIMMDDPILAGASAALANGGVKAFAKSAALELPRGIRINTVSPNVLEE

SWENYKDFFIGFNPVSAKKVANAFVKSVKGGQTGQSYEVY"

CDS 478877..479116

/gene="secG"

/locus_tag="JFBMFIFI_00432"

/inference="ab initio prediction:Prodigal:002006"

/inference="similar to AA sequence:UniProtKB:O32233"

/codon_start=1

/transl_table=11

/product="putative protein-export membrane protein SecG"

/db_xref="COG:COG1314"

/translation="MYNALLLAMLVISVLLIIVITMQPTKTNSASSALTGGAEQLFGK

QKARGFEAVLQRVTVILGIAFFVIALVLAYVTAQG"

CDS 479369..480124

/gene="est"

/locus_tag="JFBMFIFI_00433"

/EC_number="3.1.1.1"

/inference="ab initio prediction:Prodigal:002006"

/inference="similar to AA sequence:UniProtKB:Q06174"

/codon_start=1

/transl_table=11

/product="Carboxylesterase"

/translation="MKKIALPEPFFFENGPRAILLLHAYTGSSNDMRMLGRALEKEGY
TVYAPHFKGHGTLDPTDILAATPADWWLDTQNALAFLREKGYQEIAVFGLSLGGIFAT
KALEEEGLLAAGTLCSPLFLDENNHVVDSFLKYVATVQKVAGIEPTKIAESLPKIKIE
VQQQLLELGNYLPPIQMKIPEITKPFFIAQAGQDELIDSQSAYELKKALVQAAVSFHW
YEESSHVITVGRDHHALENDVLDFLNQLHWNEG"

CDS 480128..482551

/gene="rnr"

/locus_tag="JFBMFIFI_00434"

/EC_number="3.1.13.1"

/inference="ab initio prediction:Prodigal:002006"

/inference="similar to AA sequence:UniProtKB:O32231"

/codon_start=1

/transl_table=11

/product="Ribonuclease R"

/db_xref="COG:COG0557"

/translation="MTIKKTTKEAIIAYLEGSTKKSFAVKDISEGMGLTSAADFKMLV
SALAEMERKGSLKLNKKGHFKLPTKKPVLTGIFRANDRGFGFVSIEDEEDDVYIPQGH
TNFALDGDVVTVDITRVPEPWNDKGAEGVVETIVERKFHQLVGEFYAYDEAGIAETDL
YGYVLPQDKKINDMRVFIEAKGIKPVDGAIVLVEITYYPDLAFPKSMQGIVSQTIGHK
NDPGVDILAIVYKHGIPIEFPEAVLAEADAVPETISDQDRENRRDLTNEMIVTIDGED
AKDLDDAVTVRKLDNGNYHLGVHIADVSYYVTEDSALDGEAFERATSVYLTDRVIPML
PHRLSNGLCSLNPNEDRLTMSCEMEINSAGEVVNHDIFPSVIHSSKRMTYTAINQILM
EKDPEVRAEYQEFVPMFELMGELHELLVNKRQARGAIDFEAPEAQIIVNNEGHPEEIV
MRHRGIGERLIESFMLSANETVAEHYFKLDVPFIYRVHEQPDSDRMQRFMEFVTAFGI
LMKGTSGEVSPKQLQKVLRGVSGEPEEAVISTMLLRSMKQAKYDAEALGHFGLGAEFY
THFTSPIRRYPDLIVHRLIRSYATKGIGSEEKAKWAGLLPEIAEQSSKMERRAVDAER
ETDSLKKTEYMSDKIGEIFDGVISSVTKFGIFVELPNTIEGLIHISNMKEDYFTFIEN

HLMLVGERTGVSYRIGQPVKIKVTKADVETREIDFELIPDPNAPKVPVIERTKKGRGK
PEERRRGATPTRSGKKTESEPKKTHKKKGKLKVDLASKTNAGKGKKTAAKGKNPFYSA
VAKKKKKKK"
CDS 482583..483041
/gene="smpB"
/locus_tag="JFBMFIFI_00435"
/inference="ab initio prediction:Prodigal:002006"
/inference="similar to AA sequence:UniProtKB:O32230"
/codon_start=1
/transl_table=11
/product="SsrA-binding protein"
/db_xref="COG:COG0691"
/translation="MPKHEGKLVAQNRKARHDYTIIDTIEAGMVLQGTEIKSIRGARI
NLKDGYARIYKDEIYLHNVHISPYEQGNQFNHDPLRIRKLLMHKKQIARLVNETKGTG
ITLVPLKVYIKNGYAKVLIGLAKGKKNYDKREDLKRKDAKRDLDRTMKIR"
CDS 483243..484640
/gene="gmuD_1"
/locus_tag="JFBMFIFI_00436"
/EC_number="3.2.1.86"
/inference="ab initio prediction:Prodigal:002006"
/inference="similar to AA sequence:UniProtKB:O05508"
/codon_start=1
/transl_table=11
/product="6-phospho-beta-glucosidase GmuD"
/db_xref="COG:COG2723"
/translation="MVKYEFPENFWWGSAASGPQTEGVYPGDGKGESLWDYWYEAEPE
KFFNQVGPDKASRVYRKYPEDIQLMKATGHNSFRTSIQWSRLIPNGTGAVNPEAVTFY
NNLIDEMIANGIEPFMNLYHFDIPMALQREGGWVNRKTVDAYVAFAKTCFELFGDRVK
KWFTHNEPIVPVEAGYLYQFHYPMEINLRHAVQVGFHEMLASAKAIEAYHEMKLDGEI
GIILNLTPSYPRDAANPADVKAAAIADAFFNRSFLDPSVKGTFPTELVEILKEIDHLP
EYEADDLKTIAENTVDLLGINYYQPRRIKAKETPIDSTNGPMPEDYFDNYEMPGRKMN
IYRGWEIYEKGIYDILTNVRENYGNIRCFISENGMGVEGEERFINDAGEIEDDYRIEF
ISEHLKYVHQAIAEGSNVQGYHMWTCMDNWSWTNAYKNRYGFISVNLDKEGERTIKKS

GHWFKKMTDANGFND"

CDS complement(484803..486116)

/locus_tag="JFBMFIFI_00437"

/inference="ab initio prediction:Prodigal:002006"

/codon_start=1

/transl_table=11

/product="hypothetical protein"

/translation="MINIDSAFFFYASHLFEASFEAFLMFYFFKNFIGVPRLNKVFLL

ACFFCLDFVTTYFEQLHPFIYLAILISVLTLISFSFLGSTKLRLQAAALYFIMTLFSE

KVVIYVSDAIYILSSNFQHSDSLKIIFFYFCSDILQFFFVYNVVQLRKGLNKQFYLTF

LMTLSLVVINLILIYNSLLVIFLEVNKIYISLTILSIFVILFFSYFVIQLVEKMARHY

QLDFENRTLNQQLHYQKMTQDKMLKAQKEVRSLKHDLRNHLVSLSYLVKKNDLPSTEI

YLKELLAMESLADPTSFTENLVIDALLTQLEERCLIENIHLDLVVKPLNIKEIKDIDL

AILFGNAFDNALEAVSPLPEDQKKICLSMHFKKNYLFITLKNTQNNHYHFHSDYSYPT

TKENKKNHGFGLKNMEQVVSKYDGQLKIKNEKETFTLVLVLKDAL"

CDS complement(486109..486828)

/gene="ypdB_1"

/locus_tag="JFBMFIFI_00438"

/inference="ab initio prediction:Prodigal:002006"

/inference="similar to AA sequence:UniProtKB:P0AE39"

/codon_start=1

/transl_table=11

/product="Transcriptional regulatory protein YpdB"

/db_xref="COG:COG3279"

/translation="MHIAICEDETVQQEELYNLLQAYQSFFPTPLAIEVFSNAEDLIE

VCHYSGNRFDLIFLDIALPKLNGIEAAKIIRQMDAEVELVFLTSMLDYSLEGYHVKAL

RYLLKPIKAHQLDELLKTIKNSQPKASLTISNKFGQKKIALDQIYFIESRQRKLFFHT

TTESSEMYGKLNDLEEKLKFKHFARCHQSYLIHLPYVTEIIKDQVKLENQLLLPISKA

HRKTFKEAFFDYLTIGGEWLD"

CDS 486971..488119

/gene="rodA_1"

/locus_tag="JFBMFIFI_00439"

/EC_number="2.4.1.129"

/inference="ab initio prediction:Prodigal:002006"

/inference="similar to AA sequence:UniProtKB:P39604"

/codon_start=1

/transl_table=11

/product="Peptidoglycan glycosyltransferase RodA"

/db_xref="COG:COG0772"

/translation="MTKEKNNEQNFAIVLLLMILALISLATIHSATGGWHYVIMQGAW

YLVGAVIVFIVMQFDSEQLWKLAPVAYGIGIILLIAVLIVGVEINGSTRWINLGFMNF

QTSEVMKIAFILTLARLLTSHNRKVMNRTIKSDCWLLAKIVLLSLPPIGLIMLQSDLG

TTIVFIAIIAGMTLLSGVSWKILGPLIGIVSSILIGMFCLILFNREILYKIGFHSYQF

DRVDVWLDPYLDVSGDSYQSIQSMKAIGSGQIGGKGFGMSEVYVPENHTDFIFTVLGE

NFGFIGSAILLFVYFLLIYRFVLTCFKIRNEFYSYIVTGVIMLVSFHIFENIGMTIGL

LPVTGIPLPFISYGGSSLLGNMIAVGLVLSMNHHNHDYIFSEKDKNFE"

CDS 488127..489287

/gene="ftsW_1"

/locus_tag="JFBMFIFI_00440"

/EC_number="2.4.1.129"

/inference="ab initio prediction:Prodigal:002006"

/inference="similar to AA sequence:UniProtKB:O07639"

/codon_start=1

/transl_table=11

/product="putative peptidoglycan glycosyltransferase FtsW"

/db_xref="COG:COG0772"

/translation="MAVYLVKKKGERMKKIDYYLLLPYLTLIFIGSLMVYSASADVSL

VQYGTGTKYFVGQLVFIGAGMTMLIITSQLKMSFLKNKNLVLCLFFAMLLLLASVLII

GENINGAKRWIDIGFFNIQPVEISKLVLVWYTAYILSRRQSQMQRNWFKAILPPIVIV

GISSLLILLQPDAGSVIIIAIVIVVQIFASGMPFKIGMISSLAASGVIAGYLKLLTIY

GAKMPGMSTYRYARFEAFWNPFELADSSGMQLSGSYLALGRGGVFGVGLGKSVLKKGY

LPEPHTDFILAIIGEEFGLIGVCLILVALSTLILRIFYLANHTKDSFSSLLCIGIGTM

MLIQTGINIGGVIGLVPISGVTLPFVSYGGSSILILSIALGLVFNVSRRITK"

CDS complement(489345..489764)

/locus_tag="JFBMFIFI_00441"

/inference="ab initio prediction:Prodigal:002006"

/codon_start=1

/transl_table=11

/product="hypothetical protein"

/translation="MEIREVDSKKNQEMLSTLLENYSQSQNKNIPVYEKKEISLAAYD

GATFLGGITGEIFWNHLHVSLLAIHPDNQKGGVGSRLLEQIELIARENNCTLILLETM

SWQAPKFYPKHGYTLFGTVEDYPIKGEAQYSFQKRLS"

CDS 489914..490633

/locus_tag="JFBMFIFI_00442"

/inference="ab initio prediction:Prodigal:002006"

/codon_start=1

/transl_table=11

/product="hypothetical protein"

/translation="MRYLKNHLMIISIIFIGFLLLFIPIFTGAVDRELTIDGMYNFIG

SFFGIIGAILLAFSESKTQNTALREEIELNSKKERQIQAEFLYKEMLFQKIENLYAKL

NTSIYKNEEIIRFIRDTNSILANEQITVRLVQNRNETEKLIHDIELISIYFTENGNKV

EKIVSLFNYSNKYLSKLEHEFLIDQGRNFNKELSSYQAILKRLVFLLDDLSIDMITIM

KNEITKLNDLSNLNQFGEKQS"

CDS 490706..492190

/locus_tag="JFBMFIFI_00443"

/inference="ab initio prediction:Prodigal:002006"

/codon_start=1

/transl_table=11

/product="hypothetical protein"

/translation="MDILLDKLGTRKVKILELLISEDRYWRLDEIADRIKSSKKTVQL

DMKMIQHDIAEWSTDEIKLDVSSRGSFLKKSNDFYMHKIYLSYIKAGIPYHLFDALFQ

EDIESLTYFAEDQHVSYTSFYKQIKELREWLDYYAISIVNNPPAFRGTEKQIRYCGMH

IYWRVFKGLEWPFPTIDREPILEMITAAEKVYGHTFNLIEREQTAYWAACAQVRILGG

DLIDCVDEFQGIKEGNPLYKELEPLILNMFKEMPLSGDALTNEVDYIFSLFNIIPHQI

ETTELVKDTVGFLKQNQLPSYQATELILDEFLKNLHTIGLKKDNLLLIESLSSIHYHA

YLFKADSIRFYFSKFAYNERKYHRLFYKKFQPIAERLAKIPEVAPIFEQSEFLFNHYI

PFIAPYIDWSYYEKIIRVQILTENGRYTEIKLGKDIINILPHRLEIVSSYQLEPAHEA

EVDLIVTDCLITGIDTPVFICKIPPTMGDWTRLERYISDISMRE"

CDS 492342..493058

/gene="gmuR_2"

/locus_tag="JFBMFIFI_00444"

/inference="ab initio prediction:Prodigal:002006"

/inference="similar to AA sequence:UniProtKB:O05509"

/codon_start=1

/transl_table=11

/product="HTH-type transcriptional regulator GmuR"

/db_xref="COG:COG2188"

/translation="MVKYQEIANEIRQRIKMGLYPVDTLIPDQITLAKEFDVSRMTMK

KALDILAMEGLIYRQRGAGTFVMKNALAHPLDAPAKEYEGLTKQLKGQQLTSQVIYFN

IEFPSQEVMDQLMLKESQPVYKIIRLRSVNQEPYVIEHTYMPADIVTGLTEDVLLGSI

YAYLKEELGFVFGGAYRKIHADKASEYDQKYLECSEHDPVLEVEQLVYLKDGRPFEYS

RSRHRYDKRSYTVLEVSNNS"

CDS complement(493096..494382)

/locus_tag="JFBMFIFI_00445"

/inference="ab initio prediction:Prodigal:002006"

/codon_start=1

/transl_table=11

/product="hypothetical protein"

/translation="MSTKYQNQFILFCTGILLVALGGIIIVFNETIFMPLFFLVAIAF

FIDGLSHLIRFFIHKQKDWKLLQDSSIKVFVGLIIYYLPTVPIHLVMMIFGGYTAIKG

FAKIIAYRTLRKSHLAGWYQLLVSGIFLLTIGFLLLFSPFFHLNQTLNTFGLYFLMYG

IVNLYFFLLNLLERTKIDDFKRKIRITLPIFVEAFLPKMVLKETNNFLKTSSDPSSIS

EFVDKKNDLVPNLEIFIHVTEKSFGAIGHMDLYFDGEIISYGNYDEASYRLFDTMGDG

VLMTSSKEDYIPFCIKYSQKTLFGFGIILTDEQVVKVRNAISELKSTATPWRSELELA

LMDNPNINPNDYSDYASDVSKEIAASFYKFKNEPFKSYFVLTTNCVLLADTILGPSGI

DLLKLNGILSPGTYLHYLRQELKKTDSNVISYEIYN"

CDS 494650..496614

/gene="fbp"

/locus_tag="JFBMFIFI_00446"

/EC_number="3.1.3.11"

/inference="ab initio prediction:Prodigal:002006"

/inference="similar to AA sequence:UniProtKB:Q7A3I5"

/codon_start=1

/transl_table=11

/product="Fructose-1,6-bisphosphatase class 3"

/translation="MNDQQKYLKLLAKQFPTIEQTASEIINLEAILNLPKGTEHFISD

VHGEYDAFQHVLRNGSGNVKQKIKVLFKGRLNQQEMKQLATLIYYPEEKTARTISQLQ

SKAEIELWYQKTLIQLIELCEFVASKYTRSKVRKAMPQEFSYIIEELLFKSSTISDKE

DYYQEIIATIILLEQGTEFIAAMSYLIQRLVVDHLHVVGDIYDRGPFPDKIIDTLMNY

HSVDIQWGNHDILWMGAASGSAVSLANVIRICARYDNLAILEEGYGISLRPLLTFSDM

TYADDSNNWFKPKMNVKNQEYSAEEIRQITKMHQAIAIIQFKLEGAIIERHPEFDMDS

RLVLSRIDYSAGTINLNGRKYPLKHHYFPTINPKNPYQLTCEEEKLIQKLVTAFKSSE

RLQKHIAFLFQKGNMYLTYNNNLLLHGCLPLNPDGSFMQMQISGNSYSGKGLLDQFER

ELRLAYHKKDGTKEVHLDMLWYLWTGAVSPLFGKNQMTTFERYFVEDEETHIEKKNAY

YHLRNNSEICKNILREFEVDPEIGHIINGHTPVKEKIGESPIKADGKLIVIDGGFSKA

YQKTTGLAGYTLLYNSYGMELASHQPYSSKKEAIENEQDIVSTIRVIDRELKRKKVRE

TDNGKELKKQLIDLKALLNAYREGEIFEER"

CDS complement(496917..497102)

/locus_tag="JFBMFIFI_00447"

/inference="ab initio prediction:Prodigal:002006"

/codon_start=1

/transl_table=11

/product="hypothetical protein"

/translation="MEYADQIVFTVEDKEFEGYIEKVYENSFLVVALNSDTDLNEKYH

GKLIVRKTDCRLLEGSR"

CDS 497221..498090

/locus_tag="JFBMFIFI_00448"

/inference="ab initio prediction:Prodigal:002006"

/codon_start=1

/transl_table=11

/product="hypothetical protein"

/translation="MSQLGHRIKIIRKKNNMTQKILADKICSQSVLSRIETGEEVPNA

VVLHQLCERLGITVEQVMSNNMQSISNHTELIKKMRIYFDNNRYEELALFLKESNCIN

EFHEDVDLQFYYYCLGSCRYYLDKEYKEALHDLQMALEYTYSPNNHYYTNYEILILSC

IGKTYFSMGQVQKAFYYLHESLAALSSVSINENTYLLTRIFYNIATTHALNGTYEEAQ

IYLEKGIQFANQVHNSYYLSELFYEKAIISHLQRNIEVAIEEMYVASGVAKAVDNQEL

VALTQEKIDLWKE"

CDS 498296..499708

/gene="cydA"

/locus_tag="JFBMFIFI_00449"

/EC_number="1.10.3.-"

/inference="ab initio prediction:Prodigal:002006"

/inference="similar to AA sequence:UniProtKB:Q09049"

/codon_start=1

/transl_table=11

/product="Cytochrome bd ubiquinol oxidase subunit 1"

/db_xref="COG:COG1271"

/translation="MSFDIESLARFQFAMTTVFHYFFVPLSIGLALTVAIMQTMYVVK

KNEMYKEMAKFWGHIFLLSFAVGVVTGIIQEFQFGMNWSDYSRFVGDIFGAPLAVEAL

LAFFMESTFIGLWMFGWDKFNKKLHLAFIWLVVFGSMMSAFWILVANGFMQHPVGYEL

NNGRAELVDFGAVLTSHQVWYEFTHVIFSAIMLGAFVVAGLSAFRLLKKEHKAFYQSS

LNIGLVIGLVAAVLTIGAGDLQTKALVNDQPMKFAATEGLYEDSGDPASWTLIGFQDT

DKKETVWSIEIPYLLSILSFNKPEGGVKGMLTINEELIAQYGKQNYFPPVKTLFWSFR

IMAGSGVFLALVAVVGLWFSRKKKTLLEKKWLLYVIAFCTFVPFIGTTAGWLITELGR

YPWTVYGLFTIADSVSPNVSVTSLLISNTIYFLLFAGLGSVLVYLITRELNQGPYHEA

KVKDHDQKANVDPFEKEAFK"

CDS 499708..500733

/gene="cydB"

/locus_tag="JFBMFIFI_00450"

/EC_number="7.1.1.7"

/inference="ab initio prediction:Prodigal:002006"

/inference="similar to AA sequence:UniProtKB:P0ABK2"

/codon_start=1

/transl_table=11

/product="Cytochrome bd-I ubiquinol oxidase subunit 2"

/db_xref="COG:COG1294"

/translation="MADISGLQFFWFILIGVLFAGFFFLEGFDFGVGMSTRFLARDRE

ERDQVIGTIGPFWDGNEVWLITAGGAMFASFPNWYAAVFSGYYLILFAILFGLIIRGV

SFEFRHKMATEKGRHFWDWTLFFGSIIPPFFFGVLFTSMVSGMPLDADGNMMATFTDY

FTPFSVVGGVAVTLLCFLHGLNYIRIKTLGDIRERAEALAKKLYILLFVGLVAFAGLL

YFYTDFFTVHLVSTLVLLVIIILLSVMATYGAYKNKEMLSFITSGLTLIAVVALLFFG

LFPRLMVSSLDPAYDLLIATGSSSPYTLKLMTWISLSILPFVLGYQGWSYYIFRQRIS

KEKAVKY"

CDS 500769..502478

/gene="cydD"

/locus_tag="JFBMFIFI_00451"

/inference="ab initio prediction:Prodigal:002006"

/inference="similar to AA sequence:UniProtKB:P29018"

/codon_start=1

/transl_table=11

/product="ATP-binding/permease protein CydD"

/db_xref="COG:COG4988"

/translation="MKKMMVMLAGISFLQAFMIIFQARYLALSITGLWNGEGLTSQFS

HMLFFFLAFAGRHLLTLIREKVLDRFSYEKGKELRKELLTKVFSLGPNLVQKAGTGNV

VTMALEGIRQAENYITLFLSKMMNMMIIPWLVLAYVFMLDVDSGVTMIIVFPIIIIFM

VILGYAARGKADRQYETYRVLSNHFVDSLQGLETLKLLGLSKKYQKNVHDVSESYRKA

TMSTLKIAILSTFALDFFTTLSIAVIALFLGLRLLDGGLTLLPALTVLILSPEFFLPL

REFSSDYHDTLDGKNAMGAILDVLALESPTDVDILTEAEGKWTVDSQLVIKDLTVKYE

NSDTSALKNIDFNWQGFGKIGLIGASGSGKSSFIETIGGFLQPESVAEIKINNVKVPH

FAQRNWQKEMLYIPQKPYLFHDTLANNIRFYVPTASDNEVEAAAEKAGLKDFIKELPD

GIETIIGESGRMISGGQAQRVAIARAFLDTERKILLFDEPTAHLDIETEVALKEAILP

LMDNHLVFFATHRLHWTNEMDYILVIDNGEIVEAGTQEELLGKAGAYVALMNQMRGGN

HHE"

CDS 502471..504273

/locus_tag="JFBMFIFI_00452"

/inference="ab initio prediction:Prodigal:002006"

/inference="similar to AA sequence:UniProtKB:Q57538"

/codon_start=1

/transl_table=11

/product="putative ABC transporter ATP-binding/permease

protein"

/db_xref="COG:COG1132"

/translation="MNKKRGQEIRETFSQDTWVKPYLTKYRKLLYLVLFLGFMTLFCG

SALMFTSGFLISKSASMGISIRNGSNENIMLVYVPIVLTRAFGIGRPSFRYVERLTSH

NWVLKMTSDLRVKLYRSLEKDAIFFKGKYKTGDILAVLAEDIEHIQNLYLRTIFPTLI

SWGIYVVVVIALGFFSVPFAIMMLIMLAVVTILLPLVSLLVNGARMYSQKTARTGLYN

KLTDAVLGVGDWLFSGRKSDFIASYEGNEFDVRKDDAKIKQFDRTRDFIVQLIFALIA

LVVLTWTSMIFQGEHGGAANWIGAFVLAIFPLIDAFAPVPSAVSELTIYEDSVKRMNA

LPVVEETEVSPATLAAIKELETESFKELLIDDVCFSYEQDQKQILENVTLRIPKGKKI

AILGKSGAGKSTLGKLIRGDLKPQQGRVLLNNVSTYQLQDTVANWIGVINQNPYLFNT

TVLNNVRLGNITATDEEVIAALYQVGLGEMLAALPDGFHTIVEEAGGRFSGGERQRLA

LARILLQDAPIVLLDEPTVGLDPITEQVLLDVLFNVLSDKTIIWITHHLQGVDQMDQV

VFIESGKIEIEGTPKELLANNKRYQTLYHLDRGE"

CDS 504412..504615

/locus_tag="JFBMFIFI_00453"

/inference="ab initio prediction:Prodigal:002006"

/codon_start=1

/transl_table=11

/product="hypothetical protein"

/translation="MYKSKRGYLAKEDGQNYIDFARDIKVAKYLLRKKFPFQQVLDAT

NLNPKELSMVIKEVRKEANYQYE"

CDS complement(504701..505963)

/gene="hflX_1"

/locus_tag="JFBMFIFI_00454"

/inference="ab initio prediction:Prodigal:002006"

/inference="similar to AA sequence:UniProtKB:P25519"

/codon_start=1

/transl_table=11

/product="GTPase HflX"

/db_xref="COG:COG2262"

/translation="MEKNIIIVGVSGNQENFDYSMTELKELAFANQLKVVGEIRQNLA

KAHVATYVGKGKLTEIINLAAELEVDTIVTNDELTPTQLRNLEAQLELTVIDRTRLIL

AIFAERAQSKEAKLQVEIAQLNYEMPRLRTDQGTILDQQGGGSGLNNRGSGEKQIELD

RRTIKNQIKRLNQELESITKEQQTRRHKRQKNQIPLVSLVGYTNAGKSTTMNQILQHF

HSQEAKTVFEKDMLFATLDTSVREIVLPDRKKFLLSDTVGFVSKLPHQLVKAFRSTLE

EAKQADLLIHVVDYSDPNYQLMMDTTDKTLAEIGILDIPVILAYNKADLIPEISYPTY

EDNHFIYSARDEKSLSMLVEIIKSTIFKEYTKATFLIPYNQTQYVAYLNEKAAVESEE

YLDNGTKIVAEVSPIDLNKLAMFHIDSD"

CDS complement(506447..506926)

/locus_tag="JFBMFIFI_00455"

/inference="ab initio prediction:Prodigal:002006"

/codon_start=1

/transl_table=11

/product="hypothetical protein"

/translation="MSNYVLEEKESFTILGFGVELKSDYTDFVGINQEKSDFLQKVQA

DGTIDTLKSVASNSYFFIVNEAVNNKMMYYVGVMSKEELPEATRLIQFPKGEYLVVKG

EGATSEELSNNLTGMTFGQVLPEVTNVAYVGGPNTSVEMETRDGVLVGEMWIPVVKQ"

CDS 507159..508118

/locus_tag="JFBMFIFI_00456"

/inference="ab initio prediction:Prodigal:002006"

/codon_start=1

/transl_table=11

/product="hypothetical protein"

/translation="MNKVERINTMMRYINNRAHFTISEIMSEFNISRSTAIRDINEIE

TIGIPLVSEVGRSGGYFVMNNSFLPAIRFTNNEVKALFIAFMATRNQQLPYLSSRQSL

AEKLIGLIPENQQDDLVNLNQMLLFEGTNPYNPDLLDLSDLPHPILEKLIQSFLIDRY

LILTINEEKKLIQIKFYLVHLYKEKNSWHLEGLDLTSKNKRKFQVDSLEDVEIYPTKS

RLSAKKIQEHMTKIEKAPNIIIEMGLEAILQFKKYSPYRLSLFYINPFQTKALLKIVV

DVTKQAELVEMANWLLFLGEDMHIKKLPKEIEDYIQTKELVNR"

CDS 508539..508952

/locus_tag="JFBMFIFI_00457"

/inference="ab initio prediction:Prodigal:002006"

/codon_start=1

/transl_table=11

/product="hypothetical protein"

/translation="MEEVLDKGPFFHGTKAKLAIGEFLEPKHESNFEEKRISNYIYFT

GTLDAAKWGAELAVGADLKERIYLVEPLGEFENDPNLTDKKFPGNPTRSYRTMGLLKI

VGELASWERHSEESINTMLTNLEALRNSGLAEIDD"

CDS complement(509036..510022)

/gene="hepT_1"

/locus_tag="JFBMFIFI_00458"

/EC_number="2.5.1.30"

/inference="ab initio prediction:Prodigal:002006"

/inference="similar to AA sequence:UniProtKB:P31114"

/codon_start=1

/transl_table=11

/product="Heptaprenyl diphosphate synthase component 2"

/db_xref="COG:COG0142"

/translation="MPIHPMWEPFPEIKHDLAETYDVIEKKVRIRNKDIQQTINDLLH

SGGKLLRPAYFILFSRFGSLDKKNHKKIVYTAASLEILHMATLVHDDVIDDSPTRRGS

QTVQSQYGKDIAVYTGDFLFAVYFGLIADSIDSFSTLKLNAFTMKRILIGELDQMHLR

YNTEVTLRQYLRRITGKTAQLFSLSCFEGAKVGKADLAVTTLSYHIGHNIGIAFQILD

DILDYTENSETLKKPVLEDVKQGVYSLPLILAMKDHKKEFEPYLNKGASMDQADIAAI

LDLIRRYKGVELAKDLAERYTNKALKSLEKLPNQPEKEILCTLTRQLLNRDH"

CDS 510310..511218

/gene="menA_1"

/locus_tag="JFBMFIFI_00459"

/EC_number="2.5.1.74"

/inference="ab initio prediction:Prodigal:002006"

/inference="protein motif:HAMAP:MF_01937"

/codon_start=1

/transl_table=11

/product="1,4-dihydroxy-2-naphthoate

octaprenyltransferase"

/translation="MKFKTFLELVEMQAKTASILPYFMGILFAWYHYEELHLINLVLF

FIAMFLFNMAVDAIDNYMDYKKASKEHNYREEVNVIGREKIPMPLVATLIILMVVVSA

GLGLYLVKQTGIPLLYMGLYCYFVGIFYSSGPKPISSMPLGELFSGFTMGFMIYIISI

YVNAYNVMTFDLRTFLIILFASIPNMFAIANLMLANNISDLEEDRTNKRYTLPHYIGK

KNALSLFKWLYILAFAALIIAVWLGIYPKMMLFTLVAIPLVRKNTKRFLAKQVKSETF

VYAVQNLAAITSLQVLTFALGIWLNF"

CDS 511327..511704

/locus_tag="JFBMFIFI_00460"

/inference="ab initio prediction:Prodigal:002006"

/codon_start=1

/transl_table=11

/product="hypothetical protein"

/translation="MNVLRGQIQVEKYNFELATGSSAAETKVEVLINEVVPTEEADKG

ILEEGKMFRMEVPFALQLEGFKIDGQISQIIQIPEFFGVPSEIAVEDMRELSRPLIKY

IERLTYEVTEIAFDEPGFALNFE"

CDS 512038..513243

/locus_tag="JFBMFIFI_00461"

/EC_number="1.6.99.-"

/inference="ab initio prediction:Prodigal:002006"

/inference="similar to AA sequence:UniProtKB:Q2FZV7"

/codon_start=1

/transl_table=11

/product="NADH dehydrogenase-like protein"

/db_xref="COG:COG1252"

/translation="MDKMKIVILGAGYGGLRALKGLQKKHLNAEITLVNKNEYHYEAT

YLHEVASGANPPERISFAIKDVVDTKQTTFIQDTVIKVNKDDKTVELDKTGLISYDYL

IFALGFESESFGITGVDEYALPMVDINTAVAIKEHMHRQFAQYEATKDDALLSIVVCG

AGFTSIEYLGELTQQMPKLIKQYNLPADKIQLTCIEAMPTLLPMFVEKLSTYGIQKLK

DRGVKFLVGTPIKEVTADTVIYEENEERKSIKAKTIVWTTGVKGSSVVGGSGFEERRG

RVMVEADLTAPGYPEVFIIGDCSAVMNPENNRPYPTTAQIALKQADAAVANLVAKVNN

QPIVPFTFKSQGSVCSIGNNEAIGEVLGANLKGYPASMMKKVIEDRSLSQTGGLKIMF

SKGRFDLYH"

CDS 513366..513983

/gene="COQ5_1"

/locus_tag="JFBMFIFI_00462"

/EC_number="2.1.1.163"

/inference="ab initio prediction:Prodigal:002006"

/inference="protein motif:HAMAP:MF_01813"

/codon_start=1

/transl_table=11

/product="2-methoxy-6-polyprenyl-1,4-benzoquinol

methylase, mitochondrial"

/translation="MGNIDVFNRVASKYDTPERSEIAKITAKEIQKHIVNGKDKTAID

YGCGTGLVGLELVADFETLLFADASQSMVEVVETKIEQATIKNAKTLLLDIEEVSNSD

IQVDYIFLVQVLLHVQDIQPLLSNLYKLLKPNGHLLIVDFDYNEQVNSDKVHNGFKQQ

ELIQLMEKIGFVESNAEIFYHGKNIFMNQDASFFILDAVKPDLND"

CDS 514167..514835

/gene="gpmA_1"

/locus_tag="JFBMFIFI_00463"

/EC_number="5.4.2.11"

/inference="ab initio prediction:Prodigal:002006"

/inference="similar to AA sequence:UniProtKB:B4RIY7"

/codon_start=1

/transl_table=11

/product="2,3-bisphosphoglycerate-dependent

phosphoglycerate mutase"

/translation="MGKGCTFYFVRHGETYFNRYMKMQGWSNTPLTKDGRFTVIRSGR

GLSDIRFDAAYCSDLSRTKETLEILLEENFTSRNLKIQEMPEFREAFYGSFEGSDVDE

SWATISKELGYETADEMREKATLADRMDGTKAADPLHDAEDFLTFWLRVEKGMLQLIE

KHRETDQNILIVAHGNTIRNLLNGIVPELEMPTSVDNASVSVVNYHDGQYHLERFNDT

GHFR"

CDS 515030..515806

/locus_tag="JFBMFIFI_00464"

/EC_number="3.1.3.-"

/inference="ab initio prediction:Prodigal:002006"

/inference="similar to AA sequence:UniProtKB:Q5XD45"

/codon_start=1

/transl_table=11

/product="Putative phosphatase"

/translation="MKPTALVFFDLDGTLLNQHSEVEEEVIEALEAVKAKGGVPIIAT

GRTNIEFEHIADATGIDSSVSMNGQFITYEGTEVYRNVLPSESLKRLKAATDERELGL

SFYTNKWVKTSVENETLHRAYAFIHADIPEIDPKAHLVDDIFMALVLNEDPSHDDYFR

TSFPEFSFYRNTPFSMDTIIKGNSKATGIRQLQKAMGLESVPTYAFGDGPNDLEMFQV

ADYSVAMGNGIAALKERASFISASNIDGGIVKGLDYFNLI"

tmRNA 515936..516299

/gene="ssrA"

/locus_tag="JFBMFIFI_00465"

/product="transfer-messenger RNA, SsrA"

/inference="COORDINATES:profile:Aragorn:001002"

CDS complement(516712..517008)

/locus_tag="JFBMFIFI_00466"

/inference="ab initio prediction:Prodigal:002006"

/codon_start=1

/transl_table=11

/product="hypothetical protein"

/translation="MGKLKWFSGGKERSNQAENIITDLLDDLKTDLDNESLKKVLENY

LEELKQKGASVPLILSRMNLDISKAIRNDGVTLSDYQSKKLKELTSISNIRYGY"

CDS complement(517233..518099)

/gene="hcaR_1"

/locus_tag="JFBMFIFI_00467"

/inference="ab initio prediction:Prodigal:002006"

/inference="similar to AA sequence:UniProtKB:Q47141"

/codon_start=1

/transl_table=11

/product="Hca operon transcriptional activator HcaR"

/translation="MELRVLTYFLTVAREKTISKAAEVLHLSQPTLSKQLKELEEELG

VTLFIRGNRFITLTEDGIYLMNRGKEILSLVESTTTNLIKNEVISGELTIGGGESQAF

DFLSRILHGLRNDYPEINIHLYSGNADDVLEKIDKGLLDFGLVIDPVEKQKYEYIRLP

LVDSWGILVNKSHPIAYKKTVTPKDIQQTPLLISNQSFVDNQLSEWFGGNIEHLNVIG

TYNLLYNASLLVKEGVASVLCIDGIVNTANTNLTFVPFSPPLTANINIVWKKGQIFSS

ASKEFLRLLKLV"

CDS 518226..518795

/gene="dapH_1"

/locus_tag="JFBMFIFI_00468"

/EC_number="2.3.1.89"

/inference="ab initio prediction:Prodigal:002006"

/inference="protein motif:HAMAP:MF_01691"

/codon_start=1

/transl_table=11

/product="2,3,4,5-tetrahydropyridine-2,6-dicarboxylate

N-acetyltransferase"

/translation="MSNISLLQKIIGKEIVKDSPLFEEIHSVKKNNEQLLMMLNADYY

PNEEVLKHLEKITSQKIDSSVKISQPFYSDFGKHIRFGKDIFINQNVTFVDLGGITIE

DQVLIGPGSRLITVNHLISPKKRRGIKVEPIYIKKNAWLGANVTVLPGVTIGKNSIVA

ADSTVTKDVPDNVIVVGSPAKVVREIEEE"

CDS 518826..519164

/locus_tag="JFBMFIFI_00469"

/inference="ab initio prediction:Prodigal:002006"

/codon_start=1

/transl_table=11

/product="hypothetical protein"

/translation="MNIIIGKQLFQIELVSNQATKELIARLPINLKMNDLHGNEKYAY

FSEILPTQEEKVDEIKKGDIMLYGSDCLVLFYKTFSTNYSYTKIGKVKEVDQLDFISE

IEAINVILVK"

CDS 519187..519933

/locus_tag="JFBMFIFI_00470"

/EC_number="1.-.-.-"

/inference="ab initio prediction:Prodigal:002006"

/inference="similar to AA sequence:UniProtKB:Q7A3L9"

/codon_start=1

/transl_table=11

/product="putative oxidoreductase"

/translation="MSIKDKVIIITGASSGIGEATAILLAQKGAKLVLAARREEKLQT

IVEKIKENSGEAVYFVTDVTKRIDNQKLVDFTIKTYGKVDVIFLNAGIMPNSPLSALK

EDEWDQMIDINIKGVLNGLAAVLPYFGEQKSGHVLTTSSVAGLKAYPGGAVYGATKWA

IRDLMEVLRMESAQEGTNIRTATIYPAAINTELLDKITDSKTAEGMTSLYDKYGIAPE

RVASIVAYAIDQPEDVNINEFTVGPTSQPW"

CDS 520198..521178

/gene="yajO_1"

/locus_tag="JFBMFIFI_00471"

/EC_number="1.1.-.-"

/inference="ab initio prediction:Prodigal:002006"

/inference="similar to AA sequence:UniProtKB:P77735"

/codon_start=1

/transl_table=11

/product="1-deoxyxylulose-5-phosphate synthase YajO"

/db_xref="COG:COG0667"

/translation="MEYTKLGNTGLDVSRICLGTMSFGDPKNWIHKWVLEEEDSRPLI

KRALELGINFFDTANVYSLGRSEEILGQALKDYANRDEIVLATKVHQKMFDGPNGQGL

SRKAIMSQIDQSLNRLQTDYVDLYIIHRWDPFTPIEETMEALNDVVKAGKARYIGASA

MYAYQFQKANNIAEKNGWTKFVSMQNHLNLIYREEEREMIPYCLSEKIALTPYSPMAS

GRLIRSATEATKRSTTDNAQKDKYDETADKDQMIIDRVAELAAKYETNKVNIALGWLL

QKNPVVAPVIGATKMNHIETAVGAVDFKLSQADILYLEEPYIPHKVIGHA"

CDS complement(521219..522022)

/locus_tag="JFBMFIFI_00472"

/inference="ab initio prediction:Prodigal:002006"

/codon_start=1

/transl_table=11

/product="hypothetical protein"

/translation="MMNKLPQATPPKTIPFGPEAFQKSNHTEIRWLGNSGIFLNSRGY

CMMVDPVLEGFDLPLLIDMPIKTDQVPHLDSILITHSDNDHFSIPTLEKLLPLTTALH

APKYVAGLVKEKINHEAIGHDIHASFDDGPLKITLTPADHLWQNESKKYDRIFKLEDF

CGFWIETPDGTLWIPGDSKLLPSHLEMPEPNAILFDFSDNEWHIGLDNAIKLANTYPN

SDLILSHWGTVDAPQMNVFNGNPADLLHRVDNPERIQIVAAGDVFILEK"

CDS complement(522041..522358)

/locus_tag="JFBMFIFI_00473"

/inference="ab initio prediction:Prodigal:002006"

/codon_start=1

/transl_table=11

/product="hypothetical protein"

/translation="MEKITAGRDQLGEFAPKFAELNDDVLFGQVWSREDKLNPRDRSI

VTITALISGGNFEQLVFHMGKAKENGVTKEEISEIITHLAFYTGWPKAWSAFTIAKEI

YEK"

CDS 522484..522864

/gene="adhR_1"

/locus_tag="JFBMFIFI_00474"

/inference="ab initio prediction:Prodigal:002006"

/inference="similar to AA sequence:UniProtKB:O06008"

/codon_start=1

/transl_table=11

/product="HTH-type transcriptional regulator AdhR"

/db_xref="COG:COG0789"

/translation="MNISEVAAKYNMTPATIRYYESQGLMPAITRNKSGTRDFQEEDL

KWVEFIKCMRDSGLSIHSLSKYSELYQIGEETLIERKEILMDEYQKLLKKQATINGTV

AKLEKKIENYNYKIKQSSENLYTE"

CDS 522899..523792

/gene="murR_1"

/locus_tag="JFBMFIFI_00475"

/inference="ab initio prediction:Prodigal:002006"

/inference="similar to AA sequence:UniProtKB:P77245"

/codon_start=1

/transl_table=11

/product="HTH-type transcriptional regulator MurR"

/db_xref="COG:COG1737"

/translation="MYLFQRIEEAVFKQNDARRVIGEFILENRDQLKSYTMEEIAKQT

FTSKPTLVRFAKYFGYSGWKEFMYAFSHEVAYHDDNAYLDVDPNFPFSAQHDTLEIIE

SIATLKIQSIKDTVSLIVAEDLNKAAELLHRADSVVIYGTSPNYYYGELIKRNLLSIQ

KKAIMANSGESGVITQTLNDKDCAIMISYSGNSHDDNPTSNVKLLMKNSVPIISITSG

GENYLRDYSDVILNISSKEKLYSKIANFATQESILFILDALFAKVFSLDYEVNKKNKI

SNSKLLESKRQATFKELSEKF"

CDS 524034..525881

/gene="bglF_1"

/locus_tag="JFBMFIFI_00476"

/inference="ab initio prediction:Prodigal:002006"

/inference="similar to AA sequence:UniProtKB:P08722"

/codon_start=1

/transl_table=11

/product="PTS system beta-glucoside-specific EIIBCA

component"

/db_xref="COG:COG1263"

/translation="MSKKYEKLAQEIVEKLGGKENISDAYHCQTRLRFKLIDEQKVDK

EELEELDGVATYLVNAGVHQVVIGTHVKDVFEEIDKKVDVSEQNKAPSDKKSVFNIII

EFVAGTFQPIIPALSGAGMVKAVLALLVVFKIVSVESQTYYLLNLFADGVFFFLPMML

AFTVAQKLRCNPILAASVAAMMMHPNWGTLVAAGEPVKFFDIIPFNLATYTGSVIPIL

LIILVQSYVEKFLNRVIPKSVELVFVPMLTFLIMGTLAFSVLGPIGSIIGGYLAGFFM

FLSMNASWVPAVLIGGFLPIMVMFGLHNGVAPLGVMQMAELGYDSIFGPGCVCSNIAQ

ATASAVVAFRTKDKKMKQLAVSGSITAYMGITEPTLYGVNLPKKYPLVAAMIGGGAGG

LYAGLTNTHRFATGSSGLPAILLYIGDNSMVFFWNIVIALLISAVVSAVLTYILSFKF

EKEVDLPSVKEVILEDATVSNPIKGNIIPLNEVKDGAFASGALGKGFAIEPIEGKVIA

PFNGKIEAVFPTKHAIGLVSETGIELLIHVGLNTVELKGQYFDTLVEPGQTVKKGQAI

LNFDLEKIKESGYETQVPVVITNSPQYSSIELTNKGKLLNNEEVLIVKV"

CDS 525948..527399

/gene="bglH_2"

/locus_tag="JFBMFIFI_00477"

/EC_number="3.2.1.86"

/inference="ab initio prediction:Prodigal:002006"

/inference="similar to AA sequence:UniProtKB:P40740"

/codon_start=1

/transl_table=11

/product="Aryl-phospho-beta-D-glucosidase BglH"

/db_xref="COG:COG2723"

/translation="MTLDKNFLWGGATAANQAEGGVLEGGRKLSNIDVLPCGEDRKKV

ALGELEMLDWQDGYYYPAKEAIDMYHRYMEDIQLFAEMGFKVYRMSLSWSRIFPNGDD

AEPNEEGLAFYESIFKELRKHQIEPLVTIAHFDAPIHLIKTYGGWKNRQLIEFYTRYA

ETVLKRYKGLVKYWLTINEINILLHQPFVGGGIIFKEGENQEEIKYQAAHHQLVASAL

ATKIAHEVDSRNQVGCMLAGGSHYPYTCRPEDYQEAINRDREGYFFIDVQARGKYPNY

ALKKLAREGLNIVMEVGDKEILAASPVDFVTFSYYCSRTVSAHSEDYEQSTGNIFASI

KNKNLPSTEWGWQIDPLGLRNSLNQLYDRYQKPLFIVENGLGAIDIPDSNGYVEDEYR

IDYLREHIQAFKDAVEIDGVELLGYTTWGCIDLVAASTGQMSKRYGFIYVDRDDEGNG

SLARSKKKSFYWYQQVIETNGENLSSENLNIEK"

CDS 527935..529572

/locus_tag="JFBMFIFI_00478"

/EC_number="3.6.3.-"

/inference="ab initio prediction:Prodigal:002006"

/inference="similar to AA sequence:UniProtKB:Q99T13"

/codon_start=1

/transl_table=11

/product="Putative multidrug export ATP-binding/permease

protein"

/db_xref="COG:COG1132"

/translation="MISGILISWLSAATIFYFQKIIDSSSLLVVDKKAVLFYGLTLIF

VPILSYLDEYPKNYLANNLYFHYKQESLKKVSLIRYDRYVNIGFGVLLQRIENGATAG

KQVVFDFYFRIVRELIPDILFNVFFILLINKKLIMYLLIGYLVVFFTTKFLLKFLYGM

KESILVNEEQINRVLSRGIMELVTFRINKRYEKELAELNILINETVVSRTRIALIHEL

FFTLFAVLVSLIKILVVCLFIFNQLEGSIGTLVALIIYIDKIYSPIAIFNVLYVQFKM

NSVSFDRLIEFLELENEDNLINGYYEVDPIEFINLENISYSIDETLILDTIFLQFEKG

KRYALIGESGAGKSTLVKLIVGLIKPTKGTIRFNQRNITELNFNSLYEKVTYISQDSP

IFDGSLRENIIFDKKVSDDKVIEMLKTCRLEKYFNQLEHGLDEQLGEKGVKLSGGQKQ

SIAFARMAFSDSDLIILDEATSALDHLTEKAVMQNILPFFENKIVIFITHKMNFLESI

DQIICLKNGLVIEEGSYDELIEKQGYLYELKQNSKKE"

CDS 530032..531570

/gene="ybiT_2"

/locus_tag="JFBMFIFI_00479"

/inference="ab initio prediction:Prodigal:002006"

/inference="similar to AA sequence:UniProtKB:P0A9U3"

/codon_start=1

/transl_table=11

/product="putative ABC transporter ATP-binding protein

YbiT"

/db_xref="COG:COG0488"

/translation="MGLLKVENLSHSFADKVLYENGAFNLFKNDHMGIVGQNGAGKST
LMDIIIGKTIPDEGSIKWQSNVKIGHLDQYADVDRELKVADYLQLAFSKLFEIEKRMT
NLYEESAISGNEELLLQAAECQEQLEIHDFYSIESTINKIVVGLGIDVIGVNREMKVL
SGGQRAKVILAKLLLEEPNVLLLDEPTNFLDKEHVEWLANYLTGFSGAFIVISHDYEF
LEKIATCICDIEFGTIKKYHGKYSEFLKQKEHLREDYVRQYGAQQKKIKETEEFIRKN
IAGINTKMAQGRRKHLERMERLTEPSFTHKPTIKFKEVPLASHKVLTVNQLSVGYDRP
LLSHLNFSILGNQKIVITGFNGIGKSTLLKTLVGMNSSLAGGFQFSPQVKVGYYEQDL
AWENGLLTPIQVVSDAHPKLTVKEIRRHLAQCGVNDKHVTQGIATLSGGEQAKVKLCI
LTLKTVNFLIMDEPTNHLDVEAKEALKEALIKFEGTVLLVSHEEKFYRDWTDKVISIE
QESI"

CDS 531712..532026

/locus_tag="JFBMFIFI_00480"

/inference="ab initio prediction:Prodigal:002006"

/codon_start=1

/transl_table=11

/product="hypothetical protein"

/translation="MEKIFAEADDYHLKSFFEENNLNVTDFRMGTISILPGERVPKEG
LSRHEENEYSYIISGGMTGESGGEAFEISGGMGTLIPAGEEHWCVNNGDVPCEICWVL
IK"

CDS 532047..533687

/gene="dppE_1"

/locus_tag="JFBMFIFI_00481"

/inference="ab initio prediction:Prodigal:002006"

/inference="similar to AA sequence:UniProtKB:P26906"

/codon_start=1

/transl_table=11

/product="Dipeptide-binding protein DppE"

/db_xref="COG:COG4166"

/translation="MKKRIGLGLVSLAVITITLIGCGSKSDKENKNAETSNQPLNLMV
ASEVTTLDSLNMLDFPDAVTHTAVFEGLYAIGEKETVIPAVAKELPEISEDGKTYTIK
LREDAVWSNGTKVTANDFIYAWKKLADPAEGLVYSFLIQQTILNGEDVVSGKKPVDEL
GIKAVDDYTIEVQLSEPKPFFTSVLTFPVFFPQNQEFVEKQGDNYGTTNETVIYNGPF
TMGKWKQGELGWDLKKNPTYHDKKNVKSDLIHYEVIKESSTALNLFDNDQLDVATLTG

VIASENTENPDFLSVPTSIINYVRLNQKRNGQTTPLANENVRKAMALALDKETMADKV
VADGSNALNGLIPSGFVGNPETNEDFRKEAGDVMAFDKTKALDYWNKAKAELGDKIDI
ELMVTDDGVYKKMGEYIQGNLEENLPGLKISIRSMPTEAALNFARESNYDMFLIYWAP
DYLDPISTLNVFKSPNAMNYENPAYNELLEKASVTYALDLEARWKTLIEAEKFLLEDA
GTIGLSQRNDAILQKQNVKGIQYHSFGAPVVLKGAYKE"

CDS complement(533745..534557)

/gene="bmrR"

/locus_tag="JFBMFIFI_00482"

/inference="ab initio prediction:Prodigal:002006"

/inference="similar to AA sequence:UniProtKB:P39075"

/codon_start=1

/transl_table=11

/product="Multidrug-efflux transporter 1 regulator"

/db_xref="COG:COG0789"

/translation="MNSYFKIKEMADLYDIGTDTLRHYDKIGLLKPSRSSNGYRLYSI
QDLYKLNLIRDLRNLDFSLEAIGNYLEKRTIEQTTNLLNIEYELVNEKIQELEKTQAY
LKERINLMDESPKIIANEVNVLKLQSRYCIQELAPLESPIEVDYTLKKLQKRNESLIP
LLTQSAIGAFFDCENSYLKKQASFNSVFFILKENQVKPDFVLAAGSYLSIHYRGSYQQ
SFTYLSKLYTYINEHKLVALGPPFELYKIDFHETLKEAEFLTEIQIQVVEST"

CDS 534687..535172

/gene="ureI"

/locus_tag="JFBMFIFI_00483"

/inference="ab initio prediction:Prodigal:002006"

/inference="similar to AA sequence:UniProtKB:Q09068"

/codon_start=1

/transl_table=11

/product="Acid-activated urea channel"

/translation="MLGVGMLFVGVTLISNGLGGLLKIDKKSLAVMNGFTGVLSFIIN
LVYLWRGDYYAAATGFLFAFTYLFVTLVSVFNLDDRVYGIFCLFVAINTVPCAYISFA
SEGDWRFAIIWLVWGALWLTGFIETVLAKKIGIPVYYFAIFTGIFTAWIPGFLMITEL
W"

CDS complement(535216..535392)

/locus_tag="JFBMFIFI_00484"

/inference="ab initio prediction:Prodigal:002006"

/codon_start=1

/transl_table=11

/product="hypothetical protein"

/translation="MKRLIRNGLISVGAASIAVGATLITGAVAWYSSDLALKKLDNQS

QSKTQCQCRKCKHK"

CDS 535641..536075

/locus_tag="JFBMFIFI_00485"

/inference="ab initio prediction:Prodigal:002006"

/codon_start=1

/transl_table=11

/product="hypothetical protein"

/translation="MTNYSKKEFKTLGKIEHTIIKLDETLGKMEDTDSKIKHFFEQEK

AIHEIKKTIREANKLDKLEAKEIEAGAENIAFDEHEEDRIVASIGKHFEKLDGTLEKL

DDTDSKVKGYIEQKKAIHEVKKILHKAGKLEKYTEDELNTPI"

CDS 536208..536531

/locus_tag="JFBMFIFI_00486"

/inference="ab initio prediction:Prodigal:002006"

/codon_start=1

/transl_table=11

/product="hypothetical protein"

/translation="MIWYGVLGLIFFISIIGGCVSGIMLILFRIELHTVTKRKPKKKK

NIAKWEKRLKPIRLKVNYMKYLFLTSLASVITLFLILWVFTIQLQVTNQLPDLELRSE

QQAEK"

CDS 536940..537857

/gene="ydhF"

/locus_tag="JFBMFIFI_00487"

/EC_number="1.-.-.-"

/inference="ab initio prediction:Prodigal:002006"

/inference="similar to AA sequence:UniProtKB:P76187"

/codon_start=1

/transl_table=11

/product="Oxidoreductase YdhF"

/db_xref="COG:COG4989"

/translation="MNKIDLGKSGLLASEIALGCMRMADNSVSDASKVIQTAFDNGID

FYDHADIYGGGASEEIFAKALKETSIKREDILIQSKCGIRKGSFDFSKEHIINSVEGS

LKRLNMDYLDALALHRPDTLVEPEEVAEAFNELQQSGKVKQFGVSNQNPMQIELLQKY

IDQKLIVNQLQFGIMHTGMIDAGINVNMTNSRSIDHDGSVLDYSRLHDMTIQAWSPYQ

FGFFEGVFLDNDKFPELNATINEIAATKGVTNTAIATAWITRHPAKIQTIIGTMNPTR

IKDAAMASTITLSREEWYSIYRAAGNVLP"

CDS 538068..538508

/locus_tag="JFBMFIFI_00488"

/inference="ab initio prediction:Prodigal:002006"

/codon_start=1

/transl_table=11

/product="hypothetical protein"

/translation="MKKHGIAIIERKIGNQKFILIQEKAIGTPDEKGLLELPSGEIKE

DEQAIEALKREVKAMTGLSITTIQTDNDEAFFETKTVEMTEMIHFQTYICEATGVHLT

KSDQYKNIRWISLGVLKKWLQTPEKIANHHFDVLTKYTEQCHSK"

CDS complement(538552..539463)

/locus_tag="JFBMFIFI_00489"

/inference="ab initio prediction:Prodigal:002006"

/codon_start=1

/transl_table=11

/product="hypothetical protein"

/translation="MSTILLQAVGFLLIIFVAYILKKAGIFKTSDGHILSKLIVNLTL

PAAIIVGFNGVKVNSIFFLLILIGFLTNIILVTTAGFLGRKKEPIERGLLMFSIAGYN

IGNFTLPFVQGFFSAAVPILGIFDMGNALMLSGGTVALVEGITGKKEEKFSIVRSLKR

LFKSPPFTIYIVMFVLALFHLLIPEKALSIIKLFSSGNAFLSMFMIGLYLEINIDRGS

LKTVAKILITRYFFATIAACLFYFVLPFPELVRKVLVLIAFAPIASLSTINSIAFGAK

ESITGFLSSASIIISLIIMTGILLVLM"

CDS 539661..540014

/locus_tag="JFBMFIFI_00490"

/inference="ab initio prediction:Prodigal:002006"

/codon_start=1

/transl_table=11

/product="hypothetical protein"

/translation="MLILKAEIKHNLVKSNGTPAKILDIQGTNFFQPMILLNQQLFAG

RYLITSCQIVPGVTYLMEIGLEALTTNRDVEKIISKDASFDIFEKELKIGSGKLLDYW

FDQNPQLFDIEIVHS"

CDS 540211..541323

/gene="ald"

/locus_tag="JFBMFIFI_00491"

/EC_number="1.4.1.1"

/inference="ab initio prediction:Prodigal:002006"

/inference="similar to AA sequence:UniProtKB:Q08352"

/codon_start=1

/transl_table=11

/product="Alanine dehydrogenase"

/db_xref="COG:COG0686"

/translation="MLIGIPKEIKNNENRVAIVPAGVSELVAAGHKVIIETNAGTNAG

FLDAAYEAAGAEIKADAKSVWAAEMVMKVKEPLAEEYNYFYEGLILFTYLHLAPDFVL

TKTLKDAGVTAIGYETMRGADGSLPLLTPMSEVAGRMAVQVGAQFLESQKGGSGVLLA

GVPGVSRGKVTIIGGGVSGVNAAKVAIGIGASVTILDVNPKRLAELDDLFGSQIDTLI

SNSYNIAHLLKHADLVIGAVLIPGDKAPKLVSEEMVKNMKPGSVLVDIAIDQGGIFET

TDRVTTHDQPTYTKHGVIHYAVANMPGAVAKTSTLALTNVTAPYALQIANKGIVAAAK

ENPTILTGINLINHQITYQAVAKAHGFDYAVALDVM"

CDS 541525..541752

/locus_tag="JFBMFIFI_00492"

/inference="ab initio prediction:Prodigal:002006"

/codon_start=1

/transl_table=11

/product="hypothetical protein"

/translation="MARGNKEVKVEVRETERNTEDFVELELFINDEKIGTVQQEGKKN

PTVSMKDGKSFVVKTVDAGINDLIMDYNLHN"

CDS 541901..542860

/locus_tag="JFBMFIFI_00493"

/inference="ab initio prediction:Prodigal:002006"

/codon_start=1

/transl_table=11

/product="hypothetical protein"

/translation="MERVIKSKKSREKIISVFQLAQKNWENANVSSQAAQLAYFVLLS

LFPILLVAGNLIPFLPISTEEIMPYVQSAVPPDIFKLLQPILDNILHSSSGTAISFGI

VTAIWSASSGFNALQQVMNNVYGTEMRKNFIVARIFSFIIALVMIVVLGSVILIFVFG

EQVIRFVQEKLALNMDILSEFTSIKWVTTILVLLIVFMILYWLVPNVRWGIKYALPGA

VFATVGWLLSSQAFSIYVRFQGGKSIGTGTLSIFIVLMLWLYLIATILLLGGFINVMV

YHYLKEIKEPHPDDDQAIPQAKGMKQRRKLIRIKAENENLMKF"

CDS 542975..544426

/gene="arcD_1"

/locus_tag="JFBMFIFI_00494"

/inference="ab initio prediction:Prodigal:002006"

/inference="similar to AA sequence:UniProtKB:P18275"

/codon_start=1

/transl_table=11

/product="Arginine/ornithine antiporter"

/db_xref="COG:COG0531"

/translation="MKKKTATKSVLKTQQKVGFLPLMTIVIGSAIGAGIFNLSKDMAL

GAAPGAVLISWGIVGVGIIALALCFQNLSDKRPELDSGIFQYAEDGFGKFAGFVSAWG

YWLSIWLGNVAFATMLMSSLGFFFPIFKGGQNIPSIIGASILLWLLNYLVAKGVENAA

FINFVVTVCKLMPIFVFIVFAIMAFQTSIFTADFWGTMTQQFEFKDVMNQVKSTMLIT

IFVFVGIEGASILSARARNKKDVGRATIIGVLCVLVVYMLVTILSFGVMSQNQLANLP

EPAMAYVLEEIVGYWGAAFICGGLSISILGVWLSWTILPAETGLIAAKQGIFPSIFGR

INKHGAPAEALTITSMCIQLFMFTFLITDQAYQFVASLVSSVVIITYIFVTMYQVKFS

YQQPKGRQRTIQLVIGLVATAFQIWAFVSAGLKFMLLLTLLFVPGIFVYIKAQKEQHQ

RHFFSPIEKIVVALIFIGSLFAIYFLATGTITI"

CDS complement(544475..545122)

/locus_tag="JFBMFIFI_00495"

/inference="ab initio prediction:Prodigal:002006"

/codon_start=1

/transl_table=11

/product="hypothetical protein"

/translation="MEKFIQPYTYNFIQFQANELLKAHRSLNDFATIQTMKLVVIEKI

LTRFNQPTESEKELLNRIVQFSHSQKEMDAYLILLEDFIIPFEQPSDAALKKLFRKTK
KLHIPNWDTLNLADYTFYGWNDAGKQRKYLILFEQQKLIGIEGTLSPTTHKGFCAICH
TSSQVSLFMNIGKHNKDGQFTSKGNYICHDSHQCNKNIMELDELQKFIGIVKQKE"

CDS 545884..547062
/gene="rodA_2"
/locus_tag="JFBMFIFI_00496"
/EC_number="2.4.1.129"
/inference="ab initio prediction:Prodigal:002006"
/inference="similar to AA sequence:UniProtKB:P39604"
/codon_start=1
/transl_table=11
/product="Peptidoglycan glycosyltransferase RodA"
/db_xref="COG:COG0772"
/translation="MAENSNSESKIDYGIILSVMLLCIISIATIYSTTVIDMGGSIKA
TIMHIAWYVIGAVAIAVIMQFDSEQLWKLAPIAYWGGIVLLVLVLFLYDRPTEAVTGA
KSWFKIGGLTFQPSEVMKVAFILMMARVVTKHNSRFNQHQPSTDFLLLGKMFLTAAIP
LGLVLVQNDLGTTLVFIAIIGGMVLMSGITWKILLPLFTLGFSTFGGALWLVMNDRPF
LLKLGFKNYQFARIDSWLNPYHDVSGDAYQLVQSMKAIGSGKVFGKGFGIFEVYVPVR
ESDMIFSTIAENFGFIGSCILIFLYFLLIYQMIRVCFDTKNEFYTYISTGVIMMILFH
VLENIGMSIGLLPLTGIPLPFISQGGTALLGNMMAVGLILSMRYHYKSYMFSDEEKEF
"

CDS 547178..547531
/gene="spxA_1"
/locus_tag="JFBMFIFI_00497"
/inference="ab initio prediction:Prodigal:002006"
/inference="similar to AA sequence:UniProtKB:Q8DU17"
/codon_start=1
/transl_table=11
/product="Regulatory protein Spx"
/db_xref="COG:COG1393"
/translation="MIQFYEHPRCSTCKKARAWLDENKVVYNQINLLETPPTAKELAS
WIEASGLPIRRFFNTSGGKYRELGLKDKIDDMDIAEASALLATDGMLIKRPLMTDGKK
LTLGFKEADFEKNWK"

CDS 547549..547893

/locus_tag="JFBMFIFI_00498"

/inference="ab initio prediction:Prodigal:002006"

/inference="similar to AA sequence:UniProtKB:A0A0H3JT43"

/codon_start=1

/transl_table=11

/product="Glycine cleavage system H-like protein"

/translation="MTVYYSENGLWLEEVQEGVKVGISAKGQDDLGEVMFVDLTPETT

QVEKDATLIGVEAAKAVTELTSPVKGQVVAWNLELSENPERLNSTKKEDNWIVILDQV

DKELLSSLAISE"

CDS 548295..549326

/gene="metN2"

/locus_tag="JFBMFIFI_00499"

/EC_number="3.6.3.-"

/inference="ab initio prediction:Prodigal:002006"

/inference="similar to AA sequence:UniProtKB:Q831K6"

/codon_start=1

/transl_table=11

/product="Methionine import ATP-binding protein MetN 2"

/db_xref="COG:COG1135"

/translation="MIELKDIKKIFQTKKGTVEAVNGVNLSIESGDIYGIVGYSGAGK

STLIRMFNGLEAPSSGTVSVNGKVISTLKGSSLRKERQKIGMVFQHFNLLWSRTVVEN

ILFPLEIAGVPKEKRLARANELVKLVGLEGREDAYPAQLSGGQKQRVGIARSLANDPT

LLLCDEATSALDPQTTDEVLDLLLDINKRLNLTIVLITHEMHVIRKICDKVAVMESGK

IVEHGNVMDVFKRPKEEVTKRFIRQDSNDEEDTHLVLEELLAEYPDGKVVRLTFHGEQ

AKLPIISKIVKEDEVDLTIIEGNIKKTQEGAIGSLYVQLMGQPEKINQAIENLRKMRV

EVEVITDVK"

CDS 549316..550005

/gene="metP_1"

/locus_tag="JFBMFIFI_00500"

/inference="ab initio prediction:Prodigal:002006"

/inference="similar to AA sequence:UniProtKB:O32168"

/codon_start=1

/transl_table=11

/product="Methionine import system permease protein MetP"

/db_xref="COG:COG2011"

/translation="MLNKIDFSAYLDFSTVDPEKIKTATLQTLGMTAGSLLLVFILGL

VLGLVLYETNGKNTKLAKFFYWFVSILVNVFRSVPFIILIVLLIPVTKALVGKIMGPT

AALPALIISAAPFYARMVEIGFREIDKGVIEAAEAMGANKFQVIYKVLLPESLPAIVS

GITVTAISLVGYTAMAGVIGAGGLGNLAYLEGFQRNQMGVTVVATFIILVIVFAIQLL

GDFAVKKIDKR"

CDS 550051..550884

/gene="metQ_1"

/locus_tag="JFBMFIFI_00501"

/inference="ab initio prediction:Prodigal:002006"

/inference="similar to AA sequence:UniProtKB:O32167"

/codon_start=1

/transl_table=11

/product="Methionine-binding lipoprotein MetQ"

/db_xref="COG:COG1464"

/translation="MKKTRIFGLIVALVLVVTLAACGSNGAKEDKKAKDNVLKIGASN

IPHAVILEHVKPILEKEGIDLQITTYQDYILPNKALADGEIDANYFQHIPYFNLQVKE

NDYDFVNAGAIHIEPLGLYSKRVKNLDDLKDGATVIVSNSTTDWGRVISILQDAGLVK

VKDGVDITTASFDDIVENKKNLKFTYEADPALMTTFLNNDEGDIVAINSNFAVDNGLD

PLKDAIALEKTSSPYANIVAVRKEDKDNENIKKLVEVLKSKDVKEWILKEWNGAVVPV

D"

CDS 551014..552000

/locus_tag="JFBMFIFI_00502"

/inference="ab initio prediction:Prodigal:002006"

/codon_start=1

/transl_table=11

/product="hypothetical protein"

/translation="MKRLIVEEVMKQEKAKLLLKNISFDVEEAEVVGIKCSIDESHLL

FDLIEGEKIPTSGKIQRQLTSMESDRKNDGLYEKLTVEKYLTFFNRLNGQKIDLANLK

ESFALIDCWKTAIEKLSLAQKKRVGLLRIYSAQPDLVLIESPLTDLDNEGIELYIKCL

EIFKKEAIAVLVTAPYLEELILLSSKVYVKHESGLNQVDLSEEKGNDEPTYSANLQPN

VFKILCKIDDKMIFFSPTEIDYIESINGVSQVHVGGESFPSALTMTELEARLIKFGFF

RCHRSYLVNLQRVRELVSYSRNSFTLVLADEIKTKLPLSRTRLEELKLLLEI"

CDS 552154..552903

/locus_tag="JFBMFIFI_00503"

/inference="ab initio prediction:Prodigal:002006"

/codon_start=1

/transl_table=11

/product="hypothetical protein"

/translation="MSSSMRKIQTLFILKLEMILKNLTIMLGPIMTILFVFIFKTVIE

VPTPGGTQPESVEFVTGYILQIGVIFNVTMTGIMAASMPLAEEKEKQTLRVLLSSSVS

KVEFLIGSLAPVLVIMTAINFILIPISGVFVGNMLVYLLITTIVSVITIILGLLIGMS

AKNQMAVSLLSMPFMLILMMIPLLFQLNDLAGKASSFIYTGTLNEIISRLAQNDPNPV

SLKNGLVLVAWFIVASGSCLYFYKKKGLESE"

CDS 552988..553647

/locus_tag="JFBMFIFI_00504"

/inference="ab initio prediction:Prodigal:002006"

/codon_start=1

/transl_table=11

/product="hypothetical protein"

/translation="MLKLLKTKEFWLNCLFLIALLVINFIPSILFGVGIGMGLSNSPF

FPYVVSLLFLASVAIIVMLALKRKIITFKWDFLTGRNLAIIFIAFFLMKVVSVVIAMF

MTTDTTANQEAINQLTSSNSSLLMAVMIVIAAPINEEIIFRASITQLFFKKHQVAALI

VTSVVFGLFHGPTDIQSFLLYSSMGAALSAVYLKTGRIECSIALHFLNNGLSFVAMQF

I"

CDS 553666..554046

/locus_tag="JFBMFIFI_00505"

/inference="ab initio prediction:Prodigal:002006"

/codon_start=1

/transl_table=11

/product="hypothetical protein"

/translation="MNLQALHHVAIIVSDYQKSKEFYVDLLGFEVIRENYRPERNDHK

LDLKFGNSELEIFAMPNNPKRVSNPEACGLRHLAFKVDAIEEVIAELAAKGIDCEPIR

IDDYTNEKMTFFFDPDGLPLELHE"

CDS complement(554096..554821)

/locus_tag="JFBMFIFI_00506"

/EC_number="2.4.2.-"

/inference="ab initio prediction:Prodigal:002006"

/inference="similar to AA sequence:UniProtKB:O33341"

/codon_start=1

/transl_table=11

/product="Putative glutamine amidotransferase"

/db_xref="COG:COG2071"

/translation="MKPMIGIAGNSLTKHASVFHDNFVTYTPQGFVDGVKKAEGIPII

FPVGDPAEAKEYMAKVDGLLLAGGQDISPHLYGEEPSIKLEETAPKRDVFELALIKEA

FKQKKPILAVCRGMQLLNVAQGGNLYQDLSAYPEWTVQHLQASHPAIGIHTVTINEQS

HLGQLMGSNYSVNSYHHQAVKTLAADFVATAWSPDGLVEAFEAKDQDQSVVAVQWHPE

LMQETDPVMQRLFTDLIQRSTTK"

CDS 555174..555947

/gene="sufC"

/locus_tag="JFBMFIFI_00507"

/inference="ab initio prediction:Prodigal:002006"

/inference="similar to AA sequence:UniProtKB:P80866"

/codon_start=1

/transl_table=11

/product="Vegetative protein 296"

/db_xref="COG:COG0396"

/translation="MAVLEVKDLHVSIEDKEILKGVNLVMNTGEIHAIMGPNGTGKST

LSAAIMGHPSYEVTQGEILLDGENVLEMEVDERARAGLFLAMQYPSEIAGITNAEFMR

AAINARRGEDDKMSVMDFIKKLDSKMDVLDMPEEMAERYLNEGFSGGEKKRNEILQLM

MIEPTFAILDEIDSGLDIDALKVVSKGVNAMRGDNFGSLIITHYQRLLNYVTPDIVHV

MMNGVIVKTGGAELAKRLEAEGYKGIRDELGIDIELDED"

CDS 555960..557246

/locus_tag="JFBMFIFI_00508"

/inference="ab initio prediction:Prodigal:002006"

/inference="similar to AA sequence:UniProtKB:Q7A6L4"

/note="UPF0051 protein SA0778"

/codon_start=1

/transl_table=11

/product="hypothetical protein"

/translation="MKETKFTDHLDALRDFSLSHAEPEWMLDLRITMLNKIAELDLPF

FERVKINRWSLLETPEINLETEALMLADDFLEVDNDAPKIVQIGRHTALEQLPMELIE

KGVIFTDIFTALTEHPELVKEAYMQLAVKPDEDKLTAFHAAFMNSGIFLYVPKNVVIE

EPLEALFVQNSQIKENFVKHVLILADTNSEFSYVEKYQTIGNEKNTANIVVEVITKPG

SKVKFSAVDQLGENTTTYFNRRGHLLKDSTINWALGVMNTGDVVADFDSDLIGEGSHS

EVKIVAISMGKQVQGIDTRVTNYGRHSIGHILQHGVIRDKSTLTFNGIGHIIKGAKGA

DAQQESRVLMLSDKARGDANPILLIDENDVTAGHAASVGRVDPEEMYYLMSRGLRKQD

AERLVIRGFLGSVIAAIPIKAVRDELVEVIERKLSL"

CDS 557243..558481

/gene="csd"

/locus_tag="JFBMFIFI_00509"

/EC_number="2.8.1.7"

/inference="ab initio prediction:Prodigal:002006"

/inference="similar to AA sequence:UniProtKB:P99177"

/codon_start=1

/transl_table=11

/product="putative cysteine desulfurase"

/translation="MILDAEALKKDFPILFQEVNDEPLVYLDNAATSQKPQAVLDALS

DYYHFDNANVHRGVHTLAERATAKYEAAREKVRHLIKASETAEILFTRGTTTSLNWVA

RSYGEENVTEGDEIVISYMEHHSNIIPWQQLAKQKKATLKYIDLTPDGQLDMESAKKQ

ITEKTKIVAIAHVSNVLGVVNPIKELVGLAHQVGAVIVVDGAQSVPHMDVDVVDLDAD

FYAFSGHKMLGPTGIGILYGKRHLLEKMEPIEFGGEMIDFVYQNDSTWKELPWKFEAG

TPNIAGAIGLGAAVDYLNQVGLPGIHQYEQELVAYVLPKLMAISGITIYGPTDPAIRT

GVITFNLEGVHPHDLATAMDMEGVAVRAGHHCAQPLMNYLKVNSTARASFYFYNTKAD

ADRFIAALLATKEFFSHGAI"

CDS 558468..558935

/gene="iscU"

/locus_tag="JFBMFIFI_00510"

/inference="ab initio prediction:Prodigal:002006"

/inference="similar to AA sequence:UniProtKB:Q9A1G2"

/codon_start=1

/transl_table=11

/product="Iron-sulfur cluster assembly scaffold protein

IscU"

/db_xref="COG:COG0822"

/translation="MALSRLDNLYRQVILDHSSHPHHHGVLDQSTNQIELNNPTCGDV

IQIQLLVEDETVKDIRFSGSGCTISTASASMMTDAVIGKPVKEALALSEEFSLLVQGK

ELTNPDSLGDAQILSGVAKFPARIKCATLGWKALEKALIEGTNTGIDGHLASH"

CDS 558969..560381

/locus_tag="JFBMFIFI_00511"

/inference="ab initio prediction:Prodigal:002006"

/inference="similar to AA sequence:UniProtKB:Q7A6L4"

/note="UPF0051 protein SA0778"

/codon_start=1

/transl_table=11

/product="hypothetical protein"

/translation="MSEIPEVSGVPELDEYQYGFHDDVESVFTTGSGLSEEVVREISR

VKEEPEWMLDFRLKSLEQFNKMPMQTWGPDLSDIDYDALTYYKKSSGGAARDWEDVPD

KIKETFERIGIPEAERKYLAGASAQYESEVVYHNMKDEFSKLGIVFTDTDSALKEYPE

LFKEYFSKLVPPTDNKLAALNSAVWSGGTFIYVPKGVRCDVPLQTYFRINAENMGQFE

RTLIIVDEGASVHYVEGCTAPTYSTNSLHAAIVEIFVKKDAYCRYTTIQNWSDNVYNL

VTKRAKALEGATMEWIDGNLGAKVTMKYPSIFLDGRGARGTMLSIAFASEGQNQDTGA

KMIHNAPNTSSSIVSKSIAKDGGEVNYRGQVTFGRKSEGSISHIECDTIIMDDLSKSD

TIPFNEIHNSNVSLEHEAKVSKISEEQLYYLMSRGLSEQEATEMIIMGFVEPFTKELP

MEYAVELNRLIQYEMEGSVG"

CDS complement(560826..561422)

/locus_tag="JFBMFIFI_00512"

/inference="ab initio prediction:Prodigal:002006"

/codon_start=1

/transl_table=11

/product="hypothetical protein"

/translation="MTFEDLYEELIDFQHSSVKKSTISINVRYAKNQILPAFGKMKIT

DITVSYCQKMVNNWHKKYKTYDTMRKQTAQILSYGISQEYIENVMRSTLLPRRKEHEE

QRKFYTKEELKTLLDSFKNFGNTKQYAFFRLVAYTGMRKSEALALQWKDIDVFNKELH
VNKTVAIDEHKKRTSYFKHLKQKHQKELYHLILKHWQS"

CDS 563143..565641

/locus_tag="JFBMFIFI_00513"

/inference="ab initio prediction:Prodigal:002006"

/codon_start=1

/transl_table=11

/product="hypothetical protein"

/translation="MKRVFLSHSSKQKESYLRPVVKKLQADRGKERFVYDEETFEEGL
KNDELITSWLEKTDLFVLFISDEALESPWVRKEIFEAKELQKSGYLNKIYPIIIDAQI
THEDSRIPDWMRKQYNLRLVSKPSVAARKIISRMTEISWEKNPKLKEIQQLFVGRNDM
MQELEIRLDSFDKSAPNTLIATGMYSMGRRTFLKKGLTKLNFISESYSLPTIKLDAHQ
SIEDFILLINDFGISSVDISIFNLMTMKQVEKIDLAVQMLIEINDAKDILLIIDDGAI
VSPIREISKWFLEVNKAISNRSSDIYMCVVSKYRTNVKSLFDKDYIYSLHVPELSLSE
RKGLLKRYSKLINLQLELSDMNYISSFFSGLPEEIVYTCDVIKENGVGYIKGKTELIS
DYSDRKVSRVISIYDDNEEAKSLLSLIALFDFVSSDMLKDIIGEDVKYLALLNDFFAQ
GICDNIGSNGEYLVLNSAIKNYVSRQRIPLTKKFKKGIQNHVKDFVVNYEENDEGRDV
ADVFFSLKESLILGEDLNTHMLIPSHYLKSMKELYDRRNSDLEVIKLADAILEQEEFM
DNHIIREIRYFLCSSLARMKDDRFKSEVQFISGAEHNFLFGFYYRHVGKDEEAADRLN
KALNERPRFSRAKRELVTVYNSLDEYDKAYELARENYEENRSNEYHIQAYFQALSSMR
NLKMSNTEKKNIMNQLLEDMNKIESEKAKNMYLIMYAKYYLYIEEDIEMAKKYVEEAN
EDSPDNMYVLLCQFDIYEKANDIDKLGEIIKAIENNIKNSRSKYFREFKKCQALYQAK
IGNISNARSIAKSSYLPENSKQILLARIDKMVIL"

CDS complement(566499..566933)

/locus_tag="JFBMFIFI_00514"

/inference="ab initio prediction:Prodigal:002006"

/inference="similar to AA sequence:ISfinder:IS1476"

/codon_start=1

/transl_table=11

/product="ISL3 family transposase IS1476"

/translation="MSFIYYDAITHGLIDILPDRKLITLRAHFLHYSLKERSMVQSIV
VDMNAGYFTLAKELIPNPKIIIDRFHLVQLLTIALNKTRIRLMNQFKRWKPEEAKDYR
KLKRYWKLHLEDSQKVNYTDYQYHRLFRKIMPILKFPTIYFP"

CDS complement(567055..567381)

/locus_tag="JFBMFIFI_00515"

/inference="ab initio prediction:Prodigal:002006"

/codon_start=1

/transl_table=11

/product="hypothetical protein"

/translation="MSLTHCIRTLIDLKDKNISFPENYCEERVIKGRRTKLIYATLTY

KPACCKRCGCLNTQFSIVKDGFLTSKIKMEITKNYFISNRVKQSVAFELEDNSSRKNI

AKRHFI"

CDS 567688..568458

/locus_tag="JFBMFIFI_00516"

/inference="ab initio prediction:Prodigal:002006"

/codon_start=1

/transl_table=11

/product="hypothetical protein"

/translation="MTNWLIPCNPLKYDVEGVFQEISEIDWKQSNPIKYAEIGDKVYI

YISKPVQAVKYYCEVISVNKKESTIDDSSFVMEGSTYKNYGRYMTLRLIKKMNEPVRI

DELSEIGIRPPQGPHRLKPEITSLFEEYINIDEVIESVGKDFQSIVYKEGKRIGLYTY

CYERNQKLRKKAIEIHGTRCLGCRFSFLEMYGEYGEDYIEIHHTKPLSSIKEEVIVNP

DTDLVPLCSNCHRIVHRRKEILTINELKMLIKQATERF"

CDS complement(568638..568754)

/locus_tag="JFBMFIFI_00517"

/inference="ab initio prediction:Prodigal:002006"

/codon_start=1

/transl_table=11

/product="hypothetical protein"

/translation="MGLGGGNLGGNVLFQGYPIDLLEATGSYTAEHLQGYLN"

CDS complement(568816..569190)

/gene="uvrA_2"

/locus_tag="JFBMFIFI_00518"

/inference="ab initio prediction:Prodigal:002006"

/inference="similar to AA sequence:UniProtKB:P0A698"

/codon_start=1

/transl_table=11

/product="UvrABC system protein A"

/db_xref="COG:COG0178"

/translation="MGDVEQPCEKCHGKRYNEEALSVSSHARSIYDILQLIIGEAVDL

FDDPSLKEAIQNLIDVNLGYVKLGQSLDTFSGGELQRLKIVKIIHQTTDNLLILDEPS

TGLHEADVLNLLSLFKKLLKKK"

CDS complement(569165..569566)

/gene="uvrA_3"

/locus_tag="JFBMFIFI_00519"

/inference="ab initio prediction:Prodigal:002006"

/inference="protein motif:HAMAP:MF_00205"

/codon_start=1

/transl_table=11

/product="UvrABC system protein A"

/translation="MVRSAKIDGKSIADCVMMPISDLISFINQIDSPSVEIVLKDLIK

KLESLKTVGLSYLFLNRPTSTLSGGESQRIKMTKHLTSALADVLYIFDEPSVGLHPED

ILGMTTIIEGLKRERLYKIGLDLYGRCRATL"

CDS complement(569648..570364)

/gene="uvrA_4"

/locus_tag="JFBMFIFI_00520"

/inference="ab initio prediction:Prodigal:002006"

/inference="protein motif:HAMAP:MF_00205"

/codon_start=1

/transl_table=11

/product="UvrABC system protein A"

/translation="MSDYISIKGARTNNLKNISLTIPKHKITVATGVSGSGKSSLIFD

TLAAESQRLLNDTYSSYIQQLLPHYPQPVVDEIENLPVSIVINQKKIGGNARSTVGTV

TDIYTSLRLLFSRIAEPFIGYSMVYSFNHPQGMCTLCKGLGETKEINLDHLINFDKSL

NEGAINFPTFQPGGWRLTRYTETGNFDNDKKIKDFTTNERELLLYNTGSAPIHPTEQW

PKTSTYIGVIPRITKSFIEK"

CDS complement(570357..570788)

/locus_tag="JFBMFIFI_00521"

/inference="ab initio prediction:Prodigal:002006"

/codon_start=1

/transl_table=11

/product="hypothetical protein"

/translation="MTLLTNTLIDLQCELVAERNMKNPQNISWLQYDILHHLSLKEEQ

LPSEISIALGISRTKLSKALKELKLTGYIIQKPSEKDGRELITSLSDSGKQFLQTINL

GHQQLSQVADNIFTEEEKIQFAQLSSKFSKALKSERTSFHE"

CDS 570969..571157

/locus_tag="JFBMFIFI_00522"

/inference="ab initio prediction:Prodigal:002006"

/codon_start=1

/transl_table=11

/product="hypothetical protein"

/translation="MKIKEIIQKNLILRIVLYLLIGIPVGLLMIDNEIVAGLGIMLLF

LTALKTESSKKRKPKNKI"

CDS 571523..572716

/locus_tag="JFBMFIFI_00523"

/inference="ab initio prediction:Prodigal:002006"

/codon_start=1

/transl_table=11

/product="hypothetical protein"

/translation="MFQRPTYLKQLIQFKDSDFIKVITGVRRSGKSVLLMLYKEYLLQ

ENVPEDHIIYMNFESFDYQTITTEEKFREKLNELVPKDSKKIYLLFDEIQVVEGWQRV

VNGIRVSFDSDIIITGSNANMLSGELATLLSGRYIEIPIYPFSFHEFLSVKGIEQDSR

RVDAAYVEYEKYGGFPSVVIAEESIKDTILSGIFDSIVLNDVALRAGVKDPTALKSII

RFLSDNVGQLVNASKIVNTLKSEGVETTVHTVNRYLDLLENGYLFYRAKQYDIRGREY

LRTNGKYFIVDSGLRRNAVGRKDGNYSNRLENIVYIELKRRGYTVDVGRLDSKEIDFV

ARKLDETLYVQVAYELPNNTHETDNLLNIKDNYKKIVVTGRYHDVEQIDGIPIIYIAD

WLLAD"

CDS 573272..573709

/locus_tag="JFBMFIFI_00524"

/inference="ab initio prediction:Prodigal:002006"

/codon_start=1

/transl_table=11

/product="hypothetical protein"

/translation="MEFKKFTENDLVKCTKTFIEIFNDEPWNDKWTVERAEHYLSDYY

HTPGFTGVLALEENEIVGFIYGVSKVWWDGNEFYIHEMGVKRNFQNKGIGKALLNHLI

KELNSSDINNFALLTDRGVPAEEFYKRNGFKEIERLVFYSRDL"

CDS 573851..574591

/gene="truA_1"

/locus_tag="JFBMFIFI_00525"

/EC_number="5.4.99.12"

/inference="ab initio prediction:Prodigal:002006"

/inference="similar to AA sequence:UniProtKB:P07649"

/codon_start=1

/transl_table=11

/product="tRNA pseudouridine synthase A"

/db_xref="COG:COG0101"

/translation="MRNIKLTIEYDGKRYLGWQRLGDSDNTIQGKIENILKQMTTEKI

EIIGSGRTDAGTHARGQIANFKTESTMELAKMLEFMNRYLPRDIIIKKVEEVPERFHS

RYNATGKKYSYYIWNDAIPSAFERQYTFQYAKKLDIDRMNKACSLLIGSHDFIGFSSL

KKSKKSTVRTIEELSIQREGKKIHFTFVGDGFLHNMVRIIMGTLLEIGAGTMIVDSIE

EVFESKIRQYAGETVPSQGLFLDEVYYR"

CDS complement(574685..575290)

/locus_tag="JFBMFIFI_00526"

/inference="ab initio prediction:Prodigal:002006"

/codon_start=1

/transl_table=11

/product="hypothetical protein"

/translation="MNTNKLIKSLIFYMFSAFGISLTIKAMIGVSSFNSLNVALANVT

TIKIGTITTIINCCFLLLCWFLDNQRAIKPYFLMLMALLLFGTIINLFLYTFLKSVTI

SNYFLRVLLFIVGTTISGYSTGRALSLNLLKFPIEHFCVLIANRSNRSFKFYRYSIDI

FCVSFSLLLSFLYQLPIVVREGTVISLFLLSFMISWAKEKE"

CDS 575380..576042

/gene="yeiL"

/locus_tag="JFBMFIFI_00527"

/inference="ab initio prediction:Prodigal:002006"

/inference="similar to AA sequence:UniProtKB:P0A9E9"

/codon_start=1

/transl_table=11

/product="Regulatory protein YeiL"

/db_xref="COG:COG0664"

/translation="MIKTAYQVSSKKQLLQQINFFSEETLKNSFIFEFGMNEFIQQEG

NEMSFIFYILQGKAKILKTEENGKRLIIQFIERNDFIGELSLVKAEERGKDVVSLGQT

ICLGIPMSYAQSVLLNDVHFLQKISQYIGHKLLIRMEHFAANQSFDLKYRLAELMLAV

SVDDLYKEKHTEISEYLGVSYRHLLHIFSQFKEWGFIEKKGQNYQIHRKNLELFLREK

QS"

CDS complement(576130..578130)

/locus_tag="JFBMFIFI_00528"

/inference="ab initio prediction:Prodigal:002006"

/codon_start=1

/transl_table=11

/product="hypothetical protein"

/translation="MGKEYYRLNPALRSAETKQENIENWLSIQKTDFYHAQVAQERDY

CFWCRIVETAKPNSLESYTSMSYTLNDPNTLNSAAKTNFILREDEVLIIHTLAVVRNG

QVIDKLNDINVKVLDHVRDENYGNFDNEKSVTILIRDLHLNDIFLIETTIERTYASDS

IRNKFFRYLYTYPGPYWAYSNYQWELKNETGQTIEGHYRYFRDESDTILEKEKIVLED

QDSFIIEEQQYTGKELKDLEIAPFIDFATQKTYPEITGTLANLYQQFYQVEIGTFAAD

LLKDLEALPSLESKIKYAIDFVQKEIYYLYNSSEMDGHEPQAAEVTYQTKQGDCKAKT

VLLKVILDSLGVDSEIVLVNYNGDIFLPIYTPSPFNFNHAILKIYIDNHIYFVDATVA

NDQGFLEKRRKDSFMNYLEIKAGTTLQKQAPFQDQLPAIEEIIHCDVKEDVATFTLEG

TMRGGVANNTREMFKNQANKDIISRFNGSIYSNMVLYNKYEQNEIDKHFSDTSIQIIE

DNKELNELKVIYKATISEPYLKEGNKRFLHYWDRNYFLNDEAENHHHKDFPLWVDRNS

VKMEIHLTVDQSIDQQEIFTRQECDFKSKYLQHRVTKKVSEHGVSCYLNYQTYRNLTL

SGKELEDYIKINNQIRNSNWGLGIDIIEDGLFKKLGRLFKGK"

CDS 578381..578764

/locus_tag="JFBMFIFI_00529"

/inference="ab initio prediction:Prodigal:002006"

/codon_start=1

/transl_table=11

/product="hypothetical protein"

/translation="MKIEHIGLWTKDLEGMRKFYENYFNAQASELYHNTKTGFHSYFL

TFESGARLEIMYRQDITHRIVGGEILGFAHLAIALGNKEKVDALTKQLIEDGYKCLSP

SRTTGDGYYESVITDPEGNRIEITA"

CDS 578778..579617

/gene="yidA_1"

/locus_tag="JFBMFIFI_00530"

/EC_number="3.1.3.23"

/inference="ab initio prediction:Prodigal:002006"

/inference="similar to AA sequence:UniProtKB:P0A8Y5"

/codon_start=1

/transl_table=11

/product="Sugar phosphatase YidA"

/db_xref="COG:COG0561"

/translation="MNVKAIVLDIDGTLLNDEKKLTEKTKEALINAQQKGIKVVLASG

RPTPGMLKYVEELQMDRYNGLLVSYNGAHVYDVTAEKELFSQPLSVNTSQHILEHLKQ

FDVLPMIAKDEYMYVNNVFNGMLDLGEPMGIFNIIEYESRGGNFQLCEKEDLAAFVDF

PLHKILVAAQPEYLNENWKKIIAPFEAKVSGVFSAPMYFEFTDKGIDKANALDKTLTP

LGIEREHIISFGDGHNDLSLIEYAGMGVAMGNAVDELKNSADKITLTNNEDGIAKALA

ELL"

CDS 579721..580572

/gene="ybbH_2"

/locus_tag="JFBMFIFI_00531"

/inference="ab initio prediction:Prodigal:002006"

/inference="similar to AA sequence:UniProtKB:Q45581"

/codon_start=1

/transl_table=11

/product="putative HTH-type transcriptional regulator

YbbH"

/db_xref="COG:COG1737"

/translation="MNVLLKLKNLKHLTTSETVLVNFILASPEKVIYFSPKELAEASF

VSISTIYRLINKLKLDGLNDLKLELVKYLNAHTSETIIDINYPISSEDNNYAVMKNLQ

TVYDQTVQATIATNDLENLMRTSILLQEAEVIDVYTTSANLYFAENFKFQMQEIGKTV

HVPKDNYTQNLTAANSTKQHLAIVVSFEGRGTNIPQLFAILKKNNCKILLITAENSPL
LSEKVDSHFLFPSLESHYHKISSFSTRTSLMYIFDILYLSYFNKDYDKNIAYKLENYK
KMNPDLI"

CDS 580726..581034

/locus_tag="JFBMFIFI_00532"
/inference="ab initio prediction:Prodigal:002006"
/codon_start=1
/transl_table=11
/product="hypothetical protein"
/translation="MGANAPTVKDDYIRVRTNKELKKKATEIVESMQLDMSTVINMTL
DQIVRHNGLPFDVTNEDEAEIKFKKLQLEVAKGTTAAKAGKVKDIDEVRALFTKSNLD
"

CDS 581038..581364

/locus_tag="JFBMFIFI_00533"
/inference="ab initio prediction:Prodigal:002006"
/codon_start=1
/transl_table=11
/product="hypothetical protein"
/translation="MEAYGLQLSELAIQDLVQIEDYIASNLRSSIDASYFITEFLDKL
ELLKFSPKLGVCAKEKFKVPLDEKIAYRILFIDPYLAIHYIEDNDLVITVSRIFHQKQ
EYNKLF"

CDS 581683..583452

/locus_tag="JFBMFIFI_00534"
/inference="ab initio prediction:Prodigal:002006"
/codon_start=1
/transl_table=11
/product="hypothetical protein"
/translation="MDKKDLPIIHELAYTNQGLQTFEADVREESELKEKLLLNYPTVY
IVNDKEKSKKFSVYIGETSNIKRRTIEHLNTDSKKREDWEKLLLSPSSKMYVIGHHYF
NKSLTLDIENKLMLYMSSVASVEAIYNRRMNQQNDYYTSVKLDNIFSNIWKNLNKKNK
ELFPIERIIRDSAIFKASPFHKLTDEQLRTKEFILEKVMKAINSKKTGQLILVAGEAG
SGKTVLMSSLFYELNQLTPEDSENISGQDITSFLIVNHDQQLKVYEQIATKLGIKTKK
NPKVVSKPTTFISNHLPENPVDVVIVDEAHLLWTQGKQSYRGKNQLYDLLDRAKVVVA

VFDIKQILTTEQYWEDSEILDVQKKAIDEENYIILNNQLRINSDQKTVKWIRSVIDDQ
KINTIPKDTKGYELEIFNSPEELHNAIKEKAKNKKFGISRVIANFDWEYVNARSPEND
DYWRVKINNWSLPWNLQIPGVKEEKNKNLAWAEQEQTIDEVGSTFTVQGFDLNYAGVI
IGPSVKYRNGKIIFEKEFSKNKKATRKRSLKDGSKKYFAETLLKNELNVLLTRGVNGL
YIYAVDEELQQALLDAKKRGEIK"

CDS 583449..583790
/locus_tag="JFBMFIFI_00535"
/inference="ab initio prediction:Prodigal:002006"
/codon_start=1
/transl_table=11
/product="hypothetical protein"
/translation="MTQKQTSMEKVKQFREERDWRQFHNEKDLAISISLEASELLELF
QWKNSEEVTETKLERIKEELADVLIYSYMMADNLNLDIETIITEKLIKNNEKYPINKS
KGNKSKYTDLN"

CDS complement(583886..585181)
/locus_tag="JFBMFIFI_00536"
/inference="ab initio prediction:Prodigal:002006"
/inference="similar to AA sequence:ISfinder:ISSau8"
/codon_start=1
/transl_table=11
/product="ISL3 family transposase ISSau8"
/translation="MSLTHCIRTLLDLKDKNISFPENYCEERVIKGRKTKLIYATLTY
KPVCCTRCGCLNTQFSIVKNGFLTSNIKWISTTHFPTYLVLKKQRFLCRECHYSFLAE
STEIAKHCFISNRVKQSIAFELEDNSSRKDIAKRHFISDTTVLRVLVARGKSCLNGFD
SLPAHLCFDEFKSVKRVEGKMSFIYCDAITHGLIDILPDRKLVTLRAHFLRYSLKERS
KVQSIVVDMNAGYFTLAKELFPNANVIIDRFHLVQLLTVALNKTRIQLMNQLKRWKPE
EMKDYRKLKRYWKLLLQDSQKVNYTDYQYHRLFRKVMPHSEIVDYLLSLNSTLKATYT
CYQDLLYAMSQNNSDLLKSVLSSPHEQVSDYMSTALQTLRKHEERIRNTFQSKRSNGP
LEGSINKIKVIKRTAYGYTSFLNYKYRILISFQEKEKTI"

CDS complement(585368..585934)
/locus_tag="JFBMFIFI_00537"
/EC_number="1.5.1.45"
/inference="ab initio prediction:Prodigal:002006"

/inference="similar to AA sequence:UniProtKB:A4IT49"

/codon_start=1

/transl_table=11

/product="NAD(P)H-dependent FAD/FMN reductase"

/db_xref="COG:COG0431"

/translation="MKIVLLSGSTVGSKTQTMMKTIEEKIQTNYSDVELTFLNLKELT

IQFSDGRNFLDYDGDTGFVTRSLMDADIILIGSPTFQASIPGTLKNIFDLLPQNAFQG

KIIGLAMTAGSAKHFLVAETQLKPIINYMKGNLVPNYVFVEEVDFNQNEIVNDDVLFR

LDKLVEDAIVLTKAYQQMWQEQEDAYGF"

CDS complement(585931..587016)

/gene="fgd"

/locus_tag="JFBMFIFI_00538"

/EC_number="1.1.98.2"

/inference="ab initio prediction:Prodigal:002006"

/inference="protein motif:HAMAP:MF_02123"

/codon_start=1

/transl_table=11

/product="F420-dependent glucose-6-phosphate

dehydrogenase"

/translation="MKKNLNYLPQFDSTKGIEFGLYTLGDHIANPHTGAQISAGQRLK

DIREMAILSEQAGLDIFVVGESHQDYFVTQAHAVVLSAIAQATSKIKVSSGATIMSTS

DPVRVFENFSTIDLLSDGRVELVAGRASRVGLFELLGYDLQDYEELFEEKFDLLLKIN

QNEYINWEGQFRRPLTNAHIIPRPNGGQLPIWRAVGGSPGSAIRAGRAGVPMYMAHLG

GPASVFKQTIDAYRQSAAEVGYDETTLPIATAGLFYAAETSQDAMKEYYPHVNEGIQL

SNGQGFPKQAFAQATDKRSIINVGSPQQIIEKILYQHEMFGHQRYVAQLDFGGVPFDK

IMKNIELIGNEILPAVKKYTAKESNTK"

CDS 587480..590107

/gene="mngB_1"

/locus_tag="JFBMFIFI_00539"

/EC_number="3.2.1.-"

/inference="ab initio prediction:Prodigal:002006"

/inference="similar to AA sequence:UniProtKB:P54746"

/codon_start=1

/transl_table=11

/product="Mannosylglycerate hydrolase"

/db_xref="COG:COG0383"

/translation="MKKIHVVAHTHWDREWYFSDNEAFIQFSYHMDEVIYALENGELD

YYYLDGQLSILDDYLKVYPNQETKIKELVEGNKLFIGPWYTQMDEFVVAGESVVKNLQ

IGIDMSKRLGGHTALGYLPDSFGQGKDMPKIYNGFGIEDAVFWRGMPNEVTKSREFYW

TAEDGSKVLTINIRNGYYAGVALVEGEIYQKKAILDIVSEDSATTNITLPVGGDQRAV

DRNLKAIIKEANKEFAEEYQIEESNYPHIFNLLKKEATDLPTVQGEFMSGSVSKIHRS

IYSSRYDLKKINDTIENRLIFQLEPLMLMADDLGIPFKRELMDYTWKLILKNHAHDSI

GGCNTDKTNEMIMARYKEADQLSYSTVDYLVRKIAESIEGIQENDLVLFNTLPYRRTE

PYTLEVSTKKTTIKLFDDQRHEIPFQLISTEKVYAGEVRRSESDYDEAKYYYIHKISF

INELPALSYQIVSVEEQDESATQEKPVALEETMIENAAYKITFSNGQFSILAKEQNIT

YKNCLHVEESGDEGDTYDYSPAYFDSIHHLFFETAEVAIQSGKFLSTMSVTGSWFVPK

DLAARKAQKVDTEIEYTLTLSLKNDSKRVDMKLSLNNQALDHRMRLVVKTPVKSQVSY

ADTLFGIVERNNHDPHIHDWRELGWKEEPTEIYPMIHYANTHDLETSWTVMSKGIKEY

QVIDSDMYITLFRGVGFLGRPELLRRPGDASGNQFRYIPTPDSQLLGTLEMELSLIIA

TDYNPADIQREYQMYSVDTPYYQVQTLNRFTNPIQYFQSNKVDHLSTLKKSIDLSELP

LVFSAIELSRDGEWVNIRLYNPLLEETVKAEIIPLTTQAQVRFVNLNGDLITSLGLIN

EIPLGDFKPGQIKTISIKR"

CDS 590131..591459

/gene="licC_3"

/locus_tag="JFBMFIFI_00540"

/inference="ab initio prediction:Prodigal:002006"

/inference="similar to AA sequence:UniProtKB:P46317"

/codon_start=1

/transl_table=11

/product="Lichenan permease IIC component"

/db_xref="COG:COG1455"

/translation="MKINVEKLQEPLLKIANKVDNNSVLQAIKNAFISSIPFTVVGSF

SNLIKMQIDAFFGDTSIGQTASKIFGNVYLGTLGIVALLIVFSTAYNYARELMRKHPE

ANINPLLATLLAFAAYFVMVPNNVNFADSTADIIQGYANNFFSYEGMFTALIVGLLAV

FFYSVLAKRKIAIKLPGNVPPNVFDSFFALIPISIVLLGCSVVKVAIEAFDFNSFMDL

VSQLFVQPLTSVGTGLPAIVVVILLQQILWFLGLHGFNIVWGVVSALWLPVFLENVAK

FAETNSFDNISIAPNTMTNVYAMIGGSGATFGLILAILIFTPKTYSGREVAKLSFIPG
LFGINEPIIFGFPIVLNPLMFIPFVFVPIFNAVVAYIVTKIGWVVPLVVLNSGNEPIF
ISTWILGAFHLSPVILTAVLVVVDVIIYAPFVRMNMKFDQSQLLNVENQA"

CDS 591777..592574

/gene="rpiR"
/locus_tag="JFBMFIFI_00541"
/inference="ab initio prediction:Prodigal:002006"
/inference="similar to AA sequence:UniProtKB:P0ACS7"
/codon_start=1
/transl_table=11
/product="HTH-type transcriptional regulator RpiR"
/db_xref="COG:COG1737"
/translation="MESYIELTIQANEKKFTDVEKIIAQYFLDGNKELGIQELAKELS
VSNSSITRFCKKIGLANYKELIYLYKYSSKDKQKEADSSISYVNQNYSLLLNDIIEKL
NRDEVKKAALMIDAGRIIHYWGTGYNAFCGEDFQFKFSRLGKIINVIKDEHSIVMMAH
GVQEGDLVVLSSLSGENNAVLTALKIVAEKRAKAIVITANEKSPYKKFSEAFCLTSSM
GKKDMGGISPQIPVLTVIDTLYTEILAMYHEDTVKSWIKSEVILKGK"

CDS 592591..593859

/locus_tag="JFBMFIFI_00542"
/inference="ab initio prediction:Prodigal:002006"
/codon_start=1
/transl_table=11
/product="hypothetical protein"
/translation="MTIQYSKEVIETLKATVAQKSSNPRWSEVFSNCFDNTLETTVKL
TEAEDIFVITGDIPAMWLRDSSAQIKPYLVIANQDPKIKEMIQGLLERQVKCILIDPY
ANAFNETENGACYHQDITEMNGWIWERKYEVDSLCYPIELAYLLWKKTGETNHFTKEF
KQAAETIIKLFKREQRHENSTYRFERFGERPEDTLSNDGLGEPCGYTGMTWSGFRPSD
DSCTFNYLVPANMFAVVILGYLAEIFEEIYHEKTAVEEANQLKEEINRGIEEWGIVEH
EGKKVYAYEVDGLGNYVLMDDANVPSLLAAPYLGYCEIDDAIYQQTRAVLLSEKNPYY
YEGNYLKGIGSEHTPKEYVWPIALAIQGLTTNKKAEKIEMLNKLVETEAGTNYMHEGI
NVNSPNEYTREWFSWANMMFCELLLDCLEL"

CDS 594020..594670

/locus_tag="JFBMFIFI_00543"

/inference="ab initio prediction:Prodigal:002006"

/codon_start=1

/transl_table=11

/product="hypothetical protein"

/translation="MSKKYNTEETIKAILDVSTQLFVEKGYEKTTIQDIVNNLDGLSR

GAIYHHFQSKEEIIDGVIKRLVPNTKYIDAISNRKDLNGLEKMQHLLLETLLNKEAGT

SFGLTYSLFNNPKFFMLYMRNSSEVLSPQIEAYIIEGNQDGSLSVKFPKQIGEIVVLL

LNTWFTLALFPNTVDTFWDKIQASKYVLDSIGVNILSDEIISRIKHEIKEKEGKHE"

CDS 594663..595607

/gene="rutD_2"

/locus_tag="JFBMFIFI_00544"

/EC_number="3.5.1.-"

/inference="ab initio prediction:Prodigal:002006"

/inference="protein motif:HAMAP:MF_00832"

/codon_start=1

/transl_table=11

/product="Putative aminoacrylate hydrolase RutD"

/translation="MNKNKRNKPILKFLKIGALSLIGLIVIAFGSIYVAHQISLKNEE

SKIEYYGQKIDVFDGTMNVLVEGKGKETVVLLTGYGTASPGLDFKPLIKELKTKYKVV

VIEPFGYGLSSQTKRERTAENMVEEIHAVTEKLKLDSFILMGHSIAGIYGLNYVNTYP

ENVEAFVGIDSSTPNQPWPGFKDAFPNFLQKSGIFRLMVKLNPESFKVGDMDDATFEQ

IRLLTMKNMSNETMRKEGYALDQSFESAKKLSFPKELPVLLFVAEKNESTLDGWLELH

QEQIAHSVKGEVIELPGTHYLHHTQSKQIVKDLNQFLN"

CDS complement(595689..597206)

/locus_tag="JFBMFIFI_00545"

/inference="ab initio prediction:Prodigal:002006"

/codon_start=1

/transl_table=11

/product="hypothetical protein"

/translation="MKLFFDTLLERKDELTTALLQHIQISFIALLIAVLIALPLGIYL

SNHRKIAEPIIQITAIFQTIPSLALLGLLIPLVGIGTLPATIALVIYALLPILRNTYT

GIIEVDSSLIEASNAMGMNLWRRLSKVQIPLAMPMIMAGIRNAMVLIIGTATLAALIG

AGGLGSLILLGIDRNNNQLILLGAIPAALLAILFDYLLRFFERISFKKTLLTLGGITV

IMLGFILLPLLNQSQKEIVIGGKLGAEPDILINMYKELIETDSDLTVTLKPNMGKTSF
LFNALKNGDIDIYPEFTGTVLETFLEENAETHNPDKVYQQAKNGVAKKFQMSYLKPMA
YNNTYALAVSPQLANDYKLKTISDLIPLADTAKAGFTLEFTDRQDGYKGIQDLYKLTF
KSVQTMEPKLRYSAIESGSINVLDAYSTDSELAQYKLTVLKDDKELFPPYQGAPLLLT
KTIEKYPELETILSKLAGKITDAEMRKMNYAVNVEGKSPKTVAHNYLVKEKLVKE"

CDS complement(597203..598189)
/gene="btuD_1"
/locus_tag="JFBMFIFI_00546"
/EC_number="7.6.2.8"
/inference="ab initio prediction:Prodigal:002006"
/inference="protein motif:HAMAP:MF_01005"
/codon_start=1
/transl_table=11
/product="Vitamin B12 import ATP-binding protein BtuD"
/translation="MIKFENVTKEYQADTPVVSSLNLEIKAGEFFVLIGPSGSGKTTT
LKMINRLIPLTTGTIRIQGKPISEYNLNELRWNIGYVLQQIALFPNMTIEENITIVPE
MKKWPKDQLANRVTDLLNSVGLDAESYRHRKPAELSGGEQQRVGVLRALAADPDIILM
DEPFSALDPISRKKLQEDVAKLQKKVQKTVVFVTHDMQEALALGDRICIMRDGLVEQI
GTPAELAAQPKNEFVREFLQSGHIPIEEAGTVQDLLDANCYACLTTDLLNDLDSNFLV
PSDSIDSLVLKLTNASEISIKNPESSLLIGTITAEHLVAFLASKLQTGGAPL"

CDS complement(598608..599261)
/gene="glnP_1"
/locus_tag="JFBMFIFI_00547"
/inference="ab initio prediction:Prodigal:002006"
/inference="similar to AA sequence:UniProtKB:O34606"
/codon_start=1
/transl_table=11
/product="putative glutamine ABC transporter permease
protein GlnP"
/db_xref="COG:COG0765"
/translation="MQFFDAFSWINIRFLLDGLLVTVQVALFSILFSFVIGGLIGLVR
FMKVPVASKLVGILTDIIRNLPLLLIIFFTYFALPQIGIRLSIFWASVAALTIFESAM
LSEIIRAGLNAVPKGQMEAGRSTGLTYSQTLVSIIFPQAFKQMLPPIVSQFIALIKDT

SLAVIISLPELTHNAKIIYGQDTNAVLPMFAAMAFLYFIVCYALSLVSKRLELRLAN"

CDS complement(599276..599923)

/gene="glnM"

/locus_tag="JFBMFIFI_00548"

/inference="ab initio prediction:Prodigal:002006"

/inference="similar to AA sequence:UniProtKB:O34671"

/codon_start=1

/transl_table=11

/product="putative glutamine ABC transporter permease

protein GlnM"

/db_xref="COG:COG0765"

/translation="MFTIFSNYSDALLEGFKFTLYSSILALLFSLIIGTLMAIFQLSH

TKLIRNLAKAYVEFFRNIPLLVIVMFFYVVVPLYWFQIDGFTAGTIGLTIYTSAFIAE

TVRAGIDGVPRGQMEAGLSSGFTYVQAMWYIVLPQAFKIVIPPLGNQFINLVKNSSVL

AIVAGLDLMYQGDLIASETFNTFDTYIVVAAFYLILTLPLSYLMKYVERRLEVQA"

CDS complement(599963..600760)

/gene="glnH_1"

/locus_tag="JFBMFIFI_00549"

/inference="ab initio prediction:Prodigal:002006"

/inference="similar to AA sequence:UniProtKB:O34563"

/codon_start=1

/transl_table=11

/product="ABC transporter glutamine-binding protein GlnH"

/db_xref="COG:COG0834"

/translation="MKKKWNKIALLFFVLLSTSLLGACGQKSVAGEDILERIEKDQEI

VWGVKTDTRLFGLINIGTGNVEGFDIDIAKAITKEILGSKGHAEFVEVTSKTRIPLLK

NGNIDAIIATMTISDERKKVVDFSDVYFDAGQSLLVKKGSPIKGIESLNANTTVLAVK

GSTSAVNIRKKAPEAKILELENYAEAFTALKSGQGDAMTTDNSILLGIAEENPDYTLA

GDIFTDEPYGIAINKGQEPFLNRVNEALATLKANGTYDKIYDKWFPK"

CDS complement(600776..601504)

/gene="glnQ_2"

/locus_tag="JFBMFIFI_00550"

/EC_number="3.6.3.-"

/inference="ab initio prediction:Prodigal:002006"

/inference="similar to AA sequence:UniProtKB:O34677"

/codon_start=1

/transl_table=11

/product="Glutamine transport ATP-binding protein GlnQ"

/db_xref="COG:COG1126"

/translation="MIEFKNVEKYYGQFHALKNINLSIKKGEVVVVIGPSGSGKSTML

RCINGLEDITEGSLLINDIDLHSKTTNINEVRKNMGMVFQHFNLYPHKTVLENITLAP

IKVLKKTKEEANALAETLLEKVGMLDKKDSYPSQLSGGQQQRIAIARGLAMQPEVMLF

DEPTSALDPEMIGEVLEVMKNLAREGMSMVVVTHEMGFAREVADRVIFMADGQILEDE

EAHTFYEAPKEIRAQQFLSKIINH"

CDS 601874..602035

/locus_tag="JFBMFIFI_00551"

/inference="ab initio prediction:Prodigal:002006"

/codon_start=1

/transl_table=11

/product="hypothetical protein"

/translation="MSKAEKSMANFTILFVTFCHSVIFNPVLTRVESAHNWNYQLKNK

LPACLSYTY"

CDS 602492..604366

/gene="ettA_1"

/locus_tag="JFBMFIFI_00552"

/inference="ab initio prediction:Prodigal:002006"

/inference="similar to AA sequence:UniProtKB:P0A9W3"

/codon_start=1

/transl_table=11

/product="Energy-dependent translational throttle protein

EttA"

/db_xref="COG:COG0488"

/translation="MYIHIQEIEKSYGSLVLFEELSLKIPQGHKIGLVGDNGSGKSSL

FKLITGEESPDKGLVIRKKNLVLGYLEQLPEVTGTTSVQEVIEADFKELIEIQQHLKE

LEKQMEQSVEGVIIEKYGVLQEKFIQLGGYELAYQIEKMASGLGIQQLLNKPFNQLSG

GEQTKVGLVKVLLKEPDLLLLDEPTNHLDLHAIEWLENYLKSYQGTLFTISHDRHFLD

QVVTEIYDLEDGEIQVYQGNYTQYQKAKKARLLKEQQNYEEQQKKIKKLEDAIRRYRQ

WGNESGNEDMFKKAKSLEKRLARMTQLTRPVLEKKHIQLKLAVNQRSGKKVVTFSNVQ

KSYGAKQLFQQLNFQLLWAKHIGIVGENGVGKSTLIRLLLQEEQPDCGTIELGEQVKI

GYLPQKIEFSDENKSVLETFRFYVAMEEGESRGFLAQFLFYGSDVFRLVKNLSGGQRM

RLKLAILMKEETNLLILDEPTNHLDIATREVVEDVLADYTGTLLAISHDRYFLDKLFN

EILWLTPTDITLFNGNYQWAKKKKEEQATLLEKQDISERENGKKLSKVKNGKTAKSPT

LTDIENLLQNQSKIVTELDDKLAHETNLEQLYKWSQERDEVLLKVKQLEDQWIELADE

"

CDS complement(604427..604912)

/gene="ogt"

/locus_tag="JFBMFIFI_00553"

/EC_number="2.1.1.63"

/inference="ab initio prediction:Prodigal:002006"

/inference="similar to AA sequence:UniProtKB:P11742"

/codon_start=1

/transl_table=11

/product="Methylated-DNA--protein-cysteine

methyltransferase, constitutive"

/db_xref="COG:COG0350"

/translation="MLINSPIGFLHILEDQQGITNIIFVNEEEALAIKKDTTGPFGKQ

LALELERYFSGELKKFTVPLSLQAGTPFQQHVWQALQNVPYGSTQSYADIAVAIENPK

AVRAIGQANRNNPLPIVIPCHRVIGKNGSMTGYSGASEIGIAKKRYLLALEKNNCVSA

R"

CDS 605089..606471

/locus_tag="JFBMFIFI_00554"

/inference="ab initio prediction:Prodigal:002006"

/codon_start=1

/transl_table=11

/product="hypothetical protein"

/translation="MERVHIYHTNDLHSHFENWPRIEAYLAQQQKAHQEQNENVFTFD

IGDACDRVHPLTEATDGQANIQLLNEGHYDAVTVGNNEGIGNSKKQLNQLYTKANFDV

VLANLADIRTGEAPDWALPFKIYETKDGFRFGVFGLTAPFPTSYVPNGWQVSEPDSII

PEILELLTPLVDAVILLSHLGIMEDRRIAEMYPMIDLIIGSHTHHLLPDGEIVRQTLI

TAAGKFGMYVGHIELEIDDFKRLTVTKASVIETARLPVASHEAERIKEYEIEGHRLLK
TQKVAYLPNNLPVDWFASSPLVEMGLKAVKDYAKTEVSILNAGLFMKGLTAGLVSKDD
LHTLLPHPMRVMRCTLSGANIARMILEMEKNRGYLKSFPIKGMGFRGKLFGEICYDGI
EYDATTRSIKWLGEPLDEYKEYTFATVDHFLYIPYFPTIEICGVNEVIFPYFIRNVFG
QYLTRNYPIE"

CDS 606555..607292

/locus_tag="JFBMFIFI_00555"
/inference="ab initio prediction:Prodigal:002006"
/codon_start=1
/transl_table=11
/product="hypothetical protein"
/translation="MSDEKEVEVIQESSTAKEQDTTELTTTEATELTVEEENLVVRMI
DETNLIVEGRKYELVLNYRDGFNSEKLAERYSDILSKYDFIVADWGFDQLRLKGFYED
KNRKVPQEQRIGSLEDYLYEFCNFGCAYFVLKRTGGRKEKTVRPKRTRTKNTQISATK
AEATKPTREVATKNQKTARPTKQRSDKSRSDNQKNKNKSFVAKQVKEKSEAPVVSKPA
VETVKEKNGQRHFNIRRNDVEKVKNKE"

CDS 607305..608069

/gene="yutF"
/locus_tag="JFBMFIFI_00556"
/EC_number="3.1.3.-"
/inference="ab initio prediction:Prodigal:002006"
/inference="similar to AA sequence:UniProtKB:O32125"
/codon_start=1
/transl_table=11
/product="Acid sugar phosphatase"
/db_xref="COG:COG0647"
/translation="MKYKGYLIDLDGTMYRGSEPIPAATAFIKRLQAEKIPFLFVTNN
TTKSQEEVVKNLSTNFDIHVTEAEVYTGSIATAAYLKSLDKGNKVYAIGEAGLKLALS
EAGFVEEETNPDYVVVALDRNVHYHNFELATLAIHRGARFISTNKDTNLPSEKGLIPG
AGALTALIIASTKKQPTYIGKPEAIIMEEALKVIGLDKSDVLMVGDNYETDIMAGIQN
EIDSLLVLSGFTSEKDLEKVAKQPTYVVKSLAEWEF"

CDS 608069..608698

/locus_tag="JFBMFIFI_00557"

/inference="ab initio prediction:Prodigal:002006"

/codon_start=1

/transl_table=11

/product="hypothetical protein"

/translation="MKLKTIHFFGILSIFLFIISVGIALTINMFPLYWFDIDYLGIAE

YVGVPKEILVENYSALMRYLNLPWVTTLNMPDFPSSANGLLHFYEVKRLFLVDYVVLI

GSGIGSFFFIRYLKLKKLIWILIRPFQIALVAPVVVLFFVAVSFDRLFILFHEVFFNN

DAWIFNPATDPIIEALPQEFFMHCFILAFILIEVQFLLGYLLTRRELKK"

CDS complement(608780..609226)

/gene="ypjQ"

/locus_tag="JFBMFIFI_00558"

/inference="ab initio prediction:Prodigal:002006"

/inference="similar to AA sequence:UniProtKB:P54173"

/codon_start=1

/transl_table=11

/product="putative protein YpjQ"

/db_xref="COG:COG1267"

/translation="MELTDIAELVMFLQQSYVQDLTIEICLEHVNAVLKKREVQNAII

TGIQLDILAEQNLLMQPLQDIIAEDEGLYGIDEIMALSIVNVYGSIGFTNFGYIDKVK

PGILKKLNDHDSGEIHTFLDDIIGAIAASAASRLAHADPSKSDITH"

CDS 609467..609703

/locus_tag="JFBMFIFI_00559"

/inference="ab initio prediction:Prodigal:002006"

/codon_start=1

/transl_table=11

/product="hypothetical protein"

/translation="MDPIVEFCVNNAANGTQPIIEQLEKDGKVEVVSYDCLNECVVCA

KDFFALAEGKLVRADTPEELLTKIYSSLEEEGWL"

CDS complement(609869..610861)

/gene="yumC"

/locus_tag="JFBMFIFI_00560"

/EC_number="1.18.1.2"

/inference="ab initio prediction:Prodigal:002006"

/inference="similar to AA sequence:UniProtKB:O05268"

/codon_start=1

/transl_table=11

/product="Ferredoxin--NADP reductase 2"

/db_xref="COG:COG0492"

/translation="MDSIDDVFDITIIGGGPVGMFAAFYGGMRNAKVKIIESLPKLGG

QLATLYPEKKIYDIAGFPEVKAQELVDNLEKQMSRFETTICLEEEVLSVEKDAEGLFV

LETTKHTHYSKAIIITAGNGAFQPRRLELDNAIRYEGTSLHYFVDNLERFKNRKVAIC

GGGDSAVDWALMLEPVAKEVFLIHRRNQFRAHEHSVSLLDKSTVQLKTPFIPAKINGS

KEALDSITLKEVRGDQVEELVVDDFLINYGFVSSIGAIKNWGLDIEKNAIVVNSKMET

TIPGIYAAGDICTYNGKVKLIAAGFGEAPTAINNAMSYIYPDKKVQPMHSTSLF"

CDS complement(611039..611530)

/locus_tag="JFBMFIFI_00561"

/inference="ab initio prediction:Prodigal:002006"

/codon_start=1

/transl_table=11

/product="hypothetical protein"

/translation="MQILTNYPLIAALSAICFSQFIKVPIAFLLKKNTTWALAVSTGG

MPSSHSAGVTALITALILNYGWESPYVAIAVTFGVIVMFDAMGVRRQSGEQGLVLNQL

IIDLQNLKIKSNESGESPKRKQRDKHIKEYLGHKPLEVLFGIFTGIAMAYLVRAILNY

FNY"

CDS 611705..612298

/locus_tag="JFBMFIFI_00562"

/EC_number="5.2.1.8"

/inference="ab initio prediction:Prodigal:002006"

/inference="similar to AA sequence:UniProtKB:Q7A6I1"

/codon_start=1

/transl_table=11

/product="Putative peptidyl-prolyl cis-trans isomerase"

/translation="MTQFPQLSTEVAANEKLVTMKTTMGDIKLKLFPDLAPKTVENFL

GLAAKGYYDGIIFHRVIPDFMIQGGDPTGTGMGGESLWGEEFNDEFSKQAFNLNGALS

MANAGPNTNGSQFFIVSNTEVPQNMLTQLEGAGYEKEVIDAYAKNGGTPWLDFRHTVF

GHVIEGMDVVYDIQNVKRGAQDKPVNGVRIESIEIAD"

CDS 612478..613341

/gene="gmuE_1"

/locus_tag="JFBMFIFI_00563"

/EC_number="2.7.1.4"

/inference="ab initio prediction:Prodigal:002006"

/inference="similar to AA sequence:UniProtKB:O05510"

/codon_start=1

/transl_table=11

/product="Putative fructokinase"

/db_xref="COG:COG1940"

/translation="MMYGAIEAGGTKFVCAISDENFEIKERVSIPTTTPEETLSHVFE

FFDQFKLDSIGIGSFGPIDVNKKSATYGYVTTTPKVAWTNFDFLGAVKKRYEIPVGWT

TDVNAAALGELKKGAAIGLDSCVYLTVGTGIGGGAVVNGKLLAGYGHPEMGHMLVRLH

PDESYGGFCPYHGNCLEGIAAGPAIEGRYGVKGHELADKIEVWQMEAYYLAQALMNYT

LILSPERIVLGGGVMKQNQLYTLVREEFKKLMAGYVAVPPLEEYIVAPGLGDNAGVTG

CLLLAADELTK"

CDS complement(613390..614562)

/gene="patB"

/locus_tag="JFBMFIFI_00564"

/EC_number="4.4.1.13"

/inference="ab initio prediction:Prodigal:002006"

/inference="similar to AA sequence:UniProtKB:Q08432"

/codon_start=1

/transl_table=11

/product="Cystathionine beta-lyase PatB"

/db_xref="COG:COG1168"

/translation="MQANFDEIINRKGTNSVKWDMLTEKFGSEDVLPMWVADMDFATP

KTILSALKKSLDVQILGYSIPSDGLYDAIIDWQSNHHQLNLNKSDILFSPGVVPSLAL

MVTALTEPGDSVMIHDPVYPPFTEVVRSNQRQLVRSSLIIENKQFKMDFVDIEEKLKN

HSVKLFILCNPHNPGGRVWSTTELKQLAELCKKYKVILVSDEIHGDLVFAPHSFTSVV

TLDPSYQEFVVTLTAATKTFNMAGVKHSMIFVKNKELKAKIAMEQHKSEQDGINTFGY

VATEAAYNTGEVWLEELLDYLSENLDYACKFLEKELPQVKFLKPEATYLIWLDCSYLG

LSDSALRKQLVNVGRLGLNPGISFGPNGSQFMRMNIATSRATLKEGLERLKVALTK"

CDS 614757..615134

/gene="yugI"

/locus_tag="JFBMFIFI_00565"

/inference="ab initio prediction:Prodigal:002006"

/inference="similar to AA sequence:UniProtKB:P80870"

/codon_start=1

/transl_table=11

/product="General stress protein 13"

/db_xref="COG:COG1098"

/translation="MTYKIGMIVKGKVTGIQPYGAFVSLDKDTQGLVHISECKHGFVK

NLNEVLQVGEEIEVMVIDIDEYTKKISLSMRSLEQKSTMHYHYKKRKYRPDKADKVGF

ESLRQMMPKWIEEAKNDEKKRQK"

CDS 615261..615620

/locus_tag="JFBMFIFI_00566"

/inference="ab initio prediction:Prodigal:002006"

/codon_start=1

/transl_table=11

/product="hypothetical protein"

/translation="MTMDQEGRSVFLVSNKTGETANFSFPSAKIAENRTGLASILEDL

KHKLTIDVSSLHLFELTNAIVADVRIPLFVFELTENLDDLESLLKVSKDDTLSWQHTD

AIRGALSTWEISGVPQF"

CDS 615756..617102

/gene="pgi"

/locus_tag="JFBMFIFI_00567"

/EC_number="5.3.1.9"

/inference="ab initio prediction:Prodigal:002006"

/inference="similar to AA sequence:UniProtKB:P81181"

/codon_start=1

/transl_table=11

/product="Glucose-6-phosphate isomerase"

/db_xref="COG:COG0166"

/translation="MPHIQFDYSKIDKFLQPHEVAYLQQQVTTADEMLRKGTGQGNDF

LGWIDLPKNYDKDEFARIKAAAKKIQSDSEVLIVIGIGGSYLGAKAALDFLNHSFYNL

QDSKDRKAPQIFFAGNSISSSYLHDLIDIIGDRDFSVNIISKSGTTTEPAIAFRVFKE

LLVKKYGAEEAKKRIYATTDKAQGALKNEANAEGYESFIIPDDVGGRFSVLTPVGLLP

IAASGADIDSLMKGAADASEAYSNPKLEENEAYQYAAVRNALYRKGKVTELLINYEPS

LQYFSEWWKQLFGESEGKDQKGIYPSSANFSTDLHSLGQYIQEGRRNIFETVIKVDKA

RHDIKIPVTEADLDGLGYLQGKEIDFVNTKAFQGTLLAHTDGDVPNLLVTIPETDAYT

LGYLMYFFEIAVGISGYLNGVNPFDQPGVEAYKKNMFALLGKPGFEELAKELNERL"

CDS 617377..617907

/locus_tag="JFBMFIFI_00568"

/inference="ab initio prediction:Prodigal:002006"

/codon_start=1

/transl_table=11

/product="hypothetical protein"

/translation="MGNKVVLHQAKTSDLGLIEGMLYETASWLKSIGSKQWNGVLEGK

DNHNTPQAIERGEVFYCTINDEPVGMFILWSNQSEWDAQLWGAEDSSQYFYLHRLNLV

RKFSGQGIANEILAEIKAYSQKKGKSGVRLDCISSNAVLNKLYQSADFAFLKTIYDHD

AGEQVADFNLYEYDCK"

rRNA 618450..620001

/locus_tag="JFBMFIFI_00569"

/product="16S ribosomal RNA"

rRNA 620395..623312

/locus_tag="JFBMFIFI_00570"

/product="23S ribosomal RNA"

rRNA 623574..623683

/locus_tag="JFBMFIFI_00571"

/product="5S ribosomal RNA"

tRNA 623724..623799

/locus_tag="JFBMFIFI_00572"

/product="tRNA-Val"

/inference="COORDINATES:profile:Aragorn:001002"

/note="tRNA-Val(tac)"

tRNA 623800..623874

/locus_tag="JFBMFIFI_00573"

/product="tRNA-Lys"

/inference="COORDINATES:profile:Aragorn:001002"

/note="tRNA-Lys(ttt)"

tRNA 623916..623998

/locus_tag="JFBMFIFI_00574"

/product="tRNA-Leu"

/inference="COORDINATES:profile:Aragorn:001002"

/note="tRNA-Leu(tag)"

tRNA 624010..624085

/locus_tag="JFBMFIFI_00575"

/product="tRNA-Thr"

/inference="COORDINATES:profile:Aragorn:001002"

/note="tRNA-Thr(tgt)"

tRNA 624111..624182

/locus_tag="JFBMFIFI_00576"

/product="tRNA-Gly"

/inference="COORDINATES:profile:Aragorn:001002"

/note="tRNA-Gly(gcc)"

tRNA 624237..624325

/locus_tag="JFBMFIFI_00577"

/product="tRNA-Leu"

/inference="COORDINATES:profile:Aragorn:001002"

/note="tRNA-Leu(taa)"

tRNA 624328..624403

/locus_tag="JFBMFIFI_00578"

/product="tRNA-Arg"

/inference="COORDINATES:profile:Aragorn:001002"

/note="tRNA-Arg(acg)"

tRNA 624438..624511

/locus_tag="JFBMFIFI_00579"

/product="tRNA-Pro"

/inference="COORDINATES:profile:Aragorn:001002"

/note="tRNA-Pro(tgg)"

tRNA 624527..624600

/locus_tag="JFBMFIFI_00580"

/product="tRNA-Ala"

/inference="COORDINATES:profile:Aragorn:001002"

/note="tRNA-Ala(tgc)"

tRNA 624630..624705

/locus_tag="JFBMFIFI_00581"

/product="tRNA-Met"

/inference="COORDINATES:profile:Aragorn:001002"

/note="tRNA-Met(cat)"

tRNA 624730..624805

/locus_tag="JFBMFIFI_00582"

/product="tRNA-Met"

/inference="COORDINATES:profile:Aragorn:001002"

/note="tRNA-Met(cat)"

tRNA 624848..624937

/locus_tag="JFBMFIFI_00583"

/product="tRNA-Ser"

/inference="COORDINATES:profile:Aragorn:001002"

/note="tRNA-Ser(tga)"

tRNA 624987..625061

/locus_tag="JFBMFIFI_00584"

/product="tRNA-Met"

/inference="COORDINATES:profile:Aragorn:001002"

/note="tRNA-Met(cat)"

tRNA 625115..625190

/locus_tag="JFBMFIFI_00585"

/product="tRNA-Asp"

/inference="COORDINATES:profile:Aragorn:001002"

/note="tRNA-Asp(gtc)"

tRNA 625204..625278

/locus_tag="JFBMFIFI_00586"

/product="tRNA-Phe"

/inference="COORDINATES:profile:Aragorn:001002"

/note="tRNA-Phe(gaa)"

tRNA 625287..625357

/locus_tag="JFBMFIFI_00587"

/product="tRNA-Gly"

/inference="COORDINATES:profile:Aragorn:001002"

/note="tRNA-Gly(tcc)"

tRNA 625361..625436

/locus_tag="JFBMFIFI_00588"

/product="tRNA-Ile"

/inference="COORDINATES:profile:Aragorn:001002"

/note="tRNA-Ile(gat)"

tRNA 625457..625546

/locus_tag="JFBMFIFI_00589"

/product="tRNA-Ser"

/inference="COORDINATES:profile:Aragorn:001002"

/note="tRNA-Ser(gct)"

tRNA 625561..625632

/locus_tag="JFBMFIFI_00590"

/product="tRNA-Glu"

/inference="COORDINATES:profile:Aragorn:001002"

/note="tRNA-Glu(ttc)"

tRNA 625705..625794

/locus_tag="JFBMFIFI_00591"

/product="tRNA-Ser"

/inference="COORDINATES:profile:Aragorn:001002"

/note="tRNA-Ser(tga)"

tRNA 625844..625918

/locus_tag="JFBMFIFI_00592"

/product="tRNA-Met"

/inference="COORDINATES:profile:Aragorn:001002"

/note="tRNA-Met(cat)"

tRNA 625972..626047

/locus_tag="JFBMFIFI_00593"

/product="tRNA-Asp"

/inference="COORDINATES:profile:Aragorn:001002"

/note="tRNA-Asp(gtc)"

tRNA 626075..626145

/locus_tag="JFBMFIFI_00594"

/product="tRNA-Gly"

/inference="COORDINATES:profile:Aragorn:001002"

/note="tRNA-Gly(tcc)"

tRNA 626153..626228

/locus_tag="JFBMFIFI_00595"

/product="tRNA-Ile"

/inference="COORDINATES:profile:Aragorn:001002"

/note="tRNA-Ile(gat)"

tRNA 626246..626335

/locus_tag="JFBMFIFI_00596"

/product="tRNA-Ser"

/inference="COORDINATES:profile:Aragorn:001002"

/note="tRNA-Ser(gct)"

tRNA 626350..626421

/locus_tag="JFBMFIFI_00597"

/product="tRNA-Glu"

/inference="COORDINATES:profile:Aragorn:001002"

/note="tRNA-Glu(ttc)"

CDS complement(626522..627316)

/locus_tag="JFBMFIFI_00598"

/inference="ab initio prediction:Prodigal:002006"

/codon_start=1

/transl_table=11

/product="hypothetical protein"

/translation="MFQSIKNNLFYHPLFLIFINPISAILFGTLYALQFTKIHWLNFF

FFYLFILATQFIETILSNYVKKQQPIRLFPLILFELLAIILSIYFIFQLNYLVFLLMI

VYLFAIHFQFFPYNLTETLYGLILNSFFKGGIITYLSYFIQAKFISQNLYYWSISLIL

LALLVSFTKDVVLSNKTPEVNKQNHLIFIAILTLLYLSNLVIFFIFKTTYPYLWLQLL

TLPIALRLIIIMKPNRAKYSTSIKFKTLLLFQLTYILIASLTILLK"

CDS 627575..628765

/gene="metK"

/locus_tag="JFBMFIFI_00599"

/EC_number="2.5.1.6"

/inference="ab initio prediction:Prodigal:002006"

/inference="similar to AA sequence:UniProtKB:P66767"

/codon_start=1

/transl_table=11

/product="S-adenosylmethionine synthase"

/translation="MMERRLFTSESVSEGHPDKIADQISDGILDAILAKDPDARVACE

TTVTTGLVLVVGEISTSTYVDIQKVVRNTIRGIGYTRAKYGFDADTCAVMVAIDEQSS

DIAQGVDDSVEFKADEEDKLNQIGAGDQGLMFGFAIDETPELMPLPIALSHRLTKRLA

TVRKTGLVSYLRPDAKAQVTVEYDELGKPFRVDTIVISTQHHPDTEISELKKDILDLV

INEVIPSELLDAETKYFINPTGRFVIGGPQGDSGLTGRKIIVDTYGGYARHGGGAFSG

KDPTKVDRSASYAARYIAKNIVAAGFATKCEVQLAYAIGVAQPVSIAIDTFGTGTVSE

SELIKAVRANFDLRPAGIIKMLDLQRPIYQQTAAYGHFGRTDIDLTWEATDKVEALKA

SIGK"

CDS 628954..630438

/gene="bmr3"

/locus_tag="JFBMFIFI_00600"

/inference="ab initio prediction:Prodigal:002006"

/inference="similar to AA sequence:UniProtKB:P96712"

/codon_start=1

/transl_table=11

/product="Multidrug resistance protein 3"

/translation="METKKKQTNVLLVTIAIFVGTFMTAVEGTIVSTAMPTIVGSLEG

MAIMNWVFSIYLLTNAMMTPVYGKLSDMIGRKPIFIIGAIIFVIGSSLCGLAQTMDQL

ILFRAIQGIGAGAIMPVSFTIIADIYPYEKRAKVMGMNGAAWGIAGIFGPLLGGFIVD

QLSWHWIFYINVPVGIITIILIALFLHEDFSFEKKPIDFLGCFSLMAALLFLLYGFQI

VGDTGEFSASMAGVFALAVGMFALFIFAEKRAIDPIIPLSLFNNRTFVIQNIVAALVS

GFLIGIDVYIPMWMQGLLGMKAAMGGFAITPMSLTWIIGSFIAGRVILKHPVKSILSS

SLVIVGISGLMMVLAPMTTPFAFFLLVTAIIGIGMGITITTTTVTAQSVVPQDQIGVA

TSSNTLFRILGQTVMVSVYGIVLNSSIAKHIAAQTVDGVNEKMMNKLINPLTAVDLDP

KIVQPLREILYDGLHSVFILALVVVVLAFVINQFDKKNAQKISV"

CDS complement(630482..631045)

/locus_tag="JFBMFIFI_00601"

/inference="ab initio prediction:Prodigal:002006"

/codon_start=1

/transl_table=11

/product="hypothetical protein"

/translation="MLERAMHYSHTLLKKAITTGDSVIDATVGNGGDTVFLASLVGPF

GKVFGFDIQKEAIETTQQKLLLTGLTEQVELFQQGHETIDSVLPKNSQIAAAIFNLGY

LPTSDKSIITQADTTLLAIEHILPRLRKTGLVVIVVYYGHDGGLAEKDAVLNYCQTLP

QEEFNVLQYGFINQRNQPPFLLAIEKK"

CDS complement(631175..632131)

/locus_tag="JFBMFIFI_00602"

/inference="ab initio prediction:Prodigal:002006"

/codon_start=1

/transl_table=11

/product="hypothetical protein"

/translation="MNQFKFTNDPNKRYHTWNYALREQFGEKVFKVPLDGGFDCPNRD

GTVAHGGCTFCSVSGSGDFAGDRIDPLPIQFQKEVQMMHNKWPNVTKYIAYFQNFTNT

HAPVAELRHKFEQVVNEKGVVGIQIATRPDCLPDDVVDYLAELNQRYYLWVELGLQTI

HEKTSQLINRAHDYQTYLDGVEKLRKHNINVCTHLINGLPGETTEMMLESNKRMILDS

DIQGVKLHLLHLMRNTKMVRDYHEGRLEFMDMETYVNLICDQVEQIPPEIIIHRLTGD

APRDTIIGPLWSLKKWEVLNAIDRELKQRNTFQGSKNIRLHG"

CDS 632291..633505

/locus_tag="JFBMFIFI_00603"

/inference="ab initio prediction:Prodigal:002006"

/codon_start=1

/transl_table=11

/product="hypothetical protein"

/translation="MQMNKKERLPRDLWIVAIGMVLLYTGLSFIWPFNMLYMTENLGM

SDTAAGTALLVNSGIGIIGSVIGGIIFDRVSGYVSLAIGTGILVLTTGSLFLFHGHPA

FIYNIWAVSVAMGMVFAGLYTAAGLTHPSGGRTGFNTIYVAQNIGVAVGPFLAGFLAK

DGLGNVYTGSFAFALIYALFFFVYFRKIDWHSNKVTSETKHKQKGTVRGKATKIGLIS

FGLLLLTYLFCQLPHVQWQSNLSTYMTSQKGVTTAQYGNLWSINGTLILIGQVLIIPL

VARFKEKLSLQIYIGIGLFFCSFLFAMQAESYGGFLLGMILLTLGEMFAWPAIPTIAY

KLAPVGQAGLYQGLVNGTATAARMIAPIFGAVVVANLGGITSLFIAIFILLGLAIVSL

FLQQKTQKMMDI"

CDS complement(633562..634230)

/locus_tag="JFBMFIFI_00604"

/inference="ab initio prediction:Prodigal:002006"

/codon_start=1

/transl_table=11

/product="hypothetical protein"

/translation="MNHYQKKWVFASLLALLGFGFVASSVMLKATWVLTFDQNLTTLI

RDPITTGKSNFYLPITQFGSVKLIIFMGFIFCLFLIFRKKDWGNACWFVVNLAIGAGL

LNLTMKNIFRRPRPIIEHLVNENTFSFPSGHAMGSMVFYFSVAFILCQFIRSNKLQII

VCLIASFMIVSIGMSRIYAGVHFPSDILGGYLLAFSWMALALAYYEKWSQWFQSKALQ

LLAK"

CDS complement(634371..635330)

/locus_tag="JFBMFIFI_00605"

/inference="ab initio prediction:Prodigal:002006"

/codon_start=1

/transl_table=11

/product="hypothetical protein"

/translation="MKTKLIKRMVILSLSMLAIGAVGSIVTYPMAHKKVTLDLNKTVD

ATSVKQVSVKGTSMDITLADSPDNKIHVSVNGETQSADKFDIKTKQNGDTLEVTFDEP

KLIEMNFVLFFNYNNRSATVSLPKSVKQATVKTNFGEIYTNNFVGESLELKTDAGDIN

LSGLKLTDLNAKTNAGRISLSQSAITNTDLKASAGALYVNNLVSSKTKLNTSAGNIDL

NQVTGDLVATSNAGEIDLTNKTIDQNIDLQTNFGSIDVQSESKPTNLTVIAATDLGEM

DIFDQNNREQTFGAGKYKMNLKTNAGDIEVNHSAYENDDEDFD"

CDS complement(635320..635964)

/locus_tag="JFBMFIFI_00606"

/inference="ab initio prediction:Prodigal:002006"

/codon_start=1

/transl_table=11

/product="hypothetical protein"

/translation="MNKEHFMIELKLSLRDLDESERQDVMRDYLEHFENGLAEGKTEE

QIAKELGSPSQIAKEILTTLGVKPQVKGPEYSQGDWVSFENEKNPYQEPSYQPRRHNP

NSPLMTVFKFIGLGFFNLVFVLGPAVALLGCLFAGWITSIAFTLTPLGGIFMIVTAFS

TASLFQFFFTILLCGVGLLLLTLLYPITILIFKLVKRYVLWNVRTIFGGPYYEN"

CDS complement(635969..636286)

/locus_tag="JFBMFIFI_00607"

/inference="ab initio prediction:Prodigal:002006"

/codon_start=1

/transl_table=11

/product="hypothetical protein"

/translation="MNTQFKKGVLELCVLVILEKKDCYGYELIETISKHIEISEGTIY

PLLRKLTKEGLCTTYLQESNEGPSRKYYQVTEDGVAYLGKQLIEWKEFIKSVDTIIEI

GDE"

CDS 636539..636799

/locus_tag="JFBMFIFI_00608"

/inference="ab initio prediction:Prodigal:002006"

/codon_start=1

/transl_table=11

/product="hypothetical protein"

/translation="MLIDDSILKNEYGCGQIKNVDFPKITIETEEGQILELKITKKQK

RDPIFWKSIKDIWQAGIWIPFNKKLSNLIESDWLVETNPAFA"

CDS 637172..639595

/locus_tag="JFBMFIFI_00609"

/EC_number="3.2.1.-"

/inference="ab initio prediction:Prodigal:002006"

/inference="similar to AA sequence:UniProtKB:P37710"

/codon_start=1

/transl_table=11

/product="Autolysin"

/db_xref="COG:COG1388"

/translation="MEKTRKERITAKKATQRLEQFKESNVVVKKGLAFVSTTLIVGSL

SLPTFSAVASANELANSSQKKIEENNETPIEGTKEAIEPASTVDSSVADSSEAASSES

ESVDSSEVAPEAEEPAKEETVPVIETEEPLSKEEVKEQDISEVQAPVANYAVAEQMLM

SRSMAPQAFINQIASSAQTIANSNDLYASVMIAQAILESGWGNSALASSPNFNLFGIK

GNYNGQSVAMKTLEDDGHGNYYEIIDYFRKYPSYHQSLEDYASVLRNGPSWNHNYYSG

AWKSNTSSYRDATAYLQGRYATDTSYASKLNSVIAANNLTQYDTGGGGNVTPDPNPGG

ETGNGNGGSTGTETTYTVKSGDSLWGIASKHGVSVANLKSWNNLKSDMIFVGQKLIVK

GGTTTPTPKPDPTPTPDPTPNPGTSSTYTVKAGDSLWSIANKHGVSVANLKSWNNLKS

DIILVGQKLTVKGGTTTPAPNPGTGTGSNNGNGGGTTTGSTYTVKSGDSLWAIANKHG

VSVANLKAWNNLSSDTIHIGQTLTIKGGTTAPAPNPGTGSNNGNGGGTTTGSTYTVKS

GDSLWSIANSNGVSVANLKAWNNLSSDTILVGQKLTIKGGTTTPAPNPGTGSNNGNGG

GTTTGSTYTVKSGDSLWAIANKNNVSVANLKAWNNLSSDTIHIGQKLTIKGGTATPAP

NPGTGSTTGNTGGTTAGSTYTVKSGDSLWMIANKNNVSVANLKSWNKLSSDVIFVGQK

LVFKAGGSTTNTNTNTNPTSNKTYKVISGDSLWVIANKNGTTVANLKAWNNLKSDVIL

VGQTLIVNK"

CDS 639975..641438

/gene="nagE"

/locus_tag="JFBMFIFI_00610"

/inference="ab initio prediction:Prodigal:002006"

/inference="similar to AA sequence:UniProtKB:P09323"

/codon_start=1

/transl_table=11

/product="PTS system N-acetylglucosamine-specific EIICBA

component"

/db_xref="COG:COG1263"

/translation="MMKYFQRIGRSLMLPVAVLPAAAVLMGIGYWIDPSGWGGDSAMA

AFLIKAGSSIIDNMAVLFAVGIALGMSKDKDGSAALSGLVAFLVVTTLLSTDSVAMLQ

GIKPEAVNPAFAKIGNQFIGILSGLIAAEMYNRFSHVQLPQALAFFSGKRLVPIMSAV

AMIIASAILFFVWPVIFTALVSFGTAISKLGWVGAGLYGFFNRLLIPTGLHHALNSVF

WFDVAGINDIGNFWAGTGTKGITGMYQAGFFPVMMFGLPAGAFAMYQAARPEKKKVVA

SLMIAAGFASFFTGVTEPLEFSFMFVAPALYFVHAVLTGLSLAVAAFFHWTAGFGFSA

GLVDFVLSFRLPLANQPYMLILQGLVFAALYYFLFKFLIAKFNLMTPGREEGEGEEDE

DMPTEKTVDVPEGGNKFTPMATKIYAGLGGDENVTAVDNCTTRLRIQVKDMDKVDQKA

IKATGVPGINVVGKNDIQVIVGTEVQFVADEINKIKK"

CDS 641902..642684

/gene="glpF_1"

/locus_tag="JFBMFIFI_00611"

/inference="ab initio prediction:Prodigal:002006"

/inference="similar to AA sequence:UniProtKB:P18156"

/codon_start=1

/transl_table=11

/product="Glycerol uptake facilitator protein"

/db_xref="COG:COG0580"

/translation="MESFYAELIGTMVLIVFGSGVVAGNLLIKSKAYNMGWVGITIAW

ALGVTLGVYVVAGGSAAHLNPAVTLAFAAIGAFPWSEVPQYIAGQLIGAFLGAIIVYL

VYVKHWKVTEDKGAKLGVFATGPAIRSPFANLMTEAIGTFILVFGLLSIGANEFAVGL

NPIIVGLLILGIGLALGGPTGYAINPARDLMPRIAHAILPIAGKGDSDWSYAWIPVVG

PIIGALAGGFFYKVVYFESSIIGLIIFIIVLASLVIGTKEKN"

CDS 642701..644197

/gene="glpK_1"

/locus_tag="JFBMFIFI_00612"

/EC_number="2.7.1.30"

/inference="ab initio prediction:Prodigal:002006"

/inference="similar to AA sequence:UniProtKB:Q5HGD2"

/codon_start=1

/transl_table=11

/product="Glycerol kinase"

/db_xref="COG:COG0554"

/translation="MEKKYVLAIDQGTTSTRAILFNKKGEIVHTSQREFTQYFPEPGW

VEHDANEIWVTTLAVIAGVLIESDTKPTEIHSIGITNQRETTVVWDKNTGLPVYHALV

WQSRQTSDIANQIIADGYEELVKSKTGLRVDAYFSGTKVRWILDNVEGARARAEKGDL

LFGTIDTWLIWKLTGNKVHVTDYTNASRTMMFNIHQLKWDDELLDILTIPKKMLPEVR

SSSEVYGETVEYHMFGQRVPIAGVAGDQQAALFGQNCFEEGMIKNTYGTGCFILMNTG

EKAVNSENGLITTIAYGLDGKVNYALEGSVFVAGSAVQWLRDGLKMFTDAGETEEYAL

RAGSSDGVYMVPAFVGLGAPHWDTDARGAIFGLTRGTSKDVFIRATLESIAYQSKDVM

ETMIQDSGIPITEMRVDGGAAKNNFLMQFQSDILNIEIKRPKVSETTALGAAYLAGLA

TGFWESIEDIKANWLLDKSYEPKMEDAERDVLYHGWEKAVTATRSFKN"

CDS 644240..645892

/gene="glpD"

/locus_tag="JFBMFIFI_00613"

/EC_number="1.1.5.3"

/inference="ab initio prediction:Prodigal:002006"

/inference="similar to AA sequence:UniProtKB:P18158"

/codon_start=1

/transl_table=11

/product="Aerobic glycerol-3-phosphate dehydrogenase"

/db_xref="COG:COG0578"

/translation="MMKFSKQTRQDNIEKMQKAPLDLLVIGGGITGAGITLDAQDRGL

QVGVLEMRDFASGTSSRSTKLVHGGLRYLKQFEIKVVQEVGQERAIVYENAPHVTTPL

WMVLPFYKGGTFGSFTTAIGLEMYDHLAKVKKNERRYMLKPERAVEKEPYLKKNGLKG

AGVYVEYRTDDARLTIEVLKKAAEKGAYIANYVKVDRFIYDVEGRVKGVYFHDELTGE

TGQIYAKKIINASGPWVDDLRELDDSKKGKTMHLTKGVHLVIDESKFPISNAIYFDTP

FDDKRMMFAIPREGKTYIGTTDTNYKGDPKEPGVTLADVEYILAAANQMFDITPIQVA

DVESSWSGVRPLIHEEGKDPSEISRKDEIFHSESGLFTIAGGKLTGYRKMSEKVVDQV

MLELSIEEGRAYRKSATKNLILSGGNVGGGDRFNQFVAEKVEVGTHLGLDSLTAEALV

HRYGSNVDEVYSYLKASQGSALEPVDYLMLHYGLEHEMVIKPIDYLLRRSSQLLFDSE

HAKAVKELIVDEMAEYYDWDEAVRAEYLDEVEQQLTASTTFN"

CDS 646168..647571

/gene="dgt"

/locus_tag="JFBMFIFI_00614"

/EC_number="3.1.5.1"

/inference="ab initio prediction:Prodigal:002006"

/inference="similar to AA sequence:UniProtKB:P15723"

/codon_start=1

/transl_table=11

/product="Deoxyguanosinetriphosphate triphosphohydrolase"

/db_xref="COG:COG0232"

/translation="MRMNWDQLLNDERRRKTTVNYAKSRDVRSAFENDYQRIVMSASF

RRLQDKAQVFPLEKSDFIRTRLTHSLEVSTIAKSMGGMVAYHLLENQLDDAFTKEHAE

KIPEVLACAGLLHDMGNPPFGHFGEESIREWFQENSHVLVYGDRTLAEVLDKSMLQDF

FNFEGNAQVLRVISKLHYQFDEYGMNLTYATLNSIMKYPVSSTEINKKQIRSKKMGYF

HADQQLFDEITESTGALNRRHPLTYLLEVADDIAYLNADLEDGIKKDIVSIAQILNEF
EAVENPNRVTATVHQELIKRSERYKNHDDSFTGQQWIASSLRGQLINRSLEVFYENYE
AIMAGTFNESLLDASEADQLVTTLQGLSRKYLYVDKGVVETELAGSEIIGSLLDMFIP
AAIYYDSELSDRATQKSKRLMSLISDNYIGCYHKNAEGQTDEMKLYLRLLLITDFICG
MTDSYAKDLYQKLHGLI"

CDS complement(647608..648099)

/locus_tag="JFBMFIFI_00615"

/inference="ab initio prediction:Prodigal:002006"

/codon_start=1

/transl_table=11

/product="hypothetical protein"

/translation="MKLKGKKLISTYQEFLRYFFWGLIATGINLGLYELLILTVPIHY
LVINGIVWVVTVLFGYYTNRRFVFKTPPKQLKETIYEASHFVGFRLVSGFFDTFTMWL
LLTIFGINALWAKLAANLVASLINYVTSKQIVFKRGKRTTEEIQRDLEQENNLPTPDL
TNQ"

CDS 648307..649566

/gene="dctA"

/locus_tag="JFBMFIFI_00616"

/inference="ab initio prediction:Prodigal:002006"

/inference="similar to AA sequence:UniProtKB:P96603"

/codon_start=1

/transl_table=11

/product="C4-dicarboxylate transport protein"

/db_xref="COG:COG1301"

/translation="MGKVWQQYKASVLLLGGLIIGGIAGVVFGEKATVVQPLGDLFLN
LMFVMLVPLVFFSISSAIANMNGMKRLGKIMGSTFFIFFATALVAAVVGYIGVVLYNP
IKGIDVDSVKALMGTPEASSTADMGLLERIVNTLTVPDFHLLLSKSNMLQLIVFSILV
GVSTSMADEAGKPVAKLLASGNAVMMKMVSVIMYYAPIGLGCYFASVIGTLGSKILGG
YARSMILYVVIAVIYYLLFFSMYAFIAGGKAGLKIYWRNIAPPSITAIATCSSAASIP
VNLEYAKKMGITPDIAETVIPLGANTHKDGSVIGGVIKIAFLFGIFGKEMTTPSAMVT
VILGAFLVGAVMGAIPGGGMIAEMLIVNIFGFPLEAVPVIVIISTIIDMPATLINSSG
NLASGMLVARIVEGKNWLKDFLTDTPA"

CDS complement(649632..651017)

/gene="fumC"

/locus_tag="JFBMFIFI_00617"

/EC_number="4.2.1.2"

/inference="ab initio prediction:Prodigal:002006"

/inference="similar to AA sequence:UniProtKB:P64173"

/codon_start=1

/transl_table=11

/product="Fumarate hydratase class II"

/translation="MPDTHQQYRIEKDSLGEIKVANDKFWGAQTERSLANFKIGTEKI

PASVILAFAQLKRATAIVNEQNKTLDPQIKEAIVAATDAILTGEYMEHFPLVTWQTGS

GTQSNMNMNEVIAHLANQQSEGTNLTIHPNDHVNMSQSSNDTFPTAMNIAAYDAVISE

LIPECKKMIEVLEEKIEAYQNLVKIGRTHLQDATPLTFGQEISGWKTMIERGLHFIET

SSQSILELAIGGTAVGTGLNAPAGFGEAVANELSLAMGHPFKSAPNKFYALTSHSDLS

YTHGAIRSLASDLMKIANDVRWLASGPRSGIGEITIPANEPGSSIMPGKINPTQSEAL

TMVVAQVMGNDVTINVAASQGNFELNVYKPVIILNFLQSVTLLSDAMRSFRLNCLVGL

EANEAAMGTLVERSLMLVTALSPHIGYEKSATIAKKAIAENLSLRESALALNFVTEAE

FDLWVDATKMI"

CDS 651549..653963

/gene="leuS"

/locus_tag="JFBMFIFI_00618"

/EC_number="6.1.1.4"

/inference="ab initio prediction:Prodigal:002006"

/inference="similar to AA sequence:UniProtKB:P67513"

/codon_start=1

/transl_table=11

/product="Leucine--tRNA ligase"

/translation="MSYNHKTIEKKWQKYWAAHNSFNTTEDPKKENFYALDMFPYPSG

QGLHVGHPEGYTATDILARMKRSQGYNVLHPMGWDAFGLPAEQYALDTGNDPAEFTEH

NIGTFRRQINSLGFSYDWNREINTTDPHYYKWTQWIFTKLYEKGLAYEAEVAVNWCPA

LGTVLANEEVIDGKSERGGFPVYRKPMKQWMLKITAYAERLLDDLDTVDWPESIKDMQ

RNWIGKSVGANVTFKIKDRSEDFTVFTTRPDTLFGATYAVLAPELDLVKEITTPEQKE

AVEAYITEVSLKTDLDRTDLAKSKTGVFTGAYAVNPVNGQEIPIWIADYVLATYGTGA

IMAVPAHDERDYEFAKTFNLTIIPVLEGGNVAEEPYTGDGVHVNSGFLDGLDKETAIE

VMNKWLEENGHGKKEITYRLRDWLFSRQRYWGEPIPVIHWEDGTTTALPESELPLLLP

KTDEIKPSGTGESPLANISEWVNVVDPKTGMKGRRETNTMPQWAGSSWYFLRFIDPHN

KNEIASKEKLEKWLPVDIYIGGAEHAVLHLLYARFWHKFLYDIGVVPTKEPFQKLYNQ

GMILGENNEKMSKSKGNVVNPDDVVEKFGADTLRMYEMFMGPLDSAIAWSENGLEGSR

KFLDRVWRLVVDEEGKLRDRITTINNGKLDRVYHQTVKKVTEDYQSLHFNTAISQMMV

FVNEAYKTDALPIEYVAGLVQLLAPIAPHVSEELWEKLGNEGSLTYVSWPTYDESFLV

ESEVEVIFQVNGKVKAKAMVPKDIAKDDMETLATENEAIKEALVGKTVRKVIVVPGKL

VNIVAN"

CDS complement(654010..654549)

/locus_tag="JFBMFIFI_00619"

/inference="ab initio prediction:Prodigal:002006"

/codon_start=1

/transl_table=11

/product="hypothetical protein"

/translation="MSNNVIIMNFDSESKSYQAFSEIKKMHVEKQIKGEQMAILEHKP

NHQLEPKEFIDFNGPDKNMKGGLIGMLVGVLAGPLGVLLGWFTGSLIGSVQDAKEIKG

AMSVFEETIDIIHEGDTGLILIAEEDDNRFINELVFQKLEGRITRLAVEDVEKEIELA

KETQENVEDHAKKSWSDRK"

CDS complement(654657..655073)

/locus_tag="JFBMFIFI_00620"

/inference="ab initio prediction:Prodigal:002006"

/codon_start=1

/transl_table=11

/product="hypothetical protein"

/translation="MKDKNKWLHYLLFFLIMGLGVFLICYVNFRIGIFSVQNKESLWM

FFAKYITTIFLGVLIGIEFLVSQINKSGKWRINLAKLTFLGIPSILFTFFNVQIANLN

PGLNFLSTNQTFLIFWQIVLGYTLVTSFYKNDSIRF"

CDS 655443..657788

/gene="ctpE_1"

/locus_tag="JFBMFIFI_00621"

/EC_number="7.2.2.10"

/inference="ab initio prediction:Prodigal:002006"

/inference="similar to AA sequence:UniProtKB:A0R3Y2"

/codon_start=1

/transl_table=11

/product="Calcium-transporting ATPase CtpE"

/db_xref="COG:COG0474"

/translation="MEAKVQQVGLTTEEVASRLARGENNKPLEPMTKSFKSIFYDNIC

TLFNFINIVIAGFIIYTGSYKNLIFLGVIVANTCIGIFQEIRAKRNVDQLSLLNQAKI

TVIRNGSQVQIQQHELVQDDLMIVRRGEQLCVDGIVIETEGLELDESQLTGEADPVNK

WTDSEVLSGSYVISGNALVKATKVGEESYSSKIALEAKKGKEIYSELVKTLKRIIKML

TFAIIPIGGLLFFSNYMGNMITIEQAILGTAAAIIGMIPEGLILITSIALAVGVINLT

KRKVLVKTMGSIETLARVDLLCLDKTGTITDGNLKVINLLPYQKTTKEELEKIISNLV

EGLQDDNSTSLALQKAVEKNFSWNLTRAVPFSSIRKWSGATFEGEGTYIMGAPEYLFA

ELPVEIQLKINEAIESGNRVLAFAASQEPLASNHLPDDLNLIGLVFMEDTIRPEAPAT

LAYFEQQGVEVRIISGDNPKTVSHIAKRAGVANGDRFIDMSKIEEDSDLTEIAEAHTV

FGRVSPTQKRELVKAMKKAGHTVGMTGDGVNDILALKEADCSIAMAEGSDAAKGVSDF

VLLDSNFDSMVGVVMEGRRVVNNIQRVASLYLTKTVYSAVLAVIFIFLRTAYPFEPIQ

LSPINALTVGIPSFFLALRPNYSQIKGGFLKNVLKPAFSAGLTVVIYILTILVVGKIL

NLSFSTTSTLSVLLTGAVCFTALIHISRPLDRKIMAMIASLIALFLIIFIFFGSVFSL

VSLFNIQLLLFAVPLLATVVPLYHLMRRIVTRIADGKPEAI"

CDS 657941..659371

/gene="ribZ"

/locus_tag="JFBMFIFI_00622"

/inference="ab initio prediction:Prodigal:002006"

/inference="similar to AA sequence:UniProtKB:Q180E3"

/codon_start=1

/transl_table=11

/product="Riboflavin transporter RibZ"

/translation="MGNVIQSYQMDPKVQKNRWLILVAVGLFTFMSTLDGSIVNIAIP

VISERLGVPMNQSEWIVSVYLMTVCILILLFGKIGDLIGKIKVFRWGTFIFVLGSLLC

GIEINLGFLLVARIIQAIGASMTMSANYGIITEIFPIKERGKALGLMGSFVSLGSIAG

PGIGGILLAHFDWSSIFLINVPVGLITIFMGMKILPKDLFLKEEKIDYAGFLAFALFI

GTLFIGIFIGQEIGFLHGIVIGMFSIAVISFIAFIRIENKVEKPLVHFGLFKNSLFSI

SLLCAFLIFVTNFFFNVMMPFYLQGTLSLPPSQAGLLLMVFPMVMVIAAPISGSLSDK

IGAEILTFIGLAVLTLVQLAYIFIGVDTNLIWFAVLTGLMGLGSALFQSPNNAIVMSS

VPKDQLGIAGSMNSLARNLGMVFGISFATTILYWAMSSKAGFKVTTYLADQPQLFVYG

MHVTFTVSFAICLFATCLTGYRLLKK"

CDS 659400..660272

/gene="yddE_1"

/locus_tag="JFBMFIFI_00623"

/EC_number="5.1.-.-"

/inference="ab initio prediction:Prodigal:002006"

/inference="similar to AA sequence:UniProtKB:P37757"

/codon_start=1

/transl_table=11

/product="putative isomerase YddE"

/db_xref="COG:COG0384"

/translation="MKKIKVGHYQAFSQVPNEGNPAGVVFGAEELSVKEMQKIAQTVG

FNETVFVLPSEKADYRLRYFTPGHEIDLCGHGTMAATYGICQNYGVQEEFHFETNVGQ

IKVEYHIATGKIKMEQADAKFKPFTGNVKALCGALGIEVADLDDRYSIEYGNTGIWTL

LLPIKKLDSFKRMQPQNQLFSKILTEIPTASIHPFSLEAYGERVNLHGRHFSSSFSGT

VEDPVTGTASGVMGAYYLKEIAKVPIVELAIEQGHEMSRPGEVSVMAKQLGNKIEVAI

SGVACFVEELEVEY"

CDS complement(660323..661216)

/locus_tag="JFBMFIFI_00624"

/inference="ab initio prediction:Prodigal:002006"

/codon_start=1

/transl_table=11

/product="hypothetical protein"

/translation="MTTKIGEAIKKKRLVGGLTQAELAEGICTQATISNLESKGSLPT

TSILLKITDKLNIEFNEIYEYSLGNQNGYTQIFKEVRRLCSKRSHKQAYELLTTEINF

SKLDTIYEIKQFYYFMGISGLIAHENISDAIYYFNQAIDSESGNTLDFLDVSATNGIG

IAYDMANENDKALTYYEKSLEQLDELLTIIDTINNSPEIAKIYYNTAMFYSKIGEYNK

AVNLCSLGIQLLQNEDLTYYLEYLLYEKGFNLLKLNKKEEAAKFYLYALVMADIHNNN

LVLEVIKSNLKEYDIQEYKYR"

CDS complement(661540..662451)

/locus_tag="JFBMFIFI_00625"

/inference="ab initio prediction:Prodigal:002006"

/codon_start=1

/transl_table=11

/product="hypothetical protein"

/translation="MTGQIGAKIKSKRLAKKLTQADLAADICTQATISNLENKDRLPT

ISVLLKITDRLDMEFSEVYAYTSGKSANHTQIYQKIRDLCRIQKHQEAYDLITTTIQL

DKLETNSDIKRYYYYLGITGVIGYGKISDGIYYFNQALSKKENEQIDYLDISALNGLA

IAYDIKNEDDKALNYYQQSLTKLEALPLLIESIKDKIEIAKVYYNTAKFYSKIEDYSK

AIELCTSGIKLLQNENLTYYLDYLFYEKGFNLLKIKKIDEAEKYYLYAFILADIHRNK

YLVSVIINDIKLYSIQNVNVSRTLQLN"

CDS 662848..664506

/gene="murJ_1"

/locus_tag="JFBMFIFI_00626"

/inference="ab initio prediction:Prodigal:002006"

/inference="similar to AA sequence:UniProtKB:O34674"

/codon_start=1

/transl_table=11

/product="Lipid II flippase MurJ"

/db_xref="COG:COG2244"

/translation="MMPNSPINLEEAAVEEQSAQEKMIRGSAWMTGGSIFSRVLGAIY

IIPWMAWMGSAATGKAANGLYALGYTPYALFLTIATAGVPSAIAKQVSYYNALGEYNT

GRKLFKKGMGLMVVTGVVCFLIMYLLAPVIGVGEPDQIQVIRSLSWALLIIPGMSLIR

GFFQGYQEMAPSAISQLIEQIARIIFMLASVYLIMQVFNGQMVTAVTASTFAAFIGAL

FSLLALGYYWLKQKPRMDALAATSLDKIDVSTNEILVEMIKEAIPFVIIGSGITFFQL

IDQYTFERIVIATTNTTAVLAREMFAVFAFNANKLIMITISIAVSMAVTAIPLITEAF

TKNLIKEVRKQVSTNIQLFCFVMFPAAIGMAAVAEPLYTVFYQHSDLGTTILQISCYM

SIILGLYTVLSAILQSMNQNKYAIFCLVVGVLVKAAVQYPLVKMFQAEGALYATIFGF

SVSSLMLLWTLRRLTRFNVRFIFKRILLITLMTLLMAIVTILVRELCYLFVSPMSREW

ALVVTIVSAIAGGFSYLYLSLKTRLADRLLGAKVAGIRRRLRIK"

CDS 665093..666235

/gene="metC"

/locus_tag="JFBMFIFI_00627"

/EC_number="4.4.1.13"

/inference="ab initio prediction:Prodigal:002006"

/inference="similar to AA sequence:UniProtKB:A2RM21"

/codon_start=1

/transl_table=11

/product="Cystathionine beta-lyase"

/db_xref="COG:COG0626"

/translation="MKIESLIVQGIPAKNNENGAIVPPIYLASTYVQPSIEEHQTYAY

GRGGNPTRNALEELISKIEGVDYGFAFASGMAATTTAFNLFKSGDKILVNSNVYGGTY

RYLDTVFQQQELRYELVDDFNLLSEEDIDTTVKALFIETPSNPLLQVTDIQRLAAIAH

QKGILIIVDNTFATPYLQKPFELGADIVLYSATKYLSGHADVIAGLLTVADSGLAARI

KHLQNTLGNILSPFDSYSLIRGIKTLSVRMDRQEANTVKILAFLKEHEAIARLNYPGS

ASPEEAAIQKKQTRGNGSVFSIELTEEYNPLKFVEELDLFDLAVSLGGVESLVCHPST

MTHESYAIELQEKIGISAGLLRFAIGIEHVDDLINDLTQALDLAKK"

CDS 666268..667080

/locus_tag="JFBMFIFI_00628"

/inference="ab initio prediction:Prodigal:002006"

/inference="similar to AA sequence:UniProtKB:P45024"

/codon_start=1

/transl_table=11

/product="putative amino-acid ABC transporter-binding

protein"

/db_xref="COG:COG0834"

/translation="MKKNKIVWLISFLLVGIFVLIGCGQSEKAADNQENVDSLAAIKK

AGTLKVGIEGTFPPFNFHDEKDQLKGFEVELAEAVAKKIGVKAEFVETKWDSLIAGLD

VNKYDVVFNNVSVTEERQKKYDFTNPYAYSHATLAVKEDSETKTLADLKGKKSAQTVS

SNYAQDAEKLGAEVVPTDGFAQSIELVLGNRADGTVNDDVTFFYYQKEKPENKIRLID

EAIATSEIAAITNKNNPELTKAIDGALAELKKEGITSKLSITYFGKDITENK"

CDS 667124..667789

/gene="tcyB"

/locus_tag="JFBMFIFI_00629"

/inference="ab initio prediction:Prodigal:002006"

/inference="similar to AA sequence:UniProtKB:P42200"

/codon_start=1

/transl_table=11

/product="L-cystine transport system permease protein
TcyB"
/db_xref="COG:COG0765"
/translation="MSETIELLVTSFWPILKAGLLITIPLTLIAFTLGLLIAVVTALV
RLSKLRVLKFFFAGYVWIFRGTPLLVQLFIVFFGLPKAGIEFSAWAAAIITFSLNVGA
YSSESIRSAILAIPRGQWEAAYSIGMSKGMVLWRIIAPQALRISVPPLSNTFIGLVKD
TSLASSITIVEMFMVGQQIASRTYEPLLLYSLVAAIYLVFCTFLTVLQGKLEIATSKH
IRK"

CDS 667800..668543

/gene="tcyC"
/locus_tag="JFBMFIFI_00630"
/EC_number="3.6.3.-"
/inference="ab initio prediction:Prodigal:002006"
/inference="similar to AA sequence:UniProtKB:P39456"
/codon_start=1
/transl_table=11
/product="L-cystine import ATP-binding protein TcyC"
/db_xref="COG:COG1126"
/translation="MLQVKQISKKFNEQLALKEINLSFETGSTTVIVGPSGSGKSTLL
RCLNLLEIPENGTLSLGASYFDFTKELKQKDVLALRRKTGMVFQGFHLFPHLTILKNV
MEGPVYVRKESKVTAENKARALLKKVGLAEKVDRYPDELSGGQQQRAAIARALAMNPE
FLLFDEPTSALDPELEAEVLSVLCGLVEEGNSLIIVTHNLAFAREVADRILFLENGEI
LFEGTPELFFQGSGSKRIEGFISAMLPNK"

CDS 668568..670853

/gene="metE_1"
/locus_tag="JFBMFIFI_00631"
/EC_number="2.1.1.14"
/inference="ab initio prediction:Prodigal:002006"
/inference="similar to AA sequence:UniProtKB:P80877"
/codon_start=1
/transl_table=11
/product="5-methyltetrahydropteroyltriglutamate--
homocysteine methyltransferase"

/db_xref="COG:COG0620"

/translation="MTKVKSSNLGYPRLGEKREWKRALEKFWNGQLTEAELVATTKKI

RLAALEKQKKQGIDLIPVADFSLYDHILDTSIAFNVIPQRFQKEAVNESKLTTYFKLA

RGSKAGVASEMTKWFNTNYHYIVPELEHAQPELKENLALTYYLEAKEEIGIEGKPVIV

GPITYLKLGKGYQAKEFSLILKKFIPLYQQLLKELEAAGVEWVQIDEPYLATGEAKAD

IELYRKTYQDLKEAAPNLKIELQTYFESVDAYEAVVTLPVEAIGLDFIHDHGETLAAI

EKNGFPEDKILAAGIIDGRNVWASNLAEKLELIKQLLKQVTPERLWLQPSNSLLHVPI

TKKSEKELDPVLLGGLAFADEKLIELTLLTKAVNNGRESISKELSENQRNNEALNHSS

QRNNPAVKEAILNLEKLKVERKAPFSERIKVQKAWLNLPILPTTTIGSFPQSPEVRKK

RAEWLRGGLSKTDYEGYINQEIARWIKIQEELDLDVLVHGEFERTDMVEYFGQKLAGF

QATKFGWVQSYGSRAVRPPLIYGDVAFIEPITVKESVYAQSLTKRPVKGMLTAPVTII

NWSFVRDDVPKSTVGNQIGLALRKEVEALEAAGIKIIQVDEPALREGLPLKRERWKAY

LDAAVYSFKLTTTSVANDTQIHTHMCYAEFDDVIETIDALDADVISIEASRSHGEIIS

TFEKNTYDKEIGLGVYDIHSPRVPSVEEILVNIRRPLKVIPIDQFWINPDCGLKTRKE

PETIEALKKMVEATKIIRASY"

CDS 670893..672767

/gene="yitJ"

/locus_tag="JFBMFIFI_00632"

/inference="ab initio prediction:Prodigal:002006"

/inference="similar to AA sequence:UniProtKB:O06745"

/codon_start=1

/transl_table=11

/product="Bifunctional homocysteine

S-methyltransferase/5,10-methylenetetrahydrofolate

reductase"

/db_xref="COG:COG0646"

/translation="MGLKEVLGTKLLIADGAMGTLLYQYGIDRSHEELNCSHPEAILK

IHQDYLAAGADIIQTNTYGANYIKLARYGLEDQVQKINRAGITLAKEARGDKEAFILG

TIGGIYGAVDTKIHDPGMEEISRSFKEQLYCLLLGGVDGILLETYYDLNELKTVLAIA

REATDIPIIANVSIHEVGILANGQSLATALEELADLGANVVGTNCLLGPHFMAQSFES

VPLIPGKVLAAYPNASFPTMENGRIVYQKETDYFKLFGELFRKEGVGLIGGCCGTTPE

HIAAYREGLTTLDLVTQKKVKALTTYRFQAKQETGKERLLDKVQREYTVLVELDPPRT

FNTTAFFKGAARLKEVGVDAITLSDNSLASARISNMALSAMLKYEYDIQPLVHLTTRD

HNLIGLNSQIMGFHKLGIQDVLAVTGDPAKVGDFPGASSVFDVSSFELIKLIKNFNQG

IAYTGKNLREKTNFQVAAAFNPNIPRLERAGRLLKKKQEAGADYFITQPIFDSEKVKI

LKEVMKTAGVTIPIFIGVIPLVSSRNAEFLHNEVPGILLTDETRERMRMAEEEGRGLE

VGMTIAKELIDEICQEFNGIYLMTPFMNYEISAELCEYIQAKKQQEKSICSCGQVHSQ

"

CDS 672953..673684

/gene="rluB_1"

/locus_tag="JFBMFIFI_00633"

/EC_number="5.4.99.22"

/inference="ab initio prediction:Prodigal:002006"

/inference="similar to AA sequence:UniProtKB:P35159"

/codon_start=1

/transl_table=11

/product="Ribosomal large subunit pseudouridine synthase

B"

/db_xref="COG:COG1187"

/translation="MRLDKFLAHTGFGTRKSVKSIIKAKQVRINDKIAKDGKEQVKPE

VDQVFVLDEKVHYQEFIYLMLHKPQGVVSATIDNVHPTVISLLGPNEQLFDPFPVGRL

DKDTEGLLLVTNDGTLAHNLLSPKKHVAKCYLAKIQGIVTKEDQVEFEKGLTLNDGFL

CQPAELDILEVDESQQISKIQVTIHEGKFHQVKRMFEAVDKKVIYLKRLSMGPIRLDE

RLDLGEYRALTEAEMDLLQGIQKEN"

CDS complement(673730..674566)

/gene="nnrD"

/locus_tag="JFBMFIFI_00634"

/EC_number="4.2.1.136"

/inference="ab initio prediction:Prodigal:002006"

/inference="similar to AA sequence:UniProtKB:Q833Y3"

/codon_start=1

/transl_table=11

/product="ADP-dependent (S)-NAD(P)H-hydrate dehydratase"

/db_xref="COG:COG0063"

/translation="MQKLDQNIVQGILPKRKNESYKTNYGRVLLIGGNEEMGGAIIIA

ASGAVYSGAGLVTVATHPNNHAALHGRLPEAMVLNAYHSEKLINQIKKSDVIVIGPGL

GLEKEALDILKTVLATVSSHQQIVIDGSAITLMAQENLKTPKAKTVYTPHLGEWQRLS
KLKPEEQTKDLNAHFRKQLNATVVLKKHHSEIYFEDEVWQNTAGNPAMATGGMGDALT
GMLVGFLAQFPNHKTAVLASVFLHSYIADQLAKTHYVTLPTQIIQLIPKTMKDFATKF
DF"

CDS 674874..676274

/locus_tag="JFBMFIFI_00635"

/EC_number="3.4.13.-"

/inference="ab initio prediction:Prodigal:002006"

/inference="similar to AA sequence:UniProtKB:Q7A522"

/codon_start=1

/transl_table=11

/product="Putative dipeptidase"

/translation="MGIDWKKEVEARKDDMFADLFTLLKIDSVRDDSKATEDAPVGPG
PKEALLKFLEIGERDGFITKNVGNLAGHIEYGSGSETLGVFAHVDVVPVGTGWTHDPF
DPIVKDGRIYARGSSDDKGPGMAGYYALKIIKELGLPVSKRVRFIIGTDEESGWKCMD
HYLAVEEKPDFGFSPDAEFPIINGEKGILSVYLNFKGDNQGAVNELVRFDAGLRENMV
PQDASAVVISDEAEKMKSDFFDFVEQHPISGTCEVADNQVTFTVVGKGAHGMEPRGGI
NAATFLATFLNRYAFGGDAKNYLAVTAKYLHDDSRAHKLALNYVDDVMGDLTMNPGVF
TFTSEKGGQIVLNFRFPQGVTVEGIESSLTNQLADYGITLSRGKAQLPHYVPADDPMV
KVLLDVYEKHTGEKGAEKTIGGGTYGRLLERGVAYGAMFPNSIDTMHQTDEFMAIDDL
VNATIIYADAMYELIK"

CDS complement(676340..677521)

/gene="yqhD"

/locus_tag="JFBMFIFI_00636"

/EC_number="1.1.1.-"

/inference="ab initio prediction:Prodigal:002006"

/inference="similar to AA sequence:UniProtKB:Q46856"

/codon_start=1

/transl_table=11

/product="Alcohol dehydrogenase YqhD"

/db_xref="COG:COG1979"

/translation="MNTEIANFNYYNPTKIIFGKNRIEELATLVPKDKKVLLLYGGGS
VVRFGTLDKVKAALAGYQIGEFGGIEANPTYETLMKAIELVKRDGYDYLLAVGGGSVI

DGTKFIAAGALFDRDPIHIFGAGIGDKLPITKALPFGSILTLPATGSEMNSGGVVTFV

EKKAKLGFGSPVTFPQFSILDPELTYTLPKRQLANGVIDSFVHIMEQYMTYPVNAMVQ

DRFAEGLLQTLIEIGPKVIDETNHDYNLRANFMWTATNALNGILSPGVPQDWASHSLG

HEITALYHIDHARTLAIVLPSLLEIRKTEKLDKLVQYAERVWLITDGTAEEKATLAIN

KTREFFESLGAPTHFSDYDLDAEVVDILVDQLDKHGLSKLSERQDQTLEISRKIYQNA

L"

CDS complement(677600..678271)

/locus_tag="JFBMFIFI_00637"

/inference="ab initio prediction:Prodigal:002006"

/codon_start=1

/transl_table=11

/product="hypothetical protein"

/translation="MDNMNILFLSRLALAGLLGGIIGFDREFRAKEAGIRTHSLVCLG

SALLMIISQHGFSDVVGTPGFSLDVSRVAAQVVSGIGFIGAGMIIIQKQNVVGLTSAA

GIWATSGIGLAVGGGMYIVSIGATILTIIGFEFSAIFFRKIDYHTTTLTLSTTKAESI

PLVTGLLKEKQKVYQNFESKREIFEETKVYHIYFSIRTKNSTEEMELVEALQEIPHIT

VEKIN"

CDS 678431..679324

/locus_tag="JFBMFIFI_00638"

/inference="ab initio prediction:Prodigal:002006"

/codon_start=1

/transl_table=11

/product="hypothetical protein"

/translation="MMMKKILVFGGNRFFGQKTVQALIQKGYLVTIANRGVRSDDFGD

NVNRIKVDRTDPMSSGWQEISQSYWDVVIDNICYTKEEAQIAIDYLTGKVGQYLFTSS

LSVYEGEQIGFSETDFRPDTFIIDPTSELNYSEGKRQAEATFVAASDFPVAILRIPIV

LDDDDYTERLHYYIRKIRNEETILVRDLSAQMSFIKGSEVAKVMLWLIDINFKGIINA

SSKEIISMNELMNWIEEVVKVEPLVIESDQDENQSPFSVEKDWYLDCQKMSELGYPLP

DLSSWLVPLIRRLNEKMAMNA"

CDS 679340..680197

/gene="rlmA"

/locus_tag="JFBMFIFI_00639"

/EC_number="2.1.1.187"

/inference="ab initio prediction:Prodigal:002006"

/inference="similar to AA sequence:UniProtKB:P36999"

/codon_start=1

/transl_table=11

/product="23S rRNA (guanine(745)-N(1))-methyltransferase"

/db_xref="COG:COG0500"

/translation="MKKIDQGIDFLTNWKQLFQCPVCQSRFDEIKGTSFNCENGHSFD

VSKKGTLYFLTGSAKNEYDKEMLSPRFSIAQAGLFNPLLEQIYPLINTDSKEIQTLLD

VGCGEGSQLDYLTQLGLEGPKIGFDISKDAIQLASTHFSSAFWCVADLAKSPFASKQY

DTILNIFSPSNYQEFKRLLKNDGKVIKVVPNTDYLIELRKLFYQDQEAKQSYQNDAVV

NKFSQHFSKVTVEQCRYSFDLTPELFSDLMKMTPLAWGASQAAKDFALENPLKKVTVD

VSILIGMSE"

CDS 680524..681684

/gene="queG"

/locus_tag="JFBMFIFI_00640"

/EC_number="1.17.99.6"

/inference="ab initio prediction:Prodigal:002006"

/inference="similar to AA sequence:UniProtKB:P97030"

/codon_start=1

/transl_table=11

/product="Epoxyqueuosine reductase"

/db_xref="COG:COG1600"

/translation="MSGTSLSLKERIIQESQRIGIDKIGFASAEPFSNLEKKLEEQQE

KGHHSGFEHPVIKERIYPELIFDEPRSIISIALAYPTKMDESPERVKGQRRGQFARAS

WGTDYHDILREKLARLIEFIKQEAKEEVLFKPMVDTGELIDVAVAQRAGLGFIGKNGL

LITEEFGSYVYLGEIITNLEFEFDEVVANGCGDCTRCITGCPTTALLGAGRMNAKKCL

SFQTQTKGMMPVEYRKKMGHVIYGCDICQMVCPYNKGKNFHFHEAMEPVPEDVAPLLQ

PLLSISNKEFKKRFGVMAGSWRGKKPIQRNAIIALANYRDKTALPALLRCIEEDPRPV

IRGTAAWAIGQIVAKQDVTVIEFLRTAYQKEEDEEARVEFKQALQTLEEKSS"

CDS 681744..682256

/gene="trmL"

/locus_tag="JFBMFIFI_00641"

/EC_number="2.1.1.207"

/inference="ab initio prediction:Prodigal:002006"

/inference="similar to AA sequence:UniProtKB:P44868"

/codon_start=1

/transl_table=11

/product="tRNA (cytidine(34)-2'-O)-methyltransferase"

/db_xref="COG:COG0219"

/translation="MPNHIVLFEPQIPANTGNIARTCAATNSTLHLIEPLGFSTDDKQ

LKRAGLDYWNDVAIRYHSDLAAFLKVAESGQLHLITKFGHEVYSDVDLTDSKLDHYFI

FGKETTGLPEEFMRQYEQECLRIPMNDEHVRSLNLSNTAAILVYEALRQQNFENMELT

HNYGEIDKLS"

CDS 682673..683461

/locus_tag="JFBMFIFI_00642"

/inference="ab initio prediction:Prodigal:002006"

/codon_start=1

/transl_table=11

/product="hypothetical protein"

/translation="MEMVKKGLTGFQLKVLGLVLMVFDHIAEFFLEMGVPFWFHWLGR

LVAPIFLFTSSEGYIHTRNKKKYLFRLLVGFWIMSIGNYVVNRYVAEGIGIFNNIFGT

IFLGVLYMYLIDLFKAAWQKKQVLSVLGAIILMVLPIAAGFLIMWLPEVNFLAFNILF

LFVPTVLTTEGGFLLVVLAVLFYLFHGKKSLQVAALLIISALSYFSAGGNPFTENYQW

MMGLASIPIILYNGEKGRGMRNFFYYFYPAHIYVLFFIAHWLQK"

CDS complement(683526..684827)

/gene="pbuO"

/locus_tag="JFBMFIFI_00643"

/inference="ab initio prediction:Prodigal:002006"

/inference="similar to AA sequence:UniProtKB:O34978"

/codon_start=1

/transl_table=11

/product="Guanine/hypoxanthine permease PbuO"

/db_xref="COG:COG2252"

/translation="MSNFFRLKENNTTIQTEIIAGLTTFLTMAYIIVVNPVILSAAGV

PFNQVFMATILAAVVGTGWMALAANYPIGIAPGLGMNAYFVTVVASGVDYKTAFASVF

IAGLIFLLLSFTSLREKLIIAIPETLKSAITAGIGLFIAFVGLRLSGVIVADEANLVK

LGDLHSLGVILTLIGIILSVILMLLNVRGALFISMVVIAVISYFTGQIKFDGFMALPN
FGHDLMITNPITPFKDVIENGLYGAVLSFLLITIFDTTGTMIAVTKKAGLMKDGNLEN
AKQALLADAVGTTVGAVFGTSPTSAYIESGTGVAAGGRTGLTALTIAVMFAISSFFFP
VVSAISSVSAITAPALVIVGSMMMASAAEIHWDDLSEAFPAFIVIVTMPLTNSIATGL
ALGFITYPITKAVTGKFKDVHPLVYVFAVLFFIQMFFLGGH"

CDS complement(685048..685284)

/locus_tag="JFBMFIFI_00644"

/inference="ab initio prediction:Prodigal:002006"

/codon_start=1

/transl_table=11

/product="hypothetical protein"

/translation="MEQDKFGMQTISSLYYDTDDFQLIQQSIEKPIYREKFKILSYGI
PEPTDTLFLHLKKKSNSLRLQKTRAFIFRKGHYL"

CDS 685729..687717

/locus_tag="JFBMFIFI_00645"

/inference="ab initio prediction:Prodigal:002006"

/codon_start=1

/transl_table=11

/product="hypothetical protein"

/translation="MQLKKFISIGLIASLVSPVLVTGKVIAEETQTSVDTSNQEISVV
PDVEFQRLIRTTLRLPDDAIVTKEVMENLTSLSIGSSKITDFTGLEYAINVTNIRING
QALDSSLFPDLSGLSLLTHIDLDRTESDNVIIEKLANITSLQSINIQRNPQITDVSVL
ANLPNLQALNIQFNGVSDFRWVNDAPKLTRLSATGQNTGRDNAAVNLMRSKLIYDSER
KTITLPFAEFPKQLTNFDGYTPSFSTALSDTYFVFDGVEMGADRLTIDTNGLTINQIE
PDAYENATSFIYNARYNNPFGTYEIPAGFTFYAISAGTYYQNFTIENDPIAGAPVNVH
YVDEENNELAPVEELNGNVNESYQAQEKDIPDWVLKEVPNNVSGLFTTDVQTVTYVYE
KKDGEPVTVYYQDEAGNELAPAEQLSGKLDFPYSTSEKELAGWVVKEVPANAEGLFTD
KNQTVVYIYEKATAAPITVYYQDEEGHNLIPSEQLDGKLDAPYEASIKEIAGWVLKTS
PENTKGLFTDKAQTIVYIYEKANGAPVTVDYQDTEGSKLSPSEQLIGQLDATYETMAK
SFIGWELKQVPTNAKGLFTSENQSVVYIYQKQQPGTSISQIQPPFKTANPLPATAKVS
KNSLPETGEKQSLNYSLAGVGIMGLAVLAILRKRTKNK"

CDS complement(687767..689458)

/gene="argS"

/locus_tag="JFBMFIFI_00646"

/EC_number="6.1.1.19"

/inference="ab initio prediction:Prodigal:002006"

/inference="similar to AA sequence:UniProtKB:Q54869"

/codon_start=1

/transl_table=11

/product="Arginine--tRNA ligase"

/db_xref="COG:COG0018"

/translation="MDYKLLVAELLQKQIGETLTTEQIFNLLEKPKSADHGDIAFPTF

ALAKAFRKAPQQIATDLGTKITDPLIAKIDVVGPYLNFFLNKESVSSTVLKEVLDAGT

TYGNQSIGLGQTIPIDLSSPNIAKPISMGHLRSTVIGNSIANIAEKLGFKPIRINHLG

DWGTQFGKLIVAYKLWGNEESVKAEPINELLRLYVKFHAEAEEKPELDDEARAWFKRL

EDGDEEAVALWKWFREESLTEFNHIYKLLGIEFDSYNGEAFYNDKMDEVLDLLEEKHI

LTVDNGATIVDLEKYNLNPALIRKSDGATLYITRDLAAALYRHRTYNFAQSLYVVGNE

QSNHFKQLKAVLKELGFDWADQMHHVPFGLITQGGKKLSTRKGKIVLLEEVLNEAVNL

ANQQITEKNPDLADKDTTASQVGIGAVIFHDLKNDRLNNFDFVLEEVVRFEGETGPYV

QYTRARAMSILRKAADVSFDSASLALDDEYSWEVIKLIQEYPTTVLRAYDKLEPSVIA

KHAIHLAQAFNRYYAQVKVLNDDAQKPARLALVKAVTIILKEDLRLLGVGAPDEM"

CDS 689925..690380

/gene="argR_1"

/locus_tag="JFBMFIFI_00647"

/inference="ab initio prediction:Prodigal:002006"

/inference="similar to AA sequence:UniProtKB:P17893"

/codon_start=1

/transl_table=11

/product="Arginine repressor"

/db_xref="COG:COG1438"

/translation="MKKTNRQMVIKQIINNHDVATQEELLQFLKAEGVEATQATISRD

IKEMNLVKTTSSKGTVKYILFQHNKMSDKEKLEQTIGEVVIKLTRVQFMTIIMTIPGN

AHVVAALLDSIDFPEMVGTVGGNDTILVISPSEEEAIRMYDYFNKWTSF"

CDS 690451..691986

/gene="algC"

/locus_tag="JFBMFIFI_00648"

/EC_number="5.4.2.2"

/inference="ab initio prediction:Prodigal:002006"

/inference="similar to AA sequence:UniProtKB:P26276"

/codon_start=1

/transl_table=11

/product="Phosphomannomutase/phosphoglucomutase"

/db_xref="COG:COG1109"

/translation="MNESSIPVLNALQNGSDVRGIALTTEDKVMNLTDDRVERIAYGF

ASWLKEIKKLAVDDPHYPIRVAVGHDSRLSADRIKSALIEGLVNANFEVFDVGLATTP

AMFMATQYVDYDCDAAIMITASHLPYEYNGLKFFTKDGGAEKEDITYILEHADWEYVF

WGNMKGFVTPRFLLKDYANDLVDKIRVGIQDEENYQQPLLGRHIIVDAGNGAGGFFAT

QVLEVLGANITGSQFLEPDGHFPNHIPNPDNKAAMASIQSAVLAHNADLGVIFDTDVD

RSALVDKQGKTLNRNNLIGIISAIVIKENPGTTIVTNSTTSEHLKNFIESLGGKQNRY

ITGYRNVINRAIQLNKDGIPTSLAIETSGHAALKENYFLDDGAYLVAKILMTDAVLTK

EGKELSSLITNLKQPVETDEVRFKILSEDISLVGADAMEDFRGFIYKTADLEVEPRNL

EGVRVNTSGKYGTGWMLLRLSLHEPLLVLNFESDQKGAIALLRKDLKNFFEENDELDA

SLL"

CDS 692071..692931

/gene="yvgN"

/locus_tag="JFBMFIFI_00649"

/EC_number="1.1.1.-"

/inference="ab initio prediction:Prodigal:002006"

/inference="similar to AA sequence:UniProtKB:O32210"

/codon_start=1

/transl_table=11

/product="Glyoxal reductase"

/db_xref="COG:COG0656"

/translation="MELAITNSLKMANGVEIPQLGLGVFLVKEEDELNMAVKSAIYDG

YRHIDTAMIYNNEEFVGKAIKETEIPREELFITSKVWNYDHGYEETKAAFEATLKRLD

TDYLDLYLIHWASPNYIETWQAMEELYEAGKIKAIGVSNFQIHHLEDLMAHTKIKPMI

NQIETHPEFPQNELHEFMEKHGILHEAWGPLGQGKHDLLSHPILVKIGEKYNKTAAQI

VLRWHVERGIVVIPKSVTPHRIKENSQIFDFSLSKEDMAEIATLNTGTRYAKDPDDEE

FLKTSAIRPE"

CDS 693205..693381

/locus_tag="JFBMFIFI_00650"

/inference="ab initio prediction:Prodigal:002006"

/codon_start=1

/transl_table=11

/product="hypothetical protein"

/translation="MRAIRKKRNTPGFKNFLTNTVFGNIEGNQAEKQKDEKKNSSSPI

YIPKNSNTDKHKKK"

CDS complement(693460..694818)

/locus_tag="JFBMFIFI_00651"

/inference="ab initio prediction:Prodigal:002006"

/inference="similar to AA sequence:UniProtKB:P9WFP1"

/note="UPF0053 protein Rv2366c"

/codon_start=1

/transl_table=11

/product="hypothetical protein"

/translation="MSIGTGMIIFFIILGIAALFVMSEFVLVRIRPSRLDFLIENGNK

KAILLKKMTQHLDTYLSATQLGVTITSLALGWLGDPTFKRLFDNLFENFSLPSSVSSL

LSIVISFSVLTFIQVIVGELVPKNFAINKTEKIGLLIARPLQIWYLLMFPIVYLLNGI

ANGISRSFGLKSVGESDESVSEEELRIIMSESLKSGEINHDEYQFVENVFAFDNRMSR

EIMVPRTEMVTVSTDMSLKEISQLVQTERYTRYPVIQDGDKDSIVGVINTKEIFAAYV

TYVESGLSDEFMITKYVRPVISVIETIPIKEILVRMQKERNQVAILVDEYGGTSGMIS

IEDIVEEIVGDINDDYATTEAPEMTKIDDHHYLVSARMLIDDINELFGLDIDEENVDT

IGGWMMNEKYDLVEGDHIVFKSYTFTVKKAGKSTIEWIEITLSDNKAETHEVKEKNKD

EE"

CDS 695149..697275

/gene="pbpF"

/locus_tag="JFBMFIFI_00652"

/inference="ab initio prediction:Prodigal:002006"

/inference="similar to AA sequence:UniProtKB:P38050"

/codon_start=1

/transl_table=11

/product="Penicillin-binding protein 1F"

/db_xref="COG:COG0744"

/translation="MEEKTFFAKFKAQMIIFWQWLRPYLGKVHRWRKRIWKKYHINKI

ILLATLLVVLVTSIYLLYLAKTADVASLKAGLEQTTTIYDENGAEAGTLYDQKGTFVS

IDQIAPALQDAVVSTEDKRFYEHGGFDVKGILRAAVGYVVNRGNIVGGGSTLTQQLAK

NAFLSQNQTLSRKAEELFLAIEIEKKYSKADILEMYLNNAYFGNGVWGVEDATQKYYG

KHASELTVTEAASIAAILKSPSYYNPIDHMDNSIERRNLILDLMVDNQKLTPEEANVA

KSEPLLLEDTYHATSGYQYPYYFDALIDEAINDYGLKEEDILNKGYKIYTALNQDYQK

QMQETINNDALFQYSEDGTLVQTGSVAMNPKTGGVYALVGGRGEHVFRGFNRATQIRV

QPGSIMKPLAVYTPALESGYKIDSLLKDEQKAYGSDNYTPENYNGIYLGEVPMYQAVE

DSINAPAVWLLDQIGVDKGFEKVKKFGIPLKEDDRYLGLALGGLTKGVSPLQMASAYT

TFANEGKRSEGHLITKIVDATGAVIVDNTDTKTKKVTTPEVAKDMTSMLMGVFDNGTG

QSALPEGYKIAGKTGSTELTFGEKNGTKDQWIVGYTPDVVLSTWIGYDQTDENHYLNG

LSSEGVAPVFRSQMANILPATPMTPFTTESASEIVTEENKGTMDKWKDSFDEGAKYWG

DKFKEGSSYLKDKTGEFFNGLKEKIK"

CDS 697405..697749

/locus_tag="JFBMFIFI_00653"

/inference="ab initio prediction:Prodigal:002006"

/inference="similar to AA sequence:UniProtKB:Q7A4V3"

/note="UPF0342 protein SA1663"

/codon_start=1

/transl_table=11

/product="hypothetical protein"

/translation="MSTNIYDTANQLERDLRETEAYVALKAAYEAVKANPESNDMFKE

FQGIQIKLQQKQMSGEEILEEEIKEAQEMAMKTGENETIKVLMEAEQKLSQLIDDVNR

IIMSPIQELYQG"

CDS 697860..699101

/locus_tag="JFBMFIFI_00654"

/inference="ab initio prediction:Prodigal:002006"

/codon_start=1

/transl_table=11

/product="hypothetical protein"

/translation="MVQFIHAADLHLDSPFIGLKSLPPFLWEQIYQSTFKSLEKIVDL

AIDKQVDFVCLAGDIYDSDDRSVKAQAYLRNELIRLESAEIPVFICHGNHDYIENTGL

HLTMPKNVTIFNEEVETHWLTTKKNERIAITSFSYPKRWVRERMIESYPKREETADYH

IGMLHGSVDGVESEHGTYAPFTLTELKNKNYDYWALGHIHKRQILSEDPLVIYSGNTQ

GRSSKELNSKGCEWIQLTAEAKTHQFYSTATIQWKKVQLSLKGIERLGDVYQTIQEFI

QTKKNNKQSLFIELRLIDSESLALNVRKKINSGELLEAVQQVSETDDVIIWVHRIVLE

KEMVGNNQFSFMTKEWQEAINLVAEESWFNEATNFLFEEGQIEDYVDSRDQRYRELII

TEAKERIIQQLGIEGSEKNEN"

CDS 699091..701898

/locus_tag="JFBMFIFI_00655"

/inference="ab initio prediction:Prodigal:002006"

/codon_start=1

/transl_table=11

/product="hypothetical protein"

/translation="MKIESIEIYGYGKWVDKSFTDIQDLQVFYGKNEAGKSTVMSFIH

SILFGFPTKQGSGTRYEPRNSSQYGGRLRISETVYGNVLIERVKGKTSGNVTVTLANG

ETGSEDLLEKIIFGIDKNTYQALFSFDLVGLQKVQQMNQTKLNRYFLSVGTLGNERLL

RIVDKFQQEANKLYKASGRVPEINQKISQLKQKEQQLKSVKARNNEYNQLQNEKLKLS

EHLKQFERQQEDLERELAGLKRLSSNWAHFKEAQEIQNQIQKKALINLPEDGLYQLKQ

LNDELARIVTTQIKEKEQILQLSRKAGQDERFSFYLEHKDRFKEANLKIEQIKELMKK

KEIVEAKQLQDQRYLEQLLHQNGLPIYGEIPQVLSTELKAEVQNLIDLQASIQEKEVN

LQQDKQRLNFQIEVLNQQIDQLENQLWEAKDFQEYQKPEVVPQRQKRARKKQSKSFYA

VAFSSLLSLVAGYLIGTTLGVILLIVGGLGLLITGYQAFGTKREKATTKTNEKYSYEE

YIKQVEIRKQWREKLAEADDVTDALNYFTDQENQFNLEKQQFNCQHQQFSQKTMIPNE

VSLEKLVVDDPFFEIRQQISVTEKQELEIDALTKQISEWFSGVEFSQQIIGIGMENLA

TFLFSFQQFYLQQVEIENAQQHISQRVAEIRQELLSGEENQKVIEKNKKSLFASVDVS

SEEEFRKKYVLYQELQQKKARLDLLEDQIAEDLILFTNYRDKEELIEALQTQDETLQL

LKKKISSQLKQKAECEVALKNLEEGGQYSVLLQEFANLKSELQELVQSWSSLRIAAEI

IEKMLTLSRKDRLPEMIEDASHYFNLLTKKNYQRLLFKDERIQVQRQDGSIYEVNELS

QGTGEQLYVALRFAFIKNATDLLKLPLLIDDGFVNFDAERRSEIFQLLNEISQTNQII

YFTCSSESQKEFSKEQVKVLQ"

CDS 701940..702923

/gene="yhaM"

/locus_tag="JFBMFIFI_00656"

/EC_number="3.1.-.-"

/inference="ab initio prediction:Prodigal:002006"

/inference="similar to AA sequence:UniProtKB:O07521"

/codon_start=1

/transl_table=11

/product="3'-5' exoribonuclease YhaM"

/db_xref="COG:COG3481"

/translation="MDKKIFDYNVDDTFDLFLLVKNADVRVAKNGKKFIAFTFQDVSG

QIDGKYWDASDSDIEAFLAGKVVRVSGKRELYQGNPQVKLFKIRLANDNEPNSPELFM

ERAPIKKEEIAEEINQTIFEITNANMNRVVRHLMTKYQKEFFQFPAAKKHHHAYMGGL

SYHTISMLRLAKFIAEQYPEINKPLLYSGVILHDLGKVMELSGAMSTEYTLEGNLIGH

IVIVDEEITKACLALKIDDKSEDIILLKHMILAHHGQLEYGSPVRPRLREAEVLHQID

NIDASINMLNVALNRTEPGTFTERIFGMDNRAFYKPNMTKVTSEEQLELLE"

CDS complement(702988..703899)

/gene="prsA_1"

/locus_tag="JFBMFIFI_00657"

/EC_number="5.2.1.8"

/inference="ab initio prediction:Prodigal:002006"

/inference="similar to AA sequence:UniProtKB:P0C2B5"

/codon_start=1

/transl_table=11

/product="Foldase protein PrsA"

/translation="MKKLITAGAIILTSLTLASCSTNNDVATTKAGNITKEDLYEAMK

TTAGPSSLQQLIISDVLTEKFGDKVTDKQVDKDFNAQKEQYGDQFAELLKNSNYTEES

YKEVIRMNLLIEAAVKANTKFTDEDYKTAWESFVPKLTVQHILVADEATAKDLIAKIN

AGEDFSTLAKENSLDTGSKEDGGKLPEFDHNTPYDPLFIEAAAKLKDGEVTQEPVNGS

NGYHIIKMIKNPGKGKMEDHKKDLETTMIKARLTDDAYVQSILSKIMQDAKIEIKDKD

LKGAMDTFLKQDDSSKESSKATDSSKK"

CDS complement(704039..704938)

/gene="prsA1"

/locus_tag="JFBMFIFI_00658"

/EC_number="5.2.1.8"

/inference="ab initio prediction:Prodigal:002006"

/inference="similar to AA sequence:UniProtKB:Q8Y759"

/codon_start=1

/transl_table=11

/product="Foldase protein PrsA 1"

/db_xref="COG:COG0760"

/translation="MKKFLVSFTVAAALLTMAGCSNSGKTVASTTSGKITQEQLYDKM

KDQYGTTVLQTMLLSNVLEDQYGSKVDKKAVEAEYNTLKEQYGDSFEDVLKQSNTTSK

ALKENIRSQLLMDAAIADNSKFTDADYKRAFETWTPKMTAQHILVADEATAKDLIAKI

NAGEDFDKLAKENSTDTATKEDGGKLPEFDYTSSLGTEFATAASKLKDGEVSKEPVQS

QSGYHIIKMVKNPGKGKWEDHKKELKTILVTEIRNDQAKLTPILAKVIKKANVQIKDE

DLKDAMAPFTQPAATSSSSESKK"

CDS complement(705094..705492)

/locus_tag="JFBMFIFI_00659"

/inference="ab initio prediction:Prodigal:002006"

/codon_start=1

/transl_table=11

/product="hypothetical protein"

/translation="MGHSFLKGLFIGSIVGGITALLTTPRSGKENRDILLSYVDDTTV

LVDDVSTSVNALKDAIQTLTTEGLELVNEFSEDMTDTVEEFTMQNQPRLRRIEEKAQK

LTTDLEEIAEIFPEMAEESNQNIQVQNTPS"

CDS complement(705495..705923)

/gene="hit"

/locus_tag="JFBMFIFI_00660"

/inference="ab initio prediction:Prodigal:002006"

/inference="similar to AA sequence:UniProtKB:O07513"

/codon_start=1

/transl_table=11

/product="Protein hit"

/db_xref="COG:COG0537"

/translation="MEECIFCKIINREIPSHVVYEDEDVLAFLDITQVTKGHTLLIPK

LHVSDIFEYNQELAATIFSRLPKVARAIEKSDPAIKGLNIVNNNREVAYQTVFHSHVH

LIPRYDTKDDFSMRFGNHMADYTPDQLSTVAQSIKNELED"

CDS 706161..706910

/gene="ecsA"

/locus_tag="JFBMFIFI_00661"

/inference="ab initio prediction:Prodigal:002006"

/inference="similar to AA sequence:UniProtKB:P55339"

/codon_start=1

/transl_table=11

/product="ABC-type transporter ATP-binding protein EcsA"

/db_xref="COG:COG1131"

/translation="MSLKVTNLTGGYSQVPVLKDISFEVEKGKLIGLIGLNGAGKSTA

IKHIIGLMNPQKGSITIDELTLTADTEGYRKKFAFIPETPVLYEELTLKEHIELTAMA

YDVPQDIAFQRAEKLLTSFRLSNKLDWFPANFSKGMKQKVMILCAFLVEPSLYIIDEP

FLGLDPLGINALLELMNEMKEQGASILMSTHILATAERQCDGFVLLHNGEIKAQGTLE

DLRASFQMPEATLDEIYIHLTKEEDQLNDDA"

CDS 706897..708129

/locus_tag="JFBMFIFI_00662"

/inference="ab initio prediction:Prodigal:002006"

/codon_start=1

/transl_table=11

/product="hypothetical protein"

/translation="MMMLEIWKKRLKLHEKKMARYLKYVLNDHFVIVCFFIFGALSLG

YSNLLKTLDSSFIWGRWIALLIFVASIFIGKLATLIEAPDAVFLLPKEKELPRYLKAA

KAYSLLLPTFLIVLLTAFVMPLLVATTEFDFFDMTYFALILLLLKNWDLDIQLLSLKL

SGKEERLKWQLIKLGIAIFIVALAVFFSPILATCLAIILQFVSGPIFKKIENETIYQW

EMMVQDEEQRMHRIYQFINLFTDIPSLKGKVKRRRYLDVLLQRIQPVHKNTFSFLYAR

TFLRGTEYSGLYLRLSVIVVLLIIFSHSFIMAILLSILFIYLTGFQLLPLYFHYDNMA

LTRLYPVNELAKGKALKKLFSVALGGQAIAIFITSLFTLTLAESLLIFIGCLAFVFIF

TQVYVPIRLKKMKKSRAY"

CDS 708303..709163

/gene="dat"

/locus_tag="JFBMFIFI_00663"

/EC_number="2.6.1.21"

/inference="ab initio prediction:Prodigal:002006"

/inference="similar to AA sequence:UniProtKB:P19938"

/codon_start=1

/transl_table=11

/product="D-alanine aminotransferase"

/translation="MKVIWNDKIVERSDVEIDMEDRGYQFGDGLYEVIRAYNGVFFTA

DEHIDRLFKGAEKIELVLPFTKNKLKELLNELLVANDIETGNVYFQITRGIAIPRDHT

YPDPTKVPAVFTASATKVPRNQVKMDTGIPVITIPDCRWLHCDIKSISLLGNIMAKHE

AHKKGAEEAIQHRDGIVTEGSSTNMWIVKDGTIYTHPDGNLILAGITKIVLLKVAREA

GIPVKEEAFTLEQLKAADEVFSSSTTIEAMPVVEIDGVKVGDGKRGPIVKQLQDLYVA

AVEEVCGKIR"

CDS 709287..710711

/gene="emrY"

/locus_tag="JFBMFIFI_00664"

/inference="ab initio prediction:Prodigal:002006"

/inference="similar to AA sequence:UniProtKB:P52600"

/codon_start=1

/transl_table=11

/product="putative multidrug resistance protein EmrY"

/translation="METKYQNSHIVTILGVIIMGTFVTILNQTLLSTALPSIMNDFSI

TATSGQWLTTSYMLINGIMIPITAYLVERFTTRQLYLFAMITFFIGTAIAATADVYWV

LIIGRMVQAVGAGVVLPLQTIVILYMFPIEKRGAAMGLIGLGMNFAPAIGPTFSGWVV

QNYHWSMLFYFILPFALIDIIIAFFVLKNVSEVGKPKLDWLSVIYSTLGFGGLLFGFS

NASSHDFLSINVLGAILVGIVSMILLVLRCNRSEHPLLNFKVFKIRGFRLNMIISFII

LAGMYGGIILFPIYFQSIRGWSPMKSGMILLPGSIVIAVMSPITGRLFDKYGGKKLAI

TGLFLITIMTFCIGFLDLNSSVTLIVSIQLIRSLGISLTLMPLQTGAFNAVPLDMAAH

ASAMFNTQRQLSGSMGTALFVVIMTMVSNSQSAVALGSPILASLKGFQFVFMVVGLFS

LAGFLLTFLIREKRKNIEVDVLRN"

CDS 711060..711857

/locus_tag="JFBMFIFI_00665"

/inference="ab initio prediction:Prodigal:002006"

/codon_start=1

/transl_table=11

/product="hypothetical protein"

/translation="MDFEIDTGWKLLPVGGDTGQAYMGIKAEEKLFLKRNSSPFLAAL

SVEGITPRLVWTKRIGNGDVLTAQEWLNGRTLKSSEMTLPSVAKLLYKIHHSDTLRKM

LSRVGGEEVSPEKLIQSYQMELPHDLKIHPMLNEILAKLKAEIKSMESVQPRVCHGDI

YRKNWLLSDENRLYLVDWDMAVLADPALDLSMLLCQYVDKKQWRAWLENYGVVVTDEL

IKRIEWYALINFLQQTKRHHYQSRFHEMNQDILTLQSIYKNTNETYN"

CDS 711931..712575

/gene="trmB"

/locus_tag="JFBMFIFI_00666"

/EC_number="2.1.1.33"

/inference="ab initio prediction:Prodigal:002006"

/inference="similar to AA sequence:UniProtKB:P67506"

/codon_start=1

/transl_table=11

/product="tRNA (guanine-N(7)-)-methyltransferase"

/db_xref="COG:COG0220"

/translation="MRLRNKPGAPAKIASYPQYIVDTPETWKGKWHERFGNQNPIHIE

VGTGKGRFVTEMAKLHPEINYIGIELQMSVVVVALDKLIAEDLPNLQLLHVNGGALSQ

YFEKGEVNQVYLNFSDPWPKTRHEKRRLTSADFIANYESILIPEGEIHFKTDNQGLFE

YSLHSFSKYGMILEQVWLNLHESDFEGNVMTEYEEKFSNRGQRIYRVVAKFRKK"

CDS complement(712634..712972)

/locus_tag="JFBMFIFI_00667"

/inference="ab initio prediction:Prodigal:002006"

/codon_start=1

/transl_table=11

/product="hypothetical protein"

/translation="MTDRDSNYSTYALVGGFIFGAGLTCGYQIAKLVQKKKVIHGDEI

LDTVKNLFLEEGPIEGSWIELKRVPLRKFAFKTEVYYGGVSRIEEDQLMQYEFIADAH

TGSILDLYRI"

CDS 713158..714237

/gene="pepA"

/locus_tag="JFBMFIFI_00668"

/EC_number="3.4.11.7"

/inference="ab initio prediction:Prodigal:002006"

/inference="similar to AA sequence:UniProtKB:Q48677"

/codon_start=1

/transl_table=11

/product="Glutamyl aminopeptidase"

/db_xref="COG:COG1363"

/translation="MNEETFSLVKKMTELQGTSGFEHPIREVMRKEITPLVDEVVQDG

LGGIFGIRRSKAENAPRIMLAAHMDEVGFMLASITEQGLFRVVPLGGWNPYVVSAQRF

TLQTAKGNYPCVSSSVPPHLLRGTNGKAGAPDIGDILFDAGFDSKEEAMAFGVRPGDS

IVPDVETIKTANGKKIISKAWDNRYGCTVVIEALKELKGEALPNTLIAGANVQEEVGL

RGTKGAVHQFKPDLFFAVDCSAADDLTGDKSKYGHLGEGFLLRIFDPGMITLKGMREF

LLDTAETNNIPYQYFVSKGGTDAGAAHISNNGVPSAVIGVCARYIHTHQTMFHMDDYA

AAKEMVLQVARTMDRSTYETIMKNN"

CDS 714264..714578

/gene="ytpP"

/locus_tag="JFBMFIFI_00669"

/inference="ab initio prediction:Prodigal:002006"

/inference="similar to AA sequence:UniProtKB:O34357"

/codon_start=1

/transl_table=11

/product="Thioredoxin-like protein YtpP"

/translation="MRELKSIEEFQVEKNKGKTIFFFTADWCGDCVYIKPVMPEIEAA

HPDFTFIQVDRDEFIDLCGELSIMGIPSFVAFENGKETGRFVNKDRKTQAEIESFIKN

LA"

CDS 714615..715088

/locus_tag="JFBMFIFI_00670"

/inference="ab initio prediction:Prodigal:002006"

/inference="similar to AA sequence:UniProtKB:Q7A551"

/codon_start=1

/transl_table=11

/product="Putative universal stress protein"

/translation="MTVGTQQYKHVMVAVDGSDESISALNKAIETAKRNDADLLIVHV

VDNRAYTMGVASFDIEASEEETNVMNIALETYQKQAVDQGVKKVMTELVSGSPKELLA

KTLPADYQIDLILCGQSGMNRMERLMMGSVSQYIIRFAPCDVLIIRSQESDIKND"

CDS 715114..715728

/locus_tag="JFBMFIFI_00671"

/inference="ab initio prediction:Prodigal:002006"

/codon_start=1

/transl_table=11

/product="hypothetical protein"

/translation="MISCYNKEGIGDTLIIMTGNSTFADQKFESKGSVTRIFKSETNE

TVGFNFFNVSDILSVTGNGQVNLTSKEVSLLNEKLKEVGFADVLEADETPKFVVGLVK

ECVPHPDSDHLSITQTEIDNGEIVQIVCGAPNIDKGQKVVVAKVGAMMPNGLLIWDGE

LRGEPSHGMICSGKELNLPDAPLEKGILVLPTSAVVGEAFLVGA"

CDS 715999..718737

/locus_tag="JFBMFIFI_00672"

/inference="ab initio prediction:Prodigal:002006"

/codon_start=1

/transl_table=11

/product="hypothetical protein"

/translation="MSNYDGPSYQNGRNTPKKFKFPFYSDTDKVSQKEKNETLNETKK

IKDKPVKRSREGHYSAPEAVFSEFLTRNDAYSRMETKAAEKKEQREFEERKKRIEQRI

LDKENIRKSIEKTPTALTDSRSSTPFKVQKVPSPVYGFKQEKKVDSSGIDYQKMEKAL

QKEFDTFILLATEAEVQENEFEKQDNTALLTFNTEGDLVEEESIELIETDSKELDEVL

INEAILEEIVDEESKLPEEELVEVSSEEKEDKHSKRLQRSLQGMIEEEQDIHLNRGTN

VARYFGEAAENLPEETNDEIEETVVEPIEVDESDSDLEIQAALSRFNYLSQQLQEVST

PMHDEGSETLDEEEPPHDEEYLKSLEISEEASNSGWDDGEMEEEAFIIEPQLNEVELS

EEEPLPNSEEEPISEGQASELSEIPQAINPTKIELPSMNSISEKEEQDLRQYHLPPLN

LLNPPVEFENSSVDDWVLEQAELLNETLDAFGVDAQVIGWTIGPAVTQFELQLGRGVK

VNKITNLSDDLKLALAAKDIRIEAPIPGKSSVGVEIPNCNSRPVMLSEVMNSQAFKES

KSPLTVALGVNLAGEAMVTTIEKMPHGLIAGATGSGKSVFINSLLISILYKAKPSEVK

LILIDPKAVELAPYNDIPHLLSPVISEPKAAAEALKWAVDEMEERYQKLAAAGVRNIE

RFNEKAEETGEFGLKIPYIVIIIDELADLMMVASSEVQDYIARITQKARAAGIHLLVA

TQRPSVDVITGTIKNNIPTRVAFMVSSQIDSRTILDIGGAEKLLGRGDMLFMENGSGR

PIRIQGTYVENEIDTIVKHVKEQRGPNYLFEPETLLAKLELVEGQDELFETILAFIAN

EETVSVSLLQRKFKIGFNRASNLIEALESQNLISGNKGSKPRDVFLTQAEYEKNYL"

CDS 719147..720478

/gene="murC"

/locus_tag="JFBMFIFI_00673"

/EC_number="6.3.2.8"

/inference="ab initio prediction:Prodigal:002006"

/inference="similar to AA sequence:UniProtKB:P65475"

/codon_start=1

/transl_table=11

/product="UDP-N-acetylmuramate--L-alanine ligase"

/translation="MNKQTTYHFVGIKGSGMSALALVLHDKGFKVQGSDVDNYYFTQK

GIEDAKITILPFSKENIHDGLTIIAGNAFPDTHEEIKAALDMGIPVIRYHKFIGDFLN

DYTSVAITGSHGKTSTTGLLAHVLSSIEPTSYIIGDGTGHGVPDAEFFVLEACEYRRH

FLAYSPDYAIMTNIDFDHPDYFTGIEDVFSAFQTMANQVKKAIIACGEDEELKKLKAD

VPVLYYGFSDDHNYQAKNIIRTTKGSGFDVYVEKEFFGHFEIPTYGAHNVLNALAVIA

VCQLEGISAELIAENLTTFQGVKRRFSEKSMADMIIIDDYAHHPQEIRATLDAARQKY

PDKELIAVFQPHTFTRTIALLSDFAAALNLADRVYLCDIFGSAREEQGNVTIQDLADK

ITNGALVLQEENMSPLLDHHDAVVIFMGAGDVQKFEYAYETILSRTILQNN"

CDS 720710..721201

/locus_tag="JFBMFIFI_00674"

/inference="ab initio prediction:Prodigal:002006"

/codon_start=1

/transl_table=11

/product="hypothetical protein"

/translation="MIGKPRKIGKLISEISEGDNLSVTENIEDKDILLYLGLTNDANP

LFIQHDYASQTAYEKPLVPTVMLIGIMTSAISKHLPGPGSLVVNVSVNLLNPVFHYET

ITFDFEVIRVDLMKEVITITVDGKNMKDERVIDAVFMVEPPRLLGLPEEIEEQNEVEE

TTK"

CDS 721338..721631

/locus_tag="JFBMFIFI_00675"

/inference="ab initio prediction:Prodigal:002006"

/codon_start=1

/transl_table=11

/product="hypothetical protein"

/translation="METYRLKIRPYQMTDLKAAVTIFSDTEMMTYYPDPFSKQKTMEW

LEWSIEHYQRNGMALWAVCLKETGQLIGDYGIVQQVIDGEQKYELGYHIAREY"

CDS 721860..724520

/gene="polA"

/locus_tag="JFBMFIFI_00676"

/EC_number="2.7.7.7"

/inference="ab initio prediction:Prodigal:002006"

/inference="similar to AA sequence:UniProtKB:P52026"

/codon_start=1

/transl_table=11

/product="DNA polymerase I"

/translation="MTKKNKLLLIDGSSIAFRAFYAIPGSLDRFMNKNGLHTNALFSF

HRMLDNEMKKEQPTHVLVAFDAGKTTFRNAFYDAYKDGRQKTPGEFKEQMPYLRDLLE

GFGIPHYELDNYEADDIIGTLATVVDPNEFDVVVVSGDRDLTQLAKENVRVDITIKGV

SEIEENTPAYLMEKYGIEPHQIIDMKGLAGDSSDNIPGVTNIGEKTALKLLHEFGSVE

GVYENIDGMKPSKRKENLINDKEQALLSKKLATIEVDAPLEIKVDELLYTGKDVGKLI

EFYKEMDFKSFLKDLDTTEYDEENKIEKAEIIFEFVTELKADMFGPIGGLYVEMLTDN

YHTAEIVGLSWSNGEKIYVAEPDVALASPIFQEWAKDPANKKQVFDAKRTVVALQRYD

TTLEGVDFDVLLASYILNTNDNSKDLAEVAMEHDYYEVAADESIYGKGAKLAIPEDAS

VLNEHLARKIKAIEVLSIKLKQELIDSEQTALFYEMELPLSMVLAEMEMTGIKVNANR

LLEMKAEFAIRLTDIENRIYEEAGETFNINSPKQLGVILFEKMQLPVIKKTKTGYSTA

VDVLEQLRPQAPIVDDILHYRQIAKIQSTYVEGLLKVINGNTGKVHTRYVQTLTQTGR

LSSVDPNLQNIPIRLEEGRKIRQAFVPSHEGWKIFASDYSQIELRVLADISNDQHLKE

AFLEGQDIHSSTAMRVFGIEKAEDVTGNMRRQAKAVNFGIVYGISDYGLSQNLGITRK

EAQTFIDTYFEKYPGVKQYMSDIVREAKDKGYVETLFHRRRFLKDINSRNFNLRSFAE

RTAMNTPIQGSAADIIKVAMIQMDRRLKAENLKTKMLLQVHDELIFEAPEEEIAILEK

LVPEVMESAVKLNVPLKVDSSYGDSWYDAK"

CDS 724597..725451

/gene="mutM"

/locus_tag="JFBMFIFI_00677"

/EC_number="3.2.2.23"

/inference="ab initio prediction:Prodigal:002006"

/inference="similar to AA sequence:UniProtKB:P42371"

/codon_start=1

/transl_table=11

/product="Formamidopyrimidine-DNA glycosylase"

/db_xref="COG:COG0266"

/translation="MPELPEVETVRKGLTNLVEGATIQDVDVYWDRIITAPFSSEAFR

NDLLGQTIHKIERRGKYLIFLMDDWAMISHLRMEGKYEVEKTGTPLKKHTHVVFHLTD

GRDLRYLDVRKFGRMTLVPIGTEHEAPGIKGLGPEPIPELFKLEPFSVALGSKSRAIK

PLLLEQKIVVGLGNIYVDEALFEAKIHPLRPANSLTSIEIKNLHHSIIGVLGRAVEAG

GTTIRTYQNALGEAGSFQVDLLVYGKTGEPCVNCGQPIQKIKVAQRGTHFCSHCQTLT

ERNSRPQH"

CDS 725471..726085

/gene="coaE"

/locus_tag="JFBMFIFI_00678"

/EC_number="2.7.1.24"

/inference="ab initio prediction:Prodigal:002006"

/inference="similar to AA sequence:UniProtKB:P63831"

/codon_start=1

/transl_table=11

/product="Dephospho-CoA kinase"

/translation="MTYILGLTGSIATGKSTVSNIFKALGFPVVDADIGAREVVEVGA

PGLQALVDYFGQELLTHDGQLNREALGTIVFANETKRKKLNELLKPYIRSWIDREKNK

VIASGAPLVIMDIPLLYEAGGYQEMMDSIMVVAIPDELQITRLMARNQLSKSEALQRI

EAQIPIAKKVEWADSVIDNSGSLVDTRQQVEKWLQEKNLFPDQS"

CDS 726333..726791

/gene="nrdR"

/locus_tag="JFBMFIFI_00679"

/inference="ab initio prediction:Prodigal:002006"

/inference="similar to AA sequence:UniProtKB:P67318"

/codon_start=1

/transl_table=11

/product="Transcriptional repressor NrdR"

/db_xref="COG:COG1327"

/translation="MQCPRCQNNGSRVVDSRPADDGRAIRRRRECEACSFRFTTFERT

EQTPLLVVKKNGTREEFNREKILRGLIRSCEKRPVAMDQVERMVDEVENKIRSLGENE

VSTTLIGEYIMERLAEVDEVAYIRFASVYREFKDMDFFLKELQKLKNKDK"

CDS 726815..728275

/gene="dnaB"

/locus_tag="JFBMFIFI_00680"

/inference="ab initio prediction:Prodigal:002006"

/inference="similar to AA sequence:UniProtKB:P07908"

/codon_start=1

/transl_table=11

/product="Replication initiation and membrane attachment

protein"

/db_xref="COG:COG3611"

/translation="MNYPLKNLSPKDGFEVRQNALLSDMDQKILTFLYQPLTGATAYS

LYMTLWSEIGEESYWSEGILHSELLTILNCGIPELYRARVKLEALGLLKTYLKQEPEK

QFIYELQSPLTAAEFFKDDLLSLLLLETVGKRKYKSLRQRYSINKIDTEKYVEVTKGF

LDVFSFDKNKFSENQELLKDSTALIGNERPVETTQLDNKTFEFKFFYEGLATQYINRS

SITKEIENSILVLHTMYGIDELEMQKFVLQACDIDSGKVDEKKLKQVVYSNYHQTNQQ

PVELKERIVEDPTSNQNKQKFRKNDLSHQGFSKEDIVLIEISEEFTPFDFIENIKKQK

GGYVARTEEWAVENLIKQSNLPKSVINILIHYVLVIRGNPTLTQNLADSIANDWSQSK

VKSPEEAIRKVQELYRENEIKKQKRAEQQASYSKPRNNQYNSAAGNQRNRQETLPEWA

KENQAEVEEKPMSEADQKAFMDRIARIQNLGKEGDQ"

CDS 728275..729201

/gene="dnaI"

/locus_tag="JFBMFIFI_00681"

/inference="ab initio prediction:Prodigal:002006"

/inference="similar to AA sequence:UniProtKB:P06567"

/codon_start=1

/transl_table=11

/product="Primosomal protein DnaI"

/db_xref="COG:COG1484"

/translation="MEDMGKGLTKIIKDRKLTSQYEQLIQEVLKDPDVAKFIEENREK

LTDERIEKSYAKLYEFVQEKRKFEKNQGMLAPGYRPTLFMNFHVIDVTYVPTSELIAK

QKEYEIRRRVNSMDMPKDVRTATLDKFQLTDDRREALSEVLDFIDSFLKEPKQFHKAL

YLQGSFGVGKTYLLGAIAHDLAKNGYTTTLLHFPSFAVEMKQSIGKNNMSEKLDTIKK

SAILMLDDIGADSMSSWIRDDVLGVILQYRMQEQLPTFFSSNFDMKQLENEHLRTSQR

GEDEPLKAKRIMERIRYLAKEINMTGKNRRNY"

CDS 729623..731560

/gene="thrS_2"

/locus_tag="JFBMFIFI_00682"

/EC_number="6.1.1.3"

/inference="ab initio prediction:Prodigal:002006"

/inference="similar to AA sequence:UniProtKB:Q97PI4"

/codon_start=1

/transl_table=11

/product="Threonine--tRNA ligase"

/db_xref="COG:COG0441"

/translation="MSEIKITFPDGAVKEYPAGSSTLEVAKGISNSLAKKALAGKFNG

ELVDLVRPLEVDGSLEIITPDHEDALQILRHSSAHLMANALRRLYPDIHFGVGPAIDN

GFYYDTDTEAPITEEDLPKIEAEMMKIVKENNPIVRKEVTREEALELFKSDPYKVELI

TALPEDEAITVYDQGDFVDLCRGVHVPTTGRIQVFKLLSLAGAYWRGNSNNKMMQRIY

GTAFFDKKDLKEFLQMREEAKLRDHRKLGKELDLFMTSSDVGSGLPFWLPNGATIRRV

IERYIVDREVSLGYQHVYTPIMANVEFYKTSGHWDHYHEDMFPPMDMGDGEMLVLRPM

NCPHHMMVYKNDIHSYRELPIRIAELGMMHRYEKSGALSGLQRVREMTLNDGHTFVRP

DQILDEFKRTMELMLAVYDDFDISDYRFRLSYRDPENTDKYFDDDAMWEKAEAMLKSA

MDELGLDYFEATGEAAFYGPKVDVQVKTALGIEETLSTIQLDFLLPERFDLTYVGEDG

ENTHRPVVIHRGIVSTMERFVAYLTEEYKGAFPTWLAPMQATIIPVNMDLHADYAYQL

KERMTAMGMRVEVDDRNEKMGYKIRASQMQKIPYQLVVGDNEVEEASVTIRKYGEKTT

TTEDFSMFLDSVVAEIKNFSK"

CDS 731728..733158

/locus_tag="JFBMFIFI_00683"

/inference="ab initio prediction:Prodigal:002006"

/codon_start=1

/transl_table=11

/product="hypothetical protein"

/translation="MKMKNSLAKIAVSCIVGGVIFLGLISNSTAVEKNKYKKITYPEV

GAFFLEPQTTDTTAEKQLNDYLNRRLVDLKSETRLKNYLPRMTAFLLNYTKDTTNTGS

ITYKIEEKKIVKSLKVNQELETVQRKADVTTISTKTGNPLMLHELFNDEETGYQEIKT

KLTKKLEESVGLSDQQKMEIMKQVNEVGFENTTHYGLSNNELIFQFPTEQGIEIVGFA

KNQLRRQLKAEYMTEALNQQVAEEVKQEEAIRAQKEAERKAAEERQRIIESQQAKLPS

SGKVIALTFDDGPDPAVTPRVLDLLAKYNARATFFVLGKNAAAYPNLVVQEINAGHEI

GNHSWSHPDLTALPKEKALNQVNQTNQTIQELTGYTTNLLRPPYGAITNALASDVKMP

IIEWSVDTMDWKSKNGTAVYQETMKNAQNGSIVLMHDIHPTTADGLERILKDLTAQGY

KFVTISELFGTPMQPGLQYYSRGSVR"

CDS 733405..733926

/gene="infC"

/locus_tag="JFBMFIFI_00684"

/inference="ab initio prediction:Prodigal:002006"

/inference="similar to AA sequence:UniProtKB:P03000"

/codon_start=1

/transl_table=11

/product="Translation initiation factor IF-3"

/translation="MTIAKDMMVNDGIRARELRLIGSDGEQLGVKTKSEALQIAEAAN

LDLVLVAPTAKPPVARVMDYGKFRFEQQKKEREARKNQKIINVKEVRLSPTIDVNDFN

TKLRNARKFLEKGDKVKASIRFKGRAITHKEIGHKVLTRLADETEDLATVESKPKMDG

RSMFMLLAPKNDK"

CDS 733957..734157

/gene="rpmI"

/locus_tag="JFBMFIFI_00685"

/inference="ab initio prediction:Prodigal:002006"

/inference="similar to AA sequence:UniProtKB:P55874"

/codon_start=1

/transl_table=11

/product="50S ribosomal protein L35"

/db_xref="COG:COG0291"

/translation="MPKQKTHRGSAKRFKRTGGGGLKRHHAFTSHMFANKSQKQKRKL

RKAGMVSKGDFKRIRQQLSQMK"

CDS 734272..734631

/gene="rplT"

/locus_tag="JFBMFIFI_00686"

/inference="ab initio prediction:Prodigal:002006"

/inference="similar to AA sequence:UniProtKB:P55873"

/codon_start=1

/transl_table=11

/product="50S ribosomal protein L20"

/db_xref="COG:COG0292"

/translation="MPRVKGGTVTRKRRKKVLKLSKGYYGAKSKLFKVANQQVMKSYQ

YAYRDRRQKKRDFRKLWIARINAAARMNNMSYSVLMHGLKLAEIDINRKMLADIAVHD

AAAFTALAEQAKTALGK"

CDS 735142..737334

/gene="nrdD"

/locus_tag="JFBMFIFI_00687"

/EC_number="1.1.98.6"

/inference="ab initio prediction:Prodigal:002006"

/inference="similar to AA sequence:UniProtKB:P28903"

/codon_start=1

/transl_table=11

/product="Anaerobic ribonucleoside-triphosphate reductase"

/db_xref="COG:COG1328"

/translation="MVELTSDKLVSDEDASKVLNLLVVKRDGRSVRFDDKKIYEALMK

AEKEVNVGTAIQTENEIKEIMDKINQEILARFTTDIKIYEIQNIVEHILLSEKKVELA

DRYINYRATRDFERAQSIDINFTIEKLINKDRSVVNENANKDSEVFNTQRDLTAGIVG

KSMGLKMLPPHVANAHQKGEIHYHDLDYQPYSPMTNCCLIDFKTMLTEGFKIGNAEVE

SPKSIQTAAAQMAQIIANVASSQYGGCSADRIDEVLAPFAELNYKKHLATAKEWIDGE

ERQLAFAKEKTDKDIYDAMQSLEYEINTLYSSQGQTPFTSLGFGLGTSWIEREIQQAI

LKVRIKGIGKEQRTAIFPKLIFTLKRGVNLNETDPNYDLKKLALECATKRMYPDVLMY

DKLVELTGSFKTPMGCRSFLQGWQDENGVDVSSGRMNLGVLTLNLPRIAMEANGDKER

FWSLLNERLDICKDGLVYRVERVKEATPSNAPILYMHGAFGKRLNKTESVNELFKNRR

ATVSLGYIGLYEVATAFYGGTWEQNQEAKKFTLAILETMKKQTDAWSNKYDYHFSVYG

TPSESLTDRFCRLDTEKFGLVKDITDKEYYTNSYHYDVRKNPTPFEKLEFEKDYPQFA

SGGFIHYCEYPNMRQNPKALESVWDFAYDKIGYLGTNTPIDHCYKCDFDGDFEPTERG

FECPQCGNHDPKTCDVVKRTCGYLGNPQARPMVHGRHKEISSRVKHMK"

CDS 737351..737908

/locus_tag="JFBMFIFI_00688"

/inference="ab initio prediction:Prodigal:002006"

/codon_start=1

/transl_table=11

/product="hypothetical protein"

/translation="MRNPKPQEWQSEKLSKQKIADYKPFNFVDGEGVRCSLYVSGCLF

ACEGCYNKVAQSFNYGIEYTVELENQIIEDLSHSYVQGLTLLGGEPFLNTEVTIGLAK

RIRQEYGNTKDIWSWTGYLWEELMLETPDKLELLGLIDIVVDGRFELAKKDLTLQFRG

SSNQRIIDVQKSLATEKVVLWNPAL"

CDS complement(737970..739034)

/gene="graS"

/locus_tag="JFBMFIFI_00689"

/EC_number="2.7.13.3"

/inference="ab initio prediction:Prodigal:002006"

/inference="similar to AA sequence:UniProtKB:Q7A6Z3"

/codon_start=1

/transl_table=11

/product="Sensor histidine kinase GraS"

/translation="MNGFKFLKDQWILIVFWFIGLGILNLVLILDPNQTFNIDNLIYV

SLLLTVFFLFLLAGLYVYYSKWYREIEEKRDTGEDGLLSFLDGAMNEEQHFIQDYIND

ILVLHQKEINRLTKAQQDQKDFVDSWVHEIKVPLAGTKLIIESLEDDIPERKLYQLEN

ELKKMNHYVEQVLYFSRIDSFSRDYLLQEYSLEKIINTVIRDNALYFIQNHLTLEFDA

ENQKVLTDEKWLIFIVEQILSNSIKYTPVGGTITIGLSKNNLGAWLTITDRGIGIPLE

DQRRIFDKGFTGYNGRVDTSHHSTGLGLYLAKNLSEKLGHRLFVESTVGEGTTFKILF

PFLTYFNDESEESQFIPTKP"

CDS complement(739162..739836)

/gene="graR"

/locus_tag="JFBMFIFI_00690"

/inference="ab initio prediction:Prodigal:002006"

/inference="similar to AA sequence:UniProtKB:Q5HI09"

/codon_start=1

/transl_table=11

/product="Response regulator protein GraR"

/db_xref="COG:COG0745"

/translation="MFKIMIVEDDETIRESVSDSLLKWDFDCFSTTNFANTLTDFIEE

KPHLVLLDINLPVFDGFYWCQKIREISNVPILFISSRSTNMDMVMAMNMGGDDFINKP

FSIEVLIAKINAVLRRTYNYADQATDAIQHNNVILNLQDGSATVNDTHIDLSKNEYKL

LHLLMKNHGKIISREKLLRGLWDDERYVDDNTLTVNINRLRKKIDQAGAVGYIETKIG

QGYIIP"

CDS 740075..740842

/gene="bceA"

/locus_tag="JFBMFIFI_00691"

/inference="ab initio prediction:Prodigal:002006"

/inference="similar to AA sequence:UniProtKB:O34697"

/codon_start=1

/transl_table=11

/product="Bacitracin export ATP-binding protein BceA"

/db_xref="COG:COG1136"

/translation="MKKIVEVNQLKKTYGKKGNVYIALENISFDIEKGEFIGIMGPSG

AGKSTLLNIMSTIDRPTTGHVKIDGTDVTLMKESRLSQFRRHELGFIFQDFNLMDSLT

VKENILLPLALDKQKVTIMEEQVVKVAKILGIEELLEKYPAEISIGQKQRVAAGRAII

TQPKLIFADEPTGALDSKAATELLQYLSEINRLEETTILMVTHDAFSASYCSRILFIK

DGLLFAEIVRNGTRKQFFQKIIEMQATIGGGISHDLI"

CDS 740829..742814

/gene="bceB_1"

/locus_tag="JFBMFIFI_00692"

/inference="ab initio prediction:Prodigal:002006"

/inference="similar to AA sequence:UniProtKB:O34741"

/codon_start=1

/transl_table=11

/product="Bacitracin export permease protein BceB"

/db_xref="COG:COG0577"

/translation="MTLSKLAFKNMKTQFRDYFVYFVSMSFSIMVYFTFILMAYSPIL

KKVAENSIKVDTLLQVSSVMILIFVVLFMFYSNAFFIKKRKKEIGLYNLLGMRKWQIA

QLFFLENLILGAGALLIGILLGSLLSKLFAMILLAVMRLKIDFHFNFSSAAISQTCLV

FLFIFVIVAIQNASLVYRYKIIDLFKASGVGNQIPKATVFTLIRGLLGIFLIGLGYWT

ALNLISILGFSSSYIFLGIVLSIVINTVVGTYLFFNAFLVILIKFSQRQKKLYYRGLN

LLMISSLLFRLKRNANTLATIAVLSATTLCAVGGAAALYTFTEGTIDLTTSFDIHYEA

SNTKVKKIIEDTLNEFPKFKISEEATGEFKVISGKSDNSKKETANFYSVISESDYNKI

VGVSNYGKKINLKDSSSGFLISQYFLSTIFDNPIQKKIQLKNQSIDVTIQGFDDHIPF
GTRAVNGDVLVVTDELYKKIKNPAATFTRRGIMLSNYADSQALNKTLADRLESYNKSV
FLDSKEEDVFFTPRDLFVLRQPYEATVYSSVGSIMYVAIFLGLVFMFATGSIIMLKQL
SEAEEELHHYRILKKIGVSSKEIQKSIYRQTAFVFAFPIIIGSLHTYVAIHAMSKVLL
NTDLWLGYVASACFILIYSIFYLFTARAYIRIVNRKV"

CDS 743178..744866

/gene="oppA"

/locus_tag="JFBMFIFI_00693"

/inference="ab initio prediction:Prodigal:002006"

/inference="similar to AA sequence:UniProtKB:P24141"

/codon_start=1

/transl_table=11

/product="Oligopeptide-binding protein OppA"

/db_xref="COG:COG4166"

/translation="MKKGKLVSLLGIALTSTIVLAACGGGSGSDKASDSASKDSSGKM
AKKQELNLIESAEIPTMDSVMNTDTVGSIVMNNVFEGLYRQDLDNNPVLGMAAEEPKI
SDDKLTYTFKIRDDAKWSNGDPVTAGDFVFSWRRLVDPTTAAPYSYMMDGVIANASAV
IAGEKQPDELGVKAVDDKTLEIQLERAVPYFKGLLSLAMYYPQNEKFVTEQGEKYASN
SDALVYNGPFKLAAWDGTGLSWEYDKNDTYWDKDTVKLDKINVDVVKETSTALNLYDD
GQIDRMVLTGEYVQTRQDDPDLKKFPTSSVFFLKFNQERNGKATPLANENIRKALSMA
FDKQAYADTVLQNGSVPANGLVPAGLAKDPKNDKDFRKENGDLSKYDAKKAQEFWKKG
LKELGVDKLDLEFLSDDTENAKRSSEFMQGQLEENLDGLKITLRNVPFKVRIDNNDKQ
DYDIQLSGWGADFSDPINFLELFETGNGNNKSGFSNEKYDSLLKEISTTSLTDPEKRW
EQMLEAEKVLFDNGAIAPIYQRYWAVLEKPTVKDIGYHLVGADYSYKWTYLEDK"

CDS 745252..746940

/gene="dppE_2"

/locus_tag="JFBMFIFI_00694"

/inference="ab initio prediction:Prodigal:002006"

/inference="similar to AA sequence:UniProtKB:P26906"

/codon_start=1

/transl_table=11

/product="Dipeptide-binding protein DppE"

/db_xref="COG:COG4166"

/translation="MKKSKVIGLASLALFSTIVLAACGGNKDNSSTDSGSKTANSGEL
AEKQELNLIESAEIPTMDSVLNTDAVGSIVMNNVFEGLYRKGLDGENVLGMAKEEPTV
SEDGLTYTFKLREDATWSNGDAVTANDFVFAWQRVVNPETAAQYSYMMSGIIENASEI
SKGEKPVTDLGIKAIDATTLEVKLAAAVPYFKDLLSLTMYMPQNEKYVTEKGEDFAKN
SENLVYNGPFELTDWDGTGLSWSYKKNDSYWDKKAVKLDRINVDVVKETGTALNLYDS
GDIDQMKLSGEYVQTRDGDPDLISIPTSSVFYLKYNQKRGDKDTALANENIRKAISMA
FDKQAYSDTVLQNGSIPADGLVPEGLAKDPENGEDFRKENGNLLSYNQKEAKKLWEKG
LKEIGTDKVTLEMLSDDTENAKRSLEFMQNQLQENLPGLTIKLKNVPFKVRVQLNNQM
DYDIQVAGWGADYPDPINYLELFGTDNSNNRSHYSNAEYDQLLTEIGTTSLADPEKRW
DQMLEAEKILMKTAGIGPMYQRYNAVLQKPYIKDLARQIVGPEYSYKWAYVEKH"

CDS complement(746976..747557)

/locus_tag="JFBMFIFI_00695"

/inference="ab initio prediction:Prodigal:002006"

/codon_start=1

/transl_table=11

/product="hypothetical protein"

/translation="MKITYTLSKNDYLATQEFILDYSKQFKKQLRMQRNLILGIFFIT
LALLLQLTAKSGWQLTWVTTFLTILALALFVSLFFLKKMTKKMALRKLENNGDMHPEL
FTLTRKIELGNKQIKTRMGTNPPTTAQWSMVQKVIIKPDFILYFINDSQANLLYCPSM
NLSNTDYEELKQFIYSHLPNERINEIKEKASEK"

CDS 747701..748222

/gene="niaR"

/locus_tag="JFBMFIFI_00696"

/inference="ab initio prediction:Prodigal:002006"

/inference="similar to AA sequence:UniProtKB:Q9X1T8"

/codon_start=1

/transl_table=11

/product="putative transcription repressor NiaR"

/db_xref="COG:COG1827"

/translation="MKAIERREAIVVSLTKSSQAISASQLAKELNVSRQIIVGDVALL
RAEGMDILATPRGYIIPKASIEERFIGQIACQHTEKEMREELYTIVDYGGEVVNVIVE
HALYGELIGQLTIRSRSEVDDFFVKMSENQALPLSHLTNGIHLHTIACKDKKTFLKIK
AELTKLGILYQNE"

CDS 748310..748864

/gene="niaX"

/locus_tag="JFBMFIFI_00697"

/inference="ab initio prediction:Prodigal:002006"

/inference="similar to AA sequence:UniProtKB:A2RKV5"

/codon_start=1

/transl_table=11

/product="Niacin transporter NiaX"

/translation="MKTGKVQRLTISALLIAVGILIPMFSPVRFMMEPVSFTLGSHVA

IFIAMFISPSVAVAVALGTTFGFFIGGFPIIIVMRALSHVLFALVGALILKKYPTILK

SPVKSQIFSLGIGIIHGAAEVAVVAVFYFTGNAAGTFYSQGFMQSIILLVGVGSVVHS

MIDFALSYIIWRGLTARRSPLTNK"

CDS 749110..749679

/gene="spsB"

/locus_tag="JFBMFIFI_00698"

/EC_number="3.4.21.89"

/inference="ab initio prediction:Prodigal:002006"

/inference="similar to AA sequence:UniProtKB:P0A068"

/codon_start=1

/transl_table=11

/product="Signal peptidase IB"

/translation="MNGKSNSRGFIRELFSTLITLAVALTVVLLLRTFIFSPVIVKGE

SMSPTLANSDRILLLKMEKVKRFSIVTFPAPDDPSQNYVKRVIGLPGDSISYKNDVLE

INGKAYEEPYLDEYKAQLPERTLLTNDFTLEQITGDEVVPEGEYLVLGDNRQNSKDSR

MIGYIKADDIQGVADYRIWPIKTFGRIDQ"

CDS 749974..750510

/locus_tag="JFBMFIFI_00699"

/inference="ab initio prediction:Prodigal:002006"

/codon_start=1

/transl_table=11

/product="hypothetical protein"

/translation="MFTKFKPTWMVEAIYQITPEQLKKRNFKAVLTDLDNTLIAWNNP

DGTEELLLWIEEMKNAGIPVVVISNNKAVRIERVVNQLGLKYVQRAMKPLTKGFKEAQ

KLVDLPKDQILMVGDQIMTDIRGANAAGIQNVLVKPVVESDAWNTKFNRAMERRIMKY

LWKKHPEMSWRKTIDDNE"

CDS 750497..751630

/gene="yqeH"

/locus_tag="JFBMFIFI_00700"

/inference="ab initio prediction:Prodigal:002006"

/inference="similar to AA sequence:UniProtKB:P54453"

/codon_start=1

/transl_table=11

/product="putative protein YqeH"

/db_xref="COG:COG1161"

/translation="MTTNEVDLNEEIRCIGCGAVIQTNAPGELGYTPVSALEKGLENG

EVYCQRCFRLRHYNEIQDVQLTDDDFLRLLNEIGAEDALIVNVIDIFDFNGSLIPGLH

RFVGNNPVLLVGNKVDLLPHSLKRGKMTQWLRERAHEEGLRPKDVILTSAMKPKEMDD

LLKTIEKYREGRDVFVVGVTNVGKSTLINQIIKNTAGVADVITTSQFPGTTLDRIEIP

LDDGKSLIDTPGIIHRHQMAHVLGDKDLKLIAPKKEIKPMVYQLNEGQTLFFGGVARF

DYIQGGRRSFTCYTSNDLKIHRTKLEKADELYANHVGEMLQPPRKDEVGNFPELVRFE

FSVKERSDIVFAGLGWVTVNEPGAVVAGWAPKGVDVVIRKSLI"

CDS 751680..751997

/locus_tag="JFBMFIFI_00701"

/inference="ab initio prediction:Prodigal:002006"

/inference="similar to AA sequence:UniProtKB:P71376"

/codon_start=1

/transl_table=11

/product="RNA-binding protein"

/db_xref="COG:COG1534"

/translation="MKLTGKQKRFLRSQAHHLTPIFQVGKGGLNDDMVKQIGEAVEKR

ELIKVSLLQNTDEEVDDVALALEKALKVNAVQVIGRVIVLFKPSSQEKYQRISTELPR

ASR"

CDS 752026..752649

/gene="nadD"

/locus_tag="JFBMFIFI_00702"

/EC_number="2.7.7.18"

/inference="ab initio prediction:Prodigal:002006"

/inference="similar to AA sequence:UniProtKB:P54455"

/codon_start=1

/transl_table=11

/product="Nicotinate-nucleotide adenylyltransferase"

/db_xref="COG:COG1057"

/translation="MQSQTNVLTEVESLPEERIRVGIIGGTFNPPHIGHLVIADQVCQ

QLGLDKVYFMPDANPPHIDKKEAIAAEHRVAMVEKAIEDNPLFGLESCEIQRGGISYT

FDTMLELTKAHPEIDYYFIIGGDMVDYLPKWYRIDELIQMVQFVAVKRPNYADSSPYP

LIWVDVPAMEISSTGLRKKIKNGCSVQYLIPDKTLAYIKEKELYQND"

CDS 752642..753244

/locus_tag="JFBMFIFI_00703"

/inference="ab initio prediction:Prodigal:002006"

/codon_start=1

/transl_table=11

/product="hypothetical protein"

/translation="MTKSDIVYGNQYFRLDRISLLERVQMQMSERRFKHVLGVEEMAI

ALAGRYGASLEAASIAALTHDYAKERDSDEMREIIQREGFPIELLEYGNEIWHGPVGA

YLVQKELGVDDEDILNAIRHHTVGASEMSLLEKIIYVADYIEPGRDFPGVAEARKLAI

TDLDAAVAFETKHTLLYLVEKNAKICPLTIATYNSWVVKS"

CDS 753256..753615

/gene="rsfS"

/locus_tag="JFBMFIFI_00704"

/inference="ab initio prediction:Prodigal:002006"

/inference="similar to AA sequence:UniProtKB:Q9KD89"

/codon_start=1

/transl_table=11

/product="Ribosomal silencing factor RsfS"

/db_xref="COG:COG0799"

/translation="MTLSSEEVLKIAVKAGDDKRAEDIMALDVRTISILADYFVVMHG

NSEKQVEAIMNEVIDNAEAAHVEVKRVEGRDSAKWILIDLGDVVVHVFHYSERAFYNL

EKLWSDAPLVDISEMVS"

CDS 753618..754355

/gene="COQ5_2"

/locus_tag="JFBMFIFI_00705"

/EC_number="2.1.1.163"

/inference="ab initio prediction:Prodigal:002006"

/inference="protein motif:HAMAP:MF_01813"

/codon_start=1

/transl_table=11

/product="2-methoxy-6-polyprenyl-1,4-benzoquinol

methylase, mitochondrial"

/translation="MSYQTFAQVYDAIMDDSLYKDWLSFVQQEIKNEGQSLLELACGT

GALAVTLKEAGFDVTGLDLSENMLSLANERALKKGLKLPLLEGNMLDLSEIGHYKVVT

CFSDSICYMPDDEAVLKVFQEVFSILENGGKFLFDVHSTYQIDEIFPGYMYNDQSEEI

SFLWTSYEGENPHSIEHDLTFYVLNAETSLYERFDERHYERTYPIEDFLTMLKVAGFS

RVKVTTDFGRAAVTKESTRWFFTCEKD"

CDS 754592..756361

/gene="recQ_1"

/locus_tag="JFBMFIFI_00706"

/EC_number="3.6.4.12"

/inference="ab initio prediction:Prodigal:002006"

/inference="similar to AA sequence:UniProtKB:P15043"

/codon_start=1

/transl_table=11

/product="ATP-dependent DNA helicase RecQ"

/db_xref="COG:COG0514"

/translation="MEEQAVKLLNEIYGYSDFRNGQKEIISKVLHGQDALAIMPTGGG

KSICYQIPALVFSGLTIVISPLISLMKDQVDALLELGIPAAYVNSTLSTKEMNQRLRD

AADGAYKLLYVAPERFQTEEIHYLMQSVQVDLIAIDEAHCISQWGHDFRPSYLALCET

LAGMVQRPTVLALTATATPVVSEDIIKLLGISEENCVKTGFSRKNLAFQVVKGQDRNR

YLLDYLKLNDGQSGIVYASTRKEVERIHQLLLSKGIQSGMYHGGMGEQARNVAQEAFL

YDKTTVMVATNAFGMGIDKSNVRFVIHYQIPKNIEAYYQEAGRAGRDGLDSDAILLFS

PQDMMIQQFFIDQSEMNEELKKYEYAKLREMAQYGSTQMCLQRFIVRYFGEDCPDCGR

CSNCLDDREAAIITLEAQKVLSCVKRMGETFGKSMIMKVLTGSKDQKIKQWNFEELST

YGLMKGVPQKEVTQLIDYLTAEKYLSPTDGQFPILKVTTKGVAVLKGEEEVTRKQAKV

AEKVAVDDVLFDQLREVRSQLASEHKVPPYIIFSDETLREMCQKLPTNDEEMLTVKGI

GENKLEKYGNYFLEILLTAKTVK"

CDS 756411..757589

/gene="tmcAL"

/locus_tag="JFBMFIFI_00707"

/EC_number="6.3.4.-"

/inference="ab initio prediction:Prodigal:002006"

/inference="similar to AA sequence:UniProtKB:O34513"

/codon_start=1

/transl_table=11

/product="tRNA(Met) cytidine acetate ligase"

/db_xref="COG:COG1323"

/translation="MKSCGVIVEYNPFHNGHRYHIQQARLKSGADVVIAVMSGNFLQR

GEPALLDKWARAEMALAAGVDIVVELPVYSSVQAADYFAEGGIAILHALQCDSFCFGS

ENGKEKDFQQVAKWLIDEKEAFDLAFQMVKNSGSSYAAQVEALLKEVYPTLSLNLSEP

NNALGLSYAKANLAYPKPMEMYTIKREKAGYHETTIQDLEFASATAIRKSLIKKESTV

ESVMPASSLELVNQEKFVSWENYWQLLRYQIMVSTVDDLQQIYQMNEGIEFRLKEKVK

EVETFTEFIGLVKNKRYTWTRIQRLCIYILLQLKKTDVEASVEGLKAIRVLGFTEQGR

RYLKAKKTKIKLPIVTNLNAANKNFFDLDIRAGFIYQLGNPHISPQDYRRKPIYQIKS

"

CDS 757824..758384

/locus_tag="JFBMFIFI_00708"

/inference="ab initio prediction:Prodigal:002006"

/codon_start=1

/transl_table=11

/product="hypothetical protein"

/translation="MKWSLIELQKYRNEPLLFSETVELKKSLMERDSEILDVSPIKID

GTLIVEKEEVIAHLVISLGLTMPSARSLKPVLVPMLIEANEIYVPKNVTDFAANEREE

TVIYLDKDLIDLTEAVEDTILLNLPLQVFTPEEEAEDDMPSGNGWEVVSEEEYVMRLE

EQKSQSVDPRFAGLADLFSDNTDNDQ"

CDS 758459..758638

/gene="rpmF"

/locus_tag="JFBMFIFI_00709"

/inference="ab initio prediction:Prodigal:002006"

/inference="similar to AA sequence:UniProtKB:P66210"

/codon_start=1

/transl_table=11

/product="50S ribosomal protein L32"

/translation="MAVPARRTSKAKKNRRRTHYKLEVPGMNPCPNCGELKKSHHVCP

ACGQYDGKEVISKEA"

CDS 758794..759168

/locus_tag="JFBMFIFI_00710"

/inference="ab initio prediction:Prodigal:002006"

/codon_start=1

/transl_table=11

/product="hypothetical protein"

/translation="MKIPFTIAEIAFYIAPVLFLLILKSLIRKRLIIRHVPIKPPDVL

VPFLLIGIHILSEMTLKFSLLPYFAIFMLSLGIVIVCLMAYKKGEILFSRFFKTFWRF

LFLFSFLMYYILVGLNIAHHFI"

CDS 759493..759924

/gene="mraZ"

/locus_tag="JFBMFIFI_00711"

/inference="ab initio prediction:Prodigal:002006"

/inference="similar to AA sequence:UniProtKB:P65439"

/codon_start=1

/transl_table=11

/product="Transcriptional regulator MraZ"

/translation="MLMGEFKHNIDAKGRLIMPAKFREDLGEKFIITRGMDGCLFGYP

QNEWSALEEKLKQLPLAKKDARAFTRFFYSAATECELDKQGRINIPQTLREHADLEKV

CHVIGVSDRIEIWSQERWTKFSDEAEESFDDIAENMIDFGF"

CDS 759941..760894

/gene="rsmH"

/locus_tag="JFBMFIFI_00712"

/EC_number="2.1.1.199"

/inference="ab initio prediction:Prodigal:002006"

/inference="similar to AA sequence:UniProtKB:P60392"

/codon_start=1

/transl_table=11

/product="Ribosomal RNA small subunit methyltransferase H"

/translation="MTTFNHETVLLHETVDGLALKPDGVYVDCTLGGAGHSEYLLSQL

SEEGHLYAFDQDERALENAKIRLAPFVEKGMVTFIKSNFRFIKEELNQIGIFEVDGIL

YDLGVSSPQLDEAERGFSYHQDAPLDMRMDTQAPLTAKEIVNTWSYHELIRIFYRYGE

EKFSKQIARKIEAAREVAPIETTGELVELIKEGIPAPARRKGGHPAKRVFQAIRIAVN

DELSAVEDSLEAAISLIKVGGRVSVITFHSLEDRIVKSIFKEHSALPELPPGLPVMPT

EFQPELKLVNRKPIIPTEEELEQNNRARSAKLRIAEKQKEK"

CDS 760935..761312

/gene="ftsL_1"

/locus_tag="JFBMFIFI_00713"

/inference="ab initio prediction:Prodigal:002006"

/inference="protein motif:HAMAP:MF_00910"

/codon_start=1

/transl_table=11

/product="Cell division protein FtsL"

/translation="MVLTNNLARELEIDVPLRSPEIPKESPVHMPAPRKAGVTKLEKL

MLIVVGVTAFMLMAVCISQEISISSKNRGIQDTTVAISDTKNVNDNLAQEIQELSRYD

RVYDIALKADLKMNQNNVRNVTK"

CDS 761309..763498

/gene="pbpB"

/locus_tag="JFBMFIFI_00714"

/inference="ab initio prediction:Prodigal:002006"

/inference="similar to AA sequence:UniProtKB:Q07868"

/codon_start=1

/transl_table=11

/product="Penicillin-binding protein 2B"

/db_xref="COG:COG0768"

/translation="MKKNKSPQKNRKQIAMILFFLTIFLFVVFISRFSYVMVKGQING

EDLAEKVNNLYTRSSVLKANRGTIYDIDGKPIAMDAASYSLVAVLTDKWSTKENPNYV

KDKEKTAEVISQNIPLSKEAVLEILNRKGEDGKPLAQVEFGNEGKDLSYETKVTIEKT

KLPGLVFTETPKRLYPNGVFASNLVGIASLPTKTDEDTADFVPGTELVGEMGIEQAYN

KLLTGTPGKYEFKTDNFGYALPNSKVKEVKAKDGSDMYLTLNNRLQVYMESKVAEIAA

KYQPETMTATLMNAKTGAIIATTQSPTFNATTKENIDKSWRNMLVEDSFEPGSTMKVL

TLGAAINEGVFNPNETFLSGTKTIEGGQIRDHNGGVGWGHISYLEGLQRSSNVAFSNL

VEKMGNDTWKKYMTDFGLEKSTDSGLPNEAIGKIGFDYPLEKANTAFGQGLTVTDFQM

LQAFSAVANKGKMMKPYFVDRTVNPNTGEEKVTQPTVVGQPITAETAQKELEYLQEVV

YGENGTGQAYKIDGYKIAAKTGTAEIVDPATGLYATGDNNYVFSVVGMAPAEDPELIM

YITMKQPQVYDGTITGGGMIAEVFNPVMKRALQYQELGKQAEIEENNQVVMPRVMGSL

KEEARQKLEENKLNVTIIGNGDTIVQQMPLPDEPVLEGQRAMLLTNGAMTMPNMTGWS

KNDVLKVSEMTGQKFKIVGDGFVTTQSLAENSSMEGVAEIEVTLVAP"

CDS 763520..764995

/gene="murE_1"

/locus_tag="JFBMFIFI_00715"

/EC_number="6.3.2.13"

/inference="ab initio prediction:Prodigal:002006"

/inference="similar to AA sequence:UniProtKB:Q819Q0"

/codon_start=1

/transl_table=11

/product="UDP-N-acetylmuramoyl-L-alanyl-D-glutamate--2,

6-diaminopimelate ligase"

/db_xref="COG:COG0769"

/translation="MKASNLIASIQFKRLSKELPKDLEINSITQDTREITEGSLFVCI

TGALFDGHQFAQDAVEKGAVLLIAEKPIDVSVPVIYVANANRAMAIVADAFYDHPSQD

FRIIGVTGTNGKTTITHLIDQIFRDHQEVTGVIGTMYRRIGNTTFETKNTTPDSLTLQ

KTFAEMRQKNVTTCAMEVSSHSLVQGRVWGTDFDIAVFTNLSQDHLEYHHTMEEYAHA

KSLLFSQLGNIYNPEKPKYAILNMDDPVGRDFRKMTAAEVLTYGIEEAADFRANSIEI

TNHGTRFLLEFNGKEYPVHLQLAGKFNILNALGAIGAAYAAGLELETIISSLEGISGV

RGRFELVKGPQDFAVVVDYAHTPDGLLNVLTTINEFKTGDVYCVVGCGGDRDKTKRPK

MARIAMDYADHVIFTSDNPRTEDPNEILKDVVAELGSDESYLLEVDRRKAIELAVKKA

QANDIILIAGKGHEDYQIIGTTKHHFDDVEEAKKAIETFYK"

CDS 765184..766149

/gene="mraY"

/locus_tag="JFBMFIFI_00716"

/EC_number="2.7.8.13"

/inference="ab initio prediction:Prodigal:002006"

/inference="similar to AA sequence:UniProtKB:Q2FZ93"

/codon_start=1

/transl_table=11

/product="Phospho-N-acetylmuramoyl-pentapeptide-

transferase"

/db_xref="COG:COG0472"

/translation="MHWTEMLMPFVSSFALTIMVMPMFIGYFRMKQIGQVTRDEGPKW

HEVKTGTPTMGGVVFIIATLITAVWVGIWQGALTLSLGLLLFILALYGVLGFLDDFIK

VFKKRNLGLTSKQKLIGQIIGGIIFFIVFKYEGLATKIAFPFIGSIDIGWLYGIFVIF

WLVGFSNAVNLTDGLDGLVAGTASIAYGTYTVIAWHQQQTDILIFCVTIVGGLVGFFF

FNKKPAKIFMGDVGSLALGGGLAAVSILLHQEWSLLLIGLIFVIETASVMIQVTSFKL

TGKRVFKMSPIHHHFEMSGWSEWRVVLTFWSVGLLAAIVALLVIL"

CDS 766167..767549

/gene="murD"

/locus_tag="JFBMFIFI_00717"

/EC_number="6.3.2.9"

/inference="ab initio prediction:Prodigal:002006"

/inference="similar to AA sequence:UniProtKB:Q8E186"

/codon_start=1

/transl_table=11

/product="UDP-N-acetylmuramoylalanine--D-glutamate ligase"

/translation="MKKITTYENKKVLVVGLALSGVNAAKLLHELGALVTVNDYKSFE

ENPEAQELLESGIRVVTGGHPVELLDEDFELVVKNPGIMYNNPIVQRAVEKGIPVITE

VELAYEVSESQMIGITGTNGKTTTTTMIAEILNFNRKKGHAYVAGNIGMPASLVAQTT

TPEDEIIIELSSFQLMGITKMKPSIAVITNIFEAHIDYHGTREEYVAAKWRITENQTE

EDYLILNWDQEELRELAKTSKAKIIPFSRKEVLANGAYLKGDMIYYQDEEIMDKKAIL

IPGEHNVENALAAIAVAKLLGQSNENIVGLLTTFAGVKHRTQFVTEYKSRKFYNDSKA

TNILATENALKGFTDPVILLAGGLDRGNGFDELIPALRNVQALIVFGETAAKLEEAGK

AAGVKEIHVVQNVEAAVPVAYELSTEKSVILLSPACASWDQYRSFEVRGDVYIKAIEQ

LISDLKEQEE"

CDS 767577..768674

/gene="murG"

/locus_tag="JFBMFIFI_00718"

/EC_number="2.4.1.227"

/inference="ab initio prediction:Prodigal:002006"

/inference="similar to AA sequence:UniProtKB:Q9HW01"

/codon_start=1

/transl_table=11

/product="UDP-N-acetylglucosamine--N-acetylmuramyl-

(pentapeptide) pyrophosphoryl-undecaprenol

N-acetylglucosamine transferase"

/db_xref="COG:COG0707"

/translation="MKIILSGGGTGGHIYPALALIRRLKEVNPSVEIMYIGTEKGLEK

KLVEKAGIAFKSVEIQGFKRSLSWSNVKTIQLFFKSVADSKKLVKEFKPDVVIGTGGY

VCGPVVYAAAKLGVPTIIHEQNSVAGVTNKFLSKFVSKIALCFEEARGEFPKVPEKVV

LTGNPRGQEMLAVVKSDILAEYDLNPELPTLLIFGGSRGARRINEAFVEALPQLVGKD

FQILFATGEVHYQKISNELTILDVKNNNVSVVPYIYNMPEVFANVSLVMARSGATSLA

ELTALGLPSILIPSPYVTNDHQTRNAESLTHNGAAVMVPDAELTGDRLIKEVDRLMTD

TLARNKMAEAAKKIGITDASDRIIALINEVV"

CDS 768709..769605

/gene="divIB"

/locus_tag="JFBMFIFI_00719"

/inference="ab initio prediction:Prodigal:002006"

/inference="similar to AA sequence:UniProtKB:P16655"

/codon_start=1

/transl_table=11

/product="Cell division protein DivIB"

/db_xref="COG:COG1589"

/translation="MANWNKRRPYQKESKQSPQSMTPWEKMQQASNESSVEQEVQLKK

KKREKKVVSMEDKLPRLKELRRKKMYRRLYTLIFLFSFAIFLIVYFISPFSRIETISV

VGANEVTDQAVIDASRLKSGDSLWSNFFERKKIAQKIENQLEQVKSATLQFDGLNSFK

IAVKEYKTVAYLAKDNEYHNILENGKIVKESRKVSIGNPPIFVDFKEGPALDEMILQY

SQLSTNIKNSISEIQFTQSDSDDYLITLNMNDGNQVVASIPSFAEQMAYYPDAVKKTD

GQKGIINMEVGIFFKAFEEKAK"

CDS 769752..771068

/gene="ftsA"

/locus_tag="JFBMFIFI_00720"

/inference="ab initio prediction:Prodigal:002006"

/inference="similar to AA sequence:UniProtKB:P28264"

/codon_start=1

/transl_table=11

/product="Cell division protein FtsA"

/db_xref="COG:COG0849"

/translation="MGNTGIYVSLDIGTTSIKVVVAEFVNGQMNIIGVGNEKSEGLSR

GIIVDIDQTVDSIKKAIKQAEEKANIDIRNVIVGIPANNVKIEPCHGMIAVSGDNREI

TEIDVENVMAAAMVKSVPPEREIIAVIPEEFVVDGFSEIRDPRGMIGVRLEMHATMIT

GPKTILHNTKRCVEKAGLQIEEVVLQPLAISSAAMTKEEAKFGTILIDLGGGQSTAAV

MHDNQLKFTFVDPEGGEYVSKDISVILNTSFDSAERIKREYGFALPEETSPDEYFPVD

VIGKNEPVRIDEQYLSEIIEARQLQIFDKIKAELDAVGARELPGGIVITGGAAALPGL

IELAKDVFEINVNLYVPDQMGMRFPTFSTGIGLIEYVANLDEIHAIAKGRGKGSTYTS

AQPQKQTSHVEYETEEVVKEKAVKKPKNEEDKFTNKAKNFFTNFFD"

CDS 771185..772459

/gene="ftsZ"

/locus_tag="JFBMFIFI_00721"

/inference="ab initio prediction:Prodigal:002006"

/inference="similar to AA sequence:UniProtKB:P17865"

/codon_start=1

/transl_table=11

/product="Cell division protein FtsZ"

/db_xref="COG:COG0206"

/translation="MELEFDSNLNGAVIKVIGVGGAGNNAVNRMIDEGVKGVEFIVAN

TDIQALQSSNAEIKIQLGPKLTRGLGAGSNPDIGRKAAEESEEQIAEALRGADMIFVT

AGMGGGTGTGAAPVVARIAKEQSALTVGVITRPFTFEGPKRGRFAAEGVSEMKDHVDT

LVIISNNRLLEIVDKKTPMLEAFHEADNVLRQGVQGISDLITAPGYVNLDFADVKTVM

ENQGSALMGIGMASGENRTAEATKKAISSPLLEVSIDGAEQVLLNITGGPDLTLFEAQ

DASDIVSAASTTEVNIIFGTSINENLGDEVIVTVIATGIDARKGQQAQGGTNRSRGFA

NSEDTAFSRQTEAPATNQQAPQAKTEKDPFGDWDIRREPNVRENSNFQREDTSSEKAE

FDVFKRDSAAEKGHSNDEDSLDTPPFFRRRRK"

CDS 772460..773131

/gene="yggS"

/locus_tag="JFBMFIFI_00722"

/inference="ab initio prediction:Prodigal:002006"

/inference="similar to AA sequence:UniProtKB:P67080"

/codon_start=1

/transl_table=11

/product="Pyridoxal phosphate homeostasis protein"

/db_xref="COG:COG0325"

/translation="MTIKENLQQVEETLNLSLTHAQRQPEAATLIAVTKAVSNEETAE

IYHLGIKNLAENRPEGLIAKQDFLPEKDIIWHYIGNLQSRKVKQVINRIDYFHSLDRL

TLASEIEKRADHQIACFVQVNVSKEESKHGISPAELTEFIAELANYSKIQVIGLMTMA

PLGANEAEIRHYFSELRILQAEIASKKIPYAPCTELSMGMSNDYQLAVEEGATFIRVG

SLLFK"

CDS 773152..773649

/gene="sepF"

/locus_tag="JFBMFIFI_00723"

/inference="ab initio prediction:Prodigal:002006"

/inference="protein motif:HAMAP:MF_01197"

/codon_start=1

/transl_table=11

/product="Cell division protein SepF"

/translation="MSMLSNVSKFFGLDEEDDYDYEEYEVPEEAPKAVNTQPKAQVKT

FNEARQAAPTTNRVPNQKNSKVVAMNQSTIPQQSKIVVFEPRVYSEVQEVADLLISNQ

SVVLNFNRIEEDQAKRIVDFLTGTIYAINGDIQRIGDEIFLCTPNNVEIDGMLTEMMR

DKEFY"

CDS 773670..773930

/locus_tag="JFBMFIFI_00724"

/inference="ab initio prediction:Prodigal:002006"

/codon_start=1

/transl_table=11

/product="hypothetical protein"

/translation="MFELYLILARLINIYQGVMVVYILLSWLPGAYDSKLGVVLAKIC

EPYLSVFRRIIPSLGGIDFSPILAFFVLGLAQQGLRVILLGM"

CDS 773973..774752

/locus_tag="JFBMFIFI_00725"

/inference="ab initio prediction:Prodigal:002006"

/codon_start=1

/transl_table=11

/product="hypothetical protein"

/translation="MIENVYQHFRKEERPFIDALSEWITEVEDQYTPYLTDFLDPRQM

YIVESLVGKRDLKLYYFGGYEAAERKRVFICPDYYTPTQEEFKIDICEIRYPTKFAKL

SHSKILGTLMSVGVKREYFGDIVFDGERWQFFVDVGMKNYVENQVDKIGNVSVRIETR

DYTEIITPIDDWTLVQDTVSSMRLDAIIASIFKISRQRAKQLIEAGKVKLNWAEQLRP

DFELGLLDIVSVRGYGRIQIKEIEGKTKKDKWRIQFGVLFK"

CDS 774841..775509

/gene="gpsB_1"

/locus_tag="JFBMFIFI_00726"

/inference="ab initio prediction:Prodigal:002006"

/inference="protein motif:HAMAP:MF_02011"

/codon_start=1

/transl_table=11

/product="Cell cycle protein GpsB"

/translation="MVLTPLDIHNKEFPVKMRGYDQDEVNDYLDQIIKDYEMVLKDKR

ELEKKLKFSEEKVEHFNNLQDALNKSIIVAQEAADRVRENANKESRMIIMEAEKNADR

LLDEAVTKARKITLETDTLKKESRVFKQRLQLLIESQLEMVKNKEWDELLQATSLETI

EIPTLKDVLSGLPSNTEDDLVIEDEINELSSIDSYDDEKEEEDDKFHLEGTLAAIELP

EDKD"

CDS 776177..778960

/gene="ileS"

/locus_tag="JFBMFIFI_00727"

/EC_number="6.1.1.5"

/inference="ab initio prediction:Prodigal:002006"

/inference="similar to AA sequence:UniProtKB:P67509"

/codon_start=1

/transl_table=11

/product="Isoleucine--tRNA ligase"

/translation="MKMKETLQLGQTEFPMRGNLPVREEQWQADWKTVDMYGQRQKLN

EGKPTFVLHDGPPFANGNIHMGHALNKISKDIIVRYKSMSGYRSPYVPGWDTHGLPIE

QVLTNKGIKRKEMSLADYRRLCEEYALEQVNIQREDFKRLGVAGEWDNPYITLNPDYE

AAQIRVFGKMAEKGYIYKGLKPIYWSPSSESSLAEAEIEYKDVKSASIYVAFKVVDGK

GLLDNDTSFVIWTTTPWTIPANLGITVNADYSYVVIEADGAKFVVAKDLLEKVSSEIG

WENVTVLNELKGSELEYMTAQHPLYERTSLLMVGDHVTLDAGTGLVHTAPGHGDDDFI

VGQKYKLNVLSPVDSKGVFTDEAPGFEGVFYDKGNKLITEALNEKGALLKLDFFTHSY

PHDWRSKKPVIFRATPQWFASIQDFRGEILEAVEGVEWVQKWGKTRLYNMVRDRGDWV

ISRQRAWGVPLPIFYGENGEPIITPETIEHVANLFAEFGSTVWFEREAKDLLPEGFTH

EFSPNGIFTKEMDIMDVWFDSGSSHQAVLAARENLTYPADMYLEGSDQYRGWFNSSIT

TSVAINGIAPYKTVLSQGMVLDGEGRKMSKSLGNTILPSKVIKQMGADIIRLWVSSVD

AQSDVRVSDEILKQVSEVYRKIRNTMRFLLANTSDFKPAEHGIKYDDLRSVDKYLLIR

LNQLVGAVEENYENYDFSSNYQLINNFCTVDLSQFYLDFAKDVVYIEAENAYERRAMQ

TVFYEVLEKMTKLLTPILPHTAEEIWNFLDEEEDYVQLAEMPAAIEYPNQGELLDMWD

AFMTFRDHVQKALEEARNEKKIGKSFEAKLVLYPTEQTKVLFTALDSNLAQLLIVSDL

EIAGDKAAAPSEALQFDDVAIVVEKAHGETCERCRGVKKDVGSHADAPTLCDRCATIV

EEFYPAALEVEAE"

CDS 779321..779761

/locus_tag="JFBMFIFI_00728"

/inference="ab initio prediction:Prodigal:002006"

/codon_start=1

/transl_table=11

/product="hypothetical protein"

/translation="MRIRIEEDVSNDEELEVTIHCQSINEEVEALIQLLETQEESIPV

FKEKKIKQLSLKDIFYFETVDNKTFVYSATEVYQTNLKLYELEVRLTNSYFIRTSKSL

ILNILKIEHVTPAFNSRFEAKLENNETIIISRKFAPLLKKKLGI"

CDS 779779..780231

/locus_tag="JFBMFIFI_00729"

/inference="ab initio prediction:Prodigal:002006"

/codon_start=1

/transl_table=11

/product="hypothetical protein"

/translation="MENARDKFLGFIQRYCIIFTGSTLSMLAYLSIYKEPFVSIDVLL
VLMFISAIISFFQQLLFSSEENYSKKAVLIRSFLHFLIIMTSVLLSATLFNWVETTQQ
FWVLTLIIIGTYSILWLVFYFKDHLTSQKINQNLNLYKERMKKHDESN"

CDS 780215..781132

/gene="lnrL"

/locus_tag="JFBMFIFI_00730"

/EC_number="3.6.3.-"

/inference="ab initio prediction:Prodigal:002006"

/inference="similar to AA sequence:UniProtKB:P94440"

/codon_start=1

/transl_table=11

/product="Linearmycin resistance ATP-binding protein LnrL"

/db_xref="COG:COG1131"

/translation="MTNLINIQGLTKKYQQKEVVSSLDLKVEKGSLFAFLGPNGAGKS
TTINMISTAIKPTSGSIFVDGLEVGKENSKIRQKIGIVFQDNVLDDLLTVEENLLFWG
KIYSLSKNELNKQLDFLENLLHLTDILKQRFGTLSGGQKRRAEIARAIIHRPLLLILD
EPTTGLDPKTRVQVWQTITYLREKLGMTVFLTTHYMEEAAEADKIAVINHGELIALGT
PNQLRNKYSRDSLNLYKTSLEDLKIECEKRKLGYTEGIDFIRIQVRDTDEALHLLNEL
NDLYQSFEVRKGNLDDVFLALIKGGTNND"

CDS 781125..781979

/locus_tag="JFBMFIFI_00731"

/inference="ab initio prediction:Prodigal:002006"

/codon_start=1

/transl_table=11

/product="hypothetical protein"

/translation="MIRRGMKIYFRDPIGVLTSMLTVIIIIVLYAVFLGNNLTKAIEA
DTAGIEKIDLFVKAWVLAGIITVMPVTLSMGIFSIKIDDDLTGTTKGLMVAPISRFKI
VLSYLMTALIITISFTILAFIIGYFYLAQLGLKIVSWEVVVPILGVIVVNAFCSIAMM
FFVISFIKSRTSYSSISAIVGTLVGFLAGIYLPIGQLPGIVQTVMKFFPMTYSAGLLR
NNILDSSFKTIFADAPSSMVDSLKENLGVNLFIGNQLVTSRLAFFILISMGCLFLLLT
VLRKKKSS"

CDS 782261..783259

/gene="dapF"

/locus_tag="JFBMFIFI_00732"

/EC_number="5.1.1.7"

/inference="ab initio prediction:Prodigal:002006"

/inference="similar to AA sequence:UniProtKB:Q81XR2"

/codon_start=1

/transl_table=11

/product="Diaminopimelate epimerase"

/db_xref="COG:COG0253"

/translation="MQIQLTKVHGSENDFFILDETLLDRTLTEKELVQLTKIVTNRQT

GLLHGADGVLLVSKSSHHHSDVRAKMRVINNDGSEASMCGNGLRTVARYVAEKEQMDY

FKIETLHADLLVQKAKEIGKDVATYQVEISPVSFHTSTLPLNTEEDTFINQPFPELAE

GLLFSAVAVPNPHLIAFVDHETLNGPLLEKIATYVNGANPYFPDGVNVSFVEVLGKNQ

IFVKTYERGVGFTNACGTAMSASSLMYVLLKSGEFNEKISVYNPGGMVQTLVHQDQED

DYWMELIGNATFVQKIELSLEDLLNQRFDNVDIKMLNENDAYQEFVKEAQMKLVNK"

CDS 783407..783607

/gene="cspD_1"

/locus_tag="JFBMFIFI_00733"

/inference="ab initio prediction:Prodigal:002006"

/inference="similar to AA sequence:UniProtKB:Q45099"

/codon_start=1

/transl_table=11

/product="Cold shock-like protein CspD"

/db_xref="COG:COG1278"

/translation="MEKGTVKWFNGEKGFGFIEVEGGEDVFVHFSAIQGDGFKTLDEG

QPVEFEIVEGNRGAQAANVVKL"

CDS 783988..785652

/gene="alsS"

/locus_tag="JFBMFIFI_00734"

/EC_number="2.2.1.6"

/inference="ab initio prediction:Prodigal:002006"

/inference="similar to AA sequence:UniProtKB:Q04789"

/codon_start=1

/transl_table=11

/product="Acetolactate synthase"
/db_xref="COG:COG0028"
/translation="MKSEKKYGADAVVESLENLEVEYVFGIPGAKIDRVFDRLVDGGP
ELIVARHEQNAAFMAAGIGRITGKPGVVIATSGPGASNLATGLVTATAEGDPVLAIGG
QVKRADLLKLTHQSMDNAALFEPITKYSAEIQEPENISEVLANAYRAAEGAKQGASFV
SIPQDVIDSPIKNKVIAPLQIPKLGPASPVEITLLSQRIKAAKLPVFLLGMRASSPEI
TEAIRNIVRVSDIPVVETFQGAGIISRELEKSFFGRVGLFRNQPGDMLLKRSDLVITI
GYDPIEYEARNWNAESDADIIVIDSTPAEIDQYFQPEKELVGDIAQTLDFLLPYLKGY
KLDDDSTLYLNSLQEKLAQRDIPPVDENALYTHPLAVISTLQKLVTDEMTVTVDIGSH
YIWMARHFRSYEPRHLLFSNGMQTLGVALPWAIAAALVRPTTQIISVSGDGGFLFSAQ
ELETAVRRKLNIVHLIWNDGRYNMVEFQEEMKYGRSSGVDFGPVDFVKYADAFGAKGL
RVTKPSELEKVLKEAFATKGPVIVDIPIDYKDNQALGKTILPDQFY"

CDS 785690..786406
/gene="aldC"
/locus_tag="JFBMFIFI_00735"
/EC_number="4.1.1.5"
/inference="ab initio prediction:Prodigal:002006"
/inference="similar to AA sequence:UniProtKB:Q8L208"
/codon_start=1
/transl_table=11
/product="Alpha-acetolactate decarboxylase"
/db_xref="COG:COG3527"
/translation="MSENKTLFQQGTLGALMAGLYDGTLTIRELLEHGNTGIGTMDAL
DGELIIIDGEAYQAKSDGSFKKLSGLEKVPYAAVTYFDAERDYEINKTMTASQVKELI
EFELSSLNSFSAVKIKGHFKQIHVRNVPKQNKPYPRLIDVSKNQPEYQETEMDGTIIG
FYTPALFQGAAAAGFHLHFINDQKTFGGHVLDFVIERGQVEIETIETLEQHFQIHDAT
FMETEFDDSNLHKEIELAES"

CDS 786653..787591
/gene="menH_1"
/locus_tag="JFBMFIFI_00736"
/EC_number="4.2.99.20"
/inference="ab initio prediction:Prodigal:002006"
/inference="protein motif:HAMAP:MF_01660"

/codon_start=1

/transl_table=11

/product="2-succinyl-6-hydroxy-2,

4-cyclohexadiene-1-carboxylate synthase"

/translation="MKKKIIIGLSSLATITVLGLAGAGLYFYDVAVAINKKDFLESND

KKVDKKDPWAAEKKWYTEADREEIKLTSNDDLVLSSIYIAAEKPSNKVVILAHGYSGN

LEEMAPYAKLYHDMGFNILAPDARGHGSSEGNYIGFGWPERKDYQQWIQLMIDKVGSD

TEIALHGVSMGAATVMMTSGEKLPKNVKVIVEDCGYSSVTDELNVQLKELYNLPSFPL

VPITSVVTKIRAGYSFEEASAVEQVKKNKVPMLFIHGDQDSFVPTEMVYKVYEANASE

KELYIAPNSEHAKAYEKNKVEYSQKVQDFVGKYISN"

CDS complement(787663..788466)

/locus_tag="JFBMFIFI_00737"

/inference="ab initio prediction:Prodigal:002006"

/inference="similar to AA sequence:ISfinder:ISEfa10"

/codon_start=1

/transl_table=11

/product="IS3 family transposase ISEfa10"

/translation="MYWQQRFNRKNPDKQIEEEMIKIHQEHKDYVYPRMNQELRHRGI

LVNKKKVQRLMKKIGILVRSFTRKSRKYNSYKGTVGRVAKNRIHRRFYTSIVHQKMTT

DTTEFKYYEKDHSGSLRIKKLYLNPFMDMYNSEIISYTISEKPTAHAIMSALKEAIEK

TSDCTYRRTFHSDQGWGYQMKAYSHELKKHQIYQSMSRKGNCLDNSPMENFFSILKQE

LYHGVIYKSYQELKQAIEHYIKYYNHSRIKEKLGWQSPVQFRKKMSFTA"

CDS complement(788541..789065)

/locus_tag="JFBMFIFI_00738"

/inference="ab initio prediction:Prodigal:002006"

/inference="similar to AA sequence:ISfinder:ISEfa10"

/codon_start=1

/transl_table=11

/product="IS3 family transposase ISEfa10"

/translation="MTKYSVEFKMNIVQDYLNGEGGTPYLAKKYGFKGDSQVRNWINA

YKEFGKEGLLHSRRNKTYSVQFKLDAIELYLTTEMSYREVANQFGVSTFSTIANWYIA

YQKNGVDGLSQPKGRPSKMPKKKNKLPVSDSSKTELTYLEELEKENRRLKIELAYLKE

LRRLRLKEQQNNKR"

CDS complement(789194..789904)

/gene="gamA"

/locus_tag="JFBMFIFI_00739"

/EC_number="3.5.99.6"

/inference="ab initio prediction:Prodigal:002006"

/inference="similar to AA sequence:UniProtKB:O31458"

/codon_start=1

/transl_table=11

/product="putative glucosamine-6-phosphate deaminase 2"

/db_xref="COG:COG0363"

/translation="MKIIIEENYEQLSQTTAQILLGHMHQDKRVNVSVTAGNSPKRFY

EIITPLVKNKTHLANVHYYSFDEVPFKGEAVGMTEGLLRAEYFGPAEIAESNIHLLSP

DNYQEFDAEIANAGGLDVMLIGIGGDGHFCANMPGSTRFDAMTHKVDILPEYPWYDGL

KADAGDKMPDAFYTMGAASIMKVKHLVLIANGKHKADILKKALEGPVDPNIPSSILKL

HPNLTVILDQEAASALDK"

CDS complement(789992..790438)

/locus_tag="JFBMFIFI_00740"

/inference="ab initio prediction:Prodigal:002006"

/codon_start=1

/transl_table=11

/product="hypothetical protein"

/translation="MAIEQYKLQLNTPLMTVWDFISIPENWLPLLPDFIRLNQLSLTT

YHVFLYLKLGPIDREVQLEFNIQLNKQTHSVDFTFHSTTGKVTGYGFLKADAPTTTTT

NLTLAIEVKLSGKTGLLFKPALTSLKTSWSQETLNKLALLLNESEN"

CDS complement(790558..790812)

/locus_tag="JFBMFIFI_00741"

/inference="ab initio prediction:Prodigal:002006"

/codon_start=1

/transl_table=11

/product="hypothetical protein"

/translation="MLGFIWSLIVGGVLGAIGGMILGKDVPGGMIGNIIVGFLGSWIG

SSLFGTWGPVIGGFAILPALIGAIILIFIYSLIVGNRNKR"

CDS complement(790986..792212)

/locus_tag="JFBMFIFI_00742"

/inference="ab initio prediction:Prodigal:002006"

/inference="similar to AA sequence:UniProtKB:P60108"

/codon_start=1

/transl_table=11

/product="TelA-like protein"

/translation="MTEFDKNIPESSVKEVNSELEDLLANPFSDATAPLSSTPQAPLQ

ESSKEAVAPRLVDRLPKERQVQAQALAEQIDVGNAQAIMSYGAAAQQKLGEFSHSMLN

HVQNQDTGEIGDSLNDLMYRLNEANPDELRAEDNNVFKKIFGKVKRSVYEMTSKYQKI

GAQIDKIAIKLDKEKSGLLNDNMMLEQLYQKNKDYFDALNIYIAAGELKMEELQTTLI

PEAVKKAETSNDQMDVQIVNDLNQFLDRLEKRTHDLRLARQMTIQQAPQIRLIQNTNQ

ALAEKIQSSINTAIPLWKNQIAIALTLLRQKDAVTAQRQVSETTNDLLKKNSAMLKIS

AIETAKENERGVIDIETLQQTQNDLVETLQETLKIQQEGRIKRKDAEKELAVMETDLR

DKLLALTTDDNSGKRY"

CDS complement(792252..792962)

/locus_tag="JFBMFIFI_00743"

/inference="ab initio prediction:Prodigal:002006"

/codon_start=1

/transl_table=11

/product="hypothetical protein"

/translation="MNHLFKNIFGILRYTLTSVFCVLAFLVFILFFKVSFGPAVLATF

ILGGLLFSVTGLKKQSKSTDKKATIDESLHRLTEDKETFYKEAGMSKEETQFFRETMN

TAKNQILVLEQNLKQVSKLKAIEARNNTIHLTQALFKDITNEPRRLHEVDKFLYVHLP

SLVDLTEKYNEINNHEIKNKTTFETLEKSASTIDEMCQLIAVDYAAFKADDLAEMDLE

IELAKQAIERDNEEQEEQ"

CDS 793204..793767

/gene="nudF"

/locus_tag="JFBMFIFI_00744"

/EC_number="3.6.1.13"

/inference="ab initio prediction:Prodigal:002006"

/inference="similar to AA sequence:UniProtKB:P54570"

/codon_start=1

/transl_table=11

/product="ADP-ribose pyrophosphatase"
/db_xref="COG:COG0494"
/translation="MDFEEKTIKREHIYQGAIIDLFLDDVLLPNGKEAKREIIKHPGA
VAIAAFTNDDKMIFVKQYRKALEKVIMEIPAGKIDGADTEPMDAAKRELEEETGYRAQ
IFSHETSFYTAPGFADEIIHLYRAEGLTQVENPLPQDEDEFLELVELSFEEAWAAYEN
QEMCDAKTTCALLLWKLRRVAESGANN"

CDS 793767..794024
/locus_tag="JFBMFIFI_00745"
/inference="ab initio prediction:Prodigal:002006"
/codon_start=1
/transl_table=11
/product="hypothetical protein"
/translation="MSKPEMTRTELKRAKEQEEKERKKRDKERLRVEKEYQKQLEKEG
HITKSRMIENEKSRETGRFLTKAIVIVTLLLAVVLAIVFLI"

CDS 794078..794770
/gene="mtnN"
/locus_tag="JFBMFIFI_00746"
/EC_number="3.2.2.9"
/inference="ab initio prediction:Prodigal:002006"
/inference="similar to AA sequence:UniProtKB:Q5E2X3"
/codon_start=1
/transl_table=11
/product="5'-methylthioadenosine/S-adenosylhomocysteine
nucleosidase"
/db_xref="COG:COG0775"
/translation="MKIGIIGAMEEEILLLKSKMSNKKEWTEAKADFIEGQIGEVEVV
LVRCGIGKVNAALTTTLLLAKHDIDLIVNTGSAGGIGAGLHVGDVVIASEMAYHDVDA
TVFGYSIGQVPQMPARYIANQGTIEKTITAAKKTGLTPVKGLIVTSDSFIASQAQTDV
ILSNFPDALASEMEGAAIAQVCYQFDVPFVIIRAMSDVADEEAGVSFDEFIIEAGKKS
AEMVLELVASLA"

CDS 794807..795352
/gene="rutB_1"
/locus_tag="JFBMFIFI_00747"

/EC_number="3.5.1.110"

/inference="ab initio prediction:Prodigal:002006"

/inference="protein motif:HAMAP:MF_00830"

/codon_start=1

/transl_table=11

/product="Peroxyureidoacrylate/ureidoacrylate

amidohydrolase RutB"

/translation="MKALLSIDYTNDFVATDGALTTGVAGQNIESDLVSLTEKFIEAG

DLVVFAIDAHDSKDNYHPENKLFPPHNLIGTTGRLLYGELASLYERKQREKNVYWIDK

RHYSAFSGTDLDIRLRERGITELYLSGVCTDICVLHTAVDAYNLGYSIVISEKTVASF

DEVGHKWALNHFKNTLGATII"

CDS 795879..796175

/locus_tag="JFBMFIFI_00748"

/inference="ab initio prediction:Prodigal:002006"

/inference="similar to AA sequence:UniProtKB:Q8DZR0"

/codon_start=1

/transl_table=11

/product="ESAT-6-like protein"

/translation="MVQIKLTPDELEASATKYTAGASDVREVLSRLSQEQNNISQNWD

GTAFEKFEQQFYELSGKVNEFADLLDQINTQLNQVATTIRDTDAEIASKLGFQG"

CDS 796365..799718

/locus_tag="JFBMFIFI_00749"

/inference="ab initio prediction:Prodigal:002006"

/codon_start=1

/transl_table=11

/product="hypothetical protein"

/translation="MRKKLIIGTLFFVLVIALGFSLTYMGLFTNSKADKEYEEQVAKM

RIALVNEDVGTEFEGMTYSLGENYVKKIEKDANQNWYVVSRSIAESGLANDTYNLMIV

VPSNFSSNTFSLNETAPEKAEISYKINANGNKDVENEAVKVAKNTIAELNKSLVDVYV

SSILENLYTAQKNVGTVIGNQIEKIDIYGNTVYTPLEGYTSQFSTIQSAGDQSIKGID

QHKSVLTNYLESVVAYGTNQSDFDKNLQALITDQEKNQIDYLHFTESVAKHNTELGSE

ETQALYDQLVLQNSLIGAEFVKSEDTLVSQINGAESYISDVKSNILAQKAEVERQFAE

DKLVLTRKIERQLKDQFGIGNDKISIQQLLDLDPEGQDLEVNLDAIRKQEFERINKLS

TKISSLLYLKENIINASPYLDEDSKTKIKENLALIQKYIGRNNLVRILEVGEELDNPP
PYPEKNEDKEQNYKTIGSMAIKDSIEAIELDAKYEQIIYDTSGVIPDYTVLSGLKAHL
EVPTGIKVTSATLQTTNGDVPLEIENLINSSKTLNLEEVIGLEQLGKMVSIKLTYESD
FSSVEGTDLSLNLVKVANESLNLILTPVIGISITPPIIDPPGAELPPPTLVDGEPIKN
GIKVGLPTNSEFDKEAYIKLINQYSDSRYQNGLLTGEYMEEYVAVLAELEDLFGPLNG
EGNLPNLDKPRQASRLYNELINDTNSFNVFDILVRLMIVEPTEIYESYLTDFTEYEAS
ADDMELKVATLDEKIVTTTETAGKLNEEVENQLKNLSAWQKTMNELEREEGTTDSLTK
GEHSTTKQIQSELQSLMTISEGLKKNSESNIEDLKSVNAVFDAFNEDALAIQNSGLKV
TKNTKDLMNDFQTQVDDTDNFSKTFNQVLKNGQQNGVINNQFVDFLAAPVKEKDNGRI
SSGDAYYPYLIIVALFITAVFTAYVLDLKVWQAKQVNNFETEDNLFIRNLPIVLFGLI
AAIIEGIVIALISFNMQQMSPDVQLNWILVVVLVQIVFVLLASYLLRQFKMLGMFANL
FLFAAYLLLTEAVGKALDTEAALYQIRNYSPLNSAESLLGNIANQVPNTFSNLVGFII
LIPIVLILNLVVWFPKKAEVTEETPPDEN"

CDS 799740..800213

/locus_tag="JFBMFIFI_00750"

/inference="ab initio prediction:Prodigal:002006"

/codon_start=1

/transl_table=11

/product="hypothetical protein"

/translation="MLKKSIRFFILGSFFLVPSCFVFAETKLEPNGLEIKADRLEQEN
TSNNTESTATDAFFNEATMERYNEIKTRKEEKIKQEKEEFFLTPSKKVELYSATDFFT
EPEEESLMQHESTAVATNETSTTNETKSALLLSGSGLGILLTGAAVSVSNYRKGV"

CDS 800220..800471

/locus_tag="JFBMFIFI_00751"

/inference="ab initio prediction:Prodigal:002006"

/codon_start=1

/transl_table=11

/product="hypothetical protein"

/translation="MDQKYHINITVSYQDLPGKDLDLRVSTHQTVKSFIMDLDQTLHI
QRTQADIYQLKVVNKGLLLADNQKLYRYPVTTGDHIIIY"

CDS 800490..801755

/gene="essB"

/locus_tag="JFBMFIFI_00752"

/inference="ab initio prediction:Prodigal:002006"

/inference="similar to AA sequence:UniProtKB:P0C053"

/codon_start=1

/transl_table=11

/product="Type VII secretion system protein EssB"

/db_xref="COG:COG4499"

/translation="MSEISIIKFDDKTYTITKEKTDWTLHLKKSDAQLRNEEEITLLL

EAKKEFLPLSVQVEEDAFIFHFEPLALGLDYSQVLLKSQAEKIRAALNLAVFEKYVGS

YYTFFLDPENLQFDVNLVPYIAYRGLKSGLPPAELTAENFLRQYKSIVIALFSKKQTF

SSLYGGNLERAKESNFIKTIANAKSVKEITDYLEKEYQGTIAADEKNLRVVSKRKFMT

YKQLTIWFSVAIIILLIPLVYLVGFNNPHQAKLLKADTAFLKNDYEQVIQTLQPIATD

KIDATQKYELAYSYVQGKDLSDKQKAIIMKSISLKSAPTYLDYWIENGRGNLDKALEH

GKRLEDNTLILYGLQQKIEQIKNNPDLSGAERENKQLGDAEADYQKYQEAVQKADEELQ

KEDAEKANEFLDSSATDEVAAEPTKTGDK"

CDS 801770..806296

/gene="essC"

/locus_tag="JFBMFIFI_00753"

/inference="ab initio prediction:Prodigal:002006"

/inference="similar to AA sequence:UniProtKB:Q932J9"

/codon_start=1

/transl_table=11

/product="Type VII secretion system protein EssC"

/db_xref="COG:COG1674"

/translation="MNKVKLIISNRQFLHQLLLDSKEQYKIGSNPHDDLYFKDVGDFE

LELAFDVEKAKWILIQGTEEVILEVNQPQKIKVGEEELSYNLIEARLPVAYQLAIEKN

WHIGSDQDAVVQLPKAFSDKISLTKTAEGWELQPLGCALFVNYRFTAEKFLLKAGDVL

YLAGSFVTIFQDELHVEGDGALEVHLPKLATSSYGFYDEYPDYHRSPRIIYRDPEEKV

SIGTPDSEPTKPNEQLLKLIVPPLMMLGATIAMSFLMPRGPYVLITAGTTIVTTIFSI

SSYIKSRKQYKIDSAERVHIYNQYLVTKSEELYRLNQEQRVGKLYHYPDILTLTQQVA

QYNHRIYEKTPLHFDFLTYRLGLGRVPSSSDISYKGSDGQKKLNDLEQEGRNLWTESR

HVEGVPVTTELTLGPIGYIGPRPLVLEQLQLLVNQLAFFHSYHDLQFITIFPEEELEQ

WNWLRWLPHATLQDMNVRGFVYHQRSRDQVLNSLTQILKNRQNAKSEGGRDADSTIFT

PHYVVLITDVKLILDHTIMEFFNEDPTELGCSIIFVQDVMSSLSENVKTVIDIRDRNT

GVMVLENGDLKNTSFALDHFPVDFDKERLPRTLAPLNHLQNLKNSIPESVTFLEMYEV
ERFEELNVQDRWAAHSPHKSLAVPLGLRGKEDVVMLNLHEKAHGPHGLVAGTTGSGKS
EIVQSYILSLAVNFHPYDVAFLLIDYKGGGMANLFRNLPHLLGSITNLDGAQSMRALI
SINAELKRRQRLFSENNVNHINQYQKLYKDGDVSDPMPHLFLISDEFAELKAEQPEFM
SELVSTARIGRSLGIHLILATQKPSGVVNDQIWSNSKFKLALKVADRADSMEMLKTPD
AAEITQAGRAYLQVGNNEIYELFQSAWSGADYQPDKDESTAEDLTVYAINDLGQYDIL
SEDLSGLENADEVKTVPTELDAIIDGIQAITEKAGIEALPRPWLPPLAARNYLPLLDP
VSYQEAWATENKGELVPVIGIADIPTMQAQETLSLNLSKEGHMAVYSSPAFGKSTFLQ
TIMMGLARKHNPERLHAYLLDFGTNGLLPLRSLPHIADTMNSDEDEKIGKFIRRMATE
IKRRKKMLSQYAVANLAMYEKASGNEEPSILILVDGYEGFKGMKYEDPLEKILTQISR
EGAGIGLHLIISAGRTASMRANLQSNIKLQLALKMIDDAEPRNIVGRTSLAIDDLPGR
GLIKLEDPALFQAALPTEGEETLDIIDAIQAEAKEMDAYWTGERPEEIPMIPEVLDFE
EFVAKKATRNMLQTSGIPIGLDFENVLPIGLDYKKEKSTLILGDTVETVSTMTNVLMK
AVDSAAEIDFALLDNNEGAFKKEYSTKEYYGSLANEESYREFFAKLQVFYEDREDEYE
DLLDENEDLTLAEYAGRVKPFIIFVPDMPSLLEMLPKEKDFEKSVATLIQNSWKMGMH
FVLGGISNKTGKAYDAISDVFKQQKSGIVLTKLNEQHILEASNKNYNEPSLLPYEAYY
TRNNFGQKLKLPKV"

CDS 806324..806722

/locus_tag="JFBMFIFI_00754"

/inference="ab initio prediction:Prodigal:002006"

/codon_start=1

/transl_table=11

/product="hypothetical protein"

/translation="MFDFFKTPTEQKRDKYYSLYQNLKDCETYHQKKVSEAQSTFDSY
KNSVPNFSNSKIPSKDFDQKRESLTTDIATYLAEEEKKKAGLKSAKQQAKAQYEMYKA
LAISEAQEKQRKKEEKAKKDKEALEEARRA"

CDS 806756..807055

/locus_tag="JFBMFIFI_00755"

/inference="ab initio prediction:Prodigal:002006"

/codon_start=1

/transl_table=11

/product="hypothetical protein"

/translation="MAKIEILGESLEQASKGADDLKNSVKTSLDLTKKLKNYVGGAKW

SGETRDAFEAYLDIIVQYHEDLYAATKLQTTAFNHLKENIAAFDSQPEAAKVRSL"

CDS 807055..807789

/locus_tag="JFBMFIFI_00756"

/inference="ab initio prediction:Prodigal:002006"

/codon_start=1

/transl_table=11

/product="hypothetical protein"

/translation="MQTEEKIINENSFSVSELFLLAEACGTSFIFGLPDKKVQLLLNS

RMMQDAGETLQEKGILSEDETLTEGGYYVLSALNSYINSSQYTRINNCMVGFLPDDQE

NLVLLVEVEANKKYQLKIIDKLVLLKNLFDESPLLQREPLEDETEFILKKMRNSQRRI

AEKLELKTNLLNIEIFKGELYKEEVDSAEYHEQWLMYQDQGELYAVDFNEPKYYRASQ

YWLMKFVFDSLAIPYGAAASEQQVAE"

CDS 807878..808186

/locus_tag="JFBMFIFI_00757"

/inference="ab initio prediction:Prodigal:002006"

/codon_start=1

/transl_table=11

/product="hypothetical protein"

/translation="MGKEKFFVNPTEMEKIASYLDQINKEFEGNAKTAAKKIISSSFY

KEGKAKEVFSSYQDITNKSFEVTQHYNRAFQLVEMAKEEAMALDQALAAKIIGEQELT

"

CDS 808203..809798

/locus_tag="JFBMFIFI_00758"

/inference="ab initio prediction:Prodigal:002006"

/codon_start=1

/transl_table=11

/product="hypothetical protein"

/translation="MDVKTSSGGWETMRSSVGNVVGKGAWGKGVIDDLKDITKNLEKA

EEKISAKDEDGVVSFHHLDRTTTYQEIYEKMEVLYGFTNKAHDLVDETIDEKFYQEID

KYAVAMRDLTINKYSTKNITGATKQELVFTSSSMQEMQTVSKDKITVDDLFSDSSPLG

KKMKAEFDQYKLTQDKEIEYKDYKQSMLNTGAFEYTSIKDKQMKKEMWVNLGIAAVII

IASIAFPPAGMALGLVVSSLEVGSAITGKDWVSGRELGTGERVFRGILGGVGLGGELY

ALNAFSKNVRLANQIKYGGEGATSVQHLGDMVANSGKTSYGRVTEFGNSVKLNSTDSG

IKYTKNINPRNKNPRFKPQKITLNNGEIAYKAKDGTLVRSPDYLDDIGNIKWPKEQGF

VTDANGKAIIKDANVTKGQTLDRYGDSGGTFTSPLENGKPLDYDARGLPYPESMMDYH

KYEIVKDINMKNVQQGFDNLNDLDRKMLDTLMDRYNFTLEDIANPQSGKISEVFGSGG

GTQIKLGTSVEWYEKIGLIKEIK"

CDS 809798..810133

/locus_tag="JFBMFIFI_00759"

/inference="ab initio prediction:Prodigal:002006"

/codon_start=1

/transl_table=11

/product="hypothetical protein"

/translation="MLTFDEEKQIIQNKIIEKGYDSLRVSVFNNKDINREEWQTRIEY

CLEKKCYVVYSLADRASLMGSIREFQDFKKAEDAFFKVLDLTVEYNSLKVMNNESPEY

KSPLWNEKN"

CDS 810482..810865

/locus_tag="JFBMFIFI_00760"

/inference="ab initio prediction:Prodigal:002006"

/codon_start=1

/transl_table=11

/product="hypothetical protein"

/translation="MLYLFIITDELLIAYKDVDFTKPNLQMDKPGVGFQDYKDISEEY

RLITDKIKGGLKSYNSQFDSKNVRLSDNQVKLIDAAVSRTIKKLARDTGNKMKDIGDL

EIELGDVGIPEEVRKVIISQFYGTY"

CDS 810879..811460

/locus_tag="JFBMFIFI_00761"

/inference="ab initio prediction:Prodigal:002006"

/codon_start=1

/transl_table=11

/product="hypothetical protein"

/translation="MVANSGKGSYGRVTEFRSSIDANDIVEIKRIDEKTINFKFNDKY

DYDEFMRQLKNQEKGMNDLSIDEFLKNRENYKLNGRSTEANKLQKEFREIALADKIEE

LMETGVSFNDANSQAKDWLKDLAVLHDPDQVAGGNPLNLTGLGDKGVNSSIGSQWQYK

IKDLEEFVLHQSQNMTELELKETFLNIIMDLNK"

CDS 811479..812036

/locus_tag="JFBMFIFI_00762"

/inference="ab initio prediction:Prodigal:002006"

/codon_start=1

/transl_table=11

/product="hypothetical protein"

/translation="MQFEVLKDFREEKVISKETKERYLGKISEEMMEFWGNYGLGNFC

NGFFKSVNPDEYKELFETTSLRYTENGVVVFATGLADLIIENNKRLYFLQYRKGIISL

LPVKMKFFLQALSEENSVFLSDYLNSDQYFLALEKYGAVQYEEAFGYVPLLGLGGSEK

IENLDKVKIKEHIFLISQLVGPVDL"

CDS 812760..813200

/locus_tag="JFBMFIFI_00763"

/inference="ab initio prediction:Prodigal:002006"

/codon_start=1

/transl_table=11

/product="hypothetical protein"

/translation="MDQHKRTSEELNELLKKANNEFHMYYRKTNEITMDFLLQPEEAI

QIAKEYHKREKMEGVVNINLDSILFDEAYTFKINNNEDDIRSVWRIQVDLPPNDFLME

DYTLIVSDKDKKVLGVIDPNGHPIFEVNDFTDEDIKYITNDGSV"

CDS 813498..813692

/locus_tag="JFBMFIFI_00764"

/inference="ab initio prediction:Prodigal:002006"

/codon_start=1

/transl_table=11

/product="hypothetical protein"

/translation="MKAEDIKGLMPDQIKDKFALPNLPECVGEVTLPKGSNFRIGEVS

PLFSSNCDGIQFDLKVKFQN"

CDS 813689..813973

/locus_tag="JFBMFIFI_00765"

/inference="ab initio prediction:Prodigal:002006"

/codon_start=1

/transl_table=11

/product="hypothetical protein"

/translation="MKENDNYLLVRVSVTPSLELKDNILSNIYAINKLGEIQWQIKNN

TPKGNAEYICAPLVGMDIKKDGSLFVTDFMGRRFEVNQKNGVLEQMKIVK"

CDS 814197..814448

/locus_tag="JFBMFIFI_00766"

/inference="ab initio prediction:Prodigal:002006"

/codon_start=1

/transl_table=11

/product="hypothetical protein"

/translation="MVKNGLFLHLYFLLNTSLDPGYLGDTPILVDINKVDGLKVPTGN

ESGAWQGLWEPGGFTNGGIPEAVVNQIQGGTYTVTDVFK"

CDS 814468..814827

/locus_tag="JFBMFIFI_00767"

/inference="ab initio prediction:Prodigal:002006"

/codon_start=1

/transl_table=11

/product="hypothetical protein"

/translation="MEIIRGNGFAIVKKNDEYTIDWLQGPFDQEVSYPITKELADKAL

KSSQDAYEVQIYAETGLWPKKTTEEEENERVREFIRQFPELLIKVPDNQDLFTKRELD

ELLPLAKKEIAESEENE"

CDS 815062..815604

/locus_tag="JFBMFIFI_00768"

/inference="ab initio prediction:Prodigal:002006"

/codon_start=1

/transl_table=11

/product="hypothetical protein"

/translation="MIRNFHFDFAQVNWLFKGREINIKMENIIFSSIDKINDQVSVKV

GGNFISESEYYYDFSGNLLFELHKMNGEIKWCHNGELFKRTVLNIENIGFYTNKRIIL

IMYKANENEAFFVDLKNEFLHEVNKPKGFQLAYFEETKNNILIVCNGDAENTDKFGRD

QKNFILDISTGELQENGIAY"

CDS 815691..816062

/locus_tag="JFBMFIFI_00769"

/inference="ab initio prediction:Prodigal:002006"

/codon_start=1

/transl_table=11

/product="hypothetical protein"

/translation="MNISIKRATGVMGGTSKIDVLVNDKKVNKLKNNEEIVISTETED

SKIRVKQWFFGSPDTVVKSNSKIEVKINQTALYLYIGALLLVFAGFASQLILFSFAGL

IACIAVIVYSNKNWFKLEINE"

CDS 816221..817915

/gene="hscC"

/locus_tag="JFBMFIFI_00770"

/inference="ab initio prediction:Prodigal:002006"

/inference="similar to AA sequence:UniProtKB:P77319"

/codon_start=1

/transl_table=11

/product="Chaperone protein HscC"

/db_xref="COG:COG0443"

/translation="MATIGIDLGTSNSLVGYWNGESAVLIENEFGEVLTPSIISVDDN

QEILVGQIAKERLMTHPQLTAATFKRFMGTEKIYKMGTYSFTPVELSTLLLKKLKQNA

ERMLNEECCHAIISVPAYFNNVQRAATIEAANLAGLEVNYLISEPTAAAMAYGIHRLE

EDSRILVIDLGGGTFDVSLLEMFEGIMQVEAIAGDNRLGGEDFTYAILKDCLAENKLD

QQVLPAEIEASLYKKIEMIKKELSPTTPQQFEFILDEKEYTYQLTEERFRSICQPLLA

KLRAPIIRVLNDSGIKLDEIDRIILIGGATKSTTIRSFVSKLLGKLPFTQINPDEAVG

VGATIQSALKENKEMLEEMILTDVCGHTLGVNTSRFMENDYRDGYFSPIIERNTTVPV

SKIQTFCTVYDNQQAIVFDIYQGENRLVKDNLKIGSVEIKMPKVPKGEPVDVRFTYDH

DGILEVIVTVPRTKKTNRIVIENTPGQLSQIEIENRLKKLESLKIHPLDRAETRLLLA

RAERLYAESIGEKREYIQYLIGNFEAVLRKQDEKETRKASSELNKLLNKLEENFQL"

CDS 817912..819951

/locus_tag="JFBMFIFI_00771"

/inference="ab initio prediction:Prodigal:002006"

/codon_start=1

/transl_table=11

/product="hypothetical protein"

/translation="MSIWEQLEIERTTDLKQIKKAYAKKMKIVLKAGNDAEFQALKEA

YDLAVKWAKSGKAESQESRHVVLPKVEATQEFHVEKEGNHSQLPTFIEAVVSLIKTNE

RRQKITEWELLLKDKENWTIPEYVQYREFMMRFLIDWYIFIPKKIIRFLINNFELITE

TEEVASDSLTSYFRVASHQIINVPNFSFAAFSGMTEDSIFEFCRLRYTIYQKLTTDLK

DENITELYSVAIKLNEKDTDLINLYMLYLVKKELNPKWQPEEYIHELDSYLIRAKENQ
SDNEVTIFIDMYYQAITTKSGLDQKFLNAWHDYYIPTELAYLMTGYIYDYNGDQITAV
QIWKNLPNVYSVIVQKKIFAVKKTVDVEVKEKKEKKQSYNVVIWLAVFVVIGIFIAGI
TQTKKQEPDPDFVKYLQSRNDFSSIHKEYREKFQENSGGTEEETNNESEPVDEATLLD
DQKFIYYFLMTNDAEKRQMFIDQSVAEDAKAFFLEHINDAPAFPRADITEFRIQSDYI
VDFGKAQLITYSFEQSYLLQVDENDKIMNVLAKGWTETSTAEFQALVEDINVRPETST
NFFVMDYLLSENQKESLQVNSEYVTKELYGVLEKNASGATNDYDEASWQLSKFSDGRV
AIVVNDNNGEHRYILSFDRDGRLEHIYSGGWEQLDLAERQFIYDNCEEKENFYTF"

CDS 820306..820905

/locus_tag="JFBMFIFI_00772"

/inference="ab initio prediction:Prodigal:002006"

/codon_start=1

/transl_table=11

/product="hypothetical protein"

/translation="MKKLGLASLGFLTMITLAGCSPEATTQNEAENQVSQTSETDTKA
ETKITEEMVNEAQQKWADGLVAIGKAYQEGNDYEEEASKMIDDRYGFQDGVVLFKPTK
TSEENFRLTKEDALSYFVGGAHEEDEGFALAPWSNVRFENEATIIDSDSALASGLYYF
TSAETGEETEVEYTFGYYLDSDGVLKIQLQHSSLPFEAK"

CDS complement(820992..821462)

/locus_tag="JFBMFIFI_00773"

/inference="ab initio prediction:Prodigal:002006"

/codon_start=1

/transl_table=11

/product="hypothetical protein"

/translation="MKINETRDAKLMGLLGKTMQDKHIELYPDFFKPYDAEAVTKSFI
DYFKNPKQRVYLWQDENNQDAIAAYLWLEEEQMPENVYRYGYTRLYLHHILIMPSYQG
QGLSQELLKFADTYAKEHDIQHVELHYWPNNGIAKHTYQKYGYEVYTEIAQKRI"

CDS 821626..822147

/locus_tag="JFBMFIFI_00774"

/inference="ab initio prediction:Prodigal:002006"

/codon_start=1

/transl_table=11

/product="hypothetical protein"

/translation="MMIETEHLLLVPYKIAFLEATLIGDDKLTEVSGFQVAKQWPGVE
FFFYLPFVLDELKANPEMEEWTYLVVLKENQTIIGELSAQGNPRKTGIAEIGYGIVDE
YSGNGYATEASQSFIQWLVNYPILKIVAKTYQNHRKSQHILQKLGFHKTGEELLGGDE
VVYHFEYDSQLKS"

CDS 822266..822880

/locus_tag="JFBMFIFI_00775"

/inference="ab initio prediction:Prodigal:002006"

/codon_start=1

/transl_table=11

/product="hypothetical protein"

/translation="MKQSSKLTVRDLVTIGIMSILIIFFGLIVSMVGRLATGGLSIII
SGALSAFFCAPIYMLLSFRVAKRGVAFLNAILRSLMYVVMGVPHVLLIFIPAALLAEL
AMTPADTSYRSLGRTTLSWSIFSTFNALHGPLLFVMLGAKYMMEKAPHSPSAERLALM
LEYYYSPMWLVVIGGLAFIGAAAGCLVGWKLLHKHFVPAGTVKL"

CDS 822915..823601

/gene="ecfT_1"

/locus_tag="JFBMFIFI_00776"

/inference="ab initio prediction:Prodigal:002006"

/inference="similar to AA sequence:UniProtKB:A2RI03"

/codon_start=1

/transl_table=11

/product="Energy-coupling factor transporter transmembrane
protein EcfT"

/db_xref="COG:COG0619"

/translation="MESIKLDARTKTLVVLVSGFFIFFTSDFGLILLFLFLLGYSYLA
GERKVLPYITIAFIFTNFFQRFAFGYEDSVIMATFAFFAYFAVRTIPLLLGGIVLSKT
PPGELIATLQKCYMPRAILIPLAVGLRFLPTIRQETGAILDVMRLRGISPTVWNIWRH
PLLTFEYLLVPLVIRSLTIADDISMAAVTRGIENPATRSSLREIKFSTADRFVCLALV
IVVAVLLYVG"

CDS 823797..825278

/gene="ykoD_1"

/locus_tag="JFBMFIFI_00777"

/EC_number="3.6.3.-"

/inference="ab initio prediction:Prodigal:002006"

/inference="similar to AA sequence:UniProtKB:O34362"

/codon_start=1

/transl_table=11

/product="Putative HMP/thiamine import ATP-binding protein

YkoD"

/db_xref="COG:COG1122"

/translation="MIKLEQVDFAYKAEEPLLNQVDLAIKKGEFILLTGPSGGGKTTL

TRIINGLIPYFFGGVRTGTVEVAGKEISDLNSWELAKELGSVFQDPRSQFFAPIVKDE

IAFFCENYGLPTAEIKERVTRASLEVGIEHLLEHQVIQLSSGEKQKVAIASIRVADQQ

IYLLDEPSANLDSKATLELQDILTQWKAKGHTIVIAEHRLYYLNELIDRAVYLREGKM

ERIFSQADWRNLTEEELVDYGLRSPVLAWKDVPEIPKKVMDLSSGFELESLSVGFPKV

KQPVLENLTTHIGAGEIVAIIGKNGAGKSTLAKTLTGLLKEKQGQFKYKGQAIPVKKR

SKVAWYVMQEADFQLFSASVLEELTIGQKKSELIQERAKELLTSLGLWEYRHRHPASL

SGGQKQRLTIAVALMQETPLIILDEPTAGLDGANLKRIVSVIKQTAAKGTSFLIITHD

FEFLNQAVERVLYFSNGGLQADYPLNKLTATEVQENMLGITHS"

CDS 825466..826179

/locus_tag="JFBMFIFI_00778"

/EC_number="2.4.2.-"

/inference="ab initio prediction:Prodigal:002006"

/inference="similar to AA sequence:UniProtKB:O33341"

/codon_start=1

/transl_table=11

/product="Putative glutamine amidotransferase"

/db_xref="COG:COG2071"

/translation="MIGISSNLLTTPLPDTITNQRYHLSTDYIRSVAEAGGLPMILPQ

LNKKAAKEIVQKIDGLILSGGLDVHPSTYQQQIQDESYSYSVERDQYELALLKEALKQ

NKPVLGICRGAQLINSAFGGNLVADIEAELPKSKTKHLQELAPEKGTHGITICQNSIL

YRILKESSLVVNSFHHQAVNQVGKGLKASSWTEDGLIESIEGTEFKFLVGVQWHPEIM

SGNDLASQLLFQRFVEATK"

CDS 826198..827859

/gene="dppE_3"

/locus_tag="JFBMFIFI_00779"

/inference="ab initio prediction:Prodigal:002006"

/inference="similar to AA sequence:UniProtKB:P26906"

/codon_start=1

/transl_table=11

/product="Dipeptide-binding protein DppE"

/db_xref="COG:COG4166"

/translation="MKNRTKVSLFAMLAIILVIGACGNDKKESTAETSSEKVEKVADK

QEITVTTTAELATLDSALYTDVVSSDAIGQIFEGLYRIDKNNDAELGIAKAEPKINEA

KTVVYTYQIRDDAKWSNGDPVTADDFVYAYQKVVNPATGSQSSNQMDIFENAEAIRNGE

RPITDLGVKALDEKTLEITLTSPVPYLAKLLTGTPFYPQNKKVSTDLADKYGTNSENV

IGNGPFTISKWDGTNLSWAYTKNKNYWDAKNVKLEHIDVEVAKETATGANLFDGGEVQ

YTGLTDEFIQKYQGSENYHEQPKALIGYLGFNTERENTGNVHLRKALALAFDKKSYTE

GVLIDGSKPLDGFIPTDFAKDPKNGKDFRKENGNLMAFDAKEAKAEWELAKKDLGISE

LTLEVLSSDAGSAKKTVEYLQAQFEENLSGLTIQLKSVPLKNRLDLTRAGDYDVFFGT

WTPDYADPINFLEIYQSNGGINFSKYSNPTYDAGITEVKTSLATEPEKRWDKMLELEK

QLIQEDAAIATVYQGAQAYLLAPEVTGLQVLPFGRTVSYRLAQIN"

CDS complement(827909..828322)

/locus_tag="JFBMFIFI_00780"

/inference="ab initio prediction:Prodigal:002006"

/codon_start=1

/transl_table=11

/product="hypothetical protein"

/translation="MMTEEKNLCMDDLKMDLAKALAAHVDILKNVDQIEPDQIDGLAF

VMRSLGFMLEKTPCLLLRDNDEDVYYAMFQYYSLLEELKHNVKMSYPHARIHTKLLID

ILEGFPKTYSSDMIEWWELKTGLVVEHTKQTILMD"

CDS complement(828654..829109)

/gene="yueI"

/locus_tag="JFBMFIFI_00781"

/inference="ab initio prediction:Prodigal:002006"

/inference="similar to AA sequence:UniProtKB:O32092"

/codon_start=1

/transl_table=11

/product="putative protein YueI"

/db_xref="COG:COG5506"

/translation="MGEKDVNDYLTSGIYGTPQTKPEERNRYLGSLRERVYLSMSIEE

LSSLLYIDALKEELELHPDGQVLLNGEVNSRALGPYLKLCGQESVQFTIVTNQFAKKS

DTGLLFVAANAVNQEVIAVSEKYPLADPTPKEEESVPTEKKSLLKRFFS"

CDS 829722..830675

/gene="yclN"

/locus_tag="JFBMFIFI_00782"

/inference="ab initio prediction:Prodigal:002006"

/inference="similar to AA sequence:UniProtKB:P94418"

/codon_start=1

/transl_table=11

/product="Petrobactin import system permease protein YclN"

/db_xref="COG:COG4606"

/translation="MRKGIWIVLLSVIAFVSLFIGVQDIELSHVLQFNFTKNEQLILQ

STRIPRTVSLIIAGATLSICGLIMQHLTQNKFVSPTTAGTMDSARIGILVAMLFFTDA

SIFQRTVIAFLFAFAGTVAFIFMVNHLQIKNAVMVPLIGLMFGSILGSIATFLAYQNN

LVQNMASWLQGNFSLVSRGNYELMYLAIPLMIVAYLYADYFTLAGLGKEVAINAGVSY

QFVQFGGILIVALASSIVILTVGSLPFLGVVVPNLIAIARGDHLRKTLFETAVFGAIF

LLICDIISRVVIAPYEVSVSLVVGIIGSVLFIYLLLRGEKQ"

CDS 830672..831637

/gene="yclO"

/locus_tag="JFBMFIFI_00783"

/inference="ab initio prediction:Prodigal:002006"

/inference="similar to AA sequence:UniProtKB:P94419"

/codon_start=1

/transl_table=11

/product="Petrobactin import system permease protein YclO"

/db_xref="COG:COG4605"

/translation="MMQRIKKEHWVYGLLLLVILVLIGLYLVLENNGNWDYILPLRSK

KLVAFIIVGISTTVATISFQTLAQNNILTPSILGLDSLYVLFQTMILFFYGSNHLVVR

DKQINFLISVVLMVGISFVLYQIVFRKYSSNLYLLMMVGMIAGTFFRSVSTFLQVLMD

PNEFDKIQGKLFASFNNIDVALLGITLLIGMAALMFLFSQRKVLDVLHLGRDQAVNLG

VNVEGTMLKVLVLVAILTSISTALVGPITFLGFMVANLTYRIFKTYQHGILFIGGSFL

SIIVLLVGQLVVERVFHLTTTLSVVIEFLGGCYFIYLLIKERKRE"

CDS 831641..832399

/gene="yclP"

/locus_tag="JFBMFIFI_00784"

/EC_number="7.2.2.-"

/inference="ab initio prediction:Prodigal:002006"

/inference="similar to AA sequence:UniProtKB:P94420"

/codon_start=1

/transl_table=11

/product="Petrobactin import ATP-binding protein YclP"

/db_xref="COG:COG4604"

/translation="MQIKNVSKSYGNKKVISEITLPIKEGAITAFIGPNGAGKSTLLS

MMSRLIPKDTGSIYIDGGEVKSWKQDELAKKLSVLKQSNAFNLKLTVRELVAFGRFPY

SKGRLKEEDQLKIEEALSYLGLVELAEEYVDTLSGGQLQRAYIAMVLAQDTDYILLDE

PLNNLDMNYGVQMMKTLRRLVDELGKTIVLVIHDINFAASYADEIVAMKNGQLFATGT

TSEMMTKEVLDPLYEMDIRICEVEGKRFCLYFSE"

CDS 832431..833393

/gene="yclQ"

/locus_tag="JFBMFIFI_00785"

/inference="ab initio prediction:Prodigal:002006"

/inference="similar to AA sequence:UniProtKB:P94421"

/codon_start=1

/transl_table=11

/product="Petrobactin-binding protein YclQ"

/db_xref="COG:COG4607"

/translation="MKKIKLSVCLVALGFVLAACGSGNESAKKESSSASKNTEETMIT

VKDVNGDVEVPKNPERVVVFDMGMLDTIDALGESDAVVGVAKDSLPKYLSKFDSDKVE

SAGGIKEPDFEKINALKPDLIIISGRQSDSLDELKKIAPTLSLETDSKDLWESINKNV

STIGTIFDKSDEAKKKLDALSEKIDVLNKKNTGSDMKTLTVLLNEGSLSAYGKGSRFA

ILNDVFGFPLVDDKIEASTHGQSVSFEYVLEKNPDVLFVIDRTKAIGGDTSKNNLTEN

ELIKQTNAAKNDKIVMLDPEVWYLSGGGLISTEMMLEEVSNVAK"

CDS 833519..834157

/locus_tag="JFBMFIFI_00786"

/inference="ab initio prediction:Prodigal:002006"

/inference="similar to AA sequence:UniProtKB:Q99UZ6"

/note="UPF0637 protein SA0957"

/codon_start=1

/transl_table=11

/product="hypothetical protein"

/translation="MNEVTFTQEDFDVFKLEGLDTRMTAIRSQIQPKFKALATEFAQE

LASDLQEEEPLHVHIAQHIRRTKNAPNDTWCAIGGDKRGYKKYPHFQLGIYADHLFIW

LAMIDNPQAEKEIATSLLTQLDFLANLPKGYVVSLDHHFPKTTPVSEIDLKKGLERFR

DVKKGEFLIGREIWSDDTLLEDPAAAQAFILETYRTLLPIYQTAMEAQKHAK"

CDS 834350..834880

/locus_tag="JFBMFIFI_00787"

/inference="ab initio prediction:Prodigal:002006"

/codon_start=1

/transl_table=11

/product="hypothetical protein"

/translation="MKKEKVKLLVQGIILLILGVLFMMNPVRQGVLFLLILGSVFAFS

GVVIILDGIFITKGVKYKVFRILEGILLGGFGLIFFLRNPESGAVIIIYSLVWLMILM

SIFNTIAIFKVKSGIKWLSIALNIIVIWIGVQSLFDPQLALAIFYWTVAFQLIFMGIN

HITLYFVLPNEEEGIR"

CDS complement(835130..835306)

/locus_tag="JFBMFIFI_00788"

/inference="ab initio prediction:Prodigal:002006"

/codon_start=1

/transl_table=11

/product="hypothetical protein"

/translation="MSQNKFKDATKIDNATLARNTMKADPSQNGTPIPFQSGYSEYEA

NKTTQSLFETVIFT"

CDS complement(835662..836267)

/gene="rpsD"

/locus_tag="JFBMFIFI_00789"

/inference="ab initio prediction:Prodigal:002006"

/inference="similar to AA sequence:UniProtKB:P21466"

/codon_start=1

/transl_table=11

/product="30S ribosomal protein S4"

/db_xref="COG:COG0522"

/translation="MSRYTGPSWKVSRRLGISLLGTGKEIERRPYAPGPHGPNSRKKI

SEYGMQLQEKQKLRNMYGMNERQFKNLFVKAGKLKEGKHGVNFMILLEQRLDNVVYRL

GLASTRRQARQLVNHGHILVDGKRVDIPSYHVEVGQVISVREKSKNMVTIKAAVEGLF

GRPDFITFDTEKLEGSLSRLPEREELYAEIDEAFIVEFYNR"

CDS complement(836540..837007)

/gene="msrC"

/locus_tag="JFBMFIFI_00790"

/EC_number="1.8.4.14"

/inference="ab initio prediction:Prodigal:002006"

/inference="similar to AA sequence:UniProtKB:P76270"

/codon_start=1

/transl_table=11

/product="Free methionine-R-sulfoxide reductase"

/db_xref="COG:COG1956"

/translation="MTKAQDYRLTNAQLEAIIGDETNLIANLSNAAALLFENLKDINW

SGFYLFEEASGELVLGPFQGKVACIRIPVGRGVCGTAFQKKESLFVNDVHEFPGHIAC

DARSQSEIVIPLEKNGKIIGVLDIDAPIKARFDQEDQEHLETFVQLLLKNSSL"

CDS complement(837030..837512)

/locus_tag="JFBMFIFI_00791"

/inference="ab initio prediction:Prodigal:002006"

/codon_start=1

/transl_table=11

/product="hypothetical protein"

/translation="MLLLLKICGILGFIFCLVLVGFALKIKKVGSKKFQKKIGLIFFV

SLALFSFSQFKLSKQAAVQRETYTISSKSTLEGSQTFLRVLVFSKNKKDLETIFNTLE

KKEKKKGVDSLFVRFNVDNGGEIGKFIANGKIAWTKEGEKQVGLKKDKVGFDYNKELK

"

CDS 837762..839477

/gene="ezrA"

/locus_tag="JFBMFIFI_00792"

/inference="ab initio prediction:Prodigal:002006"

/inference="similar to AA sequence:UniProtKB:O34894"

/codon_start=1

/transl_table=11

/product="Septation ring formation regulator EzrA"

/db_xref="COG:COG4477"

/translation="MNVIVILIVIIVLALVLYGASYFLKKKHYHKIDELENRKLSLTE

IPVIDEINKLKKLQLTGQTEKSFKEWEKVWRNIATVHFPDIENYLFDAEQATDRMKLV

KAQQAEDAATKLMDDTQVSIDKVQAALKKLIQSEEENRLEIKKTQEIYQNIRKKLLTQ

SFSFGGALENLERRLTYLELDFTKFSELTTSGDHIEAREVLERVATDTAELDRVVGLV

PTLLKTLTVDVVDQVTELKEGHAQLLAENFVFLDSDIPAEIKTIEENSEAAKSLTETC

EVDEAQKMVQDIETNIDKLYDLMQDEIDAKAFVNDQQKMFEDYLTHVIGNNRKLLIEI

DRVAQSYTLNHDELEAAKGIETELDTLKTSFDNFMEDSKNNQVVYSVLADFFEETGDK

LTEIEEKQQSINSGLLHLRKDEMEARKKLDEYEFEMRAMKRYVEKQHLPGLPEEYLDL

FFSTTKRIEELAQQLNKLKVDMKEISRLCFMCQDDVTLAKEKTEEIVDSALLTEYMMQ

YANRYRHSNESIADAIDSTLRLFNKDYRYQEALETISTALELVEAGAYKKVEDNYYAD

KKINQ"

CDS 839669..840808

/gene="iscS_1"

/locus_tag="JFBMFIFI_00793"

/EC_number="2.8.1.7"

/inference="ab initio prediction:Prodigal:002006"

/inference="similar to AA sequence:UniProtKB:P0A6B7"

/codon_start=1

/transl_table=11

/product="Cysteine desulfurase IscS"

/db_xref="COG:COG1104"

/translation="MIYFDNSATTQTDPSVVATYVKVTEEVFGNPSSLHHLGDHADGL

LQQSRKQVASIMGVEPKEIFFTSGGTEGDNWAIKGTAIEKGKYGKHLITSSVEHPAVK

ESMAQLEKQGFEVTYLSVNREGIISLEELKEALRSDTILVSIMAVNNEVGSIQPIAEI

GEILKAYPKVHFHVDAVQAIGKIKLDLSASSRVDLATFSAHKFHGPRGIGFMYIRQGK

KIAPLLSGGGQEFGRRSGTENLAGIVAMSKALRLLYTDALVKQQKQREIKEYLSAGLE

KYSKVSVFSSPTGAPHILCFALRGIRGEVFVHAMEKVGIYISTTSACSSRSQTSSSTL

SAMHVPENLATSAVRVSLTDTNTLAEAKQFLVEFAKLEEQFRDIH"

CDS 840826..842043

/gene="thiI"

/locus_tag="JFBMFIFI_00794"

/EC_number="2.8.1.4"

/inference="ab initio prediction:Prodigal:002006"

/inference="similar to AA sequence:UniProtKB:Q81KU0"

/codon_start=1

/transl_table=11

/product="putative tRNA sulfurtransferase"

/db_xref="COG:COG0301"

/translation="MNYTEIMVRYGELSTKGKNKRTFINRLSQNVRRALHDFPEIKIL

GERDHMYLELNGADSHIVMKRLEPIFGIQNYSPVVRLPRDMDVVKKVAVEMVQSVFKE

GQTFKITTKRADHLFELDTNDINQLLGAEVLRNVEGIKVQVKKPDIEVKVEVRLEGIF

LSCQRIAGAGGLPVGSGGRAMLMLSGGIDSPVAGYLAMKRGVEVQAVHFHSPPYTSPR

ALQKAKDLTVKLTAYVGSIQFIEVPFTEIQEEIKRVVPEGYLMTVTRRMMMRLTDKIR

EQQRGLAIVNGESLGQVASQTLQSMVSINEVTNTPIIRPVVSMDKNEIIEIAQKIDTL

ELSIQPFEDCCTIFAPPAPKTKPKLEKAQKFEARLDVDGLIERAMAGLVISEIKIGDS

LEEQQEEAFSEFL"

CDS 842285..843874

/gene="nfdA"

/locus_tag="JFBMFIFI_00795"

/EC_number="3.5.1.91"

/inference="ab initio prediction:Prodigal:002006"

/inference="similar to AA sequence:UniProtKB:Q68AP4"

/codon_start=1

/transl_table=11

/product="N-substituted formamide deformylase"

/translation="MKLWKNGLFYTMSNEGETVEAVLTNNGEIIGVGTEESLKEYYPE

VLEEIDLAGSTVFPGFVDSHLHLLWYGQSLARLNLNQCTSKKTALRMIKEKVAKLREK

EWLFVEGYNENKWQDSTEAFKKSDLDALSMTHPILVRRIDYHNVVINEAFTQEIGIKK

WQKFEGGGEIVLDEKGEFTGVLKDEATNLAIDAFPPATPSEMEDYLTLAVKDLWTKGI

TGGHTEDLHYFNGFEGTLATFKACLCVDKLPFRSHLLIHNAELEAFNQSNQQFLDGDP
FIELGAMKIFYDGTVGSRTALMKNGYASDSEHHGLQIQTDDIFEKMVQSARNAGLPVA
IHILGDLAFEKVIQTLKAYPPKKGQRDRMIHTPWLNPGLLAKAKNMPLLFDVQPQFMS
SDLPWALEVLGTNHPPLAFAWRSIAEAGFPTAGGSDAPVETPNPFLGIHAAVTRTVSS
DKNGKAYFPDEGLSVFEAISLYTTGSALAGYQEKTRGQIKVGFVADFTVLEENPFTIE
KENLADIHVLKTIISEQIVFG"

CDS 844061..844720
/gene="rex_1"
/locus_tag="JFBMFIFI_00796"
/inference="ab initio prediction:Prodigal:002006"
/inference="similar to AA sequence:UniProtKB:O05521"
/codon_start=1
/transl_table=11
/product="Redox-sensing transcriptional repressor Rex"
/db_xref="COG:COG2344"
/translation="MEKVEKNKNVPKATAKRLPLYYRYLKSLEESGIHRIKSKDFSTL
TQVPSTTIRRDFSHFGELGRSGYGYEVGHVLNVFDQILNVNRVTKVALVGVGNLGRAL
MSNNFRRDDNLKITCGFETNEELCNQKIGGIPVYHISQMEEIIQHESITTAISTVPSQ
HSQEAINQLVAAGVTAVLNFAPGRVKVSNEIDVRYIDLTTELLTLIYFNEKIRKQTQL
S"

CDS 844914..845405
/gene="tpx"
/locus_tag="JFBMFIFI_00797"
/EC_number="1.11.1.15"
/inference="ab initio prediction:Prodigal:002006"
/inference="similar to AA sequence:UniProtKB:P80864"
/codon_start=1
/transl_table=11
/product="Thiol peroxidase"
/db_xref="COG:COG2077"
/translation="MEITKRGTVIKVEGTQPKTGEKAPHFTLNNLSDKKINLADYQGK
VVLVSVVPNIDTSVCAIQTKHFNQEAGQLENVQLITISNNTKEEQADWCAGNGVEMEM
LHDTDLEFAKAYGVYMPELGLLARSVFVIDQDGVLVYEEIVPEMSHEPDYTAALAATK

ALI"

CDS 846003..848648

/gene="valS"

/locus_tag="JFBMFIFI_00798"

/EC_number="6.1.1.9"

/inference="ab initio prediction:Prodigal:002006"

/inference="similar to AA sequence:UniProtKB:Q05873"

/codon_start=1

/transl_table=11

/product="Valine--tRNA ligase"

/db_xref="COG:COG0525"

/translation="MSEELKMPTKYQPQEVEAGRYEEWLKKDLFKPSEDQTKEPYSIV

IPPPNVTGKLHLGHAWDTTLQDIIIRQKRMQGYDTLWLPGMDHAGIATQAKVEEKLAQ

EGISRYDLGREKFIETVWDWKEEYASVIRAQWAKVGISVDYSRERFTLDSGLSDAVRK

VFVSMYEKDLIYRGEYIINWDPKAKTALSDIEVIHKDVEGAFYHMSYPLADGSGTVEL

ATTRPETMLGDTAIAVHPEDSRYKHLIGKMVILPLVNKEIPIIADDYVEMDFGTGVVK

ITPAHDPNDFEVGNRHDLPRINVMDESGVMNELAGKYQDMDRFAARKAVVADLKELGH

LIKIETMVHSVGHSERTGVVVEPRLSTQWFVKMAPLAKEAMDNQATDQAIEFVPERFE

TNYMRWMENIHDWVISRQLWWGHQIPAWYHKTTGELYVGMEAPVDSENWVQDPDVLDT

WFSSALWPFSTMGWPDTEAPDFKRYFPTNTLVTGYDIIAFWVSRMIFQSLEFTGKRPF

QKALIHGLIRDEDGRKMSKSLGNGIDPMEVIDKYGADALRWFLSNGSAPGQDVRFSYD

KMDAAWNFINKIWNASRFAIMNMNGLSYEEIDFSGEKTIADRWILTRLNQTVEKVTDL

FEKFEFGEAGRHLYNFIWDDFCDWYIEMSKGVLNGENEAAKQTTRSILAYVLDQTLRL

LHPIMPFVTEKIWESVPHEGESLVIAEYPVVHPELNDEAAAKGMDVLMELIRSVRTIR

SEVNTPLSKKIELLIKTNDASIETFLKENETYIERFCNPENLVISSQIEAPESAMSAV

ITGAEIYLPLAGLINIDEEIIRLEKEMAKWESEVKRVQGKLSNERFVSSAPEAVVQVE

REKEKDYLEKREAVAERIASLKAQQ"

CDS 848811..850118

/gene="fpgS"

/locus_tag="JFBMFIFI_00799"

/EC_number="6.3.2.17"

/inference="ab initio prediction:Prodigal:002006"

/inference="similar to AA sequence:UniProtKB:P15925"

/codon_start=1

/transl_table=11

/product="Folylpolyglutamate synthase"

/translation="MFKTYEEALEWIHGRLVMGMKPGLKRMEWMMKRLGHPERRFKSI

HVAGTNGKGSTVTYLREMLEAAGQVVGTFTSPYITTFNERISVNGEPISNQEILRLAN

LVYPLVMELEETSLGGPSEFEVVNTMMFIYFGEGHADVVLIEVGLGGLVDSTNVVTPV

VSAITTIGFDHMHILGGTIAEIAEQKAGIIKPGVPVVVGNVDSEALKVITKVAEENSS

PLLCFNKDYFISKWKTLPTWGETFTFEDDFICLRQVVLSMLGKHQVENAALALEVLRV

YSHETGLAIGHEQMLRGLKAAFWPGRLEKINEDPLIILDGAHNEPAMNRLVETLKNDF

EQQEIYVLFAALGDKAVDKMLGQLQVLPNVHLILTSFDYPRAASANQLTTNLTGNYDV

EEVWQEGLVKTVNEMDSNSVLIITGSLYFISEVRHYFLDETNN"

CDS 850133..850795

/locus_tag="JFBMFIFI_00800"

/EC_number="3.1.3.-"

/inference="ab initio prediction:Prodigal:002006"

/inference="similar to AA sequence:UniProtKB:Q9X0Y1"

/codon_start=1

/transl_table=11

/product="Phosphorylated carbohydrates phosphatase"

/db_xref="COG:COG0637"

/translation="MGKVKAVIFDMDGLIFDTETLYYRSMQEVADRLGLPFDYKYYLK

FVGTSDEELHENLYRDFKNDEKVATLITDSRTRLDEIVEDEGLMVKAGFIELLDFLEA

EGIKKVVASSNLKEMVANFLKRENIQHRFDYFVSGDEVKRAKPDPEIFEKAWSGLAVP

KSETLILEDSINGIRAGFDAGIRVIMVPDLIPPSPEAEEKTSAIYEDLSYVKDFIKNQ

NK"

CDS 850845..851531

/locus_tag="JFBMFIFI_00801"

/inference="ab initio prediction:Prodigal:002006"

/inference="similar to AA sequence:UniProtKB:Q02170"

/note="UPF0758 protein YsxA"

/codon_start=1

/transl_table=11

/product="hypothetical protein"

/translation="MKERSLVLEVPKYSRPRERLEEYGEKALGTHELLAILLRTGSKD
NNVLAVAMELLNNFGDLHQLKLASLEELREIKGIGHAKSIEIRAAIEFGVRIASASQL
KIGHITSSQMVGDLLYTEMKDLPQEHVVALYLNTKNHLIKKETIFVGGLNSSIAHPRE
IFRGAVRISAARIILGHNHPSGNPEPSQADISFTKRIIECGELMGIDLLDHIVVGEKG
YISLKETGII"

CDS 851888..852088

/gene="cspLA_2"
/locus_tag="JFBMFIFI_00802"
/inference="ab initio prediction:Prodigal:002006"
/inference="similar to AA sequence:UniProtKB:P0A355"
/codon_start=1
/transl_table=11
/product="Cold shock-like protein CspLA"
/db_xref="COG:COG1278"
/translation="MEQGTVKWFNSEKGFGFIERENGDDVFVHFSAIQGDGFKTLEEG
QAVTFDVEDGNRGPQAANVEKA"

CDS 852640..853653

/gene="mreB"
/locus_tag="JFBMFIFI_00803"
/inference="ab initio prediction:Prodigal:002006"
/inference="similar to AA sequence:UniProtKB:Q01465"
/codon_start=1
/transl_table=11
/product="Cell shape-determining protein MreB"
/db_xref="COG:COG1077"
/translation="MFGFGSKDIGIDLGTANTMVYLDGKGIVLREPSVVAKNTNNGDI
VAVGEEARNMIGRTPGSIVAIRPMKDGVIADYETTAAMMKYYIQKTIGKSSSKPYVMV
CVPSGVTEVEKRAVIDATRTAGAKDAFIIEEPFAAAIGAGLPVMDPTGSMVVDIGGGT
TDVATISLGGIVSSRSIRMAGDRLDDAIIHYVRKKFNLLIGERTAEAVKMEIGCASVE
KSKEYGDMSVRGRDLLTGLPKTIEISAVDVSQSLQEVVEAIIVAVKETLEETSPEISA
DVIDHGIVLTGGGALLKNIAEVIADETQVPVFVANEPLDCVAIGTGESLKHIDLFKKN
ANR"

CDS 854018..854809

/gene="mreC"

/locus_tag="JFBMFIFI_00804"

/inference="ab initio prediction:Prodigal:002006"

/inference="similar to AA sequence:UniProtKB:Q8Y6Y4"

/codon_start=1

/transl_table=11

/product="Cell shape-determining protein MreC"

/db_xref="COG:COG1792"

/translation="MGLIAYSIKERAKLPIVQQFTNDITAITGRVFAKPANAVVSFID

SIDDLKNTYEENQLLKSKIDKLYEQQVQLADLKEANKKMQEQLDLQSDISDFNKINGT

VIGRNPDTWIDQIIVDRGSQNGIEKGMAVMSGPGLVGRVSEVSPTSSKVQLITTADTK

NNRVAAEVQGGAAPVFGIINGYDKETKRLIMKQITADAVPEKGTQVVTSGLGGKTPRT

LPIGEVDEVKLDSFGLSKEVYVKPYADTNDIRYVTFIQRSAESGE"

CDS 854814..855335

/gene="mreD"

/locus_tag="JFBMFIFI_00805"

/inference="ab initio prediction:Prodigal:002006"

/inference="similar to AA sequence:UniProtKB:Q01467"

/codon_start=1

/transl_table=11

/product="Rod shape-determining protein MreD"

/db_xref="COG:COG2891"

/translation="MNMNLKKSFLPPLIIFCFFLIDGVLAALFSKTFYDGNFVLVPRL

IVISFVMMSFYLPRNKMLLYAIIFGLLYDSYYSGVLGVYVALFPIIVYITEKLKKVLN

PNVLVVGMMLIINLSMVEGALYLFYQVLGQNTMDVNSFLAERLGPTLLLNSAIFIFLY

FPLKKTILKLTES"

CDS 855393..856085

/gene="minC"

/locus_tag="JFBMFIFI_00806"

/inference="ab initio prediction:Prodigal:002006"

/inference="similar to AA sequence:UniProtKB:Q01463"

/codon_start=1

/transl_table=11

/product="Septum site-determining protein MinC"
/db_xref="COG:COG0850"
/translation="MKQSVTLKGTKDGFVLTLDESVAFETVLSDLDQLLEKLHSENKE
QKEVQKGISLEVKTGMRLFSEDEKAKITEKISSKSQFQLKKISAEVLTYQQATEWHES
NSLQMEIQTIRSGQVLQAPGDILLIGKIHPGGTIRASGSIFILGELLGLAHAGFDGDN
EAIVVADFQTDAQIRIAESVQIIENKAKNQAGTDEKQFAYINDLHILDFEGLDKLRTI
RPKMGKVTGGLF"

CDS 856085..856873
/gene="minD"
/locus_tag="JFBMFIFI_00807"
/inference="ab initio prediction:Prodigal:002006"
/inference="similar to AA sequence:UniProtKB:Q01464"
/codon_start=1
/transl_table=11
/product="Septum site-determining protein MinD"
/db_xref="COG:COG2894"
/translation="MGTAIVITSGKGGVGKTTSTANIGTALALQGKKVCLIDMDIGLR
NLDVILGLENRIIYDIVDVVEERAKLHQAIIKDKRFNDNLYLLPAAQNADKSSVSGEQ
MIEIVDQLRPDYDYILIDCPAGIEQGFKNSIAAADSAIVITTPEISAVRDADRIIGLL
EQTEMESPRLIINRIRQRMMQEGEVLDIDEITKHLSIDLLGIVFDDDSVIRSSNKGDP
IVLDPKNPASLGYRNIARRILGETVPLMSFKKEKKNFFQKIFGK"

CDS 857246..858067
/gene="glnH_2"
/locus_tag="JFBMFIFI_00808"
/inference="ab initio prediction:Prodigal:002006"
/inference="similar to AA sequence:UniProtKB:P0AEQ3"
/codon_start=1
/transl_table=11
/product="Glutamine-binding periplasmic protein"
/db_xref="COG:COG0834"
/translation="MKKLQMILVIAGVFILVAACGKSGAGNKKEAKEQDSYSQIEKNG
KLVVGLDDTFAPMSYRDNNGDIVGFDVDLAKAVGEKLDLKLEFQAIDWAMKETELNAG
NIDLIWNGYTITPERKEKVAFSTPYLDNSQAIIVLKDSEIQKKSDLKGKTVTAQQSSS

AVDAVKNDSEVTLTDLKTGDLVQYPSNNDVFNDLEAKRSEAAVADEVLARYYISLKGA
EKYRILTENFGKEEYGVGMRKADKQLKEKIDQALAELKTDGTYDKIYATWFGKEK"

CDS 858093..858872

/gene="artP"

/locus_tag="JFBMFIFI_00809"

/inference="ab initio prediction:Prodigal:002006"

/inference="similar to AA sequence:UniProtKB:P54535"

/codon_start=1

/transl_table=11

/product="Arginine-binding extracellular protein ArtP"

/db_xref="COG:COG0834"

/translation="MKKKLILVSLLCLAFVIGGCKSETKANTNEKNKIIVGLDDTFVP
MGYRDKAGKIVGLDVDLAKEFGKRANVDIEFQPIDWAMKETELNSGNIDLIWNGYAMN
PERSEKVNFSTPYLEIGQAIIVLKDSPIETKNDLAGKTVAAQQSSSAVTILSQDAPEL
LKSFKGGEMVTYASNNDVFNDLDSKRSEAIIVGETYGRYTIKQKGEDNYRILKENFGV
ENIAVGVRKADKELLKQVNQALKEMEEDGTKEKIVNKWFSE"

CDS 858916..859545

/gene="artQ"

/locus_tag="JFBMFIFI_00810"

/inference="ab initio prediction:Prodigal:002006"

/inference="similar to AA sequence:UniProtKB:P54536"

/codon_start=1

/transl_table=11

/product="Arginine transport system permease protein ArtQ"

/db_xref="COG:COG0765"

/translation="MITEIIPALLDGLKMTLLLFFIILIISIPLGFLVALARVYAPKF
ISFFIQVYIYIMRGSPLLLQLMIVFFGLPLVGITLDRFSAAIIAFSLNYVAYYAEIFR
GGIIAVPVGQFEAIQVLGIGKIRGFQRIILPQVFRVVLPSVGNEVISLVKDTSLIYVI
GLGELLRAGQIAANTYASLIPFVAVGLVYLAITGIVTLGLNQIEKKVRF"

CDS 859633..860310

/gene="glnQ_3"

/locus_tag="JFBMFIFI_00811"

/inference="ab initio prediction:Prodigal:002006"

/inference="similar to AA sequence:UniProtKB:P10346"

/codon_start=1

/transl_table=11

/product="Glutamine transport ATP-binding protein GlnQ"

/db_xref="COG:COG1126"

/translation="MILEVKNVTKKFNGLPVIQNFDFSIAAGEIVTLVGKSGTGKTTL

MRLINNLEKADSGTIKIGNQLLCETKNEQSVYASKKDIRAYHNSIGMVFQDYQLFPNL

TVLENCLEAPIAQKLDTKSNLIKKAEELLGQVGLSDKIQVMPSTLSGGQKQRVAIARA

MMLNPKILCFDEPTSALDRESTVEIGKMIQKIATDQTGILIVTHDLEFAEDIGTRVVS

SEEFINK"

CDS 860483..860791

/gene="rplU"

/locus_tag="JFBMFIFI_00812"

/inference="ab initio prediction:Prodigal:002006"

/inference="similar to AA sequence:UniProtKB:A2RLA4"

/codon_start=1

/transl_table=11

/product="50S ribosomal protein L21"

/db_xref="COG:COG0261"

/translation="MYAIIKTGGKQVKVEVGQAIYVEKLNVEAGETVSFDEVILVGGE

ETKVGAPTIAGATVEGTVEKHGKQKKVTTFKYKPKKHTHRKQGHRQPYTKVVINAINA

"

CDS 860809..861141

/locus_tag="JFBMFIFI_00813"

/inference="ab initio prediction:Prodigal:002006"

/codon_start=1

/transl_table=11

/product="hypothetical protein"

/translation="MIQALFKRDEDGNLVSFEITGHAESGPDGSDIVCAAVSALTFGA

TNSIEALAGFQPLVEMDEENGGHLYVEVIQDINREQSHIAQILLESLLLSIGTIVEEY

PEYVKIIK"

CDS 861178..861465

/gene="rpmA"

/locus_tag="JFBMFIFI_00814"

/inference="ab initio prediction:Prodigal:002006"

/inference="similar to AA sequence:UniProtKB:P66133"

/codon_start=1

/transl_table=11

/product="50S ribosomal protein L27"

/translation="MLKMNLQFFAHKKGGGSTTNGRDSNSKRLGAKRADGQTVTGGSI

LYRQRGTKIYPGVNVGIGGDDTLFAKVDGVVRFERKGRDKKQVSVYPAVAQ"

CDS 861869..862882

/gene="exuR"

/locus_tag="JFBMFIFI_00815"

/inference="ab initio prediction:Prodigal:002006"

/inference="similar to AA sequence:UniProtKB:Q9JMQ1"

/codon_start=1

/transl_table=11

/product="putative HTH-type transcriptional repressor

ExuR"

/db_xref="COG:COG1609"

/translation="MATTIKDIVERTGLSLGTVSKYLNGVSVKPKNKVLIDEAVEFLD

FKVNLFARGLRTKESKSIGIIIPSLELTFSTKIVAITEKYLAQNGYVVMICVVTGDEE

IDSEKLQNLVNQSVDGLLVIPPAKNCFLTTWLEKMNGFIPVIVLDRYLESNVVFDHVV

VDNRQAAKKAVTRLLLKGHTRIGIIAGDKGGYTSKERLLGYQDALNSQGVSLKPEYIV

YGDFSKKSGYYACQELFELQNVPTAIFVTNYDMTVGALECFNSYQKKIGEDISLIGFD

SAEISTILTPKLSMVIQPVDKMAEFVAEELLKAIREPENVKPKSTVFTCELLIEDSIQ

SIK"

CDS 863017..863931

/gene="rihC"

/locus_tag="JFBMFIFI_00816"

/EC_number="3.2.-.-"

/inference="ab initio prediction:Prodigal:002006"

/inference="similar to AA sequence:UniProtKB:P22564"

/codon_start=1

/transl_table=11

/product="Non-specific ribonucleoside hydrolase RihC"

/db_xref="COG:COG1957"

/translation="MTRRPIIIDTDPGIDDAVAIAIALNSEELDVKLISTVAGNVDVE

KTTRNALKLVTILGKETPVAKGADRPLLKELETASDVHGESGMDGYEFPESQNKELSI

HAVEALKKYILASPEKITLVPIAALTNIALLFTMYPEVKENIEEIVMMGGTLSRGNTN

SIAEFNTYVDPHAAQMVFQAGVPITMVGLDVTDTAVLTSGENEQIKTYGKVGEMFYAL

FQHYRGGSLKSGLKMHDVCAIAYLLQPEMFKTQLTHAEIQLEGPAAGAVIADLKMNYH

TTTNVSVCLEIDVEKFRHWTLKELQKNQ"

CDS 863950..865323

/gene="dcuD"

/locus_tag="JFBMFIFI_00817"

/inference="ab initio prediction:Prodigal:002006"

/inference="similar to AA sequence:UniProtKB:P45428"

/codon_start=1

/transl_table=11

/product="Putative cryptic C4-dicarboxylate transporter

DcuD"

/db_xref="COG:COG3069"

/translation="MIEIFLVILVLGVVAYLIIKNYNPILTLISGGLVLLACAVILGH

PLYPAGEGTGLGVFDIFMKFKDTIIGQVQSAGLVIMILFGYSGYMNKIGANQVAVNLL

VKPMMKIKAKALFVPIIFLIGNLMSLVIPSASSLAIILMSILYPMLQGIGISSLTAAG

VIAMTATIMPTPLGADNVIAAETLGYDLLNYVMWNAKISIPTLFIIAVAHYFWQKYCD

KKEGASAFIEMDQAKLEKIEESNTPTFYAILPLMPLLLILIVGIMSIVIKGITMDIFV

LTMIAFFIAVLIETCRYRSFKTVQLSAVEMFNGMGQGFSRVVMLVVGGSLFTTAIQSL

GVIDSLLSSVESSTSAGIVTMFIFSGATTLFGILSGGGLAMFYAVIELIPNIADKAGV

DGILITLPMQMIANLARTISPVAAVVMIVASTVGVSPVRVLKRTSVPTIVGIVSVIVL

SLLILPY"

CDS 865565..866572

/locus_tag="JFBMFIFI_00818"

/inference="ab initio prediction:Prodigal:002006"

/codon_start=1

/transl_table=11

/product="hypothetical protein"

/translation="MTINQIEQLFKLLDESSLVLKNELDSSYLDALLETGENLIDGKQ
ARQVEGVPTEAVIERLNRLYAQWEKDEVTPEEIRKAFQLALLKGAKVDNLQANHQMTP
DAIGFIMSYIIEKMIGDEATKIRIFDPAVGTANLLSTIYNHLTLKNIVVEAEGVDIDD
LLLSLASVLIGLQRQSIKLIHQDAIQDLLVDPVDVVVSDLPVGFYPLDERVTEFKTKA
KTGHSYAHHLLIEQSVHYLKPGGVGVFLVPSQLFETEEAPALTKFIQETSYLQGMLHL
PKELFKTEHSRKSILLIQKKGAEAKQAKQVLLAQIPDFKNQAAMLRFMNEVDTWKKEN
N"

CDS 866604..867794

/gene="ackA"

/locus_tag="JFBMFIFI_00819"

/EC_number="2.7.2.1"

/inference="ab initio prediction:Prodigal:002006"

/inference="similar to AA sequence:UniProtKB:P37877"

/codon_start=1

/transl_table=11

/product="Acetate kinase"

/db_xref="COG:COG0282"

/translation="MTKTIAINAGSSSLKWQLFEMPAETVIAKGIVERIGLNDSIFTI
KYGEGQKFEEILDINDHEFAVEMLLKQLIELKIIGSFDEITGVGHRVVAGGEIFKDSA
LITGNVLDQIEELSELAPLHNPANATGIRAFKKLLPEITSVAVFDTSFHTTMPKTSYL
YSIPMNYYEDFAARKYGAHGTSHKFVSERAAEMLGRPLEELKLVSCHLGNGASITAID
GGKSVDTSMGFTPLAGVTMGTRSGDIDPSLIAYLMGKLNITDINEFINILNKESGLLG
LSGISSDMRDLEVAQLTDENANVALRIFADRIKKYIGSYIATMNGVDAIIFTAGIGEN
DIHMRKEIIDGITVFGCEMDAELNNVRSDERVISTDDSKVKVLLIPTDEEVMIARDVE
RLSK"

CDS complement(867847..868311)

/locus_tag="JFBMFIFI_00820"

/inference="ab initio prediction:Prodigal:002006"

/inference="similar to AA sequence:UniProtKB:Q7A551"

/codon_start=1

/transl_table=11

/product="Putative universal stress protein"

/translation="MLQQYKRILVAVDGSEEAELAFKKAVHVANRNESALLLLHVIDT

RAFQSVSSFDGAMAEQATEQAKNTMEEYVKYAKKHDVQDVSYTIEYGSPKALIAKQIP
EEKKVDLIMVGATGLNAVERIFIGSVSEYVIRQAPCDVLVVRTDLENKIPQK"

CDS 868454..868684

/locus_tag="JFBMFIFI_00821"
/inference="ab initio prediction:Prodigal:002006"
/codon_start=1
/transl_table=11
/product="hypothetical protein"
/translation="MKKEFCLIQDVFAEPKFKLFKNYSIEHQLIYLQDISPEILENFA
KQRGVGAVKMTALIERLAGPVSTLPLDVFTTE"

CDS complement(868717..868995)

/locus_tag="JFBMFIFI_00822"
/inference="ab initio prediction:Prodigal:002006"
/codon_start=1
/transl_table=11
/product="hypothetical protein"
/translation="MTILMILFALILFATAGFLLSGKASILIINEKENQHAANQVFFK
SYGALLLLCGIFALVLIFIHSTWLLLLFLVATCAIMLLLILGLNRRID"

CDS complement(869082..870359)

/locus_tag="JFBMFIFI_00823"
/inference="ab initio prediction:Prodigal:002006"
/inference="similar to AA sequence:UniProtKB:P9WQN1"
/codon_start=1
/transl_table=11
/product="putative AAA domain-containing protein"
/db_xref="COG:COG2256"
/translation="MNQPLAFRMRPTNIDQIVGQTHLVGEGKIIRRMVESKMLSSMIL
YGPPGIGKTSIASAIAGSTKYAFRMLNAATDTKKDLQIVVEEAKMSGTVILLLDEVHR
LDKPKQDFLLPHLENGRVVMIGATTENPYITINPAIRSRTQIFELKPLEISDIEVAIE
RALVDKVNGLGDSAVEVTEEALLHFARATNGDLRSSLNGLELAVKSTHPNQDGLIIID
LAIAEECVQRKALTHDKNGDAHYDVISALQKSIRGSDVNAALHYMGRLVEAGDLPIIA
RRLMVIAYEDIGLANPAAAARTVTAVQAAEKLGFPEARIPLANAVVDLCLSPKSNSTY
VAIDAALSDIRAGKSGDVPDHLRDSHYSGAKDLGRGIDYKYPHAYPEAWVDQQYLPDK

LKNATYYVPNYTGKYEASLAGQYDKINKLKKKK"

CDS 870675..871088

/gene="lrgA_1"

/locus_tag="JFBMFIFI_00824"

/inference="ab initio prediction:Prodigal:002006"

/inference="similar to AA sequence:UniProtKB:P72358"

/codon_start=1

/transl_table=11

/product="Antiholin-like protein LrgA"

/db_xref="COG:COG1380"

/translation="MNIYKQFLIILSFSFLGEVLSKVFNLPVPGSVIGMLFLFLALEF

KILKVKDVETVGGFLLGNLSILFLPAGVGIMVYFPIIKDTWWLLLLVSIITTAFTVAF

VGVIVQAVKRRFEDSAVDLDETRKDDAKSVSRTYK"

CDS 871066..871773

/gene="yohK"

/locus_tag="JFBMFIFI_00825"

/inference="ab initio prediction:Prodigal:002006"

/inference="similar to AA sequence:UniProtKB:P0AD19"

/codon_start=1

/transl_table=11

/product="Inner membrane protein YohK"

/db_xref="COG:COG1346"

/translation="MYQELTSNPMFGLMLSIGMYLFALAIFKKFPFPLFNPLVLSTIF

VIIILKVGRISYENYFEGGTILNILITPATVALGIPLYNTFHLLKKHVRSILTGIICG

TIASTFLTGALAVALHLKKDLIVSIMPKSVTTAIALGISEKMNGIATVTLVMVIVTGI

IGSIIGPPILKRLKVEDPVAKGIALGTSAHAIGTSKALEMGHVEGAMSGLAIGVTGVV

TVFVAPLMMKFILVIFF"

CDS 871986..872957

/locus_tag="JFBMFIFI_00826"

/inference="ab initio prediction:Prodigal:002006"

/codon_start=1

/transl_table=11

/product="hypothetical protein"

/translation="MADNTTDNARFIFSYFRGKGWTAQAICGMLGNMQGESGIIADKD
EYGGGGGYGLVQWTPKSNLVNWANANGLNYRSVETQCKRIQWELDNGQQFYATGAYPI
NFRQFSQSTQSPTYLASAFINNYERPANANQPQRGVWAEEWYRTLNNGTIPPIGDGGK
KVTKYFCGINVGTDFTQVQAYRVAINNQWKIDYLDIYYYQKDSNWFIGINNLNFTGDG
GVEAYRMAFNNVFGIPFDKITYYSYEEQAWEIGYSGVSLTEYQNLRIRMMAKGLQLRE
VKIGTNPDRYDVLAINLSFRDIQRYRLQFMSEFADIIVGTRVTYKEM"

CDS 873252..874577

/locus_tag="JFBMFIFI_00827"

/inference="ab initio prediction:Prodigal:002006"

/codon_start=1

/transl_table=11

/product="hypothetical protein"

/translation="MSDTEQRERIKIQFKPQIIEDTTTITDQMVDTVKGTLNELITES
VSKVNELNIQRFEEKQAELEKEYQSKIGELIAEYEVKKNELDQKIADKEKLVLENAKL
EAESILQNAYEESNEMYQSVQNEIINLKAQLMDYQAEVEKEKQEQDKELARRLDELNL
VYQKKQAELEASLNEKEQALLRDARNEANQTKDEAKQESLLLLSEAQLKAADLRDGTA
QELLEEKAEHQAKLSDDFLRLTREKEWFSLYQEQVEQRFIEKERALYQKTQTELDELQ
LKVHELQNQSDFYQDMDQERKDKSFSIGLYICLVAGVNVVLISLFGQNSVVLPILSAF
LGSYLIYTTLTDGRFESGTKKLTKKNEWLIEKNDELAKKTTELTQSLQEVMIEKDQLA
KAKFEHEALVQNLEELKDLVRKQGTQRKLVESENIVLRKRLMDQKKRTE"

CDS 874775..875284

/locus_tag="JFBMFIFI_00828"

/inference="ab initio prediction:Prodigal:002006"

/codon_start=1

/transl_table=11

/product="hypothetical protein"

/translation="MTLASEFEQVKEFHETFSPATNKTPTAFTVEEALYRADFTTEEI
LEFLYATVEGDLVAFDQLVAKWQEGITKTVNKIKTEAKPVEDKLIGQVDALTDANYFN
YGSFVLLGVDPRPIFNFVHQANMGKLFPDGKPHYREGDGKVLKPDNWETDFAPESKIK
AEIERQKQL"

CDS 875299..877575

/gene="gshAB"

/locus_tag="JFBMFIFI_00829"

/inference="ab initio prediction:Prodigal:002006"

/inference="similar to AA sequence:UniProtKB:Q9CM00"

/codon_start=1

/transl_table=11

/product="Glutathione biosynthesis bifunctional protein

GshAB"

/db_xref="COG:COG1181"

/translation="MNDIKTRIKNEKLGPAFIKGTFGLEKEGLRVTPTGDLALTDHPA

ILGNRSFHPYIQTDFSESQLELITPPVESIDELYNWLAAIHDVTERSLPKDEYIWPMS

MPGALPEEAQISVAKLDNQEDVAYRIHLSEKYGKKKQMVSGIHYNFELDAGFLNALYQ

GQSSIKEYSQFKSEVYMKLAKNFLRYRWVLTYLFGASPTVASNFYQADQEPLKDYVRS

IRGSHYGYVNTKEIQVSFESLESYVEDIEELVNKGALSEEKEFYSAVRLRGGKKNRDL

LTDGIHYLEFRMFDFNPYAAFGMTKEDMKFIHLFLMSLVWMDKIATVEEIEAGNLMNE

KTALEHPNTVSNFQQEAIRLVDNMLEMLQSCEFSTEMKELVIQAKAQILDPQKTISAR

LIKDIAKVGSFKELGMKLSRQYKEEAFLKPYSLRGFTSMELSTQLLMFDAIQKGVEVG

ILDASEQFLKLRHLDHIEYVKNANMTSKDQYIAPLIMENKTVTKKVLAGAGYKVPAGE

EYQNAREAKGAYWNYAGKGIVVKPKSTNFGVGISIFKDGPSREDYELAIDFAFKEDKA

ILVEEFITGTEYRFFVIGDAVKAIMLRMPANVVGNGEQTIEALVKEKNKDPLRGTDHL

APLELIQLGEIEQLMLKEQGYTIQSIPTKGTIVYLRENSNISTGGDSFDYTDTIDDSY

KQAAIDMTKALGAVVCGVDLIIPDYTKPSTKTNPGYAVIEANFNPAMHMHTFPYKGQG

RRLTMAILDMLFPELGIF"

CDS 877889..879034

/gene="iscS_2"

/locus_tag="JFBMFIFI_00830"

/EC_number="2.8.1.7"

/inference="ab initio prediction:Prodigal:002006"

/inference="similar to AA sequence:UniProtKB:O54055"

/codon_start=1

/transl_table=11

/product="Cysteine desulfurase IscS"

/translation="MEKIYLDHAATSPVHPEVLEVTYEAMKNHYGNASSIHNFGRDSR

RLLDDARLTFAKSIGAKQSEIVITSGGTESDNTAIIETALSRQETGKHLITSEIEHHA

VLEPMEYLESLGFEVTYLPVDTFGRVDPEAVREAIRPDTTLVSIMYGNNEVGTLMDIA

KIGEIVAETDSYFHTDAVQAYGLEDIDVKRDQIDLLSVSAHKINGPKGVGFLYISEGI

HLPSFMRGGEQETKRRAGTENIPAIVGFKKAVEIIAAEKKERREKYQGYKGQLIKNLV

DTKIDFEINGDQQNGLAHIVNIWIKGVSSEQLLMNLDLMGIAISAGSACTAGNVEPSH

VLTAMYGPENPRISESVRISFGLGTTSEMLSELTDKLALFVSRLKIN"

CDS 879498..879842

/locus_tag="JFBMFIFI_00831"

/inference="ab initio prediction:Prodigal:002006"

/codon_start=1

/transl_table=11

/product="hypothetical protein"

/translation="MAFETTASLVGSTEKYRLTPNVKKFTLRDNGFEETSSGNYQFIR

PLDGSPENKQGIKLKIIVNKDLKTFKMSTTTSNGLRAVNIYTNENFKMSVDKFKFIMD

GFIERGVMEKAE"

CDS 879939..881192

/gene="mnmA"

/locus_tag="JFBMFIFI_00832"

/EC_number="2.8.1.13"

/inference="ab initio prediction:Prodigal:002006"

/inference="similar to AA sequence:UniProtKB:Q97T38"

/codon_start=1

/transl_table=11

/product="tRNA-specific 2-thiouridylase MnmA"

/db_xref="COG:COG0482"

/translation="MFIVIQYINLDLSLKAVIMIKTGKIRPSNRKDFQNLFEMMVKEM

TDNSKTRVVVGMSGGVDSSVTALLLKEQGYDVVGIFMKNWDDTNEAGVCTATEDYNDV

IRVANQIGIPYYSINFEKQYWDNVFQYFLDEYKLGRTPNPDVMCNKEIKFKAFLDYAM

SLGADYVATGHYAQVERDEAGVTHMLRGVDTNKDQTYFLNQLSQEQLAKTMFPLGGME

KSEVRRIAEEAGLATAKKKDSTGVCFIGERDFKKFLMTYLPAQKGKMVTPEGEVKGEH

DGLMYYTIGQRQGLGIGGGGASSEPWFVIGKDLASNTLYVGQGFHHEWLYATHLDATD

IHFTTMKEMPREFKCTAKFRYRQTDTGVTVHLNEDGTTARVEFDEPVRAITPGQAVVF

YDGMECLGGGTIDVAYNDAKVLQYV"

CDS 881359..881814

/locus_tag="JFBMFIFI_00833"

/inference="ab initio prediction:Prodigal:002006"

/codon_start=1

/transl_table=11

/product="hypothetical protein"

/translation="MEEIKNLIRQNPDKFKKNPLLSIVASMCGEEQKIQNIEENWYVV

FKETIKLVAITTVEGVEHYFNQLNPTIAYLLINYIKILEFSDLELFMEHGIKLVDPEN

PTKYTKEQKEQDYQEMYDWLLENSRLTNELSLTLVIRYHALLNQLLIED"

CDS 881956..882588

/gene="pspA_1"

/locus_tag="JFBMFIFI_00834"

/EC_number="3.1.3.3"

/inference="ab initio prediction:Prodigal:002006"

/inference="similar to AA sequence:UniProtKB:D3DFG8"

/codon_start=1

/transl_table=11

/product="Phosphoserine phosphatase 1"

/db_xref="COG:COG0406"

/translation="MAKLYFVRHGKTEWNLAMKLQGSKGDSPLLEESYFETALLGERL

KKISFSKVYVSPQKRAIETANVLMEELLIKPEIIQKEGLKEFGLGELEGQLILDAIDA

YPEQMHHLRNNPHEYDPSEFGGETFSEMIKRSVVVVEEAVMQNPNENLLFVGHGATLV

ALIQTLSGKPLEQIRKAGGLDNSSLSIIEMNELGYELILWNDTSHLDSLN"

CDS 882666..883343

/gene="cpoB"

/locus_tag="JFBMFIFI_00835"

/inference="ab initio prediction:Prodigal:002006"

/inference="protein motif:HAMAP:MF_02066"

/codon_start=1

/transl_table=11

/product="Cell division coordinator CpoB"

/translation="MDENLKAFELWDKGEYSEAIQVLFNQIEENPENMASYYNLANML

LVAKKEEDAKAVLLVANEKSPNHPEILYAFGTYYYQIEDYPQGIHAFLAVFQQDTYLK

KDSLVMIAQCYIALGNPQKALVYLLTAEEIDGTDSDVLIVMGNTFMQIKDFKQAQEYF

KKVTEVTPKNAEAWFKRGLTSLVLNEEKKLVESYFNQSMELDPVFYQKNRQQLAEIQA

FIQKNEG"

CDS 883374..885908

/gene="recD2"

/locus_tag="JFBMFIFI_00836"

/EC_number="3.6.4.12"

/inference="ab initio prediction:Prodigal:002006"

/inference="similar to AA sequence:UniProtKB:Q9RT63"

/codon_start=1

/transl_table=11

/product="ATP-dependent RecD-like DNA helicase"

/db_xref="COG:COG0507"

/translation="MTMQEDLDLFIGSEPFVVGQVVAIFYQNPTNFYKVILVRVVETD

SNFKEKEIVVTGSFGQIQEEEIYRFYGKLTDHPKFGVQFTASRYSPERPTSAAGIVNY

LSSEKFPGIGKKTAENIVEALGDRAIDEIIANPAVLKMVAGLNQKKIDLLAETLQAGD

GMEKIIIGLNGYGFGSQLAFSIYQTYQEETLEIIQENPYQLVDDIENIGFKKADAIAE

QLGFAADSPARIRAAILYALNELCLGRGDTYTLAEPLLEESLRVLEESRSVMIDPDFV

ANELIGLLEERRLVEHQEKLYLHSLYAAEWGISTSVKRLLERKKKINYDQHDIPKEIR

KLEKRLNISYGASQIEAIAEAVVSPLFILTGGPGTGKTTVLNGIVALFAELNGLSLDI

NDYHNAIFPILLAAPTGRAAKRMNESTGLPSSTIHRLLGLNGREKNSDPVSERELEGG

LLIVDEMSMVDTWLANSLLKAVPTNMQVILVGDKDQLPSVGPGQVLHDLLASKVIPSM

ELNEIYRQEDGSSIIPLAHEIKDGNLPADFTANKKDRSFFQCDTMQIENVIRQVVERA

KTKGFTAQDIQVLAPMYRGPAGIDALNKMMQEIFNPNDTGKRKELKFNDKVYRIGDKV

LQLVNNPESNVFNGDMGEIVGISYAKETDDKVDEMTIQFDTNEVTYKRNEWNKITLAY

CCSIHKSQGSEFKMVILPMVRNYNRMLRRDLLYTAITRSRDLLILCGEPQAFWTCVER

SSDDRLTTLKDRLLEDDELEVSYQVIASPYQEKEQVLDVEPKTLPEKKVTEVKTKEVN

LFELEEESVEYQVEIKDPNRYILTIEMVKNNQIDPMIGMENVTPYN"

CDS complement(886041..887408)

/gene="licC_4"

/locus_tag="JFBMFIFI_00837"

/inference="ab initio prediction:Prodigal:002006"

/inference="similar to AA sequence:UniProtKB:P46317"

/codon_start=1

/transl_table=11

/product="Lichenan permease IIC component"
/db_xref="COG:COG1455"
/translation="MMTKIEDLMTKRIMPIAEKINNNYFLGALSESFVRLTFIILGIA
LVAIVGYWPIPTAWGTWLTSVGIMEHVDAVINASTNALALYVSFSFAAAYAKKQQVNS
QNAGFLGLLSFLIISPQTIMLTTSQGITEVAKNYSTNPITKETTSKVIEAFPISAFGG
QSLLAALLVSFVASWLYIKMTKKGFNPKLPDSIPPMVAESLSPAFSSMLVVGVAFSLR
VGFGFTQAGNMLTFFNELVAKPLGLLTSTPIAFILILTLASFLWFFGIHPNVVYGAIS
PLTYTIILGNIAAYKASGGIGTDLPYGNLGIITTMITMGGAGCTLGLILSMVVAKSKR
YKAMFKLAIIPELFNINEPLVFGMPMVLNPIFFLPMVFSPLIIGLSAWGLTSFIQVNL
NPLIGLMPWTTPKLLSIPLAGGIKYILIFLICLTINTLIYYPFFKIADRQALLDEKNE
ELQAV"
CDS complement(887426..888526)
/locus_tag="JFBMFIFI_00838"
/inference="ab initio prediction:Prodigal:002006"
/codon_start=1
/transl_table=11
/product="hypothetical protein"
/translation="MTKTIGISIYPEKTSYEETIAYLKLAAKYKFKRVFTSLLQVTGD
STDVINQFKKIIHYANSLDMEVILDVNPLLLPNLGASWKDLSFFADLGVGTLRLDGGY
SGLEEMIMSHNPHGIKIELNMSAGLEYIDRIMSFSPNKKQLSASYNFYPQRQTGMLVE
HTKEITKKYHTHHLPATLFVSSKSAEVGPWQLQEGLCTLEEHRNLSLNTQLKQILLLD
LADHIIIGNCFASEKELKEMSHLFYDEPFSLDIELERPLSESEATILNSDFHFVRPEY
SEYVLRSAFSRHLHKKEKIKPVDYLGDLKFGDIIIANNRYGQYECELQIILQTTAHSE
NKNKIGTLSKDDLHLLQDLAHLPYQKFTFNSK"
CDS 888653..889744
/gene="rhaR_3"
/locus_tag="JFBMFIFI_00839"
/inference="ab initio prediction:Prodigal:002006"
/inference="protein motif:HAMAP:MF_01533"
/codon_start=1
/transl_table=11
/product="HTH-type transcriptional activator RhaR"
/translation="MENLTLFLLCKLHKANCYHVGYTPFCVKDIYTTFSKFDLSNIIL

KYEDKFKNALSEDIYIYVINELNSVVIACSKNKESYIFSLQLEGKVTKESIHNLAELF
YLEIYSAYKSSFILVDTLNFDREAIKEKVFKKTYEQSLHHNPNFEKYLLRAVQKGDAN
EALDYLDELIKLPLNASLAKNSDRHYKNLLISYVTIITRATIDGGLYSENAYILSDNT
IEKIEKQTRLNSDKAYQLAREIILEFIQLLMTKKRSRHSGYVNQTIQYIQKNTQNKIK
VSTIADLLDITPNYLATLFKKETGMTILAYSQNEKIKEAIYLIENTTISLSEIGSILS
FTDQSHFIKVFKKIKNQTLKDYQRRSKKV"

CDS complement(890010..890945)

/gene="ytlR"

/locus_tag="JFBMFIFI_00840"

/EC_number="2.7.1.-"

/inference="ab initio prediction:Prodigal:002006"

/inference="similar to AA sequence:UniProtKB:O34799"

/codon_start=1

/transl_table=11

/product="Putative lipid kinase YtlR"

/db_xref="COG:COG1597"

/translation="MKKIIYHFIVNEHSGSGNGLKIWQKILPIMEKKEISYQKYTSTF
AGETIELVEKIAKKINIDERLVVIGGDGTLHEAIQGLGETYQNLPIGYIPAGSGNDFA
RGVGISRKPLKALEQLLNAETFRSIDVIEFSEENTGKTGYFVNNIGLGFDALIVKLTN
HSTSKAWLNKYNLGSLAYLSSLIKAYFHQPNFPIVIDVDGQSHYLDKAFLVTTTNHPF
FGGGVNIAPMAKPTNGLIDVIIVSKIPIIKIAFLFVLMMLGGRHTRFKSVLHFNGKDI
RIETKMPENGQADGEELGSQLFNLHFTPTSRYFWF"

CDS 891184..891942

/gene="yxdL"

/locus_tag="JFBMFIFI_00841"

/inference="ab initio prediction:Prodigal:002006"

/inference="similar to AA sequence:UniProtKB:P42423"

/codon_start=1

/transl_table=11

/product="ABC transporter ATP-binding protein YxdL"

/db_xref="COG:COG1136"

/translation="MNESVVSIKDVRKVYGKKNENQFEALKGLSFEIEKGEFVGIMGP
SGSGKTTLLNVLATLDTATSGTIKIAGKDITHLNSDQLSDFRAAQLGFIFQDFNLLEN

LTMYENIALPLSLQDVPSKKIMPKVEKIAKTLDITRILNSYPTEVSGGQKQRVAAARA
LVHEPAILLGDEPTGALDSKNAKSLLESMKGLNEEQAVTILLVTHDAYSASYCKRILF
IQDGVIFKELKRKESREEFYHEILDVLADFGTDE"

CDS 891961..893796

/gene="bceB_2"

/locus_tag="JFBMFIFI_00842"

/inference="ab initio prediction:Prodigal:002006"

/inference="similar to AA sequence:UniProtKB:O34741"

/codon_start=1

/transl_table=11

/product="Bacitracin export permease protein BceB"

/db_xref="COG:COG0577"

/translation="MLLKLSLSGIKSKLKDYIVLLVGLVMSSSIFYMFQTLSMNKDFL
KNNAMISVLAFVFLAGSVLLGIITIVYILYANSFLLSLRQKEYGMYMTLGAKKKKIGK
MMFIETLIVGSVSLILGIIVGVGLSQVVGQLLMKQLDFNSPDYTPLYFPAISVTVIFF
LALFLFAAIVNVFKLARFSTLELINGEKQADHIVIRKNKLVFQSFIAFILLLIGYIAM
VNIVQFQMLGILVALVTITAGTFLFFQTFLPYFVQTLKNNRRISGKKINIFTFSQLSF
RVNDLTRVLAMVAMLIALAVGSIAVGIGFKNNSELMVEKAAAYDVVIHNPTAKEDKVL
AKMEFAKKINYHYKSDGTMQYFIKEELNENPLFMAENGVLKANEEKFRKVETPLELGT
IIKVEEQYNNEWASAFAQIREDRIEYGQDLPIKIVSKDEFKQLSGEEHTVLVGNTTGF
FNYLPQLKKLNELEKNREPKQEYLASRYESYSMWNIVSSGTVFMGLFLGFAFLAMMAS
CLMFKVLSGASNDIKRYEMLSKIGVRRSLLSRSIYVEMFLVFAFPALIGVIHVLVGMQ
MFKTLLLAPYYKIWIPFAIFTVIYAVYYLITVYLYKGIVLEKAKK"

CDS 894101..894967

/locus_tag="JFBMFIFI_00843"

/inference="ab initio prediction:Prodigal:002006"

/codon_start=1

/transl_table=11

/product="hypothetical protein"

/translation="MKKKLMSHRHSMKVVDVLHVLVGAFFIAIAVNGFFLPNQIVSGG
VSGLSVVLNYQFGWQPSLILLAVNVPLLILCFLTLGVSAGLKTILGSLILPFYVELTS
TMKPVTENPLLAAVFGGIVVGIGIGIVFKGKASTGGTGIIALVIHKYLRLPKGTSVAL
IDGLIVFSAFLVFDVDIVMYSLMSLFIISRVIDLVQVGFNRSKNVLVISESVLLIKEQ

ILTELDRGVTNISIRGGYGNQEQEMLMCVIPEREFNQLKEAVLEIDANAFVVVMSASE

VMGRGFSLLREQ"

CDS 895318..897963

/gene="alaS"

/locus_tag="JFBMFIFI_00844"

/EC_number="6.1.1.7"

/inference="ab initio prediction:Prodigal:002006"

/inference="similar to AA sequence:UniProtKB:P67011"

/codon_start=1

/transl_table=11

/product="Alanine--tRNA ligase"

/translation="MKKLSSAEVRQMYIDFFASKGHKIEPSASLVPVEDPTLLWINSG

VATLKKYFDGTIIPENPRIVNAQKSIRTNDIENVGKTARHHTLFEMLGNFSIGDYFKE

DAITWAWELLTSPEWFDMEPEKLYMTVNPNDEKAKEIWLSLGIPEDHIIPLEENFWDI

GAGPSGPNTEIFYDRGPEYNDVAEDDPENFPGGENERYLEIWNVVFSEFNHNPDDTFT

PLPKKNIDTGMGLERMVSIIQDAPTNFETDLFLPIIEAVEKLSGTVKYGANQTTDISF

KVIADHVRAVSFAIGDGALPSNEGRGYVLRRLLRRAVMHGKKLGIHKAFMYQLVPVVG

EIMSSYYPEILEQKEFIEKVIRTEEERFHETINDGLTILNNLVKELKEAGKEEIEGKD

IFKLYDTYGFPVELTEEYAEDEGLKVDHAGFEAEMEMQRNRARSARSDEKSMNVQTGL

LADIKIDSEFIGYDKTDAVGTLELIVAEDALVDSVKTGSEAQLIFNQTPFYAEMGGQV

ADTGVIKNKDGETVAKVTSVKKAPSGQPLHFVDVLAELKLGEEYELVVDQVARRKITR

NHTATHLLHRALKDILGDHANQAGSLVAPHYLRFDFTHFGQITAKELVEMERIVNEKI

WASLPVVTVETGINEAKEMGAMALFGEKYGEMVRVVNVDGYSIELCGGVHVSNTSEIG

IFKILSESGIGAGVRRIEAVTSEEAYLALFEEQTRLNEVAALVKAPQTKEVPTKVSQL

LMELKEVQKENESLQAKLANEQAGEVFKEIQDVSGVSVVTAQVEVKDMNGLRQLADQW

KQNNISDVLVLGSVQDGKVSLLAAVSEETIKKGLKAGDLIKEIAPLVGGGGGGRPDMA

QAGGKNPAGLADALASVNQWVEAKI"

CDS 898201..898470

/locus_tag="JFBMFIFI_00845"

/inference="ab initio prediction:Prodigal:002006"

/inference="similar to AA sequence:UniProtKB:Q836B3"

/note="UPF0297 protein EF_1202"

/codon_start=1

/transl_table=11

/product="hypothetical protein"

/translation="MSSIDETVRFNFGESHEKNVKETLTLVYDALEEKGYNPINQIVG

YLLSGDPAYIPRHNDARNLIRRHERDEIVEELVKTYLKESGRTFK"

CDS 898467..898889

/gene="yrrK"

/locus_tag="JFBMFIFI_00846"

/EC_number="3.1.-.-"

/inference="ab initio prediction:Prodigal:002006"

/inference="similar to AA sequence:UniProtKB:O34634"

/codon_start=1

/transl_table=11

/product="Putative pre-16S rRNA nuclease"

/db_xref="COG:COG0816"

/translation="MRTMGLDVGSRTVGIAISDPFGWTAQGIETIRINEDEENYGISR

VMELVQEYEVSKFVVGLPKNMDNSIGPRAEASMHYAELLKKQIDLPVIFQDERLTTVQ

AERMLIEQANTSREKRKKVIDKVAAVMILQNYLDGNPN"

CDS 898951..899259

/locus_tag="JFBMFIFI_00847"

/inference="ab initio prediction:Prodigal:002006"

/inference="similar to AA sequence:UniProtKB:Q97SX0"

/note="UPF0473 protein SP_0194"

/codon_start=1

/transl_table=11

/product="hypothetical protein"

/translation="MSHDHNHDDEHHDHEHITIIDENGNEELYEILFTFDSDDYGKSY

VLVYPAGIPEGEEIELQAFSYVEQENGDQGDLKPIETDEEWDMIEEVLNTFMADEDGE

"

CDS 899678..900835

/gene="mltG"

/locus_tag="JFBMFIFI_00848"

/EC_number="4.2.2.-"

/inference="ab initio prediction:Prodigal:002006"

/inference="similar to AA sequence:UniProtKB:A0A0H2ZLQ1"

/codon_start=1

/transl_table=11

/product="Endolytic murein transglycosylase"

/translation="MGNNNENQSEKQEKLVAESHSRMLEREKESKVVKKIVLSIISVL

LVLVVILGFVGYRYVSTSLEPLDPKSEKKISVEVPIGSTSKEIAKILEDKNIIKSALV

FNYYVKTNNAADFLAGFYQLSPSMTLDELITELQKGGSTDPSKTILIKEGYTLDQIAD

SIAKSTKFKADEVMALVQNQAFLDQMHKKYPKLLDSAMSAENVKYRLEGYLFPAVYDF

SAKMTLDEIIEKMISKTNEVLYPFYGTINAQGKTVQQVMTVASLLEKEGVSYEDRTKI

ASVFYNRIAEDMPLQTDISVLYALGEHKEFISIKETEVESPYNLYVNKGFGPGPFNSP

SLEAIKAALNPVDSSYLFFLADTDTGKVYFADTYEEHLELKEKYIDSKLEK"

CDS 900923..901552

/gene="udk_1"

/locus_tag="JFBMFIFI_00849"

/EC_number="2.7.1.48"

/inference="ab initio prediction:Prodigal:002006"

/inference="similar to AA sequence:UniProtKB:P67413"

/codon_start=1

/transl_table=11

/product="Uridine kinase"

/db_xref="COG:COG0572"

/translation="MGKNKPIVIGVTGGSGSGKTSVSRAIFNQFSGHSILLLEQDFYY

KDQSQLDFTERLKTNYDHPFAFDTDLMIQHVLDLIAYKSIEKPVYDYAAHTRSDQIVL

QEPKEVIIIEGILILEDSRLRELMDIKLYVDTDDDIRIIRRIKRDMEERGRTLDSVID

QYLSVVKPMYHQFIEPTKKYADLIIPEGGQNQVAIDLMTTKISSILNNQ"

CDS 901711..902181

/gene="greA"

/locus_tag="JFBMFIFI_00850"

/inference="ab initio prediction:Prodigal:002006"

/inference="similar to AA sequence:UniProtKB:P80240"

/codon_start=1

/transl_table=11

/product="Transcription elongation factor GreA"

/db_xref="COG:COG0782"

/translation="MVEKVYPMTLEGKEKLEKELEELITVKRKEIVERIKIARSFGDL

SENSEYESAKDEQAFVEGRITSIENMIRLSQIIDSNNDKNTVSLGHTVTFIELPDGEE

EVYTIVGSAEADPFSGKISNDSPIAKALLGKSINDKVLIATPGGNMEVKIVKIA"

CDS 902523..903266

/locus_tag="JFBMFIFI_00851"

/inference="ab initio prediction:Prodigal:002006"

/codon_start=1

/transl_table=11

/product="hypothetical protein"

/translation="MNNKWKLFILAECLLLIGLVYQIFSAVPLLIAFILGAGLFAWSL

KNKFQKKRSNSFWIVLGSLLMIFVLLSSSAFWAIIIIGVVFFIGNGSDMFTNMKSNDF

SKAPWKNKEMIIVETSGAVKKNNKRMKRPWIGNERIGNSVYEWDDINFSIFAGDTIID

LGNTLLPKDESYVVIRKGFGKTRILVPVGVGIMIEHSAISGKIKFEGEFFSLKNESVK

LYSEQYEESTRKIKIITNILIGDLEVIGI"

CDS 903263..904339

/gene="liaS"

/locus_tag="JFBMFIFI_00852"

/EC_number="2.7.13.3"

/inference="ab initio prediction:Prodigal:002006"

/inference="similar to AA sequence:UniProtKB:O32198"

/codon_start=1

/transl_table=11

/product="Sensor histidine kinase LiaS"

/db_xref="COG:COG4585"

/translation="MKKASNQKLFLSVSFFSFIIMLLISIAYGSAISKNAWYIDLISL

KILYIPFLVYLVAFSLIIGLAVSMVIYFINQKKFEQIENELQSLAEGNYESFIFEQRK

VDLENTTVYQEDIEKHILVVRDKLLELSKEVQINGNQPKLVDGETKEEILTQERHRLA

RELHDSVSQQLFAAMMLLSALNEQASVNTNELFQKQLKMVESIINESQSEMRALLLHL

RPISLEGKTLRKGIEQLLIELKSKVQILLKWEVDDIYLPNGVEDHLFRIVQELLSNTL

RHSKAKELEVYLKQIDQSVLLKVIDDGVGFDTSETKAGNYGLQNIKERVSGMGGTCKF

ISFPGKGTSIEIKIPIIKGSELND"

CDS 904332..904964

/gene="liaR"

/locus_tag="JFBMFIFI_00853"

/inference="ab initio prediction:Prodigal:002006"

/inference="similar to AA sequence:UniProtKB:O32197"

/codon_start=1

/transl_table=11

/product="Transcriptional regulatory protein LiaR"

/db_xref="COG:COG2197"

/translation="MIKVLLIDDHEMVRLGVSAYLSIQEDIEVIGEAENGQIGYEKAL

ELRPDVIMMDLVMDVMDGIESTKAILKDWPEARIIIVTSFIDDEKVYPALEAGASSYM

LKTSTAGEIANAIRSTYAGESILEPEVTGKMMERFTKKKEPVLHDDLTNREQEILLLI

AQGNSNQEIADQLFITLKTVKTHVSNILAKLDVEDRTQAAIYAFKHHLIE"

CDS 905082..905741

/gene="ktrA"

/locus_tag="JFBMFIFI_00854"

/inference="ab initio prediction:Prodigal:002006"

/inference="similar to AA sequence:UniProtKB:O32080"

/codon_start=1

/transl_table=11

/product="Ktr system potassium uptake protein A"

/db_xref="COG:COG0569"

/translation="MKKNFAIIGLGRFGGSICRTLIEAGQDVLVIDSSEDRVNEFMNI

ATHAVVANAQDESVLRSLGIRNFDHVVVAIGEDIQASILATLMIKEMGVPFVTAKAQN

EYHGQVLEKVGADHVVHPERDMGIRIGHHLVSKNVVDYLELSDKYSLVELKVTNSKFY

NKSLADLDFRAKFGLTVIGMRRGQEMIISPTADEKIQENDTLMLVGAVDDLDRLEAKM

P"

CDS 905984..906274

/locus_tag="JFBMFIFI_00855"

/inference="ab initio prediction:Prodigal:002006"

/codon_start=1

/transl_table=11

/product="hypothetical protein"

/translation="MKIEITERAKKWFVDEVGVSSGDAVRFFGKYGGSSPIQTGFSLG

IDLTEPQTPLGKIELDGVTYFVEEADEWYFRGHNLKVDYNEQVDEPSYEYVQ"

CDS complement(906468..908141)

/gene="rnj1"

/locus_tag="JFBMFIFI_00856"

/EC_number="3.1.-.-"

/inference="ab initio prediction:Prodigal:002006"

/inference="similar to AA sequence:UniProtKB:Q8K5W8"

/codon_start=1

/transl_table=11

/product="Ribonuclease J 1"

/translation="MKPTIKNNEAAVFGIGGLGEIGKNTYGVQFQDEIIIVDAGIKFP

EDDLLGIDYVIPDYSYIVQNINKVKALVITHGHEDHIGGIPFLLKQVNIPIYAGPLAL

ALIRNKLDEHGLLRDAELNQIGEDTVIKFRKTSVSFFRTTHSIPDALGVVVKTPPGNI

VLTGDFKFDFTPVGEPANLHRMAKIGDEGVLALLSDSTNAEVPNFTKSEQIVGESITS

IFQKVEGRIIFATFASNISRLQQVANAAVRTGRKIAVFGRSMEAAMVTGRQLGYITAP

EDTFVDAQELNRLPASEVTILCTGSQGEPMAALSRIANGTHRQISIQPGDTVVFSSSP

IPGNTTSVNRVINLLSEAGAEVIHGKVNNIHTSGHGGQQEQKLMLRLMKPKYFVPVHG

EFRMQKIHTTLAKQVGIPSENSFILANGDVLALTAESARMAGHFNAGDVYVDGKGIGD

IGNVVLRDRRILSEEGLVVVVVTIDYKTKDILAGPDILSRGFIYMRESGDLINEAQRV

IFRALRETTSSDENLTEQLIKETVIGSLQPFLFEKTERHPMILPIIMPV"

CDS complement(908145..908357)

/locus_tag="JFBMFIFI_00857"

/inference="ab initio prediction:Prodigal:002006"

/inference="similar to AA sequence:UniProtKB:Q5XA23"

/note="UPF0356 protein M6_Spy1605"

/codon_start=1

/transl_table=11

/product="hypothetical protein"

/translation="MIFKITYQETKGRNPKREDTKALYVETEDAVQARKLVEENTPYN

IEFVQPVEGKHLAYEQQSPEFEITEF"

CDS 909001..910731

/locus_tag="JFBMFIFI_00858"

/inference="ab initio prediction:Prodigal:002006"

/inference="similar to AA sequence:UniProtKB:P9WQJ1"

/codon_start=1

/transl_table=11

/product="putative ABC transporter ATP-binding protein"

/db_xref="COG:COG1132"

/translation="MKLMWNYTKKYKKLVFLNFICVFGFILIELGLPTILAQMIDKGI

AQNDFEVVKRYGVFMLVICLIGLVALIFLAYCGSKITTGIVRDIRDDLFEKTQSFSHR

EYDQFGVSSLVTRTTNDAFQVMQFMQMILRMGMMTPLMFISSFIMIIRTSPSLSGVVF

IAVPFLLVGVLIIGKKSEPLSEKQQANLDAINLNLRENLTGLRVIRAFVNEKFQESRF

SKVNGNYTAVSKKLFKLMAFAQPGFSLIFNLLFACILWLGTIQISQNNLQVGQLIAFI

EYIFHALFSFMLFANVFMMYPRAAVSANRIQEVLDTEAVIKENEDGVTETETHGYLTF

ENVTFAYPGNTETPVIRDVSFSSKPGETIAFIGSTGSGKSTLIQLIPRFYDVTRGRIL

LDGVDVRDYKLSALRQKIGFIPQKALLFTGTIAENMRYGKWNATTKELEEASDIAQAK

EFISLKPKQFDELLAEGGSNMSGGQKQRLSIARAIVKKPDVYIFDDSFSALDYQTDAN

LRARLRDETLDATVLIVAQRVGTIMHADKILVLNEGEVVGSGTHQELLKTCDVYYDIA

SSQLSKEELA"

CDS 910728..912506

/locus_tag="JFBMFIFI_00859"

/EC_number="7.6.2.-"

/inference="ab initio prediction:Prodigal:002006"

/inference="similar to AA sequence:UniProtKB:P9WQJ3"

/codon_start=1

/transl_table=11

/product="Fatty acid ABC transporter ATP-binding/permease

protein"

/db_xref="COG:COG1132"

/translation="MRNSLGSIKRIGSYIKPYKIGFIVALLLTVICMFANALQPFIMG

LILTEVGHNVADIANGVAGAAINFPYVIEIIIYMLIIALVYQITMYASAWLMTNVVQN

TMRDLRADIDNKINRLPVSYFDKNQQGNILSRVTNDVDAISNAMQQSLIQIVNSVLGI

VFAVGMMLVLSIPLALILIVTIPVSILISKFIVNKSQPYFKGQQKSLGELNGYVQESF

SGFSVIKLYGKEADTLKEFKEINHRLASFGFKAAFISGIMMPLVGLVSNLGYIGVAVF

GGYAVIQGTLTLGNLQAFTQYVWQINQPISQITQLSGVLQSAAAATGRVFEILDEPEE

KPDQVQRPLPEKVEGNVSFEHVQFGYNKSNPLIKDLNVEVKSGQTVAIVGPTGAGKTT

LINLLMRFYDVDQGAIKIDGIDTKEMSRADVRSQFGMVLQDAWLYNASISDNIRFGKL
DATEYEVVDAAKTANVDHFIRTLPNGYDMILNQEASNISLGQKQLLTIARAVISNPKI
LILDEATSSVDTRLEALIQKAMKRVMEGRTSFVIAHRLSTIRDADLILVMDQGEIIEQ
GTHESLLAKGGFYEKLYNSQFSEEAE"

CDS 912633..912818

/locus_tag="JFBMFIFI_00860"

/inference="ab initio prediction:Prodigal:002006"

/codon_start=1

/transl_table=11

/product="hypothetical protein"

/translation="METSEWIKSELRKLAVDEKSTFLVNETFAYIDEIKSDNESLQIA
LEGELWSPKKWNEKPKP"

CDS 912902..913681

/gene="ybhA"

/locus_tag="JFBMFIFI_00861"

/EC_number="3.1.3.74"

/inference="ab initio prediction:Prodigal:002006"

/inference="similar to AA sequence:UniProtKB:P21829"

/codon_start=1

/transl_table=11

/product="Pyridoxal phosphate phosphatase YbhA"

/db_xref="COG:COG0561"

/translation="MQKKMIFFDIDGTLLTDEKKVLPSTKEALKQLKKNGHEVAIATG
RNLFLARDVIEELDFENYIVCNGAAGYFQHELVYENTLNAAEYQRLIQVADQNRHQLV
YQSPELLRRRDAEADFMMSEAMRSIDFGVPEYDRDFYQNNTLYQSLIFYGTDDHALYE
SGQFPQFRFVRWHDFGVDILPHDGSKANTALKMAQSKGIAVEDTLAFGDGLNDLELLT
KVGVGVAMGNAFEEVKLRANKVTKNNNEDGIALALEELNLI"

tRNA 913718..913792

/locus_tag="JFBMFIFI_00862"

/product="tRNA-Ala"

/inference="COORDINATES:profile:Aragorn:001002"

/note="tRNA-Ala(ggc)"

CDS 914068..914157

/locus_tag="JFBMFIFI_00863"

/inference="ab initio prediction:Prodigal:002006"

/codon_start=1

/transl_table=11

/product="hypothetical protein"

/translation="MSTNFIIWLVSTIIITIIVGVIIFKKLKK"

CDS complement(914340..914489)

/locus_tag="JFBMFIFI_00864"

/inference="ab initio prediction:Prodigal:002006"

/codon_start=1

/transl_table=11

/product="hypothetical protein"

/translation="MRLIKLFEDHKLSLTELQHYLWNLSASEIHDELGFEIWEYYVSL

KNHAE"

CDS 914722..915312

/gene="rplY"

/locus_tag="JFBMFIFI_00865"

/inference="ab initio prediction:Prodigal:002006"

/inference="similar to AA sequence:UniProtKB:Q7A7B3"

/codon_start=1

/transl_table=11

/product="50S ribosomal protein L25"

/translation="MTILLEVEKREVRPRSIKKRLRQEGRIPADVYGYKTENIAISID

EKILQKALRKNGQNAVYTIDIDGKKMNVLLMEEQTDALSGKWIYVDFVSVDMKELTEV

EAQIVLINDAVGVKQGGMLNQTLYSTLVSATPDKLPETIEVDVSKLEIGQSITIGDLK

ENKDYTILADKEEQIAAVEERVVAPEPEAEETTTVE"

CDS complement(915343..915828)

/locus_tag="JFBMFIFI_00866"

/inference="ab initio prediction:Prodigal:002006"

/codon_start=1

/transl_table=11

/product="hypothetical protein"

/translation="MNYEQLLNYVSEEFSVYEVPLIEQTGSYGFFSNGTILIEETQSE

IEKKCILAEEYGHGIKNVGNILDQQVTENRKQELVARRYAHELLVPLIELVRAKKFGC
NSLFETAEYLEVTEDFLIEALEQYKVQYADGIIVSNHQIYFEPFLEITSLKDQTNQKK
P"
CDS complement(915841..916197)
/locus_tag="JFBMFIFI_00867"
/inference="ab initio prediction:Prodigal:002006"
/codon_start=1
/transl_table=11
/product="hypothetical protein"
/translation="MKKQLIVGEMIDYFLQQQKMTRKHLGELLGKSESAVSKWISGAN
TPLAKDLAIMSEIFNVDIETLMYGFPSEENKLNFDQINTVAAHMANTNKKLSKEDIEN
INHYIDFIISQKEQKD"
CDS 916413..916817
/locus_tag="JFBMFIFI_00868"
/inference="ab initio prediction:Prodigal:002006"
/codon_start=1
/transl_table=11
/product="hypothetical protein"
/translation="MVTLFDEINKEKTKIAAEEVLMQYRKLKMISKREIQKRMINTYN
RSSSDEESDITKRKVSAKIYAEEETERIERAVESIPNEKQRMVIEERYLKGFEKYDSE
CSFNLDMPTSSYSNYKAAGLIAVAWALGCEEY"
CDS 917283..917684
/locus_tag="JFBMFIFI_00869"
/inference="ab initio prediction:Prodigal:002006"
/codon_start=1
/transl_table=11
/product="hypothetical protein"
/translation="MFTNILQEIGAFLFGGSMPILRVYLLAVFIDLVTGYIKALKNHN
WRSAINCWGILTKLTTIFAIISAAILDAIGPFFGIQIPINIALWATGFLILYEIGSIL
ENFGEMGIKLGFIKKYLGIFTDQMNGKDEDK"
CDS 917681..918676
/locus_tag="JFBMFIFI_00870"
/inference="ab initio prediction:Prodigal:002006"

/codon_start=1

/transl_table=11

/product="hypothetical protein"

/translation="MSRILDISEWQVPTTIDYQKVAQGLDLVIVRVQYGSNYIDKYYQ

THIREFKKYGIPVAVYAWVRGVNRIDMEQEANDFYQRAKEFNPCFWWLDVEEQSMIDM

REGCSVYLQKLKALGAKRVGIYIAHHLYKKFNLNVAEADGIWIPHYGLNNGTPNSQPE

FACDLHQYTSVGKLSGYSGHLDLNRLLGSRNIEWFTDATKSNNSLPNQEAASKISVGD

QVSVQSFATHYATGESIPDWVKNSSYKVIEIRQKKQSNSEYQYLMSSINSWVLEQDIK

VEGTSQKSNVYVVQNGDNLWSIAEKVYGVGADYPKLKAKNGLTSDIIQPNQHINY"

CDS complement(918739..919155)

/locus_tag="JFBMFIFI_00871"

/inference="ab initio prediction:Prodigal:002006"

/codon_start=1

/transl_table=11

/product="hypothetical protein"

/translation="MSSKTNKIFVNLPVENLDRSVKFFTQLGYTFNPQFTDENATCMI

ISDSIFAMLLVKPYFKGFTKKEIPNTTTDSEVIVALSADTRATVDELVDNAIKAGAKI

TNEPSDLGFMYSRSFQDLDGHLWEIVYMDESTVDEG"

CDS complement(919414..919974)

/gene="def"

/locus_tag="JFBMFIFI_00872"

/EC_number="3.5.1.88"

/inference="ab initio prediction:Prodigal:002006"

/inference="similar to AA sequence:UniProtKB:Q82ZJ0"

/codon_start=1

/transl_table=11

/product="Peptide deformylase"

/db_xref="COG:COG0242"

/translation="MITMDDIIREGHPTLRESAAHVDFPLTDQELKLTAEMMEFLVNS

QDPELSEKYNLRGGVGLAAPQLDVKKRIIAVHIPGTEENPEPQLSTVMINPKIISHSV

QNSCLSEGEGCLSVDREVPGYVVRHTRITLQYFDTEGNEHKLRLKNYAAIVVQHEIDH

INGIMFYDHINPDNPYKVENDVTLIS"

CDS 920207..921553

/gene="gdh"

/locus_tag="JFBMFIFI_00873"

/EC_number="1.4.1.4"

/inference="ab initio prediction:Prodigal:002006"

/inference="similar to AA sequence:UniProtKB:P31026"

/codon_start=1

/transl_table=11

/product="NADP-specific glutamate dehydrogenase"

/db_xref="COG:COG0334"

/translation="MEKAKEYIAAVYKKVEERDPHQVEFLQAVKEFFDTIEPVLVRHP

KMIEENILERIVEPERLIQFRVPWTDDEGNVQVNRAFRAQFSSAIGPYKGGLRFHPSV

NQSIVKFLGFEQIFKNSLTGLPIGGGKGGSDFNPKGKSDFEVMRFCQSFMTELQKYIG

PDTDVPAGDIGVGGREIGYLFGQYKKLNGFGAGVLTGKPIPMGGSLARTEATGYGLVY

FTNEMLKDVGRTFKGQKVVVSGSGNVAIYAIEKVHQLGGTVIACSDSDGYILDLEGIN

VETVKRLKEVERKRISEYVNEHTSAEYIEGSIWELENEFQIALPSATQNEINGITAKK

LVAQGVYAVAEGANMPSDLEAIKVYQEHGVLYGPAKAANAGGVAVSALEMSQNSIRLS

WTFEEVDDRLKDIMATIYQQCRDVAKEYGVEGDFVSGANIAGFLKVAEAMLSQGVV"

CDS 922104..922202

/locus_tag="JFBMFIFI_00874"

/inference="ab initio prediction:Prodigal:002006"

/codon_start=1

/transl_table=11

/product="hypothetical protein"

/translation="MMEKPSQKDSSDPGSQRLQEKLIKFHCTHLSI"

CDS 922259..924877

/gene="mgtB_1"

/locus_tag="JFBMFIFI_00875"

/EC_number="7.2.2.14"

/inference="ab initio prediction:Prodigal:002006"

/inference="similar to AA sequence:UniProtKB:P22036"

/codon_start=1

/transl_table=11

/product="Magnesium-transporting ATPase, P-type 1"

/db_xref="COG:COG0474"

/translation="MMMNRKTQIKTEKKSKDNELKKLGYLQEEEVLSQFGTSLAGLTE

DEAKNRLEKYGPNEVASQKPTPWFILLIEAFKDPFVYVLMLLLVVSALTNDVEASIVM

GLMILFSAGIRFVQEYRSQKASLALKELIETTCAVTRDGETRELPIDEVVPGDIVVLS

TGDMIPADARLIWTKDLFVNQSSLTGESMPVEKFVRVDDHVADKDETALDLHNLAFMG

TDVLSGQGRVVILKTGGETFFGDIASNVSEKRGETGFDRGVTNVSKLLIRFMLVMVPI

VFLINGLTKGDWGEAFFFSIAIAVGLTPEMLPMIITSNLAKGAIAMSKKKVIVKELNA

IQNLGAMDILCTDKTGTITEDKVVLVRHVNPVGDDCERVLELAYLNSNYQTGWKNLMD

HAVIQYFSENRENFAIGEVEKIDEIPFDFSRRRLTVAVNNQGHQVMVTKGAVEEMMKI

CSFVELNDEIVPLTKDLMDRMMDVSIKMNESGMRVLGVAYKNDVHDTAIYSIEDENEM

ILAGFMGFLDPAKKSSITAIKSLHEHGVNVKVLTGDNEIVSKKVCRDVGIEVGNALLG

TQIENMSDEELTLATESTNLFAKLNPTQKARIIALLQAKGHTVGFMGDGINDAPALRK

ADVGISVDTAADITKEASSIILLEKSLNVLEDGILEGRSVFGNMMKYVKMTISSNFGN

VFSVLVASAFLPFLPMLSIQLLVQNLIYDVAQLTIPWDRMDEEDLMLPAKWDTSNLMK

FTLSIGPVSSIFDILTYLLMWFVFQANTVQEAALFHSGWFVIGLITQTVVVHVIRTKK

IPFIQSRASFGVTVSTILVIIAAVLIPSSPIGQVFDLVALPSNYWPWMIGIVIAYIVT

VQLVKMLYIKVNKEWL"

CDS 925495..926607

/gene="pdhA"

/locus_tag="JFBMFIFI_00876"

/EC_number="1.2.4.1"

/inference="ab initio prediction:Prodigal:002006"

/inference="similar to AA sequence:UniProtKB:Q820A6"

/codon_start=1

/transl_table=11

/product="Pyruvate dehydrogenase E1 component subunit

alpha"

/translation="MATKKNKPIDFDALMDSVNADFPTIQILDEDGKVVNPDLMPDLS

DEELVDLMSKMVWSRVLDQRSTALNRQGRLGFFAPTAGQEASQLASHFAMEKEDFLLP

GYRDVPQLVQHGLPLKEAFLWSRGHAGGNKYDVNLHAMPPQIIIGAQIVQAAGVALGM

KKRGKKNVVLTYTGDGGSSQGDFYEGMNFAGAYDAPAIFIIQNNGYAISTPRSVQTKA

KTLAQKAVAAGIPGVVVDGMDPLAVYAVTKEARDRAIAGDGPTLIETLTYRYGPHTLS

GDDPTRYRTKETDDIWEKRDPLVRMRNFLTEKGIWSEEKENEVIEATKEEIKEAIKAA

DQEPKQKVSDFLKNMFEEPNQTIKEQIAIFEAKESK"

CDS 926611..927588

/gene="pdhB"

/locus_tag="JFBMFIFI_00877"

/EC_number="1.2.4.1"

/inference="ab initio prediction:Prodigal:002006"

/inference="similar to AA sequence:UniProtKB:P21874"

/codon_start=1

/transl_table=11

/product="Pyruvate dehydrogenase E1 component subunit

beta"

/translation="MAQKTMIQAITDALALELGKDENVLIFGEDVGNNGGVFRATEGL

QATYGEDRVFDTPLAESGIGGLAVGLALEGFRPVPEIQFFGFVFEVMDSIVGQAARTR

YRMSGTRNLPITIRAPFGGGVHTPELHSDNLEGLIAQSPGIKIVIPSNPYDAKGLLIS

AIRDNDPVVFLEHMKLYRSFREEVPEEAYTVPLGKAAIAKEGTDISVITYGAMVREAL

KAAEKLEKEGISVEIVDLRTVSPLDIETIIASVEKTGRVVVVQEAQRQAGVGAMVMSE

ISERAILSLEAPIGRVAAPDTVFPFGQAENAWLPNANDIEEKIKEIYNF"

CDS 927727..929355

/gene="pdhC"

/locus_tag="JFBMFIFI_00878"

/EC_number="2.3.1.12"

/inference="ab initio prediction:Prodigal:002006"

/inference="similar to AA sequence:UniProtKB:P21883"

/codon_start=1

/transl_table=11

/product="Dihydrolipoyllysine-residue acetyltransferase

component of pyruvate dehydrogenase complex"

/db_xref="COG:COG0508"

/translation="MAFKFKLPDIGEGIHEGEIVKWFAKVGDTIKEDDTLLEVQNDKS

VEEIPSPVTGTIKSILVEEGTVAVVGDVLVEIDAPGYEEEEAAAPAPAKTEAPAPAAT

SGNAALFQFKLPDIGEGIAEGEIVKWFVAPGDTIKEDDTLLEVQNDKSVEEIPSPVTG

IVKNILVSEGTVANVGDVLVEIDAPDYVDHSAPASEQTPAQPAAVPTSEAPTQPATGN

GSGVVAVSDPSKRILAMPSVRQLAREKGIDISTVAPTGKNGRITKEDILNFNGSAAPA

KVETASTAPAAQNNAPAEKAAAPAQPFSSNLGEMETREKMSPTRKAIAKAMVNSKATA

PHVTLFDEVDATKLMAHRKRFKDVAADKGVKLTFLPYVVKALVSVLRKYPALNASIDD

ATQEIVYKHYFNIGIATDTDHGLFVPNIKNADAKSIFSIAGEITTHAQAAAAGKLAAA

DMRGGSTTISNIGSIGGGWFTPVINYPEVAILGVGKIAKKAIVNADDEIVVAPVMQLS

LSFDHRIIDGATAQKAMNDMKTLLADPELLLMEG"

CDS 929363..930769

/gene="pdhD"

/locus_tag="JFBMFIFI_00879"

/EC_number="1.8.1.4"

/inference="ab initio prediction:Prodigal:002006"

/inference="similar to AA sequence:UniProtKB:P99084"

/codon_start=1

/transl_table=11

/product="Dihydrolipoyl dehydrogenase"

/translation="MVVGDFAIELDTVVIGSGPGGYVAAIRAAQMGQKVAIVEKENLG

GVCLNVGCIPSKALISAGHRYQEAMHSEVFGVTAENVVLDFAKTQDWKENTVVNSLTS

GVAMLLKKNKVEVLKGEAFFVDEHDLRVMTETNAQSYSFNNAIVATGSRPIEIKGFKF

GKRIIDSTGGLALQEVPKKLVVIGGGYIGSELAGAYANLGAEVVILEGAPSILPNFEK

DMVKYVTDNFKKKNVTIETSAMAKEAIETADGVTVKYEVNGAEKSIEADYVMVTVGRR

PNTDELGLEQAGVEVGERGLITVDAQGRTNKSNIYAIGDIVPGLALAHKASYEGKIAA

EAISGKKVAVDYKAMPAVCFTDPELASVGFTQAEAKEQGLDVKASKFPLQGNGRAISL

NATEGFVRLVTTKEDNVLVGAQVAGVGASDIIAELGLALEAGMNAEDIALTIHAHPSL

AETVMDAAELALGLPIHI"

CDS 931085..932032

/gene="ldh_3"

/locus_tag="JFBMFIFI_00880"

/EC_number="1.1.1.27"

/inference="ab initio prediction:Prodigal:002006"

/inference="similar to AA sequence:UniProtKB:P13714"

/codon_start=1

/transl_table=11

/product="L-lactate dehydrogenase"

/db_xref="COG:COG0039"

/translation="MGRGHLNKVMIAGSNESAYDFIFLSMIKMQIKELVVLNLPGLEK
DVIEDLSYTESIFPNGALKIGNEKDFHDTDLLVITAIEERIEGETDHDYLKRNIKLIR
KIVNQAMANSFDGMVLVASEPSDIFTYLIWKFSGLPKERIFGIGTYFDTIYFQNMLSK
FFKISVRDVKGYIVGGSDRNQKVAIWSRAYVGGTPVLGLTVNPDNDFNQDVMFEMEEK
ILKRSDALKITESGYTIAAILSKILQFIGNNEEAIVPLVHLVDIDEYKAIPLSLPILL
GENGLSASYELGFSDSEKQEILAVAKEVREKLDFIEQGK"

CDS 932054..932326

/locus_tag="JFBMFIFI_00881"
/inference="ab initio prediction:Prodigal:002006"
/inference="similar to AA sequence:UniProtKB:Q9K9K7"
/note="UPF0223 protein BH2638"
/codon_start=1
/transl_table=11
/product="hypothetical protein"
/translation="MEKSYSYPIDLDWTQDEMIQVIDLWRMVELAYEKGVSREDFLKK
YQAFKKVVPAIGEEKKWGREFEHISGYSLYKVVKEAKESNRKSIKL"

CDS 932464..933243

/gene="suhB"
/locus_tag="JFBMFIFI_00882"
/EC_number="3.1.3.11"
/inference="ab initio prediction:Prodigal:002006"
/inference="similar to AA sequence:UniProtKB:O33832"
/codon_start=1
/transl_table=11
/product="Fructose-1,
6-bisphosphatase/inositol-1-monophosphatase"
/db_xref="COG:COG0483"
/translation="MTRDQMDKLIRSWMEEAAAMIKESFKTTLTIEIKSDRNDLVTNM
DKKIEQFLIEKIRSNFPDDRILGEEGYGDKIEDLVGRVWIIDPIDGTLNFVKQQANFA
IMIGVYQDGEGRMGYIYDVMAGEFYWAIAGEGAYLNGNPLPNVADIELREGLVALSSG
VFSSDKYKAKEIVRGSSGLRMVGSAGIETVHVAAGRLVAYITHRLQPWDMAAGKVIAE
ELGLVYTQINGEPVDLLQQNATIIATPTAHKEILTNYLVDI"

CDS 933823..936534

/gene="citB"

/locus_tag="JFBMFIFI_00883"

/EC_number="4.2.1.-"

/inference="ab initio prediction:Prodigal:002006"

/inference="similar to AA sequence:UniProtKB:P09339"

/codon_start=1

/transl_table=11

/product="Aconitate/2-methylaconitate hydratase"

/db_xref="COG:COG1048"

/translation="MTIKKKSEAVLTIGENTYHYYQLKTLEKLETIEIKKLPFSIRVL

LESVLRQAGENGITEEHIKQLAKWSGKNSNEGEVPFKPSRVILQDFTGVPAVVDLASL

RKTIADLGGDPTTINPEVPVDLVVDHSVQVDAAGTMNALQVNMKMEFQRNEERYRFLS

WAQKAFDNYRVVPPATGIVHQVNIEYLATVVTTKQLNEKEFLIYPDSLVGTDSHTTMV

NALGVLGWGVGGIEAEASMLGEPSYFPIPDVVGVRFVNELSSGATATDLALKVTQVLR

EYKVVGKFVEFFGPGLNYLTLADRATVANMAPEYGATCGFFPVDQETLNYLRLTGRES

NQIALVKRYLEENQLFYTPGKTTDPDYTTIIEIDLSEIEANLAGPKRPQDLVPLSQMQ

ADFKRALTAPEGNLGFGLSAKEVEKTVEVELDQKKVQMKTGALAIAAITSCTNTSNPF

VMISAGLVAKKAVELGLEVPTYVKTSLAPGSKVVTAYLEKAGLLPYLEKLGFNLVGYG

CTTCIGNSGPLRPEVEEVIKEENLLVSAVLSGNRNFEGRIHALVKANYLASPPLVVLY

ALAGNVNVDVINDAIGVSNQGEAVYFKDIWPTRAEIETLIEAYVTPELFKKEYEHVFS

DNAEWNAIETTDEALYPWDPESTYIANPPYFDGMSLEPKEIKPLRNLAVLGKFGDSVT

TDHISPAGSIGPGSPAGKYLLEKGIAIRNFNSYGSRRGHHEVMMRGTLANIRIRNQLV

PDTEGGITKFEPTNEILSIYDAAMKYQAEGKGLVILAGEDYGMGSSRDWAAKGVQLLG

VEAVIAKSYERIHRSNLVMMGVLPLQFKPGEGAETLGLDGTEEFAIEIEDTIGLLGDV

PVCATSKNGTRIQFMTTVRFDSEVDLTYYRHGGILPMVVRKKLKNKL"

CDS 936605..937747

/gene="citZ"

/locus_tag="JFBMFIFI_00884"

/EC_number="2.3.3.16"

/inference="ab initio prediction:Prodigal:002006"

/inference="similar to AA sequence:UniProtKB:P39120"

/codon_start=1

/transl_table=11

/product="Citrate synthase 2"

/db_xref="COG:COG0372"

/translation="MEIHKGLEGVIVSETKISSIVENQLLFAGYNIDELVEKNVSFEE

VIYLLWHLKMPTELEFSIFTQELQANMAISDTIITCLKIQTRQNLHPMSVLRSTVSLL

GVFDPKAEEINPEAVYEQSLSLQAKIPTIIAAYSRLRVGLDPIAPRYDLSFAANFMYM

LTGEEADEQAVSAMNEALVLHADHDLNASTFTARVCASTSSDSYSCITAAIGSLKGPL

HGGANERVFDMLLEIEAGNLSVEEYLDQKLSRKEKVMGFGHRVYKTEDPRKKHLKKLA

KELTMANGKEQWYFLSCQVEEYLKETKGLIPNVDFYSATVYHCLGIDSDLFTLLFAMS

RVSGWLGHIYEQKVEDCLIRPRSHYVGPQNQVYTPLNKEVSIGGAE"

CDS 937744..939033

/gene="icd"

/locus_tag="JFBMFIFI_00885"

/EC_number="1.1.1.42"

/inference="ab initio prediction:Prodigal:002006"

/inference="similar to AA sequence:UniProtKB:P39126"

/codon_start=1

/transl_table=11

/product="Isocitrate dehydrogenase [NADP]"

/db_xref="COG:COG0538"

/translation="MMSQGTKIEFEHGQLKVPNFPIIPFIEGDGIGPEIWQAAKAVFE

AAVSKAYGNERKVIWQEVLAGEKAFNSTGNWLPDETLEIIKSHYVAIKGPLTTPIGGG

FRSLNVTLRQTLDLYICYRPVRYFKGVPSPMRHPELTDMQIFRENTEDIYAGIEFAAS

SPEANKLIAFLKNEFNVDKIRFPESSAIGVKPVSKEGTERLVRSAIQHALSHKRKSVT

LVHKGNIMKFTEGGFKNWGYELAEKEFGKDVFTWTKYQKIKEEQGKNQAEEAYKQALV

EGKLIIKDKIADIFLQEILIHPADYDVIATLNLNGDYISDALAAQVGGIGIAPGANLN

EETGHGIFEATHGTAPEFAGLNQLNPSSLLLSGALLFDYLGWGEVAELITSSIEQAIA

NKTVTMDFARQMPQATEVSCSGFGEELVKLIQASSLQ"

CDS 939628..941469

/gene="typA"

/locus_tag="JFBMFIFI_00886"

/inference="ab initio prediction:Prodigal:002006"

/inference="similar to AA sequence:UniProtKB:P32132"

/codon_start=1

/transl_table=11

/product="GTP-binding protein TypA/BipA"

/db_xref="COG:COG1217"

/translation="MKRRTDIRNVAIIAHVDHGKTTLVDELLKQSETLDSHNELAERA

MDSGDIEKERGITILAKNTAVDYKGTRINIMDTPGHADFGGEVERIMKMVDGVVLVVD

AYEGTMPQTRFVLKKALEQKLTPIVVVNKIDRDAARPAEVVDEVLELFIELGADDDQL

EFPVIYASAMNGTSSMSDDKNDQEATMDNVFDTIIEAIPAPIDNSDEPLQFQVSLLDY

NDYVGRIGIGRVFRGKIKVGDNVTLTKLDGSTKNFRVTKLFGFFGLSRVEINEAIAGD

LIAVSGMEDIFVGETVTPVDHQDPLPVLHIDEPTLQMTFLVNNSPFAGREGKWVTSRK

IEERLMSQLHTDVSLRVESTGSPDAWIVSGRGELHLSILIENMRREGYELQVSRPSVI

LKQFDGKVCEPFELVQIDTPEEYMGSIIESLSQRKGEMQDMQNNGNGQVRLTFLAPAR

GLIGYSTEFLSMTRGYGIMNHTFDQYLPLAAGKIGGRRNGALVSTETGKTTTYGIMGV

EDRGTIFIEPGVEIYEGMIVGENSRENDITVNITKAKQMTNVRSANKDQTNVIKRPRT

LSLEESLEFLGDDEYCEVTPESIRLRKQVLNKNEREKSAKKKRLATE"

CDS 941747..942034

/locus_tag="JFBMFIFI_00887"

/inference="ab initio prediction:Prodigal:002006"

/inference="similar to AA sequence:UniProtKB:Q831P3"

/note="UPF0358 protein EF_2458"

/codon_start=1

/transl_table=11

/product="hypothetical protein"

/translation="MNEFVQKQTALDILMADAEKIYNLINSQKQHLCLAQCPAFEEVV

DTQMYGFSKEVDYAVKIGVLTDTEGHRVLSDLEKNLNDVYTEMYDEKKEQV"

CDS 942074..943246

/gene="ftsW_2"

/locus_tag="JFBMFIFI_00888"

/EC_number="2.4.1.129"

/inference="ab initio prediction:Prodigal:002006"

/inference="similar to AA sequence:UniProtKB:O07639"

/codon_start=1

/transl_table=11

/product="putative peptidoglycan glycosyltransferase FtsW"

/db_xref="COG:COG0772"

/translation="MKKLKNIDYYIFIPYIVLSVFGVLMVYSASSFAATIAPINAAPD

TFFKQQGVFVLLGFFVTMFVLLFKYELFKSKRLMMSAVMVILVMLLYLFFFGKELNGA

AGWLRIAGISVQPAEYAKIVIIWYFAFIFSKKQKGIVHEFRKTITPPVTIFLIFLALI

IMQPDIGGAGIILVIGVVMIFASGVSASLGISLGIAGMALIYGILELVKVYGTKLFFL

QEYQYDRFLAFWDPFSVSDTAGTQLINSYYALSRGGLFGVGIGQSVQKTGYLPEPFTD

FIISILGEELGLVGVLVVVSIFVFLILRIYLIGIRAKDAFGSLLCIGIATMLLVQGFI

NLGGVIGLLPITGVTFPFISYGGSSTLVLTISIGLVLNVSATEKINQQKRIAEKRN"

CDS 943398..946829

/gene="pycA"

/locus_tag="JFBMFIFI_00889"

/EC_number="6.4.1.1"

/inference="ab initio prediction:Prodigal:002006"

/inference="similar to AA sequence:UniProtKB:A0A0H3JRU9"

/codon_start=1

/transl_table=11

/product="Pyruvate carboxylase"

/translation="MKKILVANRGEIAIRIFRACTELHIDTVAVYAQEDAGSVHRFKA

DEAYLVGKGKKPIEAYLDIEDMIRIAKYAGADAIHPGYGFLSENLTFARRCQEEGIIF

IGPSLHHLDIFGDKIKAKEAAVAAGIQSIPGTNGPVNSLDEVLEFGETHGYPIMVKAA

LGGGGRGMRVAASKAEAKDSYERAKSEAKAAFGSDEVYVERYISSPKHIEVQILGDTH

GNVLHLFERDCSVQRRHQKVVEVAPCVSISTELRHEMCDAAVQLMKHVGYINAGTVEF

LLEDDRFYFIEVNPRVQVEHTITEMITGIDIVQSQIKIAMGMDLFKDMHLPQQKDLHM

IGAAIQCRITTEDPMNNFLPDTGKIDTYRSPGGFGIRLDAGNGFQGSVVSPFFDSLLV

KVCVQGMTFDDAVKKMDRSLKEFRIRGVKTNIPFMQNVISHPIFLSGNAKTTFIDTSP

ELFIFPKTRDRGNKTMKYIGNITVNGFPGIEKGEKKFLEAARVPSKLILPNEPYISAK

NILDSQGADAVVEWVKNQNQVLLTDTTFRDAHQSLLATRVRTQDFVNIAEETQKGIPQ

LFSEEMWGGATFDVAYRFLNEDPWERLRKLRRRMPNTLFQMLFRGSNAVGYQNYPDNV

IVEFIQQAATNGIDVFRIFDSLNWIPQMEKSIQAVRDTGKIAEAAICYTGDINDPKRA

KYTVDYYKEMALELERQGAHIIALKDMAGLLMPQAAYRLISELKATVDVPIHLHTHDT

SGNGIFTYASAVKAGVDIVDVAMSAISGATSQPSMSSLYYALVNGDRTPDIEIKNVQQ

LNHYWEDVRGFYTDFENGISAPQTEVYQHEMPGGQYSNLQQQAKAVGLGEKWEEVKQM

YSTVNQMFGDVVKVTPSSKVVGDMALFMIQNGLSEADVYEKGASIDFPDSVIGFFRGD

LGQPVGGFPEKLQKIILKDKKAITVRPGSLAKSVDFAAVKKELAEKMGEEPTHEDVLS
YIMYPQVFLEYCKMHDQYGDVTLLDTPTFFHGMRHGESVEVRIEKGKTLIIKLIEVGE
PDSEGNRILYFELNGQGREVVIKDISIKGTATLRRKAEPTNKEQVGATMPGSVLEVLV
EKGERVKAGQPILITEAMKMETTIQANFEGIVDQIYVQNEDIIETGDLLIEIKPR"

CDS 946875..947936

/locus_tag="JFBMFIFI_00890"

/inference="ab initio prediction:Prodigal:002006"

/codon_start=1

/transl_table=11

/product="hypothetical protein"

/translation="MKTFLRAIPLFIVLMIIFYATPILQSKDNPKVPANTHETGQEMD
KPKTGMVQETIPATGFAAYIHQPVSKITEKYGEPTRKDPTAYGFEWWIYNQQDRNYLQ
VGVNNGQVVTVFALGKELTVEPFEIGMNIDDIYQVTTLYPNFEINFNEDVYQIELSEA
DMNYHPLVAFDNGTFAMLMLDQVSNEILGVRYLDADVLLHLNVYEVASMTPISTIINE
QLDWELVNQANEKQTLEMINVLRRRNQLNSLIENVELTKLATSVFTTYNSRTDEEIQS
KGLSNKQISEVVTKDSSGAKDSDSLYLNHTSDATWVMSYWFSLETQRELLMNKKLRNS
GIRYQNQEVILLLDSLDKL"

CDS 948036..948470

/locus_tag="JFBMFIFI_00891"

/inference="ab initio prediction:Prodigal:002006"

/codon_start=1

/transl_table=11

/product="hypothetical protein"

/translation="MIVTEDYLDMETEAFALVQSIMESDIGQNYCAAKIALEQDEAAQ
AKIHAFNQAKKAFEEIEVYGKFAPDFKEKNREVRRLKREMDVDEFVYQYRLVETDLQT
ILDEVSLTLARAVSDTIKVPAGNPFFATGASGCGTGGGCGCG"

CDS 948486..948821

/locus_tag="JFBMFIFI_00892"

/inference="ab initio prediction:Prodigal:002006"

/codon_start=1

/transl_table=11

/product="hypothetical protein"

/translation="MELILNERQGIIVWVYSLRHLKTLKRFGLIHYVSKRMKYVVIYV

DKSEVETTEKKLNGLHFVRKVDLSMRPEINMDFGDRIGKAKNRKEVEDAEEIAMIEGA

TQLKLKEMV"

CDS 948995..949567

/gene="rsmD"

/locus_tag="JFBMFIFI_00893"

/EC_number="2.1.1.171"

/inference="ab initio prediction:Prodigal:002006"

/inference="similar to AA sequence:UniProtKB:P44869"

/codon_start=1

/transl_table=11

/product="Ribosomal RNA small subunit methyltransferase D"

/db_xref="COG:COG0742"

/translation="MRVISGEYGGRKIKAVPGTNTRPTTDKIKESIFNIIGPYFDGGV

CLDLFAGSGGLAIEAVSRGFDKAILIDKEILAIKTIKENIEMTKEPEKFQLYRNEANR

ALDALSRQNQAFDLIFMDPPYMKQEIEKQLTQIMDKQLLNPNGKIICEVDKKTDLPDK

VAGLVATRRESYGMTKIVIYQLDEKSEEDE"

CDS 949560..950051

/gene="coaD"

/locus_tag="JFBMFIFI_00894"

/EC_number="2.7.7.3"

/inference="ab initio prediction:Prodigal:002006"

/inference="similar to AA sequence:UniProtKB:Q831P9"

/codon_start=1

/transl_table=11

/product="Phosphopantetheine adenylyltransferase"

/db_xref="COG:COG0669"

/translation="MSKKALFPGSFDPLTNGHVDTIQRAAKLFDEVIIAVLTNTSKVS

LFNSNEKIDLIEKSLQHIENVQVISHVGGLTIDLAKDLEITAMIRGMRNTLDFEYETN

IALMNKQLNEEIETVILLADEKYRFLSSSLIKEVARFGGDISAFVPKVVNEAIKEKYK

KLN"

CDS 950067..951107

/gene="ylbL"

/locus_tag="JFBMFIFI_00895"

/inference="ab initio prediction:Prodigal:002006"

/inference="similar to AA sequence:UniProtKB:O34470"

/codon_start=1

/transl_table=11

/product="putative protein YlbL"

/db_xref="COG:COG3480"

/translation="MMTNKPKKTKYILTIIAILIVLGLVVPIPYFIESPGSAVHLNEL

ISVDNQKDKEDGSFMLTTVGVRQATAFTYFMRFLPFHEGLTKKELYGDVSSNKEFNQV

QDYYMTSSVNAAIELAYRTAGAEYKTHYKGVYVMSILDESKFKGKLEVGDTIVSLDGH

SFKSSEEFIDYVKSKKVGEEIDVVVNRNGSEKKVSAPLMKMDTDKKAGLGISLVNDTT

ITTTIPVDVNTEEIGGPSAGLMFSLEIYTQLKGINLRDGRQIAGTGTISPDGTVGRIG

GIDKKVVAADKEGATIFFAPDDEIDPAIKKKYPELKTNYEEAKAAAKKIGTKMKIVPV

KTFQDAVDYLEK"

CDS 951218..951892

/gene="comEA"

/locus_tag="JFBMFIFI_00896"

/inference="ab initio prediction:Prodigal:002006"

/inference="similar to AA sequence:UniProtKB:P39694"

/codon_start=1

/transl_table=11

/product="ComE operon protein 1"

/db_xref="COG:COG1555"

/translation="MEKIQQVKTWMLDHLFAFKIGLAVLGIGLIFSSLVFARSSAVSE

EIPSLAVDSTLFNEDSSEEKSRKEDVEVPDEKMVVDVKGAVLNPGVYSVKEEMRLIDA

IELAGGFLTEADSVGLNLSLRLQDQMVIYVPLIGEELKFEEVDREENVKQKDQNSEVE

AKLNINQAEANELQTLTGIGAKKAQMIVEYRQENGSFQSIEDLKKISGIGEKIFEGLK

EQITVG"

CDS 952038..952517

/gene="tadA_1"

/locus_tag="JFBMFIFI_00897"

/EC_number="3.5.4.33"

/inference="ab initio prediction:Prodigal:002006"

/inference="protein motif:HAMAP:MF_00972"

/codon_start=1

/transl_table=11

/product="tRNA-specific adenosine deaminase"

/translation="MKRIPWDQYFMAQSLLLSLRSTCTRLTVGATIVRDKRIIAGGYN

GSVTGDLHCIDEGCYIVDGHCLRTIHAEMNAILQCAKFGVGTENAEIYVTHFPCLQCT

KMILQAGIKKIHYLEDYHNDPYAIKLIEQAHVQCQKVTLSNEYFAQLDFGNGKNNVE"

CDS 952518..954863

/gene="comEC"

/locus_tag="JFBMFIFI_00898"

/inference="ab initio prediction:Prodigal:002006"

/inference="similar to AA sequence:UniProtKB:P39695"

/codon_start=1

/transl_table=11

/product="ComE operon protein 3"

/db_xref="COG:COG0658"

/translation="MRNFWIFPALLSLSSVLIFFTNAHFISVLVVLFGISRIVCIGIP

KVIVTSFIIMGFTVLFTSRFIMTNQTDLAGNEQEFVVEVNPTQSKVDGDQLQFYGTVL

KKKKYGKVKKEKIVGFYQLKNEKEQIFWKKNQNFIELTAKGSLEQPENNRNFAQFNYR

KFLKRETIHWVLKINKFEKVEKKKIAIWQIPKIVDLLRRKLLLHIESYPIDKVGEFTG

ALLFGNSNQLEETTRLNFSKLGLIHLLSISGVHVQYLVTVFRRLFRRFKLSKELTDEA

LLLMLPLYGALAGGQTSIFRAVSMRWLPILGEKIKLQCSSLDAWSLTLIISLWLKPTQ

IFSVGFQLSYLLTLFLLLFPLNLIDFLKHDVMKSLFISSMMLLMSIPILAYHFYEFSW

ATVIATSLFTLIFIYGLLPILLALLIASIFWLNQPFFQFLVEIVGILISWIESFLQKI

NSIGSFMITTGRPKFIFIFLFFSCILIFIMQLEKRKHRFLSLVTLCVSLGCLIFSTRF

DSSLKVVVLDVGQGDSILIKDRFGKGAYLIDTGGALTFEKKKWAKKKKNTSIAQNKLI

PAIKAEGISYLNGVFISHGHVDHMGALAELSESIPIKEISYATGSETKPAFRKALKML

KKAGTRLNSLLAPEEWSISSDIKLKALYPSEVGQGDNRDSLTLYAEIGQISWLFTGDL

DSEGELKLLQKYPKAKVNVLKVGHHGSSTSSSSIFLKTIEAKVGLISAGLDNQFQHPR

PETISRLKEQNMIIFRTDLDGAIYYTEPLTGQGSFKRIMEK"

CDS 954948..955973

/gene="yqeN"

/locus_tag="JFBMFIFI_00899"

/inference="ab initio prediction:Prodigal:002006"

/inference="similar to AA sequence:UniProtKB:P54459"

/codon_start=1

/transl_table=11

/product="putative protein YqeN"

/db_xref="COG:COG1466"

/translation="MNYTAEMAKITKGQFSPVYLFLGTESYLADSAKQTLIQATLAED

ERDLNFGIYDMEEVPVGVALDDAESVPFFGDKRLVIMDRPNFFTAEKSKQKIDHDLVW

LENYLKNPPDFTILAFFAPYEKLDERKKITKLLKKTATVIEVNTLSEKEVRHFLKDTI

ANEGYTMTPEAFELFIQLTDAKLSTAMSELPKLLLFSSDTKQITKSAVNDLVAKSLEQ

NIFALVEYVLKRQTANALSLYQDLLLQKEDPIKINAILMTQFRLLLQVKFLEKKGYQQ

GDSAGMLKVHPYRVKLAIQQARKFSEKVLVSAFEGLVDAEYRLKTGQGDKEMQFELFV

LQFAAVK"

CDS 956278..956541

/gene="rpsT"

/locus_tag="JFBMFIFI_00900"

/inference="ab initio prediction:Prodigal:002006"

/inference="similar to AA sequence:UniProtKB:A2RMG0"

/codon_start=1

/transl_table=11

/product="30S ribosomal protein S20"

/db_xref="COG:COG0268"

/translation="MNHLPNIESAIKRVRVNEKAALQNNAQKSAMRTAIKKFEQAVEA

GSENVASLHKEAIKAVDMAATKGLIHRNKANRDKSRLTAKLAK"

CDS 956739..957542

/gene="proC_1"

/locus_tag="JFBMFIFI_00901"

/EC_number="1.5.1.2"

/inference="ab initio prediction:Prodigal:002006"

/inference="similar to AA sequence:UniProtKB:P0A9L8"

/codon_start=1

/transl_table=11

/product="Pyrroline-5-carboxylate reductase"

/db_xref="COG:COG0345"

/translation="MKRSDEMDSIGVIGAGQMGGALLKGLIESGFSSSDSVFVSGGSG
RTAAILQKELHYNLCQSNSEVAKKVDVLILAVTPQIMPTVLNEIKEAIMPDTLLISLA
AGMEFSEYTKLLNEKIKLVRAIPNTPVSIQAGMTALSYNDQIGSGERDKTEALFQAVG
EVVEVSEAQLNSAGTVSGCSPAFIAIFIEALADGGVLNGLSRKQAYLLAEQAILGTAK
LALLSGDHPGIVKDDVCSPGGSTIKGVVALEQFGFRNAVIQAIDAATKN"

CDS 957575..959566

/gene="uvrB"

/locus_tag="JFBMFIFI_00902"

/inference="ab initio prediction:Prodigal:002006"

/inference="similar to AA sequence:UniProtKB:P37954"

/codon_start=1

/transl_table=11

/product="UvrABC system protein B"

/db_xref="COG:COG0556"

/translation="MKAVVKDTFDLVSKYQPNGDQPEAIRQLVKGINEGRKEQTLLGA
TGTGKTFTVSNVIQQVNKPTLVIAHNKTLAGQLYGEFKEFFPNNAVEYFVSYYDYYQP
EAYVPSSDTYIEKESSVNDEIDKLRHSATSSLIERRDVIVVASVSCIYGLVNPLDYKE
HVLSIRQGAEMERNELLRRLVEMQFERNDIDFQRGRFRVRGDVVEIFLASRDSEAIRV
EFFGDEIERIREIDVLTGEIKADVEHVPIFPATHFIANADKTREAVNNIKAELEARLK
VLRGEDKLVEAQRLEQRTNYDMEMMLEMGYCSGIENYSRQMDGRKAGEPPYTLLDFFP
DDSMIVIDESHITMSQIRGMYNGDQARKQRLVDYGFRLPSALDNRPLRLEEFEEHVNQ
IMYISATPGPYELERTPEVVQQIIRPTGLLDPIIEVRPIHGQIDDLIGEINARSERNE
RVFITTLTKKMSEDLTNYLKEVGIKVKYLHSDIKTLERTEIIRDLRLGEFDVLIGINL
LREGIDVPEVSLVAILDADKEGFLRSERSLVQTIGRAARNSNGKVIMYADKMTDSMKA
AIDETGRRRATQEAYNKEHGIVPQTIIKEVRDLISITKAVDDSNDYDSAAEMINDLTQ
AERKELIVKMEMEMRAAAKELDFEKAANLRDIVLELKAK"

CDS 959830..960894

/gene="ulaG"

/locus_tag="JFBMFIFI_00903"

/EC_number="3.1.1.-"

/inference="ab initio prediction:Prodigal:002006"

/inference="similar to AA sequence:UniProtKB:P39300"

/codon_start=1

/transl_table=11

/product="putative L-ascorbate-6-phosphate lactonase UlaG"

/db_xref="COG:COG2220"

/translation="MAKINEVTRESWILATFPEWGTYLNEEIEATVVKPNTFAMWWLG

CTGIWLKSDKSTNILCDLWCGTGKQSHGNGKMKKGHQMMRMSGCENMQPNLRTQPFVI

DPFAITNLDALVVTHIHSDHVDINTAAAVLQNCDASVPFIGPQGVVDIWTEWGVPKER

CIVVKPGDVIKVKDITITALEAFDRTALITATEDVVLKGQLPQDMDKIAVNYLFDTSG

GSLYHAGDSHYSNQFAKHGNQYKIDVALGAYGENPRGITDKVTSVDMLRMAESLNAKV

VIPVHYDIWANFQADPKEISMLWESKKDRLQYGFKPYIWQVGGKFVYPDDKDKREFNY

YRGFDDVFTVENDVPFPSFL"

CDS 960969..962393

/gene="ulaA_1"

/locus_tag="JFBMFIFI_00904"

/inference="ab initio prediction:Prodigal:002006"

/inference="similar to AA sequence:UniProtKB:P39301"

/codon_start=1

/transl_table=11

/product="Ascorbate-specific PTS system EIIC component"

/db_xref="COG:COG3037"

/translation="MDIFLRIFEWFANNILQKPEFFIGIIVFVGYVLLKKPLYECFAG

FIKATVGYMILNVGAGGLVTTFRPILAGLNDRFNLDAAVIDPYFGLNAVNAALENLGI

SLSWTMISLLIGFSLNIVLVLFRKITKLRTLFITGHIMVQQSTTITWIIFFLFPQYRN

LTGGIMVGILVGLYWAVSSNLTVGPTQRLTGNAGFAIGHQQMFAVWLTDKVAGKLGDP

EKNLDTIKMPKWLSIFHDNIVATGTLMMLFFGVIMTILGEDYLREINPAFTPTTSFLM

YIISQSLFFAVYLAILMQGVRMFVAELTNSFQGISNRILPGSLPAVDCAATYNFAPPN

AVLFGFIFGALGQFITIFALIIFKSPVLIITGFVPVFFDNATIAVFANKRGGIRAAMI

CSFVSGVLQVSISAFAVMFFGLYKYGGWHGNIDFELFWPWAGVIMKYLGVIGFALVCV

FFLAIPQLQYRFSKNKSAYNEGLE"

CDS 962426..962704

/gene="ulaB"

/locus_tag="JFBMFIFI_00905"

/EC_number="2.7.1.194"

/inference="ab initio prediction:Prodigal:002006"

/inference="similar to AA sequence:UniProtKB:Q9EXD8"

/codon_start=1

/transl_table=11

/product="Ascorbate-specific PTS system EIIB component"

/translation="MLKVLAACGNGMGSSMVIKMKIEKALKELAVTDYKVDYCSVGEA

KSQANGYDIVVASQHLIHELDGRTKGTLLGLDNLMDDNEIKTKLAPLV"

CDS 962773..963246

/gene="ulaC"

/locus_tag="JFBMFIFI_00906"

/inference="ab initio prediction:Prodigal:002006"

/inference="similar to AA sequence:UniProtKB:P69820"

/codon_start=1

/transl_table=11

/product="Ascorbate-specific PTS system EIIA component"

/db_xref="COG:COG1762"

/translation="MLSLKQSLIENHSIKLGESAETWQEAIAVAAEPLIASGAVERDY

PTAIIASTEKFGPYYILMPGMAMPHARPEDGVKRDAFSLVVLKNPVVFSDGKEVSVLI

TLAATSSNIHTGIAIPQIVAVFELPNIIQRLEQASEVNEILSIIDEADMSAYLNE"

CDS 963264..963902

/gene="sgbH"

/locus_tag="JFBMFIFI_00907"

/EC_number="4.1.1.85"

/inference="ab initio prediction:Prodigal:002006"

/inference="similar to AA sequence:UniProtKB:P37678"

/codon_start=1

/transl_table=11

/product="3-keto-L-gulonate-6-phosphate decarboxylase

SgbH"

/db_xref="COG:COG0269"

/translation="MGRPNLQIALDNSSLSSALQSISLAGPIVDIVEAGTILCLQSGM

EAVRCLRALYPNKLIVADTKCADAGGTVAKNCSDAGADWMTVICCATLATMEAAMKEV

KELQVELYGEWTFEQAKDWYRIGIRQVIYHQSRDALLAGDTWGEKDLEKLRRLIDMGF

KVSVTGGLTKESLLLFKELDVYTFITGRGITAAPDPALAASEFKAEIKRIWG"

CDS 963907..964770

/gene="ulaE"

/locus_tag="JFBMFIFI_00908"

/EC_number="5.1.3.22"

/inference="ab initio prediction:Prodigal:002006"

/inference="similar to AA sequence:UniProtKB:Q8XDI5"

/codon_start=1

/transl_table=11

/product="L-ribulose-5-phosphate 3-epimerase UlaE"

/db_xref="COG:COG3623"

/translation="MNTLGIYEKALPKNISWEERLQLAKSLGFDFVEMSIDETDERLA

RLDWSENSRNEVSQAIVKTGVTIQSLCLSGHRRYPLGSVDPVIRGKGLEIMAKAIDLA

SDLGIRVIQLAGYDVYYEKKSVATRSFFIENLEKSVALAARKQITLAIEIMDDSFMSS

ITKYKRIKKQIPSPWLQVYPDLGNLSAWPENDPGYELELGKGAIVATHLKDTLAVTDN

FSGKFKGVPFGEGCVDFLGCLKTMKSFSYNGPFLIEMWSEESPDFKEEIKAAKAFLLP

ILKEAGFNYEY"

CDS 964760..965476

/gene="araD"

/locus_tag="JFBMFIFI_00909"

/EC_number="5.1.3.4"

/inference="ab initio prediction:Prodigal:002006"

/inference="similar to AA sequence:UniProtKB:P08203"

/codon_start=1

/transl_table=11

/product="L-ribulose-5-phosphate 4-epimerase AraD"

/db_xref="COG:COG0235"

/translation="MNTKAKIIKEMQKNVYEANLFLPKAGLVKLTWGNVSQINRELGL

IVIKPSGVPYEKMQEADMVVTDLSGKVLQGELNPSSDLPTHCVLYQKFPEIGSVVHTH

SKWAVCWAQAGRGISAYGTTHADTFYGTVPCTRELTQTEVEVDYEVETGKVIVETFNE

KKLDPLAIPAVIVKGHGPFTWGDTPQKAVENSLILEEVAEMALHSEELASATECLLPG

YLLDKHYFRKHGSAAYYGQF"

CDS 965647..967728

/locus_tag="JFBMFIFI_00910"

/inference="ab initio prediction:Prodigal:002006"

/codon_start=1

/transl_table=11

/product="hypothetical protein"

/translation="MQFDSKFNQFLKILVKQKEISGITALANRSGLSRRMIYYYIEKL

NELLLVLDSPILEKQARGVLQVSSQQQRKIKEWLKNQKKQDFILTINERRVIVTVLIL

IENQKWQLNHFQELFLVSRNTILKDIQWVKEHFAVSEIQLKSTKAKGYHIIVDELERR

QLIYQQLYLIETGQKEACFDFLLGALGYENLEILKETIIEPIKKLIEKTKGSLGKELA

AQDIHILSKLIFILKDRNTKNCIPKWTDGEEFLIKERLEYQVAKKLLVKIELIIKINY

LEEEALYYGMLLLCIEKNTDAHFRSNPFENLIYLTENLVTLFEQIVGLYFHDRENLIK

KIQTHLKVLYYRHVFDMQMPSYGLDNVSTYYQKAFRLTEKVSNLMKSDVVFQHSFPDG

FSKEELAEVALYFEEAILKEQTKQYVPQLIIVSDFPDVLNSLLENHLRQLLPNVSIVG

ILKSDNAHFYSGKVDYCISTNTNYVHNQGETVIVSVILTKEEKMRIKKIEQSHGRYLK

MRDKIKQLMNDYQYLTNQEDFIAELSHVLEQSEKNSLETQHIELIDFLKNPFFCCIKQ

EVGSLTEMMSWLSGPLIEEKYIQPTYTQQVLKELKEERFIFLYPKVLLVHTDYRFGSM

KPGCSFLYLKTPFVLSTNEQVQFVLFLATEENMGHVPLLFELDHLLQGSFLRQLKEKN

SFKEAIASIDW"

CDS 967956..968225

/gene="rpsO"

/locus_tag="JFBMFIFI_00911"

/inference="ab initio prediction:Prodigal:002006"

/inference="similar to AA sequence:UniProtKB:A2RMV9"

/codon_start=1

/transl_table=11

/product="30S ribosomal protein S15"

/db_xref="COG:COG0184"

/translation="MAISKEDKNVIMKQYATHEGDTGSPEVQIAVLTAEINHLNEHAR

VHKKDHHSYRGLMKKIGHRRNLLAYLRNKDVARYRELIQSLGLRR"

CDS 968440..970554

/gene="pnp_1"

/locus_tag="JFBMFIFI_00912"

/EC_number="2.7.7.8"

/inference="ab initio prediction:Prodigal:002006"

/inference="similar to AA sequence:UniProtKB:P50849"

/codon_start=1

/transl_table=11

/product="Polyribonucleotide nucleotidyltransferase"

/db_xref="COG:COG1185"

/translation="MTEKQIFTKEWAGRTLTIETGQLAKQANGAVLVRYGDTVVLTAA

VASKKAKDVDFFPLTVNYDEKMYAVGKIPGGFIKREARPSERATLTARLIDRPIRPMF

AEGFRNEVQITNTVMSVEQDCSPEFSAMFGSSLSLAISDIPFNGPIAGVDVGRINGEY

VINPTIEQNELSDIHLTVAGTKDAINMVESGAKEVSEEDMLGALIFGHNAIKELVAFQ

EEIQAAVGKEKMEIQLLQIDAELEKEVNDSYQAKMVAAIQTEEKLAREENIEEVKDIA

IAFFAEKYADHDEAARIQKEIKQIVEDMEKNEVRRLITVDKIRPDGRKVDEIRHLSSE

TGILPRTHGSGLFTRGQTQALSIATLAPLGEHQIIDGLGLEDSKRFIHHYNFPQYSVG

STGPSRGPGRREIGHGALGERALSEVIPSEEDFPYTIRLVAEVLESNGSSSQASICAG

TLALMDAGVPIKAPVAGIAMGLVKNEDNYTVLTDIQGLEDHLGDMDFKVAGTKDGITA

LQMDIKIDGITQQILEEALTQAKKARLEILEELVSTIAEPRQELSPYAPKIEMIKINP

DKIKVVIGRGGDQINAIIEETGVKIDIDQEGNVSIASTEADMIKKAKTIIEELTREIK

VGEIFDGTVKRIEKFGAFIEIIKGKDGLVHISELANERVGKVEDILALGDKVMVKVTE

IDNQGRINLSRKALLKEEEAKK"

CDS complement(970812..971801)

/gene="birA"

/locus_tag="JFBMFIFI_00913"

/EC_number="6.3.4.15"

/inference="ab initio prediction:Prodigal:002006"

/inference="similar to AA sequence:UniProtKB:P0CI75"

/codon_start=1

/transl_table=11

/product="Bifunctional ligase/repressor BirA"

/db_xref="COG:COG0340"

/translation="MTTTKEKVLAFIQEAYPEPISGQLICDKLTISRTAVWKAIQSLK

EEGYDIHSQGKSGYYFTQPPVSKVAISAQLNTKFIGKHLTVLPTISSTNTVAKQLADV

TPIDGNVLIAEEQIGARGRLGRTWNSTKGKSVALSITLTPSISPTNASLITQIVAAAF

VLASEPILPVKIKWPNDIVVNGQKLCGILTEMSAELTEIHYVVVGIGINTNLDITDFS

DELQKKATSFALQLGQKIDPNQLISAFLNQFEHLYQEFIETGSTEKFLTICREHSAVI

GKDIHIISSKETRQAQAITIDNAGQLIVKFKGDTESTPLLAGEISIRGLNGYI"

CDS 972060..972872

/gene="yidA_2"

/locus_tag="JFBMFIFI_00914"

/EC_number="3.1.3.23"

/inference="ab initio prediction:Prodigal:002006"

/inference="similar to AA sequence:UniProtKB:P0A8Y5"

/codon_start=1

/transl_table=11

/product="Sugar phosphatase YidA"

/db_xref="COG:COG0561"

/translation="MIKLIAIDIDGTLLNSQHRLTQKVIDTIKRAKAQGVKIVLCTGR

PIVGILPYLEKLDLLDSTDIAITQNGALVQETASKKVLSHLTIELEQLKEIEKFSKTQ

KAQLTFFDDKKMYTLHPEPNKLLFDDAKLLHTELTVIDKKDLSSDMFLTKAMFLGDST

EIDQLLLDLPPSFYDEFYCVRSVSYNFEFLNRGASKGEALKVLAESLKLKPENVMAIG

DAENDRSMLEYAGSAVVMENALLEIQALATFVTKSNDNDGVAFAIEELVLTK"

CDS complement(972808..973644)

/locus_tag="JFBMFIFI_00915"

/inference="ab initio prediction:Prodigal:002006"

/codon_start=1

/transl_table=11

/product="hypothetical protein"

/translation="MKRRYKISLIIILTLISLALIGLLVIQNKTYSPSDEALSASKTS

ENVKVIRTKDALIFTPKKKTDNPAVLFYQGALVDETSYSIWAHQLAEAGYETYLIHQP

FNMAVLGANKAEKIIDKYAIDSYVIGGHSLGGVMASRFAKKQTSDNLKGVFFLASYPD

EKGALNATELPVLSITGSKDGVLNWESYHSSQKLLPAKTDFQVIDGGNHAGFGSYGPQ

KGDNKASISDKKQQSEVSELLLKWLQTIKEEVGKIPTSSFIWSTLILQSQMQHHRCHL

IL"

CDS 973973..975022

/gene="asd"

/locus_tag="JFBMFIFI_00916"

/EC_number="1.2.1.11"

/inference="ab initio prediction:Prodigal:002006"

/inference="similar to AA sequence:UniProtKB:Q04797"

/codon_start=1

/transl_table=11

/product="Aspartate-semialdehyde dehydrogenase"

/db_xref="COG:COG0136"

/translation="MSGYQVAVVGATGAVGTKMLEMLAEADFPIAGVKLLASKRSAGK

VLLFKDQELVIEETTAESFEGIDIALFSAGGSVSKEFAPEAVKRGAVVIDNTSAFRMD

LNVPLVVPQVNELALKEHQGIIANPNCSTIQMMVALEPVRKAYGLSRIIVSTYQAVSG

AGVSAINELKKQSQKMLNDEPVEAHILPCGGDKKHFPIAFNALPQIDVFSDAGYTYEE

WKMMNETKKIMADETIKVAATCVRIPVVSGHSESIYFEVKDELATVFGIQNELEKAPG

VVLQDDPATQLYPTALTSVGKKETFVGRVRKDLDDDLGFHMWVVSDNLLKGAAWNSIE

IAESLHRLDLVRVPK"

CDS 975057..975935

/gene="dapA"

/locus_tag="JFBMFIFI_00917"

/EC_number="4.3.3.7"

/inference="ab initio prediction:Prodigal:002006"

/inference="similar to AA sequence:UniProtKB:Q04796"

/codon_start=1

/transl_table=11

/product="4-hydroxy-tetrahydrodipicolinate synthase"

/db_xref="COG:COG0329"

/translation="MEIATAQIMTAMVTPFNENGQVDFAKTATLIEYLLANGTEGLIV

GGTTGESPTLSHSEKLELYKKTVEIVHGRVPIIAGTGSFNTAETIAFTKEVAEIAGID

AALVVAPYYNRPNQEGLYQHFKAVAKASPLPIMIYNVSARTGVNVEVQTTLRLSLLEN

VIGIKECAGLEAMSAIIEGSEPDFLVYTGEDHLAFPTKCMGGAGVISVASHVVGNEMS

EMYRLLDNGLLADAARIYRMLLPKMSIVFSVPSPAPVKAVLNQQGISVGGVRLPLVEC

TKEEEMTIFNRLEINR"

CDS 975967..977658

/gene="rnj2"

/locus_tag="JFBMFIFI_00918"

/EC_number="3.1.-.-"

/inference="ab initio prediction:Prodigal:002006"

/inference="similar to AA sequence:UniProtKB:Q8K7S6"

/codon_start=1

/transl_table=11

/product="Ribonuclease J 2"

/translation="MSDIKIIPLGGVRENGKNMYIVEVEEEIFVLDCGLMYPENELLG

IDVVIPDFSYLEANADRVTGVFLTHGHADAIGALPYFLQKFNVPVFGTELTIALAKLF

VNKDNLVKGFKDYHVIDDKTEIDFGNAVVSFFKTTHTIPDSVGIALKTSEGTIVYTGD

FKFDQSATPMYQSNFGKISDIGEGNVLALLSDSAEAEGSVENASDLKIAGEVLDTFRN

TDGRIIVACIASNIMRVQQVLDAAHKSNRKIFITGRDLEEIVKTAMTLNKIQLPSEEL

IVSVKEIDKYPDDEIVVLETGNLGEPIQSLQKMAIGKHRQVNIKEGDLVYITTTTSTA

METTVAKTEDMIYRAGGTVKQISTNLKASGHGTAKDLQLMINLLKPTYFIPVQGEYRR

LAAHADLAHEVGIPRKNIFIPGKGDVMEYTKGRMRMAGQVEAGNTMIDGIGVGDIGNI

VLRDRKLLSEDGIFVAVVTISRKQGKIISGPDITTRGFVYVRANEDLIKESNEIVKKV

VEDNLSRKEFEWSRLKQEIRESLSKYLFEKTRRRPVILPIIMESSYRNTKRNKES"

CDS complement(977714..978604)

/gene="gbuC"

/locus_tag="JFBMFIFI_00919"

/inference="ab initio prediction:Prodigal:002006"

/inference="similar to AA sequence:UniProtKB:Q9RR44"

/codon_start=1

/transl_table=11

/product="Glycine betaine/carnitine transport binding

protein GbuC"

/translation="MKKIKTLLLLMIFPFFLASCASVLSPYDAKKPVGEQINYTITGI

DAGAGIMSSTEKAISDYQLDKNNWQLQPSSTAAMTSTLAKAIKDKRPIVVTGWTPHWM

FTKFDLKFLEDPKGSFGKAENIHTIVRKDLKKDEPTAYEVLDRFYWTPEEMADVMLQV

NEGVDPEKAAADWLKANPEKLEEWTKDLKKVDGNPIKLTYVAWDSEIASTNVVALALN

EVGYDTTVQAMEIQPMWASVATNAADAMVAAWLPNTAGIFYEDYKDDIEDLGINLEGA

KLGLVVPTYMENINSIEDLK"

CDS complement(978632..979480)

/gene="gbuB"

/locus_tag="JFBMFIFI_00920"

/inference="ab initio prediction:Prodigal:002006"

/inference="similar to AA sequence:UniProtKB:Q9RR45"

/codon_start=1

/transl_table=11

/product="Glycine betaine/carnitine transport permease

protein GbuB"

/translation="MDNIPTLPIANWIDQLVESLTQFEGFFNFISIIIASIAGAFQWI

FDILPIWLFIVVVVLGVYYVNRNGKKWGLVSFSLLGLLFIWNLGFWRDMTQTLTLVLT

SSFIALIIGVPLGIWMSKSRWAEKIIRPILDFMQTMPAFVYLIPAVAFFGIGMVPGVV

ASVIFSMPPTVRMTNLGIREVPTELDEAADSFGSTAWQKLFKVELPLAKSTIMAGINQ

SMMLSLSMVVIASMIGTMGLGTKVYFAVGRNDAGNGFAAGLAIVILAIILDRITQAFT

KDKRSK"

CDS complement(979473..980666)

/gene="gbuA"

/locus_tag="JFBMFIFI_00921"

/EC_number="7.6.2.9"

/inference="ab initio prediction:Prodigal:002006"

/inference="similar to AA sequence:UniProtKB:Q9RR46"

/codon_start=1

/transl_table=11

/product="Glycine betaine/carnitine transport ATP-binding

protein GbuA"

/translation="MSKIRVENLTKVFGKKSAKALAMLKDNKSKKEILKETGATIGVD

NVSFSVEEGEIFVIMGLSGSGKSTLIRLLNRLIEPTSGDIFLDDQDLVKMDKKTLRET

RRKSMSMVFQNFGLFPNRTILQNAEYGLEIQRVEKEERALKANEALDLVGLNGYGDQY

PDQLSGGMQQRVGLARALANNPDILLMDEAFSALDPLNRKDMQNQLLDLQSSVQKTII

FITHDLNEALRIGDRIMIMRDGAVVQSGTPEEILTTPENDYVEKFIEDVDRSKVLTAA

NVMIEPTTVSLEKSGPRVALKRMRDENLSSLFVVNRHGELIGIVHANEVLAAIQEQKN

SLEGIIHNDVATTEPDVAISDLLDTVSTMNVPIAVVENKKLRGIIIRGSVLAALAGNE

VEQHG"

CDS 981139..981807

/gene="yedJ"

/locus_tag="JFBMFIFI_00922"

/inference="ab initio prediction:Prodigal:002006"

/inference="similar to AA sequence:UniProtKB:P46144"

/codon_start=1

/transl_table=11

/product="putative protein YedJ"

/db_xref="COG:COG1418"

/translation="MKLDKSLIIDKTKQFVYEELINEPSGHDWWHIQRVVNTTNTIAK

EEQKMQSVDLFICEMAALLHDIADEKLNENAAAGEKRVQEWLEKNNVSPLDQAAILII

ILNMSYKGGTNKVELKTIEGKIVQDADRLDALGAIGIARTLAYSGHHGRPIHDPRLVA

REDMTLEEYRSGEDTAIMHFYEKLLKLKELMKTETGKKLAIERHNYMLHFLDQFYGEW

DGER"

CDS 981970..982392

/locus_tag="JFBMFIFI_00923"

/inference="ab initio prediction:Prodigal:002006"

/codon_start=1

/transl_table=11

/product="hypothetical protein"

/translation="MVSIRYVQDKDKSFWFKLDKHISEVEFEKKVRDKRGYILVENKI

PIGLLRYNLFWDNTPFCTMLFIDWEYQYKGYGRTLMEFWEKDMESLGYGMVLTSTQAD

ETAHYFYRKIGYHEAGKLCIDIPDYEQPIELFFIKMIG"

CDS 982713..983408

/gene="srrA_2"

/locus_tag="JFBMFIFI_00924"

/inference="ab initio prediction:Prodigal:002006"

/inference="similar to AA sequence:UniProtKB:Q9L524"

/codon_start=1

/transl_table=11

/product="Transcriptional regulatory protein SrrA"

/db_xref="COG:COG0745"

/translation="MKEYTVLVVDDDKEIRKAIAIYLQNEEITVIEAEDGLVALKKLE

ENDVHLILLDVMMPNLDGIMTAYRIREKKLNVPIVMLSAKSEDIDKIHGLQIGADDYI

TKPFNPLELMARVKSHLRRYMTLGVYEINDDLIEVRGLSLDQNAKKITVDGREIKMTP

TEFKITALLMGHAGKVFSIDEIYESVWQEPSFNADNTVSVHIRKIREKIEVNPKNPKY

LKVVWGVGYKIEK"

CDS 983398..985599

/gene="sasA_1"

/locus_tag="JFBMFIFI_00925"

/EC_number="2.7.-.-"

/inference="ab initio prediction:Prodigal:002006"

/inference="protein motif:HAMAP:MF_01837"

/codon_start=1

/transl_table=11

/product="Adaptive-response sensory-kinase SasA"

/translation="MKNKFRTIIFWLLLTFSVCYAVVPLLNWNAEPTSNSPIGSFSQN

PDELYGNDQPTTINMIYSDHFYKKLLKDPEKNLVVSNDQMDAYRFFYGDLGTQLADID

QQYQDLLVNAVNERNQTEIERLNNEKNVKQAAIMKNFMDDDALREKIKKEKLIYLQKN

KAELTDQYKNELKDVDESFYYYLVDSNGEVYTNLPEKQVLKSIDEAQKVIKSKKTEEL

QKIDEWAYVTNNIFGNERDLLFRGDGNQVEIKGVAAVKANSPVSNDKKRMEQLNKRRF

WKIGFGVLAGLIAVVMGISNLKKAVLFPKLHKKYTKVSLDIRILALVMFSFILYMTAI

EAYWGTKIFFVVSFIVVACLTTQVRYLVESIRKKELKKQWKNSFSVKIGQFFVAVFCK

LSSPSQIIGLIFVYLATGFGIPVALLATLRYGYFSILLYGVLFCLISLPITIGVIKSY

LKMKRLSRQPRAILNQLDAEEYPLENQHLPELEADLKKIKTIVLSSAQRQVQSEKLKT

ELISNVSHDLRTPLTSIISYADLLRQGTNTEVEKEHYIEIINQKAGRMKHLIDDLFEV

SKMSTGEVILEKSEVNLGQLLQQSIAEYTEEFDKADLQLRVSKGLEPVLISLDGERIW

RVLDNIMGNIVKYALPGTRVYMKLTDGPDFVRLVFKNISKYELNEEATELVERFKRGD

DSRNTEGSGLGLAIAHSIIDLHKGKFEIIVDGDMFKLVIDLPKEKLNEEIN"

CDS 985742..986230

/locus_tag="JFBMFIFI_00926"

/inference="ab initio prediction:Prodigal:002006"

/codon_start=1

/transl_table=11

/product="hypothetical protein"

/translation="MVDIYLREAVVADGPILESYQLEDSQYTTMPMEAMTESLHIIEK

YPILILVENDLAGFFILEIGMDVQIYTDEPNTVLLRAYSIDDRFQGKGYGKLSLERLH

GFVETKFPSIKKVVLAVNHGNISAQMLYLKAGFYDTKRRIHGIKGVQFVFEMDINETS

KE"

CDS 986236..986991

/locus_tag="JFBMFIFI_00927"

/inference="ab initio prediction:Prodigal:002006"

/codon_start=1

/transl_table=11

/product="hypothetical protein"

/translation="MKFVFDLDGTICFNGGPVSPRMMNYLKELSVDHEIIFASARPIR

DLLPVIDPYFYGHTLIGGNGALISQQTKISKIASFSQSDTQTLLYLIEKYQATYLIDS

EWNYAYTGTDTHPIKLGLDPLKQAQLVYVNELTSIVKVLILTATDLAAFEKELLLLNV

VYHFHGDENVIDISPQAISKWSALEKIGIKTGEFIAFGNDQNDISMFKEAKIGIQVGT

YKNLTPYASETLNGQSQKDIEEAIIEKLDELKD"

CDS 987258..989300

/gene="copB"

/locus_tag="JFBMFIFI_00928"

/EC_number="7.2.2.8"

/inference="ab initio prediction:Prodigal:002006"

/inference="similar to AA sequence:UniProtKB:P05425"

/codon_start=1

/transl_table=11

/product="Copper-exporting P-type ATPase B"

/translation="MHEHNHSMMSHDEMDHMSHESMPGMGHMAHMGNLKVKFFWSLVL

AIPIIILSPMMGMELPFQFTFPGSDWLVLILATILFIYGGKPFLSGAKMELKDRSPAM

MTLISLGISVAYFYSLYAFIMNHFINSNAHIMDFFWELATLIVIMLLGHWIEMRAVSN

AGDALKKMAELLPTQANRLEKDGSSKKILLKEVQVGDLLLIKAGEKIPADGKIVEGQS

SINESMVTGEAKEVKKNRGDKVIGGAVNGSGTLTIEVSGTGDTGYLAQVMNLVGDAQK

EKSKVESLSDKVAKWLFYVALFVGIFTFIIWFFLTKELTTPLERMVTVLVIACPHALG

LAIPLVTARSTSLGAKNGLLIRNRNALEAAKKIDSILMDKTGTLTEGAFKVHTVESLT

KEYSEEQILAYFAALERTSTHPLAVGILTKAEAEKVTIPVATETKNMPGIGLSGKINQ

EEISIVTAAYLEKMGIVYDKKHFEELASLGNSISYLVRKGEAVGLIAQGDKIKPGAKA

MIAALIAEGIEPVMVTGDNAASAEMVANQLGINQVYASLMPEDKEKIVKDYQKNKKVV

MMVGDGVNDAPSLARADVGVAIGAGTDIAIDSADVILVKSDPNDILHFLSLAKNTTRK

MIQNLWWGAGYNIIAIPLAAGILAPIGFILSPAVGAILMSLSTVIVALNALTLRIK"

CDS 989418..989909

/gene="yafP"

/locus_tag="JFBMFIFI_00929"
/EC_number="2.3.1.-"
/inference="ab initio prediction:Prodigal:002006"
/inference="similar to AA sequence:UniProtKB:Q47158"
/codon_start=1
/transl_table=11
/product="putative N-acetyltransferase YafP"
/db_xref="COG:COG0454"
/translation="MEEIQIEKFQKKDICEIIELFYRTIESVNNQDYTKDQILAWQGK
EHEAKRKREWSDKLRSSQTYLARNSKNQLVGFLEFTQLKELNLFFVHHDYQRKGIGKQ
LFLYLMKELKKKSSEKITANVSITARPFFESLGFISIKEQVVQLNGQEFINYKMEKSL
NKH"

CDS 990030..990302
/locus_tag="JFBMFIFI_00930"
/inference="ab initio prediction:Prodigal:002006"
/codon_start=1
/transl_table=11
/product="hypothetical protein"
/translation="MKHVNECKKCESADILKIEGKQMIGETINQLRTTILESIPVTRF
LCSDCGYTEEWVENHLDLKRMKRKVSQVNESTHSIKMLLKNLEFEG"

CDS 990464..991489
/locus_tag="JFBMFIFI_00931"
/inference="ab initio prediction:Prodigal:002006"
/codon_start=1
/transl_table=11
/product="hypothetical protein"
/translation="MIDFSYAKMKQILVHYVGNKAQEQGYEISENSIEELDEEMSEVL
HEIFLSSFKEAKFHHFTHPTSLEYNESYNYIKDVFEEETSFFEGSVNLVKQLYAASSH
PNVKAGDLWLIYMSDCIVEGELTDAIGIFKVENKEVFLKNEYNNQAVSIGYEKGITKS
DIDKAAIVFNFSAKDGYRVVALDRLNRNDSVYWKQLFLAIEKIQNDQFLAESFINVCA
EFVKKDDDGIVGKSAFIKEAQEYLEVQEEINLEDFADVLVAEAKQDEFKTVVENYEKS
HDVSFPDNFKLNQENKDKFSKKVKNKIKINNNISVMVKDVNKLMENEITEGFDEVEQK
RYLKIYF"

CDS complement(991548..993170)

/gene="pucR_1"

/locus_tag="JFBMFIFI_00932"

/inference="ab initio prediction:Prodigal:002006"

/inference="similar to AA sequence:UniProtKB:O32138"

/codon_start=1

/transl_table=11

/product="Purine catabolism regulatory protein"

/db_xref="COG:COG2508"

/translation="MPILLTDILDLDSLGDSRIISGTDGLTNEVSDIMVMEAPDVENW

SSSGQLILTSLYSIQNYTEQQQFAFIEKLAHLKISGLIIKIDRFVEQIPNGFILGSQQ

FSLPIIEIPNKIRYTTILVEVMQLLSNHKARLLNRYRDVHSYFSILALNQATIGDILS

ALESYIHNPISLCTEDFNVLATTDSRLNLCDIQDEAEIEEKSEVNFTYQRKLVSHPNF

AETSFWQLAIPIYTNEVGKKLLIIHEINHPTREIDFMAIENAVIALQMDMLKQFAVRN

VKQNYRNDLFDDLLYGKCATPEQQVDYAQQIGLSANTTYRVIVWQFSNQKLTTQPSFE

KRKKLADCGQQLIDRIKQHYPNIAYRIRSNRIVFIIEDTYFQPKKSIDFSSIMKKIYQ

NWQGESILLNIGISDPCTLKTLDSESKKSLQIIRQGQAFLKTDFILFYQDLGIYRVFA

ELEKNTELTTFIPAKLSILIDTYPEWIPTLKIFLDENQNLKKTAEQLFVHYKTISYRL

TKIKELTTINFADAEELLAIQMGLRIYLLQNR"

CDS 993469..994827

/locus_tag="JFBMFIFI_00933"

/inference="ab initio prediction:Prodigal:002006"

/codon_start=1

/transl_table=11

/product="hypothetical protein"

/translation="MKKNQKKGLLLALILMITALPTIAYAAGIEAVKTPTGFMAFVTI

IPLVLVLTLLFLKVDMIIAGLAGGILAMLIGGIGLAEANTQFLETIPTMLSITVPIIN

SAIAMAVFKSGGYTAALTLAKRGTKGKVEYVSAFIVILLAAATYMSGIGGGSAMVIAP

LAFAAVGVVPELIAAMSLAAAVSFTTSPASLESSIVSKLGDVTVSEYVATMRPYWLVF

VIIAILLAFFGTKLRKVGFKEDATDEYSSLSNGQLFKITLPAIFLLFAVIFGPLVNDL

VGVAIFTPLVYMVVTILLIFICTKFNLNESVEAMVDGSTYILTRLFQVGIFLAFINII

AETGTFAVIAGVANHAPAFLVVPVAVLTGILIGIPAGAYVGSVLTLVLPVAVSLGFTP

LALGFVTIGVGLGSQMSFVNITMQALSSGFQIPILDVVKGNIKWISLASVLLLVISLV

FA"

CDS 994859..996082

/gene="atzC_1"

/locus_tag="JFBMFIFI_00934"

/EC_number="3.5.4.42"

/inference="ab initio prediction:Prodigal:002006"

/inference="similar to AA sequence:UniProtKB:O52063"

/codon_start=1

/transl_table=11

/product="N-isopropylammelide isopropyl amidohydrolase"

/translation="MDILLKQVRLNEDEELMDVGITDGKISAIEKEINEPATKIIEAN

GKVLVPGFVESHIHLDKALIASRKPNKSGTLQEAIKVTAELKPTFTKEDIYTRAKKAL

EMIIPRGVTAIRTHAEFDPAQGFTGFETIMQLKEEYKDLVDIQVVAFPQEGIFKAPGT

EKMMYEAMEMGADVVGGIPYNDSPANDHIDLIFKIAKKYNKPIDLHQDFSDEATDISI

EYLCHKTIEEGYQGRVSVGHLTALHALPKEQLDPIIALIAEAGISVMALPATDLHLGA

RNDAYNVRRAVTPIRKLRDGGVNMCIATNNIRNAFTPYGNGDILQTAMLAIPVGHLGG

ADDLPTVFPMITENPAKAIGLTNYGIGVGKQADLVLLDTLLKHDAIIDIPERVFVIKN

GKITVESHKETIIYR"

CDS complement(996571..996903)

/locus_tag="JFBMFIFI_00935"

/inference="ab initio prediction:Prodigal:002006"

/codon_start=1

/transl_table=11

/product="hypothetical protein"

/translation="MRKTIGIISIILSVFVGFQSMIAGLGNTLSQNNEIGGSTGFLLS

ILLLIAGITVLASKGAKGMLIFSMLLYAIGGLLGFVGAGSYSDLVIWSVISLLFAVLL

LLQIRKIH"

CDS complement(996917..997420)

/locus_tag="JFBMFIFI_00936"

/inference="ab initio prediction:Prodigal:002006"

/codon_start=1

/transl_table=11

/product="hypothetical protein"

/translation="MKNVKKIGLGLFIMSLAGVALGCSSDTSTKKAETKKEDKTEEVK
KDQATDSGDLGDFHVAIQDFSRGQDFEGSEVGIVQYEFTNNSEDNQMFTVAISPKVFQ
NGVALDSAVMTEDGFSDGLTEIQPGAKITIKTAYKLADTENPVSVEVSPTFSLKDEKL
TKEFTLQ"

CDS complement(997563..998759)

/gene="pknD"

/locus_tag="JFBMFIFI_00937"

/EC_number="2.7.11.1"

/inference="ab initio prediction:Prodigal:002006"

/inference="protein motif:HAMAP:MF_01957"

/codon_start=1

/transl_table=11

/product="Serine/threonine-protein kinase PknD"

/translation="MNLEKGHYKEIEALTEKENGPVLVRNIQTKEFFVRKKYALTLQN
RLEKLSLITHPHLPKIEEIVTSNNELWLYEEFIHGKNLQEYIQLNQQLPTETTLEIAK
SVTEALVVLHEKNLIHRDIKLTNIMISNDGVIKLIDFDAIRFYDGTKDTDTVNLGTLG
FAAPEQFGFAETDERSDIYSLGVVLNVCLTKNYPKDQLTTNVYLKDIVQKSIKLDPSN
RFQSAKDMQAAIHQQLTLLKIEKNHPKQNPKVSVEKKRLVPKTPIQKTVTKEQSNFIR
HYVPGFRTNQLWKKVIACFGYLCIFLLYSIMLSRGTPLDRFLEGITYTVLFILPFIIL
TNFLSIKKWIPFIHSNKLWAVCCGWLLVGICWFVLSGITIQTFENLYSPEFIEQRELN
STDSKT"

CDS 998923..999351

/locus_tag="JFBMFIFI_00938"

/inference="ab initio prediction:Prodigal:002006"

/codon_start=1

/transl_table=11

/product="hypothetical protein"

/translation="MEKDTDKLMHSLKKATDFKTLFSQHQENLIEEELKLYLTELLSK
KNVTKAEVMRKSGISEATGYQYFDGKRKPSREKLIALAIGFGMDVDETNDLLKKTGYA
KLYPKHKWDAIVIFGMSHSQSLIEIDELLFDAQLKTFSEG"

CDS 999767..1000306

/gene="bglK_2"

/locus_tag="JFBMFIFI_00939"

/EC_number="2.7.1.85"

/inference="ab initio prediction:Prodigal:002006"

/inference="similar to AA sequence:UniProtKB:Q93LQ8"

/codon_start=1

/transl_table=11

/product="Beta-glucoside kinase"

/db_xref="COG:COG1940"

/translation="MERTHEKYYLSLDVGGTFIKHALIDKSGHIICLAKVTTPINLAG

FLETIKQIVQSYLPTIRAVCFSCPGKIDTKSGTVYFGGALLYLDKFSIKTYIETEFSI

PCTIINDGKAAALAELWRGQLEGIANGGVITLGTGVGGGLVINGNLLEGTNFQAGEIS

YMLMSRDSPIALSEIAGSM"

CDS 1000415..1000702

/locus_tag="JFBMFIFI_00940"

/inference="ab initio prediction:Prodigal:002006"

/codon_start=1

/transl_table=11

/product="hypothetical protein"

/translation="MFEEYCRNIAIIISNIQTVVDLERVAIGGGISEQPLLIEEICRQ

YRLMRETSKGILEMLHPISIVACEFRNEAGLMGALYHLLIQIEEKQLDYHS"

CDS 1001133..1002239

/gene="nlhH"

/locus_tag="JFBMFIFI_00941"

/EC_number="3.1.1.1"

/inference="ab initio prediction:Prodigal:002006"

/inference="similar to AA sequence:UniProtKB:P9WK87"

/codon_start=1

/transl_table=11

/product="Carboxylesterase NlhH"

/db_xref="COG:COG0657"

/translation="MPRKYDKKLLTDIIKKQKNEVVDDVNILFKPIPDCDIKGAMDPR

LYKEMKNMALLTKFMPKRMMNMKMDMKSLPKLRKMFNSVDSTPIIEEGVKVIPDFVTA

ADGFKIPIYCFKQDKPVVDAPVLYFIHGGGFFGGSTDVVTDALKLIVANTNIVAFSID

YRLAPEHPYPTGHEDCYNGLKWIHENASRFGGDPNNIFVAGDSAGGNLTLYCTNKSLE

DKLNLVKGQLILYPTVNMGGIDDEHVRFSKDKFDIYKKQARAINLSLDMLSGATDGLG
VYLGTQDLLNKYLTPYMDVSSEMPPTFVTVGEHDFLKIETLAYARKLIMQGVDTTTIV
YKGLGHAYIDRIGFLPQSEDCAIEMGNFILKHRS"

CDS 1002245..1003072

/locus_tag="JFBMFIFI_00942"

/inference="ab initio prediction:Prodigal:002006"

/codon_start=1

/transl_table=11

/product="hypothetical protein"

/translation="MKKAKITFFVIIMTLLLICTFYDLEISKALFNTSSHYGQFFEAI
GELPMTYIGCFSAAALVVTTKKRTNWSFYVGKIGFSSLMLLFSFFSVMMSGMHISIPL
WLKLVLTLVTCLFFYLLARAVPTSQHMNLRKAAKVGLLLAISAILLITILKMLWGRMR
FREMTDPANQFTRWYLPQSLTTNNEFMSFPSGHAANATVVLWISLLPTFVKSLVGKKR
LLEVTAIVWIVLVMISRIVMGAHFATDVIVGMLISMMLFFFLKYKFMSQNSENTMLL"

CDS 1003162..1004079

/gene="rhaS_3"

/locus_tag="JFBMFIFI_00943"

/inference="ab initio prediction:Prodigal:002006"

/inference="protein motif:HAMAP:MF_01534"

/codon_start=1

/transl_table=11

/product="HTH-type transcriptional activator RhaS"

/translation="MSRTTYESVQMDENLPMRILHFSHDRPLLLPDGRSYQFDAATLQ
FVPPHWHRSIELTYVVSGTIHVRQNDKEQIYENESFFVVNSGEIHELSSVPTDNFELI
CFIISYDFVQQFIPDVEKIRFDMGTTNNGYQMLATLFREIMSLYYESQPYGHLQIQAR
LIEIIYHLCRYYQSEEAEPTSQKYRRSQTLNKHILEYIHKHYMDNLTLEQMAKTFSFS
REHFSRLFKETFGKTFLNYLNDYRLYCAFPDIVNSRKTIETISMLHGFPSSKALIKQF
KETYHETPIQYRKNRVVTILDHNTDKPKH"

CDS 1004210..1004719

/locus_tag="JFBMFIFI_00944"

/inference="ab initio prediction:Prodigal:002006"

/codon_start=1

/transl_table=11

/product="hypothetical protein"

/translation="MSMKKYVFKKIVTDFVIINIIINGIFYIFNFRKFSGQLTFNDIA

TDLFVGLLLLGGACSLFGFVNMRKELLKGKIDVSEFKTSQLYNKLPKATVLRVLLLTL

MTAIITMTFFILVPKIVGIDKINHFIGFSFKIFTAGLMAAVIGYIVVDLSISDYQHSH

ITMKDTTVV"

CDS 1005034..1005642

/gene="thiT"

/locus_tag="JFBMFIFI_00945"

/inference="ab initio prediction:Prodigal:002006"

/inference="similar to AA sequence:UniProtKB:Q037U3"

/codon_start=1

/transl_table=11

/product="Thiamine transporter ThiT"

/translation="MSKNLGVWIEGTIIAALAMALSFIPIDIGSSFSISLGMIPMTLY

CFRRGFVPGIVASFLWGVLHFVVGKVYFLSVPQVLIEYLFAFLFIGFAGLFAKNVQLA

IQNNSRKDWTKWIIIGTLVGTVARYFWHFVAGVVFWGEFAFGGMSPVVFSLVMNGASG

LATAIATIMVLSILAVKAPALFVPKDSRFLLKGARSTFLNSK"

CDS 1005769..1007217

/gene="clsA_1"

/locus_tag="JFBMFIFI_00946"

/EC_number="2.7.8.-"

/inference="ab initio prediction:Prodigal:002006"

/inference="similar to AA sequence:UniProtKB:P71040"

/codon_start=1

/transl_table=11

/product="Major cardiolipin synthase ClsA"

/db_xref="COG:COG1502"

/translation="MSTFMLVMQAIFIINTVFAILTVFREKRDIAATWAWLLVLVLLP

IVGFIAYLFIGKKISREKIFDIKTQESMGMKELVMAQKEMLLEDELLSTTQATENAKE

MASLFLESDESIVTKGNKIKLFIDGHKKFDSLVADIQNAEHHIHMLYYTIHQDELGRR

VLAALEERAAAGVEVLVIYDAMGSRSTKHSFFKRLESLGGKAEPFFGSHWAIINLRLN

YRNHRKIVVIDGKIGYVGGFNVGDEYLGKDKKFGYWRDTHLRIEGNAVLALQSRFAMD

WNAAVDKHKIEYKEEYFPIIHNKGKSNMQIVSSGPDSEQQQIKKGYIKMISMAKKSIY

IQSPYFIPDDSVLDAISIAAMSGIDVRIMIPNKPDHPFVYRATTYYAGEMVAAGAKVY
IYDNGFLHAKTMVIDGEIASVGTANLDFRSFKLNFEVNAFVYDPVVAMELKAIYEKDM
EECYLLTKEILANQSRWMKFKQEFSRLLSPIL"

CDS 1007486..1008682

/gene="opuCA"

/locus_tag="JFBMFIFI_00947"

/EC_number="7.6.2.9"

/inference="ab initio prediction:Prodigal:002006"

/inference="similar to AA sequence:UniProtKB:Q9KHT9"

/codon_start=1

/transl_table=11

/product="Carnitine transport ATP-binding protein OpuCA"

/db_xref="COG:COG1125"

/translation="MKDLLEFKNVSKVYKGGKKAVDDVSLSFEKGEFIAFIGTSGSGK
TTTMRMINRMIEPTSGQILINGEDIAKKDPVELRREIGYVIQQIGLMPHMTIRENIVM
VPKLLKWSDEKRKEIAERLIKLVDLPDEFLDRYPSELSGGQQQRIGVVRALAADQDII
LMDEPFGALDPITREALQDLVKELQQEMGRTIIFVTHDMDEALKLADRIVIMREGRVI
QFDTPDNILREPADKFVEEFIGHERLIQARPNIQTVEQVMLKTPVSITPGKSLTDAIR
LMREKRVDTLLVTDDAGVLKGYIDIESIDYNHKTATSVGDIMNKNAFFVRKDSLLRDT
VRRILKRGLKYVPVVDEKGLLVGIVTRASLVDVVYDTIWGDEEVEDTPKVESETELEA
VTESNE"

CDS 1008698..1009333

/gene="opuCB"

/locus_tag="JFBMFIFI_00948"

/inference="ab initio prediction:Prodigal:002006"

/inference="similar to AA sequence:UniProtKB:Q9KHT8"

/codon_start=1

/transl_table=11

/product="Carnitine transport permease protein OpuCB"

/db_xref="COG:COG1174"

/translation="MMNFFTTYGSDLLLKTGEHIYISAVALGLGILVAVPLGVLLTRT
NKIASVVIGIASVLQTVPSLALLALMIPFLGIGKTPAIVALFIYSLLPILRNTYLGVK
GVDPNLKDAAKGMGMTNIQSIMMVELPLAAPVIMAGIRLGAVYVIAWATLASYIGAGG

LGDFIFNGLNLYKPELIIGGTIPVTILALLADFLLGKVEKWFTPLALRTNE"

CDS 1009346..1010278

/gene="opuCC_1"

/locus_tag="JFBMFIFI_00949"

/inference="ab initio prediction:Prodigal:002006"

/inference="similar to AA sequence:UniProtKB:O32243"

/codon_start=1

/transl_table=11

/product="Glycine betaine/carnitine/choline-binding

protein OpuCC"

/db_xref="COG:COG1732"

/translation="MNTMKKIKNIVALGLVALVLSSCALPGLGGGLNKEAIKVTGGVT

TETQILAGIVKEMIEHYTDQKVEVINNLGSTTINHQAMINGDANISAARYTGTDLTSI

LQLPAEKDPKKALEIVQQEFKKRYNQNYYPSYGFANTYAFMVTKETAEKYNLKKISDL

KAVADQLEAGVDTSWLEKEGDGYKAFKKDYGFDFKRVYPMQIGLVYDALAAGKMDAVL

GYSTDGRIASYDLVVLEDDLRFFPPYDASPVASKEILEAYPEIDKVLKKLEGTISTET

MQKLNYKADNDLIEPEVVAKEFLEEHNYFEEGGK"

CDS 1010281..1010940

/gene="opuCD"

/locus_tag="JFBMFIFI_00950"

/inference="ab initio prediction:Prodigal:002006"

/inference="similar to AA sequence:UniProtKB:O34742"

/codon_start=1

/transl_table=11

/product="Glycine betaine/carnitine/choline transport

system permease protein OpuCD"

/db_xref="COG:COG1174"

/translation="MEKMNMLQQLIYYFQQNGAYVFSQFMRHFLISIYGVLFAAIIGI

PLGIWIARHYKMSGWVIGMANVIQTVPSLAMLSILMLGLGLGVNTVIVTVFLYSLLPI

IKNTYTGIRSVDHNILDSGKGMGMTKFQILYMVELPLSMSVIMAGIRNALVVAIGITA

IGSFIGAGGLGDIIIRGTNATDGTAIILAGALPTAAMAIISDLFLGFLERKLDPVRSS

K"

CDS complement(1011000..1011329)

/locus_tag="JFBMFIFI_00951"

/inference="ab initio prediction:Prodigal:002006"

/codon_start=1

/transl_table=11

/product="hypothetical protein"

/translation="MENYFAEKQTRAFNFLQKKLLVGNTKPSLKILPQMPENQIRFFL

NQAYEQRKAIIIQINKTKHILNVNEQIGLLRYSPLDQNRAILESRKDNTVHMIALQDI

RYIRLAD"

CDS 1011607..1012476

/locus_tag="JFBMFIFI_00952"

/inference="ab initio prediction:Prodigal:002006"

/inference="similar to AA sequence:UniProtKB:Q8NWR0"

/codon_start=1

/transl_table=11

/product="DegV domain-containing protein"

/translation="MSYQIFTDSCSDLPLEFVEKHKIAIISMIISIDGKEYSDDLGKT

FNRNQFFQQLKEGKQATTSQINIGTYYEAFKPFVEKSEPILYLAFSSGLSGSYNNAVS

AVQMLEDKFGTVDITVIDTKAACLGEGLLVYQAALMKEQGKTLTEVAQWVENHKMNLH

SWVTVDDLKHLERGGRISSVAATMGSLLSVKPIIVVTPEGTLEPVAKVRGRKKSLHYL

VDKTVEGLRDPENQTIIVGHVGVPEEAEEVKKELLEKVKVKEILVYPYGPTIAAHTGF

GSMAVFSFGEVRV"

CDS 1012623..1013993

/locus_tag="JFBMFIFI_00953"

/inference="ab initio prediction:Prodigal:002006"

/codon_start=1

/transl_table=11

/product="hypothetical protein"

/translation="MIESDMQQKQNPDKKTALVLGGGGARGSYQIGVWQGLLELGIDF

QIVTGTSVGALNGGLIVQGDYQAAKEMWQAIDTSHVLEFESPVDATSFKGYRKTVGTF

VFNALRQKGISALPLKNSIIDRLLDEDWMRNRGIEFGIVLTEFPAMRQVSVFLDEIPQ

GLVSLYLLGSASFFPAMQPTKIGTKSYVDGGYHDNIPIDLAISKGATNLIVVDVKGPG

ITKAIKLPAEATVHQIVSKWPLGAVLLFDGARSEFNIRLGYLETLKSYGKLAGSWYSF

KNEAIVNHEKLFYQKFVRLLNDPQDFFIAAFYQNEKNQELLLKRIRGAWRGRVGKKEL

SYAIHELTGKLFQLNPTKIYSFSEFNQAILEKYEAARKAIETASLAATFEIDMIYSGD

EWLDNYAQMIPFISNLKMVLYFVDLLEAKQGRLMVSKNLQWLIYRKPVAFMMATYIYQ

LKKMIK"

CDS 1014106..1014330

/locus_tag="JFBMFIFI_00954"

/inference="ab initio prediction:Prodigal:002006"

/codon_start=1

/transl_table=11

/product="hypothetical protein"

/translation="MAKEFSFEIMEEIAVLSENAKGWKKELNLVSWNGNQPKFDLRDW

DATHEKMGKGLTLSNDEVKALKIALENVSL"

CDS 1014346..1015230

/locus_tag="JFBMFIFI_00955"

/inference="ab initio prediction:Prodigal:002006"

/inference="similar to AA sequence:UniProtKB:P23839"

/note="UPF0701 protein YicC"

/codon_start=1

/transl_table=11

/product="hypothetical protein"

/translation="MKSMTGFGRASTSQEMVQVDIEMKSVNHRFLEVVSRIPKELNFM

ELPLKKQVSAQLKRGRVEIFLNLKKSADSQKKLVINWSLVDEAVAFATEASRKYDQIP

GLDFKSDLMELIASSDFLTVEEKEVGEEIIEPLVREATEKALKELVASRLSEGLRLKE

HVLNHLEQFQLAIHQITDLSELFEEHYRTRLEDRLQEVVGNFIDEPRLLTELAIMLEK

ADIHEELERLDSHIVHFRQLVEKPEPVGRELDFLIQEMNREVNTIGSKANQLKLSDTV

ILMKTQLEKIREQVQNIE"

CDS 1015261..1015875

/gene="gmk"

/locus_tag="JFBMFIFI_00956"

/EC_number="2.7.4.8"

/inference="ab initio prediction:Prodigal:002006"

/inference="similar to AA sequence:UniProtKB:Q8Y672"

/codon_start=1

/transl_table=11

/product="Guanylate kinase"

/db_xref="COG:COG0194"

/translation="MTDRGLLIVLSGPSGVGKGTVRQAIFENDGTNFDYSISMTTRKK

RVGETDGVDYFFRTKEEFETLIENGGLLEYAEYVGNYYGTPLAYVEETLASGKDVFLE

IEVQGALQVREKMPEGVFIFLTPPDLVELKSRIIGRGTDEMAVIEERMEKAIEEIEMM

RLYDYAVVNDQVENAVRNVKQIIESEHLRVSRVIHRYKKMIEEL"

CDS 1015881..1016096

/gene="rpoZ"

/locus_tag="JFBMFIFI_00957"

/EC_number="2.7.7.6"

/inference="ab initio prediction:Prodigal:002006"

/inference="similar to AA sequence:UniProtKB:Q5XAP2"

/codon_start=1

/transl_table=11

/product="DNA-directed RNA polymerase subunit omega"

/translation="MMLEPSIDNLLEKIDSKYSLVIIASKRAHQLHAKEMHLLESYDS

YKNVGKALEEINAGDLVIDPTTVGPEE"

CDS 1016385..1017587

/gene="coaBC"

/locus_tag="JFBMFIFI_00958"

/inference="ab initio prediction:Prodigal:002006"

/inference="similar to AA sequence:UniProtKB:P0ABQ0"

/codon_start=1

/transl_table=11

/product="Coenzyme A biosynthesis bifunctional protein

CoaBC"

/db_xref="COG:COG0452"

/translation="MLKGKKIALYVTGGIAIYKVCDLVRKLIKSGAEVKVAMTASATK

FVTPLTFQVLSKNDVYLDTFDEKVSQEVAHIHLADWSDMAVIAPATANSIAKLANGIA

DDFVSTALLATTAPVYVVPAMNQHMLENPATVRNLKTITADGRYIIEPATGFLAEGYE

GRGRMPEPADILNEIELSLLKQQKNLPLSGKKIVVTAGGTKERIDPVRFITNDSSGKM

GYSLAIAARDLGAEVHLISATTLIEAPKGVRVVNVQTAADMEKAVLAEFDAADIVIMA

AAVSDYKPAIEIKEKIKKDEDKLILNLTKTTDILAELGQKKTHQFLIGFAAETNNIEE

YALGKLKKKKANLIVANDVSKPDAGFNKETNAVTIYSEVSEPIEISVRSKLEIAKEIL
NVALSQIS"

CDS 1017623..1020037

/gene="priA"

/locus_tag="JFBMFIFI_00959"

/EC_number="3.6.4.-"

/inference="ab initio prediction:Prodigal:002006"

/inference="protein motif:HAMAP:MF_00983"

/codon_start=1

/transl_table=11

/product="primosomal protein N'"

/translation="MVSIAKVIVDVPTMQTNQPYDYGIPPEFVETLMKGMRVEVPFGR
GGRRVQGFVVDIVHSTDYTGELKYIASLMDLNPVLNEEMLALGKEMAETTFAFQITCL
QTMLPSVMRAKYEKKIRLIDEVSESIFTTIFKGRNEISWEEAEERELLPKLMELKKHE
TVEVVYEVKDKARTKKKRMIKRLLSFEQLEEAKIGLGKRAPKQQILISYLQAMENDVR
SVSEVTKNTTITAANLKEAETKGWLTFFEEETYRDPFENHEFKQTEAFTLNASQTAAI
TPILAAAKENRAETFLLKGITGSGKTEVYLQTIAETLKDGKSALMLVPEIALTPQMVN
HFKGRFGNEVAVLHSGLSVGEKYDEWRKIQRGEAKVVVGARSSIFAPVTNIGVIIIDE
EHEGSYKQDESPRYHARDMALWRSLYHQCPVILGSATPSLESRARAQKKVYTLLELTE
RANRKALPEVEVVDMRDELKKQNRSSFSLVLQEKIRERIAKKEQIVLLLNRRGYSSFV
MCRDCGFVLPCPNCDISLTLHMDTKTMKCHYCGHEEAIPHTCPSCKGHKIRYYGTGTQ
KIEEELHALIPEAKIIRMDVDTTRKKGAHEKLLTAFGNGEADILLGTQMIAKGLDFPN
ITLVGVLNADTALGLPDFRASERTFQLLTQVSGRAGRAELTGEVIVQTYNPDHYAIVL
AQQHDYDHFYQHEMLLRRRGSYPPYYYTILLTTSHEEELQAAKKMQEIVQFIRPEMQP
ETIILGPTPKAVARVNNRYYYQTIIKYKHEPLLFSKLHELLINSQKEMAKGLQVTIDS
EPMHFI"

CDS 1020068..1020562

/gene="def1"

/locus_tag="JFBMFIFI_00960"

/EC_number="3.5.1.88"

/inference="ab initio prediction:Prodigal:002006"

/inference="similar to AA sequence:UniProtKB:Q819U0"

/codon_start=1

/transl_table=11

/product="Peptide deformylase 1"

/db_xref="COG:COG0242"

/translation="MALLPIVQYPDDILETPASEVTEITDETLQLLADMYQTMVENDG

IGIAAPQVNHSLRIAIVELDEESGLFEMINPEIISQEGSSIDVEGCLSFPGVYGTVDR

ADYIVVRFTDRFGDEYEVEADGYLARAFQHEIEHLDGKLFTDKIIEHIDPQDLESYME

EHGE"

CDS 1020559..1021512

/gene="fmt"

/locus_tag="JFBMFIFI_00961"

/EC_number="2.1.2.9"

/inference="ab initio prediction:Prodigal:002006"

/inference="similar to AA sequence:UniProtKB:Q81WH2"

/codon_start=1

/transl_table=11

/product="Methionyl-tRNA formyltransferase"

/db_xref="COG:COG0223"

/translation="MTKIIFMGTPAFSVPILDELVRAGYDIQAVVTQPDRPVGRKKTL

TPTPVKVAALKHNIPVLQPEKITGSKEMTEIEALAPDLIVTAAFGQFLPQKLLDVPKL

GAINVHASLLPKYRGGAPVHYALMNGDSETGVTIMYMEKKMDAGDILSQRKLAITEKD

DVGTLFERLSILGRDLLMDTLPELLAGKIVPVKQDEALVTYSPNISREEERINWDKTA

QQIDYQVRGMRPWPVAYTLLAGNRLKLWDVTPITEETTTEAPGTIIQLGKEAIWVACG

DQTILQLNKIQPAGKGQLTAGEFLRGIGSQLTVGEMVGQDV"

CDS 1021505..1022893

/gene="rsmB"

/locus_tag="JFBMFIFI_00962"

/EC_number="2.1.1.176"

/inference="ab initio prediction:Prodigal:002006"

/inference="similar to AA sequence:UniProtKB:P36929"

/codon_start=1

/transl_table=11

/product="Ribosomal RNA small subunit methyltransferase B"

/db_xref="COG:COG0144"

/translation="MSKEQTEPKKEVQKRNTKKTSRYLAMEILDKTEKNGAYSNLLLN
ESIQKNNLSSADAGLLTELVYGVLQRRLTLDFYLADFLDESKKIDSWVRNLLRLSIYQ
MIYLDKIPQHAILFEAAEIAKKKGHVGISKFVNGVLRNAERRGFKDLTDIKDDAERLS
LEISMPLWLVEKFINQIGYSETKALGESLLIPSRASARVNQRYLTVEEALYALEEEGF
AVRRSEITLDAVISDGGHFASSPLFTSGQLTIQDETSMLVAPALQIEPHHQVLDACAA
PGGKTTHIASFLSHEAGGKVVALDLHKHKVKLVKENAARLHVDDVVEGVVLDARKVDE
VFPDESFDRILVDAPCSGLGLMRRKPDIKYTKKERDLLNLQKIQLEILESVAPKLKKY
GIMVYSTCTITEEENEKTVEQFLALHPEFEKTTVLVGETLQPCLKNEFIQIYPHDFET
DGFFISCLKKIN"

CDS 1022924..1023667

/gene="stp"

/locus_tag="JFBMFIFI_00963"

/EC_number="3.1.3.16"

/inference="ab initio prediction:Prodigal:002006"

/inference="similar to AA sequence:UniProtKB:Q8Y678"

/codon_start=1

/transl_table=11

/product="Serine/threonine phosphatase stp"

/db_xref="COG:COG0631"

/translation="MQVVFQSDIGKTRKNNQDFAGQFDNQAGLRLVVVCDGMGGHKAG
DVASEMAVSHLGHAWEETNYVTAEEVTQWMLKQITLENERIVGKSTQFSDLDGMGTTL
VAAVLFEEELVIANIGDSRGYLYSNKKLVQLTEDHSLVNELLKSGEITSEAAANHPRK
NVLTRSLGVSSEIDIDITIFSILKDDILLLCSDGLTNMVEDSAISEILSQDISIEEKV
NALVSLANEKGGADNITVLLADFAAREES"

CDS 1023673..1025697

/gene="prkC"

/locus_tag="JFBMFIFI_00964"

/EC_number="2.7.11.1"

/inference="ab initio prediction:Prodigal:002006"

/inference="similar to AA sequence:UniProtKB:O34507"

/codon_start=1

/transl_table=11

/product="Serine/threonine-protein kinase PrkC"

/db_xref="COG:COG0515"
/translation="MEIGKKLSGRYKIIGTVGGGGMANVYLAHDLILDRDVAVKVLRY
DFREDKDIIRRFQREALSATELVHPNIVSVYDVGEEDNFQYIVMEYVKGTDLKKYIGN
HFPIPYQKVIDIMEQILSAVADAHRNRIIHRDLKPQNVLIDETGLVKITDFGIAVALS
ETSITQTNSLLGSVHYLSPEQARGSMATKQSDIYALGIILYELLTGNVPFEGESAVSI
ALKHFQETVPSVKDFDGRIPQPLENVVLKATAKEASDRYQSAEEMATDIATALSPQRA
GEPKFEPASMMEETKVLTPIPGDLLETESVEAEEVKEEDEQKPVKDQKKKSKKKKIIL
FSIIGVILLFVIGFFAIAMSGPKDVKIPDLTGLSEAEAKSELINAKLKVGDVTEEANP
EVEEGQVIRSDPKTDRTVKENTKVNLFISTGKATEKFGNFVGEDFQVVKAKLTNLKYD
VKTPIEVFSPSVPKGQIIEQSIEKGQEVVPSETQVVFTISKGEEGFIMPDMLNYGKKD
VDDIASAQGLKVTYNSDYSDTVTAGLVMAQTPGAGETVFRNDSITVTLSKGPKEIPVV
SVTKKITLEYLAKTINKPASTTDSSNQSANSSSSEASISSVSSITEPVANKFEVYIED
EENNFNTPVVTKSILATESIDLTFKIKEGGVARYKVVRDGETIINEEVRP"

CDS 1025859..1026755
/gene="rsgA"
/locus_tag="JFBMFIFI_00965"
/EC_number="3.6.1.-"
/inference="ab initio prediction:Prodigal:002006"
/inference="similar to AA sequence:UniProtKB:O34530"
/codon_start=1
/transl_table=11
/product="Small ribosomal subunit biogenesis GTPase RsgA"
/db_xref="COG:COG1162"
/translation="MPRGQIRKALSGFYYVYCEGETYQTRGRGNFRKRNLTPLVGDEV
IFESGNQDEGILKELLPRKNELVRPTVANVDLGVIVMSAVEPNFSSNLLDRFLVTLES
KKIKALIYLTKIDLLDQEQYQEMEQIKVAYEKIGYPVILPKREMDHEPIEELTPYFKE
KITVFMGQSGAGKSTLLNQIAPDLALKTGVISNALGRGKHTTRHVELLPLYGGLVADT
PGFSSIEFLEVEADELPELFPDFVEVQHECRFRGCKHREEPGCQVKKDVEEGTILASR
YKHYLQFLEEVENRKPKYGKKN"

CDS 1026775..1027425
/gene="rpe"
/locus_tag="JFBMFIFI_00966"
/EC_number="5.1.3.1"

/inference="ab initio prediction:Prodigal:002006"

/inference="similar to AA sequence:UniProtKB:P74061"

/codon_start=1

/transl_table=11

/product="Ribulose-phosphate 3-epimerase"

/translation="MKIAPSILSADFANLERDVRLVESGGADYIHVDVMDGHFVPNIT

LGANIVSAIRPVTKLPLDCHLMIENPENYIEDFAKAGADIITVHVESTPHIHRAIQMI

KAAGVKAGVVLNPGTPVEAVKYVLAECDLVLVMTVNPGFGGQSFIEETLEKITELSAL

KEIKNYHYEIEVDGGIVPETAAKCKLAGADVFVAGSYVYNAENPLEQIQNLKDALK"

CDS 1027426..1028076

/gene="thiN"

/locus_tag="JFBMFIFI_00967"

/EC_number="2.7.6.2"

/inference="ab initio prediction:Prodigal:002006"

/inference="similar to AA sequence:UniProtKB:O34664"

/codon_start=1

/transl_table=11

/product="Thiamine pyrophosphokinase"

/db_xref="COG:COG1564"

/translation="MTTRVALLVGGPENMIPDFMQLENQETKWIGVDRGALRLIEKGI

VPRMAVGDFDSISTADLAVLKERIADVRISPAEKDETDTEMAVALALNELKADEVVIY

GGTAGRLDHFLANLWMVLQPRFIKHAAKIKMVDCQNSLSFYLPGSYELIKEADKRYLA

FVCLTPITKLSLVDQKYRLDEADFLYPTSLASNEFIGETGHFSFTSGVIAVIQSTD"

CDS complement(1028180..1028368)

/gene="rpmB"

/locus_tag="JFBMFIFI_00968"

/inference="ab initio prediction:Prodigal:002006"

/inference="similar to AA sequence:UniProtKB:P37807"

/codon_start=1

/transl_table=11

/product="50S ribosomal protein L28"

/db_xref="COG:COG0227"

/translation="MAKECVITGRKSKSGNRRSHAMNANKRTWKANLQKVRILVDGKP

KKVWVSARALKSGKVERV"

CDS 1028721..1029083

/locus_tag="JFBMFIFI_00969"

/inference="ab initio prediction:Prodigal:002006"

/inference="similar to AA sequence:UniProtKB:Q5XA14"

/codon_start=1

/transl_table=11

/product="hypothetical protein"

/translation="MAVKIKTQFGTIDITNDVIATVVGGAATDIYGIVGMASKNQIRD

NLNDILKKENYSRGAVVRQEENGVAVDVFIIVSYGTKISEVCRNVQEKVKYNLETMLG

VSANTVNVYVQGVRVMQD"

CDS 1029142..1030809

/locus_tag="JFBMFIFI_00970"

/inference="ab initio prediction:Prodigal:002006"

/inference="similar to AA sequence:UniProtKB:Q7A5Z4"

/codon_start=1

/transl_table=11

/product="hypothetical protein"

/translation="MEVTKLEGNQFQAMVAIGAKRLTKNAEFVNSLNVFPVPDGDTGT

NMNLSMTSGAKAVNSSTSDSVGELANALSKGLLMGARGNSGVILSQLFRGFSKSIVGK

ETLTAKEFAAAFTSGVETAYKAVMKPVEGTILTVARESAKAGEKKAKDTDDLVVVMEA

VVRGAKKSLAKTPDLLPVLKEVGVVDSGGQGLLFVYEGFLEALSGNIVEEDYQQPNEK

QMTEMVNAEHHRSVSNHIATEDIKYGYCTEIMVHIGDGETVDSEFDYDTFRNHLNDIG

DSLLVVADDEIIKVHVHTEHPGEVMNYGQKFGSLMKIKVDNMRLQHETILDHEKEAKP

VEKIPYGIIAIAAGEGVQELFKSLGAHYVISGGQTMNPSTEDILKAVAEVHAEKVIIL

PNNKNIFMAADQAAEVSDTPVVVVPSKTISQGMTAMLAFNSSNDLETNKAEMTAELDN

VVSGQVTIAVRDTAIDGVTIKKDDYMGIIEGKIKFSETSRKKVAVETLQAMISEDSEI

VTIILGEDGDMNEAKEVAETIEAEFEDVEVEIHEGNQPVYPYILSVE"

CDS 1030850..1031515

/gene="sdhB"

/locus_tag="JFBMFIFI_00971"

/EC_number="4.3.1.17"

/inference="ab initio prediction:Prodigal:002006"

/inference="similar to AA sequence:UniProtKB:P33074"

/codon_start=1

/transl_table=11

/product="L-serine dehydratase, beta chain"

/translation="MAQRYHSVFDIIGPVMVGPSSSHTAGAARIGKVVQKIFGATPAK

VDIFLYESFAKTYRGHGTDIALVGGLLGMDPADERLADSIEIAKELGIEINFIPTNEK

AEHPNSVKLVVSTPEKSLSVVGISIGGGKIQISEVNGFKIQLEENQPTFLIVHQDVPG

MIAQVSNILAEKAINIGTMTVSRESKGQQAIMMIEVDQYEVGETLAELKQVEHIKQAS

FFK"

CDS 1031716..1032588

/gene="sdhA"

/locus_tag="JFBMFIFI_00972"

/EC_number="4.3.1.17"

/inference="ab initio prediction:Prodigal:002006"

/inference="similar to AA sequence:UniProtKB:P33073"

/codon_start=1

/transl_table=11

/product="L-serine dehydratase, alpha chain"

/translation="MFQSVSELVTEANQVGSIAEVMIQLECQTTRMPRHEVIKKMEQQ

LSVMENAAKKGTAGVSSATGLTGGDATKLEAYLAKGNFLSGETILIAVQNAIATNEVN

AAMGLICATPTAGSAGVVPGVLFAAKDRLDLDRDAMVRFLFVAGAFGLVIANNASISG

AEGGCQAEIGSASAIASAALVDAAGGTPEMCSAAIAMVLKNMMGLICDPVAGLVEVPC

VKRNALGASQAMISADMALAGITSVIPTDEVIEAMYKVGRQMPSIFKETAEGGLADTP

TGREIKMRIFGQSV"

CDS 1032593..1034644

/gene="recG"

/locus_tag="JFBMFIFI_00973"

/EC_number="3.6.4.12"

/inference="ab initio prediction:Prodigal:002006"

/inference="similar to AA sequence:UniProtKB:P64325"

/codon_start=1

/transl_table=11

/product="ATP-dependent DNA helicase RecG"

/translation="MGKSIYDPIELLPQVGPKRLEALHQLGIYTILDILSHYPFRYED
IQVKDLTEIEDQEKVTLKGDVVSEAVLTRFGPKKNRLVFRLVIDHAVIAVSFFNQPYL
KSKIVAGEELAVFGKWDGKRKSLTGIKILGVATEQEASNFESIYNGNKGIKQKTIFQL
VEQAYERYQDVIPEIIPNYLREKYRLISHREAIYAMHFPTAEEQTIQARREVVFEEFL
VFQMKMQVLRKQEKASGRGTAILYEVADLRRFIQSLPFELTKAQKRVVNEICSDLRQS
LHMHRLLQGDVGSGKTIVAAIALFATVNAGFQGALMVPTGILAEQHMESLDQLFDPLE
VKVALLTGATKTKERREILEQLASGELDVIIGTHALIQEDVHFAKLGLVITDEQHRFG
VNQRKILREKGTHPDVLFMTATPIPRTLAITAYGEMDVSIIDELPAGRIPIETTWTRP
KNFEHTLSFIETQLHKGSQAYVICPLIEESESLDVKNATDIYEKLCLYYGPKNFTVGL
LHGKMKAAEKDAIMESFKNNEIQVLVSTTVIEVGVNVPNATTMVIYDADRFGLSQLHQ
LRGRVGRGSKESYCILVANPKNDTGIERMKIMTETTDGFVLSEKDLELRGPGDLFGNK
QSGLPDFKVGDIVGDFGALEAARKEAAALINQTDFFDNPNYAALRREVGLENMSALDF
D"

CDS 1034744..1035784

/gene="plsX"

/locus_tag="JFBMFIFI_00974"

/EC_number="2.3.1.274"

/inference="ab initio prediction:Prodigal:002006"

/inference="similar to AA sequence:UniProtKB:Q82ZE8"

/codon_start=1

/transl_table=11

/product="Phosphate acyltransferase"

/db_xref="COG:COG0416"

/translation="MRIAVDAMGGDNAPQAIVEGVMMAAKEFHDVEFILFGKEDAIRT
YLTDETNIKIVHTDEKINSDDDPVRAVRRKKNASMILAAQAVKNKEADALFSAGNTGA
LLTAGLLIIGRIKGIDRPGLMPTLPVVTGADQVFNLMDVGANADTKPENILQYAILAS
YYAKFVRGVENPSVGLLNNGTEENKGNDVTKRAYALLAAEKEINFIGNVEARELLNGV
ADVVVTDGFTGNAVLKTIEGTAMSMMKLLKNAVYDSGIKGKLGGALLKDSLSEMKDKL
DYSRYGGAVLFGVKAPVVKTHGSTEKESVYFTIRQIHEMLNSHVIDDLVTYFESKAEA
EAILVESKKETE"

CDS 1035913..1036155

/gene="acpP"

/locus_tag="JFBMFIFI_00975"

/inference="ab initio prediction:Prodigal:002006"

/inference="similar to AA sequence:UniProtKB:Q5HGK0"

/codon_start=1

/transl_table=11

/product="Acyl carrier protein"

/db_xref="COG:COG0236"

/translation="MSQNETFDKVAKIIVERFGVDEDKVTTELTFKDDLGADSLDIVE

LVMELEDVFGTEISDEDAEQITTVGDAVKYIDAHLN"

CDS 1036423..1037118

/gene="rnc"

/locus_tag="JFBMFIFI_00976"

/EC_number="3.1.26.3"

/inference="ab initio prediction:Prodigal:002006"

/inference="similar to AA sequence:UniProtKB:P51833"

/codon_start=1

/transl_table=11

/product="Ribonuclease 3"

/db_xref="COG:COG0571"

/translation="MNELFLKELKDKFGISFRNLSYLEEAFTHSSYVNEHRDCHIQDN

ERIEFLGDAVLELTVSRYLFDHYPDLPEGKLTRLRATIVCEASLSQFAKEYGFDTYIR

LGRGEERMNGRNRPALLCDLFESFIGALYLDQGIEAVLDFLRQTIFPKIATGAFSHVM

DHKTNLQEWLQQNGEIVIEYNLLDEVGPAHEKEFIVEVSAEGKVLGKGQGKTKKAAEQ

AAAENALFIIKTK"

CDS 1037143..1040715

/gene="smc_1"

/locus_tag="JFBMFIFI_00977"

/inference="ab initio prediction:Prodigal:002006"

/inference="similar to AA sequence:UniProtKB:P51834"

/codon_start=1

/transl_table=11

/product="Chromosome partition protein Smc"

/db_xref="COG:COG1196"

/translation="MYLKKIEIAGFKSFADKTTIEFVNGVTAVVGPNGSGKSNITEAI

RWVLGEQSAKSLRGGRMNDVIFAGSESRKQLNLAEVTLVLENEDHFLPLDFSEISITR
RLHRNGDSEFYLNKQSCRLKDIVDLFMDSGLGKESFSIISQGKVEAIFNSKPEDRRSI
FEEAAGVLKYKTRKKKAEQKLFETEDNLNRVQDIVYELEDQIEPLREQSSIAKDYVMQ
KEQLSEVEIALTVVEVEMLKEKWLANKNQAETLATEISEARQELQTAETTVADLREKR
QKMDAQLDESQARLVELVKTYEQTEAQKKVLSERSKNTKENREQFEQSKAKLEVKIQE
LDQQLADLTKDLTEKQAHERELRGTLAAAEKEQKMFNQNSSVTVESLRDDYVDLMQKQ
TTLRNEQGYLEKTFLQASQKNMKSDATVRALESDSALAKAKVQEKQLELSTVQKNLAT
KLLGHQEIQADLQKNRYDLETNEQKMYEALRLMQQAKAKRDSLADLNEDYAGFYQGVK
EILKRKREIGGVVGAVAELIDVPKETEVAIDIALGAASQNVVVEDEESGRKGITYLKQ
KRLGRATFLPLTTIKPRYLPSNVVQTVKGNEGYLGIASQLVRYPEKVATVIENLLGTT
IVARDLTSANQIARSIQFKYRVVTLEGDIMNAGGSMTGGASKKGNQGSIFARKNELSN
LNQQISSMEATLEAKEIDVRALKQQVKKQELELEELRVLGEKERLKEQQLKNDVELYE
EKEKRLTRELKAYTFEFQEAKEETEHYHQRTAEITAEMSEISTEMENINRQIDLISSE
NDKRTQLQASANHRVHEVTAQLAVAKEQVTSLVKEQKRLEDSLQVEKENLASLVEQLE
LMINSDGSHQLTKGDLEEKLAEIEVEKTAFEETILSMKEERYTLEERIDEFDRTVAAK
NNQQHILLEQRSKAEVAMNRHDVEIENRLGHLSEEYGLSFDFAKVNHVLELSLEDARQ
KVKLLKRGIEELGSVNLGAIDEFERVNERYSFLTEQRDDLLTAKASLFETMDEMDEEV
KKRFSEVFFAIRDKFSIVFPQMFGGGYAELRLTDPEDLLNTGIDIIAQPPGKKLQNLS
LLSGGERAFTAIALLFSIIQVRPVPFCILDEVEAALDEANVARFGRYLSQFEGDTQFI
VITHRKGTMEEADVLYGVTMQESGISKIVSVRLEEVEEKGLVKG"

CDS 1040890..1041024

/locus_tag="JFBMFIFI_00978"

/inference="ab initio prediction:Prodigal:002006"

/codon_start=1

/transl_table=11

/product="hypothetical protein"

/translation="MSQLLEKKMKIREDKKRPINFPYFLAGAKRLNKLFILGGTRQND
"

CDS 1041017..1041856

/gene="yidA_3"

/locus_tag="JFBMFIFI_00979"

/EC_number="3.1.3.23"

/inference="ab initio prediction:Prodigal:002006"

/inference="similar to AA sequence:UniProtKB:P0A8Y5"

/codon_start=1

/transl_table=11

/product="Sugar phosphatase YidA"

/db_xref="COG:COG0561"

/translation="MIKLIAIDLDGTLLNENKEITPETKEALTKAKAKGVKVVLCTGR

PLLGMAHFLEELNLKEEGDYGITYNGGLVQKTQTGEVLSQKTLTKAQIEELYQLTNEL

QVPLNFIDLVQVYAPPYPENRESLYGGIMKALPMVPTTMADLPEDAEFNKAVIAVDVA

ILDEAIAKIPVEFTEKYTMMKSRPVLLEVLNKEVDKGRGLAVLCDLLDIKPEEVMALG

DEENDLAMIQFAGLGVAMGNATEEVKNVAQFVTKSNHENGVAYAVEKFVLNEETFNSH

SNA"

CDS 1041902..1042897

/gene="ftsY"

/locus_tag="JFBMFIFI_00980"

/inference="ab initio prediction:Prodigal:002006"

/inference="similar to AA sequence:UniProtKB:P51835"

/codon_start=1

/transl_table=11

/product="Signal recognition particle receptor FtsY"

/db_xref="COG:COG0552"

/translation="MGFFDKIKKAFTGEEVEKTEVTEKYEKGLEKTRKSFSERMNELF

ANFRTVDEDFFDELEEVLIGSDVGFETTMEITDALRQEVKLRNAKNQNEVQEAIIEKL

VELYERGDTEIPEIKLNPTGLTVILFVGVNGVGKTTTIGKMAHQYQVAGKKVLLAAGD

TFRAGAIDQLKVWGERVNVEVVSGPAGGDPAAVVFDAVKQAKNQQADILMVDTAGRLQ

NKVNLMNELEKMKRVIDRELPDTPLEVLLVLDATTGQNAMLQAKQFKETTDVTGLVLT

KLDGTAKGGIVLAIRNELNIPVKYVGLGEGMDDLQVFDPNEYVFGLFSQLINKEN"

CDS 1043059..1043403

/locus_tag="JFBMFIFI_00981"

/inference="ab initio prediction:Prodigal:002006"

/inference="similar to AA sequence:UniProtKB:Q97QD1"

/note="UPF0122 protein SP_1288"

/codon_start=1

/transl_table=11

/product="hypothetical protein"

/translation="MELEKTNQMNALFEFYESLLTEKQQNYMQLYYADDFSLGEIAEE

FGVSRQAVYDNIKRTEHILKEYERKLHNVANFVESEAVIEELTNYVKENYPLDQTLIK

LIEALQTEKDNE"

CDS 1043420..1044844

/gene="ffh"

/locus_tag="JFBMFIFI_00982"

/inference="ab initio prediction:Prodigal:002006"

/inference="similar to AA sequence:UniProtKB:P37105"

/codon_start=1

/transl_table=11

/product="Signal recognition particle protein"

/db_xref="COG:COG0541"

/translation="MAFEGLSERLQAAMNKMRRKGKISEADVKEMMREVRLALLEADV

NFKVVKEFVKTVSDRAIGSEVLESLSPAQQIVKIVDEELTALMGGEQSTIQSAPKSPT

VIMMVGLQGAGKTTTAGKLANFLRKKQNRRPLLVAADIYRPAAIQQLETLGEQLDIPV

FSMGDQVSPVEIAKMGIEKAKEEHLDFVIIDTAGRLHIDETLMTELTDIKAVTNPTEI

FLVVDAMTGQDAVNVAESFNEQLDITGVILTKLDGDTRGGAALSIRSVTGKPIKFTGQ

GEKLEDLEPFYPDRMASRILGMGDMLTLIEKAQQDFDEKKAEEMTQKMQDNSFDFNDF

IEQMDQLNNMGPIEDILKMIPGMNNVPGLDKMQIDPKDTARMKAIVLSMTPQERENPD

LLSQSRRRRIARGSGRPIAEVNRMIKQFNESKKMMSQMSKGNFNGMDGMFGSGVGGKL

GKMAMNSMVRKNKKKLRKKNKKKK"

CDS 1045037..1045309

/gene="rpsP"

/locus_tag="JFBMFIFI_00983"

/inference="ab initio prediction:Prodigal:002006"

/inference="similar to AA sequence:UniProtKB:P66444"

/codon_start=1

/transl_table=11

/product="30S ribosomal protein S16"

/db_xref="COG:COG0228"

/translation="MAVKIRLKRMGSKKNPFYRMVVADSRSPRDGRYIEVVGTYNPVV

QPAAVKVEEEKVLGWLANGAQPSDTVRNILSKEGIMKKFHDSKNVK"

CDS 1045324..1045566

/locus_tag="JFBMFIFI_00984"

/inference="ab initio prediction:Prodigal:002006"

/inference="similar to AA sequence:UniProtKB:P9WFM7"

/note="UPF0109 protein Rv2908c"

/codon_start=1

/transl_table=11

/product="hypothetical protein"

/translation="MADVKELILTIVRPLVTHPDELGIVIEETNEYLDYQLSVHPDDI

GRVIGKQGRVAKAIRTIVYSVRVKGPKRVRLSIVDG"

CDS 1045726..1046241

/gene="rimM"

/locus_tag="JFBMFIFI_00985"

/inference="ab initio prediction:Prodigal:002006"

/inference="similar to AA sequence:UniProtKB:P66656"

/codon_start=1

/transl_table=11

/product="Ribosome maturation factor RimM"

/translation="MAELFNVGKIVNTQGLKGEVRVISRTDFPDERYKKGNKLLLEKE

GKPLIELVVASHRKHKNFDILSFKDHPSINDVEKYRDGMLQITSDQMHDLPENEFYLH

EIIGLKVIDEEGEEIGKIKEVLSPGANDVWVIQRVQQKDLLIPYIEEVVKKVDIAEGV

VTIHIMEGLLD"

CDS 1046241..1046990

/gene="trmD"

/locus_tag="JFBMFIFI_00986"

/EC_number="2.1.1.228"

/inference="ab initio prediction:Prodigal:002006"

/inference="similar to AA sequence:UniProtKB:Q6GHJ5"

/codon_start=1

/transl_table=11

/product="tRNA (guanine-N(1)-)-methyltransferase"

/translation="MRIDVLTLFPRMFDGPMKESIIGKAIEKKLLEFNAINFREFSTH

RHQNVDDYPYGGGAGMLLMAQPIFGAVEAIEAEVPETKKRVILLDPAGIPFTQSVAEE

LAEEEHLIFICGHYEGYDERIRELVTDEISLGDYVLTGGELGAMVIVDATVRLLPDVL

GNQSSAMTDSHSTGLLEHPQYTRPAEFRGMEVPHVLTNGNHKLIREWQEKESLRRTYQ

RRPDMLEEVELSKEQKNWLKEIKAEEDGSEE"

CDS 1047136..1047483

/gene="rplS"

/locus_tag="JFBMFIFI_00987"

/inference="ab initio prediction:Prodigal:002006"

/inference="similar to AA sequence:UniProtKB:O31742"

/codon_start=1

/transl_table=11

/product="50S ribosomal protein L19"

/db_xref="COG:COG0335"

/translation="MSQLMESITNEQLRSDIPAFRPGDTVRVHARVVEGTRERIQLFE

GVVIKRRGAGISETYTVRKLSGGVGVERTFPIHTPRVAQIEVVRFGKVRRAKLYYLRA

LHGKAARIKEARR"

CDS 1047740..1048132

/gene="adhR_2"

/locus_tag="JFBMFIFI_00988"

/inference="ab initio prediction:Prodigal:002006"

/inference="similar to AA sequence:UniProtKB:O06008"

/codon_start=1

/transl_table=11

/product="HTH-type transcriptional regulator AdhR"

/db_xref="COG:COG0789"

/translation="MNIKKISELSGVSADTIRYYERIGLIPPVKRAANGIREFDEEDL

RWITFSRQMRNAGLSIESLVEYLTLFQIGNETVPARKEIIANQISELKEKAEALNGAI

DRLEFKLENYDEHMLVTENSLRSFNENK"

CDS 1048287..1049066

/locus_tag="JFBMFIFI_00989"

/inference="ab initio prediction:Prodigal:002006"

/codon_start=1

/transl_table=11

/product="hypothetical protein"

/translation="MAISKTAQKNHDELFPNHQSVLAETDPEFIELFDNFTFDEVLQY
STIDVKLRMKLTLASLIAMQSVNEYKAMMVGALNVGVSPVEIKEIVYQAVPYAGLGKV
FDFLHATNEVLLARGIKLPLEKKGTTTVENRYQKGLEVVRQVADDAVDKMVETMPSNQ
KHLATFLADNCFGDYFTREGLTAQERELLIFTMLASMGGADVQLKGHIRNNDRIGNKK
ERLIETITILLPFIGYPRTLTALTCINEILPEKINYQKWRM"

CDS 1049068..1050048

/gene="yajO_2"

/locus_tag="JFBMFIFI_00990"

/EC_number="1.1.-.-"

/inference="ab initio prediction:Prodigal:002006"

/inference="similar to AA sequence:UniProtKB:P77735"

/codon_start=1

/transl_table=11

/product="1-deoxyxylulose-5-phosphate synthase YajO"

/db_xref="COG:COG0667"

/translation="MKYVKFGNTGMDVSQLCLGAMGFGDPNSGFHEWVLEEEESKVVI
KKALDLGINFFDTANIYSYGASERILGKALNEYANRDEIVVATKLFSTMKKAPNSGGL
SRKEIFHQLDQSLERLGMDYVDLYIIHRWDYNTPIEETMEALHDVVKSGKVRYIGASA
MAAWQFAKAQAVADKNGWTKFVSMQNHLNLLYREEEREMLPLCADQKIAVTPYSPLAA
GRLTRDWGASTKRFETDKTAMSKYDKTEEQDRLIVERVAKIAEKRGIERVEVALAWLL
QKEQVVAPIIGATKESHLLKAMPALDLVLTEEEIKFLEEPYLPHAIVGAN"

CDS 1050096..1050464

/locus_tag="JFBMFIFI_00991"

/inference="ab initio prediction:Prodigal:002006"

/codon_start=1

/transl_table=11

/product="hypothetical protein"

/translation="MNIKEVSKKTNTSENTIRYYERMGLIPAIKRTNAGIRWFDEEDL
KWILFVKQMMEVNLSIEGLIDYATLVVQDNAMQQAKTEILTEQKMQLQEQIVLLEKAK
LCIDCELEKQHNETNKLSDN"

CDS 1050477..1051529

/gene="adhA"

/locus_tag="JFBMFIFI_00992"

/EC_number="1.1.1.-"

/inference="ab initio prediction:Prodigal:002006"

/inference="similar to AA sequence:UniProtKB:C0SPA5"

/codon_start=1

/transl_table=11

/product="putative formaldehyde dehydrogenase AdhA"

/db_xref="COG:COG1064"

/translation="MCESCQTKVFQAVDKNYSSFERGFIERRALKPDDVAIDIKYCGI

CHSDIHQVDNDFGRGVYPMVPGHEITGIVSAVGDQVTNFKVGDRVGVGCFVDSCGECN

YCLQGEEQFCTKGVVVVFNSTNYDGSKTYGGYSQNIVVKDKFVIQIPDTLDLAEASPL

LCAGITTFSPMKHWNVGPGKKVAIIGMGGLGHIAVQFAHALGADVAVLGHSETKNNEA

QQFGADHYYLTSDPETFEKLAGQFDFILNTVAVSLDVDAYISLLKVNGAMVYVGLATE

AQSFHVGKLFGKQAIITASNVGGIPMTQEMIQFAAENNILPKIELITADQIPEAYERV

LNSDVKYRFVIDMETL"

CDS 1051615..1052499

/gene="hcaR_2"

/locus_tag="JFBMFIFI_00993"

/inference="ab initio prediction:Prodigal:002006"

/inference="similar to AA sequence:UniProtKB:Q47141"

/codon_start=1

/transl_table=11

/product="Hca operon transcriptional activator HcaR"

/translation="MELRLLRYFWTVAEEKNISKAAKVLHITQPTLSRQIKELEEELG

TALFSREKKQLRLTEEGYFLKERAIEILSLAESTEREFRNQANKINGGYFSIGCVEAD

NSDTMAMMLEELLHDYPEVAFNIVSGTSDDISDKLEKGILDLAILLEPTTVNDFGKII

LPREERWGLLVSKNSFLAKKNQIEPVDLEGIPLLCSNRIAIQSMLEDWLNKPLDSLHI

IGTYNLIFNIFSLVENRVGSALTIEGATLNRNASEFTFVPLFPEIKTNCVLVWKKNRI

QSPVVKELIQRFHHAFEA"

CDS 1052613..1053026

/locus_tag="JFBMFIFI_00994"

/inference="ab initio prediction:Prodigal:002006"

/codon_start=1

/transl_table=11

/product="hypothetical protein"

/translation="MAKFEEVKKGIIFPIGEKNEAYANYFIGQSYLKTLIADPAVNVG

VANVTFEPGCRNNWHIHHEGFQLLLVTGGEGWYQEAGKAPQFLQVGDVIVTHDGVKHW

HGATKDSWFEHIAITAGVPEWLEPVSDEEYSQLKK"

CDS 1053091..1054248

/locus_tag="JFBMFIFI_00995"

/inference="ab initio prediction:Prodigal:002006"

/codon_start=1

/transl_table=11

/product="hypothetical protein"

/translation="MHKLIKISLLSVSLLIVSAGAIAANIPAISLAYPLVPKTLVEFI

TTMPSLFIILTVPISHKIARKIGYKLTVQLGVFMVLTAGIAPIFVHSFGLLFMSRVLF

GVGIGLINPLLFSFATQLYKGQELSSVIGLQSACEGIGGMLITFSVGQLLITNWRLSF

LAYLIALPILLLFTSFVPKVTPIIEKEKNKLKKVKSTNTIYGYFILLILVVTIYMSFS

IKSTALLLEKGIGTATDGSNLLALVGLGSMLAGFLFGRTLTFTKNWTVAISFLGLGIS

LFIIAYSQTIIWISIASIFCGFSFRTFVPYLFNEVNQNQAGNSERDTALLLIGFNLGA

AFTPMSIALLERIIPFKDSSGIFFTEGLIMFLLTIGAVSIQIKSHLKMKDL"

CDS 1054666..1055010

/locus_tag="JFBMFIFI_00996"

/inference="ab initio prediction:Prodigal:002006"

/codon_start=1

/transl_table=11

/product="hypothetical protein"

/translation="MRNYYFLEYGSDKCIAVVENDLRERYDLEFNKHGDCIVGDCMNY

SGKYADQMLIKENYFGKDWQYPEHKEYKTVIAISFIEGKNKNKIQKCNTIKDWFAGME

HVHLLKEETVVG"

CDS 1055210..1055632

/locus_tag="JFBMFIFI_00997"

/inference="ab initio prediction:Prodigal:002006"

/codon_start=1

/transl_table=11

/product="hypothetical protein"

/translation="MPNITASFEGILREKQVPLAKSIDEQKQTFYSGSFQLTPTKTLP

FHVAFQSADGISDVEIVYSKLAMISNYQKKAELLELFNQLNEAETAYYRICLAGDGEV

YLRLLTRTTNEVTPIYEMLITGSGIAQQIINKIEALLK"

CDS 1055666..1056079

/locus_tag="JFBMFIFI_00998"

/inference="ab initio prediction:Prodigal:002006"

/codon_start=1

/transl_table=11

/product="hypothetical protein"

/translation="MEKSIYHTEVVNQDGGTGFAYVKNNGLSVQVSSPLSSEEGSNPE

ELLGLAYSTCLNATIQAVLKEQGKSNLSRVQINVDLIPEEIGYHFNVEALVWIDELSI

EASQKVAEAADLRCPISKLLAGSETVVMHAVADLD"

CDS complement(1056129..1056620)

/locus_tag="JFBMFIFI_00999"

/inference="ab initio prediction:Prodigal:002006"

/inference="similar to AA sequence:UniProtKB:P9WFK9"

/note="UPF0234 protein Rv0566c"

/codon_start=1

/transl_table=11

/product="hypothetical protein"

/translation="MAKEASFDIVSEINHEEVKNAVAIALKEIKNRFDFKGSISDIKI

ENNQLVLISEDDYKIGQVKDVLTSKLIKRGVPTKNIHYGESEKAFGGNVRQHAELISG

IDKENAKKINTLIKQSGIKVKSTIQDDQIRVTGKSRDDLQQVISLLRGANLPVDLQFS

NYR"

CDS 1056882..1057799

/locus_tag="JFBMFIFI_01000"

/inference="ab initio prediction:Prodigal:002006"

/codon_start=1

/transl_table=11

/product="hypothetical protein"

/translation="MAQLKTPSFYSELAKKIFFVFFAAITNAIALNNFLIPARVYGAG

LNGVSQLLSSFLFDFSHIEISTGLLIMLFNIPIALLGWYKVGRDFTLFSFLTVALMSI

FSMILPIQEVTNDPIMNAIVGGVISGVGVGLSLKYGFSTGGMDIVSMVLAKTTGRSIG

TLMFGINFIVISLAGFAYGWEYALYTLLSIYVLTKVVDTIHTSHQKVTAMIVTQHSAE

MTTAIKEKLIRGITLLPAKGGFSGADSSVLMVVVTRYELYDLEQAVKETDPFAFVNIL
QTNRVIGEFWDSDRQKSHREETRRSQENR"

CDS complement(1057837..1058649)

/gene="yitU"

/locus_tag="JFBMFIFI_01001"

/EC_number="3.1.3.104"

/inference="ab initio prediction:Prodigal:002006"

/inference="similar to AA sequence:UniProtKB:P70947"

/codon_start=1

/transl_table=11

/product="5-amino-6-(5-phospho-D-ribitylamino)uracil
phosphatase YitU"

/db_xref="COG:COG0561"

/translation="MEKKLIAIDLDGTTLNNESKISLKTKDVFKKVRDAGHTVSIVTG
RPYRISKQFYQELGLDSPIVNFNGALCHMPANRNWESQYHMTLERDVALDILSLKQHI
DIHMICAEVKDSFYADTFYEPNQHFFPEGKNSFTALTPQNLKDNPTSVCLFTTAENQK
PITNELIARYGDSIEVRTWGGNTPCLEVVSAGVQKALGVERIADVYGFKQKDIIAFGD
EDNDYEMIQYAGHGVVMQNGIEALKNISNDITRHTNDEDGLALYLENYLNLA"

CDS 1058775..1059527

/locus_tag="JFBMFIFI_01002"

/inference="ab initio prediction:Prodigal:002006"

/codon_start=1

/transl_table=11

/product="hypothetical protein"

/translation="MITIKRVVIDGNPLLEVVSQDKATEQLPTVVFYHGWTNYKESVL
VNGYELAKRGMRALLPDAYLHGERMEMPLVEEAYSEFWNIVTHNLKELPGIRDYYVEA
GLSDPNRFGVTGLSMGGITTCGALTCYPWIKAAVCLMGSPEPIQFSHWLLSSSWAEGL
SVPEKYLTKLQELEPIDLSCQPEKIAGRPVHFWHGTSDTMVPYQPTFDFYLKIKEENF
AKNVSFTTTEGVGHKVPYLTSVEMADFFAEHI"

CDS 1059607..1059948

/locus_tag="JFBMFIFI_01003"

/inference="ab initio prediction:Prodigal:002006"

/codon_start=1

/transl_table=11

/product="hypothetical protein"

/translation="MTDKETVWTEDEINAIKEHIVAALEQVIDPELGIDIVNLGLVYE

VELYNGGQCVIKMTLTTMGCPLADVITEHIIQALKEVPEVSETKVELVWYPAWTTDRM

SRYARIALGIR"

CDS 1060135..1061292

/locus_tag="JFBMFIFI_01004"

/inference="ab initio prediction:Prodigal:002006"

/codon_start=1

/transl_table=11

/product="hypothetical protein"

/translation="MKKIEIDWNYWIVRFLFVVVVILGFIFITNFQVVMDGIGRFLGV

LSPFIFGFIVAYLLNGAQKKIEILVKKINVPLIQKRSRAISVLILYTLSIFLIFLILN

YLVPLIIQNIIDVIHLLPAFYSSLLEFATGLEDKGIIELIKLEDFFKSFTSSYTPEKI

LMQSTASLASLGIFAKNISVGVLNTFLAFVISIYALIFKDSLLGFFAQVSKKILSEKT

FKRSKTLVQRTNEIFYKFIICQFIDACILGVLSTIILSLLGVKFAVTLGILLGICNMI

PYFGSIFASIVTMIITFFTGGLSLAIATIISLLILQQIDGNIIGPRIMGGALNLNPIL

IILSITVGGAYFGVLGMFLSVPVAAILKILVMNWLEATSKGDEKGQEISPL"

CDS 1061517..1061978

/locus_tag="JFBMFIFI_01005"

/inference="ab initio prediction:Prodigal:002006"

/inference="similar to AA sequence:UniProtKB:Q7A551"

/codon_start=1

/transl_table=11

/product="Putative universal stress protein"

/translation="MPQEYQRVLVAIDGSKESDLAFKKAVQVAKRNKAALISLHVIND

SDSVFSYGYAGIDLNQLIANETKESKEKLDTLLLYAKEQGVESVQSIIEFGNPKKLIA

KTIPEKEKIDLIIVGATGLNAIERVLVGSVASYVITHAACDVLVVRDEISE"

CDS 1062327..1064945

/gene="clpB"

/locus_tag="JFBMFIFI_01006"

/inference="ab initio prediction:Prodigal:002006"

/inference="similar to AA sequence:UniProtKB:O68185"

/codon_start=1

/transl_table=11

/product="Chaperone protein ClpB"

/db_xref="COG:COG0542"

/translation="MELEKLTSTMQQALGDAQQIAVTRHNQDIDIVHLWKIFLTPDHF

ARGLYQESGIDTAAFEQEVDRELDRYPSVEGGNVQYGQGMSQNLYNLLLEADKIRESF

KDEYMATETVVLALMKLKNYSLTRYLKEQGLNEKTLRELIEKMRGGDRVTSQNQEEQY

QALEKYGTDLIQAVKSGKQDPVIGRDEEIRDVIRILSRKTKNNPVLIGEPGVGKTAII

EGLAQRIVRKDVPENLKDKTIFSLDMGALIAGAKFRGEFEERLKAVLKEVKKSDGRII

LFIDEIHTIVGAGKTEGSMDAGNLLKPMLARGELHCIGATTLDEYRQYMEKDKALERR

FQRVLVNEPTVEDTISILRGLKERFEIHHSVNIHDNALVAAATLSNRYITDRYLPDKA

IDLIDEACATIRVEMNSMPTELDQVTRRLMQLEIEEAALKKESDDASKKRLNLLQEEL

SELREQANQLKMKWETEKEEVSKIRDKRAEIEQARRELEEAESNYDLERAAVLRHGQI

PELEKEIEALEAENEEKIKGDNRLVQEAVTENEIATVIGRMTGIPVSKLVEGDREKLL

KLGETLHKRVIGQEEAVDSVTDAVIRARAGLQDPSKPLGSFLFLGPTGVGKTELAKAL

AENLFDSEEHMVRIDMSEYMEKHSVSRLVGAPPGYVGYEEGGQLTEAVRRSPYTIVLL

DEIEKAHPDVFNILLQVLDDGRLTDSKGRIVDFKNTVLIMTSNIGSSLLLDGTDADGQ

VDPAVEDQVMSLLKGSFKPEFLNRIDDTVLFTPLSLEDVKGIIVKMTASLSERLVDQE

IVLEISDEAKTWIAENAYDPIYGARPLKRFLTKEVETPLAKEMIAGRVLPKTKVIVTL

LGDRLVFENIDLSEDV"

CDS complement(1065168..1065368)

/locus_tag="JFBMFIFI_01007"

/inference="ab initio prediction:Prodigal:002006"

/codon_start=1

/transl_table=11

/product="hypothetical protein"

/translation="MKYVATIFWAFCVGQATYYIGSALGNSTYDFKLATLLGLAGGVL

CIAIGELLASDSKKAKIETTTD"

CDS 1065605..1068949

/gene="dnaE"

/locus_tag="JFBMFIFI_01008"

/EC_number="2.7.7.7"

/inference="ab initio prediction:Prodigal:002006"

/inference="similar to AA sequence:UniProtKB:P10443"

/codon_start=1

/transl_table=11

/product="DNA polymerase III subunit alpha"

/db_xref="COG:COG0587"

/translation="MTFVHLQVISAFSLLKSTTSIEKLVISAKNKGYSAIALTDHNVL

YGVVDFYKCCLKHNIKPILGLTLDIAGVMDSGNNYPIVLLAENLEGYQNLMALSTEKQ

LLPDGERLDFQHIKPYSAHVIAVTPGETGEIEQALLNKNPQLADDITTKWLDLFKKEN

FYLGVQIHQKLQPIQSNLKALAETKHLKTVALHDIRYLDPNDDFSTKVLRAIDAGEKI

DLAIESTSGPFYLPEMSGIRERFATAGLEKEVEETVKLANRIQPEILLHQHLLPRYPV

PTDAKTEDYLAEQCFKGLQKRVPNADERYQKRLNYELSVIQKMGFSDYFLIVWDLMAY

AHKTKILTGAGRGSAAGSLVSYVLEITDVDPIEYDLLFERFLNEERFTMPDIDLDFPD

NRREEMLIYVKNKYGQGHVAQIATFGTLAAKMALRDVARVFGLSQNEANVWSKAIPTV

LGITLEEAFKTSSSLKKLVNETEKNKLLFDTAKRIEGLPRHVSTHAAGVVISDQPLVS

LIPLQHGSNGIHLTQYAMGNVEEIGLLKMDFLGLRNLQILDNALQLVKRENGTDVDIH

KIPMDDQAALEIFRKSDTSGVFQFESSGIKNVLRKLGPTSIEDVAAVNALYRPGPMEQ

IDLFIDRKKGQVPIEYPHDSLKEILGVTYGVMVYQEQVMQVASKMGGFSLGQADILRR

AMSKKQKNIIDEERKHFVEGALEKGYTSEVATTVYSYIERFANYGFNRAHAFGYAFIA

YQLAFLKAHYPHAFFAALLNSAINNPTRIKEYLLEAKKRKITVLPPDINQSLYMFSLK

EGKIQFGLISIKGLRRDLVKEIIASRKEHGPFKDLVDFLRRIDQKWLKDEIIRPFIYA

GAFDCFGYSRGVLVASLDGILTSVKFSGNNVGLFDILAPKYETHTADLSMEERLEMEE

QTLGAYLSGHPVESFENIRFLKQAGYIKDLQAGTNARIIGLVKSVRKIRTKKGEQMAF

VQVNDQSAECSITLFPQKLRQFGRLIEKNKIIYIEGKVEEGQQEEKQILVNQILDAVV

LGSESSKDKCFIRIQSELDSPEILKKMNQMLQEEHGNIPVILYYVATNKKMVLNESEW

VNGSEKLILKLEELVGKGNVVLQKGS"

CDS 1069182..1070144

/gene="pfkA"

/locus_tag="JFBMFIFI_01009"

/EC_number="2.7.1.11"

/inference="ab initio prediction:Prodigal:002006"

/inference="similar to AA sequence:UniProtKB:P00512"

/codon_start=1

/transl_table=11

/product="ATP-dependent 6-phosphofructokinase"

/translation="MKRIAVLTSGGDAPGMNAAVRAVVRKGIYEGMEVYGINYGFAGL

VAGDIRKLSISDVGDMIQRGGTFLYSARYPEFATEEGQLKGIEQLKKFGIEGLVVIGG

DGSYHGAMALTKHGYPAVGLPGTIDNDIPGTDFCIGFDTAINTVLESIDRIRDTATSH

VRTFIIEVMGRDAGDIALWAGIAGGAEQIIIPEKEFDMAEVAKTIQKGRDRGKKHSLI

VVAEGVMGGNEFAEKLAEHGDYHARVTVLGHVQRGGSPSARDRVLASNFGAHAVDLLK

VGRGGLCVGIRDNKIVENDIVETLEKGKHKADLSLYQLNKEISY"

CDS 1070206..1071963

/gene="pyk"

/locus_tag="JFBMFIFI_01010"

/EC_number="2.7.1.40"

/inference="ab initio prediction:Prodigal:002006"

/inference="similar to AA sequence:UniProtKB:Q02499"

/codon_start=1

/transl_table=11

/product="Pyruvate kinase"

/translation="MKKTKIVCTIGPASESVETLVKLIEAGMNVCRLNFSHGDFEEHG

ARIKNIREASKITGKMVAILLDTKGPEIRTHNMKDGKVEFTTGDVERISMTEVEGDNT

RFSVSYPELINDVNPGSHILLDDGLIDLEVTDIDRAANEIVTVVKNSGTLKNKKGVNV

PNVSINLPGITEKDAADIIFGIENDVDYIAASFVRRPSDVLEITEILEKHNATHIQII

PKIENQEGVDNIDEILKISDGLMVARGDLGVEIPTEDVPIVQKSLIRKCNELGKPVIT

ATQMLDSMQKNPRPTRAEASDVANAIFDGTDAIMLSGETAAGDYPVEAVQTMNSIAIR

TEEALVNQDAFALKAYSKTDMTEAIGQSVGHTARNLGIQTIVAATESGHTARMISKYR

PKSHILAITFSERQMRGLALSWGVYPQVAEKPVSTDDMFNLATRVSQETGFAKEGDLI

IITAGVPVGERGTTNLMKIQLIGSQLTSGHGIGRTSVIGKAVVAKNAEEANANAIEGG

ILVVPTTDKEYLPAIEKSAAIVVEEGGLTSHAAVVGIAMSIPVIVGAANATSLIKDDE

LITVDSRRGIIYRGATTAI"

CDS 1072144..1072839

/gene="walR_1"

/locus_tag="JFBMFIFI_01011"

/inference="ab initio prediction:Prodigal:002006"

/inference="similar to AA sequence:UniProtKB:Q9RDT5"

/codon_start=1

/transl_table=11

/product="Transcriptional regulatory protein WalR"

/db_xref="COG:COG0745"

/translation="MNYTVLIVENKQEANQKIAEELFASGYHIRLANSSETVDEELKK

QTPDLLLIDSCSINQQELGALLKKGQKNVVPVLVITNKESQEKVLRLLGVKKEAYITS

PFEINDLIKQICNLVGHQSSNTLNNQNQFSGTRIGDLVVNIEKMLVKKETVEIELTTT

EFKILKYLMQHPNQVVTKERIYRSIWQEDYHEGENSINVHICNLKKKIETFPSKPQYI

KTVWGKGYMLEWS"

CDS 1072930..1073391

/locus_tag="JFBMFIFI_01012"

/inference="ab initio prediction:Prodigal:002006"

/codon_start=1

/transl_table=11

/product="hypothetical protein"

/translation="MMIESWLFLGVILGIALIAKNQSLIIATVVVLILKLIPQTDKWM

TLIQSKGINWGVTVITVSILIPIATGQIGFKDLVNAFKTPVGWIAVLCGIGVSLLSSK

GVGLLAGSPEVTVALVFGTIMGVVLLEGLAAGPVIAAGLTYCILQLFQLGK"

CDS 1073501..1074382

/gene="cvfB"

/locus_tag="JFBMFIFI_01013"

/inference="ab initio prediction:Prodigal:002006"

/inference="similar to AA sequence:UniProtKB:Q7A5Q1"

/codon_start=1

/transl_table=11

/product="Conserved virulence factor B"

/translation="MNESLGKIVTGIVTDENEKAYFIQKEGVTYRLNKTETMNYQLGD

PVEGFAYIAMNKDFVMTQEIPKVRVGHYAWGTVVDTRKDLGVFVDIGLPDKEMVVSLD

ELPTEKRLWPKKGDQLMIAIKVDQKDRMWGTLAEESVFQSIARKGKIEELKNKNITGT

AFRLKLVGTYLITEDFYLGFIHPSERKDEPRLGEVIQGRVIGVRPDGLLNISLMPRAF

EAIDDDAGMLLAILERTQTGSFPYTDKSDPEAIQERFGISKGQFKRALGNLMKQRKIV

QKDGETILVEALNENEK"

CDS 1074509..1074976

/gene="fur"

/locus_tag="JFBMFIFI_01014"

/inference="ab initio prediction:Prodigal:002006"

/inference="similar to AA sequence:UniProtKB:P54574"

/codon_start=1

/transl_table=11

/product="Ferric uptake regulation protein"

/db_xref="COG:COG0735"

/translation="MKTTESPLLKIKNQLHSAGFKLTPQREATVGVLLEKEKDHLSAE

EVYMLVKEKSPDIGLATVYRTLEILTELNIVNKISFNDGLARYDVRKEGAKHFHHHLL

CIECGSIEEVHDDLLEDIERVVESRFHFIVKDHRLTFHGICKECQEKGKKDSE"

CDS 1075064..1075954

/gene="xerD"

/locus_tag="JFBMFIFI_01015"

/inference="ab initio prediction:Prodigal:002006"

/inference="similar to AA sequence:UniProtKB:P0A0P0"

/codon_start=1

/transl_table=11

/product="Tyrosine recombinase XerD"

/translation="MEDDLKEYLHFLTIERGLSKNTIESYRRDLVHYLDYLNEHQIAS

WNELDRYFVLSFLQQLKDEEKAAGTIIRMVSSLRKFHQFLKQERLSEIDPMLHIDTPK

KAQTLPKILSMKEVEKLIETPDTNTTLGLRDRAMLEVMYATGLRVTELTELRLTDLHL

SLGLIQTIGKGDKERIIPLGDLAIEWIEKYLRYSRVKLEKSGKRTPYLFLNHHGGGLT

RQGVWKNLKKLVLLAGIEKEVTPHTLRHSFATHLLENGADLRVVQELLGHVDISTTQI

YTHITKKRMTEVYKTYHPRA"

CDS 1076046..1077215

/gene="deoB_1"

/locus_tag="JFBMFIFI_01016"

/EC_number="5.4.2.7"

/inference="ab initio prediction:Prodigal:002006"

/inference="similar to AA sequence:UniProtKB:P99100"

/codon_start=1

/transl_table=11

/product="Phosphopentomutase"

/translation="MVFKRIHLIVMDSVGIGEAPDAAVFGDIGSDTLGHIAEEAGLTI
PNLEKLGLGNIRPLKAISERIDNQGYYTKLEEISVGKDTMTGHWEITGLNIQTPFRVF
PNGFPQELLEKIEAFSGRKIVCNLPYSGTEVLEDFGEHQMKTGDLIIYTSADPVLQIA
AHEEIIPLEELYKICQYVRDITLDDPYMIGRIIARPYLGTPGAFKRTSNRHDYALNPF
GKTVLNDLSEAGNDVIAIGKINDIFNGSGITESVRTTSNMDGVDQLLKVMTQDFNGLS
FLNLVDFDALFGHRRDVKGYAEAIEEFDQRLPEVLAELRSDDLLLITADHGNDPTAPG
TDHTREYVPLLAYSPKLSGHGKIPQGHFADIGATIAENFKVDGTGFGTSFLKELI"

CDS 1077241..1078062

/gene="punA_1"
/locus_tag="JFBMFIFI_01017"
/EC_number="2.4.2.1"
/inference="ab initio prediction:Prodigal:002006"
/inference="similar to AA sequence:UniProtKB:P77834"
/codon_start=1
/transl_table=11
/product="Purine nucleoside phosphorylase 1"
/translation="MTTKLQEKITEAAEFIKKQGIEDVEIGLILGSGLGELGDEVGEA
KKIKYDQIPHFPVSTVEGHAGQLVYGQLGGKKVLAMQGRFHFYEGYTLEEVTFPIRVM
KELGIHSVIVTNAAGGINTSFAQGELMLITDHINYTGVNPLMGPNDPAMGPRFTDMSQ
AYDVAYQQIVREVAKEMSLDLKEGVYIGYTGPTYETPAEIKMSRIMGADAVGMSTVPE
VIVAAHAGLRVIGISCITNLAAGMQESLNHDEVVETTQRVKGTFKALVKNTLAAI"

CDS 1078325..1079644

/gene="lysA"
/locus_tag="JFBMFIFI_01018"
/EC_number="4.1.1.20"
/inference="ab initio prediction:Prodigal:002006"
/inference="similar to AA sequence:UniProtKB:P9WIU7"
/codon_start=1
/transl_table=11
/product="Diaminopimelate decarboxylase"
/db_xref="COG:COG0019"
/translation="MENSLLTGTMKINQENHLEIGGVDTVKLVEKYGTPVYVYDVFQI
KKQARAFKQAFSAQNITAQVAYASKAFACLAMYQLMAQEGLSVDVVSGGELYTAIQAD

YPPEKIHFHGNNKTESELIEALNYKIGCFVVDNFSELHLLHKLTTERKQEVNILLRVT
PGVEAHTHEYISTGQEDSKFGFDLLNGQAKSAVNLALTMPYMKLIGLHSHIGSQIFET
EGFIMAIDKLLASVKEWQKATAFDLEVLNLGGGFGIRYIESDKPLAISEYVDRIIAEV
IAKTTEYGITMPEIWIEPGRSIVGDAGITLYQTGSEKEVPNVRNYLAVDGGMSDNIRP
ALYDAEYTGILANRGNDPVTETYSIAGKCCESGDMLIWDLPLPKAERNDILAVFSTGA
YGYSMANNYNRIPRPPVVFVENGTDYLVVERESYADLMRLDRSLPKG"

CDS 1079667..1080053

/locus_tag="JFBMFIFI_01019"

/inference="ab initio prediction:Prodigal:002006"

/codon_start=1

/transl_table=11

/product="hypothetical protein"

/translation="MLINYKNDYEKIMMGLLSFVPDLKEVSRLKAEINWYQAEENRRL
YLWSNEETTDFIGMLGIEIEPEMVLLRHISINPSFRKEGISFRMLQELDEKLPERKII
GTLETAPIIAKWEHYKENSEKPNELT"

CDS 1080074..1080853

/gene="scpA"

/locus_tag="JFBMFIFI_01020"

/inference="ab initio prediction:Prodigal:002006"

/inference="similar to AA sequence:UniProtKB:P35154"

/codon_start=1

/transl_table=11

/product="Segregation and condensation protein A"

/db_xref="COG:COG1354"

/translation="MADITVKIDVFEGPLDLLLHLIQQLELDIYDIPMAEITAQYLAY
LHTMKELELDIAGDYLVMAATLMAIKSKMLLPKQELEVDEDEEGFYEEGEDPRDALVD
QLLEYRKYKYAADLLKDKEEERSQYYTKTPMNLQDLQIDVPLQPLQVTTIDLFTAFHQ
MITKKQQKIPMQTKISSEEITIDEKMKTIVKRLKRLKKDEAILFDDFFQDPTKSEMVT
TFMALLELIKEGAIWINQEEICGDILVYGSQDENETTQGVE"

CDS 1080853..1081440

/gene="scpB"

/locus_tag="JFBMFIFI_01021"

/inference="ab initio prediction:Prodigal:002006"

/inference="similar to AA sequence:UniProtKB:C1CMI5"

/codon_start=1

/transl_table=11

/product="Segregation and condensation protein B"

/translation="MMNEIGAVETLLFVAGDEGLSLEEIATVLECTTQFAFQLLTQLQ

KNYESSKASGLMLLEVGNHYQLATKKEYAGIIKKYAVSPLSTNLSQAALETLAIIAYK

QPLTRMEIDEIRGVQTSGALQKLLLRGLIEDKGRVNGPGRAILYGTTNYFMDYFGLKN

IKELPAIDELEQDQEEVESDLFFEKFNQQFQSQEE"

CDS 1081467..1082210

/gene="rluB_2"

/locus_tag="JFBMFIFI_01022"

/EC_number="5.4.99.22"

/inference="ab initio prediction:Prodigal:002006"

/inference="similar to AA sequence:UniProtKB:P35159"

/codon_start=1

/transl_table=11

/product="Ribosomal large subunit pseudouridine synthase

B"

/db_xref="COG:COG1187"

/translation="MERLQKVIAHAGVASRRKAEELIATGHVKVNGLTVKEMGIQIGN

SDVVEVDGVPIDREQPVYFLLYKPRNTISAVSDDKERRVVTDFFGGVSQRIYPVGRLD

YDTTGVLIMTNDGELSQQLMHPKYHIPKTYVAKVKGMVDPMSIKKLERGIKIDGKKTA

PAKANLISADRVKQTSIVELTIYEGRNRQVKNMFMALGFPVQKLKRETYGSLNLEGLK

PGEWRELKSFEVNSLRNLAKESQNQKDKR"

CDS 1082295..1083017

/gene="srrA_3"

/locus_tag="JFBMFIFI_01023"

/inference="ab initio prediction:Prodigal:002006"

/inference="similar to AA sequence:UniProtKB:Q9L524"

/codon_start=1

/transl_table=11

/product="Transcriptional regulatory protein SrrA"

/db_xref="COG:COG0745"

/translation="MKTDAINILIVDDEERIRRLLKMYLERENFIITETDNGEDALSL
ALENDYDLILLDLMLPKMDGIDVAKNLRETKDTPIMMLTAKGEETNRVQGFEVGADDY
IVKPFSPREVVLRVAAILKRTQSAKPITPENPDLIVFPHIEVDNQAHRVLADGKPVNL
TPKEYDLLLYLAQAPDQIFGREQLLREVWKYEFFGDLRTVDTHVKRLREKLAQQSDKA
AKMIVTVWGLGYKFNSHYDSKE"

CDS 1083024..1084826

/gene="resE"
/locus_tag="JFBMFIFI_01024"
/EC_number="2.7.13.3"
/inference="ab initio prediction:Prodigal:002006"
/inference="similar to AA sequence:UniProtKB:P35164"
/codon_start=1
/transl_table=11
/product="Sensor histidine kinase ResE"
/translation="MTKLNSIVFRTWLVTMVIVAFCLISSTLIYSTIYQRTVEEIYVT
DFNKSISSVEKLVVKNPVFLLNNPEKFDPFDAHLFFLIEYDGKKESFFNEHYETLSTT
YADKIMTRKDVQEAAKATEDREIRGNMGGKNNSEFILNIKHFSYDGKKGTLYSYADLS
FLTSIAKKMNQWTAFIFIMLLILSVLFYYYLKLRIGNPLSRMRDIAFEYAKNDFTRQT
PIRSRDELSQLALAMNKMGKSLESIGMATRQEKELLAHILSSMTTGVLFYNRDKDLLM
SNPNGEEFLQQWRRSAQYSDVERMPYILDQKINDVIENTVDLIFELQLDDYYYKINLV
PLYSEDMVTVRGVLASVQDMTKERRLDTMRVDFINNVSHELRTPLVMIRGYSEAILDD
VAETREDKHEMAKIIWEESERMSRMVNEMLDLSRMEAGYIELQRTEVDLYSFVRDLTS
RFNQIAQSSEVKLSFEIQPDMETYYMDEDKMSQVLFNLINNAIRHTTMAHKKNGQVEI
LVHLDEVMDELLIEVKDNGTGIPKKDLPFIFERFYKADKSRVMNKENKSGTGIGLSIV
KSIVEEHEGYMEVQSIEDVSTSFIIHLPYRDKVN"

CDS 1085295..1085885

/gene="fmnP"
/locus_tag="JFBMFIFI_01025"
/inference="ab initio prediction:Prodigal:002006"
/inference="similar to AA sequence:UniProtKB:P50726"
/codon_start=1
/transl_table=11
/product="Riboflavin transporter FmnP"

/db_xref="COG:COG3601"

/translation="MKTSNTKRLVGIAMFGAIAYVLMFFAFPILPSASFMKVDFSDIP

ILLGMFLYGPVGGVLIALVRTVLHYLQTSGDMGYPIGDIASFIASIVYSYPIYFIMSK

HMKKGTLTMSNTLVANVTGVISLATIMSLANWLIITPLYLAVMGFSVGPIKEFVLLGV

LPFNIIKGLLVSSVFIFMFTKLQPWIIKNQATHINE"

CDS complement(1085965..1086222)

/locus_tag="JFBMFIFI_01026"

/inference="ab initio prediction:Prodigal:002006"

/inference="similar to AA sequence:UniProtKB:P10245"

/codon_start=1

/transl_table=11

/product="Ferredoxin"

/translation="MPKFYTKVDQEKCIACGLCQIKAPQIYEYTEDGLAYNLIDQNTG

QMPLPDALISEFKLAYTACPTNAILRKSEPFNASPSIITNN"

CDS 1086269..1087339

/locus_tag="JFBMFIFI_01027"

/inference="ab initio prediction:Prodigal:002006"

/codon_start=1

/transl_table=11

/product="hypothetical protein"

/translation="MDELSYLDYLLFSFFSTSEPRKAMTVFHIISGKRTASILYQAEI

YRLSAYFSLFNKLKKEHYQHSLKKGIALKILKKEENDYYCLTEMGQKEITAYFEKQYY

PTELNFLQNGRWIHVYWRRLVFVSQVLSEIRYKNQSYIPIEKEWSLQVWVKKWLKSKS

ESREELAEAFGIEWLQLARNQSKISAEIMIGFLTGHDYYGQTTMQLATAFLKEAVEVR

IILLDGLSKLIHEVTEQPETFPIFFSIFTDLLETHGAVSQSVRQTQKNLMEGLDLIEI

AKIRQLKLSTVSEHVIELSLIDKKFNVHSLIKKQPFLEVKQQLENQPMSSFPDIKEIF

PEVPFYVYRLMQIERWREKNAN"

CDS 1087329..1088792

/gene="recQ_2"

/locus_tag="JFBMFIFI_01028"

/EC_number="3.6.4.12"

/inference="ab initio prediction:Prodigal:002006"

/inference="similar to AA sequence:UniProtKB:P15043"

/codon_start=1

/transl_table=11

/product="ATP-dependent DNA helicase RecQ"

/db_xref="COG:COG0514"

/translation="MRTKQQIIEEALFEHFGYRQLRSGQYEAISAALSGENTLVLLPT

GTGKSICYQLTGYLSEGLVVIVSPLLSLMQDQVEQLRQKGEKKVAALTSLLDYSEKAY

IFQQFNELKFLFLSPEMLQQERVLNALKKSTIALFAIDEAHCISQWGMDFRPDYLTLG

SVLKELNSPLTMALTATANQRVKEDIFAALQLIPEETTQIIHSVDRPNIALEVVECYK

NKNEQLMDYVTKLEKPGIIYFSSRKLADEIAEMIRKMTPYSAESYHSEVESADRIRLQ

QQFIHNKIDIICATNAFGMGINKPNIRFVIHYHIPSSMESYLQEIGRCGRDGLPSLAV

LLYEKSDVYIQMRLQENDLPTESMLEYVYRKGSIVPGSCSESQYRLLESYLNLNIPLH

EAKAQLKNRSYQKNAQLKYMTDYAETTSCKRAVMLHYFEESQATKISNCCSICGIAYE

PYLAREKEPEIAEENKELKWLEKITYLFKLDKDSKNG"

CDS 1088921..1089502

/locus_tag="JFBMFIFI_01029"

/inference="ab initio prediction:Prodigal:002006"

/codon_start=1

/transl_table=11

/product="hypothetical protein"

/translation="MSKKRIDDQQKEEAWSRKFDDEGGFSEGNYSRTARKKAKGGISP

ILTTLCVFLALLIILPISVVWWYSNNKDVEDTGSANDQVTMNSSSSSKESSSSSSKES

SSESKPAESSSAVSSSSAPVEETPAPVEPTPPVEETPPPVEEPPVTNTYTVKSGDNLY

RIAVNNGMTLDQIKQLNGLSSDTVSVGQVLKIN"

CDS 1089587..1090267

/gene="cmk"

/locus_tag="JFBMFIFI_01030"

/EC_number="2.7.4.25"

/inference="ab initio prediction:Prodigal:002006"

/inference="similar to AA sequence:UniProtKB:Q97PK6"

/codon_start=1

/transl_table=11

/product="Cytidylate kinase"

/db_xref="COG:COG0283"

/translation="MTEKRLRIAIDGPASAGKSTVAKIVAKDLGYIYCDTGAMYRALT

YMAMKNNISIDDEAGLVQLLQDMTITFDPSAEIQKVFVNGKEVTDEIRQSDVTNGVSA

VSAHQAVRVELVKRQQEIAKAGGIVMDGRDIGTAVLPDAEIKIFLVASVTERAERRFK

ENQTKGITTALEVLQQEIADRDYKDSTRKVSPLLQAEDAILVDTTSLSIEGVVAKIKS

IIEETVKK"

CDS 1090367..1091575

/gene="ypfD"

/locus_tag="JFBMFIFI_01031"

/inference="ab initio prediction:Prodigal:002006"

/inference="similar to AA sequence:UniProtKB:P38494"

/codon_start=1

/transl_table=11

/product="30S ribosomal protein S1"

/db_xref="COG:COG0539"

/translation="MTENVENQETMMDAMNSVQDVNIGDIVQGEILTIQDNKQAIVGI

VGGGVEGVIPFNELSSTPFENVTDVVNVGDVVDLVVIKPIKDKENGSFLLSKRRIDAK

KVWEGIQKDFEAGTIIQAPVTDVVKGGLVVDVGIRGFVPASMVDAHFVDDFSVYKGQT

LGFKIVEIEPSENRLILSHKAVVEAEKEVAKKEIMTKLVEGDTVTGTVARLTNFGAFI

DLGGVDGLVHISQISYNHVKAPADVLTVGEEVQVKILSINEETGRISLSIKDTQPGPW

ENIEERAAVGSTLSGIVKRLTSFGAFVEVYPGVEGLVHISQISHNHIATPHEVLHEGD

EIKVKVLEVNPTDQRLSLSIKALEEKPASEKSEKEDVVDYTLPEEDSGFTLGDILGEQ

LSDMTSEDDE"

CDS 1091943..1093256

/gene="der"

/locus_tag="JFBMFIFI_01032"

/inference="ab initio prediction:Prodigal:002006"

/inference="similar to AA sequence:UniProtKB:P50743"

/codon_start=1

/transl_table=11

/product="GTPase Der"

/db_xref="COG:COG1160"

/translation="MAKPVIAIVGRPNVGKSTIFNRIVGERISIVEDVSGVTRDRIYA

DGEWLGKEFNIIDTGGIDIGDEPFLEQIKQQAEIAMDEADVIIFITSGRESVTDADEN

VAKMLYRTKKPVLLAVNKIDNPEMRNDVFDFYSLGLGEPYPISGSHGLGLGDLLDAAI

GHFPEETEEEYDDSVIKFSLIGRPNVGKSSLVNAILGEERVIVSDIAGTTRDAIDTEF

VSADGTEFVMIDTAGMRKRGKVYETTEKYSVLRALRAIERSDVVLVVLDAETGIREQD

KKVAGYAHEAGRGIIIVVNKWDTLEKDNSTMKKFEEDIRESFAYLSYAPIVYVSAKTK

QRLNTLPDVIQRVSMSQNLRIQSAVLNDVIMDAVAMNPTPTDKGKRLKIYYATQVAIK

PPTFVIFVNEPEMLHFSYSRFLENRIRDAFTFEGTPIRIIARQRK"

CDS 1093518..1093793

/gene="hup"

/locus_tag="JFBMFIFI_01033"

/inference="ab initio prediction:Prodigal:002006"

/inference="similar to AA sequence:UniProtKB:P0A3H0"

/codon_start=1

/transl_table=11

/product="DNA-binding protein HU"

/translation="MANKAELIESVATSTGLTKKDATAAVDAVFETIQTTLSSGEKVQ

LIGFGNFEVRERAARKGRNPQTGEEIQIAASKVPAFKPGKALKDAVK"

CDS 1093929..1094993

/gene="aroB_1"

/locus_tag="JFBMFIFI_01034"

/EC_number="4.2.3.4"

/inference="ab initio prediction:Prodigal:002006"

/inference="similar to AA sequence:UniProtKB:Q6GGU4"

/codon_start=1

/transl_table=11

/product="3-dehydroquinate synthase"

/translation="MKISLTRNQQVVPFYFAAPISKLVAENKFLFQETNKYLIITNQT

SYDRYYEKLRLAIGNEFNQFWYVCPNGQQSANLAEFSSVLAYCEEMELRSSISVIAFG

DEGVSRLAGFFASLYLGGVSLIDIPTTLMGLEISLLHQVALNHQRNLDILATQQAVSA

IFFEARFLQDNQLSELQEGFSCWLQLAVTLDSVFYDLLKEQFPTRKELEIKSVVPYVT

SYLNLIETSNKVKSDGLARYFGSEFQLAITLNSTEESMSSNSLAIGLSFSLLISAKYM

GASLDLDGWFKWLADLNYDLNLPESWFITDLLAKLTQNDLGKKTILMVLLDSFGKLKQ

VAVPVLTVAEAIEEYQLIKK"

CDS 1095020..1096285

/gene="bepA"

/locus_tag="JFBMFIFI_01035"

/EC_number="3.4.-.-"

/inference="ab initio prediction:Prodigal:002006"

/inference="protein motif:HAMAP:MF_00997"

/codon_start=1

/transl_table=11

/product="Beta-barrel assembly-enhancing protease"

/translation="MNYGEQMIEALQNNALEEAQELFIEALEKDQPEELYVLADTLYQ

LGFLAETKAIYEHLLIDFSHDDELKIGLAEIAIEEDDIDGAMDWLMAIEEESPAYPQA

LLVSADLYQVQGLYEVSEQKLLLAKELLPDEPILTFALAELYFVMGKYAQAIYGYEEL

SEAGLGEISGINLAQRIGSAYSAVGDFEQAIPYLELAEKENETTDLLFELGFTYLQNK

EYRRASETLFKLKELDPSYTSLYPYLAKSLEEENQLDRATEVIREGLRADQYNPELFY

YAADLFLKLGDEEQGEYYYQESLELNPDNETVQLALINLYLKQERFSEAVVKIETALE

NEEADAQFYWSLALAQEGLENYAKAAEAYQTAYPSFTRNKDFLKAYALFLREEGALEL

TKEVIEKYLEIESSDEEMIAILDEINSDI"

CDS 1096364..1096921

/locus_tag="JFBMFIFI_01036"

/inference="ab initio prediction:Prodigal:002006"

/inference="similar to AA sequence:UniProtKB:Q81SV3"

/note="UPF0302 protein BA_1542/GBAA_1542/BAS1430"

/codon_start=1

/transl_table=11

/product="hypothetical protein"

/translation="MSTKISLETKKEFLSWFLDYHQLKRREAMWILNYLMTHETVLNK

VHFVEAVRTTPRGMALSAIGTEEEPFRFFKEGTVFEDPEQGFHEIRLNWADQLYVELF

FNDQWKSPEYVAVLEDNPYHRWNDSVSDEVKQEVDEALVKFDLERKKAELTPKINKAL

EEGNRALFDELSKEWNQLMVEFEKL"

CDS 1097464..1097799

/gene="ypjD"

/locus_tag="JFBMFIFI_01037"

/inference="ab initio prediction:Prodigal:002006"

/inference="similar to AA sequence:UniProtKB:P42979"

/codon_start=1

/transl_table=11

/product="putative protein YpjD"

/db_xref="COG:COG1694"

/translation="MKKSLQDMQVEVDDYINQFKTGYFSPLGQMARLTEEVGELAREI

NHHHGEKLKKTSEPAKEVAEELGDVLFVVLSMANSLDIDMTEAFDLVMKKFNQRDKFR

FERNDGKND"

CDS 1097792..1098583

/gene="dapB"

/locus_tag="JFBMFIFI_01038"

/EC_number="1.17.1.8"

/inference="ab initio prediction:Prodigal:002006"

/inference="similar to AA sequence:UniProtKB:P63895"

/codon_start=1

/transl_table=11

/product="4-hydroxy-tetrahydrodipicolinate reductase"

/db_xref="COG:COG0289"

/translation="MIKIVVAGFKGKMGLTATKMVLDKKEFELVGVLDPFAKEENINQ

LALFSEENVPVFTTKEALASQISPDVWIDFTIPKVAYENTKFAIEHGIRPVVGTTGFT

DTQVQELIAESSEKQIGGLIAPNFAIGAILMMQFAQKAAHYFPDVEIMELHHDQKLDA

PSGTAIKTAEMIAEARGYHQQGNPEEKEIIKGARGADFEGMKIHSVRLPGLVAHQQVQ

FGSVGEGLTIRHDSYDRASFMTGVALGCEKVMELNQLVYGLENLL"

CDS 1098580..1099776

/gene="cca"

/locus_tag="JFBMFIFI_01039"

/EC_number="2.7.7.72"

/inference="ab initio prediction:Prodigal:002006"

/inference="similar to AA sequence:UniProtKB:Q7SIB1"

/codon_start=1

/transl_table=11

/product="CCA-adding enzyme"

/translation="MMQLAPEFEEALPVIQKIEEAGFVAYFVGGSVRDTILKKPISDV

DIATSAFPEEIKAIFPRTVDVGIEHGTVMVLWQKEEYEITTFRTESTYQDFRRPDEVT

FVRSLEEDLKRRDFTVNALAMSRDGEIIDYFDGLVDLNKGIIKAVGSATERFHEDALR
MMRGVRFVSQLDFTLEAKTEAAIMEHHALLEKIAVERVQVEFIKLLLGKGRSQATIKL
IETELYQYCPGLRKHGEGLYRYADLSGELPDSLTAWTLLVYFIQLDNQSIDAFMREWK
CSKKAIQEVEVAKRALDFRLTTDWSKELLYHTGLEIAVAVEKMHALLTGESDLAALET
HYKNLPIHNKKQLKVTGNELIATLNRQPGIWLGELLVEIEREVLIGRLSNQTEAILSW
AKEREI"

CDS 1099803..1100171

/locus_tag="JFBMFIFI_01040"
/inference="ab initio prediction:Prodigal:002006"
/codon_start=1
/transl_table=11
/product="hypothetical protein"
/translation="MITKLSKTFIVKEEQTAIEMKSGDLAVLATPALVAMLEETAKDS
ILELLTEGETTVGIEVNLKHVRPSRISEEIQCEAELIEQTKNILRFTLKALAGESIIA
EGTHQRAIVNSEKFMAKLAK"

CDS 1100332..1100607

/locus_tag="JFBMFIFI_01041"
/inference="ab initio prediction:Prodigal:002006"
/codon_start=1
/transl_table=11
/product="hypothetical protein"
/translation="MDYLRVSKMMKAIAEPNRLQIVDKISTGEKCACDILDDFNFTQP
TLSHHMKVLIDAGIVSARKEGKWHYYSLLSENIAEFQKLTNQIFKLQ"

CDS 1100890..1101342

/gene="bltD_1"
/locus_tag="JFBMFIFI_01042"
/EC_number="2.3.1.57"
/inference="ab initio prediction:Prodigal:002006"
/inference="similar to AA sequence:UniProtKB:P39909"
/codon_start=1
/transl_table=11
/product="Spermine/spermidine acetyltransferase"
/db_xref="COG:COG0454"

/translation="MNQISLKEVTKENIEDILQLQVKESQKSFVATTSKSLAYAYVSR
DAVSPYGIYEEEQLIGYVSLVFDENDQMYNIWHFLIDAKYQGYGYGKKAMRAVMYNIR
EKAYGLTNTVALGVDLNNKLAIHLYEEFGFIDSGETDDDGELIMLCKI"

CDS complement(1101443..1102213)

/gene="yadH"

/locus_tag="JFBMFIFI_01043"

/inference="ab initio prediction:Prodigal:002006"

/inference="similar to AA sequence:UniProtKB:P0AFN6"

/codon_start=1

/transl_table=11

/product="Inner membrane transport permease YadH"

/db_xref="COG:COG0842"

/translation="MYKLYFTALKSLAAKETNRYLRIWIQTLVPPVITTSLYFVIFGN
LIGGRIGEIEGFSYMEFIVPGLIMMSVITSSYSNVASSFFSQKLQKNIEELLIAPVPT
HVIIWGFVIGGLGRSILVGTLVTIISLFFVPLHVYSWFIVIVTLLMTAILFSLAGLLN
GVFAKSYDDVSIVPTFVLQPLTYLGGVFYAISMLPPFWQAVSKINPIVYMVSGFRYGF
LGQTDVPIAFSLSILVLFTIILYSVCWYLINKGRGLRS"

CDS complement(1102206..1103123)

/gene="yadG"

/locus_tag="JFBMFIFI_01044"

/inference="ab initio prediction:Prodigal:002006"

/inference="similar to AA sequence:UniProtKB:P36879"

/codon_start=1

/transl_table=11

/product="putative ABC transporter ATP-binding protein
YadG"

/db_xref="COG:COG1131"

/translation="MTYALELTDLKKVYATGVEALRGINLVVEEGDFYALLGPNGAGK
STTIGIITSLVNKTSGKVKVFDYDIDTDLVRAKQQIGLVPQEFNFNPFETVEQIVVNQ
AGYYGVPRNEALKRSEKYLKQSNLWEKRHERARMLSGGMKRRLMIARALMHEPRLLIL
DEPTAGVDIELRREMWTFLRELNENGTTIILTTHYLEEAEMLCRNIGIIQSGELIENT
SMKTLLAKLEFETFIFDLAPYDKKPAINGYQSFFEDDLTLAVEVERNQGLNNLFEQLT
KQNISVLSMRNKSNRLEELFLKITEEKHV"

CDS 1103489..1104088

/locus_tag="JFBMFIFI_01045"

/inference="ab initio prediction:Prodigal:002006"

/codon_start=1

/transl_table=11

/product="hypothetical protein"

/translation="MSNKYIELLKNRRSIYGIGKDVTLADDAIVSLVKEAVKESPASF

NSQTSRVVVLFNESHNKLWSIVEDALRKEVPADAFEPTLAKLNSFRSGYGTILFFEDT

DIVKNLQNQFELYADNFPVWSEQSSGIAQHSVWTALAEENIGASLQHYNPLIDDAVKA

AWDLPAAWNLRAQMPFGSIVAEAGEKEYMDDAARFRVFN"

CDS 1104501..1105532

/gene="argC"

/locus_tag="JFBMFIFI_01046"

/EC_number="1.2.1.38"

/inference="ab initio prediction:Prodigal:002006"

/inference="similar to AA sequence:UniProtKB:Q07906"

/codon_start=1

/transl_table=11

/product="N-acetyl-gamma-glutamyl-phosphate reductase"

/translation="MKISIIGATGYSGLELIRLLESHSKVEIFSLHSYSNYSENVAYL

YPHLKGICELPLEPIDVSRICQHSQVVFFAAPSGIAKDQVAPFLEAGLTVIDLSGDLR

LKNLDDYPTWYHFPAAEKGLLDQAIYGLSEWADVKKQKLISNPGCYATATLLALAPLV

IENLIELDSIVVDAKSGLSGAGKSMQTTSMYTEANDNLTIYKMNQHQHIPEIIQQLKK

WQSGVENIQFSTSLIPVTRGISTTIYAKLKSRLTTEELNAFYTSHYNDKPFVRIQAPN

KLPTIKQVVGSNFCDIGMTVNPTTNQVIIVSAIDNLVKGAAGQAVQNLNQSFGFAPND

GLKFSPIFP"

CDS 1105561..1106757

/gene="argJ"

/locus_tag="JFBMFIFI_01047"

/inference="ab initio prediction:Prodigal:002006"

/inference="similar to AA sequence:UniProtKB:Q07908"

/codon_start=1

/transl_table=11

/product="Arginine biosynthesis bifunctional protein ArgJ"

/translation="MKNIEGTIASPIGFFAAGVHGKLKYKKKDIGLIYSTFPAQAAAV

YTTNQFQAAPINITKDSLKNGQTLQAVIVNSGNANACNGQQGIEDAYQMRQLTANKLG

IPATEIAVASTGIIGEPLPMDKITAGITALDYSKGEPSDFHESILTTDTCEKLIVVEV

LLNKKKVTISGVAKGSGMIHPNMATMLAFITTDVAIDAKLLDQTLRQSVNQTFNQITV

DGDTSTNDMVLVMANGAAKNEELKSDTSDYVIFKNAFDAVCQHLAKKIAQDGEGATKL

IEVTVASAKSVLEARMIAKTIVSSSLVKTAMFGEDPNWGRIICALGYSEGTLNPENIN

IEIGPIEVLNRTKAVLYDANALREYLQNEVIRITVDVGVGNENGVAWGCDLSYEYVKI

NACYHT"

CDS 1107012..1107764

/gene="argB"

/locus_tag="JFBMFIFI_01048"

/EC_number="2.7.2.8"

/inference="ab initio prediction:Prodigal:002006"

/inference="similar to AA sequence:UniProtKB:Q9X2A4"

/codon_start=1

/transl_table=11

/product="Acetylglutamate kinase"

/db_xref="COG:COG0548"

/translation="MSGTNRIVIKIGGVSCEELDDTFFLDLKKLQQQGKEIIIVHGGG

KAITHWMENLAIPVEIKNGLRVTNEKALAVTKMVLLGEVQPSLLQQFARQKIKCLGLN

AANNKLLSASQVQQGSLGFVGEIKTVNTELLKDVLDQGYIAIVAPLGVDDNGQWLNIN

ADDTASKIAEAMKAEELILMTEVLGVKKEGEILKSLSYQELDLFIENGTVSGGMIPKL

KCAHYALRNGVKQVRVTLGLTMQGTKMIGEGS"

CDS 1107764..1108897

/gene="argD"

/locus_tag="JFBMFIFI_01049"

/EC_number="2.6.1.11"

/inference="ab initio prediction:Prodigal:002006"

/inference="similar to AA sequence:UniProtKB:O66442"

/codon_start=1

/transl_table=11

/product="Acetylornithine aminotransferase"

/db_xref="COG:COG4992"

/translation="MVSSLFQNYTKVPIELVRGLGTTVFDQEGKSYLDFTSGIAVCNL

GHCHPQLVSVLEEQSHKIWHTSNLFENSLQEEVAEALCGVNYVAFFANSGAEANEAAL

KLARRHTGKEKIITCKNSFHGRTFATMSATGQAKIHEGFGSLLPTFDYVAYNDFSALE

AAIDEETAAVMLELVQGEGGVIPAEKEYITDLVALCHKMGVLVIVDEVQTGIGRTGNF

FCYQHYDFEPDIITLAKGLGSGFPVGAMLGKKELAKSFGAGVHGSTFGGNKLAMAVAS

KTIEIINEESFLADVTNKSLFFKEILNAQLAAKKQVVAIRGMGLMIGIELTVPVVEIV

EKLRTNGLLVLGAGEKVLRILPPLTVTKTELEQASQMIQEVIV"

CDS 1108947..1109888

/gene="argF"

/locus_tag="JFBMFIFI_01050"

/EC_number="2.1.3.3"

/inference="ab initio prediction:Prodigal:002006"

/inference="similar to AA sequence:UniProtKB:Q81M99"

/codon_start=1

/transl_table=11

/product="Ornithine carbamoyltransferase"

/db_xref="COG:COG0078"

/translation="MQTTKKDTLTLLEWSSEELLSMIELAIDIKGKPEKYQEALKGKI

LGMIFDKASTRTRVSFEAGMLQLGGSAIVLFSRDLQIGRGEPIIDTAKVLSEYLDGLM

IRTFAHKTVEQLAEYGTMPIINGLTDEHHPCQAIADLLTIYEVKGTFEGVKLAYLGDG

NNVCHSLLLASALVGLTIHCGIPQGYAVDPVILKIAQDIAESTGAVISITESPIEAVT

DADFIYTDVWTSMGQEAENDSRLKDFSEEYQVNAKLVAYAKPDYHFLHCLPAHREEEV

TSEIIDGPHSLIYQQAGNRLHGQKALLVMTLGENNSI"

CDS 1110046..1110519

/locus_tag="JFBMFIFI_01051"

/inference="ab initio prediction:Prodigal:002006"

/codon_start=1

/transl_table=11

/product="hypothetical protein"

/translation="MKREVVGLKFFFRNGETWTIGKEHIGDLWIKQITKSFGRIGTSD

FQEIYPCESLRIEIAQEADHVHTDDINLGGLELGMFERATQYPDIEMMDILYKDANHP

KSDVIVDKDRVYFPYKATDSDGNDNEYQSSHVKDNHLYIVIDPTKTVKDLYPEKF"

CDS 1110646..1113444

/gene="dinG_1"

/locus_tag="JFBMFIFI_01052"

/EC_number="3.1.-.-"

/inference="ab initio prediction:Prodigal:002006"

/inference="similar to AA sequence:UniProtKB:Q6GGV4"

/codon_start=1

/transl_table=11

/product="3'-5' exonuclease DinG"

/translation="MKKTETYAVVDIETTGGNVENGDKMIQFGCVLIEDGEIVQKFAT

DINPLMRIPKQIEYLTGITTKQVANAPYFEDVATTIYNLLADCIFVAHNIQFDYQFLN

GELKQSGLPPLKLKGMDTVELAQILLPTAPSFRLTDLATQFNFKHENPHQADSDAYVT

GQLFLLLKEKAKELPLVTLEKLVPLAQHCSMDTAEFFSDRLEELKSSLPPLPENLLIK

NGIALQKKEVVIESRSYREQSDYPLGTDEKTTLFKNKLTVREAQITMMDTLNTYFSGD

SKKHMAIEAATGVGKTLGYLIPLAYHGTPEKPVMVSTYTTLLQKQLMEKDIAQLNQLL

PFKLSAAIIKSKSHYLHLAKYEAELKKETTHKNEALIKMRILTWLTITKTGDIDELNL

ANLNQPFWQKVRHRGWLVDAKEDAWKDEDFYLFAKKQAEKASLIVTNHAFLCHDLQRE

ERELPSVETLVVDEAHHLSDVAISASSELFNYYSLIQAFRTLGKVEADSSFLQQFCQL

GKRVKAIKMYQLTSLEMSILSLDEQLSDFIDYLLAWSYSLKEGSQKHQEYTEILVDPE

TIWPLEIKKAIKDIHALFVEINFIGEQIIANCLNEKEQLGLSEIYFVEEFNQLLKGIA

KNHVVFTEVFQLSEKNDVTWFTYNEKSPKNSFKIKRSKLDSGSFLQTNLVEKIPKIVY

TGATLEIKESFSYFTEQIGEAELTTLVLPSPYNYAKQARLYFSTDMKPIKSLSKKQYV

TDIISHLEQLATENVENMLVLFNSLETLQDVYRGIQQKALFTEREILAQGISGSRERM

LKRFFHANGGILLGADSFWEGVDLPGKALRMIVITRLPFDSPEQPYTKAKYQLLAQQQ

LNPFSVDALPKATLRLRQGLGRLIRSEQDKGILLILDDRLFKTNYGKHMLQALPKELP

KESLTIKEISAETKKFLR"

CDS 1113554..1114030

/locus_tag="JFBMFIFI_01053"

/inference="ab initio prediction:Prodigal:002006"

/codon_start=1

/transl_table=11

/product="hypothetical protein"

/translation="MKKGIILTLTIVMSIIVIGAGYVYYVGHQPMAKARKEAVEIAEE

HAGLKEMDQFFWYNGTETYFTVTGKNDKKENIIVIIAKKGAKTQIIQQSDTISESEAR
KLTREAKNPKKILETRIGMEKGVPIWEVAYKEENGRLGYHIITLKDGEYVRDIGNI"

CDS 1114057..1115250

/locus_tag="JFBMFIFI_01054"
/EC_number="2.6.1.1"
/inference="ab initio prediction:Prodigal:002006"
/inference="similar to AA sequence:UniProtKB:P23034"
/codon_start=1
/transl_table=11
/product="Aspartate aminotransferase"
/translation="MDLISERMKNVSPSITLATSAKAKKLLAEGIDIIGLGVGEPDFQ
TADSIKKAAIRSIENGSASYYTATAGLPELRKAVLQRVLEDEGLVYEDNEVIVAEGAK
NVLYNLFQAILNEGDEVLILAPYWVSYTEQVKLAAGQPVVIETQPENDYKVTVAQLEA
QLSSKTKALLLNSPSNPTGMIYTKEELAAIGNWAVKNNILIVSDEIYGKLIYNGHEFI
SIATISDEIKAQTILVNGVSKTYAMTGWRIGYALGNAKIIKAMIDVASHSTSNPTAAS
QYAAIEALSGDQEIVETMRQAFEERLNVAYSLLSAIPGFDVKKPQGAFYLFPNVKKAA
MACGFSSVDEFVDGLLMEAHVAVVQGSGFGAPDNFRISYATDLATLTKGIERINQYVL
EKMND"

CDS 1115283..1116581

/gene="asnS"
/locus_tag="JFBMFIFI_01055"
/EC_number="6.1.1.22"
/inference="ab initio prediction:Prodigal:002006"
/inference="similar to AA sequence:UniProtKB:P67572"
/codon_start=1
/transl_table=11
/product="Asparagine--tRNA ligase"
/translation="MKRITINEAKNYVGEEVEIGAWVANKRSSGKIAFLQLRDGTAFF
QGIVVKSEVGDDVFQLAKGLNQETSIMVKGTIQEDSRSKFGYEIVVSGVEVIGESHDY
PITPKEHGTEFLMDHRHLWLRSSKQHAIMQIRNELIRATYEFFNKEGFMKIDPPILTA
SAAEDTTELFHTEYFDEEAYLSQSGQLYMEAAAMAFGKVFSFGPTFRAEKSKTRRHLI
EFWMIEPEMAFMDQDQSLEVQEQYVAYLVQSVLDNCDYALNVLERDKELLKKYTELPF
PRISYDDAVKLLNENGFDDVKWGDDFGSPHETFIASSFDKPVFILNYPKAIKPFYMKP

HPDRDDVVLCADMIAPEGYGEIIGGSEREVDFDNLSAAIKKFGLTEEDYAWYLDLRKY

GTVPHSGFGLGLERAVTWISGTEHIRESIPFPRLLNRIYP"

CDS 1116691..1117404

/gene="dnaD"

/locus_tag="JFBMFIFI_01056"

/inference="ab initio prediction:Prodigal:002006"

/inference="similar to AA sequence:UniProtKB:P39787"

/codon_start=1

/transl_table=11

/product="DNA replication protein DnaD"

/db_xref="COG:COG3935"

/translation="MNNQVLQKWLKAGDTTVSNVLLTYYRQIGLSNEELVLIIQLKAM

IDQGSLFPDTKQIADRMGSSTDQVFQGIHELIKKQVLTIETETSPDGKSQDYYSLTLL

WDKLVIYIHQEEQIKKQSDSIDQEKNLYEQFEAEFGRPLSPIEIETIGMWLDEDHYAL

ELIELALREAVLSQVYSLKYVDRILLSWERKNIRTKDQVLKESEKFRQNSAPANQVKK

VAKNESTVQVPLHNWLKNE"

CDS 1117425..1118069

/gene="nth"

/locus_tag="JFBMFIFI_01057"

/EC_number="4.2.99.18"

/inference="ab initio prediction:Prodigal:002006"

/inference="similar to AA sequence:UniProtKB:P0AB83"

/codon_start=1

/transl_table=11

/product="Endonuclease III"

/db_xref="COG:COG0177"

/translation="MFSKAKTIHAIEVMGEMFPNAKCELTHKNPFELLIAVMLSAQAT

DISVNKVTPHLFEKYPTPEAFLAVPVEAIMDDIKTIGLFRNKARHIQGCCRKLIDEFN

GEVPKTRKELTSLPGVGIKTANVVLGDAFGVPAIAVDTHVERVTKRLGICRLKDSVGE

VEATLMKKLPESMWVEAHHRLIFFGRYHCTARSPKCDICPLLEECREGKQRMGI"

CDS complement(1118136..1120772)

/gene="ponA"

/locus_tag="JFBMFIFI_01058"

/inference="ab initio prediction:Prodigal:002006"

/inference="similar to AA sequence:UniProtKB:P39793"

/codon_start=1

/transl_table=11

/product="Penicillin-binding protein 1A/1B"

/db_xref="COG:COG0744"

/translation="MMDNNNDEMSRVAQKQKKTTTKVKKNGKPKMKLWKKIILSIVGI

GVIGLASGVALFAYYASSAPTLTEKDLRGVISSKILDSKGQVIKEIGSENREVAEGDE

IPQQLKDAITSIEDRRFYKHFGIDPIRIGGAVVANLTGGFASEGGSTLTQQLVKLSVF

STKASDQTLKRKAQEAWLAIQLERKFSKEQILEFYINKIYLANNNYGMAKASTYYFGK

PLTELSLAQVALLAGMPQAPNTYDPYTNPEEATKRRNLVLDAMYDNKKITASERDAAK

AEDVTTGLVEHQADSSDDLVIDAYLEQVIEEVTKKTDLDVFTDGLTVYTNLDMEAQKH

LYDTLNTEDYIAYPDDQLQAGVSMVDVNTGQIKALGGSRKQTAQRGLNRATDLKRSVG

STMKPLSAYGPAIDYLNYSTYQQVIDGPYTYSDGTPIANYDKRYRGQMSIRTALEDSR

NIPALKTLQEVGLDKSNTFLQGLGIDIKQPDGVTNGLVESNAIGGEVTPLQLSAAYAA

FANGGTYYEPYAVSKVVLQNDSEIDLKTKGTEAMKDSTAYMMTDMLKDVIKSGTGTNA

QIPNLPQAGKTGTTNYDDTIAAKFPDSSAPDAWFSGYTTNYSISVWVGYDDPNADNHD

LNPTSQKLPGLIYKELMSHVSANIETKDWKKPSTVVSSAVENGSNPAKKPGPNTPQSS

IVNELFVKGFAPTETSISYGEALTAPTGLTASYDKAKNSISISWDAYKLQASGTPSYV

LTVGGQSYTTSDLNYTVLSPPEGEVSISLSVSANGKTGPAASTSVQVPKTTEEPPAKD

SSSDSSSQSSSSSSSSSSSSATSSSSEPESSSSSSSSSEEVPDPEGTPPPGGTARNTNA

KSGSKETIPFLAGLTSFVKNWF"

CDS complement(1120796..1121416)

/gene="recU"

/locus_tag="JFBMFIFI_01059"

/EC_number="3.1.22.4"

/inference="ab initio prediction:Prodigal:002006"

/inference="similar to AA sequence:UniProtKB:Q5KXY4"

/codon_start=1

/transl_table=11

/product="Holliday junction resolvase RecU"

/db_xref="COG:COG3331"

/translation="MAIKYPNGKSYTNSAEFSKKKQAPKKDHSFAKRGMTLEEDINAS

NESYLAFGKAVIHKKPTPVQIVQVDYPKRSAAVIKEAYFRQASTTDYNGVYKGFYLDF
EAKETKNKQSFPLKNFHEHQITHMKQCIEQQAICFVLLRFSLSNRLFLLEATKLISFW
DLQNNEGRKSIPLEELEKSGYELYYGLSPRIPYLDAVDLIIEKADE"

CDS 1121487..1122023

/locus_tag="JFBMFIFI_01060"

/inference="ab initio prediction:Prodigal:002006"

/inference="similar to AA sequence:UniProtKB:P50838"

/note="UPF0398 protein YpsA"

/codon_start=1

/transl_table=11

/product="hypothetical protein"

/translation="MSNLIISGYRSFELGVFKEDDPKIIVIKKCLKENLINLIENGVE
WILVGGQSGVEQWSAEVVNELKTDYPEIKLGVIFPFYEFGSNWNEQNRMKLEKIKGYA
DYTDSTSHKPYENPLQLRAHQQFLLDHSDEALLVYDPEFEGKTKYLYEAIQRKADQVN
YPCSLIDFDQLQNSSSDL"

CDS 1122123..1122452

/gene="gpsB_2"

/locus_tag="JFBMFIFI_01061"

/inference="ab initio prediction:Prodigal:002006"

/inference="similar to AA sequence:UniProtKB:Q8Y614"

/codon_start=1

/transl_table=11

/product="Cell cycle protein GpsB"

/db_xref="COG:COG3599"

/translation="MANRNLTTKDILQKEFKTAMRGYSPAEVDEFLDNVIRDYESYNK
ELTQLKAENERLLSKVDELTKQATVAKPAYSAQPNTTVTNFDILKRLSNLERHVFGSK
LDDTDEL"

CDS 1123087..1124241

/gene="rlmL"

/locus_tag="JFBMFIFI_01062"

/EC_number="2.1.1.173"

/inference="ab initio prediction:Prodigal:002006"

/inference="similar to AA sequence:UniProtKB:Q9K0V4"

/codon_start=1

/transl_table=11

/product="Ribosomal RNA large subunit methyltransferase L"

/db_xref="COG:COG0116"

/translation="MKNFQLVATAASGIEALVGKEIKDLGYECQVENGKVFFKGTERD

IAKTNLWLRTADRVKIIVGEFDAYEFDELFEKTKALPWEDLLPMDAHFPVAGKSIKSK

LYSVSDCQAIVKKAIVDRLSDVYHRNTRLPETGALYQLEVALLKDKVTLTLDTTGPSL

FKRGYRTSKGGAPLKENMAAALVMLTSWRKDRPLYDPVCGSGTIPIEAALIGHNIAPG

FNRSFAAEEWEWFDKEIWSEVRTEAEGLADYDVELDIVGTDIDGYMIEIAQENAIEAG

LGDSITFKQMQLSDFTTDKKYGVIIANPPYGERLGEEEAVHTLYRQMGTTYRPLKTWS

KYILTSDLTFESFYGERATKKRKLYNGALRTDFFQYWGERPPRAPRKQED"

CDS 1124244..1125752

/gene="ypwA"

/locus_tag="JFBMFIFI_01063"

/EC_number="3.4.17.19"

/inference="ab initio prediction:Prodigal:002006"

/inference="similar to AA sequence:UniProtKB:P50848"

/codon_start=1

/transl_table=11

/product="Carboxypeptidase 1"

/db_xref="COG:COG2317"

/translation="MTELEQQIQGFLSLLKEISLLEETDALLGWDALTGMPEDSSEHR

SEVTSYIAGLAFEKSVSKSMADYLAYLSQHQEELNSELKKMVEIVQKNYDLNHKIPQA

EYQEFIKVISQADSSWRKARDAQDFTIFEPSLAKIIAFKKKFIEYWHKDEKTPYDVLL

NQYEPGMTVEILDAVFTELREGIQEIVKKINEKGTTPTTDFLSRYISIENQRAFSIKV

IEKMGYRFTAGRLDDTTHPFMESMNRNDARITTRWDEHNFKMAVFGIIHEAGHGIYEQ

NIAERFDYTPLRGGVSMGIHESQSLFYEIVMGSDKNFWQDNYELLQSYSEGKLDDIDF

ETFYKGLHKTAPSLIRIEADTLTYPLHIIIRYEIEKLIFNEGVSVSDLPQIWNEKYQE

YLGITPENDTVGILQDVHWSGGSFGYFPSYALGFMYAAQLQHSMAQEIDLAATYKTGN

YEPIRQWLTDNIHQYGSLKEPNELIKNATGEPLNPRYLLALQDKIYQEVYHY"

CDS complement(1125823..1126176)

/locus_tag="JFBMFIFI_01064"

/inference="ab initio prediction:Prodigal:002006"

/codon_start=1

/transl_table=11

/product="hypothetical protein"

/translation="MIVLLTARSASDNAGIAMLLLTSLILFILGRLIYKGKMLWILAK

EPKGNPSKLKIQQQFRLARRVGSSAIWAAVVTLFGGLSSIRVSSDSLHTLFFSAFIFS

IVGPTIYLTQKTNRS"

CDS complement(1126272..1126700)

/gene="rnhA"

/locus_tag="JFBMFIFI_01065"

/inference="ab initio prediction:Prodigal:002006"

/inference="similar to AA sequence:UniProtKB:P54162"

/codon_start=1

/transl_table=11

/product="14.7 kDa ribonuclease H-like protein"

/db_xref="COG:COG0328"

/translation="MLKLYTDASTKGNPGPSGAGVVVIGENIYDQLAFPLTNLLDNHE

AEFEALICGLTYLKEQNMQAGTLMIYTDSQIVARAVERNYAKKANYQLYLSQIRSLLA

PFELTFINWVAEKQNKGADHMARQALQERLKSIEQKSSID"

CDS 1126954..1127448

/locus_tag="JFBMFIFI_01066"

/inference="ab initio prediction:Prodigal:002006"

/codon_start=1

/transl_table=11

/product="hypothetical protein"

/translation="MKNEDKKNYRLSLEPAYQVIYWSVSSILFSISLIGILEIQRANA

ISIIFALLFVTSVYLGMGSHLIIQQNKIYLNYLRGIKKTDISIDEIGHATVYRWGQDL

EMKNDEHSVVLYFFNKKNKQLFWKKFLEMYPEVSVTKDEDTVSYIHGATVENRMDTVK

KATN"

CDS 1127598..1128167

/locus_tag="JFBMFIFI_01067"

/inference="ab initio prediction:Prodigal:002006"

/codon_start=1

/transl_table=11

/product="hypothetical protein"

/translation="MESKVNPIYFENREEWRHWLLMNADKCTEIWFLFPLKASHKSGV

SYNDAVEEALCFGWIDSTVRSFNEEFKVQRFSRRNPKSTYSQANKERLHWLMANQLIH

PTILDEVSKVLSETFVFPEDILCLLKEGQEVWENYQTFSDPYKRIRIAYIDAARKRPD

EFHKRLNNFIRKTKQGSQIKGYGGIDKYY"

CDS complement(1128259..1128468)

/gene="cspD_2"

/locus_tag="JFBMFIFI_01068"

/inference="ab initio prediction:Prodigal:002006"

/inference="similar to AA sequence:UniProtKB:P51777"

/codon_start=1

/transl_table=11

/product="Cold shock protein CspD"

/db_xref="COG:COG1278"

/translation="MENGTVKWFSTEKGYGFIEYNDGEDLFVHFTGIEGEGFKTLSEG

QHVSFVVLEGTRGLQATQVNVLEKE"

CDS 1128700..1130373

/gene="fhs1"

/locus_tag="JFBMFIFI_01069"

/EC_number="6.3.4.3"

/inference="ab initio prediction:Prodigal:002006"

/inference="similar to AA sequence:UniProtKB:Q5XC12"

/codon_start=1

/transl_table=11

/product="Formate--tetrahydrofolate ligase 1"

/translation="MSLSDIEIAQQTEMEPIEKIAQKINLESDHLELYGKYKAKIDFE

TLEKSQEGSTGKLILVTAINPTPAGEGKSTVTIGLADALSRLGKSTMIALREPSLGPT

MGIKGGAAGGGYAQVVPMEDINLHFTGDMHAITAANNALSALIDNHIFQGNELLIDSR

RVIWKRVVDLNDRALRHVTIGLGGPLQGVPREDGFDITVASEIMAILCLATSLKDLKK

RLSKIVVGYTYQRQPVTVKDLKVEGALTLLLKDAIKPNLVQTLEHTPALVHGGPFANI

AHGCNSVLATRTALHLADYTVTEAGFGADLGAEKFLDIKVPQLKKAPDVVVVVATIRA

LKMHGGVPKSNLASENVEAVEIGFSNLQKHIENMQSYGLPVVVAINEFVSDTVAEVQM

VKEKCLALGVEAALTSVWEKGSEGGVALAEAVLAEIDHTQVFSPLYDTDNETIETKVT

KIVQKIYGGKGVAFSKKALTQMKNFEENGWGNLPICMAKTQYSLSDDPSLLGRPSDFI
VTIREFVPKLGAGFIVALTGDVMTMPGLPKQPAALNMDVAEDGTVSGLF"

CDS 1130548..1131300

/locus_tag="JFBMFIFI_01070"

/inference="ab initio prediction:Prodigal:002006"

/codon_start=1

/transl_table=11

/product="hypothetical protein"

/translation="MIKLTSGQVAQLTNISKSTLRHYVDKELVTPSVSNENGYHFFTE
KDLYMVFQIKMLRTIGFSIEEIRTSFRVDYFGDHFDEIYNRTKDQIDELEAIKDKLLK
LKEIKKSITIGKTEVVFCSDRYLKKLPESYQVGMFQLDYQTLSKLKQSDFDLSAELYY
MYHKTTGETLSYYEGSSSSYDKKLEAGNYAVKHFLANSEQKFEEEVAAFMKDSLFLLG
FVATNDLYIHENSYLSLGYSDEMVYSIEVRVK"

CDS 1131297..1131902

/locus_tag="JFBMFIFI_01071"

/inference="ab initio prediction:Prodigal:002006"

/codon_start=1

/transl_table=11

/product="hypothetical protein"

/translation="MTFKLHDLKEGDIDDSVFSNLILEGFNEKFTHKLYAKHSVENFS
RALANYYYANAQIIFILEGEEVCGCIVLSNQNVKITHLLRHFFEYSLGIVDCFRALFL
VMLLSHRVAKSECYVEMLVVRSIDQGRGFGNRLIEEAQNRLATDKTLTLYVANKNKAA
YYLYKKKQFHLEKIIYSRVSEYIMGVSKWSFMRYRKDSSDD"

CDS 1131895..1132899

/locus_tag="JFBMFIFI_01072"

/inference="ab initio prediction:Prodigal:002006"

/codon_start=1

/transl_table=11

/product="hypothetical protein"

/translation="MIKKIVLSLSLVLIAILAVFLVGNDYQMKEQRVSIPTTGGTLSA
VITAPKKQKINGVVVFVHGDGPQEATQEGGYKPLMERFAKQGYASISWDKLGVGQSSG
NWLSQSMDDRAKEVEQVIKWLKENKPEMVAKIGLWGASQAGWVIPKVLNSQEDIAFSI
LVAPAINWQSQGKYNTMEELRAAGKSEQELQKAGVDFDAESELIKKNNSYQDYLKSGG

EYDLSEDRYGFIQRNINSDASKDLEGVRSKIYLVLAEKDQNVDSDETKRIYSQKIANA

SLQVKVIKNAEHMMLNPKIAHSSFLIQTIGLFLPKYFLINEDYLNYCEHSLKEQQEEF

"

CDS 1132906..1133586

/gene="udg"

/locus_tag="JFBMFIFI_01073"

/EC_number="3.2.2.27"

/inference="ab initio prediction:Prodigal:002006"

/inference="similar to AA sequence:UniProtKB:Q5SKC5"

/codon_start=1

/transl_table=11

/product="Type-4 uracil-DNA glycosylase"

/db_xref="COG:COG1573"

/translation="MKGSELVDTILTKKMIEEAKRKTEKYAVEGFLAGQGPKNPKFML

IGEAPGEAEAMETGVPFSGRAGKELMRFFDLLEVTREDVYITSPFRSRPYKIKEKLDR

KTGEITSRKYNRAPNKKELLAHAPLIDYEIDQIQPPFILTMGNIGLQRLLGKDKKVGQ

VHGELIISPILKLKNNGLDGYEWTEKEYAIFPTFHPASIFYNRQLNEKIQADLLLFKK

LIQSKNER"

CDS 1133690..1135591

/gene="ettA_2"

/locus_tag="JFBMFIFI_01074"

/inference="ab initio prediction:Prodigal:002006"

/inference="similar to AA sequence:UniProtKB:P0A9W3"

/codon_start=1

/transl_table=11

/product="Energy-dependent translational throttle protein

EttA"

/db_xref="COG:COG0488"

/translation="MKDIKVLNLTKTYGEKTLFDDISFTINEGERVGLIGTNGTGKTS

LLNVLTGIDGAEKGDIQTAKDYRIGYLKQHPDLNPEQTVFDAVFDGDTPILVAVRGYE

KALERLTLDPMNENYQNQYSQAEQEMNRHDAWIADTNAKMILNQLGVKDLEQSVATLS

GGQRKRVGLAQVLIQAPDLLILDEPTNHLDFATISWLEKYLSSYRGALLLVTHDRYFL

DRVVNRMLELSYGKLTSYTGNYESYVRERAERQLEAEKQDQKRKQLYLKELDWMRAGA

KARTTKQQARIDRFHDLEGNLNQAKSDGKVEINMDGSRLGKRVFELKDASLTLDDHVI

LNEFNLLVQTRDRIGISGLNGAGKSTLLNTLAGRFILDSGEMIVGETVKIAYYTQMTE

DMDPTKRVIQYLQEVGEEIETTNGTRVSVTELLEQFLFDRHMHGALISKLSGGEKRRL

FLLKLLMERPNVLLLDEPTNDLDIATLTVLEDYIETFPGAVISVSHDRYFLDKTVERL

LIFEGNGIITPYLGSITEYIALNKDTPETHENKKAIDPVAAANTKDSEIKKEKVKLTF

TELKEWETIEEELEALESEIEDVKVAMAESGSDFTTLQELQVKMQKLEEDLENKMNRW

DYLGQFVTD"

CDS 1135606..1136553

/gene="thyA"

/locus_tag="JFBMFIFI_01075"

/EC_number="2.1.1.45"

/inference="ab initio prediction:Prodigal:002006"

/inference="similar to AA sequence:UniProtKB:Q834R3"

/codon_start=1

/transl_table=11

/product="Thymidylate synthase"

/db_xref="COG:COG0207"

/translation="MEKEYLALGKKVMETGAVKTDRTGTGTKSLFGYQMRFDLAKGFP

LLTTKRVPMGLIKSELLWFIKGDTNIRYLLQHNNHIWDEWAFERYIKSSDYVGPDMTN

FGRRAVTDPDFNAIYQVELEKFCTRILEDEAFAAIYGELGNIYGSQWRNWKTTQGETI

DQLKDVIEMIKKTPDSRRLIVSAWNPEDVPSMALPPCHTLFQFYVADGKLSCQLYQRS

ADIFLGVPFNIASYALLTHLIAHETGLEVGEFVHTLGDAHLYSNHMEQMTEQLSRDIR

EFPTLKLNPDKESIFDFDVADIKIEGYDPHPAIKAPIAV"

CDS 1136580..1137068

/gene="folA"

/locus_tag="JFBMFIFI_01076"

/EC_number="1.5.1.3"

/inference="ab initio prediction:Prodigal:002006"

/inference="similar to AA sequence:UniProtKB:P00380"

/codon_start=1

/transl_table=11

/product="Dihydrofolate reductase"

/translation="MIAYLWAQDEQGVIGNKGTLPWSLPNDLKYFKEMTTGNAIVMGR

KTFEGMGKRPLPNRINIVLTTDLGYEAEGVTVMHTREEILDFAKNYEKDTFITGGTGV
FNAFIDDADILYRTMIKGTFEGDTIFPELDWSEWEIISANPGILDERNRYPHVFEAYK
RK"

CDS complement(1137157..1137816)
/locus_tag="JFBMFIFI_01077"
/inference="ab initio prediction:Prodigal:002006"
/inference="similar to AA sequence:UniProtKB:P67153"
/note="UPF0073 inner membrane protein YqfA"
/codon_start=1
/transl_table=11
/product="hypothetical protein"
/translation="MKETLNNQTHFSKSYLIVNEVFNAVTHGIATGLSIAGLVILLIK
GAQSGSVLETVSYAIYGSTLILLYLASTLFHSLIFTRAKRVFQIFDHSSIYLLIAGTY
TPYCLVTIGGALGWTLFGIIWFMAILGVIYKCIWLDKYQNASTVIYIIMGWLCILAMK
PLYDGLGPVGFWLLVAGGVSFTVGALIYSMRSVKFMHVLWHLFVMLGTGFMYFSILLY
V"

CDS 1138087..1138929
/locus_tag="JFBMFIFI_01078"
/inference="ab initio prediction:Prodigal:002006"
/inference="similar to AA sequence:UniProtKB:Q97QT7"
/codon_start=1
/transl_table=11
/product="DegV domain-containing protein"
/db_xref="COG:COG1307"
/translation="MKKIKIVTDSSAELGSDEIESLGIHVLPLTVMIDDVLYTDGVTI
KKEDFMGMMAEAKDLPKTSQPPIGYFVELYDELGADGSEVISIHMTEKLSGTVNAAHQ
AAQLSKTDVTVIDSDFTDRGLSFQVLEAAKLAQEGADKETILKRIEHVKEHTKLYICV
VTLENLVKGGRIGKLAGALSSFLNIKVMMELVNGQLEVCVRGRGMKPINKWIAELQET
LKNTPNLNAMSFSFADDEVYLENLKNEFQAILPDTPMRVKLTSPIISTHAGKGAFAIM
YYTD"

CDS 1139011..1139898
/locus_tag="JFBMFIFI_01079"
/inference="ab initio prediction:Prodigal:002006"

/codon_start=1

/transl_table=11

/product="hypothetical protein"

/translation="MKRIRSFLGIILFLGVVALIVFQVMMFISKDNSKTTIIENKGKE

SSSEQIKTKELNLVAIGDSLTEGIGDSTGRGGYVPLVAELLESKDEIETVSTSNYGIS

GNRSDQILKRIKKDEKLQNDVKKADVIVLTVGGNDLMKVVRSTLLKVKEDSFIKPQKE

YKERIEETFKELRSLNSDAPIYVFGIYNPFYLYFSEITEMQDIVDSWNETTQSVVEEE

QKAHFIPINDILYKGGNQPELSENQKDTIDSSVKDKKESKVFNDLLFEEDNFHPNDSG

YELMAQSLFDEMMASRKEW"

CDS 1139931..1140566

/locus_tag="JFBMFIFI_01080"

/inference="ab initio prediction:Prodigal:002006"

/codon_start=1

/transl_table=11

/product="hypothetical protein"

/translation="MKTKLTNWLKSLKKIRIDNPWKWAFLGLIAIILGLGIWGFTKIT

VPVNTITDTTASKNKKDVSFEVAAEKAEINRLVAHSLNEFIEDDGIKYELVLNDEAEL

IGTFKLFGHDVQFYLYLLPYVMENGNVQLKATNLSVGELNLPITSVMNLLAKQLKIPE

WVQIDSKKQLIVLNLNEYKTDTGMTIKAKKFDLKNDDIRFTISVPLKEIKK"

CDS 1140582..1141103

/gene="msrA_1"

/locus_tag="JFBMFIFI_01081"

/EC_number="1.8.4.11"

/inference="ab initio prediction:Prodigal:002006"

/inference="similar to AA sequence:UniProtKB:P54154"

/codon_start=1

/transl_table=11

/product="Peptide methionine sulfoxide reductase MsrA"

/db_xref="COG:COG0225"

/translation="MKEIAIFAGGCFWCMVAPFDEQPGIQQVLSGYTGGQTVNPTYED

VMSHKTGHTEAVEITFDPTIISYQELVDIYWQQTDPTDAMGQFQDRGDNYRPVIFVTG

EKQREIAEHSKSQLEASGKYKKPIVTMIEEASVFYPAEEYHQNFYKKNPARYEAEQQE

RQAYEKENRRDGV"

CDS 1141103..1141324

/locus_tag="JFBMFIFI_01082"

/inference="ab initio prediction:Prodigal:002006"

/inference="similar to AA sequence:UniProtKB:O31864"

/note="UPF0346 protein YozE"

/codon_start=1

/transl_table=11

/product="hypothetical protein"

/translation="MRQSFYHFLMTERNPHKRDEVSQFANDAFLDIAFPKQSESYHDI

SHYLEMNGEYLPSMRIFDEAWERYIEKMN"

CDS 1141361..1142071

/gene="deoD"

/locus_tag="JFBMFIFI_01083"

/EC_number="2.4.2.1"

/inference="ab initio prediction:Prodigal:002006"

/inference="similar to AA sequence:UniProtKB:Q5EEL8"

/codon_start=1

/transl_table=11

/product="Purine nucleoside phosphorylase DeoD-type"

/db_xref="COG:COG0813"

/translation="MSIHIEAQKGQIAETVLLPGDPLRAKYIAETFLTEVEQYNRVRN

MFGYTGLYKGERISVQGTGMGLPSMMIYAEELITEYNVQNLIRVGTAGGMQKDVKVRD

VVLAQAATTDSSINRNTFGGQVDFAPIADFDLLKTAYDIGTEKGLSLKVGNVLSADRF

YNAELDKVKLANYGILAVEMEAAGLYSLAAKHNRKALAILTISDHIFTGEETSSDERE

RTFTDMMIVALETGLKQK"

CDS 1142088..1143572

/gene="ctpA"

/locus_tag="JFBMFIFI_01084"

/EC_number="3.4.21.102"

/inference="ab initio prediction:Prodigal:002006"

/inference="similar to AA sequence:UniProtKB:O34666"

/codon_start=1

/transl_table=11

/product="Carboxy-terminal processing protease CtpA"

/db_xref="COG:COG0793"

/translation="MNNESEEKETQLNSSKRKTKIGLPAYIVSLLLVALIAAGGTYFV

TKQLNENATSQSATNSGAKTEQEFAKLQEVYQQLTTRFFQKTDSNKLIEGAITGMVNS

LDDPYSQYLNAEEATALNDSISSSFEGIGAEVMNQDDAITISAPIVGSPAEKAGLKTN

DIILKADDKELTGLSLTKAVSFIRGEKGTKVVLTIKRGDQVFDVTVTRDTIPVETVKS

RLDENDPTIGYIQITSFATPTYKEVTEAVEQLRKDGAKSFIFDVRQNPGGLLDQALQL

SNMFVDEGKILMQTQERGQEPQVIKADASLGDFKVTEPVTLLVDEGSASASEILAGAM

KESGNVTVIGTKTFGKGTVQTVANLSDKSELKLTIAKWLTPEGQWIHKKGLEPTIAVD

LPDYVHLLRVDPSKKYQAGDLSDQVKNLQAILKALGYTLDNTDGYFDASTTEAVNAFQ

TAHELPVDGIVTGKTADALVADLVKLIKENDTQYEKAVSYLQEK"

CDS 1143871..1144416

/gene="sipV"

/locus_tag="JFBMFIFI_01085"

/EC_number="3.4.21.89"

/inference="ab initio prediction:Prodigal:002006"

/inference="similar to AA sequence:UniProtKB:O07560"

/codon_start=1

/transl_table=11

/product="Signal peptidase I V"

/db_xref="COG:COG0681"

/translation="MENKIWRDHLWDWFKAFLLAVCVAVALRYFILLPITVQGSSMIP

TLHQGDQMIVESISKMRRFDIIVFQDSSNRTLVKRIIGLPGDTIVYKEDQLYVNDKKV

SEPFLKNSLVEKAGETWTSDFDLEQLTGTNKIPKNSYFVLGDNRRSSNDSRAFGFVDE

KDIIGKTNFIYYPMKRAGFIN"

CDS 1144475..1145140

/gene="sipS_2"

/locus_tag="JFBMFIFI_01086"

/EC_number="3.4.21.89"

/inference="ab initio prediction:Prodigal:002006"

/inference="similar to AA sequence:UniProtKB:P28628"

/codon_start=1

/transl_table=11

/product="Signal peptidase I S"
/db_xref="COG:COG0681"
/translation="MAKTNYSDTSFDEIENFEPRSRKYPSKQSGKNKKGKKNKRNEIV
STFGYIIVALVIALLVRQFLFAPVSVDGESMMPTLKDGDRIVLNKFEKIDRFDIVVFP
GPDDPSRLYIKRVIGLPGDEITIQDDILYINGKKVDEPYLDVFKAKLKENQLLTGDFT
LMGKTGESKVPEGEYFVMGDNRSNSKDSRIFGFVHADKIDGTAEFRIWPLTDFGFIKA
REK"

CDS 1145210..1146079
/gene="rbgA"
/locus_tag="JFBMFIFI_01087"
/inference="ab initio prediction:Prodigal:002006"
/inference="similar to AA sequence:UniProtKB:O31743"
/codon_start=1
/transl_table=11
/product="Ribosome biogenesis GTPase A"
/db_xref="COG:COG1161"
/translation="MTIQWFPGHMAKARREVSEKLKLVDIVFELVDARIPLSSRNPIL
DELIGEKPRVIILNKSDLADERQTKAWVNYFKEQGISAVPIVAQEGKGMQNVMSEAKL
LLKEKFDRMRSKGINPRAIRAMSIGIPNVGKSTLINRFIKRNIAKTGNKPGVTKGQQW
LKLGKELELLDTPGILWPKFEDPEIGKKLALTGAIKDTLLQMDDIALFGLDVMRTYYP
NQLAKRYSLTKEELVLELPELLILISEKRGFREDYSRASEMIVFEIRSGRLGRYTLDQ
APVTLPTEGLENE"

CDS 1146079..1146864
/gene="rnhB"
/locus_tag="JFBMFIFI_01088"
/EC_number="3.1.26.4"
/inference="ab initio prediction:Prodigal:002006"
/inference="similar to AA sequence:UniProtKB:O31744"
/codon_start=1
/transl_table=11
/product="Ribonuclease HII"
/db_xref="COG:COG0164"
/translation="MEKKQTIKEIKTILASITDANDPRLIELKNDERKGTINAIKSWE

KAQNKLVALEVKHQEMLAYELAIWQDGRNYIAGIDEVGRGPLAGPVVAAAVILPHDFH

VLEINDSKQLSESKRDELFDKIKDSALAIGIGIKDEGIIDKVNIYEATKLAMIEAVEN

LTIDPEHLLIDAMVLDLPIPQSKIIKGDTKSLSIAAASIIAKVTRDRMMKEYAKMYPG

YGFEKNAGYGTKVHLAGLDAQGITPIHRKSFSPVKELVLETTE"

CDS 1146990..1147859

/gene="dprA"

/locus_tag="JFBMFIFI_01089"

/inference="ab initio prediction:Prodigal:002006"

/inference="similar to AA sequence:UniProtKB:Q8DPI7"

/codon_start=1

/transl_table=11

/product="DNA processing protein DprA"

/db_xref="COG:COG0758"

/translation="MKKETLRDQLVHFTLCQGIGATASIQMIAALIDQPTSSLYELSQ

LVGLSASKRTAFITSYHKINFEEIKKLYQKMHIGWVTILDDDYPDYLKHIYNPPAVLF

YQGNKELLRKKMLAVVGARKNTLYGVRALKQILPGLIASDFLIVSGLATGIDFEAHQL

TLELSGETIGVVGTGLDLCYPKENQDLQKKLAKEHLVLSEYPLGTKPLKQHFPMRNRI

ISGLAIGTLVIEAAARSGSLITANLALAEGREVFALPGNITSSLSAGTNELISKGAKC

VTKTEDILEEFYQ"

CDS 1148041..1150119

/gene="topA"

/locus_tag="JFBMFIFI_01090"

/EC_number="5.6.2.1"

/inference="ab initio prediction:Prodigal:002006"

/inference="similar to AA sequence:UniProtKB:Q7A5Y5"

/codon_start=1

/transl_table=11

/product="DNA topoisomerase 1"

/translation="MAYKYLVIVESPAKAKTIEKYLGKNYKVLASLGHIRDLPKSKMG

VDVENNYEPHYISIRGKGDIIKDLKKHAKKAEKVFLAADPDREGEAIAWHLSFLLGLD

IEDKNRVVFNEITKEAVKGAFKEPRSINMDLVDSQQARRILDRLVGYTISPILWKKVK

KGLSAGRVQSVALKLIIDRENEITTFKPEEYWSIGGNFKKAAKKFKANFYGIDGKKKE

LKNTDDVQAVIKKLTSKDFEVTNVTKKERKRNPAVPFTTSSLQQEAARKLNFRTRKTM

MVAQQLYEGISLGRGGAVGLITYMRTDSTRIADSAKAEAAEFIVKEYGKEYSATTFKK
TKNAQGSQDAHEAIRPSSVLRTPAEIEKYLTKDQLKLYKLIWSRLVASQMTPAVFDTM
RVDLLQNGVMFRANGQKIKFPGFTKVYIEGNDEGTEEKDNILPDMEKGDLVKSVDIEP
KQHFTQPPARFTEATLIRSLEENGVGRPSTYAPTLETIQRRYYVKLQSKRFEPTELGG
IVNTIIVEFFPQIVDIHFTADMEQQLDFVEEGKENWVGVIDRFYKPFEIEVAKAEEGI
EKIQIKDEPAGFDCDLCGHPMVIKLGRYGKFYACSNFPECRNTKAIVKEIGVTCPVCK
EGQVIERKSKKNRIFYGCDKYPECEFVSWDKPVGRDCPKCQHYLVEKKLKGGKQVVCS
DCDYKEAVQK"

CDS 1150369..1151688

/gene="trmFO"

/locus_tag="JFBMFIFI_01091"

/EC_number="2.1.1.74"

/inference="ab initio prediction:Prodigal:002006"

/inference="similar to AA sequence:UniProtKB:P39815"

/codon_start=1

/transl_table=11

/product="Methylenetetrahydrofolate--tRNA-(uracil-5-)-
methyltransferase TrmFO"

/db_xref="COG:COG1206"

/translation="MNNEQFVNVIGAGLAGSEAAFQIAERGVKVHLYEMRPVKNTEAH
HSPNFAELVCTNSLRANQLTNAAGLLKEEMRQFNSLIIAAADATAIPAGGALAVDRDT
FSKKVTDCLKNHPNITVYSEEVTTLPEGPTIIATGPLTSEALAKEISAFTESEGLYFY
DAAAPIVDKNTIDMDKVYLKSRYDKGEAAYLNCPMTETEFNRFYDALVEAEVAPLKSF
EKEKFFEGCMPVEVMAQRGRKTLLFGPMKPVGLEDPKTGRRPYAVVQLRQDDAAGSLY
NLVGFQTHLKWGEQKRILQMIPGLEQAEIVRYGVMHRNTFMKSPDLLKQTYQSKKRSD
LFFAGQMTGVEGYVESAASGLLAGINAAKLIKGEELLVLPTETTMGSMAHYITHADAR
YFQPMNANFGLFPPLPERIRDKNLRNTAYAERSLEIVANLKKIRSIQ"

CDS 1151755..1152654

/gene="xerC_2"

/locus_tag="JFBMFIFI_01092"

/inference="ab initio prediction:Prodigal:002006"

/inference="similar to AA sequence:UniProtKB:P39776"

/codon_start=1

/transl_table=11

/product="Tyrosine recombinase XerC"

/db_xref="COG:COG4974"

/translation="MKEDQWKKTFLQYLIIERHYSELTRKAYDEDISDFHDFLAETGE

ASFFAVELADVRIYLGVLNEKQYSRNTVSRKISSLRAFYQFLLKNEVVEENPFSYIHL

KKKSLRLPRFFYEKEMESLFEAVKGDEPLDFRNEALLEVLYGTGIRVSECCGICLKDI

DFELGILLVHGKGNKERYVPFGHYAEEAIRTYLKLGREVLMTKYGKQHDYLFINHHGD

GITATGVEYVLNQVIKKSSLTSEIHPHMLRHTFATHLLNNGADMRTVQELLGHASLSS

TQIYAHVTKEHLQKDYRAFHPRA"

CDS 1152823..1153362

/gene="hslV"

/locus_tag="JFBMFIFI_01093"

/EC_number="3.4.25.2"

/inference="ab initio prediction:Prodigal:002006"

/inference="similar to AA sequence:UniProtKB:P65797"

/codon_start=1

/transl_table=11

/product="ATP-dependent protease subunit HslV"

/translation="MTTFHATTIFAIKHNGKSAMAGDGQVTMGESVIMKGTARKVRRI

YNGEVLVGFAGSVADAFTLEEKFEGKLNEYKGNLKRAAVELAQEWRTDRAMQKLEALL

IVMNKDEMLMVSGGGEVIEPDDGILAIGSGGNYALSAGRALKKHGKHLSAQEIAQESL

TVAADICVFTNHNIVVEEL"

CDS 1153381..1154799

/gene="clpY"

/locus_tag="JFBMFIFI_01094"

/inference="ab initio prediction:Prodigal:002006"

/inference="similar to AA sequence:UniProtKB:P39778"

/codon_start=1

/transl_table=11

/product="ATP-dependent protease ATPase subunit ClpY"

/db_xref="COG:COG1220"

/translation="MMNTNMQMTPKEIVAELDKYIIGQHQAKKSVAVALRNRYRRMQL

DEEMQREVTPKNMLMIGPTGVGKTEIARRLAQLVQAPFVKVEATKFTEVGYVGRDVES

MVRDLVENAIKIVEKQQYSNVYTKAENKAVERLAKLLVPGIKKEKKKAESTNPFESMM

NNMGMNMNQMAEEPEEEITEQISTNRKVIAQQIRDGLLDQRELTVEVEERKKTVNSPM

NAGLEQMGIDLNDTLGALTPKRKVKRTVTVKEALELFIQEESSKLVNPEDIHSEAIRL

AQNTGIIFIDEIDKITSKAGQSGEVSREGVQRDILPIVEGSQVSTKYGTIQTDHILFV

ASGAFHVSKPSDLIPELQGRFPIRVELDDLDASDFVKILTEPNNALVKQYVAMLGTEN

INVTFTFEAIERLAEIAYQVNHETDNIGARRLHTILEKLLEDLLFEAPDMHMGDITIT

ESYVNDKIEHIVADKDLSRYIL"

CDS 1154822..1155631

/gene="codY"

/locus_tag="JFBMFIFI_01095"

/inference="ab initio prediction:Prodigal:002006"

/inference="similar to AA sequence:UniProtKB:Q819X8"

/codon_start=1

/transl_table=11

/product="GTP-sensing transcriptional pleiotropic

repressor CodY"

/db_xref="COG:COG4465"

/translation="MNELLLKMRQINSMLQQEGGIDSAKVAKSGSLPFNSMVEILGNS

LDANTYLISEKGRLLGFNEKHEINNERVKQMLQDKKFPQDYTTSMLSITETKANIGIE

SDYTAFPIENRDLVADGLTTLVPIYAAGERLGTIILARISREFTDSDLILAEYSATIV

GMEILNQKSNQIEEETRSLAIVQMAIKTLSYSELKAVKAIFEELDGTEGRLVASTIAD

KIGITRSVIVNALRKLESGGIIDSRSLGMKGTYIRVNNSKFIQELAKEQIY"

CDS 1155815..1156693

/gene="lacX"

/locus_tag="JFBMFIFI_01096"

/inference="ab initio prediction:Prodigal:002006"

/inference="similar to AA sequence:UniProtKB:P23496"

/codon_start=1

/transl_table=11

/product="Protein LacX, plasmid"

/translation="MTIQLENERISVTILTKGAELASIKNKETGIEYLWQADPTYWGR

HAPVLFPVVGRLKEDSYQVDGKSYPMNQHGFARDGDFEIVEQDDSSVTLELGATTETK

RVYPFDFKLKIQYRLVGETVTTHYTVENPSSTEKLYFSIGAHPGFNIPLTKETVFEDY

FLSFSPRKTRTTIPLTGAYLNTPAKTLAQTNTDIALSRHLFDNDALIYEVKGENIFTI

KSEKTPHSVSVKFTDFPYVGVWSPPKTDAPFVCIEPWFGIADTFDANGNLKEKLGIQS

LEPTQQFEAEFDITVS"

CDS complement(1156764..1157378)

/gene="plsY_2"

/locus_tag="JFBMFIFI_01097"

/EC_number="2.3.1.275"

/inference="ab initio prediction:Prodigal:002006"

/inference="similar to AA sequence:UniProtKB:Q45064"

/codon_start=1

/transl_table=11

/product="Glycerol-3-phosphate acyltransferase"

/db_xref="COG:COG0344"

/translation="MLEICIMLVIAYLLGSIPSGVWIGKVFYHKDIRNFGSGNSGTTN

TYRVLGKKAGTAVLLMDILKGTLAASLPFFFHSDVNPLIIGLGAIVGHTYPIFAQFKG

GKAVATSAGALLAYNPQFFVFCALLFITLIYVTRMVSLASIIAVLIIAPASWYYHDPI

LTTIASILMVFIILRHRGNITRILDGTENKVPFGLGYKKSKKNE"

CDS 1157708..1159723

/gene="parE"

/locus_tag="JFBMFIFI_01098"

/EC_number="5.6.2.2"

/inference="ab initio prediction:Prodigal:002006"

/inference="similar to AA sequence:UniProtKB:H7C794"

/codon_start=1

/transl_table=11

/product="DNA topoisomerase 4 subunit B"

/db_xref="COG:COG0187"

/translation="MPKKINMEYNDEAIQVLEGLEAVRKRPGMYIGSTDSRGLHHLVY

EIVDNSVDEALSGYGTEITVTLHKDTSVSVKDNGRGMPVGMHASGIPTAQVIFTVLHA

GGKFGQGGYKTSGGLHGVGASVVNALSEWLVVTIVRDGVVYEQRFKNGGIPVDTLKKI

RNTKEKNGTTVHFKPDTTIFSTVNYSYDILSERLRESAFLLKGLKIELIDERTEKSEI

FHYEEGIKEFVEYLNEEKDHLNPVAYFSGENNQIEIEFAFQYNDGYSENILSFVNNVR

TKDGGTHESGMKTSLTKAFNEYARKVNLLKEKDKNLEGSDFREGLAAIISVRIPEDLL

QFEGQTKGKLGTPQARAAVDAVVGEQLGFYLIENGETSQMLVRKALKAREARDAARKA
REESRNGKKRKKNESLLSGKLTPAQSRNPKRNEIYLVEGDSAGGSAKQGRDRKFQAIL
PLRGKVINTEKAKLQDILKNEEINTMIYTIGAGVGAEFDLADCNYDKVIIMTDADTDG
AHIQVLLLTFFYRYMKPLIEAGKVYIALPPLYKISKGIGKKEVIEYAWTDDELEAKTK
KVGKGYILQRYKGLGEMNADQLWETTMDPETRTLIRVRIDDAAQAERRVSTLMGDKVE
PRRKWIESHVEFSLEDGGSILENSQMMEASNEQVALFETEGSVEDGN"

CDS 1159713..1162184

/gene="parC"
/locus_tag="JFBMFIFI_01099"
/EC_number="5.6.2.2"
/inference="ab initio prediction:Prodigal:002006"
/inference="similar to AA sequence:UniProtKB:P72525"
/codon_start=1
/transl_table=11
/product="DNA topoisomerase 4 subunit A"
/db_xref="COG:COG0188"
/translation="MEIRSDIQELTLEEVMGDRFGRYSKYIIQERALPDIRDGLKPVQ
RRILYAMNVEGNTSEKGFRKSAKTVGNVIGNYHPHGDSSVYEAMVRISQDWKLREVLV
EMHGNNGSMDGDPPAAMRYTEARLSKISAELLRDIEKETVDFVLNFDDTDKEPTVLPA
KFPNLLVNGATGISAGYATDIPPHNLSEVVDATIYLIDNPEATVEELMHHLKGPDFPT
GGILQGIDGIRQAYKTGKGKVIVRSKTHIEDLRGGKQQIVITEIPFEVNKSVMVKRMD
EIRLNKKIDGIAEVRDESDRTGLQIVVELKKDANAEGILTYLLKNTDLQVSYNFNMVA
IDKKRPEQVGIVRMLSAYIEHQREVITRRTNFNLFKAESRQHIVEGLIKALSILDKVI
AAIRSSKDKKNAKENLIKEFAFTEPQAEAIVSLQLYRLTNTDITALEKEAKELAASIS
SYRKILDSPKEMARVLKNELKEIQEKFRTPRRTIIQDAIEELKIDTEVLVSQEEVIVS
VTREGYLKRSSLRSYSASKPEEIGLKDGDLPIFVKQMNTLNHLLMFTSNGNLIYRPVH
EIADLRWKDIGEHISQTIGLAMDETIVKTIGLVDYLPEATFTFITKAGMIKQTAVADF
VAGRGYKTRATVAMKLKQEDDHLIEVVYSENPANKDVFLVTNRGFGLRYPLSEVPVVG
SKAAGVKAINLKEHDYVVNGILMATDSTRTNVVMLTQRGAVKKMNVSEFDILGRAKRG
LLVLRELKKNPHRMIFMQAVEKDTTINVVTTKGKEFIVNTDSYQVSDRYSNGSFILDE
TTDGEPQDCQIILPYPVIVKDNGKN"

CDS 1162627..1164873

/gene="pflB"

/locus_tag="JFBMFIFI_01100"

/EC_number="2.3.1.54"

/inference="ab initio prediction:Prodigal:002006"

/inference="similar to AA sequence:UniProtKB:Q7A7X6"

/codon_start=1

/transl_table=11

/product="Formate acetyltransferase"

/translation="MKQWNGFKGEKWRTSVNTRDFIQANYTEYTGDDSFLEPIAPSTD

KLWTKLQELFDVQHEKNGVYDMDNNIPATITSHKPGYLIKEEETIVGLQTDVPLKQAF

MPFGGINMANNALTANGFEVDDEMTSIFTDWRKTHNQGVFDAYTPEMRAARKNKIITG

LPDAYGRGRIIGDYRRIALYGVDFLMKQKAKDHANTGNKVMTDDVIRLREEISEQYRA

LGQIKEMAASYGFDISQPAETAKEAIQWLYFGYLAAIKSQNGAAMSIGRISAFLDIYI

QRDLEAGLITEFDAQEMIDHLIMKLRMVKFARTPDYNQLFSGYPIWATLSIAGMGLDG

RSLVTKNDFRILHTLTNMGPSPEPNLTVLYSSKEPTGFRTFAAKIAKESSSIQFENDD

LLRANWGSDDCAIACCVSATVVGKDMQFFGARANLAKAVLYAINGGVDEMTKAQVGPK

FRPMTGDTLDYNEFISRYKDIMDWLAELYVNTLNVIHYMHDKYAYEAPQLALMDSDLK

RTFATGIAGISHAADSLMAIKHGNVEVIRDENGLAIDYKPTNEFPTYGNDQEEADETA

NWILDYFMTQIKRQHTYRNSTPTTSLLTITSNVVYGKATGNTPDGRRAGKPLAPGANP

SYQDGAFLGEKNGLLASLNSTARLNYGCALDGISNTQTINPTGLGKDEETRIDNLRNV

MDGYFDNSGYHLNVNVFTNELLLDAQAHPEKYPNLTIRVSGYAVKFRDLTPEQQADVI

ARTSHDRL"

CDS 1164940..1165698

/gene="pflA"

/locus_tag="JFBMFIFI_01101"

/EC_number="1.97.1.4"

/inference="ab initio prediction:Prodigal:002006"

/inference="similar to AA sequence:UniProtKB:Q7A7X5"

/codon_start=1

/transl_table=11

/product="Pyruvate formate-lyase-activating enzyme"

/translation="MSQPVIGTIHSTENFGTVDGPGVRFIVFTQGCRMRCQFCHNPDT

WKIGGGKERSTDDILAEALRYKTYWGKDGGITVSGGEPLLQMEFLIDLFKKAKAAGVH

TTLDSCGKPFTREEPFFSQFEELMKYTDLILFDIKHIDDEQHKVLTSLGNSNILEMAK

YLSEINQPVWIRHVLVPQRTDYDEYLIRLSDFIKTLNNVKKVEVLPYHTMGLYKYQEM
GIPYPLEGIETPSTERVNNAKKILQTADYTGYLN"

CDS 1165804..1166751

/gene="hdfR"

/locus_tag="JFBMFIFI_01102"

/inference="ab initio prediction:Prodigal:002006"

/inference="protein motif:HAMAP:MF_01233"

/codon_start=1

/transl_table=11

/product="HTH-type transcriptional regulator HdfR"

/translation="MKTKSDNIFSSKTLNYFLQLADTMNYTQAAQLLGITQPALTQQI
KKLERIAGAPLFYSVGKKLHLSDAGKTMLRTTHEVYDILNAATDEIQQTTSATIGKIR
IGLLSSIEDKVFTDFIINYFNKFPDVEIALMMLPRNEIWEKLENNKIDLAIMYLPDES
IKNWKPYKSKKIMDEEILFLHHDENLDKKKKIKMKDTTGKRWVTYPDSYYINEVLKES
FKNQMADLPVVAGNYTTPAQLYKFSNATECYTALPNSFIKAHEKEAELKAIPFEPAIK
FTMSFVFRSDKDQIPRIANFLQSFDAYMSESDYISRLKS"

CDS 1166779..1167705

/gene="ppaC"

/locus_tag="JFBMFIFI_01103"

/EC_number="3.6.1.1"

/inference="ab initio prediction:Prodigal:002006"

/inference="similar to AA sequence:UniProtKB:P37487"

/codon_start=1

/transl_table=11

/product="Manganese-dependent inorganic pyrophosphatase"

/db_xref="COG:COG1227"

/translation="MSKVLIFGHLNPDTDAITSAISFAYLQKQLGVDAEAVSLGEISE
ETAFALDYFNVPAPRIVETVANETKQVMLVDHNEFQQSVADIREVEVLAVVDHHRIAN
FETANPLYYRAEPVGCTNTIILKLYKEHGIEIPKEIAGLMASAIVSDSLLFKSPTCTE
EDVKAAKELAIMSEIDLAVYGLDLLKAGTNLADKTPEQLLNMDAKSFPMGAKNVRVAQ
VNTVAIEDVFNKQAAIEATMLKEMAENNFDLFILVVTNILDSDSVILALGNPITAVEE
AFNVTLTDNKALLKGVVSRKKQVVPQLTEALS"

CDS 1167862..1169613

/gene="btsS"

/locus_tag="JFBMFIFI_01104"

/EC_number="2.7.13.3"

/inference="ab initio prediction:Prodigal:002006"

/inference="similar to AA sequence:UniProtKB:P0AD14"

/codon_start=1

/transl_table=11

/product="Sensor histidine kinase BtsS"

/db_xref="COG:COG3275"

/translation="MFSLFIMMMERVGLIILLAYLLVNVTYFKTILLNRERLSSKLQL

MAVFGLFAIISNFTGVELTANKIISTDFLTVLSDNASIANTRTLTIGVSGLIGGPFVG

LGVGLLAGMHRVIQGGGTSLFYLFSSSFVGILSGLAGARFARKKQFPSPIQAAGIGAL

MELVQMLFVFLFSGTLLEGWLLVKFIAFPMILLNSIGTFIFLSIITSTLKQEEQMRAV

QTHDVLDLAAKTLPYFREGLTESSCRQVAKIIQQYTKVAAISMTDTHQILAHVGAGSD

HHIPELEVITELSKDVLQNGQLAIAKSKNQIGCSNPTCQLSAAIVIPLISKNQVVGTL

KMYFTDPTKLTHVEEQLAEGLGTIFSSQLELGEAEIQSKLLKDAEIKSLQAQVNPHFF

FNAMNTISALMRQNAEQARTLLLQLSTYFRANLQGARQVLIPLTAELKHVEAYLSLEQ

ARFPQRFQVTFNIDPNLETLLLPPFLLQVLVENAIRHAFGNRKTDNQIVVQLEQKENF

VLVQVSDNGIGIPLDRVEKVGKEVIESEKGTGTALENLNKRLVGLFGLEASLQFSQNK

TGGTTVLLKIPLDREEG"

CDS 1169615..1170349

/gene="lytR"

/locus_tag="JFBMFIFI_01105"

/inference="ab initio prediction:Prodigal:002006"

/inference="similar to AA sequence:UniProtKB:P60611"

/codon_start=1

/transl_table=11

/product="Sensory transduction protein LytR"

/db_xref="COG:COG3279"

/translation="MHILIVDDEPLARDELAYLVETHPNVISVDKAESIEEALEKMVN

QKPDLVFLDIHLTDESGFDLADKFKKMNHPPKIVFATAYDEYALKAFEVDAIDYILKP

FEEERVRQAVEKSHSAISHLSTSEETTNLAREINGKIAIQADERIFVIALSDILYISV

DNGASHIITKQQQIDTNETLTHLESRLNSRLFFKTHRSFIVNREAIQEVQPWFNHTYQ

VTVKNGDKIPVSRSYVKQLKEEIGLN"

CDS 1170527..1170970

/gene="lrgA_2"

/locus_tag="JFBMFIFI_01106"

/inference="ab initio prediction:Prodigal:002006"

/inference="protein motif:HAMAP:MF_01141"

/codon_start=1

/transl_table=11

/product="Antiholin-like protein LrgA"

/translation="METKKIYTFLQQAFVFAFIMLLANLIVGILPFPMPASVMGLILL

FIALCLKIVKLEQVESLGNSLTGLISFLFVPSGISVMNSLGIMGQYGVQIVLIIIIAT

IILLAITGWTATALLNLKKNQSFSWDGLKSLFSIKRTHKKLDEVK"

CDS 1170972..1171655

/gene="lrgB"

/locus_tag="JFBMFIFI_01107"

/inference="ab initio prediction:Prodigal:002006"

/inference="similar to AA sequence:UniProtKB:P60643"

/codon_start=1

/transl_table=11

/product="Antiholin-like protein LrgB"

/db_xref="COG:COG1346"

/translation="MTPYFGIIVSMVAFGIGTFLFKKSNGFFLFTPLFVAMVLGIIFL

KVGHFSYTEYSSGGKMISFFLEPATIAFAIPLYKQVDVLKKYWVQIVSAIIVGSIGSV

AVVYILSGIIHVDQSIMASLLPEASTTAIAVPLSESIGGIAAITSFAVIFNAVIIYAL

GRFLLEKFKIKNPIARGLALGASGHALGVAVGIELGEVEAAMASIAVVVVGVVTVIVV

PAFAVFIGL"

CDS complement(1172233..1173669)

/locus_tag="JFBMFIFI_01108"

/inference="ab initio prediction:Prodigal:002006"

/codon_start=1

/transl_table=11

/product="hypothetical protein"

/translation="MLISLKKEEWFGNIKNDILGGLVTSIALIPEVIGFAIIAGVNPI

TALFASVTTAIVTSITSGRPAMVSAAAGSMALVMVTLIKNHGIEYMVAATILTGILQL
ALGYLGVHKLMKFISKPVMLGFVNALAILIFLAQVQQLGNKNIFTYIMVGISIVLMYM
IPKLSKAIPPALIVIIIMTLLSLFPQFGLQTVGDLGDMSGKLPFPSLPMVPFTLETLI
IIFPTALALSMVGLIESLLTLPLIDDMTESKGDSQREVKAQGLANVVTGFFGGPAGCA
MIGQAVINVKSGGRKRLSTLTAGLALLFLIVALKSLMIQIPTAALIGIMITVAFETFD
WESTKKLKKVSLTETLIMLVTVAIVVYTHNLAIGILVGVLLSIFVFIAKVSKLKFNKA
NETIYISGQLFFASANSLIEYFEKQDPQENIRIDLSHVHIWDESGIDALFATLDILDT
KYHSYKIVGLQTTNMKLNARIDSINSLE"

CDS complement(1173692..1174102)

/locus_tag="JFBMFIFI_01109"

/inference="ab initio prediction:Prodigal:002006"

/codon_start=1

/transl_table=11

/product="hypothetical protein"

/translation="MKKIVLAFDGSTHALNAVNEAEKLMQGFPDSSVTILEVLDIAKA
KDQVLDLSEDYEERKAVRIEEVKQQLIGLLDIFEITILIGDPASEIIQHVKKNDYDLL
IMGSRGLNLLQEFVLGSVSHKVMKYVSIPVLIVK"

CDS complement(1174366..1176042)

/gene="cdr_1"

/locus_tag="JFBMFIFI_01110"

/EC_number="1.8.1.14"

/inference="ab initio prediction:Prodigal:002006"

/inference="protein motif:HAMAP:MF_01608"

/codon_start=1

/transl_table=11

/product="Coenzyme A disulfide reductase"

/translation="MKIVIIGSVAAGTSVAAKARRNTEDAEICVYDQDKDISYSICGI
PYYIGEEVEDLDKLTPRNAAWFKKRYNVDIFTEHRVTAINTEKQTLEIQNLQTGEIFI
DSYDELVLATGAKPIVPEVFLNQQSKQNIFHVRNIQDARSIHEFIQNGKINTATIIGA
GFIGLEMAEQLAHKGIEVTLIQRGQQIMKQMDSDMAFRAQKELETNNVDVLLNTTITK
VIGKEEFITELVTNQGKTIKSDLVILAAGVEPNTSLTQATKIKLGTSGAIAVTKKMET
SVPHIYAVGDVAESFSVITKKPIYRPLGSTANKMGRIAGDVITGGTLEHRGILGTGIV
RAFDLTIGYTGLSEKEALAEGIDVAILYNIKPDHADYLGGKELTIKALADKSTGKILG

AQIIGRQGVDKRIDVLATAISFGAVAEDLFHLDLAYAPPFATTKDPILYTGMALDNAV

KKGTPLMTPNELMKRQVIGEKLQIIDTRSKKQYEKSSVEGAIHIPLTELRKRQSELDK

DLVTVTYCNKGVSGNAAQNILINKGFKEVYNLSGGNKNYQVIVEMENHND"

CDS complement(1176039..1177112)

/gene="acr3"

/locus_tag="JFBMFIFI_01111"

/inference="ab initio prediction:Prodigal:002006"

/inference="similar to AA sequence:UniProtKB:Q8NQC8"

/codon_start=1

/transl_table=11

/product="Arsenical-resistance protein Acr3"

/db_xref="COG:COG0798"

/translation="MESVTKKLSLLDRYLTLWIFLAMGIGVGLGYFVPDIATGINKLE

IGTTSIPIAIGLILMMYPPLAKVKYEEMWRVFKDWKVLLLSLFQNWIIGPILMFVLAV

LFLHDYPEYMVGLIMIGLARCIAMVIVWSNLAQADNEYTAGLVAFNSIFQIVFYSVFA

YIFVTVIPGWLGLETHSISIGMGEIATSVFIYLGIPFIAGFLTRKILVKRKGITWYEE

KFIPKISPITLIALLFTIIVMFSVKGEKVIELPMDVIRIAIPLLIYFVLMFFVSFFIS

KKMGTTYPIAASLSFTAASNNFELAIAVAVGVFGINSGEAFAAVIGPLVEVPVLIALV

NVALKFKQKYYKTNKNNRNEAKE"

CDS complement(1177191..1177556)

/locus_tag="JFBMFIFI_01112"

/inference="ab initio prediction:Prodigal:002006"

/codon_start=1

/transl_table=11

/product="hypothetical protein"

/translation="MDYKLIAKIFKALGDSNRVQIVDLLSCGELCACDLLEHFNFTQP

TLSHHMKVLISAEIVQTRKSGIWHNYSLNEENMTFISDVIQSLVKNNENCICHSIENG

ECSCNNDQLVNSSNSTYLK"

CDS complement(1178146..1178709)

/locus_tag="JFBMFIFI_01113"

/inference="ab initio prediction:Prodigal:002006"

/codon_start=1

/transl_table=11

/product="hypothetical protein"

/translation="MKKSFSAFLVSIFFLLFIVSGCKKENNSVNEDTIKEVLSELLNL

PNEKLFKSIQNYSDQVISGEVNNSKEPEYSSILENMFHSQFTDEGYEKFINETAMYYV

TTTQDAGFITKVKDVYVTQNKEDELRYTFTATINYTDNNNESGTTKISGNAEFKEKGK

LTIFKITTNDLLEKMKKIANEVKIPKE"

CDS complement(1178761..1179276)

/gene="paiA_1"

/locus_tag="JFBMFIFI_01114"

/EC_number="2.3.1.57"

/inference="ab initio prediction:Prodigal:002006"

/inference="similar to AA sequence:UniProtKB:P21340"

/codon_start=1

/transl_table=11

/product="Spermidine/spermine N(1)-acetyltransferase"

/db_xref="COG:COG0454"

/translation="MKVEIKKCELKDLDCIRKISIRTFKDTFEKYNTEQDLINYIDTA

YNKNKLRIELLNPNSIFYFIFSDDDLAGYMKLNSKEAQTENTFSNSLEIERIYILPAH

KRKGLGKKLIHFASQYGKDSGVETIWLGVWEKNEPALHFYKNLGFCITGEHSFMLGQD

KQTDYLMTKQI"

CDS complement(1179266..1179745)

/locus_tag="JFBMFIFI_01115"

/inference="ab initio prediction:Prodigal:002006"

/codon_start=1

/transl_table=11

/product="hypothetical protein"

/translation="MVEILRDIGTIARALDSIANVEFKNYKLSKGQYLYIVRIYENPG

IIPDKLAELVKVDRTTAARSINKLVENGLIKKMTDPTNKKIRRLYVTSKGIEVVPIIQ

KENDYSNTVALQGFTEEEALQFSSMLKKISTNIDSDWKIVKKGHKRNYLKGNDSNEG"

CDS 1180048..1181352

/gene="umuC"

/locus_tag="JFBMFIFI_01116"

/inference="ab initio prediction:Prodigal:002006"

/inference="similar to AA sequence:UniProtKB:P04152"

/codon_start=1

/transl_table=11

/product="Protein UmuC"

/db_xref="COG:COG0389"

/translation="MELELDYTNEPRSDILCIDVKSFYASVECVERNLNPLTTMLVVM

SNAENAGGLILASSPAAKQRLGISNVMRKFDLPTHKDLLIVPPRMALYIDKNKEIIEI

FRKYAIEDDILIYSIDEAFIKVTPIKKLLKATAYEIARLIQQDIYHTLGLYSTVGIGD

NPLLAKLALDNEAKDAKDFKAEWRYEDVQEKVWQISPLTDMWGIGRRTAKKFELMNIK

SVYDLAHYDYYLLKEKMGVIGEQLYAHAWGIDRSDIAKKYTPLEKSYSNAQILPRDYT

LASEIELVIKEMADQVATRLRQKQCQTQCISLFIGHSMYESTKGFNHQMKISATNNTK

KLTDFCLQLFRQHYRGETIRQIGVGCSKIVYSTDIQLDLFRESEEVEADAGLDFIVDS

IRDKYGFTSLVHASSLMTGATAIKRSHLVGGHASGKDEKDVK"

CDS 1181342..1181722

/locus_tag="JFBMFIFI_01117"

/inference="ab initio prediction:Prodigal:002006"

/codon_start=1

/transl_table=11

/product="hypothetical protein"

/translation="MLNKFNERYQDRGTIKWLGMYLSEHTASMDADKSSRSLKILQKK

QMNFLEIATILDSALIKNQAVSIQLEEIDTDGNYLPDITGKIEGYEDSGIWIDNTHIA

YEEIRNISLYTEIKWSAFEKRNQS"

CDS complement(1181780..1181977)

/locus_tag="JFBMFIFI_01118"

/inference="ab initio prediction:Prodigal:002006"

/codon_start=1

/transl_table=11

/product="hypothetical protein"

/translation="MIPKNRLKQLTTIIALILVSVALTACGGNSKKEEITPGKESKVK

IDGKEYDVKTTEEGAAIKMGE"

CDS complement(1182111..1182821)

/locus_tag="JFBMFIFI_01119"

/inference="ab initio prediction:Prodigal:002006"

/codon_start=1

/transl_table=11

/product="hypothetical protein"

/translation="MELNQVMKTLERLGTEQTKKTFLRHGAPGPLFGVKIGDLKKELL

KDVKKDQQLALDLFETGNSDAQYLAGLTINPKLIEKKQLTHWANYSNWSAISEFVVAG

VASESPYAVELAKEWMTSDQELIEDTGWSCYGKFLSFAPDTSIDQEEVLALLKIIENT

IHQAKNSVRYTMNQFVINTGCYYTPLLKEAQQVAETIGPVQVTLGSKDCKVPLASEAI

EKAIRLERIGKKRKKVIC"

CDS complement(1182838..1183731)

/locus_tag="JFBMFIFI_01120"

/inference="ab initio prediction:Prodigal:002006"

/codon_start=1

/transl_table=11

/product="hypothetical protein"

/translation="MKILGNKIKNARLTAGLSQKELAAGICNQSTISNLEKGISIPSL

LTLMIISERLNIDFDEIYQYTTKKKDSYADIFQQVRVLCAQIKHKDAYDLIKEKIDFN

RLETPYELKQYYYYLGITSLIGNKNSSDGIYYFNLALTTESGKNVEFLDILATSGMAT

VYDIRNEDNKALTYFEKVMNQLDQLTCSTPRIEDGIEVAKIYYNMAKFYSKIKDYKLA

VHLSTLGINLIQEQHISYYLDFLLYEKGFNLMKLGQVEEAERYYFYAAAMANLNDNQH

AVDGIKRNMKDYNLKGYNYWE"

CDS 1184109..1185473

/gene="mntH"

/locus_tag="JFBMFIFI_01121"

/inference="ab initio prediction:Prodigal:002006"

/inference="similar to AA sequence:UniProtKB:Q93V04"

/codon_start=1

/transl_table=11

/product="Divalent metal cation transporter MntH"

/translation="MSRKEELKEKHQSTGWLRKSHNISLEEVNNTVKIPQNAGFWRKL

FAFMGPGALVAVGYVDPGNWATSIAGGSKFGYTLLSVILISNLIAMLLQEMSARLGIA

TDMDLAQATRNSVGKKPAIVLWILTELAIVATDIAEVIGSAIALNLLFNIPLLVGVAI

TTLDVLVLLLLQKKGFRIIESIVIVLMFTIFIVFLFEVILTRPDMAALLSGYIPSSQI

VTDPSMLFIALGILGATVMPHNLYLHSSIIQTRQYERTLEGRKEAVKFAKIDSVFSLT

GAFVINSLILILGASAFHGTGSVVAEIQDAYKLLSPTVGVAAASTLFAVALLASGQNS

TITGTLSGQIVMEGFIHLRVAPWLRRVITRLIAVVPVFICTWLLGEGGTAQLLLWSQV
ILSLQLPFAVVPLVLYTSDKKKMGEFVNPTWIKILAWIATVVIIALNIFLVGYILITG
QDLG"

CDS complement(1185516..1186367)
/gene="aadK"
/locus_tag="JFBMFIFI_01122"
/EC_number="2.7.7.-"
/inference="ab initio prediction:Prodigal:002006"
/inference="similar to AA sequence:UniProtKB:P17585"
/codon_start=1
/transl_table=11
/product="Aminoglycoside 6-adenylyltransferase"
/translation="MRSEEEMYHLILQKAQLDERIRGVLLNGSRANKHVKPDRLQDFD
IMYVVTELTSFIEDSRWVDYFGPRLIMQLPDESALFPNDTSKETFAYLMQFEDGNRID
LTLISSETVKKQTKLDSLTKVLLDKDQLFTNLPESDATDYLVSLPSEAEFNDCINEFW
WVSTYVAKGLARKEITYAKAMLDGPVRDMFLLALNWKLVAQSNQPLNLGKNGKFLQSY
LTTEEWRALLTTYSNGEIQSTWEALFNMCAFFMAISDYIELKLEYKNKTKQEKILTYL
EKLKNNQ"

CDS complement(1186585..1187166)
/locus_tag="JFBMFIFI_01123"
/inference="ab initio prediction:Prodigal:002006"
/codon_start=1
/transl_table=11
/product="hypothetical protein"
/translation="MNNTKNKTYRIAILGILTAIIFIQSFVPMLGYIPIPPLNPTIIH
ITVIIAALSLGTKEGVILGGVWGLTRWAKVFLMPGPLDPIIFINPIITILPRILVGFI
AGYAFYLLRRKLKESSAMVIASVLASLTNTILVLFFIYIFNGADYAAAMKVDVSGLLK
VLGTVVLTNGLAEALAAGIIAPIVARTLKKIVH"

CDS complement(1187277..1187867)
/locus_tag="JFBMFIFI_01124"
/inference="ab initio prediction:Prodigal:002006"
/codon_start=1
/transl_table=11

/product="hypothetical protein"

/translation="MGRDKNNTYRIAILGMLTAIILIQNFVPVMGYIPIPPLNPTIIH

ITVIIAAITLGYKDGMILGGIWGVACIIRAFTFPTTPLDLLLFTNPVIAIVPRILVGL

VAGYVYQILKRTAVKDTFAMIISAILGSLTNTILVLTFIYIFYRQEYAAFVQVDVGSL

LVVLGTVVITSGLAEAIAAAVIVPLVAKPLKHFVLK"

CDS complement(1187986..1188900)

/gene="mccF_1"

/locus_tag="JFBMFIFI_01125"

/inference="ab initio prediction:Prodigal:002006"

/inference="similar to AA sequence:UniProtKB:Q47511"

/codon_start=1

/transl_table=11

/product="Microcin C7 self-immunity protein MccF"

/translation="MLKKGDTIGIIACSNGKKLSQKKKINELINLLETQFSLTVIEAA

TIYEKNEFPFSGTPAERAQALMQLYLNPSVVMIFDISGGNVANEILPYLDFKQIEKQR

KLFVGYSDLTVILNALYTKTTCSGINYQLLNLVGEEQSSQLAYFNDYFFHNKRQTSIT

AQWLSSSSPITNIIKGTLIGGNIRCLLKLAGTDYLPNCQNKLLFLEANSGDITAMATY

LAQLDQIGYLQQCQAVILGQFTEIEAQKQTTAFYKLVSSYLMNYQKPILMTHEIGHSA

SSKALSIGEIVYFDEDHGKLRMTLADDK"

CDS 1189183..1189575

/locus_tag="JFBMFIFI_01126"

/inference="ab initio prediction:Prodigal:002006"

/codon_start=1

/transl_table=11

/product="hypothetical protein"

/translation="MNTIIRKNPRTMPAPVGEYSHVTIVPKNSTLYTFSGQIGTDLSG

FISEDHEKQVEDTFKNIERALLSEELTSDNIIKVNVWSVDEINWDHFYDMWGKLFSVN

PSMTIAYVTALGLPEIKIEIEILAAKPE"

CDS complement(1189643..1190203)

/gene="rutB_2"

/locus_tag="JFBMFIFI_01127"

/EC_number="3.5.1.110"

/inference="ab initio prediction:Prodigal:002006"

/inference="protein motif:HAMAP:MF_00830"

/codon_start=1

/transl_table=11

/product="Peroxyureidoacrylate/ureidoacrylate

amidohydrolase RutB"

/translation="MADKSHSVLLVVDVQKAFNDVSWGERSNQTAESHISELITLFRQ

NEIDVIHIKHQSNNPESLFYPEHITSEFKSEATPLKNELILTKTVNSAFIGTNLEEIL

HEKGITKLYIVGLTTPHCVSTTTRMAANLGFKCYLVEDATASFELIGHTGITYSANEV

QELTVVTLNEEFAEILSTSQVQEQFE"

CDS complement(1190244..1190927)

/locus_tag="JFBMFIFI_01128"

/inference="ab initio prediction:Prodigal:002006"

/codon_start=1

/transl_table=11

/product="hypothetical protein"

/translation="MKVKIKTLFIVIGIILIGLLGYLLWHKFAPDIEMFFNPDVSKEI

LMNKVRSHGISTGVLLVLLTSIMCAIPGLPTSIIGVLIGICYGPLVGSMLNIIGNTLG

NILSIFLIRKFKFIDKSKTSNHWVKTISRAKHPDIGITFAYMIPVIPSFLVNYTTTIM

NVKAKRLIWMVLFGALPSSVLYAFGGDAFFKGNHEKAILLVASVLILILLVFLIEKDA

KSDHSKIKK"

CDS complement(1191129..1191650)

/gene="yraA"

/locus_tag="JFBMFIFI_01129"

/EC_number="3.2.-.-"

/inference="ab initio prediction:Prodigal:002006"

/inference="similar to AA sequence:UniProtKB:O06006"

/codon_start=1

/transl_table=11

/product="Putative cysteine protease YraA"

/db_xref="COG:COG0693"

/translation="MTHTNRKIIALVSDDFEDLELWYPVLRLREAGFTVDLVAEKAQT

VYHGKYGVPVTSDYSFTDIKVADYAGILVPGGWSPDKLRRFPEVLEMVRYFDTQKLPI

GQICHAGWVLISAGILDGVNVTSTPGIKDDMTNAGAIWHDTPAITDGHIISSRRPPDL

PEYMQHYLAALEK"

CDS complement(1191733..1192662)

/gene="msrR"

/locus_tag="JFBMFIFI_01130"

/inference="ab initio prediction:Prodigal:002006"

/inference="similar to AA sequence:UniProtKB:Q99Q02"

/codon_start=1

/transl_table=11

/product="Regulatory protein MsrR"

/translation="MRNQHRKKKHFFRNFILIICVILIGIGGWAFASYRGSLKEALKD

QNLPSESDITFNGEKAKDANDVNILFIGSDSRGEDRGRSDSLMIAHYDSKAKQPKLVS

VMRDMYVDIPGNGKDKINAAYSYGGAELVRQTISEDFGINIQYYAIVDFESFPKIVDT

LLPEGVEIDAEKDMSEYIDVAISAGPQKMDGQTLLQYARFRHDEESDFGRVRRQQQAL

NALAEQGAQFNKITKLPSILGKVEGYTVTNLPNSLLLSVAKDFILGDTKPMETLTLPV

EGSWENATYEGSGSVLDINVEQNREALAKFLED"

CDS complement(1193000..1193314)

/locus_tag="JFBMFIFI_01131"

/inference="ab initio prediction:Prodigal:002006"

/codon_start=1

/transl_table=11

/product="hypothetical protein"

/translation="MRIITVCGSFKFKDEMMKVAEQLELTGNVALIPIFPLNNNINAF

TEKELTMLGKMHKEKIKLSDAIVVVNVNSYIGKSTKSEIEFAESLNKEIVYYTDLISN

IK"

CDS complement(1193318..1193752)

/locus_tag="JFBMFIFI_01132"

/inference="ab initio prediction:Prodigal:002006"

/codon_start=1

/transl_table=11

/product="hypothetical protein"

/translation="MGIESGLLEQINTFYQELSELKKQVLKQHRINEVQLKILTELKI

EALSLKKLALSLTTEKSTLSRQVASLVKSGWLIKDQSADKRMQFISLTVFGKESLTKI

EEQLEQPWTSLFHTWSEEEKQLLSILLGRVARSLHQQKKVDE"

CDS complement(1193878..1194696)

/gene="ssuB"

/locus_tag="JFBMFIFI_01133"

/EC_number="3.6.3.-"

/inference="ab initio prediction:Prodigal:002006"

/inference="similar to AA sequence:UniProtKB:Q8KZQ6"

/codon_start=1

/transl_table=11

/product="Aliphatic sulfonates import ATP-binding protein

SsuB"

/db_xref="COG:COG1116"

/translation="MMTLPSVLSVKGIKKTFYPGTANQNEVLKGLDLEVKAGDFVTII

GGNGAGKSTLLNSIAGNFIIDSGSIKINDKDITKLKEDQRAGLIGRVFQDPRLGTAPR

MTVGENMAMAYRRGAKRGLRKGTSAKEQDFFKEQLSRLDLGLEDRLAAEMGLLSGGQR

QAITLLMATMKRPELLLLDEHTAALDPKTAKVVLALTDERIKADNLTALMITHNMQDA

LTYGNRLIMLDYGKVVVDLNASEKANLTVPEVMNLFQSATKDGQIKDEMILSSN"

CDS complement(1194696..1195589)

/locus_tag="JFBMFIFI_01134"

/inference="ab initio prediction:Prodigal:002006"

/codon_start=1

/transl_table=11

/product="hypothetical protein"

/translation="MNLIVTATSQGLLWGIMALGIFITFRILDLPDMTAEGSFPLGAA

VCVKLIIMGIHPIVATLIAFVTGMLAGAVTGILITKFNIPGLLAGILTMTGLYSINLR

VMGRANLSLLGKPTIDNLLQKFNLPSQFDTIFIGLFIAVFVISALVLFFRTELGQALI

ATGDNEIMARSLGISTNKTKILGLMLSNGLIAFAGALIAQDDGYADISKGVGTIVIGL

ASVIIGEVVFGNLSFKNRLICVILGSIIYRLIIMFVLFIGLESNDLKLISAFILAICL

AMPSLRTKFNLNFFTTNKEAR"

CDS complement(1195614..1196600)

/locus_tag="JFBMFIFI_01135"

/inference="ab initio prediction:Prodigal:002006"

/codon_start=1

/transl_table=11

/product="hypothetical protein"

/translation="MKKMIGFITVLALILSYAFVTESKKEDQKKSAIPVVGVLQLTSH

PALDKIYEGIVDALKEDGFIDGKTMVLDFQNAQGDQSNLKTMSERFMTKKADIMVGIA

TPSAQALANVSSEVPIVLGAVTDPKGAGLVKDNQHPGGNITGVSDQTPVEDQFNLIKT

LVPKAKKIGIIYSSSEDNSIIQGKQAAEAAKKLGLETITATVASTNDVSQIATNLATQ

VDAIYVPADNTVASAMSTLVNITDAAKIPVFPAVDTMVEEGGLATVGLNQYELGKMTG

KMTARILKGESKPATTPIEYLKTGDKIVNPKKAQELGITIPQDILDSAIIIK"

CDS complement(1197008..1197787)

/gene="trpA"

/locus_tag="JFBMFIFI_01136"

/EC_number="4.2.1.20"

/inference="ab initio prediction:Prodigal:002006"

/inference="similar to AA sequence:UniProtKB:Q97P33"

/codon_start=1

/transl_table=11

/product="Tryptophan synthase alpha chain"

/db_xref="COG:COG0159"

/translation="MTKKTLEKAIRQAKVPVVTYIMAGDGGLYQLEKDLQLLEESGVA

AIELGIPFSDPVADGPVIQAAGLRALNDGVSLEGILTTLSKSKVKVPLVIMSYFNPIM

QIGIEHFVHLAQKTFIKGLIIPDLPYEHQELIQPYLSGTDIALVPLVSLTSPLERIKT

LVSEAEGFVYAVTVNGTTGERQLFNADLDRHLETIKALSPVPVLAGFGISNLEQVKHF

NQVCDGVIIGSKIVDMLHRKKRMELEDFLKQATETKSTTER"

CDS complement(1197780..1198985)

/gene="trpB"

/locus_tag="JFBMFIFI_01137"

/EC_number="4.2.1.20"

/inference="ab initio prediction:Prodigal:002006"

/inference="similar to AA sequence:UniProtKB:Q81TL8"

/codon_start=1

/transl_table=11

/product="Tryptophan synthase beta chain"

/db_xref="COG:COG0133"

/translation="MTYQAPDKNGFYGNYGGRFVPETLMEAVKELELAYEASKTDEAF

QKELAYYLVEYVGRKSPLYFAEQLTKYAGGAKIYLKREDLNHTGAHKINNTIGQALLT
KKMGKRKIVAETGAGQHGVATATVAALFNMDCIIFMGEEDVKRQALNVFRMELLGATV
VTVTSGGKTLKDAVNEALRYWVAHVEDTHYVMGSVLGPHPFPQIVRDYQSVIGLEAKE
QHLEKENRLPEAIVACVGGGSNAMGLFYPFINDESVKMYGVEATGMGIETTLHAASMA
KGEVGVLHGSMMHVLQDKFGQITEPYSISAGLDYPGVGPEHSFFRDLGRATYHGVTDK
EAIAAFQLLCQVEGIIPALESSHAIAYAIKLAKEMQPEESMIVCLSGRGDKDVDQIRT
YLKGGSSHD"

CDS complement(1198987..1199583)
/gene="trpF"
/locus_tag="JFBMFIFI_01138"
/EC_number="5.3.1.24"
/inference="ab initio prediction:Prodigal:002006"
/inference="similar to AA sequence:UniProtKB:Q56320"
/codon_start=1
/transl_table=11
/product="N-(5'-phosphoribosyl)anthranilate isomerase"
/db_xref="COG:COG0135"
/translation="MKVKICGLKEAKDVETAIEAGADFIGFVFAPSKRQITKKDAHEL
AKKIPKRVKIVGVFVDAPLEEVLDFIKSVPLDLVQCHGNESVAYLNQLPVPAIKAFGV
QSDFALEKLKPYADHYLLLDAPIAGNGETFEWKNLAIPVDYRKQLFLAGGLTASNIQS
AIRYFHPLVVDVSSGVETNQQKDPQKIKEFIKKAKEVA"

CDS complement(1199580..1200344)
/gene="trpC"
/locus_tag="JFBMFIFI_01139"
/EC_number="4.1.1.48"
/inference="ab initio prediction:Prodigal:002006"
/inference="similar to AA sequence:UniProtKB:Q9PI11"
/codon_start=1
/transl_table=11
/product="Indole-3-glycerol phosphate synthase"
/db_xref="COG:COG0134"
/translation="MSFLEEIIAVKNKEVASMQIEEIKKIKKRPSFYQFLKEHPQHMQ
LIAEIKRASPSKGAIATDIDPLSQAMLYEKAGAGAISVLTDEQFFKGSITDLQRVAEV

VNVPLLCKDFIISKKQLIRASNAGASIVLLIVAALSVKQLTELYQAAEELGLEVLVEV

HDLAELKIAENLGAKLIGVNNRNLKTFEVSIAVSEILAKELSLKSSTCYISESGFKEK

ADVQRVAKDYQAVLVGEALMREQSPEQAARNLRIKR"

CDS complement(1200341..1201363)

/gene="trpD2"

/locus_tag="JFBMFIFI_01140"

/EC_number="2.4.2.18"

/inference="ab initio prediction:Prodigal:002006"

/inference="similar to AA sequence:UniProtKB:Q8YXQ9"

/codon_start=1

/transl_table=11

/product="Anthranilate phosphoribosyltransferase 2"

/db_xref="COG:COG0547"

/translation="MIETLLQKIYRKENLSISETRLIGSYMLSGTASSIEITAFLTAL

KMKGETIDEMVGMVEAIKSLGASVQEESATAMDNCGTGGDASESFNISTTSAFVLAAA

GIPIAKHGNRSISSRSGSSDVCQELGIELNLSIEEATQALKTVGITFIFAPHVHPNMK

YVNSIRKELKTPTIFNLIGPLTNPVPLETQLVGIYRRDLIENVALTLQQLGRKRGIVV

NGADFMDEASLSGINHYALLEDGIVSLHTVTPEEVGLKTYQLDDIKGGDAKENAAILL

SVLRGESSAYYDTVLLNSGLGLYANGKVSSIKAGVEMANKVIQSGLALQKLEEIKLFT

QGVKQL"

CDS complement(1201356..1201946)

/gene="pabA"

/locus_tag="JFBMFIFI_01141"

/EC_number="2.6.1.85"

/inference="ab initio prediction:Prodigal:002006"

/inference="similar to AA sequence:UniProtKB:P28819"

/codon_start=1

/transl_table=11

/product="Aminodeoxychorismate/anthranilate synthase

component 2"

/db_xref="COG:COG0512"

/translation="MILLVDNYDSFTYNLAQYIGEFAHEVVIKRNDASDLFDSATQAD

GIILSPGPGTPSTAGNMQELIRIFHQTKPILGICLGHEGIGQFFGAKLVIAESVMHGK

VSKISQNGGAIFQQLPDELTVMRYHSLVLLKEHFPETLTILATSMDDHEIMALKVKDY

PVYGLQFHPESIGTPHGKKMIENFIKETRKEMKVND"

CDS complement(1201943..1203316)

/gene="trpE"

/locus_tag="JFBMFIFI_01142"

/EC_number="4.1.3.27"

/inference="ab initio prediction:Prodigal:002006"

/inference="similar to AA sequence:UniProtKB:P20580"

/codon_start=1

/transl_table=11

/product="Anthranilate synthase component 1"

/db_xref="COG:COG0147"

/translation="MKKMTVLKKDSHSINQFYRALTGEKKVLLESYDNNTSKGRFSII

ARNPLHELKVYQKNFYFDGVKSVEADPLAKIQHFISRNHGENQLDLPFQGGGIGYVGY

DTLAIYENLGPLPPETRDIPDCCFYIYESFMIFDHQKKQLTLVEDNTYSGRSAEQLTN

DLAKVKKEISLFLRQDNPKKATQKFNLDYQSNLDKETFINLVNQAKRKIHEGDCFQIV

LSQRLTASFTGDSFSYYEELRLLNKTPYLYYLDFDETKIIGSSPESLVKVEGQLVTTN

PIAGTRKRGKTSAEDEYLANELLLDEKELAEHRMLVDLGRNDIGKVAEIGSVSVPVYL

TIERFRYVMHIVSVVSGKLKTNLTALDALKAVLPAGTVSGAPKLRAMERIYQWENVRR

GIYGGAVGYLSNNGDCDFALAIRTMILHKEQAYVQAGAGIVFDSVPESEYLETLQKAK

ALLEVGK"

CDS complement(1204113..1204808)

/gene="cah"

/locus_tag="JFBMFIFI_01143"

/EC_number="4.2.1.1"

/inference="ab initio prediction:Prodigal:002006"

/inference="similar to AA sequence:UniProtKB:Q50940"

/codon_start=1

/transl_table=11

/product="Carbonic anhydrase"

/db_xref="COG:COG3338"

/translation="MDWYYQGDRGPEKWKDICAIFKEAEEGSLQSPICLNQKDVTNWI

ASGLLQFDYHPTTFETSYYNHTLHLAPIANERINHVVFNHKRYVLEDIHFHLPSEHLI

DEKSFPLEFHMVHRSPKNELLVIGIMAEPTMVPLNEKMANLDRKALNPRNSARGLLVP
IDLMQLLPDELGFFHYSGSLTTPPTSGPVNWVVFKSPVYMRQGLLEAFKEQIGKTNRP
VQPRKNRPIYLSK"
CDS complement(1204915..1206987)
/gene="QRSL1"
/locus_tag="JFBMFIFI_01144"
/EC_number="6.3.5.7"
/inference="ab initio prediction:Prodigal:002006"
/inference="protein motif:HAMAP:MF_00120"
/codon_start=1
/transl_table=11
/product="Glutamyl-tRNA(Gln) amidotransferase subunit A,
chloroplastic/mitochondrial"
/translation="MKNRLRKWEQISLLILTGIMVSSFTPEGRIIASESNEPQSQTLV
IKDSSVPQEAIASPEDPEIAPPTENSEKPQDQLTSLSELPPESATEIESTFTVEDYKK
SDASELAVAVRNKQVSSVQLVQFAYQVIEENDKIYNAMISLRKEEALKEASNLQDTGQ
PFLGVPLVVKGLGHTISGGYNSNGLLFGKDSISKSTSTFVKAFQNAGFIVIGQTNFPE
MGLKNITNSKLYGPTGSAWNSAYQAGGSSGGSATAVAAGYTPIGTGSDAGGSIRIPAS
WNGIIGFKPSRGVLVGNSASERGQTSHFAETKSMADTRALFDALKNKELPQNTFTKNV
KIGYSVKSPVNTQVDPKAIQAVESAVAFLREEGFQVEEVDSPYDGVELMKNYYTIGAS
SMGIIDYLAKQQAKRPVEIADVDWTTWALFQASKDLTKLDLDQAWENVRKITDELVTF
QNNYPLFLTPTTATTAPLLNDTAMLPEHIEQIKNMENRPKEEKLNLVYEQWLAGLTHT
PFTQISNLTGTPAISLPTYVSQEGLPLGIQFTTSADNDYLLLDVGDYFENHYGFNRNA
AEIPEIPIDKETPIPNESAVTEDAEKTKNGEETKIEPIPDSENQELSKPNLTHKQQLS
ANNLVIEETKIDLQKNFPKTSEKVSQTFVLIGSFILMTTSGGLCLKFIMQKKVETSRT
GKRSKNYC"
CDS 1207310..1209019
/gene="rqcH"
/locus_tag="JFBMFIFI_01145"
/inference="ab initio prediction:Prodigal:002006"
/inference="similar to AA sequence:UniProtKB:D4FWG0"
/codon_start=1
/transl_table=11

/product="Rqc2 RqcH"

/translation="MSFDGIFTHTMVDELKELLSNGRISKVHQPYQSEIILVMRANGK

NHKVLLSAHPSYARIQITEIPYENPNTPPNFCMMMRKQLEGAILENIEQIGNDRVIHF

TFKSRDEIGDVRNVVLIVELMGRHSNIFLIEQDTKRIVDCIKHVPTSMNTYRSVMPGA

TYISPPHQDKLNPFATTEAEIAQVVTPDPETTMQKQLQTAYQGLGGDTAAELVYRSES

NPAELPAVFQAFISEVSYGKYTPTLTGNNKKEHFTPTPFLSLTGEKKLFPSLSQLLDA

YYSGKAEKDRVNQQGSELIRLVNVELQRNVKKLKKLEKTLVETEYADDYRVKGEILTA

YLHEFEKGQKEVTLPNFYDEEKPLLITLDVRKNPSQNAQKYFAKYQKLRNAVAFVGEQ

IELTLSEIDYLESVATQLSLATPKDVAEIKEELMQEGYLKKKKTKKKQMKAQQSKPDT

YLSSDGTTILVGKNNLQNDQLTLKTARKSDIWLHTKNIPGSHVIIQDADPSEETILEA

ANIAAYFSKSQLSASVPVDLVPVKLIRKPNGAKPGYVIYEGQRTVYVTPDKELVARLK

VSQ"

CDS complement(1209146..1209502)

/locus_tag="JFBMFIFI_01146"

/inference="ab initio prediction:Prodigal:002006"

/codon_start=1

/transl_table=11

/product="hypothetical protein"

/translation="MKKLFDETHEHETGDFRTIWYGYITETPDTELQNKLLTIIKEDL

TKEKKHVNTVTHWVFYHEKTSGDAIGDTVRAAFMVREKDGNFVANYTMSDFDFVISFD

ALNQFKVDMEASLNQK"

CDS complement(1209524..1209982)

/locus_tag="JFBMFIFI_01147"

/inference="ab initio prediction:Prodigal:002006"

/inference="similar to AA sequence:UniProtKB:P9WKQ3"

/codon_start=1

/transl_table=11

/product="hypothetical protein"

/db_xref="COG:COG2764"

/translation="MAKIYPYLAFDSAKEALDYYTEVFGATDIIRLPVSPEQATQFGI

SAEKLADTTMHASFTVLGAPLFCADNFMGPVQSSNQVSIMLDINSEDAQAAHEADAFY

QKIAASGQAKITMPFEEQFWGGKMGQLVDKYGITWMLHSQPYSKINLNTK"

CDS complement(1210119..1210829)

/locus_tag="JFBMFIFI_01148"

/inference="ab initio prediction:Prodigal:002006"

/codon_start=1

/transl_table=11

/product="hypothetical protein"

/translation="MDLTTIMNRLAELGDEQTKKTFMRHGATEPLFGVKIGDLKKYLV

KELKNNNELALALFNTGNSDAQYLAGLIINPKKVTKEQLQTWVTQSNWSMISESIVAS

VAAESPYAVELATEWMKSEKELIQDAGWAAYANFISITPDSLLNENEILELLKIVETT

IHSAKNRVRYSMNQFVICVGSYYAPLVEEAAQVADIIGKVSVNVGNTACKVPIATETI

EKIIKLDRVGKKRKNAVC"

CDS complement(1210911..1211807)

/locus_tag="JFBMFIFI_01149"

/inference="ab initio prediction:Prodigal:002006"

/codon_start=1

/transl_table=11

/product="hypothetical protein"

/translation="MEKIGNKIRKYRLEKNMTQSQLADGICTQATISNLENNSSLPSI

ANLLLIAERLSIKHTDLYEAILVNSSGYIDTFNKVKSLQREDNYIEANQLLKKAIDYS

DLASIYEKKEYFYYFGVSSLRGLHDFSDAHYHFNQCLALSEVDDLKTLDLLATMGIAE

AYDMCHQFDKADTYFIKAINQLEQVSSWKKSPNDRLDILEAYKKAATFYLKIAQYSLA

LSLSAAGINFYATENINQGLEDLLDIKAQSLFYLGDVELAEEYYFYAMALAKLNKNDK

QIQLLREQIIKHGLKQYDYLNN"

CDS complement(1211858..1212829)

/locus_tag="JFBMFIFI_01150"

/inference="ab initio prediction:Prodigal:002006"

/codon_start=1

/transl_table=11

/product="hypothetical protein"

/translation="MNYLNLLEEAYELDEMSEARLPLLEEVIREADLARDIETAVEAR

WLLIDTCLYVGFPKKQLIAFSWLINLYESSEGKVDVFDLLWHYKWIAEHIVRFPEVSL

SKINALTEDMQKKFKEYNYSLRPYYKVKTLQAISMGNRAMANEFFPLWQGTKSDFMND

CLACETTEIVAYYHFIGDYQAAIKAAAPILAGRQACAEVPHLTYGHTALSYYHLDKRE

EAQNSFELGYPMVKKRVSLLITVSQFITYLLLIDNQEEAVQVYQENQKIASELEGDLS

TLHFYLASAGLKHEKQEACLAQATTLAQKFDERNQNTYYQDILAKIQ"

CDS complement(1212861..1214678)

/gene="htpG"

/locus_tag="JFBMFIFI_01151"

/inference="ab initio prediction:Prodigal:002006"

/inference="protein motif:HAMAP:MF_00505"

/codon_start=1

/transl_table=11

/product="Chaperone protein HtpG"

/translation="MNPEENRFKVNLGGMIEILSNHLYSQKDVYIRELLQNATDAIRG

RKLISEDNFVGEIHVDLTIHRSGSSTLFFEDNGIGLTEDEIHQFLATIASSSKGEKNF

EEKQDFIGRFGIGLLSCFIVADEIVMVTTSAKTRESFQWQGNADGTYEIKKLSQSLEE

PGTKIYLTSKALKNHEEHTNFILEDLADALVKYGRCLEVPIFLTCDKGEKIVNQETMG

LGKFDTLESLSRDQILKLGQKILQEDFQDYLLIETANGRTKGIAYIIPHTVRMNAKKR

NTIYLNQMFVSDEIDSLLPSWAFFTRCILWTDELQPVASREDFYKNKRLKTVAKELGS

SLKQGIEMLESSALEKLIAAHYLSFKSLASEDPDFLKMVYTFLPFRTLNGEEKFADIL

SQNETIYYALSVDDFRQIADLARNQGLIVINGGYSYDSQLLTEISQILEDFPFNKLKI

IQPEDFQHTFQPLSFFESTHLQEKLKEINQQFEELNLLVIVKHFDPSSMPMVLLSNHL

TQNQRELERTAEESNQLFGGILEDLMLEADQLPPAKLYLNYDNPLVKRIFEKKQPAGI

KNIIEVLYIQALLLGHYPLKKKELNLLNSSLLGLLDQFI"

CDS 1214997..1215959

/locus_tag="JFBMFIFI_01152"

/inference="ab initio prediction:Prodigal:002006"

/codon_start=1

/transl_table=11

/product="hypothetical protein"

/translation="MKRKQSKRISYKIIGLVFLLILLIICSIGWMFYQQSKPVKDIFI

PEWQYPIKQLNFPKSSSKVISHRGYKQGGVENTLEAIEAAAHFEPAYVEIDIQQTKDD

EMVVIHDRSLKRLANKKRKIRNSTLKELKKVKLKQNGFVGQIPTLEEAIQLAQQEQQP

LLLDIKTNPTDSLDFPQQLVDLLTKYKVQNYYLIQSQDENFLKRVKQINPQIQVGLII

TALPSKDFSDFQFLSLKYKLANQNLLDSQVKEKRPIFLWTLDQPAEIELFMHKNIEAV

ITDDSSTATVLQQTIKPATLSSSYLERIKNAEQKADLKRSLSVP"

CDS 1216070..1216894

/gene="zupT"

/locus_tag="JFBMFIFI_01153"

/inference="ab initio prediction:Prodigal:002006"

/inference="similar to AA sequence:UniProtKB:P0A8H3"

/codon_start=1

/transl_table=11

/product="Zinc transporter ZupT"

/db_xref="COG:COG0428"

/translation="MIQFLMQFEPWQQALIGTLFTWGLTALGSALVFFFKDINRDVFN

LMLGFASGVMIAASFWSLLDPALDMAEANGSIPWLVVGIGFAVGGLFLYLADKLLPHM

HFGENHEVEGLPSNLRRTILLVFSITLHNIPEGLAVGVAFGAANQVSGSPEAGIAAAL

AVAIGIGIQNFPEGAAVSIPLRQEGLSRTKAFLYGQASGIVEPIAGVIGAILVTYVSS

VLPYALAFAAGAMIYVVVEELIPEAQQKQTAKGAHYATFGVMVGFLIMMILDVALG"

CDS complement(1216977..1217762)

/gene="yusV_1"

/locus_tag="JFBMFIFI_01154"

/inference="ab initio prediction:Prodigal:002006"

/inference="similar to AA sequence:UniProtKB:O32188"

/codon_start=1

/transl_table=11

/product="putative siderophore transport system

ATP-binding protein YusV"

/db_xref="COG:COG1120"

/translation="MATIETKELSIAYEKNLIVEKLNLTIPTGKITSIIGANGCGKST

VIKTIGRVLKQKMGSVYLNGEDIQKLPTKEIAQQMAVLPQEQQAPSGMTVEELVIYGR

FPHQKGFGKLTTYDKEIIAKAIQQTKLTQYKNREVDSLSGGQRQKVWIAMALAQETEL

ILLDEPTTYLDLAHQLEILELLEQLNQKEQRTIVMVLHDINLAARFSDFMVAINGGTI

IEKGSPKEVMTTEILKKTFQIDALITEDPRTKKPLCLTYNLLS"

CDS complement(1217775..1218770)

/gene="feuC_1"

/locus_tag="JFBMFIFI_01155"

/inference="ab initio prediction:Prodigal:002006"

/inference="similar to AA sequence:UniProtKB:P40411"

/codon_start=1

/transl_table=11

/product="Iron-uptake system permease protein FeuC"

/db_xref="COG:COG0609"

/translation="MKKRKNPLFILLLLSLFLIGLIIWSLNTGKMALTPNEIMQIILG

NGTAKAHLIIFEFRLPKILLGIFVGIGMGISGCLMQSLLKNDMASPGTLGISSGSGVF

VIFALMMGMREVSPLMLPIIAFIGGLLAAGLIFIFSYQRGKQLSPLTLILTGVALATG

YDALTLLFSLRMDTDKFEFAQRWMAGSLWGDQWSYLLFLIPCILILSLWAYFHSIHLN

ALHLGNESAIGLGVSVKKEFLLMAFVSILLSSVSVAIGGNFFFVGMISPHIAKKMVGV

NHKYSIPASGLVGGIIILLSDTLLRASHLSGTIPVGLLITIISTPYFLYLLARSR"

CDS complement(1218767..1219786)

/gene="feuB_1"

/locus_tag="JFBMFIFI_01156"

/inference="ab initio prediction:Prodigal:002006"

/inference="similar to AA sequence:UniProtKB:P40410"

/codon_start=1

/transl_table=11

/product="Iron-uptake system permease protein FeuB"

/db_xref="COG:COG0609"

/translation="MNSSTKNKYPFSYSTFILIGLVLLLILFILSISFGAAKIEVNTA

WQAVFSYHENEMDHKIIRTLRLPRTIADLIVGASLAVTGAIMQGTTRNPMADSGLLGI

SSGAAFAVALCLAFVPEYTYWQMLLYSCLGAAFTTTITYGMAAFGKKGLTPQRLLLSG

IAVSMLFGALSTLIALQFKLGKAMVYWSAGGTASANWTDLKVLTPVFILGVIGSIFLS

PKITILQLGEDIAVGLGLKASWIKVNSVLIVLILTGLSVILVGPISFVGLIVPHIVRF

FVGVDYRRIIPASLIYGAVFLIAADLLGRLINQPKETPLGIIFAIIGVPFFLFLSRQQ

GRDFN"

CDS complement(1219801..1220856)

/gene="ycgT"

/locus_tag="JFBMFIFI_01157"

/EC_number="1.18.1.2"

/inference="ab initio prediction:Prodigal:002006"

/inference="similar to AA sequence:UniProtKB:O31475"

/codon_start=1

/transl_table=11

/product="Ferredoxin--NADP reductase 1"

/db_xref="COG:COG0492"

/translation="MENDELYDITIIGAGPAGLFSAFYSGLREMKTKVLECHPILGGK

LNLYTEKMIWDVGGLPPLPGGKLVEHLVQQAKTFHPTIKTNEKIVSIDKNEAGHFILT

AENGAIHYSKTVVLAIGWGILKPKKIELTDVEKFEKTNLNYTIQNPQSLKGKQVLLSG

GGNSAVDWANLLEPIADSVHLVYRKDELKGHEAEVSKLLASKVKCHFNQTIEAFIPDE

SDTKIAKVQLTDSQSGATQFIEIDEVVINHGYEEESTIFKQNTVGLKQKDNYYIEISP

AGISNVPGIFAAGDVVHHEGKLHLIAGAFQDAANAVNQAKLYLDPTAHSYAAVSSHND

KFTDQNKVLVEEFFYNE"

CDS complement(1220873..1221808)

/gene="yxeB_1"

/locus_tag="JFBMFIFI_01158"

/inference="ab initio prediction:Prodigal:002006"

/inference="similar to AA sequence:UniProtKB:P54941"

/codon_start=1

/transl_table=11

/product="Iron(3+)-hydroxamate-binding protein YxeB"

/db_xref="COG:COG0614"

/translation="MKHLFTGKKIVFITIVSSLLFVIGCGNASNNGENTEEDTRQIKT

ELGEVKVPKSPKRVIVQYITGDVLALGIEPIGMSEVYDSAAYEDKVKDVKDLGQWAEW

DEEEMMDLDPDLILTIDKATYERFSKIAPTVYVPYGEMTTTERVTFIGDVLNKSSQAE

KVLKEYNDNIIDSQKKLKDVGFDQSTVSIFEGGLKEMMVIGQKFGTGLVAYDNLKLIA

PEKIQKEVLDKDEFTVSLSLEVLPEYAGDYIIRNTYDGMDNMNDSAIWKALPAIENKH

LIEIPFGLSYYTDILSANAQIDFIRDQLIEKATTN"

CDS 1222198..1222641

/locus_tag="JFBMFIFI_01159"

/inference="ab initio prediction:Prodigal:002006"

/inference="similar to AA sequence:UniProtKB:P0A8D3"

/note="UPF0178 protein YaiI"

/codon_start=1

/transl_table=11

/product="hypothetical protein"

/translation="MHIYIDADACPVKDIIISEATQRLLAVTIVTSFSHFSTVLQPAG
VDTIYVDNGAEAADYRIMRLIKKDDVLVTQDYGLASLALAKGAIVLHQTGFRYSTENI
DQLLQSRYLSAMQRKSGKRTKGPKAFTNEDREKFRALFIETISSK"
CDS 1222822..1223034
/locus_tag="JFBMFIFI_01160"
/inference="ab initio prediction:Prodigal:002006"
/codon_start=1
/transl_table=11
/product="hypothetical protein"
/translation="MTQTDTNVVAILTDLIEKKKSLDSSVYAFYFEGVKQSVRLLDTT
YELYPKFLEYTTNEGILPELALPEGA"
CDS 1223061..1223369
/locus_tag="JFBMFIFI_01161"
/inference="ab initio prediction:Prodigal:002006"
/codon_start=1
/transl_table=11
/product="hypothetical protein"
/translation="MNFVALTGAVISEVKIINLSSGAPLCRFTFEADGRSFNCLISGM
LAYDFVYEVEEGTFLTFEARINDRMQLVLVNYTLDKKGLSFHSAEAYQDTGYPHKKIS
"
CDS complement(1223416..1224777)
/gene="gltA"
/locus_tag="JFBMFIFI_01162"
/EC_number="2.3.3.16"
/inference="ab initio prediction:Prodigal:002006"
/inference="similar to AA sequence:UniProtKB:Q9RWB2"
/codon_start=1
/transl_table=11
/product="Citrate synthase"
/db_xref="COG:COG0372"
/translation="MVEVHLNVTPEILQLTALCVHNSQIDSDLYQKLDVKKGLRDING
KGVLAGLTNISTVQPQTLKNGEVVSPDGLLEYRGINIKELTTGYMHDNRFGFEEVTYL
LLFGILPTKAELADFKKILAQKRTLPTNFVRDVVMKASSRDIMNALSRSVLTLASYDK

QADDISLANVLNQSLMLISVFPLLSVYAYHAFNHYERGESLYIHYPDETLSTAENLLR
MLRPNKEYTESEALILDLALVLHMEHGGGNNSSFTTHVVTSSGTDTYSTIAAALGSLK
GPKHGGANIKVVEMFEDLKQTVADYQDEEQVASYLRKLLNKEAFDKKGLIYGMGHAVY
SISDPRAEIFKGFVQQLAEEKGRIDEFELYSLVEQLAPKIIGEERHIYKGVSANVDFF
SGFVYSMLGLPTELYTPVFAIARIVGWSAHRLEELINTNKIIRPAYDSILASTSYVPL
DAR"

CDS complement(1224919..1225548)

/gene="pyrE"

/locus_tag="JFBMFIFI_01163"

/EC_number="2.4.2.10"

/inference="ab initio prediction:Prodigal:002006"

/inference="similar to AA sequence:UniProtKB:Q8DTV2"

/codon_start=1

/transl_table=11

/product="Orotate phosphoribosyltransferase"

/db_xref="COG:COG0461"

/translation="MTLEKTIAKDLLTIEAVSLSPNAPFTWASGIKSPIYCDNRITMS
YPKVRREIAQGLADLIKTNYPDAEVIAGTATAGIPHAAWVADLLDLPMVYIRGKAKEH
GKGNQIEGRISPNQKMVIIEDLISTGGSVIEAAVAAKNEGADVIGTAAIFTYELPKGL
ANFKENKLSFHTLTNYSTLIDVALEMGSIKAEDITLLKEWKKDPASWGK"

CDS complement(1225549..1226265)

/gene="pyrF"

/locus_tag="JFBMFIFI_01164"

/EC_number="4.1.1.23"

/inference="ab initio prediction:Prodigal:002006"

/inference="similar to AA sequence:UniProtKB:P25971"

/codon_start=1

/transl_table=11

/product="Orotidine 5'-phosphate decarboxylase"

/db_xref="COG:COG0284"

/translation="MQSKPIIALDFSTSEEIKQFLAHFKEEQLFVKVGMELYYQNGPE
VVKLMKSLGHQIFLDLKLHDIPNTVERSMKGLAALDVDMINVHAAGGKQMMEAAMNGI
ISGTPAGSQRPELIAVTQLTSTTQEAMRTEQLIQVPLLQSVIHYAKLTQSAGLDGVVC

SALEAQLIKENTASDFLCVTPGIRPAGDEKGDQKRVATPARARAAGSSFIVVGRPITL

AADPVKAYHTIKNQWNGVED"

CDS complement(1226267..1227184)

/gene="pyrDB"

/locus_tag="JFBMFIFI_01165"

/EC_number="1.3.1.14"

/inference="ab initio prediction:Prodigal:002006"

/inference="similar to AA sequence:UniProtKB:P0DH74"

/codon_start=1

/transl_table=11

/product="Dihydroorotate dehydrogenase B (NAD(+)),

catalytic subunit"

/db_xref="COG:COG0167"

/translation="MNRLAVKLPGLDLKNPIMPASGCFGFGEEYAKYFDLNVLGSMMV

KAATLEPRFGNATPRVAETPSGMLNAIGLQNPGLDVIMRDKLPKLEKYNVPIIANVAG

STKEDYVTVCEKISLAPNVHAIELNVSCPNVKHGGIAFGTDPEVIYDLTKAVKEVSSV

PIYVKLSPNVTDIVPIAKAVEAAGADGITMINTLLGMRIDLKTKKPILANQTGGLSGP

AIKPVAIRMIHQVSHAVDIPIIGMGGVYTVDDVLEMFMAGASAVAIGTANFTDPTICP

KLIQELPKRMDELGISTLSELIKEVREGR"

CDS complement(1227184..1227963)

/gene="pyrK"

/locus_tag="JFBMFIFI_01166"

/inference="ab initio prediction:Prodigal:002006"

/inference="similar to AA sequence:UniProtKB:P0DH76"

/codon_start=1

/transl_table=11

/product="Dihydroorotate dehydrogenase B (NAD(+)),

electron transfer subunit"

/db_xref="COG:COG0543"

/translation="MLQEIMTVVSQEQLAPKIFEMRLTGELVKEMQTPDQFLHLLVPR

KDLILRRPISIAEIIPERQECVIIYRVDGDGTAAMSELMAGDKIDVMGPLGNGYPLDK

VKPGEKALIIGGGIGVPPLYELSKQLVAMGVKVQHVFGFATQEVIFYEEKFKKLGDVL

YSTDDGSYGLHGHVGMALDTLDSDYVPDVVYACGSRGLLMAVENRYENSNAYFSLEAR

MACGMGACYACVCHKKDDPTGATSMKVCDEGPVFKVGEVVL"

CDS complement(1228092..1231271)

/gene="carB"

/locus_tag="JFBMFIFI_01167"

/EC_number="6.3.5.5"

/inference="ab initio prediction:Prodigal:002006"

/inference="similar to AA sequence:UniProtKB:O32771"

/codon_start=1

/transl_table=11

/product="Carbamoyl-phosphate synthase large chain"

/db_xref="COG:COG0458"

/translation="MPKRTDIKKIMVIGSGPIIIGQAAEFDYAGTQACLALREEGYEV

VLVNSNPATIMTDKEIADKVYIEPITLEFVSRILRKELPDAIVPTLGGQTGLNMAMEL

AASGILDELEIELLGTKLSAIDQAEDRDLFRQLMHDLNEPVPESDIVHTVEEAVAFSE

KIGFPLIVRPAFTLGGTGGGICNNLKELKEIVTNGLKLSPATQCLVEMSIAGYKEIEY

EVMRDSADNAMVVCNMENIDPVGIHTGDSIVVAPSQTLSDYEYQLLRDSSLKIIRALG

IEGGCNVQLALDPNSFNYYIIEVNPRVSRSSALASKATGYPIAKLAAKIAVGLTLDEM

KNPVTGTTYAQFEPALDYVVAKIPRWPFDKFEHGERKLGTQMKATGEVMSIGRNIEEA

LLKAVRSLEIGTIHIETPEANAVSDEDLVGKIINAQDDRLFYLIEGIRRGYSIQDLAE

LTKIDLFFLDKLLHIVEIEQDLKDHPYDLTSLENAKKHGFSDVAVAKFWETDALRIRE

IRKENGLVPVYKMVDTCAGEFESKTPYFYSTYEQENESIVSKKESVLVLGSGPIRIGQ

GVEFDYATVHSVKAIQKCGYEAIIMNSNPETVSTDFSISDKLYFEPLTLEDVMHVIDL

EQPKGVIVQFGGQTAINLAEPLTKLGVPILGTTVEDLDRAENRDLFEQALKSIDIPQP

LGDTARSEAEAVTIANRIGYPVLVRPSYVLGGRAMEIVENQKDLENYMRNAVKASPEH

PVLVDRYLLGKECEVDAISDGTDVLIPGIMEHIERAGVHSGDSMAVYPPQNLSQKIQD

TIADYTKRLALGLNCIGMMNVQFVIFEEQVYVIEVNPRASRTAPFLSKVTGIPMTQVA

TNVILGKSLKEQGYKDGLYPESTKVHVKAPVFSFAKLLSVDTNLGPEMKSTGEVMGTD

STLEKALYKAFEGSNMHLADHGTALFTIADEDKVESGELAKRFLAIGYQLVATSGTAK

YLKQEGILVKEINKITEDEELTVLDVIREGKVQVVVNTMGRDKEVQSDGFQIRREAVE

QGVPLFTSLDTTSAILKVLESRSFDTLAL"

CDS complement(1231264..1232352)

/gene="carA_2"

/locus_tag="JFBMFIFI_01168"

/EC_number="6.3.5.5"

/inference="ab initio prediction:Prodigal:002006"

/inference="similar to AA sequence:UniProtKB:P99147"

/codon_start=1

/transl_table=11

/product="Carbamoyl-phosphate synthase small chain"

/translation="MDRLLLLEDGTIFKGKGFGAPIEVTGEVVFTTGMTGYQETITDQ

SYNGQMIVFTYPLVGNYGINRDDFESIEPTCKGVIVKEHARVASNWRSQITLDEFLKR

KNIPGISGIDTRQLTKLLREKGTIKGMITGANENLEHAFDQLRATVLPTNQVAQVSTS

RPYPSPGSGRNVVVIDFGLKHSILRELSNRNCNLTILPYNTTAQTILELDPDGVMLTN

GPGDPKDVPEALEMIRGIQGKLPIFGICLGHQLISLANGADTFKLKFGHRGFNHPVRE

IATGRIDFTSQNHGYAVDEKSIDKNELIVTHIELNDGTVEGIKHRHHPVFSVQFHPDA

APGPHDAGHLFDQFMELMDAGKERKANA"

CDS complement(1232366..1233643)

/gene="pyrC"

/locus_tag="JFBMFIFI_01169"

/EC_number="3.5.2.3"

/inference="ab initio prediction:Prodigal:002006"

/inference="similar to AA sequence:UniProtKB:P65906"

/codon_start=1

/transl_table=11

/product="Dihydroorotase"

/translation="MYTWIKNVKMVNHRNELEAIELMIKDDKIEAIGTDLSLIGIQAE

KTIDGQDALITPGLVDVHVHLREPGFTYKETIQTGSEAAARGGFTTICAMPNTNPVPD

TVEKFEELQALIKKDAVVKVLHYAPITTGLKSDELVDQPSLLAAGAFAFTNDGVGVQT

AGTMYLAMVEAAKNNTAIVAHTEDDSLLFGGVMHKGKISEKLNLPGILSITEASQIAR

DVLLSEATGAHYHVCHVSTKESVRVIRDAKKAGIHVTAEVTPHHLLLSEEDIPSDTAI

YKMNPPLRGTDDREALIEGLLDGTIDFIATDHAPHSAEEKSGSMVGAPFGIVGSETAF

QLLYTHFVKTGKFTLLQLIDWMSTKPAEVFNLNAGSLEVGAPADLALFNLTEEEEIDA

KDFKSLSSNTPFIGWKVVGNTVMTLVDGKVVYS"

CDS complement(1233772..1234686)

/gene="pyrB"

/locus_tag="JFBMFIFI_01170"

/EC_number="2.1.3.2"

/inference="ab initio prediction:Prodigal:002006"

/inference="similar to AA sequence:UniProtKB:P05654"

/codon_start=1

/transl_table=11

/product="Aspartate carbamoyltransferase"

/db_xref="COG:COG0540"

/translation="MTATIQKLQLDHLVSVEALSNEEVMTLIKRAQEFKNGASYHPKH

PKYAVNLFFENSTRTHKSFEMAEKKLGLDIIDFEVATSSVKKGETLYDTVLTMSALGV

DVAVIRHGDEAYYEELIASTGIKLAVVNGGDGSGQHPSQSLLDLMTIYEEFGKFEGLK

VAIVGDLTHSRVANSNMQLLNRLGAEVFFSGPEEWFDASYNEFGTYLPLDELVEEVDV

MMMLRVQHERHEYYGNFSKEKYLATYGLTEERESKMKPDAIIMHPAPVNRDVELEGSL

VECPRSRIVQQMSNGVYTRMAILEAVLE"

CDS complement(1234755..1236065)

/gene="pyrP"

/locus_tag="JFBMFIFI_01171"

/inference="ab initio prediction:Prodigal:002006"

/inference="similar to AA sequence:UniProtKB:P39766"

/codon_start=1

/transl_table=11

/product="Uracil permease"

/translation="MSQEKFRNENVVLDIHEKPGTIHWFGLSLQHLFTMFGATVLVPI

LVGLNPGIALVTSGIGTLLYIAITKGKIPAYLGSSFAFITPMILVIEKGGYPAAFQGA

LYAGLVYWVVALVIKLIGSDWLDKVLPPIVVGPVVMVIGLSLSGTAATQAMYEHATAA

PENYNVTYILIALATLAITIVCNMYLKGFLSLIPILIGIVGGYILSLAFGIVDLSGVQ

EAAWFQVPKFEIPFLSYKPAFSLAALATMAPIAFVTMSEHIGHLMVLNKITERNFFKK

PGLSRTLFADGAATVVASLLGGPPATSYGENIGVLALTKVHSVFVIGGAATLAILFGF

VGKVSAVILSIPGPVIGGISFLLFGVIASSGLRILIDNKINFDEKRNLIIASVILVIG

IGGAYFQFGSLTLSGMSLATVVGIVLNLILPKKAQSEIEHEAKV"

CDS complement(1236268..1236816)

/gene="pyrR"

/locus_tag="JFBMFIFI_01172"

/inference="ab initio prediction:Prodigal:002006"

/inference="similar to AA sequence:UniProtKB:F2MMP6"

/codon_start=1

/transl_table=11

/product="Bifunctional protein pyrR"

/translation="MTERKEIEVVDAAAMKRALTRITYEIIERNKGIEDLVLVGIKTR

GIYIARRIADRLKQLESVDIPVGELDISLYRDDVHTSDGREPEINGSNIPVSIEGKQV

ILVDDVLYTGRTIRAALDALMDIARPRKISLAVLVDRGHRELPIRADFVGKNIPTSLD

EQIKVEVEELDGLDKVSIQKIK"

CDS complement(1237036..1237950)

/gene="rluD_1"

/locus_tag="JFBMFIFI_01173"

/EC_number="5.4.99.23"

/inference="ab initio prediction:Prodigal:002006"

/inference="similar to AA sequence:UniProtKB:P33643"

/codon_start=1

/transl_table=11

/product="Ribosomal large subunit pseudouridine synthase

D"

/db_xref="COG:COG0564"

/translation="MRTTDEHNFTVNEEKGRLDKVLTELLPDKSRSQLQQWIKEGDAL

VNNEPSKANYKVQQGDKISIIEPEAVPLEIVAENIPLEIVYEDQDVLVVNKPQGMVVH

PSVGHMTGTLVNALMYHIKDLSGINGVVRPGIVHRIDKDTSGLLMVAKNDQAHEKLAA

QLKDKTSLREYVALVHGEIPHEKGTIDAPIGRAKEDRKKQAIIDDGRPAVTHFKVIEG

LKDYTVVSLQLETGRTHQIRVHMRYIGFPIVGDPTYGPKKTIKGHGQFLHAKVLGFKH

PTTGEFLTFEAPIPEIFEETLTELRNND"

CDS complement(1237971..1238429)

/gene="lspA"

/locus_tag="JFBMFIFI_01174"

/EC_number="3.4.23.36"

/inference="ab initio prediction:Prodigal:002006"

/inference="similar to AA sequence:UniProtKB:Q9HVM5"

/codon_start=1

/transl_table=11

/product="Lipoprotein signal peptidase"

/db_xref="COG:COG0597"

/translation="MIVYYALAAVILVIDQITKYLVVNNIDLYEVKEVIPGILSWMYI

RNTGAAWSILEGQMWFFYIITFAVVIAVIYIMQKYAKGNWLFSLGLSLILAGALGNFI

DRLRLGYVVDMIRLDFMNFPIFNVADMSLSIGVAVIILYVLLDEKNKKLS"

CDS complement(1238861..1240216)

/locus_tag="JFBMFIFI_01175"

/inference="ab initio prediction:Prodigal:002006"

/codon_start=1

/transl_table=11

/product="hypothetical protein"

/translation="MSFKKIAIIVSSIALLIVIGIYTGKALTYQTKFLPKTMINDMDI

SNKTVFQVNKELQKSYRGKTFVATEGGMELFKFNGSDVGITDDFTESLTLIKDQQDQW

SWPIRILKNKVQKEKLEKITYNKETFAGFFAGLPLVSANRIKPENAKIEKTESNFNLK

PEVMGNTFDLDKVKASLITTIESEETRISLDDYYQKPTIYKDNPELIKITNEANSLSK

LDISYLIVDQKETISAERLFQWISLDAENNIVIDEDKVTSYLTELATKYSTKTKSRKF

VSTKKGIVDVLPGIYGWTFLVNDEVNSLVADIRTKTNLQDREPKREGSGYGVDIGKTY

VEVDLTNQHMWFYKDGVVALETDVVTGKPTTPTPPGAFYIWNREPNAILRGVDYETPV

KYWMPIDWDGVGIHDSNWQPAYGGNLYLTVGSHGCINTPPGVMEKLFAIAPVGTPVLV

F"

CDS complement(1240376..1241590)

/gene="pucG"

/locus_tag="JFBMFIFI_01176"

/EC_number="2.6.1.112"

/inference="ab initio prediction:Prodigal:002006"

/inference="similar to AA sequence:UniProtKB:O32148"

/codon_start=1

/transl_table=11

/product="(S)-ureidoglycine--glyoxylate transaminase"

/db_xref="COG:COG0075"

/translation="MIKQLSVPTRTIMTPGPVEVEPRVLKALSTPIIGQYDPEFFRVM

GEVSTLLKTPFETTNAQAFVVDGTSRSGLEAAMLGLVEKGDRVLVPAYGRFGYLFVEL

AERAGGEVKLIEKTWGETFSPEEVIAAIKEFNPKVVALVHGETSTGQIQELKEIGQYC

QENGHLFLVDAVATFCGAPVKVDEWGIDIAVGGTQKCLSVPSGMSPITYNKKVEDILT
SRYQMELGLSDEHRNSDFIKSNYLDLSQIQRYWGPEHINHHTEMTSMIYGIHEGLRIV
EEETLEVRYARHSLNEKAMMAGIKAMGLNLFGNSKSKMVTVTCVEIPKGVDGEAVRST
LLNEFGVEIASSFGSLKGKIWRIGNMGYSSRKENVLHTLGALEAAILYHGGEIIKGEA
TLAAMKVYDSQK"

CDS 1241875..1243515

/gene="pucR_2"

/locus_tag="JFBMFIFI_01177"

/inference="ab initio prediction:Prodigal:002006"

/inference="similar to AA sequence:UniProtKB:O32138"

/codon_start=1

/transl_table=11

/product="Purine catabolism regulatory protein"

/db_xref="COG:COG2508"

/translation="MTTIGELLQIPRFSNIQVLNSHADLTKIVDTIEISETPDVAAYL
PKNVFLLTTAMVFKDDPKGLCDMLRSLNALPAAGIGIKLGRFINELDPMVLAVADELG
FPVLQIPSTVTLGTTSHQLLSYLWDTETEKLNFALDIQQKFSNMMIKGATLTSLTRHL
GSILKRPVLLINPFLEVVAESRHLQNDPKFSNTTIEKIKPKLREAQSSGKEYSFLLED
EEENPLLVSVFPIMSSAYFPYLLVIFKADQIPYPFSQFAIEQANTVLSYTLYKNLKLT
ENLLNQKKDFFYQLSEKKLPAEAKLINWLDYGKDYGIIKSSYYRVIIINFEETLLDQT
NQQLSDDRSYLSYEWLEKQINLHFKKALLFPIKHTHYYALLLQKSTNLLADKLVAIHN
GLIDQLPISLNFSIGNEVTDPASIHFSFREAMDALDISLKEDVQPVVTFFQSKGLNKL
IDYVPKEDIEHFCLVNLKSLAFPRSEMNRELRRTLKVYLESQCEITITAKKLFIHRNT
VKYRIAKCEELFESPINDPKLSLNLRLALVLSDTEDQI"

CDS complement(1243569..1244510)

/gene="arcC1_1"

/locus_tag="JFBMFIFI_01178"

/EC_number="2.7.2.2"

/inference="ab initio prediction:Prodigal:002006"

/inference="similar to AA sequence:UniProtKB:P0A2X8"

/codon_start=1

/transl_table=11

/product="Carbamate kinase 1"

/translation="MGKRVVIALGGNAILRPNQEATFENQLANVKTSTDLIAEVKKAG
HKVVVTHGNGPQVGNILRQNEEAKAFVPPLPIDVCSAESQGFIGYMMEQTLKNALKLA
DVKGEVVTLLTETEVDAKDPAFLEPTKPIGVFFTDEEANELEKSKGWVMAEDAGRGYR
RVVPSPQPVKIHGVSAIKALIEMDTVVISTGGGGIPVVADEKGFLKGVEAVIDKDRSA
LRLSEQVDADVFMILTDVPNVYLNYGQPDQLKLEGISLADANKYMAEGHFADGSMGPK
MEAAIAFAAQGKESIICSLDEAVAALRGEAGTRVLPA"

CDS complement(1244513..1245391)

/locus_tag="JFBMFIFI_01179"

/inference="ab initio prediction:Prodigal:002006"

/codon_start=1

/transl_table=11

/product="hypothetical protein"

/translation="MIEVWKYPIERINELIPDSIWQLHSEFTNGVNLVDEAGNLLFIG
TDKNGELPFGLHLTFRNLVHFKAELNKNTRFHYHNLTFVNQENLEYSLSLKNSTPYSV
ALEKKGQLNSDAVDSLFKWASKEEKKRNGLGEYLPTTEFILDNWMIENKQAIGYLFSE
NPVEIRHGLHYFIGRGQGLTPSGDDFLVGLLSLEKGFSIVDNQFEIILETFVSSEKLT
TDISEAYLQAALKGRFSTSINQLIDVLAGTKNKTALPDILTKIIQNGHTSGIDTLTGI
LVGLLIGTKDIKKGAS"

CDS complement(1245579..1246841)

/locus_tag="JFBMFIFI_01180"

/inference="ab initio prediction:Prodigal:002006"

/codon_start=1

/transl_table=11

/product="hypothetical protein"

/translation="MTYKTIDEANKAVAAKVVASSPFLLDVVPAKEAIPVLEGKVLLH
AGPPIKWENMPDPMKGSCIGAVLFEEWETDEESARALLESGKVEFMPCHHFDAVGPMG
GITSGNMPVFIVENKTDGNFAYCTMNEGIGAVLRFGAYSEEVVNRLRWMRDVLGPTLS
QALKTIENGLNLNVLIAKAIAMGDEFHQRNIAASLAFLKEMAPIIVGLDMDDTNRQAV
IKFLADTDQFFLNIMMAATKAVMDGARMIEEGTIVTAMCRNGENFGIRISGMGDEWFT
GPVNTPQGLYFTGFTGEDASPDMGDSAITETFGVGGMAMIAAPAVTRFIGTGGFQDAL
AISNEMLEIVIDQNPNFTVPTWDFQGICLGIDARKVVETGITPVINTGIANKKAGAGQ
IGAGTVHPPIECFEKAIVAYAKKLGMDS"

CDS complement(1246873..1248630)

/gene="fdrA"

/locus_tag="JFBMFIFI_01181"

/inference="ab initio prediction:Prodigal:002006"

/inference="similar to AA sequence:UniProtKB:Q47208"

/codon_start=1

/transl_table=11

/product="Protein FdrA"

/db_xref="COG:COG0074"

/translation="MLHTIIKENAYQDSVVLMLLTNKISTMDGVNRVSIMMGTPANKD

IMGGSGLRTPELEKASANDLAIVLDTEDASIIDGVLVEVDEFLTSQATSDNEVADETV

RTWDKAMKMGADANLALLSIPGTYAALEAEKALDEGLNAFIFSDNMTIEDELHLKQKA

HEKGLLVMGPDCGTGIINGVPMAFTNIVRPGKIGIVGASGTGIQEVSTIIDKLGAGVT

NAIGTGGRDLSEKIGGITMLDSIAALEKDPQTEVIVVISKPPAKVVRDKVVALLREVS

KPVVAIFLGEKPTHHEKNLYFAYTLEEAAKLAVDVLNGKEVTALDESITIPEVSLQPN

QKHLKGYYSGGTLASEAGMLIADALQLKDGLIKQDGFVLKTDGNEIIDLGDDMYTQGK

PHPMIDPEKRIEMIKHAADDAETAIILLDVVLGYGAHMDMASELAPAIQEVKAKAHAA

GRELVVLGTVVGTTSDPQNLMRQKEILEEAGVIVCESNNKAVRTGLGILGYQVTDTEK

KVEAADATEPVAIPEVSEKVSALLTTQPYVINLGLKSFSEAIRETGGKAVQFDWRPAA

GGDVRLQKVLYFLNNYQTK"

CDS complement(1248660..1249709)

/gene="allD_1"

/locus_tag="JFBMFIFI_01182"

/EC_number="1.1.1.350"

/inference="ab initio prediction:Prodigal:002006"

/inference="similar to AA sequence:UniProtKB:P77555"

/codon_start=1

/transl_table=11

/product="Ureidoglycolate dehydrogenase (NAD(+))"

/db_xref="COG:COG2055"

/translation="MKIPKKQLHKLIEAKIEAAGLTKEHAEIVSDVLTFADARGIHSH

GAVRVEYYSERIAKGGITNRPEFSFKKTAPSCGVFEADNGSGHVAAKLAMDEAIKMAK

ETGLAVVGVRNMSHSGALAYFVEMAAENDLVGISVCQSDPMVVPFGGSEAYFGTNPIA

FAAPSHDDRMITFDMATTVQAWGKVLHARSRNESIPDTWAVDENGNPTTDSTKVSALV

PIAGPKGYGLMMMVDILSGMLLGLPFGKHVSSMYHDLSAGRELGQLHIVINPAFFTNL

DLFKENISQMLDELKEIKPSEGFSEVNYPGERGRMREKKYEETGIEIVDDIYNYLIGD

AIHFDRYDHKNKFAE"

CDS complement(1249820..1250893)

/gene="allD_2"

/locus_tag="JFBMFIFI_01183"

/EC_number="1.1.1.350"

/inference="ab initio prediction:Prodigal:002006"

/inference="similar to AA sequence:UniProtKB:P77555"

/codon_start=1

/transl_table=11

/product="Ureidoglycolate dehydrogenase (NAD(+))"

/db_xref="COG:COG2055"

/translation="MTELEMVRVTESELHELIQKKLEKAGLSSIHANTVAETLAFADA

RGIHSHGAVRVDYYAERIAKGGSNLTPNFKFNQTGPSTGIFEADNGVGHYASNEALKE

AIRLAKETGIGVVGVHQMGHSGALAYFVKQAAEADMIALSVCQSDPMVVPFGGAEPYF

GTNPIAFAAPRKDHTPVVFDMATTVQAWGKILDARARNVDIPDTWAVDGSGNPTTNPH

EVKALLPIAGPKGYGLMMMVDILSGTLLGLPFGKHVSSMYTDITAGRNLGQLHIIINP

SFFTDLDKFKADVNQMVEELHKTKPASGFDQVLYPGELSEIVSEKYKKEGIPIVKEIY

DYLVSETIHFNRYDKASAFGEQD"

CDS complement(1250924..1251709)

/gene="allE"

/locus_tag="JFBMFIFI_01184"

/EC_number="3.5.3.26"

/inference="ab initio prediction:Prodigal:002006"

/inference="similar to AA sequence:UniProtKB:P75713"

/codon_start=1

/transl_table=11

/product="(S)-ureidoglycine aminohydrolase"

/db_xref="COG:COG3257"

/translation="MGYRNKNTGYNDDLLASRSIIRRGNFALIPPDGLVNNTIPGFEN

CELSILSSPKLGASFVDYLVTLLPNGKNTRGFGEPGVETFVYVLSGKVKISDGENEYI

HTDGGYVYLPAGKKMYLENIYGENSELFLYKKRYEAIDGYEAHVVSGNTNDIQAEEYE

GMSDVLLTDLLPKGLGFDMNFHILSFKPGASHGYIETHVQEHGAYMLSGAGVYVLDNE
WLPIKKGDYLFMGAYVPQATYAVGRDESFSYLYSKDCNRDAQI"

CDS complement(1251806..1253062)
/gene="exuT"
/locus_tag="JFBMFIFI_01185"
/inference="ab initio prediction:Prodigal:002006"
/inference="similar to AA sequence:UniProtKB:O34456"
/codon_start=1
/transl_table=11
/product="Hexuronate transporter"
/translation="MENQQVAKKKGLEYWQQIIIMLCLGWTVIWIYRSALSPIFPELN
LSLGGGISDSSLGAIASFYFFGYTGMQIPAGILVDKFGKKMVLIPGFTLFALAAIVIA
NANGITMVYAGSLMAGIGCGSYYGSAYSLSSESIPQERRGLSTAIINSGSAVGMGIGL
ILSSLLVKQLGLPWQIMMYLVAILVVLIMIVFIKVIRTTPDAVVNHPTAKAKHDPEDK
VSMSTLFAPHMIASYVLYFATCYGYYMVVTWLPSFLQQERGFEGVAIGFSSALVAFSA
IPGALFFSRLSDKFQDKKIQFIVVLELLAAAMLVLTVMAPTSGVLLVGLVLYGLLGKL
AVEPIIISYIGDTAPKKGYGTTFGVFNFFGMSSSVLAPWVTGVISDATGSKVNGFFLS
AIIMVVGTVLFLGANIMMKNKKAKAE"

CDS complement(1253267..1254505)
/gene="allC"
/locus_tag="JFBMFIFI_01186"
/EC_number="3.5.3.9"
/inference="ab initio prediction:Prodigal:002006"
/inference="similar to AA sequence:UniProtKB:P77425"
/codon_start=1
/transl_table=11
/product="Allantoate amidohydrolase"
/db_xref="COG:COG0624"
/translation="MTHLDLDKEVLESVKWLSNIGADPTGGTTRLLYSDSWLEAQNGV
KAKLEEIGMTTKFDAIGNLFGRLEGSKYPEETILSGSHIDTVVNGGKLDGQFGVLSAY
LAVKLLKEKYGQPLRSLEVISMAEEEGSRFPYAFWGSKNLFNEAKKEDVIDISDFNGI
KFVDAMHNVGFDFKDESEPIRDDIKAFVEIHIEQGNVLELEGKSVGVVTSIVGQKRYT
IVLKGQANHAGTTPMKYRKDAVYAFSKICAQSIDKAREVGEPLVLTFGKVEPKPNTVN

VVPGEVLFTMDCRHTNQAELDAFTAEIEADMKRIAEEMGMEIEIDLWMNEPPVPMDEK
IINVLKEVSEAEGINYRMMHSGAGHDSQIIAPHIPTAMIFVPSIGGISHNPAEDTKNE
DLVEGVRTMAAALYKLAYVD"

CDS 1254762..1255613

/gene="aes_1"
/locus_tag="JFBMFIFI_01187"
/EC_number="3.1.1.-"
/inference="ab initio prediction:Prodigal:002006"
/inference="protein motif:HAMAP:MF_01958"
/codon_start=1
/transl_table=11
/product="Acetyl esterase"
/translation="MLLYKDFIFHTTEKYQLEASLYQPTGTPLNQTIIYLHGGGLIWG
SRFDLPEDYIQLFLSAGYHFLTLDYPLAPEVELPEIEAAIQTGINWFLNQANQSLELK
SNEFILFGRSAGAYLGFLYQKNALAVQPKKFISFYGYHSINEAFYLRPSSFYLRYPKI
SEETKKALIQTKPLVSGPLETRYAIYIYARQTGRWIDLILQHQQNYSNYSLTNEELKM
LPPTFIAQSTADQDVPYSIGAYLNQHILNKEFITIEGLAHDFDKDPNIKEAQMTYRKL
IEWLSKD"

CDS 1255810..1257270

/gene="dtpT"
/locus_tag="JFBMFIFI_01188"
/inference="ab initio prediction:Prodigal:002006"
/inference="similar to AA sequence:UniProtKB:P0C2U3"
/codon_start=1
/transl_table=11
/product="Di-/tripeptide transporter"
/db_xref="COG:COG3104"
/translation="MKKEKTFFGQPRGLSTLFFTEMWERFSYYGMRAILIYYIYDSVA
NGGLGYSKSLAASIMAIYGSMVYMSSVIGGWLSDRVLGSRKTIFYGGILIMIGHIILA
TPFGAPALFASIAIIVLGTGMLKPNVSEIVGGLYHKNDLRRDSGFSIFVMGINIGSFL
APIILGAVQKEYNYHVGFSIAAFGMLAGLIQYYFQGKKYLGTVGLEPTNPISETEKSA
VYKNLIKGIGLLAIVILGAALLGKLTIEFVIFLLSILGVALPVYYFTKMIRSKDVNDM
ERSRIWAYIPLFIASIFFWSIEEQGSVVLAIYADERTNLDFMGFRLIPSWFQSLNPLF

VILMSTMFAWLWTKLGERQPSTTIKFSIGIVFAGLSFVILVIPALLNGVDTLVSPWWL

VLSFFLVIVGEMCLSPVGLSATTKLAPRAFQSQMMSVWFLGDAAAQAINAQIVSLYSP

ENEVMYFAIVGGFTIAVGLLLSLMNSKIKTLMQGLN"

CDS 1257468..1257707

/locus_tag="JFBMFIFI_01189"

/inference="ab initio prediction:Prodigal:002006"

/codon_start=1

/transl_table=11

/product="hypothetical protein"

/translation="MITTTIEKVWAIQGLLFKNEDYQLALELEDSGNRVQLRYIVLNE

DSPNYQINQEFGWNQSDFELYKSSRDAEENLLVIA"

CDS complement(1257823..1258377)

/locus_tag="JFBMFIFI_01190"

/inference="ab initio prediction:Prodigal:002006"

/codon_start=1

/transl_table=11

/product="hypothetical protein"

/translation="MENKKRKLDNNPQLTEASIHPDLIVTTEEVFRYFDWNQLDWEVE

KQNSEYGAYLIKLDHLLIRFRVAKTTPTKVGQFVTLWKRNVKGIIEPYTSQDKMDFVV

INTRLGEHIGQFVFPKKVLIEKGILSVNGVGGKRGFRVYPLWDQPDNPQAIQTQKWQL

NYFLDLTQGSQQLNAKSIQRLYNI"

CDS 1258621..1259385

/locus_tag="JFBMFIFI_01191"

/inference="ab initio prediction:Prodigal:002006"

/codon_start=1

/transl_table=11

/product="hypothetical protein"

/translation="MKNRKDYLKLLFLISLSVCLMFFINEHYIKADKKNPVISSTEPA

VTPKKKTKTSDLPISAQLDVPLLYQFDEPSLYNGCEVTSLAMLIQFYGSTVTKNELAD

NLVSVPLMDDDGYMGNPNQAFVGDVSGNDSPGLGVYHGPIANLAKEYVGNENVLDSTG

SDFTDVMAQVAAGNPVWIITTATFSPVDDFVTWETTTGKIDVTYSMHSVVVTGYDAES

IYFNDPYGEKDASMDKENFIAAFEQMGSQAISLFNN"

CDS complement(1259443..1263231)

/gene="addA"

/locus_tag="JFBMFIFI_01192"

/EC_number="3.1.-.-"

/inference="ab initio prediction:Prodigal:002006"

/inference="similar to AA sequence:UniProtKB:P23478"

/codon_start=1

/transl_table=11

/product="ATP-dependent helicase/nuclease subunit A"

/db_xref="COG:COG1074"

/translation="MMELPIKPQNSRFTDGQWQAVYEEGHNILVSASAGSGKTTVLVQ

RVIEKIKAGVNVDELLIVTYTEAAAREMKARIQVAIQKAITGEGDVAMKQHLVKQVPL

LPQASISTLHAFCLQVIRRYYYLIDLDPVFRLLTDETEMLLLKEDVWSDLREELYGTD

NNDFYALTATYSNDRNDDGLTRLIFSLYEFSRANPNPRLWLEHLSDLYRVDSGDLADT

KLFKELLKPQIIESLYGLIATGQKASSIAEGTEELTKQHELVFSETLFFEELLESIMG

NHYETAYNQLSAFKFERIATVKKAADESIKEAGAELKALRDQYKERYNQLVTDYFTTS

PQEMSAIMEKATPLVDEMARVTQLFTEAYSKEKRRQNVLDFNDLEHLTLEILANFEGN

TWSPTEASNYYREKFSEVLVDEYQDVNQLQENIIRWLSSEDSVNGNLFMVGDVKQSIY

SFRLADPSLFLTKYEKFGQHIDGERIILAENFRSRGEVLHFTNLIFEQLMDKSVGQLF

YDEDAKLIHGFPDFPETNCHQPELLIYEKGSNEDTDSEEVESVEWDMRIDDKTEGELL

MVAQRIQQLIAEKFPIYDKDKKVNRPISYRDIVLLTPTKKNNLILLDVFKRMSVPIHV

SDTQNYFQTTEIKIMMALLKIIDNPYQDIPLAAVLRSPIVALDENQLASIRITKKTGD

YYAALENFYKTYQGQAKASHFDTILFEKVKRFMEQLENWRRIARREQLVTLIWTIYED

TGFLDYVGGMSSGKQRQANLHALYQRAESYEVSSFKGLFQFVRFIEKMQEKDKDLAEP

TAISASEDAVRVMTIHGSKGLEFPVVFVLDLTKRFNLQDIQKSYLFNEDYGVGTSFKD

LERRISYTTLPEVALKIEKKKKLLSEEMRKLYVAVTRAEEKLFLVGSYKNEESAWLEW

GQTAHYSEAILPSSERFNASSLMKWIGMSLVRHPDGKNDYLETPAKIGSIWQHPARFK

ISFHNESSIRENIQTIDLDEDSTWLENLEVQAKNNDKHQNDEAFQLAVKRMEYEYTHE

SATRTTSYQSVSEIKRLFEEPENARMVKIDVNKPRNRFVSEKLSRPKFMSDESEPSAA

EIGTATHLIMQNIDLTQIPTKESVKDLIATFVAKGLLTDKLATKIQVDAILAFFTTEL

GELMLEKPKLVTREAPFSLLMDAEKIFTDMHEDASDKLLIHGIIDGFLEEEDGLILYD

FKTDYVDQNSTIALEKISKNYRGQMNLYREALENISGKPVKAAYLCLLSIGKTLLIEK

"

CDS complement(1263228..1266848)

/gene="addB"

/locus_tag="JFBMFIFI_01193"

/EC_number="3.1.-.-"

/inference="ab initio prediction:Prodigal:002006"

/inference="similar to AA sequence:UniProtKB:P23477"

/codon_start=1

/transl_table=11

/product="ATP-dependent helicase/deoxyribonuclease subunit

B"

/db_xref="COG:COG3857"

/translation="MGLQFVLGRGNTDKRAIMLEEIATTITNNKQAMIFYLVPDHIQF

EAEVSVLKRLADLPPFNEQKIMGAMRLQVFSFSRLAWYFLQDSDLFIKPQLTNAGLAM

LIRKLLLEHEEELTIYRGEVRKSGFVQQLTELFLELRSGRINEADVAEIIAKQGDSLK

EADFKLKLQDILLLYRGFERNLIGSYIEKEDILTALTKKIETLDLSQTTIYIESYQSF

NAQEQELIGALLKKCKDVTIALNLDQGYPVEKPALHELFYATGETYYQLYQLARNGNI

PVKQDCIISEKSAEYSEELNQLEDFWLTSSRLLPSNYSEAKELISVNEGIEVWAASDK

QAEVTHVAKEIRRLVESGKYRYQDVLVLTRHLNDYRALVNPIFKENDIELFNDQAETM

NHHPLVEFVLALLAIKKYNWRYQDIMRLLRTELLIPVSDSELSDDRKIRVAVRQQQVN

KFRSQIDIAENVILAYGYEGYHWTSNKPWHYTKFVHEENAGQTTEDQRIEEISNSVRL

FLKNLLEPLFKKLDQAKNGLAAAEIVYTFLESSGVSDQIAFWRDQAIESGDLETSKEH

EQAWDTFLQLLDEYVEVLGKTSFDLDAFYEIIMTGFEQATYSMIPPSIDQVTFSSIES

TRIGTTKVTFILGMTEVNLPAKSENASVLTETDRELFSAFLEEGKYLRPSIEAQTANE

PYLAYLAYLASTEKLIFSYPVADDSKEGPKLSPYVQRIVAGLEIIIQEKVGTVADLAP

TASIEEQLEFIGTKRSTLTQLLVLLRQRKENGEEIPPFWLSLYHRIKMDGTKTGALLE

RLKSSLQHKNVPIQLKPEIVEELYGKDLYTSVSRMENFYSCHFKHYATYGLGLKERDV

FELSSAGTGEFFHDALDQLFKSLKERNLQLENLDEASLKNVANDVLEVLYGKPKYLIL

STSNRMNYIRFQLGNTIKRVAWALANQSRRSGMTTVETEVLFGQIASQKGIDGLVLPL

ENQGQLYVRGKIDRVDTMEVAGEQYLSVIDYKSSAHTFDYRDAYYGLAMQMITYLDTA

LMNSETLIGGKAKPSGAFYLHVKNPFISNDKISNEENYQEELLKPFKLDGILLEDEAM

LRQLDKSLEPTKSSIVYPYRELKNESLKSAKFVNESEMNALRSHNQELFTKAGNKIMA

GATDLNPYYKDKQRIACGMCPFRSVCEFDVMLPENNYHRVEPIDPKEVLRRMASKEGE

TE"

CDS complement(1266922..1267470)

/locus_tag="JFBMFIFI_01194"

/inference="ab initio prediction:Prodigal:002006"

/codon_start=1

/transl_table=11

/product="hypothetical protein"

/translation="MKVIIGAGNTSFNGWISTNKEDLDLLEKESFKKFFHEKKADALF

AEHVWEHLTFAEGKIAARNCYDFLEDGGYLRVAVPDRNFRNADYQKLVQVGGPGPKEH

PAFSHKILYDVSVFTKLFKECGFEVNLLEYCDDKGTFHYNYWNPDDGRVGRSFRFDTR

NSKSKLGMVSLILDAHKPIIIS"

CDS complement(1267501..1268046)

/locus_tag="JFBMFIFI_01195"

/inference="ab initio prediction:Prodigal:002006"

/codon_start=1

/transl_table=11

/product="hypothetical protein"

/translation="MESYVVLLRGINVGKNHLLAMSDLKKLLESLNFTQVTTYIRSGN

AVMVGKKMDTVALAKEIQFKLSQLAGFLIPIVIYTGSEFKEIVEKNLSIASRLRDDQQ

LILAYQQEEIVDIEALSLSNDVESGWIVKKAVCMQITGPQLESKLLKDYQKLFKQNDW

TLRNWKTSLKLVELVKALKQI"

CDS complement(1268133..1268399)

/locus_tag="JFBMFIFI_01196"

/inference="ab initio prediction:Prodigal:002006"

/codon_start=1

/transl_table=11

/product="hypothetical protein"

/translation="MSDTIIACLYFVSVNSLLFILMGVDKYRAKKRLWRIPEKTLLSF

GILGGGLGGLIGMTLFHHKTRVPKFKVIYSLGTILMLGILYFLF"

CDS complement(1268400..1269629)

/gene="pepT_1"

/locus_tag="JFBMFIFI_01197"

/EC_number="3.4.11.4"

/inference="ab initio prediction:Prodigal:002006"

/inference="similar to AA sequence:UniProtKB:Q76HM5"

/codon_start=1

/transl_table=11

/product="Peptidase T"

/db_xref="COG:COG2195"

/translation="MYENLIPRFLRYVKTETRSDATSKTVPSTQSQVEFAQTLKEELA

DLGMSQVSYNEKNGFVTATLPSNCDKEVPTIGFIAHMDTADFNAVNVNPQFHENYNGE

EIILSATENIRLAPNDFPNLKNYIGQTLITTDGTTLLGADDKAGIAEIMTAMEILLKD

KTIKHGTIKVAFGPDEEIGIGADLFDVANFGADFAYTMDGGPVGELEYESFNAAQAEI

KIQGKNVHPGTAKNTMVNALKLALKIDAALPQDEVPEKTDGREGFFHLVSMSGEVEEA

QMTYIIRDHDHDKFIARKNQLQDIANKINEESGSERVNVTLFDQYYNMKDIIEKDLSI

VDLAEKAMINLGIKPIIEPIRGGTDGSKLSFMGLPTPNIFAGGENFHGRYEFVAVESM

RKATDVIVEIAKLNVER"

CDS complement(1269667..1270791)

/locus_tag="JFBMFIFI_01198"

/inference="ab initio prediction:Prodigal:002006"

/inference="similar to AA sequence:UniProtKB:P67272"

/codon_start=1

/transl_table=11

/product="GTP cyclohydrolase 1 type 2"

/db_xref="COG:COG0327"

/translation="MKKILGNEFIQKFEEFAPKSLAENGDPVGLQIGTLNKTIKRILV

TLDVRPEVVAEAIEQNVDLIFAHHPPIFRGAKNLVIDNPQNKMYAELLKHDIAVYAAH

TNLDVATNGLNDWLGQAIGLDNTEIMSISKQESYKKMAAYVPKSHEKQVREALTAAGA

GKIGPNYAQCSYTLDGVGRFTPINNALPAIGELNQAEEVNEAKVEVIFPQRLTESVER

ALFSSHPYEEPAYDLYTIENFVDSYGFGRVGNLAEPITVLDFATKLKTIFSVSGLRVV

TATPEKMIQRVAICGGSAGDYYLDAKKHGADVYITGDVYYHTAHDILASGMTVIDPGH

HIESICIAELADLFTEWSETNHWDIEIVQSTLNTDPFIFI"

CDS complement(1270788..1271489)

/gene="trmK"

/locus_tag="JFBMFIFI_01199"

/EC_number="2.1.1.217"

/inference="ab initio prediction:Prodigal:002006"

/inference="similar to AA sequence:UniProtKB:P54471"

/codon_start=1

/transl_table=11

/product="tRNA (adenine(22)-N(1))-methyltransferase"

/db_xref="COG:COG2384"

/translation="MDEKQLSLRLEKASNYVPNNSKLADIGSDHAYLPCALVYQKKIQ

FAVAGEVVEGPFLTAKEQVKRLGFQAMIDVRLGNGLAVIDLADQITAITICGMGGTLI

ASILDDGFHAGKLTGGERLILQPNIGEENVRRWLMDHDYQIIAEELLEEDAKLYEIIV

AEKSIVQPSYSQSELKFGLELKENYPDLFKKKWQRQLEKEYTILASLEAATSDQKEKI

ALFKTKIAEIEELIQ"

CDS complement(1271633..1273120)

/locus_tag="JFBMFIFI_01200"

/inference="ab initio prediction:Prodigal:002006"

/codon_start=1

/transl_table=11

/product="hypothetical protein"

/translation="MMKSRNERIAELKKTNQKKMIRKSAGIVNTSIIMASLAIPTFSV

LASAEENATTSERVIKAVADSSNSASDTATEIAPTPVPVDPETPIEETPEIPKEEVEV

PETKPETPETKPEVKPETPGTKPVAPIVETKPTPKVEVETTRPFSKEEVSQQNIAEVQ

VPEANHSEAEKIMFVKNQSTQEFIDKISESAAEIGAEKDLYASVMIAQAILESGSGNS

SLSSEPNYNLFGIKGTFENQSVALLTLEDDGSGNYYQITGEFRKYPSYKESLEDYSKL

LTGGTSFNASFYSGTWKSNTTSYKDATAFLTGKYATDTRYAEKLDQLIDTYDLTQYDD

GVKKVSETEVQQDVMHTIVSGDTLWDLGNTFGVSVQELMEWNQLTSDLIFVNQELIVK

KAPIAPPVVAPVENVSAAATDTKKVEDTNLTGFQQKQNGTLAFDEATSDLNQASVKEE

YQVAEGDSLYTISKKFNVSPLEIKHWNDMKHNLLFIGQTLTIHTV"

CDS complement(1273107..1273769)

/locus_tag="JFBMFIFI_01201"

/inference="ab initio prediction:Prodigal:002006"

/codon_start=1

/transl_table=11

/product="hypothetical protein"

/translation="MTENLREQVSLLEKENRRIRQKLRHSYEAYKAMELEKNQIQEES

FKEKALPAHYQDVEEKLEYYKKMVDSLASENQVLQNQLDTTPRYNEVVKEDSTRFAQD

ELLEKIKLQEIQIETLIEEKNQIKLELKNSKAEIAEVLIDAKMRARKIVETADEEVVG

YKNKAAIELALFQKKLQETQQTILRTKLDVTNLFEDIDTKINSISQIDVTTMGGNEND
EK"
CDS complement(1274016..1274729)
/locus_tag="JFBMFIFI_01202"
/inference="ab initio prediction:Prodigal:002006"
/codon_start=1
/transl_table=11
/product="hypothetical protein"
/translation="MKKKEKCIICHKDYSETDGLHLTTLSKELRELILSEHPEFSEEA
FICMDDLMDYRLHYIKDMIKTDSENIESLNKNVLDSIKEGMPIAKNTNEDVTTNLTLG
EKVADGIAKFGGSWGFIFLFLFVLVAWIIINSIALFTKPFDPYPFILLNLILSCLAAI
QAPVIMMSQNRQEKRDRQQSDSDYQVNLKSEIEIRLLHEKMDHMLTEQWEHLVNIQNI
QVDLLNELQERMEVLEKKV"
CDS complement(1274758..1274991)
/locus_tag="JFBMFIFI_01203"
/inference="ab initio prediction:Prodigal:002006"
/codon_start=1
/transl_table=11
/product="hypothetical protein"
/translation="MSECLVSLSDGEKVVLKISEKELFSKISNGDGFLRSRLIRFYND
QEDAIYINPVQIVKITFSSGNSDNALKIDTRYQ"
CDS complement(1275101..1275565)
/locus_tag="JFBMFIFI_01204"
/inference="ab initio prediction:Prodigal:002006"
/codon_start=1
/transl_table=11
/product="hypothetical protein"
/translation="MKKKKLAFSLVVAILVVVAVGCGNGADKKTESSSSITSDSEKES
ETSSTAPAVEEPIVYTAVVKQDSNSETGQVWVESLLPVDSNKETPPIFKEEVVLLTDG
SNVFDGKTNEKISLDEIKAGTNLEVTLAAEPVSTMSIPPQIPGKDVHKIVVK"
CDS complement(1275719..1276846)
/gene="sigA_1"
/locus_tag="JFBMFIFI_01205"

/inference="ab initio prediction:Prodigal:002006"

/inference="similar to AA sequence:UniProtKB:P0A0J0"

/codon_start=1

/transl_table=11

/product="RNA polymerase sigma factor SigA"

/db_xref="COG:COG0568"

/translation="MAKKEEVKNNTVTLLEQAIKKVIKDNKLKGSIHYDELTNLVVTP

YELDADQIDDVIMQVEDGGVSVVGDDGGPTDRQILAKEKDKKAAEEQEDLTAPAGVKI

NDPVRMYLKEIGRVPLLNAQEEVALALLIEQGDQEAKQRLAEANLRLVVSIAKRYVGR

GMQFLDLIQEGNMGLMKAVEKFDYQKGFKFSTYATWWIRQAITRAIADQARTIRIPVH

MVETINKLIRIQRQLLQDLGREPTPEEIGAEMDLPTEKVREILKIAQEPVSLETPIGE

EDDSHLGDFIEDQDATSPAEHAAYELLKEQLEDVLDTLTDREENVLRLRFGLDDGRTR

TLEEVGKVFGVTRERIRQIEAKALRKLRHPSRSKQLKDFLE"

CDS complement(1276884..1278770)

/gene="dnaG"

/locus_tag="JFBMFIFI_01206"

/EC_number="2.7.7.-"

/inference="ab initio prediction:Prodigal:002006"

/inference="similar to AA sequence:UniProtKB:P05096"

/codon_start=1

/transl_table=11

/product="DNA primase"

/db_xref="COG:COG0358"

/translation="MAMMIPEETVNRIRQETNIVDVVSQYVQLKKSGKNLFGFCPFHE

ERTPSFSVAEDKQIFHCFSCGKGGNVFTFLMEVDGLSFPEAVFKTAEMSQIKLDDTLL

NNRSHQNQESDSKRDKLIQAHEETADLFQHVLLNTKVGEEAYHYLLNRGLTKELIETF

KIGFAPRERTMLHQYLLGKEYTDEVLNETGLFVERDNGELIDRFYNRIMFPIRNQQGK

TIAFSGRIFQQETEDKAGPKYLNSPETYLFNKRNVLFNFDMARGAIRREKEVVLFEGF

MDVIAAWNAGVKNGVASMGTSLTNEQIHMLDRVTDRVLIAYDGDNAGIEATKRGVDLL

TTETAFDVEVVSFTEGLDPDDFIKKYGASAFVELLAHGRDTLFAFKMRYHRKGLNLQN

ENERLSYLDLILTDMLTITSAVEREMYLKQLSSEFDISLDSLNEQYQQLFKEKRAQRK

EVKNQFNFGPEPEIEPYFPEEEMVVQVDTQKRKLDLIEQTERYLLNRLFHFEEAWIQV

QSQVEDYHFIHDDYQTLYLLFESFKDTVGEYGSIDAFIDFVKEPPLKNLLVEIEMLSL

SEEMTAKEIDDYLDVIVRKSSLSEQLKHKQAAMKEAQRIGDQVVLQQLMLEIVDLSRQ

LKNK"

CDS complement(1279025..1280740)

/locus_tag="JFBMFIFI_01207"

/EC_number="3.6.3.-"

/inference="ab initio prediction:Prodigal:002006"

/inference="similar to AA sequence:UniProtKB:Q99T13"

/codon_start=1

/transl_table=11

/product="Putative multidrug export ATP-binding/permease

protein"

/db_xref="COG:COG1132"

/translation="MLKRFFSYYRPYRKLFILDFSCAIVAAILELAFPIVVNQVIDKL

LPTGNWSLIVTAALSLLFFYVVNTALQYVVVYFGHMLGVNIETDMRRELYAHLQSQPF

SYYDNQKTGKLMSRLTTDLFEISEVAHHGPEDVFITIMSLVGSFALMLSIHVKLALAT

FIMIPFITLALVFFNKRMTKVNTQIYRDLGEFNAGVEASVSGVRVVQAFSNEAHEQER

FEGLNQAYRKSKVLFYKMMGISAAYNYLLMRLINLFALFFGAYYTITNEITYGQFVGF

ILLTNVFVRPIEKVNNMIESYPKGIAGFKRFTEEIDKEPAIQDVPNARDDIVLTGDIE

YKDVSFWYDDSKKVLDKINLSIKEGETVAFVGPSGAGKTTICNLLPRFYEVNEGAVLV

DGYTIDALTMSSLRSQIGVVQQDVFLFPGSIKENVAYGKLDATEDEINHAIRLAHLEQ

VVAEMPDGLETIIGERGVKLSGGQKQRLAIARMFLKNPPILILDEATSALDTETEQVI

QESLNSLSEGRTTLIIAHRLATIKHANRIVVVNEEGIAEEGTHDDLMARNGAYKRLYD

AQFKD"

CDS complement(1280939..1283023)

/gene="glyS"

/locus_tag="JFBMFIFI_01208"

/EC_number="6.1.1.14"

/inference="ab initio prediction:Prodigal:002006"

/inference="similar to AA sequence:UniProtKB:P00961"

/codon_start=1

/transl_table=11

/product="Glycine--tRNA ligase beta subunit"

/db_xref="COG:COG0751"

/translation="MAKDLLLEIGLEEIPAKYVTPSSQQLVKRVSDFLTENKLTFGEI
VPFSTPRRLAVIVKSVADKQEDTAEIVKGPAKKIALDAEGNWSKAAQGFVRGQGATVE
DITFKEIKGVEYVHIDKFTAGKPAMDILTKLDQVITGMTFPVSMSWGNHSFKYIRPIH
WITAMLDETIIPFKILDIETSNTSRGHRFLGEDTSFAGASDYESKLEKEFVISDSQKR
KAMIVDQIQKIASEKQWQVELDADLLEEVTNLVEYPTAFAGSFEEKYLSIPEEVLVTS
MKEHQRYFEVRDEKGALAPYFISVRNGNAIHIENVIKGNEKVLTARLEDGEFFYQEDQ
QIKIADAVERLKLVTFHEKIGSIYEKMQRVSYFAETIGKVVGLTNEELSDLQRASEIY
KFDLVTNMVGEFPELQGIMGEKYALMQGEKPSVATAIREHYLPTSSDGVLPVTNVGAV
LAIADKLDSVMSFFAVGMIPTGSNDPYALRRQAYGVVRIVENKGWYFPIETLRGEMIS
AAIQHQGKLSDQLEASTPEVIDFIKARVRQHLLSQKIRYDIIDAVLKSEQEDLIKMAE
TALVLEKHIQEATFKPTIEALTRVMNLAKKGQELLSESQLEVKPELFETESEKALFEA
ILVAKEAFSGENMELNYLALETLGPKVEAFFEDNMVMTDDEKIRNNRLALLVQLGNLA
LSFASIDQLIVK"

CDS complement(1283025..1283936)

/gene="glyQ"

/locus_tag="JFBMFIFI_01209"

/EC_number="6.1.1.14"

/inference="ab initio prediction:Prodigal:002006"

/inference="similar to AA sequence:UniProtKB:O67081"

/codon_start=1

/transl_table=11

/product="Glycine--tRNA ligase alpha subunit"

/db_xref="COG:COG0752"

/translation="MSKKLTIQEMILTLQNFWSNQGCMLMQSYDTEKGAGTMSPYTFL
RAIGPEPWNAAYVEPSRRPADGRYGENPNRLYQHHQFQVVMKPSPDNIQELYLESLKL
LGINPLEHDIRFVEDNWENPSMGCAGLGWEVWLDGMEITQFTYFQQVGGLECKPVTSE
ITYGIERLASYMQEVDSVYDLEWTNGVRYGEIFFEPEYEHSKYAFETSNQELLLTLFD
AYEKEAQVQIEHGLVHPAYDYVLKCSHTFNLLDARGAVSVTERAGYLARIRNMARALA
KAFVAEREKLGFPLLQKNNDVLVKEEN"

CDS complement(1284294..1285277)

/locus_tag="JFBMFIFI_01210"

/inference="ab initio prediction:Prodigal:002006"

/codon_start=1

/transl_table=11

/product="hypothetical protein"

/translation="MAKDKLNQENIEVNQSKESGTKATSEKATIFSYLSDINRIKVDY

TKEPLKIKYKWASTHFIFPTQDSFDLEGQKLALRLEEKIEEAYSLNQSISPKERTNQK

KWRILAAVLLIIGIGGLSANSIYGTYLARKIEQNTQITMEKVKKVNPAKSEDEKQERN

AFKEQPYQPIETYLDDSDIETYLGSIAIPDVNLNLPIVKGVGEQNLYRGAATNKIGQQ

MGKGNYPMAAHKVPGNDTSLFGPLFQVTPGMKIYLSDNQNIYIYTARESYVVEPERVD

ILDDVIGQTLLTLYTCSTDAGVERLVVQADFEKQLKMTDASPEEQAIFARE"

CDS complement(1285430..1286635)

/locus_tag="JFBMFIFI_01211"

/inference="ab initio prediction:Prodigal:002006"

/codon_start=1

/transl_table=11

/product="hypothetical protein"

/translation="MKNSFKNNLVKGIVVSSLVLGVGVTAILSPIAANAATITASKEK

DSGNEYSAYYDQTIALDDTYSIGFKHTSETGFFWAAFENTDPNSAVANGSGTIGTEFT

FTITNGEDAFPFTVTLTEEEKEVGQYIGDFTLLFGGAEFSDNNFPLVNLGSAFNPLDK

ITFVNADGEAFLGTITADVSSLDTTKLGIYPVQYTVNETREASAGKSTKATTTELQTA

SFTVPVAVVNETFQTYKEETTLTIDSAKNNVDNKASYTAKDGTLRFDVWNTGGTNYTL

NVVDLLTEGQPVVKEFNFSSNTTIGYVLDVTYEDIFKQLDQYTINFAFTTSTGGTDVG

TGSVAANTGNTVAPTASTPVAKSPVAKANKPTKLAQTNAGLGMLPAVGGLTAVGGSIG

AIFKSRRKK"

CDS complement(1286955..1287740)

/gene="recO"

/locus_tag="JFBMFIFI_01212"

/inference="ab initio prediction:Prodigal:002006"

/inference="similar to AA sequence:UniProtKB:P42095"

/codon_start=1

/transl_table=11

/product="DNA repair protein RecO"

/db_xref="COG:COG1381"

/translation="MSQLEEVDGIILSVRNHRENDRLVKIFTNRFGKRMFFVKGTRKP

NSKLKSAILPFTKAQYIADIRESGLCFLRDAKEVHHYSSMQKDIFLNAYATYVLGLAD

AALEDGIVDPTLFHKIEMTLTDIDEGYDPEIMLNIFEVQLLPYFGVAPELRGCVACGT
TEGIFDYSSSYGGLLCQKDWHLDVHRYHATPRAIHFLRIFSRIDLEKLGEIKVKEETK
TELRRLLDMIYDESVGIKLKSKSFIDQMHTWGDMLLPKKKPIE"

CDS complement(1287819..1288718)

/gene="era"

/locus_tag="JFBMFIFI_01213"

/inference="ab initio prediction:Prodigal:002006"

/inference="similar to AA sequence:UniProtKB:P42182"

/codon_start=1

/transl_table=11

/product="GTPase Era"

/db_xref="COG:COG1159"

/translation="MSEHKSGFVAIIGRPNVGKSTLLNRVVGQKIAIMSDKAQTTRNK
IQGIYTTPEAQMIFIDTPGIHKPKHRLGDFMVDSALSTLREVDVIILMVNAEEKRGPG
DNFIIEKLQGNKTPIFLVINKIDKVHPEKLLEIIDNYRELLPFEQIVPISATEGNNVE
TLLNELLRYLPAGPQFYPEDQVTDHPEYFIVSELIREKVLELTREEIPHSVAVVVEGM
KRNENDKVQVQATIIVERSSQKGIIIGKGGKMLKDIGIKARRDIEVMLGDKIYLELWV
KVQKDWRDKQNYLTDYGYRKDNY"

CDS complement(1288715..1289128)

/gene="cdd_1"

/locus_tag="JFBMFIFI_01214"

/EC_number="3.5.4.5"

/inference="ab initio prediction:Prodigal:002006"

/inference="similar to AA sequence:UniProtKB:P19079"

/codon_start=1

/transl_table=11

/product="Cytidine deaminase"

/db_xref="COG:COG0295"

/translation="MTKHDVQKQLWIQKANEMLTKAYVPYSHFPVGAALVTTDGKIYS
GCNIENASYGLSNCAERTAIFKAISEGEREFSYLVITGDTDGPIAPCGACRQVIAEFC
PSTMPILLTNKKGDQMETTIASLLPGAFTPEDLIT"

CDS complement(1289143..1289568)

/gene="dgkA"

/locus_tag="JFBMFIFI_01215"

/EC_number="2.7.1.66"

/inference="ab initio prediction:Prodigal:002006"

/inference="similar to AA sequence:UniProtKB:Q05888"

/codon_start=1

/transl_table=11

/product="Undecaprenol kinase"

/db_xref="COG:COG0818"

/translation="MDWNDEREAQDKKEFDERCEKNKNFLESFKHACDGVVTVYKEEL

NMKIHLLLGALVLLLSWYLNISKNEWLWMLLTIFLVIVMEIWNTVIENVVDLATNKEF

HPLAKKAKDMAAAAVLVTASFSVVVGAIIFIPKLIQLFF"

CDS complement(1289552..1290028)

/gene="ybeY"

/locus_tag="JFBMFIFI_01216"

/EC_number="3.1.-.-"

/inference="ab initio prediction:Prodigal:002006"

/inference="protein motif:HAMAP:MF_00009"

/codon_start=1

/transl_table=11

/product="Endoribonuclease YbeY"

/translation="MELDLFDETGKITEEQTKMVKTLLEFAGSHINLPEDTEMSVTFV

DNAAIQVINRDYRGKDQPTDVISFAMEDEVPDEVAINFDLLDEKMPRNIGDIIISVDK

TAEQASDYGHSFDRELGFLALHGFLHLNGYDHMEEAEEKVMFDLQKEILDAYGLER"

CDS complement(1290048..1292219)

/gene="pgpH"

/locus_tag="JFBMFIFI_01217"

/EC_number="3.1.4.59"

/inference="ab initio prediction:Prodigal:002006"

/inference="similar to AA sequence:UniProtKB:A0A0H3GGY3"

/codon_start=1

/transl_table=11

/product="Cyclic-di-AMP phosphodiesterase PgpH"

/translation="MKRAIERIQKKMGKYYIPTLLILFSVVLFLIMFHSVQPRSLDVK

LYQVAEETVRANATVEDREKTLESQNLAKENVTPVYRFNSTIATTKISNINFLFATID
EVKSQLAERKKAEAPNATAETSEASSKEKISLIHEKMKNTNDQAEEFITALPDWALVH
LVDTESSTLKTIKENVLKLVKEAMENPIRAEALADVKQKAKDQLDYTNLTGNNQRVSV
LLIENSIVENDIYDKKATELAKAEAVERVQPALILQGQVIVQEGHIVDSNVMHQLKLL
GLLDKKTSYQPMYGLVIMLIAQALVLYFLELTQREKMVQRGKNLTLYAFMMIMGIGVM
KGLQLIQDVGADFLAMFYPAALVPLLLTIFLSRRFGIIANAFLAAFSIFIYISDTGTS
FSIIITLFYLLSGAMGTMLSRNRVRRKMYISFIWLTIFNILFLFAFIFYLNIQIASQE
GIMIIVYGSLSGLLSYLMALLLNPYIEVLFEDNAVMTMNELANPNQELLKELLTRAPG
TYHHSLMVANLSANAVGAIGGNSMFARVASYYHDVGKIRHPFFFVENLPVGMDNPHNM
LTPEESKEIIFNHVTEGVKMLEEAKMPQSIIDICAQHHGTTLMKFFYVKAKEHDDTVK
ESDFRYPGPKPQTKEAAVINIADSAEAAVRAMSNPTKEKIADFIHNLINGRIIDGQFD
ECEITLKELKIIEKSICEGLNGTFHSRIEYPSLKKDKATEK"

CDS complement(1292231..1293217)

/locus_tag="JFBMFIFI_01218"

/inference="ab initio prediction:Prodigal:002006"

/inference="similar to AA sequence:UniProtKB:P9WIA3"

/codon_start=1

/transl_table=11

/product="PhoH-like protein"

/db_xref="COG:COG1702"

/translation="MTKSDEVTKELQINNGDIAVALFGNHDKNLTLLEESLNVTINSR
GEKIDVSGQEKNVDTTVSILRQLQELLKKGISIGAPDIIGALKMADRGTLDYFSSMYD
EEIGKDREGKPIRVKNFGQRQYVAAVKKNDITFGIGPAGTGKTYLAVVMAVDALKKGE
VKKLILTRPAVEAGEKLGFLPGDLKEKVDPYLRPIYDALNSVFGSEHTSRLMDRGVIE
IAPLAYMRGRTLEDAFVILDEAQNATAAQMKMFLTRLGFGSKMIVNGDKTQIDLPRGA
SSGLVHAERTLQGIKGIGFVTFDSNDVVRHPVVASIIEAYNQETKQQFNEPE"

CDS complement(1293418..1293690)

/locus_tag="JFBMFIFI_01219"

/inference="ab initio prediction:Prodigal:002006"

/codon_start=1

/transl_table=11

/product="hypothetical protein"

/translation="MEKFIRNTTKKSFEITRNLVDTTENLTDKALDPVIMRKGFKVNT

LVNGLLLTVPVGQLVLNQKLNGWGLGIGVLSTTSLLINLYLKRNQK"

CDS complement(1293808..1294254)

/locus_tag="JFBMFIFI_01220"

/inference="ab initio prediction:Prodigal:002006"

/codon_start=1

/transl_table=11

/product="hypothetical protein"

/translation="MKEELINHVLSTLSEIQKNTTDFNTIFTSSSASDLSESQMIVLV

TLSIKSDVKITDISKMFAMTPGAATAMCDKLEEKGYLFRIRSKKDRRVVLIENTKKAT

EIVGKLFADFDLNQLEEMNTTLIKINQLFIELSGIIKPQESKKEEK"

CDS complement(1294376..1294819)

/gene="yqeY"

/locus_tag="JFBMFIFI_01221"

/inference="ab initio prediction:Prodigal:002006"

/inference="similar to AA sequence:UniProtKB:P54464"

/codon_start=1

/transl_table=11

/product="putative protein YqeY"

/db_xref="COG:COG1610"

/translation="MSLLTTLNEDMKTAMRAKDKETLSVVRMLKASLQNEQIKLGEEL

NADQELTILAREMKQRRDSVAEFKKADRQDLVDKTQAEIEIVEKYMPKQLSEDEIKTI

VSAAIAKVGASSMKDFGQVMGAVMPETKGKADGNEVNRIVKELLN"

CDS complement(1294853..1295026)

/gene="rpsU"

/locus_tag="JFBMFIFI_01222"

/inference="ab initio prediction:Prodigal:002006"

/inference="similar to AA sequence:UniProtKB:P21478"

/codon_start=1

/transl_table=11

/product="30S ribosomal protein S21"

/db_xref="COG:COG0828"

/translation="MSKTVVRKNESLDDALRRFKRTVSKTGTLQEARKREFYEKPSVK

RKKKSEAARKRKF"

CDS complement(1295360..1295779)

/gene="zur"

/locus_tag="JFBMFIFI_01223"

/inference="ab initio prediction:Prodigal:002006"

/inference="similar to AA sequence:UniProtKB:P54479"

/codon_start=1

/transl_table=11

/product="Zinc-specific metallo-regulatory protein"

/db_xref="COG:COG0735"

/translation="MTAVEQAIQKLKENGYKYTDKRENMLLLFANENRYLTAKEVQIC

LKDKYPNLSYDTIYRNIYTFVEMNILEETELNGEKIFRFGCMHTGHHHHFICTICGNT

REINMCPMNFFEDQLTGCEIESHRFEIFGKCEKCVAI"

CDS 1296012..1296635

/gene="ccpN"

/locus_tag="JFBMFIFI_01224"

/inference="ab initio prediction:Prodigal:002006"

/inference="similar to AA sequence:UniProtKB:O34994"

/codon_start=1

/transl_table=11

/product="Transcriptional repressor CcpN"

/db_xref="COG:COG0517"

/translation="MELTTRQKKIIQIVKTDEPISADNIAKQLSLSKPTLRSDLAILT

MTGILDARPKVGYFFSGQNFDPLLFDDLYEKKITEIMVPPINVKKETTVSDAVTMLFM

HDVGTLYVVENDELLGVISRKDLLRSTISSTDTHTIPVAMIMTRMPNVITIQENERIL

DAGYKIIQHEIDSIPVVDTQNPLKVIGKISKTVVLKHFINEALHLNR"

CDS 1296678..1297478

/gene="yqfL_1"

/locus_tag="JFBMFIFI_01225"

/EC_number="2.7.11.32"

/inference="ab initio prediction:Prodigal:002006"

/inference="similar to AA sequence:UniProtKB:P54470"

/codon_start=1

/transl_table=11

/product="Putative pyruvate, phosphate dikinase regulatory

protein"

/db_xref="COG:COG1806"

/translation="MNTNPVIYVLSDSIGETAQKVVSAVLVQFPTLKITDIRRHPFIE

DKMMLNEILNEALKENGVIVHTLVTAEFVDLVTNFCQDTGLSFVDVMSPLTKVLHAKT

GIAPKEEPGALHKLDEHYFRRVSAIEFAVKFDDGKDPRSFLLADYVILGISRTSKTPL

SMYLANKNYKVANLPLIPEASLPKELFEIDPAKIIGLTSSPEALMKIRTSRLKSLGLN

EDSSYSSKERVIEELAYSAKLYSQLGIKVIDVEHRAIEETAALICELP"

CDS 1297675..1298505

/gene="yqfL_2"

/locus_tag="JFBMFIFI_01226"

/EC_number="2.7.11.32"

/inference="ab initio prediction:Prodigal:002006"

/inference="similar to AA sequence:UniProtKB:P54470"

/codon_start=1

/transl_table=11

/product="Putative pyruvate, phosphate dikinase regulatory

protein"

/db_xref="COG:COG1806"

/translation="MENQTDQFVYIISDSVGETANKLSQAAMSQFPTTDFNVSRYPFV

RGEGTLLSILEQAKNENAMIVHTLITDGLSKIACDFCQKHNLFCFDLLNPMVDEIVKR

TGSTPSKEPGALHQLNDNYFHRIGAIEFAVKYDDGKDPKGFLEADIVILGVSRTSKTP

LSMFLANKNLKVANLPLIPESHIPDQLWKVNPKKIVGLTNDPELLNSIRRERMIAYGL

NPDTTYSDRDRINEELEFAQNLYEKLGCLVINVATKSIEETAAIILDTLQLDDMSYDN

"

CDS complement(1298617..1299804)

/gene="tuf_1"

/locus_tag="JFBMFIFI_01227"

/inference="ab initio prediction:Prodigal:002006"

/inference="similar to AA sequence:UniProtKB:P99152"

/codon_start=1

/transl_table=11

/product="Elongation factor Tu"

/translation="MAKATFDRSKSHVNIGTIGHVDHGKTTLTAAIATVLSKHGGGSA
QNYADIDNAPEEKERGITINTSHIEYETETRHYAHVDCPGHADYVKNMITGAAQMDGA
ILVVSAADGPMPQTREHILLSRQVGVPYIVVFLNKVDMVDDEELLELVEMEVRDLLSE
YDFPGDDTPVIAGSALKALEGEASYEEKVLELMAAVDEYIPTPQRDTEKPFMMPVEDV
FSITGRGTVATGRVERGEVRVGEEVEIVGINEAPTKTIVTGVEMFRKLLDYAQAGDNI
GALLRGVAREDIERGQVLAKPGTITPHTKFKAEIYVLSKEEGGRHTPFFTNYRPQFYF
RTTDVTGVCELPEGVEMVMPGDNVTIEVTLINPIAIEAGTNFSIREGGRTVGAGVVAE
IQA"

CDS complement(1299866..1301950)

/gene="fus"

/locus_tag="JFBMFIFI_01228"

/inference="ab initio prediction:Prodigal:002006"

/inference="similar to AA sequence:UniProtKB:Q5XDW4"

/codon_start=1

/transl_table=11

/product="Elongation factor G"

/translation="MSKREFSLEKTRNIGIMAHIDAGKTTTTERVLYYTGKIHKIGET
HDGGAQMDWMDQEQERGITITSAATTAEWNGYRVNIIDTPGHVDFTVEVERSLRVLDG
AVALLDAQSGVEPQTETVWRQATTYGVPRIVFVNKMDKTGADFLYSVGTIHDRLQANA
HPIQLPIGAEDNFTGIIDLIKMKAEIYEDDLGNNIREEDIPADYQELANEWRDKLIEA
VAETDEDLMMKYLEGEELTNDEIVKAIREATVKAEFYPVLCGSAFKNKGVQLMLDAVI
DYLPSPLDVPAITGTVPGTEEVVARPADDDAPFAALAFKVMTDPFVGRLTFFRVYSGT
LNGGSYVKNATKGKRERIGRILQMHANSRNEIPVVYSGDIAAAVGLKDTTTGDTLCDE
KQEVILESMIFPEPVIQVAIEPKSKADQDKMGIALQKLAEEDPTFRAETDQETGETII
AGMGELHLDIIVDRMRREFNVEASVGAPQVSYRETFRGSTEAEGKFVRQSGGKGQYGH
VWIEFTPNEEGKGFEFENAIVGGVVPREYIPAVKAGLEGALENGVLAGYPLVDIKAKL
YDGSYHDVDSNEMAFKVAASMALKNAAKKANPVILEPIMAVEITVPEDYLGDVMGHVT
ARRGRIEGTETRGNAQIVQSMIPLSEMFGYATTLRSASQGRGTFSMTFDHYEDVPKSI
SEEIIKKNGGEV"

CDS complement(1301992..1302462)

/gene="rpsG_1"

/locus_tag="JFBMFIFI_01229"

/inference="ab initio prediction:Prodigal:002006"

/inference="similar to AA sequence:UniProtKB:P21469"

/codon_start=1

/transl_table=11

/product="30S ribosomal protein S7"

/db_xref="COG:COG0049"

/translation="MPRKGPITKRDVLPDPIYNSKLVTRLVNRIMVDGKRGKAATILY

SSFEMIEEETGNDPMEVFEQAMKNIMPVLEVKARRVGGSNYQVPVEVRADRRTALGLR

WLVSYSRLRGEDTMEQRLAKEIMDAANNTGSAVKKREDTHKMAEANKAFAHYRW"

CDS complement(1302519..1302932)

/gene="rpsL_1"

/locus_tag="JFBMFIFI_01230"

/inference="ab initio prediction:Prodigal:002006"

/inference="similar to AA sequence:UniProtKB:P21472"

/codon_start=1

/transl_table=11

/product="30S ribosomal protein S12"

/db_xref="COG:COG0048"

/translation="MPTINQLVRKRRKSTKSMSDSPALNKGYNSFKKRQTDTKSPQKR

GVCTRVGTMTPKKPNSALRKYARVRLSNLLEVTAYIPGIGHNLQEHSVVLIRGGRVKD

LPGVRYHIVRGALDTAGVTDRKQSRSKYGAKRPKA"

CDS complement(1303464..1304297)

/locus_tag="JFBMFIFI_01231"

/inference="ab initio prediction:Prodigal:002006"

/codon_start=1

/transl_table=11

/product="hypothetical protein"

/translation="MQIKNKQLSLYLSVIVVSLMALFFFPQTINAAPTFPAATSNYYV

DDAKILSSTTEQFIQSVNNNYQQTTEKPQIAVAAVKNMQGLTREDYANQLFEKWQIGN

KKLDNGVLILFSLEERQIWIEVGYGLEGALTDSGTGAILDRNIDLLKADKFDEALKSI

FTEVAVKINGEYDFKNEEIFSGYEVDAEKYSDSDDSGILSVFILIGIIVVVSIFFGGG

GSNGPGGRRRRGNISPLLFGLSSGIFRGGSGSGGSGGFGGGGSFGGGGSSGGGGAGRS

F"

CDS complement(1304338..1304904)

/gene="lemA_2"

/locus_tag="JFBMFIFI_01232"

/inference="ab initio prediction:Prodigal:002006"

/inference="similar to AA sequence:UniProtKB:A8AVK0"

/codon_start=1

/transl_table=11

/product="Protein LemA"

/db_xref="COG:COG1704"

/translation="MKNWMKAVIGVVVVLALLAIPFITSYNSLVNAESAVDSQWANVE

SKLQRRYDLIPNLVNSVKGSMTQEKEVFGAIADARSKLSGAKTVDDKVKANNELESAV

SRLLVVVENYPDLKSNQNVQGLMDELAGTENRISVERDRYNKAVQTYNNKVKRFPGSI

IANMTGFEPRAFFTAVDGADKAPSVNFD"

CDS complement(1304998..1305408)

/gene="psiE"

/locus_tag="JFBMFIFI_01233"

/inference="ab initio prediction:Prodigal:002006"

/inference="protein motif:HAMAP:MF_01048"

/codon_start=1

/transl_table=11

/product="Protein PsiE"

/translation="MEFNENKYTLKIAKFLRLLLNVALSILGLVLAYCLAKEIVYIFL

MITQNGFHDYYELIQEILVFFLYFEFISLVVKYFESNYHFPLDYFIYIGITAVIRIII

TNHGQPIDTLILSAGIVVLLVALVIVKRNNLDTK"

CDS 1305730..1306419

/gene="srrA_4"

/locus_tag="JFBMFIFI_01234"

/inference="ab initio prediction:Prodigal:002006"

/inference="similar to AA sequence:UniProtKB:Q9L524"

/codon_start=1

/transl_table=11

/product="Transcriptional regulatory protein SrrA"

/db_xref="COG:COG0745"

/translation="MKKILIVEDDNDINKLLSEFLSAEGYETQSAFSGTEVQFYTKQT

QFDLILLDLMLPGINGEEIIQEIRQTNQTPIIVISAKSDKQSKIDVLKSGADDFVSKP
FDLDILSARIETNLRRSNTLPIDTKNMELTHKGILLQPDSRKVYLNEQLLKLTNFEFD
ILALLMSNPKKVFTKSNIFESVWQEESLVGDDGTVSVHISHLRTKFAKVSPDIVYIDT
VWGIGFKMNDD"

CDS 1306752..1307684
/gene="bcrA"
/locus_tag="JFBMFIFI_01235"
/inference="ab initio prediction:Prodigal:002006"
/inference="similar to AA sequence:UniProtKB:Q5WNX0"
/codon_start=1
/transl_table=11
/product="Bacitracin transport ATP-binding protein BcrA"
/translation="MLEPVLVTYNLSKYFKKEAVVKQVSLRINKGDIYGFVGKNGAGK
TTTLEMLSALIEPDSGEIELFGVHEKVKKRREMKRMAVLVGEPAYFPKLTAKENLTYF
CSQHGIVETELIDKTLLKMGLDPCDKKKARSFSLGMKQRLGLAYVFIHPADLIILDEP
TNGLDASSITTLRNLILELNQKYQVTFLIASHLLSELETIATKYGFIHNGALIEELDQ
QTLAEKLSPKIKIIPSDIEKTIVLLETMVDSDDFEVLPHNVIVVRSDKISVEELSLFF
FNEQLYIKEIYEIKPTLEQYFTHLLKEQQRKDGL"

CDS 1307684..1308523
/locus_tag="JFBMFIFI_01236"
/inference="ab initio prediction:Prodigal:002006"
/codon_start=1
/transl_table=11
/product="hypothetical protein"
/translation="MWLLIKADFYRYTRGKKWIGLFATLLVISITSIATYYYIPLLLD
SIAVSNTEEPLDIETIQEGETIRREVQEANAPTFLKVTGGNTFWVVLLVIFVSINFLV
EDFNSRIHKNNLSFGISRKQIYLGKYFFGLLAITIITSFYHFSSLIINCLLFNITDSF
FDYFSRTVQVVLMQLPLFFAIYAVCFGFVYLLKSKLKIYGIIFGGSLILIPIQMKLAK
FFPDAVPMIDRFKILSSIGKIGSWENHSLDRYALNLIIFSLYSLVFLTLGWIGFKNYN
LEK"

CDS 1308563..1309471
/gene="rcsC_2"
/locus_tag="JFBMFIFI_01237"

/EC_number="2.7.13.3"

/inference="ab initio prediction:Prodigal:002006"

/inference="protein motif:HAMAP:MF_00979"

/codon_start=1

/transl_table=11

/product="Sensor histidine kinase RcsC"

/translation="MTYLFIFVLGILVGSVSFRFFINQQLKTMTTNLKTITKRSTNER

LRISSGQKEIKKLVKQINLQLEKEQQTRLLIDEEFLLLRQAMTNMSHDLRTPLTSILG

YLKLAEKDTITPAENKRYLAVVKSKTINLNYLIEQFFELSRIESKILVVVKEPVDLTS

IIQEILASYYEDFIQKQTIPTITIPEHPVWGMGDKEALRRVFNNLLQNMLKHNASSVC

LSLEEQPEKQTVLLTFKNHAPNLSPEDVKQIFNRFYTADRMRSGQNTGLGLTIVKELV

IQMGGEIDARLQNQQLAISIKLAMKE"

CDS complement(1309532..1311388)

/locus_tag="JFBMFIFI_01238"

/inference="ab initio prediction:Prodigal:002006"

/codon_start=1

/transl_table=11

/product="hypothetical protein"

/translation="MKKKLVGLVVLLLALGSFDMLALADGSYEITSYDVNVDLQKDGS

GEFKEEITYDFDGSFNGVFYNLDRTGIKDVTNIEVTVKGKDGQESAPFTQNDSEKVGT

YQLTEENNFLKFKVFQKANDEQKTVVYRYKIPEVVTNYTDTAEFNGRLIGAGWEDPID

NIIIHINLPQAAEKGTLRAWAHGDLSGKIAVIKDKKVQLEVPNNPSNTFVEARVIFPT

AVTPDNPNVKNEKRLAKIMAEEKEMAEAANRKRQTIMWIFIIIAVISVVVAVVGSIWG

RKIAQKNKFKDLFVPEHLYELPEDMTPAVMYSATKNKAPRTTEITATLMDLVRKKQVT

IDEIEIGKSKKGKTTFLLKKVSNPPEITLLSHEKYLMKWLFDQVGNGEAVTLKMIEDY

GKKDQKKAKKFTDSYKKWQDKVKLDADEKGYISKTNTKALTFISVYSIFLFILLIVTL

ILMGTLNAFYPLIMILLFVVSVINAVQWIGFFPVRTLEGETAVRQWQAFSDMLRDVSN

LKMADVGSLIIWDHFLVYAISLGVSTEVIKALALQFPAEALDSMTIGHYYLYGAMFHS

NFGSSVNSGFETSFNNAMGNAVSNASSASGTGGGFSGGSSGGFGGGSGGGAF"

CDS complement(1311528..1312433)

/gene="nfo"

/locus_tag="JFBMFIFI_01239"

/EC_number="3.1.21.2"

/inference="ab initio prediction:Prodigal:002006"

/inference="similar to AA sequence:UniProtKB:Q5KX27"

/codon_start=1

/transl_table=11

/product="putative endonuclease 4"

/db_xref="COG:COG0648"

/translation="MVLIGSHVSMSGKKMLLGAAEEAASYNADTFMIYTGAPQNTRRK

AIEEMNIPAGEAFMKEHKLSNIVVHAPYIINLGNTIKPENFGFATQFLREEIVRSEAL

GAKQITMHPGAHVGAGADAAIAQIVKGLNEVLTKDQTAQIALETMAGKGTEIGRTFEE

LAKIIEGVTLNEKLSVTLDTCHINDAGYNVREDFDGVLEEFDRIIGLDRLKVIHVNDS

KNPQGSHKDRHANIGFGTIGFEALNKVVHHPKLSDLAKILETPYVGEDKKDKKAPYGY

EIKMFREQKFNPNLIEDILNQVGFE"

CDS 1312572..1313318

/gene="nfrA1"

/locus_tag="JFBMFIFI_01240"

/EC_number="1.5.1.38"

/inference="ab initio prediction:Prodigal:002006"

/inference="similar to AA sequence:UniProtKB:P39605"

/codon_start=1

/transl_table=11

/product="FMN reductase (NADPH)"

/db_xref="COG:COG0778"

/translation="MNPVIETIIKHKSIRKFKETPLSKEQVELLVTAAQHTSTSSFMQ

SYSIIGVTDPIKKQQLAEVGNQPYIAEAGHLFVFVADLNRNKQIAEKNEKDTTVLEST

DRFLVGFTDASLAAQTTLLAAESLGLGGVFLGSLLNNAEKVIEILELPTQTFPVVGLA

VGYPDQEPQLKPRLPMAHIYHENTYKVQSEPQEALADYDSVVTEYYDLRNANTRIDSF

TNQATKSMDLKHPGRMKLLEMIHAQNLLKY"

CDS complement(1313364..1314131)

/locus_tag="JFBMFIFI_01241"

/inference="ab initio prediction:Prodigal:002006"

/codon_start=1

/transl_table=11

/product="hypothetical protein"

/translation="MDREFLNKKIDSEEEKKSSTRQFSKEEFQAELVKEELSSKEPSF

ETQGIKLSSNIPTSQEKLSSPSAEEKESPLENTLSTREIRRQYWQEKQVKEDLENRPF

HRYPDYFYGGLWLRLFAYLVDLIVIQSIGRIAVNNMFTAFNLPKDTASFSYYTLAHLA

VYLLYFILMTKFTNGQTVGKMIFGLRVVCFKEQNLSWSTVLVREGFCRYFLNLSVLRA

LYLVLLFTPQKQHVGDLLSDTSVVSENLLKASQLDPV"

CDS complement(1314133..1315170)

/gene="sppA"

/locus_tag="JFBMFIFI_01242"

/EC_number="3.4.21.-"

/inference="ab initio prediction:Prodigal:002006"

/inference="similar to AA sequence:UniProtKB:O34525"

/codon_start=1

/transl_table=11

/product="Putative signal peptide peptidase SppA"

/db_xref="COG:COG0616"

/translation="MNKKRWTAIGIAVGLFLLSFASTYMMGVVKKEKDQTDSLTGTYQ

SLFGEDQVSKTIIEDGSTDKQIAVLTVDGTIVSGGTSGLFGSAGYDHAAFMQQLQWIE

EDKAVKGIVLVVNSPGGGTFESAQIKDKLVEMKNKTKKPLYVSMQNMAASGGYYISAN

ADKIFATEETMTGSIGVIMSGLNYSGLYDKLGISDTTVKSGEYKDIMSQSRPVTDGDK

AILQTMIDDSYARFVNVIVEGREMDEARVRELADGRIYDGAQAKSVGLVDEIGYKEDA

INALKKDFNLKNAQVFEYQAASFPLSSLLFSKASSALAPEKNTVSELNRLIEKFGTVE

SPKMMYLYGGE"

CDS complement(1315379..1317163)

/gene="aspS"

/locus_tag="JFBMFIFI_01243"

/EC_number="6.1.1.12"

/inference="ab initio prediction:Prodigal:002006"

/inference="similar to AA sequence:UniProtKB:P67015"

/codon_start=1

/transl_table=11

/product="Aspartate--tRNA ligase"

/translation="MAKRTIYAGQVSEELVGQEITLKGWVQKRRNHGDLIFIDLRDRE

GLVQIVFNPTFSKEALEIAEKVRSEYVIEVTGKVVKREADAINPNLATGAVELEATKL

TVLNKAKTTPFYIEDGVSVSDEKRLEYRYLDLRRPEMAKSIIMRHGITKSIRDYLDHD
GFIDIETPYMTKSTPEGARDYLVPSRVHPGHFYALPQSPQLFKQLLMGAGFDRYYQIV
RCFRDEDLRGDRQPEFTQIDIETSFLEAEEIQDLTEGLLKKVMEDVKGVKIDLPLPRM
DYDEAISRYGSDKPDVRFGLELVQLSEAMKDSSFKVFSGAIANGGEVKGINVKGAATK
FTRKEIDELGTFASVYGAKGLAWLKVEETGLTGPIARFFTEDSETIIQAMDAEAGDLL
LFVADKKSVVADTLGALRLKLGKELDLIDESQFAFLWVVNWPLLEFDEENNRYTSAHH
PFTMPKESDIELLTTDPSKVYAEAYDIVLNGYELGGGSLRIHSRDIQEKMFTALGFTK
KAAEEQFGFLLEALDYGFPPHGGIALGLDRLAMLLAGKENIREVIAFPKNGKAVDPLT
AAPSIVSPAQLDELSIKTTRIEAEEETE"

CDS complement(1317194..1318519)
/gene="hisS"
/locus_tag="JFBMFIFI_01244"
/EC_number="6.1.1.21"
/inference="ab initio prediction:Prodigal:002006"
/inference="similar to AA sequence:UniProtKB:P30053"
/codon_start=1
/transl_table=11
/product="Histidine--tRNA ligase"
/translation="MAFQKPKGTVDILPGVSKKWQYVEEISRMVMGDYQFHEMRTPIF
ESYELFSRGVGETSDIVSKEMYDFMDKGDRRLALRPEGTAAIVRAYVENKLFGPEHSN
PYKVYYMGPMFRYERPQGGRQRQFHQLGVEVFGSNNPATDVETMALAMDLFHQFGLKE
FKLVINSLGDKTSRDAYRAALIAYLEPHFDELSHDSQVRLHKNPLRVLDSKDKKDNEI
VKNAPSILDFLNEESSKHFETVKTMLEALEIPFEIDHKMVRGLDYYNHTIFEIMSDAD
GFGSLTTLCAGGRYNGLVEEIGGPETPGFGFGMGLERLMIALDSENVEIPELNELDVY
VVGLGDETNIETLKLVQNIREFGFSAERDYLNRKAKAQFKTAAKLNAKVVLTVGESEL
ENQVVNFKVMKTGKQETVSMKEIYQNFDKVFNLQTTDMTAFNDFFNKED"

CDS 1319235..1320551
/locus_tag="JFBMFIFI_01245"
/inference="ab initio prediction:Prodigal:002006"
/codon_start=1
/transl_table=11
/product="hypothetical protein"
/translation="MKSKNIPQQHKKYFLLFFIVLLSLTIFATVALANFSTIKVDAAV

VNVRTGPGLSYDVMSQVGAGDTVNVLEEKNEWYKVRLSNDRIGWVASWLVNNTEISAA
TNTVGTITGEKINVRSESNEQAEIIGSVVKGTELTVLFQENGWTQVQYNQQVAWVNSN
FIEVKAAAVQEAPQTVATKDNSNADVQTISIRTEGTRIRTSPSTDSKVVLTASQGQSF
SYIGTEGDWYHVKASDGTEGYVANWLVDLSATAEAAPTAQISSLSEATIVIDPGHGGS
DPGAEGSNYYESEITLSTAEILADRLRQSGANVILTRTTDTDVGLRDRAYISNQAKAD
VFISIHYDSTPNPNEGSGTTTYYYSDSNKALAKAVNNGLSKGPLPNNGYRFGDHQVTR
ENAQPALLLEMGYVNNDTDSALITTKSYQQTMSNHIVDALTNYFNK"

CDS complement(1320599..1321045)

/gene="dtd"

/locus_tag="JFBMFIFI_01246"

/EC_number="3.1.1.96"

/inference="ab initio prediction:Prodigal:002006"

/inference="similar to AA sequence:UniProtKB:P0A026"

/codon_start=1

/transl_table=11

/product="D-aminoacyl-tRNA deacylase"

/translation="MKVVVQRSLAASVKINEKIVGEIDKGFVLLVGVTETDSEVDVDY
LVGKISKMRVFEDDAGKMNLSIEQIGGKILSISQFTLYADTKKGNRPSFIKAAGAEQA
TALYDSLNNKLRKSGLIVETGEFGADMAVSLVNDGPVTIILDSQNK"

CDS complement(1321109..1323319)

/gene="relA"

/locus_tag="JFBMFIFI_01247"

/EC_number="2.7.6.5"

/inference="ab initio prediction:Prodigal:002006"

/inference="similar to AA sequence:UniProtKB:O54408"

/codon_start=1

/transl_table=11

/product="GTP pyrophosphokinase"

/db_xref="COG:COG0317"

/translation="MPKNKNYSAGEVIALTSTYMNSEHVAFVKKACEYATKAHEGQLR
KSGEEYIIHPIQVAAILAELKMDPATVATGFLHDVVEDTEMTLEDIAKEFSPEVAMLV
DGVTKLGKIKYKSHEEQQAENHRKMLLAMAQDLRVIMVKLADRLHNLRTLKHHRADKQ
RRIANETLEIYAPLAHRLGISRIKWELEDTSLRYLNPQQYYRIVHLMNSKREEREAYI

ADSIDKIQESVDELKLKAEIYGRPKHIYSIYRKMKDQKKQFNEIYDLLAIRVMVDSIK
DCYAVLGAIHTRWKPMPGRFKDYIAMPKANMYQSIHTTVIGPRGNPVEVQIRTFEMHE
VAEFGVAAHWAYKEGITKKIENDAVGTKLSWFRDIIELQDEANDASDFMESVKEDIFK
DKVYVFTPKGDVSELPSGAGPLDFAFNIHTEIGNKTIGAKVNGKIVPLNYKLKNGDIV
DVMTSPNSFGPSRDWINLVSTSKAKNKIKRFFKLQDRDVNIEKGKDMIEKQLIEMQFA
PKDFLTKQHIKELLERFNFTNEDDLYAAVGYGELTALIIANRLTEKERRERDKEKENQ
SLDTIEKKNDKKPEKMKVRHEGGIVIQGVDNLLVRISRCCSPVPGDKIVGYITKGRGI
SIHREDCPNIKGADDTENRLIEVEWEDAASKGQDYDAELQVEGYNRTGLLNEVLQVVN
SMTRNLTSVNGKVDNNKMATITLTVGIQNLNQLEKIVDKIKTIPDVYSVRRITS"

CDS complement(1323636..1324127)

/gene="sbmC_1"

/locus_tag="JFBMFIFI_01248"

/inference="ab initio prediction:Prodigal:002006"

/inference="protein motif:HAMAP:MF_01896"

/codon_start=1

/transl_table=11

/product="DNA gyrase inhibitor"

/translation="MIKPISITTKILEHQKIIYVRFRGSYTEFRKNSRKLFKELFDFA
TTNNLIIPDITKILTIYDDNPYITQEKDLRTSVAMTISNSINVIEKGNICVSSISGKF
GVGHFELSAKEYEEAWQYMYQEWLFKEEGKARDAVPFELYVTEPPKNLKDKSYTDIYI
PII"

CDS complement(1324160..1324546)

/gene="rhaS_4"

/locus_tag="JFBMFIFI_01249"

/inference="ab initio prediction:Prodigal:002006"

/inference="protein motif:HAMAP:MF_01534"

/codon_start=1

/transl_table=11

/product="HTH-type transcriptional activator RhaS"

/translation="MSNKISKQEEVYIRLFNKSEDYIEQHLNEAISLSNLANYSNFSD
YHFHRIFKKYSNITLKKFITRFKLERAAIFMSVNRSLSLTIIASKYGYNDSSSFSRAF
KRHFGCSPSIYRREQEMTRNIKNNMT"

CDS complement(1324850..1325986)

/locus_tag="JFBMFIFI_01250"

/inference="ab initio prediction:Prodigal:002006"

/codon_start=1

/transl_table=11

/product="hypothetical protein"

/translation="MRFFSNKLTLIITVASLLSPIFFQTDTAYAITDAEIVDVHTQVS

QIEAKIKELDEAMTQSDVSIGRFNSQIKALETKIKEQEIKIKEGLVAVKKFESQKDNL

KPLLAEKGATWSDQKRLTVNDSTPPEKEEIHFSEEAKFPMIQEQIKVTTQQKKLLAAE

LVQKKLELKRANLKKEYVVLEAAATKSDSGIATEQIEAVKNSYQQQNVLWDKRATQIS

QDIAVFSQSESTELPSIQFGFEAPVKDPISSTFGLRFGYDTNGFHKGLDFASAIGTEI

HPVMAGEVVVAQEDGPMYEGYGNVVVIRHDNGTWTLYAHQSELLVKVGDRVETKTVIG

KVGQTGQSDGPHLHLEVRTSPEGGVGHVVDPAPLIDNLNLGQEG"

CDS complement(1326176..1326847)

/gene="deoC_1"

/locus_tag="JFBMFIFI_01251"

/EC_number="4.1.2.4"

/inference="ab initio prediction:Prodigal:002006"

/inference="similar to AA sequence:UniProtKB:P39121"

/codon_start=1

/transl_table=11

/product="Deoxyribose-phosphate aldolase"

/db_xref="COG:COG0274"

/translation="MSENLNKYIDHTLLKPEATEEQITILCQEAAKYNFMSVCINPTW

VSKAAALLAESDVKVCTVIGFPLGANTPEVKAYEANDAIKNGANEVDMVINIGALKGK

DDELVERDMRGVVEAAKNRAVSKVIIETALLTEEEKIRACEIAKKVGADFVKTSTGFS

TGGATVEDVKLMRATVGPEMGVKASGGVRSLEDASAFIEAGATRLGASSGVAIMEGQT

AKNDY"

CDS complement(1326929..1327693)

/gene="rsmE"

/locus_tag="JFBMFIFI_01252"

/EC_number="2.1.1.193"

/inference="ab initio prediction:Prodigal:002006"

/inference="similar to AA sequence:UniProtKB:P54461"

/codon_start=1
/transl_table=11
/product="Ribosomal RNA small subunit methyltransferase E"
/db_xref="COG:COG1385"
/translation="MQRYFLKEMIDDYQNQLISIEGDQYHHMVRVMRMKVEDKVFLVL
PNSQAFIAEIQTIENDYLDLKWIADEHQNKELPVEVTIVSGLPKGDKLELIVQKGTEL
GASHFIPFAAKFSITKWDEKKGLKKIQRLEKIALEAAEQAQRHVVPTIKAVHSLSQLI
ETSQGYDRLLVAYEESAKVGEDQNFAKVLKNLETAEKVLVVFGPEGGLSEKEISSLTD
AGFICCALGPRILRTETAPLYVLSALSYQLELNQSK"
CDS complement(1327694..1328644)
/gene="prmA"
/locus_tag="JFBMFIFI_01253"
/EC_number="2.1.1.-"
/inference="ab initio prediction:Prodigal:002006"
/inference="similar to AA sequence:UniProtKB:P0A0P5"
/codon_start=1
/transl_table=11
/product="Ribosomal protein L11 methyltransferase"
/db_xref="COG:COG2264"
/translation="MKWTEVSVLTASEAVEAISNILVEAGTSGVSIEDMFDFDHTPDD
GFGEIWALDKEDFPENGVIIKAYFPETVFLPEILPGIKQRILELETFGLDIGANQVET
TEVSDTSWATAWKKYYHPLQVTRFMTVVPSWEAYTPKFSDERIIRLDPGLAFGTGTHP
TTRLSMQALETSITGGETLLDVGTGSGVLSIASKALGAGDVFAYDLDEVAVRAAKENM
ALNDYAKDVPVKANDLLKGITIEADIVMANILAEIILLLIEDAWRVLKPNGLFITSGI
IDSKRDEVLEHLVAQGFEIEQILQMKDWFAIIARKPAEEE"
CDS complement(1328796..1329287)
/locus_tag="JFBMFIFI_01254"
/inference="ab initio prediction:Prodigal:002006"
/codon_start=1
/transl_table=11
/product="hypothetical protein"
/translation="MAKEELLDYINETFEAANFEFDWLVQWNKRQHGIEVFFTLFVET
DSSTVIEDVDGTVAENDIIEFEDGIVFYDPLKSKLNLDDYLVGIPFDSKKGIEKGVIE

AAAKYLRIVVTEGQADLLDFATDPTIETFEMHWNDNDFAGTIATLKETGRYEDTLIAY

PKY"

CDS complement(1329304..1329918)

/locus_tag="JFBMFIFI_01255"

/EC_number="3.2.2.-"

/inference="ab initio prediction:Prodigal:002006"

/inference="protein motif:HAMAP:MF_00527"

/codon_start=1

/transl_table=11

/product="Putative 3-methyladenine DNA glycosylase"

/translation="MNPWFDSKKTTEEIAQELLGMLVVKETDAGIVSGWIVETEAYLG

EIDQAAHSYKLKKTPRLNSMYQEAGTIYIYSMHTHHMLNLVVQAKGIPEAVLIRGIEP

FEGLAIMEERRAQTGFAVTDGPGKLTKAMGIDKSDDGSSMLVPPLTISQKERRYPQEV

ELSKRIGIPNKGTWTEAFLRYSVKGNPYVSKLKGTIEPDYGWKK"

CDS 1330212..1330526

/locus_tag="JFBMFIFI_01256"

/inference="ab initio prediction:Prodigal:002006"

/codon_start=1

/transl_table=11

/product="hypothetical protein"

/translation="MKNRENSYFDGGLFELIGWNILGTLITIFTLGICAPWAITMVYG

WKINHSVIEGRRLQFNGSAVGLFGHWIKWLLLTIITFGIYGFWVSIKLENWKVSNTTF

RN"

CDS complement(1330614..1331780)

/gene="mtlD"

/locus_tag="JFBMFIFI_01257"

/EC_number="1.1.1.17"

/inference="ab initio prediction:Prodigal:002006"

/inference="similar to AA sequence:UniProtKB:Q83PQ0"

/codon_start=1

/transl_table=11

/product="Mannitol-1-phosphate 5-dehydrogenase"

/db_xref="COG:COG0246"

/translation="MKAVHFGGGNIGRGFIGEVLSQNGFDIIFVDVNTEIINALNTFG
SYKIELAAAGKEQITVKNVSGINNQLNPEEVVAALIQADIITTAIGPNILPLIAPLIA
AGIKRRFEANITTPIDVIACENMIGGSDYLKEEVEKHLSENEKKYLTTYIGFPNAAVD
RIVPIQHHEDKLFVSVEPFKEWVVDATTLKNEAIQLKGVQYVENLLPFIERKLFTVNT
GHATTAYAGKYYGFETIEEAIKDPTVKNQVQAVLKETGDLLITKWKFNPTEHQAYINK
ILSRFENPYISDEISRVARTPLRKLGYDERFIRPIRETNDSHLKNEALIKTVAMILTY
RDEKDPESIELARLIENASISEVIATVTNLTNSDLIAIIKHNYENLAEEKSIVR"

CDS complement(1331781..1332221)

/gene="mtlF"

/locus_tag="JFBMFIFI_01258"

/inference="ab initio prediction:Prodigal:002006"

/inference="similar to AA sequence:UniProtKB:C0H3V2"

/codon_start=1

/transl_table=11

/product="Mannitol-specific phosphotransferase enzyme IIA
component"

/db_xref="COG:COG2213"

/translation="MDILTIENIKLNQHFTTKESAIRAAGEVLVLAGYVEADYVDAMV
QRENDVTTYMGNFIAIPHGTDEAKKYIKKSGISIIQVPNGVDFGTPEEEKMVTVLFGI
AGVENEHLDILSQIAIFCSDIDNVVELASAQTAEEIVALLQEVD"

CDS complement(1332237..1334315)

/gene="mtlR_1"

/locus_tag="JFBMFIFI_01259"

/inference="ab initio prediction:Prodigal:002006"

/inference="similar to AA sequence:UniProtKB:P96574"

/codon_start=1

/transl_table=11

/product="Transcriptional regulator MtlR"

/translation="MYFSAREKRIVEILLHNPLGVQIDYLCEEFKVSKRTIYRELSSL
EGTLAQYQMKLIKEKGAGYKLIGNEVLLAELKMKLSKIKNEFDPKQRQSAITCLLLTT
DEEMKMEAMALDFGVSIGTIQADLASIEEILSGFTIQVQRKKSIGISVKAPESERRQI
LSGVINSELNEYDFFEFLKRLEKEQLLDTLDNYFMKYIDNESLYIANHAIQSVQKRFF
EKVTDSQLQQLIILLAISIHRLKLGKEITDIQYSIKETDIHQPIEIASELFADVEKLI

EVNVTDHEIYFLALQIRGMNGHIQRDIFLENYDVELSFKIKELIHLVSTRIEWDFSHD

TTLFYDLMAHLSAALKRSVAPIPQMNNPLLEKIKIKYTELYQILEESLASVFPETVFL

LNEVAYIVIHFASTYEKKSKTKKLSVLVICSSGIGTAKILENRLKKNIPEINEVLISR

IAQLDSLELGSFDLILSTIFLSGFRAEYKVITPLLLEEEIRDIQLYIKSNFTSTTFLT

KDTRQQINTTNDQIESFQEVYKGMKVANQILENFDIKAVRYSQTLESTINEICQSLEG

LILTDAKVVAAKLMGRLKQAPIGIPETNLALFHCISSEIKFPYFSIYEIKEPLSIIGM

DRTSIKMKRILLLLGPEPLSDYDQKVLGEISASIIESNLNMELYNSGARDEIYRLLSK

LFLIELTKYK"

CDS complement(1334385..1335815)

/gene="mtlA_1"

/locus_tag="JFBMFIFI_01260"

/inference="ab initio prediction:Prodigal:002006"

/inference="similar to AA sequence:UniProtKB:P50852"

/codon_start=1

/transl_table=11

/product="PTS system mannitol-specific EIICB component"

/translation="MAQKHGEKKGIKAIIQKMGSYLSGMVMPNIGAFIAWGIITALFI

ETGWWPNAELANMVGPMLTYLLPLLIGYTGGNMVYGQRGAVVGAIATMGVIVGSEIPM

FIGAMLLGPLGGYCAKKFDDVFQAKIKPGFEMLVNNFSSGIIGFILAIIAYFGVGPIV

TALSQAMANGVEVIVNAGLLPLANIFIEPAKVLFLNNAINHGILTPLGVEQAAETGKS

ILFLLEANPGPGLGILVAYMFFGKGSARASASGAAVIQFVGGIHEIYFPYILMKPLLF

FAAIAGGVAGTFTFSILGAGLVAAASPGSILAVLAMTPRDGYFPVIAGVAVGAIVSFL

VAMVILKADKTTDEDDFEGQVAATQALKAESKGQTIETISDLVETDVSVGTIKRIIFA

CDAGMGSSAMGASILRNKVKKAGLDMSVTNSAINNLVDEEGVLIITQEELAERAKVKT

PQAIHVSVENFLNSPRYDEIVDKLKK"

CDS 1336286..1336858

/locus_tag="JFBMFIFI_01261"

/inference="ab initio prediction:Prodigal:002006"

/codon_start=1

/transl_table=11

/product="hypothetical protein"

/translation="MAKSYLYPRKKKKFTKIKTTLLICILLILVLMSFYFYKTTIKNQ

TIVFQSPVVFVEKDQQKKTLSAPSRISTFKENSFEISTKQIVDNNETTNQNKVIVFDL

TITNNSKKDSTFSDNDFTLVQGGTHFTGNMSGLTDKETFNSEVIKPKTTLTRSVAFSV

PANIAGKNEFDLIISNDNWANQQTTIHITN"

CDS complement(1336923..1337939)

/gene="mccF_2"

/locus_tag="JFBMFIFI_01262"

/inference="ab initio prediction:Prodigal:002006"

/inference="similar to AA sequence:UniProtKB:Q47511"

/codon_start=1

/transl_table=11

/product="Microcin C7 self-immunity protein MccF"

/translation="MLNYPILKRNGTIGITAPSSGVKKELHPILKEAIERMEARGFAV

QCGETPWMHSKLKSAPAYQRAEELNRMLTDPNIDLIIPPWGGELLLEIIDLIDYSAVQ

PKWILGYSDTSMLLLAITLNTGIATAHGTNLIDLRGERTDNTTGKWLEVLGTKVNQTV

QQFSSERYQKDWNFEEPTEMIFDLTHKTQWKLVSGTKKTLNGRLLGGCVDVIRSLIGT

PYGDVKKFQKEQLLDEPILWYLENCELTLPDLRRSLLQMKLAGWFDSISGILFGRSDA

NLPQDGYTVEELYQDLEKELGVPIVYDVDCGHVPPQITWINGARATVEIKDGKATVCQ

LFKT"

CDS 1338079..1338540

/locus_tag="JFBMFIFI_01263"

/inference="ab initio prediction:Prodigal:002006"

/codon_start=1

/transl_table=11

/product="hypothetical protein"

/translation="MTDLNYLWKIKIEELPFGAEGAVLEAMHDYPKGKTILLPPADWN

GDSEEEFFFDPIGFKHKLIALFHHYATQATTIMEANLTFQERANQIIKLDATLYTILY

YYLNQEHDASTLDSSDVERLILMSTDLYFKPTTSTKNDWLNQLNQAKFLKL"

CDS 1338577..1339002

/locus_tag="JFBMFIFI_01264"

/inference="ab initio prediction:Prodigal:002006"

/codon_start=1

/transl_table=11

/product="hypothetical protein"

/translation="MNGQFNLKTLEKSQQIIALFLLIISMFIFISILIYTYLPSLFFF

SSILNDYHYTAINKFTAGPISQLLIGSGFLLASLLLLTKHLIVPAKLASVLLFFVAIS

NKYRFHFTTWFIPKPLFFFLLLLVIFGLFYSHDKKRCGF"

CDS complement(1339055..1339261)

/locus_tag="JFBMFIFI_01265"

/inference="ab initio prediction:Prodigal:002006"

/codon_start=1

/transl_table=11

/product="hypothetical protein"

/translation="MFRKTNYINQCDVALNYIKLHAIENVKVTEMNLVIEKNDRISVI

TMPKNAEMLIKILDNVKERQEQMN"

CDS 1339404..1340525

/locus_tag="JFBMFIFI_01266"

/inference="ab initio prediction:Prodigal:002006"

/codon_start=1

/transl_table=11

/product="hypothetical protein"

/translation="MKSAEELAHYYVKKKTLTGLSIEKHISIYQRISHYTIPSDVILF

SFSGFQKRLNPYHHEGFFIYVLTADYLIALDTSEKLLDQKFPLKTMTKLSSQSGLYYS

HLTLTINHQLYQIDSPVIEEFIIYLTKLLPAYTIKQATLEKNGNSEAERKALETNQSA

KENWLITLCFGQSSSKKLSLAVELAKKTATYYEENMLDNKKSFQASYKKEHYLKFIEL

YEIVGLWKSTSIKIQHVPIERSIVGGLNYCYGDKLKSNNNGFCFGASAYTNNPFGCHR

LKISVGNHPWWSFGRYNDEKNWVIDKIALKKRMLEQAQFYLYCPAFDLEYSLLVLDAL

PKFLNPKKDQENWIFYNDSVLPKRPDEERIESNNHFIDH"

CDS complement(1340615..1341028)

/locus_tag="JFBMFIFI_01267"

/inference="ab initio prediction:Prodigal:002006"

/codon_start=1

/transl_table=11

/product="hypothetical protein"

/translation="MKKKSLVLMMLLFLAVGLIACGNADSKVPKKEALTFENFEKALK

KEKYDLGEREEKMAEFIGAKEGFALPVNNKNIEIYQFEKNDKKLKEITKNKEFEIEGI

AKIPVEVNGNFILIASEHPDKDKIIEIFNNFDGSK"

CDS 1341351..1342079

/locus_tag="JFBMFIFI_01268"

/inference="ab initio prediction:Prodigal:002006"

/codon_start=1

/transl_table=11

/product="hypothetical protein"

/translation="MENKLNSFHLKVIAIIAMLINHIGSGFRLFEYSESLFFFTEFIG

KLTFPIMAFLLVEGFHYTKNVRKYAFRLGFFWIISIYPFHMLHYPNSPFDLIELVNNI

FFTLLMGLILLVLYEKTNNQFVHIGLVIIFTLATFISDWNLFGILMIFGFYVIKNDKL

KKIIPPVYTTAFLFLIMLIGYLVEPSSVPWYEMFSTLGVLMTIPLLLNYNGQRGYSPS

WIKWGFYLFYPLHLIVLVLIRALI"

CDS complement(1342188..1344023)

/gene="lepA"

/locus_tag="JFBMFIFI_01269"

/EC_number="3.6.5.-"

/inference="ab initio prediction:Prodigal:002006"

/inference="similar to AA sequence:UniProtKB:P65272"

/codon_start=1

/transl_table=11

/product="Elongation factor 4"

/translation="MDKNEMKIRQSRIRNFSIIAHIDHGKSTLADRILEKTDTVANRD

MQAQLLDSMDLERERGITIKLNAVELTYTAKDGIDYTFHLIDTPGHVDFTYEVSRSLA

ACEGAILVVDAAQGIEAQTLANVYLALDNDLEIIPVINKIDLPAADPERVRGEIEDVI

GIDASDAVLASAKAGIGIEDILEQIVEKVPAPTGDLDAPLQALIFDSVYDSYRGVILN

VRIMSGVVKSGDKIQMMSNGATFEVADVGIFSPKPIKRDFLMVGDVGYITASIKTVQD

TRVGDTITLANNPATEALPGYRKMNPMVYCGLYPIDSSRYNELREALERLQLNDAALQ

FEAETSQALGFGFRCGFLGLLHMDVIQERLEREFDLDLITTAPSVIYHVNLTDGSEVV

VDNPAEMPEQGVIDSVEEPYVKASIMVPNDYVGAVMEIAQRKRGEFITMDYLDEFRVN

VIYEIPLSEIVYDFFDKLKSSTKGYASLDYDLIGYRPSKLVKMEIMLNSEKVDALSFI

VHRDFAYERGKIIVEKLRSLIPRQQFEVPIQAAIGNKILSRSTIKALRKNVLAKCYGG

DISRKRKLLEKQKEGKKRMKQIGSVEVPQDAFMAVLKMDEDQPKK"

CDS complement(1344226..1345386)

/gene="dnaJ"

/locus_tag="JFBMFIFI_01270"

/inference="ab initio prediction:Prodigal:002006"

/inference="similar to AA sequence:UniProtKB:O87778"

/codon_start=1

/transl_table=11

/product="Chaperone protein DnaJ"

/db_xref="COG:COG0484"

/translation="MAKRDYYEALGIAKGASDDEIKKAYRKLSKKYHPDINQEAGAED

KFKEIAEAYEVLSDAQKRAAYDQYGHASTDPNFGGGGGFGGFGGSSAGFGGGFEDIFE

SFFGGGGRSQNPNAPRQGDDLQYTLNLKFEEAVFGKETSITYNREDTCETCHGNGAKP

GTDPVTCSKCHGSGTLNVERNTPLGRVMTRQTCDVCHGTGQEIKEKCPTCHGAGHVKE

RHTVKVTVPAGVEDGQQMRLQGQGEAGMNGGPHGDLFVVFRVEESDLFDRDGSEIYYD

LPISFVQAALGDEIEVPTVHGKIKLKVPAGTQTGTNFRLRGKGAPKLRGNGTGDQHVK

VQVITPKNLSEKQAKAMRDFAEASGIEVSEQEGNIFDKVKDAFKSDGKKKRK"

CDS complement(1345534..1347375)

/gene="dnaK"

/locus_tag="JFBMFIFI_01271"

/inference="ab initio prediction:Prodigal:002006"

/inference="similar to AA sequence:UniProtKB:P17820"

/codon_start=1

/transl_table=11

/product="Chaperone protein DnaK"

/db_xref="COG:COG0443"

/translation="MSKIIGIDLGTTNSAVAVLEGGEAKIIANPEGNRTTPSVVSFKN

GEIQVGEVAKRQAVTNPNTISSVKRHIGEAGFSIEMEGKKYTAQEISAMILQYLKGFA

EEYLGEKVEKAVITVPAYFNDAQRQATKDAGKIAGLEVERIVNEPTAAALAYGLDKTD

KDEKVLVFDLGGGTFDVSILELGDGVFDVLATAGDNKLGGDDFDNKIIDYMVAEFKKE

NAIDLSKDKMAVQRLKDAAEKAKKDLSGVTSTQISLPFITAGEAGPLHLEMNLTRAKF

DELTHDLVDRTKVPVRQALKDAGLTASDIDEVILVGGSTRIPAVVEAVKKETNQEPNK

SVNPDEVVAMGAAIQGGVITGDVKDIVLLDVTPLSLGIETMGGVFTKLIDRNTTIPTS

KSQVFSTAADNQPAVDVHVLQGERPMAADNKTLGRFQLTDIPAAPRGVPQIEVSFDID

KNGIVNVRAKDLGTQKEQTITIKSSSGLSDEEIERMVKDAEANAEADKTRKEEVDLRN

DVDQLLFSVDKTLAELEGKVDADEVKKAEEAREELKAAVEANDLEAMKTKRDALNEIV

QALTVKLYEQAAQQQAEENPEAAQAGADDVVDAEFEEFEEVEKDEDK"

CDS complement(1347530..1348081)

/gene="grpE"

/locus_tag="JFBMFIFI_01272"

/inference="ab initio prediction:Prodigal:002006"

/inference="similar to AA sequence:UniProtKB:Q5KWZ6"

/codon_start=1

/transl_table=11

/product="Protein GrpE"

/db_xref="COG:COG0576"

/translation="MAKEEKQDLNEELKNKTEEVEESTDTEVEELIEVDELTETKVAL

DEMEDRYLRLQAELANIRKRNQKEREDAAKYRAQNLATELLPVIDNLERAMASQVSDE

ESEGLKKGLEMVMTTFRTALKNEGIEEINPVNEAFDPNFHQAVQTQPVEEGQESDTVV

TVLQKGYVLKDRVLRPAMVIVAQ"

CDS complement(1348100..1349137)

/gene="hrcA"

/locus_tag="JFBMFIFI_01273"

/inference="ab initio prediction:Prodigal:002006"

/inference="similar to AA sequence:UniProtKB:P30727"

/codon_start=1

/transl_table=11

/product="Heat-inducible transcription repressor HrcA"

/db_xref="COG:COG1420"

/translation="MLTERQILILTTIIRLYTDYGNAVGSKTLMNEAGLNYSSATIRN

EMGRLEELGFIQKTHSSSGRIPSIKGYRFYVDHLVHPDKVTSKDIAIIKSSFGNEFHE

LDEIVAESAEVLSQLTSYTAITLGPELKDSRLTGFRLVPLSDFQVMVILVTDKGHVEN

QIFNLPKSINGYELEKIVRIFNDQLVGYPLVEVYQKLKNDIPQLLQKYVRTPIGIIDV

VDNIITRAAQDRIFVRGRMNILDFNEAMDVEKFKSIYNLMDGNTSLTSLINQSHEGIE

VKIGQELQNELFQDFSLITASYDVVGHGSGMIALLGPTSMPYSKMIGLVDVFRNELSQ

KMIEYYRSMED"

CDS complement(1349375..1349845)

/locus_tag="JFBMFIFI_01274"

/inference="ab initio prediction:Prodigal:002006"

/codon_start=1

/transl_table=11

/product="hypothetical protein"

/translation="MQIYTLCFLKKENEILLLNRQKNPWMGRWNGVGGKIEVGETPLE

CALREIKEETDISLDHAHYGGQMDWYEDGVLRGGLELYLAELPSDFHYPTPKGTREGI

LDFKKIDWIFAEKNQGMPDNIPFILNHLLTSPDKQYFSCFFESGALQKVIKVEK"

CDS complement(1349866..1350270)

/locus_tag="JFBMFIFI_01275"

/inference="ab initio prediction:Prodigal:002006"

/codon_start=1

/transl_table=11

/product="hypothetical protein"

/translation="MTESKRTIHEIQKEWGQFKYLVMAHSQQHYNQIRLLFKESCNLV

ESYVDFKRLVGEALAVEQTIGSFSNAVQHVWGYFKKIATNEEKLTFQRKLFDYQAGMA

SSEILVTYLRKLTIHYEIPYLLSSFFLFPQDS"

CDS complement(1350378..1351748)

/gene="argH"

/locus_tag="JFBMFIFI_01276"

/EC_number="4.3.2.1"

/inference="ab initio prediction:Prodigal:002006"

/inference="similar to AA sequence:UniProtKB:Q9LAE5"

/codon_start=1

/transl_table=11

/product="Argininosuccinate lyase"

/db_xref="COG:COG0165"

/translation="MSEKLWGGRFEEKSEAWVDQFGASIEFDQKLAKEDLLGSLAHVQ

MLGKCKIIPQEEVETIVGGLNKLVEKLAENKLHFTSVNEDIHLNMEKLLHDEIGPVAG

KLHTARSRNDQVATDMHLYLKNQVAEIIHAVNDLLDILIEKAESNIQTIMPGYTHLQH

AQPISLAHHLLAYHSMFKRDLERLEESLKRIDISPLGCAALAGTTFPIDRHYAADLLG

FSKIYSNSLDGVSDRDFILEFLSNSSILMMHLSRFCEEIILWTSHEFQFATLTDAFST

GSSIMPQKKNPDMAELIRGKTGRVYGNLFSLLTVLKGLPLAYNKDLQEDKEGMFDTVE

TVLQSLRIFTGMIATIQFHSDTMKKATQKDFSNATELADYLATKGIPFREAHEIVGKL

VLACTKKGIYLQDVSLAEYQKVAPIIEADIYYFLESDTAVARRTSAGGTAFSEIEAAI

AKAKLS"

CDS complement(1351775..1352968)

/gene="argG"

/locus_tag="JFBMFIFI_01277"

/EC_number="6.3.4.5"

/inference="ab initio prediction:Prodigal:002006"

/inference="similar to AA sequence:UniProtKB:P77973"

/codon_start=1

/transl_table=11

/product="Argininosuccinate synthase"

/translation="MTKEKIVLAYSGGLDTSVAIKWLLEEGYDVIACCLDVGERKNLD

FIKEKALTMGASASYTIDAKEEFANDFALIALQAHAYYEGKYPLISALSRPLIAKKLV

EVAEKEGATAIAHGCTGKGNDQVRFEVAIHSLAPSMKVIAPVREWKWSREEEIAYAQK

HEIPVPIDLDNPFSIDQNLWGRSNECGILENPWIAPPEVAYELTNSLETTPDTPDTIE

IEFLAGVPVALNGVSLSLSELIIQLNTLAGKHGVGRIDHIENRLVGIKSREVYEAPGA

VTLITAHKELEDLTFVRDIAHFKPLIEQKITEMIYNGLWFNPLTEALVAFLKTTQQYV

NGTVRVKLFKGHAIVEGRKSPNSLYDEELATYTSADTFDQEAAVGFIKLWGLPTQVSA

EVLKK"

CDS complement(1353178..1353669)

/gene="tadA_2"

/locus_tag="JFBMFIFI_01278"

/EC_number="3.5.4.33"

/inference="ab initio prediction:Prodigal:002006"

/inference="protein motif:HAMAP:MF_00972"

/codon_start=1

/transl_table=11

/product="tRNA-specific adenosine deaminase"

/translation="MIILTRESHEFYLKRCIELSQSARDNGNTPFGALLVDAEGSILL

EQENIEITEHICTGHAETSLAARASQKYDKDFLWECTLYTTAEPCAMCTGAIYWGNIG

TIVFAMTEKRLLELTGDNDQNPTFNLPSSEIIARGQKDIQIIGPFPSVEVEAAAVHEG

YWD"

CDS complement(1353681..1354829)

/gene="hemW"

/locus_tag="JFBMFIFI_01279"

/inference="ab initio prediction:Prodigal:002006"

/inference="similar to AA sequence:UniProtKB:P54304"

/codon_start=1

/transl_table=11

/product="Heme chaperone HemW"

/db_xref="COG:COG0635"

/translation="MTAAYIHIPFCEHICFYCDFNKVFLEGQPVDEYVDLLVREMKLT

MEQQGETEIETIYVGGGTPTTLNEKQLDRLLIGIKENLPFKIGNEFTFEANPGDLSVE

KLQVLHDHGVNRLSMGVQSFNDDLLKKIGRIHRVSDVYQSISNARQVGFENISIDLIY

RLPGQTEADFESSLKKALELDLPHYSTYSLILENKTIFYNLMRQGKLPLPTEDAEANM

YQMAIDQMADKGRKQYEISNYALPGYESKHNLIYWKNEKYYGFGAGAHGYLGGHRYQN

NGPIQQYLKPLREHKLPVLRSQKLSLEEKIEEELFLGLRKLQGVSIEHFFDKFQVSVF

DIYQETIEELVEAGLLEVSQQWLRLTPRGKFLGNEVFQAFLLSDGKNK"

CDS complement(1354854..1355798)

/gene="ribF"

/locus_tag="JFBMFIFI_01280"

/inference="ab initio prediction:Prodigal:002006"

/inference="similar to AA sequence:UniProtKB:P0AG40"

/codon_start=1

/transl_table=11

/product="Bifunctional riboflavin kinase/FMN

adenylyltransferase"

/db_xref="COG:COG0196"

/translation="MKVINIHHPYEASQIPNEDVVLALGFFDGVHLGHQEVIERAKKI

ADEKKLKLAVMTFNQHPSIVFQKINPEQMKYLSTIERKKCLMEDLGVDYLYIIEFTSH

FASLAPLDFVDQYMVDLHAKVVVAGFDYTYGPKEIADMAHLPGYAAERFDVVVVDKQT

TDNKKISSTRIREKLAEGEMEKVNALLGYAYQTPGRVVHGDARGRLLGFPTANIEVTS

GVRLPQPGIYAVKIKVGQNWHLGMASIGYNVTFGDDRDLTVEVYILDFNQDIYGEQVE

VAWYHYLRSELKFDSVEQLIAQLKQDEIDTATFFNESK"

CDS complement(1355814..1356734)

/gene="truB"

/locus_tag="JFBMFIFI_01281"

/EC_number="5.4.99.25"

/inference="ab initio prediction:Prodigal:002006"

/inference="similar to AA sequence:UniProtKB:P65855"

/codon_start=1

/transl_table=11

/product="tRNA pseudouridine synthase B"

/translation="MEGILPLWKERGMTSHDCVFKLRKILQTKKIGHTGTLDPDVDGV

LPICVGRATKVVEYMMETGKAYVGEITLGFSTTTEDSSGEVVETKKVSEVPTIAEIDK

LMKKFEGIITQVPPMYSAVKVNGKRLYEYARNGETVERPERQATIKKFIRSSEPVFNE

NAGTVSWRFEVECGKGTYVRTLAVDLGEALGYPAHMSDLTRILSGTFKASDCVTLAQV

QEAVDQAEIDQKLFPLEFGLKELPAVEISKDVWEKVKDGAVLAKSDYQDVASFPFVVM

YQDRACSIYDVHPTKPTLLKPVKVLRNQES"

CDS 1356931..1357773

/locus_tag="JFBMFIFI_01282"

/inference="ab initio prediction:Prodigal:002006"

/inference="similar to AA sequence:UniProtKB:Q97QT7"

/codon_start=1

/transl_table=11

/product="DegV domain-containing protein"

/db_xref="COG:COG1307"

/translation="MKNIKIVTDSTVQLSKEEIEKYDITIVPLTAMIDSTVYIDEKTI

TKEEFLEKMNAATELPKTSQPAIGSFVDTYRDLTADGSDVLSIHVTEVLSGTVQAAHQ

ASNLIKGNVTVIDSKFIDRAMAFQVIAAAKMAQKGHSIAEISEKLDTIRSKTDLFICV

VHLDNLIKGGRIGHTMGKISNFLNIKLMLRLTKDGLEPDTKGRGMRSMQKRVDKMIED

MKKTAGIKAIGITHIGLTPFTEELIANFKQQFPSAEYHIAYASPSIMTHAGKDAFSVM

YLTH"

CDS 1357810..1358703

/gene="dagK_1"

/locus_tag="JFBMFIFI_01283"

/EC_number="2.7.1.107"

/inference="ab initio prediction:Prodigal:002006"

/inference="similar to AA sequence:UniProtKB:Q6GFF9"

/codon_start=1

/transl_table=11

/product="Diacylglycerol kinase"

/translation="MVKKVMIIFNPSSGKSQSKKIAQQVQDYLEEQDSAYQVKQMGTK
SETDATKFAKEAAENNFDLVVSIGGDGTISDVVSGLSPFEHRPKLGIIPAGTVNNLAR
VLEIPLDIPAAIENLVTGDLTPLDVGQVNDSYMISTLTLGVLADAALEVSQQEKQKFG
PFAFIFKGSRILVQHKHYHLKIQHDDGKWRGSAQLLLVTMTNSAGGFPYLDPDAAVDD
GYFHVFIVPELSFTGSLRFLPYFISGKAKKIPDVTYFKTKKMTIEGAKKEIRSRIDGD
PSEKLPLNMQVLEGHIQVLAP"

CDS complement(1358724..1359398)

/locus_tag="JFBMFIFI_01284"

/inference="ab initio prediction:Prodigal:002006"

/codon_start=1

/transl_table=11

/product="hypothetical protein"

/translation="MMPFSYLATEKPLKLSKILDPSRCIIAERENMPADLSEEDFYTE
MKKSNPVLKREEVRIIEKGDSRPLFNVFSILQQVTDPLILSMFALPYVYEVTDFPFAE
TEIYHLTLTESQLNDNVLLSKMEKIYFAQLTSCEEYFQTLFIRNKKVECLLYWDPIRD
HLDNGKSRPYSFLPCGLYITGLNAEDCFQGVEKHLATNDFFREFEAINLKINQKLIIS
KKDNAR"

CDS complement(1359438..1360034)

/locus_tag="JFBMFIFI_01285"

/inference="ab initio prediction:Prodigal:002006"

/codon_start=1

/transl_table=11

/product="hypothetical protein"

/translation="MSLYSYIGTEKKMPVAEPPAENIVAMIARDTLDKGVTDEMVLSY
LQNQTGDDSLTLDQIAFFDTEKEIVGGFEILNLTNSTDIKKHFTTKFVYECSDFPVWE
PIENETNTGDEYTFEQITQYQQMMDEARAVNAVQQTSFMDVLEKCFEDTDKVELYTCW
MGEEGVREAQRRVEKWDIVKANPLLLDLGEKEFLVIEK"

CDS complement(1360177..1360524)

/gene="rbfA"

/locus_tag="JFBMFIFI_01286"

/inference="ab initio prediction:Prodigal:002006"

/inference="similar to AA sequence:UniProtKB:P65967"

/codon_start=1

/transl_table=11

/product="Ribosome-binding factor A"

/translation="MANHRLGRVTQEVQREVNDILKKRVRDPRVQDVNITDVRVTGDL

QQATIYYSILSDKASDIEKAQAGLEKAAGLIRKELGQRLTLYKTPELSFARDESVAYG

NRIDELLRDLNKE"

CDS complement(1360558..1363065)

/gene="infB"

/locus_tag="JFBMFIFI_01287"

/inference="ab initio prediction:Prodigal:002006"

/inference="protein motif:HAMAP:MF_00100_B"

/codon_start=1

/transl_table=11

/product="Translation initiation factor IF-2"

/translation="MGKKRVHEYAKEHDMSSKRVIEKAKELGIELGNHMSTIDENQVQ

KLNQVFTKKEAGTKVAANTKKSDRPHSSGQGGNTTNSKEDNQKRPAQSGGQNAGKPTQ

TGKPAQNKTTNTRPATKPSQGQSNNTNRPAAKTSQGQSSNTNRPAAKTGQGQSNNTNR

PAAKTSQGQSSNTSRPAAQGQGTTSGNNAQGANRGSGGGQGGYQGNRGGQGGNRGGYQ

GGYNNRNNFRKKKRRSGETKPVAPPVPRKFKELPEVLVYSDGMTVADISKKIYREPAE

IIKKLFLLGVMATLNQSLSKEAIELLATDYGIEAEEKIELDVSDLDVYFDQETNEEAL

VTRPPVVTIMGHVDHGKTTLLDSLRNTKVSLGEAGGITQHIGAYQIDVDGKPITFLDT

PGHAAFTTMRARGAGITDITVLVVAADDGVMPQTVEAINHAKAAEVPIIVAVNKVDKP

GANPERVMQELTEHGLVPESWGGETIFVEISAKFQQNLEELLEMILLVAEVQELKADP

TRLAIGTVIEARLDKSKGPVATLLVQEGTLKVGDPIVVGNTFGRVRVMSNETGRRVKT

AGPAAPVEITGLNNAPQAGDRFVVFEDEKTARSAGEDRAKRAAVEQRSVTNRVTLDNL

FDSLQEGELKEVNVIIKADVQGSVEALAASLQKIEVEGVRVKIIHTAVGAINESDITL

AAASNAIVIGFNVRPTPQAKQQAEQEAVDIRLHRIIYNAIDEIETAMKGMLDPEFEEK

ITGQAIVRETFKVSKVGTIAGGYVTEGFITRNSSIRLIRDSIVIFEGELASLKRFKDD

VKEVKLGFEFGFMIKDYNEVQMDDVIEAYQMVEIKRK"

CDS complement(1363081..1363386)

/gene="rplGA"

/locus_tag="JFBMFIFI_01288"

/inference="ab initio prediction:Prodigal:002006"

/inference="similar to AA sequence:UniProtKB:P32729"

/codon_start=1

/transl_table=11

/product="putative ribosomal protein YlxQ"

/db_xref="COG:COG1358"

/translation="MNNDQKVLNLLGMAQRAGKLVSGEDLSLKEIRNGTAKLVIVAVD

ASENTRKKISDKGQHYEVLVAVHFTKMELSHAIGKERTICTIIDKGFAKKFRELLSI"

CDS complement(1363383..1363667)

/locus_tag="JFBMFIFI_01289"

/inference="ab initio prediction:Prodigal:002006"

/codon_start=1

/transl_table=11

/product="hypothetical protein"

/translation="MQKRKVPMRKCVVTNEMKPKKEMIRIVRNKEGVVAIDPTGKMPG

RGAYVSIEPTVVQTAWDKHVLDRHLEATINDEFYQELLDYVTHQKARMSL"

CDS complement(1363684..1364961)

/gene="nusA"

/locus_tag="JFBMFIFI_01290"

/inference="ab initio prediction:Prodigal:002006"

/inference="similar to AA sequence:UniProtKB:P32727"

/codon_start=1

/transl_table=11

/product="Transcription termination/antitermination

protein NusA"

/db_xref="COG:COG0195"

/translation="MSKEMLNALDALETEKGIAKEIVIEALEAALVSAYKRNYSQAQN

VEVEFDMKKGNIHVYAVKEVVEVVFDSRLEVSIEDALELNKAYEIGDKIRFEVTPKDF

GRIAAQTAKQVIMQRVREAERSIIYNEFVAYENDIMQGIVERQDNRYIYVNLGKIEAV

LSKQEQIPNETYKAHDRIKVYVTKVENTSKGPQIFVSRSHPDLLKRLFEQEVPEIYDG

TVEIVSIAREAGDRAKVAVMSRDANIDPVGTCVGPKGQRVQAIVNELKGENMDIVEWN

ENPATYIANALNPAQVVDVTFNEAQGSCVVVVPDYQLSLAIGKRGQNARLAAKLTGFK

IDIKSESDMAALAEVKEETDLETVAEYVEAVEENMAVEAENDAEEVLESIEDLEESRE

VSELDSETEEDILNPEDAEELIELAEEQDGVDE"

CDS complement(1364995..1365468)

/gene="rimP"

/locus_tag="JFBMFIFI_01291"

/inference="ab initio prediction:Prodigal:002006"

/inference="similar to AA sequence:UniProtKB:Q97S61"

/codon_start=1

/transl_table=11

/product="Ribosome maturation factor RimP"

/db_xref="COG:COG0779"

/translation="MSNVVDTVKTIVQPIVDEFNFELVDVEFVKEGKSWFLRMYIDKP

GGIDIEDCALVSEKISEKMDAINPDPIPQAYFLEVSSPGAERPLKKEQDYQNAVGSYV

NISLYEPVDGEKMYEGTLEEVNEDSLILTIRIKTREKKMEFDRKKIAKARLAIKF"

CDS 1365968..1366171

/locus_tag="JFBMFIFI_01292"

/inference="ab initio prediction:Prodigal:002006"

/codon_start=1

/transl_table=11

/product="hypothetical protein"

/translation="MDQDFLFMCGIIVFFTGAFSLSLSGMCYRWRAFLNKKAWDGLTL

PFLYFGLPLVSIGLILIYFYYPY"

CDS complement(1366219..1370568)

/gene="polC_1"

/locus_tag="JFBMFIFI_01293"

/EC_number="2.7.7.7"

/inference="ab initio prediction:Prodigal:002006"

/inference="similar to AA sequence:UniProtKB:P13267"

/codon_start=1

/transl_table=11

/product="DNA polymerase III PolC-type"

/db_xref="COG:COG2176"

/translation="MSLSQEELFQKLLEQINLHQELDYQPYFEKASIRKVTVHKQSKL

WHFDFYFQDILPFEIYQNFSEKLQIAFRSIAAISFSIETENPELTIEKLEHYWPEAVK

RSGVSSPIINQLFRQQLPVLNLKKVQLYVENEVVKGHLTNQYLPPVEEHYQKLGFPKF

RIEPVIDEEANTAKLAEFKAKKEEAEQLLAERAADNLQKAEEERKRNGGDNKVANLPK

GKLVLGRAINDKEEIKPMAEIIEEERRVTIEGYVFDVEVRVLRSERQILILKITDYTS

SFAVKMFSNSPEDEAVFAALKKGMWVRARGAVQEDNFMRDLVMSARDITEVSRELRKD

KADEDKKRVELHLHSNMSQMDATNNITDLVEQAAKWGHPAVAITDHSGAQSFPDAYHA

GKKNGVKILYGIEANIVDDGVPIAYNPKHIALSEATYVVFDVETTGLSAVYDTIIELA

AVKMYKGNIIETFEQFIDPGHPLSQTTINLTGITDDMVRGSKSEEEVLRLFKEFSGDD

ILVAHNASFDMGFLNTSYGKYDIAEAENPVIDTLEMSRFLHPQLKSHRLNTLAKRYGV

NLEQHHRAIYDSESTGQLCWIFIKEAREEHDMHYHDELNMHIGEGDSYKRARPFHATI

IATTQDGLKNLFKLVSMSMVNYFYRTPRIPRSKLTEYREGLIVGSACSSGEVFEAMMQ

KGYDEAKNRAKFYDYLEVMPKPVYAPLIERELVKSEKDLEEIIRNMVRMGDELGKLVV

ATGNVHYLNPEDSTYRKILIHSQGGANPLNRSILPEVHFRTTDEMLKEFSFLGKEMAE

RIVVENTHKVADLVADDITPVKTDLYTPKIEGSEDEIRKLSYDEAHRLYGEVLPEIVE

KRIEKELKSIIGNGFSVIYLISQKLVHKSVSEGYLVGSRGSVGSSFVATMTGITEVNP

MPPHYRCPECQYSEFFEDGSVGSGYDLPEKACPKCGARLFKDGHDIPFETFLGFHGDK

VPDIDLNFSGDYQAKAHDYTKVLFGEDYVFRAGTIGTVADRTAYGFVKGYERDMNLNF

RAAEVDRLAKGSTGVKRTTGQHPGGIIVIPDYMDVYDFTPIQFPADAQDSEWKTTHFD

FHSIHDNVLKLDILGHDDPTVIRMLQDLSGIDPKTIPTDDPDVMQIFGGTEILGVTPK

QIESNTGTLGIPEFGTRFVRGMLEQTKPTTFAELLQISGLSHGTDVWLGNAEELIRTK

DIPLSQVIGCRDDIMVYLIHNGLDDGLAFKIMESVRKGKGIPEDWQKSMRDEKIPTWY

IDSCLKIKYMFPKAHAAAYVLMALRVAYYKVHFPILYYAAFLSVRAADFDLVAMSQGK

EALKAKMKEINDKGLDASTKEKNLLTILEICNEMVERGFEIKMIDLYKSDANDFIIEG

NALIAPFRAVPSLGANVAKQICIAREEKKFLSKEDLAKRGKVSKTLIEYMTENNVLKD

LPDENQLSLFDMM"

CDS complement(1370686..1372392)

/gene="proS"

/locus_tag="JFBMFIFI_01294"

/EC_number="6.1.1.15"

/inference="ab initio prediction:Prodigal:002006"

/inference="similar to AA sequence:UniProtKB:Q831W7"

/codon_start=1

/transl_table=11

/product="Proline--tRNA ligase"

/db_xref="COG:COG0442"

/translation="MKQSKLFVPTLREVPNDAEVISHQMLLRAGYIRQISSGVYSYLP
LANLVIEKLKTIIREEFEKIDAVEMLMPSLLPKELWEESGRYETYGPNLMRLKDRNGR
DFLLGPTHEEAFTTLIRDEINSYKRLPLSLYQIQTKFRDEKRPRFGLLRGREFIMNDA
YSFHDSDESLDVGYRQFEQAYNRIFERCGLEFRSIIGDGGAMGGSDSKEFMAISDIGE
DTICYSDASDYAANLEMATNFYMRKKSHEIEKELEKVATPGVKTVEEVANLLEVEESR
VIKSILFIADEKPVLVVVRGDHEINDVKLKNHLNADFLEMATDEQASELLGASFGSLG
PINVPEEIQVIADRYVQDLGNAVCGANEEGFHFINVNPDRDLKVEEYLDLRFVLEGEL
SPDGQGVIKFTKGIEIGHIFKLGTRYSESMNATILDQNGRSIPVIMGCYGIGVSRLLS
AIVEQQADDRGVNWPRNIAPYEVHLIPVNMKAEDQTSLSDDLYTELKQAGLSVLMDDR
NERVGVKFADSDLIGLPVRITVGKKASEGIVEIKLRKTSETIEVKKEELIDTLNILLN
AN"

CDS complement(1372517..1373788)

/gene="rasP"

/locus_tag="JFBMFIFI_01295"

/EC_number="3.4.24.-"

/inference="ab initio prediction:Prodigal:002006"

/inference="similar to AA sequence:UniProtKB:O31754"

/codon_start=1

/transl_table=11

/product="Regulator of sigma-W protease RasP"

/db_xref="COG:COG0750"

/translation="MVTTIITFLIVFSILVVVHEYGHYYFAKKSGILVREFAIGFGPK
IFSYRKNGTTYTIRILPIGGYVRMAGYGEDETEIKPGMPIGLIINSEGIVTTINTSKK
TQLMEAVPMEVVSIDLEKDLHVEGYLAGNEDELVRYPVLHDATIIEEDGTEVQIAPID
VQFQSASLPKRMMTNFAGPMNNFILAILAFTVIAFMQGGVVSHSTQLGEIASDSVAAK
VGLVQGDTILAVDGKKMSDWTMLATTIQAHPAKEIKLDVETKAGKKETINVTPKSVKV
EDKKIGQIGIGPAIDTSFGAKIAYGFTQTWFIMVQIVTVLGSIFTKGFNIGMFSGPVG
IYAATEQVVQIGFMGVFNFLAVLSINLGIVNLLPIPALDGGKLLLNIIEGIRRKPLDP
DKEGIITMVGFGLMMLLMILVTWNDIQRYFF"

CDS complement(1373802..1374944)

/gene="dxr"

/locus_tag="JFBMFIFI_01296"

/EC_number="1.1.1.267"

/inference="ab initio prediction:Prodigal:002006"

/inference="similar to AA sequence:UniProtKB:Q9RCT1"

/codon_start=1

/transl_table=11

/product="1-deoxy-D-xylulose 5-phosphate reductoisomerase"

/db_xref="COG:COG0743"

/translation="MKKICLLGATGSVGENTVAVVTAHPDQFQIVAFSFYQNKEKGRK

LIQKLQPKIVSVATKEDATGLSLEFPEIRFTYGIEGLSEVVADSEVQLVVTALSGSVG

LLPTLTAIELGKTVAIANKETLVMAGALVMNLAKEKNVELLPVDSEHSAIFQCLQGEN

ISEVEELIITASGGSFRELSRKELVNVTLSDALNHPNWAMGQKITIDSATMMNKGLEV

IEAHWLFGLTYDKIKVVLHKESIVHSMVSFVDGAVMAQMGASDMREPIQYALSYPKRI

PMIQPKPFDLAKIGALHFAEMDFERFPLLALAYETGKMGGTAPTMMNAANEIAVAAFI

KGQISFLEIENYVEKAIKLDNYVKNPDLETILNVDKETRKLVTSWL"

CDS complement(1375004..1375789)

/locus_tag="JFBMFIFI_01297"

/inference="ab initio prediction:Prodigal:002006"

/codon_start=1

/transl_table=11

/product="hypothetical protein"

/translation="MKQRVITAVVALVFFIPVLYIGSWTLEIVAAAMGLVGLFELFRM

KGNSFFTIEGLLASIGMLLLLVPTERLSFLPSNYSPLYLFYICGILLLVCTVFSKNSF

SFDDAGVAILGALYIGYGFHYFIATRAAGLDLLMYILFVVWATDIGAYMFGRKIGKHK

LAPAISPNKTIEGSLGGILSALVVAALYLTFYPQDYSMGIMLALTVLFSIFGQLGDLV

ESAFKRHYDVKDSGKILPGHGGILDRFDSLLFVLPMLHLVALI"

CDS complement(1376054..1376830)

/gene="uppS"

/locus_tag="JFBMFIFI_01298"

/EC_number="2.5.1.31"

/inference="ab initio prediction:Prodigal:002006"

/inference="similar to AA sequence:UniProtKB:Q88VJ8"

/codon_start=1

/transl_table=11

/product="Ditrans,polycis-undecaprenyl-diphosphate

synthase ((2E,6E)-farnesyl-diphosphate specific)"

/db_xref="COG:COG0020"

/translation="MKHLFKRKGKQQAGTNPHPFQENEVIPNHIAIIMDGNGRWAQKK

FLPRVAGHREGMNTVKKITKHANKLGVKVLTLYAFSTENWKRPTDEVSFLMQLPVDFF

DTFVPDLIKENVKVNVMGFVDQLPDHTKKAVEKAIEDTKHNTGMILNFALNYGSRSEM

LEATKIVAEKVQNNQLKLDEITEEVYAEALMTHTLGDYQDPDLLVRTSGEERISNFLL

WQVAYSEFYFSKVLWPDFDEDVLETAIGVYQTRHRRFGGL"

CDS 1377123..1377392

/locus_tag="JFBMFIFI_01299"

/inference="ab initio prediction:Prodigal:002006"

/codon_start=1

/transl_table=11

/product="hypothetical protein"

/translation="MDDFTEFTDSETKKVAALQEIADTVDRLDTILEELKGTDNKLKA

WYEQKKAVYEIKKILHDATHYERYNKAEADEFLSEYNSFVRPKQP"

CDS complement(1377558..1378115)

/gene="frr"

/locus_tag="JFBMFIFI_01300"

/inference="ab initio prediction:Prodigal:002006"

/inference="similar to AA sequence:UniProtKB:Q5XDH3"

/codon_start=1

/transl_table=11

/product="Ribosome-recycling factor"

/translation="MSQVLLNEAKEKMTKAEQALQRELGSIRAGRANASILDRIQVEY

YGAPTPLNQLASITIPEARVLMVSPFDKTSLQDIERALMQSDVGITPTNDGNVIRLVI

PMLTEERRKELAKRVKKEAENSKVTVRNIRRDLIDELKKAEKNKEMTEDELRNYETEA

QKLTDDSVKNLDKIAADKEKELLEV"

CDS complement(1378117..1378839)

/gene="pyrH"

/locus_tag="JFBMFIFI_01301"

/EC_number="2.7.4.22"

/inference="ab initio prediction:Prodigal:002006"

/inference="similar to AA sequence:UniProtKB:Q831V1"

/codon_start=1

/transl_table=11

/product="Uridylate kinase"

/db_xref="COG:COG0528"

/translation="MNKPKYKRIVLKISGEALAGEEGFGIKPPTIREIAKEIKEVHDL

GVEIAIVVGGGNIWRGQVGSQMGMDRAQADYMGMLATVMNALALQDNLETVGVPTRVQ

TSIEMRQIAEPYIRRKAMRHLEKGRVVIFAGGTGNPYFSTDTTAALRAAEIDADVILM

AKNNVDGVYSADPKVDVTATKFEELTHLEIINKGLQVMDTTASSLSMDNDIPLVVFNL

NETGNIKRVALGETIGTTVRGK"

CDS complement(1378950..1379831)

/gene="tsf"

/locus_tag="JFBMFIFI_01302"

/inference="ab initio prediction:Prodigal:002006"

/inference="similar to AA sequence:UniProtKB:P99171"

/codon_start=1

/transl_table=11

/product="Elongation factor Ts"

/translation="MTQVTAALVKKLRDMTGVGMMDAKRALVAVEGDIDKAVDHLREN

GMAKAAKKADRVAAEGLASVFVEGNTAAIVEINSETDFVSKNDQFQTLVAEVTRQVAE

ANPATLEEALEIKTASGTISSEIMEATTVIGEKISFRRFERLTKDDNSAFGAYLHMGG

RIAVLTLIEGTTDEEIAKDVAMHIAAINPKYVSRDQVSQDEIAHETKILTEQALNEGK

PANIVEKMIQGRLNKYLAEISLVDQPFVKDPDQTVGQFLASKGASVKTFVRFEVGDGI

EKREDNFVEEVMSQVNK"

CDS complement(1379998..1380792)

/gene="rpsB"

/locus_tag="JFBMFIFI_01303"

/inference="ab initio prediction:Prodigal:002006"

/inference="similar to AA sequence:UniProtKB:A2RNV0"

/codon_start=1

/transl_table=11

/product="30S ribosomal protein S2"

/db_xref="COG:COG0052"

/translation="MAVISMKQLLEAGVHFGHQTRRWNPKMKRYIFTERNGIYIIDLQ

KTVKLVDEAYNYMKDVAENGGIALFVGTKKQAQEAVKDEAIRSGQYFVNHRWLGGTLT

NWDTIQKRIKRLKEINKMEEDGTFEVLPKKEVGILVKQRERLQKFLGGIEDMPRIPDV

MFIVDPRKERIAVQEAHKLNIPIVGIVDTNCDPDEIDVVIPANDDAIRAVKLLTAKMA

DAMIEGRQGEDEVVEETFVAEVVAEEGTETASIEEIVEVVEGDNAE"

CDS complement(1381005..1381457)

/gene="bltD_2"

/locus_tag="JFBMFIFI_01304"

/EC_number="2.3.1.57"

/inference="ab initio prediction:Prodigal:002006"

/inference="similar to AA sequence:UniProtKB:P39909"

/codon_start=1

/transl_table=11

/product="Spermine/spermidine acetyltransferase"

/db_xref="COG:COG0454"

/translation="MKTEIRRVTKENWREIVQLKVAKGQENFIESNAESLLEAVFEGE

DWVPVGLYSENQLVGFAMYGCYDSTNRSIWLDRFMIGEANQSHGLGKLFLEKICQYLP

TEYELKQILLSFYPENQNARKFYEAYGFITTTKVDENGEEIYYLDVSH"

CDS complement(1381546..1382793)

/gene="pepT_2"

/locus_tag="JFBMFIFI_01305"

/EC_number="3.4.11.4"

/inference="ab initio prediction:Prodigal:002006"

/inference="similar to AA sequence:UniProtKB:Q76HM5"

/codon_start=1

/transl_table=11

/product="Peptidase T"

/db_xref="COG:COG2195"

/translation="MYPDLVQRFMTYVKKETRSDPTSSTVPTTTSQTDFAKDLVVELT

SLGLADVQLNVKNGFVTGTLPSTTSYKVPTIGFIAHIDTADFNAVNIQPQIHPNYDGK

ELVLHSDLDIKLKPTDFPNLKNYIGETLITTDGTTLLGADDKAGIAEIVTAIDYFIQN

PEIEHGMIKVAFGPDEEIGRGADLFDVVNFGADFAYTIDSGTVGRLEYETFNAAEAKI

VIKGKSVHPGVAKNTLINALKIALEFDAALPQNEVPERTAGYEGFYLLNNLSGDVEDA

QLTYIIRDHDQQKFSERKQFFENLVDRMNLDLGYEAISLVLKDQYYNMGEIIQKKPEI

IQLAIEAMKNIGIKAIVEPFRGGTDGSKISYLGLPCPNLFTGGENFHGRYEFISVESM

EKATATIIEISQLNVTKNKNSQL"

CDS 1383371..1385029

/gene="dppE_4"

/locus_tag="JFBMFIFI_01306"

/inference="ab initio prediction:Prodigal:002006"

/inference="similar to AA sequence:UniProtKB:P26906"

/codon_start=1

/transl_table=11

/product="Dipeptide-binding protein DppE"

/db_xref="COG:COG4166"

/translation="MKKWKLGLSLVITASILAACGGSNGKDTAATKESDTNKSANVAQ

VLRASVPAEITTLDTTKATDRNTFTVIEQIFEGLYRFNGDNELELALAKEPAKISDDG

LTYTFKLKEDAKWSNGEPVTANDFVYAWSKIVNPASAAPNAYLFANIKNAKAITAGEK

ELAELGVKATSDYELEVNLELPQPSFLSLVAIGWFYPQNQKFVEEQGEGYAMDTSKIL

YNGPFTLTDWKDGATNWSYEKNKSYHDQKNVSLDKVDISVVKETSTGVQLYEANELDV

TTLVGDYAKENEDNPDLVRKQELALQYLSYNTVEGSPLANTHLRKALSLAIDRSAITD

NIVANGSDPLGGLIPTKLAKNPESGEDFRKENGSFLDYDLKEAKKEWDLAKKDVGEQL

ELELVTSDDAMEKKLGEYIQSTISENLKGITIKIRSVPKNVALEERRAGNFDLATAGW

IAGDIDPTGFFVLFETDGAYNYGKYKNDKFQQLITDSKTIDANNPEKRYEKFLAAEKI

LFEDAAFEPLYQRTTNYLQRSTVKGLESHLLGSDFTFRNVTISK"

CDS 1385274..1385987

/gene="gph_1"

/locus_tag="JFBMFIFI_01307"

/EC_number="3.1.3.18"

/inference="ab initio prediction:Prodigal:002006"

/inference="protein motif:HAMAP:MF_00495"

/codon_start=1

/transl_table=11

/product="Phosphoglycolate phosphatase"

/translation="MINAIVFDVDDTLYDQQAPFKNAIANCFPAYFSSLDLTRAYLRF

RYHSDETFCKYISGEWTLSYMRFYRIREVLKEQGYSLTEDQGLLFQENYETELNSIQL

LPEAKELLNALASKSDIQIGIITNGPTDHQSKKIQQLQLEQWIPAEKMIISESSGFAK

PDRRIFDLAARQFKFEPSTTLYIGDNYDNDVNGAKEAGWQAFWFNHRKREKPVSLWSC
DEEITEFHEFPSKVLSLLD"

CDS complement(1386043..1386786)

/locus_tag="JFBMFIFI_01308"

/inference="ab initio prediction:Prodigal:002006"

/codon_start=1

/transl_table=11

/product="hypothetical protein"

/translation="MDDHDISLLMAHLGDSDASLRDEGLYTLVALAVEKNCLNTVQLR
RMKNQLLSKKYLFKGMQEDETDDIFWRSFSNLYLAVIVEYDRTKEHFMSTKELSEIAL
NLCCYLTLENDNRGFIEGKGWAHAFAHAGDLANELAKHPYLESSDLSLLTVTIFRKVT
NFGKMFSFGEEERLIQSLLTILKKQPDFSVSIICLIEKQQIEIDKNKSKQELIHQNNY
YNTRRFYQQFLLSSNTPSIVKQNLEKWSI"

CDS complement(1386888..1390115)

/gene="rapA"

/locus_tag="JFBMFIFI_01309"

/EC_number="3.6.4.-"

/inference="ab initio prediction:Prodigal:002006"

/inference="protein motif:HAMAP:MF_01821"

/codon_start=1

/transl_table=11

/product="RNA polymerase-associated protein RapA"

/translation="MKWSIPERVIDRARIYVAEKRVLVITPFLEKKIWTAEVMGSEIY
HVELDGTTRESDFCECPYWKDHGYCKHTVAVELALKEQGVSRVMTAENAEKMTAVLPD
PGQELTESFTRVFLKENREIFLEVEDKLELMMEYKVEIKSTSNSLIRNNDEVLALSLR
AGLDKLYIVKDVASFFESYHNQGTFDLNTNQTLDFKQIKITAEDSLILDFLGQQAASN
EMMGVGSKEPKSIYSNKRYLVISPILAEETIQLIQENGKLQFFVGENKHKRMIFVPNQ
LPISFELFQQNQKISLYINNQTDARLKAYHWFIAKHAIFQPSKEQLRAYQPLQNFLQR
YDGKEVVIDPVNMPDFTAYVLPLLAKIGEVSIDEAIQDTFIQEPLKTKIYFTYHQDAV
HAVVDFSYKNLVLSTDEKNNQLGDSGVQVIRDSQQELKILNSFKQFDYHRDETSYAKR
MVRDDDFYTLFTKEIPFLEMDAEVYVDDLLDSMFLDQIDPETSIDVQNEGSLLDVRFD
IKGITPDEVDQVLKSLVEKRSFHKLDNGVLLSLETDAFKHVSDVLAELRITKDFQNGK
ITLPSYRGLEVQETIGLEESNKRKLSRKFKNLIEDLNDPAHFEAEVPKNLEAELRPYQ

ITGFKWLKMLAKYGFGGILADDMGLGKTIQVITFILSEIEDYGKNEPFLIVSPASLTY

NWHHEIKKFAPSLESFVVSGTAEERQKRIEEVGPNQILITSYPSFRQDVEFYKKKKFS

TLVLDESQMVKNYHTKTAQALRELTIRKRFALSGTPIENKIEELWAIFQLIMPGFFPP

MKQFKTMPYPQIARMIQPFVLRRIKKDVLKELPDKIETDLYSSMTKEQKTVYLAYLQR

IQDSVRSMSGDDFKRNRIEILAGLTRLRQICCDPRLFVDNYTGDSGKLEQLKELLQTA

KENGQRVLLFSQFTSMLSIIEEELAQEGIETFYLSGQTKPKERIEMVDRFNAGEKEVF

LISLKAGGTGLNLTGADTVILYDLWWNPAVEEQAAGRAHRLGQKKVVQVWRLIAEGTI

EEKISQLQQEKKALFDQVITSEATDTKQLTQLTESDIREILNIGV"

CDS complement(1390280..1393426)

/locus_tag="JFBMFIFI_01310"

/inference="ab initio prediction:Prodigal:002006"

/codon_start=1

/transl_table=11

/product="hypothetical protein"

/translation="MKPIKLNLQAFGPYLNETIDFSSFYEHHIFLIAGKTGAGKTTLF

DAMSYALFGKTNGLNREPKEMRSTFAKPSEETKVAFTFSAQNKVYQIERIPEQVLAKK

RGDGVREVKAKATITIFDEFGKELNHFTKTTEVNQIVEEVVQLEDEQFRQIIMLPQGD

FRRFLNANSNEKEKILRKLFGTEPYRLFSEKLKEQKKLISVAIKDQEKELITTLQHAR

WVTELPEILELGQARGKKELSYLKAQQENMHNQLKKNEKRIQLLKEEENKRQLELQKV

QAEILLFNEKEQLYSQQTIHKSATERIMTLKEKLNKLKEAELIQPLLVRQLELKNEMA

ELATEVHSLEDQKKLAEKEKNYWETLYQEKLAESPEQERREMKLIELKQQKIVLDGYS

RVRQEAERLAEKINLLDREKVACANQLQEEQKKEMAVVQKLDQLKEVEQQLNQLDKEQ

TRVQTIDQKLVEFSQIEEKIQQLKNQEITVRGSLLLAEEKVAETEKNYLKAKSDWAKI

QIRRLALDLEDGAPCPVCGSVEHPYLHSQVEDMDEFETMSQAENQLDFWENELTTVKE

TVASFKTRIEGLHEQLADSESQLEKRMENDPLATRLEKSSLDQLKKEVKVQLEKIETQ

IMELKQQHSKETELLEEQNKLKSFIEKKSAELNIIEQDYQNVWQEQLEKKGELTQIKQ

QLPLELLDETIFKGRLSQLELEVAAFKQELSERELKKVAAQDQLKEIISNQAHLKKQS

AKAEKLELELTEKLNDAINQSSFENQKSIEATLTELEEVVDWEKEIQQYEKEEYRITQ

RIAELENHLLSVEKPNIEIAESRLKEVAIERETMQEEQTRNQQIHMQNKKVLKEATEI

IYSIQEQWDLLDEVTNLSAVANGDGDQSKMGFERYVLTYFLEEVLTIANGRLQQLSNN

RYLFDLNREEGSRKTDTGLEINIYDDNAGGLRNVRTLSGGESFIAALALSLSLSEVVQ

QHAGGVQIETMFIDEGFGSLDEDALEEAMDALLQIEGSGRLVGIISHVKELKERIPDK

LLVESNGTGKSKIKISHG"

CDS complement(1393423..1394574)

/gene="sbcD"

/locus_tag="JFBMFIFI_01311"

/inference="ab initio prediction:Prodigal:002006"

/inference="similar to AA sequence:UniProtKB:A6QGP7"

/codon_start=1

/transl_table=11

/product="Nuclease SbcCD subunit D"

/translation="MKFIHTADWHIGKKIHGFQLLKEQREVFEQMLEVAIREEVDAIV
VAGDLYDRAVPPSEAVALLNQLFIELNIENRFPILAISGNHDSPTRLETGGPWYQTLN
FHLHTKISQSLKPIVIGNTQFFLLPYFEPIDARLYFKDEKLVTHELAAKRVVEEMEKQ
FNSEYNQVLVAHLFVAGSLRTESETEVMVGGLDQVSASIFNTFDYVALGHLHDPNATR
HAKLQYSGTLLKFSISEANQQKGFKLIELSDEGELTNQFFPVSPEKELRVLEGFFADL
IEPTYYEAQKRDDFIYVQLKDTKMIPDAMNRLREIYPNVLGMERLDRSGLMATMNLTT
REKLKTKDPSELFKDYYEEVMGEALSSKQETLLNELLQNLDVTNKGGRN"

CDS 1394775..1395392

/gene="plsC"

/locus_tag="JFBMFIFI_01312"

/EC_number="2.3.1.-"

/inference="ab initio prediction:Prodigal:002006"

/inference="similar to AA sequence:UniProtKB:Q8DNY1"

/codon_start=1

/transl_table=11

/product="1-acyl-sn-glycerol-3-phosphate acyltransferase"

/db_xref="COG:COG0204"

/translation="MFYRFIRQVARFVVFLLNGNARYTNKERLPKEEAYILVGPHRTW
FDPIYFALAGTPTIFSFMAKQELFKNPILRWILIHANAFPVDRENPGPSVIKKPVKFL
KEGNLSLIMFPSGTRHSNDLKGGAATIAKLSGAPIVPAVFQGPLTIKGLLSRKRVTVN
FGEPIYLDKKVKLTKETLKDIEDQMQAAFDQLDAEIDPNYKYEAK"

CDS complement(1395476..1396507)

/gene="namA"

/locus_tag="JFBMFIFI_01313"

/EC_number="1.6.99.1"

/inference="ab initio prediction:Prodigal:002006"

/inference="similar to AA sequence:UniProtKB:Q5KXG9"

/codon_start=1

/transl_table=11

/product="NADPH dehydrogenase"

/db_xref="COG:COG1902"

/translation="MVNLDHSFSIKGLTLKNRIVMAPMCQYSVEKEDGKPNDWHFVHY

VSRAVGGVGLILIEMTDVEPNGRITNNDLGLWTDEQIPAFKKIIDDVHRQGAKIGIQI

AHAGRKAQDTEEPVGATNQPIEDGVSRRGVPRALTTEEVGAMVDKYKDAARRAVEAGV

DTIEIHGAHGYLIHQFISPLVNTREDQYGQDLARFPVEIIKAVKSVMPDDMPLLMRLS

AIEYVENGYDIQHSLEIAKKCKEAGVDVFHISSGGEGTPGSLKPGNYPGYQVPMARKY

REMLNVPVIAVGKLESPELAEAVIANQDADLVAIGRGLLDDPYWGIHALKKIGKSVVP

PKQYERGIR"

CDS complement(1396746..1398536)

/gene="yheH"

/locus_tag="JFBMFIFI_01314"

/EC_number="3.6.3.-"

/inference="ab initio prediction:Prodigal:002006"

/inference="similar to AA sequence:UniProtKB:O07549"

/codon_start=1

/transl_table=11

/product="putative multidrug resistance ABC transporter

ATP-binding/permease protein YheH"

/db_xref="COG:COG1132"

/translation="MEENKSEWLKSIPVKEQVAIVKRMFVFAKPFKKQFFIAIFFGVI

LAIINILLPRILQIFMDSYLTPRKATTEIIITFASIYFAVTLAKIVVWFCQMYIFNMA

AEKTVQNIRNQLFEKLHTLGMRYFDQTPAGSIVSRVTNDTETIKEFWAVFLTVLQGLF

AIISAFTAMFLLNSQIAMWCLLFLPILMVVVWYYQHFSSKVYRGMREKLSQLNTKLNE

SISGMGIIQQFRQEKRLQAEFNETNNDYFQSRISMVKINALLLGPIISLLYTFSLAVI

LGFFGYNALTEPVSIGVIYAFISYTQSFFNPMTNMMDYLSIFQDGIVSSSRVLKVMDE

TSLTPQQDSLATGEIQDAEIEFKHVSFSYDGEHDVLTDISFTAKPGETVALVGHTGSG

KSSIINVLLRFYEFNRGDILIDGKSIKEYPITEIREKMGLVLQDSFLFYGDIKRNIRL

MNQTITDYQIEEAARFVQADSFIEELPGQYNAKVIERGASYSSGQRQLISFARTIVTD

PKILVLDEATANIDTETETLIQEGLKKMRQGRTTIAIAHRLSTIRDANLILVLDHGKI

IERGTHEELIELGGIYYDMYRLQNSENATG"

CDS complement(1398536..1400287)

/gene="yheI"

/locus_tag="JFBMFIFI_01315"

/EC_number="3.6.3.-"

/inference="ab initio prediction:Prodigal:002006"

/inference="similar to AA sequence:UniProtKB:O07550"

/codon_start=1

/transl_table=11

/product="putative multidrug resistance ABC transporter

ATP-binding/permease protein YheI"

/db_xref="COG:COG1132"

/translation="MGIFKKLGWFFKQEKKSYITGIFFLILVALVQLVPPRVIGVVVD

EIAAGKMTGASLSRWLLILVAAGIGQYTFRYIWRLNIWGGAAKLEQTLRNNLFKHFTQ

MDHSFFQKYRTGDLMAHATNDLSAIQMVAGGGILTLADSMITGGATIIAMAIFIDWRL

TLIALIPLPLLAVASRVLGSKLHERFRGAQAAFSSMNDKTQESISGIKVIKTFGEEAA

DVADFKEQTDKVVAKNKRVYLIDSLFDPAITFIMGISYVLTIIIGGSYVMSGTISIGQ

LISFISYIAMLIWPMFAIGRLFNILERGSASYNRVEELLSESSSIIENPNAVQELVNG

RLDYRIDEFHYPEDDRVALNHVHFTIKQGDTLGIVGKTGAGKTTLFKLLLREYDEYAG

SIRFGGHDIRDYSLNALLHGIGYVPQDQFLFSTTIRENIRFANPNLTEEDVINAAKLT

AIHDDILGLPNGYDTLVGERGVSLSGGQKQRISIARALITNPELLILDDALSAVDAKT

EEAILSALKAQRRDQTTIIAAHRISSVMHAEEIIVLDDAEIIERGNHQELIEIDGWYQ

DMFDRQQLEDKLEGGAN"

CDS complement(1400471..1400707)

/locus_tag="JFBMFIFI_01316"

/inference="ab initio prediction:Prodigal:002006"

/inference="similar to AA sequence:UniProtKB:P67291"

/note="UPF0154 protein SA1178"

/codon_start=1

/transl_table=11

/product="hypothetical protein"

/translation="MNMGIAILLIVLALVGGLVGGYFIARKYMMDYFKKNPPVNEDML

RMLMMQMGQKPSEKKIKQMMASMQVQTEKANKKK"

CDS complement(1400925..1402916)

/gene="tkt"

/locus_tag="JFBMFIFI_01317"

/EC_number="2.2.1.1"

/inference="ab initio prediction:Prodigal:002006"

/inference="similar to AA sequence:UniProtKB:Q5XAK5"

/codon_start=1

/transl_table=11

/product="Transketolase"

/translation="MFDTTDQLAVNTIRTLSMDGIQKANSGHPGLPMGAAPMAYTLWT

KHLKVNPKNSSWTDRDRFVLSAGHGSMLLYSMLHLSGYAVTMDDLKNFRQWDSKTPGH

PEVHHTDGVEATTGPLGQGIANAVGMAMAEAHIAATYNKEGHTIVDHFTYALCGDGDL

MEGVSAEAASMAGHLELGKLVVLYDSNDISLDGPTSKAFTENVGKRFEAYNWQHILVK

DGNDLAEIEAAIEAAKAETKKPTLIEVKTVIGFGAPNAGTSKVHGAPLGVDGIEAAKK

AYGWDYPAFTVPTAVAERFEETMIQKGAEAEAAWNEKFAAYKTAYPELAAQFEKIISG

DLPSDWNSELPVYEVGQSAASRVTSSETIQALSKRLPGFWGGSADLSASNNTMVAGEK

DFEPGQYEGRNIWYGVREFAMAAAMNGIALHGGSRIYGGTFFVFTDYLRAAIRLAAIS

KAPVTYVFTHDSIAVGEDGPTHEPVEQLSSLRGMPNLSVIRPADGNEVVAAWEQAITS

TDHPTILVLTRQNLPVLPGTKENARKNVAKGAYVISPQAGEKPAGILIATGSEVALAI

EAQKALQAEGQDVSVVSMPSFDLFAKQDKAYQESVLPNDVRKRVSIEMGATFGWERFV

GLDGAMIGIDKYGASAPGDIVIEKYGFTVENVVQTFKNL"

CDS complement(1403415..1403654)

/locus_tag="JFBMFIFI_01318"

/inference="ab initio prediction:Prodigal:002006"

/inference="similar to AA sequence:UniProtKB:Q97PW7"

/note="UPF0291 protein SP_1473"

/codon_start=1

/transl_table=11

/product="hypothetical protein"

/translation="MLEKNKMSRINELANKAKKEGLTITEQAEQKQLREEYLTAFRGG

MRHHIEGMKVVDDEGNDVTPEKLKDIQKEKGLHGR"

CDS 1403824..1404447

/gene="lexA"

/locus_tag="JFBMFIFI_01319"

/EC_number="3.4.21.88"

/inference="ab initio prediction:Prodigal:002006"

/inference="similar to AA sequence:UniProtKB:P31080"

/codon_start=1

/transl_table=11

/product="LexA repressor"

/db_xref="COG:COG1974"

/translation="MRKTPESRQIEVLQYIHEQVQLKGYPPTVREIGTAVSLSSTSTV

HGHLSRLEKNGYIQRDPSKPRAIELTSLGLSKLGVPSDKIPLLGTVTAGEPILAVEEA

IDYFPIPPHLSYDPESLFMLKIRGESMINAGILDGDDVIVRKQTNADNGDIVIAMTNE

DEATCKRFYKEKTHYRLQPENDALEPIILNEVSILGKVVGLYRSHIS"

CDS 1404825..1405118

/locus_tag="JFBMFIFI_01320"

/inference="ab initio prediction:Prodigal:002006"

/codon_start=1

/transl_table=11

/product="hypothetical protein"

/translation="MVAFMFYWFSLLVQVAWIVLSVGLCAMYLNNKENGNLWAFSALN

ILSMIYSLVVIWIYNTWDFGVTTSSWFIIIVGILAILTVLEFILGREPKTKTA"

CDS 1405304..1406200

/locus_tag="JFBMFIFI_01321"

/inference="ab initio prediction:Prodigal:002006"

/codon_start=1

/transl_table=11

/product="hypothetical protein"

/translation="MKNVKVVLVFLFLLNGSLFPAFVNADEAKNDFITVEKSTVIANK

NGEAKVSGTTKKKTKVNYGKFKSEKSDSKGKFTLTLKNKTKEKKAFTLKVKINGETYS

KKVSVKPYSKKEAEQKKLEAEAKKIKKEKLAKELKTATNAVKLAERKPTRASYDKANA

LVVALTNENKELSNRLIKIMDTVEANEALAQKKAESESKEKERIANEKLEAERKIAEE

QKALEEKRIADEQARIAQEQEQQQAQTAAEVETGQGLIKGSESGIYHTPGSTYYDRTT

NVVQWFNTIEEAVAAGYRAPKR"

CDS complement(1406546..1407046)

/locus_tag="JFBMFIFI_01322"

/inference="ab initio prediction:Prodigal:002006"

/codon_start=1

/transl_table=11

/product="hypothetical protein"

/translation="MKHDLRLSQSSKEGLLYGMIICGITAFLMTFLNIYLQFYTVNKE

VLFIILKAFPLFFIVAMLLENFVISRFANKLVEKYSATQDSFNAHILFSIFFTVVGMS

FCMTLIGDFIGHGFVLEKGIVARFLMAWPRNFGVVLFIELLIAQPLARKVMSTIHREK

SESNLA"

CDS complement(1407371..1407883)

/gene="apt"

/locus_tag="JFBMFIFI_01323"

/EC_number="2.4.2.7"

/inference="ab initio prediction:Prodigal:002006"

/inference="similar to AA sequence:UniProtKB:P63544"

/codon_start=1

/transl_table=11

/product="Adenine phosphoribosyltransferase"

/db_xref="COG:COG0503"

/translation="MDLKKYIADVPDFPEKGIIFRDISPLMADGDAYRYATNQITQYA

KDKGVEMIVGPEARGFIVGCPVAYELGIGFAPVRKKGKLPRETIEVSYGLEYGSDILQ

VHKDAIKPGQKVLVCDDLLATGGTIAATIELIEKLGGVVVGTAFFIELTDLNGRDKIK

GYDIFNLMEY"

CDS complement(1407901..1410231)

/locus_tag="JFBMFIFI_01324"

/inference="ab initio prediction:Prodigal:002006"

/codon_start=1

/transl_table=11

/product="hypothetical protein"

/translation="MLKSRMNWKLINQVVEHNKITELSESLSSSPLFAGLLLNRGLDT

KEKAEAFLAPDERWIHDPFLLVDMDKVVERITTAIEAGEKIVVYGDYDADGVTSTSVL

KETLEMLGAEVDFYIPNRFTDGYGPNVRVFEQLIEEGAGLILTCDNGVSGHEAIQKAN

ELGIDVIVTDHHELPETLPEAYGIIHPRHPAGAYPFGDLAGVGVAFKLATALLGEFPV

ELLDLVAIGTIADLVSLTGENRALVKQGLAVLKSSERIGLSALIKAAGVTQETVTEET

VGFALAPRLNAVGRLGEAKPAVELLTTFDEEEADSLATFINKKNEERQAYVAEITEEA

FQMIADLTEEPSVYILARQNWHEGVLGIVASKVVEKTGKPALVLNIDETGSYVKGSGR

SISSYHLYESINEVRGLTTSFGGHHMAVGITLPMENIELLQEQLNLFWERQNNTSVAG

IELSIDEKITLSEITIATIDEIDRLAPFGTGNPKPSFLIEHVSGQDIRKIGGNNAHLK

MKAVDKETSLDVIGFQFGPVADEMNKDAEVSLVGKLSVNEWNGNKKPQLMLEDIAVEG

LQIFDMRGTHISPTIWQEDACFVFFNKKIQEKQQTNIPAGSQVEFIENLAEASAVTTT

KEKIVFVDCPNQTELVTEILRNTQAEKIIACFYSGDDSYLNGMPSRQQFGQLFKFAAS

HRDVDIRHKLKVLSDYLKIKENNLIFMINVFFEVGFVTIENGIMNFVENAPKSELSEA

VVYKNRLEKIEAEKLLVYSHFAELETWLKAQLTNED"

CDS 1410384..1410821

/gene="elaA"

/locus_tag="JFBMFIFI_01325"

/inference="ab initio prediction:Prodigal:002006"

/inference="similar to AA sequence:UniProtKB:P0AEH3"

/codon_start=1

/transl_table=11

/product="Protein ElaA"

/db_xref="COG:COG2153"

/translation="MTWKVKNFNELTTTELFYIYQQRIKIFVVEQNCPYQDIDELDLT

SLHVFKQSESGEIMAYGRIIAQDPRLHFGRIIVAQKYRKTGLGKELLSTILKTIEEKF

PNREIEIQAQAYLEKFYGSFGFLPISEIYLEDDIPHIDMLRQV"

CDS complement(1410891..1411529)

/gene="satP"

/locus_tag="JFBMFIFI_01326"

/inference="ab initio prediction:Prodigal:002006"

/inference="similar to AA sequence:UniProtKB:P0AC98"

/codon_start=1

/transl_table=11

/product="Succinate-acetate/proton symporter SatP"

/db_xref="COG:COG1584"

/translation="MEKKYYEVKTVTIDPTAIGLFGLAIVTLVASSQKLGWTEGTSGV

IPWAIFLGGVAQLIASAYDAKHNNLFGATAFAAYGLFWLGTGMSWMIQSGLFGTALQE
SFDVKDLGFAFIGYFIFTVFMTIGALETNKVLFIIFFLIDFLFLGLFMSTLGIAPEFF
HMMAAIAEFLIAVFSFYGCGAAVLNGHFGYTFLPIGKPFGIITKPKSTKKKK"

CDS complement(1411780..1412172)
/locus_tag="JFBMFIFI_01327"
/inference="ab initio prediction:Prodigal:002006"
/codon_start=1
/transl_table=11
/product="hypothetical protein"
/translation="MKNQWRLVVGIILVLIIVLFAIFNVDAVPVNFGFIKVDWPLIMI
ILGSLFIGAIATVLVSTSSSIQVKKELKKVKKELDDKDNQTKEQLSKVQAEYEQELLE
KEVEIEAKQKKINSLEDELVSKMTNPIV"

CDS complement(1412500..1413303)
/locus_tag="JFBMFIFI_01328"
/EC_number="1.-.-.-"
/inference="ab initio prediction:Prodigal:002006"
/inference="similar to AA sequence:UniProtKB:Q7A3L9"
/codon_start=1
/transl_table=11
/product="putative oxidoreductase"
/translation="MLDNRRDLTDKVVVITGGSGGLGEQIAYACARQNARVVVCARRI
DKLGKVKQNCEAISGKEAYAFQLDIANPEIIKEVVERIYDEVGTVDVLVNNAGFGFFE
EALKFDMDLAEKMFRVNVLGMMYMTQLVALQMAERKVGHIINVASQAGKMATPKSSVY
SATKFAVLGYSNALRLELKPLGIYVTTVNPGPIATNFFDIADKEGGYLDKVDRLVLDS
EVVANRIVGVMGIPRRELNLPVLLEVASKFYTLFPHIGDYIAGNVFNNK"

CDS complement(1413333..1414271)
/gene="rnz"
/locus_tag="JFBMFIFI_01329"
/EC_number="3.1.26.11"
/inference="ab initio prediction:Prodigal:002006"
/inference="similar to AA sequence:UniProtKB:P54548"
/codon_start=1
/transl_table=11

/product="Ribonuclease Z"

/db_xref="COG:COG1234"

/translation="MELLFLGTGAGVPAKHRNVTSIALKLLEERNSIWLFDCGEGTQQ

QILHTTLKPRKIEKIFITHLHGDHIFGLPGLLSSRSFQGGDEPLVVYGPKGIREYVLT

SLKVSGTHLKYALSFHEITEEGVLFQDQKFKVTCLELDHGIQSFGYRIEESDHQGMLQ

VEALKAVGVPFGPMYGRIKNGETVTLENGQVIDGRDFVGEDQKGRIVTILGDTRKHPN

SVLLAQNADVLVHESTFDGEDRKIARDYHHSTSLDAAEVAKEANAKRLLLTHISARYL

GKEAYALEKEAQSIFKKSHIVKDFEEIEIQLVRTDK"

CDS complement(1414381..1416255)

/gene="oatA"

/locus_tag="JFBMFIFI_01330"

/EC_number="2.3.1.-"

/inference="ab initio prediction:Prodigal:002006"

/inference="similar to AA sequence:UniProtKB:Q7A3D6"

/codon_start=1

/transl_table=11

/product="O-acetyltransferase OatA"

/translation="MEKAKRLKSSRYMSGLDGLRALAVIGVIFYHLTPHILPGGFLGV

PIFFVLSGYLISDLLIQEFEQNGKISLKQFWKRRLNRLYPALVTMLILVTGWITIFER

SLLLNIRNMILTSLVYLNNWWQISQGASYFDRFTTPSPFVHLWSLAIEGQFYLIWPVI

VVFAIVFIKRNDWIFYTFIGVSILSALLMAILYVPGADPSRVYYGTDTRAFSLLIGSA

LAFIWPSTKLKKNLPNAGRVTLDAVGVLSLGLLIWLMVQLMDSDPFTYYGGIFIFSLI

SGILVAVVAHPATWLAKLLSLQPLKWIGQRSYGLYLWQYPVMILYEVKVGDTSLHPLR

HLIIQITLIILISEASYRFVEMPFKKLNIREVLNWNVIVDHGKSLWRNQTITGKMHKV

GIVLLSLILIISLVGFTVAPVGDSPGKIRLAAQLKANQTKIDKQKQDLIEQKNKEVEE

SAVREPLFGLTPEQMEEAGELKVTLIGDSVMLSVVNPVEQLFPKAVIDGVIGRQLYQT

VPVVESLKQEDKLGNPVVIALGTNGAFTDKQMDDLLAAVGDSKKVYLVNTQVPKNWKN

SVNQGISSAAKRHSNVEVIDWNSYSQNHSDWFYEDGVHPNETGAEEYTKLIATSVLKN

"

CDS complement(1416476..1417789)

/gene="obg"

/locus_tag="JFBMFIFI_01331"

/EC_number="3.6.5.-"

/inference="ab initio prediction:Prodigal:002006"

/inference="similar to AA sequence:UniProtKB:P20964"

/codon_start=1

/transl_table=11

/product="GTPase Obg"

/db_xref="COG:COG0536"

/translation="MSMFLDQVTINVKAGSGGNGMVAFRREKYVPDGGPAGGDGGDGG

DIVFVVDEGLRTLLDFRFTRHFKAQEGEKGMSKGMHGRGAGDTIIKVPPGTTIKDTDS

GMVLGDLVYHDQELVVAKGGRGGRGNIRFASARNPAPEIAENGEPGQERNLELELKVL

ADVGLVGFPSVGKSTILSIVSKARPKIGAYHFTTLVPNLGMVQAEDGRSFVMADLPGL

IEGASQGIGLGTQFLRHIERTRVILHVIDMSGSEGRDPFDDYLAINKELETYNLRLME

RPQLIVANKMDMPEAEENLKIFREKLNAGKTDEFTDDIPVFPISAISHKGMNNVLSAT

ADVLDVTSEFPLYELVDEDETVLYKHTPEEKGFEITRDPDAAWVLSGDKLEKTFKMTN

FEHDESIMRFARQLRAMGVDEEMRARGAKDGDIVRIMKYEFEFVE"

CDS complement(1418155..1418913)

/locus_tag="JFBMFIFI_01332"

/EC_number="3.5.1.-"

/inference="ab initio prediction:Prodigal:002006"

/inference="similar to AA sequence:UniProtKB:P59745"

/codon_start=1

/transl_table=11

/product="Carbohydrate deacetylase"

/db_xref="COG:COG3394"

/translation="MKLIVNADDFGLSKGVNYGIIEAHRSGIVTSTTLMVNMPGFSSA

VALAKENPSLGVGIHLVLTAGQSIAENVSSLTDQKTNNFYKKEDYSEKVLVTEVEKEW

EAQIQQFKQTGLSMTHIDSHHHVHMNPSLLPLVLKLAEKYQVPLRQSHHDLNTGAKIE

ELTPTVRHTTNFDAGFYASEATVEKLNQLLQSYQAVESLEIATHPAFIDNDLLALSSY

TKKRLDELDILTSNEVKNKIASLGIERINYSQLT"

CDS complement(1418969..1419658)

/locus_tag="JFBMFIFI_01333"

/inference="ab initio prediction:Prodigal:002006"

/codon_start=1

/transl_table=11

/product="hypothetical protein"

/translation="MKSSTLLGVIVLGTIGLGLAATAVASFADLGGSSPESQVNVSSS

KSSSEASTTKNNDSQNKNSKTNSEEVSSSEEKESSSSLASNSSEVDSSNKESTSSLDS

SSSEKNEPTKEDSSSSIADVSNGNTDLEDSTSSESNLNSAVSEQNPATQAEQEATIET

PAVNPPAADANNSENKVMDEVTYVTDVFPQTGEQKSQLPLIMGIILVILVILFLVFMR

KKNKDEQNKKN"

CDS complement(1419893..1420984)

/locus_tag="JFBMFIFI_01334"

/inference="ab initio prediction:Prodigal:002006"

/codon_start=1

/transl_table=11

/product="hypothetical protein"

/translation="MGKLGVSIYPERSTFEKDQAYLDLAHKHGYTRVFTSLLEIDGDA

DQVLGSFNKVVAYANSLGMEVMVDINPGLFTQLNISYDDLSFFHKMGAYGIRLDIGFT

GQEEARMTRNPYGIKIEVNMSSGTKYVDSIMAFSPNRENLLGSHNFYPHRYSGLEYNH

FVYCSNQYRNHNLNTAAFVNSHEATFGPWPTQDGLCSLEDHRDLEIATQVKHLVLTDL

IDDIIIGNAYASEAEIIAMAEAFNADYPSVKVDLDQEISDVERKVVLDELHSYRGDRS

AYILRSTMTRIKYKNEEFPPHNTIDIKRGDLLVDNVDFGQYKGEMQIALKEMENIGRV

NVVGRISDDELFLLDFLKPWSNFKLIENK"

CDS 1421331..1422101

/locus_tag="JFBMFIFI_01335"

/inference="ab initio prediction:Prodigal:002006"

/codon_start=1

/transl_table=11

/product="hypothetical protein"

/translation="MKNKKLILGILLLIVAIVAIIFGINYVNKDKKDDVKTENSSSSE

QIESSSEAEAPVDFSKLTLPQLSNEVTENEAVVEIVTTMGTIKAKLFPEFAPKAVENF

ITHSKDGYYDGTIFHRVIEDFMIQGGDPDGTGMGGESIWGKPFGVEISPELYHIRGAL

AMAKTNQPISIGSQFYVVQNPADMSAGLSSSTTPEKIIEAYKNGGYPSLDGGYTVFGQ

VIEGMDVVDKIAAVEVGQSDKPKEDVKIEKINVIQEAK"

CDS 1422171..1422734

/locus_tag="JFBMFIFI_01336"

/inference="ab initio prediction:Prodigal:002006"

/codon_start=1

/transl_table=11

/product="hypothetical protein"

/translation="MAEEAELKAIIQNDQEMLRILKIVKSLNLPDWWICAGFIRNKIW

DVLHGYPSDTPLNDIDIIFFDPVDTSIETEENLNEKLHVLDSTMPWSVKNQARMHHKA

DFPPFKSSQDGIAHFPELPTSIGVKLTQDGQLLIAAPWGIKELMNLIVSPTPYYQLNS

QHYSIYQERLKEKDWQKTWPLLKFRDC"

CDS complement(1422800..1423060)

/locus_tag="JFBMFIFI_01337"

/inference="ab initio prediction:Prodigal:002006"

/codon_start=1

/transl_table=11

/product="hypothetical protein"

/translation="MECDKKILNRLKRSEGQMRGILKMMEDGKECKEIIVQLSAVRSS

IDKVMGLMVAENLIQSIEYENGTNPEKDEAVRDAVELIVKTM"

CDS complement(1423050..1423355)

/locus_tag="JFBMFIFI_01338"

/EC_number="2.8.1.-"

/inference="ab initio prediction:Prodigal:002006"

/inference="similar to AA sequence:UniProtKB:D3RPB9"

/codon_start=1

/transl_table=11

/product="Sulfurtransferase"

/db_xref="COG:COG0607"

/translation="MFETISMPEFEQLIKKKSASIIDVREAHEYEQGHIADVLFIPMQ

SIPEKLDTLDYDENYYVVCHSGVRSNAVCQYLAQEGYHVTNVMGGMSAWKGESVYGM"

CDS complement(1423358..1425007)

/gene="cdr_2"

/locus_tag="JFBMFIFI_01339"

/EC_number="1.8.1.14"

/inference="ab initio prediction:Prodigal:002006"

/inference="protein motif:HAMAP:MF_01608"

/codon_start=1

/transl_table=11

/product="Coenzyme A disulfide reductase"

/translation="MKIVIIGGVAGGMSAATRLRRLNEQAEIIVLEKGPYVSFANCGL

PYYVAGEIENRSDLLVQTPEQLKARFRLDVRPNSEAIKIDTVGKVIVVASESDTYTLA

YDKLILSPGAKPFIPPIKGLEMAKNSFTLRNVPDVDEVMTYIESHNPKKAVIIGAGFI

GLEMAESLVNRGIHVTIVEKAPHVLPTIDEEMAAFITKELSKNQINVITGTSVIGIEN

EGRSVIIENGAKIETDFILLSVGVQPENRLAVDSGIQTGLRGGIVVDDNYQTSAKDVY

AVGDAILVKQYSTQKDTLISLASPANRQGRQVADILSGLKRKNLGSLGTAIVRVFDQT

IASTGLTEQQLKSEEKNYQVVHIQGKNHAGYYPNASMILLKLLFNPISGEIYGAQAVG

KEGVDKRIDILATAIKGNLTVEDLPELEFTYAPPFGSAKDPVNMAGYAALNSMEGITE

SIQWHELQSYLEKGALLLDVRGEKEIAANGYFPNALTIPLDSLRGRMHELPQDKEFIV

SCQSGLRSYIAERILKQNGFRVKNLDGAFSLYSTVRPEEVK"

CDS complement(1425010..1425315)

/gene="glpE_1"

/locus_tag="JFBMFIFI_01340"

/EC_number="2.8.1.1"

/inference="ab initio prediction:Prodigal:002006"

/inference="similar to AA sequence:UniProtKB:P0A6V5"

/codon_start=1

/transl_table=11

/product="Thiosulfate sulfurtransferase GlpE"

/db_xref="COG:COG0607"

/translation="MFLFKKIPSISIKELERKLTEKIVLIDVREPNEFRNGHILSAKN

IPLNKIGNYKPKTEVYVICQSGMRSKAATKKLINAGYDAINVKGGMLAWNGSIKGGN"

CDS complement(1425429..1426169)

/gene="COQ3"

/locus_tag="JFBMFIFI_01341"

/EC_number="2.1.1.222"

/inference="ab initio prediction:Prodigal:002006"

/inference="protein motif:HAMAP:MF_00472"

/codon_start=1

/transl_table=11

/product="Ubiquinone biosynthesis O-methyltransferase,

mitochondrial"

/translation="MKENKYDNPKFFEQYSQMSRSVEGLKGAGEWHELKKMLPDFKGK
RVLDLGCGFGWHCIYAAEHGASSVIGIDLSERMLAEAKKKTSFPQIQYIQQGIEEYEY
PKDSFDVVISSLAFHYIESFEQICKNVRQCLVSGGDFVFSVEHPIFTAQGMQDWSYDE
AGNRLHWPVDRYFDEDQRSSIFLDEEVIKYHKTLTSYLNTLLKNNFEITSIVEPEPAE
ELLDIPYMQDELRRPMMLLIAAKKKNSV"

CDS complement(1426303..1427433)

/locus_tag="JFBMFIFI_01342"

/inference="ab initio prediction:Prodigal:002006"

/codon_start=1

/transl_table=11

/product="hypothetical protein"

/translation="MILFIWIMFGTIFMLGIINGITPYFSRRSSQFGVSIPVEYQDDP
FIKKQQRFFLSMNVSLSFILGLPILYAMRLDVSEQIAQFISFYLLFALVAFMGIVGLL
FLKIRKNIIEFKSTIPKEIVADESHDIVVDTTYRQRRLIVPSYFIIISNLIIILAVVG
ITLINYEQMPDRIITNWDINMKPSVTVAKSYTSALALPVMQLFMMGTMYVANLSFQKA
KQKVDPRNPAVSIEKNRAFRYAWSIYGLVMSILLQLLLGGLQLIMIFDWMNKVNFPLM
IIIFVVLTIGGTLILSLKYGQGGERLKLKGRKGDKIETVAGYDDDISWKLGVIYFNSK
DPSIWVEKRFGVGNTMNMARWQSWAFLIAILAIPLLIGILFN"

CDS complement(1427430..1427819)

/gene="ytrA_1"

/locus_tag="JFBMFIFI_01343"

/inference="ab initio prediction:Prodigal:002006"

/inference="similar to AA sequence:UniProtKB:O34712"

/codon_start=1

/transl_table=11

/product="HTH-type transcriptional repressor YtrA"

/db_xref="COG:COG1725"

/translation="MFIRIQPESQTPIYTQLIYQIKRGILKKELLPGEGLPSVRSLAG
DIGINMHTVNKAYKLLVEEGVLVQQKKGFMVNPTMKIKMQEKELALYRERLEELIIDT
HIFSISEEEIEKMRESIEKMLKESEQI"

CDS complement(1427835..1428620)

/gene="yxlG"

/locus_tag="JFBMFIFI_01344"

/inference="ab initio prediction:Prodigal:002006"

/inference="similar to AA sequence:UniProtKB:P94375"

/codon_start=1

/transl_table=11

/product="putative transmembrane protein YxlG"

/translation="MKGLLVLLKKEYQQSYKEFKLFWLPIVFLFLGISQPITLYFLPE

ILKKLGGTEGISIIPIQQSGSEVLSATLSSQFDQLGMMVVVVATMGTILSEKNNGMLD

FILTKPVSEWNYIISKWLANFSIVALGIILGYYASIYYTMIYYSAVPLTTSFWALICF

LLWVGWIISLILFCSTFFKSQGMVALITLGVFFLLKLVASFDLAINIINPAMLSQQAI

NFLVTEKWNATVTGSLIFLISTAAILIYSSVIWIKHRKYTIHA"

CDS complement(1428617..1429522)

/gene="yxlF"

/locus_tag="JFBMFIFI_01345"

/EC_number="3.6.3.-"

/inference="ab initio prediction:Prodigal:002006"

/inference="similar to AA sequence:UniProtKB:P94374"

/codon_start=1

/transl_table=11

/product="putative ABC transporter ATP-binding protein

YxlF"

/db_xref="COG:COG1131"

/translation="MEISIENISKKFKQKKAVVDFSLKIQANECVALIGPNGAGKTTL

LKIIVGILEASEGTIYLNDQKSTTQKKKIGYLPQTPQFFQWMTAEETLFFMGSLSNMS

AIELDREIPLVLKKVGLHDSGKIKISQFSGGMKQRLGIAQAILHKPSFLVMDEPVSAL

DPIGRREVIAILNEIKKETAIIFSTHILNDASEICDRFCLIKKGHKVLDTTLDELYDA

NSKNQFVVKYKGDGSRWLEHVRQLSFVIEVSEKKQEAIISVTDSAQNKIKILSSVVEV

NLDVISFEVRQESIEDIFFKEVLAE"

CDS complement(1429512..1429736)

/locus_tag="JFBMFIFI_01346"

/inference="ab initio prediction:Prodigal:002006"

/codon_start=1

/transl_table=11

/product="hypothetical protein"

/translation="MNTDFSQISQIDLRPFIPIIVLATLLSVVALIDLIRYRKERKNF

WIWFIIILFVNTIGSILYFIFGRKDKYYGN"

CDS 1430029..1431108

/gene="recA_1"

/locus_tag="JFBMFIFI_01347"

/inference="ab initio prediction:Prodigal:002006"

/inference="similar to AA sequence:UniProtKB:P16971"

/codon_start=1

/transl_table=11

/product="Protein RecA"

/db_xref="COG:COG0468"

/translation="MAKEKMAPRDISQKMGDGKQAALSKALSKIEKDFGKGAIMKLGE

KTSMKIETISSGSIALDVALGVGGYPKGRILEVYGPESSGKTTMALHAIAEAQKAGGT

AAFIDAEHALDPKYAAALGVNIDDLLLSQPDTGEQALEIADALVSSGAVDILVVDSVA

ALVPRAEIEGEMGQSHVGLQARLMSQALRKLSGSINNTKTICIFINQIREKVGVMFGN

PEVTPGGRALKFYATIRLEVRRAEQIKTGTDILGNRTKLKVVKNKVAPPFKIAEVDIM

YGEGISRAGELVDMASDSDIIDKAGAWYSYNGERIGQGRENAKKYLLDNPELYAEIEK

KVRDNYFAKEVAVEPEKTEAPIEKE"

CDS 1431268..1431801

/locus_tag="JFBMFIFI_01348"

/inference="ab initio prediction:Prodigal:002006"

/codon_start=1

/transl_table=11

/product="hypothetical protein"

/translation="MKLNKSLEQAICIITLLATQDKAIPVTSSVINHRLNGSPTYIKK

LMRKLVVNNLVTSVSGNNGGFTLVHSPDDISILQIVEAVEGKVSTYPNSGLIDVVFQD

MQVAANQGEVVLAETFEQADSLWSQYLAKQSVADLLYKTLGRNAIPVLNWNELDEHKS

ILKKVFNSVSSAEVDEK"

CDS complement(1431928..1432410)

/locus_tag="JFBMFIFI_01349"

/inference="ab initio prediction:Prodigal:002006"

/codon_start=1

/transl_table=11

/product="hypothetical protein"

/translation="MKKIQIVGFVCISLMVLGGCGTKNEDVKKESTSEMSAISSEEKK

DKNSKNETNSESSSEVSSEVSSSIVEEESSSEVVVEEVETKITDAKSATEYLLKQLNM

GPDIQGSGEENSVTELEKDDKGDFYTIRLVSLELKNNGGSGTIGRYKVYLDGNYELLN

"

CDS 1432885..1433637

/gene="lacR_1"

/locus_tag="JFBMFIFI_01350"

/inference="ab initio prediction:Prodigal:002006"

/inference="similar to AA sequence:UniProtKB:P67744"

/codon_start=1

/transl_table=11

/product="Lactose phosphotransferase system repressor"

/translation="MLTEERHAFIIKKLTESDIVKSQDLMMELNCSESTIRRDLALLE

ESGELIRVHGGAKRNYHLDEELDIVEKSAKNIVQKKAIAQLAASLVHNNDTIYIDAGS

TTIEMIPFLTQKNITVVTNGVQHASLLADYKINTILIGGQLKNTTKAIIGSTSIAELA

RYQFDKTFLGMNGVHPKYGYTTPDPEEASLKSMALKQGFETFILLDKSKFDKVNFTQV

ASIESATILTETLNQIDYQSYFTKTKIIEVKI"

CDS 1433634..1434548

/gene="lacC_1"

/locus_tag="JFBMFIFI_01351"

/EC_number="2.7.1.144"

/inference="ab initio prediction:Prodigal:002006"

/inference="similar to AA sequence:UniProtKB:Q833W9"

/codon_start=1

/transl_table=11

/product="Tagatose-6-phosphate kinase"

/db_xref="COG:COG1105"

/translation="MIYTVTLNPSIDYIVHLEKIELGELNRMDSDLKLPGGKGINVSR

VLHQLEHDTTTLGFIGGFTGHFISDWLVKEGVKTNFISIQDDTRINIKLKAISETEVN

GLGPTITSKEASALLTQLDQLTSDDVVILSGSKPPSLPDDYYHKMIELITKSGAEFVI

DTTGTDLMNALPYHPLLVKPNHHELAELFNVTFSSVEEMLPFGKKLIELGAQYALISM

AGDGALFFTPDAIYTSTVPKGTVKNSVGAGDSMIAGFIGTYVATNDPIKAFQMGVATG
SATAFSDDLATKENILALVNDVKITQQY"

CDS 1434569..1436458

/gene="fruA_1"

/locus_tag="JFBMFIFI_01352"

/inference="ab initio prediction:Prodigal:002006"

/inference="similar to AA sequence:UniProtKB:P71012"

/codon_start=1

/transl_table=11

/product="PTS system fructose-specific EIIABC component"

/db_xref="COG:COG1299"

/translation="MNIKDLLVKDVMIMDLKATTKEAAIDEMIAKLAASGRISDAEIY
KEGIMKREAQTSTGLGDGIAMPHAKNKAVNQATVLFAKSAQGVDYDSLDGQPAFLFFM
IAAPEGANDTHLQALAGLSKLLINPDFVASLKEATSPDEVEELVAQAEIAYAEAEKAE
LEANKNSVPSEDKPFVVAVTACPTGIAHTYMAEDALKKKAAEMGVDIRVETNGSEGIK
NKLTDDEIARAAGVIVAADKNVEMNRFAGKAVLERPVSDGIRKSEELIRKAVASDAPI
YHASGEKSSETTEKSGGSIWSKIYKDLMNGVSHMLPFVVGGGILMAISFLVENTMGDK
SQAFQFLNSIGGNAFSFLIPILAGFIAMSIGDRPGLMPGMVGGFMAVESGAGFLGGLA
AGFIAGYIILGLRKLFSGLPKSLDGIKPILLYPVFGLLIIGAVMFFIIDPIFGSINTA
MLNFLENLGTGNAVLLGALLGGMMAFDMGGPFNKAAYTFSIGVFTDTGDGKLMAAVMA
GGMVPPLAIALASTIFKNKFTADERKSGLTNYILGLSFITEGAIPFAATDPIRVIGSS
VIGAVIAGGLTQFWGISIPAPHGGIFVITLANHALLFLASLAIGTVISALILGIWRKP
VDLID"

CDS complement(1436678..1437286)

/gene="betI"

/locus_tag="JFBMFIFI_01353"

/inference="ab initio prediction:Prodigal:002006"

/inference="protein motif:HAMAP:MF_00768"

/codon_start=1

/transl_table=11

/product="HTH-type transcriptional regulator BetI"

/translation="MKEEREQRILDAAMSEFATHSYYEAKTDRIAEIAKVSKGLIFHY
FGSKEKLYLQTVQAALTILINAVDLDQAPKKDLVQLVVWSTTKKIFLSQEYPLAFRLV

LQSYGNAPKKLQSQLQEFYQEQQKKSYEVIEHVLNQMTLKPEVSKEVAVSLIQSVFEN

IQIEAQRYLKANPEATATDFEFLFTRTKEMLYFIQYGMCEVN"

CDS complement(1437283..1438098)

/locus_tag="JFBMFIFI_01354"

/inference="ab initio prediction:Prodigal:002006"

/codon_start=1

/transl_table=11

/product="hypothetical protein"

/translation="MTIFKIEWKTQYKSVLAWCGVIVLILSLFMAVFPTMQSAGMTDI

VNTKLSAMPQDVLKIFNLNDGSSLLEVMGYFAYVFQYVFIASAIYAVLIGAQALIKEE

SDGTIEFLYAQPMTRKELVGFKMVANLAILATFWVITFIFAAIIILIFRNETDKLGDI

FFKLIQLFLNDGLILVALLGIGFFFSTIIKSSKQATGLALGFVFGTYVIGILSELNDK

VSFLKYISLLHYGVPANLLKEWMGWTYLIILIVVITVFSLLSIFMYQKKDLKI"

CDS complement(1438095..1438982)

/gene="btuD_2"

/locus_tag="JFBMFIFI_01355"

/EC_number="7.6.2.8"

/inference="ab initio prediction:Prodigal:002006"

/inference="protein motif:HAMAP:MF_01005"

/codon_start=1

/transl_table=11

/product="Vitamin B12 import ATP-binding protein BtuD"

/translation="MKAIEINGLTKMYKDKAAVKNVHLEVNEGEIFGFIGPNGAGKST

TIKALLNFIYPTSGSAKILGLDSVKDSKAIKREVSYVSSDVRFYPAMTASEIIGYAAE

FHQIQDAEAKIKHYYDRFEIESNKKLGDMSLGNKKKVAIVAGLIAEPKLMILDEPSSG

LDPLMQHRLFEILTEKNKSGMTVFLSSHDLMEVQNYCSKAAFIKDGELIRIEDIDKDK

ADGKVVQLRGKNLIGTEFIKKGATIIESTASSLKFIYNGDLQLILPLFVNSSITDIII

KNQDLEDKFMAMYEGGKKA"

CDS 1439174..1440298

/gene="vioD"

/locus_tag="JFBMFIFI_01356"

/EC_number="4.2.1.145"

/inference="ab initio prediction:Prodigal:002006"

/inference="similar to AA sequence:UniProtKB:Q84CG1"

/codon_start=1

/transl_table=11

/product="Capreomycidine synthase"

/translation="MKIAHFGVEEWLNEREKSAIYDLAGSSIASFTLEEIISIDGTQP

QDFFSELLPKKMNYGWIEGSPEFKNEVSKLYKNMPVDNILQTNGATGANLLALYALVE

PNDHVIAMHPSYQQLYDIPKSLGADVSFWEIKAENAWQPQLADLKKLIRTDTKLICLN

NANNPTGTLLSEEFLQKIIEIAKSVDAYILVDEVYKPLDDTHSTPSIVDLYEKGIATN

SLSKTYSVPGIRIGWTASNHETANLFRKYRDYTMICAGVIDDALAVHTLKNKKLVLNR

NKKIVSTNLAILEKWVANEPLVSVILPKSVSTSFIKLDIPLNSEEFCIQLLEETGVLL

VPGSRFDLEQHARLGYCTHTETLLTGLEKLSHFLTRYTEK"

CDS complement(1440349..1440699)

/gene="adhR_3"

/locus_tag="JFBMFIFI_01357"

/inference="ab initio prediction:Prodigal:002006"

/inference="similar to AA sequence:UniProtKB:O06008"

/codon_start=1

/transl_table=11

/product="HTH-type transcriptional regulator AdhR"

/db_xref="COG:COG0789"

/translation="MSYKINQVAQKIGVKPQIIRYYEHEGMLSNISRNPNGYRSYSEE

DISYIRFLESVKKLKRSGLPLIDLREYAKLINSENKECTVEKKALEDEYEKINTYIEN

LVEAKEYIKKKLKK"

CDS complement(1440989..1441714)

/gene="artM"

/locus_tag="JFBMFIFI_01358"

/inference="ab initio prediction:Prodigal:002006"

/inference="similar to AA sequence:UniProtKB:P54537"

/codon_start=1

/transl_table=11

/product="Arginine transport ATP-binding protein ArtM"

/db_xref="COG:COG1126"

/translation="MIKIAHLKKSFGDVEVLKDINEEVNKGEVVVIIGPSGSGKSTLL

RCLNLLEEPTSGDIIFEDTTITKVGQDKLNQLREKMGMVFQSFNLFPHMTVVENLKIA

PMKVKGLTATEAEEKAKDLLAKVGLADKADAYPASLSGGQQQRVAIARALAMDPDVML

FDEPTSALDPEMVGEVLKVMKDLAESGMTMVVVTHEMGFAKEVSDRVLFMDAGYIVEQ

GTPTEVFEHPKNERTQDFLAKVL"

CDS complement(1441707..1443182)

/gene="mltF_2"

/locus_tag="JFBMFIFI_01359"

/EC_number="4.2.2.-"

/inference="ab initio prediction:Prodigal:002006"

/inference="protein motif:HAMAP:MF_02016"

/codon_start=1

/transl_table=11

/product="Membrane-bound lytic murein transglycosylase F"

/translation="MKQKKWINLILFLCVSLSIWGGTKTVEAAETSLADVKSSGKLVI

GTSADYPPYEFHAKLDGKDQIVGMDILIAEKIAKDIGVSLVIRDMDYDSLLPALEAGK

VDMVIAGMNPTDERRKSVDFSDIYYQGGQFIIIRNEDKDIFTQKSDFSGKILGVQKGT

MQETVAKEQIEDVNMMGLTKIPDLILALTTKKIDGIVLEEPSALAYVSNDKNLAFIDG

KFDLDANEQGSAIAFRKGSESLVTAVNQSIAEIKEQNLIPQFIKKAGEQLIEGQGGNE

EGDVKPIFSFWEYFAKGTGYTIFIAFISVIIGIILGAVLALMRLSKSKISRGIAGVYV

EFVRGTPMMIQVMFIYFALGVVINIPALLAGIIAVSLNSGAYICEIIRSGLNSVSKGQ

MEAARSLGMGKVDTMRYIIFPQALKNIWPALGNEFITVIKESSIVSIIGVGDLIYQTK

VVTAITYRPVAPLAVTLIIYFILTFSLTKLLNYYEGKMNHD"

CDS complement(1443418..1443633)

/gene="copZ_1"

/locus_tag="JFBMFIFI_01360"

/inference="ab initio prediction:Prodigal:002006"

/inference="similar to AA sequence:UniProtKB:O32221"

/codon_start=1

/transl_table=11

/product="Copper chaperone CopZ"

/translation="METIKLKVTGMSCEHCVKRVEESVLTINGVEKVKVHLKKGIAKV

KYNQQLATPAKINDAVIAAGYEASIVE"

CDS complement(1443661..1444116)

/gene="copY"

/locus_tag="JFBMFIFI_01361"

/inference="ab initio prediction:Prodigal:002006"

/inference="similar to AA sequence:UniProtKB:Q47839"

/codon_start=1

/transl_table=11

/product="Transcriptional repressor CopY"

/translation="MTTAEKVKISDAEWEIMRVVWSKKQASSQEIVSVLATKMDWKPA

TIKTLIGRLVKKELLTTEADGNRFIYRANVSETESVKSATESLFAHVCSKKVGSTIGN

LIEEAQLSKSDIQMLEKLIQSKKATAVEEIVCNCVPGQCECQEHQNVTI"

CDS 1444271..1444426

/locus_tag="JFBMFIFI_01362"

/inference="ab initio prediction:Prodigal:002006"

/codon_start=1

/transl_table=11

/product="hypothetical protein"

/translation="MGLTHKTETNLDDLFSRFAVEPEPEKRKEDDDDEKDDDKKTKAK

KTEDKKK"

CDS complement(1444479..1445051)

/locus_tag="JFBMFIFI_01363"

/inference="ab initio prediction:Prodigal:002006"

/codon_start=1

/transl_table=11

/product="hypothetical protein"

/translation="MLSEKEFFKKQKIYQTSRLKLRQFKVDDADSMYTYTSNPNVAKY

CSWPVYQTIEEAQKYIQFILTCYEKQVLAPWAIEIVETGEMIGVIDFVSWSDMHENVE

VGYVIAEEHWGKGYVTEAFSKLIQIAFEEINVHRLTAKCCSVNQASAAVMKKNGLFFE

GKTRESFYKDQQFYDMDHYGMVRNDYFARI"

CDS complement(1445163..1445483)

/locus_tag="JFBMFIFI_01364"

/inference="ab initio prediction:Prodigal:002006"

/codon_start=1

/transl_table=11

/product="hypothetical protein"

/translation="MEFNDYQKLANRTLYGNEQVLTNCALGVASETGEVVDLIKKYTF

HGHDLDKKALTKELGDVLWYLSQIAEWADIPFENVATQNIEKLEKRYPNGFSKEASQN

RVDD"

CDS complement(1445511..1446806)

/gene="hemL1_1"

/locus_tag="JFBMFIFI_01365"

/EC_number="5.4.3.8"

/inference="ab initio prediction:Prodigal:002006"

/inference="similar to AA sequence:UniProtKB:P99096"

/codon_start=1

/transl_table=11

/product="Glutamate-1-semialdehyde 2,1-aminomutase 1"

/translation="MRSTEHSEAIFSEAVELMPGGVSSPVRAFKSVDVPPIVMDHGKG

SRIVDVDGNSYIDYVLSWGPLILGHAEPHVVAAIQQTASLGTSFGAPTLMENKLAKLV

IERVPSIEMIRFVNSGTEATMSALRLARGYTGRDKILKLEGSYHGHDDALLVKVGSGV

ATLGLPDSPGVPKSTTSNTLVAPYNDSEAVRRIFKEYQGQIAAVIVEPVAGNMGVIPP

VPGFLETVREVTTKDGALLIFDEVMSGFRVGYYSAQGHYSVTPDLTCLGKVIGGGVPV

GAYGGRRDIMEHMAPAGSVYQAGTLSGNPLAMAAGYATLSNLYEADYTHFEKLGDALE

TGIAKLSLKYNLPITVNRAGSMIGLFFNSGPVSNYQESKASDTEFFGNYYREMANRGI

FLPPSQFEGLFLSKAHTLSDIEETLVAMDESFTALIMNK"

CDS complement(1446803..1448191)

/gene="hemA"

/locus_tag="JFBMFIFI_01366"

/EC_number="1.2.1.70"

/inference="ab initio prediction:Prodigal:002006"

/inference="similar to AA sequence:UniProtKB:P16618"

/codon_start=1

/transl_table=11

/product="Glutamyl-tRNA reductase"

/db_xref="COG:COG0373"

/translation="MKILLYGVSHQTTPIEIRERYTIKEDDVPKQLTEIKAFNGVSEV

VILTTCNRTEYYLYIDQTEFMHGDMLRYIGEYTGYDVSDVISTSYGKSNSDVATHIFK

VATGLDSLMVGETQILSQVKFALKNAQEVATAGPILSSLFNKAVSFSKRVHTHTKIDQ
LSFNPSTAAVELLKLEWENLKEKRILLLGAGKMIRLAAKSLLENGAQHITVVSRNSLK
AEAFAHDMNEWVQTNYHPEKLKRYVYSAEYENLPMALAGADGVIVATKASEYIVTTES
IIKMQAIRSGSVKELCLIDLAVPRNVDPDLNLVKGIRIFDMDQIAVQIDHFKEEREKI
IKGILADIDEAVIKFNRWYQERRAVPYIYQIRKDTMAIREKTIDSLAKKLPDLDAREM
KLIDKHMQSVVNQLIKTPIQSMKEFARMNPSKDMNDALELFVRSIGLKQSEEQTEVPI
EDLQLLEKEGDL"

CDS complement(1448325..1448912)

/gene="engB"

/locus_tag="JFBMFIFI_01367"

/inference="ab initio prediction:Prodigal:002006"

/inference="similar to AA sequence:UniProtKB:P38424"

/codon_start=1

/transl_table=11

/product="putative GTP-binding protein EngB"

/db_xref="COG:COG0218"

/translation="MDVHKADIVISAVAPAQYPSTGLPEIALAGRSNVGKSSFINRMI
NRKGLARTSSKPGKTQTLNFYIIQDQFYFVDVPGYGYAKVSKTERAKWGQMLETYLTK
RDTLKAVLSVVDLRHAPSKEDVQMYQFLKYYDLPVIVVATKADKIPRSKWNKHIKIVK
DTLEFDKNDEFVMFSSETGEGKDEAWRLIEKYLAL"

CDS complement(1449079..1449459)

/locus_tag="JFBMFIFI_01368"

/inference="ab initio prediction:Prodigal:002006"

/codon_start=1

/transl_table=11

/product="hypothetical protein"

/translation="MYISQITNLQTELNQKEAKVSDSGWIKAVTSGVTHNANQPFFYR
KIGNRVEFRGGGNFPIAGGYTFLIMPSGFYPSQEYRASATVQDSDTTKKFIFQVLTNG
TCKIIHNTVSSNIVWMDGQHYYVD"

CDS complement(1449531..1449935)

/locus_tag="JFBMFIFI_01369"

/inference="ab initio prediction:Prodigal:002006"

/codon_start=1

/transl_table=11

/product="hypothetical protein"

/translation="MAKLEEIYDGMKKAGTVIDENFKKLEIAIYDSGWVDVTGPVKGI

QVRRIGKEVKLKGTSIVNESTSATVFVLPKGLRPSDGFAYDLGFQESSTGHRCILFFN

KDGNVVVLYNVKDNAPVIFNGISWFTDEELPV"

CDS complement(1450042..1451064)

/locus_tag="JFBMFIFI_01370"

/inference="ab initio prediction:Prodigal:002006"

/codon_start=1

/transl_table=11

/product="hypothetical protein"

/translation="MEQGLFFPSKNGDRKYKASDFTGYFSHLFSNGVFSNNSENLQVL

ASKTNGLAVVVGAGYGNINGYLYQLTEAKTIVFKVADVSGTAKKGAVVLRMDLNERLM

SVETKGTTELTRTNSIYELMLAEVAIPGGGLPITQSMIKDTRGNGELCGYVSSLIDID

PTTLWLQFEADWKKWYENKQNEMNLNTEELKKYLVEAKKEFRDWFNYLKEMLDENTAT

NLQNQLNELIADKKELKATEVVLTIEHNLKEFPQISVLAWRYGIGTVPLNTQPNDINF

DGTDVSSIVAEVRYYSRNKLSVLVPKNYSMEHPTIIKLTSNKYLLIEDINSLEIRIND

NINNIK"

CDS complement(1451072..1452175)

/locus_tag="JFBMFIFI_01371"

/inference="ab initio prediction:Prodigal:002006"

/codon_start=1

/transl_table=11

/product="hypothetical protein"

/translation="MILWVLNQELERINGIEQFSSFILTKRYSVPSEFELHLPMTEEN

LQLIQDNQLSLGNILMKESELVGYLIEEMNSNFEKNGGEIVIKGRDLRVYLDRRIILG

EKAYFGTPQKLIHDWLESCVINPKDNQRKMKQFKLGDFPDSTRKMSVELKYTNLLESI

STLCQELEWGFSVEIELSTKQLVLSIYEGVDRTNGQSKNTKVLFSQDFENILTQSVIV

NNLNLKTTALVESQWEEQKYLLEVANSTSDILRREIYVDAKNIGQPTGEDKLTETQQK

ELVKQLGTKELLDFQIIQTVEADVVMNGSTRYQEDFMLGDRVTILSESLGLELDTRIE

TVEEVYEERGLEIRLSFGNQVPTLVDKIRKKVK"

CDS complement(1452178..1453122)

/locus_tag="JFBMFIFI_01372"

/inference="ab initio prediction:Prodigal:002006"

/codon_start=1

/transl_table=11

/product="hypothetical protein"

/translation="MECEIYNQVLGQKLMIKDYITETFATDGNPKGNYFLLKLDGLGE

VQADRHTMDAHGDGSIYINSNLAERELQIELVILADESKNMEELRREISKIVNPKAGL

LELRYKEEQKTYRINVASDHVPTFTTEDYLGKKRQRVTLTLVASDPFWYAENSEVHYL

SNWQPNLEWELEFPLTENKDLGIELETFLKDGLVTLENDGDEFTGAIFLLSASGDVLN

PEIIRIMPDGSSTQKMKLLTSMKAGDRIRISTISGQKRVEKWSDDRQKWLNIFNTLTM

DSTFIQLDVGENYLRSTAATHSDQLEIKVQYESRYVGV"

CDS complement(1453122..1454714)

/locus_tag="JFBMFIFI_01373"

/inference="ab initio prediction:Prodigal:002006"

/codon_start=1

/transl_table=11

/product="hypothetical protein"

/translation="MDMNLSGMSGNLNDILGTVGLIQDDFSALSGTIGQLMEPFGAVH

SAVEDIKNSAGSIGDIFSGDIGIIDGLSGAFSGVVSSAENISGALSGFSEIKGTFTDL

QEMLSVTGADFSDLGGLMSSIPKPNFSAILPLLSTLGTGAMAAAASVWALIAPFLAIA

LPIIAVVAGVALLMNHLGIFSDISKVFSGDMSLGEFFITLKDKLFGFVSGIIEAVPQI

IENGKNLIIGFIKGLSEKIPEVIQSGIEMLTNFVSTIISAVPGLIQAGITLLTNFISG

LVTIIPTILATALSLMVGFVAMIISAIPNLIAAGIQILIALIGGITSMIGNLLKIGGD

FISTIISGIATVIGQLKDKGAEIFNKLKDGIVEKMAGMLDIGKNIIQGLIDGVGAMKN

AVVNKIKEIGNSMLDGIKGLFGIKSPSRVMKGIGGYMSIGLANGIESESDKVKRATDH

LAEAAMIDPKDLKMKMSDMRSLSKPLSFSNMIDTQGLEASNSNRKMISNQQTIQFNQP

IARPSELLRKMRQANQEMGWNM"

CDS complement(1454736..1454969)

/locus_tag="JFBMFIFI_01374"

/inference="ab initio prediction:Prodigal:002006"

/codon_start=1

/transl_table=11

/product="hypothetical protein"

/translation="MQLRVIDSYEMMAKSAMANRYAHHAKQAKERKIFDKEKALRSLS

VGSHENKLNPNLISNASRALKYYEVKFIADAQV"

CDS complement(1455053..1455487)

/locus_tag="JFBMFIFI_01375"

/inference="ab initio prediction:Prodigal:002006"

/codon_start=1

/transl_table=11

/product="hypothetical protein"

/translation="MFTIDINEKAYEVKGNLRFARDIETYLSTKKDGINQLNGLALLY

MGLQSDSINALLNFLYYGIKTSERPSMDQIETALDKLLEQDGEALDVLFLKALGVMEL

SGFFAKTRKTVMENLKKEASNKELVKQMEQKRKKAISLLSKL"

CDS complement(1455567..1456091)

/locus_tag="JFBMFIFI_01376"

/inference="ab initio prediction:Prodigal:002006"

/codon_start=1

/transl_table=11

/product="hypothetical protein"

/translation="MTVQPIEGKKVVYLVQATNAAKGAKALLPGFQTEGTWTREHEAL

DEQTKSGRILGYGAKTETFELTLYSAPGDAGQEAIANAYDNEEQVKVWRVELQQNTDG

SYPARFGYSIIQSVAISDADSFAELTVTLPVIGKTQPGTLTDIPEELINLALYGFENP

GEKTGELGEEAAKE"

CDS complement(1456426..1456770)

/locus_tag="JFBMFIFI_01377"

/inference="ab initio prediction:Prodigal:002006"

/codon_start=1

/transl_table=11

/product="hypothetical protein"

/translation="MGEVYVKQQSKKLVDERLRNIPKLTAMVKLSERVIAEPALKKRL

EEAKLELETIKATISSLPDELQKDILTKRYIVQNKYETDIQVYMELNMSESYYYRLKK

DAFELLSFLWGF"

CDS complement(1457002..1458246)

/gene="clpX"

/locus_tag="JFBMFIFI_01378"

/inference="ab initio prediction:Prodigal:002006"

/inference="similar to AA sequence:UniProtKB:P50866"

/codon_start=1

/transl_table=11

/product="ATP-dependent Clp protease ATP-binding subunit

ClpX"

/db_xref="COG:COG1219"

/translation="MFNDEETKGPVNCSFCGKSQDQVKKIVAGPGVFICNECIELCKE

IIDEEFNEAGYGEFLDVPKPQEIRSILNDYVIGQDQAKKALSVAVYNHYKRVNQLNGQ

EDEVELQKSNISLIGPTGSGKTFLAQSLARILNVPFAIADATSLTEAGYVGEDVENIL

LKLMQAADYDVEKAEKGIIYIDEIDKIARKSENVSITRDVSGEGVQQALLKILEGTVA

NVPPQGGRKHPHQEFVQLDTSNILFIVGGAFDGIEEIVKNRMGEKVIGFGTNTKKIDE

SQSLMQQIIPEDLLKFGLIPEFIGRLPVMAALEKLTEEDLVHILTEPKNSLVKQYKKL

FDLDDVELVFEDQALREIAKKAIERNTGARGLRSIIESIMLEIMFDLPSRPEITKCVI

TEDTVIGSGKPELYDTEGSAVV"

CDS complement(1458502..1459614)

/gene="sigA_2"

/locus_tag="JFBMFIFI_01379"

/inference="ab initio prediction:Prodigal:002006"

/inference="similar to AA sequence:UniProtKB:P0A0J0"

/codon_start=1

/transl_table=11

/product="RNA polymerase sigma factor SigA"

/db_xref="COG:COG0568"

/translation="MSTQAKGDKKTMKKATDKFILKHKLKAMVHIDEVTKELALPNHL

TVEEIDTLIQRIEDAGVSVVGDDGGPTERQLLVPAIEKIEIKKADLAPPAGVPMDDPV

RMYLKEIGQVDLLTGAEEVTLAEQVLEGNQDARNHLAEANLRLVVSIAKRYMGRGMQF

LDLIQEGNLGLMKAVEKFDHTKGFKFSTYATWWIRQSITRSIADQARTIRIPVHMIET

INKLVRIQRQLIQDLGREATPEELGAEMDLSTERIREILKISQEPVSLETPIGEEDDS

QLGDFIEDHGAADPEEQASYELLKLQIEDALDTLTDREENVLRLRFGLDDGRIRTLEE

VGKVFGVTRERIRQIEAKALRKLRHPSRSKQLKDYL"

CDS complement(1459949..1461229)

/gene="tig"

/locus_tag="JFBMFIFI_01380"

/EC_number="5.2.1.8"

/inference="ab initio prediction:Prodigal:002006"

/inference="similar to AA sequence:UniProtKB:P80698"

/codon_start=1

/transl_table=11

/product="Trigger factor"

/db_xref="COG:COG0544"

/translation="MTAKWEKKGTNDGVLKFEIKEEKIKEGLEKAFNKVKKNLNVPGF

RKGKVSRQVFNKMYGEESLFEDALNILLPEAYTIALEESGIDPVDQPKIDVESMEKGQ

PWIITAEVTVKPEVKLGEYKNLTVEKQDREVTDEDVAAKIKEKQEAQAELVLKEDAIV

NGDTAVIDFEGFKDGVAFEGGKGENTSLEIGSNSFIPGFEEQLIGAKAGDTVEVKVTF

PEEYHSEDLKGADAVFNVTIHEVKAKELPELDDEFAKDVDEEVETLAELEAKIKAELV

EAKTAAAEEAVQEAAIRQAVDNAEIVELPHVMVHDEVHRQMDQFLNDMQRQGVSPEMY

YQLTNTTEEDLHKQMEEDADIRTRTNLVLEAIVAAEGFDPSEEEVEKEIKELAEQYNM

EEKAVRAALSVDMLKHDISMKKAIDAITETAVEK"

CDS complement(1461427..1462386)

/locus_tag="JFBMFIFI_01381"

/inference="ab initio prediction:Prodigal:002006"

/codon_start=1

/transl_table=11

/product="hypothetical protein"

/translation="MGEKIIFPKNYEQYVKKAVAAFQLGRMEESLPYFKEAYQLKQEE

KINTFYATALYQVGRYQEAKEVVEDQLAFYEKENQLYAFYVSILIRSHYFLQAEAIIK

KQLMRKGNDSEKITWQSLLGFSKEEQKNKQVSQEKKIADIGKRVLSMMDKSYQEQSDL

MKEMDTIPFQSYLKLAKLLLQNPFVSGLIKATIIERLVNEQLDEAIEITWFDQERIIR

PKELTSIEENATVRQVQQLLEEKIGLNDPTLFASVSQEAQLHFIFLYPFIEEVITSPE

TWVELYRQQYELDYIGQTEKLPHFDDLKLWQNRLNELILELMS"

CDS 1462603..1462836

/locus_tag="JFBMFIFI_01382"

/inference="ab initio prediction:Prodigal:002006"

/codon_start=1

/transl_table=11

/product="hypothetical protein"

/translation="MTSFSKINLHELLTIKDLSQLDQLTRRRLKDVGITEGSIVEIKR
RYPFGGPCMIESDGQRVGIRKHTLDFIFGDLVL"
CDS 1462833..1464836
/gene="feoB"
/locus_tag="JFBMFIFI_01383"
/inference="ab initio prediction:Prodigal:002006"
/inference="similar to AA sequence:UniProtKB:Q8GNS3"
/codon_start=1
/transl_table=11
/product="Fe(2+) transporter FeoB"
/db_xref="COG:COG0370"
/translation="MKIALLGNPNTGKTTVFNHLTDSYAYVGNWNGVTVEKKIGKIQN
SKIKIVDLPGVYSLNPLTKDESVATSFLLKESFSEILNIANAAQLKRNLLLTIELLEY
GKPVTLILNMMDIVKKQGVELDLKLLEHALQTKVLASDARTNQGLPEVRQELLEHENQ
PLKNNSFFLDYGPVIEGLIKNGQDILSQHYQFNPEISRWFMIQFFAGNKMIDEFIHAN
NFTLLDNLKQKASDKTAYKDAEDAIYSVKLLFIEETLNKAITKEANQKITKLTQRIDK
ITTHPFLGIPLFLTIFFFMFKLTFDWVGGPLSDFLDTLLSGPISEGIDGILTYLGVAP
FIHSLIVDGLIAGVGGVLVFIPQIFVLFACISFLEDSGYMSRAALVMDRLMESVGLNG
KAFIPLLIGFGCNVPGIMAARTIEQNNERLITTLISPFMSCSARLPVYSLFIAAFFDE
HRALIVLSLYLLGIFVAILLAKVYSLIFKTKDSSVFIVDLPEYNLPKLNVLWHSTWDK
GKGFVKKAGTVIFCGTVIIWLLSYTGPTGIAPSIDTSFMAVIGHSLVPFFAPLGFASW
QAISAIMAGVLAKEVVGSTMIILFHVSGETALISELSSVFTPLAAYSFLVFVLLYIPC
FAVLGAIKAETGSYKWAVYSAFSSLGIAYLFSFLVYQIGLLFS"
CDS 1464949..1465134
/locus_tag="JFBMFIFI_01384"
/inference="ab initio prediction:Prodigal:002006"
/codon_start=1
/transl_table=11
/product="hypothetical protein"
/translation="MSIFSLLVTISLGILIFGWTFYQLIQLFKNSKQGTCSACSSGCE
AKQLADAAKKKKDTIKF"
CDS 1465275..1465568
/locus_tag="JFBMFIFI_01385"

/inference="ab initio prediction:Prodigal:002006"

/codon_start=1

/transl_table=11

/product="hypothetical protein"

/translation="MNQGYVRDLSKEDQIELQTISDLIFVETIVNGFYELKTIQVPLP

ADIPLGRIYTREKIGDLLLNENHFSILIETNDDKYLYQSSTVKIPSYVLRDRD"

CDS complement(1465663..1466445)

/gene="accA"

/locus_tag="JFBMFIFI_01386"

/EC_number="2.1.3.15"

/inference="ab initio prediction:Prodigal:002006"

/inference="similar to AA sequence:UniProtKB:Q9FBB7"

/codon_start=1

/transl_table=11

/product="Acetyl-coenzyme A carboxylase carboxyl

transferase subunit alpha"

/db_xref="COG:COG0825"

/translation="MKNASEIVALSRKTTRLTALEYMNFLFKDFIEFHGDRSYRDDKA

VVGGIATLNAQPVTVIGIQKGHTLEENMLRNFGSPHPEGYRKALRLMKQAEKFNRPIV

TFINTAGAYCGVEAEERGQGEAIARNLMEMSQLKVPIISIIIGEGGSGGALALALGNQ

VWMMEHTMYAILSPEGFSSILWKDASRSKEAAELMKLTASDLLGLDVIDLLIPETKEG

VLLAQETIAKEMQLQLIEALETLNKLTPEELIEDRYNRFRKY"

CDS complement(1466474..1467325)

/gene="accD"

/locus_tag="JFBMFIFI_01387"

/EC_number="2.1.3.15"

/inference="ab initio prediction:Prodigal:002006"

/inference="similar to AA sequence:UniProtKB:P0CC08"

/codon_start=1

/transl_table=11

/product="Acetyl-coenzyme A carboxylase carboxyl

transferase subunit beta"

/db_xref="COG:COG0777"

/translation="MRLFKKREYIPIDPNRQRIQAEEKNDPKVPDGMWEKCPSCKKAI
YTKDLGEERICPSCGYCFRIGALERIQLTVDEGSFEEWDRGIENKNLMDFPGYPEKIA
SLQQKTGLDEAVVTGKALIDGQPLVIGVMDASFIMGSMGTVVGEKITRAFEKATKEKL
PVLIFTASGGARMQEGILSLMQMAKISGAVAKHSAAGLLYVVVLTDPTTGGVSASFAM
QGDIILAEPQALIGFAGRRVIEQTINEALPDDFQKAESLLEHGFVDKIVPRPELRNTL
SLLLRLH"

CDS complement(1467359..1468723)

/gene="accC"

/locus_tag="JFBMFIFI_01388"

/EC_number="6.3.4.14"

/inference="ab initio prediction:Prodigal:002006"

/inference="similar to AA sequence:UniProtKB:P37798"

/codon_start=1

/transl_table=11

/product="Biotin carboxylase"

/db_xref="COG:COG0439"

/translation="MFTKILVANRGEIAVRIIRACKELGIRTVAVYSEADKDALHMQL
ADEAICIGPAKATDSYLNMQSILSAAVVTKAQAIHPGFGFLSENSVFATMCGECNITF
IGPDAETIDMMGNKANARALMIEADVPVIPGSDGFVTSVEMAVEIADQLGYPVMLKAA
AGGGGKGIRKVMKPEELEAAYLSASSEAGAAFGDDRMYMERIIQHARHIEVQILADNY
GHVIHLGERDCSLQRNNQKIIEESPSVAISDKERQILGETAVRAAKHVGYKNAGTIEF
LVDQNGFYFMEMNTRIQVEHPVTEMATGIDIVKEQLLIASGKKMTLEQSDIHLTGHTI
ECRINAENPAFHFAPSPGRIDYLLLPSGGLGLRVDSAMFAGYEIPPYYDAMIAKIITK
GENRSEAIAKMKRALSELVIDGIVTNQFFQEDLLQDERFVNGEYDTNFLQDTYLPEWT
LKNS"

CDS complement(1468728..1469159)

/gene="fabZ"

/locus_tag="JFBMFIFI_01389"

/EC_number="4.2.1.59"

/inference="ab initio prediction:Prodigal:002006"

/inference="similar to AA sequence:UniProtKB:P0A6Q6"

/codon_start=1

/transl_table=11

/product="3-hydroxyacyl-[acyl-carrier-protein] dehydratase

FabZ"

/db_xref="COG:COG0764"

/translation="MSVLTAQEVMELIPNRYPIFFIDYVDELIPGEHVVATKNVTINE

DFFQGHFPGNPTMPGVLILESLAQAGSIPLLKLDKFQGQTAYLGGMNKVKFRKKVVPG

DVLKLHVDIIKLKDYAGIGKAVAYVDGKKVCEAELTFIIGR"

CDS complement(1469185..1469655)

/gene="accB"

/locus_tag="JFBMFIFI_01390"

/inference="ab initio prediction:Prodigal:002006"

/inference="similar to AA sequence:UniProtKB:Q5XAE6"

/codon_start=1

/transl_table=11

/product="Biotin carboxyl carrier protein of acetyl-CoA

carboxylase"

/translation="MNIAEVKELLTIVNDSKLTEFDLQMDNVVLHMSKNTHQAPKETA

APQAPTQQATAVSTVPQTATEVQVEEVVKTTVSEEGNLIKAPIVGVVYLAASPDKPNF

KKVGDTVEVGETLCIVEAMKLMNEITSDVAGTVTEVLIENEEVVEYNQPLFRIV"

CDS complement(1469655..1470899)

/gene="fabF"

/locus_tag="JFBMFIFI_01391"

/EC_number="2.3.1.179"

/inference="ab initio prediction:Prodigal:002006"

/inference="similar to AA sequence:UniProtKB:O34340"

/codon_start=1

/transl_table=11

/product="3-oxoacyl-[acyl-carrier-protein] synthase 2"

/db_xref="COG:COG0304"

/translation="MNRVVITGMGAVTPVGNTVETYWDSLKNGKSGIAEIKRFDASET

GITLAAELKDFDATLYMPRKETKRTDLFSQYGIAAAVQAMDDSQLDTEKIDVDRFGVI

VSSGIGGMNTIQEQVIKMHEKGPKRVAPFFVPMVIGNMAAGNIAIRVGAKGLCTSIVT

ACASGTNSIGEAFRSIKHGYSDVILAGGTEATICEIGIAGFGALTALSKSTDPARGSV

PFDKERNGFVMGEGAGVLVLEELQHALDRGAKIYGEVVGYGSTCDAGHMTAPSIDGSG

AGKAMIQAMKEAGISASDVDYINAHGTSTPANDSAETTAIKYAMGEEIAKQVPISSTK

SMVGHLLGAAGAIEGIACVKALQDGFLPPTIGYQVVDEACDLDYIPNVGRAATEAKYA

LSNSLGFGGHNAVVCFKKWEDA"

CDS complement(1470917..1471654)

/gene="fabG_1"

/locus_tag="JFBMFIFI_01392"

/EC_number="1.1.1.100"

/inference="ab initio prediction:Prodigal:002006"

/inference="similar to AA sequence:UniProtKB:P99093"

/codon_start=1

/transl_table=11

/product="3-oxoacyl-[acyl-carrier-protein] reductase FabG"

/translation="MTLKGKTVIVTGSSRGIGKSIAIEFAKEGANIVLNGRKPISEEL

IAEIEGFDVKCHTILGDVSNFDMAKQLIDETKEVFGSVDVLVNNAGITNDKLLMRMSE

EDFDATLNINLKGTFNTIRHATGIMLKQRSGSIINLSSIVGQIGNVGQANYAASKAGV

IGLTKSAARELAARGITVNAIAPGFIETDMTDELSDKNKDLMKQQIPLSRFGQVEDVA

KAAVFLSQNSYITGQVINVDGGMVMNG"

CDS complement(1471694..1472629)

/gene="fabD"

/locus_tag="JFBMFIFI_01393"

/EC_number="2.3.1.39"

/inference="ab initio prediction:Prodigal:002006"

/inference="similar to AA sequence:UniProtKB:P0AAI9"

/codon_start=1

/transl_table=11

/product="Malonyl CoA-acyl carrier protein transacylase"

/db_xref="COG:COG0331"

/translation="MKIAFVYSGQGAQYQGMGQEFYEANQTVRELFDEATEVLGFDMA

ALCFNENDKLNETTYTQPAILTVSVAIDTLLKEHGIEPEVVAGLSLGEYSALVKAGVL

DFKEALLLVKKRGQFMTEAVPSGAGAMSAIMGLDRETVKAACLEASQLGVVTPANYNM

PGQIVIAGMKAAVERAGEILTEKGAKRVIPLQVSGPFHTALLEPAAKQLEKALENVSI

HEPKLPIISNTEAKVIGNQAEIAPLLVRQVMSPVLWEDSVRTMIDDLGVTTFIEVGPG

KALSSFIKKIDRSVTVLNVENQKSLEKTLEKLKAE"

CDS complement(1472797..1473750)

/locus_tag="JFBMFIFI_01394"

/EC_number="1.6.5.11"

/inference="ab initio prediction:Prodigal:002006"

/inference="similar to AA sequence:UniProtKB:Q9I4V0"

/codon_start=1

/transl_table=11

/product="NADH:quinone reductase"

/db_xref="COG:COG2070"

/translation="MQSNICEKLGIDYPIIQGAMAWVANAELASAVSNAGGLGIIASG

HAPEEVVRDQIRQAKEKTNRPFGVNIMLLSPHVDAVVKAVCEEGVKVVTTGAGSPGKY

MKLFKEHGITVIPVVASVALAKRMEKDGADAIIIEGMEAGGHIGKLTTMVLVPQIVDA

VSIPVIAAGGIGDGRGMAAAFMLGASAVQLGTRFLVAHECTIHQNFKDAVLKARDIDT

TVTGQITGHPVRTIRNKLTRIYDQAEKEETSKDKPDFERLEELGKGTLRLAVVEGDIK

NGSMMSGQIAGLVSREQSCSDIILELMTEYQTVIKEKAQAL"

CDS complement(1473775..1474005)

/gene="acpA"

/locus_tag="JFBMFIFI_01395"

/inference="ab initio prediction:Prodigal:002006"

/inference="similar to AA sequence:UniProtKB:P80643"

/codon_start=1

/transl_table=11

/product="Acyl carrier protein"

/db_xref="COG:COG0236"

/translation="MTFEKIQAIIVDQLDKEEAEVQLTTNFREDLEADSLDLFQIIND

IEDEFDIKIETEEGLNTVQDIVNFVEAELKNK"

CDS complement(1474072..1475049)

/gene="fabH"

/locus_tag="JFBMFIFI_01396"

/EC_number="2.3.1.180"

/inference="ab initio prediction:Prodigal:002006"

/inference="similar to AA sequence:UniProtKB:Q820T1"

/codon_start=1

/transl_table=11

/product="3-oxoacyl-[acyl-carrier-protein] synthase 3"

/db_xref="COG:COG0332"

/translation="MGTKIVSTGSYVPKKQVSNEMLESFMETNDEWIKTRTGIHHRHL

AEGENTSVLCGKAAKAMLDKADVEASEIDLIIVATMTPDYLSPSTACLVQEYIGANKA

MAFDLNAACSGFVYALSVAEKMMASGKFHYALVIGGEVMSKVIDWTDRSTAVLFGDGA

GGVLLEANSEKNTFIAEDLHADGGRALSLTAGSMAVNNPYHKTEETNGYYLKMDGRGI

FDFAIRSVPKSVRQVVENAGLELEDIDYILPHQANYRIVEAIAKKLKLPMTKFKTNMA

EYGNTSGASIGILLDELVSNNELVLGSNEKVILTGFGGGLTWGSILIEL"

CDS complement(1475055..1475519)

/locus_tag="JFBMFIFI_01397"

/inference="ab initio prediction:Prodigal:002006"

/codon_start=1

/transl_table=11

/product="hypothetical protein"

/translation="MLPNSFETINDYLVDVFNEILTIEETALQNSSFEDVSIKEMHTV

EAIGMYQRRTTSEVAKKLQITVGTLTVAVNNLVRKGYVERIRSEDDRRVVKLGLTKRG

RLLFRLHDKFHKEMVKETIEDMQPEEIDILIKGLRNLHGFLDKTQKKLKEQG"

CDS complement(1475817..1476164)

/locus_tag="JFBMFIFI_01398"

/inference="ab initio prediction:Prodigal:002006"

/codon_start=1

/transl_table=11

/product="hypothetical protein"

/translation="MGFKITDEIQSVVEIELRKGASKSRIATLLGVNYDEALVIIDEV

KESFRPDVGDEIRFTFRDEKMAGVIQKLLTNSAVVEIFWEQSSSIMKDVCEDKTIVNF

KDIIEVTNLNVVS"

CDS 1476527..1477198

/gene="hssR_1"

/locus_tag="JFBMFIFI_01399"

/inference="ab initio prediction:Prodigal:002006"

/inference="similar to AA sequence:UniProtKB:A6QJK3"

/codon_start=1

/transl_table=11

/product="Heme response regulator HssR"

/translation="MKTILVADDDQLIKNMVHFFLTSEGFNVLTASDGDEALEIITKN

QIDLAILDVMMPGKTGYQVCQQIRENSSIPVILLTARGEMVDKEAGFQAGTDDYITKP

FELKELLFRVNALLRRSQTPNETVIQIGQLKIDSQNYLVTLGKKELYLPLKEFEVLHK

LASYPKKTFTRDQLIDSIWGFDYEGNDRTVDVHIKRLREHLTPESGVKISTIRGLGYR

LEVQL"

CDS 1477195..1478583

/gene="hssS"

/locus_tag="JFBMFIFI_01400"

/EC_number="2.7.13.3"

/inference="ab initio prediction:Prodigal:002006"

/inference="similar to AA sequence:UniProtKB:A6QJK4"

/codon_start=1

/transl_table=11

/product="Heme sensor protein HssS"

/translation="MKTLYKKIVGATFLVLVCSFFLAFLLSNLYYQTHLKPINDSKVT

KIAEEMRDYFAKNTELDLTSYLTHITSLGYEVYQVSENGEETAYGDSFRKTSLPQESI

DLVRSGEIYHGIANFPQNILITGFFDNDLRNTIGVPIKTANSTEAIFIRPDPTQQFGE

LRIYFALLLLLTVLIGFCFIFLSSSFIVKPIKKLTESTKQLISGQFTEINSTSRQDEI

GQLTNSFATMATEINKSEKARQEFVANVSHEIQSPLTTIQGYTSILKTNQHSLEENFN

YLTIIESETARLSDLTKQLLALSYLDNENQLVQKKSVDIAAQIRHYIQFTQWNWQEKN

IYLSVELIPAFINGNENFLYQIWQNLINNALNYTPEYGEIQIKIEDTTENFIITIFNS

GPTIPDSDIDHLFERFYQVDKNRTRQNGTSGLGLAITEKIVHLHNGTISVLNKTHPKG

VLFSVQLPKAHL"

CDS 1478654..1479742

/locus_tag="JFBMFIFI_01401"

/inference="ab initio prediction:Prodigal:002006"

/inference="similar to AA sequence:UniProtKB:P9WG17"

/codon_start=1

/transl_table=11

/product="putative ABC transporter permease"

/db_xref="COG:COG0577"

/translation="MFLAWREIKHTKVRYALIAFIMILIIWLVLFVTGLANGLASDNA
SAIKESPASYYVLEKGADNRFARSTVTSDEWQMIQQKLKADATPLSIQMSTISNSKGE
EKTDISYFLLEPSSFQEPQLSSGKFQKTDPKKVIVDEKLQRNGYKIGDSIKDTVSETV
FTISGFTKNQTYSHTGVVFLSPEQWKLIKQPQKTDAITYNAIALDISKSKAETLSFKG
LDVVPQNDIVQNIPGYSEEQGSLTMMIAFLYIIAAFVLTVFFYVITLQKMDQFGMLKA
IGAKTSYLGRSLFMQITLISIFSLLIGNGLTYGTAAILPASMPFKLTGTTALLSSILF
LAVALIGALLSLYKVAKVDAIEAIGGTN"

CDS 1479742..1480416

/gene="hrtA_1"

/locus_tag="JFBMFIFI_01402"

/EC_number="3.6.3.-"

/inference="ab initio prediction:Prodigal:002006"

/inference="similar to AA sequence:UniProtKB:A6QJK1"

/codon_start=1

/transl_table=11

/product="Putative hemin import ATP-binding protein HrtA"

/translation="MTAKLILEDVSKYYQDGSEQIEVLKNLSLSVAEGEFVAIVGPSG
AGKSTFLSIAGALLSPSSGNIQIGGTSLNSLSQKKWTQLRLEKIGFIFQSGNLIPYLN
VLDQLLLIPELNGTKKQNRQKALDLLEDLGLSHRLTQYPEKLSGGERQRVAIARALMN
EPDIILADEPTASLDSERGYQVVKMIAAEVHKKNKAAVMVTHDERVLDLVDRVLRIED
GRLYTV"

CDS complement(1480514..1482418)

/gene="mutS2_1"

/locus_tag="JFBMFIFI_01403"

/EC_number="3.1.-.-"

/inference="ab initio prediction:Prodigal:002006"

/inference="similar to AA sequence:UniProtKB:P65496"

/codon_start=1

/transl_table=11

/product="Endonuclease MutS2"

/translation="MNLTTIEKIQFNQVKEQIERHCVSSLGKKRFKKLQLNAKPVVVT
TRYNETEEARALLDAKLTMPFMGLSSIDVFMEQLEKGLLLEASSLIDVADFLRSGRMI
RRFMEKHESLAPTLTMYARSITEFTEIEDEIYFSIKNGQVADEASKELRRIRRLIAEK

ESKIEESLSKFLKNKDNQKQIQEFFVSKKNERFTIPIKAAYKNQVAGTIIETSSKGTT
VFIEPTAVTKLNDELAMLKVEESTEIYQILATLSGLILENLVPIQLNLETIAEYDMIF
ARGKYSRLIQGVTPKLNQRGYIHLVNAVHPLIEANAVPLNFTVGEDYRGLIITGPNAG
GKTVVLKTVGLLALMTLLGIQLPADEGTEMGLFEGIFVDIGDSQSLENALSTFSSHIQ
NIADIMQVAPRNSLVLFDEIGSGTEPNEGAALAIAILEEFYQRGNIVVATTHYGEIKR
YSEIHPDFINAAMAFDQATLTPLYQLLMGESGESNALWIAKKMNLKEHVIQQAQRYME
DKDYSLTKASIPKNKTMIDSQTERAESVSLLKKGDRVLLLDYNENGLVFEDNPLRETV
TVFYRNDYHEVVPKRLKLEVLATDLYPPDYDLSTLFTSYTERKLDHDINRGSKKALKK
IHREIKQQDR"

tRNA complement(1483382..1483455)

/locus_tag="JFBMFIFI_01404"

/product="tRNA-Arg"

/inference="COORDINATES:profile:Aragorn:001002"

/note="tRNA-Arg(tct)"

CDS 1483753..1483965

/locus_tag="JFBMFIFI_01405"

/inference="ab initio prediction:Prodigal:002006"

/codon_start=1

/transl_table=11

/product="hypothetical protein"

/translation="MYYLYANLIGEWTCLNLDPNARIDGIHPDLWLKQHPDFLFDTPF
VEIFYASVHYQIHPSQIQTFKMSTNK"

CDS complement(1484017..1484493)

/locus_tag="JFBMFIFI_01406"

/inference="ab initio prediction:Prodigal:002006"

/codon_start=1

/transl_table=11

/product="hypothetical protein"

/translation="MALIKNTDKNIQLSLKLLNNKFLEIKESSHESWIPFALKLDVAG
DCYEYEAKRGAVFTLYEINRLIGELEKNLGTKEAVNTFEEIEFYSSQAYFGMIFIDPL
EADLVEVEFWFNMDVLSQSETKGYDQGYRFAVEVTDLKSFIAEIKHELKEILIKEG"

CDS complement(1484705..1485598)

/locus_tag="JFBMFIFI_01407"

/inference="ab initio prediction:Prodigal:002006"

/codon_start=1

/transl_table=11

/product="hypothetical protein"

/translation="MENIGGKIKALRIQKEMRQKEFAKGICSNVTLSNIENNKQNPSM

EILIKICDKLEIALQDLYGEPIDTEEVKNLTANFDQLQWLCGNHRHREAYILLMEEIA

EEQLITICFQKKYYYFLGLTQVIHLEKPDEAIFNFSRTLNTENEAKTETIEDVLALNG

LGIAYTFKGEMDKAEFYFMKSLEHMKKFETEVLSEQKEMGKIAFNTAKHYSDKKEYKK

AVDLCLEALSILINLDSRYHLSALYFLLGYNLFELGDRVNSSKNYFIAKALAYVDNNQ

HVLDAVDEELEKHKIVFDALI"

CDS complement(1485907..1487241)

/gene="glnA"

/locus_tag="JFBMFIFI_01408"

/EC_number="6.3.1.2"

/inference="ab initio prediction:Prodigal:002006"

/inference="similar to AA sequence:UniProtKB:P99095"

/codon_start=1

/transl_table=11

/product="Glutamine synthetase"

/translation="MPEFTKEEIKRIAKEEDVRFLRLMFTDIMGIIKNVEVPISQLEK

VLSNKMMFDGSSIEGFVRIEESDMYLRPDLDSWLIFPWGTGKGKIARLICDIYNPGGT

PFAGDPRNNLKRVLKEMEEFGFTEFNLGPEPEFFLFKLDENGEPTLELNDKGGYFDFA

PTDLGENCRREIVLQLEEMGFEIEASHHEVAPGQHEIDWKYANAIDACDYIQTFKLVV

KTVARQHGLHATFMPKPLFGVNGSGMHFNMSLFNKDGNAFYDEEGEMQLSETARQFMA

GILKHARAYTAICNPTVNSYKRLVPGYEAPVYVAWSGRNRSPLVRVPESRGLSTRIEL

RSVDPTANPYLAMAVLLKSGLDGIKNQMKVPDPVDRNIYVMTEEERHERGINDLPSTL

HNAIKYLRQDEVVKEALGSHIYNNFVEAKRIEWAAFRQQVSEWEREQYMELY"

CDS complement(1487324..1487716)

/gene="glnR"

/locus_tag="JFBMFIFI_01409"

/inference="ab initio prediction:Prodigal:002006"

/inference="similar to AA sequence:UniProtKB:P37582"

/codon_start=1

/transl_table=11

/product="HTH-type transcriptional regulator GlnR"

/db_xref="COG:COG0789"

/translation="MKEKELRRSMSVFPIGTVMKLTDLTARQIRYYEEQDLIHPERNE

GNRRMYSLNDIDVLLEIKDYLSDGINMAGIKRIYELKQQEKEKKRIEQKRVMTDDDVR

RILHAEFLSVSGLNANGPHQSVDPKRMI"

CDS complement(1487987..1489237)

/locus_tag="JFBMFIFI_01410"

/inference="ab initio prediction:Prodigal:002006"

/codon_start=1

/transl_table=11

/product="hypothetical protein"

/translation="MSWKSPYTVELKQKIQEVEEKIKPLHDLVHQIALENQNKVLSSF

RENQVSDQHFNPTTGYGYDDFGRDTLEKVYADVFKAEAGLVRPQIISGTHAISTALFG

VLRPGDDLLYITGAPYDTLLEIVGETGNGIGSLKEYQIGYQHVDLLADGEVDFDAVKN

CITPKTKMIAIQRSRGYASRPSFTIEKIEAMIQTVRQWAPEAVIFVDNCYGEFVEERE

PIEVGADLIAGSLIKNPGGGLAKIGGYIVGREDLIEACGYRLTSPGIGREAGASLYSL

QEMYQGFFLAPHVVGEAVKGAIYTAALLAECQIESTPKWDDPRTDLIQMISLNDPEKM

VRFAQTIQKYSPINAHVMPIGAYMPGYEDDVIMAAGTFIQGASLELTADGPIRSPYTL

YVQGGLTYEHVKLAVTAAVSELFF"

CDS complement(1489234..1490481)

/gene="hflX_2"

/locus_tag="JFBMFIFI_01411"

/inference="ab initio prediction:Prodigal:002006"

/inference="similar to AA sequence:UniProtKB:P25519"

/codon_start=1

/transl_table=11

/product="GTPase HflX"

/db_xref="COG:COG2262"

/translation="MNTKEARERVIIVGVQTTESDQAFVYSLKELAQLTETAKGEVVA

ELTQKRPRMDSKTYLGKGKLQELVRLAEETEADVIIFNHGLTPGQTRNIQAEIDLKVI

DRVQLILDIFAMRAQSKEGKLQVGLAQLHYLLPRLAGQGKNLSKLGGGIGTRGPGETK

LETDRRHIRDQITDIKRTLKETEAHRERSRERRNDGTTFQIGLMGYTNAGKSTLLNQL

THAETYEQNQLFATLDPLTRKLVLPTGMNVTLTDTVGFIQDLPTQLIDAFHSTLEETK

NVDLLLHVVDAAAEDMLGHEKTVLELLKELKMDRIPMLTVYNKRDLITGSFLPDLFPS

VLISARNDDDIVELYHKIMEIMKETLVPYQFEIAADQGGALVKLKKETLIISEEYNEE

KNSYFVKGYAKEQSKWIGERELE"

CDS complement(1490498..1491448)

/gene="miaA"

/locus_tag="JFBMFIFI_01412"

/EC_number="2.5.1.75"

/inference="ab initio prediction:Prodigal:002006"

/inference="similar to AA sequence:UniProtKB:Q9KAC3"

/codon_start=1

/transl_table=11

/product="tRNA dimethylallyltransferase"

/db_xref="COG:COG0324"

/translation="MTDKTKIVIIVGPTAVGKTNLSISLAQAIDGEIISGDSMQIYRR

LDIGTAKVTKDEQAGIPHHLIDCVDVTQGYSVSDFQKDARKAISAIEARGKVPIIVGG

TGLYIEALLYDLSFGGTVEETDLAYRHLKELEAREKGNLALWSELQMNDPKAAENIHP

NNVKRVIRALEVYHLTGKPFSSFQKEHTIKEPFYDAKIIGLTTDREVLYQRINERVLN

MFEMGLLEEAHWLYQQNIPNSQAALGIGYKELTAYFNGKINLEEAQKQIQQNSRRYAK

RQLTWFRNRWAVVEWWDLIKNPNQKATLIETIKTFLSNQK"

CDS complement(1491450..1492190)

/gene="glpQ_1"

/locus_tag="JFBMFIFI_01413"

/EC_number="3.1.4.46"

/inference="ab initio prediction:Prodigal:002006"

/inference="similar to AA sequence:UniProtKB:P37965"

/codon_start=1

/transl_table=11

/product="Glycerophosphodiester phosphodiesterase"

/db_xref="COG:COG0584"

/translation="MKTKIIAHRGSKSTRPENTLTAFREALHVGSDGIELDVHLSSDG

EVVVIHDETVDRTTNGTGLVSELTLQELKSLDAGSWFDPLYSKVTIPTLKEVLDMLVT

EGFYGLLNIELKTDKIVYPEMSRKVYRLVQETAPTYDIVYSSFNYDTLIEMKKINDKN

QVALLFKKVGRAQTRLNGEYFVEAWHVPVDWAKARLILGKPRLPLRVWTVNEKSSIQY

FIRKKVAAIITDYPEIALMIRNHWDKGE"

CDS 1492379..1492609

/locus_tag="JFBMFIFI_01414"

/inference="ab initio prediction:Prodigal:002006"

/codon_start=1

/transl_table=11

/product="hypothetical protein"

/translation="MYLTIEETAEYLNLPISDIERLIREKQIRTLSDGETLLIYKEQF

NLFIKEMEKAKKELEDYLAEPIPEDIDIKDED"

CDS complement(1492690..1493091)

/gene="glpE_2"

/locus_tag="JFBMFIFI_01415"

/EC_number="2.8.1.1"

/inference="ab initio prediction:Prodigal:002006"

/inference="protein motif:HAMAP:MF_01009"

/codon_start=1

/transl_table=11

/product="Thiosulfate sulfurtransferase GlpE"

/translation="MKLSGNLIVNILLALVLLGMIGYEVYGYFNRKRAAVELSEEDFK

KDMRKVQVIDVREKAEFDAGHILGARNIPYSAFKTRMVEIRKDIPVYLYDQKKAMSGR

AAVRLKKAGYTEIYRLKNGYQGWTGKVKKKS"

CDS complement(1493180..1494298)

/locus_tag="JFBMFIFI_01416"

/inference="ab initio prediction:Prodigal:002006"

/codon_start=1

/transl_table=11

/product="hypothetical protein"

/translation="MKWTKQALYGTIILVFLSIIGLGGCGKTVENHEEKKEETAISYK

KIPIELDSIKKVMGWLSNEEVLVHCGHDFKDVLYKFNVVTGELKILYKPNALILTSAI

SPDSKKIFIQLAEEQKSKLQVIDLEGKILKENTVDTQGYLNVSWNGSNNAELFLAYYE

TENVLKVLDWKVSENTLKDVPNDSLTPVWYSDNLYVYVDNLGEFLLTKGELYLGDVRT

GKKTYLRNQVSGFYLNNDALITFSPSDFNEYELLLSYQYPFMVDKGFLEVPKMSMNDR

LLFPYLSQAQRKAPIYGVLPKKAVHLELEMGEYQLAELDFEERKIIPILDLPDNAPIQ

VSPNGKRVLYGWRFENVADLKTKKIYPLLNIPEKTPKS"

CDS complement(1494446..1495414)

/gene="glcK"

/locus_tag="JFBMFIFI_01417"

/EC_number="2.7.1.2"

/inference="ab initio prediction:Prodigal:002006"

/inference="similar to AA sequence:UniProtKB:P54495"

/codon_start=1

/transl_table=11

/product="Glucokinase"

/db_xref="COG:COG1940"

/translation="MSKKLIGIDLGGTTVKFAILTEAGEVQQKWSIETDSDDEGSKIV

PSIVASINQHLERYNMTKADFIGIGMGSPGTVDREKGTVIGAYNLNWKTLQFVKKDIE

EGTGIPFAIDNDANVAALGERWKGAGENGDDVTFITLGTGVGGGIIAEGHLLHGIVGA

AGEIGHITVDPEGYECTCGKKGCLETVASATGVVRVARDYADNFSGDSRLKYMIDDGQ

QVTAKTVFDLAKDGDVLAIKVIDKVSFYLGLACGNIGNMLNPSDIVIGGGVSAAGEFL

LDQVRHYFEQFSFPQVRQSTRIKLAQLGNDAGIIGASSLARQFSKK"

CDS complement(1495435..1495638)

/gene="yqgQ"

/locus_tag="JFBMFIFI_01418"

/inference="ab initio prediction:Prodigal:002006"

/inference="similar to AA sequence:UniProtKB:P54494"

/codon_start=1

/transl_table=11

/product="putative protein YqgQ"

/db_xref="COG:COG4483"

/translation="MRTLYDVQQLLRRFGIYVYVGKRLWDIELMQIELKSLNDEGLIP

NETYIAAVLVLKQEHRIEEGKEK"

CDS complement(1495657..1496367)

/gene="gluP"

/locus_tag="JFBMFIFI_01419"

/EC_number="3.4.21.105"

/inference="ab initio prediction:Prodigal:002006"

/inference="similar to AA sequence:UniProtKB:P54493"

/codon_start=1

/transl_table=11

/product="Rhomboid protease GluP"

/db_xref="COG:COG0705"

/translation="MNYQTEMKLKRFSNKPFMTYTFLTIQIVLFLMMTLFFGGSENSD

TLVFFGAKENSLIMSGEWWRLITPMFLHIGWLHLAVNSVSIYYIGAHLEKILGHWRFA

LIYLLSGVAGNVASFAFNDSISAGASTSIFGLFATTLMLAETFKGNTYYREIAKTFGI

LIVLNFITGFLSIGAGNVDNAGHVGGLVGGFLIATAISVPNAPVDLKTKRIIAGVAYT

IALLFFIAIGFKRTTIGV"

CDS complement(1496445..1496978)

/gene="yqgN"

/locus_tag="JFBMFIFI_01420"

/inference="ab initio prediction:Prodigal:002006"

/inference="similar to AA sequence:UniProtKB:P54491"

/codon_start=1

/transl_table=11

/product="putative protein YqgN"

/db_xref="COG:COG0212"

/translation="MDKSIVRVELIERLEKLSEVEKAKIEKRLVEKLLAHSWWENAST

IGLTLAQALEIDTKPLIKAAWQSDKKVVVPRTKKAGQMDFVYYTPETKMERSSFGILE

PIKSLPAVPKEQIQLLIVPGVGFNSAGYRIGFGAGYYDRYLADYQGKTISLALPQQLT

NDWTVDSYDIPVQLILT"

CDS complement(1497086..1497235)

/gene="rpmGA"

/locus_tag="JFBMFIFI_01421"

/inference="ab initio prediction:Prodigal:002006"

/inference="similar to AA sequence:UniProtKB:P56849"

/codon_start=1

/transl_table=11

/product="50S ribosomal protein L33 1"

/db_xref="COG:COG0267"

/translation="MRVNITLECTECKERNYHSKKNKRNNPDRVEFKKYCPRERRMTL
HRETK"

CDS 1497506..1497856

/locus_tag="JFBMFIFI_01422"

/inference="ab initio prediction:Prodigal:002006"

/codon_start=1

/transl_table=11

/product="hypothetical protein"

/translation="MDIWWIVIILLIISVALIVWSFYKKDNGEKVQEEFEELSLQLMQ
DIYHLKNRVAALENELNIQSEETSSSMKIHDVLKNHVVTLYTQGVSLENIASQTQLSL
ATVQVVVNEYVEQN"

CDS 1497877..1498443

/locus_tag="JFBMFIFI_01423"

/inference="ab initio prediction:Prodigal:002006"

/codon_start=1

/transl_table=11

/product="hypothetical protein"

/translation="MNRPALRFLAVGFLVSALVLSGYRLFLYEPSTSTAKSSETAKKE
KTLSADEKSYKEKYEELLTKVELEKVTGDSSSTDSSKTDSSSKTADDKKATDAAAAAA
KKKADEEAAKKKAEEDKVKKYTLVIGQGDPTSAAVDQLASQGIIKDANEFTQFLSAND
YEMYIRDGSYEVNSSMSYEQIAKIITHR"

CDS complement(1498569..1500623)

/gene="pbpH"

/locus_tag="JFBMFIFI_01424"

/inference="ab initio prediction:Prodigal:002006"

/inference="similar to AA sequence:UniProtKB:Q796K8"

/codon_start=1

/transl_table=11

/product="Penicillin-binding protein H"

/db_xref="COG:COG0768"

/translation="MAESNGSMKKKKSHIPFRLNLLFFIVFLLFASLIVRLGYLQIVK
GEEFEAEVKRTEMTTATGTVPRGMIYDSQGRQLVGNTALQAITYTRGAQVSGKDMMKI
ATKLGTFIQMTPDSIEDLNERDLQDYWAIVHEKELNKRLSKKELELKGSELYQVQLSK

ITPEDLAPITEQEKQVAAIFKRMNGAYALTTTYIKSKDVSDTEIAAISENLVDLPGVD
TSTDWQRVYPEGEMLRSILGSVTTEKVGLPEDQASALLAKGYARNDRVGNSYLEKQYE
TVLSGSKSKSETETNANGDIINTIAKYEGSKGDNLVLTIDIEFQKQVEEITKNALNGI
LGGLTDRMYVMASDPHTGEILAMTGQKYNFETNQIEDDALGVMNSQYTMGSSVKGATV
LAGYMDGVISLDNNMILDQPLLFKGTALKSSWFNRNGSNELMSDETALEVSSNSYMMQ
IAMRMGGQNNYVPNGSLDIDMNLVFSKLRSYYAQFGLGVPTGIDLPGESTGLPGEQSN
AGLALDFAYGQFDTYTTLQLNQYISTIANGGNRIAPRVVKEIRETDEDGKLGALQTEL
QPNILNRVNVGNEEIKRVQTGMYNVVHGSHRNKTGTGSLVASPNNIAGKTGTAEAFYY
GPQTAMNGTSVYNLTFVGYAPADNPEIAVTVVVPFMPNDKNTINKDVASAVFNAYFDM
KK"

CDS 1500825..1503629

/locus_tag="JFBMFIFI_01425"

/inference="ab initio prediction:Prodigal:002006"

/codon_start=1

/transl_table=11

/product="hypothetical protein"

/translation="MSNLLQAIKKFFQSHWPLLLAFFGPFIIMATIYGFQGVYPFGNS
TLLTVDLGQQYVDFYAYYRETLLHNPLSFFYSFSKAIGGDMIGLWAYYLTSPFNLILI
LFPPKLITLAVTTLTLIKISTAGLTFGILLKKAFDGKGFILAAFSISYALMGYTIVNQ
LNIMWLDGLVFLPMVILGIEKLLITKKGSFYTLFLAIMLVANYYIAYMICIFTVVYFF
FRLTGIHFPNKIALKKKIIYFFTTFLRFAWYSLLAGGLSAFLLIPTFNALLASKASYT
NFKLDWNFNYPIQEIIAKFYLGAFNFDQMPDGTPNVFIGSLALISFGCFFFNRAFPIR
ERFGALLVSIFLILSMNIKALNLMWHGMQYPIWYPYRFSFVFCFFMILLGFRSFMQLK
KIPIWGIILTLSLTALASVYLLKAQFDFIYEAQIILTSLFVVLIVLLLILKAQNYFWL
PLAFFLVSVSEMTINATVDLSRLSYVTNSSFLDYKKEVGSAINQIQENDSDFYRIEKT
FLRSKNDSFQFGYPSVTHFSSTFEKEIPTLFGNLGFPVGNGFVTYSNGTLFTDAFFGI
KYFASENNTLYQLPGNYLDSSSELATDSLPDNPLNENVLENFTKSESNLTKQDLTLNV
MSNKPDLRYYLKQNSHEFISIFKNPNALPIAFGADEQLLQEKLLIGQPIQLQENLLQS
MDQKDERIRYFTPLAFNSTVFQNVTSNGNMQNITYKKQVANKKATVDFQFTPQTDDPY
YITLGPSIKEKNATIYLNGKVLNQYKTYRDSIVINVASRQKGETITISFELLEDTLWL
DNFHLYRFNEAAFQSVITNLKANQLTIDSYGDTYFKGHVTIQNDNQILMTTIPDSPGW
TVKIDGKPVKSKKVLDTLMAVPISKGKHQVEMRYRTPYFKEAVIISLLSGGLLIASHL
FFTKGKHSHNQVQLKETKPK"

CDS 1503626..1504261

/locus_tag="JFBMFIFI_01426"

/inference="ab initio prediction:Prodigal:002006"

/codon_start=1

/transl_table=11

/product="hypothetical protein"

/translation="MKPIKKELPRIPKQLILADFDEIEDEAFYENCLFENCTLSNRQL

DKVCFKHSRFINVTFENMTFHQLELLDVEFHKCNISNTEMIGVILHRSLLKGSKFVGC

NFSEGMVLDTSFEDCFGNYSFFSYTTFKRVVFEATSLIESDFFEVKWQKLQFDQCNLD

GANFMNTDLRGLDFSTSTFEKIALSFDLLKGCTINPQQSLVLVAALGVNIS"

CDS complement(1504663..1504998)

/locus_tag="JFBMFIFI_01427"

/inference="ab initio prediction:Prodigal:002006"

/inference="similar to AA sequence:ISfinder:ISSpn6"

/codon_start=1

/transl_table=11

/product="IS200/IS605 family transposase ISSpn6"

/translation="MKKDNQSLSHTVWKCKYHIVFAPKYRRQIIYGKYKQSIGKILRE

LCERKGVEILEANACKDHIHLLVSIPPKLSISSFIGYLKGKSSLMIFDRHANLKYRYG

NRKFWCRGY"

CDS complement(1505177..1505785)

/gene="sodA"

/locus_tag="JFBMFIFI_01428"

/EC_number="1.15.1.1"

/inference="ab initio prediction:Prodigal:002006"

/inference="similar to AA sequence:UniProtKB:P00449"

/codon_start=1

/transl_table=11

/product="Superoxide dismutase [Mn]"

/translation="MTYTVPELPYAYDALEPNIDAETMHLHHDKHHVTYVTNLNAAIE

KHPELGEKTIEDLVSNLDAIPEDIRTAVRNNGGGHANHSFFWEILSPTGGGEPTGEVK

DAIDATFGSFAKFQEDFAAAATTRFGSGWAWLVLDGGKLAITSTPNQDTPLSEGKTPI

LGLDVWEHAYYLNYKNVRPAYIKAFWNLVNWEQVAKHYAAAK"

CDS 1505990..1506814

/locus_tag="JFBMFIFI_01429"

/inference="ab initio prediction:Prodigal:002006"

/codon_start=1

/transl_table=11

/product="hypothetical protein"

/translation="MTSFFKRNNQSQFETMGVSIIQLTKLFIASLSKPEQLTAAATLK

KGKVFLYFLFIAFISAIPAFLQSYQLLTDFNRDGQTIISEMPEFSIKNGEFITNEPID

SFSKKTHSINFVFDPEGTKPELEEDLSENLVGIGFLKDGLHFNNASQPISLTYQQMDG

LSKEFFVNILASMQRMTGFILVIAFILMLLLSLFLTLLFNFLYTVFANLVTALTRRRL

PFSHVFKMTLFASTLPTIIFTLFNLFGLRIVYQTELSLIVTVYLYYLALRTLPKPE"

CDS complement(1507040..1508389)

/gene="cshB"

/locus_tag="JFBMFIFI_01430"

/EC_number="3.6.4.13"

/inference="ab initio prediction:Prodigal:002006"

/inference="similar to AA sequence:UniProtKB:Q8Y755"

/codon_start=1

/transl_table=11

/product="DEAD-box ATP-dependent RNA helicase CshB"

/db_xref="COG:COG0513"

/translation="MEHTFEKFGLQPFLIDAIQEIGFTKPTEVQEKLIPEIMKSESVI

GHSQTGTGKTHTFLLPLFNNIDPLKEEVQVVITTPSRELAEQIYQAALQLVKHAPNKI

IVQSFVGGTDKKRQLTKLAGTQPHVVIGTPGRILDMINENALRVHTSPVLVIDEADMT

LDMGFLTEVDQIASRLPNNLQMLVFSATIPTKLKPFLKKYMENPHYEHIQPKSVISPT

IENWLISTKGRDRVDVVYQLLTTGTPYLAIVFANTKQKVDELADGLKSRGLKVAKIHG

DIPPRERKRVMKQVQNLDYQFVVATDLAARGIDIEGVSHVINAEIPRDLDFFIHRVGR

TGRNGMKGTAITLYAPSDEGLVNDIEKLGIEFVPKTIKNDEIVDTFDRNRRNHRDKTT

KETLNPSMRGMVQKAKKKVKPAYKKKMSRTIKEHNEQKRRVERRSQARAIKKANKKK"

CDS 1508733..1510214

/gene="zwf"

/locus_tag="JFBMFIFI_01431"

/EC_number="1.1.1.49"

/inference="ab initio prediction:Prodigal:002006"

/inference="similar to AA sequence:UniProtKB:P54547"

/codon_start=1

/transl_table=11

/product="Glucose-6-phosphate 1-dehydrogenase"

/db_xref="COG:COG0364"

/translation="MIEEKIALFTIFGGTGDLARRKLYPSLYRLYKKGFLNKKFAVIG

TARREWSDEYYREIVLDTIAEITESKEDAIEFASHFYYQAHNVTDTEHYASLKTLSQK

LNEKYKLEENHIYYLAMSPQFFGTITEHLKDQGLINDTGFNRLIIEKPFGHDYESASI

LNNQIRSSFKEEQIYRIDHYLGKEMIQNISAVRFANAIFESMWNNRYIDNIQITLSES

LGVEERGGYYDTSGALRDMVQNHILQVVSLLAMEPPTELKDEAIRDEKIKALRSLRIF

SPEEVRNYFVRGQYGPSTVNGENSLGYREEENVADDSTTETFVAGKVLIDNFRWAGVP

FYIRTGKKLREKGTQINIQFKNMPLNLFNDDHSVPLEPNVLTIYIQPTEGFSLQLNSK

KIGQGLETELLNLKHKNSAETSANSPEAYEKLILDCLNGDATNFTHWDEVASSWRFVD

VIREAWDQETPEFPNYPSGSMGPVCSDQLLASDGFKWFWNPQK"

CDS complement(1510271..1510894)

/locus_tag="JFBMFIFI_01432"

/inference="ab initio prediction:Prodigal:002006"

/codon_start=1

/transl_table=11

/product="hypothetical protein"

/translation="MRILRKIMLFLVITMVSLGIGYYYGSQSQIGSESSKTTQPVSNK

TTEDTKSLKTSEEINEEAKSTPPDQSESELEIIEAEIHRLVNEEREAQGVPSLAINPQ

LVKASRKRSEEIVTDFSHERQGISSFSIFKEPEYEYIYQAVGENIVMATYQGSSKEMG

AYFFKLWKESPGHYKNMISPNFNEVGTGIYKQDGILYGTQLFGTPRN"

CDS complement(1510996..1511325)

/gene="ppa"

/locus_tag="JFBMFIFI_01433"

/EC_number="3.6.1.1"

/inference="ab initio prediction:Prodigal:002006"

/inference="protein motif:HAMAP:MF_00209"

/codon_start=1

/transl_table=11

/product="Inorganic pyrophosphatase"

/translation="MKKFTVYVNIDRPIGYKDKLGTVYPVNYGFVEGVIAGDGEEQDV

YIISKEINEELVVFEGEVVAVIYRKDDIEDKWVATKIGEKLSYKEIKKSVEFLEQYFD

SEIKMLE"

CDS complement(1511596..1512552)

/gene="nrnA"

/locus_tag="JFBMFIFI_01434"

/EC_number="3.1.-.-"

/inference="ab initio prediction:Prodigal:002006"

/inference="similar to AA sequence:UniProtKB:Q8DTN6"

/codon_start=1

/transl_table=11

/product="putative bifunctional oligoribonuclease and PAP

phosphatase NrnA"

/db_xref="COG:COG0618"

/translation="MSQEVFDQILNEIKHYKTIIVHRHKRPDPDALGSQGGLVEILKA

SFPDKQILKAGGPVDGLQFLSEMDPVTAEDYKNSLVIVTDTANSPRISGDDYTLGEKL

IKIDHHPNDEPYGDIVFVNTKASSCSEIIVDFYDYHKAELKLTDEGARLLYSGIVGDT

GRFLYPATTSHTLQVAAELMTYNFSATDLNNELNVMSPAVALLSGYVLQNIEVNELGV

GRVILTKECLAEFGVADSETAAVVSLPGSIEGVLLWGIFVEQPEGYFRCRLRSKGPVI

NEIAKRHHGGGHPLASGANAKDLAEVAEIMSEFDEAAKNWNV"

CDS complement(1512582..1513913)

/gene="IMPDH_2"

/locus_tag="JFBMFIFI_01435"

/EC_number="1.1.1.205"

/inference="ab initio prediction:Prodigal:002006"

/inference="protein motif:HAMAP:MF_01964"

/codon_start=1

/transl_table=11

/product="Inosine-5'-monophosphate dehydrogenase"

/translation="MPTKHDQILAYIETLPVGERISVRLIAKNLQVSEGTAYRAIKEA

ENVGLVSTIQRVGTIRIERKMKEHFEKLTFGEVVKIIEGDILGGIAGLDKSLNKFIIG

AMTEEAMLRYITPGSLMIVGNRVGAQRLALKNGAAVLITGGFDTCDEIVELANQFELP

VIRTTYDSFTVATMINRAMTDQLIKKDIMLVGDIYTNLEKTMYLNSDATVADYRKLND

KSRHSRFPIVNKKMRLIGIVTAKDVVGKPDNLGIDRVMTKDPSCVKTHMSVASVGHLM

IWDGLEVMPVVKDDLTLMGIVSRQDIMKAMQLAQRQPQVGDTIADQIAEQLELTSDDP

TQWDAAIPSFKFSVSPQMTNSVGTISFGVLSELVANASQRTLLAYQKRNAVIEQMNLH

YFKMIQLESELEIKPRVLEIGRRSSKLDIEVFIENTLVAKAIVICQLMERT"

CDS complement(1513937..1514617)

/locus_tag="JFBMFIFI_01436"

/inference="ab initio prediction:Prodigal:002006"

/inference="similar to AA sequence:UniProtKB:P99149"

/note="UPF0173 metal-dependent hydrolase SA1529"

/codon_start=1

/transl_table=11

/product="hypothetical protein"

/translation="MKISWHGQSCIRIITNTDQTILFDPYITGNPTSDLNAETVEADV

IIITHAHNDHIGDTEAIAKRTNALIIANVEIANYFSNKGYRTHGMQMGGKHQFDFGLI

KMTPAIHGSTYEIDGLQLTLGLAAGIILSIENKTFYHAGDTALFSDMQLIGAFKPIDI

AFLPIGDNYTMGPEDAALAAELLAAKKVVPIHYNTFPLIHQDPEEFTKLLAKDQGIIL

EVGAQLTL"

CDS complement(1514819..1515949)

/gene="dinB"

/locus_tag="JFBMFIFI_01437"

/EC_number="2.7.7.7"

/inference="ab initio prediction:Prodigal:002006"

/inference="similar to AA sequence:UniProtKB:Q47155"

/codon_start=1

/transl_table=11

/product="DNA polymerase IV"

/db_xref="COG:COG0389"

/translation="MDGVIQFAEPVRDTSRKIIHVDMDAFYASVEERENPDLKGKPLV

IARHPKDNGGRGVVTTANYEARKFGIHSAMSAQKAYELCPHAIFVPGRHELYSEISAE

IREIFKHYTDLIEPLSLDEAYLDVTENKKNLKSATQIAYLIQKEIWETLHLTCSAGVS

YNKFIAKLASDYKKPAGITVIPPEKALDFLKALEIGKFYGVGKKTAEKMYERNIFNGA

DLYKLSELELIHLFGKMGYSLYRKVRGIDNSAVQPSRERKSVGREHTYGTPLTTEEEV

LSELRFLAGKVHSSLTRNQKHGKIVVLKLRNTDYETMTKRISLPAYVKGSEEIFFHAR
NLWEEVGSLDKGIRLVGITITGLDPITFENIILPLWDKNKFY"
CDS complement(1516376..1517023)
/gene="mntR"
/locus_tag="JFBMFIFI_01438"
/inference="ab initio prediction:Prodigal:002006"
/inference="protein motif:HAMAP:MF_00732"
/codon_start=1
/transl_table=11
/product="HTH-type transcriptional regulator MntR"
/translation="MTPNKEDYLKIIFELGGAHAKINNKQIVTGLNVSAASVSEMMSK
LLKDGYVEHTPYQGVQLTDAGLLKAGILIRKHRLWEVFLVEHLGYSWNQVHDEAEVLE
HVTSGTLADRLENYLEFPDICPHGGVIPDKDGTIVEQTLPTLIEAKVNEMIELKRVLD
DRELLDYLVAAGLKIGDRCSVYEVGAYEGPITLIKDDEKIQISYKAACNMFIQIL"
CDS complement(1517694..1520303)
/gene="adhE_1"
/locus_tag="JFBMFIFI_01439"
/inference="ab initio prediction:Prodigal:002006"
/inference="similar to AA sequence:UniProtKB:P0A9Q7"
/codon_start=1
/transl_table=11
/product="Aldehyde-alcohol dehydrogenase"
/db_xref="COG:COG1012"
/translation="MAKTKTEKKVAVVEENDVQQMIDTLAAKGQAALKVLETFDQEKI
DYIVHQMAMAGLDQHMPLAKMAVEETGRGIYEDKCIKNMFATESIWNSIKHNKTAGVI
SDDQQTQITEVATPVGVVCGVTPVTNPTSTTMFKAIISLKTRNPIIFAFHPSAQNCSA
ASAKVLYDAAIKAGAPEGCIQWVEKPSIEATNSLMNHPDIAIVLATGGSAMVKSAYST
GKPALGVGPGNVPSYIEKTAKIKRAVNDVIASKSFDNGMICASEQAVIVDEEIYDEVK
AEFVKHQVYVVKQKEIAQLEAVVMNEGKYAVNPAIVGKSAYEIAKLAGIDVPERTKIL
IAEIAGAGEKYPLSREKLSPVLAMIKATSTEHAFELCDTMLELGGLGHSAAIHTLNDN
LAREFGIQMKACRILVNSPTAQGGIGDLYNNMIPSLTLGCGSYGKNSVSKNVSTINLM
NVKTIAKRRNNMQWFRLPSKIYFEKNSLQYLQKMENVERVFLVCDPGMVQFGYADLVI
KELNKRNNAVQIEVFSQVEPNPSTNTVEKGTEAMLAFNPDTIVALGGGSAMDAAKGMW

LFFEKPETEFFGAKQKFLDIRKRTYKIPALTKTQFVCIPTTSGTGSEVTPFAVITDSE

THVKYPLADYALTPDVAIIDPQFVMSVPKSVTADTGMDVLTHAIESYVSVMASDYTRG

LSLQAIKLVFENLKNSYELANEEAREKMHNASTMAGMAFANAFLGINHSIAHKIGGEY

NIPHGRTNAILLPHVIRYNAKDPSKHALFPKYEFFRADEDYADIARFMGFKGNTTAEL

VESLVQEIIKLGKSLNIDMSLKGQGVTQETLDTTVDNMAELAFMDQCTTANPKEPLIS

ELRQIIIDSYIGE"

CDS complement(1520563..1520844)

/locus_tag="JFBMFIFI_01440"

/inference="ab initio prediction:Prodigal:002006"

/codon_start=1

/transl_table=11

/product="hypothetical protein"

/translation="MESRQILSYERIEPWLTLKVKEFTKEGYPTITEDDLWRFLVSFS

WKRTTPEHYYQQIKQIVRITVNDYLDFASLEAQVYQVESLSEMDLEGLL"

CDS complement(1520876..1523449)

/gene="mprF"

/locus_tag="JFBMFIFI_01441"

/EC_number="2.3.2.3"

/inference="ab initio prediction:Prodigal:002006"

/inference="similar to AA sequence:UniProtKB:C0H3X7"

/codon_start=1

/transl_table=11

/product="Phosphatidylglycerol lysyltransferase"

/translation="MDKKFKNVVAWFQKHLTWIKLFFIGFILLFVLSQVLKIASDINY

QDLKNNLFSQSPLAIIVMLVGGLIAILPMLIYDFTIVKLLPGEFTKEHIFKSGWITNT

FTNIGGFGGFLGASLRATFYGKSANHKEIVLAVSKIAIFLLSGLSIYSMMALGSFTFG

DYGIVFRRYWPWLLGGSLYFPLVFIFTKIKSKTLFEDLPLSRELRLVFASLLEWGAAM

GFFILIGYLLKEPVHLTEVFPIFVIASIVGIVSMVPGGVGTFDIFMIYGLGQIGVSKE

LAVIWLLFYRIFYYIIPFFIGILFFVHDGGSKINQYLDGLPKILLQKLAHVFLVSFVY

FSGFMMIIISTVPNLAFDNPIFVRLYPFTFFFLTQLSNVIFGFLLLGLARGIESKVKK

AYLPTIIILGFGIINTLQKDFSWGLTIFLGIVLVAVYVSRHEFKRVKLTYSWGKILLD

GVVIFGTLVLYIIIGYLNSPRFHHRKPVPHYFLFPSHTVWIAGFIGVIAGIASFMLIL

AYLRRSKEKIGTSFDETRLMRLLSQFGGNEVSHLVFLRDKRYYFYQEENEDQIVFQYR

EEADKLIIMGEPIGNQAFLEKAIVHFMDEANRFGFSLVFYEVSGSLVTILHELGYDFM

KVGEEGHVNLPDFTISGKKRRAERALMNKLERENFHFSILEPPFTQDTFTELKAVSDN

WLNHRQEKGFSLGFFDEYYLNQAAVAVVTNEAGNIVAFASMMPSGTDKLISIDLMRHH

LVDAPPGVMDYLFVQLFESNRELGYESFNMGMAPLSKVGVTRYSFLSERIAGVIYRYG

TKFYGFQGLRKYKEKYVTNWIPKYVAFRKKSSILFTMLQLVIVVGKKKSLTNHSNDQI

E"

CDS complement(1523629..1524006)

/locus_tag="JFBMFIFI_01442"

/inference="ab initio prediction:Prodigal:002006"

/codon_start=1

/transl_table=11

/product="hypothetical protein"

/translation="MGNLIGLLPMVAVVGVFYFLMMKPQKKAADEKKKMMNALKKGDS

IVTIGGLHGEIDEINESDKTVVLDCDGIYLTFERAAIARVTNSAPMVAETIVTEETVI

EAPADSVEEVVVEEKTETIDETK"

CDS complement(1524109..1525254)

/gene="tgt"

/locus_tag="JFBMFIFI_01443"

/EC_number="2.4.2.29"

/inference="ab initio prediction:Prodigal:002006"

/inference="similar to AA sequence:UniProtKB:P66905"

/codon_start=1

/transl_table=11

/product="Queuine tRNA-ribosyltransferase"

/translation="MTEPAVKYRLIKKEKHTGARLGEIITPHGTFQTPMFMPVGTLAT

VKSIAPEELDAMGAGIILSNTYHLWLRPGEDIVEEAGGLHKFMNWDKGILTDSGGFQV

FSLSDMRRIEEEGVHFRNHLNGSKMFLSPEKAINIQNKLGPDIMMSFDECPPFDESFD

YVKKSVERTSRWAERGLKAHANPDYQGLFGIIQGAGYRELREQSARDLVSMDFPGYSI

GGLSVGEPKHLMNEVLEYTTPLIPEHKPRYLMGVGSADSLIDGVIRGVDMFDCVLPTR

IARNGTCMTSAGRLVVKNAQYERDFRPLDEKCDCHTCKNYTRAYIRHLIKADETFGLR

LTSYHNLYFLQNLMKNVRQAIMDDNLLEFREAFFEEYGFNKPNAKNF"

CDS complement(1525287..1525898)

/locus_tag="JFBMFIFI_01444"

/inference="ab initio prediction:Prodigal:002006"

/codon_start=1

/transl_table=11

/product="hypothetical protein"

/translation="MEGVSPIDEKKMTGTQQRIIDAALHLISQVGYKSTTTKLIAQEA

EVNETTIFKNFKSKEALMNVAFKQHANQIKEEVDSFFQQEFENSRDLLKRTGHFIQDT

YEKHRYVVIGSIKEVGNEKAKTIFNYKQEYINAALSEKLQEFPDADHFCQKDYETISF

IYNNAIVALLLQKIQQNGEQKKSAITLENIIDMTLRPFDEEQV"

CDS complement(1526563..1527594)

/gene="queA"

/locus_tag="JFBMFIFI_01445"

/EC_number="2.4.99.17"

/inference="ab initio prediction:Prodigal:002006"

/inference="similar to AA sequence:UniProtKB:A8AYK5"

/codon_start=1

/transl_table=11

/product="S-adenosylmethionine:tRNA

ribosyltransferase-isomerase"

/db_xref="COG:COG0809"

/translation="MLTTKDFDFDLPEELIAQTPLEDRSASKLLVLDRETGAIKDKHF

TDILDELSEGDALVMNDTRVLPARLYGTKPETGGHLEVLLLKNTTGDQWETLVKPAKR

AKVGTEIEFGDGRLKAVVLEELEHGGRIIEFSYTGIFLEVLESLGEMPLPPYIKERLE

DSERYQTVYAKENGSAAAPTAGLHFTEDLLEKIKAKGVEIVFLTLHVGLGTFRPVSVD

SIEDHEMHAEFYRLSEESAETLRKVKANGGRIVAVGTTSIRTLETIGTKFNGEIEADS

GWTSIFITPGYQFKVVDAFSTNFHLPQSTLVMLISAFAGREKILAAYQHAIDEQYRFF

SFGDAMFIK"

CDS complement(1527611..1528633)

/gene="ruvB"

/locus_tag="JFBMFIFI_01446"

/EC_number="3.6.4.12"

/inference="ab initio prediction:Prodigal:002006"

/inference="similar to AA sequence:UniProtKB:Q97SR6"

/codon_start=1

/transl_table=11

/product="Holliday junction ATP-dependent DNA helicase

RuvB"

/db_xref="COG:COG2255"

/translation="MVNKMNDEERILSGESESYEEQSYEKSLRPQRLNQYIGQTKVKK

ELTIYIEAAKTREEALDHVLLYGPPGLGKTTMAMVIANEMDVGIRTTSGPAIEKAGDL

IALLNELEAGDVLFIDEIHRMPRMVEEMLYSAMEDFFVDIIVGQGPSAHPVHFPLPPF

TLIGATTRAGLLSAPLRDRFGIVSHMEYYTVKDLSDIVLRSADVFETRIDGMGALEIA

RRSRGTPRIANRLLKRVRDFAQVEGNGAVSQEIADEALALLRVDQAGLDYIDQKMLKT

MIELYRGGPVGLSTIAANIGEETETVEDMYEPYLLQMGFLQRTPRGRIVTKLGYDHLG

YPYQTD"

CDS complement(1528663..1529283)

/gene="ruvA"

/locus_tag="JFBMFIFI_01447"

/EC_number="3.6.4.12"

/inference="ab initio prediction:Prodigal:002006"

/inference="similar to AA sequence:UniProtKB:P40832"

/codon_start=1

/transl_table=11

/product="Holliday junction ATP-dependent DNA helicase

RuvA"

/db_xref="COG:COG0632"

/translation="MYEYIKGTLTFVGPAYIVIENGGIGYQLLIANPFRFSSKLNQEV

QVYVYQAVREDAITLFGFKDFSEKQLYLKLISVSGIGPKSSLAILASDDHAGLVQAIE

GDDVAYLTKFPGVGKKTASQIVLDLKGKLDDLMTPADGVTSGALQEKLTLPTNHGHVE

ETLEALSALGYSGKEIKRIEPQIRSLNEHSTDAYLRAALRLLMKKK"

CDS complement(1529391..1530596)

/locus_tag="JFBMFIFI_01448"

/inference="ab initio prediction:Prodigal:002006"

/codon_start=1

/transl_table=11

/product="hypothetical protein"

/translation="MNKKKITTLLLASSGISSIGEWIYFIALNLIVLNRGATPAAVGV

LYILRPIATVLTTSIMSHFLDDLKKRKWLIILDITRACMIGSLVIINELWWIYLIVFL

IQGAVSIHSPLLITYTVSLIPAEKRKKYNALSSLVHSGGFLLGPAIAGLLLMVGTPKL

AIVVNSTALFLSAYFIFLLPNCETVPREKKPNTGITFSETWFEVKSFSLKYGNETLIY

SSFLLLMSFAAGLDSIEASFAKGMLALTDGEYGLLVSIAGLGILCGSGIVTIFSEKLS

VAHLLQSGSFFFVLGYFIYAISTHFISAAVGFFLLSFALAFANTGFYTFRQNYLPISK

MGLLISFYDLVEALLSIFIVGVVSLLAIYVHLRKVSLIVVFCMWLVLLFFYKKVKNEK

VAKKLVRKR"

CDS complement(1531460..1533736)

/locus_tag="JFBMFIFI_01449"

/EC_number="3.2.1.-"

/inference="ab initio prediction:Prodigal:002006"

/inference="similar to AA sequence:UniProtKB:T2KMH9"

/codon_start=1

/transl_table=11

/product="Putative beta-xylosidase"

/translation="MQKKWSQLIENGLVTIKNENGATLRFSTESGIGIIEADGYAFKD

LNRNGKLQPYKDWRLPISERIEDLANQLTISEIAGLMLYSGHQAVTSSKNSFASMFAG

TYDGLALEDSTAVVSDLTDQQKEFLTKDHLRHILVTVVESPEISATWSNNLQAFVEGL

GHGIPVNISSDPRHGADANSEYNAGAGGDISKWPEALGLAATFEPELVRDFGHIAGRE

YRALGIATALSPQVDLATEPRWMRFSGTFGEGVKLVTDLSEAYCSGFQTSEKASEIHS

GWGYTSVNAMVKHWPGGGSGEAGRDAHYAFGKYAVYPGGNFKEHLKPFTEGAFKLKGG

TEKAAAVMPYYTISTNQDLKNRENVGNGYSHYLVQELLRDEYGYDGVVCTDWGITKDH

QEMDSFLSGKDWGVEELTVAERHYKVLLAGVDQFGGNNEVEPIMAAYELGKAEFGKEF

IEARFRRSAKRLLRNLFQVGLFENPYIDSAKTKKTVGHPDFMKKGFEAQQKSIILLKN

QNNVLPVAKKQTVYIPKRRLKASKDWFGQEIPAREFLPVEAALVEKYYHLTTDPAQAD

FALCFMESPQTSGFSLEDLAKGGTGYVPISLQYRPYTARLGRKESLAGGDPLEAFSNR

SYYVKSNEAINEADLDIVIETREKMGNKPLIAIVAMKNPTILHEFETLVDGILVDFGV

QTQALLSLLSGEVEPSGLLPFQIPDKMESVEKQLEDVPFDMTCHRDENHNSYDFAYGL

NWTGVITDQRVEKYGLKS"

CDS complement(1534005..1534448)

/gene="msrB"

/locus_tag="JFBMFIFI_01450"

/EC_number="1.8.4.12"

/inference="ab initio prediction:Prodigal:002006"

/inference="similar to AA sequence:UniProtKB:E6ESW1"

/codon_start=1

/transl_table=11

/product="Peptide methionine sulfoxide reductase MsrB"

/translation="MTEKNKEELKQKLTDIQYEVTQNNGTERPFTGEYDDFYENGLYV

DIVSGEPLFTSKDKYDAGCGWPAFTKPVSEKEIIEKTDQTFGMKRVEVRSKEANSHLG

HVFTDGPKELGGLRYCINSAALRFIPVEELEAQGYGDYQKYFKEE"

CDS complement(1534520..1535047)

/locus_tag="JFBMFIFI_01451"

/inference="ab initio prediction:Prodigal:002006"

/codon_start=1

/transl_table=11

/product="hypothetical protein"

/translation="METAKLKQRTNQLRQQLNSENALVFEEVVEYLRASPVNTKKTEE

ILAELLDHLIELEQIGKTSKDLFGENVHGYCRQLVKGLHKDTFIEKLNFALLIVALSA

IFELVFAFINGVIDWRLSTAHLICNLFFAQLAFWAYQKIDHNSEIQKKVYYFAFSTLF

IVIYVALGLVLSENR"

CDS complement(1535087..1535422)

/locus_tag="JFBMFIFI_01452"

/inference="ab initio prediction:Prodigal:002006"

/codon_start=1

/transl_table=11

/product="hypothetical protein"

/translation="METSQMLKGLLEGCILSIIKDQEAYGYEMVAKLADYGLDMVSEG

SIYPVLLKLQKQDYVSSRMLPSKEGPKRKYYSITKQGHTYLEEFQKQWSKVATSVNDI

IEFSDNHER"

CDS complement(1535599..1536177)

/gene="maf"

/locus_tag="JFBMFIFI_01453"

/EC_number="3.6.1.9"

/inference="ab initio prediction:Prodigal:002006"

/inference="similar to AA sequence:UniProtKB:Q02169"

/codon_start=1

/transl_table=11

/product="dTTP/UTP pyrophosphatase"

/db_xref="COG:COG0424"

/translation="MKVILASGSPRRKELLSKIISNFEVVVSDVDETIDESLPGNVIV

SELAKRKALVVAKNFPTDLVIGSDTIVYLDGQVLGKPSSEQEAAQMLGKLSGTKHHVY

TAVYMTNGSLEVTEVVDTVVTLYSLTEKEIKNYIASGEPMDKAGAYGIQGLGALLVSE

IQGDFYAVAGFPVAVVYRMLAKFDGIEIDFAR"

CDS complement(1536201..1538324)

/gene="mutL"

/locus_tag="JFBMFIFI_01454"

/inference="ab initio prediction:Prodigal:002006"

/inference="similar to AA sequence:UniProtKB:P49850"

/codon_start=1

/transl_table=11

/product="DNA mismatch repair protein MutL"

/db_xref="COG:COG0323"

/translation="MGKIVELSEKLANQIAAGEVIERPASVVKELVENAIDAGSTQID

ILIEEAGLKKIQIIDNGAGIAADDVRNAFKRHATSKIHSRDDLFRIRTLGFRGEALPS

IASVSEITLETATGVGAGSYLALKGGDIVEERANPARKGTSITVENLFFNTPARLKYV

KTIQTELATIGDIVNRLAMSNTQVAFRLVHDGNQMLRTMGNGDLKQTLAGIYGVSVAK

KMREVEKEDLDFKLKGYVSLPELTRASRNYMSIMINGRYIKNYLLNKAIVAGYRSKLM

VGRFPFVCLDIQMDPLLVDVNVHPTKQEVRISKEKELMELIETAIHECLSHEQLIPES

LENFSFKRPYDASRFQSEQTKIDLTASKTSSDELFSKNQAEDSKENQVHSIEKKSTED

KNQDYFKTIPFKSTPSPIPAQREASPFERGIGYEAAEGQFYLKKNRVEEEFIEDPFAP

IPENLMNEVTVETTHNLQSEPPEIESNQNQSELQELHTESHLKAAEKLIKEEAQLKNK

AIAHLPNLEYVGQMHGTYLFAQNENGLYILDQHAAQERIKYEFFRKKIGEVSRDLQDL

LVPIMLDYPNSDAIKIKENNEALEEIGLFLEPFGQNSFLVRSHPVWFNRGQEEEIIKE

MIDLLLEQGSISVAKFREATAIMMSCKGSIKANHHLNDQEARALLVDLAKTENPYNCP

HGRPVLIQFTNKDMERMFKRIQDPH"

CDS complement(1538391..1541012)

/gene="mutS"

/locus_tag="JFBMFIFI_01455"

/inference="ab initio prediction:Prodigal:002006"

/inference="similar to AA sequence:UniProtKB:P0A1Y0"

/codon_start=1

/transl_table=11

/product="DNA mismatch repair protein MutS"

/db_xref="COG:COG0249"

/translation="MPQKTKHTPMMEQYLAIKANYEDAFLFYRLGDFYEMFYDDAIKA

TQLLEITLTARNRNADEPIPMCGVPYHAARGYIDILIEKGYKVAICEQVEDPKTAKGM

VKREVVQLITPGTAIETKNMDAKKNNYLSALLVQEGNKFDLAYVDLSTGELKATQLDS

VEDVINELSSIQTKEIVFNGESNEEFQQELKNKLDVMISNQKEIIENAEFSYLTSDLE

ETSTLSVLKLLLSYLSVTQKRSLAHIQKAETYVPTHFLKMDHYSKYNLELTTSIRNGQ

KKGTLLWLLDETKTAMGGRLLKQWIDRPLIQEQKIKGRQAIVESLMNHFFERTDLNEA

LTRVYDLERLAGRVAFGNVNGRDLLQLKTSLLQIPQLIEIIAIINDTEWDTLLGALDP

VTEVVDLIELAIHEDAPLSLKDGGVIKDGYHEKLDQYRDAMRNGKKWMAQLEAEERAK

TGIKGLKVGYNRIFGYYIEITKANLANLPEGTYERKQTLANAERFITPALKEKETLIL

EAEEKSMALEYELFAEVRETVKNYIERLQRLAKNVATIDVLQSFATVSEKYHFIKPTM

TTGKHEINLLEGRHPVVEKVLGQQTYVPNSVEMTTETDILLITGPNMSGKSTYMRQLA

LTVIMAQMGCFVPAEKADLPIFDQIFTRIGAADDLISGQSTFMVEMMEANQALRHATD

RSLILFDEIGRGTATYDGMALAEAIIEYIHQHVHAKTLFSTHYHELTVLEDSLKGLEN

IHVGAVEENGEVVFLHKMMPGPADKSYGIHVAKLAGLPEELLERAATILEKLEQQDTV

VLDYQPTYATAAPKITEEKTKQTNIVEESDGQLSLFGERNPNEADVIDAINQLNLLAM

TPLDAINTLSQLQQKLK"

CDS complement(1541033..1541422)

/locus_tag="JFBMFIFI_01456"

/inference="ab initio prediction:Prodigal:002006"

/codon_start=1

/transl_table=11

/product="hypothetical protein"

/translation="MRGTNESLLDEATKAHLQRLTDLLKENELIQDYQMIEEKAQNHA

HLNQLIADIKIKQKEAVAFNHYGKPEAEKIAIKEAEAFTKEFNEHMVVTSYQDALVEA

NDILQHLVATVQDELKEQINQTLEDNR"

CDS complement(1541419..1542228)

/gene="ymdB"

/locus_tag="JFBMFIFI_01457"

/EC_number="3.1.4.16"

/inference="ab initio prediction:Prodigal:002006"

/inference="similar to AA sequence:UniProtKB:O31775"

/codon_start=1

/transl_table=11

/product="2',3'-cyclic-nucleotide 2'-phosphodiesterase"

/db_xref="COG:COG1692"

/translation="MRLLFVGDVVGSMGREMVHDYLPKLKRKYRPQVTILNGENAAAG

RGITEKIYKSFLQDGVDIVTMGNHTWDNREIFDFIDDAKKMIRPANYPEDTTPGKGMA

FIKVNQLELAVINLHGRVFMADLDDPFRKIEDLVDEARQRTPLIFVDFHAETTSEKQA

MGWFLDGQVSAVVGTHTHVQTSDARILPQGTAYLTDVGMTGPYDEILGMAKEQVINRF

LTQMPTRFEVPKTGRKTLSACYIELDDSTGAAKKIEPILINEDHPFGGDFE"

CDS complement(1542411..1543976)

/gene="rny"

/locus_tag="JFBMFIFI_01458"

/EC_number="3.1.-.-"

/inference="ab initio prediction:Prodigal:002006"

/inference="similar to AA sequence:UniProtKB:O31774"

/codon_start=1

/transl_table=11

/product="Ribonuclease Y"

/db_xref="COG:COG1418"

/translation="MNTIIISVAFAIVALVVGLIVGYIVRKSTHEKELAGARNTATGI

LEEAKREAETLKKEALLEAKDENHRYRTEIESELKERRSDILKQENRLVQREENLDRK

DDGLEKRERTLEAKEEKLSSRQQLIDDQEQKVTALVEEQQLELERVAALSRDEAKDMI

MKETEEDLSHELAVMVKESEQKAKEEADRKAKNLISLAIQRCAADQVSETTVSVVTLP

SDEMKGRIIGREGRNIRTLETLTGIDLIIDDTPEAVILSGFDPIRREIARMTLEKLIQ

DGRIHPARIEEMVEKSRKEMDERIREIGEQATFDVGVHSLHPDLIKILGRLRFRTSYG

QNVLNHSIEVAKLAGILAAELGEDVTLAKRAGLLHDIGKALDHEIEGSHVEIGEEIAI

KYKENATVINAIASHHGDTEATSVISVLVAAADALSAARPGARSESLENYIRRLEKLE

GISNSFTGVERSFAIQAGREVRIMVQPEIIDDLTAITLARDIRKRIEDELDYPGHIKV

TVIRETRAVDYAK"

CDS complement(1544365..1545519)

/gene="recA_2"

/locus_tag="JFBMFIFI_01459"

/inference="ab initio prediction:Prodigal:002006"

/inference="similar to AA sequence:UniProtKB:P16971"

/codon_start=1

/transl_table=11

/product="Protein RecA"

/db_xref="COG:COG0468"

/translation="MADDRKQALDAALKKIEKNFGKGSVMKLGEKIDTQVLTIPSGSL

ALDVALGVGGYPKGRIIEVYGPESSGKTTVSLHAVAEVQKQGGIAAFIDAEHALDPKY

AAALGVNIDELLLSQPDTGEQALEIADALVSSGAIDIVVVDSVAALVPRAEIEGEMGD

SHVGLQARLMSQALRKLSGSINKTKTIALFINQIREKVGVMFGNPEVTPGGRALKFYA

TVRLEVRRAEQIKNGTDVVGNRTKIKVVKNKVAPPFKVAEVDIMYGEGISQVGELLDM

GSEKDIVNKSGAWYSYEGERIGQGRENAKKYFMENPELRVEIEQKVRAAYGIGEEVVE

EEKTEPVKNEKKAVENKVSEAESIAVDKIAKENENQTEIVLDLPEIDPEK"

CDS complement(1545825..1547084)

/gene="cinA"

/locus_tag="JFBMFIFI_01460"

/inference="ab initio prediction:Prodigal:002006"

/inference="similar to AA sequence:UniProtKB:Q8DRX2"

/codon_start=1

/transl_table=11

/product="Putative competence-damage inducible protein"

/db_xref="COG:COG1058"

/translation="MKAEIIAVGTELLLGQIVNSNAAFLSQELAVLGINVYHHVVVGD

NPERLDQVLTEAEKRSDLIILSGGLGPTKDDMTKQTIAAHLNRSLITDAATLKTIVEY

HKKTKRPMPENNKLQAVVIEGSTVLKNPTGLAAGMFIKENGKMYVLLPGPPSELKPMF

LQEARPLLLANSDKPELLVSRVLRFFGIGESKLVTILDDLIENQKNPTIAPYAGVHEV

TLRLTANGGSEKACNELLDRLEATIQERVGTYFYGYGDETTLVEVVVDLLKEKKLKIT

AAESLTGGAFQSLLASVSGVSATFDGGVVTYSNESKTKVLGVSSDTIKQYGVVSEACS

IEMAEGVKQLYGTDIAISFTGVAGPETLENQPAGTVWIGLAVIGQKTIAKCFHFGRNR

NGNREQAALSGLEMVRRILKNIPLDEE"

CDS complement(1547138..1548532)

/gene="cpdA"

/locus_tag="JFBMFIFI_01461"

/EC_number="3.1.4.53"

/inference="ab initio prediction:Prodigal:002006"

/inference="protein motif:HAMAP:MF_00905"

/codon_start=1

/transl_table=11

/product="3',5'-cyclic adenosine monophosphate

phosphodiesterase CpdA"

/translation="MKKKKTLLLALGIFFLFLSGCSQKNEATQKGTNQISESNIKNQV

VEPGFKLWVITDIHYISPSLYDDGEKFKFIIQTSAGKDIIYQEETLEALVYKAQREKP

NVLVVSGDLTLNGEKQSAIELAVYFAEIEKTGTNVYVIPGNHDISDGWAKKYRAEKAE

ATDQILPKDFSRIFQDFGYNEAISVDSNSLSYLVAPRENLWIAMIDTNKYSWQGSTRG

PITSGSLRDETYQWLQECFTLAAEKGAKVIPVIHHNLLDHNQLVNQGFTLDNAEEMQK

LLSQEKVSFTLSGHIHAQDIASKQLNGEPIYDIVTGSFSMLSNPIGELTFENGQMEYQ

RISTDVDIWAQATKNKNPDLLNHNEYLRSLMQKDGEAFAISQIYEEQWPDKSQAESIG

KFVGEANIRFFGGQSFVEKAEVEKVTRELEQNPAYQALQKQPKSSLTSYIESIYIDQD

TNNIQLKIPFQFLK"

CDS complement(1548644..1549222)

/gene="pgsA"

/locus_tag="JFBMFIFI_01462"

/EC_number="2.7.8.5"

/inference="ab initio prediction:Prodigal:002006"

/inference="similar to AA sequence:UniProtKB:P63756"

/codon_start=1

/transl_table=11

/product="CDP-diacylglycerol--glycerol-3-phosphate

3-phosphatidyltransferase"

/translation="MNLPNKLTVLRIFMIPIFMIVVLGSFDWGQVTWLGSTIEITQLV

GAIIFAVASITDWLDGKIARARGLVTNFGKFADPLADKMLVVTAFIVLVGQGVVPSWV

VAIIICRELAVTGLRLLLVGDGEVMSAAMPGKIKTATQMIAIILLLLNNFPFAGMNFP

FAMIMLYICLFFTVYSGIDYFVKNKHVFSGPM"

CDS complement(1549360..1551435)

/gene="nhaK_1"

/locus_tag="JFBMFIFI_01463"

/inference="ab initio prediction:Prodigal:002006"

/inference="similar to AA sequence:UniProtKB:O32212"

/codon_start=1

/transl_table=11

/product="Sodium, potassium, lithium and rubidium/H(+)

antiporter"

/db_xref="COG:COG0025"

/translation="METLFLFLIMIVGIIFSAVISKYVPKVPLALIQISIGILMVLLP

LNRELHFEPEIFMVCVIAPLVFYEGQKVSRKELWELKGPILLLAFGLVLLSVVLGGIV

IHWLIPSMPLAISFALAAIISPTDTVALKSIVKNVKLPTNIMGVLEGESLINDAAGLV

SFKVALGVALTGVFSVKEASVSFVVAAIGGVVLGAFIGVMFVKLRLRLRKMGLEESEL

LILIQLATPFVIYILAEKLEFSGILAVVAAGIVHGFEHDKLQKTTTKLKLVSNNVWST

LVYFLNSLVFLLLGSILPSVIQEIWDAADVHVLDLVLASILIISFLMFLRFIWVYVLY

NHFVDPNYGSFEDYLAQLAGKKSVEKEPEISRFKYAFITTLAGVHGTISLATALSIPL

ILTNGEAFPLRNELLFITACVILISLVSATILLPLILPKEKNELMLVPILSEASAYQK

VLQQTIVRLEQNRTDENHYVLNEIIMEIGSKLTEIETGYVRDENRKIIAEVLKTAQNE

ESRQINELLENNEISPMVQRLYQVYLENSRQFSETNFFKKVWFRLKMGLMKKRLKKMQ

KEKFTEQFTERFQARFHENSELIQEFTHSQRLITESVIGTIQKQITPENRKESLIVME

RYNRRLKLAKLSSGISKNEVRSYASLALQLEREEIQRLLDVGEIDYATANQLGERVTY

DELSELSIN"

CDS complement(1551515..1552399)

/locus_tag="JFBMFIFI_01464"

/inference="ab initio prediction:Prodigal:002006"

/codon_start=1

/transl_table=11

/product="hypothetical protein"

/translation="MNEIGEKLQEARKAKGYTLDDLQQMTKIQKRYLIAIEEGNFDVM

PGKFYARAFIKQYADTVGLNGDQLLEQYTDAVPQTHDEEYVEKVNTNQTRSENHVTNE

LVEKIKNMLPTILIVLLVFAIIGGIWYAATKTGNKDDGSIISKDADSVSITSTSESKE

SSQEKESQSSSSKEPEKEPEKPKQAVAVASSTGATTEYTVTNATEPGTITLTAQGGAS

WVGVEVNGAPADQKTMQSGESLEVPLPAGTTSVSVRIGNAAATSITLNGEAVAFAPEA

AGVITQSLTFTITPVVAQ"

CDS complement(1552497..1553222)

/gene="fabG_2"

/locus_tag="JFBMFIFI_01465"

/EC_number="1.1.1.100"

/inference="ab initio prediction:Prodigal:002006"

/inference="similar to AA sequence:UniProtKB:P50941"

/codon_start=1

/transl_table=11

/product="3-oxoacyl-[acyl-carrier-protein] reductase FabG"

/translation="MKFALIMGASGDIGAAIAQDLAKAGWSLYLHYHTDFSSVEKQVE

AYQVHYPQQDFFTLQLDMTNETEIPSFLDSIFQLDAFIIASGFTQYQLLTDTESQVMD

RMWQVHIKTPILLVQKLQKKLAASANGRVIFISSIYGEVGSAMEVLYSTTKGAQLAFV

RSYSKEVASLGITVNAISPGAIATKMNQSFSEAEFNALKSEIPLGRLGSTAEISFWVQ

QLVEPLSQYMTGQSLIVSGGWLK"

CDS complement(1553219..1554520)

/locus_tag="JFBMFIFI_01466"

/inference="ab initio prediction:Prodigal:002006"

/codon_start=1

/transl_table=11

/product="hypothetical protein"

/translation="MEKKEYQQLDETLYTETLPNGLKVTLLPKNEFHKTYALFTTNYG

SIDNQFIPLGKSEMVTVPDGIAHFLEHKMFEKEDGDIFNTFGKQGASANAFTSFTRTS

YLFSSTSHVLENVATLLDFVQEPYFTKETVDKEKGIIAQEIQMYDDEPDWRLFFGIIG

NMYPLHPLHIDIAGTVDSIMDITAENLYESYETFYHPSNMNLLVVGKMDPTEMMEWIR

QNQAKKDFPQATEIVRHFPTETVSEIKAFDQIEMPVNRAKSIVGVKGVAPAPVGKAAL

VYKTTMNLLLSLLFGSTSENYLRLYNEGTIDDSFSYEFNLERTFHFVDVGGDAKDPKA

LSDAVKVILINAKTSPELTQENLEMVKKRMIGSVLQSLNSLEYIANQYSQESYGEASL

FDLVPIIEAISLTEVVQLAENFMVPEHMSVFHIVPKGASKA"

CDS complement(1554522..1555778)

/locus_tag="JFBMFIFI_01467"

/inference="ab initio prediction:Prodigal:002006"

/codon_start=1

/transl_table=11

/product="hypothetical protein"

/translation="MTIQLEEGVHLHVIPTKKYKTVRFVLKFRAPIDAETITKRALLS

SILETNSKKYPTQTQLRSELANLYGASFGLSVTKKGTQHIFTAGMNLVNEKYLSGEPA

VLMNGIALLKEILLNPNAADGKFDEATFQREKENLFDYYESIFDDKQAYASLALQALY

FENEDQKMPSIGTAEELEKITATSLYTYYQQLIKEDTIDIYVLGDVDALEMKAAFADF

NFTPRKKATGSIYYKETLRNNSKSEVEKQEITQAKYNLAYETNGYYLEKDYFALQVFN

GLFGGYPHSKLFMNVREKESLAYYASSSLDTFRGMMTVQTGIESNKVQQVSEIIALQL

AEMQEGNFSEEAMNQTKEMLKNQILQSEDNSGALIERMYIYDVVDKKLSVEEWQNAVD

AVKKEEIIAVANQVHLKATFFLTGEA"

CDS complement(1555957..1556919)

/locus_tag="JFBMFIFI_01468"

/inference="ab initio prediction:Prodigal:002006"

/codon_start=1

/transl_table=11

/product="hypothetical protein"

/translation="MDAFISTVSIVVSSALIYAAPLIFTALGGTFSERGGIVNVGLEG

IMVMGAFSSIVFNLAFAEQLGSWTPWIALLVGGGIGVIFSLIHAVATVNLRANHIVSG

TVINMLAPALAVFLTRVLFEGKGQTDIIQQNFGKSSIPLLKDIPIIGKMFFTNTSAMA

FVAMFVAILAWFLLFKTRFGLRLRSVGEHPQAADTLGINVYLMRYAGVMISGLLGGIG

GAVAAQSISLNFSHSTIAGQGFIALAAMIFGKWNPLGAMGAAIFFGFAQSLSVIGSYI

PLIKDVPSVVLQTAPYILTIIVLVGVIGKSEAPAADGETYIKSK"

CDS complement(1556921..1558018)

/locus_tag="JFBMFIFI_01469"

/inference="ab initio prediction:Prodigal:002006"

/codon_start=1

/transl_table=11

/product="hypothetical protein"

/translation="MNHKSERLINILVPVLSVVLGLLLGAIIMWSFGYDPVLGYTSLV

EGAFGGPFYIGETLRQATPLILTALGFAVANTAGFFNIGVAGQALFGWVGSVTMALMM

PDLPKLLLVPLCLIVGALCGAIWAGIAGFLRAYFDTSEVIVTIMLNYTALYIANHMVR

NVLTKGDDATPRISENASLRSELLAQISDNSTLHYGLLLAIIMCVVVWIMMQKTTLGY

ELRAVGLNPYASEYSGMSTKKNIILAMVISGGLAGLGGSMEGLGNFQNLFVQGSMPSI
GFDGMAVSLLGAGNPFGILLAGLLFGALKIGGISMPMGSDVPTEVVDIVIASIIFFVG
SSYLIRYFMNKTKRPNRKIAATNSEAKKGVN"

CDS complement(1558008..1559576)
/gene="mglA_1"
/locus_tag="JFBMFIFI_01470"
/EC_number="7.5.2.11"
/inference="ab initio prediction:Prodigal:002006"
/inference="similar to AA sequence:UniProtKB:P0AAG8"
/codon_start=1
/transl_table=11
/product="Galactose/methyl galactoside import ATP-binding
protein MglA"
/db_xref="COG:COG1129"
/translation="MTGTDYVIEMSGITKQFGDFKANDNINLQLKKGEIHALLGENGA
GKSTLMNILSGLLEPTSGDIKINGKKVTISSPSMANKLGIGMVHQHFMLVKKFTVTEN
IILGSEPSKLGFIDKLAAKKIIKELSGKYGLAVDANAVVEDISVGMEQRVEIIKTLYR
GADILILDEPTAVLTPQEIGELIEIMHELVKEGKSIILITHKLDEIKQVADRCTVIRR
GQSIGTVNVKETSQQQLADMMVGRSVSFKTTKTAAKPKEVVLSVENLIVKESRGLDAV
KDLSLEVRAGEVLGIAGIDGNGQSELIQAITGLSKVEKGKITLNGREIQNLPPRKITE
TGLGHIPEDRHKYGLVLPMSLEDNIALQTYYQKPLSSHGFLNQQAINTYAKKLIAEFD
VRCHNERVPASALSGGNQQKAIIARELDRNPSLLIAAQPTRGLDVGAIEYIHKRLIEQ
RDNDKAVLLMSFELDEILNVSDRIAVMYEGNIVAVVKPEETTREELGLLMAGSSLEKA
RAAESAGKEHSIES"

CDS 1559852..1560232
/locus_tag="JFBMFIFI_01471"
/inference="ab initio prediction:Prodigal:002006"
/codon_start=1
/transl_table=11
/product="hypothetical protein"
/translation="MLAMKRIYGDYDATDGTRVLVDRLWPRGIKKTSAHLDYWAKELA
PSTQLRKWFNHEEEKMPEFAAAYRKELFSSTTAKNKMQELSKKSQHETLTFLFAAKSY
EVNHVVVLLSIMKKEYGAKLTSTK"

CDS complement(1560279..1561331)

/gene="tmpC_1"

/locus_tag="JFBMFIFI_01472"

/inference="ab initio prediction:Prodigal:002006"

/inference="similar to AA sequence:UniProtKB:P29724"

/codon_start=1

/transl_table=11

/product="Membrane lipoprotein TmpC"

/db_xref="COG:COG1744"

/translation="MKKRNLMGLLALGVGVSMTLAACGGNSGDKGKDGGDDKKTTHTA

AMVTDVGGVDDKSFNQSAWEGLQAWGKEHGLKKGKDGFNYLQSNSDSDFVTNLNSAVK

GNFNTVFGIGYKIAPALEDVAKQNPKTNFAIIDSVVEGDNVVSIVFKDNEAAFLAGVA

AAKTTETKKLGFIGGQEGEVIGRFEAGFIAGVKAVDPDIDVKVEYAGSFGDPAKGKQL

AAAMYNSGIDIIYQAAGDTGNGVFSEAKDIMKADESKKVWVIGVDRDQDAEGEYDGGN

LTLTSTLKGVAATVIDISNRAMDDKFPGGETLTYGLKEDGVGITEGNLSKEVLKDVDD

YKAQIIDGKVEVPEKP"

CDS complement(1561593..1563932)

/gene="spoIIIE"

/locus_tag="JFBMFIFI_01473"

/inference="ab initio prediction:Prodigal:002006"

/inference="similar to AA sequence:UniProtKB:P21458"

/codon_start=1

/transl_table=11

/product="DNA translocase SpoIIIE"

/db_xref="COG:COG1674"

/translation="MVQKKKKKKNTKKQQGRNLSIEIIGLIFICSAILAGIKLGFVGR

LMANLFRFFVGNTYLVSVLIFGLYGTYLLIRGKEPVYKNKKIIGFSVIYTSVLILFHA

FLFGPIMDTKVNVISATIRYFMTDMSASTTSQSLGGGMIGALLYSLSYFLFSQWGTYL

ISGLIFVIGIFLFFNLSFKVALEYVRNYLRKMYALLKEKWIVYKEQQAIKKTQQEKSQ

AEAVAKNKVKPISAGKKLAEEVKEPEKGEAQQLNLEIDSYQSRVQKPEVKVEQATEVM

DVSDANEEGLSELEFEIQAEPENRDYQLPPTSLLNEIEALDQTNEYALIKLNVKKLEE

TFASFGVEAKVTKANLGPAVTKYEVQPAVGVKVSKIVGLSDDIALALAAKDIRMEAPI

PGKSFIGIEVPNSEVSTVAFRDIIEGQTIHPDKLLEVPLGRDITGSVAVADLSKMPHL

LVAGSTGSGKSVCINGIITSILMRAKPNEVKLMMIDPKMVELNVYNGIPHLLTPVVTN

PKKAAQALQKVVIEMEQRYEKFAATGMRNITGYNAMVIEHNLENGENNPTLPYIVVIV

DELADLMMVASNEVEESITRLAQMARAAGIHMILATQRPSVDVITGIIKANVPSRIAF

AVSSGTDSRTIIDGSGAEKLLGRGDMLFLPMGENKPVRVQGAFISDAEVERVVEFVTE

QQGANYQEEMMPTEIPETVNGEVQDELYDDAVAMVLEMQTASISLLQRRFRIGYNRAA

RLVDEMEMRGIVGPSEGSKPRKVNITHLPQRESEESNPT"

CDS complement(1564090..1564914)

/locus_tag="JFBMFIFI_01474"

/inference="ab initio prediction:Prodigal:002006"

/codon_start=1

/transl_table=11

/product="hypothetical protein"

/translation="MEIKVIKKEAKSYLKGNWGTAIGSMFIMGLIVMAINFVVSMVSG

LSTMLQMIPLSYMENSEEAMLALIPAFIGSLGVIIVFSIVVALVQQLLSVGYKWALLD

LIDGKNYSVGSLFQGFNRTSFKTIGLIIMMGIFTGLWSLLFIVPGIIKSYSYSQALNI

LKDNPEIGIMDAITASRKLMDGKKGQLFVLQLTFILWYVIPVIVLILVIVLLGALGGS

NESNGFFLAFALLGYFALIIYILVINFYIYPYLTTSEQNFYRHLTDVKIDEKIAEI"

CDS complement(1565074..1567152)

/gene="topB"

/locus_tag="JFBMFIFI_01475"

/EC_number="5.6.2.1"

/inference="ab initio prediction:Prodigal:002006"

/inference="similar to AA sequence:UniProtKB:P14294"

/codon_start=1

/transl_table=11

/product="DNA topoisomerase 3"

/db_xref="COG:COG0550"

/translation="MKILVIAEKPSVGKELARVLGATQKSKNYIEGKDYIVTWALGHL

LGLKMPEDYKKEWATWDMESLPLIPPKMATKPLKNTGHQLKAIKQLATRKDVSSAVIA

TDAGREGELVARWILEYVHFNKPVKRLWISSQTDKAIKDGFKNLKPSQAYDHLYDSAI

ARSQADWLVGLNVTRALTVKYQDSLSAGRVQTPTLAMVRQQEEKIEKFVPETFYTVSL

LSNGEKAQVTEGAATKFSDRKEAESLATRLKNQAVKVVEVKEKQQIEYAPLPYDLTEL

QRDANKRYQFSAKKTLGLMQNLYERHKVLSYPRTDSKHLTTDMAATMKDRLLAIQVFD

PERVKIALKNGGKTTNKSVYNNSKVTDHHGIIPTEERPRAEKMDNDELRIYRMVVERF
LGLFLPAYQAVKHTYTLGVDGISFKLTQEKVIEKGWKAEAVVSSQTSYKKGEVLGNPS
FTVNKHLTEAPSRLTEASLLQKMEKTGLGTPATRAEIIEKLISSDLMERNQNKLSVSP
KGRQLLTLVNPALVTPDLTAQWEKSLEEIANGKLKKDQFIKQIESETSRLVKEIKTSN
RNYTDHSLTNKVCPECGEKLKEKSGRDGKILVCSSSECSYRRRKDPKVSMHRCSQCHK
KMEVHEGKNGAYFKCKYCNISEKIGDKKNKKMSKHEEKKMLKKYSQPTEEVESPLALA
LKAAMADSED"

CDS complement(1567165..1567479)
/locus_tag="JFBMFIFI_01476"
/inference="ab initio prediction:Prodigal:002006"
/codon_start=1
/transl_table=11
/product="hypothetical protein"
/translation="MYQVVTLFGEYEPWWFFEDWKEEIVSFEQFDSFSEANQNYHEKY
IKLIGDYENYVTKKHYLTAFWNEEEKRYCEDCEDDIQLFHSIMLLKEEKPMEINHQVG
SV"

CDS 1567618..1567893
/gene="acyP"
/locus_tag="JFBMFIFI_01477"
/EC_number="3.6.1.7"
/inference="ab initio prediction:Prodigal:002006"
/inference="similar to AA sequence:UniProtKB:O35031"
/codon_start=1
/transl_table=11
/product="Acylphosphatase"
/db_xref="COG:COG1254"
/translation="MRTIKMNASGRVQGVGFRYMTKMVADQIGIGGIVCNQNDGSVYI
EANGTEDEIDLFIEKIKKSPSPSGRVDHLDIIDDPNIMIRNKFSVTN"

CDS 1567959..1568873
/gene="yidC"
/locus_tag="JFBMFIFI_01478"
/inference="ab initio prediction:Prodigal:002006"
/inference="protein motif:HAMAP:MF_01811"

/codon_start=1

/transl_table=11

/product="Membrane protein insertase YidC"

/translation="MNKLKKLVLTSGVLSLVLFLSGCMKMDKQGNPTGFIYEYLVVPT

GNAIIWLADLFNGSYGLAIIFITLIVRIIILPLNLSQSKKSMVQQEKMAFIKPELDVV

QQKQKTATTAEEKAAAQQELMDVYKRNNMSMMGGIGCLPILIQMPVFTAMFQAIRLSP

QIAESNFLGINLGDRNAYLAIAAGLVYVIQAFISMIGLSPEQKKQMRTMLFMNPVMIL

VFTWSSPAGLGLYWLVGGVFACFQTLITNLYHKPKIKAAIAEELKDRPVVQPAAPIKQ

AEVVSPSSKKSTSNNKKKRNDGKQQRRN"

CDS 1569284..1569490

/locus_tag="JFBMFIFI_01479"

/inference="ab initio prediction:Prodigal:002006"

/inference="similar to AA sequence:ISfinder:IS657"

/codon_start=1

/transl_table=11

/product="IS200/IS605 family transposase IS657"

/translation="MIFDRHANLKYRYGNRKFWCRGYYVDTVGRNKKQIEEYIRNQVQ

EDYVADQLTLFEEYDPFTGNKNTK"

CDS complement(1569654..1571168)

/gene="arlS"

/locus_tag="JFBMFIFI_01480"

/EC_number="2.7.13.3"

/inference="ab initio prediction:Prodigal:002006"

/inference="similar to AA sequence:UniProtKB:Q7A5N3"

/codon_start=1

/transl_table=11

/product="Signal transduction histidine-protein kinase

ArlS"

/translation="MIKTTKEREKEIGVRRRVSLKWKWTIGASMAIFFTYTLFSVAIY

LGFTQMMISQENSNVNDIMSGVAAKLKVGSSNLTKETVKNSLTPNYYEIDSTVDNRFL

MTEGLDTTADALFSKINEIGIVVAVFDPSGSKLYESRPSHVTFEKKPKPEIKEIKING

ARGLSGVTPIMSSQNQNIIGYVQVTNNLTRYHEMSNTVLLAMLLMGFLALVVSGVLGY

LLAINFLRPIKQMTQTMNDVRKDTQSDSRMIVPDSNDELSHLTELFNDMLDKMQKYIE

QQKQFVEDVSHELRTPVAIMEGHLKLLDRWGKEDPEILEESLTASLQEIERMKSLVQE
MLDLSRAEQVDIHYKHELTDVKNIINQVFNNFSMIHPDFVFNLDDDLAGNVMVQIYRN
HFEQILIILLDNAVKYSTNRKEIHISVAEDLYDIQIAIQDFGEGIAPEDLQKIFNRFY
RIDKARSREKGGNGLGLAIAKELLDGYQGNITVESVLDFGTIFRIRLPIYREGK"

CDS complement(1571165..1571851)

/gene="arlR_1"
/locus_tag="JFBMFIFI_01481"
/inference="ab initio prediction:Prodigal:002006"
/inference="similar to AA sequence:UniProtKB:Q9KJN4"
/codon_start=1
/transl_table=11
/product="Response regulator ArlR"
/db_xref="COG:COG0745"
/translation="MNRILIIEDEKNLARFVELELKHEGYETEVQNNGRKGLETALDS
DWDAILLDLMLPELNGIEVCRRIRQVKNTPIIMMTARDSVIDRVSGLDHGADDYIVKP
FAIEELLARLRALLRRIDIEGEQNVTKQTTVTYRDLTIEKENRVVRRGDKVIELTKRE
YELLLALMENINVVLARDVLLNKVWGYETEVETNVVDVYIRYLRNKIDVPEEESYIQT
VRGTGYVMRS"

CDS complement(1572278..1573705)

/gene="gnd"
/locus_tag="JFBMFIFI_01482"
/EC_number="1.1.1.44"
/inference="ab initio prediction:Prodigal:002006"
/inference="similar to AA sequence:UniProtKB:P96789"
/codon_start=1
/transl_table=11
/product="6-phosphogluconate dehydrogenase,
decarboxylating"
/db_xref="COG:COG0362"
/translation="MSKQEIGVIGMAVMGKNLALNIESRGYTVSIFNRTGSKTEEVIA
EHPDKKLVPTYTIEDFVASLEKPRRILIMVKAGAPTDSTIQALLPHLDKGDILIDGGN
TFFEDTIRRNQELDQSGINFIGTGVSGGEEGALKGPAIMPGGQKEAYDLVAPILEKIA
AVAKEDGEPCVTYIGPNGAGHYVKMVHNGIEYGDMQLIAESYALLRDVAGLSVEETAE

VFAEWNKGELDSYLMEITSDILTRKDPETGKPIVEVILDRAGNKGTGKWTSQSALDLG

VPLPLITESVFARYISAMKDERVTASKILPKPAGFILEEDKKEFIEKVRQALYFSKLM

SYAQGFAQMRIASEEHDWDLQYGDIAKIFRAGCIIRARFLQKITDAYVREPELKNLLL

DEYFIGITKSYQEAVRDVVGIAVKAGVPIPTFSSAIAYYDSYRSATLPANLIQAQRDY

FGAHTYERTDKEGVFHYDWYGQENK"

CDS complement(1573817..1574941)

/gene="pepT_3"

/locus_tag="JFBMFIFI_01483"

/EC_number="3.4.11.4"

/inference="ab initio prediction:Prodigal:002006"

/inference="similar to AA sequence:UniProtKB:Q81WU4"

/codon_start=1

/transl_table=11

/product="Peptidase T"

/db_xref="COG:COG2195"

/translation="MLEVNEDRLVSRFLELIKINSETRHEAMIQEYLKKEFSLLGLTV

EEDQTSGETGFGANNLICTLNGNPEIEPFFFSCHMDTVAPGNEIKPVIKDKTIYSDGT

TILGADDKAGIAIMFEIIHLLKERKLAHGTIEFIISVGEESGLVGANAFDVQQLSSSF

GFVLDTGGPVGSITVGSPTQYRIEAILTGVTAHAGVAPEKGVSAAQIAASAIHQMKLG

RIDAETTANIGFINGGVATNIVMDRLEIIAEARSVNADSCEAQVAHMIETFERVATEM

GGKAAVITEKKYNGYRFTPETNVVKIAAKALTNIGIKPNYQVSGGGSDANVFNGKGKE

TTNLAIGYEKIHTVHEYLPISEFTKAAAMAYQIVEEITLN"

CDS 1575158..1576114

/locus_tag="JFBMFIFI_01484"

/inference="ab initio prediction:Prodigal:002006"

/codon_start=1

/transl_table=11

/product="hypothetical protein"

/translation="MRQGYHTNESGVLVPNSEDYVGGWIHVSDPSEEEVNQLVKEFKF

PKDYITSVLDAHEVSRQEMLKEEDTNSAILLVLQYPFLIESELGYKEYMTYPISIILT

DHTIITASKHLPQFISEITHNNPNFAVDTTHQEQFVLRLFWYISASYMRFLQEIDIET

RRLELQLTQSTKNEQLYALMSLQKSLIYFSTAINSNGPIFDIMKETDRFSKDERTRSF

LHDVIVENNQAQIMTSQYQKILDQVSTVFSAVVSNNLNNIMKILTSITIVLTIPTILG

GIYGMNVEIPFIDTPGAFWLIMVIMIISSVLTVWLLKKKDFF"

CDS 1576187..1576333

/locus_tag="JFBMFIFI_01485"

/inference="ab initio prediction:Prodigal:002006"

/codon_start=1

/transl_table=11

/product="hypothetical protein"

/translation="MKNKDLNKVTYKKAPTKMEKITKIVVWIMLFAMVGSGLLTVLYT

LFIN"

CDS complement(1576382..1577626)

/gene="macB_2"

/locus_tag="JFBMFIFI_01486"

/EC_number="3.6.3.-"

/inference="ab initio prediction:Prodigal:002006"

/inference="similar to AA sequence:UniProtKB:Q2EHL8"

/codon_start=1

/transl_table=11

/product="Macrolide export ATP-binding/permease protein

MacB"

/translation="MIINILISTFLSLKAHKLRVFLTMVGIIIGITSVVTISTLGEGM

KQQVLSATSSTSANNVKIMYSMEQEDNTGMGYVDYSNSDFSFSMVDLKKIKDTEGLES

IFPDYGQQFMGDSIDQELDYFGTKAYLMVAPNTGGIKVKYGRDFQPSDANRDRIILSH

TVFDDSISMENPADMINQAVSIGGYMYKVIGITEPVDFDSMSMTDMMGGFDYMSAYKS

YISKASYNELAKSKAISGIKLKLKDDVDRTQVIGSVISTLQELHPDVKGIFKEDDSNQ

QMQKQNEAAIGGMTGFLMAITAISLLVGGIGVMNIMYVSVTERKREIGIRRAIGAKPR

TILIQFLLEAAFITLLGGLIGIGLGYGLSTIVGGFVQIKPVMTPQIFLISSLVSILTG

VVFGIIPAINAARMDPIKAIYR"

CDS complement(1577623..1578330)

/gene="yknY_2"

/locus_tag="JFBMFIFI_01487"

/EC_number="3.6.3.-"

/inference="ab initio prediction:Prodigal:002006"

/inference="similar to AA sequence:UniProtKB:O31711"

/codon_start=1

/transl_table=11

/product="putative ABC transporter ATP-binding protein

YknY"

/db_xref="COG:COG1136"

/translation="MTETNEKAPLIVLKDINKYFPVGKEKLRVLKSLDLTIEEGEFIM

IMGKSGSGKTTLMNIIGFLDRISEGTYLFKGKDVSTLSENKKSEYRNEFFGFVFQQFF

LIDSLNVMQNVELPMVYQGVKKSSEKHEKAKKYLELVGLGTKTKSKASELSGGQQQRV

AIARALVNDPLLIMADEPTGALDSETGREIMETLKKLNEEGKTIVMVTHDADMTKYAT

RVVHMKDGLFVEEEVHQ"

CDS complement(1578347..1579543)

/locus_tag="JFBMFIFI_01488"

/inference="ab initio prediction:Prodigal:002006"

/codon_start=1

/transl_table=11

/product="hypothetical protein"

/translation="MKKKWIVGIVIVVVAVIAIVVAINMSKPKQPVKSQSKDDGIDYF

VVPDVDQVYINGAVTPDQTESFTKKEANDSEPDIKVNNGDVVDAGTVLFTYEDKLVTK

EMEEGNRGINKGYTQRANAIAKRDRDLANVKAETMTDPETGETTTSDTTKEKNNISSQ

AQDTIDSIDQEIQALNEQVVAAAEKQYITTTAKFRGRVSIPEVKDASAPILRLTSEGF

YVAGKVNEKDLSKIKLDQKADIKVVSNGNTVTGKISFIDDNPPDVASNNDASADASGG

AAAGMSSYLVKLSLDSLEGIKNGYHVQATINLGDELIKIPTAAIRKDGAVNYVLVNDF

GSVIRRELVLGEEDGKETTIQSGLESADKVIVSSKKEIKEGDILEGGEESVDSSNSGG

PVVTAN"

CDS complement(1579814..1581529)

/gene="recN"

/locus_tag="JFBMFIFI_01489"

/inference="ab initio prediction:Prodigal:002006"

/inference="similar to AA sequence:UniProtKB:P05824"

/codon_start=1

/transl_table=11

/product="DNA repair protein RecN"

/db_xref="COG:COG0497"

/translation="MLQELAIKNFAIIHDLSLSFETGMTVLTGETGAGKSIIIDAVGL
LAGGRASSDFIRHGENKCVLEGLFTLNKDALTFELLKEFNIDFEDETVLIQRYIHRNG
KNVCRINGRLVTIATLRLIGETMIDIHGQNEHQELMNPERHLDMLDQFGDQELVQLLT
DYDSTYVEYQKVKKAYDKWQSSEQELAQRMDMLVYQTNDIELAELISGEEEALIEEKN
LLVNYQRIVGALSVSYDALQGEEGSGVDLIGTAMTEMSTIDSLDESYRQISENIANSY
FQLQEAASEILREMDQLAYDEERLNEIEKRLDFIQQMKRKYGESVQEVLDYYEKIIVE
LDQIQNRETHMNELISQLGMLSKSLSEKAETVSLKRQEIARLLEKEIHEQLKELYMEK
VIFEVRFLATEKAGEMKTFNEKGQDIVEFYIATNPGEPLKPLAKVASGGELSRMMLAM
KTIFSKSQGITSIIFDEVDTGVSGRVAQAIANKIYLVAIHSQVLCITHLPQVAAMADH
HLFISKTQVDERTETHVTALQQKEKVQEVARMLAGTEITKLTIEHAKELLKLAKTEKE
KYNSI"

CDS complement(1581570..1582019)

/gene="argR_2"

/locus_tag="JFBMFIFI_01490"

/inference="ab initio prediction:Prodigal:002006"

/inference="similar to AA sequence:UniProtKB:P17893"

/codon_start=1

/transl_table=11

/product="Arginine repressor"

/db_xref="COG:COG1438"

/translation="MKKKERHQLLKELIQEHVIEKQEDFVRVLEEKGIEVTQATISRD
IKELHLVKVPAQTGGYRYSLPPDIQFDTAKKLERLIKDAFVSIDYQDYFLVLKTIPGN
AYALGKLIESSNFDGVFGTIAGDDTILIICRSATAANQIQDQLLSLV"

CDS complement(1582219..1583040)

/gene="tlyA"

/locus_tag="JFBMFIFI_01491"

/inference="ab initio prediction:Prodigal:002006"

/inference="similar to AA sequence:UniProtKB:Q06803"

/codon_start=1

/transl_table=11

/product="Hemolysin A"

/db_xref="COG:COG1189"

/translation="MKKERVDVLLVEQGLFETREKAKRAIMAGQIYNQNEERLDKPGE

KIPTETSLKIKGETLRYVSRGGLKLEKALEVFEVTVEDKIMLDIGSSTGGFTDAALQN
GAKMSYALDVGYNQLAWKLRQDPRVEVMERVNFRHSTPEDFTKGQPTIASIDVSFISL
RLILPVLKNILAPGGDVLALIKPQFEAGREGVGKKGIVRDPAVHKQVLEDMVHFVIGI
GYDVMALDYSPITGGEGNIEFLMHLKWNQKTTGTIAYTVNIDHTLASAYDTLKSK"

CDS complement(1583061..1583942)
/locus_tag="JFBMFIFI_01492"
/EC_number="2.5.1.10"
/inference="ab initio prediction:Prodigal:002006"
/inference="similar to AA sequence:UniProtKB:Q08291"
/codon_start=1
/transl_table=11
/product="Farnesyl diphosphate synthase"
/translation="MDLNEFKSIALPQLEGTLLEELEIGVPTKGSLFEAMTYSVKAGG
KRIRPLLLLATIQSLGGNIKSGLLAASALEYIHTYSLIHDDLPAMDDDALRRGQPTNH
IIYGEALAILAGDGLLTLGFELLAKSPLTEKQKVRLIIALSKAAGANGMVVGQVSDME
GESQKLTLTDLQNIHKKKTGELLKFAAYAGAVIVDADQETETQLVKFASHLGLAFQIR
DDILDVIGTTAELGKETGMDAVHQKSTYPGLLTLAGAKKELAEELAKAQTSLANLDNK
SVADTQLLEDFITLLEI"

CDS complement(1583958..1584194)
/gene="xseB"
/locus_tag="JFBMFIFI_01493"
/EC_number="3.1.11.6"
/inference="ab initio prediction:Prodigal:002006"
/inference="similar to AA sequence:UniProtKB:P0A8G9"
/codon_start=1
/transl_table=11
/product="Exodeoxyribonuclease 7 small subunit"
/db_xref="COG:COG1722"
/translation="MSAKAKLKFEEAMQQLEEIVTNLERGDVPLEEALEQFQKGVGLS
KICKETLQNAEETLTKMVDEKGNEAPFENPSENE"

CDS complement(1584191..1585531)
/gene="xseA"
/locus_tag="JFBMFIFI_01494"

/EC_number="3.1.11.6"

/inference="ab initio prediction:Prodigal:002006"

/inference="similar to AA sequence:UniProtKB:P04994"

/codon_start=1

/transl_table=11

/product="Exodeoxyribonuclease 7 large subunit"

/db_xref="COG:COG1570"

/translation="MSPEYLTVTALTKYIKRKFDHDPYLERVYLTGEISNFRMRPNAH

QYFSLKDNQAKISALMFKGAFQQLKFTPEEGMKVLIIGRISLYEASGNYQIYVEHMEP

DGVGALYQALEEMKKKLQLEGLFDAPKQLISTFPKRIAVITSPTGAVVRDIMTTIKRR

YPIVQLVIFPTLVQGERAAQDIVKSIQMVEEKGDFDTMIIARGGGSIEDLWPFNEESV

ARAIFEATTPTISSVGHETDTTIADLVADVRAPTPTAAAELSVPLLRDEILKIEQMRM

RLMKSYAGKIDVLKQRLMRSLGSYIFRQPERLYEGYAQNLDLAIERLIRSLEYRITQQ

NHDLTMLSTKLKAFDPSQLVKQRKEEVQFLEQQLTVQMERYMADKTKDIHNLMQSLDY

LSPLKIMDRGYSYVTKNGEVIKESQQVQLDDVLKIHLGTGQIEAKVIEKVEETT"

CDS complement(1585546..1586403)

/gene="folD"

/locus_tag="JFBMFIFI_01495"

/inference="ab initio prediction:Prodigal:002006"

/inference="similar to AA sequence:UniProtKB:Q0PA35"

/codon_start=1

/transl_table=11

/product="Bifunctional protein FolD protein"

/db_xref="COG:COG0190"

/translation="MSAIIMDGKGLADEMQVEMFKTVEGLKEKGITPGLVVLLVGENP

ASQTYVKNKEKRAVALGFNSLVKRLPESISEKELVNEIEFYNQNPDFHGILVQLPLPN

HIDAETILNTIDPSKDVDGFHPVNMGKLLIGQPDMIPCTPYGIMKLLARYKIDIAGKN

AVIIGRSNIVGKPMAQLLLMEHATVTIAHSKTANLAELAKTADILVVAIGRGHFVTKE

FIKPGAVVIDVGMNRDQNGKLIGDVASAEVAKVAGYLTPVPKGVGPMTITMLLYQTIK

NAEKQVKSF"

CDS complement(1586679..1587107)

/gene="nusB"

/locus_tag="JFBMFIFI_01496"

/inference="ab initio prediction:Prodigal:002006"

/inference="similar to AA sequence:UniProtKB:P65578"

/codon_start=1

/transl_table=11

/product="Transcription antitermination protein NusB"

/translation="MSLTRRDIREKALQALFQLSANEGLTIDQAIQQALTSEDELQDE

VEVVEVPKYLDLVVTGVLQNQAIIDEKIQANLENWSMNRLAKTDLIIMRVAIFEMLYV

TDVPGRVALNEALEITKLYSDEKSRKFVNGVLAKIVDQEN"

CDS complement(1587104..1587532)

/locus_tag="JFBMFIFI_01497"

/inference="ab initio prediction:Prodigal:002006"

/inference="similar to AA sequence:UniProtKB:Q5XA93"

/codon_start=1

/transl_table=11

/product="hypothetical protein"

/translation="MAEETKLTLQDNKASLGEIEIAPEVIEVISGIAASKIEGVYAMH

GNLTSGVVELFGRTSHKKGVHLTVDESGLKIDVYCLINYGVSVPKVASEMQEKIRQQL

LFMTDIELAEVNIHVVGIVPEKTVPQELLDLDLDEDGEDA"

CDS complement(1587591..1588148)

/gene="efp"

/locus_tag="JFBMFIFI_01498"

/inference="ab initio prediction:Prodigal:002006"

/inference="similar to AA sequence:UniProtKB:P99066"

/codon_start=1

/transl_table=11

/product="Elongation factor P"

/translation="MISVNDFKTGLTIEFDGGIWRVVEFQHVKPGKGAAFVRSKLKNL

RTGAAQEKTFRAGEKVAKAQIDNRRMQYLYESGGAHVFMDSETYDQLELQESQIEDEL

KYLKENMEVQIIMYGAETLGVELPNTVELRVEETDPGIRGDTSSGGSKPAKMETGVYV

NVPFFVNVDDVLIVNTQDGSYVSRA"

CDS complement(1588300..1589367)

/gene="ypdF"

/locus_tag="JFBMFIFI_01499"

/EC_number="3.4.11.-"

/inference="ab initio prediction:Prodigal:002006"

/inference="similar to AA sequence:UniProtKB:P76524"

/codon_start=1

/transl_table=11

/product="Aminopeptidase YpdF"

/db_xref="COG:COG0006"

/translation="MSRIEKLRKGMQQRGLDALLVTSPYNLRYVSNFTGTTGLSVITL

TNAFFVTDFRYTEQVAEQAKGFTVVQNHGPIYDEVVKIVTEQKIEALGFEQDFVTFSI

FDMLEEIIPETTDLVPVSGLIEGLREVKEPEEIETIKKACQISDAAFDYILGVIKPGM

SEIEVANLLDFHMRSLGATGVSFDTIVASGLRSAMPHGVASQKLIEKGDFVTLDFGCY

YNGYVSDMTRTFAVGEPIAKLKEIYQVTLEAQLRVIDAAKPGISGIELDAVARDYIAS

KGYGEAFGHSTGHGIGLEIHEGPNVSKLAEKAFVPGNVITDEPGIYLPGIGGVRIEDD

LLITVGGNEILVHSPKELIIL"

CDS complement(1589598..1589978)

/locus_tag="JFBMFIFI_01500"

/inference="ab initio prediction:Prodigal:002006"

/codon_start=1

/transl_table=11

/product="hypothetical protein"

/translation="MNQKGAILPTVIMFVALLLLLALGTITIYQGQMRQLMILNQHYE

AKSLMLIAKKQLEIKRGAVKREVKAMKLEYSTGDVTIKQGQVGEFQFFVKLQNDYILE

DVFTLSDLPLKLDTSEMNSEIISP"

CDS complement(1589979..1590464)

/locus_tag="JFBMFIFI_01501"

/inference="ab initio prediction:Prodigal:002006"

/codon_start=1

/transl_table=11

/product="hypothetical protein"

/translation="MKNNLKIVHSSTKNNFIAGFTLLEALVATFILFICVSILSLSLQ

NYQRIREVTFKDRQLEWHLFLNQFEEEVKNTSLVKIEVEKVVFNRDNSPEKDQFFYEK

YRNMLRKRTNKGAGGHQPVLMKLETIKFKKLNDFLEIKVTFTNQESYEALVKVNYMGD

S"

CDS complement(1590454..1590687)

/locus_tag="JFBMFIFI_01502"

/inference="ab initio prediction:Prodigal:002006"

/codon_start=1

/transl_table=11

/product="hypothetical protein"

/translation="MKKTSKESGYLVLEGLVAFVLILSTISLYFPLLTTILQRLQIEK

VNVEKNRRRYEQIQLGSDEVSEIQLKGIEWDEE"

CDS complement(1590671..1591108)

/locus_tag="JFBMFIFI_01503"

/inference="ab initio prediction:Prodigal:002006"

/codon_start=1

/transl_table=11

/product="hypothetical protein"

/translation="MINARGFTLIETILILSIIMVLFGLPTVIANKTYEKVQKMLFFE

AFQSHLLATQNYALLANKKTSLTIFKSGTVRYQVLSEAKGVVNQELTIPKSLTVDKNN

SIRYAAKTGRPSSFKTIKFKSLDKSYTFKFQMGSGRYIFEENE"

CDS complement(1591105..1591440)

/locus_tag="JFBMFIFI_01504"

/inference="ab initio prediction:Prodigal:002006"

/codon_start=1

/transl_table=11

/product="hypothetical protein"

/translation="MKNKRVSHKKWKLNNKGFTLVEMILVLFVISVLLILVIPNVVQQ

KKKIDNQGTEALMTVIETQIELFLLEKEPGIEVSFAALKADNYLKQKQIDNAINKGLA

IENNQVVQR"

CDS complement(1591437..1592504)

/gene="epsF_1"

/locus_tag="JFBMFIFI_01505"

/inference="ab initio prediction:Prodigal:002006"

/inference="similar to AA sequence:UniProtKB:P45780"

/codon_start=1

/transl_table=11

/product="Type II secretion system protein F"

/db_xref="COG:COG1459"

/translation="MGLLRKKVIDNTKSNKKIKETQQSFFLTKLAVLVAEGFSLKESL

LFLKIMLPKQAVWLNQALNQLEEGQEFFQVLNQLGFSERISSQVYLAQIHGQFSQVLA

DSGAFLEVNGKRKKKLKQLLQYPMLLVIFMFGILFGIRLLLLPHFNDLVQQNGSFTSL

VSGIAIGLIYYFPYVFMGLLFSLLIIKISLTNYFKKQTAIAKLNFIVALPFFGNLIKL

YYTYYFSYEWAQLFKSGYSMLRIIEVMKAKETTKIMQEVANEMEKGMKNGIGLHVVMK

QLPFLKTELGAIIFHGELTSQLASELNLYGQICQNEFVQKIEKFMGWIQPLVFILVAF

FILCIYLALLLPMFTMMEGIL"

CDS complement(1592458..1593522)

/gene="comGA"

/locus_tag="JFBMFIFI_01506"

/inference="ab initio prediction:Prodigal:002006"

/inference="similar to AA sequence:UniProtKB:P25953"

/codon_start=1

/transl_table=11

/product="ComG operon protein 1"

/db_xref="COG:COG2804"

/translation="MKIEQNVIQLMGEAEKRGVNDIHLLPRENKYQIYFRVSGHLVEH

SVVSLKKGAQLIAYLKFLSNMDVGEKRFPQSGAKNMLINGSNKSVRFSTITDFLHQES

LVIRFLNQTQKRKLVQTCYFPNQVTVLKQLIEVKSGLILFSGPVGSGKTTTMFQLIKE

FVGKENCQVFTIEDPVEIQETLFLQTQVNPKAGIGYDLLIKASLRHHPDIIVVGEIRD

QETAKMVIRAALTGHLIVSSIHAKNGEGVVKRLLELGVSAQQIKQSLIGVISQKLVPQ

YCPFCQGECHLYCSFLAVYAKRSVIYETLTGRELESLMEGGDAQINLEKQTKSFNYYL

RKAYSYGFITKKSYRQYKIQ"

CDS complement(1593670..1594497)

/gene="cysL"

/locus_tag="JFBMFIFI_01507"

/inference="ab initio prediction:Prodigal:002006"

/inference="similar to AA sequence:UniProtKB:P39647"

/codon_start=1

/transl_table=11

/product="HTH-type transcriptional regulator CysL"

/db_xref="COG:COG0583"

/translation="MLRLIETFKVVYEEMNFTLAAEKLFIAQPTVSMHIQKLEEELST

TLFIRDGRKKVIPTQSADILYIQGKAMLTLWQETHEKIQQVHGQIRHTCKIGASNTIA

VYVLPLILKELKQDFPMIDFEIYMSNSDEVLKNVRNMKWDFGLIETTLNSGELERIQV

LQDELVLAGKTEEDFWLLRESGSGTQHYTHDYLMEFNLLTPKKMLVESNEMIVKLLEK

GIGKSIISKRAVGNIPYQSLPSHYQRSFYLIRKKDEIEWQRAVSNQFITRLKELKID"

CDS 1594654..1595646

/locus_tag="JFBMFIFI_01508"

/inference="ab initio prediction:Prodigal:002006"

/inference="similar to AA sequence:UniProtKB:P62723"

/note="UPF0324 inner membrane protein YeiH"

/codon_start=1

/transl_table=11

/product="hypothetical protein"

/translation="MNKIKQFLPGVIICVGVAVLSKILAIFLPTLGAATLSIFLGIFL

GNTFLKHERYSRGTKFSESTLLEYSVVLLGGTITFQTISVLGISGFLFIILQMTGTIL

CAIFIGRKLKFSENITLLMASGNAVCGSSAVAATAPVLDADEEEVGLVITIVNLTGTL

LMLLLPVLSYFFYNYEHLKTSALIGGILQSVGQVVASGSMVNSQVLTTAMIFKIMRIL

MLVVIVYLFGRYKTNETANKQATGKKSVKIPWYVIGFFIICLIGSLGFIPEFLQNVMH

DTSGWFEIIALAGIGLRLNLADLIKQGSRFALYGLGIGVSQILIAVTLISIFFH"

CDS 1595923..1596768

/locus_tag="JFBMFIFI_01509"

/inference="ab initio prediction:Prodigal:002006"

/codon_start=1

/transl_table=11

/product="hypothetical protein"

/translation="MDKKTIFNYLKIVVGAFIFSLAVNMFAIPNELGEGGVTGITMML

YYLFTWSPALTNLMFNSVLLIIGYKYLDKATIFLTIFAVALTSIFLRLTTGWGFSADE

TIVAALAAGAMMGCGMGLIMLGGGTTAGSAILAKLANKYLGWNTSYALLFFDLIVVIP

SVFIIGLENMLFTVLSLYVSTKVLDFLLEGFNPKKSVTIISDQYELIARDIDKELERG

ITIFHGQGYYLKQDRKILYTVVNRQQLLHLTKIVNKYDPKAFFIINDVQRVVGEGFTK

QVTSE"

CDS complement(1596812..1597366)

/locus_tag="JFBMFIFI_01510"

/inference="ab initio prediction:Prodigal:002006"

/codon_start=1

/transl_table=11

/product="hypothetical protein"

/translation="MIRVGRKADVYEVVPLIVEMIVEAELSILATMSSEELTQLLQLG

FQLEDYRYSYKNAIVLTNNLDETVGVAFGYPGELGPIINQPFTPILVSHGFSEEFVFF

PDRETFQEEWYLDIMIIKANQRGKGYGKELLQAILNSKKGTIGLNCAQNNPQLLQFYA

SFNFKTVGSLVIGGHRYNHMQVEV"

CDS complement(1597402..1597746)

/locus_tag="JFBMFIFI_01511"

/inference="ab initio prediction:Prodigal:002006"

/codon_start=1

/transl_table=11

/product="hypothetical protein"

/translation="MKKGVLVYSGTPMMGRSVIRSSAVGLFDVGNPQKYVEKMKAVIQ

EKNLDWSFKLDETESDLEELLKEQTDLIVLLPGLERKFSHGNFPVEKYIQLNSLDFHS

YNLAPVLAFMEK"

CDS complement(1597825..1598550)

/gene="yebC"

/locus_tag="JFBMFIFI_01512"

/inference="ab initio prediction:Prodigal:002006"

/inference="similar to AA sequence:UniProtKB:P0A8A0"

/codon_start=1

/transl_table=11

/product="putative transcriptional regulatory protein

YebC"

/db_xref="COG:COG0217"

/translation="MSGHSKWSNIQGRKNAQDAKRGQIFQKISREIYMAVKSGGPDPS

TNPQLRMMMDKAKSANMPNDNVKRAIAKGSQSGEGENYEEITYEGYGPNGIAVLVHTL

TDNRNRTGTNVRVAFNKNGGSLGETGSVSYMFDRKGYLAIERRDLAVDEDTMLMSVLE

AGGEDMETSDEVFEIYTEPSDFISVRDALEASGYKLAQAEITMVPQTTNELPADKRAL

FQTMLDKLEEDDDVSEVFHNAEM"

CDS complement(1598650..1599174)

/locus_tag="JFBMFIFI_01513"

/inference="ab initio prediction:Prodigal:002006"

/inference="similar to AA sequence:ISfinder:ISCac2"

/codon_start=1

/transl_table=11

/product="IS1595 family transposase ISCac2"

/translation="MNRKIILYIASSLDGYIAKPDGDLDWLINSENDGDNGLFAFMGQ

IDTTIMGRTTYDEVLGFDVPFPYVGYENYVFTNHPPVNDENGVHFIKGDIKAFISTIK

KTAGKDIFLIGGSQIITEFLALDLIDEIILSIAPVLIGKGIPLFAGDYLEKRYKLDQV

RQYKELVQIHYKSL"

CDS complement(1599224..1599745)

/locus_tag="JFBMFIFI_01514"

/inference="ab initio prediction:Prodigal:002006"

/codon_start=1

/transl_table=11

/product="hypothetical protein"

/translation="MKMKASTKDNLFIVLAIFMMGVLFYSSSQPYGKQSVTPLLDQIL

VNQPFKELLSGVNFHYAGSEVSIAAQGYSSFIEFFIRKGAHFGTYFLLGLFWFLGLKN

KLSSSSLAALIAWLLAAGYAAFDEFHQSFTADRTPLFQDVILDSVGALTGILVALLFI

QLFTKKKRKKTRR"

CDS complement(1599964..1600266)

/locus_tag="JFBMFIFI_01515"

/inference="ab initio prediction:Prodigal:002006"

/codon_start=1

/transl_table=11

/product="hypothetical protein"

/translation="MSRNFKVFLAIEGILALSFIQDLVRNPILVTLLIGSFIIMAVAN

KNNNHDLGKKALYLCIGSLLVGLFTSGVFAAMLIVVLIWWIIFGLDELSYHIQKRN"

CDS 1600451..1602262

/gene="pepF1_1"

/locus_tag="JFBMFIFI_01516"

/EC_number="3.4.24.-"

/inference="ab initio prediction:Prodigal:002006"

/inference="similar to AA sequence:UniProtKB:P54124"

/codon_start=1

/transl_table=11

/product="Oligoendopeptidase F, plasmid"

/translation="MKYTMNWDLESIFPGGSASLALKEKINLMTEQIIDYTAEVTSWN

PQLDQPTYTHLKTILEKEEKIKYGLTQSFSFINAVQSANVKDTQSGAIFGQLFELNSQ

FATSETIFIKKMVEIPDEEWQKLIALPDFSPIKFNLSENRQHGKELLSEAEEALINAL

SIDGFEAWSKHYDTLVGTIEIPFEEKDGTTTLLSAGQAFNKMTGDPDPLVRDQLFSQW

EATWSKLAPVFADTLNHLVGFRLATYKAHGVTDFLKRPLEYNRMKPETLDAMWQAVSS

HKTAFVDYLNRKAKLFGKEKLDWQDLDAPIIIPGSKAQVYPYDEGAEFIIDNFKKFSP

KMADFAQMAFDNRWIEAEDRPGKRPGGYCTSLPESGESRIFMTYSESPSEVSTLAHEL

GHAFHSYVMKDLPGVSQEYAMNVAETASTFAEMIVADATVKEAKDDAEKIALLDTKMS

SALAMFLNIHARFLFETNFYTERQEGIVSFERLSELMESAQKEAYQDSLGSYHPYFWA

SKLHFFISDVPFYNFPYTFGYLFSLGIYARSIEEGAGFEDKYIALLRDTASMTTEELA

MKHLGVDLTKPDFWVAGIKIMEQDVQTFMDLTDSYSK"

CDS complement(1602422..1603747)

/gene="cwlO_1"

/locus_tag="JFBMFIFI_01517"

/EC_number="3.4.-.-"

/inference="ab initio prediction:Prodigal:002006"

/inference="similar to AA sequence:UniProtKB:P40767"

/codon_start=1

/transl_table=11

/product="Peptidoglycan DL-endopeptidase CwlO"

/db_xref="COG:COG0791"

/translation="MNKRLATIAIIGSMTFSSVLLPAIATADTVDSKIEQQDNKINTL

KANSSSTQADLEAIETSIATNETSAKELLAEIKAANNEMQQLDQEIGVLQTKIEQRNE

QLQNQARSVQVNGDSANYVEFVINAESVTDIIGRIDVVSKLVTANRDLVKEQVRDQES

VKTKKTETEKKVNQQNTLAGQLEATQAKLASQKFEKEAVVAQLASETATAEGEKAGFL

AQKAEAEKQAALLATAKEESAKAVQVAAATKETATIATNTASNQNSAADSSSSVDSST

PAPVNPTPAPNPGNGNNGGNGGGGNTTPPDPTPTPIPTPDPPSGGGTSWSNLQPIAES

LIGIPYQWGGTTTSGFDCSGFTQYVFGKAGVTIPRVASAQYSASQKVSNPRAGDLVFF

SQPGSGGVIDHVGIYVNSSTFIGSQSTPGVAYASYSGYWGAYIVGYGRY"

CDS complement(1604044..1604592)

/gene="tag_1"

/locus_tag="JFBMFIFI_01518"

/EC_number="3.2.2.20"

/inference="ab initio prediction:Prodigal:002006"

/inference="similar to AA sequence:UniProtKB:P05100"

/codon_start=1

/transl_table=11

/product="DNA-3-methyladenine glycosylase 1"

/db_xref="COG:COG2818"

/translation="MRRCDWAKSELDKAYHDEEWGKPLHGDDAIFELLILETMQAGLS

WSTVLVKRENFRQALDGFDYHLIATYDENKYTELLENKGLIRNKLKIKSIINNAKAFL

NVQKEWGSFDDYLWSFVDGKPILNEFKEISQVPAKTELSEKLAKDLKKRGFSFVGPVT

CYAFMQAAGLTNDHLMDCDFRN"

CDS 1604864..1605829

/locus_tag="JFBMFIFI_01519"

/inference="ab initio prediction:Prodigal:002006"

/inference="similar to AA sequence:UniProtKB:P67330"

/note="UPF0176 protein SP_0095"

/codon_start=1

/transl_table=11

/product="hypothetical protein"

/translation="MSKDIRVLLYYQYVPIEDAEQFAKDHLAFCQEIGLKGRILIADE

GINGTVSGDFETTQRYMDEMHADPYFAETVFKIDDENQDAFKKMHVRYRPELVSLNLE

DDVNPLELTGAYLSPKEFREAILDDDVVVIDARNDYEYDLGHFRGSVRPDIRSFRELP

QWIRDNKEQFMEKRVVTYCTGGIRCEKFSGWLVREGFKDVGQLHGGIATYGKDPEVQG

DLWDGQMYVFDSRISVPINHKEHVIVGRDWFDGTPCERYMNCADPKCNRQILVSEENE

AKYLRGCSHECRVSPENRYVKEQNLSLVEVAERLAVINEKLPTLN"

CDS complement(1605893..1606696)

/gene="btuD_3"

/locus_tag="JFBMFIFI_01520"

/EC_number="7.6.2.8"

/inference="ab initio prediction:Prodigal:002006"

/inference="protein motif:HAMAP:MF_01005"

/codon_start=1

/transl_table=11

/product="Vitamin B12 import ATP-binding protein BtuD"

/translation="MVKILELKGIHKSFEVGTVNENHVLKGIDLTLEEGDFVTIIGGN

GAGKSTLLNSVAGSFGVDQGEILLDNINVTQQKTAKRAKYIGRVFQDPRMGTATRLSI

EANLAIAYNRGKKRGLSRGVKDKQRAEFKEQLKELDLGLENRLKMEVGLLSGGQRQAL

TLLMATLVTPKLLLLDEHTAALDPKTSQSVLNLTEKIVNEKNLTTLMITHNMEDAIRY

GNRLIMLYNGKIVVDIKGPEKNNLTVADLLALFQKNSGQTLTDDALILA"

CDS complement(1606698..1607609)

/locus_tag="JFBMFIFI_01521"

/inference="ab initio prediction:Prodigal:002006"

/codon_start=1

/transl_table=11

/product="hypothetical protein"

/translation="MDILLSSVSQGLLWSIMAIGVYLTYRILDIADLTAEASFPLGAA

ICASQIVAGMSPVLATLLGLVGGMVAGLVSGLLHTKLKIPALLTGILTMTALYSINLR

IMGQANVSLLGKETVMRMLTTMGMDSQMAVLAVGGIFVAIVIIFLYLFFSTEIGLAIH

ATGDNNEMSEANGINTDTMKIMGYMISNGLIALSGALMAQNNGYADISMGVGTIVIGL

ASIIISEVIFNNVSFVKRLFVIIIGSIIYRLIIAGVLELGVAPTDLKLFSAIILTICL

ASPLLRSKLRGLLKLKSKTASVKKGVQ"

CDS complement(1607688..1608683)

/locus_tag="JFBMFIFI_01522"

/inference="ab initio prediction:Prodigal:002006"

/codon_start=1

/transl_table=11

/product="hypothetical protein"

/translation="MKKLTTKKYLVAGLTGALALGLLVGCGNKEKDSGTATDKDAVSV

GILQYMEHNSLDAAREGFLAELKDAGYEEGKNLKVDSLNSQGDQANLKSMSERLVKEK

NDLILTIATPATISVANETDKIPVLFTAVTDAVAADIVKSNEKPGGNITGTSDMVPID

DQTNLLLSIVPKAKKIGMIYNSSEENSVIQADLAKKALEKAGVDVKVATVTSTNDVQQ

VMTSLVKDVDGIYIPTDNTLANTMETVGEIAKSNKIPVVAGSTEQVEVGGLATYGIDY

NELGRQTGKLAVKILKGEAKPGDLAIETSKNLKLVVNEDMAKALDIDPASIKVEK"

CDS 1609339..1609668

/locus_tag="JFBMFIFI_01523"

/inference="ab initio prediction:Prodigal:002006"

/codon_start=1

/transl_table=11

/product="hypothetical protein"

/translation="MEQLLIKQILSLSKKYGNQKIYLAPNYYRIKREEDIYRISQLMA

DSCGSYLHNPRITVRIDTEKEKLTLLNYYNDYKTPVQLIYYTPLENDFFIDELTKILK

EMTSIDY"

CDS 1609812..1613114

/gene="mtgA"

/locus_tag="JFBMFIFI_01524"

/EC_number="2.4.1.129"

/inference="ab initio prediction:Prodigal:002006"

/inference="protein motif:HAMAP:MF_00766"

/codon_start=1

/transl_table=11

/product="Biosynthetic peptidoglycan transglycosylase"

/translation="MNDPQNNSPSFIERCKKGFNHILRTVKEKWISFKDKRASTKSNL

NETTLSPNLTEEEEQTPVDTNKETIDTIGSSQQTEPLADTTDLQEENSNEKKQFDENL

STESVASKEVEPSLEEKSSDSVTDKPKNKVIAILFATQVIILANWNSLKRHWSKLLPT

PTDETATPSSKVEDSPSVESESAMSLTDIAPKEPTEKEAGTEMKYELAIIPTQPEEVI

SENEDKKEKLIFTFNVAMGVIKNLALSLLVLLLVGGALAGGIGLGYFAYLVSTEKPPT

YEEMRADISNLEQVSNMYFADNQPIGAVQSDLVRKVVPLSEMSKYLQSAIVATEDEYF

YEHNGVVPKAVARALVQEFTGSKNQTGGSTLTQQLVKQQILSSEVSFKRKANEILLAI

RLENYFTKDEILEAYLNTSTFGRNSSGQNIAGVEEAALGIFGIHASELNLPQAAFIAG

LPQSPSQYTPYNQLGELRTNLEPGMERKNTVLFRMYREKFITKKDYDEAMKYDLAKDF

IQPTESVDNHDYSFVYNSVLKEATLLLMKQNYKKDKLTDADIADSDTLYNKYYFEAQD

QLQNNGYEIHSTIDKGVYDAMQNVVANYGDRFGTTYYRNKIDEFTGAVVLDETTGEPV

QEAEPVQNGSVLMDNASGKIIGFVGGRDFSISQVDHAFGTHRQPGSTIKPLLVYAPAL

EQGLIQPTTMIPDTVINYLQPDGNLWNPSNYGGGISGKFVSAREALKRSYNNPTSKIY

LEMLKNSPAGMSPKDYMKKLGFTSIADEEYERPALSLGGTETGPTVLEQTNAFAAVAN

NGNYVEGYMIENITDKKGNIIYEHETKSEPVFTPQTAYLTIDMMRSVVESGTAAGILN

ELNFSPDLAGKTGTTNDFIDVWFIASTPQVTLSSWIGYDNDGDERRQLQYYDNGADFD

ASGANESYWAELANAIYSVNPTIMGADKSFVRPNGIVSSTVLAQTGMKPGKMTLPDGK

TLDVTGPTTTDLFNSNFLPGTTVFNFAVGATSSELNTFWFDTYLKAREEEEKKKIEDA

KDKEQKKKEEENKKKSEEDKASEEKKKKEEEEKKKKEEEEKKKAEEEANKPPTEGTAP

GTGTGTIPPPAN"

CDS complement(1613221..1614225)

/gene="ccpA_2"

/locus_tag="JFBMFIFI_01525"

/inference="ab initio prediction:Prodigal:002006"

/inference="similar to AA sequence:UniProtKB:P25144"

/codon_start=1

/transl_table=11

/product="Catabolite control protein A"

/db_xref="COG:COG1609"

/translation="MEKQTITIYDVAREANVSMATVSRVVNGNPNVKPTTRKKVLEVI

DRLDYRPNAVARGLASKKTTTVGVIIPDVTNLYFASLARGIDDIATMYKYNIILANSD

QNDVKEVQVLNTLLAKQVDGIIYMGHKITDEMRAEFSRSKTPVVLAGTVDPDEQVGSV

NIDYQAATEKVITQLIAKGNKRVAFITGSLLEPINGVYRLNGYKNALKKAGIKFDESL

IFESEYSYKAGEALWDKLAGAKATAAFVGDDELAIGVLNGAIDHGVKIPANFEVITSN

NSKLTEMVRPRLSTITQPLYDIGAVSMRLLTKLMNKEEVDEKTIVLPYNIADRDTTKK

"

CDS 1614490..1615590

/gene="pepQ"

/locus_tag="JFBMFIFI_01526"

/EC_number="3.4.13.9"

/inference="ab initio prediction:Prodigal:002006"

/inference="similar to AA sequence:UniProtKB:Q9S6S1"

/codon_start=1

/transl_table=11

/product="Xaa-Pro dipeptidase"

/db_xref="COG:COG0006"

/translation="MKKQLIDLKNWLKENQGDVAFINDPANVAYLTGYESDPHERVLA

LLVFPDADPFLFTPALEMDDARNGAWEYDVFGYLDNENPWEIIAEKVKAAVPTVSNWA
VEKEFLTLARFEALQNEFPTATFKQDLTSFMQRLKLIKTPTEIKTMIEAGKWADFAFD
IGFNAIKEGVMEQEIVAEIEYQLKKKGIMQMSFSTIVLAGDNAASPHGNPGQRKIQKD
ELVLFDLGVVYDGYTSDATRTVAYGEPSQKAQEIYAIVLKAHQAALDAVKPGITAGEL
DTIARDIITDAGYGPYFNHRLGHGLGSSVHEFPSIMAGNPLVIEEGMCFSIEPGIYVP
GVAGVRIEDCLYVTKDGCEVFTHTPKEYTVIG"

CDS complement(1615637..1616170)

/locus_tag="JFBMFIFI_01527"

/inference="ab initio prediction:Prodigal:002006"

/codon_start=1

/transl_table=11

/product="hypothetical protein"

/translation="MSKNGGFLLGAFIGGTAAAITALLFAPKSGQELREDLANKTEEF
KGAAKDYTDLAVDKGTEIKNAAVDATSDIKINLKETANQFASQLKQTSKEVGSDLDDI
RAELKDASADVADSAADVVDVATDKGEEIKDTVTETAKDATKEAKSVAKDAKEKATDV
KDEAKKQVEEVKSEVKK"

CDS complement(1616204..1616647)

/locus_tag="JFBMFIFI_01528"

/inference="ab initio prediction:Prodigal:002006"

/codon_start=1

/transl_table=11

/product="hypothetical protein"

/translation="MSGGEIAAIIAAVAFAVLVIFIVVNLMKLSKIAGEIQQTVKEAN
NSISVITKDVDSLSIEVEGLLNKANALVDDVNGKLGKTDPLFQAIGDLGVTVSDLNHS
SRNLVGHVTGATKKTAQVSVASKIGRTAMAMNRNRKAKKTAKQEF"

CDS 1616879..1617781

/gene="ykuT"

/locus_tag="JFBMFIFI_01529"

/inference="ab initio prediction:Prodigal:002006"

/inference="similar to AA sequence:UniProtKB:O34897"

/codon_start=1

/transl_table=11

/product="putative MscS family protein YkuT"

/db_xref="COG:COG0668"

/translation="MDSSSTVDSSVTSEVAQKVEKSTNFLLNYWDNIKWGDIIAIFIT

KGIQLLILAILFLILNKIGRVFIEKSFNNYKLKKQVSESRMDTLSSLSLNVFYYIMFF

FFLYGLLSILGVPVATLLAGAGVVGIAVGLGAQGFISDIVTGFFIILEKQLDVGDYVR

LDPIEGTVIAVGLRTTQIKSVNGTLNFIPNRNITIVSNLSRGNMRALIDIRMVPNADI

NLVTEVIEKVNNELVPEHSEIVQGPTLLGLTDLGNGQLAFRVVIFTLNGDQYHVQNEF

LKHYVQALTEAGVEIPASPLNILK"

CDS complement(1617825..1618850)

/locus_tag="JFBMFIFI_01530"

/inference="ab initio prediction:Prodigal:002006"

/codon_start=1

/transl_table=11

/product="hypothetical protein"

/translation="MKDLGYNEENGIYQCEFCRFTTEKGQVYPIEDENKFFEAWRFME

HHIKTEHHSVLMALLAKGKEEHGLSQQQVELISLFYQGKKDSDIQKELGIASLSTLRN

HRFNLRKKERQAQNFLRIMAFLKDDEQQKNPTKFTKEQDYFTHNGQLIRYPKGKKVEF

VILEKISLHFQVGKKYSEKEINQVLSKIYPEDHALLRRRLIDMNFLQRDLAGTWYEKI

EGEKHQMDYKKELKMQAKEVKIEGGIYQIKNTKNGKRLIASTPNLKSLNGLKFSLKNK

SYINKQLQEEWTAFGEEVFEFKVLEVVTEKELEKKPMKKILEQKLETWLEKEQPYDET

GYITKKE"

CDS complement(1619007..1620143)

/locus_tag="JFBMFIFI_01531"

/EC_number="3.5.1.47"

/inference="ab initio prediction:Prodigal:002006"

/inference="similar to AA sequence:UniProtKB:D5E0A1"

/codon_start=1

/transl_table=11

/product="N-acetyldiaminopimelate deacetylase"

/db_xref="COG:COG1473"

/translation="MLNENPFVKIRRELHLIPEIGLEEYKTHEYLMKKIMDLPQEHLE

IKTWNTAIVVRVKGTEGAKTIGWRTDIDGLPVTEETGLSFQSIHEGKMHACGHDMHMA

IALGLLTKFSHQPGKDNFVVLFQPAEENESGGKLVYDHGLLDEWMPDEIYALHVQPDL

PVGTIGSRSGTLFAGTCTIRAKFKGKSGHAAYPHKSNDMIVAASQFVTQLQTIVSRSV

NPVEGGVITIGTFNAGTAENIISGEANLSGTIRTLTTEMSQLMMERVRQIKVGIEQSY

QCEIELILQQGGYLPVVNAEKETNEFIAFMENNPAVDFVLSPTAMIGEDFGYLLDKIP

GTMFWLGVNSPFELHHAKMNPNEAAIPFAIEQVGAFLAAKLNQV"

CDS complement(1620349..1621050)

/gene="dapH_2"

/locus_tag="JFBMFIFI_01532"

/EC_number="2.3.1.89"

/inference="ab initio prediction:Prodigal:002006"

/inference="similar to AA sequence:UniProtKB:Q81MQ2"

/codon_start=1

/transl_table=11

/product="2,3,4,5-tetrahydropyridine-2,6-dicarboxylate

N-acetyltransferase"

/db_xref="COG:COG2171"

/translation="MDANEIIKFIAESEKKTPVKVYIKGSLKEVTFPETIQNFTNCKS

GVLFGDWKDVEPFLKENAAQIIDYVVENDRRNSAVPLVDAKPLNARIEPGAIIRDRVE

IGNQAVIMMGALINIGAIVGDNTMIDMGAVLGGRATVGKNCHVGAGAVLAGVVEPASA

QPVVIEDDVLIGANAVILEGIRVGKGAVVAAGSIVVNDVAPYTVVAGVPAKKIKDIDE

KTKSKTGLIEELRKL"

CDS complement(1621130..1621630)

/gene="ykuL"

/locus_tag="JFBMFIFI_01533"

/inference="ab initio prediction:Prodigal:002006"

/inference="similar to AA sequence:UniProtKB:O31698"

/codon_start=1

/transl_table=11

/product="CBS domain-containing protein YkuL"

/db_xref="COG:COG0517"

/translation="MIGKEIGEMLLENKEGFLIPAEMVAHVQVNNHLDHALLVLTKIR

YSVIPVLDNEYKIKGLISMPMVIDAIMGLEEIDFDKLSDICVHEVMQTEFPIVRNPYD

LEDVLHLLVDNAFLCVASEDGSFTGIVTRREILKATNRIAHEFENEFEVVRKAEKKAS

NKSAIR"

CDS complement(1621764..1622186)

/locus_tag="JFBMFIFI_01534"

/inference="ab initio prediction:Prodigal:002006"

/codon_start=1

/transl_table=11

/product="hypothetical protein"

/translation="MEKNATLNFSIFHSMRARLTSFLMGLLIEKKIKIRNGRKRHINF

SYFLAGVNRLNELFIRLAFMSQSFEKKIKIRDGKKRHINFSYFLARPNRLNELFIRLA

FMSQSFEKKIKIRDGKSAISIFPIFLPGLTGSTSFLSG"

CDS complement(1622415..1627133)

/locus_tag="JFBMFIFI_01535"

/inference="ab initio prediction:Prodigal:002006"

/codon_start=1

/transl_table=11

/product="hypothetical protein"

/translation="MSIKKSILRSLITVSALCSLAIILPLEESVTAVGTSQESPTEVA

STSIPEIKEAPYASHFFPKELLKWSPETDANASHNRSNVPLAKRVQGSKTNQDQSTEA

KVVSLAITNRNTSGTPSQGSSDKAVYNFSYWQYIDTMVAWAGSAGEGIIVPPSGDVID

SAHRNGVPVLGTVFFPPAAYGGKSEWVQQFVEKDENGRFIVADKLLELADYYGFDGWF

INQETKTDPATAKAMKELLLYLQEHKKAEMDIMWYDSMLENGSISWQGALNDKNSAYF

QTGDTRVSDTMFLDFNWKNTLSKISKSAEAAKDLNRSPYDLFAGIDVQAQGFNSRPKP

SAIVENGKPIVSLGLYCPDWTLRDGANYDVTKYWQNEEKFWVNAAGDPRDTTLGANEW

QGISRYFVEKSPVTSLPFVTNFNVGNGDNVYQNGELVKEGTFNNRSLQDIMPTYRWIV

ANEGNQLTPSVDYSTAYNGGSSIALTGSTTVGGTSTVKLYKSELALTGKESGTILYQG

EAELELVLGFKDKPEEMVSLVGETTTVGDWKKVEVALDAYQNKTVDTISVKVKGTTAN

PATKIALGQIAIAENKTSLVSPVKNVTISGQSIEEDLFANLRLNWESAAENTSNYRIY

RVDQSGARHYEGTSGNQSYYVSGQKRPADNILRYQIVPVDNQQNEYSEQAGEVEFNFP

ELQAPLANFSASKSFIKVGEEITLTSHSALSAEKLEWLIEGGTPENAEGETVKTSFSK

AGTYTVRLTASNQAGANEAIKENYITVYDETFTEPLVNLSLSAVDTDSSGYTNQNETP

MQALDGDLLTKWCDNANDQPWMSVDLGREVSLTSFKLFHAEAGGENAEWNTRDYEILV

SHDNKNWTQVVHQDKNTKGITEDSIPLTEARYVKLKLNKAEQTGKVARIYDFEILGYD

KTGIEVGKNQAALSELRSLYYQTNVNEADYTADSFANLVAARLAVKDVLDKEAPNLDE

IETTKQQLKGAFDSLVTVKDSARLTLENEINAAKKIEGTLYTEASFERFTSEIEKAEL

LLKQSDTTIDALVVQIKQLQEAKINLVLKTDQLEQTRNELLNLIVEAKKIQPEDYTAE

SYQELTKSIQTAEELANNEVASLSMLEIAKFQLTKTMNSLVTLEADKLGKARQELEEF
LQSIELLNTDNYTPESLAILFENIKIAKENLQKNELTVEMIQADKEKISQSLAELVPS
ESAILEKAIEELQANQKIAKALNEKEYTVATYQKVKEALESAEVILEQPNGKLQDIEL
INVTLIQTMNNLKTVKEEAKEVALQKLIDYVQALEALNELNYTSDSFAKVTEQIKLAK
QVIENPMVTLEELEETLGNLENSVNQLISLEEATLTEAREKLTKLLETAKAIVASDYT
TDSYSVLTKAMEEATGLLFLRKPATLAEIEGAHLNLATAIDQLVTQESVDRNALKIQI
TELLTKAKEAISSGKYNELSSQKLEQVVNQVEGVVADPVATIENLVAAKTNLTNAINE
LVLKEEVPAEVPATKPEKETLIPKGTNPKEQVVSTGNQGKSEFPQTGEANQINRGAVG
IVSLFVSFFILTSKIWKRVV"

CDS complement(1627362..1627877)
/locus_tag="JFBMFIFI_01536"
/inference="ab initio prediction:Prodigal:002006"
/codon_start=1
/transl_table=11
/product="hypothetical protein"
/translation="MKVLVVSDNHGDRDVLVNLINTYEGKVDKFFHCGDSELEATDSV
WDTMLTVRGNCDYDSAYPDTRVTKVGSETILQVHGHLHDVKFTMNTLLYTAKEVGANF
AFFGHTHELAVEYVDGILFLNPGSIRLPRGQYASIKTYAIVETTPEEIDVTYYDPTHK
PVGGLNFKFNR"

CDS complement(1627893..1629251)
/gene="rph"
/locus_tag="JFBMFIFI_01537"
/EC_number="2.7.7.56"
/inference="ab initio prediction:Prodigal:002006"
/inference="protein motif:HAMAP:MF_00564"
/codon_start=1
/transl_table=11
/product="Ribonuclease PH"
/translation="MRSDGREYDEGRKVTIETNYLKHPEGSVLISVGDTKVICNATLE
TRVPPFMRGEGRGWVNAEYSMLPRATESRSIRESAKGKVKGRTMEIQRLIARSLRSVV
DLEVLGERSITVDCDVIQADGGTRTASITGAFVALKLAIQTLLDSGELTQDPIKENLA
AISVGILPSGEAVLDLNYPEDSSALVDMNLVMTASGRFVEIQGTGEEATFSAEELNQM
LALGKNGIATLIELQNSVIKDTASPVKRHAEIPTNEILVATNNPGKAREFEALFAKKG

WQVKTLLDYPEIPEVEETGTTFAENALLKAETIAKKLNMLVLADDSGLKVDALDGQPG

VYSARFAGEPKSDAANNAKLLHELSSTKPEDKTAQFHCTLALAMPGKESLVVSGEVAG

LIIAIPRGENGFGYDPLFYLPEKEKTMAELTSDEKNKLSHRAIALQNLEKVWDTWIET

KE"

CDS complement(1629274..1630086)

/gene="murI"

/locus_tag="JFBMFIFI_01538"

/EC_number="5.1.1.3"

/inference="ab initio prediction:Prodigal:002006"

/inference="similar to AA sequence:UniProtKB:Q836J0"

/codon_start=1

/transl_table=11

/product="Glutamate racemase"

/db_xref="COG:COG0796"

/translation="MEKQAIGFIDSGVGGLTVVKEAMRQLPNESIYYVGDTARCPYGP

RPEAQVRQFTWEMTHFLLDKDIKMLVIACNTATAAALEDIKKKLAIPVIGVILPGSRA

AIKATNTNAIGVIGTAGTVKSDMYRQMIRSKDRLAQVSSLACPKFVPIVESHKYNSAI

AKKVVAETLKPMQKQGVDTLILGCTHYPLLRPIIQNVMGSKVTLIDSGAETVSEVSTI

LDYFAIAEDSKSKLVSERHFYTTGSAQMFKEIGSEWLNLPDMQVEQIKLGND"

CDS 1630299..1631363

/locus_tag="JFBMFIFI_01539"

/inference="ab initio prediction:Prodigal:002006"

/codon_start=1

/transl_table=11

/product="hypothetical protein"

/translation="MKNEEQAKPIDLENLTGSISMFGYELIRDSLIPNLLGKETNEIL

YWAGKELARQYPVANYSDLVLFYKKACFGDLVLIKEKKHQKLFTLTGPTVTKRMEQHN

PNFSLEAGFLAEQMQMLQELYAEALSEINLRTKTVTITLQWDAKEPVSIDQPLETVVL

SEDYLSDEELEAEYQELDLSKKISDEPEFSEDEPEEIASFPDFQEFNKTQELDNEEIS

YPELEVKEVELEETSLPKETEELPLEEKELDDDEEEDIIVIESIPPFIEVEPAIFDDL

AETLADETFSDLEHSDSVIVEPTIPTEHLTAEEALHEFDALSQVFSDINNEAAPIEEE

ELSIFDSLPSRSSRHNKKKK"

CDS complement(1631416..1632675)

/gene="yhaP"

/locus_tag="JFBMFIFI_01540"

/inference="ab initio prediction:Prodigal:002006"

/inference="similar to AA sequence:UniProtKB:O07523"

/codon_start=1

/transl_table=11

/product="putative protein YhaP"

/db_xref="COG:COG1668"

/translation="MNKFWIIVGEVYKKNVKSGAFLSMVLSPVIIIAVIGIVAFFVNS

SSEVPKIAIVSDNQEIVQLLKTDKKSYKVNAKITTTADAEKALKKDDIAGYLVINESK

SAITGEFIKTPTSADVDTTLMLQTLTALQTNQIASNLGLSQEEVGSLTQPADIKVKTI

KFDGDKQQTNNDTDTMIKVFGAYFVSFVIYMLMIYYSAIIAQEIASEKGTRIMEIILS

SVSATEHFFGKLVGILFVCLTQIVAYIVMGTIAYRFGKSLDFVQNLMNGIDLVEILKG

LISYSLIFFILGMLLYSVLSAFLGSLVSKVEEASKAVTPLTFVIMIGFFAGIYGMSSP

NAPLVKIGSYVPLFTPFIMPFRIANDTVSSGGVLTSILITFVFTGVVTYLSLMMYRSN

VLVYSDTGLFKTMKNSWVIMRNERTKK"

CDS complement(1632668..1633600)

/locus_tag="JFBMFIFI_01541"

/EC_number="7.6.2.-"

/inference="ab initio prediction:Prodigal:002006"

/inference="similar to AA sequence:UniProtKB:O86311"

/codon_start=1

/transl_table=11

/product="Multidrug efflux system ATP-binding protein"

/db_xref="COG:COG1131"

/translation="MNKFVEWGRRTMLEVKHLKKTFGNLTAVDDLSFTIKDGEILGMI

GQNGAGKTTTFRLILKFLTQDSGEVLWNGHPLKKTDYDIIGYLPEERGLYPKVSIEDQ

IYYFARLRGKSKKEIKPLIDGWMEKFQVKGKKTDKVKSLSKGNQQKVQLICTLIHEPK

LVILDEPFSGLDPVNADLLQKGIIELREKGSCVIFSSHQMENVEEICDHLVMLRNGEM

VLNGKVKDIREQFGRTKIFIDSKLNQNELLALPGVNEVSTTTEGLSVLHLTDPAYGQE

IFNIVTKDGYIPTFSQMPPTLEEIFKMKAGDLHE"

CDS complement(1633851..1635122)

/gene="dltD"

/locus_tag="JFBMFIFI_01542"

/inference="ab initio prediction:Prodigal:002006"

/inference="similar to AA sequence:UniProtKB:Q2FZW3"

/codon_start=1

/transl_table=11

/product="Protein DltD"

/db_xref="COG:COG3966"

/translation="MTMKRKLFMAVGPLVVAVVVLLGLLFLPVTILPTGQKQINNSAT

AISTNIMKGEKVYQAAMEEKRMIPFFGSSELSRFDAFHPSVLAGKYDRNYTPYLIGNA

GSQSLTHFLAMEGMGESLTNKKAVFIISPQWFVKGGAPSMMFDSHFSPLQTYEFILSD

EVDSPQRRYAAERFLEYKAVCSDQNMEKMLHKIEKGESITKGQKGYAKLKRQVLRNED

KLFGAYVSGKNAKKVQNQLKNLPNTYDNEQLDQKAYEVGKKSSTNNPYQIKNGFYKTR

IQPIEGELAGSQKKFDYEFSKEFSDFQVVLDKMEANNMNVRFVIPPVNQKWLEYTGLS

QEMLNNFSKKITHQLTSQGFQVIDLSGDGSEDYFMQDTIHIGWRGWVALDKQLDPFLS

AEKPDKSKITLNNYFLSKEWQDLPAKDIPTK"

CDS complement(1635122..1635355)

/gene="dltC"

/locus_tag="JFBMFIFI_01543"

/inference="ab initio prediction:Prodigal:002006"

/inference="similar to AA sequence:UniProtKB:P55153"

/codon_start=1

/transl_table=11

/product="D-alanyl carrier protein"

/db_xref="COG:COG0236"

/translation="MDKKATLLDILEDLTGSDEARENMDVALFDEGLLDSLGTVQLLI

EIESTMGISVPVSEFDRTEWATPNLILQQLENM"

CDS complement(1635411..1636592)

/locus_tag="JFBMFIFI_01544"

/inference="ab initio prediction:Prodigal:002006"

/codon_start=1

/transl_table=11

/product="hypothetical protein"

/translation="MIQPYDAPSYFIIIGALLLPIIIGLLRGKRYLVYQNIVTLIMLF

LTFGGKSWHQGISIIIYLIWQWALVYSYFNYRQKKNATNIFVGAVLLAIAPLAIVKIV
PFTSGHVSIIGFLGISYLTFKAVQMVMEIRDGLIKEFDPKMFLQFLLFFPTISSGPID
RYRRFEKDFKQPPVKEEYIALISKGIWMIMLGSLYKFIIAHYIGTFAVPYTERLALDV
GGFSISLLMYMYSYSLYLFFDFAGYSLFAVGTSYLMGIQTPVNFNKPFSSPNLKEFWN
RWHMSLSFWFRDYVFMRMVFILMKKKIFKSRITVSNIGYMTLFLLMGIWHGLTWFYIA
YGLFHGVAMCINDAWLRYKKKHKNLPSNWATKALAIFITFNTVCFSFLIFSGIFAKML
H"

CDS complement(1636589..1638112)
/gene="dltA"
/locus_tag="JFBMFIFI_01545"
/EC_number="6.2.1.54"
/inference="ab initio prediction:Prodigal:002006"
/inference="similar to AA sequence:UniProtKB:P35854"
/codon_start=1
/transl_table=11
/product="D-alanine--D-alanyl carrier protein ligase"
/db_xref="COG:COG1020"
/translation="MKQTIIQRLNDLAIADPTAICYEKAGKTYTYLDLKEQSDRIAVY
FQSINLTPKSPILVFGGLEFEMVATFIGAIKAGHAYIPVDSHTPSERLEQIIAIAEPE
VVVTLSELPHQLSEMTKEITAEELEGIRQNPTQKDLDANIAVKGSDNFYIIFTSGTTG
APKGVQISHDNLLSFSEWMLSDFDLIKGQRFLAQAPYSFDLSVMDLYPALLSGGTLVP
LEKEITNNFKQLFEVLPTLKVNVWVSTPSFIDICLLDKNFSSEEYPGLTTFLFCGEEL
TNSTATLLMQRFPNAQIFNTYGPTEATVAVTQVQIKPEALSKYSRLPIGRVKSDTKII
LVDENLKEVPPGQVGELVITGPSVSKGYLNNPEKTNAAFYEIDGVGAYLTGDTGKYEG
DLLFYQGRLDFQIKLHGYRIELEDIDHNIEQVSYVKAATVVPKLKEHKAHSLVAYVVA
EPHEFEKEYQLTNAIKKELAPLIMDYMMPNKWIYVDQLPLTSNGKVDRKGLMNEVNK"

CDS complement(1638161..1638310)
/locus_tag="JFBMFIFI_01546"
/inference="ab initio prediction:Prodigal:002006"
/codon_start=1
/transl_table=11
/product="hypothetical protein"
/translation="MFAKLQKLKENQIFIFCAKTVFYFVVLLILVYLYDYTNTNSGNF

IYNEF"

CDS complement(1638829..1640637)

/gene="uvrC_2"

/locus_tag="JFBMFIFI_01547"

/inference="ab initio prediction:Prodigal:002006"

/inference="similar to AA sequence:UniProtKB:Q5KWH6"

/codon_start=1

/transl_table=11

/product="UvrABC system protein C"

/db_xref="COG:COG0322"

/translation="MKNEHIEHKLALLPNQPGCYLMKDKNGTIIYVGKAKVLKNRVRS

YFRGSHDTKTERLVSEIADFEYIVTGSNIEALLLEINLIKKNDPKYNIMLKDDKTYPF

IKITNEKYPRLLITRKVLKDKATYFGPYPDVKAANETKKLLDKLYPLRKCKVLPNRVC

LYYHLGQCLAPCIQEIPKETYTEMVEEIKRFLNGGYQEVKQEIEEKMQQASLNLEFEK

AAEFRDQINSIVTTMEQQKMTSADFVDRDVFGYAVDKGWMCVQVFFVRQGKLIERDVS

LFPFYNEPEEDFLAFIGQFYQKNHHFIPKEILIPKDSDAKSVEALVTANVLQPKRGQK

KELVELANKNAAVSLSEKFSLIEKKEERTYGAVKKLGDAMNIPLPSRIEAFDNSNTMG

VDPVSAMVVFLDGQPARNEYRKYKIKTVVGPDDYASMREVIYRRYARVLKEDLPLPDL

VVIDGGKGQVDAAREVLENQLGLDIPIAGLAKDNKHRTSELLFGPELQTIPLKRNSQE

FFLLQRIQDEVHRFAITFHRQLRNKNSFASKLDGIDGLGPTRKKNLLKAFKSMKNIQE

ASIEELKAIGLPKNVAENVYEKFKQPELNPELDSDK"

CDS complement(1641066..1641803)

/locus_tag="JFBMFIFI_01548"

/EC_number="3.1.1.2"

/inference="ab initio prediction:Prodigal:002006"

/inference="similar to AA sequence:UniProtKB:P22862"

/codon_start=1

/transl_table=11

/product="Arylesterase"

/translation="MKSVYIPVEGTEIFFRINGKGSPLVLLHGNGEDSRIFEKQILFF

SDHYQVIAIDTRGHGRSEHGTDILNFTRIALDIVAVLDYLSIEKADFIGFSDGGNSAM

YLTVKHPSYVKSLILIGANLEPKGMKKTSFLGVKLAYRTTAFLANFSSKFNQRKQIID

LMLKQLNLTTDQLKTINVPTLVIAGEKDMIEEEHTRLIAESIPNAQLAIIPAADHFLI

MKHPEMFNKLALEFLLFNSDKTEVNPH"

CDS complement(1642010..1642321)

/gene="trxA_1"

/locus_tag="JFBMFIFI_01549"

/inference="ab initio prediction:Prodigal:002006"

/inference="similar to AA sequence:UniProtKB:P14949"

/codon_start=1

/transl_table=11

/product="Thioredoxin"

/db_xref="COG:COG0526"

/translation="MVLAVTDKTFETETKEGLVITDFWATWCGPCRMQSPVLDELETE

IGDKVKIVKMDVDANPVVPADFGIMSIPTLLVKKDGEVVEKLVGYHAKEQLEEIIAKY

A"

CDS complement(1642476..1644842)

/gene="mutS2_2"

/locus_tag="JFBMFIFI_01550"

/EC_number="3.1.-.-"

/inference="ab initio prediction:Prodigal:002006"

/inference="similar to AA sequence:UniProtKB:P65496"

/codon_start=1

/transl_table=11

/product="Endonuclease MutS2"

/translation="MNTKIMQTLEFDKIKKALIAYTASELGRVEVEELMPATDLTKVQ

QWQEETEDGAKLLRLKGGMPIPKLQNIKPHLKRLEIGAMLNGIEIAQIGKVLRTTKEV

TRFFDDLSEIGVELFRLYELVAKLVTLPTLNQMIREAIDEDGHVMDDASQALKGIRTG

MKRSESNIREKLDSLIRGKNAQYLSDALVTMRNDRYVIPVKAEYRNHFGGVVHDQSST

GQTLFIEPQSVVDLNNKLRQLQIEERQEIDRILAELSNEIAPYGAEILNNMFLLGKLD

FIGAKAAYGKNIKATRPLVNEENDVRFIQARHPLIDEKQAVANDILIGGDYQALVITG

PNTGGKTITLKTLGLLQLMGQSGLQLPVAEGSQMGIFTEVFADIGDEQSIEQSLSTFS

SHMTNIVSILNKIDNKSLILFDELGAGTDPQEGAALAIAILDQVGAVGSYVMATTHYP

ELKAYGYNRPGTINASMEFDVETLSPTYRLLIGVPGRSNAFEISKRLGLDTAVIDSAR

SLIDGESQNLNEMIADLENRRKMTEMEYHEVRQYVEEAETLYQDLTTALEQFFGERED

LVKKAREKANQIVSEAEEEASKIVSDLRKMQLTGQFEGGIKEHELIDAKSKLANLHHE

ETLTKNKVLKKAKAKQQFKKGDEVIVASYGQRGTLMEKVEGNNWLVQLGILKMKIKES
DMTLAQPEKEPTRKMVATVRSSNDSHVSTQLDLRGERYESALSEMDQYLDAALLAGYP
QVTIVHGKGTGALRQGVTEALKNHRSVKKFETAPQNQGGNGATIVYFS"

CDS complement(1645218..1645763)

/locus_tag="JFBMFIFI_01551"

/inference="ab initio prediction:Prodigal:002006"

/codon_start=1

/transl_table=11

/product="hypothetical protein"

/translation="MLSLGIILIIAIGFYSGARRGLVLQIVLTIGYLLSYLVARIYYI
RLGSQLELFVPYPSATENSTFVFFDHSLGLELDKAFYNAVAFMLIIFVGWLLTRFVGS
FLNSLTFFPIVRQANYLGGGMLGFVVSYTAVFLILSILAMIPMESIQNALGQSGLAQM
IVKNTPILSNQIYNWWIGTIK"

CDS complement(1645798..1646091)

/gene="zapA"

/locus_tag="JFBMFIFI_01552"

/inference="ab initio prediction:Prodigal:002006"

/inference="similar to AA sequence:UniProtKB:P94542"

/codon_start=1

/transl_table=11

/product="Cell division protein ZapA"

/db_xref="COG:COG3027"

/translation="MSGGKKRFKATIAGKSYTIVGSRPIEHLQLVANTVDEQIHQIKA
LTTGLDLEEIAVLAAVNAVSDQLEMQIKMENMQLEMEELQEKINQLEMAETKE"

CDS 1646340..1647293

/gene="rnhC"

/locus_tag="JFBMFIFI_01553"

/EC_number="3.1.26.4"

/inference="ab initio prediction:Prodigal:002006"

/inference="similar to AA sequence:UniProtKB:P94541"

/codon_start=1

/transl_table=11

/product="Ribonuclease HIII"

/db_xref="COG:COG1039"

/translation="MANEVILVDKKTVLEMKNYYQSVLKSQTPPGAVFAAKKNTVNVT

GYNSGKVLFQGASASQEAQIWSSKVIDTGSTTKKKASSSKKPIDTPLPSGFSNWSVIG

SDEVGTGSYFGPLTVVAAFVSKEQIPLLKELGVKDSKDLKDPQICAIAKDLLTFLPHS

LLNVMPEKYNKIQPTMTQGKMKAVLHNQALGNVLAKIQPEIPDAILIDQFELPSTYYK

HIADQPNQIKDKVYFQTKGEGHHLAVAAASILARYAFLKGLEEMSKDAGTTIPSGAGS

TADLVAARLLKRGGINLLGKYAKLHFANTEKAKKIAGLNKK"

CDS complement(1647340..1648650)

/gene="steT"

/locus_tag="JFBMFIFI_01554"

/inference="ab initio prediction:Prodigal:002006"

/inference="similar to AA sequence:UniProtKB:O34739"

/codon_start=1

/transl_table=11

/product="Serine/threonine exchanger SteT"

/db_xref="COG:COG0531"

/translation="MSERQLKKEVSSLTALTVVVGTVIGAGIFFKPTAVYGAAGSPGL

GLIAWILGGIIAIAGGLTVAEIGTIYTETGGMMIYLEKVFGKWLGFLVGWAQMIVYFP

ANIAALAIIFATQVVSLFDLKDGIIVPIAIIVAAGVMGLNLLGTKYGGFVQNIATFLK

LIPIAVIIIAGLVYSGGGVVRLFPISATDHPFVTSLGSALVATVFAYDGWMNVGTLAG

EMKNPGKVLPKVIIGGLSIVTAIYILINVAYLFVLDAGQLAATDTPAAAVAHLLFGNF

GGKLVTIGILISVFGGINGYTISGLRIPYTLASQNLLPFSKWFGKLGKTGNMPVNGGI

VMLLISIIMIISGQFNQLTDLIVFVIWIFNTLTFVAVIKLRKTAPSIERPYKVPFYPV

VPLIAILGGFYIILNTLYVQPANALLGLGLTLLGLPVYLYSQKK"

CDS complement(1649101..1651518)

/gene="pheT"

/locus_tag="JFBMFIFI_01555"

/EC_number="6.1.1.20"

/inference="ab initio prediction:Prodigal:002006"

/inference="similar to AA sequence:UniProtKB:Q5XCX3"

/codon_start=1

/transl_table=11

/product="Phenylalanine--tRNA ligase beta subunit"

/translation="MNVSYQWLKEYLNLDEITPDALADKMSRTGIEVEDVFYGETGLK
KIVVGHTLSVVDHPDSDHLHICQVDIGEGEPTQIVCGAPNIATGQKVIVALPGARIVG
NAKIKKSKMRGEVSNGMICSLQELAFSENVVPKKYAEGIYILPKDAIPGESVFPILAM
DDAILDLSITPNRADALSMRGVAYEVGAIYDQKPEFKDFALNEAADDTIENYLTVDVL
AKEDAPIYTIRVIKDVKIAESPMWLQTKLMNAGIRPINNVVDVTNYILLEYGQPLHAF
DYDRLGSKEIVVRRGNTGEELVTLDDKKQELTSEMIVITNGEKPVALAGVMGGLDSEI
IDQTVTVALESAVFEPISIRKTAKALNLRSESSSRFEKGINKATVIEASKHAASLIAE
LTGGTVVSGVASKGELKAIDVALNITLERINGSLGTDITSSEVVAIFQRLGFGVNHAD
GLFEVVIPPRRWDISIEADLIEEVARIYGYNNLPSTLPKGRTTASSLSPKQRLVRHTR
HYLEGAGLSQAISYVLTTTEKAQQFMLQESQPTLLDLPMTEDRSTLRMNLLSGLLDDV
HYNIARKNQDVALYEVGRVFYHEEGRVLPIEEEHLAGVLTGLQVNNDWHGNGKPVDFF
FLKGIIEGLLDTYGFTADISYQADQNRDGMHPGRTATILVGETEIGYLGQIHPLRAKE
YDLKDTYGFEINLQKVLDSEKKPTIYQTIPKYPGMTRDIALLVDEAVTNQEITQLIEA
KGGKYLADVHLFDIYNGDKIEAGKKSMAYKLSYLNPEATLVEEEVTLAFDKVQKALVE
TFHVVVR"

CDS complement(1651525..1652571)

/gene="pheS"

/locus_tag="JFBMFIFI_01556"

/EC_number="6.1.1.20"

/inference="ab initio prediction:Prodigal:002006"

/inference="similar to AA sequence:UniProtKB:Q4L5E3"

/codon_start=1

/transl_table=11

/product="Phenylalanine--tRNA ligase alpha subunit"

/db_xref="COG:COG0016"

/translation="MELKAKIDALKEEALAKIKASENLDSLNQVRVAYLGKKGPLTEV
LRGMKDLSAEERPKIGALANVVRDEITTMLEEKKALLEMEKINQALAAETVDVTLPGN
HVGVGQPHVLTQIIEEIEDLFIGMGYSIIEGPEVEEDRYNFEMMNLPKDHPARDMQDT
FYITEEVLLRTHTSPVQARTMEKHDFSKGPLRMISPGRVYRRDSDDATHSHQFHQIEG
LVVDEQITMGDLKGTLELFAKHLFGAEREIRLRPSYFPFTEPSVEVDVSCFKCGGSGC
NVCKHTGWIEILGAGIVHPNVLEMAGIDAAKYSGFAFGLGPDRVAMLKYGIDDIRHFY
QNDVRFLNQFKVKE"

CDS complement(1652987..1653568)

/gene="azoR_2"
/locus_tag="JFBMFIFI_01557"
/EC_number="1.7.1.17"
/inference="ab initio prediction:Prodigal:002006"
/inference="protein motif:HAMAP:MF_01216"
/codon_start=1
/transl_table=11
/product="FMN-dependent NADH-azoreductase"
/translation="MKPTILFVHPWEGSYNKALLTAFEEKFKQEKTEYQLIDLYADDF
QPIMTKGDLALYNKGETTDPLISKYQAILNQTNELIIISPIWWYELPAIFKGFIDKVM
LKDFAYDEKATHLVGKLTHIKRTHLITTATSPKWYLKYIVGNYIQKVLINRTLKDNGI
RRVKWHHLGRIKTISQEQRTAFIKKRVEDWFHL"

CDS complement(1653565..1654002)
/locus_tag="JFBMFIFI_01558"
/inference="ab initio prediction:Prodigal:002006"
/codon_start=1
/transl_table=11
/product="hypothetical protein"
/translation="MYTIKGLAQEVLLPISTIRYYERVGLIQPKRSENNYRCFDEVDS
LKIKYIKVMKHAGFSIDEMKQFIQLLDVPSTPNCSLLSDQLITEKERQITQRIKQDQL
LLGLLNSLKPNIIAANYAENEKALVQQVTAIYQDIVEEEERIK"

CDS complement(1654152..1654397)
/locus_tag="JFBMFIFI_01559"
/inference="ab initio prediction:Prodigal:002006"
/codon_start=1
/transl_table=11
/product="hypothetical protein"
/translation="MNNFFKRGFLVEATGEVYPSSDQSVCQSIEEFISPSQKLEFLSQ
KKPVTFYLDGILYQAEVNEAQSRGGYFINCTEVKETK"

CDS complement(1654664..1654996)
/gene="yodB_1"
/locus_tag="JFBMFIFI_01560"
/inference="ab initio prediction:Prodigal:002006"

/inference="similar to AA sequence:UniProtKB:O34844"

/codon_start=1

/transl_table=11

/product="HTH-type transcriptional regulator YodB"

/db_xref="COG:COG1733"

/translation="METKTCEEKQATICPKFEQTFSILGKKWMGLIIDVLLEGPQRFK

DMAAKIPSVSDRVLVERLKELEQCGIVTRTVYPDSPVRVAYSLTEKGESLKPVMDEVQ

CWADKWVG"

CDS 1655291..1655905

/locus_tag="JFBMFIFI_01561"

/EC_number="1.6.5.2"

/inference="ab initio prediction:Prodigal:002006"

/inference="similar to AA sequence:UniProtKB:Q9RYU4"

/codon_start=1

/transl_table=11

/product="NAD(P)H dehydrogenase (quinone)"

/db_xref="COG:COG0655"

/translation="MSKTNVAVIYYSSTGGNTQMARWAAEAAKEAGAEVRLVKAHELA

PDVAIDSNPIWRKNVTDTADIPDATSADLEWADAIIFSSPSRFGVMASQLKQFIDLQG

GLWAAGKLANKAVTAMATAGNPHGGQEEVIQSIYTVMQHWSTIIVPTGYINQSTFAAG

GNPYGTSATIGNEGNVVDAENIKPAVYDQAKRVTAVATALKNNL"

CDS complement(1655943..1657046)

/locus_tag="JFBMFIFI_01562"

/inference="ab initio prediction:Prodigal:002006"

/codon_start=1

/transl_table=11

/product="hypothetical protein"

/translation="MDNLDKKIKEEFEKIDVPTDKVFQAIEAGLSRSPKSKPFWLKKS

FITVACVGLLAVGGVALYVNKNSEYTSSESQTELNSKAMLENTPKTNQSEDTKVQEKA

EYTTDEPEAETNNEASSVTDPNRKIISTVSMELESKEFSKSVKEIESLVKKVNGYIEN

YSLYGNPSSVSPEVKPFGETNESPSFNNRSADFVLRIPEEKVAEVLPLIEKSGRIISK

NTTNQDITLNYQDTESRKKVLLLEQDKLMELLGKATNVDEMIVLEKRLSEIRVEIENL

ESQLRSFDNQINYTTIYVSLRDVKAYTESVKNEVISERISEGFKNSLIQIQTFFINLI

VWILANSLYLLLVGLIGFGSYYLVKKKKLKKNN"

CDS complement(1657048..1657581)

/gene="sigV"

/locus_tag="JFBMFIFI_01563"

/inference="ab initio prediction:Prodigal:002006"

/inference="similar to AA sequence:UniProtKB:O05404"

/codon_start=1

/transl_table=11

/product="RNA polymerase sigma factor SigV"

/db_xref="COG:COG1595"

/translation="MEIETEKLVRKAKKGNRKALELLLKENYQQMYKTALIYVKNEQD

ALDVVQETAYKVTMKLDTLKENQYFKTWLIKILIRCAYDVLDKKKPVEDLNQVSQTKF

SSESENLNQQQIEHLTLFEAIEKLPLNYQNVVILFYFQDFSIKEIATIVMYPEGTVKY

NLHEARKTLKKLLGGAE"

CDS complement(1657756..1658271)

/locus_tag="JFBMFIFI_01564"

/inference="ab initio prediction:Prodigal:002006"

/codon_start=1

/transl_table=11

/product="hypothetical protein"

/translation="MSEAKWKTDTEYLGYIEDLIHREEVQKLAEYTQHHFTTRLEHSI

SVSYRSYRIAKKFGWNARSTARAGLLHDLFYYDWRDTKFDEGTHAFVHPRIACENAEK

LTPLTDLERDIIVKHMWLATIAPPRYKESYVVTFVDKYCACAEVFSPLMGKARTSVKQ

KWSKLKLSVQS"

CDS complement(1658399..1659160)

/locus_tag="JFBMFIFI_01565"

/EC_number="2.1.1.-"

/inference="ab initio prediction:Prodigal:002006"

/inference="similar to AA sequence:UniProtKB:Q7A794"

/codon_start=1

/transl_table=11

/product="Putative TrmH family tRNA/rRNA

methyltransferase"

/translation="METILSTKNERVKNWKKLQTKKGREQAGAYLIEGIHLIDEAIKN
QASISELIISEDKQLNLDIDYPVEKQILVSAEISKQLSETETSQGVFAVLQMLVPEND
PDLTKPLLFLDNVQDPGNVGTMIRTADAAGFGGVVLGKGSVDLYNSKVIRSMQGSHFH
LPIYQGDLQEWFDLMKATKIPVYGTELNEAAASYLTMTRAKQFALVMGNEGNGLSEEF
LKQTTKNLYIPIVGQAESLNVAVAAGILMFALTEA"

CDS complement(1659223..1660515)

/gene="smc_2"

/locus_tag="JFBMFIFI_01566"

/inference="ab initio prediction:Prodigal:002006"

/inference="protein motif:HAMAP:MF_01894"

/codon_start=1

/transl_table=11

/product="Chromosome partition protein Smc"

/translation="MKKRITLGVITTLLAFNPMALGSVVQAESVDEINKKAKEIDEKN
NEVTNKITESTNSLENLTTEKKALETDVTNLQKQIDEIIIKLQKQEETLLKTEEKIED
LKKEIKILEERIEKRTDKLDNQARYVQTNGDAANLASVVLTAESFSDLIGRVSAVTTL
VSANKAIVSEQERDQQELEVLEENAQSEKVTATAMKNEIESSKNNLFAQKSEIDDKIV
QIATKYQLTENEKTDLINEKVVLASQASQLSNKLQAEEARIVAEQQAKIAADLQAEAM
NLAAQQQQTNTQSTPTPNVNGTGFIVPANGPVTSPFGYRSDPFTGATTFHKGIDIAGG
GAIVASKAGVVEYSNYNNGGYGYLVIIDHGVINGINYKTYYAHMATGSLTKVPGQTVN
QGEQIGVMGTTGSSTGIHLHFEIRENNNPVNPGPFIGL"

CDS 1660693..1661589

/locus_tag="JFBMFIFI_01567"

/inference="ab initio prediction:Prodigal:002006"

/codon_start=1

/transl_table=11

/product="hypothetical protein"

/translation="MAIRLNKQQVIYNFSKEHPPALIVENGATIQVETADCFTDQIDS
ANYAFSGLNWDQINPATGPIFVKGAEPGDVLKVKIKKIELDSMATMITGPDMGVLGSD
LTQTTIKKCPIKNDFVQLTSTLKVKVNKMIGVIGTAPKDEAISCGTPDAHGGNMDCKE
ITEGADLYLPVHTKGALLALGDVHAAMGDGEVAVCGAEVASLVTIEVHILKNSSLPTP
LLVNSEKIMTLSSFPTLDEASVDATKKMVSLLQEKSQLSSADAILLLSLAGDLRICQI
VDPNKTVRMELDKHYFKLPLAD"

CDS 1661678..1662628

/gene="ccpB_4"

/locus_tag="JFBMFIFI_01568"

/inference="ab initio prediction:Prodigal:002006"

/inference="similar to AA sequence:UniProtKB:P37517"

/codon_start=1

/transl_table=11

/product="Catabolite control protein B"

/translation="MTSIRDIARLSGFSVSTVSRVLNDHPYVSAEKRAHIQKIIKELD

YTVNQNAVNLSIGKTKTIGIILPYANNPGFDKIIQGILTTAIKHKYNLMLLPTNYSKD

EEIKHLNLLKNKRVDGIIITSKANSWDVLNDYAKYGSIVTCEETPLLTISAAYTDRLS

SYLEVFHYLKKRGHTNVAFTSVRSAEKSNSTKSVLAAYEQVFAPPLEKNYRTNCFTYE

DGLRAGEYFLNQANPPTAIYANGDEIAAGIYHVAIEKGLRIPSDLVLIGEEYLPVGKV

LELSSMDHQLELLGQVAFELAIQQRIKKKKIPHILRCAPN"

CDS 1662925..1664400

/locus_tag="JFBMFIFI_01569"

/inference="ab initio prediction:Prodigal:002006"

/codon_start=1

/transl_table=11

/product="hypothetical protein"

/translation="MYDFLDKYTKRKIKLVQLLLSEDRYWSLNEVAAYLNCSKKTVQA

DIRNLKNELLLWTPEISLESSSKGYYLTKVPHFNSQKIKIHYLQTSVSFSLFYHLFLG

DIVSFTHFAKKQFISYSLMNKEVNLLKKELMFYALSISANPPTLNGLETQIRYYAQCF

FWNSYSGIDWPFSETNREELLGLILEIETLFEHPFNQIEREQIAYGIAIARSRISRGF

LIQEEIHFQNLTALAPFDNQLIPLYKKMLANLELTPQQLEREIEHLFSLMAIIPTATQ

PTQIIKKLLMNYEIQNPIYYQATKAFLNAFNQNVKTIDFDFQDPTLQFNLTSIHTHAY

FFIGLPTHHISRTIIPASQEAKDTLGKLYDDFFDTIKDDYELKKIFQQKEFLLSSYAP

HLMPYLDRNYYEKKIRIQIISNNGRHYESKLKRDLIDYYNFNIEIVSSFENTATIDLI

LTDVMTTQSLNSLNCFLWSSTPSRGDWDRLTNHLKKLIDHA"

CDS complement(1664439..1665959)

/gene="clsA_2"

/locus_tag="JFBMFIFI_01570"

/EC_number="2.7.8.-"

/inference="ab initio prediction:Prodigal:002006"

/inference="similar to AA sequence:UniProtKB:P71040"

/codon_start=1

/transl_table=11

/product="Major cardiolipin synthase ClsA"

/db_xref="COG:COG1502"

/translation="MKNKSIQLVIIISLTIGLAYFLSSWSQQLFFLISFGIELLGVFI

GLRLLLVDQRSTNSKVAWIAIIFVIPVFGVISYLFFGRNPQNRIFSTNQNVERAKLIE

RIHQVSCESEPNETPKLSQRIEALTGIHAMRGNRLTLLTDGQETFSAILEALQGAKDH

IHIQYYIFKQDTISTKIRDILVEKAKEGVTVRFLYDGFGSKKLTKEFLAPLVEAGVHF

YAYDPIYSIWFARTANLRNHRKIIVVDGDIGFTGGLNVGDEYLGITQRFRFWRDTHLK

MEGKGVLELQEAFLYDWVYMENRENAASEFISDEGVKRYLKSDSVGNEWIQVVYGGPY

DKEKLIRDALLDLIDSADESVWIMSPYLVPDEESLAVLRRVAMSGINVKLLIPGKADM

SLSFHGSNAYIKTLTEAGAEVYSYRNDSFIHGKLLIIDGKRAAIGTANFDVRSFRLNH

ELMVFVYEPSEAINKMKMDFENDLKESQLNTVESLNNKSRSQKIKETLASLFTPIL"

CDS complement(1666130..1666708)

/gene="pdxT"

/locus_tag="JFBMFIFI_01571"

/EC_number="4.3.3.6"

/inference="ab initio prediction:Prodigal:002006"

/inference="similar to AA sequence:UniProtKB:Q5L3Y1"

/codon_start=1

/transl_table=11

/product="Pyridoxal 5'-phosphate synthase subunit PdxT"

/db_xref="COG:COG0311"

/translation="MLKIGILAFQGAITEHQKMLEQLGVEVVLVKKPTDLYDLAGLII

PGGESTAIGRLLRSNQMIEPIKQFFKEGKYIFGTCAGLILCGKKTEQTQFDEVRLGII

DIEAERNGFGRQKDSFETQVWMEKIKTDIPAVFIRAPYIRSMGDSVTALVSFEGTVVA

AESKTVLVTSFHPELTDNLQVMNYFIEKIQSN"

CDS complement(1666711..1667604)

/gene="pdxS"

/locus_tag="JFBMFIFI_01572"

/EC_number="4.3.3.6"

/inference="ab initio prediction:Prodigal:002006"

/inference="similar to AA sequence:UniProtKB:P37527"

/codon_start=1

/transl_table=11

/product="Pyridoxal 5'-phosphate synthase subunit PdxS"

/db_xref="COG:COG0214"

/translation="MMEEKVIGSQLVKRGMAQMQKGGVIMDVINAEQAKIAEAAGAVA

VMALERVPSDIRAAGGVARMADPTIVEEVMAAVSIPVMAKARIGHIAEARILEAMGVD

YIDESEVLTPADEEFHLLKSSYTVPFVCGCRDLGEALRRIGEGASMVRTKGEPGTGNI

VEAVRHMRKVNQQIRSLSGKNEDELMTFAKDIGAPYELIREVKQLGKLPVVNFAAGGV

ATPADAALMMSLGADGIFVGSGIFKAEHPEKFARAIVEATTHFEDYALLAELSKGLGT

PMKGIDISQIPATERMQERGW"

CDS 1667743..1669158

/gene="norG"

/locus_tag="JFBMFIFI_01573"

/inference="ab initio prediction:Prodigal:002006"

/inference="similar to AA sequence:UniProtKB:Q2G1P1"

/codon_start=1

/transl_table=11

/product="HTH-type transcriptional regulator NorG"

/db_xref="COG:COG1167"

/translation="MWDLSPYQSKKIPLYQKIMLLIQDGIKNGELVAGSLLPSERKLA

QLLQVNRSTVIRAFDELSDRGILIRKKGSGTYINAEKWGLKTQPIINWQATVSPLGDM

ESSFYQKQVEQIKHTQISSSYLDLGNGDLPTDLLPALKTPELSWSQLIQEEKELYSNE

LGLTSLRKSIQSYLKKTYHLTVPLSEILVTSGTQQALFLITQGLLKPGDTVGIEAPSY

FYSLKLFQAAGVRLIPLPIDNDGLLLNRLEELNLNFPLKMIFLNPIFQNPTGSVMSYE

RKKKLLDYCKLKRILIIEDDAYGGLAFSPTVDTSPIKKIDQDQQVLYVGSLSKLVGQD

IRIGWMVGPSTILKQLAKIRSQIDSGLSALPQLLADSFLKNDQPTQQEHLKQTLKTRA

HDLEEWLTGRFGEEIRFQPAKGGYHLYTYFPNYSTAQFSQLLQDLLNEGIFVAEGRLF

GEKQQAIRFSFGHFSTQFFLS"

CDS complement(1669228..1669860)

/locus_tag="JFBMFIFI_01574"

/inference="ab initio prediction:Prodigal:002006"

/codon_start=1

/transl_table=11

/product="hypothetical protein"

/translation="MAVQALTENKVLSLLDWAYGKIIDGSMPGMTSAESLAQDYLTKH

KTVKKSAQALIRMQNGKSMTTGFVTNLGGLLTLPVAMPANISAVLLIQLQMIAALAVM

GGYDLKSDQVRTFVYLCLVGTSMAEVVKSTGIRVGNQAAMMGIQKIPSAVLLKLNQKI

GFTLVVKFGEKGAVSLAKLVPLAGGVIGGTIDGVSTNLIGKNAIRMFIQE"

CDS complement(1670379..1671698)

/gene="licH_1"

/locus_tag="JFBMFIFI_01575"

/EC_number="3.2.1.86"

/inference="ab initio prediction:Prodigal:002006"

/inference="similar to AA sequence:UniProtKB:P46320"

/codon_start=1

/transl_table=11

/product="putative 6-phospho-beta-glucosidase"

/db_xref="COG:COG1486"

/translation="MKKGLKIVTIGGGSSYTPELVEGFIKRYEELPLTELWLVDVEAG

EEKQSIVGEMAKRMFKAAGIDCEVHLTLDRKSALKDADFVTTQLRVGQLDARILDERI

PLSHGMIGQETNGAGGIFKALRTVPVILDIVEDMKVLCPNAWLVNFANPAGMVTEAVL

RYGKWDKVVGLCNIPVNAEVEEAEIIGRKQEELFFQFAGINHLHWHTVVDNQGNDLTD

ELIAKMYGKDADGKSIVANIKDNNLIFEQVENLHMVPCPYHMYYYMTDEMLKDELEDF

KNNGTRAEKVKAIEKELFELYKDPELDHKPQQLAERGGARYSDAACNIINSIYNDKRL

IMTVSTKNNGTVTDLPADSAVEVTCMITGNGPVPFQFGGFAPAERGLLQLMKAMEELT

IEAAVTGDYGTLLQAFTTNPLITSGNEGKQVMDELLEAHKQYLPQFN"

CDS 1671952..1672704

/gene="glvR_2"

/locus_tag="JFBMFIFI_01576"

/inference="ab initio prediction:Prodigal:002006"

/inference="similar to AA sequence:UniProtKB:P54717"

/codon_start=1

/transl_table=11

/product="HTH-type transcriptional regulator GlvR"

/db_xref="COG:COG1737"

/translation="MLFLDKTPDLTSLDLTLYKYISEHSEAVTKMKIRELAEATHTST

TSILRFCKQFECTGFSEFRIKLQLYLKEQKQLKTSSKISDETSYIDFLQRTTEPFYQS

KIKEAVDLLKDKELILFIGEGSSNVMALYGALYFSSIFNMALHIEDPTNHPVDFLSSS

LSDKVCIVALSVNGETPEIIHYISHLNFSKSSVLSITNSSKSTVAQLSDVNIPYYITR

EVKNTADITSQLPALYTIEYLAKAVGNQLKKD"

CDS complement(1672765..1673232)

/locus_tag="JFBMFIFI_01577"

/inference="ab initio prediction:Prodigal:002006"

/inference="similar to AA sequence:UniProtKB:Q7A551"

/codon_start=1

/transl_table=11

/product="Putative universal stress protein"

/translation="MKQEYKHILVAIDGSTESELAFQKAVQVAIRNEATLLLVQVIDP

IAFQSFSGSEELMNEQVIVQISEQVKGNMEDYLKTAKELGVKNVSYTIEYGSPKRIIA

KDLAEEKKIDLIMIGATGLNALERFFMGSVSSYVIREASCDVLVVRKNSENKV"

CDS complement(1673388..1673852)

/locus_tag="JFBMFIFI_01578"

/inference="ab initio prediction:Prodigal:002006"

/inference="similar to AA sequence:UniProtKB:Q7A551"

/codon_start=1

/transl_table=11

/product="Putative universal stress protein"

/translation="MLQQYKRILVAVDGSDESEAAFRKAVHVANRNQSSLFLLHVIDT

MSFQSVSGYEGLITENVTDQVKETLEEYKKYAELQGVEEFQYLIEYGSPKILIAKDVP

KEYQVDLIMLGATGLNAVERLFVGSVSRYVIQNASCDVLVVRTDLENQPIQF"

CDS complement(1673962..1674213)

/locus_tag="JFBMFIFI_01579"

/inference="ab initio prediction:Prodigal:002006"

/codon_start=1

/transl_table=11

/product="hypothetical protein"

/translation="MKIEENWADVFIYIIEFKPLSNFRELKKITRTIVIEEELSIEEV

KELFLKKVSGIDTITFIDFLDDGWHLKKKSCSKEMLLTE"

CDS complement(1674439..1677060)

/locus_tag="JFBMFIFI_01580"

/EC_number="7.2.2.10"

/inference="ab initio prediction:Prodigal:002006"

/inference="similar to AA sequence:UniProtKB:Q73E41"

/codon_start=1

/transl_table=11

/product="Calcium-transporting ATPase 1"

/translation="MLWVKESIDEIVKKLGSNEDKGLDDASILEKQKKYGANEFEEGK

KETTLEKVGHHLAEITTIILLVAAAISAYLALTTGYGWAKVIVILAIVVLNIVLGIYQ

ENSAEKALDALKNMNAHLTTVIRSGVRKPIDAKELVPGDIIELQAGDLVPADARIIES

SSLQVEESALTGESLPVEKDGNLTVTEDVPLGDRLNMVYSGCLVTNGRAKAIVVETGM

DTEMGKIASLINNTTKLKTPLQIRLKELAKRLSLVAIVAGILIFGIGVLIHGETMIEM

LMVAISLSVAAVPETLPVIVTLTLTYGVKNMVKKNTIIRRIPAVETIGNTSVICSDKT

GTLTQNKMQVQKIWAANALPKSVNELFTNEENQLLELLSISNNATVELIEEKEQIIGD

PTESAIIGLLQAKGITKASLEEKYPRVFELPFDSERKLMTTIHKTDNGYIAITKGAFD

RIPVTFSPELLNEAKRVHDEFAEDALRVISAAYQTFDTMPTELTAEALEHDLIFLGMV

GMIDPPRPESRPAVLAAKKAGIKTVMITGDHIVTASAIAKEIGILEEGDLAITGQDLD

KMNQAELEAKVRDVSVYARVSPEDKIRIVQAWQANGEIVAMTGDGVNDAPALKAADVG

IAMGITGTDVSKNAADMILTDDNFATIVDAVAEGRTAYENIRKTIYFLLSTNFSQIFI

MLIAVILGWGVPVVAVQLLLINVVSDGIPGFWLSLEKPDADIMNRKPIRKDAGIFANG

LGKKVGTQAVVFTIVTLIGFYIGQFVTVSSSIGASYEVGMTMSFVILAWSSVAHIFNV

RSDKSIFKVGILSNPRLFYSALGSMLIILGLALIPSLATIFFLVPMSLTHWIISFVLA

MSPLVFVEIQKNFFTKK"

CDS complement(1677289..1678179)

/locus_tag="JFBMFIFI_01581"

/inference="ab initio prediction:Prodigal:002006"

/codon_start=1

/transl_table=11

/product="hypothetical protein"

/translation="MKDYQFIKDYKEQEHYRNSFNQLVERTYGFNFESWYQLGAWNEN

YQCYSYLAKNEVIANVSINHMQIRYQGESYPMIQIGTVMTHPDYQNQGLIRELIKKIE

ADYQDKMGGFYLFANEQVLDFYPKFGYKKQIESCYTLNYQSEKKINSSSVEKVDSQNQ

AHHQLIQRLIANRVSINQEFQILTMEHLEWFYYHTALADAIYYLPSLDSIILASCEAG

KLDIFDILTTGKLKLDEFLHEIEQEFTFEKVEFHFMPDEDWMDKIDVTPSVDNDDTLF

VKPLQRNWPSQFKFPQISHA"

CDS complement(1678504..1679133)

/gene="yodC_2"

/locus_tag="JFBMFIFI_01582"

/EC_number="1.-.-.-"

/inference="ab initio prediction:Prodigal:002006"

/inference="similar to AA sequence:UniProtKB:P81102"

/codon_start=1

/transl_table=11

/product="Putative NAD(P)H nitroreductase YodC"

/db_xref="COG:COG0778"

/translation="MKTNTSLKEVIKGRKSVRAYDPSVKISKEEMTEILTEATLAPSS

VNMQPWRFVVAESDEAKAKLHPLVRFNTRQNETSAAMIVIFGDLECYEYGPEIYETAV

AEGKMMEEVKEQQLAAIMPLYQNLSKEKMNEIVQIDASLAAMQLMLVARTHGYETNPI

GGFEADQLAEALDLDPERYVPVMIVSIGKAVETGYDSIRLPIEKFTTWK"

CDS complement(1679163..1679594)

/locus_tag="JFBMFIFI_01583"

/inference="ab initio prediction:Prodigal:002006"

/codon_start=1

/transl_table=11

/product="hypothetical protein"

/translation="MNNQEKQSEELFFLLKNLSNQMNLCFEKRTECSLTRYEIMHILA

KKERVLQGDLQRALKIDPAAVTRHLKKLEEEKCIVRERNSQNNREVYVQITPIGLTRM

QACQSKTVDTLEQVYQGITNEQRKELIGIVEKISQNISTIN"

CDS 1679811..1680290

/gene="ylaC"

/locus_tag="JFBMFIFI_01584"

/inference="ab initio prediction:Prodigal:002006"

/inference="similar to AA sequence:UniProtKB:O07627"

/codon_start=1

/transl_table=11

/product="RNA polymerase sigma factor YlaC"

/translation="MKIYQYEDRLIQLSKELIFYLIKTGSTKEDAQDIAQEVFVKILE

TELILPVDKVRAWMYRVAIRKYLDLYRRKKRYSEIIEENFLSLPNWQLQPEQNELLYT

ALNQLDPSSASLLIMKYEQELSIKEMMSILDASESKIKSDLFRARKKLVQILEKEGY"

CDS 1680291..1681259

/locus_tag="JFBMFIFI_01585"

/inference="ab initio prediction:Prodigal:002006"

/codon_start=1

/transl_table=11

/product="hypothetical protein"

/translation="MENTDNLDQQFKTLARKTKRKRWLITILISFIVSLFVVFGSYKL

TQFFAKKSYLKLDQQFELVADVASPNIHSDSRYISLSNVLGGTLVSNRYKNVNGYQIP

WEQKQGSYDWITYSADSGTDAVVEISNDQQIVGYRNKATRNKIPVFFNPHAKDSLIPA

SNEAQSLKDMPNHLAEVGITFDKPYTYQEIQKMIPKNLLINWYWIGTNAHFDTTVLSS

YLGINANEQNGSLTKSDYNHFIKQLNKANKQMGYQLNNDAVSYSLYEDGVTVTEEHST

LETAKFSGIILSGSTENFKSLENKPWLYASSVGIVIEKVPFIQPTK"

CDS complement(1681344..1682906)

/gene="yfkN_2"

/locus_tag="JFBMFIFI_01586"

/inference="ab initio prediction:Prodigal:002006"

/inference="similar to AA sequence:UniProtKB:O34313"

/codon_start=1

/transl_table=11

/product="Trifunctional nucleotide phosphoesterase protein

YfkN"

/db_xref="COG:COG0737"

/translation="MELTILATSDLHGYILPSNFGSRQQDLPFGVAKVATILKEEQKK

AQGPVILIENGDFIQGSPLSYYIAKRRKTEDPTDLMATLNTIGYDVGLLGNHEFNYGT

DYLKKAIQAVNYPVICANILNEAGKPAFGNAYEIIEKEGVKIAILGLTTQYIPNWEHP

DHIKSLSFKSIVETAKEYVPKLRQLADVVIVSYHGGFEKDLENGKPTEVLTGENEAYQ

LLMEVSGIDALITGHQHRVIATQINGVPVIQPGFRGEYVGEIKLDLEKNATGFKVKTG

EASLIATGEALPDETIMKQIASISEEVEDWLDQPLGKVSGDMTIKDSNQARMVEHPYV

EFIQKVQMAATGTDISGTALFNNEGKGFGEIITMRDVVTNYIYPNTLAVLNVSGADLK

AALERSAAFFQVEKDGSIGINPEFINPKPQYYNYDMYEGVDYTINVKNPIGQRIEKLM

YHGQPVRPTDSLEVTANQYRAVGGGDYGMYDASKIIREVQTDMTELIADYLVEHPVIE

ATTNQNFHVIID"

CDS complement(1682972..1683760)

/locus_tag="JFBMFIFI_01587"

/inference="ab initio prediction:Prodigal:002006"

/codon_start=1

/transl_table=11

/product="hypothetical protein"

/translation="MLPTQPKKNRYKTAALIIMIALIYFWAFTGTSFDGIKDSAGTVT

KSILDGLFHPDWAYVYTGDGEDLISMLIQTIAIAFLGTFISAILSLPFAFWAARNKKS

NRVISTSGKFVLTAIRTFPEIVLAIMFIKTVGPGSFAGVLAVSIHSIGMLAKLYSEAI

ENMDQGPGESVTSAGGSKVNVLFFATLPQLVPEFISYTLYRFELAVRSASILGIVGAG

GIGTPMIFAIDARSWDRVGIILIGIIVMVTCIDLISGALRKKLV"

CDS complement(1683760..1684548)

/gene="phnE"

/locus_tag="JFBMFIFI_01588"

/inference="ab initio prediction:Prodigal:002006"

/inference="similar to AA sequence:UniProtKB:A0QQ68"

/codon_start=1

/transl_table=11

/product="Phosphate-import permease protein PhnE"

/db_xref="COG:COG3639"

/translation="MNTESNLLPKGKSKLKNLIWLGLFLVAFIYSANLSGVDMSKLFT

NGNQMTVILKKMASPDWSYVEVVIEPLLETIRMAILGTTFGAIVALPFSLLAARTIVK

NKFINGILRFILNIIRTIPDLMLAAVFVAIVGIGPVAGIITLSIFSFGMISKLFYEAI

ETIDEGPVEALTASGANKMQIIRYAIFPQVSSHFFSYLLYAFEINVRASTVLGYIGAG

GIGIYLQRSLSQFRYDRTAIIVLVIFAVVLVIDTISNKLRERLL"

CDS complement(1684545..1685342)

/gene="glnQ_4"

/locus_tag="JFBMFIFI_01589"

/inference="ab initio prediction:Prodigal:002006"

/inference="similar to AA sequence:UniProtKB:P10346"

/codon_start=1

/transl_table=11

/product="Glutamine transport ATP-binding protein GlnQ"

/db_xref="COG:COG1126"

/translation="MAATAEQKTSIISFKNVSKVYPNGVQGLKDLNIEIEKGEFVIVV

GLSGAGKSTMLRAINRLHEITSGDIEIDGQSITRAKGKNLRLIRRDIGMIFQSFNLVK

RSSVLRNVLTGRVAYHSTLSTMFGLFPKEDKQLAYEALQRVNLAEKVYTRADELSGGQ

QQRVSIARALTQEPKIILADEPVASLDPLTTEKVMDDLSRINQELGITIVVNLHSVEL

ARQYGSRIIGLRDGQVVFDGSVAEATDETLRGIYGEKILEANEEAVV"

CDS 1685732..1686484

/gene="ydjF"

/locus_tag="JFBMFIFI_01590"

/inference="ab initio prediction:Prodigal:002006"

/inference="similar to AA sequence:UniProtKB:P77721"

/codon_start=1

/transl_table=11

/product="putative HTH-type transcriptional regulator

YdjF"

/db_xref="COG:COG1349"

/translation="MLKSQRIQKIKEYVNAHQTVSLDKLMEEFQVSKNTIRRDVQYLV

RNEELKKVYGGVSTIHSELIPYTERADKNQSKKQKIAELAATFIKNGDVIFLDSGTTT

SELIPFIKHLDLTIVTNNITIIVECLAYENLTVFSTGGMLERKTNSFAGFKSLELLKS

YNINKAFMAATGISISNGVTNSSPSESSIKTTVVERSSEVFLLVDDSKFDRYALTTYC

DLKTIPMIITNEEPNEHYMTYGKEHGVTFLYD"

CDS complement(1686893..1687255)

/locus_tag="JFBMFIFI_01591"

/inference="ab initio prediction:Prodigal:002006"

/codon_start=1

/transl_table=11

/product="hypothetical protein"

/translation="MKLYKLFGVTENWREEKKEYKAYLKRVDAMPDDYKFVFKEIQDY

MWTFSDFTGQKILEALSDLLDMFEEGAANNVDVFTLTGDDVGEFADGIVREVASTWIE

DRKSKMNKKIKKFREKQK"

CDS complement(1687257..1687589)

/locus_tag="JFBMFIFI_01592"

/inference="ab initio prediction:Prodigal:002006"

/codon_start=1

/transl_table=11

/product="hypothetical protein"

/translation="MDKLTEMLKGILEGLVLEIISHKETYGYEITKTLNELGFTDIVE

GTVYTILLRIEKMGFVTVEKKPSEKGPARKFYSLNEKGQEELASFWQRWAFISEKMSS

IKEYLGGN"

CDS complement(1688080..1689474)

/locus_tag="JFBMFIFI_01593"

/inference="ab initio prediction:Prodigal:002006"

/codon_start=1

/transl_table=11

/product="hypothetical protein"

/translation="MINKREIVLGKVNEPVLFSNVAPTLLTDETIVERKSKLLAVMKK

EGFQTLVIYADKEHGSNFEYLTGFIPRFEEGLLIVEDSGECTVILGNENLKMAAFSRV

PVTLKHSPLLSLPNQPMDNEQPLEDLFTELGLDKNKKIGLVGWKMFTTKNSNPSTYFD

LPYFIVEALKKVKHPEAELLNATYLFIGGEKGVRTTNNANEIAHYEYGANLASTCILN

AMNAVDSGVTESELGNLLTAEGQFNSVVTIAAAGQRFAKANLYPTHKKVQLGDPLSLT

TAFKGGLSSRAGFVIHSEKELPENQKDYLDRVAKPYYQAVVSWLEGIKIGMEGTELYD

LVEEVFPKASYNWHLNPGHLVSDEEWMSSPVYSGSTEKLKSGMIFQIDIIPSIAGYTG

VSAEECIAIADQELQMEIKEQYPALWERIATRRSYLENELNIHLSPDVLPLSNTVAYL

RPFYLAKDQAFYCK"

CDS complement(1689478..1690245)

/gene="glcR_1"

/locus_tag="JFBMFIFI_01594"

/inference="ab initio prediction:Prodigal:002006"

/inference="similar to AA sequence:UniProtKB:P94591"

/codon_start=1

/transl_table=11

/product="HTH-type transcriptional repressor GlcR"

/db_xref="COG:COG1349"

/translation="MLIEERLEKIATIIEENRSATIDYLAENLSVSKDTIRRDLIKLE

QKNIVRRIHGGAVLANREALIMGYQQRASQLNHVKEEIAQKVAEVVKDHSSILFDSSS

TVEATISKLGNKDIHAITNSLTQAIELAKYEKTSVTLLPGKLHKDQLFLSGTETVKKI

EQYNPDYTLLGVFALSEEGLFIHTEEEGLVKRQMIAQGRKVIALCDHTKMDTTGFFKI

CSLSEIDILVTDRQPKELLMDCLNKSNVEVLLTREEE"

CDS complement(1690448..1694602)

/locus_tag="JFBMFIFI_01595"

/inference="ab initio prediction:Prodigal:002006"

/codon_start=1

/transl_table=11

/product="hypothetical protein"

/translation="MVDNEEEDKSIKARQLIFLEEIEELKNKKYLGAYVSQQVTEAYL

RYVEDQKDKVSSEVEIPNKEVLKKQAISKEVPKRRSQIGAVGPKEPLESIDRKKKVQT

KYSSTKEEERRAFLNSLVGKTNSHQIEPDESQVDFEAKAEKIIQEPRQNISVNHEKTP

SKAEPLTKEQQRSRNLSFILSTGVVLLLLGGLVLATTSWFMFAPVIKVLFILSVSIVF

GGMGVVAQKLKIKQTSFAFMALAFLFLPISVVSSAYYELFGSFFSLTGEGRDWLGVIV

TGIFIAIYGSVAKKYESKLFKWITLLFVYSFMVFLVLALTGYTRYRLFSLGLLLLVIS

LSRIIKQKKIQTLIHFFEKELIVFQISSLSISSIWLLTTIQANFLTVVNFGLYGILFF

SLSLQLKKDNLALQIIGNIFLNIGILISLLLSYPVSPSIAVVVMAVLSSTFLWLSKIK

RLQVISVIFIKRQVWASGLVTLIYGTLYGFSLLALNLKEPFIDQLFLGVAALIFLGGS

YQFMKLTNSKFTCFYFYGMSYLTIASCLMLMQVDVIFGQRLLLISSIIGYSLIFLRKP

FIQSKLMLGSASLIWYLSTLTKILWLLTAWEQTFWLVILWSVILGTTIYFKLVEKEKS

RPLLEWGIPTSLVISLLPLVKNVENYGLAIAGFSLVLLILSFVLTHFKQTILAASSLV

VASVIYLVSLISLVVQLNLIEGTWLNIGVLLLGILFSYLLRSHLSVKVSAIGIVLFSG

LSSLYPLHLIKVTPIGIILYSQVIAVLFGIVSWKLKGNAPLIKELKQSVHWGMHSLFF

ISLGSIVSFNQWLTAPIIYLLPLICYGWLLTVTINGGSIQSKFSYLGINCSLMTLFLM

SLGQLASFSWLDGPVISLIMALLLYGCYRGIPQLKIVASWLGMGATLIALAINTPIYF

EHIMWGAFIVSLFLWFLLCDYLKDYPLKGLKIGQLLGWFTLIAWHVHVLEFKWYFLTL

LLFLVINGILTWLKKAALLSVNPLQFSPNLIVTLVGISYLRIQLYIEGAVLLEACLIL

FFMCYTYLFARQTMGKVAKAILQTAFLVSVLDFCYWGIHYGQKFVTIPAVLTLKVTVS

LIVVSLILRYVWHLSEKQLKVEWAIVALGLMILLQEALWTGTIKQFLSFTVFSLLCYL

IGYKFHFKGYEYSGLIGLFVGVLSLRQLVWSDNKVIYELAIIIFTTVFLYMLTKQKQQ

PMILLITKTAFYLSISDLSYWMLRYFEASLYQPLYKTAQIAVAFIILSLILRRVWQLS

QQYLLLEWGYVIVGMGILMVQALEVGTLSQLLVFSVAALICMITGFIFKFKSYFIVGM

TTIAISLVYNQRHFWSSLPWWFYLILGGVLLIAIASSTEWRQRKKEEGGLSLVKKVVK

IFDQWH"

CDS complement(1694819..1695673)

/gene="iolJ"

/locus_tag="JFBMFIFI_01596"

/EC_number="4.1.2.29"

/inference="ab initio prediction:Prodigal:002006"

/inference="similar to AA sequence:UniProtKB:P42420"

/codon_start=1

/transl_table=11

/product="6-phospho-5-dehydro-2-deoxy-D-gluconate

aldolase"

/db_xref="COG:COG0191"

/translation="MGLVPMTELVKEAKQKHYAIAQFNINGYQWAQAIIAAAQEENAP

VILAASDRVIDYLGGFKMVVTLVRGLVQTLNVTNPVVLHLDHGQSVARCFAAIDAGFS

SVMFDGSKYPIEKNIKLTKQVVDYAHLKGVSVEAEVGSVGGVEDGITGGIQYARLTDC

CRMVEETQIDALAAALGSVHGAYHGEPVLGFTEMLEISQATSIPLVLHGASGIPKEQI

KKAIELGHAKININTELNQVWAQAVKASFMKNPESYNPQAILLPAKEALGKVVKEKIR

EFKNNRVF"

CDS complement(1695704..1696720)

/gene="iolG_3"

/locus_tag="JFBMFIFI_01597"

/EC_number="1.1.1.18"

/inference="ab initio prediction:Prodigal:002006"

/inference="similar to AA sequence:UniProtKB:Q9WYP5"

/codon_start=1

/transl_table=11

/product="Myo-inositol 2-dehydrogenase"

/db_xref="COG:COG0673"

/translation="MSENIVIGVIGAGRIGRLHVENMLKMAGVRVKTVADPYSEHAKD

WAEKLGIEQLVSNHEIIFNDAEINCVFICSPTDTHTTMIKAAAKAGKNIFCEKPISFS

DEETIEAFKAVEKAGVKLQIGFNRRFDKNFDRVKKCVATGKIGELHILKITSRDPEPP

GLDYVKSSGGLFMDMAIHDFDMARFVSGSEVEEVYVQGAALINPEFASLGDVDTAIIT

LKFANGALGVIDNSRKAVYGYDQRVEAFGSNGAVEIGNETETLAKLSTNSGVELDQPL

HFFLERYNKAYIREVQEFFEAIQKDKAVPCSFEDGIMAQRIAQAAKLSLTTGKPVRVT

KLIK"

CDS complement(1696750..1697646)

/gene="iolE_2"

/locus_tag="JFBMFIFI_01598"

/EC_number="4.2.1.44"

/inference="ab initio prediction:Prodigal:002006"

/inference="similar to AA sequence:UniProtKB:P42416"

/codon_start=1

/transl_table=11

/product="Inosose dehydratase"

/db_xref="COG:COG1082"

/translation="MTNQPKIKLGIAPIAWTNDDMPELGSENSFEQCISEMALAGFTG

TEIGNKYPKDPKALQEKLKLRNLEVASAWFSTFLTTKPYTETAEAFIQHRDFLWAMGA

KVIVVAEQGHSIQGKMDTPLFKDKPQFTDAEWVKLTEGLEALGELAHEKDMEIVYHHH

MGTGVQTTAEIDRLMKETDPTKVSLLFDTGHLVFSGENPLQIYKAYQDRIKHIHFKDI

RDTVTQEVKAENNSFLSAVKQGAFTVPGDGVIDFKPIMSEIEKANYTGWIVVEAEQDP

AVANPFEYALKARNYLKKECKI"

CDS complement(1697662..1698684)

/gene="iolG_4"

/locus_tag="JFBMFIFI_01599"

/EC_number="1.1.1.18"

/inference="ab initio prediction:Prodigal:002006"

/inference="similar to AA sequence:UniProtKB:Q9WYP5"

/codon_start=1

/transl_table=11

/product="Myo-inositol 2-dehydrogenase"

/db_xref="COG:COG0673"

/translation="MRTIKVGIVGLGRLGKIHAQNLAQNVPNCQLYAACSLNEEELIY

AKTELGVEKGYANYAEMIADSELDAVAIASPSGYHCQQINAALEKGLHVYSEKPIGLD

LEEIQATMDVINRHPKQIFMLGFMRRYDDSYRYAKELVEAGELGELTVVRCYGIDPSS
GMESFVKFAGASNSGGLFADMSIHDIDLVRWFTKAEVAKVWAIGKNAAYPELDQVNEL
ETGAAMLQLTDKTMGLLVAGRNCAHGYHVETELIGTKGMLRIANFPEKNLVTVLNSNG
VVRPCSQDFPERFAGAFITEMKEFIECILTEKQPEVTSYDGLQATKVALACKESFEKN
QLIELK"

CDS complement(1698713..1700626)

/gene="iolD"

/locus_tag="JFBMFIFI_01600"

/EC_number="3.7.1.22"

/inference="ab initio prediction:Prodigal:002006"

/inference="similar to AA sequence:UniProtKB:P42415"

/codon_start=1

/transl_table=11

/product="3D-(3,5/4)-trihydroxycyclohexane-1,2-dione
hydrolase"

/db_xref="COG:COG3962"

/translation="MKKTIRLTTAQALVRFLNQQYLSVDGKESLFVEGVFHIFGHGNV
LGIGQALEQNPGSLKIIQGKNEQGMAQAAIAFSKEKLRRKIFAVTTSVGPGAANLVTA
AGTALANNLPVLLLPGDTFASRQPDPVLQQIEQESSAGITTNDALKPVSRYWDRITRP
EQVMSSLIRAFEVLTNPVTAGPVTICLAQDVEGEAYDYPAEFFNKRVHYLDRKAPTER
ELIGAIELIRNSKKPLIIVGGGAKYSDCREELMTISEKFGIPLVETQAGKSTVEAAFK
NNLGGIGVTGTLAANKAAHVADLVIGVGTRYTDFTTGSKSIFANEEKQFLSINVSRMQ
AYKLDSFQVVADAKETLRLLLEQLEEYHAEFNHEIEALKKEWQMERQRLSQIEFKQQD
FVPEISEQFTQDKLNDYAQSLKTELPQTTALLAINDFVQEDSIIVGAAGSLPGDLQRI
WQPKKPNTYHVEYGYSCMGYEIAGTLGAKLAHPNQEVYAMIGDGSFHMLHSEFITSIQ
YQQKITILLFDNSGFGCINNLQMGNGSQSFGTEFRKPDDSLMKIDFAMVAAGYGAKSY
TVRTLNELKAALEDAQKQTVSTLIDIKVLPKTMTDGYESWWNVGVAEVSEGNTTKSYY
QKKQEMLKQARDY"

CDS complement(1700665..1701630)

/gene="iolC_1"

/locus_tag="JFBMFIFI_01601"

/EC_number="2.7.1.92"

/inference="ab initio prediction:Prodigal:002006"

/inference="similar to AA sequence:UniProtKB:P42414"

/codon_start=1

/transl_table=11

/product="5-dehydro-2-deoxygluconokinase"

/db_xref="COG:COG0524"

/translation="MKKKNFDMIALGRACIDLNAVEYNRGMEETMTFSKYVGGSPANI

AIGSAKLGLNVGFIGKIPDDQHGRFIENYMKKSGIDTSNMILDTEGHKAGLAFTEIKS

PEECSILMYREQVADLYLTPNEINEEYLKSAEMLVVSGTALAQSPSREAVLKAVSLAK

KNQLKVVFELDYRPYTWKNPAETAVYYSLVAEQADIVIGTRDEYDSMENIVNGTNEAT

IAYLFQHSPELIVIKHGVEGSYAYTKTGEIYQGKAYKTQVLKTFGAGDSYAAAFLFAL

NSGKEIEKALKYGSASAAIVVSKHSSSEAMPTVAEIEQLIQEQED"

CDS complement(1701651..1702469)

/gene="iolB"

/locus_tag="JFBMFIFI_01602"

/EC_number="5.3.1.30"

/inference="ab initio prediction:Prodigal:002006"

/inference="similar to AA sequence:UniProtKB:P42413"

/codon_start=1

/transl_table=11

/product="5-deoxy-glucuronate isomerase"

/db_xref="COG:COG3718"

/translation="MSKLLYPPKPKELTEGVTLLQEISEKNAPLTYIRLKIIEMESGA

SYEESLTQFECCLVALTGKITVTDFAETFSEIGTRESVFERIPTDSVYLSTQQKFRVT

AKTAAKIVLCYAPSEKKLPTQLIKASDNSIENRGMYQNKRLVHTILSDTSSISDALLV

VEVLTDSGNWSSYPPHKHDQNDLPRESLLEETYYHEMNPRQGFVFQRVYTDDRSLDET

MTVENGDVVVVPKGYHPVGVPDGYESYYLNIMAGPIKKWQFHNDPAHEWILNRK"

CDS complement(1702670..1704133)

/gene="iolA"

/locus_tag="JFBMFIFI_01603"

/EC_number="1.2.1.-"

/inference="ab initio prediction:Prodigal:002006"

/inference="similar to AA sequence:UniProtKB:P42412"

/codon_start=1

/transl_table=11

/product="Malonate-semialdehyde dehydrogenase"

/db_xref="COG:COG1012"

/translation="MVEVRRLKNYINGEWVNSQTTNYEDVLNPATKEIICQVPLSTKE

DVELATKASLAAFEKWKNVAVPRRARILFKFQELITRNKQELARLITIENGKNLKEAL

GEVQRGIENVEFAAGAPSLMMGDSLASIATDVEAANYRYPVGVVGGITPFNFPMMVPC

WMFPMAIACGNSFILKPSERTPLLTEKLVELFTEAGLPAGVFNVIYGAHDVVNGILEH

PEIKAISFVGSKTVGQYIYRKGSEHLKRVQSLTGAKNHTIVLNDADLKETVPAIVAAA

FGSAGERCMAAAVVTVEEGIADQFMEQLVAETKKIKIGNGLEEEVFLGPVIREENQKR

TFQYIETGIKEGAKLVTDGRENVPTDGYFVGPTIFDQVTTEMTIWKDELFAPVLSIIR

VKNLKEAVAIANQSKFANGACLFTNNAGAIRYFRERIDAGMLGINLGVPAPMAFFPFS

GWKDSFYGTLHANGKDSVDFYTRKKVVTARYPQASFE"

CDS 1704463..1705221

/gene="srlR"

/locus_tag="JFBMFIFI_01604"

/inference="ab initio prediction:Prodigal:002006"

/inference="similar to AA sequence:UniProtKB:P15082"

/codon_start=1

/transl_table=11

/product="Glucitol operon repressor"

/db_xref="COG:COG1349"

/translation="MKMKRIQKIEEYIQEHKHVSLNELCTYFNVSKNTIRRDIQYLET

EGLLEKVYGGVIAANSQLIPFESRTIENKLQKELIAAKAASLIEENDLIFIDSGTTTS

HLLDSVSPAIHFTVLTNNLDIINSAATMTNINLVVVGTTFKRRTRSFVELSEETFLSR

YNINKAFMAATGVSIAHGLTNSDPLEYEIKKWISAKTNQLYLLADLTKFDKSTLLTYL

ELSNIDGLITTNMLPHEYQHFCKQHHIPIYYPLT"

CDS 1705236..1705625

/locus_tag="JFBMFIFI_01605"

/inference="ab initio prediction:Prodigal:002006"

/codon_start=1

/transl_table=11

/product="hypothetical protein"

/translation="MPLIQIDLIEGRTDSEIKLLLDTIHEVLVETFEIPINDRFQILH

QHPANEMIIEDTGLGFKRSQNVVVLSITSRQRKEKQKVDFYQKVVQALTNRCGIAPED

VMISITINEQADWSFGFGRAQFLTGEL"

CDS complement(1705703..1706728)

/gene="alsC"

/locus_tag="JFBMFIFI_01606"

/inference="ab initio prediction:Prodigal:002006"

/inference="similar to AA sequence:UniProtKB:P32720"

/codon_start=1

/transl_table=11

/product="D-allose transport system permease protein AlsC"

/db_xref="COG:COG1172"

/translation="MAQQQNHEMKEPNVVDRKGNRKAFIQKYLIIFVLIGMVIILTGV

NPNFISFNNILNLLTQTSIYGILALGLFIVLVSKGIDLSVGSTLAFAGIVAGTISQTK

DAVDKVFPQLGQAPFIVTILIAILIGALIGLINGSLIAYTGIPPFIATLGTQIAVRGA

ALMVSKGKPISNINPAIDFLGDRIFGFLPVPVLIYGVIIFLMWVLMNYTSFGKSIYAI

GGNVEAAEISGINVKRNLVLVYVISGVLAGISSLIYIGRTGGSIQPAAGTMYETTAIA

AATIGGTSHSGGIGTVWGVVVGALILGTLTNGFTLLGVDAYVQQIIQGGIIIGAVVLD

MRKNRRV"

CDS complement(1706759..1708276)

/gene="mglA_2"

/locus_tag="JFBMFIFI_01607"

/EC_number="7.5.2.11"

/inference="ab initio prediction:Prodigal:002006"

/inference="similar to AA sequence:UniProtKB:P0AAG8"

/codon_start=1

/transl_table=11

/product="Galactose/methyl galactoside import ATP-binding

protein MglA"

/db_xref="COG:COG1129"

/translation="MEADNYLLRVEDIEKEFPGVKALNGVQLKIAPGEVHALLGENGA

GKSTLMKCLIGMHTPSKGKIWFDGRYIENYTITEALEMGISMIHQELSPVREKSIMEN

IWLGREPKNKFGLIDHKKMYQMTLDVLDRIQTKLDPKMLIKNLTVAKMQMIELAKAIS

YNAKLIIMDEPTSSLTEKETLQLYKIIRQLKAENCSIIYISHKLDEIQTICDTLTVLR

DGQYVTDRLVKDTSTQEMINLMVGRDINQLFPKQKTEIGKVKFEIRDFSDGKHFNHIS
FDVREGEILGFAGLVGAGRSEVMESLFGVRSRISGEIYMDGKKISINNAGDAVKNKLA
FLTEDRRKTGIFPMLSVGYNIVSSSIDSFKNKIGLLDNKRLADEAENYIETLKVKTPS
ANAAIMNLSGGNQQKVLIAKWLLTKPEVLILDEPTRGIDVGAKAEIHRNISTMAYTGK
CIIMVSSELPEILGMSDRVVVMHEGKITGILENKDLTQEEILEYASGEKDDFPIK"

CDS complement(1708365..1709312)

/gene="xypA"

/locus_tag="JFBMFIFI_01608"

/inference="ab initio prediction:Prodigal:002006"

/inference="similar to AA sequence:UniProtKB:A0QYB3"

/codon_start=1

/transl_table=11

/product="Xylitol-binding protein"

/db_xref="COG:COG1879"

/translation="MKKMWKVLLMSFTLLFLVACGADSSSNKEKTIGYAINNLNDTFQ
TYILEAAKETAKENKIDIKVENAKEDLISQQDQVNTLIQNGVSALIVVPVDTSAMGPI
TKAAQNAGIPLVYVNRNPYAGKEKEMPKDVYYVGSDEITAGIMQMDYIGEQLNGEGGI
TVLMGILGNEGAVQRTKGVSDTAAGKFPKNKILAKETGEWQRDKALSIAENWISTYGD
DVKAIIANNDEMALGAVQAAKKNGRSDILITGIDAIPDALDAVESGDLATTIFQDAKG
QGGGAVDTILAVFDNKPPKEAIKYVPFKLVTPENVAEFK"

CDS complement(1709748..1710677)

/locus_tag="JFBMFIFI_01609"

/inference="ab initio prediction:Prodigal:002006"

/codon_start=1

/transl_table=11

/product="hypothetical protein"

/translation="MKNFKKLFLVGLGVSLAVTLAACGGGEKASEKDKAYVPEKLTVQ
FVPSQAAETLEAKAKPLEKLLSKELGIPVEVSVSTDYNTIIEAMDSKQVDVGFLPPNA
YVLAHEQSGAKVLLQAQRYGIEQPGGKKTDELVDSYKSMIVVKKGSKIKELKDLKGKK
IATQDVTSSAGYVWPAAEMKKAGVDIEKSDITTVQVKGHDQAILSVLNGDVDAAFVFE
DARNNVVKDVPTIFDEVEPMYTTAPIPNDTVTVRADMSDDWNTKIQDAFINIGKSEEG
KAIISAIYTHEGYVKSKDSNFDIVREYAKEVGQ"

CDS 1711143..1712390

/gene="nrgA"

/locus_tag="JFBMFIFI_01610"

/inference="ab initio prediction:Prodigal:002006"

/inference="similar to AA sequence:UniProtKB:Q07429"

/codon_start=1

/transl_table=11

/product="Ammonium transporter"

/translation="MIDTGDTAFIILCGALVFLMTPGLAFFYGGMVRRKNTLNTMMSV

LFVCGLASVMWIAFGYSLAFGEDHGGFIGSLNHLFFNGVTGSPNPTYSETIPETLFAS

FQMMFSIITPALIYGAVAERMKFSTLFVFIAIWLIIVYYPMAHMVWGSGGFLAELGSV

DFAGGNVVHISSGITGLVLALFLGKRRDYGRLNYRPHNIPFVVLGASLLWFGWFGFNA

GSALGANALAVHALLTTNTAAATAMLSWMLVEQVTQGKPTVMGAITGAVVGLVAITPG

AGFVPLWSAFIIGGVVSPLCFFFISKLKHKWGLDDSLDAFGCHGLGGIWGGIATGIFA

KKSINGVAQWDGLFYGETRLFVAQIISIVITIAFSAIATGLILIILKQFMPLRVEDKE

EVLGLDLAEHDETAYPTFMGMDS"

CDS 1712418..1712759

/gene="glnB"

/locus_tag="JFBMFIFI_01611"

/inference="ab initio prediction:Prodigal:002006"

/inference="similar to AA sequence:UniProtKB:O66513"

/codon_start=1

/transl_table=11

/product="Nitrogen regulatory protein P-II"

/db_xref="COG:COG0347"

/translation="MKKIEAIIRQEKLEDLKTALYTKASISGMTVAQVLGFGKQLGWT

EYYRGNEVMINLVPKVEIKLVVASETVDQVIDIIVETCRTGDVGDGKIFVTALEDCVR

IRTGERGIDAV"

CDS 1712922..1713656

/gene="cpnA"

/locus_tag="JFBMFIFI_01612"

/EC_number="1.1.1.163"

/inference="ab initio prediction:Prodigal:002006"

/inference="similar to AA sequence:UniProtKB:Q8GAV9"

/codon_start=1

/transl_table=11

/product="Cyclopentanol dehydrogenase"

/translation="MNRLKNKVALVTGGTSGIGEKITDCFIAEGATVVVCDINQEALN

KAKGKENVVTKKLDISSEAEWTQVVQEVIEQFGKIDILANNAGISSDKDLETTTVEEW

ELQHKINALGPFLGMKAVVPYMKKAGQGSIVNTASYTALVGAGINGYTGSKGSIRAVS

KAAAADLGYFNIRVNSVYPGVIETPMSAAVSEYKEAMAQLIQATPLGRIGKPEEVANA

ILFLASDEASFINGAELVIDGGYSAH"

CDS complement(1713746..1715233)

/gene="rssB"

/locus_tag="JFBMFIFI_01613"

/inference="ab initio prediction:Prodigal:002006"

/inference="protein motif:HAMAP:MF_00958"

/codon_start=1

/transl_table=11

/product="Regulator of RpoS"

/translation="MYRMLLVDDEYMILAGLQKLIPWQELGIEIVGTAKNGQEALDFV

RNNVVDIVISDVTMPLLSGIEFIRQAQSEDIYFHFLILSGYQEFDYVKEGLRMGADNY

LIKPVDKVELIETLEKIIKELNSEAEQLQTQSVLFDYLLQGWTHDDIDYIELRKMIKR

YGYRLTDRPYTALIIAYPMEAEEALIEFIADQKQGMYYKQNDDQSYTVIFDGSKEELK

AFIKDYQQFSQSLLGDFLIGVGETVNEIVEVASSYEHALHALQIRNFYQIKGLELTKQ

IETGMIPQLSFTKFNQALYIRDFDTIREEVDMMLEKLRKHGALPEYSRQMIFLIFMDL

YRQFENLDESFYQVNFEKIATAQSFEGLESVIEETLLEIKHVKKERSYSQNTQHMLEI

ITKRYSEDLSLKVVADELFLNVMYLGQIFKKETQKSFSQYLNQYRIKKAQNLLVQSDG

NINEIAIAVGYTSAGYFYKNFKKICGISPKEFRERFENSYDPIDS"

CDS complement(1715234..1716949)

/locus_tag="JFBMFIFI_01614"

/inference="ab initio prediction:Prodigal:002006"

/codon_start=1

/transl_table=11

/product="hypothetical protein"

/translation="MKQKNLIYSLLKVYSLVMILVITSFAVTVSYATFDRNRKDTAKV

TQQTASRLADILSENENKINRIAIELTNNQEKVANLFDYFQLSYSDYLSQSLSREGIE

GGISSIYLPQKIANLYYADDKIESIAIDLKNYQEILYSNEQKKMGSKEKRMPDLKNQL
TLSKTLMNPNTSEQIGTLYVTFSKQELNTIIDNSGAKNQLQTFIFSDRMKQLYANKIP
SNNGLLQAEIEKGITKNSEIDFLSLSQQYYIYGSETASGMQVLTAVSKKSVAIQSFNA
IFFILLGSALINLFLLYFLYRIFRKYAYQVEDIVASVSQVSSGKLDWRITTADKEAEL
KDISTGINQMLDGINQYIEDIYTLEIKQKDAHMRALQSQINPHFLYNTLEYIRMYAVS
EGAEELADVVYTFATLLRNNTSQEKTTTLKKELEFCEKYVVYLYQMRYPGNIAYSFAID
SAIENLVIPKFSIQPLIENYFVHGIDYMRIDNVISVKANIEEDKITILIRDNGKGMSS
EKIKDLNQSLMESHSKFGGSIGILNVNERLRSYFGESYRMCIQETQAHGVTISISFEK
EKEEV"

CDS complement(1717010..1717627)
/locus_tag="JFBMFIFI_01615"
/inference="ab initio prediction:Prodigal:002006"
/codon_start=1
/transl_table=11
/product="hypothetical protein"
/translation="MLGKYLETFFNRCYLFIKLSLIFWGLSFTGGLIFGIGPALVTIA
SLFLEYRWEYKQMNKATWWKIYKENFKKSTILFYIFGAISVFLAYNLYLSVQITGLLF
LMIDFLIIFALVILSSAFWFAILIQSQYDCSLKDTLKLAAILSFMSFKNTLVLTIGGV
VLIMITYKFPGLILFGSIGFWIIFVANVSQPLFTKLDEQLVSLES"

CDS complement(1717743..1719215)
/locus_tag="JFBMFIFI_01616"
/inference="ab initio prediction:Prodigal:002006"
/codon_start=1
/transl_table=11
/product="hypothetical protein"
/translation="MKKSWKKLAVGSALGLILAGSLAGCSGLKGDATKKKEGASDSGA
PTLLMYQIGDKPDNYDELMKVANKKIEAETGVQLNLQYIGWGDYEKKMSVITSSGENY
DIALANNYVTNAQKGAYADLTDLAPKLAKDAYDMLDPAYIKGNLIDGKLYAFPVNANV
FAEQVLTFNKQFLDKYNLDISNVKSYQDIGELLKVVKEKEPTVTPLAAGQGFKVESNM
DYPITNGMPFAIDLLGDDSKIINQYDNDRMKANLKTMHEYYQAGYIAQDAATSNTDHP
LEGKTWFIRQETQGPYDYGDTILTTAAGQPLVSKPVTLPIKTTGAAQMANFVVSSNSK
NKEKAVEVLGLINSNPELLNGLVYGIEGEAWEKVGDNQVKLLDGYQPKTHMSAWNTGN
NKIIYVQDSITEDQIAERDKKIGEAEQSPILGFNFNTESVKSEITNIANVMNQYIDGL

NTGTLDPDKAIPEMNAKLEKAGLDKVQTEMQKQYDAFRKE"

CDS complement(1719239..1720156)

/gene="araQ_1"

/locus_tag="JFBMFIFI_01617"

/inference="ab initio prediction:Prodigal:002006"

/inference="similar to AA sequence:UniProtKB:P94530"

/codon_start=1

/transl_table=11

/product="L-arabinose transport system permease protein

AraQ"

/db_xref="COG:COG0395"

/translation="MKKKKVTQLTVRTFGKKADFLFNLLVGIFAFSCVFPFIFVIIIS

LTSETSLVTNGYALIPQEWSFDGYRYLAEMKDQILQALFITVFVTVVGTLINVTFTST

YAYAISRPSFKYRRFFTIFALITMLFSPGMVPNYIVMTNMLQLKDTVWALILPMALSP

FNIIVMRTFFKRSVPDAIIESAKIDGASELRVFLQIVLPLAVPGIATISLFAALGYWN

DWFNALLYIQSDNLIPLQYLLMKIQSNIEYMTQNAGMSAQLSGGAAAIPKEATRMAMV

VISTLPIACSYPFFQRYFVSGLTIGGVKE"

CDS complement(1720175..1721122)

/gene="yteP_1"

/locus_tag="JFBMFIFI_01618"

/inference="ab initio prediction:Prodigal:002006"

/inference="similar to AA sequence:UniProtKB:C0SPB3"

/codon_start=1

/transl_table=11

/product="putative multiple-sugar transport system

permease YteP"

/db_xref="COG:COG4209"

/translation="MKGKTKKKTLWKDFMKNKAFLLMALPGAIWLVLFFYIPVLGNVV

AFKDFQYSDGGFFKSLSQSPWVGFENFKFLFSSSNAYLITRNTVLYNVGFILINLLFA

IVFAVILSEIRSKKMIKVYQTSMLLPYFISWVIISYFVYAFLSPDKGLLNAIITGNGG

EAINWYNEPRFWPFILLFLGVWKGIGYNSIIYFASIMGIDPTYYEAAMVDGATKWQQI

KNVTIPQLVPLITILSILAVGNIFKADFGLFYQIPRNSGALYEVTSVLDTYIYNGLTS

SGDIGMASAAGLYQSVVGCILVVGANLFVRRFDEESALF"

CDS complement(1721324..1722721)

/gene="rcsC_3"

/locus_tag="JFBMFIFI_01619"

/EC_number="2.7.13.3"

/inference="ab initio prediction:Prodigal:002006"

/inference="protein motif:HAMAP:MF_00979"

/codon_start=1

/transl_table=11

/product="Sensor histidine kinase RcsC"

/translation="MIRTIKGQFISSFILIACLIIGVISLTTLILVNRHFDRYIVERQ

EDRLAEIKHSLEFSFVNEEGNWDLAEIEKIGESALDSGIDITVYSNENERIWQTTKQS

MGHMRMNGKHHKMMNQENQNTDLEYVEKNVVLKDGKQAIGQVQFGYYGPLTYSDHDVA

FISAMRRSLILIAIFALTFSLFFATWVARKLSLPLIQVNRFTQAIAKGHYNEKTPPKS

STKEVNELIISVQDLSNQLEQQEELRNRLSSDISHEIRTPLTTLKGHIEAMLDGVWEP

TPERLKSCYEEVNRLSRLIGDIDKITALEAQQEVVRKSRFDLYQLAQTVALTFEPLIF

QKGIQFELTGEKTFIDADQDKISQIILNLLSNALKFTESGGKIQVKVKIKDQFAVLMV

EDSGCGIDSTDLEHIFDRFYMADDSRKQNNEGQGIGLAIVKSIVKAHDGTVSVSSSVN

LGTRFIIQLPLANTK"

CDS complement(1722718..1723401)

/gene="walR_2"

/locus_tag="JFBMFIFI_01620"

/inference="ab initio prediction:Prodigal:002006"

/inference="similar to AA sequence:UniProtKB:P37478"

/codon_start=1

/transl_table=11

/product="Transcriptional regulatory protein WalR"

/db_xref="COG:COG0745"

/translation="MKILIVDDEPKILDVIEAYLVVNGHLVYRAETGSQALEKYRVVG

PDLIILDWMLPDSSGMEVCQEIRLESSVPIIMLTAKAGEKNIISGLKMGADDYVVKPF

SPKELVMRVETVLRRTGYQAKTQKIKFNDGKLVIDVLGKQVFQSNQLVCLTGTEFDLL

HIFAESPEQIFSREQLIEHVKGIEFDGMDRVIDSHIKNLRQKIEDNPKKPEFILTVHG

RGYRFGGKK"

CDS complement(1723576..1723758)

/locus_tag="JFBMFIFI_01621"

/inference="ab initio prediction:Prodigal:002006"

/codon_start=1

/transl_table=11

/product="hypothetical protein"

/translation="MNHCVNQMGNSNVGEVGGFTERLGHSFGMGMFSSSIIDFLLLIL

LIFVVVKVVQGIRSKD"

CDS complement(1723934..1724875)

/gene="oppF"

/locus_tag="JFBMFIFI_01622"

/inference="ab initio prediction:Prodigal:002006"

/inference="similar to AA sequence:UniProtKB:P24137"

/codon_start=1

/transl_table=11

/product="Oligopeptide transport ATP-binding protein OppF"

/db_xref="COG:COG4608"

/translation="MTSKEKILQVKGLKQFFNVGQSNEVQAVNDISFDVFKGETFGLV

GESGCGKSTTGRAIIRLYDPTSGEIIFQDKEVHDIKGKKDMLSFRRDMQMIFQDPYAS

LNPKMKVRDLIAEGIDIHGLAESTADRNKQVDELLKTVGLNPDHSTRYPHEFSGGQRQ

RIGIARALAVQPKFIICDEPISALDVSIQAQVVNLLMELQKEKGLTYLFIAHDLSMVK

YISDRIGVMYYGRLVELAPADEVYNNPLHPYTESLLSAVPLPDPNYERSRIRIPYQPR

EATGAEEKLREITPGHFVYCTEADIPALQAKAASYKK"

CDS complement(1724865..1725974)

/gene="oppD"

/locus_tag="JFBMFIFI_01623"

/inference="ab initio prediction:Prodigal:002006"

/inference="similar to AA sequence:UniProtKB:P24136"

/codon_start=1

/transl_table=11

/product="Oligopeptide transport ATP-binding protein OppD"

/db_xref="COG:COG0444"

/translation="MSNILEVKDLTITFDTFAGTVQAIRGVSFDLKKGETLAIVGESG

SGKSVTSRSIMRLLAKNANVEKGEILFNGKDINKRTEKEMQGIRGKEIAMIFQDPMTS

LNPTMTIGKQIAEPIIKHQKVSKKEALERALELLKLVGLPDPERRMKQYPHQFSGGQR
QRIVIAIALACNPEVLIADEPTTALDVTIQAQILDLMKQLQKKIATSIIFITHDLGVV
ANVADRVAVMYAGKIVEVGTVDEIFYNPQHPYTWGLISSMPTLDIKGKSLYAIPGTPP
DLLDPPKGDAFAPRNEFAMKIDTELEPPFFKVSDTHYAATWLLHPDAPSVTPPGEIRA
RRELFAKDATNTASEATFEPINDADAGKGVKLDDK"

CDS complement(1725989..1727029)

/gene="oppC"
/locus_tag="JFBMFIFI_01624"
/inference="ab initio prediction:Prodigal:002006"
/inference="similar to AA sequence:UniProtKB:P24139"
/codon_start=1
/transl_table=11
/product="Oligopeptide transport system permease protein
OppC"
/db_xref="COG:COG1173"
/translation="MSNLNEELKNLSPEMFEPAQDSGLVDREKIAAPSLNFFQDSWRR
LKKNKAAVICLFILIFMISASIAAPLISPHDPNKQTVAYANLPPRIKGVNINGLNGTA
KIGNTDKRVDKYAQKNVPDDVNYIFGTDGLGRDLFSRILAGMRVSLLIALIAAFFDLT
IGVSYGLISGMSGGRVDNFMQRILEIIGGIPNLVVLILMLLVLQPGILSIGIALAITG
WITMARVVRAQTLKLKNQEYILAAKTLGESNLKIAIKHLIPNLSGVIIINTMFTIPAA
IFFEAFLSFIGIGIPAPQASLGTLLSDGYKTFRFLPHLMWYPAAVMCILMISFNLLAD
GLRDAFDPKMKD"

CDS complement(1727029..1727970)

/gene="oppB"
/locus_tag="JFBMFIFI_01625"
/inference="ab initio prediction:Prodigal:002006"
/inference="similar to AA sequence:UniProtKB:P24138"
/codon_start=1
/transl_table=11
/product="Oligopeptide transport system permease protein
OppB"
/db_xref="COG:COG0601"
/translation="MKNYAKFLGKRIFFMLITLLLIASITFFLLKLLPGTPYSNQEKL

SQEQIYIMNEKYGLNKPVIEQYWVYITGLLKGDLGTSFQFNNTPVSTLLSARVGPSLQ

LGLQAVILGTFLGIILGVIAAMNQNTWIDTSATFAAILGRSIPNFVFAVLLQLIFGVY

LKWFPIAMWKDGFASSVLPTIALAISPLADSARFIRTEMVEVLSSDYIELARAKGLAR

WVVSFKHGVRNALIPLVTLLGPMAVGLMTGSLVVENIFSIPGIGEQFVKSIMTNDYPT

IMGVTMLYSAMLVAIILLVDILYGIIDPRIRVAGGSN"

CDS 1728238..1728591

/locus_tag="JFBMFIFI_01626"

/inference="ab initio prediction:Prodigal:002006"

/codon_start=1

/transl_table=11

/product="hypothetical protein"

/translation="MKPKKIVSFLIILPLSVVFLWQVIKFQKVTFLETSNLSFIAAGI

LLIIGIFWYTLSSGVFDFFNYSLKKSAQALKRNTSDAEIEPLSKAVGKGYRSPLVAGA

VLLIFSLITLFGYYL"

CDS 1728698..1729699

/gene="trpS"

/locus_tag="JFBMFIFI_01627"

/EC_number="6.1.1.2"

/inference="ab initio prediction:Prodigal:002006"

/inference="similar to AA sequence:UniProtKB:P21656"

/codon_start=1

/transl_table=11

/product="Tryptophan--tRNA ligase"

/db_xref="COG:COG0180"

/translation="METIFSGIQPSGIPTIGNYIGAMKQFIALQENYDCYYCIVDEHA

ITVPQDRIKLREQIRGLTALYLAVGLDPAKSTIFIQSEVPAHTQAAWIVQCNISLGEL

ERMTQFKDKSAKSNRAGVSAGLLTYPPLMVADIILYQSNFVPVGEDQKQHLELTRDFV

DRFNNKYGNGNQVLTLPEVMIPKFGGRIMSLQEPTKKMSKSDDNQKGFISMLDDPASI

RKKIKSAVTDSSGVIEFDVENKPGISNLLTIYSAFSGETIESITPRYEGVGYGKFKED

LAEAVIAVMEPIQERYYALLKSDELDEILDAGAKHANAVANKTLKKMQNAVGLGRKR"

CDS complement(1729753..1730760)

/gene="lplJ"

/locus_tag="JFBMFIFI_01628"

/EC_number="6.3.1.20"

/inference="ab initio prediction:Prodigal:002006"

/inference="similar to AA sequence:UniProtKB:O07608"

/codon_start=1

/transl_table=11

/product="Lipoate-protein ligase LplJ"

/db_xref="COG:COG0095"

/translation="MYFVNNNEITDPRINLAIETFLLKNLTLDEPILLFYINEPSIII

GRNQNTIEEINIDYVEEKKIHVVRRLSGGGAVYHDFGNLSFSFIMPDDGNSFRDFAKF

TEPVIASLHKMGVEGAELKGRNDLVIDDKKFSGNAMYATSGRMFAHGTIMFDSDIEAV

VGALKVRKDKIESKGIKSIRSRVTNIKPFLSDAYQYISIKEFRDQLLLSIFNVTDRSQ

VKEYHLTESDWEKIHEISAEYYGNWDWNYGKSPKFELNRRQRFPIGSVEFKMNVEKGY

IKDVRIFGDFFGLGEISDVEKRLEGIKYDRSEIEKALEDVDVQNYFGNIEKEDLLNLI

Y"

CDS complement(1730782..1731528)

/locus_tag="JFBMFIFI_01629"

/inference="ab initio prediction:Prodigal:002006"

/inference="similar to AA sequence:UniProtKB:P9WGC1"

/codon_start=1

/transl_table=11

/product="hypothetical protein"

/db_xref="COG:COG1234"

/translation="MKLTILGYWGGYPINNEGTSSYLVESEGYHLLIDAGSASLISLE

NHLDPLELDAVILSHYHHDHVADIGVLQFMRLLKRKNNGEDRASLLPIHAHTENAAGF

AALTLERVSEGVAYKETDKLNIGPFEITFMRTLHPVPCFAMRIKEVKTGKVLVYTADS

GFLPDFVPFSEKADLLLADTNFFEGMENHQVHMTSLEVARIAKKAEVKTLVLTHLPQI

GDLELLKAQAINHAEGIEVLLAKKDLVIDL"

CDS complement(1731659..1733065)

/gene="hemY"

/locus_tag="JFBMFIFI_01630"

/EC_number="1.3.3.4"

/inference="ab initio prediction:Prodigal:002006"

/inference="similar to AA sequence:UniProtKB:P32397"

/codon_start=1

/transl_table=11

/product="Protoporphyrinogen oxidase"

/db_xref="COG:COG1232"

/translation="MERAKKRIAIVGGGITGLTAAYEIKKFIEKEQLPFELILLEGST

RVGGKIHTLQIGTRFFDTGAESIDVRYSGAMTLIKELGLEEELVYSSGDKPDVYFYNQ

LHQLTYPTYKGIPIHRTDIWKNKLLTFHGKLASYKDLVVPKTKIEEDLTVSSFLKRRL

GEEQVEHVVEPFFSKIYAGDLDEMGVRSSNEFIYDVEQNHRRLSKGLSNYPEFFDGDG

NYVTFEKGLEVLPKKLAETIEPHIQYGKKVFEISKGQEGTYVIDINRKEQMRVGAVIV

ATPATEYSRLFKDEKLTSFFNKTVTASIGFILLSFPKGAIKNEPKGFGFVTPRRNDSH

VTSVILLDKKWPTLKQNDEEVLIGANFGRRGEESLVSLSNLEIEEYILKDLNQILGIE

VPPNYSKIARWPNAIPQYTIHHEERKQKLVAILRADFPGIYIGGNGFGGFGINQCVAQ

ANRISKATIEYMKKQNCI"

CDS 1733523..1734023

/gene="hmoB"

/locus_tag="JFBMFIFI_01631"

/EC_number="1.14.14.18"

/inference="ab initio prediction:Prodigal:002006"

/inference="similar to AA sequence:UniProtKB:P38049"

/codon_start=1

/transl_table=11

/product="Heme-degrading monooxygenase HmoB"

/db_xref="COG:COG2329"

/translation="MSLYVYTTTGTRPYLTKYLHDEHPMHNFTLMQKTTASDIYLLLD

ESNAASVFNAPFGYENRFQYGLKPHDGIAVFEHIGLAPENEKSFLLQFDRIKEDLDAQ

PGLEALYLLRSEKKVAYVILTFWKDDSYFNKWTKSAHFRPLVPYITDHQTRSSDITYA

DIFQLR"

CDS complement(1734098..1734865)

/locus_tag="JFBMFIFI_01632"

/inference="ab initio prediction:Prodigal:002006"

/codon_start=1

/transl_table=11

/product="hypothetical protein"

/translation="MRSSDYRAAARKSLEGQWGTFALLMLSSGILSMLVAGGIGSIFG
SDNSFVSTLTELLLTFAFGYGIFYASLYAVRGGKAEVGMIFSIFNGGRYLPLLLVNLV
EWLVGLISSFVLALPFIGILGFGVISTMLLTGGSGNLVSSVVGFGGFSAIALLTLLFV
VIIAGVGTLVDGYFQIVVLIRLDFPEMKLGDVFSYAFTLLKGRLWDLFVLQLSFIGWY
LLGIFVIPLLWIVPYKIVAISEFYDVAKENNELKELI"

CDS complement(1734879..1735961)

/gene="ysdC_1"

/locus_tag="JFBMFIFI_01633"

/EC_number="3.4.11.-"

/inference="ab initio prediction:Prodigal:002006"

/inference="similar to AA sequence:UniProtKB:P94521"

/codon_start=1

/transl_table=11

/product="Putative aminopeptidase YsdC"

/db_xref="COG:COG1363"

/translation="MDKKELQLLKDLTDAKGVPGNEGQVRDVFVKYAEKNADKVFYDG
LGSVIAKQVGDKEGPKVLFAGHLDEVGFMVTGITEEGFLKFQTLGGWWSQVMLAQQVE
ITTGKNKVIHGVIGCKPPHVLTVEVRNKAYDIQDMFIDIGATSKDEAMEWGVKPGDMV
TPYIEFKRLNDSKFLLAKAWDNRIGLAVTLRVLENLKASGHPNIAFGAGNVQEEVGLR
GAKTATYLVDPDIAFALDTGTAGDTPGMTAKEADSKLGQGPQIIIYDASMVAHKELRD
FVVSVAEELDIPFQYTVIARGGTDAGSQHVTRNGMPALAITVATRYLHSHTSVIHEDD
YLNTVKLVTEVVKRLDRKTVEKIKFG"

CDS complement(1736303..1736491)

/locus_tag="JFBMFIFI_01634"

/inference="ab initio prediction:Prodigal:002006"

/codon_start=1

/transl_table=11

/product="hypothetical protein"

/translation="MRQTNSIWIYILAAVGFIMVFGFLAAAFSAILFLIIKILVPIAL
VVWLINFISRQVNDRNRR"

CDS complement(1736641..1737831)

/gene="lysC"

/locus_tag="JFBMFIFI_01635"

/EC_number="2.7.2.4"

/inference="ab initio prediction:Prodigal:002006"

/inference="similar to AA sequence:UniProtKB:Q59229"

/codon_start=1

/transl_table=11

/product="Aspartokinase"

/translation="MKILVQKFGGTSVKDDESRAAAEKHIVQAVKDGYKVIVVVSAIG

RLGDPYATDSLLSLIDGDESQLDLREKDLLLSVGETISATVFTNQLKKNGLPAAALTG

QDAGIITNDDFSNAKVLRVETEPIFEFFKKVDVVVVTGFQGVTETGHITTIGRGGSDT

TAALLGKAFKAERIDIFTDVSGMMTADPRLVEQAKFLEVVSYQEVSNMAHEGAKVIHP

RAVELAMQATIPLRIRSTYLPVTDLGTLVTHSELQVGHYRLVTGIAHVSNLVQFTIET

TKQSPETIFSLLANAGISVDFINIFPHQVVFTLVKHEAELAKIIFSENNIAVSTLEDC

AKVAVVGAGITGVPGVTAKIVTSLAAEDIAILQSADSYTTIWILVSGENLQNAVCTLH

DVFL"

CDS complement(1737909..1739804)

/gene="asnB"

/locus_tag="JFBMFIFI_01636"

/EC_number="6.3.5.4"

/inference="ab initio prediction:Prodigal:002006"

/inference="similar to AA sequence:UniProtKB:P54420"

/codon_start=1

/transl_table=11

/product="Asparagine synthetase [glutamine-hydrolyzing] 1"

/db_xref="COG:COG0367"

/translation="MCGFVGCIHGSDLNDHDLDYEKKIKEMNKLIVHRGPDDEGFFFD

DHISFGFRRLSIIDVEKGHQPLSYENERYWIVFNGEVYNYIELRDELIADGLTFETDS

DTEVIIATYAKYKEKTAERLRGMFGFVIWDKVEKSVYGARDHFGIKPFHYAEEDGNIY

FASEKKSIYEILKKKEINPVALQNYLTFQFVPDPETLTENIHRLPPGHYFTKPLNGEM

EITRYWEATFAPIQKSEDEFAKAIKDVLYDSVEKHMRSDVTVGSFLSGGVDSSIIVAI

AREFNPKIKTISVGFEREGYSEIDVAKETADRLNVENISEIITAQQFMDEFPRFVWHM

DDPLADPAAVPQFFLAEIARKHVKVALSGEGADEVFGGYTIYNEPNSLKRIDSLPSGL

KKGLNQLAKIMPEGMRGKSFIQRGTTPMEQRYVGNAKIFEEAEKKKLMVNYLAGHPYQ

NVTKPFYDRSKGYDPIDRMQYIDIHTWLNGDLLLNADRTTMAASLELRTPFLDKEVFK

VARELPSDIRIANGTTKYILRKAAESFVPDNVLYRKKLGFPVPIRHWLKDEMNSWVKG
IIADSQTDHLINKSYVLGLLEDHCAGKLDNSRKIWTVITFMVWHKLYVETGEQFLVLP
NSKAVIQ"
CDS complement(1740001..1740594)
/locus_tag="JFBMFIFI_01637"
/inference="ab initio prediction:Prodigal:002006"
/codon_start=1
/transl_table=11
/product="hypothetical protein"
/translation="MKKKLILYKKVNLIENRPLLQLLSRLSLIIFLLLLLVPVGYIAM
NDSVLKNELSFLDFNAETSLVRIILLLSEKLISIILLFFISLVLHEAIHGLFFKVFGA
KKVTFGFKRGMAYAEASGSYFRAWQFKVIALSPFVGLSLLYFILAQYIPISAFLLFAL
HTSGCVGDFYFIYLLIKFKEIDWIEDTETGINLYKKN"
CDS 1740704..1741345
/gene="cutC"
/locus_tag="JFBMFIFI_01638"
/inference="ab initio prediction:Prodigal:002006"
/inference="similar to AA sequence:UniProtKB:Q830V2"
/codon_start=1
/transl_table=11
/product="Copper homeostasis protein CutC"
/db_xref="COG:COG3142"
/translation="MLLKEVCVENFTLIPEALSRGANRVELCDNLTVGGTTVSTGVME
ETIHYCGEKHVPVMAIIRPRGGNFVFHDTELKIMKTDLMEAKKLGVDGVVIGCLTADN
WIDEDAITDLLDEANGLQVTFHMAFDEIPADKQFAAIDWLVEQGVQRILTHGGPSHLA
IESTLPRLKELVAYADKRIIILPGGGITNENADFIAQELGVNEIHGTKIVGTL"
CDS complement(1741399..1742451)
/locus_tag="JFBMFIFI_01639"
/inference="ab initio prediction:Prodigal:002006"
/codon_start=1
/transl_table=11
/product="hypothetical protein"
/translation="MRKWKRVLFFLLIIQFINLSMKSVDAQAENSETHKVQKGETVWR

IAKSYGITIAELKEWNDMGSDTVYAGQIIKVKNPTLTAKELPKTGPVILENQMEYIVQ

PGETLWRIATRNKMTVDEIKSWNQLSSDKIKDGQILKIKKQVGIPPVVKNKVIALTFD

DGPSASVTPRVLDILKKQDVKATFFVTGQSAKANPELIRREISEGHEIGNHTLSHPKL

TSLSIEEAQYQVSATNEIIQNIANYQIKLLRPPYGLIDDKLIAVYQMPIIEWSVDTED

WKSKNADMIYEEVMRSSTSGAIVLMHDIHPTTADALETTIISLKKQGYSFVSISDLYG

GVLSGEKQYYQASDVR"

CDS complement(1742710..1742922)

/locus_tag="JFBMFIFI_01640"

/inference="ab initio prediction:Prodigal:002006"

/codon_start=1

/transl_table=11

/product="hypothetical protein"

/translation="MEITGEEIKKYVQKIKNQEVDRVSALCWLIANYRVLCLHDLSLI

AALIDRPEIHYSLEVEKYSTFLNSLD"

CDS complement(1743126..1745201)

/gene="ltaS1_2"

/locus_tag="JFBMFIFI_01641"

/inference="ab initio prediction:Prodigal:002006"

/inference="similar to AA sequence:UniProtKB:Q797B3"

/codon_start=1

/transl_table=11

/product="Lipoteichoic acid synthase 1"

/db_xref="COG:COG1368"

/translation="MKEKRKNMTIISRILNKRMGFFIVAVLLFWLKTYVVYKTKFSLG

VSGSFQQVLLFVNPISFTLLLFGISLFSNGRKSYIVLLVTDFIMTLWLFANVVYYREF

SDFMTINIIKSASAVSGNMDSSFKSLVHISDFFVFLDLLLLASLLIFKIVKIDQTVIK

KRVAFSVTAIAMAVFATNLSLAEMDRPQLLTRTFDRNYIIKYLGLNAYTVYDGIKTAQ

ANATKANADGSQMDEVKEYIQSKQLTPNVDYFGKAAGKNVFVIHLESFQQFMIDYKSN

GVEVTPTLNEFYHDSNTVSFDNFFHQVGQGKTSDAEMMLENSLYGLSEGSAMVSNGTE

NTFQAAPAILNQKGYTTAAFHGDVASFWNRDNTYKSWGYNYFFDSSYYPKGKDYDLGY

GLKDKIFLSQSAKYLEQLPQPFYAKMITVTNHYPYPLDEANATIPKTTTGDSTVDGYV

QTARYLDEAIKEFLAYLKDSGLYDDSLILMYGDHYGISSNHGNAVSQLLGTKEYTDFD

DAMFQKVPFMIRAPGLKGGINHTYGGEIDVLPTLLHLLGEQTTDYIQFGSDLLATDNQ

QIVPFRNGDFVSKNFTKVHGTIYNTATGEEITDLTEEQKVIFDQEKQHVEKELALSDR

VITGDLLRFYTPKGFNVVDKEKYSYTFKSGIKQLEEAAKEVKSKTLLQEHNNQSTVNL

YQTDAPELK"

CDS complement(1745379..1746251)

/gene="thrB"

/locus_tag="JFBMFIFI_01642"

/EC_number="2.7.1.39"

/inference="ab initio prediction:Prodigal:002006"

/inference="similar to AA sequence:UniProtKB:Q8Y4A6"

/codon_start=1

/transl_table=11

/product="Homoserine kinase"

/db_xref="COG:COG0083"

/translation="MLRIKVPGTTANLGPGFDSCGLALQLYLTLEVGAEQDSWEVEHQ

LGSGIPTDSKNVIVTTALSLAPDLAPRKLKMTSDVPSTRGLGSSSAAIVAGIELANQL

ADLQLTAAKKVEIATLIEGHPDNVAPAILGDFVVATKINQEVFAVKHAFPETGIIAFI

PQKELLTSESRTVLPSELSYSEAVKASSIANVMIASVLEGNLFLAGQMMEQDLWHEIY

RKKLIPHLEKIREISQENGAYGTFLSGAGPTVLVLVPKDKLKLLMSLLKEVDNEAKIE

AFDIDRFGVQISHQ"

CDS complement(1746360..1747646)

/gene="hom"

/locus_tag="JFBMFIFI_01643"

/EC_number="1.1.1.3"

/inference="ab initio prediction:Prodigal:002006"

/inference="similar to AA sequence:UniProtKB:P19582"

/codon_start=1

/transl_table=11

/product="Homoserine dehydrogenase"

/db_xref="COG:COG0460"

/translation="MKKQIQVGILGFGTVGSGVIRILKDHNEKIGQVTGEEISIKKVL

VRNIEKNRGLISEGIELTTNADDILNDSEIAVVLEVMGSIDVAKEYISRALKAGKHVV

TANKDLIALHGKELVALAKENQCDLYYEASVAGGIPILRTIVDSLASDNIKKVLGIVN

GTTNFMLTKMTAENKSYEEVLKEAQDLGFAESDPTNDVDGIDAARKMVILTRLAFGMH

VDLDQIETRGIRHVKSIDIETAKQLGYKIKLIGSAEEANGSVVVDVGPVLVPQDHPLA

GVHNENNAVVVQGAAVGETMFYGPGAGELPTATSVVSDLITVAKNIRLGTTGNVFNSY

QLETKLTPDEEVYAKYYLAIEMLDKTGLFLELTKIFAASDVGFDKIIQEPQAGQTSKV

VIITHKMNKKQQKEILKQVELAKDMDLLVHFKVMEG"

CDS complement(1747964..1748197)

/gene="ykuJ"

/locus_tag="JFBMFIFI_01644"

/inference="ab initio prediction:Prodigal:002006"

/inference="similar to AA sequence:UniProtKB:O34588"

/codon_start=1

/transl_table=11

/product="putative protein YkuJ"

/db_xref="COG:COG4703"

/translation="MKSSQLVAIIHRLEAMQENTDSEVQVRRFEKEGQERCLISYDPK

AETYELEEVSDHSVYQFDDIDLIAIEIYELLQD"

CDS 1748590..1749771

/gene="dapX"

/locus_tag="JFBMFIFI_01645"

/EC_number="2.6.1.-"

/inference="ab initio prediction:Prodigal:002006"

/inference="similar to AA sequence:UniProtKB:P16524"

/codon_start=1

/transl_table=11

/product="putative N-acetyl-LL-diaminopimelate

aminotransferase"

/db_xref="COG:COG0436"

/translation="MTVLSEKFNQQTYKIEVSDIRRFDERVSVIEDMLKLTLGEPDFN

TPEHVKLAGISAIENNDSHYTGMAGDLELRKAVATFMQKKYQVSFAPENEILVTVGAT

EALSASLLAVLNPGDKIIVPTPIYPGYEPLITLARAEPIYIDTTSNGFVLTPEMIEAA

MLEHGDQVKAIILNYPSNPTGVTYNREEVKAIADAVKKYSIFVISDEIYSELTYGETH

VSIAEFARDQTILINGLSKSHAMTGWRIGFILAPQELIGQIVKVHQYLVTSATTMAQK

AAIAALTAGADDALPMKIEYMKRRDFLYEKMKNLGFEIARPNGAFYIFAKIPDGYTQN

SMNFCVDLAEKNKLAIIPGSAFGAAGEGFVRLSYAASMEKLELAMERLTAYMATNKPT

P"

CDS complement(1749837..1750730)

/gene="glxR"

/locus_tag="JFBMFIFI_01646"

/EC_number="1.1.1.60"

/inference="ab initio prediction:Prodigal:002006"

/inference="similar to AA sequence:UniProtKB:P77161"

/codon_start=1

/transl_table=11

/product="2-hydroxy-3-oxopropionate reductase"

/db_xref="COG:COG2084"

/translation="MSKQKIGFIGVGVMGASIVKHLLKDGYEVNVYNRTKSKADEVVS

LGAIWQDTPKAVTIESDIVFTIVGYPKDVEEVYYSEDGIFAGATSEKILVDMTTSTPS

LAQKIFATGRERKIEVLDAPVSGGDLGAKNGTLTIMVGGTKEAYQIVEPIFNVFSGKV

KLQGAAGSGQHTKMANQIMIAGTMVGMSELLVYANAAGLELESVLDTVGGGSAQNWSL

TNYAPRILREDFTAGFFVKHFVKDLKIALAEAEKMDIQLPGTSLAKELYEKLENQGHG

NDGTQALIKLWWPAGVQPKRN"

CDS complement(1750821..1751849)

/locus_tag="JFBMFIFI_01647"

/inference="ab initio prediction:Prodigal:002006"

/codon_start=1

/transl_table=11

/product="hypothetical protein"

/translation="MDRRNKWALGAILVVGLAIFAWEFRKLSFAQVYQELLQLNWFWL

LAALGCMLIHWGIEAKIIQGLLQRRNSNYSFINAYRIPLIEHLFNAITPFSSGGQPAQ

LMALKKTGVDFGVGSSVLLMKFVVYQGMIVLNFVCCVFFGYQALSAELSKLSTFVFIG

LAVNLGVITILLLITFKASWLKKVIHWVIGWLSRFISSDKLGKFEISLLEKVDTFYEE

SLYIRSQRSLLIKTSILTFIQLVFYFVVPYFILRSLGLTDISILTIISLHAFIILIVS

LFPIPGGAGGAEYSFTLLFGSFILSPEKLIIALILWRLITHYTGIFMGVAALGIKDTK

IAVAKVRT"

CDS complement(1751822..1752880)

/gene="cpoA"

/locus_tag="JFBMFIFI_01648"

/EC_number="2.4.1.-"

/inference="ab initio prediction:Prodigal:002006"

/inference="similar to AA sequence:UniProtKB:Q8DPV9"

/codon_start=1

/transl_table=11

/product="Alpha-galactosylglucosyldiacylglycerol synthase"

/db_xref="COG:COG0438"

/translation="MIKVNMFSSADKVKGQGVGSAYLELIRLLQEQAKDEIEMTINRY

QKTQLSHYHTIDPLFYLTTFFRKRFGRRIGYVHFVPNTLKESIKIPKFAEKILDWYVV

SFYKRMDHLVVVNPSFIPELVALGIDEANITYIPNFVAQSTFYAYSPNEIAELRKEEG

LADDKFVVFGAGQVQERKGVFDFATLAEEMPDVQFIWAGGFSFGKITDGYERYKKLMD

HPPENLKFVGIIDRKKMVDYYNLANVFLLPSYNELFPMCILEAFGTETPVLLRDLPLY

KPVIDGYYAACEDRQAMAAEIRKLKDNQEDYQKLKERAIAGNAAYSEERLTTIWIRFY

QEQALKGENKWIEGTNGL"

CDS complement(1752899..1754074)

/locus_tag="JFBMFIFI_01649"

/EC_number="2.4.1.337"

/inference="ab initio prediction:Prodigal:002006"

/inference="similar to AA sequence:UniProtKB:Q8CWR6"

/codon_start=1

/transl_table=11

/product="Alpha-monoglucosyldiacylglycerol synthase"

/db_xref="COG:COG0438"

/translation="MNIGLFTDTYFPQVSGVATSIKTLKEELEHKGHQVTIFTTTDPN

AEEDEPTIIRLTSIPFLSFKDRRIAVSGAYVALRKAKKLNLDIIHTHTEFSVGFTGKY

VANQLDIPLVHTYHTMYEDYLHYIAKGKILRPNHVKLLSRYFCNQTVGVIAPSERVLN

QLADYGVTESIEVIPTGVKLQKFERLENRDIRTELGLTQEDKVILSLSRLSKEKNTEA

ILLAMPEIIKNEPNAKLIIVGKGPQRPTLDALAAELEIEEYVQFLGEKPSEEVAGYYQ

MADLFVSASESESQGLTYIEALASGTSIIAKENAYTRGLVGANEFGALFNQDEELAET

IISTLATLNDQKNNEWKRQELLYDISATAFGERVLQFYEDSNRKYQVETATHLNVAK"

CDS complement(1754470..1756191)

/gene="ptsI"

/locus_tag="JFBMFIFI_01650"

/EC_number="2.7.3.9"

/inference="ab initio prediction:Prodigal:002006"

/inference="similar to AA sequence:UniProtKB:P23530"

/codon_start=1

/transl_table=11

/product="Phosphoenolpyruvate-protein phosphotransferase"

/db_xref="COG:COG1080"

/translation="MVEMLKGIAASDGVAIAKVYMLIEPDLSFNKITVEDSASENERL

ANALNKAKQELEMIRQKAAESLGEEEAQVFDAHLMVLSDPELIGSIEGNIKDNKVNAE

SGLKEVTDMFISMFEGMEDNPYMQERAADIKDVTKRVMSHLLGVKLPNPSMIDEEVIV

VAHDLTPSDTAQLNRQFVKAFVTDIGGRTSHSAIMARSLEIPAIVGTKEITAKVKEND

AIIVDGLEGDVIIHPEASDVTKYEEKATAFAAQKAEWDKLKNEKTLTADGKHIELAAN

IGTPKDLVGVKNNGGEAVGLYRTEFLYMDSPDFPTEEAQFEAYKAVLEGMEGKAVVVR

TMDIGGDKELPYLTLPHEMNPFLGYRAIRICLAQPDMFRTQLRALLRASVFGQLRIMF

PMIATLGEFRQAKSMLLEEKAKLVSEGVEVSNDIEVGIMIEIPAAAVIADKFAKEVDF

FSIGTNDLIQYTMAADRMNERVSYLYQPYNPSILRLIKNVIDASHKEGKWTGMCGEMA

GDQTAVPLLMGLGLDEFSMSASSILKTRSLMKRLDTTKMAELADKAINDCDTAEEVVA

LVESYTK"

CDS complement(1756191..1756457)

/gene="ptsH"

/locus_tag="JFBMFIFI_01651"

/inference="ab initio prediction:Prodigal:002006"

/inference="similar to AA sequence:UniProtKB:P07515"

/codon_start=1

/transl_table=11

/product="Phosphocarrier protein HPr"

/db_xref="COG:COG1925"

/translation="MEKREFHVVADTGIHARPATLLVQTASKFNSDINLEYKGKSVNL

KSIMGVMSLGVGQGADVVITAEGADEAEAIAGIEETMKKEGLAE"

CDS complement(1756628..1756828)

/locus_tag="JFBMFIFI_01652"

/inference="ab initio prediction:Prodigal:002006"

/codon_start=1

/transl_table=11

/product="hypothetical protein"

/translation="MSHEEMEKQVTELVDGQIQELLVKKEDFMTFRTIWLSHPRKDEI

IGEAKLHGEVVYRLVPIEKSNM"

CDS complement(1756881..1757741)

/locus_tag="JFBMFIFI_01653"

/inference="ab initio prediction:Prodigal:002006"

/codon_start=1

/transl_table=11

/product="hypothetical protein"

/translation="MKEKLVTILFSLFIFWTSIGLLFQLFSSDNRTYFILYIIGIILI

GGLCVSGFLLLVYPTWLALFIISIVIGINFVIERFSLFNSRVGLLESSAVHADSFFIR

EATIYLLPFNLAIFWLINIVSCHLLWKQITRYIRGLKSQGVIYVVGATMASMSIHRLL

TPSLLNLKRYSWFQGESFIYGEGSYRFYFNWLLIVGLLHLFIWSVLVLSENWKQTISA

EYSYQTLSVYSLIVFCTMIVGFYTLNWLTVFLYILFNGFTYFLFTQSKMGYEIDLNQL

MPSFFKKKLF"

CDS 1757934..1760180

/gene="clpE"

/locus_tag="JFBMFIFI_01654"

/inference="ab initio prediction:Prodigal:002006"

/inference="similar to AA sequence:UniProtKB:Q9S5Z2"

/codon_start=1

/transl_table=11

/product="ATP-dependent Clp protease ATP-binding subunit

ClpE"

/db_xref="COG:COG0542"

/translation="MLCQNCNQHDATIHLYTSMNGQRGQIDLCQNCYQELKAAKERGD

LKMNRSNNTPDPFGLGGLDELFKAFQAGQSPQGQPNPQMIPPTQSGGGGKRPNGRQGG

LLAEYGTNLTDMAKNGAIDPVIGRDNEIKRVIEILNRRTKNNPVLIGEPGVGKTAVVE

GLAQKIIEGDVPQKLMDKEVIRLDVASLVQGTGIRGQFEERMQQLMDELKKNPQIILF

IDEVHEIVGAGSAEGSMDAGNMLKPALARGELQMVGATTLKEYRTIEKDAALERRMQP

VRVDEPTAEETITILKGIQKKYEDYHQVGYTDEAIESAVTLSSRYIQDRFLPDKAIDL

LDEAGSKKNLTIQTVDPKIIEDKIADAASQKQAALQQEDYEKAAYYRDQAAKLAMRD

QQQPDTEKPIVTEKDMEQIIEMKTNIPVGDIKEKEQAQLRNLAEDLQQHVIGQNEAVE

KVSKAIRRSRIGLNKKNRPIGSFLFVGPTGVGKTELAKQLAVELFGTADSIIRFDMSE

YMEKHSVAKLIGSPPGYVGYDEAGQLTEKVRRNPYSIVLLDEIEKAHPDVMHMFLQIL

DDGRLTDAQGRTVSFKDTIIIMTSNAGTGNVEASVGFGATNNATQNSVLDQLTNYFKP

EFINRFDAIVEFNQLDKTHLIKIVDLLLLDVNHMLVDQGISIVVDEAAKEKLVELGYD

PKMGARPLRRVIQEQIEDKIADFYLDHPEIKELAATTDANGDLIVTEKSNEPVVSEEV

VLTKKEDE"

CDS complement(1760230..1761603)

/gene="ntpJ_1"

/locus_tag="JFBMFIFI_01655"

/inference="ab initio prediction:Prodigal:002006"

/inference="similar to AA sequence:UniProtKB:P43440"

/codon_start=1

/transl_table=11

/product="Potassium/sodium uptake protein NtpJ"

/translation="MQKLKKIMRQLVGRFSSIQMIVIFYFIAVVVAAILLSLPIFRIP

GQELSFLDLMFTAVSTVSVTGLTTIDLGSTYNRAGVGLLMVLFQLGSLGIMMISTSFF

ILSKRKISLKQRQLIMVDMNQPNLSGIVRLIRNALAIIIWFQLIGGLILSVYFYYNYY

TIHDALFYGFYTAVSAVTNSGADITGTSLIDFSQDYFIQTIVMFLIVVGGIGFPVLIE

LKAFFNYKKKKHDLPFRFSLFAKLSMWAFLILFVVGSVLIWLLEFDDFFKGMNFTESA

FYSMFFSVSTRNAGLLTTPLGNFSDGTLLLFAALMFVGASPSSVGGGIRTTTLAIVVL

YLFSFIRSRENINIFGRRIHNDDVKKSIVVLNLSIALCFFSVLVLSVTEKHSLVSLVV

EVASAFGTTGLSMGITPSLSIFGKIMIMILMFIGRVGMLYMLMLLVPKEKRDANYLYP

TEKIIIG"

CDS 1761842..1762123

/locus_tag="JFBMFIFI_01656"

/inference="ab initio prediction:Prodigal:002006"

/codon_start=1

/transl_table=11

/product="hypothetical protein"

/translation="MKLIDVTNSHSSLVAEQLGNTDAVFIKVYSLGQTTVIFSGAATH

KDVVLTNKLRNIKNNEINYAITEVLKTTPDQVDILKAPNLVEISVNLTD"

CDS complement(1762876..1764453)

/gene="prfC"

/locus_tag="JFBMFIFI_01657"

/inference="ab initio prediction:Prodigal:002006"

/inference="similar to AA sequence:UniProtKB:Q99V72"

/codon_start=1

/transl_table=11

/product="Peptide chain release factor 3"

/translation="MKQTLKEEVASRKTFAIISHPDAGKTTITEQLLLFGGAIRQAGT

VKGKKSGKFAKSDWMEIEKQRGISVTSSVMQFDYQGKRVNILDTPGHEDFSEDTYRTL

MAVDSAVMVIDSAKGIEPQTKKLFKVCRMRGIPIFTFINKLDRDGKEPIDLLAELEEV

LEIDSYPMNWPIGMGKGLMGLYDNYNKRIEIHRPEQLGGERFIELNAEGQIEGDHPIK

ISNVYDQALEDIELLNEAGNEFSEERIANGELTPVFFGSALTNFGVQTFLDTYLKFAP

SPTAHKTDDGEKISPYTEEFSGFVFKIQANMDPAHRDRIAFLRICSGEFERGMDVTLS

RTARKMKLSNSTQFMAESRETVQHAVAGDIIGLYDTGNFQIGDTLFSGKMNVSYEKLP

QFTPEMFMKVSAKNVMKQKSFHKGMNQLVQEGAIQLYKTYHTEDYILGAVGQLQFEVF

QHRMLGEYNAEVIMTPMGSKIARWIRPEDLDENMSSSRNLLVRDRYDQPLFLFENQFA

MRWFHDKYPDIELTALL"

CDS complement(1764799..1766109)

/locus_tag="JFBMFIFI_01658"

/inference="ab initio prediction:Prodigal:002006"

/inference="similar to AA sequence:UniProtKB:P9WFP1"

/note="UPF0053 protein Rv2366c"

/codon_start=1

/transl_table=11

/product="hypothetical protein"

/translation="MTPDPESQSIVGQLILILALTLLNAFFASAEIALVSLNKGRMES

QANDGDRKARRLVKLLENPNGFLATIQVGITLAGFFSSASAATSIATKLQPVFGDVSW

AKEVSIIVVTLILSYVTLVFGELYPKRIAMQKAEEVSKFSVGFISIIEKLMKPFVFLL

SFSTDVLVKLTPMEIDAEEEKLTREEMRFMLESGQKDGILEASEVHMLNGVFSLDTKM

AREIMVPRTDTFMIDLEDSYDENIDLLLDCRYSRVPIYEEDKDNIIGIIHLKDVLKEA

KRVGFEQINLKEILNAALFVPETIFIDDLLFELKKTQNQMAILLDEYGGVVGIVTLED

LVEEIVGEIEDEYDEISDLYQKIDESTYLVQGRMPIDKFNELFHLNVEGKDVDTIAGY

MLTELGTIPENNENLSLKVDSVELISKEVENTRLMNIEVKVLTS"

CDS complement(1766248..1766805)

/gene="nucH"

/locus_tag="JFBMFIFI_01659"

/EC_number="3.1.31.1"

/inference="ab initio prediction:Prodigal:002006"

/inference="similar to AA sequence:UniProtKB:P43270"

/codon_start=1

/transl_table=11

/product="Thermonuclease"

/translation="MKNSKKIMSGIGALVVLLIGTFFVAENQQITEPAKDTQRIAIEL

ERVVDGDTIIMKENGERKRMRLLLIDTPESNTNKTGKTQPFGKEAKEFLTDYLKGKEL

SIVYDENHEKVDQYDRTLAYLYANDQLVEEVLLKEGLARLGYYNEKELYFNELKQSES

EAKKAKKHIWSLTNYVGEKGFKDNQ"

CDS complement(1767042..1768397)

/gene="nox"

/locus_tag="JFBMFIFI_01660"

/EC_number="1.6.99.3"

/inference="ab initio prediction:Prodigal:002006"

/inference="similar to AA sequence:UniProtKB:P37061"

/codon_start=1

/transl_table=11

/product="NADH oxidase"

/db_xref="COG:COG0446"

/translation="MKVVVVGCTHAGTAAVKTILTEHPEAEVFVFERNDNVSFLSCGI

ALYVGGVVKDPAGLFYSNPEELASMGAKVKMEHNVKNIDTENKTVLVEDIKSGEAFDV

SYDKLVMTTGSWPIIPPIEGIKSKNVLLCKNYNQANEIIKEAENAEKIVIVGGGYIGI

ELVEAFAESGKKVTLIDGLDRILNKYLDKEFTDILEHDLQERGVTLALNQSVEAFVPD

ASGAVKSVKTPVGEYDADLVILCVGFKPNTELLTGKVKMLGNGAIVVDEYMRTSDPSI

FAAGDSCAVHYNPNGGSAYIPLATNAVRMGSLVGKNIVSEKVKYRGTQATSGLYLFGF

NIGSTGVTVSGAHHFGLNVRSVIVKDNYRPEFMPTTEEVLMQLVYEVGTNRIVGGQLM

SKYDITQSANSLSLCIQNKMTIEDLAYVDFFFQPHFDRPWNYLNILAQAALEQERKLA

K"

CDS complement(1768677..1769804)

/gene="yhhT_1"

/locus_tag="JFBMFIFI_01661"

/inference="ab initio prediction:Prodigal:002006"

/inference="similar to AA sequence:UniProtKB:P0AGM0"

/codon_start=1

/transl_table=11

/product="Putative transport protein YhhT"

/db_xref="COG:COG0628"

/translation="MEVFKKSKLMFWSVWLLVIAVFIFMCTKIDFLFAPVGTFISTIF

APLVIAGFFFYMLNPVINLLGKFNVKRKYAIGIVFLLLIGAIIFLAVTVIPNLVEQIS

ELMNSLPTYLKELEKMSNDIFNQQWLKELGIEDKFKSSDISIGAIATNVFSGVTKSLG

SIIGAVTNTTLVVLTVPVVLFYMFKDGHKFGPSATKFFPEDYRGKVLELLGQMSNTIA

SYISGQALVCLFVGTFTFIGYLIIGLPYGFLLGFIAGVTNIIPFIGPYIGMAPAIIIG

LTVSPLQAILVCVVVLVVQQIDGNLISPNVIGKSLAIHPLTIITILLVAGNIAGLIGM

ILGVPFYAVSKTIILYIRDILKLRKDHLATIGNSSENQVGK"

CDS complement(1769970..1771007)

/gene="ysdC_2"

/locus_tag="JFBMFIFI_01662"

/EC_number="3.4.11.-"

/inference="ab initio prediction:Prodigal:002006"

/inference="similar to AA sequence:UniProtKB:P94521"

/codon_start=1

/transl_table=11

/product="Putative aminopeptidase YsdC"

/db_xref="COG:COG1363"

/translation="MSKSTIEFVSDLTNIASPTGNTYEIIAYLKNILEGLGYSPQKNR

KGSLMVTVKGHNHEEQRFVTAHVDTLGAMVRGIKPDGRLKLDLIGGFRFNAIEGEYCT

IQTSTGKTYTGTILMHQTSVHVYKDAGTAERNQENMEIRIDEKVTTAEETAALGIQVG

DFISFNPRTEITASGFIKSRHLDDKISVAVLIKFLEKIKNEKIELPYTTHFFISTNEE

IGYGGNSNISSKVVDYLAVDMGAMGDDQQTDEYTVSICVKDGSGPYHYHLRKQLTELC

ETNGIPYKLDIYPFYASDASAAMRAGADVRHALIGAGVESSHAYERSHIESIQATEQL

VEAYLLSELCE"

CDS 1771186..1771620

/locus_tag="JFBMFIFI_01663"

/EC_number="2.3.1.-"

/inference="ab initio prediction:Prodigal:002006"

/inference="similar to AA sequence:UniProtKB:Q5HH30"

/codon_start=1

/transl_table=11

/product="Acetyltransferase"

/translation="MKFIWTTDLTSTTYHDALAVRKEVFIVGQGVDAAIEIDALESST

EHVTGYLNGEPVAAARLNRIQNDTYKVQRVAVLSTYRGQHLGEKLMQEIERYAIENDV

ANLVLGAQDHAIGFYEKLGYTVDGDGFMEANIPHHMMRKQKN"

CDS complement(1771631..1771885)

/locus_tag="JFBMFIFI_01664"

/inference="ab initio prediction:Prodigal:002006"

/codon_start=1

/transl_table=11

/product="hypothetical protein"

/translation="MKMVLEEIEGNIARLIPDSGAAPFYLEIKYLPEKCQLGDVLNVY

YQKENNQTRRLIDKIPNEGEQRLALMKAKREALLKRKQKN"

CDS complement(1771891..1772892)

/locus_tag="JFBMFIFI_01665"

/inference="ab initio prediction:Prodigal:002006"

/codon_start=1

/transl_table=11

/product="hypothetical protein"

/translation="MAQKKRKKRTKQKQLNQRYLVIFSVMLFCFFIGKMVGQNENSLT

DLTKDISEQIDSVFKSNTQKKTKTDAKITPEQTENLTFRFLDVGQGDATLIQSEDGTT

ILIDTGRYDDKNKRIIEYLNTYIGVGGKIDLLIFTHNDSDHIGHGDLVMDYFDVKEVW

LNGHDSTTKVYEKLLDSIAAKDSLYEEPKSGEVRKIGPFSIEVLNPTEESKSNQNDDS

IVTRISTSNFSSMFTGDAASRVEKEMLAKNIPLESTLLHVGHHGSKESNSPEWLAAVN

PKIAIYSAGINNSYHHPSSETIERLKSLNIPFYGTDKSGTITVSVAKDGTYSVKTEE"

CDS complement(1773237..1774610)

/gene="mgtE"

/locus_tag="JFBMFIFI_01666"

/inference="ab initio prediction:Prodigal:002006"

/inference="similar to AA sequence:UniProtKB:Q830V1"

/codon_start=1

/transl_table=11

/product="Magnesium transporter MgtE"

/db_xref="COG:COG2239"

/translation="MNEAQLEIEKQMELLKELLNQGNITEFREEFLATHTYEQGQFYL

GLTKEERQKMYHFLSPNEMGDMFEIIEEDEESVEIYLEEMDLQYAADMLGAMYADNAV

DILKQLEKQQIKEYLALMDEESANEIKELLHYKDETAGAIMTTEFVSIVANQTVRSAM

AILKSKAPEAETIYYVYVVDTDDTLVGVISLRDLIINEDDALISDLMSERPVFVTVDD

DQNDVAKTIRDYDFLAVPVVDDGDHLLGIITVDDIIDVIDDEATSDYSGLAGVDVDET

TENPFIAASKRLPWLITLLFLGMSTASLISRYEVMVSDASILAVFISLITGTAGNAGT

QSLAVAVRKLATKEDGQTNFGKMILSEIMTGLVTGLVTGVTIFFVVGIWKHNFILGFV

IGMAMLCAITVANLAGSLIPILMDRLGFDPAVASGPFITTLSDLTSVLIYFNIAALFM

SYFIGTN"

CDS complement(1774639..1775541)

/gene="rluD_2"

/locus_tag="JFBMFIFI_01667"

/EC_number="5.4.99.23"

/inference="ab initio prediction:Prodigal:002006"

/inference="similar to AA sequence:UniProtKB:P33643"

/codon_start=1

/transl_table=11

/product="Ribosomal large subunit pseudouridine synthase

D"

/db_xref="COG:COG0564"

/translation="MEFSWIYQETESHQVKQFLRTKGVSRGLLAKVKFQGGKIEVNGQ

IQNAIHYLAFGDQVTITVPDEKGYETTVPVENPIEIVYEDQHLLIVNKPAGVVSIPSQ

LYPEQTMANRVKGYYCRQNYADQVVHIVTRLDRDTTGLMLFAKHGFAHALMDVLLREN

RLTKRYVALVTGSISGSEHQKIELPIGRTPDSIITRMVKPEGKYALTEFWIKNRFALA

TLVDIRLHTGRTHQIRVHFAHLGHPLIGDDLYGGSHNQWIKRQALHCQELEFVHPFTQ

ENMHIKAPYPTDIRQWLDAEKELV"

CDS complement(1775531..1776343)

/gene="nadK"

/locus_tag="JFBMFIFI_01668"

/EC_number="2.7.1.23"

/inference="ab initio prediction:Prodigal:002006"

/inference="similar to AA sequence:UniProtKB:P65779"

/codon_start=1

/transl_table=11

/product="NAD kinase"

/db_xref="COG:COG0061"

/translation="MKIAIIYNNNKKSVTLAEELRKLCQKNQLVIDEKKPNVVITIGG

DGTLLSAFHRYAHLLDEVRFVGVHTGHLGFYTDWRDYELEELVASLVTDEGESVSYPL

LDVEVCYQNQPEKARFVALNESTLKRVGGTMVCDVYIKDELFERFRGDGICVSTPTGS

TAYNKSVGGAVIHPRLEAIQLTEIASINNRVFRTLSSPLIVARDEWIKLKPATEEGFI

LTIDQLTSAEKNIIEINYRIAKERIHFARYRHTHFWSRVEDAFIGAKKNHGV"

CDS complement(1776411..1777064)

/gene="yjbM"

/locus_tag="JFBMFIFI_01669"

/EC_number="2.7.6.5"

/inference="ab initio prediction:Prodigal:002006"

/inference="similar to AA sequence:UniProtKB:O31611"

/codon_start=1

/transl_table=11

/product="GTP pyrophosphokinase YjbM"

/db_xref="COG:COG2357"

/translation="MIENWDDFFAPYTQAVDELKIKLKGIRKQFRQESQHSPIEFVTG

RVKPKDSILAKAQLRSIPINRVEQDMQDIAGLRIMCQFVDDIHAVVALLRSRHDFRIV

EERDYITNNKESGYRSYHIVLEYPVQLIHGEKKILMEIQIRTLSMNFWATIEHSLNYK

YQGEFPEDINIRLQRAAEAAFQLDEEMSKIRVEIQEAQQLFSHNKGYQDPKNKKSSS"

CDS 1777233..1777814

/gene="yjbK"

/locus_tag="JFBMFIFI_01670"

/EC_number="3.6.1.-"

/inference="ab initio prediction:Prodigal:002006"

/inference="similar to AA sequence:UniProtKB:O31609"

/codon_start=1

/transl_table=11

/product="Putative triphosphatase YjbK"

/db_xref="COG:COG4116"

/translation="MSEEIEIEFKNLLTEKEYQTLLYSFNATENELFTQINSYFDTPD

WDLKKLHAGLRIRLLPDSAELTLKTPFENHLLETTDSLSLMEAEKLISDGKIKADGAV

GAKLKSLNISPERLRLLGTLKTTRFERQTSQGLFVLDKSYYGNEVDYELEFEVPDYSI

GKIHFDNFLEKHQIPLRPAKNKIARMIDATFKK"

CDS 1778004..1778696

/locus_tag="JFBMFIFI_01671"

/inference="ab initio prediction:Prodigal:002006"

/inference="similar to AA sequence:UniProtKB:O31606"

/note="UPF0413 protein YjbH"

/codon_start=1

/transl_table=11

/product="hypothetical protein"

/translation="MLAKPNDTTHSNSNFKRIIEIYLFVNPIGSKCYTSEKEVLSFVE

GLEQKVHFRFIPFHNFQTVTKYMQFHNLPEKNLAFRNQICTSTYQASLAYKAALMQGK

KKGRAYLMQLQSELIEDNSLFSENLLIQVAEKVNLDVEMFIEDKNSDFAKASYEEDQR

IAREMQIESNPSMVVFDNQNDDYGLLVEDCISVDLLKKLCNPKSSLKENHKKMFIQTE

HTPLQKKNLRVL"

CDS complement(1778774..1780591)

/gene="pepF1_2"

/locus_tag="JFBMFIFI_01672"

/EC_number="3.4.24.-"

/inference="ab initio prediction:Prodigal:002006"

/inference="similar to AA sequence:UniProtKB:P54124"

/codon_start=1

/transl_table=11

/product="Oligoendopeptidase F, plasmid"

/translation="MAETKKLPTRAEVPEELTWDLEVIFKSDAEFNQSYQELEEKLTK

VNSVKGKIGQSSEDLLKGIDYLLDISNQLETIYVYAHLKNDQDTTNSEYQAMYDRANN

LATKSSEAISWFEPEVLEIPEETLAKFFEENKKLDIYRHFIDQMTSSRAHILSANEEA
LLAGAGEIFGASSRTFSVLNNADIQFPVIKDEEGNDIQLTHGVYGQLMESTNREVREA
AFKNLYKTYEGLKNTFASTLSAHVKYHNYNADVHHYSSARAKALAANHIPEAVYDTLL
EVVTENLPLLHRYVALRKELLDVEELHMYDLYTPITGEATVSYTYEEAKAETLKALAP
LGEEYLSIVEEAFENRWVDVVENSGKRSGAYSSGAYETNPYILMNWNDTLNQLFTLVH
EMGHSVHSYYTRKNQPYVYGDYSIFLAEIASTTNENLLTEYLLETQTDPKIRAYVLNH
YLDGFKGTIFRQTQFAEFEHFIHEKAAAGIPLTTEFMSDYYRKLNARYYGPDVVEDPE
IAIEWTRIPHFYYNYYVYQYATGFSAASALAAKILAGEEHALEHYLDYLKSGSSNFPI
EVMKKAGVDMTEKAYITDAMHVFEARLNEFEALIAELKA"

CDS complement(1780733..1781899)

/gene="coiA"

/locus_tag="JFBMFIFI_01673"

/inference="ab initio prediction:Prodigal:002006"

/inference="similar to AA sequence:UniProtKB:O31604"

/codon_start=1

/transl_table=11

/product="Competence protein CoiA"

/db_xref="COG:COG4469"

/translation="MLIALNNQNKHVLAHEEGKENKVHYLCPVCKGSVCLKKGEIKLP
HFAHLQSEKCQVFSEGETEEHLIGKRVLYNWFIQQGIPCQLEAYIPSLKQRPDLVIWL
SSDQPCAIEFQCSPLSIKRLKERTKGYNNAGYTVYWILGNAFFPKKSFTASQRAFFTY
RQDLGFQLYYLDTLKKELLCLVHIMEEEPAKKLSYQIIYFSLLKPKNSVSKLIGILNN
LKKGQQLNTNEKGAQHLLYCHFHLNQNRFYGTTEMKKFQHYLYEQGESLVTIPKEIYF
PFYKNSGIKTSSYYWRYLIIEWLALKGEGAVFLESQYKDFLLKLINEQKIELHQLAQI
STETIISICKAYLSTLKSFGLIEKVGNQQWLILKKPYFYKNENEKMTAFKQYQN"

CDS complement(1782104..1782772)

/gene="mecA"

/locus_tag="JFBMFIFI_01674"

/inference="ab initio prediction:Prodigal:002006"

/inference="similar to AA sequence:UniProtKB:P37958"

/codon_start=1

/transl_table=11

/product="Adapter protein MecA 1"

/db_xref="COG:COG4862"

/translation="MEMEHINENTIRVLIENADLEERGITFLDLLGNHKQIESFFYSI

LEEVDVDEQFHESDAITFQVLPNGNGLELFISKGGGLNEQFDLSELPENSTPEDFTEF

VKKQILNSSNELDEIDSYLQDPELLTSEVILKLNQFEDMISLANMMYLDNAVSNLYSY

QGSYFLQLVFFVEEMTETSVEDEIAIALEFTEETQITADVLGEYGKLIMEKSALELTR

HYFK"

CDS complement(1783017..1783415)

/gene="spxA_2"

/locus_tag="JFBMFIFI_01675"

/inference="ab initio prediction:Prodigal:002006"

/inference="similar to AA sequence:UniProtKB:O31602"

/codon_start=1

/transl_table=11

/product="Regulatory protein Spx"

/db_xref="COG:COG1393"

/translation="MVTLYTSPSCTSCRKARAWLEEHNIPYKERNIFSEPLNIPEIKA

ILRMTEDGTEEIISTRSKVFQELEVDLEDMPLQDLFDLVQKNPGLLRRPIMLDEKRLQ

VGYNEDEIRRFLPREVRALELQQAQLLVSY"

CDS 1783875..1784399

/locus_tag="JFBMFIFI_01676"

/inference="ab initio prediction:Prodigal:002006"

/inference="similar to AA sequence:ISfinder:ISEfa10"

/codon_start=1

/transl_table=11

/product="IS3 family transposase ISEfa10"

/translation="MTKYSVEFKMNIVQDYLNGEGGTPYLAKKYGFKGDSQVRNWINA

YKEFGKEGLLHSRRNKTYSVQFKLDAIELYLTTEMSYREVANQFGVSTFSTIANLYIA

YQKNGVDGLSQPKGRPSKMTKKKNKLPVSDSSKIELTYLEELEKENRRLKIELAYLKE

LRRLRLKEQQNNKR"

CDS 1784474..1785277

/locus_tag="JFBMFIFI_01677"

/inference="ab initio prediction:Prodigal:002006"

/inference="similar to AA sequence:ISfinder:ISEfa10"

/codon_start=1

/transl_table=11

/product="IS3 family transposase ISEfa10"

/translation="MYWQQRFNRKNPDKQIEEEMIKIHQEHKDYGYRRMNQELRHRGI

LVNKKKVQRLMKKIGILVRSFTRKSRKYNSYKGTVGRVAKNRIHRRFYTSIVHQKMTT

DTTEFKYYEKDHSGSLRIKKLYLNPFMDMYNSEIISYTISEKPTAHAIMSALKEAIEK

TSDCTYRRTFHSDQGWGYQMKAYSHELKKHQIYQSMSRKGNCLDNSPMENFFSILKQE

LYHGVIYKSYQELKQAIEHYIKYYNHSRIKEKLGWQSPVQFRKKMSFTA"

CDS complement(1785402..1786757)

/locus_tag="JFBMFIFI_01678"

/inference="ab initio prediction:Prodigal:002006"

/inference="similar to AA sequence:UniProtKB:Q97ST4"

/note="UPF0210 protein SP_0239"

/codon_start=1

/transl_table=11

/product="hypothetical protein"

/translation="MKSEQISETIRMIDEENLDIRTITMGISLLDCADSDGEVAREKI

YTKITTMAKDLVKVGEQIESEYGIPIINKRISVTPIAIVAGASADKDYVAFAKTLDAA

AKAVGVNFIGGFSALVQKGYTKGDQILIQSIPEALANTESVCASVNVGSTRAGINMDA

VRDMGEVVKKTAELTAETSGLGCAKLVIFANAVEDNPFMAGAFHGVGEADCVINVGIS

GPGVVKRAIEKVKGQPLDVVAETVKKTAFKITRMGQLVGRVASERLGIPFGIVDLSLA

PTPAIGDSVAYILEEMGLEMVGTHGTTAALALLNDAVKKGGVMACEHVGGLSGAFIPV

SEDAGMIAAVQAGVLNLEKLEAMTAICSVGLDMIAVPGDTPATTLAAMIADEAAIGVI

NNKTTAVRVIPAFGTKVGDLVEFGGLLGRAPVMPVNAKSSAAFIERGGRIPAPIHSFK

N"

CDS complement(1786782..1787051)

/locus_tag="JFBMFIFI_01679"

/inference="ab initio prediction:Prodigal:002006"

/inference="similar to AA sequence:UniProtKB:P67382"

/note="UPF0237 protein SP_0238"

/codon_start=1

/transl_table=11

/product="hypothetical protein"

/translation="MKAVLTVIGKDNVGIIAGVSKELVALTINILDVSQTIMEGNFTM
MMMCDLSETTTEFDQVKETLKATGDKLKVKISIQREELFSTMHSL"

CDS complement(1787217..1787546)

/gene="trxA_2"

/locus_tag="JFBMFIFI_01680"

/inference="ab initio prediction:Prodigal:002006"

/inference="similar to AA sequence:UniProtKB:P0A0K6"

/codon_start=1

/transl_table=11

/product="Thioredoxin"

/db_xref="COG:COG0526"

/translation="MITNLTQDNFQTEISNGLTLVDFWGEWCSGCQRLSAVLTSVAPL
FSDNQIKMAKLNVNQNKQIAEAYQIMSIPTMILFKNGEPIEKITGYLPREVLIDYLNN
RTSQPSD"

CDS complement(1787536..1787799)

/locus_tag="JFBMFIFI_01681"

/inference="ab initio prediction:Prodigal:002006"

/inference="similar to AA sequence:UniProtKB:Q97NX8"

/codon_start=1

/transl_table=11

/product="Putative membrane protein insertion efficiency
factor"

/db_xref="COG:COG0759"

/translation="MLKKIWLAPIRFYQKFISPMLPPRCRYYPTCSTYALTAIEKHGI
IKGTIMGTSRILRCHPFVKGGLDYVPTTFSLKRNPGEECHHDH"

CDS complement(1788426..1790540)

/gene="nhaK_2"

/locus_tag="JFBMFIFI_01682"

/inference="ab initio prediction:Prodigal:002006"

/inference="similar to AA sequence:UniProtKB:O32212"

/codon_start=1

/transl_table=11

/product="Sodium, potassium, lithium and rubidium/H(+)

antiporter"

/db_xref="COG:COG0025"

/translation="MFPVLEGTLILIALVIASNILSHYLIAIPTALLQVGLGLLVALV

FNVKIELDTEWFMLLFVAPLLYNDGRHYPKKDLWKLRIPILANSILLVFLTTVIGGFT

MYWVMNGKIPLAAAFALAAILSPTDPVAVNGIAQQVKLPVGVLRLVRGESLVNDASGL

IAFKYAIAATVTGYFSLTAAISDFFYMAIMGLLMGILLAWLIIGLKEWLSIQGIKDVI

LHTLIQILIPFLIFIIVEEWLHASGVIAVVAAGVVANNHRKANESRMAEVQIVTERTW

DVIIYLLNGIMFLILGIELPFAMGTALENSINGNNYQLFGYVILLWFIILIIRVLWTY

SYMWFDYSFGKFKRETVPSFKIALMSGLTGVRGAVTMAGILSIPYLLNTGFAFPGRSS

ILFIASGVIVFTLVAATISLPLLTKSKRRFETSGDDFVDFLEPITDGDEVTDFEEAKA

RIRIMNTAISIIEQESQPQNRMAAYDLLHEYDHLIRRMQMEYNSKETIVQFLKDEADI

RIIGINAEIMQTEKMIINQSVSTKTGHLYIQQLNRRKRGLRNLWVARFNRLVVVGKRV

YRQLIKENLPIKKREVYRQEHQEKLNLERETAKLAIRTLSAHMKQNQQKEIPIDKTIA

YHLIVEYRNKIERIKRFGEDYQAEYEEQLQELRLKALNAERGDIQKLYENGDISVNLA

IQLRRFVNYRESSIMELGDEDD"

CDS 1790889..1791329

/gene="cueR"

/locus_tag="JFBMFIFI_01683"

/inference="ab initio prediction:Prodigal:002006"

/inference="similar to AA sequence:UniProtKB:Q93CH6"

/codon_start=1

/transl_table=11

/product="HTH-type transcriptional regulator CueR"

/db_xref="COG:COG0789"

/translation="MGKRIKATATEVRISQEALRYYEKKGIIKPERLANGYRSYTPEQ

ITELKYVSVMKYAHFSLTEIATMTNLFSKPPTPECNQTSKKILSEKIEYLEDTIKHYQ

EIVQLLSSLPSSDDSASYLQKKPEIDQFIETIFEQIKQTEGIDF"

CDS 1791329..1792030

/gene="yflN"

/locus_tag="JFBMFIFI_01684"

/EC_number="3.-.-.-"

/inference="ab initio prediction:Prodigal:002006"

/inference="similar to AA sequence:UniProtKB:O34409"

/codon_start=1

/transl_table=11

/product="putative metallo-hydrolase YflN"

/db_xref="COG:COG0491"

/translation="MKTLEKKDFWQLTDMPVLFPINCFLIAENQELTLIDTGLNRSAK

GILNAINKIGLPLTKIILTHAHTDHVGSLTKINAAFPDAKILIGKQEWSILQEQLNPS

NSHTYSNYPDLTPLAVEPTGFLEDGDRINSLLIIETPGHTLGSISIFDQRHGYLVVGD

LLQTRGGPAIAGDIRPLFPFPGKATWEPKIAIQSTEKILTFQPSLIACGHGDWLTDPN

QQLSSLVTRAKKKLN"

CDS 1792172..1792738

/locus_tag="JFBMFIFI_01685"

/inference="ab initio prediction:Prodigal:002006"

/codon_start=1

/transl_table=11

/product="hypothetical protein"

/translation="MNTMEKELELWGQELEKFQLPRWNLLPDIDLYMDQVLTMIEKYL

SPLMIHEDQKIITAAMINNYVKLGLIPAPVKKRYTRKHIAFLIAISILKQVFTIQEIK

DGILFQASVVGINEAFNLFCDEQEKSLYVVAAQVTGTKSIPILEEQIPIDFLAVKLAT

LSFATKLITEKTIALGKEKISTKKEAEE"

CDS 1792742..1793599

/locus_tag="JFBMFIFI_01686"

/inference="ab initio prediction:Prodigal:002006"

/inference="similar to AA sequence:UniProtKB:Q8DP17"

/codon_start=1

/transl_table=11

/product="DegV domain-containing protein"

/db_xref="COG:COG1307"

/translation="MTTDKIALLVDSCTDVPDDMIEKYGMFVIPLKIIYKDRVYTDKV

DINAEKVYAHLDTEIPTTSLPDGQEITTIFDKIKNEGYTKVVVVTISSGLSGTNNLVR

LIAEDYSGLDVFVVDTKNIGIGSGMQGIQAGQLIASGADWNTIKTTLTANVAKSKVFF

CVDTLLYLQKGGRIGLVSSILGSALNLRPIISCNEDGVYYTVAKVRGRKKNLEKAVEL

ATKFIGSHQRFNLAIVDGAAKEEAKEITAKLKQLFPQAEAIFEGAVGPALGVHTGPGL

IGIGIQLLD"

CDS complement(1793652..1794257)

/locus_tag="JFBMFIFI_01687"

/inference="ab initio prediction:Prodigal:002006"

/codon_start=1

/transl_table=11

/product="hypothetical protein"

/translation="MSEVSKEKLEKVMQPILDWQTEFEQTKEYAQLLDATKEISGSII

YTFAKVQYTEFSRDAKKWTAGSLEEVLVDYFPAKFILAADDFKQIEQVLEAYFTFLEM

TNKIKNAPTLLKRVKKVAPEMIERAANPVNWSPAKKAEMEAVIKTSQPEAPTAKAPVR

RMTNRRPASPVKQQPVLSTKIGRNQPCPCGSGKKYKKCCGA"

CDS 1794552..1795736

/locus_tag="JFBMFIFI_01688"

/EC_number="2.4.1.337"

/inference="ab initio prediction:Prodigal:002006"

/inference="similar to AA sequence:UniProtKB:Q8CWR6"

/codon_start=1

/transl_table=11

/product="Alpha-monoglucosyldiacylglycerol synthase"

/db_xref="COG:COG0438"

/translation="MNIGLFTDTYLPQVSGVATSIKTLKDELEQQGHNVTIFTTTDPN

ADKNEENIIRLASIPFFSFKDRRIAISGSHSAVKKAKELNLDLIHTQTEFSLGLTGKQ

VANHLKIPCIHTYHTMYEDYLHYIAKGKIVKPYHVKILARYFCNQTNGLIAPSERVLK

QLRDYDVVRPIEIIPTGVVLEDFNLTTKVNIRQELGISTDTPVLLSLSRLSQEKNITA

LLLAMPELLQAKPNLQLVIVGKGPQCSELKALATRLDITDHVIFVGEKKRQEVPAYYA

MADLFVSASESESQGLTYIESLACGTNIIAKQNEYTESLIGNGDFGKLFVQDEDLAST

ILAELDKPKDLADLEKKRTQLLYDISSEAFGKHVFEFYQTVIAEYQYKAPRGLKIVKF

YR"

CDS 1795858..1797150

/locus_tag="JFBMFIFI_01689"

/inference="ab initio prediction:Prodigal:002006"

/codon_start=1

/transl_table=11

/product="hypothetical protein"

/translation="MSVMRLNQLYLFTSGLLLLLVGSIIITENVSLFLPFKFFIVGIL
LFDGLSHVTQFIQKKKSGKETLLNTSFDLILAAIFFFSDIPFYLLAVAFGGYIALKGA
ALLVNYQTYRDNQLPNRLNLLLGGLSQVIIGILLFFSPRLTLNQILIIIGLYFIFYGI
SNLFFTIQAFISPETKNRLKRKIRFTLPVFIEAFIPRAVLKETNALLKPESIASKTNE
MTSSKKEATVVPDLEIFIHVTEKSFGSIGHMDLCFEGEIISYGNYDEDSYRLFDTRGE
GVLISTNKESYIPFCIEHNKKTLFGFGIQLNEQQRAGIRQEIKKLKEDCYSWKSHLEL
AEKEIPNSIVENEYLDYASCLYKATHCQLYKFKKGPFKHYFVLTTNCVLLADTILGPS
GIDLLTINGILTPGTYLDFLNHEFKRKNSNVITYQVYN"

CDS complement(1797195..1799486)

/gene="helD"

/locus_tag="JFBMFIFI_01690"

/EC_number="3.6.4.12"

/inference="ab initio prediction:Prodigal:002006"

/inference="similar to AA sequence:UniProtKB:O32215"

/codon_start=1

/transl_table=11

/product="DNA helicase IV"

/db_xref="COG:COG3973"

/translation="MEKNTDEFNYEQERVDWTVAQMVKREAILTSSVENVREEAQAIR
SNFWKDVTVNVSEPDDVIETEVSIRQQELLLQERELNYKHVEKQLKAISKLKDAPYFA
RIDIREGEEPIEEIYIGIASFMSDDDQFLVYDWRAPISSAYYDGALGAVNYLTPDGVQ
TVDVSLKRQFLIKAAEIEAMFDTSETIGDEMLQDILGNQSNTQMKSIVSTIQQEQNKI
IRDTTSQLLFVQGAAGSGKTSAILQRIAYLLYHFRNGLDSNQMLIFSPNKLFNHYISN
VLPELGEKNMIQTTFLDFAQSRISGLKVESLFEQFENKKNQKRQAIMNLKEEPIFFKA
LDRYAEYVSKGGMIFKEVKLKQEVLFSKKEISTIFYSFPEHYTLAQRFTYTTEELLKR
LKPIIKKESKKDWVEDYLELLSEEEYKALMNNQKFTSFKEEKEFLANKVVTKQFKKVR
KALNKKSYFHYKAQYANFLKVVPKLIKIENYGVSLAEWQAEEAFVIKQLNRKQIMMED
TVPYLYLQDLIVGKETMRTIKFVFIDEIQDYSAQQIRYLKSLFPNSRFTMLGDLNQAI
FKNKQKEKTLLDSIKPLFDEDKISQIDLTKTYRSTADITNFTKGILLDSQMIEAFDRK
GELPQIVVRSSYKEIIAEITQVLKKTNDVSTITAIIGKTKKECEEAYAALKDYFDLTL
ISKENQKLADGLIILPSYLAKGLEFDTVIVLDASANNYQLESERTLLYTICSRAMHRL
IVTSHGAISPLLADIPSDLYQLT"

CDS complement(1799811..1800047)

/locus_tag="JFBMFIFI_01691"

/inference="ab initio prediction:Prodigal:002006"

/codon_start=1

/transl_table=11

/product="hypothetical protein"

/translation="MQSSNKAQLEQKMIYDVRFIAERKVSLNIKEEVEIHKTLILPSE

LSHKKLKQIIQTRFINVKSVSCINDFGKGIYLEE"

CDS complement(1800103..1801266)

/locus_tag="JFBMFIFI_01692"

/inference="ab initio prediction:Prodigal:002006"

/codon_start=1

/transl_table=11

/product="hypothetical protein"

/translation="MKRAKFYFIICLITSFISNYSLLKVEAADNDSVPVSARVVLPEN

QFNKEASYFYLKMTPNQKQDLEVVLSNTSDTVQKVEVSLAAGITNDNGIIDYPDGQKK

FDASLKYPFSKLASTEKNVTIDPKSDKSVMIKVKMPAEEFDGLIIGGINIRLNQADDT

KKESEGGMQIKNIIRYNFGVVLVENETVIVPEMKMNKAFPSQVMGVNTVKANLSNTEP

GIIDGLEIVAKVTKKGSKEVLYEATKQGLRMAPNSTFDFGVSMDNKAYQAGDYTMSVV

AKAENPDKEWSWNKDFTVDRETAQKLNAKAADLEKDYSMYWIVGGIVIGVLLVVVILC

IIFYRKKKKKAEAERRKRQAQKRKRKKAPTNRPNKKRPIAGEISTKRSEKNKK"

CDS complement(1801465..1802250)

/locus_tag="JFBMFIFI_01693"

/inference="ab initio prediction:Prodigal:002006"

/codon_start=1

/transl_table=11

/product="hypothetical protein"

/translation="MTKNKVALVGLASIVLSLVGGTNLASAASVPTKSDVIFEEDKDP

NTIGEIIRPGTPSEVIEPDEGGHTTGSLRINHVPNIHFGNQKISTASKEYNAIVEGYK

KDGVANYIPNFVQVADARGSMTAKWKLSVVGTIFSPTVKTDSPKLTNTHITLNQEKIT

NNVYDELTPNTTLDRVAGFGTTAVKINTDGTSGIDILNVKANQNTNSTITSSVFNANY

TKDATYTADQENTGISLHVPASDIKVDGETYEANLIWTLADVI"

CDS complement(1802477..1803883)

/locus_tag="JFBMFIFI_01694"

/inference="ab initio prediction:Prodigal:002006"

/codon_start=1

/transl_table=11

/product="hypothetical protein"

/translation="MKKNKFALVGLCTIALSLATGVSTVIATETNQADGVIIAKEEAP

ATVDATAPVAPEPISAAVTFAAPVNYTNVSTIAEFKAALLDVKVTAIKVTKSIKFTGN

ITNIPNRDVVIDGSADTGVVIDSNIYSIYGKQNTKGTNVFAVQNATITGDAGCGRFFT

GGAGNGPSSYGWDVNAKGVTYTGARFVHLSEGTLVFEGTNKIDTGAENAWVHDLVFKK

GTVYNGTSASKGQFSAFYFNGALIKGKATGKVTIEDNATINVKISPNDDKNYYYPVFY

DKVYQVNVGNGVAFNVDAAGVAFQFIPRADFPEEPSLNISGGSSLNFNGRGGGSYATM

KIQQYGTNINLDEGAELIVTGNAKHGVIESVYNYVDFNLNAATNFEVTNKLANAPLFN

SSKTEIKGVNLSEVLTWTKTGGDYSRAPETAFGDLRLFNTSITKNTNGEVQSTSASAV

SDFQIANYGKVKFQGGGN"

CDS complement(1803930..1804745)

/locus_tag="JFBMFIFI_01695"

/inference="ab initio prediction:Prodigal:002006"

/codon_start=1

/transl_table=11

/product="hypothetical protein"

/translation="MKLTKVAVIALVSTVGLSGLAATANAAVNESVPTKGKVEFKAPT

NPTGPVVKPDTEDTEIETEGGHTTGTLRIEHAPHINFGKVIYRAGNQTFQANNESYTY

TSGAVTETQYIPNFLQVTDERGIEAEWAVTVSSTVLTASATDKLDNTKIVLKEQKLTN

NVFDYVVPASTSDRITGFSGSAIEIPTDGTNSVEILKTKAGKSTNGSKSSIVFNSAYT

ESTNYVAPQDPANETDAPIGKNSGITLVKPQSDSVNVGKVYQADLVWTISSAI"

CDS complement(1804775..1805587)

/locus_tag="JFBMFIFI_01696"

/inference="ab initio prediction:Prodigal:002006"

/codon_start=1

/transl_table=11

/product="hypothetical protein"

/translation="MKKQILATALLSGLILVTFVPAASAIDKTADTKGSVQFEKVGEN

EVGEVVKPDTEDKPENIIVPEGGGNTTGPLRLQHVPDIKFGTVTFKTGNTKHQAILEN

YTKDGVAGDQAIPQFVQVTDERGDDTGTWKVTVSGTTFKEKVAGGGPATVPELVNTKI

VVKQQKFFNTVTDYATPVVSAETLVSGFTGTELTIPTNGTDSVLVMQTKAGKSTNGSK

TSVVFNSDYTDASSYVVGEKNAGIILDKANGDNIVVDKAYESDLTWTLSDSI"

CDS complement(1805610..1806425)

/locus_tag="JFBMFIFI_01697"

/inference="ab initio prediction:Prodigal:002006"

/codon_start=1

/transl_table=11

/product="hypothetical protein"

/translation="MKLKQVRKIGLISAVSALTLFSSALPAFAVDKSVTTQGDVGFEK

DESGTTDPLDPEKPDPLEPFVPVDPPTPTTGALRLDVTPFFHFGTNNKIVATKQNYYA

LFARGVKFGETEESDVPNYLQVTDARGGNKGFKVSVKTNGTFTSGTDKIENTKITLKN

FSVNSGSEIKDSTVFPTVSATPIVISDNQAHDLLSAAVGQGYGIWTMPMGSSTEKTSG

KGKDGTGTEGTVDATKEGRNPAVQLTIPAGQVIKDNTATPYVTDLNWTISDSI"

CDS complement(1806501..1807562)

/locus_tag="JFBMFIFI_01698"

/inference="ab initio prediction:Prodigal:002006"

/codon_start=1

/transl_table=11

/product="hypothetical protein"

/translation="MSIKFNSLLSKGVLFLGLLALVGFLSGSAGKQAMASEMESTVGI

EFTGEGTKIKLPDTGGDKIDVEVPNSDTSTGDNYLPQTGESKSYYQWFLFGLLMVVIA

LYRLIKSRKMNIKNLNATKFSLFALLLLLLLSPRQVEAVDGGQMKTEGDVGFRKNTDI

TPPVDPLNPDKPLEPFKPPTAGPLSLNYVSDIQFGSYDLADSETIFYASLDTIKYADS

SKQRFKRPNFVELTDNRGLNTGWRLTVKQNGQLKNADGAELNGAKLAFNNTSIVSLDE

ITATPTGFTKDAVLLNDNQNVLVMAAENGTGMGSWSVNFGQDDTEGERSISLTVPKDS

VKEKGHYTTSLTWTLSDSI"

CDS complement(1807583..1811167)

/locus_tag="JFBMFIFI_01699"

/inference="ab initio prediction:Prodigal:002006"

/codon_start=1

/transl_table=11

/product="hypothetical protein"

/translation="MIRYKKRILVLTIAIFLSIGLGIGAVNQFISSSGGRVEADSGVK

LVADKKELALGTAAVLTIKREFRDESKTDNILSVKLPEGITFSESKTKELNDEKGVKS
IAWNAEKQQIEVSLIETKDEVNIQLSIIGNKVGNYQLNAELSGEQSNVIDLAVIENQI
DFAKQGTDESESLLDNDLLLNQITPFIATGYTDIHQYPGIKVIPKTESTSTAPYVIPG
KNSFVMQLSEGTTYTAVGEGIYDNTDNKYKGSLRGYVFEDSSKLTNPTAPRYILATKV
GIYKGNWIDVRVVVDEVRVRRKNFNNQNPNENGLSESAEFGVATVTYEDRGINYPESY
SSTKYGDYFMAVGTLKNSSNDDYYSYHYEFYDNQTGTKLNDISGMWNFQRQNKFKQTD
IRNDAKHMSSIFASDAAAKPSTVTYKTEPIQAGFDRFFGDAGSVATEDTYLTTLFDNI
ADYPIGMTAQGNTSETSTANGMILMYNQTPLARIYPSKPEVIGERTTDNPMRMKYQIL
QNIPAQKSAYYSTDFQISSELPVEYDFDLSTVKVQNVLDNTEITNQFTASYDGSNKNK
LILKAVNPLDDAFNNKMYRILVEALPNEKFDLNEYKNKVGHREKQYDLDTSYIQVPLT
AKNTFNTGNVLLGTVTQTAEVETDPTKDKAISYMLYRGIPDADPLDGKKVAIGADWDK
LDPVDFIVPGTLRTDTGSPNDEPIKITGFYNTPDTSKKGEADVWVVIETAQGVEAKIK
VKILIENGDPIPNVQIQEMIKNTTQSLDFSTNQTAHIQDELTFQGKITKAVEGSIWNK
AIISITVPEGVELPTVSSVKLSDGTSEIPATITIDGRKITAKLTNSVTATEAMYLKFD
TKVLDAALGQSYTSTTEATGEDSNQDPVISSSSANLAVLDYSDPMITFEPKVEKKITG
GDWEENKNVKPGNAVRFTIQSSLKNAYTVWKNQKIVVDLPEGLDSIVLSTSVTIVRPG
KPDVELTNGGLTKFEKIGNQIIFETTDPQYQFTEMDSRIRFNYTADVMVEAVTKSPLE
TLVHMSGLNSRNEAVGLPNQLISLDVTEGILDFSATDQVDFKTNDLISTGTVIHDPKA
DFVVNITDSRGIANAPWSINAEVSKEFKNGDRLLNGNLFFVDQAGNSLPLSKGAAQPI
IQNDSSLTSQQIVWKNNEGKGLFLKQRAGMNVSGSYSGEITWTLSVGP"

CDS complement(1811500..1813032)

/locus_tag="JFBMFIFI_01700"

/inference="ab initio prediction:Prodigal:002006"

/codon_start=1

/transl_table=11

/product="hypothetical protein"

/translation="MNVFLERISLRKMYLLSLLDSEKRGFSIKELEQKLGHNSKTITK
MVQSLKIELAPWQNSITLVTNNDRTLSLKKKASFSLETINLYYLKESFIFKACDAIFN
EEFIDIATFSSANYISYSTLYGRLNEIKPLLEHYSIEFKANNMASFEGEENQIRYFFY
HFYWSTHWGMEWPFQKIDKNQFCEIIKRIEGLRKTTTLYISEQESIAFWLGVITTRIN
LGHTIEQVGLYNSIIDDNSAFTYFKNALLEEFKQLFPTISESDLLNEIQFLYVVMYSN
DYFEMDDPQISETLIFSQNRNGEIFGATNHWLKVCSDFFEVSLSAAEYGAIYANLIHL
HAEIAFFKGNISVFFNDYLEVSHIKDETNDFYDSLMDFFYQKLIENKKYKKIFINKER

LLPRYKMIARKFINIDSQKNSIKVHLISVYGNKKLAYLQNLINRSNEEHLEFSEDYNN
VDLIVSDRLYAGISQLNTPMIIWGEEPDENDLAEVNKVIKKISFDKMHKKLVKYTHFP
AI"

CDS 1813622..1814641

/locus_tag="JFBMFIFI_01701"
/inference="ab initio prediction:Prodigal:002006"
/codon_start=1
/transl_table=11
/product="hypothetical protein"
/translation="MSKKNRYFLGIVLAIGVGIFIWEFRKLSFTEVWRELQQLNWGWI
LVAFLSMLVYWGFKAKIIQSLLRRRYKSYKFSNAYRVSLIEDFFNAITPFSTGGQPAQ
LVALNKTGVDIGVGSSVLLMKFVVYQGMIVVVFGICVLFGYQALSSQISQLAGLIIFG
LLIHIVVIIVLLLITFKANWLKSIIHWILKGLKRFKDQETIARWEEGLVEKIDAFYEE
SVYIRNEKKLLLEISLLTLIQLFFYFIVPYFILLSLGVSHISILTIISLHAFIVLIVS
MFPIPGGAGGAEYSFTLLFGGFIANQEKLVIALILWRIFTYYLGIFLGAFALKLKKSE
PLEKD"

CDS complement(1814686..1815444)

/gene="fabI"
/locus_tag="JFBMFIFI_01702"
/EC_number="1.3.1.9"
/inference="ab initio prediction:Prodigal:002006"
/inference="similar to AA sequence:UniProtKB:P54616"
/codon_start=1
/transl_table=11
/product="Enoyl-[acyl-carrier-protein] reductase [NADH]
FabI"
/db_xref="COG:COG0623"
/translation="MNTLLKDKKIVIMGVVNKASIAWGCAKAMNDCGATVIYTYQNDR
VKKQLEKLVGTEANLVECDVATDEQVEEAFNQIHKTYGTIDGLVHSIAFARKEELGGN
VFDSTREGFAIAHDISSYSLLLVSRYASKIMNPGGSIITMTYIGSERAIANYNIMGLA
KASLEAAVRYLALDLAKQDIRVNAISSGAIKTLAASGIKGFNALLDEQAARTPSGKQV
TTEEVGNTAAFLMSDMSRGIVGEIIYVDKGTHMT"

CDS complement(1815670..1815969)

/locus_tag="JFBMFIFI_01703"

/inference="ab initio prediction:Prodigal:002006"

/codon_start=1

/transl_table=11

/product="hypothetical protein"

/translation="MQKNYRTLEYRQFEELKNRVKLIDFYWMRYKSQHRQKDYSEEVL

DHIEVIEDFIYKKRYEELRQVKINFRRTKVKLPEKNYQKLKQYSELSNLLQNSLK"

CDS complement(1816515..1816694)

/locus_tag="JFBMFIFI_01704"

/inference="ab initio prediction:Prodigal:002006"

/codon_start=1

/transl_table=11

/product="hypothetical protein"

/translation="MGETRSVVMTILSIIGFFVVLGIAIKFLGLALIVGFKFLLPLAL

VILLIQGISKKTKNA"

CDS complement(1817075..1817416)

/gene="ziaR"

/locus_tag="JFBMFIFI_01705"

/inference="ab initio prediction:Prodigal:002006"

/inference="similar to AA sequence:UniProtKB:Q55940"

/codon_start=1

/transl_table=11

/product="Transcriptional repressor SmtB"

/translation="MEESNKIVQPEIVQRVMNKLPEADIVAKLSDFIKVLGDGTRIKI

IWILEENEMCVNDLAVALNMSQSAVSHQLKTLKTANVVKSKREGKNIFYSLSDDHVKD

IFLKTLEHIQE"

CDS 1817616..1817978

/locus_tag="JFBMFIFI_01706"

/inference="ab initio prediction:Prodigal:002006"

/codon_start=1

/transl_table=11

/product="hypothetical protein"

/translation="MNPSQDKIESIDAYISQFPLDIQLILKKLRKVIKEAAPESSEKI

SYQMPTFVLNGNLVHFAAQKKHIGFYPTPSAIIAFEEALKDYKTSKGAIQFPFDKPIP

YELVREIVLFRVAENKLK"

CDS complement(1818057..1818479)

/locus_tag="JFBMFIFI_01707"

/inference="ab initio prediction:Prodigal:002006"

/codon_start=1

/transl_table=11

/product="hypothetical protein"

/translation="MEPKNDRAIKQEIHKLAKNADHQLLAIWAAECAERVLPIFNENY

QEDSRPSEAIRANRNWIKGKCTVSEARAAAFAAHAAARDAVEDNCCYAARSAGHAAAT

VHVATHAVYASDYAAKANPTERIWDYQRLVELLQEEIV"

CDS 1818691..1820130

/gene="bglH_3"

/locus_tag="JFBMFIFI_01708"

/EC_number="3.2.1.86"

/inference="ab initio prediction:Prodigal:002006"

/inference="similar to AA sequence:UniProtKB:P40740"

/codon_start=1

/transl_table=11

/product="Aryl-phospho-beta-D-glucosidase BglH"

/db_xref="COG:COG2723"

/translation="MTKKSIFRDDFLWGGATAANQFEGAYLEDGKGLTIADVSPGGKE

RRALLASEDYPLEIDETKYSYPNHLGIDFYHRYKEDIALFAEMGFKTFRMSISWARIF

PNGDDATPNEAGLAYYENVIDECLKYNIEPTITISHYETPLNLVKAYGGWKNRELITL

FEKYARTILERFHSKVKYWMTFNEINTGVMGSFFTLAMRNESLQNNFQALHNQFVASA

KAVKIAHELNPDLQVGCMSIYATMYSIDSNPDTVMSTVEKNRMFNYFCNDVQVRGAYP

TYALKFFEQNNITLDIAEGDLEVIKNHTVDYLSFSYYMSATNSIEPQDATTEGNFFGG

VKNPFLEASEWGWEIDPVGLRIALCDLYGRYGVPLYISENGLGAVDQPDADFYVADDY

RIAYLQKHISEMEKAVELDGVDLFAYTPWGCIDLVSASTGEMAKRYGFIYVDLDDNGQ

GTLNRYKKKSFDWYKQVIASNGMDLSSNN"

CDS 1820203..1821015

/gene="yokD"

/locus_tag="JFBMFIFI_01709"

/EC_number="2.3.1.-"

/inference="ab initio prediction:Prodigal:002006"

/inference="similar to AA sequence:UniProtKB:O32003"

/codon_start=1

/transl_table=11

/product="SPbeta prophage-derived aminoglycoside

N(3')-acetyltransferase-like protein YokD"

/db_xref="COG:COG2746"

/translation="MTELDTIQKLAHPITKDSLVTDFKNLGIKKGDILTVHSSLSSIG

WVSGGAIAVILALFEVLGENGTLIMPAHSGDLSDPSGWGNPPVPEIWWQTIRDTMPGY

HPKYTPTRSMGAIAEAFRSFPEVSRSSHPQVSFSGWGQYAADILANHSLNYGLGECSP

LGKIYQFSDAKILLLGVNYENNTSFHLAEYRQSDVKPIKYGAPILENDKTIWTEYTDI

ELFTDNFLTIGTDFEAENKCINGTVGNSKVKLFNQKKAVDFAESWLKNHSQK"

CDS complement(1821043..1822326)

/locus_tag="JFBMFIFI_01710"

/inference="ab initio prediction:Prodigal:002006"

/codon_start=1

/transl_table=11

/product="hypothetical protein"

/translation="MTQLLGVRIANLRKQKKLSTKKLATGIISISYLSNIETCKKIPS

LETLLFLADRLNVSHQQLVFEEVVNSDLETLFIDLFNCLVRNQMLEAKQKLDEIEHNY

SIQMESPIAEISYYLLVCIYQLKSWQFNEAQEIRELFIHSFAKLIDKSFLPIELQRYY

YFYEGLRLNLATSYLESINSYQKYLALEHSDAKMNVMVEGYIALNYICLSQYESALKL

IDSALDKTKKLPLNQQGNKIQLLYFKGFVYYFIQFYNHAVTSLEAALISLEDYPEMEA

EFRLIIHYRLVCTLQEMKAESEKETYLDAFYFSLIAKLQSNRPILKNEYLPIAELFVL

FSEKGELEKAELLLATLNGLEARPQEMDYFIQFGSALCAIHAGNEKAYVEQITQLLKV

IHLSNDDSFIQKVKKQASSYFAKQQKYKKSYEILK"

CDS complement(1822418..1822981)

/gene="sutR"

/locus_tag="JFBMFIFI_01711"

/inference="ab initio prediction:Prodigal:002006"

/inference="similar to AA sequence:UniProtKB:P77626"

/codon_start=1

/transl_table=11

/product="HTH-type transcriptional regulator SutR"

/db_xref="COG:COG1396"

/translation="MTNQLNENDQMVLSLAGNLKRLRKANKLSLEGLADQTGVSKLTI

NKIEKGKTNPTIGVLWKIANGLGVTLMELLESEEVVLLRGDENFTIFSDDNQWALAPL

VTNLNKSETFRARLAPTSRYTPESHPNGSKEVITVLEGELTLIVGGTTYLLKKSDTIR

FAANLPHVYQNNSDQPVELHLTMTSDY"

CDS 1823110..1824045

/locus_tag="JFBMFIFI_01712"

/inference="ab initio prediction:Prodigal:002006"

/codon_start=1

/transl_table=11

/product="hypothetical protein"

/translation="MRAILVGILASFFFSATFIINRVMSTGGTSWAWTASLRFLFAIP

FLLLLVIGRKGLKELLAEMQKNIGTWLLWGTCGGVGFYSLLSFASAYSPSWLTSGTWQ

ITILAGALLSPLFYVSIHTNGSTKKIRQKIPFKSLGFSLIILLGVILMQVKEANQLAF

SDFLFGFIPVVVAAFLYPLGNRKMMEVCQGKLDTFQRTLGMALSSIPVALLLALFGYQ

QTGLPTAPQFLQILLLAIFSGVIATILFFVATDLAKSNMALLAAVESTQAGAMIFTIL

GEVIILNGPFPQGLSLIGMILVILGMLITSIANRR"

CDS complement(1824367..1824522)

/locus_tag="JFBMFIFI_01713"

/inference="ab initio prediction:Prodigal:002006"

/codon_start=1

/transl_table=11

/product="hypothetical protein"

/translation="MSSNKQRKKDAKNLKENTVIARGTKRCVECGEPVTDPRKGTVCD

VCWKRKG"

CDS complement(1824681..1825673)

/gene="lacD2"

/locus_tag="JFBMFIFI_01714"

/EC_number="4.1.2.40"

/inference="ab initio prediction:Prodigal:002006"

/inference="similar to AA sequence:UniProtKB:Q8DWE5"

/codon_start=1

/transl_table=11

/product="Tagatose 1,6-diphosphate aldolase 2"

/db_xref="COG:COG3684"

/translation="MKVLSKNKREALERLSDSNGIIGALAIDQRGSLKKMIEKDSPKE

LGDAGIIQFKELVSEELTPYATAILLDPEYGLPAAKVRDSDAGLLLAYEKTGYDVNEV

GRLPDLLPEWSVKRLKENGADAIKFLLYYDVDEDPKINQLKHVFMERVGAECLAEDIP

FFLELVSYDADNSDVTSAEYAKIKPHKVIEMMKEFSKENYYVDVLKMEVPVNMSFVEG

YGAQDEAPVYTQEEAAAYFREQSAATHLPFIFLSAGVSAELFQETLVFAKRAGSNFNG

VLCGRATWKNGVAPYSKLGEEAAREWLKTEGKKNSQELMTVLKETAQPWFTKIK"

CDS complement(1825698..1826633)

/gene="lacC_2"

/locus_tag="JFBMFIFI_01715"

/EC_number="2.7.1.144"

/inference="ab initio prediction:Prodigal:002006"

/inference="similar to AA sequence:UniProtKB:Q5HE12"

/codon_start=1

/transl_table=11

/product="Tagatose-6-phosphate kinase"

/db_xref="COG:COG1105"

/translation="MILAVTMNPSVDISYQLSDFQLNDVNRCDEISKTAGGKGLNVAR

VIKLLQGNVLATGIIGGTLGTFITQELTKSKIPHDFSKTEKESRNCIALLHGGQQTEI

LEAGPSLTEAEGEVFLNKFTELLEKVQLITISGSLPKGLSVSFYQQMLDSANKKGVPI

VMDCSGSTLETVLKNQSKPLLIKPNLTELNQLEGQIFSEKDYTQLKTILQQERYQGID

WIVVSLGKDGAFVKYKEQFYQVKIPKIDVVNPVGSGDATVAGLAVALAENKSVEDTLK

TAMTAGILNTLENKTGWIDLAYFDDYYKKIKVENY"

CDS complement(1826634..1827803)

/gene="agaA_2"

/locus_tag="JFBMFIFI_01716"

/EC_number="3.5.1.-"

/inference="ab initio prediction:Prodigal:002006"

/inference="similar to AA sequence:UniProtKB:Q8XAC3"

/codon_start=1

/transl_table=11

/product="N-acetylgalactosamine-6-phosphate deacetylase"

/db_xref="COG:COG1820"

/translation="MDKVIYAEKFFLASGMTGSGYLKINESKFGEFSKELPKGEYQLL

DYATSWIAPGLVDTHIHGLLGHDVMDNDFAGIKAISTGLLACGVTSFLPTTLTDSTER

LDKVIETIGKLHREVAGARIQGIFLEGPFFTEKHKGAQNTAYFSDPLIEKLEKWQKLS

GGLIKKIALAPERKGSAEFIQYATSKGIKVALAHSDASYEEAKNAVDHGASIFVHTYN

GMSPLNHREPGMVGAALTLKGVFNELICDGQHVHPVAAKILMDVRSREEVVLITDCMR

AGSMPDGSYTLGEFPVEVKQGAARLKNGSLAGSVLQLKDAIKNVVDWGIATPLEAIRM

ASAIPAESVGIADKCGKIEPDAPADFIVLSKTLELEATYLAGELRYQSDDLKGRA"

CDS complement(1827837..1828685)

/gene="manZ_1"

/locus_tag="JFBMFIFI_01717"

/inference="ab initio prediction:Prodigal:002006"

/inference="similar to AA sequence:UniProtKB:P69805"

/codon_start=1

/transl_table=11

/product="PTS system mannose-specific EIID component"

/db_xref="COG:COG3716"

/translation="MASNESIEKVDLISNDYKDPTVTKVITKKDLNKMVWRSLLLQAS

FNYERMQGGGWTYSLIPGLKKIHKNEEDLSSSLLDHMQFFNTHPFLVTFIQGIILAME

ENKEKRDTIRGIKVAMMGPLGGIGDALFWLTLLPITAGIGAALSSDGNPVGPILFLVV

FNVVHFGLLFFLMHYGYNTGTKAIASLKEGTKKISRAASIVGLTVIGALVATYINFNL

GLTIHAGEIKVNLQEGVIDQIMPKLLPLLYTFFCYWMLKKGKSPLLLIGITVIVGVLG

SFIGLF"

CDS complement(1828675..1829469)

/gene="agaC"

/locus_tag="JFBMFIFI_01718"

/inference="ab initio prediction:Prodigal:002006"

/inference="similar to AA sequence:UniProtKB:P42910"

/codon_start=1

/transl_table=11

/product="N-acetylgalactosamine permease IIC component 1"

/db_xref="COG:COG3715"

/translation="MLTQALLIGIWAGIAGIDLFNGLTHIHRPLVTGAVVGLILGDFK

TGLMAGATLELVWAGMVPLAGAQPPNVVIGGIIGTAFAILTKQDPKVAVGIAVPFAVA

AQGCVTLLFTAYSPVMHKMDDYAAKADTKSIDRLTYMGPLILFVFYFIISFLPIYFGA

DQAGKIVELLPTWLIGGLGVAGGIMPAVGFAMLLKIMFKWTYAPFFAIGFVAAAYGKL

PILAIAIIGISFAAYDYFIGNKSDGESGNHKIKVLNDEEEDYSNGI"

CDS complement(1829618..1830112)

/gene="sorB"

/locus_tag="JFBMFIFI_01719"

/EC_number="2.7.1.206"

/inference="ab initio prediction:Prodigal:002006"

/inference="similar to AA sequence:UniProtKB:Q9RGG4"

/codon_start=1

/transl_table=11

/product="PTS system sorbose-specific EIIB component"

/db_xref="COG:COG3444"

/translation="MTKPNILLTRIDNRLVHGQVGMTWTNTLGANLVVVANDEVATDS

VQQNLMDMVLPETVQIRFFTLEKTIRIIGKAAPHQKILLVVKTPEDALTLVEGGVPIE

FLNIGNLHFSEGKQQLSSTVSVDKNDVETFRKLNQLGVKMEVKGIPSERGQDLMNLLK

EATF"

CDS complement(1830139..1831311)

/gene="agaS"

/locus_tag="JFBMFIFI_01720"

/EC_number="3.5.99.-"

/inference="ab initio prediction:Prodigal:002006"

/inference="similar to AA sequence:UniProtKB:A0KYQ7"

/codon_start=1

/transl_table=11

/product="D-galactosamine-6-phosphate deaminase AgaS"

/db_xref="COG:COG2222"

/translation="MYELTTEELTQLGAVITANEIKQQPELWQEAFENYRSKENEINR

FLTDLSNQFKKMRIIFTGAGTSAYVGDTVLPYLKGQLNGKKWDLMSIPTTDLVSNPEA

FFANEIPTLLVSFARSGNSPESLAAVSLGKQLVKDFYQVTITCAFDGKLANQAEGDAK

NLLLLMPEKANDKGFAMTGSYSCMTLTALLVFDLTSLSQKAKWIEQLKVMGEDVLQRS

TEIQKTIDLDFNRVIYLGSGSLAGMTREAQLKILELTAGQIATVFDSSLGFRHGPKSF

VNEQTLVFVFTSNDPYTRQYDLDILNELKQDQIACYVSGLSVAGETNFGGNNFTFLGN

GKDIPDAYLVLPYIIFAQTIAVLSAIKVGNQPDTPSPTGTVNRVVKGVEIYPYQVD"

CDS complement(1831334..1831759)

/gene="manX_1"

/locus_tag="JFBMFIFI_01721"

/EC_number="2.7.1.191"

/inference="ab initio prediction:Prodigal:002006"

/inference="similar to AA sequence:UniProtKB:P69797"

/codon_start=1

/transl_table=11

/product="PTS system mannose-specific EIIAB component"

/db_xref="COG:COG2893"

/translation="MIGMILCGHGNFASGMYSAIKLIAGEQEDFATVDFSAGMSSEEL

KEQLLVQIAKTENGNGVVIFTDIPGGTPFNQAVLLSTENESVKIVSGTNLPLLLESSF

KRDLSLTEFVSQALKSGAEGLKTFKIKETEPIREDSFGI"

CDS complement(1831952..1832680)

/gene="nagR_1"

/locus_tag="JFBMFIFI_01722"

/inference="ab initio prediction:Prodigal:002006"

/inference="similar to AA sequence:UniProtKB:O34817"

/codon_start=1

/transl_table=11

/product="HTH-type transcriptional repressor NagR"

/db_xref="COG:COG2188"

/translation="MINKNSSVPLYDQLVDILVNEINTKLKTNEKMMSEREICQKYDV

SRTTVRLALSELENMGYIYKRHGKGTFVAALSQGKQNLMDNYSFTDQMRESGKAPQTK

VLFFKIVLSNQYISDKMGVDLGTEVYRLKRLRLADGIPMMVETSYIPVKEFQGLEESR

IAKKPLYEIFKEDYNELVKVADEEFSASLLTDKEALLLESEDDGACLRLKRTSYNKAN

IVIEFTLSTARSDQFVYKVRHIQQ"

CDS complement(1832795..1833913)

/gene="glxK"

/locus_tag="JFBMFIFI_01723"

/EC_number="2.7.1.31"

/inference="ab initio prediction:Prodigal:002006"

/inference="similar to AA sequence:UniProtKB:P77364"

/codon_start=1

/transl_table=11

/product="Glycerate 3-kinase"

/db_xref="COG:COG1929"

/translation="MKVVIAIDSMKGSLTSSEANQIVATVFKAAGYEVEQVAIADGGE

GTVEAYLTNQEGEWIEVETLDPLGEICKSFYGWYPGKKEAVIEVAASSGLGLIQNRKH

NHPSQTSSFGTGQLILSAMDKGAKSIVIGLGGSGTIDVGIGILKALGVEFFDENGLEL

AGIGNDLAKIKRIDRNKMDQRLKTIEITVASDVSNPLTGSEGAVAIFGEQKGLWSSEL

ASYDQAMANFLKIATGNSISSLGDGAAGGMGFALRHFLNAKVIPGFTLIAAENELAAK

IKIADLVITGEGQIDQQSLYGKVPVAIAKLAKKNNIPVIAFVGSIKGDMTEAYQAGIS

EIIPIVQSVSTLENALKNASENLEKSVKQTVKLLHLFN"

CDS complement(1834090..1835622)

/gene="ifcA"

/locus_tag="JFBMFIFI_01724"

/EC_number="1.3.5.4"

/inference="ab initio prediction:Prodigal:002006"

/inference="similar to AA sequence:UniProtKB:Q9Z4P0"

/codon_start=1

/transl_table=11

/product="Fumarate reductase flavoprotein subunit"

/db_xref="COG:COG1053"

/translation="MRKKGLKVLFLMMALALIIVGCGTKENAKEENAKKESSKNESSE

VTTGASEGSSYTPLAELKDSYDIVIVGSGGAGMSAALEAKEKGLNPVIFEKMAVVGGN

TLKSSSGMNASETKFQKEEGISDSNDLFYEETLAGGHGTNDPEMLRFFVDHSADAIDW

LDSMGIRLNNLTITGGMKEKRTHRPEDGSAVGQYLVNGLIKNVQEKDIPVFVNADVKE

IMEKDDQVNGVKVQFDHKEDKTIKADAVVVTTGGFGANMEMISETNPELKDYVTTNQI

GSTGDGIKMIEKLGGYTVDMKEIQIHPTVQQEKSYLIGEVVRGEGAILVSSTGERFVN

ELDTRDAVSSAVNQTPDKFAYLIFDSGVKERAKAIEQYEKLGFVVEGQTLAELAKEIN

VPDTALEATVAKWNEDVAAKSDTAFNRTTGMEHDLSKGPYYAIKIAPGIHYTMGGIKV

NPETEVLTKEGPSIKGLYAAGELTGGLHGENRIGGNSVAEIIIFGRQAGIKSAEFVHS
LK"
CDS complement(1835785..1837728)
/gene="zosA_3"
/locus_tag="JFBMFIFI_01725"
/EC_number="3.6.3.-"
/inference="ab initio prediction:Prodigal:002006"
/inference="similar to AA sequence:UniProtKB:O31688"
/codon_start=1
/transl_table=11
/product="Zinc-transporting ATPase"
/db_xref="COG:COG2217"
/translation="MEASKEKVHNHEHEHENGGGHNHSHGDSKSAVVLFFAGLTAFIV
ALFLTDSSLLQTILFISAMLLSGYHIMLEGITDTIEATKRKRKFSPNVHILMTVAAIG
ATIIGNYEEGALLIIIFAAAHFMEEYAEGRSKREITNLLKMNPTEARLIEADGSVKVV
DVSVLKIGDKLQVLNGDQIATDGVILSGVTSIDESSINGESIPREKTVGDEVFGSTIN
GNGTFTMEVTKDSSDTLFAKILQLVNQSQSNLSKTATKIQKIEPYYVTAVLIIVPIFI
LLGPSIFSWTWNESFYRGMVFLISASPCALAASAVPATLSGISNLAKRGVLFKGGSYL
ANLAGIKAVAFDKTGTLTRGKPSVTDFYFDEIDEATTEQNYIDLIVAMEKTANHPLAN
AILGKFEAKESIELEVENEIGKGLVAQYNGAVYQIGKPTVFKNVSSKIENHNEQYSKD
GKTVVYFAKDNQVIGLIAMMDVPNANAIEVISYLKSQNIHTTMITGDSEVTGQAVGRQ
LGIDEVIGNVLPENKAQVIKDQQACYGTVAMLGDGVNDAPALVTADIGVAMGDGTDIA
IDVADAVLMQNDLTKFSYAHKVSKRLDRVVWQNIIFSMLIVAILVTLNILGKMDITIG
VIAHEGSTLIVILNGLRLLLPSK"
CDS complement(1837989..1839161)
/gene="fieF"
/locus_tag="JFBMFIFI_01726"
/inference="ab initio prediction:Prodigal:002006"
/inference="protein motif:HAMAP:MF_01425"
/codon_start=1
/transl_table=11
/product="Ferrous-iron efflux pump FieF"
/translation="MITYLINRFERKHTGSKEALRPKLGAFAGRIGLLSNLVLFLAKF

VIGLLSGSVSIMADAINNLSDTISSILTLVGFYISGKPADAEHPYGHERFEYISGMLV

SILITFVGFQFLITSIERIRNPQSVTVTWLVLIILLVSIGIKIWQGLFYQKVAKKIDS

DALVASAKDSLNDVFTTVTVLVSAMVEGLTGLKIDGYVGLAIAIYIIYSGYKMIMGFV

NELMGMRPAEEEINQIKERLSAVDNIIGYHDLLIHNYGPNKTFASVHIEIDDTWNLRK

AHEKTDLIEREFKKELGIELVCHVDPVSIHDQEQNRIHLLLKEIINGIDDTLKMHDLQ

IEKEKNEAGIMHFDIVIPKGFGLDDQELTQKIQLAVTRKIGDYQVQITFDHVYLLV"

CDS complement(1839403..1840191)

/locus_tag="JFBMFIFI_01727"

/inference="ab initio prediction:Prodigal:002006"

/codon_start=1

/transl_table=11

/product="hypothetical protein"

/translation="MYYPIQIPYILSPIFKEHVSFEEEIIPEFEDFVIFFWETKSISD

SKEVANNIIIPDGCIDLIVDYQTQQIGFSGMSQTDFHYEIINPPNFIGARLKPGAFYQ

LTGIPATEAMDNFLPLSIVDPTVNYDDFFQLDPTAGKSVLKKLIEELSGEQKPGDFIQ

LFDELFEADSVITVNELAVNMAKSTRQTQRLFLKHYGLTPKMILSVIRFQRSLEILLD

IGGSNRKPADIDYLGYYDQPHLIKEIKKNIGITPVELLKNYQKD"

CDS 1840358..1840996

/locus_tag="JFBMFIFI_01728"

/inference="ab initio prediction:Prodigal:002006"

/codon_start=1

/transl_table=11

/product="hypothetical protein"

/translation="MEHFKNNRSIIDYLNSFESSTLNCQQLTLTQDEYIITRYEKSEY

LYLLTNGLASVEILEDSNNYISAFIFENDFFGLDSFSSFPTKNHAIKVISSSAEIVKI

KKDFLLNALNQKPELYKLLLTNFADIFQRHYYFFNFLSLPPVERVKTTLLYLSDFISE

PTEASHLEIPKEITQQIVARFCRTSQSRVSICLRKLHETGFLLTKTAPFIIN"

CDS complement(1841053..1841571)

/locus_tag="JFBMFIFI_01729"

/inference="ab initio prediction:Prodigal:002006"

/codon_start=1

/transl_table=11

/product="hypothetical protein"

/translation="MLKKKSTRIILIVLLGALVIAGGGLAYYRLSRIQGTVETSIINE
KLANKEVPKVVVFTQDRTFKNEVMKKITNDIKKKDVYLEIQPIEEITDELSEWDKVVI
FTTIQSSEPPENVLPIITKYKEDKRVGVFLTAESGEWAHQPDDVEAFSAASRNLDNVD
EFTKKILDFINE"

CDS complement(1841651..1842334)

/locus_tag="JFBMFIFI_01730"

/inference="ab initio prediction:Prodigal:002006"

/codon_start=1

/transl_table=11

/product="hypothetical protein"

/translation="MKSLSKFYFTQTKEIFFKSVLAVILINLLRGVLLYLYDDSWFTF
NNGSCLPILVVSICSIVILWSLNRMDTSDETRTPASFFKVEESQKMLVRFLVSYAEIA
ILVILEGVFMIIYHNMVIDLVRIFLFFPLLACYCIAFVSYFILLKQFIKNSNSTNQVL
TICYYSSALFFVFIGNGFSRRYFSAIDSLPFFTDIYFYLSSFLFNVSICSGIIWLYLQ
KNNKLNNRG"

CDS complement(1842331..1843014)

/gene="ytrB"

/locus_tag="JFBMFIFI_01731"

/inference="ab initio prediction:Prodigal:002006"

/inference="similar to AA sequence:UniProtKB:O34641"

/codon_start=1

/transl_table=11

/product="ABC transporter ATP-binding protein YtrB"

/db_xref="COG:COG1131"

/translation="MTLSIRSVTKNYGLNKPILFDSNFTVDKGSVIGILGRNGIGKST
LLRSLAQMEPIQKGEILWEGNWLTKADVSFVNLSDYYYKQKTLETVLNEFQLLFKDFD
IEKTKQLVADFGISLAEKVTQLSNGMRQKFEIALTLGRTVHIFLFDEPFSHLDYSSRN
EIKQMIQDSVSEKNMILFTTNILENMDMLMSDVLIFTNQYQIASYNLEEIREMNNQSL
KQLYMEVTQ"

CDS complement(1843049..1843402)

/gene="ytrA_2"

/locus_tag="JFBMFIFI_01732"

/inference="ab initio prediction:Prodigal:002006"

/inference="similar to AA sequence:UniProtKB:O34712"
/codon_start=1
/transl_table=11
/product="HTH-type transcriptional repressor YtrA"
/db_xref="COG:COG1725"
/translation="MEFNENLPIYLQIISYVEEQILTNHWSTEKKVPSVRELAKELQV
NPGTVQKAYANLEQRKILITMRGIGKQVTTDQQIIKTLRESRLEESLRLFCDEMKQLN
MEKKIVIEKIIGQQW"
CDS complement(1843449..1843952)
/locus_tag="JFBMFIFI_01733"
/inference="ab initio prediction:Prodigal:002006"
/codon_start=1
/transl_table=11
/product="hypothetical protein"
/translation="MIQFVTSDDYIHVSLAFDEELSECFSFGRKNPYFALPAGLIQEN
LTNCFFNYHKDTPCTLYELKVSESVFMQARREVQQMVIEKKQYRYNIIGLVCCKLSIL
YQREKYYFCSQFVAEILEKSQAVILPKPAELIRPIDCISLIPGNCLFTGKISELVASV
NSQVRVS"
CDS complement(1844172..1845950)
/locus_tag="JFBMFIFI_01734"
/inference="ab initio prediction:Prodigal:002006"
/codon_start=1
/transl_table=11
/product="hypothetical protein"
/translation="MNKIKKFYSISMLVVLIFSILGSFQFVSADNTKTMENKQVISDI
VTNINMTRNDGSDLGTIDQWTDILVTIDFTLPNNKISAGDTTTISLPKELELQNDSTF
EVKDDAGNVVATAYANQNDKTVKLIYTDYAEKNSNISGKLWFSVQVDSYTITETGKIP
LEFDIDGQTIIKEIEYEKGTGDQDEVLYKSGVFDPNGDPQVINYSLRVNVSSKDLTDV
VVSDLLGQQDILINQDSIKIQEIDYTWNNVDEDWNMVPNSLRDVTDAFSNKITMSNGN
KGFSIGLGNINRPYLITYDGIIGHIPAQGESFDNKAVITANEIETTTSEKNFVYSDEG
GSAAGNTYQIHLIKQDKENATLLNGAEFDVIRVSTNRKVGTIATVNGEGQIKGLLQDS
YQLIETKAPVGYTLDPTPILVTPADFKESNSLFVATITATNEKEKTEISGIKMWDDFN
NKYNTRPESITVILLQNGQIFEKQEVKADDQGKWTFNFTNLPKSDTNGVAYRYTLDEV

EVAKYKKSIDNTTITNTLTELPENPVIPVVPEEPSRPELPSKPVAPQAKTESFPKTGE
EETVFAMLLGLLFVTGAAFSFIKKLN"

CDS complement(1846124..1846474)
/locus_tag="JFBMFIFI_01735"
/inference="ab initio prediction:Prodigal:002006"
/codon_start=1
/transl_table=11
/product="hypothetical protein"
/translation="MDKVDKVKENIHELYNNLIKRSGSASSLLDITDVLLQVYIKIDT
TENPEALVNRLVNYIYSVGLKGRIKLSPVEENLLSELGVFAQRAGLNGLYRADFSDKS
QFYSYFDKNQMPRH"

CDS complement(1846783..1847328)
/gene="udk_2"
/locus_tag="JFBMFIFI_01736"
/EC_number="2.7.1.48"
/inference="ab initio prediction:Prodigal:002006"
/inference="protein motif:HAMAP:MF_00551"
/codon_start=1
/transl_table=11
/product="Uridine kinase"
/translation="MYNSKIIVVSGITAGGKTSLVKELIENVDNACALSFDDYSIDAL
PSAPPMELLLEDFKTAVNQYDITQLMDDFFLTTKKRVFEFVFIDFPFGYEHDSLKPYI
DTVIYVKTPLDVSFARQVIRDYSDRDKEEIINWSKTYLNYARPIFLEHEKIVSSTADY
ILDGTISMTEQVNQLRYNDVI"

CDS complement(1848025..1849641)
/locus_tag="JFBMFIFI_01737"
/inference="ab initio prediction:Prodigal:002006"
/codon_start=1
/transl_table=11
/product="hypothetical protein"
/translation="MAYKNISLSKIDNYFKNPRHDIGVNEEDTLKKLFEKTGFQKMIN
LAEDIYLNGLVNANLITVVENPDNDRYTVYEGNRRIACLKLIDNPSKFPFLSKNQIDR
MKELKKENPSTVDFNNIACLVTDESDAYFIMDRIHSGEDKGRGVKSWESYEKDNFKHR

TSPEEKRSISNIIANKYLEYLGEDIKNLMAYTNIQRLFNNREVKSAIGIDINIPSSFS
KENILIIKSVIEKVNIIVHEKETSISREFNKSRIIEDILLPIIEELKMGGLGNQMNNE
ENTSGNNSINSNNEPIVNSDNEPMDNSNNNGVEPNKEEDDSGNNNSSGHDESQSNNNG
TNSENKDEPKEIQDEIENDDVKKGKSNKNVLKKLGAENILTDARPYKNLYQKNIRIKN
LVWEINKIKYKDFKLSTQFLLRTMMETYGHEYVDYFANLDYTDPKKMKSISKERKSRN
QTVNEIYYDFIQIHIKKNFPKFHEQAELINVSLSTNNNSSLMEILNFHIHSQNHIPDT
RELLEAWIKIAAIVESIDEILYENDLTSID"

CDS complement(1849646..1850503)

/locus_tag="JFBMFIFI_01738"

/inference="ab initio prediction:Prodigal:002006"

/codon_start=1

/transl_table=11

/product="hypothetical protein"

/translation="MNPSPLRYPGGKHKMYKYVRELVQDNNVSTYIEPFCGGAGLALE
LLINGDVNKLIINDYDYSIYCFWKSAIFYSEELIKKINSTETNIDEWRKQKEIRSNIY
NYTFLEVGFSTLFLNRTNRSGIIDKAGPIGGYEQNGNYLIDCRFNKESIINKIKKIAS
FKNRIELYNMEALDFIDEVIKNTRKSFTFFDPPYYVKGKGLYTNFYSHGDHIALSNKI
LKDLKNRKWIVTYDNSPEICNMYYHVDKVKFNLSYTLQEKKKGIEYLFFSKKINRIDD
KKYIKIVEE"

CDS complement(1851501..1851677)

/locus_tag="JFBMFIFI_01739"

/inference="ab initio prediction:Prodigal:002006"

/codon_start=1

/transl_table=11

/product="hypothetical protein"

/translation="MNKFIKYPILVSILCILLLIYKIWSNYNFQLAYTVFDYVWILLI
ALLLIFSIIKIVKK"

CDS complement(1852164..1852562)

/locus_tag="JFBMFIFI_01740"

/inference="ab initio prediction:Prodigal:002006"

/codon_start=1

/transl_table=11

/product="hypothetical protein"

/translation="MEQPVIEFQGYKIKKYNYEKTSIKDNINATEVESDGNEPFGISV
EPSLTKDLKNGLITVRVEYNGDGISISITVEGFFKINDNKDSEAIINYLVVNGTAIIF
PYIRSMISMLSSLDSENAILLPTLNTNNLI"

CDS complement(1852586..1852981)

/locus_tag="JFBMFIFI_01741"

/inference="ab initio prediction:Prodigal:002006"

/codon_start=1

/transl_table=11

/product="hypothetical protein"

/translation="MLTETYYHKEDLTIDDLNRVFDNNLDRNKQAYEFLKEDNRSNEI
NFIKTMTEKIIIKKISQGIQKNSKNKEKIQSYFFINGKLHNFDIDSLKMRVIDNMVNV
KDEGFSNNIKNNKELEDVISSNSLIYGIM"

CDS complement(1852983..1853615)

/locus_tag="JFBMFIFI_01742"

/inference="ab initio prediction:Prodigal:002006"

/codon_start=1

/transl_table=11

/product="hypothetical protein"

/translation="MRLYHSTLNSEEIIKKNQIKPNTSFNYINYVNHILDNSSILKAG
ELMGKYPGSRTQAPFMGNGVYCFNDVEKAKIYQSNAEVVIIDCEPTDIYDFDDPEWLF
GTFNYLNNEVTEKITNLYKENESMKFGWLTLVEFLIRCIQQNFKKNEPALGIVIFILN
EFMRKERPDLIINTFYDTIELSKGNEIEVKYFLIKNTNKITGLSSYERGE"

CDS complement(1853737..1854363)

/locus_tag="JFBMFIFI_01743"

/inference="ab initio prediction:Prodigal:002006"

/codon_start=1

/transl_table=11

/product="hypothetical protein"

/translation="MDNRVKMMTFVVLLALIIGCGIGLVSYTKQQAVSVSGIDKRIEK
LKKENDALRLQKEQLEEEQSELINEAVDSSGNSSSIKEETSPNQEELKQFITDYLEVM
FTYGSNAERNKAVRPFASDKYFEFINQYNSDTPIQSTFIDSKIYEISEDESRAVVRLT
YSYGVVDVTPNKYEILVNLSISKNSHGAYQIDSQENEVIQYQTQKKVW"

CDS complement(1854378..1855328)

/locus_tag="JFBMFIFI_01744"
/inference="ab initio prediction:Prodigal:002006"
/codon_start=1
/transl_table=11
/product="hypothetical protein"
/translation="MKKIILFGIFLILLPIIMVLTIVSSVFIMGDVSTTTIDSGNTTN
IGLSPEVLSYKPLVEKYCKIENIPDQVNLILAIMMQESGGRVPDVMQSSESLGLPVNT
LQPEESIAQGVKYYASLLKGTKDEDTAIQSYNYGGGFIGWLADKGGKYSKDLAVQFST
IMAAKMGWSNYGDKEYVPHVRRYIGNAHPMLDSEFANKVMEEALKYQGLPYTFGGVPP
LAFDCSSLTQWCYSAAGVSLPRTAQMQYDTMNIITNESEAQAGDLIFFKDTYDTPDFI
THVGIYLGNGKMFHAGDPIGYADITTQFWQSHFVGYGRAK"

CDS complement(1855395..1857335)

/locus_tag="JFBMFIFI_01745"
/inference="ab initio prediction:Prodigal:002006"
/codon_start=1
/transl_table=11
/product="hypothetical protein"
/translation="MNYDEYKLFLEPTGSLDVMGKVFSVMQTFFIEIPFAFLRIITSC
ITFVLDLLDVSAKIKGERSVFFEQSKDIYLKFIGGSNGRISTGSLGFILLMMALMYLT
WQFFFGKGGFAKKALHVIVVVCLGFAYFGNFTAQGTQTSGGMYLFNTVDNVSEQLKSQ
MITTFSVGKSDESLTSQDGYGEYYKNYIIMNTFNYINSGNTNGEYVTGEKLDHSKLIP
AENLSTKDKKSFYKEREGYLKEIAEENPYVQNNADKLVDKMVMVALSYANAVTLGFPI
VYANVTLSAFQFIFLVLILLFPLALLLSFIPFFRNAAMRILKMMLGILFMPVLIGFML
GIFFYLNGVVDNFILSKAADVLGVPVAMAALGGTSLIVVMLVLIVTKAVLFTLIWKNK
GKLLKLVSDNKIDDTVVNAPMNKIQEVGEKAVDTGTNVVETVAGAYTGNPQMMLSGVG
GLMDDGGGTAPSLENDPFMNEKKSLDDADTEDALINEDKSVLPTSLDKFMGEEPELDE
KDKIQDVHVMNSDDLVSDNELEDKMEEDVNVQLNDLDGDDFTEESNPVGNSDLSETSN
VDGIQGEDFVETIESENNETLGQSDELVGNPLFYDSVPDDSLFTVQEIPDPEQFQQND
NEGTMELNDSDGFYQEAFNSAQ"

CDS complement(1857347..1859857)

/locus_tag="JFBMFIFI_01746"
/inference="ab initio prediction:Prodigal:002006"
/codon_start=1

/transl_table=11

/product="hypothetical protein"

/translation="MKKKFEYPVQKMQGNLVLTKENEAIAYFSVPSISVAVTDDEKKK

KTKQDVAKVLKRLFPQKWFEIALVPKDYRLFEKMKDVESTLATESRPLGVQVLNKTVS

GLTKEMEIPYQYEWVIGVPLNKNDSSLDLKKALADGFNHVSETVMEKFGYEVAVKEDW

YEDWGAEEAEVYQTLSSIRAKRLSEEELYYHQRYQFLWYMPHTQEEVVANRGIENVTD

TQITSKLGHLRLSTEYGQSYVDVLPIGKIPTLMNFQHIGEIVQNFNFQVGLRIKAEFT

DVNGSMGVRAKMERSRTRTSNIIKEAHNTGSTQQDRIIEGKIALDDLSKKIGNKEPYV

EYGMYLIVAAKTKGQLKQRRKTVLSAFQNIHIHVSRATFDTPYLFQSVLFGNKLEKTT

RKWIHSSTARGLSEQMLFTTTFGGTNSGFYLGRVDNVYNKWDSLPEAIQASRNIVLFN

PTIANKEGIAGKVTKNPHIIMTGATGSGKSVAAQDIFFQMSLTNVKLLYVDPKKELKA

HYLALCNDPIFRNKNPELVKHIESFIFVTLDVRDKKNYGVLDPIVILEPTDAIETAKS

MIDYLGNGEWEMTQKTAISKAVKKIVNMRSQGEKVGFKHVIEELRQNVEKEIQEAGEY

LFEMIDGTILDLAFSDGSTKGLNYDDRVTILEVADLTLPKEKSNKISEHERNSIVLMF

ALGNFCKRFGEKNRHEETMEFFDESWILTASLEGQKVIKSMRRVGRSQNNTLVLITQS

VNDARSDDDTTGFGTILAFYEESEREQILEHVGLKVNDRNLKWIDNMSSGQCLLKDVF

GNVNRISIHIFLESWLELFSPMKDTVASSIENKYARAS"

CDS complement(1859914..1860321)

/locus_tag="JFBMFIFI_01747"

/inference="ab initio prediction:Prodigal:002006"

/codon_start=1

/transl_table=11

/product="hypothetical protein"

/translation="MDEKYYDYSVGLNTPYWIQEIRDGKGRLLWWFSTPMELSFFVTF

FLVFLLCLLLAPVIQLISNYSFGLGMLIYLYAPYRLARLYCEFEPDDKKMHVYLSDVF

QYGIDFLLNKTIIYQGERVEVEEEEIVFEKIKL"

CDS complement(1860331..1860549)

/locus_tag="JFBMFIFI_01748"

/inference="ab initio prediction:Prodigal:002006"

/codon_start=1

/transl_table=11

/product="hypothetical protein"

/translation="MSFANLNSFMSNEFPILVGALFCFFALQAWKKQDWLKFAGSLIF

AAVIIAVAKGSDIWSFVNGILKWFGLNL"

CDS complement(1860576..1861559)

/locus_tag="JFBMFIFI_01749"

/inference="ab initio prediction:Prodigal:002006"

/codon_start=1

/transl_table=11

/product="hypothetical protein"

/translation="MKFTKKEKKVKSPKLKQVSQKKANMVIVFTVIIFVGVLAVGTIK

NLSSVTAIDKLNKEIVTLKKDNLSTETEITQIDYPLVERYMGGFVSAYMNVDLSKDEI

VQQRLKNLEKYFAFDVSKLNDNLDKKTTRKLTSLNLLSVQTMKQYDLAKYKIEYEITE

MSLNEKTKKEEKRSSKVTSILCIPYVQSDGLITVVSLPYFSSEQDVYGKAPALEMSES

SDTTEAMAKVKPSIEKYLPTFFKKYAESNKADLTLLMKKVELMGGNYELDKVDVSQAR

YSFVGENVLVQVYVSFKNKETDFVHTEPFTLQLAKQEKSWFVVDMQHVFIK"

CDS complement(1861596..1861730)

/locus_tag="JFBMFIFI_01750"

/inference="ab initio prediction:Prodigal:002006"

/codon_start=1

/transl_table=11

/product="hypothetical protein"

/translation="MEVQQLNTLLNLLNEFNMENSVDMDSVDSTIEAVKLAIRESEGN

"

CDS complement(1861765..1861947)

/locus_tag="JFBMFIFI_01751"

/inference="ab initio prediction:Prodigal:002006"

/codon_start=1

/transl_table=11

/product="hypothetical protein"

/translation="MKCKITNLAILRENGLRPATIYDLKIIRGYLENGIEVYYESNNL

QVYDEFDNHTADLEKC"

CDS complement(1861981..1862397)

/locus_tag="JFBMFIFI_01752"

/inference="ab initio prediction:Prodigal:002006"

/codon_start=1

/transl_table=11

/product="hypothetical protein"

/translation="MYHKKVAQFIERLDEIEHELKSPESMKMSDVNAWCRKILSYEEK

IPIMKKYVFQVDLDFYEKKVTESDFSMHTNRFLQEMDLQEQAESRLYHFCLFIDEKIN

VAKTYVLDFEKSLDEYHELKSNVLRLSEMHWLHDRV"

CDS complement(1862423..1862653)

/locus_tag="JFBMFIFI_01753"

/inference="ab initio prediction:Prodigal:002006"

/codon_start=1

/transl_table=11

/product="hypothetical protein"

/translation="MLTFINVGTMEEKEWWLKVTLEEFLENKEKYDCLDYYHEKYLKE

DGVIFMNRYGGCNSFIGSQAIEEIQSDWFPAK"

CDS complement(1862692..1863972)

/locus_tag="JFBMFIFI_01754"

/inference="ab initio prediction:Prodigal:002006"

/codon_start=1

/transl_table=11

/product="hypothetical protein"

/translation="MTGEELKKLRKKLGYSLRRFGEKTGVDYTLLSRYEKGTRLISEN

KEKQLLTALGMSFEEKGEYDLHVHLDYLRFTFFDSEVSIIMNHVLGIQKGYFTFQPSN

AKNYQGIYSCGAIKIYVSTVMKQGLLLELSGQGLMELEEYLEEYGLTLDDWLLRVTNE

EYYLKKGLYSRIQSSRLDTAIDELYSEEKGNYDLHELKLKMEHPEKELISTQLAADKN

IEANFHGEPQGLTLYWGSANGHFLIRMYEKAKERAKKERKDYDMVLEEYGVVNRYELQ

LREHYAEFVIEELARGVPLQKISIDLLLSKIEVYDEIKNNNKVEYTFSERFYAVFGQF

QKIKVNGKKTETSIERSMRWVETQVIGTLKMLREIFGEKWLYEWLELCMSKIEFSEKQ

ENVINGELARLDKNENSAFLYWNKEMAEEKEKVS"

CDS complement(1863969..1864265)

/locus_tag="JFBMFIFI_01755"

/inference="ab initio prediction:Prodigal:002006"

/codon_start=1

/transl_table=11

/product="hypothetical protein"

/translation="MLPMIRISDELNKVDNFRKNLNPSVCFFKSGVDLFENEEINLYL
NLLVVRSKRVDVNMKMIEVDPKGIKNKKMLEDGYTVKTISLSDVFEDETVRLIL"

CDS complement(1864669..1865043)

/locus_tag="JFBMFIFI_01756"

/inference="ab initio prediction:Prodigal:002006"

/codon_start=1

/transl_table=11

/product="hypothetical protein"

/translation="MNQELTYTVSEAVVALSDPIKTQTLYSWVRKIEKYTPKYFLRRI
DKDNPYFVHGEPTPQLLIREKEILQFEEIIQLRKRGVRLEKAIFEVFLRKDDYEFLRE
NNWNYKLLKEKVLANYKDKEVQ"

CDS complement(1865087..1865359)

/locus_tag="JFBMFIFI_01757"

/inference="ab initio prediction:Prodigal:002006"

/codon_start=1

/transl_table=11

/product="hypothetical protein"

/translation="MSTSEQDAIEGIKEKVREFLREKGYVAETFDGDFETWVAVNARP
ANMPSSLDPSDWEEAALQGKYSRNGFTQDFSEYFEWKIKGTELKEF"

CDS complement(1865396..1865674)

/locus_tag="JFBMFIFI_01758"

/inference="ab initio prediction:Prodigal:002006"

/codon_start=1

/transl_table=11

/product="hypothetical protein"

/translation="MNTSEQDNIEIVKDKIRNYLKRKGFLIDSPFEGDFIKWVGVYAR
PMNRPTYLDANDSEDAEEQDRFSIGGSKQDFAEWFEWVIVGTDIVDFD"

CDS complement(1865693..1867234)

/locus_tag="JFBMFIFI_01759"

/inference="ab initio prediction:Prodigal:002006"

/codon_start=1

/transl_table=11

/product="hypothetical protein"

/translation="MLKKLFVYRGQRLKMRHTHLLRNYGIVLMVPVLVSLSLYYYFRV
YPLFSKGMDLKDYTVYIEPVSVTLGAFIIGMIGSLFLIRMSVLRQGFFFYRKKKQMLA
RFLKGNNFVEKIEKKTEKGKKVTEKFTKVYYRSKKELDVFTFEIGNKFQRNFLEISKD
LENLFLADLINIEREMGFVSFSYLIDNIQKRLNFEDVKAENGRITIMKGIEWFYKKLP
HMLIAGGTGGGKTMFIYALINAFGQVGRVHIADPKKSDLSQLAHFPAFEGLVVSETID
IFEMLEEANELMDKRYMYMLNHPDFVIGEDFEYYGLKPEFYIIDEFAALMSELENSKE
KGYQWWDFYGLLTPLVLKARQAGIFLIIATQRAGTDVIKGIIRDNLSCKVSLGILSED
GYGMLFGNENKNKAFVNKPKLIGRGYINAGTGVPVELYAPFFQKSFNPNEFWKAFPAM
PFSDLSEVRVNETDKELLKELEPSLISEEAQLIERNKQTVLLQEKEQKQALAEELAKK
AGLDY"

CDS complement(1867228..1867617)

/locus_tag="JFBMFIFI_01760"

/inference="ab initio prediction:Prodigal:002006"

/codon_start=1

/transl_table=11

/product="hypothetical protein"

/translation="MILLNVLMSILSYCLIGSIGFWAFKIKLKYCQNLYEACRLYLNQ
EKRILILKESYDDYGKRKKHINTINRMLFESKSNVLKSRKYTRSLDRMFILDDLSRLY
LEKLVFDLRCNTFRNYIQEEKSGVGLC"

CDS complement(1867614..1867964)

/locus_tag="JFBMFIFI_01761"

/inference="ab initio prediction:Prodigal:002006"

/codon_start=1

/transl_table=11

/product="hypothetical protein"

/translation="MQYFSMSNAMRGMGFMVLSKKTEQCFLLDYGKNLSLVVESMKRD
NPKDTSKIKYEFYKLIVNKKQTQILKVYCTEVSAMACVTKVASHLLFMMNKGHLERFQ
ENYDEALKYRNLKG"

CDS complement(1867993..1868244)

/locus_tag="JFBMFIFI_01762"

/inference="ab initio prediction:Prodigal:002006"

/codon_start=1

/transl_table=11

/product="hypothetical protein"

/translation="MMYFDLELKNFLSDFKELAKNKNVNSERLIENMREIARICKNDG

SFTVTLIESEQIHNERTIFAFVIEENEDGRNVIRYEGHN"

CDS complement(1868309..1868542)

/locus_tag="JFBMFIFI_01763"

/inference="ab initio prediction:Prodigal:002006"

/codon_start=1

/transl_table=11

/product="hypothetical protein"

/translation="MSEYSGVEMYLVNIEKNLNQIRELNTNEFSHISRKFFNDYEEKL

EEFKKNFSSFEEELGEIDGYVSQLTSYLVYDCS"

CDS complement(1868575..1868871)

/locus_tag="JFBMFIFI_01764"

/inference="ab initio prediction:Prodigal:002006"

/codon_start=1

/transl_table=11

/product="hypothetical protein"

/translation="MMNYILKNPIRIEKYIEFYGKKNSTFIFDKVSESLVSDAIEETQ

HFNKNDFYRKQKSFIFMFNTIDDPKITKLSNGVAVIPYIVVLDDDSQTVGIYID"

CDS complement(1868881..1869687)

/locus_tag="JFBMFIFI_01765"

/inference="ab initio prediction:Prodigal:002006"

/codon_start=1

/transl_table=11

/product="hypothetical protein"

/translation="MANTITQIPLKAGALGKIITLTEDTKVRVQGMQNEVVKYAATSE

AISELEIFVQNPKKGKHSEAMQELEVNDLKVMPMLPQQRGGFQQNSISFVKLVLLADK

VDVKGAADLNNFKVQENSITDIKTNAGSKLEFISKHVDYEKMLGDIRFVNAVAKEEYF

DGQKTGEYEGYEVTVSSDKLNATFTFLVSDLEINYAQFRPLVDLVEFVDPQISSLFED

VSSVERGGINISLKAETVRKKVNAAKPEHAQPQKPQEKQPEQNPKSDKKP"

CDS complement(1869968..1871872)

/locus_tag="JFBMFIFI_01766"

/inference="ab initio prediction:Prodigal:002006"

/codon_start=1

/transl_table=11

/product="hypothetical protein"

/translation="MKQNKFKNKVIHSVLTLGIVSGSLLPVASVFAEGIEQTSAVKHT

ISIIEKDVTYLKSDIDGFTPVRMVDRLRDENGAIKFCINFDLPSPNGLEYESYEQLDN

ATTYLMDAYKNGNANLTGDQAIDEYIVQATIHNIKSPDSFSLDRNFVDDYGILPRIKA

LKAEALNAPASNVPVFDNQLSFDKASLSFTLVGDEYVSDYVVTNVKGNIVSTSNVVEN

ATPNTKLVDENGNEVVTFSNGTKFKVVVPEEELEGKALTPSVNVKGEYINGYNVAVRY

GGHAGFQDVASYELKEFSEAKQATISGNIEAVKGSVKFVKMGTDEKLLDGVQFQILAS

DGETVEREIVTENGEGKASDLAYGVHYLVETGTLDSYVLNGEKIPFTINHNQEVIDLE

TIYNTLKTGKIVVHKEDQNGNPLAGAEFTKTDNEGIEEVKTTDEFGFVEFDIEANNVY

AVEETKVPEGYTGSFKQENITIENDGQLFEYTAVNTLKTGKIIIHKEDQDGKFLAGAE

FTTTDAEGKKEVKLTDEAGLVEFDIEANNVYAIEETKNPEGYTGSFKQENITIENDGQ

EISYTVKNTKDKIIEKIFTKETVKTEKGTFPQTGEKSNVLFLSGVVFVLLSGICIYFI

NSKKQFKRTK"

CDS complement(1871987..1872142)

/locus_tag="JFBMFIFI_01767"

/inference="ab initio prediction:Prodigal:002006"

/codon_start=1

/transl_table=11

/product="hypothetical protein"

/translation="MKDMVTIILDIATAQAENKKQLTEANGVLQRAELAGKELAFQEV

LEIINRG"

CDS complement(1872222..1873673)

/locus_tag="JFBMFIFI_01768"

/inference="ab initio prediction:Prodigal:002006"

/codon_start=1

/transl_table=11

/product="hypothetical protein"

/translation="MKFENLNKKLLIGSICAVSLLLGGGLVYASVSKQQEKESAQAEI

SLRQSTIKDVEKSVIGLYSNDKKEDFSKDLTNQKIDNVKIEVKKVDDKKVKDKFNTEL

NQIQYMLETKNSIQSFIPNDILKDDVTSNDINELSKKYDKSLTYNKKITEKNKLYLDG

ISKQFNDIKTAIEKTNALFLDDKKEQLADTVNEDLLNESQILITLIKNEKAKNEVVAT

HEIAMRLFTEKQEAEKVAEETAQIEKQETEKSSATEKTVAENSGGTNVEGNNGSPSNP

SSNGGSPSAPAQQGGIEGIIASSPSAQYSDQIIGVVASGSSAQVYLFEKSGGQWQTIL

STGGQVGSGGVGQASEYVSNTPKGSYGMSLAFGTGGNPGSPLPYRQITPNSYWISNVN

DPQYNTWQERATSDPADEHLISYSQQYQYAIALDYNGGVGGGSAFFLHVSNGAPTAGC

IAVPLGVMQQLITRIHGGARIINVNSQAELANY"

CDS complement(1874098..1874283)

/locus_tag="JFBMFIFI_01769"

/inference="ab initio prediction:Prodigal:002006"

/codon_start=1

/transl_table=11

/product="hypothetical protein"

/translation="MNQFLNAKEVAEILGRSENTARIRIKALNAELVKQGKRVERGLV

NKKFFFEKYGIEEPVSL"

CDS 1874459..1874830

/gene="immR_1"

/locus_tag="JFBMFIFI_01770"

/inference="ab initio prediction:Prodigal:002006"

/inference="similar to AA sequence:UniProtKB:P96631"

/codon_start=1

/transl_table=11

/product="HTH-type transcriptional regulator ImmR"

/db_xref="COG:COG1396"

/translation="MNFATRLKQLRENKDLKQKELADLLHLQQSTISAYERNKIQPTP

ETINILADFFGVTSDYLLGRQDTSKTKLEQTVNSTIEELKQENTLLFLKDDNFDEETA

RLIKIAMKNAMRTVDDMKKKD"

CDS 1874857..1875351

/gene="immA"

/locus_tag="JFBMFIFI_01771"

/EC_number="3.4.-.-"

/inference="ab initio prediction:Prodigal:002006"

/inference="similar to AA sequence:UniProtKB:P96630"

/codon_start=1

/transl_table=11

/product="Metallopeptidase ImmA"

/db_xref="COG:COG2856"

/translation="MKDKANEIIKFYNSNNVTRIIDYLGIILLKSNLGNVKGFLQFYN

NRYIIHVNHNIENESEIDMVIAHELAHYFLHKNMNCFEFNSNSLYFEDKIEKEANLFA

SELLLTDNMLLEEFIYLESFTIKDISNYFNLSYELVELKFETLKLDDSFFKEYKEHTF

EGEF"

CDS 1875351..1876478

/gene="xerC_3"

/locus_tag="JFBMFIFI_01772"

/inference="ab initio prediction:Prodigal:002006"

/inference="protein motif:HAMAP:MF_01808"

/codon_start=1

/transl_table=11

/product="Tyrosine recombinase XerC"

/translation="MVIRKDKNGKYMVDVSNGFDPITNKQKRFQKKNISTKEEAIQIE

MNARLFKLNDISKNDNLTIDNLFNLMLKENIKEDAKQSYVSSQQYNFKAHIQPYFEHS

SIKKVQRDDIIKFRDYLQSNEKKLSNNTVNKIMILLKKIFDSAIKVDLITDNPCLHLK

KLKVDKKKMQFWTITEYKDFINSIEDKDKHYKYLFTTLFFTGMRIGEALALTWNDINF

ATGEINVTKTTSIIRGEIIISEPKTKASIRRITINRKLVDELFFWKEKQQHILESYLM

TVDNNTQVYQFQPKATNKDMVHKKFKYYLKKSPHIKVIRIHDLRHSHVALLINLSNGR

EEYDTIKERLGHSSITTTIDTYSHLYPNKQKSVSDPLDDFF"

tRNA complement(1876562..1876647)

/locus_tag="JFBMFIFI_01773"

/product="tRNA-Leu"

/inference="COORDINATES:profile:Aragorn:001002"

/note="tRNA-Leu(caa)"

tRNA complement(1876682..1876752)

/locus_tag="JFBMFIFI_01774"

/product="tRNA-Cys"

/inference="COORDINATES:profile:Aragorn:001002"

/note="tRNA-Cys(gca)"

tRNA complement(1876808..1876881)

/locus_tag="JFBMFIFI_01775"

/product="tRNA-Gln"

/inference="COORDINATES:profile:Aragorn:001002"

/note="tRNA-Gln(ttg)"

tRNA complement(1876891..1876965)

/locus_tag="JFBMFIFI_01776"

/product="tRNA-His"

/inference="COORDINATES:profile:Aragorn:001002"

/note="tRNA-His(gtg)"

tRNA complement(1876995..1877069)

/locus_tag="JFBMFIFI_01777"

/product="tRNA-His"

/inference="COORDINATES:profile:Aragorn:001002"

/note="tRNA-His(gtg)"

tRNA complement(1877094..1877167)

/locus_tag="JFBMFIFI_01778"

/product="tRNA-Trp"

/inference="COORDINATES:profile:Aragorn:001002"

/note="tRNA-Trp(cca)"

tRNA complement(1877194..1877277)

/locus_tag="JFBMFIFI_01779"

/product="tRNA-Tyr"

/inference="COORDINATES:profile:Aragorn:001002"

/note="tRNA-Tyr(gta)"

tRNA complement(1877283..1877358)

/locus_tag="JFBMFIFI_01780"

/product="tRNA-Phe"

/inference="COORDINATES:profile:Aragorn:001002"

/note="tRNA-Phe(gaa)"

tRNA complement(1877394..1877470)

/locus_tag="JFBMFIFI_01781"

/product="tRNA-Asp"

/inference="COORDINATES:profile:Aragorn:001002"

/note="tRNA-Asp(gtc)"

tRNA complement(1877536..1877610)

/locus_tag="JFBMFIFI_01782"

/product="tRNA-Val"

/inference="COORDINATES:profile:Aragorn:001002"

/note="tRNA-Val(tac)"

tRNA complement(1877622..1877693)

/locus_tag="JFBMFIFI_01783"

/product="tRNA-Glu"

/inference="COORDINATES:profile:Aragorn:001002"

/note="tRNA-Glu(ttc)"

tRNA complement(1877711..1877802)

/locus_tag="JFBMFIFI_01784"

/product="tRNA-Ser"

/inference="COORDINATES:profile:Aragorn:001002"

/note="tRNA-Ser(gga)"

tRNA complement(1877861..1877936)

/locus_tag="JFBMFIFI_01785"

/product="tRNA-Asn"

/inference="COORDINATES:profile:Aragorn:001002"

/note="tRNA-Asn(gtt)"

rRNA complement(1877952..1878061)

/locus_tag="JFBMFIFI_01786"

/product="5S ribosomal RNA"

rRNA complement(1878323..1881240)

/locus_tag="JFBMFIFI_01787"

/product="23S ribosomal RNA"

rRNA complement(1881634..1883185)

/locus_tag="JFBMFIFI_01788"

/product="16S ribosomal RNA"

CDS complement(1883819..1885447)

/gene="malL_1"

/locus_tag="JFBMFIFI_01789"

/EC_number="3.2.1.10"

/inference="ab initio prediction:Prodigal:002006"

/inference="similar to AA sequence:UniProtKB:P29094"

/codon_start=1

/transl_table=11

/product="Oligo-1,6-glucosidase"

/db_xref="COG:COG0366"

/translation="MEKWWQSSVVYQIYPRSFQDSNGDGIGDIAGITSRLEYLEKLGI

DVIWLSPVYKSPNDDNGYDISDYEAILPEFGTMADMDELIREAKQRGIQIIMDLVVNH

TSDEHPWFKEAKKSKTNPYRDYYIWRNPVNGGPPNDLQSIFSGSAWEFDKETNQYYFH

LFSKRQPDLNWENPSVQEEVWKLMNFWLAKGIGGFRMDVIDLIGKIPDKGITGNGPKL

HEYLKKMHKETFGKYDVLTVGETWGATPGIAKLYSNPDREELSMVFQFEHISLDELPG

KSKWDLQPLDFIQLKAILSKWQTALGDEGWNSLFWNNHDLPRIISRWGNDSPKYRVKS

GKMLATILHLMKGTPYIYQGEEIGMVNTPVSSIEELDDIESLNMYRERLAAGYDKADI

LASINKKGRDNARRPMQWSNHNHGGFTTGTPWLELSPRYSEINVENELADSESLFYHY

QRLVQLRKNNPLVVWGTYQELVPNDTQLFVYERSYENETWLIVANFYEEEADFSHESH

KVKEIILSNYSDSSVDLVNLHLRAYETVVYRLEK"

CDS complement(1885465..1886124)

/gene="yvdM"

/locus_tag="JFBMFIFI_01790"

/EC_number="5.4.2.6"

/inference="ab initio prediction:Prodigal:002006"

/inference="similar to AA sequence:UniProtKB:O06995"

/codon_start=1

/transl_table=11

/product="Beta-phosphoglucomutase"

/db_xref="COG:COG0637"

/translation="MIKGFIFDLDGVITDTAEYHYLAWRELANKLGISIDRKFNEQLK

GISRTDSLEKILNYGGKSQTYSEAEKNELADEKNKEYQKLIQSITPADLLPGMAEFLA

EIKAANLKLGLASASKNGPFILERLGIANLFDTVVDPESLKKGKPDPEIFTKGAKQLG

LTISECVGIEDAEAGIESINAAGMFSVGVGSPEAMRFADIYVAKTAELNLEMIQKQIK

S"

CDS complement(1886193..1888463)

/gene="malP_2"

/locus_tag="JFBMFIFI_01791"

/EC_number="2.4.1.8"

/inference="ab initio prediction:Prodigal:002006"

/inference="similar to AA sequence:UniProtKB:E6ENP7"

/codon_start=1

/transl_table=11

/product="Maltose phosphorylase"

/translation="MIQRLFDIDSWKVATTKLDKEHKRLQESLTAIGNGYMGMRGNFE

EGYSGDSHVGTYLAGVWYPDKTRVGWWKNGYPDYFGKVINATNFIGVDIFVNQHKVDT

FVDEIKDFHLELDLQTGILHRHYTWVSGEIEIKFSFKRFVSVAQKELCIVEVAAEVLK

GEASIQFNAKLDGNVTNEDSNYDEKFWLEMDRGLGEYSYLTTKTIPNDFEIPQFTVTT

MMKNRVSDEKKGHTTDQEMLVQEQFDYQLKAGEKAKLTKLIAVVTSRDIEPNQQVAKA

EELLRVAEDAGISKLEREHKIVWGKRWHKADVVIAGDPESQQGIRFNIFQLFSTYYGE

DARLNVGPKGFTGEKYGGATYWDTEAYIVPMYLSVTEPEVSQQLLKYRHDQLPGALHN

ARKQGLKGALYPMVTFTGVECHNEWEITFEEIHRNAAIAYAIYNYTNYTGDEAYLLSD

GIDVLTEISRFWADRVHYSKRNEAYMLHGVTGPNEYENNVNNNWYTNTMATWVLRYTL

ESLKKVSKEKLVDLQITETEKDQWQDIIAKMYYPKNQELGIFVQHDTFLDKDLRAVET

LAATERPLNQHWSWDKILRSCFIKQADVLQGLYFLNNEYTIEEKKKNFEFYEPMTVHE

SSLSACIHAILAAEVGLEDKAVELYARTARLDLDNYNNDTEDGLHITSMSGSWLAIVQ

GFAGMRTYENQLQFKPFCPKGWDGYEFKISYRGRLLKIIVTKTTASVILVEGEALDLT

LCGENVRVTDQVQQAI"

CDS complement(1888460..1889302)

/locus_tag="JFBMFIFI_01792"

/inference="ab initio prediction:Prodigal:002006"

/codon_start=1

/transl_table=11

/product="hypothetical protein"

/translation="MKKMPFPLNYFNSIATPKRVFKGRKNLSWIQNIVIFIFLNALLM

FPVSLYFSQSTNFQLQEIMPHTLRLVTDEFATKLQAVEFENGKLISGEPFTIVEDTGV

VGVEIPKSVKKSEKNLISFEKDHLLLKDESGYQFEVRYTENFSLKNSQTKSALKEQIK

EQWFKQNKAFVSFTMMLMTGIIISFSNVLVMFIAAFFIWMTKRSALSSIRTYKESFNL

ILNASGIGTLLAVLIGLVHFDMTIMIGFQSLGLVIMLTAVFAVTHFSDTPIKNKRKRN

EEIK"

CDS complement(1889351..1890202)

/gene="malG"

/locus_tag="JFBMFIFI_01793"

/inference="ab initio prediction:Prodigal:002006"

/inference="similar to AA sequence:UniProtKB:P68183"

/codon_start=1

/transl_table=11

/product="Maltose/maltodextrin transport system permease

protein MalG"

/db_xref="COG:COG3833"

/translation="MKNLAKSQKNSRLISRFLTYLFMIVLSIIIIYPILITISSAFKS

GNIAAFNLDFNSKWTLLNFSRLFNETLYGSWYKNTLIIAISTMIVQVTVITLAGYTYS

RYRFVGRKNSLLFFLIIQMVPTMAALTAFYVMALLLGALDQYWFLTLIYIGGGIPMNT

WLMKGYFDTVPIDLDESAKLDGAGHFRIFAQIVLPLVRPMIAVQALWAFMGPFGDYML

AKFLLRSPENLTVAVGLQTFINDSQNQKVSLFAAGAVLIALPISILFFMLQKNFVSGL

LAGGTKG"

CDS complement(1890204..1891508)

/locus_tag="JFBMFIFI_01794"

/inference="ab initio prediction:Prodigal:002006"

/codon_start=1

/transl_table=11

/product="hypothetical protein"

/translation="MDSEKRENQNVKKAMLLSIIPGLGQFYNGQKFKGFIFLGLAIIF

VVEMILFGFSAFNGLITLGSTPMEDHSLFLMIEGTLQLIITILFIFFYVININDAKRV

AKIWRSGGKVNQTAKEIIANMLDNGFPYLLTFPAYIAMIFTIIFPVLVTVFMAFTNYD

FYHIPPASLIDWVGFKNFFSIFFLSAYRDTFVSVFSWTLIWTLCASTLAIVLGVFTAV

IANQDFIKGKRFFGVIFLLPWAVPAFITIMSFSNMFNDSIGAINSQVIPMLNHLPFVE

IGAVAWKTDPFWTKVAIIMIQGWLGFPYIYVMTTGILQSIPGELYEAAKIDGANAIQR

FTQITMPMILFVAAPIFVTQYTGNFNNFSMIYLFNAGGPGSVGGGAGSTDILISWIFK

LTTGTSPQYSMAAAVTLIISTVIITVSLIIFKKTNAFNMEEL"

CDS complement(1891802..1893070)

/gene="mdxE"

/locus_tag="JFBMFIFI_01795"

/inference="ab initio prediction:Prodigal:002006"

/inference="similar to AA sequence:UniProtKB:O06989"

/codon_start=1

/transl_table=11

/product="Maltodextrin-binding protein MdxE"

/db_xref="COG:COG2182"

/translation="MNKRNLKKIGLGFLSAGVILSLAACGGGSDSKKDAAKEDNKSLT

ISVAEGYVDYVNDIKADFEKENDVKIKVVEKDMFDQLEALSLDGPAGKAPDVMMSAYD

RIGPLGQQGHLAEVKLGNDSDYDETDKAQVKIDGKIFGEPAVIETLVLYYNKDLITEA

PKTFKDAEALAKDPRFEFAAEAGKNTGFLAKWTDFYYSYGLLAGYGGYVFGKDGTDPS

QVGLNNKGAVEGITYATKWFQDVWPKGMQDTTGAGDFVTNQFTTNKTAAIIDGPWQAQ

AYKEAGVNFGVAEIPTLDNGEPYQPFGGGKAWVVSNYSKNKEVAQKWLDYVTNKDNQV

KFYEATNEIPANQAAREVAKGKNDELTNAVIAQYANAQPMPNIPEMAEIWSGAENLMF

DAASGKKTPQETADAAVKQIKENIEQKYQK"

CDS complement(1893592..1895373)

/gene="bbmA"

/locus_tag="JFBMFIFI_01796"

/EC_number="3.2.1.-"

/inference="ab initio prediction:Prodigal:002006"

/inference="similar to AA sequence:UniProtKB:Q9R9H8"

/codon_start=1

/transl_table=11

/product="Intracellular maltogenic amylase"

/db_xref="COG:COG0366"

/translation="MMETAAILHRPDSEYAYLYEADNFHIRLRTKKDEVSQVGLIGGD

PYLFDVDEWYKNEEPMKLLASTDVHDYWLIETTAPYRRLQYAFHVVGKDGTEVFYGDR

GIFPYTAETLATPGYFFRMPYFQEIDRFKSPDWVKETVWYQIFPERFANGNPAITPEN

ALPWGSKEHPGREDFFGGDLQGVIDHLDYLVDLGINGIYFCPIFKASSNHKYDTIDYY

EIDPDFGDKETFKKLVDEAHKRGIRIMLDAVFNHTGDISPLWQDVVKNQEKSKYVDWF

HVHSFPVDIGKNGNFEGERTVSYDTFAFTPHMPKLNTANPEVQKYLLDIATYWIREFD

IDAWRLDVANEVDHHFWKEFFKATTDLKKDFYILGEIWHSSQSWLQGDEFHAVMNYAY

TETIEDFFVKKKIDVSKMVAGLNEQLMLYRQQTNEVQFNMLDSHDTARLLTICGDDKE

LAKATLAFTFLQHGSPCIYYGTEIGMTGDNDPDCRKCMIWDQEKQDLTMFDFTKKLIS

LRKEKQEVLSYGSLEWSLVADQEKCVGFKRVGQNEVLTCYFNQGSQETTIDKIETEEV
LLSNLTTNNPTSITIEPNGFIILSSKI"

CDS complement(1895383..1897080)
/gene="malL_2"
/locus_tag="JFBMFIFI_01797"
/EC_number="3.2.1.10"
/inference="ab initio prediction:Prodigal:002006"
/inference="similar to AA sequence:UniProtKB:P29094"
/codon_start=1
/transl_table=11
/product="Oligo-1,6-glucosidase"
/db_xref="COG:COG0366"
/translation="MSRTNWWQSAIVYQIYPRSFQDTNNDGIGDIKGIIQRLDYIKSL
GVNTIWLNPIFISPQIDNGYDISNYYGIDPIFGTLKDVQELIYEAHQRELKIIFDFVL
NHTSDQHPWFQEALKGPDNLYRDYYLWADGKKNGAEPPNNWASFFGGSTWEKDLHSQQ
YYFHLFAKEMPDLNWENPEVHVALLDIAKFWLNEGIDGFRLDAFIHMAKEKGYPALPN
PTNEKYVIAEEYYANLPKVQEYLAKFIQALREVKPDVFIVGEAASAEVKQAVEYTDPT
KNQCDAVITFRYFPEREELKDSRLPLGMQPGTLDRQQFKQVMAEWQKEMASVGGPTLY
WNNHDMPRVVSRFGDDGLYRETSSKMLATLMYLQKGLPFLLNGEEIGMKNRIIQSIKD
YEDPGAEAFYHQALALGYTEIEALELLNASSKDASRGGMQWTKEPYSGFSTVKPWSSV
NQEENYTVEAEEADEGSILHYYRRLLLLKKRELFTKGSFSLLETGDETFCYVRNWDSE
EAVVLTNMTNEPVQLTCLEETLFNGKILLSAGDHELTKKQVTLAPYSCVVIQTKMKK"

CDS complement(1897354..1898316)
/gene="degA_1"
/locus_tag="JFBMFIFI_01798"
/inference="ab initio prediction:Prodigal:002006"
/inference="similar to AA sequence:UniProtKB:P37947"
/codon_start=1
/transl_table=11
/product="HTH-type transcriptional regulator DegA"
/db_xref="COG:COG1609"
/translation="MATLNDVAKKANVSKMTVSRVINHPEQVTDELKELVFKAMKELD
YRPNVAAKALANNRTQIIKFFILEEMDTTEPYYMNLLTGIAKELDKHHYTLQLVTKNN

FDIGACDGYIITGMRESDYAWIERATKPVVLFGENHHGHDFVDTDNKSGTKKATEHAI

AQGYESIIYIGIDVKEPFEYSREAGYINTMQQAGRVPEIKRFSNRSRYSAQFILENWS

DFPTNTVFICSSDRLAIGIERSILKLGGQIPKEYGIIGFDGVFLDQIASPPLTTVKQQ

VIEMGVACARMLLNKIDQSGAPQGNLLFEPELIIRESTVERKDS"

CDS complement(1898466..1898930)

/gene="perR"

/locus_tag="JFBMFIFI_01799"

/inference="ab initio prediction:Prodigal:002006"

/inference="similar to AA sequence:UniProtKB:P71086"

/codon_start=1

/transl_table=11

/product="Peroxide operon regulator"

/db_xref="COG:COG0735"

/translation="MKNSMVAHSIEKLKASNIRITPQRYAILEYLIESESHPTADEIY

RALAERFPNMSVATVYNNLRLFTKIGFVQEMVYGDSSSRFDFTSTQHYHAICEECGKI

VDIYYPGLDDVEVVASNLTGFEVSHHRMEMYGTCPDCLAKRQAETKEKTEAI"

CDS 1899235..1900527

/gene="hemL1_2"

/locus_tag="JFBMFIFI_01800"

/EC_number="5.4.3.8"

/inference="ab initio prediction:Prodigal:002006"

/inference="similar to AA sequence:UniProtKB:Q81YV0"

/codon_start=1

/transl_table=11

/product="Glutamate-1-semialdehyde 2,1-aminomutase 1"

/db_xref="COG:COG0001"

/translation="MLDHTESNRLAEQAKKVIVGGVNSPSRSFKGVGGGNPVTMVKGD

GCYLYDVDGNKYIDYLAAFGPIITGFNHPHIVKAIKKAADTGVLFGTPTEHEITFAKM

LTDAIPSMDKVRFTNSGTEAVMSTIRIARAFTNRELVVKFSGAYHGHFDLVLVEAGSG

PSTLGASDSAGVTNGTAQEVITVPFNQIEPYKAVMEKWGHKVAAVLVEPIIGNFGMVA

PVPGFLEAVNEITHEHGALVIYDEVITNFRFHYGAAQDMLGVIPDLTAFGKSIGGGLP

IGAYGGPKHIMDTVAPLGPAYQAGTMSGNPLSMQAGIACLEVLQEPGIYERMAGFAEQ

LRDALLSAGKKNNIPTVVNQIGGSLTVYFSERPIFNYDDAKNTDSIRFGKFFKLMLDE

GINLAPSKFEAWFLTIMHTQADIDATIEAINRVFAKLD"

CDS 1900756..1901856

/locus_tag="JFBMFIFI_01801"

/inference="ab initio prediction:Prodigal:002006"

/codon_start=1

/transl_table=11

/product="hypothetical protein"

/translation="MITMKLGARTLKTGLAVAIALYVSKWVGIPSVTFAAISAIFAMQ

PSVKRSLNTLRDQTIGNILGAGVAIITVLTLGKQYVLIGFAAIILIAILHQLELDQVI

GLATVTLIVVMTTTGDDFMLYAILRFSSTMVGILTAFVINSVFFPPKYEEKLYHVVDF

ATSEILKWIRASVRNNTEYPFLKKDLQWVRNQMMKMDFYYSLFKEEGSYSKKKRFQNL

RKIVVYRQMISTTKASYNLLKTMHKNENSFSNFPKELRVLIRERLETLLSAHEQILLK

FNGRVPPNSVNFIANNRSLRKEFMDSFFEAANTDENFKDGYLQSNSVIHILSSILNYE

EYLEHLNTLVTSYKGNNWNPTTDISNIENVEH"

CDS complement(1901911..1902777)

/locus_tag="JFBMFIFI_01802"

/inference="ab initio prediction:Prodigal:002006"

/codon_start=1

/transl_table=11

/product="hypothetical protein"

/translation="MNKKSLILLFIGLILTGAIIFLGKNYSITKKNELLRQGEAQESL

VQGQEEKRTMNEKNDLEAKTTEFQSNRANLSINDYLVLINAKNSKATLAFYGDNVESE

QWTKNIKTATIEKTGLANLEMNRYYFPGYDSLRLTNENTSEKLVDLKPDVVFFFIPVV

GNQAVDISLDASSQAILASYLALKEQLPDSLIVAVTPVPSSSLMNKFNSRTLDYTSYV

TDAVTVLTENEVPLLDLHTLYLSELETTNQLLASTLEADGIQLNAQGVTIATTLFLNQ

LASPRDTTSGID"

CDS complement(1902947..1903480)

/locus_tag="JFBMFIFI_01803"

/inference="ab initio prediction:Prodigal:002006"

/inference="similar to AA sequence:UniProtKB:Q97NV6"

/note="UPF0374 protein SP_1903"

/codon_start=1

/transl_table=11

/product="hypothetical protein"

/translation="MRIPKEGEYITIQSYKHDGSLHRTWRDTMVLKTSEQSVIGCNDH

TLVTEADGRRWLTREPAIVYFHKHYWFNIIAMIRENGVSYYCNLASPYTMDEEALKYI

DYDLDIKVFPDGEKRLLDVDEYEAHRKKWQYPNDIDHILKENVKILVEWINEEKGPFS

EEYVKLWYERYQQLSRK"

CDS complement(1903628..1905130)

/locus_tag="JFBMFIFI_01804"

/inference="ab initio prediction:Prodigal:002006"

/codon_start=1

/transl_table=11

/product="hypothetical protein"

/translation="MNYFLDKLQTRKIKLIKLLKNEYPNRSIAEISKELDCSNQTLMT

TIESLIEDLKSFEINTARIRIVSKRIEADFDVNFSVDCLIHEYVRKSFEYQVILDCFF

NTTKSISDYAKDFFLSQASTYRKINGVKNILKNFNIELQQSTTLQIVGEEKMIRYFYF

SFFWETHSNINWSFEFSKEFIASFKKVIHSNAIKADHTLVNQNKIDLWFGIILTRIAS

GYSVGVIPQIKNIGDFNVNYLEKITESQAFFNQINPKLSRTKSLDELNFCYSIEIINT

HYLVYNPTMEGLLNKIKANDQIYKVASQQFIESLDKYLSQALDKEAQAILLANLFSVH

LSVFLFDNVLSPFSEKLKLHRFDRIYPSMYKMLNKAYQDFIRHKAIAKVYHDKELLFP

SYLLILYLSVDVKNYTEPIIIYLYSANSTLLEFLLKKELQKMSQYNLIFTETVTAETD

LVISDTFNTPFCLSQESTLIWESVPSDSDWMNLRNKLNEIKKEKEFSRLE"

CDS complement(1905341..1905457)

/locus_tag="JFBMFIFI_01805"

/inference="ab initio prediction:Prodigal:002006"

/codon_start=1

/transl_table=11

/product="hypothetical protein"

/translation="MSTLVEQLIKSVVEKNKEYATNSKKAAVKNAKSHQKNS"

CDS complement(1905828..1907024)

/gene="mutY"

/locus_tag="JFBMFIFI_01806"

/EC_number="3.2.2.31"

/inference="ab initio prediction:Prodigal:002006"

/inference="similar to AA sequence:UniProtKB:O31584"

/codon_start=1

/transl_table=11

/product="Adenine DNA glycosylase"

/db_xref="COG:COG1194"

/translation="MTEEKLRVDTQKTEITMWSEKKITDFRSHFLSWYDLEKRTLPWR

ENNDPYRIWVSEIMLQQTRVDTVIPYYLNFMKLFPTIADLAAAEEDILLKAWEGLGYY

SRVRNLQKAAIQIMNEFSGEMPTNPEDIATLKGIGPYTTGAISSMAFQLPVPAVDGNV

MRVVSRLFEISEDIAKPASRKTFEAIMNQIIDPYKPGDFNQAMMDLGSSTCTPTTPTC

NLCPINEFCQAFEAGTMTQYPVKSKKKKAKPVYYAGLLIQNERGEFLIEKRPAEGLLA

NMWTFPLLPEELLTVGTGLKIGKLNDKLPETIETQVLNNLSVESRQRYQIEPIWLKKP

FGEVVHLFSHLKWFITSYYGKMSSEMLTEAPENCLWVHPSDFENYTFPGPQQKMWKSY

LTKFEK"

CDS complement(1907021..1907881)

/gene="recX"

/locus_tag="JFBMFIFI_01807"

/inference="ab initio prediction:Prodigal:002006"

/inference="similar to AA sequence:UniProtKB:P66003"

/codon_start=1

/transl_table=11

/product="Regulatory protein RecX"

/translation="MAEKNKLEQLTQKKENTFPLITKIEVQKKAKERYNIYLNGEYAF

PVDEAILVKYILHKGMEISPEFRAELEEQDGFHKAYNRALNYLNYGLRSEKEVRDDLI

KHEITFATAQAVVDKLKMQNYINDSTYAESYTRTGANLGGKGPRVITQELKKRGIGDE

TINQALEQYPFEQMLENGLALAEKVVKRTARSSSRETKNKIQQNLMQKGFSSDIIQEV

LQNIDTQKDDEDEYAALKDQGDKLWRKHSRLDLSKKKQKVKTSLFQKGFPGDLISQFV

MEKEMEEEDE"

CDS 1908212..1909588

/gene="rlmCD_1"

/locus_tag="JFBMFIFI_01808"

/EC_number="2.1.1.189"

/inference="ab initio prediction:Prodigal:002006"

/inference="similar to AA sequence:UniProtKB:O31503"

/codon_start=1

/transl_table=11

/product="23S rRNA (uracil-C(5))-methyltransferase RlmCD"

/db_xref="COG:COG2265"

/translation="MNPQHTIEIGQRFPLTIKRLGINGEGIGYYKRTIVFVKGALPEE

EVVAEVTEVMPKYATATIKKIRKKSVSRVVPPCDVYEACGGCQLQHLAYDKQLDFKRD

MVLQSLEKFKPAGYEKFDLRPTIGMDDPWRYRNKAQFQVRMTEENQIIAGLYGEGTHK

LVDINNCIVQRPETTKVIQVVKGLLAELEIPIYNEIKHSGIVKTIVCRVGIQTGEVQL

VLVTNSKKLPKKRQFLEQIKILLPEVVSIMQNVNQEKTSLVMGEETIHLAGKNSITEK

LDDFAFDLSARAFFQLNPEQTAVLYDEARKALKLAPNETLVDAYCGVGTIGLSLAKEA

KEVRGMDIIPEAIEDATKNAKKLGLDHVRYEVGKAESLLPLWMKRGFKPDALVVDPPR

TGLDRELIKAINKTKPKKMVYISCNPSTLARDLVDLSKVYTIDYLQSIDMFPQTARCE

VVVRLTAK"

CDS complement(1909653..1910762)

/gene="yhhT_2"

/locus_tag="JFBMFIFI_01809"

/inference="ab initio prediction:Prodigal:002006"

/inference="similar to AA sequence:UniProtKB:P0AGM0"

/codon_start=1

/transl_table=11

/product="Putative transport protein YhhT"

/db_xref="COG:COG0628"

/translation="MRELKKSKILSIIIHLLAAATLILVCTKISFLFKPVGIFISTLF

APIIIAGFLYYVMNPVVGLLEKFTKKRSRAILIVFILIALGFVFFIGIIIPNLLSEVT

QLASSMPRFMREQQMAVTELLNRPELSNVDIQRYWNELNISTGRVINGIVNGFTTSIG

SVISVLSRSIILMVTVPVILFYMFKDGEQLPSAIRWFIPEKQQKNAEELIQTVNKTLS

SYISGQALVCLYVGTGAYITFKLLGIPYAFLLACIAGMMDIIPYIGPWIGVAPAFIIA

FANSPQSALILALSIIVIQLGESYLVYPLIMGKSLNMHPLTIILILLVAGNLAGLVGM

ILALPTYAVVKIIVLSVAKIFKLGKYKEKIVEESI"

CDS complement(1910784..1911110)

/locus_tag="JFBMFIFI_01810"

/inference="ab initio prediction:Prodigal:002006"

/codon_start=1

/transl_table=11

/product="hypothetical protein"

/translation="MNTKKNNNLINGLSYISILFAPILFPLIVWLVSPITDVKQHAKS

ALWLHILPTILTVGALFILITTGLLTNSQATLGTLTIILFFAIIIIDFALVIWNIFKG

IKLFLS"

CDS complement(1911189..1912361)

/locus_tag="JFBMFIFI_01811"

/inference="ab initio prediction:Prodigal:002006"

/codon_start=1

/transl_table=11

/product="hypothetical protein"

/translation="MNAVLAFTIIMLVWTVSDFVSKKTKSLLSSLLIASLIFLIGFKT

SIFPEDLLTSSSLLTLGTTVVGFIIVHIGTMISIAELKQQWKTVIIGVSAIIGIAVAL

LVFGPLFESRNYAIAAIGAVSGGTISIILVQEAALALGLVSVAVLPVLISAFQGIIGF

PLTTLILRKEAKNIQKEYRAGTLKVAEEVKVDKAGKEPKTKLPEAFQTTAGTLFVVGV

VVLIATYLSGLTGGIVNTFIIALLFGVILRATGVFKANILTGIDAYGLMMLGILIIIF

GPLATISPQDLFDLIVPLSLSFLVGVSGSVIFAMIAGKLLGYSMSMSIAVGLTSLYGF

PGTMILSQEAAKSVGENEEEIKVIEGQILPKMIIAGFSTVTITSVFVTSILAGFIQ"

CDS complement(1912401..1913240)

/locus_tag="JFBMFIFI_01812"

/inference="ab initio prediction:Prodigal:002006"

/codon_start=1

/transl_table=11

/product="hypothetical protein"

/translation="MKSVIKQIPLVTLSLALIGISINMFLAPHHIAAGGVSGIGVLVE

QAFGINRATTVLVLNLLMLILTFFFLGRPVFIKTVIGSMLLPISLAVVPEIKVVEDPF

LAVIFGSAIFAVGVAILYKIGASSGGTTIPPLIFQKYFGISTSLGLLLTDAVIVIFNI

FVFGTEAFFFAILSLVLTSIVMNYIETGMKRRRAVMIMSENHIDAIKVALLENLNRGI

TVFSVSGGYTGNEKNMLMIILTNQEYQSILKVIDEIDKTSFIIAYNVSEVHGLGFSYQ

PVV"

CDS complement(1913276..1914391)

/gene="pepT_4"

/locus_tag="JFBMFIFI_01813"

/EC_number="3.4.11.4"

/inference="ab initio prediction:Prodigal:002006"

/inference="similar to AA sequence:UniProtKB:Q81WU4"

/codon_start=1

/transl_table=11

/product="Peptidase T"

/db_xref="COG:COG2195"

/translation="MAVERVVATFIELVKIDSESKNEGKFQAYLKTKFEKLGLDVYED

DTIAQTGLGANNLVCRLAGNTAADALFFSCHVDTVTPGVGIKPVIKEGAVYSDGTTIL

AADDKAGIAIMIELIEQLKENNEPHGTIEFVLSPGEEIGLVGASAFDVGVLEADYGFV

LDNGGPVGSITVGSPTLYGIEVTIKGITAHAGLEPEKGVSAIEIAAKAIAKMKLGRLD

ADTTANIGTINGGTASNIVADEVKVFAEARSISHEACEAQVKHMTDLFIETAKELGGT

ATVVTDIKSVGYRFTKESKTVKLAAEAITKINRTPNYDISGGGSDANVFNAKGKETAN

LSIGYEKIHTVHEMIPIIELKKAVELAYQLVKDVGQK"

CDS complement(1914415..1914936)

/locus_tag="JFBMFIFI_01814"

/inference="ab initio prediction:Prodigal:002006"

/codon_start=1

/transl_table=11

/product="hypothetical protein"

/translation="MSKAVNYFFLAVGIIMVSFGLWLIFNPRTTLSIVVVLMGIVLLL

NGISEIISYVSERKTWSISVWYLFDGLLSTIFGLLVLFNGQVGKNFLILLFAIWILAS

AIFRILLAFSVKSVSNWFIILIVGVLGLIVGIVSLFHSIFVSVALAVVLGGFFIFQGL

TCIFISSFLRRNH"

CDS complement(1914949..1915266)

/locus_tag="JFBMFIFI_01815"

/inference="ab initio prediction:Prodigal:002006"

/codon_start=1

/transl_table=11

/product="hypothetical protein"

/translation="MNSNKLVGGLCYLSILFAPIFFPLIVLFISTDKEVSKNASTALG

LHIAPFIVTIVGVLAMILFGVISSSDDIFRGVGLVVLILIAIFDAVIFIVNIVKAIKL

FLS"

CDS 1915663..1916313

/locus_tag="JFBMFIFI_01816"

/EC_number="2.7.1.113"

/inference="ab initio prediction:Prodigal:002006"

/inference="similar to AA sequence:UniProtKB:Q74HC2"

/codon_start=1

/transl_table=11

/product="Deoxyguanosine kinase"

/db_xref="COG:COG1428"

/translation="MKGADNAVIVLAGMIGAGKSTYTKLISDALGSEAFYESVDDNRI

LEKFYEDPKRWAFSLQIYFLNTRFRSIKDALQHQNNVLDRSIYEDALFTKINFEEGNM

SDAEMDTYLDLLDNMMEELASMPKKSPDLLIYLRGSLDTVLSRIKKRGRSFEQIDGNQ

DLLNYYTHLHSRYDGWFEEYDQSPTLVIDIDNHDLENPADAEKVIALINETLKNVR"

CDS complement(1916390..1917538)

/gene="mnaA"

/locus_tag="JFBMFIFI_01817"

/EC_number="5.1.3.14"

/inference="ab initio prediction:Prodigal:002006"

/inference="similar to AA sequence:UniProtKB:P39131"

/codon_start=1

/transl_table=11

/product="UDP-N-acetylglucosamine 2-epimerase"

/db_xref="COG:COG0381"

/translation="MKKIKVMTIFGTRPEAIKMAPLVLELERQPERFESIVTVTAQHR

QMLDQVLDIFQIKPDYDLDVMKDRQTLADITANVLIGLESVMKEAKPDIVLVHGDTTT

TFAAGISAFYNQIKVGHVEAGLRTWNKYSPFPEEMNRQVTDVLADMYFAPTIESEANL

LQENHPADKIFITGNTAIDALQETVKEEYQHEVLTKIASGNRLVLMTMHRRENQGVPM

ERVFHAIRQVVDKHEDVEVVYPVHLNPVVQEAADRILGNHPRIHLIAPLDVIDFHNLA

SRSYMIMSDSGGVQEEAPSLGVPVLVLRDTTERPEGVAAGTLRLVGTETDAVLEAMTE

LLEDPSAHQKMAEASNPYGDGLASKRILDAIAYEFGLSTEKPASFQVK"

CDS complement(1917663..1917758)

/locus_tag="JFBMFIFI_01818"

/inference="ab initio prediction:Prodigal:002006"

/codon_start=1

/transl_table=11

/product="hypothetical protein"

/translation="MEFYRGIRNAFLLAIPCWIIIIALLIHYLAN"

CDS complement(1917850..1918725)

/gene="gtaB_1"

/locus_tag="JFBMFIFI_01819"

/EC_number="2.7.7.9"

/inference="ab initio prediction:Prodigal:002006"

/inference="similar to AA sequence:UniProtKB:Q05852"

/codon_start=1

/transl_table=11

/product="UTP--glucose-1-phosphate uridylyltransferase"

/db_xref="COG:COG1210"

/translation="MKKVKKAIIPAAGLGTRFLPATKAMAKEMLPIVDKPTIQFIVEE

AKAAGIEDILIITGKGKRPIEDHFDSNPELEANLRAKEKFELLELVEETTGLNLHFVR

QSYPKGLGDAVLQAKAFVGNEPFVVMLGDDLMEDKIPLTKQLMDKYEKTHASVIATMK

VPYEETYKYGIIDPEIEIEKGLYNVRKFVEKPKVEDAPSDLAIIGRYLLTPEIFTILE

NQEPGAGNEIQLTDAIDTLNKTQRVFAHEFKGVRHDVGDKYGFLTTSIEYGLTHPEVK

KDLRAYLIALAKELS"

CDS complement(1918797..1919720)

/gene="tagU_1"

/locus_tag="JFBMFIFI_01820"

/EC_number="2.7.8.-"

/inference="ab initio prediction:Prodigal:002006"

/inference="similar to AA sequence:UniProtKB:Q02115"

/codon_start=1

/transl_table=11

/product="Polyisoprenyl-teichoic acid--peptidoglycan

teichoic acid transferase TagU"

/db_xref="COG:COG1316"

/translation="MSKTKKILIGVLCVFLLAVGAIGVYAFKVYKDVTQTTDKMYEKL

DKKEEVREKPVNIDKGQDPFSVLLLGVDTGDLGRDEQGRSDSIMVMTVNPNTNESKIV

SIPRDTYTEIVGYGKTDKINHAYAFGGVSMSVNTVQKLLNIPIDYYIEVNMQGIKDIV

DAIGGIDINSPLDFSQDGIKFTKGPMHLDGKQALAFSRMRYEDPTGDYGRQGRQRQVI

EAVVKKAATFSTLTNYKEILNTMQNNMKTNLTFDNMYDIQAKYKAAAGNIEQVQMQGT

GEMINDISYQIIAPDELARVSGILRNNLELK"

CDS complement(1919928..1921142)

/gene="wecC_2"

/locus_tag="JFBMFIFI_01821"

/EC_number="1.1.1.336"

/inference="ab initio prediction:Prodigal:002006"

/inference="similar to AA sequence:UniProtKB:P27829"

/codon_start=1

/transl_table=11

/product="UDP-N-acetyl-D-mannosamine dehydrogenase"

/db_xref="COG:COG0677"

/translation="MINVVGLGYIGLPTALILAASGTKVVGTDYDEKLIGVLNAGKTT

FDELGIEKLYNEAVDSGIVFSNEYIETDMYIITVPTPYEKENKKINPNYILSAVKSVL

KVCKKETILVIESTVSPGTIDKFVRPLIIEQGFEIGTDIHIVHAPERILPGNMIHELK

HNSRTIGADNKEVGEKVKTIYSRFCESDIIVTDIRTAEMTKVVENTFRDINIAFANEL

TKICRSDNMDVNEIIKIANMHPRVNILNPGPGVGGHCISVDPWFLVGDYPGLTNIILT

AREINDSMPEFVLERIHAIMQENSMTDLKKVGLYGLTYKQDVDDVRESPTLQLLEKME

KHLAEPLKVYDPMVKSKIVKNQYMEFNEFLNGIDLVVVMVSHSHILNHLKDLNGKIVL

DTQNIIKDAYKL"

CDS complement(1921171..1922589)

/locus_tag="JFBMFIFI_01822"

/inference="ab initio prediction:Prodigal:002006"

/codon_start=1

/transl_table=11

/product="hypothetical protein"

/translation="MKSSSIIKNVALTLSSNIFSLLISTLVILVIPKLIGIEEYGYLQ

LYIFYTSYVGFLHFGWNDGIYLRYGGELYENLNKRIFNSQFYLLVISQVVIAIAVYLV

ASYLQLEQDKLFVINMTIISLIFTNLRYMFLFILQGTNRIKDYSMVTILDKLIYIVVI

LVFLLIGKKNYQFLILADLTGRAASLLYSMSLCKDIINSNPIIKKNDFKETFNNINAG

SKLMFSNIASMLIIGVVRLGIERRWSIETFGKISLTLSISNLMLIFINAIGIIMFPLL

RRIEESKLVPIYTTIRSILMIVLFSGLLFFYPLRSILLMWLENYKESIMYMTLIFPIC

IYEGKMSLLINTYLKTLRKEKSMLFFNLITLLMSVVMSFITIKVLNNLTLTVLLILGL

LMFRAIITEVYLARILKIDVSKSVVSEIAMTASFVISGWYFSGITAFIIFFLSYLIYW

IIFKNEIKKSLVVLKNYFYQEK"

CDS complement(1922609..1923748)

/gene="mshA"

/locus_tag="JFBMFIFI_01823"

/EC_number="2.4.1.250"

/inference="ab initio prediction:Prodigal:002006"

/inference="protein motif:HAMAP:MF_01695"

/codon_start=1

/transl_table=11

/product="D-inositol-3-phosphate glycosyltransferase"

/translation="MKILEVATFKLGACGPTEVLIPIIKNINQKNEHSVDVFSTLTFD

EEKEKKLTELNAKYFYKKKSKVLSKKEILSVIPDFATYDLIHIHGVYNIDNIMIAKLA

YKMGIPYVITTHGNLMEHSLQHSKIKKKLALFLFIKRFLRNAESIHALSNQEEKDIKK

IFKHKNISIIPNGIELLKEKDQLKNTIKNDKVSLLFIGRIDVHHKGIDILIDSLLSLT

EKGRGKYELYLVGPYDGNFDEEYIKNKIKENSILKNIIKIEGPKYGDDKEKYYEKCDL

FVHTSRYEGMPIAVLEAIAHGLPILITPETNLADIVNQSENGFITSTEINTVTNSLTE

ISELTKADLKGLGLNGKRWATSNLSWEKISKEYLVMYTRSMEKQD"

CDS complement(1923735..1924835)

/locus_tag="JFBMFIFI_01824"

/inference="ab initio prediction:Prodigal:002006"

/codon_start=1

/transl_table=11

/product="hypothetical protein"

/translation="MKVLVMTRLPEVDASSRQRVYQYKEILEKKHGIYLTIVPFYSKE

IFLKYRQGKLSKLKLAQLLLNRKKELKQFENFDMVWILRSLSPLENPSFLKKITRKNI

PIIFEMDDAIYLGEKANRLRTKLTRSWNHVEKYISNANAVIVGNTELEAWSLQYNKHA

FVIPTGLDVASYKLKTTDDSDLFTIGWIGSESTAPNLDLVIPAINKFAKDKKIRVIFI

GLGNYSTKLKFNDNIEFINLEWSEEIQHNYLQKFDIGIAPLYNTPFNRGKCAYKVIQY

MATGIPTVASPVGVQTELFSSKEIGFNAESEEEWIRIFDLLLNDKKLRLKLGRNSRKI

AENTFDVDILANKIAGVLYTIFEEKDKMDENT"

CDS complement(1924838..1926112)

/locus_tag="JFBMFIFI_01825"

/inference="ab initio prediction:Prodigal:002006"

/codon_start=1

/transl_table=11

/product="hypothetical protein"

/translation="MWFFISLIILISGLFLTKKLFNTLRSPVGLFTIVWMGLFTLSNL

KLVNYYDVSTSAGILIISVYYLFFFGALVASKVKVGTNKNIDSPIKYEFRRKNLLVGL

FFSIILSNTALVLWLIFLAREIGIGALLSSNMYLNRIVMSDISTIFSYLMYIPSISGS

LLAGILIAKGEKFKLIYLTTFLPTIANAIFTGQRNLTIISILLFMMPLFLIKVRVKKK

YLSKQQNMMILFLIVSLVVFFVVIGGKRGSYTDITIESKFSNPVFIKTYMYLTGSFVA

FSEQLKMWNQELMYGVNSFLPVFKILNALGVTSYDGDTFSLIESGREFIPIPMSFNVY

TFLWDVISDWGYLAIFVYPTILGFLSNLFWKKSNLHDYPSYSDILLSFIALFLSMSFI

YSTSSMTTILYVFIYTSVIFLVFKNKIIFRKG"

CDS complement(1926136..1927392)

/locus_tag="JFBMFIFI_01826"

/inference="ab initio prediction:Prodigal:002006"

/codon_start=1

/transl_table=11

/product="hypothetical protein"

/translation="MKKIWIWNHYATEMYRNSGGRHYWIAKNLKENYEPTIFCANTFH

SSDEKIDTFRKKFIKIKLNDIPFIFVKTIQPNGNGIKRVLNMLLFFFNLIIVAFKYKK

IDGKPDVIIASSVHPLTMVAGILVARRFKVPCICEVRDLWPEAIFFFTKVKENSLFGK

ILIKGEYWIYKNADKIVFTKEGDKDYIIEKGWDSEHGGEINLDKCFYINNGIDLSDYK

ENITKNIFEDEDLVMENKFKVIYCGAIRPVNNVGNIVECAELLKTHSEIMFLIYGEGI

ELSKLKELAKTKNLTNIIFKGQVNKKYIPYILSKSSLNILNYSQSQYNWSRGNSSNKL

FEYMASGKPIVSTVKMGYCLLKKYNCGVSIEENTPEKLASKILDIQSLSQIEYEKMSK

NSIEAAKDFDFKVLTKKYIEIIDMVL"

CDS complement(1927413..1928573)

/gene="epsN_2"

/locus_tag="JFBMFIFI_01827"

/EC_number="2.6.1.-"

/inference="ab initio prediction:Prodigal:002006"

/inference="similar to AA sequence:UniProtKB:Q795J3"

/codon_start=1

/transl_table=11

/product="Putative pyridoxal phosphate-dependent

aminotransferase EpsN"

/db_xref="COG:COG0399"

/translation="MVAENKRILLSSPHMSGNEQNYINNAFKENWIAPLGPNVNQFEL

DIAAYAGVKGASVTSSGTGAIHLALDILGVKQGDSVFCSTFTFVASANPALYLGADIT

FIDSEIETWNMSPFALERALSDAKKMGKLPKAIIVVNLYGQSAQMDELLEIANSYNVP

IIEDAAESLGAEYKGKKSGTLGKIGIYSFNGNKIITTSGGGVLISDDEEIIARSRFLA

TQARDEAPHYQHSVVGYNYRMSNILASVGIAQLQVLDERVTRRREIFNTYYQELAGIE

GVQFMPELNGTKSNRWLTTLTIDQKIIQLNPYEIMDKMNEANIETRALWKPLHMQPLF

KENKFYKDKENEESIASELFANGICLPSGSNMNQQDQMRVIEAFKKITVKSC"

CDS complement(1928587..1929204)

/gene="epsM_2"

/locus_tag="JFBMFIFI_01828"

/EC_number="2.3.1.-"

/inference="ab initio prediction:Prodigal:002006"

/inference="similar to AA sequence:UniProtKB:P71063"

/codon_start=1

/transl_table=11

/product="Putative acetyltransferase EpsM"

/db_xref="COG:COG0110"

/translation="MGTILVIGNGGHSKVIRMMIDRSSNKRLAILADDTIENQFEENK

IIYSPLSEIKNNKNQFEESIIAIGNNQIRKKIYERFSEINYATLIDPSSMIAHDTIVG

TGSVVMPKVIINPNVRIGMHSIINSGAIVEHDCKIGDFCHISPGAVLTGGVKIDNLVH

IGANATILPGIHIGEGAVIGAGAVVTKNVHKNQVMVGNPAKELGN"

CDS complement(1929206..1929820)

/gene="pglC_1"

/locus_tag="JFBMFIFI_01829"

/EC_number="2.7.8.36"

/inference="ab initio prediction:Prodigal:002006"

/inference="similar to AA sequence:UniProtKB:Q0P9D0"

/codon_start=1

/transl_table=11

/product="Undecaprenyl phosphate

N,N'-diacetylbacillosamine 1-phosphate transferase"

/db_xref="COG:COG2148"

/translation="MYKSFFKRLIDFSCSLLLIIILSPVIILTAFFVAIFLGRPIIFK

QQRPGLNGEIFTIYKFRSMNEKRDSNNKLFSDEIRLGKFGKYLRKLSLDELPQLFNVL

KGDMSFIGPRPLLVEYLPLYSQIQKKRHEVKPGITGWAQVNGRNAITWEQKFKLDVYY

AENSSLWLDIKIIFLTFYKLAKRADIASENHVTVAKFTGSKDCE"

CDS complement(1929834..1931642)

/gene="pglF_2"

/locus_tag="JFBMFIFI_01830"

/EC_number="4.2.1.135"

/inference="ab initio prediction:Prodigal:002006"

/inference="similar to AA sequence:UniProtKB:Q0P9D4"

/codon_start=1

/transl_table=11

/product="UDP-N-acetyl-alpha-D-glucosamine C6 dehydratase"

/db_xref="COG:COG1086"

/translation="MILTRNVKKIILISLDSLAIILSSIAAYIFLNPYIDPSNSFYIG

VVGICLFSYMGLATYFKLFSKINRYTSLKEILNVFICVSLAFFSASLVSLLAINNISW

RFLVLMYVFSLFIIAGSRIVWRILIEMRNKHLDGEQKVNKIRTLVVGAGEGGSVFIKS

SRKNDNELKIIGIVDSDQTKQNTYLYEIPVIGLIEDIPELVHEYDIAQITIAIPSLKA

IEYEYILDICNQSGVKFNLMPSVEDVLNGKLSVSRFRKIDVVDLLGREEVKLDMQQIS

KKLHGKTVLVSGAGGSIGSEICRQIMKFHPRKLILLGHGENSIYLIHKELGQIFGSDT

EIIPVIADIQDRERIFEIMKEYRPDRVYHAAAHKHVPMMECNPKEAVKNNIYGTKNMA

EAAKASDVGSFIMISTDKAVNPPNVMGATKRIAEMIVTGLNEPGKTKFAAVRFGNVLG

SRGSVVPLFKEQIENGGPITVTDFRMTRYFMTIPEASRLVIQAGALAKGGEIFILDMG

EPIKIVDLAKKVVKLSGYTEKEIAIIETGIRPGEKLYEELLVDGEKTNEKVYDKIFVG

KVSSTSIKEVEQFIAELSRSDESKIRKDILNFTNVK"

CDS complement(1931659..1932426)

/gene="ywqE_2"

/locus_tag="JFBMFIFI_01831"

/EC_number="3.1.3.48"

/inference="ab initio prediction:Prodigal:002006"

/inference="similar to AA sequence:UniProtKB:P96717"

/codon_start=1

/transl_table=11

/product="Tyrosine-protein phosphatase YwqE"

/db_xref="COG:COG4464"

/translation="MIDLHCHILPGIDDGAQSLEDSLAMAHMAVAEGITHILVTPHHK

NGVFINEKNEINQKLIELQNEFDLREIPISLFPGQEVRINGELMEEIADGKIQFIDEE

DQYLLIEFPTMSVPAYSENLFFELSKKGIIPIIVHPERNQVFMDNPNALKPFIERGAL

AQLTAGSYLGKFGKKIQKVSKQMLEANLVHFIASDAHNTTSRSFLMKAAYDKLGQDFG

NRKAESFKQLTKDLINGEIINVNAPKNVKEHKIFGLF"

CDS complement(1932426..1933151)

/gene="ywqD_1"

/locus_tag="JFBMFIFI_01832"

/EC_number="2.7.10.2"

/inference="ab initio prediction:Prodigal:002006"

/inference="similar to AA sequence:UniProtKB:P96716"

/codon_start=1

/transl_table=11

/product="Tyrosine-protein kinase YwqD"

/db_xref="COG:COG0489"

/translation="MFKFKKTKEQLIESQKNGASLVTVTKPNSVVAEQFRTIRTNIQF

SMIDRDLKSLIFTSSGPGEGKSTTSANIAVVFATQGKRVLLVDADMRKPSVNKTFKLS

NHEGLTTLLTEKEVVLGDVVHETNTENLFILTSGPIPPNPSELLDSKKMNRVIEILEE

TFDLVIFDMPPIVSVTDAQIMASKTDGTVFVIRNGIATKEAVLKAKQLLDIVNANVVG

TIFNGLEKRKDKAYKYYGIEGEN"

CDS complement(1933167..1933904)

/gene="ywqC_2"

/locus_tag="JFBMFIFI_01833"

/inference="ab initio prediction:Prodigal:002006"

/inference="similar to AA sequence:UniProtKB:P96715"

/codon_start=1

/transl_table=11

/product="putative capsular polysaccharide biosynthesis

protein YwqC"

/db_xref="COG:COG3944"

/translation="MEESISLTELFEVIRKRLALIIGLGLAGIALAAVVTFFLITPKY

SATTQLLVNRANEEGQSTNQLADLQTDVQMINTYKDIIKGPVILDDVRKELKTNLTTD

QLKSQIDVLTQQNSQVFTVQVTDADPYVAADIANAVATVFQNKIGDIMSVKNVTPISK

AVPNSSQISPNSTINMLIGLIVGLLVGIGLAFVLEFLDKTVRDEKFINDGLGWTNLGA

VSEMDEEELTAILPAGVNTRRAARSRV"

CDS complement(1934377..1935315)

/locus_tag="JFBMFIFI_01834"

/inference="ab initio prediction:Prodigal:002006"

/codon_start=1

/transl_table=11

/product="hypothetical protein"

/translation="MTSSKKMNKYFNKKEWLRFFETIQRKYQEAEVTNSSVVIAYYLL

LSFFPIVIIIGNLLPLLNLDINTILPYFSTIIPEAIYQEIVETIHRLLTSSSSGMLSF

GIVAAFWAASKGMNAMQVSMNKAYGVEPRKNMFVIRLASLAFTLILVLGIVALVLVFS

FGQVVLNYLTPLLQLPEELVAIFQSVKWPVTLFVLLFVFTLVYYMVPNAQVKVKFVLP

GAIFATIGWVLLSQAFAIYVRYFSGRTLSYGSVGIFIVLMLWLNGSGIVLTLGAVINA

SIDEFRHGKIENNSSRIGDYFERQINKGKDKLTNKK"

CDS complement(1935609..1936364)

/gene="map"

/locus_tag="JFBMFIFI_01835"

/EC_number="3.4.11.18"

/inference="ab initio prediction:Prodigal:002006"

/inference="similar to AA sequence:UniProtKB:P19994"

/codon_start=1

/transl_table=11

/product="Methionine aminopeptidase 1"

/db_xref="COG:COG0024"

/translation="MITLKSPREIEAMAESGALLANVHIALRDFIKPGITSWDIEEFV

DKFITDHGAIAAQKGFEGYKYATCVSINDEICHGFPRKEVLKDGDLIKVDMCVDLKGA

LSDSCWSYVVGKSTPELDHLMEVTKKSLYLGIEQAQVGNRIGDIGHAIQTYVEGENLG

VVREFIGHGIGPTIHESPAVPHYGEAGKGLRLKEGMVITIEPMVNTGTWKSKMDDNGW

TARTKDGGISCQFEHTLAITKDGPRILTSQGDQ"

CDS 1936650..1937090

/locus_tag="JFBMFIFI_01836"

/inference="ab initio prediction:Prodigal:002006"

/inference="similar to AA sequence:UniProtKB:Q01095"

/codon_start=1

/transl_table=11

/product="Flavodoxin"

/translation="MPSAKIVYASMTGNTEEIADVVAEALENLNIDVETVECTNAEPD

DFENVDLCIVATYTYGDGDLPDEIVDFYEDLADVDLTGKIFGTCGSGDTFYDDFCKSV

DDFTERFIETGATKGAESVKIDLAAEEEDIQKLEAFAKELVSKL"

CDS complement(1937166..1937828)

/locus_tag="JFBMFIFI_01837"

/inference="ab initio prediction:Prodigal:002006"

/codon_start=1

/transl_table=11

/product="hypothetical protein"

/translation="MKIRYQQNLIRLFFIAYMFAIYFISYRYQLMLLNGLLAYIPIEL

SFWLTNKKLKSTSLFFIISAVWLVFFPNLPYLLTDLVHLSWLRPYIPDSYALLSAPNI

WKDFYLLLAGVIGFLIVGYHSLKAFGDTLAIRFNFKKNYYVHLFYLFICGISSFGIYL

GRFSRLHTVYLITDPIESIRAIIDAFEPNMYLFILGFTILQLILFYAVSFIRLDNTSF

KA"

CDS complement(1938030..1938593)

/locus_tag="JFBMFIFI_01838"

/inference="ab initio prediction:Prodigal:002006"

/codon_start=1

/transl_table=11

/product="hypothetical protein"

/translation="MARKKTITKQQILNAAYDVVRTEGFGGFTARNIAKKMKCSTQPI

YLEFKNMDDLKNELFEKIKGYLKTEIYSHEHTGDPLLDACLNYIYFADTEKVLFRALY

IENHLGIEKMHKISLDFAMKLMNEKEETKNLSDADKFQVFTKVWIVAQGIASLLSSGL

LPMDSEQVETSLRESLSDMILGARYDA"

CDS complement(1938659..1939219)

/locus_tag="JFBMFIFI_01839"

/inference="ab initio prediction:Prodigal:002006"

/codon_start=1

/transl_table=11

/product="hypothetical protein"

/translation="MTRKKVIMKNQILDAAYSIAKRDGLSLITARNVAKEIGCSTQPI

YLEFENMDELKSVLLDKIQRQMSDKLMERKVTGNLFTDVSLNYIDFSKNEKKLFEILF

NANLLIVDLTKTESYQFLLESLVEDITEQLTNEEIKMLFCDLWIVIHGLASLTTVGQV

NNDDAANEKYIKRAKSGIIEAIKGSK"

CDS 1939515..1940753

/gene="pepS"

/locus_tag="JFBMFIFI_01840"

/EC_number="3.4.11.-"

/inference="ab initio prediction:Prodigal:002006"

/inference="similar to AA sequence:UniProtKB:Q9X4A7"

/codon_start=1

/transl_table=11

/product="Aminopeptidase PepS"

/db_xref="COG:COG2309"

/translation="MVLPNFQENLQKYADLIVSTGVNVDKGQTVVLQIDVEQAPLARL

ITRAAYKKGATEVIVKWTDDEINREIFLGTPEERLTDIPQYKIDESLDQLEKNASRIS

VRSADPDALSGVDSTKVAAYQSAAGKALKAMRIATQSNKVSWTVVAASGEKWAAKVFP

NLATSEEQVDALWDQIFKTTRIYTPDPVAAWNAHDQLLESKADELNKEQFDALHYTAP

GTDFTIGLPKGHRWEGAGSFNARGEKFMANMPTEEVFTAPDANRADGVIKSTKPLSYA

GTTILDMTFTFKDGQVVDVTAKEGEDVLKHLIATDNGSKRLGEVALVPDPSPISQSGI

VFYNTLFDENASNHLALGSAYAFSLEGGTEMTEEELIAAGLNRSDVHVDFMVGSNEMD

VDGIRLDGTRVPIFRKGNWA"

CDS 1940952..1941887

/locus_tag="JFBMFIFI_01841"

/inference="ab initio prediction:Prodigal:002006"

/codon_start=1

/transl_table=11

/product="hypothetical protein"

/translation="MKNWKVILWLVLFFPLGAYFMWRYSSWNIHLKYGISIFLGLLTA
LFTFFGGLFILFWYSGFGFLLYFIATFKSSPKKQKISFLTASVLLFGSSLALSPTTES
VESDQSQIQVVKKDTSASDIKKQKEKKASEEKLAAENKIQAEKELQAEKLKEATDALI
LAEATPTRENYNKASTLVVALTTKDSTIVSRLDTVKQTVEAEEKIVAEAKEKAETERI
AAEEARIAEEQRVQEAQRVAEEQRLANERAVAEENRVAQAAAAAPQQDNTGQRVLVTP
TGSKYHIRKCGNGTYTDASLEDALSRGLEPCSKCF"

CDS complement(1942410..1943774)

/locus_tag="JFBMFIFI_01842"

/inference="ab initio prediction:Prodigal:002006"

/codon_start=1

/transl_table=11

/product="hypothetical protein"

/translation="MKLTHSKNKERIIVLLSIYISFLLFNFFMTIFKADDLAYLKSVS
EIGYIQSSIRNYNNWSSRIVIEFFLMFLSKHFVLWKVLNATVMLGTVVIICKYVFKEI
ESRKLIVTFAVYSLIPLTLMGETGWIATTMNYHWPVFFAMVAFYSFYQLLINERINLT
IYYFSLPLLIFAVNQEQVNACFFVLTTLLSFYLWSQRKYDYKLSLISAISFLGLVFSV
TAPGNKIRAARSIINWFPNYDNLNVINKLDLGISSFGKPFFFDFNILFLLLFLLIFVL
SYTKTKNYYLRLISAMPLFFNLMIYFGNTTKQGFLNILGGNRGMIWNSENLDKLFSVN
GTGLSVFHIGTWVATLSILGLLTCLIIGIYLSFNNKNKALLVILLLLMAFCSRIIMGF
SPTVWASGMRTYYISYVVVAISVLLLFKELANVLGYKKMELVQFSISATGICTFILSI
LTKG"

CDS complement(1943900..1944937)

/locus_tag="JFBMFIFI_01843"

/inference="ab initio prediction:Prodigal:002006"

/codon_start=1

/transl_table=11

/product="hypothetical protein"

/translation="MTIKTTHAGHGGIDPGAVGNGYKEADVARLYNAKILQLTGAINA
TDDSAVSVPDNLAKIVAKVNAVSGNDDWNISIHLNAFTSSATGVEVYAYSQDAAGMAK
AAEISAKLATVYGIQNRGAKAGDELYVIKNTKGHMLLIELGFISNANDLNQILSKIDQ
ACTAIVTSFGYTGTPAPPQPVGPVLKIINLWDYTGADWLPKIQKSYANVVSADRAFKV
KTGSSWRVRLTYINYAQAVIICDELRKVYPKYTATGYRINANDPIQDTTPFAVEVDSI

PEADIENVLRHVQQTYAGIITADRIFLTPQLGTNPAQVTLEMYNFKESQRATIISQLK

ERYGISDNQFK"

CDS complement(1944937..1945137)

/locus_tag="JFBMFIFI_01844"

/inference="ab initio prediction:Prodigal:002006"

/codon_start=1

/transl_table=11

/product="hypothetical protein"

/translation="MIELIEQSTQSLIVVVAVVTALGVRGMKSTEVVQTKYLPMASMV

LGSFVSGGYDAIKSILTGKDDK"

CDS complement(1945295..1945441)

/locus_tag="JFBMFIFI_01845"

/inference="ab initio prediction:Prodigal:002006"

/codon_start=1

/transl_table=11

/product="hypothetical protein"

/translation="MNQILEIELVILIPSDSILVKQAEYEDMPKDSLECRKFKFKELS

KRNR"

CDS 1945855..1946193

/locus_tag="JFBMFIFI_01846"

/inference="ab initio prediction:Prodigal:002006"

/codon_start=1

/transl_table=11

/product="hypothetical protein"

/translation="MHLNEFVGNKIKTFREARGMTQDMLADKLNTTRQTISRYENGDR

KANQDILFELSVIFGIKVDDFFPSRDNISEESPLATIAAHMADSEKKLTKSDIDKIND

YIDLILDSKK"

CDS 1946206..1946313

/locus_tag="JFBMFIFI_01847"

/inference="ab initio prediction:Prodigal:002006"

/codon_start=1

/transl_table=11

/product="hypothetical protein"

/translation="MNSYEVLLDEIYSNINVVEEELINANGLIEVYKEA"

tRNA complement(1946877..1946949)

/locus_tag="JFBMFIFI_01848"

/product="tRNA-Arg"

/inference="COORDINATES:profile:Aragorn:001002"

/note="tRNA-Arg(cct)"

CDS complement(1947118..1947528)

/locus_tag="JFBMFIFI_01849"

/inference="ab initio prediction:Prodigal:002006"

/codon_start=1

/transl_table=11

/product="hypothetical protein"

/translation="MELIKELNVSAEYFYGRVMESVLYDIRQHTEKSMSKDQLSGFSY

SKQFAPKSSGKIVITEILENQVYAFKTITSRNEFDAKYVIDAIDETHCRVTYTEKMES

NGAFQTMNDRVVGTLLSYFRKKNMKKMLLSIEQL"

CDS complement(1947529..1947714)

/locus_tag="JFBMFIFI_01850"

/inference="ab initio prediction:Prodigal:002006"

/codon_start=1

/transl_table=11

/product="hypothetical protein"

/translation="MQKNGLFVMSIGMLTIMSSAFGAEMKINYFILITGLFLIGTGFF

IFRKGKSQGNEKTEGKK"

CDS complement(1947720..1947947)

/locus_tag="JFBMFIFI_01851"

/inference="ab initio prediction:Prodigal:002006"

/codon_start=1

/transl_table=11

/product="hypothetical protein"

/translation="MIVFNAPVQQDKIIETLTSFNEEGISYEFVEKKGIKLYFKSATD

DQEAAAKKAKEIIKGTPYGGVLYFQVTVEEG"

CDS complement(1948110..1948439)

/gene="licA_3"

/locus_tag="JFBMFIFI_01852"

/inference="ab initio prediction:Prodigal:002006"

/inference="similar to AA sequence:UniProtKB:P46319"

/codon_start=1

/transl_table=11

/product="Lichenan-specific phosphotransferase enzyme IIA

component"

/db_xref="COG:COG1447"

/translation="MGDQENLETIMGLIINGGNAKSDAMEAIQAAKKGDFELADQKLI

DSDKALVEAHHSQTGMLTQEAQGDHVAVTLLTVHSQDHLMNAITFRDLAKEMVDLYKK

MAGIPVA"

CDS complement(1948472..1948789)

/gene="celA_2"

/locus_tag="JFBMFIFI_01853"

/EC_number="2.7.1.205"

/inference="ab initio prediction:Prodigal:002006"

/inference="similar to AA sequence:UniProtKB:Q45399"

/codon_start=1

/transl_table=11

/product="PTS system cellobiose-specific EIIB component"

/translation="MAEKTIMLVCSAGMSTSLLVTKMEKAAEARGLDAEIFAVSASEA

DTHLESKNIDVMLLGPQVRFMKAQFEPKVAAKGVPLDIINMQDYGMMNGEKVLDQALS

LMK"

CDS complement(1949057..1949383)

/gene="ptcB_1"

/locus_tag="JFBMFIFI_01854"

/EC_number="2.7.1.204"

/inference="ab initio prediction:Prodigal:002006"

/inference="similar to AA sequence:UniProtKB:A2RIE6"

/codon_start=1

/transl_table=11

/product="PTS system galactose-specific EIIB component"

/db_xref="COG:COG1440"

/translation="MAKQTIVLVCSAGMSTSLLVLNMERAIEARGLDIDVFAISGSDV
ADFLEKRPINVMLLGPQVRFMKRQFGPILEPRGIPVEVITMKDYGLMDGESVLIWAEE
LMKSAN"

CDS complement(1949456..1949773)

/gene="celA_3"
/locus_tag="JFBMFIFI_01855"
/EC_number="2.7.1.205"
/inference="ab initio prediction:Prodigal:002006"
/inference="similar to AA sequence:UniProtKB:Q45399"
/codon_start=1
/transl_table=11
/product="PTS system cellobiose-specific EIIB component"
/translation="MAEKTIMLVCSAGMSTSLLVTKMEKAAEARGIDAEIFAVSASEA
DSNLESKNIDVMLLGPQVRFMKAQFEPKVAAKGIPLDIINMQDYGMMNGDKVLDQALS
MMK"

CDS complement(1949971..1951221)

/gene="licC_5"
/locus_tag="JFBMFIFI_01856"
/inference="ab initio prediction:Prodigal:002006"
/inference="similar to AA sequence:UniProtKB:P46317"
/codon_start=1
/transl_table=11
/product="Lichenan permease IIC component"
/db_xref="COG:COG1455"
/translation="MDGFINFMEKHFIPTASKIGAQRHLVAIRDAFMVTMPLMILGSL
AVLLNNLPIPGFQELMNSIFGGEAWKGFGGSVWNGTFAILSVLIAFLIAHNLLKGYGK
DGVAGGVVSVASFFALGGATGMSSNGLFIALIVGITSAEIFNLLVGNPKLIVKMPDGV
PPAVAKSFAALFPAMITISIYGLIAAIFAGFGVTDIIASFYELVQKPFMGLASTWPSA
LLLAFITPFLWFFGLHGANMIEPLMQSINLPAITANQEAIAAGDAAPYIVNKPFFDSF
VNLGGTGATLGLIIAIYLFGRRNKAHMVVTNLSAAPGIFNINEPMMFGLPIVLNPIMF
VPFVLTPMILVTVAYFATSTGLVPAAIAMPPWVTPPIIGGFIATNSIAGGVLAAVNLV
IAIVIYAPFVKIAEIQELKKEQAI"

CDS complement(1951440..1954136)

/gene="norR_2"

/locus_tag="JFBMFIFI_01857"

/inference="ab initio prediction:Prodigal:002006"

/inference="protein motif:HAMAP:MF_01314"

/codon_start=1

/transl_table=11

/product="Anaerobic nitric oxide reductase transcription

regulator NorR"

/translation="MTNRKATVFSFLESRSEKMTAVEIAAGLDLDRANVSRYLNELYK

EKKIEKINGRPVLYQRLESSPIISQEEEPEESFSHLIGSNESLKVMIQQAKAAILYPP

RGLHTIIFGKTGTGKSLFAECMYQFAIEAQTLPKDAPFISFNCADYAQNPQLLFGHIF

GIKKGSYTGASEDRVGLMSKADGGILFLDEIHRLPPEGQEMLFTFIDKGTYRPLGESS

QVYEASVQIIGATTESSDTLLSTFNRRIPMMITLPPLDQRSLDERYELVATFLQKESN

RLGQKIVVERDVLLAFMLYHADGNIGQVQRDLKLVCAKAFLHYRTHQEEYLKISQKDL

PLQVQKGLLRVKEAPEKMERLIDNKVNDFTYIPVDQKGQSENDLSADMSVYNVIEEKV

GEIMAEGGIQDIDLEELMATDMTKYFRDYVEKLSMSDAHQDLIPSQLRNLTDRLYDVA

EERLDRKYNPKARFAFALHLQSSLERIKENRQIVHPDLNNVRKKYSKEFQVAIDLSGM

IEEEYQIEVPFDEIGFITMFLTLDISDTVVPKAELVSIMVLMHGKSTATSMLETVQDL

LDTNVGGAIDMPLTMEVQKMYEKVKKTITENPETFKNGLLVLSDMGSLTSFGNMISEE

LGIRTKSLSMVSTPIVLEAVRMASVGRTLEDIYQSCQLTFENMVKSSLKTEKPQKKAV

LVTCFTGEGVAKHLNERISPVIDQSRTKIIQLQFLHREAFKQHIDELMEEFEIKAIVG

TVEFDYQNIPYFSAYDIFDNEKLNILKRIVDEDIPIEQMIQSLEGTIRNVGSVHKLVM

QSQKIVHQLQTDMHIIVEPGVDTGIIIHLAFLVERLKVGSMIREFPNLENYVKKYRLE

VDLVKAALMTLEKQYRVVMVEDEVAYIVQMFIDNQVQLTINK"

CDS complement(1954260..1955549)

/gene="yfmL"

/locus_tag="JFBMFIFI_01858"

/EC_number="3.6.4.13"

/inference="ab initio prediction:Prodigal:002006"

/inference="similar to AA sequence:UniProtKB:O34750"

/codon_start=1

/transl_table=11

/product="putative ATP-dependent RNA helicase YfmL"

/db_xref="COG:COG0513"

/translation="MKLEAKELQAYWEKLGYQEPSVIQEKAYEPMKEGVDVVGISPTG

TGKTVAYTLPILAKTVVKGGLQTVIITPSQELAVQVAAVVKEWAKEVGITVQPLIGGA

SIKRQLEKLKQKPEIVVGTAGRILEISEMKKLKLHQVATVILDEADQLLQQDQLATVR

KVVAKIPNKPQLAFFSATSNELMQDLPKWFNVEPLWLDVTAEDTSAGKVLHAYIETPN

RKRTEMLKRLTQISDFRALVFINNVANLVTVAEKLSFEGVSVAVLHGEKYKTERQHAL

QQFRNGKVKLLLTTDVASRGLDIQGLPYVIQYDLPVAKESYVHRSGRTARMGQAGTVL

TLVNERDLRDFKKVIAPLELELTRLYLFGGELMNERPETITDEVTTKTTEKSKAKAKE

KNTEQLKPSTKPAAVVKKKHKKKDQKNKGARKKKIDK"

CDS complement(1956067..1957071)

/gene="iolU_2"

/locus_tag="JFBMFIFI_01859"

/EC_number="1.1.1.371"

/inference="ab initio prediction:Prodigal:002006"

/inference="similar to AA sequence:UniProtKB:O05265"

/codon_start=1

/transl_table=11

/product="scyllo-inositol 2-dehydrogenase (NADP(+)) IolU"

/db_xref="COG:COG0673"

/translation="MLNLGIIGTNWITNQFVDAAIATGEYQLVGVYSRKLEQAEKFGA

PYQATIFENDLEKFAQHPEIDVVYIASPNSLHFQQAVTLMEAKKHVIIEKPVFSNPSE

WELAVEVAEKNNVLMLEAARHIHEENFAIVKDEISKLGTIAGATFTYMKYSSRYDQVL

AGDEPNIFSLRFSGGALSDLGVYSVYAAVGWFGMPESCHYFPQKIATGVDGMGTIILR

YPTFNVTLITGKIVQSYLPSEIYSLDKTIVMDGVNAISSIEVVDWKNDQTMQLAAAPK

ENPMVDEAKAFADVINAPDTEENKEKYASWLKLSREVNQVLKALRDDAGIVFEADTEK

"

CDS complement(1957098..1957802)

/gene="sfsA"

/locus_tag="JFBMFIFI_01860"

/inference="ab initio prediction:Prodigal:002006"

/inference="similar to AA sequence:UniProtKB:P0A823"

/codon_start=1

/transl_table=11

/product="Sugar fermentation stimulation protein A"

/db_xref="COG:COG1489"

/translation="MAEYAAIIPAIFIERPNRFIALCEVDGEQKVVHVKNTGRCKELL

IPGVTVYLNYVPSKTRKTDYDLIAVRKGPLLINIDSQVPNTIAVEGLLAGQIKLPQVK

GKIIQLKREVTYLHSKFDIYFETDLNEKIFVEVKGMTLEDSGIVSFPDAPTLRGLKHV

NELSLAIENGYQGAVCFIVQMSQANYGTINRNMQPELAVAIKKAQEKGVAIVVYTCHV

TPSQITIKEEIPFKLE"

CDS complement(1957910..1958266)

/locus_tag="JFBMFIFI_01861"

/inference="ab initio prediction:Prodigal:002006"

/codon_start=1

/transl_table=11

/product="hypothetical protein"

/translation="MKKIISIVVLVLLVIGGSYYFKNYTGQDLYTKVNAPIEELKTKD

NKGNTVLDYRYKLKAIDEKGKITMIDFTGSIGRPLREGAYLKVKYNHKKGVLSWEETS

KSSIPKKALLEIESNQ"

CDS complement(1958363..1959205)

/gene="ypbG"

/locus_tag="JFBMFIFI_01862"

/inference="ab initio prediction:Prodigal:002006"

/inference="similar to AA sequence:UniProtKB:P50733"

/codon_start=1

/transl_table=11

/product="putative protein YpbG"

/db_xref="COG:COG1408"

/translation="MWTYSIILIVLILAVLAIYLYNQNHWVGLTRFQLTIPYLATSLK

NKKIVHISDLHIPRNNVSLEKIINLTRDEKPSLVVLTGDLVDVRGELPKIELAQFAKE

LVEIAPTFAVTGNHDLNSGHLQEWENILTAAGVRVLIDEAEWLQIENAGLVVMGLSEK

ENFKTLSVPLLNGIQLNEGMQEQPKLLLAHHPEFFEQYYLDKTKAPDITFTGHAHGGQ

VRVPFIGGLFAPGQGRFPKYTSGVYYHPEMKSKRMVVSRGIGNSSFPFRINNRPELVV

VTLN"

CDS complement(1959465..1960253)

/locus_tag="JFBMFIFI_01863"

/inference="ab initio prediction:Prodigal:002006"

/codon_start=1

/transl_table=11

/product="hypothetical protein"

/translation="MTKEVVQIYYLNKDPRKNPTKIIAMIGVGLVFIVFLYLIISKVW

GLSFFPQQEIKDFPIKGVQVSADEGHLDWQSIEESKLSFGYIQATEGSSYQDDQFMVN

WDRIKGTNLRHGASHYFSFDSPGATQAENFLQVYQPTSSDLPPAIIIEFYGNKQQNPP

DKTNVETELKAFISTVSEATQQQLVLCTNRDIYERYINGKFPETLIWLVDTTSSPDIK

EPIKWSFWEYTEDGTLGNKAIKQNSLDLVVYRGSEAEFKALGQK"

CDS complement(1960607..1961983)

/gene="rlmCD_2"

/locus_tag="JFBMFIFI_01864"

/EC_number="2.1.1.189"

/inference="ab initio prediction:Prodigal:002006"

/inference="similar to AA sequence:UniProtKB:O31503"

/codon_start=1

/transl_table=11

/product="23S rRNA (uracil-C(5))-methyltransferase RlmCD"

/db_xref="COG:COG2265"

/translation="MKEERIIPVKKNEKHTVKVEDLTHEGMGVAKIEGYPIFIENALP

GEQMEIKIHKTGKSFGYGKMMKRLTSSQSRVEIKDENYTRVGITPLQHMTYASQLVFK

RQQLRNVMERIAKMPEVPVFETIGMAEPWGYRNKAQIPVRKQNDKLTTGFFRKNSHDL

IAMEDFVIQDPKIDQAVVIVRDIMREFNVKPYNEAANTGNLRHIVVRRGYHTGEIMIV

LVTRTNKLFPTSKILPAITEALPEVVSIIQNVNPSRTNKILGPENIVLWGQDYFTDTL

LGNTFQISASAFYQVNPTQTEKLYQTVLEYAELTGKETVIDAYCGIGTISLSLAKEAK

EVYGIEVVEAAVENAKNNAKLNGIENVTFEAGLAEEVMVEWAEKELAVDLLVVDPPRK

GLEGSFIEAALTMKPNKIIYVSCNPATLARDLALLADGGYKVEKVQPVDLFPQTSHVE

SVTLLVKK"

CDS complement(1962199..1963230)

/gene="dagK_2"

/locus_tag="JFBMFIFI_01865"

/EC_number="2.7.1.107"

/inference="ab initio prediction:Prodigal:002006"

/inference="similar to AA sequence:UniProtKB:O31502"

/codon_start=1

/transl_table=11

/product="Diacylglycerol kinase"

/db_xref="COG:COG1597"

/translation="MRARVIYNPTSGREVLKKSLVDILEILEAEGYETSAFATTAEPN

SAKKEAERAALDGFELIVAAGGDGTINEVVNGIAHLENRPKMAIIPAGTTNDYARALH

VPRNDVVAAAKVIQKRQTIKMDIGKANETYFINIAGGGNLTELTYDVPSQLKSIFGYL

AYLVKGAEMLPRVKPIPMRIEYDEGVYEGKASMFLLGLTNSVGGFEQIAPDAHLDDGL

FSLMIIKTSNVVEILHLVALMLNGGKHIDHPKLLYVKSSKVKAEPMDGSRMMINLDGE

YGGDAPMTFTNLHQHIEMFGDLDAIPDDAVIGEEEIEAAEEAFIKEMEHLTNEDIDGD

GVISKPDKK"

CDS complement(1963267..1964697)

/gene="gatB_1"

/locus_tag="JFBMFIFI_01866"

/EC_number="6.3.5.-"

/inference="ab initio prediction:Prodigal:002006"

/inference="similar to AA sequence:UniProtKB:O30509"

/codon_start=1

/transl_table=11

/product="Aspartyl/glutamyl-tRNA(Asn/Gln) amidotransferase

subunit B"

/db_xref="COG:COG0064"

/translation="MNFETVIGLEVHVELKTDSKMFSSAPAHFGAEPNTNTNVVDWGY

PGVLPVVNQRAIDFGMRAALALNCTISQHTKFDRKNYFYPDNPKAYQISQFDQPIGHD

GWIEIEVEGVTKKIRIERVHLEEDAGKNNHGSDGYSYVDLNRQGTPLIEIVSEADMRS

PEEAYAYLEAVKQIIQFTGVSDVKMEEGSMRCDANISLRPIGQEEFGTKAELKNLNSF

NFVRRGLAHEEIRQAKVLLSGGVIQQETRRYDEVTGETILMRVKEGSSDYRYFPEPDI

PNLEIDDAWVERVRQSIPEMPKARRLRYVNELGLPEYDAMVLTQTKEMSDFFEEMLNK

KADAKQASNWLMGEVSAYLNSEKLELAETKLTPENLAGMITLIEDGTISSKIAKKVFR

ELILNGGDAKKVVEAKGLVQLSDPAQLLPMINEVLDNNAQSIEDFKNGKDRAVGFLVG

QIMKATKGQANPGVVNQLLNQELAKR"

CDS complement(1964712..1966166)

/gene="gatA"

/locus_tag="JFBMFIFI_01867"

/EC_number="6.3.5.7"

/inference="ab initio prediction:Prodigal:002006"

/inference="similar to AA sequence:UniProtKB:Q97SE6"

/codon_start=1

/transl_table=11

/product="Glutamyl-tRNA(Gln) amidotransferase subunit A"

/db_xref="COG:COG0154"

/translation="MSLLDKELIELHNLLVSKEITAEDIMRETFSRIHETEDKVGSFI

TLNEEVALQLAKNMDAKGITESNVLAGLPIGIKDNIVTKDLRTTAASKMLEDFMPIYD

ATVMNKVYEADLIPVGKLNMDEFAMGGSTETSYFKQTKNPWDLTKVPGGSSGGSAAAV

AAGQLLISLGSDTGGSIRQPAAFTGVVGMKPTYGRVSRFGLIAFASSLDQIGPFTRTV

KDNAYVLNAISGYDANDSMSSGTSVPDFTANLTGDIKGMKIGVPKEYLQEGIDEEIKK

SVLAAIDTYKELGATVEEVSLPHSKYGIAVYYIIASSEASSNLQRFDGVRYGYRSPEA

ESLEELYVMSRSEGFGMEVKRRIMLGTFSLSSGYYDAYFKKAGQVRTLIKQDFANVFA

DYDLILGPTTPTTAFNLGENMDDPLTMYMNDILTVPVNLAGVPAISIPCGFAENGMPI

GLQLIGKHFDEETIYKAAYAFEQATDFYQKKPNL"

CDS complement(1966163..1966468)

/gene="gatC_1"

/locus_tag="JFBMFIFI_01868"

/EC_number="6.3.5.-"

/inference="ab initio prediction:Prodigal:002006"

/inference="similar to AA sequence:UniProtKB:Q5XAC6"

/codon_start=1

/transl_table=11

/product="Glutamyl-tRNA(Gln) amidotransferase subunit C"

/translation="MAINETEVKHVAKLSKLAFKDSEIAHFTEQMDEIIGMVEQLEQV

DTTGVTVTTHGLEIVNAMRKDVAVPGTERSELFKNVKAEKDGLIEVPAIMDNGEAGA"

CDS complement(1966682..1967824)

/locus_tag="JFBMFIFI_01869"

/inference="ab initio prediction:Prodigal:002006"

/codon_start=1

/transl_table=11

/product="hypothetical protein"

/translation="MNKKLMVLLAMCVVALAACGNGATPNEASKSESSKGTEKTTKTQ

LSTDYYRTVMKNDKEYQTSKSRGISLSLNSGYNMKAFETGLISLSQEPFPTDSYYFRE

GQMLDSGTIKSWIARQAAEEDPNSNNGPNGLNPADNGQVEPDTRAPRYLAQILEQNYM

VKEGDDFKLAGVSIGLAMNSVDYYQKVLYGAEFQTEISRADLEAQGKAMANEIIARMR

KMEGMGNVPIMISLFEQSPRDTLAGGVYFASAVSKDGSTTVGDWTAINEKKVIFPNNE

DDSNEEKSKFADFKSSVQEFFPNLSGVTAEAYYKDDVLTKMTVNITTQFYGASEIIAF

TQFVADKTNLLPSGIPIEISINSMQGMEAFLSREPNSNEFKYHVFD"

CDS complement(1967821..1969857)

/gene="ligA"

/locus_tag="JFBMFIFI_01870"

/EC_number="6.5.1.2"

/inference="ab initio prediction:Prodigal:002006"

/inference="similar to AA sequence:UniProtKB:Q837V6"

/codon_start=1

/transl_table=11

/product="DNA ligase"

/db_xref="COG:COG0272"

/translation="MSEPISFEEAKKRAVELRGELDQYSYQYYVKDNPSISDHDYDVL

YHELVQLETEYPDLITSDSPTQRVGGQVLPGFTKVVHEIPMLSLGNAFDREDLISFDE

RIKRLLPDEDIQYICELKIDGLAISLKYENGQLVQAATRGDGTIGEDITQNIRTVKSV

PLRLQKPYSIEVRGECYMPKSSFIALNKEREENGEDVFANPRNAAAGGLRQLDPKLAA

KRNLNTFLYTIADFGEITATGQDEALNELDQLGIRTNHERHLCNSIDEVWEYVENYHE

KRVDLPYEIDGIVIKVNNFATQEALGFTVKAPRWAIAYKFPAEEAITVVREIEWTVGR

TGVVTPTAIMDPVHLAGTTVQRASLHNVDLIKERDIRLLDTVVIHKAGDIIPEVTRVI

TDKREPNSQPYLMPTHCPACQSELVHLEEEVALRCINPKCPAQITEGLSHFVSRNAMN

IDGLGNRVIVQMYKKELVHDVADLYQLTFEQLLTLEKIKEKSANNILTAIDNSRQNSM

ERLLFGLGIRHVGAKAAKLLSERFESMAGLSQATKEDIIEVDGIGEIIADSVVTYFEL

PEVAELLAELEQCQVNLTYLGKKRAELAESDSYFNGKTIVLTGKLTHFTREEAKERIE

NLGGKVTGSVSKKTDLVVAGAEAGSKLTKAEKLEVAVWNEDQLLEQLEGEEEST"

CDS complement(1969889..1972135)

/gene="pcrA"

/locus_tag="JFBMFIFI_01871"

/EC_number="3.6.4.12"

/inference="ab initio prediction:Prodigal:002006"

/inference="similar to AA sequence:UniProtKB:O34580"

/codon_start=1

/transl_table=11

/product="ATP-dependent DNA helicase PcrA"

/db_xref="COG:COG0210"

/translation="MVSRDDLISGMNPRQKEAVIATEGPLLIMAGAGSGKTRVLTHRI

AYLIEEKQVNPWNILAITFTNKAAKEMKERVNRLNVRGGNDVWVSTFHSMCVRILRRD

IDKIGYNRAFTISDPSEQQTLMKRILKERNIDPKKYDPRSILSQISNAKNELQTAEEY

REYASSIFEKIVADCYDDYQKELRRNQAVDFDDLIMLTARLFKDSPETLDFYQNKFHY

IHVDEYQDTNHAQYTLVNTLAKRFKNLCVVGDADQSIYGWRGANMENILNFEKDYPDA

KVVLLEQNYRSTKMILKAANDVIGNNSNRRDKNLWTDNADGEKITYYRGQSEHDEARY

IVSKMQEEMRTRKLNYGDFAVLYRTNAQSRVMEENLLKSNIPYKMVGGHKFYDRKEIR

DILAYLRLIANPEDNMSFERIVNVPKRGIGPGTVDKLRTAANQYGWSLLETALNVTIT

PITGKAAGELEGFAFMLKDFHKMQEYLPVTELVQEVLKRSGYLKALEIEKTLESESRI

ENIQEFLSVTQQFEKDSSEDKSLLTFLTDLALVSDLDNLEEEQTSEVTLMTLHAAKGL

EFPIVFLIGVEEGVFPLSRALMEENELEEERRLAYVGITRAEQKLYITNAYSRMLYGR

TQANAASRFIAEISEEALESGNQMSGNLPFGRSGSSMGGSSLLNRNQTQRATASTYKS

PVSQNKVESGAEKLPWTVGDKAMHKKWGVGTVVKVSGDADNLELDVAFKEQGIKRLLA

AFAPIEKA"

CDS complement(1972509..1973375)

/locus_tag="JFBMFIFI_01872"

/inference="ab initio prediction:Prodigal:002006"

/codon_start=1

/transl_table=11

/product="hypothetical protein"

/translation="MSNQKVRPVNDILFKKVFGSPKYRHILIGFIKDILDLDIEEVTI

ENPYNIDTFYQATKEKQLLQTEVDVLARLKDQSLVTIEIQLRAQAYFKERALYYAAEK

YISNYGREDVARLEKKKTGAKYSSLYPVYGINILEFTLFKEDANPLHSFTLYDNHQEL

HFVDGNNSRLFTLAFLELKKHTTKTQKHLRYWIQYFTGEKVVESAPSYIKEAYQIVDY

QNLEREERTMIDAAEKAREDLKATIIYAEREGKHLKQLEIAKKMLEKGLTLDLISEVT

GLDQKIIRSLNE"

CDS complement(1973579..1974229)

/locus_tag="JFBMFIFI_01873"

/inference="ab initio prediction:Prodigal:002006"

/codon_start=1

/transl_table=11

/product="hypothetical protein"

/translation="MERGNNLETVAYLTIKEKIITKEWPPQAHLTEVKTAKLLGISRT

PVRKAFFKLESEGYLMIEAHKGAKILEQKSDVRGYLERLEFLELLFVQQIHFIELKEI

QLDFSKLDASLNELFQLVELNDPEVYQGKERDLLKDFLGYGRNQFKTALVIDTLRNLH

LQKNHEIETLMKRTLAKKLQHYSKTLDLLRKKEYGLARKQVRIMINQLMLSAVTEE"

CDS complement(1974255..1975526)

/gene="purD"

/locus_tag="JFBMFIFI_01874"

/EC_number="6.3.4.13"

/inference="ab initio prediction:Prodigal:002006"

/inference="similar to AA sequence:UniProtKB:P12039"

/codon_start=1

/transl_table=11

/product="Phosphoribosylamine--glycine ligase"

/db_xref="COG:COG0151"

/translation="MDILVIGSGGREHVICKKISESPLVENVYCAKGNAGMTIDGIQL

VDIKEDNHDGLIEFAKEKQIAWTFVGPEVPLLNGIVDDFEAAGLKIFGPRKNAALIEG

SKNFAKEIMMTYAIPTAYYETFTEYEQALAYVTKQGTPIVIKADGLAAGKGVVVAEDL

NEATLALKDMLIEQRFGTNSQKVVIEEFLSGEEFSLLAFVKNDKVYPMVISQDHKRAY

EGDEGPNTGGMGAYTPVPQISESIVNEAIETILKPAAIGLLKEGRPFTGILYAGIIAT

ESGPKAIEFNARFGDPETQVVLNRLTSDFALIIDDLLEGREPKISWKTTGFEIGVVVA

ADGYPTSYPTDIPLPEFTQFKDVKVYTAGVKEKGGHLVSDGGRIYLVSASGNTLEEAR

EKVYSNLEQAETDQTFYRMDIGSKAINMHPF"

CDS complement(1975665..1977200)

/gene="purH"

/locus_tag="JFBMFIFI_01875"

/inference="ab initio prediction:Prodigal:002006"

/inference="similar to AA sequence:UniProtKB:P67544"

/codon_start=1

/transl_table=11

/product="Bifunctional purine biosynthesis protein PurH"

/translation="MKRALISVSDKNGIVEFAKALIENDIEIISTGGTKKVLEEAGIH

TKGIEEITGFPEMLDGRVKTLHPLIHGGLLGRRDLASHLEAMKEHNIQPIDYVCVNLY

PFKETIMKADVTTAEAIENIDIGGPSMLRSAAKNYAAVTVVVDPLDYPVVIEEIQKEG

ETTLKTREKLAAKVFRHTASYDALIAGFLTNLVGETEPEKVTLTYDRQQGLRYGENSH

QEASFYKEPLTVDFSITNAKQIHGKELSYNNIKDADAAIRIAREFSEPVAVAVKHMNP

CGVGIGATIFEAFQRAYEADSVSIFGGIIVLNREVDLVTAEKLHGIFLEIILAPSFSE

EALALLTKKKNLRLMTLDFDQAAVKGKEMTSVLGGLLIQDQDLLSPTETPKWQVVTER

QPTKEELKAMEFAWKVMKHVKSNGIVIANDQHTLGIGAGQMNRVGSVKIALEQAAGQL

DGAVLASDAFFPMSDSVEYAASQGIKAIIQPGGSIKDQESIDAANESGMTMIFTKTRH

FRH"

CDS complement(1977227..1977808)

/gene="purN"

/locus_tag="JFBMFIFI_01876"

/EC_number="2.1.2.2"

/inference="ab initio prediction:Prodigal:002006"

/inference="similar to AA sequence:UniProtKB:P99162"

/codon_start=1

/transl_table=11

/product="Phosphoribosylglycinamide formyltransferase"

/translation="MKIAVFASGNGSNFEAIANQISAENNEAELVLLFCDRKQAFVIE

RSRQLGIPSYSFSPADFTSKTEYEEAILALLQENQIELIVLAGYMRLIGTTLLMAYQQ

KILNIHPALLPDFPGLHGIRDAFEAQVSQTGVTIHLVDDGVDTGPIIAQEAVSLEVGE

TLESLERKIHQVEHRLYPIVIQQFIEELEMSKN"

CDS complement(1977811..1978854)

/gene="purM"

/locus_tag="JFBMFIFI_01877"

/EC_number="6.3.3.1"

/inference="ab initio prediction:Prodigal:002006"

/inference="similar to AA sequence:UniProtKB:Q97TA2"

/codon_start=1

/transl_table=11

/product="Phosphoribosylformylglycinamidine cyclo-ligase"

/db_xref="COG:COG0150"

/translation="MTNAYAKAGVDVSAGYETVERIKKHVKRTERLGVMGALGGFGGC
FDLSITGVKEPVLVSGTDGVGTKLMIAIAMDQHQTIGIDCVAMCVNDIIAQGAEPLYF
LDYIATGKNKPARLEQVVAGVAEGCIEAGAALIGGETAEMPGMYEEDDYDLAGFAVGV
VEKKNLLNSNDIQAGDCLIGLASSGIHSNGYSLVRKIFFQQNNFTYDSQLAEISGKSL
GSELLTPTKIYVKPLLKALQADLVHGISHITGGGFVENIPRMLPEHLAAEIQLGTWEV
LPIFKALEKYGQVPTTEMYEIFNMGIGMVVAVSAENVDEALALFHEAGERASVIGKVT
EKQEESIIFKAEV"

CDS complement(1978881..1980326)

/gene="purF"

/locus_tag="JFBMFIFI_01878"

/EC_number="2.4.2.14"

/inference="ab initio prediction:Prodigal:002006"

/inference="similar to AA sequence:UniProtKB:P99164"

/codon_start=1

/transl_table=11

/product="Amidophosphoribosyltransferase"

/translation="MLTEVRSLNEECGVFGVWGHHNAARLSYFGLHSLQHRGQEGAGI
VANQAGKLKGHRNVGLIAEVFKDEADLQRLEGHAAIGHVRYGTAGSDGINNIQPFLFK
FYDEEMALAHNGNLINAKSLRFELEREGAIFHSNSDTEVLMHLIRRSKEATFLDKLKE
SLNTVKGGFAYLILTPEALIGALDPNGFRPLSIGQMQNGAYILASETCAIDVVGAKFV
RQIMPGEIVIIDDSGYRIETYTDETTMSICSMEYIYFARPDSDIAGVNVHTARKNMGR
RLADEAPIEADIVIGVPNSSLSAASGYAEMSGIPYEMGLVKNQYIARTFIQPTQELRE
QGVRMKLSAVRGVVAGKKVVMVDDSIVRGTTSRRIVKLLKEAGAAEVHVRIASPPLRY
PCFYGIDIQTRQELIAANHSVSEIETLIGADSLAYLSQDGLIDSIGLNYDAPYTGLCM
AYFNGDFPTSLYDYEAEYLASLEEDPAITKI"

CDS complement(1980302..1982533)

/gene="purL"

/locus_tag="JFBMFIFI_01879"

/EC_number="6.3.5.3"

/inference="ab initio prediction:Prodigal:002006"

/inference="similar to AA sequence:UniProtKB:P12042"

/codon_start=1

/transl_table=11

/product="Phosphoribosylformylglycinamidine synthase

subunit PurL"

/db_xref="COG:COG0046"

/translation="MIMVHEPTPEEIKNERIYLEWGLTDEEYQLISVDILKRLPNYTE

TGLFSVMWSEHCSYKNSKPVLRKFPTSGPQVLQGPGEGAGIVDIGDGQAVVFKAESHN

HPSAVEPYEGAATGVGGIIRDIFSMGARPIAILDSLRFGELDNERTKYLVEEVVAGIA

GYGNCIGIPTVGGEIAFDSCYAGNPLVNAMCVGLIDHKDIQKGQAKGTGNSIMYVGAK

TGRDGIHGATFASEEFNDEEEAQRSAVQVGDPFMEKLLLEACLELIYDYQDILVGIQD

MGAAGLVSSSSEMASKAGSGLVLNLDDVPQRETEMTPYEMMLSESQERMLICVKKGSE

KRVEELFQRYELEAVTIGHVTDDGMYRLYHEGNLVADLPVDALAEDAPTYYKKYEEPA

RIQAFSALEEYQPKTTNPAETLKKLLQQPTIASKRSIFEKYDSMVRTNTVVGPGSDAA

VVRIRGTKKALALTTDCNARYLYLNPEKGGQIAVAEAARNIVCSGGKPLAITDCLNYG

NPDKPEIFWELWTSADGIAKACEVLDTPVISGNVSLYNEFNGQAIYPTPMIGMVGLIE

DLAHVTTQEFKQAGDHIFVVGTTKADFNGSELQKMELGRIEGKLMDFDLVVEKAHQDK

VLAAIKAGLVTSAHDLAEGGLAVALAESAFGNDLGIEVAVDMPLNWLFSETQSRYVIS

VKTEKTSAFKQLFDDAATEIGKVTNDGKFNLTAKDGNLQLETKQLRELWEDAIPCLLK

SEV"

CDS complement(1982530..1983210)

/gene="purQ"

/locus_tag="JFBMFIFI_01880"

/EC_number="6.3.5.3"

/inference="ab initio prediction:Prodigal:002006"

/inference="similar to AA sequence:UniProtKB:P12041"

/codon_start=1

/transl_table=11

/product="Phosphoribosylformylglycinamidine synthase

subunit PurQ"

/db_xref="COG:COG0047"

/translation="MKFAVIVFPGSNCDMDMLSAVTDILGEKAEYVQHYETSLAGFDG

VLLPGGFSYGDYLRCGAIARFSAIMSEVIRFANEGKPVFGTCNGFQILLEAGLLPGAL
RRNESLHFVCKTVGLKVVTNKTNFTSEYAKGEVIQIPIAHGEGNYYCDAETLEGLKAN
NQIIFTYESENPNGSLANIAGITNQAGNVLGMMPHPERAVEELLGSNDGLRFFKSIVA
NYGKVTNT"

CDS complement(1983211..1983459)
/gene="purS"
/locus_tag="JFBMFIFI_01881"
/EC_number="6.3.5.3"
/inference="ab initio prediction:Prodigal:002006"
/inference="similar to AA sequence:UniProtKB:P12049"
/codon_start=1
/transl_table=11
/product="Phosphoribosylformylglycinamidine synthase
subunit PurS"
/db_xref="COG:COG1828"
/translation="MYLVKVYVMYKQSVLDPQGEAVKGAVHRLGYSEIEDIRIGKYFE
IKVAKSETKIETTIEEICDKLLANVVMESYHYEIQEVS"

CDS complement(1983475..1984188)
/gene="purC"
/locus_tag="JFBMFIFI_01882"
/EC_number="6.3.2.6"
/inference="ab initio prediction:Prodigal:002006"
/inference="similar to AA sequence:UniProtKB:P12046"
/codon_start=1
/transl_table=11
/product="Phosphoribosylaminoimidazole-succinocarboxamide
synthase"
/db_xref="COG:COG0152"
/translation="MEKKALIYEGKAKVLFETDHEKQIWVSYLNQATALNGAKKDQIS
GKGQLNNQITSIIFEKLKKKGVESHFIQQLSATDQLIERVTMFPLEIVIRNVAAGSFS
KRLGIVEGTPLTFPILEIYFKEDKLDDPLINDDHVAVLGLAKKDQLVVIKEKAHQINL
ELLDLFQEIGIRLVDFKLEFGQRDDGTILLADEISPDTCRLWDKETNEHLDKDIYRRD
LGDLVPVYEEVLQRLKNKK"

CDS 1984448..1984828

/gene="gloA"

/locus_tag="JFBMFIFI_01883"

/EC_number="4.4.1.5"

/inference="ab initio prediction:Prodigal:002006"

/inference="similar to AA sequence:UniProtKB:P44638"

/codon_start=1

/transl_table=11

/product="Lactoylglutathione lyase"

/db_xref="COG:COG0346"

/translation="MSKKMLHTCVRVQNLEKSMDFYTKALNLKEMRRKDFPDYQFTLV

YLAFEEGGYELELTYNYDQAEPYEIGNGYSHIAIGVDDLKATHAAHKSANYEVTDLKG

LPGSEPSYYFIQDPDGYKIEVIQN"

CDS complement(1984883..1986070)

/gene="tuf_2"

/locus_tag="JFBMFIFI_01884"

/inference="ab initio prediction:Prodigal:002006"

/inference="similar to AA sequence:UniProtKB:Q5XD49"

/codon_start=1

/transl_table=11

/product="Elongation factor Tu"

/translation="MSKQHYERTKEHVNIGTIGHVDHGKTTLTAAITSVLAKKGLANP

QDYASIDAAPEERERGITINTSHVEYETEKRHYAHIDAPGHADYVKNMITGAAQMDGA

ILVVSATDGPMPQTREHILLSRQVGVNDLIVFMNKVDLVDDEELLDLVEMEIRELLSE

YNYPGDDIPVIRGSALKALEGDEKAEAAILELMDTVDSYIPTPKREMDKPFLMPIEDV

FTITGRGTVASGRIDRGVVKVGDEVELVGLFPETKKTVVTGIEMFRKILDVGEAGDNV

GILLRGIGREDIERGQVIAKPGSITPHTKFKGEVYILSKEEGGRHTPFFTNYRPQFYF

RTTDVTGVIDLPSGTEMVMPGDNVTIDVELIAPIAVEVGTKFSIREGGRTVGAGVITE

IEK"

CDS complement(1986293..1986916)

/locus_tag="JFBMFIFI_01885"

/inference="ab initio prediction:Prodigal:002006"

/codon_start=1

/transl_table=11

/product="hypothetical protein"

/translation="MKYEWRKKDKFIYIPKAVPTQITLPSFNYLTIEGKGNPNSVEFQ

EVVGALYAYSYAIRMLPKKGIIPEGYFEYTVFPLEGFWTSEEKPKPGEAVDKDTLIYQ

LMIRQPDFVSKELVASLNEEVAKKIGVELAEQVQFQQIEEGQVLQMLHVGSYDTESIT

FAEMEAYCEENNLEKIGKWHKEIYLSDPRKVIPDKQKTTLRFPIRPI"

CDS complement(1986920..1987750)

/locus_tag="JFBMFIFI_01886"

/inference="ab initio prediction:Prodigal:002006"

/codon_start=1

/transl_table=11

/product="hypothetical protein"

/translation="MFKIGDFSRLATTSIRMLRYYDKIKLLTPAIIDEATGYRYYTAA

QLSQINKIKKLKEMGFSLAIIKELVAIEQDPRQVENYLKTREKELQVELDELNQQQNL

LKSSLKLVKEDELEMDYHVSVKLIPARQVISVRRILTSYHEEGQLWKIVGKALNEYQL

KPENPSYSLAIFRDEEYKDEDVDVEIQMAISETDPLKTNVFEIKKSPSIKVASVMMNG

SYDQLPAVNEAVAKWFEVTGEKLKGPMFNIYHVSPGQDANPVNWVTEVCYPLNKKEEV

"

CDS complement(1987956..1989260)

/gene="eno"

/locus_tag="JFBMFIFI_01887"

/EC_number="4.2.1.11"

/inference="ab initio prediction:Prodigal:002006"

/inference="similar to AA sequence:UniProtKB:Q8DTS9"

/codon_start=1

/transl_table=11

/product="Enolase"

/db_xref="COG:COG0148"

/translation="MPFITDILAREVLDSRGNPTIEVEVYTESGAFGRGMVPSGASTG

EHEAVELRDGDKSRYLGKGVLKAVDNVNNIISEAILGFDVRDQMAIDKTMIELDGTPN

KGKLGANAILGVSIAVARAAADYLDVPLYQYLGGFNTKVLPTPMMNIINGGSHSDAPI

AFQEFMIVPVGAPSFKEALRMGAEVFHALKSILHDRGLETSVGDEGGFAPRFEGTEDA

VDTIVQAVEKAGYKIGDDVRLGFDCAASEFYENGVYNYAKFEGEGGAVRTSAEQVSFL

EELVNKYPFIITIEDGMDENDWDGWKLLTEALGDKVQLVGDDLFVTNTAKLSEGIEKG
IGNSILIKVNQIGTLTETFEAIEMAKEAGYTAVVSHRSGETEDATIADISVATNAGQI
KTGSLSRTDRIAKYNQLLRIEDQLGDLAVYRGLGSFYNLKNK"

CDS complement(1989385..1990914)
/gene="gpmI"
/locus_tag="JFBMFIFI_01888"
/EC_number="5.4.2.12"
/inference="ab initio prediction:Prodigal:002006"
/inference="similar to AA sequence:UniProtKB:P39773"
/codon_start=1
/transl_table=11
/product="2,3-bisphosphoglycerate-independent
phosphoglycerate mutase"
/db_xref="COG:COG0696"
/translation="MSKSPVAIIILDGFGLRNETVGNAVALANKPNFDRYWETYPHGQ
LKAAGLDVGLPVGQMGNSEVGHTNIGAGRIVYQSLTRIDKAIEEKEFQENEALNNAFT
HTKENNSDLHLFGLLSDGGVHSHINHLIALVETAKAKGVKNVYIHAFLDGRDVAPDSG
HKYVEALEKALADLHFGEIATVSGRFYAMDRDKRWERVEKAYNAIAHGTGAKFDSAKA
AVESSYADGKMDEFVLPTVIEKDGKPVATVKDNDAIIFFNFRPDRAIQLSNAFTDTEW
EHFDRGARAENVKFVTMTLYNPSVVAEVAFAPIEMKNVIGEVLSNEGLKQLRIAETEK
YPHVTFFMNGGRNEEFPGESRILINSPKVETYDLKPEMSAYEVTDALVADIEADKHDA
IILNFANPDMVGHSGMVEPTIKAIEAVDENLGRVVDALIAKGGYAIIFADHGNSETMT
TPEGNPHTAHTTVPVPVIVTKKGVSLREGGRLADVAPTMLDLLGVEKPVEMTGESLIQ
K"

CDS complement(1990915..1991670)
/gene="tpiA"
/locus_tag="JFBMFIFI_01889"
/EC_number="5.3.1.1"
/inference="ab initio prediction:Prodigal:002006"
/inference="similar to AA sequence:UniProtKB:P50918"
/codon_start=1
/transl_table=11
/product="Triosephosphate isomerase"

/db_xref="COG:COG0149"

/translation="MRKPIIAGNWKMNKTAAEALAFAEAVKTKIPANSAVDSVIGSPT

LFLQELVQAAKGTDLKISAQNCYFEESGAFTGETSPAALNDLGVDYVIIGHSERREYF

HETDEDINKKAHAIFKNGMTPIICCGETLEQREAGETNAWVSGQIEGALKGLTAEQVA

VSVIAYEPIWAIGTGKSSTSADANDTCGVVRQTVEKLYSKEVAEAVRIQYGGSVKPEN

IAEYMAQSDIDGALVGGASLEVDSFLALLEAVK"

CDS complement(1991698..1992882)

/gene="pgk"

/locus_tag="JFBMFIFI_01890"

/EC_number="2.7.2.3"

/inference="ab initio prediction:Prodigal:002006"

/inference="similar to AA sequence:UniProtKB:Q6GIL7"

/codon_start=1

/transl_table=11

/product="Phosphoglycerate kinase"

/translation="MAKKVVTDLDLKDKKVLVRADFNVPMKDGVITNDNRIVAALPTI

EYILEQNGKVIVFSHLGKVKTEEDKADKTLRPVAERLSELLGKPVTFVPETRGAELEA

AIAAMKDGEVVMFENTRFEDIDGKKESKNDPELGKYWASLGDVFVNDAFGTAHRAHAS

NVGIASNLESAAGFLMEKEIKFIGGVVDAPERPLVAILGGAKVSDKIGVIENLLKVAD

KVLVGGGMTYTFYAAQGREIGKSLVETDKIDLAKSLLDQANGKLILPVDSVCAKEFSN

DVPTETHDDVPADMMGLDVGPKTVELFTKELQGAKTVIWNGPMGVFEMSNFANGTIGV

CEAIANLEGATTIIGGGDSAAAAMQLGFADKFTHISTGGGASLEYLEGKVLPGVAAIS

EK"

CDS complement(1993013..1994020)

/gene="gap"

/locus_tag="JFBMFIFI_01891"

/EC_number="1.2.1.12"

/inference="ab initio prediction:Prodigal:002006"

/inference="similar to AA sequence:UniProtKB:P68777"

/codon_start=1

/transl_table=11

/product="Glyceraldehyde-3-phosphate dehydrogenase"

/translation="MTVKVGINGFGRIGRLAFRRIQEVEGIEVVAINDLTDSKMLAHL

LKYDTTQGRFNGDVEVHDGFFNVNGKEVKVLANRNPEELPWGDLGVEIVLECTGFFTS

KEGAEKHLKGGAKRVVISAPGGNDVPTIVYNTNHDILTGEETVISGASCTTNCLAPMA

DVLNKEFGIAEGLMTTIHAYTGDQMTLDGPHPGGDFRRARAAAENIVPNTTGAAKAIG

LVIPELNGKLDGAAQRVPVATGSLTELVTILDKKVTVDEVNAAMKAAANESYGYTADP

IVSSDIIGMTYGSLFDETQTKVMSVGDNQLVKTVAWYDNEMSYTAQLVRTLEYFAKLG

K"

CDS complement(1994062..1995099)

/gene="cggR"

/locus_tag="JFBMFIFI_01892"

/inference="ab initio prediction:Prodigal:002006"

/inference="similar to AA sequence:UniProtKB:O32253"

/codon_start=1

/transl_table=11

/product="Central glycolytic genes regulator"

/db_xref="COG:COG2390"

/translation="MQDVLSIIEKVAPDIMDTLRERYQILRNIYLLGPVGRRVLASKM

DVTERVLRTETDFLKKRNIITSSKVGMELSASGEVLYHELDKVMGQLLGMREKENQLA

TYFQIEHCIIASGDSDEQQKVMDELGRATMESLNYLLPEGDNIIAVMGGTTMAEVADH

MTTILSNKRHLVFVPARGGLGEAVSIQANSVSAQMAKKTGGENRVLYVPEQVSEETYK

PLLQEPAVYNTLKMVDKANCVLHSIGDARLMAERRGMSSDVLALIEKEAAVGEAFGYF

FNETGKVVYKIPRIGLQLKDLARVPCVLAIAGGKSKAKAIEAYMKNAPNQTWLITDEG

AANQILKGVTL"

CDS complement(1995502..1996857)

/gene="rpoN1"

/locus_tag="JFBMFIFI_01893"

/inference="ab initio prediction:Prodigal:002006"

/inference="similar to AA sequence:UniProtKB:P30332"

/codon_start=1

/transl_table=11

/product="RNA polymerase sigma-54 factor 1"

/db_xref="COG:COG1508"

/translation="MNFEQKFSQQQKQVQKMAMTQQLQQSIQMLQYNADELISFLEQK

ALENPLIEVTVERDFAEEAALYPIQKTNYQNSSGQDEQNYLNQVPDTSTSLFEFLLEQ

IHLTMRDTYLRELVLLLTENIDDNGYLKIDLVEVAKSVGAEEIQMLDALTLLQQLDPP
GVGARNLQECLMLQIERDDRAPNMAYVIMEEEFENFANRKWKEITKNFDVSLAEIQEI
SDYVQTLTPRPGAVYDGTREQYIRPDLVVRIENESIKVLSTKTGLPVVNFQKEYYQEM
SQFKDKDVTHFMKEKYNEYEWIKRSIMQRGETIVKVGTAIVERQKEFFINPEHPIKSL
TLKEISEKLGIHESTVSRSVNGKYLETSFGIFELKSFFTNSLGNQNDGTETEEDISGD
SVRKKLLVIIEEEDKGKPLSDQKIVELMKADGIDVSRRTIAKYRDILAIPSSSKRKRF
D"

CDS complement(1997179..1998333)
/locus_tag="JFBMFIFI_01894"
/inference="ab initio prediction:Prodigal:002006"
/codon_start=1
/transl_table=11
/product="hypothetical protein"
/translation="MVVNYAWPVVKGTPISSGYGPRWGTMHNGIDFAGNSGAPIYASK
DGEVVGSMPTSQSGGFGEYIVIKHNDGHLTGYAHMSQRMKNVGDKVVQGQQIGTIGST
GDSTGPHLHFSIQTGLWGPYKDPALFLNEEVTKPLDPKRNFGVQITYFKENEVLDRLK
EFRQAFPSYSSCLQDVGGGRYAIVIQPFSYLEVKGKKMEIQGKFPHWAMKVAELVEFT
GYKGLLVAPFSGKEVGEKYLDLFKRYGFSMSIVPSPYGDAGRIDYTNDLAKKAVVISP
FNTKEALEKLIEFRKAFPSYASGSFLQQVPTNAAQYSLVVQPFTVQEVNARYAEINQR
FPTWMKNINNFHNVGDPKGVLIARFKTEEIVEKYLEVQRGTGYAMKIVDA"

tRNA complement(1998768..1998839)
/locus_tag="JFBMFIFI_01895"
/product="tRNA-Arg"
/inference="COORDINATES:profile:Aragorn:001002"
/note="tRNA-Arg(ccg)"

CDS complement(1999235..1999906)
/locus_tag="JFBMFIFI_01896"
/inference="ab initio prediction:Prodigal:002006"
/codon_start=1
/transl_table=11
/product="hypothetical protein"
/translation="MALNYILPVDNGTPISSSYGPRWGTMHNGIDFAGSIGAPIYASK
SGDVVGSMPTSQSGGFGEYIIIKHNDGHFTGYAHMSQRMKYNGDKVTQGQQIGTIGST

GDSTGPHLHFSIQTGIWGPYKDPAPFLSGATNPPVGEEETNRTKGEVTMQCIYWKPSA
TNPNPSNAYYFNGVDTVYIPSLDTISILKTIYKDNNGKDMPEYKWGNNAPWHVRLEAV
APNRK"
CDS 2000587..2001183
/gene="clpP"
/locus_tag="JFBMFIFI_01897"
/EC_number="3.4.21.92"
/inference="ab initio prediction:Prodigal:002006"
/inference="similar to AA sequence:UniProtKB:Q9RQI6"
/codon_start=1
/transl_table=11
/product="ATP-dependent Clp protease proteolytic subunit"
/db_xref="COG:COG0740"
/translation="MNLVPTVIEQSSRGERAYDIYSRLLKDRIIMLSGAIDDNVANAV
IAQLLFLDAQDSEKDIYIYINSPGGSVTAGLAIYDTMNFIKADVQTIAMGIAASMGSF
LLTAGTKGKRFALPNAEIMIHQPLGGAQGQATEIEIAARHILKTRERLNKILADQTGQ
PIEVIEKDTDRDNFMSADEAKAYGLIDEIMVNSKSLPK"
CDS 2001298..2001828
/locus_tag="JFBMFIFI_01898"
/inference="ab initio prediction:Prodigal:002006"
/codon_start=1
/transl_table=11
/product="hypothetical protein"
/translation="MDISKIKASEVGTTFGIKIGEDSAPVKVIEFINLRCPYCRKWHD
LSKDVLAKYVADGKIQRIVKHFDKEKPSLQKGNVAHHHIDYSDPQKAVTTIDYLYDHQ
DDWGDLENTEVANYVEKTLGLADQPNEKEIKGIIAEAEAANIFFVPTVIIGEHIFDEH
ITTAELTELIEAELSK"
CDS complement(2001889..2002836)
/gene="whiA"
/locus_tag="JFBMFIFI_01899"
/inference="ab initio prediction:Prodigal:002006"
/inference="similar to AA sequence:UniProtKB:O06975"
/codon_start=1

/transl_table=11

/product="putative cell division protein WhiA"

/db_xref="COG:COG1481"

/translation="MSYASDVKKELTTLEVHKEHAKAELAALIRMNGAVSLLNQKFIL

NVQTENAAIARRIYVLLKDHYEVESELLVRRKMKLKKNNVYIVRLKQGTKEVLSDLSI

MDGLLFHAHVADEVLYNPQKIRSYLRGAFLAGGSVNNPETSRYHLEIYSIYEEHNNDI

CQMMNHFGLNARTLERRNGYITYLKEAEKIADFLALIGASTGMLKFEDVRIVRDMRNS

VNRLVNCENANLNKVINAAGKQIDNIMYIDEMLGLDKLPEKLREIAVARVENPDISLK

ELGEIVPSGAISKSGINHRLRKLNEIADDLRGKELVNKV"

CDS complement(2002881..2003876)

/locus_tag="JFBMFIFI_01900"

/inference="ab initio prediction:Prodigal:002006"

/inference="similar to AA sequence:UniProtKB:Q97PN8"

/codon_start=1

/transl_table=11

/product="Putative gluconeogenesis factor"

/db_xref="COG:COG0391"

/translation="MTTIKRNRKPKMVVIGGGTGLPVILKGLKAQNADITAIVTVADD

GGSSGTLRNYVNVVPPGDIRNVLLSLSDIPEMQQEIFQYRFDTEDDFLAGHAIGNLII

AAISEMRGNVFEAIQVLSQMMRVEGHVYPAAEEALILHAIFEDGTQVSGESKIALDRK

KIKRVFVTPTENNHEAKASREVIAAIMDADMVVLGPGSLFTSILPNLMISDLGKAVVE

TNAEVVHICNIMTQLGETENFSDAEHIRVLHEHLEAKFISTVLVNTEHVPEGYLDKER

YDEYLVQVSHDFEGLRNEGCKVVSADFLELRDDGVFHDGDKVVEELFRLAFGAKV"

CDS complement(2003873..2004760)

/gene="yvcJ"

/locus_tag="JFBMFIFI_01901"

/inference="ab initio prediction:Prodigal:002006"

/inference="similar to AA sequence:UniProtKB:O06973"

/codon_start=1

/transl_table=11

/product="Nucleotide-binding protein YvcJ"

/db_xref="COG:COG1660"

/translation="MVDSLQLVIITGMSGAGKTVAMQSFEDMGYFCVDNMPPSLLPKF

WELVKESGKLTKIALVIDLRSRAFFEEIMSAIGTMDNTSFITTKILFLEASDEELVSR

YKETRRTHPLAMDGRIMDGIRTERALLADIKGRAQMVIDTSDVTPRQLREKIMSSFKT

DDTQLFRVEVVSFGFKYGLPIDADVVMDVRFLPNPHYIDNLRPLTGMDKPVYDYVMQQ

PETELFYRKFIDLLEYVLPGYKKEGKNNVTIAIGCTGGQHRSVALSERIGRQLIADDY

KVNISHRDKGKRKETVNRS"

CDS complement(2004989..2006716)

/gene="pgcA"

/locus_tag="JFBMFIFI_01902"

/EC_number="5.4.2.2"

/inference="ab initio prediction:Prodigal:002006"

/inference="similar to AA sequence:UniProtKB:P18159"

/codon_start=1

/transl_table=11

/product="Phosphoglucomutase"

/translation="MTWKTTYETWGNFEDLETNLKEELVSLAENEKMLEDAFYAPLEF

GTAGMRGVLGVGVNRMNIYTVRQATEGLARFMDSLGEETKKRGVAIAFDSRHQSPEFA

MEAAKTLGAHGIPSYVFESLRPTPELSFAVRFKNAYAGIMITASHNPAEYNGYKVYGE

DGGQMPPKEADALTEYVRGIKNSLTIDVLSEEDLKKSNLLTILGEDVDAPYLELVKTV

TVNSDLVKEMSKEMKLVFTPLHGTGQMLGERALKNAGFASISVVPEQAIPDPNFPTIK

SPNPEEHSAFEYAIRLGEKENADVLVATDPDADRLGIAVKVPAGHYEVLSGNQIASLM

LHYLLTAQKEAGTLPSNGVVLKSIVSSELATAIAKSFSIKMVDVLTGFKFIAEKIKQY

EMDHTQTFLFGFEESYGYLVKPFVRDKDAIQALVLVAEVAAFYKKQGKTMYDGLQDIY

ATYGYYEEKTISVTLAGIEGSAKIKALMAKFREEAPIEFAGISVASMEDFDASQRIYS

DGKVETIDMPAANVLKYSLADGSWIAIRPSGTEPKIKFYIAAVASSAIEVDKKVAEFE

KTIQTLINA"

CDS complement(2006902..2007867)

/gene="trxB"

/locus_tag="JFBMFIFI_01903"

/EC_number="1.8.1.9"

/inference="ab initio prediction:Prodigal:002006"

/inference="similar to AA sequence:UniProtKB:P80880"

/codon_start=1

/transl_table=11

/product="Thioredoxin reductase"

/db_xref="COG:COG0492"

/translation="MEWGTKMEVIEKIYDVIIIGSGPAGMTAALYASRSNLSTLMLER

GVPGGQMINTAEIENYPGFLSILGPELSDKMFEGAKQFGAEYAYGDVKEVIDGVAYKT

IIAGNKEYRTRAIIIASGAEHRKLGVAGENEYNGRGVSYCAVCDGAFFRNKELIVIGG

GDSAVEEGVYLTQFASKVTIVHRREELRAQKILQDRAFKNEKVDFAWNSVVEEIHGDD

MKVTGAVLVDTKDGSKREISADGAFIYVGILPQTKAFLNLGITDDEGWIVTNEEMETK

LPGIFAVGDVRQKSLRQVTTAVGDGGQAGQAVFKYIEELMETLEK"

CDS complement(2007945..2008376)

/locus_tag="JFBMFIFI_01904"

/inference="ab initio prediction:Prodigal:002006"

/codon_start=1

/transl_table=11

/product="hypothetical protein"

/translation="MSDIEQNRAPIINFFPSSDYYFNLGIEAFQKNDMKRAKKYLSRA

VTLCKTEEEKIFALCQLAICHQHVGEFQESILILTDLIAESGDIFSEAYYFQANNYAF

LEDLPKALELVETYLKLDPLGDFSEEAKELLETIQSELNEF"

CDS complement(2008529..2009452)

/gene="gtaB_2"

/locus_tag="JFBMFIFI_01905"

/EC_number="2.7.7.9"

/inference="ab initio prediction:Prodigal:002006"

/inference="similar to AA sequence:UniProtKB:Q05852"

/codon_start=1

/transl_table=11

/product="UTP--glucose-1-phosphate uridylyltransferase"

/db_xref="COG:COG1210"

/translation="MKKVKKAVIPAAGLGTRFLPATKAMAKEMLPIVDKPTIQFIVEE

AINSGIEDILIVTGKSKRPIEDHFDSNPELEANLREKDKLDLLKLVEETTGLNLYFVR

QSYPKGLGHAVLQAKAFIGNEPFVVMLGDDIMEDKVPLTKQLMDRYEKTHASNIAVMK

VPHEETSKYGIIDPEGQVDKGLYNVRNFVEKPKPEDAPSDLAIIGRYLLTPEIFDILE

NQEPGAGNEIQLTDAIDTLNKTQRVFAHEFTGIRYDVGDKFGFLKTSIQYGLKHPEIK

DSLRQYIVKLGAELALADKKVVEKKATPEKK"

CDS complement(2009477..2010481)

/gene="gpsA"

/locus_tag="JFBMFIFI_01906"

/EC_number="1.1.1.94"

/inference="ab initio prediction:Prodigal:002006"

/inference="similar to AA sequence:UniProtKB:Q97NF1"

/codon_start=1

/transl_table=11

/product="Glycerol-3-phosphate dehydrogenase [NAD(P)+]"

/db_xref="COG:COG0240"

/translation="MVKKIAVLGAGSWGTALSMVLNENGHEVRLWGNHANQMQEINEK

HSNSHYLPDVLLDENIKGYTDLKAAVKNVDVVLFVIPTKAIRSVAHDLAKVLEGPVVI

AHASKGLEQESHKRISEILAEEIPVEKRKAIVVLSGPSHAEEVAVKDITTITSASSNE

DAAKLIQQLFMNHYFRVYTNDDIIGVELGAALKNIIALGAGALHGLGYGDNAKAALMT

RGLAEISRLGVAFGAEPLTFIGLSGVGDLIVTCTSIHSRNWRAGNLLGKGHNLEEVLA

NMGMIVEGVSTTKAAYELAQQKGIEMPITEAIYAVLYKKADVKLVVSNLMTRDGKSED

"

CDS complement(2010495..2011145)

/gene="ppaX"

/locus_tag="JFBMFIFI_01907"

/EC_number="3.6.1.1"

/inference="ab initio prediction:Prodigal:002006"

/inference="similar to AA sequence:UniProtKB:Q9JMQ2"

/codon_start=1

/transl_table=11

/product="Pyrophosphatase PpaX"

/db_xref="COG:COG0546"

/translation="MAIKTLLFDFDGTLADTNALISHSHLHVLEEYYPGAYNLDSVRA

FNGPSLEMVYGSLNPQEKDQMIKKYRAYNERHHDDMVKLFPKVSENLAKLQKHGIQLG

VVSTKYNEILTRGLTVLGIKDYFEVIVGGLDYTHPKPNPEPIFVAVEKLSANLNETIM

IGDNSHDIESGKNAGIPSVFVAWSEKTLEEIKPYQPDLIVHSMDELTHFVLEENQK"

CDS complement(2011161..2012000)

/gene="lgt"

/locus_tag="JFBMFIFI_01908"

/EC_number="2.5.1.145"

/inference="ab initio prediction:Prodigal:002006"

/inference="similar to AA sequence:UniProtKB:O34752"

/codon_start=1

/transl_table=11

/product="Phosphatidylglycerol--prolipoprotein

diacylglyceryl transferase"

/db_xref="COG:COG0682"

/translation="MSLQLGAIDPIAFSLGGLSVHWYGVIIASAVLLAIFLGTNESEK

RGIKGDDIIDMMLWALPISIIGARIYYVIFEWRYYIQHPAEIIAIWNGGIAIYGGLIA

GGLTVYWFTKKRGLPFWLVLDIAAPSVIIAQAIGRWGNFVNQEAHGEATTKAFLEGIH

IPDFIVNNMNIEGVYYQPTFLYESLWNVLGFILLLILRRRKNLLKRGEVALSYVLWYS

FGRFFIEGLRTDSLMLAQTIRVSQLLSILLFVGAILLWIYRRKKYPENPYYLTGAEFA

QKN"

CDS complement(2012018..2012956)

/gene="hprK"

/locus_tag="JFBMFIFI_01909"

/EC_number="2.7.11.-"

/inference="ab initio prediction:Prodigal:002006"

/inference="similar to AA sequence:UniProtKB:Q9RE09"

/codon_start=1

/transl_table=11

/product="HPr kinase/phosphorylase"

/db_xref="COG:COG1493"

/translation="MTNSVTVKELVEVLRLSVHNGEEQLDRKIVTSDVSRPGLELTGY

FNYYPQERVQLFGRTEISFSERMTSEERLIVMRRMCRPNTPAFILSRGLVPPEELIRA

TTEAGIPLLLSSLTTTRLSSNVTNYLEGKLAERISMHGVLIDVYGLGVLIMGDSGVGK

SETALELIQRGHRLVADDRVEIHQQDEQTVVGESPDILRHLIEIRGIGIIDVMNLFGA

GAVRSSKTIQLIVNLELWSKEKKFDRLGSGDESTRIFDVDVPKISIPVKTGRNLAIII

EVAAMNFRAKSMGYDATATFEKNLEILIEKNSKENN"

CDS complement(2013141..2013494)

/locus_tag="JFBMFIFI_01910"

/inference="ab initio prediction:Prodigal:002006"

/codon_start=1

/transl_table=11

/product="hypothetical protein"

/translation="MKLWQGVVINALLFLALSGLFRSTFYVESIWIALAASLVLAILN

MAIKPILVILALPITFLTLGLFSIVINAGMLSLTSAIVGSGFHFASFGTAMWVAILLS

LSNAVIGKQTTSERY"

CDS complement(2013494..2013808)

/locus_tag="JFBMFIFI_01911"

/inference="ab initio prediction:Prodigal:002006"

/codon_start=1

/transl_table=11

/product="hypothetical protein"

/translation="MKRLEKSRSNRVISGVLGGIGEYFNIDPTIVRIIFVILTVFGVG

SPFLIYIVLAIVMPEAPRKNNDWNGYQGGNPYSNHDFGNTKKEPKRKEAEKVDDNDWS

DF"

CDS complement(2013912..2015498)

/locus_tag="JFBMFIFI_01912"

/inference="ab initio prediction:Prodigal:002006"

/codon_start=1

/transl_table=11

/product="hypothetical protein"

/translation="MKERERILELVKEGVISTEEALVLLENSAHKEGKAAVKKEQANI

QKPILKKAPVAPIPEIKEEKSAEPVLSDIEDAAEEGEARLEQEQNQDRERLEAILEEL

ANEASGYSAKLDETNKVIDDLKEQINAKKEVQMVFETKEELDELSAEEEAEVNILENE

IDALEDEVAILEDEKEELEEKLKTVKKQQWGTHKKNITEKFEIPDDWKETATETLNTV

GDKVTEAGTQFGKFMKETFSSVIENVDWKDVNIRVPGLATTKFDHDFVYPASEATILD

VKVANGDVVFKNWDSEDIKIEAKIKIYGRLDVENPFEAFLERSTIEVNDEHLLFHVPN

KRIRSDLTFYLPERTYDHTAVKLLNGNLRFEEFEAKDIYAKSTNGNIVFDRLTATMLE

TEGVNGNVSVLDSQIRDLLISSINGGITARGDIKSGNLSTVNGTVKATLTGTDLKRLE

ASSVNGAVKVAIPSNLSVEGTAKTNLGSIQSRLENTEIIKEKKDRTNQFQEFRRVASE

NILVVKLSTTTGSIMLKDSE"

CDS complement(2015683..2016300)

/locus_tag="JFBMFIFI_01913"

/inference="ab initio prediction:Prodigal:002006"

/codon_start=1

/transl_table=11

/product="hypothetical protein"

/translation="MSQKERILNLVKEGLITAEEALVLLENEAKAPEENKTETFVEEP

KESEDWKETVNETVNQVTEKVTEASAHVSKFAKKTLDSVNEHLDWKDLTVKVPHLTST

TFTHQFSYSEVESKVIDLKVFNGDVIFKNGFSSDLKIDVDVKIYGKVDVEEKPLELFL

ERSTIETKDGILTFHVPSKLVRCNVVVYLPQREYEVRFNRNPITK"

CDS complement(2016597..2019446)

/gene="uvrA_5"

/locus_tag="JFBMFIFI_01914"

/inference="ab initio prediction:Prodigal:002006"

/inference="similar to AA sequence:UniProtKB:P63383"

/codon_start=1

/transl_table=11

/product="UvrABC system protein A"

/translation="MVNDKIVVHGARSHNLKNIDVTIPRDKLVVLTGLSGSGKSSLAF

DTLYAEGQRRYVESLSAYARQFLGQMDKPDVDSIDGLSPAISIDQKTTSRNPRSTVGT

VTEINDYLRLLYARVGHPICPNDGTEISSQSVEQMVDRVLELPEKTKLQILAPVVVGK

KGQHKKVFEMIKREGYVRLRVDQEMYDISEEIELDKNKKHNIEIIIDRIVIKEGIRSR

LFDSFEAALRLAEGYAIADIIDGEEMLFSEHYACPYCGFTVGELEPRLFSFNAPFGAC

PDCDGLGMKLEVDLDLVVPDPNLTLREGAIAPWNPISSNYYPQMLEQACQYFKIDMDK

PYKKLTDHQKDFILNGSQGKKFHFHYENDFGGVRDVDIVFEGVVRNLSRRHDETNSDF

TRDQMRTYMTELPCQTCHGLRLNRQARSVKINGTDIGEVCELAIGDAVSFFDKITLSD

QEKLIAKPILKEVHSRLNFLENVGLEYLTLSRTAGTLSGGEAQRIRLATQIGSNLSGV

LYILDEPSIGLHQRDNDRLIGSLKKMRDLGNTLIVVEHDEDTMREADYLIDIGPGAGE

KGGEIIASGTPEEVANNPKSLTGDYLSGKKFIPVPEKRRKENLGKIRITGATENNLKN

VTVDFPLGRFVAVTGVSGSGKSTLVNSILKRALAKELNRNKHKPGKFKKMTGFEKLEK

VINIDQSPIGRTPRSNPATYTSVFDDMRDLFAQTNEAKIRGYKKGRFSFNVKGGRCEA

CRGDGILKIEMHFLPDVYVPCEVCHGTRYNSETLEVHYKGKNISEILNMTVEEAVEFF

EHIPKIHRKLATIVDVGLGYVTLGQPATTLSGGEAQRMKLASELHKKSNGDNFYILDE

PTTGLHTDDIARLLVVLNRLVETGNTVLVIEHNLDVIKTADYLIDLGPEGGAGGGMIV

AKGTPEDVAKVAESYTGRYLAPILKRDKDRTTKLS"

CDS complement(2019580..2020221)

/gene="yfbR"

/locus_tag="JFBMFIFI_01915"

/EC_number="3.1.3.89"

/inference="ab initio prediction:Prodigal:002006"

/inference="similar to AA sequence:UniProtKB:P76491"

/codon_start=1

/transl_table=11

/product="5'-deoxynucleotidase YfbR"

/db_xref="COG:COG1896"

/translation="MGMHQYIKSLSDLENIIRCPGKFKYEDHSVASHSFKVTQIAQFL

GTVEEQAGKEVNWRSLYEKALNHDYTELFIGDIKTPVKYATPALREMLADVEESMMEN

FVQREIPEEFQAVYLNRLKEGKDGSLEGQILSVADKVDLLYESFGEIQKGNPENVFTE

IYEESLKTILEFKHLKSVQYFLEAILPDLLSGDFTNQAQLQKISQKILTDTKI"

CDS complement(2020282..2021157)

/locus_tag="JFBMFIFI_01916"

/inference="ab initio prediction:Prodigal:002006"

/codon_start=1

/transl_table=11

/product="hypothetical protein"

/translation="MQNNLSLPNEEIIYRGDIYLGKEGAKFWQRPVVVLQNQTLNNLL

NTVVVLPLSNLIPRKILATQFILTPKETGLTTVQLLDLAKIVSIPKADLDQRVGTLSP

EILKQINTGLSFLTGIAEESEFERQIEQTFRYLYLKEVPYFEDLDHEFKDVTRQTPKN

AIRNLSSYATAFLNSRGGSIYYGIDDDGRVTGFKAQRSELDELKKDMYSILETIEPKI

SGDNYLIRFHEVYLNEEELFPDMYVLEVVVPNRSRLNEVFFDHGSDVYLKLDGIRKKL

KGSEIVDFILRKKMP"

CDS complement(2021298..2021966)

/gene="phoU"

/locus_tag="JFBMFIFI_01917"

/inference="ab initio prediction:Prodigal:002006"

/inference="similar to AA sequence:UniProtKB:P0A3Y7"

/codon_start=1

/transl_table=11

/product="Phosphate-specific transport system accessory

protein PhoU"

/db_xref="COG:COG0704"

/translation="MRRVFEEELNDLHLRFSEMGMMVNEAIYKSVKAFINHDKELASE

VIKSDQAINARELDLEKRSFEMIALQQPVTTDLRIIVTVMKASSDLERMADHAVSIAK

STIRVKGNKRIPEIEAEIAEMADKVKVMVEDVLEAYVKNDAKRAKKIAAMDSEVDIYF

KKIYKNCIEEMKSNSEIVVGASDYMLVAGYLERIGDYVTNICEWIVYLSTGKVSELNS

TNKK"

CDS complement(2022005..2022769)

/gene="pstB3_1"

/locus_tag="JFBMFIFI_01918"

/EC_number="7.3.2.1"

/inference="ab initio prediction:Prodigal:002006"

/inference="similar to AA sequence:UniProtKB:P0A2V9"

/codon_start=1

/transl_table=11

/product="Phosphate import ATP-binding protein PstB 3"

/db_xref="COG:COG1117"

/translation="MDQKEYVIESSDVHLYYGKNEALKGVSLNFEPKGITALIGPSGC

GKSTYLRTLNRMNDLIPDVTITGNVLLKGENIYGPKMDTVKLRKQVGMVFQQPNPFPF

SIYDNVTYGLRIAGMNDKEKLDQIVEGSLRKAAVWDDVKDKLNKSALSLSGGQQQRVC

IARVLAIEPEVILLDEPTSALDPVSSGKIENMLLELKEYYTMIMVTHNMQQASRISDR

TAFFLQGNLIEAGDTRQIFVNPKEKETDDYISGRFG"

CDS complement(2022781..2023587)

/gene="pstB3_2"

/locus_tag="JFBMFIFI_01919"

/EC_number="7.3.2.1"

/inference="ab initio prediction:Prodigal:002006"

/inference="similar to AA sequence:UniProtKB:P0A2V9"

/codon_start=1

/transl_table=11

/product="Phosphate import ATP-binding protein PstB 3"

/db_xref="COG:COG1117"

/translation="MTDNEQVESHIYRMNQGQNVALSTDDIHVWYGQNEAIKGVSLEF

EKNKIASLIGPSGCGKSTYLRSLNRMNDEIANTNVTGKIMYQGIDVNADNVDVYEMRK

NIGMVFQRPNPFSKSIYENITFALKRHGIKDKHELDEIVETSLKQAALWDQAKDILHK

SALALSGGQQQRLCIARAIAMKPDILLLDEPASALDPISTSKVEDTLLALKEHYTIII

VTHNMQQAARISDYTAFFYMGNVIEYDETRKIFTRPKIQSTEDYVSGHFG"

CDS complement(2023841..2024725)

/gene="pstA"

/locus_tag="JFBMFIFI_01920"

/inference="ab initio prediction:Prodigal:002006"

/inference="similar to AA sequence:UniProtKB:P07654"

/codon_start=1

/transl_table=11

/product="Phosphate transport system permease protein

PstA"

/db_xref="COG:COG0581"

/translation="MNAKKADKLAVGILYGISGIIVLILFSLLAFILWRGVPHLSWEF

LTSPAKTFTKGGGIGIQLFNSFYLLILTMLISVPISLGAGIYLSEYAKKNWITDIIRT

AIEVLSSLPSIVVGLFGFLIFVVQMGFGFSILSGALALTLFNLPLLTRTVEDSLRAIS

PTQREAGLALGLSRWETVTKIITPAALPGILTGMILAAGRVFGEAAALIYTAGQSAPA

LDFSNWNPLYISSPLNPMRPAETLAVHIWKVNSEGIMPDAAAVSAGASAVLIISVLLF

NFLARFLGKWIHKKATSA"

CDS complement(2024727..2025647)

/locus_tag="JFBMFIFI_01921"

/inference="ab initio prediction:Prodigal:002006"

/codon_start=1

/transl_table=11

/product="hypothetical protein"

/translation="MEEIQERLLKKSKKAKLESIGKFISFICIAFIVVVVASILYFVA

SKGIATFTTNKVSVWAFLTGTVWNPSSTGADGQPLVGALPMITGSFIVTLLSAIVATP

FALGAAIFMTEISPKTGKRVLQPVIELLVGIPSVVYGFIGLTVIVPFVRNYFGGSGFG

ILAGTFVLFVMILPTVTSMTVDALNSVPRYYREASLALGATRWQTIYKVVLRAAIPGI

LTSVVFGMARAFGEALAIQMVIGNAALMPSGLVSPASTLTSILTMGMGNTVMGSLENN

VLWSLALILLLMSLFFNIAIRLIGKKGAMK"

CDS complement(2025772..2026674)

/gene="pstS1_1"

/locus_tag="JFBMFIFI_01922"

/inference="ab initio prediction:Prodigal:002006"

/inference="similar to AA sequence:UniProtKB:Q8DPB1"

/codon_start=1

/transl_table=11

/product="Phosphate-binding protein PstS 1"

/db_xref="COG:COG0226"

/translation="MKVTNLTKVILTIGLGFTLAACGNNDNATDKNASSDKGSEVATN

KTITAVGSTALQPLVEAAAEEYKNANPESSITVQGGGSGTGLSQVATGAVDIGNSDVF

AEEKEGVDAKAIVDHKVAVVGMGPVVNKEVGVKGITKKQLIDIFTGKVTNWKEVGGAD

QSIVVLNRASGSGTRASFEKWGLDGASAIQSQEQDSSGAVRKIVSETPGAISYLAFSY

FDDSILTLSLDDVEPKEKNVADNSWPIWSYEHMYTKGEPTKPVKAFLDYMLSEEVQEG

IVKQLGYLPISSMKVERDHNGVIK"

CDS complement(2026836..2027693)

/gene="pstS1_2"

/locus_tag="JFBMFIFI_01923"

/inference="ab initio prediction:Prodigal:002006"

/inference="similar to AA sequence:UniProtKB:Q8DPB1"

/codon_start=1

/transl_table=11

/product="Phosphate-binding protein PstS 1"

/db_xref="COG:COG0226"

/translation="MKIKKALLLSMLAIPLLLTACGGVDRGESITAVGSSALQPLVEA

AGEEFSAQHLGKFINVQGGGSGTGLSQVAAGAVDIGNSDVFAEEKEGIDANALVDHRV

AVVGITPIVNKDVGVTGITKEELQKIFTGKITNWKEVGGKDLKIIILNRASGSGTRST

FERWALDGQESKQAQEQDSTGMVRQIVGDTPGAISYVAFSYVNDTVKSLKIDGVKPID

ANVKTNKWPIWSYEHMYTKGEPTGLTKQFIEFILSDEIQKTIVGQLGYIPSNQMQVER

SVDGVVTPI"

CDS 2028253..2028372

/locus_tag="JFBMFIFI_01924"

/inference="ab initio prediction:Prodigal:002006"

/codon_start=1

/transl_table=11

/product="hypothetical protein"

/translation="MENNKSFIDELIESVVKKAPLYAKATKKNSSNSTKISAK"

CDS complement(2028549..2030318)

/gene="phoR"

/locus_tag="JFBMFIFI_01925"

/EC_number="2.7.13.3"

/inference="ab initio prediction:Prodigal:002006"

/inference="similar to AA sequence:UniProtKB:P23545"

/codon_start=1

/transl_table=11

/product="Alkaline phosphatase synthesis sensor protein

PhoR"

/db_xref="COG:COG0642"

/translation="MKKLQLRIILIFVLIFSILSVCIGTFSSKLLRDHAFSSQKEQLE

ENATLISSLLPLKSLESTQVQDLIAPLSELQQPNNQRVTLVSLDGTVIYDSKVIKDNL

GNHGNRIEIKKVLEGAKIGTAERKSDSTNETLYYVGVPLNNQDGQLIGVLRLSKPINK

MANVDQQIRLSLIMSIVFSLILGILLTSLTTKQIAKPIEEVMEVAEDLSNKLYHNRYH

GKGYGEIAELGEVMNHLAESLEGQMYEIQQNEERINGLINHLVIGVMLLDEQRHIQIV

NPAMSVMLGQDNSYLIGKSYVEVTKSYGLTHMIEKAYQKGIPQNEEIYFYYPKDRILD

ANIVPITGKHKGELNLIVLLYDITEIKKLEKIKTDFVTNASHELRTPVTALKGFSETL

LDGAMDDKVILKQFLEIMYAESSRLDLLVNDILELSKLEQKQVPLEQELIEVQEAVRS

SFRLVKHKADEKNMNLLLNDADPIYLLGDSGRLKQIITNLLTNAVSYTEAGGKVEVFV

EQSETEATIKISDNGMGIPEAELDRIFERFYRVDKARSRNSGGTGLGLSIVKYLVENF

NGTIQVESKLGLGTTFTIILPLK"

CDS complement(2030315..2031034)

/gene="phoP"

/locus_tag="JFBMFIFI_01926"

/inference="ab initio prediction:Prodigal:002006"

/inference="similar to AA sequence:UniProtKB:P13792"

/codon_start=1

/transl_table=11

/product="Alkaline phosphatase synthesis transcriptional regulatory protein PhoP"

/db_xref="COG:COG0745"

/translation="MKKVLIVDDEESILTLLAFNLEKAGYEVQTAMDGLIGYQLALEN
QYDFIILDLMMPSMDGMEVCKKLRQEKIETPIMILTAKDDELEKIIGLELGADDYMTK
PFSPREVLARMKAIMRRIKPESKEKITESYEASPEEEVVIGELQIFPELYEVKVRGEL
IELTPKEFELLLYMMKRVNRILSREQLLNAIWNFDYAGETRIVDVHISHLREKIEVDT
KNPAYIRTVRGFGYKFEAPKV"

CDS complement(2031072..2032265)

/gene="minJ"

/locus_tag="JFBMFIFI_01927"

/inference="ab initio prediction:Prodigal:002006"

/inference="similar to AA sequence:UniProtKB:O34375"

/codon_start=1

/transl_table=11

/product="Cell division topological determinant MinJ"

/db_xref="COG:COG0265"

/translation="MNYLDSVAGIIFKAIILFFAQPLFWLGALVALLSSILRIKRERK
IYRVAIHKGFNEVRNYLISGLLPGLIISAIVILLGITINIEWLWVYPIVVAFCFILLG
YRFIHPMFTIPITIGILFITSYLIKSDSGFSVGMLDFNMDFINTGLVQNSLILTILLI
LATYFLFKQALKYLSPNFRKTSRGKWIASYLVKPLTFLPLFLLVPGQQFTELFSWWPV
FSIGNETYTLVAIPFLIGLRIKIETQPLKVAIKGIQKDIVLVSIVGIVGTVLSYYVYY
AAEVTLLLIFIGGVATLIRHNIREKKWQFLYGPVGDGIKIVGIRPDTPATKMQLEIGE
TIVVCNNQAVKNDDEFYQALLINSAYCHLKIRGLDGEYRLTETAIFADSPHELGIVTI
PDKIN"

CDS complement(2032549..2033712)

/gene="cwlO_2"

/locus_tag="JFBMFIFI_01928"

/EC_number="3.4.-.-"

/inference="ab initio prediction:Prodigal:002006"

/inference="similar to AA sequence:UniProtKB:P40767"

/codon_start=1

/transl_table=11

/product="Peptidoglycan DL-endopeptidase CwlO"

/db_xref="COG:COG0791"

/translation="MNKKMLTLVVVGTVGLSSLALPFSAAAAVDDDINRTSQKISEIS

GKKDAAQTEIGTITDTIAKNEENSVKLVAEMKETQATLKTLTTEVTALNEKIAQREDK

LKEQARTIQVNGDTQNYIDFVLSAKSFGDVVGRVDVVSQMVSANQDLVKEQKSDKEEV

ASKQKETETKSQEQALLAAKLEATKADLEQQKLEKEAIVATLASEQSGAESEKASFLA

KKEDAEKAAKAIATANAAPVVAVQTSTTAPAATPVAENNAPATPAAPPVNNSGGVLGA

AYSVIGTPYLYGGTTTAGFDCSGFTQFAFAAIGVSLPRVASAQYSATTRISQAEAQPG

DLVFFNQTGSIDHVGIYLGGGQFIGSQSSSGVAVASISPYYWAQYLVGFGRVN"

CDS complement(2033764..2034651)

/gene="ftsX"

/locus_tag="JFBMFIFI_01929"

/inference="ab initio prediction:Prodigal:002006"

/inference="similar to AA sequence:UniProtKB:Q81X30"

/codon_start=1

/transl_table=11

/product="Cell division protein FtsX"

/db_xref="COG:COG2177"

/translation="MKLRTFKRHLVESLKSLKRNGWMSVAAISAVTVTLLLVGSFISI

LLNINKLATDVENDVSVRVYIDLAATKDQKTQLKEELQKLSNVDSIDYSSRESELEKV

VGSYGSEFTLFGGDDNPLYDVYVVSTESPQDTEKVAKAAEKLTYVAKVNYGGSDAKKL

FKFVSTIRNVGSVIIVALLLTAIFLISNTIRITILSRSTEIEIMKLVGATNGFIRWPF

LLEGAWIGFFGAVLPITILSFIYVAIYNFSTGALQGTYFALLTPNPFLIQIGGLLLGI

GIIIGAFGSLISMRRFLKV"

CDS complement(2034641..2035327)

/gene="ftsE"

/locus_tag="JFBMFIFI_01930"

/inference="ab initio prediction:Prodigal:002006"

/inference="similar to AA sequence:UniProtKB:O34814"

/codon_start=1

/transl_table=11

/product="Cell division ATP-binding protein FtsE"

/db_xref="COG:COG2884"

/translation="MIEMLNVYKKYPNGITAANGLTVRIEQGEFVYVVGPSGAGKSTF

IKMMYREENATKGSIKVGDFDLVTMKDRDVPFLRRHVGVVFQDFKLLPRLTVYENIAY

AMEVVEKNPKTIKKRVLEVLDLVGLKHKVRMFPNELSGGEQQRIAIARAIANMPRVLI

ADEPTGNLDPDTSWEIMNILEEINNQGTTVVMATHNSQIVNVVKHRVLAVENGRIVRD

QLEGDYGYEA"

CDS complement(2035687..2036748)

/gene="prfB"

/locus_tag="JFBMFIFI_01931"

/inference="ab initio prediction:Prodigal:002006"

/inference="similar to AA sequence:UniProtKB:Q7A6R4"

/codon_start=1

/transl_table=11

/product="Peptide chain release factor 2"

/translation="MLVSGGLFDLESMERDIAEFDELMGEPGFWDDGIKAQSVINEAN

IIKEKYNQFQQLAETKEELDLLLEMVKEEEDAELEKELEENLTTFLAVLDQFELDMLL

SEPYDKNNAIIELHPGAGGTESQDWGSMLLRMYTRWAEKKGFKVEYLDYQDGDEAGIK

SVTLLIKGHNAYGYLKAEKGVHRLVRISPFDSAGRRHTSFVSIDVIPEMEDNVDIQIN

TDDLRIDTYRASGAGGQHINKTDSAVRITHLPTGVVVASQAQRSQMKNKDQAMGMLKA

KLYQLELEEKEKQMAEIRGEQKEIGWGSQIRSYVFHPYSMIKDHRTNYETGNVQGVMD

GDLDPFIDEYLKSKIVTKS"

CDS complement(2036890..2039409)

/gene="secA"

/locus_tag="JFBMFIFI_01932"

/inference="ab initio prediction:Prodigal:002006"

/inference="similar to AA sequence:UniProtKB:P28366"

/codon_start=1

/transl_table=11

/product="Protein translocase subunit SecA"

/db_xref="COG:COG0653"

/translation="MANFLKQLIENDKKEIKSLEKVADKIDAYGDRMAALSDEELQAK

TPEFKKRYQAGETLDDLLPEAFAVVREAAKRVLGLYPYRVQLMGGMTLHKGNIPEMKT

GEGKTLTATMPVYLNALAGEGVHVVTVNEYLASRDATEMGELYTFLGLTVGLNLNSKS

SEEKREAYNADITYSTNNELGFDYLRDNMVVYREQMVQRPLNYAIVDEVDSILIDEAR
TPLIISGQAEKSTALYTRADFFVKSLTAEADYTIDIQSKTIALTEEGMVKAEKTFKVE
NLYDIDNTALIHHIDQALRANYIMLRDIDYVVQEGEVLIVDQFTGRIMDGRRYSDGLH
QAIEAKEGVEIENESKTMANVTFQNFFRMYKKLSGMTGTAKTEQEEFREIYNIQVVEI
PTNKPIIRDDRPDLLYPTLESKFNAVVEDIKTRHANGQPILVGTVAVETSELLSDLLT
KAKIHHEVLNAKNHFKEAEIIMSAGQKGAVTIATNMAGRGTDIKLGAGVIEAGGLCVI
GTERHESRRIDNQLRGRAGRQGDPGVTQFYLSLEDELMKRFGSERIQAILERLRVQEE
DAVIQSKMISRQVESAQKRVEGNNYDTRKNVLEYDDVMREQREIMYGQRLEVIMATES
LKKITMAMIQRTVNRMVSVNTQGNKEEWNLQGIHDFATSAIVHEDSLTVQDLENKTPE
EIEALLMKRVEDIYTTKEQQFNEQMLEFEKVVILRVVDSKWTDHIDTMDQLRQGIGLR
AYAQTNPLVEYQAEGFKLFEEMIAAIEYDVTRLLMKSEIRQNLQREQVAQGSPARSTG
DGDVVEAAKHKPVKNDDKIGRNDPCPCGSGKKYKNCHGKDK"

CDS complement(2039791..2040330)

/gene="hpf"
/locus_tag="JFBMFIFI_01933"
/inference="ab initio prediction:Prodigal:002006"
/inference="similar to AA sequence:UniProtKB:A0A0H3GEZ8"
/codon_start=1
/transl_table=11
/product="Ribosome hibernation promotion factor"
/translation="MFKYNVRGENIEVTAAIREYVEKKVGKLERYFSDVPDATAHVNL
KVYSDKTAKVEVTVPLPYLVLRAEETSPDLYASVDLVVDKLERQMRKYKTKINRKSRE
KGFDFVVPNAAELDVATGELDIVRTKRVSLKPMDSEEAVLQMDMLGHNFFIFEDAETN
GISIVYRRTDGKYGLIETN"

CDS complement(2040473..2041171)

/locus_tag="JFBMFIFI_01934"
/inference="ab initio prediction:Prodigal:002006"
/codon_start=1
/transl_table=11
/product="hypothetical protein"
/translation="MKKCLMCQEGLNELITLRKILFFIKLQQESICKDCRDKFNPLTN
QIVCQGCGRISFEKGYCNDCINWKKIEPTATMNHQSLFEYNDWMKEWIEGFKFKGNYQ
LGCVFSEELKKFIYLKKQQGFLVAPIPSSQHSYANRGFNQVEALLDFAKVTYQSILLN

ESQEMKQSSKNRQERLLLKQPFSIKKEWQGKLEGKSILLVDDVYTTGRTILYAKKLIE
DSGASLVRSVSLAR"

CDS complement(2041168..2042505)
/gene="comFA"
/locus_tag="JFBMFIFI_01935"
/EC_number="3.6.4.12"
/inference="ab initio prediction:Prodigal:002006"
/inference="similar to AA sequence:UniProtKB:P39145"
/codon_start=1
/transl_table=11
/product="ComF operon protein 1"
/db_xref="COG:COG4098"
/translation="MKQLMGRELLANEFNSNTNEMNQQIEVRPGFLIKGSQISCQRCG
CKSNKLRIEAPCYCQNKCFYCLNCLQMGKIRRCSLLYSLVELNQFDSLSSPIMTWQGI
LSKQQEKASKEITESVIKKETRLIWAVTGAGKTEMIFAGIEQALKNGERVCIASPRVD
VCLELAPRVLSAFPNVPIALLHGTSQERDTYKQLIIATTHQLMRFKEAFDVLIIDEID
AFPFTADKTLAFAAEKDRKKRSCLIYLSATPSKKMQKELQNKELIASILPARYHGHPL
PEPKCIFLGEKMEGKKANKRLLNILHHFVNQKRRFLLFSPNIKKMQELEKIVQAEFFT
KTIASVHSGDPERQEKVLAMRKGELDFLLCTTILERGVTFTDIDVLVLGAEDRTFTEA
ALVQISGRAGRHRDYPTGQVVFLYHSLTRDMKRAIKQIKKMNQLARQKGFILS"

CDS complement(2042592..2043458)
/gene="degV"
/locus_tag="JFBMFIFI_01936"
/inference="ab initio prediction:Prodigal:002006"
/inference="similar to AA sequence:UniProtKB:P32436"
/codon_start=1
/transl_table=11
/product="Protein DegV"
/db_xref="COG:COG1307"
/translation="MKIAVVTDSTAYLTHDQYQKNNIFMLPLSVIIGEDVFREEVDIT
SAEFYEKVRGMESLPTSSQPTTGEIVSLLERLSKEYDAVISIHLSSKISGTYQNVSVA
ASMVESIKVYPYDSGISCMAQGYFALEAARMAKHGATPEEILGTFDEMQTTLRAYFMV
DDLNHLVRGGRLSNGAAILGSMLKIKPILHFSDKEIVVFEKIRSSKKALKRIEGMLEE

DTHKNYPIVATIIHANAEEEALKWKKQMEKKMPEVRFELSYFGPVIGTHLGEGSLGMA

WVEDRAKSHPTA"

CDS 2043769..2044419

/gene="yigZ"

/locus_tag="JFBMFIFI_01937"

/inference="ab initio prediction:Prodigal:002006"

/inference="similar to AA sequence:UniProtKB:P27862"

/codon_start=1

/transl_table=11

/product="IMPACT family member YigZ"

/db_xref="COG:COG1739"

/translation="MLLLKQYYTIKSSGSHEIEIKKSRFICHLKEVDTEADAQQFIQK

IKKEHWKANHNCAAYLIGDKDQIQRALDDGEPVGTAGIPMLEVLKKRQLKNVVAVVTR

YFGGTKLGAGGLIRAYGKSVSSALDAIGIVERSLQQEINVSISYTAVGKLENFLSHSP

YSIKETLYTENVIFVCFVLEDQVDTFKEEITNLLNGQVSFENGPKNYQETVVLTES"

CDS 2044520..2045638

/gene="tagU_2"

/locus_tag="JFBMFIFI_01938"

/EC_number="2.7.8.-"

/inference="ab initio prediction:Prodigal:002006"

/inference="protein motif:HAMAP:MF_01140"

/codon_start=1

/transl_table=11

/product="Polyisoprenyl-teichoic acid--peptidoglycan

teichoic acid transferase TagU"

/translation="MKKNKRLTTSHKVTKKNNRNKIILMILIPIMILVLAGVTYGAKL

YAEAKKTVDDSYYELDRDKTSTSKGNEIKVNPIEDTISVLVMGIDDDSARQLGSARTD

ALIYLTINPKEHKINMVSIPRDSYTTIVSKKYNGKDKINSAYTYGEEQATIETVEKLL

NVPINYYVTFNFDSFLEIVDALDGIDVDVPVSFTDTNTLGNGEVHLEKGKQLLNGEEA

LALARTRHIDNDIKRGERQQLILQAIVDKAMNVGSINKYSDVIKAAGKNMRTNLKFNE

MLSIAQTGLDGRYTFNSYVFDWTDFELEDASMVELYQDSVDFISHRFRVSLGLDEPNE

QDAADYQFVTNGYSQYKSTNSWSNSNDNTENSGSNNNW"

CDS complement(2045686..2046777)

/gene="tagO"

/locus_tag="JFBMFIFI_01939"

/EC_number="2.7.8.33"

/inference="ab initio prediction:Prodigal:002006"

/inference="similar to AA sequence:UniProtKB:O34753"

/codon_start=1

/transl_table=11

/product="putative undecaprenyl-phosphate

N-acetylglucosaminyl 1-phosphate transferase"

/db_xref="COG:COG0472"

/translation="MYNMFHIVVMLVGTMILSLILTPLVRKLAFKIGATDKPDARRVN

KKEMPTIGGLAVYLAFFIAIFFMLPIPFEQAFPLFLGATVIIITGLIDDIKELSPKMK

LLGIIIAALIIYFMADIKMDMFTIPFFGTFKLGWLSFPVTLIWILAITNAVNLIDGLD

GLATGVSIIALTTMGIIGYFFLTVASVNIPIMIFALVAALIGFLPYNFFPAKIFLGDT

GALFLGFMISVMSLQGLKNVTFISVIIPVVILGVPITDTIYAMLRRKLNNKPISSADK

MHLHHRLMALGLTHRQTVLAIYSLAVIFSFTALLYKVSTLWGSVFLTVGLLFGLELFV

ELIGLVGENHQPLLSRFKKFAKKNNQEEK"

CDS 2046916..2048289

/gene="tagU_3"

/locus_tag="JFBMFIFI_01940"

/EC_number="2.7.8.-"

/inference="ab initio prediction:Prodigal:002006"

/inference="protein motif:HAMAP:MF_01140"

/codon_start=1

/transl_table=11

/product="Polyisoprenyl-teichoic acid--peptidoglycan

teichoic acid transferase TagU"

/translation="MRSDKHSSNKKNTEKEISPDIKSRSSRINNKKRLTKKNKTILLT

ILFTLIIALISYATYFALAANRAVENIQGEDLSSTNANPRSKPVALKNKEPFSVLLLG

VDERPEDAGRSDSILVATVNPIEDSVKLVSIPRDTLVTIPGYGKDKVNAAFAYGGINL

AVETVENYLNIPINFYTKINMEGMVDLVDAVGGINVDNKYAFELDGVELDVGNFDLNG

NQALQYARMRKQDPAGDFGRQERQKEVISKIVKNALSINSLTNFNKIFNAVGKNVETN

FTGSELWELAKNYASTANNITNLTLEGPDGFLYYIPSYGQDVYVWQPSSESLQEVSNQ

LRKHLGLTAESIPTTDASTDKKTIDSTTENNSVDTYSYTEEPYVPPVQETPRWIPEEQ
QPAAPPVENPPITETPTPEVPSTPEPEIPETPDPPVDENSSSGSAGTGGIQPPGSTNE
GNAPSNP"
CDS complement(2048342..2049055)
/gene="nagR_2"
/locus_tag="JFBMFIFI_01941"
/inference="ab initio prediction:Prodigal:002006"
/inference="similar to AA sequence:UniProtKB:O34817"
/codon_start=1
/transl_table=11
/product="HTH-type transcriptional repressor NagR"
/db_xref="COG:COG2188"
/translation="MGANTPVYIQIHNQIRKEIEAGKWEIGAKIPSERELATQFNVSR
MTLRQAVQTLVDEGILERKIGSGTFVARKKVQEKMSGIESFTDIMLSQGRKPTSKTIS
YHVKPASTSESEKLQLDEEALVLRMERIRYADGIPICFEVATIPYHLVESLSKVEVTR
SLYKTLEQEKGFKIGQAEQTISAMLASERISDYLAIKRNEAVLRLKQISYFSDGQPFE
YVRTQYVGSRFEFYLEKNN"
CDS complement(2049067..2049771)
/gene="nagB"
/locus_tag="JFBMFIFI_01942"
/EC_number="3.5.99.6"
/inference="ab initio prediction:Prodigal:002006"
/inference="similar to AA sequence:UniProtKB:Q8DV70"
/codon_start=1
/transl_table=11
/product="Glucosamine-6-phosphate deaminase"
/db_xref="COG:COG0363"
/translation="MKIIVVKNQEEGGKKAFELIKSGMEKGAKVLGLATGSTPVTLYQ
EMVSSELDFSDMTSINLDEYVGLSGDDSQSYRYFMNEKLFNKKPFKETFVPNGLADAD
VECARYDKIIAEHPVDIQILGIGGNAHIGFNEPGTPFDVTTHKVALTEETIEANKRFF
DKAEDVPRFAYSMGIKSIMEAKEIILMAYGESKAEAIKATVDGEITEDVPSSILQKHD
NVVLIVDEEAAKLIQK"
CDS complement(2049844..2050977)

/gene="nagA_1"

/locus_tag="JFBMFIFI_01943"

/EC_number="3.5.1.25"

/inference="ab initio prediction:Prodigal:002006"

/inference="similar to AA sequence:UniProtKB:O34450"

/codon_start=1

/transl_table=11

/product="N-acetylglucosamine-6-phosphate deacetylase"

/db_xref="COG:COG1820"

/translation="MSTKVLKHATIYTGEEKIEDGFVRFDKEVLAVGEMKDFIDAPHE

EIIDAHGKIIVPGFIDIHSHGGYGWDAMDGNADEIDAMVKDMQKEGITSYFATTMTQS

HENIAKAMVAIKEAAERNPVIQGIHLEGPFVSKVFKGAQPEEYIEVPDVEVFDEWNKL

SGNRIRLVTYAPENSDASAFEEYCVEHNIVPSIGHSNATRAQLMDSKASHITHLYNAQ

RGLHHREPGVTGHAFLEENIYTEMIVDGYHIHPDMVKLAFMLKGPERIELITDSMRAK

GMPEGESELGGQKVIVKDKQARLETGNLAGSVLEYQDAFRNMMAFTGCSIADVVKMTS

VNQAREFGLTKKGAIEVGKDADMVIFDQELQLENTISLGKLIK"

CDS complement(2051115..2055392)

/locus_tag="JFBMFIFI_01944"

/inference="ab initio prediction:Prodigal:002006"

/codon_start=1

/transl_table=11

/product="hypothetical protein"

/translation="MKKEIRGGIDVGSTTVKLVIIDELNTVLFSKYERHYSAIKEASC

RILKDAYQVLGDCQLRLMITGSGGMGLAELLEIEFVQEVIACTRTVEELIPETDVVIE

LGGEDAKITFFDGALEQRMNGSCAGGTGAFIDQMASLLKTDAAGVNELAKNYQTIYPI

ASRCGVFAKTDIQPLINEGARVEDISASIFQAVVNQTIAGLASGRKIRGKVAFLGGPL

FFMSELRNRFIETLKLKEEDVIFPDDPQLFVARGAAYFSEDKKLTTFNCLIEKIESAK

PENLQPTDSLPPLFKNEAELEQFRTRHRQANINEKKLSDHKGVAFLGIDAGSTTTKIT

LINLAGDLLFKFYGNNEGQPLEKTIEILKTLYKQMPDDVFIGKTAITGYGEHLIKHAL

KIDIGEVETVAHYKAANRFQSDVDFILDIGGQDMKAMTIKQGVLSSIQLNEACSSGCG

SFIETFAKSLNVTVAEFAEAAVHAKQPVDLGSRCTVFMNSKVKQVQKEGATVGDISAG

LSYSVIKNALYKVIKIKRPEDLGKRIVCQGGTFYNEAVLRSFELISGCEVIRPNIAGL

MGAYGCAILALEAYEEGNNSTILGVKELDLFNSKKEFTHCGLCENNCLMTVTFFSDGR

QFTTGNRCERGARVKLNKSDKKVNLIDYKYKRLFSYKSLRKKEATRGVIGIPRVLNMY

ENYPLWHTFFTKLGFRVELSPRSNKKLYEQGMETIPSDTVCYPAKLAHGHIEALIKKD

VSAIFYPAVVFEQKEFSQAENNFNCPIVQSYPDVIRNNVDEIREGKVNYYNPYLNLAN

KESMIKVLFKTFESTDITLAEVTEALELGYQELEKFKLDIRNKGEETLLMLNLKNEKG

IVLAGRPYQIDPEINHGVADVITQEGFHILTEDSISHLGDVANLRVVNQWVYHARLYA

AANVVKKSPQLEMIQLNSFGCGLDAVTTDQVAEILEQAGKIYTVLKIDEGSNLGAIRI

RIRSLKAAVKERSKMQFKTIDTSSISSEERIIFTKEMKKKHTLLMPMMSPIHQNGLFD

VALQASGYNVAVLPELDKKAIDEGLKFVHNDACYPAIITIGQLIKALQSGNYDLDNVS

VMMTQTGGGCRATNYIPLLRKALKDAGFSKIPVVSISMGNQGVESNPGFTYNLKMLKR

ILVAALYGDLFERVVYRTRPYEMKSGSVDDLHQKWLSAVKRNVKNGSMTEFNRTIKGI

IKDFDTIPLTNEIKPRVGVVGEILVKYSPTANNDLVRLLEAEGAEVVVPDIIGFMNYS

LYNQIWKYENMGVPYKTKLVSEVAIKLIELCEKPMDKLLRASNRFNGIDSIYDIAEGA

EKVLSIGNQTGEGWFLTGEMIELLKAGVNNIVCMQPFGCLPNHVVGKGAIKELRRQYN

NANITAIDYDPGVSVVNQLNRIRLMLSTANKNLAQDPESRKKRIKLR"

CDS 2055661..2056278

/locus_tag="JFBMFIFI_01945"

/inference="ab initio prediction:Prodigal:002006"

/codon_start=1

/transl_table=11

/product="hypothetical protein"

/translation="MHYSAKKTDLRILRSKKMIFEAFVKLVKLKGYEAVTIQDIATEA

MINRATFYAHFKDKNDLYDEVFSYALDTFTKILDSELLENGNQIQINKLEHMITEIFY

VIRENRTFYLILTEGNSANSLKKKVHTLIEQRYAEIFNQLKITENDVEVPIDFIIDYM

SSIFISMVHWWLTSESDFPPEQMAHLLIKLVGNGHLTVLGIEVIK"

CDS complement(2056336..2058246)

/locus_tag="JFBMFIFI_01946"

/inference="ab initio prediction:Prodigal:002006"

/codon_start=1

/transl_table=11

/product="hypothetical protein"

/translation="MKPKKIYFKKIKVHELKKEKKLLSSKVDATILILLFLGSALINN

AILQLFQNNLDWDLVNKFIFEWHTILFLLGSSVIFIFTLWLYAILGNRYLTVGLTFLI

SLGLGVATWQKMINRSEPLYPSELSMIKELSFMVSMMESKVVLGFLLIVLVVMALAYV

FTCLQTRFIIKYNRHKSYLIRFILFITTSFLLVYIANFNQPGNLLKKAYDPNAYWIPY
SQQMNYYNNGFIGGFLFNLKVEAMAEPKGYSKEKIAELTQKYQEIAEEINQNRSGPIK
NTNIIYIMNESFSDPLALNGMEIAKDPIPLTRHLMENNRSGSILSQGYGGGTANIEFE
ALTGFSMEPMNPQLTTPYTQLLAKVPNFPSIVSYLNEQNYQTTAIHPYNTSMYKRQNV
YQSLGFQNFINEDTMKNTEKMESNPYISDKAAYQEVLDTLEASKDSNFIHLVTMQNHM
PYGSKYVSSEYEVSGTENDQITSNYLKDLSYSDQALDNFIKQIDQLAEPTIVVFWGDH
LPSVYGDVIFNQNSLDKMHETPLLIYSNVNKKDKELGMISPIYFAPELLEVANADITG
FYALLNQLQSNLPAFEKEMYLEKNAEKMKKSVNELSQETQALLADYYMIQYDITTGKK
YSLQTNLFAHNK"

CDS complement(2058359..2059306)
/gene="csbB"
/locus_tag="JFBMFIFI_01947"
/EC_number="2.4.-.-"
/inference="ab initio prediction:Prodigal:002006"
/inference="similar to AA sequence:UniProtKB:Q45539"
/codon_start=1
/transl_table=11
/product="Putative glycosyltransferase CsbB"
/db_xref="COG:COG0463"
/translation="MKTELISIVVPCFNEEESIPLFYAALEKEREFLVNADIEYIFVN
DGSNDNTLNVLRSLAKEDKERVKFISFSRNFGKEAGLYAGLQQATGDYVAVMDVDLQD
PPEMLPEMLQIIREEEYDCVGTRRVSRDGEPPIRSFFARQFYRIINNISETEIVDGAR
DYRLMTRQMVNAILSMEEYNRFSKGIFSWVGFDTKYLEYKNQERVAGETSWSFWSLFK
YSLDGIVAFSELPLAFASFVGFFSFLAAMIAMVVIVIRTLLLNDPTSGWPSLVCFILA
IGGLQLFCLGILGKYLGKTYLETKKRPIYIVKETEKDKK"

CDS complement(2059342..2059761)
/locus_tag="JFBMFIFI_01948"
/inference="ab initio prediction:Prodigal:002006"
/codon_start=1
/transl_table=11
/product="hypothetical protein"
/translation="MKKIIELATKYREVLMYLIFGVLTTIINIFVFYIFKDTLTIDYR
TSNVIAWFLSVLFAFLTNKYFVFASSGKNKQAFFKEMGLFFLYRLLSLVIDMIMMIVM

VSGLGIDALIAKLITQVVIVVINYVFSKLLIFNDKQA"

CDS complement(2059790..2060617)

/gene="nadE"

/locus_tag="JFBMFIFI_01949"

/EC_number="6.3.1.5"

/inference="ab initio prediction:Prodigal:002006"

/inference="similar to AA sequence:UniProtKB:P18843"

/codon_start=1

/transl_table=11

/product="NH(3)-dependent NAD(+) synthetase"

/db_xref="COG:COG0171"

/translation="MSSLQKKIISEMHVQPEIDVDNEIRKSIDFIKNYMLKHPFLKSV

VLGISGGQDSTLTGKLAQLAITELRNETSNSDYQFIAVRLPYGEQADEEDAMDAINFI

GADKVLRVNIKPATDANSQTLEEIGVPISDFDKGNIKARERMIAQYAIAGAYQGTVLG

TDHSAESVTGFYTKYGDGGTDINPIFRLNKRQGRALLKALQAPTHLYEKTPTADLEDN

KPGLADEVALGVTYDDLDDYLEGKDVPTEVADKIEGWYLKTQHKRHLPITIFDDFWK"

CDS complement(2060728..2062206)

/gene="pncB2"

/locus_tag="JFBMFIFI_01950"

/EC_number="6.3.4.21"

/inference="ab initio prediction:Prodigal:002006"

/inference="similar to AA sequence:UniProtKB:P9WJI7"

/codon_start=1

/transl_table=11

/product="Nicotinate phosphoribosyltransferase pncB2"

/db_xref="COG:COG1488"

/translation="MNKIYPDDSWSLHTDLYQINMMKTYWELGKADKHAVFECYFRVN

PFENGYAIFAGLERIVGYLERLTFSESDIDYLREAEEYPEAFLTYLANFKFKATIRSV

EEGELVFSNEPLVQVEGPLADCQLIETALLNIVNYQTLIATKAARIKSVVGTDPLLEF

GTRRAQEMDAAIWGTRAAYIGGCDATSNVRAGKIFGIPASGTHAHSLVQTYRNDYDAF

KAYATTHKDCVFLVDTYDTLKSGVPNAIRVAKEFGNKINFLGVRLDSGDMAYISKRVR

QQLDAAGYEDVKIYASNDLDEKTILNLKMQGAKIDVWGVGTKLITAYDQPALGAVYKL

VSIEDENGKMVDTIKLSGNAEKVSTPGKKQVWRITANEDGKSEGDYITLWDEEPDKAE

ELYMFHPVHTYINKTVTNFNARPLLKDIFINGKLVYALPNLAEVKKYATTNLESLWDE

YKRNLNPEPYPVDLSKICYDHKMANIELARKQVKEQEAYFNK"

CDS complement(2062327..2063211)

/gene="wbbL_1"

/locus_tag="JFBMFIFI_01951"

/EC_number="2.4.1.289"

/inference="ab initio prediction:Prodigal:002006"

/inference="similar to AA sequence:UniProtKB:P9WMY3"

/codon_start=1

/transl_table=11

/product="N-acetylglucosaminyl-diphospho-decaprenol

L-rhamnosyltransferase"

/db_xref="COG:COG1216"

/translation="MKKSVYFIVLNYNNAPDTIECIKSIENLNYQNVQVVLVDNLSTD

DSEEILKKELPNYTLIQTGSNAGYAAGNNRGIKYAIEDGADYVCILNNDVIVDPNFLT

VLVDYLENNSQVGVVGPRICEYEDPAILESAGSTVDFNKGKVTRLYYGEKEDKVFGKV

IPCDYVGGACMLLDVKLIKEVGYIPENYFLFYEENEWCVTIKKAGYEVVCVADAKVIH

KGSASINKVSGLSEYFMYRNLVVFMQRNATFKNKIIFYPYLFLFSLKSGFFKENGWRF

ASYFYDGLTGKNKYKNKL"

CDS complement(2063208..2064113)

/gene="arnC_1"

/locus_tag="JFBMFIFI_01952"

/EC_number="2.4.2.53"

/inference="ab initio prediction:Prodigal:002006"

/inference="protein motif:HAMAP:MF_01164"

/codon_start=1

/transl_table=11

/product="Undecaprenyl-phosphate

4-deoxy-4-formamido-L-arabinose transferase"

/translation="MNPLVSIIIPVFNGEKTIVRAITSCLEQSYKNFEILIIDNCSND

NTSKLVQEFSDKQVNYIYTKDKGRALARNIGLNKAKGSYIQFLDADDSLEKDKLKVSV

NYLEKDRTSQAYSTGIKYRNTDNQQLKVVYPKSHYEDELLAHNIFPIHSLVFRATDAS

VFPEELDYCEDWLFWVRTLLGSKIYFNDNYIGGTVYIHGENTMTQSNLMREYELYVQQ

RLKGEYKPSNLNLALKKNEISLLIIHYFNKKKLSLTKKSVSKNSKYTYLVVKVICNIP

FLKKKLVKKVNSILAKDVYTGEEIK"

CDS complement(2064541..2065731)

/locus_tag="JFBMFIFI_01953"

/inference="ab initio prediction:Prodigal:002006"

/codon_start=1

/transl_table=11

/product="hypothetical protein"

/translation="MIISLILSILFFIGLYLPTIPTDGNWMQLEIIYLAIAIIVIVVL

LFKQKKIDIKQITLGVIFNGLALYFTKISNPNSNPKEMLLYYFIFFIALTLVLQLDFT

NVNSESVMKYLKIFLLCISILSIIISISIIAQIGPVTGRLITYYSSFYPELVPRMISL

GKPVNTFGVHSLAGVFNFLFFLLNFATFYVLKAKEHFIISLFFLFFLVRLQSTTSLVC

LLIALMIIGIYIYTENKYIFFGLLALVITFLVLSWNELSIVQILKRNLFNEDSTNGLM

VRFGPKGVLYGSLQQIIQYPFKGTGYVSVPDTYYIDSGIIHFALRTSVIGMLAFYGML

LNFFVKNLNWLMGIALFTLFMFFEIGFSNFLYFRIIYFLPFFIFYLKQLLLIKKNQKE

LKLR"

CDS complement(2065728..2067089)

/locus_tag="JFBMFIFI_01954"

/inference="ab initio prediction:Prodigal:002006"

/codon_start=1

/transl_table=11

/product="hypothetical protein"

/translation="MLKNISKVFSANLVNMLISILTAFLLPYFLTVDSYAELKTFTFY

VSYLGILQLGFVDGMYIKYGGKKISEISTKVFFDERKTFVLLQIVLAVIALSISLFFQ

NTIFILLAISVIPINLFFFYRQFFQAVGEFSLFAVTTTMYAAIPFVLNLILMLVFKTQ

NPVYYCLSTILGYVVIDYYFVFKSKFFENKKGHLNYAVMKENIQVGVFVLFGNLSLNF

ITALDRWFISIFYDKSAFAFYSFAVSMLNIVNVVISSVSITFYNYLVGNRNEKQIKEI

KNILLVVGIIFMTAYFPLKFIVELFIPEYIPSLDVIAISFMSFPFSIVINTLYVNLYK

ANKNERKYFTIVVGVLVISVIINTIMMQIVDGFVSVTIGSLVVYILWYLYSQKDFSYL

RSTLKELVFIASSLIIFYLCSRHLNVYSGFLVSLILLIGTIFIFMRSTLIKIISFARG

GSK"

CDS complement(2067133..2068143)

/locus_tag="JFBMFIFI_01955"

/inference="ab initio prediction:Prodigal:002006"

/codon_start=1

/transl_table=11

/product="hypothetical protein"

/translation="MKIIAYTQGKEHPCARFRVRQYLPYLKDANIDVTEKYTKFSAYP

PRNKLLRPLWGIETIAERMIDVPKSKNYDLTWLQKTFISKYPTIEKYTKGPKLFDIDD

AIYMGNPKLLTTMAQISDGVICGNEFLAEKYGEFNKNIHIIPTGVDSDRFIPREKRNS

ELFIIGWSGSSVGLRYVYQHEKAIASFLEKHKNTRIRIVTDMAPKFTMINPKQVEFVK

WTAENEVYDIQNFDVGIMPLFDTEFEKGKCSYKMLLYMACGVPVIVSPVGMNNQVLKL

GSMGVSANTSDEWFDQLETMYQYSDSQRKEIGRNGRNVVINNFDSKVIAKELATVFQQ

YK"

CDS complement(2068147..2069295)

/locus_tag="JFBMFIFI_01956"

/inference="ab initio prediction:Prodigal:002006"

/codon_start=1

/transl_table=11

/product="hypothetical protein"

/translation="MEIQKKNRILFSMIVLLGIFFPTSILHNVISPLNVVYNTIFILC

AFFIIVINKKIVLSDAILASIISIIVFSSTLGTIFFNRENVDMISWGILSQYLVIIAY

SLMNERIFEDPFFSKLFKVVSYIIIIVGLISMLRIAFFTNIFTSFYSFSYKDLVSNMI

GRLKPVTFFGSHSIAGFIYFMFLEVWLKRLFVSKDKRNIIPVVFFGFFLIMLQSSTSY

FLLGLLLVSTLFKIFQLFGKKHLKRIIIIGTVSIVLFGGVTLKFLLPMLLSKTNGILS

RFTTGLFSDNILYLKEHPFLGSGFLSVNHLYYTDFGYMVTYLRGGIFLIISIAVLIFK

KMKPVSLFIVLGFILFEVGYPVSLYFRLYPVLLLISLENNNIVKNQGV"

CDS complement(2069313..2069906)

/locus_tag="JFBMFIFI_01957"

/inference="ab initio prediction:Prodigal:002006"

/codon_start=1

/transl_table=11

/product="hypothetical protein"

/translation="MNKTISFEEITKIYQKKWKFFLLVSLMLLLLSCFLFIREKQKTP

LMVTEVNMQFMLKHNKVKMNDVFQYRTLLLSSSFIEEVNEIAATDKKASSILARGKKG

ELQLNVSVLPNATLNVNVASSDKNEAIRFSKALKDTFRDEVRKVNYRLYITENNKTLY

EANQVLRKPLGFIIILFSLFALTFSVSLILILFKMLK"

CDS complement(2069929..2071029)

/gene="epsF_2"

/locus_tag="JFBMFIFI_01958"

/EC_number="2.4.-.-"

/inference="ab initio prediction:Prodigal:002006"

/inference="similar to AA sequence:UniProtKB:P71055"

/codon_start=1

/transl_table=11

/product="Putative glycosyltransferase EpsF"

/db_xref="COG:COG0438"

/translation="MNPPIRILHVLGIMNRGGAESFVMNLYRTIDRTKVQFDFVVHTN

EEGAFDHEITSLGGHIYRVPKFNVKNIFTYQKAWDDLLKMLPHKIIHCHIRSTASIIL

KQAKKNNYITIAHSHSTSSGSGIQGMVKNALQSKINKYADYRFACGKEAGEWLFKGAD

FTVLENGIDLEKYLFNQSVRKMMRTELQVDQSFVMGHVARFSEVKNHKFLIDIFSSIK

KKNNKAKLLLIGTGDLEDSIKEKVKSLDLESSVLFLGARDDVNHLLQAMDCFVFPSLF

EGLPVSLVEAQTASLSVFASNTISAEAKLTNLVHFMSLNDSPDEWAKVILTEALPEDK

RKMIEVKSEIQSEGYDIIQTTNKLTDFYLCLS"

CDS complement(2071030..2072004)

/gene="wbgU_1"

/locus_tag="JFBMFIFI_01959"

/EC_number="5.1.3.7"

/inference="ab initio prediction:Prodigal:002006"

/inference="similar to AA sequence:UniProtKB:Q7BJX9"

/codon_start=1

/transl_table=11

/product="UDP-N-acetylglucosamine 4-epimerase"

/translation="MGYTNVEFPKNSLFLITGGAGFIGSNLCEALLKLGYKVRCLDDL

STGKEENIAPFLSNPNFEFIKGSICDLETCMSACKDVDYISNQAAWGSVPRSIELPVV

YEEKNIKGTLNMMEAAVKNKVKKFVYASSSSVYGDEPTLPKVEGKEGNVLSPYALTKK

VNEEYASLYSKLYHLETVGLRYFNVFGRRQDPNGQYAAVIPKFVSDILEDKSPIINGD

GEHSRDFTYIENVIEANLKAMASSSEVSGEAFNIAFGSQVNLKELVDKINELLEKDVS

PTFGPERAGDIKHSNACIDKAKNMLGYQPDYSFSDGLELAVDWYKENL"

CDS complement(2072006..2073316)

/gene="wbpA_1"

/locus_tag="JFBMFIFI_01960"

/EC_number="1.1.1.136"

/inference="ab initio prediction:Prodigal:002006"

/inference="similar to AA sequence:UniProtKB:G3XD94"

/codon_start=1

/transl_table=11

/product="UDP-N-acetyl-D-glucosamine 6-dehydrogenase"

/db_xref="COG:COG0677"

/translation="MINFDDIKTGNAYISLIGLGYVGMPIAVAFAKKAKVIGFDLNQE

KIDIYKSGIDPTNEVGNEVIKRTSVEFTSNEEDLKRASFHIVAVPTPIKKDKTPDLTP

VISASELLGRNLEKGSIVVYESTVYPGVTEDICIPILEKVSGLKAGIDFKVGYSPERI

NPGDKVHRLESIIKVVSGMDEDSLSTIASVYELVVEVGVYKAESIKVAEAAKVIENSQ

RDINIAFMNELSMIFNRLDIDTKAVLKAAGTKWNFLNFEPGLVGGHCIGVDPYYLTYK

AEEVGYHSKIILSGRRINDDMGKYVVENAVKELINADTLVKTSRVGIFGFTFKENCPD

TRNTRVIDIYTELSEYGVTPLVYDPEADQEEVEKEYKIKLATIEEMENLDAIIIAVNH

TKFSEYTTDKWLSFFKKDKKIILDIKGMLDKEEFLDKNVSYWRL"

CDS complement(2073309..2074415)

/gene="pglA"

/locus_tag="JFBMFIFI_01961"

/EC_number="2.4.1.290"

/inference="ab initio prediction:Prodigal:002006"

/inference="similar to AA sequence:UniProtKB:Q0P9C9"

/codon_start=1

/transl_table=11

/product="N,

N'-diacetylbacillosaminyl-diphospho-undecaprenol

alpha-1,3-N-acetylgalactosaminyltransferase"

/translation="MKKVLILANDATNLYKLRKEIIVGLINKGYEVYVALGNDDSVEK

LVSLGCKFIEIDVDRRGLNIVKDIKLFLRYVKLVKDINPDLVMTYAIKPNIYGGMTAR

LLKKKYIINITGIGTAFQKENMLKKIVTLLYRISAKNAVVFYQNQENKSIFLKNNIVG

TKEILVPGSGVNLEQFTYCEVADTKHDKFLFIARVMEEKGVDEFLYTSKMLHKKYPEV

EFHIIGDIEEDRYESILKEYIDAGIIHYHGYTENVHDFIRNSTCVVLPSYHEGMSNAL

LESAAIGRPLIASNISGCIEIIEDGVNGYLVEPKDKESLLLGVEKFIALPRETKELMG

KAGRKKIEKEFNRKIIVDAYTKCIDGLTGGTKND"

CDS complement(2074543..2075388)

/gene="rmlD"

/locus_tag="JFBMFIFI_01962"

/EC_number="1.1.1.133"

/inference="ab initio prediction:Prodigal:002006"

/inference="similar to AA sequence:UniProtKB:O66251"

/codon_start=1

/transl_table=11

/product="dTDP-4-dehydrorhamnose reductase"

/translation="MKKILITGANGQLGTELQNLLTKKNIPYVAADIKVLDITNANSV

TTFIEEMKPDVIYHCAAYTAVDKAEDEGKEKNQLVNVVGTKNVAQAAEKVGATVVYIS

TDYVFDGTKKEEYNVDDKPNPKNEYGRAKYEGELEIQKNCSKYYIIRTSWVYGQFGAN

FVFTMQKLAQTHSKLTIVNDQYGRPTWTKDLADFMLFLVNEEAEYGIYHFSNDDSCTW

YEFAQEILKDEDISLTGVTSSEYPQKAKRPKYSILNLTKTKSLGYEVPIWQDSLQLLL

KELEK"

CDS complement(2075390..2076670)

/gene="rfbX_1"

/locus_tag="JFBMFIFI_01963"

/inference="ab initio prediction:Prodigal:002006"

/inference="similar to AA sequence:UniProtKB:P37746"

/codon_start=1

/transl_table=11

/product="Putative O-antigen transporter"

/db_xref="COG:COG2244"

/translation="MEKIKNNKIIHNFLSLASVQVLNYILPLITLPYLLRVVGVSHFG

LISFAQAIMQYFILFTDYGFNLIATRDISKVRDDKEKVSLIFCTVMAAKLILLLLSFA

VLIILILAVPKFREDTLLYLFTFGMVVGSVLFPTWFFQGIEEMKVISLLNIFSKLIFT

IGIFIVVKTPEDYLNVAILNSLGFILIGVISIFIVFFKYKVKLKLPEVHQIVSQMKEG

WDIFLSNMFSSLYTTSNTVILGFLASNTVVGYFSAADKIIKACSSVISPIIQAVYPHI

SNLIEKSKEETLILIRKIGIYITCVIGAGCLVLLFGAPYLLPLLAGNSYDNSILLIQI

MSFLPLIIGWANIFGILTLINFGYQKELSRIYIYSSLFSIITTIVLVPAFKEVGTAIN

VTLIEIIVTVAMYYVLKKKGIDIFRAKINLKDGK"

CDS complement(2076690..2077565)

/locus_tag="JFBMFIFI_01964"

/inference="ab initio prediction:Prodigal:002006"

/codon_start=1

/transl_table=11

/product="hypothetical protein"

/translation="MKIMAVVVTFNPDKQAYENIRKLSQQIDFILVVDNSLKSSRFVL

QQEGIEVVYNSENLGIGKALNLGLDYAIQHDFDWLFTFDQDSQITDNFVRNMISTNEL

YEKKEKIQPVLICPTYIHPDDAIVYDNAKETDNEFYVEVETAMTSGNALNISLLKDKN

IRFKEAYFIDFVDHEFCFHLRKAGYKIIQSLDAPLIHSLGEVKEKIIFGKTLTSTNHN

AVRRYYITRNRLDVYRNYFSQEKKWIKIDFVNSIMEFVKIILIEDQKSLKVLNIFRGT

KDALLNNWGPYRYTK"

CDS complement(2077654..2078637)

/gene="rffG"

/locus_tag="JFBMFIFI_01965"

/EC_number="4.2.1.46"

/inference="ab initio prediction:Prodigal:002006"

/inference="similar to AA sequence:UniProtKB:P27830"

/codon_start=1

/transl_table=11

/product="dTDP-glucose 4,6-dehydratase 2"

/db_xref="COG:COG1088"

/translation="MNILVTGGAGFIGSNFVHYMLENHPDYKIINLDLLTYAGNIHNL

DDVMENPNHVFVQGNINNRELVRNLVKTYDINQFVNFAAESHVDRSILNPEIFVETNI

QGTLALLDVAKELNINKYLQVSTDEVYGSLGAEGYFTEETPLAPNSPYSASKTGADLL

VRSYFETYGMNVNITRCSNNYGPYHFPEKLIPLMITNGMDNKELPIYGDGLNIRDWLH

VQDHCQAIDLVLHKGRKGEVYNIGGHNERTNNQIVEIIIEKLGLSNDLIKYVEDRLGH

DKRYAIDPTKLETELGWKPKYTFDTGIVETIDWYQANEAWWRPLKERANLN"

CDS complement(2078640..2079200)

/gene="rmlC"

/locus_tag="JFBMFIFI_01966"

/EC_number="5.1.3.13"

/inference="ab initio prediction:Prodigal:002006"

/inference="similar to AA sequence:UniProtKB:Q9HU21"

/codon_start=1

/transl_table=11

/product="dTDP-4-dehydrorhamnose 3,5-epimerase"

/db_xref="COG:COG1898"

/translation="MKVIDTKLQDVKIIETPVHGDHRGFFTESYTEDKFNEAGIPNKF

IQDNHSLSVEPGVIRGMHYQTNPKAQTKLVRVTTGAIYDVLVDMRKGSPTYGQWEGYI

LSEYNHRQLLVPKGFAHGFCTITGNVNVQYKVDELYSPENDRGIAFDDPDVGIIWPMN

NPIMSEKDTKHPQLKDADNNFVWEEK"

CDS complement(2079216..2080088)

/gene="rmlA"

/locus_tag="JFBMFIFI_01967"

/EC_number="2.7.7.24"

/inference="ab initio prediction:Prodigal:002006"

/inference="similar to AA sequence:UniProtKB:P26393"

/codon_start=1

/transl_table=11

/product="Glucose-1-phosphate thymidylyltransferase"

/db_xref="COG:COG1209"

/translation="MKGIILAGGSGTRLYPLTKAVSKQLMPIYDKPMIYYPMSTLMLA

GIKDILIISTPTDTPRFEQLLGNGNELGLNIEYKVQESPDGLAQAFILGEDFIGDDSV

CLILGDNIYYGGGMSKMLQRAAAKTDGATVFGYHVNDPERFGVVEFDEEMRAISIEEK

PTAAKSNYAVTGLYFYDNQVVEIAKNIKPSERGELEITDVNKAYLEAGKLDVELMGRG

FAWLDTGTHESLLEAATFIETIQKRQNLMVACLEEISYRMGYITKNQLLELAQPLKKN

GYGQYLLRLAETKI"

CDS complement(2080119..2080826)

/locus_tag="JFBMFIFI_01968"

/inference="ab initio prediction:Prodigal:002006"

/codon_start=1

/transl_table=11

/product="hypothetical protein"

/translation="MISVCLATFNGQEFIERQLDSIIEQVTSEDEIIIVDDGSNDRTI
DLIKAKYSEQVFLHINTSNLGPIKSFEKAISLATGDYIFLCDQDDIWLPNKVQVVIKA
FKEQKADIVVHDAQVVDGNLKELSPSWNIRNKNKLDQTLLGNIKKNAFTGCMMAFSQE
MVPLILPFPDSIEMHDQWIALVGMIEKKKIVSIDNILMKYVRHGSNVTAIKSRSLKEQ
LIGRTGTLRAVLQYKYR"

CDS complement(2080823..2082178)

/locus_tag="JFBMFIFI_01969"

/inference="ab initio prediction:Prodigal:002006"

/codon_start=1

/transl_table=11

/product="hypothetical protein"

/translation="MNRFEKGLLTVFFLELFIGGGGRLLEFGPLSIRQVIFLTLIATY
VTRIFYTKSFFNSNVNVFISKKYATYSVYALLFWFVVSSIIGILNQHSFSIIMMDFLR
VSFLGLYFPLAYYISEERFKLSWVINLLKIATLIIAIFTIGIDVTGRFILKDNFNEFY
IAINNAFPNELFFRPSRGVFYKGHFFLLFGLIISTNQWLNRKQTKLDMINVVLAIVSI
IWSETRGLLIGYMVAIFFIALLDYKVLLTPIIGFFNRLKAMVSKPNRKKLILYFCILI
SVPLLYQNMTLSRFNDMPLASNQTNSTQKKNSKVNDVSINARLYLLQASKDIVLEKPQ
NMIIGNGYGTKIGDRIDGIEMSFIDILVEQGLVGVLLWLNLCLLPFIYYFKVYKKEKR
LLYTQISLLACVVAMVLLTNINPFLNSPIGLGFLLFVIADAMNSKASFSSKFDKVEGI
K"

CDS complement(2082212..2083003)

/gene="wbbL_2"

/locus_tag="JFBMFIFI_01970"

/EC_number="2.4.1.289"

/inference="ab initio prediction:Prodigal:002006"

/inference="similar to AA sequence:UniProtKB:P9WMY3"

/codon_start=1

/transl_table=11

/product="N-acetylglucosaminyl-diphospho-decaprenol
L-rhamnosyltransferase"

/db_xref="COG:COG1216"

/translation="MNKTLATVSIVTYNSKHIFTVLDNLRKEFENDERFRFVIFDNNS
SLDYQKKLKSYEDLVILYFNDENKGFGYGHNSNFKKFAEDSEYFLVFNPDILVEKNSL

LKMIHLMDQEKDIGLCFPKVLNEDGTKQYLIRNRLTVFDFALRFIPFSFVKKMFHKRL
ATYECQDLSDKHNSDVKIGSGCFMLLRSTVFEEAKGFDERYFMYFEDSDLSLTIGKLG
ARLVYTPFSTVIHFYEKGAHKNKQLFKIFMQSMRKFFNKWGWNII"

CDS complement(2083209..2084042)
/locus_tag="JFBMFIFI_01971"
/inference="ab initio prediction:Prodigal:002006"
/codon_start=1
/transl_table=11
/product="hypothetical protein"
/translation="MKLTTVIVIYNMEIEKTKTIESLKNNKLFFECENMDSQVIIYDN
SNSPQDCPQIFKNQVYIHDERNIGIVTAYNKAWEIASNKKNDWLLLLDQDTEITEDYL
KRTIAALSNNQQEENIVAIVPTVYSNEKVVSPVFLDSLRPLKNTMPSKGLQEKKVMAI
NSGAVIRVSFLNEINGFNKEFPLDYLDHWLFHTIYEKNRKIFLSDAKLNHDLSVMNYN
TISLGRYKSIVDSEVRYYNKYLPDKKIQFKKHLFLRMMKQAISVKNKRIFLYTAKQIF
K"

CDS complement(2084411..2085730)
/locus_tag="JFBMFIFI_01972"
/inference="ab initio prediction:Prodigal:002006"
/codon_start=1
/transl_table=11
/product="hypothetical protein"
/translation="MKKIKRKKIMTFLLASTMLTTAMPLNVLAEVTDKSSEFEDSSEP
EVSTDSEESIDSSTISSENQDSSEQTETEDNKVDTNSDNGEDKEVIEEKNVETTPSIK
ENSAMGPGFYDTDMGNQRMARSTNVFLDNIKKGAIDGWSRYKILPSISAAQAVLESGW
GKSTLAINANNLFGIKGRYNGSYVVMPTLEYINGQWITVNAEFRKYPSWDASVEDHGS
FFHENPRYSNLIGVTDYRTVARLLQEDGYATDPEYAAKLISIIEYNGLADWDVQALGN
HSSKNSVKIIDLDLPTAQRWLDEIQKRYVGLITPDRVFGMKKGNDWSVKMTDVTYEQA
VGICRIFRTIFPQYEIYGTRRNVNDSIVASTPFNVEITGIARGDADSVIREVRNQYYW
LLTSDRIFITPLSSSYMIEVNSVPTNSVNGLKQELKTIYQLMDYQVQ"

CDS complement(2086082..2086972)
/locus_tag="JFBMFIFI_01973"
/inference="ab initio prediction:Prodigal:002006"
/codon_start=1

/transl_table=11

/product="hypothetical protein"

/translation="MNKKINRLFVGLSLLFIVVFAFGCGKKNTINKSQIENTAIIEKY

NRYMDLFKFTNVELVGLNNQLVETINKDTTEENEYQLQVLDEDFLTDYDKLITQTRIA

IDLTPKFDADKNARQLIDVLEDEKEILEKIHSYYLKKDSIEDNSSKRKELELQFLETA

GTVSKKSEKFNQTMVKISEELIGKDKEENIKNNKSVNYNVILIVSDVIKIVNMDLDKE

KNKEKLLIVQNNLAEQVEQLKSTSISDLEKEGFDEKSIKNYIENIELFTTTINDPHQD

ENTVQQNYYKILIAYNLATR"

CDS complement(2087171..2088154)

/gene="galE_2"

/locus_tag="JFBMFIFI_01974"

/EC_number="5.1.3.2"

/inference="ab initio prediction:Prodigal:002006"

/inference="similar to AA sequence:UniProtKB:Q7WTB1"

/codon_start=1

/transl_table=11

/product="UDP-glucose 4-epimerase"

/db_xref="COG:COG1087"

/translation="MTVLVLGGAGYIGSHAVDQLITKGYDVAVVDNLKTGHKESLPDK

ARFYQGDIRDKAFMEDVFTKENIEGVIHFAASSLVGESMEIPLDYFNNNVYGTQVVLE

VMEKYNVKSIIFSSSAATYGEPKVIPIEETAATNPESTYGETKLMMEKMLKWCDKAYG

MRFVALRYFNVAGAKLDGTIGEDHNPESHLLPIILQTALGQREKFTIYGEDYETPDGT

CIRDYVHVVDLIDAHILALEYLQAGNSSNTFNLGSSTGFSVKQMLEAAREVTGKEIPA

TVVSRRAGDPSTLIAASDKAREVLGWKPQYTDVNKIIESAWNWHGKHPEGY"

CDS complement(2088347..2089336)

/gene="yxeI_1"

/locus_tag="JFBMFIFI_01975"

/inference="ab initio prediction:Prodigal:002006"

/inference="similar to AA sequence:UniProtKB:P54948"

/codon_start=1

/transl_table=11

/product="putative protein YxeI"

/db_xref="COG:COG3049"

/translation="MCTGISLKTVEQTIFWGRTQEFDLDLPYQGVIIPKDYLIEDTLT
AFETKYAVMGIGVKGMSTMIDGVNQAGIAGGSFYFAHYNNYSSEKSIKDQGKKALRGQ
EVVLWALSNCATLEEIKTRINSEIGVAEGELSLPQHYVFQDTKGNSIVVEPSIENGFK
IFDNPIGIFTNNPPFDWHLINLQNYVQLSSQTVEGIQLDNESVFSPGKGSGLLGIPGD
YTPQSRFVRATALKFLSAKVTKEEAVGHLFHLFNSFDIPKGVVQSKGNATSQTTHYTV
VYDLAEKIIYFHNYENRQIQTLKFKEEAQQSKSITRFELEKIPQILEMKEIKN"

CDS complement(2089715..2091346)

/gene="groL"
/locus_tag="JFBMFIFI_01976"
/inference="ab initio prediction:Prodigal:002006"
/inference="similar to AA sequence:UniProtKB:P0A335"
/codon_start=1
/transl_table=11
/product="60 kDa chaperonin"
/db_xref="COG:COG0459"
/translation="MAKDIKFAEDARSAMLRGVDILANTVKVTLGPKGRNVVLEKSFG
SPLITNDGVTIAKEIELEDHFENMGAKLVSEVASKTNDIAGDGTTTATVLTQAIVREG
LKNVTAGANPVGIRRGIELATKTAVEELHAISKVVNSKEAIAQVAAISSDDERIGQLI
ADAMEKVGNDGVITIEESKGIETELDVVEGMQFDRGYLSQYMVTDNDKMEAVLENPYI
LITDKKISNIQDVLPMLEQIIQQGRPLLIIADDVDGEALPTLVLNKIRGTFNVVAVKA
PGFGDRRKAMLEDIAILTGGTVITDDLGLELKDTTIEQLGHAGKAVVTKDATTIVEGA
GAKENIEHRVALIKAQAADTTSDFDKEKLQERLAKLSGGVAVVKVGAATETELRERKL
RIEDALNATRAAVEEGIVAGGGTALINVQKKVSELEAEGDIATGIRIVLRSLEEPLRQ
IAENAGLEGSVIVDKLKHVEAGIGFNAANGEWVNMIEAGIVDPTKVTRSALQNAASVA
ALLLTTEAVVADKPAPEAPMGGGAGMDPSMMGGMM"

CDS complement(2091389..2091673)

/gene="groS"
/locus_tag="JFBMFIFI_01977"
/inference="ab initio prediction:Prodigal:002006"
/inference="similar to AA sequence:UniProtKB:O50304"
/codon_start=1
/transl_table=11
/product="10 kDa chaperonin"

/db_xref="COG:COG0234"

/translation="MLKPLGDRVIIEVAKEQEKTIGGIVLTSSAQEKPQTGTVIAIGE

GRVLENGTKVDIAVKVGDTVLFEKYAGAEVKYDGKEYLVVKEHDLVAIVD"

CDS 2091994..2092644

/gene="MroQ"

/locus_tag="JFBMFIFI_01978"

/EC_number="3.4.22.-"

/inference="ab initio prediction:Prodigal:002006"

/inference="similar to AA sequence:UniProtKB:A0A0H2XEK8"

/codon_start=1

/transl_table=11

/product="Membrane-embedded CAAX protease MroQ"

/translation="MKLTIKHTSIAMLFTYLLAQLLPVLLAQIAPKQLKIEFVVYGTL

LFFALGTLIMIQLNRKSTIRNSITLQKSLSLKQVITWGIIGVFAAIIMQQLAQFIEVH

LFQQTIASQNSAEIFAIINKYPLFLLTTVLFGPIMEEFVFRKVIFGFFFDLTGAVGAA

VISSLLFAFVHMDGHFLVYSTMGLVFCFLYTKTKNIATPIIAHVLMNTIAVILSLI"

CDS 2092702..2092908

/locus_tag="JFBMFIFI_01979"

/inference="ab initio prediction:Prodigal:002006"

/codon_start=1

/transl_table=11

/product="hypothetical protein"

/translation="MSKNGFFIQIIFNYILAAVFIWFAVDFVSTSGWGFFTILCVLIA

TNDFVRATKMLQIYLQISKSNKPK"

CDS complement(2092934..2093587)

/gene="rex_2"

/locus_tag="JFBMFIFI_01980"

/inference="ab initio prediction:Prodigal:002006"

/inference="similar to AA sequence:UniProtKB:O05521"

/codon_start=1

/transl_table=11

/product="Redox-sensing transcriptional repressor Rex"

/db_xref="COG:COG2344"

/translation="MSTQKIPKATAKRLPIYYRYLRFLNDAGKDRVSSTELSDAVKVD
SATIRRDFSYFGALGKRGYGYDVEDLLNFFSKTLNQDRLTNVALIGVGNLGHALLNYN
FHQSNNTRISAAFDIDEKLVGTIASGVPVYPMSEMVEQLKLQQIEVVILTVPAPVAQE
TVNQLVDANVKGIMNFTPLRISVPDDIRVQNVDLTNELQTLIYFLDHYTADDMTEEE"

CDS 2093912..2095876

/gene="yheS"

/locus_tag="JFBMFIFI_01981"

/inference="ab initio prediction:Prodigal:002006"

/inference="similar to AA sequence:UniProtKB:P63389"

/codon_start=1

/transl_table=11

/product="putative ABC transporter ATP-binding protein
YheS"

/db_xref="COG:COG0488"

/translation="MILLQTQQVARHFGADVLFENIQLEIQDTSRIALVGRNGAGKTT
LLKMIVGLEDPDAGKIAKGKAVTVGYLAQHTGLDSDKTIWDEMLTVFEPVLQMEKRMH
LLEEKLGEPELLANSEAYQKVLDDYDQLQHDFSSANGFGFEAEIRSVLHGFRFFEQDY
TKSITQLSGGQKTRLALARLLLEKRDLLVLDEPTNHLDIATLNWLEDYLQTYKGALLI
VSHDRYFLDKVVNEVYELSRQKMTHYKGNYTKYLDQKAANLEREWKEFEKQQTEIAKL
EDFVARNLVRATTTKRAQSRRKQLEKMDRLDKPKGDEKSARFKFQPAKESGNVVLTVN
DAAVGYDNKIISAPIQLDIRKLDAVALVGPNGIGKSTLLKSLVDDIPLIQGEKRFGAN
VELGYYDQEQANLNSTKTVLAELWDAHPLTPEKDIRSILGSFLFSGEDVEKSVTSLSG
GEKARLALAKLSMEKDNFLILDEPTNHLDIDSKEVLEDALIEFPGTLLFVSHDRYFIN
RIATTVVELSETGSKLYLGDYDYYLEKKAEEEELRLLAEEEQLKNISQAAPTELNAGT
KKHYQIGKEEQKLIRQLTRRIEALETEMAELESTIQDLENQLTLPEVFGNHVKVQEIN
QILQELKVKNETSMNEWEEQSLALEELGDV"

CDS complement(2095931..2096845)

/gene="tagU_4"

/locus_tag="JFBMFIFI_01982"

/EC_number="2.7.8.-"

/inference="ab initio prediction:Prodigal:002006"

/inference="similar to AA sequence:UniProtKB:Q02115"

/codon_start=1

/transl_table=11

/product="Polyisoprenyl-teichoic acid--peptidoglycan

teichoic acid transferase TagU"

/db_xref="COG:COG1316"

/translation="MKKTNKIIVIIGVLIVGVLGGMGAFAYKMYSDVTNVTDKIHNEV

STEKKREEKVKVSREDPFSIALLGIDTGDFGRVDQGRSDSVTVLTVNPKTKQTKILSV

PRDTYTEISGFNKKDKIAHAYAFGGIETALNTLQNLLDIPIDYYISIDMGAAEKIIDA

IGGVDVNSPLAFELDGIAFKKGPAHLNGEEALAYTRMRYDDPKGDYGRQARQREVLEN

IIKEGATLSSLMKYKDVLTVIEDNMKTNLSIDEMFEIQKNYRSAADSIEQVQLVGEGE

MLNEVSYEMIPQDEIERVSTILKNELDI"

CDS complement(2096995..2098020)

/gene="tsaD"

/locus_tag="JFBMFIFI_01983"

/EC_number="2.3.1.234"

/inference="ab initio prediction:Prodigal:002006"

/inference="similar to AA sequence:UniProtKB:O05518"

/codon_start=1

/transl_table=11

/product="tRNA N6-adenosine threonylcarbamoyltransferase"

/db_xref="COG:COG0533"

/translation="MPIKRNLILAIETSCDETSAAVVENGHILLSNIIASQIKSHMRF

GGVVPEIASRHHVEQITQCIEEAMVEADVDYSDLTAVAVTEGPGLVGALLIGVNAAKA

IAYAHNLPLIPVNHMAGHIYANRLIQPLVFPLLALVVSGGHTELVYMSADGEYQIIGE

TRDDAAGEAYDKVGRVLGLTYPGGKRIDEMAHLGEDTFHFPRGMIREDNYDFSFSGLK

SAFINTVHNAEQKAELLNKNDLAASFQASVVDVLLTKTLRAAKELDIKQLVLAGGVAA

NRGLREGLVDMMAKELPDVELIIPPLSLCGDNAGMIGAAAYVSYQKGEFADYQLNAQP

GLSFEMV"

CDS complement(2098026..2098517)

/locus_tag="JFBMFIFI_01984"

/inference="ab initio prediction:Prodigal:002006"

/codon_start=1

/transl_table=11

/product="hypothetical protein"

/translation="MENEKYTKKQIEIDYRFSSIEETSYTAKDLFDLAEVSYPHGSPW
STETFKKDLKQPYVQYEIICLGTELLGFIGYRQLFDEVELTNIAVHPKVQGQGLSQAF
LVQWIKKLHQARVIHLEVRKSNQVAIHVYKKVGFKLINIRQDYYDYPVEDAVIMNLEI
KEE"

CDS complement(2098507..2099112)

/locus_tag="JFBMFIFI_01985"

/inference="ab initio prediction:Prodigal:002006"

/codon_start=1

/transl_table=11

/product="hypothetical protein"

/translation="MLKKFRNWLTASADEEEAENQRKRWTRLTERVELQQTDFPLNEE
EVAVLSIGTAEDIPDLLEIERLSYHGETPWNQKAFEHEMKNNRNALYLVLRVYDVAIG
FIGSWFVEGEAHVTNIAIIPNYRRYGLASFLMEQMRHLAEADHNQLFSLEVRMSNTGA
QELYRKLGFKDGKIKKAYYSEDHEDALEMSLVLKGHKKSGE"

CDS complement(2099096..2099824)

/gene="tsaB"

/locus_tag="JFBMFIFI_01986"

/inference="ab initio prediction:Prodigal:002006"

/inference="similar to AA sequence:UniProtKB:O05516"

/codon_start=1

/transl_table=11

/product="tRNA threonylcarbamoyladenosine biosynthesis
protein TsaB"

/db_xref="COG:COG1214"

/translation="MKILAIDTSNQVMSVAAIENQRIVGEFTTNIKGNHSQRLMPAIT
DLMEAVEWTPAELHRIAVAKGPGSYTGLRIGVTIAKTLAWTLKKELVGISSLEVLAGN
CQGANHYIVPMMDARRGNVYTGLYRWDQEAEQLQQVEPDTHIALADWVKFLKTKEGTF
EFVGTDWAIHEETIKDALGERASEVAAQNQLPRAGVLGLLAEKAEPVDSHTFVPDYLK
LAEAEENWRAAHPNQLEESYVEKI"

CDS 2100127..2100291

/locus_tag="JFBMFIFI_01987"

/inference="ab initio prediction:Prodigal:002006"

/codon_start=1

/transl_table=11

/product="hypothetical protein"

/translation="MTDFKDKAKGLKDEVAGEAKELFGKVTNNLEKEAEGKAQKLKGK

VEKKVGDVKK"

CDS complement(2100375..2100794)

/locus_tag="JFBMFIFI_01988"

/inference="ab initio prediction:Prodigal:002006"

/codon_start=1

/transl_table=11

/product="hypothetical protein"

/translation="MTIGIYLNFKDNTKEVIHFYEKVFGSKCTDLMTYGQQPDESTHE

LDEKTKGLVMNASLEIEGDTVMFSDVPDFMGMDFIPGNNVTLVLETKDSEKLTAYFNG

LLEGATKVMPLQKTFWSDSFGQLTDKFGISWSFNLTK"

CDS complement(2101105..2101317)

/locus_tag="JFBMFIFI_01989"

/inference="ab initio prediction:Prodigal:002006"

/codon_start=1

/transl_table=11

/product="hypothetical protein"

/translation="MKIEQHWDDVDLYVVVYTSHLDESEVQSTMIIVPPESEDKLIRE

KIHATFSEIKEIRQLEYFSEALYLKN"

CDS complement(2101314..2101556)

/locus_tag="JFBMFIFI_01990"

/inference="ab initio prediction:Prodigal:002006"

/codon_start=1

/transl_table=11

/product="hypothetical protein"

/translation="MKKPVVELEKYIIYNIRFIVESEFIRQIEKNIVCPVDYNHEDVE

QLIYKKFNQVKEVVKINKLEECTKLDSKIALVGDRV"

CDS complement(2101579..2103039)

/locus_tag="JFBMFIFI_01991"

/inference="ab initio prediction:Prodigal:002006"

/codon_start=1

/transl_table=11
/product="hypothetical protein"
/translation="MLAETDKIIYRKIQLLKVLDASNDYLKTVDLANFLDLSLKTVQK
ELESLIEDLKNSSYAVKLEKVGNLYRFIKKSSVNMDLIYLDFKRESIYFYLMRKAVFR
KTIKEKKEIDYFYSASHLYKNKRIFKTYLMNYDLELDLSTLSIEGNEINIRFLYFRFF
WENYRGVEWPFDTIDRQELISEIEQLDTFLFKSMTEIEKEQFLYWVAIIKIRASNDQL
LIENRVKLNQVMFKDMQEFQQIVAKTIGVIEKTTLHSETVYLYFAYTLTTESTFYKQQ
LLADLEKDNKNFQIHKMVRELTTKFSQKFSLNTHTNYVDVYFSIYKLFVCESIYQGSL
REYESRNLFESGIIKQYEKEFALFYPEVMKNYGKKYSENRYLYQRVLFLFSTLIEESS
YRPQINIKILSSGGAIEELLLTKKVQSLANNIAIKDSNAKDLDIIVSDCMVNNTENDL
VFYWNLQPTEKEWEALARRIAEIEQDKELRIPLVTN"
CDS complement(2103064..2103618)
/locus_tag="JFBMFIFI_01992"
/inference="ab initio prediction:Prodigal:002006"
/codon_start=1
/transl_table=11
/product="hypothetical protein"
/translation="MKWRQSKFRFLKNCILYSGIFSMTILYSTELSPPAFADPVNESS
QLIESQSEQEKHPTISGAVGPLNPLNPEESVEPIDSVDRQKKAVEEKRSEPSEIELIH
PESDLEGIPDGSKAIKITDLDDLHKNKKKKNVHLNQVYIDENMPLPSEPFPNGKGKKT
TEMAYIEEVFSEFSGVVFFGVKQR"
CDS complement(2103615..2105096)
/locus_tag="JFBMFIFI_01993"
/inference="ab initio prediction:Prodigal:002006"
/codon_start=1
/transl_table=11
/product="hypothetical protein"
/translation="MRNLLNREDGKKVSLFRFIEECTQQTASFSAIMHELEISEFVLL
RTAENLSKDIELNDLTPHFSLTISRTHKTITLKKDSDASISMLYIIYIKNSLSYNILV
DILNGKFVSMTDFGDFNFVSYSAVHKKIQEVKKELANYQVRLSSKYELVGDEIKIRMF
FYHLYYPRFNQLNFPFEAKYEKLAQQFIRLLENHLDHSIQETLKTKINFFLSVSLKRI
NMKNYLTSNARIERTRYTFNHEHPLFQMVYEFLEKEYRLLKREALISESESIVAFLVG
ESGVRRTEYSPLEDIVEVQDNLTPLFVHEFEDQFKNKVEKAQHDLLIQELSVLHFKLY

YFKKVQPVFGDLLNMEFLEETYADAFKFCVEFIGRLPDKKEYKLLTSNENFVFHQYLF
LIVQIFPSKFFMETIYVCLDFSLGNYYTNIIETNLKSFNFFNIEVTSYVHEKTDLFIS
DYIYKNIKLPSLVWNMPPTAKDWANVGEFLVRIKNEKAEKVIK"

CDS complement(2105116..2105982)

/locus_tag="JFBMFIFI_01994"

/inference="ab initio prediction:Prodigal:002006"

/codon_start=1

/transl_table=11

/product="hypothetical protein"

/translation="MKKKRTRWQEIILVISSILLLIAGLTLVSGPWIKSALISNYSYD
YTRETITSSKIQENQSTLGEFDYAAVVPPSLVDVLAAMNTDYSDQVIGQLTIKSLSMN
LPILKGTTNDNLLIGAGTMQENQDMGMGNYTLIGHHMQDKSLLFGPLLKIKEGAIIQL
NDQEILYTYQVQTTKIVSETDMSVLADVGDNRLTLITCDVSSATNQRFVVVATLVEKN
NLNRKNVNTHEPKNNTEIMPKNVQEYQEVVSLTQKHEKRSISVILIGILILLFLGCLL
FFIRKHYRQLEH"

CDS complement(2106001..2107089)

/locus_tag="JFBMFIFI_01995"

/inference="ab initio prediction:Prodigal:002006"

/codon_start=1

/transl_table=11

/product="hypothetical protein"

/translation="MKKYLNIVAILTVVTMSFFSLGGITADAAELNFAVTPTIPENQV
DKTKTFFDLLMEPEKEQTVEVQLRNDTDKDVIVETQVNSATTNLNGVVEYGKTKAKQD
STLKYNLADLVEVDSEVTIPAQGGVTLPLKIKMPKESFSGILAGGISFKEKDTGEKQE
ADGEGIAIQNKYAYIVGIVLRVDPAEIQPDMLLTKVAPAQVNARNVVNANLQNPTAMF
INQLKVSAQVTRQGSKDVLFSSKKENMQMAPNSNFDYPIALSGKKMEAGKYTVKLTAE
SKGEKWEFEKDFEIDADTAKKFNAQDVTIEEDNTWLYILIGVGLILLAIVLIFIIMYR
RKKKKEAEARRKAANRKRRKRKAQSKPK"

CDS complement(2107226..2107963)

/locus_tag="JFBMFIFI_01996"

/inference="ab initio prediction:Prodigal:002006"

/codon_start=1

/transl_table=11

/product="hypothetical protein"

/translation="MKMTKLVTSGAVLISVIALGSATVSAATAGTKATNGTVKFITDT

DITNPVDPLDPEVPVNPVDPVDPEKPVDPGTAGPLSIDFASPLYFGEQKISTVDKVYT

ADAQPLSDATTRPNYVQVTDKRGGEQGWTLQVKQDGQFATADDNELVGASITFANGEA

ITGSASAIPSILTTGFSLVPDGTGAAQVVMAAKAGEGAGTQIYRAGDDATKASSVSLS

VPGKTIKYQDTYTTSLTWTLTDVPGAE"

CDS complement(2108022..2108774)

/locus_tag="JFBMFIFI_01997"

/inference="ab initio prediction:Prodigal:002006"

/codon_start=1

/transl_table=11

/product="hypothetical protein"

/translation="MKLTKLVTTSAIVLATLVGSSSAALAAEYKSNGAVEFIENTDPT

DPVDPTDPTVPVDPIDPTDPEGPNPGTPGPLSIDFASSVVFGKNKISNANQTYFADAQ

ELKDGRFVPNYVQVTDTRSGNLGWTLTVQQEGQFTNATAANKVLTGSVIKLANGAVSS

SSTSPAAVAAPTITLDPSGASSTVMSATAGSGWGTWLNYFGDVTEETIAEDEVVQKNK

AITLDVPGSTPKDAVQYKTELTWTLSDTPGNV"

CDS complement(2108771..2109130)

/locus_tag="JFBMFIFI_01998"

/inference="ab initio prediction:Prodigal:002006"

/codon_start=1

/transl_table=11

/product="hypothetical protein"

/translation="MKKRVMTFGMILVFLLTLVPVTQVFGAESENQYKSNGEVSFYGE

YIYPSEPDKEKPPISGNGTSNLPDTGKTPQQTLPQAGDRFEVFNLVLGICLIGLAGQV

VYYLTKKNNIAHGGKIT"

CDS complement(2109142..2113245)

/locus_tag="JFBMFIFI_01999"

/inference="ab initio prediction:Prodigal:002006"

/codon_start=1

/transl_table=11

/product="hypothetical protein"

/translation="MKKKKGIWLTSFALICFGMFVFALQLSGISTNQKLKALESNSKN

SLIELATDSEDLEVGSEFNLNVFVSPNVTKAIELPLPAGLRFNPEQIESPDYQISYDS
ASSIITIQPTLIEAKLAESDSAIEPEVTDETEQLTIPIALQVEKAGEFLLEAKTVYQE
DPLSSNQLNFDAAETSEKPVVETSAELEKPIAAASEADVVEPVLPKMSARAAGADVGT
WAEFIAAIADTSVSEINLTSDITRTATTAANNPGTLTRSLTIHGNNFTINFGSGGTTS
NGILLGIAPVGTQLTLDNVQLVKTQTPTTPIFNAVTGATGLGTNWVINVSNTTTTDAN
ESGLVGAVNATVNLQGTNNLSLKASASHLNVRHVIFEENSIFKSESKATNSSSAIGIT
NGTVLVKENAKVTINNVGTSGNTAGNAAQSISSSSAIAGSISDFTMEPKSEVLTTANI
YGYRTTIQNNFTMTGGAKFEAIGNTSSTVVLAQDFGDGVGVPATINISGAGTSFKVST
NSVQQANTGAAMRVQGDGSIFNISDGAEFSGHAKNGTALQIQSQGSQFNVKNKAKLNL
TQDGDNGYSLGATLRFRIRGIQTFNVTGEAEVNITKFSGRTPAVRMYGDDNVINVNTG
GHVNVRNYGDGTRNDGGGDNGNQGLLYTNGNRAVFNLEDAGSSVDVRADFGPALRSTG
SSEITAGEGTIFKMRGNTNGQTGGIFNTAVTTITVDRPLFFDFRNDRPAGGYVFNVNN
GSTFKSIQSDLSVWARGDNLDGDPVKNWSLFDYQLSGANFVRIDSTNVPAEFNTGADS
YGTTGASAYARMSGNNARAVVDELRVPTNADKSIFGHVTIPVGGEDGRDAWTDEAYVV
VKLTKADGSSQELTGSTVGKNNNSPGLSVYGEESRAGMFKIPVPNNVFLETGDKLEVV
KAWRGSADPNSNRVHVSLPEDLIAPNRTTFKITPPTPAVITNSRVVDNATKQIMGTSD
EPGSEVEISVNGVALNATAIVAADGTFTFNLPHYLEKGDVVQVKLRDHAGSASAAGVD
NPPSTNNEVGNINPTTDLPFHDTIFLAAPSVTVADILPDTNTAVKSVVVDNDKNVTQV
GSILTYTIDVANNKAAAISTNWAAVTIEDQLDEGLDPDLNSFTLNGTSIPASDIQFDE
ATRRLKVKVGNLTTQEDARLTFDARVNSAGIDRTILNKATAIGNSPREVEPFELGPEN
PTAEKQTYQAVSNEIPNPGGNVFGVLELVSAPTDISFGENLKVSAKNEIYPVADMSGG
LTVQDSRAVKSSWTVTARMSAVLESPSGKTLPNAVYYTYKGTDNVLGTDSILVYQHTS
TNEDPLTISDSWNTQEGLSLRTQVGKVYPENYQGQIEWALQDTPENE"

CDS complement(2113637..2117569)

/locus_tag="JFBMFIFI_02000"
/inference="ab initio prediction:Prodigal:002006"
/codon_start=1
/transl_table=11
/product="hypothetical protein"
/translation="MSKKKKIGFSSFVLICIGLFVLAVQFSANQRLKALETNEDKNPL
IELVMDSEELLKDNEFNLTVLISPDVTKDIVLPIPEGLTFMTEQMESEDYQVTYDSTS
RLMTIELSPIEEVESENNSATEALVQNKAERKPLVIPLAFKVENTGEYTLEAKTIYQE
NSLSSNQVTFSVTETQEKLELETDEELEEPIIDAPTVDENAPTLLRSPKSAVANVGTW

DEFGAAINDPSVSEINLTANIYQQSWNLTKLNILTRSLTINGNNFRLDLESKGIQLGI
VPVETQLTVDNVKINKTVSAHIPIFNAVTGAAGSGTNWVINISNVQTDEENESGLIGA
VNATVILRETNNLHLRAATFQLNVKKLIFEENSIFKSRSRLANGIQPTTLSAIAITNG
TILVKKNAKVSIINDGKSKEKAGKPVQSSSSAIAGSISNFTMEPDSEVLTEANIYSYR
TTIKNTFTMTGGAKFEAIGTANSIFVSAENVGKGAGVPATINISGARTSFILSSHRIG
PSEDGAVIRIQGDGSVFNVTDDAEVNVTKRISSSKLKGSAPVVRMDGKDNHININTGG
KFILHNTGSDGPNDGLGSSGNQGLLFTGGNGAVFNLKDAGSSAEIRSNYGPALVSTGS
SKITAGEGTIFKMRGRTKSKAGALFNTATSEITVDRPLFFDFRNDQYDGAKGSIFNAT
AGSTFTSIQSDLSVWPHETNLNASPAKNWSLFDYKLSGANFTKIDSTNVPNEFNTGAN
SFGSTGASAYSRMTGGKVRAVVDELRVPTNADKSIFGHVTIPLDGENGRDAFTDEAFV
VVKLMKANGKSQELTGRTIGKNNNNLGLSVYGEASRGGMFKIPVPNDVFLETGDKIEV
VRAWRGGADSNSNRVHHSLPEDLVAPNRTTFDITPPNPAVITNSRVVDNATKQISGKA
NEPGSEVEISVNGVDLATTTTVQPNGTFVFNLPHYLEKDDLVQVKLRDHAGLASTVGV
DHPPSTNNAVGNGNPTTNLTFHDQVFIAAPSVIVKDVLPDTNKAVKSVVVDNSKNATQ
IGSILTYTIDVENNKPAAIATNWKAVTIEDQLDKALDPDLNSFALNGILIPATKVEFN
EETRLLKVKVGDLGSNKKAKLTFNTRVNKTGINRTIFNKATIIGNSPREVEPFKVGPE
NPTEAKQKIQTISNKIANPGGPVFGVLELVSTPTTISFGEDLKIAPTKQRYPVTEMSG
GLTVQDSRAVKASWSITARMTSILESAEGKKLPNAMHYTYNGIDQILGTDSIVVYQHT
STNEDPLSISDSWNVQEGLNLQIPAGRVYPGRYRGTIEWTLQDTPETK"

CDS complement(2118001..2118327)

/gene="licA_4"

/locus_tag="JFBMFIFI_02001"

/inference="ab initio prediction:Prodigal:002006"

/inference="similar to AA sequence:UniProtKB:P46319"

/codon_start=1

/transl_table=11

/product="Lichenan-specific phosphotransferase enzyme IIA
component"

/db_xref="COG:COG1447"

/translation="MRDERTLNLTEQALKIILHAGDGKLLTKEALKHLENGESMQAEK
KLDQAQESLRIARGTQMDVIQMETNGQKVPHLHLFAHAQDILMTATSEWTMATHIAVL
VNKEDT"

CDS 2118735..2119106

/gene="immR_2"

/locus_tag="JFBMFIFI_02002"

/inference="ab initio prediction:Prodigal:002006"

/inference="similar to AA sequence:UniProtKB:P96631"

/codon_start=1

/transl_table=11

/product="HTH-type transcriptional regulator ImmR"

/db_xref="COG:COG1396"

/translation="MTLGEKIKMLRQKKGLTQAQLADLLFLKQNAISAYEKDVNQPSL

EMIKKMATIFNVSADYLLGIDEANKNSSLEKTFQHILEEVIYEESYAYIKDGDVTDET

AEILKLMIKKDFKLLDDIYKH"

CDS complement(2119690..2121072)

/gene="sau3AIR"

/locus_tag="JFBMFIFI_02003"

/EC_number="3.1.21.4"

/inference="ab initio prediction:Prodigal:002006"

/inference="similar to AA sequence:UniProtKB:P16667"

/codon_start=1

/transl_table=11

/product="Type-2 restriction enzyme Sau3AI"

/translation="MIVYETEDELLFKAKQANGKTFGEIDKTGRILNEKNKGGLGHIV

EEGFFGYEINSDARPDFENLNIELKVTPIKVNQNKSISAKERLVLNIIDYMKEPQNDF

TTSSFWKKNEKILMMFYQWFPEAARKDYKILGSILHQYSENDLLILKKDWETIVNKIK

LGKAHELSEGDTMYLGACTKGANKNSLREQPFSDIPAMQRAYSLKQSYMTALVRSIFD

GEVLTKLATPEELKEKSIEEILAEKFNPFIGMTDDEIAKELGIQVDKKNKSYIPQMIS

ALLGIKGTNLDKIEEFSKANIKFKTVRLEPNGKPKENMSFEKIDFDEWLDESWEESKI

YKQFEKTKFLFIIFEYKETLKENSKRELYFKGFKLWNMPEEIINSKVKELWDEVRKIT

REGVQIEYKKHGKRVRETNNFPKLNFNGVTHVRSKAKDGNDKVILPDGQKITKQCYWL

NRNYVGEILK"

CDS complement(2121356..2123944)

/locus_tag="JFBMFIFI_02004"

/inference="ab initio prediction:Prodigal:002006"

/codon_start=1

/transl_table=11

/product="hypothetical protein"

/translation="MLKKLQGQLKSSYSSIKDTVSEDFYNPVLSESISYKRVSGYFSC

KALSLYAEGLDRIAQNNGYVQFIISQNISEKDFKEIQEGYFERSKNETLTEIDKQRLG

NLAYLISQGKVDVKFGLVKNGLFHTKWGLFEDIQKDIVYFNGSLNETANAIENNYDSF

DVDVSWDVSTNVRNRIIRKTEEFNQLWSNNYSGVQVIDATDIIYPLLEEFDMGKIQKI

PNPEYNSVVFEMNDEHFFLVDKSEEGVISKKTFKNKFSFYVDQEKGYPFFRGNLTYRE

IEKIIDSLSKQAIKRDFNLVISSRVDNFIISQKYSIEEYRKSGLTLKNKDNRWQEEFR

EFKEVVESEVVRPLKPLQLQAGMYMLTQKRAANFSVPGAGKTAMLLGVFAYLNSKKKG

EPIKRLLVVSPINAFMSWKEEFDEVFKTNKKLRVLSIHDDSINGNKFAFEAQWASANL

ILINYESLPKYQDSIIKCLSNDSNTMLVYDEVHRVKGVQAKRALSALEIADKVDYRYV

LTGTPIPNGYLDIYNFLHILFKKEYSAYFGFEQNMLKNPDNWQIEEINEKLAPYFWRT

SKEDLGVPPADSDNIIKVLPSAEQIRLAEIIYTTAQNPLAALIRMIQLSTNPEIINKT

INYSDLGYSEDDDNNADSYDQVSLKVRKELEKSIHDAVIGEVNEWDLAKVGSPKFDAG

INLVMDIIKKHGKVVVWGLFVDTLKKITRVLNENGINAKIIFGGTPRDEREDIIHTFK

KETGEIQVLVSNPNTLGESVSLHNIVHDAVYFEYNYNLTFMLQSRDRIHRLGLKENQS

TRYYYLMTTSDREMYNFIDQKIYDRLAEKEERMKEAIDGEILIPEFVDNEIEEMKEII

KSERRF"

CDS complement(2123948..2125219)

/locus_tag="JFBMFIFI_02005"

/inference="ab initio prediction:Prodigal:002006"

/codon_start=1

/transl_table=11

/product="hypothetical protein"

/translation="MDEVNLFKLFEKGIVKDGGHNVSVPIKKASGEKYDGKTYAIPLK

YLYYNEQNGRIGVSLSDYESSSGRLIPGHNEEYNLIIQNMLADDDDEKTKKEMEALKR

NIATKGQDEPGYVLSDGRVIDGNRRFTARRLLEQDSAVTEQQYFEAVILDDLSVQNYD

DQKKIKSLELQIQFGKLDKVDYNPIDRAIDAYKTIVVSNIMSVAEYTDFAGLTVNETS

KRILEAELIVKFLEFANTNPDNYALAKQLDLDGPLQEMIPQYKKIKDSDNLDQLLNSL

FAKIVQMRASKEDFKLEFRQIVKSVIGTKNEEKFIEDMEDATDSIVEALDQKEVIKNN

IDLFSTLNSNQETINAMAEVQSISTNHSEKAKNLKEQTMPVKLVEKAFNSIEAIDKKT

VLGLPKLEMKKLENSLEKLKLQIDKLFSVEE"

CDS complement(2125232..2125942)

/locus_tag="JFBMFIFI_02006"

/inference="ab initio prediction:Prodigal:002006"

/codon_start=1

/transl_table=11

/product="hypothetical protein"

/translation="MNPNKELMISAKIEIDSMVDRYKGKKVSDLKEIFDMPTKTTKAS

FVKLARKMLAIPNNKFELCENKVDAVLKTVRLTGMKTPAESMSFMPVDFDEWSNQLVW

ENSSLYKYFDKKVLVLFIFQQYPTGKRVDDSEMTFLDVKIWKMSDYDLNHELKEVWEQ

VRYLINENKLEIVPIEQKNGKIVNKNNLPSSKFNALGHLRPGGVNGDDKVKLPTGQSI

VKQRFWFNTEYIKEILDL"

CDS complement(2126175..2127590)

/locus_tag="JFBMFIFI_02007"

/inference="ab initio prediction:Prodigal:002006"

/codon_start=1

/transl_table=11

/product="hypothetical protein"

/translation="MTMTLNIFSMFDGVGGFIVGLNDANRTIEKEVFKTTYSNQFEPS

RKSQDAFEVGTYRFPDMNHIPDDIMTVSEEKFQEMRDNGVNMIVGGFPCQDYSVARSL

KHEKGITGKKGVLFWEIIRAIKVIQPKYLILENVDRLLKSPSSQRGRDFAIMLGAFNK

LGYSVEWRVINAAEYGRSQRRRRVFFFVYKNDTDWAKKMDATYEENISIDGALEDIVI

NSTQYDDYIFHEGLFAREFPVNTEVKYDGKRPRHASHYLGKDTDYDDYILNISDNFTG

TVWNTGIMRHGKYFTVDTTPIEETPINLGEVIEIAKQSFINQYDNTELGEQEYTQYIS

RYIISDTDKIEKFKKLRGAKKIPRKRPDGSEYFYAEGAMSPYDSIDLPARTMLTSEAS

VNRSTHLLFENDQYRLITPNEAEVLQDFPVDWTRYKKDEITGEITEVSDRMRYFFMGN

ALVTGIVKRIGIRLAQIDNEN"

CDS complement(2127593..2127793)

/locus_tag="JFBMFIFI_02008"

/inference="ab initio prediction:Prodigal:002006"

/codon_start=1

/transl_table=11

/product="hypothetical protein"

/translation="MIQLRINEILEARNMSQKDLCALSGLRPTTVSEMCRGVRTAINL

KHLETLIEALEIDDFNQILKRD"

CDS complement(2128109..2129002)

/locus_tag="JFBMFIFI_02009"

/inference="ab initio prediction:Prodigal:002006"

/codon_start=1

/transl_table=11

/product="hypothetical protein"

/translation="MMLLGDVIKAKRLKNGLTQAELAAGICTQASISNLEKNQRIPSL

MALLAIGNRLNLNFDELSDYAIEQENPTFDVSNRVRTLRKQFKLKEAYDLIIEELPLD

KLKTYHKKKQYYYYLGITGLLGYNKIVEAHYNFNLALSSESEPNLDFYDILTINGIGL

AYYYESEEEKARTYFEKSLVQLEQFMSLMVSLDESIEITKIYYATAKFYSEIKEYSKA

VDLCSLGIQVQLDQHVNYELEMLYYEKAFNLAKLNKKSEAEECYYYATALAKMINNTM

VVETIKENIIKFRLNSTNDLL"

CDS complement(2129083..2129973)

/locus_tag="JFBMFIFI_02010"

/inference="ab initio prediction:Prodigal:002006"

/codon_start=1

/transl_table=11

/product="hypothetical protein"

/translation="MQTLGEIIKERRLAKGLKQGELAEGICTQATISNLENKSGMPNL

SILLAIASKLDIEFSELSDYALTNVNYTTAIFNQVKQLCATYKHKEAQDLLVKEIDVK

KLEKGYEIKKYYYYLGLTELIGNKSFTDAHYYFNLVLSDKSETTIDLIDVLATNGVGI

AYVLDSEIDKAKTYYEKALNQLEELIENFGEIRDSLDITTIYFNSAKFYSQIENYQKA

VELCNLGITLQQMNHTNFELDRLFYEKAYNLAKLGRTEEAEEFFFHAASVARLNKNII

VLEAIKKNMAEFKISGYKYW"

CDS complement(2130380..2131711)

/gene="noxE_1"

/locus_tag="JFBMFIFI_02011"

/EC_number="1.6.99.3"

/inference="ab initio prediction:Prodigal:002006"

/inference="similar to AA sequence:UniProtKB:A2RIB7"

/codon_start=1

/transl_table=11

/product="NADH oxidase"

/db_xref="COG:COG0446"

/translation="MKKIVIIGSNHAGLAAINVIVNSGIEAELVVFDKNSNLSYLGCG

TSLFVGKQVESVNNLFYTSKDAYETKSSKIHINTEINKIDFIEKVVYAQNQEKGEIKT

EYDELILATGANPIELKLEGRDLKNIHTVKIFQDCQVVDQLLEDEAIQNVVVIGAGAI

GIEMAEAVKRRGKSVDLIEKKATCMSNYFDPWFSEDMDACLMDNDIHLHYSEEILAFQ

GEEFVEQVVTNKAVYQADLVLIAIGFQPNTGLGKNKIRLSEQGAFKVNLKQETNLPGV

YAIGDCATIYSNALEKDTYIPRATNAVRSGMIAGSTISGIDLESTGTQGTTGISIFGY

NMVATGLNLSEAKKARFDVLYTEFEDLQKPVFMEENHSVKVRIVYDKNTRRILGAQLA

SYHDISMGIHFFSLAIEEKVTIDKLKLTDIFFLPHFNQPYNYITMAALGAK"

CDS 2132040..2132420

/locus_tag="JFBMFIFI_02012"

/inference="ab initio prediction:Prodigal:002006"

/codon_start=1

/transl_table=11

/product="hypothetical protein"

/translation="MNLLLTLGAGILAFFIGALWYTVFFGKSWIIESGMTEEKIKEQG

GAGISMVSTLVMEILVAFLVTYLIRQTNLPIMTSGLLITGIAILSSLKNYVFEKKSIK

LILINESYKAICILIMSASVFFFN"

CDS complement(2132438..2133334)

/locus_tag="JFBMFIFI_02013"

/inference="ab initio prediction:Prodigal:002006"

/codon_start=1

/transl_table=11

/product="hypothetical protein"

/translation="MSSYGETIRKIRLDKGISQKALYTGILSKSYAIEFEKGKHEISL

KRIEQILDQLIVPLDEFLFIYRGYSLSEKEWFSARYAEYGNSNNLTGLYSLLKEYEHR

KDSLSEIRSAEIRSRIRQLNEFNQERHYKKSAILVEDQLIIQQYLSNLESWTLHEIQL

FANTLDFIDYQQKATYFKALTPSFEKYRNYDRGKQILCGFLTNIIYELIQDNELLLAE

HLVNELHSLSTQLTEIFFKIIAKFYQGLILIGQLNPEKGQAMTQEAVGILLALDQNHQ

AKLFEVIANDFTQKIKVAHECK"

CDS 2133558..2134283

/locus_tag="JFBMFIFI_02014"

/inference="ab initio prediction:Prodigal:002006"

/codon_start=1

/transl_table=11

/product="hypothetical protein"

/translation="MTTKKNFLERGNDDFPFYQDASSPLNFKGMLIIILSSLIGFMLF

TTTNIPFIAPFLNVIFPLGAFIFVTKNQWTKIFRKLVPNDGLVIIGTLVANIVITFMI

GLLVTTITGTGDANPASTQFDGNFSHAVPFFLKTIPMLFGEELLTILPFLIILKLGVQ

QFNLSRKSSLLLAWLLTSLLFASLHFPTYNWNILQTVLTIGTARLILTFSYIKTKNIW

VSFIVHLLNDWVLFGLAFLASTR"

CDS complement(2134353..2135279)

/gene="opuCC_2"

/locus_tag="JFBMFIFI_02015"

/inference="ab initio prediction:Prodigal:002006"

/inference="similar to AA sequence:UniProtKB:Q9KHT7"

/codon_start=1

/transl_table=11

/product="Carnitine transport binding protein OpuCC"

/db_xref="COG:COG1732"

/translation="MTINKKLKLILSLFFVLLLTSSCTLPGLGNGKSSEGVIHVTGGV

TSESQILASLVGGMIEHDLNQPVEIINNLASANINHQALLGGDADIAAARYSGTDLTT

LLQLPAEKDPKKAYDIVKKEFEKRYNHVYFPSYGFANTFAFMVTKETAEKYKLKTISD

LAAVANKLEAGVDTTWAKREGDGYPDFVKAYGFDFKRVYPMQIGLVYDALAAGKMDVV

LGYSTDGRIGSYNLQILEDDRQFFPPYDASLVATKEILEKYPKLTPVLERLDGKITTE

TMQKLNYQVDNNLLEPSIVAKKYLEEQNYFEE"

CDS complement(2135479..2135943)

/locus_tag="JFBMFIFI_02016"

/inference="ab initio prediction:Prodigal:002006"

/codon_start=1

/transl_table=11

/product="hypothetical protein"

/translation="MKLELKIVEVTNPDFEELVTELNAFFDQQWGDIAANYQEHHNLA

KMKQAIVAYVDGKPVGCGCWKLLTEDSLTGEIKRMYVKEESRGLGIAAKLLQKLEESI

AAEGYHEAVLETGADMLGTIKFYEKQGYRLIPNYGEFVNDEVCVCMKKKFSR"

CDS complement(2136196..2137077)

/locus_tag="JFBMFIFI_02017"

/inference="ab initio prediction:Prodigal:002006"

/codon_start=1

/transl_table=11

/product="hypothetical protein"

/translation="MIILGAKLKNARIAKGLSQKELAEGICTQATISQMENHNKVPTM

SIFVDICKRLEISQAEITSDDDSEINHTIFLEVSLLCKQYKFIEAHERLTKSIKIHEL

KSNSNKKKYYYFLGLTYLFGLANDDEALFYFNLGLTLQSKELTVIDLLLTNGIGRVYE

IKKEYEKARIYFDKSILDISKLKVPVDTILVELISLYFNTAKFYKNYQRYSTAIEFLE

KGIQLSRTYSNYYLLEFILYEKGVNLYLLENKVSEEVRKLYTLAYSFSLINSNALLLE

TIEQDAKNYGIQATQFF"

CDS 2137426..2137827

/locus_tag="JFBMFIFI_02018"

/inference="ab initio prediction:Prodigal:002006"

/codon_start=1

/transl_table=11

/product="hypothetical protein"

/translation="MQLSAKKIANLLYDNCSTFLINTIDSAGFPYTEIIDKPIYRDET

MNFYLFTKANSLTLENLSTSSNSNFSFFSTNLYKKVVLKGHLKTIPDNLISLASGETI

CIPNEHQVLYFSGVEGILYSDYQTHLFYNLD"

CDS complement(2137939..2138775)

/gene="ywqG"

/locus_tag="JFBMFIFI_02019"

/inference="ab initio prediction:Prodigal:002006"

/inference="similar to AA sequence:UniProtKB:P96719"

/codon_start=1

/transl_table=11

/product="putative protein YwqG"

/db_xref="COG:COG3878"

/translation="MFHSELLTIFPNSQHKALENSRKEAIALSLENNGELGLLISKVG

GFGYFPKDETYPVNSDGQPLRLLAQLNFSELPPLKDYPTKGLLAFYIDEFDDLLGCDF

ENPLNRVGYKVLYFPECDEELAYSKGELEDLFRRFSEIERYPVVNEEYQLIGTLENQY

VTGDSVEFEATYKSEFYNFFEKAYPDPDEAESKMEELFDIDISSPNSIGGYPSFTQTD

PRIRMPEFKDDQLLFQLSSTDYGNDVQVIWGDSGIGNFFIHPTDLVNQDFSKTWYNWD

CY"

CDS complement(2138983..2139465)

/locus_tag="JFBMFIFI_02020"

/inference="ab initio prediction:Prodigal:002006"

/codon_start=1

/transl_table=11

/product="hypothetical protein"

/translation="MDRMENKKTELVYFKIKQKLANLIGIIKGIQVKPLVVKRKAVNF

SEHYASLFKELLSQVLRQLRALHSMNQDFVLTGDTFTLQERDIFYSCGTWSKENLGYL

FKLEEIQFCKNCDGKLTRLYNDGKYNLGQKGNYYKCVKCAKMYSQEAVEKLKRSEIFK

"

CDS 2139901..2140518

/locus_tag="JFBMFIFI_02021"

/EC_number="3.-.-.-"

/inference="ab initio prediction:Prodigal:002006"

/inference="similar to AA sequence:UniProtKB:Q5XD24"

/codon_start=1

/transl_table=11

/product="putative metallo-hydrolase"

/translation="MLEIKRILTGAIQENCYLIYNEKECLIVDPGADADKIIAAIQDL

DIKPIAILLTHCHYDHIGALEEVRNYFTIPVYVDPLEQSWLTDPELNLSSALGQPPVS

AQPAEFEFSYQNYTLGDFNFKVVPTPGHSVGSVSFIFDDFVVSGDALFKGSIGRTDLP

NGNFEQLIASITTQLFTLPDSLAVYAGHGEVTTIGNEKATNPFFN"

CDS complement(2140591..2141484)

/locus_tag="JFBMFIFI_02022"

/inference="ab initio prediction:Prodigal:002006"

/codon_start=1

/transl_table=11

/product="hypothetical protein"

/translation="MSHLGKRVKEQRLKKGLTQIELAEDICTQATISNLENETSLPSL

SILLAVGDRLNIDFSDIYEFVSANSTSYSDIFKQVQKYCETEDYKEAYKILKEQIDFS

KLESVYELKQYYYYVGITSLLGYQRFSDAHYNLNLALSSDSGRNLDFFDVMATNGIGI

AYDLISDKEKAQTYFEKTLVLLDEVLFTVKSSQNRFEIVKIYFNIAKFYSSIEDYQQA
IDLSDLGIRLLEEEKAVFYLDHLFYEKSLNLSKLGQQEKATENYFYAAALAKISKNTE
LYGTIKKDMAEFKIEGYNYWA"

CDS complement(2141961..2142779)

/gene="punA_2"

/locus_tag="JFBMFIFI_02023"

/EC_number="2.4.2.1"

/inference="ab initio prediction:Prodigal:002006"

/inference="similar to AA sequence:UniProtKB:P77834"

/codon_start=1

/transl_table=11

/product="Purine nucleoside phosphorylase 1"

/translation="MEKLQEKIKQAASFIREKGVGEIEIGLILGSGLGELGDEVENAI
QIPYETIPNFPTSTVEGHAGQLVYGTLGGKKVLAMQGRFHYYEGYSLEMVTFPVRVMK
ALGIHSVIVTNAAGGLNLDFTPGELMLITDQINFTGVNPLIGPNDNEMGVRFTDMSQA
YDKEYQEIVRNVAKEMNLDLKEGVYMGFTGPTYETPAEIKMARVIGADAVGMSTVPEV
IVARHSGLRVIGVSCITNLAAGMQASLNHAEVVETTERVKESFKTLVKNILASI"

CDS complement(2142804..2143967)

/gene="deoB_2"

/locus_tag="JFBMFIFI_02024"

/EC_number="5.4.2.7"

/inference="ab initio prediction:Prodigal:002006"

/inference="similar to AA sequence:UniProtKB:Q818Z9"

/codon_start=1

/transl_table=11

/product="Phosphopentomutase"

/db_xref="COG:COG1015"

/translation="MFKRVHLIVMDSVGIGEAPDAAAFGDVGSDTLGHIAEIAGLTIP
NLEKLGLGTIRPLKGVSDQATSGYATKLEEVSVGKDTMTGHWEIMGLDIKTPFRVFPN
GFPAELLEKIEAFSGRKVICNQPYSGTAVIDDFGPEQMETGALIVYTSADPVLQIAAH
EEIIPLEELYKICQYVRDITLDDPYMIGRIIARPYLGTPGNFKRTSNRHDYALSPFGK
TVLDSLKENGKDVIAIGKINDIFNGAGVTDSIRTKSNMDGVDQLLTVMKQEFTGISFL
NLVDFDALFGHRRDVPGYAQAIEEFDARLPEILANLKEDDLLLITADHGNDPSFPGTD

HTREYVPLLAYSPSMKNHGNLKQGYYADISATIAENFAVPATENGTSFLAELK"

CDS complement(2143986..2144948)

/locus_tag="JFBMFIFI_02025"

/inference="ab initio prediction:Prodigal:002006"

/codon_start=1

/transl_table=11

/product="hypothetical protein"

/translation="MENFISTLSIVVSSALIYAAPLIFTALGGTFSERSGIVNVGLEG

IMVMGAFSSIVFNLSMGDVFGNWTPWVGLLVGGGIGLIFSLIHAVATVNLRANHIVSG

TVINMLAPALAIFLTRVLYEGRGQTDFIKFNFGKSNIPLLEHIPILGDIFFKGTSAAA

FVAILIAVLAWYILFKSRFGLRLRSVGEHPQAADTLGINVYLMRYAGVMISGFLGGIG

GAVAAQSISLNFSVSTIAGQGFIALAAMIFGKWNPLGAMGAAIFFGFAQSLSVIGSYI

PIIKDVPPVALQISPYILTIIVLVAFIGKSQGPAANGETYIKSK"

CDS complement(2144950..2146044)

/locus_tag="JFBMFIFI_02026"

/inference="ab initio prediction:Prodigal:002006"

/codon_start=1

/transl_table=11

/product="hypothetical protein"

/translation="MNHKNETLRNISVPVLSVLLGLILGAIIMLIFGYDPILGYKSLI

EGSVGSSFYIGETLRQATPLILTALGFAVANTAGFFNIGVAGQALFGWLGSVTLAIMF

PDLPRMLLIPLCLIGGAVAGAIWAGIAGYLRAYFGTSEVIVTIMLNYTALYISNHMVR

NVLTNADDATPRISDNATLRSGFLANITDNSTLHYGLFIAIIMCIVMWIFMNKTTAGY

ELRAVGLNSFAAEYAGMSAKKNIVMAMVISGALAGLGGSMEGIGNFQNIFVMSSMPSI

GFDGMAVSLLGAGNPFGILVSALLFGALKIGGISMPMGSDVPTEVVDIVIAAIIFFVG

ANYLIRYFMNKFNQPKIKITQAVTAKKEVN"

CDS complement(2146034..2147617)

/gene="xylG"

/locus_tag="JFBMFIFI_02027"

/EC_number="7.5.2.10"

/inference="ab initio prediction:Prodigal:002006"

/inference="similar to AA sequence:UniProtKB:P37388"

/codon_start=1

/transl_table=11

/product="Xylose import ATP-binding protein XylG"

/db_xref="COG:COG1129"

/translation="MVDENYVIEMLNITKAFGTFKANDNINLKVKKGEIHALLGENGA

GKSTLMNILSGLLEPTSGEIKIHGKPVVINGPTVANKLGIGMVHQHFMLVKKFTVTEN

IILGNEPSKAGFIDKKVAIKTIKDLSERYGLKVNPSKVVEDISVGMEQRVEILKTLYR

GADILIFDEPTAVLTPQEIDELILIMRELVKEGKSIILITHKLDEIKAVADRCTVIRR

GKSIETVDVKSTSQQELADLMVGRSVSFKTKKGPSHPENVVLKVENLVVKESRGLEAV

KNLSLEVRAGEILGIAGIDGNGQSELIQAITGLRKIESGSIELEGKAIGNLPPRKITE

TGLGHIPEDRHKYGLVLKMSLAENIALQTYYQKPLSNHGFLDYPAIKEYARKLIEEFD

VRTPNELVPASALSGGNQQKAIIARELDRNPSLLIAAQPTRGLDVGAIEYIHKRLIEQ

RDHKKAVLLMSFELDEILNCSDRIAVMYDGKIAAIVDPKQTTEQELGLLMAGSSLEKA

RAALQAEEEEKKEAELIES"

CDS complement(2147633..2148697)

/gene="tmpC_2"

/locus_tag="JFBMFIFI_02028"

/inference="ab initio prediction:Prodigal:002006"

/inference="similar to AA sequence:UniProtKB:P29724"

/codon_start=1

/transl_table=11

/product="Membrane lipoprotein TmpC"

/db_xref="COG:COG1744"

/translation="MRKRKLLATLTVGLGLTLTLAACGGKDKASSADGAGDIKGNTVA

LVTDVGGIDDKSFNQSAWEGLQAWGKEHKVKKGKDGYNYLQSNSDSDYVTNLNTAVNN

KFKTIFGIGYKLQPALEDVAKQNPDTQFGIIDTVIEGQDNVVSVVFKDHEAAFLAGVA

AAMTTETKHVGFIGGQEGEVIGRFEAGFRQGVAEVDPEIKVDVQYVGSFGDPAKAKSQ

AAAMYNNEADIIYVAGGDSGNGVFSEAKDIMKEEDKRKVWVIGVDRDQTEEGEYSGGN

LTLTSTLKGVGAAVQDIANLSIEGKFPGGETLAYGLKDGAIDLTDGNLTPEVKTAIKD

YKDKVIKEEIVVVEKPSQLK"

CDS complement(2148968..2149360)

/gene="cdd_2"

/locus_tag="JFBMFIFI_02029"

/EC_number="3.5.4.5"

/inference="ab initio prediction:Prodigal:002006"

/inference="similar to AA sequence:UniProtKB:P19079"

/codon_start=1

/transl_table=11

/product="Cytidine deaminase"

/db_xref="COG:COG0295"

/translation="MENEKWIQQAILARDTAYVPYSKFKVGACLVTTDDRMYTGCNIE

NASYGLSNCAERTAIFKAVSTGERTFKHLVITGDTDGPIAPCGACRQVIAEFCSPEMP

VTLCNLKGDIKITTVAELLPYAFTNKDL"

CDS complement(2149379..2150662)

/locus_tag="JFBMFIFI_02030"

/inference="ab initio prediction:Prodigal:002006"

/inference="similar to AA sequence:UniProtKB:A0A2A5K485"

/codon_start=1

/transl_table=11

/product="Nucleobase transporter PlAzg2"

/translation="MDVFKLNEKGTSVKTEIIAGLTSFFAISYIIIVNSIILKDAGIP

PELSVFATIFVSIIGCLLMGFFANAPIVLTPGMGVNAFFTYTLVGTLGLSWQAAVAVV

IISSGVYCLVAFSKLSELLSRAVPEALKHGITCGIGLFLVVIGLEKGHLIEAGKDTFI

VLGDLASPHALLAIFGLVLTVILLIRNVQGGLFIGILVTTILGNILQIRDASEASFAL

SHLADYPSIFAQGDFSSLFSAKFLMAVFSLTMILVFESMGLLQGLLPDAKEKDFKKAF

KASAVTTFISGFFGTSPTIAAAESASGIEEGGKTGLTAVVAGALFTVSLFFIPLLSYV

PQAAIAPVILITGALMMKNLQHIQFEDFTEWFPAFLIFVIIPLTSSIADGLAVGFVFY

PLVKIVAGKRQAVHPAMYLISGLFLAYLIMNTVMG"

CDS complement(2150690..2151340)

/gene="deoC_2"

/locus_tag="JFBMFIFI_02031"

/EC_number="4.1.2.4"

/inference="ab initio prediction:Prodigal:002006"

/inference="similar to AA sequence:UniProtKB:P39121"

/codon_start=1

/transl_table=11

/product="Deoxyribose-phosphate aldolase"

/db_xref="COG:COG0274"

/translation="MELNRRIDHTCLKPEATKADILQLIAEAKKARFYSVCVQPVWVE

LASQSLKEDPVSICTVIGFPLGANTTETKVFEAQNALQNGADELDVVLNIGALKTGEH

EAVLEDLKAVILVAKNRALVKVIIEAALLTEEEKIVACQLAKEAGANFVKTSTGFAAT

GATPSDVRLMRKVVGETMGVKASGGIKTAKEVRLLIESGASRIGTSASVAILAETI"

CDS complement(2151376..2152677)

/gene="pdp"

/locus_tag="JFBMFIFI_02032"

/EC_number="2.4.2.2"

/inference="ab initio prediction:Prodigal:002006"

/inference="similar to AA sequence:UniProtKB:P39142"

/codon_start=1

/transl_table=11

/product="Pyrimidine-nucleoside phosphorylase"

/db_xref="COG:COG0213"

/translation="MRMVDIIEKKRDGKELTKAEINFVVTGYTKDEIPDYQVSALAMS

IFFNDMTNEEIANLTLAMAESGEMIDLSAIKGIKVDKHSTGGVGDTTTIVLAPLVAAV

GVPVAKMSGRGLGHTGGTIDKLEAIPGFNVEISNEDFIEFVNRDQVAVIGQSGDLAPA

DKKLYALRDVTGTVDSIPLIASSIMSKKIAAGADAIVLDVTTGDGAFMKNEKDAERLA

RTMVQIGKLANRQTMAIISDMSQPLGLAIGNSLEIKEAIDALNGQGPEDLMEMVYVLG

SQMVVLAKKAETLEEARKMLEEAIQSGAAIEKFKVMVRNQGGDASVIDHPEKLPQAKY

VIDLPAKKSGVVSEMVADQLGIAAMILGAGRRTKEDQIDFSVGLMLRKKVADSVVEGE

TLVTIYANSPNVEDVKAKIYESITIAETADEPVLIHQIITE"

CDS complement(2152940..2153947)

/gene="degA_2"

/locus_tag="JFBMFIFI_02033"

/inference="ab initio prediction:Prodigal:002006"

/inference="similar to AA sequence:UniProtKB:P37947"

/codon_start=1

/transl_table=11

/product="HTH-type transcriptional regulator DegA"

/db_xref="COG:COG1609"

/translation="MKITIKDVARLSGVSITTVSQILNHKGERFSAETKKKVLDVVAE

YDYKADYFAQNMISKRTKTIGMVVPDVTDLFFSKVIEGVENYLNELGFMILLCNSNHS
SEKENIYIDELLHRSVEGIILASPNPIHVPKLIDDRGRKKIPYILIDRGLNERDEGKL
ITNEFQGAYEAVELLINQGHTKIGMLGNETSFYEITDRFLGYQKCLMNHGIDYQERFV
TNEPLTIKGGYEGAQKILKQDVTALFCGNDQMAVGAYRAINEVGKKIPEDISVIGYDG
LELTDYMVPALTTVRQPIFEIGYFAAKFLVDNINNPKEKIPNKYFDTKLMKKNSTKKL
D"

CDS complement(2154604..2154972)
/locus_tag="JFBMFIFI_02034"
/inference="ab initio prediction:Prodigal:002006"
/codon_start=1
/transl_table=11
/product="hypothetical protein"
/translation="METVAFILVLIVALEHVYIMALEMFFSTSENAQKTFGLKKEFLQ
QKNVKTLMANQGLYNGFLAAGLLWGLFFVPAEFALSVQLFFLGCVIVAAVYGSLTANK
GILIKQGLPAILAFIFVLIS"

CDS complement(2154991..2155929)
/locus_tag="JFBMFIFI_02035"
/inference="ab initio prediction:Prodigal:002006"
/inference="similar to AA sequence:UniProtKB:P99173"
/codon_start=1
/transl_table=11
/product="Zinc-type alcohol dehydrogenase-like protein"
/translation="MKAVIIENYGGKEELKLAEVPTPKAEANQVIIKEEATSINPIDW
KLREGYLKQMMPWEFPIILGWDVAGTISEVGANITDWKVGDRVFARPETTRFGTYAEY
TAVDENLLAKIPENVTSSEAAAVPLAGLTAWQALFDHGKLKAGETVLIHAGAGGVGTY
AIQLAKQAGAKVITTASQKNHDLVKSLGADQVIDYRTENFVELLKDVDVVFDTMGGAS
QIDSFKVLKPKTGRMISIVGAAEEGLAEKYDVYFDSIWLKPNGEQLSKIAELMEAGKV
KSIIGATFPFSQEGLYDAHALSETHHAVGKIVIEFK"

CDS complement(2155969..2156400)
/gene="slyA"
/locus_tag="JFBMFIFI_02036"
/inference="ab initio prediction:Prodigal:002006"
/inference="protein motif:HAMAP:MF_01819"

/codon_start=1

/transl_table=11

/product="Transcriptional regulator SlyA"

/translation="MTIECGRYSRLFYKMNLLYKEMNGAFEEETGISYTKLEILYQIS

QTANMCQQDIQQKLGIDAASITRHLKKLEEEKLITRIKDQENKRFLRIALTQDGQNQL

KKLIAEKNQYEEQIFANLSLEQIEVLEGTFHKISRNITNKN"

CDS complement(2156559..2157530)

/locus_tag="JFBMFIFI_02037"

/inference="ab initio prediction:Prodigal:002006"

/codon_start=1

/transl_table=11

/product="hypothetical protein"

/translation="MSKKVFVLGGTGFLGYYTTKELLKRGYQVVTAALPPLPEEGLLP

AEVEITLGNINEMSDDEVLALLKGCEGFIYGAGADERMVPKKPASKFFYEANVLPTQR

LANLAKKAGVKKFVVFGSYFAEFAERLPEEKLKAEAYPNTRLLQEQIAFAEGDGSMVV

TSLRLPYIFGTMPGRMPLWKMFTDQIKGQAVFPALKGGTAMVTVEQVAEAAVGALENG

VHRTTYAIGDTNMKYQEFYQMMVDALGQDTQVPVVSYEDVKAIYEGIDLEADKAGLEH

GIHSTKSAKMQEYDLYLNPEDTFPILKVKRYDVIASIQETLKRCAED"

CDS 2157659..2158831

/gene="rhaS_5"

/locus_tag="JFBMFIFI_02038"

/inference="ab initio prediction:Prodigal:002006"

/inference="protein motif:HAMAP:MF_01534"

/codon_start=1

/transl_table=11

/product="HTH-type transcriptional activator RhaS"

/translation="MNQLPEFSMSDFIVTKEIYRQPTTKASQMYIQFIFVTQGQGSMQ

VNDQEYLLKKGSLLWLFSYHVYHLQEVEEELHFITFQLSLNLILFSTFTTQTTEKHLR

FFEYWDTPALTSHGEDFLYLNQVFSHALTEYQQQQNFYELSLVSDLYQVIVWFERSVR

QQREAEKELRKTSSDILLYLSLHFSEELTLTKIANQFKTTPQTINRRLKLLTGKTFTE

NLASIRMNNAVALLKFDELTIAYVANYVGYSSIPSFNKQFKALIGCSPSKLKQRAFTH

ESNSFRFQNTMSYEIYTYITTNYMKDISVKSAAKYFYASPTSINQILLNEFSMTFNSL

LEFTRMLFARSLLLCSTKKIEAISEACGYLSIRTFNRSFLAFWGNSPKKYKESLTK"

CDS complement(2158834..2159229)

/locus_tag="JFBMFIFI_02039"

/inference="ab initio prediction:Prodigal:002006"

/codon_start=1

/transl_table=11

/product="hypothetical protein"

/translation="MSSRIFIFFSRSWLIRTSLDKKFVEPLRPVRKIENSNKSVFLLF

WEFIFFPKDWFKRTSLDKKFVEPLRPVRKIGNSNKSVFLLFWEFIFFPKDWFIRTGLI

KSLLSHQPILSIHSIQYIKYHLKKIGTFW"

CDS 2159308..2159871

/gene="yvdD"

/locus_tag="JFBMFIFI_02040"

/inference="ab initio prediction:Prodigal:002006"

/inference="similar to AA sequence:UniProtKB:O06986"

/codon_start=1

/transl_table=11

/product="LOG family protein YvdD"

/db_xref="COG:COG1611"

/translation="MKRIAVYCGSKLGNQAVIAEDAKKLGALLAEENIELIYGGSNVG

LMGIVADAVLENGGSVIGVLPTFLKDREIAHDHLSQLIIVDNMHQRKEKMLELADGFI

ALPGGPGTLEELFEAFTWGQIGLHQKPCGILNSAGFYTHLEGHFKQMVETGFMTAESR

KGISIATSPAELLEHFTAYQAPPVKTY"

CDS complement(2159937..2161289)

/gene="gor"

/locus_tag="JFBMFIFI_02041"

/EC_number="1.8.1.7"

/inference="ab initio prediction:Prodigal:002006"

/inference="similar to AA sequence:UniProtKB:P06715"

/codon_start=1

/transl_table=11

/product="Glutathione reductase"

/db_xref="COG:COG1249"

/translation="MTKKYDYIAIGGGSGGIASINRAASYGMKCALIEAKELGGTCVN

VGCVPKKVMWYGSQIAEAMKLYAPDYGYDVTVDNFDFKKLIASREAYIDRIHQSYDRG

LANNKVDVIKGYATFVDEHTIEVNGELLTAEHILIATGGKPTWPTIPGAELGIDSDGF

FALEELPKRVAVVGAGYIAVEVAGVLHGLGSDTHLFIRHDKPLRSFDPYVVDGLVESM

AKEGPTLHPHSLIKEVTKNKDNSLTLHLKNGESYVVDQVIWAIGRTPVTETLKLENTA

VTLTEKGHIKVDKFQNTSVAGIYAVGDITGHLELTPVAVAAGRRLSERLFNNKPNEHL

DYRNVATVVFSHPPIGTVGLTEPAAIEKYGENQVKVYESSFTSMYTAVTEHRQLCRMK

LVCAGPNEKVVGLHGIGYGVDEMIQGFAVAIKMGATKADFDDTVAIHPTGAEEFVTMR

"

CDS complement(2161444..2161824)

/locus_tag="JFBMFIFI_02042"

/inference="ab initio prediction:Prodigal:002006"

/codon_start=1

/transl_table=11

/product="hypothetical protein"

/translation="MKLDMIGIIVKDMEKSLDFYRTLGFEFPVTANQEDYVEINQGDI

RLSFNTEKMVTPIFGEQEEPKGQRVELAFLCESSDELNQLHEKAIAKGYKSVKAPWDA

FWGQRYCILEDCDGNLISLFFPLS"

CDS complement(2161965..2162336)

/locus_tag="JFBMFIFI_02043"

/inference="ab initio prediction:Prodigal:002006"

/codon_start=1

/transl_table=11

/product="hypothetical protein"

/translation="MKPEYQWIEKLIATLPGAEIVYKDAWQAFTFLIGGKIFAILGTS

TGENLLTIKGDPARNEELKELYSDIIPGYHVNKTHWISAKLASPEIPQALLENSLEAA

YFLVLGKLPKKVRAQFETEKD"

CDS complement(2162554..2162940)

/locus_tag="JFBMFIFI_02044"

/inference="ab initio prediction:Prodigal:002006"

/codon_start=1

/transl_table=11

/product="hypothetical protein"

/translation="MQRKLTEKKVYGITGRANNSNPAPIGIAWQKFVKIKPAGECYGI

YTNYASDYRGDYDFILASPVQFETAEPVVLVAGDYLEFEVLEGQAGVAKMWEKIWSMD
INRAYTTDFEHYQNDGKVKIYIALKM"
CDS complement(2162998..2163909)
/locus_tag="JFBMFIFI_02045"
/inference="ab initio prediction:Prodigal:002006"
/codon_start=1
/transl_table=11
/product="hypothetical protein"
/translation="MLKIERMFAILIRLLHKKIIPAEELALEFNVSKRTIYRDMDSLS
FAGIPVVSLPGKNGGFTLMENYQISHFTFTETEKLSLLAGLKMQEELLQVPYLGDLIQ
KVALLTTEKAADSSPISLASATQHRPEIDAFVKERLEQILRALTEEKELMIDYISGTA
ETSKRKIQPLEIRFINGSWYLDAYCYLREGERQFKLTRIMELEEVSGVDRFQPKSRET
NRQEETAKEQAIFIFDGNQLGKLVDFYVEEELTELPDGRIQVTTLISPSQNYIPFLLT
FGKHLQIIQPEWLREQRQVEIKAMLES"
CDS 2164104..2164586
/locus_tag="JFBMFIFI_02046"
/inference="ab initio prediction:Prodigal:002006"
/codon_start=1
/transl_table=11
/product="hypothetical protein"
/translation="MRLYELTRQYEQLLGLAEEEEIATQVLEDTLEALEETIYLKVEN
TAKVIKNLEAELIGFQTEIRRLTKRKQTLEKMIKRLKLYLQEEMERMTLDKVNGELFN
VRIQNNPPSVVVMDIDKLSAYYLPQEPKLDKETLLKDLSAGKEILGAKRQQTRSIRIT
"
CDS complement(2164678..2165160)
/locus_tag="JFBMFIFI_02047"
/inference="ab initio prediction:Prodigal:002006"
/codon_start=1
/transl_table=11
/product="hypothetical protein"
/translation="MKIKRLQAIEFRQNGDYAASCQLLKELIAQNPDNAELLYQCAWS
HDTAGLEVEAVPYYEKAIQLGLEESDLSEAYLGLGSTYRTIGAYQKSKQLYLEALKKF
PENKVYPIFLAMTYYNLAEYSQAMELLLRTISEIETDSEIKTYQKAIAFYADKLDQIW

"

CDS complement(2165161..2166252)

/gene="ddlA"

/locus_tag="JFBMFIFI_02048"

/EC_number="6.3.2.4"

/inference="ab initio prediction:Prodigal:002006"

/inference="similar to AA sequence:UniProtKB:P0A1F0"

/codon_start=1

/transl_table=11

/product="D-alanine--D-alanine ligase A"

/db_xref="COG:COG1181"

/translation="MNKKINVGVVFGGKSTEYHVSLQSAKNILESLDRDKYSVHLFAF

SNEGKLLNFDESEQLLIGDGETVNNHGNETTLSHLISQPFDNQANEIDVYFPIVHGNL

GEDGSIQGFFRLLNKPFVGSSVLSSALCMDKDFSKQVLNYNGFKTAKSITVKTGEIAP

NLVSQVENELGFPVFIKPANQGSSVGVSRAVDQISFKNGLIEAFKYDSKIIIEEELIG

KEVECAVLGNGNPLASNLGSISTDESNFYSYEEKYLDESGAVLEIPAKISEESTLKIK

KLALAAYKLLECKGLARIDFFLTNQEEIYINEVNTLPGFTNISMYPQLWQASGISYGG

LVERLIQLALEEFAKEAKIQRKIDLNQVN"

CDS 2166428..2167834

/locus_tag="JFBMFIFI_02049"

/inference="ab initio prediction:Prodigal:002006"

/codon_start=1

/transl_table=11

/product="hypothetical protein"

/translation="MIKGHTKIISISILGLALFLGASLLFKNHLTTKKIHAEKIKTFK

QIKQAESKTGNPFTYQKKQVKEGTLHFFIPTDEKQQPNELIEKEMIKLSHQLLKESRK

KNELTDSEVYLTFQTKKTQEPTQNFTVSGCLFAKKEGSFRKMNQKKNAPIVLTNKTGK

QFVLGDIFKSNDSLADELKQRGEEIIMSNQTISLEKIEEIGNLDYPNDWTDSNFTYDA

DNLTIPLKKNSLGLEKISYPIETIANLVNPEFLPLNKTLPPTTNSHFRPGKKIALTFD

DGPKSEVTPAILNSLKKYNAHATFFVLGQEAANHPDLLNQILADGHELGNHSWNHSLL

TGLNPNNLDEELLKTQFAVYQATKQFPKYFRPPYGGINSSVAKSLYKPIIQWSVDTED

WKIKNPKRITNRALSGAYDGAIILMHDIYPTTQQSLDNTLKGLQQKGYQFVTISELLE

NPAKPLHQYFGSEDERKI"

CDS 2167955..2168272

/locus_tag="JFBMFIFI_02050"

/inference="ab initio prediction:Prodigal:002006"

/codon_start=1

/transl_table=11

/product="hypothetical protein"

/translation="MFEVHNSTDLLTAFDNKEEEFVIKGDYCKDIREYAKSQLSETEL

LGIELGGAGIPSLFGYLIEAVMDFFSHESKEKLKIEKKLRLYKIESIDKTAIKLRLRQ

LDY"

CDS complement(2168436..2168705)

/locus_tag="JFBMFIFI_02051"

/inference="ab initio prediction:Prodigal:002006"

/codon_start=1

/transl_table=11

/product="hypothetical protein"

/translation="MTYKVTFHTIDGKAIVVDDVEAENQNEAWQEACEQDRESFIIYV

ASKETTYKVYKQHLVSIETKDKTAAARKTSETTMTAIDALSNMGF"

CDS 2168891..2169853

/gene="dinG_2"

/locus_tag="JFBMFIFI_02052"

/EC_number="3.1.-.-"

/inference="ab initio prediction:Prodigal:002006"

/inference="protein motif:HAMAP:MF_02206"

/codon_start=1

/transl_table=11

/product="3'-5' exonuclease DinG"

/translation="MSDFMTKKYDVLAIDFETATNNNYSACSVAVALIKDLEIIDSHY

WLIQPPHNRYSAANTAIHGLSSSDTRDALQFPDIWPELQTLIRSSAFVSAHNAQFDMS

VLHEVLHYYQLPIEDFSYFDSINYSTKACAGERIPSGLKERCQRFNIIIEEHHNALSD

ALACGKLIIAATKEKNKASSLEYLNAYSTVTSKKFSELKASKFFKKPSTSTANFNRVD

LNQINAIQENSQLSHPDFSEKSFVFTGEFKKAKDELMQAVVERGGIIKSAVSGKTDYV

VEGVQDLSIVGADGFSSKQRKARELIAKGSSLTLLTEKQLEELL"

CDS complement(2169958..2170221)

/gene="rpmE2"

/locus_tag="JFBMFIFI_02053"

/inference="ab initio prediction:Prodigal:002006"

/inference="similar to AA sequence:UniProtKB:Q88Z52"

/codon_start=1

/transl_table=11

/product="50S ribosomal protein L31 type B"

/db_xref="COG:COG0254"

/translation="MKQGIHPDYHQVVFMDTTTGFKFKSGSTKHSQETVEWEDGQTYP

LIRVEITSDSHPFYTGRQKFTQADGRVDRFNKKYGIKDKNAAE"

CDS complement(2170360..2171655)

/gene="rho"

/locus_tag="JFBMFIFI_02054"

/EC_number="3.6.4.-"

/inference="ab initio prediction:Prodigal:002006"

/inference="similar to AA sequence:UniProtKB:P0AG30"

/codon_start=1

/transl_table=11

/product="Transcription termination factor Rho"

/db_xref="COG:COG1158"

/translation="MGDLLTLAELEKKTLKELYTFAKDFKIPYYSQMNKKELSLAVLR

AQEEKQGFFVAEGVLDIVSQEGFGFLRPINYTPSQEDIYISVSQIRRFGLRNGDKITG

KARPPKPSERYYGLMHVNAVNGKNPEEAKERSHFPALTALYPERQMKLETEQVKISNR

MIDILAPVGFGQRGLIVAPPKAGKTILLKEIANGITKNYPDAELIILLIDERPEEVTD

IERSVKGDVVSSTFDQQPENHVRVTELVLERAMRLVEDKRDVIILMDSITRLARAYNL

VVPPSGRTLSGGIDPAAFYRPKRFFGAARNIEEGGSLTILATALVDTGSRMDDIIYEE

FKGTGNSELHLSRELSERRIFPAIDIKKSSTRKEELLMPGERLEEIWKLRRLMTGDSL

EFTDKFIKILRKTKTNADFFASFSEEAFGKGRTNRTIRK"

CDS complement(2171671..2172939)

/gene="murA1_2"

/locus_tag="JFBMFIFI_02055"

/EC_number="2.5.1.7"

/inference="ab initio prediction:Prodigal:002006"

/inference="similar to AA sequence:UniProtKB:B1IBM3"

/codon_start=1

/transl_table=11

/product="UDP-N-acetylglucosamine

1-carboxyvinyltransferase"

/translation="MKKLIVNGGRPLSGEITISGAKNSTVALIPAAILADSPVTLEGV

PDIQDVHSLIEILEIMNVKVDFDGSTLVIDPTKIVSIPMPSGKIKSLRASYYFMGALL

SKFGQGVVGLPGGCFLGPRPIDQHLKGFEALGATVDNELGAMYLRSGDEGLTGARVYL

DVVSIGATINLMLAAVRAKGKTIIENAAREPEIIDVATLLNNMGAKVRGAGTDIIRIE

GVTELKGCRHTIIPDRIEAGTYLSMAAAMGTDVVVKNVIFEHLEGLIAKMEEMGVPME

IGEDSIRVLEAKNLKMVSIKTLPYPGFATDLQQPLTPLLLKAHGEGMIVDTIYPKRVK

HIPELVRMGAKARVETDMILLEGPTKLKGVEVEASDLRAGACLVTAGLMAEGMTTITG

VENILRGYDHIVEKLTALGADIKMIEVEDK"

CDS complement(2173198..2174793)

/gene="pyrG"

/locus_tag="JFBMFIFI_02056"

/EC_number="6.3.4.2"

/inference="ab initio prediction:Prodigal:002006"

/inference="similar to AA sequence:UniProtKB:O87761"

/codon_start=1

/transl_table=11

/product="CTP synthase"

/db_xref="COG:COG0504"

/translation="MTKYIFVTGGVVSSIGKGIVAASLGRLLKNRGLEVTIQKFDPYI

NVDPGTMSPYQHGEVFVTDDGAETDLDLGHYERFIDINLNQYSNVTTGKVYSEVLRKE

RKGEYLGATVQVIPHITNEIKEKIMRAAQTTEADIVITEVGGTVGDIESLPFLEALRQ

MKADVGSENVMYIHTTLIPYLNAAGEMKTKPTQHSVKELRSLGIQPNILVVRTEKPVP

QNLKDKLASFCDVEPEAVIESRDVETLYSIPLALQAQGMDQIVCDHLKIDAPVADMTE

WIQLEEKVLNLKKKTKIALVGKYVELPDAYLSVVEALKHAGFDYDTDIEIDWVYAEQV

TTENVAILLADADGILVPGGFGDRGLEGKIAAIQYARENKVPFLGICLGMQLACVEFA

RNVVGLKDADSAETNPTCENNIIDLMSDQENVENLGGTLRLGLYPCTLEPGTVAAKAY

DNVAVVEERHRHRYEFNNDYRETLEEAGLVFSGLSPDGRLVEIVELSDHPFYVACQFH

PELISRPTRPQKLFKAFIGASVK"

CDS complement(2175141..2175755)

/gene="rpoE"

/locus_tag="JFBMFIFI_02057"

/inference="ab initio prediction:Prodigal:002006"

/inference="protein motif:HAMAP:MF_00357"

/codon_start=1

/transl_table=11

/product="DNA-directed RNA polymerase subunit delta"

/translation="MELKQFDGMKKEELSMIEVAHAILDEKGDVLEFSELVTAIQKYL

GITVGKMKKNTPQFYTDLNIDGSFISLGENRWGLRSWFPIDSIDEEVSHSNEEEDEVP

RRKKRKKVSAFVPNTDDDDVIDYNDDDPEDEDVVLVDEEGVVIDDEEESENDLVAYKS

DLTEIGADEDEEDELPDGIEGSLTLIEEDDEDEDEFADEEEDKI"

CDS complement(2175800..2176231)

/locus_tag="JFBMFIFI_02058"

/inference="ab initio prediction:Prodigal:002006"

/codon_start=1

/transl_table=11

/product="hypothetical protein"

/translation="MDLKAGTDVKIHLETQVTQYAEPEKHVFDVMGQMVQMGSGLYLR

YIEVEGDIEIPVTIKVEADGTITLIRAGETRTRLRFGKGERYVTNYNTPQGVVALETM

TNKMQVSLRDQPFSGEIEIHYDLYLGEEKLGEYKLQLLFTA"

CDS complement(2176232..2177206)

/gene="deoR"

/locus_tag="JFBMFIFI_02059"

/inference="ab initio prediction:Prodigal:002006"

/inference="similar to AA sequence:UniProtKB:P39140"

/codon_start=1

/transl_table=11

/product="Deoxyribonucleoside regulator"

/db_xref="COG:COG2390"

/translation="MKYTEDRRKILKVATMYYSEGLTQAEIARKIGISRPVISKLLRE

ARDLGIVEIYIKDENAYSISLALEIEKKFQLKDVIVVPDGQDNSPATLKKNVARAAAL

YVANKLDQVSSVGVSWGTTVAEVIDEMPFLSYPNVTIHPLVGGVASHHLFLDANHLAF

LFSQKCSAKCTYFYAPALAESKELKQILETSSLALEALKKAKQVDMAIIGVGNPVTSS
TWEELAYIHSAELKKLKELGVVGDAVASFFDATGKELSTDLTQRLMGVTIEDLRKVKN
VIAVASGLDKILSIKALLMAGILDTIVIDQRMAEELSQSTEIENKGVE"

CDS complement(2177441..2178439)

/gene="bfmBAB"

/locus_tag="JFBMFIFI_02060"

/EC_number="1.2.4.4"

/inference="ab initio prediction:Prodigal:002006"

/inference="similar to AA sequence:UniProtKB:P37941"

/codon_start=1

/transl_table=11

/product="2-oxoisovalerate dehydrogenase subunit beta"

/db_xref="COG:COG0022"

/translation="MRELTYIEAINEALDEALAEDQTVFLLGEDIGVYGGGFGATKGL
IEKYGSQRIRCTPISESAIAGAAVGAAITGLRPIIELQFSDFITIAMDQLVNQAAKIH
YMYGGKAQVPLVMRTAGGSGTGAAAQHSQSLENWTAHIPGLKVVQPATAYDAKGLLHA
AIADNNPVMFYEHKLCYKTVSDVPNEKYIIPLGVADIKRAGSDITVVATGIMVHKTLD
VAEELAKKGLSLEVIDPRTLVPLDTETIINSVKKTGRLLVVHEAVKRSGFGGEVASVV
AESSAFYQLKAPIKRLGGAAVPMPYQKDLEKNAIPQVEDIYQAALELMEASLVKEK"

CDS complement(2178452..2179441)

/gene="acoA"

/locus_tag="JFBMFIFI_02061"

/EC_number="1.1.1.-"

/inference="ab initio prediction:Prodigal:002006"

/inference="similar to AA sequence:UniProtKB:O31404"

/codon_start=1

/transl_table=11

/product="Acetoin:2,6-dichlorophenolindophenol
oxidoreductase subunit alpha"

/db_xref="COG:COG1071"

/translation="MTSHIQNLAGLTDNQLKEIYQVMWEIRFFDEQVEALFRAGEIHG
TTHLSIGQEATAAASGYLLKTTDWITSTHRGHGHTIAKGTSMTAMMAELFGKKNGTNH
GKGGSMHIAELATGNIGSNGIVGAGYPIAVGAALSAQMQGTGAVTICYAGDGSTNEGS

FHEALNMASIWNLPVIFFIENNQYGMSSKIDQMVNIPRLAMRADSYGFPGVTIDGNDI
IAVLDATQTAIKRAQDGHGPTLIEALTYRFKGHSKSDKRVYRTKEEEEQWQIERDPLK
LTATLFEQEGVASEIELAEIKHKAQVKVAEAVAEARLGENPTLADLYTDVYAE"

CDS 2179706..2180578

/gene="lipL"
/locus_tag="JFBMFIFI_02062"
/EC_number="2.3.1.204"
/inference="ab initio prediction:Prodigal:002006"
/inference="similar to AA sequence:UniProtKB:Q9K6A7"
/codon_start=1
/transl_table=11
/product="Octanoyl-[GcvH]:protein N-octanoyltransferase"
/db_xref="COG:COG0095"
/translation="MSEVKQIDYLKEQSYTLYDSDTMPFSNRFLSHFALGDSLIKRVG
EAQKEPVLHFWTADELVILGMMDTKLPHFSTGLDSLARYNKNYIVRNAGGLAVVADAG
VLNLSMIFPDNPTHKISIDEGYHYMFRLIHNTFKAYGKSIEAYEITESYCPGDFDLSI
DGKKFAGIAQRRLRNGIGVMIYLSVNGNQEKRAKMLHDFYHEGLQGEETKWKFPHVNP
AVMATLEELLDTSFSVSDVKKMILKTLSDNNNTLIKGQYDSDLVADYQVAFGKMVQRN
EQMLQENLNKELLL"

CDS 2180575..2181960

/locus_tag="JFBMFIFI_02063"
/inference="ab initio prediction:Prodigal:002006"
/inference="protein motif:HAMAP:MF_01212"
/codon_start=1
/transl_table=11
/product="Deoxyguanosinetriphosphate
triphosphohydrolase-like protein"
/translation="MNFVPYKDQLLPVEKVFRDPVHDYIHVQYQVILDLIDSREFQRL
RRIKQLGTSSYTFHGAEHTRFTHSLGVYEIARRICDKFSRNFPMVVPGDGGWDDSERL
VVLCAALLHDIGHGPYSHTFERIFETDHEAITVAIITSEETEVNRILKQVSFDFPEKV
ASVIQKTYPNPQVVQLISSQIDADRMDYLLRDAYYTGVNYGTFDLTRILRVIRPYNEG
ISFQISGMHAVEDYIVSRYQMYMQVYFHPVSRSMEVILNHLLKRAKELYLSPDYEFQS
PVQLLAPFFEKNFSLTDYLNLDDGVLNTYFTQWRTEKDSVLSDLAHRFLDRHVFKSVS

FDLATDLDIIEKMKMLITEAGYDTNYYTALNNSFDLPYDFYRPETVNTRTQIELVQAD

GSFIELSKASDIVAAITGKTRGDERFYFPKELLTPKVTTEINLFQAIFEEFTHYIKNG

AIVHPKNELEE"

CDS 2181968..2182777

/gene="yidA_4"

/locus_tag="JFBMFIFI_02064"

/EC_number="3.1.3.23"

/inference="ab initio prediction:Prodigal:002006"

/inference="similar to AA sequence:UniProtKB:P0A8Y5"

/codon_start=1

/transl_table=11

/product="Sugar phosphatase YidA"

/db_xref="COG:COG0561"

/translation="MSIELIAIDLDGTLLTPERVVSPRVKATIAEAKEKGIKVVICTG

RPLPGVTHLLKELNLEEEGDYVITYNGALVQTAHDGEAIAHHTLDFDNFLEIEGLSQE

LGVHCHAIDRDSIYTTNKDIGYYSVYEAMLTNMSLKYRSVEEMDPNIEISKMMMIDPP

EILDPAIAKFPAGFTEKYTTLKSEPFYLEVLNKDASKGQAVRDLAGILTIPRENIMAI

GDNENDSDMLVYAGIGVAMGNAVPTVKAISDYVTETNVNDGVAVAIEKFAF"

CDS complement(2182892..2183071)

/gene="ywhB"

/locus_tag="JFBMFIFI_02065"

/EC_number="5.3.2.6"

/inference="ab initio prediction:Prodigal:002006"

/inference="similar to AA sequence:UniProtKB:P70994"

/codon_start=1

/transl_table=11

/product="2-hydroxymuconate tautomerase"

/db_xref="COG:COG1942"

/translation="MPFVHIEMLAGRTAEQKAQLVKDVTEAVMKNTGAPGENIHVILR

DMEPSDYAQNGQLKG"

CDS 2183325..2183762

/gene="ohrA"

/locus_tag="JFBMFIFI_02066"

/inference="ab initio prediction:Prodigal:002006"

/inference="similar to AA sequence:UniProtKB:O34762"

/codon_start=1

/transl_table=11

/product="Organic hydroperoxide resistance protein OhrA"

/db_xref="COG:COG1764"

/translation="MPDSKQLYQTKAINTGGRNGESHLPDGSFSVKISTPKDMGGKGQ

GSNPEQLFALGYSACFNSALELVMGQEKVSGKSQVTATVELLSDPSDNGFKLAVELDV

AIEGKELDQVQELADKAHHVCPYSKATSGNIDVTVKAVAFQEI"

CDS complement(2183801..2184247)

/locus_tag="JFBMFIFI_02067"

/inference="ab initio prediction:Prodigal:002006"

/codon_start=1

/transl_table=11

/product="hypothetical protein"

/translation="MDVKRFGTKIDEQDYHIRIGVYILILNQAKDQLLLVSPPNGAYL

LPGGEIEGNETNEETLHREVMEELGCEIEIGTYLGEAEDFYYSTFRKKYYHNPGYFYV

AKSWHKKCAPLEDFNGLEWMDIPTAIEKLKRGSHKWAVEQYLVLEK"

CDS 2184405..2185448

/locus_tag="JFBMFIFI_02068"

/inference="ab initio prediction:Prodigal:002006"

/codon_start=1

/transl_table=11

/product="hypothetical protein"

/translation="MVLYYLTLFFFIIFAVSYIQDRRKIINGFLLNLFLFSGCMLLAF

QAYETQNPILMTIFIILVAIFSFFALFGAVLLMGFSFWNARTVIKKEGRNFSNLLTLF

LGFGILFMIILSFFDLNALFPSYVVAIISFIYLVIFYFFFIFVNFLVASLVYQLYRPR

LNKHFVIVLGSGLIDGHIVPPLLANRIKKALEFYDRQAAKTSPPKIIFSGGRGDDEKL

AESVAMRAYAIEHGLPADDALIEDRSVNTLQNMLYSKEIMDTRMKGQKYKVIFSTNNF

HLFRAGIYAKKAGLNAQGIGAKTAFYFWPNAMIREFIAIIVMHKKFHLLLIGSMALVG

ILLQLALWYSNTH"

CDS complement(2185499..2186806)

/gene="pepV"

/locus_tag="JFBMFIFI_02069"

/EC_number="3.4.13.-"

/inference="ab initio prediction:Prodigal:002006"

/inference="similar to AA sequence:UniProtKB:P45494"

/codon_start=1

/transl_table=11

/product="Beta-Ala-Xaa dipeptidase"

/translation="MHEQIKAAVKGKEVEFLAGLKKVMKVDSVKGLAETGAPFGKGPK

KALETALDLASALGFETKIVSDAVGYAQYGTNNDEYIGVVGHLDVVEAGSGWSYPPFD

LTLEAGVLYGRGVLDNKGPAISTIYALAVLKELNIPLSKTIRILFGTDEESGSADIPL

YLATEKPPIYGFTPDCKYPAVYGERGLVGIEIVTVMPEKELKQISNFKGTFTRSSVPD

HLEVTLKNGEEIQIEGKRAPSNAPEMGENVLTKFAEIATNEHFFTGKLADYFSWLAAA

LHLKHDGSGLGIDFSDQASGKLMLTPYAFKIEKNKVHLTLSIRYPISVTEENILVELK

QHLPMDSQLQIIRKMPATSFPLDHPMLKIMQEVYEECTGMDGTPVTTTGATYARTMPN

IVAFGPSFPGQKGIAHNKDEYMDLTDLMKNMEIYAVTMAELAK"

CDS complement(2186834..2187352)

/locus_tag="JFBMFIFI_02070"

/inference="ab initio prediction:Prodigal:002006"

/codon_start=1

/transl_table=11

/product="hypothetical protein"

/translation="MIKVVLILNHVQAGMGSDENALLAPGGKKSPIGPGMTLEPLIES

FGGTIVATLYCGDCYFLENEAEVKKKFIGFSKKFEADAVLCGPAMHYPNFGEMCGSLA

KAMNENGIPAIAAMSAENPATANYQHDIPIVKMPKKGGIGLNDSFKHMAQLVVAKGKL

EDTARLEAEFCF"

CDS complement(2187365..2188723)

/gene="licC_6"

/locus_tag="JFBMFIFI_02071"

/inference="ab initio prediction:Prodigal:002006"

/inference="similar to AA sequence:UniProtKB:P46317"

/codon_start=1

/transl_table=11

/product="Lichenan permease IIC component"

/db_xref="COG:COG1455"

/translation="MKKVLDGMERVLMPLAKIIGENKYLIAIRDGFLISTPMLIIGSL

FLLIANFPWTPFGDFMASFLGADWASKMSVPASASFDVMAILAVVGIGYSLGKQFDID

AIAAAVLSLVTFFMVTPFFTNYTLEGTEEVVQVTSLPLEWMGSKGLFLGIIVALLGTR

IFAAVIKKGWVIKMPPGVPPTVVKSFEALIPGFIVIFIFFVISWVFTLTSYGSLQLFI

FQFLQTPLLSLGNTLGAMVIAYLFLHFFWFFGINGSSVVGAVFNPVLRALSVENLDAY

KNGLPIPNIITGQFQDMFATFGGAGSTLSLIVVMIFVCKSQRIKKLSQLSLVPGIFGI

NEPIIFGLPIVLNPILLIPFALVPTINIVIAYFAMSWGWVPFTNGIQLPWTMPIIISG

FLVSGWQGAVLQGLLFILGMFIYYPFIKVLDDQYLIDEQVATVAEEEDDISFDDFDFE

DL"

CDS complement(2188746..2189075)

/gene="ptcB_2"

/locus_tag="JFBMFIFI_02072"

/EC_number="2.7.1.204"

/inference="ab initio prediction:Prodigal:002006"

/inference="similar to AA sequence:UniProtKB:A2RIE6"

/codon_start=1

/transl_table=11

/product="PTS system galactose-specific EIIB component"

/db_xref="COG:COG1440"

/translation="MSKKKIYLFCDAGMSTSIMVNKMQAVANEHGMPLEISAYPVARA

AEVVETEKPICILLGPQVRFLLQKTKDKFEPLGIPVGGIDPEIYGMMDGEKALKEALK

LIKSQKK"

CDS complement(2189161..2189886)

/gene="nagR_3"

/locus_tag="JFBMFIFI_02073"

/inference="ab initio prediction:Prodigal:002006"

/inference="similar to AA sequence:UniProtKB:O34817"

/codon_start=1

/transl_table=11

/product="HTH-type transcriptional repressor NagR"

/db_xref="COG:COG2188"

/translation="METNKQGIYIEVAEQLHERIQNETYSAAQKLPSEYELAKEFKVS

RLTIRKAVDELIKKDILIKYKGKGTYIMTQQQKIQSGRGGLQGFTEAAEAYGLSSKTH

VIEFELVETAPDSILKALELSEGDKLYHVKRLRLANDEPMTVEDMYIDEKYLPTINQE

LASQSLFGLIEKEADIAYSHQEVEAVLVNEEMSQLLDVSIGDPMLLVHSVTYSVTGKP

LLHDTSYYRADKYTFKNTLHRFK"

CDS complement(2189925..2190878)

/locus_tag="JFBMFIFI_02074"

/EC_number="3.5.1.26"

/inference="ab initio prediction:Prodigal:002006"

/inference="similar to AA sequence:UniProtKB:Q47898"

/codon_start=1

/transl_table=11

/product="N(4)-(Beta-N-acetylglucosaminyl)-L-asparaginase"

/translation="MTYGMIATWRMAYDGVASMTEALKKGEGAGTALVEAIKQVEDYP

FYKSVGYGGLPNEECILEMDAAYMDGDTFDIGAVAGIQDVKNPIAVAEALSHERFNSF

LVGAGATKFAKQAGFDCRNMVTARAKRLWELRLEEIQKHNLSPYDGHDTVGAVALDLN

KKMVAGTSSSGLFMKKSGRVGDSPLSGSGFYVDSEIGGATATGLGEDLMKGCLSYEIV

RLMGTGMHPQAAADQVVYGFSDLLEKRKGKAGAFSLVCMNKNGEWGVATNVEFSFVVA

TEKEAAEIYLAYPAENKTTRIEVADKAWLDAYEARIKKPVD"

CDS complement(2191068..2191256)

/locus_tag="JFBMFIFI_02075"

/inference="ab initio prediction:Prodigal:002006"

/codon_start=1

/transl_table=11

/product="hypothetical protein"

/translation="MLTKNIQGKQECPYCERPVEVVFIPDRTTAELEYRECPYEDCGL

DILQMDKGSNEAEILKAR"

CDS complement(2191389..2191967)

/locus_tag="JFBMFIFI_02076"

/inference="ab initio prediction:Prodigal:002006"

/codon_start=1

/transl_table=11

/product="hypothetical protein"

/translation="MEIDKLLTFKKREELREWLLANSTTEKYCWVIVTIKPKLDTLLY

LDVVEEALCFGWIDGVKKKLSETQTAQRLSPRAKKSPWTELNKERVRRLEKLGLMTDE

GRKILPSDFHQPFKIDSVIEEKLKEDQQIYENFMAFPDLYRRIRIDTIQTNKKDAVLF

ESRLAKLIEQTKANKQYGAWHDNGRLIEDSIE"

CDS complement(2192098..2193201)

/locus_tag="JFBMFIFI_02077"

/inference="ab initio prediction:Prodigal:002006"

/codon_start=1

/transl_table=11

/product="hypothetical protein"

/translation="MKLTVRNKRIAAFVGVVIIGISIGIGFSLTNKEKFVTNLEKMVH

EKNISSLQKSIVVGTEEVKVSKDEIKSLNSYLKSDNKIKESFFADLKEQAVEKQQEEQ

TAKTFEIISKGRKWLIFPNYRLKVRPNYLTVSTDATNVIIKNQENKTIPYNKQKKQYG

PIFPGMYDFKIELLSDMPKIETNYDKVVVWDKNKNLETYLAKSAEKDKQFTENLATVI

DLFSDEYAAFMGGGFVDAENKALKDLSADLMEIRPLIESLYYEYKGITVNNDSIKLSQ

KNNQWTLNVMVFLDSNSYIESSLLPGNKMELEESKVYEYHLIYNKKGRKWEVDSRDEG

FGKSTEWKNSTEIASPKPKVYTWKGEGEQLNSL"

CDS complement(2193276..2194340)

/gene="ycdA"

/locus_tag="JFBMFIFI_02078"

/inference="ab initio prediction:Prodigal:002006"

/inference="similar to AA sequence:UniProtKB:O34538"

/codon_start=1

/transl_table=11

/product="putative lipoprotein YcdA"

/translation="MKKNVLLVLVALLAIVVAGCGGDTSSNKEKKAEVSKSKIAEMSI

ENGEYVIPFSEDGEGGSDTYIAFDVKVKNTGKEQMYVTPDSFSLFEKGEDGKISPDTS

YRTGIEDFEGGKLSSGKSLTGTVLFNIDSDKEYTLSYAPSSISEDSKNDVEVAVDLSK

YEKTKKNLDEPKKALDAYIDVVLLSKENPDYDKYISTDSEVAKEAVKKAFTKNFKDYL

VYNYKPTDEELTTFFKNFQEIEAKRATYETKVIGNVGKKAMVEVTMKGLSNEEISDIF

SEYKEEYLDQSDEYDSEKAEQYALTKYTEVLEKADLGEPRNELKVVLTKKDDKWDVNL

KSNMDGSNEYLLRAFIGGVE"

CDS complement(2194606..2196003)

/gene="nhaC_2"

/locus_tag="JFBMFIFI_02079"
/inference="ab initio prediction:Prodigal:002006"
/inference="similar to AA sequence:UniProtKB:O07553"
/codon_start=1
/transl_table=11
/product="Na(+)/H(+) antiporter NhaC"
/db_xref="COG:COG1757"
/translation="MLKREVQKGRAKMKKDVTIKEALFLLVVLLAMIGTLIIGFKLPA
HVALLFALAVVFLFAGYKKVSWKTIQEGIQEGITPGLVPIIIFILIGALISVWIAAGT
IPTIMVYGFSVLSVQFFLPTVFLICGIVGATVGSSFTTISTVGIAFLGMGQIMGLNPA
ITTGAIVSGAFLGNSISPLSDTANLSAAIAEVDLFQHIKHEFWTVIPAAFLSLLGYTF
LSFRATQTVAGDGIETISQTLHEFYTISPVTLLPVVVLFFAAWRKIPAIPTLLTSIVL
SILVRYLYHPTTSLASIGSWIQDGYISKTGVANVDLLLTRGGMQSMMWSVSLILLALS
LGGLLVKLNIIETILSQVEELMQTKSRLILMTALSSIGINLLIGEQYLSIILPGKAYK
ENYRKISLPDKYLSRTLSDAGATFNPLIPWGVSGVFIAGTLGISTLSYLPFAFFCYLV
PLITIGAGFMKTKEN"

CDS complement(2196091..2197950)

/locus_tag="JFBMFIFI_02080"
/inference="ab initio prediction:Prodigal:002006"
/codon_start=1
/transl_table=11
/product="hypothetical protein"
/translation="MMKNTFNTDDTNLKALFLGDKGENVDLFKEILNKMVDEHVGWRQ
NYMPQDLPVITPQDRSSKEFQATADNMRSVFNVLSSRLRTESLPWHSAGRFWGHMNSE
TLMPAIIAYSTAMLWNGNNVAYESSPATSQMEEEVGMEFATLMSYKNGWGHIAADGSI
ANLEGLWYARNMKSLPFAIQEVAPEMVAGKSDWELLNMSTEEVLNMLDQLQDQFDEIK
ARSARSGKNLEKLGKWIVPQTKHYSWLKAADIIGIGLDQVIAGEVNSEYRMDIDKLEA
QIRDLAAQEIPILGVVGVVGSTEEGQIDRIDQMIALREKLAKEGIYFYVHVDAAYGGY
GRSIFLDENNEFIEWDQIEAVYAKNNIFMEKNDWLTREVYESFKAISFAESVTIDPHK
MGYIPYSAGGVVIKDIRMRDVISYFATYVFEKGADIPALLGAYILEGSKAGATAAAVW
TAHKVLPLNVTGYGKLMGASIEGAYHFYHFIDGKEFKVGDKTIEIHALTKPDFNMVDY
VFNEKGNTDLVKMNKLNHDFYDYASYAKGGLYNNEFITSHTDFAIEEYGHSPFEFVNN
LGFSRSEWDRATKVTILRASGMSPYMNDREVFDDYAVKIESAIQKKLEAIYAE"

CDS complement(2198015..2199436)

/gene="gadC_1"

/locus_tag="JFBMFIFI_02081"

/inference="ab initio prediction:Prodigal:002006"

/inference="similar to AA sequence:UniProtKB:O30417"

/codon_start=1

/transl_table=11

/product="Glutamate/gamma-aminobutyrate antiporter"

/db_xref="COG:COG0531"

/translation="MKQSKKLSLFGLIGITMAFFGTVRSVPTLASTGWAQIFYMVLAA

CVFALPIALMSAELSTGWPEEGGPQVWVRNALGEKWGFVTSWLLWVQMFFGMVMVAST

VGVLIGYVINKPELGTNNIFIFAMILISYWGITFLNLKFDMVKIAGDWGAIIGVYIPF

LALVILGVLYMAKHGINADSYLGHFEASKLLPDLSDLGSLPTLTGIIFIFAGVEISSV

HANNIDNPKRNYPIAVIASVLLLVVFNLVAGLSVADSVPAGKMELANITQPFVIMTQD

LGIPAIFNNIISLMILIGVLVQLSAWVLGPSKSMIKVADEGNLPPFFQKRTAKGIPIS

FVIIQAVVISLVSFLYVVIPDISAAFLIITITTTILYCVVYLLIAISAIKLRYKMPEV

ERPFRLGSKGNGLMWFISGLSILSVIVTILVSLIPPSSISQSGHVGYIIYQVGATVIM

IGIALLIFKFKKPEWKKQDTLDD"

CDS complement(2200267..2200587)

/locus_tag="JFBMFIFI_02082"

/inference="ab initio prediction:Prodigal:002006"

/codon_start=1

/transl_table=11

/product="hypothetical protein"

/translation="MQIDKIEAKMIDEFFDGILALESKEECFAFFDDLLTLNENKTII

QRYQVAKMLYEKKTYQTIEKETHASTATIARVKRSLFDGNNSYDMLFKRIEQANKLTE

NEEE"

CDS complement(2200792..2201310)

/gene="folT"

/locus_tag="JFBMFIFI_02083"

/inference="ab initio prediction:Prodigal:002006"

/inference="similar to AA sequence:UniProtKB:A0Q1J7"

/codon_start=1

/transl_table=11

/product="Folate transporter FolT"

/translation="MNKTKLDARAITIIGLLMALEIILTRVFSFEVPFFRIGIGFLPG

VMIAMMYGPWIAGVTAVSTDIIGMVLLPKAMFFPGFTLSTFLTALIYGFVLYKKPKTL

TRIIVAVLLVSIIVNLGLNSLWLSMMYDKAFIAIFPGRVISNIIGVPLRVALIYLVVK

SRVLERFLQPVL"

CDS 2202323..2203399

/gene="metN"

/locus_tag="JFBMFIFI_02084"

/EC_number="3.6.3.-"

/inference="ab initio prediction:Prodigal:002006"

/inference="similar to AA sequence:UniProtKB:O32169"

/codon_start=1

/transl_table=11

/product="Methionine import ATP-binding protein MetN"

/db_xref="COG:COG1135"

/translation="MITLKDIEVTFENKGQKVVAVDQVDLQVEKGDVFGIVGYSGAGK

STLVRTINLLQRPTAGSVTVAGQHLLNLKPKELRAARKKIGMIFQHFNLMASRTIAEN

VAYPLKNSGLSKKAIRDKVNQLLELVGLTDKHQAYPSQLSGGQKQRVAIARSLANDPE

VLLCDEATSALDPKTTLSILKLLKDLNKKLDLTIVIITHEMQVVKEICNKVAVMEDGK

VIEAGSIVSIFTQPKKVLTKDFINTATHVDQGLETILSHPTILDLKKNDILAKISYVG

ATTSEPLIAHLYSQFSVSTNILFGNVEILQEVPIGNLIVVLSGEPAKREKALAYLASK

DVKVDLIQHKQNVVPYQKKESQIS"

CDS 2203422..2204102

/gene="metP_2"

/locus_tag="JFBMFIFI_02085"

/inference="ab initio prediction:Prodigal:002006"

/inference="similar to AA sequence:UniProtKB:O32168"

/codon_start=1

/transl_table=11

/product="Methionine import system permease protein MetP"

/db_xref="COG:COG2011"

/translation="MSNFIETYFKNVPPLRDEFIESTLQTLYMAGVTALIAGVLGIIL

GVILVVTDQGGILENKYLYSVLDKIINIFRSIPFIIMLAAIVPFTRWIAHTSIGTTAA
IVPLVVGTTPFFARQIQNALVEVDAGIIEAAQSMGSSPLGIIFRVYLKEGLNGIIRAS
SLTIINLIGLTAMAGAVGGGGLGNLAISRGYNRFNDDVTFVATLIILAIVFITQAIGN
YLIRKTSH"

CDS 2204165..2205007
/gene="metQ_2"
/locus_tag="JFBMFIFI_02086"
/inference="ab initio prediction:Prodigal:002006"
/inference="similar to AA sequence:UniProtKB:P28635"
/codon_start=1
/transl_table=11
/product="D-methionine-binding lipoprotein MetQ"
/db_xref="COG:COG1464"
/translation="MKKTKKVIFSILFVLSIFTLTACGGNAAKSDEKKVEVVKLGVVG
DDTRVWDSVKDRLKKDNIDLQIVKFTDYTQPNSALNEGELDLNSFQHQIFLDSYNKDS
GTDLVSIGETVIAPLGIYSEKIKDVKELKENDIVAIPNDVTNGGRALLLLQTAGLIKV
DPAAKQTPTVKDITENKLKLEIKELDAAQTARALADTTISLINSGMAVDAGLIPNKDA
IFLEPIDGTSKPYINIIVANAKDKDNKTYKKVVEAYQSPETIKVIEETSKGSSIPVWE
TDSK"

CDS complement(2205056..2205478)
/gene="mgsA"
/locus_tag="JFBMFIFI_02087"
/EC_number="4.2.3.3"
/inference="ab initio prediction:Prodigal:002006"
/inference="similar to AA sequence:UniProtKB:P42980"
/codon_start=1
/transl_table=11
/product="Methylglyoxal synthase"
/db_xref="COG:COG1803"
/translation="MNIALIAHDRKKELMIELAIAYREILKEHTLYATGTTGKRVIEA
TGLPVHRFKSGPLGGDQQIGALISEDKMDMVIFLRDPLAAQPHEPDVTALIRLSDVYE
IPLATNIGTAEILLRGLKSGFADWRNLKNKSEGEILDI"

CDS complement(2206113..2207342)

/gene="ydiM_1"

/locus_tag="JFBMFIFI_02088"

/inference="ab initio prediction:Prodigal:002006"

/inference="similar to AA sequence:UniProtKB:P76197"

/codon_start=1

/transl_table=11

/product="Inner membrane transport protein YdiM"

/translation="MSENSYFSTVMALYINYILQGMAAIILAQNMVPLMGQLNTNAAG

ISIVISGIGFGRILILYFAGRMSDRYGRKKIVWLGMFSYVIFFGGILISQNMWMATFF

TLFAGFANALLDTGTYPALMEAYPEANGFMSVLNKAFISLGQLILPIVVGFLIANNLY

FGYSFILCLVALFANIFFMQKRRFPKLSQTSLAVEKEQKATKFNQKPVFLVEGLALTI

FGFTSVSTFNIIVLWLPRYAEELAGMAKSSSLMLVSLYSVGSMISVFITAFLIKKWMQ

PITMILSCSIASLIVLILVILFPTPLGCSIAAFGIGIFSSGGIWQLALAILLEFFPEN

KGRMTSYYTLMTSFSVMLIPLITGNLSERNILYVFIFNCGITLASVIVAVLVAIRYRK

LTNLKKAVEVPSVFQEI"

CDS complement(2207398..2208159)

/gene="aroD"

/locus_tag="JFBMFIFI_02089"

/EC_number="4.2.1.10"

/inference="ab initio prediction:Prodigal:002006"

/inference="similar to AA sequence:UniProtKB:P58687"

/codon_start=1

/transl_table=11

/product="3-dehydroquinate dehydratase"

/db_xref="COG:COG0710"

/translation="MKTVTVKDVILGEGAPKICVPMVGKTLTELKEEATELVRLDLDI

VEWRVDFFNDVEEIEKVIEAATEIRTILKQKPILFTFRTLKEGGEREISETYYFELNQ

KMMASGLVDLTDIELFMGDTAILAAVDWAHQHNVKVVMCNHDFDKTPDKEEIISRLKK

MQTLNADICKIAVMPNSSQDVVVLLDATATMQAEFADRPIVTMSMGGLGVVSRLAGET

FGSAMTFGAAKKASAPGQVPIAELRSVLDLLHKSK"

CDS complement(2208192..2209397)

/gene="ydiM_2"

/locus_tag="JFBMFIFI_02090"

/inference="ab initio prediction:Prodigal:002006"

/inference="similar to AA sequence:UniProtKB:P76197"

/codon_start=1

/transl_table=11

/product="Inner membrane transport protein YdiM"

/translation="MKNKYMPTAIGLYINYFVHGMGVIILAQNMDALGKQWGTDSAGV

AIVISSLGIGRLIVLMASGALSDKFGRRPFVYLGMFTYALFFAGILFSPSIAVAYIFG

ILAGIANSFLDSGTYPALMESFPESPGTANIIIKAFISAGQFALPLMVGLIVSTNMWY

GWTFILAIIIFVVNGIYLFNKPFPDKEASISDKPEESTNLAQPVRKVKFAVEGVCFIL

YGYIAQATFYLVSQWLTKYGNEIAGMGDAASRALISYYSIGSLVCVFFTSALVKKKYK

PVQFLVIYTFISFVALLVLWLFPTPLVCIIAAFVIGFSAAGGVLQLGLTVMAEMFPAG

KGKVTGIFYTAGSIASFTIPLITGQLSKTNIANIILFDVGIAFIGFLLALVIYVRYNK

VFTKSNELN"

CDS complement(2209479..2210348)

/gene="aroE_1"

/locus_tag="JFBMFIFI_02091"

/EC_number="1.1.1.25"

/inference="ab initio prediction:Prodigal:002006"

/inference="similar to AA sequence:UniProtKB:Q8Y9N5"

/codon_start=1

/transl_table=11

/product="Shikimate dehydrogenase (NADP(+))"

/db_xref="COG:COG0169"

/translation="MTERLSGHTELIGLMATPIRHSLSPTMHNEAFAKLGLDYAYLAF

EVGTDQLENGIQAIRALGMRGSNISMPNKQVVTKYLDKLSPAAEMVGAVNTIVNDNGI

LTGHITDGTGFMRGLKQSDVSIIGEKMTICGAGGAATAICIQAALDGVKELSIFNRQD

EFFKNAERTVALINEKTDCKATLYDLKDQESLRQQIAESVIFTNATGVGMKPLQEESL

ITDPTMLRKELVVADVVYVPSETKLLKLAKEQGCKTVNGLGMMLWQGAAAFEMWTGQE

MPVEYIRELLFEK"

CDS complement(2210493..2212529)

/locus_tag="JFBMFIFI_02092"

/EC_number="1.-.-.-"

/inference="ab initio prediction:Prodigal:002006"

/inference="similar to AA sequence:UniProtKB:P32382"

/codon_start=1

/transl_table=11

/product="NADH oxidase"

/translation="MEQYAALFEPLVIKRMTLKNRVMMPPMGTNFANLDGSFNNEHIT

YYEQRAKGGTGLITLENACLDFPMGTNGTTQLRIDNDQYLPGLWRFNEVMHAYGACTS

VQINHAGASAYGLRLNGQQPVSASNIPSKTGNAIPRPLEKDEIMEIVKKYGEAANRAQ

RAGFDCVEIHAGHSYLISQFLSPLYNKRQDEFGGSPENRARFAQLVVAEVRQAVGPFF

PICIRISADEFLEGGNSLEDTLELLTYFSEEVDILNVSAALNDSIQYQIDQMNLADGW

RSYMARAVKEKFGKVTITSGNIRSPQVANDILVRGDADLLAMGRGLIAEPNWVNKVAN

GQENLLRKCISCNIGCADHRIAKSRPLRCTVNPDVIHEDDFKQNQVKNNLSMVVIGGG

TAALEAATTAAEVGVQVTLFEEKSYLGGLAREIARLPDKKRIDDYVVYLEERAKQLPN

LTIHLNTRADLKTIEMLAPDIVVNATGAKPLLPPIKGLKEQLENPNRQVFSIFDLLNN

MENFEEFEGKRIAVIGGGAVGLDVVEYYAERGAAEVSIVEMQEKLGKDLDLITRISMM

DMIKRHGVQEYTETALTEVCQDHFKVKQAGKESEIPFDIGFVCLGMRGDSPMMEALLE

YGKATDTAVVNIGDSKVARRIIEGTREARDILTTIHQVDRSKPLSQLKLFSGNF"

CDS 2212695..2213606

/gene="gltC"

/locus_tag="JFBMFIFI_02093"

/inference="ab initio prediction:Prodigal:002006"

/inference="similar to AA sequence:UniProtKB:P20668"

/codon_start=1

/transl_table=11

/product="HTH-type transcriptional regulator GltC"

/translation="MNLKQLYYFKMLAKTEHMTQAAEQLSITQPSLSHSMTELEKELG

TFLFEKNGRNIRLTKYGHFFLSYVERSLNELEHGEKALKELVSPDRGHIDLAFIYTLG

PYFAPLLLKEFQTIDAHQNISFSFYQGNTKNIIKSLKAEQSDIAICSMITPDDQLHFE

PIAEEEIVLIVPLDHPLAYFDEIDLKNTAGYPFISFNKKSGIRPMIDAMLAEVNVVPS

IVCEVEEDHTMAGFVAFGHGIGIVPRIAALDYYKVKILHITNPTYQRTIYATTLKNHY

LSPATRLFRDFIVDYGQQHFLASGKKI"

CDS complement(2213695..2214273)

/gene="gpmB"

/locus_tag="JFBMFIFI_02094"

/EC_number="5.4.2.-"

/inference="ab initio prediction:Prodigal:002006"

/inference="protein motif:HAMAP:MF_01040"

/codon_start=1

/transl_table=11

/product="phosphoglycerate mutase GpmB"

/translation="MTRLYLVRHGQTLFNAQHKIQGFCDSPLTEIGIRQAQIAREHLE

KEGIRFSEAYSSTSERASDTLELLTDSPYKRIKGLREWNFGSYEGEGEHLNPPLPYND

FFVQFGGESQVELEERISSTVETIMRNTQGENILIVSHGAAIANFYLHWEHTSAVKKH

KKIQNCSLFSYQYVDNQFVLEEIIEHDFTTLQ"

CDS complement(2214413..2215264)

/locus_tag="JFBMFIFI_02095"

/EC_number="1.-.-.-"

/inference="ab initio prediction:Prodigal:002006"

/inference="similar to AA sequence:UniProtKB:A0QV10"

/codon_start=1

/transl_table=11

/product="putative oxidoreductase/MSMEI_2347"

/db_xref="COG:COG0656"

/translation="MQTVKLINGVEIPILGFGVYQIPQEETAQAVKDAIQAGYRHIDT

AQSYMNEREVGKGIAESGIAREELVVTTKIWVQNVNYDGVIKSFDESLERLGLDYIDL

LLIHQPYNDVYGAWRGMEELQKSGKVRAIGVSNFSVDRVVDLAEFNEVKPQVNQIEIN

PFQQQAQTIADLHAEGVAVEAWAPFAEGRNNLFSNPVLTKIGEKYNKSVAQVIVRWLI

EQNVIVLAKSVKPERMAENIAVFDFELTSEDQQQIATLESGESQFFSHKDPAMIKMMA

DRKIEGI"

CDS complement(2215329..2215757)

/gene="adhR_4"

/locus_tag="JFBMFIFI_02096"

/inference="ab initio prediction:Prodigal:002006"

/inference="similar to AA sequence:UniProtKB:O06008"

/codon_start=1

/transl_table=11

/product="HTH-type transcriptional regulator AdhR"

/db_xref="COG:COG0789"

/translation="MNSKEVSELFNLSIDTLRYYEKAGVIPPVTRSENGYRDYQIRDL

NWIYLAKTLRKAGMSIESLQEFARLAQLHDAANENVEIKQKQILIDQLEAVDEKLEEM

QQARDLLAYKVKTYDDHIAKFKNGELLEKNSEKLWELNPK"

CDS complement(2215855..2216292)

/gene="yjaB"

/locus_tag="JFBMFIFI_02097"

/EC_number="2.3.1.-"

/inference="ab initio prediction:Prodigal:002006"

/inference="similar to AA sequence:UniProtKB:P09163"

/codon_start=1

/transl_table=11

/product="Peptidyl-lysine N-acetyltransferase YjaB"

/db_xref="COG:COG0454"

/translation="MLYHQAKSSEYEKIISIWKRSVLATHHFLKPEDMSSIEAEIRYW

LPKMNVQVWSQSEKMIGFSAVSEDTLEMLFIGPDQIGKGYGKQILKELLSAFEVTKVD

VNEQNEAAVTFYLKNGFQIAARDELDSEGRAYPILHLELTQNS"

CDS complement(2216485..2217414)

/gene="rbsB"

/locus_tag="JFBMFIFI_02098"

/inference="ab initio prediction:Prodigal:002006"

/inference="similar to AA sequence:UniProtKB:P02925"

/codon_start=1

/transl_table=11

/product="Ribose import binding protein RbsB"

/db_xref="COG:COG1879"

/translation="MKKLVLLLGASLLLMAGCGAATLDGENSSSDKVVKKEEKELVVG

VSLSTLNNPFFVSLENGIQKLADEKGTKLKIVDAQDDTAKQSNDVDDLIQQGVDILLI

NPVDSSAITPAVESANAANIPVIAIDRDSEGGKLLSLVASNNIEGGKMAAKYIEEVVG

SEAKVVELQGVPGASATRERGKGFDEYAKGKLDIIAQQAANFDRAKGLTVMENMLQSN

PEVQAVFAQNDEMALGAVEAIGAAGKTDAITVVGFDGTEDGLKAIKAGKMSATVAQQP

EEMGKLALQAAFDHFAGKKVESKIDSPLELIKK"

CDS complement(2217437..2218405)

/gene="rbsC"

/locus_tag="JFBMFIFI_02099"

/inference="ab initio prediction:Prodigal:002006"

/inference="similar to AA sequence:UniProtKB:P0AGI1"

/codon_start=1

/transl_table=11

/product="Ribose import permease protein RbsC"

/db_xref="COG:COG1172"

/translation="MKDELKVELPKKKYTGKELIGKLGPLLALLVLIIIVTAMNPSFI

APINLLNLLRQVSVNALIAFGMTFVILTGGIDLSVGSTLALSGALVAGMITSGIDPLL

AMIIGVMIGGILGAINGLLITKGKMAPFIATLATMTIFRGATLVYTDGNPITGIGDSF

IFKFVGRGYLFGIPFPVILMIVAFAILFLVLHKMTFGRKTYAIGGNEKAAFVAGIKID

RVKTMIYALSGMMASISGIIITSRLNSAQPNAGQAYEMDAIAAVVLGGTSLSGGRGRI

FGTLIGALIIGTLNNGLNLLGVSSFYQQIVKGIVIIIAVLLDRKKK"

CDS complement(2218408..2219889)

/gene="rbsA"

/locus_tag="JFBMFIFI_02100"

/EC_number="7.5.2.7"

/inference="ab initio prediction:Prodigal:002006"

/inference="similar to AA sequence:UniProtKB:P04983"

/codon_start=1

/transl_table=11

/product="Ribose import ATP-binding protein RbsA"

/db_xref="COG:COG1129"

/translation="MEVIMKNIYKAFGTNTVLEGVNFDTHGGEIHALMGENGAGKSTM

MNILTGMHKKDQGTILINGKEHSYTNPKEAEEHGVSFIHQEMNTWPQMTVLENLFIGK

EMKNKIGFIRSKEMKTLALKTFKELGITMDLDADVQTLSVGQQQMIEIAKALMTDCQV

LIMDEPTAALTDREIRMLFKIIQHLKTKEVAIIYISHRMEEIFEISDRITVMRDGMTV

DTTLTKETNVDDVVRKMVGREISDYYPKKNAEIGETVFEVKNLSGTSGFENISFSVKS

GEIVGFSGLMGAGRTEIMRGIFGIDPISQGEIILEGKKVQLKNPSTSIKAGVGFLTEN

RKEEGLVLDFSIKDNISLPSIDEFRVRGLIDTKTEDEFVQLLMKRLTVKAQNEDISAG

SLSGGNQQKVVLAKWIGIGPKVLILDEPTRGVDVGAKREIYQLMNELAERGVAIVMVS

SDLPEVLGVSDRILVVHEGKIAGELNRTEATQEKIMNLATGGK"

CDS complement(2219997..2220392)

/gene="rbsD"

/locus_tag="JFBMFIFI_02101"

/EC_number="5.4.99.62"

/inference="ab initio prediction:Prodigal:002006"

/inference="similar to AA sequence:UniProtKB:P36946"

/codon_start=1

/transl_table=11

/product="D-ribose pyranase"

/db_xref="COG:COG1869"

/translation="MKKHGILNSDISKVLSDLGHTDQITIGDAGLPVPEGVLKIDLAL

ALSDPEFIKVFGLVLEDMVVEEMVLAKEIGVSNQKQLKQIKELAPMIPIHYLSHEEFK

EQTKKSKVIIRTGEATPYSNVILQAGVIF"

CDS complement(2220367..2221272)

/gene="rbsK"

/locus_tag="JFBMFIFI_02102"

/EC_number="2.7.1.15"

/inference="ab initio prediction:Prodigal:002006"

/inference="similar to AA sequence:UniProtKB:P0A9J6"

/codon_start=1

/transl_table=11

/product="Ribokinase"

/db_xref="COG:COG0524"

/translation="MSKITVIGSLATDFVVSVDKRPVVGETIIGNDFKTTFGGKGANQ

AVAAARLGSHVAMIGKVGADSFGTEIIANLKENQISVSGVEPVTHLPSGSAHITLADG

DNSIVVISGANNAVDIQQLTKNQERIKTSDLVLLQQEIPSETVEAIVDFCYQHQIPTV

LNPAPARSISQNVIDKVTYLTPNEHEFEELFPQLTVSEGLAKYPNKLIITVGSKGVLF

NNGQEEILVPSYQVTPVDTTGAGDTFNGAFSVALTNQLSVAESIRFGNLAASLSIQKF

GAQGGMPKLEELKEQPGYEKTWNFK"

CDS complement(2221269..2222249)

/gene="purR_1"

/locus_tag="JFBMFIFI_02103"

/inference="ab initio prediction:Prodigal:002006"

/inference="similar to AA sequence:UniProtKB:P0ACP7"

/codon_start=1

/transl_table=11

/product="HTH-type transcriptional repressor PurR"

/db_xref="COG:COG1609"

/translation="MTTIKDVAKHAGVSVATVSRTINNSGYVGVESRKKIELAIKELN

FYPNEVARSLFQKKSKFVGLLLPDISNPFFPLLAKGVEDKMNQMGYQLILGNVQEDMD

KEKEYLKAFAQNNVAGVLSAINGGTKELKNIPLVMLDRVDTDKEYGVHSNDFEGGQLA

AQAIAERNPKEIVVMVGPRSIHGSLERLRGTEKVLNNFKLNYHLLETSSFQFDATQAT

TDELFKQFPKVDSIIASNDVHALSILQEALRRGIKVPEELQIIGYDDIPFSQMLFPSL

ATIAQPAYEIGFRGAELLCDRIENKLVKEKTIQLPVKIKIRESLRKKVLE"

CDS complement(2222437..2223051)

/locus_tag="JFBMFIFI_02104"

/inference="ab initio prediction:Prodigal:002006"

/codon_start=1

/transl_table=11

/product="hypothetical protein"

/translation="MKKVTVRKEYFQDNNVFSADTTLDGEDVLRVIKDPVILADDEAT

YARLQNFEFYNGTIEVKVLSRLLPDAPDHARGFIGVSFRIDENNSHFEGIYIRPTNGR

SEDQVRRNRSVQYFSYPDFKYDRFREENPGMYESYADIGLNEWIDMKIEVENNKAKLF

LNNAEQPVLIVNDLKLGAMQKGGIGLWVDIGTEGFFKDLKINSL"

CDS complement(2223119..2224108)

/gene="aes_2"

/locus_tag="JFBMFIFI_02105"

/EC_number="3.1.1.-"

/inference="ab initio prediction:Prodigal:002006"

/inference="protein motif:HAMAP:MF_01958"

/codon_start=1

/transl_table=11

/product="Acetyl esterase"

/translation="MKKFFLWLAGLIILLSIGLTIVFNLTPLPMISLARQVLFSVPTD

TRKPEFTEGSVTVLTNTSYTGKYKQSTFDLYLPDIPLPNEPTIAWVHGGGYIGGDKLE

EKEYATKLAENGYSVAVMNYELVPETTYPIPVRQVGEFIKHLEANQEVYGLNMANLFI

AGDSAGAQIASQFIAAQTNPAYGKQIQVEAVPMLKEIQGALLYCGPYNLIDIVEDSTS

QIVSFSFDKIGWSYFDERNWENSEKAKLSVVPNYVNKDFPPTFITDGNTSSFEQQGRE

FASKLEEVGVPIKTRFFDSKEVETRHEYQFKLTTPPGKIAFEDTLNFLKAYQK"

CDS complement(2224133..2224711)

/gene="mhqD_2"

/locus_tag="JFBMFIFI_02106"

/EC_number="3.1.-.-"

/inference="ab initio prediction:Prodigal:002006"

/inference="similar to AA sequence:UniProtKB:O34842"

/codon_start=1

/transl_table=11

/product="Putative hydrolase MhqD"

/db_xref="COG:COG0400"

/translation="MNYFITDKNNKEFFVLFHGTGGNEYSLLSVSGDLNPEAGVISFL

GNSGTGTTRRFFNPLIEGTLDRADFNQNVATFLDTWTEIKPQNAKLTFIGYSNGANFL

LGILEKAPEIADKIILMHPANLGYSFKEQTNTPILLTYGANDYMVPPGDVLALANQMK

PFFPKLEAKLLDSGHEVTDLEVKTIKNFLNQH"

CDS 2224754..2225728

/gene="mhqA_3"

/locus_tag="JFBMFIFI_02107"

/EC_number="1.13.11.-"

/inference="ab initio prediction:Prodigal:002006"

/inference="similar to AA sequence:UniProtKB:O34689"

/codon_start=1

/transl_table=11

/product="Putative ring-cleaving dioxygenase MhqA"

/db_xref="COG:COG0346"

/translation="MKNTLIHHVSVINRDIQKSFHFYHNTLGLNLLLKTVNQDDNEMY

HTFFGDTAGRPGTEFTVFEMKNGVDHKFGTNAIDRTVFLVPSEESLLFWEKRLNSLGI

FNCEIESYNNSKILRFEDFDGVQLGFMPLTEKQTTHYLPRITDEISGENAILGIHSIH

LRVRYLEATKQLFKKFFDWTLLTKLTTEPTPIAILGNHNELFYQEVHLIEDKKSPLEV

MGIGGTHHVAFSAKDEHELQEIQEEISLKNFLNSGIKNREFFKSLYFREANGILIEVA

TLDTHFVKENYEGLDLDQVPLYLPTFLAPKRSAIEASLASSHPQTESR"

CDS 2225734..2226273

/locus_tag="JFBMFIFI_02108"

/inference="ab initio prediction:Prodigal:002006"

/codon_start=1

/transl_table=11

/product="hypothetical protein"

/translation="MTYQDILNFWFNEIDQELWFKKDDAFDQELLRRFGTVHTQAKNG

ELSQWRDCLEGCLAEIIVLDQFSRNLFRDKPEAFAYDGMALVLAQEAIRHFNVSDLPV

EQQAFLYMPFMHSESLVIHEEALTLFAGEGLENNYAFELKHKVIIERFGRYPHRNQVL

GRESTPEELEFLAGPDSSF"

CDS 2226397..2227665

/locus_tag="JFBMFIFI_02109"

/inference="ab initio prediction:Prodigal:002006"

/codon_start=1

/transl_table=11

/product="hypothetical protein"

/translation="MSKTENSFDLTHWRKNFYLFLSGQFLSGITSMVVQYSIIWYLTK

TTGSATVLSFATILGMLPMVILSPFVGSFVDKWNKKTLLIVTDIIVAIFALILAITGT

IASDFPLWLVFVSLFIRSVAQTFQMPTIQSILPTMVPEEELTKVNGQLGMVQSANFII

APALGALLFSIVPMNHLILLDVLGAVFGVGLLLFVTIPRILSEGETIHLLADSKFGLK

KLTENKGLWYITIVGAIFTLIFMPAASLYPLMTIGYFNGTVGEAGLIEVVYSIGMLLG

GAVIGIFGKWKDRMKLVFMAYFVIGITIGLSGILPPTRTGFFYFIILNSFAGFATPYF

NTLLMAMIQQSYEPNVLGRVLGVLNSLMSITGPVGLIFAGPLADKFGVEKIFLFAGIG

TIICGIINFMIPVARNYDKQLQKKLEKPSK"

CDS 2228099..2228836

/locus_tag="JFBMFIFI_02110"

/inference="ab initio prediction:Prodigal:002006"

/codon_start=1

/transl_table=11

/product="hypothetical protein"

/translation="MNIGDKIKEQRLRKELTQEQLSALLNVSRSTVSSWEVGRNYPDL

ETIVAISDLFDISLDNLLREDTTMTKNLSNKLRWNGRYKKILLVIGILFLIYAGFNLK

LRLDEKRYLTNISAHSWKNDDIRYLGGYVLKEKDITYSTYIMENGFNPIPLKENSPWV

IARKDPLVVKVKQGNKLYIVISKANDPKVTYDATVEVDAQVNLVKIDPKWSAENQKSL
EDYLTEHQKEYKDLLDGAITKRLDIIN"

CDS 2229302..2230516

/locus_tag="JFBMFIFI_02111"

/inference="ab initio prediction:Prodigal:002006"

/codon_start=1

/transl_table=11

/product="hypothetical protein"

/translation="MNKLALSIRIILYGALLSTFIGAISFAFIYLESNISHYLWHILL
TNNTFKTLTILLFCLLGGLIVGSLRGKWGDYPQTAHHTIQQLKEHKTVNYRPVFKSLL
TALLILIFGAGVGPEAALLGAIVMLSVWQADKIRYLFFNRESFAALNPLERLGHMFHP
TRYLLTYNPKNTNEKLAATKKYVIIFYILNGLFAFIVLMKYTNQPSFISKMGMTYWEL
KDFWLFVPLVIFGVLAGKLYNLFKRKMADWMNFWSDQPIKKALIGSFAIFIVGMFTPN
LLFSGQVTLGAVPKEYLHFSTLLLVCVVIIKLVFLQVCLNTGWIGGDIFPIVFSAILL
GFGLSQLLPNFDTVFIVATVATAMTISILQSPLGLAIFIALFFPVQILPIILLTALLL
KVIQTKRGKQVV"

CDS complement(2230643..2231005)

/locus_tag="JFBMFIFI_02112"

/inference="ab initio prediction:Prodigal:002006"

/codon_start=1

/transl_table=11

/product="hypothetical protein"

/translation="MWWIVLLIIAYMLFSRLDDIIELSKSKSPDYQKEVEQRQYEHQQ
ERKEITQQLRALIGSECRIKSSDLIYMSNGSSEIKAKISQVDDDWVEVLTLRKKKHLK
VYIKIESINSLSKIVAEE"

CDS complement(2231007..2231444)

/locus_tag="JFBMFIFI_02113"

/inference="ab initio prediction:Prodigal:002006"

/codon_start=1

/transl_table=11

/product="hypothetical protein"

/translation="MTKFNERIYALRKANNFTQEEIAQELKVSRQTISNWETGTAQPT
IDKVIELANVFDVSMDELIGRSKNKTKKASGILLSLLNQVVTLYLTPVTDVLLSIGRT

EIKNCKIIEVNPTSIRVRMEDKNQQVEKLIFIKDISGFEKEAD"

CDS complement(2231751..2232377)

/locus_tag="JFBMFIFI_02114"

/inference="ab initio prediction:Prodigal:002006"

/codon_start=1

/transl_table=11

/product="hypothetical protein"

/translation="MDISEQLAEVVKVSNGFKPMKELADRLANQQTDKELAVLAHKLF

ESTDYQIRMVAVFLFGKLAAKDANSLSFLQEIVAKDQNWRVQEILAMAFDNFCKDTGY

EESLATIKSWLTAEDLHTRRAASEGLRIWTSRPYFKENPEVAIHLLALLKNDDSEYVR

KSCGNALRDISKKFPEKVLHEISKWGTSKNESQVKKLILKNKKLLILD"

CDS complement(2232406..2233380)

/gene="cbh"

/locus_tag="JFBMFIFI_02115"

/EC_number="3.5.1.24"

/inference="ab initio prediction:Prodigal:002006"

/inference="similar to AA sequence:UniProtKB:P54965"

/codon_start=1

/transl_table=11

/product="Choloylglycine hydrolase"

/translation="MCTAVTYVTNDHYFGRNLDLDFSYNETVTVTPRNFVFPYRHVSD

ISQHYAMIGMATVVNNYPLYYDATNEKGLSMAGLNFPENAVYFPEATGKDNIAPFEFI

PWLLGQCGTIKEVRELLEKINLVNTNFSDELPLSPLHWMITDQQESIVVEQTKNGLAI

YENPVGVMTNNPTFDYQLFNLNNYRHISPITPNNLFSKEIDLDIYSRGMGGIGLPGDL

SSASRFVKVAFTKLNSVSGTSESESISQFFQILGSVTQQRGLCDVGDGKYEYTIYSSC

CNMDKGIYYYTTYENSQITGVDMHKENLDGTKLVNYPLVSGQQINFVN"

CDS 2233749..2234708

/gene="asnA"

/locus_tag="JFBMFIFI_02116"

/EC_number="6.3.1.1"

/inference="ab initio prediction:Prodigal:002006"

/inference="similar to AA sequence:UniProtKB:P00963"

/codon_start=1

/transl_table=11

/product="Aspartate--ammonia ligase"

/db_xref="COG:COG2502"

/translation="MYDTQTAIGFIKRCFEEALTGSLKLKRVSAPLFVHQQSGLNDNL

SGVERPVQFDVPSLKNNGEVVHSLAKWKRVALKQYDFHAGNGLYADMNAIRRDEVLDN

LHSIYVDQWDWERVISKEQRTIDYLKEVVQGLVNAMAETASRLHTKYPSVNIPIQTEV

SFITSQELADLYPGLSSKDREHAYVKEHPTTFIMQIGAALNDGESHDHRAPDYDDWSL

NGDLLFWHEPLKCAMELSSMGIRVDDKSLLEQLEKANATDRLAFDFHKQVANNELPLT

IGGGIGQSRMCMLLLGKAHIGEVQVSLWDDETIEACDGKIFLL"

CDS 2235440..2236291

/gene="licT_1"

/locus_tag="JFBMFIFI_02117"

/inference="ab initio prediction:Prodigal:002006"

/inference="similar to AA sequence:UniProtKB:P39805"

/codon_start=1

/transl_table=11

/product="Transcription antiterminator LicT"

/db_xref="COG:COG3711"

/translation="MKIIKVLNNNVIIALNQEDEEVILMGNGLGFQMKNDSPIRTEKI

EKIFNLKNNVERKQIEKLFTEIPEDIIRISYEIISYATKKLNKDLTDSAFIAIADHLH

SAIQRDKKALHVKNFLLWDIKRFFPVELMIGQKAIDIVNEKFNTTLPEDEAGFIALHI

TNASLDSQSDDAVSLTQLIEEILTVIKYTLQLTFSENDIYFQRFITHLKFFSQRVLTN

QTYPDGENQVENELFKLVTKQYPEAFEVTKKVSEFLKTRRNYIISKDEQVVYITIHLAR

IIDKTRN"

CDS 2236415..2238313

/gene="bglF_2"

/locus_tag="JFBMFIFI_02118"

/inference="ab initio prediction:Prodigal:002006"

/inference="similar to AA sequence:UniProtKB:P08722"

/codon_start=1

/transl_table=11

/product="PTS system beta-glucoside-specific EIIBCA

component"

/db_xref="COG:COG1263"

/translation="MGKYEELAKNIVKEVGGKENVNSLTNCITRLRFKLKDESKANTD

VLKNMDGVVTVMQAGGQYQVVIGNHVPDVKKDVDAVLGVLEAPTTSGPKGNLFDQFVD

MISAIFQPILAPLSAAGMLKGVNAILIFALGAGFADSPTSAVFNAMGDGLFKFLPIFI

GYTAMKKFGGSPFLGMLIASSLVYPGFLEGAATAQFAEAGGLNFFGIPFSIPPAGYGS

TVMPIIAAAAFAAFLEKQLRKVIPDVIKLFIVPFLTALVTIPLTFLVIGPIMNIVSDA

LGNGLLSLQSFSPILFGAILGFAWQILVMFGLHWALIPFAIIALSQGDPTSLLTPSGS

VGFAQTGAVLAVMLKTKNAKLKELSIPAFISGIFGVTEPAIYGITLPRKKPFWASCIV

GGVTGAIAMGLGMQSYQMGGLGIFRYPSSISPDGDVKFAIYAMILDVCALIAGFIVAY

ALGFKDDEPTIAISKEEAKLATTGQSKKLSKEDIFSPIAGKVLPLSDAKDGAFSDGIL

GKGVVIEPDNGEVVAPFDGTVMTLFPTYHAIGLISENGTEVLIHIGIDTVQLNGKGFE

AFIKQGDTVKKGQKLVQFDIDRITSEGYSTQVPIIITNTPDFVDVISTSEPTIKANDL

LITTVISD"

CDS 2238342..2239775

/gene="bglH_4"

/locus_tag="JFBMFIFI_02119"

/EC_number="3.2.1.86"

/inference="ab initio prediction:Prodigal:002006"

/inference="similar to AA sequence:UniProtKB:P40740"

/codon_start=1

/transl_table=11

/product="Aryl-phospho-beta-D-glucosidase BglH"

/db_xref="COG:COG2723"

/translation="MSFPKDFLWGGATAANQCEGAWNVDGKGDSISDHNRAGSRTSST

HRTFDLEISDNYYYPSHTAIDFYHHYKEDIAMFAEMGYKTYRLSINWTRIFPKGDETT

PNEAGLTFYDKVFDECAKYGIEPLVTISHFELPFHLGKEYDGFLDKRVIDFYENYAVT

LFERYKDKVKYWLTFNEINFGTLPHGLRINGLFNRKYTESEQYQALNNVFLASARAVI

AGHNINPEFKIGCMLAYITMYPKTCKPEDALKTQQMNDKLNFFCGDVQAKGKYPYYMK

RYFEQNNIQVKITPEDEKLLQKGTVDYYTFSYYMTTCIADDLDKRDDKSGGNLFGGVS

NEYLETSDWGWQIDPVGLRFTLNQIYSRYELPLMVVENGLGAFDELTAEGTIEDDYRI

DYFKQHIIEMGKAIDDGVDLIGYTPWGCIDLISAGTGEMSKRYGFIYVDRDDEGKGSL

KRYPKKSFYWYKKVIESNGEDLGSIEG"

CDS complement(2240321..2240677)

/locus_tag="JFBMFIFI_02120"

/inference="ab initio prediction:Prodigal:002006"

/codon_start=1

/transl_table=11

/product="hypothetical protein"

/translation="MSLNVKSITVGLPVSNLEKSASWYEKILMSDEKLIPVEGVIEYL

IGSVWIQLFEEKINVSENVLRLEVEDLETEFERLKILGVIVDEVIEDVPGIIRYFDFS

DPDGNKLSFYWLYAQE"

CDS complement(2240742..2241515)

/locus_tag="JFBMFIFI_02121"

/inference="ab initio prediction:Prodigal:002006"

/codon_start=1

/transl_table=11

/product="hypothetical protein"

/translation="MKTKMSEILSFISEEAVSRKMTSEEIAVHFGYDKHHFSRKFKEI

NGFSVVEFLSSLKVEKAIIGLDEEKRVIDLQEHSGFESSGSFTNTFKKYTGSSPRAYK

TEMNELFYDIKSFENNDKDKSIAHFEEKSHSFCTVTIEVPDGFEKGIIFIGLFRTLIP

NHTPISGLATKNLIGNKLKNIPNGDYYLLSCAIDRSNNILSYFNLNNSLRGKEDGRLS

FPKCSGNSYTIKLREPIPEDPPILVNVGKLLISRLKNTI"

CDS 2241706..2242272

/locus_tag="JFBMFIFI_02122"

/inference="ab initio prediction:Prodigal:002006"

/codon_start=1

/transl_table=11

/product="hypothetical protein"

/translation="MKKTLLLFYGNYQFNGRKNETDVIYYFNQVLTSESDHSIDFIDV

SAMNGIGIAYSLQDEDEKALTYFEKSLDQLDELFSLIDTINDSPEIAKTYYNSAKFYS

KIGKYTKAVNLCNLGIELLIKEGLAYHLDFLFYEKAFNLMKLDEKNNSAKFYLRAMVM

ADINNNEIVMEIIKNNIIQYDIEEYKYY"

CDS 2242481..2244142

/locus_tag="JFBMFIFI_02123"

/inference="ab initio prediction:Prodigal:002006"

/codon_start=1

/transl_table=11

/product="hypothetical protein"

/translation="MKSDWKGSFIEYCSVKHTEIYDYTDSLSKFLEGTNIKKDKIPLE

FLNWIDMFYTKEPCIYYSKDAKIEIENNLEIMNTENDNNLIITTEMLENLYVALEAYK

QITEIIRDLKRMNYSEDIKNRMYRLPTYTNIVEGCLSNLYRFLILCINQNSEKDFYNQ

KKLGAMIPILIKYNFILSTQPVDVNLRNAINHGGVYFTSEGKKIHFKYSKGQETILDE

KSYFDFDELIEQNYDLIGGIILGLFNFITAHQDNIYFEEINKQEISLKNIAFFLSSPT

LTCLDINDSSIDHRQLNCVFHTKETNRGILVQYAIIYSVLLYSQFPNYQDYFINFDVP

YLLLSWVRFSKDDVEKMIKNDSNFDLPKLITEVTNQGNIIISDPSAEKISDNNNKTHF

FKPIENNIYQVRDIENVSLENEKRISAKMYVGNSINRDDLLRTIDSAVSDLKTLENNP

FSSHDSKYGTMDADAVLIAVYEEDERKNKGINLENKNFICFVEFRPSAKFTMKIGPDW

VNFNHEFIYPYKISWRKKRFYKENQLGRNDPCPCKSGKKYKKCHG"

CDS complement(2244241..2245797)

/gene="guaA"

/locus_tag="JFBMFIFI_02124"

/EC_number="6.3.5.2"

/inference="ab initio prediction:Prodigal:002006"

/inference="similar to AA sequence:UniProtKB:P99105"

/codon_start=1

/transl_table=11

/product="GMP synthase [glutamine-hydrolyzing]"

/translation="MANATDMTTIEKIIVLDFGSQYNQLITRRIREFGIYSELLSHKI

TAEEIKGMNVKGIIFSGGPNSVYDEGAFRVDPAIFELGVPILGICYGMQLMTDVLGGK

VEPANNREYGKAEIVIEDASAPLFAGLEKEETVWMSHGDLVTEVPAGFTISATSAHCP

IASMYNNELNFHAVQFHPEVRHSVHGNEILKNFAFNVCGAAGNWSIANFIDLEIAKIR

NTVGDKKVLLALSGGVDSSVVGVLLQKAIGDQLTCIFVDHGLLRKNEGDQVMESLSGK

FGLNIIRVDAKKRFMDKLAGVSDPEQKRKIIGNEFVYVFDDEATKLKGIDFLAQGTLY

TDVIESGTDTAQTIKSHHNVGGLPEDMQFKLIEPLNTLFKDEVRALGTELGMPDALVW

RQPFPGPGLGIRVLGELTEEKLEIVRESDAILREEIASAGLDRDVWQYFTVLPGIRSV

GVMGDGRTYDYTVGIRAVTSIDGMTSDFARIPWDVLQKISVRIVNEVSHVNRIVYDIT

SKPPATIEWE"

CDS complement(2245912..2246835)

/gene="coaA"

/locus_tag="JFBMFIFI_02125"

/EC_number="2.7.1.33"

/inference="ab initio prediction:Prodigal:002006"

/inference="similar to AA sequence:UniProtKB:Q83EV9"

/codon_start=1

/transl_table=11

/product="Pantothenate kinase"

/db_xref="COG:COG1072"

/translation="MKESATYHIIEREEWMEFYQNSVAPLTKEELEQLKGLNDQISLR

DVEDVYIPIAHLFDIYLTQYEALQEKKLRFLKKEVQKKPFIIGIAGSVAVGKSTTARL

LQMMLSRVYKDKQVELITTDGFLYPNAILEERGIMDKKGFPESYDMARLITFLGDVKN

GASAVKAPVYSHEVYDIVEGEYEVLDQPDILIVEGINVLQLPQNEQIYVSDFFDFSIF

VDADPELIEQWYLDRFGVLLKTAFKDPTNYYYSFAIGDREEAFSMAKNVWRTVNLTNL

EEFILPTRNRADLIIHKTDNHRIDRLFLRKY"

CDS 2247219..2248166

/gene="galK_2"

/locus_tag="JFBMFIFI_02126"

/EC_number="2.7.1.6"

/inference="ab initio prediction:Prodigal:002006"

/inference="protein motif:HAMAP:MF_00246"

/codon_start=1

/transl_table=11

/product="Galactokinase"

/translation="MTKLDSVGYGVATGKIILMGEHAVVYGEPAIALPFPAVRINAQV

EKIAQSIVELDCYFYQGPLTEAPQQLTNLITAIKESLAVLQQPQQGFKLSITSTVPAE

RGMGSSAAVAVATIRALFSYFKQPLADSVLLELTNQAEKIAHGNPSGLDAAMTSGSVP

LYYVKNQPFAPISLNLKAYLIVADTGITGQTKKAVADVAALLESHPQEIGDKIVTLGN

LVKQSKQAIEENNPVLLGGYMNQAQQILVDLTVSSPEIDLLVAAANQAGALGAKLTGG

GRGGCLVALATTKSAAEQIEAALIEAGATITWIYQIGEK"

CDS 2248168..2249157

/locus_tag="JFBMFIFI_02127"

/inference="ab initio prediction:Prodigal:002006"

/codon_start=1

/transl_table=11

/product="hypothetical protein"

/translation="MANTGKARAYTNIALIKYWGKKDDDLILPMNSSLSLTLDAFYTE

TTVHFDETLKEDQFYLNQTLQNPTQTKKTTRFLDLVRHEAGITTPAIIESTNYVPTAA

GLASSASGFAALAGAASLASGLELDLQALSRLARRGSGSATRSIYGGFVEWQMGSNDL

DSYGVPIDDASSWDLAMLFVVVNQKEKVISSRDGMKQTVATSPFYSGWLESTAKDLIT

IKEAIKEHDFQKLGETMESNGLKMHATMLGATPPFTYWEPDSIQAMQFVAELRQEGIP

CYFTMDAGPNVKVLCHKSDLPKIKAKFATIFQETQLITAYPGPGIQLSKGDFY"

CDS 2249157..2250245

/locus_tag="JFBMFIFI_02128"

/EC_number="2.7.4.2"

/inference="ab initio prediction:Prodigal:002006"

/inference="similar to AA sequence:UniProtKB:Q9KWG3"

/codon_start=1

/transl_table=11

/product="Phosphomevalonate kinase"

/translation="MIEASAPGKLYIAGEYAVVEAGHPAIIVAVDQFITVSLEQNEEY

GSIRSFQYGELPILWTRQDGQLVLDKRENPFHYILAAMELTEQYAQEQGKELSFYELS

VTSELDNSTGKKYGLGSSGAVTVATVKALCKYYQLDVTKKQVFKLAALAHLSVQGNGS

CGDIAASVYGGWIAFTTFERQWVADKFKNETLTTLLALVWPELSIQPLTPPRDLRLVI

GWTGSPASTSSLVDKVTKQREMDQASYQRFLQDSKECVEALIQAFEENNIPEIQEKIR

LNRQLLLGMSKKTGVVIETPALKKLCDLAETYHGAAKSSGAGGGDCGIVLFRQKEGML

PLITAWEKEEITNLPLHVYFDKETELGG"

CDS 2250252..2251316

/gene="fni"

/locus_tag="JFBMFIFI_02129"

/EC_number="5.3.3.2"

/inference="ab initio prediction:Prodigal:002006"

/inference="similar to AA sequence:UniProtKB:Q9KWG2"

/codon_start=1

/transl_table=11

/product="Isopentenyl-diphosphate delta-isomerase"

/translation="MTIENRKNQHVDLAESMFHQPKPSDYDALRFVHHSFPEMAVSDT

SLATSFLGLNLTSPFYINGMTGGSERTKIINGDLALIAKETGLAMATGSQSAAIKQPE
LTDTFQIIRQNNPNGLIFANLGTGNSVEAGKKAVAMLDANALQIHVNAPQELIMPEGD
RDFSNWLTEIEKMVQEIDVPIIVKEVGFGMSRETIELLIERGVQAVDVSGRGGTNFAA
IENMRRKTRELDYLETWGQSSIISLLEAQPLSQSIELLASGGIRNPLDVIKALSLGAK
AVGISGLFLHLLLENGVSATITEIQRWQEQMQTILTLLGKTTIADLQKTDLIIGGEVR
EWCLAREIPYQTFAKRSTNG"

CDS 2251580..2252578

/gene="yhfP"

/locus_tag="JFBMFIFI_02130"

/EC_number="1.6.5.-"

/inference="ab initio prediction:Prodigal:002006"

/inference="similar to AA sequence:UniProtKB:O07615"

/codon_start=1

/transl_table=11

/product="Putative quinone oxidoreductase YhfP"

/db_xref="COG:COG0604"

/translation="MTEYFNALQVDLVDSKATATVEKLTFADLPEGDILIEVHYSSVN
YKDALAFQTKGGVVRTYPMVPGIDLSGIVKESTNPKFKAGDPVLVTGYKLGIQHFGGY
SQFARVPSEWVVPLPAGLSLREAMIIGTAGFTAALSIHQLELNGLTPSGGDILVVGAT
GGVGSMAIAQLKALGYSSITAGTRKQTESDFLFSIGASQVSPLSEIQLDPKKPLAKQR
WQGVVDPVGGDLLPDLFAQINYNGALALSGNAGGINFSASVFPFILRGVKLLGIDSVD
CPFQLRQELWSKLASTMKPANLEGMLDNEISLTELPLALEKILAGKMRGRTLVKLN"

CDS complement(2252602..2253261)

/locus_tag="JFBMFIFI_02131"

/inference="ab initio prediction:Prodigal:002006"

/codon_start=1

/transl_table=11

/product="hypothetical protein"

/translation="MMKKKKKIILGGGSPPPSSEIVAYATEGLKPTSKIVYLTIRHGG
ALKYEKQIREQFYAAGFFHVQTIIGNRISKKELLKGIKSADLILIGSGITLKYARIFV
KGRVKEALIKAYHQGVPLMGFSAGSLIMPETMLISYKDNPFGIRVKKGLGLLKDTVIS
AHYSKWREHRMLRSGLKKAGVSVGYGIDDDSYLVFEDNQPRYFGTIYIEHNHEIKKME
A"

CDS complement(2253283..2254065)

/locus_tag="JFBMFIFI_02132"

/inference="ab initio prediction:Prodigal:002006"

/codon_start=1

/transl_table=11

/product="hypothetical protein"

/translation="MFVKCTAKDEEEILAYLSSRPALNLFIIGDIETYGFDSEDQDIW

CYREDDHKISGVLLRYKNNIIPAHEANFSGLEAFIACIKEQKNSRFISGRKEALDQYA

TAFPEFKKDLSYFAECQKLRMKSLQTDYVTDLEVADIPKYIALQEAAFNHSVVTIDDL

EKEIASGSAVIKVIKNQQGELVSGGKIAVESQSSGMIIGIATLESARGNGYGSAVVTS

LVEHCHNQKKSACLFFTNPAAGSIYHRLGFVDLDRWVLMQAK"

CDS complement(2254085..2254645)

/locus_tag="JFBMFIFI_02133"

/inference="ab initio prediction:Prodigal:002006"

/codon_start=1

/transl_table=11

/product="hypothetical protein"

/translation="MENKEKRKVKKCRESRVVQTHRVFPYDLNQHKTLFGGKLMSLID

DTASISVTRHSREVCVTASTDSLDFLHPIHEDHSVCVETYVTGVGKTSIEVFAKVMGE

ELLTGERYLAATSFMTFVALPTEEKPVILVPLVEPETLEERMVCAGYEKRRGKRVQTR

AFNEEFAETVTLAVPWMTKDWSAFAD"

CDS 2254814..2255371

/locus_tag="JFBMFIFI_02134"

/inference="ab initio prediction:Prodigal:002006"

/codon_start=1

/transl_table=11

/product="hypothetical protein"

/translation="MLKKIILFGDSITAGYLDGYVSKALTDRLAAFLPEDKIINVAIP

GDTTNGGLERFEQHVLKRDPDLVTILFGSNDVTLAENISLTMYKNNILTMIEQVGAEK

VLLITPSFSNPLVQHDERPNSRILAYGNAIRELAKEHQTLLADLQIAFLDSPNYVDFL

QKDGFHLNEKGYDLLAELIAHTLQS"

CDS complement(2255512..2255739)

/locus_tag="JFBMFIFI_02135"

/inference="ab initio prediction:Prodigal:002006"

/codon_start=1

/transl_table=11

/product="hypothetical protein"

/translation="MQIEDHWTDVVVYQVEIKVGHKEVRTLHKLLVFSAELTLDEIKA

NIKNRFNHVLEITRLDEIDEGLYLHGKTIAG"

CDS complement(2255766..2256224)

/locus_tag="JFBMFIFI_02136"

/inference="ab initio prediction:Prodigal:002006"

/codon_start=1

/transl_table=11

/product="hypothetical protein"

/translation="MDKKETYKLTNKLLLISCLVTFYLFYTEIVQADEPKQPNETIGQ

SYDPLDPAILIEPVLIEEDDDLNLTISFEDSDEPEELENIPDGTEEYLVSSERRKEKQ

GNQPLNQPDAIVVGNDSGGSDKDPKETSYLAELCFQLSGTVLFGTRYNEC"

CDS complement(2256217..2257686)

/locus_tag="JFBMFIFI_02137"

/inference="ab initio prediction:Prodigal:002006"

/codon_start=1

/transl_table=11

/product="hypothetical protein"

/translation="MEHFIDKLESRKLAVLKYIEDSYQQKVSILRIEKELNISSFVLA

SVVESLALDFETYDVTSYFQIKKSDTEIQLITNGEANSKLLMHIYIMESPAFKILDQI

FHGTFVNVMDFAEANFLSRAGVYKWIKKLKEELRSHGIHLSSKFQLVGSEPKIRGYFF

NMYYCLFNQMDYPFSKFLYKDARKLVRIVMENTPSPIAETMKTKLFYFFSIVLFRQKQ

KNKMCDNDLKPLALASKSQGANKMADAIKSFFTSKSIKPLESIDNEVTFIMAFIICEN

ILSDVLPNFSSQPRTNYLTTKFIDEFQQHFPRKLTLENQVKFQNELDIIHFKADYFEK

VDSIFDEIVNVVFIQENYPEIVIFCEKFIENSKNVQKRQLIQANKKYLFNQYLFLIVT

FLPVSFFMDEITVCIDFSNGLHYNRLISQNLKNLSNLNLKIEHHLTKKTDLLLTDYFI

KKKSDSLNYLVWNAPPSPKDWANVANILIKIRNESDTIG"

CDS complement(2257785..2258885)

/locus_tag="JFBMFIFI_02138"

/inference="ab initio prediction:Prodigal:002006"

/codon_start=1

/transl_table=11

/product="hypothetical protein"

/translation="MRKYNIIITLILFLSILVECKTVQASELNFAAIPTIPENQLNKE

KTYFDLVMKENTSQTIEVKLRNDTDKEVIVEPSVNSATTNINGVVEYGKVKGKADSTL

LYNLKDMVSVAKEVAIPAKSEVTVPLTITMPKESFKGILAGGITLQEKESNGKKDAKN

GEGLAIENRYAYVIGLVLRLSEEKIAPQMTLQNVKPDQVNARNVITANLQNPTARFIN

KLGVKAQITRKGNSEVLFKEDKEGMQMAPNSNFNFPVALNGKKLEAGTYVFKATATSK

DDKWDFEKEFTIDSKTAKELNDKDVSIESSYTWLYLLVGCVLLILLLAIITLVLILRK

KKKEEEIRRQKARLRKKRQNRNKAQKENLKKT"

CDS complement(2258961..2259701)

/locus_tag="JFBMFIFI_02139"

/inference="ab initio prediction:Prodigal:002006"

/codon_start=1

/transl_table=11

/product="hypothetical protein"

/translation="MKMTKLVTTAMVAVSTLVLGGAIASAATYSTGGTVKFVTDTDIT

TPVDPLDPGEEVTPVDPTDPEKPIKPGTPGPLSIDFASPLYFGEQKVSPKDQVYPANA

QPIVGGETRPNYVQVTDKRGGELGWSLQVKQNAQFETAEKNILTGAAISFENGTAVTD

SESLIPSIVTKTFSLVPGADQVIMTAAEGEGAGTQLYRAGDDTTKANSVKLAVPGKTT

KYKGTYETNLTWTLSDVPTNEVDPDPEA"

CDS complement(2259759..2260538)

/locus_tag="JFBMFIFI_02140"

/inference="ab initio prediction:Prodigal:002006"

/codon_start=1

/transl_table=11

/product="hypothetical protein"

/translation="MKLTTKMTTSAIILGALALNGSSVLAAEKASYESNGIVEFTESN

DITKPIDPTDPGEEVDPIDPTDPEGPNPGTPGPLSIDYASSLTFGKNKISNKNETYFA

NAQKIKDGSHRPNYVQVTDTRTGNLGWNLSVKQEGQFSNATATNKTLTGTTLQLASGT

ANSASTSPAAIAESVVTLDPAGATSVVMSAEKGSGWGTWASYYGTVENMEIAGETPEE

NETVAVNKAVTLSVPGATPKDAVKYSTKLTWILSDIPTEEE"

CDS complement(2260535..2260894)

/locus_tag="JFBMFIFI_02141"

/inference="ab initio prediction:Prodigal:002006"

/codon_start=1

/transl_table=11

/product="hypothetical protein"

/translation="MKKWIFLFTAVFFLVGFSGTGEASEARSEAQYQSNGEVSFYGVY

EKITEPENELPGTETHPNTGTGTVKLPLTNGQKYFPQTGEKAYLTTLLTGLFTIVLAT

LLYNSNNKKQKNGGITQ"

CDS complement(2261178..2263574)

/locus_tag="JFBMFIFI_02142"

/inference="ab initio prediction:Prodigal:002006"

/codon_start=1

/transl_table=11

/product="hypothetical protein"

/translation="MAKRKKGLKLTLLFCALFLFSTMLNTSLKKDQTIKAFDNEAKLV

LSSKVGSEKNSIQLDLEIPSTFTSEIKIPITDNSQLIDYSLNESNQNGFSITSTATEL

IIQPEIPTDEMINEESEQPLSSNLVTLNVSIQHSGTVEYVAETFQDGVKSTSNSETVT

IVEESDEVELEQDTTSEADEKTDTPSIEAVEEIKNIGKIQSRSAVTRGINDNMPQKRL

IINEKFRNPIGTGANFIEPTILELSKTKSQTGSIWSKNKLDLTQDFSLKSYIYLGDSG

KSAGDGVTFTLQNDERMATNPESVIGSSGFAMGAYSSKSGANYVHNALSIEFDTYYNN

GSSNRVDRELNRNSDYGHIAMVRPKANNNNYTGEHSYVQYSPGYLSNGKWRSFTVSWN

AETKTLNYQLEGFDQISAPIDPQTEFKGQEAYWGFTSSTGGNWAQNAIAISELPQELE

ADQEVLMKNVTQNGEYSSEINVNTGDIVEYQVTNKYLSEVFGASDWDNVATHATLPAG

IDYEVNSAIIDGVAISDQEITTDGTAMIFPKATLSFDKLTQKLVFRGKVTGADGPKKL

TTNFEAIGDNATIQDKQTIANLAEKKAGQVTFNYVDESNQPIAESISKTGYLGESYSA

ERKEFPDYLLVEIPSNENGTFTEAPISVTYRYELRRLKFISAPSQISFGESLKISTKS

EVYPISEMTGSLAVQDTRTVKSKWSITAKMSKFLESSSGHTLKNAIYYRHGSQNQLLG

AEAISVYDGLSQNDDVLAISDEWTGEEGLSLQIKTGAAFPEAYSGEISWTLLDAPANE

"

CDS complement(2263909..2264238)

/locus_tag="JFBMFIFI_02143"

/inference="ab initio prediction:Prodigal:002006"

/codon_start=1

/transl_table=11

/product="hypothetical protein"

/translation="MSLTSQQLYLILGMAVVTYLPRMLPMLLLNNREIPEKIVRWMSF

IPVSIFAALIFSDIFFWENQFSINPLENLKLIPAIVVFFIAYKTKNIMWSIVFGVVGI

SLMVWWF"

CDS complement(2264235..2264945)

/gene="ygaZ_1"

/locus_tag="JFBMFIFI_02144"

/inference="ab initio prediction:Prodigal:002006"

/inference="similar to AA sequence:UniProtKB:P76630"

/codon_start=1

/transl_table=11

/product="Inner membrane protein YgaZ"

/db_xref="COG:COG1296"

/translation="MKKEVWLDSFKVVYPVTLGFIPLGIACGMVLYDAGFSPLGIGLM

SFLVYAGASQFMVASMLVMGATVPAMIIMTFFLNLRHVLMSSSMSTYFKKSSVPFLLI

FTHSLADESYAVNYNQFLNHKWDQKHAMATTFIPYVTWGLSTIVGGIVGSQLTVNTTI

MNYVLVAMFICLLVMQFVSPLFIFVGCLSGILSVILMILLKHNIALVLGALIASIIGF

LVDEYILSKPDKKEVDSL"

CDS complement(2265115..2265798)

/gene="gpmA_2"

/locus_tag="JFBMFIFI_02145"

/EC_number="5.4.2.11"

/inference="ab initio prediction:Prodigal:002006"

/inference="similar to AA sequence:UniProtKB:B4RIY7"

/codon_start=1

/transl_table=11

/product="2,3-bisphosphoglycerate-dependent

phosphoglycerate mutase"

/translation="MKLVFVRHGLSDWNALNQFTGWVDVDLSEAGLAEAEEAGRKIKE

AGIEFDLAFTSVLKRAIKTCHIVLEGSDQLWVPEVKSWRLNERHYGALQGLNKQETAE

KYGADQVQLWRRSYDTLPPLLDPTDPQSASHDRRYANLQKSRVPMGENLKVTLERVIP

FWEDQIAPAILDNKTVLVAAHGNSLRALAKYIEGISDEDIMGLEIPTGQPLVYELDED

LTVKTKYYL"

CDS 2265968..2266174

/locus_tag="JFBMFIFI_02146"

/inference="ab initio prediction:Prodigal:002006"

/codon_start=1

/transl_table=11

/product="hypothetical protein"

/translation="MLKRIVYGSWFVSLVYFIVYLTMPFLEKAVRSGGIMVYIHVFMD

LIFIGGLFFIIVSIIRYFFVDPNK"

CDS complement(2266226..2266984)

/gene="focB"

/locus_tag="JFBMFIFI_02147"

/inference="ab initio prediction:Prodigal:002006"

/inference="similar to AA sequence:UniProtKB:P77733"

/codon_start=1

/transl_table=11

/product="putative formate transporter 2"

/db_xref="COG:COG2116"

/translation="MYTPDEILDITIDMGTKKIAKPFVSKLLLGFVGGAMISLGYLAY

IRVASSMIEDWGSIASFIGACVFPVGLIVILLGGGELITGNMMAVAAAWFDKKVNTKD

LLLNWVTITLANAVGAIFVAYFFGHIVGLTSSGVYLHETITLAQGKIAATPLQAFVSG

IGCNWFVGIALWLCYGAKESSGKILGIWFPVMVFVAIGFQHSVANLFVIPAAIFEGQA

TWLELLQNFVPVYLGNIVGGAVFVSGLYYKALKH"

CDS complement(2268330..2268536)

/locus_tag="JFBMFIFI_02148"

/EC_number="2.7.7.-"

/inference="ab initio prediction:Prodigal:002006"

/inference="similar to AA sequence:UniProtKB:Q7DDR9"

/codon_start=1

/transl_table=11

/product="Protein adenylyltransferase NmFic"

/db_xref="COG:COG2184"

/translation="MKPTTIEEIVEKYVEMNVANPLRDVNGRSTRIWLDLILKNELKK

VIDWNLINKESYLSAMERSPVNDL"

CDS complement(2268537..2268662)

/locus_tag="JFBMFIFI_02149"

/inference="ab initio prediction:Prodigal:002006"

/codon_start=1

/transl_table=11

/product="hypothetical protein"

/translation="MRNVFKGKQFDKRVIIEAVGLYCRFYLSYRDVSKILAHRGI"

CDS complement(2268940..2271579)

/locus_tag="JFBMFIFI_02150"

/inference="ab initio prediction:Prodigal:002006"

/codon_start=1

/transl_table=11

/product="hypothetical protein"

/translation="MKKLVKTFYLFYLICSIYFFCDNLVAEASKGDSLFSNTTKHLLN

PNENEIPFVNRERISIPLIKKVPIGEEITNFNHDLADEVQGKRVELRNSSVMNTSKIG

FQTVKVAICSEDSEEITVPISVNAYNPKTTIFNDQDVLALDVKNIQVTKAEIEVAMQK

NKLNELIINKSEADSWQMTDGEKLDISITSQTAQPQIGKYEATIRGTKKGTNKIVEKK

ISLLVVNSKLENGWEIGSSSDSIGKVGKKINWNSGNWWYTSNGASWIYNTSIEATLII

NGSFLAGTNENTKGGFLWANPSDSLYTKNLITNTLKRSFLYKDYQIDISQKILADGAV

EVSYQVINNSLVAQKIGISQYVDLVDVSPPSVSVINDFKGFNMVDKQPVVVMPDPETM

PNWVVSPQSTLKNFKQYSSQTVDGVGWETKKQYWNGVKHLDPPLELKEGQPKKDLGDL

GVAMKNPGVMVQPGEGTTFKQIIKFGSLIAPELSLDQEVGTMYTDESMNITGIVSDAD

SQSYRLYLEMDDTNETLIPLQDYSYIPNGAKQTFNIKIKGEIFNQGEHLVSIVAIDEY

GSRSEAKKINLTIKEFSATPKIQKIAVGKPLSNIVSELFTNIKGVNIELKDKLTLDTA

NIGFYLVEATLIANQEKELTLKIPVNVYDPISTVYDEKNNIMLDAKNTKFNVVDVREA

EKENKLFDLVMEASVPKSWDMKTGVENEVTLTLNGLSSKVGTYKSRITAKNRTTNQMI

QKEVESTVGGDLKFKSVPSEVVFLSRKVSDSEGYIQRKQENLNLEIENTLGSNWSLYI

SADPLINEQGDILKNSMIFKSLGQADQFINSSNLKIQTGEAAVVYPKIQWTSKEGLLI

KISPGLKAGNYSGELSWILSNAP"

CDS complement(2271620..2274280)

/locus_tag="JFBMFIFI_02151"

/inference="ab initio prediction:Prodigal:002006"

/codon_start=1

/transl_table=11

/product="hypothetical protein"

/translation="MKKIDNKKLCAVIFFFALSMLSKDFFNKQVYAESNQEPTKIQNN

DNSLFKLNSKLRTHHELGLIPIIQKVSVGGAITQNVTSLFQNTSGIKVKLKDEVVIDS

SKIGFQWVAVTLVDESLNEFTRSVPVNVYNPKTTTFDDSANIAINVEENEIPASELQE

SRSKGTTDQLIIEKMNITSWSMNDGSQTPIELDTSNLNLSNSSQHVIIKSRNQKNRTS

SIQKILQLKIATVQENGWIAGFSTDKIERNGFEIGWKDGLWWYTYNGNKWLYSNSIEA

ALIINGNFYPGSSDRNGLGFLRENSTTSSYAINKETSSLKRTFTYQNKFKIDIIQKLL

QNNAVEVTYQVTNLGSQTVKIGLSQFADVFVGADTVPVTPINNFKGINLTFDQSSLVI

IPDPKTLPNWAAGYYTTVSMFGQYNIQSADGIGWETGSRFRGNFGLLLTPPQILKENI

PILVGDSGVAVKNPGVNVEPSGSTTFKQTLKFGKLSKPEVTIEQNKGYLYQDEEINIA

GTIVVAENQNYRLYLELDDEQKTLIPLKEYVNIPYGQKQIFNVKVKGELFKAGVHDVS

ILGIDEYGTRSAPQKLSLSINELSALPAIQKVKIGEQLTDDLAVLVENLKGTNIVLKN

KSQLDSSKVGFSWVEVTLEANKEKEIQLKIPVNVFDPSSTIYDEKNNIMLDVKDSHFT

PVDIKVAEEMGKLKDLIVQKTQPKSWNMDTGLENEVVLTSNNMISEVGIYKSLFVAQN

KETQATIQKETKLIVDGELKFKSVPDTLLFSPTKLSQKENYVQREDSTWEIDIENTVG

SNWSLFISGSPLTNQIGESMKNSLIFKDSDAKDQPINENLQKIVIGEKKVSYPKVQWK

ANEGPLIKMNQGLKIGEYTGELYWTLSDAP"

CDS complement(2274509..2275186)

/locus_tag="JFBMFIFI_02152"

/inference="ab initio prediction:Prodigal:002006"

/codon_start=1

/transl_table=11

/product="hypothetical protein"

/translation="MKTLKKMSLFLIVGGGMASALFGSKVQAAEEEESANKTAETTTS

FSLEAGDDETIPGIIDPEVKPPTNQKGKLTINAVSNIVFPNTKLGNESGAPIKAQVPV

GEKLGVQITDIRGTGAGWKLAVAATEFQNAEKTLTLRGAKVTIPGTGNTVTEEGNDLT

FEPTSQKVVLGPDKNILLSATKDKGLGKWLHYFENDSDKVTIEIPSGNKVATYISDLT

WTLEDAP"

CDS complement(2275188..2276360)

/locus_tag="JFBMFIFI_02153"

/inference="ab initio prediction:Prodigal:002006"

/codon_start=1

/transl_table=11

/product="hypothetical protein"

/translation="MKKVLALIAIILLGSQFAPEIGQAENSMAYSIKADIPENQIDKT

LTYFDLKMEPSQNQTITLTVSNSSDKEATIVISPNVALTNQNGVIDYSKSDVKKDSSM

KYSIDSILSKKQEVLLKAGETKKVPFTLQMPDEAFDGLILGGFYVSKKEDSSKEKETE

KNVQIKNKYSYVIGIKIRSTMNEVQPKMELNQIKPALLNYRAAVTANLQNTEATIIPD

LDVEAKVMKKGRKEILHETTKKGMSMAPNSNFDFPISWDNQSLEPGNYMLKVVAKSGE

NQWKFEKEFKIASKDSADLNKEAIEIEKTAPNWTFIILETLLGMLLMILLIVYFIYRH

KKKKEAEKRARLARQRKRKKLANARQKKVQSANNINKKSQNDRQRQSQKKQNKKEY"

CDS complement(2276445..2277146)

/locus_tag="JFBMFIFI_02154"

/inference="ab initio prediction:Prodigal:002006"

/codon_start=1

/transl_table=11

/product="hypothetical protein"

/translation="MKKKTIVLASMFLISFAIGGKEASALVSSSSKSENKIKFIPGEG

VVTPPVDPVNPSNPNPTNPIDPTDPENEGTGQKGPLSIDYVSNLKFGEHKISGKDNIY

KALNANPFVQVTDLRGAGDGWSLSAKMTPFTSKNNQVLKGATLAMADSVIKAGSVSNV

SVAPVKSDVLFDNTDSKLIMNAKDKGGRGTWLNVWSGTDQANEAIKLTVLAGTPEANT

EYTSSITWELEDAPK"

CDS complement(2277202..2277879)

/locus_tag="JFBMFIFI_02155"

/inference="ab initio prediction:Prodigal:002006"

/codon_start=1

/transl_table=11

/product="hypothetical protein"

/translation="MKSMKYVLTGALILTHLTGMNGIVAYAEDEVPAEEPASTGTSVA

NFELQAGDENTVPELLDPEVQPPTGNKGPLSLDAVSSFNFPTKKLGTESKLPLEASPV

EGTKLGVQVTDERGKDLGWNLKVSATPFQTSDKTLELKGAIMTIPEGKLTTKQGVDPL

LTPSAFKVDLSTNPTSIMSATTTQGRSTWLNTFEEKGEKVTLAVPSGNKVASYSSTIT

WSLEDAP"

CDS complement(2277903..2278889)

/locus_tag="JFBMFIFI_02156"

/inference="ab initio prediction:Prodigal:002006"

/codon_start=1

/transl_table=11

/product="hypothetical protein"

/translation="MNYIIKSEYILFFLICFITIASFPKIVLAKSATSQAGIYFTEKE

SDVNDTSQIEMNGLPNTIDRGESTYLPQTGEKNNQKLLIIALVSIVLSLLIRIKVKKG

ELKMKFNKIISGALVSGSILCGMGNSVFAAPQDEITGSEDGKSGTSHGYIKLTPGDTS

TGPTDPVKPTEPPGGTGNEGSLTIDNIAPLLFDTHKLEGKEQVYTSTVVDANVQVTDK

RGEEEGWTLQISQTPFKDVEDDKKVLKGTKLVLPLGVLETVGVNVSKAPVVNSVEVTE

AASILMSATAGSGAGTWISTFDKDEIKLTVPAGNKNGEYMSTVTWSLTNAPK"

CDS complement(2279012..2279221)

/locus_tag="JFBMFIFI_02157"

/inference="ab initio prediction:Prodigal:002006"

/codon_start=1

/transl_table=11

/product="hypothetical protein"

/translation="MRIEDFWEDRKAYYISFVNVNNQLIDRMILLPPDYVDASEIEKI

ILTKFNNVKKVRHINEWGDALLLKS"

CDS complement(2279218..2279916)

/locus_tag="JFBMFIFI_02158"

/inference="ab initio prediction:Prodigal:002006"

/codon_start=1

/transl_table=11

/product="hypothetical protein"

/translation="MNALLEIFTKVHTKSNLCSYRILKKNDEVIRYEYKKNKADKIYF

VIDGTLIAECEFEYSRNKFSSLVTETQVFGLESFVLSKYGSPPINYALTAITDSVILE

INSEYFLDHLYSNPKLYHEIFSDTIKRYFLLAQSYQNSHQPPMVKVIYAFVNIMKILD

LQPDCDNKITFPRYINQKFLSKYIQSSESNISIAVNYLEKQKLIQKKPFILLKEKDLL

MYLSKLLNKKEGLN"

CDS 2280291..2281796

/locus_tag="JFBMFIFI_02159"

/inference="ab initio prediction:Prodigal:002006"

/codon_start=1

/transl_table=11

/product="hypothetical protein"

/translation="MSIENILSSSYLRQLHLVRFLITNGPVSQKVILNKFNYSNATLY

RDISNLNNMLDPIKILTNDLIKISIPIHYNNRFIFSSFLKKSTEFNLIQEIYFREDHS

LLSLADMFFVSESTVRRMIRKINKELLPYGIKIGSTPIKLFGNEKNIYNLMVRYFEET

YPSHDYPFSSLKLNVLQKIILATTDKQNDNFNYPDVERLKLIIIVIIIRLQNGNTIDT

SLADPPTISHNIISNPVYTKLFKAIFKFELNKKSLNQILYPFSTTNFATNQKQLKQMA

SKNEQIRNKILLLTQLVKKISTRYSISLDNTDDLVLHLFNTSELKQTFTSILYDKNKE

FVDEATLYNTEFIDYVKNEVQQLKNDPNGEEENIFSLCYILITHWKNLAIQLKKSSFK

LKVGLFFDSDLEHMYFINDSLNEELNHILDIVPLDFLTSKELKVTSKEFDFIITTIPD

LNYSSVPVVCIPMYLRSTDFENIIHLYNTIQRTKKSHKKLSSIPKMIKSKE"

CDS complement(2281904..2284372)

/locus_tag="JFBMFIFI_02160"

/inference="ab initio prediction:Prodigal:002006"

/codon_start=1

/transl_table=11

/product="hypothetical protein"

/translation="MNKNKVTFIFLLLVVYFFCFIETTANNLYEDREIHAEQSNSEQI

NLNRAAKERETSWIGSNAGTEIVEEANKTKLSFFPGSLRQKIVTQKGVVYNSRGTDNY

LGIYAASGSLATYRPQIPLGNAWASANNSEGDVYQVVKNVQYFRRSDNQAIKAVSKDP

KFPFEYEITLELDPIEGFYKTIKVTNLSSSNYNILVSEATELVSGMTAYSLGKNQGVF

KRNDQDTASMLVRLGDLAGNPYGDFSNYVTGNYVDGWILGGFKSDPYGIGQESMYDEG

DLISGSNSSFIYKSQPKNLGLNESITGKMYMYFGDPNPPELTLDNQDFNIYKGDEKIS

LTGSIFDKDSVQSSVYVEYPMDLIARKLKAIPIEHGVTKAVDYEIDVSMLQIGNYHIK

TWAQDPVMTKSDISDLAVNVFDLTATPITKKILKGADFAFTPDELIKNVESVNPAISN

IKKKTDTDKIGFDYAVVSLQDSIRTDKTTDISIPINVYGPNTTMDDTTNLALDAQEVW

LSQGKVKSATDLNQLILSQINPTAWNMETGTSYKSEVTSHNVKVAMGTYQAEITAKNE

KNQAIKKNVKVNVVEGIPMLKSEAQGQSLIVKLGESYKLSGTALDQDSQNYSVAYQLD

DDEPVTILDQYDNSETYNKEVSFSASISMKKLTLGLHSVKLTIVDSEGNKSKDVDFQL

NVKGELAFKEVPSTKIEFPTEKIPDVSKLIETVNPIEMIVEDYRGVGTNWKMVGTLVN

EMEDSKNNQVLKNSLVFIDEHEVEKPFILNEGMVLARGETSNSIYEFPIKWEKKQGIL

LRIPLSAKKGSYEGVMNLTLVDAP"

CDS 2285147..2285599

/locus_tag="JFBMFIFI_02161"

/inference="ab initio prediction:Prodigal:002006"

/inference="similar to AA sequence:ISfinder:ISLmo3"

/codon_start=1

/transl_table=11

/product="IS6 family transposase ISLmo3"

/translation="MNPTTIMRWVHQYGKLLFVIWKKKNKPPSTSWRMDETYIKIKGK

QHYLDMCLRKHRDTSAAYAFMKRLIRCYGEPRALVTDKCAATIAAVNKLKNEGFLKSV

DYRLSKYLNNVIEQDHRQIKKRFSKSLGFQSMTSTATTIQGIEVVNAL"

CDS complement(2286106..2286222)

/locus_tag="JFBMFIFI_02162"

/inference="ab initio prediction:Prodigal:002006"

/codon_start=1

/transl_table=11

/product="hypothetical protein"

/translation="MKTYHLGVEATLDISGGKWKPLIVCVVMHGTKRTGRIN"

CDS 2286363..2287031

/locus_tag="JFBMFIFI_02163"

/inference="ab initio prediction:Prodigal:002006"

/codon_start=1

/transl_table=11

/product="hypothetical protein"

/translation="MKNVLVLGATGRTGNFIIKELGSLHNVHITVGIREMEDFKRIPN

TSKPLAVSVLDINDEASLMQALINVDIVVNALRMRNGTSSVLKSTALVELDKQLRQAT

KKARTPLIISVGGAGSLNYPDKTNFWQDPLFPKKTFPRGKAQARLRRYLKALPDTRSW

SYLIPPPIYDFYGLRTGSYLRTTPSADERFFLNKSISYQDFAIAVRDSVSEEWTGTHL

VGNA"

CDS complement(2287559..2287798)

/locus_tag="JFBMFIFI_02164"

/inference="ab initio prediction:Prodigal:002006"

/codon_start=1

/transl_table=11

/product="hypothetical protein"

/translation="MDSVYDDKWLNINNVTTLSIDKLRKLETINANDRLKWFTINSVL

VNVNELKINPRTCMKTQKQIEILNYQESPIRFFAL"

CDS complement(2287973..2288302)

/locus_tag="JFBMFIFI_02165"

/inference="ab initio prediction:Prodigal:002006"

/codon_start=1

/transl_table=11

/product="hypothetical protein"

/translation="MLPTNDTIIFMFFNLIFCVSMGYYINRVVTYKNDERGISILAKA

SMITFSSILLLFALFMSYLVIIDIFDLHQTNQLFSLIVIRNFVSIIMVFIGVVNVFAI

KYFEKHI"

CDS complement(2288303..2288509)

/locus_tag="JFBMFIFI_02166"

/inference="ab initio prediction:Prodigal:002006"

/codon_start=1

/transl_table=11

/product="hypothetical protein"

/translation="MVIQNRIKVLRAEREWTQETLATKLDITRQSVASIEKGKYSLSL

ELAFRIARTFNVDLIEVFQDEEEK"

CDS complement(2289466..2289885)

/locus_tag="JFBMFIFI_02167"

/EC_number="3.1.-.-"

/inference="ab initio prediction:Prodigal:002006"

/inference="similar to AA sequence:UniProtKB:O67466"

/codon_start=1

/transl_table=11

/product="Putative esterase"

/db_xref="COG:COG0824"

/translation="MKLEPYYRKVFFYETDQMGIVHHSNVIRWFEEARFDLLDQLGYG

SGFLEEEGLGSPVLDVACQYKTMVRFNDELAIRIGVKTNSSARLTFNYLVLHGDKIAA

SGETTHCFINKAGKIISLKRQYPVLYQTLEQLKEENK"

CDS complement(2289985..2290410)

/locus_tag="JFBMFIFI_02168"

/inference="ab initio prediction:Prodigal:002006"

/codon_start=1

/transl_table=11

/product="hypothetical protein"

/translation="MLKKRNGLAVVVSLVVLLLVGCTSTSSDVLSDKFDQKEVQKQAE

TAIELFNNQDYRALIQLFPEDLQKEMSQDSFKKAFEKFVADKGELKEFGKTTMRNEKN

TTGDGEVATTILPVEYQKGSLNYMISINEQGELTNFLVK"

CDS complement(2290505..2291152)

/locus_tag="JFBMFIFI_02169"

/inference="ab initio prediction:Prodigal:002006"

/codon_start=1

/transl_table=11

/product="hypothetical protein"

/translation="MDQIRSYIEAIFSELPRTKEIVDMRLTMLENMTEKYEELLADGV

GENEAIGTVIRSIGSAEDLKKELELMEPTELEEKEAELVDTKNRGFGIAFSIFLYISS

PIVYLIWQNISETMAIIGCLFIVALATANLIYWGVRGSKAKKAKHEDEFREDGQKSPK

QKKSEALQSIIWTTATIIYLVLGFLFNLWHPGWLIFLVATGINSYLHYLSGEEEA"

CDS complement(2291283..2291606)

/locus_tag="JFBMFIFI_02170"

/inference="ab initio prediction:Prodigal:002006"

/codon_start=1

/transl_table=11

/product="hypothetical protein"

/translation="MISSDVIRGHNDTIILAILVKGDSYGYQISKEIALRSENMYQIK

ETTLYSSFNRLEKNGYIASYHGDETNGRRRTYYQITNSGLKYYHEKCKEWETIKQIID

QFVKG"

CDS complement(2291717..2292541)

/locus_tag="JFBMFIFI_02171"

/inference="ab initio prediction:Prodigal:002006"

/codon_start=1

/transl_table=11

/product="hypothetical protein"

/translation="MLWYQNWLHVVFYVVKFCLTIFLAIVVYQALFATILTKEIHIVT
YILFWFFSAYVVLPRLNRLLARFYVPDYFIGRAKTSDGLLGDPINLAFYGSEEDLLVA
FEKAGWHQADPLTLQSSIKIVTSSVFGKSYKKAPVSSLYLFGAQQQDLAFEREIDGNP
RKRHHVRLWKTPEDWHLPGGKQADWIGGATFDKNIGLSLFTGQITHKIDANVDKERDF
LIETLQTSGCIKDIYLAEHFTTSYHSRNGGGDRIHTDGALPFITIQSHQIMKGESE"

CDS 2292689..2293516

/gene="rhaS_6"

/locus_tag="JFBMFIFI_02172"

/inference="ab initio prediction:Prodigal:002006"

/inference="similar to AA sequence:UniProtKB:P09377"

/codon_start=1

/transl_table=11

/product="HTH-type transcriptional activator RhaS"

/translation="MDIENRKIIYDDDLDIEAYHFEGIIQAFPAHFHDYYVTGYTESG
KRQLSVNQQDYRLETGDTLLLNPLDTHACETLENYQLNYRALNLKTEVLQKAVYEITG
SYELPIFQHSIVKNEQLGELLRDVHIAIFQKKSSFEKEECSYFFIKQLLSASNLTLSP
NQANEAPTSLEKMKHVLAFIKEHYAETIHLEDLSTIADLSKYHFLRNFTKSQNLTPFQ
YIQTVRIQAAIHLLKKGIPLVEVAGLTGFHDQSHFSNTFKKCIGLTPKQYQNIFKLS"

CDS 2293529..2293942

/locus_tag="JFBMFIFI_02173"

/inference="ab initio prediction:Prodigal:002006"

/codon_start=1

/transl_table=11

/product="hypothetical protein"

/translation="MENKKCVLLIDETLPIGLIANTATVLGVSLGKELPSVVGVDTLD
AVGEKHAGITALPFPILKSTSEDIKKLRQKATEMSPNLLIIDFTDVAQTALHYQDYIE
KSKKTSTDDFNYLGLALYGDKKQINKLTGSLGLLR"

CDS complement(2294022..2295152)

/gene="metE_2"

/locus_tag="JFBMFIFI_02174"

/EC_number="2.1.1.14"

/inference="ab initio prediction:Prodigal:002006"

/inference="protein motif:HAMAP:MF_00172"

/codon_start=1

/transl_table=11

/product="5-methyltetrahydropteroyltriglutamate--

homocysteine methyltransferase"

/translation="MTNLTEEKNLVRTIPPFRADHVGSFLRPQRLKEARKAFSNQEIT

QTELRAIENAEIIQLVEQQKATGIQSITDGELRRSWWHIDFLEQLTGIEGYESETSYD

FNGVKVRPYNIRVVGKVDFNPNHSFLEDFKFLKEAVGSEGTAKQTIPSPNMLIHDGIR

NEAIYPNIDDYFSDLAKTYQKAIQAFYDVGCRYLQIDDTHWALLAAENKKELFEKNGV

DPIELAKRCAATLNSALANKPADLVVTMHVCRGNFASSWVYSGGYEKIAESLFSVTVD

GFFLEYDNERSGDFAPLRHITRPNLQIVLGLVTSKEATLEDKSQIKARIKEATEYVPL

ENLCLSPQCGFASTEEGNKISEEDQWAKLRHVVEISEEVWGK"

CDS complement(2295584..2296702)

/gene="yhhT_3"

/locus_tag="JFBMFIFI_02175"

/inference="ab initio prediction:Prodigal:002006"

/inference="similar to AA sequence:UniProtKB:P0AGM0"

/codon_start=1

/transl_table=11

/product="Putative transport protein YhhT"

/db_xref="COG:COG0628"

/translation="MGKLKESRLFFWSIELLTLATLLYVCTKIGFLFQPFGILISTLF

APILVAGFLYYMMNPLVHLLERFKIKRSLAIILIFIVLIVLLSFLVVTIIPSLIDQVS

QFAQNFPSMLKEGQNQLDLLAKRPELQNLDMQEYINDLNLSTSKLVTGVVNGFTSSVF

SIIGVISSVTLVIVTVPIVLFYMFKDGDKLPNAIVRFVPKSYKQRVLDLISDIDSTLA

SFISGQALVCLYVGTCAFIGYLVIGMPYALLLGVIAGVMDIIPYLGPWLGVAPAILIA

LTRSPLEALLVAVIIIVIQIGESNLVYPLVMGKSLDMHPLTIIFILLVAGNLAGLVGM

IIGIPAYAVIKIILHHLYRAVDDKKGIPQLNRSEIEEI"

CDS complement(2297283..2297663)

/locus_tag="JFBMFIFI_02176"

/inference="ab initio prediction:Prodigal:002006"

/codon_start=1

/transl_table=11

/product="hypothetical protein"

/translation="MITKIGQVMIYANDQEAMSDFWTKKIGFVKKAENDKGAAERWIE
IAPTKDSETIFVLQDKVAVAKAQPEMNLGTPSILFFCENIAEMYKEYQEKGITVGDLV
DVPNFGRVFNFADIESNYFAILEK"

CDS complement(2297743..2298042)

/locus_tag="JFBMFIFI_02177"

/inference="ab initio prediction:Prodigal:002006"

/codon_start=1

/transl_table=11

/product="hypothetical protein"

/translation="MIRVDFLYRVKTKNLPIMMDKFKASADKKFQSNPSNIQIEMGQQ
EIGDETLISLNIYYNCIEDYEERTKFERSQKEWLDIWFQPGDIFTQELIQVFHLV"

CDS complement(2298039..2298317)

/locus_tag="JFBMFIFI_02178"

/inference="ab initio prediction:Prodigal:002006"

/codon_start=1

/transl_table=11

/product="hypothetical protein"

/translation="MAKTNLCQSCGIPLKTEKDFGTEKDKTLSTKYCEDCYKDGEWTK
SDLDFDGMYDYNLKKFRESDMNKIEKFFLNKMYTKKFMKKLERWSANK"

CDS complement(2298412..2298858)

/locus_tag="JFBMFIFI_02179"

/inference="ab initio prediction:Prodigal:002006"

/codon_start=1

/transl_table=11

/product="hypothetical protein"

/translation="MVLISYLNFEGNTEEVIDFYEKALDGTEIKKFKFKDFPQNPNNL
IPEKELDLIMNASMKFADGIIMFSDLLPSMKEMAGNFIVGNNMSLAIVIKDKSKIEKY
FNGLSVGGTVTMPLTETPWSESFGMLVDKFGVSWQLNCTTDDYYYS"

CDS complement(2299428..2299652)

/locus_tag="JFBMFIFI_02180"

/inference="ab initio prediction:Prodigal:002006"

/codon_start=1

/transl_table=11

/product="hypothetical protein"

/translation="MQLVIGDIITAISIQGNNVTGKVTAILENTVILFCELSNHVVSK

KELKKQGYKFTKVVKGTKSIVVSSNRNLQK"

CDS complement(2299722..2299964)

/locus_tag="JFBMFIFI_02181"

/inference="ab initio prediction:Prodigal:002006"

/codon_start=1

/transl_table=11

/product="hypothetical protein"

/translation="MNNIVEFPITNSQKKRNAIGKGFVPCEISGNYLDIKKNDYGDFL

TIKVMNDQGISGEIDYEICELVVSKSELLQAINQKG"

CDS complement(2299999..2300217)

/locus_tag="JFBMFIFI_02182"

/inference="ab initio prediction:Prodigal:002006"

/codon_start=1

/transl_table=11

/product="hypothetical protein"

/translation="MIEQFWDDIIIYFVEYTSLSSENKVQSHQKAVIFNSVIIEKELE

HIIMQHIDNIIQVISIDEITDGLFFKKM"

CDS complement(2300474..2300761)

/locus_tag="JFBMFIFI_02183"

/inference="ab initio prediction:Prodigal:002006"

/codon_start=1

/transl_table=11

/product="hypothetical protein"

/translation="MEVHGESMVQLQGEKKRLQAELRYEQAYLSSNSILHVEKASDHE

VGSLSSRECLYCARALDSKITLAGLKVAIEQVEIEMEKFNRKYEKSRRKKK"

CDS complement(2301139..2303535)

/locus_tag="JFBMFIFI_02184"

/inference="ab initio prediction:Prodigal:002006"

/codon_start=1

/transl_table=11

/product="hypothetical protein"

/translation="MLKKVKLVILPIIFMALFVLGDHVSQAADTTTYVPAVNLVTIEG
QPLQLPKKIHVSNDTYNLFEESVTWNSIDSAIFDQPGVYEVTGTTVNGKLATAHITVF
SKEKKVNISVIGDSITYGMNVENMLINAYPKQLNYRLGNQYTVSNFGNIGKTLLEKGN
DPYIRTNEYTRSLTSKPDVVIIQLGTNDTKAVNFTKNSDFVADYLRLIKKYQGLETKP
LIYISLPPKIFSTAYGISQLNLEKIMPMIVETAKKADLDVSIINNQEATQDASALIPD
GVHPNAKGATLLANNVYKEIIGSKELSGKIAANEYTKTFGAINSKLATGPTNFYLSGI
SKDNWVSYENVNLVDNKGSIQFMSTSPLAGTSVEVRLDSLEGPIVGQSTLHKTTNNTT
WMINTIPIEKVTGKHTVYLTFKHNTAIATAELVRLDWIDFAYDATKPAAADSAAELEA
LLGSGLTTIKLTKDITLTKPIVLKSDITLDLNGFSLNAVGYNLTKNESINKRINVNIF
NGYVTGTNVYGTIYAANSENENYGMDIIVSDINFDGVLFIRNNVKGSVVSFNGHNEVK
STRGSNVYARNITIKAGSTYSGSTQGGGGSNESGSTVFTFGIANTDKVLTIEKEAVVE
LYPGSTGTNYAQNAVYGFSKIDIGKNATFIAKGKRPMLRTEHVASNAVVYAQPGSTVD
IQTTEAIEGVSFSYGIDYTFDHVNYLNLESQNKSKYSFMFAYRTSYLTVFGGTMSVWN
KELNSDEMPPSKTWDFETFKLNNFTNNTNMGTVESDSITLKNEFGTINNYNRISINPQ
"

CDS 2303871..2305331

/locus_tag="JFBMFIFI_02185"

/inference="ab initio prediction:Prodigal:002006"

/codon_start=1

/transl_table=11

/product="hypothetical protein"

/translation="MYDLTYNLLVDRVAKRKAQIFSMLATSPIPLDIHYLIKHTKFSK
RTISDYITAFNDERPYNMELSQNSKNEFTLKTKDPSSISLYLNEITENNPLFSIIELI
YGNTFETVESLSDHLYLSDSSVKRYLAHLKKELNQYDLNLTLTPHIDIVGNEITIRYF
FFRYFRYVFDNSFLPIEEHQTNSVYQTISKLISDSGFALSIDYTRVSNLISIFEQRIK
TGHTVLLSESVKKLHRDSDSFARFKRAFSPNFETIPDIGVLSEDELMYAYILRLDALT
YENHSTFFYADYGAQLANFEPIVSNFFTKYQLHPGLFINLKVTLQAFLMNTTFLTDLA
PYYQELDKELHKQVQLNYPEMLADWLKILDTNQMNFRYIKDVAVSLTLITTSFLQMSS
KQDLKVLFSFSGDPASIGYYKSTALKVVPRDAEVIFLSNKELNDELLTLHSIDVCIVN
FRIQAPISVCKVVKLSPIPLEVEWFSLLSTLYNHEI"

CDS complement(2305396..2308374)

/locus_tag="JFBMFIFI_02186"

/inference="ab initio prediction:Prodigal:002006"

/codon_start=1

/transl_table=11

/product="hypothetical protein"

/translation="MNKRKLAHLIGVTVLICSVLIPSVIDFSREQKTASATENSTEVA

IKAVESSENEMEQKPSFHFIETQFNEEIHGVVKIQIESDMPVTEVAVQLPSEAKIQDA

LLLEGQTHQVNEKGQTIFSQTMETKSLELPVVFDTAGEYQVSIVSDDESIVSATIKVT

ANETPIVAEEPLESETDASSEILEVEAGIENASIPETDTPLNNEKISRADENGVQDVA

TWVEFIRAFVDPTVTTINVIDDFATPSNPRLDLTTVTTGDTGNFTGGLSGVYLSVPNI

SRSLVIEGNNHQIDFKAVALCFLDTTANVNSPWDVELKDLEIYHGNYYGPLTFFNLST

NNQRLSTITYNNITNIGNQLIHSPMSHVQIKGVTSSIQQENYTSNFGAWRIYGTNQTN

IFVSKMTMLKDSELVLATIGAGNIDLGYDSNGELLLEDGSKITATANGTSGEANGVNI

LIRSGSVTAGENSEISLVPQLNSSAISLLATGASLNIGKNANVEIQSNGRSTNTNNNN

TNILWMAAGSSLLVDEGGRFAVDAVNQGASGSNVVHVNGAATFRVGKDSTLDIKSDST

SQTQNLLYFASASSIFQFSDAQEINLQRTQAMSGTATTNGLINIAGSTGLLDVDVQAA

KQWSRGNLSEEPDYDWTPIFNLNVRYTGTATRLDSVSSISQETIDSFSANFTTRNVQR

VLFEKIPDVSVTIDELSEDKTLEISHVITGIANPGSVIRFTGDTAIPAGTIPSPDINS

AEMYHVEADSNGEYRYELPADSYFTVGNTVTAYAFLEGKSDTASTIVQERIAPPNPLD

PLNPDVEVDPENKPNLPEEQGQLSIDFISQFNFGTQSISTKDKTYYANSQRLLNGDGT

VNETDERPNFIQVSDRRSSDQRGGWELSLSQESQFSTSDGKELIGAQLQLANAELISP

QENSEPNFVESLQNLIPGVRQPLIRAEGDEGQGTWIYRFGDSTTGSESVKLQVPKGSN

ANAESYSTTLNWELSAIPGN"

CDS complement(2308433..2309470)

/locus_tag="JFBMFIFI_02187"

/inference="ab initio prediction:Prodigal:002006"

/codon_start=1

/transl_table=11

/product="hypothetical protein"

/translation="MNTYPMSKQKKISILIVLSFFIFTLFPAISLAEEAELNFNVTPH

FPTSQVEDSTSYFDLNLPANAKDTLGLTLTNSSTEEVTIKITAHTAYTNVNGIVEYGK

TASEKNPTLTYAMEDLIEVPKPLTLAAGENRTIDIPIQMPKESFEGLLAGGLRIEEIR

EETESEDDNQGLAIKNKFSFVIGVLASNNRSTIVPELELLDVFPDQLNYRNVFSATIQ

NTTATFVNQLEVDAVIRAEGKDEILYQSETEKMQMAPNSHFNYPISLNGDAFRNGDYI

ATITARSGEHEWTWDQKFTINQETASRLNRADVTVDTSMNWWMIAASGLFILLLIVLI

YLFIKRKKDKK"

CDS complement(2309539..2310291)

/locus_tag="JFBMFIFI_02188"

/inference="ab initio prediction:Prodigal:002006"

/codon_start=1

/transl_table=11

/product="hypothetical protein"

/translation="MKLLNLATVAALSATILAGGATAFAEEAREVQTQGEVTFIPNSD

EETVVTPPETEPEVVIPPVVPSTGPLTIAYAPSMNFGVQPISNQDQTYSMIAEMQELA

DGTGDTPYVSFAQVQDTRGNNEGWTLSVTASEFTSGTQNAELTGAQISLLDPTIDYDG

NNALNAPTAHTSTLNLVPGAATTIMEAAEGQGAGSSSVVWGNQVALNGSTDAEVLNDA

IQLFVPGTTAKDATTYTADLSWELTATPANEI"

CDS complement(2310323..2310685)

/locus_tag="JFBMFIFI_02189"

/inference="ab initio prediction:Prodigal:002006"

/codon_start=1

/transl_table=11

/product="hypothetical protein"

/translation="MKMKGVFFLIRSTGVFMIILLMNQAFVAQAESVQSVETEGSLSF

YGTYESEIEPQPGPPNGSKPVVPNEVNKKPIQGKLPKMGELITQNWIWLIIALLVIVL

FERYRTYRKHLRVEMNHQ"

CDS complement(2310780..2311013)

/locus_tag="JFBMFIFI_02190"

/inference="ab initio prediction:Prodigal:002006"

/codon_start=1

/transl_table=11

/product="hypothetical protein"

/translation="MGKIEQHMNDTIIYYVEFTSQNQDKEKTIRKHRKLFAFRSFIYE

EEVAELVKTYLANIIEITSVSKFLEGSFINNKT"

CDS complement(2311765..2314431)

/locus_tag="JFBMFIFI_02191"

/EC_number="7.2.2.10"

/inference="ab initio prediction:Prodigal:002006"

/inference="similar to AA sequence:UniProtKB:Q73E41"

/codon_start=1

/transl_table=11

/product="Calcium-transporting ATPase 1"

/translation="MDKKAQLKETFYTQETDQVLEKLGSSLKGLTSSEAEKRLSEYGE

NQLDEGKTKSLFVKFLEQFKDFMIIVLLAAALISGAFGDIADAVIILVVVIINAILGV

AQEAKAEQAINALKEMASPEARVRRDDHIVSLKSDELVPGDIVLLEAGDVIPADLRLI

EVASLKIEEAALTGESVPVEKSLSLPDGEAGIGDRRNMAYMNSNVTYGRGIGVVVGTG

MNTEVGKIAGMLQATDENITPLQENLNQLGKYLTIAILVIAVVMFGIGMANGREWLDM

LLVSISLAVAAIPEGLPAIVTIILALGTQKMAKRKALVRKLPAVETLGSTEIICSDKT

GTLTLNQMTVEKIYYNGEVRDAKEQLDLTEPVLRIMTFSNDTQISSDGSLIGDPTETA

MIQYALDKGLDVKAELAKEPRVGEVPFDSERKLMSTIQKLADGRFLVTTKGAPDELLK

RCLKIDKAGQVIEMTEAERAAILATNHSLAKQALRVLAMAYKFIDAVPEKLTTETVET

DLIYAGMIGMIDPERKEAAEAVRVAKEAGIRPIMITGDHRDTAEAIAVRLGIIAEGDD

AAVITGADLNKISDEDFAKQVKDYSVYARVSPEHKVRIVKAWQKAGKIVAMTGDGVND

APALKTADIGIGMGITGTEVSKGASDMVLADDNFSTIVVAVEEGRKVFSNIQKAIQYL

LAANLGEVLTLFIATILGWSILAPVHILWINLVTDTFPAIALGLEPAEKDVMKRKPRG

RNSNFFSNGVLGSVIYQGILEGGITLFVYWWAVTYPVHANDVAMHLDAKTMAFATLGL

IQLFHAFNVKSIHQSIFTVGIFKNKTFNYAIVLSFVLLAATIVVPGLNDVFGVAHLDS

HQWGVVVAAAFSIIPIVECVKFVLRKTKYKAA"

CDS 2314812..2315273

/locus_tag="JFBMFIFI_02192"

/inference="ab initio prediction:Prodigal:002006"

/codon_start=1

/transl_table=11

/product="hypothetical protein"

/translation="MIIIPSGKPKIWDKSPDIGSISANRAYTGTFHKLCQAYAKEFDS

DYLILSPYYGFLKPDDFISETYDVRFTLKGVNQNTIQLAELKKQWQQLNRPETTLTIL

GGKKFQALLREIVGPTLLLNFPLAEAGGIGIMQKFLKDAVATKTELPIIKK"

CDS complement(2315397..2315837)

/gene="bcrR"

/locus_tag="JFBMFIFI_02193"

/inference="ab initio prediction:Prodigal:002006"

/inference="similar to AA sequence:UniProtKB:Q5WNW9"
/codon_start=1
/transl_table=11
/product="HTH-type transcriptional activator BcrR"
/translation="MEFSAQIKKIRTDNQLTQEQLAQQLNVSRQTISSWENNRNIPDL
EMVVSIAKLFNISLDQLILGDDIMREKLVKDSSEVKKAKINMVSVILFLLGVLSLTAK
SIIGAKVGKDGVLHEPFVLLPLGYLLVAVGIIIFVVTLIKNRKK"

CDS 2316383..2317141
/locus_tag="JFBMFIFI_02194"
/inference="ab initio prediction:Prodigal:002006"
/codon_start=1
/transl_table=11
/product="hypothetical protein"
/translation="MKKHKLNLILLLSFLAICLLISFLYEPVLSNWMTVRVPSLKSQN
GLSFPPYSPNTVKPFGTDLVGFTLLSKLIQGFKYTFIIVFFLSLAQLTISFIISSFTF
KYQKKRWVGYVLTYLDKLLTLFPKPFLLLLLIIPIYFTTIFNTEPNPSSNQVLLIKQL
IVLLLISLPNLIQLFHNELIYLFQTDYAKASLVLGSTPRRMMLVALRPQLTILSFALF
FKTVTQNLALFVYLAYFNYFLGGTLLIELDLGTQ"

CDS 2317312..2318160
/locus_tag="JFBMFIFI_02195"
/inference="ab initio prediction:Prodigal:002006"
/codon_start=1
/transl_table=11
/product="hypothetical protein"
/translation="MKLSKSLIKQSLYSLLLILGVIVLVCYFLNANQLDSNFSVSFQR
TIQQLLSFSFWQLFFQTYQTTGQNYTLLLISSSLLFVALFFISYAYFYRFLKPKHLHF
LQKFFANLETLPVVTLVLFLQWSFISLHKATGLRLLTTIGTPRQPIFLVPFFILTIFP
CIFLINYLTPYIKDAYHSDYFLFARSLGFSHSRLFFTYLLPNLIRPLKSVIGIVYLEM
VSVMIFIELQFNTGGLITSLYRTLLDFSRPQDAYVFISAYLLVLLLPYVVLKTGFNFL
TLRRST"

CDS complement(2318281..2318409)
/locus_tag="JFBMFIFI_02196"
/inference="ab initio prediction:Prodigal:002006"

/codon_start=1

/transl_table=11

/product="hypothetical protein"

/translation="MSKKWARWIIGNKTETKAVKKQKSMKSIFTNADFMLFYLGLA"

CDS complement(2318372..2319058)

/gene="pspB_1"

/locus_tag="JFBMFIFI_02197"

/EC_number="3.1.3.3"

/inference="ab initio prediction:Prodigal:002006"

/inference="similar to AA sequence:UniProtKB:D3DFP8"

/codon_start=1

/transl_table=11

/product="Putative phosphoserine phosphatase 2"

/db_xref="COG:COG0406"

/translation="MSGEKQPTIIYLARHGQTWFNTMGRVQGWSDTPLTPAGIEGVEK

LGRGLADHHFVSAYSSDSGRARETAQIVLAANKKGKDLRLKERKDIREACFGNFEGDT

DENMWTAMAQHVGLETPEEMMAEMNVEGFKKTMNAVSEIDPLGIAEDFPTLQNRLKKA

FSEIAEEADQNGGGNVFVVSHGMSIVALTAGLTQDTPIFGVENASITKLYYEAGEFNV

KEMGSMDYWK"

CDS complement(2319149..2321032)

/gene="bglF_3"

/locus_tag="JFBMFIFI_02198"

/inference="ab initio prediction:Prodigal:002006"

/inference="similar to AA sequence:UniProtKB:P08722"

/codon_start=1

/transl_table=11

/product="PTS system beta-glucoside-specific EIIBCA

component"

/db_xref="COG:COG1263"

/translation="MDNKQIAQDVLELVGGEKNINSVVHCATRLRFKLKDSSVANRAG

LEAHDEVITVVESGGQYQVVIGSHVNEVYKELMAITSLDTNTGGKEESKEKGSVVSTL

IDVISGIFTPFLGAMAGAGVLKGFLALAIVMEWLTPESGTYMVLFAAADGVFNYLPIF

LAFTAAKKFKTNPYVAVAIAAALMHPVFNAAIAEGARLTFFGIPFTAASYASTVIPII

LAIWLQSYVERFFTSFIPEFVKIILVPLCVLLVMVPVTFLAIGPLGGLFGTWLGMVYS

GIYNFSPIIAGAFMGGFWQVLVIFGMHWGFVPIMMTNLTEFGFDTMVPMLLPAVLAQG

GAAAAVFFKTRNTKMKALAGSSTITALFGITEPTVYGVTLKLKKPFIAGCIGGAIGGA

VVAFAGVKNFTFGLVSLLSLPGFISTDPNVSSSITMAIIGTIIAFVIGFVLTYILGFE

DVPEDNTSIPVAKKDEPQAGDAGTYIEKEVIESPLKGQIIPLSEVKDEAFASGALGKG

LAIQPSVGELTSPVNGIVTIVFPTGHAIGIISDDGTEILMHIGMDTVQLDGEGFTTHV

KQGQHVKIGESLVSFDIEKIQAAGFPTVTPIVITNSKDFLDVLTTEKEDVEPKDYLMT

VVI"

CDS complement(2321227..2322081)

/gene="licT_2"

/locus_tag="JFBMFIFI_02199"

/inference="ab initio prediction:Prodigal:002006"

/inference="similar to AA sequence:UniProtKB:P39805"

/codon_start=1

/transl_table=11

/product="Transcription antiterminator LicT"

/db_xref="COG:COG3711"

/translation="MNRGELSLIIEKILNNNVVLTLTEHQEEMVVMGRGLAFKKKVGD

EIDPTYIEKTFVMEGKEITNQLAELFGEIPPEEIEVTNEIIKIAQEKIDNKLSNNIYL

TLTDHIHFAITRTKEGLEIKNPLIWEIKKFYQKEYQIGVQAIQIVKEKLGIELSIDEA

AVIALHIVNASQGGYNLNKTYQITHIVQDILNIVRLHFGLVYDENSLNYTRFVTHLQY

FAQRMLNEEIPNTGDDFLFEQVQLKYKKAFECSIKINEYLEKAHHKELSIDEQVYLTI

HIHRVTEN"

CDS 2322508..2323134

/locus_tag="JFBMFIFI_02200"

/inference="ab initio prediction:Prodigal:002006"

/codon_start=1

/transl_table=11

/product="hypothetical protein"

/translation="MKKIIYVYLLDTMADWELSYVLSAINMRGMQNKISHKYTIKTVG

YTKDSIQTMGGLTVTPDCSIAEIDSNQMAALLLPGANTWDAPQHQALLKEVPDYLEQG

ILVAAICGATLALANLAVLDTRKHTSSALEFLTGFSPTYKGQALYQQALSMTDNNLIT

ASPAGALLFAKQILTALAVFPTPMIEAWYNYYLTGEVDYYMELVALSN"

CDS complement(2323248..2324819)

/gene="expZ"

/locus_tag="JFBMFIFI_02201"

/inference="ab initio prediction:Prodigal:002006"

/inference="similar to AA sequence:UniProtKB:P39115"

/codon_start=1

/transl_table=11

/product="Nucleotide-binding protein ExpZ"

/db_xref="COG:COG0488"

/translation="MLIEATNIKKAIGEKVLFEIPKWIIYPGERIGIVGLNGTGKTTL

LTILAGKEEADTGSVVINSPIGFIEQLPENQETISLSGGEETKQRIQEAFQMETGLLF

ADEPTSHLDQDGRAYLEKRLKHFAGGALIVSHDRAFLNAVCTKIVELENGVVHFYSGN

YDAYREQKEIEKMTAESEYITYEKEKRRLKKVAEATEKRAAKVKKAPSRMGNSEARLH

KMGDQRAKKKLGENVKNVEQRLQHLDVKTKPIELAPIKIKLDKGREIHQKIVLSGSKV

SKGFSTKQLLENTEFTLYNHSRTALIGANGVGKTTLIKMIIEQDKHIQQAKNVEIGYF

SQKLDGLDESQSILGNVMKESIHEEDFVRMLLARLLFKGQDVYKKVAVISGGEKNKVS

MAKLLVSSANVLIFDEPTNYLDLASIEAVESALKSYEGTILFVSHDSQFVENIATHYW

QISDKKIKMWEPETLEVSPELVKDAGATLLTSEEKMLIENRLSALLGRISMPSKKDNV

LELEKEYQELTQKLK"

CDS complement(2325177..2326307)

/gene="aes_3"

/locus_tag="JFBMFIFI_02202"

/EC_number="3.1.1.-"

/inference="ab initio prediction:Prodigal:002006"

/inference="protein motif:HAMAP:MF_01958"

/codon_start=1

/transl_table=11

/product="Acetyl esterase"

/translation="MELNEIVKALKTQQAVSVEDGIQVINKHNPDFIPGEIDPIVIQD

QMKESKTGTNETIDQATAIMPDFSDSEVARQFALIMRQTMGSSNVDISEGIETKVQKI

NGPHGEIPIRIYQSGAINKTKPAIIFFHGGGFVGGTSQVTENFCKVMAEQLEGIVLNV

EYRLAPEFQAPVASDECYAVVKWAHECASELGINPNQIAVAGDSAGGNLAAVCSYLDR

QQENNLIAFQALLYPTIYLDEETLAAKQWPIEKFGANPTLSAKLAPGFVGMAASHEMI

RTVYAGELDVHSPLLSPGHASKAELEKMPPTLIVVASYDYLRPFALDYGRKLETAGVE
VKTICYEGMFHAFIDKIGLYPQAEDCANEIVKTFKESMERNE"

CDS complement(2326425..2327492)

/gene="rlmN"

/locus_tag="JFBMFIFI_02203"

/EC_number="2.1.1.192"

/inference="ab initio prediction:Prodigal:002006"

/inference="similar to AA sequence:UniProtKB:Q7A600"

/codon_start=1

/transl_table=11

/product="putative dual-specificity RNA methyltransferase
RlmN"

/translation="MKPSIYGLKNDELTEWFVENGQKAFRATQVWDWLYVKHVTSFAK
MTNLSKELIQLLEENFIVQPLIQKVVQESNDGTIKYLFELHDGLLIETVLMRHEYGLS
VCVTTQVGCNIGCTFCASGLLKKQRDLTAGEIVAQIMQVQQYLDEKETGDRVSHIVVM
GIGEPFDNYDNVMSFLRIVNNEKGLSIGARHITVSTSGLAPKIKEFAENGLQVNLAIS
LHAPNNEIRTSIMRINRNFPIEKLMDAVDYYLEKTNRRITFEYIMLRGVNDQKEQALE
LAKLLADKRHLAYVNLIPYNAVSEHDQYSRSKKADVLAFHDTLKKKGINSVVRKEQGS
DIDAACGQLRSKQMKKESTEA"

CDS 2327786..2330419

/locus_tag="JFBMFIFI_02204"

/EC_number="7.2.2.10"

/inference="ab initio prediction:Prodigal:002006"

/inference="similar to AA sequence:UniProtKB:Q73E41"

/codon_start=1

/transl_table=11

/product="Calcium-transporting ATPase 1"

/translation="MDFKTKKRNDLLELLKTDENSGLTDNQVEKARETYGYNQFNEEE
KESLFTKIMHQLKEITTIILLFAAAISLYLTITIHPDDFAEPLVIISIVILNIFLSIR
QESNAEKALDSLKSLNAPRANVIREGQTEEIDAHELVPGDIISLETGEMIPADARILT
SSGLKVEESALTGESVPVDKDPEMDIPSDAPLGDQFNMVFSGCLITNGRAKAVVVATG
METEMGKIAGLLNSTKKATTPLQMRLHELGKKLSLIALLAGALIFVIGYLQGETMAEI
LMTAVSLAVAAVPETLPVIVTITLAYGVQNMVKRNAIIRNIPSVETLGSASVICSDKT

GTLTQNKMTIKELWAVNHQPIKAKDDYNEDEEQLLRLLSLANNATIEDRDGSEKQSGD

PTETAIIRLLQERGQEKTELEQKYPRVHEIPFDSERKLMTTVHELDDGFISITKGAFD

RIPVDFSSQIGEEAIKVHDEFAEKALRVIAVGYKKYDTLPEDLTPEELESGITFAGII

GMIDPPREESQEAVRAAKSAGIKTVMITGDHIVTASAIAKEIGILEEGDKAITGAELA

KLSDDELQATVRDYAVYARVSPEDKIKIVKAWQANGEVVAMTGDGVNDAPALKAADVG

TAMGIAGTDVSKNAADMVLTDDNFATIVHAVEEGRRVYENIRKAVYFLLSCNVSEIFI

MLIAVSMGWGVPVISVQLLLINVVADGIPGFSLSKEKADADIMEQKPTPKNASIFSGG

LLQKIGVQAVIFTIITLIGFYVGSFIDINGTIAASHDVGQTMAFIILGWSSVIHIFNA

RSKESIFKIGFMSNPLLFWSALFSIAIVLFVAIVPPVASIFSLVQLSGAHWLLATGLS

IIPLVVIEIQKAVLRKMGRSF"

CDS complement(2330612..2331925)

/locus_tag="JFBMFIFI_02205"

/inference="ab initio prediction:Prodigal:002006"

/codon_start=1

/transl_table=11

/product="hypothetical protein"

/translation="MNKNIAKELNHFSLPLVANNFFSILISSLLAAIIGRISINSIAA

TEVVNTFIYSLIGILGVGSLSFNIYSSRIRKSNPEMFKNFFKSIIQLNVLIGGISTVL

VIFFSTYFLEILYGFRNEILTIAVIYAQISAFQILFNMIIFSLSNQMKVKKQTKNILA

IGVVGSIFQIVISVFLVYSIFNNNEYSIVGIGLANSCTQLFELCCYLFILREDLSALR

SVKSSKKFFLLKKSIPLFAQEILEGSIFNVGITALLARLGIVNFSAYSVCRRLADLCL

TPMFMYCNGVVVLTGEYMTEKNKKKLLNLPIIALIIILVFYVIIGTILYVIRPLSIAF

FSNKPEIIETASSILIIVLFTSLAQPFFEVAKFNLQAIGKEKLALIITGVVNLFVLGV

LIYLKQANQLNLVTVLLMLACNYLVLYILFTIFYRLEIKKIVHNS"

CDS complement(2332869..2333819)

/locus_tag="JFBMFIFI_02206"

/EC_number="3.2.1.96"

/inference="ab initio prediction:Prodigal:002006"

/inference="similar to AA sequence:UniProtKB:B7GPC7"

/codon_start=1

/transl_table=11

/product="Endo-beta-N-acetylglucosaminidase"

/translation="MTKKANVLLLASLLLFSCLIFPIGAKAENNQQEPIMMAYYRTWR

DVTMPHDANSNLPDANVTAMTDIPENLDIVSVFHYVKPGTDQMKFWNTLRDTYVPTLH
QRGTKVVRTIDISELLKVPHVGAKPTEAEYDRYADELIATNLTPWNLDGLDVDMESTL
TSSQEEMAAGVFKALGKKLGPTSGTGKLLIYDTNKDNHSLYKKVAQYTDYLFLQAYGR
NPSLLDKTWDTYKNTISPQQFLPGVSFPEEQDKNRWDDTIDPYETSRAYSYAKWNPKE
GPKGGMFVYAIDRDGKKFGDDTITKTDFSWTKRLTDTMKE"

CDS 2334163..2335383

/gene="eis_2"

/locus_tag="JFBMFIFI_02207"

/EC_number="2.3.1.-"

/inference="ab initio prediction:Prodigal:002006"

/inference="protein motif:HAMAP:MF_01812"

/codon_start=1

/transl_table=11

/product="N-acetyltransferase Eis"

/translation="MSLEDQTKQFKMKSVDMQHLEQFNELLRYVFQVTNQDLQEVGYE
DDDLVKAKRPVLRKADVIGWFDDEKLISQLAIYPCEVNIRGKIFKMGGLTGVGTYPEY
ANLGLMHHLMKESLEMMRKNGQWISYLFPYSIPYYRRKGWEIISDKMTFTFKDTQLPK
TVPVTGYVERLELDHPDVIAVYDAFARITHGALIRDELAWEEYWRWENEGERIAAVYY
DENDEPTGCLFYWIAEDVFHMKEMFYINQEARKGLWNFITAHFSMIDKIEGHLYKNEP
IAFILEDSEIVETIQPNFMARIVDVAEFIKEYPFEMVYVPDFHFVVEDPMLEWNQGVF
GLSFNEEGSLTLTNEPIGSPVKLDIQTLTTMLMGYRRPSYLARLERIDTDSRTLRSLE
QVIPQGEPYFSDYF"

CDS 2335410..2336228

/gene="proB"

/locus_tag="JFBMFIFI_02208"

/EC_number="2.7.2.11"

/inference="ab initio prediction:Prodigal:002006"

/inference="similar to AA sequence:UniProtKB:P39820"

/codon_start=1

/transl_table=11

/product="Glutamate 5-kinase 1"

/db_xref="COG:COG0263"

/translation="MVELSRAHLNTLKRIVIKVGTSTLMYPTGAINLQRIEKLAFVLT

DLKNQGKEVILVSSGAIGVGCHRLKLTERPTTIPQQQAVAAVGQSELMSLYSNFFNNY
SQVVGQVLLTRDVIEFPESRTNVTNTFNQLLAMDIIPIVNENDTVAVDELEHQTKFGD
NDSLSALVTELTQADLLIMLSDIDGFYDKNPTVHQDACLFSEINEITADLLSLAGGNG
SKFGTGGMITKLNAAEHVLNQDSQMILANGAEPTIIFEIMHGENIGTHFLKKKS"

CDS 2336276..2337520
/gene="proA"
/locus_tag="JFBMFIFI_02209"
/EC_number="1.2.1.41"
/inference="ab initio prediction:Prodigal:002006"
/inference="similar to AA sequence:UniProtKB:Q9WYC9"
/codon_start=1
/transl_table=11
/product="Gamma-glutamyl phosphate reductase"
/db_xref="COG:COG0014"
/translation="MDTLIQLGEQAKKASRYQAQASTQEKNAALRTMSAALLAHSAEI
IAANHWDIEQAQANGIPEPMIDRLLLTKERIQDMADGILEIAELPDPIGEVNGMWKNE
AGLMIGQQRVPLGVIGIIYESRPNVTTDAGALCFKSGNAVILRGGKEAIHSNQILVTI
LQDALATTAFPPAAIQLVTDTSRETARELMRLNRYLDVLIPRGGARLIQTVLESATVP
VIETGTGNCHVYVDSAAQLKMATDIIVNGKCTRPSVCNALETLVIHEAVAAEFLPVIE
KALVEYNVELRADERALALLETAIPATESDWETEFSDFILAVKVVDSLDEAIQHINHY
STGHSEAIVTDNYFHGQQFHREIDSAAVYINASTRFTDGFEFGFGAEIGISTQKLHAR
GPMGLTELTSTKYIIFGEGQIR"

CDS complement(2337562..2339181)
/locus_tag="JFBMFIFI_02210"
/inference="ab initio prediction:Prodigal:002006"
/codon_start=1
/transl_table=11
/product="hypothetical protein"
/translation="MTSWEILEIEQTNDKKAIKKAYAKKLKKMNREEDVASFQELKEA
FDRAINWTKNQKEENTNYIKKEALYNNDADSMEYKLSAENQKELSEWIDSKKAYWFDF
NLNSSQNNVIKNNLVTQLSEFILMTGLTKWLFMSGDQKEILLSKQLEKLIFQSEEVVD
EVQLELVYYEVNKLYGDFKRRIKIENWEKLFENKIIRLTKDYEEQQAFFTDFLLTNYY
YLPQPVVSFLWKTFYLEEYTSISMKSYSHGEITDRIEQIKKIPNFSFDLLIDLEEKAR

QKFLEARLVAYFYIEKGQFVLGERKISEAQRYFDSDTELLLMKTYFQIKDSKNGYVWL
IKFMAYLPTILILNKIIKLDPTNPVARIYRAYFKTSLKFTVNKEDLTYLNAAEECDFF
EKNYILGWLAVERHSYQEAYDWLLKLDDYREAYSRKRITKAIRKLKKQEQSKTKKNRY
KQELRFYSLISFLPLYLKRKPLVKLVCVFLYFYLIGAVVLTGTLASFFLYCILCLLGI
SMFSYPIRNRYVRRMHEYYKQNAPDFYKNQS"

CDS complement(2339356..2339637)
/locus_tag="JFBMFIFI_02211"
/inference="ab initio prediction:Prodigal:002006"
/codon_start=1
/transl_table=11
/product="hypothetical protein"
/translation="MDGSEALNYLIEKTKNWPEMVIFVNWGMDSNTREVTLVAAYSHL
ITEKASFLKDGTEHGIQGTFFALTEKIFSKKRLNQWLEEIHLEKADARG"

CDS complement(2339659..2340600)
/locus_tag="JFBMFIFI_02212"
/inference="ab initio prediction:Prodigal:002006"
/inference="similar to AA sequence:UniProtKB:P9WKP3"
/codon_start=1
/transl_table=11
/product="hypothetical protein"
/db_xref="COG:COG2220"
/translation="MSQEKISLVDTGIYILKKKIKGENKLKPRNPLPAETIDVKKLDL
ATDQVFWLGHSAALLILNQKVILLDPMLGKHASPLPIAYPRFEGQRPLDLAEFEWIDL
VLFTHNHYDHLDKSSILKIKNKVELFLVPVGMKNTLVRWGIAPKRVKEISWNQDVLLD
ELTITATPTQHSTGRGLFDQNKSQANSWVLKSPKTSLFLSGDSSYADHFKEIGNQYGP
FDLALMECGQYNPKWKESHMFPDETVSAVIDSQAKKFIPIHWAGFALSFHSWQEPVEK
ASQEAVKHGIQMDTPMIGQQYKIHAENVTNKWWRKVD"

CDS complement(2340904..2342199)
/gene="rcsC_4"
/locus_tag="JFBMFIFI_02213"
/EC_number="2.7.13.3"
/inference="ab initio prediction:Prodigal:002006"
/inference="protein motif:HAMAP:MF_00979"

/codon_start=1

/transl_table=11

/product="Sensor histidine kinase RcsC"

/translation="MKNKIEKKQQRRFFIYNMVAFALIFIAFGFIIFFQVQTSLYSRV

DEDLLKQAELRGSFEPNGAFNGAPPGELSRLPDKDVTLKGQGRFVPIEWDSEGNILNK

EAIGTQLYDELSTGFKLATSKLETVHSVTLDSGSSFRYVVYKAPSSNSSDVTYLQFIS

RVDAEKQLAENFGRILIICSIGSWILSIFASYYLSQKAMSPVLASWRKQTEFVANASH

ELRTPLTIIQNKLELLLTKPNDRIIDQMEPIALSLSEIRHLSKMTSDLLLLSRADSNA

TVLTEEMVDLNRFVTEVIAPYKEIAESKEMTIWTQLDSNRVSKIDPVRIQQLLVILLD

NSLKYSQAGDSIGITTEVKDNYWIVEVRDTGQGIAEENREKVFERFYREEKSRNRETG

GTGLGLAIAKWIVDLHKGKIEILNNLPKGTIFRVKIPIK"

CDS complement(2342196..2342879)

/gene="arlR_2"

/locus_tag="JFBMFIFI_02214"

/inference="ab initio prediction:Prodigal:002006"

/inference="similar to AA sequence:UniProtKB:Q9KJN4"

/codon_start=1

/transl_table=11

/product="Response regulator ArlR"

/db_xref="COG:COG0745"

/translation="MLRLLVVEDDERLSANIIEMTEDFLKGDQAYDGADGLYQAEQNI

YDVIILDLMMPEMNGYEVLKELRRRKVETPVLILTAKDGLDDKIQGFEVGADDYLVKP

FHREELIMRLKALLKRNHSGFMENQINHQELVLNLQNKTAKIGEKILVLNGKEFDLLE

YFIQNPEMIVTKEQIFDRIWGFDSETSLSVVEVYMSNLRKKLKPYGYDEGIKTLRNVG

YMLTAVTSV"

CDS complement(2343038..2344051)

/gene="iolC_2"

/locus_tag="JFBMFIFI_02215"

/EC_number="2.7.1.92"

/inference="ab initio prediction:Prodigal:002006"

/inference="protein motif:HAMAP:MF_01668"

/codon_start=1

/transl_table=11

/product="5-dehydro-2-deoxygluconokinase"

/translation="MNILAFGEVMMRLTPPHYKLIEQTDTVDLSFTGSGVNLLSSLAH

FGYETTLLTTLPANQVGRAAAGSLRKLGIRDHLIGYQGNHIGLYFLEMGYGNRPAEVT

YLNRLASSFGESTIVDYDLDKALNHAEIVHICGIALMLSEGTREVAFALAEKAHQLGK

KVCFDFNYRPSLNEANGHEWVKAQFERILPHCDLVIGGIRDLVELLDLPDAVATATEI

ERLEEVSLRFIEKYQIRDFTGTIREKEGKNLGIRGFLRRNEQFSVSSVFDLAIYDRIG

TGDAYGAGIITGLIEEWDSQKTVTFATSNAVLTHTTFGDSPLVRKEIVEGFSQGILGD

VIR"

CDS complement(2344155..2344895)

/gene="dgaF"

/locus_tag="JFBMFIFI_02216"

/EC_number="4.1.2.14"

/inference="ab initio prediction:Prodigal:002006"

/inference="similar to AA sequence:UniProtKB:D0ZLR2"

/codon_start=1

/transl_table=11

/product="2-dehydro-3-deoxy-phosphogluconate aldolase"

/translation="MKKTPNYLEDRICLNVLANSVQNAIDCYKAADKHVVLGVLSKNY

PTDEEAIRDMELYQAATENALSVGLGAGDPNQSQMVSRISAKLQPQHINQVFTGVGAS

RALVGQDDTIVNGLVSPTGKVGLVNIATGPLSSQEPAGEVSIETAIALLKDMGGSSIK

FFNMKGLAHIEEYKAVAKACAENDFYLEPTGGIDLENFEEIVQIAVDAGVKKIIPHVY

SSIIDPETGDTRPEDVKTLLSLIKKTLA"

CDS complement(2344964..2346061)

/gene="dgaE"

/locus_tag="JFBMFIFI_02217"

/EC_number="4.3.1.29"

/inference="ab initio prediction:Prodigal:002006"

/inference="similar to AA sequence:UniProtKB:D0ZLR3"

/codon_start=1

/transl_table=11

/product="D-glucosaminate-6-phosphate ammonia lyase"

/translation="MDLYKKYDLKEVINASGKMTILGVSKVSEEVLAAQKFGGQHFFE

MSELTLKTGKAIAQLIGAEDAIVVSSASAGIAQSVAAFIGAGDNYHAYHPFTNRIKKR

EIILPKGHNVDYGTPVEVMVELGGGEVIEAGYANMCTPEHLAMMVTENTAAILYIKSH
HTVQKSMLTVSEAAKVAAEHQLPLIVDAAAEEDLVKYLDEGADLVIYSGAKALEGPSS
GLVIGNRQFIDWVRLQGKGIGRAMKVGKENILGLTAAIEQYVAEGSESGEAMKTRLAP
FLTDLNQVPHIVASYEQDAAGREIYRAKITIADSAPLTAKEVVVGLKQGNPAIYTRDY
RANNSIIEFDIRAVNKAEMEKIVSALQTLLG"

CDS complement(2346106..2347212)

/locus_tag="JFBMFIFI_02218"

/EC_number="3.1.1.-"

/inference="ab initio prediction:Prodigal:002006"

/inference="similar to AA sequence:UniProtKB:Q837K0"

/codon_start=1

/transl_table=11

/product="Deacetylase"

/db_xref="COG:COG3964"

/translation="MIDLYIKNGKTVAGTKIDIAIKDGKIVEVAETLNISNAAKTIDL
MGESYVSAGWIDDHVHCFEKMTLYYDYPDEIGVKKGVTTVIDAGTTGAENIGDFYSLA
EKAETNVFALVNISKWGIVEQDELADLKKIKEDLIAAAIDKYPDFILGMKARMSKTVV
GDNDIIPLELAKKMQRANQLPLMVHIGSAPPRLDQVLDQLEQGDIVTHCYNGKPNGIL
DSSGKIKDFVWDAYRRGVIFDIGHGTDSFNFKVAETAKAAGLICETISTDIYHRNREA
GPVYDLATTLEKMLVLGYSLEEIIPMITKNPAAAFKLTDKGQLKIGFDGDVTIFKMQT
GVKELTDSNGFTRETREQIQPTYAIVGGKVHELA"

CDS complement(2347223..2348311)

/locus_tag="JFBMFIFI_02219"

/inference="ab initio prediction:Prodigal:002006"

/codon_start=1

/transl_table=11

/product="hypothetical protein"

/translation="MMRRLGVSIYPDHSDVKTDEEYLTKAAKYGFSRIFMSMLEVTEG
KEAVAEKFTHIIQFGKNLGFEVMLDIAPTIFDDLGISYDDLAFFHQLGADGIRLDVGF
DGNKEAMISFNPYNMIIELNMSNDVAYLDNILTYQANRPFLYGCHNFYPQNGTALPFD
FFMKCNERFKKQGLRTAAFINSQVGEIGPWDINDGLPTLEMHRNLPIEVQAKHLFATN
QIDDVIIGNAYASDVELMALSKVNRYQVELAVEFVSDANPVEKEIVTTVQHFRRGDIT
SQVVRSTEVRKRFKAEANPAHDNTHEFKRGDIVIGNDGFGKYKNELQVVLESHTDERK

NLVGTVTEAEKILLDFIYPWSKFKFEEK"

CDS complement(2348308..2348469)

/locus_tag="JFBMFIFI_02220"

/inference="ab initio prediction:Prodigal:002006"

/codon_start=1

/transl_table=11

/product="hypothetical protein"

/translation="MLYGILAILASVVIGLLLHIAVLLVTTVTLFVASVIAAKFRKVF

TKMEAGKKV"

CDS complement(2348662..2349969)

/gene="licC_7"

/locus_tag="JFBMFIFI_02221"

/inference="ab initio prediction:Prodigal:002006"

/inference="similar to AA sequence:UniProtKB:P46317"

/codon_start=1

/transl_table=11

/product="Lichenan permease IIC component"

/db_xref="COG:COG1455"

/translation="MDKFVDILESKVLPVATKIGSQRHMTAIRKGIIATMPLTIVGSF

FTILLNLPIESLALMIEPYKAILDVPFRFTVGILSLYATFGIASSLAKSYKLDSLTSG

ILAVLAFLVATVVPVQVVDPIDGVITAGRYINIASLSSASLFGAIVTSIISVEIYRFM

KEKNITVKMPDGVPPEVSNSFIALLPAAVILILFWVIRHMLGFDISSALSTVLMPLKN

TLAGNSLFGGLLTVFLITFFWVLGIHGPAIMGPVIRPFWDISIAENTDAFVAGVDANH

LPNIFTEQFLQWFVWIGGAGTTLALVVLFLFSKSEYLKSLGRLSFLPGLFNINEPIIF

GAPIVMNPILGIPFIVAPLVTTTLSYFLTVTGVVPMMMARLPFTVISPIAAWMSTNWS

IMAGVLVVINFFIALAIYYPFFKVFEKQQIDRELEAKAAEATE"

CDS complement(2350117..2350770)

/locus_tag="JFBMFIFI_02222"

/inference="ab initio prediction:Prodigal:002006"

/codon_start=1

/transl_table=11

/product="hypothetical protein"

/translation="MSEKLEKPNFWYADWSFPIFVGLLAAGVFAGTHMYYVYGVGAFN

EVAIVAMLKAGMDGGSYGAAAAFGASFLFARILEGSLVGILDLGGAILTGIGIGIPAI
FLSMGMVAPVENFALAIVTGLILGLLVGGVIILIRKFTINQSNSTFGADVMMGAGNAS
GRFLGPLIILSACGASIPIGIGATIGAVIFYLWKKPIAGGAILGAMILGAIFPIALT"

CDS complement(2350791..2351573)
/locus_tag="JFBMFIFI_02223"
/inference="ab initio prediction:Prodigal:002006"
/codon_start=1
/transl_table=11
/product="hypothetical protein"
/translation="MTIMIILLKSLLIGGLVGFAAGAGAARMFHAPTTQGLGAFRTLG
EMNACEGDAASHFSFGLGFFFNAWASTVGAGAFTQDITHRVIPNWATAALMIKNKNVE
ETMHNPKKMGIAGAVIGMVLVAFLNTTASAIPPALQVTAVAVLVPAANLLINTVMPVL
FWLAAIDAGKRSGLWGTIFGGLSAMIMGNATPGVVLGILIGKGVDEIGWNRVTKSMMV
AIALLFIFSAFFRGFDLEMLESFRLQIPQWLESIHGVFNLGA"

CDS complement(2351574..2351948)
/locus_tag="JFBMFIFI_02224"
/inference="ab initio prediction:Prodigal:002006"
/codon_start=1
/transl_table=11
/product="hypothetical protein"
/translation="MTDVKQKKRQIVRVEGKAEKKNQAFSNALNQIHGKVLKESDDVI
LRIEPLHTAIVLAEEESYVEKFLFFFLKRTRVTYKVIMDVEVEISWIPVESVQFVTTH
TQGPDYVPTPLFKKKWLQKEEI"

CDS complement(2351988..2352341)
/locus_tag="JFBMFIFI_02225"
/inference="ab initio prediction:Prodigal:002006"
/codon_start=1
/transl_table=11
/product="hypothetical protein"
/translation="MLTVVIADRLGKGQNVAKGVEAAGGRAVVVPGMGADMRLGDVMQ
QENADLGISFCGSGGAGALTAATKYGYPERHGMRSVEEGITAINDGKTVLGFGFMDQE
ELGKRLVEAYVKKNA"

CDS complement(2352361..2352705)

/locus_tag="JFBMFIFI_02226"

/inference="ab initio prediction:Prodigal:002006"

/codon_start=1

/transl_table=11

/product="hypothetical protein"

/translation="MEHKLITEAVKGILAESQNPVATEKILYSALAKMEEKAIYPTEV

QLVVLTNHLGEMVRRSKEGEAIMKIDPDMFNEVSIEALAISEAITQEIGNLADDEKYV

LSIHFETAKQNN"

CDS complement(2353083..2354954)

/locus_tag="JFBMFIFI_02227"

/inference="ab initio prediction:Prodigal:002006"

/codon_start=1

/transl_table=11

/product="hypothetical protein"

/translation="MQVQLSKREIKLILTLLNNEASTTTTKELAAEFQVSVRTIKYDL

DNVKIWFEQHGTPLLAQRNKGFWLELTDSMRIELKNEVLQVERYELYPDQEIRIHQII

TLLCLRTEFSTTIELAESLHVSKNTIVADLEKVEQFVGAYQLNLIRKNYFGYTIEGSE

HQIRLLLEAIIQKEITDYDIYSIMNYITDKSAGDYRSLKLAMQPEMLQIYQKTVYTVS

KIMNQKMMEQFNYSEILAIILRVTIATARMSINRTINSYKILSNQDLLTKSQELPYLL

MKQVFESYHFPILEDEYIYIFSDILMVYEEKNIAELTRNIIEFVSEKEKIAFNEDKQL

FTNLFAHLSLKLHKKYLFVNEYNPFIEDIKSRYPVLFRSIEEASANEISKSISITNDS

FIAYIALHFLVSYEKLSMNRSVARIVYVCSTGLGVTSLIQQRIMEEVANVEIASFASV

LKAKEIIQLENPDLVVSIFPIEELEVPFIKVNPIPTKSDIQAIKKVVAEIVSNQLTGS

TKFPKLVARRQTPMKNNDEEQSRELILKGFVVYEELKKQLGLKLQVGYEEAFLLHVFM

MVHRIFYDSQYENEGNVALDTLDDFKEDVRLIKEIFAKNELSINKAEITALVQYISN"

CDS complement(2355349..2356053)

/locus_tag="JFBMFIFI_02228"

/inference="ab initio prediction:Prodigal:002006"

/codon_start=1

/transl_table=11

/product="hypothetical protein"

/translation="MKPKGLLYIEIAETLKKDILEGIYPVGTQIPTENELEVKFSVSK

ITVRKAIEILANEGYLEKKSGKGTTVLSDRLFNKLSKAASFSAMIEERGHHLSKEILA

IEKIKTNSTTPEIAAAFGKEAYKLTRLYRLDYEPYIYFEHYLPVLGDESSLEQMEKLS

LYKWLASYQKVVGKFQDTFAVAPAEAAIQKLLNTQSPYLLKRIRTSYSQSEEIIEISH

AQYDTDKFPYLIEYEI"

CDS complement(2356121..2356891)

/locus_tag="JFBMFIFI_02229"

/inference="ab initio prediction:Prodigal:002006"

/codon_start=1

/transl_table=11

/product="hypothetical protein"

/translation="MFLDNTAELSSFDLHIYKYILDHSDQVIYMRIRDLAAATHTSTA

SILRFCKKMKCSGFSEFKVKLQLSLAEKPIEPVADVDEMSYMDFLTRARQSSFKQKIQ

EAVDLLKDKELVVFLGSGSSESIAQYGSTYFSNLFTLALRIEDPSNYPIEFLAKKFSN

KICLIALSVSGETQEVINYLKHLNTSHCQVISITNSENSTIAQLSDVNIPYYINRETV

KKDHNHPEKTLELTSQLPAVYTIEAIAKAVRKTLVAES"

CDS 2357097..2358482

/gene="gmuD_2"

/locus_tag="JFBMFIFI_02230"

/EC_number="3.2.1.86"

/inference="ab initio prediction:Prodigal:002006"

/inference="similar to AA sequence:UniProtKB:O05508"

/codon_start=1

/transl_table=11

/product="6-phospho-beta-glucosidase GmuD"

/db_xref="COG:COG2723"

/translation="MTKNYSFPPHFFWGASTSATQSEGRVADDGKGENIWDFASNEYN

HRFYEGVTTEKTSRFYEDYQTDIGLMAELNFNSFRTSISWSRLIPNGVGPVNEQAVTF

YNQVIDELIAKGIEPFINLYHFDMPLELQKIGGFENKIVVQYFKQYAETCFDLFGDRV

KYWFTFNEPMIPAEAGYLHDRHYPYVVDFKRAATVLHHIVLAHCEAVKSFRERNLASK

IGIIMDIIPVYPRSQHPADLKAAEMADLFYTRSINEPLLLGRYPAELVGILTEYGQMP

VVITADLALIAETKVDILGINYYKPRRVKALDYEPNRSGVFSPEWFFANYEMPGRRMN

TSRGIEIYPQGIYDIAKMIQAKYGNIDWFVSENGIGIQGEEQFMENGMVMDDYRIDFL

KEHLIWLHKAMAEGSNCLGYHMWTFVDCWSWINAYKNRYGFYRLDLATGEKTVKKSGL

WFKDVIKEHGF"

CDS 2358536..2359855

/gene="licC_8"

/locus_tag="JFBMFIFI_02231"

/inference="ab initio prediction:Prodigal:002006"

/inference="similar to AA sequence:UniProtKB:P46317"

/codon_start=1

/transl_table=11

/product="Lichenan permease IIC component"

/db_xref="COG:COG1455"

/translation="MEKLLERFALKILPIANAIGQQRHLQAVRNGLISILPLTIIGSF

FVILLNVPIAGYAEMIAPYSAALDIPFRYTVGIMALYSAFTIGSFLGKSYKLDGVTSG

FLSMLATILMVVPINLKEGIDTAGNAVNAGRYIPIAPLSSQGLFGAIVASLLAVEIYR

FTKERKLEIKMPDGVPPVVSSSFAALLPTLFIVLVFWIPRHFFDFDLNGVLTVLISPL

KTFLTGNNLLGGVLTQFMICLFWAMGIHGHAVLGPIIRPFWDQAIIENADLFQGGMSA

FQLPNIFTEQFYQWYAQMGGTGATLALCFLFLFSKSSYLKQLGKLSILPGIFNINEPL

IFGAPIVMNPLLAVPFILVPIVNTILIYIVTILGWMPRMMVKPPFTLPAPFGALVTSN

WNIVAFIMVFVCFIASLAIYYPFFKLFEKNQLKQEADTVAQANMTEV"

CDS 2359862..2360122

/locus_tag="JFBMFIFI_02232"

/inference="ab initio prediction:Prodigal:002006"

/codon_start=1

/transl_table=11

/product="hypothetical protein"

/translation="MEILLLSLVVGTIFMTHLTLKNNQWIATSSLLKLICLGILTIGF

SIVLASLLGNFYLLVILTTLVWASILQIRFQQRVTHYNEQVN"

CDS complement(2360247..2362394)

/locus_tag="JFBMFIFI_02233"

/inference="ab initio prediction:Prodigal:002006"

/codon_start=1

/transl_table=11

/product="hypothetical protein"

/translation="MKIRIKLVTLLFILGLSIAFTTTAFAQGDNKKEGLEVQATEKET

LDRLFANSYEVFRDLRNDLGVYRDSILLEPGENYHPSSVASTGVGLMSLTIADKKNWE

EGGLEKAKQTVATMTDQLVGFSADRTANGFYRHFINMETGAQEWNSEYSTIDTAIFLA
GALFAEKYFNDAELSEAVAKLYHSIDFEAALADSDTGGIYLTMNQDGTGVSNSITLPY
NEYIIVAWFAYNQNRGNPESKAVKLWKKHYETPKNLLTKPVHDINLLTDNSDHYLSSF
TFLFPYYMVNMFSKSDEYKPYIENAYQADKLWSELTNKTNPYEWGNGAGASSEGYGYH
ADAINNNQPIIISPHIISGFLPVNPSGSQDLLDLYANKKGIYQLQSDKSKEILWRYSI
EDPTWKANSIQGIDYSTFLFGLATLDTDLGLNFFQENNNFFTNVAFDLNGGTSVTPPA
QNLAVRDILKEPTPKPTRPGYTFIGWNTSLDGSGATWDFTTTEMPSNDLTLYAQWQAN
TRTIHFDLNGGTSVIPPAQNVAVGAPVMEPTPTPIRPGYTFTGWNSSITGSGIVWDFS
TNRMPDNDLLLYAQWQINTYTVIFDSNGGTSAEYPSQSIVFGATLKEPNPAPTRQGYT
FIGWYDEANNIWDFRKMTMPDRELKLVANWEKLSSNSTESEDSGISKTENKPSAKNSV
FPKTGEKSSLMTTLGLLLIGCLAIGLYLRKYKI"

CDS complement(2362774..2364132)

/gene="npr_1"

/locus_tag="JFBMFIFI_02234"

/EC_number="1.11.1.1"

/inference="ab initio prediction:Prodigal:002006"

/inference="similar to AA sequence:UniProtKB:P37062"

/codon_start=1

/transl_table=11

/product="NADH peroxidase"

/db_xref="COG:COG0446"

/translation="MKVIIVGASHGGHQSALEMLDRYEGADVTIYEKGDFVSFLSCGM
QLYLEDKVTQVDDVRNFKPEDIEKRGGKVKSQHEVLTFDADKKEVTIKNLATGELLVD
TYDKLILASGVTPAALPVPNADLENIFFMRGRAWALQIKDKLSDDKVKNVVVIGSGYI
GVEAAEVFAKAGKNVTIIDMIDNVLGNYLDQEMTDILAPVFKENGVNLELGAQIKGFV
GEKQVTGVEHQNGVVDADLVIVAAGVTPNTSWLEGLVDLEERGQIKVDEYLRTSAKDV
YAVGDAILPLNVASQKHAPVALATTARREARYVVRNLTKDQPTEKFQGVLGTSALAVF
GYNLAMTGLNEFSASRSEVQIKGNFYKDTLRPTFVKNDGNTEVYVKLNFDEKSHKILG
GQVLSTYDITAHINTLALAIKTGLTLEDLAEADFFFQPGFDRQWSLLNLAAQSALGET
KF"

CDS 2364614..2365534

/locus_tag="JFBMFIFI_02235"

/inference="ab initio prediction:Prodigal:002006"

/codon_start=1

/transl_table=11

/product="hypothetical protein"

/translation="MRKYGETIRKIRVGKGIKQSELYKDLISKSYAINFEQGKHDISF

SLLEEILDRLLVSVDEFQYIDRDYNPNHINHFFSLFSNYANSNNIEKLEELLLESEPY

EDKLSLIKTALVKSRISQMKVFNETGSLVKAQISRHNQEVILNYLSSIQTWTYFEIIL

FTNTVEYMNSKEKNTYFELISRFVSKYKHYESAHSCLCTLYVNSIYDLLTESSITQSR

SVLDLLMRELKELSQNPTYGFHRIIYNYYEGIILMNDSKNDPPNFDKGEHKSRFAIQM

LQELEQTFIASIFETTLTNFLDSHHLNHSN"

CDS complement(2365647..2365919)

/locus_tag="JFBMFIFI_02236"

/inference="ab initio prediction:Prodigal:002006"

/codon_start=1

/transl_table=11

/product="hypothetical protein"

/translation="MKLKFTIFFNVFLFGIFIMESIPAKTEDVQLNVNNSLSEVDSKK

RVKEKEYKNNLVPKTGGEWLLLIVFIMWFVSFLLLYLFNMISHVLK"

CDS complement(2366115..2366360)

/locus_tag="JFBMFIFI_02237"

/inference="ab initio prediction:Prodigal:002006"

/codon_start=1

/transl_table=11

/product="hypothetical protein"

/translation="MLIEEHWNDGLEYYIEFEATTGMIVRKVIIVPATFEIEQVKAMV

VQRFTRVKKIICIEEVNEVLLMNDYFDMKNGSNQIIF"

CDS complement(2366708..2366977)

/locus_tag="JFBMFIFI_02238"

/inference="ab initio prediction:Prodigal:002006"

/codon_start=1

/transl_table=11

/product="hypothetical protein"

/translation="MAKTNLCQSCGMPLKDEADFGTEKDGTLSTKYCEKCYQDGEWTK

TDLDFDSMYAYNLKKFQESDMNKIEKFFLNKMYTKKFMKKLERWN"

CDS complement(2367177..2369186)

/gene="kup"

/locus_tag="JFBMFIFI_02239"

/inference="ab initio prediction:Prodigal:002006"

/inference="protein motif:HAMAP:MF_01522"

/codon_start=1

/transl_table=11

/product="Low affinity potassium transport system protein

kup"

/translation="MEANHNRQNHLNKVTMGGILVALGVVYGDIGTSPLYVMKAIVKG

NGGLAGVSEDFILGAVSLVFWTITILTTIKYVMITLKADNHGEGGIFSLYTLVRKQSK

WLIIPAMIGGATLLADGMLTPAVTVTSAVEGLREIPVFREFFGANQHVIIIIVITIIS

ALFLIQRFGTEAVGRIFGPVMLLWFTTLGIFGLVNIVKDVTLIRALSPHYAIAVLFSD

ENKMGFFILGGVFLATTGAEALYSDLGHAGRKNIYATWGFVKFSLILNYFGQAAWILE

AKNDPKMVAMQDMNPFFQMMPKPFLIYGVIIATLAAIIASQSLISGSYTLVSEAIKLN

LMPRLHSLYPSTSKGQVYIPAVNTILWAVCIGVVLHFRSSDRMEAAYGLAITVTMLMT

TILLFNYLIKRKTPMWLAVTMLLFFGAFEMSFFISSAVKFMHGGYITVLIAVAILFVM

YICNKSHSIKMRLLEYVPLADYRDQLGQLCEDGDRQKYATNLVYLTNSREKGEVESKI

MYSILDKRPKRADVYWFVNVVVTDEPYTAEYTVDNIGGTDYMIKVQLKLGFRVSQKLN

VFLRQIVTELVENGEVDIQSRNYTTMPDRKVGDFRFIIIQEQLSMEMELSGYETFIMQ

AKLFLKRHTVSPEKWFGLNASDVEIENVPLFLGKKRARVLKRVDK"

CDS 2369599..2371788

/locus_tag="JFBMFIFI_02240"

/inference="ab initio prediction:Prodigal:002006"

/codon_start=1

/transl_table=11

/product="hypothetical protein"

/translation="MLQYVDTRQGTNNAHSYSNGNTLPYTTVPFGMNHYVVQTNHEGS

WFFNPNHRVFQGIRVSHQPSPWMGDFASFLMTPIAGFHMKGDIFSYQSSYRPEEAIYQ

PHYLKIKQARYNITTELTATERGGQMRMTYATKEQPGFILNAKGNSWYKLDSETSTLT

GFINNFSGCLDEKFGIYFTLTFDHKIDLSQTGYFSENEFLTASEMTGADQHFIIRFLD

ATEKTVIANFSTSYISIEQAELNFSRENQLGFNEMKEQAANRWNHYLTKIIVTDSDTE

KVKTFYTCLYRMFLFPQKFYELDENNDPIHYNTTAKKVDKGILYTNNGFWDTYRTVYP

LYSLIAPDEYREMLEGFLTSYHEAGYLPKWLSPDERGMMPGTLIDAVIADAAVKGVIS

REMTESLLEAMLKAATKQSTSSIYGRQGTADYLKYGYVPTTHHESVNHTQDYAYSDYC

ISQVADVLGKRELANTYRKSALNYRNIFDANTGFMRGKDVDGNFRSEFNLLDWGLDYT

EGSAWQNSFAVYQDFQGLINEYGSKEAFTKKITELCNTEPRFDVLGYGFEIHEMSEMA

AVDYGQVAISNQPSFHIPYLFNYAGEPASTQVVVKQIMSRLFNSGFDGYPGDEDNGSM

AGWFVFSSMGFYPVTPGSGEYVRGIPLFDSVEIQLANGKQVSITTENNNPQSNFVADF

KLNQSEYQKLFITHDDLMAGATLDIKLGLAPVYREYSKDELPFSIQK"

CDS complement(2372436..2373650)

/gene="deoB_3"

/locus_tag="JFBMFIFI_02241"

/EC_number="5.4.2.7"

/inference="ab initio prediction:Prodigal:002006"

/inference="similar to AA sequence:UniProtKB:Q818Z9"

/codon_start=1

/transl_table=11

/product="Phosphopentomutase"

/db_xref="COG:COG1015"

/translation="MGKFIVIVLDSFGVGYMADTVKVRPRDVGANTARHILENQKMLR

LPVMEKLGLINAIGEEFTSHVFSKEAVSGSANLAHQGADSFLGHQEIMGTTPKIPLNQ

PFNEKIGEVEAQLIKCGYQVRRVGEIGQPQILVINECATVGDNLETDLGQVYNVSACL

DQLSFKEITKLGKAVREVVEVSRVIAFGGEQINLENLLAARKIKQEMFAGIDAPESGV

YRQGYQVIHLGYGIDPEVQLPTILDHAGITVSLLGKVADIVQTSSNRLFPGVDSLLLF

EQLKAEIKEIETGFICLNIQETDLAGHAEDVVGYANRLILSDQQIGEIIPLLTEEDIL

IVMADHGNDPTIGHSNHTRERVPLMIYRKGLSSIDVGSRETMSDVAATAAEYFGVKAP

QHGVSFLDLLNN"

CDS complement(2373655..2374812)

/gene="yhfX"

/locus_tag="JFBMFIFI_02242"

/inference="ab initio prediction:Prodigal:002006"

/inference="similar to AA sequence:UniProtKB:P45550"

/codon_start=1

/transl_table=11

/product="putative protein YhfX"

/db_xref="COG:COG3457"

/translation="MFMEVTKRRNAKLIEAAIQLHQTGAILPDTYVLDLDSIEANGKQ

MQAAAVEHGIELFYMTKQFGRNPLIAQKLVELGITHAVVVDFREAQVMMDHHLPLGNV

GHLVQIPQHLLETVINYGSKYITVYSLEMLNKIDEIAKRLGKKQDVLIRIVSDEDQLY

PGQYGGFKIEELIDQLPEIKKYQSAQVKGLTSFPCFLFNSETKELEATHNVQTIQKAQ

AIFSEAGVPLRELNIPSATCSETIPFIKTLGGTQGEPGHALTGTTPMHALVDLPEKPA

MVYVSEISHQFEEHSYCFGGGYYRRGHLENVLLDNGEERYQAKMEPFADENIDYYLGI

KGKHRIGATAIMAFRTQIFVTRSQVAVVSGIQVGKPKIEGIYDSQGKLISR"

CDS complement(2374826..2375176)

/locus_tag="JFBMFIFI_02243"

/inference="ab initio prediction:Prodigal:002006"

/codon_start=1

/transl_table=11

/product="hypothetical protein"

/translation="MIQEKLTVLKTAAIIDDAVYEYIQAVIVYLEEKGLMEDSQKAEV

FLTHLAMASARQQKGESINELDKVIRDEIKTEVQFGDAKILWQELAELAPVTFHENEI

DYLYLHICTMLAKI"

CDS complement(2375195..2376118)

/locus_tag="JFBMFIFI_02244"

/inference="ab initio prediction:Prodigal:002006"

/codon_start=1

/transl_table=11

/product="hypothetical protein"

/translation="MNEHFYQKTGIVITRLAKDLMQLKKGDRLPIISEYQEQFGIARG

TVQNALQFLKDKEAIVCESKGHMGTFLTDVNYDLLQQYALSDDIVGTMPLPYSRLYEG

FATGLYEAFHQRHIRLNMAYVRGSKDRIQSVVNGIYQFAVVSRFAAKEAAKNKAPITI

VQDFGDHTYLSRHIILFAEDAKTDVEDGMRVGLDSSSFDQQTLTKELTKNKNVTFVEM

PGHQLIYALKEGQIDTGVWNYDEIVDKNYQDLNYRFIEDSQLQRDMSGAVIICQTDNA

MYQTILKKNIDKTALLEIQKQVVEGKMIPRY"

CDS complement(2376187..2377284)

/gene="yhfS"

/locus_tag="JFBMFIFI_02245"

/inference="ab initio prediction:Prodigal:002006"

/inference="similar to AA sequence:UniProtKB:P45545"

/codon_start=1

/transl_table=11

/product="putative protein YhfS"

/translation="METYPLKSITVEEATELQFALVDAVTKEFKGSEILTRGDLGIVP

GFNQPKTTNQVEKVLAHFFDGEATMLVRGAGTMAIRLAIHSMMKAGETILVHDAPIYP

TTKTSFEMMGLTVITANFNCLEEIRAVVEKETIQGVLLQFTRQKPDDSYDCGEVIAAI

RAVKPQLPIVTDDNYAALKVPKIGSQLGGTLACFSTFKLLGPEGIGCIVGSASHIQKL

KKENYSGGLQVQGFEAIAVLQGLIYAPVALAISAKVTEDVCKRLNAGEIKGVKKAYIA

NAQSKVLLVELEESNAVDVLIEAEKLGAAPNPVGAESKYEFVPMFYRLSGTFRAANPM

AEKNTIRINPMRAGSETVLRILAQSIQQTQN"

CDS complement(2377301..2378179)

/gene="php"

/locus_tag="JFBMFIFI_02246"

/inference="ab initio prediction:Prodigal:002006"

/inference="similar to AA sequence:UniProtKB:P45548"

/codon_start=1

/transl_table=11

/product="Phosphotriesterase homology protein"

/db_xref="COG:COG1735"

/translation="MPLVNGMTFTHEHTTIDLSGVKQNEDCNLNCFAETIEEYKKLYK

QGVRNIVDVTVMGMKRNPEYVRQVAKASGINIIQATGCYTERFLPEFVEVETIEEIAE

RFIKEIEVGIAGSEVKAEIIGEIGSSKNHMTIKETKVFEAAVLAHKKTGKPITTHTTL

GTYGHEQVEFFKARQVDLSRVVIGHVDLSGDSDYVLQLLRQGVYVEFDTIGKENYMPD

QTRVDMLLAIQEAGFTDKVFLSLDITRKSNLNFRGGIGYSYLIETFIPMLREGGITEK

FIQKILIDNPQTFFNL"

CDS complement(2378181..2379173)

/locus_tag="JFBMFIFI_02247"

/inference="ab initio prediction:Prodigal:002006"

/codon_start=1

/transl_table=11

/product="hypothetical protein"

/translation="MKKTVQEFANATISAYQNPYKSVSYIDPEAFNKSCVQLGKQLNP

FYFGIKETSILEPMKQAFLEAGFIFHTIDKMSANGRAIDPTLVNPITGGRMTGSSSGT

AINVLIGINDIGIGSDGGGSVLAPAMALQLYSMIHPCFPVQMVTKQSTDGLSFSPSSG

IITRDFPTLEQTVELFSFKPLRRKLTIAVVKDFPSHLKQQLSQYHTIHQIDFPNVAQK

REPLIAWLKTTIKEYDCLLSLEGPIDVESYGDSVIGSFSPISKAQQEKSGKGLLRIVN

MLDLVGVSVPYGDLAKGLLIIGEKADTNALLGLAKSIEPQIPELVERYFHKGRC"

CDS complement(2379183..2380493)

/locus_tag="JFBMFIFI_02248"

/inference="ab initio prediction:Prodigal:002006"

/codon_start=1

/transl_table=11

/product="hypothetical protein"

/translation="MDKMIELIVVGALGGLCAILANRGIAVFNDGLRPVVPEFLEGKI

GKKEIAATSFALSFGLVIGFGIPVSIGASIILVHSILLATDIIGSWSPVGKKGIVIAG

VVGALYGVGITIGLQVVVDLFAKMPVDFLSSLSLVGSPVVVAFSIFPAVAIAYQHGFK

KGAISLGATALTLFAVKKFGTFELGNGTTFTLSAEGMSLLVGMIFMIIFAIGVKSEGS

DANQNLISIFLERVNRIKKNWLILAGVGGLVSAATSLLIIAGDPISLSLLGEGKYSEA

ALAAFARGIGFIPLVFSTAIVTGVYSPAGTTFVFVVGILFRGNPIVAFIVGALVMFIE

IMLLNGMAKGLDKFPGVREMGEHIRTAMNKVLEVALLIGGALASEAIAPGIGYFWVIG

LYLLNRTAKKPLVDLAVGPIAAIALGIIVNILYLLGLFSPVVAG"

CDS complement(2380511..2380879)

/locus_tag="JFBMFIFI_02249"

/inference="ab initio prediction:Prodigal:002006"

/codon_start=1

/transl_table=11

/product="hypothetical protein"

/translation="MKTIVVGGQIDKPEVKALVEKYSAGYFTIEVKSDLEAAMAVKAG

TADYYVGACNTGGGGALAMALALLGHDQCVTVSMPGKIMPEEKIRAEVASGKKAFGFT

AQDKEKVIPILIDELKTITN"

CDS complement(2381256..2382131)

/gene="bglK_3"

/locus_tag="JFBMFIFI_02250"

/EC_number="2.7.1.85"

/inference="ab initio prediction:Prodigal:002006"

/inference="similar to AA sequence:UniProtKB:Q93LQ8"

/codon_start=1

/transl_table=11

/product="Beta-glucoside kinase"

/db_xref="COG:COG1940"

/translation="MAILAFDMGGTAVKYGVWSDGELMAQSNFPTPDTWEQLQAKLLE

VKNTLRNEFTLTGVGISAPGSVDTEKGEIGGISAIPYIHHFPIQVAFEELLELPVTIE

NDANCAALAEIWQGAAKGKKDILFVVIGTGIGGAVIVDGKLHKGKHLYGGEFGIMVLD

DGRSFSDLGTAANMAKRYSEHKGSIFTGKEVFDLAEKGETDAVEAVREFYHYLSLGLF

NLQFTLDPEMIVIGGGISEKEELFEEINQRIQTRFEEKELKDFLPVIVPCTHKNDANL

VGAVAVFKEKHPAIG"

CDS complement(2382374..2385052)

/gene="mngB_2"

/locus_tag="JFBMFIFI_02251"

/EC_number="3.2.1.-"

/inference="ab initio prediction:Prodigal:002006"

/inference="similar to AA sequence:UniProtKB:P54746"

/codon_start=1

/transl_table=11

/product="Mannosylglycerate hydrolase"

/db_xref="COG:COG0383"

/translation="MKKVSIIAHSHWDREWYMAYEQHHMRLVELMDDLLELFETDPDF

DSFHLDGQTIILDDYLQVRPEKRDLLQKYITAGKLRIGPFYILQDSFLTSSESNTRNM

LVGMEESKKWGVPVQLGYFPDTFGNMGQTPQMMLQAGFDVAAFGRGVKPTGFNNVVIN

DEKYASQYSEMWWEGPDGSKILGLLFANWYSNGNEIPVEKEAAKKFWDWKLKDAEAYA

STDHLLMMNGCDHQPVQKNLSAAIRTANELYPDIEFVQTGFDEYISAMRADLPADLST

VTGELTSQETEGWYTLANTASARIYLKQWNTLVSRQLENIAEPLATMAYESTGEYPHD

TLNYAWKTLMQNHPHDSICGCSVDEVHREMVTRFEKAHEVGKYVADEAAEKLVRSIQT

AEFSDKEAIPFVVLNTSGSKKTGLMEVRLEIKRLPFSEGKPDELYHQLKTETLPNFIV

KNAAGEEVSAEIIDEGVEFGYDLPKDKFRQPFMARYVTVQLIMEQMAPLSWETFALVY

GETSDTTEKPIISDNGYQLENDYLDVKVAKNGLLTVLDKVTEETYVDLLNFEDVGDLG

NEYIFKKPNHDQGIFSKDGQAKVNILHNSANYGEIEIIHELEIPVAMEDLLLEEQKAV

IEFRYRQAERSKETATLQLKTKVLLEKGSQQLKFTTIFDNQMRDHRLRVLFPTGIETA

VHYADSIYEVAERPNKVDPAWQNPTNPQHQHAFVNVHNENTGVTVANFGLNEYEILND

RTTIAVTLLRAVGELGDWGYFPTPEAQCLGESVVQYGISFHGAKDSTRTYHEAMAAQV

PFSSYQTTLHTGDLPTNHQYAVVAGEDVALTAFKRKENGTDIVTRVYNLTGEPAEFRL

AIANQTAQLCNLLEEPMAGAVKSTALPYEIISYRWK"

CDS complement(2385068..2386354)

/locus_tag="JFBMFIFI_02252"

/inference="ab initio prediction:Prodigal:002006"

/codon_start=1

/transl_table=11

/product="hypothetical protein"

/translation="MVYTEIPTSVQKLMNTITEKCETYPRWAENFNACFANTLLTTVN

RHQDGTTFVLTGDIPAMWLRDSTAQVRPYLVVAKEDSDIGDMIAGLVEKQLSYINLDP

YANAFNEEANNAGHQDDHTKMNPWIWERKYEIDSLCYPLQLSYLLWKATGRTEQFNQT

FDEAVKNVLEVWETEQNHEQSDYQFVRDTTREEDTLVREGQGSPIAVTGMTWSGFRPS

DDRCIYHYLVPSNMFAVVVLDYLKEIYTDILKEETLVPRIEKLRAEIQAGIEKYALVK

NKAGEIIYAYEVDGLGNYSVMDDSNVPNLIAAPYLGYCSKEEETYLATRKTLLSKENP

FYYEGEFAKGIGSSHTPENYVWPIAMAMEGLTTNDKATKERILNTLVATDAGTNLMHE

GFDVNNPDNYTREWFSWANMMFCELVMDYFDIKIEK"

CDS 2386732..2387817

/gene="araR"

/locus_tag="JFBMFIFI_02253"

/inference="ab initio prediction:Prodigal:002006"

/inference="similar to AA sequence:UniProtKB:P96711"

/codon_start=1

/transl_table=11

/product="Arabinose metabolism transcriptional repressor"

/db_xref="COG:COG1609"

/translation="MKKEPLYQLIYEHLKQDILNQRYPFGSQVPTEKELTATYQVSRI

TAKRALTELESDGFIQRTPGKGSFVSYQNQLIPTTYEIAFMLPFSQAAGLERYVQGAT

DYLDSTPYRLTIHSTEGNRQRQRELLASLTKEKWDGLIIYPENLGNSLDLVVQLQLDA

FPIVLLDKKLEGTTIPTVTSDNFSGGFLAAQHAIALGHTKITYLSTVDLLENTSVRDR

YLGYLNALHEQRLQDYSANFGGQPLLRSLETVAVQEYLKEVKRSGVTCIIAENDVVAL

RLLSEAKKLKVNIPEDISLIGFDNIQMTDLVSPQLTTIQQDFYQIGYLAAESLLTQIN

QQQNSGKHLSKIVPIELIIRESVKDCL"

CDS complement(2388183..2389319)

/locus_tag="JFBMFIFI_02254"

/inference="ab initio prediction:Prodigal:002006"

/codon_start=1

/transl_table=11

/product="hypothetical protein"

/translation="MLKFELLKIYREKTIYILSAVLALVIFAPFLIGNSQLDILEYYE

NNYQANITTIEDIKDDPTAAGTIKDIKEVNGYLGEMIKAIKNGNDKENVNSELKYENK

NLEDMTAGKLDAGPLVDQKAKVVILQYLNDKNIKKISNNVKDIGSINYLSMIFSTPQI

VLIILILISFHIVYIFNLDYRKNNFMVYNSSPKSYLQIFFTKFSANMLSVIVNMASVF

TLVLSIVGVKNGLGASRYPIATIQNNSDVSIISTSDFLLKVFTFLFLFLIFIGLLALF

VSLLSSNLILNISLIILPLILGQYDLLNTFLSENVKPFIILSYIDISLIIMGGNGFHP

ITNPSITFNNGILLLSLSVGLLLIVLFYLLSNFPKKLIYMKFLK"

CDS complement(2389323..2390012)

/gene="lptB_2"

/locus_tag="JFBMFIFI_02255"

/EC_number="3.6.3.-"

/inference="ab initio prediction:Prodigal:002006"

/inference="similar to AA sequence:UniProtKB:P0A9V1"

/codon_start=1

/transl_table=11

/product="Lipopolysaccharide export system ATP-binding

protein LptB"

/db_xref="COG:COG1137"

/translation="MKERIIIEDLSVSIKKENLIQNLSLKLKEGTITCIVAPNGTGKT

TFFKLIANVLPMKNGNITINSKSFENRVEFNRGLFFLENAEQLFQNLTAYENLKFISK

LWNDQSDINKIVDFVGITKYKDKKLKFLSLGMKQKVLIAASIASGADFIIFDEPLNGL

DIENIENISNIFLELKKEGKTLLLSSHNIFETSKICDNIFFLVEGSLKEASLDYEILK

EQYNSAFKVRR"

CDS 2390101..2390427

/locus_tag="JFBMFIFI_02256"

/inference="ab initio prediction:Prodigal:002006"

/codon_start=1

/transl_table=11

/product="hypothetical protein"

/translation="MKKKLILSITLITLFGFSFLSYPVSTNEIGNTQASVKPRAYIGW

VVKYFHQYQTRATGNWAPSLHGYEYGNISVRNGYAENGYTHLGTTSRSTWFTLEHKFS

QNYRFW"

CDS complement(2390821..2392470)

/locus_tag="JFBMFIFI_02257"

/inference="ab initio prediction:Prodigal:002006"

/codon_start=1

/transl_table=11

/product="hypothetical protein"

/translation="MNSVFKRLKNYQEVYYLGENSVFQKSKIKGKFHSQYTRYGQPNE

VAGGGFAKRELLELLPSDTEEQTDITITHRYDRNLKKDAFHLVITKGTGVELTASNER

GLKYGMEALEKAIYLSNQQVVVPELTIHHEPSFPIRGVIEGFYGIPWTHDSRLDCIHF

LGKHQMNTYMYAPKDDELQRKLWREKYPAEKIEDFEELLVASQNALVDFYYMISPGND

IVYTKEDEVGVLQQKLKQMIEIGVSHFGLLLDDIDYYLKGEAKQKFGTPGLAHAYLIK

QIYSYLKNELAHSQLVICPTEYDTCYDSVYLHELSDNLPPEVQIFWTGPETLAHEIPT

SDIAKMSEIFHHPTIIWDNTPVNDFEDDKELLFLSPYYNRSYRLNQFGVVGIVANPMS

QWELSKLTIGNMAQLMWNAPGFDLTTSWQETLADYAGSEYVESLEVFTSHNENKRMIQ

PLPLELKEALEMRDKDNLNQSLTELNQAVEKLKTLPNKTFQKEIAPWFKRMEQDYQLW

QAILAEDHELVRKLGTELKGAKVRIGTNLPLTAALLWGLVD"

CDS complement(2392676..2393389)

/gene="yydK"

/locus_tag="JFBMFIFI_02258"

/inference="ab initio prediction:Prodigal:002006"

/inference="similar to AA sequence:UniProtKB:Q45591"

/codon_start=1

/transl_table=11

/product="putative HTH-type transcriptional regulator

YydK"

/db_xref="COG:COG2188"

/translation="MKYEAFVELLEQDINNGRYDQDGKLPSEDVLIESYQVTRYTLRK

AIAFLVERGQLYQVQGSGIYLRRRAKKGYFNLDDTQGITSESRGINQNVETKVVSLEE

IQFKDAAFVPEGLSLSPETILYFVKRLRLLDGKPFVVEHSYYIKSLVPYLNHEIVEQS

IFHYLKEVVKLKFGFADKIIRTEKLTQEVATLLELKKDDPTLVIIDEAYLSNGALFNY

SKLYYHYENSEFFMHATMK"

CDS complement(2393481..2394995)

/gene="bglA_1"

/locus_tag="JFBMFIFI_02259"

/EC_number="3.2.1.86"

/inference="ab initio prediction:Prodigal:002006"

/inference="similar to AA sequence:UniProtKB:Q46829"

/codon_start=1

/transl_table=11

/product="6-phospho-beta-glucosidase BglA"

/db_xref="COG:COG2723"

/translation="MTKSVTSNSPIFLTSLTGSTNFLYKERENMKKDFLWGGAVAAHQ

IEGGWDQGGKGVSIADVMTAGANGVPREITNGVIPGKNYPNHEAIDFYHHYKEDIALF

AEMGMKCFRTSIAWSRIFPNGDDLEPNEAGLQFYDDLIDELLKYDIEPIITLSHFEFP

YHLYTEYGGFRNKKLIDFFVKYADVVMNRYKDKVMYWMTFNEINNQAGGHEEIHNWTN

SAVKIEAGENPEEVIYQAALYELIASAKVVKLGHQINPEFKIGCMMAYVPIYSYSCNP

ADMMASVKGMEQRYFYNDIHARGEIPTYTLKEWEAKGYHIEYTPEDLKALKEGTVDYI

GFSYYMSAAITTLEEIAGHKLTETGPEKIVTNPYVKASDWGWQIDPIGLRYVLTTVYQ

RYNLPLFIVENGFGAYDKIAADGTINDDYRIDYLKAHITEMKKAVDEDGVDLLGYTPW

GVIDIVSFGTGEMEKRYGFIHVDKDNQGQGTLKRTKKKSFEWYKKVISSNGEIL"

CDS complement(2395059..2396354)

/gene="licC_9"

/locus_tag="JFBMFIFI_02260"

/inference="ab initio prediction:Prodigal:002006"

/inference="similar to AA sequence:UniProtKB:P46317"

/codon_start=1

/transl_table=11

/product="Lichenan permease IIC component"

/db_xref="COG:COG1455"

/translation="MSGMLDNLLNKFLKFANTKTITALKDGFVLTMPITLVGSLFLLI

ANLPFQNYGAFMARTFGENWNIGLNQVSANTFDILAMVIAIGIGYAYATNEKVDGISC

GILSFVSFLIVSASSTVTESNEIVTGIIPRAWTGGNGVIAAILVGIFVGKIFCFFMKR

NITIKMPAGVPVGVANSFAALIPGFVIILISMVVYQLCMMIANLSLTELIFKVLQTPM

QNLTDTWVGGILIVLLMSVLFWAGLHGPNIIMGVMAPILTANSIANQGIVESGKALTM

ANGARVMTPQLIDNFVKFGGTGITLGLLIAALLAAKSKQMKEISKLSIVPGFFNINEP

VIFGLPIVFNPIMLIPFILVPLIGLIITYGAITVGFLTPFNNVQVPWTTPPIISGFIL

GGFKGALIQVIILVLSVVIYFPFMKKQDQQNLLAEAEEK"

CDS 2396525..2397649

/gene="thiO"

/locus_tag="JFBMFIFI_02261"

/EC_number="1.4.3.19"

/inference="ab initio prediction:Prodigal:002006"

/inference="similar to AA sequence:UniProtKB:Q88Q83"

/codon_start=1

/transl_table=11

/product="Glycine oxidase"

/translation="MKNKRIGIVGAGIVGATTAFYLAKAGYSVTLYDDGNGQATAAAA

GIICPWLSQRRNKEWYQLAASGAAFYPQLMHDLGEDYSDSEIYQQVGALIFKKNPATL

LKLEKLALKRRETAPEIGELQILSPSEIEAKIPLLKTTESALFASGGARVDGHLLTKK

LIEMLLDNGGTFIAGKITLHQAEDHSFKIHAPTGVESYQQIILTVGAWLPELLTPLGY

SVDIRPQKGQLAHLHLSTVDNSQQWPVVIPHGEKDIIPFSHGHILVGATHENDLGYDL

SPTSPEIKAMITEASDLAPELSTADLIETKVGTRAYTSDFAPFFGPIPDNPQLYVASG

LGSSGLTTGPLIGKMLAQLVLEQETALAVEDYPVKNYVKR"

CDS complement(2397692..2398462)

/locus_tag="JFBMFIFI_02262"

/inference="ab initio prediction:Prodigal:002006"

/codon_start=1

/transl_table=11

/product="hypothetical protein"

/translation="MSIGWLYFIVIILANTVGSLSGMGGGVIIKPVLDAIGAHSVGTI

SFYSSIAVFTMAIVSTIKQVKNGVTVKPKTVLFLSLGSVLGGIIGNVTFEGLLRIYSD

EQVVQKIQISLTIVSLLFAIVYTHYKWSSWQLTKIHWYFSAGLFLGFLATLLGIGGGP

INVALLMLCFGFPIKEATVYSIVTILFSQFSKLIATGLSTGYQIFDLSLLSFIIPAAI

FGGFLGAQLSGILSDKRVLQVYQFVVVAVIGLNLWNLF"

CDS complement(2398588..2399649)

/locus_tag="JFBMFIFI_02263"

/inference="ab initio prediction:Prodigal:002006"

/codon_start=1

/transl_table=11

/product="hypothetical protein"

/translation="MINLVINESRKLIFRRSFIVYLIIIFGLVALVGGINKYAYSLNS

NEYFEQKAGDEGEPVKKTIKKGIPVFTYSEDGKPVTNLEEAVKISRSNLLAAQAKEKE

DYPNEIAYAQKELDYYEAYLKKGVTPITTNNGGESAGSFFSSLGGILSIVNMLVVVVA

SMMVASEFSDGTIKLLLTRPHKRSQILLSKLIVCLLFAAFVTLFTMVAAGIVGAILFP

VQSFMLPASTMLGTMSALKAGFVLAGTNYLLMVLYISVALMISAVFRSQALAVGIAML

MVFSSSIINTFLSLLIPKWEPLKWIVFNLLNINELGRGNSIPGDISLVAAGIGLLLYS

ILIYMLTVYLFKKRDVALT"

CDS complement(2399646..2400572)

/gene="btuD_4"

/locus_tag="JFBMFIFI_02264"

/EC_number="7.6.2.8"

/inference="ab initio prediction:Prodigal:002006"

/inference="protein motif:HAMAP:MF_01005"

/codon_start=1

/transl_table=11

/product="Vitamin B12 import ATP-binding protein BtuD"

/translation="MEEEVLLAKGLTKQVGRKILVKEVDISIGKGEIVGLLGPNGAGK

TTIIRMLTGLISRNAGTVKINSNDLDGNFEGCMEDVGAIIENPEFYKYLSGWENLKQF

ARMSRKTITDEEIKKVVERVRLTESIQNKVKTYSLGMRQRLGIAQAIIHHPSLLILDE

PLNGVDPKGMHDFRQLMREIVQDGTSILISSHLLSEMELLADKIIIIEEGKLTHIEDL

TKADEKPENSLLHITIETPEQSLLQKEVALMGLTVIAHSANSLTIEIEKNQIPAFIEH

GVQKGIPFYGITPHVSTLEERFLELTKGGSLR"

CDS complement(2401057..2403453)

/locus_tag="JFBMFIFI_02265"

/inference="ab initio prediction:Prodigal:002006"

/codon_start=1

/transl_table=11

/product="hypothetical protein"

/translation="MKKKYLGGVLLLNSLLGRTIAPEEAKAIKDIDESVQQVNEASNG

EKSPDGSWLLTTLDGHSLGYDINQNVFVVDLSSQVKVNYLGTNTGEMAYRILTSDSTE

ELEIGEYVDGDIIDLSAYTDGLYYAEISKKKSQGSQLNPDTIYVLMNKESLLVSIEIV

GPHGMINPEKVTYVVEPEISELQWTIKDEEGNFVLDGEGSSIEDEILTTFSGSTYLIN

ATVKAQSKYGNQLKNQSESRFMIRHPKSDMKMDQTYNPTSIVGIKNIDESRLEWKVSE

TSGRLIESGEGEILSEEFLRLLPENTYEISFTETSPENETHISKKVFNIRFPESTTTI

DRPFNPRVITGEKKIPESTLEWSIKDVFGTSIESDSGAEVPQMILLNLLPGEYIIYFT

ETSPEGDAHTSNGTFSIRHPESTIAIDRPYNPRYIVGEKSIPESLVEWVITDMGGRES

AWGNEESISEDILMNLPEGTYLVDFTEISPEEERHTSQGTFTIRYPEAILTMDRPYNP

RSICGEQKILTSSLTWVIKDESGSVIEHGSGDEIPQELIQNLPESIYVAGFKETSPEE

ETHTVWEEFRVRYPESSTMVNRVYNPTVVTGGKKIAESRLEWIIKGDSDEIVDSGEGS

QIPYEQIAHLEEGNYVVEYTEISPEEETHTSNGRFIIRYPESVTTIDRVYNPKSVTGK

KKIPDSRLEWAIRDEEGNVIESNEGNKIPQRIIVRLPEGDYLAEFTEKSLEDKIHVSN

GRFTIVRLVDPEESEESSKKDLPTKHDPEDDPSTLIVYYQEVRILSTGEGSPKEMIVS

"

CDS complement(2403525..2406704)

/locus_tag="JFBMFIFI_02266"

/inference="ab initio prediction:Prodigal:002006"

/codon_start=1

/transl_table=11

/product="hypothetical protein"

/translation="MNKLRNKLVGATLLSMLAVPMIIGVLHVPIGLKAITEADISAEK

ESISMVNSVPRPGENIVVTKENFLEHFTLSGTAAYSSDTGIVTLTPDLPSSAGSFTMD

SQIDLASSFYLTGRVNLGNKAEQQGGADGMGFGFHSGDVHSIGRHGNGMGLAQLPNVF

GFKLDTYYNGSHQNSEGIAADPWLNNPTGSRVAAFGAFVHSPNAILTTEQNSMLEIPA

PSNNQFRDFYMAYDTIEDKVKIVYNGQIWERDISTWHTGLDALSFIVSASTGQYHNLQ

QFEFTSMEYTPKVFEGNAVQQQVTLGESLATRDLQSFVTNVTYGDIPLNPNEYNVTLK

DIENDLVGTGKATVIVEHNFLGVRTEIEVPLDVRWGDTILAKGWGDLSVGAYTYHPAE

NKVTANQGTINSSTQVHPNFTNRYYSVNLYNLTSGAQKITDARNYVSHSVTGQVSPFD

AINQFSNGTRVASTQVGDIIELTHEESPNRLRQIVSGQEQPLRTNKNTSFLELTVSGY

QQLHFNLLNVKSNGEISKWTTNDEIDGMLGEFLTNSDFETVRIIGFSEYPDRSTVGNS

IGKILVEESLSTGNKVQYEYEVNFEVAAVPESIEVSPKIATIKTGETQQLSAKVLPEN
SVNTDLKWTSSNEELATVDEEGIVFGKRRGEVEISVETTNGLTDRATIQIVNIEIPSI
DPSNPSLEVDKENPNEGSLAIRYASSLNFGETTVVGNGQELIALPSKDQDGRDILNMV
TIQDIRPEAQRDGWQLQVRQTRELIGGLQIIMTPFVHESHLAGDSISVYEGDFIPNTD
SQIFASASEANSNQIISFGMHNPQEEGVRLLIPPGMGAGSYQTNLHWNLVSGPNQKRE
DFSFRFSGTEGNSEASVTGYNGSGKNVTIPREVTNTAAGWTTPTPVTSIGDGAFRNKG
LTSLQIPNSITSIGYQALRENQLKTLILPNSLISIGNGALEHNQLTEIEIPDTVTSIG
EWAFNDNQLATVGMSKSVETFGNLVFRENPLAEINVESESEAERLTHLLTPTVMNGVT
ELTVLRTNKQKFYQLDALNRRLWLNLENE"

CDS complement(2406747..2408183)
/locus_tag="JFBMFIFI_02267"
/inference="ab initio prediction:Prodigal:002006"
/codon_start=1
/transl_table=11
/product="hypothetical protein"
/translation="MEIPKLLEIFGEGIGRKMLLFSMLFDKEPLTKKKLEVLPYSYKT
LQSDLEQLAEILKKWDGDIRLSKYYNASLVYYKLEREAFFAPQTVISYICQKTLEFKI
LELFLYGNLKKVHQFLLEHGIGYTTYYRVLRKISGLLQKYGISINTNSLELVGKESEI
RLFYFQFLWTLCEGFGWPFKNSDEKKMIERAAIITKKHGIGVIETRKLTYWLAICEVR
ENRKITIKTEEIPNEIIKLAEEPIYENLVETLFTESQHKLGNVQRSETECYFIFWMIF
VDCVTDIRIDGASFEQISSRFIPIINEISIDRKLYIDFLKLIKWQENDGVKVSVSLLL
HSIKINSFISAFSQERIVEELRYFMTKFLNHKIELMLHDTSTLSLLKSEMKNLVAKIQ
HVKINIFLISKYGDFMKNRILKQLSSDLKKQVNWLALNEEISETTIIFTDSPIAMNPP
YKKIIYFSHPVCIKEIEKLIKLNLNYIM"

CDS complement(2408213..2409322)
/locus_tag="JFBMFIFI_02268"
/inference="ab initio prediction:Prodigal:002006"
/codon_start=1
/transl_table=11
/product="hypothetical protein"
/translation="MIKIMCIFNLIVGFTFFTTSLAEANNAPKFSVSPIIPENQINDV
SSYFNLRMEAGKEQNIEVELTNNRDSSIEIEIKAVTAKSNQNGVIDYSQLDEDLDPSL
LISFSEISEVEQLIVLKANESKKVPITIKLPSVKFDGVILGGLNFSEKIKAGEEHTQI

SNQFSYSLAVMISQTDEEVLPILNLEGISAAQSNYRNVINAEIQNSQAVIVTDMSIDA

QVYRKNEKEPLFNRRVSNFQMAPNSRLPFYIHTGSAPLVAGDYRLEMQATVNGENWEW

EEEFTIDEKMAVALNKTAVDLDSKQNGLIYLLVGSGLTIGLLSFGVLINSYVKKEKEK

KALEEKRKARREKKQLLERKKRNALKKNQIKRIQD"

CDS complement(2409589..2409807)

/locus_tag="JFBMFIFI_02269"

/inference="ab initio prediction:Prodigal:002006"

/codon_start=1

/transl_table=11

/product="hypothetical protein"

/translation="MGIEDTWEDCTIYLVGFLVKQDEKSKLIHKTLIFSSCHSINNVK

EFIHEKFKGVTHIDYIDIWNDDALRLKH"

CDS complement(2409821..2412784)

/locus_tag="JFBMFIFI_02270"

/inference="ab initio prediction:Prodigal:002006"

/codon_start=1

/transl_table=11

/product="hypothetical protein"

/translation="MKKNIYCLIAVLLVGMPVVLPSLSQTFLQGIQAEEKEFEMYEES

EVNGLKNEEFSGTNLHEEGELESPILEIGEANIEAQIKDEYSVELEPEIAPANVAIIT

NEVELRAALANPNVLEIRISGGGVGADIFEGALEVNRRVIFRGGTLNFYNDSYIKVTQ

TGELVVRRERMDSALTLNARSGSTVDSLIVIDGGKLDNQLTSDMDQLTIRNYSDNKMA

AVQTLNDGEFYNFATLRIGQTTNLDSHGRAEFGIGIKMDSGKFVDRPGGNQITAGYIY

ASKAFVSSSGSNMMQFEYKTQGAGSTMISSEVDSPIFTNLDSATVSISLDSIFTSLVS

IESISENYQFGMVNVQFIATQTGRDSPVISYLNDDRYVHMVTSGQNILGRFRSARVQT

TVANIPPVNLPTNIEIIPNSVRMLPNDSVGLAIDFTPNINELDNTSIFWWSDNDQVVT

VNQNGVITGKSVGEAEIYAETINGLRAVSSVIVEEFTFAFSGTDGNSTAIVTGYHGTD

TEIEIPSTAVNREAGWTTTSTVIGIDDRVFMDKGLTNVLIPGSITEIGEAAFYKNKLS

TLIIPDTVKSIGQQAFLENNLTELYIGSGVSRLENQVFGLNNLSSIVIPDTIQQIETL

AFHSNNLAEVTIPKTVTHLGLRSFENNSLEKVTFEGEITGLGAQVFAQNPLKEIYVEE

SSLVHYQRELSPNIIRGVTERTLLTVDEPRYSDDTQLINNLMVGDELSFSVLSKNRYQ

LDNLEAFVWEEFIPNVEWFKDAQALPTETKQQFRIAEVEESDSGSYYAIVDGSELPSL

SVEVSPLIQPDIPAINPEDGILDLENTNPVIEGLSLRYVSDLYFDATTFSSSNQTVYA

NDNDGIPKVTVQDMRSASKRNGWELQVRQNQLFMDGAELIFNPFVHEINQDLLNIKSH
NGALTVNTEAQRFAGTTTTGNPSGIATMGMGSVDGSGVSLQIPGGVGVGIYEATLIWN
LVATPSTANEHIAGK"

CDS complement(2412808..2413833)

/locus_tag="JFBMFIFI_02271"

/inference="ab initio prediction:Prodigal:002006"

/codon_start=1

/transl_table=11

/product="hypothetical protein"

/translation="MNKKIRWAFLLALMIIGWFGFELKSSATAPEFSVKTILTENQRD
ASVSYFDLLLDKGATEELQVEITNSSERELTVELQATTAVTNINGLIDYSIDVEEPDD
SLKIDFKEITSIKESEITIPRGESRILTALVQLPKEQIFDGILLGGLTISEKESTKDS
SETENQITNRFVYSIAVMIAQSENAVQPDLSLKSVEVEQRNRRNFIVGNIQNRASTIV
NHFDVEATIFKEGETTPLFHEERVDMRMAPNSTLAYGIGTNNQPLSAGTYEMHMKATN
GEEEWKWVESFTITNEKAREMNNSAVELEKDNTRLYLIIAGIVIVILFIVILVLMNRE
KFFRKKK"

CDS complement(2413928..2414608)

/locus_tag="JFBMFIFI_02272"

/inference="ab initio prediction:Prodigal:002006"

/codon_start=1

/transl_table=11

/product="hypothetical protein"

/translation="MLRKNLSIALVATTVLGMGATTRMASAVEYNGATNATTDVQAEI
KLPDSTEIEIPPVDPENPVPPEIETNPDLGDGLAINYVSDLNFGTADFNQTAGQTLVA
NPDSVGGAEFPNMVTITDIRNEDTRNGWELTVTQDADLFSGGIIKMNPTVNQANTNGV
TAATQLSLNNQAQRFASANNSNPGQAGTTSIGMGEVSLEIPAGTGVGSYDSTLTWNLV
DGPVAPPV"

CDS 2415106..2416548

/locus_tag="JFBMFIFI_02273"

/inference="ab initio prediction:Prodigal:002006"

/codon_start=1

/transl_table=11

/product="hypothetical protein"

/translation="MNDLGYKLLVSKSVRRKTTILSMLSESSTPIPLESLWIACQSTK
GTVQQDISHLIEMFPNDIELITHNFLPSLKKKATSNVITSYIDLLARDNPLFYVIEQI
FQGKKESIEDYAESLYISESTLRKFLTILKDVLAEYDLVLTMNPVDIIGKEINIRYFY
FQYFRYSHESSSLSVRDNQFNFIHDTLKKISTSYGLVLNVDYSRIISWLTIFEQRITL
GHKINLEQKLIEKYKYKPSFLKFEAAVRLHFRKINFSLDLSEEELVFAYLTRLDAIIY
EKDKPFFTDDVFDTLQEFDHLTTTFLQLANLDICLNINLKAILQAFLSNLTSLTELSP
LFQLNHNRLKKIVETNHRDTLDTWLEILDNYSTFNNSYDVAISLTLLTESQVQNKKKI
LFALTGEPSSITYYKVLALQCVPRGMEAVFIFNRPLNNALLEQLDIDLCVYNLTPFEE
LTESELFRLSDIPLNSEWVELLALLHNISH"

CDS complement(2416645..2417664)

/locus_tag="JFBMFIFI_02274"

/inference="ab initio prediction:Prodigal:002006"

/codon_start=1

/transl_table=11

/product="hypothetical protein"

/translation="MKNSFLLVVTILTISLSLVVQPFTVTANSADFSVKAILPENQRT
ANISYFDVKLLPKEEMTFQVEIENHSEATKTYEIKLNAATTNMNGVINYGEPGEDEER
DSSLKINFGEIANTEPLVSIEAGAKKNISIHVSMPNEVIDGVILGGITIFEAEEIGEK
KEQITNRFSYSVGVLISQNDERQPIDLNFKGVNVTQENRRNMISAHIQNTSARIINGL
SVDTRIYRENQENPLFHREANDLRMAPNSNFNFGVETNNQALRAGRYTMSTKAQIQDE
VWEWSEEFEITADQARELNSTAVDLETNNMRLYFYIALGVIILLAISLVILILVWKKR
SKAKN"

CDS complement(2417739..2418440)

/locus_tag="JFBMFIFI_02275"

/inference="ab initio prediction:Prodigal:002006"

/codon_start=1

/transl_table=11

/product="hypothetical protein"

/translation="MKIKLVSAAILTAMGITALGTVAHAADYPDAKTGISDVNVSVEI
GEDGGPTIPPIDPENPEPPVDPGPGNPHIGELSIRYISNLVFPTLPASTETTVVYADQ
DRNENDETFDNMVTIQDFRNDANRDGWELSVKQDTNSFIPGSFITMTPYVHPTIAAEY
QIQTPSASGLRLNNAEQVFARTESQENPSGIVSIGFANPEMDGVELSVPANTPVGDYK
TTITWNLSSAPLEDN"

CDS complement(2418437..2418790)

/locus_tag="JFBMFIFI_02276"

/inference="ab initio prediction:Prodigal:002006"

/codon_start=1

/transl_table=11

/product="hypothetical protein"

/translation="MNKQKQYLVILVLLLFSTSMQSDYADAANYIDSVNANTEVTISI

VEASESEIDGGISDNQENINENTKSSPKFNLPQTGERRTQVWFSILIGSCICLFAKAF

QSKFGKKKILGEINQ"

CDS complement(2419244..2419972)

/gene="srtB"

/locus_tag="JFBMFIFI_02277"

/EC_number="3.4.22.-"

/inference="ab initio prediction:Prodigal:002006"

/inference="similar to AA sequence:UniProtKB:Q8Y588"

/codon_start=1

/transl_table=11

/product="Sortase B"

/db_xref="COG:COG4509"

/translation="MMRKVFNLVMLAILLFSGYQIINYWVENSHNKTILSDIQTVYQQ

RKNEPHDANQLRPAFVELQKKNPDMIGWISIEGTEVDYPILQSLDNEYYLKHNYQSEV

ARAGSIFKDYRNQSEDEKNTIIYGHNMKDGSMFASINNYLNPDFYKEHPTFEYETETK

SYTVEVFAAYETTTDFYYIETNFQEPGSFTTYLDSVKLKSTITTNVEVSENDQILTLS

TCDSGLDYGKGRFVVQGKLVEKSK"

CDS complement(2419962..2420969)

/gene="isdF"

/locus_tag="JFBMFIFI_02278"

/inference="ab initio prediction:Prodigal:002006"

/inference="similar to AA sequence:UniProtKB:Q8NX64"

/codon_start=1

/transl_table=11

/product="putative heme-iron transport system permease

protein IsdF"

/translation="MNNKVYGFLFVVIALVGTLIWALLSGSLHVPLSELIQGLITGEN
EKVLIIKDLRFPRIILAFFAGAALSVSGLLLQAILRNPLADAGIIGISAGAKFFTLLI
LAIAPQFYFWTPFFAFLGGVVACCLVFGFSFKVGLKPLHLIVVGVAVNAIFTGLNDIL
TSSMVQISSQTAGNAINFGMKTWGDVQLLVSYVAIGLLVTFFIAKWCNLLALEDTTLY
SLGVPVLKMRFFIALLAVLLASVTTAIVGVISFVGLLVPHLARRLLKTNDYRILIPYS
SLLGGLLVLFADTLGRNLMAQMEIPAAIIMLIIGGPFLLFLMWRSTSIAGKNGGEKYD
A"

CDS complement(2420959..2421861)

/gene="isdE"

/locus_tag="JFBMFIFI_02279"

/inference="ab initio prediction:Prodigal:002006"

/inference="similar to AA sequence:UniProtKB:Q7A652"

/codon_start=1

/transl_table=11

/product="High-affinity heme uptake system protein IsdE"

/translation="MKKQLMLVLTLLVLLLAACGNEKNPNEKENSARVDDTVTQEKKE
QRVIATTVASAEIMAKLDYPLVGIPTTSKELPKQYKDVVEVGSPMGPDLEIMRALKPD
LVLSTSTLQADLEDGLTAAKLKTNFLDFRSIASMEKEIKTLGEQLNRQSEAEQLTQSI
DQKVAKAQTKVIQDKKPKVLILMGIPGSYLVVTEKAYIGDLVRLAGGENVITGQEQEY
LASNTEFLQQANPDIILRAAHGMPKEVVEMFDEEFKTNDNWKHFNAVKNNRVYDLDEN
LFGMTGSLNAPTALTKLVELFYEQ"

CDS complement(2421930..2424071)

/locus_tag="JFBMFIFI_02280"

/inference="ab initio prediction:Prodigal:002006"

/codon_start=1

/transl_table=11

/product="hypothetical protein"

/translation="MKKMFKKLNSCLVITLLFFSLVPSYTAIAEEPLLKDGGEYQVQL
NFYKDQTGGSTKESSAANAYLDHTGTIKVENNQPYLYVTVKNSGWWKNFATSKSGERP
ATPVPAENYQGTYQDVEVISENPADNTRKVRIKMESLDKIIFSYMHINVDTVPGFNYD
NWYQVDLTIDPSSLVVVSEPVIEEPSIPVVPTELADGTYKVPFTAKHATEEKASSMQK
YFDNPVWLKVENGKKTIAMNLNDSKTVTSFQTEVGGSLVETGIVSKDEAQNSRIVTFD
VPNLTTDLNAHVAYQVDMGGGKLYNGQANFRLLFDPTQAVAIPSNEFPSAPEPEKPTE

PTELADGTYKVPFTAKHATEEKASSMQKYFDNPVWLKVENGKKTIAMNLNDSKTVTSF

QTEVGGSLVETGIVSKDEAQNSRIVTFDVPNLTTDLNAHVAYQVDMGGGKLYNGQANF

RLLFDSTQAVGIQSSEFPSAPEPEKPVTPVDPEKPTDPEKPINPVKPADPTNLENNHQ

YSVNFSVMKADESEKSMMDQYTLKPGIIKVEKGQPYLYLTLKSSHWIKTFKTEQNGLW

SDAAVVSNDTEKDYRVVKYPIANGFAKTKAQTRVTITDMPGLNYDEEYTVSINLDATS

LKDITGQTPPVQLPTSPTTNGKVEPANEGTPEKLAKPNFLSANVASAQESKPVTKTKN

VQTSDSASIAIYSFIFILASGYFMRRYQLKK"

CDS complement(2424447..2425097)

/gene="hbp1"

/locus_tag="JFBMFIFI_02281"

/inference="ab initio prediction:Prodigal:002006"

/inference="similar to AA sequence:UniProtKB:Q8Y585"

/codon_start=1

/transl_table=11

/product="Hemin/hemoglobin-binding protein 1"

/db_xref="COG:COG5386"

/translation="MNKVNLAWLAVFALLGSFFISPKVAFAALDDGTYSINYTVVQGD

GGSASMANDYFDKPATLVVANGQSSIQLQLNHSKWITGLWVDAGNGLQSEQIISTDDS

LDTRKVSFQTGDLSAPIAAKIKVDIENSDLSYHHEYKINLSFDLQSASLIAGSAVEKT

TETQGDVKAPEVAGASTTEKVPNPKSGDQTPIYLYSLLFIGALGSLFVFRKTAKVK"

CDS 2425461..2425688

/locus_tag="JFBMFIFI_02282"

/inference="ab initio prediction:Prodigal:002006"

/codon_start=1

/transl_table=11

/product="hypothetical protein"

/translation="MKKDYGDFIANKVYEFRVLKKMSQKDLADKIGVSKQTIFVMEKG

NYVPSLLLAFRIAEYFNVEITDIFIYKKEEK"

CDS 2425688..2426008

/locus_tag="JFBMFIFI_02283"

/inference="ab initio prediction:Prodigal:002006"

/codon_start=1

/transl_table=11

/product="hypothetical protein"

/translation="MYFIEIIDNQLGEINNFNIFIGITLLLIITSVGVVYFIGKKMGS

DERSESILTKMGSIGFIVYILLNSLIINTNVLHYKQFLTMNLSLSLIVATAYLIIRYR

KDLK"

CDS complement(2426448..2430665)

/locus_tag="JFBMFIFI_02284"

/inference="ab initio prediction:Prodigal:002006"

/codon_start=1

/transl_table=11

/product="hypothetical protein"

/translation="MKNKEQTRLKMNAKSLKKMMVVSTLTLMLASSSIPSIAYAVETE

ETTDSSISTELEGEQTSILTEGIPVISKEEAEHEVPETETEDSTKEESQVIPEASVNE

ESQGTPETATKEEGQVAPEADSKTTSEENSNTRANTSSVLVAERVEEVSTETELRDAL

LGNASKIKLLQSISLVKYISIDKNITIDLNNNLLNVGSQYLWLKAESVRFENGRINGT

RPSYGTIAGDVKATNQKITFNNILFSGTNMTASTSANFDFTFEGKNEIRSQINEMKNI

VVKKNATLTVVGGGIKFLNTTESGGFILEDNAIVDMSNEYDNTIPVTWAKNIDIGENA

SFTASGKYMIFNSALPPYGVIIPLNINAKKGSTFNVNSNSKDAKAAVFSKGNYNFTFD

HLQHLNMGGSVAQPIFGAGSIGNITIKDSELNAWLIKDSATNGKPNSTWDIQDRFEIS

NFRDGYRLIYCNNPVVEKEFGQLRDYKRISNGAVTAKPTFVGIQNKIVVQVGTEFNPL

NGVTATDKIDGDLTDKIVYTVEDGKSIDTSKPGNYTIQYTVKNSNGNEAQAVTELIVE

EKKIVQTTISDLDTTSTTVSGLGEPNGLIEIKANQQVIAKGTVGSDGKYTIQIPKQST

GVTVTTSVKANNQTSEASTIVKNGEIAKTTISDLDTTSTTVSGLGEPNGLIEIKVNQQ

VIATGTIGSDGKYTIQIPKQSIGVTVTASVKANNQTSEASTIVKNGEIAKTTISKIDN

ATTFVSGTGEPDGAITLSANGNILASGKVDSAGKYSFTIAKQAVGVTVLAKVTLNGKE

SEASTIVTKAPEKVVAPVIHDYYTTDINAKGTIGGSAKQVAIYVNGVKKRTAAVTNGS

FTIYTGDLGLTVAGQSFQIAGLYDGVEGPKTTKIVEARNQLIAPTINDYYTTDANVSG

TITGSAKQVAIFVDGVQKRTAAVKNGKYVIYTGDLGLTTPGKKFQVAGIDGIMVGPKT

EATVKNKQQLVAPTINDYYTTNVNASGTIGGTAKQVAIFVDGVQKRTAAVKNGKYVIY

TGDLGLTTAGKTFEIAGIDGSAIGPKTKMIVKQKEQLAVPKINDYYTTDVNASGTITG

SVEQVAIFVDGVQKRTAAVNGGKYVIYTGDLGLTTAGKTFEIAGIAGSAIGPKTKMIV

KQKVQLVVPKINDYYTTDVNASGTITGSVEKVAIFVDGVQKRTAAVNGGKYVIYTGDL

GLTTAGKKFQIAGISSLTVGPKAEMTVKQKVQLAAPQINPYYTTDTTASGTISGDVEK

VAIFVDGVEKRKTTVVDGKYTIYSGDLGLTTAGKVFEIAGVLGLTIGPKTKMVVQQKS

QLAPPQINSYYTTDTFVSGTVTGEAERVAIFVDGIQKRTTTVLGGKYTIYSGDLGLTT

AGKTFEIAGIKDSIVGMKAKMTVLEKL"

CDS complement(2430815..2431768)

/gene="wbgU_2"

/locus_tag="JFBMFIFI_02285"

/EC_number="5.1.3.7"

/inference="ab initio prediction:Prodigal:002006"

/inference="similar to AA sequence:UniProtKB:Q7BJX9"

/codon_start=1

/transl_table=11

/product="UDP-N-acetylglucosamine 4-epimerase"

/translation="METILITGGAGFIGSHLVNHYGTYAKVVVVDNLSMGHRENILPS

ENVVFIKEDIGNKKLLNQLFKEFTFDYVFHLAAVANVAESIEFPWSTHLINQDATLLL

LEQVKKQKQLLKRFVFASSASVYGDSPHAVQTEGDAVQPLSPYALDKYASEQFTLMYH

RLYGVKTTAVRFFNVYGENQNPNSPYSGVLSLLNQGLKTNGNSEYFEFTKYGDGEQTR

DFVYVQDVIQALLLVSEKEKAIGEVYNIGTGAKTSLNQLLVLSQSLSQKKFCIQTKLA

RKGDIMNSLANITKIKELGYQPFYSIDKGIVCYWKRSIERR"

CDS complement(2431788..2433107)

/gene="wbpA_2"

/locus_tag="JFBMFIFI_02286"

/EC_number="1.1.1.136"

/inference="ab initio prediction:Prodigal:002006"

/inference="similar to AA sequence:UniProtKB:G3XD94"

/codon_start=1

/transl_table=11

/product="UDP-N-acetyl-D-glucosamine 6-dehydrogenase"

/db_xref="COG:COG0677"

/translation="MRDQFITKINNRTATIGVIGLGYVGLPLALSFAEAGFYVTGFDK

SEVKIALLKQGKSDILDISSETVQMAFSTGHFQVSSQKESLKGLDCFIICVPTPLTDS

YEPDISYIQEALDTIHSEWESQTLVILESTTYPGTSKELIHEPLIQQGYLADQDFMVA

YSPERVDPGNKKFKIKNTPKVVGGTTENSLEISTLLYQSIVEQVVPVTSTEVAEMSKL

LENTFRSINIAFINEMAILCEQMGIDIWETIQASETKPFGYMKFLPGPGIGGHCIPLD

PMYLNWKAKKSNHSSRLIELAQEINREMPKHVASKAKEALLQHNKVLKGSKILLMGMA

YKENSNDLRESPSLQIFADLQKAGSMVEFCDPLTTYFIDKHGYKQESIELDYTLMETY

DLVILLVNHDCFDRNKIANKSQLILDTKNSLNNQYPEKTIRLGDKAK"

CDS complement(2433107..2434534)

/gene="rfbX_2"

/locus_tag="JFBMFIFI_02287"

/inference="ab initio prediction:Prodigal:002006"

/inference="similar to AA sequence:UniProtKB:P37746"

/codon_start=1

/transl_table=11

/product="Putative O-antigen transporter"

/db_xref="COG:COG2244"

/translation="MSHKLLRNYGSIIVYQFVTILTPLVTTPYISRIFGPKGIGLEAY

LYSFIQLFSLLILLGLPMYASKKIAETDERNMNQLFSELFSFQIIMTGATASLYGLFI

FYFNQHQFLLILYLFTLISTGLDTSWYFVGKEKISAIMWRNIAVRVCNIGAIFLFIQT

NNDLWKYILINGLTLFLGQFLTGSIALSEVGGFTFVVRTIGKHISPIVLLFVVPSMLT

LTLSINKLLLENFNGAVEVGVFNQAYKLYVIAISFISALTSVLMPKMSKYYTTGDTER

FKKYLNFSLRFISGLALPMTIGVLVMAPQFVSWFLGEEFSEVMNPLVIISFSFVFKGL

SDVIGIQYLVVSNKNKEYALSVIVGAVVTSSCCFLFLQLNFKAQAPALALFLGTAVTL

LIELYQVRKVVSFMFVGKYLIKYAVFSLIMGGCMWGASRSIEIERTSLYLVTQVGVGV

ISYSICFILSKDPLIQLIKRKKREI"

CDS complement(2434524..2435687)

/locus_tag="JFBMFIFI_02288"

/EC_number="2.4.1.11"

/inference="ab initio prediction:Prodigal:002006"

/inference="similar to AA sequence:UniProtKB:P9WMY9"

/codon_start=1

/transl_table=11

/product="Glycogen synthase"

/db_xref="COG:COG0438"

/translation="MKKKEILFVSYDYFPNIGGVAVYTHELSQALAKRGHQITILTCY

FSTSSKVEIEWDEQVKIFRIPVPKIKKIGDFIYRRRMSTFINDLQAEKKIDIIHWQTL

NKDAKMMQYVKKTGVEVYTNHLSWFRTLYKQKKFKKITKMIGQPDKIICPSFEVEKMS

AELFGSERCVFIPNGVNFEEYQKAISHLSLLRKELGIEEGDKVIVTTNRMEPVKGMTY

FIQVIPRLLEEHPNLFICLVGDGSQEQQLKNWLEEQKINIEKVKFIGRQAHHQIKQYL

DLADIYVQPSLMEGCSIGILEAMACGNPVVACAIGGNTDILEHKKTGLLIPDQSSSAI

YEAVNYLVCHPAEAREMGRRAKSKIEHELNWGHLAKKVEQIYDAALEVSANES"

CDS complement(2435689..2436327)

/gene="pglC_2"

/locus_tag="JFBMFIFI_02289"

/EC_number="2.7.8.36"

/inference="ab initio prediction:Prodigal:002006"

/inference="similar to AA sequence:UniProtKB:Q0P9D0"

/codon_start=1

/transl_table=11

/product="Undecaprenyl phosphate

N,N'-diacetylbacillosamine 1-phosphate transferase"

/db_xref="COG:COG2148"

/translation="MIDKNKRQQIIFNCLKRVFDIVFSCICILILFPIMLVVSLLIWK

EDRGSILFIQERTGLNGVRFKIFKFRSMKEEIPEKTYMFNPVYGVPSDFIFKSESDEK

EHITKIGAFIRKTSIDELPQFFNVLKGDMSIVGPRPEIPEITCGYNKEQKMRLEKKAG

ITGLAQVNGRSDLKNGDKMKYDLYYVKNQSIKLELEILWKTIRTILTTKGAI"

CDS complement(2436345..2437091)

/gene="tuaG"

/locus_tag="JFBMFIFI_02290"

/EC_number="2.4.-.-"

/inference="ab initio prediction:Prodigal:002006"

/inference="similar to AA sequence:UniProtKB:O32268"

/codon_start=1

/transl_table=11

/product="Putative teichuronic acid biosynthesis

glycosyltransferase TuaG"

/db_xref="COG:COG0463"

/translation="MFLSERVSVIMPIFNPDCTIVDSIKSVLEQSVSNIELHLIDDGS

TIEYIHYIENFLKLDNVYFTRLNKNAGPAYARNLGLEKSTGRYVAFIDCDDIWKQEKL

AIQLSCFEKKQTALSFSEYYMVKSTGVKKHVQVPVSLTYKKLLKNTIIGCSTVVVDRK

KTGPFLMPLIQGGEDTATWLNLLRKGYEAEGTQQALVYYRIKENSFSKNKWKMLIRTW

RMYRKTQQLSLVQTSYYFCHYVVNAIKKRI"

CDS complement(2437078..2437968)

/locus_tag="JFBMFIFI_02291"

/inference="ab initio prediction:Prodigal:002006"

/codon_start=1

/transl_table=11

/product="hypothetical protein"

/translation="MIKILIGIIVSLGSVFLVIACLYFFFYPSKGILRIKELEQIKQT

FSDNQKGAKRFIIGGSDVLYSFNTEKMNQELTIPTVNLGTNVGLGLGYLVDYGKKLAK

PGDKIIACLAYSLYYNPPYHIFSFEYYRMFDKKKLTRFSVPAFIYYAIANLRYNFSYV

QREFVIGESGCYLDVTGSQLAKEKEKALYFPENFNVTNSIRQLEEFKSYCLSHEIELY

ITYPSTLGFESYRNSHYLERLTSYLTENYQVIGKPEDYFVPLNHIFNSVYHINEVGQN

FRTSRLINYLREKEKLYVSE"

CDS complement(2437928..2439307)

/locus_tag="JFBMFIFI_02292"

/inference="ab initio prediction:Prodigal:002006"

/codon_start=1

/transl_table=11

/product="hypothetical protein"

/translation="MMIISASLFVLYPLYVGILIIGSLLLLSMTYGSEQFVLKISFVF

FTISAFLGAYLSFPGYESIFLFRILLLAQLFILVLYKDSLMPLNKGLKFQGYIGLLLL

IWNIGCFITLFWAQHKIPALRHIYYIFEASCLLYVPSYFIQTRKAVDAILDIISTIYI

VSLAIGFFEVLMGWHLKLSGSLVYETTTSQFQPTGFLFNTNDYALFLAMFLPLVFYRF

YSVATHKFMRFSGILIWFSSIYLVIMTYSRLGIVSIIIVSLVIYWVYFKRITFLILFF

GFLSLLIKELFSLTFLTTTFHTIQVSFTQKGASNTDRLNLYNTIWTIVKKRHFLGVGT

GGIPIEIATFRQGYESSSLNFNSAHNFFLESLGEIGLFTIFLVLAIGMLGYKFFMLLI

KKTNKTILEYSPFLMWGIFLISSVALSTIVEQRFLWLGLGMALAISFVEEESKEDDQN

FNRDNRELR"

CDS complement(2439351..2440088)

/gene="yveL_2"

/locus_tag="JFBMFIFI_02293"

/EC_number="2.7.10.2"

/inference="ab initio prediction:Prodigal:002006"

/inference="similar to AA sequence:UniProtKB:P71051"

/codon_start=1

/transl_table=11

/product="Putative tyrosine-protein kinase YveL"

/db_xref="COG:COG0489"

/translation="MSSKIKKNKHVKENNLKKRKKKSKKNKENAKIRNKEFLNEQFRT

IQTTLTFTNSIHKENINVIGLTSAEKGDGKSFCALRLARTFAEGGKSVLLIDSDMYNP

RLSRQLLVAKKRGLSNYLASDEQFEKYIKSTIYTNLFVLPSGPMHPNINELYQTNKME

KLIKKANKIFDIVIVDTPPALILNYPRLIGRICDGMIIVTSAGKTKVTKLKEVVNILE

LANNTILGVILNKKKYSRKELRDYTYY"

CDS complement(2440128..2440778)

/gene="cap8A"

/locus_tag="JFBMFIFI_02294"

/inference="ab initio prediction:Prodigal:002006"

/inference="similar to AA sequence:UniProtKB:P72367"

/codon_start=1

/transl_table=11

/product="Capsular polysaccharide type 8 biosynthesis

protein cap8A"

/db_xref="COG:COG3944"

/translation="MSKIKTRIFHDFFRVCKKYYFIILLVPCLVTSIVYLLTVYVIPE

NYEAKTQLLVSIQNENNEQRLDELRSSIQLLGTFSSITQSQRIMNQAAMQLNQQVIHE

KLSVSIDENSLIVTLKARGENKKSAVLVVNTIADLVAKDFSNLFKGADVTILEKANTA

NQPSILFQLILAMITGVFSALAFLFFLSISNLVISNEEQIKELGLIYLGDVTLIKK"

CDS complement(2440771..2441937)

/locus_tag="JFBMFIFI_02295"

/inference="ab initio prediction:Prodigal:002006"

/codon_start=1

/transl_table=11

/product="hypothetical protein"

/translation="MKKVKAPLDKNNVFMFSSVHNWMDTRILKKEAFSLIKSGKSIEF

YAIGNQEEAPFIEGIQFHLMKKGKKWSRPFKWFFLMKKALRSRAKYYHFHDAELLCLA

PLIRKKKTNAVIIYDMHEYFAAQISTKDWLPAPVRRPISKLVCVLEKKLMRYCDGVIF

AETSYKQYFTEYKGLTKDILNYPLPIEQNKMQKEEVFTLIYVGDITEDRNIMGMIEVA
KIVMERGRFQFQLKLIGPIAQSLKDKVNRKIIEYGLESYIFWYGRIPYEDIWNHYFSA
DVGLCLLKPIPNYKNSLATKLFEYMAAGLPIIASDFPDWKSLLIENNCGLFANPLDSN
EIAQLIEHLEHDKVLAQKLGTNGREAFETQYSWKTEEEKLAVFYRFLEKVRIGE"

CDS complement(2441934..2442437)

/locus_tag="JFBMFIFI_02296"

/inference="ab initio prediction:Prodigal:002006"

/codon_start=1

/transl_table=11

/product="hypothetical protein"

/translation="MNLKKVLFVLFTLSLIITATTGCSTKGSKEKSEPTLMKEKKILI
DDLGKEKVTNHIKINLVKGEYATDSGIADDQGLVVLSFEIDNENYLEEVGVGAGDFQI
FSDGKTYQMYGEKDNFGEVIKSKGSAKGTGAYLIPANISKGIIRYQPVNPNWPNMEKL
EWSFTIK"

CDS complement(2442549..2442773)

/locus_tag="JFBMFIFI_02297"

/inference="ab initio prediction:Prodigal:002006"

/codon_start=1

/transl_table=11

/product="hypothetical protein"

/translation="MRIEDCWEDKKSYYVSFLSSENKIINRNILLSPDIQDKYEIETI
VYQNFNQVKEVLAIDELDDALLLKEVSLGL"

CDS 2443180..2444646

/locus_tag="JFBMFIFI_02298"

/inference="ab initio prediction:Prodigal:002006"

/codon_start=1

/transl_table=11

/product="hypothetical protein"

/translation="MKQLLDSQFVPNLLLLEHLYKQTSQETITYFSEALGMDRRLILK
TVHSIKKDIDNNGWQDMITINLIEKNLVARIGPLFSLQHFYAYYMSRSLCVDFVLHLF
NNQTDPIEKLTDRFYVSKATLYRRLAPLKLVLADFDLTLDFTNGKKQLIGSEKQIRYF
YFTLFWEVFRSFPYSKNLLIDFENDFFQSFYNNHTLPIQLITVFELHFEIALHRIEQG
YMMENFPTYELPEIYFSFSSFQDVAYSYFSPYSISESQIENELLALYFSIVTSTLYSK

KECLNISLNNIPWSSYRIPIIQKSISYFTTFFELELSNEEYLYLLVNVYYLQLKRSVF

NGGTLHFEFNSVDEVLAEDNQYVLNQANRFFLFLDTKKPQFHVSKFYKLYYTLLLRRI

IFNASPSLQVLICSKVGLEEQRWLEKRISDISTVPIQFHDTWTPDTAFDLIISDFQLH

QKYIPKDPDNYLLLLAFPDSSEWVKIIVRLEKIYFNKK"

CDS 2445572..2445778

/locus_tag="JFBMFIFI_02299"

/inference="ab initio prediction:Prodigal:002006"

/codon_start=1

/transl_table=11

/product="hypothetical protein"

/translation="MRIEDFWTDVHAYYVTFLTINDAEITNIILSPENYLDNDDICKI

IKNKFNNVAQILLIDDLDPGLVLK"

CDS complement(2446034..2446450)

/locus_tag="JFBMFIFI_02300"

/inference="ab initio prediction:Prodigal:002006"

/codon_start=1

/transl_table=11

/product="hypothetical protein"

/translation="MNWKEKLDRNMRKFLVGRYGVMDQLNRGLLLISVGLIFLSIIFP

QRFLTTIALLILAVSYYRIFSKKISRRYKENQRYLEFIRPVQRFYHLQMKKIKERKEY

RYVKCPECHKTTRVPKNRGKIVITCPNCHHKFEKTT"

CDS complement(2446531..2447598)

/locus_tag="JFBMFIFI_02301"

/inference="ab initio prediction:Prodigal:002006"

/codon_start=1

/transl_table=11

/product="hypothetical protein"

/translation="MKTKKSRFSLMFVLVMSIFVISACGNESNGASSSKGNEYDTAMR

EGKQSIAEKDYAGAEEAFNEALGFKEKDAVATTFLNQTKDYIAAENKYTNGKLEEAKV

AVEKVVTEKNGSKDLVKVGKELETKINKFETQQKEYQALYDSSQKQFDAGKLDDAAGT

IEELLAKDLSNSLFADLKQQATELKTKINQAQAVKVKEEQASVPDSKYKTERNSELAG

KEFTAATGKDIKTATDEEIAAWLATKNATQSSGTTATDSSANAESSSTDSSQPEVSLD

TEKNNVLTAVEQITGLSSQDNQYYITKESADLFQVEIRHAHTVDGTEISNMVGMFQYT

PSTKGLTKMDPITGAYAPYKK"

CDS 2447908..2448315

/gene="mscL"

/locus_tag="JFBMFIFI_02302"

/inference="ab initio prediction:Prodigal:002006"

/inference="similar to AA sequence:UniProtKB:P0A742"

/codon_start=1

/transl_table=11

/product="Large-conductance mechanosensitive channel"

/db_xref="COG:COG1970"

/translation="MFKNMLAEFKEFALRGSVLDLAVGVVIGGAFSAIVTSLVTNIIT

PIIVALTGGSNISDLSIKILNAKLMYGAFLQSIIDFLIIAFSIFMFIKVINTFVAKMK

KPVEEVEEEVEINATEEYLKEIRDLLAQQNKEI"

CDS 2448451..2451153

/locus_tag="JFBMFIFI_02303"

/inference="ab initio prediction:Prodigal:002006"

/codon_start=1

/transl_table=11

/product="hypothetical protein"

/translation="MRHFKNVFELYRLDWRRIFKNPFATALIIALLIIPSLYAWFNIA

ALWDPYSNTSELKIAVYSADQTADFEDTSINIGDKLIDSLHDNHSLGWVFVKSKAELD

KGVKDGTYYAGIYVPKSFSSDLLSFVKGDLKKPKIEYSVNEKINAIAPKITDKGATTL

QETISKEFVATVSQTVVSAFNTIGVDLETNLPSIRKVSSLVLDLENNLPQIDTYTQEI

LDLQTKMPDIKAKLTKANEFVEYLPQVNQMTAKISEVNSLMPQLDQTGSLILDLQKNI

PQIQNAGRQIAQIDQDFDGIAATLNQGIDEANQALTVIQEVQTIMPDVIELGQDASQT

VESTKEVIAKIQSALEPVKQAIDTGLTILKSVASSIGKLADNLSSTVITEDNKAAIIA

SLTSLSTHLDTANTMLSGLIDNLEKLQEASGSNELTTTINQLKDLQTRNNELNQKVTA

LLNELPNLSVAEIQTRLNEISATAKGFVNDLNAIDNAAISQGIQNVLTKLNEALSHAD

TILNSANTTILPNVESLLASTTGTIKNAVTYLEKYQKELPALKNEIHTANTLLNGNMD

LIVGGINKAADFYQNDFPTLKQKLATADDFVKNDLPGIEDDITKTLAMVNEKMPDVEN

ALNSASSLIKNDWPMIRSGIEKAGNFIRDQQENIDLNELIKLLKADAQSESDFLSNPV

TMKQKAWYPVPNYGSASAPFYTALCLWVGGLLFSNIASTDFVLSDKDKKRFSKREQYS

ARMLSFLSVGFFQALIVSLGNLYLLHTYAFEKTWFVAMTIFVGLVFMSILYILAAVFG

NLGKGIGIILLVLSISAGGGNFPIQLSGKFFQFINPLLPFTYAVNLLREPVGGIYWPN

AWFYALVLLLFGLVFGIFGIIFQPYISVYIKKLQAKINESHFFH"

CDS 2451341..2452096

/locus_tag="JFBMFIFI_02304"

/inference="ab initio prediction:Prodigal:002006"

/inference="similar to AA sequence:UniProtKB:P9WLN9"

/codon_start=1

/transl_table=11

/product="hypothetical protein"

/db_xref="COG:COG2513"

/translation="MLKTKYDQFKQLHNEKEALILYNCWDVATALALEESGASAIATS

SYALAQSWGFPDGQKMTFTEFYWFISRIASHVTKPFTVDIEAGYAVELKQLEANMMQL

FTLDIVGVNFEDQDLSTEVPQLWTIAKQSARIQTIKDAAKKQNKTIFLNARTDIFFQN

RVVNQQLMIEALNRTTAYANAGADCIFIPGLVDKKWLAYFIHHSPLPVNVMILPGLPS

SSELQNMGAKRISYGPASYFSTMNYFQQNARKS"

CDS 2452124..2453170

/gene="ada"

/locus_tag="JFBMFIFI_02305"

/inference="ab initio prediction:Prodigal:002006"

/inference="similar to AA sequence:UniProtKB:P06134"

/codon_start=1

/transl_table=11

/product="Bifunctional transcriptional activator/DNA

repair enzyme Ada"

/db_xref="COG:COG0350"

/translation="MITDQKEIDTYYEMLVEKNSNYEGIFFVGVKTTGILCRPTCPAK

KPLKENCEFFETAKEALLASYRPCKRCHPLTHPSNLSDDVKLLVTAIEKDPTKKWTDK

DFDNLAISANTARRQFKKEFGMTFIEYARSRRMGLAFKHIRNGESLIDTQLASGFESS

NGFRDAFSKTMGDVPQRSKKIQVLSSAWIETKLGSMLAISDEHHLFLLEFVDRVGLET

EVKKLRVRLNAAILPGKTAILEMIEKELTLYFDGELTVFKTPIRYLGSPFQTEVWDAL

QRIPAGTTTTYKKIAENLNNPLAYRAVARANGANQLSIIIPCHRVINTNGALGGYGGG

TARKEWLLKHEMNE"

CDS complement(2453281..2458467)

/gene="prtP"

/locus_tag="JFBMFIFI_02306"

/EC_number="3.4.21.96"

/inference="ab initio prediction:Prodigal:002006"

/inference="similar to AA sequence:UniProtKB:Q02470"

/codon_start=1

/transl_table=11

/product="PII-type proteinase"

/translation="MMKKKKFQNLIKWILIYTIIGGVGSQFSPIISSAIESENLSSDN

YTKLVKALESNEQNVNALNEKAFDENMAAQLEIKGLKFEDFSNTEKNEQEIRVVIQLL

NESASDTSQPPTGTKKSIEKIEQATDDVIQNQQIIKTKVEKITGNKAQRSFGYLLNGF

SIDVKYKDIDTIRDLPGVADITVATVYYPTSVEANQLANINQVWEEYQLKGEGIVVSI

IDTGIDPSHKDLRLSDTTKEKISLEESQTNIAEMGHGKAFTRKIPYGYNYADNNTTII

DENPTTNMHGMHVAGIAAANGIGADSTTAVLGVAPEAQLLAMKVFSNSSGAVALNDDV

IAAIEDSVKLGADILNMSLGSVAGNRDSNDPVQISIREAAEVGVLSVIAAGNSGLSTS

NDTNVAPQNKFGTIDTGTLGSPGVTDEGLTVASLESSVQISGRLAIYTVNEAGAKVPY

LMETPASSEGKIPYFKGVTANITGLSTPHSMIDLGTGTEADYQNKDVAGKIVLVRRGS

IPFLDKQVLAKKYGAAAVFIYNNLPNLAALEMPIADPNFITLGLTKEDGDKFAELISE

NPAINYSFEADKFLTENPKAGKISDFSSWGPTPSLEFKPEITAPGGQIYSTANQNSYQ

TNSGTSMAAPFVAGTEALIYQALKAEQSPLTGLNLIEFAKASLLNTAIPVMDQKHSDV

IISPRRQGAGLLQADQAIKNKVYLTDAKTGKASIALKQINQSTKISLQLKNAGKQDAS

YNFHDFGGVYTEDRTATAEVYEKKIDGADIKIASSEIKLKAGETKTIQLTLDLPDTFS

KNQFVEGYIGLASETQPDLVIPYMGFYGDYSEAAILDTPVYSGNSIQGKGFFTDKSNT

FLGLEKGTINPNLVAISPNKDGRKDEAKPTLYFLRHAKTATYEIVDSNKKVIRRLNED

HNVIKDYFNSVSSAFSSHTVTAATWNGMIYNPKTGGNDVVPDGHYQMKITVQGMSEKA

STQETFLPIKVDTKNPVISQAKFVTPDSLSLKIEDQLSGADLRSVAIAVNGQIETYDL

SATENEELTVPLKNTQQAANGKNQVEVVVSDYAGNIGHFNEFISFGTKEELVLFNLSD

NALITTASNSYSTEKKSYTVKGYYPAGKSIYVNGSLIDNQAGYFDMEVPLTENSTELI

FSQEANQQTIIKRVAIRAKVTPPELTIDSPTESVVIDENHYLVTGKTGSSTKVLEVVN

QSNGVKINSTDAIQADGQFSTEISLVNGQNSLMVTALDEFGNKTIKIISLRTTGYNQP

TILALSNLNVVGFTQVGVGNPNYDEISKEYTIKGRLKEPVEQFTIDGKDVAYDPIELT

FSYPVPLKQGKNTVAFYLQDSAINAEKPLVDEGYTLLLDTVLPTLDVENLQTDSQGNY

QIYTNQNPFLLKGLISDNFSGYRLYINNENVLTDANYFIFDEKFFANRLAAPFEYPVN

VTEGENSVQIGLLDTLNNLTKKAISIFYRQTSPLAPIVTADTTSLTNKAVNLTATSEA
ETLIYYCLSGEEYQLYTDQIAVTLNQKVSFKTLDKYGNYSEPTIYEVKNSQQVIASQP
KIDVSPREKLTEAVQISLGYQKELTEREQTFTHLRYSLDNGFTYSEYQIPFKLEKSTD
IYAQSYDDAGNQSEIIKETVLFEEIVEEKEKPETETTNPTIPILKPGQEEVNSIIEST
KVKESATNLVQPTMANELNDKDQLPKTGIDVKQSYSILGLWISFYSIYLLLKNKKNSS
NV"

CDS complement(2458654..2459901)

/gene="yjhB"

/locus_tag="JFBMFIFI_02307"

/inference="ab initio prediction:Prodigal:002006"

/inference="similar to AA sequence:UniProtKB:P39352"

/codon_start=1

/transl_table=11

/product="Putative metabolite transport protein YjhB"

/translation="MNKQATHSQQSMSSYQKKVLASSSAGLGLESMDIMFLTFALTSI
IADLNVSGAAAGLISSITNVGMLLGGVTFGILADRFGRIKIFTYTILIFAFATGAMYF
ASNIYLVYLFRFLSGIGAGGEYGIGMAIVAEAFPKEKLGKMTSIVAITGQVGSIIAAI
IAAIIIPRFGWNALFLFGLLPVVLTFFIRSHLDESEEWKASQIKESSQPSASLKELFK
TPNLARQTISLMVMAVIQIAGYFGLMNWLPSIVQKQLGLSVSGSSLWMIATIIGMSLG
MLLFGRILDTLGARLAYSVFLLVSAVSVFLFVFATNQWTMLLGGAVVGFFANGMFAGY
GAIVSRLYPTHIRSTANNLIINTGRAIGGFSSVVIGFLLDKYNLMAVMLFLATLYLIS
FCIMLSVKGLKRENYFNPDFTGE"

CDS complement(2460006..2460974)

/locus_tag="JFBMFIFI_02308"

/inference="ab initio prediction:Prodigal:002006"

/codon_start=1

/transl_table=11

/product="hypothetical protein"

/translation="MSQKNYQKSVREDIQGYYFKGFNSFRKTVENTKKGVFKETDYAE
EANFKFAEYQLSSLTIELKNHATFISESEEEEFVLIGKNTLYIQNISYLEGTYQTIIH
RIGQKPVKTREKTADIMTRYFNQQGINYETIQSIGKLLEITKYCPYLLGDICFLPDFG
RKQAGTSWLALHHVITGQSHSVTNQTYLFCRNNYRIIIPISYKSFKKRVENATLLYHV
QKSAFNLFSQFFEIEWLMKKEQPLNIIHQSLLKIQYKLPNYSLPELIEYLKYSWISDS

LKKNFGVEDPYLHQVRALLDIPLRLGRKEKESQRTNYLEEKEEFKM"

CDS complement(2461154..2464711)

/locus_tag="JFBMFIFI_02309"

/EC_number="1.2.7.1"

/inference="ab initio prediction:Prodigal:002006"

/inference="similar to AA sequence:UniProtKB:Q2RMD6"

/codon_start=1

/transl_table=11

/product="Pyruvate:ferredoxin oxidoreductase"

/db_xref="COG:COG0674"

/translation="MRRQKTMDGNTAAAYISYAFTEVAAIYPITPSSTMAELVDQWAS

DGKKNLFGQEVKIVEMQSEAGAAGVVHGSLKTGTMTTTYTASQGLLLMIPNMYKIAGE

LLPTVFHVASRAVTTNALNIFGDHGDVMAARQTGFAMLAEGSVQEVMDLSAVAHLSTL

TSSLPFMNFFDGFRTSHEIQKIDVLEYDELKGLMDKDALTAFRHNGMNPNHPTVSGTN

QNPDIHFQQRETINSHYDRLPEIVEKYMHEINQLRGTDYELVNYYGAADATDILIAMG

SVTPVIEQTIDYLRQSGKKVGLLNIRLYRPFPVEHFLKAVPETVERVAVLDRTKEPGA

GGEPLLLDVQSAFYDSKFRPTIIGGRYGLGSKDVTPADILGIFDHLATETELKSRFTI

GIEDDVTYLSLPKGEEVDLTSAGTYQAKFWGFGSDGTVGANKSAIKLIGDHTDKYVQG

YFSYDSKKSGGLTVSHLRFGDNPIKSTFLIEQADFIACHTAAYLSKYDLVKGLKKGGT

FLLNTVWDDEKLAQMLPYKLKKYLAENEIKFYTINAVQLANQIGLGRRINTIMQTAFF

KVTSIMPIAEVVELLKADAMKTYGRKSQEIAEKNCQAIEMAAEMVHQVEVPNTWLTEP

EISEERDTSHLPKFVFNILEPMNRQEGDSLSVGTLIEHGMTSGEIPVGMAAYEKRGVA

LEVPEWIPENCTMCNECAFVCPHAAIRPFLADEDEMEEAPAGYIVRDIRGADGLQYRI

QVAVDDCTGCGLCVAACPAKEKAIKMRPYEEQKEQAVNWAFSMTLKAKKNPFKKNTVR

GSQFEQPLVEFSGACSGCGETPYVKLLTQLFGDRMMVANATGCSSIWGGSSPATPYTT

NSTGCGPAWSNSLLEDNAEFGFGMHLAHQTRREGIALKMGQAIETGTISSELEEAFTE

WLTGINESDGTQERAANLKFSLIQEKTGNPILEAIYEEKDLFVKPSQWMIGGDGWAYD

IGFGGIDHVLASGEDVNMLVMDNEVYSNTGGQTSKATPTSAIAKFAASGKYVSKKDLG

MMAMSYGNVYVAQIALGANQAQTIKAIEEAEKFPGPSLIIAYTPCIVHGLIGGMGNAL

KETKEAVESGYWSLYRFNPSLAEKGKNPMSLDYKKPTFADMKDFMLTQTRFTSLFKAN

PTVADHLFDKTVNDAKIRFYNYARLSGQEEKIRAKLEKE"

CDS 2465017..2465586

/locus_tag="JFBMFIFI_02310"

/inference="ab initio prediction:Prodigal:002006"

/codon_start=1

/transl_table=11

/product="hypothetical protein"

/translation="MKLLKRLLITLLGLGLIFLLIIGGLIYSGTQQTPKKNADTMIIL

GAQVRGNPAVPSAILKERLDAAVPYLLENPQTKVVVTGGQGPDETDTEANVMARYLME

QGIDSQRIIKESQSSRTEENISNAKKLTDLGKTVIVTSDFHMYRGLMLARRENVDASG

LPAVSKSSATFKSYAREMLALPYGLLFDW"

CDS 2466003..2468579

/locus_tag="JFBMFIFI_02311"

/inference="ab initio prediction:Prodigal:002006"

/codon_start=1

/transl_table=11

/product="hypothetical protein"

/translation="MKKIMVGLLLSTYFLCGTSLHVFAESETPPEPILIRPIDSEDSK

GSLNSNEPNVPQSKLGPKKRLSLPDKLDPRGTNQEIPVRNQVGDLCWAYSSTDIITAN

YKKQTGTQQILSPNFYNFYSAENAFSDGFNPNTIILNKTKDAITNRKLNNGGFHNYPL

FQNILGNRGTAEIDFAMPSVVSQNQPLLKEKFDGLEANKTDVAINEIKQIPFNSYEIT

DSERKLKIEKIKQFIQESGAVTYNYNSETISHSSFKKYYNNTTKASYVPNADFNKIPN

YPTLGRPVIDHTVTIVGWDNAFSKTNFAIQPEQEDAFIVKNSWGTSSGDQGYFYVSYD

DIFVTTGAFYSVTTAEPTDHIRGYVNSTSDAWGAVKQIENKNDKIYLAANFQTKNTEE

LLKTVAFLTVQNNIDYKIYYLDRKLESESFNSAILTDLIAEGKQEEAGMKNIPIKEKT

LPVNKIYSIIIELTYPTDLAIQNFTAQTVKKEDQEDRAPVLQPGDTLVSLNNNRWRNT

SKGEYWHNNFNLYITVGTNDSPNEENTVSSIIINDKIEPKLLYQNKPLQLDTTIMPES

VKKNELVWSSNDPDIAEVDQLGIVTAKAHGRTTIRVSSKQNPDIFDELSIITDDHGCT

TETSTRLTIGEPMVGMIDIDRDEYVPENAWYNRGDVFTYLIPEDGSYTVHGYGMKPDQ

TQDLIGTDFMTNGKSRYIAVANQFFTQQLKKGDLVEISVFPSMLGMNNPLGDWGKTTV

GTSYSILFDKTSNFDYGKLTWANSGGETKESLKTTFKIGEKYQFKGSVIDSNIQDPFY

TNNNSFYFASTWKSSKPKVATINEKTGELEALSSGETVLYNTYNLHWWSKIEDSITIT

VE"

CDS 2468590..2469366

/locus_tag="JFBMFIFI_02312"

/inference="ab initio prediction:Prodigal:002006"

/codon_start=1

/transl_table=11

/product="hypothetical protein"

/translation="MIPIQKFILIMGVGLLVLPPLSVQADDTTSSEALPPQTNTSNSS

VGLTPGEPTEPHELILDIWHPTKHVGSLTIDAVTGFYFSDITLSSYGEEAYAIVARDE

INGEKPPIDTGLLDRGYTLGAQVTDVRGTGVGWILTASVSEFTAGEKKLKGAIFSFPV

SAVHSDKYIEEEESPPISKEAIFDTPNVELPILVAEPDTGLGSWIISFDTDDRGIGYP

WLMDKNGNFHRYGGPSLYVPQGNLSGNYVAAITWSLRDAP"

CDS 2469384..2470160

/locus_tag="JFBMFIFI_02313"

/inference="ab initio prediction:Prodigal:002006"

/codon_start=1

/transl_table=11

/product="hypothetical protein"

/translation="MIPIKKFILTMGVGLLVLPTLSVQADDTTSSETLPPQTNTSKSS

VGLTPGEPTEPHELLLNIWHPTKHVGSLTLDAVTGFYFSDITLSSYGEEAYAVVARDE

INGEKPTIDTGLGDRGYTLGAQVTDVRGTGAGWTLTASVSEFSAGEKKLKGAIFSFPV

STVYSDKFSDKEESAPVSKEAVFDIPNVELPLLVAEADTGLGSWLISFATDDRGVNYP

WVMDKNGNFYRYGGPNLYIPQGNLTGNYVAAITWSLRDAP"

CDS 2470178..2470954

/locus_tag="JFBMFIFI_02314"

/inference="ab initio prediction:Prodigal:002006"

/codon_start=1

/transl_table=11

/product="hypothetical protein"

/translation="MILIQKFILIMGVGLLVLPPLSVQADDTTSSEALPPQTNTSKSS

VGLTPGEPTEPHELILDIWHPTKHVGALSIDAVTGFYFSDITLSSYEEEAYAIVARDE

INGEKPPIDTGLLDRGYTLGAQVTDVRGTGVGWTLTASVSEFTAGEKKLKGAIFSFPV

SAVHSDKYIEEEESPPISKEAIFDTPNVELPILVAEPDTGLGSWIISFDTDDRGIGYP

WLMDKNGNFHRYGGPSLYVPQGNLAGNYVAAITWSLRDAP"

CDS complement(2471030..2471119)

/locus_tag="JFBMFIFI_02315"

/inference="ab initio prediction:Prodigal:002006"

/codon_start=1

/transl_table=11

/product="hypothetical protein"

/translation="MIDSDAVNFLMMKISLNIKAVITSNIKEI"

CDS complement(2471248..2473053)

/locus_tag="JFBMFIFI_02316"

/inference="ab initio prediction:Prodigal:002006"

/codon_start=1

/transl_table=11

/product="hypothetical protein"

/translation="MRKKISILLIIVCVLPLINLKLVIAETTSKLVGIELKYPENQDF

IVATADTVDVVMNLELKAMKKGETTLVEFSNKDFNYSLESFEISGNYKVVPNELGIEI

IALKDVPANTSKITIPMRYSTNVEYDQNQKIEFSIDGSSISVDIMVNKYKSFVDESEL

IKKVAYGINAKGNAVWGVFLNYNEKPLTGTKDIPFVLGDIVGEGHELIANTIAVYNVN

KPINEDGSRNLNNDGYNYDISALLQQKSTITANGFTYTDDDFNGFPKLDGLKEKNTKA

YYIIYETKVVDSDQEWLKNDVYLEGIIAEPGNGGDPLPFTENSSVSISTISSGGGGTE

VGSIEFIKVDSETKAPLANAEFVIKDGEGNIVQTILTNEDGKGTALDLLYGSYTLEET

KAPIGYTLDETPYPFEINDSEKAINLGEVTNQKIMLPAPTGSIEFIKVDSETKAPLAN

AEFVIKDGEGNIVQTILTNEDGKGTALDLLYGSYTLEETKAPIGYTLDETPYPFEIND

SEKAINLGEVTNQKIMLPAPTGSIEFIKVDSETKSLDGLTIKNTKTTNLQMNLPQSGE

NNKVGQRIKIVGLLLVFISIYLYVSRLKKRVNLKY"

CDS complement(2473099..2475030)

/locus_tag="JFBMFIFI_02317"

/inference="ab initio prediction:Prodigal:002006"

/codon_start=1

/transl_table=11

/product="hypothetical protein"

/translation="MKQNRKHKFLLFIVVLFMIVPSVNEPFKVFANSNEEELDVPSVE

DVTQNEAKELAVNSAEQIELLQKKATMDASIDLLNVPNDQENSEEQAVQTPDASELKQ

LEIQQVEIEKEQKSSVESVDSWMPDKNLQTAVAKTLKITVSEITKEQMQRLGSEASTG

QITAKNVASLQGMEYTTGGTGIVLQTTNGTINDLSPIENVRLLQLIITNNDVSDLSPL

KGQSLGLLSAENNNISDLSQLNFSNMKILAVDDNHISDLSMVPNYVGMVLARNQKIVN

AEIPRNENIKIDSVKHVLNYDFSTKNFSNISNNGLFIPNASPYFGSITWENLDLSVSS

IQYEFDRQYSFSTDSRKNNKVYYSGVVVQPFSNEIKAAPVTVKYLDEKGTELALSELL

TGKVGEPYETNGKQVNGWEIIDIPGNANGVYTEEAQEVVYLYERKKAAPITVKYQDID

GNIVEPTIKLAGDIGEPYTVEKKEIEGYSIKEVIGNPVGVFSEEEQEILFIYKKIQEQ

SANIVVRYQDNRGNKIEEDTIVEGLIGEVYNIESKAIDGYTFKEAKGDVSGLFSKDKQ

EVTFIYVKDNANLALTPTYPAKLVTKNSLIPSLDKGMNNKTTFPATGTKKNNSFMIGF

IMLAGTILIYYRKKYTKMK"

CDS complement(2475072..2475335)

/locus_tag="JFBMFIFI_02318"

/inference="ab initio prediction:Prodigal:002006"

/codon_start=1

/transl_table=11

/product="hypothetical protein"

/translation="MIEEHWIDLICYSIHFTNNSGSSFCRSIALPNNFPEDKISTFFK

NHFEGHDIKIIDISEINDVWLSKHAHSPFMRQPSECDELKWNL"

CDS 2475782..2477299

/locus_tag="JFBMFIFI_02319"

/inference="ab initio prediction:Prodigal:002006"

/codon_start=1

/transl_table=11

/product="hypothetical protein"

/translation="MLYALLKKQDFNKLQLFNHISRHEQVEINYLADTFKLSKTTIRR

HIADLNSLLKENGKTSYFQINQTDSSHYYLTPLTQEKFAHLSFKLQSHYLNISPQFQL

LALFFSSHSLTIYQICEALIISENYCYKLIKQLKAILKTVSISINKSVENQSFSLHGS

ESTIRFMYIWLFLPSYQNISWPFSHLTQDSVRLAYSKADTGPLEHSTRSAKTRIEFIL

AIIDKRVRHNQLNKPFDEPLYSIVQIFMKQHDISKNIPHFPATFPLAVGSEKEVNERE

HFNLIIRTINSSIDTQQDKLNIGEQFTLLNNPITNFYSGFIPAFFKKYNIKKSNDSLF

EFMYYTVFTHSYSIYSHFNPTMMTQLNHLDSEIQIITDNSQTFKNDLDDAFSTYSMPP

DLITDLTTELVSSVLVTLSQIHKEKVLYLYVQYSRDIVGGSFIQSEIHHFFSAKSIKF

VSSSHEADIIISDSNELSATSEFHFFEDTTRNSSWINLFSFLQRKLYELYFLTNL"

CDS complement(2477400..2478356)

/gene="yxeB_2"

/locus_tag="JFBMFIFI_02320"

/inference="ab initio prediction:Prodigal:002006"

/inference="similar to AA sequence:UniProtKB:P54941"
/codon_start=1
/transl_table=11
/product="Iron(3+)-hydroxamate-binding protein YxeB"
/db_xref="COG:COG0614"
/translation="MKNQKIKGLVFILGVLFVLTGCGSNTSDTEKAQEKEATGTHLFK
AANGEIEVPNNPKRVVVRTYMGHVLALDVPVVGATEWDLASPFIDKKVLDKIKDVGVP
MNAEEVLALEPDLIITDTEDEVASLEKIAPTVLLPYDTVRNINESVDLFGELLNRKTE
AEAWKKSFKENADKERARLAEVNFPMDVTVGLYELQDSKLFVFGSNFGRGGQVLTTGL
GLKQQDNIQKVSDGDGWKELSLEALPDYAADFMFFTSYTANGTESAELTALKANPIWK
TIPAVEKNQVIELPFEKMYYYDPIAIQAQLKLITDKLIETQK"

CDS complement(2478384..2479388)
/gene="feuC_2"
/locus_tag="JFBMFIFI_02321"
/inference="ab initio prediction:Prodigal:002006"
/inference="similar to AA sequence:UniProtKB:P40411"
/codon_start=1
/transl_table=11
/product="Iron-uptake system permease protein FeuC"
/db_xref="COG:COG0609"
/translation="MNKHKVKTSLFVGGLLLISLILIQLNLGAIRISPLETIQTLMGN
GSVRNQLILFEFRLPRIVMAMLVGMGLAISGAILQGITQNDLADSSILGINAGAGLGV
VLYLFFSTGSTAKLGVPQFLFIPLVALGGAFFAASLIYLGAWKKGIHQLRLLLVGIAV
TALLNAFILIFQLQMDEFNFEKAMVWLSGNLWNVNWSFILIIACWLAILLPLVYWFTM
RLDILRLGDQIGQSLGLEVEKTKAILLFLAVALAGISVAGGGGIAFVGLLAPHIAKRL
VGGRSQSYLPVSAVVGMILVLGADIVGKNLFAPNEVAVGLVVAIIATPYFIWLMMRTV
"

CDS complement(2479391..2480419)
/gene="feuB_2"
/locus_tag="JFBMFIFI_02322"
/inference="ab initio prediction:Prodigal:002006"
/inference="similar to AA sequence:UniProtKB:P40410"
/codon_start=1

/transl_table=11

/product="Iron-uptake system permease protein FeuB"

/db_xref="COG:COG0609"

/translation="MKQLHVKETRSARQLSLIMVLIVLFAGLILVSLLSLKYGTETIS

LKTIGESFTHYDVQDKLHQTVRYLRLPRLLGTLLVGALLALSGQLMQAVTRNPLADPS

ILGVTGGASLAVAITYGLGFNLGSPLRSVVAMLGSLLAIGLIVSMTGSRNKGINPIKM

ALAGTAMSGFFSSLSIAIGLQTKTSRNLSFWLAGGFSGTTFLDVRILLTILLVCLLVT

WWISVEINLLSLGDEVASSLGGSLLKARYLGLLIMVVATGASVAIAGNIGFIGLCIPH

IVRLILGDRFQGTFFVTCLTGGLLLSLADLVARTINPPFEIPIGAITAIIGAPFFILL

ARNYKGGR"

CDS complement(2480504..2481286)

/gene="yusV_2"

/locus_tag="JFBMFIFI_02323"

/inference="ab initio prediction:Prodigal:002006"

/inference="similar to AA sequence:UniProtKB:O32188"

/codon_start=1

/transl_table=11

/product="putative siderophore transport system

ATP-binding protein YusV"

/db_xref="COG:COG1120"

/translation="MEAVKATGITMQYEKKRVIEDLDIVLPLGKITALIGPNGSCKST

LLKGVTRYLKPVAGEFFVKEKLVQEYDPKEMAKLVAMLPQNPLAPSDITVLDLVKYGR

FPHQNTFGRFTKEDQMQVDWALKETELEELKNEPVESLSGGQRQRVWIAMTLAQNSDI

LLLDEPTTFLDLAHQLDILKLLTRLNKEMGKTIIMVIHDLNMAARFADYMVCLSDGKL

LYQGETADVFTHEMLVDVFQIDAEITTDKRTGKPMMLSYDTM"

CDS 2481506..2481883

/locus_tag="JFBMFIFI_02324"

/inference="ab initio prediction:Prodigal:002006"

/codon_start=1

/transl_table=11

/product="hypothetical protein"

/translation="MGLFSGLINNASDANINDVRKKLADVLLPTEDIELAYKLVRDMV

IFTEKRLIIIDKQGVTGKKTSYKSYPYRSISRFTVETTGHFDLDAELHIFISSALEPA

ATLTFSKDRHIVEIQQALAKAVL"

CDS 2482232..2482522

/locus_tag="JFBMFIFI_02325"

/inference="ab initio prediction:Prodigal:002006"

/codon_start=1

/transl_table=11

/product="hypothetical protein"

/translation="MDEQIRKEILDFIWSERLLDEAELQSTPDAEDCSIRIVNKKGDI

MDVKLALEAGEMHLLAPKKSLFAFVKDEAGKWKPKYFEKLNALGCSEERFMF"

CDS complement(2482606..2482938)

/gene="yodB_2"

/locus_tag="JFBMFIFI_02326"

/inference="ab initio prediction:Prodigal:002006"

/inference="similar to AA sequence:UniProtKB:O34844"

/codon_start=1

/transl_table=11

/product="HTH-type transcriptional regulator YodB"

/db_xref="COG:COG1733"

/translation="MNEELMDKATSQKCTQVEHVFSILDKKWMGLIVEVLLEGPQRYK

DIAAKIPTISDRILVERLKELADNDILVRDVYPDSQVRVEYSLTEKGYSLKNVMSEVH

IWADKWMD"

CDS complement(2483059..2484153)

/gene="yhaZ"

/locus_tag="JFBMFIFI_02327"

/inference="ab initio prediction:Prodigal:002006"

/inference="similar to AA sequence:UniProtKB:O07541"

/codon_start=1

/transl_table=11

/product="putative protein YhaZ"

/db_xref="COG:COG4335"

/translation="MATALKEVYNNEFINHFSALVQEKATSFNATKFKQEVLNEDWED

LKLKERMRRISTVLGRCLKGSYSDQIALLFQLEQECQGFPYLFFPDFVEVYGQNESDW

ELSMEAIKRFTKGSSSEFAIRTFILKNPIKVMSLMKEWATDENEHIRRLASEGCRPRL

PWGQALGLFKVNPGPVLDILELLKNDDSLYVRKSVANNLNDIAKDNPQKIIELVKAWQ

GQSQQTDWILRKGCRTLIKQADPEIMRLFGYPEVENQINMAEINSQLSELEMGEESEF

SYQFTTKGDSDLHVRIEYGIYFIKANGKATRKLFFLSEKNVSGGQTISGRRTHKWKDL

TTRKHYAGEHRVVLLLNGVEFAETSLFLKG"

CDS complement(2484283..2485614)

/gene="noxE_2"

/locus_tag="JFBMFIFI_02328"

/EC_number="1.6.99.3"

/inference="ab initio prediction:Prodigal:002006"

/inference="similar to AA sequence:UniProtKB:A2RIB7"

/codon_start=1

/transl_table=11

/product="NADH oxidase"

/db_xref="COG:COG0446"

/translation="MSKIVLIGANHAGIAASNTILDNYPENELVIFDRNTNLSYLGCG

TALWVGRQIETPDQLFYTSKDAFVEKKAKVHMETTITKVDFSDKKVYAVDTANNEICE

SYDKLILATGSVPIKPAIPGIELENVQFVKLFQDGQAADLALNDPAIENVAVIGAGYI

GVEMAEAAKRRGKNVRLFDIDTTSLAGYYDSWFTEDMDKNLMDNGIELEFGQKVLELK

GTDKVQEVVTDKGSYAADLVLLAIGFNPNTELGKDALKLFSNGAYEVNLKQETSLPDV

YAVGDCATIYSNALEATTYIALATNAVRSGIVAGHNVCGTPLESIGVQGSNGISIFGY

NMVSTGLNLKAAKQAGFDVAYTEFEDLQKPAFIKKNNEVKIRIVYDKTTRRILGAQLA

SKEDISMGIHMFSLAIEEKVTIDKLKLLDIFFLPHFNQPYNYITMAALSAK"

CDS complement(2485905..2487020)

/gene="menC"

/locus_tag="JFBMFIFI_02329"

/EC_number="4.2.1.113"

/inference="ab initio prediction:Prodigal:002006"

/inference="similar to AA sequence:UniProtKB:O34514"

/codon_start=1

/transl_table=11

/product="o-succinylbenzoate synthase"

/db_xref="COG:COG4948"

/translation="MEIVKVTLYHIELPLLTPFITSYGAIKTKAFYLVEIENKLGNKG

YGELEAFEIPDYTEETRETAGLILVNHLLPLIKGVIIEHPQEVRALLAPIRGNEMAKA

ALETAIWDLYGKEQNQSIQHLIGANRLRIPVGVSIGIQPSIEVLLQLVEGYVATGYQR

VKLKIKPGFDQEPIAAVRKAFPELTLMADANSAYTKEDLPLLKALDKYDLAMIEQPFG

FRDFVDHAWAQSQLKTRVCLDENIRCLGDVQLAHQLGSCQSINLKLARVGGLQEALEI

VDYCQSHDILVWCGGMLEAGIGRAFNLALASRNEFTFPGDISASNRYFAEDIIKTTFD

LVEGEITVPSGDGIGVEVDWQVVKNYCLKQTDYVLID"

CDS complement(2487039..2487860)

/gene="menH_2"

/locus_tag="JFBMFIFI_02330"

/EC_number="4.2.99.20"

/inference="ab initio prediction:Prodigal:002006"

/inference="similar to AA sequence:UniProtKB:P37355"

/codon_start=1

/transl_table=11

/product="2-succinyl-6-hydroxy-2,

4-cyclohexadiene-1-carboxylate synthase"

/db_xref="COG:COG0596"

/translation="MKIQIRGVEYSYTVKEGTDPKRIPWLMLHGFTGTKATFSEVTEA

LVTETIISLDLVGHGETAYHVAEKRYSMEEQILDIALFLEELKLPKVLLLGYSMGGRV

ALGFAANYPDKVNKLILESSSPGLKTNREQEMRREHDAKLAKSIIKDGLTKFVESWEN

LPLFATQKQLSTEIKSRIRDERLSQNPIGLAISLEQLGTGSQPSYWQELKQIQFPVYL

LIGEADLKFKQLAEEMGTLFPHSKLVEFAGAGHAPHIEQPMLFAKCLQEIADNQL"

CDS complement(2487857..2489596)

/gene="menD"

/locus_tag="JFBMFIFI_02331"

/EC_number="2.2.1.9"

/inference="ab initio prediction:Prodigal:002006"

/inference="similar to AA sequence:UniProtKB:Q71YZ2"

/codon_start=1

/transl_table=11

/product="2-succinyl-5-enolpyruvyl-6-hydroxy-3-

cyclohexene-1-carboxylate synthase"

/translation="MNEKNQALTDYLVTFVEELVQAGVKEAVISPGSRSTPLALLMAE

HPTLKVYVDIDERSAGFFALGIAKASKNPVVLLCTSGTAAANYFPAVAEANLSSIPLI
VLTADRPPELRGVGAPQAMEQVRLYGEHVKSFVDMALPENSLEMLRYAKWHGSKTVDI
AMTVPRGPVHLNFPLREPLVPILDPSPFTRQNINEHVHIYYSHKVIPTGTIDKIIEAC
AGKKGLIIAGPLAKKEFPAKLAAFAKQIGWPIVADPLSGLRTYGGIDNTVIDQYDAFL
RNPLEVTDLVPEVIIRFGAMPVSKSLMKWVESFAGIPYYLVDPGEEWKDPMKAATDLI
HCEEHFLVDEFTERFTQVTDSSWLTSWQTVNQLTAEIVQQSIDVAESIDEGELIHLLR
KHLPNKSGLFIGNSMPIRDVDTFFAKTEKELTLLANRGANGIDGVISTALGCGTFIQP
MYLVIGDLSFFHDMNGLLMAQKYGINLTILVVNNNGGGIFSFLPQADLPKYFETLFGT
ELNLDFAKVADLYSGGYQQVSDGEQLHEALDHAHFHKGLDIIEIKTNRYENVAVHQEL
FAKVTEELKASKR"

CDS complement(2489589..2490977)

/gene="pchA"

/locus_tag="JFBMFIFI_02332"

/EC_number="5.4.4.2"

/inference="ab initio prediction:Prodigal:002006"

/inference="similar to AA sequence:UniProtKB:Q51508"

/codon_start=1

/transl_table=11

/product="Salicylate biosynthesis isochorismate synthase"

/db_xref="COG:COG1169"

/translation="MWTLPSDISEAAKLKLEEHSSVLVSYVRELNELDALTLFKKGAK
KYTGQRIFWQNPSKTVTLAGIGCTERYSSKAGNESYTELNQFKDKLKKQTVSNEIILG
TGALLIGGFSFDSLAKADANWEEFQDAYFSLPTYLLTTTAEKSYITFNFYVTQADLIE
KPLRIMKQWEELLATPFVTETEIAAITKKEELATDSWLGAVSETVDAIKSSKELNKVV
LSRQMLLTHETDVAIEAVLEKLRKTQQNSYFFVVENGEKTFLGATPERLLSAEGNKFY
SACVAGSAKRGATFAEDQAIGDRLLADHKNVHEHQLVVEMIRHTMEGFTTDLAISGGP
TLLKNRDIQHLFLTLAGTKKEHFSFLEAVKGMHPTPALGGVPTDLALEVIRQKEPYNR
GFYGAPIGWVDSSDQGEFAVGIRSALIMGQQSLLFAGCGIVAGSDPTEELNETAIKFQ
PMLRALGGMENE"

CDS 2491332..2491925

/locus_tag="JFBMFIFI_02333"

/inference="ab initio prediction:Prodigal:002006"

/codon_start=1

/transl_table=11

/product="hypothetical protein"

/translation="MIKEIKKTPMILLGLIFISIGLNWFLLPHDIAAAGVGSIGHLVE

INFSINRSLTVWSINLTMLVMAALFLGKLVFIKSVIGSLLFPIILGFVPMFALLSSHF

ISLIIGSLLFSLGVYSLYSVGASNGGITIPPIIFNKYFRLSISKGLLLTNLIIIFFNY

ITFGLLETAFVILSIGISTLFITILTNFKPVVHDLAK"

CDS complement(2492096..2494771)

/gene="mngB_3"

/locus_tag="JFBMFIFI_02334"

/EC_number="3.2.1.-"

/inference="ab initio prediction:Prodigal:002006"

/inference="similar to AA sequence:UniProtKB:P54746"

/codon_start=1

/transl_table=11

/product="Mannosylglycerate hydrolase"

/db_xref="COG:COG0383"

/translation="MKKIVHVVPHSHWDREWYFTTSRSKIYLMHDLKKVLEQLEGENG

YDTFVLDGQASLLDDYLKWRPQDKNRIEALVKSGNLIIGPWYTQTDQLVISGESIVRN

MQYGMQVSEEFGPYMNVGYVPDSFGQAASMPQIYQEFEIEDTMFWRGVSDDDVEQTEF

KWRGEDGSVVTAFQIPSGYYIGGAIPEEKEALAKFLHEEPFKTTWKRSSTNQVYFPNG

FDQAPARENLPQLVKEMNELYKGEYELHFSTIERYIQAVKNQHPELEEIAGELINGKL

MRIHKTIFSSRPDLKALNTQVQNYLVNVLEPILTISMKLGFDYPVETIKEVWKLMFEN

AAHDSIGSCVSDTTNEDVYMRYKQARDIAMNLAELTMREIATQIHQPEEREISLTIFN

PYAKKRNEVIEAEVYLPQETFSILDANGAPLEYTILAMEDQTAYVLNQGNILNPTKES

YLPKKVYKAKIAFQSSVIPSMGYTQLTIDLAGDSYTKFATKETNQIENEFYQITVNED

GSFDIIEKTTDTVYKKQGIIEENGDNGDSFNYSPPEKDLVIQSTEFSPTINVKESDIF

KSIAVHFTMTVPADLIEREKGIRSKELPLSLRIALKKNSEIIDFTFQVDNRLVDSHRV

CVLFDTGIQSKFSTADQQFGTIQRPVVFEKEMALWNAHKEKWNEMPIAIETCQSFASL

FNEEHGLAVIPKGVREYEIIGEDFDTIRLTLFRTYGFMGKEDLLYRPGRASGETVIET

PDAQCHKVMTFEFSAYYFSDSFNQAAVANKAKKATSSLQVYQYAEFLNSRLIFTLGDE

PKGLPTHYSLFEIKGDLTLSIVKKAENRPGYIIRLYNGHVSETLNTEVVFNHPVQLVE

QVNLKEKRKRELSISDQKVLLEDISHAKFITLYVE"

CDS complement(2494857..2495960)

/gene="fruA_2"

/locus_tag="JFBMFIFI_02335"

/inference="ab initio prediction:Prodigal:002006"

/inference="similar to AA sequence:UniProtKB:P20966"

/codon_start=1

/transl_table=11

/product="PTS system fructose-specific EIIB'BC component"

/db_xref="COG:COG1299"

/translation="MKKKLQEIKGHLMSGISFVLPLIIGASLVVAIPKMIALGMGITS

LDPYADAEGFKHILYAIEQVGWVGIGLINTVLAGFIAYSIGDKAALGAGFIGGAVATN

TNAGFLGAVIAGFLAGYLVKWSKNNIKLPESFSGVMPLVILPLIATLSVAIVMGALLS

EPLAWINTSLVEWIRNMIANDVNKVLLALIMGAMIGSDLGGPVNKAAWMAGNVLLTEG

LYLPAIIVNVAICAVPLGYALATVFHKKRFNAELLDAGRNNFIMGFIGITEGAIPFTL

VSPLKLVPINMIGGALASGLGILLGMYDKMPPVGGIYGFFTVGNGWAYLIGLFAGAAF

IGFVAPLFVNFNKDEELEAQDTDSIDVEISFEN"

CDS complement(2495981..2496307)

/gene="manP_1"

/locus_tag="JFBMFIFI_02336"

/inference="ab initio prediction:Prodigal:002006"

/inference="similar to AA sequence:UniProtKB:O31645"

/codon_start=1

/transl_table=11

/product="PTS system mannose-specific EIIBCA component"

/db_xref="COG:COG1299"

/translation="MKIVGISACPAGLAHTPMAAKALEKAGKKLGYDVKIEQQGSLGQ

VNKISQQEANEADFVLLATDQKVIGLERFDGKPQLRVSINTCIKAPEAVLKKCIEAVK

ERQTAN"

CDS complement(2496304..2496765)

/gene="fruA_3"

/locus_tag="JFBMFIFI_02337"

/inference="ab initio prediction:Prodigal:002006"

/inference="similar to AA sequence:UniProtKB:P71012"

/codon_start=1

/transl_table=11

/product="PTS system fructose-specific EIIABC component"

/db_xref="COG:COG1299"

/translation="MDFTEENILLDVVVASQSELFKSVARYAEKLGFVKDVEKVYEAF

VERESEYSTGLQDGFAIPHAKSPYVLAPTVLFIRLENELEWETFDESRVKNIFALLVP

KEDEGTLHLEMLSRLATALMEDEFISQVQKNTNKDQLVTVIKNEMIGESTL"

CDS 2496990..2498927

/gene="licR_1"

/locus_tag="JFBMFIFI_02338"

/inference="ab initio prediction:Prodigal:002006"

/inference="similar to AA sequence:UniProtKB:P46321"

/codon_start=1

/transl_table=11

/product="putative licABCH operon regulator"

/db_xref="COG:COG1762"

/translation="MSKLSYQRLDDILEMLLKHSDSISMKELTSTFSVSDRTIRTDIN

NLNDTLKNVGATVSLLRGKGYSLDIQNQKAFTTWRNDSLTSNDFNLTSLEERQSFLLF

TLFKAAQPVSLDRFLEQLFVSKNTFYSYLKSIRDALIPYGLKIVNRPNIGFEIIGHEF

SKRKAINELLIVKDLQEYLVGFTEMELSLFDTIDLELLQALELESLAALELLDSDYYH

KTILSNFALALSRIVDNKTLTTFPVRIPVLKKNAQVIMNQFLTKIEHSFELKLTKEER

NYFNYCLSINAPRLVETEKEVEQSSTIAHEIVAELLEIIQKTANYDWREDVTLIKDLT

SHVEGFINMNLIETGRSNPLLDTIKNSFPLAFDLCLTHLETIGMHYGLYFSEDEIGYI

ALHIAGAIERSATINHRKHRVILICGTGRTMSRIIEAKINKRYQDKIDIVDRLSYIEL

QQYDLTEIDFVITTVPLEKMETPSVYIHMGQLDKEIEKVEPFMEKIKTQTSTIFDLFS

KDFFLYNPSVQSKKELLQKMIEPLKLQGIVPDDFYESVWIREKISQTNINRVLAIPHP

MSLTSIKSKVAVAIMPNGIDWGNGEKVHFIFLFAIKKEDYEDTGDIYDLLLEFIDRED

SQKIILNTPTFDSFIEILKEI"

CDS complement(2499156..2500427)

/gene="gmuC_1"

/locus_tag="JFBMFIFI_02339"

/inference="ab initio prediction:Prodigal:002006"

/inference="similar to AA sequence:UniProtKB:O05507"

/codon_start=1

/transl_table=11

/product="PTS system oligo-beta-mannoside-specific EIIC

component"

/db_xref="COG:COG1455"

/translation="MNGFIDKLGEKIMPVASKLGQNKYLTVLRDAFMLSFPITMFGSI

VVVLNNLPFFNDATKGTLNHLFSNGQSATMGIMSIFVTFGIGYYLSKSYEVEAIFGGA

VALSSFLILTPFFMILENGEEVSGVLALDRLGAKGMFIGMLAAFIATEIYVRFVKKGL

VIKMPDGVPPAVARSFAAMIPAILTLTIFALLNAFVIGVFNTNLHDVVYEVIQKPLVG

LGSGLPATLIALFFVQFLWFFGLHGQILVNSVMDPIWNTLMLDNLEAYKAGQPLPHII

TKPFMEIFTVGMGGSGMTLMVVILMAFFMKSRQLKDVGRLALGPGIFNVNEPVIFGMP

MVLNAAIFIPWIITPLIVTTFNYLVMAAGIVPAPTGVSVPWTVPIFINGILATNSILG

GVLQLVDIAIVGVLWFPFLKLIDRSNLNQTL"

CDS 2500819..2501553

/gene="ywqC_3"

/locus_tag="JFBMFIFI_02340"

/inference="ab initio prediction:Prodigal:002006"

/inference="similar to AA sequence:UniProtKB:P96715"

/codon_start=1

/transl_table=11

/product="putative capsular polysaccharide biosynthesis

protein YwqC"

/db_xref="COG:COG3944"

/translation="MEETISLIEIINLLRKRFFLILGTTLLGALCAFIFSFYFITPTY

TASNQLLVNPIKSDEQTANQFNDLQTDIQMISTYKDILKGPIILNEVQKRLETPLSTQ

AIASKITIETQPNSKVFAIKIIDSDAKRAATLANLISDVFQEKIGELMRIDNVHSISS

AEVNLQPVGPNKILNILIGTIIGLMMGIGFSFLLEFLNLKVRDEIYLINHLDLTHLGT

LSVIVEDGAGQQSSNLALTRSTRFKK"

CDS 2501565..2502272

/gene="yveL_3"

/locus_tag="JFBMFIFI_02341"

/EC_number="2.7.10.2"

/inference="ab initio prediction:Prodigal:002006"

/inference="similar to AA sequence:UniProtKB:P71051"

/codon_start=1

/transl_table=11

/product="Putative tyrosine-protein kinase YveL"

/db_xref="COG:COG0489"

/translation="MLNFRSKKQTRVVKAPKLITSLYPNSIISEQFRTIRSNLQFSEN

GMNLKTIGVTSSGPGEGKSTVSANLATVFADQKHRVLLIDADLRRPSIHRVFKKPNTK

GLTSLLNNPNLSVEEVIHRSKIENLFLITSGPTPTNPSELLSSNGMDRLIKLFQKNFD

LIIFDLPPTVAVTDAQIMATKVDGTILVVRNDVAYKDAVDKAVDLLRTVGANLVGFIF

NGAERKTTDPYSYYGKD"

CDS complement(2502324..2503637)

/gene="licC_10"

/locus_tag="JFBMFIFI_02342"

/inference="ab initio prediction:Prodigal:002006"

/inference="similar to AA sequence:UniProtKB:P46317"

/codon_start=1

/transl_table=11

/product="Lichenan permease IIC component"

/db_xref="COG:COG1455"

/translation="MDKFLDVLQEKMGPIAYKLDSNRYLSAIKTGFFGAMPILIIGSV

FLLFANLPIPGYADFMASIMGENWTTYFMVPYDMTMNIMTIFVVFGIAKDLAHHYKVD

DLAAVVIAVVAFFILTPTIADTGGANGIPTSNLGASGLFVGMITAILAVEISRWVEQR

GWTIKMPDSVPSNVARSFTALIPAFFVIVIFDLIRIGFSFTAYDTAQAFIFQILQTPL

TALGSSLPATIVVLLFEGLLWSFGIHGSNIVGAVMQPIWLSLTADNAAAFSAHEALPH

IVNYQFYSNFIKVGGAGGTLGLAILCVFVAKSAQFKTLGKLAIAPGIFNINEPLIFGI

PIVLNPVMMIPFIITPVVLAIVAYFAMATGLVDFTNGTNLPWTTPPIVSGFLLNGWRG

ALLQVVQIGLSMAIYFPFFKLEDNKAYQLELEGAHENQTPEEALA"

CDS complement(2503666..2503965)

/gene="licB"

/locus_tag="JFBMFIFI_02343"

/EC_number="2.7.1.-"

/inference="ab initio prediction:Prodigal:002006"

/inference="similar to AA sequence:UniProtKB:P46318"

/codon_start=1

/transl_table=11

/product="Lichenan-specific phosphotransferase enzyme IIB

component"

/db_xref="COG:COG1440"

/translation="MIKITLACAAGMSTSLLVSKMEKYALANEIDVEIIAVPEKQFEK

YADSTDILLLGPQVSFLLKKCKAAYEPKGIKVAAIPVVDYGRMNGEKVLKLALSL"

CDS complement(2503962..2504303)

/gene="licA_5"

/locus_tag="JFBMFIFI_02344"

/inference="ab initio prediction:Prodigal:002006"

/inference="similar to AA sequence:UniProtKB:P46319"

/codon_start=1

/transl_table=11

/product="Lichenan-specific phosphotransferase enzyme IIA

component"

/db_xref="COG:COG1447"

/translation="MEEHLENEEIIMSLIMNGGNARSLAMSAIESASAGDFEKAKNDL

KEGGAAISQAHQTQTELIQEEIRGNGTKISLLMVHAQDHMMNAMTVMDMAEQFIRLYE

ENAKLKELVTK"

CDS 2504704..2507367

/gene="norR_3"

/locus_tag="JFBMFIFI_02345"

/inference="ab initio prediction:Prodigal:002006"

/inference="protein motif:HAMAP:MF_01314"

/codon_start=1

/transl_table=11

/product="Anaerobic nitric oxide reductase transcription

regulator NorR"

/translation="MRRIELILQELEKSAGNTGLSTQEIAESLGLTRANVSSDLNQLV

AEGRATKEIGKPVLYFPLVKVAQLEKFSFDNPSLQMMIEQARAAILYPPKGLNMLFYG

ETGVGKSMFANLIYEYACSVKKTTKHYPFIHFNCSDYANNPQLLMGQLFGVAKNAYTG

ATEEKKGLIEEANGGMLFLDEIHRLPPEGQEMFFTFIDRGLYRRLGESNSERSAEVQI

LAATTEAPESALLKTFQRRIPMVIKIPNLLERTIEERASLITLFFNQEADRLGIPIKV

SQNSMRALLSYNCPNNIGQLKNDIQLACAKVYADFIVAHKKELIIHSPELPTHVLNGL
YLQVETRDIWTKLSQLHGKYLTFNGTNKVKLLQENKPVQSVYQLIDSTSQEMQHLEIN
QEALAKIIDTNIEEYFQQFSTVIDKPVDFQQLKNIVPVSVLEASQLMLELSEKLLKTK
ISTKVKHALVVHIYTTYTRVRSGEHLENTKLAEIKKKHPEAFKVALECVVLLNASLDI
DIPMDEAGFIAMFFVYNEEEPSAKQKFVRVIIMTHGASTATSMSTLVNSLLGVQHAIG
INMQIDESPATVMARLKTFLIESNTKEDCLFLVDMGSLNNISNEIQKELAIRCRTLPL
VSTLHVLEATRKAVMGHSLDEIYRDTKQVSLYLQPYAEKEPSFIRHSEQKTVILTICL
TGEGTAKLMKNILEKKIQFQEDYIQILPITLLHAESIEEHIKKLQQQYTILCVVTSLT
VETDLPQFHLYDLFQTNTIARIQEIVDIELSYATIGETLKEDLKYVDSDTIVQDTKLF
IQTVADTMEINLNVKVVFGSIFHISCLIDRLISGAALEPFPEKTSFQTLYQQEYQTLQ
QLVKPLEEKYSILIPEDEICYLLSLFIHHNG"

CDS complement(2507413..2508195)

/gene="glpQ_2"

/locus_tag="JFBMFIFI_02346"

/EC_number="3.1.4.46"

/inference="ab initio prediction:Prodigal:002006"

/inference="similar to AA sequence:UniProtKB:P37965"

/codon_start=1

/transl_table=11

/product="Glycerophosphodiester phosphodiesterase"

/db_xref="COG:COG0584"

/translation="MLCVAIGWTKEQPESREPKTALSIISHRGANDRQPEHSISAYQQ
AIQDQADYIEIDLRMTKDRQLVALHDETVNRTTNGTGKVENFRLAELEKLTLVSTQKE
QEKIPTLVEILEKFGKSTRYYIELRESTKGLDMVEPLIQLLEKNDLDDENFVLLQSFS
SESLIRARQLAPNLSLTWLMKAGDFDLNSAVKSDFATIGIESREVNDNVVRQIHQAGK
QVHVYFINKKSEKAEQKRLLNSQVDGYFTDFNLFTKKLINRE"

CDS complement(2508239..2508997)

/gene="glpQ_3"

/locus_tag="JFBMFIFI_02347"

/EC_number="3.1.4.46"

/inference="ab initio prediction:Prodigal:002006"

/inference="similar to AA sequence:UniProtKB:P37965"

/codon_start=1

/transl_table=11

/product="Glycerophosphodiester phosphodiesterase"

/db_xref="COG:COG0584"

/translation="MSKTKIYAHRGASGEYPENTLLSFRKAIETGVDGIECDIHLTKD

EELVVIHDEEVKRTFKGEGFVNDYTLDELRALTLAERYQKFPLYDRSWELEKIPTLEE

VLTLLQGTEIELNIELKTTMFPYFGLAEKAVKLVKKYQYEEQVVYSSFHYPSLAAIKE

VDTSAKIAWLVAHPVPRIWEYIPVLSLDGLHLSKEIGLSSDYAQLKGACQNLPIRLWT

INSSQDMKASIGQDSTALMTDFPRKALEIRALFS"

CDS complement(2509189..2510547)

/gene="ugpB"

/locus_tag="JFBMFIFI_02348"

/inference="ab initio prediction:Prodigal:002006"

/inference="similar to AA sequence:UniProtKB:P0AG80"

/codon_start=1

/transl_table=11

/product="sn-glycerol-3-phosphate-binding periplasmic

protein UgpB"

/db_xref="COG:COG1653"

/translation="MKFKKMMAVALIGLGTASVLAACDSGKDTTTASEKESSEKTTEK

TTVNFWHSMSGVNQEALNKIVTGYNNSQSKVEVKAEFQGTYEESLPKFQSVGGTKDAP

TIVQVQEIGTKMMIDSGFIEPMQKFIDADNYDTSDLEENIANYYKVDGKFYSMPFNSS

TPVMYYNKEAFKKAGLDPENPPQTFEEIEKAGLAIKKSNPAMKGFALQAYGWLYEELL

ANQGSLLMNNDNGRSKTPTKVAYDNAAGRSIFEWAEQMIKDETFANYGTNADNMVAGF

INGDVAMFLQSSASAGQVIDGAKFEVGEAYLPYPEKAEREGVVIGGASLWMSKGKETA

EQEAAWDFLKYLATPEVQAEWHVATGYFAINSKAYDEAIVAEAYKKKPQLKVAVEQLQ

ATKTSAATQGALMNMLPEERKIMETALEQVYNGAEIEPTFKAAVEQVNQAIEQANRAN

KK"

CDS complement(2510565..2511383)

/gene="araQ_2"

/locus_tag="JFBMFIFI_02349"

/inference="ab initio prediction:Prodigal:002006"

/inference="similar to AA sequence:UniProtKB:P94530"

/codon_start=1

/transl_table=11

/product="L-arabinose transport system permease protein

AraQ"

/db_xref="COG:COG0395"

/translation="MNKTFSTKAIVYLFLIIGSLVVFTPIILAFFLSFMTSQDYMAGK

IIPTEWTFDNYSTAFQRLPLVGYLLNSLIVSTLIVIGQLLFSSLAAYAFVFIEFKGRD

AIFYLFIATMMIPFEASIIPNFQTIKAWGLIDSYSGLALPFLATAFGTFLLRQTFKQI

PKELREASEIVGIGPFRFYWQVVLPVAKTSLVTLAIYGFLTSWNMYLWPLLASTNDSV

RTIQIGLKQLQSQETLNEWGVIMAGAMIVAIPTLILLFISQKRIQQGLAEGAVK"

CDS complement(2511380..2512321)

/gene="ugpA"

/locus_tag="JFBMFIFI_02350"

/inference="ab initio prediction:Prodigal:002006"

/inference="similar to AA sequence:UniProtKB:P10905"

/codon_start=1

/transl_table=11

/product="sn-glycerol-3-phosphate transport system

permease protein UgpA"

/db_xref="COG:COG1175"

/translation="MESQEIAPLYFEQVENFQRYKKIKRIKNSLIGFSFLLPSILLFG

IFIFYPLIKTIYLSFYLTNPAGETTLFVGIENYTRLFQSPLFLQSLKSTGLFVLYTVP

GTILFGFILAILANEKLKGIGIFRTLFSSTMGISVAASSVFWIFLFNPTNGYLNKIVH

LFGGTDLGWLTDPKWALISVAVSTIWMNSGFTFLIILGGLQSIDTQLYESANVEGAGY

FYQLRRITVPMLSPTLFFIFTVSIINAFQTFGQIDMLTKGGPSNETNLLVYSIYREAF

VNYQYGSGSAQAVILFILILVITACQFKFNERKVHYQ"

CDS complement(2512324..2513439)

/gene="msmX_1"

/locus_tag="JFBMFIFI_02351"

/EC_number="7.5.2.-"

/inference="ab initio prediction:Prodigal:002006"

/inference="similar to AA sequence:UniProtKB:P94360"

/codon_start=1

/transl_table=11

/product="Oligosaccharides import ATP-binding protein
MsmX"
/db_xref="COG:COG3839"
/translation="MKQVELQNISKTYHSQAKVLDEIDVTIQAGEFFVLVGPSGCGKS
TMLRMIAGLEDISDGTLKIDGEVVNHLPPKERDLAMVFQNYALYPHLTVEQNILFGLA
AKKVKKEEQQKRLAEAAEMTGLGLFLKRKPKELSGGQRQRVALARAIVSQAKLCLMDE
PLSNLDAKLRGQMRIEIKQLQRKLGMTMIYVTHDQVEAMTMGDRIMVLNAGKVQQIGD
PLTLYNAPVNEFVASFIGTPKMNFSVAHVTQENQLYVTEGLSIPLQKEQLELVDSFKE
VKVGIRPEGVQLATLDQAMGLVKVLNVEHLGDETQVIFEVNESLWTAKWAGQHMIQVG
ESLPIKLNLAALKFFNSVTGELLTTNSMIEASREGVR"
CDS complement(2513730..2514971)
/gene="csbX"
/locus_tag="JFBMFIFI_02352"
/inference="ab initio prediction:Prodigal:002006"
/inference="similar to AA sequence:UniProtKB:O05390"
/codon_start=1
/transl_table=11
/product="Alpha-ketoglutarate permease"
/db_xref="COG:COG0477"
/translation="MIGKITNRIGIPASLFYGYIGLVFFMIGAGIETSWFSAFLVSSG
YEIQLVSVIFSLYGLFVAIFSWLTSFFVNIFSVRKVMIAGLITYLVSAVILIAGIYFE
LLPVIAVAYTLRGASYPLFAYAFLIWITLRSEFKNLGKATSWFWFSFNLGLTIISPLL
ASLLLKFSNSINILAVGMVMALLGSFLSLKVNRDHLPTFNNNKSILYEMQEGIMILFE
YPRLAIGLVVKAINNIGQFGFVIMMPIFLVNHGYSLSQWGIIWATTYVVNSFAGILFG
NLGDYYGWRKIVCYFSGTLTALSCFLIGSVVFYFPGNFFLLMLAFIVFSFGIAAFGPL
SALIPAMALEKKTTALSVLNLGSGLSNFLGPVLVTVLFQKFDGFFVLAVFAVLYLLAS
ILAIFLKTPEELKKVYSLKEG"
CDS complement(2515036..2517636)
/gene="mngB_4"
/locus_tag="JFBMFIFI_02353"
/EC_number="3.2.1.-"
/inference="ab initio prediction:Prodigal:002006"
/inference="similar to AA sequence:UniProtKB:P54746"

/codon_start=1

/transl_table=11

/product="Mannosylglycerate hydrolase"

/db_xref="COG:COG0383"

/translation="MTNVHIVNHTHWDREWYFTSMDALVLSDQLFSDVLTELSCNPEA

SFVLDGQLSILDDYLDLYPEKLPEIKALIEAQQLFIGPWFTQSDAFFTSGESVLRNAM

IGIFESKKYGDFMPIGYLPDTFGFNAQIPVILNEAGLENIILWRGVSLGKHVNSPYFK

WESLGGGNQVYALNFPQGYGTGMLLEPTLEYVEGRLDKAVDFIKQFTDSKEIMIPSGN

DQLGIISDFNNKVAEINQLGKYDYQVSTYQNFLGLMKEMDLETYRGEFREPVLARVHK

TIGSVRMDLKQEIFHLEHKLIRRTEPLLVIAQQLGIDLSNRLLLRAWKKLLECQAHDS

LAGCVSDAVAEDIAHRLKEANEICDSLENIVLKRISESLALSENEFLMLNTSPVAFSG

EKVVKLLSRTKDIEIPNCAVVILEAEYIESRENIMEETPAGNRFITEAGYYILTLRVT

CDLPGLGYKVFAFENSQQKTDTFQAVSGTEIVEGNYKLTFENNQLVYQDPQRSITNFI

ALIDDGNAGDTYDFSPLLDDKAIELVFSECKCEESKLSSRMILTGKLNLPYDLQERMN

QAKTTNFAYQLSLELNATGEIKGTIQFENTILNHRIRLKVALNQTINRALSGVPYGFI

RKENQIVVDWEKHFSEMPVNIEPFEKTISVFTQEDSCSVFTHDSKEYEYQDSELLLTI

LATTDTLGKPDLLYRPGRASGDTTKKGHIMMATPLAQLKDKQVKSSFVLKFDQKKISE

KEIADWRYQLEQTSVSYQRQNLNFFIYRIDNKIQKRIEPLGLKEREFSVLSFSEHAGV

LVSGIHNSYYGNGFVIRFENPTATEIELNLVSLFPNRQVERVNAVEMIQDFSGKVPAY

GVASFLVKIK"

CDS complement(2517636..2518751)

/gene="fruA_4"

/locus_tag="JFBMFIFI_02354"

/inference="ab initio prediction:Prodigal:002006"

/inference="similar to AA sequence:UniProtKB:P20966"

/codon_start=1

/transl_table=11

/product="PTS system fructose-specific EIIB'BC component"

/db_xref="COG:COG1299"

/translation="MRKVGNSIKNHVLTGISYMIPLVIAGAVIMAISRVGGSIYGITD

IWDSGYATSSSGIVRLLHSLDGFGGIALGLMFPVIAAFIAYSVVDKLGIAPGLVGGML

VKELNAGFLGAIAAGLIAGYVCLLIRNHVKLPKSMASVVPVFLIPVLGTLVTVLMINY

VIGVPFAALNVGLENWLNSLSGGNQILMAAVVGAMVGFDLGGPVNKAAVTTAMALLTS

GVYAPNTAAQVAIIIPPIGLGLATVIGKHKYNTELKEAGKSSIIMGLVGISEGAIPFA

VESPLKVIPSTVIGSAIGSAMAVGLGAVNQAPISGFYGWFTVEKWPIYILSIAVGSLF

VAISSVLLRNKDAGIEEVSMSLDEEFDEDAWEEEWQS"

CDS complement(2518767..2519090)

/gene="manP_2"

/locus_tag="JFBMFIFI_02355"

/inference="ab initio prediction:Prodigal:002006"

/inference="similar to AA sequence:UniProtKB:O31645"

/codon_start=1

/transl_table=11

/product="PTS system mannose-specific EIIBCA component"

/db_xref="COG:COG1299"

/translation="MKRKIIAVTACATGVAHTYMAAQALKKGAKGLGDMIKVETQGAT

GIENELTEKDVQIGEVVIFAVDTKVRNEERFVGKKILRVPVAAPIKDAEKIIKEALAL

VDIDS"

CDS complement(2519106..2519552)

/gene="mngA"

/locus_tag="JFBMFIFI_02356"

/inference="ab initio prediction:Prodigal:002006"

/inference="similar to AA sequence:UniProtKB:P54745"

/codon_start=1

/transl_table=11

/product="PTS system

2-O-alpha-mannosyl-D-glycerate-specific EIIABC component"

/db_xref="COG:COG1299"

/translation="MSVEDVLDIKQVVIDLVASSQAEVIERLANELDKKGVLSNKEAY

IQSVLEREEHSTTGVGNGVAIPHGKSDTVTEAAIAFAKLNTPVEWQSLDDQPVVIVIM

LAIPNSEKGETHLRLLSEIAMKLMDDEFIVALKEETDPANIIKILK"

CDS complement(2519553..2521460)

/gene="licR_2"

/locus_tag="JFBMFIFI_02357"

/inference="ab initio prediction:Prodigal:002006"

/inference="similar to AA sequence:UniProtKB:P46321"

/codon_start=1

/transl_table=11

/product="putative licABCH operon regulator"

/db_xref="COG:COG1762"

/translation="MVLERREKEIVTILLASELTTISKIAETMHLSSKTISQSLKIID

NYFAGSGVSLIRKPKVGISLVGDRDQLNQLMQLTNQQAIPTTKEERVQYLCFKIIEQT

SYFTRQELQDSLFIGKTTLEKDMNKVNEIFALFDVTIEWIPGKGSFLNLMEHEKRKLA

VDLIYYFWSQNWQVVKTEESFTHTIEGVPDFAKGFVNLQMLKEIDELLQDYFSKSQPE

MSDMSYHSLLLHLLVAIERIKNGQLLMDEALENQFPSNSELAKFIKVLENHFEIQLPV

SEIMFINSHLNMNKLTASELIYESPNDNFVREIIKDTIVNYDEASLVGLVTHIKSVIE

RIRKDLPIANPFIADVKQSFPISFEEAIQLKQKLEKTFELIIPEDETAYLAVHIQAFK

ERKKEENIDKIKVLLVCSSGKGTSQLLAARIRRKFPKLEISRILSIQELARTKITEDL

ILSTVNLEIENHTILYVSPVLSQIDQNKINKFIDEKKEVAKGTKTFARLIHQDLVFLD

QPLANFEAVIHFIGKQLVRKGYVKKGIIQSAIDRESLSFTSFGKFATPHGAPELVLKS

AIAFLRLKKEIKWGDAKVKYIFFICIKDESSHDLEAIYDHLLEIIDSGDKSFLAKGNQ

EQLLSYLKEGK"

CDS complement(2521680..2523074)

/gene="licH_2"

/locus_tag="JFBMFIFI_02358"

/EC_number="3.2.1.86"

/inference="ab initio prediction:Prodigal:002006"

/inference="similar to AA sequence:UniProtKB:P46320"

/codon_start=1

/transl_table=11

/product="putative 6-phospho-beta-glucosidase"

/db_xref="COG:COG1486"

/translation="MSRNALKIVTIGGGSSYTPELIEGYIKRKDELPIKEIWLVDIEA

GKEKLAVVGEMAKRMVKAAGLPWEVHLTLDRRAALKDADYVSTQFRVGLLDARIKDER

IPLSHGILGQETNGAGGMFKAFRTIPVILDIINDMKELCPDAWLVNFTNPAGMVTEAA

IKHGGWKKTAGLCNVPIGHRKQAAEKLGLPEEELFFKFAGINHFHWHRVWDKEGKERT

AELIDLIYGPESNTESHLKNIHNVPFPYEQIKDLGLLPCGYHRYYYIEDEMLAHSVEE

FKNGETRAQVVKETEARLFELYKDPNLDYKPKELEQRGGTHYSDAACELIASIQNDKR

TDMVVSTENNGTITDLPYDCIVEVSGPVTGHGPEPYNWGSFPPAARGIIQVMKGMEET

VIRAAIDGDYGAALHAFTINPLVPGGTMARTLLDELLVAHKEHLPQFADKIKDIEENQ

PETVAYVTELMKSN"

CDS complement(2523265..2523936)

/gene="yfkO"

/locus_tag="JFBMFIFI_02359"

/EC_number="1.-.-.-"

/inference="ab initio prediction:Prodigal:002006"

/inference="similar to AA sequence:UniProtKB:O34475"

/codon_start=1

/transl_table=11

/product="Putative NAD(P)H nitroreductase YfkO"

/db_xref="COG:COG0778"

/translation="MNTTDKQQELLTIMDSRRAIKSFDPTKKISDEDFEFILEVGRRS

PSSVGYEPWRFLVLQNEEIREQIREVSFGAKGQLPTASHFVIILARKDARYDSNYIDH

LLKNVKKISPDIVEQMKGAYKNFQEVDMKILDDERNLFDWASKQTYLALANMMTMAQQ

IGIDSCPIEGFNYEAVTKILVENKLIDPEVFGVSVMAAFGYKENDPEFKSVRQPLNDI

VQWIN"

CDS complement(2524054..2524398)

/gene="yybR"

/locus_tag="JFBMFIFI_02360"

/inference="ab initio prediction:Prodigal:002006"

/inference="similar to AA sequence:UniProtKB:P37486"

/codon_start=1

/transl_table=11

/product="putative HTH-type transcriptional regulator

YybR"

/db_xref="COG:COG1733"

/translation="MEETQFDYSVNAGCPVTYTLEIIGGKWKSVILYHLTSGTKRFNE

IHRLVPRITTRILTLQLRELERDGVIQRKVYPEVPPKVEYTLSEFGTTLNPIVFALKK

WGDENQPENYYC"

CDS 2524559..2525092

/gene="polC_2"

/locus_tag="JFBMFIFI_02361"

/EC_number="2.7.7.7"

/inference="ab initio prediction:Prodigal:002006"

/inference="similar to AA sequence:UniProtKB:P13267"

/codon_start=1

/transl_table=11

/product="DNA polymerase III PolC-type"

/db_xref="COG:COG2176"

/translation="MNFVALDFETANHERHSACSVAMTVVRNSRIVDNYYTLIKPETP

FFWRNIQIHGIHEADVQDAPKFPEIWANMQPFFIENRLIVAHNLPFDQGVLNACLDYY

GIERPHFQTLCTVQSSKRLLTQLPNHKLNTVCDYLGINLENHHHALDDSNACANILLH

LEDQFGTDPLKKLVKHV"

CDS 2525236..2526540

/gene="ntpJ_2"

/locus_tag="JFBMFIFI_02362"

/inference="ab initio prediction:Prodigal:002006"

/inference="similar to AA sequence:UniProtKB:P43440"

/codon_start=1

/transl_table=11

/product="Potassium/sodium uptake protein NtpJ"

/translation="MALGFLSTIFIGGILLSLPFASQANQATNLLDAFFTATSAVCVT

GLTTLNTAVHWSLAGKLIIMLLIEIGGLGFMSFTIIILLASRKKINFRTRIIMKEALN

TNQLSGGVNFIIYILKFSVGLQLLAACLLSIDFIPRYGLAKGAFFSLFHSVSAFCNAG

FDLFGNSLESFQKNPLVLGVLSFLIIAGGLGFIVWRDLLTYRKNKRLSFHSKLTLRVT

FILLIGGFLFFLLTEQNLSHLKDLTFGERLMNTFFLTVTPRTAGFNSVSFSGLSQASI

LLSCFLMYIGGSSGSTAGGIKTTTLGILVMHAYSVFKGEERTQFSGRSINPTLINRAF

VLLFITMTVITTAIITLSITETIPPDFGIEYIVFEVFSAFGTVGVTLGLTPQLTAIGK

IIIMALMFTGRIGMFTFLLSIIKRTNQANTKIKYPEENVIIG"

CDS complement(2526587..2527120)

/gene="aaaT"

/locus_tag="JFBMFIFI_02363"

/EC_number="2.3.1.-"

/inference="ab initio prediction:Prodigal:002006"

/inference="similar to AA sequence:UniProtKB:P46854"

/codon_start=1

/transl_table=11

/product="L-amino acid N-acetyltransferase AaaT"

/db_xref="COG:COG0454"

/translation="MTQEKIDIVLREALPEDAGEIIRFLESAAIETGFLSMGAEGLNI

SSAEEAINLERILESENNILLVALLGDKVIATGSVSASNKPKLRHIGEIGVAVAKDYW

GFGLGTNLVDELIYWSQETGIIRRLELTVQARNQRAIHVYQKLGFNEEAIMPRGVFED

GEYLDVCLMSKMIDGID"

CDS complement(2527136..2527600)

/gene="tsaE"

/locus_tag="JFBMFIFI_02364"

/inference="ab initio prediction:Prodigal:002006"

/inference="similar to AA sequence:UniProtKB:O05515"

/codon_start=1

/transl_table=11

/product="tRNA threonylcarbamoyladenosine biosynthesis

protein TsaE"

/db_xref="COG:COG0802"

/translation="MKLNANNEQETRAIAAKLAEYLTAGDVILLEGNLGAGKTTFTKG

LAEGLGIKRVIKSPTYTIIREYTEGRLPLYHMDVYRLEETGGTELGLEEYFQGDGVSI

VEWATFIPEDLPKEYLKINLIPTGANLEQRELIFTATGTHYSQMLAELEKVF"

CDS complement(2527623..2528117)

/locus_tag="JFBMFIFI_02365"

/inference="ab initio prediction:Prodigal:002006"

/codon_start=1

/transl_table=11

/product="hypothetical protein"

/translation="MELGSEDLTVQVGDFTFNHRAAGILIQEAAILLEYYEKEDYWVL

PGGRVKVGEDSKIGVEREWLEELGLEVNGTRLIALIENFFVNHRTGSQRHFHEISMYY

QLESSSKIPFQIGQKFHGAESDKLNYSWVPLTELVNLNFRPEILTNYLVNLPECPVHL

INHE"

CDS complement(2528134..2528817)

/locus_tag="JFBMFIFI_02366"

/inference="ab initio prediction:Prodigal:002006"

/codon_start=1

/transl_table=11

/product="hypothetical protein"

/translation="MKFNELNPSQKAKGKMCFSYSGGKDSMLALHELIEIGYQPVCLV

TAINLDVERSWFHGISEPLLEAVAESLGIPLLKIRSDARSYQTEFIKGLEEAKALGAE

YCGFGDIDGDENRKWDEDTATEAGLIPLLPLWQRDHEKCVADFLNKGYSAVIKTISKS

YSIPQELLGKTLTTEIVTFLRGNELDVCGENGEYHTFVVDGPLFKKPVEFETIGIYEN

EYCYSLNIQ"

CDS complement(2528807..2529685)

/gene="rsmI"

/locus_tag="JFBMFIFI_02367"

/EC_number="2.1.1.198"

/inference="ab initio prediction:Prodigal:002006"

/inference="similar to AA sequence:UniProtKB:P67087"

/codon_start=1

/transl_table=11

/product="Ribosomal RNA small subunit methyltransferase I"

/db_xref="COG:COG0313"

/translation="MQSQKSFQDESRGMLYLIPTPIGNLEDMTIRGIRLLKEVDLIAS

EDTRNTQKLLNYFDITTSQISFHEHNTQERIGQLIEKLEAGLSIAQVSDAGMPSISDP

GHELVAACIEADIPVVPLPGANAGLTALIASGLAPQPFYFYGFLPRKKKEQLAALEEL

NQRPETLILYESPHRLKEVLKAMIGVFGEDRKIVCCRELTKKFEEFIRGTLVEALEWT

TENEVRGEFCLIIEGNHQGSLLAETDRSWEALSLKEHVELLMTELDVPSKTAIKEVAK

IRELKKQEVYGAYHEI"

CDS complement(2529657..2530016)

/locus_tag="JFBMFIFI_02368"

/inference="ab initio prediction:Prodigal:002006"

/codon_start=1

/transl_table=11

/product="hypothetical protein"

/translation="MESKISYFYVLCCKDGSFYGGYTTDLSRREKEHNDGIGAKYTKP

SYRRPLKMIYAEGFSSRSAATKAEYAFKKQSRAKKVRFLQAAGVEFPLNPKQKCLVNL

DGIEEESSNAKSKELSG"

CDS complement(2530000..2530755)

/gene="yfiC"

/locus_tag="JFBMFIFI_02369"

/EC_number="2.1.1.223"

/inference="ab initio prediction:Prodigal:002006"

/inference="protein motif:HAMAP:MF_01872"

/codon_start=1

/transl_table=11

/product="tRNA1(Val) (adenine(37)-N6)-methyltransferase"

/translation="MEKWLKEDERIDRLVRANLDIIQSPTVFSYSLDAVLLAHFANLP

YNNRGKIVDLCAGNGAVGLLLSEKTQSPIIGIELQERLADMAQRSIQLNQLEEQVSII

QADLKDATKWIAKDSVDVVTCNPPYFSSPVTSKKNPNPHLAIARHEIHTNLNEVMAVT

SDLLKMNGKAYFVHRPDRLIEILEAMKRNRLAPKRMQLVYPKVGREANILLIEGIKDG

KETGFRVMPPLVVYSEENTYLPEISEIMYGIEN"

CDS complement(2530889..2531869)

/gene="qorA"

/locus_tag="JFBMFIFI_02370"

/EC_number="1.6.5.5"

/inference="ab initio prediction:Prodigal:002006"

/inference="similar to AA sequence:UniProtKB:P28304"

/codon_start=1

/transl_table=11

/product="Quinone oxidoreductase 1"

/db_xref="COG:COG0604"

/translation="MQAIKMEKPGSYDVLKVVDIDMPIAKTGELLIEVKATAVNRTDI

VTRENKNLTAPYPILGVEIAGVVIENHSQDQRFQPGAKVCGLVNHGGYANYAVIPSER

AMLIPENLDFEEAAGLPEVFLTAYQTLYWLGELKENETVLIHAGASGVGTAAIQLAKQ

LTNAKVIVTAGSQEKLDFCLRLGADYGINYRTEDFAKEVLAKTDGQGADVILDFVGAS

YWDQNLASIGTDGRWVLIGTLGGTMIEQINLRDLMQKRISLKGTLLTPRSDAYKAKLT

NEFMEKVFPYFMDKKIKPIIDTVFPLTDVQHAHQYMENNQNIGKLILKMY"

CDS complement(2532427..2533209)

/gene="ylmA"

/locus_tag="JFBMFIFI_02371"

/EC_number="3.6.3.-"

/inference="ab initio prediction:Prodigal:002006"

/inference="similar to AA sequence:UniProtKB:O31723"

/codon_start=1

/transl_table=11

/product="putative ABC transporter ATP-binding protein

YlmA"

/db_xref="COG:COG1119"

/translation="MFEFQNVWFKKQGKWILKNINWQVKAGERWGILGLNGSGKTTLM

QLINGYMWATKGQITVLDEVFGKTSLPDLRRSIGWVSSALQQRLYEHETAEEIVASGK

FATIGLHVLATEELMNEAKTQLIASGGEHLIGKKYEVCSQGQRQLILIARALMAEPKL

LILDEPCTGLDLVAKRNLLNHIDILARHAPQTTLLYITHHTEELLPSFDKMLLLKSGE

IFAKGATQELITKDSLSAFFEEEINVELQKDGQVTTYLNRRL"

CDS complement(2533317..2534261)

/gene="prsA_2"

/locus_tag="JFBMFIFI_02372"

/EC_number="5.2.1.8"

/inference="ab initio prediction:Prodigal:002006"

/inference="similar to AA sequence:UniProtKB:P0C2B5"

/codon_start=1

/transl_table=11

/product="Foldase protein PrsA"

/translation="MRNVFKGKNVIAISVICLTMLILISCSGNKDIATTTIGNITKEE

LYEKMKTTAGASSLQKLIMLKVLTEKYGDKETDNQVNKKFEEQKEHYGDKFPGLLKNS

NYTEKSYKETIRMNLLIEAAVTAETKLTDEDYKIAWESFVPKLTAQHILVADETTAKD

LIAKINAGEDFDTLAKENSLDTGSKKDGGKLPEFDHNTPYDPLFIKATAELKDGEVSK

EPVKGSKGYHIIKMIKNPGKGEMEDHTKELEKIAIKKRLVDDAYVQSIVSKIIKDANI

EIKDEFLKKAMEDFLKQVDSSKVNSTDSSKKEKSSESK"

CDS complement(2534470..2536896)

/locus_tag="JFBMFIFI_02373"

/inference="ab initio prediction:Prodigal:002006"

/codon_start=1

/transl_table=11

/product="hypothetical protein"

/translation="MKKIMAKIIFLGVIISLYPYKTEEVHAENSSEQLTEMWDATSTE

LFGEYALISNAETDQSSNLQYIIDESARNNKSIYIPSGSYILNSNVKLRSGTHIVGDT

KNATILKNTTGKNVVLEDENYSESQNIKIEHLFFDGVGIFTRLAHTISIHHNIFYHPV

SLFPINLHASKGAVMEGNIFMRDKNHATPDTENRAIFIGGFATFGRYEYIEDVTIENN

LFGIKLDELDAIKSFSNESIIKTMNQLQHALETDQLKIEENEQNFLSTGINSYNNAKN

VLIKDNLFYQMYENDDRYGVVGDHAIYLRGSQDVQVVGNHVRGLHNGPYGGFKFKSGR

NITIMNNYLRNTGIIMYETPEFGLGDSFAEGTVAELSGWLVANNIFDFKAWQDRYAIG

IEYNRHTGIDNVYNGVFMDNHFVNYHNIPQNRRRELLIQNGAGEGFKGESTVVLGNTR

DDREANQLDVEYWSDKDYQMMPKDWKSLVDSNLYERYKNHPTPIRNTLPISKNSTIKL

SETVNPYDLVDQINDADEEKPMITILNPEVLEQVGEHKVFLKLVYQDTTQVNLTAIVT

VEGKEKARIDETTGQITFLASDKNESIVIPPEGGPGNPDVDIEPGNPGVVGPLAIVKA

PTMNFGTQVISNHDKNYNMLAEMQNLKIPLEDGTAEVPYVSFAQVVDTRGTNAGWNLK

VSLTNFTSTSHNNSLKGVRIYLRNPRIEYVSNNLNDAPLSHVDKLVLEPSTGGVSLMS

AAQSKGSGTSSIVWGNQKELNEQFENSTIELVENDAIQLFVPRETTADVGTYRSTLSW

ELSDIPGPDA"

CDS complement(2537074..2537946)

/gene="crtB"

/locus_tag="JFBMFIFI_02374"

/EC_number="2.5.1.32"

/inference="ab initio prediction:Prodigal:002006"

/inference="similar to AA sequence:UniProtKB:P37269"

/codon_start=1

/transl_table=11

/product="15-cis-phytoene synthase"

/db_xref="COG:COG1562"

/translation="MINDKQYSFDSQVDDFNYCKNIIKKHSKTFYFAFSQLSKKKAKS

IYAVYAFCREADDLVDNQKDTLKLDELLSKLKLFDKGFIPDEPIWRALSVVFSEYFLD

IQPFYDMLMGQKKDLNFTQPKTYDDLKEYSYFVAGSVGLMLLPILSQHSNEIKFPAKQ

LGEAMQLTNILRDIGEDLDMNRIYLPQEMMVRYEVTEEVLKNNIPTENFIKLWENLAN

KAEELYDSSMSMLPLVDEDAREPLLLALYVYREILNEIRRNNYHVFKEKQHVGKIRKL

QIAKKVKKQVNLIE"

CDS complement(2537936..2539423)

/gene="crtN"

/locus_tag="JFBMFIFI_02375"

/EC_number="1.3.8.-"

/inference="ab initio prediction:Prodigal:002006"

/inference="similar to AA sequence:UniProtKB:Q7A3E2"

/codon_start=1

/transl_table=11

/product="4,4'-diapophytoene desaturase

(4,4'-diaponeurosporene-forming)"

/translation="MKKIIVIGAGVAGLSAAVRLQNLGYEVHLYEKEETIGGKMNQIN

KDGFSFDVGPTIVMMPEIYREIFEVCGKNPDDYIPMEKVDPMLELFFGQEGSLLFSND

LIELTKMLEEISEEDAQGYFAFLADIYKRYLIAKEHFITKSFRGFWDFYNPRSLWAGL

RLRTFSDAYSSISKFVNDDRLRKSLAFQTLYIGVSPYQGPSLYTIIPMIELFYGVHFI

KGGMYTLATALSNLFTELGGKIHCGVAVDEIVVDNKRVIGIRVGEQIIEADAVFCGAD

FPYAMQNLIPNEKDRGKYTNKKIENMSYSCSCFLLYLGLDRKYSGKTLHNIYFAKDFK

KNINDLFEEGILPDDPSFYLYRPTLMDESLAPEGKEGVYVLVPVPELSKYDGWSEETT

NNYRDKIITLIKEKTQFKDIEEHIIVEECYTPEKFKNDFNAYYGATFGLKPILSQSNY

YRPHNKFKYADNLYFCGSSTHPGAGVPIVMQSAKLAVEELIKDDQ"

CDS complement(2539517..2539705)

/locus_tag="JFBMFIFI_02376"

/inference="ab initio prediction:Prodigal:002006"

/codon_start=1

/transl_table=11

/product="hypothetical protein"

/translation="MDKEKIILAIIILVLDVAGTVYVQYNRKLHVDQSNSTLQQVNRE

YGHYANNSVSYVYEKIVG"

CDS complement(2539998..2544497)

/locus_tag="JFBMFIFI_02377"

/inference="ab initio prediction:Prodigal:002006"

/codon_start=1

/transl_table=11

/product="hypothetical protein"

/translation="MMKIITHKKIAFLTIVVLILQIVLALESVVYSEDDVSEQSLDEV
ETTQLLNYMNAFNFKEQESNPRFFFSSSSMRGTVEDPVQVTFFSNQEVSEARVFLPEE
ATLIKEQLSAGISVDQGEQSREWIIKSERAQTTFVLPLMFDKVGNYELAVEEATAHLE
IREQEETSEEVPVEETESSKEENRIDEEQENEQLAEEVREPVSEAPQEQQTNEEETDE
ASQVVEPTVFDGETAEVATMAEFREAVGNPDIGIISVQANLTAATANVLTVDRPLLIQ
GNGYTLTFGINGFYFQLEEVTQASTIRLENATLTKVGATPLINATVESSKNWTVELED
ITEVNANTMRLASLPEGSIHFTGGVSNFTRTTSAQTFIEAKEVLATNQAEVTISRGNA
TVFFSSATVANPKFTVEQGATVTITTTAGSANTIDLRGENSEIALRGNGQLTVNTIGP
ATATTPTNTTNNAIALTGLSPKISISEKSRLSVRSTLSKSGIHLAGTNAQIHVANSEL
TVVSATQTAMNISGEAPLFAAENSTVQLTSTTGQRMSLIGANPVLNLKNSQLNMNAST
GTGINLQGATPKVLMDNSQLLMTDTGASFGIILTGADALFSLSNQSEVHLTGAGTGTT
ENIRIGNNNARPELSVTGGSTLSVTTTSGITAATDTANNAINIRGLNAQLNVIDSTLN
VNIRSGNRRALLVRGDSSVGEIYDSKINLDTLNSGGMEFIGDMPNVKISGSKTEVIIN
SAISEDAFKGNIYLGGVSNVSPGRNAELEIVENATVELNGGNFATLMINSTEAKVNIY
SEASLAVNGRNNDGLNAPIRFRAGNGYQFIIDGGHFFAGKNGGNSPVIRMSGSNNKIS
VLNGGVFEVNNPGNGVANDGGVAGGNQGVFYPSATDIGPNTFEVKGQGSYIVIDAKSG
PGIDLGRSARGQVIGSNGGYLSVSGRTATATGGIFNANILHVVFDNPLFMDYRNNRSG
GGRIFNVSNGSTLSGINSDLSLWRSGTDLNNNPTFTFDALDFEFTGSNYQNLYSTSKP
EELNTSVIGTNGLSQFSRLSSNNARWVIADELHLPTDADKHIYGRISVPVGLHDSRPS
WTNEVKVTVELDRVDGTKRNFSGYTVGHSDDSPGISIYGEEARGGLFEIELDDYLVEG
DKVRIVELEQTNSNSDDFENINLTKTLTTVPVMPPKPASFSGSIIPNHIREIVGYSEN
PQVTIEANYNGNALDTTDVEVDQDGYFSIFVGDLELKEDDEIQVFLRDKKGSAESAGI
LKPPTTNNEQGNINPKELYEYHDAIFQPATILKVVSDISPLDPLDPEIEVEPENKPEL
PEEQGLLSIDFVSSFNFGSQAISVQDQNYYAQPQRLLNGDGTVNEEEERPNYVQISDR
RSETDRNGWQLSVTQNNQFSTENGKELSGARMRLTNQQLATAHGGTAPSLQQTEPLEL
VPGAKRVLLMAQGNEGTGTWIYRFGDTNTAGNSVVLDVPKGATPEATSYSSTFTWELS
AVPGN"

CDS complement(2544494..2545558)

/locus_tag="JFBMFIFI_02378"

/inference="ab initio prediction:Prodigal:002006"

/codon_start=1

/transl_table=11

/product="hypothetical protein"

/translation="MNNANQRNSQWKLILGLFVIVLAFLRPTVVLGEETLNFHVTPEL
PQSQIEGTASYYNLNLNPGETETLVLMLQNDKEEPIEVKVTAHTAFTNVNGVVEYGKT
AEQADPTLLYSIDELIEPIDTIELAAKESKRVELTLKMPTDSFEGVLAGGLRIEEVRQ
EEENVDNGESIAIKNEFSYVVGVLARNSRTTVQPDLELLDVFADQVNYRNVISATLQN
FTPTFVNRLEVEATVQREGEDEVLYEASQSQMQMAPNSNFNFPISLEGDRFQSGEYVL
NMTARSGEEEWKWTKKFTIEADEARKLNRADVTIDTSTNWWMIGAIGLFLMILAIALY
VLLKKRKQKKGNSETGKGLK"

CDS complement(2545642..2546469)

/locus_tag="JFBMFIFI_02379"

/inference="ab initio prediction:Prodigal:002006"

/codon_start=1

/transl_table=11

/product="hypothetical protein"

/translation="MKLLNLATVGLLSATILTGGITAFAEEAREVQTEGQVTFTPNTD
EDTVVIPPETGPDAPDVEIDPEVPGTTGPLTIAKAATMNFGSQVISNQDQTYNMVAEM
QKLANPVADGPTHVPYVSFAQVQDTRGTNVGWDLKVSLDNFTADSHNGVLTGAEVSLL
DPRIQYEGNTATNAPTAHTSGLKLIPGAGAVSVMTAEDKQGAGSSSVVWGNQADLNTQ
LADDKVEVVENGAIQLFVPGSTAKDAATYTSTLSWELSDTPGEDGEDGEDGEDSDDA"

CDS complement(2546514..2546858)

/locus_tag="JFBMFIFI_02380"

/inference="ab initio prediction:Prodigal:002006"

/codon_start=1

/transl_table=11

/product="hypothetical protein"

/translation="MQVKWFFFLIQVTGIILLFLWLNSSFVAKAESVSSIKTEGSIGF
YGSYESESEPQPGLPDGVESTPPNEGINKPDSGKLPQLGSILSNYWLRIGLLLLAILL
LKRKQINLMRVV"

CDS complement(2546949..2547173)

/locus_tag="JFBMFIFI_02381"

/inference="ab initio prediction:Prodigal:002006"

/codon_start=1

/transl_table=11

/product="hypothetical protein"

/translation="MGTVEDYWEDVTIYYVEFVAFTGSNSRRKHRKTLAFNSSVSKED
VYHLVSVYFNHIDEVLYVDELTDGLLLKEQ"

CDS 2547352..2548830

/locus_tag="JFBMFIFI_02382"

/inference="ab initio prediction:Prodigal:002006"

/codon_start=1

/transl_table=11

/product="hypothetical protein"

/translation="MESAQIQLLIDKRNIRWFQILAILERSSSISTKNLSQSLNISIR
TIRYDMKEIKNYFNQTITINPSTNGYTFNINNKIDYIEKKKDLLEYEPILVILSGIFY
GKECSILDWADKLFLTDRTLISHLKKLNPMLARYQLTMDSPILSLKGKEINIRNFFLA
FFYEEDTIPHYVFPPILINNIITDMSTTKFEHFHLDVPRFKLSYLLYITYQRVSNRHT
LEISKEIVDTLATSHLRNTMNQMSLSFKKHTSIHLEPSDLVYLILMIVDSNSYDDTLN
LAPIYQEDVNSKWLPVIDGFCNELDVSDDQQARATIESFFHLNYLKNAASNTCLFLPP
SLLHYYEINYKEELKTMLSFLDKYPNAFNIDKSTQFEYTLLLFSMRLMYELKQKSYNI
AFLFEGSVESTILLENLARNYTPSNHKTYFLHAHNLPETLNKLAIDLVVTNFSEYIFE
LPTEIDSLLFNYNATAEDWNKFIKFIDPRLNKKYKIIFDSEN"

CDS 2549159..2549770

/locus_tag="JFBMFIFI_02383"

/inference="ab initio prediction:Prodigal:002006"

/inference="similar to AA sequence:ISfinder:ISBth20"

/codon_start=1

/transl_table=11

/product="IS6 family transposase ISBth20"

/translation="MRNVFKGKQFDKRIIIEAVGLYCRFSLSYRDVSEILRHRGIQVN
PTTIMRWVHQYGKLLFVIWKKKNKPTSTSWRMDETYIKIKRKQHYLYRAIDSHGNTLD
MCLRKHRDTNAAYAFMKRLIRRYGEPRALVTDKCAATIAAVNKLKNEGFLKSVDYRLS
KYLNNVIEQDHRQIKKRFSKSLGFQSMTSAATTIQGIEVVNAL"

CDS complement(2550352..2550708)

/locus_tag="JFBMFIFI_02384"

/inference="ab initio prediction:Prodigal:002006"

/codon_start=1

/transl_table=11

/product="hypothetical protein"

/translation="MTTREEVLSYVENNYQTKPEMTFKKFPTYCILRHKDNNKWYGLI

MNVSKRKIGIASDEKIDILDVKVEPELIGALLQKEGYHPGYHMNKEHWVSIDLNSISE

FTEIKEMIDNSFKMTS"

CDS complement(2550755..2551264)

/locus_tag="JFBMFIFI_02385"

/inference="ab initio prediction:Prodigal:002006"

/codon_start=1

/transl_table=11

/product="hypothetical protein"

/translation="MKQIQLLVDILVRAQERFEDTLSQMTLDEANTVPTPLIKSVTWL

MWHTARELDYQVSELNKSQPLWLNDGWTAKFGLDLPDDTEDWHHTPEEAAKVQISDKK

LLLDYLNASVVFTTRYLKTLNEESLGEIIDRNWTPAVTREIRLVSAIDDAVMHSGQAV

YTRRLVIGK"

CDS complement(2551521..2553737)

/gene="copA"

/locus_tag="JFBMFIFI_02386"

/EC_number="7.2.2.8"

/inference="ab initio prediction:Prodigal:002006"

/inference="similar to AA sequence:UniProtKB:P32113"

/codon_start=1

/transl_table=11

/product="putative copper-importing P-type ATPase A"

/translation="MENTQKKQTTMETLAITGMTCANCSQTIEKTLNKESGITLANVN

LATEKATVTYNPAETTLPKIIKSIEAAGYGAIVYDEEHQKEVAALKEKKSQRLKIDFI

VSTILSAPMVISMIFMLAGWHNPITGFFEIPVVQFILATPVQFIIGWRFYKGAYKAIK

AASPNMDVLVAMGTTAAYALSIYNAFIANRPHDLYFESSAVIITLILLGKYLEHNAKN

RTGSAIKQLMALQAKTAVVIRDGEEITVPIESVQLYDLIRIKPGEQIPVDGKIIEGTT

SLDESMLTGESLPVEKTVDDTVFGGTVNTNGTLVLQATKIGSETALSQIIRMVEEAQG

SKAPIQQIADRISAIFVPIVLVVALGTLIVTGWLTGDWELALIHSVAVLVIACPCALG

LATPTAIMVGTGLGAKNGILIKGGEALEGAAKIQAIILDKTGTITKGKPEVTDYEVLP

GENSEELLSFIMALEKNSEHPLGKAIYNYGKKKLNGEEQTVENFKALVGSGISGDIKG

ETMYMGAKRLMDENHSDYSHFSEKITALEGSGKTAMLFAKGDQCVAMIAVADQIKETS

KEAIQKLKQMNVEVYMLTGDNRLTAESIGSEVGIDAAHIFAEVLPADKANHVVSLQEK
GLSVAMVGDGINDAPALATANVGLAMGTGTDIAMEAADITLMNGDLNNIPKTIHLSKV
TLRKIKQNLFWAFIYNTIGIPFAAIGLLNPIVAGGAMAFSSVSVLLNSLALNRTKV"

CDS complement(2553739..2553948)
/gene="copZ_2"
/locus_tag="JFBMFIFI_02387"
/inference="ab initio prediction:Prodigal:002006"
/inference="similar to AA sequence:UniProtKB:Q47840"
/codon_start=1
/transl_table=11
/product="Copper chaperone CopZ"
/translation="MKNTFKITGMTCAHCVAKVEKGIQELAGVDKVKVHLKKGTATVT
YQEKQLSDETISQKIAEVGYQAEVV"

CDS complement(2554144..2554494)
/locus_tag="JFBMFIFI_02388"
/inference="ab initio prediction:Prodigal:002006"
/codon_start=1
/transl_table=11
/product="hypothetical protein"
/translation="MKKPRIFTMSFASVYPLYIKKAEKKERTKAEVDEIIFWLTGYNQ
ESLQKQVDKEVDFETFFDQAPQMNENVSLIKGVICGYRVEEIEDELMQKIRYLDKLID
ELAKGKKMEKILRT"

CDS complement(2554543..2555643)
/locus_tag="JFBMFIFI_02389"
/inference="ab initio prediction:Prodigal:002006"
/codon_start=1
/transl_table=11
/product="hypothetical protein"
/translation="MKGKVKIGMLVIVGIVILMGCYHKKSDYSSESNVKLESNQTPAI
YPENQEQANVLKDFPDLFKAAVESNESVKDIGMYVIPGLLNTTSVTSNEANTIVDCKS
MTPQGVTVVEGYVLISAYCHDHEHNSVLFVLDKDSHKYLKTIVLQDQPHAGGVTYDPK
GKNIWVCGRYEDKAEIVAISLANLEKYDLNQKVQPIEYSQRVELPQLKRASVITYWNQ
AIYVGYFADHEEGRLETYPVDEDGKMAGKLTKEQRIRTTIDLDKSNEKDTVVRELQGI

TFFKNQFFITQSYGALKDSKLLRFTYDAEKEVFLDSDVSKISDFPAHAEQISTYQNEL

FIIFESAAKPYRGIEKVWVDRVLSADLNKLMD"

CDS complement(2555753..2556517)

/locus_tag="JFBMFIFI_02390"

/inference="ab initio prediction:Prodigal:002006"

/codon_start=1

/transl_table=11

/product="hypothetical protein"

/translation="MTELKTLVNFRDLGGYSTGDGRVLQPKRLLRSGEVVGLSNQDAN

ILTTNFKLEHIIDLRSAEEISEKPDDSLENVAYTNIDIMKKEENNVASEKGMLHNLNP

DASITRMHDLYKDMVINDYAIKGYQEFVNLSIENEQGSILFHCFAGKDRTGMGAAILL

GILGVSRQDIMQDYLKTNIQRVAANQLILAEAKEKGLNDSQLEGLNNLMSVKQDYLEF

AWRTIDQEFGSFQEYILNGLKVSPSDQAALKHNYLD"

CDS 2556741..2557013

/locus_tag="JFBMFIFI_02391"

/inference="ab initio prediction:Prodigal:002006"

/codon_start=1

/transl_table=11

/product="hypothetical protein"

/translation="MISNKKMEQKAKLTTLSILLVSILTITVTILFTFPANTVLIIYG

FICLIILGMYLLNVYKHKGKSERLIYLFAFLIILLFALNTIKSFTN"

CDS 2557226..2557612

/locus_tag="JFBMFIFI_02392"

/inference="ab initio prediction:Prodigal:002006"

/codon_start=1

/transl_table=11

/product="hypothetical protein"

/translation="MNGYILKSMERVDFQLSSQVSMLDEANEAEIVLTKVAESKPKIQ

WKIVSYGELCDLLAENPENATIFLTLYSLPYKNQLKKTYPLITILEDSVKDIPTSNKA

IVKYENGIIGIIVATLLILIALSLIN"

CDS complement(2557719..2558867)

/gene="gerN"

/locus_tag="JFBMFIFI_02393"

/inference="ab initio prediction:Prodigal:002006"

/inference="similar to AA sequence:UniProtKB:Q9KI10"

/codon_start=1

/transl_table=11

/product="Na(+)/H(+)-K(+) antiporter GerN"

/db_xref="COG:COG0475"

/translation="MEFLFTLFIILVLTKVAGHFCSRMGIPSVVGDLLIGIIIGPALL

GWVLPTTFLLDFAEIGVILLMFMAGLESDLGILKKYMKPSVIVATSSVFVPITLCFAI

GMLWGFVPENALFLGLLFGATSLSISVQVLKEYKKLDSKEGATILGAAVLDDVLVVIL

LNIVISMFDENSATYPLWLLLTKKIVFFIVIFIAYKWLVPTIMRLSEKMLSSEAVIAA

GISICLLFAYFADFMGMAAIIGAFFAGIAVGQTKSRKIIDEKIEAVSYALFVPVFFVS

IGLNMTFVGINEHITFILVLTLIAVLSKLIGGGLGAKLSGFSWSSATIIGAGLVSRGE

MALIISKLGLDSSLLSNEYYSSIIMVIILTTIIAPFFLKYTIIQQNKN"

CDS 2559208..2560368

/gene="dapE"

/locus_tag="JFBMFIFI_02394"

/EC_number="3.5.1.18"

/inference="ab initio prediction:Prodigal:002006"

/inference="similar to AA sequence:UniProtKB:Q99SN6"

/codon_start=1

/transl_table=11

/product="putative succinyl-diaminopimelate desuccinylase"

/translation="MDKKEAITLLKDILKIDSTNGNEKAVADYLTTTLDRYGIASEQV

EYAKGRNQLIASLKGKQQQNSKKLGFTGHMDVVPVGEIPWKYPPFSATEEDGKIYARG

SSDMKAGLAAQVVAMIELKEQGLPFAGEIQLLATVGEETSAIGAGQLVELGYGSDLDA

LVIGEPTNNLIVIAHKGALWLRITTLGKTAHGSMPSEGINAVEHMLKLLTRFQEKFDF

SLEVDELVGASTSSIDVIHGGNGTNVIPDTCTVEIDIRTIAKQDHQLILKDVQSMITE

LQETIPNLNATIEVINDLESVRTEKDDAFVELVYSTVESVTGNQIKPFGGPGYTDGSQ

FTKAKKKYPIVILGPGVGEMAHQPNEYVEIDAYLQSIEIYKKLALSYLTEAN"

CDS 2560631..2562091

/gene="gbpA_1"

/locus_tag="JFBMFIFI_02395"

/inference="ab initio prediction:Prodigal:002006"

/inference="protein motif:HAMAP:MF_01905"

/codon_start=1

/transl_table=11

/product="GlcNAc-binding protein A"

/translation="MKMTKSLLIIAAVGAGLMTFAQQASAHGYVMQPESRAYKGTNKI

GLNTNVGQAQWEPQSIEAPKGFPNATNSPKDGKIASGDLAAFSPLDEQNQNRWHKSDI

TTGKLNVTWFLTAQHRTEKWTYFMTKQGWNPNEPLKRDSLEKITTKLDNGAKPNELMT

QEVNIPTDRSGYHVIVAVWDIYDTANAFYQVMDVNVKGSGEIVEDKEAPTTPQKLAAT

HTAFNQVSLAWDAATDNVGVTNYEILRDGKVIGETKGLTFSDTTVAPNTNYNYQIRAL

DAAGNKSALSANLAVKTPVKPVVDTEKPTIPGGLMPHMTTDTSTMIHWTAATDNVGVS

HYLVLRNGSVVGNTKELMYTDKGLTPATAYVYQIVAVDAAGNKSDASAKLTITTKEAE

GTPETNWEQNKTYVGGEVVRYNGLEYKAQWWTKGDKPSASGVWKLMTPNVVTEWNTSM

VYNGGDKVTFNKVTYQAKWWTQGDKPGSAAVWSVVK"

CDS complement(2562174..2563046)

/locus_tag="JFBMFIFI_02396"

/inference="ab initio prediction:Prodigal:002006"

/codon_start=1

/transl_table=11

/product="hypothetical protein"

/translation="MENKFAPCLWVDNQVEEMTELYTKVFENGKPLKTLYFLEDAHGK

IGDILTQSVQLANQEFILLNGGPEFKATPSISYMITCTSETQLQDLWQELSEGGKLLM

NLAIYPGVGQFGWLEDQFGISWQFSLDQSSSSQKITPCFMFSGEQYGNASIAVAEWIE

VFQSGEILDHYSNEDSTTKLAKFTLHQQEFMAMDSAVDHDFTFSLANSFYVYCENQKE

IDRLWTAITSKGTEMPCGWMGDRFGVAWQTVTRDMDTMLDRKNLTKALAVTQAVYGMM

KIDSEELRRIYNEA"

CDS complement(2563197..2564693)

/locus_tag="JFBMFIFI_02397"

/inference="ab initio prediction:Prodigal:002006"

/codon_start=1

/transl_table=11

/product="hypothetical protein"

/translation="MSYEIGKGLSRQYLLLRQLHNSSVPLTKNDLSKELNCSRPTLNN

TLILADNLIDRYGSIEKSSDGIILKEKKSINDAFIKAIILKETVLYQFVYSILFEKIV

SIESWAFDHFMSTSYVYSKMKSIRAFLRENKLELVTGGEFITIEGSEHQIRFLFFQLF
KHTSLIDEEWLFSKYNVKAIAQLSQDLAKELGIYFSPVCEYQFCIAVAINLQRSCNDY
HGEPSERQWESWKYYQTQFNFNNAFSNFERKIGKKIEEAERINILYSIIQLPFRYSSY
ENAMSRIKNIKALVPESYQLAKELFAVFHENPKTNYFGFAYLLDMFAVFSAIELYQDI
SVVKLFSDEENEQLFANKELVKQVEDLISEYEEKSDFYFLKVNRQYLLKSIFQMNQFS
ERLRGISKVIRVTIISEKGYSQELKQGAKFLQQFTQNRIELINSLEVYFDEAHLNVDL
ILSDYYPVNISSDLPIFLWNSEPTEKDFRMLEKQIELLLEDKPDLANE"

CDS complement(2564867..2565316)

/locus_tag="JFBMFIFI_02398"

/inference="ab initio prediction:Prodigal:002006"

/codon_start=1

/transl_table=11

/product="hypothetical protein"

/translation="MSKETIYNFLYNHATAFVDYWVDTYYVHTEEHKLRIDEKNYLTG
YKRECLYLFQAQQEAMLNDSDINSLCYGIGEDRAAMSTPYKEVYLNFFKFNDTVIEFL
INAQKSNQIVISDADVIDYMKIQKKHEVDNHYALFTGYMGYTTSLFD"

CDS 2565627..2566220

/gene="lytG_2"

/locus_tag="JFBMFIFI_02399"

/EC_number="3.2.1.-"

/inference="ab initio prediction:Prodigal:002006"

/inference="similar to AA sequence:UniProtKB:O32083"

/codon_start=1

/transl_table=11

/product="Exo-glucosaminidase LytG"

/db_xref="COG:COG1705"

/translation="MKKKLIITQKNKKIMLSLLLSVSIIGAFLFLNRQPKEYEYVAAE
EYSTKYQTVFIDKIADEAQNLQAQTNLFASITIAQAILESDWGNSELAQESNNLFGIK
AQESDQSTTLPTDEYEDGERITIDAAFKKYDTVQESMVDHIVFLEGASYAPVKTAKNY
IEAAHALKAGGYATDPKYAEKLIELIEQFKLDKFDTI"

CDS 2566301..2566939

/gene="walR_3"

/locus_tag="JFBMFIFI_02400"

/inference="ab initio prediction:Prodigal:002006"

/inference="similar to AA sequence:UniProtKB:Q9RDT5"

/codon_start=1

/transl_table=11

/product="Transcriptional regulatory protein WalR"

/db_xref="COG:COG0745"

/translation="MNVLIADDNPEMLQILCAYMKKAGFTTFTATNGEEALEVFYQEQ

LDIAILDWMMPKLDGLEVAAQMKKESPIKILMLTAKNQGEDEFHSLSNGVDEFVTKPF

HPQVLILRVKKLLNLTDSIHLADLTIDPMTMKVWKNEVPLDLTKKEFDLLMLLYKNRG

HILTREQLLVGVWGMDYSGVDRTVDTHIRRLREKVGDTVIHTKRGVGYSIEA"

CDS 2566929..2568332

/gene="rcsC_5"

/locus_tag="JFBMFIFI_02401"

/EC_number="2.7.13.3"

/inference="ab initio prediction:Prodigal:002006"

/inference="protein motif:HAMAP:MF_00979"

/codon_start=1

/transl_table=11

/product="Sensor histidine kinase RcsC"

/translation="MKHKPYKISTKLLLMLTTLIVSSFLLVILVNTLFLPRYYLHQME

DKVATVTSTLKKSTSTERTPKKLKELTENEQVTIIAADFNGLSTDDLNESLHTALLRE

RVALNRFWVTDDTIQQLQASPKSIQKLYDQGKQKSSFLVHLQILDNQLYLVGISLVDF

SETATIINTFTFISLGLILTLALLIIYISTKKITEPLSELKRVTEQIAKLNFIKVEDI

PPNEIGELAIHINKMSADLEHYQISLLNKNQQLKQFTADLTHELKTPIALIKAYGNGI

IDDLDDGSYLATILKQADLLSLTVDQMLDYAKLEQQDLQVEKIDVKQLFKERLSNFQL

AADTQKITFNLTDKVPAHRLIDADRILLARAFDNLLSNALKYATAPEITITWEKNDTD

LQFSIQNKTSLSDTFEPRKLLEAFYVEEKSRNKNLAGTGLGLAIVNTIIEQHQFELDV

SIKKQMIQFKLLFPFAD"

CDS complement(2568416..2569051)

/locus_tag="JFBMFIFI_02402"

/inference="ab initio prediction:Prodigal:002006"

/inference="similar to AA sequence:UniProtKB:O31533"

/note="UPF0702 transmembrane protein YetF"

/codon_start=1

/transl_table=11

/product="hypothetical protein"

/translation="MDLYYSAIIKLGLGFICLIIQINLLGKGNLAPSSAMDQVQNYVL

GGIIGGVIYNESITILQFFLVLVIWTILVLVMKFAKNNNVYIKRLIDGKPITLISNGE

VSVKECLRCGISANDLSFKLRAAGIYEVSTVKKAVMEQNGQFTIIQYGDESIKYPIVV

DGQVNEDVLDLIQRDQQWIEGELNQRGYSMGEIYMAEYISGEIRIYPYAEK"

CDS complement(2569067..2569522)

/locus_tag="JFBMFIFI_02403"

/inference="ab initio prediction:Prodigal:002006"

/codon_start=1

/transl_table=11

/product="hypothetical protein"

/translation="MEFYTFDYLVNQSQSNNYLKYSLIFCILLFLAFVIIKYMRNRIQ

TKYRDLSLILFLSLVFIIGVQFTNYSQGQSSISQSGQMVHFITQVAINEDTETKEISV

NSTSLTDEMVIKLKNEYYQVDFNSDFSAYSLMKVHIVDSDIKINGTNQK"

CDS 2569776..2571830

/locus_tag="JFBMFIFI_02404"

/inference="ab initio prediction:Prodigal:002006"

/codon_start=1

/transl_table=11

/product="hypothetical protein"

/translation="MTNKTSLKKYFNLLLVLILTLPLLLPIEGHAVTNRANSDVLGSQ

NWQGTTVTDQNGNDVTDLNTSFIGLAKYEAATNRYEFFDAATGLSRGDSGIYFLTQDQ

KKRVLISQTRNYNVVVDILTLNNELFTYRRMGKLADGSEGLVDVAHIPYQGTLDFTTP

LTDLVTETGEIDKSKSGRDILADTKWQGTVVYDENGLDVSAYNGGFLGLARYEASTNR

YEFFDKVSGQTRGDYGYYDVIRENKIRTHVSLGMNYAAAFELTEINSQRFTYTRMGKN

AEGADIKVTVEHEPYLGEYPLNFTFGETNPEPEIPEIEKPEESKPGQDLLGSTNWQGT

TVTDQNGNDVTALNAGFIGLARYDATTNRYEFYDAATGLSRGDSGIYFLTQDQKKRVL

ISQTRNYNVVVDILTLNTDLFTYQRMGKLLDGSEGLVDVAHVPYAGELEFTTPLSELV

TETGTIDQNLPGRDILANTKWQGTVVYDENGQDVSAYNSSFLGLARYEATSNRYEFFD

KATGQPRGDFGYYDVLRGNKLRAHVSLGMNYAAAFELTELNNQRFTYSRMGKNAQGED

IKVTVEHEPYQGEFALDFNFGQPIPVPEVPEVPETILPPTETPETTVPETQLPKVEQL

TNDPKQKLATDAAIFPKLESGSTDASDFPQTGEKDSNLPILLGFISTLLGTIGFAYRK

FR"

CDS 2571959..2574307

/locus_tag="JFBMFIFI_02405"

/inference="ab initio prediction:Prodigal:002006"

/codon_start=1

/transl_table=11

/product="hypothetical protein"

/translation="MNFKKIVTSLALLSLTMTGCGITGQSKTNKPSLKVTEQEFSKNK

SYAKKDFDYKVDPETFQLSLTTNGKTEIVAQPQQPRKIADYQETASEISWSYPDEHVK

ITLKKEKDHLAVKILNQAKKDNTFSWPKIDAENYTFPIAEGKSIPNNQPEWLKYFLGN

NEIDLLEAFSMSFFSINQPNFATTFVLDNTFNSQMIATTEPHLSFETSHTFVGFDPNK

TLNYRIYLTDNDPVAIAKTYQKDRVALDKFKTLEQKEQENPEINKMNGALQIYFWNNR

LLTSDDVKWQALAAKIDEPIFKWIGELLAKYGEDGDEEYQSILPAIKAGEAYKYEQNV

FLNSLNYVLLYPEFYNSEIFTKPDEVASKLIEKGVDKLTEQERYTLNQHLMAGTLGTD

VTKPLEQWGQKASTDVLTDMKKSGINKAWIGLPNWANGLMNPKMVAEANDKGYLVGPY

DSYQSIQEDASIDWNTASFPNPTLYEDATITNKKGDKIPGFLGKGRKLNSTLIFPDVK

ERVTGILANNIPFNSWFLDTDAAGEIYNDYDPTHKTTQADDVDARLKRMNYLNSLGMV

VGAETGNDFASEGMVYAHGLETPVIMWSDPDMRQNKESEYYVGDYAAVDGGIPSKYNK

VVPIKDEYKPIYTDPLYSVPLYKLVYNRSVITSHHWEWDSYKIKGLTGQRRLQEYLYN

TPPMVHLDQATWQDRKEDIVQNAKTWTPFQEKALQHEMTNFSYLSEDRLIQRTDFGAD

LSVVANFSKTDFQLGKEIIPPGTALIIEGNKTTLIDTRTVEK"

CDS complement(2574405..2575862)

/gene="katA"

/locus_tag="JFBMFIFI_02406"

/EC_number="1.11.1.6"

/inference="ab initio prediction:Prodigal:002006"

/inference="similar to AA sequence:UniProtKB:P26901"

/codon_start=1

/transl_table=11

/product="Vegetative catalase"

/db_xref="COG:COG0753"

/translation="MTKNLDSHKSNLTTDAGAPIGDNQNSMTAGNRGPVLIQDVALLE

KLAHFNRERIPERVVHAKGAGAHGYFEVTNDLTEYTKADFLSEIGKKTPVFTRFSTVA

GESGSADTLRDPRGFAVKLYTDEGNYDIVGNNTPIFFIRDAIKFPDFIHTQKRDPQTH

LKNHNAIWDFWSLSPESLHQVTILMSDRGIPATFRHMHGFGSHTFKWTNAAGESVWVK

YHFKTEQGIKNLTAEVAEELAGSNPDYHTEDLFNAIENGDAPAWKLCVQIMPLADADH

YRFNPFDVTKVWSQKDYPLIEVGRMVLDRNTDNYFAEVEQVTFTPGNIVPGVDFSPDK

LLQGRLFAYGDAHRHRVGANSHLLPINRPKNEVKNYHRDGAMRSDANGGSSVYYEPNS

LGGPTETLANKQASFEVHGMADSVAYDDDDHYTQAGDLYRLLSEDGKTRLIANIVGHM

QPVEDEAIKVRQIQHFLKADPEYGARVAAGLGINI"

CDS complement(2576103..2576942)

/locus_tag="JFBMFIFI_02407"

/inference="ab initio prediction:Prodigal:002006"

/inference="similar to AA sequence:UniProtKB:Q5XAQ1"

/codon_start=1

/transl_table=11

/product="Putative bifunctional

phosphatase/peptidyl-prolyl cis-trans isomerase"

/translation="MTKIVFFDIDGTLLDKKRRIPASAKLAILELKKQGIIPAIATGR

PPFLIKEILEELAIDTHISLNGQYVVHKGEIVYQNPMEAHLVERLAQAAEKNKQGIAF

AGSETIAGNSLTTTGSRKWQKKLSRILPFSPPTFLINAAFKRSDKIHGNRPVLKEYYE

NRLIYQCMLHATEEHDAYYSQEFPDCSFMRWNPYSVDVCPNNGSKAVGITKLMQFLEF

PIEASVAFGDGLNDQEMLKIVGTGVAMENGREELKAIADYITDKPEDDGILNGLRLLE

IIK"

CDS complement(2577058..2577279)

/locus_tag="JFBMFIFI_02408"

/inference="ab initio prediction:Prodigal:002006"

/codon_start=1

/transl_table=11

/product="hypothetical protein"

/translation="MKIEDKWKDAYLYYVTFRVEQCNLFRVIDKTLVLYEELEIDEIA

KIIKQNFNDVHEVLSVDVYQEVLVIKECR"

CDS complement(2577477..2578754)

/gene="mvaA"

/locus_tag="JFBMFIFI_02409"

/EC_number="1.1.1.88"

/inference="ab initio prediction:Prodigal:002006"

/inference="similar to AA sequence:UniProtKB:P13702"

/codon_start=1

/transl_table=11

/product="3-hydroxy-3-methylglutaryl-coenzyme A reductase"

/translation="MDSSISKFYQKNRSERIQLLVERGFLTQEVGSQLLNNPLLSDAI

ADSMIENQIGQFPLPLGVALNFLVDQKDVIVPMVVEEPSVIAASSNGAKVIRQLGGFT

TTIKERAMIGQLIFQNLVDVLAAEKVVQARSAEIIEIANLAYPSIVKRGGGVKRVETR

VILNENSEPEFLTVHLIIDVQEAMGANMINTILEATVGPITEWIQGDVLMEILSNYGD

CSLVTATCEIDPNQLTTSSYDGQWIAQRIVEATRYADLDPYRAATHNKGIMNGIDAVV

LASGNDFRAIEAGAHAYASRNGRYQAMSNWSINEVGHLVGELTLPMPVGMVGGAISVL

PLVKVNQQLLAIEKASELASVIVSVGLAQNFSALKALVSEGIQKGHMSLQMKSLAIHA

GATESEIEEVVAQLKKSKNQNLAQAQKILAEIR"

CDS complement(2578823..2579992)

/gene="thlA"

/locus_tag="JFBMFIFI_02410"

/EC_number="2.3.1.9"

/inference="ab initio prediction:Prodigal:002006"

/inference="similar to AA sequence:UniProtKB:P45359"

/codon_start=1

/transl_table=11

/product="Acetyl-CoA acetyltransferase"

/db_xref="COG:COG0183"

/translation="MREIVIIDAVRTPIGKYRGSLKDSSAVDLGVTVVKGALERANID

PESVDQVIFGNVLQAGLGQNVARQISIKAGIPYQAVAMTINEVCGSGLKAIMLGRQAI

QLGEADVVVVGGTENMSQAPSLVASTAVGMELKSEELVNSMFHDGLTDAFGEYPMGVT

AENVAEKFKVSREEQDLFAYQSHMKAAAAQEKGLFKSEIVPVQLADGTWFTEDETIRG

NSTVEKLATLKTVFKENGTVTAGNSSGINDGASALILMSKERAIAEGIPYLATIKATS

EVGIAPEIMGYAPYYAVKKVLEKGAYTIDQIDLFQLNEAFASQSIAVARDLEVPAEKL

NIYGGAIALGHPIGASGARVVTSLLQELQQTNTKVGIASLCIGGGLGVAMVVERD"

CDS 2580366..2581538

/gene="mvaS"

/locus_tag="JFBMFIFI_02411"
/EC_number="2.3.3.10"
/inference="ab initio prediction:Prodigal:002006"
/inference="similar to AA sequence:UniProtKB:Q9FD71"
/codon_start=1
/transl_table=11
/product="Hydroxymethylglutaryl-CoA synthase"
/db_xref="COG:COG3425"
/translation="MKIGIDKLSFYTPAFYVDMTELANARGIDPNKFTIGIGQDKMAF
APITQDAVTMGANAALNFLTEEDRQKIDLVILATESGIDQSKSGAIYIHRLLKLQPFA
RSIEFKEACYGATAGINLAKDYIAQHPDSKVLVIGSDIARYGLDTPGEATQGAGAVAM
LLSTNPRCIALENDQVFLTEDIMDFWRPTYSEYACVQGKYSTEQYLHFFETIWAEYQR
KFSQPLEKFAALCFHLPYTKMGKKALDLIIETAPSDVQEQLTENYRLSTLYSRNIGNI
YTGSLYLSFISLLDHQPNLAAEDKIGFFSYGSGAVGEFFSGTLQPGFKEFITTAEHQD
LLTNRKKLSISEYETRFKEELPKDGSTLEISSTDEDPAPIVLTGITEHMRQYKRKQ"

CDS complement(2581593..2584022)

/locus_tag="JFBMFIFI_02412"
/inference="ab initio prediction:Prodigal:002006"
/codon_start=1
/transl_table=11
/product="hypothetical protein"
/translation="MQMGYYIPILVICSYLIALFTAYNVVSVNRLLDDKSSLYRNGWI
LVKAIVMSVGIGAMQYVGLMGYLNHTNFSYTPFFAWFALGLLFLGCYLAFLLMSRKKT
TKNDLFIAGLLLTGGLLGVHYMGMRAIVSNGISYHPLFLMGSVLLSFISGMYTIWIVI
TQNKLLTGQLRIIQDAKKAIVLALLLSAVFYFGLVELQFETDYFLQPEMGLEATYLVV
TISLFTILLQLSFLGVSAVSFAFDTNRREISKLKDMSYSLLKSNPDPVLLINAAGEIL
SLNDAAQLLFSTELEQGITRIEEVMEPQQYCRSVSYFTEAINERKNVQFETKVTTNDG
TVKEFKIQIVPIIIEELKITEIFLVGADITKENQVEKMIRSLAYHDGLTKLPNRYFMK
EYIEDCLNELRQRYAFQKKTILSIYYIDMTELKKINDRYGHNIGDLYLTKVAQRIQRY
IGKEYFLARVGGDEFVLTFIEEYAGETEDVGENLLKVFGKAMVIEKIRLFPAASIGLV
KAYEDGSEFEELLTKVDIAMYSAKQQVRETGKTLIISYSQYEKQLQNEYQKELELMKG
IEKKEFLLHYQPKVHADTEELYGVEALIRWQVPWEDDLRYPDQFIPLAEQTGHIIQLS
EQIIEMACHQIKVWLNLGFTIPVSLNLSANDFHGDALVCLVAKQLAKHQIPPNLLELE

ITETVLMADLAESQKNLKQLHKMGVQLSIDDFGTGYSSLRYVIDFPIQSLKIDRSFTE
ALNLDAKVTAVTETIIQLADKLHLQVIVEGVETKEQLATLKTMGNFIIQGYYFSKPVS
AKDVTDKWLKG"

CDS complement(2584228..2585073)
/locus_tag="JFBMFIFI_02413"
/inference="ab initio prediction:Prodigal:002006"
/codon_start=1
/transl_table=11
/product="hypothetical protein"
/translation="MKNNKLLSDLALLGFLLLMFIIVVFTTFDQSSFVQNIIFMNVAL
LLAIITYFTTITAGLILNLIFIFLQGSYALYQSISTSETFGASLYFWLVMTPLLSVTL
YMFSYRTQKLQAENSQLERKNRQLGILDDETRLRTIRAYKEDAQVFMSTSRRFNLPVT
MVVIQVKHWNELQRMLSEDQISAVIGRVTSTMKQAIRDNDILYVLERENLTWGLLLFT
DQAGARVVTERIKEFFEESISDFSAKNQVQIEIKSGAAEYVQETIKSPYDFVEAAIKE
LEYDV"

CDS complement(2585063..2587144)
/locus_tag="JFBMFIFI_02414"
/inference="ab initio prediction:Prodigal:002006"
/codon_start=1
/transl_table=11
/product="hypothetical protein"
/translation="MKKWFLMGFVVIFSIVSSSVVVYGAEAEPQTFITKFDNTDSSLT
GVSATTNLYFQILDYWEVDQVRLNLDYQISQLTKNDVSSVTLSINGMKFNSFRPTETS
LEKQHLVIDIPKELIKQGSNVLKIEGAIMTTNGEDQCSLTETPADWLHIFKGSNVGVN
YTKKEMPETIQAFYERFSGMDSVVHKEVAVIVPEKATPTELETATYTLSGSAKANGQD
DQQIPIGTLNDSHLAQLPLVVLVAEYDHLPADYQTKIDKNSVKEQAVLKVIKKENQTL
LVITSSDKKALIKAGRYSANEELMSETQLASKEVSKDLDTATSHLKIEEDFQMTSTGD
ELKGPFHQEQTYFMSLPANRSLAANSQVNLAFRYAKNLDFDRSLMTVSIGGIPIGSKK
LTKEYADGDTATFTLPPDLDVSGNFAVTVSFDLEIKDLQCTPRQDETPWAYISPESMM
RVNTKDRTDLLFENYPFPFLKDGSFNQIAVVLPEEMNADYYRGLTNIFNLLGKYAQDN
TGEVVFYSSSATKEELKSSNIITMGSALDNTFIKGINEKLYFKYDKDGAGFLSNEKLS
IESNYGQQIGTGQLLHSPYTASNGLLVMTGATPQSVYLASKELSTQDKVQKHSGDAFT
VDKDNRVNSYRFKKKGDIGEATTFVDKVKKESNLLLYLGLVGLVLASILIALILAFRK

NRLDKEDNDEK"

CDS complement(2587166..2588416)

/gene="icaA"

/locus_tag="JFBMFIFI_02415"

/EC_number="2.4.1.-"

/inference="ab initio prediction:Prodigal:002006"

/inference="similar to AA sequence:UniProtKB:Q5HKQ0"

/codon_start=1

/transl_table=11

/product="Poly-beta-1,6-N-acetyl-D-glucosamine synthase"

/db_xref="COG:COG1215"

/translation="MTLADYLLLVAIFSIWGLLLVNVALIIAGYIYYIKIEGKELPEI

KGVPPLVTIMVPAHNEGVVIVKTVESLLRFDYPQDRYEIIVINDNSSDNSAELLAAIQ

AKNPTRQMHIINTDAVVGGKGKSNALNIGFKQAKGDLIAIYDADNTPEKTALRYLVAE

LSNDEKLGAVVGKFRTRNRNASLLTRFINIETLSFQWMAQAGRWQLFKLCTIPGTNFI

MRREIIEAIGGWDEKALAEDTEISFRIYMMGYKIKFQPKAVTWEQEPQTLKVWFRQRT

RWVKGNIYVIVKNAPLLFNAKARKIRFDILYFLSIYFLLMTSLVVSDVLLLLNLMGYV

TTTLAGFSGLLWLLAIILFVVGTFITITTEKGEMTFGNLGIILLMYVTYSQMWLAVAA

YGMVMFIKETVLKREVKWYKTERF"

CDS complement(2588427..2589995)

/locus_tag="JFBMFIFI_02416"

/inference="ab initio prediction:Prodigal:002006"

/codon_start=1

/transl_table=11

/product="hypothetical protein"

/translation="MIKWYKLMAIGCLFWLVSCFSYPTSVFADTKPDESEMKLLLVYD

SLDTSGSGEEKISTLQRLLTSFNVVVTTISVAEYHENEISDFNGIITLINAPDLAIEN

PFYLKDRDAFTGLKLHIGGNLPMKWQKQLGIELEQINGERGNLVSLSEKANEVMETEL

TVEVAAGISEAAEEFGRIDFENHNELKPYGILNGNSAYLPYLKPDGLSFILTSQLIQK

WLKQTGTFNPMLVFTDVTPFSNLPLIKYLADQLYDAGIPFALSATSVWQNVDLKAMKN

YTDMLRYVAARNGGLLLKTPEVMGVSREQRDLAAIMPKTIDELVADNVFPVGISAPAY

WNQDTEYQQDGLSYSSTVLLLPNPKKLQYRDKTTTAPIYDTTYFGLPVSSLRAIEWGA

IERHKFNTPTALTYKIPDTRKEWDSINKELNQLPFLFRGINLREHNMVTETNQIKVEN

AILTVNGEREYPETMVKSVAGKQAKTVKDTGSLTDFFNLQDKIFIVIISATLFVLSIF
FVIGYNLYKGKYRK"

CDS complement(2590022..2591518)
/locus_tag="JFBMFIFI_02417"
/inference="ab initio prediction:Prodigal:002006"
/codon_start=1
/transl_table=11
/product="hypothetical protein"
/translation="MKQLKKLLLNSGITISTVISPSLLAVFLAAIVAGIALFIQPLIG
IADNGDFLRMLYSNGLYQLPKYSDQYFSYFIKEYGIMHYYNEHGASLFSSEPLFIHLA
VVLNKLFYSQTIFDIRFLSFIYYVLFLGSIYLLTEALTYRIKGWKGYFIALFVVIIFT
DTAYIAYFNSFYSEPIMFIMCLYIVSSTLLMCRKRYNDGVLLVLLTLSSMLLITSKQQ
NAPLVVFIAFIYIGLLFIKKTVISRMMVVFSLVLVLVTGVATYKLITSEFNKINQFQS
MTRGVLLESENPEATLSDGGISEQFALLKENIYFQEYTAIDVNSPYMVDNFYEKYGFG
WILKNYLAHPKEFYDILDVAAHDIHEVQTVEMGNYEKKVGKKAKSQATYFTLYSRLKR
TVYPKTLGFLIIWTLVYLALYFPSTYRAYQQNNPRGLLRYWLILASIGMIFGILLISV
VGDGDADLAKHLFLIAVLFDFLTIMTLSDILFNRLWQQETEFDSLKLK"

CDS complement(2591685..2592857)
/locus_tag="JFBMFIFI_02418"
/inference="ab initio prediction:Prodigal:002006"
/codon_start=1
/transl_table=11
/product="hypothetical protein"
/translation="MRLKKAIGLCGLSGLFFFLLSCQPIQSNKNFIGEQHSQAESSPV
EIKIKKTNQYGANRKGAKDNEQKLETFVAEKLTGDYGIYTNYLDTDQNEEVATGHEVL
SESAGLMLRYAALKQDETLFKKTWETTVATFNESTQFSYRFSPKLQKRYPVNASVDDL
RIIRGLAEAQQFIPGATSENLVTQYGSRFYKKMVNQNKLVDFRDSQYDITNQSITLCY
IDLKTLSLLPIAVEDKKALIDAQEMILTEGYLGDQFPFYQTRYDYTQKAYIASEEINV
IESLLSILHLSEAGKTKSQSIAFIKENVEKGTLYNRYSKTGEVLDQNQSAAAYGICAM
IASEVGDEELYNRAMDRMEASQIHDPASPLNGGYGDSQTNALFSFNNLIALLAYQY"

CDS complement(2592872..2593312)
/locus_tag="JFBMFIFI_02419"
/inference="ab initio prediction:Prodigal:002006"

/codon_start=1

/transl_table=11

/product="hypothetical protein"

/translation="MKKGVLIGSGVVVLLSTVFFIFIMMNKTETKENKATLTSASGME

IMGLIDEKKDGIIYIGRPTCDKCQAFQPKLEKALAKNNQAILYYNTDEGKKTDIKNFE

KVVNVTDIQSVPAVLVVKKGQVTNRLSNFKNQNEINQFLLENKG"

CDS complement(2593731..2595158)

/gene="celB_1"

/locus_tag="JFBMFIFI_02420"

/inference="ab initio prediction:Prodigal:002006"

/inference="similar to AA sequence:UniProtKB:Q9CJ32"

/codon_start=1

/transl_table=11

/product="PTS system cellobiose-specific EIIC component"

/db_xref="COG:COG1455"

/translation="MMEALSAWLEKHLLPIAAKIGAQKHLVALRDAFIGTMPATMAGS

VAVMINAIIRDLPPQFFSGYVGGDIPIIKEIISINGYIWNGTLPIAGLIFAFSWGYNL

SRAYGVNDLAGGIVSLAASIAGITFSFTGNLATSVPAKMVETINAESATTGWSATSQG

ITATGWGWIPLNNLDSNFFFTAMVIGFVATMIFCKLMLRDMTIKLPDSVPPAVSKAFA

AIIPATVALYSVAVFYWAFAKLIPNMTFLEWIQETIAKPLLGFSQGFGAVVVVTIAIQ

LFWFFGIHGMNVLAPVLEGVFGVAQLKNLNLFQEGGVELVKAEGYKWVRGSFDAYSMF

GGSGGTIVLIVVILIFSKRADYRTVANLSLGPGIFNINEPVMFGLPIVLNPILFIPFM

LAPVVSVSIGYFATMLDLVNPVSQSIVWVTPPFLLSFLATGADWRAPIVTLVCVLASF

AIWAPFVIAANKMDPNLGESENQEQ"

CDS complement(2595532..2596479)

/gene="mntA"

/locus_tag="JFBMFIFI_02421"

/inference="ab initio prediction:Prodigal:002006"

/inference="similar to AA sequence:UniProtKB:Q8Y653"

/codon_start=1

/transl_table=11

/product="Manganese-binding lipoprotein MntA"

/db_xref="COG:COG0803"

/translation="MKNKMIYLVGLAVVLLTVAGCSAGSSNQDKKKADSTGKIQVVAT
YSIIADMIENVGRDKVDVYSMVPVGTDPHIYDPTPRDTEAVEKSDIVFYNGLNLETGK
GWFDKVITNSRKEDVTFAVSKGVEPMYLSEAGQETEEDPHAWLNVQNGIKYVENITDE
LSKISPENADYFEKNKEAYVAKLTELDTQAKEKMLSIPEENRILVSSEGAFKYFSKQY
GLQAEYIWEINTDSQGTPGQMKRIVERIKKDDVKALFVETSISPKTMEAVSRETGVPI
AAKIFTDSLADKGEDGDTYYDMIKWNIDKIYEGLSGKTN"

CDS complement(2596476..2597330)

/gene="mntB"

/locus_tag="JFBMFIFI_02422"

/inference="ab initio prediction:Prodigal:002006"

/inference="similar to AA sequence:UniProtKB:Q55282"

/codon_start=1

/transl_table=11

/product="Manganese transport system membrane protein
MntB"

/translation="MDILFSISDATGLSVTMINALLSSIILGLISGIIGSFIVLRQLS
LMGDALSHAVLPGVALSYMFGVNMMVGATFFGLLASILIEFIIKKSKIKTDTSIGIIL
STFFALGIILITKAHSGLDLNHVLFGNILAVTPQEIGTSAIILILVLVTVTLLYKELL
ITSFDPVMSKAYGLKNNFYHYLLMLLLTVFTVSALSQVGIVLVVALLITPAATAYLWS
NKLLHMMIMASALGTISGIIGVLISFSYDLPTSAAIVLVGASFFTISFILSPKNGFLK
RRKEEITI"

CDS complement(2597333..2598067)

/gene="scaC"

/locus_tag="JFBMFIFI_02423"

/EC_number="7.2.2.5"

/inference="ab initio prediction:Prodigal:002006"

/inference="similar to AA sequence:UniProtKB:P42360"

/codon_start=1

/transl_table=11

/product="Manganese import ATP-binding protein ScaC"

/db_xref="COG:COG1121"

/translation="MIQLKNINVSYFGHNALSDITTTIPTGQSIGIIGPNGAGKSTLL
KSMLGVIKKDSGEVYINDQPISGFKKKIAYVAQRSEIDLTFPITVFDTVLTGTYPSLK

LFVFPGRKEKERAKLALEKVGLSNLAKKQIGNLSGGQLQRVFIARALAQDADYYFLDE
PFVGIDMVSEKIIIDILNELKAQGKTVLTVHHDLHKVVDYFDHLVILNKKLIVSDQID
KAFTAKFIQEAYGDSLGDIVIKGVEG"

CDS complement(2598690..2603831)

/locus_tag="JFBMFIFI_02424"

/inference="ab initio prediction:Prodigal:002006"

/codon_start=1

/transl_table=11

/product="hypothetical protein"

/translation="MKNKIITLFLVTMLVFQVSSPALIYAEGTATETNSSQHEAEIPA
TAESSLPTGIAPVEEPTTQVENEENSTEVDTPTKPEEVPTSTQAEENQPVESNEVKPE
TTNKQPETLIEKAPKLAITDNLFTNIKMYKINGDEVKENDIIPNMSGIKLNMTFSFTN
KNYQTGDTFTTQLPPQIAIAKDLSGDFSPMTSAKWAIDAATKKLTITFLEDNIASEEY
KLSLITSLEKVSNSAEENQEIIFTTTPNETIYHLEVTSNIDSGKSSTAITVGDFNPKT
AKIESIFNLDRTDSANRRFTVEPYQSDSKLVFDSVKVSTSDVDFNGVLVGEKTELTEG
VDFQVTKKNSGTYQATAEIKLTNKLNKKAIIVESSLSGINGKSYIDDSTSGNEYNYFY
SYSYTYEDGKNSQYSFASQNFYTVQPLTTSGKINKETGFIDWTIDYNFNEQPLTTMSK
LTTDLANQGVEYIPDSLKIEKIGFNYISGNNYTVIEQGDGADQWGTPSINTNGTIQMN
ANSATNQAYRVTYSTKITDPTERVITNKISDGTTQKEAKISLIPNLLQKESGKIDVFN
QTMEWSITINAEKYTMKNPVIHDYFIDSVVSHTSLTVKKKLSETQFENLTEGIDYKVT
LFDETGSPVGAQPNVNGAPASFNGGVRIDLLGEYAQLKDTLVVTINTKLNTSEEETEI
KNKATLNYGDVPGVIEYEAKGTFIDPYYTGGQKMGEAAVSDGQYLYQNWLILVNSKGS
NFNQTSLSDTLPLGSELVPGSLRFEEVTSQSMLDNMRNYLRYDYNLVPVGDDVYPTKI
DTENNALNLEFNKLGSKRVYVKYKTRVKKDWYTYNQLKNKAVVKYDTKTAEYETTVYA
YNYEMALTKAVTLDSAKENVANWTVTTKSISTNLPVENPVISDTLDNGSTNAAYDPIS
FVVKNTATGEKISSDHYSLKITGNTFTITFTDYKAESNIQVTYNTISEFPGGVKSKAQ
VDSASYVGLNAYYRQAEAAINLGFSSGSGSGIVKTADLTILKVDNLDESTLLAGATFE
ILKADGTETGLKGDTDAQGELTFTGLPLGDYQLKETKAPTGYDINPEYKTGKLISLTE
SMLPIKVTNERTFLNSVTLKKTAKESNESLAGAVFELQDETGTPLESALTTDVNGTLT
LQDLKVGSYQFVETKAPTGYKLDETPLTFEIKKGQTESVEVAMTNELLPGGVVLTKTD
EQSGETLQGAVFELQKNNKEVIQSGLTTDNLGKLAVDELKPGSYQFVETKAPTGYELD
TTPVEFTIAKNQSEATEVTMTNKLTPSGIVITKTDDQTGEVLAGAVFELQDSEGKQVQ
STLTTDESGKISVNDLAPNAYQLVETKAPVGYQLDATPVPFKIGKGQTEALKIAITNR

ANPSSVLLTKVDSQTGTVLQGAIFELQDQSGKVLQRDLVTNASGKLAVSGLKPGSYQF

VETKAPTGYQLNQKPVVFTLEKDQTEAVNITVSNELIAGVVVLTKIDERSGETLAGAE

FDLQDSQGNKLIQHMVTDAAGKLAINDLKANDYQLVETKAPAGYQLDQEPVIFTVEKG

QSSALQLSKTNKAKQQTVRVQKKDAKTDELLAGAIFDLQDKDGKVIQKNLETGENGTL

EISDLANGYYQLVETKAPTGYKLEQTPMNFSIAQGSDLITVDKYNDQLNNKTLPRTSV

SKNNTYTLPKTGENPSVKWMIIGSLVVMTAMLAYIVQRRKGELLK"

CDS 2604320..2605162

/locus_tag="JFBMFIFI_02425"

/inference="ab initio prediction:Prodigal:002006"

/codon_start=1

/transl_table=11

/product="hypothetical protein"

/translation="MITIGLTNWKGHESLLVDKNKELENYTAHFPFVEMDTSFYSIQP

ETNIVNWMGKTPDGFQFIPKAFQAMTTHREWGDYFPSEKAMFQAFIASFTPMLEANRI

KAFLFQFPPYFACTKENVDYLRKIRYWMGELPVAIEFRNNSWFSENHYGATLKFLTEL

QFIQTTIDQPQTQTNSIPMVLNVTNPSLTLLRLHGRNFTGWLESSSPDWRKKRTLYNY

SPTEIKELKGYVKTLALDAKEVAVIFNNNSGGHAAPNAKALQKELDLSFENLAPQQLG

LDLF"

CDS complement(2605553..2606926)

/gene="celB_2"

/locus_tag="JFBMFIFI_02426"

/inference="ab initio prediction:Prodigal:002006"

/inference="similar to AA sequence:UniProtKB:Q9CJ32"

/codon_start=1

/transl_table=11

/product="PTS system cellobiose-specific EIIC component"

/db_xref="COG:COG1455"

/translation="MNGLMNWLEKYIIPVASKIGSQKHLVALRDAFIATMPATMAGSV

AVLLNAFFRDFPTEWGWLGFVEFMKPVVAVNGFVWTGTLAIMAVVFSMTLGYNLAKAY

NVDALSGAVVSLTAFFMGLTQTASTALTLDGELPKKAISMITAAGGTVTDGNVISAEA

WGFFNFGKHMGGTGLFTAIIFGFIATIIFSKLMLAKITIKMPDSVPPAVAKAFAAIIP

ATVALYICGIINYGFTTLTGQPIIDWITETIQTPLLALSQGFGAVILIVFLVLHLLWFF

GIHGTNVMAPVLQTIYGSAMTTNMNAYQRKEMIPYKWVAGSFEAFVWPGGAGVTLMLI

VAILIFSKRADSRTVGKLALGPGIFNINEPVMFGMPIVLNPLYMIPFILAPMATASVA
YVATMAGLVNPVVVSVIWVMPAGISGFLATGGDWRAIILTLVNLGVALVIWMPFVLAA
NKVPLED"

CDS complement(2607084..2607404)
/gene="celA_4"
/locus_tag="JFBMFIFI_02427"
/EC_number="2.7.1.205"
/inference="ab initio prediction:Prodigal:002006"
/inference="similar to AA sequence:UniProtKB:Q45399"
/codon_start=1
/transl_table=11
/product="PTS system cellobiose-specific EIIB component"
/translation="MEKKTIMLVCSAGMSTSLLVTKMEKAAIARGMEAEIFAVSASEA
DGNLETKPIDVMLLGPQVRFMKAQFEPKVAAKGIPLDVINMQDYGMMNGEKVLDQALA
LMNK"

CDS complement(2607689..2608585)
/locus_tag="JFBMFIFI_02428"
/inference="ab initio prediction:Prodigal:002006"
/codon_start=1
/transl_table=11
/product="hypothetical protein"
/translation="MSTLGEIIKEKRLAKGLKQSELAEGICTQATVSNLENKSRMPTM
SILLAIANRLNIEFSELSGYALTSVNSQTSRIFNKVKQLCAAYQHEEAYTLLKNEIDI
KKVETAYERKKYYYYLGLTTLAGHHNFIDAHYYFNLVLSDKSDSKLDLIDVLATNGIG
IAYELEDELDKALTYYEKSLVQLDELIEDLGEINDSAEITIIYFNSAKFYSHIGNYEK
AVNLCTLGITLQQMKHTNYELDRLYYEKAFNLAKLDRIEEATEFYFHAASFAKLTENK
IISEAIKKNMREFKLAGYCYWE"

CDS complement(2608849..2609751)
/gene="murB"
/locus_tag="JFBMFIFI_02429"
/EC_number="1.3.1.98"
/inference="ab initio prediction:Prodigal:002006"
/inference="similar to AA sequence:UniProtKB:Q8Y776"

/codon_start=1

/transl_table=11

/product="UDP-N-acetylenolpyruvoylglucosamine reductase"

/db_xref="COG:COG0812"

/translation="MILNEMIKEFPTVLIKANEPLSFYTYTKTGGPADILAFPKTEDE

IVKLVAWIKAQQLPLTVLGNASNLIVKDGGIRGVVMILTEMNQMQVKKEKLTVQSGAK

LIDTSYRAWSEGLTGLEFACGIPGSIGGAVFMNAGAYEGEISEVLESALVISETGEVT

RLSNEDLDFSYRYSSIQKHHGIILEAVFQLKKGNKATIKTRMDELTALREAKQPLEFP

SCGSVFKRPTGYFTGKLIQEAGLQGKIWGGAQISEKHAGFIVNINEATATDYIELIAY

IQEVILTNTGVKLETEVRIIGEDK"

CDS complement(2609865..2610896)

/locus_tag="JFBMFIFI_02430"

/inference="ab initio prediction:Prodigal:002006"

/codon_start=1

/transl_table=11

/product="hypothetical protein"

/translation="MTKKWRISMFVVSSFFLLAACGKNEQKNSENQTTIEPKESQVVN

DSSNIEENEKNQVSFDNYEYDYDVVTSATKTTFGTTPAAKMTYEEKLDKMFWSTQPPL

GLIEGNYYRNERRFDGDYRGIVEVVTDDDSKILHVEFNEFASENYYEPKYAGKSKRLS

DYAFFQAQNTRTDETLVTVVNGITFLEKQMREENRVDGAFKTVKGSSTSARNGFMPIA

AEMADWIRTPYKSYYYGYAEDFGNGIVGRLQVTTEDGKIDTVRYDEYFADKKESISEE

KLQPLYRQSKYYSLDYQKISGDNFVAFSDQLGEEIIKSQNFEQLDESLMQHPSYENYQ

KIAKNIQLN"

CDS complement(2610912..2611607)

/gene="srrA_5"

/locus_tag="JFBMFIFI_02431"

/inference="ab initio prediction:Prodigal:002006"

/inference="similar to AA sequence:UniProtKB:Q9L524"

/codon_start=1

/transl_table=11

/product="Transcriptional regulatory protein SrrA"

/db_xref="COG:COG0745"

/translation="MKKVLLIEDERSIAELQQDYLEINGISSVISVDGQEGLELALND

NFDLILLDLMLPTLNGFEICKEVRKVKDIPIIMVSAKKEEIDKIRGLGLGADDYLTKP

FSPGELIARVKSHLARYERLTASHQPTETIEIDGINVDKRARKVFVLEEEQFFTTKEF

DLLVYLMEHPNQVLSKEQLFQQVWGMEIYGGDVATVVVHIKKIREKMKQTGSSPLYIE

TIWGSGYRFNQQK"

CDS complement(2611614..2613104)

/gene="rcsC_6"

/locus_tag="JFBMFIFI_02432"

/EC_number="2.7.13.3"

/inference="ab initio prediction:Prodigal:002006"

/inference="protein motif:HAMAP:MF_00979"

/codon_start=1

/transl_table=11

/product="Sensor histidine kinase RcsC"

/translation="MTIKMRFLISYLGGIIVTLLSLFLISSITLFVVTGSVPSPNNLY

KMITRERSLSLEEEGAFLELRLLAKNEPDSLKNLENPELVKIIQEIEQQNLAVVIRQG

EDISYYSNGLVEKSLLAHFPIYEPNNLKVNGTIDNAGRLYRYMKFDFKDSSQMANSVM

VLKKENTFVEFLQKWGLLISGLIILIALIFVQLFNRSITKTIIEPLDQLKKSTETMKT

GNLESIEVPLDNEYAANEIKELADSFEEMRLQVKQSAIEQKRYEENRKELIANISHDL

KTPITSIIGYVEGLQDGVATTKEKEAQYLATIHKKATSLNHLIEELFLYSKLDLQKIG

FNFEEVNIRQFLVHISEEYQMELANQGTHLSTEFGEEHDAYCLIDREQVNRILGNLIQ

NSLNFRDMGKEFNQVILRLTQTESKVWIEVEDNGIGIDEEQQAFIFDRFYRGESSRNI

AMGGSGLGLAIVKQLVEAHGGTLQLNSIKGQGTKIAFCLNKLVEKE"

CDS complement(2613427..2614341)

/gene="yceM"

/locus_tag="JFBMFIFI_02433"

/EC_number="1.-.-.-"

/inference="ab initio prediction:Prodigal:002006"

/inference="similar to AA sequence:UniProtKB:P37168"

/codon_start=1

/transl_table=11

/product="Putative oxidoreductase YceM"

/db_xref="COG:COG0673"

/translation="MKKIGIIGLGGISQKAYLPVMMAMGQEVEWHLYTRNQERLTEIS

QQYRVEHTYPSIEALIESGITAAFVHTATETHGAIIRQLLEHNLHVYVDKPISENIAE

VKELIQLAESKNLQLVTGFNRRFAPMVQKLKAVPNKNMVFVQKNKENGGGSVERGIYD

MFIHVLDTATYLLDEPILKSSYHLVEEKGQLKNCIMQLITASTVCVASMNYVSGANSE

VMEVMSPTGTHRVLNLTEYSSNELGKETIQHFGDWTPTLEKRGFAPLIREFISGLSTG

EQPVSTESAYISHQLCHQIVMGDIKHTL"

CDS 2614549..2615304

/gene="exoA"

/locus_tag="JFBMFIFI_02434"

/EC_number="3.1.11.2"

/inference="ab initio prediction:Prodigal:002006"

/inference="similar to AA sequence:UniProtKB:P37454"

/codon_start=1

/transl_table=11

/product="Exodeoxyribonuclease"

/db_xref="COG:COG0708"

/translation="MKLISWNVNGLRAVVKKGFIDIFNQLDADFFCLQETKLQAGQID

LELPGYHQYWNYAEKKGYSGTAIFTKHEPDEVFYGLGKEEHDQEGRVITLSYPDYYVV

TCYTPNSQNELKRLDYRMTWEEDFLAYLNQLDAEKPVIVCGDLNVAHENIDIKNWKTN

RKNAGFTDEERGKFTEFLENGYIDTFRYFYPELEGAYSWWSYRFNARANNAGWRIDYF

IVSERLQEKLVSAAILNEVVGSDHCPVELQINL"

CDS complement(2615374..2617329)

/locus_tag="JFBMFIFI_02435"

/inference="ab initio prediction:Prodigal:002006"

/codon_start=1

/transl_table=11

/product="hypothetical protein"

/translation="MKKSLSSTLKLLFLSSVFLFTYINIVEAAPAPPVKGVPVSGVFS

VPSGSNSFVDPGGKMVTITKAQTSQVGSIFSTPNNLLDLNDDFSASMFIYLGNSNTFA

GDGMTFVMHNDIESTKKFSKGVGEQLGVYAKPNSGSYIMTQQLQRSFAIEFDTYYNGG

GMDANVWRMDGQGHVAYSFPDQQSSYSFNPGLKSLNHGSVYYPQENLSNGKWKSFEVR

WNATSKVLSYKFDVASWINVPIDVKKIFWDSKVYWGFTGSTGANIAETAVVFNKVPGL

VNVTENLNLVNQKGETVKDKQVAEKESITATFDINYLNGKQNLLLPTVELALEDKIHY

KKGTLKYNGIQMDDSKWIGKTFVVPPIINLSSANPKASISFDVETEDVGRHDVIKTGA

SATIKADNYTGDTTKLAFSVKGKGDTLTLISDKPSINLTSSEIEEVNVLKLTNSQILQ

RFITNLNIVAHDPEGNTADGITIQAVDETVYDRIRNLKSGVVNLELIAKKADNSSSIL

SIQVIFQNGVLSFSSVPETIDFIEGDPAVSLGLVQAVPNKSLSINDTRVSGSPWKLQV

TATNLKTDSGQALKGELSYVNSKGANLAIGSTIQTIENGTKPIGNSVVDISKVWSKDK

GLRLTIANDNYLGTYKGTLNWTLTDVP"

CDS complement(2617419..2618048)

/locus_tag="JFBMFIFI_02436"

/inference="ab initio prediction:Prodigal:002006"

/codon_start=1

/transl_table=11

/product="hypothetical protein"

/translation="MKTMKRLKGILLTLIAIVLVYVPTTVATAGVVSSNTNVVIKAGT

GKTNPEIPILPATGQLGPLSIDAVSTFNFASIEIGKKGIAQVPQGEVLGIQVTDTRGL

GTGWNVMVKISEFQNLKKDRILKADVSIPAGKVASQKSDILNAPTATGVVLNEFDSAI

FSAPKGKGMGTYTNSFEGNGEQVSITVPSDALLDSYSANLSWTLQDAPS"

CDS complement(2618123..2619211)

/locus_tag="JFBMFIFI_02437"

/inference="ab initio prediction:Prodigal:002006"

/codon_start=1

/transl_table=11

/product="hypothetical protein"

/translation="MKKILIMVAFFFLSSQLVPYIAQADDNMAYSVSAVIPENQIDKT

LTYFDLKMEPNQKQDIVLNVSNASDKEEIIVITPNNAKTNQNGVIDYSQSKGEGDSSL

TVPLTSVISEAQEVSLAPNESKQVTFTLQMPEKEFDGVILGGFYIAKKENKDKAEESE

KNVQIVNNYSYVIGIQLSENTAEVKPNLKLNQIKPALLNYRTAVTANIQNTKATKIKD

LSVDGKVMRKGSSDVLHETVKENLSMAPNSNFDFPINWDNQTLEAGTYTVKLNAKSGE

DEWKFEKDFEISAKGSNKLNKEAVELEKKPVNWLMIILIVIGSFILLISLIFYFIYRH

KKKLAAEKRARLARKRKRQAHKRKMTRS"

CDS complement(2619281..2619976)

/locus_tag="JFBMFIFI_02438"

/inference="ab initio prediction:Prodigal:002006"

/codon_start=1

/transl_table=11

/product="hypothetical protein"

/translation="MKKKISAIIALTLGLILVGGQPVAGISIGTSDNKISFTPGDLIV

SPPVNPLDPENPNPTLPIDPVDPGNPGTGQPGPLSVDYVSNLKFGVNKISGKNINYKV

LNPNPFVQVTDLRGDGDGWTLSAKMSLFTSSKQQVLKGATLNMKNSVVKAGSTGNVSL

APVKSDVVFDNLESKVVLSAKNKSGRGTWLNVWSGTDQANEAIQLNVLAGTPEANTEY

SSTITWELEDAPK"

CDS complement(2620020..2620691)

/locus_tag="JFBMFIFI_02439"

/inference="ab initio prediction:Prodigal:002006"

/codon_start=1

/transl_table=11

/product="hypothetical protein"

/translation="MKMKNGLVSVGIVAISCSFFLAEQKVEAVEVNTAKSTAKFQLEA

GDPTVPPEILDPEIGPATGNKGLLTLDSVSSFTFETKKIGAETSAPINATPTTGTKLG

LQLTDNRGLDLGWNLKVSGTSFTEKIGQGPETIELKGAVVTIPEGALTTKAGVDPLLT

PVANKVSLTATPATIMSASTLQGRSTWTNSFEGKGEKVTLAIPSGNKAGNYSSTITWS

LEDAP"

CDS complement(2620716..2621384)

/locus_tag="JFBMFIFI_02440"

/inference="ab initio prediction:Prodigal:002006"

/codon_start=1

/transl_table=11

/product="hypothetical protein"

/translation="MNLKKVVLVGFLGSAILGATTVVKAAPQDEILGSTNGLGGTSHG

YIKLTPGDVTVGPVAPVKPTTPKGDTGNAGPLTIDNVAPLLFDTHKLEGKEQVYTSSV

VGSNVQVTDNRGEEAGWSVQVSQTQFKDVVDPLKILKGAKLTLPVGTIETAGTNVSLQ

PMVYGVEVNETPVTLINAAKGSGAGTWVSTFAEKDLKLTVPAGNRNGEYMSTVTWSLT

DAPK"

CDS complement(2621755..2621961)

/locus_tag="JFBMFIFI_02441"

/inference="ab initio prediction:Prodigal:002006"

/codon_start=1

/transl_table=11

/product="hypothetical protein"

/translation="MRIEDFWTDVVLYRAEFQTENGRKITKILTFPATFNQESQISEV

IKTKFRNVDKVLFIEEWGDGLYLK"

CDS 2622502..2624034

/locus_tag="JFBMFIFI_02442"

/inference="ab initio prediction:Prodigal:002006"

/codon_start=1

/transl_table=11

/product="hypothetical protein"

/translation="MDLLLDKRNYQKIELIKYLSKQYEFISIDELCELLDLSKVAMRR

LLDSLEEDLDELFHHTVKIDTNESSQYYLVIDSQYYKSANVINMLTLHFLKQSLSYQF

LLTLFYTKYPSSNSLMDELLISSSYFYKLNKSLSKILKQWYLELDFSINDSGFNYMGK

TEFIRLFTYYFFWNSFKEQKWPFSKITLDTTKRYLATEPELVSPNLSLSKATRVEYFF

KITMNMIVNEKKYVSLDKEYQDFLTCIQDVHDLSEPMNQIFNKRLLVLPQENKASEKL

FFNFALRLILASIDSPAQRIEIGQKFLLLDNSSIDFNKKFFHRLLQFFNLTCNDIQYA

DMIYYVTLYFLSIEKLNINLLSFAREHPQVSWFGIDRIESKELSDNIKKFSNEFISEQ

PTYDSSYYTENNLDIMYNLVYFIISSLQESKFNIYIQYSKNIHSEAFIKKRLHAVFGD

QILTIVTSIEEADLIISDCIELTDYSKQEFFYFNFTYDIDTWTQLTNYIQKKIMILAF

NV"

CDS 2624138..2624587

/gene="tadA_3"

/locus_tag="JFBMFIFI_02443"

/EC_number="3.5.4.33"

/inference="ab initio prediction:Prodigal:002006"

/inference="protein motif:HAMAP:MF_00972"

/codon_start=1

/transl_table=11

/product="tRNA-specific adenosine deaminase"

/translation="MQMEQDFMKRALELASTAAKSGNEPFGAILVKDGKIVFETQNSI

HKSSDPTAHAEMNLIREFCHTNQISDLSDYTLYTSCEPCCMCSGAMVWSKLGKLVYSA

SHSQLQEIAGFNIMISSEEVFEKSPFRPTTHGLILNNEGTKILADYF"

CDS complement(2624696..2624953)

/gene="veg"

/locus_tag="JFBMFIFI_02444"

/inference="ab initio prediction:Prodigal:002006"

/inference="similar to AA sequence:UniProtKB:P37466"

/codon_start=1

/transl_table=11

/product="Protein Veg"

/db_xref="COG:COG4466"

/translation="MPNTLASIKKNLDCQLGRKIMLTAQTGRKRKTERKGVLRETYPS

VFVVDLDQDENAFERVSYSYTDVLTQSVEIQFFDENSRQYA"

CDS 2625734..2626168

/locus_tag="JFBMFIFI_02445"

/inference="ab initio prediction:Prodigal:002006"

/codon_start=1

/transl_table=11

/product="hypothetical protein"

/translation="MLTIKEVSEIMKISPYTLRFYDTKGLFPFVKRNERNIRIFTEND

LEWVYVVQCLRDTGLPLLRIRQYIDMCVAGDQTIPDRLDLLKKQQCAVKMQQIELQER

LDMVNYKILSYENQIEHKTDRIFNPFEEKLAKKMDKKVVNFK"

CDS 2626187..2626417

/locus_tag="JFBMFIFI_02446"

/inference="ab initio prediction:Prodigal:002006"

/codon_start=1

/transl_table=11

/product="hypothetical protein"

/translation="MTNKTFSLFCTIINALIMTAIISYTLTIVNVGLQNFNFPIWLRS

WFIAFLLVFALSFYLPKWIRLLISKVIVIKEG"

CDS 2626447..2627112

/gene="gloB"

/locus_tag="JFBMFIFI_02447"

/EC_number="3.1.2.6"

/inference="ab initio prediction:Prodigal:002006"

/inference="protein motif:HAMAP:MF_01374"

/codon_start=1

/transl_table=11

/product="Hydroxyacylglutathione hydrolase"

/translation="MNLIKISNSIYTCEFPTGNVVNGVTIKVNIWLVLNGTDIYIIDT

GFDYMTKELIPYLNALGTPKALLVTHGHLDHILGAHRLVREFGIPRYANQLEIDEIDR

GVAPYPTKEHELTCFFTAFDEKVLEEAGLTLYTTPGHSPGHTIFYHEKDNVLMVGDLF

TTTEKQLLPPINKFTPNMTESIDNGAVIDSIKPALITSAHGENIVYNETLYPQLVLLF

REE"

CDS 2627189..2627941

/locus_tag="JFBMFIFI_02448"

/EC_number="1.-.-.-"

/inference="ab initio prediction:Prodigal:002006"

/inference="similar to AA sequence:UniProtKB:Q7A3L9"

/codon_start=1

/transl_table=11

/product="putative oxidoreductase"

/translation="MNNIQGKVVIITGASSGIGEATVRKLTQNGAKIIMFARREERLQ

SIKSTLLNYEVEYKVGDVTKFSDMSELVQFTLERFGKIDAMFHNAGIMPMGPLANSDE

KEIAKWATAVNVNIMGVVNGLAACLPVMVEQKFGHMIAMDSVAGHLVYPNSAVYCGTK

YAVRAIMEGVRQEHLNDAIRSSIVSPGIVATELINSVGNSDIEAWIAGEVQDSTTSLS

ALDVAEAVAYILSTPDNVAISEVLMRPSKHTL"

CDS complement(2628755..2629648)

/gene="rsmA"

/locus_tag="JFBMFIFI_02449"

/EC_number="2.1.1.182"

/inference="ab initio prediction:Prodigal:002006"

/inference="similar to AA sequence:UniProtKB:P37468"

/codon_start=1

/transl_table=11

/product="Ribosomal RNA small subunit methyltransferase A"

/db_xref="COG:COG0030"

/translation="MTQRKDIATPSRTKEILAKHGFSVKKSLGQNFIVDPNILVNIVA

AAVLDKDTNVVEIGPGIGALTEHLARASKEVVAFEIDDRLLPVLADTLSPYDNVEIVH

TDVLKANLAEILTPRLNLEERLMVVANLPYYITTPIIMHFLESEVRIDGLVIMTQKEV

GDRITAAPGTKAYGSLSIAIQYYMEAEIAFIVPKTVFVPQPNVDSAIIKLTRREQPSV

IVKNEKIFFQVARAAFVQRRKTLWNNLLVRYGKEEEIKEKLVKALELADIDPKRRGET

LSLAEFGRLSDAIVEFFPDNN"

CDS complement(2629635..2630201)

/gene="rnmV"

/locus_tag="JFBMFIFI_02450"

/EC_number="3.1.26.8"

/inference="ab initio prediction:Prodigal:002006"

/inference="similar to AA sequence:UniProtKB:P37547"

/codon_start=1

/transl_table=11

/product="Ribonuclease M5"

/db_xref="COG:COG1658"

/translation="MDKIKEIIVVEGRDDTRRIKEAVVADTLETRGSAIDNSILALIA

KAQETRGVIVFTDPDFPGEKIRKTISRAVPGVKHAFLKVADAKPKGKGSLGIEHATPE

AIREALGKAYTETISKESLISRELLIDAGLLAGPFAKKRREKLGESLNIGYTNGKQLE

KRLQMFQITPEQVIEAMQRILEDENDPA"

CDS complement(2630201..2630968)

/gene="dtd3"

/locus_tag="JFBMFIFI_02451"

/EC_number="3.1.1.96"

/inference="ab initio prediction:Prodigal:002006"

/inference="similar to AA sequence:UniProtKB:P73335"

/codon_start=1

/transl_table=11

/product="D-aminoacyl-tRNA deacylase"

/translation="MLFDTHTHLNDEAFATEIPEAIARAKENDVTKMAIVGFDEKTIE

KSLELSRNYDGLYSIIGWHPTEAYLYTDKIEEKLVEQLQLPKIVAMGEMGLDYHWDTS

PKNVQKDVFRRQMRIAKELKLPISIHMRDAIEDTYQILKEEHVEDIGGIMHSFSGDVD

WMERFLDLGMHISMSGVVTFKKAFEVHEVAKQVPVDKLLIETDAPYLAPVPFRGKRNE

PAYVKYVAEKIAELRECSYQEVATQTTENAEKLFRLR"

CDS complement(2631071..2633086)

/gene="metG"

/locus_tag="JFBMFIFI_02452"

/EC_number="6.1.1.10"

/inference="ab initio prediction:Prodigal:002006"

/inference="similar to AA sequence:UniProtKB:P67579"

/codon_start=1

/transl_table=11

/product="Methionine--tRNA ligase"

/translation="MTEKKPFYITTPIYYPSGKLHIGNSYTTIACDVMARYKRMQDFD

VFYLTGTDEHGQKIENKADELGITPKEYVDTMADHMQETWRLLEISNDKFIRTTDPVH

EKVVGDIFERLLAQGDIYLGEYTGWYSVSDEEFFTETQLEEVYRDAEGNVIGGKAPSG

HEVELVKEESYFFKMSKYADRLLDYYNAHPEFIQPESRKNEMINNFIKPGLEDLAVSR

TTFSWGVKVPSNPEHVIYVWIDALANYITALGYGTDDTTLFDRYWPADVHMVGKEIVR

FHTIYWPIMLMALDLPLPKKIFGHGWLLMKDGKMSKSKGNVVYPEMLVEHFGLDALRY

YLMREVSFGSDGVFTPEDFVSRVNYDLANDLGNLLNRTVAMINKYCGGVVPTYVGDVT

AFDAPLKAVADEVVKNYQTEMDQMQFSTALAEVWRLISRANKYIDETEPWKLIKEEDK

KAELDSVMVHLAESLRVSAILLQPFLTHAPKEIFAQLGVTGKEAGEFKTVDFGQFPTD

VKVVEKGTPIFPRLDLEVEANYIKEQMAAGKPETTEEEAGVWNPEETELVSLKEKEIK

YEDFDKVELKVAEVIDCRKVEGADKLLKFRLDAGDKDHRQILSGIAQWYPEPEALIGK

KVVIVANLKPRKMRGEVSQGMILSAEKDGVLQVIEAPISAPNGSEIA"

CDS complement(2633236..2634663)

/gene="phrB"

/locus_tag="JFBMFIFI_02453"

/EC_number="4.1.99.3"

/inference="ab initio prediction:Prodigal:002006"

/inference="similar to AA sequence:UniProtKB:P00914"

/codon_start=1

/transl_table=11

/product="Deoxyribodipyrimidine photo-lyase"

/db_xref="COG:COG0415"

/translation="MSVIGMWFRKDLRLTDNTALIAALKEAQLKNDKLVGIFHLNPLQ

FKATSFNHDYFFTAVAHFVEVAKAAGLPIHFLYGEIEAAFEELTQKIPTLNQIYFNRD

ERGFGAERDRKMESFFAEKGIECHHFQDSHLHGATENRKQDQTAFKVFTPYYKSWEKL

RKSNFQKIDSQKLKEFALDKQELFSEGQAEFTQLMQAVDEKWASEVGALKARKRLRQF

ISSGIEQYDQTRDFPAQKGTSRLSQFLRTGELSIREVYHRIKAEAPDSIGKETYLKEL

CWRDFYNMIYFENPQQQVLEIKEQYRNLQWNNNEQLLQAWKTGQTGYPLVDAAMRQLN

QTGWMHNRLRMIAASFLVKDLLMDWRLGEAYFAEKLIDYDAASNIGGWQWAASTGTDA

VPYFRIFNPTTQSQRFDEAGDFIYKYVPELKGIPHAYLHEPSKMPLNIQEEKQIIIGE

DYPQPIVQHKLMRQKALELFKGIDQ"

CDS complement(2634737..2636179)

/gene="menE"

/locus_tag="JFBMFIFI_02454"

/EC_number="6.2.1.26"

/inference="ab initio prediction:Prodigal:002006"

/inference="similar to AA sequence:UniProtKB:P23971"

/codon_start=1

/transl_table=11

/product="2-succinylbenzoate--CoA ligase"

/db_xref="COG:COG0318"

/translation="MENWLTKRVHLTPEKTALVFKQKKWSFAELQVEVNQRAKRLLTL

SYPEGSRIGVLGSNSPELYFTILALQQMGHTIVFLNHRLTSYEMEQQLIDSTVAGVLY

QASFVGKIKQLSIHLQECSHSFQQVTELKEQTNFKPVPDFDLKKVTTIMYTSGTTGQA

KGVQQTFDNHWWSAIGSVLNLGLTDEDSWLCPVPLFHISGFSIMMRSLIYGMPVYLFE

KFDEKEINQTLLSGAGSIISVVSVMLKRLLVDLGNRHYPPQFRCMLLGGGPIDSGTLT

NCQKLGIPVIQSYGMTETASQVVALSNDMAVKKSGSAGLPLFPVQLEVVMPSHEVCLP

YQHGEVRIKAPNVTVGYLNQPRRSETEWFYTGDIGYLDNDGYLYIVSRLADLIISGGE

NIYPSEVEQVLMAHPSINDVAIVGKKDTQWEQVPIAFLVLEQDKELDIPGLIHFCQSQ

LAQYKIPKEYKVVSQLPRNASGKVLRRHLV"

CDS complement(2636261..2637082)

/gene="menB"

/locus_tag="JFBMFIFI_02455"

/EC_number="4.1.3.36"

/inference="ab initio prediction:Prodigal:002006"

/inference="similar to AA sequence:UniProtKB:Q5HH38"

/codon_start=1

/transl_table=11

/product="1,4-dihydroxy-2-naphthoyl-CoA synthase"

/db_xref="COG:COG0447"

/translation="MMTENWETIKEYREILFEKTDHIAKITINRPEVHNAFTPLTVSE

MIDAFTLSRDDADIGVIILTGAGDNAFCSGGDQRVRGNGGYVGEDNIPRLNVLDLQRL

IRVIPKPIIAMVKGWSIGGGNVLQLVCDLTIAGDNAKFGQTGPNVGSFDAGYGSGYLA

RVIGHKKAKEVWFMCRQYTAAEALEMGWINTVVPVADVEKETMDWAREMLKKSPTALR

FIKAAMNADTDGLAGLQQFAGDATLLYYTSDEAKEGRDAFNERRDPDFNQFPKFP"

CDS 2637224..2637592

/locus_tag="JFBMFIFI_02456"

/EC_number="3.1.2.-"

/inference="ab initio prediction:Prodigal:002006"

/inference="similar to AA sequence:UniProtKB:P45083"

/codon_start=1

/transl_table=11

/product="Putative esterase"

/db_xref="COG:COG2050"

/translation="MTLMDHLGIHYKNISKDSVELELTIEEQHKQPYGIMHGGISAVL

AETAASLGANAQLDTTKEVAVGLELNLNHLRAVPNGTIIAVATPLHIGKKTQVWEIKI

TNEQKNLVSAGRCTLFIQEL"

CDS complement(2637674..2638159)

/locus_tag="JFBMFIFI_02457"

/inference="ab initio prediction:Prodigal:002006"

/inference="similar to AA sequence:UniProtKB:Q7A551"

/codon_start=1

/transl_table=11

/product="Putative universal stress protein"

/translation="MLSRYKNILVAVDGSKNAQLAFQQAVEIALEQEKATLYVLEVVD

NQTHFVAPPMLNSQTQTYSPEAVEMFQEVVQKEVEWVENEVHELVEEAKRKGVPNAVA

VVTTGNHKHAIAHTIPTEKKIDLLIIGATGKGRIKSAILGTTTSYVVQHAPCNVLVVK

E"

CDS 2638478..2639035

/gene="bioY2"

/locus_tag="JFBMFIFI_02458"

/inference="ab initio prediction:Prodigal:002006"

/inference="similar to AA sequence:UniProtKB:A2RI45"

/codon_start=1

/transl_table=11

/product="Biotin transporter BioY2"

/db_xref="COG:COG1268"

/translation="MHTSKLKQMILNAQFAVIIAIIAQITIPLGPIPLTGQTFAVGLA

ATILGGWNSMIAVCIYLLMGLIGVPVFAGFSAGIGALLGPTGGFLIGFIFNAFITGWI

LEKTKFDLSWAIVANLIGALITLLFGTIWLKYGTGIDWSAAFTGGFLPFILPGVVKAL

LAAFCGIAVRNRLKKGTFLKKDSLN"

CDS 2639604..2639801

/locus_tag="JFBMFIFI_02459"

/inference="ab initio prediction:Prodigal:002006"

/codon_start=1

/transl_table=11

/product="hypothetical protein"

/translation="MDYTQEIVRFVRFYGIKNIKSYETSKCKYFIYVADNGQKQLYYY

DKQTKNFYFKTAKKQEKLSWL"

CDS 2640382..2643054

/gene="mgtB_2"

/locus_tag="JFBMFIFI_02460"

/EC_number="7.2.2.14"

/inference="ab initio prediction:Prodigal:002006"

/inference="similar to AA sequence:UniProtKB:P22036"

/codon_start=1

/transl_table=11

/product="Magnesium-transporting ATPase, P-type 1"

/db_xref="COG:COG0474"

/translation="MAKKKTINYKVTDQIYKFAATSHRKKVVDHFSSSLDGLNTNEVL

TARKKYGSNEIQHEKPESIPAKLVRAFINPFTLVLLLLAAISFVTDYWIAAPADKDLT

GVIIIVAMVTISGLLTFVQSVRSTNAAEKLRTMVKVTATVTRRTSGKAEIPMEELVCG

DIVHLSAGDMIPADIRILKSKDLFISQAAMTGESNPVEKYEIDLAPNTKNSNVMDYEN

VAFMGSNVVSGSAMGIVIAVGPYTQFGRIAHDVSRKKGDTSFEKGINSVSWLLIRFMA

LMAPAVFIINGLTKGDWLEAFLFGLSVAVGLTPEMLPMIVTTNLVKGTLSMAKEGTII

KNINSIQNFGAIDVLCTDKTGTLTQDKIVLEYHLDVLGKENDRVLKHAFFNSYYQTGL

KNLMDLAIIEASQNELNIPINEYEKIDEIPFDFNRRRMSVVIRNQDGKTQMITKGAVE

EMLSISSHVEYMGEVTVLTETLKNKILKTVDDLNEDGLRVIAISQKTNPAAIGEFSVK

DESDMVLMGYLAFLDPPKETTKPALEALAKNGVAVKILTGDNEKITRTVCKQVGIKVK

NVVFGTDIDSLDDAQLTAVVEENNIFVKLSPQQKTRIVKTLRNNDHTVGFMGDGINDA

SAMKEADVGISVDTAVDIAKESADVILLEKDLLILEKGLISGRKIFGNIIKYIKMTAS

SNFGNMFSVLVGSAFLPFLPILPIQVLFLNLIYDISCISIPWDNVDKEYLEKPRKWDS

SSIGSFMKWIGPTSSVFDITTYLLMFFVICPQVLGGSYSSLPPEKQLIFAALFNAGWF

VESLWSQTLVIHALRTPKVPFLQSRASFIVTTVTGLGIAVGTVLPYTNLGAHLGMVPL

PLNFFGWLALTIIAYLTLVTFMKKIYIKRFGELL"

CDS complement(2643099..2643932)

/gene="hisK"

/locus_tag="JFBMFIFI_02461"

/EC_number="3.1.3.15"

/inference="ab initio prediction:Prodigal:002006"

/inference="similar to AA sequence:UniProtKB:Q02150"

/codon_start=1

/transl_table=11

/product="Histidinol-phosphatase"

/db_xref="COG:COG1387"

/translation="MVYADYHVHSDYSDDSWYLMEDVVKDAIQLGLEEICFTDHVDYG

VKPDWQPEDVFQIGANKEVKNVHYDLYFSELERLTKKYADQIRIKTGLEFGMQVHTIE

PFEKLYHSHSFDFILLSIHQIEDQEFWLGEFQKGRTHEEVYDCYYNEMLQLVTAYQDY

SVLAHMDLVRRYLDKEVDLFHYSKAKIEKILKIVIANNKGIELNTSSERYEINGLTPS

IEILKLYHELGGKIITIGSDSHKPEHLAWKINESKELLKSIGYTQFCTFDKMEPIFHD

L"

CDS complement(2644057..2645352)

/locus_tag="JFBMFIFI_02462"

/inference="ab initio prediction:Prodigal:002006"

/codon_start=1

/transl_table=11

/product="hypothetical protein"

/translation="MQKKVIKLVLLGLLSTSVLNMPLNVWADELDNKITSQETKINEV

AQQEQKVEAELKSVQTDMATIENTAIELATKEKGLNQDKQRLNENIEKLTTIIDQREV

EIANQARAAQVNGDPANYLRMITEATSISEVLGRIHAVWTLISANTSILKTQEQDKKL

VEASQFEIDMTIKKLNETILASEKKKSELQNKQLQQVVLQSDLAARRSTEEEQKNKYI

GEKEAAQLQQAENERLKKVADEQKLAEEKALAAATNKKTEDEKVQASNPTPVSVVEPE

KPLEVTPPAQPQVPAPTPDPPTVGFNVPALLAEAQKWIGTPYSWGGGNTNGPSLGFGE

GANTVGFDCSSFVQYVFGRLGIGLPRTTYQQEYQGTYLELSQLQAGDLIFWGGRGSTH

HVGIYMGGNQYIHAPETGDVVKISSVSSYNPPSFGVRVR"

CDS complement(2645536..2645733)

/locus_tag="JFBMFIFI_02463"

/inference="ab initio prediction:Prodigal:002006"

/codon_start=1

/transl_table=11

/product="hypothetical protein"

/translation="MEAFRKFIRKNRLTFFIGNFIAAIFIVAIADLVFGSQPTIIQTV

VYAFVIATAFSIFYKPDQKDK"

CDS 2645850..2646668

/gene="yidA_5"

/locus_tag="JFBMFIFI_02464"

/EC_number="3.1.3.23"

/inference="ab initio prediction:Prodigal:002006"

/inference="similar to AA sequence:UniProtKB:P0A8Y5"

/codon_start=1

/transl_table=11

/product="Sugar phosphatase YidA"

/db_xref="COG:COG0561"

/translation="MYQLIACDLDETLIGEDKQVSKENKLAIHQAQQKGVKFVIATGR

GYRTVQDTLVELGLADLPAEYVISFNGGVITENKNNQVLEFNGITFEQGEQLFNFGLN

YDVCMHIYTEDDVYIYNFHEEEKAYLNGRINNCIELIEPSIEFLRDIPLVKVLYQNLD

KHYLEQIHADITPIVQDAFDISYSSNRYIEFNHQGVNKGSALLRLADRLGIPHNQTIA

IGDNTNDLTMIKAAELGVSVQNGTPDVKKAANYIAPETFREHAVAAVINKFVLK"

CDS complement(2646869..2648569)

/gene="ohyA"

/locus_tag="JFBMFIFI_02465"

/EC_number="4.2.1.53"

/inference="ab initio prediction:Prodigal:002006"

/inference="similar to AA sequence:UniProtKB:C7DLJ6"

/codon_start=1

/transl_table=11

/product="Oleate hydratase"

/translation="MSKKTIGLIAAGATVLGAAYGTKKLQEGKVQADEKKVDEEIKAR

YYGDKQVYFIGGGIGSLSGAAYLIRDANFAGKNIHIIEGMDILGGSNDGSGTMKDGFL

CRGGRMLNEETYENFWELFASIPSLEMPNQSVTEEILNFDHLHPTHAQARLIDKDRTI

LDAHSMGFDNNDRALMTKLLAMPEDKLDNKTIEEWFGPHFFETNFWFMWQTTFAFQKW

SSLFEFRRYMNRMMLEFSRIDTLEGVTRTRFNQYESLILPLKDYLDKHQVDFSMNKTV

TDIDFKDGEGITAETLHFSDGSTIDLNAGDVVMMTNACMTDSTTEGDFNTPAPVPEER

PISGELWYKIAQKKANLGNPEPFFAHESETNWQSFTVTNRGDELLKRIERFTGNIPGS

GALMTFKDSNWLMSIVVAAQPHFKAQDANTTIFWGYGLYPDRIGDYVKKPMKECTGEE

ILYELLCHLKWEEDWEDIKADVVNVIPCYMPYIDAQFQPRAMSDRPQVVPEGSTNFAM

ISQFVEIPKDMVFTEEYSVRAARIAVYTLFDINKKIIPVTPHNKDPKVLAKATQTMFR

"

CDS complement(2648706..2649266)

/locus_tag="JFBMFIFI_02466"

/inference="ab initio prediction:Prodigal:002006"

/codon_start=1

/transl_table=11

/product="hypothetical protein"

/translation="MKDSFITKKAISDALIELCHYKRFDKISIADITDKCRLNRQTLY

YHFSDKYDLLEWTYQVGAFSYLAEGVTLENWEEHVLKMLIEIKEKADFYQNTVSSDSE

ILSACFSKVTSTLFMELFERLDLENHVSQADRCFYARFFSYGCCGVLVDWIKVGMKET

PDTIATQFVRLANDTELLAYQRLREG"

CDS 2649473..2650192

/locus_tag="JFBMFIFI_02467"

/inference="ab initio prediction:Prodigal:002006"

/inference="similar to AA sequence:UniProtKB:P67182"

/codon_start=1

/transl_table=11

/product="putative transcriptional regulatory protein"

/translation="MGRKWANIKDKKASKDTNTSRIYAKFGIEIYVAAKQGDPDPHTN

QKLRFVIERAKTYNVPKHIIDRAIEKAKGNDEDNYSELRYEGFGPNGSMIIVDALTNN

VNRTASDVRAAYGKNGGNMGVSGAVAYMFDNTAVFGVEGKDADELLEILMEADLDVRD

ILEEDGQAIIYAEPADFHAVQEALKASGIEEFTVAETEMLPQNEVTLEGDDLVNFEKL

IDALEDLDDVQKVYHNVDLGE"

CDS complement(2650242..2650631)

/gene="catD"

/locus_tag="JFBMFIFI_02468"

/EC_number="1.-.-.-"

/inference="ab initio prediction:Prodigal:002006"

/inference="similar to AA sequence:UniProtKB:P54720"

/codon_start=1

/transl_table=11

/product="Putative oxidoreductase CatD"

/db_xref="COG:COG2259"

/translation="MGANKNKVSISLLLIRIMLGITMALHGIQKFMNLSDTTDFFVSL

GLPSFMPIIIALIEVVGGIFMIIGLLVPLVSLGFIALLGTAIFMLKSTSGFVNGYELE

LLLIVMSIASGYAHLNKKIIQFMPPSA"

CDS complement(2650657..2651607)

/locus_tag="JFBMFIFI_02469"

/inference="ab initio prediction:Prodigal:002006"

/codon_start=1

/transl_table=11

/product="hypothetical protein"

/translation="MIKKALIYISISVMAVFLVACGATSKMDKSSKNEKNQTESKQKK

KEEKQTNYQKITGENVTYYVMNPDPVFGSTAVLIEKEGKGLLVDTQFSKDDADKTIQL

IKSKKIDLETIYVSYSDPDYYFGTAFIQEAFPTAKVVATEGTIDRIKSTYENKLTVWA

DTLKDKAPKEVIIPEAIDNKVALGNEKFQITGTDKPVLYNATDQLILGGIPVSTDGHL

FMADTKTVESQENWINDLTGLEELQAKTVIPGHFGQGNKFTADNITFTKDYIQKFIEV

EQSSQTSAEIMQKMKAAYPDLAEGSLEMSAKVVTGEQAWD"

CDS complement(2651819..2652454)

/locus_tag="JFBMFIFI_02470"

/inference="ab initio prediction:Prodigal:002006"

/codon_start=1

/transl_table=11

/product="hypothetical protein"

/translation="MKIGIIAASGKAGTMIMKEAISRGHEVTAIVRNAAKITDSQVTV

LERDILEISYADVKEFDILVDAFNAPHGQEELHQTTLAHLTKILAGHKTPRLIVVGGA

GSLYVDPAKTIRVMDTPDFPDAFLPTASNMGTAYDALKANTEITWTYLSPSAMFLPDG

ERTGSYTVGLDNLLANAAGASEISYADYAIALLDEAEAGKHINQRFTVGSK"

CDS complement(2652550..2652981)

/gene="ywnA"

/locus_tag="JFBMFIFI_02471"

/inference="ab initio prediction:Prodigal:002006"

/inference="similar to AA sequence:UniProtKB:P71036"

/codon_start=1

/transl_table=11

/product="Putative HTH-type transcriptional regulator

YwnA"

/db_xref="COG:COG1959"

/translation="MSISTKFPVAVHVLSVLALNRQTTVYSDFIAASVNTNPVVIRRI

VGLLKKAGLVDSAPGVGGISLIKEPTDITLYDIYEAVNPTSKDLFTLHQDTNPNCIVG

KNIQHSLEGVMQTAEEALENELKQMTLADTIEEIQQLDQAT"

CDS complement(2653252..2655186)

/gene="licR_3"

/locus_tag="JFBMFIFI_02472"

/inference="ab initio prediction:Prodigal:002006"

/inference="similar to AA sequence:UniProtKB:P46321"

/codon_start=1

/transl_table=11

/product="putative licABCH operon regulator"

/db_xref="COG:COG1762"

/translation="MMKKVTENQLIHYLFSQNGWTKASIIASHFQFTERTIRNRISGI

NQKRVKPVIISSRQGYKIDADAYRLQNKKQEEIVNAIPVTVKERQFFLIRTFLSSAKG

GVNLFDLAEMLCVSEATIRRDIKSVKKHLTKFDIQVVSKEEDYQIKGSEAQKRKAMTY

LIFEESKKTMDQRKMVQQLLGDIDLLILQEIISDTLEQYDYFINQYSLNNILLHFAVT
LQRMREEKAIEAVEEKELDSVEYRMTKDITQKIAQQFGIVFSNAEIYNLFLLFIGNTT
LTTYESLSPEHLKEYIGNQTVDIVKEVLADIGQMYFIHLSDEAFTTKFMIHVRNLISR
SNLSKLSKNPLREDMKRTYPLIYDVAVSIASELEKRLLIQVNEDEIAYIALHLGAYLE
SVTKESDAVSCVILCPRYYDIHQGLVKKIKETYREQINIQKIVTDMSSNWQIMKPELI
ISISPFSEEVSAHFVLVHPFLTDKDYKNISDAISKVKQNRKQIKMTGYIKEFFRPEFF
YRGLSFKHKNKAIYYMTEQLFKAGYVSSGFTEKVLAREKLSSTAFSDGVAVPHALKMN
AIKTAVSVAIFDEPIDWDGKVVYIVAIFAVNKENRPIFSPMFENFIRVLSEGDNVRSL
SESENYEDFTSNLIQLMGSE"

CDS complement(2655253..2656686)
/gene="bglH_5"
/locus_tag="JFBMFIFI_02473"
/EC_number="3.2.1.86"
/inference="ab initio prediction:Prodigal:002006"
/inference="similar to AA sequence:UniProtKB:P40740"
/codon_start=1
/transl_table=11
/product="Aryl-phospho-beta-D-glucosidase BglH"
/db_xref="COG:COG2723"
/translation="MTGFPKGFLWGGAVAANQIEGGFGLDGKGLSVADVHLYNPELDI
KTASQESEMTLAEVKRAMLDEEGYYPKRHGIDFYHTYKEDLKLLAEMGFTTFRTSIDW
SRIFPNGDDAEPNELGLQFYDCLIDECLALGMEPIITMLHYETPLAITLNYGGWNNRE
VIELFSKYGEVLLKRYKDKVKYWIVINQINLVHLESFNSIAICEDQVENNEEAKYQAI
HNQLVASALIVKKAREINPIFQMGTMLADCTASPFSCSPDDVVLAMKRNRMQYFFTDV
QFRGAYPVYMRRYFAENQLDLQEEPADEILLRENTMDYLAISYYYSQTVSAKKNGMDP
TDVMENPHLKANPWGWAIDAKGFYNCLSQYYDRYQVPLMIAENGFGMYDKVEENGEIN
DDYRIAYLSEHLMQLKEAIKDGVEVIAYCAWGPIDIVSCSSAEMEKRYGFIYVDLDNF
GKGTGKRLKKKSFDWYKTVIASNGEIL"

CDS complement(2656706..2657041)
/gene="licA_6"
/locus_tag="JFBMFIFI_02474"
/inference="ab initio prediction:Prodigal:002006"
/inference="similar to AA sequence:UniProtKB:P46319"

/codon_start=1

/transl_table=11

/product="Lichenan-specific phosphotransferase enzyme IIA

component"

/db_xref="COG:COG1447"

/translation="MKGTKNMNDEMNQVAMQIILHAGNARQVIFEVFDEIGDEHFDHA

KELLKKAKKEIVLAHKAQTETIQAEASGIHHDFSLLFAHAQDTLMTISSEWNIANKMV

SLVEKLTKK"

CDS complement(2657041..2658426)

/gene="licC_11"

/locus_tag="JFBMFIFI_02475"

/inference="ab initio prediction:Prodigal:002006"

/inference="similar to AA sequence:UniProtKB:P46317"

/codon_start=1

/transl_table=11

/product="Lichenan permease IIC component"

/db_xref="COG:COG1455"

/translation="MKFRGDKKRHIKFPYSSVEPVRTQQAFDRVNRINKIFIEEDEDM

QKIIDFMTNSFAPKVNKITKNVWVASLQDSVMAILPFILVSSIVTMVSLINEIADIIP

DLSIINTFTFGLSGMFVAFLLPYFIMEKKKRGDKKLLAGLTGLALYLLLIFPEMSTDG

GSITFTLARFGAVGMFVSIVVGIFVAIIMNLFSKWSFFNKDETSLPDFIIVWFDSLLP

ITVIIVSGWLCYSIFQIDVFDLIAILFEPLQMLGQSFWGFVLMGFLMAFLYSFGISTW

VLTPVFMPIALKGIADNAALVEKGMDAVYINTQETFYSGWCAFGGVGYTMILVILLLL

AKSTRLRAIGKATILPSIFNINEPLVYGAPIVFNPMLMIPFWLNGAIIPAITYLVLST

GWISIPAKAFQLWYLPYPISTYLVSGDIKGIFLFALLAGVSLCIWYPFFKAYDNAEVK

NELEEELAVIN"

CDS complement(2658554..2658859)

/gene="celA_5"

/locus_tag="JFBMFIFI_02476"

/EC_number="2.7.1.205"

/inference="ab initio prediction:Prodigal:002006"

/inference="similar to AA sequence:UniProtKB:Q45399"

/codon_start=1

/transl_table=11

/product="PTS system cellobiose-specific EIIB component"

/translation="MKTILLICGGGASSGFMAANMRKAAKKKGLEVSISARSESELEE

HLDTIDVLLIGPHLAYMEEEAKEKAASSGREILVKVIPQKVYGMMDGVGALELLDSK"

CDS complement(2659440..2659637)

/locus_tag="JFBMFIFI_02477"

/inference="ab initio prediction:Prodigal:002006"

/codon_start=1

/transl_table=11

/product="hypothetical protein"

/translation="MNQMTANEIIEFLQRQKETTKFTFNMVNPDNFMIVIELKNEPAA

FTFINENTEATFELTDANELL"

CDS complement(2659821..2660165)

/gene="yabA"

/locus_tag="JFBMFIFI_02478"

/inference="ab initio prediction:Prodigal:002006"

/inference="similar to AA sequence:UniProtKB:P37542"

/codon_start=1

/transl_table=11

/product="Initiation-control protein YabA"

/db_xref="COG:COG4467"

/translation="MDKRTLYDSFTKLELDTDATLHQITALKKGVEELVEENAVLQIE

NQHLRDRLDDIEKQKRIDEEVVEPEMTKSRMNLEKLYEDGFHVCNVFYGSRRVGDEPC

AFCLDVIYGERR"

CDS complement(2660226..2661038)

/locus_tag="JFBMFIFI_02479"

/inference="ab initio prediction:Prodigal:002006"

/codon_start=1

/transl_table=11

/product="hypothetical protein"

/translation="MKTVIGVRFKHAGTIYYFSPGSWECHVGDQVVVETQHSIEMGNV

VIDKKEVLEEDLIHPLKEINSIATEKDFQKEEQNKIDAAKAYDIATEKITHHQLEMKL

INVEYTFDRSKMIFNFSAEGRVDFRELVKDLASVFKTRIELRQIGVRDEAKLLGGIGP

CGRMLCCSTFLGDFMPVSIKMAKDQNLSLNPTKISGLCGRLMCCLKYENDEYEAAKRE
LPDYGKKVVTPDGEGTVIGLNLLSRIIKVRLNGKETPLEYTLEELNEATANM"

CDS complement(2661069..2662064)
/locus_tag="JFBMFIFI_02480"
/inference="ab initio prediction:Prodigal:002006"
/codon_start=1
/transl_table=11
/product="hypothetical protein"
/translation="MDNENNLEWLQPQLFNRFKKNLQHGQLAHAYLFEGVPGVGKKEM
AIWLSQAIFCLEPEDGLPCGKCQNCTRILEHQHPDVTELAPDGLSIKVDQVRELKTEF
SKSGVEGRQKVFIVEDVEKMTVGAANSLLKFLEEPEGTVVAFLLTTAKQKILPTILSR
CQLVHFSPLSKRMLLTELKKLEISEKQGALLVHLTNSLESAVEMNRNEWFNEARDIIW
KWFQLISQNEKQAFIFVQTDVMPHFKEREQQMLAFELLLLAYRDALMLLYGSVDQLAY
PQHQKILQDFLARSRGKDVTAAIETILFSRKKLESNVSAQGVFEQMVLLLMRNSI"

CDS complement(2662082..2662411)
/gene="darA"
/locus_tag="JFBMFIFI_02481"
/inference="ab initio prediction:Prodigal:002006"
/inference="similar to AA sequence:UniProtKB:P37538"
/codon_start=1
/transl_table=11
/product="Cyclic di-AMP receptor A"
/db_xref="COG:COG3870"
/translation="MKLLLAIVQDKDSARLSNEFVDAGVRATKLSTTGGFLKSGNTTF
IVGIEDERIQEVLDIIRETCQAREQFMTPPISLDVSMDTHMPYPIEVQVGGATVFVMP
VEQFHQF"

CDS complement(2662413..2663060)
/gene="tmk"
/locus_tag="JFBMFIFI_02482"
/EC_number="2.7.4.9"
/inference="ab initio prediction:Prodigal:002006"
/inference="similar to AA sequence:UniProtKB:Q97R91"
/codon_start=1

/transl_table=11

/product="Thymidylate kinase"

/db_xref="COG:COG0125"

/translation="MDGIFITIEGPDGAGKTSVLKELLPLLEATVQMDVIATREPGGS

RIAEEIRELILNPVNTEMDIRTEALLYAAARRQHLIEKIKPALESGALLMCDRFVDSS

LAYQGAGRDIGVEAVREMNLFATEGLEPDLTLYLDVEASVGIERITRNKDSRQFDRLD

QEKIEFHEKVRTAYLKLASENPKRIKMIDASGSISEVVAACCTLITEKLKLKEVE"

CDS complement(2663343..2664578)

/gene="tetA"

/locus_tag="JFBMFIFI_02483"

/inference="ab initio prediction:Prodigal:002006"

/inference="similar to AA sequence:UniProtKB:P02981"

/codon_start=1

/transl_table=11

/product="Tetracycline resistance protein, class C"

/translation="MSKFKENNDQNSEKRIDKHALAFGLISAFLCGVGFSIIMPVVPF

LVHPYVNSPQNQTIIVTLLTSVYAVCVFFVAPGIGVLSDRYGRRPVLIICLLGSAIGY

LIFGIGGALWVLFTGRIVEGVTGGSISTIFAYFSDIIPAQQRTKYFGWMSAVVGVGTA

IGPALGGLLARFDYSLPLYFGALITVINVVYGFIYMPESLDKKDRIVAIPIVRLNPFT

QLITILSMKNLKWLLISAFLLWLPNGSLQAVFSQFTIDNFNWKPAIIGLMFSIIGIQD

ILSQGLIMPKLLLKYTDKQIAIIGMTAEIIGYSLIAGSALFSFYPPFFIIGMFVFGFGD

SIFGPSFNGMLSKSVDASEQGRIQGGSQSIQALARIVGPIIGGQIYIILGHAAPAFMG

VILIALAILFLYKRGLVET"

CDS complement(2664693..2665166)

/gene="pchR"

/locus_tag="JFBMFIFI_02484"

/inference="ab initio prediction:Prodigal:002006"

/inference="similar to AA sequence:UniProtKB:P40762"

/codon_start=1

/transl_table=11

/product="HTH-type transcriptional regulator PchR"

/translation="MNKEEQIKVGFRELYDKMSWLNRRKMEDSLKGYTSSEVHCMEYI

EKNKDSNVTKLADSFYMTRGAISKLTKKLIKKELIESYQKSDNKKEIYFRLTEKGREV

YDIHEKLHNEFSERDKVVFEQVTDEQFNSMLQFVELYSQHLDKEIKKVTLNTKSE"

CDS complement(2665296..2665754)

/locus_tag="JFBMFIFI_02485"

/inference="ab initio prediction:Prodigal:002006"

/codon_start=1

/transl_table=11

/product="hypothetical protein"

/translation="MLSFEEKKAIFDEYDELTVHEVSMERLNYHFEDSAVAKTIVVKF

LHPKSGNAFIYAGYLPDEDTNKGYISIRDEDAGTIHKYVQQAIEYLKKTEDGYEEGYE

EEWHDDHNDQLTLKYGNGMWSIYMSHGGLEAIFKTKEAAEGYLADEGFSY"

CDS complement(2665846..2666190)

/locus_tag="JFBMFIFI_02486"

/inference="ab initio prediction:Prodigal:002006"

/codon_start=1

/transl_table=11

/product="hypothetical protein"

/translation="MNEERETAQKILQELVGYFLENGCEETQMNLTVNGEGIWLELEG

LVPRKPGNLENLKTLLEVKRQPEMDDYYEHLMGMHREQEDYSLVGMLVDSAEITCQEG

LLNIRIYRENLY"

CDS complement(2666187..2667521)

/locus_tag="JFBMFIFI_02487"

/inference="ab initio prediction:Prodigal:002006"

/codon_start=1

/transl_table=11

/product="hypothetical protein"

/translation="MKGKLEKKNSETKLEVTRDEVTNKAMQPQITEDSKYYLLVSFSL

ALMAIAFLFQSPIEIFDGMKVIMRSSGKLLTDYMELTSIGAAFFNSGLLTLLSVLMVR

SQKITISGPLVAGILTVCGFSLFGKNLFNSIPITLGVMLYAKFERRPFSHFVLISLFG

TALGPVVSEFAFGMGLEPWKGIALGYLIGIGIGLILPPLSSHFLSFHKGFSLYNIGFT

SGIIGMFITAISRMFGHEIESVSILSSGNNLSLSIVIIGFCFVFFGLGFYYNGMSIGG

YPKLLKHSGKLVTDFFLLEGFGTTLINMGLQGIVATSYVLLVGGELNGPVIGGILTIM

GFSAFGKHPRNSIPIFIGVYLAAIFNIYQPNSTNALLAALFGTTLAPISGYYGSFAGV

VAGFLHMALVMNIGYLHGGMNLYNNGFSGGFTAAILVPIYDAILNLKKEKKA"

CDS complement(2667531..2668976)

/gene="speA"

/locus_tag="JFBMFIFI_02488"

/EC_number="4.1.1.19"

/inference="ab initio prediction:Prodigal:002006"

/inference="similar to AA sequence:UniProtKB:P21885"

/codon_start=1

/transl_table=11

/product="Arginine decarboxylase"

/db_xref="COG:COG1982"

/translation="MGEKKNFDQKQRPLFDALKKHNKQEKISFHVPGHKNGLNWLEDM

AAFRGILPFDQTEVTGLDYLHEAEGAIKKSQDLLTNFYQSQQSYYLVNGSTVGNLAMI

LGTTQKGDRVFVDRNVHQSIIHGLELNELRPIFLAPEIESKHQSPVGISLATLQKALL

AYPDVKALILTYPTYAGDIYPIEELLDLAKAHNCLTLVDEAHGAHFILGEQNASFPKS

ALFLGADIVVQSAHKMLPAITQSAYLHIGNNLTEEQKSKVEHYLHMLQSSSPSYLLML

SLEYARWFLASLTAADLEASLVLVKNWKYFFEEQGLNAEISGDPLKLILRYQGLTGIE

LGKKLEKSGLFPELVDHEKIVLTFPLIKENQDLGAFSEFDKYQLGLEKSSEKRNNFIV

NGHYSRLSELELTYAAQVGLMSEKIMWRTSIGRIAAENITPYPPGVPLALKGEKIQQE

HITQLAGWLSQGARVVGLTEQGEIQVYLEEK"

CDS complement(2669078..2669314)

/locus_tag="JFBMFIFI_02489"

/inference="ab initio prediction:Prodigal:002006"

/codon_start=1

/transl_table=11

/product="hypothetical protein"

/translation="MKMLFGKNKKGLLRKEYDESLLELMYKTKDEWEYKKDIEDSVFD

KDGLLFAQTKLAEAKYFYLFKEARIRKLKSTKVR"

CDS complement(2669325..2669921)

/gene="recR"

/locus_tag="JFBMFIFI_02490"

/inference="ab initio prediction:Prodigal:002006"

/inference="similar to AA sequence:UniProtKB:P0CB76"

/codon_start=1

/transl_table=11

/product="Recombination protein RecR"

/db_xref="COG:COG0353"

/translation="MHYPEPISKLIDSYQKLPGIGAKTAARLAFFTIDMKEEDVTDFA

KALISAKRDLHYCSICGNITEEDPCEICKDTTRDRSVVLVVEDPKDVISLEKMREYHG

LYHVLHGVLSPIEGTGPEDINIPSLIKRLQSDEINEVIIATNATAEGEATAMYLSRLI

KPAGIKVTRLAHGLSVGSDIEYADEITLLKAVEGRREL"

CDS complement(2670026..2670337)

/locus_tag="JFBMFIFI_02491"

/inference="ab initio prediction:Prodigal:002006"

/inference="similar to AA sequence:UniProtKB:A3DHB8"

/codon_start=1

/transl_table=11

/product="Nucleoid-associated protein"

/db_xref="COG:COG0718"

/translation="MRGMGNMQGMMKQMQKMQKEMAETQEALNEKEFIGSASSDLVTV

TMTGDKKMKDIVIKPEAVDPDDIDMLQDLILMATNIALDKVDNETQATMGKFTKGIPG

F"

CDS complement(2670435..2672321)

/locus_tag="JFBMFIFI_02492"

/inference="ab initio prediction:Prodigal:002006"

/codon_start=1

/transl_table=11

/product="hypothetical protein"

/translation="MSYQALYRVWRPQRFQDIAGQQAITQTLRNALIQGKSSHAYLFT

GPRGTGKTSAAKIFAKAINCQFLEDGEPCNECETCKAITSGQLNDVIEIDAASNNGVE

EIRDIRDKAKYAPTSADYKVYIIDEVHMLSTGAFNALLKTLEEPPKNVIFILATTEPH

KIPLTIISRTQRFDFKRISVKDICARMVYILNQEKIGFEDAALPVIARVAEGGMRDAL

SILDQVISYGDDMVTVANAMSVTGSLTQELLLSYFDAIVMNNTEKGLSILQSILAEGK

DAARFVEDLILFSRDLLVYQQAPEMLDLLEEASADNGFKELSQKIPAKTLYSVITILN

ETQGEMRFSNHPDIYLEVATVKMTQLRNEAPAVIQEVPTKQVDRVQESSGNANTAYLE

SELAALKKQLQEMKEQGFSGGASKTAPAKVQKKVVGGQFKPNTTGIYSVLKEATKDNL

VQLRDLWPDLLNMLSVTQRAIMKASTPVAASPNGLIVSFEYDILCQKATNDTELIEAV

SEYMRRLIGHTPKMICVPSEQWPTIRTQYLSQNKPDTVTQNKSEGSKDSIQANQAAGD
SQQQNVPTNHQISQETEQRVEASPFDGLDDLAPPPDESKTDNSVVSEALELFGESIVE
VVND"

CDS 2672555..2673322

/locus_tag="JFBMFIFI_02493"

/inference="ab initio prediction:Prodigal:002006"

/codon_start=1

/transl_table=11

/product="hypothetical protein"

/translation="MKNKKILVGVLFMSLLVLASCGPQKGASVESSNIENKSEKKEEH
LDYKNQEEWNFEAGDAQSPINIETSTAVPMVDAGEITINYNDLVIDEVDNGHSIQIDD
TGTAVINGRNFDLNQFHFHAKSEHTINSEYFPLEAHFVNKAQNGRLAVLAVLFKEGQA
NPAFETILQNIKKGEKATVSDPINTSQLFPTDFSYYHYLGSLTTPPLSENVEWYVMEH
PVEVSKEQLKTFNTYYDGNNREIQPLNERIILKHENQ"

CDS 2673634..2674446

/gene="proC_2"

/locus_tag="JFBMFIFI_02494"

/EC_number="1.5.1.2"

/inference="ab initio prediction:Prodigal:002006"

/inference="similar to AA sequence:UniProtKB:P0A9L8"

/codon_start=1

/transl_table=11

/product="Pyrroline-5-carboxylate reductase"

/db_xref="COG:COG0345"

/translation="MKIGFIGTGNMARAIIKGIIKSKQLPNENIFVFNRDSDKTTSFA
IETNTHPCTSSQKLAESVDVLVLAVKPGVIPTVLKELHTTFLKHQPLLVSIAAGTTIA
AIEDTLGSMQKHPIIRVMPNLNALIGKGMAAVCSNQFASKQQISFVLDLFNSIGIAIE
LEEHHFSTFTAIAGSSPAYTFLFIDSLARGAVKNGMPKDLATKIATQAVLGSAEMLLA
SDKSPWDLIDQVSSPGGTTVAGLVALEDEAFISTVIKGVDATIKRDHELMND"

CDS complement(2674521..2675042)

/gene="tadA_4"

/locus_tag="JFBMFIFI_02495"

/EC_number="3.5.4.33"

/inference="ab initio prediction:Prodigal:002006"

/inference="similar to AA sequence:UniProtKB:P21335"

/codon_start=1

/transl_table=11

/product="tRNA-specific adenosine deaminase"

/db_xref="COG:COG0590"

/translation="MYTHVEKEKFMREAIIEAKKAGDKLEVPIGAVIVLDGKIIARGH

NRREETNDATTHAEMIAIREANQVMESWRLEEASLFVTLEPCPMCSGALIMSRMKAVY

YGAADPKGGTAGTLLNLLTDTRFNHQVAVEKGILEEECGKLLSDFFRELRKRKKIEKT

KQKIATDEINTTK"

CDS 2675184..2676050

/locus_tag="JFBMFIFI_02496"

/inference="ab initio prediction:Prodigal:002006"

/codon_start=1

/transl_table=11

/product="hypothetical protein"

/translation="MDFQQLKTIYPTAKLSDTRQLDTALLNFPYSNQWIQIESQDLST

SEIKLLNQLFKYSETAANSLNTHFWYQFLWENKQISTTKHGDYRVIQFEIVHPSQNEN

ATSWLEAFQNIFEGCEDAFFISENYGIIIEEKQKTELSTKEIVGVLQALEDDFSIKSI

CYIGQYWTITPDFPSLFNEEYTIFQKQKGQNKGLAVLSLSQLALSHYIKSALEESELL

SELKKIFSDQAEWRELVKALWETQGNISMAAKSLYVHRNTLQYRIDRFNEATGFSLKD

KDDLMLCYLLLL"

CDS complement(2676126..2676476)

/locus_tag="JFBMFIFI_02497"

/inference="ab initio prediction:Prodigal:002006"

/codon_start=1

/transl_table=11

/product="hypothetical protein"

/translation="MKNATKGLLIGLGATVVVGTIIVSSSEKAVKKIESFMNRKRAKD

FIKEKLNGNNDALEVVDHLSDEEITNLLQIVDKVNSLKGRINVYGDNLKDAAEEFKST

LHGYKDTVKEKFEN"

CDS complement(2676579..2679071)

/gene="clpC"

/locus_tag="JFBMFIFI_02498"

/inference="ab initio prediction:Prodigal:002006"

/inference="similar to AA sequence:UniProtKB:P37571"

/codon_start=1

/transl_table=11

/product="Negative regulator of genetic competence

ClpC/MecB"

/db_xref="COG:COG0542"

/translation="MDELFTEKAKLVLILAQEEAKNFRHHSVGTEHILLALVVEQDGM

AGKTLRQFAVNEQDVREEIEHFTGYGTMKNVPKNALLPYSPRAKQVITFATDEARRLG

APLVGTEHLLLGLLREEEILSSKILSNLDVSLNKTRQLLLKKIGVSDVNGTKGARRGR

QSPKQATGGTPTLDSLARDLTALAKEGKMDPVVGRHKEVHRLIQVLSRRTKNNPVLVG

EPGVGKTAIAEGLAQKIINGEVPKDMAKKRLMMLDMGSMVAGTKYRGEFEDRMKKVIE

EIYQDGQVILFIDELHTLIGAGGAEGAIDASNILKPALARGELQTIGATTLDEYQKYI

EKDAALERRFAPITVDEPTPEEAEEILLGLRSRYEEHHGVEITDEAIHAAVQFSTRYI

TSRQLPDKAIDLIDESAAKVRLDVSDKPTPVAVAVAELDQLVNDKELAIQMQDFEKAA

AIRTKEMRQKKKIETLIVKEGKQHSASKLQVTEFDVAEVVSLWTGIPVQQMEQKESER

LLNLEKVLHSRVVGQEDAVSAVSRAMRRARSGLKDPNRPIGSFMFLGPTGVGKTELAK

ALAESMFGSEDALIRVDMSEYMEKYSTSRLIGSPPGYVGYDEGGQLTEKIRQKPYSVV

LLDEVEKAHPDVFNILLQVLDDGHLTDAKGRKVDFKNTILIMTSNLGATSLRDEKSVG

FSTKDTKKDHKAMEKRILEELKNTFRPEFINRIDETIVFHSLEKNELNEIVKLMAAII

VKRLKDLDIHVKITPAAIEVIAKAGFDPEYGARPLRRALQKEVEDRLSEELLNKTVKI

GDHVTIGAAKGKINIQVKELKKKAKPETVEVK"

CDS complement(2679102..2679563)

/gene="ctsR"

/locus_tag="JFBMFIFI_02499"

/inference="ab initio prediction:Prodigal:002006"

/inference="similar to AA sequence:UniProtKB:Q88XZ6"

/codon_start=1

/transl_table=11

/product="Transcriptional regulator CtsR"

/db_xref="COG:COG4463"

/translation="MQNQNMSDIIEAYLKQVLTTHEQIEIRRSEMANQFNCVPSQINY

VINTRFTIQQGYLVESKRGGGGYIRIIKVKLLDKAEMLDAMIQIIGDKISQKDAYSIV

QKLYEDDMLTKREATLILSAMEKPVLTLCGKLENELRAQILIAFLNNLRYE"

tRNA complement(2679787..2679860)

/locus_tag="JFBMFIFI_02500"

/product="tRNA-Pro"

/inference="COORDINATES:profile:Aragorn:001002"

/note="tRNA-Pro(tgg)"

tRNA complement(2679908..2679983)

/locus_tag="JFBMFIFI_02501"

/product="tRNA-Arg"

/inference="COORDINATES:profile:Aragorn:001002"

/note="tRNA-Arg(acg)"

tRNA complement(2679986..2680074)

/locus_tag="JFBMFIFI_02502"

/product="tRNA-Leu"

/inference="COORDINATES:profile:Aragorn:001002"

/note="tRNA-Leu(taa)"

tRNA complement(2680129..2680200)

/locus_tag="JFBMFIFI_02503"

/product="tRNA-Gly"

/inference="COORDINATES:profile:Aragorn:001002"

/note="tRNA-Gly(gcc)"

tRNA complement(2680237..2680312)

/locus_tag="JFBMFIFI_02504"

/product="tRNA-Thr"

/inference="COORDINATES:profile:Aragorn:001002"

/note="tRNA-Thr(tgt)"

tRNA complement(2680331..2680415)

/locus_tag="JFBMFIFI_02505"

/product="tRNA-Leu"

/inference="COORDINATES:profile:Aragorn:001002"

/note="tRNA-Leu(tag)"

tRNA complement(2680457..2680531)

/locus_tag="JFBMFIFI_02506"

/product="tRNA-Lys"

/inference="COORDINATES:profile:Aragorn:001002"

/note="tRNA-Lys(ttt)"

tRNA complement(2680532..2680607)

/locus_tag="JFBMFIFI_02507"

/product="tRNA-Val"

/inference="COORDINATES:profile:Aragorn:001002"

/note="tRNA-Val(tac)"

rRNA complement(2680648..2680757)

/locus_tag="JFBMFIFI_02508"

/product="5S ribosomal RNA"

rRNA complement(2681019..2683936)

/locus_tag="JFBMFIFI_02509"

/product="23S ribosomal RNA"

tRNA complement(2684249..2684321)

/locus_tag="JFBMFIFI_02510"

/product="tRNA-Ala"

/inference="COORDINATES:profile:Aragorn:001002"

/note="tRNA-Ala(tgc)"

tRNA complement(2684357..2684432)

/locus_tag="JFBMFIFI_02511"

/product="tRNA-Ile"

/inference="COORDINATES:profile:Aragorn:001002"

/note="tRNA-Ile(gat)"

rRNA complement(2684542..2686093)

/locus_tag="JFBMFIFI_02512"

/product="16S ribosomal RNA"

CDS complement(2686870..2687076)

/locus_tag="JFBMFIFI_02513"

/inference="ab initio prediction:Prodigal:002006"

/codon_start=1

/transl_table=11

/product="hypothetical protein"

/translation="MKNMDQKKVFSMLSSIYSTLIILFLVLYFFTKDTNSLLIDICLI

GLLVIAIIFAVILFINYRKQKSTK"

CDS complement(2687168..2687941)

/gene="murR_2"

/locus_tag="JFBMFIFI_02514"

/inference="ab initio prediction:Prodigal:002006"

/inference="protein motif:HAMAP:MF_02108"

/codon_start=1

/transl_table=11

/product="HTH-type transcriptional regulator MurR"

/translation="MLFLDKTVELNDTEMEIYHYISGNIDKVIFMRIRDLADEVHYST

TTILRFCRKFDCQGFAEFRIKLRMFRKNQKQIPIDTTDETTYIDFLNRTAQPEFQRSV

QEVVTILAESELVIFVGVGSSKLMAEYGALYFSSLFLMSIHIDDLFNHPLYYLSNQVS

KKSCLFIVSVDGENKEIIKNIHHLKMQNIKVISITNSAKSTIARLSDANISYYINKEQ

FQEANITSQLPALYTIELVGKEMRKQLNRTKDSIGNDLF"

CDS 2688141..2689520

/gene="celB_3"

/locus_tag="JFBMFIFI_02515"

/inference="ab initio prediction:Prodigal:002006"

/inference="similar to AA sequence:UniProtKB:Q9CJ32"

/codon_start=1

/transl_table=11

/product="PTS system cellobiose-specific EIIC component"

/db_xref="COG:COG1455"

/translation="MQGLMNWLEKYIIPIAAKIGNEKHLVALRDGFIGTMPATMAGSI

AVLLNAFLRDFPTTWGWTGFVTAMEPIIGINGYVWLGSLAIVAVIFAVALGYNLAKVY

HVDPLSGAVVALAAFFMGLTQTATTNLALSEKIPGNMVKLINEAGGSVTDGNTIVTSA

WGFFNFDAHMGGSGLFTAIIFGFISVVIFSKLMLANITIKMPDSVPPAVAKAFAAIIP

ACVALYTCGLINYGFSKLTGMPMIDWITSTIQEPLLNLSQGFGAVLLIVFLVHLLWFF

GIHGDNVMAPVLSTIWGNAMNQNMNAFQNGQDIPYKWVSGTFQAFVWPGGSGATLMLI

VAIFVFSKRPDSRAVANLSIGPGIFNINEPVMFGMPIVLNPLYMIPVILAPCVMAGVA

YFATMTGLVAPVVVSVIWVMPPIVNGFLATGGDWRAIILSIVNLAIAFVIWAPFVIAA

NKIKLEDVD"

CDS complement(2689590..2690552)

/gene="nrdF2"

/locus_tag="JFBMFIFI_02516"

/EC_number="1.17.4.1"

/inference="ab initio prediction:Prodigal:002006"

/inference="similar to AA sequence:UniProtKB:P9WH71"

/codon_start=1

/transl_table=11

/product="Ribonucleoside-diphosphate reductase subunit

beta nrdF2"

/db_xref="COG:COG0208"

/translation="MTESEQYAAINWNSFEDILDKKTWEKLTEQFWLDTRIPISNDLD

DWRNLSSIEKTLVERVFGGLTLLDTLQSQDGISALRQDVRSPHEEAVLNNIQFMESVH

AKSYSSIFSTLNTPREIEEIFNWTNSNVYLQEKAAIMNDIYQTGSPLQKKVASVFLET

FLFYSGFYTPLYYLGINKLPNVAEIIKLIIRDESVHGTYIGYKFQLGFNELSEDEQKS

LQGWIYELLYKLYENEVKYTELLYDTVGWTEEVKVYLRYNGNKALMNLGMAPLFPDTA

EDVNPIVMNGMSAGTSNHDFFSQVGNGYLLGVVEPMQKEDYEIL"

CDS complement(2690622..2692796)

/gene="nrdE2"

/locus_tag="JFBMFIFI_02517"

/EC_number="1.17.4.1"

/inference="ab initio prediction:Prodigal:002006"

/inference="similar to AA sequence:UniProtKB:P0CH00"

/codon_start=1

/transl_table=11

/product="Ribonucleoside-diphosphate reductase subunit

alpha 2"

/db_xref="COG:COG0209"

/translation="MNITKIPKEQNNPSYFKLNNQLNIPVNGKIPMHKDKEAARAYFL

EHVNPNTVFFHTLREKLDYLLENDYIEEEFISKYSFEFIKKLFQKTYDHKFRFRSFMG

AHKFYTQYALKTNDNQRYLERYEDRIAFNALYFADGNEELAMDLAEEMITQRYQPATP

SFLNAGRKRRGELVSCFLLQLTDDMNSIGRGINSALQLSRIGGGVGISLSNLRAAGDP

IKGYEGAASGVVPVMKLLEDSFSYSNQLGQRQGAGAVYLSVFHPDVVAFLATKKENAD
EKVRVKTLSLGLVVPDKFYELAKNDDYMYLFSPYDVERIYGEPFSYIDITEKYDELVN
NPDIKKSKIKARDLEIEISKLQQESGYPYVINIDAANRSNPIKGKILMSNLCSEILQV
QKPSIINDDQSYEEIGTDISCNLGSSNIVNLMASPDFGRSVRTMTRALTFITDSSSVA
VVPSVKKGNDQAHTIGLGAMGLHTYLAMNQIHYGSPESVDFINIYFLLLNYWTLVESN
NIAKERNETFVDFAESKYATGEYFDKYIEKEWQPESQRVRELFEGIFIPTTEDWEKLR
DNVKEFGLYNQNRLAVAPNGSISYVNETSASLHPITQRIEERQEKKTGKTYYPAPFLS
DETMPYYKSAYDMDMRKVIDVYAAAQQHIDQGMSLTLFMRSTIPAGLYEWKGENEKQT
TRDLSILRNYAYHKGCKSIYYIRTFTDDTDEIGSNACESCSI"

CDS complement(2692774..2693154)

/locus_tag="JFBMFIFI_02518"

/inference="ab initio prediction:Prodigal:002006"

/inference="similar to AA sequence:UniProtKB:Q97T03"

/codon_start=1

/transl_table=11

/product="Putative NrdI-like protein"

/db_xref="COG:COG1780"

/translation="MKIVYFSLTGQTRRFVKKLDLDSYEVTETNPFYRIEEDYVVVAP
TYDIEVTELINDFIEYSDNQMHLKGVAGGGNLNFANLFVFTAKDLAKKYNVPMLFAFE
FSGTDLDVINFKKEVDKLEHNKNS"

CDS complement(2693157..2693390)

/locus_tag="JFBMFIFI_02519"

/inference="ab initio prediction:Prodigal:002006"

/codon_start=1

/transl_table=11

/product="hypothetical protein"

/translation="MNKIHLYSKPNCPQCDMTKFLLKNENIEFHEKNIMEDESHLNYL
KDLGYMSVPVVMVEGIEPIVGFQPERLKELKAI"

CDS complement(2693703..2694533)

/locus_tag="JFBMFIFI_02520"

/inference="ab initio prediction:Prodigal:002006"

/codon_start=1

/transl_table=11

/product="hypothetical protein"

/translation="MEKILISTLLIFSLSLFVLSLYLHIQLKKKIKRLMTKKPNTRQR

KAQLRWNQNQKMLKKRLAKRLVFVICFGLVSLMVGLTLPFYSENRFKASSENTFKTEV

NADQNHLILDTPEQSIEKDEVPEKTGTQENPQPFSQPVRVAANFLDLNTMTFFPAELE

VTVLNSIRGEESWRLLVGENQFNPMAPAGKEYILNRLKCKIVAPENPTAIAKFSEFDF

NYYSKEGNPYERVTVITPDRLGSELEVNVEKEGYLYALIDSGDVPVINYQNNLFLATE

"

CDS complement(2694624..2695178)

/locus_tag="JFBMFIFI_02521"

/inference="ab initio prediction:Prodigal:002006"

/codon_start=1

/transl_table=11

/product="hypothetical protein"

/translation="MKKWRKINIFALMLGLLLLVGCGMRKEAEHDVSKDFTYDKKVEM

DQVVEGEEYSVRVTGYKVIRDSENMPALMMYYEFVNSSEEEISPYDLYMDAYQTSKIA

DGDTELYEADIYDGLLDEEDLTLLNNTFELVEPESQLKCATVWKLRNNKYPLEVEVYS

YEEEYLGMIQIDMQKGIAEGSTEI"

CDS complement(2695388..2695585)

/locus_tag="JFBMFIFI_02522"

/inference="ab initio prediction:Prodigal:002006"

/codon_start=1

/transl_table=11

/product="hypothetical protein"

/translation="MTLISKKNNILSYPKFIIQADADLKEQGVKTHNKSLYQILVEAN

ILNQSGEPTEWIMENGHLKEA"

CDS complement(2695704..2697020)

/locus_tag="JFBMFIFI_02523"

/inference="ab initio prediction:Prodigal:002006"

/codon_start=1

/transl_table=11

/product="hypothetical protein"

/translation="MIQEVLPNIPRHYTALAEWLTCVVFIFPLAAKRERWQFLVLLMG

VGQYALQMMVGSWPLPLWIPGMVVNVCWMFLTIVVSSKISLKNASYLCAKAFIVAEFT

ASFTWQMYCQLILNRVAKGAFISIWFPVLLYAIILTTVFLVEKRVERTDVNLTIQNKN
VFIAMLTAVIIFSISNIGFIISRTEYSFGDPLSVFLIRSLINFCGICILYMQENQQYE
HHLQNELKAVNNIFQNHYEQYVAYKESASLIERKVHDLKHQIETIRSEENIEKKEAYL
EEMTKAINTFSSDIHTGNGILDTILSRKNSYCLEQDIRFTCIVEGKLLSFLDTMDICS
LFGNALDNAIEAVLRNAELEQRLVNLRVSKRGDFVIILVENYVPDLDELSEGLPETTK
TNKDYHGYGLKSIDYIAGKYEGNMNISVKNNWFLLKVLLPLPATLN"

CDS complement(2697022..2697723)

/gene="ypdB_2"

/locus_tag="JFBMFIFI_02524"

/inference="ab initio prediction:Prodigal:002006"

/inference="similar to AA sequence:UniProtKB:P0AE39"

/codon_start=1

/transl_table=11

/product="Transcriptional regulatory protein YpdB"

/db_xref="COG:COG3279"

/translation="MKIAIVEDNEANYLTLKQHIDSFFKAHNLIGTIDYYPDGLALVN
SFKNNYDIIYFDVEMELMDGMTAAKKIRQIDEEVLIVFVTNYVQWAIEGYTVNATDFL
LKPLSYFNFSEHFKKIQKRLNHNEQKYLTIKVGSGFTKIKLSELFFIESEGHYLKIHT
HEQTFTLLESMKNMEKKLLEEDFFRCNNCYLVNLKYVTGIEKSTAKVGHYNLQISRPR
KKEFLEALTAYIGGE"

CDS 2698020..2700428

/gene="bglB"

/locus_tag="JFBMFIFI_02525"

/EC_number="3.2.1.21"

/inference="ab initio prediction:Prodigal:002006"

/inference="similar to AA sequence:UniProtKB:P14002"

/codon_start=1

/transl_table=11

/product="Thermostable beta-glucosidase B"

/db_xref="COG:COG1472"

/translation="MNNTELINKMTLQQKAFFMSGKSTWETVSYEELGIPSIFLSDGP
HGLRKQEGAADHLGLNASLKATCFPTAATIANSWDEELGEEIGRSLGAEARNSDVHVV
LGPGLNIKRNPLCGRNFEYFSEDPYQAGKMAAAYTRGIQENGVVACPKHFAANSQETR

RMTVDSIIDERTLHEIYLTGFEIAVKEGKAKSIMSAYNKVNGIYANEDKELLRDILVD
KWGFSGFVVSDWGGSNDHVLGVKNGSHLEMPSTGVNGAAEIVAAVQNGQLSEAILNER
VDELLTIILETSRHATNKSDRTFELHHEVAKKAARESIVLLKNEKNILPLQADESIAI
IGDFAQTPRYQGAGSSVVNPTKLDSTLELIDSYPLNFKGYAQGYKRSSTSDQSLISEA
VALAKKVDTVLLYVGLDEISESEGLDRTTLSLPENQLALIAAIAEVNPQIVTILSAGS
VIDMSWDTASKGVLHGYLAGQAGAGAMLDILTGKYSPSGKLSETYPVDYWDLPSAKDY
PSSTNQAIYQEGPYIGYRYFETANKSVKYPFGFGLSYTDFEYSDLQISETGISCLIKN
SGTVAGAEIVQFYVSCQSDAIYRPSKELKGFKKLFLEVDEEQLVELPFDDKTFRYYNT
ATKAWEIESGTYRLLVGTNVQDIRLTGSIYQSGKQVPLPYNPTAISSYFTAAVENYSM
SEFELLLGRAVRITPFTKGQTLTINDSISQLAYAKSWLARAVYQILNRILKQSKKKGK
PNLNVLFIYNMTFRAVSKMTDGQMSFAMMEGLMVMINGRFWFGTKQLLKATRMNRKRQ
KQGI"

CDS 2700461..2700976

/locus_tag="JFBMFIFI_02526"

/inference="ab initio prediction:Prodigal:002006"

/codon_start=1

/transl_table=11

/product="hypothetical protein"

/translation="METKKQWGLVTAIKNFKAKHPDLFEFILFNIMSNVATIVNFIVL
WIGTGFLFSQLASINFHWFIFNYSTTEGGLSSFLSFLLAYVCAQIVNFFVQRKFVFSA
TIEIRKVLPLYILTVVFAGLISVWLPPHIIKLTQPFIHGLAPTLANVVNIVLQVVINY
PMMKFKIMKKE"

CDS 2700980..2701990

/gene="arnC_2"

/locus_tag="JFBMFIFI_02527"

/EC_number="2.4.2.53"

/inference="ab initio prediction:Prodigal:002006"

/inference="protein motif:HAMAP:MF_01164"

/codon_start=1

/transl_table=11

/product="Undecaprenyl-phosphate
4-deoxy-4-formamido-L-arabinose transferase"

/translation="MKALSVVVPCYNSENYMKHCLDSLLVGGTDIEIIIVNDGSTDQT

ATIAQQYQERFSTVIKVIHQENGGHGMAVTSGIQQATGRYIKIVDSDDWVDAEALHSV
LTFINNQSAMNEVDLIINNYVYEKVGARYKKRIHYASALPVNREFSWDEVKLPVGKYL
LMHALIYHRSVLQKSKLTLPKHTFYVDNLYAFEPLPFVKKMYYLDVDLYRYFIGRNDQ
SVNEQVMIARIDQQLFVNRRMIDFYTNLPEIDNDQRNYMRQYLEIITTVSSVLLIKEG
SVENLAKKATLWKYIAEQNHPLYRELRKSLIGYSVNLPGKTGRYTVIGVYRVLQKIYG
FN"

CDS 2702012..2703139

/gene="glf"

/locus_tag="JFBMFIFI_02528"

/EC_number="5.4.99.9"

/inference="ab initio prediction:Prodigal:002006"

/inference="similar to AA sequence:UniProtKB:P37747"

/codon_start=1

/transl_table=11

/product="UDP-galactopyranose mutase"

/db_xref="COG:COG0562"

/translation="MTEKFDYLVVGSGLFGAVFAYEAAQRGFKVKVIEKREHIAGNIY
TERIADIDVHRYGAHIFHTNNETIWQYMSQFTAFNHFVNAPIANYQGKLYNLPFNMNT
FYQLWGTRTPQEAQDKIAEQRKILGTKIPENLEEQAIALVGTDIYTTLIKEYTKKQWG
KDPKELPAFIIKRLPVRFTFDNNYFSDKYQGIPVDGYTAIIEKMLASDAIQVETKTDF
FADKEMYLSTYPKIIYTGMIDEFFDYQLGILEYRSLRFETTLLPETANYQGNAVVNYT
DAATPYTRIIEHKHFNFGEQPETIITKEYSTAWHKGDDAYYPVNDQVNNQLYKRYVQL
AKNEPTITFGGRLGTYQYLNMDQIVASALALVKREFQQENR"

CDS complement(2703368..2704273)

/locus_tag="JFBMFIFI_02529"

/inference="ab initio prediction:Prodigal:002006"

/codon_start=1

/transl_table=11

/product="hypothetical protein"

/translation="MITERKFQNGINTWININSDTVGNDSDLYKQYGIDSELISYALD
RNERARADYDDEVDAFTLIYNVPKKKGQNQYYETAPMTFLIQNQRLLTISNHTNEYII
PQMERYLEKNPTDSVFKFLFASLFIISDNYFPLIEEMNKERNYINDMLKQKTTKKNLL
ALSDLETGTTYFISGTKQNAVLLEQIKTNAIYRRLNEEESEQLEDTLIEAKQLVEMTQ

LTAQVLQQLSGTYNNILNNNLNDTMKILTVLSLLLTVPTIVTGYFGMNMPLPLEQNIF

GWLIVIGISLVGWFGLAFILHFILK"

CDS 2704468..2704857

/locus_tag="JFBMFIFI_02530"

/inference="ab initio prediction:Prodigal:002006"

/codon_start=1

/transl_table=11

/product="hypothetical protein"

/translation="MTPFESSEVKAVFNQYSPQCKEALLTIRQLIIDTSAELTPHEKL

VENLKWNQPTYTAKAGTPIRIGIFEETKIALFFHCQTTLIEQFRELFTDTLVFSKNRA

IVMDPTKPLPINDLKLCIQMGLTYHSS"

CDS complement(2704905..2705888)

/gene="dus"

/locus_tag="JFBMFIFI_02531"

/EC_number="1.3.1.-"

/inference="ab initio prediction:Prodigal:002006"

/inference="similar to AA sequence:UniProtKB:P67717"

/codon_start=1

/transl_table=11

/product="putative tRNA-dihydrouridine synthase"

/translation="MTTNFWAELPKPFFILAPMEDVTDVVFRHVIKEAGSPDVFFTEF

TNSDSYCHPDGIESVRGRLVFTEDEQPVVAHIWGDKPEFFREMSLGLAKMGFKGIDIN

MGCPVPNVAGRGKGSGLILRPEVAAELIAAAKTGGIPVSVKTRIGYKEISEMENWITH

LLKQDIANLSIHLRTRDEMSKVDAHWEIIPQIMAIRDRIAPQTLITINGDIPDRETGL

KLAEQYGVDGIMIGRGIFKNPYAFEKEPREHTSQELLGLLHLQLDLQDKYAELVPRSI

VGLHRFFKIYVKGFPGASELRVKLMNTKSTEQVRQILADFDAAKATDSLLE"

CDS complement(2706058..2706441)

/locus_tag="JFBMFIFI_02532"

/inference="ab initio prediction:Prodigal:002006"

/inference="similar to AA sequence:UniProtKB:P44839"

/codon_start=1

/transl_table=11

/product="RutC family protein"

/db_xref="COG:COG0251"

/translation="MGRKIYTGKNVGASGPYSHVVDAGEYLFFSGQTAYNAIGYNGEK

YDIATQTQKCFSHLSDVMAEANVTLDDVVKVNVYLTSMKYFDEMNAVYKELFKHPFPA

RTCVAVLELPLEADIEIECTVKRQA"

CDS complement(2706643..2709564)

/locus_tag="JFBMFIFI_02533"

/inference="ab initio prediction:Prodigal:002006"

/codon_start=1

/transl_table=11

/product="hypothetical protein"

/translation="MKKFKMNKKKWAILGIWIAIVGGIYLYLLNHGKASGDNPSFGIN

VQFLVGWITKIIVLLSISLVAYSIYRIVKIRKTRKFLYVLSSLLLGILIIFNLAVNQY

AMIINTYFSASQINQSEVKKTTEQAMALTEKIEGEGAVLLENKNEVLPLQNQKVNVFG

YASRNVVYGGTGSGGGKEDNNVDLQQGLKNAGFQVNDQLTTFYDDRYVPREKVNIHKL

VGGDFNIHEPKASEYSESLLTSAKEFSDTALVVFSRNSGEGADITNEMKGYAGGTAGK

HYLELSDDEAATLDLVKENFDKVVVILNSSFPMELGFLDDEKIAGALWIGGPGSVGFN

AVGEILAGEINPSGRLVDTYAYDETSAPSFNNIGEFPYSNSEFDYEKDTWHYKYVNYA

ESIYVGYRYYETRYIDNQTGKMDEAAYEKAVQYPFGYGLSYTNFKQEITNFKVDGNEA

KMTVKVTNTGKKAGKDVVQLYYTPPYTIGGIEKSHVVLSGFAKTQELAPNQSEEVNLS

FDVENMASYDEKQNKAYVLEKGNYEIKLMNNSHDVIDSKNYEVAKDIVFNQENKRESD

ELSATNQFEDAKGDVQYLSRWNWEETMPKEMAQPKEASKELLKALKDQSVNEGKADPI

KFEKHGLELYDLKGVDYDDPKWDQLLEQLSVKDMENLITYGGFQTPAINSVKKPATID

ADGPAGINHLVSQAHGNQYTSEVVVASTWNTELVEEMGAALGKEAKASNITGLYLPGV

NIHRSPFGGRNFEYYSEDALLSGKIGSSLSKGAMDQGSYVFMKHFAMYDQETRRYEFP

TGAATWSTEQAMREVFLKPFEIAVKDSGITGIMSSYNRIGPKWVGESEALLQNVLRDE

WGFRGTVISDFYKPQYMNVDQGLSAGNDLVLYLLPTKLNNVTTDTDWGQQNMRKASHN

ILYTVVNSHAYEVAETNVPNWIYLLVTINIAVFTFLLFCFTKTTHRKKPARKFKLFTK

K"

CDS complement(2709791..2711497)

/locus_tag="JFBMFIFI_02534"

/inference="ab initio prediction:Prodigal:002006"

/codon_start=1

/transl_table=11

/product="hypothetical protein"

/translation="MNKNPLVGGKHNPLLPLNTEITLPSELAARIDLYPENRRRPVNK

TLFRNDYFQLCYNMKEKSMAVSSRGDLVYSAIPYDTARNYIQRAAIQYLLHPEEARRY

IESHKVNLETYLVESIFQVTKKRLAKNSFEVTQAYNAIFQNPLFEIQILGRLLQHDDT

GALSANLAGKFLFSENVKTGIAGYVSEYLNFVIQCTSSLPITTDGKEIQLLDSNVQEG

ILSKQNMNTELTFDSDINQALDLLPQEVKQQIESSMAIAKKTIATNNKVLLRSEYGKE

LSEVRNKYVAHSIQPKEIEHHFENVHKTENRNLVNVVSDVHALDGKLPFINENFNILV

GDVSDSRVTNKSIEGIYVIGNHDLVDVLPTNDNLQNRQWDKWTPFFKNKWFEQLLENP

DESWYLLPTGDHTYYDIVKVELEKRFPKITVLNNSSVIHNGIRYIGLTIPVVLVKRKQ

EQQKFILKWLNKLLNNDYDTPTVIVSHAPLFNELSMLSPKSKAYNKDYGCSEPRIEKL

VEEHNIIGAIHGHHHIPASSGRYKIMNFAGKELFVVCSIYSKMNTGYELMNLLPFDSL

NN"

CDS complement(2712139..2712303)

/locus_tag="JFBMFIFI_02535"

/inference="ab initio prediction:Prodigal:002006"

/codon_start=1

/transl_table=11

/product="hypothetical protein"

/translation="MEQRNTLDELFSDIEDMEFVEINEATALPETAATSGSMISNGCS

TCGSSSCCSS"

CDS complement(2712324..2712488)

/locus_tag="JFBMFIFI_02536"

/inference="ab initio prediction:Prodigal:002006"

/codon_start=1

/transl_table=11

/product="hypothetical protein"

/translation="MEQRNTLDELFSDIEDMEFVEINEATALPETAATSGSMISNGCS

TCGSSSCCSS"

CDS complement(2712509..2712673)

/locus_tag="JFBMFIFI_02537"

/inference="ab initio prediction:Prodigal:002006"

/codon_start=1

/transl_table=11

/product="hypothetical protein"

/translation="MGQHSILDELFSDIEDMEFVEINEATALPETAATSGSMISNGCS

TCGSSSCCSS"

CDS complement(2712743..2713537)

/locus_tag="JFBMFIFI_02538"

/inference="ab initio prediction:Prodigal:002006"

/codon_start=1

/transl_table=11

/product="hypothetical protein"

/translation="MGNFLIMTQYQIKLLFRNFKSLILGFSLPIVMFFIFGNLLSKYQ

VYGDVGLTSLLVPAYIPIIIINGVLVIYGQNFLVYKEQGNLIKYKLLGLKQITISASI

YLATLVFQILAVFVLIIFAYFTKGVEFPFDQIGSLIGTFVLINILEYSIAFLLTSVIN

KSVTYQSISLLIFYFQIFLGGLTFPPEMFPKFLKEIVYIFNPIIYGLEMMRDIWMGNK

SFFDEGKNISILLLWSLFFFILGFFFSKNNITKKKSSKRERVRLNI"

CDS complement(2713530..2714429)

/gene="btuD_5"

/locus_tag="JFBMFIFI_02539"

/EC_number="7.6.2.8"

/inference="ab initio prediction:Prodigal:002006"

/inference="protein motif:HAMAP:MF_01005"

/codon_start=1

/transl_table=11

/product="Vitamin B12 import ATP-binding protein BtuD"

/translation="MNIIEIRNLTKIKQEKTILNSISFDVKSGEILAILGHNGAGKTT

LINSIMKLTNYEGIINYSFQISNLYKEIAYQMQSTSFENEAKVFEICNLYKNLLKTHI

DIEGLLKQFNMEDSKNLFIKDLSGGQRQILAVLLTLIGEPKVLIFDELTTGLDSINRR

RIWDILKKLNQEKNITIILTSHFLEEVEYLANRVVILKRGEVEKIGYVNDIINEQFSD

MKKIQFETINLDQAKKLFGSQIKINGQMVSFVYNEEDEMDIFRKVKEIKGENIVMKRF

SFEDAFLQMLGYQLAEEGEIVHG"

CDS complement(2714430..2715242)

/locus_tag="JFBMFIFI_02540"

/inference="ab initio prediction:Prodigal:002006"

/codon_start=1

/transl_table=11

/product="hypothetical protein"

/translation="MYLILKSEYKISIEKNKVYFFNVFTKSSFHTDYIETIDWILLEE

LCYRGTTIEFLKENRSSTLDFLKYLLKLKRESFILAESIVDVKKAINCRSIIALGIAT

GSSEEILEKYMKVYEETTLTILGDLNGTLKEFLIQNGLKCNSKLNQHATQSKLVIINK

DHQEINDFEGYKNIIAIPYSIANMTMGPCDFDLSRPNLKLQQEIPESELNLASFANIT

SLYSVMLNCLLYLIGDIHEVLYIDTGLPLQREFKFHIPSLKLTATPVYKGGK"

CDS complement(2715243..2715965)

/locus_tag="JFBMFIFI_02541"

/inference="ab initio prediction:Prodigal:002006"

/codon_start=1

/transl_table=11

/product="hypothetical protein"

/translation="MKPKALNIEQIWTTDVDRSILRQLFHNNSISKRDLTSVQIKKVL

KNKQWESKQGINLKIYEPKTSLEKILYSRRSRRKISEKKITFNEVSKLLQFSAGITDK

ENGYFSYPSPGALYPCTLFLSLELPDFKNVLYRYNPYQHKLEEYCYDSERVIDNAIID

DELRKFPIKIFFTSDFQLLEDKYGELSYRLLNQEIGHIAQNLSLYAEELGINTTCIGG

FYECEFKKNIHGYDLLYVMVVG"

CDS complement(2715970..2716767)

/locus_tag="JFBMFIFI_02542"

/inference="ab initio prediction:Prodigal:002006"

/codon_start=1

/transl_table=11

/product="hypothetical protein"

/translation="MWISFHAYIHDYQLLDQYLSEEFNQFLDKHKNAYDEYFFIRYWL

GGPHVRLRVKLNKFFNQDQFLSLFQKSVTNFLANSKIQLVDPETFYSKEMLEGEGIKE

VFWKENGIVEIDQYQPEFLRYGGVENIEIAETMFNISSDLAKRVNSKPFLTRLYIAID

LYYFSFLQLSIDISSVSEQYSIMWSGYNITNINNSQIVKIVEQRINYIDNHKSSLVKI

YNEYLKNLSKCPKSTVFSQIHMTNNRMGIYPEIEYRIAKYMGETERC"

CDS complement(2716788..2718077)

/locus_tag="JFBMFIFI_02543"

/inference="ab initio prediction:Prodigal:002006"

/codon_start=1

/transl_table=11

/product="hypothetical protein"

/translation="MLTSNFDYYKRYSADRYNLTQVTHGTSFLKKQYGFTGNKIEPNN

TAVFNFIQGLMDYSLKIVRFDVLSRYQDHNFSPSTRNMHSAQVLFIIDKKYIYYDIYE

DVFFAAGHCSIPEIFKDKIYIFGFSDLINISRYYGEFSFYLSTLDAGHVLGNLKNYLN

MKGIEWSQVFKIKPIQILNEVCFYNHEMFGTFLLSTNRPKGELTIKNLVEHKRIAEDK

IFNELAATEYLKFMLPNVNNGWLSNENDKRQSNLSNFPYSFQMRNSAHNMVGNFSLNR

EETISDLALKNYINNLNTFQKNLTSIKQNYCFLRKNKVFYGNGEVKNKNIDFEKILYN

DHEFFDLNTFNLICVIYSKDADVRINGIINSLLSCGELMQVVGLAFSNNGKSFRPMKN

HNDTYLKSILELNSDVEINYIGVECSNPIEQFTEFYE"

CDS complement(2718095..2719798)

/locus_tag="JFBMFIFI_02544"

/inference="ab initio prediction:Prodigal:002006"

/codon_start=1

/transl_table=11

/product="hypothetical protein"

/translation="MKWVCFVEEWMLVLMVSDKIAVTKVGSTIIVSTEEDWKNYIHNI

IDSKKQLEITKNTTINDIEQDLVDITKNLIEYGIYYTFDLNSLNITKRYILNTKILTT

SKISETKAINANNFRSLESIEIQQRITKYFTSLVDPKMGVFNGHYRGISDSMPLIAFD

GKLGSRDYDAYGRANTYKDSYYTGILEALERFHGLGSSTVKSVVKTENELESENKSFI

PFKKFYHYDVFNFEQKYFDFPIYSLDKQLSWVKTQSVCEKKSVLIPEQIAYFSSEQIV

NARNEPRYISETSNGTALGSNWYESSIAAILELIERDSFLVHWYTKSKPKLIIDNINI

NDQEIQMILGYLEYIDYKTHIFDITLETGIPAYWILLEYKGEDDKALSFYTAAGSNFN

ELKAIKSALIEASTSIKVFKEYMRIKYTDEELSLLQKDFFQVKKLEDHLYLFSDNSMR

QYLDFALIKPNEISFYDSIANHRNIFSFFGLNQKELFESLVERIKIITKNIYITDLST

IFLNEIGFSCVKAHIPDFQNISFGMQYQNINKNRLQQALNQNSLNGNLKEILKCPHPF

P"

CDS complement(2719746..2721314)

/locus_tag="JFBMFIFI_02545"

/inference="ab initio prediction:Prodigal:002006"

/codon_start=1

/transl_table=11

/product="hypothetical protein"

/translation="MSDNMKVLNRQNTMIYRKGSFTSLKTSYRSIKFEISESDHDKIL
RILRLSKDSVELNQLIEDKVDMKLLKLLSDQGSLFLFNKDDDIEKYFDRKWFSIVKQY
LSPNAHLGTCLKKIIKTKYYIRKNLDAQMPRIIEIFQKYGVDLDIVNGNLVKDSDIVL
TMDRSDSNGKTLLIQNYGEGIVGTFLKDALYEDLQISDKRLVSLFAPLYILIFTIKQA
CGSENDTFFFNEVGKFSEYSLNKSEINVLSTSQTPIEVKELKTKIERIEIFEKSNVLD
KVSLSIANHNSDYANMRQSGFSTYGIISKIDSSALYVIAATTFEEAALQTIKFSLKSQ
FESLNGGTWLVSNRDDYYLNKILMLIENVEEEGILLKLSNELLLKIHVYHSYQNVFPE
VSIYMIYFPTTCTYILYLMDDKEKVLSSGKKVFSFNDELEGLLINYLLYLSNSERKYY
SPYDFDHKIDDFNYDVLSELPNQIEEKAFIENALNLFKQMDIRYEEFVWDREFELSEM
GVLCRRMDVGSYGK"

CDS complement(2721314..2723899)

/locus_tag="JFBMFIFI_02546"

/inference="ab initio prediction:Prodigal:002006"

/codon_start=1

/transl_table=11

/product="hypothetical protein"

/translation="MTKMNYNPYILTRKNSLSKESIEHINSGEIYNFVEDVQLVVTQH
EKVSQIMIDLLEKKIGGNYDNRYLNLKRAIFNNKIMKARKLIEELLKEECDKNLLEYF
DKYLLCAKNYQMINQSIDFFYKNSIEMARETLYNFANKTEIQEYLMMIQPNIYEKLSS
YLEKKSLDHRNKEKKLDGTLYKVASRAALKTSPFANITKVGRIEFGNKIYDENIPVGA
AVDEKYVKINFTYLNRVAFAYLYQSNKFYKEVQYKTPPLSTKKSGTFHYVSFVATKDD
LNNSKVFETSEILKTLKIIPELSDFLDSNSVRNHISFEQFNAILSNYITESEAFNLIK
KYVEIGLLIPTVGFKSKTDVELCTEIIYTCKKFLSEEISDDQNIFISQILETKEKLSI
CDSLIERKHIYQDFQRKININPIINSIKMNINNIFYEDGIIKNTTIKHDLLTNQQLED
LKSIQTFTLLFDVGVRMRLELGETLKSKDIHTLNNDFFTVLFDTSKKILPYWSSPEYL
GNSLNSKYVSILDQLKLSFLKEYSEFIENQKENEEVDISYIVKKYQSFIPEELIQEVD
LSTSYFFQKNNSNIILNSIYDGQEKYKARFMDYFEEYLECNSDYKGYVNDYYLDQNYI
EYTETFGFNGNVKKIELPKRAMTIGTGRKRFALVEEDSVNVEDLNIEASEKTHMINFI
NQSSKQRVNIVYRGSLIPTAMPGYISTLMQLFTSGRLTFKFSDLVKFNKLPRLKFNEI
ILARKKESLSMYANYFYSSKKDTEAQFYYKILTLCSKNIVPSNFFVVAKRDLSSGEFK
FIDFKPFYVDLLNPISVKQFLKEIIWKYKDEEYKDLFIEETMSNSGDFAEEYDLEIYK
KEGEK"

CDS complement(2723901..2724932)

/locus_tag="JFBMFIFI_02547"

/inference="ab initio prediction:Prodigal:002006"

/codon_start=1

/transl_table=11

/product="hypothetical protein"

/translation="MTIRIYDYSKSKIPLMNYLNNEILNVLQNNYYLDTDWLGGPNIL

IQLEDESKERELCHQIKKSIEHFKNDNPIPIELINKKKHFYKHEQQRLIDLELRDHVG

NMNLEEDGTVIIKNRKTGIYNSSYHKFVLDQNKYYLQKIQNKILKIIPDMCEEELISF

FVEQFKHIAIFYNGKYEEGYISFLSHVVGFFSRAKYEGKNVPYKDKFETMYTNHYKGT

SILSNKSLSVLGEWKNAWVQVSDNLRSNLNEMVEEDSQYMNLDEQHNIFVENVSKIES

PFHDRLIEKNNVKEFMMSQQMLHYRNVINLFYSTLPLFEQSMLYKHFYGFCVIKDVQN

KYNNLLLEV"

CDS complement(2725116..2725262)

/locus_tag="JFBMFIFI_02548"

/inference="ab initio prediction:Prodigal:002006"

/codon_start=1

/transl_table=11

/product="hypothetical protein"

/translation="MKNKVFVKFLKVQVLQSIVVVAMFIANITLKSNCQIFLYEPELP

KELK"

CDS complement(2725263..2725847)

/gene="agrB"

/locus_tag="JFBMFIFI_02549"

/EC_number="3.4.-.-"

/inference="ab initio prediction:Prodigal:002006"

/inference="protein motif:HAMAP:MF_00784"

/codon_start=1

/transl_table=11

/product="Accessory gene regulator protein B"

/translation="MEDKSMEEKLSRKFCEKYLKSSNLNKVEKVKVEYGLSVILINLV

KLIIVYGVAFLLGGTIDTLIVHISFLFVRRYSYGYHASKSFNCTLITIFLFAIFPYYI

NEIAYNYNVSLMYIILNTSLVSTIIFVMSPQVTKNNIVKDYRAMKIKSIIATSIIALL

ILFISDKTKKLLLLYSLSIVAFTLVASKVKKGGK"

CDS complement(2725974..2726699)

/gene="agrA_1"

/locus_tag="JFBMFIFI_02550"

/inference="ab initio prediction:Prodigal:002006"

/inference="similar to AA sequence:UniProtKB:P0A0I7"

/codon_start=1

/transl_table=11

/product="Accessory gene regulator protein A"

/db_xref="COG:COG3279"

/translation="MLNIYICEDDSKQAKTIEEYVNKYLMMMDYDATMVLNSRDGSQI

LKKLYTDKDSALFFLDIDLQDTLNGIELAAEIKKIAPNSRIVFISSHVELTYLTFIYK

VEAMDFITKSSMGDLQKRVTDCIDIAYRRQHEVGTRKEDTITIKSGEIDVFLPLDTIL

FFETTSIPHKIEVHCENRILGFYSSMKEIESMSEYFVRCHKSLIVNTKCIESIDHSNR

VIKMKDGQKRAISVRNLKKMIEN"

CDS complement(2726708..2727982)

/locus_tag="JFBMFIFI_02551"

/inference="ab initio prediction:Prodigal:002006"

/codon_start=1

/transl_table=11

/product="hypothetical protein"

/translation="MNLTLNLLLVSLIQIIMIYAMNALFIEELRNSKYVKMMFLMFIL

YFISYAYLSVLATGIIWLSLVFITIYFSKKIVRSILSVTVSLLLYIFIDYLSSYILEY

VSISIDSNYRTFISASLFFLAGYVLNNLFKRKKVFYQKELKIITIVSVLTFVVFFVTI

VVERFATDSADIENTNFFFISLYSIISALVAILFVKALRDRDNAKKKDIEIKQLKNFS

IFMESNFDEMKKFQHDYKNILLSMEGYLVDNDIDGLKKYYYTTIAKTSKNFDERLLGF

ASISKLEIPELKGILIEKMIFSLNTGTKVNIEIPEKVNHINMPILDLVRIAGILLDNA

VEATSEVDNAILKIAILKINNYTYFIIYNTISDAIPPLYKLMEIGFSTKGKNRGFGLS

NIKEIIYHYPQVDIETKIESDSFTQILKVKNS"

CDS complement(2728394..2729044)

/locus_tag="JFBMFIFI_02552"

/inference="ab initio prediction:Prodigal:002006"

/codon_start=1

/transl_table=11

/product="hypothetical protein"

/translation="MNFKTLKLPLITLGAVLVLGACGKTDKAEIVNDSETETTYSDSS

GEVIEVKGTLKDDGYFKELVTDGINPKVKDTISYETDYKNSDWDGTTLDIEHVRMVSV

ADFKDRDDMAYKELLSFKYKLTNEGSDDKHITPDNAVLILKDGTEVDAKVFMNYWDDE

VLTKNEHKDGFLYFKVKDEEALKEIASVKLTFKAKDSADEEITHAYTVDLPLEAAK"

CDS complement(2729089..2729586)

/locus_tag="JFBMFIFI_02553"

/inference="ab initio prediction:Prodigal:002006"

/codon_start=1

/transl_table=11

/product="hypothetical protein"

/translation="MKKAALITYEEQAKAYEAMSVLKQLGKTNSFGLKEVAVVQKSHD

GTKFSIKDSLDYESGKRIATGSIIGMIVGILGGPLGVLCGWIVGDLAGMGVNYVKSKK

EITVFDKIAERLAPDEAGLLILMNETNEDLLNTMVVEKLDGKIERFDYEEVKKDVEMA

KKHLS"

CDS complement(2730293..2730427)

/locus_tag="JFBMFIFI_02554"

/inference="ab initio prediction:Prodigal:002006"

/codon_start=1

/transl_table=11

/product="hypothetical protein"

/translation="MNAKDKAKMLEIIKNKQSGGRSKQMAPPKNDQKNMRKGPKIYNK

"

CDS complement(2730637..2731029)

/gene="rpsI"

/locus_tag="JFBMFIFI_02555"

/inference="ab initio prediction:Prodigal:002006"

/inference="similar to AA sequence:UniProtKB:A2RP61"

/codon_start=1

/transl_table=11

/product="30S ribosomal protein S9"

/db_xref="COG:COG0103"

/translation="MAQVQYTGTGRRKNSTARVRLVPGTGKIIMNKKDINEYIPFPYL

HEVVKQPLATTETLGSYDIYVNVNGGGFTGQAGAARHGIARALLQVDPDFRAPLKAAG
LLTRDPRMKERKKPGLKKARKASQFSKR"

CDS complement(2731043..2731486)
/gene="rplM"
/locus_tag="JFBMFIFI_02556"
/inference="ab initio prediction:Prodigal:002006"
/inference="similar to AA sequence:UniProtKB:Q7A473"
/codon_start=1
/transl_table=11
/product="50S ribosomal protein L13"
/translation="MRTTYMAKPNEVERKWYVVDATDIPLGRLSTTVATILRGKNKPT
FTPNVDTGDFVIVINADKIKLTGKKATDKIYYHHSGFQGGLKQVSAGELRATNSRRLI
ETSVKGMLPKNTLGRKQYMKLNVYGGSEHEHQAQKPEFLDITNLI"

CDS complement(2731663..2732142)
/gene="pitA"
/locus_tag="JFBMFIFI_02557"
/EC_number="2.3.1.-"
/inference="ab initio prediction:Prodigal:002006"
/inference="similar to AA sequence:UniProtKB:Q9HUU7"
/codon_start=1
/transl_table=11
/product="L-methionine sulfoximine/L-methionine sulfone
acetyltransferase"
/db_xref="COG:COG1247"
/translation="MGIEIRLNQVEDYVGLMEIENSVWNDENTPFVHVYENVIEYQLR
YPVGSMLVAVDGKEVLGLVNFNNPSPLAAHSETWLLGIGVASQAQGRGVGQKLLEALK
AKAREEGIHKLSLRVMGTNLGAQRFYLRNGFEIEGNLKEEFKIGDKYVDDIFMGIIL"

CDS complement(2732132..2732950)
/gene="truA_2"
/locus_tag="JFBMFIFI_02558"
/EC_number="5.4.99.12"
/inference="ab initio prediction:Prodigal:002006"
/inference="similar to AA sequence:UniProtKB:Q5SHU9"

/codon_start=1

/transl_table=11

/product="tRNA pseudouridine synthase A"

/db_xref="COG:COG0101"

/translation="MTTTRYKVTIQYDGTNYSGFQIQPKDRTVQGDIEKALKTMTKGI

AIKLQASGRTDAGVHALGQVIHYDYPAMIPPERMQQALNSLTTDEIAIVKVEIVDETF

HARYFTAEKTYQYRVNTNQVVDPFRRNYALHHPYPVHLDDLKAALTDIVGEHDFSSFC

AVKSGRENKVRTIYEADVTWNEATGDLIFTFRGNGFLYNMVRILIGTLLQIGNGRRPV

HDLARIIAAKDRQQAGPTASPSGLYLLKVTYKSQAEIQQILKESEQWRRENNGD"

CDS complement(2733025..2733822)

/gene="ecfT_2"

/locus_tag="JFBMFIFI_02559"

/inference="ab initio prediction:Prodigal:002006"

/inference="similar to AA sequence:UniProtKB:Q035B4"

/codon_start=1

/transl_table=11

/product="Energy-coupling factor transporter transmembrane

protein EcfT"

/translation="MMDKLIFGRYIPGDSFVHKLDPRVKLLASFYFIGIIFLANNWQS

YLFLFAFTLFSIAMSKIKFKFFINGVKPLIWLILFTVILQVLFSRGGEVYFEWGILVV

SSGGLKNGLFIFCRFVLIIFMSTLLTLTTAPLSLTDAIEYLLRPLKVIKFPVHEIALM

LSIALRFVPTLMDETEKIMNAQRARGVDFGEGNVFQQMKSIVPLLVPLFVSSFNRAEE

LATAMEARGYQGGEGRTKYRLLKWELTDSTVIIAYAIVTVALVYLRS"

CDS complement(2733819..2734700)

/gene="ecfA2"

/locus_tag="JFBMFIFI_02560"

/EC_number="3.6.3.-"

/inference="ab initio prediction:Prodigal:002006"

/inference="similar to AA sequence:UniProtKB:Q035B3"

/codon_start=1

/transl_table=11

/product="Energy-coupling factor transporter ATP-binding

protein EcfA2"

/translation="MDITFEKVGYTYQKGTPFQNKALYDIDLEIKTGSFTALVGHTGS
GKSTILQHLNALMKPTEGKVTIGDREILPETNNKNLKGIRKKVGIVFQFPEAQLFEET
VEKDICFGPMNFGVPEEDAKVLAKEMLTLVGLDDTYLERSPFDLSGGQMRRVAIAGVL
AMEPEVLVLDEPTAGLDPKGRKDMMEMFHQLYVTKGLTIVLVTHQMDDVADYADQMIV
LEGGTIVKKGLPTEIFKETEWLEEKQLGVPTAVSFGNLLKEKKGIDLGELPITTELLA
DLLVAEIEKASKAGAAQ"

CDS complement(2734676..2735515)

/gene="ecfA1"

/locus_tag="JFBMFIFI_02561"

/EC_number="7.-.-.-"

/inference="ab initio prediction:Prodigal:002006"

/inference="similar to AA sequence:UniProtKB:Q035B2"

/codon_start=1

/transl_table=11

/product="Energy-coupling factor transporter ATP-binding
protein EcfA1"

/translation="MEKIIKLDDISYKYHPNEEVEALKNVSLSIEKGEWVAIIGHNGS
GKSTLAKTINGLLAPTNGTVTVGNQLLTEETVWDVRRMVGMVFQNPDNQFVGSTVQDD
VAFGLENSGVPRDEMVKRVTDAIDKVKMSDFIEKEPARLSGGQKQRVAIAGVVALRPD
IIILDEATSMLDPQGRQEVLATVKAIKEEENLTVISITHDIDEAASANRILVMKNGQL
IREGTPQEIFSFGDELIEMGLDLPFPEKLKSALKKRNIDVPKDYLTEEGMVDWLWTLL
SKK"

CDS complement(2735764..2736933)

/gene="scmP"

/locus_tag="JFBMFIFI_02562"

/EC_number="3.5.1.-"

/inference="ab initio prediction:Prodigal:002006"

/inference="similar to AA sequence:UniProtKB:P54955"

/codon_start=1

/transl_table=11

/product="N-acetylcysteine deacetylase"

/db_xref="COG:COG1473"

/translation="MNKLKQQINKYEAEMIAFRRDLHQHPELQWEEFRTTQKVADALD

LLDIPYRKTEPTGLIAELVGGKPGETVALRADMDALPVQELNQNLAYKSLEDGKMHAC

GHDAHTAMLLTAAKALKELQPEIHGTVRFIFQPSEENAKGAKAMVQQGAVEGVDNVFG

IHIWSQMPSGKASCVVGSSFASADIFTVDIKGQGGHGAMPHDCVDAAVVASAFVMNIQ

AIVARETDPLDPVVVTIGKMDVGTRFNVIAENARLEGTVRCFSVETRSRVQKAIERYA

EHVAASYGATATVNYEYGTLPVVNDETDALFAQQIIREHLGEDVLMHERPTTGGEDFS

YFTENTKGCFALVGCGNAEKDTQWAHHHGRFNVDEAAMKVGAELYAQYAYNYLNQ"

CDS complement(2736956..2738155)

/locus_tag="JFBMFIFI_02563"

/inference="ab initio prediction:Prodigal:002006"

/codon_start=1

/transl_table=11

/product="hypothetical protein"

/translation="MSQMAAFTLLMGILYIGDVVSTRTKASVSSVFVCAVLFIIGYWT

IFPKNIVEIAGIPSIVATLLMYLLITNMGTLLSINELIKQWKTIVITLSGIAGIVILL

LTIGTLIFDLETVLVAIPPLVGGVVSSLIMSEAAQTAGLTSLSILAILIYVMQGFAGY

PLTSIMLKKEGKHMLEKYRKGEWIPDNQKEGSKKQEELDTPKLFTKIPKAYHTDFSRF

FRLGLVGMLAYYVSVLCSPFVTISPFVLCLLFGVIASSMGFLEKQPLQKANGFGFAIM

GLMLFIFDGLKNATPDMIKELAVPMIGIIVIGVIGMYIFSGIVGKLLGVSKEMAFAVS

LTALYGFPADYIITNEVIKALTDDKDEQEALTSHMLPPMLVAGFVTVTIVSVILAGIF

SSILINL"

CDS complement(2738735..2739115)

/gene="rplQ"

/locus_tag="JFBMFIFI_02564"

/inference="ab initio prediction:Prodigal:002006"

/inference="similar to AA sequence:UniProtKB:P20277"

/codon_start=1

/transl_table=11

/product="50S ribosomal protein L17"

/db_xref="COG:COG0203"

/translation="MGYRKLGRTSSQRKAMLRDLTTDLIINERIVTTEARAKEIRSTT

EKMITLGKRGDLHARRQAASFVRNETAAIKEDGDKIVVESALQKLFSDIAPRYAERQG

GYTRIMKTEPRRGDAAPMVIIELV"

CDS complement(2739143..2740087)

/gene="rpoA"

/locus_tag="JFBMFIFI_02565"

/EC_number="2.7.7.6"

/inference="ab initio prediction:Prodigal:002006"

/inference="similar to AA sequence:UniProtKB:P20429"

/codon_start=1

/transl_table=11

/product="DNA-directed RNA polymerase subunit alpha"

/db_xref="COG:COG0202"

/translation="MIEIEKPRINTIEISDDAKFGKFVVEPLERGYGTTLGNSLRRIL

LSSLPGAAVTTIQIDGVLHEFSTIDGVVEDVTSIILNIKKLALKLYSGEDKTIEIDVK

GPAVVTAADITYDSDVEILNPDLYICTVAEGARFHVRLTAKQGRGYARAEHNKHDDMP

IGVLPVDSIYTPVSRVNYQVENTRVGQKDNFDKLTLDVWADGSISPEDAVSLAAKILT

EHLDIFVNLSDEARKVEIMVEKEETHKEKMLEMTIEELDLSVRSYNCLKRAGINSVQE

LTDKSEAEMIKVRNLGRKSLEEVKFKLAELSLGLRQDD"

CDS complement(2740161..2740550)

/gene="rpsK"

/locus_tag="JFBMFIFI_02566"

/inference="ab initio prediction:Prodigal:002006"

/inference="similar to AA sequence:UniProtKB:P66357"

/codon_start=1

/transl_table=11

/product="30S ribosomal protein S11"

/translation="MAGKKVVRKRRVKKNVEVGIAHIRSTFNNTIVMITDAHGNAISW

SSAGALGFRGSKKSTPFAAQLAAETAAKACMEHGMKTVEVAVKGPGSGREAAIRSLQA

TGLEVTAIRDVTPVPHNGCRPPKRRRV"

CDS complement(2740576..2740941)

/gene="rpsM"

/locus_tag="JFBMFIFI_02567"

/inference="ab initio prediction:Prodigal:002006"

/inference="similar to AA sequence:UniProtKB:P20282"

/codon_start=1

/transl_table=11

/product="30S ribosomal protein S13"
/db_xref="COG:COG0099"
/translation="MARISGVDIPRDKRVVISLTYIYGIGKNTAIKILKAADVSEEIR
VRELTNDQLDRIRAEIDSIKVEGDLRREVNLNIKRLIEIGSYRGMRHRRGLPVRGQNT
KNNARTRKGPARTVAGKKK"
CDS complement(2740972..2741085)
/gene="rpmJ"
/locus_tag="JFBMFIFI_02568"
/inference="ab initio prediction:Prodigal:002006"
/inference="similar to AA sequence:UniProtKB:P07841"
/codon_start=1
/transl_table=11
/product="50S ribosomal protein L36"
/translation="MKVRPSVKPICEKCKVIRRNGRVMVICENPKHKQRQG"
CDS complement(2741120..2741338)
/gene="infA"
/locus_tag="JFBMFIFI_02569"
/inference="ab initio prediction:Prodigal:002006"
/inference="similar to AA sequence:UniProtKB:P20458"
/codon_start=1
/transl_table=11
/product="Translation initiation factor IF-1"
/db_xref="COG:COG0361"
/translation="MAKDDVIEIEGTVVETLPNAMFKVELENGHVVLAHVSGKIRMHY
IRILPGDKVTVELSPYDLTRGRITYRFK"
CDS complement(2741587..2742234)
/gene="adk"
/locus_tag="JFBMFIFI_02570"
/EC_number="2.7.4.3"
/inference="ab initio prediction:Prodigal:002006"
/inference="similar to AA sequence:UniProtKB:P27142"
/codon_start=1
/transl_table=11

/product="Adenylate kinase"

/translation="MNLILMGLPGAGKGTQAEQIVDTYKIPHISTGDMFRAAIKNETA

LGLEAKAYMDKGNLVPDEVTNGIVKERLAEADTDNGFLLDGFPRTLNQAEALEQILTE

LGKKIDAVINIHVDKDILMERLTGRIICRTCGATYHKVFNPPTVAGTCDRCGGHDFYQ

REDDKPETVENRINVNLELTQPLLDFYEARQVLNTVKGDQDIQDVFKEVQAIIAK"

CDS complement(2742294..2743586)

/gene="secY"

/locus_tag="JFBMFIFI_02571"

/inference="ab initio prediction:Prodigal:002006"

/inference="similar to AA sequence:UniProtKB:Q7A468"

/codon_start=1

/transl_table=11

/product="Protein translocase subunit SecY"

/translation="MFKMLSSALKEKDIRNKILFTLGVLIIFRLGTHITVPGVNAKAI

SEFSDSGIFSMLNTFGGGALSQYSIFAMGVSPYITSSIVVQLLQMDIVPKFVEWSKQG

EVGRKKLNQVTRYLTIIMAFVQSIGISYGFNALSGMGLIKNPGASTYLSIALILTAGT

MLVMWMGEQITVKGFGNGVSMIIFSGIVARVPADVMDYYNAQIRNAGPDLWKAILFTL

ALIVAIIAVVILVVYFETAKRKIKISYSKRAAASDQSTFLPLKINSAGVIPVIFASSF

IVTPQTIMGFFRGTQGDTQWFQILSNVFDYQKPIGAALYTLLIVVFTFFYAFIQVNPE

KVAENLQKQGGYIPSVRPGKGTEDYISSVLMRLSTVGAVYLGLIALLPIVAGILWNLP

QSIGLGGTSLLIVVGVALESTRQLEGQMIKRSYLGFIQ"

CDS complement(2743586..2744026)

/gene="rplO"

/locus_tag="JFBMFIFI_02572"

/inference="ab initio prediction:Prodigal:002006"

/inference="similar to AA sequence:UniProtKB:P0A0F6"

/codon_start=1

/transl_table=11

/product="50S ribosomal protein L15"

/translation="MKLHELKPAEGSRKVRNRVGRGTSSGNGKTSGRGQKGQNSRSGG

GVRLGFEGGQTPLFRRLPKRGFTNINRKEFAIVNLDTLNRFEDGTEVTPALLVESGII

KNEKSGIKVLGNGAVERKLTVKASKFSEAAEKAIVAAGGSIEVI"

CDS complement(2744069..2744248)

/gene="rpmD"

/locus_tag="JFBMFIFI_02573"

/inference="ab initio prediction:Prodigal:002006"

/inference="similar to AA sequence:UniProtKB:P19947"

/codon_start=1

/transl_table=11

/product="50S ribosomal protein L30"

/db_xref="COG:COG1841"

/translation="MANLEITLKRSVIGRPQNQRDTVKHLGLTKINSTVVKPANDAIK

GMVNTVSHLVDVKEV"

CDS complement(2744266..2744766)

/gene="rpsE"

/locus_tag="JFBMFIFI_02574"

/inference="ab initio prediction:Prodigal:002006"

/inference="similar to AA sequence:UniProtKB:P21467"

/codon_start=1

/transl_table=11

/product="30S ribosomal protein S5"

/db_xref="COG:COG0098"

/translation="MVYIDPSHLELEDRVVAINRVTKVVKGGRRLRFAALVVVGDRNG

HVGFGTGKAQEVPEAIRKAIEDAKKNLIEVPMVESTIPHEVIGRFGGGNILMKPAVAG

SGVSAGGPVRAVLELAGVADITSKSLGSSTPINMVRATVQGLTQLKRVEEVAKLRGKS

VEEILG"

CDS complement(2744787..2745143)

/gene="rplR"

/locus_tag="JFBMFIFI_02575"

/inference="ab initio prediction:Prodigal:002006"

/inference="similar to AA sequence:UniProtKB:P46899"

/codon_start=1

/transl_table=11

/product="50S ribosomal protein L18"

/db_xref="COG:COG0256"

/translation="MITKPDKNKMRQKRHARVRSKISGTAECPRLNVFRSNKNIYAQV

IDDVAGVTLASASTLDKEVSGESKVDAAAAVGTLVAKRAVEKGVKEVVFDRGGYLYHG

RVQALAEAARENGLEF"

CDS complement(2745273..2745809)

/gene="rplF"

/locus_tag="JFBMFIFI_02576"

/inference="ab initio prediction:Prodigal:002006"

/inference="similar to AA sequence:UniProtKB:P02391"

/codon_start=1

/transl_table=11

/product="50S ribosomal protein L6"

/translation="MSRIGKKIITIPAGVTVTENGNEITVKGPKGELTRSFSPVITMH

TEGNEITFTRPNDDKENRALHGTMRANLNNMVIGVTEGFEKALELIGVGYRAQLQGKK

LVLNVGYSHPVEFTPEEGITVEVPSNTSVIIKGANKERVGELAANIRGVRPPEPYKGK

GIRYVGEHVRRKEGKTGK"

CDS complement(2745841..2746239)

/gene="rpsH"

/locus_tag="JFBMFIFI_02577"

/inference="ab initio prediction:Prodigal:002006"

/inference="similar to AA sequence:UniProtKB:P12879"

/codon_start=1

/transl_table=11

/product="30S ribosomal protein S8"

/db_xref="COG:COG0096"

/translation="MVMTDPIADFLTRIRNANMVRHESLELPASRIKKDIADILKREG

FIKDVEYIEDDKQNVIRVFLKYGKNNERVITGLKRISKPGLRVYAKTGEVPKVLNGLG

IAIVSTSEGVITDKEARAKNIGGEILAYVW"

CDS complement(2746273..2746458)

/gene="rpsZ"

/locus_tag="JFBMFIFI_02578"

/inference="ab initio prediction:Prodigal:002006"

/inference="similar to AA sequence:UniProtKB:A2RNP2"

/codon_start=1

/transl_table=11

/product="30S ribosomal protein S14 type Z"

/db_xref="COG:COG0199"

/translation="MAKKSMIAKNKRPAKFSTQAYTRCERCGRPHSVYRKFKLCRICF

RELAYKGQIPGVKKASW"

CDS complement(2746477..2747016)

/gene="rplE"

/locus_tag="JFBMFIFI_02579"

/inference="ab initio prediction:Prodigal:002006"

/inference="similar to AA sequence:UniProtKB:P08895"

/codon_start=1

/transl_table=11

/product="50S ribosomal protein L5"

/translation="MNRLKEKYVKEITPSMMEKFSYKSIMQTPQVDKIVINMGVGDAV

SNAKNLDKAVDELTVISGQKPMITKAKNSIAGFRLREGMPIGTKVTLRGERMYEFLDK

LVSVSLPRVRDFHGVSKKAFDGRGNYTLGIKEQLIFPEVDYDTVDKVRGMDIVIVTTA

KTDEESRELLTQLGMPFQK"

CDS complement(2747044..2747355)

/gene="rplX"

/locus_tag="JFBMFIFI_02580"

/inference="ab initio prediction:Prodigal:002006"

/inference="similar to AA sequence:UniProtKB:P04455"

/codon_start=1

/transl_table=11

/product="50S ribosomal protein L24"

/translation="MIVKTGDKVKVITGKDKGKEGVILKAFPKKDRVIVEGMNMMKKH

QKPSSMNPQGGILETEAPIHVSNVMLIDSKTGEPTRVGFKVEDGKKVRVSKKTGEVLD

K"

CDS complement(2747389..2747757)

/gene="rplN"

/locus_tag="JFBMFIFI_02581"

/inference="ab initio prediction:Prodigal:002006"

/inference="similar to AA sequence:UniProtKB:P12875"

/codon_start=1

/transl_table=11

/product="50S ribosomal protein L14"

/db_xref="COG:COG0093"

/translation="MIQQESRLKVADNSGAREVLTIKVLGGSGRKTANIGDVIVCTVK

HATPGGVVKKGEVVKAVIVRTKTGARRTDGSYIKFDENACVIIRDDKSPRGTRIFGPV

ARELRDSNFMKIVSLAPEVL"

CDS complement(2747812..2748078)

/gene="rpsQ"

/locus_tag="JFBMFIFI_02582"

/inference="ab initio prediction:Prodigal:002006"

/inference="similar to AA sequence:UniProtKB:P12874"

/codon_start=1

/transl_table=11

/product="30S ribosomal protein S17"

/db_xref="COG:COG0186"

/translation="MSEERNQRKVYQGRVVSDKMDKTIVVEIATYKKHGIYGKRVKYS

KKFKAHDENNVAKTGDVVKIMETRPLSATKRFRLVEVVEEAVIV"

CDS complement(2748110..2748304)

/gene="rpmC"

/locus_tag="JFBMFIFI_02583"

/inference="ab initio prediction:Prodigal:002006"

/inference="similar to AA sequence:UniProtKB:P12873"

/codon_start=1

/transl_table=11

/product="50S ribosomal protein L29"

/db_xref="COG:COG0255"

/translation="MKANELNALSTAEMVEKEKEYKEELFNLRFQLATGQLENTSRLS

EVRKSIARIKTALRQSELQK"

CDS complement(2748294..2748728)

/gene="rplP"

/locus_tag="JFBMFIFI_02584"

/inference="ab initio prediction:Prodigal:002006"

/inference="similar to AA sequence:UniProtKB:P0A475"

/codon_start=1

/transl_table=11

/product="50S ribosomal protein L16"

/db_xref="COG:COG0197"

/translation="MLVPKRVKYRREFRGKMRGEAKGGKEVTFGEWGLQAVDSHWITN

RQIEAARIAMTRYMKRGGKVWIKIFPHKSYTAKAIGVRMGSGKGAPEGWVAPVKRGKI

MFEIAGVSEEVAREALRLASHKLPVKTKIVKRTENGGESNES"

CDS complement(2748731..2749387)

/gene="rpsC"

/locus_tag="JFBMFIFI_02585"

/inference="ab initio prediction:Prodigal:002006"

/inference="similar to AA sequence:UniProtKB:P21465"

/codon_start=1

/transl_table=11

/product="30S ribosomal protein S3"

/db_xref="COG:COG0092"

/translation="MGQKINPIGLRVGIIRDWDAKWYAEKEYANYLHEDLRIRSYIAK

NLSDAAVSTVEIERAANRVNVSVHTAKPGMVIGKGGSEVESLRKNLNELTGKRVHINI

VEIKKPDLDAKLVGEGIARQLENRVAFRRAQKQAIQRTMRSGAKGIKTQVSGRLNGAD

IARSEGYSEGTVPLHTLRADIDYAWEEADTTYGKLGVKVWIYRGEVLPTKKNAEKGGK

"

CDS complement(2749391..2749747)

/gene="rplV"

/locus_tag="JFBMFIFI_02586"

/inference="ab initio prediction:Prodigal:002006"

/inference="similar to AA sequence:UniProtKB:A2RNQ0"

/codon_start=1

/transl_table=11

/product="50S ribosomal protein L22"

/db_xref="COG:COG0091"

/translation="MSEQITAAKATANTVRISARKVRLVIDLIRGKSIGEAISILKFT

PNGASVPVEKVLMSAVANAEHNYDLDVENLVISEAYANEGPTMKRFRPRAKGSASPIM

KRTSHITVVVSEKKEG"

CDS complement(2749771..2750049)

/gene="rpsS"

/locus_tag="JFBMFIFI_02587"

/inference="ab initio prediction:Prodigal:002006"

/inference="similar to AA sequence:UniProtKB:A2RNQ1"

/codon_start=1

/transl_table=11

/product="30S ribosomal protein S19"

/db_xref="COG:COG0185"

/translation="MGRSLKKGPFVDEHLMKKMNALAESDKKHVVKTWSRRSTIFPSF

VGYTIAVYDGRKHVPVYVQEDMVGHKLGEFAPTRTYRGHAADDKKTKR"

CDS complement(2750108..2750941)

/gene="rplB"

/locus_tag="JFBMFIFI_02588"

/inference="ab initio prediction:Prodigal:002006"

/inference="similar to AA sequence:UniProtKB:P42919"

/codon_start=1

/transl_table=11

/product="50S ribosomal protein L2"

/db_xref="COG:COG0090"

/translation="MGIRKYKPTTNGRRNMTGSDFAEITSTTPEKTLLEPTKKTAGRN

NNGRITVRHHGGGHKRQYRVIDFKRNKDDIAGIVKTVEYDPNRSANIALIHYTDGIKT

YIIAPKGIEVGQRIFSGKEADIKVGNALPLENIPVGTVIHNIEMKPGKGGQLVRSAGT

SAQVLGKEGKYVLVRLNSGEVRMILATCRATIGSVGNEQHELINIGKAGRSRWMGKRP

TVRGSVMNPNDHPHGGGEGKAPIGRKAPVSPWGQPALGYKTRNKKAKSDKFIVRRRKT

K"

CDS complement(2750979..2751263)

/gene="rplW"

/locus_tag="JFBMFIFI_02589"

/inference="ab initio prediction:Prodigal:002006"

/inference="similar to AA sequence:UniProtKB:P04454"

/codon_start=1

/transl_table=11

/product="50S ribosomal protein L23"

/translation="MDARDIIKRPVITEATMLITDDKKYTFEVDVRANKTQVKQAVQE

IFGVKVKNVNIMNVRGKLKRMGKYAGYTKKRRKAIVTLTAESKEIQLFEA"

CDS complement(2751263..2751886)

/gene="rplD"

/locus_tag="JFBMFIFI_02590"

/inference="ab initio prediction:Prodigal:002006"

/inference="similar to AA sequence:UniProtKB:P42921"

/codon_start=1

/transl_table=11

/product="50S ribosomal protein L4"

/db_xref="COG:COG0088"

/translation="MANVALFKQDGTQNGEVTLNDAIFGIEPNENVVFDAIIMQRASL

RQGNHSVKNRSAVRGGGRKPWRQKGTGRARQGSIRSPQWRGGGVVFGPTPRSYSYKLP

KKVRRLAIKSVLSTKVAENNLIVVDALNFDAPKTKEFAQVLKNLNVDQKVLVVVESDN

DFATLSARNLPGVKVVTHDGITVLDVVSNVKMILTQTALTKVEEALK"

CDS complement(2751912..2752547)

/gene="rplC"

/locus_tag="JFBMFIFI_02591"

/inference="ab initio prediction:Prodigal:002006"

/inference="similar to AA sequence:UniProtKB:P42920"

/codon_start=1

/transl_table=11

/product="50S ribosomal protein L3"

/db_xref="COG:COG0087"

/translation="MTKGILGKKVGMTQVFTENGELIPVTVIEATPNVVLQLKTMEND

GYEAVQLGYQDKREVLSNKPAKGHVAKANATPKRFIREFNDVTLGEYEVGQEIKVDVF

EAGDIVDVTGTSKGKGFQGVIKRHGQSRGPMSHGSRYHRRPGSMGPVAPNRVFKRKLL

PGRMGGDRITIQNLEIVRIDVEKNVILIKGNVPGAKKSLIQIRTALKSAAK"

CDS complement(2752598..2752906)

/gene="rpsJ"

/locus_tag="JFBMFIFI_02592"

/inference="ab initio prediction:Prodigal:002006"

/inference="similar to AA sequence:UniProtKB:P21471"

/codon_start=1

/transl_table=11

/product="30S ribosomal protein S10"

/db_xref="COG:COG0051"

/translation="MAKQKIRIRLKAYEHRILDQSADKIVETAKRTGASVSGPIPLPT

ERTLYTVIRATHKYKDSREQFEMRTHKRLIDIVNPTPKTVDALMKLDLPSGVDIEIKL

"

CDS complement(2753327..2754346)

/gene="ilvE"

/locus_tag="JFBMFIFI_02593"

/EC_number="2.6.1.42"

/inference="ab initio prediction:Prodigal:002006"

/inference="similar to AA sequence:UniProtKB:P9WQ75"

/codon_start=1

/transl_table=11

/product="Branched-chain-amino-acid aminotransferase"

/db_xref="COG:COG0115"

/translation="MKKVDIDWENLGFTYIKTDLRYLSYWKNGAWDAGVMTEDNQVHI

SEGSTALHYGQTAFEGLKAYRCKDGSINLFRADENAKRLQDSCRRLLMPEVPTDVFIK

ACVDVVKANEHFIPPYGTGGTLYLRPYIVGVGNNIGVNPAPEYLFSVFCMPVGAYFKG

GLTPTNFVVSEYDRAAGHGTGAAKVGGNYAASLLPGEEAHQRQFSDCIYLDPITHTKI

EEVGAANFFGITANNEFVTPLSPSILPSITKYSLLHLAEKRLGMIPVEGDVYIDKLER

FVEAGACGTAAVISPIAGIQYGEDFHIFYSETEVGPVTRKLYDELCGIQFGDKEAPEG

WIIKI"

CDS complement(2754663..2755850)

/gene="tuf_3"

/locus_tag="JFBMFIFI_02594"

/inference="ab initio prediction:Prodigal:002006"

/inference="similar to AA sequence:UniProtKB:P99152"

/codon_start=1

/transl_table=11

/product="Elongation factor Tu"

/translation="MAKETFDRSKPHVNIGTIGHVDHGKTTLTAAIATVLSKHGGGSA
QNYADIDNAPEEKERGITINTSHIEYETDTRHYAHVDCPGHADYVKNMITGAAQMDGA
ILVVSAADGPMPQTREHILLSRQVGVPYIVVFLNKVDMVDDEELLELVEMEVRDLLTE
YDFPGDDTPVIAGSALKALEGEAAYEEKVLELMAAVDEYIPTPQRDTEKPFMMPVEDV
FSITGRGTVATGRVERGEVRVGEEIEIVGINEAPTKTTVTGVEMFRKLLDYAQAGDNI
GALLRGVAREDIERGQVLAKPGTITPHTKFKAEIYVLSKEEGGRHTPFFTNYRPQFYF
RTTDVTGVCELPEGVEMVMPGDNVTIEVTLINPIAIEAGTNFSIREGGRTVGAGVVAE
IQA"

CDS complement(2755997..2758081)

/gene="fusA"

/locus_tag="JFBMFIFI_02595"

/inference="ab initio prediction:Prodigal:002006"

/inference="similar to AA sequence:UniProtKB:Q839G9"

/codon_start=1

/transl_table=11

/product="Elongation factor G"

/db_xref="COG:COG0480"

/translation="MANREFSLEKTRNIGIMAHVDAGKTTTTERILYYTGKIHKIGET
HEGASQMDWMEQEQERGITITSAATTAEWKNYRVNIIDTPGHVDFTIEVQRSLRVLDG
AVTVLDSQSGVEPQTETVWRQATDYRVPRIVFCNKMDKIGADFLYSVNSLHERLQANA
HPIQLPIGAEDDFTGIIDLVKMKAEIYTNDLGTDIQETDIPEEYLEAATEWRKKLIEA
VVETDEELMMKYLDGEEITEEELKAGIRQATINVEFFPVMAGSAFKNKGVQLMLDAVL
DYLPSPLDVEAIKGIDVKTEEETTRPADDSAPFASLAFKVMTDPFVGRLTFFRVYSGV
LESGSYVLNASKNKKERIGRILQMHANTRKEIEKVFSGDIAAAVGLKDTTTGDTLCAL
DSAVILESIEFPEPVIQVAVEPKSKADQDKMGIALQKLAEEDPSFRVETNAETGETVI
SGMGELHLDVLVDRMRREFNVDASVGAPQVSYRETFRGSTQAEGKFVRQSGGKGQYGH
VWIEFTPNEEGAGFEFENAIVGGVVPREYIPAVKAGLEASLDNGVLAGYPLVDIKAKL
YDGSYHDVDSNETAFKVAASMALKAAAKKANPVILEPMMKVIVTVPEDYLGDIMGHIT
ARRGRVEGMEAHGNSQIVNAIVPLANMFGYATTLRSSTQGRGTFMMVFDHYEDLPKSI
QEEIIAKNGGNN"

CDS complement(2758195..2758665)

/gene="rpsG_2"

/locus_tag="JFBMFIFI_02596"

/inference="ab initio prediction:Prodigal:002006"

/inference="similar to AA sequence:UniProtKB:P21469"

/codon_start=1

/transl_table=11

/product="30S ribosomal protein S7"

/db_xref="COG:COG0049"

/translation="MPRKGPITKRDVLPDPMYNSKLVTRLINRLMVDGKRGKAATILY

NAFDTIKEQSGNDPMEVFEQAMKNIMPVLEVKARRVGGSNYQVPVEVRAERRTTLALR

WLVNYSRLRGEDTMEQRLAKEIMDAANNTGSAVKKREDTHKMAEANKAFAHYRW"

CDS complement(2758732..2759145)

/gene="rpsL_2"

/locus_tag="JFBMFIFI_02597"

/inference="ab initio prediction:Prodigal:002006"

/inference="similar to AA sequence:UniProtKB:A2RP74"

/codon_start=1

/transl_table=11

/product="30S ribosomal protein S12"

/db_xref="COG:COG0048"

/translation="MPTINQLVRKPRKAKMDKSDSPALNKGYNSFKKAATNTSSPQKR

GVCTRVGTMTPKKPNSALRKYARVRLSNLLEVTAYIPGIGHNLQEHSVVLIRGGRVKD

LPGVRYHVVRGALDTAGVTDRKQSRSKYGAKKPKA"

CDS 2759497..2760237

/gene="comC"

/locus_tag="JFBMFIFI_02598"

/inference="ab initio prediction:Prodigal:002006"

/inference="similar to AA sequence:UniProtKB:P15378"

/codon_start=1

/transl_table=11

/product="Type 4 prepilin-like proteins leader

peptide-processing enzyme"

/translation="MLHIFSLFLLWFVGCCLGSFFMVIGTRIPIHQSIIVPRSHCPNC

QIPLHPYQLVPLISYFVQKGRCVTCRIKIPFLYPIIEGLSGFCYLVAYLSFGHSPTQF

RIILLLISFSLIFIVSDIFYQLLPDSVMFCFFCLTILVQIRNFPTHFLGGFSLFSLFF

LVAYLSPDGLGGGDIKLAGVLGWLLGFKLGLLAILLACLAGFTYFLFLFNYKKADLTT

KVPFAPFILFGALIAYFVTNQSSLAAIF"

CDS complement(2760298..2763945)

/gene="rpoC"

/locus_tag="JFBMFIFI_02599"

/EC_number="2.7.7.6"

/inference="ab initio prediction:Prodigal:002006"

/inference="similar to AA sequence:UniProtKB:P37871"

/codon_start=1

/transl_table=11

/product="DNA-directed RNA polymerase subunit beta'"

/db_xref="COG:COG0086"

/translation="MIDVNNFENMQIGLASPDKIRSWSYGEVKKPETINYRTLKPERD

GLFCERIFGPSKDWECACGKYKRIRYKGIVCDRCGVEVTRSKVRRERMGHIELAAPVS

HIWYFKGIPSRMGLVLDMSPRALEEIIYFASYVVIEPGDTPLEKKQLLTEREYREKRM

QYGQGFQAAMGAEAIKQLLLDVQLEKEVAELKEELKSAQGQKRTRAIRRLDILEAFRN

SGNDPSWMVMDVVPIIPPDLRPMVQLEGGRFATSDLNDLYRRVINRNNRLKRLLDLNA

PNIIVQNEKRMLQEAVDALIDNGRRGRPVTGPGNRPLKSLSHMLKGKQGRFRQNLLGK

RVDYSGRSVIVVGPNLKMYQCGLPKEMAIELFKPFVMKELVERELANNIKNAKRKIDR

QDEAIWDVLEDVIREHPVLLNRAPTLHRLGIQAFEPILVDGRAIRLHPLVCEAYNADF

DGDQMAVHVPLSDEAQAEARMLMLAAQNILNPKDGKPVVTPSQDMVLGNYYLTMEEEG

REGEGMVFKDLTEAITAYQSGYVHMHSRIGVQTDNIYRESNHPIEKPFSDWQKERILI

TTVGKLLFNEIMPPEFPYLNEPTNFNLEDSTPDKYFVEAGTDIPAHIKKQDLILPFKK

KNLGNIIAEVFKKFHITETSKMLDRMKDLGYKHSTRAGITVGIADIVVLHEKQEVLDD

AHNQVDNVTKQFRRGLITDEERYERVIAIWNKAKDTIQIKLMESLDAKNPIFMMSDSG

ARGNISNFTQLAGMRGLMAAPNGQIMELPITSNFREGLSVLEMFISTHGARKGMTDTA

LKTADSGYLTRRLVDVAQDVIIRETDCGTDRGLDIAAIKEGNEVIEPLEERLLGRYIR

KTVFHPETGKKIITENNIITEDIAKEIIDAGIEKVTIRSVFTCNTKHGVCKHCYGRNL

ATGSEVEVGEAVGTIAAQSIGEPGTQLTMRTFHTGGVAGDDITQGLPRIQEIFEARNP

KGQAVITEVTGEVIVIEENAAERTKEVTIKGATDTRSYPVPYTARLKVVEGDYIHRGE

ALTEGSIDPKQLLRVRDVLSVENYLLREVQKVYRMQGVEIGDKHIEVMVRQMLRKIRV

MDPGSTDILPGTLMDISEFTDKNLKTLVAGDVPATGRPVLLGITKASLETNSFLSAAS

FQETTRVLTDAAIRGKRDPLLGLKENVIIGKIIPAGTGMAKYRNMEPKSIGVVSENVY

SINDTVDTSAE"

CDS complement(2764083..2767655)

/gene="rpoB"

/locus_tag="JFBMFIFI_02600"

/EC_number="2.7.7.6"

/inference="ab initio prediction:Prodigal:002006"

/inference="similar to AA sequence:UniProtKB:P37870"

/codon_start=1

/transl_table=11

/product="DNA-directed RNA polymerase subunit beta"

/db_xref="COG:COG0085"

/translation="MAGHLVNYGKHRTRRSFARISEVLELPNLIEIQTDSYQWFLDEG

LREMFKDISPIADFAGNLSLEFVDYQLYSPKYTVEEARSHDANYSAPLHVKLRLQNRE

TGEVKDQEVFFGDFPLMTDMGTFIINGAERVIVSQLVRSPGVYFHGKVDKNGKQGFGS

TVIPNRGAWLEYETDAKDLSYVRIDRTRKIPLSVLVRALGFGSDDQILEIFGDNESLR

LTLEKDLHKNANDSRVEEALKDVYERLRPGEPKTADSSRSLLNARFFDPKRYDLANVG

RYKVNKKLNLKTRLFNQTLAETLIDPETGEIIAEKGTLLDRDMINKLSPFIDAGINSV

TLYPSDEGVVPEPFTIQVVQVVSPKDPDRIVNVIGNGDVSTDIKNVTSADIISSMSYF

FNLQEGIGNVDDIDHLGNRRIRSVGELLQNQFRIGLSRMERVVRERMSIQDVATVTPQ

QLINIRPVVAAIKEFFGSSQLSQFMDQTNPLGELTHKRRLSALGPGGLTRDRAGYEVR

DVHYSHYGRMCPIETPEGPNIGLINSLSSYAKINKFGFIETPYRRVDRETGKVTKHID

YLTADEEDHYVVAQANSVLLEDGSFESDIVMARFISENLEVPISRVDYMDVSPKQVVA

VATSCIPFLENDDSNRALMGANMQRQAVPLIHPQAPLVGTGMEYISAHDSGAALICRN

PGVVEYVDAKEIRIRQDNGALDKYSITKFRRSNAGTCYNQRPIVALGDRVDASEILAD

GSSMENGEMALGQNVLVAFMTWEGYNYEDAIIMSERLVKDDVYTSVHIEEYESEARDT

KLGPEEITREIPNVGDDALKDLDEFGIIRIGAEVRDGDLLVGKVTPKGVTELSAEERL

LHAIFGEKAREVRDTSLRVPHGGGGIVHDVKIFTREAGDELSPGVNMLVRVYIVQKRK

INEGDKMAGRHGNKGVVSRIMPEEDMPYLPDGTPVDIMLNPLGVPSRMNIGQVLELHI

GMAARQLGIHIATPVFDGASEEDVWATVQEAGMANDAKTILYDGRSGEPFDNRISVGI

MYMIKLAHMVDDKLHARSTGPYSLVTQQPLGGKAQFGGQRFGEMEVWALEAYGAAYTL

QEILTYKSDDVVGRVKTYEAIVKGEKIPKPGVPESFRVLVKELQALGLDMKVLDANDE

EIELRDLDDDDDVVNIDALSKYAEEQEKIRAEAAEKERLANEEN"

CDS complement(2768016..2768471)

/locus_tag="JFBMFIFI_02601"

/inference="ab initio prediction:Prodigal:002006"

/codon_start=1

/transl_table=11

/product="hypothetical protein"

/translation="MQEEQPRPLFTYGSLMEGLFNYQLYLAGKVMGKPRRARIKGELY

HLTEKGYPALLVGDDWVYGEVFELCDFHPTLKELDTLENYYGSNHSENEYERKVVTVW

LYNEQTQLFDEKLEVYCYPYRIDNDASFAQHSLYLPEGDWHSQALQKNK"

CDS complement(2768471..2769118)

/gene="pcp"

/locus_tag="JFBMFIFI_02602"

/EC_number="3.4.19.3"

/inference="ab initio prediction:Prodigal:002006"

/inference="similar to AA sequence:UniProtKB:Q5XDD4"

/codon_start=1

/transl_table=11

/product="Pyrrolidone-carboxylate peptidase"

/translation="MKILVTGFDPFGGEPINPALEAVKGLAPVINGAEVIQLEIPTVF

GKAAEVVKKAIEEHQPDVVLNIGQAGGRFTVSPERVAINIDDARIADNEGNQPVDEVI

QADGAPAYFTQLPIKAMVTAIKEAGIPGSVSNTAGTFVCNHIMYQVQYLIEKEYPNLK

GGFIHVPFIPAQVLDKANQPYMSLPDITKGLTAAISAIVEYDGKADLKAVGGAIH"

CDS complement(2769181..2770113)

/locus_tag="JFBMFIFI_02603"

/inference="ab initio prediction:Prodigal:002006"

/codon_start=1

/transl_table=11

/product="hypothetical protein"

/translation="MENFASTILEFFYILIGLILIMTAVRAFRDQTNPARIGTAAFWL

LLGVVFGAGNYIPFIVDGIIILLMGVLTLFKQVRLGKVADLNEKESEQAAGKLGNLVF

VPCLVLALVAVVVAQTAPFGPLSGQIGIGIAAVISLVVAMFITKAKPKVVLDDTDRMI

QQVGTTGILPQLLAALGVIFNAAGVGTVISNGISGFVPEGNRLIGVIAYCLGMMIFTM

IMGNGFAAFTVITAGIGVPFVIAQGADPVIAGALAMTAGFCGTLLTPMAANFNALPVA

LLEIKDQNAVIKAQAPLAIIMLVVHVVLMYFWAF"

CDS complement(2770117..2770824)

/locus_tag="JFBMFIFI_02604"

/inference="ab initio prediction:Prodigal:002006"

/codon_start=1

/transl_table=11

/product="hypothetical protein"

/translation="MEIIKLIGILIILVGFIFKFDTIAVVLVAALATALVSGISFTEF

LALLGEAFVSNRLVTLFLLTLPMIGLSERFGLRQQAVVIIEKIKNLTPGRFLTIYMII

REIAGTLSIRVQGQTQFIRPIVNPMAQAAAVNKYGDVSEEDQDKIKARAAATENFGNF

FAQNTFIAAAGVLLIQTTFDSLGYEVSNVSIALASVPVALIMLVMVAIYNMIFDKKMA

KKYGVNVGAASKEKGER"

CDS complement(2770972..2771583)

/gene="rsmC"

/locus_tag="JFBMFIFI_02605"

/EC_number="2.1.1.172"

/inference="ab initio prediction:Prodigal:002006"

/inference="similar to AA sequence:UniProtKB:P39406"

/codon_start=1

/transl_table=11

/product="Ribosomal RNA small subunit methyltransferase C"

/db_xref="COG:COG2813"

/translation="MADHYYTNKPSAVSKEAAWTYTLKGHEFKFVTDQGVFSKKTVDF

GSRLLIETLDLKELVDGPILDVGCGYGPMGLSLAKADPERQVEMVDVNERALGLAKQN

ASNNRISTVDIHVSDVYDQVKGKDFAAIVSNPPIRAGKEVVHSILTGAYHYLKEGGSL

TVVIQKKQGAPSAKAKMEETFGNAEIIAKDKGYWIIQSIKQVN"

CDS complement(2771676..2774024)

/gene="dinG_3"

/locus_tag="JFBMFIFI_02606"

/EC_number="3.1.-.-"

/inference="ab initio prediction:Prodigal:002006"

/inference="protein motif:HAMAP:MF_02206"

/codon_start=1

/transl_table=11

/product="3'-5' exonuclease DinG"

/translation="MVKEKIAVRQLVSFILRKGSIDQRFTSSAHTAIEGTRIHKKIQK

AAGENYQAEVVLKTKRMLNQKEYTIEGRLDGIVREENQPVLIDEIKTSETPFDLLNEA

EQALHWGQLKCYGYLLCLEEDLDEVCLQLSYFQTLTEELTKTQEIWTRKELIQFFDDL

LKKYEDWIIFKDNWTKLRDQSIKELEFPYGSYRNGQRELAVAVYKTILTEQELFCEAP

TGIGKTMSTLFPSIKAIGEGKAEKLFYLTAKTITRQVAEDAVSEMERKGLQLKNVTLT

AKDKICFLDERICNPDHCIYARGYYDRLNEAMFDLLTKENQLTRDVIETYARKYTICP

FELSLDVSLWCDTIICDYNYLFDPVVYLRRFFTEEKNDYVFLIDEVHNLVERSKSMFS

ASLSRSKIHQLKKALQNQDEDKKLLRAVNRLDKDLAAFKKNCEGREFLIQTEPADTFL

NHVWKFTEAAKIWLPLNMQSEVQGELLAVYFEALTYLKISELYDERYVTYIQVKGFEV

TIKQLCLNPSYLLNQTLKMGKASILFSATLSPMSYFSQILGGEKEALCYQLPSPFAPE

KQLLLVSDYVDTKYQNRDSSIEPIIDSLAALIEGKNGNYLFFFPSYHYLDQVYTQFSI

KYPKFKTIIQESMMDESERELFLASFQDNPSESLAGFCVLGGIFSEGIDLKGERLIGV

AIIGVGLGQLNVEQDLVEKYYDHENGQGYQYAYQIPGMNKVLQAAGRVIRGRGDKGVV

LLLDQRFHSTRYQMLFPEHWSNAKRVTNTKEVSTELEQFWQK"

CDS complement(2774200..2774994)

/gene="fabG_3"

/locus_tag="JFBMFIFI_02607"

/EC_number="1.1.1.100"

/inference="ab initio prediction:Prodigal:002006"

/inference="similar to AA sequence:UniProtKB:Q9KQH7"

/codon_start=1

/transl_table=11

/product="3-oxoacyl-[acyl-carrier-protein] reductase FabG"

/translation="MDMYLKGQTALVTGSTKGIGKAIAFELAKEGVDVIVNGRSTGTV

NEVVAEIKTAYPMTNPSAAPFDITTQEGTKQMTDAFPKVDILVNNLGIFTNSDYFQLI

DADWQHYFDVNVLSGNRLASYYLPQMLERDAGRILFIASEVAVMPDAEMPQYSMSKTM

QLSLSKTLAKLTQGSKVTVNTVMPGSTMTEGVVEMLENWFPGGNETLAEKGQRFMKEN

RPNSLIQKFIAPEEIGRMVVFVSSPMASAINGAALRVDGGLVPTLY"

CDS complement(2775063..2775905)

/gene="ytbE"

/locus_tag="JFBMFIFI_02608"

/EC_number="1.-.-.-"

/inference="ab initio prediction:Prodigal:002006"

/inference="similar to AA sequence:UniProtKB:O34678"

/codon_start=1

/transl_table=11

/product="putative oxidoreductase YtbE"

/db_xref="COG:COG0656"

/translation="MNINFEDQVTLNNEVNMPWVGLGVFRVEDHEELVEAIKLAIVQG

YRSIDTASIYGNEEAVGEGIRQGIVAAGISRKELFVTSKVWTADMGFEEVEKAYQVSL

DKLGLDYLDLYLIHWPVAGKFIPTWKKMEELYQDGKIRSIGVSNFQIHHLEELFAEAS

IKPVINQVEYHPKLTQLELQNYLKQHNIQMEAWSPLMNGEILTNPVILEIAERHGKTA

AQVILRWDLQNGVITIPKSTNEQRLAQNKDIVDFNLSEKDMQQIQQLNENYRIGPDPD

NFDF"

CDS 2776051..2776416

/gene="ytcD"

/locus_tag="JFBMFIFI_02609"

/inference="ab initio prediction:Prodigal:002006"

/inference="similar to AA sequence:UniProtKB:O34533"

/codon_start=1

/transl_table=11

/product="putative HTH-type transcriptional regulator

YtcD"

/db_xref="COG:COG1733"

/translation="MKTITYNIGVEATIDVIGGKWKPIILCHLKHGTKRTSELKRLMP

SITQKMLTQQLRELEADGVVTRIVYEQVPPKVEYSLSSYGQSLAPVLELLCLWGEKHI

EQRVSLYKEDIVLENTELT"

CDS complement(2776466..2777401)

/gene="ccpB_5"

/locus_tag="JFBMFIFI_02610"

/inference="ab initio prediction:Prodigal:002006"

/inference="similar to AA sequence:UniProtKB:P37517"

/codon_start=1

/transl_table=11

/product="Catabolite control protein B"

/translation="MKLNIKDIAKLSGCSVSTVSRVLNQHPYVSEEKKERILKVIAEH
DYAPNSQARDLQSGESKTIGVIVPAISLIYFDQIINGILENAFEKGYMLTLLPSNYDK
AKEKYYFTQLKNKRFDGIIVTSRVHEMAEIEKFTKYGTIVCCEDTGDYLVSGVYVDRL
AAYNQVFAELKAQGKNKIALTLGRSQLISNSARLTFEAYQKNFGPLNPDYLVDNCYDG
PGGILAGDYFSQLKDVPEVVFTNNDETAVAIFQVFQRKGLAVQVIGQSNMYVSEAMGI
STIDLQLSEIGRLAFDLVCTKEYQKICLKSEYIKR"

CDS complement(2777469..2777771)

/locus_tag="JFBMFIFI_02611"

/inference="ab initio prediction:Prodigal:002006"

/codon_start=1

/transl_table=11

/product="hypothetical protein"

/translation="MRGIIEVIDSEGLRYCYVELKLVEDQIHIQQKSVFEHGYSCIIK
SNQIKEINLGEEEGVRKVTLTLENQLIHIVENGSLFIPVIANFILKDYFVPTGQLV"

CDS complement(2777977..2778342)

/gene="rplL"

/locus_tag="JFBMFIFI_02612"

/inference="ab initio prediction:Prodigal:002006"

/inference="similar to AA sequence:UniProtKB:P02394"

/codon_start=1

/transl_table=11

/product="50S ribosomal protein L7/L12"

/db_xref="COG:COG0222"

/translation="MALNIEQIIADLKVSTVLELNDLVKAIEEEFGVTAAAPVAAAGA
AGPAAEEQSEFTVELTAAGDQKIKVIKVVREATGLGLKEAKGLVDGAPAPIKEGVSKD
DAEALKTQLEEVGASVTVK"

CDS complement(2778417..2778920)

/gene="rplJ"

/locus_tag="JFBMFIFI_02613"

/inference="ab initio prediction:Prodigal:002006"

/inference="similar to AA sequence:UniProtKB:Q5XCB5"

/codon_start=1

/transl_table=11

/product="50S ribosomal protein L10"

/translation="MSQAAIAKKQAIVEDVTVKFTDAASVVIVDYRGLTVDQVTELRK

QLREAGVEMKVIKNSILSRAAEAANLPGLGDVFKGPTAVAFSNEDVIAPAKIINDFAE

KAEALEIKGGIIEGKVSSKEEIEALAKLPNREGLLSMLLSVLQAPVRNTALIIKAVAE

QKEEEVA"

CDS complement(2779313..2780002)

/gene="rplA"

/locus_tag="JFBMFIFI_02614"

/inference="ab initio prediction:Prodigal:002006"

/inference="similar to AA sequence:UniProtKB:Q99W68"

/codon_start=1

/transl_table=11

/product="50S ribosomal protein L1"

/translation="MAKKSKQYQTAAKQVDVTKAYSVEEAVALVKSIDFAKFDATVEV

AYRLGVDPKKADQQIRGAVVLPNGTGKTQRVLVFAKGDKAKEAEAAGADYVGESDLVQ

KISGGWFDFDVIVATPDMMAEVGRLGRVLGPKGLMPNPKTGTVTMDVTKAINEIKAGK

VTYRVDKAGNVQAPIGKVSFDDAKLVENFKTINDTMLKVKPSSAKGQYIKNITVTTTF

GPGVKVDQGSF"

CDS complement(2780107..2780532)

/gene="rplK"

/locus_tag="JFBMFIFI_02615"

/inference="ab initio prediction:Prodigal:002006"

/inference="similar to AA sequence:UniProtKB:Q06796"

/codon_start=1

/transl_table=11

/product="50S ribosomal protein L11"

/db_xref="COG:COG0080"

/translation="MAKKVVKLVKLQIPAGKANPAPPVGPALGQAQVNIMGFCKEFNA

RTADQAGLIIPVVISVYEDRSFTFITKTPPAAVLLKKAAGIESGSGEPNTKKVATVKR

DKVREIAETKMPDLNAASVESAMRMVEGTARSMGITIED"

CDS complement(2780879..2781796)

/locus_tag="JFBMFIFI_02616"

/inference="ab initio prediction:Prodigal:002006"

/codon_start=1

/transl_table=11

/product="hypothetical protein"

/translation="MKKIILSLVGLLLLVGLIGGAYFWFSPPKNQPTLPAKKSIVEKD

TASSADSASPSAESETTLNVPTIFVHGYSGGKGSFGGMIKRFSQDKEGTPSLVATIDS

DASIHYEGEYDSKAKNPLIQVLFSDNKSTEENQSWWLQELFQSLKANYGIETLNAVGH

SMGGVSLTNYITKFGQDVSYPSLDKLVLIGAPINGLEIGVDGVTDFDLTDEGPLMATE

RYQNFMNLRDNLPTGLAVLSIAGDKEDGTKSDGSVSVASALSARFIFEGKVADYREMT

FTGLKAAHSLLHENQAVDQAVAQFLWKED"

CDS complement(2781866..2782414)

/gene="nusG"

/locus_tag="JFBMFIFI_02617"

/inference="ab initio prediction:Prodigal:002006"

/inference="similar to AA sequence:UniProtKB:Q06795"

/codon_start=1

/transl_table=11

/product="Transcription termination/antitermination

protein NusG"

/db_xref="COG:COG0250"

/translation="MEQIESEKQWYVLHTYSGYENKVKQNIESRSNSMGMEDYIFRVV

VPEEEETETKNGKEKVNMKKTFPGYVLVEMIMADDSWYVVRNTPGVTGFVGSHGAGSK

PAPLLNEEIEVILRRLGISTRHQEIDFEVGDTVTIIEGAFSGLTGKITEIDNERAKLK

VNVEMFGRETSTELDFEQIDTL"

CDS complement(2782545..2782718)

/gene="secE"

/locus_tag="JFBMFIFI_02618"

/inference="ab initio prediction:Prodigal:002006"

/inference="protein motif:HAMAP:MF_00422"

/codon_start=1

/transl_table=11

/product="Protein translocase subunit SecE"

/translation="MGKFFSGVKSEMKQVTWPTGKELRKYTMTVFGTVFFFVIFFMIV

DFGITSLLGLFIK"

CDS complement(2783018..2783959)

/gene="menA_2"

/locus_tag="JFBMFIFI_02619"

/EC_number="2.5.1.74"

/inference="ab initio prediction:Prodigal:002006"

/inference="protein motif:HAMAP:MF_01937"

/codon_start=1

/transl_table=11

/product="1,4-dihydroxy-2-naphthoate

octaprenyltransferase"

/translation="MSIKTFLKLVEIQTKLASLFPFLLGALFAALYFKEFNLGNTLLF

FAAMLIFDMTTTAINNFMDFLKAKDEGYKDQVNIIGQEKIPEKLVRAIILTMLVIAAA

LGLWLVFRTDILVLFMGGACFFIGIFYTYGPVPISRMPVGELFSGVTMGFGIFFIAVY

VNLASGTLLNLQFLPNYQFALVGNYLQILVILLVSIPSIFTIANIMLANNLCDLDEDI

VNHRYTLVFYIGRKRGVQLFNVLIYLSYVAVVLAVVLKIYHPIMLAVLLTLFPVSKNL

SIFNQKQVKTETFVLSIKNLILINGALVCLMLISLLF"

CDS complement(2784095..2785204)

/gene="apbE"

/locus_tag="JFBMFIFI_02620"

/EC_number="2.7.1.180"

/inference="ab initio prediction:Prodigal:002006"

/inference="similar to AA sequence:UniProtKB:O83774"

/codon_start=1

/transl_table=11

/product="FAD:protein FMN transferase"

/db_xref="COG:COG1477"

/translation="MKVANKKKITVGLIFLLAFVAVGCSKGTDGKETSKKDSTTNTIK

EPYSDTQFLMGTVVKVSIYNEGKEAVLEKAFDRIKELAAKITVNEKGSEIDAINDNAG

VKPVEVTDDMYYLLKEAQHYSKTSEGGFDMAIGAVTDLWRIGFPDARKPSQAEIDAVL

PLVDYHKVIFDDKKQTVYLSEKGMQLDLGAIAKGYITDEVATVLKDNDVDTAIIDLGG

NIFVMGTSPRREGADWNVGIQDPNQTRGTTIGSIPESNRSIVTSGIYERFYEDPDTGI

KYDHLMNGKTGYPFDNDIAGVTIVSDKSIAGDGLSTAVFSKGVKGGLEFIDDIDGVEA

IFVTKENKVYISKGLKDTFKLTSKDYTLEKNETLK"

CDS complement(2785448..2786386)

/locus_tag="JFBMFIFI_02621"

/inference="ab initio prediction:Prodigal:002006"

/codon_start=1

/transl_table=11

/product="hypothetical protein"

/translation="MKFKKAMSVASLVFASTVVLAACGGNEDKKDSSSESSAASSEVA

KDSSSKEETKEMKQVAGGELKDGTYKLVEKDYDDKGWKVEFAMTVKDGKITESDYNYI

NEKGDKKSDDKEYQKAMEAKTKTGPAMFIPELNEALVAAQNAADVEVVSGATHSSEAF

RNYAQQLVQAAQAGNQETIEIANTADLQDGTYSLATKNASNGWTETFEMTVEGGKITK

SNYDGTNDKGEIKSENKEYQEMMKKTAGVGPADFFPTLNKALVEKQDAGAVEVVSGAT

HSSEAFKLWAAQLINAAQKGDTAKIEVDNIVMEEVK"

CDS 2786741..2788603

/gene="cdr_3"

/locus_tag="JFBMFIFI_02622"

/EC_number="1.8.1.14"

/inference="ab initio prediction:Prodigal:002006"

/inference="protein motif:HAMAP:MF_01608"

/codon_start=1

/transl_table=11

/product="Coenzyme A disulfide reductase"

/translation="MTGKNIVVIGAGYAGVAATKKLAKKYKKNKDVTITLIDRHSYHT

MMTELHEIAGARVEPTAVQYDLQRLFCRLKNVNLVTDNVTNVDHDTKTVTTEHGSYSY

DYLVLGMGGEPNDFGTPGVKEFGFTLWSWEDAMKLRGHIEETVAKAAKVRDAAKRKAM

LTFTVCGSGFTGIEMVGELLEWKDRLAKDNKISADEISLIVVEAAPTILNMLDRNDAG

KAERYMTKKGIKILTDAPIVEVKEDHIVLKSGETIPTSTLIWTAGVKANSDTAEFGME

AARAGRLVANQYMEAKDLENVYVIGDLVYFEEFENTPTPQIVQAAECTAHTAAANLIA

DIEGTEKHAYKGNYQGFMVSIGSRYGVACLFNKIHLSGFLAILMKHIVNLKYFFDIRS

GYYAFQYIMHEFFHIKDNRNIFRGHLSRYGNVLWSVPLRIFYGSMWLLEAGKKTFGWF

GGESWFTDSVKLPFAWLQAETTSGASEAVEQVKEVAKPIFGFSYAYGEEPMMVFDKAP

KWFEWLMEKMLPTPDVALFFQKAMTIIELLIALAIIAGLFTWLANAATIALVVSFALS

GMFYWVNIWFIFAALALMNGSGRAFGLDYYVIPWVQKVAGKWWYGTPKSLYGDK"

CDS 2788811..2789293

/locus_tag="JFBMFIFI_02623"

/inference="ab initio prediction:Prodigal:002006"

/codon_start=1

/transl_table=11

/product="hypothetical protein"

/translation="MKTKFKALIPYFKMIRRWDIIIILVLTIGSFLPLGLFYLNQATA

ETDSPSGERQLIAHISADGKEVKQIVLTGHTGTDTYRYEDEDGDFNLLEIKDETIRMI

DADCNDLVCVRSFGAISKPGETILCLPHKLIVEVRSTDGINDGGMVSFGGLDTLLANK

"

CDS 2789344..2789901

/locus_tag="JFBMFIFI_02624"

/inference="ab initio prediction:Prodigal:002006"

/codon_start=1

/transl_table=11

/product="hypothetical protein"

/translation="MTKNQKLVYIALLAAQGVVIGLIERSIPFPFAFAPGAKLGLANL

ITIIAIFTLPFKDSFTVVWMRLLLTTLLGGTLSTFLYSCAGALLSYFGMLIVKQLGPK

RVSIIGISATGGILHNIGQLAVASWIAQTWTVMLYLPVLSFMGILAGVAIGIAANYLL

EHVETLRNFRAVYGRFDQQTKGEHK"

CDS 2789898..2790878

/gene="hepT_2"

/locus_tag="JFBMFIFI_02625"

/EC_number="2.5.1.30"

/inference="ab initio prediction:Prodigal:002006"

/inference="similar to AA sequence:UniProtKB:P31114"

/codon_start=1

/transl_table=11

/product="Heptaprenyl diphosphate synthase component 2"

/db_xref="COG:COG0142"

/translation="MKIHPMWDTYPELQMELEKTVELIEKNIKIRNKEIEATILALIH

SGGKLLRPAYSLLFSRFGPNQDAEKARAVAAALEILHMATLIHDDIVDDSPMRRGTPT

IQAKYSKDVAVYAGDYLFTVCFKLLARYSSSLESIQIDTRGMERILMGELDQMHLRYN

QKITIRQYLTQISGKTAQLFALSCYSGALESGTSEKFARSCYHIGNHIGMAFQIMDDI

LDYSQDANIFGKPVLEDVRQGVYSAPLIFALRKYPEKFKPYLDKKDQMTNDDTQIVHK

LVLDLGGLAAAQELAEKYTTKALKELTKLPETPEKAIIIQLTRSLLTRYD"

CDS 2791273..2791458

/gene="cbnBM1"

/locus_tag="JFBMFIFI_02626"

/inference="ab initio prediction:Prodigal:002006"

/inference="similar to AA sequence:UniProtKB:P38579"

/codon_start=1

/transl_table=11

/product="Bacteriocin carnobacteriocin BM1"

/translation="MKSVKELNKKEMQQINGGAISYGNGVYCNKEKCWVNKAENKQAI

TGIVIGGWASSLAGMGH"

CDS 2791473..2791739

/locus_tag="JFBMFIFI_02627"

/inference="ab initio prediction:Prodigal:002006"

/codon_start=1

/transl_table=11

/product="hypothetical protein"

/translation="MIKDEKINKIYALVKSALDNTDVKNDKKLSLLLMRIQETSINGE

LFYDYKKELQPAISMYSIEHNFRVPDDLVKLLALVQTPKAWSGF"

CDS complement(2792042..2792623)

/gene="sigH"

/locus_tag="JFBMFIFI_02628"

/inference="ab initio prediction:Prodigal:002006"

/inference="similar to AA sequence:UniProtKB:P17869"

/codon_start=1

/transl_table=11

/product="RNA polymerase sigma-H factor"

/db_xref="COG:COG1595"

/translation="MTNEEKKKVPDAELIKGIKGGDTEFFEQLFQRYRFLTLKISRKY

NVIDYDQDDIMQEARILFFQVIHEHQVEKGMSFGNLYKMKLQQFYFNLIRRQNAIKRK

GNKFLGSLDEVRETSSSENYLYAPQKDATPEALLLAREGEHRYYQLLSLYEKQVLLAH

TKGYEIEEIATYYDKSIQSVKNALSRCRTKFND"

CDS complement(2792684..2793226)

/locus_tag="JFBMFIFI_02629"

/inference="ab initio prediction:Prodigal:002006"

/codon_start=1

/transl_table=11

/product="hypothetical protein"

/translation="MKKDLLIIDGYNMIGAWPELVKLKDQDQMEDARDSLLHQLSNYQ

RYEGIEIWVVFDAQLVPGIQKSYQRYTVTVIFTKEGETADSFIERIVSEKNNRLTQVT

VATSDLAEQWLVFSRGALRKSANELYKDLKESNKSIKMETEKFHSQTIRRNSPWDLEQ

LVDLEQLLSQLSEKKDNGNL"

CDS complement(2793265..2794140)

/locus_tag="JFBMFIFI_02630"

/EC_number="2.1.1.-"

/inference="ab initio prediction:Prodigal:002006"

/inference="similar to AA sequence:UniProtKB:Q7A794"

/codon_start=1

/transl_table=11

/product="Putative TrmH family tRNA/rRNA

methyltransferase"

/translation="MSNQKSNFGKKVTQQNRRPSKAHKPKRDENRGRKEIEQVMDQSE

IEEDRDLVAGRIPAIEVLKSERDINKIFLQEGLSGSKISEILGLAKKRNIQVQMVPKS

KLDTLVDGMNHQGIVVAAAAFQYAELDDLFAAAALKKEDPFFIILDGLEDPHNLGSIM

RTADASGAHGIIIPKRRAVGLTATVAKASTGAIEHVPVVRVTNLVQTINELKDRGVWL

YGTDMKGQDYRQWDTKLPIAVVMGNEGKGISRLVKEQMDGMVTIPMVGHVQSLNASVA

ASLLMYEVYRGRNAL"

CDS complement(2794121..2794582)

/gene="mrnC"

/locus_tag="JFBMFIFI_02631"

/EC_number="3.1.26.-"

/inference="ab initio prediction:Prodigal:002006"

/inference="similar to AA sequence:UniProtKB:O31418"

/codon_start=1

/transl_table=11

/product="Mini-ribonuclease 3"

/db_xref="COG:COG1939"

/translation="MILPKERDGDENNMTTEKDWSLLNGLALAYIGDAIYEVYIRDYL

IQSGKTRPNQLHKSATKYVSAKAQNALMQQMLAVDGLLTENEITMYKRGRNSKSHTSA

KNADITTYRVATGFESLMGYLHLSKQTERMEELIQWCIQQTEEDQNEQSKK"

CDS complement(2794545..2795954)

/gene="cysS"

/locus_tag="JFBMFIFI_02632"

/EC_number="6.1.1.16"

/inference="ab initio prediction:Prodigal:002006"

/inference="similar to AA sequence:UniProtKB:Q06752"

/codon_start=1

/transl_table=11

/product="Cysteine--tRNA ligase"

/db_xref="COG:COG0215"

/translation="MLKIYNTMTREKEVFVPLTEGQVKMYVCGPTVYNYIHIGNARST

VAFDTVRRYFEFRGYQVQFVSNFTDVDDKIIQAAKELNITAPDVADRFIEAFYTDVTA

LGVEKATKNPRVMENMPDIIEFIQVLIDKGYAYESDGDVYYRTRKFKNYGKLSDQSID

ELEIGASNRLEKIENERKEDPVDFALWKSAKADEISWESPWGAGRPGWHIECSVLATK

YLGDTIDIHGGGQDLTFPHHENEIAQSEAKTGHPFANYWMHNGFVTMDEEKMSKSLGN

FVLVHELIKQVDPQVLRFFMATTHYRRPISYNEATISEAKNNLNKVQMAASNLHYRLS

DATIELPTDQDYFQQLAEFKQTFITEMDDDFNAANGITVIYELAKVMNIYSEQAEVSK

AVIEAMLWLFKELLQVFGITLENQAELLDENIEQLLSERTIARAEKNFARSDEIRDLL

KAEGIIIDDTPQGTRWRRE"

CDS complement(2796106..2796624)

/gene="cysE"

/locus_tag="JFBMFIFI_02633"

/EC_number="2.3.1.30"

/inference="ab initio prediction:Prodigal:002006"

/inference="similar to AA sequence:UniProtKB:P67765"

/codon_start=1

/transl_table=11

/product="Serine acetyltransferase"

/translation="MSIREEISVIKKNDPSVKSTLEILLTYPGFHAVLNHRLAHFFYR
HKLYLLAKILATISRFFTGIEIHPGAKIGRRLFIDHGMGVVIGETAIVGDDVIIFHGV
TLGGTGKETGQRHPKVGNHVLLSAHVQVLGPIEIGDYAKIGASAVVLKSIPAHTTAVG
IPAKVVKSALTE"

CDS complement(2797295..2798767)

/gene="gltX"

/locus_tag="JFBMFIFI_02634"

/EC_number="6.1.1.17"

/inference="ab initio prediction:Prodigal:002006"

/inference="similar to AA sequence:UniProtKB:P99170"

/codon_start=1

/transl_table=11

/product="Glutamate--tRNA ligase"

/translation="MAKKVRVRYAPSPTGHLHIGNARTALFNYLFARHNGGDFILRIE
DTDAKRNIADGESSQLDNLTWLGMDWDEGPDRPGNYGPYRQSERGSVYNPLIEQLLLS
NRAYKCYCSEEELEAERDAQKSRSEMPHYSGKCASLTPAEQAEKEAAGLTPVIRFRVP
RDTTYSFDDLVKGPISFESSSIGGDFVIAKRDGIPTYNFAVAVDDHYMEISHVLRGDD
HIANTPKQLMIYEAFEWTPPTFGHMTLIINSETGKKLSKRDESILQFIEQYRDLGYLP
EAMFNFIGLLGWSPVGEDEIFNQADFIKMFDPERLSKSPAAFDGKKLEWINNQYVKAT
DLDTLTDLSLVHLVKAGLVEANPTPEKIEWVRQLVSLYHEQMSYGAEIVELSELFFTD
TPKLDDVAKEVLAGETVPVVLNAFHKVLGEIELFDVASIKAGIKTVQKETGVKGKNLF
MPIRVAVTGQSHGPELGETIELLGREKAKAHLEAAIANLG"

CDS complement(2798916..2799389)

/gene="ispF"

/locus_tag="JFBMFIFI_02635"

/EC_number="4.6.1.12"

/inference="ab initio prediction:Prodigal:002006"

/inference="similar to AA sequence:UniProtKB:Q06756"

/codon_start=1

/transl_table=11

/product="2-C-methyl-D-erythritol 2,4-cyclodiphosphate
synthase"

/db_xref="COG:COG0245"

/translation="MRIGQGYDVHQLVEGRDCIIGGVKIPYELGLLGHSDADVLLHAI
IDALLGAAGKGDIGHFFPDTDAQFKDANSRELFREVWAILKTEGYFLGNIDATIIAEA
PKMQTYLEEMKQNIAADCESDSSQINIKATTSEKMGFVGRKEGIAVMVVCLLGKK"
CDS complement(2799389..2800213)
/gene="ispD"
/locus_tag="JFBMFIFI_02636"
/EC_number="2.7.7.60"
/inference="ab initio prediction:Prodigal:002006"
/inference="similar to AA sequence:UniProtKB:Q06755"
/codon_start=1
/transl_table=11
/product="2-C-methyl-D-erythritol 4-phosphate
cytidylyltransferase"
/db_xref="COG:COG1211"
/translation="MSQLFGKKINSQKSKKTLFFEFPIFLPELTSSTNFLFKGVILVG
YELVLLAAGQGRRMKASKNKILLHLLGKPVIEYALTTFVNDADCQHIVLVVEKKEKEL
IEAVLAQNEFAGKVPITVVIGGKERQDSVYNGLQYLKKQTGVVLIHDGARPFLEANLI
KKLYQKAQRTNATIVGVPVKDTIKKVVEGKVDQTIPREELWQIQTPQAFQISFIQEVH
ERARQEGFFGTDDASLVEHYGHAVEVTEGSYMNIKLTTPDDLVIGEAILKMKERGN"
CDS complement(2800428..2801543)
/gene="yacL"
/locus_tag="JFBMFIFI_02637"
/EC_number="3.1.-.-"
/inference="ab initio prediction:Prodigal:002006"
/inference="similar to AA sequence:UniProtKB:Q06754"
/codon_start=1
/transl_table=11
/product="putative PIN and TRAM-domain containing protein
YacL"
/db_xref="COG:COG4956"
/translation="MTKKIITAVFAVVGGSIGASFLPDIWSLFNVTNSFLNNTFTNII
IGAIIFLLFSLVLVKYVERGVKKAEVYLSQQSAAYLLFGSLGTIIALLLAFLVTMPLM
GLKWPVLSTILPIIIYMVFAYLGFRIGTTRREEWRKLIQPRAKKSNEEDANGKVLERK

FDDDFRKYKILDTSVIIDGRIYDIAKTGFIEGTLMIPNFVLMELQYIADSADSLKRVR

GRRGLDILNALQKESDIQVEMYDGDFEDITEVDSKLIKLAKLLDGVVVTNDYNLNKVS

EFQNVPVLNINELANAVKPVVIPGENMNVMVVKAGTERNQGVAYLDDGTMIVVEDGQY

YMNKSIEVVVTSALQTAAGRMIFAKPVHAQRGIKEKE"

CDS complement(2801614..2802990)

/gene="radA"

/locus_tag="JFBMFIFI_02638"

/EC_number="3.6.4.-"

/inference="ab initio prediction:Prodigal:002006"

/inference="similar to AA sequence:UniProtKB:P37572"

/codon_start=1

/transl_table=11

/product="DNA repair protein RadA"

/db_xref="COG:COG1066"

/translation="MAKKKATKFVCQACGYESPKWMGRCPNCGAWNQMMEELEVDKND

RQNRVSMTGQTAKAEAIQDITVSKVPRVKTELNELNRVLGGGVVPGSLVLIGGDPGIG

KSTLLLQVSAQLNIAGGKVLYVSGEESASQIKMRADRLGVKGADFYIYPETDMGAIRQ

TIEDLKPEYVIIDSIQTMSQPDVSGVVGSVSQVRESTADLMKIAKSNNIAIFIVGHVT

KEGAIAGPRMLEHMVDTVLYFEGDRHHTFRILRAVKNRFGSTNEIGIFEMREGGLVEV

LNPSELFLEERLDGATGSAVVAAMEGTRPILVEIQSLITPTVFGNAKRTASGVDHNRA

SLIMAVLEKRAGLMLQNQDAYLKAAGGVKLNEPAIDLAIAVSVASSYREKETRAKDCF

IGEIGLTGEVRRVSRIDQRVQEAAKLGFTRMFIPKNNIGGWTFPKDVEIVGVSTVADT

IRKALGDK"

CDS complement(2803473..2803727)

/locus_tag="JFBMFIFI_02639"

/inference="ab initio prediction:Prodigal:002006"

/codon_start=1

/transl_table=11

/product="hypothetical protein"

/translation="MKKQRKVVAKVIVNKKKFVISDMNHSEEKNILNESEEKEPQPYQ

VEKITKGVFYNGWEIATASGFIEKIAITLVRYTVFPEKQR"

CDS complement(2803684..2803827)

/locus_tag="JFBMFIFI_02640"

/inference="ab initio prediction:Prodigal:002006"

/codon_start=1

/transl_table=11

/product="hypothetical protein"

/translation="MLKNNKKLAALLFSISLFCGIVYRNVEFLKGRIFEETAESSSES

NSQ"

CDS complement(2803922..2806420)

/locus_tag="JFBMFIFI_02641"

/inference="ab initio prediction:Prodigal:002006"

/codon_start=1

/transl_table=11

/product="hypothetical protein"

/translation="MKIKNFLLVLFGAMGLVVSLIPTVAASTLPPLSLPIDNVFAVPS

GANSYVDEHITVITKDSKSQVGSIFSTESNKLDLTKSFHAEMYVYLGNKRDGAADGMA

FVMHTDKNRTKIFTGGSGDQLGVFAKVGSGPSGLLEQIERSFAIEFDTYHNGDTMDAN

VERNANKGHIAYAFPEFKSSYNMSSDKVVSLKHQGLYYPNDYLSNGEWHPFSVDWDAD

TKELNYKFDDAPTVSVPINPLSVFGSTSVYWGFTGSTGDASQESKVAFKQIPGLVNIS

SNMKVTKDGKNIDNTIIPGGDGDVKVQYDLKYDSGKQNLLKPVFNLDLDEFLSFKPGT

LTVNGTKISDDYFVDGKLTYNLLGNLTELDNNLTISFEAIPKVVTDKNAKTIINYSIN

AENYFGNSLNTSFSIEKVTIIKSSYFENQSWVINEINRQLAPKKIDVSVYDRDLSKVT

KIDLTSAPKIIGEYIPKTIDSLSNLSHLRLANQKLSGVLPEELGNLSKLTYLSIYGNT

FDGEIPLSIGKLQQLTLIALDDNNLKGLVPTNIASLPKLNQLYLNGNKLSGQLPDFRM

NMGLIKIHNNQITYNLATVPLFLSSAKSKDYTKTFIQGLKLTGNSKVSSQNKQIKPFD

EMDQGYFNLKGMQGVQTIDLVDEHTYVIKNAVDGTVYYRGKKDKTVTIPYQKDISYQI

VLDDAEKNPNSVFTILGKERELKFKEIPVFLSLKIKVGSENQPVIPEGNLIIFDDREN

KNWKLSVTVSELTQGQRKLQGGYSYTGKDGISQPIFTGQKFLLETGQSDSVNEDILIS

NAWGSNYGLKYTASNSNHIGNYQGDVIWTLEDTP"

CDS complement(2806454..2807611)

/locus_tag="JFBMFIFI_02642"

/inference="ab initio prediction:Prodigal:002006"

/codon_start=1

/transl_table=11

/product="hypothetical protein"

/translation="MKKIISILVVCLIGLQHMPMIGEAAETMAYSVKANIPENQINKT
LTYFDLKMEPNQRQDITLTVSNSSDEKATILISPNVAMTNQNGVIDYSKADEKVDSTL
KNPVTSIISKAQEVTLEPKETKEVPFTIQMPEKEFDGLILGGFYISKKEDDSNTKDKE
KDVQIKNKYSYVIGLQLRENTNEVKPVMELNDIKPVLLNYRTAVTANLQNTEATIMKD
VSINAKVMKKGSEKVLHETDKKGLSMAPNSNFDFPINWDNQSLDAGTYRLQLIAQSGE
DKWEFDKEFTISAKDSTDLNKEAVELEKTEPNWILIISVVAGITIFLILVIGFFIYRH
QKKKAAEKRARLMKQRKRKKQQELRKRTDSSEPLNKRKKVNKRPINQNRKN"

CDS complement(2807696..2808151)

/locus_tag="JFBMFIFI_02643"
/inference="ab initio prediction:Prodigal:002006"
/codon_start=1
/transl_table=11
/product="hypothetical protein"
/translation="MKKSSYIMATVVLASLASGGVQAAVSSTHTSENKIDFTAEGWHL
SAKMSEFKSGNKVLRGATLAFKDGVVKAGSSSNISVAPTKSDVLFDNTDSKPFMNAMD
KGGRGTWVTVWSGTDQANEAIQLNVVAGTPEANTEYTSSITWELADAPK"

CDS complement(2808343..2810226)

/locus_tag="JFBMFIFI_02644"
/inference="ab initio prediction:Prodigal:002006"
/codon_start=1
/transl_table=11
/product="hypothetical protein"
/translation="MIKKRIPRFGLMFLTSIVILFFFNISRVQAAYDSNISINPLYAN
GITIKNGQLNLSFPTVGSEGTLTYVPSGSNIPFHARYANEKYVGIDIIRDEKVIFTTS
VNGDTKQEVLTNALSNLKVKDTDILHFYLAEPTKEGVTNFEKETSLPADKFKGYFQKD
MYYMMTYGGFIPVNNGISHLMYDENKIRTSATQMSSLFSGNILNFGYSGSKDYNFFGI
VEPDTQFHTFYRGYDYYAISHYEITDGHQLGSTKITASALGETSYSDMGKQLNKLPVK
DGTIYKIFSAEPRKIRLWKNGVSRQIQDKLAKETYVELTKNQGLVELDFNRVTSKAVT
VELGSNPEKLNVEDFTNVSDYPNLSSSFKTNVKTDMLGTYNQDISIRQNLVTAANYFL
STVSSKVTVVDTTPPTATGKANVEIPIYESLPTNPAELLENLKDNSGIENLKTEYLDE
NGDTSIPGVKIVKIRLTDVSGNFSDISVPITVVPGSLAIARVPNLDFGQFKIGVATKS
MNQVTSEIEIHDFRGTKEGWTLQASKSDFSTQDGKKINADITFINGIVSSLEQQINGV
NTYNITLNDSPQPIMQAQKNSGMKKWVGTFKTENVYLDNISPDARVGSYESTINWTLL

NAP"

CDS complement(2810440..2811582)

/locus_tag="JFBMFIFI_02645"

/inference="ab initio prediction:Prodigal:002006"

/codon_start=1

/transl_table=11

/product="hypothetical protein"

/translation="MKKIISILALCLLGIHYMPMTGEAAETMAYSVKANIPENQINKT

LTYFDLKMEPNQRQELTLTVSNSSDEKATILISPNVAMTNQNGVIDYSKMDEKLDSTL

KNPVTSLISKAQEVTLEPKESKEVSFTVQMPEKEFDGLILGGFYISKKEDDSASKDKE

KDVQIKNKYSYVIGLQIRENTNEVKPVMTLHDIKPVLLNYRTAVTANLKNTEATIMKD

LAVDAKVMKKGTTKVLHETKKEGMTMAPNSNFDFPVNWDNQSLDPGTYMLQLVAQSGE

EKWEFEKEFTISAKDSTDLNKEAVELEKTEPNWILIISVVIGVMFLLILVVVFFISRH

QKKKAAEKRARLIKQRKRKKQQELRKRKSISEQTKRPNRNSRKNKV"

CDS complement(2811669..2812367)

/locus_tag="JFBMFIFI_02646"

/inference="ab initio prediction:Prodigal:002006"

/codon_start=1

/transl_table=11

/product="hypothetical protein"

/translation="MKKSSVIMATVAFASFSFGGAEAKAEVSSTHTSENKINFTAGEG

VVTPPVDPTNPDNPNVPNPVDPTDPTNPGTGQTGPLSIDYVSNIKFGEHKITGKDIAY

KAKNANPFVQVTDLRGASDGWNLSAKMTEFKSGNKVLRGATLAIKDGVVKAGSSSNIS

LAPTKSDILFDNTESKTVMNAKDKGGRGTWLTIWSGTDQANEAIQLNVVAGTPEANTE

YTSSITWELEDAPK"

CDS complement(2812415..2813071)

/locus_tag="JFBMFIFI_02647"

/inference="ab initio prediction:Prodigal:002006"

/codon_start=1

/transl_table=11

/product="hypothetical protein"

/translation="MKINKYSILGLSVLMSVAILGSQSVEAAEASAGTSKANFELQAG

DDTTVPEILDPEIQPPTLNKGPLSLDAVSSFNFPTKKLGSESQAPLEATPVEGTKLGL

QVTDSRGQDLGWNLKVSATAFETADKKLTLKGAVMTIPEGKLTTGEGVDPLLTPSAFK

VNLSPTATSIMSAATTQGRSTWVNSFEGKGEKVTLAVPSGNKVASYVSTITWSLEDAP

"

CDS complement(2813099..2814088)

/locus_tag="JFBMFIFI_02648"

/inference="ab initio prediction:Prodigal:002006"

/codon_start=1

/transl_table=11

/product="hypothetical protein"

/translation="MKIIKHVLSWGLSVCLLFNVTTTSLAVTTTSQVGIYFTEKEDKA

EVENGNIVFQKNKELPKTEGDRTSSKLPQTGEHKDRHLFIIGAILLVASMLVKKLRTK

RGGRRMKLKTMTMLGLIGSGMLVGTGLVHAAPQDEVTGSTDGKGGTSHGYINLTPGDT

DTGKTDPIEPTDPPGGTGNEGVLTLDNVAPLLFSTHKLEGKEQVYTSVVENSNIQVTD

KRGEEAGWNVQVSQTPFKDIADETKILKGAKLILPVGVVKADEGNVSLAPSVRSVEVN

ADLAVVMNATAGTGAGTWRTVFDKDEIKLTVPAGNKKGEYMSTVTWTLMDAPK"

CDS complement(2814182..2814397)

/locus_tag="JFBMFIFI_02649"

/inference="ab initio prediction:Prodigal:002006"

/codon_start=1

/transl_table=11

/product="hypothetical protein"

/translation="MRIEDKWDDGVLYHIGFQVSLDGELRNIRKNLVLPVELDRLELE

KLIKENFHLVTKVQFIEYYDELLILKK"

CDS complement(2814381..2815928)

/locus_tag="JFBMFIFI_02650"

/inference="ab initio prediction:Prodigal:002006"

/codon_start=1

/transl_table=11

/product="hypothetical protein"

/translation="MLEFLDLEYIKMVKILDFVNKSVEPVSIDEIARHIKTVRKTANT

LLVLIKSELKDFDFEIRMDSKKKYYLKNKYNAQKKTDYVNLDAYVLACGKRSIVFSMV

EELYHRGYINVTKFCTDKYISSATFSRAKKQLTRTLNKSNLKLATHLKGGIIGEEYYI

RLFYFQFFNTFYNSIEWPFQQNQKIEMERFYQKKIAKITNELNFIQKSKLYYLISITR

KRLLQNNTLLENTYSFNEFKNYNEIYKICRDYLNDHGIWSEKDVANETNFFMYMIYSE
RIFSIDEEFLGNFILFSQDNNECLKINQIWIEEFKSSFKQKISLKDEFQIYQDLYILN
QRHDLSLNRMSVFFKYENNLNSTVLNQKIYQQTKDFFYKLIENKEFSDFIKNGELNIQ
ENVVIEKYYHYIYTYLFNKKEIESIKIFVSNTFDVLSEKFIKKKLLLLFGGNIEIQSQ
LNDQTNLIITNSSMDQNYSEELLISSYKDTASLNKIIYIIREKINIQIDNCEMNKMEE
NKVENRR"

CDS complement(2816327..2817868)

/locus_tag="JFBMFIFI_02651"

/inference="ab initio prediction:Prodigal:002006"

/inference="similar to AA sequence:UniProtKB:Q97P44"

/codon_start=1

/transl_table=11

/product="Putative trans-acting regulator"

/translation="MYKDIEKNFQLIFSTSFKKKRIKKRELVGMRRILSPQDSRILHL
VEFLYDKDELFMRDLGDELNVSFKTLKRDIEYARLILYPIDIKVSGNKGVKLVIPSNY
SITYLYTALLSASLEFRFLEKIFLCETYTVDELAEELFISSSSLRRMVGRMNEVLQEE
EMEINLNPVQIIGNELRIYNLIAYYVLERYFFKKTIFTKAQIEAVKDLIAFALKQSYV
ELDYRSRERLELYILVQLTRMKHDHFVVSEPNLNRKNIFLDVDTTVIKKKLKDSFGKE
FSNQDLLQLFAIFLDGKWTFGYNQLLEYSKVNPNLKQAKEKFEELFESIAFKYKIEQT
SREELLVHLCNIRMQDDGAYHTLYDNLSPIVTHFKHDYFEGIEFIRLKLIEIFPDEEL
KEYKLNAYISILIIHWDKFFFELERKVIPLKIGFVYIVEEKYRKLILDEISYRFKNRF
TIKVINDDSLGDIEKVKNQCDLILTNIPGIDIPGLEVICFPIYPKEKDWSRLEYFYSN
FNFPK"

CDS complement(2817873..2818352)

/gene="bsaA"

/locus_tag="JFBMFIFI_02652"

/inference="ab initio prediction:Prodigal:002006"

/inference="similar to AA sequence:UniProtKB:P99097"

/codon_start=1

/transl_table=11

/product="Glutathione peroxidase BsaA"

/translation="MTIYDYTATLENGETYSMEKYRGKVLVIVNTATKCGLAPQFEDL
ETIYETYHEKGLEVLGFPSNQFKQELSTSEEAGEACRLTYGVTFPMHEISKVNGDDAN

PIFSYLVKNTSGVLGSKIKWNFTKFLIDQEGNIVKRYAPKDSPKNMIEDIENLLARK"

CDS complement(2818458..2819126)

/gene="hrtA_2"

/locus_tag="JFBMFIFI_02653"

/EC_number="3.6.3.-"

/inference="ab initio prediction:Prodigal:002006"

/inference="similar to AA sequence:UniProtKB:Q99RR8"

/codon_start=1

/transl_table=11

/product="Putative hemin import ATP-binding protein HrtA"

/db_xref="COG:COG1136"

/translation="MNLLELKQVTKKFGQGHTEVTALKKTDFAIKAGEFVAIIGPSGS

GKSTLLTIAGGLQSPTEGEVFINGKNFSNVKEKQRSALRFKELGFILQASNLIPFLTV

NDQLKLVNKVEKKAFDQKATDKLLEDLGVLSLKNSYPKDLSGGERQRVAIARALYHHP

SVILADEPTASLDSEKAFEVVDILAKETKEKQKATIMVTHDQRLIERCDKVYEMRDGI

LKLL"

CDS complement(2819131..2820207)

/locus_tag="JFBMFIFI_02654"

/inference="ab initio prediction:Prodigal:002006"

/inference="similar to AA sequence:UniProtKB:P9WG17"

/codon_start=1

/transl_table=11

/product="putative ABC transporter permease"

/db_xref="COG:COG0577"

/translation="MFLAWNEIKYSKTRFALIVGVMFLVSYLVFFLTGLAYGLAQDNR

LVVDKWDAAGIVLSSEANNNLSASTISSDDVKDIKAKETAELGQLAGVITKKGTSEKI

NINVFGVNQDEFINPNVTEGRTFEKEGEVVVDDSLKIKGLKLGDKIRLSTNGATLKIV

GFTTNSKFNTAPIVYMDMAEWQAAKFGTTDPSATQRISGVVVKSGNMKDLGLDKDKVS

GITSKTFISNIPGYTAQTLTFSIMIGFLFVIAAFVIGIFIYVLTLQKSSMFGVMKAQG

IATSYIAKSIVAQTFLLATLGVGIGLVLTLGTALVLPEAVPYMTNYLFLAGFTVVLIS

VAILGALFSVRTIVKIDPLEAIGG"

CDS complement(2820347..2820943)

/locus_tag="JFBMFIFI_02655"

/inference="ab initio prediction:Prodigal:002006"

/codon_start=1

/transl_table=11

/product="hypothetical protein"

/translation="MEKLTRKEEMQLTRKKILKEAQILFMQKGYKSTSTREIAQQSKI

TQPALYHHFKDKETLYIEVIRELTTSVKKELDKILKKETDPETKLSGMILTLIDLHPT

NIMLMIHDILNEMQPENQYLLFQLSQDTYSRPFDDFFTEMQEKGLLRPEVSARAAAKF

VVTSISPMFNGNQKFASKTPMSEQVRELTDFMLHGVFK"

CDS complement(2821078..2822397)

/gene="triA"

/locus_tag="JFBMFIFI_02656"

/EC_number="3.5.4.45"

/inference="ab initio prediction:Prodigal:002006"

/inference="similar to AA sequence:UniProtKB:Q9EYU0"

/codon_start=1

/transl_table=11

/product="Melamine deaminase"

/translation="MKTLIQHVQLVTMNATRDVFPDGYLIIQDTRIVALGAMENLTEE

LSHFDKVIDGEQGILMPGMINTHTHIGMVPFRSLGDDVPDRLRRFLFPLEQHMTKELA

YHSGKYAIAEMQLAGVTSFMDMYYFEDELAKATAEMGSRGILGETVIDFPTCDAQEPH

GGLAYAENYIPKWLGNPLITPAIAPHAPNTNSQEALEKTTMLAEKYDVPVSLHVAEMD

YEVQYFMDKYGLTPVGYLHKIGFLSPRVIAAHCIFLTDDDIELLKITGTKVAHCIGAN

TKSAKGVARIQDLLAAGVPVGLGTDGPSSGNTLDLFTQMKLFANFHKTTLKDRGAFPA

VEIVALATNGGAEVLGLSKEVGSLEVGKQADVVLVETKSVNMFPNFDPYAVLVYSGNA

SNVEMVYINGRCVVEKKQLVQQSLSSLQQNLQAEMSEFKKRTMELAD"

CDS complement(2822394..2824106)

/gene="ade"

/locus_tag="JFBMFIFI_02657"

/EC_number="3.5.4.2"

/inference="ab initio prediction:Prodigal:002006"

/inference="similar to AA sequence:UniProtKB:Q72EX7"

/codon_start=1

/transl_table=11

/product="Adenine deaminase"

/db_xref="COG:COG1001"

/translation="MQVDLLVENAQVFNTFIQKFEAKHVAIAGERFFYISEENLKYLE

PKATIDAGGRHMIPGLIDCHMHIESSMTIPSIFSKAALYHGVTTIIADSHEVANVFGL

KGVEAFLQAETELDIFYAIPSSVPSTTVDLETTGGVIGLPEVRELLKSPKVMCLGEAM

NFKGIISEPDSLIRQIIRECQTTRPTMPIEGHVPKITKEDLATFIYHGVTADHTHQSP

ASIYEKICNGMFLEMQGKSITKENIQTIVDNHFYEYVALITDDVMADDLLEGHLNQIV

KNAIKCGMPAEWAIYTSTYTPAKRMGFQDRGAIAPGQMADFILLDDLAQLDIYQVYKK

GQLVHTKGENIQAPEATENFPAEFYQSVHCKMAELVDFQLTVSADATEASCNVMQIQE

VGTFTKPVQRILPVKNGIVDWENSGLALVVVMERYGKNGNIAYGLVENTLTKKGAVAT

TWAHDHHNVMVLGTSAADMVVAQQRLVEIQGGYIVVDQERVVAECPLPIGGILSAAPL

SVLGTELKAVRKAMVDLGYKNMNEIMSFSTLSLPVSPAIKITDIGMMDTRTQAIIPVI

ARFK"

CDS complement(2824204..2825262)

/locus_tag="JFBMFIFI_02658"

/inference="ab initio prediction:Prodigal:002006"

/codon_start=1

/transl_table=11

/product="hypothetical protein"

/translation="MKKGLGFVAVALLGALVVSGCGNKAADKSSNAPKELVVSTFGLS

EDIVQKDIFKPFEEANNVKIVVETGTSSERYTKLENNPNSKVDVIELSQSNAAKGLDE

GLFAKIDAEKVPSMANLTDAAKEIATDGAGPAYGVNSIGIIYNKEAAGMEIKEWSDLW

NPALKGKISIPDIATTYGPSMVYLANDYKGGDIEKDKGAAAFKGLKELAPNVVKTYGK

SSDLANMFKSGEIIAAVVGDFAVPIITQADADVAYVVPESGTYANFNTINITKNSKNK

ELAEKFIDWRISQELQEVTAKSLNEAPTNAKVVLDDATAENKTYGAVADRAKVIDFSF

VNGQLASWIDQWNKTLNQ"

CDS complement(2825289..2826329)

/gene="potA_1"

/locus_tag="JFBMFIFI_02659"

/EC_number="7.6.2.11"

/inference="ab initio prediction:Prodigal:002006"

/inference="similar to AA sequence:UniProtKB:P69874"

/codon_start=1

/transl_table=11

/product="Spermidine/putrescine import ATP-binding protein

PotA"

/db_xref="COG:COG3842"

/translation="MSFVDLKDIRVSYDGKTNILEELNISMEKGELVSLLGPSGCGKT

TTLRVIAGLIEPNDGNFVLDNEDLTKVPVHKRNFGMVFQSYALFPHLTVRENVAFGLK

LRKESKENIATRVTEMLKVCGLEDFGDRYPKQMSGGQRQRVALARALVIEPKLLLLDE

PLSNLDAKLRINMRMEIKRIQRQLGITSVFVTHDQEECFSISDKVAVMNKGIIEQYDT

PEVIYNNPKTEFVARFIGFENFFQLQKKSEFSYVAENGTIFETTRSSENNKAIGTIRP

DDIEIVHPEQENRTNLLAGEISVRTFLGKSYQYEVKTAIGKLLVNGSDKQVYHSGDRV

QLCLPEHKVVLV"

CDS complement(2826353..2827132)

/gene="ydcV_1"

/locus_tag="JFBMFIFI_02660"

/inference="ab initio prediction:Prodigal:002006"

/inference="similar to AA sequence:UniProtKB:P0AFR9"

/codon_start=1

/transl_table=11

/product="Inner membrane ABC transporter permease protein

YdcV"

/translation="MRKQRGLTLFVGLVFAFLFLPLVLIAITSFGENATIQFPIEGFT

FKWYQNIFVSEAFVDGFLLSLEIGLLATVLALIIGVPAAYALARHGIKGRKWLKSFFL

SPTIVPGIVVGYSLFQFVVIQLQLPVFQGLLLGHFLISLPYIIRVVGSSLEQLDFSIE

EVAWTLGCTRTRAFIRIVLPNITSGIFASFMLAFINSFNNIPVSMFLSGPGVTTLPTS

LLNYIEYNYDPTVSAISVLLMIGTIVLMYIIEKTLGLAAIA"

CDS complement(2827125..2827958)

/gene="potH"

/locus_tag="JFBMFIFI_02661"

/inference="ab initio prediction:Prodigal:002006"

/inference="similar to AA sequence:UniProtKB:P31135"

/codon_start=1

/transl_table=11

/product="Putrescine transport system permease protein

PotH"

/db_xref="COG:COG1176"

/translation="MKKSIPYLILAPGLILLIFFLVMPLVSILLPTVFEGGFSLTAYQ

QFFSDSYNISIFIRTIRVSLIVTLISALCGVPTAYYIARSPKKWRSLLMALTLFPLLT

NSVIRSFAWINILGQNGVINNFLLSIGVIKQPIVMLYTEFSIIVGSIYLFLPIMVMTL

VGVVENIDPEIMEAAETLGANRFSAFMKVILPLSIPGIIVGSVLVFTGTLTAYTTPQL

LGGNKNMLLATFLYQKAMALGDWSGASVIAFVMIVTTIVVMRVFNWIAGKADKRGELN

A"

CDS complement(2828395..2829840)

/gene="rcsC_7"

/locus_tag="JFBMFIFI_02662"

/EC_number="2.7.13.3"

/inference="ab initio prediction:Prodigal:002006"

/inference="protein motif:HAMAP:MF_00979"

/codon_start=1

/transl_table=11

/product="Sensor histidine kinase RcsC"

/translation="MRIRTKNFLFTSGIIIIVVTLAFGLLYWIMPGYYQQKKESELSK

IMEDVAKQIEGKTISETTEILDKIGLETGAVWTLINGEDQLIYPDGQLLSVTQAVNTN

EVVVRDDSSFGNVTESGAAQPLITAMNIQTNDLGNWGDVIQKYKYFVDNEGQVYKLNA

IRTLQPIDEAKGVLLDIYPWILAVSFLIGGIGAFIYSYYSTKRIYQLSMITKQMTEMN

ETTRCPVVGKDEVSTLATDINYLYGNLLTTIEQLNQEIELTAEIERSKAEFMRIASHE

LKTPITAMSGIVEGMLYNVGEFKDRDHYLAICKEILATQSQLVKDILYISRLEMIEGA

SEREIFSVSELVEKEVLPVFELMAKTKKYKLETKIEPVQIEGVREDLKRVLMNLLSNA

LQYTSENGRIVVTLNEAGFALENECEPLTSDDLSKVFAAFYRPDYARARKDGGTGLGL

YIVGQLLERDHLTYSFEPTKQKNGMVFKIKW"

CDS complement(2829837..2830508)

/gene="srrA_6"

/locus_tag="JFBMFIFI_02663"

/inference="ab initio prediction:Prodigal:002006"

/inference="similar to AA sequence:UniProtKB:Q9L524"

/codon_start=1

/transl_table=11

/product="Transcriptional regulatory protein SrrA"

/db_xref="COG:COG0745"

/translation="MQKRILVVEDDEMTNKVITNFLAEQNFTVFSALDGQAGWDIFQK

ETLDLIILDIMLPKIDGLALLAQFREVSQIPIIMLTALDDEYTQVVSYSNLIDDYVTK

PFSPTILVKRVEAVLRRNVQEEENSGKIKYGELTIDLDACTVENQSETVNLTKKEYDI

VTQLISRRGKVVTREQLMDQIWGYDYNPSDRIIDTHIKNIRKKLPGIEIRTVKGTGYA

IEAEL"

CDS complement(2830587..2831849)

/gene="yknZ"

/locus_tag="JFBMFIFI_02664"

/inference="ab initio prediction:Prodigal:002006"

/inference="similar to AA sequence:UniProtKB:O31712"

/codon_start=1

/transl_table=11

/product="putative ABC transporter permease YknZ"

/db_xref="COG:COG0577"

/translation="MLENLSLSLQSIWAHKLRSILTMLGVIIGIAAIISIFSIIEGNT

ANMKKQIMGGPNNTMNVDFGPASQFSGSMGMGGNGNEKKPNYLPIFTAEQMDQVRQTP

GIKNASLTYQKSASIFRGSKSVNSEIKAMDKNYFDMLPQKLTAGRFFTETDYKGQNQV

VVLDKTAYENLFPEEDGIGKIVELNGTPFKVIGVVEDPEANNELSSGGGMMTMAMGGG

ENGKAYVPLTQWPKVAGEINPTPAVIVQGEDTDGLKPAAEKVAMLLNGFIPQSDYVFG

IMNLEDFEKQMDEFNRSEFYLLAGIASISLLVGGIGVMNIMLVSVTERTREIGVKKAL

GARRKVILLQFLTESVTLTFIGGITGILFGLIAGKGITSALNYPYMVSWLAIIGSMAF

CSIIGIVFGLMPAIKASKLDPIEALRYD"

CDS complement(2831833..2832531)

/gene="yknY_3"

/locus_tag="JFBMFIFI_02665"

/EC_number="3.6.3.-"

/inference="ab initio prediction:Prodigal:002006"

/inference="similar to AA sequence:UniProtKB:O31711"

/codon_start=1

/transl_table=11

/product="putative ABC transporter ATP-binding protein

YknY"

/db_xref="COG:COG1136"

/translation="MLKLTNIYKSYWQADSEVEILKNINLSIEAGEYVSIMGPSGSGK

STLMNILGCLDKPTRGDYLIENTDVSSLKDYELSDLRNESIGFVFQNFNLMPRLTALQ

NVELPLVYGKVSKAERKERGIAALKQVGLEERMHFKPAELSGGQKQRVAVARALVTNA

NFILADEPTGALDSKTSEQIMDLFSELNKNGKTIIVITHEQEVADYTKRKIILRDGLI

TKDERSVTAYVGKS"

CDS complement(2832515..2833780)

/gene="yknX"

/locus_tag="JFBMFIFI_02666"

/inference="ab initio prediction:Prodigal:002006"

/inference="similar to AA sequence:UniProtKB:O31710"

/codon_start=1

/transl_table=11

/product="Putative efflux system component YknX"

/db_xref="COG:COG0845"

/translation="MKKKKPIKLISSVVIVLAIIAGSIFYFTRSQSNANSLSQKLIPQ

TVKELSGDGNKGSNFLLTGSVAPNQISKLTLDNSKGTVSEVHVKEGDSVVKGQKLYTY

QNPDGELALKEAQLTVTNQANDLEQKRSDASLKWEQHNKKQADLDKLTTKYNQAGAEE

KEALKQEKTQLETEVAQAKSDARTADSLIGTAEIELEKAQLGVTNAEKKYGSNDALSE

VDGVVKKVDTDQMNKSVSEGNKETFMEITDTSSLYVNGQVDEFNKDQLAIDQPVKIID

RTDEKKVWSGKISKVGNLAAESGGDDEKKEEENPNLSKYPFKVIFDPTDTPPPLGRHM

YVEVLPKGDEANKVKLPLDFVIQEGKESYVWLVKKGKIEKSKIEAGEPDKETNTVEIT

KGLTEEDQIVYPYSTLKAGMEVGSDVKIN"

CDS complement(2833800..2834030)

/locus_tag="JFBMFIFI_02667"

/inference="ab initio prediction:Prodigal:002006"

/codon_start=1

/transl_table=11

/product="hypothetical protein"

/translation="MKKNILASLILVVGMTSLLAGCGGSKKDSEAENMGATVTNGKGV

VTETKEGGTVFKAEDGGEAQFNESPQEAQKAK"

CDS complement(2834186..2835088)

/locus_tag="JFBMFIFI_02668"

/inference="ab initio prediction:Prodigal:002006"

/codon_start=1

/transl_table=11

/product="hypothetical protein"

/translation="MQKQIGDVVKEARKAKRMTQKELANGICTQATVSNLENNNSVPA

VRTLLAIGERLKIDFSDLSEYLPVSENGHRELFYQVKELFKKLKYKEAKDLLLKKIKV

KDLTESIDVKEYNYYMGMASLMGDKNFSDAHYHFNLSMQASTNKNVDILDSLTTNGIA

VAYFMANELDKSKYYFEKSLAQLEEIRKSDVQFSDIAESIKIYYNSAKYYSAVKNYTK

AIELCSAGIKLQKEDGSIHGLELLLYEKGINLCKQGKIEDAAHIFYLAVALCELNGRK

DLIQTIKKDSEEYKIESLVFSYKI"

CDS 2835419..2836003

/locus_tag="JFBMFIFI_02669"

/inference="ab initio prediction:Prodigal:002006"

/codon_start=1

/transl_table=11

/product="hypothetical protein"

/translation="MKQSISDTIISRRTSKTSLDTPISPEEIFTLLEKASYAPYHGKN

EPWVAKIVTTEAEKKWLYERIIASYERNQVIIDEQTRERMTTKMTRLILGAPATILFA

REIFVDNPRKNYDSIEATSALIQNFSLLAWEQDLVGFWATSPFILDSTLANELGFPSN

YELIANYRLGHRDLEQPLRIATRQPIENWASSLL"

CDS 2836317..2837741

/gene="bglF_4"

/locus_tag="JFBMFIFI_02670"

/inference="ab initio prediction:Prodigal:002006"

/inference="similar to AA sequence:UniProtKB:P08722"

/codon_start=1

/transl_table=11

/product="PTS system beta-glucoside-specific EIIBCA

component"

/db_xref="COG:COG1263"

/translation="MAKYTDLATDILDKIGGKENVKSVFHCVTRLRFKLKDESIAKTE

EIKKMDGVVTVMQSGGQYQVVIGTHVPEVFAEVLKVGGLSTDDSMDTSDADDGPKGSL

FDRFIDMISGIFTPILGVLSATGMIKGFVALFVALKWLDSTSGTYIILQATGDALFYS
LPVFLGYTASKKFGGNPFIGMSIGVSMLYPAIAAVMGADMPVLYTLFQGTLLESPIKI
TFLGIPVIMMSYASSVIPIILAVFVSSKVEKFFTKVIPSVVRNFLVPFCTLLVIVPLT
FLVIGPIATWAGMLLGAATGAIYDLSPVVAGLLLGGFWQVFVIFGLHWGLVPIAMNNI
ASGNGDPILAMTLAASFAQIGAVLAVWMKTKNQKLKTLAIPAFISGIFGVTEPAIYGV
TLPLKKPFYASCAAAAIGGGILGFFNTTGYIMGGLGIFAFPSYINPAGLDMGLWGAVI
ACVVGMILGFVFTYVLGFKDEEFA"

CDS 2837814..2839271

/gene="bglH_6"

/locus_tag="JFBMFIFI_02671"

/EC_number="3.2.1.86"

/inference="ab initio prediction:Prodigal:002006"

/inference="similar to AA sequence:UniProtKB:P40740"

/codon_start=1

/transl_table=11

/product="Aryl-phospho-beta-D-glucosidase BglH"

/db_xref="COG:COG2723"

/translation="MSIKTQFPADFLWGGATAANQLEGAFDVDGKGLSTFDMVTFTPK
EERTGPLLSDYTKEQIEDALAGKMGDNFPKRRGIDFYHRYKEDIALFAEMGFKTFRLS
ISWPRIFPNGDETEPNEAGLAFYDKVFDECLKYNIEPLVTLSHYEMPLHLATEYGGWA
NRKVIDFFVHYAETVFKRYKDKVKYWLTFNEINVMAMSPYIGGGILAGTYENHMQAAY
QGTHHQFVASARAVKAGHEIIPDSMIGCMLARMESYAETCNPDDVQRALEEDHANLFY
TDVQVRGYYPSYMDRFFEEKNITIEMEPGDEELLLLHTVDFLSFSYYMSMVQSGDPTK
QQVVGNFMSGLKNPYLESSDWGWQIDPKGLRITLNKMYDRYQVPLFIVENGLGAYDTV
EEDGSIHDSYRIDYLRQHIEQMSEAIKDGVDLIGYTPWGCIDLISASTNEMSKRYGFI
YVDQDDYGNGTLERSRKDSFNWYKQVIASNGTDLA"

CDS complement(2839326..2839886)

/gene="maa_1"

/locus_tag="JFBMFIFI_02672"

/EC_number="2.3.1.79"

/inference="ab initio prediction:Prodigal:002006"

/inference="similar to AA sequence:UniProtKB:P77791"

/codon_start=1

/transl_table=11

/product="Maltose O-acetyltransferase"

/db_xref="COG:COG0110"

/translation="MSLQEQVNSGKPYDDFSTEAVLARDVAKGLTRKYNNKINLEGTY

DLSILTELLGKHGQQCMIESNFRCEFGFNISIGENFFANYDLIILDCNQVTIGNDVLF

GPRVGLYAANHAQNPIARAAGEVYSKPIVIGNKVWLCGDVSVAPGVTIGDNSIIGTGS

VVTKDIPANVVAAGNPCRVIKSIVDE"

CDS complement(2840008..2840568)

/locus_tag="JFBMFIFI_02673"

/inference="ab initio prediction:Prodigal:002006"

/codon_start=1

/transl_table=11

/product="hypothetical protein"

/translation="MKMKKIAFIASVLLLSIGVAACGNGSKDSKDSSSKAASSASSEE

VVTGASISDKPEDIIHGLGPDGNWIFALTGDAKLTEDLVVSGTFYDKNDKSSDVYRKL

ALYTQDADKKVTGEFKLTVPKMIVESPNFKIQNGTIVGDVQVSAEGFELADSTVEGNV

TFDTQELKDSAKLDTGKVTGTVEVAK"

CDS 2841009..2841359

/gene="isdG"

/locus_tag="JFBMFIFI_02674"

/EC_number="1.14.14.18"

/inference="ab initio prediction:Prodigal:002006"

/inference="similar to AA sequence:UniProtKB:Q81L50"

/codon_start=1

/transl_table=11

/product="Heme-degrading monooxygenase"

/db_xref="COG:COG2329"

/translation="MFIVTNTIKIEKEHVDQVVTRFNGGHAQASIAGIEGFLGFELWR

KKDTHPDYSEIVVTSRWDNEANQKAWLKTDGFKKAHGRTADTREQHAHRTGIISNEIA

EFETVLSQEPVLEG"

CDS complement(2841479..2842618)

/locus_tag="JFBMFIFI_02675"

/inference="ab initio prediction:Prodigal:002006"

/codon_start=1

/transl_table=11

/product="hypothetical protein"

/translation="MKITKKMVLSVMSIGLLFISFSHVSAEENPSNVAISAVIPDNQI

DKNKTYFDLLMTPGEEQELEVVLTNSSDEAITMESSVNSAITNDNGVVDYSQAKPRYD

KTLKHPLSSIATFEKEQVIPANSKKTMKIKLKMPETEIKGIIAGGVYLSAKDNEDKAK

NDDSGVQIKNKYVYVVGIQLRTREDISDVQPDLVLDKKLIQPKQVNYRNYLGINLQNK

EPVFIRDLTVEAKIYKKGSNEVFVESKNENLKMAPNSNFNYGVNWNNKEFEAGTYRAK

VTAIAKDYDNKTWNWDEEFTITAKDAKNLNEKAIDLEVEKTPIWIYFAIAGGILLLLI

VIIYLIKRHIDKKKEAQRKAQLRKKRELKKKKEAQKKRVTRKEND"

CDS complement(2842782..2843468)

/locus_tag="JFBMFIFI_02676"

/inference="ab initio prediction:Prodigal:002006"

/codon_start=1

/transl_table=11

/product="hypothetical protein"

/translation="MEQDTQKNVEIQNEELYRKLTKRNEQYMMSLDKALDAANLTEDR

KREIYSEMLPVLVEGQKTGQTARQLYGTVSEQIGKVLDGPRKAEANAPSKDWMIFVDG

GLMMVALMSLVMGATGLIGKSQGGSEMGIATLALNFVGGGLVVLLIAKNTPNRDGSNK

KKGGIFRYILVVGAAMMAWLLIMTFSMAFLPASINIILPPIGYLIVAAVAFAGKMYFK

KTFNVRGGIF"

CDS complement(2843575..2844675)

/gene="ychF"

/locus_tag="JFBMFIFI_02677"

/inference="ab initio prediction:Prodigal:002006"

/inference="similar to AA sequence:UniProtKB:P37518"

/codon_start=1

/transl_table=11

/product="Ribosome-binding ATPase YchF"

/db_xref="COG:COG0012"

/translation="MALTAGIVGLPNVGKSTLFNAITKAGVEAANYPFATIDPNVGMV

EVPDYRLARLTELVTPKKTVPTTFEFTDIAGIVKGASKGEGLGNKFLSHIRQVDAICH

VVRCFDDENITHVDGRIDPIADIETINLELVLADLESVEKRHARVGKLARTKDKDALA

EFAVLEKIKPVLEEGKSARTIEFNEEEAKIVKSLFLLTIKPVLYVANVSEDEIGDAEE
NEYVKQVREFAATENSGVNVICARIEEEIAELDDDEKSEFLTELGIKESGLDQLIRSA
YDLLGLATYFTAGVQEVRAWTFVKGIKAPQAAGIIHSDFEHGFIRAETVSFADLDKYE
NMAAAKEAGRVRLEGKEYIVQDGDVMLFRFNV"

CDS complement(2844708..2844902)

/locus_tag="JFBMFIFI_02678"
/inference="ab initio prediction:Prodigal:002006"
/codon_start=1
/transl_table=11
/product="hypothetical protein"
/translation="MKMYQLNDSVEMKKPHPCGTNRWLIIRMGMDIRIKCEKCGHMVL
MPRREFEKKMKKVLQTADTK"

CDS complement(2844930..2845823)

/gene="spo0J"
/locus_tag="JFBMFIFI_02679"
/inference="ab initio prediction:Prodigal:002006"
/inference="similar to AA sequence:UniProtKB:P26497"
/codon_start=1
/transl_table=11
/product="Stage 0 sporulation protein J"
/db_xref="COG:COG1475"
/translation="MANKNSKGLGRGIDALFGDYSELDEIDVTNEMVKEIPLEEIRPN
PYQPRKTFDEEALNELADSIRQSGVFQPIIVRESTIKGYEIIAGERRFRASKLAGKAT
VPAIIRAFDEERMMEVAVLENLQREDLTSLEEAEAYDMLMKKLKLTQEEVAKRLGKSR
PYIANYLRLLGLPESVKKMLQSDEISMGQARTLLGLKDKRMIIKIANKVVRDNLTVRQ
LEQLVNQLNQPKEVEQKSNKAEKKPYYIRESEDRLMDKFGTAVQISDKGNKGKIEIEY
LSTDDLTRILDILEIQFDDEE"

CDS complement(2845813..2846577)

/gene="soj"
/locus_tag="JFBMFIFI_02680"
/EC_number="3.6.-.-"
/inference="ab initio prediction:Prodigal:002006"
/inference="similar to AA sequence:UniProtKB:P37522"

/codon_start=1

/transl_table=11

/product="Sporulation initiation inhibitor protein Soj"

/db_xref="COG:COG1192"

/translation="MARIISVANQKGGVGKTTTTVNLGACLAYYGKKILLVDIDAQGN

ATSGLGVRKADVEKDIYDILVNETPVDEVVLETSRENLWIVPATIQLAGAEIELTTQM

ARESRLKQALAKVSDDYDYILIDCPPSLGHLTINAFTASDSILIPVQCEYYALEGLSQ

LLNTVRLVQKHFNPDLKIEGVLLTMLDARTNLGYEVVDEVKKYFRERVYKTIIPRNIR

LSEAPSHGLSIIDYDARSRGSEVYLELAKEVLAHGE"

CDS complement(2846737..2847645)

/gene="noc"

/locus_tag="JFBMFIFI_02681"

/inference="ab initio prediction:Prodigal:002006"

/inference="similar to AA sequence:UniProtKB:P37524"

/codon_start=1

/transl_table=11

/product="Nucleoid occlusion protein"

/db_xref="COG:COG1475"

/translation="MALSDLFGSGKSKKKTEEKLDKVVPEVVNDTIVNEEAHQTIQRI

EAKQIIPNRFQPRKIFNEEKLNELARTIHIHGLIQPIVIREYAPNEYEIIAGERRFRA

MMLLEWTEVPAIVQEMTDTETASVALIENLQREELTSIEEAKAYQGLLELNGITQEAL

AQRIGKSQSFVANKLRLLKLSTPAQQALLTQEISERHGRSLLALDEENQGIVVKTIIS

EKLTVKETEELVKKIIAEKAGPEQPKKKKRKKGISKDVRLALNTIKKSITMVTDTGID

MKTEEEDLEDVYRITIEIPKSKTNDK"

CDS complement(2847734..2848450)

/gene="rsmG"

/locus_tag="JFBMFIFI_02682"

/EC_number="2.1.1.-"

/inference="ab initio prediction:Prodigal:002006"

/inference="similar to AA sequence:UniProtKB:P25813"

/codon_start=1

/transl_table=11

/product="Ribosomal RNA small subunit methyltransferase G"

/db_xref="COG:COG0357"

/translation="MNPEEFKEALLEKGIPLTDKQMHQFDQYLKLLQEWNEKINLTAI

TEEEEVYLKHFYDSILAGLYVDFKTGVQSLCDVGAGAGFPSIPLKIVFPSLSVTIVDS

LNKRIQFLNTLANELELEDVHFYHDRAETFGQNKQFRESFDFVTARAVAKMSVLTELC

LPLVKKGGLFIALKASNSDEEMKEGDRAIRLLGGKVREDIVCQLPREAGERHIILIDK

KKETPKTYPRKPGTPNRKPL"

CDS complement(2848698..2849471)

/locus_tag="JFBMFIFI_02683"

/inference="ab initio prediction:Prodigal:002006"

/codon_start=1

/transl_table=11

/product="hypothetical protein"

/translation="MDTKMKAKIKKSLALIEKEHDIKILYAVESGSRAWGFPSEDSDY

DVRFIYVHTKEWYLSIAPKRDVIEYPIDDELDISGWDLQKALKLLRKSNPSLIEWLNS

SIIYLETGDLASQLRTLAQTHVSQKQLMYHYYHMARGNFRDYLQGEHVKIKKYFYVLR

PVLACLWLEKYQEAPPVLFDQLLTLPDLDSELIAEVKALLIRKKRGDELDLENQNPIL

NQFIEEQLTQLEVILKSEKDQPSMLSYALLDELFTKYML"

CDS complement(2849600..2850769)

/gene="yycB"

/locus_tag="JFBMFIFI_02684"

/inference="ab initio prediction:Prodigal:002006"

/inference="similar to AA sequence:UniProtKB:P37482"

/codon_start=1

/transl_table=11

/product="putative transporter YycB"

/db_xref="COG:COG2807"

/translation="MQKNERRLVGLIFVVAFNLRVGISSVPALLELIKKDLGISNFQS

SLITSIPVICMGVFALTVPYFQRTLGRRRTIFFSLLILGLGTLARFFVTSYNLFLVTA

FMIGFGIAIIGPLLSGFIKSNFPFRSGLMIGVYSLSMGLGASLSSGLVLPLTEHFKGN

WGVSLALWGVFALIAALIWQMFSPVESQRKSAKSTSFSLPLKNKKAWKYTLFFGIQSG

IFYGMTTWLALTALEKGATTLQSGYFLTLYTVIQMLCSFIIPTLMDQFGKMKQWMFFS

SGLVFIGAGMIALAPSVVFFVLGIILAAIGLGGLFPIAVLLPIRNTKTAEETSLWTSM

IQSFGYILGGFMPVLMGAVKDTTGSTIAPFLIMVLLSSVLLILSFSEKNEPSIDL"

CDS complement(2850756..2851397)

/gene="lutR"

/locus_tag="JFBMFIFI_02685"

/inference="ab initio prediction:Prodigal:002006"

/inference="similar to AA sequence:UniProtKB:O07007"

/codon_start=1

/transl_table=11

/product="HTH-type transcriptional regulator LutR"

/db_xref="COG:COG2186"

/translation="MSNRNLVNQTIQKLENLLGEGTYQVGDKLPVEKVLAEECGVGRS

TLREAVKILEHAGVLTVKQGSGTYVNQHRVATYSTEQLLEIREMLEVQSGKLATSRFT

TEELMELKEVLFERNEALEKGQFAHYVEADLNFHWLIVKASRNPFLIRWYQEILPDLK

QTLSSFVLRTDNYSDNTELHQVIFQALVEQNEATVVAATLENNQRELVRYAKK"

CDS complement(2851614..2852150)

/locus_tag="JFBMFIFI_02686"

/inference="ab initio prediction:Prodigal:002006"

/codon_start=1

/transl_table=11

/product="hypothetical protein"

/translation="MEIRTIETEDYAEIKVLIQKAFENEEHSDGDEQLLVERIRKEKS

YQKEFEVIALINSEIVGHGLLSPVIIEQKESSLIEGAVMALAPLAVKKEYQGKGIGRA

IIEELENRAKTAGYQGLSILGDPGYYQRFGYFPAKIKQIEAPFEIESNYFLIKELTEN

SLENCQGIVRYLPAFGLN"

CDS 2852307..2852945

/locus_tag="JFBMFIFI_02687"

/inference="ab initio prediction:Prodigal:002006"

/codon_start=1

/transl_table=11

/product="hypothetical protein"

/translation="MNKKIVLGMISIATVALLGACGNGNTKETSTNNNNQATSSSQVA

SSTTESSPSTTSTSSLLNQEFKVSLDDAIQLFSKEHPDVAITSIEFDTNFGAYGYKIE

GVDDAKEYELFIDADTKEISKQKEERLDSEDANGAARKNDALLLDNIISPQKAMEAAF

SEANKQGDITEWKLSQEVQKTFYEVTIKNGSNETEVKIDSVSGDILQTEQDD"

CDS 2853213..2856557

/locus_tag="JFBMFIFI_02688"

/inference="ab initio prediction:Prodigal:002006"

/codon_start=1

/transl_table=11

/product="hypothetical protein"

/translation="MKKFNCFLSKLLIVGLVSSSISSFTPPVLANETVVTTEETTSLP

DESTTSPEVSAPKEEAITVPPAETPITPPIPETEQEPLKQDVATPTITDAPTIADPQP

EAAPEKKTIPLQLLGINDFHGALSTTGTAYIEGTAFSKAGKAALLATYLNNAQTNFQS

EKPDGTTIRVQAGDMVGASPANSGLLQDEPTIKVLNKMNFEIGTLGNHEFDEGLGEFN

RIMTGQAPLPGEFNQITEDYTHEASTQDIVIANMTDKNGNIPYGWEPYTIKTVKDGDK

EVKVGFIGIVTTEIPNLVLKQHYQDYNFLDEAETIAKYDKILQDQGVNAIVVLAHVPA

VSTNQVVSGEAADIMDKVNTARPDNSVDAFFAGHNHQYTNGVVGKTRIVQSTSQGKAF

INLKAELDPETNDFVAVPDAEVTPVQAGVTEDPEVKAIIDDADTRVTKVTSEKIGTAT

TNQTISREVEKDSPNKESAVGNLVTDGQRVMAQKQGFNVDFAMTNNGGIRADLLVQED

RSITWGAAQNVQPFGNILQVVHMTGQQIENVLNEQYDEEGKYFLQLSGLTYQYTDTGD

ANQPYKVHQIRKTNGELLDPNASYLVVVNDFLFGGGDGFKTFTEATFEATMTQDTDTF

IDYIKAQEADGKTIEAQIEGRKSYISPEQIKAEEEAAAIASIKAATVLPDIKEGITTF

TGKTIPNGTVTVQLKDSKARTILSTTANEKGDFTLDLQSLNLAKDHVVTFVVTDANGY

SAAFDKTVLAKEENGESAAIEAIKAATVLPDIKEGVTTFTGKTIPNGTVTAQLKNSKA

RAILSTTANEKGDFALDLQSLNLAKDHVVTFVVTDANGYSVAFDKTVLAKEENGEAAA

IEAIKTATVLPDIKEGITTFSGKTIPNGTVTAQLKNSKARAILSTTANEKGDFALDLQ

SLNLAKDHVVTFVVTDANGYSAAFDKTVLAKEENGEAAAIAAIKAATVLPDIKEGITT

FTGKTIPNGTVTAQLKNSKARTILSTTANEKGDFTLDLQSLNLAKDHVVTFVVTDANG

YSVAFDKTVLAKDAVTNGNGTTTGNGNNGTLTGTTNNPLSPPTVAASFPQTGENESSV

STLAGLILLGSAAFYLTKKNKHKSIA"

CDS 2856814..2857410

/locus_tag="JFBMFIFI_02689"

/inference="ab initio prediction:Prodigal:002006"

/codon_start=1

/transl_table=11

/product="hypothetical protein"

/translation="MTTLPNIGKPATNALQNIGITDLEQLTHYSEKTLLQIHGVGPKA

VRILKETLESQNLSFKNTELPKVNFAVLCTLNCENAPKKKIILDYLIAAAAANQAVLE
TLLVDTLSWIKPGESQLEGKTPFIKAVINHRQDLSSLEIQSILTHGREASAHGIITDK
KGNKTYFSDIFLFSSSKKEAKIKQITSFVKTEKNYHTN"

CDS 2857581..2859089

/locus_tag="JFBMFIFI_02690"

/inference="ab initio prediction:Prodigal:002006"

/codon_start=1

/transl_table=11

/product="hypothetical protein"

/translation="MELDSLLSTSTLREITLIKLLNQRYPNWISKEEISSQLYISNRT
LKSTVIVINSFFKEKNYEQYIETDAALGYKLSASTNAISNLIYLERLKSSTSFNLLLS
IYNKKFISSKHFTDYYFISLTSFYKDVRTINLILDKFGIQFNSREGTLSGESSQIRYF
FCKFFWFSYGMSEWPFQQVDEGETMEMSKKIGAQINPYLSPIQTRQLAFALSVCLNDL
NTKKFIEPTFKTLESDNLLYQEIQDTIHQKYYFFKKNILKHEVNFIYSFIVSNPIFIQ
TEKMFSFIKTSFQEKDSANFAYSNQLLTELINNSSHLMDVNFLDKQNFLAHILCLNFQ
MDTFHDTHKYIEDLQLDELKQLNPDLFNGYLLIFSAIEKQGIYPTYPIETRSYRLKAY
FFFLFSLSDSAVEINQISIQVLSDKSIYRKILKNDLIKNIPYTIDFTDNNDQNNGPDI
IVTDTPTFFNQLDIPIFYISFPPTSNEWLELNSLVEQEIQKKRTLQNFRPFN"

CDS complement(2859146..2861194)

/locus_tag="JFBMFIFI_02691"

/inference="ab initio prediction:Prodigal:002006"

/codon_start=1

/transl_table=11

/product="hypothetical protein"

/translation="MKKRVRVLMLFILFFSAVNPAYATVTSTIETSGIEEISGTQEQE
SAENTAPDKLVDPTTEAPELQEDSATKESEPTPTVTPISEAETNDPAATLKNEDPVVR
AVVDYGSQFFNDVSFSDMAGNTLAKVKDTDKIKVNYTFDITTPVKEGETMSIALPEEL
KLVNYTEFPLKDNDGNTIALVTTDKNTGLITLIFTDYVNGKSAISGELFFWAKLDKTK
VADGNNTISMPVNGVTTDLTLKVSKTTDSTSGTTNPTVLFKSGAYDKNDPTLINWTIT
LNNANQNLFQPTIIDTIGPGQTLVKTSFVVNYRDAAKKSKMKYALPTTLTNDRTEVSF
TDAGFDLKLENLGSFSIINHYSSAVITYKTKVDNSGTQYINSASSADENNDSQTRNAT
VKFYGSGGEASGSVNEAIDEISNTIDEAESIDPNELTPSENTQLEESKNTAQTTLDNE
DATIEELDKAKENLGEAIQEVTPAKPEENQEVKEAIEKLKEILVVIEGKAATDYTQES

WDVLEEVKEKAEVIVGKGTDDSHSVTVEEVTEIQKQLEEGLTQLELIPTPETILELEN
LVTEAENKQKEEYTNETWVPFEESLELAKESLAEFKNDPQSVKNSDMEQITIQLEQAM
SQLKKVESQAVELPIEPSENPHEQQGQFPATGEKHSYLIGLLGILVSTGSILIYRRRV
"

tRNA complement(2861469..2861543)
/locus_tag="JFBMFIFI_02692"
/product="tRNA-Val"
/inference="COORDINATES:profile:Aragorn:001002"
/note="tRNA-Val(gac)"

CDS complement(2861655..2864297)
/locus_tag="JFBMFIFI_02693"
/EC_number="7.2.2.10"
/inference="ab initio prediction:Prodigal:002006"
/inference="similar to AA sequence:UniProtKB:Q73E41"
/codon_start=1
/transl_table=11
/product="Calcium-transporting ATPase 1"
/translation="MNKTFVGLTKEEVEESRQKHGSNALTEKESETFFDKLKGNFDDP
IIKILLVALVLNLIFAFLGYAEWFEAFGIAIAVLLATLIGTWSEFSNETAFQKLQEDA
SKITVKVFRDGHVTEISIDDIVQGDEIILQTGDKIPADGILIDGSLRVDQASLNGESD
EAKKETLPEGATYEDGDTFNKHALFRGAVITSGEAVMKVTKVGDASFFGQMAAELTVE
ERESPLKLKLADLANKISKAGYTAGILIFIAFMINKIFVANNFNGAEIAKYFSDIGPV
GNDVVEAFILAVIVIVVAVPEGLPMMIAMVLSQNMKKMLRDNILVRKLEGIETSGSLN
MLFSDKTGTITKGQLDVVSYINGANTSFDKLGAIPEELKKIVSTTILNNTNAVISEDD
QGAVVIIGGNPTEKAVLAFVEKDEKSFGTLEHVSAIPFSSARKFSATQVAGEWNTTLI
KGAPEKILPNCSTYYDEQGQKQPFTKLAELDNEINELAKKSIRVLILATTDEIVTEES
LPSDYTLVGILAIRDDVRPEAVEAIHDVQQAGVQVVMITGDRKETAIAIAKDAKILQS
PDDIVLTSQELNDMPDEEVKSLLPNMRVIARALPTDKSRLVRLSQEIGLVVGMTGDGV
NDSPALKRADVGFAMGSGTEVAKEAGDIVILDDNFKSIAQSILFGRTIYRSIQKFIVF
QLTLNVGALLISFIAPFIGIDHPLTITQMLWVNLVMDTLAALAFGAEPTLKRYMKEKP
KRRDENIVTRSMFSAFAFQGVVITIMGLWMLKGGFLNQFFSSELALTTGFFSFFILYA
VFNGFNVRTEKLNIFDNLNRNKMFMRIMLLIIVVQIIMTFVGGPILRTTALSVSEWAI
VLGLSVLAIPIDMARKLVVKAMGK"

CDS complement(2864535..2865116)

/gene="yceD_1"

/locus_tag="JFBMFIFI_02694"

/inference="ab initio prediction:Prodigal:002006"

/inference="similar to AA sequence:UniProtKB:P80875"

/codon_start=1

/transl_table=11

/product="General stress protein 16U"

/db_xref="COG:COG2310"

/translation="MAINLSKGQKIDLTKTNPGLTKTIIGLGWDTNQYDGGAAFDLDA

SAFLLDSTGKCLNEKNFIFYNNLTSEDGSVVHTGDNRTGEGDGDDEQIKIDLSKISPA

VDRIAITVTIDDAVARNQNFGQVSNAFVHILNEETGEEILNFDLGEDFSVETAVVFCE

LYRHNGEWKFNAIGSGYQGGLAALVQAYGLDAG"

CDS complement(2865220..2865801)

/gene="yceD_2"

/locus_tag="JFBMFIFI_02695"

/inference="ab initio prediction:Prodigal:002006"

/inference="similar to AA sequence:UniProtKB:P80875"

/codon_start=1

/transl_table=11

/product="General stress protein 16U"

/db_xref="COG:COG2310"

/translation="MAVSLAKGQKVDLTKNNPGLKTVRVGLGWDTNKYDGQGDFDLDA

EVFLLAANEKVKSDADFIFYNQPTSPDGAVQHLGDNRTGEGDGDDESVTVELGKVDSA

IEKIRFTVTIDSADERNQNFGQVSNAFIRILNADNEEELIRYDLGEDFSIETAIVVGE

LYRHNGEWKFTAIGSGYQGGLAALGRDFGVNIG"

CDS complement(2865825..2866427)

/gene="yceC"

/locus_tag="JFBMFIFI_02696"

/inference="ab initio prediction:Prodigal:002006"

/inference="similar to AA sequence:UniProtKB:P81100"

/codon_start=1

/transl_table=11

/product="Stress response protein SCP2"
/db_xref="COG:COG2310"
/translation="MAINLSKGQKIDLTKSNPTLNNITVGLGWDEAAQSGGGGLLGGL
FGSKPAAIDCDASVILLQGDRFVDKKDVVYFGHLKSEDGSIAHQGDNLTGEGDGDDEV
VKVSLNKVSPKYNKIVFVVNIYQAQKRKQDFGMIKNAFIRIMDDSGVEIARYNLTDNY
QGLTGLVAGELYRHEGEWKFAAVGSGMNVASLSEMLQTYK"

CDS 2866676..2868301
/locus_tag="JFBMFIFI_02697"
/inference="ab initio prediction:Prodigal:002006"
/codon_start=1
/transl_table=11
/product="hypothetical protein"
/translation="MQELRPIPQDLNKIDWQMEFVLPISLRSGFSKTEDSMTYPQVAG
AVLGVPMDEFSYSEWLYDIVYQDNSTIHLLAGNLDKQIDNQIFQGIQKLFNIHNEKKG
LSPNRFVAFMQGNNLLPLRDHPDYYRYLTVKFIDLIKIFEENHGDLLHADFRRVVVDC
IKWGFNYIDKWLQEIDLATEVPKVIWYGNATKSEQYFLCLLYLLGFDVLVFNSEKKNI
FKDIKGLSISTFEYPSEMPLEPFPTEKPTRKSTTARKASMELNNIINADGSLLLKPFQ
LRDYLPESLTLQTTYDEIRIIGKEKAFVRPNFKVKKGAVEIPVLFSKVLGVSVDRGKY
WTDFYEVKQSTPLVEVFKTFPIKPEIKGNQQFHYQFALENGTLNPEKIITANWWMHKE
VSKGMQLALANGISRYVEKAHFLPQGKETKADVQMYLFSQAMTIPPLITKLLQQFDYA
QDIPKIMIYNDGNNGTLTRSDAALLLLLNEIGFDVLLYNPTGQNDIELYIDESKFDSH
WLEEVSFTEVFQTSSMAAKGNNAIKSIFNKFLK"

CDS 2868320..2869423
/locus_tag="JFBMFIFI_02698"
/inference="ab initio prediction:Prodigal:002006"
/inference="similar to AA sequence:UniProtKB:P60108"
/codon_start=1
/transl_table=11
/product="TelA-like protein"
/translation="MENQNETGLEEVQIVSVEKRISQAEAQKAELKTAPDVINISKNI
DIKDQISILEFGKEPAEGISQFSGRILNNMSVAETQKSTVMLNQLGKIMDKFEPEDFA
EEKKGFFGKIFGKGKDIMAQIMAKYQTMGGEIEVVYTEIKKYEVKMKNNVAMLNQLAD
ENIKYYFELEKYVAAGELIAEDLRNNKIPQLQERVNNGDQMAGIELSKTSAALEMVEQ

RIYDLEMAKQVSLQTGPQIDMLQKGNVRLIGKINSAFVITIPVFKSALIQAVAIKQQK

MTAEAMTELDNRTNEMLLKNAQNISQNSIEIAKMSGSPSIKIETMEQTWSTIMNGLEE

TKQIEAQNIVEREAGKKRLLELQKDYEAKKHTM"

CDS complement(2869804..2870565)

/locus_tag="JFBMFIFI_02699"

/inference="ab initio prediction:Prodigal:002006"

/codon_start=1

/transl_table=11

/product="hypothetical protein"

/translation="MKKVLFTDLDGTILFSKNSLPKELKIENCVVAETYRNGHFGYME

KELVAFLQEWTQEQLFIPVTTRSTEQYERLADCFSTFNLPFALASNGGNLYRYGRLDL

EWQAATKQELRKELNQKELVLELIQKMIPATSIRKIKDSDELYYCVLTIERIWEEKFI

LEVNKELAKWGWCAYFQHKKLYFLPKALSKEHGITRLKEELAESAHYLAMGDTEMDYG

MLKQQDQYLYFSDFSEKNLETTTYSLEKIHMLLEG"

CDS complement(2870562..2871722)

/gene="stiP"

/locus_tag="JFBMFIFI_02700"

/EC_number="3.4.22.-"

/inference="ab initio prediction:Prodigal:002006"

/inference="similar to AA sequence:UniProtKB:Q6FAX7"

/codon_start=1

/transl_table=11

/product="Cysteine protease StiP"

/translation="MKPFIKGSYSDEDVIFLLQDISPYVAEIATPEREVLIQNGTHYS

EMLPVEYQPTAEYIALFKETLQESADRIALSVGIVAEQIIASKRKYYQGDLGKTLENL

VLVSLARAGTPIGVLIKRYLKLVYQVDVPHYSISIIRGRGIDEVALDYIYRQSEQADI

QFIDGWTGKGAITKELTEACVKYNQEHPKLVVPLSDSLAVLADTGYCTTLYGTREDFL

IPSACLNSTVSGLVSRTVLNEQFIKPGEVHGAKYYKEFEALDLSNEYIETIMEAYLVN

KVEMDREIAEAMVNWLPEEEPSWAGMKAIETIKADFGYESIHFIKPGVGETTRVLLRR

VPEKLLINPEKTNDLRHILLLAKELNVPIEEYTKMPYSCCGLIKQKVGEKEI"

CDS complement(2871723..2873108)

/locus_tag="JFBMFIFI_02701"

/inference="ab initio prediction:Prodigal:002006"

/codon_start=1

/transl_table=11

/product="hypothetical protein"

/translation="MTEIKEYQLTEELKVQVSIIKNPLQIEPDALFQLAARINKKRSF

LFVSPVLGKHLPVNPGIPLLIGRALALTYADKMDDQKELIQATLNTEFGSQNFEFNLA

QLPKVELVKPVTIIGFCETATALGQAFYASFSSPAEYIHTTREILGADITPLLAFEEE

HSHATSHKVYAENQDFFQNENEIILVDDEVTTGKTNLNIIRDIIKEFPHKKEFTIVSI

LDWRNESDQKAYQQLEKELGITIKEVSLLKGEVTELGVSSSLFEEEISNEEEQDIELC

ELPIFQKEIEFSKILITSEDNFGNESKLPFLSSTGRFGLTNKIDTLTSQAKILGAKLK

SLREAGECLVLGTGEFMYVPLRIATFMGADVFFQATTRSPILSRNDVSGYPIFHKRQF

KSIEQPVADNYVYRIPKNHYRDIFVMVERVVDEANLEGLIKQLTSDQTNVYVIDFSRN

RIYQSRKTGGN"

CDS complement(2873111..2874340)

/locus_tag="JFBMFIFI_02702"

/inference="ab initio prediction:Prodigal:002006"

/codon_start=1

/transl_table=11

/product="hypothetical protein"

/translation="MQHFNFLTQEEENQIFYKKPQPINIQQDRSLAMISASEKARIAN

SLGAVLYMPGTREDIAQIITKSRYSATTYILCLEDSIGDVSLEIAEENLLNQLRQIQE

YKKTQSQREQDKLPMIFIRIRTPQHLADFGEKLGTLANEVNGFALPKADNRNLEDYLV

TLIQVAEKADTWLWAMPILESKELMVRQTRESELNKIYALLTQPKFEQVILNIRLGAT

DMCGLFGLRRSIDSTIYDVSVMRDFMTDVLNYFSLDCRFVVSGCVWEFFSKRPRVMKP

LLRKTPFATKLGEDGQILREELISDANDGLIREVLLDKLNGISGKTCIHPTHIVPVNS

QYVISKEEYYDAKRILDSTNGQVGVFKSEKGNKMNEVKPHLLWANQVMRRSEIYGVFN

EQKDFVDLLIAEYLSED"

CDS 2874745..2875593

/gene="gpmA_3"

/locus_tag="JFBMFIFI_02703"

/EC_number="5.4.2.11"

/inference="ab initio prediction:Prodigal:002006"

/inference="protein motif:HAMAP:MF_01039"

/codon_start=1

/transl_table=11

/product="2,3-bisphosphoglycerate-dependent

phosphoglycerate mutase"

/translation="MMKKHWGKLAIVAFGALLMVGCGTNNTAADKKESSSASSEVATT

KDGEVTFYVVRHGKTMLNTTDRVQGWSDAVLTPAGEEVVGFAGKGLEDVEFKTAYSSD

SGRAMQTARIILDENKKSSKVALQTDERFREFNFGTYEGDLNETMWTDIAQSQGKTLE

EWQSAGISPKDFANSVAALDKEKVEEGINWPAEDYATISARLKAGITELAEKESKKGN

HNVLLVSHGLSMGALVDTLFDDFKLPDGGFKNASVTTITYKDGEFKLGDINDLSYIEA

GEKASK"

CDS complement(2875641..2876714)

/locus_tag="JFBMFIFI_02704"

/inference="ab initio prediction:Prodigal:002006"

/codon_start=1

/transl_table=11

/product="hypothetical protein"

/translation="MKKIVVIGGYGHVGGQVSRQLAKRYSTVVVAGRSEEKALKFSRN

SSEKLSYLSVDLTNEDDWDFLQNTDLVIICIDQTNTLFLERCLELGVDYLDISANYAF

FQQLMYLDKEKHNSTAILGVGLAPGLTNLVTKESLKLMPHSEKIDISILLGLGDTHGK

EAMSWTIKNMNTRYTLADSTLVTSFTGGKKIRFSGDRRLRKAYFFPFPDQLTLPSSLA

IPIVKTRLVFDSRSTTGIFAALTKLNLIRFSEKKRVKSLVLALFQKIKIGQPTYQIKI

DAYKKTEHVGLFLKGKNEAQITADMAIATAVLLLEGSHSKGIYQIDELFYLKEQDNNL

ILVVEQSDNQIQINQEPIEYGRL"

CDS 2876847..2877899

/gene="malT"

/locus_tag="JFBMFIFI_02705"

/inference="ab initio prediction:Prodigal:002006"

/inference="protein motif:HAMAP:MF_01247"

/codon_start=1

/transl_table=11

/product="HTH-type transcriptional regulator MalT"

/translation="MNETMLKKQLQKIALTSQDLVSYQKSISLFLKKESLFSSICFHL

IDSETLLTRQATTDKKIQAIHPQLMSYEYLDESVHTYRQMLKTQTDISILSQLANPIL

IDNKKFTEILAPANIADEMRIALIYQDICWGFLTLFKATDDGFFTKAEADLLTYIAPF

IAAYLGKSTQFQLTNQPLENQELASFIMLNKQLELVGYTQSGESWLHRLQVELQLSSN

QIPIPLQNLATRLFFSKEPSEKLLLSLDDGELVSVVASRYVIPLDSPKIQQVSITFQE

PSLSEKINYQMLQKQLTAQEQKVVGYILSGLSNKLIASELAISVYTVQDHIKSIFTKL

AINSRNELLPQLIQKK"

CDS complement(2877945..2878796)

/gene="mshD"

/locus_tag="JFBMFIFI_02706"

/EC_number="2.3.1.189"

/inference="ab initio prediction:Prodigal:002006"

/inference="protein motif:HAMAP:MF_01698"

/codon_start=1

/transl_table=11

/product="Mycothiol acetyltransferase"

/translation="MVTEINQVSTLKNPEISEVNQLITTLQKKKNLSFKLNKGMIAEE

NPYVNHILYRNQGVLLGYMLLSAYDPQELEVTTVFDGTQQEFMLMYTCLKQYGKEKNN

KNCLFIVDQEDTLLTSIIKELEIPYAFSEYHMRFNQDHLTTFNKAKSLELLDAQESDR

EIIVRLNEERFDNQEITTKELMEVELEGIQLARVNGEIVGKLKVDNYQGEVGIYGFMI

APHLRGQGVGRATLEAVITTIYKNKIDKLYLEVESTNARALHLYQSMGFRIEAKFDYY

DEKYSIT"

CDS complement(2878944..2879315)

/gene="fra"

/locus_tag="JFBMFIFI_02707"

/inference="ab initio prediction:Prodigal:002006"

/inference="similar to AA sequence:UniProtKB:Q797E6"

/codon_start=1

/transl_table=11

/product="Intracellular iron chaperone frataxin"

/db_xref="COG:COG5646"

/translation="MDTFTEFIAEIEQPEQRARVEEVLTWATTKFPTLEKKIAWNQPM

LTDHGTFIIGFSVSKKHLAVAPEALGIEVFSDQIKEAGYGQTKNLMQIPWESPIDYGL

LERMIEFNISDKANYSSFWRK"

CDS complement(2879331..2879732)

/locus_tag="JFBMFIFI_02708"

/inference="ab initio prediction:Prodigal:002006"

/codon_start=1

/transl_table=11

/product="hypothetical protein"

/translation="MAELVLDFQFTSSINQVWTALTDSDMLAKWMWKNDFKPVVGYKF

TYRAEPNEWWDGIVEGQVLEVDEPHSLSYTWGTGPEVHTVVWTLTALEDGGTQLHLDQ

TGISEQPGALEGAKYGQMELVGKLEKLLTEA"

CDS 2879945..2880748

/locus_tag="JFBMFIFI_02709"

/inference="ab initio prediction:Prodigal:002006"

/codon_start=1

/transl_table=11

/product="hypothetical protein"

/translation="MELKLDVLISEMQTTIQLNQQEGTIKIVKAQEIPKTEHTLYFLL

KNNEFVASGTTNFPANLKDVEFNQVMTITPPWEIEPEHLEQLFLAQASDKGVNLVSLS

NPEGQVPTNAKKTVNAYLETLFFILEKFGYQIKPKTDLPKSKVKPAKARHRWTKEVSQ

IEFFIDTRESTATALWVKRNEMLLKAGAKMMPTVPLNKDGSVGFSARLGEKLRYDYSD

KIKDFTTTEDLTFKSVNEIGIFLYFGGTNSWLEMLDANGKSINEWTVVE"

CDS complement(2880808..2881032)

/locus_tag="JFBMFIFI_02710"

/inference="ab initio prediction:Prodigal:002006"

/codon_start=1

/transl_table=11

/product="hypothetical protein"

/translation="MRIEKNWDDCFVYTINLEIPATADRKNWFINRIIVLKNELDLSE

MKDFIKQTFGPEVILVHADLWDEGLLIKEK"

CDS complement(2881078..2882184)

/locus_tag="JFBMFIFI_02711"

/inference="ab initio prediction:Prodigal:002006"

/codon_start=1

/transl_table=11

/product="hypothetical protein"

/translation="MLHVKRKLIIGLIFLLSLVSVIPQANASQLKFSVEPVIPENQKD

TSHSYFDLIMKSSEQQTLKVHMRNDTDNEVTVIPSVHAATTNINGVVEYGESNTKLDK

TSPYNIEDIVKPTVEEVKIPAKGSTDLELKIEMPKDGFDGILAGGITLKEKDATEESE

KKNAQQGLAIENKYAYVVAVVLQETDVKIDSELKLGAVEPNQVNARNVINANLQNTQA

KYLNQLSVDTKITKKGESEVLYTSKKEDMQMAPNTSFAYPVSLNGEKLKAGDYTLVMT

AKSMDKTWKFKKDFTIKADVAKELNEKDVTVKKDYTWLYIVIGIVLILSALVLIWFIM

RKKKQEKQKQAELRRRQAKRKKRAQRAKIEKNND"

CDS complement(2882512..2883297)

/locus_tag="JFBMFIFI_02712"

/inference="ab initio prediction:Prodigal:002006"

/codon_start=1

/transl_table=11

/product="hypothetical protein"

/translation="MKSLKLATVGTLLFSTILISGVGSVSAAPGPIPANRDTNAQVKF

IEDESPTDPTDPTDPDPEKPVDPVDPTDPEKPVDPGTNGPLSLDYASSLDFGEQLIST

KNQTYFAKPQYLRGEDGKADLNNPVPNYAQVTDKRGGEKGWSLSVKQNGQFKSVKEGK

ELVGAEVTFKNGQVASVSNSKIPSVVKTSFSLKNDGTGVAENIMSAAAGEGFGTWVYR

AGDVSNMAESIALSVPGSSVKDADTYKTTLTWTLTDTPANSGE"

CDS complement(2883335..2884132)

/locus_tag="JFBMFIFI_02713"

/inference="ab initio prediction:Prodigal:002006"

/codon_start=1

/transl_table=11

/product="hypothetical protein"

/translation="MKLIKYGTTAAMLIGSLATTALPALAAEEDYTRTYQSGGMVEFV

PDEGPTDPVDPVDPDPNKPVNPWDPTTPEHKPNPGTDGPLSIDFASSIDFGKNKITNK

DETYFANPQYLWNEDMSDFDPATARPNYVQITDKRGNNGGWSLSVKQEGQFKNDKTLN

KELTGSQITFTDGVSASNIENVIAPKTFDMALTPNTATKVMTAVKGAGAGTWVDRFGT

LEDVEVEGKTVKKNKAITLDVPGTTAKDAVRYDTKLTWTLTDVPGNE"

CDS complement(2884119..2884538)

/locus_tag="JFBMFIFI_02714"

/inference="ab initio prediction:Prodigal:002006"

/codon_start=1

/transl_table=11

/product="hypothetical protein"

/translation="MKNIWVPSNGHYNLYQEMNKGGAEMKKRIVLVLLFLGCLSISWT

VVDASEKQTYESNSGIGFYGKYEYPETETEVVTPNQILPAGRPSPSLSNHGSLPQTGT

TNTGYLNLIGMLILSGSLLLILLNKNKKQEEIYNEIN"

CDS complement(2884480..2887611)

/locus_tag="JFBMFIFI_02715"

/inference="ab initio prediction:Prodigal:002006"

/codon_start=1

/transl_table=11

/product="hypothetical protein"

/translation="MRIRRNKKQISMWISALFVLGLILSGIHYATKFKESGIKAEELN

LTITSSQQEFYENDVFQFDITINDSLSNREFYLEVPQEFKLNTADILQANQGVIEKIE

PVGLKQKITLANDGNAPTKINISGNLTVAGDFKVKVTDETGLEKQFPLAVKAHPSIET

LIDPEEKEESEPNPAAPETSIEQPKAEELAPPVAEIPVENEKLMESISPRSAIQPRLN

LLPSGTLLVNEPGVIKIPKLNPPQTISGEYGLVLSRFKESKVTYFVHGVNKGTPAASQ

RFTRAQSGEDTYIYYTKTGIYKGRVIDVKEVIVNTASGGSTFGTATPTAGGTNNMLSA

WVGGGSRYNPATIRVEFYDSETGEKVKVKGVWNLSDIDHDESAIMYSDSIQEVYARED

SDLLGKFGNNQLTVESPRNTNASETDETRWVSIAYGETDELTFDYYSSNSWNGYDVKS

LVPIAFPDPSKIGTVEDQVTKKVNYKTYVSVPYRQLKNFEPQLIIEDPIIDELSVQSF

NIIDTLTGQNVNNLFTLSQQGNNIRAEAKDASLKRADFYNKMYMLEVVATVKDGADLS

KYPYEEGYRLLPNTSQIIVTNSDASDKIVKSNEAFAKLKDERLEVKEEVFHLDGSAAD

IATPGEQLTYRGTSLSAYPSETETINYATLSFATTVDEQLEAITNITLKDETGQVVGT

GSYDAGTRKITASITKPVNRKQAVYLEYNGTVKKGVPTGTLVKGKTTVEGKYSNGFEI

PLSYSNEVTTLIEDYGVLEESVTHQDGSVADDAQVNDILHYKIIYRTPYKQADTKYTT

ISINSDITELTNYTKDSNNLVAHTESGKPLAVPTIVGTTLSLSDSSGTIAANETIIVE

FDVTVDAGKVSDGTIIKNSATAAMTFSDNFKTVGLQSNLVETRIKGVLEFISAPKELN

FGNDLKISTKDQVYPIQNKDEGLIVQDSRGEGNQWSMKATLLKELTSESGHKLIDSLH

YYNQGKELAFTVGDSIPIRDQKTADNQPVEISNDWQDVQAGPVLKVKAGQPYNEKYMG

AIQWTLQSVPGNE"

CDS 2888041..2889480

/locus_tag="JFBMFIFI_02716"

/inference="ab initio prediction:Prodigal:002006"

/codon_start=1

/transl_table=11

/product="hypothetical protein"

/translation="MNFLLNKEYQHRMAILNHLELTPLQTTSIQEVCDSLSLSSFIVK

KSIKQIIEDLNEHHITGIQIKLDSKKISLNSDGTDSILTLQRIYLKDSNLFLLLDGIF

KEKEFSLEQFSANQYLSLSAAYLLRNELEVILNNYSISIDSDLQLSGLESDIRLFLFE

VYFHSFNSLEYPFSKELVELSRLFIKEISTFLGISFSSTQEIKITFFLCILFQRLFFG

NTLKKQEMSIHFKDIQLAIKPMVINFFLDTTTLTQAQIKLETSFLCSFLFAGNLIPKI

STNASLKFIESSCKPALDLTALFLKQLNKQFDIQTDATYYPLLRKELNLIHYKFLKFN

HSDLMIYFQANPEFLKENYPDFFLFSESFIQTNFKPTEETTILFYDYLFSLIRFLPAT

LLNNPIYVCVDFSHGKSYNHFISTSIKSFKFLNILIEPSLSNKTNLYLSDFKPTQAHC

DYIIWKNPPLPTDWEYFGNKIIELKHQER"

CDS 2889485..2889973

/locus_tag="JFBMFIFI_02717"

/inference="ab initio prediction:Prodigal:002006"

/codon_start=1

/transl_table=11

/product="hypothetical protein"

/translation="MNHKLVHFTQSSIICGFLFSGLFFTVSSEVTANNSDIVLIADNS

TVKPVNPLNPAITVDPIEAPPKSTDAIEQIQPNKELENIHPTTRRKAKPIKKKPKEKK

VASKKESALKITELTPYSFQTDAVLPPDPSSSGGNNAYHQTNTGQLLANLSGFILYGT

KK"

CDS 2890107..2890676

/gene="hin_1"

/locus_tag="JFBMFIFI_02718"

/inference="ab initio prediction:Prodigal:002006"

/inference="similar to AA sequence:UniProtKB:P03013"

/codon_start=1

/transl_table=11

/product="DNA-invertase hin"

/db_xref="COG:COG1961"

/translation="MAILGYARVSTHYQQLDSQLAALKHYGCDRIYTETESGRNEKRV

ELNKVLNELQSGDTFVIFKLDRLSRGTKHLLILMEEFKQRNIHFISIQNNIDTTTSMG

RFFFTIMSAFAEMEAELISERVISGLNAARNNGKQLGRPPVNKNIEMVLDLYTNTDLS

IAKIARTAEVSRSTVYRYLEKNNVPLKCK"

CDS complement(2890811..2892799)

/locus_tag="JFBMFIFI_02719"

/inference="ab initio prediction:Prodigal:002006"

/codon_start=1

/transl_table=11

/product="hypothetical protein"

/translation="MKKLKIIQALLVFSLLITTISPLLVVAETSENNEVSQATDIVTR

ADLGDQDWLINEVNKQLAPKTIGVDTTFEDLDKIVRIRITTNGNNNPPIVGQIPEEIV

KLKNLARLELFSNELTGKIPQAFSQMPKLDYLRIDYNQMTGTIPEGLGNIGYINFTNN

KFVGQLPKSLYENRTGENEVNISGNQVTINDMLAIPKSIYNVATFLYTGGYDGHIETK

NNYFNNLKSTDVFTPFLVGSPTFLDLFVSQSFDRELFPEHVLTIEDTSLGKIIYEGEI

NSNLSFPLEQWESGSHLLKFTLDEAFNNPNNSTFVTLDIAATETVNVRYLDEQDNEIH

EANLIKGYSGEAYDATTPEYKLDIPTYKLDETQLPLNAIGTIGSQYQEVIYRYEKLEG

APVKVNYQDEEGNELAPAEILTGKLGVSYSANEKSIKDWLLKIKPENEIGVFTDQPQI

VTYIYEKVEGAPITVNYQDEEGNELAPTEILTGKISTPYSVKEKPIKDWLLKIEPENA

KGTFTDQPQTVTYVYEKVEGAPITVNYQDEEGNELETSEILTGRVGTAYNTNKKNIKG

WTLKSEPKNATGAFTEEPQTVAYIYEKEKLSTNEILPSPINKPNVNNPLFIHSITEHS

GIFPKTGEEKNYSLIIGILIFISFIVTVDKKKYLGNKD"

CDS 2893206..2893532

/gene="hin_2"

/locus_tag="JFBMFIFI_02720"

/inference="ab initio prediction:Prodigal:002006"

/inference="similar to AA sequence:UniProtKB:P03013"

/codon_start=1

/transl_table=11

/product="DNA-invertase hin"

/db_xref="COG:COG1961"

/translation="MATIGYMRISTAHQKFDSQEQALKRFGVNKLYQEQDSGRNPHRL

VLKQVLKKLKKGDTLVIFKLDRLARSTKQLLHLLEDFDKRGIHFVSIQNNIDTSTPMG

KFFLPL"

CDS 2893529..2893771

/locus_tag="JFBMFIFI_02721"

/inference="ab initio prediction:Prodigal:002006"

/codon_start=1

/transl_table=11

/product="hypothetical protein"

/translation="MSAFSEMEASLINERIMAGLEAAKLNGKSLGRPPLVEKNQLALQ

LYYENRFSVKEIATISGLATSTVYKIIKQEKEKPKN"

CDS complement(2893960..2894793)

/gene="carC"

/locus_tag="JFBMFIFI_02722"

/EC_number="3.7.1.13"

/inference="ab initio prediction:Prodigal:002006"

/inference="similar to AA sequence:UniProtKB:Q9AQM4"

/codon_start=1

/transl_table=11

/product="2-hydroxy-6-oxo-6-(2'-aminophenyl)hexa-2,

4-dienoic acid hydrolase"

/translation="MGYVEASNLFIKDAHGKKFAYREVGKKTNMPLILLTHLSANLDN

WDPKIVDGLARKFWVIAFDGQGVGLSGGSTPKKILEMTNETVSFIQALGLNKVHILGL

SMGGMVAQEIVLSHPKLVEKLVLVGTGPKGGQGIGRVAKVTNYSMLRALITFKDIKYY

LFFTSSQSSRTKAKEYLIRLKSRKKNRDKSIRLHSYNQQLKAIKRWSLGEIDPLEEIK

QPTLIINGDTDIMVPTANSYDLAQRISDSKLIIYKDSGHGSLFQYPEEFVVDVGTFLM

V"

CDS complement(2894813..2895817)

/gene="adh"

/locus_tag="JFBMFIFI_02723"

/EC_number="1.1.1.1"

/inference="ab initio prediction:Prodigal:002006"

/inference="similar to AA sequence:UniProtKB:P42327"

/codon_start=1

/transl_table=11

/product="Alcohol dehydrogenase"

/translation="MKAAQINKYTKKVHAKITEISIPEISDNQVLIKTKAAAVNPLEI

LNITGAVKLIQDYQMPLTIGNELTGVIDKVGKNVQGFKVGDAVYARLPIEHIGAFAEY

VAVAADAIWFLPDHLDFITGAAVPLTGLTAYQGLHEKLNAQAGQTIFIPGGSGSFGQM

AIPIAKAMGLTVIVSGNAAAKERTLAVGADQFIDYKTENYWEVLDSVDFVIDTLGAAE

FERELSIIKPGGRLLSLITGPNKRFAISQGLPKWKQFLFGLVGSKYDKKAKEKNIDYH

FIFVRADGNQLKLVSEIIEKNKIIPAIDPSKFRLEDINQALELVEKGHPQGKVVIRFD

"

CDS complement(2895854..2896912)

/gene="nemA"

/locus_tag="JFBMFIFI_02724"

/EC_number="1.3.1.-"

/inference="ab initio prediction:Prodigal:002006"

/inference="similar to AA sequence:UniProtKB:P77258"

/codon_start=1

/transl_table=11

/product="N-ethylmaleimide reductase"

/db_xref="COG:COG1902"

/translation="MENLWNPITIKEQVVKNRLVMAPMTRSRAKENGVPSKLMSKYYQ

QRASMGLIITEGIQPSAVGQGYLYTPGIYTEEQIIGWQEITNAVHQQEGTIFFQLMHV

GRLSHPDNVKGLQAQAPSAIAPTEEIFTGNGMQAIPVPLEMTKEDIKMTVDDFRLAAK

NAIQAGADGVEIHSANGYLLHQFLGENSNIRTDEYGGSIKNRVRFVLEVAQAVAAEIG

ADKTGIRISPMNQLGGVDEGNAGNQLYSYLVSELAKLDLAYLHMMYVSHEDSLLKDIR

TAWPNVLIINRPSRPVDQIDFDILNGNAEMVSVGTLALANPDLVERLKTGKPLNQPHS

ETFYGNEGEVGYTDYPFL"

CDS complement(2896967..2897707)

/locus_tag="JFBMFIFI_02725"

/EC_number="1.-.-.-"

/inference="ab initio prediction:Prodigal:002006"

/inference="similar to AA sequence:UniProtKB:P9WGR7"

/codon_start=1

/transl_table=11

/product="putative oxidoreductase"

/db_xref="COG:COG1028"

/translation="MSKKYTVITGASSGIGYETALAFAGKEKNMILIARRTEQLTELQ

KRIQKQYPTLDIIVKISDLSDKKQVYGLYESLKQYDIETWINNAGVGASGALMNNDLK

QIERIIQVNIEATTILSTLYVKDYANVQGAQLINVSSAMGYVIALGNVAYSASKFFVS
VLTEGLAAELKDAELQAKVLAPALTATAFIQQSSDSELDYKNYQLRSPQEVASYLMAL
YESTQTVGIVNEKNEFILSNAIFPVFSI"

CDS complement(2897723..2898298)
/gene="comR"
/locus_tag="JFBMFIFI_02726"
/inference="ab initio prediction:Prodigal:002006"
/inference="similar to AA sequence:UniProtKB:P75952"
/codon_start=1
/transl_table=11
/product="HTH-type transcriptional repressor ComR"
/translation="MGRAKEFDQEVVLDKAMQIFWEKGYEKTSMQDLVDHMGIHRRSI
YDTFGDKHELFLKTLDCYEYRLNHIIKQRLTQDMTIKEKLEMLFLIVVSTDDEYPKGC
LIVNTATELSLLDKQIEHKIQELFEKSETYLYMLLVEAKNKGEISKSENLRELASYLH
NAWVGIRVLVKTTDDQMKLHSIVKTTLSIIK"

CDS complement(2898499..2899881)
/gene="sacA"
/locus_tag="JFBMFIFI_02727"
/EC_number="3.2.1.26"
/inference="ab initio prediction:Prodigal:002006"
/inference="similar to AA sequence:UniProtKB:P0DJA7"
/codon_start=1
/transl_table=11
/product="Sucrose-6-phosphate hydrolase"
/translation="MTVDKWISESKESVNTMYKPLHHFSAPIGWINDPNGFVYYKGEY
HLFYQFHPYSAKWGPMHWGHAKSKDLLNWEHLPVALEPNQPYDEGGCFSGTALVQEDQ
LILMYTGVSEEDGKSRQIQCIAMSTDGITFEKSPHNPVIDERHVKNTTDFRDPKIFRK
NEKYYSLVASTKDGIGNVLLFESDDLVSWVFKSTFLTAEPHQGKIWECPDLFELDGKD
VLIVSPIAFTSEGARYTNVNSSVYFVGKVDWDTYKFIPESYEEIDSGLDFYAPQTLKD
DRNRRILIAWQQMWGRNIPTDDLGHHWAGSMIIPRELRLVDGKLIQTIVSEYNEALTL
ASSQNVDRLYRGQLEPFVQLETTEPFSIEIGADEDYLKFDYDGESLMIDRTNLQQSIQ
GEEEQSDKRSKCYSSENLIVKIYVDKACCEIIVNDEVTFSTTFYLESVDAEIRVKGKD
LKIDMYNKKI"

CDS complement(2899886..2901505)

/locus_tag="JFBMFIFI_02728"

/inference="ab initio prediction:Prodigal:002006"

/codon_start=1

/transl_table=11

/product="hypothetical protein"

/translation="MKTKKMIIIGFSAVVLLALGSCGKKDNSSATASKDYELTNVEFP

LKEKVELKMLVSENSTAPKDPNDMLIFKRLEEQSNVHISWTNYANDFAEKRNLDIASS

DLPDAIFNAAASEQDLLTWGQNGVIVPLEETIEKYMPNLQKVFEEAPEYKQMLTAPDG

HIYSLPWIEELGEGKKSIHTVNSLGWINQAWLTKLGLEQPKNAEELYNVLKAFKEQDP

NGNGKADEIPLSFISNDDGNDLKMLFAAFGDGTGDNGDHLVVDDDGKVQFTANTESYK

KAVKFFNKAYEEGLIDTESFEQDWARYVAKGNDQKYGLYFTWDPHNVTGGTEDYVMYQ

PMANESGKINVTRTNNFGFSRDRFVVTSANKNIELTAKWIDQMYDPIQSIQNNWGTYG

DKGQNIFKYDEEKNQLTHEPLDGAAPVEVRQKTAVGGPLAILDTYYGNYTTMPDDAKW

RLDLMDKFIFPYVQNENNYPNIFLSAKDTKRLADIEADMKDFVLRKRDEWLTKGNIDQ

EWNDYLKQLKAFGLDEWLEIKQRNYDEYSKG"

CDS complement(2901549..2902433)

/gene="ycjP"

/locus_tag="JFBMFIFI_02729"

/inference="ab initio prediction:Prodigal:002006"

/inference="similar to AA sequence:UniProtKB:P77716"

/codon_start=1

/transl_table=11

/product="Inner membrane ABC transporter permease protein

YcjP"

/db_xref="COG:COG0395"

/translation="MLNKQTRFDRNVLIVNRVLIGLLVFITIVPLAYVLAASFQDPDT

LMSQGISFNLKNWTLAGYQKVLANDSILRGFVNSMFYSFAYAAFSTIITMLCAYPLSK

KEFVGRKVIMSFFLLTMFFGGGLVPTYMVVKNLGILDTPWAIILPGAINIWNIILART

YFQSLPDELEEAAKIDGANSMQVFFKILLPLAKPIMFVLFLYAFVGQWNSYFDAMIYI

KDPDLQPLQLILRNILIQNQIDTNMVGSQAAMAEMEKVAQLIKYATIIISSLPLIIMY

PFFQKYFDKGVLVGSMKG"

CDS complement(2902433..2903347)

/gene="yteP_2"

/locus_tag="JFBMFIFI_02730"

/inference="ab initio prediction:Prodigal:002006"

/inference="similar to AA sequence:UniProtKB:C0SPB3"

/codon_start=1

/transl_table=11

/product="putative multiple-sugar transport system

permease YteP"

/db_xref="COG:COG4209"

/translation="MKQKVKNNLFYMRKNYLLYLLVLPAIVLTFIFKYVPIYGLLIAF

KNYNPLQGIIGSEWVGLEHFTRFLSSPNFMILLVNTLKLSVYGLLLGFFPPIILAISF

NLLASDKLKKRLQLILYAPNFISVVIIVGMIFLFFSNKGPVNHIYEFFTGKTVPFLTE

PDFFRPLYIFSGIWQGIGWSSILYTATLSNVSPELIDAAKIDGANILQRIWHVDIPAI

KPVMVISFILAAGGIMSVGYEKAYLMQTTMNLPSSEIIATYVYKVGLQSGDYSYSTAV

GLFNSLINLVLIIAVNQTVKKLNDGEGL"

CDS complement(2903480..2904337)

/gene="gmuE_2"

/locus_tag="JFBMFIFI_02731"

/EC_number="2.7.1.4"

/inference="ab initio prediction:Prodigal:002006"

/inference="similar to AA sequence:UniProtKB:O05510"

/codon_start=1

/transl_table=11

/product="Putative fructokinase"

/db_xref="COG:COG1940"

/translation="MQLYGSIEGGGTKFVCAIGDEQLEIVEKVVFKTTTPEETLKKVV

QFFEEKRVSAIGMGMFGPLEQRVNHEKYGYITSTPKQGWANVDVVSQLKEVLNVPIYF

TTDVNSSAYGEFISRGAVPGLVYYTIGTGIGAGIVLNGEIIGVTGHPEMGHVSVKRRL

DDADFEGVCPYHKDCLEGLASGPTLQARLGILGERVALDHPVFELIGYYIAQACVQAT

LTLRPSVIVLGGGVMSSNLLEIVKKEFKKTLNNYIEIENIDTYLSLPTVVENGSATIG

NFALAKKII"

CDS complement(2904365..2905336)

/gene="degA_3"

/locus_tag="JFBMFIFI_02732"

/inference="ab initio prediction:Prodigal:002006"

/inference="similar to AA sequence:UniProtKB:P37947"

/codon_start=1

/transl_table=11

/product="HTH-type transcriptional regulator DegA"

/db_xref="COG:COG1609"

/translation="MKATMKDVAKLAGVGVGTVSRVINGVRVKDSTLKKVNQSIQELG

YEPDIYARGMKTNRSNTIALIVPTIWHPFFSEFGFHVEKELKALGYKIFLCNSTGDPA

VEDEYIQMIKQNKVDGIIGITYTDIDKSVSSDIPFVSIDRHFTSKVTIVAADSKQGGE

IAFNELNKRGCQHFAFVGTHQDKESETKNRRIYFEKSARRAGKKISILDFEEPIIDFE

NQVQKFFIENPTIDGLFGINDFLAMDVMKELEKLGKRIPEDIQVIGFDGVKLSEEREY

LMTTIRQPLETMAKVSVEQIMNAINGDPIDYKTIIPVTFIEGSTTKN"

CDS complement(2905621..2906670)

/locus_tag="JFBMFIFI_02733"

/inference="ab initio prediction:Prodigal:002006"

/codon_start=1

/transl_table=11

/product="hypothetical protein"

/translation="MEKRKTGFRTYLTDGEAGINISWGSIFAGVITFLSIFITLSLIG

SAIGFGTLKLTTDQPFEGVGTGLIIWSIIMLIISLMSAGFVAGIAARRIGLLHGFLTW

ATSMLLVVILVSFTAVGAFSAVGSLLGNVASGVGSGVQTIASGTGDVISKGIDKVSDE

VGTVNTSELQDNIDKYLKDTDVPELQPDYLKDQLSQATDTIKDAGKEALKNPDDSDKI

FETTADSLKEQAKTIGDSVDKDAIANAVAKNSDLSPEEAQQATDNIYNELQKASKETQ

EQIDTVRENLEETKADLKESIDDARKAADDAADATAKASIWGFVAMVLGMIITSLAGL

WGSNLVKDPQREVKM"

CDS complement(2907745..2909643)

/gene="mnmG"

/locus_tag="JFBMFIFI_02734"

/inference="ab initio prediction:Prodigal:002006"

/inference="similar to AA sequence:UniProtKB:P64230"

/codon_start=1

/transl_table=11

/product="tRNA uridine 5-carboxymethylaminomethyl

modification enzyme MnmG"

/translation="MERFEAGTFDVIVVGAGHAGSEAALASARMGQKTLLLTINLDMV

AFMPCNPSVGGPAKGVVVREIDALGGEMGRNIDKTYIQMRMLNTGKGPAVRALRAQAD

KFLYANEMKHTIEREDNIILRQGIAEDLIIEDGVCLGIVTNTGAVYRSKSVILTAGTS

SRGQIIIGELKYSSGPNNSQPSIKLSESLLRNGFELARFKTGTPPRVKASTIDYSVTE

EQPGDEKANHFSYETPDSAYVKDQLSCWLTYSNEKTHDIIKANLHRAPMFTGIVEGVG

ARYCPSIEDKIVRFSDKPRHQMFLEPEGRNTEEVYVQGLSTSMPEDVQLDMIRSVAGL

EKAEMMRTGYAIEYDVVVPYQLRPSLETKIVENLFTAGQMNGTSGYEEAASQGLMAGI

NAALKNQGKEPFVMKRSEGYIGVMVDDLVTKGTMEPYRLLTSRAEYRLLLRHDNADFR

LTEIGHEIGLVSDERYETFLAKKALVEDEIKRLMKTRLKPTAELQAFLVDVKGASPLK

DGILAADFLRRPEMTYEEVVSFAPATIELPLAVKEQVEIQIKYEGYIQKAIEKVEKMK

RMESKRIPERIDYEAINGLATEARQKLIKIQPETIAQASRISGVNPADISILMVYVEQ

GKIAKVAE"

CDS complement(2909668..2911056)

/gene="mnmE"

/locus_tag="JFBMFIFI_02735"

/EC_number="3.6.-.-"

/inference="ab initio prediction:Prodigal:002006"

/inference="similar to AA sequence:UniProtKB:Q8YN91"

/codon_start=1

/transl_table=11

/product="tRNA modification GTPase MnmE"

/db_xref="COG:COG0486"

/translation="MSLEFDTIAAVSTPPGEGAIGIVRLSGEEALQVADKVYQAGTKK

LVDVASHTIHYGHIRDPKTSETIDEVMVSVMRSPKTFTREDVVEINCHGGITSVNQVL

QIVLQNGARLAEPGEFTKRAFLNGRIDLSQAEAVMDLIRAKTDKAMYLALQQLDGNLS

TLIRNLRSEILDTLAQVEVNIDYPEYDDVETLTARLLVEKAYHVKASINQLLQTASQG

KILREGLATAIIGRPNVGKSSLLNFLLQEEKAIVTEIAGTTRDVIEEYVNVRGVPLKL

VDTAGIRETEDIVEKIGVERSRQALQEADLVLLVFNQNEPLTLEDKLLIEATADNHRI

IILNKMDLPNQLDLAELEGLVDPASIVKTSILTKSGIDVLEEKIAALFFAGATGDRDA

TYVSNVRHIALLHDAEAALDEVINGIEADMPVDLVQIDMTRCWDLLGEITGDSVQDEL

LTQLFSQFCLGK"

CDS complement(2911569..2912204)

/locus_tag="JFBMFIFI_02736"

/inference="ab initio prediction:Prodigal:002006"

/codon_start=1

/transl_table=11

/product="hypothetical protein"

/translation="MTKKEDILMQLNNPPTSLGDLRKIAKDIKKDHALAMELWDTEIY

LARLLSILIMDKNMLTQELIDKLDKDSEEYNTSERNQLIDWLMANQLAKSKKTISLME

SWQNSQSSLQRRVFWYYQGRLRWMGKTPPPNTEELLLLIEAQIENEAPEVQWAMNFTA

AQIGIFQKEYRPRCIQLGERTGLYKNQKVAKNCTPDYLPLYIAMQVDKLNK"

CDS complement(2912342..2912764)

/locus_tag="JFBMFIFI_02737"

/inference="ab initio prediction:Prodigal:002006"

/codon_start=1

/transl_table=11

/product="hypothetical protein"

/translation="MKHIKKEFLLNCKPEEAWQLVVDRNKYEKWAFAFQEGSTYTGDM

GLNETISFVDGSGDGLVSKVVVFEPEKEIKFSFLGEVTSGKYVEVPAFADMLEHYIFE

PVGNQTKMFVDVAMDDEYYDMMNEMWDQAGIELIKLSN"

CDS complement(2912828..2913232)

/locus_tag="JFBMFIFI_02738"

/inference="ab initio prediction:Prodigal:002006"

/codon_start=1

/transl_table=11

/product="hypothetical protein"

/translation="MSEQTKKISTFLTFSGNAEKAMNLYVSLFEDAKILSLTHFGEGD

RGVVGQVLNGEFEINGQSFMVMDMEKQYAPELNWGVSIFVRCQDEAEFDRLFTTFSEG

GSVMMGPEAVNHLRKVAWVTDSFGITWQLIWE"

CDS 2913426..2914418

/locus_tag="JFBMFIFI_02739"

/inference="ab initio prediction:Prodigal:002006"

/codon_start=1

/transl_table=11

/product="hypothetical protein"

/translation="MKLSVLDQIPVTKGNTSVAALDKAKELALLADELGYHRMWFAEH

HGSASVASAAPEIIVAHLAALTKNIRLGTGGTMIMHYSPLKMAETFKTLSALSPGRID

FGAGRAPGGDMKAIYALAQSKRPQMRELYEKLETTLALINDKVPVDELYQTTIASPTP

VTLPEAWLLGSSGDSALQAARMGVGYSFAQFFSGSMTKEILSLYKDRFIPSYFMEKPE

INVAYLATVAESIEEAEFEARPTDIQRLMMTKGQLTGIITPEEAQNFSLTEMDRLKIQ

ESRKIHFVGTAKAVADRLVEESEYYRFQEAMIISTPHSQEKRLNVYRLLAKEML"

CDS 2914564..2915535

/gene="ypuA"

/locus_tag="JFBMFIFI_02740"

/inference="ab initio prediction:Prodigal:002006"

/inference="similar to AA sequence:UniProtKB:P31847"

/codon_start=1

/transl_table=11

/product="putative protein YpuA"

/db_xref="COG:COG4086"

/translation="MKLKKIITAGLVGLLATGFLVSSTTAFAQTPINETWGKPAFTRG

VTLSDAQIQETRDLLGIKKDDSVDEFVVDGTVIGKYLQNSNNPGAQVFSSSLIQRTSA

GDGVKVEIVTPKNITLITTEQYRNASITAGVTDATIKVASAVPVTGEGALAGVFMAFD

ENGQALDKEAIAVGQEELGVISSISDANSDNSKFSDANLNVAFAEIKAELASIRKKQD

ELATKADVERIVNDALKNNSLENVVTPEQKTEIVNFMIRFQNSSAIDNKELINQLNRF

GEDVMNNAKNWLGEARNSLNSEQAQGFLQNVRTNWDNFVNWLTGFFN"

CDS complement(2915618..2916166)

/locus_tag="JFBMFIFI_02741"

/inference="ab initio prediction:Prodigal:002006"

/codon_start=1

/transl_table=11

/product="hypothetical protein"

/translation="MLLCWYKIEGSVRSGMLDLKKFKIGLRTLKTGISVFLCMLIFTI

FDRGTPIVATLTAVFALREDMNNTLKFGRHRIVGNTFGAIFAVVVILLYEWVGNIVLV

QLVGIPLLIMIMITFCVATGHSEGVVGACGTFLTIIFMIPENESFGYALQRVVDTFIG

TGIALGVNHFIRPKKVRASSIE"

CDS 2916747..2917721

/locus_tag="JFBMFIFI_02742"

/inference="ab initio prediction:Prodigal:002006"

/codon_start=1

/transl_table=11

/product="hypothetical protein"

/translation="MTEKKTNEEEIKKNEEEVVVTPENEVLKDSEETVVSTETEATPA

KEDEIAPSDTETVVEDDVVTEEVIVDEEVPSKDLNEVDATTETTATNTAEEVILEEGI

EALASSGVVEGVSIEGEEIDGEFDEEDLEDEDLEDEEPKGKLAKHMGKILYSLLGIMI

VAVLIFGYYVFPFFSKIGGTWQGENTAGTFKIVNKGSKSEFSFVDMNGVKGMNLVFTS

ELTRKGVNQFGATDTSVKIVLDKKTIDEANIKAFKELTDQVKVKDETDKEVVLVYTKA

AYETAFKDTNLDEYFNYRLTNFNFALQGKDLMLKNKDFATPEVNFTRK"

CDS 2918022..2918738

/gene="treR"

/locus_tag="JFBMFIFI_02743"

/inference="ab initio prediction:Prodigal:002006"

/inference="similar to AA sequence:UniProtKB:P39796"

/codon_start=1

/transl_table=11

/product="HTH-type transcriptional regulator TreR"

/db_xref="COG:COG2188"

/translation="MNKFHEIYLDLEKAILEKKYQPGQLLPSENELTKEYSVSRETIR

KALVLLLESGYIQKKQGKGSIVLDVNRFDFPVSGLTSFKELQESQQMKSETIVTKNEV

QTIPEKLAERLKLPTDTKVIYIERQRRFNGEVVILDKDYLLLSIIAEMPQAAAQDSIY

NYIEHTLGLAISYAQKEITVDPITPEDQELMDLGDDTHIVVVRSDVYLEDTTLFQYTE

SRHRLDRFRFVEFARRKSPV"

CDS complement(2918790..2919251)

/locus_tag="JFBMFIFI_02744"

/inference="ab initio prediction:Prodigal:002006"

/codon_start=1

/transl_table=11

/product="hypothetical protein"

/translation="MQLPVEKNSKLAKNLTLASALSLTVVTLLSVLQVLYLVALSNTD

LSKLKELTAEQAAVIRESITLPTILFFAGIALIYLIVTVFMYLFVMKIKKGKAISVIP

YYVSILMLAYSFLQSAIAVNIFNAALYFIVGVVVGFAVFYGYKYKKSIVSE"

CDS complement(2919497..2921269)

/locus_tag="JFBMFIFI_02745"

/EC_number="4.2.1.53"

/inference="ab initio prediction:Prodigal:002006"

/inference="similar to AA sequence:UniProtKB:B9E972"

/codon_start=1

/transl_table=11

/product="Oleate hydratase"

/db_xref="COG:COG4716"

/translation="MYRSNGNYEAFARAKKPVRSDEVSAYLVGSGLASLSAAAFLIRD

GQVKGDKIHILEELPIAGGSLDGIYKNEYGFVIRGGREMESHFECLWDLFRSIPSLEV

EDASVLDEFYWLNKEDPNSSKCRLIHNQGEQFPTDGQLTLSEKAIKEILALCLMKETQ

LDDKKITDVFTKEFFSSNFWTYWCTMFAFEEWHSAMEMRRYLMRFIHHIDALADFSSL

KFTKYNQYESLVLPMLAYLKENGVHFQYDTKVENVLVTSENGNKKAERLILNVKGQEK

IVKLSENDLVFITNGSITESSTYGNNTTPAPPTKELGGSWNLWKKMVEQDSDFGKPEK

FCENIPDESWFVSATVTTLDKKIAPYIEKISKRDPYAGRVVTGGIVHAKDSNWKMSYT

INRQPHFKNQPEDQLVIWVYALLSDKPGNYIKKTITECTGNEIASEWLYQMGVPTDLI

DDLAKNSCNTVPCYMPYITSYFMPRAKGDRPLVIPDGYKNLAFIGNFAETERDTVFTT

EYSVRTAMEAVYTLLDIDRGVPEVFASSYDIRMLLNASYYLNDKKGIKEVKVPLLEGI

IERKGLKKIKGTFIEELLEDAKLL"

CDS complement(2921516..2922862)

/gene="gadB_2"

/locus_tag="JFBMFIFI_02746"

/EC_number="4.1.1.15"

/inference="ab initio prediction:Prodigal:002006"

/inference="similar to AA sequence:UniProtKB:Q9CG20"

/codon_start=1

/transl_table=11

/product="Glutamate decarboxylase"

/db_xref="COG:COG0076"

/translation="MTKKDFSTQKLPSKTFFEDGIGSAVAYDAIKSYLIDEGNSRQNL

ATFCQTYMDDEAIKLMSETLDKNAIDKSEYPRTAKVEASCVNMLANLWNAPSENFIGT

STVGSSEACMLGGMAMKFRWRHLAKKKGIDTTKRKPNLVISSGFQVCWEKFGVYWDVE
LREVPVDPDHLSLDLDRVFDYVDEYTIGIVGILGITYTGKFDDIEALDKMVEEYNKKN
NANLVIHIDGASGGMFVPFVNPELVWDFRLKNVVSINTSGHKYGLVYPGVGWVIWKDR
EYLPEELIFDVSYLGGHMPTMAINFSRSASQIIGQYYMFLKLGYNGYRQVHTETKEVA
MYLAKSVEETGLFKIYNDGSNIPIVCYTLKNDAQVKWSLYDLADRLLMKGWQVPAYPL
PKNMDNIIIQRFVCRADLTMPMAEEMVEDFKEAIKELATATVLGHDSGDKVHGFTH"

CDS complement(2922978..2925404)

/gene="smc_3"

/locus_tag="JFBMFIFI_02747"

/inference="ab initio prediction:Prodigal:002006"

/inference="protein motif:HAMAP:MF_01894"

/codon_start=1

/transl_table=11

/product="Chromosome partition protein Smc"

/translation="MKMTKQEWINLFRNKILLISVIAIAFIPILYSSIFDKSVWDPYG
RAKDLPVAVVNQDKPTELLGQKMNVGQQVVDNLKKDHQLDWNFVSKEEAEKGMKDLKY
YMIVTITEDFSKNAASIINHTPQKMEIIYTTNDSLNYIANEISTVGATALETQVREQV
IEAYATAVVDAGQKLVGALGQAANGANQLADGGGELQTGLKQYTEGVFQADSGSNQLA
DGTGQLANSIGPLASGVSQLDSGANQLSSALNQANQALGPIENNVAGIDAGLTQLATG
TQDLANALTTFENNLDPNVKIVLENDLQNIRNEINSIITNKDKLLGISNDATGVADQA
KVASASLSGANLEMQKIQTDIDSYVADLLAGTTISDNEKADIVSSISSKVDAILDEQI
SSITAQINTSLSAVESELNTLSAKASDLSASANDVSNVTNAMAASAQQLSSSEASIHA
GINSFDALLAQVPNSSHAVGLTSGLYAISSDLNLAATKLPTALAGVNQLTTGSSQLST
GLDQLQSQIPTLSNGVNQLNTGANQLQAGLAELNENSPELMSGIGQLEGGAKELSSAL
DEGVSESSTLKITKKNINQFAAPTSLKNNEYSKVANYGEALAPYIMSLALFVGSMLFN
FIYPIRKISLEGQSSGAWWLSKVSLGFVVSSIMALIQASIMLLIGLQVDNIFQFFLTA
FVSAWSYMAITMFLAMTFDNPGRFVAMILLVLQLGGAGGTFPIQLQAPFFKAIHPYLP
MSYSVYAFREAISSGIGAPLFRKSILILALLTIIFVALLRYSMHVLQKKNLQNVSELN
DNQKLQGLEN"

CDS complement(2925376..2926809)

/gene="gadC_2"

/locus_tag="JFBMFIFI_02748"

/inference="ab initio prediction:Prodigal:002006"

/inference="similar to AA sequence:UniProtKB:O30417"

/codon_start=1

/transl_table=11

/product="Glutamate/gamma-aminobutyrate antiporter"

/db_xref="COG:COG0531"

/translation="MNKKKNISKFGFFAMTASLFITVYEYPTFADSGKTLIFFLLLCG

IFWFLPVALSSAELATIEGYQDGGIFSWVGEPLGEKFGFAAIFFQWFQITVGFVTMIY

FIIGTVSDVLNVPVLNTNVGVKFILVILIFWLLTALQFKGTKITEQVAKYGFSIGIVI

PVLLMLVLAIKYIASGNQISKNFTDTHFFPDKSNISALVSFVLAYMGVEASAPHIADL

ENPKKDYPKIMFALVIVGVVLSTIGGSVVAMVSHGNISANAGVVNALKEIISPGKESP

LVIILGILVSFGVMAQVSSWIVSPTEGLQFVASKGLLPKKYEEVNKHGVPVPLLIVQG

IIVTAWAAILTFGSGNSGGNVSFQTAISLTVIIYLSAYILFFIAYLVIVFKKKELKRE

YQIPGGSKVKIFVAGSGLLLSIAAIMTAFMIPDTMSKTEGKTYITTLIFSYIVTVVAP

FLFYQFYSKNHSLKGATHHEDDQTRMD"

CDS complement(2927038..2928222)

/gene="yxeI_2"

/locus_tag="JFBMFIFI_02749"

/inference="ab initio prediction:Prodigal:002006"

/inference="similar to AA sequence:UniProtKB:P54948"

/codon_start=1

/transl_table=11

/product="putative protein YxeI"

/db_xref="COG:COG3049"

/translation="MCTSIFLETKDNKHLLARTMDFSFPLDFDVVYLPKKNEWVSEAD

KEKHQSKYGMLGAGRLLGTSYFVADGVNEHGLAIAELYLPQKAVYQKELDSEKINLAP

HEFITWALGEFKSISELKKELSKVNLLEVPAPLIDTVTPLHWILTDTTGNCMVIEPTG

KMLHLKENPVGVMTNTPLLDWHIDNLSNYLNVRPKQYEPTKFGKYTSHAFSQGTGTLG

LPGGYTPPERFVRAAFFKEYIDQAGIEIEAVTNAVRILATVQIPKGIVVTDDDKEDYS

QYIGMMCNESKTYYYTDYNNTRISQITLTDELLERNSPKVFVTKKTEDIDILNNEILS

PEEKISSMGQKGEAGISTLDILRQELALGLKSANNGSLNNDAHQTIDQAIEQLKQLKN

NV"

CDS complement(2928734..2929999)

/gene="ilvA"

/locus_tag="JFBMFIFI_02750"

/EC_number="4.3.1.19"

/inference="ab initio prediction:Prodigal:002006"

/inference="similar to AA sequence:UniProtKB:P9WG95"

/codon_start=1

/transl_table=11

/product="L-threonine dehydratase biosynthetic IlvA"

/db_xref="COG:COG1171"

/translation="MSKNKLVTKEDVLKARDVLRSVVKETPLEKDLYLSEKYQCTVYL

KREDLQWVRSFKLRGAYYAISQLNELQLAQGVTCASAGNHAQGVAFTCAKLGISATIF

MPTTTPQQKQNQVKFFGKDKVEIVLIGDTFDECSKEALAFSEKMGSAFIHPFEDPDTI

AGQGTIAVEVFEELAREEAQADYLFAAIGGGGLVSGLLSYTTAVSPETKVIGVEPSGA

ASMALSLDAGEVLSLDTIDKFVDGASVGKVGGLNYQHCVEMVHDVIDVEEGLVCSTIL

DMYTKQAIVAEPAGALSVAALEPYREEIKGKTVVCIISGGNNDINRMQEIEERSLLFE

GLKLYFLVNFPQRPGALKEFVSDVLGPDDDITKFEYTKKVNRNSGPVIIGILLKYKED

SVSLIERLSQFDPTYISINDNQMLYTLLV"

CDS complement(2930018..2930608)

/gene="leuD"

/locus_tag="JFBMFIFI_02751"

/EC_number="4.2.1.33"

/inference="ab initio prediction:Prodigal:002006"

/inference="similar to AA sequence:UniProtKB:Q8DTG5"

/codon_start=1

/transl_table=11

/product="3-isopropylmalate dehydratase small subunit"

/db_xref="COG:COG0066"

/translation="MEAIKVHEGQIVALMNDNIDTDQIIPKAFLKRIEKTGFGEFLFD

EWRYLPDRSLNPDFSLNDPKRQEATILITGENFGCGSSREHAAWALLDYRFRVIIAGG

YSDIFYMNCTKNGVLPIHLPKDVREKLAALTPEQRVTIDLPNQLVKSPVGDYPFDIDQ

VWKHKFIHGLDDIGITLGSHQDALQGYEDKYDQQYN"

CDS complement(2930595..2931983)

/gene="leuC"

/locus_tag="JFBMFIFI_02752"

/EC_number="4.2.1.33"

/inference="ab initio prediction:Prodigal:002006"

/inference="similar to AA sequence:UniProtKB:P80858"

/codon_start=1

/transl_table=11

/product="3-isopropylmalate dehydratase large subunit"

/db_xref="COG:COG0065"

/translation="MGKTMFDKLWDRHVIYGEVGEPQLLYVDLHLIHEVTSPQGFEGL

RVEGRTVRRPDKTYGTLDHNVPTEDIFNIQDLVAKKQIEALQKNCAEFGITLADNGSE

NQGIVHMVGPETGLTQPGKVIVCGDSHTATHGAFGSIGFGIGSSEVEHVLATQSIWQQ

KPKTMGIEIVGELPQGVYSKDIILHLIATYGVAFGTGYAVEYYGETVRNMTMEERMTI

CNMAIEGGAKMGMMAPDETTFEYIRGRAYAPKNFEKAVADWKTLPSDPDALYDLDIKV

DVSELAPYVTWGTNPEMGVPFTENFPAIENMNHERAYRYMGLEPGQKASEIELGYVFI

GSCTNGRLSDLKEAARFVAGQKVKAGIRAMVVPGSRQVKLAAEKIGLDQVFKDAGFEW

REPGCSMCLGMNPDQVPDGVHCASTSNRNFEGRQGKGARTHLVSPAMAAAAAIHGHFI

DIRQEVEIYGSH"

CDS complement(2931983..2933041)

/gene="leuB"

/locus_tag="JFBMFIFI_02753"

/EC_number="1.1.1.85"

/inference="ab initio prediction:Prodigal:002006"

/inference="similar to AA sequence:UniProtKB:P12010"

/codon_start=1

/transl_table=11

/product="3-isopropylmalate dehydrogenase"

/db_xref="COG:COG0473"

/translation="MEYKITALPGDGIGPEIMESGLSVLKAIEEVFGHQFQVTKRPFG

GAGIDAEGDPLSDKTLNACKESEAILLGAIGGPKWQKASKTPEMGLLQLRKALGLYAN

IRPIQVPTSIAHLSPLKAEIVSGSDFVVVRELTGGIYFGEPKHWNDEEALDSLVYTRE

EIRRICVQAFEIAQGRKKKVTSVDKANVLSSSKLWRKVVAEVAQDYPDVTLEHLYVDA

AAMLIIQKPTTFDVIVTENLFGDILSDEASVITGSLGMLPSASHGIEGPSVYEPIHGS

APDIAGKNCANPMSMILSVAMMLRESFSLISEAIAIEAASNTVMEAGILTTDLGGTAT

TTEFTEAVRKQIIEGGRR"

CDS complement(2933045..2934571)

/gene="leuA"

/locus_tag="JFBMFIFI_02754"

/EC_number="2.3.3.13"

/inference="ab initio prediction:Prodigal:002006"

/inference="similar to AA sequence:UniProtKB:Q71Y35"

/codon_start=1

/transl_table=11

/product="2-isopropylmalate synthase"

/translation="MSYIQFFDTTLRDGEQTPGVNFNQKEKIQIALQLENWGVDVIEA

GFPISSTGDFESVQKIANTLKKATVCGLARCVEADIDCAYEALKGAVSPQVHIFLATS

PVHLEYKLKMTQEEVLESIAHHVAYARTKFEKVQFSPEDATRTPIDFLTKAVQTAIDA

GAIIINIPDTVGYTNPTEFGALFRHLRDNIAQFDDVIFSTHCHDDLGMSVANALAAIE

NGARRVEGTVNGIGERAGNTSLEEVAVALHIRHDYYQAESGITLAETKRTSDLVSRLS

GMAIPKNKAVIGANAYAHESGIHQDGVLKNPDTYEIITPALVGVEKNSLPLGKLSGRH

AFNTKVEEMGYHLSDEENKIAFKRFKHLADVKKQVTEEDIHAIMLGQVAEDTNYELTH

LQLQYVSGGLQGAIVSIAEEKGTAPLEDAATGAGSIEAIYNTIDRIMEASVELTDYRI

QAVTKGQDAQAEVHTTVKDGNGRTFHGIGIDFDVLNASAKAYIQASGKAKDQVEKGAN

"

CDS complement(2934709..2935704)

/gene="ilvC"

/locus_tag="JFBMFIFI_02755"

/EC_number="1.1.1.86"

/inference="ab initio prediction:Prodigal:002006"

/inference="similar to AA sequence:UniProtKB:Q2FWK4"

/codon_start=1

/transl_table=11

/product="Ketol-acid reductoisomerase (NADP(+))"

/db_xref="COG:COG0059"

/translation="MTKVYYDNTVTEDALKDKKIAVVGYGSQGHAHSQNLRDNGYDVV

IGIREGKSAEAAREDGFTVLSVADAAKQSDVIMVLLPDETQGTIYENEIAPYLEAGNS

LVFAHGFNIHFDVIAPPENVDVFLVAPKGPGHLVRRTFVEGAAVPALFAVYQDATGKA

RDLALSYAKGIGATRAGVLETSFKEETETDLFGEQAVLCGGVTSLIQAGFETLTEAGY

QPEVAYFEVLHEMKLIVDLMYEGGMGKMRHSISNTAEYGDYVSGPRVITADTKAAMKE

VLTDIQTGAFAKAFVDDNKNEFKEFKQMRASQQGHQIEEVGARLREMMPFVKPNN"

CDS complement(2935755..2936240)

/gene="ilvH"

/locus_tag="JFBMFIFI_02756"

/EC_number="2.2.1.6"

/inference="ab initio prediction:Prodigal:002006"

/inference="similar to AA sequence:UniProtKB:A0QUX7"

/codon_start=1

/transl_table=11

/product="Acetolactate synthase small subunit"

/db_xref="COG:COG0440"

/translation="MRRVITATVRDATGVMNRFTGVISRRQYNVSSISVGRTIEPEVS

RITIVVEIETEEEAQQVIKQLYKQIDVLKVKDITDCPHLERELALIKVDAPLSVRPEI

NAILEPFRGQIIDVGTDSVVVQVTGTSEKVEGFIDLISPYGILQIARTGVTGFTRSPK

T"

CDS complement(2936241..2937932)

/gene="ilvB"

/locus_tag="JFBMFIFI_02757"

/EC_number="2.2.1.6"

/inference="ab initio prediction:Prodigal:002006"

/inference="similar to AA sequence:UniProtKB:P37251"

/codon_start=1

/transl_table=11

/product="Acetolactate synthase large subunit"

/db_xref="COG:COG0028"

/translation="MTNETKMKTGAQLLVDALEKQEVEMIFGYPGGAVLPLYDAFYDS

DIPHILTRHEQGAVHAAEGYARVTGKPGVVVVTSGPGATNALTGIADAMSDSIPLVVF

TGQVHRPGIGKDAFQEADMIGLTTPITKYNYQVRDAKELPRIINEAFHIATTGRKGPV

VIDIPKDVGIMQVAESSPAIIDLPGYTTSYPVDMKQIESVMAALAKAEKPLILAGAGI

NHAGAGLELLEFSERYQIPIVNTLLGLGSFPYHHPLFTGMGGMHGSYTANMSLTECDL

LINFGSRFDDRLASCPKEFAPEAFIVHVDIDPSELGKVIKTDIPIAGDVKEILDYLLQ

QPLLEKADTSEWQELTQARKKRYPFGYKESDEEIKPQKVIELIGEITHGEALVATDVG

QHQMWTAQYYPYKNRYQIVTSGGLGTMGFGFPAAIGAQLAFPDKTVVCIVGDGGFQMT
NQELSIIHEYNLNVKTVILNNGVLGMVHQWQQKFHGARFSHSEFDSQPDFVKLSEAYG
VKAISLSDPATLKADLTAAFNEPGAIVIDVHISPDEVVLPMIPSGQPNHAMEGVE"

CDS complement(2938015..2939706)

/gene="ilvD"

/locus_tag="JFBMFIFI_02758"

/EC_number="4.2.1.9"

/inference="ab initio prediction:Prodigal:002006"

/inference="similar to AA sequence:UniProtKB:P51785"

/codon_start=1

/transl_table=11

/product="Dihydroxy-acid dehydratase"

/db_xref="COG:COG0129"

/translation="MRSDTIKKGVEQAPARSLLHATGQIKSSGDMDKPFIAICNSYID
IVPGHVHLRELADIAKEAIREAGGIPFEFNTIGVDDGIAMGHIGMRYSLPSREVIADA
AETVINAHWFDGVFYIPNCDKITPGMLLAAVRTNVPGIFCSGGPMKAGLSATGKALTL
SSMFEAVGTFKEGKMTQEEFLDMEANACPTCGSCAGMFTANSMNCLMEVLGLAVPYNG
TALAVSDERRELIRQSAFHLMDLVKNDIKPRDIITKDAIDDAFALDMAMGGSTNTVLH
TLALAHEAGIDYELERINDIAARVPYLSKIAPSSSYSMHDVHEAGGVPAIINELIKVG
NAIHPDRITVTGKTLRENNEDKEILNKDVIHPKENAFSPVGGLSMLFGNIAPKGATIK
VGGVDPSVKVFEGEAICFSTHDEAVAAIDDHRVTRGHVVVIRYEGPKGGPGMPEMLAP
TSSIVGRGLGKEVALITDGRFSGATRGIAVGHISPEAAAGGPIALIQDGDRITIDLPN
RTINVNVSDEVLEERRTHLPRFKAKVRTGYLARYAALVTSAHTGGVLKIPDELLD"

CDS complement(2940872..2941099)

/locus_tag="JFBMFIFI_02759"

/inference="ab initio prediction:Prodigal:002006"

/codon_start=1

/transl_table=11

/product="hypothetical protein"

/translation="MKKIIISVSLGLFIIGGTQPIFASELDIHTDIEITNNLNRATLK
PWFKKIPPLKYKGRTRINYYPNSGGYIGVYL"

CDS complement(2941183..2941923)

/gene="agrA_2"

/locus_tag="JFBMFIFI_02760"

/inference="ab initio prediction:Prodigal:002006"

/inference="similar to AA sequence:UniProtKB:P0A0I7"

/codon_start=1

/transl_table=11

/product="Accessory gene regulator protein A"

/db_xref="COG:COG3279"

/translation="MLSIFICEDNDIQRSNIEAIIKNYIMIEEFDMELTLSTSNPYDI

INFLDENPRVRGIYFLDVDLGYDINGIQLGSQIREKDIDGKIIFITTHSELLILTFTY

KVEAMDYILKDDTDIIKKRIYEALKQAQKHYHTETMIEKNRIKLKVGSQIRIFPTEDV

MFIETSPIPHKLTLHLTSSTLNFYGKINEMTALSPVMFRSHKSYIINIENIKSIDKTN

REITMNNGETCLLSVRQLKKLEITIMNK"

CDS complement(2941931..2943232)

/locus_tag="JFBMFIFI_02761"

/inference="ab initio prediction:Prodigal:002006"

/codon_start=1

/transl_table=11

/product="hypothetical protein"

/translation="MTELLINYISFSHLIEFTLAYFLLDQLYMQNKITYLLVTLALLF

SVYIGGSFTIIDTLLLFIFFFFLSIKNSKSKNIIHSLLTVSIAYLLEIFTTILMRPLF

LKSIESTKLTNTFFMFICIVVGTFSLIFLSSWYIRKWILSRINFGKKETALSYLLTLC

LLAYQTYWLLAIYSGHTPLLKIFIIVFYAILIIPILSFIQTLKKNEELKFQAHQRKIE

YDMMSKYAEEVKEQYQKIRKFRHDYVNILSSIENYLEQNKIEELKVFYHAHVKQTKSL

FKSNMLRLDDLQKIEAIEIQSILATKLIMAQEKKIDVQIEVSENISKNIPIDPVILIR

ILGILLDNAIEELETLTYGKLLVGIFTINSDLVFIIQNNVREDIESVQLLKKQGYSTK

GETRGIGLANVEELISLEPRLLLETTIKDKLFIQKIIIMKG"

CDS 2943553..2944077

/locus_tag="JFBMFIFI_02762"

/inference="ab initio prediction:Prodigal:002006"

/codon_start=1

/transl_table=11

/product="hypothetical protein"

/translation="MKNKKIKKVAITLTAFLIVGGNIIPSMTAFASENNVKIEDTVTQ

NNVFDENYKMNEDELKAFGLSETEIAKFNQYKVSGIQLVDGKAFQNGQEIITMERGKF
TWAVKAIRKGYNALPSKVKSFIAQYTGLNAFLGLIEHYTGALEDGIYKACIRVGMPSS
VANFVTKTIMLFVF"

CDS 2944102..2944296
/locus_tag="JFBMFIFI_02763"
/inference="ab initio prediction:Prodigal:002006"
/codon_start=1
/transl_table=11
/product="hypothetical protein"
/translation="MEFKEICKIIFTLMLSLAFIIAGYNLFFSDYTTIKRIIILVTTA
LIGIQVLVNWFDLYNKYKNK"

CDS complement(2944353..2944706)
/locus_tag="JFBMFIFI_02764"
/inference="ab initio prediction:Prodigal:002006"
/inference="similar to AA sequence:ISfinder:ISLmo3"
/codon_start=1
/transl_table=11
/product="IS6 family transposase ISLmo3"
/translation="MNPTTIMRWVHQYGKLLFVIWKKKNKSPSTSWRMDETYIKIKGK
QHYLDMCLRKHRDTSAAYAFMKRLIRCYGEPRALVTDKYAATITAVNKLKNKGFLKSV
DYRLSKYLYNVTQKT"

CDS complement(2944942..2946600)
/gene="treA"
/locus_tag="JFBMFIFI_02765"
/EC_number="3.2.1.93"
/inference="ab initio prediction:Prodigal:002006"
/inference="similar to AA sequence:UniProtKB:P39795"
/codon_start=1
/transl_table=11
/product="Trehalose-6-phosphate hydrolase"
/db_xref="COG:COG0366"
/translation="MSNFHDKVVYQVYPKSFKDSNGDGIGDLRGVIEKLAYLADLGVD
MLWLNPFFTSPQNDNGYDISNYKEIDPLFGTMADFEELVHKAEALGMEIMLDMVLNHS

STEHEWFQKALAGDKEYQDYYYLRPAQENGDLPTNWESKFGGPSWSKFGQTDLYYMHL
FDPTQADLNWHNPKVREELHQVVNFWLNKGVKGFRFDVINLIGKSTTELVDDLTGNGK
PLYTDQPIVHDYLKEMNQATFGRVADTVTVGEMSSTTIENCVLYSNPTREELSMAFSF
HHLKVDYVDGEKWSLMPFDFLALKNILNDWQTGMSEGDGWNALFWNNHDQPRAISRFA
DPVNYHSESATLLAQTIHLLRGTPYIYQGEELGMTNPDYEAIEDFMDIETHNAYRELQ
EKGLSESEAMAIIREKSRDNNRTPMQWTDEEHAGFTTGTPWLKVVDNYREINVAKEVA
KGSIFSYYQRLIQLRKELPIIANGSYEGLLLEHPSVYAYARHLNGETVLVFNHFYAEP
VTIQVPEEYLNRSSRYLIGNGQERELTSSLTLAPYETIAFYLDA"

CDS complement(2946734..2948176)

/gene="treB"

/locus_tag="JFBMFIFI_02766"

/inference="ab initio prediction:Prodigal:002006"

/inference="similar to AA sequence:UniProtKB:P36672"

/codon_start=1

/transl_table=11

/product="PTS system trehalose-specific EIIBC component"

/db_xref="COG:COG1263"

/translation="MADYKNDAQLLLEDIGGKDNISAVTHCATRMRFVLVDPKAADKE
KIEKIPAVKGTFTQAGQFQVIIGNDVATFYNEFVKLSGTEGVSKDAVKSVAKQNMNPV
QRAMAVLAEIFTPIIPAIIVGGLILGFRNVLEGVEMGFLNGQTITEVSVFWSGVNDFL
WLIGEAIFHFLPVGITWSIAKKMGTTQILGIVLGITLVSPQLLNAYSVASTKAAEIPF
WDFGFFTIDKIGYQAQVIPAMLAGFMLAYLEIWLRKFIPQSISMIFVPFFSLIPTVIA
ANMILGPIGWWVGDKVSMIVNAGLTSSVNWLFGALFGFLYAPLVITGLHHMSNAIDFQ
LVADFGGTNLWPMIALSNIAQGSAVLAIIFLHRGNKEEEQISIPAAISCYLGVTEPAM
FGINLKYVYPFIAAMIGSAIAGLVSTIFNVTANTVGVGGLPGILSIQPPYWGVFLVCM
LITIVVPFGLTVLFRKYGIFNQLDPIDESV"

CDS complement(2948378..2949241)

/locus_tag="JFBMFIFI_02767"

/inference="ab initio prediction:Prodigal:002006"

/codon_start=1

/transl_table=11

/product="hypothetical protein"

/translation="MIPISKGIFLLASLLLLGGCTGGSSDSMKKKTSEITNTSSENSV

SKSDQSSESSKKAEDSSVTPENGLTLDQVMDAVKNQLETQVVIRLPTSIPLSNPNLHV

SAATVSDATSYHVIFFESKEPIPINNNALNDPNQASQIVTISALRYPSVSEANVAVGY

QKADAGYENGASGIDLGHGLTGYENAGAGSQFIGWNEGNWYLDVRSSLANTTQPGRQM

ATKIVEKLEKELLPAPNSVGKIQADVTDDKPNQTSIVWQEEDSVYTIESSIDVLSTIG

IATSFDDGATD"

CDS 2949406..2949972

/gene="dhaS"

/locus_tag="JFBMFIFI_02768"

/inference="ab initio prediction:Prodigal:002006"

/inference="similar to AA sequence:UniProtKB:Q9CIV9"

/codon_start=1

/transl_table=11

/product="HTH-type dhaKLM operon transcriptional activator

DhaS"

/translation="MHDSLITKKVIAHSLKELMQLTPFQKISIRDIMGHADIRRQTFY

YHFQDKYELLAWIYNQEASENIEDYLDYEHWSKVITRLFYYLKENKTFYLNALEVTEQ

NSFDLYFFEHTKSLILTVVTDMQNNAQVVIPEESIQFFCTFYAHAFVGMTKEWLFSDC

KTPVELLSQNIQLLLEESFSTVIERFKS"

CDS complement(2950304..2951293)

/gene="dhaK_1"

/locus_tag="JFBMFIFI_02769"

/EC_number="2.7.1.121"

/inference="ab initio prediction:Prodigal:002006"

/inference="similar to AA sequence:UniProtKB:Q9CIV8"

/codon_start=1

/transl_table=11

/product="PTS-dependent dihydroxyacetone kinase,

dihydroxyacetone-binding subunit DhaK"

/db_xref="COG:COG2376"

/translation="MKKIMNHPDKIVSQMLSGLLYANSDKLAGIKGTGVIYRQTKSEN

KVAVVSGGGSGHEPAHTGFVGTGMLAACICGPIFVPPTAEEILSGIKVADQGAGVFLV

IKNFEKDVASFLKAKELAEAEGYQVSYVIVNDDCSIEENSFKKRRRGVAGTILVQKIV

AAAAEEGYSLEQLTELATKVVRSMNTLGLALSPSVVPGEDQAQFQLAENEISFGIGIH

GEPGYRTEILHSSERLAIELVNKLKSQFHWKKGEKFAMMINGLGGTPLMELFVFSNDV
RRLLELEGLQVEFKKVGDFVTSNDMSGLSLTFLRLEDEKWLEWLKVPVDTYAW"

CDS complement(2951395..2952138)

/locus_tag="JFBMFIFI_02770"

/inference="ab initio prediction:Prodigal:002006"

/codon_start=1

/transl_table=11

/product="hypothetical protein"

/translation="MENKSRKFSLQVIMYLSFFAMVAVNALANILPFNGMNTGEISNL
YNVLFTPAGYVFSIWGVIYIAVFIWLVNLSLNKEPIRKISASLFILSCLLNGSWIFFW
HYQFVGTSVFVITALFVTLLLLYLYERGQKGLVWPFSIYLGWVTVATLANISYYLVAE
QQISMSSQAVSASLFLLLGLVIGALMLWIYQDRIFNLVFIWAYLGIFFRDYDSSRLVS
LIALFSGIGLILLVGISFLKREKNLSKIK"

CDS complement(2952271..2953404)

/gene="gldA"

/locus_tag="JFBMFIFI_02771"

/EC_number="1.1.1.6"

/inference="ab initio prediction:Prodigal:002006"

/inference="similar to AA sequence:UniProtKB:P0A9S5"

/codon_start=1

/transl_table=11

/product="Glycerol dehydrogenase"

/db_xref="COG:COG0371"

/translation="MRKAFIAPSKYVQGEGELLNLGYFVKTFGKSALLIAHKDDIGRV
QSQLDQTAEKFGIEFISSNFNGECSREEVARLQEFAKGHNCDCVIGLGGGKAIDTAKC
VAAGEALIIVPTIAATDAPTSHSAVLYTPEGEFDDYAYFKQSPSVVLVDTKIISQAPT
RFLVSGMGDALSTYFEARATANSFSNVNAGLPCGANSGECPPAKGTNAAYGMAKLCYT
MLLENGLNAKIACDNNLVTPALENIVETNILLSGLGFESAGLAAIHAVHDGLTALEGT
HHYFHGEKVAFSAIVQLVLENAPTEELYEVLDFSLSIGLPVCLADIGVESISYEEALA
VAEKACIPEESIHSMPLPITVEAVASALLAADKIGANYKANLV"

CDS complement(2953505..2953876)

/gene="dhaM"

/locus_tag="JFBMFIFI_02772"

/EC_number="2.7.1.121"

/inference="ab initio prediction:Prodigal:002006"

/inference="similar to AA sequence:UniProtKB:Q9CIV6"

/codon_start=1

/transl_table=11

/product="PTS-dependent dihydroxyacetone kinase,

phosphotransferase subunit DhaM"

/db_xref="COG:COG3412"

/translation="MSKLGVVVVSHVPAIAEGVVTLMKEVAKDVSITFAGGTDDNEVG

TSFAKIEAAFEENTGETLLAFYDLGSAKMNLEMVMELTDKEVHLYDTAFIESSYTAAA

LIQAGAELDAIEEQLAPMKVK"

CDS complement(2953869..2954453)

/gene="dhaL"

/locus_tag="JFBMFIFI_02773"

/EC_number="2.7.1.121"

/inference="ab initio prediction:Prodigal:002006"

/inference="similar to AA sequence:UniProtKB:Q9CIV7"

/codon_start=1

/transl_table=11

/product="PTS-dependent dihydroxyacetone kinase,

ADP-binding subunit DhaL"

/db_xref="COG:COG2376"

/translation="MNVENIIKWLDLFTEKVNENKDYLSELDSAIGDGDHGMNMARGT

TAVKEVLDAKKPTTVVDIFKLTGMTLVSKVGGASGPLYGSAFIGMAKEASATDDLGEI

LQAGLDSIEKRGKAVAGEKTMIDTWVPVIEAIKTNELTTEVIESAVNATKDMKATKGR

ASYLGERSIGHIDPGAASSGYLFETMMEAGVCNE"

CDS complement(2954480..2955469)

/gene="dhaK_2"

/locus_tag="JFBMFIFI_02774"

/EC_number="2.7.1.121"

/inference="ab initio prediction:Prodigal:002006"

/inference="similar to AA sequence:UniProtKB:Q9CIV8"

/codon_start=1

/transl_table=11

/product="PTS-dependent dihydroxyacetone kinase,

dihydroxyacetone-binding subunit DhaK"

/db_xref="COG:COG2376"

/translation="MKKIINQPEAVVEEMLQGLVYAHGDSIKRLPESGVIAKKNKVNG

QVGLVSGGGSGHEPAHAGFVGQGMLSAAVCGPVFTSPTPDQILAAIQEVDAGAGVFLI

IKNYSGDVMNFDMAKELAEMEDIKVDFIVVDDDIAVEDSTYTAGKRGVAGTVLVHKIL

GAAADQGATLTEIKQLAEKIVPAIKTLGVALSGATAPEVGKPGFVLADNEIEFGVGIH

GEPGYRKEKIAPSKELAQEIVSKLKLAFDWKSGERYGLLVNGLGSTPLMEQYIFTNDV

GLLLEEEGLVVEFKKVGDYMTAIDMAGLSLTLIKLEENEWLDHLNYETKTIAW"

CDS 2955714..2956340

/locus_tag="JFBMFIFI_02775"

/inference="ab initio prediction:Prodigal:002006"

/codon_start=1

/transl_table=11

/product="hypothetical protein"

/translation="MLEIGKKIKELRLSKKLTQKDLAKILNVTPQAISKWERNESNPD

IQMLVTLSKYFNISVDDILGNKNENFFGSFISKIKGNKKMEKVTKKNQSEDFENTENE

KKVLIFDIVFSLISDEGQIQTQFLTSKLELFLKKSNENITVKTYSSDKVDQYGAQADV

LLLVPTFGYAKDELEKKFPGTPILVISKKDYGMLNVEELSNKIIDLLG"

CDS complement(2956631..2957188)

/locus_tag="JFBMFIFI_02776"

/inference="ab initio prediction:Prodigal:002006"

/codon_start=1

/transl_table=11

/product="hypothetical protein"

/translation="MFIKDSISNISLRRIETKDIFDLWKMAHDGDLEWTKWNGPYFNN

PILTFEEFKKVAEENYLNNPYRAVIVYENKVVGQVSAFWEDGELKNWLEFGILLYDQN

TWSKGMGSIVTKLWITHLFEITPHIHRIGFTTWSGNKGMMKVGEKIGMTKEAQIRKVR

YYQKKYYDSIKYGVLRNEWLLLQES"

CDS complement(2957505..2958188)

/locus_tag="JFBMFIFI_02777"

/inference="ab initio prediction:Prodigal:002006"

/codon_start=1

/transl_table=11

/product="hypothetical protein"

/translation="MNKFILTTIFFSLVLFTTACSTNKTNEAPVASSQNQSSESVEHE

HSHEQTKASQGIFEDSEVKDRELSDWEGSWKSIYPYLVDGSLDEVFEKKAKDGDKTFE

EYKEYYREGYQTDIDSIDINKGIISFHMNDKWETAEYNYDGYEILTYESGKKGVRYLF

TSVENNSKAPKFIQFSDHIIEPQKSGHFHICMGNESHKELLSEMTNWPTYFPESMSKK

DIIHDMLYH"

CDS complement(2958584..2959372)

/locus_tag="JFBMFIFI_02778"

/inference="ab initio prediction:Prodigal:002006"

/codon_start=1

/transl_table=11

/product="hypothetical protein"

/translation="MNKNLLKVVINFFAGVLIVSSALMYFPTLSNKSAEATDIGNIGD

AIRNYKYEGISVDSEDPVVSEKEVIETEKMLNEDGNSVQIEKNASSLIEITQNEKTTE

PAVYVSEHKIIGENIKDSSSLSIVYNQKGEVLKKMTLLGSQMNDDIRVQYYVNAEKKI

NMVVDTEGTIISGESFSDGIATKLNAGKENSRNKRSVASWGSCMGSCLKSKGVESWVI

SAVTTLCSLGCFLTAGAGCIGCIVAAAAMKGSQASTCAVSCAGK"

CDS complement(2959386..2959532)

/locus_tag="JFBMFIFI_02779"

/inference="ab initio prediction:Prodigal:002006"

/codon_start=1

/transl_table=11

/product="hypothetical protein"

/translation="MKLKKEQIVLLLFLVLLFIDALFPFKLSTMVLLGIMFFVCIGMF

YKKK"

CDS complement(2959555..2959986)

/locus_tag="JFBMFIFI_02780"

/inference="ab initio prediction:Prodigal:002006"

/codon_start=1

/transl_table=11

/product="hypothetical protein"

/translation="MKKNIVIFVLALITFGTVLTVKYSDKLIYFYNVSSLKKLDTNDL
KTLTKQEGTHIIYIGRPTCEYCFDFVPKLNKVVKETNLEVNYYDTDKNRKDLQMTELL
TELNVETVPAVTVVKDKTVVKQINPTKLSAIELENEFLKYN"

CDS complement(2960717..2961238)

/locus_tag="JFBMFIFI_02781"

/inference="ab initio prediction:Prodigal:002006"

/codon_start=1

/transl_table=11

/product="hypothetical protein"

/translation="MGTIDKAIDFLNNPLNKGVLWAGGIISFILLLCNVLLSSDNLKL
LFIDSLLDKYGWTLPLIFLFCIVFLIVGFVSGKIKQKEDKKFEDHMNTTREDLFTDKD
ALLYLNRLFKNHPNPCKLPQLNQKVKLLEQYGLIAKASNQILVYSQDEIENPYFPYIL
QPIAEKRLKKMQN"

CDS complement(2961503..2961910)

/locus_tag="JFBMFIFI_02782"

/inference="ab initio prediction:Prodigal:002006"

/codon_start=1

/transl_table=11

/product="hypothetical protein"

/translation="MSMYSGVKDTIKKDIAREEAKDHPNKMLLDAWKDQYKAYELAEK
KMAQQSALKKAIKAKFDEMIEETKKLKDKNSSSFESVSNQITAVKDESRNCFKGTAGS
KYRVKVTNKNKTIIGVKNDHSSLVKLSGRRVGL"

CDS complement(2961907..2963064)

/locus_tag="JFBMFIFI_02783"

/inference="ab initio prediction:Prodigal:002006"

/codon_start=1

/transl_table=11

/product="hypothetical protein"

/translation="MNVSESQYWGLSNEIYFDNTLKVNIPYKNKEDGSWWVTVSSVNN
PSGLQAAAVVPLSEWEAQKNKNPKKYDTIVVVARGSESNNALDVVQDWLVADVYQGTG
IGQFYTPNLGINQTNEFVEFSQKVFRDQLAPDGVGSFTGHSLGGKLATVGAIRSGKLA
TTFAAANPFHELNTVEKAKALLGMYNAQILDYAHEGDVVPFSPPGPRIGTKRYIKNNI
KKTGNFFKDMMNAHIQAGFNSTEFSSAFKNGSAEQILYTPERIKELSGRFRHIESELD

SIKRNLFDYQWFEGDELYYFEKSLKNEAMPGGLYSELSEYEIEDMIRDIAKFSRNGQP

YLHDPDKMDDVLSGIEQAKKEMDELGGDTYQAAVEMEAKDKELAEKRMNFK"

CDS 2963138..2963614

/locus_tag="JFBMFIFI_02784"

/inference="ab initio prediction:Prodigal:002006"

/codon_start=1

/transl_table=11

/product="hypothetical protein"

/translation="MKNKKYILLALLILTCGGLFMNYKIKENEKIEIEYLDSLADKQE

PYIKKYLTYYFNGIETVTLTTKERIPTGTVGIHGYINNDPSLGFTATFDSNKPGSNMG

LLPTFDITPMRKPESETPAKNMEEILKEERKKKELKKKPVQHKTGLISKKHPTKLY"

CDS complement(2963948..2964505)

/locus_tag="JFBMFIFI_02785"

/inference="ab initio prediction:Prodigal:002006"

/codon_start=1

/transl_table=11

/product="hypothetical protein"

/translation="MSAVLFFSDQTEFIDWLDIHHQIASEIWVVFFKKKTNKGSLTWS

ESVDCALAFGWIDGIRKTVDEDSYKIRFTPRKPNSLWSKVNVQKVHKLIELNQMRTEG

MAAFKQRKDKIGYSSVHRNVTLVKEYEDEIRKNLSSWEFFNQLSPSYKRDSIWWIMSA

KKEETRLRRLNRLIVSWEKGEKFRY"

CDS complement(2964502..2965434)

/gene="ephA"

/locus_tag="JFBMFIFI_02786"

/EC_number="3.3.2.10"

/inference="ab initio prediction:Prodigal:002006"

/inference="similar to AA sequence:UniProtKB:I6YGS0"

/codon_start=1

/transl_table=11

/product="Epoxide hydrolase A"

/translation="MTINYEFPTPTLISVNGVELEVFEAGQKNWGRPIVLCHGWPEHA

FSWRYQVTPLVEAGYHVIIPNQRGYGESSCPKEVIKYDIEHLTGDLVALLDHYQYKDA

IFMGHDWGANVVWSMALLYPERVSKMINLSLPYQDRGEKPWLDFMEDVFGDEYYFVHF

NKQPGVADAILDENVEQFLRNLYRKNAPSQGPSEGMEMIHLAKATKPLGEPIMSAEDL
SVYIAAFNKTGFTSSINWYRNLNRNWHLLATVSPILQQPTLMVYGEKDLIPPLPNITD
FVPNIDIKSLDAGHWIQEERPEELNQLILEWLGK"

CDS complement(2965804..2966196)

/locus_tag="JFBMFIFI_02787"

/inference="ab initio prediction:Prodigal:002006"

/codon_start=1

/transl_table=11

/product="hypothetical protein"

/translation="MKYILMGFSITCLIFILFQPKASAVKFEENKHKQKTNEYSKLQV
ISVEKSLDVAERTEKKERLDSVGKWRDKNMENSKNKEKRKRIKKSPNEAKMSILKKIS
NIWFRRMYCKVKKFLEKIGWSWYRDEKI"

CDS complement(2966361..2967098)

/locus_tag="JFBMFIFI_02788"

/inference="ab initio prediction:Prodigal:002006"

/codon_start=1

/transl_table=11

/product="hypothetical protein"

/translation="MEKYTATGTTVEEAVSNGLKKLGLKKDEAQITIIFEGKKGILGF
GKKDAIVEISPKLVKETPSVTESTDVPELEVEKQTQEVNPVVEVEERNDEIAFKAISD
YLTSIATEMGAPSTVQVDVVQDQVIFHMETQKPGLVIGKHGKVLNALQSLAQVLMHRH
AKSKLTAIVNVGDYRERREAVLKQLADRTSEKVLRTNQAVFLEPMPAFERKQIHFYIS
KNDKLSTHSEGNEPHRYLVVEPTKKRF"

CDS complement(2967145..2967981)

/gene="misCA"

/locus_tag="JFBMFIFI_02789"

/inference="ab initio prediction:Prodigal:002006"

/inference="similar to AA sequence:UniProtKB:Q01625"

/codon_start=1

/transl_table=11

/product="Membrane protein insertase MisCA"

/db_xref="COG:COG0706"

/translation="MRKRKRLLIVLASIVLMVVLTGCGTSPITADSTGFWDQYVVWNF

SRAITSLSNIFFGSYGLGIIVFTIIIRIILLPLMHHQTKSTRKMSEMQPQLKALQQKY

ASKDTETQNKLKEETQKLYSAAGVNPVMGCLPMLVQMPVLMAMYQAISRTDVLKTGQF

LWMDLGSRDPYFILPILAAILTFATTKLSTMSQAESNPTTSAMLYMMPALILFMGISL

PSALSLYWVVGNAFSVGQTLLLNNPFKIKREREDKIKAERDYQKALEKAKNPKKKRNK

KR"

CDS complement(2968018..2968359)

/gene="rnpA"

/locus_tag="JFBMFIFI_02790"

/EC_number="3.1.26.5"

/inference="ab initio prediction:Prodigal:002006"

/inference="similar to AA sequence:UniProtKB:P0A0H5"

/codon_start=1

/transl_table=11

/product="Ribonuclease P protein component"

/db_xref="COG:COG0594"

/translation="MRKAYRVKKEADFQKVFHKGKSTANRQFIIYTLNKENQEHFRVG

LSVGKKVGNAVARNAVKRRIRQSLTELKSELKADKDFIVIARVPAAGMSTKEVKKSLT

HVLKLAKMINK"

CDS complement(2968576..2968710)

/gene="rpmH"

/locus_tag="JFBMFIFI_02791"

/inference="ab initio prediction:Prodigal:002006"

/inference="similar to AA sequence:UniProtKB:P23376"

/codon_start=1

/transl_table=11

/product="50S ribosomal protein L34"

/translation="MKRTYQPKKRKRQKVHGFRKRMSTKNGRRVLQSRRRKGRKELSA

"

CDS 2969456..2970796

/gene="dnaA"

/locus_tag="JFBMFIFI_02792"

/inference="ab initio prediction:Prodigal:002006"

/inference="similar to AA sequence:UniProtKB:P05648"

/codon_start=1

/transl_table=11

/product="Chromosomal replication initiator protein DnaA"

/db_xref="COG:COG0593"

/translation="MDDLQSLWNYLKERFKESLAEVSYNTWVKSAHPIRITEQNIIIE

VPSSLHKDYWEKNLTTRIVENIYEYSGREISPLFIIKNEQDSTDNSSFENNSSISKKD

GPRKDALLNDKYTFETFVIGKGNQMAHAAALVVAEEPGTIYNPLFFYGGVGLGKTHLM

HAIGHQMLLINPDAKVKYVSSETFANDFINSIQTKSQEKFRNEYRSVDLLLVDDIQFF

ADKEGTQEEFFHTFNALYDERKQIVLTSDRLPNEIPKLQERLVSRFAWGLSVDITPPD

LETRIAILRKKANAERLEIPGDTLSYIAGQIDSNIRELEGALVRVQAYAAIESRDITT

SLAADALKSILPSNKPTTLSILEIQSVVSKYYQIALSDLKGKKRVKSIVVPRQIAMYL

SRELTSNSLPKIGAEFGGKDHTTVIHAYEKIDHALTKDEELRRDIEEIKNKLSS"

CDS 2971020..2972162

/gene="dnaN"

/locus_tag="JFBMFIFI_02793"

/inference="ab initio prediction:Prodigal:002006"

/inference="similar to AA sequence:UniProtKB:P05649"

/codon_start=1

/transl_table=11

/product="Beta sliding clamp"

/db_xref="COG:COG0592"

/translation="MKFSIKRSEFLKSLADVQRAISSRTTIPILTGLKIMASEEGLTL

TGSDSDISIEAFISQEDEKNNLTIDQAGSIVLQARFFSEIVKKLPDEMMEVEVLDHFQ

TLITSANASFTVNGLDANNYPHLPVIDTQETFVLPVNLFKQVIGQTVIAISTHESRPI

LTGVNMVIEHGKLLAVATDSHRLSQRIIPLELNSALADQKYNVIIPGKSLIELSRTLD

DSHDNIEMMITENQVLFKMENMLFYSRLLEGYYPDTARLIPETASTEIVFNASSLLGS

IERASLLSHEGKNNVVKVSITPDLVEISGNSPDVGNVKEELAYISVEGEPIDLSFNPD

YMKDALRTFGQTDIRIKFTSPVRPFILVPSEDDKTFIQLITPVRTF"

CDS 2972487..2972729

/locus_tag="JFBMFIFI_02794"

/inference="ab initio prediction:Prodigal:002006"

/codon_start=1

/transl_table=11

/product="hypothetical protein"

/translation="MKETVYIDSEFITLGQLLKHVNIISSGGMAKWYLAEHTVLLDNE

VENRRGKKIFPGSIVEIPDEGTYFIQDASQSQKAED"

CDS 2972729..2973850

/gene="recF"

/locus_tag="JFBMFIFI_02795"

/inference="ab initio prediction:Prodigal:002006"

/inference="similar to AA sequence:UniProtKB:Q8RDL3"

/codon_start=1

/transl_table=11

/product="DNA replication and repair protein RecF"

/db_xref="COG:COG1195"

/translation="MFLEEIKLTQYRNYEAAEVQFSEGINVFLGENAQGKTNLMEAVY

VLAMTRSHRTSNDKELIFWNQEFAKISGRIQKKNSSFPLEIVVSNKGKKAKFNHLEQK

KLSDYLGKLNVILFAPEDLSLVKGSPAIRRKFLDMEMGQMNPIYLHNLVNYQHILKQR

NQYLKQLMMKKAKDLTFLEVLTEQLATYGAEILLERLSFVKKLEKWAQPIHHEISRER

EDLVIEYQCSLPLTNELDKASIYQDLLAAFEKGRKRELDQGSTIVGPHRDDLKFLVNN

QNVQTYGSQGQQRTTALSIKLAEIDLMREITGEYPILLLDDVLSELDDERQTHLLKAI

ENKVQTFLTTTSLDGIKKNILKAPRVFQVSSGHVEMESE"

CDS 2973853..2975790

/gene="gyrB"

/locus_tag="JFBMFIFI_02796"

/EC_number="5.6.2.2"

/inference="ab initio prediction:Prodigal:002006"

/inference="similar to AA sequence:UniProtKB:Q839Z1"

/codon_start=1

/transl_table=11

/product="DNA gyrase subunit B"

/db_xref="COG:COG0187"

/translation="MSEETRDMDALAQEYDASQIQVLEGLEAVRKRPGMYIGSTSGQG

LHHLVWEIVDNSIDEALAGFASEINVIIEEDDSITVIDDGRGIPIGIQAKTGRPAVET

VFTVLHAGGKFGGGGYKVSGGLHGVGSSVVNALSTILEVMVHKDGKIHYQKFHQGKVG

EDLKIIGDTDRQGTTVHFVPDPEIFTETTVFEFEKLSTRVRELAFLNKGLKISIEDKR

EGQETLKEFHYEGGIKSYVEHLNHNKTVLFDDPIYIEGEQQGIVVEAAMQYTDGYHTN
LLSFANNIHTYEGGTHESGFKTALTRVINDYARKSKLMKENEENLTGEDVREGLTAVI
SIKHPDPQFEGQTKTKLGNSEARTITDRLFSAHFDKFLMENPQVARRVIEKGILASKA
RLAAKRAREVTRKKSGLEISNLPGKLADCSSKDPSISEIFIVEGDSAGGSAKQGRSRL
FQAILPIRGKILNVEKATLDKILANEEIRSLFTAMGTGFGGDFDVSKARYHKLVIMTD
ADVDGAHIRTLLLTLFYRYMRPVVEAGYVYIAQPPLYQVRQGKKMVYLDSDAELEAYL
KEIPAQPKPVIQRYKGLGEMDAEQLWSTTMDPERRRMLQVTVDDAIEADQVFEMLMGD
RVEPRRKFIEENAVYVQNLDI"

CDS 2975940..2978489

/gene="gyrA"

/locus_tag="JFBMFIFI_02797"

/EC_number="5.6.2.2"

/inference="ab initio prediction:Prodigal:002006"

/inference="similar to AA sequence:UniProtKB:Q8DPM2"

/codon_start=1

/transl_table=11

/product="DNA gyrase subunit A"

/db_xref="COG:COG0188"

/translation="MAEEFKENISEINLTKEMRTSFLDYAMSVIVARALPDVRDGMKP
VHRRILYGMNELGVTPDKPYKKSARIVGDVMGKYHPHGDSAIYESMVRMAQPFSYRNT
LVDGHGNFGSVDGDGAAAMRYTEARMSKIAMEMLRDINKDTIDYQDNYDGSEREPEVL
PARFPNLLVNGATGIAVGMATNIPPHNLSEVISALHILMNNPDATTADLMEVLPGPDF
PTGGLVMGKSGIRRAYETGRGSITIRAKVEIEILKNGKERIVVHELPYMVNKAKLVER
IADLAREKRIEGITDLGDESDRDGMRVVIDVRRDVSASVVLNNLYKLTPMQTSFGFNM
LAIVKGVPKVLSLKSILENYLEHQEIVIRRRTEFDKRKAEARAHILEGLRIALDHIDE
IIKIIRGSKTGEIAKNELMERFSLSEKQAQAILDMRMVRLTGLERDKIEAEYNDLVEL
IKDLTDILASSTRIHTIIETELLEIQDRFADPRRTELLVGEVLSLEDEDLIEEEDIVI
TLTHNGYIKRLPNNEFRAQRRGGRGVQGMGVHDDDFIEKLISCSTHDTLLFFTNNGKV
YRAKGYEIPEYGRTAKGIPAINLLGIDSGEKIQAIINVKGQAADNHFLFFTTLKGVVK
RTSVSAFANIRSNGLKSIVLREDDELMNVCVTDGDQNVIIGTHLGYAVTFNENTVRDM
GRSASGVRGIRLREDDYVVGMDVMKPDSEVLVITENGYGKRTKASEYPIKGRGGKGIK
TANITAKNGNLAGLTTVNGEEDIMLITNTGVIIRFDVDTVSQTGRATLGVRLIRVGED
AIVSTMAKVDAEPIEEEVETETTPIVSEASNELDDSNTAEEIENDTDLDQE"

CDS 2978732..2979031

/gene="rpsF"

/locus_tag="JFBMFIFI_02798"

/inference="ab initio prediction:Prodigal:002006"

/inference="similar to AA sequence:UniProtKB:P21468"

/codon_start=1

/transl_table=11

/product="30S ribosomal protein S6"

/db_xref="COG:COG0360"

/translation="MSQTAKYEINYIIRPNIEEAAKAALVERFDTILKDNGAEIIESK

DWSKRRLAYEIKDFREGIYHIVKLTATDAAAINEFDRLAKISDDIIRHIVIREEV"

CDS 2979082..2979633

/gene="ssbA"

/locus_tag="JFBMFIFI_02799"

/inference="ab initio prediction:Prodigal:002006"

/inference="similar to AA sequence:UniProtKB:P37455"

/codon_start=1

/transl_table=11

/product="Single-stranded DNA-binding protein A"

/db_xref="COG:COG0629"

/translation="MINRVVLVGRLTKDADLRYTSSGTAVASFTVAVNRQFTNQSGER

EADFINCVAWRKTAETLANFTRKGSLVGVEGRIQTRSYDNQQGQRVYVTEVVVDTFSM

LESKSTNEQRQSHDTSGSNGGATSPSKPSQQSGGYQQNSYNSNANNQSSAPQQQYQNS

NRNEDPFASSGQPIDISDDDLPF"

CDS 2979663..2979899

/gene="rpsR"

/locus_tag="JFBMFIFI_02800"

/inference="ab initio prediction:Prodigal:002006"

/inference="similar to AA sequence:UniProtKB:A2RNZ2"

/codon_start=1

/transl_table=11

/product="30S ribosomal protein S18"

/db_xref="COG:COG0238"

/translation="MAQQRRGGRKRRKVCYFTANHIDNIDYKDVELLKRFVSERGKIL
PRRVTGTCAKHQRKLTIAIKRARIMGLLPFVSAE"
CDS 2980140..2982143
/gene="gdpP"
/locus_tag="JFBMFIFI_02801"
/EC_number="3.1.4.59"
/inference="ab initio prediction:Prodigal:002006"
/inference="similar to AA sequence:UniProtKB:A4ITV2"
/codon_start=1
/transl_table=11
/product="Cyclic-di-AMP phosphodiesterase GdpP"
/db_xref="COG:COG3887"
/translation="MKKNLPHEKLPVFLRDKKLKIASLVLGIILFSVIVVAFFANFWI
GVTLFFLFIVISLLVLYALKKITVETNKYIADLSYRIKRGEQEALIKMPIGILLYNEE
YELQWANPYLQLYFGKQEVLGRKIADIDKELAGIIKKNQESGLTNIKWGNKDFQMIVQ
DDIKVVYLMDITQYAKIKEQYIEEQIVIGNIFIDNYDEIVQSMNDRSVSNLNNFVTTQ
LTNWAREHHIYLKRVSDDRFIVLMYMKSLDKIESEKFSIIDRIRERTSKQNFPLTLSI
GLAYGDKDLSDLAKLAQSNLDLALGRGGDQVVVKSADEEARFYGGKTNPMEKRTRVRS
RMISQALQEIMKQTDLIFVMGHKYPDMDAIGACLGIRRIAEMNNKEAYIVVNPEEFSN
DIARLMQEVEKDPNISRYIITPEKAQEMISPASLVIMVDHHRPTITIAPDLLKVTNQV
VVIDHHRRGEDFPENPVLVYIEPYASSTAELITELFEYQSNEATPINKIEATAMLAGM
IVDTRSFSLRTGSRTFDAASYLRSCGADAVMIQRLLKENVDVYLLRSHLIESIEFLSE
NMAVATGEEDRVYDTVVAAQAADTMLSMADVDASFVITKRSDGRIGISARSLGEINVQ
IIMEKLGGGGHLSNAATQIEGCTVAEAKEQLRKVIQGQIKEEV"
CDS 2982144..2982599
/gene="rplI"
/locus_tag="JFBMFIFI_02802"
/inference="ab initio prediction:Prodigal:002006"
/inference="similar to AA sequence:UniProtKB:P02417"
/codon_start=1
/transl_table=11
/product="50S ribosomal protein L9"
/translation="MKVIFLQDVKGKGKKGESKNVADGYAQNYLIKNGLAKEATSSSL

SELQGQKKAQEKHDAEILKEANDLKAFLEDEKTVITMPAKAGDDSRLFGSITSKQIAD

AVKKQFDVKLDKRKIDLTTPIRNLGAAKIDVKIHPEVVATLTINVIKED"

CDS complement(2982663..2984174)

/locus_tag="JFBMFIFI_02803"

/inference="ab initio prediction:Prodigal:002006"

/codon_start=1

/transl_table=11

/product="hypothetical protein"

/translation="MTTFLDKETIKKLEIIEFLVENNGIALDSTISTALAMDKRTLLK

LIKLIQNDLLFFNVENKVTLEINHSNKEINIIFKSNFTLSELNLQYHKKSLNYIITKD

IFFQNFPNPHHWANENYLSTSTLVRRLKEYTEFLTQYELVYDRAQPDTISGSEHQIRY

FYFIFFWETHTTLEWPFPSIEKNKLIHFFSENQEIFSYLSVIDLERFMYLISISIHRI

QSNNMIDYKYIYEEIENYSVSYYEFEKIIRELLESFLGDFRNIDNELIFLYFISNIIG

TFSMDSFKTIDVSFLTSSHSSLSVPAQSSLLWIDSFTKYFEVNLTPKQMLFLTTNLVS

LHTQVLYFTGSINIYGTNNSQNYVQEKYLLASTTIKKFTEHLNKTTDFPFNIAEFTYL

NTQYTLLTLDILKKKITPLNVCLFSSINNEFPEILKKELSYHIFFPLIITNEVLPETD

LIIADFFIDEALAEGKKSFQWNNFPTKQDWGKLIHIIDLFYMHQLDLELNVLS"

CDS 2984607..2984828

/locus_tag="JFBMFIFI_02804"

/inference="ab initio prediction:Prodigal:002006"

/codon_start=1

/transl_table=11

/product="hypothetical protein"

/translation="MKIEDYWEDVMIYFLAYTTIESPEKVIEETVITPMIADIKLEEI

KEILKKQNGILVDRIILFELWEDGLLLKT"

CDS 2984845..2985288

/locus_tag="JFBMFIFI_02805"

/inference="ab initio prediction:Prodigal:002006"

/codon_start=1

/transl_table=11

/product="hypothetical protein"

/translation="MKEVKVFYVKFSTKEEKSRIINKTIVVPIDSSKLEVEKIVQTKF

RAVKNIELIDYFSDALVFGQTVNSEPIKKDRISKKEKVAGNWQDVNIYTVQFETLDNH

KIEKNMIFPLDYTDEKAEELIKQGLTNLKNIRNIQKTKNGWLKKE"

CDS 2985437..2987674

/locus_tag="JFBMFIFI_02806"

/inference="ab initio prediction:Prodigal:002006"

/codon_start=1

/transl_table=11

/product="hypothetical protein"

/translation="MTKKNDSTKMKKGLTATLAVATIASQLAFAAPSIAKAEETETTE

PTATAATPRALALWTGAKLKSASITPLNLDTQVGGSLALDFSINAWGAGLAEDFYMTT

NIPVEFRPLVNEAFKQGILFQNLIQTGKFAGPLGMFPTKLTFDERSLTYDEETGLMSI

RSDQKFTVVGQASASYQMNLDYKTLYNTLKVNIPDATSGNGYYIQTAESSSGIISIEL

GDRGAEAFISEKQAIVGTGGLPEIKGADAITTQVNEEFNPLAGVTASDKEDGDLTGKI

EVTSDVNVNKPGVYKVSYSITDSDRNVTKVDRVVTVSDEHIIGNIAPKAYKIGQESVT

GAYYGDVATIALVVNGKEYNKIPVKADGSFSYYAEDKIKNNDDDVRMVAYSAYGKQLD

NKPVKITSSQGTMTPKVYKVGETNISGTYTGDVASARLYVNGVVQSSGGTFANGGFNY

YVGAGRIKAGDIVTMDAFSKDGTLIEQNIKVTVEGVSTSGSIKANDYKVGDTNITGTY

TGDVKRLRLYINGVSQSWGGIVENGTFKYYVGSNKISATDRVTMNVYDKDDNLLQENA

PVKVINASATGSISPAEYAIDDNEITGTYTGDVAKARISINGKAQAWGGSFSNGRFSY

YVGNGKIKAGDKVTITAYDKNDNVLDANKNVSIKVDTVKGTISPSNYSVGDVNVTGTY

TGSVAKARLSINGKEQAWGGSFSGGTFSYYIGANKIKAGDNVTITAYDSNDKALDSNK

KVVIN"

CDS 2987837..2988331

/locus_tag="JFBMFIFI_02807"

/inference="ab initio prediction:Prodigal:002006"

/codon_start=1

/transl_table=11

/product="hypothetical protein"

/translation="MKKLLISFSLVALLFLGACSGTDSKSNQNNKNQDKSSETKQETG

SALTEPVVVNGMELTIGQLKTTAVSSGEGKENKNLYGFEINGKNISSTKSGLGAIDFV

LTTSDGKEHAIDDTIVNFGNEIEPDKSITGKMYFAIDKNQKITKLSYKPEDKVLNSWD

VKTK"

CDS 2988353..2989627

/gene="wecC_3"

/locus_tag="JFBMFIFI_02808"

/EC_number="1.1.1.336"

/inference="ab initio prediction:Prodigal:002006"

/inference="similar to AA sequence:UniProtKB:P27829"

/codon_start=1

/transl_table=11

/product="UDP-N-acetyl-D-mannosamine dehydrogenase"

/db_xref="COG:COG0677"

/translation="MKIVTIGLGYIGLPTSIMFANHGQEVIGIDVQSDVIKSLQNNQV

HIEEPGLQSALLKARNSGGFRAQMEIEKADVFIIAVPTPNNADKYQSCDLTYVLSAVR

SIIPYLEKGNTVIIESTVAPRTTEDIILPLVESTGLEVGKDLFLVHCPERVLPGKILH

ELKHNNRIIGGVTKACVAAGKKIYGLFVEGDLISSSAAAAELSKLMENTYRDVNIALA

NELAKIGDELAIDSLEVIEMANKHPRVNIHTPGPGVGGHCLAVDPYFIVAEAPTITPL

IQQAREINRSMPKFIVEKTTALMEQVNGKKITILGVTYKGNVDDIRESPALEIAEILQ

DNNEFEVILYDPHVQAEWIEPDFNKAVMSSDLLLVLTDHDEFKEIKAEQLDDMTQKNV

FDTKSIFLKSSEINLVTMGNLAAIKKTIAVGK"

CDS 2989645..2990328

/gene="ywqC_4"

/locus_tag="JFBMFIFI_02809"

/inference="ab initio prediction:Prodigal:002006"

/inference="similar to AA sequence:UniProtKB:P96715"

/codon_start=1

/transl_table=11

/product="putative capsular polysaccharide biosynthesis

protein YwqC"

/db_xref="COG:COG3944"

/translation="MDESMGLKKLLATIKQNKWWLMGGIVFSVFLMSLYLWFVATPIY

QSNTQLLISQTEAKETPIQSQDIQANLELINTYSAIVTSPRILEEVGKELNNRYSEEH

LVEAIKVSNASNSQIIEIIVEDRNPNATVEIANTTANVFKDQILEIMKVNNITVLSSA

KKVKNPKPIRPQKTLMLTLSLFIGIVVGIICVFVQTFFDRSVKGPNDIQYVLGINLLG

TVNTIKEND"

CDS 2990467..2991135

/gene="ywqD_2"

/locus_tag="JFBMFIFI_02810"

/EC_number="2.7.10.2"

/inference="ab initio prediction:Prodigal:002006"

/inference="similar to AA sequence:UniProtKB:P96716"

/codon_start=1

/transl_table=11

/product="Tyrosine-protein kinase YwqD"

/db_xref="COG:COG0489"

/translation="MNDRRQLITQTNPNSIISEQFKTIRTGIDFAGVDEKIKTLLITS

PEVGTGKSTISANLAIVFAQKGLRTLLIDGDMRKPTVHKTFDKNMNIGLSNSLTEDID

IVNCCQRTTIRNLFILTCGVIPPNPNELLGSRRMKVALDRLKKNFDRIIIDTPPVMVV

SDALVLAPNTDGVILVIRDNETLKDRAKQALEQIKITKTPVIGAILNGVSEKENSPYY

HVYK"

CDS 2991146..2991790

/gene="wcaJ"

/locus_tag="JFBMFIFI_02811"

/EC_number="2.7.8.31"

/inference="ab initio prediction:Prodigal:002006"

/inference="similar to AA sequence:UniProtKB:P71241"

/codon_start=1

/transl_table=11

/product="UDP-glucose:undecaprenyl-phosphate

glucose-1-phosphate transferase"

/db_xref="COG:COG2148"

/translation="MENRQLMIQLNGKKRYLKIKRLTDILLALIGLILLAPIFLSVYL

SIKISEPKAPVFFKQIRAGKEGQEFEMYKFRTMQVNAEKKLAELLQYNEIEGAMFKMK

KDPRITKIGGFLRKTSLDEFPQLVNVLKGEMSLIGPRPPLPREVEKYSAYDSQRLTIM

PGCTGLWQVSGRNELTFNQMVELDLSYIQNLSFILDLKILIKTFFVILVPKNAY"

CDS 2991831..2992538

/gene="tagA"

/locus_tag="JFBMFIFI_02812"

/EC_number="2.4.1.187"

/inference="ab initio prediction:Prodigal:002006"

/inference="similar to AA sequence:UniProtKB:P27620"

/codon_start=1

/transl_table=11

/product="N-acetylglucosaminyldiphosphoundecaprenol

N-acetyl-beta-D-mannosaminyltransferase"

/db_xref="COG:COG1922"

/translation="MNETLEVVDEIIKKRVPTQHVVINASKINLMAKNEWLRTIVNSC

PLINADGQSIVWAGRFLGIHVPERVAGIDLFSELVEKAAKEGLRLYYFGGEEEVVQKV

IQLHQEAYPQLKIVGFRNGYFKEDESSKIAQDICDSQADILFVAFSSPKKEFWIHKYK

EQMNIPFMMGVGGSFDVIAGKTHRAPKWMQKLGFEWFFRLIQEPRRMFSRYLKGNTLF

LVKVLKEKVNRKEQNND"

CDS 2992531..2993793

/locus_tag="JFBMFIFI_02813"

/inference="ab initio prediction:Prodigal:002006"

/codon_start=1

/transl_table=11

/product="hypothetical protein"

/translation="MISEKRVVYFFLLLMASFPIIDVFNGYVLSMNYSIPIGVIYRLF

CFFFLVLMILCKGLNQNTYTFYTVLFLLIFLLILGIQSVLFQNPLTLIFEDSTVLIKF

FLWVLIPYFIYQHRVFMEKNKYEKIFIYIDFFFTIGLLIPYFLKIGNQTYANSNAGYK

GFFFATNDITLAFIVSATFTGWYLVKHLDKHFSFRLLGLIILYFANMLCLLLLTTKTG

VFYGIFLTIGLLIYFLFFQTKIQASYRLLVSFFLIVAIFSLLIVNKDFIFSALSGTVD

RILYFHNLYNGNLIRLLTSSRSEYLQGGWDYFTQSNHPVLVPLFGFGFQYRLVNFGRI

GLIEMDFFDLLFGFGLVGILVTSSLIIYFFILAFKKKNWSIYTLMYGVVLVYSFFVGH

VFFSALSTTLLGLICGGIILEDQKEDLE"

CDS 2993790..2995211

/locus_tag="JFBMFIFI_02814"

/inference="ab initio prediction:Prodigal:002006"

/codon_start=1

/transl_table=11

/product="hypothetical protein"

/translation="MRPLVRRLIQFSIGSIGAAFLNLILIPITTYYLSPEEYGKTSMF

LLAQTLLIYVIYLGFDQAFTREFYEYKDKHRLLGNALIVPLVSAIFLIVGMVLFAPTI

SQLLFASSKYVGAVNLLALSVIFLIFERFILLYIRMGNQALTFSMYTILIKLAILIFT

VLFLILFKPVFITVVYGTLVGQIIGDFILIISHLKLLDFRTVKFDFKLVKSLAKFGIP

VVIATFIYSLFVVIDKLFLRYFTDFSQLGLYTAAFKIASALLILQVSFSNFWIPTAYE

WYQQKKTIKYYKFVSDSMMLAISLLFIALLLSKELILIILSPAYSEAKYIFPFLCFYP

LMVTVSETTNLGIVFSKKSYLNIVVSLLALVVSIGLNLWLTPLLGAIGTAIATGTAYI

AYFGARTYFSMRIWEGFSVKKHFIVTGLLYSASLYSIFGSDGLSQYVVILLILGLILF

LYRSLIKFCLMKIKAEMLIKKEY"

CDS 2995213..2996232

/gene="bshA_1"

/locus_tag="JFBMFIFI_02815"

/EC_number="2.4.1.-"

/inference="ab initio prediction:Prodigal:002006"

/inference="similar to AA sequence:UniProtKB:Q81ST7"

/codon_start=1

/transl_table=11

/product="N-acetyl-alpha-D-glucosaminyl L-malate synthase"

/db_xref="COG:COG0438"

/translation="MKILMVGPNANSKGGIASVIANFKAYYQGNSIEYLDSWQEKRRF

ITGIRAFFKIKRKVEKEQFDVVHFHVAQRGSFFRKALLATRVRKQTKVLFHMHASQFD

TFYSDASPMIKKYIRKTLNQLDGLVVLSKEWSDFYENLTDTPITVIENAVLVPAEINY

NSQSTKIITFGRIGVRKGSYDILEIAKKIEVLFPEITFILYGDGEVEKVREKLKEQQI

KNVQLGGWINKNEQEYLMKDAVLHFLPSYQEGLPMAILETMSYGIPNLSTNVGGIPQV

LKDMKNGMVASPGDNEKMIEKLIYFLSNEDIRLNYSRASYETILHTFSINPYFEKWSI

YYDEL"

CDS complement(2996317..2997708)

/locus_tag="JFBMFIFI_02816"

/inference="ab initio prediction:Prodigal:002006"

/codon_start=1

/transl_table=11

/product="hypothetical protein"

/translation="MLDIATQKKSRYSLFLTLFILFQPLLDLLTTFSLLVLKSSITPG

ILIRFLVALTSVIYLILMKKNEKNRLFLIYLLSLGVILSLNLLTTYLFKDLYILSEEV

KSVAKISYYVIMLLAYITAFKELQLANKLNNYFPKVIIYATILVNLVMIIANLTSTSV

SSYMYMKAGKSGWFYAGNELGSLLAIAFPVVLWYTLKKSSQLKKLYFWIPTLLTVYSL
LIIGTKVGYGAIILTLPIAFLTIFIEIYFNKQYNLKMYILNASITLALFIGVLIITPM
ISVSTNTTIHLDYLAEQKTITEVDSSSENTQEAAVEAEVPSSPDIPADKDTELNELIY
SGRTVYLAQYKTFFKEANLSQKAFGMGYGGNYKDQPKTIEMDFYDIFYQFGFIGAFVL
LLPLLYYGFNLIKVMWANKKMILQLKYILILASLAIALGIAYIAGHILTAPAVSIYFV
SILAYLIVELNLD"

CDS complement(2997751..2999190)

/locus_tag="JFBMFIFI_02817"
/inference="ab initio prediction:Prodigal:002006"
/codon_start=1
/transl_table=11
/product="hypothetical protein"
/translation="MKTINAKSTNHVFINILHLFFGTIVTKFIGAIATILYANYTDVK
DYGVLSIALSFSAILTFLTDSGISHTAIREGTKKNSDIGKIMYSYVTVRVILFFLVSL
FSTIFIYFVYRNDLQMQQAILLLVIPSLVGSLLQGVGGTFFQIIEKMNYVSYINIILG
VGNSLALFLGILFQLSIGTICLVYGLSSVLAGIYGLAISVRAISFKKEFDRKILYQLL
SFTTNSAIIILTPQLGPIILAKVLSVTDVAYYSAAFKIPAMLYQFPALIATAFYPKLF
SLGNDKNSSEHRKTSSLELQLMTILGVGLALPFILNPEYWILTLLGQKYLPSVNVLLI
LSFLVFIQAIKYPLADFLTTYGLQWQRTLIMLIGLAISIIGYIVLGQRFGIVGGALAP
VFAEVIMIIGCALFITNGLSFLFKNIALILLSGILTSFFFIAFLTQMNYLFAIVIVEF
IFIFLTILSNKNVQKIINLAITKLKMSFR"

CDS complement(2999252..3000199)

/gene="wbgU_3"
/locus_tag="JFBMFIFI_02818"
/EC_number="5.1.3.7"
/inference="ab initio prediction:Prodigal:002006"
/inference="similar to AA sequence:UniProtKB:Q7BJX9"
/codon_start=1
/transl_table=11
/product="UDP-N-acetylglucosamine 4-epimerase"
/translation="MNKILITGGAGFIGSHLVDQLIETHQIWVIDNLSMGVMANLPNH
KNLTFIYGDICDKALLSKLLCSHKFDYIFHLAAIANVQDSVMEPVKTHQVNFEATLTL
LDLIKKEQPTLKRLVFASSAAVYGDLEELPKKESSSVLPKTPYAIDKYAAERFVLSYF

SLYNVPTSAVRFFNVYGPRQNPTSTYSGVISIITNKLKNNKEKKETFTVYGTGQQTRD
FIYVKDVVQSLILVSKSEKSLGEVYNIGSGEQISLNRLIKTYTSLTGLSLNLTFKTKR
DGDIQDSYASITKIKSLGFKQTIDLKTGLNEYWQATFQK"

CDS complement(3000256..3001347)

/gene="bshA_2"
/locus_tag="JFBMFIFI_02819"
/EC_number="2.4.1.-"
/inference="ab initio prediction:Prodigal:002006"
/inference="similar to AA sequence:UniProtKB:Q81ST7"
/codon_start=1
/transl_table=11
/product="N-acetyl-alpha-D-glucosaminyl L-malate synthase"
/db_xref="COG:COG0438"
/translation="MKILHVNAGNETGGGKSHIVSLLSQFNPEEVDLLVFEEGPVAEH
ARKYNINVYVIENKFNLLFTIKSIEKHINKKNYDVIHTHGPRANFFIGLTKKKSHLIW
TTTIHSDPLLDFENRGIKGAIFKKLNIWSYSKIDCFLLITESFKTQLTQMNVPENKIV
TIYNAIDYSDKNELTPLPKQLNSNFILLIVARLQPVKNHALLLQALSELELNNVTLLI
AGDGPLEDNLKSISQDLNLTDSIEFLGHQEDVDNLYQLADVCLLTSYSEGFPTVLLEA
AKFKCPAIATNVGATKQIIPHSDFGWVIDNNKDSLKIALTEAYIAWKENRLAHKGELF
HKYAKTNFSMPKLAKDIKAIYCQLLQTKK"

CDS complement(3001376..3002095)

/gene="tarA"
/locus_tag="JFBMFIFI_02820"
/EC_number="2.4.1.187"
/inference="ab initio prediction:Prodigal:002006"
/inference="similar to AA sequence:UniProtKB:Q7A714"
/codon_start=1
/transl_table=11
/product="N-acetylglucosaminyldiphosphoundecaprenol
N-acetyl-beta-D-mannosaminyltransferase"
/translation="MNFKTIDVLGIPFTCTNQSNFIFQLEKDLFENKSTFVVTANPEI
VMYAKASPNFNSLLHSADYITADGIGIVKAAQFLGTPLPGRITGYDLLCSLLEVASQK
NYSIYFLGAKEETLNLALKNITNSYPNLTIAGSHHGYFEISDPTIIENIKKTQPDFIF

VALGFPRQEQWIASAQKVIGGGIFIGVGGSFDVLAGTVKRAPLFWQNLHLEWLYRLIK

QPTRWKRMIALPKFVYHIFKD"

CDS 3002541..3004034

/locus_tag="JFBMFIFI_02821"

/inference="ab initio prediction:Prodigal:002006"

/inference="similar to AA sequence:UniProtKB:Q97P44"

/codon_start=1

/transl_table=11

/product="Putative trans-acting regulator"

/translation="MQSILNSIDSRQISFIEVLFFQDDWITLGTIAEKLDCSEKILRS

NINIINNIYAPFQITTSVKRGIRILYPENYNLDFVYSVTLGNSYEFNFLELVFFNESL

KKGEMVESLFISPSSLNRIVKKCTAFFSQFGIEIRTSPYQMVGDELKIQNFYVHYFLE

KYLLDSSPFEDKELGLINTILDFFLKYYQDDSGFSNKKKLLFSAMTALTRLKNGHVRK

IGKISKENEEIIHEILSHTNLVQSFRQLMGIELTKNMLIQLFYVFFHDDYILSTNIFP

EKLHNPKVEEDFAIIDQFLESLSIKLGIELQNKELLVLKIYNARNFLFGKNYILHNKK

IGFSQHIEQEYPLFSKILKKEIKAASFSTIEKENPSFFNEVLYELIIHWTNLTMELTK

SVVPISIGIISDQGTEHAEFIRKLIDYRFGKYISASIIASENLKFNQNKYAMVIATMS

YFYGDFFDIPVISINSVPNEHDWKNIQKKLQELSCFEGEMGTKYIKI"

CDS 3004398..3004907

/locus_tag="JFBMFIFI_02822"

/inference="ab initio prediction:Prodigal:002006"

/codon_start=1

/transl_table=11

/product="hypothetical protein"

/translation="MGLLFFLSACAVKEERSQDKESEGIIIDTPYLLEEGSIITSGEN

VTIGAINYSLSKVSTIAGVNNTRLAIIYFKVTNKTKQEIIPNEFWFRYVKVTQNNEDP

LPIGALPYENREDNNGQLIKQANQGLKKDTTVDVAVAYILDGTADLNVIFMNTEYKEL

AKNSYSVKE"

CDS 3004951..3006903

/locus_tag="JFBMFIFI_02823"

/inference="ab initio prediction:Prodigal:002006"

/codon_start=1

/transl_table=11

/product="hypothetical protein"

/translation="MNKKMKFIGMSAAVLLAISPVVTPILGVSSAVVYADSSYDAILR

NALKASVRVDGWNNRVDPADTPVVDKNLKQGLMELVNSGAKNTIYTGEETNVFFTQIF

TKVIAKEKKLKEILGAAGENSEAQFLVNGKSDVTLDDLSSSVLTLTVKVFGTDGKTAE

ADITLNTDAPEIALKEGISLTRFVGEEDGYIDFVNQTSEFIELTNADDSWSILEGSTN

KKTMPSKEGIKGEGFIDDEGVYLKAGSYTQLVTVTLMNSETELREKRTISVTVNVIPD

HYDESEFEFQELNGNSSFKSGSTILNAKLGSMDISADGKRLSEMLIQNNPDYFNFDSK

LINRAQLDEDLKTRFLELFRIWAEEGVVEFDTRPQDKLRLSVDSSNIDFSKENSMALV

PFTYVTEFGKTMTVTLELLVKQPNYPIIEFNTNQDMTINVGTPFNLYDFKAYQDSAAN

LLTNRVSISGSVNTSKVGTYELTYSVVNKYNLKTTLIRTINVVDPNGSPNNKPTITSL

ETVGYVKYVAGYGIRVWSTPDGNGSNQYLPHGTAWKIDQKATFKDGSIWYRVGKDQWV

DGSYIKFTPVSSPGMKELKGVGTINYVQGYSVNVYRGAEASGENWTGNQLKHGTKWRV

YGEKDGFYNLGGDQWVSTKYISFQKD"

CDS 3006951..3008822

/locus_tag="JFBMFIFI_02824"

/inference="ab initio prediction:Prodigal:002006"

/codon_start=1

/transl_table=11

/product="hypothetical protein"

/translation="MNKLKYVTLNAVLILGLLPVASTIAAVETVHADELSATTRQNSG

MASLLDDKLKREASISIDDLNGHASVLADIVNQESNEAIISGYPAGPKNVALAALFKA

ILKVDDSSFTLQSLTNQISRGQLVINGKTAITAEEIKSSERLIIEFKLFSLGSTAPSI

SQPVTLNVKHYSAVNPDLVKKVTEVYNVRGDLKVKDPEGLEITDYTIKIAGTNTELMS

DAKPGYLIEAGLIDAGTGKFINEGSYVEKFDIYVGDLLVKANVERTLEISIGDAQPSF

EIRGGKYDSGSTITNTSHFLTPIEGDSVDSLEASIKRVVLDNIKVSDMNVNVSATSDY

SALTAENSVIEAPVVFDLSKTATYKVKVTYKKAGSNITSVITVPVIVKAATAPVIRFT

QGQNLTIPVGTSFDLAKNIKVYENKVATVEGQNVVWKVDGAIDTATPGVYRLRYIATN

PKGLSTELTRIITVEGKATEKPTIEKFVSVGYVNYVPGYGIRVWGEPQSNGSDRVLAH

GTAWKIDQKATMKDGSVWYRVGTNQWVDAQYVSLTPINEANEENVSGVATINYVEGYS

VNVYSSPASSSASWTGKQLKHGTKWLTYKRATVNGKTFYNLGGSQWVDSQYITFSAN"

CDS 3008904..3013184

/locus_tag="JFBMFIFI_02825"

/inference="ab initio prediction:Prodigal:002006"

/codon_start=1

/transl_table=11

/product="hypothetical protein"

/translation="MNTKKSNISMNFLLIGILFYLALLAPTASAAPIATEGLIDVGED

LQLSYYFGKKPGNVEIEPNRVQVANVNFLTKGKRVEHLYQNGSNKGTISIGFNGANSY

VDPTNITVRSITGGGINSAITATYTANYAGNQFEITSTLTPRGNNIIVSDTIKNISGK

DLKNVWIARSYDTMLDGNDAVPVKYLGSNNGLYIAQNDSKLSYNFHMPIGPEGWAADQ

YNRQIVPAVFSTPTNLGQASLNKPAGDIAFSNGDSGIFMKWSPKDFPKDSSREIGYIV

GVTPENVDAPEVTVARESFEYLGGDYDLRGTVLSKDPSVTDMKMYYQVDRESPKEFKN

VANSPLGSAHSWSTVIPESELTGGYPKQISVYAIDSNGYYSDAKTVDLTEPKSQPVTV

KYLNQEGKLLKETKITGRYGESYEAEVLDFTNYTVENQPENSIGTISNKPQEVVIKYV

GQYVGETNGKVTARYVNQNGKEIADAVTASGNGLFGSPYTTEQKDIEHYEFVGIDAEG

ATKNGFYTDSIQTVTFIYQGKNLGEKGKITVEYQNQEGKLLGEDFIIGRYGDSYDVSE

KLLKEFRYYTRDPLPNNSKGTITDQPQKVIITYTGEPFGNSSGKVTVKYINQNGDEIA

DAIQANGSGLFGGSYTTEQKKIPNYEFKEVDSSNSGATGFYTESPQTVIYKYQGKEVG

ETGKITVEYRNQEGKLLDQAFITGRYGDIYDVSLNHLKEFDYYTRDTLPTNAKGTINN

QPQTVIVTYTGKQVGNAQGKVKAKYINQEGLEISTEEDATGSGLFGSPYTTEQKEIPN

YEFKEIDSSGAKQTGVYKEAPQMVVYKYKGKSVGENGKITIEYRNQVGALLGIEFIIG

SYGDKYDIERDNDGALVKNFDHYTINSLPENKKGLVSNEPQLITVTYTGELVGEDKGK

VTATYVNQAGEVLSEATKANGDGRFGSPYTTEQKEIANYDFTGVGPTGAPQNGTYSMK

PQTVIFKYTGQKGGNIIVKYVNQSGKELKTEILKGNVGNAATINPPTTGLFSNYNLIP

NQNLVATFSPEEQVITVNYDGKPAKSLTVKHLGTDGYVFSSEVIANKKYGDSYQLEPS

EQDGYSFDRVVGNKNGTYSNENQQVTFYYKKILVQTGTITVKYLEVNTGKVLAPSIKL

TGVVGDGYSTTAKEIKGYVTPTDQSGTFQEGFKDVIYEYAKEPILGANITVKYVDKYG

KAIKVLGQKIIPGGIIGTQVPVEVVTPTLGYRIISSKPDQTSLKYQAYKQVITCTVAM

GLTTSGRAEVRKAAPSSSGATIRYVNDSDPENASEIAPSLSVSGFVGENIWQPNYLPE

FIEDYELVSDIESFNTELFGDESKIINVVYHFAPTTDMKKEVKIKIVDASGDLLKTVT

QVVTVGEAYTIEVPVIEGYNYQEDTSINLTGTWEDGMPEEYTLTYDLI"

CDS 3013368..3013610

/locus_tag="JFBMFIFI_02826"

/inference="ab initio prediction:Prodigal:002006"

/codon_start=1

/transl_table=11

/product="hypothetical protein"

/translation="MKIEDNWTDVEIVNVEFLGFNNEVYSRTMIVPSGHSEDEIKIML

VEEFPPVKKYLAIDFSEEGFYYKGNYFTTSCSCVDE"

CDS 3013814..3015202

/gene="dnaC"

/locus_tag="JFBMFIFI_02827"

/EC_number="3.6.4.12"

/inference="ab initio prediction:Prodigal:002006"

/inference="similar to AA sequence:UniProtKB:P37469"

/codon_start=1

/transl_table=11

/product="Replicative DNA helicase"

/db_xref="COG:COG0305"

/translation="MANEAFQDRLPPQSIEAEQAVLGAIFLDPETVVGALEFLEPQDF

YRRGHQLIFQTMLDLNDRNEAIDIVTVMNNLESQNQLEDVGGMSYLAELAVAVPTAAN

MEHYAKIIEGKSILRKLIHTATDIVTQGFEEGEELSAILDQAERSILEVSERRNSNGF

LAISDVLNSSIAKIDQLYQNDEEITGLPTGYQALDQMTAGLQKEELIILAARPAVGKT

AFALNIAQNIGTKTDETVAIFSLEMGAESLVNRMLCAEGNIDASHLRTGNLNEEEWQN

LIVAMGSLSKASIYIDDTAGIRIAEIRAKCRRLAQEKGGIGLILIDYLQLIEGSGKES

RQQEVSEISRQLKKLAKELKVPVIALSQLSRGVEQRQDKRPVMSDIRESGSIEQDADI

VAFLYRDDYYDRGEDGDDDDRDSDRDEAGEDNIIEVIIEKNRSGARGTVKLLFVKEYN

KFTSISYRPEPM"

CDS 3015441..3016733

/gene="purA"

/locus_tag="JFBMFIFI_02828"

/EC_number="6.3.4.4"

/inference="ab initio prediction:Prodigal:002006"

/inference="similar to AA sequence:UniProtKB:P65887"

/codon_start=1

/transl_table=11

/product="Adenylosuccinate synthetase"

/db_xref="COG:COG0104"

/translation="MSSVVVVGTQWGDEGKGKITDFLSENAEVIARYQGGDNAGHTIK

FDGVTYKLHLIPSGIFYKEKISVIGNGVVVNPKSLITELAYLKDKEINTDNLRISDRA
HVILPYHIKLDQLQEDSKGDQKIGTTIKGIGPAYMDKAARVGIRIADLLDKEIFEERL
RINLEEKNRQFVKMFDSTELKFDDIFEEYYQYGQELKQYVCDTSVILNDALDAGKRVL
FEGAQGVMLDIDQGTYPFVTSSNPVAGGVTIGSGVGPSKIDKVVGVCKAYTSRVGDGP
FPTELFDEVGNQIREVGKEYGTTTGRPRRVGWFDTVVMRHAKRVSGITNLSLNSIDVL
SGLETVKICTSYERNGEKIFHYPASLKELAECTPIYEELPGWSEDITGCRTLEDLPQN
ARNYVHRVSELVGVRISTFSVGPDRNQTNVLESVWAQI"

CDS complement(3016779..3017477)

/gene="dcuR_1"

/locus_tag="JFBMFIFI_02829"

/inference="ab initio prediction:Prodigal:002006"

/inference="similar to AA sequence:UniProtKB:P0AD01"

/codon_start=1

/transl_table=11

/product="Transcriptional regulatory protein DcuR"

/db_xref="COG:COG4565"

/translation="MFRVLIVEDDPMVLSINQRYLQKIPNFMVAGTADSYEHAIQLTS
QYFYDLILVDIHLKSGNGLDLLKTWRKREYPAEIIMITAASQQEALRISKHYGVTDYI
LKPFQFKRFKQSIELFQQNQLLLSSNSVLTQKEIDSLHHSDPLSISEIELENKLEKGL
TQTTLDLIVSLIKQQKAGFTVSDITQATELSHVSVRKYLHYLESTQQIEMRLEYGTVG
RPTSVYYLIENFDK"

CDS complement(3017480..3019015)

/gene="dcuS_1"

/locus_tag="JFBMFIFI_02830"

/EC_number="2.7.13.3"

/inference="ab initio prediction:Prodigal:002006"

/inference="similar to AA sequence:UniProtKB:P0AEC8"

/codon_start=1

/transl_table=11

/product="Sensor histidine kinase DcuS"

/db_xref="COG:COG3290"

/translation="MKTPRKGQRLWMKLTLMVLMTVIVTLITSYILLNQQITKSVKKN
EEIHLLKVARTISEYPLVHQSLEQKKTSSELQEYANQMKTNYDLDFVVVMTMDHIRLT

HPDPTKINQAFQGGDEESALKGVESTSIAEGTLGPSLRAFVPVFSTDNTEIGVIALGV

KTATIKLISQETMKPLTLSLIIAFSFGLGAAIFTAYTLKKQMYDLEPKEIARLLEERN

AMLDYTKDAIVVTDKENTISLANFEAQRIFSLISEKEIIVGSSIQDFLPNTTAISDES

IDKLYHTNGLDYLVSSAPIIVRKKIIGQIFIIRDATELHLLTDRLFNTTAYATTLQAQ

SHNFLNKLHVIYGLTDLEDYEQLMEYLEKILEPEQEFAQRMVYLVHNPVIAGFLIGER

SKFSEKNSPFMIEVYPDIPVTDQQNAAHYWMSLIRVLNQFILEHELAEKLQIRFGFWN

QHLQTTYELELDTRLQSNLLNEIEGAYFKKILLRANGQMNIEKNKNWLKITFSVAYSE

ELL"

CDS 3019240..3020577

/gene="cimH"

/locus_tag="JFBMFIFI_02831"

/inference="ab initio prediction:Prodigal:002006"

/inference="similar to AA sequence:UniProtKB:P94363"

/codon_start=1

/transl_table=11

/product="Citrate/malate transporter"

/db_xref="COG:COG3493"

/translation="MRVANEEVKKVSEVPKTWIEKIKTAKVGDIPLPAYLGMAFVILL

SAYFEELPVNMLGGFAIILSLGWLLGGIGGSIPGLKNFGGAAIFSLMIPSILVFFNFF

NTNTLEATDMLMKQANFLYFYIACLVCGSILGMHRKILVQGLMRMIIPMACGMILALV

VGTAIGTLLGLGFEHTLFYIVTPVISGGIGEGILPLSLGYSAITGIPSEQLVGQLIPA

TIIGNFFAIISSGFLNRLGEKKPDKCGQGQLVKLNADDDDMAEAMKEDKSPLDVKLMG

AGVLVACTLFIAGGLLQKFTGFPGPVLMILVAALLKYLSILPGDIEKGSKQLYKFVSA

NFTFPLMVGLGMLYIPLKDVVGMLSWQYFVVVISVVLTVVLTGYFVGGKMNMYPIEAA

IITACQSGMGGTGDVAILSASNRMNLMPFAQVATRLGGAITVISMTALMRFFF"

CDS 3020629..3021783

/locus_tag="JFBMFIFI_02832"

/EC_number="1.1.1.38"

/inference="ab initio prediction:Prodigal:002006"

/inference="similar to AA sequence:UniProtKB:P16468"

/codon_start=1

/transl_table=11

/product="NAD-dependent malic enzyme"

/translation="MTDVKAKALAISKEKGGKLEVVPTVPIETMEDLSIAYTPGVAAV
CTAIAENPEKVFDYTTKRNMVAVVTDGSAVLGLGNIGPEAAIPVMEGKAALFKRFGNV
NAVPISLATQDVEEIISHIVAISPSFGGINLEDISAPRCFEIERRLKEQLDIPVFHDD
QHGTAIIVLAALYNGLRLTGKKIDEIHAVVNGGGAAGIAISKMFLAAGVKNLMIVDKT
GIISATDDNLPTHHAEMAKITNLEHRTGSLEEALKGADVFVGVSAPGALKQEWVRHMN
DKAMVFAMANPTPEIFPDEAKAGGTYIVGTGRSDFPNQINNVLAFPGIFRGALDARAS
DITIEMQIAAAQGIASVVTDEQLAPDYIMPDVFTPGVADIVANSVRKAVK"

tRNA 3021951..3022025

/locus_tag="JFBMFIFI_02833"

/product="tRNA-Lys"

/inference="COORDINATES:profile:Aragorn:001002"

/note="tRNA-Lys(ctt)"

CDS 3022540..3022908

/locus_tag="JFBMFIFI_02834"

/inference="ab initio prediction:Prodigal:002006"

/codon_start=1

/transl_table=11

/product="hypothetical protein"

/translation="MSNLTQEWLNRTREQKKNLELLETRLIEESNITLNEYYVLYFLN
ISEEKEMRLNDLQVEIGLSQSAMSRMISRMENKNCSLIERHCCMTDRRGIYIGITKSG
YLKLKSSQKIVDSIVRKFLN"

CDS 3022967..3023602

/gene="yhfK"

/locus_tag="JFBMFIFI_02835"

/EC_number="4.-.-.-"

/inference="ab initio prediction:Prodigal:002006"

/inference="similar to AA sequence:UniProtKB:O07609"

/codon_start=1

/transl_table=11

/product="putative sugar epimerase YhfK"

/db_xref="COG:COG0702"

/translation="MKIFIVGASGRVGTKLASILAKMGHQVYAGTRHIEKDSGHENII
PIYIDLHSSQEELIEPLKGMDVIYFVAGSKGKDLLQSDLFGAVKVMQAAESNGTKRFI

HLSSIFALEPNRWNESFLKNLEDYNIAKFFSDHWLVHNTNLDYTILQPGALTEDLGTG

KIDVNVTDLGSNSIEDVVDVLVTILDYPNTIKKVIKMHEGTSPIKEAIKTI"

CDS 3023624..3024730

/locus_tag="JFBMFIFI_02836"

/EC_number="1.-.-.-"

/inference="ab initio prediction:Prodigal:002006"

/inference="similar to AA sequence:UniProtKB:P32382"

/codon_start=1

/transl_table=11

/product="NADH oxidase"

/translation="MTTLTETFKFKRGLEIKNRIVMAPMTTKMSFFDGVITTDELNYY

GLRTGEVGAIITAAANVQANGKGWEGELGVYDDKFIPGLSKLAATIKQNGTKAILQIF

HGGRMTDSKVLRGEQVVSASAIAAEREGAEVPKELTEIEILEIIESFKNATKRAIQAG

FDGVEIHGANTYLIQQFFSPHSNRREDKWGGTLEKRFNFINELVNGVTDVVDQSGIQG

FIVGYRFSPEEYEVPGISFSDTLYLVDKLSEKSLDYLHISLNQYDRISSNHSFVEKTL

LAYVHEKINNRVPLIGVGNIQTLADVNETLLYSEFVAVGRALLIDPHWTNKIINGQEH

LIRRTLSHYDQEELIIGNGVWGFLEQMMPERLIK"

CDS complement(3025195..3025290)

/locus_tag="JFBMFIFI_02837"

/inference="ab initio prediction:Prodigal:002006"

/codon_start=1

/transl_table=11

/product="hypothetical protein"

/translation="MYINMNIQGKLWELLTTASTIDIFTPLYLYL"

CDS 3025750..3026622

/locus_tag="JFBMFIFI_02838"

/inference="ab initio prediction:Prodigal:002006"

/codon_start=1

/transl_table=11

/product="hypothetical protein"

/translation="MSTKLQNDGHEVYGELIRKIRLDKRQSLEEISDGIMSYSLLSKF

ERGETMLTIDKFFKILDKLNIGFDEFELMLNGYQDNPVKMFFSIIKYYYKNKDVFKLK

EKMIDEMKEFKLSGDKKNKLNYIVIYILLDSLEEEEEVPQAYLTELSDYLFSIEDWTQ

YELLLYSHAIEKLNIQIVLLFSKELLGKLEIYTENIELKKIVIKAILNTILKCLKYGF

LEDAYFFMEAIEKVLTDDFLWEKSILYYIKGRYYMLIGDESTGKFMMDEALNIMKHFD

DTQIEDYVKGFFII"

CDS complement(3026923..3027870)

/locus_tag="JFBMFIFI_02839"

/inference="ab initio prediction:Prodigal:002006"

/codon_start=1

/transl_table=11

/product="hypothetical protein"

/translation="MVKRLLSSSASDVAKMSPSELKQAILASEGRTICSENVVMAPPV

AEDITNAEVASAYGADLILLNAFDCLNPIILGLPGFSNFNEIVAAMQDTSKNTRNPVA

EVKKLVGRPVGVNLEPVDLEADMAEDLLIISEGRICSEATLKRADELGFDFICLTGNP

GTGVTNRQIAKAVKLAKKHFSGLVIAGKMHGAGVAEPVASSEAIHEFIEAGADIILLP

AVGTVPGFTDSELIEAVKYAKSKGALTMSAIGTSQESADKETIKQIAIRNKVAGVDIQ

HIGDAGYGGLAPAENIYAMSLAIRGMRHTMNRISRSVNR"

CDS complement(3028005..3028286)

/locus_tag="JFBMFIFI_02840"

/inference="ab initio prediction:Prodigal:002006"

/codon_start=1

/transl_table=11

/product="hypothetical protein"

/translation="MSTIIEITDKIGLDYYFVTLTLNHDKLEIKNTHSKIKKIQRELP

ISKIEKVTLTQRFGAKTLNFHYKNEFFSIVDCGTSIVPFLEAVFINKIV"

CDS 3028440..3030086

/gene="dcuS_2"

/locus_tag="JFBMFIFI_02841"

/EC_number="2.7.13.3"

/inference="ab initio prediction:Prodigal:002006"

/inference="similar to AA sequence:UniProtKB:P0AEC8"

/codon_start=1

/transl_table=11

/product="Sensor histidine kinase DcuS"

/db_xref="COG:COG3290"

/translation="MKTKIKKGLVNVPKRKKLSLQSTIIVVVTAATLVSLVVSAVLIR
NYVIAKEFENTKEKIANIANLAAESSIVKDGLLGLVEQDEVQNYAQMVVDTTNVDFVV
VLDTNMIRLSHPDETVIGETFSNVKDAKKALSGKSHYSEQIGVLGDGYRYFTPVFNSS
GEVIGVVCVGLTMDTINRDVSDAQWKILVGLGLGLIVGVIGAIFLAQKIKSILFGLEP
SEIATRLSEKEIIENEVSEGILAISSNQEIVLVNREAIKMFAQSDIQQEIKLGVHLDE
VLYSVLFEQVFETKEKLKDQALYLSGIEVIASVAPIFIEGDFAGAVATFRDQSEMQNL
IHELSGTEQYIDSMRAQTHEFMNKMHVILGLIELEKYSEVTSFIRQLNNDYQEEVGYI
TEKIKVPAIAGFLLGKINEAKEQGVEIILEEQSFLPELKTGESIHLLLQIMGNLLDNA
KEAVAHTLDKRVTLLVSYDLEGQILIIKVTDSGQGIAADIQAKMFERGFSTKGEHRGY
GLNLIQTIVTNHQGFIEVKSQQNQGTTFYIELPYEMEEAT"

CDS 3030083..3030811

/gene="dcuR_2"

/locus_tag="JFBMFIFI_02842"

/inference="ab initio prediction:Prodigal:002006"

/inference="similar to AA sequence:UniProtKB:P0AD01"

/codon_start=1

/transl_table=11

/product="Transcriptional regulatory protein DcuR"

/db_xref="COG:COG4565"

/translation="MTNVLIIEDDPMVAMLNQQFIEKIADVHIVGNVRNIPDARELLA
EESVDLLLLDVYLPGMTGIEFLAELHDKKINVAVILITAANDVSTVKEAIYYGVVDYL
IKPFTFERFKLAFEKFEKLTDVTQELQSTDQSQLDKYFNSGTIPAAPVSSGKVEDLPK
GLSKLTLKKVYRQIQKIKGSFSTEQLAGEIGLSRISTKKYLLFLVDIQILHEEMEYLE
IGRPLTLYSLRDTHQNEIEKYLKN"

CDS 3030834..3031226

/locus_tag="JFBMFIFI_02843"

/inference="ab initio prediction:Prodigal:002006"

/codon_start=1

/transl_table=11

/product="hypothetical protein"

/translation="MGLDYQEIEKIINNETNAGGFAELIKNFKEIGVSHYEYRVAAGM
YRYYDEDSSIDIQFNGIPKPVVPIESAEKIKQTVKRAQAGEITFEQFTELAGEAGIAY
WATDISKMVVNYYGIQGSLLLSEPISEV"

CDS complement(3031288..3032301)

/gene="hgdH"

/locus_tag="JFBMFIFI_02844"

/EC_number="1.1.1.399"

/inference="ab initio prediction:Prodigal:002006"

/inference="similar to AA sequence:UniProtKB:D2RJU7"

/codon_start=1

/transl_table=11

/product="(R)-2-hydroxyglutarate dehydrogenase"

/db_xref="COG:COG1052"

/translation="MFKIACYGVRPNEVPFFQSLNKYNYELTLVEELLTHQNIQTAQH

HDAVLLRGNCIADRQNLSKMAEYGIKYVFTRTVGFNHIDLEAAAEYKMEVARVPSYSP

NAIAELSLTLAMMLLRNTAYTTNRTSFKNFIVDSQMFSREIRNCTVGVIGTGKIGLTE

AKLFKGLGAKVIGYDVFESDAAKEVLEFKSLDALLAESDIVSIHVPYFPGKNDKMINA

EFLSKMKEDAILINTARGELQDNEAILEALETNQIGAFGTDVFANEKDLFFKKFSPWQ

ALPDPTVQKLVDLYPRVLVTPHIGSNTDEALANMVETSFENFNEILTTGKTANAVELP

VLA"

CDS complement(3032347..3033405)

/locus_tag="JFBMFIFI_02845"

/inference="ab initio prediction:Prodigal:002006"

/codon_start=1

/transl_table=11

/product="hypothetical protein"

/translation="MDILEIIQTTVTDMTIISAITSTVFIIMLGFFCRKKGIFSAEVG

KILSKVVLTVALPALAFNAFMQDIDPDTLKQGMNVLIWGIAIYIILIFISKPFFLSYK

GDKQDTLRVLTIFGSTTFFGTPIVSAIYGPVGVMFSSIFNIGYRIFLYSYGYIKMSGL

KMELKNIKTMFLNPIVIATFAGLFIWIFQGSMPQVTVAGIDGAAAHQVAFLRIDQTAL

WLFKPMTYLAGLASPLAWLSIGSTLGEISFKKAASDKTSWYYSVVKVILVPAINIVFL

AILTVTNILPVSFVALATIVIMMATPTATVAAAYAISFDKDALLASNASLLSTVMAVI

MTPVWIIILEVISKTGLF"

CDS 3034347..3034844

/gene="yncA"

/locus_tag="JFBMFIFI_02846"

/EC_number="2.3.1.-"

/inference="ab initio prediction:Prodigal:002006"

/inference="similar to AA sequence:UniProtKB:Q8ZPD3"

/codon_start=1

/transl_table=11

/product="L-methionine sulfoximine/L-methionine sulfone

acetyltransferase"

/db_xref="COG:COG1247"

/translation="MIRLATEIDIPVILAIYNQAILKTTATYDEEIHTIEEQLAWFNE

KRQKEIPLIVFEEEGIVAGYGTYGPFREKSAFRYTIEHSIYVGEAHQGKGIGEKLLLE

LIKLAKVADYWVMIGGIDHGNPGSIHLHKKLGFTYCGTLKAVGYKFNQFLDLDYYQLD

LRADA"

CDS 3034953..3035492

/gene="hpt_1"

/locus_tag="JFBMFIFI_02847"

/EC_number="2.4.2.8"

/inference="ab initio prediction:Prodigal:002006"

/inference="similar to AA sequence:UniProtKB:Q5XEL6"

/codon_start=1

/transl_table=11

/product="Hypoxanthine-guanine phosphoribosyltransferase"

/translation="MIQNDILKVLYSKEELAQRTTELGKELTEDYRNKKPLLVGILKG

AIFFTTDLAKAMDINVEIDFMDVSSYGNSTISSGEVKIIKDLSASAADRDIIIVEDII

DSGRTLEYLVSLFKRRGANTVKLVTLLDKPEGRVVDIKADYVGFLVPNEFVVGYGIDF

AERYRNLPYVAVLKPEMYQ"

CDS 3035649..3036959

/gene="npr_2"

/locus_tag="JFBMFIFI_02848"

/EC_number="1.11.1.1"

/inference="ab initio prediction:Prodigal:002006"

/inference="similar to AA sequence:UniProtKB:P37062"

/codon_start=1

/transl_table=11

/product="NADH peroxidase"

/db_xref="COG:COG0446"

/translation="MKIVIVGGSFAGVTAAVTARKKFPKAEIVLLEKKNTIGFIPSAL

HLILNGEIESLQQAYFISEAEIEEHNIHLILAAEVKKMEIENQIISYENQNILKNIAY

DKLILATGSTQNSYRIKGIDSERILKYKSIEKAQEALALVNENQTFTIVGGGQVGVEM

ADCLVRQNKKVQLIESMSGILVKYFDQEMLAPLLLEMKNKGVISYFNETVQEIKEIEN

RVILTTNNQEIESECAVFALSVKPNLPYLDPTIRCHEDGSIFVDEYLKTSVENIFAIG

DCIQIPSSLSSETFYVSLTNNAVRAGVVVVDNLIEPQTVFIGSARTIGTKVFDYFIAS

TGMTEAESIFFDEEISTTYVTQSSSLFNQEPIKGKLLYNKATRKILGAQFISKSNILE

KINTLSLGIQMGITVEELAQKDYYFHPAWSPVYDVTNQLGLMKD"

CDS 3036970..3038436

/locus_tag="JFBMFIFI_02849"

/inference="ab initio prediction:Prodigal:002006"

/codon_start=1

/transl_table=11

/product="hypothetical protein"

/translation="MRIEDFLEKKEGLQIAILKKLVLENGKISYEDLRNYLGISKASL

ESYLEELESYLSEYQGDCRVKSDGSKIELFVSNHFSIVTVYTDYVKRSLKFQLIDYAF

KHQKFTAVKITNDLSISESSLFRKIKESNDLLKEFNIQIKNGSLHGEELQIRYFYFQL

YWLVTPNEKFQETVLPAQIHNLINGFEKELDIKISDFSRIRVHLWFAISKKRANISEK

IYKNSQKKLKLYENDRLYKQVRQLILLYFSRYSIEVGEEESMLHFIFLTTQSILSEKD

FINYDLIRSRRTPTALVDTLLRETVLHYYLPVKPSLALERKISYYLSQINGTLYFFEG

ALEIYDREDFLAQESKVWRKNLKNLSEELLTTALANFNQKATSLNTLQGLTLLEYSSV

LSIVDFKISKELHIGIDLNLMEMHTEILTQMLELNLKNVTGITVEEYKKQRNYDLVIT

NNLAFSEYPKGTEIYVTSDFFSSYDLKQIKSRINELKI"

CDS 3038655..3040163

/gene="glpK_2"

/locus_tag="JFBMFIFI_02850"

/EC_number="2.7.1.30"

/inference="ab initio prediction:Prodigal:002006"

/inference="similar to AA sequence:UniProtKB:O34154"

/codon_start=1

/transl_table=11

/product="Glycerol kinase"

/db_xref="COG:COG0554"

/translation="MTEAKYIMSIDQGTTSSRAIIFDKHGKNVGNSQKEFTQYFPNDG
WVEHNANEIWNSVQSVIAGAFIESGIRPEQIAGIGITNQRETTVIWDKKTGLPIHNAV
VWQSRQSSPIADQLKADGYADFFHKKTGLIIDAYFSATKIRWILDHVEGAQERAEKGE
LLFGTIDTWLVWKLTDGGSHVTDYSNASRTMMFNIHDLEWDQEILDLLNIPKVMLPKA
CSNSEIYGYTKNFHFYGSEVPISGMAGDQQAALFGQMAFEPGMVKNTYGTGSFIVMNT
GEVPTLSKNNLLTTIGYGINGKVYYAIEGSIFVTGSAIQWLRDGLKMIQSAADTEKIA
MESTSADDIFVVPAFTGLGAPYWDSAARGAIFGITRGTTREDFVKATLQSIAYQARDV
ISTMQHDTGIDIPLLKVDGGAANNNYLMQFQADILDTAVQRASNLETTALGAAFLAGL
AVGFWKDLDELKEFYEEGEIFEPTMPAEKRENLYEGWQQAVAATQMFKHTSK"

CDS 3040196..3042025

/gene="glpO"

/locus_tag="JFBMFIFI_02851"

/EC_number="1.1.3.21"

/inference="ab initio prediction:Prodigal:002006"

/inference="similar to AA sequence:UniProtKB:O86963"

/codon_start=1

/transl_table=11

/product="Alpha-glycerophosphate oxidase"

/db_xref="COG:COG0578"

/translation="MAFSNETRQKIVKDLKTKQLDVLIIGGGITGAGVAVQASASGMQ
TGLIEMQDFAEGTSSRSTKLVHGGIRYLKNFDVEVVSDTVTERAVVQGIAPHIPKPAP
MLLPIYNEASSTFNMFSVKIAMDLYDQLANVTGSKYANYTLTADEVLEREPYLKKEGL
LGGGVYLDFKNNDARLVIENIKQAAADGGAMLSRVKAIGFLYNESGNVNGVKAQDLLT
DEIFDIHANLVINTSGPWVDKVRELDQSKKITPQIRPTKGVHLVIDSSRLTVPQPTYL
DTGKNDGRMFFVVPRENKTYFGTTDTDYQGDYAQPTVEQADVDYLLECINYRYPSAKI
TLADIESSWVGLRPLISTNGGSDYNGGDNGKLSDNSFSQVIDVVTKYQNKEVDRFAVE
DVLNNLESSLAESATTPSTVSRGSDLTREEDGLITLSGGKITDYRKMAEGAMQLIQKT
LEAEFHKVFTLIDSKTYQISGGKMDPGKVEETLETLAKEGIEKGLSKEEAIYLADLYG
SNVTALFANIDKLKAFPGMGLGESLSLDYALNEEMTLTPADFLVRRTNHMLFMRDSLD
EIKKPVVDVMAERLNWSEEEKSNYINELAQVINESDLIELKKG"

CDS 3042277..3042993

/gene="glpF_2"

/locus_tag="JFBMFIFI_02852"

/inference="ab initio prediction:Prodigal:002006"

/inference="similar to AA sequence:UniProtKB:P18156"

/codon_start=1

/transl_table=11

/product="Glycerol uptake facilitator protein"

/db_xref="COG:COG0580"

/translation="MSGDTLQIFSEFLGTLILVLLGDGVCAAVNLKKSKAEASGWIVI

ALGWGAAVTIAVYVAGFMGPAHLNPAVTIAMAIAGNFSWSLVVPFILAQVAGGVIGAT

LVWLTYLDHWQATEDKGAILGTFATGPAIRNYPANVLTEMIGTIVLVLGLLSFSQHTF

ADGMNPLVVGVLIVSIGLSLGGATGYAINPARDLGPRIAHQILPIANKGDSDWSYSWV

PVVGPVLGAIIAAGIFLVIM"

CDS complement(3043076..3043534)

/gene="luxS"

/locus_tag="JFBMFIFI_02853"

/EC_number="4.4.1.21"

/inference="ab initio prediction:Prodigal:002006"

/inference="similar to AA sequence:UniProtKB:Q9ZMW8"

/codon_start=1

/transl_table=11

/product="S-ribosylhomocysteine lyase"

/db_xref="COG:COG1854"

/translation="MEKMNVESFNLDHTKVKAPYVRLAGRKDGENGDVILKYDVRFKQ

PNKEHMEMKSLHSLEHLTAELIRNHADYVVDWSPMGCQTGFYLTVINHDNYDDILSVL

EATMKDVLVATEVPASNEVQCGWAASHTLEGAQQLATEFLAKRDEWSEVF"

CDS complement(3043553..3044194)

/locus_tag="JFBMFIFI_02854"

/EC_number="2.1.1.-"

/inference="ab initio prediction:Prodigal:002006"

/inference="similar to AA sequence:UniProtKB:Q6HDF0"

/codon_start=1

/transl_table=11

/product="putative methyltransferase"

/translation="MGREFLDVFSEWANDYDTFVEGQDPAYQPVFKDYSLILDTIVTK

SGSSVLEFGIGTANLTLRLLAAGKSVFPIEPSPEMRQLAKAKLPAEIVVYDGDLETYP

RPAMPIDTIVSSYVFHHLTDVEKAEALKDYATLLPKNGKLVFADTMFQTKKALDEKVQ

YALEKNYADLAADLVREYYPLIPTMEEAFRTAGFTANFIQMNDYVWIVEATKI"

CDS complement(3044204..3045349)

/gene="metB"

/locus_tag="JFBMFIFI_02855"

/EC_number="2.5.1.48"

/inference="ab initio prediction:Prodigal:002006"

/inference="similar to AA sequence:UniProtKB:P56069"

/codon_start=1

/transl_table=11

/product="Cystathionine gamma-synthase"

/db_xref="COG:COG0626"

/translation="MKMKTKLIHGGISRDSHTGALSVPIYQVSTYAQEKVGKHKGYEY

SRSGNPTRFALEELIADLEEGTRGFAFGSGLAAIHTVFSLFGQGDHVILGDDVYGGTY

RLLNSVMKKQGLTFTIVDTSNPEEIIAAIKPTTKAVYLESPSNPLLKISDIALIARLA

KENNLLTIVDNTFATPYFQQPLVLGADIVVHSGTKYLGGHSDVVAGLATTNNEAIADE

IGFLQNAIGGVLGPQDSWLLQRGIKTLGIRMEELAANALLVAQLLEKHPSVARVYYHG

LPTHKGHEIAKKQMTGYSGIVSFELNPGLSAETFVESLQLFTLAESLGAVESLAEIPA

LMTHASIPADLRLKAGIKDELIRLSVGLEDSEDLITDLEQALLLASK"

CDS complement(3045361..3046272)

/gene="cysM"

/locus_tag="JFBMFIFI_02856"

/EC_number="2.5.1.47"

/inference="ab initio prediction:Prodigal:002006"

/inference="similar to AA sequence:UniProtKB:P56067"

/codon_start=1

/transl_table=11

/product="Cysteine synthase"

/db_xref="COG:COG0031"

/translation="MLFHTIHELIGQTPLLEIQGFTLPQGTKIFAKLELCNPGGSVKD

RLGVKLLETAFAEGAINGDSTIIEPTAGNTGIGLALAAQQYGLKVIFVVPEKFSLEKQ

VLMQALGATLVHTPTEAGITGAIEKARELAQTIPNSFVPMQFENPNNPLTYYETLGPE

LLQDHPEPFTSFVAGAGSGGTFAGTARYLKEQQPTIRTCIVEPEGSILNGGPAHGHDT

EGIGMEFIPKFLDTTLFDEIYTISDDDAFYYVKELAKRTGLFIGSSSGAAFAACLKEA

ALLPAGSHIVTIFPDSSERYMSQKIYR"

CDS 3046544..3046969

/locus_tag="JFBMFIFI_02857"

/inference="ab initio prediction:Prodigal:002006"

/codon_start=1

/transl_table=11

/product="hypothetical protein"

/translation="MVLDNWKVYVAKIDGFSTLVRINMTAKEQGWANEFPLCVVLTLQ

VLAKEENGFPTEAELARLETIEQAILPVIDKHGKLAVSLLSQEGTQDLIMFTDKPTEL

SSAVDKKLISLDIPEAEFQLGSPDGDDQWAWYDFVYPKD"

CDS complement(3047150..3047485)

/locus_tag="JFBMFIFI_02858"

/inference="ab initio prediction:Prodigal:002006"

/codon_start=1

/transl_table=11

/product="hypothetical protein"

/translation="MSYKHLLEILIFALDAASILIIVWGVLLASKDLIKSEFSHKSRQ

DIIRNNNIIKNHLGSYILFGLEILIAADIIESILNPSFQDLIVLASIVVIRTAISYFL

TKEIEAIKE"

CDS 3047656..3047976

/locus_tag="JFBMFIFI_02859"

/inference="ab initio prediction:Prodigal:002006"

/codon_start=1

/transl_table=11

/product="hypothetical protein"

/translation="MEITEFQDWVRDYYLARGWSDLNIFIRIGFLSEETGEVARAIRA

IEIGRDRPDEADQNDVELKENLKEELGDVLGNLLVIANKYEISLEDILISHKEKLSKR

FADE"

CDS complement(3048182..3049126)

/locus_tag="JFBMFIFI_02860"
/inference="ab initio prediction:Prodigal:002006"
/inference="similar to AA sequence:UniProtKB:P99173"
/codon_start=1
/transl_table=11
/product="Zinc-type alcohol dehydrogenase-like protein"
/translation="MKAIGFTHFGGPEVFEEITTKELPTYKENQVLIKTLKAGVNPYD
ALLRSGEMAKFRPLAFPIIPGTDVVGEIIAVGNDVTDFSIGDIVIANPSIGGYSQYIA
ISHKRIMKKPEKMSLSIAAGFASVSVTAYWAIMGFAPVKNGATIIIQGASGAVGGVAV
QIAKDNGLYVIGIGNSRNKEYVLSLGADEFVAYNEEHSTSTLANRADIVLDASFGGKG
APSGLDLVKDGGYYLSLTAVPGAQPDKNVTIVSIQRTKDMTTGKALTYLSDLYQRKEI
IMETALEFPLTAVGASKAHQAIETKKQAGKIILSVENN"

CDS 3049551..3050063
/gene="crr_2"
/locus_tag="JFBMFIFI_02861"
/inference="ab initio prediction:Prodigal:002006"
/inference="similar to AA sequence:UniProtKB:P60857"
/codon_start=1
/transl_table=11
/product="PTS system glucose-specific EIIA component"
/translation="MGLFFNKKKKEEAVASLYEELVAVADGKVLPISEVPDPVFAEKM
MGEGFAIDPTSDVIVSPISGTLVQVADTLHAYGIQSDLGVEVLVHVGLDTVNLQGKGF
EAKVKIGDAVKKGDPLVKIDREYLIANAPSIVIPVIVTNGNMEAYNYDFKGSGNAVAG
ETVAMVVSKA"

CDS 3050104..3050940
/gene="sacY"
/locus_tag="JFBMFIFI_02862"
/inference="ab initio prediction:Prodigal:002006"
/inference="similar to AA sequence:UniProtKB:P15401"
/codon_start=1
/transl_table=11
/product="Levansucrase and sucrase synthesis operon
antiterminator"

/db_xref="COG:COG3711"

/translation="MKIKKVFNQNAVLVVDGTTEKVAIGKGIGFNKKKNDLVFDYDIE

QLFIMENEQENFQQLLSQIDESYFFASERIIEHAEHALKEKLNEHIHIALADHIAFAM

DRLKNGIVVRNKLRKEIEVLYAEEFLIAEWAIEYLSTQFGSVFTLDEAAYIAIHIHSA

RTGEYGNSKSIREITMVSEMARVVGEELVLDFNQADLSLSYSRLVTHLRFVLERFYTK

KYHTMDLEVLAIIKKNYHASYQIALKVAEMMKQDFNVVLPDDELGYITIHIERLSGLK

NI"

CDS complement(3051009..3052544)

/locus_tag="JFBMFIFI_02863"

/inference="ab initio prediction:Prodigal:002006"

/codon_start=1

/transl_table=11

/product="hypothetical protein"

/translation="MKSQKSAINITVGILAQIIIVLLGFVTRKVFIDYLGNDANGLNN

YFTNIVAMLSLVELGLGNSLLFSLYKPLAEKNHDLINRLLSVYKKIYYWIIAIIGFLG

IILASFMPTLIGSDSGFSNSYIIAVFSLFILSTMTTYLFTYKRLLLTANQDGYYATLA

DIIYSIIRALLQIFIIMKTQNFILFLVVDIFLRLIENYVLSLFIQKKYAYLKTLKNSR

LEPALKETIIDNTKSLAFHRIGTFIMTGLDSILINAFYNLGTVSIFSNYTLISSTLLN

LVTQFSTGISASFGLLLAEGKLKRLFETYRMANFINFMIVNFSAVTLLALFNPFMQLW

LGDKFLFDNSIVFILVLNFTIASLTTINGSIRGAAGLFKPDKYLHIFYALLKIAFSLL

LNQYLGLIGIFLATTICYLIKDLIVLPIIVYRGIFKKSARVFYQEFFGYGSVILVSGL

LISLTKQFIYSWSLAPFSAFLCLALMCLIIPNLLAISVYHRSQKYKDSKELIFSTLFK

KKS"

CDS 3052797..3053504

/gene="walR_4"

/locus_tag="JFBMFIFI_02864"

/inference="ab initio prediction:Prodigal:002006"

/inference="similar to AA sequence:UniProtKB:P37478"

/codon_start=1

/transl_table=11

/product="Transcriptional regulatory protein WalR"

/db_xref="COG:COG0745"

/translation="MKKILVVDDEKPISDIVKFNLTKEGYEVSTAYDGEEALKMVPEV

EPDLIILDLMLPKIDGLEVCREVRKNYDMPIIMVTAKDSEIDKVLGLELGADDYVTKP
FSNRELVARVKANLRRHGNAATPVVEEDDNNEIEVGALTVHPDAYIVSKRGEKIELTH
REFELLHYLARHLGQVMTREHLLQTVWGYDYFGDVRTVDVTVRRLREKIEDNPSHPGW
LVTRRGVGYYLRNPEQE"

CDS 3053511..3055340

/gene="walK"

/locus_tag="JFBMFIFI_02865"

/EC_number="2.7.13.3"

/inference="ab initio prediction:Prodigal:002006"

/inference="similar to AA sequence:UniProtKB:Q7A8E0"

/codon_start=1

/transl_table=11

/product="Sensor protein kinase WalK"

/translation="MNKRIKIFQSIHFKIVIVFVFLLIVVLEIIGAYFVGQLETKMVN
VFKDDISERVNFLGTNLQPILKNPDSKTYHEDINRLVSDFSRRNISKTEIIDVDHYIV
GTNSNPNTIGRISKDNDVKQALVIGKQSERPYPDENNDRVWKVVMPIISDDNKILGVI
SVESNIESVYKQISSITEIFFKASMIAAGVTVVLALFISRAITKPISEMKKQATQMAE
GDYSGQVKIYGQDELGQLSLAINDLSTKVEEAQESTEAERRRLDSVLEHMTDGVIATD
RRGKVVIINETALELLNLTQDKAVGYSILEILKIQEHFTLRHLLETQEELILDFSTED
NEVTLRGEFSLIQRETGFISGLVCVLHDITEQEKIERERRDFVSNVSHELRTPLTSMR
SYLEALNDGAWKDPDIAPRFLAVTQEETDRMIRMITDLLNLSRMDAGKDTFELEYVNI
NELFSHVLNRFDMMLQSADKPVKPFVIKRDFTKRDLWVEVDADKMIQVLDNIMNNAIK
YSPSGGTITCRLMETHNNIVISIADEGLGVPKKDIPHVFDRFFRVDKARARSMGGTGL
GLAISKEVVQKHGGKIWLESIENKGSTFFISLPYVPYEEDEWI"

CDS 3055337..3056680

/gene="yycH"

/locus_tag="JFBMFIFI_02866"

/inference="ab initio prediction:Prodigal:002006"

/inference="similar to AA sequence:UniProtKB:Q794W0"

/codon_start=1

/transl_table=11

/product="Two-component system WalR/WalK regulatory
protein YycH"

/db_xref="COG:COG4863"

/translation="MKIRYFVRIALVFLVALSLVLTWAIWTMPNKIENSSDSNSKKTG

INSTLQENEIFSANRVVFHTDNNPKLASNPDLMARLDKEFYEWKFSNIKDAKTYNDED

YTSKLGEGNSLEFIYPTARPFSTLTKSFSKLASNYRNQTFQRIVIPLNESGEIHFFDD

NSRTVYSAEIEGFNKSTLSDVLTSNEKYFVDVSIQKLATRFVYLPKEEVELSSPSYIA

ELKPSLEIRNRLFEDLSEITNSTTADKRFEQYYDNVSKVTVDKETNILTYNRSSTSVK

LDLQDVLKYSISELKDFEVWPGKIRFFQYNNTKQQVVYRRYIEGFPVFGRGGMENDFG

GTYLTIAKDGVLSRMQMSLVIAQTPVFDEMAEKRLLNGSELIAHLDSIGYPINTIDDI

ELGYCWTKNLESDGLESEESGANTIVDFEPSWYIFTNGIWYNIDELSDNQDGEAK"

CDS 3056682..3057584

/gene="yycI"

/locus_tag="JFBMFIFI_02867"

/inference="ab initio prediction:Prodigal:002006"

/inference="similar to AA sequence:UniProtKB:Q45612"

/codon_start=1

/transl_table=11

/product="Two-component system WalR/WalK regulatory

protein YycI"

/db_xref="COG:COG4853"

/translation="MDFKRIELIFLLTFLSLNIFLLYSYFGKATTLTYESAETSTVVP

FDEMKKDGIKFDAKQFSDEKLLVPYVKAKNDNLLKQKVDQLPKDTTISMEDKHTIYRP

LTEPLELFPVGEKFNSKDIKKYAEKIDAFIAKEHIFFGESYNFFKYSATQNAIIYTQK

AEGFRILDGTGRITFHMDGGKIISYIQTYAGKIEVTGKSRPLISEKNAIESLYQNNEI

PADSVINGRPKLGYYRTLWLPKDNLSIYGPVWYVSLKIGNEEKIKLVSGVDGTIIKHT

SSTTNDSDSSSDGAATEEELAPLE"

CDS 3057729..3058541

/gene="yycJ"

/locus_tag="JFBMFIFI_02868"

/EC_number="3.-.-.-"

/inference="ab initio prediction:Prodigal:002006"

/inference="similar to AA sequence:UniProtKB:C0SP91"

/codon_start=1

/transl_table=11

/product="Putative metallo-hydrolase YycJ"

/db_xref="COG:COG1235"

/translation="MITEETDALKVSILASGSSGNVTYIESGKKKLLVDSGLSGKKVT

ELLKQINRDIADIDGILVTHEHRDHIHGVGVLARKYKMDVYANEKTWQAMSPIIGEVK

TEQKHIFEMGTTLSIGDIDIESFGVSHDAVAPQFYCFHKNNKRFAMITDTGYVNDRMR

GVVENANAYLFESNHDLEMLRMGAYPWSLKQRILGDKGHLSNEDGAIAMAEVLGDATK

RIYLGHLSRENNLKELAHMTAVSVLREKGTGVEEQFKIYDTDPDKAAPLFIV"

CDS 3058851..3060425

/locus_tag="JFBMFIFI_02869"

/inference="ab initio prediction:Prodigal:002006"

/codon_start=1

/transl_table=11

/product="hypothetical protein"

/translation="MRYDENGNLISSEENHAVDQELSSEDVMGTGMTNETFDEYTNEN

QERFPEEEILADRKAVDDAIAADKREEKPQPTVAPTPSEPIVEKTVEPRRKTQKQPVE

ASAGNGGSGGVRPPKNEKKDPQPPTPKKGGFKSGLVGGLVGGLVMVVLGGGALYGYTQ

SQDTNVGSTNNNTAKNVQTSNISANVTTDTTEAVAKVEDAVVSVVNMANSQGNFLQGG

GNSSQSSDSSDLQTQSEGSGVIYKKDGKTAYIVTNNHVIDGSDAIEVILKDGTKVEAK

LIGADQWTDLAVLSIPADKVKTVATFGNSDDIKVGEPAIAIGSPLGTNFATSVTQGIV

SAKDRSVAMDIDGDGVEDWDMTAIQTDAAINPGNSGGALINLAGQVIGINSMKISQDT

VEGMGFAIPSNDVVKIINELQQNGKIVRPVLGVVLRDLSQISEQQQQSVLKLPEDVTE

GVVITNVQKDSAAAKGGLEQYDTITEIDGQKITDSVSLRKVIYNLKVGDTVEVKFYRD

GKLETAKVTMEAANSI"

CDS complement(3060554..3061363)

/locus_tag="JFBMFIFI_02870"

/EC_number="3.1.1.23"

/inference="ab initio prediction:Prodigal:002006"

/inference="similar to AA sequence:UniProtKB:A0QNZ7"

/codon_start=1

/transl_table=11

/product="Monoacylglycerol lipase"

/db_xref="COG:COG2267"

/translation="MAEFIDRYTDFAEFLTSHGFLVVGNDHLGHGRSVAASSDFGYFS

KKNSKDYVIEDIYTLHQLVKQDYPTLSYFLMGHSMGSFIVRNYLQKYGASVDGAIIMG

TSGPKLETALILPILEVLNKLQPRKQNKWVDQLAFGSFNNYFPEDAAEFAWLSENQEN

VQRYMDHPQTGFIFTNNGFLTLFTLLQDATKPGWANPIPRELPLLIISGEDDPVGQMG

VGIRKVFRELEELDFVDITFCSYPTMRHEILMETNHLLVYSDILDWLSKHL"

CDS 3061972..3062451

/gene="rlmH"

/locus_tag="JFBMFIFI_02871"

/EC_number="2.1.1.177"

/inference="ab initio prediction:Prodigal:002006"

/inference="similar to AA sequence:UniProtKB:Q45601"

/codon_start=1

/transl_table=11

/product="Ribosomal RNA large subunit methyltransferase H"

/db_xref="COG:COG1576"

/translation="MNIKIITVGKLKEKYLKMGIDEYTKRLGAYCKIELIEVPDEKAP

EKLSEAEMLQVKEKEGERILAKIPENAYVFALAIEGKQRTSEEFSKEIEQLGIQGKSN

LVFVIGGSLGLSQAVMKRSNTPISFGKMTLPHQLMRLVLVEQVYRGFRIMKGEPYHK"

CDS 3063569..3065362

/gene="tpl"

/locus_tag="JFBMFIFI_02872"

/EC_number="4.1.99.2"

/inference="ab initio prediction:Prodigal:002006"

/inference="protein motif:HAMAP:MF_00543"

/codon_start=1

/transl_table=11

/product="Tyrosine phenol-lyase"

/translation="MYEIREFKTYDTESIIDFIIKIQRNEYNINISLDDQTDLLKIDE

SYIDNGGNFWVAQDQFGAIIGTLGVQRLSKNRVALKKMFVHSAYRNKKIGKSLINYLF

QFCEDNKINEIYLGTTDKFKDAQIFYKKNGFKEISINELPKDFPILEVDNHFYKKKIS

SRLYSPYQLHISQRIPELGRKDRIEALKDCGNNALALVPSKIKFDLFTDSSRSAISIY

QFSEALVADEGPLGTGQTYSELLENLKKYFGFEHNILFTQGRAAEAAFCNKMLGENTV

IMSNKTFSTTQYHQNLNGAKIVSVHSEESEDFSSPYLFKGNVDLIKLERELYSAERNT

LIWIELCSNYIGGHGLSYKNLKKIKALSLRFNVSLVMDATRILENAILIKKYEMPENS

INSIIKKITSFADYLVMSLKKDFLCSSGGLISCRNKEDYLVLSDYQIIHGGEISGYEK
SLVSIGLMEAFSNDTHIEYRYYLTKILYDALKISNIPVLEPNSVHGVWIDIDTLVENT
LHKNDENILIGVQNTIYIETGIRVGVQQAFGRNLVRLALPHRLYDESQTRYIATAISK
IFENSIEIKRYTQNDIDDSITSNMWVTYLEK"

CDS 3065380..3071073

/gene="ppsB"

/locus_tag="JFBMFIFI_02873"

/EC_number="2.3.1.-"

/inference="ab initio prediction:Prodigal:002006"

/inference="similar to AA sequence:UniProtKB:P39846"

/codon_start=1

/transl_table=11

/product="Plipastatin synthase subunit B"

/translation="MIPNEHTIHGYFEKQANYSGKSIALVDEKKSISYQDLSNLANKY
ANHLMENDFQNKKIGVLMEPSIDIVVVILAILKVGSCYVPLSSQLPDKRKEYIINDSE
LSVVVCDQLNANFGTDDVPTLLYTENTSVKDALSFKSTNHLADSEAYIIYTSGTTGVP
KGVPIKHKSIMNTLFWRIHEYKMNSNIVQLQTFSFIFDGSISSLFSVLLSGGTVVIPT
EKTKRNPETLINLIKYKKITHLFMIPTLFVSLLETKQVESFYSLKVVTLGGEKIPVSI
LKNPIVKDTSFEIVNEYGPTENSVTSTFSRNVTEGNVSSIGKPISNCDTVVVTESLAE
ATFGEIGELCVAGVGLTEGYLNNSTLNQEKFVIFDSKLFYKTGDLVRLDNLGNIIFIG
RKDFQVKIRGYRIEIEEIEKYVTSFEKISEAVVIVQRFPKVGEVLIAYYSSEIEIEES
EIKNFLNKSIPDYMIPLKFIKVNSFNTTPSGKINKSALPKIVSLIEHKQLIMPSSELE
QQLIDIWRKIFGQEKIGVQDNFFSLGGHSLLIGSLLNEIDFQLKKQLTFEEVYSNPTI
QEMAVLLSTKIVQKSSRLSPTSLLHYPLSPSQKGVYSYTQRRPESLAYNISMLFHFTK
IYETTQIVDALNYLIEKYSCFRTRFDVIGDSIVQIVDAYKPIKYNIEENGNSERTLPE
FLKSNIKPFNLKDDYSFRAKIFKLNNKQSLLIDTHHIVFDGISSTILQNDLAEFLRDK
YKIEIDRLEFKDYIIEEKNTSIKKRQQDYTWWEKYLENGLPNTIIPLNDIPKSDSINT
FKAEKIDFDIEENVFSDLSDIAGNDKSYSVIVLSAFIMLIQKQTNENELVIGIPFAGR
NNKSIVNEIGMFVNTLPFKYNVNKEHNFKEIVQSINKLMMNLHDKQSIGPNDILEILS
RKNKGISNNVLFDIVFSVQIESDGDKNDNFKFELVEPFDAKFPITFTIEKQGSSLKCA
FEFEKNKYTSQQIKQIIENFRNLLLNITKKPLAPIKEIEMISHEKREEILSRNHVSGF
KNDYTIHERFESIVNMFPNKNAIREQNKSVTYQELNFRAEKIANKLTSKGLHSEDFIG
IHMDKSSEMLATMLAVLKIGCAYVPIDPKYPEDRKKYILKNSKLNWVISTENEELKLL

NVSSPKENMNYPADFIKSKNNSKVESPAYVIYTSGSTGKPKGVVVNHKNVMSLLFPKK
NNFDFSEKDCWTAFHSFCFDFSVWEIYGAILYGGTLVIVSDEIARNPKEFMNLLISEK
VTVLNQTPSAFYTLIETEEPNTEYPYLKWVIFGGEALSPSKLKNWNKIHPHTKLINMY
GITETTVHVTYKELTSETLNESISNIGKPINTLDYVVMDTNMNIVPAGVVGELYVRGH
GVSMGYLNQPELTIERFIQNPIKPEETMYRSGDLVFETPYGELIYVGRIDDQIKIRGY
RIELGEVEKVIQKIQSIKTCRVLVKEDNSGEKSIIVYYETNSADEIDLMYETKKLLPN
YMIPSSFIKISNWPRTSNGKLDQKKLLKINQSDDSKFKNNIKASYDEIRLLELFKKVL
NIDNILVTDNFFDIGGHSLRVPIIVTMINEEYSSNITFSEFYLAPCVKDLSALIREEK
RTETSRIIKLKSSSDLYAKNMFLIHGGNARVNTYSEFSRNEHTGWNYLGIELFKNKLV
DIEKLDCNKLAKVYVKLIKEKQPKGPYYIFGTCIGGTIAYEIALQLEKEYLENVHLSL
ASVEAPNINQRKITFNHFRKKYIEKVSNLFNYPEEITNTYEEHWEKIIDTLGKRKTHI
HEICKTLEFSINNLESNSIRELFKSNNLTQSLTLLRDNYKPTEIFEGPVNYYNATEQE
LPSRYTWSHLCKDITYHDVSGNHDSIFYKENVTEFHNQIKYNFKLNSREKENDTI"

CDS 3071070..3072293

/gene="rizA"

/locus_tag="JFBMFIFI_02874"

/EC_number="6.3.2.48"

/inference="ab initio prediction:Prodigal:002006"

/inference="similar to AA sequence:UniProtKB:B5UAT8"

/codon_start=1

/transl_table=11

/product="L-arginine-specific L-amino acid ligase"

/translation="MKTLIFLYDYPSLNEAIRLLVDHHNCILIAQKGQVTSTRKELEE
TFNQIIEMDDLFNPNSVYEVVKELSQTNEISKIITTYEPAVEVAGYVREKFGITGLSL
EQSELVRDKYKMKTFLKKFDIRSANICKVNNEKEKIAFLEQNNFPLIMKPFDGAATAK
TFTVNNFEDIDSIKMNYPEVSFVLEEFVHGEEYHLDAIVQDGKVILNSVGKYLDNIID
CIEGGATVGSVIYPASHKFSKNLKEMLEMNLRVIDFLGLENCICHTEFFITPYGEVIF
SEIAARIGGGKLIGPAIQHIYGINLFEALIAVELGKNIPINKKNDHDFCGFVTFDTTK
ENVGTVTKIASEKDFHTIDGLIANKILYSIGDEVKKAENSAERTGYIIISGKSEKDVR
KKISQSREIYHLEVS"

CDS 3072305..3073504

/gene="mdrP"

/locus_tag="JFBMFIFI_02875"

/inference="ab initio prediction:Prodigal:002006"

/inference="similar to AA sequence:UniProtKB:A0A1C7E424"

/codon_start=1

/transl_table=11

/product="Na(+), Li(+), K(+)/H(+) antiporter"

/translation="MFKEIDKNIKIRLFLNFISKLAMSTILPFIVVYYSQVFNQRIAG

IFVMITMACQIVSTIYSGYVTDKIGRLPLMKIGEICKLFCFLLMIVFSNPLIFFLLIT

FVSIAQGIINPAMEAMLIDNSTVETRTKIVSMNYWINNLAILLGTIIGGALFYQHFLL

IRVLLFLSSIFICYVLYMKLKETAGTVSKDESKGISLNYLSILKNKKYLLYFLGSICI

FSVELQRNNYIVLSLPKSIEVSGVIFSNVSLMSFLTSINTILVILFLPLVIKKFSKKN

LKTYLFTSVIIFSLGYSVLSLSNIFFVLLIATIAISAGELLYTPASQTVFLESIEDTK

KGMYLAINSLGIQIAQILAGGFLIVTSYFSRSINSSLIFMIGIMGLLLLMTSYTTKNM

ESVKETI"

CDS complement(3074998..3075675)

/gene="sfp"

/locus_tag="JFBMFIFI_02876"

/EC_number="2.7.8.7"

/inference="ab initio prediction:Prodigal:002006"

/inference="similar to AA sequence:UniProtKB:P39135"

/codon_start=1

/transl_table=11

/product="4'-phosphopantetheinyl transferase Sfp"

/db_xref="COG:COG2091"

/translation="MLRIWIVSIEREIEETKWIQLVNEIDSTRKLSINGFKLHSDKKR

ALIAGLLIKAMVKRYHPNVPYKKISKNRYGKPYIQGINDFHFNISHSGSYVCCAVSDS

EVGIDIEKISSSIIIDNYKPFFSVAEWEQINSDEKKRLDNFYSLWTLKESYLKKIGTG

LSKDPSTFTIHIGSNIKVIDQNIIREEKFLLPEFSKYYKLAVCSKKSFEYSHKKISCQ

DFINKYY"

CDS 3077646..3077939

/locus_tag="JFBMFIFI_02877"

/inference="ab initio prediction:Prodigal:002006"

/codon_start=1

/transl_table=11

/product="hypothetical protein"

/translation="MSSHFRKEYFCDWLMINEGLIVEEDCVKESVEILLHEELPFILQ

SKVPAAFYHQASFQNNDHEWEMHVFLTEYTQEPLYFYVEKDKKRIEEKWAKEE"

CDS 3077944..3078273

/locus_tag="JFBMFIFI_02878"

/inference="ab initio prediction:Prodigal:002006"

/codon_start=1

/transl_table=11

/product="hypothetical protein"

/translation="MFMDKKVRYMTKAIQEEVPAAIQLALWGLIDARVEENAKLDYFQ

IFELSPSEDGKEQIVLHRQEVPLWEKKSQFVLSSPQTGSEVKPFTGKIWVLDQQEYQM

MLFPSDY"

CDS 3078311..3078769

/locus_tag="JFBMFIFI_02879"

/inference="ab initio prediction:Prodigal:002006"

/codon_start=1

/transl_table=11

/product="hypothetical protein"

/translation="MNEKKKPAKRVSLVSVKLVRESSILYSNRKIRNPEDAYLLLKDF

LEDKDREHLIVVGLDSKTQPTMLTVAHIGAIGSCIVSGREIMKPLILSNATSFIVGHN

HPSTDTTPSNADIECTKRLKKVGELMDIQLRDHLIIGENSYVSLKEEGVF"

CDS complement(3079122..3080693)

/locus_tag="JFBMFIFI_02880"

/inference="ab initio prediction:Prodigal:002006"

/codon_start=1

/transl_table=11

/product="hypothetical protein"

/translation="MTKNYLPYNKRPARVLDINKEEYLDSTIAQFDDSSLINKNPILN

TEEVEDKKNRSTYSNYFSGKIMNRYYLRYLPEQGEKGFYLLKKSLLEKRSAFFFIFEI

QDILAALVITEAYYPLADTSGTLLPFQLEKYLQHNKFLVNIIDEIEETPVDVHSSNTP

YGYGLTRLKNTDAYTDTALMNTFYPFFNECLNKLTQLLSSTSNELKNGKLLLYLTHYY

NSILEELMLNGGQNFLKNIKDGVSADVDDKVQKFKLDDEQNLTLDEQIVKFITIENLH

GFLYLDAITDIDKEIDFNENRKDKSSAAVTLSKKRFTDFEHIKEDPINFLLNFMKKRP

YYEKSESPSFKEHLEFEELIDFKNEQKELIAKLSDPTTSDNDKDILAKKFLILFDKKL
SECQIDAPRQKELQKSISTGSITNQLTLSDTMGLPDRVGQMRIDEKEAFSVLFKDSRI
QKKEKELTILNQKIQRVRKELDELKKEEPISLLTKSVENINKAKKEMDTQSTNPNIKA
LVRSVEELVLDNSTH"

CDS 3080907..3082718

/locus_tag="JFBMFIFI_02881"

/inference="ab initio prediction:Prodigal:002006"

/codon_start=1

/transl_table=11

/product="hypothetical protein"

/translation="MKEKAQRSIGRTAVVFQDSINKEATTEPLKEPVQSKTVSKEALN
NGQEFTLGLDPVFHLTRHGLYKEGSKAKDYLCQALFVSEVVCNSKNNELNLKVTYFYQ
GKWNVTDWLKPIQITQNKIMELVNVGVDIPQTKAPSISLFLLCQYRKLTPVTSYSTLG
WSKWNNEKGQEEIVFLQDNITSQNGEKSGVYIGERFDIQPKGSFKTWSNLIKSEVIGN
TAMETAFLLSLSSITIAYFKMIPNAKDISIGLIHYNSPSTSGKGTMLALGLSIFGNPS
QNHPKSLLRSHSSTYRALIKLLSDTHGLLIGLDEISATRGDKYEAKSKANLIYDVSNG
RDSDRLNSSSELQEASVHSSLFLSTGEESLSDFTNNNQGVQVRTFELKGLVYTSSANQ
ADHIKAICNQNYGFLAKKVSAVLLSTLPNVLEQEFLMWLQKFEKEFSSSNVESRMCRF
LAAVMLGTFLVQKSMGIQMNADKVFDLLCQQNRMQLSNMNLAENAYQSLTRHLSSVEN
YFKSQRNQKTYSKNYGFITDKGNKRYYNINLSSFKIIMSELKFTNEEGIRKKWKELYV
NGDCRFQFEKDRLVNRVIVEGKREEVVTLTFDLETEE"

CDS 3082819..3083448

/locus_tag="JFBMFIFI_02882"

/inference="ab initio prediction:Prodigal:002006"

/codon_start=1

/transl_table=11

/product="hypothetical protein"

/translation="MKKIAYYAVREGRTRGVYENWPDAQAEISGYSGAVFKKFNTKEE
AESFIQKGNEAKEIVVSDEEVQTNINISDFQIYVDGSWNPKKQQYGWGYVAVLYGKEI
GRGYGKGDNKKYLSNCQVAGEVVAVLQALDYAISKEFKSVEILYDFDGIKHWATGTWG
AKKLISQAYKYFYDQKSSEIKVTFVKVQAHSGNYFNTMADTLAKKGVNL"

CDS 3083445..3083546

/locus_tag="JFBMFIFI_02883"

/inference="ab initio prediction:Prodigal:002006"

/codon_start=1

/transl_table=11

/product="hypothetical protein"

/translation="MSQQLEEPENTMIFFEILVKTLLPYIIARGADD"

CDS 3083635..3083886

/locus_tag="JFBMFIFI_02884"

/inference="ab initio prediction:Prodigal:002006"

/codon_start=1

/transl_table=11

/product="hypothetical protein"

/translation="MIRRNSQLHKLYNYITLNHPSGTFTTEKIYQNAYLIGLNPTSIS

GSLYQLKQKGILSNSGGRMTSRGRVQKQWRLNKTAIITN"

CDS 3083905..3085317

/locus_tag="JFBMFIFI_02885"

/inference="ab initio prediction:Prodigal:002006"

/codon_start=1

/transl_table=11

/product="hypothetical protein"

/translation="MSLAYIRTSISKKEKISLDVQQEWIIKKAHELNLTTPLFYEDCG

VSGHQMGNRKGVSQLIQHLKQVTNETLLVYRYNRLSRNMLELLDLLELCERQNIVVVS

VMEPLPAGIGTSSAIQKVFVQLIGLVSEFQRSVTVENVRSGLKQKRRNGLPLASNPPY

GYSYTQEKLLPQKEEGAVVQWLYDEYVNESKGYTKLAQLLTQKGIRYRGTTFSSLHVQ

TILKNPVYYGVLKGGSFGSYLGTHHCLITKDQFDAVQKVRGKRQVTQKQPTSHPLQKK

IQCPFCGRALGFRKMKKNNKTYCYFTCSKPTCVARYQRVSPIEQEIKRVIQAFLNQAD

VKQRLIQEVENQRGSNMKQLKLDLSQLQKKKQALFQDFERGKVAESEFLQRLDQLKQE

KSLVHQPQASSQNQELEAFQAFLTATQKETILPVPDDFYFDLVERVEVTKDYQVSEVY

LTHLEQNLVEGNPISLENNS"

CDS 3085343..3086983

/locus_tag="JFBMFIFI_02886"

/inference="ab initio prediction:Prodigal:002006"

/codon_start=1

/transl_table=11

/product="hypothetical protein"

/translation="MTEQTERKRVVGYIRVSTEEQAEEGYSLDAQRENILRFCDQHHY

ELVDVYADEGISGKTIGKRPGIKCLMADASKNKFDLVVVWKLTRLGRSIKDVLGIAEL

LFANNVEFRSISENFDISTSTGRLMFQMLGVFGEFERNQISENVSMAMTSLVKHKKRF

AGGRMLGYVSAVDENGVKILQVEPNEAEIVRLIFGQYLQGKGYRAIANFLNKQGYQTV

KGNSFTTIAVKGILMNPTYAGYLRYGKYINWEEKRRKGLNPHPIQVEGSHEPIIEKKV

YHHIQEKLKANHAQPKWTHSGENVLTGLLKCPECGAPMAASNTTNTLKDGTKKRMRYY

SCSVFRSKGSSICHANSIRAEVAEAFVEERLKEVVLLPAHLKEIIKGLNKELKEQRAP

LELEQKVVQQQEQDIQAKITKWKSLIEDTPELAKELEARMKELEIELAVTRQRRQTIA

SFLEKEGVQLNPAEVEKMLHYVNALLAGRNKKDIKAIYRTFIESITFNKETKGDIQIR

MLFDAPIINQLNQVYQAGGTSQVGVSPVSLSFPIQITI"

CDS 3087240..3087521

/locus_tag="JFBMFIFI_02887"

/inference="ab initio prediction:Prodigal:002006"

/codon_start=1

/transl_table=11

/product="hypothetical protein"

/translation="MLDNERNDSSRVYPLEEDLLVPVYKELYHLVGEAGTVAIFNAFK

GRQIQFPMRFYTKEAIVQQIKNSDRQVTNKELAKRYDVTERFIRSVRSQ"

CDS 3087518..3087853

/locus_tag="JFBMFIFI_02888"

/inference="ab initio prediction:Prodigal:002006"

/codon_start=1

/transl_table=11

/product="hypothetical protein"

/translation="MKKNLVEWASEEFKRVKKLEVSSEEIGYESIELEREELDENLLP

TNVKDFRLPDKLLIHFFQVDGNPEGKAETEVCLQLYILANPVSNKWLLVGFLTDHQLT

SFYEPEDCR"

CDS 3087952..3088485

/locus_tag="JFBMFIFI_02889"

/inference="ab initio prediction:Prodigal:002006"

/codon_start=1

/transl_table=11

/product="hypothetical protein"
/translation="MFEKVSETMKLEMMVNPERSHRYVLSRIWDKKKELATVVTIYPS
GLEALQGDLTSLLIVNQLVKLGYGGFHSVNLFSKVGLKSTNAGSLHQAWDEGTDEVIQ
ICAEKSAVIIFAWGSIGETNKIAGARIEQVKEKLQKDAKKWRFLSDAEGKAYFHPLAS
KVRSQWNVVPYKEASKK"
CDS 3088697..3088963
/locus_tag="JFBMFIFI_02890"
/inference="ab initio prediction:Prodigal:002006"
/codon_start=1
/transl_table=11
/product="hypothetical protein"
/translation="MYLELAKLVGEENAIVMYREYRGFQLNMPMRLVSSQHIRHIVET
EYDGTNGEEIARFYGYTERHIRRLIAENNRGNQKKMEGKENESN"
CDS 3088950..3089993
/locus_tag="JFBMFIFI_02891"
/inference="ab initio prediction:Prodigal:002006"
/codon_start=1
/transl_table=11
/product="hypothetical protein"
/translation="MKATKVFGKKAIGIVVVTLFMLAGCGNKGQEAYDTAMEKGIQSV
VSEEYAKAEVSFELALESSKEDKKATLYLNQVQNYVQAKTEFEKKDYSGAQKTLEKVL
KSKDGLKEILKAADKLNQEIEAIQKEVSENEEKLTEATRLTEAKSYQESNGILDQILK
QDLGQMASEFKEKCEALKQKNDTALEEASQQAQEKEAKKAVEASAQVPAKLISNKDEA
FNYVQVKMPQAGNLTYISYEAGVYHFSADSGQAGWHITVDEAGNLTEEKVEDQSPVAN
SDTDGWAPGVKESAILSIKENGYLAQNAVEGVNYYFSNRDDTYTQLIGELPGGSEKLP
IVTINKKTGDYHG"
CDS 3090119..3092341
/locus_tag="JFBMFIFI_02892"
/inference="ab initio prediction:Prodigal:002006"
/codon_start=1
/transl_table=11
/product="hypothetical protein"
/translation="MAGAIVLALIVVGIYYGSQKMKGIDVGKIKKYIKGFFIFYVSSV

LIGFIAKLILRGQMSSGGSVNGILYSIYRHPMIAELIFIGIGFFLYKKYKRYQEEKEY

KEVFEEWTHQDFPTYDRWDFIHSVDRFVKGELQFVQEEIPYGRAAVFLQEFSLDETYD

LEFLAYEPIRSKEKIELKEYGCLITTEGIAIKHQAVYPKKKAVQLENGKEKNQDYFPV

ESYYLPYEGMMKITGHPTCFLMLYSDGKRVKLPYDQLLIPAQLLQKIFEEIILRGYSQ

TLYRESLEMKSKVDERKDKNVGEQDIPVNEIDEDELRESFEKGQHNEFQKKVAGTLVG

SQVVNPSSTNSLASQLKEQQFNQMVNGRQGHGVAAEYGNHVVDKVMGKTAIQLGGENS

KNGADRLVNGQALQTKYCQSANESIGAAFKDKNGRAVSFKYTDQMIEVPRDQYVKAVE

LFEKRIEKGQVPGESNPANARKYVKKGHLTYEHAKQVGKAGTITSLGVDTVGALKSTL

PSASLLLAVSYFNCRGQGMSHHESMKQSGAVFGRSLGVGVVINVSAIQAAKYMAGKSL

VKDPGVLTSRIGTGIMMSLTFGPDTVDVLRGRISIQQLFKNSVVSVSGILGSTAGSSV

GGLVGGMAGGSTASSSVKKLLDSFIEDDAEEMYQLMKEEFIDVVMSASITAIEYQEVV

SLTFANKKFPGMLKSMYASRDPRTFAREKICEEAVITQFKKRPPILDEEWEEVICEFE

"

CDS 3092590..3093153

/locus_tag="JFBMFIFI_02893"

/inference="ab initio prediction:Prodigal:002006"

/codon_start=1

/transl_table=11

/product="hypothetical protein"

/translation="MKLKNIVGINVGQNVSRIKDKEDKVSRIYTNEDLLNDLHEKHIG

LVRKTKIGTARNEKYFIYAGDILYSFISSTAGIVSQENDGKIINQNFARLTLNTKEID

PRYLCYVLNESISVDKQMSILMQGSVVPKMTPAILSEINIEFPELSKQLEIGNSYFDL

NRYYYLSKMEIELQKKTHFEILKKLDQ"

CDS 3093173..3094768

/locus_tag="JFBMFIFI_02894"

/inference="ab initio prediction:Prodigal:002006"

/codon_start=1

/transl_table=11

/product="hypothetical protein"

/translation="MSVELNQRLFSAADNLRSKMDASEYKNYLLGLIFYKYLSDKLVQ

KVVELADESLENYDTVDKQAELYKELLSDETDKADLISTLIDTIGYDIEPNYLFNVLS

NQAKQNVFQLNDLNKAFINLSSKYSQFNNLFDDVDLQSKKLGSDEQQRNVTITEVLKK

LNDIDVLGHEGDVIGDAYEFLISQFASEAGKKAGEFYTPHMVSDMMAQIVTLGQEDKN

LFSVFDPTMGSGSLMLNVRNYLTYPDNVKYHGQELNTTTYNLAKMNLILHGVDAEDMQ
VRNGDTLNKDWPTDEPYTFDSVVMNPPYSAKWSADDTFLDDSRFNRYGKLAPKSKADF
AFLLHGFYHLKDTGTMAIVLPHGVLFRGAAEGAIRQKLLEDGSIYAVIGMPANLFFGT
SIPTTVIILKKNRTARDVLFIDASSDFVKDKNQNKLTEENITKILSIYKERKAVEKYA
HVASFDEIKENDYNLNIPRYVDTFEEEAPIDMATIGTEIKEIRQEKRALEKNLVETIS
SLQFSSEDAEWIKGALEVFDYEE"

CDS 3094758..3096008

/locus_tag="JFBMFIFI_02895"

/inference="ab initio prediction:Prodigal:002006"

/codon_start=1

/transl_table=11

/product="hypothetical protein"

/translation="MKNNQLPEVRFPEFKDAWELRKAKAIFKSIVEKGKENLPVLSVT
QKNGVVYREDVGIDIKYDPKTLNGYKVIHPNNFVISLRSFQGGFELSDKLGITSPAYT
IFEPVDKNLNDNLFWKSKFKTFDFIESLKTVTFGIRDGKSISFTEFGDLKIIFPSKEE
QEKIGIFFKSLDDTIALYQHKLNILKQTKRGLLQKMFPKEGEKVPEVRFPEFSDDWEL
RKLDETFDFPVSTNSLSRSQLNYDKGEIKSIHYGDILVNYDSILEIAKDRIPFITDGV
LDKYKPNLLENGDLIFADAAEDETVGKAVEVAGKTNECIVAGLHTIVARPKEKMAKFF
WGYYINSDIYHSQLLRLMQGTKVASISKTNLKKTNVSYPKSFVEQQKIGAFFKTLDNT
IALHQRELEILKNTKKAFLQKMFV"

CDS 3096022..3099183

/gene="hsdR"

/locus_tag="JFBMFIFI_02896"

/EC_number="3.1.21.3"

/inference="ab initio prediction:Prodigal:002006"

/inference="similar to AA sequence:UniProtKB:Q7A801"

/codon_start=1

/transl_table=11

/product="Type-1 restriction enzyme R protein"

/translation="MNKIPHRDEAEVEAQLIQVLGEGHNQWTYRPELKSEEDLWNNLR
DKIVHNNLSEIGEAPLTDKEFEAIKTEFLLRTQTPFDAAKWLKGENGIARITVEREDP
ALGSMSLVLYSNQYNSGGFFSYEVVHQIAKQKSSSEGRDRRFDVTLLINGLPIIQIEL
KQVTAKDGYYQAFNQIKKYAEEGVFRNNIFSTLQLFVVSNEQTTRYFANGMPKELHQK

FLFSWRTKENRKVDNLYEFCKQVLNIPHAHRLIANYTIVSEDQDNKTLMVLHPYQVHA
IEALFTAANKHQSGYVWHATGSGKTLTSFVSTKLLARKSGIDRTIMLVDRKDLDNQTT
SEFTKFASEFNTGISSGNAKSNSLIVGTGNAKELRETLLADANSNVVIVTTRQKLDAA
LKSAKKHEEKKGTKRFQKLLGQHIVFIVDECHRALGSENMEEIKEFFPNSTWFGFTGT
PIFDENKKQVKGQLARTTHDQYGEVLHTYTIKNALEDGAVLGFQVEHEDTIEPTSLDN
QIHRKLREVEKYAAFSPERINQVIDTMDGSKKEEYIDPSIYEKDEHIQKVIHKIFRPD
NAYMKFDFSNGRPQKSAILTTSSIANAKRYYKAIKELAKEENWLQKEFSHHPIREGRT
MNDPDFPRIAITYSLDENTENAKEQQDEMEEIIQDYNEYYGTAWTLADIDRYNGDINN
RLARKRAEFKQFGRQVDLVIVVDRLLTGFDAPTIQTLFVDRNLEYAGLIQAFSRTNRT
YPEKTKGLIVTFRKPHTMEKNVAAATKLYSEAKEESGLVYPTYDESKKRFKQAYKKMN
EFVLVPGEVDEHTSLETRVEYVKAFQELNNAYEALVTYDDYNDDMEQSKTLQNQVGIL
EEQVGVYHTVKGSLVEQNEENPEENIDFSGIEFYGDNSIKLYDIDSTYIDQLLGTYLA
NSPDIREEIEKALQKLNKIEGVKQVYREILNAMDNGTLDKNEDIFAVKRHFFTQSRDQ
AIEDFSKEWFVSELELHSSAIQYMMGTERIPNMKAIIESKKYEKYKEIHSKAKPFSYA
QAIKREWREVLDEVIIPLDDELR"

CDS 3099345..3100088

/locus_tag="JFBMFIFI_02897"

/inference="ab initio prediction:Prodigal:002006"

/codon_start=1

/transl_table=11

/product="hypothetical protein"

/translation="MKKHEALVDLVVGMLSSYFDSENVYKPRLVTNQIIKGKAMENHV
DIYLEFTQMNNTERTVINVVSGREVNTQDVLKLANTLDDLEFKSKGVLYYDLCISEEA
IAVAEYKLKTSKFVFQEEVIKSMIKRFGMMLPDETVIGDPFWVIMETDADVGNKGNYY
ICGDMIPLFLSYKQAKEIADGNHGYEVCGLSQNHLRTLSGITSATSHELGLLIPRYGS
LKSDEYEPLIYAPGNKNILKSYFRGENYV"

CDS 3100081..3100821

/locus_tag="JFBMFIFI_02898"

/inference="ab initio prediction:Prodigal:002006"

/codon_start=1

/transl_table=11

/product="hypothetical protein"

/translation="MFSKFKNRGRVLEDIIEETCKKIAEDERIDAKISKRIPLIGSDK

ATHEFDVLYEYEHFGLQYRVAIECKSWNKSINKSQLTDFAYKLKSVGNINGIFLSESN
LQRGAKLVSEHEDIKFIRYSDFRSFSLSENEKYLFPNYRTIGDPFWMFINKAGKGFLE
QNVLFKDILFLFESKYYAMEFQKLYLEEENKDIELVGVSQRHLKDIQHSQIKNKFQIS
LFNPIEELEQSMFPFSSIGYEELDWFIR"

CDS 3101357..3102535

/locus_tag="JFBMFIFI_02899"

/inference="ab initio prediction:Prodigal:002006"

/codon_start=1

/transl_table=11

/product="hypothetical protein"

/translation="MSENENVNVYQGRLKQMKTIGEAQKIISQALQMENLSVLMGAGC
SSFFIGNKEAAISTMAGLFSEFIKENPNFRVLGTNIEGKVNNNLEELMDFMNAIKQAN
HLKKIDDDIEDNIILVKEFITTKIIEGMKCDELADTYKRFYLKTISSNRKNPINVVTT
NYDMYNERALDELNFIYNNGFTGSYKRTFNPNMYKYMYVDNMNLNKDVWNRVDHFYNL
YKIHGSISWKKEEDVISEVSIDQIKSETYENVMIYPTPLKDRSTLMVPYTDLMRSFQD
NITRKNSVLITLGYSFGDDHINRIILNNLSIPSFRLIILGETEDKDGKKTNIGKIKEM
DDSRITIINSDTKIHYFNNFVEKLMPSISDEEEEKRKISDQISQLFNEFEHTGGSGNE
"

CDS 3102528..3104372

/locus_tag="JFBMFIFI_02900"

/inference="ab initio prediction:Prodigal:002006"

/codon_start=1

/transl_table=11

/product="hypothetical protein"

/translation="MNNRSIGRLVLIKNSIVIAEVFNGIDTHINTMDGTRFVGEIGTY
VSIYELGRVLIAEVLSAESSQHLSSAPLAKPNSNRIISLKLIGEIVSDDFNFGVSKMP
LLYSEVHIVTNKELDIMLAVKDSEINVDNENTRISTFSLGQSVLFPEYKVKVDLDQFF
GFHTAIFGNTGSGKSNSIAKMIQNIYRKKNYSAVGSRLILIDSNGEYEKAFSEINEIN
SEIQCDFVKPLSKDKNIEIPIWLLSVDDWAILLNASEKTQIPVLRKTLEVVNIFFSKN
SKSEGIKNHMLAATIIGLLNSSESSPSKSDKISSLLLNFGTEGINLNSSINSKMTFKK
CIEVSYGQLEDIEVVIEYLKQFNEPALMEAFKLEKTTLFSSDEFLSAMKFAVLYEGSL
NSQRVQEYTAPLVSRMQALVESDFSKVFTKTMFKNKESFFEETIDNNNVLNIDVSSMD
DSMAEVLVRVLSKIILDYQKNHDKKADKPISLIIEEAHRYIKDTQISQGMYEFDIFQR

IAKEGRKYGMLMILSTQRPSDLSKSVVSQCSNFIVHRIQNPDDLNYISRMVPYIDKDT

IDRLTYLQTGNALVFGSAIRIPMLTLFDEAKPNTDGNSAKISEKWYVQ"

CDS 3104697..3105524

/locus_tag="JFBMFIFI_02901"

/inference="ab initio prediction:Prodigal:002006"

/codon_start=1

/transl_table=11

/product="hypothetical protein"

/translation="MEIIKLIKVFIASPSDVKEKRDEIENLIHYWNSLSNSRGEKIFL

LPIRWEKNTSSEYKEDLSGQKVINNQILLNSDILIAVFGGSLGQPVDGYESGTVSEIE

TFYNVNKRGVGVFFENKPCSNDILTNPNYHKVEDYQKELQNEKKGLYKVYEQREILAF

LDKEVSIVKNIPLKGEDDYYEMTVSDIFINNRANFFQLEVKIPLNDPAMKHIGGYEKN

ETHWIADWNKNIDGIKKGDTVKFKIKKISEFYRQGMTHNGEKVYPRNITTSDFKILK"

CDS 3105578..3106384

/locus_tag="JFBMFIFI_02902"

/inference="ab initio prediction:Prodigal:002006"

/codon_start=1

/transl_table=11

/product="hypothetical protein"

/translation="MTIGVCWVRKKGNSEELIVASDSRLCGGHRWDQCPKIITFPRGD

CFLSFAGTANYAYPLMMQIYFSSLMTEKVRDRAIDVTVFNTLVLEGINNLQSSIFDEV

SPEDIKENEFIFGGYSSEKKKYCLWIYKFNPGEKRFQKNEIKGKFLNIGKIAIVGDVK

SEYKKQLYKYISSENSYVDKEYFELEPLQILINMLRVSNNTSTIGGAPQIVKIYQHLQ

ARPIGVYWPKKEEDLKLNRTLIGRRLSTDENSSFSFLNPQTLQTNRLPIW"

CDS 3106419..3107078

/locus_tag="JFBMFIFI_02903"

/inference="ab initio prediction:Prodigal:002006"

/codon_start=1

/transl_table=11

/product="hypothetical protein"

/translation="MKKNKKGIKNILVKRNIEHLVHFTNVKNVESIMQNGIIPVKNHK

EYNVTAFVNDRIRDDELMEMTSFSVEFPNYKMLSKLKLNYPEEEWVIFFLRSSIILDK

TSLFCYDNASNKEIKELSKTSRQEYEMFEKMFSDIDGYPLRAKTRLKRYFTTSPQAEI

LIEGNIDLNYIEKIVFLTAESKNKNKKLVPSFIKQEVIPDYFFAREDIEFLKGEKDEI

K"

CDS 3107065..3108621

/locus_tag="JFBMFIFI_02904"

/inference="ab initio prediction:Prodigal:002006"

/codon_start=1

/transl_table=11

/product="hypothetical protein"

/translation="MKLNEDSIEWAIIHNKIVKDTDLFPLPKEIEIFEDNLEESVEVF

KDIELTTYNWKTARRFFIPKKELSYRLATQLDPLDSILLSAIVHQYGEGIEKRRIPLE

KEKVFSYRLSPNKLGHMYTLEDSWKKFWEVSLRKAEKYSYIVYVDIADFYNQIYHHTV

ENELIACSFPNPVKKSLINLFGGLTSKTSRGLPVGPHSTHIFAEMTLIPLDGFLDLIS

KEFSRYADDIIVFCDSKKEAEIMIYEIAKFIDSNQRLVLNSEKTRIYKNNEFKELCIE

MTRDNPINDIELEMVETLNKYDVNLYTTEQTITLDENDIKIFSQKRVNQCLEAYLQEK

SPNFDRIKWLYRRLSQIQVGSAMEFTVENLTRLTPALNEVIQYFISIGTSNNNEINLE

KYGDEIFNLLDDDLVKSSNYLQLELISLFSSSNNYNHMDKLISRFESSSESIKREILL

ACYNSENPKNVYGWIYGLKEQVGNFDPWTKRAYYIASTTMIAENKKFFYKDIQPQNEI

ERYIINWAKR"

CDS complement(3109017..3109169)

/locus_tag="JFBMFIFI_02905"

/inference="ab initio prediction:Prodigal:002006"

/codon_start=1

/transl_table=11

/product="hypothetical protein"

/translation="MSLLKSAIKFKAAKTVTNKAFGNGTISTLAAAKMTQKSNARGNA

RRAGRK"

CDS 3109621..3110031

/locus_tag="JFBMFIFI_02906"

/inference="ab initio prediction:Prodigal:002006"

/codon_start=1

/transl_table=11

/product="hypothetical protein"

/translation="MYFQTAEYKEKRDAYETLKTKLSNRFNKHTEYMMSIQSMVSNAT

KKNFSAESEGLPNQDSVDKFMELTGELSNLMDIQYRQAESLASASTVASEKYEEYSGL
YQVEKKREEEYKAEEARKAEEKKREEREKREKMN"

CDS 3110054..3110359
/locus_tag="JFBMFIFI_02907"
/inference="ab initio prediction:Prodigal:002006"
/codon_start=1
/transl_table=11
/product="hypothetical protein"
/translation="MSDVRIKEGALADAIATSKELKEALHQSLKTINTLEGISSGSDR
TWTGESKSIYLMYLNILLKSHQKLEDIVNTQDKALRTLKKDIASMDKGATMTTIRSI"

CDS 3110400..3111080
/locus_tag="JFBMFIFI_02908"
/inference="ab initio prediction:Prodigal:002006"
/codon_start=1
/transl_table=11
/product="hypothetical protein"
/translation="MKKDVFSVQEVYVLMDILGGTELFGFPELHLLGLTALESSEVGV
ERLQKKQIVTEKNQLTSVGMTLLKILERYCESETYVMVENCFLVPDEKEAICLEKVQE
GYRLSVLPGKALLEKLYKEHELIRREATEEELTFKKQRLRRSELRQLGLEEAEFQVVP
LSIGKVSPTEESGLTKEEWLFFELEKQLYAVHPSEERVYKMSQYWLTQWLVKELKVSV
ELPEGVTV"

CDS 3111080..3111397
/locus_tag="JFBMFIFI_02909"
/inference="ab initio prediction:Prodigal:002006"
/codon_start=1
/transl_table=11
/product="hypothetical protein"
/translation="MGKIQVNPDELADIQTFLKKAVTQLEVAYKAAEKLERIDFYEAG
DAMGQIGMYPILTQHVAGLLGHYQRVDEYVKFTGETFVELDESLARGIEEPGKKVPGG
RIL"

CDS 3111398..3112732
/locus_tag="JFBMFIFI_02910"
/inference="ab initio prediction:Prodigal:002006"

/codon_start=1

/transl_table=11

/product="hypothetical protein"

/translation="MDVRYNEGDWLNKIQATQLLGSDGANEEGLSVVGLLKKAAREVE

KAEDDIARYDKDYAIRLDVRQYPSQYDGWGEKMYWLQQAVTKIPDELSSSVDYPFLQS

MDQFVEKLSHFDIASETTPAKVAIPNPNYSADGMDRNGSLQQVSLTDLMYRKSPLTKY

MKAEYFALQQESGIGCTYEEYEALAFASTAFEYTSFNEKKTDFVVNTVVNGGILALGA

ALPWTIAMGLGLVSGGVNSYEALSGKAIGGRELSGKERILRGVFGALDLALVGYSGVK

GFKNRGSKVPEIEALNENEITRQRVLKNIEESKKARESSNFKEFSKKSNSIVDDVAKN

ASGAKVPSISNKKLNDLPDNVKNSFEKYDKNGWGGNVEGQTPGTKAGGTYKNRDGKLP

SVDENGNKITYGEFDVNNKLSNSRRDSERFVRGSDGSLYYTDNHYDSFIKIE"

CDS 3112744..3113139

/locus_tag="JFBMFIFI_02911"

/inference="ab initio prediction:Prodigal:002006"

/codon_start=1

/transl_table=11

/product="hypothetical protein"

/translation="MIKNEILHISKSDVLELDKKMKKNDCFIVNIQGDDIQTKNEFLI

SMTEHFMLPDSLGWDSFTDWMTDLSWINNKCFCIIINNYADFLKEDSESKEIVLEIFE

EDILPFWEKDVTKVVVNGQVCPFNVYLVD"

CDS 3113287..3113760

/locus_tag="JFBMFIFI_02912"

/inference="ab initio prediction:Prodigal:002006"

/codon_start=1

/transl_table=11

/product="hypothetical protein"

/translation="MSIDNSKKAKVIIEENEDLVDDFGGASTDIIAQAQTILEVEFPE

DYKLFLTEFGALTFGSIEIYGVFREDFENSGVPDTVWTTLNERKLVSMPNHLIVIYNT

GMGELYCMNFNDLNNNNEPKITSYFPGFDEKAQKNEVLYENFGEFLLDMVHEEVN"

CDS 3113849..3114193

/locus_tag="JFBMFIFI_02913"

/inference="ab initio prediction:Prodigal:002006"

/codon_start=1

/transl_table=11

/product="hypothetical protein"

/translation="MSINNLDYGGFVVSNNVIDGKQIRYTFREKSDIPQLNGWTIYST

EDSEEYVNEADNFQILGASSLENLAPVMLEIFNAPYNTDLCWLYEDGVHVGFYDLKSE

EEKDITQIIGGY"

CDS 3114196..3114354

/locus_tag="JFBMFIFI_02914"

/inference="ab initio prediction:Prodigal:002006"

/codon_start=1

/transl_table=11

/product="hypothetical protein"

/translation="MEENKTFYSIVFKDLEEINQRKQQKVVELDDKKKEVALKNILDI

MAFFGDKS"

CDS 3114772..3115656

/locus_tag="JFBMFIFI_02915"

/inference="ab initio prediction:Prodigal:002006"

/codon_start=1

/transl_table=11

/product="hypothetical protein"

/translation="MKSLGDAIKSGRLKRNMTQQELAEGICTQATISNIEKSGKLPAI

NLLLAIADRLDIEIEEFYYLIGENTTENGKIMKKVKLLCSQSRHQDAAILLKEINEVE

LESVSEKKEFYYYKGITSLIASNNFSDALCYFNLSNDIHEEGYTSIYDVLALSGISIV

YSMHEEDDKALVYTDRTLQLLDEFVAEGYEKKDTNDIVRIYFNSAKIYSKTKKYEKAV

SLSSMGISLQQLNDSMNGLEHLMYEKAYNLQQLEQVSEAEKFYFYAAAMAMINKNNEM

SKTISSDMEVYKVQHFMY"

CDS 3115694..3115831

/locus_tag="JFBMFIFI_02916"

/inference="ab initio prediction:Prodigal:002006"

/codon_start=1

/transl_table=11

/product="hypothetical protein"

/translation="MKNFFKITNSLDLLKVTVTCIVGGIAIFYFAQFIGGFIGKMLPL

F"

CDS 3116463..3116735

/locus_tag="JFBMFIFI_02917"

/inference="ab initio prediction:Prodigal:002006"

/codon_start=1

/transl_table=11

/product="hypothetical protein"

/translation="MDNGTLDKNEDIFVVKRHFFTQARDQAIKDFFKEWLELRSLAIP

YTIGIERIPNMKAIIESKKYENYKEIHSEAKLFSYAQAIKRECVKY"

CDS 3116907..3117650

/locus_tag="JFBMFIFI_02918"

/inference="ab initio prediction:Prodigal:002006"

/codon_start=1

/transl_table=11

/product="hypothetical protein"

/translation="MPIYTGKTKDEAIENGLKDLGVSKKNVKISIIQEPKNGFLGKFG

KEAKVEITVLTDDYLEKRKKIIIYGVPSIIVILLIIFFVYFAREDISSSVTKKSDNEI

AVSQISSEISESEIESQSELRDKASSSETTEIETHAKLEYAVVKKDKKNIVSGDVSLT

GGDGTQVNVEINEESIKPGTYQVNWTPGTFNGSDPDFGYGLIWINGDSNTIQIMPEET

NFVTFNEGDTITFQFAGKGENDRIRLIEE"

CDS 3117777..3118145

/locus_tag="JFBMFIFI_02919"

/inference="ab initio prediction:Prodigal:002006"

/codon_start=1

/transl_table=11

/product="hypothetical protein"

/translation="MIDFKNSSYVKLKMVNEIPPNIDQLLISEEDIIGTYRGIRDYVT

FTNKRVIAINIQGITGKKKDYTSLPYSKIQVFSVETGGTFDLDSELELYFSGLGRVKF

EFTGKSDIVSIGKFISDYVL"

CDS 3118271..3120079

/locus_tag="JFBMFIFI_02920"

/inference="ab initio prediction:Prodigal:002006"

/codon_start=1

/transl_table=11

/product="hypothetical protein"

/translation="MATTIEVNKQSVKQLLETGKNKKFVIPEYQRSYAWSDDQIQTLF

DDLVEYTENNDESTYFLGTVVSYENDKNEQEIIDGQQRITSLFLLLRALYSKLTSMAE

TPESKNFKLQIASALWEQDELTAEVDYGKTLIISRVMGDEGNKIFSNILITGKTQADA

KDNYSQNYVMFVELIEKYATAQPELFYWFIRNVLNKAILLPITADSQDTALTIFSTLN

DRGLALSDADIFKAKMYNHLSQDEKLTFIEKWKQLDEDASDVNESIQKLFYYYMFYLR

ALENDRNTTTPGIRKYYSRNNFEILYRSGVIADLNKLINLWLVVNNRTEIENENWSKN

IQIKKVLDGLSSYPNEFWKYPVVIYYLRYNEVSDFENEFLVFLNKLLAVLSARYILTP

TINAVKRGILNLNAEIINSSTPKFDFNQIDEKELKEKIKYAHRNTVRMILKMLAYQHQ

DELLPEKWEIEHIFPQKWQSSYFPTVSEKEVRDIVENIGNKIPFEKKLNIIASNGYFT

KKQISYEKSHIQILLELSSSYKDWGLDEIRERNIRISDELYDLLKRWGLNENDSDKED

FHFISIPDNRIYDYQNFLKSLKWEDSDENRQKFLEM"

CDS 3120419..3120712

/locus_tag="JFBMFIFI_02921"

/inference="ab initio prediction:Prodigal:002006"

/codon_start=1

/transl_table=11

/product="hypothetical protein"

/translation="MLGGKGALGSKARDFNFQQANSNLAKQLNDSPSLAKTFGMESGN

IKPRDIEKYRVQNNLTWHELNNGTTIQLVPSEINSTFGHIGGVGEINAGAFVK"

CDS 3120750..3121217

/locus_tag="JFBMFIFI_02922"

/inference="ab initio prediction:Prodigal:002006"

/codon_start=1

/transl_table=11

/product="hypothetical protein"

/translation="MKINDEVFGELEFDYSWFKKSKVDFYNKEVEIVILVAGDEEADF

EKEQYDAYKMLIKNWLDIQKTFLSPILDYYQNKREELGYDIELNDNYPEIVTIEEILE

HITLVGIKVPYGELYGGRSIGISFDCTWDSENGIGIRLSDEKVIEVGYQDVAI"

CDS 3121375..3121749

/locus_tag="JFBMFIFI_02923"

/inference="ab initio prediction:Prodigal:002006"

/codon_start=1

/transl_table=11

/product="hypothetical protein"

/translation="MNKGNEIEQNLASFDFTDSIITKLYYEENLTDLTIEVDYFWTEL

ETNELIKLEIFNCLSLNYEIPESLYESNNGILNWSQFTIQKIEFSIEKNPTLKIFTYD

YDNPLLVITFMNLSISRSEIQP"

CDS 3121847..3122248

/locus_tag="JFBMFIFI_02924"

/inference="ab initio prediction:Prodigal:002006"

/codon_start=1

/transl_table=11

/product="hypothetical protein"

/translation="MEELKKFEQKNDISLPSEYEEFLRQYNGGYPDKSIFKIKGNEEK

YESVLNVFYGIGDMYDNLQKNIDFSDELLEVGFLPIADDPGGNQICIGISEKYYGKIY

FWEHELGNENELENLFYVSDSFINFLNSLHN"

CDS 3122333..3122920

/locus_tag="JFBMFIFI_02925"

/inference="ab initio prediction:Prodigal:002006"

/codon_start=1

/transl_table=11

/product="hypothetical protein"

/translation="MDYNFKKENDKKTIILFKENKYIGLQELINTIDLYNITLEDIVL

KIELVKDNSSAKEVIGSERALVEILDEVVEISDLFEGIIDDEDLFPSIQIPIDKFKEI

ILDTICAKISNDNLHEKIEDIINKWDPLEVFPFAPKDEYENEINKIAKLVNEFQSTEE

LALAIEKIFFDSFGAEIINVPKEVYISLADNILKK"

CDS 3123531..3124586

/locus_tag="JFBMFIFI_02926"

/inference="ab initio prediction:Prodigal:002006"

/codon_start=1

/transl_table=11

/product="hypothetical protein"

/translation="MGICSGILIPSYATLKRTSLSQSINSPSLKWKIGGILCSILYLI

SLAKLIQLNSAIVFIVLGIALIWLYQHQSENLVFDVENKNDYPEYAIFESLFLFIVIF

ASVFVLKEDKKEGITNLLPGLICTIFIIMIFYSIYRYKKHPERRISFKLSSVIIYKGM

LTNFILAFLTFYQVIKNGESELMVIYYIYLVGMLVKGAFISFLKKIFYGISEQQLIMT
GLLVSFIFLLFQPTFYVGILCLSLFVSELNSELDQYVYNHVDLPKDFRLIAKARLTNI
GSVINQLIMFTTLYIAALYTNTTVLNVLKAYHSQKESLDFLSVLNVTKNSMLVVFVIY
LYGLQKILRKVTTINNE"

CDS 3124841..3125323

/locus_tag="JFBMFIFI_02927"

/inference="ab initio prediction:Prodigal:002006"

/codon_start=1

/transl_table=11

/product="hypothetical protein"

/translation="MAIYKTHLGVYGVCIREKKLLCINKNSGPYKNRYDLPGGSQKEF
ESLIDTLKREVAEETIFSVTQYSNVRCYDSFVQCENTIVHHIFVLYNIDIKLEENNHL
ELEDEKNDSLGSSWIEIKSLNLDNSSPIILKLLEEIENKFSQTLLDITSYKNWNVREQ
"

CDS complement(3125717..3127210)

/locus_tag="JFBMFIFI_02928"

/inference="ab initio prediction:Prodigal:002006"

/codon_start=1

/transl_table=11

/product="hypothetical protein"

/translation="MDTLLMKKDLAKLTLFRELVYNQPKELSLDYFSELLNISKRSTL
RTVDELAHDLEKDFEDMEIKKNKYSYSIMNNSLMNNEYFIVSLQLFYLKNSIQFNIIY
SLLTKYFDSMTQLSEYLYISTPHLYRQMPEIKRFLAGFKIDIIFTEAKKKTNFVGSPK
HLKTFYYFFYWGISQGIAWPFEEFFPLKKFNETFQLAFTDPSHIPSKTKKLEVLFLLS
YLDKVNPPFELSEDIKEIATIFKDINDVSTYSHNYVKHEDERLFINLLSRISMADLDN
EEEKVFIYQRVLSSDSPIVTHSIKITESIFTHFFPDIFISIEDRAVTFYYIFIFHIYI
HYFGIDLSVLFTNPVSLKRFSTKSEQFKLIEKNIVHFFDSITTEHNIKVDEKYLNGYY
FLIASIIDYVDPMFLKIYIQYSKHIAGKNIIKSKLYTMFGKENIIIVDDINEAKIIIS
DCFENENQGQILFLIAEISRPEMWQNLMLLILKEKFQKSFRFPLMLD"

CDS 3127489..3127722

/locus_tag="JFBMFIFI_02929"

/inference="ab initio prediction:Prodigal:002006"

/codon_start=1

/transl_table=11

/product="hypothetical protein"

/translation="MIIDDNWTDVFIYNVEFQVLVKRGTHEFEKSISKSLVFNSELGE

SKVATIIKEKFKNVVKVSYVDYFGESLELKNKN"

CDS 3127742..3128044

/locus_tag="JFBMFIFI_02930"

/inference="ab initio prediction:Prodigal:002006"

/codon_start=1

/transl_table=11

/product="hypothetical protein"

/translation="MPVQKQVLLLFPLILLFFCFPVVSLASTTTSQAGIVFTESDESA

KKKELDEKKIPSTGSEEVKKDRYLPQTGEKHQTSVLIVGSLLLVGSVVKINRVKRR"

CDS 3128051..3128722

/locus_tag="JFBMFIFI_02931"

/inference="ab initio prediction:Prodigal:002006"

/codon_start=1

/transl_table=11

/product="hypothetical protein"

/translation="MNSKKLLVTGLISTSFLFNIGTVAFAAPGDTIKGSENGKSGTSH

GYIKLTPGDNTGGPTVPTKPTVPPGGTDNTGSLTVDNVAPLLFDTHKLEGKEQIYTSV

VTDSNVQVTDNRGEEAGWNLQVSQTAFTDSTDATKILKGAKLILPVGVIETAGTNVSL

SPIVSEVELNEKPTVLMHALAGSGAGTWTSVFDKDEIKLIVPAGNKNGEYLSTVTWSL

LDAPK"

CDS 3128749..3129414

/locus_tag="JFBMFIFI_02932"

/inference="ab initio prediction:Prodigal:002006"

/codon_start=1

/transl_table=11

/product="hypothetical protein"

/translation="MKMKNKLIGLSLNTGILVSVVLLGTTQVNAAEVSKGSSTANFEL

QAGDSTTVPEIIDPGIEPGLDNKGPLSLDAVSSFNFPTKKLGAESKLPLEASPVLGTK

LGLQVTDERGEDLGWNLKVSATNFKTADDKLELKGAVMTIPEGTLTTKEGVDPLLTPT

AFKVNLSTTPTSIMKASTTQGRSTWVNSFEGKGEKVTLAVPSGNKVATYSSTITWSLE

DAP"

CDS 3129437..3130129

/locus_tag="JFBMFIFI_02933"

/inference="ab initio prediction:Prodigal:002006"

/codon_start=1

/transl_table=11

/product="hypothetical protein"

/translation="MKKRIILIALTIVIPLTGGFKVKATSNGISESKIDFIAGDGVVT

PPVNPKDPDNPIIPTPIDSSDPENKGTGQIGPLSVDYVSNLKFGQQKISGRTIAYKAL

NADPFVQVTDLRGSGDGWSLSAKMSPFTNKNKQELKGATLSMKNSVVKAGSTSNISLA

PVKSDLLFDNQESKLVMNASNKGGRGTWLNVWSGTEEANESIQLNVLAGTPEANTEYT

SSITWELEDAPK"

CDS 3130206..3131300

/locus_tag="JFBMFIFI_02934"

/inference="ab initio prediction:Prodigal:002006"

/codon_start=1

/transl_table=11

/product="hypothetical protein"

/translation="MRKLYSLIVILLIGGLISPIIGRAEGSMAYSAKANIPENQINKT

LTYFDLKMEPGQEQEITLTVSNSSDKQTTIILSPNIATTNQNGVIDYSQTKGKLDSTL

KTPLSSVISKEQEVTLAPNETKQVPFTLKMPDKSFNGLLLGGFYVTKKEEAEDSKEAE

KNVQIKNNYSYVIGIQLRETLEEVKPQLKMNQIKPALLNYRTAVTVNLQNTEATIIKE

FDVHAKVRKKGNGTVLHEATKTDMSMAPNSNFDFPISWDNQSLEPGTYTLDMTAKSGE

NQWTFEEDFTISAKESKTLNTDAVELEKKAPNWTLIILSVIAAMTLLIGGMLYLIYRH

KKKKEAARKARIRMQKRRKKLNKKKPEVGK"

CDS 3131346..3132011

/locus_tag="JFBMFIFI_02935"

/inference="ab initio prediction:Prodigal:002006"

/codon_start=1

/transl_table=11

/product="hypothetical protein"

/translation="MKKSIGWLVVTIFGFGAPVFADSTGVITFEDDTNVVRPVNPMDP

SKLGVEDPNNPATNNPGPLSLNVAPLQFDFGKQTTKDAILSYSAKDIGTQFIQVTDNR

TDADGWALTIQRNELVSKGNAEIRGAKLTIPKGVIRNSLHVPATDSAENKHLKTQAVD

VYGDNTPVTILSADQLNNNGKATTISVWESSKVRLTFKTLVTKKGAYISTVNWSLVTG

PTS"

CDS 3132061..3133206

/locus_tag="JFBMFIFI_02936"

/inference="ab initio prediction:Prodigal:002006"

/codon_start=1

/transl_table=11

/product="hypothetical protein"

/translation="MKKIISIMILALLLLGRNVYAENSEATLQQKAEVSYSVKKIKAA

NEINETSSFYDLGVTKGENKEIQAIIANSSNQPIYVKSQIFTAFTNENGTITYNAKPK

SISKSLKFKLSDFATIEASDKRIKVPPHSEKTVTAFIDVPKDVPDGVMLGVWYFEKEN

QVVENKEEQGIGIANKYSYSLAIKVTVNQEIEKPELKLKGVTTGLSNYRKAIQATIEN

ESPGIISKLTVNAQILNEKNGELLYERKQEDMIMAPNSNFSYPIFLKDQEMKPGDYVL

NVEASTNDPKWPSQNWSWSQKFSITKEKAQQINQDALNDNVSSENNNNLVLIGVLFLI

VSGLVTLFVLIRSNQKKKRKKLKMMAKRKKREETKLKQKRLSKRQDK"

CDS 3133235..3135478

/locus_tag="JFBMFIFI_02937"

/inference="ab initio prediction:Prodigal:002006"

/codon_start=1

/transl_table=11

/product="hypothetical protein"

/translation="MKSFFKRLSLFASLSLILCIWNPLHASAEIEQVSLIGRPYPTAS

KVTDTTTLTKYNLLQGKQAQPYTGYTSWSLATLNQYGDILYNNNRIGTTRFSDMSFYQ

SLNGSVSNLLTQRAFEVVKGHQYQVSYQLKNSSYGTAVPQFKMSIRQGSGFDGEIIKQ

LSVPLDSMTYQNFKTVDFSGAVSFQTRLYSEQVFVVMSGSNLSIIDLDQNIIEARRDV

EQLFTDTSYTKLNEFVTQKELDNVKKTVDLVVNSTERAFLLQYTNLAQEFLDAIQMSL

ITTELGDNRENLNSMMIKGTTYPHAYVRVTGNSNLPATSLVSPFKDEPENFTLQADEN

GDYTVNLENDGHFIAGEKVVVVANRHGKRLSETVTVKDQTIPTGKAKTVHSILKDAIP

IPDKFVSDLNDSNSTNKKITVEYSMKNNSEKITEMLQKSGKYLVYVTIKDDASNSTEI

QSELIVYDSNSLLESTNFQIDQEELISLSNAEMLDRFLKDSQTQAYLISDTNKIDLVN

KLQIRGLDSLKKEVGTYSLSYFLAKEDSGLTKELTSEFTVTVTQPGASKPIDPTNPQP

GNETDAENEGTGNIGYLRVDYAPSNFDFGKVTTSFLNKTYQANQPLSVSGKTLTQQWI

QVSDTRLSDNGWTLSVSQSTPFIGSDGSTLEGAQLKIPKGKIYNTASNSEPIIDNSII

SRGVSLSETPTAILTTPKANASGKNITTNVWDPTQVSLYLPGGSAKNKVRYQTTINWS

LVTEVPN"

CDS 3136121..3137227

/locus_tag="JFBMFIFI_02938"

/inference="ab initio prediction:Prodigal:002006"

/codon_start=1

/transl_table=11

/product="hypothetical protein"

/translation="MDKHKVSWLELFYDLVFVVAISSTNHLLTTVNNHPQHFFTIIGE

YLLMIIPMWWAWAGQTMFLNRYRLIVKKPHYYTFVQMFFVMLMTASFNLNFDETYFTF

LLGFIGIRVLTILQYHSVKRCIDSQTEKKVIQLLTNYFTFSLILSTTSLFFTGGTRYF

ILFLGIFLDIIIPLIKRKELVASPIDVGHLAERLGLFTLICFGETVVALITILNGETM

DFTTLFYVMIAFILISLMWTSYYARLDMRIDKKQVTNGQLLLYSNLFMLIAITSFAAA

LHLGYKPSMPWQQINLLFIFSFFLFYSTKHFIFIYHPRKKVSKNTLQKIIGLGILIIL

LGCVFLIVAIQPIWTLIVLICFSALDNIILYKES"

CDS 3137316..3138149

/gene="msrA_2"

/locus_tag="JFBMFIFI_02939"

/EC_number="1.8.4.11"

/inference="ab initio prediction:Prodigal:002006"

/inference="protein motif:HAMAP:MF_01401"

/codon_start=1

/transl_table=11

/product="Peptide methionine sulfoxide reductase MsrA"

/translation="MKITQEEVVRELYNLILNSGTREWERSLLITTRNALENQANFED

QLAKLESELRPLASRNNLTPDVTDFYLKLTGDPVGEMKFDMSHHLGKDLAYQERAVFG

GGCFWCMVEPFETKPGIISVLSGYTGGHVDHPTYDQVSGGYTGHVEGVEIIFDTRMVS

YEELVELYWQLTDPTDALGQFQDRGTQYRPIIFVQNTKQKQIAETSKQQLIESGKYKR

PIVTEIQPASIFWPAENYHQQFYKKQPKRYKSIKRARHQLLSYLRLKDKIRSPFNKDT

K"

CDS complement(3138511..3139437)

/locus_tag="JFBMFIFI_02940"

/inference="ab initio prediction:Prodigal:002006"

/codon_start=1

/transl_table=11

/product="hypothetical protein"

/translation="MKFYHVKTDQDLLNLDQNERSFILCDQEKFKHIYESFNIMKRKE

EANEKNLDTIHFESHFDYDVTSFSFFEWQENKLEFEKVYLYFSKDYLIFVCNQNETLF

NEIVADIEIDKEIQPNGIDTLTYSYYKSLDHILAKMFVSLENSEQTIADLEMRLLEQM

EEGNFETIMQLKSMSFEAKRHLRLLTYIGDQILSNDNNLISEENLKYFKNISTKMSRL

YEFGSSLHEKANHLLELYNTSVSEKSNGFLNKLTIITVFTTPIAIFSGIYGMNFVNIP

ELKFEYGYFVLLGVLLAIITGSYFYLKKSKIL"

CDS 3139770..3142580

/gene="dgaR"

/locus_tag="JFBMFIFI_02941"

/inference="ab initio prediction:Prodigal:002006"

/inference="similar to AA sequence:UniProtKB:D0ZLR9"

/codon_start=1

/transl_table=11

/product="Transcriptional regulatory protein DagR"

/translation="MKRIEKIYLFVSKQSKALSREEVASGAGITTKEVAEAFGIQRTN

ASKDLNELVKDGLLAKIDGRPVRYLECGLVSQTKQSQLKKTPIPTRSKKEYVDIFQKV

IGGNGSMKVPIEQAKAAILYPPRGLNCLITGATGSGKTHFAHIMFEFAKDRGLINGHE

ELVVFNCADYAHNSELLMSHLFGYAAGAFTGANKEKEGLIAKADGGMLFLDEIHRLPP

EGQEMIFYFMDNGKYNRLGETAKERQADVRIICATTEDATSYLLSTFIRRIPISIQLP

SFNQRQTKEKIDLVKLMVGIEAERIQRKISLTEDVVKALIGSVTYGNVGQLKSNIQLI

CARSFLSHMDKEEIVITLEDLTEEVKIGLSLLANNRQFLAELAQDLEPIMEVSPDQNL

MLPLVDSYELPYNLYEIIDEKAALLKDDGVDQDSINQFIMTDINVHLKSFYKDHRLTF

DIEKKLAEIVEQKVIDLTKKIYDVAKAKLNDSFQVNFIYAMSLHISSFLKRHQLDNQG

EMNSNDSIRKMVKEYPAEYEVALDIKALILDAYQIEIPLEEVEYLTVLLVSLKKEKPT

GRIGIVVAAHGNSTATSMVQVVKQLFGSDNLVAVDMPVEMKPRVALEEIINAVEQVNN

GSGVILLVDMGSLGTFSEEIHRRTGILVRTVDMVTTALVLEVARKTELLDTDLDRLFH

SLKNFSGYGNNLNQESEQEVPQPNQSLKKAIVAICASGQGTAQRMKDIIEKYLAEVGE

KELEVLPISIVEMAAVLKKLQTNYQLIAVTGIVDPKIGVRYIPMELLFSGEAKSILQE

LVLDAEDIFEPVVLDDQQSWEICQDYLRTNFTFINAEKVMEPFWAFTQKLTEVFLGGL

TNQAFTINMIMHLGGMVERIVRQDCLTVPEDQRSDYNLERFQQVKEIAKIVEEALLIK

IPPEELYYLVQILNNTLETEVK"

CDS 3142738..3143232

/gene="manX_2"

/locus_tag="JFBMFIFI_02942"

/EC_number="2.7.1.191"

/inference="ab initio prediction:Prodigal:002006"

/inference="similar to AA sequence:UniProtKB:P69797"

/codon_start=1

/transl_table=11

/product="PTS system mannose-specific EIIAB component"

/db_xref="COG:COG2893"

/translation="MAIDIRLVRIDDRLIHGQVATVWAKRLDIQRMIVVSDAVTKDNL

RKTLLQQAAPPGIKVNVITVAKMIEVYSNKLFDNVRVILLFTNPVEVAKMVQAGIYFP

TVNIGAMGYTTGKKMISNTIAIDDDDLKAFTYLDSRGIELEIRKVITDSQQNLMDVLK

KAKL"

CDS 3143333..3144325

/gene="manX_3"

/locus_tag="JFBMFIFI_02943"

/EC_number="2.7.1.191"

/inference="ab initio prediction:Prodigal:002006"

/inference="similar to AA sequence:UniProtKB:Q5XAF5"

/codon_start=1

/transl_table=11

/product="PTS system mannose-specific EIIAB component"

/translation="MVGIILASHGEFAEGILQSGAMIFGEQENVKAVTLMPSEGPDDV

KAKMQEAIASFDNQDEVLFLVDLWGGTPFNQASSLFEEHKDKWAIVAGMSLPMVIEAY

ASRFSMTSAQEIATHIIGTAKEGVRVKPEELEPEEATSAAVTEDALPKGALAPGTVVG

DGKLKMVLVRVDSRLLHGQVATAWTKSTQPNRIIVVSDAVSKDDLRKKLIEQAAPPGV

KANVIPVEKMIEIAKDPRFGNTKALLLFENPQDVLRAVEGGVDIEEVNVGSMAHSVGK

VVVSKVLSMGQADVDAFENLKAKGVKFDVRKVPNDSSANMEDILKKAKSELANA"

CDS 3144358..3145161

/gene="sorA"

/locus_tag="JFBMFIFI_02944"

/inference="ab initio prediction:Prodigal:002006"

/inference="similar to AA sequence:UniProtKB:P37082"

/codon_start=1

/transl_table=11

/product="PTS system sorbose-specific EIIC component"

/db_xref="COG:COG3715"

/translation="MSIISMVLVILIAFLAGMEGILDEFQFHQPLVACTLIGLVTGNL

TAGIILGGTLQMIALGWANIGAAVAPDAALASVASAIILVLGGQGEKGIPSAIAIAVP

LAVAGLFLTMVVRTAAVPIVHMMDTAAEEGNFKKIEALHIFAVCLQGIRIAIPAAALL

FIPAETVQSFLESMPAWLTEGMAIGGGMVVAVGYALVINMMATKEVWPFFIIGFVVAA

ISQLTLIALGALGLALAFIYLNLSKMGGSSNGGGGGNSGDPLGDILNDY"

CDS 3145189..3146112

/gene="manZ_2"

/locus_tag="JFBMFIFI_02945"

/inference="ab initio prediction:Prodigal:002006"

/inference="similar to AA sequence:UniProtKB:P69805"

/codon_start=1

/transl_table=11

/product="PTS system mannose-specific EIID component"

/db_xref="COG:COG3716"

/translation="MAEKMAEKIVLTKKDRLAVAWRSTFIQGSWNYERMQNGGWAFSM

IPAIKKLYSTKEDRAAALKRHLEFFNTHPYIASPILGVTLALEEERANGAPVDDVAIQ

GVKVGMMGPLAGVGDPVFWFTVRPMLGALGASLAMGGNILGPIIFFVAWNLIRWGFMW

YTQEFGYKAGSKITDDLSGGLLQDITKGASILGMFVLAALVQRWVSIKFLPTVSEVKL

DKGAYIEWGNLPGGGEGMQKAFEQVNSGMALSPIKVTTLQDNLDQLIPGLAALMLTFF

CMWLLKKKVSPIVIILGLFVVGVLGHVIGLL"

CDS 3146259..3146636

/locus_tag="JFBMFIFI_02946"

/inference="ab initio prediction:Prodigal:002006"

/codon_start=1

/transl_table=11

/product="hypothetical protein"

/translation="MVESINTKVDLVIDATAFTGLTDYGKIMIGDKGFEFYNARDARK
FIQIPWEEVNHVIASVMFKGKWIPRYAIETKKNGTYTFSSKKPKTVLRAMREYVDPAD
MIQSLSFFDVMKRAVKSISWKKK"

CDS complement(3146806..3147765)

/gene="lacC_3"

/locus_tag="JFBMFIFI_02947"

/EC_number="2.7.1.144"

/inference="ab initio prediction:Prodigal:002006"

/inference="similar to AA sequence:UniProtKB:Q833W9"

/codon_start=1

/transl_table=11

/product="Tagatose-6-phosphate kinase"

/db_xref="COG:COG1105"

/translation="MILTITLNPSMDFIYTTNHFQTGHLNRFKNPARVVGGKGINSGR
TSAILGSDVIVTGVLAGMNGEHIAKLLETEKFISTFRFIGGETRNAITIMHDNNCHTE
LVEEGPHVSKSVEKEIVSDILAICQKYPLIQTICLSGSANTGNESFYAEVIQILKNEL
HPSVRILADISRHQLKNVLSATEKPFFIKPNIHEFSELISKEVHSKADVVENLDYQGL
ATIPLLMVSCGAEGAIVRYQGKIYDLTIPQIKLVNPTGSGDATVGGIAYGLDQGLPID
DTLKYGMACGIANAMETAVGFVQLENVEALLKEIKINEMFIPK"

CDS complement(3147786..3148541)

/gene="lacR_2"

/locus_tag="JFBMFIFI_02948"

/inference="ab initio prediction:Prodigal:002006"

/inference="similar to AA sequence:UniProtKB:P67744"

/codon_start=1

/transl_table=11

/product="Lactose phosphotransferase system repressor"

/translation="MLKAERRSEILRLLDERNFMTVSDVADAMGVSSMTIRRDVNELG
EENQLIRLYGGIQKIDIKEKELTTSEKINLHKDRKEFIGKVMNSLIKDGDVVYVGAGT
TILYALPFITKKELFVVTNSLIAFNYLVQNTDYKLLLTGGDYAPVTEEFIGEHAEKAF
DTLNLDIAFAATNGIYNNNLTTSNLLQGSIQKAAFAHAKVKVAVADSTKFNVSDVYTL
YHLSDLDYVITDDKIDSETFDYYSQYVPLLNKK"

CDS 3148820..3149314

/locus_tag="JFBMFIFI_02949"

/inference="ab initio prediction:Prodigal:002006"

/codon_start=1

/transl_table=11

/product="hypothetical protein"

/translation="MSIKELFQPELIDLQVQANSEEELFAVIAERLLELGYVYSDYLT

GITLRERNFPTGLITQHLNIALPHSDTEYVKKPFIYIVRLKQPVIVRQMGDNQEMLVK

DIFFLGIKEPTKQVGLLQLLITLFQEEAFMEALQNVEESEAMYALFITKLAEMEEERV

WQKS"

CDS 3149299..3149601

/gene="gatB_2"

/locus_tag="JFBMFIFI_02950"

/EC_number="2.7.1.200"

/inference="ab initio prediction:Prodigal:002006"

/inference="similar to AA sequence:UniProtKB:P37188"

/codon_start=1

/transl_table=11

/product="PTS system galactitol-specific EIIB component"

/db_xref="COG:COG3414"

/translation="MAKKLIVACGSGVATSTTIAEKIKAKFEDDGIDYPVEAVDYKSI

MNELPSASIYVYIAQPDIEVLDRAAELSIPVFAGIPFLTGMGMDAIYAEIVEETGK"

CDS 3149636..3150994

/gene="gatC_2"

/locus_tag="JFBMFIFI_02951"

/inference="ab initio prediction:Prodigal:002006"

/inference="similar to AA sequence:UniProtKB:P69831"

/codon_start=1

/transl_table=11

/product="PTS system galactitol-specific EIIC component"

/db_xref="COG:COG3775"

/translation="MEIFQSIINYILDLGSAIFVPLIILLLGLIAGMAFKKAFMSAIT

LGIAFTGMSMVIGFMSEAVGPASEALAKNTGISLPALDLGWTGAASITWSWSYAFVFF

AVVIGVNFLMLVFKLTKTLNVDMWNVWGKALTAYLVYFISGSLIAGFITAIVQVILEL

KMGDMFQKHIQDLTGIPLVTVTHLMNISAVLLLPFNVVMDKIPFFNKRADTNALKKKI
GIFSENSVMGFIIGLLLGLAAAYGISGSLNLAIQIATAMALFPMISKLFMQSLSPLAD
AMSEMMKKRFKDREVYIGLDWPILAGRSEIWVTAILLVPVFIGYAMILPGNAVLPLAG
IINYSIAVGGLLLTGGNLYRMLTLGVISMPLYLYSATYLAPVLTGLAEKTKAVELAKG
QQITWSSIEGPEFRILFAEAFKGNWLAIAGGIAFIGLFIWLYRYMTKAEVPSKRYSEV
AP"

CDS 3151014..3151280

/locus_tag="JFBMFIFI_02952"

/inference="ab initio prediction:Prodigal:002006"

/codon_start=1

/transl_table=11

/product="hypothetical protein"

/translation="MKNNAEKAKWIGLLFVALGLFILALLTHNTFLNLIVIGLAVYIY
KYGNPILFKEYDAKRQAKYQHYQIVQKAASEATRNGLLFQSKKR"

CDS 3151300..3152052

/gene="mtnB"

/locus_tag="JFBMFIFI_02953"

/EC_number="4.2.1.109"

/inference="ab initio prediction:Prodigal:002006"

/inference="protein motif:HAMAP:MF_01677"

/codon_start=1

/transl_table=11

/product="Methylthioribulose-1-phosphate dehydratase"

/translation="MSDGRILFEREREDMAKIIKLIFDRKNTNVAGGNFSFKVTDKMG
KEFIIMTPTMMSEAYLGVVTPSQVLVVEPHTRKVIAGEGKLTREINLHEAVYDANPEI
KAVLHAHAPNSMFWATSGLDMPNLTEATQKVEYIEVLPFEPNCSEELAELVSQHIKQN
KLKVPHELLLNSHGVLINATGKNGMAAIHKALSILDTVEWNAEIAYKQTLFQKLNLLD
GYYSKGIKVGSLEDLMEGRPIWNQAVADNGGD"

CDS complement(3152523..3153998)

/gene="bglH_7"

/locus_tag="JFBMFIFI_02954"

/EC_number="3.2.1.86"

/inference="ab initio prediction:Prodigal:002006"

/inference="similar to AA sequence:UniProtKB:P40740"

/codon_start=1

/transl_table=11

/product="Aryl-phospho-beta-D-glucosidase BglH"

/db_xref="COG:COG2723"

/translation="MTNSTSIFPKNFLWGGAIAACQTEGGYGKNGRGMAVSDISFYDS

QIDRQDLAKHRNITTEKIEAAMEDPNTKRYPKRHGIDFYHHYKEDIALCAEMGFKVFR

FSMAWSRIFPTGDELTPNQEGLAFYDAVLTEIEKYGMEPLVTISHFEMPVALVTNYCG

WTDRKVIQLFTRFAETLFTHFGQRVTYWISFNEINAGRFSTFKSTGVVADKTDQYVQD

CYQAVHHQFVAAALITKRLHEINPKAQMGCMIARFTTYPATCNPDDVLQMMHDDQYDN

FFYTDVMIRGAYPGYMNRFFKENDVAIQWADGDKELLATHTADYLAFSYYMSNISSAN

PNDLDQTDANLKKGLKNPYLESSAWGWQIDPKGLRYTLNNLYDRYQVPLFIVENGIGA

EDHVTTDGKIHDTYRIDYLQKHIEQMREAIVDGVELIGYTMWSSLDIVSSGTSEMSKR

YGFIYVDQDDEGNGTLSRSKKDSFYWYQKVIASNGADLSLN"

CDS complement(3154015..3155874)

/gene="bglF_5"

/locus_tag="JFBMFIFI_02955"

/inference="ab initio prediction:Prodigal:002006"

/inference="similar to AA sequence:UniProtKB:P08722"

/codon_start=1

/transl_table=11

/product="PTS system beta-glucoside-specific EIIBCA

component"

/db_xref="COG:COG1263"

/translation="MDTNQLAQTILENVGGEKNVTSLVHCATRLRFKLKNRDIVDKAQ

VEAIPGVVTVMESGGQFQVVIGNMVPEVYEAIGAISNLTNDDSTETTTNKDETIFGKF

IDLISTIFTPLLGVMAGAGILKGLLSLALNFKLVMPDSSTHIILNAIADSLFYFLPML

LAITAARKFKANIYVAVAIAGALIYPTIIELAASPKAVHFFGIPIVMVKYTSTVIPII

LAIYVMSIVEKFLNKRLHQSIKNFITPAILLVTIVPLTLMIFGPFGVYVGNGIASILT

AIIAFNPIIAGAVIAASWQILVIFGLHWGIVPVMINNIATMGKDPLKPSTAISVFAQA

GASLGVMLKTKNKEFKALSASAALTALFGITEPAVYGVTLRLKRPFLIGILSAAVGGG

IAGYAGSAGYASGPSSILMIPAFYGPNGEGFVGFLIAIAVSFTLAAVLTYLIGFEDIP

SETTTETNNKDTIQPTTSVQSETIASPVNGVLLPLSDVKDKAFASGILGGGIAVVPST

GEIVSPVDGFVTVAFKTGHAIGLLSDEGAEILIHIGLDTVQLDGQYFDLKVEQNQHVK

KGDLLVAFDLDAIVAAGFDVTTPIIITNSTNYEDVIPTEKQETQMGDRLITLL"

CDS complement(3156258..3157112)

/gene="licT_3"

/locus_tag="JFBMFIFI_02956"

/inference="ab initio prediction:Prodigal:002006"

/inference="similar to AA sequence:UniProtKB:P39805"

/codon_start=1

/transl_table=11

/product="Transcription antiterminator LicT"

/db_xref="COG:COG3711"

/translation="MNIHKILNNNVLIAIDDNGIEQVMMGKGIGFKQNAGDPVDITRA

DKIFHLENNGLKQHFNSLIDEVPYPILKVTEEFIDISKQRLKQKLNESLHVSLVDHIY

HALKRHENEQTISNSLVWEINRLYPAEFGLAKEFLTMIQTEIAIELPIDEAGFIAMHL

INAEMNEEMNATVASTKEVAAILKIVKYHLGVEFDEESLNFYRFLTHLRFFVQRVSKN

HLLENEDPELYLMMKKKYPQAYTCTTKIAEYIYATFHIDLTSEEMLYLLIHLKRLKIR

DKALTNKD"

CDS 3157300..3158298

/locus_tag="JFBMFIFI_02957"

/inference="ab initio prediction:Prodigal:002006"

/codon_start=1

/transl_table=11

/product="hypothetical protein"

/translation="MANIITKPFFNIDEQIRLLSGDRNLIIKDVVTAKKHLLEKNYFD

LINGFETLLLEDPNDKAVGYKNKSFIDFLSLYNFDSELRKELLTVISSFEIKLKSSIA

YHFCEKFCRNQLNNLAYLDKNNYVVPSGLGTKHPISQAFYKDSFAFFKQPYNARYANY

VEYARNKHSYIGRYTNPPLWVIIKQLMLGDILFMIWYLDKDVLKNVLLDFNLTMSDVD

LFKNSVEIIKDIRNSCAHFELINRFRTSSSISINAALATRLNLNVLQQQTNRTRYRIK

LYDTLKVLGMYEDIFQVRRVIVRFYDRNISIRKKYLIMPLLDRMGASDIKDWKKLK"

CDS 3159014..3161779

/gene="yloB"

/locus_tag="JFBMFIFI_02958"

/EC_number="7.2.2.10"

/inference="ab initio prediction:Prodigal:002006"

/inference="similar to AA sequence:UniProtKB:O34431"

/codon_start=1

/transl_table=11

/product="Calcium-transporting ATPase"

/db_xref="COG:COG0474"

/translation="MKYYTKEKHEVLKEFDVTIESGLTDASSAANREKFGENKLKEEK

ADPYWRIFLRSFKEPIVIVLMGAIVLSFFSAYYDLQIKGDIKHGTEAIYEGTAILILI

LINATLSFWQEISAKKSLDALKQLSNRKVSVMRNGNWGHYDSVDLVPGDIVKVNVGDF

IEADIRWLETSELQIIESHLTGEADAIQKQIDALEGDVGVGDQTNMGFSGSTVSNGSG

IGIVVATGQQTELGKIAELLQNVESKPSPLQMTVGKLTRSLMLISGVVVVFTLVVGLF

QSYQATGVLTFSAVGSVLSTAIALAVASIPDALPAVLSIVLTIGASKMAKNKGLIKSL

SSVETLGATSYVCSDKTGTLTKNEMTVIKFFANGTIYSVSGKGYGPEGEITSTDSNAP

SYHDFVKGAVLCNEAEVKFIDGKYKPFGNPTEVALTILGEKAQLKKESLLEQGIEIYR

VLPFTSSRKMMSVIVKENNEYKLYTKGAPDILIEKSRFTLQNGNLVDTSDVKELLDTT

TLSFANEALRTLAVAEKVISKEEAETGSVEELETGFTVTGIAGIIDPPRDEVRASVEL

LKEASVKVVMITGDHEATAKAIAYDLKIIDSVDAPSIKGAEIEKMSDEELFQRVKDTQ

VYARVSPEHKQRIVEQLQKHGEIVAMTGDGVNDAPALRAADIGIAMGIAGTEVTKDSA

DLILLDDKFTTIEKTVESGRTIYANIKNFIRHELTTNVAEVLSLLIGLLFFTTGIGQV

SASTPTLTALMILWVNMVSDAIPSFSLGYDVAESDIMNEKPRDPKESVLANYTWSRVL

IRGTVMGLTVFLAFVWAAKSGLSGNQAQTVAFLTLVYGQLWHVFDARSSKTLFRRNPF

QNKHLIAAVLFAGISSYLVTIIPFFNTVMGTAPLSLNIYLLVLFVPAIPTLVLSGLKE

IFGIKIW"

CDS 3161861..3162163

/locus_tag="JFBMFIFI_02959"

/inference="ab initio prediction:Prodigal:002006"

/codon_start=1

/transl_table=11

/product="hypothetical protein"

/translation="MKKFWFLGGLLVAPFIVGWIANQVWQFPTLLVSGFVYIFLFLCL

TPSGDFLSASANYETKKINPNHSVETVSKRSMDRLEILRLILLFLLGAACFYYSFN"

CDS 3162297..3162602

/locus_tag="JFBMFIFI_02960"

/inference="ab initio prediction:Prodigal:002006"

/codon_start=1

/transl_table=11

/product="hypothetical protein"

/translation="MIKELIEGWSIQMNANTICYEDHEANYVVLPKMEEAPFGDLIPV

IFKISQGELVIQQMSHLVKSLTINQQKQLVICWRDEFIGQHDEVLATTVPFVIEDTE"

CDS complement(3162879..3163592)

/gene="gmuR_3"

/locus_tag="JFBMFIFI_02961"

/inference="ab initio prediction:Prodigal:002006"

/inference="similar to AA sequence:UniProtKB:O05509"

/codon_start=1

/transl_table=11

/product="HTH-type transcriptional regulator GmuR"

/db_xref="COG:COG2188"

/translation="MDKTYKYIEVYQDIKTKIACNEYVIGQKIPSGSELAIRYGCSNL

TVKKGLDMLVKEGVLRRRSGFGTEVLRKPIERSLVSGPNIGLLNVLGEEHVDSKIHSF

AIEQPSKKIAETLKISTSDYVYHIVRSRYIDSNPYSIEEIIMPLDLIPHLQPKHLKAS

IYKYIENELKLTIKTSHIRIRGDKANEFDALVLEIKPEQFIIEVEKIVYLDSGMPFEH

SITRHLYNDFEFEAVFVEN"

CDS 3163839..3165254

/gene="gmuD_3"

/locus_tag="JFBMFIFI_02962"

/EC_number="3.2.1.86"

/inference="ab initio prediction:Prodigal:002006"

/inference="similar to AA sequence:UniProtKB:O05508"

/codon_start=1

/transl_table=11

/product="6-phospho-beta-glucosidase GmuD"

/db_xref="COG:COG2723"

/translation="MENKRIQFPDGFWWGSAWSAEQAEGRGETGKAETIWERWFKEEP

NRFYNRVSSEIATDHIHRYKEDVQLMKETGHNSFRVSISWARMFPDDGVGLVNPLAIE

FYRSLFTEMNQNGIAVFANLYHFDMPAALQDQGGWESREVVEAYVRFAKTCFEEFGDL

VSQWFTFNEPLGPILGTYLECFHYPNIVDFKRGAQAAFNTIFAHARAIEEFKKLGLSS
KIGVILNLSPTYPRSQHPADVEAAEIADQFYTRSFLNPMVLGHFPKKLVALLKEHDQL
PVYSASDLEIIQNNTAQLLGLNYYEPRRVKARLTSINPAGPFLPDWFFEPYIMPGRKM
NHYRGWEIYERGIYELCMDIKDNYGNIESFISENGMGVADEERFMDENGYVEDDYRIE
YIQNHLAFVWKAIEEGANIKGYHLWTFIDCWSWINAYKNRYGLVSLNLATQERTIKKS
GEMYKKMSEENGFEFDLSQLY"

CDS 3165269..3166579

/gene="licC_12"
/locus_tag="JFBMFIFI_02963"
/inference="ab initio prediction:Prodigal:002006"
/inference="similar to AA sequence:UniProtKB:P46317"
/codon_start=1
/transl_table=11
/product="Lichenan permease IIC component"
/db_xref="COG:COG1455"
/translation="MENKMSFFDRFGMVAQKMGNQIHLRTLRDAFATFMPFMMLAGFV
TLFNYVIFDPAGFLSSVISSGILEKIQSIGTPIASATLSITSLLIVAAISYHMCQNRN
YENTIAAILVSISSLMVLTPMMTTFTPEGAKNALEIPNVIPLDYIGASGMFVGIFVGL
IATELFIKLSKNKKLQINLSGNIPPAVMKSFNVMIPIMITIVSISIVGFLVKELFHSD
VNTLITTLVTAPLSKVTTGLPGFLLITSVANLFFGFGIHQAVISGSLLDPFLIQNMQE
NMAAYANHEEIPHIINMAFKDTFAVMGGSGNTIALLIAIFIFSRRQDYKDFAKLSVTP
AVFNISEPIIFGLPIVFNISLIIPFILAPIFSLTIAYFATAMGFINHVVVQIPWTTPP
VISGFLATAGDWRAAFLQIIIIAVSVFIYLPFLRIDEKVTATKQ"

CDS complement(3166679..3167269)

/locus_tag="JFBMFIFI_02964"
/inference="ab initio prediction:Prodigal:002006"
/codon_start=1
/transl_table=11
/product="hypothetical protein"
/translation="MKKFKLFIDMKKEEQYLKEMAEKGWGLVKYSAYNRYTFEKIHPE
SLSYRIDYQMFKKKGDYTDYLTLFEDSGWKHISGSQSSGFHFFLPENDNNQDLDIFSD
SQSSRARYKRLYNQATLWVALMIVYFILLQPSFENISSWYLAPTIWEYSGLQLVGMIV
MQTFFVLIQLLPMLIFMSGAVIYALIGTKAKKLATM"

CDS complement(3167279..3167593)

/locus_tag="JFBMFIFI_02965"

/inference="ab initio prediction:Prodigal:002006"

/codon_start=1

/transl_table=11

/product="hypothetical protein"

/translation="MNVNKILPLTETTFYIMISLLEPSHGYAIMQRVEELSNGRVRIA

AGTMYGATENLLKQKLIHEVPSPNEDKRRRVYLLTDAGKEVLKLEVERLKQLLLIADE

LL"

CDS 3167879..3168736

/locus_tag="JFBMFIFI_02966"

/inference="ab initio prediction:Prodigal:002006"

/codon_start=1

/transl_table=11

/product="hypothetical protein"

/translation="MAKIILTTESCSDLSKELLEEKQILSVPFSVNFPDRTVYDGEIP

IQEIYDYYQETKKIPKTNAVSPHQYTEFFEEAAGENPTSEIIHIGYSSACSCSFQNAM

IGVEECEKAKVHLIDSKNVSGGLGNLTLKAAEIIANNPQDSVAELVLKIENFVQKTHT

SFVADTLDFLLAGGRVSNAAAIGAAILRLKPRIDIVEGKLIASKKYRGRMKKIAPQFV

KDFVESQDFNRDNVYLFYALGTDTEVIELLKTSLEKQGFRQIHVDMIGSVMTVHGGKG

AIGLSATTY"

CDS 3169480..3173997

/locus_tag="JFBMFIFI_02967"

/inference="ab initio prediction:Prodigal:002006"

/codon_start=1

/transl_table=11

/product="hypothetical protein"

/translation="MFKVTNKIVSFFLIFSMVFPTLIGGLFSTGTQVFAAELGGGAII

DSVKMDKTDLYNGERVGISVTFSEKEGIGIKNGDTITMSLPPELIGIVSSAELKDPAT

GKVLGEAKIVNDQVICTFNSTAEELINVKGYFYFKVRVDSQTEGTISKTTDFGVNIPD

IDYTVTYEKGEAGNDDDYPFFSKYGHMSEDGSNKITWQVIINQPKKELVHNGTNQKLI

EVDDTSAADQTLLANSFRYFIEDKDKNFTWLSQAEFSQYGTLEIDPANGNHFNVKLFA

KEISNHKFGLVYDTHINDLSKKEFANDYTANYQITGEEQTIEKNTSYTENNSAGGGGS

GDLPEKGSARLVKSLENKADVLLPGITFELFNAANQSLGSYQTDDQGQINVKNMELGQ
YYFKEVSAPEQFDFDQTKQYPFEIKENSATGELIPVTNKIKKTSLNLTKVWVGPETTS
IDVRIYADGQDINRQVTIEKASNWQLLVTNLDKYNVDGSLINYTVQEVNVPAGYESVV
TGNNDTGLTITNTNIEKLSIPVIKKWQGKATDSVEVSLKRNNKITDKTIQLSPANNWR
AEFTDLAKYDSKGKEISYSVVEAELAGYVATYTGNAQDGFTITNTRSTSITIPVTKKW
VGPVGKPVTVELFSNGVTTGRSVKLTANQNWTSAFTNLPEYDENGQEINYTLKEIQQD
NYDATITGSAKEGFTVTNKNNEKVTIPVKKEWFGPVGEKVIIRLLAEGADTSKSLELT
SQKNWQGAFTDLPKYTETGEEISYTIKEDKLENYFSLIDGNAKEGFLVTNITSEQVDL
PVKKKWQGEKKDSVKIYAQVNGLLLPDVFIVLSEENNWEGTIDYLPKYDSAGELFRYT
VVEEELSGYVTTYAGDQDTGYTVTNTRADSTSIPVTKKWVGPVGGDVVVKLFANGKDT
AISLTLQANQNWEGKFTNLPTYDDKGIEIKYTVEEIAQENYESSIKGNSTEGFVITNT
NKEEISIPVKKVWNGPEKDAITVFLYNDKNVKIKEMTINEAMNWQASFDHLPKYNSDG
KAINYFIREENMENYRPLIQGSKEAGYTINNINTEKVAVSVTKKWVGPIAGRIQVNLV
KNGQMLSNYLTLDQTTNWQGTFSELEKYQADGTENEYTIVETVGYKNYDASISGTAET
GYRVTNTNSETITIPVAKEWVGPIGESATIELFRDSETTPLKLVLNADNDWKGEFADL
PKYDDAGNEYNYQIKEVTVANYEGEVTGDSETGFVVTNTNIEKIQIPVTKKWVGPAAE
NVTIQLKQNDVLMNSELLLNAENNWQGTFVDLPKYDKFGEEYQYTLTEKSLENYRSEI
TGTPETGFVVTNTNCEKIQLPVKKKWIGKKADEVQIKLMKNGVPIEEMLSLNEENQWQ
GKFTDLPKYDDAGVENQYTVIEEELPGYQSEVTGDVSTGFIVTNRYLGTEENPPNPSR
PELPNAGDLSGKPELVRELGNQMSVGDQDHQLPKTNSKQENYLWLGVLSVSLGMLSYG
MCYRKMGSKTK"

CDS 3174194..3174502

/locus_tag="JFBMFIFI_02968"

/inference="ab initio prediction:Prodigal:002006"

/codon_start=1

/transl_table=11

/product="hypothetical protein"

/translation="MVNMRVFLSLLVSIISFSTPPLSANVVSGNVKQPQNRAVQYYVV
DRKSQPQVLRDSLWFNGSPWYTYKYISVPNGYRPVGRVSSAVTASTGSDKRTIVSYCS
"

CDS 3174555..3174938

/locus_tag="JFBMFIFI_02969"

/inference="ab initio prediction:Prodigal:002006"

/codon_start=1

/transl_table=11

/product="hypothetical protein"

/translation="MHKIKIAILLFLLFPLMVACTPTKKFELEGNWTSVSKDTTDVNS

SYSIIKKIEFSSNGLAFITYEDGNQTKIGYEFDSEKANDEKYGILKLNFTDTDVTSTD

VTKVKNKENQIKLDIGYTAVFEREN"

CDS 3175138..3175383

/locus_tag="JFBMFIFI_02970"

/inference="ab initio prediction:Prodigal:002006"

/codon_start=1

/transl_table=11

/product="hypothetical protein"

/translation="MNLSEHTASLEAEKKRLLKVVPVKPRMSSEDIEKSIDTAILRSS

LVAIQIEAVDDDGNNYCFAMQTLGLIKPFNISITYKQ"

CDS 3175406..3175879

/locus_tag="JFBMFIFI_02971"

/inference="ab initio prediction:Prodigal:002006"

/codon_start=1

/transl_table=11

/product="hypothetical protein"

/translation="MKNKRTLAIASIILLTIALIVSMIVITPKIASMFKEDSNYRAPD

TTTSGNLIKIDDTFHVDTTSAIVSSLYYDEENGERYNRLVEYVYKLNYDDDYIIIQSI

TNNFYSLFFKQFEYVIVNKENKEVTILKTKDEFLKECKKRNIKVELKSKDQFDWY"

CDS complement(3176207..3176446)

/locus_tag="JFBMFIFI_02972"

/inference="ab initio prediction:Prodigal:002006"

/codon_start=1

/transl_table=11

/product="hypothetical protein"

/translation="MKKNSIVFSILFILTCGGLYMNHKIKENNEKQQEWLNKVVSEQE

PYINKYLNYYYNGIESVTLTESEQIPTGAVSIEGM"

CDS 3177343..3178212

/gene="yddE_2"

/locus_tag="JFBMFIFI_02973"

/EC_number="5.1.-.-"

/inference="ab initio prediction:Prodigal:002006"

/inference="similar to AA sequence:UniProtKB:P37757"

/codon_start=1

/transl_table=11

/product="putative isomerase YddE"

/db_xref="COG:COG0384"

/translation="MNTLYEVSSFTVAGEGGNLAGVKLLKEDENLSTNEMQVMAKALN

YSETAFVKPLNKGEFQLRYFTPLAEVPLCGHATIAAFSLMSQLNQLAMGNYIMHTKAG

RLSISVLAEATIFMEQSLPIFYDEKPDRGLIAHSLGIETSDIPSDYPIEIVSTGLKDL

IIPVKNTTILQNIKPNMNKIREISDQLDIIGYHVFALDDENQHLIHCRNFAPLVGIDE

EYATGTSNGALACYLFKHDTLQGNVEYTFSQGRQSIQQKGKVLVELKTTGATIEQVFV

GGQAVVKRSLDNF"

CDS 3178259..3178786

/locus_tag="JFBMFIFI_02974"

/inference="ab initio prediction:Prodigal:002006"

/codon_start=1

/transl_table=11

/product="hypothetical protein"

/translation="MQKEFRKMVYNDLSTIKKYKEEFIKSEENMDGTGGLSAAVNIEE

WFCRSVENENENSISDKLVPAIQYIYIDHSTNKIIGMLQLRLKLNQYLTSIGGHIGYS

INPTERKKGYGKQMLKQALIEAKKQGLEKVLLTCDSDNQGSRKIIESNRGILEETIFN

EERVKTINRYWIYLE"

CDS 3179031..3179879

/locus_tag="JFBMFIFI_02975"

/inference="ab initio prediction:Prodigal:002006"

/codon_start=1

/transl_table=11

/product="hypothetical protein"

/translation="MKKYGVGILLMASLLLVTACGNGGNTKETKKEDTSTSAKSAKDK

KAKTSDKTSSEASTSKESEVVTPTKNELSIDGFWLATGKEDQKGSSWKFENNVLTVNG

QENWTYSVADNLDKNGYTVITITNDQSENHALLVKKTANGFEGITVEGDAYQSYLNDE

NASVGNQVITFVPQTEIDNLTWSGIDNAIDFYEGVYKNTANEESKDINWDNYRRDLWT

IVPDGTQGNTITLHWTNIGGAGGSYVQLIKGQDTTEMIQYDGNAAYPDSPSKKMTIRN

SDFKVL"

CDS complement(3179947..3180999)

/gene="sasA_2"

/locus_tag="JFBMFIFI_02976"

/EC_number="2.7.-.-"

/inference="ab initio prediction:Prodigal:002006"

/inference="protein motif:HAMAP:MF_01837"

/codon_start=1

/transl_table=11

/product="Adaptive-response sensory-kinase SasA"

/translation="MKKLFSRFSLTMSLVLFVFLMMLCSTVIVALIILLLFKTGMLQY

FPRFPEKYRLFGPILLLLFISILTGTMLTGISSKRATRPYKMAIEAFNRVASGDFTVE

LKFKHVPYELEELTKSFNRMTKELSGIETLRSDFINHFSHEFKTPIVSIQGFAKLLEN

PNLSEEERQKYVAIIIQESGRLTTLSSTILHLSKVENHEIISEKRAIQLDEQLRQTIL

LLEPKWQKKRIVWELELDDSILNSDEDLLQQMWINLLDNAIKFSPENGVVKVKLMNLT

DTVVVKITDQGSGMSSETQQRLFDKFYQGDASHSKEGNGLGMSLVKNILRICDGEIGL

KSSLGNGSSFTITLKK"

CDS complement(3180996..3181670)

/gene="hssR_2"

/locus_tag="JFBMFIFI_02977"

/inference="ab initio prediction:Prodigal:002006"

/inference="similar to AA sequence:UniProtKB:A6QJK3"

/codon_start=1

/transl_table=11

/product="Heme response regulator HssR"

/translation="METILIVEDDPHTRNLMEIILKNNGFQTVTATNGIEALDVLDKR

MISLIILDIMMPEMDGYQLTQNLREADFQLPILMVTAKETPSEKKKGFLVGTDDYMTK

PVDEEEMILRILALLRRSKIAIEHKIVLGEVTLDSDSLTVSRGTLQQTLPKKEFYLLF

KLLAYPDKIFTRLQLMDEIWGYETETDERTVNVHINRLRERFVEYPEFTIVTVRGLGY

KAVKNI"

CDS 3181823..3184189

/gene="btuD_6"

/locus_tag="JFBMFIFI_02978"

/EC_number="7.6.2.8"

/inference="ab initio prediction:Prodigal:002006"

/inference="protein motif:HAMAP:MF_01005"

/codon_start=1

/transl_table=11

/product="Vitamin B12 import ATP-binding protein BtuD"

/translation="MLQLKNIFKKYTTGEFTQIALDGVSLNFRKNEFAAILGPSGSGK

TTLLNIIGGLDQYDEGDLLINGQSTKKYKDGDWDAYRNNSVGFIFQSYNLIGHLTVLD

NVKMGMTLSGVPKAEQNQRALDVLERVGLKEHTHKKPNQLSGGQMQRVAIARSLVNDP

DIILADEPTGALDTQTSQQIMDLIAEIAKDKLVIMVTHNPDLAEEFADRIIEFRDGEV

MSDSNPMTASEPSKNYQLKKTSMSFWNALTLSGKNIWTKKWRTALIAFASSIGIIGIA

LVLSLSNGFSKQIDSIENDTLSGFPITITETANDMTFGPSNRLATEEDDKANEKQRKT

EAVFPEQPAEEETVHENSLSKEYIAYLADMDSNLISGMSFTSNVNMNLIHQNDGEVAA

VQTDSLNLTSYPTNGEAKGSSFLEQNYDLLAGSYPTGKNDLILVVRENNQLASGMVDA

LGLDSSQKEINFDQFIGQEMTSIFNDDFYTETNGFFKLNPDLTSLYEGESGTDLKIVG

VIRGKEDLSIAGLSEGIKYSDTLAQEFIGNAQKSAIVLAQEAADFNVMTGEVFSTNTG

GAVDTRSFSPENNPLTATVSTVTKDQLLTRFGAIEKPSGITIYPADFEKKEQVLEHLD

QWNKDQPEEKQVVYTDMAATVTSLMGNTLDAITVVLVAFAAISLVVSLIMIGIITYIS

VMERTKEIGVLRALGARKKDITRVFNAETFIIGTFSGLLGIGIAYLVTIPANIVMENV

TGLADVAQLNPLHALALIIVSVLLTMLGGLIPARLAAKKDPVEALRSE"

CDS 3184190..3184537

/locus_tag="JFBMFIFI_02979"

/inference="ab initio prediction:Prodigal:002006"

/codon_start=1

/transl_table=11

/product="hypothetical protein"

/translation="MGSRGTQFLAEIKQMLHSIDEKYPTHILDDPEHFIACFKKQEQT

IEEISLRLTNFRNSYELMDTKEQQSVEELKKIMKEQEEMRLVFHDWGNPLAIFSQQQA

VLKEIKTTLSFET"

CDS complement(3184611..3184811)

/locus_tag="JFBMFIFI_02980"

/inference="ab initio prediction:Prodigal:002006"

/codon_start=1

/transl_table=11

/product="hypothetical protein"

/translation="MNGKNRSNLKVGQLVDIVLKKDQRTGTLTRGHIKRILTNSSSHP

HGIKVMLVESDQVGRVQHIVEE"

CDS 3184971..3185495

/locus_tag="JFBMFIFI_02981"

/inference="ab initio prediction:Prodigal:002006"

/codon_start=1

/transl_table=11

/product="hypothetical protein"

/translation="MTNNDILIRLRYALNIKDSDMVEIFRMGGIELTKAEVQQMLINP

KKNTTTEVEKSEPIVNEELKVCDNFMLESFLNGLIISQRGRKETTGDDTQKPELMIKN

DRSVNNVLLKKVKIALSLTSDDILDMLDEAGVRISNSELSAVLRKEGHRNYKECGDRY

ARNFLKGLALRYRG"

CDS 3185686..3186537

/gene="ywtE"

/locus_tag="JFBMFIFI_02982"

/EC_number="3.1.3.104"

/inference="ab initio prediction:Prodigal:002006"

/inference="similar to AA sequence:UniProtKB:P96741"

/codon_start=1

/transl_table=11

/product="5-amino-6-(5-phospho-D-ribitylamino)uracil

phosphatase YwtE"

/db_xref="COG:COG0561"

/translation="MKIVFSDIDGTFHQMGAEIPQINLDAIDAMHQQGDRFVFVSGRS

CTQIEKMQEELGKEGDIIFGNGAGFKLVGQPAQYENCLSLETCVNIMKFLDQENIFYH

IHTSEEVYLKPVQYFTKHFKELRKTFLPMGEQGQQIMDFKENYFTNDCVHHEDLGTFF

KENPQIKLLKIEVMESDEVKTTKIVKMLANENVYAFSSFFTGLEIVNPSSNKGSAINQ

YMKLFPDATSYGIGDAENDLPMLEVVDIAVAVDNATEIVKEACQEFTLDCLNGGVGHY

IYEKIIV"

CDS 3186562..3187401

/locus_tag="JFBMFIFI_02983"

/inference="ab initio prediction:Prodigal:002006"

/codon_start=1

/transl_table=11

/product="hypothetical protein"

/translation="MDITVDLVKKLIFEQFPQWSHLEIKPVKNSGHDNRTFHLGDDLT

IRLPSGKEYEPQIQKEAKWLPVLTQHLSLSITAPVAKGKPTPEYPLAWSINRWLVGET

VTHTNVDLQKFAIELARFLKELEAINAENGPQAGAHNFYRGGDLAVYIEEFEQALTQL

PAGPQRNHYQDIWTTALATKWEKKPVWVHGDIAVGNLLVNDGHLSGVIDFGILGTGDP

ACDLVMAWTFFDSKTRKAFKEEMGLDDTTWQRGKAWALWKALVTPDDAVSKKVLVDML

AAD"

CDS 3187430..3188476

/locus_tag="JFBMFIFI_02984"

/inference="ab initio prediction:Prodigal:002006"

/codon_start=1

/transl_table=11

/product="hypothetical protein"

/translation="MDNQEIRTLRLANQQISSTNFKKPEELVQYLGAMQAQEYAMAKW

AIGLRIPNLTVQDVDAAIDAGKIIRMHLLRPTWHFIVPEDSRWMLALTAPQIHKKIKS

FYSKFDLTPKKMHQACSIIEEALVEKELTRKELATIVAEKGIQTTSQSFSFIILFGEL

EGLLCSGKMRGNQAVYTLLSKFVAPSLTSFIREEALAKLALRYFTSRGPATVQDFAYW

SGLSIKDARLGTQFLSNDFSSFLNDGKEYWYLPTNNAYVPNDCGTFLFPDFDEYGISY

KNRDIFLNKEYPISPFMEHKHWLMVGGMIEGTWQSDKSDLSEVRTELFNPKKRVNQHA

IHKAVANYQQFHLK"

CDS complement(3188535..3189065)

/gene="tag_2"

/locus_tag="JFBMFIFI_02985"

/EC_number="3.2.2.20"

/inference="ab initio prediction:Prodigal:002006"

/inference="similar to AA sequence:UniProtKB:P05100"

/codon_start=1

/transl_table=11

/product="DNA-3-methyladenine glycosylase 1"

/db_xref="COG:COG2818"

/translation="MKKNSTTSTGIQFYQNQWGTPTHDDQLLFELLTVGTFQVGLGWK

MVVNKRDVFLRNFQQMDILKVAAMMPDDVERIMEDPAMIRNPRKINATITNARAIIGI

QKEYGSFAAYLWQFVDNQTIEYEYTEASEVPTSSPLSEKVAKELKKKGFKFVGPIVTY

MFMKASGLVHDTIIDR"

CDS complement(3189119..3190639)

/gene="fcbA2"

/locus_tag="JFBMFIFI_02986"

/EC_number="6.2.1.33"

/inference="ab initio prediction:Prodigal:002006"

/inference="similar to AA sequence:UniProtKB:P86832"

/codon_start=1

/transl_table=11

/product="4-chlorobenzoate--CoA ligase"

/translation="MYNKYEPLNLYTNFANAAKEFPETPIYFDEPLLAFPELNLQTTY

SECTAAILKKATQLKKIGVKKADKVIVYKSAKFDTYLAAVAISYLGAVPIMVSAHLPA

ETIDVFVNRLDQPWVLLDSETSSKCAQLQNLPKNKLIAVDELLAVEIDEICQQEELEK

DVISYMTHTSGTTGIPKLIAHSANSMGWRTKWQKNIFDFIKEKELVAFHISPVHSRFN

IGISSLMAKGFPLLAIADSSAENVTKVLGDYQPGVLETHPNHFVQWASLAREKPEVFS

SIKYYHSTFDAINKETLATFLRCSTFKKPVFLQVYGQSECGPMIMRVHNLSSIKKINA

RDMGVGMTGLTEVRIVDQAGNPVAANISGNIQMLSKGRALTYYKEETRFEDNLYGDWW

DSGDYGFKDSHGKLHLQDRQVDLVDTIASTLAIEDKLLDELTFLEEVVIIRGKNGSPQ

PVLAVYPTHEMDWDRWWASVSDLPHLNEPIMMTFDELPRTATMKIQRLALEQQLKG"

CDS 3190886..3192394

/locus_tag="JFBMFIFI_02987"

/inference="ab initio prediction:Prodigal:002006"

/inference="similar to AA sequence:UniProtKB:Q6NEC9"

/note="UPF0371 protein DIP2346"

/codon_start=1

/transl_table=11

/product="hypothetical protein"

/translation="MKKIGFDPQQYIEEQSKYILERVHDYDKLYLEFGGKLIGDKHAK

RVLPGFDEDSKIKLLQKLKDQAEILICVYAGDIERNKIRGDYGITYDMDILRLIDELR
GYGLEINSVVITRYNGQPATKLFINKLERRNIKVYKHTAIEDYPTNIDKIVSEEGFGK
NSYIPTTKPIVVVTAPGPGSGKLATCLSQLYHESRHGKAAGYSKFETFPVWNVPLKHP
LNIAYEAATVDLKDVNMIDSFHFDKYQTVAVNYNRDVETFPVIKRIIERITGKESVYQ
SPTDMGVNRVGFGITDDDVVQEAAKQEIIRRCFATECDFKKGLVDEETVNRIKLIMEE
VELKKEDRGPVKRARHYSEKLKEQNETNETPGVIAFELQDGRIVTGKTTSLMDSCSAA
ILNSLKILANISDEIFLLSPLVLETIQNMKTNDLHSKITSLNANEILIALAISAVTNP
TAQLAYDKLAELADVQAHSTVMLSKNDEQILRELGIDITCDPIYSSENLYYI"

CDS 3192510..3193280

/locus_tag="JFBMFIFI_02988"
/inference="ab initio prediction:Prodigal:002006"
/codon_start=1
/transl_table=11
/product="hypothetical protein"
/translation="MKISVRHRFIKKIPVLEVVMDELKLAPLPLVIYYHGWQSSKELV
LTQARKLAKKGIRVLLPDAMNHGERRVGAISTIPAMTFWSSVQFNLAEFSQLTHFYQK
QGLIRNEKIGVGGVSMGGITTCALLTQHPEIQVAACIMGTPAPTSYLQLVLRTVAERD
IFVPQDLPLLLNWLDNYDLALNPSKLAKRPLLFWHGKEDEKIPFESAYDFYEEIKEQE
YAENVLFLVDEKERHLVRSEVMDQVTEFFTEELIGINE"

CDS 3193647..3193868

/locus_tag="JFBMFIFI_02989"
/inference="ab initio prediction:Prodigal:002006"
/codon_start=1
/transl_table=11
/product="hypothetical protein"
/translation="MTFEEILPHLKNGKKIIRSGWGGFELHVFLEPTSTHKGSTLNPY
FLIKTADEGYTMFSPTVCDILAEDWEIVQ"

CDS 3193941..3194705

/gene="bacC"
/locus_tag="JFBMFIFI_02990"
/EC_number="1.1.1.385"
/inference="ab initio prediction:Prodigal:002006"
/inference="similar to AA sequence:UniProtKB:Q8KWT4"

/codon_start=1

/transl_table=11

/product="Dihydroanticapsin 7-dehydrogenase"

/translation="MIIDEFKEKVVFITGISSGIGLAQAEAFLKQGATVFGMDIVEPD

VLTNAVFQNSIGQNFAYLTGDVAQQKDVKEAVKTALSTFGKIDILLNTAGILDNYTPT

LETDEALWDRVLNTNLKGMYFVANAILPGMLEQKHGVIVNMASIAGLVAGGGGAAYTT

AKHGIIGYTKQLDYDYAKHGIRCNAIAPGAIETPMNKADFAGTGEMAKWVAKETPAGR

WAQPKEVADLTLFLASSLADYIHGTVLPIDGGWLEK"

CDS 3194898..3195407

/gene="queT_2"

/locus_tag="JFBMFIFI_02991"

/inference="ab initio prediction:Prodigal:002006"

/inference="similar to AA sequence:UniProtKB:A2RM05"

/codon_start=1

/transl_table=11

/product="Queuosine precursor transporter QueT"

/db_xref="COG:COG4708"

/translation="MNKFEKNQSAKWSAASLTKMALVTALYVTVTLVFSVISFGAIQF

RLAEMFNYLAVFNKRYIIAVTLGVMLANLASPLGMIDVIVGGLSTFCVLVLVYFVTRK

IKNQFVKLAVTALICSFSMFTIAAQLTLLLGIPFWFSWLTIGLGEFVSMTIGGVLIHW

ISKKIDLTK"

CDS 3195525..3196484

/gene="prs2"

/locus_tag="JFBMFIFI_02992"

/EC_number="2.7.6.1"

/inference="ab initio prediction:Prodigal:002006"

/inference="similar to AA sequence:UniProtKB:Q5XC85"

/codon_start=1

/transl_table=11

/product="Putative ribose-phosphate pyrophosphokinase 2"

/translation="MTTSYKDPSLKIFSLNANQPLAEKIAEVVGIELGKSSVRHFSDG

EIQINIEESIRGDHVYIIQSTSNPVNDHLLELLIMIDALKRASAKTINVVMPYFGYAR

QDRTAKPREPITAKLVANMIQKAGATRILTLDLHTVQLQGFFDIPVDNLFTLPLLARY

YHECGLNNEEVVVVSPKNSGVGRARTLSEYLDTTLAIVDQHVDENGEESGYVIGNITG
KTCIMIDDMINTGETLAKAANVLMDSGAKEVYACASHGLFSANATEILENAPIKKICV
TDSIDLTEKRTPENLEIITCSQLMGEGIKRIHENVPMSSLFKF"

CDS 3196970..3197806

/gene="metQ_3"
/locus_tag="JFBMFIFI_02993"
/inference="ab initio prediction:Prodigal:002006"
/inference="similar to AA sequence:UniProtKB:P28635"
/codon_start=1
/transl_table=11
/product="D-methionine-binding lipoprotein MetQ"
/db_xref="COG:COG1464"
/translation="MKKRIGLITLAAAFIFILGACGAKGTGDKADKVEKVKIGVVSES
AIEIWKDVAKRLKDQNIDLEVVEFTDYNQPNIALKNGDIDLNAFQHVAFLEDFNKNND
ADLTPIGFTFVSPLGIYSEKIKDIKELKDGDSIAIPNDVTNGGRALLLLQALDLIKLD
TKAASPTVNDITENTKNIQIKELDAAQTARSLQDVAAAVVNTNYAVDAGFSPKKDALY
LDTDNISEVKDRYKNVIAARKADKDNKVYKKVVAEYQTEATKKLIDSTTNGTDIPIWD
AK"

CDS 3198333..3199199

/gene="fba"
/locus_tag="JFBMFIFI_02994"
/EC_number="4.1.2.13"
/inference="ab initio prediction:Prodigal:002006"
/inference="similar to AA sequence:UniProtKB:P0A4S2"
/codon_start=1
/transl_table=11
/product="Fructose-bisphosphate aldolase"
/db_xref="COG:COG0191"
/translation="MALVSAAEMTKKAREGKYAIGAFNTNNLEWTKAILEGAQASNAP
VMIQTSMGAAKYMGGYEVCYNLVKNLIDSMGITVPVALHLDHGDYEDALECIRVGYTS
VMFDGSHLPFEENLEKAKEVVKQAHAKGVSVECEVGSIGGEEDGIIGTGELASPEECK
IIADLGIDFLAAGIGNIHGSYPSNWTGLSFETLSAIADITDGMPLVLHGGSGIPVEQV
KKAISLGVSKINVNTECQEVFAAATRKYIEEGKDLEGKGFDPRKLLAPGTTAVKELVE

QRIEWFGSNGKA"

CDS 3199343..3200062

/gene="rsuA"

/locus_tag="JFBMFIFI_02995"

/EC_number="5.4.99.19"

/inference="ab initio prediction:Prodigal:002006"

/inference="similar to AA sequence:UniProtKB:P0AA43"

/codon_start=1

/transl_table=11

/product="Ribosomal small subunit pseudouridine synthase

A"

/db_xref="COG:COG1187"

/translation="MRLDKLLSETGYGSRRQVKKLIKGKQVRIDGELILTDNYNADSQ

LQEILVAGKKVTHNTHVYYMLNKPNGVVSAVRDEENQTVLDLLAPEDIRAGLFPVGRL

DKDTEGLLLITDNGQLAHQLLVPHKEIFKTYEVKVNGLTTTEDQEAFAQGIIFHGDIQ

CKPAKLTILAQSPIESHVLLSISEGKFHQVKKMFLSVGKKVTYLKRLTMGPLTLDEDL

AVGTYRPLNQRELDLLKPYFK"

CDS 3200459..3201658

/locus_tag="JFBMFIFI_02996"

/EC_number="3.5.1.47"

/inference="ab initio prediction:Prodigal:002006"

/inference="protein motif:HAMAP:MF_01692"

/codon_start=1

/transl_table=11

/product="N-acetyldiaminopimelate deacetylase"

/translation="MTTTNQKTLKNFIETRKDTYQALALDIHDHPEVSNYEYYSSNAL

VQQLKAEGFDVKVDVAGHRTGFDARYQAKKSGPTIVFLAEYDALPGIGHACGHNLFGT

TSVLAASALKQIIDEVGGEIRVYGTPGEEGGENGSAKGSFVREGFFKDVDVALCVHPS

YENGLTAHSLANDPVDIEFFGKASHAAAAPEKGINALDAVLQVYNSINALRQHLSDDV

RIHGIITHGGDVANVVPEYASARFYLRAAARSTLNDVYQKVENIVKGAAFATGTTYKF

GLFQNSVDDTVPTYRFDEIYLKHLNEAGFDVAEKFSPAGGGSTDVGNVSQVIPTIQPT

ISISDEYIAGHSIEFKAAARSQKGLDSILLGADLLANTALDLILDDALLAEVKAKHQE

SLAQQPK"

CDS 3201961..3203040

/locus_tag="JFBMFIFI_02997"

/inference="ab initio prediction:Prodigal:002006"

/codon_start=1

/transl_table=11

/product="hypothetical protein"

/translation="MDYSELKKISDFTHDPAVTISLRTHRTSPKNQKDAIVLKNLINE

TEERLLELYDKRKVWGVMDNLRALETELNHQKNLDTLILFASEDFKEAIKLPIEIEKD

TVKIDKKFSTRNLIRAVKQTEHYYVLTLSQQEVRVLEFFNDTFVQEFETDDFPFKNTE

FYETDPTQNSIGAVQNNLIKEFFNRADNAFYKIYRENPLPVILAGVQRNIEYYHEIAN

QDEWFIGSIHGSFDRTKITNHEIVELAYPVIEAYVSDQLDQSLADLETAENENKLVNE

LSNIYKAVTEGRGKKLYVEKDFFQSAVIEEGTLIVTEDTSQAEVVDDIVNEIIHTVTE

FGGEVVFVPNDYLAKYHHIALILRY"

CDS 3203262..3203612

/locus_tag="JFBMFIFI_02998"

/inference="ab initio prediction:Prodigal:002006"

/codon_start=1

/transl_table=11

/product="hypothetical protein"

/translation="MENLPNCPECNSEYTYEDGNLLVCPECAHEWSADAAEKAEEANA

VRDSNGNILTDGDTVTVIKDLKVKGYLTAIKLGTKVKNIRLIDGDLNAGHDIDCKIDG

FGAMKLKSEFVKKI"

CDS 3203968..3207999

/gene="yfkN_3"

/locus_tag="JFBMFIFI_02999"

/inference="ab initio prediction:Prodigal:002006"

/inference="similar to AA sequence:UniProtKB:O34313"

/codon_start=1

/transl_table=11

/product="Trifunctional nucleotide phosphoesterase protein

YfkN"

/db_xref="COG:COG0737"

/translation="MKQKKHIQRHLFKSINVVAVLFLLFGGVAPAALAVESLNQTDAN

QEQVVLEENIEQIEMKADTETPVVESPVIEEPPEKASDVTKIEETSVPEEVAPVEKEQ
APLTSEADPVPTAPEPEERKLTILGTTDVHGSLWDWSYEDSAPKDGGLAKISTIVNET
RAIDPDTILVDAGDNIQGTLLTDDLYNVKPELLAQTHPMIKAMNYMQYDSMSLGNHEF
NFGLGLIEKIRNDANFPLLSANTYKKVDGTNLVDAYKIKEVKGVKVGVLGLTIPHVPM
WDGAKVESLEFRSLREEAKKQVRILKDVEQVDVIVAAIHAGLKNSDPEAAAENVINEV
PEIDAFVIGHDHKEVAQKMVDYTGKEKPVGAAKDTGTGVVKIELELNKPDTKWEVTGS
SVEVLQTANKVADPAVKELTQDAHETVVEFIQDPIGTAKADFLPEAEIPGIPEAQLQP
TAMISLINNVQMKVTGADIAAGALFKVNSHLDAGPITFADVFDIYKYPNTLIGVEIKG
DQLKRYMESQANYYNQYKKGDVTIGFNEKIRVYNYDMFTGVNYKIDISKPTGSRIVDL
TYQGKPVADSDTFKLAINNYRYEGLVSDGIITAEPYYVSDPITLRSSIVDYIAEKGEI
DPEVENGKNWEIIGADLEHPLRSYIIEQIKLGTNDQIKIVDSDTGRTPNVKTLNADEL
IAAGAIPDDVLASLEKLTIMHTNDMHGRMLPDSKNNALGLAKLKTYKKQVNPTLMVDS
GDAFQGLPISNYSKGFDMAKAMNEVGYDAMTVGNHEFDFGYDVAMQYKEKLNFPIVSA
NIYKDGVRSFDPYTIVEKNGKKYAIIGLTTPETATKTHPNNVKGVTFKDPIPEAKAVL
AELKGQADAYVFLTHLGIDETTPTQWRGDTLAKTLSEDPDFANANIVVLDGHSHSELP
AGKQFGKVMLAQTGNHLNNVGLVEAKFSDPVAIKASLASASSLADLEEDAAVKAIVDQ
AEANFKEGTSEVIMENNPYLLNGERDNVRARETNLGNLISDALSDYGQTGFANKSDFA
VINGGGIRANIPAGKVTKGDVISVLPFGNIISQVAATGDQVYAMFEHSLRSLPKMDEA
GQVILDENGLPALGANGGFLQTSESIKVYYDSNKKGANPEASTAGERILRVKILNETT
GKYEDLKRDKTYYVTTNDFLAAGGDGFTMLGGSREEGPSMDEIVIDYLREASQLRLVR
AAEKVVDLSKYAKELPGERIISMTEADFNKMEKPVEPGKPVEPGKPVEPGKPGEGNGG
VGEEGQGSGNGSGGTGTGSGNVGTNTGTNTGTGGSQATNGKKPTTNFPATGESEFSWF
FSGFVLIVVGGAASYVTRRKKVS"

CDS 3208247..3209308

/locus_tag="JFBMFIFI_03000"

/inference="ab initio prediction:Prodigal:002006"

/codon_start=1

/transl_table=11

/product="hypothetical protein"

/translation="MMIKEIIANLAIIISAIYLSSKGYGLPNEKNSSVLIRLLYGLFA
ASIGVILMNFSIRLLGDVIIDLRTVPIIIVTTFLGGLPGLVASVLIGVARLFISVSQV
AQNTAATYFILGIVLYFVDLTIKNKSIQNRLWVIYGIVMSLISVNFYINVIEKNRVFI
LLLVWVYTALGTIIAYGFAKEIQKNKNNAQMLEELSKIDHLTNISNRYSFDKIIMDYH

EKKEEYILLLLDIDKFKNVNDTFGHDVGDRVLRQVATTLSNTTIQHAIPYRIGGDEFA

ILIRGSYARQEIVDLIKIVKQDILFNEVKVNETESTKIRVSIGSSHTMKNKKFLSIEE

MYKEADVNLYEDKHSKILG"

CDS 3209476..3210219

/gene="hexR_1"

/locus_tag="JFBMFIFI_03001"

/inference="ab initio prediction:Prodigal:002006"

/inference="similar to AA sequence:UniProtKB:Q88P32"

/codon_start=1

/transl_table=11

/product="HTH-type transcriptional regulator HexR"

/db_xref="COG:COG1737"

/translation="MFNYEQIKKLSGLELTVYHYIIENVKAVQKMTIRELSEKSHVST

STILRFCSKMNCEGFSELKYKLKEETGDSVIEQLYDPSFQVASFFKKITEASFEEALK

QATKLICDAERVVFLGIGTSGILGAYGQRYFSNVAINSYSINDPFLPAPSKGVENSLI

IALSVSGETTEVVGQVIDLKKSGAKILSITNAESSTLAKIADLNISYYMPDEHARQYD

SAVNLTTQIPVVSLIEILAHRAEQELLAR"

CDS 3210515..3211813

/gene="gmuC_2"

/locus_tag="JFBMFIFI_03002"

/inference="ab initio prediction:Prodigal:002006"

/inference="similar to AA sequence:UniProtKB:O05507"

/codon_start=1

/transl_table=11

/product="PTS system oligo-beta-mannoside-specific EIIC

component"

/db_xref="COG:COG1455"

/translation="MGNWINTKLIPQILKFINVKPIVALKNGMLYTMPFTIVGSVFLL

LANLPVESWAKWVTDSGFGAYFNQAYGASFAIMAIFAVMGIAYSYVKSEGYEGMAAGM

IALVIFILTMSSSITDPETSVTIGNIINKDWTGGKGMISAIIIGLIVGSVYSWFMKRD

IRIKLPESVPENVANSFTALIPAAVLITGSLGVYIFFDKVFNLTMIEWIYKVIQTPLQ

GITDSFGGALMIAFLVPFLWFFGVHGSTIVGGIMGSLLQTNSLENQAIIDSGKELTLA

NGGHIVTQQFMDQFLTVTGAGMTIGIVVFMVFFAKSAQFKELGKMSLAPAIFNINEPI

IFATPIVMNPLMVIPFIATPVVSATITYFALYSGLVPLFTAVQVPWTTPPIISGLLIG

GWRAAVLQLVVLVIGFFIYLPFIRKVDSMNIDVEAGKPIV"

CDS 3211858..3213288

/gene="bglA_2"

/locus_tag="JFBMFIFI_03003"

/EC_number="3.2.1.86"

/inference="ab initio prediction:Prodigal:002006"

/inference="similar to AA sequence:UniProtKB:P42973"

/codon_start=1

/transl_table=11

/product="Aryl-phospho-beta-D-glucosidase BglA"

/db_xref="COG:COG2723"

/translation="MSSLRKDFLWGGAVAAHQLEGGWNKDGKGVSVADVMTVGANGVP

REITNGVLQGKNYPNHEGIDFYTHYKEDIKLFAEMGFKCFRTSIAWTRIFPKGDESEP

NELGLKFYDELFDECLKYGIEPVITLSHFEMPYHLVTEYGGWRNRKMIDFFVRFAEVC

FTHYKDKVKYWMTFNEINNQANYNEDFAPFTNSGIAYQPGENREKIMYQAAHYELVAS

ALAVKIGHEINPDFQIGCMIAMCPIYPLSCKPEDMMMSVSAMHKRYWFTDVHVRGYYP

AYLEKYFQRKGFNLDITVEDKLLLLEGCVDYIGFSYYMSFTTESKFDNPQYDYDESKD

LVRNPYVKASDWGWQIDPVGLRYAMNWFYDRYQLPLFIVENGFGAIDQLNSDGTIDDD

YRVDYLKAHIKAMKTAVEEDGIDLLGYTPWGCIDLVSAGTGEMKKRYGFIYVDKDNEG

QGTLKRSKKKSFDWYKQVIATNGEQL"

CDS 3213318..3213635

/gene="ptcB_3"

/locus_tag="JFBMFIFI_03004"

/EC_number="2.7.1.205"

/inference="ab initio prediction:Prodigal:002006"

/inference="similar to AA sequence:UniProtKB:Q9CIF0"

/codon_start=1

/transl_table=11

/product="PTS system cellobiose-specific EIIB component"

/db_xref="COG:COG1440"

/translation="MEKKTIMLACAAGMSTSLLVKKMLEAAKNQGIDADIFAVSATEV

DDNISTKPIDVVMLGPQVRFLKDDMTKKLEPKGIPVAVIDMADYGMMKGDKVLATALA

MLG"

CDS complement(3213681..3215048)

/gene="allB"

/locus_tag="JFBMFIFI_03005"

/EC_number="3.5.2.5"

/inference="ab initio prediction:Prodigal:002006"

/inference="similar to AA sequence:UniProtKB:P77671"

/codon_start=1

/transl_table=11

/product="Allantoinase"

/db_xref="COG:COG0044"

/translation="MSYDLLIKNGLVILENEAIETDVAVKDGKIAAIGNNLSAASETI

DATGLIVSPGMVDAHVHITDPGGGYRDQWEGYLTGTKACAKGGVTSFMEMPLNQVPAT

VDGKSLQIKYDAGKNKLTSDVGSFGGLVPFNLEHGIEELNDGGVAAYKCFMATCGDRS

IDGDFMNVDDYSLYEGMKHIAKTGKVLAIHAENAAITDKLGELAYKNGETTLAAYVAT

RPVFTEVEPIKRAILFAKETGCRIHICHVACPEGVDEITKARNEGVDVTCETCTHYLY

FDTSELDAIGPVVKCSPPIRDKENQNGMWEKVLAGEIAFVTSDHSPCTPDLKATDNAF

EAWGGISGVQNNVDVLFDEGVQKRGMSLTQFADIIATAPAKRYDLDAKGSISIGKDAD

FVLIKPNAPYTLKAEDLEYKNKISPYIGREIGAQVAQTILRGISIYSQETGVTEAHPG

EFILK"

CDS complement(3215089..3216555)

/gene="ybbW"

/locus_tag="JFBMFIFI_03006"

/inference="ab initio prediction:Prodigal:002006"

/inference="similar to AA sequence:UniProtKB:P75712"

/codon_start=1

/transl_table=11

/product="Putative allantoin permease"

/db_xref="COG:COG1953"

/translation="MAEKDVFAHEADIEKYKERGYNDDLLPKTKDRRNMNAKNYFTLW

MGSVHNIPNYTAVGGFLFLGLSPINVILALIISSMAVAAFMVYNGRAGSKYGIPFAMH

LRSTYGDLGAKLPGFLRGCVAAIAWFGLQNYAGSLALLILIGKIWPSFLTLGGDFNFF

GLSLPGLIAFTIFWAANLLIGIGGGGALNKFTAILNPLIYVVFGGMAIWAIKVGGGIG

PILAFTPSGADVQNNAPLFVYLIIITSVLSVWAAPGASVSDFTQNATSTRAQTIGQTA
SFLVAYIIFAFSSVAILIGGSIHYGVQEWNVLEIVEKWDSLPAICLAMLVFLLTTIST
NATGNIIPAAYQLSALFPKTINYKKGVIIASVISYLIMPWKLMENANSIFAFLNIIGA
VLGPVAGVMLAHFYFVKKQKIDLNALYMDTKADNSQNHYRGINKGAYVATIVALLVSI
SGQFIPALQVISSLSWLIGFGLAFILYLVLKKFIPEKV"

CDS 3217316..3218797

/gene="guaB"
/locus_tag="JFBMFIFI_03007"
/EC_number="1.1.1.205"
/inference="ab initio prediction:Prodigal:002006"
/inference="similar to AA sequence:UniProtKB:P0C0H6"
/codon_start=1
/transl_table=11
/product="Inosine-5'-monophosphate dehydrogenase"
/db_xref="COG:COG0516"
/translation="MSNWETKFAKEGYTFDDVLLVPAESHVLPNDVDMSVKLAKNITL
NIPLMSASMDTVTDSKMAIAMARQGGLGVIHKNMSTKQQADEVRKVKRSESGVIIDPF
FLTPDHLVSDAEELMGRYRISGVPIVNNMEDRILVGILTNRDLRFVTDYSIKIDEVMT
KDQLVTAPVGTSLKDAEKTLQKHKIEKLPIVDAEGRLSGLITIKDIEKVIEFPNAAKD
EHGRLLVAAAVGVTTDTFERAEALLDAGADAIIIDTAHGHSAGVIRKIKEIREHFPEA
TLIAGNVATGEATRALYDVGVDVVKVGIGPGSICTTRVVAGVGVPQLTAIYDAAEVAR
EYGRTIIADGGIKYSGDIVKAIAAGGHAVMLGSMLAGTDESPGEFEIFQGRRFKTYRG
MGSLGAMEKGSSDRYFQGGTNEANKLVPEGIEGRVAYKGGVSDIIFQMIGGLKAGMGY
VGAADLKYLRDDAQFIRMSGAGLRESHPHDVQITKEAPNYSLQ"

CDS 3219429..3219644

/locus_tag="JFBMFIFI_03008"
/inference="ab initio prediction:Prodigal:002006"
/codon_start=1
/transl_table=11
/product="hypothetical protein"
/translation="MKIEDFWMDMYSFYVIFITNEDVQIRKLLFLQENHIEHDQICAI
IKSKFHNVNRVLSIEEWDAGLALKQSR"

CDS 3219681..3220658

/locus_tag="JFBMFIFI_03009"

/inference="ab initio prediction:Prodigal:002006"

/codon_start=1

/transl_table=11

/product="hypothetical protein"

/translation="MKTIKHVLLWGVSLFCFFSFTVTSFANTATSQAGISFTEKEDMG

AGNTSLPKNKDLPNTGVDRTSSKLPQTGETAEGNLLITGLVFLGAAIAVEKLRTKRGG

RRMKLKTMTMLGLIGSGMLLGASTSTAAPQDVVTGSEDGKGGTSHGYINLTPGDSDTG

KTDPTDPTDPSGETGNEGILTIDHVVPLLFSSHKLEGKEQVYTSVVDRPNVQVTDKRG

EEAGWNVQVSQTAFVDQTDATKTLKGAKLVLPVGTLKDVGNVSLAPELYAVEVNDAPA

TFMNAKTGSGAGTWTNFFDKDEIKLTVPAGNKKGEYMSTVTWTLMDAPK"

CDS 3220684..3221340

/locus_tag="JFBMFIFI_03010"

/inference="ab initio prediction:Prodigal:002006"

/codon_start=1

/transl_table=11

/product="hypothetical protein"

/translation="MKLAKYSIVGLGLLMSVSILGNQSVKATEASAGSSKANFELQAG

DDTTVPELLDPEIQPPTLNKGPLSLDAVSSFNFPTKKLGSESQAPLEATPVEGTKLGL

QVTDSRGQDLGWNLKVSATAFETADKKLTLKGAVMTIPEGKLTTGEGVDPLLTPSAFK

VNLSPTATSIMSAATTQGRSTWVNSFEGKGEKVTLAVPSGNKVASYVSTITWSLEDAP

"

CDS 3221376..3222074

/locus_tag="JFBMFIFI_03011"

/inference="ab initio prediction:Prodigal:002006"

/codon_start=1

/transl_table=11

/product="hypothetical protein"

/translation="MKKSSLIITTFALASFAFGGAKVQAEVSSTHTSENKINFTAGED

VVTPPVDPTNPDNPNVPNPVDPTDPTNPGTGQTGPLSIDYVSNIKFGEHKITGKDIAY

KAKNANPFVQVTDLRGAGDGWNLSAKMTEFKSGNKVLRGATLAFKDGVVKAGSSSNIS

VAPTKTDVLFDNTDSKPFMNAMDKGGRGTWLTVWSGTDQANEAVQLNVVAGTPEANTE

YTSSITWELEDAPK"

CDS 3222159..3223295

/locus_tag="JFBMFIFI_03012"

/inference="ab initio prediction:Prodigal:002006"

/codon_start=1

/transl_table=11

/product="hypothetical protein"

/translation="MKKIISILVVCLIGLQHMPMIGEAAETMAYSVKANIPENQINKT

LTYFDLKMEPNQRQDITLTVSNSSDEKATILISPNVAMTNQNGVIDYSKADEKVDSTL

KNPVTSIISKAQEVTLEPKETKEVPFTIQMPEKEFDGLILGGFYISKKEDNSNTKDKE

KDVQIKNKYSYVIGLQLRENTNEVKPVMELHDIKPALLNYRTAVTANLQNTEATIMKD

VSINAKVMKKGSEKVLHETDKKGLSMAPNSNFDFPINWDNQSLDAGTYRLQLIAQSGE

DKWEFDKEFTISAKDSTDLNKEAVELEKTEPNWILIISVVVGMVLVLILVLVFFINRH

QKKKAAEKRARLMKQRKRKKQQELRKRKHLSEQIKRPNRKIRKE"

CDS 3223431..3225953

/locus_tag="JFBMFIFI_03013"

/inference="ab initio prediction:Prodigal:002006"

/codon_start=1

/transl_table=11

/product="hypothetical protein"

/translation="MRINKILLALVGAGVLMISLNQVVKAVSPAPPLSLPLDNVFTVP

KGANSSVDKNITIITEDTKSQVGSIFSTEGNKLDLTQSFHAEMYVYLGDKRETAADGM

TFVMHADKARAANYTGGRGSQLGVYAAQGSGGVYGLKEQIERSFAIEFDTYHNGDFNA

DKMDIEVGKNENKGHIAYAFPDLLSSYNLKDDKVVSLKHQGLYYPTDYLSNGKWHLFS

VDWDAEKRIFAYDFDDAPTVSVPINPSTIFGTNSVYWGFTGSTGGFSQESKVAFKQIP

GLVNVSSNMKITKDGKDITDTGVSGTEGDIKIQYDLKYHDGKQDMLRPVFNLDVSDFL

SFTPGTLTVNGVVVSDDNFVDGKLSYSLPTNLNKIEDTLTILFEGTPKIVTDKDIKAS

LNYSITADNYFGNNMKSTFPIKKVDIVTRADFENQSWLINEINNQLAPKKLDIDVYEV

DLAKITSIDNTNTSGTVYPTEYIPASITKLPNLATLKIENLNLVGRLPAELADLIKLK

NLSISGNNFVGGIPSSFGKMSQIELLNLNNNHLKGTVPLNLGLLPNLKKISLHDNPLS

GQLPDFPMNMEQITLNNTQITYNLATIPSFLGKMEGNNYSNTFIAGLKLSGNVEVNSK

NAQIKPFNKSDSGYFNLKVNQEDTTQVLYDEHIYTIKNTVDGTVYYKGKKDVEATIPY

KKDISYTVILDDAEKNPNNVFIVLGKKRELKFQEIPVFLSLKIKLGAEKKAVMKEGNV

LIFDDRENSKWKLSITPTELTQGERKLHGEYIYTNKSGVSYSILNGQKFLLETGESDS

VNEVIPISDTWDNKYGLSYKAYRSNIIGEYQGDVTWTLEDAP"

CDS 3225974..3226165

/locus_tag="JFBMFIFI_03014"

/inference="ab initio prediction:Prodigal:002006"

/codon_start=1

/transl_table=11

/product="hypothetical protein"

/translation="MVLKKSQTQDVVINGTSQKIAIGSETFPTIQWAEAAELLLKVSP

DAKVGSYQGEVTWLLSDAP"

CDS 3226796..3230521

/locus_tag="JFBMFIFI_03015"

/inference="ab initio prediction:Prodigal:002006"

/codon_start=1

/transl_table=11

/product="hypothetical protein"

/translation="MSKHKIKIIFIFILSLATATVYGNYAFFKGHIFAETNESTPRSE

ATNVEKKEEQPTNQEIGTELDTGWEIGTAADYFEKKGNKISWEKGAWWYTYDKGSWVF

RDSIEATLIVNGNFLSGTSENTNGGFLWANANESIYTVNHEKSMLKRSFPYKTYNIEI

IQQLLEDGSVEISYQVTNNNLEAQKIGVSQYVDIGDNSPIRVLNDFKGLNVSNVKSLA

MIPDPETMPNWAASSYGSSLMNFTPYSSNTADGLGWESGKRYRNILAVLTPPVKLKEN

QPMDLSDSGAAMKNPGIIVLPKETAVFKQRLKYGKFIPPALTVNQKSGDLYTSESFEI

TGTVSAVSSSNYRLYAELDDAWKTLVPLKDFKDIPLQEVQSYKASIEGGKISAGNHTV

TIVAIDEYGARSDVQKIDFLIREVSGTPIVQKVKLGEELTQDLTKLFKDVKGTGTKLK

QLAPFDSTKIGFQWVDATLIDDNLKEEIVKVPVTIYDPQSTIFNDSDNLILNVKNAKL

TIAEINTAIERNSLDQLILEKSDANAWQMEDGKETNLSITTQNSKPQLGRYEATIRAT

KKGTAKFVEKKISVIVTKEPLESGWELGASTDFVEKNGNKINWNKGNWWYTYNGSPWV

YTTPTMSTIEATLIINGSFLPGTSEERTKGFLWGNQVESLYTRNTATNTLKRSFIYQT

YQIDLIQQLLEDGTAEVTYQVTNNNPEAQRIGVSQFVDLVDASPPVVSVLKDFKGFHM

LHYVQPVVVMPDPETMPNWVVSAQSTLKNFQQYNSQTVDGVGWESKKHYWNGSKPLNP

PLELQEGQAVNNLGDLGMAMKNPGVTVLPGEATTFKQRIKFGGMVAPEITLDQKIESL

YKNESIDITGTISDINNQSYRLYLEMDDKNKSMIPLLDYADVPFNEVQNYQAKIEGKL

FSAGKHIVSILGIDEYGSRSVAKQIELTINELSGEPKIQKVKLGEAISNDLKNLFKVV

KGTNVTLKSPLSIDSSVIGFQWVEATLIDNQQKEVTEKIPVNVYNSESTVFDDVTNSA

LDAKNTSFDLVDVRQSDQEGTLDELVYKKVAPKAWNIADGTELPIELIANEIKPILGS

YVATFKATREDSSTNIQKNSDIVVGGVLKFKEVPETLDYKSAKINQKIQYIERKQPNW

NMSLENTIGSNWSLFASVTPFENQSKDKLNSSLVYKNEKMEEVAINDTSQKIATGNET

YSTIQWAESAGMLLKVNPDAKIGRYQGEINWVLSDAP"

CDS complement(3230571..3232010)

/locus_tag="JFBMFIFI_03016"

/inference="ab initio prediction:Prodigal:002006"

/codon_start=1

/transl_table=11

/product="hypothetical protein"

/translation="MKKLLDKPFHLILKLLEHFYKKTPQETINYYSDFLNVDRRTILK

IITDLERDIADCQWENQLTLEVTETKIIATFSTNFSLENFYRYYMERSLCVELVQSIF

KEAEISLDQIIENFFVSRTTFYRRITPLKEVLAEFDLELDFTKKQFLIGEEKQIRYFF

SVFFWEIFRSTGEYKHPDLKDTEYLNRVKQDLNLSIPHFLYFQLYLNISLTRISQGYL

VSEVIPYPIKEINYSYAQFKNLTAPYFNKLMPAQQSLEIHSLYFLCVTSTIYPKKVTN

LIPIQKIPELKFAQQWILYYTNYFDTILSNEDYVYLYLNLSLIYEKNKTFHGGTSSFG

VNSVTEVLREENPDVLRYSMQFFSYLSTQEEEFHIAPFQVLSFTLLIRRLLINGAPPL

RLLVCSKVGQEETDWIQSSIKKISSVPVEFYSSTSPDLDLIISDFPLPPDLISVSLDK

LFLWLTFPSPKEWRSLLKKLEEIYYTKLN"

CDS 3232496..3233827

/locus_tag="JFBMFIFI_03017"

/inference="ab initio prediction:Prodigal:002006"

/codon_start=1

/transl_table=11

/product="hypothetical protein"

/translation="MNRNQKISRGVFILLGTMIVLGGCGANNNKNETKKEVPSESSVA

KKEVELKVGNWEKTNYAALKTAISEHGTLSEDYQKSPKPYAVFDWDNTTIINDIGEAV

LTYQLMNLRFKMNPTELETALKTNIPADDFKEEWNNKAGGAVNIEKIAADIGSSYQAL

YQNYEGLAGNQSLEEVKKFPEYVDFSSKLRYLYEAIGSSFSSDISYPWVTYLVSGMTS

KEVQDLSQEAADYWLEQDLGEETWTSPADLKGQAGELAVTFKTGIRTVPEMQDLYQTL

MANGIDVYVVCSASFIDVIVPFATHAKYGYQVPKEQVYAMQLKKNAEGIIQAEMDPDYD

QTQGKGKTDTIIKFIAPKHENVTPILVAGDSNGDVAMLSDFPDLQVGLIFNRVKDGGI

GKLAKEATESAGNKDQRYFLQGRDENKGQLIPSSKSILLGETEEKLLNVAK"

CDS 3234039..3235589

/gene="murE_2"

/locus_tag="JFBMFIFI_03018"

/EC_number="6.3.2.7"

/inference="ab initio prediction:Prodigal:002006"

/inference="similar to AA sequence:UniProtKB:Q97PS1"

/codon_start=1

/transl_table=11

/product="UDP-N-acetylmuramoyl-L-alanyl-D-glutamate--L-

lysine ligase"

/db_xref="COG:COG0769"

/translation="MTKSISMKEIIQLLKQYNQFLGFMANNEMNHSKLMTSVFEQIHF

DSRKVVENTLFICKGINFQSDYLIQAIHAGATGFLMEEKQRKELDMTLEIPGILVKHS

QKSMALIAMHFYDYPQKKLRIIGVTGTKGKTTTSFFIKRILDQQNGNKTALFSTVFTD

VGLGQTPAELTTPESLDLYRLMDQAVGKGYQDLVMEVSSQAYLMQRVYQLEYDLGIFL

NISPDHINENEHVDFADYLKHKLKIFKHSKAVVIHTDTLALSEIKKTAEESTPNVIWA

GEAEGVAGTNYSLAKVEHQAESVQFQIVEKKLFEAEQRRLATRFCLNIVGAVNLENAL

VAIAATRYLGCSEAEIKAGLASAKVPGRMEIIPHKEAMIIVDYAHNAISLQKLLMWTK

ARYPDGQQIVVFGCPGNKGFSRRQDLGETAAQLADYVILTEDDPGFEDPRAICEEIGS

HLNGFVSYEFILNREAAITKALKRVQAGDVLLVVGKGSDAFQLRNGVRESYVTDSQVI

KDQLQFVL"

CDS 3235713..3236402

/gene="srrA_7"

/locus_tag="JFBMFIFI_03019"

/inference="ab initio prediction:Prodigal:002006"

/inference="similar to AA sequence:UniProtKB:Q9L524"

/codon_start=1

/transl_table=11

/product="Transcriptional regulatory protein SrrA"

/db_xref="COG:COG0745"

/translation="MKILVVDDDKEIVELLSIYIKNEGYEVEKAYNGKEAMTKIVTNP

DIDLMVLDVMMPKMDGIEVVKELRKESQMPVLMLSAKTTDMDKIQGLITGADDYVAKP

FNPLEVMARIKSLLRRSNYQVTNDEPDILEIGPLVIQKDSHEVTTISGKSIQLTALEF

GILYLLASHPNRVFSADEIFERVWQQESLVSAKTVMVHVSHLRDKIEEATGGEKVVQT
VWGVGYKIEAR"

CDS 3236455..3237588

/gene="sasA_3"

/locus_tag="JFBMFIFI_03020"

/EC_number="2.7.-.-"

/inference="ab initio prediction:Prodigal:002006"

/inference="protein motif:HAMAP:MF_01837"

/codon_start=1

/transl_table=11

/product="Adaptive-response sensory-kinase SasA"

/translation="MKLTGKEKSELIFEGFITAGLIYLLYLAVLIIFDQLIVVSPDVR
DSIWIFKTSVTIGNTQILSYKIIFIAFLVITGIITIFWRLKRRYKQMQLRHVISELHY
IAEGHYDHRIPFELPGDMNKVVDSINVLVDSTVTAMEEERKIEQSKDELITNVSHDIR
TPLTSIIGYLGLIEDQQYQSQEELLKYTHTAYIKAKQMKILVEDLFEYTKVRQHTTPL
NLTEFDMLKLLDQLAADFELDAEKKGMTIEVEPGSAAIPMEADAEKLVRVFNNLISNA
LKYGVGGKKITIEAQKVGKEVIIAVNNDGPIIPEEALNQLFDRFYRVEESRSQETGGT
GLGLAIAQSIVTLHGGYIYARSSQELTSFVLHLPIRQQPYEKG"

CDS 3237721..3239040

/gene="dacA_2"

/locus_tag="JFBMFIFI_03021"

/EC_number="3.4.16.4"

/inference="ab initio prediction:Prodigal:002006"

/inference="similar to AA sequence:UniProtKB:Q05523"

/codon_start=1

/transl_table=11

/product="D-alanyl-D-alanine carboxypeptidase DacA"

/translation="MKKNNKLTLVFALILAVGTVFPSLLIGGKEAFAAEAPTINAAAA
FAIEPKTGKVLLNQNGDAQLGIASMTKMITEYLVLEAIKEGKLTWDQKLSIDDYSYNV
SQNNELSNVPLRKDSQYTVKELFEAMAIYSANAAAITLATAVSGSEPAFVDAMREKVK
SWGAKDFYLVNATGLTNSDLHGNIYPGSADTDENTMTARDMAIVAQHLLNDFPEVLDT
AKIPRMDFRKGTEDEIHMENWNWMLPGLIYGRDNVDGLKTGTTDIAGACFTGTAEENG
MRVITVVMNAGDGETNKGARFEATSQIMDYAFANWEMKDIVKKGDSNKALKPLTVDKG

KEDSVKLIIDEDLKMLVQKDTDVKNLNVTFTMKDGLLTSDNKIEAPVKKGLEVGTAQI
VPANDTLGYVDGSQGKSVKVVTENAVEKAGFFSLMGRNISEFFSNLF"

CDS 3239255..3241867

/locus_tag="JFBMFIFI_03022"

/EC_number="7.2.2.10"

/inference="ab initio prediction:Prodigal:002006"

/inference="similar to AA sequence:UniProtKB:Q73E41"

/codon_start=1

/transl_table=11

/product="Calcium-transporting ATPase 1"

/translation="MEQTKNSIYSSDISKKLEVNVNQGLSNEDVQQRLLKYGKNELVG
IKEKTLWDHLLHSLKDITIIILLVATGISTYVAFTSHPDDFTEPVVIFLIVLLNIFLG
IREQVKAEKSTKALKSFHVQEAKVVRDGKLGLYETGELVPGDIIQLETGDKVPADAVI
LDEMNLFVDESLLTGESEPAAKDSSYQPSGNEGIGDRKDKLFSGSLVVSGKATAIVVE
TGMTTEIGKISSLLETETTLAPIQIRMQKLGKVLSIVALLAALSAVGIGWVRGETVPE
LLMIAISLAVAAIPEVLPVVVTISLSYGISTMAKKNAIIRTPTAVETIGHVSVICSDK
TGTLTQNKMEVEKIWAGSEAAKSVKHELNQTEKELIKYFYLASSANQNQKIGNPTELA
ILDLALQEIEDKQALEQNYQKIHEIPFDSSRKRMTVVYQTVEGYLSLTKGAFDRLDLA
LKNKEEDEKIKAVHDEFANQALRVLGLGVKKFKQLPADLSDEFLESNLEFAGFVGIID
PPRKESYAAVKKASEAGIKTIMITGDHLITAKRIAEDIGILKAGLKVMDGTELASLTD
QQLEQVIDEYRVFARTSPEDKIRIVKALQNKNEIVAMTGDGVNDAPALKAANVGIAMG
SGTDVAKEAADMILVDDNFSTIVAAVQEGRRVYSNIRKSIYAMLGCNLSAVMIVLISL
LMGWGAPVTAIQLLIIKVVADGIPGFSLCVEKSESESMKQPPVVNGTSIFHDGLLKKI
GTISLVFTVVTLIAIYIGKFVSFNGRVTPSLEIAQSMTFVVLGLTTIVHMYNCRSRHS
VFKTNFFSNKLLVTTTISGAMIIILLPMIPFTATIFGLVPLSSYHWLVIGILSMIPLI
FIEIQKGMNRFGRL"

CDS 3242283..3243566

/gene="serS"

/locus_tag="JFBMFIFI_03023"

/EC_number="6.1.1.11"

/inference="ab initio prediction:Prodigal:002006"

/inference="similar to AA sequence:UniProtKB:P99178"

/codon_start=1

/transl_table=11

/product="Serine--tRNA ligase"

/translation="MLDIKKLRSDFATVEAKLATRGVKKELLENFVELDEKRRELIVK

AEKLKKYRNTVSGDIALLKRNKENADDKIKEMREVGDEIKVLDDELVAIDTALSEIAA

TLPNLPHDSVPVGADEDENVELRRWSTPKTFDFEPKAHWDIAEELDILDFERGAKVAG

SRFVYYKGLGARLERAIYNFMLDLHTGEHGYQEMITPYMANAKSMYGTGQFPKFKEDV

FQIENEDLTMISTAEITLTNYYRDEILADEQLPIYFTALSPSFRSEAGSAGRDTRGLI

RLHQFNKVEMVKFSKPENSYDELEKMTANAEAVLQKLDLPYRVLSLCTGDMGFSAAKT

YDLEVWIPAQDVYREISSCSNCEDFQARRAMIRYRNEETGKPEHVHTLNGSGLAVGRT

FAAILENYQQADGSVKIPDVLIPYMGGVTEITKAK"

CDS 3243704..3244234

/gene="msrA_3"

/locus_tag="JFBMFIFI_03024"

/EC_number="1.8.4.11"

/inference="ab initio prediction:Prodigal:002006"

/inference="similar to AA sequence:UniProtKB:P54154"

/codon_start=1

/transl_table=11

/product="Peptide methionine sulfoxide reductase MsrA"

/db_xref="COG:COG0225"

/translation="MTENHEEKAIFAGGCFWCMVKPFDTQPGIKSVVAGYTGGHTVNP

TYEEVCSETTGHTEAVEITFDPTIFSYQKLVEIYWQQTDPTDASGQFADRGSSYRPAI

FYLTEEQREIAEASKKALAESGRFKQPIVTEIVPAMPFYPAETYHQDYYQKNPMHYNR

YRQGSGRAGFIEKAWK"

CDS 3244417..3245049

/gene="pspB_2"

/locus_tag="JFBMFIFI_03025"

/EC_number="3.1.3.3"

/inference="ab initio prediction:Prodigal:002006"

/inference="similar to AA sequence:UniProtKB:D3DFP8"

/codon_start=1

/transl_table=11

/product="Putative phosphoserine phosphatase 2"

/db_xref="COG:COG0406"

/translation="MAKLYFVRHGKTEWNLEGRFQGGYGDSALLEEAIEAAKETGKRL

SEISFAHVYTSPQKRAKDTAEYIIEESRLNLPLTEVDGLREIGFGDWEGQPFSYAEEN

HLEAYINLKAHPEKYDPSAFNGETYEELIERSQKAVEKAVANHPGEDLLFVAHGVTLL

TIMHTLIGKETGDIRSKGLLSNTSISILEIDEKAGYSIVSWNDTTHLEVK"

rRNA 3245644..3247195

/locus_tag="JFBMFIFI_03026"

/product="16S ribosomal RNA"

rRNA 3247589..3250506

/locus_tag="JFBMFIFI_03027"

/product="23S ribosomal RNA"

rRNA 3250768..3250877

/locus_tag="JFBMFIFI_03028"

/product="5S ribosomal RNA"

CDS 3251184..3252539

/locus_tag="JFBMFIFI_03029"

/inference="ab initio prediction:Prodigal:002006"

/codon_start=1

/transl_table=11

/product="hypothetical protein"

/translation="MSQKKTIIGLIAAIVVVIAGIYIFKSIGYQNKFLPNTVVDGLAI

ENKTVSEANNELKNHYQNKEFSATENGKELFTFKGADIGITDDFTKDLTKLKNDQNGW

SWPVRMLKKTSTKSELKDVTYDQATFDQFVENLPLTNESRVKPENAKVEKTAAGFTIE

KEVMGDTFDLDKVKKVLAESINSGNTKIKLETSYQKPTVMSDDAGLKERLAKLESLSK

LTITYNISGQKETVPPATLLSWLSESPEGEVTVDQAGVKVYLQGLSDKHSTYEKTRTF

KSTRRGEVQVPAGTYGWTLQVDKEAPALAADILTKTDLQDRKPIVAGSGYGVDVGNTY

IEVDLVNQHMWYYREGALVLETDVVTGKPATPTPAGVFSVWNKERNATLKGEDYATPV

DHWMPIDWNGVGIHDSPWQPAYGGTLYQTVGSHGCINTPPAVMVQLYEKADLGTPVLV

F"

CDS 3252684..3254120

/gene="bglH_8"

/locus_tag="JFBMFIFI_03030"

/EC_number="3.2.1.86"

/inference="ab initio prediction:Prodigal:002006"

/inference="similar to AA sequence:UniProtKB:P40740"

/codon_start=1

/transl_table=11

/product="Aryl-phospho-beta-D-glucosidase BglH"

/db_xref="COG:COG2723"

/translation="MTNESNFPKNFLWGGAIAANQAEGAYLTDGKGLSPVDILPDAAH

GRWEALSNPKKAIETKYDFYPSHESIDFYNRYKEDLKMLHEMGFKTFRTSICWARIFP

KGDEKEPNEAGLKFYDDLFDECHKYGIEPLVTINHFDTPVALFENYGGWKNRQLVDFY

LNYCDVIFKRYKGKVKYWMTFNEINMILHIPFFGGGMDVSEEENPAQVKYQAAHHQLV

ASAMATKLGHEIDPDNQIGCMLAAGATYPNTCNPNDVWAALEADREGYFFIDVQARGY

YPSYSKRFFKENNIKLEIADGDLQVLKENTVDYVSFSYYSSRLTSADPEVNAETEGNV

FATLKNPYLKASEWGWQIDPLGLRVTMNQIYDRYQKPLFVVENGLGAVDTVEADGSIN

DDYRIDYLREHVREMGEAIADGVECWGYTPWGCIDLVSAGTGEMKKRYGFIYVDKDND

GNGTLDRKKKKSFDWYKQVIASNGVTLD"

CDS complement(3254328..3256640)

/gene="chiA1"

/locus_tag="JFBMFIFI_03031"

/EC_number="3.2.1.14"

/inference="ab initio prediction:Prodigal:002006"

/inference="similar to AA sequence:UniProtKB:P20533"

/codon_start=1

/transl_table=11

/product="Chitinase A1"

/translation="MKKNNRKLFGTVSALALTLTLATTTIGSLAAPLPAKAAEATATT

APYRNVMYYGDWSIWGGEGNFYPKDIPASQLTHLNFAFLDFDSSGNLKFTDKDAAVGA

PVGQEGVQWGGANAGVINALQDLRVANPNLKLGVSIGGWSKSANFAPVAANPTARANF

VANVMKFIKYTNMDFVDLDWEYPASVREPDKVDNKNDEGTPNATPADKANYITLLQDL

RTALDKQGTDIGKRYELTVALPASQNTLANGVDIPALFKIVDFANMMTYDMRGAWSPT

SGHHTSLYGNPADPDYAQGLSVDQTVNYLTKQGVDSSKIVIGSAFYTRGWNKVAPGTN

TAQPGLFQAAEKNNKDADLSPTYGAPNEKALATGDGGRAGGVWAYRSISTLLTKSDGL

TEYWDDVAKAPYLYSKKTGEFYSFDNLRSIDFKTKYVKDQKLGGVISWMQSQDKPTTS

TKRDELTTGIKKGLFGDATLPTSTTIYAPLDIAVTVTPYSENGVGYEITVKNNEKANE

SNEVLKATELSFETIKLPKFTIPTNSNEVLSAGDYKAGQVSVANGNTVIDLSGVYDAQ
QIPQGSSYTFRLKSSAATADVARISEINLAQRMAKNSGELGKQLIFKNDQTTPQPDPS
DKEAPSIPTGLKTGTITGNQIDLTWTASTDNVKVAGYKIYRDGIEVGTTVEPKFSDTK
LTASTKYSYQVTAFDASGNSSAKSGTLAATTTADTTVPDPGTAPAWDAAKAYNAGDSV
TYGGKTYKAKWWTQNNIPGTEQWGPWELVN"

CDS complement(3256684..3258153)
/locus_tag="JFBMFIFI_03032"
/inference="ab initio prediction:Prodigal:002006"
/codon_start=1
/transl_table=11
/product="hypothetical protein"
/translation="MEKRIYLKGFLAGAVILAGLSFNGIKSHAATDDASVMPDISNKQ
ILMGFYHSWQSKGNDGYQRGTSKDLDLREVPEAYNVVAVAFMKGTGIPTFKPDGPTDA
EFRAQVGELNKQGRPVLISLGGADAHIELRSGMEQAFANEIIRLVEVYGFDGLDIDLE
QLAITAGDNQTVIPAALKLVKDHYRAEGKHFIVSMAPEFPYLKPNAPYAKYISSLEGY
YDFIAPQLYNQGGDGVSVGSDWIAQNNDSKKFEFLYYMADSLIHGTRGYIQIPANKLV
LGIPANNDAAATGFVKDPQAVYQAFDKLKTDGNPVKGLMTWSVNWDQGKNSAGVGYNG
GFANSYADLIGTGSTTPIPDTTPPTQPTALKADKVEFNQVSLSWTASTDNVRVAKYLV
FANGQLVGDTRSANYLATSLKANTAYDFTVQAVDAAGNKSTISAPLKVTTAAEPQTTW
SATAIYVGGDKVIYQGKTYQAQWWTQGETPGTAQVWKLI"

CDS 3258571..3260625
/locus_tag="JFBMFIFI_03033"
/inference="ab initio prediction:Prodigal:002006"
/codon_start=1
/transl_table=11
/product="hypothetical protein"
/translation="MLTNESIFLINSLTEQNGKSVKELARRYQISQKKLWYEIDQINI
ELAFLQLPKIQLETGRLAISNQLKEGWKAERERLKRRDIQFQEERIYLIILYTFIAQE
AISNSHYQDLLQVSKNSVLLDLKKVKSLCSSYHLEFSYSRKMGYHFIGSEQNIRKLAE
YAISKLLALSIGEWTLTLIFQLWQIIPSQHEISQELLALSKKYQVTFVQERLTEFIWL
LCLLKVRKQRKVILFNTKELVFIKKQPLHNLGQEVAKQLLDSDSEMEGAFVTTRLLSA
LQGTKQFHQDKKFKELTTAIIERVQVLTGTIYPDKNSLSRSLFEHLVPAYFRIFFEIP
LENPYGSQIKRDYSELYYLVEKGLKPLAESLGKAIPSHEIAYFTIHFGGQLPVIKRLV

PSLRALTICPNGISSSLMMNAQLKELFPQIKFHPVHSLDQAKLIPEIEYDLVFATTYF
PTTKKVYLAKPMLNPVEKEMLKRTVLDDFENQTGSQAVDVAALMKIVKKHTEIKNEKA
LYESLSSALYGIDSEQPQGGRTLTELLEKRFIQFSDASLDWQSAIALATEPLLKANFV
TEAYVTAMIENVKEMGAYIVLAPKVAVPHARPEDGVQQLGISLLHLSQPVNFNLTQEY
DEEREVQLIFVLAAIDNVGHLTALKQLSQILEDDEKIAALIATKDQDQLYQKLKDEGT
AN"

CDS 3260641..3260928

/locus_tag="JFBMFIFI_03034"

/inference="ab initio prediction:Prodigal:002006"

/codon_start=1

/transl_table=11

/product="hypothetical protein"

/translation="MIKVVTVCGNGIGSSLMLKLKLEEIAKENQIEIDAESIDSNAAA
GKVADLFVTVKEFADVFKDNQKVVFTRSYMNKKKIKEDVLPTLLELTETKK"

CDS 3260941..3262311

/gene="ulaA_2"

/locus_tag="JFBMFIFI_03035"

/inference="ab initio prediction:Prodigal:002006"

/inference="similar to AA sequence:UniProtKB:P39301"

/codon_start=1

/transl_table=11

/product="Ascorbate-specific PTS system EIIC component"

/db_xref="COG:COG3037"

/translation="MGILEFLKDVLSEPAILMGLMAMVGLIALKSPLHKVLTGTLGPI
LGYLMLAAGAGVISSNLEPLSKMIEVGFKIKGVVPNNEAITSVAQDLLGVETMTILIF
GLLFNLLIARFTRYKYVFLTGHHSFFMACLLSAVLGAVGFSGWQMILVGGFFLGAWSS
ISPAITQKFTLKVTDGDEVAMGHFGSLGYFVAGAIGQAVGKGSASTEDIKVPEKWSFL
RNTTISTALTMVVFYLVAAIAAGPEFVATLSNGKSPFLYAILCGLQFAVGVAIVYAGV
RMILADLIPAFQGIATKVIPNAVPAVDCAVFFPYAPTAVILGFVSSFIGGLLGMFILG
YAGSVLIIPGLVPHFFCGATAGVYGNAMGGRRGAIIGAFVNGLGLAFLPALLLPVLGS
LGFSNTTFGDIDFGVIGIVLGKVGEWMGAVGIYGLVAILLIVLILPTIIRPSKIAINN
GEMDVD"

CDS 3262344..3263057

/gene="tal"
/locus_tag="JFBMFIFI_03036"
/EC_number="2.2.1.2"
/inference="ab initio prediction:Prodigal:002006"
/inference="similar to AA sequence:UniProtKB:Q9WYD1"
/codon_start=1
/transl_table=11
/product="Transaldolase"
/db_xref="COG:COG0176"
/translation="MTQELTIKIYADGAEIDKMVEAYQSGKITGFTTNPSLMKKAGIT
SYTEFAEKAVAAIPDLPISFEVFADDFVTMEQEAEKIASFGKNVYVKIPISTTNGESS
IPLIKKLSEKGLSLNVTAILTIEQVTETVAAFKEGTENIVSVFAGRAADTGVDPMPLM
RKAAEICHSKQGTELLWASSREVLNIFQAQEAKADIITCTPEILAKLPMIGMDLKELS
LDTVKLFNRDIRALGYSIL"
CDS 3263395..3264663
/locus_tag="JFBMFIFI_03037"
/inference="ab initio prediction:Prodigal:002006"
/codon_start=1
/transl_table=11
/product="hypothetical protein"
/translation="MTKKVIQTALLIALFATPIVTTTTVFAEDFDTKIQEQDKKINEL
KNESQNTKTDLEKITTNVTQNEEKSKQILVDMQATQNKLDTLQQENQELTVKIDQRED
QLEKQARVVQVNGDTQNYIDFVLEAKSMSDIIGRVDVVAQMVSANRAMVKQQADDKAQ
VVKQEKEVAKKSDEQKVLAADLAKTQEKLQTQKLEKESIVAQIAADTATAEGDKNKFL
AQKVAAEKEAEDLRIAKVAADKKASEDAEAARVVQLANAKAAEAAKNTTVAAAPPAEN
PQGGGTPPVSTGAYGRPTNAPVSSSYGPRSGYDANGFHKGIDFGGAVGTPIYASMSGR
VVIAQYDGMPVSGYGIATVIQHDNGTWTLYAHQSSQSVKVGDVVTKGQQIGAIGATGQ
VDGAHLHFEIRTAQDGGMGNVVDPAPLLGL"
CDS 3265242..3266105
/gene="aroE_2"
/locus_tag="JFBMFIFI_03038"
/EC_number="1.1.1.25"
/inference="ab initio prediction:Prodigal:002006"

/inference="similar to AA sequence:UniProtKB:Q8Y9N5"

/codon_start=1

/transl_table=11

/product="Shikimate dehydrogenase (NADP(+))"

/db_xref="COG:COG0169"

/translation="MSERITGHTELIGLFATPIRHSISPQMHNESFARLGLDYAYLAF

EVGKEELPAAIQSIKTLGMRGANLSMPNKQLACQYMDHLSPAAKMAGAINTIVNDDGV

LTGHITDGTGFMSSLEMNGISIIGKQMTILGAGGAATAILIQAALDGVGKISVFNAKD

AFFATAADKIASIKAETGCDIQLHDLADEDLLRQEIAASAILVNATGVGMKPLEGQSL

ITDASLLRSDLVVADVIYTPSETALLKLAKAQGCQTLNGLGMLIGQGAAAFKLWTGKE

MPVAEIKELLF"

CDS 3266170..3267180

/gene="aroF"

/locus_tag="JFBMFIFI_03039"

/EC_number="2.5.1.54"

/inference="ab initio prediction:Prodigal:002006"

/inference="similar to AA sequence:UniProtKB:Q9WYH8"

/codon_start=1

/transl_table=11

/product="Phospho-2-dehydro-3-deoxyheptonate aldolase"

/db_xref="COG:COG2876"

/translation="MIIIMKSGATLEEINAVVSRVKEEGLNVQMNDGEERMVIGVIGD

TSRAHDLPFASYDGVDRTVQISSTYKLTSREFHPSPTVIDVGGVKIGDGNLVVMAGPC

SVESREQIIETAKIVKAGGAQFLRGGAFKPRTSPYAFQGLKEEGLKYMREAADLTGLK

VITEVMDVENLPLVCKYADMLQVGARNMQNFQLLQAIGKTGMPVALKRGISGTIDEWI

HAAEYIANEGNFNILFVERGIRSYEPYTRNTFDLSAVPAIKKLSHFPIMVDPSHGTGR

WDMVTPMAMAGVAAGADALMVEIHPEPEKALSDGPQSLTPENYMDMMNKVTKLSAMLK

EI"

CDS 3267209..3268276

/gene="aroB_2"

/locus_tag="JFBMFIFI_03040"

/EC_number="4.2.3.4"

/inference="ab initio prediction:Prodigal:002006"

/inference="similar to AA sequence:UniProtKB:Q9KNV2"

/codon_start=1

/transl_table=11

/product="3-dehydroquinate synthase"

/db_xref="COG:COG0337"

/translation="MEIKVTLPHHSYLLQIQAGLLEQCGDWVKSLWAPQRIVIVTDEN

VQKLYGQKVLESVNAAGFEASLAVVPAGETSKNLAVAEELYEAFLTAGLTRSDGVIAL

GGGVIGDLAGFVASTYLRGLHFLQIPTTLLAQVDSSIGGKTAVNLKQGKNMVGTFSQP

DGVLIDPLTLNTLEPRRVREGIAEIVKSAAIADLGLWNLLASFKDETELLEHAETVIA

ACCEVKRKVVEEDELDTGNRLKLNFGHTIGHGLEQVAGYGVCTHGEAVAIGMIQMNQV

AEMKGLTATGTTEKLKQMLQKFHLPIEWTPWEPEKLMAAMVHDKKASGDTVRIIQLKD

IGEATIVKIPLSELDEYVNKV"

CDS 3268329..3269495

/gene="aroC"

/locus_tag="JFBMFIFI_03041"

/EC_number="4.2.3.5"

/inference="ab initio prediction:Prodigal:002006"

/inference="similar to AA sequence:UniProtKB:P31104"

/codon_start=1

/transl_table=11

/product="Chorismate synthase"

/db_xref="COG:COG0082"

/translation="MRYLTAGESHGPVLTAIIEGLPAGTPLLTEDINKELARRQAGYG

RGGRMKIEKDQAIITAGIRHGEALGSPIALTVENKDWKNWKTVMSIEPIEEEKVGQRR

VSKPRPGHADLVGGIKYGHRDLRNILERSSARETTVRVAVGAIAKKLLHELGIEVVGH

VVEIGGVQANLKNKYTIDEIREGSEKSPVRCLDPEVEQEMMDKIDEAKKKGDTIGGVV

EVVVGGVPAGLGSYVQWDKKLDAKIARAIVSINAFKGAEFGVGFDMARKPGSEVMDEI

LWDENTGYTRGSNNLGGFEGGMTNGMPIVVRGVMKPIPTLYKPLMSVDIDTKEEYKAS

IERSDSCAVPAASVVAEAVVAWEVAEAVLDKFDGDSFERLKAEVAEYRRYANNF"

CDS 3269895..3271130

/locus_tag="JFBMFIFI_03042"

/inference="ab initio prediction:Prodigal:002006"

/codon_start=1

/transl_table=11

/product="hypothetical protein"

/translation="MSQLFEKKIKIREDKKRPINFFYFLARAKRLNKLFIKGVELLEK

QQVLIVGLGLIGGSIALDIKKEHPNTEIIGLDIQTDSLVMGHKLGIIDTIGTDLANEA

PKADLIIFCCPVKQTELLIKELVRLPLKASVLVTDTGSTKSSIVETAEILGAAGIAFI

GGHPMAGSHKSGVIAAKIDLFENAYYLLTPASMDEQNKLAVAQLQEWLQGTNAKFMVL

SPLEHDEITGMLSHLPHIIAAGLVNQTTEFSDIHPQARRLAAGGFRDMTRIASSDPVM

WTDILLTNKTVLLELLDDWKSSIGEMMGYLKDENHTAIYRFFDEAKETRDAMPIHKDG

ALPAFFDLFVDVPDYPGIISEVTGYLAEEEISVINLKILETREDINGILQLTFQNQSD

LERGKACLKNKSSYRCYEK"

CDS 3271142..3272428

/gene="aroA"

/locus_tag="JFBMFIFI_03043"

/EC_number="2.5.1.19"

/inference="ab initio prediction:Prodigal:002006"

/inference="similar to AA sequence:UniProtKB:Q9S400"

/codon_start=1

/transl_table=11

/product="3-phosphoshikimate 1-carboxyvinyltransferase"

/db_xref="COG:COG0128"

/translation="MKLVTNKNNLKGTIKVPGDKSMSHRSIMFGSIAEGTTRIKHFLR

ADDCMSTIAAFRALGVEIEETPEEIIVYGKGWDALKAPTEAIDIGNSGTTIRLLMGIL

AGRDFESTLFGDESIAKRPMNRVMLPLNQMGAHCVGSHETEFPPVTVRGTTSLHPISY

QMPVSSAQVKSAILFAALQADGETTIIEKKKTRDHSEHMIRQFGGEIFVDDLTIKVKG

KQKFTGQTMTVPGDISSAAFFIVAGLIASNSQICLTNVGLNPTRTGIIDVVEQMGGNL

TISDASRDTGKLAGTITVETSQLKGVEIGGDLIPRLIDELPVIALLATQAEGITIIKD

AAELKVKETNRIDAVVRELTTLGAKIEATDDGMIIYGKTKLHGGKVTSYGDHRIGMML

QVAALIIDDGEVELEKAEAVSVSYPEFFKDIVSLCN"

CDS 3272452..3272955

/gene="aroK"

/locus_tag="JFBMFIFI_03044"

/EC_number="2.7.1.71"

/inference="ab initio prediction:Prodigal:002006"

/inference="similar to AA sequence:UniProtKB:P0A6D7"

/codon_start=1

/transl_table=11

/product="Shikimate kinase 1"

/db_xref="COG:COG0703"

/translation="MDQIILTGFMGAGKTTVGEILSELTALPQIDIDSEIIAEQGCPV

SDIFEQKGEQGFRDLEHQKLKEVLQKKAIISTGGGIVLRPENREVLKSFSPVIYLKTD

PAIFLERLEGDTTRPLVQEKTPAEIRAIFEPRIQLYEETADFIIETDELNQAEVAAAI

LKVLAEQ"

CDS 3272998..3273846

/gene="pheA"

/locus_tag="JFBMFIFI_03045"

/inference="ab initio prediction:Prodigal:002006"

/inference="similar to AA sequence:UniProtKB:P27603"

/codon_start=1

/transl_table=11

/product="Bifunctional chorismate mutase/prephenate

dehydratase"

/translation="MKVAYLGPAASFTHMASEKAFPNAELIPSVTIPDCIKALEDGTV

ESAIVPIENTIEGTVNVTLDYLFHQANIPIQAELVLPIAQHLMVHPDNVSKWREAEKV

LSHPQALAQCETYLSDELDKAIREATPSTAYAARRVGEQVEPVLAAIAPRLSAKEYGL

TIVAEDIQELELNQTRFLVLSHQPVKIELPSTKEKISISVTLPNNMPGALHKVLAAFS

WRQIDLCKIESRPLKTTLGEYFFLIDLYVGDQEKLIDNAIEEIQLTGCKTKIFGRYSV

HTIPIS"

CDS 3274080..3275768

/gene="dppE_5"

/locus_tag="JFBMFIFI_03046"

/inference="ab initio prediction:Prodigal:002006"

/inference="similar to AA sequence:UniProtKB:P26906"

/codon_start=1

/transl_table=11

/product="Dipeptide-binding protein DppE"

/db_xref="COG:COG4166"

/translation="MKVNKKWFGIGLLTCSVILAVGCSNNKADDKGTTASSDTSEGKL
AKEQVLNLTEVAELPTGDTALSTDTVGFTVFNQVLEGLYRLDKDSKPVPALAKEEATV
SEDGLTYTFKLREDSTWSNGDKVTANDFVFAWQRVANPETGSQYAYLLEGVENAEAII
SGEKTPDTLGVKAVTDYEFEVKMEKPVPYFISLMAFPTFFPQNEKFVTEKKDKYGTSA
DTMIFNGPFIFKDWDGTNLSWKYEKNPNYWDAENVKLTEINVDVVKETATALNLYDAG
KLDRVLLTGEYAKQFRDTPEYKITTEARSAYMQYNQLREGKKTIFANENMRKAVAYSY
DQDLLANEILANGSQILTGLVPADLAANPKTGEDFRKESGDYLQFNQKEAKKYWELAK
KDLGVDKVEFDLIADDDESNKKISAFLKDQIETSLPGTTVNVRSLPFKVRLEVATNQE
YDLQQGGWGADFADPVNFIDLLQTDGAYNRSGYSNKEYDALLADSKGKNANNPEARWE
NLLDAEKILLDDAGVSPMFQRAAASLQKPYVKGIYSHQVGAQFTYKNAYIEEHE"

CDS 3275954..3276418

/locus_tag="JFBMFIFI_03047"

/inference="ab initio prediction:Prodigal:002006"

/codon_start=1

/transl_table=11

/product="hypothetical protein"

/translation="MRLINIETYTPGTNTVPTDLIYPLDHTQATKTELEEISLAIVAF
YQKIATELGLTDFASLQGVAVFPKTITDPRADIHLHFIRGGTRVAPVLQPEQQATNLD
EEKGWLIEKMGDKELTIYLTIPTNNIYRQLRYLSLTECLYFFKANIIKQLQQ"

CDS complement(3276524..3276736)

/locus_tag="JFBMFIFI_03048"

/inference="ab initio prediction:Prodigal:002006"

/codon_start=1

/transl_table=11

/product="hypothetical protein"

/translation="MDKKQYICEKCGCHSYEHDQFQATGGNFAKIFDVQNKKFITISC
HDCGFTELYKAQTSDGWNVLDFLIGG"

CDS 3277010..3278440

/gene="mtlA_2"

/locus_tag="JFBMFIFI_03049"

/inference="ab initio prediction:Prodigal:002006"

/inference="similar to AA sequence:UniProtKB:P50852"

/codon_start=1

/transl_table=11

/product="PTS system mannitol-specific EIICB component"

/translation="MLKKTNKIRFQLQQVGSYLSGMIMPNIGAFIAWGLITALFIPAG

WFPNVKIAALIEPIMVYLLPILIGYTGGKLVFGTRGAVVATLATTGLIVGAEMPMLMG

AMVMGPVAAWLLKSFDKLITGKVPTGFEMLVNNFSAGVMGMLLAITSLFAVGPAMEYL

NGLMVNGINYLVHHQLLSLISLLVEPGKVLFLNNAINHVALGPLGIEQATETGKSILF

LIEANPGPGLGILLAYTFFGVGSMKQTAPGAVIIQLFGGIHEIYFPYILMKPALILSA

ISGGIVGILTFSLFNVGLVGPVSPGSILAILTLTPRGDYLGIIAGILTAATVSFSISV

LIIKFSKVSLEIDLSTKKQMGELKRVSKPSKSETETTNEAKNLKQTSEFPEIVNKIIF

AYDVEIGTSAMGASILRKKMQEAGMEIEINHALIDQISKDSGTIIITQQELTLQALKK

FPESYHISVVSFMKNKEYDALIKKLS"

CDS 3278464..3280536

/gene="mtlR_2"

/locus_tag="JFBMFIFI_03050"

/inference="ab initio prediction:Prodigal:002006"

/inference="similar to AA sequence:UniProtKB:P96574"

/codon_start=1

/transl_table=11

/product="Transcriptional regulator MtlR"

/translation="MYLSARARLILEFLLMNKANVTNAVLASELDVSERTVRRDLHEV

EAILDTFQLKLSKENSQLSIIGTEINRQNFKWQLLDLAHNEFTPLERQNFILKTLLRE

TEPLKLMALATDLSVTISTISSDLVKLEEVLGKDVSIERKRGSGIRLKASENKKREMM

SDLFGSALPKNTLYHYFNQQMEEAEVQSMIEDKLLNLLDANLLNRVEQVVREWRTGLK

NSVTDDAYLTLIVHITISIERLLNGQHLVTVPANLVEAIDYPEYQIAKALLAECLEME

ESVVPLGEVAYVTMHLRGVKFQTEKIDLAGIEGIQAVTLANHLITRIADEIHYPLTDP

ALFKGLVAHLRSALRRLDQDMRIQNPLIDSIKKDYPDLFQLVRNAFDEGYPRKNAPDE

EIGYLVLHFGSAILQVKKQEVFSGLVVCSSGIGTSKMLMTRLQQALPQLKKLKSTSLF

EMLHHEVASSYNVIVSTIDLGKVDFDYFLVSPILSEAEIIQIDVYLQKKQGVLPKYPI

KEAEKFNLQGSIHHFEKLEEQVGTVLALLKTFQVFPIQQVVKNIEEVSEVITKVIYQN

QPKSRMDYLINALTVEEQQGGVGIPGTTLALFFIRNEMISRPLFQIFTLRNHVRLQAT

DKTEIEISTVLVLLAPEKLATGSIDILSRLSTLMIESDEVIQSFESGDRNKITSLVIQ

KLHQFIDS"

CDS complement(3280627..3281403)

/locus_tag="JFBMFIFI_03051"

/inference="ab initio prediction:Prodigal:002006"

/codon_start=1

/transl_table=11

/product="hypothetical protein"

/translation="MIFLTHQHYFDYSKGQNLSQIEHEILAFLLSDSQNEQPCSIRMV

AKELTTSTATIIRLCKKLGFSGYTAFTEKLRLEYTDHSPTEATQLLSANLNNHFTMYM

KDYQDTLDALSIRDVTVFTQSLTTSHVVQIIGDESDAFITYIAAKLQSKGFRVLVSSS

KDTLLNFEKKLNETMLILVFNRHSATESLLKKIDYVKEKKVIMIGFLGSKQSVNFRQL

LDAQFEIRDDHSQSLNHDAYDSAVIMIFDVLMSLVSDSSK"

CDS 3281640..3282032

/locus_tag="JFBMFIFI_03052"

/inference="ab initio prediction:Prodigal:002006"

/codon_start=1

/transl_table=11

/product="hypothetical protein"

/translation="MAMKEQDVLNHLEKYGFKGEFGKSIWAMPSSLLNGFNYINVNFR

YQILNFSEDGVYVIDVGQATNKIVDVVPQLIPASALVSITLKKGFLTNKAYISTIYGD

ISFKVMKNIMNKKWHKQNYENLQEKYPG"

CDS complement(3282220..3283176)

/gene="prsA_3"

/locus_tag="JFBMFIFI_03053"

/EC_number="5.2.1.8"

/inference="ab initio prediction:Prodigal:002006"

/inference="similar to AA sequence:UniProtKB:P0C2B5"

/codon_start=1

/transl_table=11

/product="Foldase protein PrsA"

/translation="MKKKLIISGAVFLGLLTTGCTNSSATVASTTAGKVTQEELYQEM

KANYGESTLQTMLIEQVLAKKYGDSATTKKVNAKFNEVAEMYGGTEAFESILVQYGYA

NGAAYKATIKQNLLIEAAVKDKTNLTDADYKELWENSLYFQTILVADEDTAKEVITKL

DAGEKFKDLAAEYSTDTTTSENGGDADFYNISTGTTYDTTVTDAAAKLNDADFTKTAI

STDAGFYVVNMVVNPTSTSKTWQDYQSELTEMAVTANLADSTFTTEILTTLLKAANVQ

IKDSDLENALSAYLTPTTTSSSAVTSDSSTDETTTTSTTDSE"

CDS complement(3283294..3284832)

/locus_tag="JFBMFIFI_03054"

/inference="ab initio prediction:Prodigal:002006"

/inference="similar to AA sequence:UniProtKB:Q97P44"

/codon_start=1

/transl_table=11

/product="Putative trans-acting regulator"

/translation="MKELLSTKEKVYLDITSALLGEPIDIYTLADELFMSVRNLKKYI

DDLNVLIHPISIYFIDTNSVNIHYPDSLNYQHIYKSIYVNNLNYSLLELLFLEENNTL

ETLEEHFFLSESTLRRTISFINQRLAPFDIIIDTKNFNIIGDEKNIIQFFVSYFQEKY

TFQDIKLGNSLVQFLDYIYSDFTKFLNFPTNFPTKNRFIFWVGVGLKRIERNHSLPIN

NNSEYLTQFTYFFDALLKEQHNQKLKLEHNLTSRSFAEALTFFIETGFLFSNDGLNQL

IEKYPDLRDNYQKISYILLELEEIFTISMTESAKTNMQLKLMNGFTFYQKLIFKGGLL

NDVPLQFKQRTLFIINRLPEIIQRTFKKYFPNEDEWMANYFIYIIITHWDTFIPNMLK

KAPIIHVGIVVETDLEHALYLKNKLAYYYPFNLDAMLIPDITTERIDNKKLDIILTTF

PLHELSTTTKIISINHALSKQNVNDLYSSFGIFFEKKLEEKLFSGEFGNISILDEAPR

IPIK"

CDS 3285059..3287596

/locus_tag="JFBMFIFI_03055"

/inference="ab initio prediction:Prodigal:002006"

/codon_start=1

/transl_table=11

/product="hypothetical protein"

/translation="MNLKKLVLSSLIAIIMPISAFSESRTALDEAPLGLQLDDIFTPT

EMQNNSAKVYRNGNGTDVVIITDDGGQQGGLWSTEAMKLDLSQDFHASMKIYFDDVGS

NSADGAAFVMHSDPKGLSAFSFDGGQTLGVYGYKSDSNPERGAVQNSFAIEFDTYRND

SGENQFDNVDGLGDNHVASSYPGLSSSYERVGSPKRNKIYHDKYSESINLEKGALSNG

KWHDFSFNYSAETHEFTYIFDGIERQLYDGLDTSVFGTNSVYWGFTGSTGKKESKQAV

IFENIPGLVDAKVTEQVLNKKNESLLETEVTEGETVSYQTDIAYIKGKQSWRNLKVTS

NGNQKVTPIAESINILLPDGSKYTPKENPFTEEGNLNLDLTGVELSDPNQHIQISYDA

VIKKGNEAKTRVLDSIQVAGSNNTIKSQEIGYWSIPTKYEFVFDEVADISTEITGKLL

NTGDFEQVDLQLSNAYLSDGNQINSKAEFDPATQLFKLVLEEGVHLLAEEELVAQLTV

DNEVIPITKTQVVDKMPPTAEGREVYMSINEDFPSSSKFVRNLMDTNKHLQPEDFNYE
FNTNLNEIDVSKAGEHPIELTVADKAGNKSEMIHSTLKILEANKQLESKKQLELKSKD
LIQKTTIEKNEFLLKQLAATAWEINAEAEKIDLTEQIIIQNSDEITENPGEYTIKLAV
PNKNLYREVPLIVTDGELKLEVDEFPEMDAQIVSRSKKYSFPTTTKFSITDERVTESG
WKVSVSQSGFTIPKGYTGSQQALNYLSLEMNNQESTSIASQGSKEIVLNDADSDLYFS
FIVKKGANNYLSKNDKYSNEITWTMMKDNTLQLSKLNVTTPLKKENV"

CDS 3287608..3288399

/locus_tag="JFBMFIFI_03056"

/inference="ab initio prediction:Prodigal:002006"

/codon_start=1

/transl_table=11

/product="hypothetical protein"

/translation="MKNTIKMLMTSTVILGGFGSTVAIADQATELNSTSSIKFEAPIN
EEGTKPVDPTDPEQELPEGGGENGEKPGEENTASGPLRIDYASNFNFGKQKISSKQET
YLSNLSIKNQEQNYLPNFVQVTDNRGLHDGWKLSVVASELKDEKGHSLKGSIIDLSNI
SANHSVFKNDLTIDKSNEVSLDGKTPTVIVEDAHGSGTNNHGEGTWAIAFGEKATESQ
DDDVTLTVPKEVGVLANASTNYQSVLTWSVEPATVSNTKAMLPSI"

CDS 3288432..3289451

/locus_tag="JFBMFIFI_03057"

/inference="ab initio prediction:Prodigal:002006"

/codon_start=1

/transl_table=11

/product="hypothetical protein"

/translation="MTSLKRWLIGMMSTLLLIFIAPVAQAGGMNFTVEPLFNEHQIAE
NQSHFEMKVEPNSSEKVSVQITNGSDKDKKFSLQINNATTSKNGEISYEKKLNVPDDS
AQVDLQTITTIPHELSLKAGESRIVDISFEVPEQAFEGVLLGGLQVMEQNEAAEQADE
KISFKNKYSFIVPIVMYETDAQLKADVKLKKVFPTLTNAYPSLEVQLQNPVATTLPTK
DVTVKITPKNKKEVLKEQQLKEVTFAPNSTWNNQLDWEKEEFKAGDYTAHIKIKSEYG
DWKWDQDFTIAGTKAKELNKKAVGIKSENPSTFLIVGILLLFLVTVVLIYMLTKEKRK
NQLEK"

CDS 3289659..3290210

/gene="maa_2"

/locus_tag="JFBMFIFI_03058"

/EC_number="2.3.1.79"

/inference="ab initio prediction:Prodigal:002006"

/inference="similar to AA sequence:UniProtKB:P77791"

/codon_start=1

/transl_table=11

/product="Maltose O-acetyltransferase"

/db_xref="COG:COG0110"

/translation="MKSEKEKMIAGEMYRAGDAELRASRENARKLIRQFNQSEDKVER

FNLIKNLFGNFEEGSFIEPNLRVDYGYNIHVGANFYANFDCTFLDVCPITIGDNVMFA

PGVQLYTATHPIDPVERNSGLEYAKPITIGNNVWIGGSAIVVPGVTLGDNVVVAAGAV

VTKSFPDNVVIGGNPARVIKEIG"

CDS 3290579..3290959

/locus_tag="JFBMFIFI_03059"

/inference="ab initio prediction:Prodigal:002006"

/codon_start=1

/transl_table=11

/product="hypothetical protein"

/translation="MKIEHVAIWVKDLETTRKFYQKYFKASVNDLYHNATKGFTSYFL

TFESGARLEIMQRTDIVIGHEADSLGWAHIAFSVGSKEQVVALTERLVADGYACLNGP

RLTGDGYFESVVEDPEKNLIEITE"

CDS 3290987..3291262

/locus_tag="JFBMFIFI_03060"

/inference="ab initio prediction:Prodigal:002006"

/codon_start=1

/transl_table=11

/product="hypothetical protein"

/translation="MLDKQRAEIDQIDRELVALFERRMAIVTEIGEIKKANSLPIFDE

AREINVIERAEERLANSDYAPYVGQLFKDLMNVTKEYQKNIVKKREL"

CDS complement(3291257..3291820)

/locus_tag="JFBMFIFI_03061"

/inference="ab initio prediction:Prodigal:002006"

/codon_start=1

/transl_table=11

/product="hypothetical protein"

/translation="MQTNYLIVFYAIILYYTTIVSFRVLFPASFLPNLTTFMLFSTTR

ERNSPKKKSSSKTSQTTTSKKKRKVPKKLPPKPKTPLERILFSIKKTGAKLIYYGIPT

KKQVLVITIIAFFSYLITFSNETFVITYLYLCVIYLISLIYLAYIDTKEENELLKKQL

YQLTKHRKKRVKKRPNTPTEKKRERNL"

CDS 3292018..3292803

/locus_tag="JFBMFIFI_03062"

/inference="ab initio prediction:Prodigal:002006"

/codon_start=1

/transl_table=11

/product="hypothetical protein"

/translation="MLAFAWFKKKKDKYDEFDDEEYDEDYDEDYDDEDYDDEDYDDEE

DDEDDEDTSKYDYEAIAGNFERENTRLFKDNERLKRKLAMAEEELKMTALEKNQLLDK

AELAEKDNQLDNQSTKKQLSDTIALYTKISEENKSLKNQLEKLNTYGFDVIETEKKLA

KYHVIERENTELKAQLMNVKVDLADAVLNAKEMAKDIVNAASEEANNYQIGIETKRAA

VTEEINDIYHELDHSRLKLNNLFNEVRSQVDLLKDMAEGKERR"

CDS 3293254..3294492

/gene="arcA"

/locus_tag="JFBMFIFI_03063"

/EC_number="3.5.3.6"

/inference="ab initio prediction:Prodigal:002006"

/inference="similar to AA sequence:UniProtKB:P63554"

/codon_start=1

/transl_table=11

/product="Arginine deiminase"

/translation="MENPIHIMSEIGKLKTVLLKRPGEEVENLTPDIMGRLLFDDIPY

LPIIQEEHDYFAKALTDNGTEVLYLEKLTAEAIDAGGIREVFIDRMLSESEISSPKIA

SALREYLLSMETFPMVTKIMAGVRTRDIDVTTSNLVDISNKEHYPFFMDPMPNLYFTR

DPAASLGNGLTINSMHYTARRRESMFMEIIIQYHPRFANKGVEVWLDRDHPESIEGGD

ELVLNERVVAIGISQRTSAKAIEALAKALFSRNSNFEKVVAIKIPNVRAMMHLDTVFT

MVDYDKFTIHPGIQADGGKVDTYIIEPSTVPGEIRMTERNDLQEVLREVLNVPELILI

PCGNGDEIVAPREQWNDGSNTLAIAPGVVVTYNRNYVSNELLRSYGVKVIEVISSELS

RGRGGPRCMSMPLIREDLKK"

CDS 3294519..3295535

/gene="arcB"

/locus_tag="JFBMFIFI_03064"

/EC_number="2.1.3.3"

/inference="ab initio prediction:Prodigal:002006"

/inference="similar to AA sequence:UniProtKB:Q5XAY4"

/codon_start=1

/transl_table=11

/product="Ornithine carbamoyltransferase, catabolic"

/translation="MNESVFEGRSLLAEKDFTKEELTYLVDFSAHLKKLKKAGVPHHY

LEGKNIALLFEKTSTRTRSAFTTAAIDLGAHPEYLGKNDIQLGKKESVEDTAKVLGSM

FDGIEFRGFSQKVVEQLAEFSGVPVWNGLTDEWHPTQMIADFLTVKENFGYLEGINLV

YVGDGRNNMANSLLVTGAILGVNVRICTPKSLYTDAKIVELAEGFAKKSGSQLVISDN

VAETVSGADVLYTDVWVSMGEEDKFAERVELLQPYQINMEMINQTGNENMIILHCLPA

FHDTETEYGRDIAARFGISEMEITDEAFRSKYGRQFEQAENRMHSIKAIMAATLGNLF

IPRV"

CDS 3295702..3297135

/gene="arcD_2"

/locus_tag="JFBMFIFI_03065"

/inference="ab initio prediction:Prodigal:002006"

/inference="similar to AA sequence:UniProtKB:P18275"

/codon_start=1

/transl_table=11

/product="Arginine/ornithine antiporter"

/db_xref="COG:COG0531"

/translation="MSEQTQEKKLGIIPLSALVVGAIVGGGVFNLMSDMAREASLGAI

IIGWLIAGLGMAMLAFSFQNLIEKRPDLDAGIYSYAKEGFGNYMGFNAAWGYWLSALL

GNVAYATLVFSSLGYFFKLFGNGQNLASVVAASILLWLVHYLILQGVESATFINTIIT

AAKLIPLAIFFVAMIVAFKLGIFTTDFWGTLSGNFELGEVMSQVKGTMLITVWVFIGI

EGAVVFSGRAAKKSDVGKATILGLVTVIAIYLLTTILSLGVMTRPELADLKQPAMAYL

LESVVGKWGAILINIGVVISVLGAWLSWTMFAAELPYQAAKSGAFPKRFAKENRNGAP

VNSLIFTNVLIQVFIFSFLISDRAYNFAFSLASSAILIPYAFTAFYQLKISIQEKADT

PHRTRNLIIGVLASLYGVWLIYAGGIQFFLLTMLLYGPGIIIYAWVQKENKKKLFSKG

EWICAGVVILLFIVCVIQLINGSIKIG"

CDS 3297164..3298108

/gene="arcC1_2"

/locus_tag="JFBMFIFI_03066"

/EC_number="2.7.2.2"

/inference="ab initio prediction:Prodigal:002006"

/inference="similar to AA sequence:UniProtKB:P0A2X8"

/codon_start=1

/transl_table=11

/product="Carbamate kinase 1"

/translation="MSKRKIVVALGGNAILSTDASDKAQKEALKATAAYLVEIIKQGN

ELIISHGNGPQVGNLVLQQQAAASKSNPAMPLDTCVAMTQGSIGYWLQNALENAFKKE

GIEKSVISVVSQVVVDQNDVAFIHPTKPIGPFLTQSEAHEQMLLSDDTYQEDAGRGWR

KVVPSPKPVSILEYPIINQLVENGVVTISVGGGGIPVIEAENEFVGVEAVIDKDFASQ

KLAELVEADLLVILTGVEQVYINYNQPNQKALTTVTTKELYQYIQENQFAPGSMLPKI

EAAISFVEHNPKGKAVITSLENLGNFNTENAGTTIVRG"

CDS 3298335..3299045

/locus_tag="JFBMFIFI_03067"

/inference="ab initio prediction:Prodigal:002006"

/codon_start=1

/transl_table=11

/product="hypothetical protein"

/translation="MQKNEVQLEELGSIRNNQVFSGFTDDEFEKIQDHMYLRQYKKGQ

VLFDNGDYRNRIYFLIEGLVRVERYDSSGTFCYLDYVKKEHLFPYGGMFFDEFYHFSA

YAVTDIEVYYIPTDIFEKISKGNTAQLLYFYQALSTNLESHELKIQYCLASSATVRVV

ETLAILMKHLGENYFSGTIRIPYPITLKEIAINSGTTRETASNVIKTLKKEKKLDYCQ

KHLIFQDVGFFLENGEIT"

CDS 3299332..3300627

/locus_tag="JFBMFIFI_03068"

/inference="ab initio prediction:Prodigal:002006"

/inference="similar to AA sequence:ISfinder:ISSau8"

/codon_start=1

/transl_table=11

/product="ISL3 family transposase ISSau8"

/translation="MSLTHCIRTLLDLKDKNISFPQNYCEERVIKGRRTKLIYATLTY

NPVCCTRCGCLNTQFSIVKNGFLTSNIKWISTTHFPTYLVLKKQRFLCRECHCSFLAE

STEIAKHCFISNRVKQSIAFELEDNSSRKDIAKRHFISDTTVLRVLVARGKSCLNRFD

SLPAHLCFDEFKSVKRVEGKMSFIYCDAITHSLIDILPDRKLVTLRAHFLRYSLKKRS

KVQSIVVDMNAGYFTLAKELFPNANVIIDRFHLVQLLTVALNKTRIQLMNQFKRWKPE

EMKDYRKLKRYWKLLLQNSQKVNYTDYQYHRLFRKVMPHSEIVDYLLSLNSTLKATYT

CYQDLLYAMSQNNSDLLKSVLSSPHEQVSDYMSTALQTLRKHEERIRNTFQSKRSNGP

LEGSINKIKVIKRTAYGYASFLNYKYRILISFQEKEKTI"

CDS complement(3300701..3302752)

/locus_tag="JFBMFIFI_03069"

/inference="ab initio prediction:Prodigal:002006"

/codon_start=1

/transl_table=11

/product="hypothetical protein"

/translation="MKLHNFLDKKNALMDALQSEDSAENPTTISSKNDDSFKSDLVFN

QIKSEERVIEPISEIKRAPKTVREPISRRAVTPFQVTQKTNPNPSQAAESEYKARLQE

LETNFKEKQLHFEQEMLEKEKEAQQNRQDLERNLKEKLEQQYNEQVATIKQEKEQNRE

QLQKQFNEQIAEIEHLSLEKRIEIETKYQEKLKMLEGIYAEQQDLSEAKYAEKEANLE

KAYQDKQAQFDKQQQAKIAEIEAQKRKQDEEYERKYLEKIEKLDLFYKERQTVLEKDI

AEKEKEIIRNAETLAEEKLDRAAREAEQLLSEIKAKVALQQQNLEHSIIETEEKNSKA

IEDAKKMASNIINSANGEAKKKLEKAELQAKTALESARSDSQLLIENTKNEAENMLKI

AKANVFSAKDAAEKDIILEKAKHENEMADERYQLKTEREAFLVYKEEFQKFKTGIEEN

LARKEHILATKTQEDIAYLENKAIELDNKLDLRSTLAEKQFSLVKWASLTISIVTIAF

FVMAILTQASYVSIGLAFVAVVASLYLFLASLLNHRNVDEEMAQLGSDDDDDEFDDDD

FDDDDDEFDDDDFDDDDDEFDDDDFDDGDDEFDDDDFDDDDDEFDDDDFDDDDEFDDD

DFDDDDDEFDDDDFDDDDEFDDDDFDDDDEFDDDDFDDDDEFDEKPAKKSIFRKKNKK

R"

CDS complement(3302875..3304017)

/locus_tag="JFBMFIFI_03070"

/inference="ab initio prediction:Prodigal:002006"

/codon_start=1

/transl_table=11

/product="hypothetical protein"

/translation="MKIVVVDPGKNEVKSFCFSNNGTLELVNSFPSKTEEIIEERFLE

EDSSLRQYRLELNGHYYMVGEGVDTTYNTELTKNTLHHKLCIYATIADFVTDPDEEIL

LVLGYPSTDFQNNFRREDYQRLIYGDKDGFIEFVQNGEPVSFQLANVIVLPEGQAMLP

RMTEVDDYYSLYVLDIGGQNVNFREFDNKGNAKNAFSIDVAGVNFLERALEAELRRAI

ENPNVNFRGIDYNQAILEGKITDIGAIEGFSDSTSFITHVVQNFVKKEIMDELSVRGY

NINARGQHIIFTGGGSLLLQPYIEDLLKGNKTNLIFSKSAKWDNCLSYTIKAFQDQFT

SNEQFTGFIKPIYSELMRNNFADFYKGVNQGASQEQDDTMDFNRIL"

CDS complement(3304632..3305216)

/locus_tag="JFBMFIFI_03071"

/EC_number="1.14.99.53"

/inference="ab initio prediction:Prodigal:002006"

/inference="similar to AA sequence:UniProtKB:Q838S1"

/codon_start=1

/transl_table=11

/product="Lytic chitin monooxygenase"

/db_xref="COG:COG3397"

/translation="MKKRFFKAILVVGLLVGGLTAFATPSFAHGYISSPGSRAFLGSS

AGGNLNTNVGQAQWEPQSIEAPKNTFIPGKLASAGLAGFAPLDQQSPTRWYKTAISKG

NLDLTWTLTANHKTATWDYYMTKQGWNQSQPLDIKNFDKIGSVNDNGTVPPKTLKHTV

SIPNDRTGYHVIYAVWNVYDTTNAFYQAIDVNVN"

CDS complement(3305646..3306251)

/gene="pspA_2"

/locus_tag="JFBMFIFI_03072"

/EC_number="3.1.3.3"

/inference="ab initio prediction:Prodigal:002006"

/inference="similar to AA sequence:UniProtKB:D3DFG8"

/codon_start=1

/transl_table=11

/product="Phosphoserine phosphatase 1"

/db_xref="COG:COG0406"

/translation="MTTTTLYFVRHGQTEWNLDERMQGHLNSDLTAHGIQQTLNLAKG

LADMGLTHCYTSDSPRTQQTTDLLTSELNLPIKAETGLREINMGLWEGENKTTIQSRY

PESWTNFWHHPHLYQPNGNGETFADLQTRSVQALNEIIHQHPGATILIVSHRITIKVM
LAYLLNQPLAELWKTGDIAPTSLNCVEITADQAILVSAGKV"

CDS 3306365..3307261

/gene="cmpR"

/locus_tag="JFBMFIFI_03073"

/inference="ab initio prediction:Prodigal:002006"

/inference="similar to AA sequence:UniProtKB:Q55459"

/codon_start=1

/transl_table=11

/product="HTH-type transcriptional activator CmpR"

/translation="MNLHGLRLFYEVAKQKSVTQAAKKLAISQPAVTAQIKKFQEEEN
VMLLIPDGRGIQLTSIGQEVYQEAQKLFAIEKTIENRIQAFTLDEQSLIKLSGNYVTI
NYLVPQWLAEFKNQYKAAVIQVDMLNTADAVGKLVSNQVDFAFIGEEGEKYAEFVELR
KIGIDEFYFVVAPEHKYANKTISLVELAKESFIGREAGSYTRTELASLFEEAGVAEPI
PTLHYNGVHEAVTAAALGYGIHFCSGLVVAKQIKNGELARIYTEEPTKKRALYLCTRK
KDKLNGMEQLFLDFLIEKLPND"

CDS 3307398..3309725

/gene="ctpE_2"

/locus_tag="JFBMFIFI_03074"

/EC_number="7.2.2.10"

/inference="ab initio prediction:Prodigal:002006"

/inference="similar to AA sequence:UniProtKB:A0R3Y2"

/codon_start=1

/transl_table=11

/product="Calcium-transporting ATPase CtpE"

/db_xref="COG:COG0474"

/translation="MKQSEEILGLSANEVKERQNNGQQNDYQENVAKSTKMIIKDNVW
TLFNFLNLAIGVCLAAVGAFSNMVFLVIILVNILIGIYQEIHARNLVAKLAIISESRV
TVIRSGKKVEIAATELVMDDIVLLAAGEQVPSDMKVVQGRAEVNEALLTGESELIEKT
ASDELLSGSFLSSGQCYAQVIHVGKDNYATRIAEEAKVHKPLQSELMNSVAKVAKFTS
FVIIPLGLVLFFEAFFMRSDSTQVAVVSSAAALLGMLPKGLVLLISISLATAVTKLAN
KRILVQDMYSVEVLAHVDTLCLDKTGTITEGKMKVQKVEMLNDVTVTDFETIMGSYLA
ASQDNNITMQAIRDYYQENNQYTATDILAFSSERKWGAMYLENIGTVVLGAPERLVNA

ENLPTEVLGAQEQGYRVLMLAVAKNQKMVDAILPELEPLAVFEIDDPIRPKANETLAY
LKAEGVDLKVISGDNPVTVSNIARRAGLEDYANYIDLSKGFNDDEVRESASRYSVFGR
VSPQQKKILVKALKDDGHVVAMTGDGVNDVLALREADCSIAMAEGDSATRQIANLVLL
DSDFTSLPDVLFEGRRVVNNLTKVASIFFIKTIYSFLLALICIVTNLAFPFIPIQITL
IDLAIEGYPSFFLSFEGDKTKVHKGFLKTVLTNALPNALLVILNIVVVYLYSQSANLS
AIETTTVMYYLLVGVSLLAVIRACMPFNPLRIFLAVTTTVGVYVAAFLFQSLLHIDTL
TSNTLPLFLIMITISAIIRIIVELVKKRKVVPQLN"

CDS complement(3309755..3311290)

/locus_tag="JFBMFIFI_03075"

/inference="ab initio prediction:Prodigal:002006"

/codon_start=1

/transl_table=11

/product="hypothetical protein"

/translation="MEELLSSSEQRELKIIHLLYQEEKLWTVEQLANYLQCSIDTCYR
YIDRIKQVFYDYGNEFELISKKTKGVLLKKTEHASLSKYESIYIEETIDFKLLSELFH
STYLTTEKLADHLFISKSTLYRKLKKIAILLRKNGIHLNISTLQLTGNEVWIREFFYL
VYWSTSDSGFWPFESVPKHVLTHRVENIISSQNSYFSTIEKLKLTYRMAISFIRVQQK
NFITHSIGDSFIDPFKEEYFEFITLNLMKTVPSNYQKNEKDYLSLIFCTYPYLDKSDL
NFCGIVSWHSINNTIPYQLTDKLLSSLSTIYPTQNLLTNKKLFYQLLCISIYATYFQA
SFSKTSEFLKLSTLLKQTHTCFYLNTKHALATICQEEPFKRILLQPNFYIHSTIILLS
SYVDVLGYCNEIHVRLISSHDRISESLLRNELLRHFPEKISITTSAEFNHQTAALHYD
LIITDLLSSVSVDDFSSQYYFWSYPPMKRDWENIATIITTIDQKKHANHFHESLILKE
API"

CDS complement(3311391..3312884)

/gene="thrC"

/locus_tag="JFBMFIFI_03076"

/EC_number="4.2.3.1"

/inference="ab initio prediction:Prodigal:002006"

/inference="similar to AA sequence:UniProtKB:P23669"

/codon_start=1

/transl_table=11

/product="Threonine synthase"

/db_xref="COG:COG0498"

/translation="MSLNYRSTRDAENVVTASQAILQGLAVDGGLYVPTELPTLNLDF
QDLATKSYQEVAFIVLKAFLTDFTDEELMSCINGAYDAKFDDSAIAPLKKVGDIHYLE
LSHGATLAFKDIALSILPYFMTVAAKKNNVDREIVILTATSGDTGKAAMAGFSDVPGT
KIIVFYPKSGVSPIQEKQMVTQKGDNTFVVGIKGNFDDAQTNVKNIFNNSELKAQLAE
NGYQFSSANSINIGRLVPQIVYYVYSYAQLIKRQEIKLGDKINFVVPTGNFGNILAGY
YAKQIGVPINKLICASNENNVLTDFFTTGIYNRNRPFHVTTSPSMDILISSNLERLIY
HIAGNETLETKNRMSDLATSGEYTITDSMRDQLSEFYGAEASEHEVKETIAAIFSEHD
YLIDTHTGVATVAYQKYLAQEKDSTPTVIVSTASPYKFPQHVMAAFKEISSETSDFST
VQQLNTLSKVPIPASVTELFTAPILHDTVIESADMEETVKAILHLKD"

CDS 3314263..3314955

/locus_tag="JFBMFIFI_03077"

/inference="ab initio prediction:Prodigal:002006"

/codon_start=1

/transl_table=11

/product="hypothetical protein"

/translation="MSSNILDYSLYVSGNFKEHYAKKQVKKNEIIIDQKNPFNTPENN
LYIVISGQVLVEVSNSFGKNCYYDLVSNNQLFGTESILETHPYPRGISYQARAMTDVV
YLEINSQFFLDHMYINPKLYHYILEDVTKRYFSVTQSYQLMNETPVVRVSNALLNLAR
VLDLKADSSKRKKLPLYINQTFITKYIHSSKSRVSEAFSYLEEVGTIERKPITIINEE
KLNQVLLEKLHA"

CDS 3315009..3315239

/locus_tag="JFBMFIFI_03078"

/inference="ab initio prediction:Prodigal:002006"

/codon_start=1

/transl_table=11

/product="hypothetical protein"

/translation="MKQRKGNLIMQTDGNWNDVCVYAVNFSTLDGRHIEKILIFPEKF
TKEIIEKAVLKRFRQIDKVERIEAWCGALSLK"

CDS 3315560..3316612

/locus_tag="JFBMFIFI_03079"

/inference="ab initio prediction:Prodigal:002006"

/codon_start=1

/transl_table=11

/product="hypothetical protein"

/translation="MEKRKEKSAGWIPKILKKEDEYDEYDDYDEEYDDEYEDDYEELE

EKIELLTKENATVKAKLMDCVKALREISSQKDQLESQLGQEKALPQHYQTLEKKVIDY

RSKILESNQIIEQLKVQLEEERTAKKAESPLLEKHLAQSKEQLSQLTTLNTQLEKRVQ

NLTEEVLGTSQEVSKNKELTALVTQLKEEKMVKEEEYEIQIKHLRSELAEWKTELSQK

QHENEQLTNQLFDEQTTHYRGKEFENQLIQAKERIQKLVLENEQLRSSMEGSKSEIAD

VMIEAKIQSKQMLDQAHVEAARIHNKTKLELQQHNREAKVLVNQVDRIQMESEILFTQ

LKERLTSVTAADTTQL"

CDS 3316856..3318595

/locus_tag="JFBMFIFI_03080"

/inference="ab initio prediction:Prodigal:002006"

/codon_start=1

/transl_table=11

/product="hypothetical protein"

/translation="MKSQTIYYPKESIKMERKSAFFAVYKNESIEEVFFRAFTQLSYL

EKRNYEEKHIENILNTLPRYSLTRQLVIELSDISVERDSISWEVFGKEFERANKQMLL

YQFFHQTQEGIEHLVFNFLPVYKSNEGFPLQIGMDKALIDQNITGTMNEEERVKKWEE

IEQIRIEESGLPIILTLQMEKEIEKLKMVDDAEYSEEFPIDVNAELMDLEITEIKTKK

NKLDKVYQAFEPKAVELQQNLSEVTQFIQLLQEQIKIEEQDITIQKKRMDEAVKQVNY

LREQTLAVTEEQESVKERLQLLTNQSEEIVKESQEYYAELKSHSQSQLNKATEVLEEI

QGLVQAKKSVDGYRKEYEQILNEVHEREKSIEERIKLVETTTIEKFKAKLLLTRDEKE

QLEISQQKKELMIRDLEEKLARAESYLLEDIKVEKEAKRNGEYVKNRKIKIALEEYDA

LYSKVKYLEHTWRVNQELVVRKEKLTITDSELRYEKEEIIQIKQTAKEVELFVIMDEC

RTINERVIIRKWPFIRPKAVLLTKDYDALKEKSTYFDLIERENRSLERWFSESVLNKK

QVSHKNLTNKIEE"

CDS 3318789..3320222

/locus_tag="JFBMFIFI_03081"

/inference="ab initio prediction:Prodigal:002006"

/codon_start=1

/transl_table=11

/product="hypothetical protein"

/translation="MQKLLKKQDQLALELLALLYLYNGESLSIVWCMDTLGETNRRLL

VAIIENLSNKKNMNFHIQYSKKNVKAIFFDEFSLDDIYHDYYKNSVEIILLDRIFEGK

VKNLVEYSTTHFYSYSTLYRKIQKLNLYFSSDFKITVGKNKNIPGKEHSIRCYYASLY
NVLYGNRKMKFQKEIKKSEKYLKSIQINSEYLYSKLTFSEQISVIYYFTISRLRMEEY
PIKLRPSSLKNSMFEEIVDNFDCFTEGLEVTKKQKKIERNWLLFYFLTTMQLNQKDNL
SGYFNQKIFESLYKNESKPAEWLTHFNQCVGSNFSESEQTVMEFRLYQKIYQHVYFSK
VVESLYQNPEKREVSGRVKSVTYKVQSWYENAPFKKQLIHHGITIDVIVDLVLEHTDF
LKRSKKIYIGTKKGRHWDQYLAKQLLTTELAEQITVVENWGKTTDFVIVDQKIPVLPA
YKTFIISKNPSLKELEKIQKELQKLLA"

CDS 3320264..3320494

/locus_tag="JFBMFIFI_03082"
/inference="ab initio prediction:Prodigal:002006"
/codon_start=1
/transl_table=11
/product="hypothetical protein"
/translation="MELKLLYFIMIVIIIIELVILVDLLRLRYQFNNFKKMTLEKKNV
RKKEVHSKPKRVKEKSKVEDYFDENDDFSSYL"

CDS 3320531..3321103

/locus_tag="JFBMFIFI_03083"
/inference="ab initio prediction:Prodigal:002006"
/codon_start=1
/transl_table=11
/product="hypothetical protein"
/translation="MSKKKVVSRKKFRIEKSKTKKNISPVLMSLCIFLLLLIILPIAT
IMWYSGGTEIDVEYGIEKDSVSIEKEENYVDTSSMNSRDAMKSPAIYKSRQSSSSINQ
EDLAANEAAIPEEETTKEDQEVPIETTQDTNEMNPVPIKSVPEYHTVEEGEGLWRIAK
NNNLTLEEIKTLNNLTSDVIQVGQMLLVKN"

CDS complement(3321317..3322717)

/gene="hsrA"
/locus_tag="JFBMFIFI_03084"
/inference="ab initio prediction:Prodigal:002006"
/inference="similar to AA sequence:UniProtKB:P31474"
/codon_start=1
/transl_table=11
/product="putative transport protein HsrA"

/translation="MNTETTKLEKKVLLAIVSIAMLTFMGIVSETALNVAFPILMKEF
NVVGATIQWLTTGYLLIVAILVPISPMLVKRFPTKRLFQTAACIFTIGTIFCGLATSF
PLLLVGRLIQATGTGISLPLMFNIILALVPMNKRGVVMGMAGLVTSFAPALGPTYGGI
IVTTFGWQWIFILLLPIAIVAITLGSFNITTINELKKIPFDFISIGLSALAFSGIIYG
FSTAGEIGWQAPVVIGSLATGFVSLVIFSWRQLTIEKPLVDLKVFKFPMFVVGLSNVF
LVMMIILSCSFLLPIYTESVFGVNATTAGMMMLPASVLSAIMAPIAGRILDINGPKKL
ITIGTFISLMAITGFLFFTSNLVPLTVILLHMLLLLGASLTFIPSQTNGLNQLPEKMS
SDGTATISTLQQVAGAVGTSLVAGIVTTSQIKINNPSNTLSKAELTTAGVHNSFILLF
ILAGIGFTLSFFIKKK"

CDS complement(3322698..3323213)

/locus_tag="JFBMFIFI_03085"

/inference="ab initio prediction:Prodigal:002006"

/codon_start=1

/transl_table=11

/product="hypothetical protein"

/translation="MKLSDISQINMKTKRLNRLHKKHADQALASLGIHIGQSLILEVL
LAKEACTQKEIAAYLQISTSSITNPIKRLEEKNLIQKKQSSTDLRYNSITLTADGMAT
AKTIITTIEDIDQTMTSDFDPTEIKLLNKLMDRMIHNLEPDFPVTELKKDEYSNMYKN
YLEVNNEHRNN"

CDS complement(3323377..3324483)

/gene="msmX_2"

/locus_tag="JFBMFIFI_03086"

/EC_number="7.5.2.-"

/inference="ab initio prediction:Prodigal:002006"

/inference="similar to AA sequence:UniProtKB:P94360"

/codon_start=1

/transl_table=11

/product="Oligosaccharides import ATP-binding protein
MsmX"

/db_xref="COG:COG3839"

/translation="MVEIALNHIHKKYDNAESYSVTDFNLQIKDREFIVFVGPSGCGK
STTLRMIAGLEDITEGELFIGDTLMNDVAPKDRDIAMVFQNYALYPHMTVYDNMAFGL
KLRKYDKAEIKKRVEEAGEILGLTEYLKRKPAALSGGQRQRVALGRAIVRDAKVFLMD

EPLSNLDAKLRVAMRAEIAKLHRRLETTTIYVTHDQTEAMTMADRIVIMKDGFVQQIG

SPKEVYDTPINVFVAGFIGSPAMNFFNVTLNGDVISNGLGLKLTIPEGKRKMLAEKGY

DGKKLIFGIRPEDIHSEQIALDSNPNSVVHAEVVVSELLGAETMLYTRVDDTEFISKV

DARDFHRPEEFVDLAFDLNKGHFFDTETQLVIKA"

CDS 3324702..3325352

/gene="gph_2"

/locus_tag="JFBMFIFI_03087"

/EC_number="3.1.3.18"

/inference="ab initio prediction:Prodigal:002006"

/inference="similar to AA sequence:UniProtKB:P32662"

/codon_start=1

/transl_table=11

/product="Phosphoglycolate phosphatase"

/db_xref="COG:COG0546"

/translation="MSKRIYLFDFDGTLGDSEECSIVATQGAFKEVNLPIPNKNQIKK

AMGIPIEKSFKLMGADGLKENQFESLLHIFRERYKENEVDHLRLFPYVEETLSKLEER

SVVMYVLSSKKSDVLQRNLEELGIDAYFKDYYGSDKVEHYKPHPDGIYKILEKYQHLK

SETLMIGDASYDLQMARRAGVSNCGVTWGSFSEADLRIEKPDFIVHDFRDLLDLTE"

CDS 3325536..3326333

/locus_tag="JFBMFIFI_03088"

/inference="ab initio prediction:Prodigal:002006"

/codon_start=1

/transl_table=11

/product="hypothetical protein"

/translation="MSRVKTRAELKAEAKDLLRGRWGDGILLNLVPTLLQIALFVFIA

FISAAAIALAVIFFRDTDFMSQTSTYTDGYQGNSGGGYGGGGIFGLFISLITIGISFT

FLDWLRQPTKKIQPFKDAFQVYTSKNFLTVLVISILSSIFTFLWSLLFVIPGIIKALA

YSQSYFIYKDLSSHGGNEGMRYTNYITESRQLMDGHKGRYFLLQLSFIGWHILALLTL

GIGYLWLNPYMSATYAAFYKDLAQDRYLSGAKVYEDVEDTTNEWTND"

CDS 3326523..3326765

/locus_tag="JFBMFIFI_03089"

/inference="ab initio prediction:Prodigal:002006"

/inference="similar to AA sequence:ISfinder:ISSpn6"

/codon_start=1

/transl_table=11

/product="IS200/IS605 family transposase ISSpn6"

/translation="MKKDNQSLSHTVWKCKYHIVFAPKYRRQIIYGKYKQSIGKILRE

LCERKGVEILEANACKDHIHLLVSIPPKLSISSFIG"

CDS 3326787..3326993

/locus_tag="JFBMFIFI_03090"

/inference="ab initio prediction:Prodigal:002006"

/inference="similar to AA sequence:ISfinder:IS657"

/codon_start=1

/transl_table=11

/product="IS200/IS605 family transposase IS657"

/translation="MIFDRHANLKYRYGNRKFWCRGYYVDTVGRNKKQIEEYIRNQVQ

EDYVADQLTLFEEYDPFTGNKNTK"

CDS complement(3327109..3328002)

/locus_tag="JFBMFIFI_03091"

/inference="ab initio prediction:Prodigal:002006"

/codon_start=1

/transl_table=11

/product="hypothetical protein"

/translation="MKKWVPLLCLVFLAIIGSFYLAQSKKESSIEQSKKDLKNQIPII

LLHGSGGDEYSFSPFSEELIQKEDSSSEVLPVTINEDGLLDYQGSLNSDTNRPLISLG

FTENNAPIEDWSTWLKILMIDLKKHYGFQQADIVGYSNGGLAATYYAETIGHDSDVPT

LRRLVLIGAPFNDLDEEENAGNANFEELVGQSPELGNYLAIKQHLSKDLAVLSIAGDD

ETETYTDGIVPLRSALSSRFIFSKYVHTYFEKITTEGPSDHLSLLSNQQVIDWTYWFL

FDYKLAKEREIIFASQSKPPV"

CDS 3328185..3329276

/gene="hcxA"

/locus_tag="JFBMFIFI_03092"

/EC_number="1.1.1.-"

/inference="ab initio prediction:Prodigal:002006"

/inference="similar to AA sequence:UniProtKB:P45579"

/codon_start=1

/transl_table=11

/product="Hydroxycarboxylate dehydrogenase A"

/db_xref="COG:COG0371"

/translation="MQNTLIVRGAPQEYICEVGCWDQLEERLIQRGLTHVLIVHGELS

WQVAQAKFPKLTHVKASFDVYHKECTYAERDRLMALVTEIGADGIIAVGGGKVTDVVK

ATAAQLVLPAIILPTLASTCAAYTPLSVMYNEQSEMVGLDIFPTSNSLVLVDPAIIIG

SPKRYMVAGIGDTLAKWYEAKVIIEQLEDPPVEVEIAYFAANLCQKNLMRYSTDALKA

MDEKKINTAFVKVIETTILVAGMVGGFGDEYGRTSGAHSIHDALTLIPEAHNQLHGNK

VAYSIFVQLAIENKWTEIDELVPFYQELGLPISLADMKLSKISKDIFYKVAEAATQEN

ENIHLLPGDITAEKVFNAFFELEKYSKTK"

CDS 3329522..3330994

/gene="mltF_3"

/locus_tag="JFBMFIFI_03093"

/EC_number="4.2.2.-"

/inference="ab initio prediction:Prodigal:002006"

/inference="protein motif:HAMAP:MF_02016"

/codon_start=1

/transl_table=11

/product="Membrane-bound lytic murein transglycosylase F"

/translation="MVKKSWVKKKLNSLLALVIFCSIVIPVTQVSAETTDTKLKAVQD

KGFLVVGLSADYPPYEFHQTVDGEDTIVGFDIDIANKIAEDMGVKLAITEMNFDSLLG

ALQTGKIDMIISGIAPTPERLKQVDFSDPYMDVNQKVVVRKGDADKYKKVTDFKGVKV

GVQKQTTQEALAKNELYGSKPVSLQKVPDLILNLQEKKVDALVLEGPVADAYLSQNED

MAYADVKFTDGAKQTAVAIEKNNPAFKAQINASIDEIKTKGLLADYQKKATELMFHEA

SFFEQYGSYFAKGTMYTVLLALIGVIFGSVLGSLLSLMKLAKTKLLRWPAAIYIEYVR

GTPLLVQTFIVFFGGTALFGLELSAFVSSCIALSLNSAAYVAEIIRAGIMAVNKGQME

SARSLGMSQGQSMRFIILPQAIKNILPALGNEFVTVIKESSVVSVIGVTELMFQTGVV

QGASFKPFLPLIITSLIYFALTFTLSRLLGVAERRMQASD"

CDS 3331096..3332247

/locus_tag="JFBMFIFI_03094"

/EC_number="2.6.1.1"

/inference="ab initio prediction:Prodigal:002006"

/inference="similar to AA sequence:UniProtKB:P23034"

/codon_start=1

/transl_table=11

/product="Aspartate aminotransferase"

/translation="MDSKKLAKKHQVIPSNILADIGTLAKTTPNLIDLSIGDPDLITD

DRIIQQAFLDVKKGHTKYTESGGSSDLIEAIQDFYHRFYQLDFAKNQIRATVGALHGM

YLTLQTILDPGDEVIIHEPYFSPYKDQVIHSGGVPVLVPTYEKDGFMLDVNLLEAAIT

SRTKALILNSPNNPTGAVFSPETFKKIAELAKKYDFFILSDEVYDGFSFYAPFVPMAT

YAPDHTVTFGSLSKNFAMTGWRIGYMVAPSYLNNAAKLLNEGITYSAPTPSQRAAIYA

LNHAEELIPKVSTVFKERLEYIEKRVAEIEYLSLHPVKGSMYAFINIQKSGLDSREFS

QYVLKETSVLVIPGLAFGESGKNYVRLAATQNLMVLKEAFDRLAKLRFN"

CDS 3332631..3332909

/locus_tag="JFBMFIFI_03095"

/inference="ab initio prediction:Prodigal:002006"

/codon_start=1

/transl_table=11

/product="hypothetical protein"

/translation="MSSIDKYWSDRLIYHIGFIANVSKTNSLVEKKEFHRTFIFPSSL

TEIEIKKMIFHKLDNVIEITYIEEFVEALELRTGNEPHGIAPGDYNIN"

CDS 3333296..3335677

/locus_tag="JFBMFIFI_03096"

/inference="ab initio prediction:Prodigal:002006"

/codon_start=1

/transl_table=11

/product="hypothetical protein"

/translation="MKNGVRFIVALFLVFSMVAHLPINLTIAMNDDVVPLENKGKDGC

SLTSESAENEESLPPAIENTESPIPIGTENKEEVSRTHETNEEEGVITTEDVDVTSEL

ATESQAGEIAPRTVVLGNSVLTADYQTGACGQGVGHIAFTYTAVLGLTINDNPLIILG

LPAELASQLNPSPTKQAAFLASLTGTVTYPSSVLSNTTIDLHAATTEFTLSYDVTNSA

VVLTFKKNTLSLGIASKWATDVKFDTVALYKKGISIPPASNGTSYAVKGNFTDLGGGI

NLLPGNQLKSGVISSATMPLGTCPVLPIQAPIISSLVNNQSSVSGSVNQTQDSNYVYT

VNLNFGRVDGTAIPVAINGVAVAANGTFTTAIPAALEYLDTVSAVVIATAKVGTDLYQ

STSSTATVSWPISPPILSNLAAGANQVVGTAIQAVGGNYKMQLQINNDPTRTQSSPLP

GNGSFNFSLSQVLQGGDSVTVKTQGYSNRTGLLLVESSAISQTVAYTKPSLTIVQVIE

KMNSQGTWETATAAVTGQTIRYTITTTLTNTNAKWNQSSVRNWIPAGLSNLTSAQMSK

KDSVGTITSLGAPQLVTDGASPSTQAWNYQGAPASYLQQSGESLILQYVATISGSALN

TSLLNSTFINGTDGGGTAIPQQSSQSSLPVGNGTLRFIQAPATIEFQNLKVPAKQTIY

NATTVPNPLIVADGRVIKSQWRVYVKEAKALTSASGKVLTGAFIYSKNGGNQSVNQDN

LEIFSYTSLNDNNVPVNWQANEGIRLNLTPSPNLNVNESYQGKLDWVLSDGPI"

CDS 3335784..3336548

/locus_tag="JFBMFIFI_03097"

/inference="ab initio prediction:Prodigal:002006"

/codon_start=1

/transl_table=11

/product="hypothetical protein"

/translation="MTRGDVMKVGTLCLLAPLVLVSLGGAVVDAATAGTMNSISDVTF

TTNTDPTNPVNPTDPTKPVLPVDPLDPADPHEPGTAGPLSIDYVSNFHFGNKVIQVTD

ATYYASMDSVKDVTTDQVVAVPNYLQVTDKRGSNVGWKVTVTQNGQFKTANTTPDVLD

NAQLKLTNATSNSTMNPILAPTASALVTIDPLGASSSLVVSAAEDKGMGTWTTAFGDA

VSGGQSISLSVPGTTDKVKNVQYKASLTWNLEDGPL"

CDS 3336579..3337601

/locus_tag="JFBMFIFI_03098"

/inference="ab initio prediction:Prodigal:002006"

/codon_start=1

/transl_table=11

/product="hypothetical protein"

/translation="MGRLLKWIGIIGFMIGLLFSGAGYVFANEMNFSVQAILPENQRD

KKQSYFDLKMEPKQEQVIQIELKNDTGEDIIVETTANTAVTNDNGLADYTKSAGKKDS

SLKISFAEIANVPKETLVPKQSSTLLDVQLKMPDEVYDGVLLGGLYFKEKEVESEKTE

KEEGAVAIENRFAYSVGVLLSETDVVVQPKLALNEVKADQKTGHNIISLNLQNQHAAL

VKKLKVDAKIYEQGNKTILYEKKQDDLRMAPNSNFNYAIDVVDTELKPGTYEAEVVAN

DGYRDWKMKKTFVIESAEAEKLNKSSIDDPPKTFDSRWYILIGGIAVIFVGGIAYWLG

KNGKKT"

CDS 3337761..3344627

/locus_tag="JFBMFIFI_03099"

/inference="ab initio prediction:Prodigal:002006"

/codon_start=1

/transl_table=11

/product="hypothetical protein"

/translation="MSSQSRDGEKKRFIFNRGTLKKAFIISAASVLLFSPAAPIFQNG

QFLGFQDQTASAAGLAEVSILGSSTLVSNTGTPTSNGTILSPNLDGNYDVDLTFEGQA

LANVGLADPKVVVFSLPPELEGKVVGGATIDIDAKLLPITPGDLPAVSVLIGAVDTAL

NTLKAAATPLGVDLTSLTTAFGAIRNAQDLGSYQASLPGVVSADGKSISVDFTDGLGL

FVRQAFVDTFQPLRDAVNQLSFPFPASITLNPLLAALKLALNPVFNLVDTLVEDSSGL

LTNFLSVNLLAGTSYDVKFAVSKPDAAQATIHAAAVKNPIIDASLLGAINSEGANATL

IFANPIWDNYVIPAPSVNQVYVGDVAITGTVALTNPIPPGSTFEAIVTLADGSTVTAP

VNAVDGSFSIDVSGKTLTANDVLSTIIQGTNSGVPKDSLSTETTVLAVPNPEWDAYVV

KEPTISPVHVGDTNVTGTVTLNTPIPAGTTFTAVVTLPDGSKVNAPVGVDGVITVPLN

GYVLQAGDTLSTIIQATNNGATKDSTPTVSTVLPEVNTEWENYVVTPPVISDVTVGDT

NVTGTVSLATPIPAGTTFEAIVTLPNGSQVTAPVGIDGVITVPLNGYILAADDVLNTI

IQATNAGETKDSTNVATTVKAVTWGTYTVAAPSLQPIFTTATAIDGTVTLATPIPLGA

TFEAIATLPNGSTVSVPVNGDGSFSLPLGGVQLAAGEQVSVIVQGTLNGETRDSQIIT

ATVQAVSNPEWDNYVVAAPVLDPLRVNDASLTGNVTLNTPIPVGTTFTAQVTLPDGST

ISGIVAPNGAISLNLNGYPLVVGDTLSVIVQAKNGSQGKNSPAVSVTVLPVLVDPEWT

NYVVATPTINPVRVGDTTATGAVTLNTPIPAGTTFTAEITLPGGTVVTAPVNPDGVIS

VDLGGANLVVGDTLTTVIKAKNGTDGKVSQSVTTTVIAAAVDPEWTNYVVATPVITPV

YDDATSVTGMVTLNTPIPVGTTFTAAVTLPNGSVVTAPVNPDGAITVDLGGYTLVAGD

TLTTVLQAKNGVDGKNSPTVTTTVLARDTNPEWTNYAIVAPVIDPTYDGATVINGKVT

LTTPIPVGSTFVAIVTLPNGTTATADVQPDGTISVTNLGGYVLQEGDSFTTLVQGTNN

GVIKNSPTVTTVVQAEPGNTEWENYVVAAPVIDPVHVVDATVTGTVTLATPIPVGTTF

EALVTLPDGSVVSAPVNGDGNISVPLNGYVLQVGDTLSTLVQATNNGETKNSNAITTT

VLGPIVDPEWTNYVVANPVIAPVYDDATTVTGTVTLATPIPVGTTFSAIVTLPNGTKV

SAAVQPDGVITVDLNGYVLVAGDQLSTVVEAKNGTDVKTSSSVGTTVLARDTNPEWTN

YVVTAPTVNPIHVGDVALTGTVALAQPIPAGTTFTAYTILPDGSKVSAPVNPDGSISI

DLTGKTLVAGDDLKTYIEATNGGQTKDSAQITTTVLAKEGNPEWDNYVVAAPVVDPIR

AGATEITGAVTLNTPIPAGTTFTAIVTLPDGSTLTALVNPDGSITVDLGGKTLVAGDT

VVTYVEAANAGQTKDSAQITSTVLPADGDTEWTNYIVATPTVNPVYEGDTTVTGTVVL

NTPIPAGTTFVAIVTLPDGSTVSAPVNPDGTISVDLGGKTLVAGDTISTLVEATHGGE

TKDSSTVTSTVLPKVVDPWESYVVADPTINPVHADDVAVTGTVTLTQPIPDGTTFVAI

VTLPDGTKLTGTVAADGTITVPLNGATLEPGDVISTIVQAQNDGKTKDSNPVDSTILD

PTGESDWDKYIVATPVLDPIYVGATQITGKVTLIQPIPDGTTFKATLVLPNGTKLTGT
IEADGTITVDLDGNILTAGDTITEYIEATNENRVKEGPSASGQVQPREVDPWENYVVK
EPTINPVHAGDKTVTGSVTLNQPIPAGVTFTAVVTLPDGSQVSGTVAEDGTITVDLGD
VVLKEGDTISTVVVAHRGDETKESTPVESTILPGEIDPAWANYAITLPSIDDVYVSDT
KVTGKVQLNTPIPNGTTFKALITFADGRTETVDLADDGTFKIDWTLLRAAHALTEGDS
ITATIQGTNAENLKTGPTATTIVLADKVPTSEWDSYVVAPPTIDPVHAGDQTITGKVT
FNTPIPAGTTFVANVTLPNGTVIEAPIAVDGSYKVTVTGPQLKVGEKVSATVQAKNGT
ETKDSSAVSTTVLAKTGGSNGGNNGNSGNGGNTGNTGNTGNSGTGATTTNYGNKTYSK
SDTFPQAGETQNSVFGLLGVLAIGLAGLFLFSKKNRKMTK"

CDS 3344790..3345776
/gene="strB1"
/locus_tag="JFBMFIFI_03100"
/EC_number="2.1.4.2"
/inference="ab initio prediction:Prodigal:002006"
/inference="similar to AA sequence:UniProtKB:P08078"
/codon_start=1
/transl_table=11
/product="Inosamine-phosphate amidinotransferase 1"
/db_xref="COG:COG1834"
/translation="MIKNYVESEFAPLKRVVLAQSQFYIPKEKENEETNFLSEDLPKL
EENGKEVFGDVTELYPNLQKQWEQEKNEMNRLLVSYGIEVVRPRLLTDYEKELGIQTG
DGYANFFSRDPFFTVGNLIIEGCLRFPHRRLEILAMRNLLIAESQSKESLYFATPQPD
ISDGETSEAGPFLEGGDILVYGKTIFVGYSGLASNIVGIKWLESVLVHWDYKVVPVRL
HKDILHLDCALSLVKEGLMICCEEAFLDGIPKELKSWSSIKISLKEASLLMANGLPIN
ESVYITDSSFTRIIKELESYGIKVETLDYQVSRRFGGSFRCTTQPLLRNNNE"

CDS complement(3345821..3346273)
/locus_tag="JFBMFIFI_03101"
/inference="ab initio prediction:Prodigal:002006"
/codon_start=1
/transl_table=11
/product="hypothetical protein"
/translation="MIFTLIVQFSFSFLTTAAFAIITNVPKRSLIACGLTGMLGWMVY
WSADSFGSGDVFATFLGAFTVALASYYFSKHKRLPVTIFNIPGIVPLVPGSLAYQSVR

NLVLGDYVEAVSFAVRALMIAGAIAAGLVLSEVFNHNIRNFREKKEKF"

CDS complement(3346432..3347184)

/locus_tag="JFBMFIFI_03102"

/inference="ab initio prediction:Prodigal:002006"

/codon_start=1

/transl_table=11

/product="hypothetical protein"

/translation="MTNKTIDLLLDTCLLAGKIMMESGAEMYRVEDTMNRIAANLNGH

TGISFVTPTGIFMGLEGETGLKMQQIPSRTINLEKVSKVNDLSRDFAAKKIDLTELSI

KLKHLEKDTNFFPIWLQTISAAVVSGTLMILFGGSWPDLAATCLIGAIGFLVFFYSAD

FLKIKFLAEFFASFIIGILAIFTVRFGLGINLDTIIIGCVMPLVPGVPITNAVRDLLA

GHLLSGMARGTEALITACMIGVGIAVVFQLFY"

CDS 3347540..3348742

/gene="ydhC"

/locus_tag="JFBMFIFI_03103"

/inference="ab initio prediction:Prodigal:002006"

/inference="similar to AA sequence:UniProtKB:P37597"

/codon_start=1

/transl_table=11

/product="Inner membrane transport protein YdhC"

/translation="MKKSIETKVKAPALLLLIVLVGFPQISETIFTPSLPDIAVAFQT

TMGSVQLTLSIYFFAFALGVFFWGLISDYIGRRPAILYGVVLYGVGSCLCLWADSIEF

LLFARFVQAFGASTGSVVTQTILRESTTGNQRHALFAQISAALAFTPAIGPLIGGFVD

QYWGFSAVFLVLVSMSIGVFGYAYFGLAETLDASSRSSVALRPIFKRFLKNKKVLTYG

FLIGAINGVLFSYYAEAPFIFMEYFHLSAAIYGLLGIVIALALIIGSMLSKRWLAIWQ

PEKIIFKGIQIMLVGAVILLTLSFFNFIPGVIQFSCYLIGIFITLIGTGTALPNCLSL

ALVDFQDVIGSAGGIFSLYYYLLVSLITLGMSVLHNGTLWAMPLYFIGIGIFMLILTK

RFIYLLKE"

CDS 3348902..3349930

/gene="add"

/locus_tag="JFBMFIFI_03104"

/EC_number="3.5.4.4"

/inference="ab initio prediction:Prodigal:002006"

/inference="similar to AA sequence:UniProtKB:P63907"

/codon_start=1

/transl_table=11

/product="Adenosine deaminase"

/db_xref="COG:COG1816"

/translation="MNVLEQKVVEKLPKVELHCHLDGSVSRKTIRKIAEAQDYSLPES

EAELRKLVQAGDECQSLLEYIGKFDTVLDCLQVEEAITEAAYDLIGDVKKENVTYIEV

RFAPMLSTHKGLSADQVVQATLNGLKKGEADFGVTSRAILCMMRHHDDQKNHEIIELT

KEYLGQGVVGIDLAGDEAKYPAGDYKSLLKVALEYNLPITLHAGECGCAGNVRESIDM

GATRIGHGIALKDDPEILAYCIENGITVEMCPNSNLQTKTVTEWSDYPYLKFKDAGLK

ISLNTDNRTITDTNLTKEFMTLDNLYQIGYAGMQELTENSIKASFVDEQTKKALLQTV

KTAYQEVR"

CDS 3350137..3350754

/locus_tag="JFBMFIFI_03105"

/inference="ab initio prediction:Prodigal:002006"

/codon_start=1

/transl_table=11

/product="hypothetical protein"

/translation="MESNYTSLGKVKTGLTTVQGIFIVGSIYSLLNIIVQFMMSTQSM

VMPEGGEYTPSMWDQFSGIFNQITSYIGVPGSLLFSILAVMIATFGKEIITRLDQQEP

LYSTENAATIKKMSLVTISIGAVASILVLVGIVATFLQQQAVMNMDMEAASSGMYEMK

WYDSYQWVFGSEIAGGFVLIIGYIGLYLALVGLANFFTDKITKEA"

CDS 3350981..3351520

/gene="dut"

/locus_tag="JFBMFIFI_03106"

/EC_number="3.6.1.23"

/inference="ab initio prediction:Prodigal:002006"

/inference="similar to AA sequence:UniProtKB:P06968"

/codon_start=1

/transl_table=11

/product="Deoxyuridine 5'-triphosphate

nucleotidohydrolase"

/db_xref="COG:COG0756"

/translation="MSKKRGFEVIEKYNQAGINLPTRATKQAAGYDFEAAEDIVIPTF
WKTLFKTVIWEVTGAFKEVQVAEVDQRFLKPILVPTGIKSYMGADEYLQLANRSGNPL
KSFLILANGVGVIDADYYNNEANEGHIMFQFLNFGLTDKVIKKGERIGQGIFLPFLKA
DNETDIVGERTGGFGSSGK"

CDS 3352221..3352445

/locus_tag="JFBMFIFI_03107"

/inference="ab initio prediction:Prodigal:002006"

/codon_start=1

/transl_table=11

/product="hypothetical protein"

/translation="MVSNGIEAYWQDAVIYVVCFQVRNSNGNYRDIKKNLIFKEELSE
QEVADIITRKFSNVQKVKYVDCWSEALQLK"

CDS 3352585..3353922

/locus_tag="JFBMFIFI_03108"

/inference="ab initio prediction:Prodigal:002006"

/codon_start=1

/transl_table=11

/product="hypothetical protein"

/translation="MIGTSKKTTHIQLNQIKQLVQTKYQGNLEIKSDRQGFSLERNVG
FNIQEFTIQILKEEILFVLINTALKNETQDVQLFLEKNYISLSLYYSYQKRLNQYLKK
YSIQLIANPYIHFEGEEHKIRLFLYQFYWEFFQGNEWPFEKVSKLDTHKLTDKIQNDM
QDGMGVTQKIQLSYAVAICASRIKSGCYIEKEHSLSFKRTDVFLNIFNFYGNFIKTWS
HKVPNIENEIVCLWYLSGMFATVSLKDWENADESDANQQAVQLINDLVQNLDFIFKEK
NKEQELVCQLLFSIYQEQNYFELVFETNSKELQKQVPSSIFKSINNCVEHLAGLMKKD
KAQSDRLLKRFFDLLLPMIQESLNKVKVKLYTVGTPYDYFLQKKELEMRDKSLMVVEN
SANEELDLIISDFYLEEEYQVPIFIRSFPSTDNDWELLSNLVSKIKLNLKGVI"

CDS 3353980..3357882

/locus_tag="JFBMFIFI_03109"

/inference="ab initio prediction:Prodigal:002006"

/codon_start=1

/transl_table=11

/product="hypothetical protein"

/translation="MKNTRNKIIILAGMLVLLIGSGVFAINQFFTAGINASEALSIRA

NKETILVDEKFDITLNEQGTLQTDSEQLDSSEPTADTDSQADKKVLEKEIASTKIQLP
IGLEFDAEKTKQLNEQSDKFTTTWDSDTRTIEIKWLEYQTKNELILALEAKTPGTYEL
EAIRQFNDEKETSEKLMIQVKELLQDSTAPVVEESIVNKSTESLIQPVITRQTRAVGV
DEPPVASEGTVANVTDQATFIAAMKDTNVTQINLMNDILITTGVVNHYPGYALGPAMR
NGKTLVIEGDPDGTGVKRLDFQGLSMQFQFTSANQSMNLVMRNLKLNGTNYYGPFSNA
TNNVNQNNFNMYYHNVDYTGSQLTASYASKAYFSGDVKVDTAPSYVSLSGATMTTQDR
GTLYKPDGTTAAVTNQQNMEAGNVTFMENSHFWGRTTGSANFHVYGGGNFDVGKNAVI
DLEVGGPNQWGESGSANGWPAVYLLAGAEFRVRDGAKVNITTNKTTGIQSTGRGGIGM
ENNAKLIVESGAELNIDLDKTLHRNYNGAYGDAIRMGTGGSVLVQSGGKLNLAMSNIS
NGYANASYLSMINMTGSGSFNVEDGGSLTMKRTGTTSTIPYNGINVQGAGGSVLIGRR
ATLDAELDGAGISNVINMASGSTFNFSDAESVDLRLMNTNTASRLIFMNGTLNVDIQN
IMAWNRGQDILNETAARQWNPMYGIKTVFSGGNVTSTTGNSITSSVTTDFQANFRTQN
FTRLKFAFIPDVDLVINRPLTDNKTNTSSHIITGISYPGSFIRLSLVDASGNPVANQS
LLPTATIPSQIESEQGNVANNFHTNADSEGNWSVSLPAGSYLEAGMRIKGYGFLNGKD
ASDTALVLDRTAPDAPVLNPIKDQDTTITGTAEVGSTVRAYSVVGDIQLGSDVIADST
GKYTITIPADKRPLVPYLEYYTTSSDHATDEEGNATPNTSAKSNLQAVVDTTPPTADP
NPKEFNLADGPVILDQNPQNYVQNILDNQDPIPGKTFSFEFVAGKGPDQIDTTQIGGH
SVVVRVFDQAKNYIDVTVGISIIDDSVVSGVNIFLKATDFSMIVPEFNDLTTTELLHA
ELIKRSQAVAWNKVTGEPVQTEIGTDGGISGVVGSYNLSVRALDVTPATYLSKSVVGT
VTGGTIEFFQAPKDVDFGSKEISSRKKVYTPTEDVSMIIEDSRGKTANWELYLKVGKD
LTLDNNEAISLPGALRFVNSSNEEVEINGQSGLVHSQTGSLSPMKTTVSWNRSLNQGL
LLRVNPGQATKGSYTGELVWTLMDTAENEKSSSATTPD"

CDS 3357922..3358245

/locus_tag="JFBMFIFI_03110"

/inference="ab initio prediction:Prodigal:002006"

/codon_start=1

/transl_table=11

/product="hypothetical protein"

/translation="MKIKIGFLLLLLGLSFNFLAVSTNVYADGAESDVGIFFSDIEEV
PSKPDLPNLKPNPTPEKPNTGGTTFPQTGEKAGFSSYISGLGVVVVAIFLVNKNKKRS
QNGES"

CDS 3358247..3358993

/locus_tag="JFBMFIFI_03111"

/inference="ab initio prediction:Prodigal:002006"

/codon_start=1

/transl_table=11

/product="hypothetical protein"

/translation="MKVTKLVLGSSLILGSLVIGQIGAFAGETGDSARSTADVEYTVN

TNPINPTDPTDPGNEIKPDPENPGTGNAGPLSIDYISNITFGQKVASGNAATYNAALS

RVINTDDSTKPVPNYVQVTDNRGTISGWNLAVKQEGQFKNTKGSLLGAAELSLLNGTA

NSANAVVGAIPSTNKNIALKLNASGNGVNSTVMTATSGQGSGTWTTMFGKDTTEGAES

VQLHVPANTKIEKGAYTTTLVWTLSTDAAQ"

CDS 3359017..3359781

/locus_tag="JFBMFIFI_03112"

/inference="ab initio prediction:Prodigal:002006"

/codon_start=1

/transl_table=11

/product="hypothetical protein"

/translation="MKNWKLSLVGLASISGFLLSGVEVEAATTATLNGSGEISYLENT

MHPPLVDPENPGEPGGEIVTVPPKEGTAGPLSIDYVSDFQFDTQLISGNDERYYSKLT

TIADKDGSNQREVPNFIQITDNRGNNMGWSLSVKQNGQLTSQTTNAELTGAAITIHNL

TSDAKNNDGSNGPVLASSAMNNQFTLNPNGAVTPLLVASENQGFGTWVTLFGERDNPL

IPADTAIELTVPGAAKKEKAHYTTELAWILSDEPSN"

CDS 3359795..3360499

/locus_tag="JFBMFIFI_03113"

/inference="ab initio prediction:Prodigal:002006"

/codon_start=1

/transl_table=11

/product="hypothetical protein"

/translation="MKLTKLAVGTILLSSSIFVSTTSAFAALEDGQAAKVESNGSITL

REKPVDPENPDNSGPLRIKSITNIEFGEQFISGDDQVYSAKYKTDLNEEGNALPDSLT

VIDDRGTNNGWELQVKNDGFHSADKSITLANAELVIATKSALNLSANTLPSQLKTVTL

ANDGFHSVVNASQNEGVGTTTHLFGTAKADAAGKTENTDVQLKIPGRSTKVKDTTYET

TLTWVLLDDPSANTNP"

CDS 3360525..3361259

/locus_tag="JFBMFIFI_03114"

/inference="ab initio prediction:Prodigal:002006"

/codon_start=1

/transl_table=11

/product="hypothetical protein"

/translation="MKTTKLVALTILASTGVALFSQTAFAVETNQASELKSNGVIQFK

QDRDPDPEGNEGPLRIESIAPINFGIQDISGDTKTYNSIYTEEVGGQYLPLNVVTVDD

RGTNEGWQLQVKETRPFSELDTDGTTVKPNGSVLTGSVITLKSATVERPLDKITEPIT

SRPGNATATTAVNSTGFQTLVSADANQGMGTWQTLFGEKVDAATDAGKAVENSAVTLT

IPGDTAKVKDALYQAELTWILLSTPE"

CDS 3361287..3361991

/locus_tag="JFBMFIFI_03115"

/inference="ab initio prediction:Prodigal:002006"

/codon_start=1

/transl_table=11

/product="hypothetical protein"

/translation="MNQTKKVLVALTLTTGFFVAPAALAADVPSKGYVDFKNEDEQLG

GSLRIDRVSAINFGTVGLKGDTAVYSAIYTADETGTPGTFLPLNVQTTDNRGTNAGWQ

LQVKHSRQFSEIDAEGNLVPDGSVLTNSTLEFTSTSAVDTAAISEAGKLGPSGFKSET

VLNDSFKTVVNAGEGEGVGSWKLLFGAVSDTASTESTPVHSDTVKLTVPGSTQKKENV

KYEAELTWTLLATPEI"

CDS 3362036..3362845

/locus_tag="JFBMFIFI_03116"

/inference="ab initio prediction:Prodigal:002006"

/codon_start=1

/transl_table=11

/product="hypothetical protein"

/translation="MKLTKLTLISVVTAGAILGAASPALADVTYPQTVDTKGVVQFKD

SDGEPAPVDPTDPDPNPVEPEIPAGTSGLVRLDQVPTLDFGTATIRGTKETQGAKYVR

LNNVGGTLRHFVPAYVQVTDERGTNAGWNVKVKASEFKAYDGDTLSEDQAGLVGATIS

FKNAHVNNHSGMTAPEITQYSPVANEVIVDASATSTDKVLLDAAATKGMGIWSTSFFD

APAGTAIPTSTDIANVPQDASIELAIPGTAQKSKDFRYESTLTWTISDTPA"

CDS 3362903..3363733

/locus_tag="JFBMFIFI_03117"

/inference="ab initio prediction:Prodigal:002006"

/codon_start=1

/transl_table=11

/product="hypothetical protein"

/translation="MKLTKKSIIGFVTVATLLAGSAPAVFADVVGTAEPKGKVNFVEG

EIDTEPPTVISPETEPPEEIKPEEPNFTTGLLRIVDAPVFDFGDVAISSSNALYSVKT

NKYSKVVDGVVTATPVYKAPMVQIEDKRGETTTQTWSLAVSATEFKTADDETLTGAEI

RIKDKGIVFNNTRTPDRVAPGSGINTTAAGFTIGSTAKVVLSSEAGKGNALTSLALDP

QYKEGGFDSGSGTVQAYTADTKVDNVQLFVPVTATKITDALYTSTISWTLSDTPDAIN

"

CDS 3363814..3365043

/locus_tag="JFBMFIFI_03118"

/inference="ab initio prediction:Prodigal:002006"

/codon_start=1

/transl_table=11

/product="hypothetical protein"

/translation="MKKMKKNIVLTLLATITLFIPIMKPVMAEEGDNGANNVSVSTNI

PDNQIDKNQTYFDLLMEPGKEQELEVVLRNNTDKEVTMLADVNTAITNDNGVVDYSWN

VASGLEQAKINNKDEANTKIDLKSIGYDSTLKQPLSEMASIDKEIKIPAKSQVTAKVK

VKMPDEQIDGVIAGGIYLTQKEDTSSDEGKSQGVQIKNKFVYVVGIQIRQNADISALV

PDMKLDPKKIVPTQINHRNYLGINLQNIEPVFIRKLTVDAKVYKKGSAEVLHEATQEG

MKMAPNSNFNFGVNWENQEFKAGDYRVKVTAKAEDYNKEWSWDEEFTITKDKADEMNA

KAIELEKSEIAWWVYALIGIGLLLLVLLIAYLIKRHIDKKKKEEELKRKRALARKRKK

KNAAASHKKNEQTRKKD"

CDS 3365070..3366506

/locus_tag="JFBMFIFI_03119"

/inference="ab initio prediction:Prodigal:002006"

/codon_start=1

/transl_table=11

/product="hypothetical protein"

/translation="MDKIINRKIKLIQLLYSYTTYTGVQEIADSMGLTIKTIQLELAE

IIEQLNLNNYGIKLVNEGATYYLEREAINHIDLLYFSLEKESLFFFFTNKLFYNQLDP

KKLYEIYHYSYSHFHKRKIEYGNHLRKYELSFSSKSLSIVGSETMIRFYFFSFYWEKY

GKIEWPFSHVEKEWVLSLVRKIEEDTQLSLSEIEQLKLAYWLAVILNRTLNGHFIADS
QFVEKESDFITHQNNVAWRELFFDDINKVDSNTSNQELTYLMSIVLLVLDGKKIRGLI
AQNQVDYKVGDVMDLSLKFCQSVQEAFKLKIEWELMLNSVYLVFYSEMKLSGKVRSQK
EKYNVNKNASTYKLFIEKYQKFSLDLIETTEQFRSFINVSELWEELMLIVFHVADMDS
YRPCLTAQIKLAAGNLEERLLMNCLNQLDYKIKYETEGKSPVDLVITDIYFEQSDDVT
TYFPWNRTSTQDNFKRLSHFLKEFKFTR"

CDS 3366818..3367327

/locus_tag="JFBMFIFI_03120"

/inference="ab initio prediction:Prodigal:002006"

/codon_start=1

/transl_table=11

/product="hypothetical protein"

/translation="MARPQSKSELIQLSTENYQKLKKLISSFSDAERIGKFPFEDRDK
TIRDVLAHLYEWQLMMEEWYKVGMEGGTPDVPAKGYTWRTTPELNQLIWKKYQKMNLV
EVEERLETSHKRMLVLINAHTNDELFMRNCYSWTKTTTLGAYFISATSSHYEWAMKKI
RKYQRMIKK"

CDS 3367947..3368681

/gene="yocH"

/locus_tag="JFBMFIFI_03121"

/inference="ab initio prediction:Prodigal:002006"

/inference="similar to AA sequence:UniProtKB:O34669"

/codon_start=1

/transl_table=11

/product="Cell wall-binding protein YocH"

/db_xref="COG:COG1388"

/translation="MNFNKSALSTLMIFSMVFLSLFGLRASSVKADKLPNGNWEIKDG
DTLSEIGLQTGLSVDIIRLLNPTTDPFFLQIGYELKLQESADELAQKQAEQVKLAEGA
SEEAVVAVEAATEVVIPAPEPEVVPEPEVVAEVAPTNETALGTFEATYYTAFDGTQIG
ITANGTDVRNGQTTTAEGYRIIAADPSVLPLNTVVRVTTGNGESFLAQVCDTGGAIIG
GRIDILVGSVSEATALGRTVADLTII"

CDS complement(3368751..3369899)

/locus_tag="JFBMFIFI_03122"

/inference="ab initio prediction:Prodigal:002006"

/codon_start=1

/transl_table=11

/product="hypothetical protein"

/translation="MVLILNQIIINLALIVSTVLTCYFLGLKISSNQRFKEYSSDFST

TPPLLIILFGIFIGCSSLVLSSNQIVVSQIMKIDMRYVFVFFSVIYGNRRLGEIATGI

LIFGKTIEYILAPENNALNYFNNLVLTIFLLIVSIIIKKYKLSLKKSVSYFILVFIST

RIIIFSIYFKPILELPKLISMAIYFSIFTSIFLITIIIVNMAVSVASTMNLYRTGSIT

DHLTNLYNRRMFTLDLNDSFHGSQNSSSTFCLAIIDIDDFKQINDTYGHHIGDQVLKH

FSTILKENLPQTKGELYRIGGEEFSFLTYLPREEAYATLQEFRERLIQTPYNHNTNKL

FISASIGMTEFNSEVDVDNYEGSNAVYTRADQALYEAKQTGKNKLIFF"

CDS 3370118..3371422

/locus_tag="JFBMFIFI_03123"

/inference="ab initio prediction:Prodigal:002006"

/inference="similar to AA sequence:UniProtKB:P9WFP3"

/note="UPF0053 protein Rv1842c"

/codon_start=1

/transl_table=11

/product="hypothetical protein"

/translation="MILFIRFLVIVLLIGATALFVAAEFALVKVRTSRLEQMVVEGVK

NAPLAKLVHEHLDAYLSACQLGITLTSLALGWIGESTVEAAIHPIFVWLNIPEAVTKI

VAFAIAFSIITFFHVVVGELVPKSFAISKTENVVLAVVKPLHVFYKTMYPFIWVLNSS

AAGISRMLGFEFAGEGDEAHSEEELRLIASESFKHGDINQSELLYLNRIFDFDNRLAN

EVMIPRQEMAVMTTDMTVEEAAKLSLDEQFTRYPVMKDSKDDIVGVINSRDLFNVFVK

KTLAPTDSIESLIQPILQVMETTPISDLLEKMKKEQRHIAILLDEYGGTEGLVTAEDI

LEEIVGEMRDEFDADEVPDIRKIDVGKYLISGKVLLETVEKLLHIKFEEDSAVSTIGG

WILNRKYDVEQGDTFSYKNLEFTVVELENHTIQMVEVAVIES"

CDS complement(3371577..3371954)

/gene="alkA_1"

/locus_tag="JFBMFIFI_03124"

/EC_number="3.2.2.21"

/inference="ab initio prediction:Prodigal:002006"

/inference="similar to AA sequence:UniProtKB:P37878"

/codon_start=1

/transl_table=11

/product="DNA-3-methyladenine glycosylase"

/db_xref="COG:COG0122"

/translation="MSFAYTLKRRLIEAYGESIDYEGVKYWSFPKPDLIASLAASDLT

SLQLSQKKGDYLIGIAQLIASKKLSKEKLLAMDNFAASEKNLTDLYGIGPWTANYVLM

RCLRYPEAFPVTDVGLLRAIQFS"

CDS complement(3371979..3372398)

/gene="alkA_2"

/locus_tag="JFBMFIFI_03125"

/EC_number="3.2.2.21"

/inference="ab initio prediction:Prodigal:002006"

/inference="similar to AA sequence:UniProtKB:P37878"

/codon_start=1

/transl_table=11

/product="DNA-3-methyladenine glycosylase"

/db_xref="COG:COG0122"

/translation="MKPLNYSETIEQQIHIPLPPDFNYELNLDYLSREKNESLHTIHE

KKIRRIIQVEQVDTLIEISYHPDHFLICIFLQNTRPTQLEEHLKIINYIYDWFDLHRD

LTQFYSLAQKDSVLKQSVSKFYGLRLIGIPDLFEALS"

CDS 3372653..3373216

/gene="adaA"

/locus_tag="JFBMFIFI_03126"

/EC_number="2.1.1.-"

/inference="ab initio prediction:Prodigal:002006"

/inference="similar to AA sequence:UniProtKB:P19219"

/codon_start=1

/transl_table=11

/product="Bifunctional transcriptional activator/DNA

repair enzyme AdaA"

/db_xref="COG:COG2169"

/translation="MGREITDAQWQAIVTNDSQFDDVFFYGVKTTKIFCRPSCKSRIP

NRENVEFFLDKEEALKAGYRSCKRCKSGGERVPDDEWTDQIKDFIEKFYREHLTLDII

ASECHGSPYHLHRVFKKQMSITPLSYLHQIRIEKAQELLVHHQLDIQKIGETVGIPNS

AQFSTLFKKITKMTPSEYRRLKVERNE"

CDS 3373218..3373730

/gene="adaB"

/locus_tag="JFBMFIFI_03127"

/EC_number="2.1.1.63"

/inference="ab initio prediction:Prodigal:002006"

/inference="similar to AA sequence:UniProtKB:P19220"

/codon_start=1

/transl_table=11

/product="Methylated-DNA--protein-cysteine

methyltransferase, inducible"

/db_xref="COG:COG0350"

/translation="MSEIKHIYWSQLNFLEWQMIVAKTEKGLCFIGSNQGTAKELEKW

MRKRYSDFKLIQDDDKLAASIEALTNYLKGKQRDFKIELDIIGTDFQKDVWESLKAIP

YGETRNYSMIADSIKRPKAVRAVGTAIGANPLLIIVPCHRVLGKNKRLTGYRGGLAMK

ERLIALESAK"

CDS 3374274..3375194

/gene="rlhA"

/locus_tag="JFBMFIFI_03128"

/inference="ab initio prediction:Prodigal:002006"

/inference="similar to AA sequence:UniProtKB:P76104"

/codon_start=1

/transl_table=11

/product="23S rRNA 5-hydroxycytidine synthase"

/db_xref="COG:COG0826"

/translation="MIELIATAESIEQAQALIEAGVDTLYIGEDFYGLRLPTSFNLEE

IEKITKIAHAGKTQICVAVNAIMHNDRIDFVGPYLKKLAEIGVDRVAIGDPGVIHILK

TEKIPLPFVYDAQIMVTSSKQINFWVKRGATGAVLARELPYDELRILAPKIDVPAELL

VYGATCIHQSKRPLVENYFNFIEKPEDTSKDRGLFISEPKKPETHYSIYEDINGTHIF

ATNDLNLVSGLEKLVEIGLTQWKLEGIFTTGQDFVEIVKLFVVAKEALLAGIWNESLL

EELNQKIVALHPEKRELDEGFFIKDPDEVK"

CDS 3375205..3376437

/locus_tag="JFBMFIFI_03129"

/inference="ab initio prediction:Prodigal:002006"

/codon_start=1

/transl_table=11

/product="hypothetical protein"

/translation="MRKILKKPEVLAPAGTLEKLKTAIYYGADAVYIGGDAYGLRSRA

GNFTYDEMREGVEFAKKYNAKVYVAANMVTHEGDEIGAGEFFRTLRDIGISAVIVSDP

ALIETCAMEAPGLPIHLSTQASATNYQTLEFWRNEGLERVVLAREVGMAEIKEIREQT

EVEIEAFIHGAMCISYSGRCVLSNHMSQRDANRGGCSQSCRWKYDLFDLPISGERESL

VGGQPLEEFSMSAVDMSMIHHIPELVENGVDSLKIEGRMKSIHYVSTVSNVYRQAVDT

YCADPDNYVFKQEWEDELWKVAQRELSTGFYYGVPTEDEQLFGKRRKIPAYGFIGQVL

DYDPLTQIATIQQRNNFGVGDDVEFYGPGFTHSHQVIETLWNEEDEAIDRAPNAMMTV

KTKVTTPVKRYDMIRKLR"

CDS 3376702..3377691

/gene="yxeI_3"

/locus_tag="JFBMFIFI_03130"

/inference="ab initio prediction:Prodigal:002006"

/inference="similar to AA sequence:UniProtKB:P54948"

/codon_start=1

/transl_table=11

/product="putative protein YxeI"

/db_xref="COG:COG3049"

/translation="MCTSIVLRTMDHKNLLSRTMDFAFDLDARPTICPRNYQWISGVD

ELSYTGKYAFVGAGKDVEHLLFADGVNEHGLSCAALYLPGEAVYGPAAKPNKVNLAPH

DFLLWILSNCKSIADLQKKLGTINLVDVEVPLLGITTPLHWIMTDSTGQCMVIEPRDS

LLKMQENPVNVLTNTPKLEWHISNLRNYIGIKPEPFTSKMFGEFEAKPFSQASGTSIL

PGGYTPPERFVRVAYLKEYIKKSKNEEEAITNIWHILNSVCIPNGVVIQENGSPDYTQ

YVASMCSESQTYYFSTCKNNQINSVKLTKELISTLTEPKTFDIKENQKFNSLN"

CDS 3377710..3379113

/gene="gadB_3"

/locus_tag="JFBMFIFI_03131"

/EC_number="4.1.1.15"

/inference="ab initio prediction:Prodigal:002006"

/inference="similar to AA sequence:UniProtKB:O30418"

/codon_start=1

/transl_table=11

/product="Glutamate decarboxylase"

/db_xref="COG:COG0076"

/translation="MFNKKIEKEIMEETNCPIFGSLEAGQILPNYKIGKESVDPRVAY

QLVKDQLVDEGNARQNLATFCQTYMEPEAEQLMAETFEKNAIDKSEYPQTAKLESSCV

NMIADLWNASEDEKFMGTSTVGSSEGCMLGGMAMKFRWRNLAEKRGLDIQAQKPNLVI

SSGFQVCWEKFCVYWDIEMREVPMDEEHLSINLDKVMDYVDEYTIGIVGILGITYTGK

FDDIAALDTLVESYNQINEHQLVIHVDAASGGMFVPFVNPELAWDFRLKNVVSINTSG

HKYGLVYPGVGWILWRDEEYLPKELVFDVSYLGGHMPTMAINFSRSASQIIGQYYNFL

RFGFEGYRKIHTRTKEAALYLAKVVEETGLFEIYNDGGNLPIVCYKLKEKASVEWNLY

DLADRLQMKGWQVPAYPLPEEMNNVIIQRYVCRADFGQSMAEEFASDLNESIHDLNNA

HILFHKEEANKTQGFTH"

CDS 3379449..3380924

/gene="gadC_3"

/locus_tag="JFBMFIFI_03132"

/inference="ab initio prediction:Prodigal:002006"

/inference="similar to AA sequence:UniProtKB:O30417"

/codon_start=1

/transl_table=11

/product="Glutamate/gamma-aminobutyrate antiporter"

/db_xref="COG:COG0531"

/translation="MDNEKSGKSAKSLTLFGFFAITASLFITVYEYPTFATSGLQLVF

FLLLCGFCWFLPVALCSAEMATVEGWQEGGIFAWVGKTLGKRFGFAAIFFQWFQVTVG

FVTMIYFILGSLSYIFNFKALDNNPMVKFIGVLVIFWLLTFSQFKGTKFTANVAKIGF

VFGITIPVLTLFGLSIAYIASGNPLQLHVSAGSFIPKLKDMSALVVFMLAYMGVEASA

PHINEMKNPKKDYPLAMIMLILVGIVLNTIGGASVAAVVPLKELSLSSGVIQTFEVLI

LKYGSGLGWLVKLIAGLIAFGVMAQVCSWIVGPTRGLLTVAEEGILPAKFKEVNKNNV

PVPLILVQGLIVSIWAAVLTFGGGGNNVSFLTAISLTVVIYLIGYVLFFIAYFILVLK

KDNLDRTYHVPGGKGVKLTIAAIGLIMSILAILTAFIPPASLTGHSAHSYELILAISF

IVTLLLPFIIYMFRNKSEVGKKEDIKGLVAKIESDEASNVI"

CDS complement(3380981..3382471)

/locus_tag="JFBMFIFI_03133"

/inference="ab initio prediction:Prodigal:002006"

/codon_start=1

/transl_table=11

/product="hypothetical protein"

/translation="MIDEKYDIVGQATRNIKQILAIISEEKRWYTILEISERSDMITR
TVQRYIHELDARIRNFNDDNIQLLVTKNKGVFLELHPGADLRGFSLFLLEDNITIELL
KSLFFENFHSVKKFAYDNFMSETTIRRLLKMFQEIVEPYQIYLTRETFIIDGKESQIR
LFIYIIFWKVYRGATWPFETISQKLINKHVDKVATILNLNLTAIHKQQIAYILAINII
RVRKKHFIEKEDRWDHYINIEEDYSGYKKFKAIFEDMNMGSDGEISFFYLIMETRPKI
YEVKEISDRIMNYHKKQNSSIWLATELFLIRFSENFLEIPEDQRSIFITNSFCAHLFC
DLFDNFSLDINGYEYLDNHEKFYPILNTKLDSLIDELYAETKNKIFLEKEFLLTRYAL
LFSLLKPLTYFEKEIQIVLDTDLPKLAEINLKNQILDTLKYRYTIVFLDKNNAEEADL
FLTTTPTPLLINQYLDGKVLYIGSQLSPRDFINIETIVADTIKEKN"

CDS 3382755..3383750

/gene="yxeI_4"

/locus_tag="JFBMFIFI_03134"

/inference="ab initio prediction:Prodigal:002006"

/inference="similar to AA sequence:UniProtKB:P54948"

/codon_start=1

/transl_table=11

/product="putative protein YxeI"

/db_xref="COG:COG3049"

/translation="MCTSIVMESLDGKHFLSRTMDFGFDLEATPSLCPRKYHWKSATD
GSEFIGDYSLVGAGRNLGQMLFTDGVNEHGLSCAALYLPGESTYEPTTKAGKINLAPH
EFLLWILTSCKSIEDLSDKLDSINLVDTKVPLLGIVTPLHWIMTDNTGKCVVIEPHGS
PLEIITNPVKVLTNTPKLEWHISNLRNFIGIKPEQFASRKFGDFEATPFSQASGTSLL
QGGFTPPERFVRAAYLKEYIDEAKNEEEAITNIWHVLNSVRIPRGVVIKNDGNPDITQ
YVASMCSESKTYYFTPYQNSQISCVTLTQDYIENGTAPISCGISKNQEFKWLNKS"

CDS 3383773..3386223

/locus_tag="JFBMFIFI_03135"

/inference="ab initio prediction:Prodigal:002006"

/codon_start=1

/transl_table=11

/product="hypothetical protein"

/translation="MNMVKKEWKNIFTNKVLLISVIAITFIPILYAGFFSKSVWDPYG
RAKNLPVAVVNEDRNVMMAGQKVNVGEQLVDNLKSNHDLDWKFVSAKEAEKGMENLDY
YMIVTIPEDFSKNATTLLDEKPQKMDIIYKTNGSLNYIGEEISTIGATTLESQVRENV
TESYVEVAGKVGKQLVSGITQASDGAKELADGGLQLQTGLGQYTSGVTQADSGASQLA
DGTNQLSSSVAPLSSGVSELYTGSQQLDTGINELNSKIPELQSGVSQLDSGLQQLSTG
SGQLTDGLAQMSAQLNDPTTAAQLNQLTSGLAEFRGIMDEIDFSAHSKIALDAAKRAD
EISIKAAELQTKLDTIAAELNPEQLKLEITEKINQSSLPSEEKNSLINNINPLIEEHT
KRQQETINTLLKEISGGLVEIGNDLHELSIAAKSVSELPNDASQLDQGFIQIQDGTIQ
LMNSMNQIKQGVGTANNPASLYGGAVALNSGIDAAYAGGQEMNSQIPTLASGVSQLSV
GGDQLATGLGQMYGQMPALASGVNQLDDGAIQLSSGLDLLSSNSPQLLDGINQLDAGS
NELADKLGSGAAEGNNLKLNKATVSMFAAPSKLKHEEYSNVSNYGEALSPYIMSLALF
VGCLVFNFVFPIRKVSMAGQSSKNWWLSKLSIGVGVALAMAIIEATVMLMLGLTVEFI
PQFYLIAIVSSLSYMFIVMFFAMTFDNPGRFIAMVLLVLQLGGAGGTFPMPLTNGFFN
AIHPYLPMSYSIYGFRQAISGGIGTDLYTKSVVILAIAFVVFVALLRLSMDHLQKKHL
NGISQLDNNQELQAVEAQ"

CDS 3386293..3386553

/locus_tag="JFBMFIFI_03136"

/inference="ab initio prediction:Prodigal:002006"

/codon_start=1

/transl_table=11

/product="hypothetical protein"

/translation="MTNYDMKKDHTLEKIAETLEKLDTQLAKISEETESESKFKNIKL
WTEEKKAVHEIKKILHEVGKYEKYNEKECDQERKALAKSEGA"

CDS 3386639..3387682

/locus_tag="JFBMFIFI_03137"

/inference="ab initio prediction:Prodigal:002006"

/codon_start=1

/transl_table=11

/product="hypothetical protein"

/translation="MLEIWGNMLQRKRFRQVLIFCGIALILYLSKGIFTLIVLTYIFS
FLLSRGVKKIKKIVPISHTIITLTIYAGIAGLICLFFMKSVPILANQISNLYDLATSF
YQTKDFSNVPLAKELEGYVQNFDVISKVESNISTFSHFVKSISELSVNMAWAFVLSLF

YVLEIKQIKHFFALFKKEPFTILYNDLSEMFSRFNRIFSSVMTVQFKIVLFNTLLTIT

SLIFLGFPHIISLSIMLFILSWIPVFGVMLSFIPLSIIAYMQSGLNQVIYILVITICI

HLAETYILRPKLMSSQTELPTFFIFVILLLSPEVIGVWGLLLGIPIFLFILDILGIKK

IEKDNLKERTQVV"

CDS complement(3387794..3389254)

/gene="nylA"

/locus_tag="JFBMFIFI_03138"

/EC_number="3.5.2.12"

/inference="ab initio prediction:Prodigal:002006"

/inference="similar to AA sequence:UniProtKB:P13398"

/codon_start=1

/transl_table=11

/product="6-aminohexanoate-cyclic-dimer hydrolase"

/translation="MSHLDGLALAEKIRNSEVTPTKLIEEAFKKIKSENPTLNFVTST

RFSKAIEEAQVRDFSTLPFGGVPILIKGLGQDLAGEPSTAGAKLLRNNSALTTSHFVS

ALEKAGFIVIGQTNTPEFGFKNITDPELYGPTSNPWNPAYSAGGSSGGAAAAVASGTL

PIAAASDGGGSIRIPASFSGLVGLKPTRGKTPVGPGTGRNWQGAAISFALTTSIRDTA

AMLDALQVIEPSAAFQASLYEPGYLTSLGTLAKKKFRIAYSLASPVHTPVSTAAKNAV

LDAVTFLKAQGHHVVEKEPDIDGIALMESYYVMNAGETAAMFKNLEKQLARSLTIEDM

ELMTWTLFNAGKHISAADYSNSLALWDQAAEKMAAFNQEYDLYLTPTTATTAPKIAAE

LQTPEQIQQMKQVEKLSSTEQMQLVWDMFEKSLALSPFTQQANLTGQPAISLPTHIAE

NGLPLGIHFTAPKNREEWLLEIGLEFEQARLFKMRE"

CDS 3389460..3389837

/locus_tag="JFBMFIFI_03139"

/inference="ab initio prediction:Prodigal:002006"

/codon_start=1

/transl_table=11

/product="hypothetical protein"

/translation="MTEEKNSSQKEEQAKNSGWKKINKKFVLLSFFFLFIGFISGYQV

SGMNNQAGTRQPRADQVENYDDQNNNTMENGRPDQRGLPSDRSNGEESDAISGATESE

EDQLESGSTTNQSNNTSATEDVI"

CDS 3390032..3390796

/gene="ygaZ_2"

/locus_tag="JFBMFIFI_03140"

/inference="ab initio prediction:Prodigal:002006"

/inference="similar to AA sequence:UniProtKB:P76630"

/codon_start=1

/transl_table=11

/product="Inner membrane protein YgaZ"

/db_xref="COG:COG1296"

/translation="MLKKNQAIWRETLKVAMPLCLSYIPVGLACGVLLQKVGFNGFYT

ALISFLVFSGGAQFLAAAMLVDAAPIASIILMTLFLELRYILLSSSLSTFIKQEKKGF

IALFTRTINDENYAVNYLKFSTDKNWTSKKALMVNRYSLASWLISNVIGSMLGSTIPL

DTHIVDFALTAMFIYMFTMQLQNKLIIFVGLLSGVLSVVTMLVFQSTIGLIIATVIAS

LTGYFIEKKLKVAKIVDKPMLMDRKPIETEVTASNE"

CDS 3390789..3391106

/locus_tag="JFBMFIFI_03141"

/inference="ab initio prediction:Prodigal:002006"

/codon_start=1

/transl_table=11

/product="hypothetical protein"

/translation="MNNQLILLILGMAFVSYLPRVIPMMYFSKREIPQWFHDWMKYVP

AALFAALFFKDVFIVDHSFSLITNIKVLAAVLVMIVAYKTKSMGLSVVAGLGSLLLLN

YLM"

CDS complement(3391468..3391974)

/gene="paiA_2"

/locus_tag="JFBMFIFI_03142"

/EC_number="2.3.1.57"

/inference="ab initio prediction:Prodigal:002006"

/inference="similar to AA sequence:UniProtKB:P21340"

/codon_start=1

/transl_table=11

/product="Spermidine/spermine N(1)-acetyltransferase"

/db_xref="COG:COG0454"

/translation="MIGLKKATNKEEAILLNKLAREIWEEYYTAIIGAEQVEYMLTNL

QSTEKIYQDIVNGMNYFLINFDGKTVGYTGFQLENDYLFLSKLYVKSTARQHGIGKKV

FQQIQNIARENDLLKIQLTVNKYNDKSIAAYKKMGFVTVKEQVVDIGGGYIMDDFVME
YSLEDNTK"

CDS complement(3392048..3393307)

/gene="tyrS1"

/locus_tag="JFBMFIFI_03143"

/EC_number="6.1.1.1"

/inference="ab initio prediction:Prodigal:002006"

/inference="similar to AA sequence:UniProtKB:P22326"

/codon_start=1

/transl_table=11

/product="Tyrosine--tRNA ligase 1"

/db_xref="COG:COG0162"

/translation="MNIIEELEWRDAINQQTDAEGLKELVTEKKISLYCGVDPTGDSM
HIGHLIPFMMMKRFQLAGHHPYILIGGATGTIGDPSGRTSERQLQTMEQVQQNVDALT
AQMQNLFDFGGNDDVTMVNNYDWTHDLTLLDFLRDYGKNFNINTMLAKDIVSSRLDTG
ISFTEFTYQILQSMDYLHLFKHHDVQLQIGGADQWGNITAGLDLIRKKEGSEAKAFGL
TIPLMLKADGTKFGKTAGGAIWLDPKKTTPFEFFQFWLNQDDRDVIKYLKFFTFLTQD
EISALAEKVATEPHKREAQKTLAREMTHFVHGQKALDEAEKITSALFTGDVKNLTADE
IEEGFKNMPTFEAPKEEKNIVDWLVELGIEPSKRQAREDITNGAISMNGEKVLSLEAI
ASPSNSFDERFILIRKGKKNYSLVKLV"

CDS complement(3393774..3394151)

/gene="yabJ"

/locus_tag="JFBMFIFI_03144"

/EC_number="3.5.99.10"

/inference="ab initio prediction:Prodigal:002006"

/inference="similar to AA sequence:UniProtKB:P37552"

/codon_start=1

/transl_table=11

/product="2-iminobutanoate/2-iminopropanoate deaminase"

/db_xref="COG:COG0251"

/translation="MLEKIYTDQAPAAIGPYSQAIKIENTLYTSGQIPVDPTNGKVIS
TEITEQTQQVMKNLQAVLEEGGSNLNSVIKTTCFLTDMADFAAFNEVYGSYFGDHKPA
RSCVAVKELPLNVRVEVEAIAVI"

CDS complement(3394145..3395350)

/gene="malY"

/locus_tag="JFBMFIFI_03145"

/inference="ab initio prediction:Prodigal:002006"

/inference="similar to AA sequence:UniProtKB:P23256"

/codon_start=1

/transl_table=11

/product="Protein MalY"

/db_xref="COG:COG1168"

/translation="MKQTNFDEVTDRTGTYCTQWDYIEDRFGEKNLLPFSVSDTDFKV

PTAIIDTLKKRMDHEIFGYTRWNHLSFKGAIQQWYQNRFQSTIETDWVLYSPSVIYSV

AKLIELKSERGDYIVMQTPAYDAFFKTITDNQRIIHENPLLFNDGHYTIDFIDLEKRL

SHPKTKIFLLCSPHNPTGRVWTKVELLKIIALCQKYDVFMISDDIHMDIVRADFTYLP

LTSCTTDLKNIAICSSASKTFNTPGLICSYLLIPDNKLREEFLVTLKNRDGLSSTSIF

GMLSTISAYHECAPWVDELNLYVTNNLVLLQEFLTTELPEIQLVKPEATYLAWLDVSR

LSYTSKQLQEALIHYGKVAIMPGNTYGTAGENFIRMNVGCPREKLIDGLQRLKLAVNQ

LKSQEEFTC"

CDS complement(3395368..3396921)

/gene="malX"

/locus_tag="JFBMFIFI_03146"

/inference="ab initio prediction:Prodigal:002006"

/inference="similar to AA sequence:UniProtKB:P19642"

/codon_start=1

/transl_table=11

/product="PTS system maltose-specific EIICB component"

/db_xref="COG:COG1263"

/translation="MEKKKFDFWEFFQGLGKTFMLPVALLAFMGLLLGIGSSFSSPST

LETLPFLNQPWLQIIFKFMSTIGGFAFTYLPLLFAMAIPLGLARYEKGVAALSGFVGY

VIMNLSINFYLTETGRLADLDNLREAGQGMVMGIQTVEMGVLGGIIVGIIVYLLHSNF

YDIQLPDAFAFFGGARFIPIITSLVLAIVGILIPMIWPIFAMAITGIGTLIQKSGIFG

PFLFGAGERLLLPFGLHHILVSMIRFTEAGGTEIVNGQSISGALNIFYAQLQSGTPIS

PAATAFLSQGKMPTFMFGLPAAALAMYQTALPENRHKVKGLLISGVIATFVTGITEPI

EFLFLFIAPALYGFHVIMTGLGFMLMALLGVVIGNTDGGILDFIIFGVLQGNYTKWYL

VVVAGIIWFVIYYSVFKYVILKFNLKTPGREVLQEDFSEAELTHKKKGKYDGARILAA

LGGSQNIDSLDNCITRLRLVVKDMSLIDDAELTACGALGVMKLNDTNLQVIIGTQVAS

VKNQIEKIL"

CDS complement(3397221..3398006)

/gene="hexR_2"

/locus_tag="JFBMFIFI_03147"

/inference="ab initio prediction:Prodigal:002006"

/inference="similar to AA sequence:UniProtKB:Q88P32"

/codon_start=1

/transl_table=11

/product="HTH-type transcriptional regulator HexR"

/db_xref="COG:COG1737"

/translation="MQTFYKRLLQKRETLSLLEKQVLEYILSHPEKIIQMNLSQVSKE

TFVSTATISRTCKQLGYAGFQELKYTLSQYVKTEKQQLPLSFSSITGIEEMAERIRVE

VDQTLSHLNKENLSKGADYLIKSNRVEFFGVGASLPSCLDAARKLTFSGRIANAREDW

DELRAVAHSLTPDDVAILVSYSGETIHIIEFATILKERNVPIIGIIGTDNSQLEQLAS

LTYQAKITNCYYGDVDMSSRIPLNLVLEFLIIHYLNISTLLKD"

CDS 3398238..3399350

/gene="frmA"

/locus_tag="JFBMFIFI_03148"

/EC_number="1.1.1.284"

/inference="ab initio prediction:Prodigal:002006"

/inference="similar to AA sequence:UniProtKB:P25437"

/codon_start=1

/transl_table=11

/product="S-(hydroxymethyl)glutathione dehydrogenase"

/db_xref="COG:COG1062"

/translation="MKSRAAVAFGPDQPLKIVEIDVEAPKKGEVLVKITDTAICHTDA

FTLSGDDPEGVFPAVLGHEGGGIVIEVGEGVTSVDVGDHVIPLYTAECGKCKFCLSGK

TNLCQAVRETQGKGLMPDGTTRFSYQGEPIYHYMGTSTFSEYTVIPEISLAKINPEAP

LEKACLLGCGVTTGIGAVHNTAKVQAGDTVAIFGLGAIGLAVIQGAVQAKAKRILVID

TNPDKFELAKEMGATDFINPNDYPQPIQEVIVEMTDGGVDFSFECIGNTNVMRSALEC

CHKGWGQSIIIGVAGAGQEISTRPFQLVTGRVWKGSAFGGVKGRSELPAMVEQAMRGE

IQLDPFITHTLPFDQINEAFDLLHAGKSIRTVLSYD"

CDS 3399371..3400222

/gene="yeiG"

/locus_tag="JFBMFIFI_03149"

/EC_number="3.1.2.12"

/inference="ab initio prediction:Prodigal:002006"

/inference="similar to AA sequence:UniProtKB:P33018"

/codon_start=1

/transl_table=11

/product="S-formylglutathione hydrolase YeiG"

/db_xref="COG:COG0627"

/translation="MGNVQIETIEEHKVFDGTQYKFRHYSTLLNCSMTFSLFLPNKEI

FANPPLLWWLSGLTCTDDNFTHKAGAQKVAAKKGLALIMPDTSPRGANVPNDDAWDLG

QGAGFYLNATEEPWQTHYKMYDYITEELTGILRDKFNLIGPEAIAGHSMGGHGALVIG

LRNPKRFSSISAFAPIVNPSEVPWGIKAFSTYLGQDKTTWSQWDAVELLAAAPTGEAS

PILISQGESDPFYKEQLQPEKLQTVAQRKKIPLTLKLEPEFDHSYYFVSTYIEEHIEF

HLQHLEK"

CDS complement(3400271..3400669)

/locus_tag="JFBMFIFI_03150"

/inference="ab initio prediction:Prodigal:002006"

/codon_start=1

/transl_table=11

/product="hypothetical protein"

/translation="MKKTIKIALTILVSGVLLLSLASFLLPKQQQTKNDDLSQQLDRF

NPFVSKEDVYVLTSSEHGTKQSPGGYIYSQDAYKSDGNHYEVSFYAGDELREGAYLKL

DAKGVYINDWEEVSQEDLPEKVKEAFTSKE"

CDS complement(3400898..3401590)

/locus_tag="JFBMFIFI_03151"

/inference="ab initio prediction:Prodigal:002006"

/codon_start=1

/transl_table=11

/product="hypothetical protein"

/translation="MKIDIWSDFVCPFCYIGKRHLEEALGDREDVEVVFHSFELDPNG

AIEHDEDIHQLIATKYGISYEQSKANNDSIGQTAAAAGLTYDFDNMKNTNSFTIHRLA
QFAKEVGKGNEFVEAGMHAYFTDGAFLNDEETLVAIATSVGLDEKRTREIIHSQEYLA
PVRVDETKAMELVIQGVPFFLIDDQYAVSGAQPVSAFENILAQVDAKNATSQQTLVTE
DGANCKDDSCTI"

CDS 3401783..3402184
/locus_tag="JFBMFIFI_03152"
/inference="ab initio prediction:Prodigal:002006"
/codon_start=1
/transl_table=11
/product="hypothetical protein"
/translation="MNYFVKLFIASALIFLVIDLIWLIVIARQTYQNQIGSLLGPTKV
VPAAIFYVLYIIGIIFFVLQPALDKGSLSYALIAGGFLGLLCYGTYDLTNLATLKNWS
TVVTVIDLAWGAFITATTSGLVYGLAKYFNW"

CDS 3402207..3404039
/locus_tag="JFBMFIFI_03153"
/inference="ab initio prediction:Prodigal:002006"
/codon_start=1
/transl_table=11
/product="hypothetical protein"
/translation="MMRERGGDLTSDHLVVAFQAGAQRVIQQKNHLNGINVFPVEDGD
TGSNLASLMSSLLEEALRQMQTTQEVFTNMADAALIGARGNSGIIFAQYLNGLASHLT
DSTEKFSTSAFATSAKFAVEDAYQAIEKPVEGTMITVIREWAETLFMHKETERDIEPL
LEKGLEAAKKALSETPKQLKILKKKSVVDAGAKGFVLFIEGFTEAICDSNFQLENKEE
GTEYVTLMQRDSTQHESNEEPIFRYCTEILLAKITATSTAIKEQLAGLGDSLIVANSP
SRTRIHIHTNQPEEVVHRLRQVGEIQQQKADDMLMQYQLKKGPKHAIALVTDSIADLP
QEFIFNNQIQVLPMNLLIDSVSYLDKVTLQPSYFYKLNRLAKENPTSAQPNSKTVENL
FSFLGSHYQEIIVVTVAGKLSGTYQMIKEVAKKFQTNNLKIAVIDSKQNSAAEGLVVM
NAAEWIAEGLPFNQVVENVELATQTTEILVSVNELDAMVRSGRLPGIAGKIAKFINLK
PIVGLDQAGGGKINGVAFSTAGNEKKIMNKIKKRRKAGEILRYAVIHANDLERATKLS
QEFTKEIGFPPTYLMEISTVVAMSAGEGCVAIALSWTEGRGEKG"

CDS 3404036..3404815
/locus_tag="JFBMFIFI_03154"
/inference="ab initio prediction:Prodigal:002006"

/codon_start=1

/transl_table=11

/product="hypothetical protein"

/translation="MNTTYLIVAGLLLCYFTLLFIIAQVIENNSIVDLAWGPGFIIVA

VSSYWLMSEKSLAATWVTILVTIWGTRLFIHLAKRNIGKPEDYRYVNMRKRWGTKLPR

LKAFLNVFVLQGVLLYIVSLPILMINTSDVMNFYWWNYIGIAVWLIGFFFEVIGDWQL

TAFKKDKSNHGKLLTTGLWSLTRHPNYFGEATSWWGIYLITLTSLSALAGIVGPLVIT

LLLLFVSGVPLLEKKYKDRPDFIHYAEKTAKFFPLLGKKGL"

CDS 3405112..3405777

/gene="aqpZ"

/locus_tag="JFBMFIFI_03155"

/inference="ab initio prediction:Prodigal:002006"

/inference="similar to AA sequence:UniProtKB:P60844"

/codon_start=1

/transl_table=11

/product="Aquaporin Z"

/db_xref="COG:COG0580"

/translation="MLKKGIAEFIGTFVLVLFGTGAAVLGGGIEGIGTLGIAMAFGLS

IVAMAYSIGTISGCHVNPAVSIAMYVNKRLSISELVYYIVGQVLGAVVATGVLKIILS

TSDMAVTNLGQNSFGALGAGGAFLVEAILTFIFILVIIVVTGKHGSPEFAGLVIGLTL

VLIHLLGIPLTGTSVNPARSFSPAIFAGGEALSQLWVFIVAPIVGGILAAITSKLLLD

SEA"

CDS 3406078..3407271

/gene="cwlO_3"

/locus_tag="JFBMFIFI_03156"

/EC_number="3.4.-.-"

/inference="ab initio prediction:Prodigal:002006"

/inference="similar to AA sequence:UniProtKB:P40767"

/codon_start=1

/transl_table=11

/product="Peptidoglycan DL-endopeptidase CwlO"

/db_xref="COG:COG0791"

/translation="MKKRILTLVLAGTLSMTALATPLTALADDYSDKINSQNEKIKEI

ETQEKDVTTKLEGVTKEIVVAEEKARVLVEQSQATHAEMEKLTKEVDSLNAKIEKRTA
QLEKQARAVQVSASSEGYVDFILSADSLSDVVGRVDVVAQMVSANRELVKAQAEDKAT
VESNKKKTETKLTEQHEVAGQLEKLKGELEGKKLEQESVVATLAASKASAEGERDGFI
AQKEEADRKAADLKAAEEAAKKAPVLQTSTEDKKAPVTTTNQESNPPAPIGGGTSGTV
TAAEGQAIVAEAANYLGTPYVWGGKSPGGFDCSGFTGYVYSRVLGKSIGGYTVPQESA
GVEVSMGALQAGDLLFWGARGNTYHVAIYVGGGTYIHAPTEGDVVKYQSMGGYAPSFA
VRVVK"

CDS 3407852..3408742

/locus_tag="JFBMFIFI_03157"
/inference="ab initio prediction:Prodigal:002006"
/codon_start=1
/transl_table=11
/product="hypothetical protein"
/translation="MKKLGEKIKNIRRSKGLTQNELAEGICTQATVSNLENGASLPSV
TTLLAIADRLKINFSEISDYMPTNDNSFIEIFNQVKALCNKGEYKKAQTLLKKSIKLD
KLERDYEIKEYYYYLGLTSLVGEGNYSDAHYNFNLALFSETGKKLDIIDVLTTNGIAV
AYFLNDELDKAHVYFTKSIAQLEELRSAGDHLLDSMEVIKIYFNTAKYYSATENYKKA
IEFCTIGIKLQQKEDKMHGLENLLYEKAHNLFKLENVKEAEDYYFHALSVSMLNKNDE
LTETLKKELAKNKINSYSYK"

CDS 3409086..3409943

/gene="salL"
/locus_tag="JFBMFIFI_03158"
/EC_number="2.5.1.94"
/inference="ab initio prediction:Prodigal:002006"
/inference="similar to AA sequence:UniProtKB:A4X3Q0"
/codon_start=1
/transl_table=11
/product="Adenosyl-chloride synthase"
/db_xref="COG:COG1912"
/translation="MGKYLVLQTDFGLGDGAVSAMYGVAHMVSDEVVVEDLTHDIPPY
DIWVASYRLLQTVKYWPKGTVFVSVVDPGVGSDRRSIVCETESGHYILTPDNGSLTHI
RHYQGIREVRAIDEVVSRLPHSEESHTFHGRDIYAYNGARLASGEINFSELGDVVSQD
SLTYLDLIDAKEENGILTGSIDVLDIRFGSLWSNIPLALFKQAGIVHGDSIQVTIFHQ

GKKVYQNIMMFAKSFADVNIGEPLVYVNSLVNVGVAVNQDSFSRLYHIGTGTAWTIQL
RKAPKVIFE"

CDS 3409973..3410521
/locus_tag="JFBMFIFI_03159"
/inference="ab initio prediction:Prodigal:002006"
/inference="similar to AA sequence:UniProtKB:Q97SA4"
/note="UPF0397 protein SP_0482"
/codon_start=1
/transl_table=11
/product="hypothetical protein"
/translation="MKKDFSVKTIVAIGIGSAVFVILGRFAVIPIGIPNTNLETAYPF
LALMGVLYGPFAGALIGLIGHTLKDLTTYGPWWSWIICSGIIGAVYGFIGSKINLSAG
EFGKKAIIRFNLYQVLGNVIVWGLIAPTLDVLIYSEPASKVYTQGVGAVITNSLSVGI
LGTLLMVAYAATRTKKGSLKKD"

CDS 3410638..3412347
/gene="ykoD_2"
/locus_tag="JFBMFIFI_03160"
/EC_number="3.6.3.-"
/inference="ab initio prediction:Prodigal:002006"
/inference="similar to AA sequence:UniProtKB:O34362"
/codon_start=1
/transl_table=11
/product="Putative HMP/thiamine import ATP-binding protein
YkoD"
/db_xref="COG:COG1122"
/translation="MKKPLIIFDNFSFQYHSQAEPTLYDLNLTIYEGEKILILGPSGS
GKSTFAQCINGLIPHSYEGEITGSVIVNGKNLQETSLFELSFDVGTVLQDPDGQFIGL
TVAEDIAFSLENDCVPQNEMIPAVEKWAKVVDIAHQLSQRPQDLSGGQKQRVSMAGVL
IDEVPILLFDEPLANLDPAAGKQTIELIDQIHQQSETTVLIIEHRLEDVLHREVDRIL
VFDEGRIISDSTPDELLKTEILSETGIREPLYLTAMKYAGVDLEELADLADIHKVAGP
QLKEAMEQWLAAIPEFNTVTHNEEFFKVENLVYRYNKDRPLVLNQVSARFHKGEMLSI
VGKNGAGKSTLSKAICGFIQPQSGTMEWQGTDFSTLSIKERADKIGYVMQNPNQMISK
TLLFDEVALGLVLRGVEEAEIKQRVEHVLKICGLYTFRNWPISALSFGQKKRVTIASI

LVLNPAMIILDEPTAGQDFKHYTEMMSFLEELNRGGVTVAMITHDMHLMLEYTNRALV
IADGEILADTESVKVLTNQELIKEASLKETSLFTFAKALGMQDPFAFTRKFIAYDREV
RLR"

CDS 3412344..3413174
/gene="ecfT_3"
/locus_tag="JFBMFIFI_03161"
/inference="ab initio prediction:Prodigal:002006"
/inference="similar to AA sequence:UniProtKB:Q035B4"
/codon_start=1
/transl_table=11
/product="Energy-coupling factor transporter transmembrane
protein EcfT"
/translation="MSQQQLLGYLPNTTPIHRLNGASKLICLILLSVACMTTYDTRFL
LFMCAFSVLLFALSKIRWRQISFVVKFILIFSLLNILAVYIFAPEYGVELYGSRTVLW
EGIGRYTITSEQLFYEFNLILKYICTIPLALVFLLTTNPSEFASSLNRIGVSYKISYS
VALALRYIPDIQEDFFNISQAQQARGYEMSKKGKLFSRLKGTARIVLPLIFSSLERIE
VISTAMELRRFGKSKKRTWYAEKPYHTSDYFAVAIALILTIISFTLIYLNGSRFYNPF
"

CDS complement(3413222..3413689)
/locus_tag="JFBMFIFI_03162"
/inference="ab initio prediction:Prodigal:002006"
/codon_start=1
/transl_table=11
/product="hypothetical protein"
/translation="MPTNKKEGIFFTVVMCSMMVLGMTIYNIALVEGISTELIKLVAF
GFFPGFIVALIIDVAIVSPVAKKLAFKLPINKEKPWQIILAISGCMVCGMVLFMSIFG
LVTEGNFSGNIFMNYLVVVRNNIVMALPLQWLIVGPLARKILGMYQNWQLKTA"

CDS complement(3414106..3414804)
/locus_tag="JFBMFIFI_03163"
/inference="ab initio prediction:Prodigal:002006"
/codon_start=1
/transl_table=11
/product="hypothetical protein"

/translation="MKIEFFHDVICSFCFPMSYRIRQLKADMPELEIVHRSFALVKTP
TDFIAMFGSREQAKAEIMSHWKHANQNDDLHRFNIEGMKKETFLFPTSMNGLIAVKAA
EIIEGSNAGWDLFDALQKAFFVENKNIESPDIIKQEVVKLGLDLNTWEEAFQATSTKK
QVEEDFNLANNYSIKSIPTLIVNGKYEISGAQPLAKIKDYLKIIQEKENTKVVLQDIG
GESCNLEDGKWNCD"

CDS 3415006..3417141

/gene="nhaK_3"
/locus_tag="JFBMFIFI_03164"
/inference="ab initio prediction:Prodigal:002006"
/inference="similar to AA sequence:UniProtKB:O32212"
/codon_start=1
/transl_table=11
/product="Sodium, potassium, lithium and rubidium/H(+)
antiporter"
/db_xref="COG:COG0025"
/translation="MYKKEVNTLLPILEGAIILFVMVVLSNIISHYIVSIPTALIQIG
LGLIVALVFGTQIELETSWFMLLFVAPLLYNDGRHYPKRDLWNLRIPIFGNSILLVFL
TTVIGGYIMYFVTDGKMPLPAALALAAILSPTDPVAVNGIAERVKLPTGVLRLVRGES
LVNDASGLIAFKYAIAATVTGVFSLKTATIDFFYMAIVGVLLGIVLQLVLLAIKEWLS
AQGIKDVVLHTLIQILTPFIIYIIVEELFHASGVIAVVAAGVVANNKKTVTENRMAEV
RIVTERTWDVIIYLLNGMMFLILGIELPFAMRTALENPLMSNYLLFGYVILLWLVILV
SRILWTYSYMWFGFSFGKNKSGVKPDFKIALMSGLTGVRGAVTMAGILSVPYLLESGA
AFPQRSVMLFVASGVIVATLIAATIALPILTDSKKRFETSGDELIDFPENSVLEDPKE
EELKEAQARIRIMQIAIRTIEQESRPENRMASYDLIHEYNHMIRRLQMEYNSKESIAQ
FLKEEIAIRLVALDAEVVRVKEMRDNGEVEREIAEQYLKALARRRNALTSDWSSKFKR
LWILARRFYRRNLGLFFTLGTREAYQEEYRKRLALEKEAAKSAIRSVSAFMKKDRKKG
SRTDKAVAYHLIVEYRNKIERIKRYGEDYAEEYDQQLQELRLKALNAERTDIQKMFEN
GDISRDLATQLRRFVNYTESSVMDIGEEE"

CDS 3417361..3417903

/gene="puuR"
/locus_tag="JFBMFIFI_03165"
/inference="ab initio prediction:Prodigal:002006"
/inference="similar to AA sequence:UniProtKB:P0A9U6"

/codon_start=1

/transl_table=11

/product="HTH-type transcriptional regulator PuuR"

/translation="MEIGNRIKNLRIQKNLTQEELGERTDLSKGYISQLERDLSSPSL

ETFFDLLEVLGCSPKDFFDEGQEEQRVVYTENEMTNFEDEEKGYKIQWLVPESNENEM

EPIQLTFKEKGEFKEFPPSLAETFAYVTEGAVCIEIGLKRYFAKKGESIYFHATDHHQ

IKNEFNGISNLLVVVTNSYL"

CDS 3417922..3419016

/gene="potA_2"

/locus_tag="JFBMFIFI_03166"

/EC_number="7.6.2.11"

/inference="ab initio prediction:Prodigal:002006"

/inference="similar to AA sequence:UniProtKB:Q7A679"

/codon_start=1

/transl_table=11

/product="Spermidine/putrescine import ATP-binding protein

PotA"

/translation="MTKNTIITFENIIKQFDDDEPVLKSIDFEIERGKFYTLLGPSGC

GKTTILRLIAGFTEATSGTIMLDGQRVNDIPANKRKVNTVFQDYALFPHMNVFDNIAF

GLTIKKMNKKVIEEKVKDVLKMVQLPGFEERDISEMSGGQRQRVAIARALVNEPEVLL

LDEPLSALDLKLRTEMQYELRELQRRLGITFVFVTHDQEEALAMSDEIFVMNKGEIVQ

SGSPVDIYDEPIDRFVANFIGESNIVEGQMVADYEVRFVNHTFECVDAGMRQNEKVEI

VIRPEDLTITTVEKGKLSATVATQLFRGVHYEIVCKDMDENIWVIHSTKKATVGAEVG

IFFEAEDIHVMRFGETEEEFDARLDSYDED"

CDS 3419029..3419844

/gene="potB"

/locus_tag="JFBMFIFI_03167"

/inference="ab initio prediction:Prodigal:002006"

/inference="similar to AA sequence:UniProtKB:P0AFK4"

/codon_start=1

/transl_table=11

/product="Spermidine/putrescine transport system permease

protein PotB"

/db_xref="COG:COG1176"

/translation="MAKQTKLFYFIPYVMWLALFVVAPLILIIYQSFFDVNGQFTLEN

YKIYFTSGTYLKMTFNSIWYASLITFFTLLISYPTAYFLNKTKHKQLWLMLIILPTWI

NLLLKAYAFIGIFSMNGSINHFLEFIGIGKQQILFTDFSFMFVAAYIELPFMILPIFN

AIQELNPSYISASRDLGANNVETFRRVIFPLTLNGVKSGVQAVFIPSLSLFMLTRLIG

GNRVITLGTAIEEHFLVTQNWGMGSTIGVVLIVAMIIIMLVTGEKKRKGAKHK"

CDS 3419841..3420668

/gene="ydcV_2"

/locus_tag="JFBMFIFI_03168"

/inference="ab initio prediction:Prodigal:002006"

/inference="similar to AA sequence:UniProtKB:P0AFR9"

/codon_start=1

/transl_table=11

/product="Inner membrane ABC transporter permease protein

YdcV"

/translation="MKNFKWSTIYLTFVFLLLYIPIFYLIFYSFNAGGNMNEFTGFTW

EHYAEVFADTRLITIVLNTLLVAFLSALIATLIGTFGAMGIYYTKRRQARNALLSFNN

ILMVSPDVIIGASFLIFFTFLGLGLGFGSVLLSHIAFSIPIVVLMVLPKLQEMNDSMS

MAARDLGANTFQVISKIVLPSITPGILAGFFMAFTYSLDDFAVTFFVTGNGFTTLSVE

IYSRARQGVSLEINALSALMFVFSLILVAGYYFIQQHSQTKKQKNRHKRHLEAVEIK"

CDS 3420665..3421738

/gene="potD"

/locus_tag="JFBMFIFI_03169"

/inference="ab initio prediction:Prodigal:002006"

/inference="similar to AA sequence:UniProtKB:P0AFK9"

/codon_start=1

/transl_table=11

/product="Spermidine/putrescine-binding periplasmic

protein"

/db_xref="COG:COG0687"

/translation="MKKLVTFTALILILSSGLYFMTKQLEKAQGFAGNNVLTLYNWGD

YIDPDLIKKFEKETNYKVSYETFDSNEAMFTKIKQGGTAYDLTIPSEYMIQKMIKEEM

LIPLDKSKIKGLENIDSRFLDLDFDLNNKFSIPYFWGTLGIIYNDKFVKEEEMQHWDD

LWNPKLANNVMLIDGAREVIGLSLNSLGYSLNSKNMQQLTAASDKLSTLTPNVKAIVA

DEIKMYMVQEESAVAVTFSGEASEMLDNNEHLHYVIPSEGSNLWFDNFVIPKTAKNKE

AAYAFINFMLEPKNAAQNAEYIGYSTPNKAAMAYLPKELVEDEQFYPSDETMAHLEVY

EDLGAKYLAIYNDLFLEFKMYRK"

CDS 3422151..3423047

/locus_tag="JFBMFIFI_03170"

/inference="ab initio prediction:Prodigal:002006"

/codon_start=1

/transl_table=11

/product="hypothetical protein"

/translation="MEELEYYSSNQVARELDIELALLQRLSGLIEKTLMNTDYFYRDK

RNRRLYTHANINELEAILTIKKKNKLTYAAAIFGNYANTQHNLSSEEVKKEELSDVLK

KLQNQEVVLKQQSEKIDVLTNTIEKLLNLENQNYEALLGLSEKIDTYTSQKKTRPTAS

KVNVKEKVDSSFVSENERNTKKQTLQTTTATMEIAIQNPSNTKTGTTMIKKAEFLELR

KKLKYLPFKWSESRKSLNLSKKDAKHYSQYVNDSKQFLEEVMNPRKLGNYYFIKREQK

ELLNSYGNFYDTLELTAFPEKK"

CDS 3423148..3424056

/gene="rarD"

/locus_tag="JFBMFIFI_03171"

/inference="ab initio prediction:Prodigal:002006"

/inference="similar to AA sequence:UniProtKB:P27844"

/codon_start=1

/transl_table=11

/product="Protein RarD"

/db_xref="COG:COG2962"

/translation="MNQEQVQNQKKGLISGLIAYILWGVLPLYWKSVGEVSALSILCY

RIFWSFIFMVVILIISGKTKAFIAETKALLQDHKRVIAIILAALLVSANWFIFIFSIG

SGHVVEASLGYYINPLVNVVLATVFLKERLGRAEFIACLLAATGVLVLAIESGTIPWA

SLAMALTFSLYGLIKKIAQVSSFTGLTLETLVMTPFAIIYLLFFSKEGFMTFDLPINL

LLIGSGIVTAIPLFLFAEAAKNISYILLGFLQYIAPTLMLISAVFLFNESFELPQLLA

FGSIWIGIAIFTTSNILALKRNRMRN"

CDS 3424160..3425011

/gene="ispE"

/locus_tag="JFBMFIFI_03172"

/EC_number="2.7.1.148"

/inference="ab initio prediction:Prodigal:002006"

/inference="similar to AA sequence:UniProtKB:P9WKG7"

/codon_start=1

/transl_table=11

/product="4-diphosphocytidyl-2-C-methyl-D-erythritol

kinase"

/db_xref="COG:COG1947"

/translation="MEIIEKAPAKINLSLDVLYKREDGFHELEMVMTTVDLADRIALK

SLPEDQIVIRTTNGVLPLDRRNHAFQAAKLIKETFAIETGVEITIEKKIPIAAGLAGG

SSDAAATLRGLNRLWDLNLTLEEVAELGSKVGSDVPYCVHGGTAFVSGRGEKVEPIGE

MPQCWVVLVKPRVGVSTGSVFSVLSFDTVTHPDTAGMVAAIKAKDYAKMTQKVGNLLE

EVTIARHPDIERVKEKMLKFGADAALMSGSGPTIFALCDKYSRAQRVYNGLKGFCDEV

YLVRTLK"

CDS complement(3425067..3426221)

/gene="nagA_2"

/locus_tag="JFBMFIFI_03173"

/EC_number="3.5.1.25"

/inference="ab initio prediction:Prodigal:002006"

/inference="similar to AA sequence:UniProtKB:O34450"

/codon_start=1

/transl_table=11

/product="N-acetylglucosamine-6-phosphate deacetylase"

/db_xref="COG:COG1820"

/translation="MKTIISNVRLGEERDKTTSSVYIENQKITTIAPFDSLKEEAQTT

YNIINGHGHLLIPGMIDVHIHGANNYDMMDGTTKSIQEVSKKCAETGCTSFLVTSVSS

SLEDLLAMIHSVKKVIGHEEGAKIVGLHLEGPYLNVEKKGMQNPKFLRHADFDELDRI

FAEAGDLIKMMTIAPELPGGIELIAYLKQKGVVVAIAHSNATYEEAQTAFKAGASHIT

HCFNAMPTIHHRSPGLVTAALEEDEVSLQAIVDGVHLHPGIVRLMHKIKGPDKIVLTT

DALQAMGVGDGSYLFGGHHVTVTDGVARLKDGTLASSTVTMNKSLQHSVNFGIPLTDS

IAMASTTPATILNLTQLGKIAPGYDADLVLLDGEFDVVKTLIKGNICKVS"

CDS complement(3426218..3426994)

/gene="glcR_2"
/locus_tag="JFBMFIFI_03174"
/inference="ab initio prediction:Prodigal:002006"
/inference="similar to AA sequence:UniProtKB:P94591"
/codon_start=1
/transl_table=11
/product="HTH-type transcriptional repressor GlcR"
/db_xref="COG:COG1349"
/translation="MDQKQRLAKILELLEEKKKLSQIEIAEFFSISKDTARRDILLLA
ESNLVERYRGGITLPYTKAKIEKYTERLITHAPEKERLAKLAITRFMKNNMTIMLDVS
TTVNFIAQHIRQEHILLVTHSIDNALACSQTNEKNRIFLLGGFFNPASHMLAGPSIVE
QLDQFDFDVAFIGALGINSTGIFYSELDDLYMKKRMIQNARKVCLLVDSSKVNQTSSF
KLDFTGIDCIITTTPFPNEIEKVLSTHNIEVIYEKEGGMK"
CDS 3427185..3428210
/gene="aes_4"
/locus_tag="JFBMFIFI_03175"
/EC_number="3.1.1.-"
/inference="ab initio prediction:Prodigal:002006"
/inference="protein motif:HAMAP:MF_01958"
/codon_start=1
/transl_table=11
/product="Acetyl esterase"
/translation="MKQEKNETRKALRYVAPFLFAAIAIPTVVMQFTPTPVSRLLRRR
FSKEKSQYIPQNVAEIVAKTKSINDLNYESSLANGFLDVIMPKEAEKKSPLIIWIHGG
AYVGGDKRDVTHYAATIASFGYVVANINYALAPEQPYPGPLIQLTESYLYLKEHAEEF
NIDLTQVFFAGDSAGAQIVSQFFAIQTNPKLADAVSIQAVVPKETLKGGLLFCGPFDI
PKLGTDSKSTLMNILMNQVAWAYVGEKKWLLDERLEQVSIQNHITNDYPPCLVADGNT
NSFEDHGRDFVALLEANGIEVEGIFFSTDENRTEHEYQFILDSEAGRYTFERMMEFIE
KHVTKID"
CDS complement(3428304..3429272)
/locus_tag="JFBMFIFI_03176"
/inference="ab initio prediction:Prodigal:002006"
/codon_start=1

/transl_table=11

/product="hypothetical protein"

/translation="MRILIFGAGTIGLSYAWLLSDKHDVSVYVRPEKQENAYETYSIS

AQDLRKEKSYAFKFSPNLVTDLTSEYDLIIVTVNRCQLLKSLPILKTNKKNANILFML

NHWDITTEIEKYFTKEEYLLGFPSQVGGGKQDDKLDIVVFTEGTILGIQTPEQKKLIY

DYKEEFEAADLHVTIQEHLVDWLKVHYLQLAIRAGAILKAGGFEAFSTNSKAISEMIV

ACREGLKVCEASGVATKKLLPARIFYYPKAIVTPFMKHLFQNNETTKLIEYYMQNGLS

EWIYGYQEVLKAGEDLMIPMPTWRSYEAYVADYISQHPKLEAVLQK"

CDS 3429521..3430723

/locus_tag="JFBMFIFI_03177"

/inference="ab initio prediction:Prodigal:002006"

/codon_start=1

/transl_table=11

/product="hypothetical protein"

/translation="MLKKSLARRLTIQYGLLQSMYWMGFCVIMGFATVFLLYKDFEGQ

QIGVILAVSNICAALLQPVIASFADRSKHVTLKTIISFLAFIVILLTILLVLIPVNKV

IIALLFILIGTVVLTIQPLLNSLIFEYINRGIKINYGLARGLGSLSFAAISFVLGFIV

NRFSPAVLPYLYILFYSFILLIAYSFKMPRVLKEEPDLSICSSPSKKTFKLAELTTFF

KRYHNLYLLLISVCCLFIFHNIISTYLIQIMQNVGGSDTDFGISLALAASVELPTMMG

FAYLVRKIKGSTLLKVSAIFFTVKALVFLLAPSVGVIYLGQILQALSFALYIPASVYY

MNELMEPTDRIKGQAIIMVAMTLGGVFGNLIGGILLDNFTIFVMLVAGVIFSLIGTLL

LCYSVKEI"

CDS 3431292..3432134

/gene="mltF_4"

/locus_tag="JFBMFIFI_03178"

/EC_number="4.2.2.-"

/inference="ab initio prediction:Prodigal:002006"

/inference="protein motif:HAMAP:MF_02016"

/codon_start=1

/transl_table=11

/product="Membrane-bound lytic murein transglycosylase F"

/translation="MKKRGMFGWSILLVALVIMTGCGSKDKAAGGKESETFTVGMEAG

YAPFNWTQSDDANGGVKIDGTKEYAGGYDVAIAQKVADGLGKKLVIVKTEWDGLIPAL

TSGKIDAVMAGMSPTEERKKSIDFSDSYYKSNLVMVVKNGSKFQSATSIQDFEGAKVT

GQLNTFHYSVIDQIKGVKKETAMDNFSAMRVALQSGVIDGYVSELPEGISAQSANSDF

KLVEFKDGFKTSDEDTAIAVGLVQGSPLTAKINKILAEISPEEQAEIMEAAIKNQPAA

NLKD"

CDS 3432159..3432917

/gene="glnP_2"

/locus_tag="JFBMFIFI_03179"

/inference="ab initio prediction:Prodigal:002006"

/inference="similar to AA sequence:UniProtKB:P0AEQ6"

/codon_start=1

/transl_table=11

/product="Glutamine transport system permease protein

GlnP"

/db_xref="COG:COG0765"

/translation="MSFEWIVKIISENGPMFLRGAGVTLLIALIGTVLGSIIGLLIGV

VRTIPKPELKINRIILKIVNGILSVYIEIFRGTPMIVQAMVIYYGSALAFGIDIDRIV

AAIFIVSINTGAYMSEIVRGGIVSIDKGQFEAAQAIGMNHIQTMTNVVLPQVFRNILP

ATGNEFVINIKDTSVLNVISVTELFFQTKSIAGNNFRYFESFFVACVIYFVMTFTVTR

ILRYVERRLDGPDNYIMYANQMQVAKIENDIQNN"

CDS 3432936..3433670

/gene="glnQ_5"

/locus_tag="JFBMFIFI_03180"

/EC_number="3.6.3.-"

/inference="ab initio prediction:Prodigal:002006"

/inference="similar to AA sequence:UniProtKB:O34677"

/codon_start=1

/transl_table=11

/product="Glutamine transport ATP-binding protein GlnQ"

/db_xref="COG:COG1126"

/translation="MEKVIEVQHLMKSFGNHEVLKDIDFTVNKGEVVCVIGSSGSGKS

TLLRCINLLEKLSGGDILYKGKNIMQDTHDINEYRKHLGMVFQQFNLFNNHNVLNNCM

VGQIKVLKRTKEQAETVARKNLQLVGMEEYINAKPSQLSGGQKQRVAIARALSMDPDV

LLFDEPTSALDPEMVGEVLKVMKELAGLGLTMLVVTHEMDFAKDVADRVVFMDKGVIV

EEGPPAEIFNHPKEERTKEFLKRTLN"

CDS 3433891..3434832

/gene="znuA"

/locus_tag="JFBMFIFI_03181"

/inference="ab initio prediction:Prodigal:002006"

/inference="similar to AA sequence:UniProtKB:O34966"

/codon_start=1

/transl_table=11

/product="High-affinity zinc uptake system binding-protein

ZnuA"

/db_xref="COG:COG0803"

/translation="MKSLKKGSLLIASLAILFIMVGCGNQEATKDSTKLQIVTSFYPM

YDFTQNVAGDNAEVSVLMKAGTEPHDYEPSAKDIAKIADSDVFVYNSKEMETWVSSAL

TNIDTKKTVVVDASQGIDLLEGNHSDDETEAEHEGHSHAHDPHIWLDPVLAQKQVDTI

KEGIIKADTKNKETYEKNALAYKEKLAALNEKFEMGLKNAENRTFVTQHAAFAYLANR

YDLEQVAIAGLSPDQEPSPAKLAELNDFIKENNIKIIYFAETASPKIAKTVANETGAK

LEVLSPIEGITQEEQEKGVDYIKVMEKNLEALEKAIK"

CDS 3434868..3435575

/gene="znuC"

/locus_tag="JFBMFIFI_03182"

/EC_number="7.2.2.-"

/inference="ab initio prediction:Prodigal:002006"

/inference="similar to AA sequence:UniProtKB:O34946"

/codon_start=1

/transl_table=11

/product="High-affinity zinc uptake system ATP-binding

protein ZnuC"

/db_xref="COG:COG1121"

/translation="MHYIEVKDLTFYYEQEPVLENISFHVDPGEFVMLTGENGAAKST

LLRNVLGLLKPAKGTATISPVNVNGEKLSIGYIPQQIASFNAGFPSTVLELVRSGRYQ

QGRWFKRLTPEDHEHVRRALLSVGMWEMRDRKVGELSGGQKQRICLARIFATDPDLFV

LDEPTTGMDIEARADFYKLLRHNSEVHGKGILMVTHDHDDIKDYADRHIQLVRKEDSP

WRCFAMNSCNMPSKPHS"

CDS 3435521..3436351

/gene="znuB"

/locus_tag="JFBMFIFI_03183"

/inference="ab initio prediction:Prodigal:002006"

/inference="similar to AA sequence:UniProtKB:O34610"

/codon_start=1

/transl_table=11

/product="High-affinity zinc uptake system membrane

protein ZnuB"

/db_xref="COG:COG1108"

/translation="MEMFRHEFMQHAFQASFLIAVIAPLLGLFLVLRRQSLMADTLSH

ISLAGVALGMFVNINPTVTTLIVVVIAAVAIEYVRSLYKTYSEVSIAVLMSAGLSIAL

VLMSLDQGSGTASVQQYLFGSIVTISREQVYLLAVLFAIVVTLFFLFRKPMYVLTFDE

DTAFTAGLPTRLMSLLFNVITGVTIAVIMPIAGALLVSAIMILPAAIAMRLSKSFNWV

MLMGVVVGLIGMFGGLTTSYEFGTPPGATITLIFIVIFIFSGVIGKLIAKFKKKNTSN

"

CDS 3436531..3437352

/gene="purR_2"

/locus_tag="JFBMFIFI_03184"

/inference="ab initio prediction:Prodigal:002006"

/inference="similar to AA sequence:UniProtKB:P37551"

/codon_start=1

/transl_table=11

/product="Pur operon repressor"

/db_xref="COG:COG0503"

/translation="MKVKRSERLIDMTRYLLERPHKLVSLTYFANRFDSAKSSISEDL

TIVKKTFKERGTGTLETVPGAAGGVKFIPEIAKTEADEFVNEMCIRLSEPDRLLPGGY

VYLSDLLGEPSILRQVGKIIATQYLDQKIDAVMTVATKGVPIAQAVSSYLNVPFVIVR

RDSKITEGSTVSINYVSGSSERVEKMELSKRSLKRGSRVLIVDDFMKGGGTVNGMKSL

IEEFEAELVGITVFAESTFSGNRMIDDYSSLLCVDEVDIRDKTIHVSPGNYYKTK"

CDS complement(3437405..3437914)

/locus_tag="JFBMFIFI_03185"

/inference="ab initio prediction:Prodigal:002006"

/codon_start=1

/transl_table=11

/product="hypothetical protein"

/translation="MTNQDATYWIKQLELEAHPEGGYYKRMFESKELFETTDKRARHH

YSSIYFLLNQASPSHFHRLKSDEIWYYHTGSPLTVHLLHPDGHYEKIKLGLDLANGEV

LQAVVPKNVIFGSSIEENGDFAVVSCMVSPGFDFKDFELFTQAELLLDYPDHQEIIEK

LAYEALPLD"

CDS 3438223..3439473

/gene="cycB"

/locus_tag="JFBMFIFI_03186"

/inference="ab initio prediction:Prodigal:002006"

/inference="similar to AA sequence:UniProtKB:O07009"

/codon_start=1

/transl_table=11

/product="Cyclodextrin-binding protein"

/db_xref="COG:COG2182"

/translation="MKMKNKIILLSSIALVSIGLLAGCSSGKEDSKKDSAEKVTFWYM

GDGSKEIQPIIDDFEKESGVKVDLQSIPWSASHDKLLTAVASGDGPDVVQMGTTWMAE

FVDAGALKDISSYIDSEDTLKSDNFFDGSVTTTKFDDKYYAVPWYTETRALYYRTDLL

KEVGYDKAPATWEELQDAATKLAARGKDMYGFGADLAEPTSAFMFARQNGSELFDKSG

KPLFDKKPFVEAVDYLDSFIQNGSAPKADLGLDVSQTFGSTGIVPMFISGPWMITAIT

KDAPDIEGKWATAVLPKKENNMSSTGGANLAMFESSKNEKNGMKLIEFLARPENQLTF

FKNSNSLPTSHKAWEDPILAEDEKIAVFGEQLKNSEPMPMMKEWEEISQAYLKVWEQI

YANGADTQKEMTEFNKQTETILGK"

CDS 3439716..3440609

/gene="lacF_2"

/locus_tag="JFBMFIFI_03187"

/inference="ab initio prediction:Prodigal:002006"

/inference="similar to AA sequence:UniProtKB:P29823"

/codon_start=1

/transl_table=11

/product="Lactose transport system permease protein LacF"

/translation="MKAFLNKKIPYLFVGPSLILLALFSLIPIVIALFISFTDINLAG

LANWSRISFIGFDNYKNIVSDPIFLKSIFNTAFYVVLGVPLVIVCSLAIALLINFGQN

RFFAFMRLVFYTPSITNVVAVAVVWTYLFNPSIGLINHMLAYINIGPIPWLQDPVIAK

VSLVILALWRSIGINMIIFLAALQGIPKEYYEAAQLDGASSWNQLRFITVPMLRFSIF

FVTITTMIGWLQFFEEPFVMTKGGPLDSTTSVALFIYRNGFQLSKFGYAAAGSFILFL

AIIVITLLQFRLQKKDADNQM"

CDS 3440633..3441463

/gene="araQ_3"

/locus_tag="JFBMFIFI_03188"

/inference="ab initio prediction:Prodigal:002006"

/inference="similar to AA sequence:UniProtKB:P94530"

/codon_start=1

/transl_table=11

/product="L-arabinose transport system permease protein

AraQ"

/db_xref="COG:COG0395"

/translation="MGNFQARQKRQKLIVGLFLAVWGFITFVPFIWMILSSFKTNPEI

MQLNPSILPKNFTFDNFIKLFRDMNFAVYLKNTLIITGFSFFGLFLNAMAGYGFAKFK

FKGKEKYFMLVLATMMIPGQVTMIPVYLLLNVAGLTNTMAGIILPGLVGAFGIFLFRQ

FMSTISDEIIEAARLDGASEFYIFTRIILPISKPILAVQGILTFIGGWNSFMWPLIIA

NDEKFYTLSVGLSLLKGQHGNDYALQMAGSTFMVIPIIIIFMMFQKYILQGFNVSGLK

"

CDS 3441582..3443732

/locus_tag="JFBMFIFI_03189"

/EC_number="3.2.1.-"

/inference="ab initio prediction:Prodigal:002006"

/inference="similar to AA sequence:UniProtKB:T2KMH0"

/codon_start=1

/transl_table=11

/product="Beta-xylosidase"

/translation="MQTAELEGLLSQMTREEKVTQLLQLAAGFYIDLDAVTGPIEEMG

LTEKMIENAGTVLGISGAEDVIRTQKSYLEKNRLGIPLIFMADVIHGYKTIFPIPLAI

GSSWNTELAEETARIAALESAVSGLHVTFSPMVDLVRDPRWGRVMESTGEDAFLNAEL

AKAFVRGYQGTDLKNDFERVAACVKHFAAYGAPIAGREYNTVNMSERQLRESYLPGYR

AALDEGAKLVMTAFNTVDGVPATANKWLMRDLLRNEWDFNGVLISDWGAVKELVPHGV
AADEKEAAILALNASVDIEMMTMCYMDTLEASIEAGEIEEALVDEAVLRILNLKNDLG
LFENPYRGADAEKEKALVLSQEHRSLARKAAEESIVLLKNEGVLPLKQNQKIALIGPG
AESKDILGAWSWQGESEKAISLAEGLAQHVPAEQLLIAKGCDILSGTQAEIDAAVAVA
KEADVIVLALGENSDMSGEAASRSDIRLPKIQLELLAQLKSVGKPIIVTLFNGRPLDI
HELEVAGIVEAWFPGTEGGAALANILMGAVNPSAKLSMSFPYNVGQVPVYYNPDNTGR
PELGRSVDEKYVSKYLDSPNEALYPFGFGLSYTSFSYSDMTLSTTELKDSETLTVQVT
VKNSGKYAGKEIVQLYLRDIVGEVVRPVKELKGYQKIELAAGAEKTVSFTVTEEQLRY
VHQDCSFSSDSGVFEVMVGPSSAEVQSARFNLIK"

CDS 3443752..3447096

/locus_tag="JFBMFIFI_03190"

/EC_number="2.4.1.333"

/inference="ab initio prediction:Prodigal:002006"

/inference="similar to AA sequence:UniProtKB:Q92AT0"

/codon_start=1

/transl_table=11

/product="1,2-beta-oligoglucan phosphorylase"

/db_xref="COG:COG3459"

/translation="MTTTQNLIQFQAGKTTVQFLKSGDIYEINHAGTMYNQVHTNMID
GSLNNIYLRKLENGKIQFFPLVGVQSQSALYQGDNQLKWTGIAGGVEYEVIFSLTEDD
CWFWEVHLQGNQVEVDVIYGQDIGLAGIGALQSNEAYVGQYIDHAVFKDEETGFTVCS
RQNQPQSDNQFPYFQQGSLTKNNGYSTDGFQFYGLSYKETNSPFALTQENLANEIYQY
EMAYTALQSERVELNGKATFVFYGFAAENHPLAITKVEYQEKIQQAWQELKEKDSSDF
ILQETVKRAVNFGEPVQTESLTKTEITHLFPSRQAEEYDGEQLLSFFTDREAHIVLKE
KELQMERAHGHILLSGEHLTISDEIITTTSYMYGLFNAQVVLGNTSMNKLMSNARNAL
NVMKTSGQRIYIELDGSYHLLTMPSAFEMSFNYAKWYYKLADDMLIITNYTTVADAEL
KLEVRSQSGHVYKFIVTNQITMNENEYNVPFNMKKDGNTLTFTPDNQSTMAKNYPNLA
YYMQIDKADFIVADDHLLVESQLDSAYPLVVIALAETAEFDLTIQGKTQGNEFQFVQR
DFATEVNKYNRHFEKIMNQFKLTHENKDIQTEMSRLNTLAWWYTHNMLVHYLMPHGLE
QYGGAAWGTRDVSQGPVEYFFATDKAEIVRDIIQTLFANQFENDGNWPQWFMFDKYDQ
IKAEESHGDVIVWPLKVVGDYLKRTGDYSLLESQIPYTDRKTFKKTTKTVSLLEHLRK
EIDYIKGNFLAGTYLSCYGDGDWDDTLQPYDSRLKKNMASSWTVALTYQTLKQLAEVL
AEKAPEFSLEIAELVTGIEKDFKKYMLQTDVIPGFVYMEDPEHVELMIHPTDTKTGIQ

YRLLPMTRSMIGELLTPAEAEAHYQLICQELYHPDGVRLMNRPATYQGGVSTNFKRAE
QAANFGREVGLQYVHAHIRFTEAMAKLGKADEVWRGFSVINPVQITDVVANAERRQSN
AYFSSSDGNFKTRYAAQEHFAELKTGDVTVKGGWRIYSSGPGIYMNQLISNGLGIRKE
SDHLIFDPILTKELDGLVFKYECLGVPVEIHYHVGQQEVKGIKVNGELLPSEIETNRY
RDGGIKVANDVLKAALLKSQKIDIYC"

CDS 3447204..3448175

/gene="rbsR"

/locus_tag="JFBMFIFI_03191"

/inference="ab initio prediction:Prodigal:002006"

/inference="similar to AA sequence:UniProtKB:P0ACQ0"

/codon_start=1

/transl_table=11

/product="Ribose operon repressor"

/db_xref="COG:COG1609"

/translation="MVGIKDIAKKAGVSISTVSYALNGSPKVTEATRNRIRAIADELH
YVPNMAARTLKRQQTNIIGVYLTNYGGSFYGQLLEGVMDTLFAKGYEMIVCCGDKSHL
FLPERMIDGAIILDATFSDEEIMRYAKRGHQIVVLDRELEHQNIRQVLLDNKAGATLA
IDCLKYKSERPFYIVTGPVDSQDSRERLKACQIELDRSATPYQLIEGDFTEKSGRLAA
EKIHREYQEPVAVFSLNDEMAIGMYKYFAKTELNIGKEIDLVGFDNTTVSEYLVPSLT
TIEYSKHKWGAVAASKLLELIQGEKTENELIYTSLIRGNSVGEKSDL"

CDS 3448190..3448939

/locus_tag="JFBMFIFI_03192"

/inference="ab initio prediction:Prodigal:002006"

/codon_start=1

/transl_table=11

/product="hypothetical protein"

/translation="MAVLSSRFTSKTLDLAMSFQVIIPQEFKEPLPVLYLLHGLSDDD
HSWLDNTALVRYASDMQLVIVMPQVHRSFYTDMVEGGAYWTFLTEELPDLVARWLPIS
LEKNKTFVAGLSMGGYGALKWGLNYPDKFAGIASLSGATDLVTMWKDDANRDLEFKRL
FGSIEKLEGSTNDLSYLINQVKGQANVPEILQICGTEDFLYDYNLNFSKRLLELGFNY
SWVEEKGTHDWVFWDQQIQTVLDWISKKITN"

CDS complement(3449435..3450820)

/gene="gbpA_2"

/locus_tag="JFBMFIFI_03193"
/inference="ab initio prediction:Prodigal:002006"
/inference="protein motif:HAMAP:MF_01905"
/codon_start=1
/transl_table=11
/product="GlcNAc-binding protein A"
/translation="MKKLTASKAMKLGLSSAILGGALFFSTNFASAHGYVEQPVSRGY
AGELAKNSIGWGPALEKYGNVITNPQSLESPKGFPEAGPADGKIASADGGLGQIGDFV
LDQQSLNRWDKQEMTGGVNSFVWKYTAEHKTTKWHYYITKKGWNPNAPLTRSEFELIG
TVNHDGSMPSNNPVHKINVPTDRTGYYVILAVWDVADTPNAFYNVIDVNLKNDGSGEE
GETPNQPQNLKSDEVTNKSIKLSWTAPTNTDVKEYNIYRDTIKVGTVGGTTFTDIDLQ
EKTTYGYQVEAVGFDGKVSEKSSVVSVKTKETPAEDTEKPSVPSKVHSMKTTSNSVDL
MWTESTHPIAVKEYTVYRDGKKVGTTKTNRFLDTGLNAETNYVYTVKAVSIGGNISDK
SDSFKVTTAKEMNEHREWKVGSFSNPVSYTANELVSYQGKNYKVLNTHVNYGDLTWAP
GIPNSLFEEVK"
CDS 3451364..3452185
/locus_tag="JFBMFIFI_03194"
/inference="ab initio prediction:Prodigal:002006"
/codon_start=1
/transl_table=11
/product="hypothetical protein"
/translation="MNKRKIQIAVISIIAVLTLGGAFLNNKYAHVATPQQNKTQVVKK
ETKKDTVANSKKTAKEKKESLESEKKNESSKESSKDSTKETDTEKKIADKNVEKATST
VIESADDVQEEVANTTTNNETKNPVVEHKEEPNAPPATPSQPEPTQPTPENKPTPEPK
NEVTFSIKGTATNSSSYFISPQKVEMKEGQSVMDVLSDYCRNNGIQVGIRGGTYVAGI
NNLYEFAKGAESGWLYRVNGVFPSYGAAQYAVQVGDTIEWMYTENMGKDVGAPQV"
CDS 3452388..3452786
/locus_tag="JFBMFIFI_03195"
/inference="ab initio prediction:Prodigal:002006"
/codon_start=1
/transl_table=11
/product="hypothetical protein"
/translation="MNKIIRLVLSIGLITLIVGCGNATVTKEDSSSSKTKDVTVTVIL

KENHKEFDQKKIEVAEKTDLQTAMEKNFDVVTDKGFIKSIEGKEQDNAEQNTKGSYWM
YDINGAPATVGASDTKLKDGDEIVWDLSGQ"

CDS 3452776..3453306

/locus_tag="JFBMFIFI_03196"

/inference="ab initio prediction:Prodigal:002006"

/codon_start=1

/transl_table=11

/product="hypothetical protein"

/translation="MDNNTISSRFTVRRIAFLALMVALCQVSRMIFQFLPNVQPVTVI
LILLTLHLGIWDGIVVATLSILTSNLMLGMGVWTIAQIVSFVMIVLLTGLVIRPYIHK
IPFLVMVFYCGLMGFFYGFIISLVQAPFFGIQAFWPYFLAGVPFDAMHAIGNSGFYFI
LAPILTPLLKKFVAVN"

CDS 3453886..3455265

/locus_tag="JFBMFIFI_03197"

/inference="ab initio prediction:Prodigal:002006"

/codon_start=1

/transl_table=11

/product="hypothetical protein"

/translation="MKQRLITLLATFTLMIGFVAVPVSSAYAAVTVDEAIDGATAKML
ADGFISSDWEALAIARSNTPASKELRQDYYDSIVSSISHSSYFSATDYERTIIGLVSI
GADPTNISEATQRNNLVDDLYHMDVANSGTNGIIYGILALQTKDYEVPADAVFSIQDL
IDKLISLQKVSGGWALFGSASDIDITGMAMTALAKYKDMPEVAEALDRSVDYLSTVQL
ESGGFKGWSNENSNSLSQAIMGLTMTGNDPTGPRFTKNFNAIDALMTYRTDDGGFKWL
LTDAGSNSMALDQAAYTLAQYKAFLNGGASIYDFVNNPVPQLIETPVDPDPEVTDPEV
TDPETTDPEVTDPETTEPEVTNPDPGVTEPETTNPEKEKTPDPTPTEKPTVVEPIAVK
EADKPANLIKAPISNTAENTKSDETNYFPKTGESSEFGIFTALLGTSIVLIGLIVWYE
NKKKQTAKA"

CDS 3455383..3456264

/gene="ecfT_4"

/locus_tag="JFBMFIFI_03198"

/inference="ab initio prediction:Prodigal:002006"

/inference="protein motif:HAMAP:MF_01461"

/codon_start=1

/transl_table=11

/product="Energy-coupling factor transporter transmembrane

protein EcfT"

/translation="MRTVLNSVHPFTSFCYYIGVILLSMMFLHPIFLIGELIVLIMFN

LYQNNGAKMKKMIQGSWIFILAIAVINPLLNHRGSHFLFYIGRNPITLEAVVYGILMA

LSFSCLLIIFISYNSVISSHKFLYLFSRISPQLALLTMITMRFVPLFIRRLTTITAIQ

KTRGIQMETGKIKTRAYNGMRLVQILLVCSLEEALQTADSMEARGYGVKKRTTYLSYQ

LEMRDILSLIFGALILISCIYGKLNGFGNLKIYPELGTLGLSIIQNQVLLVLTLLFVS

YPLILEGRELLWWRWQK"

CDS 3456243..3457883

/gene="ykoD_3"

/locus_tag="JFBMFIFI_03199"

/EC_number="3.6.3.-"

/inference="ab initio prediction:Prodigal:002006"

/inference="similar to AA sequence:UniProtKB:O34362"

/codon_start=1

/transl_table=11

/product="Putative HMP/thiamine import ATP-binding protein

YkoD"

/db_xref="COG:COG1122"

/translation="MVALAEIRNLSFTYPEEKKEALVMNSLVIEEGDFLVLAGSSGSG

KTTLLRHFKKELWPIGTRTGALFYQNENYQELSDIRSATEIGMVFQNPENQIVMDNVM

GELAFALENIGCPPKIIEKRIAELISFLGFQDLLEQSIHTLSGGQKQLVNLASVLILQ

PKLLVLDEPTAQLDPIATRDFLGLLKRIHEELGITIIMSEHRLDEVIPLATKLVMMDA

GEIIENSPPMIAIQNLWKIAGKQLFIPQVPRLFLEMGSSSLPFSVLSGQRALPPLSTL

ENEQKNESEKRDENRFLVAKSIAFQYEKNGRFILRELNFSIKKGEWIGIVGKNGTGKS

TFLMVLAGLLNCRRGKVTLEGNRLDKIDLQERYERIGYVSQNPAYHFAYDTVFDEFYQ

RSIQIKLNNPELAAKEMLMELEIEHISARNPLDCSGGEQQLVSLGLALLSNPEILLLD

EPTKGLDPVRKHHLGTLLKKLKEEGTTIVMATHDMEFAAAYGTRAALLFDGKIISEGF

VQDFFSDNFFYTTAINRLVRKQLPRALTWEDVIPYVKS"

CDS 3457870..3458559

/locus_tag="JFBMFIFI_03200"

/inference="ab initio prediction:Prodigal:002006"

/codon_start=1

/transl_table=11

/product="hypothetical protein"

/translation="MLRAKKNLWGLILFLIFMLVLSVAISDKYYLLVSFLFLIATMVP

LYRRFETKKIRSREIVFIAVLGAIAATSRVPFAMIPSVQPTTFVIIVSAMVLGSESGF

VVGATAALVSNMFLGQGPWTPWQMFCWGMVGFTAGLLKDSPILKDMWSRLVFGFIWGF

LFGWIMNLWYVVAYINPLNWIAFVQAYAASFYFDLAHALSNVFFLWLFSPSWLKIIRR

FQKKYGLINLE"

CDS 3458771..3459199

/gene="pduV"

/locus_tag="JFBMFIFI_03201"

/inference="ab initio prediction:Prodigal:002006"

/inference="similar to AA sequence:UniProtKB:Q9XDM6"

/codon_start=1

/transl_table=11

/product="Propanediol utilization protein PduV"

/db_xref="COG:COG4917"

/translation="MKKVMFIGSIGCGKTTLCQRIKGVELSYHKTQAVQFHADMIDTP

GEFIQHRQYYSALTVTAAEAEVVALLASVSEKEQVFAPLFATLFAKPTIGIVTKIDLA

ENEADILAAEKRLYLAGAKVVFRVSSYEDAGIKELVDYLQ"

CDS 3459218..3459793

/gene="yvqK"

/locus_tag="JFBMFIFI_03202"

/EC_number="2.5.1.17"

/inference="ab initio prediction:Prodigal:002006"

/inference="similar to AA sequence:UniProtKB:O34899"

/codon_start=1

/transl_table=11

/product="Corrinoid adenosyltransferase"

/db_xref="COG:COG2096"

/translation="MKIYTKTGDKGMTRLVGGAQVSKDSARVTAYGTLDELNSLLGYI

VSQMNDSENPDIKAELAEIQQYLFDCGTDLATPEGIRDYRMTKEPTKWLESRIDFYTE

LPPQIEEFIIPGGTPVASQLHMARTVARRGERHIVSVAWTAEINSHVLKFTNRLSDYF

FAVARVVNFRANCPDVSYKRSGKVFHNGPTK"

CDS 3460461..3461609

/gene="adhE_2"

/locus_tag="JFBMFIFI_03203"

/inference="ab initio prediction:Prodigal:002006"

/inference="similar to AA sequence:UniProtKB:P0A9Q7"

/codon_start=1

/transl_table=11

/product="Aldehyde-alcohol dehydrogenase"

/db_xref="COG:COG1012"

/translation="MLIDMEKISFKTDLFIGENALDRLKEYENKKIFIVTDPFIVSSG

MIDAVTSRISAKNEFAIFSDIIPDPPIENVVAGISALNEFDGDMMVAIGGGSAIDAAK

AMKFFGQKLGTVKAMPFVVIPTTSGTGSEVTNFSVITNQEKAMKYPIVTDAILPDEAI

LDAELVRTVPPAITADTGMDVLTHALEAYVSTKANDYSDALCEKVVMLVFDYLERAYR

NGDDMEAREKMHNASCLAGMAFNITSLGLNHGIAHTAGAKFHIPHGRMNTLLLQHVIR

YNAGITDFQSIPTSEAAKRYTALAKILGLPASNTRTGVRSLINEIKQLQKKLNMPTTL

SECGITKEVFTKEKHAIAVGALKDGCTATNPRIPKELEIEEILETMLV"

CDS 3461817..3462161

/gene="pduU"

/locus_tag="JFBMFIFI_03204"

/inference="ab initio prediction:Prodigal:002006"

/inference="similar to AA sequence:UniProtKB:P0A1D1"

/codon_start=1

/transl_table=11

/product="Propanediol utilization protein PduU"

/db_xref="COG:COG4810"

/translation="MEEKQRMIQEYVPGKQVTLAHIIASPDQTIYEKLGLLESNYNAI

GILTITPSEAAIIAVDIATKAANVQIGFIDRFSGSVVITGDVASVESALSEVLSGLET

ILGFTGTRITKT"

CDS 3462264..3462836

/gene="pdtaR"

/locus_tag="JFBMFIFI_03205"

/inference="ab initio prediction:Prodigal:002006"

/inference="similar to AA sequence:UniProtKB:P9WGM3"

/codon_start=1

/transl_table=11

/product="putative transcriptional regulatory protein

pdtaR"

/db_xref="COG:COG3707"

/translation="MNGRIVIVDDEPITRMDIRDILEAGGYDVVGEASDGFEAIELCK

SQHPDLVIMDIQMPLLDGLKAGKKIASENLAGGIILLSAFSDPTNTERAKNFGALGYL

VKPLDEKSLIPTVEMSIAKGKETQKLEEQLNKLTKKLEERKIIERAKGILMIENKITE

EDAYQMIRTLSMDKRSPMIEIAEMIVMTDD"

CDS 3462829..3464256

/gene="pdtaS"

/locus_tag="JFBMFIFI_03206"

/EC_number="2.7.13.3"

/inference="ab initio prediction:Prodigal:002006"

/inference="similar to AA sequence:UniProtKB:P9WGL5"

/codon_start=1

/transl_table=11

/product="putative sensor histidine kinase pdtaS"

/db_xref="COG:COG3920"

/translation="MIKTQIAELCQKYSDLTEEDIAEISFQASKIEMSPMYSEQDAFI

DIINIYTSEAVVIYHKPPLTIPSLYKETVVGKAAKRENEPGVLRTFETALNSEGLLAR

NQEDKLIRQKVYPIRNHKRNIAVLIVEEDVSQTIKESFEVSNKYQDFVEVSSVLQAID

SFNESIVDNLNDGILIFDEDGYLLQKNKAADSYYEAFGYIGNIIGLHYDNLSLDLSTF

EQLVYLRSIGKWSNSEIKEIKFDTYYFAMKQIFVDKENVFIMMLQDKTEIKNKEAEII

SKSVAIREIHHRVKNNLQSVVSLLRIQARRCESQEAKTALNESVSRILAISATHELLS

KQVEDGIQLKTVLESVVYNIQRCFLDRNHITVVSDVSPDIVIDSDRTVAIALIVNELL

QNSYDHAFGNEQVGLIKLTAQAEEKVITISVIDDGTGFDVKKVSTTSLGLQIVNSYVK

DKLRGKIKIKSKEETGTSTRFFFKN"

CDS 3464485..3465915

/locus_tag="JFBMFIFI_03207"

/inference="ab initio prediction:Prodigal:002006"

/codon_start=1

/transl_table=11

/product="hypothetical protein"

/translation="MTEKILSVGIDLGTSTTQLVLSELFIENMASAFSIPRIVISDKK

VIYKSDIRFTPILADNLIDVEEIKQFVEEQYRKAGIQKEEIQMGAVIITGETARKENA

SKVLTALSGFAGDFVVATAGPDLESIIAGKGAGAHTYSKENRMSVVNLDIGGGTTNLA

LFEDGEVSDTGCLDIGGRLIKVDSTTKKITYIAPKLQEIIAKQNLPIQLNQPTSVEQL

QPIIKEMVRLLENSVGLGMQSNFYERILTNKGLRLDQRIKGISFSGGVADCIAKNLPE

NPFKYGDIGLLLGQGIANSRLVEEKEVIPSVETIRATVVGAGSHTAEVSGSTITYTKE

ILPIQNIPILKLALADEVGTSQEIASKISEKLQWYQLENEFQMIALGLSGEKSPSFQT

VLNLAEAIVKGLDTLIQRKDPLIVMVQEDMAKALGQSIHSLLPKDYPFVCLDSVRVEN

GDYIDIGNPIANGTVLPVVVKTLVFN"

CDS 3466015..3467382

/gene="eutB"

/locus_tag="JFBMFIFI_03208"

/EC_number="4.3.1.7"

/inference="ab initio prediction:Prodigal:002006"

/inference="similar to AA sequence:UniProtKB:P0AEJ6"

/codon_start=1

/transl_table=11

/product="Ethanolamine ammonia-lyase heavy chain"

/db_xref="COG:COG4303"

/translation="MILKTILFGKTYQFKTVMEVMAKANEVKSGDSLAGISATSAEER

VAAKVVLAQLKLSDLFNNPAVPYEEDEVTRIIVDGVNKRTYEQIKNWTVEELREWLLD

LRTTDHDIKILGRGLTSEMVAAVAKLMSNMDLIYAAKKMVVMKTANTTIGMPGHFSAR

LQPNHPTDNIDGIMASLMEGLSYGIGDAVIGLNPVDDSTESVKRILHRFEDFRQEWEI

PTQTCVLAHVKTQMEAMRQGAPTGLVFQSIAGSEKGNTAFGFNATDIAEAKKLALEVG

AAAGPNVMYFETGQGSELSSDAHYGVDQVTMEARCYGFAKKFDPYLVNTVVGFIGPEY

LYDSKQVIRAGLEDHFMGKLSGISMGCDVCYTNHMKADQNDAENLAVLLATAGCNFIM

GIPHADDVMLNYQTTGYHETATLRDMFNLRPTTEFDQWMEKMGFSINGKLTERAGDAS

VFLKK"

CDS 3467401..3468312

/gene="eutC"

/locus_tag="JFBMFIFI_03209"

/EC_number="4.3.1.7"

/inference="ab initio prediction:Prodigal:002006"

/inference="similar to AA sequence:UniProtKB:P19636"

/codon_start=1

/transl_table=11

/product="Ethanolamine ammonia-lyase light chain"

/db_xref="COG:COG4302"

/translation="MVDEKNLKTIIENILSEMADGNVDLSNDVQSAIAVKVTETVAQN

DVEDGCIPDITEVDIKKQFLVPHAEDPEGYQKMKQFTPARLGLWRAGTRYKTQSTLRF

RADHAAAQDAVFSYVDEALVKEMNFVSVETLCHDKDEYVTRPDLGRQFSPEMCEIIKD

NTTHGAKVQVVVGDGLSSAAIGANIKDIIPSIKQGLKMFNLDFDSVVFVKHARVPAMD

KIGELTDADVVCLLIGERPGLVTAESMSAYIAYKPTVGMPEARRTVISNIHSGGTPAV

EAGAYIAELIKNMLDKKKSGIDLKEAE"

CDS 3468328..3468981

/gene="eutL"

/locus_tag="JFBMFIFI_03210"

/inference="ab initio prediction:Prodigal:002006"

/inference="similar to AA sequence:UniProtKB:P76541"

/codon_start=1

/transl_table=11

/product="Ethanolamine utilization protein EutL"

/db_xref="COG:COG4816"

/translation="MKNDRLGASVLSVKVISNVDAAMAKSLNLGPNQRSLGLITSDCD

DVTYVALDEATKAAEVDVVYAKSLYAGAANASTKLAGEVIGIIAGPSPAEVKSGLEVA

IQEIESGASFISSNEDDSIPYFAHCVSRTGSFLSKEANVTEGEALAYLIAPPLEAMYA

LDAALKAADVDIAAFYGPPSETNFGGALLTGSQSACKAACDAFAQAVEVVASNPTAY"

CDS 3469002..3469550

/locus_tag="JFBMFIFI_03211"

/inference="ab initio prediction:Prodigal:002006"

/codon_start=1

/transl_table=11

/product="hypothetical protein"

/translation="MKYEALGMIEVTGFLGAIEAADTALKAANVELLQAEVISGGLTT

VQVVGDVGAVQAAVEAAVSVTEKLGCLVGSHVIPRMDAATAEMVFSAIAKQDVAEPIE

NVVKQTETSLVKEAKESLKKEIQEVIEESLEQELAQMKVVDLRKLAYQMELTSLSKKE

IKFANKKALIDAIQAEKERNDK"

CDS 3469550..3471016

/gene="adhE_3"

/locus_tag="JFBMFIFI_03212"

/inference="ab initio prediction:Prodigal:002006"

/inference="similar to AA sequence:UniProtKB:P0A9Q7"

/codon_start=1

/transl_table=11

/product="Aldehyde-alcohol dehydrogenase"

/db_xref="COG:COG1012"

/translation="MTIQDKDLVSIQEVRDLIGHAKKAQKELANMSQQQIDTICEAIA

KAGYDNRVKLAKMAHEETGFGIWQDKVVKNAFGSKGVFEAIKDMKTVGVLSDDEQTKV

MEVAVPVGVVAGLIPSTNPTSTVIYKALISIKAGNSIVFSPHPNALNSILETVKVIEE

AAVAAGCPVGAISSMQVPTIQGTDQLMKHKDTSLILATGGSAMVKAAYSSGTPAIGVG

PGNGPAFIERTANIPMAVKRILDSKTFDNGTICASEQSIIVEEINREKVINELEKQGA

YFLNAKEAEQLEKFIMRANGSMNPQIVGKSVQRIASLTGLSIPEGARVLIAEESRVGS

KVPYSREKLAPILAFYTAPNWSAACELSMDILNHEGAGHTMIIHSEDKAVIREFALKK

PVSRLLVNTGGSLGGIGASTNLMPALTLGCGAVGGSSTSDNIGPANLFNIRRVAYGVR

ELEELRGSDAPCTTTVGMDASKEDLIDVLVEKVLAQLQ"

CDS 3471074..3471358

/gene="pduA"

/locus_tag="JFBMFIFI_03213"

/inference="ab initio prediction:Prodigal:002006"

/inference="similar to AA sequence:UniProtKB:P0A1C7"

/codon_start=1

/transl_table=11

/product="Propanediol utilization protein PduA"

/db_xref="COG:COG4577"

/translation="MANANALGMIETKGLVAAVEAADAMVKAANVTLIGKEQVGGGLV

TVMVRGDVGAVKAATDAGAAAAERVGQLLSVHVIPRPHSEVDAILPKGAL"

CDS 3471490..3472224

/locus_tag="JFBMFIFI_03214"

/inference="ab initio prediction:Prodigal:002006"

/codon_start=1

/transl_table=11

/product="hypothetical protein"

/translation="MAILTEETVRRLIKTTEIQETKVVEVEKGTIITPSARSFLSDHQ

IKLQEIPSCETNREDNQKNKAEVTGNQEVKIVQNPFLYKTLDGGKFVEKPVYMSVLKG

NIIVPNDHTEIKLRGQLDTLHGEVLKVQLLASEAGFSDLIEELELIARCSQFMQESRL

PSKLAYPSETEVVTFSIDYQMGALVIALNQLRLMIEQTSLMAYESLKEADGTIKEVEL

LEVLTKLARFCWKLMDNVNKGKYKKR"

CDS 3472235..3472876

/gene="pduL"

/locus_tag="JFBMFIFI_03215"

/EC_number="2.3.1.222"

/inference="ab initio prediction:Prodigal:002006"

/inference="similar to AA sequence:UniProtKB:Q9XDN5"

/codon_start=1

/transl_table=11

/product="Phosphate propanoyltransferase"

/db_xref="COG:COG4869"

/translation="MNQEDLNRLIDEVVKRVENQNGVEVEASGRHIHLDRATIDALFG

QGYELKVAKYLSQPGQFASKERVTLIGPKGVLHNVIVLGPERNQAQAEISATDALAIG

VKVPVRESGDIAGTPGILVMNGSKSVQLSEGLIVAKRHIHMTVEDAVKANLQQGEIVQ

VKINAGRPLIFDDVVIRVSSKFATVMHIDYDEANACGWSKGTRAQIIKKSKSL"

CDS 3472873..3473481

/locus_tag="JFBMFIFI_03216"

/inference="ab initio prediction:Prodigal:002006"

/codon_start=1

/transl_table=11

/product="hypothetical protein"

/translation="MSGVDELVQKVMEAVLVKLTNETQPKLKLIGTSDSNVLNLIARL

PKVSIADCSIEEADILFISQLSVDQMGRIANACPQTLEESSILKHFLKGKKVLLLQEG

IEFYAYRQSASYGLRQKLEEFELQWRRYGAEIVSEEEKTWQLPRSQNEQLDQGMVTKK

VWTEAKIKELNLVSGTSFKLENNALLTALAKDYLREKNIHWS"

CDS 3473531..3473800

/gene="ccmL"

/locus_tag="JFBMFIFI_03217"

/inference="ab initio prediction:Prodigal:002006"

/inference="similar to AA sequence:UniProtKB:P72759"

/codon_start=1

/transl_table=11

/product="Carbon dioxide concentrating mechanism protein

CcmL"

/translation="MIIGKVTGSLWATRKDEKLNGLKFLIVETETDEHQSAPQSLVAA

DNAGAGFGDLVLVTTGGAARLSLDKEGVPVDAVIVGIIDSVEREG"

CDS 3473893..3474996

/locus_tag="JFBMFIFI_03218"

/inference="ab initio prediction:Prodigal:002006"

/codon_start=1

/transl_table=11

/product="hypothetical protein"

/translation="MSINEIIMYIMVFFMILGAIDKCIGNKFGLGEQFEEGIMAMGAL

ALSMIGIITLAPVLAKVLSPVVVPLYSALGADPAMFATTLLANDMGGFALAQQLAIDP

QAGLFAGTILGAMMGPTLVFTIPVALGIIDKNDHKFLATGVLSGIITIPIGCLAGGLV

AGFPIQMILMNLIPIVLVAAIIMLGLWFKPEAMIKGFTVFGKFVVIVAIIGLVVGAIQ

LLVGVTLIPGIEPITSGIEVVGGIALTLAGAFCFVFVITKVFKTPLMKLGKVLGMNEI

AAAGLVATLANSIPMFQMMKDMDDRGKIINVAFAVSAAFVLGDHLGFTAGVAKEMIFP

MMVGKLIGGVTAILVAVFMANRMLGKKTTEGAK"

CDS 3475000..3475455

/gene="eutQ"

/locus_tag="JFBMFIFI_03219"

/inference="ab initio prediction:Prodigal:002006"

/inference="similar to AA sequence:UniProtKB:P76555"

/codon_start=1

/transl_table=11

/product="Ethanolamine utilization protein EutQ"

/db_xref="COG:COG4766"

/translation="MADIDKNMLERIVREVIMEQMTKGKADTKTVDPSGVLSIKLPQL

DVSEEDRLDTGKAGDVVYCKDLVNLSESPRLGCGLMVMKNTTFDWTLEYDEVDYIIEG

QLDVLVGGRKISAGPGEIILIPKGSGIQFSVTGEARFIYVTYPADWQSQ"

CDS 3475882..3476541

/locus_tag="JFBMFIFI_03220"

/inference="ab initio prediction:Prodigal:002006"

/codon_start=1

/transl_table=11

/product="hypothetical protein"

/translation="MKKIIIACSLVLLMASGCSLTQKDESKKIKETDSELVHSSKKKR

DRKKKSTTNENEKKDSSFQNQKESDSTQTSEVLWNTFRPNPAEADEEVMYESDGEGKM

SAVIPDYSHYTKEQVIELLGEPSSVVTDSSEIQSRLEGNEWQLIKAEFEKGKLTESQA

KAFIFASADLGIAVGFNRDIELLVYEDQDKPNVYLTNGQVDFITPLTDYIEFNGKLQM

D"

CDS 3476679..3477698

/gene="yhhX"

/locus_tag="JFBMFIFI_03221"

/EC_number="1.-.-.-"

/inference="ab initio prediction:Prodigal:002006"

/inference="similar to AA sequence:UniProtKB:P46853"

/codon_start=1

/transl_table=11

/product="putative oxidoreductase YhhX"

/db_xref="COG:COG0673"

/translation="MLTIAYIANGKSTNRYHLPFSTKLPNQIKVKTIYSRTDKNEWDK

IEGVHYTTNIDDIFNDPEIQLVVVTTPSSLHYYYGKMILEHGKNALIEKPFTETSAEA

KELFALAKEKNLLVQCYQNRRFDSDFLTVQKVIESGVLGEILEVEMHYDYFRPEVPES

VTNYSKDTSYLYGHGCHTIDQVLSYFGNPEKIHYDVRQLLGKGRMNDYFDLDFYYGAM

KVSVKSSYFRLKERPSFVVYGKKGSFVKQTKDRQEEHLKMFYMPNHADFGIDLPEHYG

TLTYIDDEGIYHEEKVVSEVGDYSRVYEGLYETIINGKEKSVKDEETIRQLEILEEGT

QSISN"

CDS 3477947..3478396

/gene="sbmC_2"

/locus_tag="JFBMFIFI_03222"

/inference="ab initio prediction:Prodigal:002006"

/inference="protein motif:HAMAP:MF_01896"

/codon_start=1

/transl_table=11

/product="DNA gyrase inhibitor"

/translation="MNIKQIPKKKIIYMRRTGAYGSENKSLMEHFKKWLKDNNLLNSE

SVILAIPRDNPQTTNPKNCRYDVALVVNSFDDIEQLDINKDVLRAGKYAIFTTEHTEE

AITKAMKNMFSEMNDKGCSFNARQPIIERYAVKMVENNKCEICVPIL"

CDS complement(3479730..3480257)

/gene="rhaR_4"

/locus_tag="JFBMFIFI_03223"

/inference="ab initio prediction:Prodigal:002006"

/inference="protein motif:HAMAP:MF_01533"

/codon_start=1

/transl_table=11

/product="HTH-type transcriptional activator RhaR"

/translation="MTKIPKITYQLKKEKSNESSTIKKNPITQELEDFAFNQVDPLSV

LSSYYLNHQSADPIDSINEVPKLIEKKEITDAKRYIKMNLHRTITLDETAKKVFLSPY

YFSKLFKAETGITFVAYVSQQKMVQATELLRHSDISISQIAKSLGFNQTSYFSRIFKK

EYAMTPKEFRNFSKL"

CDS 3480669..3482036

/gene="glmU"

/locus_tag="JFBMFIFI_03224"

/inference="ab initio prediction:Prodigal:002006"

/inference="similar to AA sequence:UniProtKB:Q7A7B4"

/codon_start=1

/transl_table=11

/product="Bifunctional protein GlmU"

/translation="MTQRYAIILAAGQGSRMKSKLYKVLHPVAGKPMVEHVVGQVEAA

GSDRIVTIVGFGAEQVKEHLGDRSEYALQAEQLGTGHAVLQAESLLKDKDGITLVICG

DTPLLTAETLNDLFDYHQEKGAKATILTAVAEDPTGYGRVLRDDLGIVTRIVEQKDAT

QEEARVQEINTGTYCFDNKMLFDALTKVGTANAQGEYYLPDVIEIMKSKGQIVAAYRM
KKLEEALGVNDRIALAEAAETMRNRINRKHMENGVTLLDPKNTYIDVDVEIGRDTIIE
AGVSLKGKTVIGEDCYIGSNSEISDSILGDRIKVTSSNIESSEMASDSNIGPYSHLRP
ASKIGERVHLGNFVEVKNSTIANDTKVGHLTYVGDADLGENINVGCGTIFVNYDGKNK
HRATVGDNVFIGCNANLVAPVAIEKDSYIAAGSTITSDVPTGSLAIARARQENKPDYF
DRLPH"

CDS 3482181..3483164

/gene="prs"

/locus_tag="JFBMFIFI_03225"

/EC_number="2.7.6.1"

/inference="ab initio prediction:Prodigal:002006"

/inference="similar to AA sequence:UniProtKB:P14193"

/codon_start=1

/transl_table=11

/product="Ribose-phosphate pyrophosphokinase"

/db_xref="COG:COG0462"

/translation="MSEHYFDPKLKIFALNSNRPLAEKIANEVGVELGKLSVDQFSDG
EIRINIEESIRGDHVYIVQSTSSPVNDNLMELLIMIDALKRASAKTINLVIPYYGYAR
QDRKARSREPITAKLVANMITAAGADRILTLDLHASQIQGFFDLPVDHLMGAPLLANY
FLNHDIANDDVVVVSPDHGGVTRARKLAEFLKAPIAIIDKRRPKANVAEVMNIIGNVE
GKKCILIDDMIDTAGTITLAAGALAEAGATEVYACCTHPVLSGPALQRIQDSAIKQLI
VTDSIYLPEDRKCEKIVEISVGVLMGDAIKRIHENKSVSPLFEKKFKRDKN"

CDS 3483538..3485187

/gene="dppE_6"

/locus_tag="JFBMFIFI_03226"

/inference="ab initio prediction:Prodigal:002006"

/inference="similar to AA sequence:UniProtKB:P26906"

/codon_start=1

/transl_table=11

/product="Dipeptide-binding protein DppE"

/db_xref="COG:COG4166"

/translation="MKKMVFVVFLSILSITMLTACGGNDKDNSQSSSSADEKLEVLNL
SVPAELPTVDPALVQDAISFNALNQVMEGLYRLDQKGNPIPAAADGEAEVSEDGLTYT

FKLKPNLVWSNGDAVTAADFEFGWKRVVDPKTAANYAFIMKDVKNAQKIMDGTVASEE
LGIKAMDELTLEVKVEKPTENFLTTITRGTFFPQNEKFVKKEGEQFGTNSERAIYNGP
FVLTEWDGTGLSWNYEKNPSYWDKKNVSLDKVTNQVIKEATTGINLYENDTIDLVRLT
GEQAKQYIDNPEFKAPLEARSVYLELNQIDHPALKNQKVREAIALTINREDLVKMIIA
NGSEALGGLVTNDLAKNTETKEDFRKASGSYLDYDAKKAKALWNEAQKELGSSTAEIE
LVTDDDETSKKVSQFIQSSIEAELEGIKVKIRNVPFKNRIELGDTGQFGLLLSGWGAD
SNDPDAFLNLFLSDSVFNGGKYKNQEYDKLVIAATGEDSSDPNKKWADDLAAEKLLMQ
DVGIVPLYQKADSMLLKSKVKDYIQYQIGSPNLKYVSIQED"

CDS 3485612..3486949

/locus_tag="JFBMFIFI_03227"

/EC_number="3.4.13.-"

/inference="ab initio prediction:Prodigal:002006"

/inference="similar to AA sequence:UniProtKB:Q7A522"

/codon_start=1

/transl_table=11

/product="Putative dipeptidase"

/translation="MSIQLTEEQKEASVKSLTRLINCPSFNTSDGLTNPPFGTGVQLC
LEEALAICEELGMTTYLDPNGFYAYADYGTGTDLIGVLCHLDVVPAGDLTLWNTDPFS
AVVKDGIIYGRGSQDDKGPTIAALSAFKAVVEAGYTFKQRIRFVFGADEETLWRCMAQ
YNKNEEAPTMGFVPDGAFPLIYAEKGLLQVTLTGPGSEELDLDCGDAFNVVPGNASYT
GSDSKALLPHFMKLGLPVKELAGTLTVEGKAVHTSLAGSGVNAINLLAQGLVANYPHP
MIHFLAEQVGSQTNGATIFGEVKDDVSGELTFNVGMLTINKNQSEIKIDMRIPVTADK
KQLVDKLQATAKEYNLKYHEFDYVPSLYVPKDSQLVETLLGVYREATNDLTEPITSGG
ATYARTMKNMVAFGAHFPTSVGLAHQANEGLVLEELYKATEIYAAAIKKLCCE"

CDS complement(3487100..3487762)

/locus_tag="JFBMFIFI_03228"

/EC_number="2.1.1.6"

/inference="ab initio prediction:Prodigal:002006"

/inference="similar to AA sequence:UniProtKB:O07431"

/codon_start=1

/transl_table=11

/product="Catechol O-methyltransferase"

/db_xref="COG:COG4122"

/translation="MEELFSAVDAYFIDKLIEKEAIFDQVLANNQKHGLPPHDVSPSQ
GKFLYLLSKIAKAQRILEIGTLGGYSTIWFAKALPQNGKIISLEFDQTHAKVAQENTK
LAGFSDKIDIIVGPAAESLAQLVNENSLPFDLIFIDADKENNPLYLKYAQQLAKSGTI
IIGDNVVRNGEVLSTESNDGRILGVRQFVDELATNQKLTSTAIETVGVKGYDGFTISI
VE"

CDS complement(3487834..3488721)

/gene="tfdR"

/locus_tag="JFBMFIFI_03229"

/inference="ab initio prediction:Prodigal:002006"

/inference="similar to AA sequence:UniProtKB:Q46M57"

/codon_start=1

/transl_table=11

/product="HTH-type transcriptional regulator TdfR"

/translation="MDIRQLRYFIAIAEEKNITQAAARLHMAQPPLSQQLKQMEEKLG
TILIERTSRKTHLTTAGEVLYQEALKILQQLEETEQLVKETSLGLKGQLRLGVNTLSA
EELAPSLQSFQNKFPDVTYAIHQNESKLLAEMVRNHTIELALVRFPLELHGFDITYLN
VEPFYFICDGKKDALQPGPNPNDYYGIANSKLLLPSTEGLGVYHSIIEYLAKFQLNPT
SISTCSDLSLLFNLVESGFCTSIVPKSVLQLHADRKIEAHLLDDPLFNSSYGLIWSKN
RTLTKVAEHFLAEFVGIHD"

CDS 3488836..3489654

/locus_tag="JFBMFIFI_03230"

/inference="ab initio prediction:Prodigal:002006"

/codon_start=1

/transl_table=11

/product="hypothetical protein"

/translation="MKKNYVLFFSLIGIVLISIGIFSVTLLKDSSTRSKDQSTSEKQP
TSQTSDSKQIESSQELESKENEVMKTDNPQLNQDLITATANQNLSEIKRLLTVGANVD
AMDEKQQTGLLVATHKNNVEMAEIFLLNGANVNQQDAIQDSPFLYAGAEGRTEILQMM
LAYNPDTTLTNRFGGTALIPAAEKGHLDNVRLLLEQTDIDVNHVNQPGWTALLEAIVL
TNGDTTQQQIVQVLLEHGADPNLADANGIRPLRHANQMGYGKIAEELRNAGAYE"

CDS 3489671..3490891

/gene="atzC_2"

/locus_tag="JFBMFIFI_03231"

/EC_number="3.5.4.42"

/inference="ab initio prediction:Prodigal:002006"

/inference="similar to AA sequence:UniProtKB:O52063"

/codon_start=1

/transl_table=11

/product="N-isopropylammelide isopropyl amidohydrolase"

/translation="MTRKKWLQNVRVETGYEYEGETIVGTKTELVDLLIQEGLIKQIL

PSNSVIREDAEYYDAKGLLALPGFIEKHSHLDKSRLGTNWQAVTKVPSIIQRFESEMV

ELEKLPLSVSQRAEVLLNRYLSNGVTKIRSHVDVHPTAGLHYFSSVKEVLDNYQEKLS

SEIVAFPQHGLLRSNSIELMQEALTKGATIVGGVDPTAVDGDMKKSLEATFSLANQNE

AGIDIHLHERGEMGLKTVEYFLSLVENHNLQGKVAISHAFALGDVSGSQKAELFQSLA

ENQVEIMTSVPITGVIPPVMDLKAAGVKVSVGCDNTFDNWSPYGNGDILERVGRLGEV

FGQVTEQELSQTLGFITDGVTPLNPNGKQVWPVVGDKADFVFVEASCSAEAVARNSKK

VIVMHQGEPTHGSF"

CDS 3490990..3491829

/locus_tag="JFBMFIFI_03232"

/inference="ab initio prediction:Prodigal:002006"

/codon_start=1

/transl_table=11

/product="hypothetical protein"

/translation="MAYWRALSLKNKCLFVGSPVFLISVWYLLFNQAKIVTDIITAPF

NLSWVNLFKFFLNEQWLDVLIKNFKTILINLSIVISAQLLFFIIGIVLILVLQWKSSK

ATKWYFGNKLIFAGYLFMLVSTISLAGILGNVSYQLVDTINSSLQKLSVAELTKLSDE

VTGVVSQFTWSLDSIISEATTLMNTVKHIIATTKEIANIPDLITVWFDNLTIYRNYLL

GFVGASTVVIISGHLVELYRIFELNKHLIRRNKKVSVDERIITLLEQQQKLLEKLSEK

EES"

CDS complement(3492358..3493323)

/gene="ldh_4"

/locus_tag="JFBMFIFI_03233"

/EC_number="1.1.1.27"

/inference="ab initio prediction:Prodigal:002006"

/inference="similar to AA sequence:UniProtKB:P00343"

/codon_start=1

/transl_table=11

/product="L-lactate dehydrogenase"

/db_xref="COG:COG0039"

/translation="MKQTEIKDHQKVIVVGDGAVGSSYAFALVTQNIAQEIGIIDIDK

DKTEGDAIDLSHALAFTSPKKIYSATYQDCHDADLIVITAGAAQKPGETRLDLVNKNL

KIFKAIIADIMASGFDGILLVASNPVDILTYATWKFSGLPKSRVIGSGTSLDSARFRQ

AIADLVHVDARNVHGYILGEHGDTEFPVWSHANIGGLQIYEWVKDNPEVDEEALVKVF

FKVRDAAYEIIEKKGATFYGIAVALARITKAILNDESSILPLSVYLDGEYGQEDIFIG

APAVINRQGIQHVIEIPLTDAEMDKMIHSASTLKQILNDAFDALD"

CDS 3493646..3494215

/gene="pth"

/locus_tag="JFBMFIFI_03234"

/EC_number="3.1.1.29"

/inference="ab initio prediction:Prodigal:002006"

/inference="similar to AA sequence:UniProtKB:B5XIP6"

/codon_start=1

/transl_table=11

/product="Peptidyl-tRNA hydrolase"

/translation="MKIIVGLGNPGAKYTATKHNIGFITMDEMAFQHKLQFNKSKFDA

VYAEAFIGTEKVLLVKPQTFMNDSGQAVRPLMDYYDLGIEDLVVVYDDLDLPVGKIRL

RQKGSAGGHNGIKSLIQHLGTSDFNRIRIGIDRPFPGQTVINHVLGAFPKEHHEDILF

AVKDAAEALNYWIEGHTFLETMNQFNRKS"

CDS 3494455..3497988

/gene="mfd"

/locus_tag="JFBMFIFI_03235"

/EC_number="3.6.4.-"

/inference="ab initio prediction:Prodigal:002006"

/inference="similar to AA sequence:UniProtKB:Q7A7B2"

/codon_start=1

/transl_table=11

/product="Transcription-repair-coupling factor"

/translation="MVDIKQLLADTSDIENLLESLEENQTQLVTGLSGSARTLVISTI

LEKKKKPIILVAHNLFHASQLIEDFSGFVPEDQLHLFPVDEMIQAEMSISSPEYRAER

VAALDFLLSGKKGIVIIPLSGVRKLITPKEVWKKARFKITKGGELDPTNLAQDLVDMG
YTREHLVGKPGEFSMRGGIVDIYPLTEEYPVRVELFDTEVDSLRYFEADTQRSIASID
KITILPATDTIYTSSMLKAGAQDFSEAVERNSDLILDAATKGLFIKNLTPIIDAFDKG
EPMDSLALYTDFIYPQHTSVLDYMHKNSLLIMDEYPRIMETERRLIEEEAEWITSKLE
ERKILQHQTFANDLRGVLKDVQQGTLYFSLFQKGMGNIRFKQIHAFQYRNMQQFFGQM
PLLKTEMDRWVKQKNTVLVIVSDEDRAKKVNQTFKDFEIQSKIVKPKKIETGKVQIMM
GAIHNGFELPKEKIVIINEREMFNKVTKKVARRQTLSNAERLKSYTELNPGDFVVHVN
HGIGKYTGMETLEIGGVHQDYMSVLYKDDAKLFIPVTQINLLQKYVSSDAKTPKINKL
GGTEWAKTKKKVAAKIEDIADDLIELYAAREAEVGFAFSPDSPYQQEFENAFPYTETD
DQLRSTAEIKHDMESKKPMDRLLVGDVGYGKTEVAMRAIFKAVQDGKQAAFLVPTTIL
AQQHYESLVQRFEDFPVEIGLLSRFRTKKQQNETMDGLKKGLVDVVIGTHRILSKDIE
FLDLGLLIVDEEQRFGVKHKEKLKQLKAQVDVLTLTATPIPRTLHMSMLGVRDLSVIE
TPPANRYPVQTYVMEQNPGAIREAIERELTRGGQVFYLYNRVETIGKKVEELQMLVPD
AKIAYAHGQMTEAQLESILYQFVEGEYDVLVTTTIIETGVDIPNVNTLFVENADHMGL
SQLYQLRGRVGRSNRVAYAYFMYQPDKVLTEVSEKRLQAIKDFTELGSGFKIAMRDLS
IRGAGNLLGAQQHGFIDSVGFDLYSEMLSEAVVRKRGLEKKDEKTVVEIDLGINAYLP
STYIEDERQKIEIYKRIRELTDRDEYTTLQDDLIDRFGVFPDEVADLLAIGAIKMESE
RALFETIRRVDDCVHLTLSTSGTVSLPAEEIMKALGDIPLQATMSVKKEKLVVTMHLK
DINASFEWLGYIEKFVANIAKYRSKLVAGQL"

CDS 3498048..3499652

/gene="murJ_2"

/locus_tag="JFBMFIFI_03236"

/inference="ab initio prediction:Prodigal:002006"

/inference="protein motif:HAMAP:MF_02078"

/codon_start=1

/transl_table=11

/product="lipid II flippase MurJ"

/translation="MGNKQMKQMMKGAVVLSVASLIAKVLSAVYRVPFQNMVGNTGFY
VYQQVYPIYGIGMTFALSGFPIFLSKLIAETKHNGERKIILKRASLILTLFAIVLFLG
IYTFSDVIANGMGDSQLAPIVKAVSWMFLLMPGLVVWRGYFQGTENMIPTAISQVVEQ
IVRVAVILIAAALFLKSDWSEYRMGTYAMSSAWIAGIAALFVMMYYYYLKGGNQALKE
VPSLNYGEVTNAPSYSKLFLRFLTEGSAICILSALLILLQLIDSFTLFNGLMDSGVLA
DEAKNLKGIYDRGQPLVQLGMVVGTGFASSLIPMLTKHFVHRQEGEFYRTAVSLLRIT

TAFAMVATAGMLTLMPFINDLLFGDRDGNAVLSVYILAIIVASLIGAYNTILQSRNQH
RMAMLALVFGLLLKWGFNQFLIGPFGTMGASIATVFSLGGILLVLWLAMPTRLKGSLL
KGSFLLKLVICCWIMTFVITIVFLFLGQWAFIATRSGSLVAIIIGISIGVPLFVWLIF
KFKLFTIREWLYMPFGKRIVRIRNRK"

CDS 3499662..3500723

/locus_tag="JFBMFIFI_03237"

/inference="ab initio prediction:Prodigal:002006"

/codon_start=1

/transl_table=11

/product="hypothetical protein"

/translation="MGKILVVGLGPGDLAQLPFGVYQLLKKGLPIYLRTKLHPVVENL
KSEGLMFEDFDEVYEANEQFETVYEKIVGDLLKEATISKEDIIYAVPGHPMVAEKSVQ
LLLENTAGIVVEIKGGQSFLDDFFTAVKVDPVEGFQLLDGLDLDQNAVQMGQHVIVMQ
IFNEYIASDVKLTLQEIYPDDHQVALVHSAGSEAEQIDWLPLYEMDRMEGVHNLTSLY
VPPLALDDRAKSFQTVQTYMDEIGGENGDIWLLEQTHETLIPYLKEETAELISAIENE
DIDNIIEELGDVLMQVMYQTNYGERTGYFNLEDVLETLNHKLRRRHPHVFDGVKVETV
EELDALWQKIKAEEKRNLT"

CDS 3500720..3500995

/locus_tag="JFBMFIFI_03238"

/inference="ab initio prediction:Prodigal:002006"

/inference="similar to AA sequence:UniProtKB:O25966"

/codon_start=1

/transl_table=11

/product="hypothetical protein"

/db_xref="COG:COG1188"

/translation="MRLDKFLKVSRIIKRRTIAKEVADKGRIEVNGITAKSSTDVKIG
DELSIAFGNKTLVVKIDRIIETTKKDEAAHMYTIISETYKNDPEMKA"

CDS 3501272..3501751

/gene="ftsL_2"

/locus_tag="JFBMFIFI_03239"

/inference="ab initio prediction:Prodigal:002006"

/inference="protein motif:HAMAP:MF_00910"

/codon_start=1

/transl_table=11

/product="Cell division protein FtsL"

/translation="MNKKRGAMNVTSLGNEYTQEKNLQSHREKKHKRQTRRRLFAILF

VGLLIIGGLGFKIMANQQALAKMKSEEVQVAKELKKANLDQRLLNNQIKQLKDDSYIE

KLARAKYDLSKDNEIIFNLSGEATTKSLEESKKKIDADKKAETQESGKENEAPEPNS"

CDS 3501885..3502388

/gene="pnp_2"

/locus_tag="JFBMFIFI_03240"

/EC_number="2.7.7.8"

/inference="ab initio prediction:Prodigal:002006"

/inference="protein motif:HAMAP:MF_01595"

/codon_start=1

/transl_table=11

/product="Polyribonucleotide nucleotidyltransferase"

/translation="MSIEVGSKISGKVSGITNFGAFIDLGEGKTGLVHISEVSDTFIK

DIKDVLKVGDEVLVKVMSIGDDGKIGLSIRRAVDKPASETTPRRESTGGYQGRREGGS

GGGRDSNRSGGYQNQRNTRNTRNEPPKKEDFDSLMSSFIKDSDDRLTSLKRNTEGKRG

GRGGRRS"

CDS 3502469..3503869

/gene="tilS"

/locus_tag="JFBMFIFI_03241"

/EC_number="6.3.4.19"

/inference="ab initio prediction:Prodigal:002006"

/inference="similar to AA sequence:UniProtKB:P37563"

/codon_start=1

/transl_table=11

/product="tRNA(Ile)-lysidine synthase"

/db_xref="COG:COG0037"

/translation="MSVKQTFLKACQKDSYWSPESRILLAVSGGVDSMVLLDLVSHLP

PSIKPWFAVVHFNHQLREVSDIEEQFLQSYCLEREIPFYSKRWESVDLPQNGVEAAAR

SARYTYFQLIMEQVGATHIVTAHHGDDQVETILMRLVRGSHLIGISGIKTKRPFAKGI

LVRPLLSSTKKELVSYSQQYAVPYYEDESNQNLMYTRNRYRNEIVPLLRKENTNLVEH

FMDFSDDLSDIISLVQPLINEVFKQVVKIRSPEYYEVDFPELITHPKEMQRQVVSQLL

SQLADQNPFEYKREHINQIVDLANDSKSNSYLTLPEGFICRKGYQLIVISKELNEPKK

AIDFTVILEMNQWQKLSNGDRIGLFSSHVLADSVPGLKIYLDCHSVQLPLTVRHRQDG

DRMSIKGLNGGTKKIKDILIDQKIPIEERNSAYLVTDLTKKIIWLVKYKESQLSIGKE

TDKIQYILVYQNHELY"

CDS 3503888..3504430

/gene="hpt_2"

/locus_tag="JFBMFIFI_03242"

/EC_number="2.4.2.8"

/inference="ab initio prediction:Prodigal:002006"

/inference="similar to AA sequence:UniProtKB:Q5XEL6"

/codon_start=1

/transl_table=11

/product="Hypoxanthine-guanine phosphoribosyltransferase"

/translation="MQNDIERVLFSREDLAEKTVELGKILTEEYEGKNPLVIGVLKGA

TFFMSDLVKEIDTYLEMDFMDVSSYGNEMVSSGEVKIIKDLDTNVAGRDLLIVEDIID

SGRTLAYLVDLFKYRQAKSVKIVTLLDKPEGRAVKMEADYVGYLVPNEFVVGYGLDYA

ERYRNLPYIGILKPEIYQEK"

CDS 3504545..3506680

/gene="ftsH"

/locus_tag="JFBMFIFI_03243"

/EC_number="3.4.24.-"

/inference="ab initio prediction:Prodigal:002006"

/inference="similar to AA sequence:UniProtKB:Q88Z31"

/codon_start=1

/transl_table=11

/product="ATP-dependent zinc metalloprotease FtsH"

/db_xref="COG:COG0465"

/translation="MKKNGLFKNGIFYAIVFLGIIGIVSWAGNGASSGQSSEISASKF

VKQLKEDEVKEFSIQPSGGVYKITGEYRKAQKVEGSTGGTGFSLFGSKKEAESKNFTT

AVLSNDSTINQINEIAQENKVTMTPLEEATMGFWLSLLISFAPILVLVLLFYLMMGQS

QGGQGGGGRGVMNFGKSKAKESDNKANKVRFSDVAGAEEEKQELVEVVEFLKDPRRFI

ALGARIPAGVLLEGPPGTGKTLLAKAVAGEAGVPFYSISGSDFVEMFVGVGASRVRDL

FETAKKNAPAIIFIDEIDAVGRQRGAGMGGGHDEREQTLNQLLVEMDGFSGNEGVIII

AATNRSDVLDPALLRPGRFDRQILVGRPDVRGREAILKVHAKNKPLADDVDLKVVAQQ
TPGFAGADLENVLNEAALVAARRNKKKIDALDVDEAQDRVIAGPAKKDRVISKREREM
VAYHEAGHTIVGMVLNDARVVHKVTIVPRGKAGGYAIMLPKEDRFLMTKTEMFEQIVG
LLGGRTAEEMIFDVQSTGASNDFEQATGLARSMVTEYGMSDALGPVQYEGNHQVFVGR
DYGQTKAYSEQVAYEIDQEVRKILTDAHKEARRILEEHRAEHKLIAEKLLELETLDEK
TIKSLFETGEMPRGKEAKEEYPREDGASFEEAKKALEAKEAEKMKEEKAELEARKKAE
EEATVEEEKGAEEASKEIKLEKKEEDSHE"

CDS 3506788..3507675

/gene="hslO"

/locus_tag="JFBMFIFI_03244"

/inference="ab initio prediction:Prodigal:002006"

/inference="similar to AA sequence:UniProtKB:P99082"

/codon_start=1

/transl_table=11

/product="33 kDa chaperonin"

/translation="MSDYLLKSVCYDGQIRAYAVCATETVGEAQKRHDTWSASSAALG
RTMIGALLLGATMKGQDKMTVKVEGDGPAGTIIVDSNGAGETKGYISNPKVHLPPNEA
GKIDVRGAVGTEGTLTVIKDMRMKETFSGQVPLVSGELGEDFTYYMANSEQVPSAIGL
SVLVDTDNSIKAAGGFMIQVMPGATDATLDAIEKNIAEIPLVSKLMDEGESPEEILFR
LLGGEENVKILEKMPVTFKCDCSKERFANAIITLGPTEIQDMIDEDHGAEANCHFCGN
SYHYSEEDLAELKIEAAGL"

CDS 3507980..3508915

/gene="cysK"

/locus_tag="JFBMFIFI_03245"

/EC_number="2.5.1.47"

/inference="ab initio prediction:Prodigal:002006"

/inference="similar to AA sequence:UniProtKB:Q5XAQ3"

/codon_start=1

/transl_table=11

/product="Cysteine synthase"

/translation="MVKIVSSVTQLIGETPIVKLNRVVPEDAADVYVKLEFFNAGGSV
KDRIALNMIEVAEEEGILKPGDTIVEPTSGNTGIGLAMVAAAKGYQAILVMTDTMSIE
RRKILRAYGATLILTPGAEGMKGAIAKATELAAQPGYFMPMQFENLANPAVHEVTTGP

EILEAFDGKGPDAFVAGVGTGGTLTGVGHVLKQANPDVLIYAVEPAESPVLAGGSPSP
HKIQGIGAGFIPEVLDRTVYDGILEVASEEALLMGREVAAKEGILVGISSGAAIKAAI
EVAKKLGKGKSVLAVVPDNGERYLSTALYPTVEGE"

CDS 3509110..3510105

/gene="dusB"

/locus_tag="JFBMFIFI_03246"

/EC_number="1.3.1.-"

/inference="ab initio prediction:Prodigal:002006"

/inference="similar to AA sequence:UniProtKB:P0ABT5"

/codon_start=1

/transl_table=11

/product="tRNA-dihydrouridine synthase B"

/db_xref="COG:COG0042"

/translation="MWKIGDVEIPNRVVVAPMAGISNAAFRVTVKEFGAGLVVCEMIS
DKGIQTRNKKTLDMLHIEENEYPLSVQIFGGDKANLVEAAKFVEANTQAAIIDINMGC
PVNKIIKAEAGAKWLLDPNKVYEMVAAVADAVKIPVTVKMRIGWDSDHVFAVENALAA
ERAGAGAVAMHGRTRVQMYEGQADWDVLRQVKQELTIPFMGNGDVRTPMDAKRMLDEV
GADGVMIGRAALGNPWMIYQTKHYLETGILLPEPSAREKIKTAKLHLERLVNLKGEVI
ATREFRQHAAYYLKGISRAAKTKVAVNQATRPEEMARILDDFVDATEEREMKLAK"

CDS 3510287..3511786

/gene="lysS"

/locus_tag="JFBMFIFI_03247"

/EC_number="6.1.1.6"

/inference="ab initio prediction:Prodigal:002006"

/inference="similar to AA sequence:UniProtKB:Q9RHV9"

/codon_start=1

/transl_table=11

/product="Lysine--tRNA ligase"

/translation="MSNETNQQEELNDQLKVRREKMEQLREKNIDPFGERFERTHLSS
ELHEAYDRFTKEELVEQKETATVAGRIMTKRGKGKVGFAHLQDRKGQIQIYVRKDAVG
DDAYDIFKNADLGDFIGVTGEVMKTDTGEVTIKPTSLVQLSKALRPLPDKYHGLTNVE
QRYRQRYLDLISNKESFDRFTKRSEIIREVRNYLNEQDYLEVETPTLHNLAGGAAARP
FITHHNALDMELYLRIALELHLKRLIVGGMEKVYEIGRVFRNEGVDTTHNPEFTMLEV

YTAYTDYKDVMDLTEGLIKTVANRVLGTGEITYGEVTVDLDTKWKRQHMLDAIKEYSG
VDFWPVMSDEEARALAKENNVTITEHMQYGHVVNEFFETFVEEKLIQPTFIYGHPVEI
SPLAKKNPQDGRFTDRFEVFIVGNEYGNAFTELNDPIDQRERFEAQMRERAGGNDEAH
QLDEDFLEALEYGMPPTGGLGIGIDRLVMLLTDASAIRDVLLFPTMRNH"

ORIGIN

1 aaaaaagttt taaaaagtgt tgacaacaaa ttaaccatgt gttaatattt agaagttgtc
61 aaaacgacaa acgaaacaac ttagaccttt gaaaactgaa caaagtaaga cgaaccaaat
121 gtgtaggatg actcaacaag gttgagtcaa acaagcaaga aagtgaataa ttattcgcta
181 gcaaaaacta atgagcttca agcatcattt tatatgagag tttgatcctg gctcaggacg
241 aacgctggcg gcgtgcctaa tacatgcaag tcgaacgcac aaagttgaag agcttgctct
301 ttaaccaagt gagtggcgga cgggtgagta acacgtgggt aacctgccca ttagaggggg
361 ataacattcg gaaacggatg ctaataccgc atagtttcag gaatcgcatg attcttgaag
421 gaaaggtggc ttcggctacc actaatggat ggacccgcgg cgtattagct agttggtgag
481 gtaatggctc accaaggcaa tgatacgtag ccgacctgag agggtgatcg gccacactgg
541 gactgagaca cggcccagac tcctacggga ggcagcagta gggaatcttc cgcaatggac
601 gaaagtctga cggagcaacg ccgcgtgagt gaagaaggtt ttcggatcgt aaaactctgt
661 tgttaaagaa gaacaaggat gagagtaact gctcatcccc tgacggtatt taaccagaaa
721 gccacggcta actacgtgcc agcagccgcg gtaatacgta ggtggcaagc gttgtccgga
781 tttattgggc gtaaagcgag cgcaggcggt tctttaagtc tgatgtgaaa gcccccggct
841 caaccgggga gggtcattgg aaactggaga acttgagtgc agaagaggag agtggaattc
901 cacgtgtagc ggtgaaatgc gtagatatgt ggaggaacac cagtggcgaa ggcgactctc
961 tggtctgtaa ctgacgctga ggctcgaaag cgtggggagc aaacaggatt agataccctg
1021 gtagtccacg ccgtaaacga tgagtgctaa gtgttggagg gtttccgccc ttcagtgctg
1081 cagctaacgc attaagcact ccgcctgggg agtacggccg caaggctgaa actcaaagga
1141 attgacgggg acccgcacaa gcggtggagc atgtggttta attcgaagca acgcgaagaa
1201 ccttaccagg tcttgacatc ctttgaccac tctagagata gagctttccc ttcggggaca
1261 aagtgacagg tggtgcatgg ttgtcgtcag ctcgtgtcgt gagatgttgg gttaagtccc
1321 gcaacgagcg caacccttat tactagttgc cagcatttag ttgggcactc tagtgagact
1381 gccggtgaca aaccggagga aggtggggat gacgtcaaat catcatgccc cttatgacct
1441 gggctacaca cgtgctacaa tggatggtac aacgagtcgc aaggtcgcga ggccaagcta
1501 atctcttaaa gccattctca gttcggattg taggctgcaa ctcgcctaca tgaagccgga
1561 atcgctagta atcgcggatc agaacgccgc ggtgaatacg ttcccgggtc ttgtacacac
1621 cgcccgtcac accacgagag tttgtaacac ccgaagccgg tgaggtaacc ttttaggagc

1681 cagccgtcta aggtgggata gataattggg gtgaagtcgt aacaaggtag ccgtatcgga
1741 aggtgcggct ggatcacctc ctttctaagg aatattacgg aaatcccaca cattcgtttt
1801 tactttgttc agttttgaga ggtctaactc tcaaaacgat tcatctctat tcattagagg
1861 tcaatcacat tttgttcttt gaaaactgga tactgtttaa aaagtaatta aagtaagaaa
1921 ccgagaaaac accgcgtttt aaatgagttt tttaattagt tcttatcgta agataatgaa
1981 taaaatcatt gactaacatc atgagattcc ttcgggaata ccatcgataa aaatttagtt
2041 gtagtggttt ctaattcgag gacatgaagg gtttgagaag cagagcgtac tagcgtacgt
2101 gagcatcgca gaacacgaaa tggacgaaga agtaggaagc aatacagata aattaggtta
2161 agttaataag ggcgcacggt ggatgccttg gcactaagag ccgatgaagg acgggactaa
2221 ctccgatatg ctttggggag ctgtaagtaa gctttgatcc aaagatttcc gaatggggga
2281 acccagcatt ttttatagaa tgttactgct agctgaatac ataggctagt agaggtagac
2341 gcagagaact gaaacatcta agtacctgca ggaagagaaa gaaaattcga ttccctgagt
2401 agcggcgagc gaaacgggaa gagcccaaac caaagagctt gctctttggg gttgtaggac
2461 acgacattaa gagtcataaa tgagttttgt agaagaagcg atctggaaag gtccgccgaa
2521 gagggtaaaa gccccgtaat cgaaacaaag ttcactctgt tgtgtatcct gagtacggcg
2581 gaacacgaga aattccgtcg gaatccgcgg ggaccatccc gcaaggctaa atactcctta
2641 gtgaccgata gtgaaccagt accgtgaggg aaaggtgaaa agaaccccgg aaggggagtg
2701 aaagagctcc tgaaaccgtg tgcttacaaa tagttagagc ccgttaatgg gtgatagcgt
2761 gccttttgta gaatgaaccg gcgagttacg attacatgcg aggttaagtt gataagacgg
2821 agccgtagcg aaagcgagtc tgaatagggc gaatgagtat gtggtcgtag acccgaaacc
2881 aagtgaccta cccatgtcca ggttgaaggt gcggtaatac gcactggagg accgaaccca
2941 cgtatgttga aaaatgcggg gatgaggtgt gggtagcgga gaaattccaa tcgaacttgg
3001 agatagctgg ttctctccga aatagcttta gggctagcct cggaattaag aatcatggag
3061 gtagagccac tgtttggact aggggccctt ctagggttac cgaattcaga taaactccga
3121 atgccattga tttatatccg ggagtcagac tgcgagtgat aagatccgta gtcgaaaggg
3181 aaacagccca gaccaccagc taaggtccca aagttactat taagtggaaa aggatgtggg
3241 gttgcttaga caactaggat gttggctcag aagcagccat catttaaaga gtgcgtaata
3301 gctcactagt cgagtgaccc tgcgccgaaa atttaccggg gctaaatagt acaccgaagc
3361 tgtggataga accttagggt tctatggtag gagagcgttc taagggcgtc gaagctagac
3421 cgtgaggact agtggagcgc ttagaagtga gaatgccggt atgagtagcg aaagacgggt
3481 gagaatcccg tccaccgtat gactaaggtt tcctggggaa ggctcgtcct cccagggtta
3541 gtcgggacct aagccgaggc cgataggcgt aggcgatgga caacaggttg atattcctgt
3601 accagtttct tttgtttgaa caatggaggg acgcaggagg ctaagaaatg cgcactgttg

3661 gatatgtgcg tctaagcaat gagtcttgag gtgagttaaa tgcttgcttc tgtacggaca

3721 agttgtgatg gggagggaaa ttacagtacc gaagtttctg atgtcacact gccaagaaaa

3781 gcttctagtt agaaagaaat tgcccgtacc gcaaaccgac acaggtagtc gaggagagta

3841 tcctaaggtg tgcgagagaa ctctcgttaa ggaactcggc aaaatgaccc cgtaacttcg

3901 ggagaagggg tgctgatcga aagatcagcc gcagtgaata ggcccaagcg actgtttatc

3961 aaaaacacag gtctctgcaa aatcgaaaga tgacgtatag ggctgacgc ctgcccggtg

4021 ctggaaggtt aagaggattg gttagcttcg gcgaagctaa gaattgaagc cccagtaaac

4081 ggcggccgta actataacgg tcctaaggta gcgaaattcc ttgtcgggta agttccgacc

4141 cgcacgaaag gcgtaacgat ttgggcactg tctcaacgag agactcggtg aaattatagt

4201 acctgtgaag atgcaggtta cccgcgacag gacggaaaga ccccatggag ctttactgca

4261 gtttgatatt gagtgtttgt acagcttgta caggataggt aggagccata gaagtcagga

4321 cgccagtctt gatggaggca ttggtgggat actacccttg ctgtatgacc actctaaccc

4381 tagccactaa tcgtggctgg agacagtgtc agacgggcag tttgactggg gcggtcgcct

4441 cctaaagagt aacggaggcg cccaaaggtt ccctcagaat ggttggaaat cattcgtaga

4501 gtgtaaaggc agaagggagc ttgactgcga gacctacaag tcgagcaggg acgaaagtcg

4561 ggcttagtga tccggtggtt ccgcatggaa gggccatcgc tcaacggata aaagctaccc

4621 tggggataac aggcttatct cccccaagag tccacatcga cggggaggtt tggcacctcg

4681 atgtcggctc atcgcatcct ggggctgtag tcggtcccaa gggttgggct gttcgcccat

4741 taaagcggta cgcgagctgg gttcagaacg tcgtgagaca gttcggtccc tatccgtcgc

4801 gggcgcagga aatttgagag gagctgtcct tagtacgaga ggaccgggat ggacacaccg

4861 ctggtgtacc agttgttctg ccaagagcat cgctgggtag ctatgtgtgg aagggataaa

4921 cgctgaaagc atctaagcgt gaagcccccc tcgagatgag atttcccatc acgtaagtga

4981 gtaagacccc tgagagatga tcaggtagat aggttggaag tggaagtaca gcgatgtatg

5041 gagcggacca atactaatcg gtcgaggact taaccaagaa ataaacggtg aagaacgttt

5101 ccaaaacaat tactttttaa acagttccag ttttgagaga tcaaactctc tacaactaaa

5161 taattgaatc gcagtttagt gtcgatgtaa ccatcaacct aaacaagcat tttttaacaa

5221 attgcaagct aatcaaggat gtcactgagc gaaccccgga atgcactagt gtgcatgagg

5281 agtggagtaa aggaaacaga cgcagagtag cgccgcaagt tgtaaaaaaa attgtgtggt

5341 ggcgatagcg aaaaggatac acctgttccc atgccgaaca cagcagttaa gcttttcagc

5401 gccgagagta gttgggggtt tccccctgtg agggtaggac gttgccacgc aaaagattca

5461 atcgaaaaaa aggactattg aaaaatagtt ctttttttat gcaaaatttt aaaaatgatc

5521 attatgtaga atttcttatt acttttctca actattcgaa ttatgaactg agtactctga

5581 acacggcatc cttacagcgc tgtttaccga ttgcttcggc tggcatctac atctgaaaag

5641 cataaaaacc ttgattttta ttgaaactta aagtttttttg aagtaatcaa tagagtttga
5701 cttatggtga tatatgaaca gtttattaaa taggagaaat tatagatgga aaaaaataca
5761 attatagaaa aagtttgtta cacggattta gtttttgagc gagtaaataa aaagcttagt
5821 aagactatga cacaaaatga aataaaattc ttagtgctag ctattttaaa tgataacaga
5881 agtcaatttg aaaaaatagg taaaaattac tatatatcta atataccaaa aggaataaaa
5941 ttagtcatta actcttcaac gtaccgtttg ataactgtag ataaaattta actaatgagg
6001 agtactataa aattacatat ataaagaata taagtgttag tttacttgtc tttgctttgt
6061 tgttgagctg tcaaaaatct ctaatggtag tagaaccaac tttggttagt taatttgtta
6121 actattttaa ctgaaacttg ttatgttatt gaagcaattt cacttacaaa aaataaaaga
6181 cgtttcagtt aatattaact gaggcgtctt tttatgcgta ttctagtata agggcttgtt
6241 tttgtaattg acgcaaataa tttgaaattg ttccagtata gatatgagta aaaatagcta
6301 attcttgtaa atcttgaggt gtaaccatat gtggcttttt gctaaattca taaagaccac
6361 tatcttctaa aatagtcgct ataaattcgg aacaaaagaa ggctcgatta cgttgataat
6421 ctatatttac ggctaaagta attaagccta ataaattata ccgcaagtct tctttatgtt
6481 gatgaaaata gtttatttgt tcttgaatat gtttgtattc atccaaggtg atttcacaag
6541 aataaattga gcaacgagca cttaaaaaga aatcatgtga aatatcttct cgaacaaaac
6601 ccccaataaa cgggttattt aagtcttttc gaccaaaact agctaaatca ttcaaagaag
6661 gcatcagagc aatcgaagcg tgattgtagc tagctctagt atataattta atcgtttttg
6721 ataataaact acttgttgca gttaaaacta ggtataaatg catgaataat ggtcatctcc
6781 tgttcattca ttaattaaat actactattt ttttattata cagtaaaaca cagcatatat
6841 atatggcact taggtctgat tattaaaaat tgatgtaaat atgaaaaaat aatgaatgaa
6901 agaaaaagaa ttttttagag atgaaaatag cgaaaataaa gagttttctt attttttttg
6961 atatatgaag aaagttttga taaattaaga taaaaaaaac acagcgtttt atttcttttt
7021 atgatatatt tgtgttatct taatggaata atcgtttatt ttatttgttt ttaatccttt
7081 aaaatcaaag gtaatttgaa attgatgaaa aaattattat tttaaaagcg ttatttagtg
7141 accgtaaaac aaaaacagat aggagaaaca atatatgagc caaatgcaat taaaatgttt
7201 tatcgagatt atgcttaatt taattgaaga gaatgaatgg gaagatacct gtgaaatatt
7261 aggtattgag gatcaagatg agtttcgtat tattaatgaa atgcgtcagg atcaaatgaa
7321 tatcttcact gaaacagcat ctgcctatat ggaagtgtgc atgaaaaagc atgacggaat
7381 tgattacgaa gatgaacatt ttaaagaaat gactttgcat gaatggctaa ttcacaatcg
7441 tccagaagtt ccattcttat atatttaaat ttaaaagaaa aagcagcgcc taatatggcc
7501 tgcttttttt aattgtcttc ttcgaaaagt gtcccttttt ttatttgaat taattgggaa
7561 tgatagatgc ccaggtttcc tgaaaataaa tagctaataa cacaaattaa gaataaataa

7621 ggcaaaccag ctgttccaaa aagctctaag cccatgacaa aacatgcaat aggtgtgttt

7681 gttgctccag aaaatacacc gataaatcct aaaccagcta agaatgatac tggaagagct

7741 aggaaaggtg ctaacgtgct tcctagagta gccccaatga caaatagtgg tgtaacctct

7801 cctccttgaa aacctgctcc aagggtcaca gatgtaaaaa ttaatttaat tagaaaagat

7861 aaaggtggaa ggtcaccatt aaatgattgt tctaacatcg gtaaacttaa tcctagataa

7921 gcacgactac caacaacgat gactaagata ataattataa tcccaccaaa aaaactttta

7981 aaaatagggt taggaaacca ttcactaaat tttgttttta gataagccgt tagtcgacta

8041 aagagacgtc cagttaagcc gaataaaatt gctgccaaaa ttaattttaa gattaggagt

8101 ggtgtgctag ttggaattag tcccatttga tagatgtgat gagtggcgcc aagagccagt

8161 gtaacccagt ttgctactaa agcagaccat aagctaggaa atagtgcttc ttcgcggaaa

8221 tagcctaata cgagtacttc catagcaaat aaagttccag ctataggtgt gccaaaaacc

8281 gaactaaatc ctgcactcat tccacagata atgagaatgc gtctctcttt agcattaatt

8341 ttaatgattc gcccgacaaa atcagcagta gcaccaccca tttgtacagc agttccttcg

8401 cggccagcag aaccaccaaa aagatgcgtg gctaaggttc ctatcaatgt taaagggatt

8461 aggcgtaaag ggatttttc ttccccacca atcgcttgct caatgactaa attattgcct

8521 ttatgggcat tctttccaaa gtctttatat aaataactaa taaaagcccc agctaaaggc

8581 agtaaaaaaa gtaaccaaga gtaatgcact tgtaaagctg ttactttttc taaagatttc

8641 aagaaaaaag cagataaagc gcctgttaaa ataccgacaa aaccagcgta aataaaccac

8701 ttaatcaggt aaaagagaat ttgagtaaat tctaactgtt taattttatg acaaattgag

8761 tgcacgataa ttctccttct ttccatctaa gtaaatagaa tgaacaaatt catcttattt

8821 taccacaaat tattctatta gtaagataaa gtcaaaaaag actattggag ctagccaaat

8881 agtcttttta ttaactggtg cgttttagaa atgctgaagg tggcaaacca taatattttt

8941 taaataactt gctaaaataa taggcatcat tatagccgac taaatttgcc gcttctttaa

9001 ctgtgatatc tttttcattt tccattaatt cttttgcacg atttaatcgt aatttaatca

9061 gataattgat gggtgattct ccagtttctt ctttaaagac tctagaaata tacgttgggc

9121 tgatatacaa tgtttgagat aaagagctta ggctaacttc ctctgtatga tgattttcta

9181 aataatgaat aatgtcattg acgatggttt gcttttcttg ctcttcatag gaaagtttta

9241 gagcattgat ttctaattga ctagagtcgg catcacgtaa aatatagata atcaatttca

9301 tcaccaatgc ttttaacatc aagtcatatc ccgcttcacc tttggctttc tccattaaaa

9361 tttcttggca gacatcaaaa aattctgttt taaattttgt taaatgaaga atagatgtgt

9421 taaaaggaaa atgatcacgt gaaaatcctt gaagtgcgaa attgcgaaaa ccaatgtgta

9481 tttgtgtact ttccattcct tgttgatagc gctcatgatg ataaacacca ggattaaaca

9541 agagtattgt ttctttttct aaaaaaacgg tttcatcatt gattacataa tctacggcac

9601 catcaataat aatcgacaat tcgagaaagt catgatggtg gtgttgtcct tctgaaggac

9661 cattattgta acaatcaaag atatataaaa tttctgggtt aaatgttttt gtatttagtt

9721 gtaacatccc ctattcacct ctttcaagcg ctttctatta tacgttaaaa aatgaggaac

9781 cgcaacagaa ctagtctata tatataggta aatagaaggt gatataactg aatatgtctc

9841 gttttttgat gtgaataggc aagctaagtc tcttaaattg tcctctttta ttttaggcaa

9901 gaataaacaa gtttatccat cttaattaat aaaaatgacc attctttttt tgaactatat

9961 ccagtaatct ataacaataa taacgaatat gaaaggttag attttaggct agtttcactc

10021 gcaagccatt ctagaaaaag ataaattccc aaaaaagtaa agagacattt ttctgccatg

10081 gtaaaaactg cgagttcaac atattataat taggaggaat ttatatggga agcattaaat

10141 gggtttggca atatgctaag gtctatcgat ggaagacaat actcgcaact accttagtat

10201 tgattacctc aattttaagt attgtgtatc cacttttggg tggaaggata gtggacgaag

10261 tcatagatca agggaaatca gaattattac tgccattatt aggtgccatg attggtgtca

10321 catttattcg aacagtatta cgttatagtt atcagatttt atttgaaagg attggacaaa

10381 attcactatt tcaaattcgt gaagatttgt atatgaaatt gcaagaacta gattttaatt

10441 tttttaatca gacgcgtgtt ggagatatta tggcgcgaat gactggcgat acggatgcga

10501 ttcgtcattt tgtttcatgg gtattttaca atatcttaga aaatttacta ttatttattt

10561 gtgccgttgt agtgatggct agaattgact ggcgccttat gttagcttta gttgcaatta

10621 ctcctttaat tggtttttta acaatgagaa tgtctcaaaa agcacaacct attttctatg

10681 aaatcagaga aagtttttca cgcttgaatt caatggtgga agaaaatata agtgggaatc

10741 gtgttgttaa agcatttaca agagaagaat ttgaaattga aaaatttaat caccataatg

10801 atgattttaa gcaacgaaat attgattcag cagaagtatc aaaacagtat ttaccaattt

10861 tagattcatt agcaagcagt ttgacaataa ttactttagt tttaggtggc tattttgtta

10921 ttattggtca aatgacgtta ggggatttag tcgcatttaa tggtttttttg tggatgttaa

10981 atatgccaat gcgcatgagt ggttggttaa ttaatgatgt ccaacgattt atcgcatcct

11041 cctttaaaat tcgtgaaatg ctaggttcta aatctcggat tccaattgtc tctgaaaaac

11101 ctgcaacagc tttaaaaggc tatgttgaat ttgatgatgt gtcctttcat tttgatgatg

11161 acccagaaac agaagtccta agtcatattt ctttgaaagc tgaacctggt caaacgattg

11221 gtattctagg cgaaacaggt tcaggaaaat cgacgttagt taatttgatt gcacgtttct

11281 atgatcctac ctctggaact gttttaattg atggcgttga tgcaaagaaa tggcatgtac

11341 gtgaactacg tgaccatatt gcgatcgtaa tgcaagatat attcttattt tcggatacga

11401 ttgaagaaaa tatttcattt ggtgtaccag gcgcagatag ccaagaaatc gagcgtatgg

11461 ctcgcatagc agatgctaac cattttatcg agaagatgcc tgacgggtat gagacggttg

11521 ttggtgaacg tggtgtcgga ttatctggcg gacagaagca aagaatttct ttagccagag

11581 ctttaatgaa aaatccggct attttaattt tagatgatac aacttcagct gttgatatgg

11641 agacggagac aaagattcaa ggagaactag atcagattac tgtcgacaaa acaacattta

11701 ttattgcaca tcgagtatct tctgttaagg atgcagatga aattttgatt atggatcatg

11761 ggaaaattgt agaacgagga actcatgaat cgctcttggc atataagggc tactattatg

11821 aagtgtataa taaacaactt ggaaactttg atgtaacgga ggtggaacca aatggctaga

11881 aataaatttg atgtcgatga agaattagaa caagagttta ataaagaaca ctatcaacgc

11941 ttatttcaat atgtaaaacc ttataaaaaa ccaatgctaa ctacattgtt tgtcatttta

12001 attgcaaata ttgcgacaat gctaggacct tattttacta aattagttat tgatgatgta

12061 attccaaata aaaatatgac attgttaggt tggttagctg ttgcttttat agtctcagta

12121 tttgtaactg gttggtgtat gcggtatcga atccaatcca ttacgttaat cggtcaagat

12181 atgcttaaag atatgcgttc agatattttt gaacatttac aaaaattgcc attctcttat

12241 tttgacagcc gaccacatgg gaaaatcttg atccgagtag tgaattatat taacacatta

12301 agtgatttgc tttcaaatgg attaattaat ttaatttcag atgtgattag tgtggtcatt

12361 acgttgattt ttatgtttgc gattgacttt aaattaacgc tgtatagctt ggctttgtta

12421 ccagttttat tcatttttgt aatgatcatt aaaaataaac aacgaaaagc ctatcaagtt

12481 ttaagcaaca agcaatcgaa tctaaatgcg tatattcatg aaagtattgc tggaattaaa

12541 gtaacacaat cattttctag ggaagatgag aattacaaaa tttttcaga ggttagcaat

12601 gaatatcgct cttcttggat gcatgctgta aaagttcaat ttcttttatg gccgggtgtt

12661 caaaatatcg ctgtgattac gacctgtcta atttatttcg tgggaatccg aggcttaggt

12721 gtcgaggttt caaccggtac attaatcgct ttcataggtt atgtaaataa tttttggaat

12781 ccggtcatta atattgggaa tttttataat tcattaatta cagcaacagc ctatttagaa

12841 agaatttttg aaacattgga tgtagaacca gatattaaag atgcaccaga cgcatttgaa

12901 ttgcccccaa ttaaggggga tgttcgtttt cgtcatgtaa ccttcagata tgaagaaggc

12961 aaagatattt taaaagatat ttcatttcat gttttaccgg gtgagtcaat tgctttagtt

13021 ggaccgactg gagctggtaa gacgaccgtc attaatttgt taagccgctt ttacgatatc

13081 aacgaaggcg aaattttaat tgatggtgtg gatatcagac gagtgacatt acattcttta

13141 agaaaacaaa tgggtgttat gctacaagac acatttattt tttcaggaac tattttagac

13201 aatattcgtt atggaaaatt agatgcaacc gaagaagaag tgattgccgc tgctaaggtt

13261 gttcgggctc atgattttat tgttgattta aaagaaggat accacacggc tgtaaaagaa

13321 cgtggtagta cattgtctgc aggacaaaga caattaattt cttttgcacg agctttgtta

13381 gctgatccca aaatattaat tttagatgaa gcgacttcaa gtattgatac aaaaacagag

13441 acattgttac aggatggttt agataagttg ctagaaggcc gaacttcatt tattattgcc

13501 catcgcttat ctaccattaa aaatagctca agaatctttt atattgatca aggaacggtt

13561 caagaggcag gtagccatga tgaattaatg gcagagaaag gctattatta tcaattatac
13621 caatctcaat ttgatttatt aaaagaaata tagatatgtg ctcaaaaagg ctagcagacc
13681 agattttacg ctagcctttt tgttatctag agcgttctaa gttgctaatt taataaaaat
13741 aagtaaacta aagatagaaa gaaaggggtt ggaaaagttg gaattaattg aagatgagaa
13801 tggtctcgag ttagtgcata gaaatgggaa ttgggtacta aagaaagaag ttgggttttc
13861 accagtccaa acaatggtag caagtgtggc agcatgcagc acatatgtgt atcaaagtgt
13921 tttagaaaat tccaatattg cgtaccaact tcatggagtt gaatttgatt atgaaactgc
13981 atcagaagga actgttcatc cagtgacaaa aataaacatt caatttaagg tgacgattga
14041 tgaaggagca agagagcgag caactcgcgg tttgaaatta atcgccaaac attgcccagt
14101 catccaatcc ttggctgatt ctgttgttgt tgaagaaatt ttagttttta gttaaaaaaa
14161 gacaataaat gagttattta gtcgatttta aaaagaattt gtaaaataat gaaagaatag
14221 tagagtattg tttgcatatt taagaaatcc atgttatagt tagtttattg atttttgtat
14281 cggattattt tggtttttgt tgttttatga acttcttcca agtgccattc aagaactata
14341 aaaaatttag caacatcaca aggaggaaac atattatgac aaaaggtaac gtaaaatggt
14401 ttaacgcaga aaaaggattc ggttttattg aaagtgaaga cggcggagac gtattcgtac
14461 acttttcagc tatccaagga gacggattca aatctttaga cgaaggtcaa gctgttgagt
14521 ttgatactga agaaggcaac cgtggaccac aagctgtaaa cgtaactaaa gcttaatttt
14581 aattaattat gtttattcaa aacttagaca gaaatgtcta agtttttttt atttgaattg
14641 aactaaaaag cttcacacct tttgtaaaaa tgggtgtgaa gcttttttat taaatttgat
14701 caattaaagg aatgcctgtc tcttttgcat aaagtgtcat gacttttgtc cagtttgttc
14761 ctaatgggtc accagattcc ttaaataata atttcatctt gtcattctga gcttggctaa
14821 tttgctgatc aatagctaat ccagattcag taaggtaaat ttttttgctg cgtttatcgg
14881 tttcatgaga tttttctca atccatccta attcggttag ttcttgcatt ggacgattca
14941 atgcttgttt ggatatatct aatacaatta aaagctcttt aattgttaat cctggaaaac
15001 gatgaacaaa aaataaaatt cggcggtgta cttttgtaat gccgtatttt ccgactaatt
15061 catcagaagt tttactaaat gctcgatagg caaaataaaa aagtgccaaa tttttatcaa
15121 aattaatatc ttcttgctcc attgaaattc cccctatggc tttaaaagta aaaccgatta
15181 ttcatgatgt tagtatagca caattattgt tataaaaaaa taaataggtc aatcttattg
15241 acttattttc ttgagaggtt tatactacaa aacgtaaaga agataaagtg agtttcaagg
15301 atacaaaatt agtagtagga ggatttttat gtacactgtt ggaaattatt tgttagaccg
15361 attgactgaa ttaggaatta gagatatttt tggagttcct ggggattata atttgaaatt
15421 tttagatcat gtaatgactc ataaagaact taattggatc ggaaatgcca atgagctaaa
15481 tgcggcttat gcagcagatg gatacgctcg aacaaaagga atagcagcgt tagtgacaac

15541 atttggagtt ggagaattaa gtgcagctaa tggaacagca gggagttatg ctgaaaaagt

15601 tcctgtagtt caaattgttg gaactcctac aacggctgtt caaaatagtc ataaattagt

15661 tcatcatacc ttaggggatg gtcgttttga tcactttgaa aaaatgcaga cagaaattaa

15721 tggagcgatt gctcacttaa cagctgataa tgccctagct gaaattgatc gagttttgcg

15781 gattgcagtt actgaaagat gcccagttta tattaattta gcaattgatg tagctgaggt

15841 agtagctgaa aaaccactta aacctttaat ggaagaatcc aaaaaagtgg aagaggaaac

15901 aactttagtc ttaaataaaa ttgaaaaagc gttacaagat tctaaaaatc cagtagtttt

15961 aattggaaat gaaattgcaa gttttcatct agagagcgca ttggccgatt ttgtcaaaaa

16021 atttaattta cctgtgactg ttttgccttt tggcaaaggt ggttttgatg aagaagatgc

16081 gcatttcatt ggcgtttata caggcgcacc tacggctgaa agcattaaag aacgcgttga

16141 aaaagccgat ttaattttaa ttataggagc gaaattaaca gattcagcta cagctggttt

16201 tagctatgat tttgaagata gacaagtgat ctctgtgggc agcgacgaag tttcttttta

16261 tggtgaaata atgaagcctg ttgcctttgc tcaatttgtg aatgggttaa attctttgaa

16321 ttatctaggt tatacaggtg aaattaagca agttgaaaga gtagcggata tagaagcaaa

16381 agcttcaaat ttaactcaaa ataattttg gaaattcgtc gaaaaatatt taagcaatgg

16441 ggatacttta gttgcagaac aaggaacctc tttttttgga gcatcattag ttcctttaaa

16501 atctaaaatg aaatttattg gtcaaccatt gtggggatcg ataggatata ctttccctgc

16561 tatgttaggt agccaaattg ccaatcctgc aagtcgtcat ttattgttta ttggggatgg

16621 ttccttgcaa ctgacaattc aagagttagg tatgacattt agagaaaaat taactccaat

16681 tgtatttgta atcaataatg atggttatac agttgaacga gaaattcatg gtcctaatga

16741 gctttataat gatattccta tgtgggatta tcaaaattta ccttatgtat ttggtggaaa

16801 taaaggaaat gttgcgacct ataaagtcac tactgaagaa gaattagtgg cagctatgag

16861 ccaagctcgt caagatacaa ctcgattaca atggattgaa gttgttatgg gaaaacaaga

16921 ttcacctgat ttattagttc agttagggaa agtctttgct aaacaaaatt cataattgga

16981 caaactatta aaaaatcaaa aagtagctcg catttatgac gaggtgcttt ttgatttttt

17041 atctatatag agttcaatat cggcttgacg ataaagctca tcgatagttt gtgagggttt

17101 cgatgaactg ctaccaattg aagttgaaat tcgaagaaca aattgattat ctaattggat

17161 agttagttgg ctaatttgct gcttaatttt agcttttgtt tctatgatag ctttatttga

17221 aagtgctgat gggataacaa gtacaaattc atctccgcca atgcgataaa ctttatgttg

17281 aggaaaggaa gcgttaagta aagaactaga tacttttttt aagataagat cgccaatgtc

17341 atgtccataa gtatcattaa aatgtttaaa attattaatg tctaaaagaa atatcgtaac

17401 taatttttct tgtttaaaaa gagcagttaa atctttatca aatgagtagc gattgaatac

17461 actagttaaa taatcatatt ttgaaacaga ttcataatgc aaaaaggctt ctttctcggt

17521 ttcgatatct tttgaaaatt gataactaat aagcattcct aataaattgt atataataaa

17581 gactagggta atcggagtat tattctggta aggtgagtga ataagaaaat taaggataac

17641 taaaacaatg ctactagtaa aaataaatgt gagacgtaaa taaacttttt tatcctgtac

17701 ccaggagcta ataaaatatt ggcttaaccc gattaaacag taagtgagac agattccaat

17761 aatcggtggt gaaagtccat aaaaaagacg tgagattcca ataataactc cagcaatcag

17821 gggtgttatg ccaccgtatg aaaaactagc cattacaagt ggaactaaac ggatatcaat

17881 taagaaacta tcttggtaag agattgaaaa attcattaga atgattcctg taatcccact

17941 cccaagtccc ataactaatt tatgtattaa aggtgattga tgagttaaaa gagtttttga

18001 aattttagat aataagtacg cgctgctgat taggatggag agattggcaa ggacttcttt

18061 aaccatttgt aacatgtaga tcctccttaa ttgattaaca atgatttaat agtaacataa

18121 caatggtagt tatttaaagc ttggatggaa cttattttaa aagtaactaa aaaaaagtat

18181 taaattagag agtaaaaggc tcttttttgt cagaattgtg atactcttaa gagagcttaa

18241 aattatgaaa agaggttact agtatgagtg gtttaattta cgaacaaaaa catagaatta

18301 agtattacga gtgtgatgcg actaaaaaaa ttagtgtacc aatgctatta aatattatgc

18361 tctatgtttc aggaaaacaa ggtaaactgt taaatgttgg tgatgacgtg gttgcgcaac

18421 atggtttatc ctggattatt ttgcagacag agattaatgt agagcgtttg ccaaaggcta

18481 atgaagaaat tactgtggtc actcagcctc aatcttacaa taagtttttt acctatcgtg

18541 aatttaaagt ttatgatgaa gcgggagagt tatgtgtaac aactcattgt acctttgcta

18601 tgatggattt taataaacga aaaatggttc gaattgtaga cgaattagtt gccccttatg

18661 aggtagaagc aacgaaaaaa ttggttcgca cgccgaagcc agaaattgta gatcaagaag

18721 cggctaaaac aattgattat cgcgtacgat atttagatat tgatgcgaat cagcatgtta

18781 ataatgcgaa gtatttagat tggttcttag attcactggg gcttgagttt attaaaaatc

18841 atgagctaaa aagtattaat gttaaatatg aaaaagaagt gtcttatgga aattgggttc

18901 aatgtgaagt gagtcaaaaa gtacaagaag atggtcggat tttgactgct catcaaatta

18961 ataatgatgg aattccgtca tgtgaggcga gtatgacttg gcttcctctt taagtagtgg

19021 gtttttcta aaaatataaa aggtgctgac aatcaatggt tacaataaat gatattgcac

19081 gattggctgg ggtagctaag agtacagttt cccgttattt gaatggtgga tctgttagtg

19141 caaaaacaaa ggctaagatt gataaaattg tagcagagac tgggtataaa ccaaatacat

19201 ttgctcaaag cctcaaagct aagaatactg ccatgatagg aactattgtt cctcgtttag

19261 attcctatgc tacgactcaa ggcttaaaag ggattgatga acaattacgt gctgtagatt

19321 atcagttgtt gattacaaat agcaatcaag atatccaaag ggaaattgaa gctatttatg

19381 cttacgcaaa gcaaaaagta gcgggaatca ttttatttgc aacagttatt acagatgagc

19441 accgcaaagc gattgattct agcaaagttc cagtcattat cttaggtcaa caagctgatg

19501 ggtatcacag tgttgttcat gatgattatc aagcaggtta tatgctaggg aaatacgcag

19561 ttagtttagg tcaccggatc tttacctatt ttggggtatt tgaagatgat attgctgttg

19621 gacaaaaacg taagcaaggg atcttagatg ggattaaacc taatgggaaa ataaagatta

19681 ttgaaacaac ctttgcaatt gaggccgcat accaaaaagc gttacaaatt cttcctaaga

19741 tggaatctag ctatgtaata tgcgcaacag ataatattgc actaggtgtt ttaaaagcgg

19801 cacatgaatt gaaaatggtc ataccagatc aattttcgtt atctggtttc ggagattatg

19861 atgtaacgac agcggttttc ccaatgatta caacgattaa atttccgtac tatcaatcag

19921 gtgtaatagc tgcagaaaat ctttatcaac ttattgaagg ggaaaagatt ccagaaatca

19981 ttaaattaga taatcaatta gttgaacgtg aatcaacaaa aagacttaaa taatccaaaa

20041 taggctaatt aggcggggcc tcctaattag tctattttgg gttgaaacaa ttaagaagaa

20101 aaaaattctt gacaatgtaa acgaaatcat ttattctaaa aatagaaacc ggttccaaat

20161 acaacgttga tttggatagg ttaaaaaatt tttagaatga aaaggaaccg gttcctttaa

20221 aattaaaagg aggtatttaa agtgaattat aaagaaattg cgcaagatct tttagttaat

20281 ctaggtggaa aagaaaatat tgtagcagca acccattgtg ctaccagatt acgtttagtg

20341 ttacaggacg aaacaaaaat aaatcagata gaaattgaga ataacgctag tgttaaaggg

20401 actttttcca caggtggcca gtatcaaatt attttaggca gtggagttgt ggacgaagtt

20461 tataaggagt ttgtgcagct agcgaatatt gagtcaatgt ctactcaaga cgtaaaaaac

20521 ttaggaacaa aaaaattaaa cccaatccaa caatttgtta aagtgttatc agatattttt

20581 attccaatta ttccagcgat tgttgcttgt ggattaatga tgggaatcaa taatgtactt

20641 acagctgaag atttatttat cagtggcaac tctttaattg gagcttatcc acaaatggca

20701 ggtttagcta gcatgattaa tacattctcc aatgcggcct atgtcttttt gcctgtttta

20761 attggttttt ctgcagctca gaagtttggc ggaaatgctt atttaggagc agtgctagga

20821 atgttaatgg tacatcccga tttattaaca gcttttagtt atccacaagc ggtagcagaa

20881 aatagtgtcc cttattggaa tgtatttgga ttacacattg ctcaagtggg ctatcaaaat

20941 acagtcctcc cgattttagt tgcaacgtat attttggcaa aaatagaaaa attatgtcat

21001 aaagtaattc caacagtttt agataattta ctaacgccat tattcacttt atttataaca

21061 gcctttttaa cctttacagt tgtgggtccg attactcgaa cagctggaga tatgctgact

21121 aatggattag cctggttata tgattcaacg ggttttattg gaggcggaat catcgggcta

21181 ttttatgcac cgcttgtgat tactggaatg catcatagtt ttattgcagt agaaacacaa

21241 ttattagctg atattgcaaa aactggcgga acatttatct ttccaatagc atccatgtca

21301 aatgtagcac aaggggcagc ctgtttagca atgatcttta ttactaaaaa tagtaaaaca

21361 aaaagtttgg caacagcttc ctctatttca gcgtatctag gcattacaga acctgctatg

21421 tttggtgtga atttgaaatt gaggtatcct ttttatgcag ctatgattgg ttctgctgtt

21481 ggtagtgcat atgtaacctt ctttaaagtt aaggcagttg ctttaggcgc tacagggtta

21541 ccaggtatta tcgccattac acctggctat atgacttatt tcattatagg gattataatg

21601 acaacaattt taacgtttgt cctaacgtat atttttagta tacgtggagc caaattaact

21661 ataaatcaat cagagtagat aaataggaga agataaatga aggaatggac acgaaaagaa

21721 agataccgaa aaattgaaga agtatcgcca gaatatttgg taaatttggc taatcaagtt

21781 gagaagtcag tttataggca gacgtatcat attcaaccac caacaggttt attaaatgat

21841 ccaaatggct ttagttactt taatggtcaa tggcatttat tttatcaatg gtttccttta

21901 ggtcctgttc atgggttaaa gtattggtat catgttgttt cagacgattt agttcactgg

21961 gaaaattgtg gtgttggaat tagtcctagc actaagtatg attctcatgg tgcgtactca

22021 ggaagtggtt tggttgacaa aaaccagtta cacttaatgt atacaggaaa tacacgatct

22081 aagaactgga gccggacacc gcaccaatta atggcaatta tggataagaa tggacaaata

22141 aaaaaaatcg aagagcctgt cattgaggga attccagagg gctatacaga taattttaga

22201 gatcctaaaa tgtggaaaca cgaggggtat tattatgcca ttatcggtgc agaaagaaag

22261 aatcatactg ggactgcact tgtgtaccaa tcagaaaatt taacagattg ggtgttgcta

22321 ggtgagttta atcctcaggt ccaagatttt ggctttatgt gggaatgtcc agattttttt

22381 gaattagatg gtagtccttg ttttttattt tctccacagg gtatagagcc tattggggat

22441 tcctatcaaa atatctatca aactggatat tttctgggga caaaatttaa ttatgaaaca

22501 ttgcgactag aacataatgg atttcatgaa ctcgacttag gttttgattt ctatgctgct

22561 caaacgacaa ttgcacctga tgggcgtcgt attttgattg cgtggatggg attacctgaa

22621 attaagtatc ctagtgatca agatggttgg gcacactgtt taacgattcc acgagagttg

22681 tcggttctgg aaggacaatt aattcaaaga ccaattacag agctaaccac actaagagga

22741 gcatatgtaa aacgccaaga ctcgcaatac ggaagagtac atttaggaaa tctattagga

22801 acaagttatg aagtgaaaat aaagctacgt gtggaagaaa aaacaaaatt tgggctttgt

22861 ttatttgaag actcggtaac aaataaaggg ttgtatttag aagttgattt aaataaaggg

22921 aattttaaag tggagcggaa agattgtggg attccttttg ctacagaata cggaacatcg

22981 cgacaaatga agtataataa aaaagaaatt agtctgcata tatttgtaga tacaacatct

23041 gctgagattt ttgcgaatga tggattagct gtatttacta caagagtttt tccagaaaat

23101 caacaaagtg gaatcgggct atttgttgaa aaaggcttaa tggaatatga attagaagga

23161 tggaaattaa ataaaatgga tataaaaagt acggagaaat aaaactccgt actttttcgt

23221 ttttttttag taaaagtagc gttccaattc atcatgctca ttggtactat ttaaggcgac

23281 taataattta attcgagctt ttggcccagt taaaccatta gtaaaaatta cgcccatctc

23341 tctcaaacgt tttccgccac cttcataatc atagacatct tgtgcaacac cgttgaaaca

23401 gcgggaaact aagacgattg ggatattacg tgcaacgagt tttgataagc ccgaaagagt

23461 tgctggaggt aaattaccag ctccaagagc ttcaacgacc agtccatcag ttgtaggatt

23521 atcaagtgca tcaaaaatcg tactatccat tcctgcataa gcttttaata aataaacgtt

23581 tttatcaaca gaagttaaat caaatttttc ttcctcaatt aattcttgga aaaacaagac

23641 tttattttta gcaatcaagc caattgggcc aaaagttggt gtgcgaaaag tagcaacatt

23701 ggttgtatga gttttggtta catagcgtgc tgtatgaatt tcatcattca ttacgactaa

23761 gacaccctta tctttagcct catcagaagt ggccacccaa actgcactct ggaagttata

23821 aaggccatct gacccaatct cattgctaga acgcatagct ccagtcataa cgataggaat

23881 atgttgttcc aaggtaatat ctaggaaata tgctgtttct tctaacgtat ctgtcccatg

23941 agtaatgatg acaccatcaa ttgcttcaga aagaaccgcc tgttcaattc tattttttaa

24001 ctgtagcatt tctgtcggag tcatatgtgg agaaggatgt tggaagatat cttcaacaat

24061 taaatgagct tccttttcaa ataaatgacc ttgagttaat aaaggatttt cagaatttgg

24121 agcaactttc cctgttgttt gatcctcgct cattgcgatg gttccaccag tgtgtaaaac

24181 taaaatattc ttcaagaaag tacctcctta taataaaaag ttcgttgagc tgtttagacc

24241 tgtcagaaaa atagggaatt ccatgaaaag tgttaattta cttttttggg aattcatctt

24301 ttttccgaag gactagctca aaagaactag cctagataaa aagttcgttg agtcggctaa

24361 atactcaatc atctcgatac tattttatca caaattaagg aaggaatctc actttgaaaa

24421 aatatagtta gttagtttag tttcttagtt cgtatgaatt ccatccaatt tttaaacaaa

24481 ttttttccat gctcattcca agtattgata ggcgtattta aaggattatt tcctgggaaa

24541 tagtataagg gtaaaattgg tttagttaaa gtttgtacat cacgtaggta ttctttttct

24601 aaggtttctg tagcgtattc aggatgacca aaaacataag tttctaagcc attataattt

24661 gtagctaaat agagacctgc agcatcggat tgagctacag gaatcaattc aggatgctgg

24721 ctaactagac aggaatcaac agttgtgttg cgggagtgag gtgcatgaaa atcagtttta

24781 gtagtatttg ctaaccagtg ttcagctaca caaaaacgat gctgaaaaat acctactaat

24841 ttagtgggta agtgaatttt tggtatttga taatactcat ataaggcggc ttgagctccc

24901 caacaaataa agagagagtg agtgtgagtc gcttttaaat gagtaaaaat agtcttcaat

24961 tcaagccagt aatcaacgtg ttcaaaaggc aattgttcaa cgggagcacc agtaataata

25021 aagccatcaa atagttggtc ccggacatca tcaaaagtaa ggtaattttc ttgtaagtca

25081 tcgggaatag ctttagcttg atagtgtgtt ttagggtaaa gcaaagtaat ttcaacttta

25141 aataggttgt tttgacttaa taaggatgcc aattgagtag cggttgcttc tttagtcggc

25201 attaagttta aaatagcaat ggaaagaatt ggtttagaac tcatataaat tcctccttaa

25261 taaaaagctc gttgagccgg ttagctccgg taagaaaata ggaaaaattg ataggacgct

25321 ttttgtcctc tcgaattttt atctttttca cgaggagttg gttcataagc gctggcctta

25381 attagttagt gaacagtctg taatgcctga gctaaatcct gaataatatc atgtgtgtct

25441 tcaattccaa ccgataatcg aattaaatct tctgttactc cactctcaag taattgttcc

25501 gaatttaatt ggcgatgagt cgaactttct ggatgtaaaa tggaggtacg aatatcagca

25561 caatgaatga ctaattgaat gaagttaagg tgattaataa attgtttagc agccgaggcg

25621 ccacctttaa ttccgaatgt aaccatccca ctagctccat ttggcaagta cttttgtgct

25681 aatggataat gggaatctgt ttctaatcca ggatagcgaa caaatgaaac gtctttttgt

25741 tcttgaagcc acttagcaac tgctagggca ttttctgaat gttttttgga gcgtaagacc

25801 aaggtttcag aaccaagatt gctcaaataa gcattgaaag gactttgaat attgcctaag

25861 tcgcgtacta attgaactct gagctttgtt gcataagctg cttgcccaaa atcagccaca

25921 tagcgtacgc cgtgatagct atcatcaggt aaaactaact cgtcaaattt accattatcc

25981 caattgaaat tcccaccatc aatgacaatt cctcccagtg cagtagcatg tccgtcaatg

26041 tatttcgttg tagaatgaac cacaatattt gcaccatgct ttaaaggatt acataaagcg

26101 ggtccagtta aagaactatc gatgattaat ggcaaatcaa gtattttgca aatggaatga

26161 aatttttcaa aatcaaaaac atttaagccc ggattagcaa tcgtttcccc aaaaactaat

26221 tttgtattct gatcagctaa agcaataatc tcttcaggag acgcatcctg atcgaaaaag

26281 cgagtagtaa tcccaaattt tggtaacgta acagcgaata aattggttgt tccaccataa

26341 attgttttgg agcttaagat attatcacca gctttagcga tatttagaat gctgttaaaa

26401 attgcagcca tgccagaaga agtagcgaca gctgcaacac catcctctag taaagcaatt

26461 ttttcttcaa aaacagcaac agttggatta ctgatacgag aatatagatg tccttcagca

26521 gataaatcaa aaagagcagc gacatcctca gcctgttcat aagcaaatgt tgtactttga

26581 taaagtggta aaactcgagg ttctccattc tttggttgat aagtcccttg tacagccaat

26641 gttccttttc cgtatgcttg tcttgctttt gtcatgtgta attcctcctt tatgaaataa

26701 aaagtttgtt gagttgttta gctccgacag aaaaagtagc aaaaaagcac tcgcccttta

26761 ccaaatccca attaagggat tgataaagga cgagcgcgat agatatcgag tcgtgttacc

26821 accttttatt tgtttatttt tcacaaaata aacctcttca agtaccgtgc ttaagttagc

26881 aagagactct atcgctataa caggcgaacc ctgtaggatt taaagacaaa actagtcgtt

26941 caatcactaa aactccgagg ccatgttcat taatttgtga attgcctttt tacaccaaaa

27001 caaggctctc taagaattca tccattaatt acttttctca tcacggtttt atattttctt

27061 tgattgttcc tatcataaat gataaaaaat aaaaatgcaa gaattaaatg aaaaaaaact

27121 catctaaatt aaatctaatt ctcataataa gggttaagag gactaaattg ttattttat

27181 agttaaatca aactatcaag gcagttatca tgctataatg aaggaatcaa agaactggaa

27241 gaggttatta tgggaaaaaa agaatttgca gattatggca ttggcgaaga tgtactaaaa

27301 gctttaaatg gtttaggtta ttttcaacca acaaaagttc aagaggaagt aattcctgtg

27361 gctttggcag atatggatgt gattgtaact tcacagactg ggagtggtaa aacggctagt

27421 tttggtattc cattatgtga aaaaatcctt tgggaagaaa atcgaccaca agcattggtt

27481 ttagttccaa cgcgtgaatt ggctttgcaa gtaaatgaag atattgcaaa tattggacgt

27541 tttcgtcgta taaaagcgac atctgttttt ggtaaagcat cttttgagcg tcaaaaatca

27601 gagttaaaac aaaaaagtca tattgttgta ggaacacctg ggcgtgtttt agatcatttg

27661 aaaaaaggaa cgtttcctgt ggataaactc gaatatttaa ttctagatga agcagatgaa

27721 atgttaaata tgggctttat tgaccaagtc gaagaaataa ttggcttctt accaaaagag

27781 cgtcaaacct tactattttc ggcaactatg ccagtagagg tggaacgttt agccagtcac

27841 tatatgaagg acgaagcagt atcaattaag attgaaacca cagaagattc aaaacctaaa

27901 attttaaaat catttttaat ggtatctggg gataaaaaaa tgccaacctt gttagactta

27961 ttaacagtgg aaaacccaga tagttgtatt gttttctgta atcgtcaaga aacagtgaat

28021 aatgtctatg aatacctaaa taaagctggt ttacctgtgg ataaacttca tggaggtatg

28081 attcaagaag atcgttttga tgtaatggat gattttagaa aaggaaaatt ccgttattta

28141 gtggctacgg atgttgctgc tcgcggaatt gatgtggata acattactca tgtagtcaac

28201 tacgatgtac ctgtggaaaa agaaagtttt gttcaccgaa ttggtcgaac aggtcgagct

28261 ggaaaaaccg gcgtagcttt aactttagtt gagccttatg aaaaagagcg ttgggctgaa

28321 attaaaaggt acagtgcttc agatgttagc gaaattacgg cgccaagtgc acgttttgtt

28381 ttacgtcata aaccagcctt tgaaaagaaa attgctgaac gtccgcgttt aaaaaaagat

28441 aaaggcacag tattaagcca agatattacg aagctgtatt ttaatggtgg aaagaagaaa

28501 aaaattcggg cgattgattt tgttgggaca atttctaaaa tacctggcat tcaggctgat

28561 gatattggga ttattacaat cggagaccaa ggtagttatg ttgaaatttt aaatggcaaa

28621 gggcctttgg ttttaaatgc aatgaaaacg aaaaatgtta aagggaaaac tcttaaagta

28681 aatatcgcac gtcgcaaata agttatatcc aaataaaaaa gtgtggtttc aagttctgaa

28741 tgaagtgaac ttgaaaccac acttttttta tttgtttgct attattttaa aatgaatatt

28801 aatttatttt ccataaaaat tgaatatgtt atttattttc aagaaaggct ttacattttc

28861 ctttaattaa gataataagt tcttgattca tccgacgata cccataattc ttatgttctt

28921 gatgaatctt tatcatctct tcttcaattt gtttatctgg atttttacga ttaaaacgct

28981 gttgccaata catataggtt gatctaggaa acccaagggt ttcaagaata tcgattagtc

29041 tgaatttttc tcggaggtta tgaatgatta gcgcttgttg ttctgttgtt ccttcaaacg

29101 caacctcctt aattctttta aataggcgag ttcaattttt aatcgccgat tttctttttc

29161 aagctcttct aaataggtta actctgtttt tgaagaatca ctaactggta atttattctt

29221 ttttttaggc attttagatg gacgtccttt cggttgtgag agtccatcta cgccattttt

29281 ctggtaggct atataccaat tggcaattgt cgaaaaagtg cttactccaa actgattggc

29341 aacttctcga taggacatct ctgttgttaa atataactct atcgcatcta gcttgaattg

29401 aacagaataa gttttatttc gacgactatg aagtaggcct tccttaccaa attctttata
29461 cgcatttatc cagttacgca cctgggaatc ccccttaaat ccatattttt tagctagata
29521 gggggttcct ccctcaccgt ttaaataatc ttgtacaatg ttcattttaa attctacact
29581 atattttgtc atacaaaaag acctccaaaa aattagattt ttaggtctaa cttttggggg
29641 tcggtacaaa gaaataaatt cttgtactta gctttttctc gatttttagt tggaaaatct
29701 attttacaat aattgttgta gatgggttgt taaaatctcc atagatttgg acaccgtaat
29761 caactgtaat cacatcgcct gcttttgttt gttggattgg agatgaacca taaagacctt
29821 caaaccaagt tttagcccat attgagaatg cacccgttac ggcattaatt cctgcgtctt
29881 cttcaatgat tggtccatta ttcactacac gcattgtttt accattgaca gagatacgca
29941 cttgattcgc accgtttggc acacttcctg tcaaatactc atcgccttct gtaaaatcgt
30001 cgacgattgg agccgtatag ggttgaacga cttgaacacc ttcataattt tcaccattct
30061 catctgtaaa ggccactttg atttgatctt tattgttcaa taagaagtct ctcgtatctt
30121 tgacaaaacg ggattcaata gcaaaatatc ctgtctctgc agctggtgtt gcggtgtatc
30181 cagtatctac ccattcatcg gtaccgtctt ttttaattag taaactcact tgacttgttt
30241 ttactccttc aggaacaaat ccgacaactt gttgttgttg aacgccgaca tagttgaccg
30301 caaatggttt tgcatttttt ttataaacaa acgttactgt ttgtgcttct tcagtaaata
30361 ttccagttgc attttcagga gtttctttta atgcataacc tttaatggtc ttagcttctg
30421 attcaaaaga cgctccaagt tctccagtta gtacttctga ttccgcaagt gtatttccca
30481 ctgtatctac ataatttact gtaactggcg cagctttcac tacttcccaa gtgtaagttc
30541 ccgcatcagt tgcaccacta taggtagaca ttaaatctgt acttgatgaa aaagtttttt
30601 cactctcttc acttgcccat ttaccagtgt aaacagaagt gatttcaatc tctggtaagt
30661 ttgtattttc tccaaaagta aatttacttc ctagtgataa ggtagttaaa tttgtcgttt
30721 gaccgagcat attcgtcata ttcgttacat tactagtatc aaatccgcta aggtttaagc
30781 tggttaaact cgtacattta ttgaacatac ctttcatagt cgttacactc gttgtagtga
30841 actgactaac atctaggttc tttaaattcg tacactcttc aaacattcca ttcatttgaa
30901 tggcttgaga ggtatttaat ccgctactat ctaattctgt taaactacta caattagaga
30961 atatatcttg catatttgtt actttagacg tatcaaaatg actggtatca aagcttgtca
31021 agctgctaca cttataaaac atggagttca tgtttgttac tttagacgta tctaaatttt
31081 ctatcccttc aatggtttca agatttgacc agttagaaaa gagagagcta gagtcttcat
31141 ccgctactat atccccttta aagaccacct tagatacatc tgcttttttg atgcctgcta
31201 ccgtagcagt cgaactattc gtccctttaa ctaaatttcc tgactcaatc gtcaagacac
31261 catcttctaa aacccaagga caagttcccc actttggacg ctcataaaca taggtcacgg
31321 tttgattgaa ggtagtaaaa gtacctgtcg catttgcagg ggtttctttt aacatatacc

31381 ctgtaatatc ttttgcactt gtggtgtaat cctctcccaa ttcacctatt aaggtatctg

31441 atgtagccaa ttcagctcca gcttcatcga cataattcac tgttacagga gaaacttctg

31501 tcgtttccca cacatacgtt ccagcatctg tctgttgatt atactcattc ataaaggttt

31561 tactgtcttt aaatatttta gaaccatctg tttttatcca agcccctgta tacaaattac

31621 ttgttgtatc aatatccggc agagacgtac cctgtatacc cgtatactct cctagtgtta

31681 aagttttcat atttgaagtt tgtcctatga aacttcccca tccatatggg ccgcaagaac

31741 gcatatcaaa actagttaaa tctaatgcag ttaaacttga acatccagaa aacattgctg

31801 caaagtttat tactttagat gtattaaatc catctaaatt tatagtttct aaactgctac

31861 agtattggaa cattccatac agatccgtta attttgaagt atccatgtga ctaagatcaa

31921 tattttttaa atttttatta tctttgaaca tggcacgcat actctctact ttagacgtat

31981 ctagattctc aatattttca taactttcag cggtactacc cataaatcta gaatttccag

32041 ctagaagtac aatgggtcca tcaaatataa ttttagttgc ttcattgtct ggagttgtgc

32101 ccctatctcc taaagttcct gcaccaattg tcaagacccc atccgaatcc agttcccacg

32161 aacaggtacc tgactttccc gttttaggtg ttacagcacg attttcttcg tcagtctttg

32221 ttggtacttc ttgttcaccg gctagttcgc ttccgttttc tacaaatgca gactccgcaa

32281 atactatgac actaccactt atacttgata atactaaact cgataataaa agatagggta

32341 ctaattttct ttttttcatt tttttacctc tttttctttt tagtttacta aattactaaa

32401 ctattcatac taggattaaa taccagtatg aataacttct agttttttca tgttaacacg

32461 gatttcctga ttattctttc attttttgca ccttacgtta ttttttttga cagaatccag

32521 ctattacggt atttattcaa ctgtaaattt tcacttttaa caataaaaca agcgcaaatc

32581 aattaatatt ttgctaaatc caattatgta atggaatgaa taaccttaaa aagttaaaaa

32641 taatcttcga taaattggtc tttcaaattt ttaaatgtct atattaatct aaggtaccag

32701 aatgataatg aataagacaa actgattctt ataaaccacc ttttatgata taatttgtca

32761 tgaaaaggtg gttatttttt gtcactttaa aagataaatt gcgcattatt taatcgtttt

32821 ttttgcacaa ttataacaag cttccaacct agattatcta gttggaagct tgtttcttta

32881 attcctacta ctagactatc acttgtttct tctgtaactt ctatcacaat aatggttaga

32941 ttatttattg taaggcatat tcaatctgag aaaacaaacg ttgaattaat ctgaaattac

33001 cgttcgttat cttagtaaag gtttcatagt ctgagaaata ttcaatcgtg attgaaagtt

33061 ttaattcttt ccatttatgc cctaatatac gatgtatttc atctttattt aacatatcaa

33121 actcatggtc aatcctattc tcgaataaag ttgaggatac cgtgtgatct ttttttaaat

33181 acgagacgtt cggattaata taactgtaaa atatgctgtt catataacgt ttttatttgt

33241 tccagtgaca gaagtttcaa gcaatctatt tcgtccacta ttaataaata aacatcctca

33301 aaacttttcg ttagtggata tttatactcg ctatctttag attgctgaaa ctcacaaata

33361 gactttagtg cacatagaac tgaattttca tctagttcaa aaaatcacaa gaataaaatg

33421 tctctgtcgt ttatttttat acgatttaaa tagtaatatt taaagaattt tttagtagaa

33481 tgcatttaaa aaaattattt ctattcattg attttgcaac agcttcttaa aattaaagtt

33541 tagaaaattt ttatcggtat ccgtaaaaat tctggccata gtattgaatt gatctaaata

33601 aataatttta ttatctaaat ttaccataaa aaacctatga gtactttcat cgcgtttaaa

33661 ttcatatgga cattcagata ataggactaa ttttcactct acatattgag gatcactata

33721 cttaattttc ataaaataat tttcccttct aaattttatt ctagctgatt atagcgtatt

33781 tttatttatt ttaaaacagt acatagaatt gtaatgattt aagaaaacgg ttacatatcc

33841 aatttaaaca acttttataa ttttttcaa attaataaaa taacataatc ataaagaaga

33901 attaaaaaaa tgtcttgaaa aagattatcg tactaagtag aaaaataata aaaagtatat

33961 gaacatcttg acataaagag cattcataag atatactttg ttggtagaca gaaagtgaat

34021 cataaacatt tgtttagcgt acatcttgaa ttacataata gtaaataatt aaatcattga

34081 aaaataaggg gtttaaagtc ttgtttttct ttatttcaaa gtgactcgta aacaaatgtt

34141 tacgagtcac ttttttgta attataagtg gaaattactc gaattctcta gagatttaac

34201 atcctatcaa gtctgacttc catggtatat tacaactttt ttaaagtgat ttgtaaataa

34261 aagtgacaat aagttacttg gattaaagaa ttgaaccttt taaggaggat taaaatgagc

34321 agtattgaag agtattggaa agatactatt gattaatagg atatggaaaa aaaagctttt

34381 acataaagct gtaaccgttt tggtatggca acccttttc gaacaatttt tttaaggtgt

34441 ccatttttta taactgccat ttcaagcgca gcttcagata agcgttcctt tgcttcataa

34501 taataagtgc ttcgagaaat gtttaaggta cggcacatcg ctgatactgc atatttgtga

34561 cggttcatat ggatcacttt gactttcgtc cgaatatcag cgccgcttgc tttaaaatat

34621 cattctccat ttttagttgt ttattttgtt tacggagttc tttcagttct acttgttcag

34681 gtgttaaatt gtctttttct ttaaaagaac cggaggttcg ctgctgtaaa agccatttat

34741 caaaagcgga aggagtcaat tcgtactcac ggattagttc ttgccttgat tttccagcca

34801 gatataagtc aaccatttgt tttttaaaag cttctgaata agttctacgt tttcgtttag

34861 acatataatt tccctccagt gtgattgtta tttactagtt tacacacctt aattttttg

34921 ttcagttaag tatagcctat ccaattgttt atgagtattt tttaggagga aataaatatg

34981 aagagaattg aagattgttg ggtcgatact gtatcttatt atattcaatt tgaaacgact

35041 aaaaataaaa aagtatccaa aatgattatt ttagatgtaa cttacacggt aaaagaagtt

35101 gaagagatta ttttgctcaa ttttgaccaa gtaaaaaaag tatgttttat tgaagaagta

35161 gaagactgtc tttctttaaa aaaatgaaaa aaggggaggt tttatggtcg caaagaagag

35221 aggaaataag aaaaaagcctg cctcgactaa aagagaacag aaaaaagctt tgaaacaagg

35281 caatcatttg atgactgaat acaatcaatg gttagccaaa ggctactcct tagaggggga

35341 gatgcgagaa acagttaaaa atcctattct tcctgaaaag tacaaagact attggaatga

35401 gattgcctct cagacgtcta aacacgttag ggagggacaa cgtttaaaag ggaaattacg

35461 acgttctcgc tttcttgatc cctccttact agaagaccca atgagacaac ctttagttgc

35521 taagaaaaaa aactggattg aatcctttgt attttatggg attattgtgg tcgtcatagt

35581 acttattagt actttatttt ctggtcctgg agatagtggt gttcctcgaa atattatggg

35641 ctacgctccg ttaaccgttt taacgaagag tatgcattct gtctatccga aggattcttt

35701 cttattgatt caagtagaag attccaaaaa gttaacgatt ggagacgaca ttacgtttct

35761 gaaagaaaat aacaccactg ttacgcatag aattattggc attcaagaga attttgagaa

35821 tcaaggacaa cgtgggtttg aaacgaaagg ggtggacaat cctctggcgg acgaggacat

35881 tgtcattgca gacaatgtga ttggaaaagt gatctattct aattacacca ttggtcgact

35941 cttattgatt gttcgaaata atcttttgat ggtagctgta ttgatgattg tcagtatctt

36001 tttcctagat gccttgatca cattaataaa atcttatcgt aaaacgaagt actgaggtaa

36061 gtaaaatact tggaaagagg aggcattctg aatggaaaag aggacgaaag ttgctttagc

36121 gaccgcactg atggttggaa taatgggcgt ttctggtacg tttgcctgga cctctattag

36181 tcaacgagcc attaatgaag tcagtatgac gaagcagcct ggtgggcgtg ttcatgatga

36241 ttatgaaggg attagaggat tggacacaac cacagaaggc gatattaata agaatatcta

36301 tgctgaaaat tacagcaaca agcccctcta tgttcgaata aagctcacag aatttttga

36361 atcaggttta ggggcaggtc agtatatggt agacagtacc gatcatatta tgaaaccaac

36421 atcggataac caagcgaccg ttttgcccaa ttcaggttta gacaatgccg ctttatcaga

36481 tagaacctca tggccagcat acttaccaaa cgggttgttg tcaaatggct ctccatctga

36541 gttaagacaa gtgattcaat ggtcattagg agataccaat gatgaccgaa aggtcttcat

36601 gccgaccttt aaccaaaaca atatggatat ttctgcggat gtgacaggtg aagccattga

36661 cgagttgact tggaaaaaga atctctatta tgaggatttg tcaaaagtac tgaatggaac

36721 ctttgagcaa tggtcgttag gtgaaaccta cacaagtacg ttgatcaaaa ataacggtga

36781 cacagcggca ccagtagaaa caccgggtgt gacccacact gccaaagaga ccgttgttcc

36841 gactaatgga ggttatatga cgatgacaga ttggctgagc caaggcaaac caaccggtta

36901 tttttgggta catgactcag atggttggtt ctattgggca actgaattag agccctacag

36961 cgcgactagt ttagtgttgg atcgtttcca tgtcgtggtg ccacaagatg atttttatta

37021 tgcgattaat gtcatcagtg atttttcaac agaagaggat gtggttcaat ggtcaggaaa

37081 gacattgaac gctgatgact tactaacagc gattcaaaaa gcgaagtaag gatattgaca

37141 agcgtgtgtc aatccacgat atttttataa cgttcgttat cgtattgatg catcgtaaga

37201 cccgtttaac ttgattcaat gttaaatggt taaattagtt atttatctaa cgtagatttt

37261 atagcgtgtt tctacataga ataacatcct ataaaatcaa acgttatgat ttatataaaa

37321 aatataagaa aaaaaggaga gttaaataat gacgaaggta acgaaaagaa atcgtaaaaa

37381 gactgcaatt acagtcgcag gtttaggggc attattattg acgacaggga catttgcatg

37441 gacgtcaatt agtcaacaag cattaaatga gaagtctgga aagatagaag caggtggacg

37501 tgttcacgat gattacaata aagaatcagg aaacaaagac atctacgcag aaaactatca

37561 aactcaaaat gttatcgttc gtattaaatt gtcagagtat atggaagaca atttaacccc

37621 aatggttgca ggtacagtta aaacagacaa gagtacttgg actcctttta cagtaccagg

37681 ttcaaaaaat tctgaagcct atagaaatta tgtaacatgg gatttaggag gagaaaaagt

37741 attcttacca acatttaaca aagtcgagac agataaatcc gttgatgcgt caggtgatgc

37801 cattgatgtt cttacgaatg gacaaacagc accaggaact ggagaacaca tcgattttac

37861 tgcaggaacg gagtataacg ctactggagc tgtagtcacg actcctggca caggtcatgt

37921 agcgaaatca acgttgacgc aatcaaaagc gccactgtct atggctgact ggttagcttt

37981 accagctgca gaccaaaaag gtgatttctg ggttattgac gaagatggtt gggcctactg

38041 ggcaaacatc ttagcaccag gtgagtcgac tagtttactc ttatcccaat tgaattggga

38101 tcaaactgct ttaggcgatt tggatgattg gtattatggg attgatgtgt taggtgagtt

38161 tgcgacaaaa tctgatgttg gcaaatttgc gacagattca gcatatggtg caccaagtac

38221 gaatgcgaac caaattttgg atatgcttac agtggaacca gtatcattga ccattacagc

38281 aagtggtgtc aatgcagact cgaatacaat tgacgcaggt acgtctcgtc agtttactgc

38341 tacggcagct acacgtgcaa caactgaatg gtcagtggaa cccgcaagtg caggtacgat

38401 cgacaacaca ggtaaattaa cgattaatag gaatgctgta aaaggaacaa aagtaacgat

38461 taatgcgaaa caaggaagaa atacagggtc taaagaatta actgtgacga gtatgaatgg

38521 tgcagtagtt ggagatacca tccaatttaa tggccaagac tatatttacc taaaagacct

38581 acaagacggg aaccgtttaa tcatgagtaa agctactgtt ggaactgcgg caacatctct

38641 aacttacaat ggtagtgcat tagatacatt gatgaaatct cactatgcaa gtttatcaga

38701 agctgcaaaa acagaagttc aacctgtcca agcatcattt gatgtgggag cacctaaagg

38761 tacaacggca ataggacgtg atgccgatgg attcttagtg acttcaccaa ctcctgatcg

38821 tgctgttgtg accgaaggtg gagagaaaaa agctttcgcg ttgtcagtgg atgaattaaa

38881 tgatgtttca ggaccaggaa aagtctttga gactgtagct tcaagagaag cactcaatgc

38941 gagtggggct gtgacgtatt ggtggttgcg ttctcctgtt acgtctacgc atgcgtggta

39001 tgtgagcacc ggctccaatg ctggctcgtt gtacgccagc acgatgacga ctgcgagtgg

39061 ggcccgcccg gcctatatta tcaaccgtta atcctctgtc gcgcttgcgc ttgacagaac

39121 cgagctttcg ctcgtgcttt aggccgtttt taaacggtta aggttttaga ttatttcctt

39181 caagagtagt gactcttgga ggaaattaaa aatgaaaaag gagctatcat gagttattca

39241 cagtttaaag tcagtgggca agtcactcaa ctttgcgaag aagttcttcg attgacgagc

39301 aaagcccta aaaaatttcg tttttcgttg tgtcagcgaa tggattcttt agcactatcc
39361 tgtttatcag acatttatag agcgaattct ttatcattgt caaacgaatc ttctcaaatc
39421 caacgacaaa gttttcagag taatgccctt gtaacgttaa aaatactggg gacaatggga
39481 agaataggga cagaaaatca gtgttttacc attggtgaat atggggtttt atctaaaaaa
39541 ataaccgcct gtgagcaatt gattcgacgg tggattgaat cggacaatca aagagtacac
39601 taaagttttt taaatgcgta tttaaaaaac tttagtgtgc ccctttgggg acaaaaggat
39661 cagactatga agcaaactca aaagcgacga attggtggtt gcgttctcct aatacgtcta
39721 cgaatgcgaa tggggcccgc ccggactcaa tagaggaatt cattattctt acctgataaa
39781 aaaagaaact ctcttttttt atgtgtatcc tattgaggga gtctgtacaa gagttatgtg
39841 tggcaactcc ttttctttct tgatatttca gaagaaagaa ttacgagtga tgtgaactgc
39901 ctaaaataga aagaaaggca acgaccattg ttttttattt cgtttcgtcg gaaaactttg
39961 agaagagatt tcttacactc gtcttatttc aaagtaaggg tgataaaaaa tgaatcaaaa
40021 gataattatc tctcaactag tcgaaagtat ctttgaatta aatcaatctc aatccaagga
40081 aacagtttct caagttttat ttggttcaaa aagaaaaaaa taaaagaatt aggttttgac
40141 aactgtaagt gttatggctc tttttttatta gaatctatta cgtacccaga aacgctgaaa
40201 ctaatagata ttttgatcaa gcaaggttat ctatacgaaa ataaagtcag taattaccgt
40261 tttcttgtgt tgactcgcga agggataaaa cttatgacca aacatcatga tatcgagatt
40321 caagggttat ttgaagagct tcctaaagaa acagtcacta aaagagaact caagatacta
40381 agaaaaaaag tagccaatga atgtaatgtg agtcctttta ttattttttaa taatcttgta
40441 ctagagaata tagaacaaaa aaatccgaca actcgcgatg aatttttgac tatcaatgga
40501 ttagcagaag caaaatgggg ccagtttgga gaacaagaag gtaatctatt aggtattaaa
40561 agtgagttaa caaacacacg taaccaccgt ggctgtttta ataagtatcg ctttatatta
40621 ttcaaaaaag agtgaaaact gagaatttaa gaatagttgc acataggagg aggataatta
40681 atgaggacca taaaaaaagt aatgacaaaa atgaatccca ttgtcaaaag acaaagggtg
40741 ttgtttattg gcgtcgtttc tcttctctta accattctaa tagtaaccaa tactctggga
40801 tggcaatctt ctcaacaaaa agccctaaac accttgacgg ttaaccgaga ccctcaaagt
40861 gtcacgttaa tcaaattcgc aagagacaaa gaggaccaag aaacggatca cgtgttagaa
40921 ggtgctgttt tcgcgttgta tcaggttact aaagatggag acaaacaaat tggtgggtac
40981 tacacaacga atgacaaagg tcaaattaac gtcgacgtgc caccagggga gtactatttt
41041 gaagaaatca ctccaccttc agggtatacc tttgatctgg ataagaatgg caaagtgacg
41101 aaacactatt tgtttactgt gtcctcagaa gataaagaag cagctattga ggtaaaagtc
41161 tttaaccgac tctctaaagg acagctagaa gtggtgaaaa ccgttaaaaa tgaagacgat
41221 tccctcttgt ctgatcaaca aaaacaacaa gatttcctct tcactattac gtatagtgat

41281 aaaggaacct acagctatca aatagacgac agtgtcaatc gatatgaagt gaaaagtgga

41341 aacacgatta cgttgaaaca tggacaacaa gccattttca aagatatccc tgaagggatt

41401 cactatacag tgacggaaca cccccttctt ggtgtggtat ctgaggcgtc ccattcaagt

41461 ggaacaattc atagtaaggc gtcacaagca gcgtttacga atacctttt aacgactact

41521 agtgagttac gtctccaaaa acaagtggtt tctgggagtg gaaaacctct cacagaagaa

41581 caactccaac aagagtttac ctttagattt actttttcgg ataaagaaac agcttttcct

41641 tatgaaacga ccactggtcg aacaggggaa ttgaaatctg atgacacgct taccttaaaa

41701 catgatgaaa ctgtgacgtt taaaaacatg tccattcgta ctacttatac ggtgacagag

41761 gaaccgacag taggctatat cagtgggcaa acgtattggg aaggacaatt agtgaaaact

41821 gatggagtac ttattgtcgt gaccaatgag gtagatacag attctaccgc aacgaatgac

41881 ttaacgttta ccaaacaagt tgttgcagaa aaacctgatg aagacatcct tttctcattt

41941 gaagtgacgt tttcagattc taagaactat gagtatcaaa tagacgatgg tctggtactt

42001 tcccataaaa gtggggatgt aattcaactt aaagccaatc aaaaagtaac gtttaaggat

42061 ttgcctgtgg gagtagggta tcaaattaaa gagttagaga cacctcatta tcgaggtgaa

42121 ctgacagagg taacaggtac cattctttct tctgaagata cgaactatac gtttatgaac

42181 cattttgaag gaaatccttt tttacagata aaaaaagtgg gagaaggaaa cgagtttgat

42241 ccaactaaag agtttacgtt cactgtttac gtgaatgatc aagcgttaga agagaaagtg

42301 gtgttgaaag caggagaaat gagtgacgct gttccgttaa ctattggaga tacttggcgc

42361 gtagtagagg atatcagtga agaccctact taccaacaaa cagggattgt caatagtatt

42421 ggtcaaatca ctcaagtaga tcagaatgtg ttagtaactc aaaccaatac ccatatcact

42481 cagaaacaag tgaccattgc gggacaaaaa cactggatca tccctgcgga atatcaagag

42541 aaacaaccgg agtccatcat tgttcgattg atgaatggcg accgattggc cgctactcaa

42601 gaggtgaagg gtccggggtg gtcgtatgaa tttaaggttc cggaagttga ttcagaagga

42661 aatctcattg tctataggat agaagaagag tctatccctg gttttgcgat gagttcgatt

42721 gaggattctt atgatattac caacacgtat gtgacgcccg ttcaagtggc gccactcaca

42781 gtagtcaaaa aaattaaagg ggatacccca ctctcgtcaa gtaccttcga atttacgctg

42841 tctcctggtg acgaaactgt aagcattcaa ggaagtggtc gtgtggacat cccttctccg

42901 acgttcgact ctcctggtac gtatcactat accattcgag aaaaaaattt aggattagtg

42961 ggatatacct acgatatagc tgtctatcag tggacgattg tggtcgaagc tgaaggtgat

43021 gctcttacaa tcacctcaga gacactgact aaagatgggg aagcctacaa cagtcgagag

43081 ttagtctttg tgaatcagtt caagacatcg ttagatgaaa aacaaacgat tagaggaacg

43141 aaaacatggc atcatggaac caattctacc aagtccaccc ctacatccat tatcgttcta

43201 ctgttggctg acaatgaagt catccaacaa aaagaaatca tggaaccaac ggattggagt

43261 tatcaattta cggtacctgt gtacactgtt tcaggggaaa aaattgttta ttcgatccaa

43321 gaagtccccg tgtcggggta ccaaactaaa gtgtctggtt acgatctcat caatacctat

43381 atcgaatcaa cacctaaacc acctggtaag aaaaatcctg acaaaggaat ccttgcccaa

43441 accggtgaaa aaattagtat gatcgcattt attgctggat tgattcttgt gattggagtg

43501 tctggtttct acctccatca aagaaatcat aaaaaacaaa aaaataaaag agtaaaaaat

43561 ataagaagta aaaaaaggcg ttaattttaa ctaaccagtg ggaaaagaag cttatggaca

43621 acataggctt ctttccctat taaaatgcgc cttctgaaac caaaaaattc acgttgaacc

43681 accacttaag aataaaaata ctaactaaaa agagggattt taccaatgaa aattaataga

43741 caagaagcga ttgtgcaaaa ctttgaatat aaaacgttaa ctaaaattca taaagcccaa

43801 actagttttc aaaccgtgat cgatccttta gatctatcca aactaaatac atcattctca

43861 ggaaagcatt ctgtctttgg actaagaatg acgtataatt tagtgattgg caaacggatt

43921 gtgattgttg gaaatatcac ccaattggtt gaaatagaag agaaggtcat cgataagatt

43981 gaagattttt ctaaaaccga actgaataaa ctatttcaac ccttatttag gatgcttgaa

44041 gaattggtgt acgaagtgac tcaaattaca ttagacaaaa aagggattgc cttaaacttt

44101 acagcagatt atgactagtt ctttagtcaa gaatagatta gtcgatttga agcatccaaa

44161 attgttaaaa agaacccctg ctacaatcag ttgggaacta tgcagagatc caggagatta

44221 gtatccataa tctccgaaga aatgatcaaa tcgatttaat cgtctctaat aaaaagaatt

44281 taatagagcg atttaaaccc acttacactc tattgtttaa acagcgatgt aacagacata

44341 tcggtctggc aaagtaaccg aatcgattct ctaaatgtcg atgacttttt ttagtcgtca

44401 tcataaaaaa tctataatag atgattggga ccattttgtt cataatccta ttataggaga

44461 gtaacgatcg atcaggagga gaaaaaatgg agaagaaaaa aaactaaaac atttgggtca

44521 ttgatgaaga tggttgggct tattgggcga acatcctacc accaggtgaa tctacttcat

44581 tattactaaa ctctctcttg gaataaatct actatggctg atttagaaaa atggtattac

44641 ggaattgatg tcgttgggga attttccagc aagtctgatg tctctcaatt agccacttct

44701 gactatggaa gtccgtcttc gaatggaaaa aagataatat atgttattct tagttccgtt

44761 tcttctttgt ccattcgatc taaagaggag gatttaacca cgatatctgc tggaacctct

44821 agagagtttt atgctgaggt taactatcga gcgtcattag attggtcaat gactccagaa

44881 atcaattcac tctatgaaaa atatgaggac ttaaacccat tttcactttc aaaaaaactg

44941 aatatcgaaa ttaaattcgt tccattcaaa gaccgtcctt atggaaacat aaaaataata

45001 tttggagaac caattatatt tattaacatg agtaaagaaa atgaacactt tttcatttgt

45061 gcgcatgaac tttatcatgc aatagcgcat aaacagctta gatataatta ccaaacaaat

45121 attgttgcaa aagcaaaaat ggaaattgaa gctaacttat ttgcatccca tttaataatg

45181 aatgattact tactaaaata caaaactcca cctaaaaatt ttttaaagtt acaaactgtc

45241 tatgggttat ttgatgaaat gaaagatttt ttctagttta atgttctatc actatgtaat

45301 attttacaga atatctatta tacttctttt tgtacgtatg cccacgtaat aaggagataa

45361 aaatatgtca actgtagtat attcaaaaaa atacgattat gtatatagtt atcaaacaaa

45421 aaaaggaaaa ttgtggggtt atagatttaa ttattatagt tctagcaata taagaaaaga

45481 gattggaaaa agaaagttca tcactgaaaa agaagcctat cgtgatttgc tgactgttca

45541 agagcaagtc gaaaaaggaa atttcaaggc tattgaaaat aataagctaa caatcaagga

45601 atattgttat gtctggttgg aatctcaaca aagtaaaaac ccttctataa atactattcg

45661 aggtgcaaaa acaactattg aattacacat tatacctaga atcggtcact taaaaataca

45721 ggctatctct aggtttgaat ataagcgcct attcttagaa gacttattaa aaactacttc

45781 tgctcgtagc gtagaaacac accacaaacg atttatagct attatcaatt ctgctgttaa

45841 aaatgaaatt attttagcaa acaattttaa aggatttaac ttttcagatt acgaagtaga

45901 aaatattaaa aataaatact tttcaaaaaa gaagccgcta aatttctaaa taagttagag

45961 ttgctagatt atcggaagaa aacagttttt tatttattat tttcaactgg tatgaggaaa

46021 ggtgaattac ttgctcttcg atggcaagat attgatttaa aaaaggtgaa attgatattt

46081 caagaactag aggtgaccaa aaaaactcta caccaaaaac taaatccagt ataagaaaaa

46141 tacagattga tcccagcact atcgaaatgt tgaataaata taaaataaac gaacgtaaat

46201 ttttgttatc taatggtcaa aaattaaatg aaaccgatct ttttgcaaca aatagatatg

46261 gtaacccaat ttcatattgt ggaatcacag aacaattccg attgttttgc aatgataatg

46321 atcttcctta tattacttta catgggctac gtcataccca tgcaacattg ttacttaaag

46381 caaatgctaa tatcaaatat atatctaggc ggcttggtca taaaaatatt gaaataactt

46441 taaacacata tagtcacgtc cttatagaaa aagaattaga aactgcggaa attttttcaa

46501 atgttgtaac taatttagct aaattaggat agtgcgggaa aaattgcggg aaataactag

46561 aaatttcaaa aatatatgtc aaataacaaa actgttaatg aacattttat catttaaacc

46621 tcttataaca tcgcatttaa cccgaattta tgttaaaatg atttagatat actatagaat

46681 agaggtaaaa aatcaaatga ttacagttag taatgtaagt ctgttatttt cagatagaaa

46741 tttatttgaa gatgtaaata tcaaattcac tcctggcaac tgttatggac ttatcggcgc

46801 aaatggcgca ggtaaatcaa cgttccttaa aattttatct ggcgacatcc aaccaactac

46861 cggtgatatt tcaatgtcat ctggtgagcg tttaaccttc ttaaaacaaa accattttga

46921 ttatgaagaa tttggcgttt tagaaacggt tattatgggc cataaacgtt tatacgatat

46981 catgaaagaa aaagatgcta tctacatgaa agaagaattt actgatgaag atggtattcg

47041 cgctgctgaa cttgaaggcg aatttgctga attagatggt tgggaagctg aaccagaagc

47101 ggctgtttta ctgcaaggtc ttggcattga tgaaagtctt caacaaaaaa caatgagcga

47161 attaactggt ggtcaaaaag ttaaagtgct tttagctcaa gctttattcg gtaaacctga

47221 tgtattacta ctagatgagc ctaccaatgg attggataaa caatcaattg aatggttaga

47281 agaattttta attaatttct ctaatactgt tattgttgtt tcccatgacc gtcatttctt

47341 aaacaaagtg tgtacgcata tggccgatgt tgattttggt aagatcaaac tttacgttgg

47401 gaactacgat ttctggttag aatcaagtca attagcttca cgtttagcaa atgactctaa

47461 ttctaaaaaa gaagaacaaa ttaaagaatt acaagacttt attgcgcgat ttagtgcaaa

47521 tgcctctaaa tcaaaacaag caacgtctcg taaaaaaatg cttgaaaaaa ttacacttga

47581 tgatattcaa ccatcttctc gtcgttatcc attcgttgga tttacacctg atcgtgaaat

47641 tggaaatgat ttaattcgtg tcgaaaatgt ttctaaaaca attgatggcg tgaaaatttt

47701 agacaatatc agcttctcgt taaaccgtga agataaaaca gcatttatca gccaacatga

47761 tattgcgatt acaacattgt ttaaaattat catgggcgaa atggaacctg attctggtag

47821 cgttaagtgg ggtgtcacta ctactcaatc ttatttacca aaagataata cggctgaatt

47881 tgaaaaaggc gacttaaccg ttgttgattg gttacgccaa tttgcttcaa aagaagaaag

47941 tgacaatact ttcttacgta gtttcttagg ccgtatgtta ttctctggtg aagatgtgat

48001 gaaaaaagta tctgtccttt caggtggaga aaaagttcgt tgtatgcttt ctaaaatgat

48061 gttaagtaaa tcaaatgttt tggttcttga tgatccaaca aaccatttag acttagagtc

48121 aattacagct ttaaatgatg gcttgattgc tttcaaaggt gctttactat ttggttctca

48181 tgatcatcag tttatccaaa caattgcaaa tcgcattatc gaagtttcat ctaatggtgt

48241 agtagatcgt gctgatacaa cttacgatga tttcttggaa gataaaaacg ttcaagaaca

48301 agtagcaaaa ttatacgcaa actaatttaa aaagtgtggt ttcaagttta cgaacttgaa

48361 accacacttt tttctaaatt ttcctctaga tatatacata tctaaaaatt taaaaatcct

48421 aattagtttg tagattccac taattggttg aattcatcct ctatctcatt atttctaatt

48481 tcctcttcat taataacact tctacgtgat tttggacttt cttcgtatcc ttcttctggt

48541 aactctcgtt caaatcgatt tgtttctgga ttttttaaa tcttgaatta tattctccca

48601 taaatgaaga aaaagagaaa tcagtttcca tagttcccat atcatccgtt aaaaatccaa

48661 caatttcctc accaactcgt gtattttcaa ttccatctga taggacagta atctcttcct

48721 ttaattcttc ttcagacatt tcaggaaatt tttctttaaa acctagctct tcttttgtaa

48781 tttctccgcc ggattctaat atgattatcg tttgtcctac taagttagca tcaccagata

48841 acaccttcgt tatttctaac gtaagcttaa tataaggagt gcttggacct ccttcttgat

48901 cataaacata gggttcttct gcaactacaa ttccctcaac acctatagtt gaactatttt

48961 ttattgtttc taatgaatca aaatcgataa ccattgcatt gcttgtttta gcaattttct

49021 aatttgcaca ataattcata agccaagaaa gttattagag aggattctaa gaagtttggc

49081 attaacttag aaattgattt cacctaaaag cagatgagac gaactcttag aataatagaa

49141 accgcatgaa atacattttt gcaaatgtca gtttcatacg gtttttttat tttgattaaa

49201 tttatcttgg ttaatgcttt tataattgtt ttgtatatat gcccactaaa aattgccaca

49261 gaatttgccg cattttataa tgattcttaa tgtaccttta ggaatattaa atttttgaat

49321 atgttaaaag tgcttattta gaggtgtttt gttattcttt caaaagaggt tttattacta

49381 actgtaatca tttgattttt tacttctatt ctatagtata tctaaataac tttagcataa

49441 atgcgatgtt ataagtcttt ttctgaaaag aatgtgagaa aattgtggca ctcaggataa

49501 agaaagtaat tgataagtta acggatttgt ttgtgtaagg ttgattataa ggtaacagta

49561 ctatctttta ttgttacgga ttgaagtatt taaaataagt tgcgtacttt caattaagtt

49621 acaaatttct aatttgagca ttaaaccaac aataagttaa caaaaagcaa tttttgttcg

49681 gttatttacc tctattttac ataaataacg ttgtaaacaa gatgttattc tattatacta

49741 gaataagaca cataaaaaaa cttggagggt acttaaaatg aaatttgtta aaaatggttt

49801 tttgcttgtt tttggattgg ttttagtcgt aagtttagca gcatgtggag cgaaaaaaga

49861 gtctggagcg aaagaagaga gctatactcc aaaagaatta accgttcaat ttgttccaac

49921 tattgaggca aacacgattg aagcgaaagc taaaccgtta gaaaagttgt tatcagatga

49981 actcggaata cctgtgaaag taactgtatc aacagattat aatacaatta tagaagcaat

50041 ggcttcaaaa caaacggatg tagggattat gccgccgaat gcatatgttt taggtcatga

50101 gcaagaagca gctgatgcga ttttacaagc gcaacgcttt gctattaaac aacctggtgg

50161 cgaaacgtca acagaattag ctgatggcta tcgagcaatg gttgttgtga aaaagggtag

50221 cccgattaag aacttaaaag atttaaaagg gaagaaaatt gcagctcaag atgttgtttc

50281 ggctagtggg tatgtttttc ctgtagctga aatgcaaaaa gctggaatca atgttgataa

50341 ggagattcaa tttgcaacag ttaaggggat tgatgctggc ttaatggctg ttttagatgg

50401 tagcgtggat gcagcattta gttttgagga tgggcgtaat ttattgaaga aggatttacc

50461 agatgtattt gataaattag atgcgatgta ttttacagag gctaaaatac caaatgatgc

50521 gattgcagta cgcccagatt taagtgataa gtggaaaaca aaagttcaag atgcttttgt

50581 agccattggg aaatctgata aaggtcgtga attaatcagt tcgttatatg ctcatgaagg

50641 gtatgtacct gctgacgata gtagttttga tattgttcgt gaatatgcaa aagaggttgg

50701 acaataataa ataaacaaat taagagaggc tgagaccgag gtcttagtct ttctcatttt

50761 agaatgaaaa ggtgacgata tggaagtaac tttactggta acaagtgata ttcatggcta

50821 tattcggggt acaaatgagt tgactggaga agcaacagcc gatggttttg ctcgtgttgc

50881 aacctatata aagaagcgca aaaaaataga tccaaaccta attctaattg aaaatggcga

50941 ttttttagaa ggatctatgt tagctaatac tttagcaacg catcaagata aatggagtat

51001 ccatcctttc atccagctaa tgaatcaact caactatgac agtgctgtac ctggaaatca

51061 tgaatttaac tatggaataa attatttaaa aaaggcttta aagggattaa aggcacctta

51121 tttagctgca aatattgttt tagagaatgg caaaacttgt tgggaagcct atcaaattgt

51181 ggaacgcaaa gggattcgaa tcgctatagt tggactagta acttcttatg taaccagatg

51241 ggaaaaggtt gaaaatatta aaggattgcg ctttttagat ccaattttaa cagccaaaaa

51301 atgggtccct tttattcaag agacagaaca gccagactta attgtgctag cctatcatgg

51361 aggattagaa cgtgatttag tgacaggtga agcgacagaa tatatgactg gagaaaatgt

51421 tgggtaccga ttagctactg aaatccctgg aataaacgga gtcattactg ggcatcagca

51481 ccggacctta gccggagcag taaatggggt tcctttactt cagcctggga cacgcggagc

51541 aagtattggc gaaatgattt ttgagcttca acaaaaagag cagaaatggc atgtgaaaag

51601 tgctaaagcc caaacaattt cgttggcttc tgaaaaagaa gatactgctg tcttaaaaga

51661 gattgaagaa tgggagcatt taagtgctaa tgatcgaaaa aaaccaatta taaaagggct

51721 agattcggtt gaaccagatg cttatcagtt tttgcacaaa gttcaaaaag ttgcctcaag

51781 acgtaaaata tcagttgtct cccttgtgaa aagttcagtt ggttcaacgg ctttaacaat

51841 ggagatgatt cttcaaaatt atacatttcc ttgtacattt gcgattctcc gttttacagg

51901 acaggaatta aaacatttga taattaaagg aagagactat taccaattga accaagcggg

51961 ccgattatcg ccttataaat tagggcgaga tgagcaaaca gaaaatgaat tgattgatgg

52021 attaacctat cgtttctatt ttgattcaaa atcggattcc tatttagtcg aattagttga

52081 tgtagcagat gatgagctga ttgagttaag ctttaaccat tatttgggtg ctctgaatgg

52141 agaacgtgtg attggaatgg aaaaaatcat acaagaaata atgatttata tgcctgattt

52201 aattcgtgag tatcaattag aagagtgtgt agcgattccg gtccatggtc aaggatttta

52261 tccggagatt agctaaatag acatgaaaaa agaacgaact gactatccat ccatgatagt

52321 cagttcgttc ttttttttt agacttagtg aatcaactct gtaaacaaat aaagtataca

52381 ggcattattt gttaagacat gtagttgatt tcggtaatat aaggttcgat cataaacgcg

52441 ccatttagta aaatccatct ttatttgtaa aataggctgg tcatttaatt gagcagttaa

52501 tttgtgctgc ttttctttta tataaattgt atttaacgaa aaaggcggtt gttgattaag

52561 ctcagacaag gctgccttga cagttaatga tttcgagcga tactgctttt cttttttaa

52621 ttgacgattt aaaggattaa aagaaaataa gagtccttca tcaattaacg gttttgtcat

52681 ttctaaagat aagggtaatt cttcaatgga aacatgttct ttgtaggtta attcattggc

52741 cgcctgaact ttttccaaaa taatttgttt ttcatgtaaa tgaaagaaac agatatagtc

52801 agctttttcc agatgtgcac cgaattgaat cagatcgtta cgactccatt ctgaacggct

52861 agcatactga tttgttaatt gataaataga tggagcaact tcatctgctg tatggtcagt

52921 aaaacgtttg gtattatcgt attcataaag accattagct ccacaagcta gtgccatacg

52981 tccgttttta gcaatattaa tttggagcac aggcagatcc cataattttt ttttagtggc

53041 ttttaattgt gctagtgaat aactgtaaaa gccggttgaa tcagaaaaat agagttggtg

53101 attaaagata gcagtgtcta ttggagcttc ttcgaaagcc aaactccttt ctaaataagg

53161 ggtaagatcc tttaagtcaa actgcatagc ctcagaagga cgaggttcaa aatagacagg

53221 acgttcaatt tcattcaagg caagcatcca gttgttccaa ttataaatag ctagctgatt

53281 ggtttctaac cataaataaa gttttccact atataattgt gagtcataaa aggtaccatt

53341 aattgttatt ttagttgtaa gtatgtgctc tttttccata agtgtcaccc tttccccact

53401 aaacttgttc tttcttctat tttagcattg aatacgtcaa aagtgggctt ttttattgta

53461 aaagaataaa aaaggtagaa cttttctaaa ctagacaaat ctgttctaaa ttagcattga

53521 aagcgctttt tatcatgcta tactgatttt attatgaaag aggaggagtt aaaatgaaaa

53581 agtttgtcag tttaatggtt gtattaacag ttgctctagt aggattatcc gcttgtggga

53641 gcggtgagaa gacaaaaggc gcaagtaaaa aagaagaaaa taccttaatt gtctattcgc

53701 caaatagtga ggatatcgtc aatacattaa ttccaatgtt tgaaaaagaa acaggaatta

53761 aagttgaatt ggtttcagca ggtactggag agttattgaa acgaatttct tctgagaagg

53821 aaaacccgta tgctgatgtg atgtttgggg gcttaaataa atctaaatta actaatgaag

53881 atttatttga aaaatatacg tctaaaaatg acaaagatat gatcgaaggt catcaaaata

53941 ttgctggagt attaacacct tatattactg atggcagtaa tattttaatt aatactgatt

54001 taattgggga tataaaaatt gaaggttatg aagatttatt aaatgcacaa cttaaaggca

54061 agattgcagc tgccgatcca gccagttcta gttctggttt tgcacaattg acgaatatgc

54121 ttttggcaat gggtggcgat tatgaatcag ataaaggctg ggattatgtg ggtagtttaa

54181 ttaagaattt ggatggaaaa gttgcaaatg gatctggtgc tgtgcacaaa ggagttgcag

54241 atggtgaatt tgttgttgga ttaacttatg aagatccatc agctagctat atccgtgatg

54301 gtgctccagt taagattgtc tatcctaaag aaggtgctgt ttatctagat gctgctttag

54361 cgattattaa agatgcaaaa cacaaagaaa atgctgaaaa atttattgat tttatgacat

54421 caaaagaagc acaagacatt tttggcgagg aactgacaaa tcgtccgtta cgaaaagatg

54481 cggcattagg gaatcatatg gtcccaatga aagacattaa aatccttaca gaagatgaag

54541 attatgtgca aaaaaacaaa gataaaatcg taaaccaata tacagattta tttacagaat

54601 tacaataaga aaattgtagg aggcgagatg gacaaatgag tacaacaatt aaaattaaaa

54661 atgttcttaa aaaatatggt gaaagcgttg tgattccaga tttatctgtt gaaattaaac

54721 ccagtgaatt ttttacttta ctgggtccat ctggttgcgg gaaaacaact ttattgcgga

54781 tggtggcagg ttttaacagt attgaaggag gcgatatttg ttttaatgaa acgaaaatta

54841 atcatgtaac tccacaaaat cggaatattg gcatggtttt ccagaattat gctatttttc

54901 cgcatttaac agtgaaacaa aatatcgctt ttggtttgca aaatagaaaa ataaaaatag

54961 ccaaagatga gattgagaag aaagttgaaa aaatgatgga tgtcgttcaa attcaaaaat

55021 ttaaagaccg cttacctgaa aatttatcag gaggtcaaca acaaagaatt gcattagcac

55081 gagcaattgt cattgaacca agtgttcttt taatggatga acctttatcg aatttagatg

55141 ctaagttgcg agttgatatg cgaaatgcga ttcgaaatat tcaaaaagaa gttggtatta

55201 caactattta cgtgactcat gatcaagaag aagcgatggc tgtttctgat cgaattgcgg

55261 tgatgaattt agggagtatt caacatgttg gaacaccgca aaatatttat catcgtccgg

55321 ccaatgtatt tgtggctaat tttattggtc gaaccaatat tattgaacgt gaactgaata

55381 tggaaggaac agtaggctgt ttacatttag atcaagacaa tagtgtaaag ataacgaact

55441 tagcggatcc aaagaataga caatctggta aggtgaaagt ttcgattaga cccgaagagt

55501 ttataattgt cgaagatggc ttgggaattc gggctaaggt cttaaataat atgtttttag

55561 gtttaaatac ccactataat gtggaattag ctagtggtga acaagttgaa attattcagg

55621 aatcaacact agaaaaagtc attgaacctg gaacttggat tggcttacac attaaaaaag

55681 ataaaattaa cctttttgat agtcaaacgg aagtgaattt gacaaaagga gtgataaatg

55741 atgtttggta aagggcaaaa aaaagatgtt tggacgtata ttactattgg catcttctta

55801 ctttatttaa ttttattggt tctgccatta tttaccttat taaaagcagg ctttacaaat

55861 cctgatggga ccggattttc cattgcttat tttactaaat tttttggtaa aaactattat

55921 tacgatgctt tatttaacag tttaaaagtc acgatttcag ttacgctatt agccgtggtt

55981 ttagcaacac cgttagctta tattatgaca acggtgaaaa ttaaaggcaa agtatttttg

56041 caaattctaa tcttgatttc ttcaatggct ccgcctttta ttggtgctta ctcttggatt

56101 ttattattag gccgaaatgg tgcaattaca aaatttattt caagtatgtt tggtgtgaag

56161 gtccctgata tttatgggtt tacaggaatt gtaattgtgt taactctaca attagttcca

56221 ttaatctata tgtatttgat gggagctcta aagagcattg ataattctat tattgaagcg

56281 gctgaaagca tgggttgtac aggaattaaa aaaatggtga aagttatttt cccattaatt

56341 atgccaactc ttttagccgg ggcgttatta gtctttatgc gtgctctagc tgattttgga

56401 acaccgatgt taattgggga aggctatcgc acagtgccag ttttaatatt taatgagttt

56461 attagcgaaa tgggtggaga tgatggtttt gcagcagcaa ttagtgtaat tgtcattgtt

56521 tttgcgattg cggtcttctt gattcaaaaa tatattagca atcgtaaatc cttttcaatg

56581 aacgctttgc atccgatgga agctaaaaaa gcaaaaggaa tccgaaatat tcttgctcat

56641 acctatattt atggctttat tgtggcggcg attgctccgc aattgtatgt gatttataca

56701 gctttttgga aaacttccgg acgaatattt atcaaaggtt attcgctaga tagctttagg

56761 ttagcctttg atcgttctgg tgacgcgatt aagaatactt tttcattggc gattgcttcg

56821 attgtgattg tcgtgttctt agctatttta attgcgtatg taaccgttag aagaaaaaat

56881 accttaacaa atgggctcga tattgttaca atgattccct acatcgttcc aggttctgtg

56941 atgggaattg ctttgttatt gacttttaat aaaccacctt tattattaag tgggacagca

57001 ttgattttaa ttgtgtcgta tgtaatcaga cgtttgcctt atacgattcg ttcaagcgct

57061 gcgattgtgc atcaaattag ccctagtatt gaagaagctt ctgtcagttt aggagcatcc

57121 aatttaaaaa cattctttag ggtgactttt ccaatgatgc ttccaggtgt tatttcaggt

57181 gctattttaa gttgggtaac cattatcaca gagttaagca caacgattat tttgtataca

57241 gctaaaacac ggacgatgtc tgtggctatt tatacagaag ttattcgtgg gaattatgga

57301 actgcagccg cgttgtcaac gattttaaca ctaattacag ttagttcgtt attactattc

57361 ttcaagttaa cgggtaaaaa agaaatttca atttaaatat agataatgag ggattgatga

57421 tggataagaa acaacataaa ttcaaagaaa aaacccgact tcttttttg aagtatacgt

57481 ttattccgat tgttttctta ttcgttattt ttgggattag catattctct tatgctagtt

57541 ggaaagttcg taatgatacg gtaaaggcta gccaggaaac aatgctagct tttaatcgtg

57601 tcttagctac atatgaaaaa gaaatgatac gcatggcgaa caatgaatat gttttagatt

57661 atttaacgca acagaagaag agtaatttag tttttgaaga gttttacgga tttaatttgc

57721 aacaagaagt taaaagtatt ttccatcttg ttgatatgaa aaggttttct ctaacgtcat

57781 cttcatctga ggattacctt gcgaatgaaa ttccaagtta tcgtttaaag cgttggaaaa

57841 atccgaagca gatggtaatt gatgttcaaa caaattattt ttcttataat aaagtcactg

57901 gattaacctt aatccaacct gtcggaaagg atgggaaaat tgttggttat ttaatttatc

57961 gtttatatga tgaggatttt caaaaactat tatttggaaa gaatgttgat attgttgtga

58021 tagcagatca atatgaacgc attattgcca caacgagtaa tcgtgtcaaa gggctgatgg

58081 gcaaattcag tgtagacgaa agagaaggca atgagatttt acttgatagt aaaaagtata

58141 ttctggcaga aaatcagcac tctctctatg gtgttcaggt aaaaacctta atccagaaac

58201 cgaattatca agaagtactt gtttcttatt tattactgac gactattagt agtttagttc

58261 tttatttcct agtcaattat ttagctaaaa aaatgtcaga gcgcaatagt cagtcggtga

58321 gtgttttaat gtcgggttta gatagtttaa aaacaggtga gttaactaat tttgttgaaa

58381 tcaagacagg tgatgaattt gaagaattag cggatcacta taataaaatg cttaaaaact

58441 taaatcaatt gttattacga aatgaagagc tcacctatgt tttacagcgg gatgaaataa

58501 aatttttaca aagccaattt aatcctcatt ttttatttaa tatgctagaa actattcgct

58561 atacgatggt ttctgatcaa gataaggcac aacggatcat tttaacttta gcccgtttgt

58621 tgcgttatag tattcaccaa ggtcaaaatt gggaaatttt gcaaaaggat atggactata

58681 tttctgatta tttagctttg catcaatacc gttataacaa tcgattaacc tatcaaattg

58741 acctcgatcc aagtttgacg aaagttgaaa ttcctaagct gctattacag ccaattgtag

58801 aaaattcgat taaatatggg taccaaaatc aaactaattt aacaattaaa atatctgttc

58861 aagcgcaaca agacaaaatt aggattacag tagaagatga tggaccaggt attgctacta

58921 aacaattatc cgaaatcgaa gctaatttgc gccaagaaat aatcaactta agtagtatag

58981 ggttatacaa tacggaccgg aaaattaaac tgatttatgg cgaaaaatat ggactgaaaa

59041 ttaagagcac attgggtttg ggcacaattg tccaaattga attgccgtta ataaaggatg

59101 atggggatgt ttaaagtatt gattattgaa gatgaggata ttatccgaga aggcttgaaa

59161 tttagtttag actggggaaa agaaaactgt cagattgtcg gagaagctgc taatggagta

59221 gaagcactgg aaaaaataac taaattgaaa cccgatattt tattactaga tttaaatatg

59281 ccgttaaaaa atggcttgat ggttctaaaa gaaagctatc aaatagcgtt atacagcacg

59341 attattatct caggatatga tgattttgaa aaagcgaagg aggcaattaa gtacggggtt

59401 acagagtatt tattgaagcc agtagatcat gaagaactga ttgttgcact gagaaaagcg

59461 cgagaagcaa ttgagttaaa gcaacaatat caatttttac aagagaaatt aaatggagta

59521 gaagatgtag acattcttga ttttaagcga attacaaatc gtcataaatt atcaaaagaa

59581 gttgcacgtt taatcagttt tattgaagaa gagtacagtc aaaaaattcg attacaagac

59641 ttagtttttg agttgaatcg tagtaaaact tatttaaatc aaaagtttaa gttagaaaca

59701 ggctatacat tcaatgagta tttaaatcgt tatcgagtgt atcaagctat ttatcttctg

59761 aaaaatagtg atgagaaaat tagtagtatc gcgcttgagg taggttttag caattatcgc

59821 tattttattg atatattcaa aaaatataca caagtattgc ctagcgattt tgcaagttat

59881 tcaaaaggct atgtgccaga agcataaaaa aggagtcaaa acatgccaaa taatgacaat

59941 gttttgactc tttttattca ttcatcaagc ttttttagcg acttcttttt tagcaatcgc

60001 tggtaaaatc atacctagtg caataaaaat aatcggtgtg atgatgttta aagtaaattg

60061 gaaaacccat gcttttggat ctgcagcata atcgattttc ggcaccatac ctaagataca

60121 agcaaaggcg gtaaagagga aacaccaagc gccaatgaca tatccaattt tatgattttt

60181 tacaaatttg tactcagaag agaaattttt atgcaatttg tttaacatca tataggcaaa

60241 gaatacccat aaataacgaa gtggcatcac aacagcattt aaattcagca accatttata

60301 taaatcattc atactaccaa tcccaatagc aggcacaata attagaatac taactaaaat

60361 tccggtcata gcgtagccat tgactggagt acctttgcta tttaatttag ctaatttgcg

60421 tggaatatat tcttcatcag catcgccaag taagattttt agcggagcat cgattgagaa

60481 ggctaaggct gatatttgag ccattgtatt ggcaatcgca tataaaatca taaagaagtt

60541 tccaactccg taataagcac ctaaacgttg aaaggcgtca tatgcaccat tagccattaa

60601 atcatcagga atgttgttac tatcaaatag catccccatt gcaaatgaac ccaataaggc

60661 acaaacagca accatcgctg ctaaagctaa catcccttta ggaaattctt tagaaggatt

60721 ttttgtgttg tttacgtaag gtgaaatttt ttcagctcca ccaactgcaa agactaacat

60781 tgatattgtt gtgaaataag caaaattaaa agtcggtaaa taggttttta tatcgcccat

60841 atttggtgtg gcaaaagatg caccacgaag agcaggagcg gttacagcta ataaaataaa

60901 taataaagac atgacaaaca ttgccattcc tgcaatacta ccaattcttt ttaaagtggt

60961 taaacccttt gatgaaaccc atagaaagac taagaaaatg actaaactaa ttagagatac

61021 agttagggct ggtgtatcat tcactagatt tccattttgt ttaaatgccc aaccaagagc

61081 gattaaaatt ccttgtggtt tttgtgctaa ataaggaata tgcaccaccc agtatgtcca

61141 agcagcgtaa taagccagct tcttagtggt tgtttcttta atccaagtac taactccccc

61201 attgccatct ttaaaagtcg aacctagttg accgacaatt aatgcatatg gaacaaagta

61261 taaaagcata attaaaatcc atgatgtaat aactgaaatt ccttgagttg catagttgtt

61321 tacaacattt cctaggcccc aaaccgaaac aaaggcaatt aaggccacgt tataccaacg

61381 taactttttc tcttctttca ttcaatgaat cgtcctctct gtttacaatt aaaatgaatc

61441 atataagttc aaaggatatc aaaagatacc gtttttatca taaaccgatt tatataagga

61501 aacaactatt ttctttggtt tctaacagtt tgattagtct ttgattaagg gattgttatt

61561 taatgaacat gagtattaat taaacaaaat gagagtgttt ctctttcgta ttggggttat

61621 aagtgggata atgagaataa ggcaaaaata agctagtttt tcagaatatt ttatagagga

61681 taaatgggaa tttggggaga taaaatcaat taatatcgtt ttatctgtca ctattcttaa

61741 taaaaagcgc tgattagtgt ataggctagc ctttgtaatt agagctaacc ggctcaacaa

61801 actttttatt aggctagttt ttatgagcca actcctcgag aaaaagataa aaattcgaga

61861 ggacaaaaag cgtcctatca atttttccta ttttcttgcc ggagctaacc ggctcaacaa

61921 actttttatt taaggaggaa attatgaaga ataaaggaat atggttactt caagaaacaa

61981 ctccagaaca attgcaacga gtgaaggatt tagcgccgaa ttatgaggtt attgatgggt

62041 tttcagactc aactttaaat tttcctgcag ctgatatcga aatagtttat ggctggagtt

62101 cagcaaaagc tgatttttta ttagaaaata aggatagtca cttaaaatgg attcaagcaa

62161 aatctgcagg tgtggatacc atgaatctgt ctttactaaa cgaaaaaaat attattttga

62221 cgaatgctag cggaattcat ggtgttccta tcgctgagtc agtttttggt atgctgttag

62281 ctgatactag agggattaaa aaggctatca atcaacaaac aaataaagtc tggtcacaaa

62341 cagaaagttt agtagagcta aagggcaaaa ctatgatgat tattggaatg ggccaggttg

62401 gaaaagaagt tgctcgtcta gcgcaggctt ttggattaaa tgtgattggt gttaatcgaa

62461 gtggaaatcc tgtaacagaa gtctctgaaa ttattaagca agatcaaatt ccaaaacata

62521 ttaaaagagc cgattttgtt gttaatattt taccgttaac ttcagaaaca accaattact

62581 atgatgactc ttttttaca agcatgaaaa aaggtgctgg ttttattaat gttggaagag

62641 ggccgtctgt agatacagat gctttaattc gccagataaa aaatgggcag attggttttg

62701 ccggtttaga tgtttttaaa gaagaacctt tggcaaaaga tagtccatta tgggatttac

62761 cagaagtctt gattacgccc catattagtg gagtagctga acatttcaag aaacgtttat

62821 ttgctatatt tgaagaaaat cttacggcat atgttgccgg agaagagttg ccccggaatg

62881 tcattgatta taaacacaat tattgaaaaa tcggtagtaa agtagaacag aaactcgttt

62941 tctgttctac ttttttatca tggttcttgt tgctttagca acttagaacc atgatacgca

63001 tccatcaaag atggactgtg gtcctaaaat gttggtttaa tgaggttctt gttgctttag

63061 caacttagaa ccatgataca catccatcaa agatggactg tgatcctaaa atgttggttt

63121 gttgaggttc ttgttgcttt agcaacttag aaccatgata cgcatccatc aaagatggac

63181 tgtgatccta gaatgttggt ttactgaggt tcttgttgct ttagcaactt agaaccatga

63241 tacacatccg tcaaagatgg actgtggtcc taaaatgttg gtttatttaa ataaatacaa

63301 tattgtacat ggcttcgtga agcgtaatgc ttttaaataa gggttttaaa gaaatatgtt

63361 tctacttcat ttgtattttt tatccagata tagaggttaa attgacacag attttttcg

63421 ctgttatatt tctaaaagag ccttacttaa tacaacgcaa ggaatgtgtc taatagaaag

63481 ataatgagaa ggagcaaata aataatgaag aaaaagagta gttggttagt gattagtttt

63541 attgcattaa gtttagttgt ggcaggatgt actggaggaa atagtgataa agccactagc

63601 gctgataaga aagaaaaaaa tgggtgggag caaatcaaag aaaaaggtgt gttaactgta

63661 gcaacatcag gaaccttatt ccctacttct tattatgcaa aagatgaaaa ggaattaact

63721 ggttatgaag ttgaaatcgt taaggaaatt gctaaacgct tagatgtaaa agttaaattt

63781 gttgaaatgg gttttgatgg catgttgtca gctattaata gtgggcaggt cgatacagca

63841 gcgaataata ttgatttatc agataagcgt aaagagaaat tcgctttttc tgatccttat

63901 aaatattcat ttgccagtat ggttgtacgt aaatcagatc aatctgggat tcattctcta

63961 gaagatttga aaggtaaaaa atctgctgga gcagcaacaa ccacttatat gaaaattgct

64021 gaaaaatttg gagcagagag tgtcgtgtat gacaatgtaa ctaacgatca atatttatta

64081 gatgtagcaa atggtcggac agatgtcatc ttaaatgatt attacttgca aaaaatggcg

64141 acagaagcac ttccagaaat cccagtcgaa attaacccag gattatttta taatcctagt

64201 caagctggct tagtgatgaa aaaagaaaat actgaactga agactaaagt ggatgatgca

64261 attactgaaa tgaaaaaaga tggcactcta aaaaaattat ctgaacagtt ttttggtgga

64321 gcagatgtgt caaaagaagc agatgtgaaa attactaaag tagtcgaggt agaataggag

64381 tcgagaatat gagcggaatt caatggcagt atctctttaa tccgagttta gcgctagaaa

64441 gcttaccgtt tattttacaa ggtttgggtt atacgctttg gatttcagta gtaagtatga

64501 cttttggaac aattttaggg ttctttttag cactatttag attatctaaa tttaaagtat

64561 taaacttgtt aagtcgtata tatatttcct ttatgcgagg aacgcctatg ttagttttct

64621 tatttattct ttattttggc tttcctttta ttggaatcaa gtttgacgct gttactgcag

64681 ctgtgcttgg ctttagtttg catagtagcg cctatatcgc agagatatta cgtgcttgtt

64741 taaactcgat tgataaaggg caatgggagg catcatatgc attaggtatg ccgtattttt

64801 tcattatgcg caaaatcatc ttaccgcaag ctttgcggac tgcaattgga cccttaagta

64861 atgttttgct agatttgatt aaaggaactt ctttagcggc aatgattaca gttcctgaaa

64921 tatttcaaca agcaaagatt gttggtggtc gagaattcga ttatatgaca atgtatatac

64981 tagtggcctt ggtctactgg ggaatttgta gtctattcac cattttacaa actattttgg

65041 agaaaaagtt ttctctttat acaaactgac tagctgttaa aaaaagtaat tagtaaaggt

65101 agctaaaaga aaggactatg aattgttttt tcatagtcct ttcttctatc tagtggtttt

65161 acgtttaatt aaatcaaatt ctaaaggcaa taaaggtagg tttttatata atagttgatt

65221 aaagatgatt gtaaaggcat tttgagcttg tttagcaatt gggtaatcaa tcgtggtgat

65281 atccaataaa cgtgaaattt caatattatc aaaaccaacg actgaaaaat ccgttggaat

65341 gttgtagcct cgtcgacgag attctgcgac taatcctgca gcaacagcat ctgaactagt

65401 tgcaaagcca tctggtttgt cagattgttc tgcataccaa tcaagaatcg cttccccgtc

65461 ttcgatggag tttaagcctt taaactgttg ttcaagtttt ggatctaatt tatatttttg

65521 acaaaagtca gcgtaagctt gcatgcggct cttggtattt aatcctcgtg tactgccata

65581 taaattggca atgttacggc aaccagaagc gtaaaggtgt tctaaaccta acatgtaccc

65641 ttggtaatgg ttcataaaga cagaaggaat gtgctttgaa tcaactcgtt gccaagtgac

65701 aatggggcca tattttgtga aaggttcaag ttgatcccaa tcatttaaac gaatgactag

65761 cactaaacca tctaattgtt tatggcgtag catttcaaaa gcatctaatt cttttttctt

65821 atcgccacca gtaataaata aagtgacgtt atagccttct ttttgagcgg ctaacgtaaa

65881 gcttcttaga aacacagtca aagagtctga aaaatcaggt gcgataatgc ctaacatttt

65941 agtcatacct gtttttaaaa atacagcatt gcgattaggt acataatcta attttttgat

66001 aacagcttca actttagctc gagtgtcatc gctgacataa ccgttgcgat taattacacg

66061 agaaacagtt gctgaagaaa caccagcaat tttagcaatc tcattaatat tagccatttt

66121 tcacccatcc cttcaaacta tctctagtat agcaaaaagc gagctttaaa tgtagggagt

66181 taggcagaat atatgggaat aagcctttaa attgtgacaa ctttttatga atctttcata

66241 taataataaa aaattcttgc cacaacaaaa taaaacagct atcatctaat agatatagta

66301 aaaagttaaa ggagtgcttt atggataaac gtttgtcatt atcatcttat atatttattg

66361 gatcaatgtt atttggacta ttttttggag ctggaaattt aatttttccc gtacatatgg

66421 gacaggaagc aggcacaaat attttgccag ctacgctagg ttttcttgtt actggcatcg

66481 gcttaccttt tttaggggtt gttgcgattg gtgtttctaa aagtgacggg ctgtttgatt

66541 tagccagtcg tgttcatcca atctatggta tttttatgac tgttgccctt tatatgacga

66601 ttggaccatt ctttgctttg ccaagaacgg gaactgtttc gtatgaaatt ggaatcgcac

66661 cttatttacc aagccaatac caaacagtag gtttactgct atttacagtg attttctttg

66721 cgattgcgtt agctttctca atgaaaccga ctaaaatttt aatttgggtt gggaaaattt

66781 tgaatcctct ctttcttgtt tttctagcta ttttgattgt tactgctttt ctaaaaccaa

66841 tgggcgtaat ttctgaagca gcggtacatg gcaattatgt taccgagcca tttattacag

66901 gattcaccca agggtataat acaatggatg cattagcatc attggctttt gggattattg

66961 ttgttcaaac aatcaagggt ctaggtgttc gaaatccttc taatattgcg attgatacga

67021 ttaaatctgg aattgtcagt gtcattttaa tggcagttat ctacggtagt ttagcttata

67081 ttggagcgac tagtgtaggt caatttgaag tatctgaaaa tggtggaata gcgttggcac

67141 aaattgccca acattatttt ggttcgtttg gtagtgtatt gttggctatt attgtaacag

67201 tcgcctgttt aaaaactgcg attggactaa tcaccgcttg ttcagaaaca ttttgcgaaa

67261 tgtttcccaa ttcatttagc taccggactt atgtcatttt atttacactg ttagcttgtg

67321 gaattgcaaa tgtgggctta actaagatta tttcgttatc aatacctgtt ttaatgttct

67381 tatatccttt agcaattacc ttgattttct tggcattatt gtctccatta tttaaaaacc

67441 gtcaagttgt ttatgtaacg acaacgatat tcactatttt tgtcagtatt gctgatggct

67501 taaatgcttt gccagctggt attaaatcaa ttagttttat cgataatatt ctagcattct

67561 acagtcgcta tttgccttta tttgatatcg gtatgggctg ggttttccca gcaattcttg

67621 gtttaattat aggttggatt attagcttag taaaaaaaca agagctacgt ttttaattag

67681 taaatcaacg tatggagtct acatttgaga aaatggcctc catacgtttt tgattttatc

67741 cgttttaata taaaaagtcc ttgagcttta cgtaacgtat aggtgtaaga taagttttat

67801 caggaggtgt tggagttgga atatacagtt aaaaaattaa gtgaactagc tggaatcagt

67861 ggtcggacat tacgctttta tgatcaaatt gatttattaa aacccaatag aatcaattca

67921 tcaggttacc ggatatacgg tggcaaagaa gtcgatcgtt tgcagcaaat tcttttttat

67981 cgtaacttag ggttgccact tgaagaaatt aaagaagtat tggatgcacc aaacttcgat

68041 agtgaaacag ctttaaccgc gcattaccaa cacttactag aacaacaaca gcaactgaat

68101 aaattaattg caacggttga aaaaacgtta caagtgaaaa gaggaatgag tagtatgaca

68161 gataaagaaa aatttgaagg ctttaaaaaa gagttgattg agaaaaatga gacgcaatat

68221 ggtgatgaaa ttcgcgaaaa atatggcaat aaagctgtag aagaatcaaa tcaaaaaatg

68281 gcaagtatga gtaaagaaaa atacgaagct tttgaaaaat tgggaaatga attgttagaa

68341 gagcttgaag ttgcaatgaa cgaccagaat cctcagggta cagcaggaca aagagttgct

68401 gccttacaca aggaatggtt aagctttact tggggaagtt attctaaaga agcacatcgc

68461 ggtttggccg aaatgtatgt ttcagatcca cgatttacgg cttactatga tgaaaaggct

68521 ggagacggcg cggctagtta tttacgtgat gcaattgtag cctacacgaa ataaaaaaaa

68581 cagagtagaa aatctactct gttttttga ctagatatac ctaagtcctt gtgattactt

68641 ttaaattagt tagaattttt attgatgaaa gctacaaatg aatctgcaat agtttgtagg

68701 aagccaactg atgcaggtac tacttttcca tcttcgccaa ttgctttaga tgcatcaata

68761 taaagttcag gttgagccat aactggacta tcgccaaaca ctaatgattg acggatttgg

68821 tggttggcta atgcaccaga aatacctgct ggagaaactg aaccaacaag aactggttta

68881 cctggccaaa cactttcgcc ccaaggacga gaaccaacgt cgatagcatt tttcaatgat

68941 gctggcatac cgcggttata ttctggagtt ataaatatat atgcatcttg ctctttcatt

69001 tctttacgga aacgattata gctttcgaca ccatctattt ctaaatcttc attgtatact

69061 ggtaaatcac caatttcaat aaaagatact gtgtaatcag ctgggaataa ttcagctaat

69121 tgttttgcta caattttgtt taaagaatct ttacgtaaac ttcctactaa aatcccgatt

69181 ttttttgtca taattaatta cctccgaatg aattattttt gtttttatct tgtaaatgaa

69241 ttatacctcg tttacttact ttttacaagt gaaatgtttt aaaatttttt cctttgggaa

69301 tttcagactg atttagcacc tattttctta agttgagttt atgttagtgg aactgtcaaa

69361 acttaaagga gaacataaaa atggaattaa agacagatga cttaaaagaa attatttaag

69421 actaagcgtg gaatcataaa gctgttgaaa tatattttga ggaagaagga tataaagtgg

69481 gagatattag ttcatctgaa ggatttgaaa atagttcaat aaaaataatc attaaatgaa

69541 tggtttattt acagatgatg aaactaaaaa cttcttttct aaactattaa accatacaaa

69601 tatcactatt ttttgtattg gtagacgaat tataatgtca ttttagattg ataggcgagt

69661 aaagttggta gaaaggtcca taaaaagttg ttttatattt gttgaaattt tctagtttta

69721 aatataaact gggtaaatag aggtgatagt tttggtgaaa aaatttaagt atacacttat

69781 atttttatct ataggagtac tcatttcgtt attatttgta tttaagattg atgaaaaagg

69841 atttttaata tctattacaa atattttatc gttattgatt tttaaaacgg attctttgta

69901 taatctggtt tctagtcaat caatagcggt acagaatatt atttatggag tgaccaatgt

69961 actgtcctta atgatctttg gactattaat tgattttttt agagacgtaa aaaaaagata

70021 gaaaaaagtg gtgaaaatca attgtcgatt ttcaccactt ttttcttttt aataggagcg

70081 tcgctacaca agaagttcca ttcaaggata cattttataa cgcaataaga ataatatacc

70141 ctcctttatt gcgaaacaaa tcgcaagcgt atttcatcag ttttagttat tattatcaac

70201 tcatatatta attatattgt taagagtatt ttttataatt cagattgaac ctaatttaga

70261 acttatttta ttcaattaaa tattcggtca atgtataaaa aataaaaggc atgcaatgat

70321 atcaactatc aaacatacct ttttcgtatt tagatggttt aaattgctta ccagtgccct

70381 gtattgccaa atgtagaaat agatggtgga ttaagaatca aatcgtcgat attcataata

70441 ttatcctcct ttttaaataa aaaaacattt ctctttccta ataataaaac ataagctaca

70501 attaggcaag gagatataaa gaataagggg gaaaaaaatt gaaaagttta ggggaagcta

70561 ttaaagcagg tcgattgaaa aaaaacatga cgcaacaaga attagctgaa ggtatttgta

70621 cgcaagcaac aatcagtaat atagaaaaag ctggtaagat tccagcaatt accttacttt

70681 tagcgattgc agatcgtttg gatattgata ttgatgagct ttattattta atgggcgaaa

70741 atacaacgaa aaatggcaaa ataatgaaga aagttaaagt tttatgtagt caatcaaatc

70801 acaaagaagc ggcaacttta cttaaagaaa ttaatgaagc tgaattagaa acaataaatg

70861 aaaaaaagga atattactac tataaaggca ttaccagttt agtagcgttt cataatttct

70921 cagatgcact tttttacttt aacttgtcta atgatactca aggagaaggc tacatatcta

70981 tttatgatgt cttgggattg agcggagttt caattgctta ctcgatgaat gacgaagatg

71041 aaaaagcctt ggtatacaca gaaagaaccc tgaatattct tgatgagttt gtagcagaag

71101 gctatgaaaa aaacgataca aatgatatcg ttagaaccta ttttaactct gcaaaaatat

71161 attccaaaat gaaaaactac gaaaaagctg tgagtttaag ttcaatgggg attgctttgc

71221 agcaattgaa tgatagtatg aatgggctgg aatatttaat gtatgagaag gcttataatt

71281 tacaacaatt agaacaagtt actgaagctg aaaagtttta tttctatgca gcggcaatgg

71341 ccatgatgaa taaaaataat gaagtgatag aaacagttaa aagtgatatg aaactttaca

71401 atgtctccca ctttatgtac tagtcgtact tgtataattg tatagaaaca agtaaaagga

71461 ttaacactgt cgctacatat gtttttcctt ttattatttt tattagttat atgtatacat

71521 ataactatat tgtagcgtat tagagatgag gtggatttca tgcaaaaagt aaaagttaga

71581 cgtataggga attctcttgg agtgatttta ccaaaggaca gaggggttaa agttggggaa

71641 tgtttaacat accaaaaaaa tggtagtatt attcaattag atttatcaga agctcaaaaa

71701 tcacaggata gaaaactaat tgaagaaagc tttagagacc ttgaggaagg taatacgtta

71761 actgaaaaag aaatgataaa agaatttgga aaatatggat ggggtaattg ataaatgaca

71821 gattatcccg ttgaatatac aaaaaaattc caaaacagtt ttagagaaga tatcaaagaa

71881 tggtctgaga agtatttttt tctgactaga aaatcacaaa atttgttcgt tcaatttata

71941 aatctatcga attaacaaaa atatttcctg aaatgcatga ataaatttca agggtttatg

72001 gttttgatag acctaagtat agaatcttga ttggaaaaaa ctatgctatt ttttatcgga

72061 ttgataaagt gcaaaataaa attttagttg gaaatcttta taatcagaaa caaatgcagc

72121 tagattttta aatatacaaa ttagataact tatcgagtac caatggcgag cttgctgttg

72181 gtactttcaa atttcataaa aagtttggtt ttcttgaaag ttggataaga cacagttata

72241 aaagtctttg aataaacctg tttgccagta tattttctaa cccctagttt tacttctaat

72301 aaaatcaaac tatttttcaa gagcaattag ttatattcgt gataaatagt gacaatttaa

72361 aataaccagc ctgtcataat ttctattagc aaagcctagc taagtataca aacaaaatga

72421 gcttcactac ataaaaaaac agagtagaaa atctactctg taatttagaa tcactacaaa

72481 aacgcgactg tttttcaatt ttttttgcag tttattcaaa ttgtgcagca tacaaacgat

72541 gatagtaact gtctattttt agcaattcgt catgagttcc agtttcaaca atcatacctt

72601 tatccatcac taaaattaaa tctgcttcac gaatcgtcga caagcgatgt gcaatgacaa

72661 aactggttcg ccccttcatt attcttagaa aagcagtttg aatctgttgc tcagttaagg

72721 tatcaacgga actggttgct tcatctaaaa ttaacattgg cggatcgcta agcatggttc

72781 tagcaatggt taaaagttga cgctgaccag aagaaataga aactccacca ctgccaataa

72841 ttgtgtcata cccttgaggc atttttataa taaaagcgtg agctaaagca gcttttgctg

72901 cctggataat ttcctcttca ctagctgtcg gatgaccata agcaatattt tctttaatgg

72961 atcctgaaaa taaccaagta tcttgcagca ccataccaaa ggaacgtctt aaactgtctc

73021 gtgtatattt tttgatatct gtaccatcaa ttttaatgct cccttgatct aactcataga

73081 aacgcatcaa taagttaacc aatgtcgatt ttcctgcacc agtctggcca acaattgcga

73141 ttgtttcacc tgcagcaaca cttagattta agtcttggat tagtgggcga tcttgttggt

73201 aggagaaata aacatggtcg aatgccacat ttccgttaat tgttgttaga gttgtggcat

73261 ttgtagcatc tgggatttct ggaacagcat caagaacttc aaaaacacgc cttaatcctg

73321 cagcagctgt ttgaatctgt gttgtaatcc cagataactc aatgaaaggt ttagaaaatt

73381 gagaagagta aataataaaa cttgagacga caccgaccgt gatactacca tttcctgtta

73441 caactaaaat accaccgatt gttccgatta aaagatacgc aaggtgatcg acaaaacgag

73501 cagaaggatt ggtgatggac gaagtgaatt gtgctttacg tccccaaatg tataagcggt

73561 tattaatttg ttcgaaattt tcttgaacgt agtcttcttg attaaaagct ttgacgactt

73621 tttgattggt aaccatttca gaaacaaatc cagaaatttc accgacaatt cgttgttgct

73681 gggtgaagtt tttttgtgaa agtcgagcta caatccaagc aatcagaaaa attaaaggcg

73741 ttgtcactaa aacaattaac gttaaaagtg gactaagata aagcataaag cctagtgaag

73801 caaagaccac gatcacccca gaaaataaat tgtttaaagt tagactaacc gcatctgaaa

73861 tactatctaa atcatttgta aagcgactga taattgtccc atgtggggta ttatcaaaaa

73921 agcttaaggg aagttcgttt agtttatcaa aggttttaat acgcaagtct ttaatagcat

73981 tataggaaac ttgattagaa aaataattga tcatccattg ggtaattatt gctaacaagt

74041 aaacggcagc caataaccaa agaacatgga aaagcttgct aaactcaact tgaccttttc

74101 caacaatcgc gtcgattcct tttcctgtgt agtaagtcgc agcaactgta gcgctacctc

74161 cgattaagcc acaaaataaa gcagcgaaaa taggcatttt ataatgagtc ataaaaggta

74221 aaagtcgttt taaatcactt ggattgattt ttttattttg catattaatg aacctcctta

74281 acgtcagttt cattctcact ttgagagtca taaatttgac gataaatatc tgattcagct

74341 aataattgct tatgagtacc actattagcc agctttccat cgtttagtac aaggatttga

74401 tcggctgttt gaatcgaact aatacgttgc gaaacgatga caacagtagt atttgttaag

74461 cggctagcta gagctcttct caaatttaaa tcagtttggt agtccaaggc acttaaggaa

74521 tcatctaaaa ttagaatatc cggttggcta actaatgctc gggcaatcgt tagacgttgt

74581 cgttgtccac cagaaaagtt tttaccacct tcaaaaacag gagagtttaa gccgcctttt

74641 aagcttgcaa caaaatcagc actttgagca atttctaaag ccatttgtaa ttctgcttct

74701 gtcgcatccg ctttacccca tcttaagttt tcagcaattg ttccagtaaa taaactacta

74761 ttttgtggta cataaccgat tgagcggcgc aaactttgaa gatcaaactc attaacattg

74821 gttccgttaa tcagaagagt tccagcagta acatcataga atcgtggaat taaattaatt

74881 agagtacttt tcccacttcc tgtaggacca gttacgccta aaatttgtcc tttcgataaa

74941 ttaaattcaa tattttctaa ggcatttgcg ctagcagcag catatttaaa ggatacattc

75001 tcaaattgaa tagcagtctg attttcgata gatgtcagat aggttggaga ttcattttca

75061 acaacactaa catcagtatc taagacttca ttgattcgtg cagcagatgc cgctgattta

75121 gtaaatataa caactagatt ggccaaaata ataagtgcta atagcatttg cataatatag

75181 ttgattaaag ctaaaacttc accttgcatc aaactacctg tattaacttg gaaacctcca

75241 acataaagga ttatcaaaat acccgcattc atgattaacg aggtagcagg ggtcattaaa

75301 gccgaaagat ttgctacacg gatataagat tttgccaact catcactgac atcttcagct

75361 ctttgctttt cttttttgct acgagcaaag gcacgaatca ctcggacacc gcttagattc

75421 tcagaaacaa cttgtgttag acgatcgacc ttcgtttgaa caattttata aagaggaatg

75481 gtttttgtca taataaagta aagaattaga caaaatagag gcaatagaac ggcaaaaaca

75541 agagctaatt taggactaac ataaatcgcc ataatcagcg aaccaatact taaaaatgga

75601 gcacgaatca ctaaacgaat taacatagca agagctagct gcatttggtt aatatcatta

75661 gttgctcgcg taatcaaagt cgctgtacca aattggtcaa tttccttgtg agaaaatgta

75721 ttgattttgc gcattaaggc attccgaagt tcagtcccaa aaccctgtga agcaatcgaa

75781 gcggaatatt gacaaataaa aacggaaatt aaaccaataa ccgacattgc cagcatcagg

75841 ccccccattt tataaatata agccgtatct cctttgttaa tcccattatc aattaatttg

75901 gccattaaca gaggtaaaaa taactcgaaa acggcttcga caaatttaaa gaaaggacca

75961 attaccattt ggagtcggta ctttttagca taacgtaata aagaaatcaa atgaatactc

76021 ctttctatac tcaagggatt taatttaatt gtatccctaa actttttatt caataagtat

76081 aacataaggg gacaaattag ttgatactag attctcatta gaatataagt taaaaagagt

76141 catccttgat aaaaaatgaa caagcgcgta tgatgaaaat aaagcaacac gaaaggagca

76201 aggggatgga aattaccttt gaaaaaatag ttcttttatt ctttatttat gcctttatag

76261 gttggttatg ggaaacagtt tattgttcaa ttaaagccaa aaagtttgtt tatcggggtt

76321 ttttaattgg tccatattgt ccgatttatg gatttggggt attagcagtt ttatctttaa

76381 ttagtcctta tcaacaaaat attatccttt tatatgtatt ttcagcctta gtagttaccc

76441 ttttggaata tgtaactagt tatggtttag agaaattatt tcataccaca tggtgggact

76501 atcatgacgt tccattgaat ttgaatggtc gcattgcgtt accaatttct ttattttggg

76561 gagttgggtg tgtttttatt gtgaaagttg tgcaaccgat tgtcagtgag tgggttcaat

76621 ttttgataaa tcaatttggg attttcttac ctgttgcaat tgttgttttg atgacagttg

76681 atttgatcta ctctgtattt actttagcag ctttccaaaa agagttggca aaactaagtg

76741 ttgaaattga agaacgaaaa aataatttag tagaaaaaat aacggaaaca agccaaacac

76801 ttatggaaga attaaagcaa agtaccgaat taaaaaaagc ttttgccaaa ctaaatttta

76861 atcaaaagag aatgctgcga gcttattcta aattaaatat gccaacaatt aagaatttca

76921 aagaattaaa agagattcag gaaagattta gaaaaaaagg ctaaaaataa gtgtatttgg

76981 ggataaaata ctctatttag aagcgagtgt tacgatttcg tttctttaac gttacttttt

77041 ggttactaaa aatgatttta caagtcattt tgacataaaa ctttatggaa ttccttctgt

77101 tttctataag ataaaaaaga atggagcgaa tccattcttt tttatttagg caaagcctta

77161 gtactttccg atagcggtga ttaaatcaga gcttgtgcta tggatagcgc cgttcaactt

77221 aactaaaccg actgtataca agtttacata ggaaaattgc gattcagcca cttcttgcaa

77281 tgctgctagt ttgtgtgggt ttgttgctcc ttgttgacga gagtctgtat agagaccgag

77341 aataggaata ccagcttggt aagccactcc gatttcagtt gcgacaccaa catcaattgt

77401 cgcaccatct aaaacagcaa tcattaattg actgtttaaa agtgccttcg tatcatattg

77461 ggctatcgtt ttagagtctg cataggcatt tttatcatta atttccattt gttcttgggg

77521 aacataaagc tctaattgag ggtacgccgc tctaatttgt tggacaagtt gttggttgta

77581 gtgtaactcc atttcagaaa ataacgggct agcaaaataa ctatttttga tcattttttc

77641 tcctcctaat atcttttatt acactcttag tataccttta aaatgcgatt gtaacaaaaa

77701 gtaatctttg tgaaatatac caaggctgat tattcgttat aatgtgggga gcaattaagt

77761 aagatcgtat tactaaatga agggtgttgt cgaaataagt gaaattacga aaattggttg

77821 taagcgtggt tcttacattt ctaagcgtaa atctaatagc aaatgttgga tacgcagcgt

77881 cacttgacga aattaataaa gagcaacaag caaaacaagc aaaaatggca actgtagata

77941 gtgaaattag ccaaacttta gtttcattaa atgataaaaa taaggaaatt gaagctttaa

78001 accaacaagt ttctgaaaaa caagaaagct tgaaggatac agcagaaaaa attaaagcaa

78061 aacaagcaaa cgttgatgaa cgtgttgctc aagctaaaaa aagattacaa tcgcttcaaa

78121 catctgaagc aagcaaaagt atggtattaa tgattcttga atctgaaagt ttaactgact

78181 ttttaaaccg tgcttatgtt atcaatcgat tgcaatctgc tgataacgat aatttagaag

78241 aagctaaaaa agatcaagaa gaacttacaa atttagaagc taaattacgt gaagatgcag

78301 caactttagc tgaacaaaaa gagcaagtca ataaagatac aactgagtta aacacaaaaa

78361 tggctagctt acaaaaaaca atggatgaaa ataaagatgc tttaaatgct ttagatgaac

78421 aaaaacgtac agaacaagct cgtatagatg atgaagcagc gaagaaaaaa caagctgaag

78481 agttagctaa aaaagctgaa gctgctaaag tttcaactac ggcagttgcg caaaatacag

78541 ctcaaaatga tgctgttgct ccagcagctc caagcaaccc tactgatcaa tcaggtgcag

78601 gtggcgctgg taaaacaata attgttgaat caactgctta ttcatatgct gaaaatggtt

78661 caagcttctt tacagctaat ggaacagatt tacgagtgaa tccaatggtt attgctgttg

78721 accctcgtgt tattccaatt ggttcacgtg ttgaggtttc tggttatggt gttgcgattg

78781 cagctgatac aggtggtgca attaaaggaa ataaaattga tgtccatttc tcttctgtag

78841 cagaatgttt acaatgggga cgtcgtacag ttacaattaa aatcttagat taaaaatatc

78901 taagatagaa ataggtgcaa acgatggcag ttgatattga tttttatcat gaaaaagatg

78961 agcaggcatt tctagataaa tgggaagctg aaaaaggctc aattgaagat attgatttat

79021 tttactctga gttagctgag gtagttgaag aagcctatca aaatggaaca gtaaaacttg

79081 gtaagaaatt tgtttatcaa gatgttgtgg ttggctatgt tgattacaat acattcaata

79141 atctatttct attcagtcta gctaaacaat aaattcttaa ctgactagta tgaaaccggt

79201 gatgaaccgg atttgtacta gtcagttttt tttgtggata tctaggtact atttttgctc

79261 aaaggttcta tatggatgga tattaataaa aaagttgctt tcaatagttt ttaattagcc

79321 atccctctag tttagtgtac caaggttatg acatggataa taaaactata aggtatggaa

79381 actgaatttt atacttctgg tttaagaatt gtttaaagaa ttttatcaaa gtatctaaag

79441 ataatcttgc ctactttatg cccttcaagt ttgaatatct aagggaagtg tgtcagaata

79501 aggccagagg agggaagctg aataagattt tacatgtgta aaaatagttt attggaggta

79561 gaaaagatgc gatctgttaa gaagaagacc atgggttttg tgataagcca taaagagaac

79621 gaacatcgcc gtgctttatt accaaaagat atcaaaatta ttcaaaatcc tactcaatta

79681 ttttttgaaa agaactatgg aaacgtttta aatattgccg atgaggaata tttggcttta

79741 ggttgtcaga ttgtttcccg tgaagtggcg ttgcagcaag atattatttg tgaacctaag

79801 attggagatg ctgaattttt acagaatttg ttaccaggac aactgatatt tggttgggtt

79861 catgcagtcc aaaatcgaga cattacagat cttttaattg aacaaaaaag ttctgcttac

79921 gcatgggaaa aaatgtatta tatgaatcgt cattcttatt ggcgcaataa tgaattagca

79981 ggagaaagtg gaattgttca tgcttattta cttaatgggc ggatgcccta tgacacaaaa

80041 gtggctattt taggtagagg aagtacagct caaggagcca atcgtatttt aactaaattg

80101 ggagcagatg tctgtattta caatcgaaac caagaagcgc tatttaaaaa agaattaggt

80161 gattatgatg tcattgttaa cgcaattctt tgggatacaa ctcgacatga ccatatcatt

80221 tatgaaaaag atttaaaacg aatgaaaaaa ggtgcgatga ttattgatat cagttgtgat

80281 gaacatggcg ccattgaaac aacatcacca actaccttcg aagaccctac ctactatgtt

80341 gacggtattt tacattatgc tgttgatcat accccaagta ttttttatca gacagctact

80401 gctgcaattt ctgaagaaac tagtaagtat atcgatgatt taattgaagg aaatccgaat

80461 cctattttaa gagatgcatt aattattgaa gatggaaaaa taatggatca agatatcaac

80521 aagttccaag accgttaaat gtaaataaac gaaaaaaggg ctttcccata aagaaagcct

80581 tttttcgttt tatgcattta tctaagctaa atcactacca tttgtatcaa tcactttttt

80641 ataccagtta aatgattttt tctcataacg gttgccagtt ccttggccat tgtcatctaa

80701 atcaacatag ataaaaccat agcgtttaga catctcacca gttgaagcac taaccaaatc

80761 gatacagccc caaggagtat accccattaa atccacacca tcttcgattg cttcgcccat

80821 tgcgctaata tgactacgta aataatcgat tctataatca tctgcaacat agtgattttc

80881 gtcaggagta tcatatgcac ctaaaccatt ttcaacaata aataatggtt tttgataacg

80941 tccatataat tcatttaaag ctaaacgcaa gcccagtggg tcaatttgcc agccccactc

81001 tgatgcttct aagaaaggat tgctaacacc agacaataga ttgccagaag actgcgctaa

81061 tttgtcagca tcagtcgtca caacagttga catataataa ctaaagccaa tgtaatcaac

81121 aggatgtgct tttaaaattt ctaaatcgcc aggttgaata tctaaagtaa cattattttc

81181 ttcccaaata cgttttgcgt aatgaggata ttccccttta gcttgaacat ctgcacaata

81241 gaagttgaag cgttgattat attcaagagc ctctaaatga ttgactggat ttgaatcgta

81301 cgcataagtt gtcgcataaa taatcataca acccacctgt aaattaggat ctaactcatg

81361 agcaatttta gttgctttac tacttgccac aaactgatga tgcaacccttggtaaatcgt

81421 ttgtttatca gcagcgccag atttcattgt taaaccttga ctcataactg gaaagtgaag

81481 tgcactatta atctcattaa aagtcatcca gtatttaacc tttgcatggt aacgctctaa

81541 aaccacttga gcatagcgtt cataaaaatc aatcaatttg cgattgcccc aaccaccata

81601 ttctttagct aagtgtaaag gcatttcata atgagaaata gttacaacag gctcaatgcc

81661 atatttcaaa cattcatcaa aaattttatc gtagaaagct aaaccggctt cattaggtgt

81721 agtttcatcc cctttaggaa aaatacgaga ccaagcaatt gataaacgat agactttaaa

81781 gcccatttta gcaaataaag caatatcctc ttggtaacgg tgataatggt caatcccgtt

81841 gtgatttgga taaacatatt tagtttcatc aatttcaaag tcaaaatcag gttgtgcaac

81901 aattttaaag cgctctttgc caccaggtag cgcatccgcg acagaaagac ctctgccatc

81961 ttctaagtag ccaccctcta attgattcgc tgcagttgct cctccccaaa ggaagttttt

82021 aggaaaaatt gatttcgttg ttgtcataaa aaaagcaccc caattcattt aatactgaaa

82081 gtataatttg tgagatgcat tccatgtcaa gccttagtca aaatttaact ggaaaaggct

82141 atttttagtg ctacactaga aataatatga agtgttaaaa gagaggggga agtttgatgg

82201 aagttgaaaa aataatcaag aaaattcgcc gagaattaat agctggcaac cgtctccttg

82261 gtgttgcaac tggatctggt atgagcgcta gtcaggtgca aaaaggtgga gcagatttta

82321 ttttagcttt gaactctggg cgttttaggg tacgaggtag aagttcatta gccggttttt

82381 taccatttga aaacagcaat caagaggttt taaattttgc aacaaaagag attttacctt

82441 tgttgcagca tacaccgatt atttttggtt taaatgcaac agatcccacg attgatttag

82501 ttaatttcat tggtacgata aaggcaaacg gtttttccgg ggtaaataac tatccaactg

82561 tcggtttgtt agatggccag tttagtgagg ctttggaaga aagcggctgt tcgtatcaat

82621 tagaagttga ggcaattaag ttagcccatg atcagaagtt atttacaatt gcttttgtct

82681 ttaaccaaga acaagcccgt caaatgacag aagttggtgc agatgttatt tgctttcact

82741 gtggactaac aagagggggc aaactaggag ctaaaaaagt actaagctta gccaatgtaa

82801 ttgcctctgc taatgaaata gtcaaagtga gtcgagaaat acgcccaaat gttattacta

82861 tgatttatgg tggaccagtg agcagtttaa ttgatattcg ttatatttat aaccagattc

82921 cggggttaaa tggttatatt ggtggttcca cctttgatcg aataacgcca gaagaattaa

82981 ttattcaaaa agcgaaagaa tttaaaagcc caagagatag ccaagatact agtttgatgt

83041 ctcaaatgtt agatggtatc gataaacatt atgattatgt agtttttgta gaaaaatata

83101 tagaacaaaa ttaccatgag ggtatttatt tagaggattt agcacaagta gctcatgtga

83161 caagtagcta tcttagtaca ttatttaaaa agaaaacggg agcaagtttt actgaatatt

83221 taattaaatt tcgcttaaat aaagcgattg aactgatggc taaaccacaa agactttctt

83281 tcaaagaaat tgccttctta gttggttatc cggattatgc tcagtttaat aaaatattta

83341 aaaagtatat gggaacttca ccttctgaat atagcaatgc tagaacaaag gagtgaagat

83401 aaacactagt taaactgttt tgaaaacgtt ttattccttg gctgttactc ttataattta

83461 aagtgtaatc attaggaaag gagctcgttt ataatgaaaa aaactgttgc attagttgga

83521 acactagata caaaagggga agaatattta tacgtaaaaa aattgcttca acaaaaaggt

83581 ttggaagttc taacagtcca tacaggtgtc tttaaagcac agtttaaacc agatattgat

83641 aatgcggtga tagcagaagc tgcagggatt acaattgctg aattagttga aagaaaagat

83701 cgagcatttg cgactgaaaa attagcgcaa ggtctaaaag aagtacttcc agaattatac

83761 aagaaagata aatttcaagg tgttttatca tttggtggct ctggaggaac ctcattagtg

83821 acgccagcga tgcaaaaatt acctattggt gtaccaaaga ttatggtctc tacgatggca

83881 gcaggagatg tgaccccata tgttgggact agtgatatta ttatgattcc atcaattgtg

83941 gatgttgctg gtttaaattc aatttcgacg aagttattta caaatgctgt ttacgcgatt

84001 agtggtatgt taacgactaa ttttaaagaa gaagtaaggc ataaaccctt aattgcagct

84061 agtatgtttg gcgtaacgac tccagcagta acagtagcta gagaattttt agaacaatca

84121 ggttatgaag ttttagtgtt ccatacgaca ggtacaggtg ggaaaataat ggaatcgttg

84181 gttcaggatg gctttattga aggcgtttta gatttgacga ttactgagtg ggcagatgaa

84241 ttatttggtg gggtattaag tgcgggtcca actcgtttag aagcggcagc tttaacaggt

84301 actcctcaag tagtttcagt tggtgcttta gatatggtca attttggacc tatcgaaact

84361 gtgccagaaa aatataaaca acgcaatttg tatcaacaca atccgacaat tacattgatg

84421 cgcacgacaa aagcagaaaa tcagcaatta ggtgaaaaaa tagctgagaa attaaattta

84481 gctactggaa aaacggtact tatattgcct ttaaaaggta tctctgctat tgatgtagag

84541 ggtcagcctt tttacggtcc aatagaggat caacaactat ttaaatcatt aaaagagaat

84601 ctaagaaatc cactagtcaa gattatccaa cgggatgaag caattaacga ttctaccttt

84661 gcgcaatatg cagcagaaca attaataaac ctaatcgaaa ataaataaaa ggagttgtga

84721 agaaaaatga acaagcaaac aagaaacgaa atcatgaata gtttttataa caaaagaaaa

84781 aaaggtgagt atttagtcgg tgttggtgca ggcacaggaa ttacagccaa gagtagtgaa

84841 gctggtggtg cagatatgtt aattatctat aattctggtc gttatcggat ggcagggcgc

84901 ggttctcttg ccggcttact ttcctatggt gatgcgaatc agattgtagt ggaaatgggg

84961 caagaagtct tacctgttgt ggagcacaca ccagttttag caggtgtgtg tggaacggac

85021 ccattccgtg taatgagtgt ttatttacag caattacaag cacaaggctt taacggagtg

85081 caaaatttcc ccactgttgg attaattgat ggtaactttc gccagaattt agaagaaaca

85141 aatatgggat acgaactaga agtcgaaatg attcgtgaag ctcatcaatt aaatttactc

85201 acaacgccct atgtatttga tgaaaagcaa gcacaggata tggctgaggc aggtgctgat

85261 attttagtag cacatatggg attaactact aaagggagca ttggggctaa aacagcttta

85321 acattagatg attgtgtgga gcgaatcgaa aaaattatcg ctgctggtaa agctgtaaat

85381 cctgaaattt tagttatttg tcatggcgga ccaattgctg aaccggaaga tgcccagtat

85441 gtattaaata aaaccaaaga tttagatgga tttttggag catcaagtat cgaacgtttc

85501 gcggccgaaa aaggaattcg tgagcaaact gagaaattta aagcaattaa gtaggctagc

85561 tcttatgagc caactcctcg gaaaaaagat gaatggtaaa aatgacaaaa agcgtcattc

85621 ctcccattcc ctatttttct accagagcta accggctcaa caagcttttt attaggctag

85681 ctcttatgag ccaactcctc ggaaaaaggt gaatggtaaa aatgacaaaa agcgtcattc

85741 cttccattcc ctatttttct actagagcta accgattcaa caagttttta aataatggag

85801 gtaatggata atgaagtatt ttgttaagat tgaagatgtt gaaacacaag gtttttcatg

85861 gggaacatta caatggttaa atgaacctca agtaacaggt agcaaggtaa tggtaacggg

85921 ggtagtgaat ctatttccag gtgaagggca tcaacgtcac aatcatgagg gctgcgaaga

85981 agtgctttat gttctaaaag gaacaggagt gcaaagttta gagttagata caggtaaagt

86041 agaaaaagaa attgaagcag gggagttaat tcgaattcca gcgggtgttt atcattccac

86101 tattaataat ggcaaagatc cattagaaat tctagctatc tatcactatt caggtcctga

86161 aatagccatg aaaaatgatc cggattgcca aattactgca cctcaaaaaa attaattact

86221 aaaagccttc acctgccttt tctaagtagg tgaaggcttt ttttgttcgt ggtataataa

86281 gtaaagaata gctaaaataa gtataggtta acaaggggga gactgtagtg tcaaatatta

86341 atgagattgc gaaattggca ggtgtttccc gagcaacagt ttcaagagtt attaatagca

86401 aaggctatgt taaagaagaa acacgacaaa aagttcaaaa agtcgtggat gatcttgatt

86461 atacaccaaa tcaaaatgcg atttacttga aaaatggtga aacaaaaaca attggcctcg

86521 tgtcgccaga cttttcagat atactctcac attttttaag tagttttgca acaattgcta

86581 aaaaatatgg ctatcaggtc acacttttat taacggatta tcatcaagaa agtgagattg

86641 aagcttttca aaaactaaaa caaaaacaaa ttgacggaat ctttcaaatt gtacgttcaa

86701 atgaatggga agtcattgaa aaatatagca agtatggacc gattgtaact tggcaaagag

86761 ttacgtcacc caaaatagaa tccgtcttta tggatcatta tgaaggcttt aaactaggtt

86821 tggatcatct ttatgaaaaa ggtcaccgtg aaatcgtccc ttttttggc attgaaaaaa

86881 gcttaaatac tgttgctaga tataaggctt ggcaagattt ttgtcaagaa aaaggggtag

86941 cgttacaagc agaaaaaagt cgctataatt tgcatacaag taccgaggga cgagaattag

87001 ctcgatggtg ggaagctgaa aaagaaaaac caacagctct acttttcccg acagataatg

87061 ttgctgttgg ttttttagca gaggcacgtg aacgaggtat ttcagttcct aaagatgttg

87121 cagttgttgg atttgataac tcttcgattg cagaactatt taatttaacg acaattgatt

87181 atccgatgca cttacaagct gaaaatgcct tttttaagtt atataatcaa ttaaagcaca

87241 aaaatttgcc tttacatcct ttatcattta aactaatcac tcgcggaaca acttaaattg

87301 acaagctctt ttgaatgaat ttttcggaaa aaaagatgaa aagagggaat cctgtgtcaa

87361 aggaacaatt acgcaataaa ttaaccctat taccagaact tcctggttgt tatattatga

87421 agaataaaac cgatgatatt atttatgttg ggaaagccaa aaatttaaaa aaccgagtga

87481 cttcttattt tcgtgggact aaagacggaa aaacattttt attagtagaa gaaatcgttg

87541 attttgaaac cattattacg tcaaccgata aggaagcctt attgttagaa attaccttaa

87601 ttcaaaaaca tcatcctaaa tacaatatta aactaaaaga agggtcaagc tatccatata

87661 ttaaaataac tggagaccga gacccacgcc ttatcattac atctgatgtt gaaaaggatg

87721 gtgggcatta ttttggacct tacccgaatg tttatgcagc gacagaaacc atgcatttta

87781 ttgaaaaagt ttatccatta aaacggtgtg ttggtagaca aagacgagcg tgtctttatt

87841 atcatatggg acaatgtcta ggtccttgcg atcatgaagt acctgttgaa gtttatgagg

87901 cacaaattaa acgtattaaa gcttttttaa atggtgatac agctaaagta aaagctgaat

87961 taaaagaaaa aatgctgaaa gctgcagaag aacaagttta tgagcgagca gcagagtacc

88021 gtgatcaatt acgctacatt gaagcaactg tggaaaaaca aaaaaattatt tcaacggact

88081 acacaactcg agatattttt agcttttatg tagataaagg ctggatttcc attcaagtct

88141 ttttaattcg ccaagcgact ttaattaaac gtgaagccgc attattccct tgtattcaaa

88201 gtagtggtga agaagattta gcaacattta tcttacaatt ttataatgaa aaaaatcatt

88261 tgctgccaaa agaaatccta gttccagaag gtgttgaaaa agagctaatc agtgatattt

88321 tatctgttgc cgttcgagta ccagttcgag gacaaaagaa aaatttatta gatttagcca

88381 cacaaaatag ccagatttct ttaaatgagc gttttaattt aattgaaatg gatgagcgta

88441 aaacgattgg cgctgctgaa gaattaagcg aagcactagg tttgccttca ctaaaacgga

88501 tcgaagcttt tgatcattct aatattcaag gaacaagtcc tgtatcggcg atggttgtat

88561 tcgaaaatgg acgtccttca aaaaagaatt atcgtaaata taaaattaag acagtggtgg

88621 ggagtaatga agccgcaaca actgaagaag ttattcgaag acggtataca cgtttattaa

88681 aagaaaaaca agaattacca ggcttaattt taatggatgg gggaattgta caggttcgcg

88741 ctgctttaaa tgttctagag aatgaactag gattaactat ccctgtagct ggaatggtta

88801 aggatgacaa gcatcgaacg tctaatttat tatttggtga agaattaact gttgtcccaa

88861 ttgattcaaa aagtccagca ttttatttag ttcaaagaat tcaggatgaa gtccaccgtt

88921 ttgctattac ctttcataga caagtaagaa gcaagaatag tttgtcttct ttactggatc

88981 aaattccagg agtcggccct aaaacacgaa ccaaattatt aaaacatttt ggttcattaa

89041 aaaaattaaa agaggctgat ctaaaagata ttcaagcgct aggcgtttct aaaaatgtcg

89101 cattattaat aaaaataaga ctatagaaga accatttgct atttagtata gcagatggtt

89161 ttttgattaa aatttaagag aaataaaaag aacctattgt ctttatggta ggggaagctt

89221 atgataaaga tacatagttt atttattttt atatagggag tgtgtgtaca catgaaaaaa

89281 ttagtagtat tttgtataat agtgccagtt tttttactta gtggctgtag tgaaaatcga

89341 gaagtggagt acgagaacga gatcaaggaa ttaaaaacag aattgaaaga gttgaaaaaa

89401 gaaaatgctg atttaatggc aaccttgcgt gaactggctt tagcgaatac agaaacggct

89461 caaaaagaaa atgaagaaag ccaagttaat aatcaagaag atgttcctaa gcaagaaaca

89521 acagaagaag tagagccgaa tatagatgat aaaaagcgtg atttgaaaga agagattatt

89581 gaattgaaaa aacaattagg tggcgactta acaagagaac aacgtatgga aattaaaaag

89641 aaaattaaag aacttgcaga acaattttaa gtaacaaggt gtgcataaag ctgttctgct

89701 ttgtataaac tggaggcaag agccagcaat cggcagtttc gattgaagga tcgtgctgaa

89761 gtttactaga agcaagcttt aggaaccaaa ttataacaag gcgtgaataa agctgttcta

89821 attcatatct taactatgta aaaaagctga gaacaaaagt tctcagcttt ttagcctact

89881 ttggaagagc tgactagtgc tctacaactc tattttacac aattacttaa ttattttcaa

89941 tcactattta tatagcgaac tatttttaaa ttccctaaaa atagttattt tttgaaaatt

90001 agaacctatt tctattaaag ggtagggcta aaggaacaat tagatacaga aatacgccct

90061 aatagtacta agggcgtatt tttgttaaat taaaacttta tctaagaaat tttttgtgcg

90121 ttcattttta ggttgattaa agacttcttc aggtttgcct tcttcaacaa ttaagccacc

90181 atccataaag acaacacgat ctgcaacttc ttttgcaaag cccatttcat gagtcacaac

90241 aatcatggtc atgccttctt ttgctaattg ttgcataaca gctaagacat cgcccaccat

90301 ctcaggatct aaggcacttg taggctcgtc aaacagcata atgtcaggag acatagctaa

90361 agctctagca attgcgacac gctgtttttg cccacctgat aaagaacttg gataagcatc

90421 ggctttttct cttagtccaa cagtttctaa taagtcaaaa gccttttgac gggcttcttc

90481 tttagttaat ttttttaatt cgattggtgc taacgtaata ttttctaaaa tcgttaaatg

90541 tggaaataaa ttaaaatgtt gaaaaaccat tccgatattt tcacgtactt tattaatatc

90601 taggctcgta tcagttaaat catagccatc aataataatt gttccaccat taatttcttc

90661 taaatggttc atacaacgta aaaaggtact tttccctgaa ccagaaggtc cgattaaaca

90721 aacgacttct ccttcttgaa cttctaagtt taaacctttt aaaacttcta atgaaccata

90781 acttttcttt aactcttgaa catttaattt aaccattgtt aactctcctt tcaatcctat

90841 tagagattcg tgttaaggca gtgataacaa caatgtaaat aaaggcaatc acaaaccaca

90901 tatttgaatc gtacgttcta gcaacaacaa ttttaccaga ttgagttaat tcaactagcc

90961 caattacaga aagaatcgtt gtatctttta gggtaatgac aaattgatta ataaatgaag

91021 gaatcataat tttgattgct tgcggtaaaa tgattttacg catggcttta ctataaggta

91081 aacctaaact acgagcggct tcaagttgac ctttgtcaac ggcgttaatt cctccacgaa

91141 ctaactcagc aatataagct ccagcattta aacttaaaac aattattgca gcaatagttg

91201 ccaccatttt aattcctagc agttgtggaa tcgtaaagaa tacgaagaaa gctaaaacca

91261 tcattgggat accacgcatg atatcgacat aaaccattga gattcctctt aaaatcttac

91321 taggagcgac actcattagg ccaaataaaa tacctaagac tgatgcaatt gctattgaaa

91381 ctagagtaac aaataaagtt gtccctagac cacttaaaag ttgtttgtaa ttttctttaa

91441 ctaaagcaaa aatactttgt ttttctgttt tttcttcacc tagatatttt tcagtaatct

91501 ctttatattt tccactattt tttaaattgg ttaaaccatt gttaaattta gttagcaatt

91561 ccggattttg tcccttatta acagcaaaac catattgccc gcctttttct ttttctccaa

91621 ctaattttag tggcgcattt tgtgtaatcg cgtaggccat tactggataa tcttcaaagg

91681 ctgcatcagc acttccagct ttaacagctt cgtacatatt agcagagtca tcaaaagttg

91741 aaactttaaa accgtattta tctttgattg agttagcaaa agtagctcct tcagttcctg

91801 ttttgattgc aactgttttc ccttttaaat cctcataaga tttaattgta tcattggtat

91861 cagctacagc cataacgaca ccactttcaa aataaggatc agaaaaatca aatgtttgtt

91921 ttcgttcatc tgtaatgctc attccagcaa tgacagcgtc gacctgatct gcttgtagag

91981 cttgaactgc ggcattgaag cccaagggtt taatctcaac ttcaaaacct tgatcttcag

92041 caatagcgtt aagtaaatcc atatcaattc caacgaaatt gttatttgca tcttggaatt

92101 caaagggagc aaaagtaacg tctgtaccaa taatatattt ttcttttgtt tcagcatgag

92161 catcagtagt taatgtaaag tttaataaaa cacctaaagt taataataat gaaaatagaa

92221 ataagtatgt tttacgtgtg ttcaatgaaa tctcctccta ttcaaggttt tattttattg

92281 gaaaagccaa aaacttgcaa ctaatatgta taccactaat gttactattt ctaacatgat

92341 gtgttagaag taaaacgttt tctaatttaa aatgcttttt ttaaaactat gtaaacgtaa

92401 aacaaaataa aaaataactg agttattcga gtgtgtttat gattgacatt tagtgtttaa

92461 taagataaaa tgaatgatag tcagtcatgt tttttgagtt aagaacatgt ttagttcaaa

92521 ttaaggggtg caaaatgaaa cttgataaga aaaatagatt aatttcagcc gccatagaag

92581 cttttaatga aaaaggttta gagcaaacta aaattgcaga tatcgttaaa atcgcaggtg

92641 ttgcccaagg aacgtactac ttatatttcc cttcaaaatt agctattatg ccagctatag

92701 ctgaggtagt agtagaaaaa aacattgcgg agttgcatca aggaatggat tcaactttac

92761 cttattcaga acaattaaat caattgattg aaattagttt tagtattaca gaaaaatatg

92821 cagatgtgat tgcgttaacc tatgctggtt tagcttccag tgagcattta aatgaatggg

92881 aaaagattta tgaaccttac tatctatatg tgacagattt tcttgaacaa ggcatagcta

92941 aggaagccat taaagctaag atgaatccta aagcgacagc ccgtttatta attggtttaa

93001 tcgaaaatgc agcagagcaa ttatatttgt atgcagaaac agatgataca gccgtagaag

93061 atcaaaaaga agaagtttac caatttgcta aaagagcatt agggataatt taaagaatta

93121 atttaaagat tggaagagtg aaatgaatga ttagagaagc aagaaaagaa gatgcaaaag

93181 caatagcgcc attaattatg gtaattttag aagatatgga gcttgaagta tttaaagaat

93241 attcaaaaga aacaattgaa gaattattag ttgaaggtat tcaaactgaa acataccgct

93301 atagttaccg ccatgctcac gtgtgtatgc gtgatggtga aattgccggg gttgtctttg

93361 gctatcgtgg tgaaaaagaa ccagtgattg atgaaccctt aattgatatt ttagaagcaa

93421 agggacttaa ctctgatagt agactcttta ttgaaaagga gacttttgca ggtgaatggt

93481 acttagattc acttgtaacg gcagaaaaat tccgtggtcg tggagtaggt acggaactat

93541 tagatgcatt aggagagttt gctaaagaag acggagaaga aattatcggc ttaaattgtg

93601 atcaatctaa tgctggtgcc caaaagttat atgaaaaaat gggatttaaa aagactggtg

93661 aagttacaat cagtggtcat gaatacaatc atatgcaaaa acaagtttaa acgataaaaa

93721 aagtcttttc tattcagaaa ttatgaatag gaaagacttt tatgattgtg tcattaatag

93781 gcaggtaaat taagaggtcc atctttagct ggtgcgcgtg ggattaccca atgatctaat

93841 ataacagcaa gacagacgca gagaggtaaa tcttgctctc ttttaataga aagagcataa

93901 aaatctccag aggctaaata aactttatcc attgtcatga cagttttagt caagtggaga

93961 attttataat gatgatttgc aaaatcacca ctaataatcc aatgaattcg actgacacga

94021 aaataaggat tgtgaatacc aggatacttt gaaacagaac aaactttttt accgcttaaa

94081 tagatatcaa atttcggcat taaagaaagt tttgtttgtt tgacttcagc gactaaagtg

94141 ccgtctatct tgtacaaata taagccatct ccaacattac cccatttgcc tgtgattaaa

94201 taaatatctt ttccggcttc atcttttata ataatgcgtt cttttctagc gacatattgt

94261 tccttcatat agatatgaat catgtccttc acctctagtg atagagtagc acaaattgta

94321 aataaattca ttcattaaat aggcagtaat ttattaaatt aatttaaagt ctccattttt

94381 tttgagcaaa acagttgcta gttttttagc tggatgctat tatatgggta agatatctga

94441 tgtgtacgat atatccttta cagtggtgac cgaacaagta ataccttta gggatccaaa

94501 ctgaaatcaa actgataaca agccttttca cttggtagtt agagatgttt gacagcggag

94561 cccaccttgc tataagcggg gcaacacatc gggatgctga accggcacaa tcggatttct

94621 tttaaaagta aattattttt aagatcgcct agtcctcatg actaggcgat ttttttgcat

94681 tttgctttag aaataaaagt ttttacacgc ataaaatttg aatttacaca agtggctttt

94741 tatagatgta aaagagataa catcttataa tttcaaaaaa aaacaaaaat gataaaaatg

94801 tgaaaaaagc tatagtagtt tctgtggtat cctaaaaaag ttgcttttta ggatctttat

94861 tttgattcaa taagcagtta gatgattgtt caatagttag catgtttatg tattagcttt

94921 atttatagat gattctataa aagcaaccca gtcctaaata tgaataaaag cattatcata

94981 gtcattttag aaaatgtatg agataaaaaa tgtacttctg aaacgacaaa agaaaatagt

95041 tatgcaatca ataactactc tatactaata attgttaaca actaattgaa tctaaaaata

95101 tgctttggga ggtttttgaa tgatggatca caaagcaaaa aatacttttg ccttaagaga

95161 aataaaaaaa ataatagaga atcatgaaga caaaatagat tttgtagctg aggaaagaaa

95221 aacgatggat gaaattaaaa gaattattga agaagtttat atgtgggaca ataaagccag

95281 tgaatatgaa gaaaaacaaa gtaatcaaaa atttgaaaat gaaatgaaaa ttttatttaa

95341 aagaaattaa ttataatcat tcagataaac taaaaagtgt agcgagattt aatcaaggag

95401 gagaagcatg aatattgaac aatttttagg caaaagacaa tgtattaaat actcgatagt

95461 aaaagaatta cttacacttc aagaacacca aatatctaaa aatcaattac taagctgttt

95521 gtcaatttcc acaacaatgc tcaatcagat agttcaagaa attaattcag atataagttt

95581 tttaaaccta gaaaatgaaa ttaagttagt tgaatatcga aagtatggtc agaaatttta

95641 tgagttaaaa ctaacaaaaa gtgtttcact agatcatttt ttattgcatt atttaacaac

95701 ttccccttct ttttttattc ttgttgatat cttttttgaa aaaataagta gtatttctga

95761 aggtgcgagt agatattatt tttctgagtc tacgtttcga agagaattaa atctactcaa

95821 taaaatacta aaaccctatg gtcttagtat taatatacag aataagattt atttaatggg

95881 cagtgagtgg gatttacgtt tattttcaac ttcttttta tcaaaatctt ttggtacgaa

95941 aaggtggcca tttaaattta ttagtttatc agatagccaa aagtatattg atttaattcc

96001 tgataatttt tttgatacta atccattaaa aaataatttg tctgtatcct atttgatagc

96061 gatatctatt tatagaatgc gtaaagaaaa aaacacaaat tttattatgg aaagcgacta

96121 tctatattct gaagaaagta cttgttttaa agagttttta atcaaaggga gtcagttttt

96181 tgaaaattat agtcctaaaa cagcaagaga tagatttgaa atagaaaaca gaattatttt

96241 tacctcatta cttcttaatg gtaactttac taatgtgaaa aaattaccat acttttttta

96301 tagagatcct gcttttcaaa aggaagagct atttcagctt ggttttacat ttcttacgca

96361 attggagtct tgtttatcta gtccactaac gatggaagaa tatacgctat taacttatca

96421 gtttagtttg attgcatatc gacatttttt tcttaatcag attgattcaa cccagatgat

96481 accaataaaa aagattgata gctgtacagt tgataaagta gaagtaatga caaaatattt

96541 gttagaacgg gaagacaata atttttcgga aaaaaaacaa caaatgcttg aaaaagaata

96601 tagcacgatc ttgttaaata cgattaattg gttaagatat aagccgattc ttaaattata

96661 tttaataagt ggtcaggatg ttgaacagtt aaaaaaaacc attttagcgc tacatttaga

96721 agttacattt aatttaatta tcgtagataa ggtagataag gaagtagatt taattgtaag

96781 tgatgcggcg tatattgaaa actacttaag tgagtatcca aatgtaccag ttttttactg

96841 ggaatcgctc tcgtcttcaa ataacattcg cttatatgaa aaactttcta ttttaaatga

96901 aaaaaattgg gataaactga aaatcacact ctgaaaaagc tttttataac tacatttttt

96961 caaaaaaaag gtaaattgaa gttaaaattg aaatagatag taattgtttt tatgttagtt

97021 ttataaagct gactgtttat tatttgaatt tagatgatag tctttgatgt gtgaaatgat

97081 gaggaaatgt tctttatttt ataaatcaat ggatattggc caagaatgat tagtagaaaa

97141 taaatagttt gaaaaaaata gagaaaagag gaaataaaaa atgaagaaaa gaaaaatagt

97201 tccttatatt ttagtatcca gtttagtctt atcaagttta agcggaagtt taactgtatt

97261 tgcggaagaa gctctcacag gaaatgaaat taatgctacg attgattcaa gagattctga

97321 aaaccaagaa gttgagagtt ttgttgaagc agaaactttt gcaacaagcg gaacttgggg

97381 cacgtgccca tggacaatag attcaaaagg tgtgttgaca attaaatcgg gaacattagg

97441 tcggggacaa agtgggagta ctccaactgc acctggttta aacagacagg atgtaacgaa

97501 ggtaattatt gaagagaatg taaaaactgg taccagtgcg gcggaactgt ttatgaactg

97561 gggtaaactt acagaaatcg agaatctaat taatttagat acttcaaata ctagagatat

97621 gagtaggatg ttttcaggct gtcaaaaatt aactactata gatgttagtg gatttaatac

97681 ttccaaagta acaaacatgg actccatgtt tagttcactt acttcagtaa actctttaga

97741 tttgagtaat tttgatatgt ctaatgtaac caataccaat acaatgcttt atacagcatt

97801 ggagtcatta acacttggtg aaacatgtca attcccaaat gatacaaatt tagctagcat

97861 taaaacaacc tcgggtatct atacaggagg ttggacacaa gaaaatggca caaaagtatt

97921 tccttctagt agtgcattca tagcagacta taacggagca acggatgccg ggacttatgt

97981 ttgggaaaaa gtaaaatctg ctccagttat agtgaagtat atagatgaac aagggaaaga

98041 actggcagaa tcagatacct tatcaggtgg tctaggagat gcctatataa ctaaagcgaa

98101 agacatcaaa ggatatgaac taaaagaaac gcctattaac gcaactggaa gttatactag

98161 taaagaacaa acggtgaact atgtctatga aaaaagtgtt gccaaatggg gaacgtgccc

98221 atggatatta gaagatggtg ttttgacgat tgaagcagga atcttaggta aaggtacagc

98281 taccaatata gtcgcagata ttccaaagaa tgaagtcact aaggttgtct ttgaagggtc

98341 tatagaagca caaagtgatt ctacaaattt attcagttcg tttcctaatc ttcagtcatt

98401 cgaaaatatg aaaaattttg acacttctaa tgtaacaatg atgcagggga tgtttgaaaa

98461 ctgtactagt ttaaaaacga tagatgtcag taactttaat acatctaaag tcatcactat

98521 gaattctatg ttttcaggta caggcttaac taatttagat atgagtcagt tagacatggg

98581 ggaaattcaa tacaaaggtt ctatgttttc tggttctaac aatttaagta cattgacatt

98641 aagtgataaa aatcagatag atggaacatc actaccagct attaatacag attctggtaa

98701 atacactggt gcttggataa gtactgatga gaagaaaata ttcccttcta gttctgattt

98761 tatgtccacc tataatggga aaactgatgc agggacctat atatgggaag tagcaaaagt

98821 tgctccagtt accgtgaatt atattgatgg aaagggaggg acattggcgg aaccagatac

98881 attgatcggt gaaatgggag atgtatatac gactaaagcc aaagcaatta aaggatatga

98941 gctaaaagag acacctgaaa atgcgactgg aactttttct aaagaagcac aaacagtgac

99001 ctatgtctat gaagctatcg aaggcggaga agtgaccgtt aattacgagg atacagaagg

99061 taataaactt gctgaatccg atgtactgac agggaagttt ggaacatctt ttgagtcaaa

99121 cgcggtcgaa attactggat atgagttaaa ggaaacacca gagaatgcaa ctggaacatt

99181 tagcgaaaca gctcaaaccg taacttatgt ctatcaagca gtccagggtg gagacatcac

99241 ggttaattat gtggatacag caggaaaagt gttagacact caaacagtaa caggtaaatt

99301 taatgaaagt tatacaacag aagcgaaaat gatagaagga tatacgttaa aaacgacacc

99361 taaaaatcat acaggtctct ttaccttaga agcgcaaacc gtaacttatg tctatcaagc

99421 gagtgtcgga gctcctgtga cagttcgtta tatcaaccag gtaggaaatg aaattgcagc

99481 ttcaacgagc ctaactggaa aagtgggtca aactgttacg gcaacggcaa aaacaccagg

99541 tggttataca ctaacaagtg catcaaatac gcaagaagct acaattggaa caaccgaaca

99601 aacgattacg tttgaatatc aaggaaatgc tgcttctaaa gtcacaacgg aatatgtaga

99661 tactgaaggg gtccgtatcg ctccggttca aacgattact ggtggaaata tcggggatgc

99721 ctacgcaaca gaggcggaaa cgattcaggg gtatacgctt caaacaaccc cagaaaatgc

99781 atctggtttc cggacacaag gtgcgattac cgtaacctat gtatacaaaa agaaagattc

99841 aaaaccattt atggtgaatt atgtgggcgt tcaacaacaa caagtagttg ggtttgtccc

99901 tgaagggatt acgacaacta aagtgagcct gttgattaaa aaagacggaa gcaatgaatg

99961 ggtagacaca ggttatactg gaacaccaga cgcagatact ggttactttg ctattgaatc

100021 tcgttttgtt aaaggcacga aagacttttt attaaataat aaagatcaaa tcaaagtagt

100081 ctttaccgat ggaactggtg aaaattacga aggcgttcaa gtagtacaac cttatactgc

100141 accggttgta aatgacttta cagaaggcga tgactatttg actggaagtg tgccgaatgg

100201 agcgaatcaa gtacgtgttt ctattaatgg caatcgactg cgtgtcgtaa atagtggtcc

100261 aattattacc gatgatgccg gaattaatgc agtaaccgga gagttttcaa tctgggctaa

100321 aacttggtgt gaaggtagtt atggatcaag tccaatgcat caaacaaaag caggcgatgt

100381 gattacagta gattatggcg ctcaaattta tggagctttc aacaatccgt ctacgaccat

100441 tgttgtaaaa taatttttca aactaaaaat tggaagagat actcttaaca taatagatac

100501 tagaaaaata aatattctag gctattatgt tgggagtttt tttattctat cgaaaagact

100561 aatgatattc acgttatttt acttaacaaa ataacgtgaa tattcaatct agttatgact

100621 cttttagtaa gtaatgtaat tgatttatac cttattttat gaaatagata atgacaagta

100681 tgaaatgaaa aaaagaaaca tctgggacga caaaaagaag caattagaaa agccctaatt

100741 agtttatact tataattgtt aacaaatgac ggcgatatga atatttctga tggtaatttt

100801 gagaaaagag cagccaattt ctagaaaaat aaatagctta gagattagag tcaaattaga

100861 aaatttaaac tctgctagtc aaaactattg tagttaataa attttatcga ttatattttt

100921 gaggatagaa atagagggag aaaagttagt agattcgcta tgtttttcaa tattaaagaa

100981 aaaaacatga aaaatagttg aatagaaaaa atattatgta taaacagcaa tataaccaaa

101041 ttttcaaaaa aaaacaaata ataaatttaa attgaaaaaa aacaataaga attcaaggta

101101 caataatcta gagttgtaaa taaaaaataa aaagaaaagg aaaaagatga gcagaaagta

101161 aatatcttaa tagagggagt aattgaaatg caaattgatg attattggca agacaaagat

101221 ttatatgaag tgatttttaa agcaaaacgt gatggtggag ataaacagtt tacaaaatat

101281 tttatattcc cagatgtaga taaagttgaa aaaataaaaa aatgtattga aaaaaaactg

101341 cctggaaaaa ttgaagtaat tagtattaat catttattta tggcttttga ggaaaggaaa

101401 acctctaata gatgattgca tatccaaagc atagatggat ttctaagtga tatgaatcta

101461 tccataacta aagaaatgaa ggtgcaaact agtaatgaat gaaataaaga aaatttttca

101521 agtgtttaaa gcaatacgta aacgattcat tctattttta ctatttgtaa ttttgggagt

101581 tagtctggtt gcaggataca gcatattcgt agtaaatcct aagtatagct caacagtaca

101641 aatgattgtt caagcagatg aagcagatac tcctaattct gtgaatacaa ttaatgcaaa

101701 tgtgctattg attaacacat ataaagactt aattaaaggg gacgtcgttc tggacgaagt

101761 tcaaaggaat ttaggaaaag aggggtttac aatctcaacc tcagaactaa aaaatgttct

101821 tcagattaca cagtcagcaa attcacagat gtttatgatt ggagcgacta gtgattcacc

101881 acaacatgca gcacaaatag ttaatgctac tgctcaagtt tttaaagaga aggcttttga

101941 tatgttgagt gtgaaaaaag taacaatatc ctcttatgga aaagaaaatg tgaatcctac

102001 atcacccaat ataagaaata acatagttat tagtttattc ttagcggtat ttataagtgg

102061 agggatttgt ttgctcttag aattattagg agataatttt aagggtaagg aagccattga

102121 agatgaatta ggtttgtctt tattgggaac agtaacgtta attgagaaaa aataacggtg

102181 atgaatagct taaagttggg aggaaaaaac atgcatcaag gtaatggaaa tgaaactagt

102241 ccaaaagtta agagtaaaga aaaagataaa agtaggataa aaagaaatcc agaaaaaaaa

102301 aaggctggaa ataaaaatta tagaccaccg tcaatgattg taacagataa tagaatttta

102361 gaacagtatt cgttaattcg ttcaaatata aaatttaagc tagaagaata cgaaatgaaa

102421 actgtatctg ttacttcttc tattatggga gaaggaaaaa caacgacgtc tttaaatcta

102481 gcgacttctt ttgcccgaga cggatacaaa gtattattaa ttgatgcaga tttaagaagg

102541 ccgaatattg gacgttcttt aaaaatatct gaagttcgtg gattgagtac tttgttgacg

102601 gaacgtttga aaaatgttgg agcatttata gtcaaggttc ctaaaattga aaatttatgg

102661 gtattaccta gtggtctggt tcccaataat ccagctgagt tattaggttc taaaagtatg

102721 aaaagtttgc taaaagaatt gaaattacat tttgatcttt taatttttga tttacctcca

102781 gttggaattg tgacagatgc tcaaattctt agtgctcaaa cagatgctac agtgctagtt

102841 gttcgagatg attttgtaaa aaagcaagaa gttctaagag caaaagaatt attagaaaat

102901 gcgaatgcta acttaattgg agttgtttta aacggcgtta aaaagtcaga ggatggcact

102961 aactataatt cttattattg atgtaaaaga aaggtgtgga agaggatgat tgatttacac

103021 tgtcatttat taccaaatgt tgatgatggt ccagaaagtc tagaagagtc tatcaaacaa

103081 ggtcaattag caaaaaaaaa taatatcagc catattgttt gtacgccaca tcatcttgat

103141 ggaaattata ataaccctaa agagcaaatt ttagattctt tttttaactt aaaagctgaa

103201 tttgagcgaa acgctattga tgtatctttg atattgagtc aagaagttcg tttaactcca

103261 gacttgatac ttgcattaga aaaagatgaa cttgttttta ttgattcagc tagcaggtat

103321 atccaaattg aatttcctag tcatcatatt cctagatttt cacagcagat aataactgac

103381 ttagttttga gaggtcacat accgattatt gttcatccgg aaagaaatag tggttttcaa

103441 aaaaatccta atgctctaat tgagttttta gaaataggcg cattagcaga agtaaccgtt

103501 ccaagttact tgggttattt tggtaaaaag attcaacaac ttgctgttag atatgttcat

103561 catggattag tacaactaat ggcctcagat acccatggat ataaacgtca aatagtatta

103621 ggatctggat atagcctaat gaaaaaaaaa gaaggaatag aaatggttca acaatttaaa

103681 aaaaatgctc gctgtgttat tgagggagaa gaattaactg aacttaatta tagtaagctg

103741 aaaaaaagaa attggcaatt ttattaggag gtgaaaaagt gacaagaaag aaaaaaacag

103801 ttttattagc gattatagat ggtttgataa taatttttgg aaatattttg actactatca

103861 tactgaaaaa tgaaggaatt ggtaattatt ttatatttag tacatgtttg atgattagca

103921 tttattttat tttggttcat tatactaatt tttataaaac ttcctacaga tatatgaaaa

103981 taaaagaaag tagcattttt ttcatagcta ttctaacaac tggatttatt gaatggttag

104041 cttcattgtt tctcacaata cactatggat tttatatagt tatttccaat ttaatcgttt

104101 ttctgttttt agttggaagt cgtttagcat ggaaatttat ttttgagctg agacatgatg

104161 ttagatactt aaataaaaat aaaaaaaaga atgtcttaat tattggtgct ggaaatgcag

104221 gtactctatt gattcagcat attcaacagg atacaaaaac aacaatgaac attaaaggtt

104281 ttattgatga tgatagtgag aaacaacaga tgcgatatag aggaattaaa gttatcggta

104341 caacgaaaat gattcaagaa atttgccgac aagaaaaaat acaaacgatt attcttgcga

104401 ttccgtcact aaatgaaaaa agaaaaaaag aattagttga ctctttgtct aatgaaccaa

104461 tagagctttt acaaatgcct tcctttgaga aaatgttgct agaatctgtt gaaaaaaagc

104521 agccacaaaa aataaatatt ttagatgtac ttggccgcga agaggtgcaa ttagactgtg

104581 aaattttgca taaaaattta aaaggaaaaa caatcttaat tactggagct ggtggatcga

104641 ttggatcaga aattgttcgg caagtttgtc attttcgacc aaaaacaatt cttttattag

104701 gtcatggaga aaactcaatt taccaaatat tacatgaagt taaaagtata gatgttgctc

104761 cattttctac cataaaattt attcctatta tagtatccat ttccgacaaa acgatgataa

104821 ataaagttat gaaaatgtat caaccagaga ttgtttatca tgctgcagct cataaacatg

104881 tccctttaat ggaatgcaac attgatgaag ccataaaaaa taatatttat ggaactctta

104941 atgtagctga agctgctaag agaaataatg tagaaacttt tgtaatgata tcaacagaca

105001 aagcagtacg tccaacaaac gtgatgggtt caactaaaag gatagctgaa atgctagtaa

105061 cagaattaaa tgacgttgga aaaactaatt tttgtgcagt ccgctttgga aatgttttag

105121 gtagtcgagg aagcgttata ccactattta aaagacaaat tcttgcggga ggaccagtca

105181 caattacaga caagaaaatg acgcgttatt ttatgactat tccagaagca agtcgtttag

105241 tcattcaagc aggtgctctt tcaataggag gagaaatatt tgtattagat atgggagagc

105301 caactaaaat cattgattta gctaaaagaa tgattgaact ttcagggtat tcagaaatgg

105361 aaatcaccat taaagaaact ggaattcggt ctggagagaa gttgtacgaa gaactactag

105421 ttactaaaga aaaaaagcct aagcaagtat ttgaaaaaat atttgttgga gaaataaaag

105481 ggtttcttag agaggaaatt gatgtgtttt taaatcaatt gatgacgaca gacgatcaat

105541 tgaaaaaaat aaatttaatt cagtttgcta atgagtccct caactaaaaa agaagagaaa

105601 ggtgtcatat atgactcgaa atatattatt agcctcacca catatgagca aagaaggata

105661 cgaggaaatg tttgttaaag aagcatttac tagcaactgg attgcaccag tcggagaaaa

105721 tattaatcaa tttgaaaaaa aagtttctga acgaatagga agtaaaggag ctatagcgct

105781 atcttcgggg acagcagcgt tacatttagc tttaaaagct gcaggagtga aggcaggtga

105841 tattgttttt tgttcttctt taacgtttgc agctagtgta aatccgataa actatctagg

105901 agcaatacca gtgtttattg gaagtgaaga aaaaacatgg ggaatggacc caaaagtttt

105961 aaagaaagct tttcaaaaat atccagagtg caaagcagtc attgttgttc acctctatgg

106021 actagttgca gatctagatc caattttaga gatttgcgat caacacaatg ccatagtaat

106081 tgaagatgct gctgaagcat taggtgcaaa atataaagaa aaagcggttg gttcctttgg

106141 agattacggg atattttctt ttaatggaaa taaaattata acaacttcat ctggtggaat

106201 gattgtatcg gaagatagtg aaaagttaga aaaagtgaga ttttggtcta ctcaatctcg

106261 ggaggttgaa cgccattacg aacacaaaga aattggttat aactatcgaa tgagcaatgt

106321 tttagccgga attggaagag gacagttaat ggtattagat gaaagaatcc aacaaaaacg

106381 tgctatatat caattttatc aaaaagcatt agcggatatt gaagggattc agtttatgcc

106441 ggaaataaat ggaatttatg caacctattg gctaagttgt gtaactttta cagattccaa

106501 attaccgagt aagataattg aaacgttagg aaattgcgga atagaagccc gtccaatttg

106561 gaaaccaatg cacagtcaac caatatttaa ccaatatgat tctctaggaa tagaatttga

106621 tgattggata ttcaaacacg gagtatgcct tccttctgat acgaagatgg aagaactaga

106681 tttattactt atttctaagc ttattcatga aatttgtcgt aaagaaagtg aggggaaata

106741 aaaatgtggc tctttctttt aagtttagtc ttgattgtat acacaatggt gggttaccca

106801 ctactgttaa aatttttagt taagcatgtg aaaaaaaagt taatttatag aaatacaaga

106861 ttttttccaa cagtaacttg tatcataacc gcccacaatg aagaagcggt tattcttaag

106921 aagttacaaa atttagaatc ttgtgtatat ccagacgagt tgttagatat tattattgca

106981 tcagacttca gtgaggataa aacaaattca attgttcaaa aatatataga tgagtacaaa

107041 ggaaaaagaa ggatttattt acaccaaact aaagaacata aagggaaaac aaatgcacaa

107101 aatgaagctg ttgctttagc taaaggagag attcttgttt tttcagatgc aaatgcatta

107161 cttgattcaa gaagtatttg tcgtttagct caagcctttt cagatgattc aataggttat

107221 gtaacaggga aattagttta tacgaacgca gtggagtcaa ctactagtaa ctcggaatgt

107281 aactattggg attatgattt acaaatgaga aaatgggaat cagatatagg ctctataaca

107341 gcaggaaacg gtgctttata tgctattagg cgttctatct atacaaatat tcctttgaca

107401 tattctcatg atagctattt tccaccctat gtagtagtac aaggaaaacg tgcaattttt

107461 gatgaacaag ccattgctta tgaaaaagct ggtgagacaa atgaagatga atttaaacgt

107521 aaaataagaa tgtctcgtaa tattttttct ttaaattata ttctccctca aaaatacaat

107581 gtagtgagaa atggctggtt tacgattttt tatttgtcac accgtacatt tcgaaataac

107641 ctatatcttt tccacattat attactccta acaaatattt acctagtaaa tcaatctcca

107701 ctattcagtt ttattttaat aggtcagtgt ttagtttacg gttgtgcctt attaggaatc

107761 gtttggaaaa acccaattct tcatatggtt tattactatg tacttacttt atgtgctcaa

107821 cttgtaggtg taataaagga aataacagga cagtcaaaac caacttgggg aaaagcagaa

107881 agtacaagga aataaaagct gttgaaagga gcaacatgaa atgataaagg gaacaatagt

107941 aatcctaatg attgtatcaa taggattaat agtaaataaa tattttcaaa ataaaaattg

108001 gctaacagta gaaaatattg caattgtttc ttttcttatt ttgtttgatc tccctatatt

108061 tttacaaaca ccaaattggg caatggatga acaattttta atccttattt taattggaaa

108121 tgttggtttt attggagctt tcttatttat gcagccgaca aaagagatgg cttttatagt

108181 aaaaacagag aaagcaacct tttctctaaa aaaagatgga attcgaatgc taacacttct

108241 tgttttctgt tatttagcat atgaattata tcaactacaa gcttataatt tttcaattgt

108301 tcgttctaca ttgttgaatg atcgagttgg agaatatttt gaagatgctg gaactcaaaa

108361 tttaggattt attaagcaag gttttacaac attaaattta ctcttgattt cttattattg

108421 gaaacataaa aatcacctcg gaacactttt atggatttgt tattttatta ttacattact

108481 gtctgctcat acacggtttg tagtagtagt cgcggcggtg attccattag tttattataa

108541 ctatcaaata aagccaatta agctttctac tttaattggt ttaggaatta ttttcttgtt

108601 atttattacc attagcaact atgcacgagg tggagtatta ggtaaaggag attctcgatt

108661 tagtattgat gaaatcatga ctggtcacaa tatgaaagat caattattcc gagcctctgc

108721 agggagtacg gagcactttc atacattatt tactagtgat gttcctaatg attggttaag

108781 gcaatattat tactttattc ctctaagttt tgttccaaga gttttatgga gcaataaacc

108841 tgttgttagc tatttttgga gagtaacaca atctatcact ggtgcgtatc caaatgggaa

108901 aatgaaccct gttttaacga caacctattt aggggaagcc taccatcaaa tcggctggat

108961 aggtgtattg attgctacgt ttatttattt taaattgtta tttttttgtt taaatacatt

109021 taaaaaattt gaatatagtg agttgctgat ttatagcagt attttatgga ttccaatgag

109081 tctacgaggt ggatttagtt cattaatttt aactcaagtg ccaacttttc taattttata

109141 tggtttaaaa gttatccatg tttatgaagt tggacaaata aagtataaga aagaaggaca

109201 attatgaaaa ttttagcact tagcgatagc tatccagttc atccacggct aaagaaagtg

109261 agtcggtatt ttgggaaaac agaaacgaca actttttttc tttggaatag aggtgatgac

109321 gaaattctag gtgatagaag tgagaccgtt tttaatttgc caagctctct aggaaataag

109381 tggaaaaaag ctcttaattt aattcggttt cgtaaggcag ttaaagtaca tttaagcgag

109441 ttcacatatg atttagttat ttgtcgacat tggcaacagt tagttctgtt atcaacgatt

109501 aggaataaac agaaatataa aattgtttat gatgtttgtg atatgccaaa taataaatgg

109561 gtgcgtcact tggaatctag gttaatcaat aaaactagta tagtagtatt ggcatctcgt

109621 ttctttattc ttgactatac atcaaaaata gatgaactag ttgttttaga aaatcgatca

109681 tggagtcatt ttagtaaaaa aaaatttgtt ccgactaaag tagaaacacc aataaatgtg

109741 actatccagc taacctttct tggtcgtata cgttatggag aaattttagc tcgattgatt

109801 gaatgtgttc aaaaagatca tgagattagt ttgaattttt atggtacagg accagatgaa

109861 tcaaagctta ttcttctagt caatcaatta gaaataaaaa atgtatattt tcacggagaa

109921 tatatagaag aaaaaattcc taatatatat gaaaaaagtg actatatttg ggcagcctac

109981 gataatcagt tattgaatgt taaaaaagct atttctaata agttttttga aacattgacc

110041 taccaaactc caggtatttt ttctgaagat accgaattgg gtgagttgat tactactcag

110101 gaaataggat ttatagtcaa tccttactca acggaatcta ttgtagagtg tttcgaaaaa

110161 atgaaagata aatcaatgtc tagaaaaatt aaaaataatc aaaaaaaatg gaatgaacca

110221 ctttattttg acgattatct tgtggaatct ccttatttaa aaattgatga aatgaaggtg

110281 gaaaaatgaa aattagcgta gtgggtttgg ggtatgtagg actttcgaat gccttgttgt

110341 tagctcgcaa taatgaagta acggcatatg atatatcagc tgagaaaata aaacagctcc

110401 aaaatggaaa aactccaatt ctagaaaaag aaattattga ttttatgaac aatttaaaat

110461 taacaattga gtatacatgt gatgaaaaat acctttctga tttaggggag cttgttattt

110521 tagccgttcc aacaaattat gatgaagaca cgagaaactt tgatacatct attcttgaag

110581 aaatgattca aaaaattatg aaagtaaatt ttaaagggat ggttattatc aaatcaacgg

110641 ttccaattgg atttacaaaa aaaatgagac ttttgtataa aacaaatcaa attatttttt

110701 gtccagaatt cttaagggaa gggaaatctc tttttgataa tttacatcct tcaagaatta

110761 ttattggaga tgaaagtaat tccggtaaat taattggtca attattttta gatgctgcta

110821 cagagaaaaa tatacctctg ctttacatgt ctgctactga agcggagtca gtcaaacttt

110881 tttcaaatac gtatttagct atgcgaattg cttattttaa cgaattggat acgtattgtg

110941 caatagaaga attgaataca aaaaaaatta tagaaggaat gtctcatgat gagcggattg

111001 gatctaaata taataatccg tcttttggct atggtggata ttgcttgccg aaagattcga

111061 agcagcttgt tcatacatta aaaggtgtac caaatcaact aatatcttcc attccaacat

111121 caaatgaggt tcggaaaaat tttattgtat ctgaagttct aaaagaaaaa ccaaaggtta

111181 ttggaatata taaactaagt atgaagaaaa attctgataa ctttagacaa gcatcgattt

111241 tagacattat cactgaatta aagcaaaaaa aaatgaaagt gattatttat gaaccaggat

111301 tagatgatgt agaatatgct ggagtcgaaa tcagaaaaaa tctagctagt tttaaagatg

111361 aatgcaattt aatattggct aatagaatgg ataatgaatt aaaaagtgta gaagaaaatg

111421 tatacaccag agatatattc gggaaggact tatagagatg attaatgtaa tagggttagg

111481 atatatcggt ttgccaacag cattaatttt agcagcaaat ggacaagaaa tagttgggac

111541 agatataaat aaggatacaa tagcacaatt aaaaaataaa gaaattcctt ttgaagaaga

111601 agggcttagt gagttatttc aggatgcctt agaaaataat attcattttc aggtgaatta

111661 cgctcaaaca gattgttaca tcattacagt tccaacccct tatttggaag aaagtcgaaa

111721 agttgattca tcttatttaa ttgcagcagt tcggtcagta cttgaaaatt gtttaccaga

111781 aacaactatt attatagagt caacgatttc accaggaacg attgatcgtg tggtaacccc

111841 tttgattgaa caagagggat tatcgttaga tagggacatt tatttagtcc atgctccaga

111901 acgaatttta cctggtcaaa tgataagtga attaaaaaat aactctagaa caattggttc

111961 agataatcca gaaagtgcca tagaagtgaa aaaaatctat caatcatttt gcaaaggaga

112021 aattatatgt acaacaatta aaatagcaga aatgacaaaa gtagtagaaa atacgtatcg

112081 agcagtaaat attgcttttt caaatgaatt gtctctgctg tgtaaagaag gtcaaatgaa

112141 tgtttacgat attatagaaa ttgcaaatat gcatccaaga gtaaatattt taagtccggg

112201 accaggtgtt gggggacatt gcattccagt tgatccttgg tttttagttg gcgattatcc

112261 agaacagaca gaactcatat gcaacgcatt gaaaattaat gagaatcggc ctaaatacgt

112321 atttgaaaag gcaactgaaa ttgcaagaga gcatggatta agtcaaacag aaattggtat

112381 atatggaatg acgtataaaa aaaatgtcga agatataaga gaaagtccaa caattcaatt

112441 acttgattat ttacaagaaa aaaggaatga tccttttata gtatacgatc cattagtgaa

112501 aactaaaatt ttagacagcc aaataatgaa ttttgaagaa tttcttaaaa aagcaaaatt

112561 ggttattgtg atggtagatc atcaacatct attggatcat aaagtttttt tgaaagaaaa

112621 gcttatttta gatactagaa attgcctagc acagagttat attttataaa tacaatagat

112681 taatagaagg agttcattta tggcaaggaa tgaaaacagg gatagactaa ctgaagccac

112741 tcaagttgga atgaaaaaac aaatgatttc gggtatgatt tggacaattg cagatatctt

112801 cggaaatcaa gtagcaaagt tgattgtaca aattatctta gcacgactac tacttcctaa

112861 agattttggg gtcattagta ttatttctgt attcattgct ctttcacagt cgttgatgga

112921 cagtggattc caaaatgctt taattcgaga agaaaaaact tgtgaagaag atttagcaac

112981 tgttttttat tttaattttt ttatgagtat cgtactatat ggagcattat tcttcgctgc

113041 tccttcacta agtttatttt tccatgagcc aaaaattagaa ctgattctaa gggtagtagg

113101 aattatctta attattaatt catttggatt aattcagaga acgattttaa gcaagcaatt

113161 aaaattcaaa actcaaatga taatcaatat gctatcttca attatatcag gcctaatagc

113221 aattttttgt gcatttagag gatttgggat ttggagtctg gtgattcaaa caatagtgat

113281 gcaattttta caggcaagct tatttacaat gaaaaatcgc tggtggccaa aaaccggatt

113341 ttattggaaa tcatttaagc ggctatataa atttggatgg aaaatcatga tctcagggtt

113401 acttaataca gcataccaaa atatctattt tgtattaatt ggtcgactat atagtgcttc

113461 ggaccttggc tactatacga atgctcaaaa gctaagagat gcggcagatt ttactataac

113521 aggggctgtg cgaaaagtaa gctatccagc attaagtaaa gttaaagatc agcaagaggt

113581 tcttaaaaga ggatatcgta aaatattaag atacactgtt ttttttactt tgcctatttc

113641 acttggatta gctgtaattg caccgcaact tttcccagta ttatttggaa ctaaatggga

113701 ttattctatt ccttatttcc agatactttg tttggccgga atatttttcc cgttacatgc

113761 cattaattta aatatacttc aagttaaagg tcgttctgat ttggtgttga aactggagct

113821 tataaataaa ggttttgctg tagctttaat tgttctttca ttgatttttc atggagggat

113881 gatgggatta gtttggatga gtgttttaag ttcttttgtt tcatatctta tcaattcgta

113941 ttattctgga ttctttttat cttattcaac aaaaaaccaa ataaaagaca gtggaggtag

114001 ttttatatca gcaattttaa tggcagtaat gctattgatt ttttcaaatt ttataaaggt

114061 atctggttta ggtggagttt tacttttaag ttgcagtggt ttttttagtt atctattctg

114121 tcacatattg ttccaaacgg cagaaagcaa agaaattatg aattatgtaa aacagtttgt

114181 aaaacatgag gtgaaagtaa atgaagatag tgtataaaaa aggtatgtat caagggagga

114241 taaaacgcac cttagatgtc atcctatcgt taagtgcatt ggtaattcta tttccgtttt

114301 tgttagtagt tagtttgttc atccgagtga aaataggttc accattattt ttttgtcaag

114361 aacgccctgg gaaaaatggt gaagtattta ctatgtataa atttcgtagt atgacaaatg

114421 agcgagatgc aaaaggaaat tttttacctg actctgagcg tctccatttt tttggaaagt

114481 gtttgagagc ttcaagtata gatgagctac ctgaactggt gaatattttg aaaggtgata

114541 tgtctattgt tggaccaaga ccactactag ttcaatattt accactttat acagatgaac

114601 aaaaaattag acattttgtt agaccagggt tgactggatt agctcaagta aatggccgaa

114661 atactcttag ttgggaagaa aagtttctgc tagatgcgca atatgtagga aatatttcgt

114721 ttagtcaaga tatgagaatt attttattaa caatgaaaaa agttttccaa aaagatacta

114781 ttcattcaga taaccattgt actatggaag agtttagagg aagtaaggca ggtgattgac

114841 agtgaagcaa ttagttattt taggcggcgg gggtcacggc caagtttgtg cagaaattgc

114901 ttatttaatg aacaaatggc agacaattgt atttttagat gatttagaaa taccgaatac

114961 aaaaatgtat aaatgggatg gacaatttca cgaagttcaa aaatatgata gtgagaaaac

115021 tgaatttttt gttgcagtag gaaattcttc cattcgaaaa aaagtagttg aagatctact

115081 cgttaaactg aagaaatttg caactttaat tcatccaagc gctattatta gcgccaattc

115141 aagtatagag catggctgtg tacttatggc tggagtgatt gttaacccta atattaagat

115201 tggaacattt tgcatcttta acacaagcta cacggttgct cacgattgta tactttcttc

115261 ttatgtgcat ttatcgccag gtgttcatct ggctggaagt gtcgtaataa tagagaagcg

115321 tggattggaa taggttctac tgtcagtaat aatgtattta tttgtgaaga aacaactata

115381 ggaccaggtt ttctagtcgt gaaggataat aattaaaaag ggttttattt tggacaacca

115441 tgtaaaaaaa tagtttaact tgcagtatta tgtacaaact ttataatgca gcattaaaaa

115501 taaggtgttg ttcaaaagga gaagtatgaa ggaaaaaatt tacaaccaaa ttattttaaa

115561 attagaagaa cacaaaaaaa tactgttaaa agaattaaac gaagaccttt tagttttctc

115621 acaccaggtg gaaaagcatt tgaaaactaa taaaggctat gataaaagga agtgtaattg

115681 ttatttttgt agtaaaatac tagattgtca aaaaaatatc aatttaacaa ttaatgaagt

115741 gaaagatatt gaacaaagga tagctaatat tgatagtgag aaatgaagag ctaaaatgaa

115801 tcaaataatt aagctagctt atgcttaaaa ttttagagaa actccaatac tttgagtttc

115861 tctaaaattt ttaaacctat aaataaaaaa attatatttt ttcttgtttt gataaacgat

115921 agccaccatg aaaaacagga cctacatatt cattgaaggc aaagccacta cgaatagctt

115981 ctgagacaaa ttctttagcc tttaagacag aatctctaac tgtaagtcca tttgctaaac

116041 cagccgtgat agccgctgca aatgtgcacc cagctccatg attataagca ggagtgattt

116101 tttgattttc caagactaaa aattctgaac cgtcgtaaaa taggtcaatg gctttatcac

116161 ctgctaaggc ttttccacct ttaatgacaa catttttagc acctaagaga tgaatttttt

116221 tagcggcagc tttcatatct tctaaagttg ttagttttcc taaaccagat aaaacacctg

116281 cttcgaataa attaggtgtt gcaaccgttg ctaatgggat taattcatca cgtaaagcct

116341 cagcgctatc tggatttaag acttcatctt cgcccttgca aaccattact ggatcaataa

116401 cgatattttt aagttgttgt tctttgattg ttttcgctac tagatcaata atagaaacac

116461 tcccaagcat accagttttc agagcagcaa tttcaccacc agctagaatt gtttttaatt

116521 gttcctcaac aatagccaca tcaattggat gcacctgatg atgccaaccg ttgtctggat

116581 ccatcgtcac gatagttgta agtgcagcta aaccgtaagt actatattct ccaaatgttt

116641 tcaaatcagc ttgaatacca gcgccaccac ttgagtcgct accagcaata gttaatgttt

116701 ttggaattaa tgtcataaaa tttcctcctg tgtaataaaa agtttgttga gctgtttagg

116761 cctggcagaa aaataggaaa ttccaataaa agtgtccttt tactttttg ggaatttatc

116821 ttttttccga aggactggtt caaaagaact ggcctagata aaaagtttgt tgatccgttt

116881 aggcctatca aaagcttgtt gagtttcgct cagccccaaa agaagagtta aagcccgaaa

116941 cactaatcta aaatagactg tgttgctcat gaatagcttg gcgtttctag tttagtttta

117001 cgttaattaa gtctatttgt caattaagaa aagctgaact caagtaagtt cagccacgtt

117061 tttataaatc ttcattgata caacgcataa tagcctcagc aacaccgttc tcattattgc

117121 tagatgttag atattttgca tggcttttaa cttcttcttc agcattttcc atagcaaagc

117181 ttacacctgc aacttggagc atcgacacat cattaaaatt atcaccaatt gccattacat

117241 tttccatagg aatacctagc ctttttgcag ttcttgctaa agcaattcct ttttgtgcat

117301 ctttgtgatt aatttcaata ttattaatga aagaagaggt aatcgctaaa gaacctaatg

117361 tttcaagctt ttctttaata ggagcgaggg tatgttgacc atcatcacta aaagcaatta

117421 atttcaggac ttcaatcgag tggtcattca ctaattcttt atagtcatca atgtaattga

117481 tattcattag ctccaaacgc gccgctgcta aaacaacgcc catcttataa gtggtttcgg

117541 gatttaaatt gacaagtaag gacgcaacag attcaatccg tttgacttta ttatcagaat

117601 aaataccttt agaagtgact acttcagcat aaatattgtg ctcttttaac agttgtaaaa

117661 ttgttctaac cgtatctttt tcgattccga tattatcaat aattttacct tggccatcat

117721 atacttgagc accatttaat gtaatcatgg ggcaattaat gccaacctct gttaacaatg

117781 gatgagcttc agtgtacccg cgacctgttg aaaccataaa cttaatacca cggcgctcag

117841 cttcagcaat tgcctgagca tttgcctttg aaataaccat cttctcgttc aataacgttc

117901 cgtccatatc tgatgcaatc agctctatca ttatttttcc tccaatattt tgtgatcctc

117961 tttagtttac cattaatatg gaaaaatggt aaagaaaaga tttaataaat cagaaaacgg

118021 gaaaatctat ttttattcta ttgtttgtaa cttgcttcat aagaaagagt tgagtacaat

118081 agaaagaaga attggaaatt aacgttactt agagagatta agttaaaagg agacaaaagc

118141 aatgttggag agtagtcaac aatccggtca atctttggcc cagcaagtag ctaacaaaat

118201 agagtcttta attaaagagc aacgttataa aactggagat aaattaccaa atgaatttga

118261 attggcaaca gaattaactg ttggaagagg taccatccgt gaagcgataa aattattagc

118321 agcgagaaat gtcgttgtaa ttcagcgtgg taaaggcact tttgtatcta aaacacctgg

118381 tataactgaa gatccattag ggttaagctt tattaatgat aaaaaaagat tgagtagcga

118441 cttaatggat gtccgtgtga tgattgaacc agaaattgct aaattagccg ctgaaaatgc

118501 aacaagcgaa gaagtagaac aaatggaaaa aatttgtgat gagattgcaa ctttaattca

118561 tgcaaatgaa aatcatgaaa tgaaagatat tgagttgcat tcagctattg cgaagtcgag

118621 taaaaatgtt gtcgtgccca gtttgattcc aattatccaa acagctatat cgttatttat

118681 taatctaacc aatcgcagtt taaaagaaga aacaattgaa actcatcgtc aaattgtaga

118741 ggcaattaaa aataaagatg gcgaatctgc aaaaaaagcg atgcaacggc atttaggtta

118801 caataaagaa gaattaaatc tataaaaggg tgaagcaagc ttggattttg tctctaagct

118861 tgcctcaccc ttttattctt aatataagct aaattaattt atcaaaaaat tagttcaata

118921 aaaatgaaaa aagtgggaat atacttaata aaaatagagg aaaattaggg ttggagtttt

118981 ttttctccat atttttttga aataacataa aactgattag aactaaaaat aaaactagag

119041 cattgagtac tctcggtcca gatgcgtata ttgttggtga aaaaaacatt aatacacagg

119101 aaagtaagcc agctaaatag caaaaaaatg taaaaatagg atttctagtc acaaggcatg

119161 aacaaaagat aatatagata agaacaatgc tccaaaaaat ataaggaaat actcctatta

119221 taaaatttct tgtcaataaa ttttcaaaac tatcaaatgg aacgaaattt attctattta

119281 agtcagtcaa aagagttggt tttactattg ttaagcttaa gataattgtt cccattatat

119341 agaatatttt ctttaatttt tcagattgta gaagatttat tgttagaaga gaaattagta

119401 agataaataa tttaaaatga gtaataattt gttcgaataa ccaaaatata cctgttttca

119461 tatgtcctaa aaaggaaagt tcattaaatt caggaaacca tctgactatt tcagattcga

119521 accttactgt atttcctggt gcaaaagtca ttagtagtat ccctgcaaat actatcaagg

119581 ttagaattat caagtaaata gatggctttt ctttaattgt gaataggtag atatgataaa

119641 caaacatgat tccaaaagca cagagaacta attgttcatt agaaatagca aaaatgcacg

119701 taataattac tcttactaat tgttttaaag aaaattcagt cgtttttttt ctgaaaaatt

119761 tgtcagcaaa aggaattaag acataagcac ctaatgctaa tggccataaa taattaacag

119821 aacctgtcat ccaaaaaaat ccgctattaa taacgtcaaa tgatgaataa ccaaacaaaa

119881 gaataaccac cacgacattg tatatcttta tttccccttt aaatattcga acaattgaag

119941 tagctaacaa aaatataaaa gccgtattta gcattctcca gagattgatt gggatgtgga

120001 atatataata taacatcgtt tccggaaaaa ttcgggacga ccatgtattg tatcgtaagc

120061 ttaaataatc agctaatgaa tatttttcct tgattaaagc gaaccaacca tcatcactac

120121 cttcgataaa attaaatcga agttgataaa gaaatacgat gaaaatcaag gtgcaccata

120181 agaaaattgt tctttgcttg tgattaattt ttaggtccat gtcatttttc tcctaccttt

120241 tataagtttt taattatctt aatgatttat tttattaatt atagtatcta agtaaattaa

120301 gtaatctaat ccccatttaa gcataattat tgtgaagagt aaagactttg tctatgtaat

120361 acttaaaatc taagtttaat acaaaatttc attagcgttc attgtatgtt gtttcaaatt

120421 aaaaagataa attaaattaa aagttattga taaatgatta aaagaacaga tcccttgatg

120481 ttaaataata ataattgttg acatccacta aagaaaacgt tatcatgaaa ccgtagtcat

120541 aagacgtatg acgtccgatg tatttcaaaa tgctaaggag tggttagatg aaaattggaa

120601 ttattggttt aggtaaaatg ggtttaaatt tagcagaaaa ctgcgtagat gctggtcaca

120661 aagttgttgg ttttgatttg aaaccacctg tgttaaatca aaagcaaaaa aatattgcaa

120721 tcgttactga tttagtagaa atgattaaac aattaccgaa accacgtatt atatggtcaa

120781 tggtacctag tggcaaacca acagaagcta tttttcagga aatgaatgaa cttctggata

120841 caaatgatat ttttatagat ggcggaaatt caaattataa agagtcggtt gttcgtggca

120901 agataatgga agctaaaaag attcatttct ttgatgttgg aacctcaggc ggtatagaag

120961 gtgctagaga aggcggttgc tttatgatag gaggaaatca agacgtattt aaacaaattg

121021 aacctctttt tgtagatatc gctgtggaaa atggctatta ttatgcaggt gctgttggga

121081 gcggacatta cttaaagatg gttcataatg gtattgagta cggtatgatg caagccatcg

121141 gcgaaggttt tgatcttttg gagcatgctc catatgaatt tgattatgaa aaagttgcaa

121201 aagtttggaa taacggttcg gtgattcgct cgtggttgat ggaattaacg gaatcagctt

121261 ttagaaaaga tcaaaaatta gaagagattc aaggaattat gtattcgtct ggggaaggta

121321 agtggacagt tgaagaatca ttggaacaac aagtggcaac acctattatt accatgtcac

121381 taatgatgcg ttatcgttca ttatatccag atacatttag tgggaaagtg gtagcagctt

121441 tgagaaatga atttggcgga cacgatgtag aaaagaaata gaggtgaagg aaatgaaaga

121501 ttatttaatt ggtgttgata ttgggacaac tagtacaaaa gcagttttat atgatgcgaa

121561 aggaaatgtg aagggctact cgaataaaga atatcctttg tatcaagaat taccagatat

121621 ggcggagcaa gatccagatg agatatttga aacggtcatt gatgtattaa cagctgttgt

121681 ccgtaaaagt ggagcagatg ttactagaat tgcaggagtt tctttttcaa gtgcgatgca

121741 tagtttgatt ttgttagatc aagaaaaaca attgctgaca cgttgcatta cttgggcaga

121801 taacagagcc tatcatcaag cggaagaatt aaagaaccat aaggatggtt tagctattta

121861 tcatcgtacg ggaacgccga ttcatccaat gtcaccttta agcaaaattt tatggttgaa

121921 aaaagagcag cctgaactta ttgcaaaaac agctcatttt atcggaatta aagaatatgt

121981 attttataaa ttatttggcc aatttaaagt ggatatctca attgcttcag caactggatt

122041 attcaatatt catcaacttg attgggacca agaagtttta gccctactag atatatctgt

122101 ggaccagtta tctcaattag ttgaaacaga ttatcaagtc acgggcttaa atgaatctta

122161 tgcagaggtc attggcttac ctgtggaaac gccatttatt gtaggtgcaa gtgatggatg

122221 cctatcaaat ttaggagtga acgccattga tggcaaaacg ttagctctaa cgattggaac

122281 aagtggcgct gttcgaatgg tgacagataa acctgttact gatccaaaag gacgaacttt

122341 ctgctatgct ttaacgaaag acaaatgggt cattggtgga ccagtcaata atggtggaat

122401 tgtttttaga tgggttagag atcaattatt cgctccagaa aagataacag cagagcaaat

122461 gcaagtagac agttatgaaa ttttaactca aattgctgaa aagataccag caggatcaga

122521 tggattgctt tttcatccgt atttaggtgg agagcgagct ccactttggg atgctaatgc

122581 taaaggtgct tttctaggtt tgacaacaag gcatactcga gctcacatgg ttcgtgcatc

122641 tctcgaagga attgttttta acctgtattc cgtgatgttg attttagaag aactagtgga

122701 acgtcccgag agaattcatg cgacaggtgg atttgctcgc tcagcattat ggcgtcagtt

122761 actggcagat atttttgagc aagaagtatc tattccagaa agttacgaaa gttcttgttt

122821 aggagcagca gtgataggaa tggaaagttt aggcttaatt gattcgattg aagaggtctc

122881 taaaatgata ggtgtaacaa atcagcatac acctaatgaa gctcatttcc cggtttatcg

122941 tgaattactg cctatattta tacggacaac acgattatta caaaatgaat ttcaagcaat

123001 tgctgagttt cagcggaaat atagtaaata gagcaactat tattcttcat tcgagcctga

123061 acgaactcac caagcttttt attaggctag ctcttatgag ttagtctctc ggaagaaaga

123121 tgaaatttcg aggtggcaaa aaccgccaca tcaaatttcc ctattcttca ttcgagcctg

123181 aacgaactca ccaagctttt tattaatcta gtttttatga gccaactcct caagaaaaag

123241 ataaaaattt gagctgtcaa aaagtgtcat ctcaattttt cttatctttc ttgttggagt

123301 taactggcgc aacgaacttt ttatactagg aggaatttga tggaattatt aatagtaggt

123361 atcggcgttt tgattctttt gtttttgatt atgaaggtta agttaaatac gtttgtttct

123421 ctgattattg tttcaatttt ggttgctatt ggtttgggaa tgcctttaac tcaaattatt

123481 actactgttg aagagggggt tggtagtcaa ctcggtcatc ttgctttggt ttttgggttt

123541 ggagcaattt taggaaagtt agtctcagat gctggtggag cctatcgtat ttctacaaca

123601 ttgattaata aatttggacg taaaaaaatt caaattgctg ttttaattgc ttcttttatc

123661 attggaattg ctttgttttt tgaagtaggt cttgttttat taataccaat catttttaca

123721 attgctagtg aactcggtat ctctattcta tatttaggaa ttcccatggc agcagcactt

123781 tctgttacac acggtttttt accgccacat cctgcgccaa cagccatcgc aggcatctac

123841 ggtgcaaata tcggaatggt attgtcattg ctgttccaac gagtattctg gcaggtccta

123901 tgtatactaa agtagctcag aaaattgttc cagatgcatt taatcgtgtc gggaatataa

123961 aagcattggg ggaacagaaa aaatttacac tagaagatac tccaggtttt ggaattagta

124021 cactcacagc tttatttcca gttatattga tggcgcttgc aacttgttat gaattgctaa

124081 gtggtgggaa aataaccgat aaatctccgt tgtatgaaca agtcattgct ttgataggta

124141 caccaggtat ggccatgtta ttatccttat tatttgctat ttatacaatg ggagttcgtc

124201 gccaacaaac aattccagca attatgaaat cagcagaaga agcgattaaa caaattgcta

124261 tgatgttatt gattattgga ggcggtggtg cttttaaaca agtattacta gatggtggag

124321 tcggagatta tgtcgcacaa ttatttgcgg gttcaacttt atctccttta attttaggtt

124381 gggtaattgc agtagtttta agagtttgtt taggttctgc tactgtggct gctttaacag

124441 ctgctggact tgcagctccc ttgatggcag caacaggaac agatccagca ctaatggtaa

124501 ttgctacagg agcaggtagt ttgtttgcct ctcatgtaaa tgatgcgggt ttctggatgt

124561 tcaaagaata tttcaattta tcgattaaag aaacttttgc aacatggaca gttttagaat

124621 caattatttc agttgcggga ttattaggag ccttagcttt gaatttaatc gtttaatttt

124681 aaagtcggat tttctttatg aaaatctgac tttttctttt aaattaaacc tatcaactgt

124741 tatacttgaa atgtaaacta ttattagttg gttacgacat aataattaaa tatttagctg

124801 aatttagcta taatggaatt ttaggatagg acaacaaaat ttttatataa ggagcgatat

124861 gataatcatg aaaacaatta ttcataacga ttggcaagat attttacaag aagaatttga

124921 atctacttac tatcaagaat taagagaatt tttaaaaaca gaatatgcgg aacaaactat

124981 ttatccttcg atgaatcata tttgggaagc ctttgaatgg actccatatg agcaggtgaa

125041 agttgttata ttaggacaag atccttatca tggtcctaat caggcacatg ggttaagttt

125101 ttcggtccaa ccaaatgtaa aggtccctcc ttctttagtc aatatatata aagaattagc

125161 agatgactta ggtatcacac cagttaatca tggttattta aaatcgtggg ctgaacaagg
[truncated: 4,217,075 more chars]
