# Supplementary material for: Reference Whole Genome Sequence Analyses and Characterization of a Novel Carnobacterium maltaromaticum Distinct Sequence Type Isolated from a North American Gray Wolf (Canis lupus) Gastrointestinal Tract
Source: Vet Sci. 2025 Apr 27;12(5):410. doi: 10.3390/vetsci12050410 (PMC12115997; doi:10.3390/vetsci12050410)
Supplement: Supplementary file 1 [file vetsci-12-00410-s001.zip › KlewsEtAl_Supplementary Table S2 - AntiSMASH results.pdf]

Table S2. Results from the antiSMASH Bacterial Version for the ClWan1 Genome.

Region	Type	From	To	Core Biosynthetic Gene(s)
1	Terpene	3,512,202	3,512,202	Phytoene synthase
2	Type III PKS	2,560,366	2,601,538	HMG-CoA Synthase
3	Thiopeptide, cyclic lactone autoinducer	2,705,243	2,735,847	LAP, LanC, YcaO;
4	RiPP-like	2,786,273	2,796,458	Bacteriocin (type II)
5	Non-ribosomal peptide	3,045,380	3,091,073	NRPS (Tyr, Thr) Loading

NOTE: HMG-CoA: Hydroxymethylglutaryl coenzyme A, NRPS: Non-ribosomal peptide synthetase, RiPP: Ribosomal synthesized post-translationally modified peptide product, LAP: Linear azol(in) containing peptide
