# Supplementary material for: Reference Whole Genome Sequence Analyses and Characterization of a Novel Carnobacterium maltaromaticum Distinct Sequence Type Isolated from a North American Gray Wolf (Canis lupus) Gastrointestinal Tract
Source: Vet Sci. 2025 Apr 27;12(5):410. doi: 10.3390/vetsci12050410 (PMC12115997; doi:10.3390/vetsci12050410)
Supplement: Supplementary file 1 [file vetsci-12-00410-s001.zip › KlewsEtAl_Supplementary Table S3 Type Strain Genome dDDH_ClWan1.pdf]

Type Strain Genome Server

Query strain	Subject strain	dDDH (d0, in %)	C.I. (d0, in %)	dDDH (d4, in %)	C.I. (d4, in %)	dDDH (d6, in %)	C.I. (d6, in %)	G+C content difference (in %)
'ClWan1'	Carnobacterium maltaromaticum MX5	76.1	[72.1 - 79.7]	90.6	[88.4 - 92.5]	81.4	[78.0 - 84.3]	0.05
'ClWan1'	Lactobacillus carnis DSM 20722	81.7	[77.9 - 85.1]	90.6	[88.3 - 92.4]	86.1	[83.0 - 88.8]	0.17
'ClWan1'	Carnobacterium piscicola ATCC 35586	81.6	[77.7 - 85.0]	90.1	[87.8 - 92.0]	86.0	[82.8 - 88.6]	0.02
'ClWan1'	Enterococcus phoeniculicola ATCC BAA-412	12.8	[10.1 - 16.1]	25.5	[23.2 - 28.0]	13.2	[10.9 - 16.0]	1.91
'ClWan1'	Carnobacterium viridans MPL-11	13.3	[10.6 - 16.6]	25.2	[22.9 - 27.7]	13.7	[11.3 - 16.5]	0.68
'ClWan1'	Enterococcus lactis DSM 23655	12.8	[10.1 - 16.0]	25.1	[22.8 - 27.6]	13.2	[10.8 - 15.9]	3.65
'ClWan1'	Enterococcus alcedinis CCM8433	12.8	[10.1 - 16.1]	24.3	[22.0 - 26.8]	13.2	[10.8 - 15.9]	3.1
'ClWan1'	Enterococcus wangshanyuanii CGMCC 1.15942	12.8	[10.1 - 16.1]	23.4	[21.2 - 25.9]	13.2	[10.8 - 15.9]	2.77
'ClWan1'	Dellaglioa algida DSM 15638	12.8	[10.1 - 16.1]	22.9	[20.7 - 25.4]	13.2	[10.9 - 16.0]	1.55
'ClWan1'	Enterococcus silesiacus LMG 23085	13.0	[10.3 - 16.3]	22.8	[20.5 - 25.3]	13.3	[11.0 - 16.1]	1.93
'ClWan1'	Carnobacterium gallinarum MT44	18.5	[15.4 - 22.1]	22.4	[20.2 - 24.9]	18.2	[15.6 - 21.2]	0.2
'ClWan1'	Tetragenococcus muriaticus DSM 15685	12.7	[10.0 - 16.0]	22.3	[20.0 - 24.7]	13.1	[10.8 - 15.8]	1.47
'ClWan1'	Enterococcus faecalis NBRC 100480	13.0	[10.3 - 16.3]	21.9	[19.6 - 24.3]	13.3	[11.0 - 16.1]	3.04
'ClWan1'	Enterococcus camelliae TISTR 932	12.7	[10.0 - 16.0]	21.7	[19.5 - 24.2]	13.1	[10.8 - 15.9]	4.14
'ClWan1'	Carnobacterium divergens 66	16.1	[13.2 - 19.6]	21.7	[19.4 - 24.1]	16.1	[13.6 - 19.1]	0.62
